# The intracellular immune receptor Rx1 regulates the DNA-binding activity of a Golden2-like transcription factor

**DOI:** 10.1074/jbc.RA117.000485

**Published:** 2017-12-07

**Authors:** Philip D. Townsend, Christopher H. Dixon, Erik J. Slootweg, Octavina C. A. Sukarta, Ally W. H. Yang, Timothy R. Hughes, Gary J. Sharples, Lars-Olof Pålsson, Frank L. W. Takken, Aska Goverse, Martin J. Cann

**Affiliations:** From the ‡ Department of Biosciences,; § Biophysical Sciences Institute, and; ** Department of Chemistry, Durham University, South Road, Durham DH1 3LE, United Kingdom,; the ¶ Laboratory of Nematology, Department of Plant Sciences, Wageningen University, 6708 PB Wageningen, The Netherlands,; the ‖ Donnelly Centre, University of Toronto, Toronto, Ontario M5S 3E1, Canada, and; ‡‡ Molecular Plant Pathology, Swammerdam Institute for Life Sciences, University of Amsterdam, Science Park 904, 1098 XH Amsterdam, The Netherlands

**Keywords:** cellular immune response, DNA-binding protein, fluorescence resonance energy transfer, host-pathogen interaction, Nod-like receptor, plant biochemistry, plant defense, plant virus, .

## Abstract

Plant nucleotide-binding leucine–rich repeat (NLR) proteins enable the immune system to recognize and respond to pathogen attack. An early consequence of immune activation is transcriptional reprogramming, and some NLRs have been shown to act in the nucleus and interact with transcription factors. The Rx1 NLR protein of potato is further able to bind and distort double-stranded DNA. However, Rx1 host targets that support a role for Rx1 in transcriptional reprogramming at DNA are unknown. Here, we report a functional interaction between Rx1 and *Nb*Glk1, a Golden2-like transcription factor. Rx1 binds to *Nb*Glk1 *in vitro* and *in planta. Nb*Glk1 binds to known Golden2-like consensus DNA sequences. Rx1 reduces the binding affinity of *Nb*Glk1 for DNA *in vitro. Nb*Glk1 activates cellular responses to potato virus X, whereas Rx1 associates with *Nb*Glk1 and prevents its assembly on DNA *in planta* unless activated by PVX. This study provides new mechanistic insight into how an NLR can coordinate an immune signaling response at DNA following pathogen perceptions.

## Introduction

Plants possess an innate immune system that enables cell-autonomous defense responses upon pathogen perception (1, 2). Plant NLR^3^ immune receptors detect strain-specific pathogen effectors to mediate the immune responses to an invading pathogen (2–4). NLR proteins belong to the STAND (signal-transduction ATPases with numerous domains) loop ATPases of the AAA superfamily and have a multidomain structure that allows them to function as a sensor, switch, and response factor (5, 6).

The NLR N terminus typically consists of either a CC or TIR (Toll-interleukin 1 receptor) domain (7). The NLR NB domain, also referred to as the NB-ARC domain, is proposed to function as a molecular switch in NLR activation (6, 8–10). The C-terminal LRR domain is required for pathogen recognition specificity and for maintaining the NLR protein in an autoinhibited state. The NB-ARC domains of tomato I-2 and Mi-1, flax M and L6, and barley MLA27 are ADP-bound in this autoinhibited state (7, 11, 12). Pathogen recognition via the LRR domain is proposed to permit the exchange of ADP for ATP, allowing the NB-ARC domain to adopt its activated state. ATP hydrolysis to ADP is proposed to re-establish the inactivated state. For example, mutants of the tomato I-2 NLR with reduced levels of *in vitro* ATP hydrolysis are autoactivated *in vivo* (11). Nucleotide hydrolysis in NLR inactivation may extend further than ADP. For example, the NB subdomain of rice Os2g_25900 and the NB-ARC domains of maize PSiP and *Arabidopsis* Rpm1 possess a nucleotide phosphatase activity in which all phosphates are removed from the nucleotide triphosphate to leave the nucleoside base (13).

A crucial question concerns the nature of the downstream signaling component(s) for plant NLR proteins and how these are activated or inactivated by NLR proteins upon pathogen perception. Several NLR proteins, including N, MLA10, and Rx1, have a dynamic nuclear-cytoplasmic distribution, whereas RRS1-R is restricted to the nucleus, dependent upon the presence of the PopP2 immune elicitor (14–18). Several NLRs, including barley MLA1 and MLA10, *Arabidopsis* RPS4 and SNC1, and the tobacco N protein, show a nuclear localization (17, 19–21). Redirection of MLA10, N, RPS4, and SNC1 from the nucleus to the cytoplasm reduces immune activation suggesting a signaling component resident in the nucleus (14, 17, 20, 22). One of the most important and earliest consequences of immune activation is transcriptional reprogramming (23–25). The association of MLA10 with Myb and WRKY transcription factors suggests that plant NLRs themselves might regulate transcription in the immune response (26, 27).

Biochemical data suggest that at least a subset of plant NLRs are directly active at DNA. Rx1 of potato, I-2 of tomato, and the orphan NLR PSiP of maize interact directly with DNA *in vitro* (28, 29). The *Rx1* gene, introgressed in potato from the wild species *Solanum tuberosum* subsp. *andigena*, confers resistance to PVX upon recognition of its coat protein (30, 31). The Rx1 protein binds to genomic DNA *in situ* dependent upon immune activation (29). In addition, Rx1 induces ATP-dependent bending and melting of DNA *in vitro*. Analysis of Rx1 binding to a variety of DNA structures demonstrated a preference for topologies resembling transcription bubbles. Rx1 therefore binds, bends, and distorts DNA in a manner reminiscent of the formation of the transcription initiation complex (32–35).

*In vitro* analysis demonstrated no sequence specificity in the Rx1 interaction with dsDNA. Therefore, there is a question regarding how the non-specific interaction of Rx1 with DNA can be reconciled with a specific role in immune activation. The Rx1 CC domain is responsible for the nuclear accumulation of Rx1. Furthermore, the Rx1 CC domain diffusion rate in the nucleus is low, pointing to complex formation with nuclear components (18). We therefore set out to identify nuclear interactors of the Rx1 CC domain and investigate their role with Rx1 in immunity at DNA. Here, we demonstrate that Rx1 interacts directly with a Golden2-like TF (*Nb*Glk1). This Golden2-like TF mediates immune responses to PVX and has its activity at DNA regulated by Rx1. The findings therefore provide new insight into the action of NLR proteins at genomic DNA in controlling immune responses.

## Results

### Rx1 CC domain interacts with a DNA-associated protein

**Figure 1.**
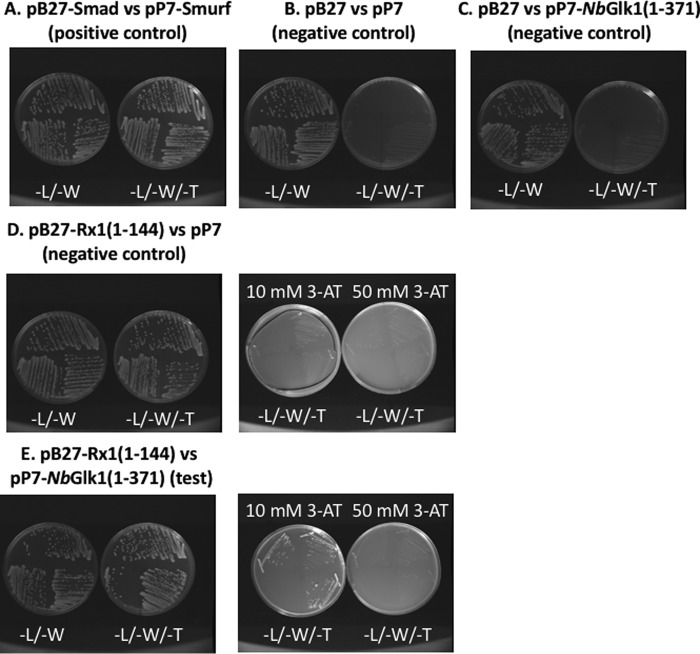
**N terminus of the Rx1 NLR protein interacts with the *Nb*Glk1 transcription factor.** Yeast two-hybrid analysis of Rx1(1–144) bait fragment against a prey fragment of amino acids 1–371 of *Nb*Glk1. Rx1(1–144) was fused to the Gal4 DNA-binding domain, and *Nb*Glk1(1–371) was fused to the Gal4 activation domain. Plates were grown on medium lacking leucine and tryptophan (−L/−W) and medium lacking leucine, tryptophan, and histidine (−L/−W/−H), supplemented with 10 or 50 mm 3-AT. *A,* Smad *versus* Smurf positive control. *B,* empty pB27 bait *versus* empty pP7 prey negative control. *C,* empty pB27 bait *versus Nb*Glk1(1–371) in prey negative control. *D,* Rx1(1–144) containing bait *versus* empty pP7 prey negative control. *E,* Rx1(1–144) in pB27 bait plasmid with *Nb*Glk1(1–371) in pP7 prey plasmid.

We set out to identify Rx1 CC domain interactors and investigate their role in immunity at DNA. Amino acids 1–144 of Rx1, encompassing the CC domain, were used as bait in a yeast two-hybrid screen using a random-primed *Nicotiana benthamiana* mixed tissue cDNA library. We identified a total of six clones with sequence similarity to a GLK TF (here called *Nb*Glk1) (Fig. 1). The six clones corresponded to three individual cDNAs isolated twice each. Individual clones were presumably obtained multiple times due to amplification of the cDNA library. The full-length *Nb*Glk1 cDNA (Fig. S1*A*) encodes a 402-amino acid protein with a predicted molecular mass of 44,531 Da and carries a single Myb-type helix-turn-helix DNA-binding domain. GLK TFs are classified into the GARP TF family (36). *Nb*Glk1 possessed a C-terminal GCT box specific to GLK-type TFs and an AREAEAA hexapeptide sequence within the DNA-binding domain typical of GARP family TFs (Fig. S1*B*) (37, 38).

### Rx1 interacts directly with NbGlk1 in vitro and in vivo

**Figure 2.**
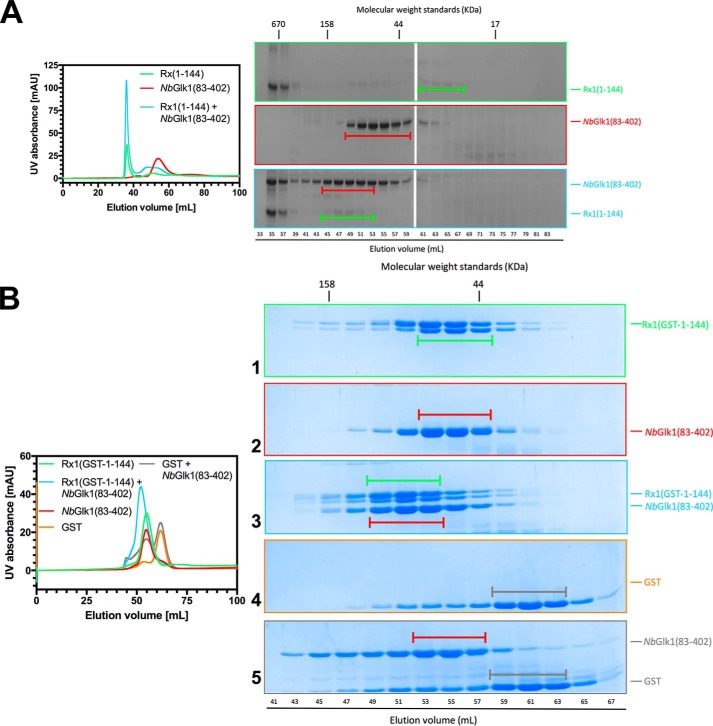
**Rx1 binds *Nb*Glk1 *in vitro*.** *A,* interaction of Rx1(1–144) with *Nb*Glk1. On the *left* are representative gel-filtration chromatograms of Rx1, *Nb*Glk1(83–402), and Rx1 incubated with *Nb*Glk1(83–402). Peak fractions were visualized by SDS-PAGE. *B,* interaction of Rx1(GST-1–144) with *Nb*Glk1. On the *left* are representative gel-filtration chromatograms of Rx1(GST-1–144), *Nb*Glk1(83–402), GST, and Rx1(GST-1–144) incubated with *Nb*Glk1(83–402) and GST incubated with *Nb*Glk1(83–402). Peak fractions were visualized by SDS-PAGE.

The *Nb*Glk1 yeast two-hybrid clones all encompassed the Myb-type DNA-binding domain. We therefore assessed Rx1 binding to *Nb*Glk1 before analyzing their interactions with DNA. Unfortunately, we were unable to express full-length *Nb*Glk1 as a recombinant protein. We therefore expressed amino acids 83–402 of *Nb*Glk1 (*Nb*Glk1(83–402)) and examined its interaction with the Rx1–CC domain (Rx1(1–144)) by size-exclusion chromatography. Rx1(1–144) ran predominantly in the void volume (Fig. 2*A*, *upper SDS-PAGE panel,* elution volume of 35–37 ml). We hypothesize that this peak consists of a higher order oligomeric Rx1–CC complex but its relevance to Rx1 biochemistry, if any, is not known. We noted a shift in the peak bands corresponding to Rx1(1–144) (Fig. 2*A*, *upper SDS-PAGE panel*, *capped green bar*) and *Nb*Glk1(83–402) (Fig. 2*A*, *middle SDS-PAGE panel*, *capped red bar*) when incubated together (Fig. 2*A*, lower SDS-PAGE panel) showing that *Nb*Glk1 and Rx1–CC interact directly *in vitro*.

**Figure 3.**
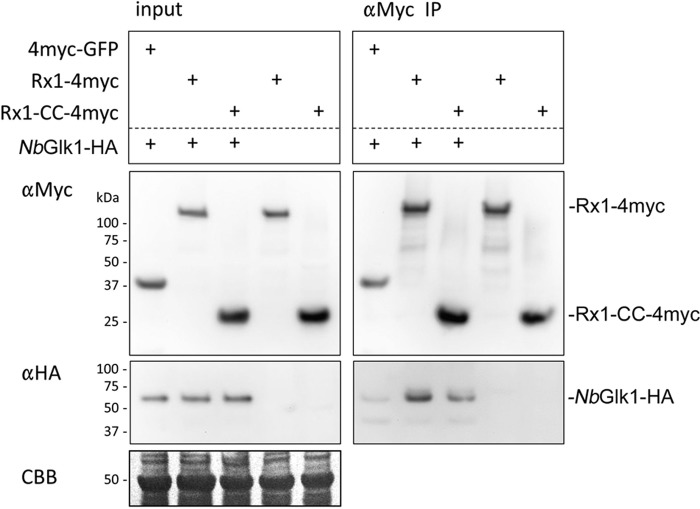
**Rx1 binds *Nb*Glk1 *in planta*.** Shown is co-immunoprecipitation of 4×Myc-tagged full-length Rx1 or Rx1–CC when co-expressed *in planta* with C-terminally HA-tagged *Nb*Glk1. The labels on the figure are as follows: *Input* denotes the constructs agroinfiltrated into *N. benthamiana* leaves; α*Myc* denotes an immunoblot probed using an anti-Myc epitope tag antibody; α*HA* denotes an immunoblot probed using an anti-HA epitope tag antibody; α*Myc IP* denotes immunoprecipitation of the denoted input samples using an anti-Myc epitope tag antibody; *CBB* denotes Coomassie Blue stain loading control for the denoted input samples. Immunoblot bands corresponding to Rx1-4myc, Rx1–CC-4myc, and *Nb*Glk1-HA from the αMyc and αHA immunoblots are indicated.

It is possible that the observed interaction between Rx1(1–144) and *Nb*Glk1(83–402) was influenced by an unknown property of Rx1(1–144) that also causes a proportion of the protein to run in the gel filtration void volume. To enhance protein solubility, we developed a new Rx1–CC protein with an N-terminal GST tag. We assessed binding of the Rx1–CC domain–GST fusion (Rx1(GST-1–144) to *Nb*Glk1(83–402). Rx1(GST-1–144) was completely soluble with no detectable protein in the void volume (Fig. 2*B*, *UV trace*). However, Rx1(GST-1–144) was susceptible to some proteolytic cleavage during purification at the extreme C terminus and resulted in the protein running as a doublet by SDS-PAGE. We noted a shift in the peak bands corresponding to Rx1(GST-1–144) (Fig. 2*B*, *SDS-PAGE panel 1*, *capped green bar*) and *Nb*Glk1(83–402) (Fig. 2*B*, *SDS-PAGE panel 2*, *capped red bar*) when incubated together (Fig. 2*B*, *SDS-PAGE panel 3*). GST protein expressed alone (Fig. 3, *SDS-PAGE panel 4*) showed no shift in peak band when expressed with *Nb*Glk1(83–402) (Fig. 2*B*, *SDS-PAGE panel 5*). *Nb*Glk1 and Rx1(GST-1–144) therefore interact directly *in vitro*.

We next assessed whether full-length Rx1 and *Nb*Glk1 interact *in planta*. We performed co-immunoprecipitation experiments using full-length *Nb*Glk1 fused to a 4×HA epitope tag with either full-length Rx1 or the CC domain fused to a 4×Myc epitope tag. *Nb*Glk1-4HA, when co-expressed with full-length Rx1 or the CC domain, was immunoprecipitated using the anti-Myc antibody and could be detected with the anti-HA tag antibody (Fig. 3). A very faint immunoblot band for *Nb*Glk1-4HA was observed after immunoprecipitation using an anti-Myc antibody when co-expressed with a fusion of GFP to a 4×Myc epitope tag. This signal was significantly lower than those obtained on co-expression with Rx1 indicating that its immunoprecipitation was specific. Full-length *Nb*Glk1 therefore interacts with the CC domain and full-length Rx1 *in planta*.

### Rx1 modulates NbGlk1 interactions with DNA in vitro

We investigated whether *Nb*Glk1 showed sequence-specific DNA binding. We analyzed the DNA-binding properties of the *Nb*Glk1 DNA-binding domain compared with the known binding properties of GLK TFs using a protein-binding microarray (PBM) consisting of ∼41,000 35-mer probes in which all possible 10-mers occur once and all non-palindromic 8-mers are represented 32 times, allowing for an unbiased assessment of sequence preference for all possible 8-mers (39). Values for individual 8-mers were obtained as *E*-scores (representing relative rank of intensities ranging from −0.5 to + 0.5) and *Z*-scores (scaling approximately with binding affinity) (Fig. S2). The DNA-binding sequence for the top scoring 8-mer for *Nb*Glk1 was 5′-AGATTCCC-3′ and 5′-AGATTTCC-3′ for *E*-score (0.49648) and *Z*-score (37.25190), respectively. Both identified DNA motifs are similar to the AGATTCT core palindrome recognized by the GLK TF encoded by At2g20570 of *Arabidopsis thaliana* (40).

**Figure 4.**
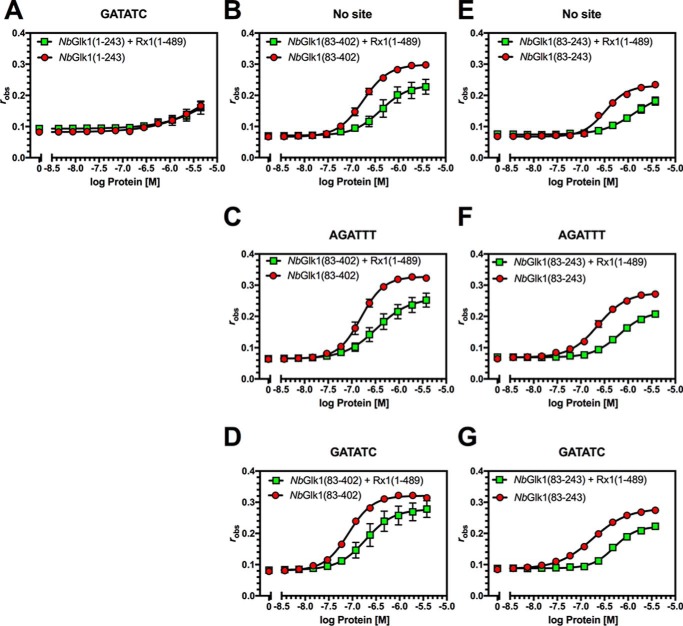
**Influence of Rx1(1–489) on *Nb*Glk1 DNA binding.** Fluorescence anisotropy values are plotted against log protein concentration for DNA motifs in the presence or absence of Rx1(1–489). *A, Nb*Glk1(1–243) with the GATATC motif. *B, Nb*Glk1(83–402) in the absence of a specific motif. *C, Nb*Glk1(83–402) with the AGATTT motif. *D, Nb*Glk1(83–402) with the GATATC motif. *E, Nb*Glk1(83–243) in the absence of a specific motif. *F, Nb*Glk1(83–243) with the AGATTT motif. *G, Nb*Glk1(83–243) with the GATATC motif (means ± S.E.; *n* >3). Statistical analyses of the curves are provided in the text and Tables 1 and 2.

We measured the *K_d_* value of *Nb*Glk1 for dsDNA substrates lacking the hypothesized *Nb*Glk1-binding site (No site), with a concatenated AGATTTCC-binding site (from the *Nb*Glk1 PBM, labeled AGATTT) and with a concatenated GGATATCC-binding site (from the GLK1 consensus site (40)) and also identified in the PBM analysis of *Nb*Glk1 (*Z*-score = 29.62117, labeled GATATC) by fluorescence anisotropy (Table 1). A recombinant protein consisting of *Nb*Glk1 residues 1–243 (*Nb*Glk1(1–243)) encompassing the Myb-type helix-turn-helix DNA-binding domain displayed only a weak affinity (*K_d_* >1 μm; Table 1) for the GATATC-binding site (Fig. 4*A*). This low affinity prevented drawing conclusions on the influence of Rx1 on *Nb*Glk1(1–243) DNA binding (Fig. 4*A* and Table 1).

**Table 1:** **Influence of hexahistidine-tagged Rx1 proteins on the dissociation constant (*K_d_*) of *Nb*Glk1 for DNA-binding motifs** Values are in μm (±S.D.) ND = not determined; – = no additional Rx1 protein; *, *p* < 0.0001 compared with the No site motif; #, *p* > 0.05 compared with the No site motif (*F* test).

	*Nb*Glk1(1–243)			*Nb*Glk1(83–243)			*Nb*Glk1(83–402)		
	–	Rx1(1–144)	Rx1(1–489)	–	Rx1(1–144)	Rx1(1–489)	–	Rx1(1–144)	Rx1(1–489)
No site	ND	ND	ND	0.33 ± 0.02	0.85 ± 0.27	1.34 ± 0.42	0.18 ± 0.01	0.40 ± 0.02	0.42 ± 0.09
AGATTT	ND	ND	ND	0.23 ± 0.01#	0.76 ± 0.52	0.65 ± 0.04	0.16 ± 0.01#	0.46 ± 0.06	0.34 ± 0.04
GATATC	>1	>1	>1	0.18 ± 0.01#	0.40 ± 0.15	0.50 ± 0.03	0.08 ± 0.00*	0.30 ± 0.07	0.19 ± 0.04

Analysis of the *Nb*Glk1 ORF revealed a pI of 3.8 for the N-terminal 82 amino acids. We hypothesized that the acidic N-terminal 82 amino acids could interfere with analysis of the interactions of the *Nb*Glk1 Myb-type domain with DNA. We therefore measured *Nb*Glk1 DNA binding with a protein lacking the N-terminal 82 amino acids. *Nb*Glk1(83–402) possessed a similar *K_d_* value for both DNA containing the no *Nb*Glk1-binding site (Fig. 4*B*) or a AGATTT-binding site (*F*-test, *p* = 0.41) (Fig. 4*C*). However, *Nb*Glk1(83–402) possessed a lower *K_d_* value for the GATATC-binding site (*F*-test, *p* < 0.0065) (Fig. 4*D*). In comparison with *Nb*Glk1(83–402), a C-terminally truncated recombinant protein consisting of amino acids 83–243 of *Nb*Glk1 (*Nb*Glk1(83–243)) showed a similar *K_d_* value for DNA irrespective of whether it contained no *Nb*Glk1-binding site (Fig. 4*E*) or the AGATTT site (*F*-test, *p* = 0.507) (Fig. 4*F*) or GATATC sites (*F*-test, *p* = 0.0827) (Fig. 4*G*). There was also no significant difference in affinity between *Nb*Glk1(83–402) and *Nb*Glk1(83–243) for the No site (*p* = 0.3659) and AGATTT (*p* = 0.2171) DNA-binding motifs. However, there was a significant difference in affinity between *Nb*Glk1(83–402) and *Nb*Glk1(83–243) for the GATATC (*p* < 0.001) DNA-binding motif. In conclusion, *Nb*Glk1 shows a higher affinity for DNA carrying a GLK family-binding site than for a random sequence. Furthermore, both the N and C termini of the protein contribute to DNA-binding affinity. The acidic N-terminal 82 amino acids significantly reduced DNA binding. Amino acids 243–402 also enhance affinity for the GATATC motif compared with the No site DNA. Furthermore, amino acids 243–402 enhance affinity for all DNA sequences examined. For example, *Nb*Glk1(83–402) showed a higher affinity than *Nb*Glk1(83–243) for the No site DNA (*F*-test, *p* < 0.001), the AGATTT motif (*F*-test, *p* = 0.0196), and the GATATC motif (*F*-test, *p* = 0.0436). We therefore hypothesize that a region(s) within amino acids 243–402 contributes to DNA binding.

**Figure 5.**
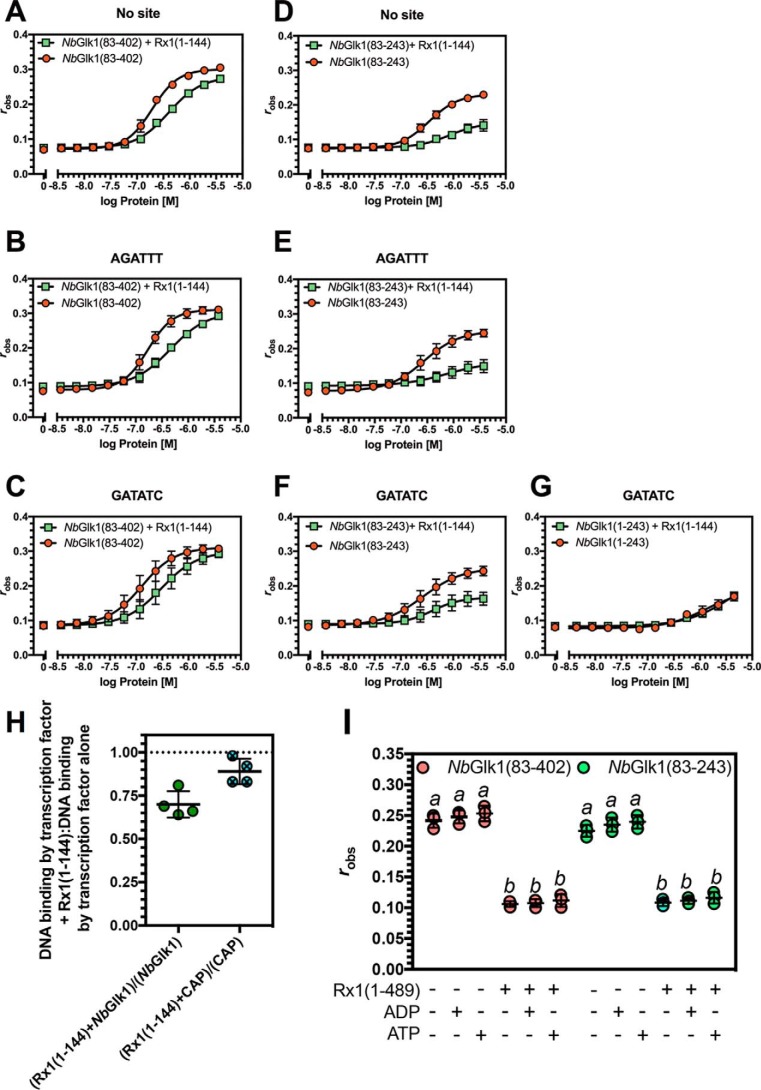
**Influence of Rx1(1–144) and Rx1(1–489) on *Nb*Glk1 DNA binding.** *A–G* shows fluorescence anisotropy values plotted against log protein concentration for varying *Nb*Glk1 constructs binding to different DNA motifs in the presence or absence of absence of Rx1(1–144) (*n* = 4). *A, Nb*Glk1(83–402) binding to DNA in the absence of a specific motif. *B, Nb*Glk1(83–402) binding to DNA with the AGATTT motif. *C, Nb*Glk1(83–402) binding to DNA with the GATATC motif. *D, Nb*Glk1(83–243) binding to DNA in the absence of a specific motif. *E, Nb*Glk1(83–243) binding to DNA with the AGATTT motif. *F, Nb*Glk1(83–243) binding to DNA with the GATATC motif. *G, Nb*Glk1(1–243) binding to DNA with the GATATC motif. Statistical analyses of the curves for *A–G* are provided in the text and Tables 1 and 2. *H,* ratio of fluorescence anisotropy values for 10 μm *Nb*Glk1 or CAP binding to dsDNA with or without Rx1(1–144) (scatter plot ± S.D.). The means are significantly different (*p* = 0.0286, Mann-Whitney). *I,* DNA binding for *Nb*Glk1(83–402) and *Nb*Glk1(83–243) was measured in the presence or absence of 10 μm nucleotide (scatter plot ± S.D.; *bars with different letters* are significantly different (*p* < 0.05); one-way ANOVA with post hoc Holm-Sidak multiple comparison).

Because our underlying hypothesis was that *Nb*Glk1 interacted with Rx1 at DNA, we investigated the influence of amino acids 1-489 of Rx1 (consisting of the CC and NB-ARC domains; Rx1(1–489)) on *Nb*Glk1 dsDNA-binding affinity. Experiments were performed using conditions under which only *Nb*Glk1 and not Rx1 contributed to DNA binding. These conditions were established by using a concentration of Rx1(1–489) that gave no change in anisotropy when incubated with DNA alone. *Nb*Glk1(83–402) and *Nb*Glk1(83–243) showed a higher *K_d_* value for DNA containing no *Nb*Glk1-binding site (Fig. 4, *A* and *D*), the AGATTT-binding site (Fig. 4, *B* and *E*), and the GATATC-binding sites (Fig. 4, *C* and *F*) in the presence of Rx1(1–489) (*F*-test, *p* < 0.0001). Similar data were observed for the influence of Rx1(1–144) (CC domain only) on *Nb*Glk1(83–402) and *Nb*Glk1(83–243) DNA binding (Table 1 and Fig. 5, *A–G*) (F-test, *p* < 0.0001). We confirmed that the increased *K_d_* value was specific for *Nb*Glk1 by using the CAP TF of *Escherichia coli*, which has a similar molecular weight and pI to *Nb*Glk1, as a negative control. Rx1(1–144) reduced *Nb*Glk1 but not CAP binding to dsDNA (Fig. 4*H*). ATP stimulates Rx1(1–489)-mediated dsDNA distortion (29). We therefore investigated whether ATP and/or ADP modified the influence of Rx1(1–489) on *Nb*Glk1 DNA binding. We observed no effect indicating that the switch function of the NB-ARC domain does not influence *Nb*Glk1 DNA binding (Fig. 5*I*). In conclusion, the Rx1–CC and –CCNB-ARC domains reduce the affinity of *Nb*Glk1 for DNA. The data are consistent with the observation that the Rx1 CC-binding surface overlaps with the *Nb*Glk1 DNA-binding domain. Rx1 may therefore restrict access of *Nb*Glk1 to DNA whether binding is to a consensus or non-consensus binding site.

**Figure 6.**
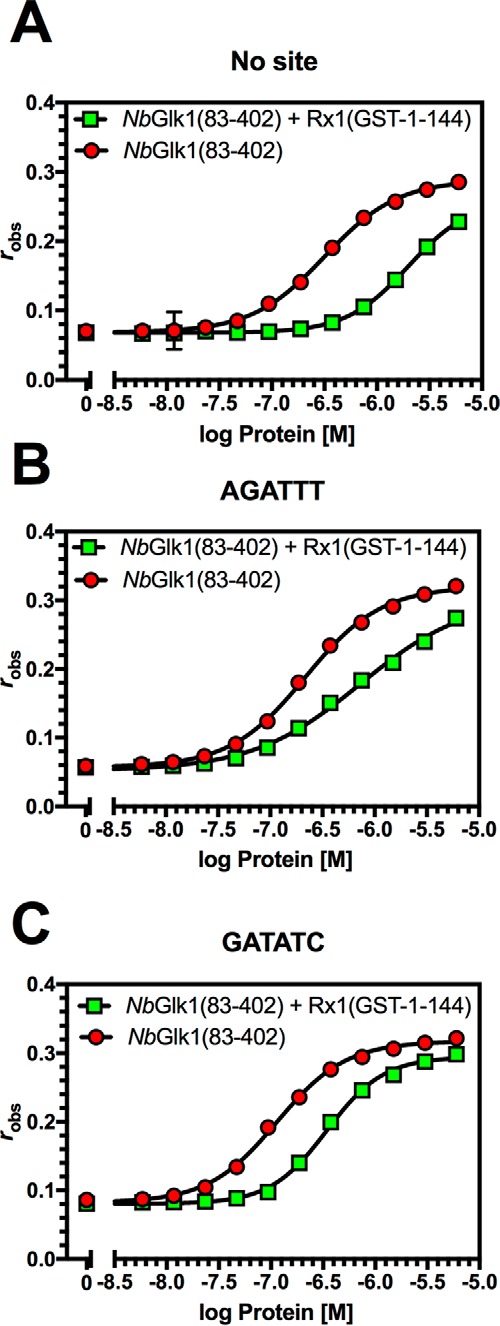
**Influence of Rx1(GST-1–144) on *Nb*Glk1 DNA binding.** The panels show fluorescence anisotropy values plotted against log protein concentration for *Nb*Glk1(83–402) binding to different DNA motifs in the presence or absence of Rx1(GST-1–144) (*n* = 4). *A, Nb*Glk1(83–402) binding to DNA in the absence of a specific motif. *B, Nb*Glk1(83–402) binding to DNA with the AGATTT motif. *C, Nb*Glk1(83–402) binding to DNA with the GATATC motif.

Although both Rx1(1–489) and Rx1(1–144) reduce the binding affinity of *Nb*Glk1 for DNA, a potential alternative explanation for the reduction in *Nb*Glk1 DNA-binding affinity caused by Rx1(1–144) is that the higher ordered Rx1–CC complex observed by gel filtration analysis (Fig. 2*A*) is sufficient to reduce the concentration of soluble *Nb*Glk1. This might therefore manifest as an apparent reduction in DNA-binding affinity. We therefore assessed the influence of Rx1(GST-1–144) on *Nb*Glk1(83–402) binding to DNA containing no *Nb*Glk1-binding site (Fig. 6*A*), the AGATTT site (Fig. 6*B*), or GATATC site (Fig. 6*C*). *Nb*Glk1(83–402) showed a higher *K_d_* value for DNA containing no *Nb*Glk1-binding site (Fig. 6*A*), the AGATTT-binding site (Fig. 6*B*), and the GATATC-binding sites (Fig. 6*C*) in the presence of Rx1(GST-1–144) (*F*-test, *p* < 0.0001) (Table 2). The Rx1 CC domain therefore reduces the affinity of *Nb*Glk1(83–402) for DNA, and this observation is not an artifact due to Rx1(1–144) complex formation.

**Table 2:** **Influence of GST-tagged Rx1 protein on the dissociation constant (*K_d_*) of *Nb*Glk1(83–402) for DNA-binding motifs** Values are in μm (±S.D.); – = no additional Rx1 protein; *, *p* < 0.0001 compared with the No site motif (*F* test).

	*Nb*Glk1(83–402)	
	–	Rx1(GST-1–144)
No site	0.32 ± 0.03	1.99 ± 0.09
AGATTT	0.22 ± 0.00*	0.73 ± 0.07
GATATC	0.11 ± 0.00*	0.34 ± 0.00

### Rx1 and NbGlk1 interact at DNA in situ

**Figure 7.**
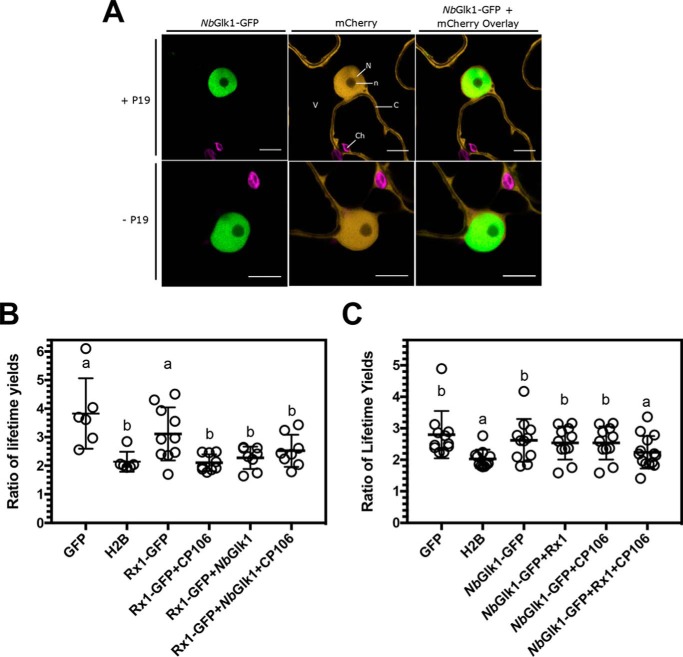
**Binding of Rx1 and N*b*Glk1 protein to DNA *in situ*.** *A,* representative confocal images of nuclei and surrounding cytoplasm of *N. benthamiana* epidermal cells transiently co-expressing *Nb*Glk1-GFP with mCherry. Images are of the *Nb*Glk1-GFP channel (*left-hand panel*), the mCherry channel (*center panel*), and an overlay of the *Nb*Glk1-GFP and mCherry channels (*right-hand panel*). Co-expression was performed in the presence (*upper panels*) or absence (*lower panels*) of the p19 silencing suppressor. *Scale bar* represents a width of 10 μm. Subcellular structures are indicated by *N* = nucleus; *n* = nucleolus; *C* = cytoplasm; *V* = vacuole; *Ch* = choloroplasts. *B,* ratio of the long to short GFP lifetimes for a Rx1-GFP full-length construct alone and upon co-expression with *Nb*Glk1 and the avirulent CP106 allele of the PVX CP (scatter plot ± S.D.; *bars with different letters* are significantly different (*p* < 0.05); one-way ANOVA with post hoc Dunnett's multiple comparison). *C,* ratio of the long to short GFP lifetimes for *Nb*Glk1-GFP full-length construct alone and upon co-expression with Rx1 and the avirulent CP106 allele of the PVX CP (scatter plot ± S.D.; *bars with different letters* are significantly different (*p* < 0.05); one-way ANOVA with post hoc Dunnett's multiple comparison).

We next investigated Rx1 and *Nb*Glk1 interactions with DNA in the plant cell. We first used confocal laser-scanning microscopy to confirm the distribution of *Nb*Glk1 to the nucleus. The subcellular localization of *Nb*Glk1 *in planta* was determined by transiently expressing *Nb*Glk1-GFP in the epidermal cells of *N. benthamiana. Nb*Glk1-GFP was expressed either with or without the tombusvirus p19 silencing suppressor to enhance protein accumulation. In line with its role as a transcription factor, strong fluorescence of *Nb*Glk1-GFP was observed exclusively in the nucleus (Fig. 7*A*). This pattern does not resemble free mCherry, which has a nucleocytoplasmic distribution. The pattern of *Nb*Glk1-GFP localization was not affected by p19 overexpression, minimizing the likelihood of an overexpression artifact. Upon closer examination, the fluorescence of *Nb*Glk1-GFP was restricted to the nucleoplasm, with a lack of signal in the nucleolus. A very small amount of GFP signal was observed in the nucleolus in the absence of p19. This was the most likely background due to the longer opening of the pinhole of the microscope (pinhole of 1 for imaging with p19 and pinhole of 1.5 without p19).

Having established that *Nb*Glk1 is localized exclusively in the nucleus, we studied Rx1–*Nb*Glk1–DNA interactions using Förster resonance energy transfer–fluorescence lifetime imaging microscopy. FRET-FLIM has been used previously to demonstrate Rx1 binding to DNA in response to immune activation (29). GFP (negative control), histone H2B fused to GFP (GFP-H2B; positive control), full-length Rx1 with or without an N-terminal GFP tag, or full-length *Nb*Glk1 with or without an N-terminal GFP tag were transiently expressed in *N. benthamiana*. The constituent fluorescence lifetimes of the GFP tag were examined in leaves counterstained with the nucleic acid stain LDS 751. GFP showed two distinct lifetimes at ∼0.5 and ∼1.5 ns. Different extents of energy transfer from GFP to acceptor LDS 751 modulate the relative contribution of these two lifetimes. A drop in the ratio of the ∼1.5 ns (long) to ∼0.5 ns (short) GFP lifetimes indicates an interaction with DNA in the cell (29). First, we monitored the interaction of an Rx1-GFP fusion with DNA with or without *Nb*Glk1 (untagged) in the presence or absence of the avirulent PVX coat protein (CP106). Rx1-GFP expressed without *Nb*Glk1 only bound DNA in the presence of CP106 as expected (Fig. 7*B*) (29). In contrast, Rx1-GFP co-expressed with untagged *Nb*Glk1 bound DNA irrespective of the presence of CP106. Overexpressed *Nb*Glk1 is therefore able to recruit Rx1 to DNA. Second, we monitored the interaction of an *Nb*Glk1-GFP tag with DNA with or without Rx1 (untagged) in the presence or absence of CP106. Surprisingly, *Nb*Glk1-GFP only bound DNA in the joint presence of Rx1 and CP106 (Fig. 7*C*). We noted variation in the ratio of lifetime yields for GFP between the two sets of experiments (Fig. 7, *B* and *C*). This variation might have a biological (*e.g.* time of year for sampling and expression) or technical (*e.g.* fitting) cause. Regardless, the value for the ratio of lifetimes for GFP is distinct from the positive control within and between experiments.

### NbGlk1 reduces susceptibility to PVX

We hypothesized that *Nb*Glk1 plays a role in Rx1-mediated immunity as it interacts with DNA in response to PVX. We used virus-induced gene silencing (41, 42) to investigate the *Nb*Glk1 requirement for Rx1-mediated immunity. Virus-induced gene silencing with independent *Nb*Glk1 clones gave a bleaching phenotype consistent with a previously described role in the formation of the photosynthetic apparatus (43). The silenced leaves were too fragile for further infiltration and precluded an analysis of immune function by this method. We therefore used *Nb*Glk1 overexpression to investigate a role in immunity.

**Figure 8.**
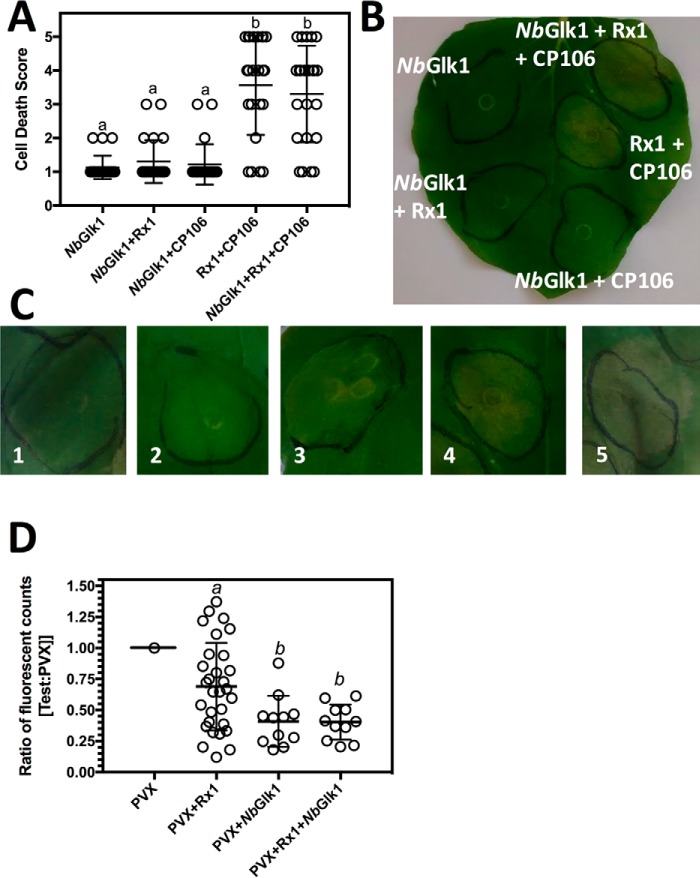
**Influence of Rx1 interactors on the immune response to PVX.** *A,* average cell death score for *N. benthamiana* leaves expressing N*b*Glk1 expressed in combination with CP106 and Rx1. Leaves were agroinfiltrated with *A. tumefaciens* transformed with constructs pBIN35S-CP106, pBIN35S-Rx1, and pBIN35S-*Nb*Glk1-4HA. The pBIN35S-CP106 construct expresses only the PVX CP106 coat protein (scatter plot ± S.D.; *letters* indicate statistically different data sets (*p* < 0.05) calculated using Dunnett's multiple comparisons test). *B,* representative *N. benthamiana* leaf of the results provided in *A. C,* representative images of *N. benthamiana* leaf areas for each cell death score. The *number within each image* indicates the assigned score for the associated degree of cell death. *D,* ratio of fluorescence compared with a control of GFP expressed from a PVX amplicon for *N. benthamiana* leaves expressing GFP from a PVX amplicon in combination with *Nb*Glk1 and Rx1. Leaves were infiltrated with *A. tumefaciens* transformed with pGR106 with and without constructs pBIN35S-Rx1 and pBIN35S-*Nb*Glk1-4HA. pGR106 encodes a PVX amplicon in which GFP expression is driven from a duplicated coat protein promoter (scatter plot ±S.D.; *bars with different letters* are significantly different (*p* < 0.05); one-way ANOVA with post hoc Dunnett's multiple comparison).

Rx1 mediates two forms of immunity: (*a*) a cell death response promoted by expression of CP106, and (*b*) symptomless virus resistance (called extreme resistance) (30). We first investigated a role for *Nb*Glk1 in CP106-mediated cell death. CP106-mediated cell death is activated through the sole expression of the PVX CP106 coat protein in the absence of other viral proteins. *Nb*Glk1, Rx1, and CP106 were transiently expressed in *N. benthamiana*. Rx1-mediated immune responses were assessed by scoring cell death within the infiltrated areas. Cell death associated with the hypersensitive response was observed in the presence of Rx1 and CP106 and was not influenced by *Nb*Glk1 (Fig. 8, *A–C*). Cell death was not as extensive as previously reported for Rx1 and CP106 co-expression (18) as agroinfiltration was performed at the same low *A*_600 nm_ that was used for the FRET-FLIM analysis (Fig. 7, *B* and *C*).

Next, we investigated a role for *Nb*Glk1 in PVX-mediated extreme resistance. PVX-mediated extreme resistance is activated by the virus following detection of CP106. *Nb*Glk1, Rx1, and a PVX amplicon in which GFP expression is driven from a coat protein promoter were transiently expressed in *N. benthamiana*. The Rx1 expression construct possessed an out-of-frame second start codon introduced upstream of the genuine Rx1 start codon reducing translation efficiency and increasing the sensitivity of the assay to immune modifiers (18). We monitored virus resistance by visualizing GFP expression within the infiltrated region of the leaf. Rx1 suppressed PVX-directed GFP expression as expected (Fig. 8*D*). Surprisingly, overexpressed *Nb*Glk1 was sufficient to suppress PVX-directed GFP expression independent of Rx1. Rx1 co-expression with *Nb*Glk1 did not further reduce GFP levels. Overexpressed *Nb*Glk1 is therefore able to bypass the requirement for Rx1 in extreme resistance to PVX.

## Discussion

A yeast two-hybrid screen identified the GLK-like TF, *Nb*Glk1, as an Rx1-interacting protein (Figs. 1–3). GLK-like TFs are involved in defense signaling in *Arabidopsis*, providing resistance to cucumber mosaic virus (44) and the fungal pathogens *Fusarium graminearum* (45) and *Hyaloperonospora arabidopsidis* (46). *Nb*Glk1 therefore links Rx1 with a TF class known to function in biotic stress (47). Rx1 binds and distorts DNA *in vitro* but without an apparent sequence specificity (29). *Nb*Glk1 could therefore provide the sequence selectivity for Rx1 at DNA. *Nb*Glk1 bound dsDNA non-specifically but showed a higher affinity for specific DNA motifs bound by the related GLK1 TF (At2g20570 of *A. thaliana*) (40). TFs generally show a lower affinity for non-specific DNA sequences (48), and this may assist TFs to scan DNA in searches for their cognate motifs (49). *Nb*Glk1 therefore has the properties of a TF that can target Rx1 to a specific DNA motif.

The *Nb*Glk1 N terminus reduced the affinity for DNA *in vitro* implying an autoinhibitory role of this domain in DNA binding (Fig. 4*A* and Table 1). In agreement, full-length *Nb*Glk1 overexpressed in the plant does not show an interaction with DNA by FRET-FLIM (Fig. 7*C*). Co-incubation of Rx1 with *Nb*Glk1 decreases the affinity of *Nb*Glk1 for DNA *in vitro* (Figs. 4, *B–G*, 5, *A–G*, and 6, *A–C*, and Tables 1 and 2). We propose this arises from the Rx1–CC-binding surface overlapping the *Nb*Glk1 DNA-binding domain.

*Nb*Glk1 is not bound to DNA *in planta* unless Rx1 is activated through PVX-derived CP106 (Fig. 7*C*). Rx1 does not interact with DNA unless it is activated by CP106 or when co-expressed with *Nb*Glk1 *in planta* (Fig. 7*B*). A caveat with the interpretation of the FRET-FLIM data is the possibility of false-negative results. If the expressed GFP fusion protein has saturated all available DNA-binding sites, the accumulation of an increased pool of non-DNA-bound protein will shift the ratio of the long to short lifetimes to the GFP negative control. In the current analysis, however, the *in vitro* data are entirely consistent with the FRET-FLIM analysis. In the absence of an available alternative method not susceptible to the same issue of false negatives, however, the interpretation of the data should be viewed with some caution.

**Figure 9.**
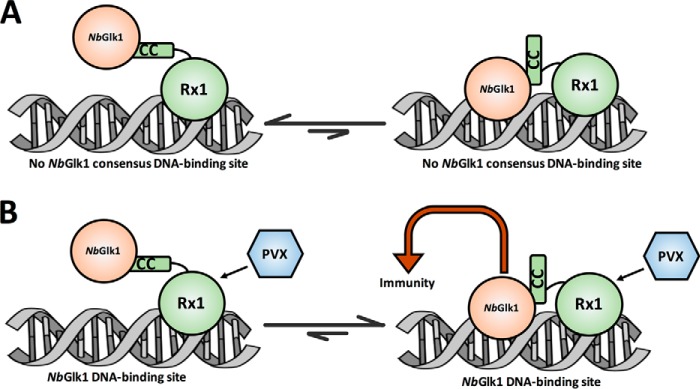
**Model for interactions of Rx1 with *Nb*Glk1.** *A,* in the absence of PVX Rx1 interacts with *Nb*Glk1 at DNA, and a non-DNA-bound state is favored for *Nb*Glk1 at a non-*Nb*Glk1 consensus DNA-binding site. *B,* Rx1-activation by PVX with a DNA-bound state favored for *Nb*Glk1 at a *Nb*Glk1 consensus DNA-binding site.

One interpretation of these data is that co-expression of Rx1 and *Nb*Glk1 permits complex formation at DNA. However, this complex might form at amounts escaping the detection level of the FRET-FLIM assays (Fig. 9*A*, *right-hand side*). Following co-expression of Rx1 and *Nb*Glk1, the DNA-bound state of the complex might be stabilized by the combined intrinsic DNA-binding activity of Rx1 with the low affinity of *Nb*Glk1 for non-consensus sequences. The joint affinity of both proteins for DNA shifts the equilibrium to a DNA-bound state detectable in our setup (Fig. 9*A*, *left-hand side*). The Rx1–*Nb*Glk1 complex could be arranged such that specifically Rx1 contacts DNA, and the autoinhibited *Nb*Glk1 stabilizes this complex by its weak interaction with non-consensus sequences. Immune activation via PVX permits an uncharacterized structural change in the complex releasing the negative regulation on *Nb*Glk1, permitting it to identify and directly and strongly interact with its consensus sequence (Fig. 9*B*, *right-hand side*). Once *Nb*Glk1 is stably bound to its consensus sequences, immune signaling is activated. This model is in agreement with the observation that overexpression of *Nb*Glk1 alone overcomes the need for Rx1; apparently, in this situation a sufficiently large pool of active *Nb*Glk1 is present to identify and interact with these consensus motifs and to trigger immunity. Overexpressed *Nb*Glk1 was not observed to bind DNA *in planta* in our assays, but it should be noted that the fluorescent techniques used to monitor DNA binding (FRET-FLIM) are less sensitive than those monitoring immunity (steady-state fluorescence). Overexpressed *Nb*Glk1 complex will be in a thermodynamically-coupled cycle of four states: non-DNA-bound or DNA-bound at either a non-consensus site or a *Nb*Glk1 consensus site. Hence, even if the bound state at a consensus site is thermodynamically non-favored in the absence of Rx1/CP106, sufficient binding may occur on overexpression to permit an immune response.

The function of Rx1 may therefore be to enable the specific activation of *Nb*Glk1 in response to PVX by releasing autoinhibition allowing *Nb*Glk1 to scan for and interact with its consensus sequences *in planta*. In conclusion, we identify *Nb*Glk1 as an immune activating protein acting at DNA and regulated by Rx1. These observations provide a direct and unexpected link between NLR-mediated perception of PVX and transcriptional processes at DNA. More generally, the findings suggest that nuclear-localized TFs involved in immunity are inactive until de-repressed by an activated NLR following pathogen perception.

## Experimental procedures

### Oligonucleotides

All oligonucleotide sequences used for this study are provided in Table S1.

### Plasmids

The *Nb*Glk1 (Niben101Scf06721g00011.1 (https://solgenomics.net)^4^ open-reading frame was amplified by PCR from cDNA synthesized from *N. benthamiana* whole leaf material, and the DNA was sequenced on both strands by Sanger DNA sequencing (Fig. S1*A*). Several differences from the computed open-reading frame for Niben101Scf06721g00011.1 were noted. The cloned open-reading frame is shown in Fig. S1*B*. A PCR product spanning *Nb*Glk1 residues 83–402 of an *E. coli* codon-optimized synthetic cDNA (Genscript, Fig. S1*C*) was cloned into the NdeI and XhoI sites of pET14b (pET14b-*Nb*Glk1(83–402) and fitted with a hexahistidine tag for affinity purification of recombinant protein. The oligonucleotides used to construct pET14b-*Nb*Glk1(83–402) were *Nb*Glk1-1 and *Nb*Glk1-2. Similarly, PCR products spanning residues 83–243 and 1–243 of the *Nb*Glk1 synthetic cDNA were cloned into the NdeI and BamHI sites of pET14b (pET14b-*Nb*Glk1(83–243) and pET14b-*Nb*Glk1(1–243)) and fitted with hexahistidine tags for affinity purification of recombinant protein. The oligonucleotides used to construct pET14b-*Nb*Glk1(83–243) were *Nb*Glk1-3 and *Nb*Glk1-4. The oligonucleotides used to construct pET14b-*Nb*Glk1(1–243) were *Nb*Glk1-5 and *Nb*Glk1-6. A PCR product spanning residues 82–244 of the *Nb*Glk1 synthetic cDNA was cloned into the AscI and SbfI sites of pTH6838, a modified T7-driven GST expression vector (pTH6838-*Nb*Glk1(82–244)) (39).

A PCR product spanning residues 1–144 of Rx1 (GenBank^TM^ accession number AJ011801.1) was cloned into the NcoI and NotI sites of pET28a (pET28a-Rx1–CC) and fitted with a hexahistidine tag for affinity purification of recombinant protein. The oligonucleotides used to construct pET28a-Rx1–CC were Rx1-1 and Rx1-2. A PCR product spanning residues 1–489 of Rx1 was cloned into the NdeI and XhoI sites of pET22b (pET22b-Rx1–CCNBARC) to give an N-terminal hexahistidine tag for affinity purification of recombinant protein. The oligonucleotides used to construct pET22b-Rx1–CCNBARC were Rx1-3 and Rx1-4. A PCR product spanning Rx1 residues 1–144 was cloned into the BamHI and XhoI sites of pGEX-6P-1 (pGEX-6P-1-Rx1(1–144)) and fitted with a GST tag for affinity purification of recombinant protein. The oligonucleotides used to construct pGEX-6P-1-Rx1(1–144) were Rx1–9 and Rx1-10.

A PCR product encompassing the full-length native *Nb*Glk1 cDNA was cloned into the NcoI and NotI sites of pRAP35S-YFP-4HA to make pRAP35S-*Nb*Glk1-4HA. pRAP35S-YFP-4HA was initially constructed by cloning YFP into the NcoI and KpnI sites of pRAP35S. A linker encoding a NotI site was synthesized by annealing the oligonucleotides *Nb*Glk1-7 and *Nb*Glk1-8. Two copies of a 2×HA tag synthesized by annealing the oligonucleotides *Nb*Glk1-9 and *Nb*Glk1-10 were inserted into NotI and XbaI sites of this vector to make pRAP35S-YFP-4HA. The oligonucleotides used to construct pRAP35S-*Nb*Glk1-4HA were *Nb*Glk1-11 and *Nb*Glk1-12. An AscI/PacI fragment from pRAP35S-*Nb*Glk1-4HA encompassing the 35S promoter and *Nb*Glk1-4HA fusion was cloned into the corresponding sites of the binary vector pBIN+ to make pBIN35S-*Nb*Glk1-4HA. A PCR product encompassing the full-length native *Nb*Glk1 cDNA was introduced into Gateway donor vector pDONR207 (Invitrogen) to make pDONR207-*Nb*Glk1. The *Nb*Glk1 gene was then recombined into the Gateway destination binary vector pK7WGF2 (50) to make GFP-*Nb*Glk1. The oligonucleotides used to make pDONR207-*Nb*Glk1 were *Nb*Glk1-13 and *Nb*Glk1-14.

GFP expression was driven from a PVX amplicon by a duplicated coat protein in pGR106 as described previously (51). An AscI/PacI fragment from pRAP35S-Rx1 (18) was cloned into the corresponding sites of the binary vector pBIN+ to make pBIN35S-Rx1. pBIN-35S-based plasmids corresponding to GFP-H2B, Rx1-GFP, Rx1-mCherry, and CP106 are as described (18, 29).

To make pBIN35S-Rx1-4myc and pBIN35S-Rx1–CC plasmids, the oligonucleotides Rx1-5 and Rx1-6 were annealed to make a dsDNA segment with a 5′ NotI and a 3′ XbaI overhang, which encodes a double c-Myc epitope (AAASEQKLISEEDLGEQKLISEEDLT). This segment was introduced between the NotI and XbaI sites in pRAP-YFP to create pRAP-YFPmMyc. A 4×Myc tag was created by fusing an AscI-SpeI fragment of this plasmid with an NheI-PacI fragment from this plasmid in an AscI-PacI-digested pRAP plasmid. pRAP:Rx1-4myc was created by replacing the YFP in pRAP-YFP-4Myc with the Rx1 sequence from pRAP:Rx1-GFP (18) via the NcoI and NotI restriction sites. pRAP:Rx1-4myc (Rx1 CC) was created by replacing the YFP gene in pRAP:YFPmMyc with the Rx1–CC sequence amplified with the oligonucleotides Rx1-7 and Rx1-8. Amplification with these oligonucleotides introduces an NcoI site overlapping the start codon and an NotI site immediately following the sequence coding for amino acid 144 of the CC of Rx1. The expression cassettes were transferred from pRAP to pBIN+ (52) via the restriction sites AscI and PacI.

### Protein expression and purification

Protein corresponding to Rx1–CCNBARC was expressed from pET22b-Rx1–CCNBARC in *E. coli* Rosetta2(DE3) pLysS. A 20-ml culture was grown overnight in Luria broth supplemented with 100 μg ml^−1^ ampicillin and 34 μg ml^−1^ chloramphenicol at 37 °C. This culture was diluted into 1 liter of Luria broth supplemented with ampicillin and chloramphenicol and grown at 37 °C to *A*_600 nm_ = 0.7. The growth temperature was reduced to 22 °C, and protein production was induced when cells reached 22 °C for 16 h with 100 μm isopropyl β-d-thiogalactoside. Cells were harvested by centrifugation (4000 × *g*, 20 min, 4 °C). Pelleted cells were washed with 50 mm Tris-HCl, pH 8.5, 1 mm EDTA, and the pellet was resuspended in 50 mm Tris-HCl, pH 8.0, 200 mm NaCl, 5 mm EDTA, 5 mm DTT, 1% (v/v) Triton X-100. Cells were lysed by sonication (150 s) and centrifuged (42,000 × *g*, 60 min, 4 °C), and inclusion bodies were washed three times in 30 ml of 50 mm Tris-HCl, pH 8.0, 1 m NaCl, 5 mm EDTA, 5 mm DTT, 2% (v/v) Triton X-100, 1 m urea and twice in 50 mm Tris-HCl, pH 9.0, 100 mm NaCl, 1 mm EDTA, 1 mm DTT. Sonication (60 s) was used to aid resuspension and ensure complete cell lysis. The final pellet was resuspended in 5 ml of 50 mm Tris-HCl, pH 9.0, 100 mm NaCl, 1 mm EDTA, 1 mm dithiothreitol, 8 m urea. Material was incubated at room temperature with rocking for 2 h prior to centrifugation (42,000 × *g*, 30 min, 4 °C), and the pellet was discarded. The supernatant was filtered through a 0.2-μm filter, aliquoted, and stored at −20 °C. To refold, protein was added dropwise slowly to a final concentration of 1 mg ml^−1^ into 50 mm Tris-HCl, pH 8.5, 9.6 mm NaCl, 0.4 mm KCl, 2 mm MgCl_2_, 2 mm CaCl_2_, 0.5 m arginine, 0.4 m sucrose, 0.75 m guanidine HCl, 1 mm glutathione, 0.1 mm reduced glutathione and incubated at 4 °C for 1 h. Refolded protein was centrifuged (42,000 × *g*, 30 min, 4 °C) and supernatant dialyzed into 20 mm Tris-HCl, pH 7.5, 50 mm NaCl, 2 mm MgCl_2_ overnight at 4 °C, concentrated, and stored at −20 °C in 20% (v/v) glycerol.

Protein corresponding to the Rx1 CC domain (Rx1(1–144)) was expressed from pET28a-Rx1–CC in *E. coli* BL21(DE3) pLysS. A starter culture was grown overnight at 37 °C in Luria broth supplemented with 50 μg ml^−1^ kanamycin and 34 μg ml^−1^ chloramphenicol. The overnight culture was diluted 1:50 into fresh Luria broth with antibiotics and grown with shaking at 37 °C to *A*_600 nm_ = 0.8. The growth temperature was reduced to 25 °C, and protein production was induced with 0.5 mm isopropyl β-d-thiogalactoside for 3 h. Cells were centrifuged (4000 × *g*, 20 min, 4 °C). Pelleted cells were washed with 50 mm Tris-HCl, pH 8.5, 1 mm EDTA and centrifuged (5500 × *g*, 20 min, 4 °C). Cells were resuspended in twice their volume of lysis buffer (50 mm Tris-HCl, pH 8.0, 200 mm NaCl, 40 mm imidazole, 5 mm β-mercaptoethanol, and SIGMAFAST^TM^ protease inhibitor mixture tablets). Cells were lysed by sonication (150 s), and the lysate was cleared by centrifugation at (42,000 × *g*, 60 min, 4 °C). The supernatant was loaded onto a 5-ml HisPrep HP nickel-nitrilotriacetic acid column (GE Healthcare) on an AKTA Pure chromatography system at 2 ml min^−1^ (GE Healthcare). The column was washed with 5 bed volumes of lysis buffer, 20 bed volumes of wash buffer (lysis buffer + 1 m NaCl), 5 bed volumes of lysis buffer and eluted with lysis buffer supplemented with 500 mm imidazole. Peak fractions were assessed by SDS-PAGE, pooled, concentrated, exchanged into storage buffer (50 mm Tris-HCl, pH 7.5, 500 mm NaCl, 1 mm EDTA, 1 mm DTT, 20% (v/v) glycerol), and stored at −80 °C.

Protein corresponding to the Rx1 CC domain fused to GST (Rx1(GST-1–144)) was expressed from pGEX-6P-1-Rx1(1–144) in *E. coli* BL21Tuner (DE3) pRARE. A starter culture was grown overnight at 37 °C in Luria broth supplemented with 100 μg ml^−1^ ampicillin and 34 μg ml^−1^ chloramphenicol. The overnight culture was diluted 1:50 into fresh Luria broth with antibiotics and grown with shaking at 37 °C to *A*_60 nm_ = 0.8. The growth temperature was reduced to 22 °C, and protein production was induced with 0.1 mm isopropyl β-d-thiogalactoside for 15 h. Cells were centrifuged (4000 × *g*, 20 min, 4 °C). Pelleted cells were washed with 50 mm Tris-HCl, pH 8.5, 1 mm EDTA and centrifuged (5500 × *g*, 20 min, 4 °C). Cells were resuspended in twice their volume of lysis buffer (50 mm Tris-HCl, pH 8.0, 200 mm NaCl, 1 mm DTT, and SIGMAFAST^TM^ protease inhibitor mixture tablets). Cells were lysed by sonication (150 s), and the lysate was cleared by centrifugation at (50,000 × *g*, 60 min, 4 °C). The supernatant was loaded onto a 5-ml GST-agarose column (Thermo Fisher Scientific) on an AKTA Pure chromatography system at 1 ml min^−1^ (GE Healthcare). The column was washed with 5 bed volumes of lysis buffer, 20 bed volumes of wash buffer (lysis buffer + 0.5 m NaCl), 5 bed volumes of lysis buffer and eluted with lysis buffer supplemented with 10 mm reduced glutathione. Rx1(GST-1–144) was further purified by size-exclusion chromatography using a Superdex 75 16/60 column (GE Healthcare) in 50 mm Tris-HCl, pH 8.0, 500 mm NaCl, 1 mm DTT, 1 mm EDTA at 1 ml min^−1^. Peak fractions were assessed by SDS-PAGE, pooled, concentrated, exchanged into storage buffer (50 mm Tris-HCl, pH 7.5, 200 mm NaCl, 1 mm EDTA, 1 mm DTT, 20% (v/v) glycerol), and stored at −80 °C.

Proteins corresponding to *Nb*Glk1(83–402), *Nb*Glk1(83-243), and *Nb*Glk1(1–243) were expressed from pET14b-*Nb*Glk1(83–402), pET14b-*Nb*Glk1(83–243), and pET14b-*Nb*Glk1(1–243), respectively, in *E. coli* BL21(DE3) pLysS. Protein expression and purification were identical to Rx1–CC except that protein was eluted in lysis buffer supplemented with 250 mm imidazole. Protein corresponding to the CAP TF was expressed and purified as described previously (53).

### Yeast two-hybrid analyses

Hybrigenics Services SAS (Paris, France) performed the yeast two-hybrid screen using Rx1 (amino acids 1–144) cloned into pB27 bait plasmid as a C-terminal fusion to LexA (N-LexA-Rx1-C). The screen was performed against a random-primed *N. benthamiana* mixed tissue cDNA library constructed into pP6 prey plasmid. A total of 96.6 million clones (∼9-fold library coverage) were screened following a mating approach with Y187 (*MATα*) and L40 Gal4 (*MATα*) yeast strains as described previously (54). To confirm protein–protein interactions, freshly transformed yeast colonies were resuspended in 1 ml of sterile deionized water, and 10-μl aliquots were spotted onto medium lacking leucine and tryptophan (-L/-W) and medium lacking leucine, tryptophan, and histidine (-L/-W/-H), supplemented with 10 or 50 mm 3-amino-1,2,4-triazole (3-AT). Growth was scored after 5–7 days of incubation at 28 °C.

### Protein binding microarray

PBM was performed with protein derived from plasmid pTH6838-*Nb*Glk1(82–244) essentially as described (55, 56). *Nb*Glk1(82–244) was analyzed in duplicate on two different arrays with differing probe sequences. Microarray data were processed by removing spots flagged as “bad” or “suspect” and employing spatial de-trending (using a 7 × 7 window centered on each spot) as described (56). Calculation of 8-mer *Z*- and *E*-scores was performed as described previously (57). *Z*-scores were derived by averaging the spot intensity for each probe containing the 8-mer, subtracting the median value for each 8-mer, and then dividing by the standard deviation to yield a distribution with a median of zero and a standard deviation of one. *E*-scores are a modified version of the AUROC statistic, which considers the relative ranking of probes containing a given 8-mer, and range from −0.5 to +0.5, with *E* >0.45 taken as highly statistically significant (58). A position weight matrix (PWM) was derived using the PWMalign algorithm, which aligns the top 10 8-mer *E*-scores and tallies the frequency at each position to generate a PWM (56).

### Gel filtration analysis

Gel filtration analysis of protein was performed at 4 °C using an Sephacryl HiPrep 16/60 S200 HR column (GE Healthcare) on an AKTA Pure chromatography system (GE Healthcare). Protein was dialyzed overnight against running buffer (20 mm Tris-HCl, pH 7.4, 140 mm NaCl, 1 mm DTT). Proteins were incubated on ice for 30 min individually or together and then centrifuged at 12,000 × *g* for 30 min. Protein loading concentration was ∼75 μm. Columns were run at a flow rate of 0.5 ml/min in running buffer. Thirty μl of each eluted fraction was subjected to SDS-PAGE and visualization with Quick Coomassie stain (Generon). Molecular mass was calibrated with gel filtration standards (Bio-Rad): bovine thyroglobulin (670 kDa), bovine γ-globulin (158 kDa), chicken ovalbumin (44 kDa), and equine myoglobin (17 kDa).

### Co-immunoprecipitation

*N. benthamiana* leaves were infiltrated with *Agrobacterium tumefaciens* strains (GV3101, MOG101) transformed with combinations of pBIN35S-*Nb*Glk1-4HA, pBIN35S-Rx-CC-4myc, and pBIN35S-Rx1-4myc, and leaf material was harvested 2 days after infiltration. 100 mg of leaf material was ground in liquid nitrogen and resuspended in 1.5 ml of extraction buffer (10% (v/v) glycerol, 25 mm Tris-HCl, pH 7.5, 1 mm Na_2_EDTA, 150 mm NaCl, 0.6 mg/ml Pefabloc SC, 20 mg/ml polyvinylpolypyrrolidone, 0.1% (v/v) Tween 20, 5 mm DTT). The supernatant was passed through a 5-ml G-25 Sephadex column after pelleting the cell debris. The resulting sample was incubated at 4 °C with 50 μl of magnetic beads (Miltenyi μMACs) and conjugated antibodies (Sigma) for 1–2 h. Unbound proteins were removed by washing five times with washing buffer (10% (v/v) glycerol, 25 mm Tris-HCl, pH 7.5, 1 mm Na_2_EDTA, 150 mm NaCl, 0.15% (v/v) Nonidet P-40, 5 mm DTT). Captured proteins were released by heating beads in 1× NuPAGE LDS sample buffer (60 mm DTT). Start material (before incubation with beads), the unbound fraction, and the captured proteins were separated on NuPAGE Novex 12% bis-tris gels in MES buffer (50 mm MES, 50 mm Tris base, 0.1% SDS, 1 mm EDTA, pH 7.3) and blotted on polyvinylidene difluoride (PVDF) membranes for immunoblot analysis. Affinity-tagged proteins were detected using peroxidase-conjugated antibodies (c-Myc: goat anti-c-Myc (Abcam 9132) and donkey anti-goat peroxidase-conjugated (Jackson ImmunoResearch no. 705-035-147), HA: rat anti-HA HRP-conjugated (Roche Applied Science 12013819001)). Peroxidase activity was visualized with the SuperSignal^TM^ West Dura and Femto Substrates (Thermo Fisher Scientific) and imaged in a Syngene G:BOX Chemi HR-16 gel documentation system.

### Fluorescence anisotropy

Double-stranded DNA substrates lacking a putative *Nb*Glk1-binding site (No site), with a concatenated AGATTTCC-binding site (AGATTTCC), and with a concatenated GGATATCC-binding site (GGATATCC) were made by annealing synthetic oligonucleotides. FA-1 and FA-2 were annealed to make No site. FA-3 and FA-4 were annealed to make AGATTTCC. FA-5 and FA-6 were annealed to make GGATATCC. FA-1, FA-3, and FA-5 were end-labeled with 6-carboxyfluorescein. Double-stranded DNA was annealed by mixing 10 μm concentrations of complementary oligonucleotides in 150 mm NaCl, 15 mm sodium citrate, heating to 95 °C, and cooling to room temperature over 5 h. Changes in anisotropy were measured using a Synergy^TM^ H4 fluorescence spectrophotometer (BioTek) fitted with polarizing filters (λ_em_ = 528 nm, λ_ex_ = 485 nm, bandwidth = 20 nm, averaging time = 10 s). Anisotropy was determined using 10 nm fluorescein end-labeled oligonucleotides (Eurofins MWG) with variable protein in 20 mm Tris-HCl, pH 7.4, 140 mm NaCl, 1 mm DTT. Anisotropy was calculated using Gen5 software (BioTek).

### Time-resolved FRET in situ

*A. tumefaciens* strain GV3101 (pMP90) was transformed with constructs pK7WGF2 (GFP negative control), pK7WGF2-H2B (GFP-H2B positive control), pBIN35SRx1-GFP (Rx1-GFP), pBIN35S-CP106, pBIN35S-Rx1, pBIN35S-*Nb*Glk1-4HA, and pK7WGF2-GFP-*Nb*Glk1 (GFP-*Nb*Glk1), and the experiments were performed as described previously (29).

### N. benthamiana hypersensitive response assay

*N. benthamiana* leaves were infiltrated with *A. tumefaciens* transformed with constructs pBIN35S-CP106, pBIN35S-Rx1, and pBIN35S-*Nb*Glk1-4HA at *A*_600 nm_ = 0.1–0.5. Plants were grown for 4 days at 25 °C with 16 h of light. Leaves were harvested, visually inspected, photographed, and scored 1–5 for cell death: with 1 being no visual sign of any cell death whatsoever in the infiltrated region and 5 being complete cell death throughout the infiltrated region (Fig. 8*C*).

### Overexpression transient PVX resistance assay

*N. benthamiana* leaves were infiltrated with *A. tumefaciens* transformed with pGR106 with and without constructs pBIN35S-Rx1 and pBIN35S-*Nb*Glk1-4HA at a *A*_600 nm_ = 0.1–0.5. Leaves were grown for 4 days and then harvested. Three different 10-mm diameter leaf discs were excised, and each disc was placed into a 96-well plate for each infiltrated area. The fluorescence intensity of each leaf disc was measured using a Synergy^TM^ H4 fluorescence spectrophotometer (BioTek) (λ_em_ = 550 nm, λ_ex_ = 410 nm, bandwidth = 20 nm, averaging time = 10 s). An average of the fluorescence intensities for the three leaf discs was calculated to give a value for each infiltrated area. The fluorescence intensity of each averaged area was normalized to an infiltrated area on each leaf with only pGR106.

### Confocal laser-scanning microscopy

Imaging was performed using a Zeiss LSM 510 confocal microscope (Carl Zeiss) using settings as described (18). *Agrobacterium* harboring the appropriate constructs was infiltrated on *N. benthamiana* leaves. Imaging was performed 48 h post-inoculation.

### Statistical analysis

Error bars represent either the standard deviation or standard error of the mean with the number of replicates as indicated in the legends. All replicates are independent biological experiments. Statistical comparisons (*p* values) were obtained from one-way ANOVA with the indicated post hoc test unless otherwise indicated. *p* values in statistical comparisons are indicated in the figures and indicate compared data sets as described in the figure legends. Calculated values for *K_d_* were compared using the extra-sum-of-squares *F* test.

## Author contributions

P. D. T., C. H. D., E. J. S., O. C. S., and A. W. Y. data curation; P. D. T., C. H. D., E. J. S., O. C. S., A. W. Y., and M. J. C. investigation; P. D. T., C. H. D., E. J. S., O. C. S., A. W. Y., G. J. S., and L.-O. P. methodology; P. D. T., E. J. S., T. R. H., G. J. S., L.-O. P., F. L. T., A. G., and M. J. C. writing-review and editing; T. R. H., A. G., and M. J. C. supervision; T. R. H., A. G., and M. J. C. project administration; G. J. S., L.-O. P., F. L. T., A. G., and M. J. C. conceptualization; M. J. C. formal analysis; M. J. C. funding acquisition; M. J. C. writing-original draft.

## Supplementary Material

Supporting Information

## Footnotes

4 Please note that the JBC is not responsible for the long-term archiving and maintenance of this site or any other third party hosted site.

## Footnotes

3 The abbreviations used are:

NLR: nucleotide-binding leucine–rich repeat
CC: coiled-coil
dsDNA: double-stranded DNA
FLIM: fluorescence lifetime imaging microscopy
GARP: maize GOLDEN2, ARR B-class proteins from *Arabidopsis*, and *Chlamydomonas* Psr1
GLK: Golden2-like
GST: glutathione *S*-transferase
NB: nucleotide binding
NB-ARC: nucleotide binding, Apaf-1, R-proteins, and CED-4
LRR: leucine-rich repeat
PBM: protein binding microarray
PVX: protein virus X
CAP: catabolite activator protein
TF: transcription factor
ANOVA: analysis of variance
bis-tris: 2-[bis(2-hydroxyethyl)amino]-2-(hydroxymethyl)propane-1,3-diol
PWM: position weight matrix
3-AT: 3-amino-1,2,4-triazole.
