# Supplementary material for: The intracellular immune receptor Rx1 regulates the DNA-binding activity of a Golden2-like transcription factor
Source: J Biol Chem. 2017 Dec 7;293(9):3218–33. doi: 10.1074/jbc.RA117.000485 (PMC5836133; doi:10.1074/jbc.RA117.000485)
Supplement: Supporting Information [file supp_293_9_3218__index.html]

The intracellular immune receptor Rx1 regulates the DNA-binding activity of a Golden2-like transcription factor — Rx1 regulates transcription factor DNA-binding — The intracellular immune receptor Rx1 regulates the DNA-binding activity of a Golden2-like transcription factor — Rx1 regulates transcription factor DNA-binding — Supporting Information 

# The intracellular immune receptor Rx1 regulates the DNA-binding activity of a Golden2-like transcription factor

## Supporting Information

- Supplementary Data - Supplementary figures and tables
- Supplementary Figure S2a - Supplementary Figure S2a
- Supplementary Figure S2b - Supplementary Figure S2b
- Supplementary Figure S2c - Supplementary Figure S2c
- Supplementary Figure S2d - Supplementary Figure S2d
