# Supplementary material for: The intracellular immune receptor Rx1 regulates the DNA-binding activity of a Golden2-like transcription factor
Source: J Biol Chem. 2017 Dec 7;293(9):3218–33. doi: 10.1074/jbc.RA117.000485 (PMC5836133; doi:10.1074/jbc.RA117.000485)
Supplement: Supporting Information [file supp_RA117.000485_133217_1_supp_23412_zzzjtw.pdf]

The intracellular immune receptor Rx1 regulates the DNA-binding activity of a Golden2-like transcription factor*

Philip D. Townsend[1,2,7], Christopher H. Dixon [1,2,7], Erik J. Slootweg[4], Octavina C.A. Sukarta[4], Ally W.H. Yang[5], Timothy R. Hughes[5], Gary J. Sharples[1,2], Lars-Olof Pålsson[3], Frank L.W. Takken[6], Aska Goverse[4], and Martin J. Cann[1,2]

Figure S1. A. cDNA sequence for *Nb*Glk1. **B.** *Nb*Glk1 open reading frame. The AREAEAA motif typical of GARP family transcription factors is indicated in red. The GCT-box typical of GLK-like transcription factors is shown in blue. **C.** *Nb*Glk1 *E. coli* optimized open reading frame.

A.

ATGCTAACTATATCACCTTTGAGCTACAAAACCACCAAAAATGACAGGGATAATCACATGGAAAGTTTCACGATGCGAGG
AGTTGATGATTTTCCAGAGTTTGGTGATGGGAATTTGCTCGAAAGCATTGATTTTGATGACATTTTCTTGGGGATTAACG
TCGACGATGGTGTTCTACCAGATTTGGAGATGGACTCAGAGATTCTTGCTGAATTATCAGTTAGCAGTGGCGACGAATCT
GATCGTATGAATAGTTATAATACTACTACACCACCACTCCAAGTAGAAGAACGCAACTCTTCTACAAAAGAAGAAGTGGA
AAAAGTAGTATCACATTTGAATAAGAAGAGTGAAAAAACTAAACCTAAGTCAAAGGAGAAGTCCAAGAATCCAGAAGGGA
AGAGAAAAGTGAAGGTGGATTGGACGCCAGAGCTACATCGAAGATTTGTGCAAGCAGTAGAGCAATTAGGTGTAGATAAA
GCAGTCCCATCTAGGATTTTAGAACTTATGGGTGTTGAAGGTCTCACTCGCCATAACATAGCTAGCCATCTTCAAAAATA
TAGAGCCCATCGGAAACATTTGCTTGCGAGAGAAGCGGAGGCGGCAAGTTGGAGCCAAAGAAAACAAATGTACGGCAGTG
CAGCCGTGGTTGGAGGTGGCGGAAAGAGAGACATAAACCCATGGCCGTCAGCACCAACCATGGGTTTTCCGCCTATGACG
GCACCACCCATGGTGCCGCCTCATTTTAGACCTCTACATGTCTGGGGTCATCCGCCCACCGATCAATCTATGATGCATAT
GTGGCCGAAACATATGATGCCTCTCCCACCAGCATGGGCACCGATTGTTTCTCCTCATTCTTCACCACTTACAGATCATC
CTTCCTTTTGGCATTCACACCATCAAAGAGTACTAAACTCTCTTGCACCAGGCACCCCTTGCTTTCCTTCACCAGTAGCG
CCCCCGAGATTTCCAGTTCAGGGCATCCCACCCCCTGCCATGGTCAAAGCTGTTCCCACCGGAACACGACAAGATCTGCC
AAAACCTCCTTCTGATTTTCATCCTTCAAAGGAGAGCATAAATGCGGCCATTGGAAATGTTTTAGCAAAGCCATGGCTAC
CACCACCACCCCTCGGATTGAAACCTCCCTCATTCCACAGTGTGTTGAATGAATTACAACGTCAAGGGATTAATAAAATA
CCTCCA

B.

MLTISPLSYKTTKNDRDNHMESFTMRGVDDFPEFGDGNLLESIDFDDIFLGINVDDGVLPDLEMDSEILAELSVSSGDES
DRMNSYNTTTPPLQVEERNSSTKEEVEKVVSHLNKKSEKTKPKSKEKSKNPEGKRKVKVDWTPELHRRFVQAVEQLGVDK
AVPSRILELMGVEGLTRHNIASHLQKYRAHRKHLL**AREAEAA**SWSQRKQMYGSAAVVGGGGKRDINPWPSAPTMGFPPMT
APPMVPPHFRPLHVWGHPPTDQSMMHMWPKHMMPLPPAWAPIVSPHSSPLTDHPSFWHSHHQRVLNSLAPGTPCFPSPVA
PPRFPVQGIPPPAMVKAVPTGARQDLPKPPSDFHP**SKESIDAAIGDVLAKPCLPPPPLGLKPPSIDSVLNELQRQGINKI**
PP

C.

ATGCTGACGATCTCCCCGCTGAGCTACAAAACGACGAAAAACGACCGCGACAATCACATGGAATCATTTACGATGCGTGG
CGTGGACGATTTTCCGGAATTTGGCGACGGTAACCTGCTGGAATCCATTGATTTTGATGACATTTTCCTGGGCATCAATG
TTGATGACGGTGTCCTGCCGGATCTGGAAATGGACAGCGAAATCCTGGCGGAACTGAGCGTGAGCAGCGGCGATGAAAGT
GACCGTATGAACTCCTATAATACCACGACCCCGCCGCTGCAAGTGGAAGAACGCAACAGTTCCACGAAAGAAGAAGTTGA
AAAAGTGGTTTCACATCTGAACAAAAAATCGGAAAAAACCAAACCGAAATCAAAAGAAAAATCGAAAAACCCGGAAGGCA
AACGCAAAGTTAAAGTCGATTGGACGCCGGAACTGCACCGTCGCTTTGTGCAGGCAGTTGAACAACTGGGTGTGGACAAA
GCTGTTCCGAGCCGTATTCTGGAACTGATGGGCGTTGAAGGTCTGACCCGCCATAACATCGCGTCTCATCTGCAAAAATA
TCGTGCCCATCGCAAACACCTGCTGGCGCGTGAAGCGGAAGCCGCAAGCTGGTCTCAGCGCAAACAAATGTATGGCTCAG
CAGCTGTCGTGGGCGGTGGCGGTAAACGCGATATTAATCCGTGGCCGAGCGCCCCGACGATGGGCTTTCCGCCGATGACC
GCCCCGCCGATGGTTCCGCCGCATTTTCGCCCGCTGCATGTCTGGGGTCACCCGCCGACCGATCAGAGTATGATGCACAT
GTGGCCGAAACACATGATGCCGCTGCCGCCGGCATGGGCTCCGATTGTCAGCCCGCACTCATCGCCGCTGACGGATCATC
CGAGCTTTTGGCACTCTCATCACCAACGTGTTCTGAATAGTCTGGCACCGGGCACCCCGTGCTTTCCGAGCCCGGTTGCT
CCGCCGCGCTTCCCGGTGCAGGGCATTCCGCCGCCGGCAATGGTCAAAGCTGTGCCGACCGGCGCGCGTCAGGATCTGCC
GAAACCGCCGTCTGACTTCCATCCGAGTAAAGAATCCATTGATGCCGCAATTGGTGATGTGCTGGCAAAACCGTGCCTGC
CGCCGCCGCCGCTGGGTCTGAAACCGCCGTCAATCGACTCCGTGCTGAACGAACTGCAACGCCAAGGCATCAATAAAATT
CCGCCGTAA

Figure S2. Oligonucleotide sequences and associated *E*- and *Z*-scores for PBM with *Nb*Glk1 DNA-binding domain.

See attached files.

Figure S2a

Oligonucleotide sequence and associated *E*-score (first experiment)

Figure S2b

Oligonucleotide sequence and associated *E*-score (second experiment)

Figure S2c

Oligonucleotide sequence and associated *Z*-score (first experiment)

Figure S2d

Oligonucleotide sequence and associated *Z*-score (second experiment)

Supplementary Table 1. The oligonucleotides used for this study.

**Primer Name**	**Orientation**	**Sequence**
*Nb*Glk1-1	Sense	5'-GGC CAT ATG AAC TCC TAT AAT ACC ACG AC-3'
*Nb*Glk1-2	Antisense	5'- GGC CTC GAG AGT TGG GGT ATT TTA TTA ATC CC-3'
*Nb*Glk1-3	Sense	5'-GGC CAT ATG AAC TCC TAT AAT ACC ACG AC-3'
*Nb*Glk1-4	Antisense	5'-GCC GGA TCC TTA CGG CGG GGC GGT CAT C-3'
*Nb*Glk1-5	Sense	5'-GGC CAT ATG CTG ACG ATC TCC CC-3'
*Nb*Glk1-6	Antisense	5'-GCC GGA TCC TTA CGG CGG GGC GGT CAT C-3'
*Nb*Glk1-7	Sense	5'-CTA CAA GTC AGC GGC CGC ATA AGG TAC-3'
*Nb*Glk1-8	Antisense	5'-CTT ATG CGG CCG CTG ACT TGT AGA GCT-3'
*Nb*Glk1-9	Sense	5'-GGC CGC TAG CTA CCC TTA TGA TGT GCC AGA CTA TGC TGG AGG TTA TCC TTA CGA TGT ACC TGA TTA TGC TAC TAG TTA AT-3'
*Nb*Glk1-10	Antisense	5'-CTA GAT TAA CTA GTA GCA TAA TCA GGT ACA TCG TAA GGA TAA CCT CCA GCA TAG TCT GGC ACA TCA TAA GGG TAG CTA GC-3'
*Nb*Glk1-11	Sense	5'-GGC TCA TGA TCA CTA TAT CAC CTT TGA GC-3'
*Nb*Glk1-12	Antisense	5'-GGC GCG GCC GCA GTT GGA GGT ATT TTA TTA ATC CC-3'
*Nb*Glk1-13	Sense	5'-GGG GAC AAG TTT GTA CAA AAA AGC AGG CTA CAT GCT AAC TAT ATC ACC TTT G-3'
*Nb*Glk1-14	Antisense	5'-GGG GAC CAC TTT GTA AAG AAA GCT GGG TCA TGG AGG TAT TTT ATT AAT C-3'
Rx1-9	Sense	5'-CTC GAG TTA GCC AAC CAT TAT ATT CTC GGG-3'
Rx1-10	Antisense	5'-GG CCA TAT GCA GAT CTT CGT TCA AGA C-3'
Rx1-1	Sense	5'-GGC CCA TGG CTT ATG CTG CTG TTA C-3'
Rx1-2	Antisense	5'-GGC GCG GCC GCA CCA ACC ATT ATA TTC TC-3'
Rx1-3	Sense	5'-GCC CAT ATG GCT TAT GCT GCT GTT AC-3'
Rx1-4	Antisense	5'-GCCCTCGAGTGCACATGAATTTGATCACTC-3'
Rx1-5	Sense	5'-GGC CGC TAG CGA GCA AAA GCT CAT TAG TGA GGA AGA CTT AGG TGA ACA GAA GCT AAT CTC TGA AGA GGA TCT TAC TAG TTA AT-3'
Rx1-6	Antisense	5'-CTA GAT TAA CTA GTA AGA TCC TCT TCA GAG ATT AGC TTC TGT TCA CCT AAG TCT TCC TCA CTA ATG AGC TTT TGC TCG CTA GC-3'
Rx1-7	Sense	5'-TTT TTT GGA TCC ATG GCT TAT GCT GCT GTT ACT TCC C-3'
Rx1-8	Antisense	5'-GTG GTA CCT TAA GCG GCC GCA CCA ACC ATT ATA TTC TCG GGC TGC-3'
Rx1-9	Sense	5'-GGA TCC ATG GCT TAT GCT GCT GTT AC-3'
Rx1-10	Antisense	5'-CTC GAG TTA GCC AAC CAT TAT ATT CTC GGG-3'
FA-1	Sense	5'-CAC TGT GAC ACA CTG TGA CAC ACT GTG ACA CAC TGT GAC ACA CTG TGA CC-3'
FA-2	Antisense	5'-GGT CAC AGT GTG TCA CAG TGT GTC ACA GTG TGT CAC AGT GTG TCA CAG TG-3'
FA-3	Sense	5'-CAG ATT TCC ACA GAT TTC CAC AGA TTT CCA CAG ATT TCC ACA GAT TTC CC-3'
FA-4	Antisense	5'-GGG AAA TCT GTG GAA ATC TGT GGA AAT CTG TGG AAA TCT GTG GAA ATC TG-3'
FA-5	Sense	5'-CGG ATA TCC ACG GAT ATC CAC GGA TAT CCA CGG ATA TCC ACG GAT ATC CC-3'
FA-6	Antisense	5'-GGG ATA TCC GTG GAT ATC CGT GGA TAT CCG TGG ATA TCC GTG GAT ATC CG-3'
