# Supplementary material for: The intracellular immune receptor Rx1 regulates the DNA-binding activity of a Golden2-like transcription factor
Source: J Biol Chem. 2017 Dec 7;293(9):3218–33. doi: 10.1074/jbc.RA117.000485 (PMC5836133; doi:10.1074/jbc.RA117.000485)
Supplement: Supporting Information [file supp_RA117.000485_133217_1_supp_23413_vzvjtw.pdf]

#8mers	all
AAAAAAAA	0.290610112187
AAAAAAAC	0.147253497596
AAAAAAAG	-0.0871898978976
AAAAAAAT	0.320754579925
AAAAAACA	0.167701266768
AAAAAACC	0.0541528046141
AAAAAACG	0.029248577156
AAAAAACT	-0.0286641858219
AAAAAAGA	0.0259686487586
AAAAAAGC	-0.0785751918074
AAAAAAGG	-0.0504249415251
AAAAAAGT	0.0360335885404
AAAAAATA	0.233454631276
AAAAAATC	0.450610898842
AAAAAATG	0.0812104860219
AAAAAATT	0.244080474862
AAAAACAA	0.162893215848
AAAAACAC	-0.000946782585787
AAAAACAG	0.101339473799
AAAAACAT	0.0511207710378
AAAAACCA	0.14291546206
AAAAACCC	0.046593104309
AAAAACCG	0.0813258175584
AAAAACCT	0.082653669025
AAAAACGA	0.0110293310501
AAAAACGC	0.0909816900481
AAAAACGG	-0.332132349311
AAAAACGT	-0.0299256643288
AAAAACTA	0.0842226838871
AAAAACTC	0.0119716497191
AAAAACTG	-0.152398230268
AAAAACTT	-0.0829121461067
AAAAAGAA	0.161872379196
AAAAAGAC	-0.190014818474
AAAAAGAG	-0.101639668115
AAAAAGAT	0.21628434036
AAAAAGCA	-0.123047035775
AAAAAGCC	-0.11522989452
AAAAAGCG	-0.102747247958
AAAAAGCT	-0.123388490985
AAAAAGGA	0.0661403104349
AAAAAGGC	-0.118942175247
AAAAAGGG	-0.00843704728039
AAAAAGGT	-0.223258070514
AAAAAGTA	-0.108548225415
AAAAAGTC	0.0223116774781
AAAAAGTG	0.0472462336379
AAAAAGTT	0.123674690919
AAAAATAA	0.202686211556
AAAAATAC	0.276200369009
AAAAATAG	-0.0851640928043
AAAAATAT	0.333787898091
AAAAATCA	0.352105821336

AAAAATCC 0.440706711454
AAAAATCG 0.380038529935
AAAAATCT 0.446812773058
AAAAATGA 0.0578223451667
AAAAATGC -0.0344573975279
AAAAATGG 0.00683055610442
AAAAATGT 0.025659389509
AAAAATTA 0.166551406178
AAAAATTC 0.136212091277
AAAAATTG 0.0195146984566
AAAAATTT 0.251492897758
AAAACAAA 0.00913246164366
AAAACAAC 0.108535244152
AAAACAAG 0.00356079206195
AAAACAAT 0.11982479334
AAAACACA 0.111058969461
AAAACACC -0.0499048421542
AAAACACG 0.050233089021
AAAACACT 0.0170572254587
AAAACAGA 0.122050469978
AAAACAGC 0.102406199917
AAAACAGG 0.104699780628
AAAACAGT -0.0239986634318
AAAACATA 0.184246926163
AAAACATC 0.0234774638302
AAAACATG -0.103886124015
AAAACATT -0.0403238386422
AAAACCAA 0.189348284265
AAAACCAC 0.0211889645955
AAAACCAG -0.0365682158267
AAAACCAT -0.0209361788677
AAAACCCA 0.086365551407
AAAACCCC -0.244427437362
AAAACCCG -0.0111380025684
AAAACCCT -0.104747714226
AAAACCGA 0.036170150945
AAAACCGC -0.144097908229
AAAACCGG 0.110300292011
AAAACCGT -0.0228860677512
AAAACCTA 0.00623102291052
AAAACCTC -0.15357346679
AAAACCTG -0.101348154541
AAAACCTT -0.168903269007
AAAACGAA 0.176894662891
AAAACGAC -0.0735032653915
AAAACGAG -0.04806473387
AAAACGAT 0.0428028057696
AAAACGCA 0.158527834183
AAAACGCC -0.286618765211
AAAACGCG 0.100979165295
AAAACGCT -0.105110704955
AAAACGGA 0.032191474537
AAAACGGC -0.196865204672
AAAACGGG -0.251154320988

AAAACGGT -0.0970109113263
AAAACGTA 0.229637313662
AAAACGTC 0.0185623086042
AAAACGTG -0.138329052229
AAAACGTT -0.109580020085
AAAACTAA 0.00313948681416
AAAACTAC -0.01309029836
AAAACTAG 0.0117483369558
AAAACTAT 0.0970952921998
AAAACTCA -0.0509217962315
AAAACTCC -0.0865705364037
AAAACTCG -0.110551975408
AAAACTCT -0.0876613934296
AAAACTGA 0.18738831665
AAAACTGC -0.0163744134354
AAAACTGG -0.0796614634789
AAAACTGT -0.0671903688382
AAAACTTA 0.186883905245
AAAACTTC -0.0849345517412
AAAACTTG 0.012327848223
AAAACTTT -0.0694414475309
AAAAGAAA 0.200284309205
AAAAGAAC -0.0958500751019
AAAAGAAG -0.123835569485
AAAAGAAT 0.247653625766
AAAAGACA 0.0203571977431
AAAAGACC -0.241593964335
AAAAGACG 0.112229599175
AAAAGACT -0.193156187748
AAAAGAGA 0.00860112899805
AAAAGAGC -0.0782919321119
AAAAGAGG 0.00921397835298
AAAAGAGT 0.172636671364
AAAAGATA 0.145648093262
AAAAGATC 0.309457946387
AAAAGATG -0.202140701211
AAAAGATT 0.380323936859
AAAAGCAA 0.0848948385255
AAAAGCAC 0.0308556968498
AAAAGCAG 0.0471854486377
AAAAGCAT -0.159122804469
AAAAGCCA -0.103965827432
AAAAGCCC -0.0661086223438
AAAAGCCG -0.119414944705
AAAAGCCT -0.0343174383631
AAAAGCGA -0.0251115017506
AAAAGCGC 0.101650371188
AAAAGCGG -0.0926779306437
AAAAGCGT -0.254022314076
AAAAGCTA -0.0761820780866
AAAAGCTC -0.0777228945419
AAAAGCTG 0.145875869942
AAAAGCTT -0.113209239448
AAAAGGAA 0.0670925816154

AAAAGGAC -0.0809624946491
AAAAGGAG -0.111256985512
AAAAGGAT 0.0152906309474
AAAAGGCA -0.0994566559644
AAAAGGCC -0.266495014537
AAAAGGCG -0.0693170426975
AAAAGGCT -0.185081842176
AAAAGGGA 0.0413130329929
AAAAGGGC -0.249295709199
AAAAGGGG -0.0599512685007
AAAAGGGT -0.135929602739
AAAAGGTA 0.0650343586028
AAAAGGTC -0.119345120506
AAAAGGTG -0.186799109004
AAAAGGTT -0.0428371378185
AAAAGTAA 0.063158631364
AAAAGTAC -0.0948209620409
AAAAGTAG 0.174089462903
AAAAGTAT -0.0453343599727
AAAAGTCA -0.221850399419
AAAAGTCC -0.0590733848733
AAAAGTCG 0.0967666688621
AAAAGTCT -0.0899348923152
AAAAGTGA -0.0562690376225
AAAAGTGC -0.0592437090091
AAAAGTGG 0.103319666057
AAAAGTGT -0.06457863667
AAAAGTTA 0.0295570438598
AAAAGTTC -0.0437607344121
AAAAGTTG -0.0310534191449
AAAAGTTT -0.171908496732
AAAATAAA 0.111686255139
AAAATAAC 0.1872081705
AAAATAAG 0.0645664143652
AAAATAAT 0.170323984639
AAAATACA 0.260771722865
AAAATACC 0.18238490259
AAAATACG 0.238806011109
AAAATACT 0.155082987552
AAAATAGA 0.0604376605414
AAAATAGC -0.014532273764
AAAATAGG 0.108719006223
AAAATAGT 0.112771411008
AAAATATA 0.288999462467
AAAATATC 0.411376772218
AAAATATG 0.191453227376
AAAATATT 0.31541897524
AAAATCAA 0.307691078407
AAAATCAC 0.381786352476
AAAATCAG 0.394591538782
AAAATCAT 0.359657463203
AAAATCCA 0.465624046606
AAAATCCC 0.423010765185
AAAATCCG 0.439277255036

AAAATCCT 0.413205077546
AAAATCGA 0.392900207749
AAAATCGC 0.398963204461
AAAATCGG 0.353305827147
AAAATCGT 0.394243013019
AAAATCTA 0.4608571644
AAAATCTC 0.452079079672
AAAATCTG 0.480021210756
AAAATCTT 0.433568794046
AAAATGAA 0.182805879383
AAAATGAC -0.154135775211
AAAATGAG 0.186888556361
AAAATGAT 0.107414648858
AAAATGCA 0.074892856624
AAAATGCC -0.139937942876
AAAATGCG 0.0451193468249
AAAATGCT 0.0511048479654
AAAATGGA -0.107302590782
AAAATGGC -0.128157844186
AAAATGGG 0.0220065204505
AAAATGGT -0.061022195877
AAAATGTA 0.0927984148939
AAAATGTC -0.0391901829717
AAAATGTG 0.0886507924978
AAAATGTT 0.0342735296055
AAAATTAA 0.160906386529
AAAATTAC 0.233323866443
AAAATTAG 0.0050440185295
AAAATTAT 0.200626857367
AAAATTCA 0.0325928578485
AAAATTCC 0.21253613819
AAAATTCG 0.294951590595
AAAATTCT 0.203882083031
AAAATTGA 0.0832318800575
AAAATTGC 0.13829849055
AAAATTGG 0.0292804751556
AAAATTGT 0.0978726206942
AAAATTTA 0.267061296626
AAAATTTC 0.279442999724
AAAATTTG 0.263425102637
AAAATTTT 0.260195905028
AAACAAAA 0.085716439991
AAACAAAC 0.019791379135
AAACAAAG 0.0190504554501
AAACAAAT 0.17868668906
AAACAACA 0.0208170499615
AAACAACC 0.00697017096128
AAACAACG 0.0539174523848
AAACAACT 0.0398805805355
AAACAAGA 0.0760736533026
AAACAAGC -0.0950075392433
AAACAAGG 0.0640558628865
AAACAAGT 0.108495795736
AAACAATA 0.193725393343

AAACAATC	0.190471140296
AAACAATG	-0.0532666017753
AAACAATT	0.0339963555731
AAACACAA	0.100159807069
AAACACAC	0.107404248902
AAACACAG	0.0874159601606
AAACACAT	-0.0267023084858
AAACACCA	-0.200571209418
AAACACCC	-0.118810744846
AAACACCG	0.0779545105271
AAACACCT	-0.0267851176035
AAACACGA	0.0922440668291
AAACACGC	-0.068116204527
AAACACGG	-0.226008604416
AAACACGT	0.0713736854816
AAACACTA	0.153039257235
AAACACTC	-0.159772991723
AAACACTG	-0.116941037009
AAACACTT	-0.0796173347086
AAACAGAA	0.03927089508
AAACAGAC	-0.167418604141
AAACAGAG	-0.212512072523
AAACAGAT	0.297720586621
AAACAGCA	0.0354288787899
AAACAGCC	-0.169368191721
AAACAGCG	-0.129466809363
AAACAGCT	0.0858774134499
AAACAGGA	-0.0738371965407
AAACAGGC	-0.130595323866
AAACAGGG	-0.127710507356
AAACAGGT	-0.0483577172473
AAACAGTA	0.0662167855534
AAACAGTC	-0.227684446291
AAACAGTG	-0.118171839412
AAACAGTT	-0.117538110221
AAACATAA	0.184628092823
AAACATAC	0.0513342141658
AAACATAG	0.0575610535644
AAACATAT	0.158332485872
AAACATCA	0.0448549387648
AAACATCC	-0.0650284020746
AAACATCG	-0.0460636613252
AAACATCT	-0.142437265363
AAACATGA	-0.0303996629524
AAACATGC	-0.0263221437469
AAACATGG	-0.0521689487767
AAACATGT	-0.137727010404
AAACATTA	0.138082065468
AAACATTC	-0.0340490752982
AAACATTG	0.0082726843619
AAACATTT	0.0457584140157
AAACCAAA	0.255759592056
AAACCAAC	0.0387532143013
AAACCAAG	0.0121484042699

AAACCAAT	-0.00091247886891
AAACCACA	0.0743759467826
AAACCACC	-0.14477914952
AAACCACG	-0.0711734010717
AAACCACT	-0.0541423820343
AAACCAGA	0.17607686228
AAACCAGC	0.0224323356547
AAACCAGG	-0.178890096141
AAACCAGT	-0.0241827482491
AAACCATA	0.104343846701
AAACCATC	-0.107913836284
AAACCATG	-0.167401597676
AAACCATT	-0.0317778820508
AAACCCAA	0.0061743640405
AAACCCAC	-0.0758604157889
AAACCCAG	0.0957884596384
AAACCCAT	-0.182174690992
AAACCCCA	-0.195270837448
AAACCCCC	-0.355881619225
AAACCCCG	-0.17268672892
AAACCCCT	-0.303007778693
AAACCCGA	-0.0463493474706
AAACCCGC	0.00316272718028
AAACCCGG	-0.0238499432071
AAACCCGT	-0.140971604816
AAACCCTA	-0.0372539334331
AAACCCTC	-0.221723367603
AAACCCTG	-0.131565082815
AAACCCTT	-0.160046639232
AAACCGAA	0.151391015509
AAACCGAC	-0.16246323368
AAACCGAG	-0.207192576754
AAACCGAT	0.118342334629
AAACCGCA	-0.0793149053632
AAACCGCC	-0.15568089056
AAACCGCG	-0.0597218106386
AAACCGCT	-0.183041239809
AAACCGGA	0.181428965758
AAACCGGC	0.0854683966717
AAACCGGG	0.0309429313561
AAACCGGT	-0.108386023842
AAACCGTA	-0.0365000960457
AAACCGTC	-0.209867854223
AAACCGTG	-0.0193425058684
AAACCGTT	-0.0651523633889
AAACCTAA	-0.102907763916
AAACCTAC	0.0374046289463
AAACCTAG	-0.0328333964428
AAACCTAT	-0.115478255312
AAACCTCA	-0.223027716692
AAACCTCC	-0.405534470292
AAACCTCG	-0.0064628202595
AAACCTCT	-0.183005406222
AAACCTGA	-0.0854644127235

AAACCTGC -0.242035644991
AAACCTGG -0.106753597418
AAACCTGT -0.13182854632
AAACCTTA 0.0286010669828
AAACCTTC -0.176271575455
AAACCTTG -0.145915287156
AAACCTTT 0.0464152368777
AAACGAAA 0.202704772256
AAACGAAC -0.199432277109
AAACGAAG 0.0263638080572
AAACGAAT 0.226116833496
AAACGACA 0.124023360888
AAACGACC -0.200063619699
AAACGACG -0.173342202418
AAACGACT 0.0693983990237
AAACGAGA 0.19979225081
AAACGAGC -0.226048926163
AAACGAGG 0.0665974169366
AAACGAGT 0.0947394418375
AAACGATA 0.109560583108
AAACGATC 0.1777301585
AAACGATG -0.0683930672788
AAACGATT 0.132141560008
AAACGCAA 0.245895137507
AAACGCAC -0.278832941641
AAACGCAG -0.0740987727502
AAACGCAT 0.0147792483257
AAACGCCA -0.120330477507
AAACGCCC -0.2453321921
AAACGCCG -0.286226874712
AAACGCCT -0.0432577218956
AAACGCGA 0.0210189879854
AAACGCGC -0.0103564666173
AAACGCGG -0.111465055534
AAACGCGT -0.0282443083273
AAACGCTA -0.040298015971
AAACGCTC -0.18027090501
AAACGCTG -0.0576248579756
AAACGCTT -0.0739505806933
AAACGGAA 0.084742783258
AAACGGAC -0.112773908892
AAACGGAG -0.0598250495272
AAACGGAT 0.247115743485
AAACGGCA -0.0306115927208
AAACGGCC -0.151052982421
AAACGGCG -0.134508990318
AAACGGCT -0.129309217884
AAACGGGA -0.00548873851625
AAACGGGC -0.016924318006
AAACGGGG -0.104788400912
AAACGGGT -0.183315127711
AAACGGTA 0.0636685891941
AAACGGTC -0.14721739153
AAACGGTG 0.0563014635339

AAACGGTT -0.206112834326
AAACGTAA 0.117848996847
AAACGTAC -0.00209918008496
AAACGTAG -0.0280576684558
AAACGTAT 0.193225633054
AAACGTCA -0.105996354815
AAACGTCC -0.0373469120916
AAACGTCG -0.0940025916521
AAACGTCT -0.222958956883
AAACGTGA -0.00517848909965
AAACGTGC -0.10648526143
AAACGTGG -0.0788189641924
AAACGTGT -0.189600037438
AAACGTTA -0.0430023214462
AAACGTTC -0.150071317974
AAACGTTG -0.164707528687
AAACGTTT 0.024247960819
AAACTAAA 0.0854226896983
AAACTAAC 0.0543782519924
AAACTAAG 0.0699164635887
AAACTAAT 0.0442505774846
AAACTACA 0.0221532069565
AAACTACC -0.134392358287
AAACTACG -0.0108682527922
AAACTACT 0.113789897288
AAACTAGA 0.153484021535
AAACTAGC -0.111421216712
AAACTAGG 0.0272616332282
AAACTAGT 0.00486015060704
AAACTATA -0.0170979036218
AAACTATC -0.127234030189
AAACTATG -0.00687315677073
AAACTATT 0.0787572821176
AAACTCAA 0.220825374871
AAACTCAC -0.189972165889
AAACTCAG -0.0621922201982
AAACTCAT 0.0327367230894
AAACTCCA -0.209272666
AAACTCCC -0.153596869615
AAACTCCG -0.0809129601656
AAACTCCT -0.179061728395
AAACTCGA -0.108619858098
AAACTCGC -0.136616979572
AAACTCGG 0.0430856712437
AAACTCGT -0.11131967771
AAACTCTA -0.160845996752
AAACTCTC -0.000306508734773
AAACTCTG -0.0185496174827
AAACTCTT -0.0486205744338
AAACTGAA 0.0852267114492
AAACTGAC -0.00757049670943
AAACTGAG -0.14809881679
AAACTGAT 0.227505702207
AAACTGCA -0.0273687850511

AAACTGCC -0.0944713359017
AAACTGCG -0.0989072207952
AAACTGCT -0.198048188937
AAACTGGA 0.0715049467552
AAACTGGC -0.193345534141
AAACTGGG -0.0451469498642
AAACTGGT -0.146286783672
AAACTGTA -0.169366655142
AAACTGTC -0.165518163014
AAACTGTG 0.0839611814101
AAACTGTT -0.0679558102012
AAACTTAA 0.129244693679
AAACTTAC 0.117089562888
AAACTTAG -0.130557556883
AAACTTAT 0.06679761945
AAACTTCA 0.0347696599015
AAACTTCC -0.109791495199
AAACTTCG -0.155481329645
AAACTTCT -0.00197040549738
AAACTTGA -0.00127700376273
AAACTTGC -0.00915985065303
AAACTTGG -0.216174985732
AAACTTGT -0.0218885156644
AAACTTTA 0.0463600307992
AAACTTTC -0.048251897342
AAACTTTG -0.0217779643716
AAAGAAAA 0.200147094338
AAAGAAAC 0.00988495905508
AAAGAAAG 0.0674080746154
AAAGAAAT 0.246262267009
AAAGAACA -0.254530640462
AAAGAACC 0.0622693807346
AAAGAACG 0.0937355628841
AAAGAACT -0.0157659882764
AAAGAAGA -0.080302706025
AAAGAAGC -0.105032056775
AAAGAAGG -0.331018861403
AAAGAAGT 0.066536399692
AAAGAATA 0.127911699488
AAAGAATC 0.436900371029
AAAGAATG 0.0936077172287
AAAGAATT 0.0710022808827
AAAGACAA 0.0138436505746
AAAGACAC 0.141624953347
AAAGACAG -0.035445344574
AAAGACAT 0.0432501262377
AAAGACCA -0.129139436241
AAAGACCC -0.292562168317
AAAGACCG -0.115565208548
AAAGACCT -0.056201240684
AAAGACGA 0.225929767942
AAAGACGC -0.121965406734
AAAGACGG -0.102007077651
AAAGACGT -0.122388801071

AAAGACTA	-0.106506701065
AAAGACTC	-0.168559857764
AAAGACTG	-0.0225058759613
AAAGACTT	-0.148160493827
AAAGAGAA	0.100614173747
AAAGAGAC	-0.202823378351
AAAGAGAG	-0.170025889497
AAAGAGAT	0.264351401217
AAAGAGCA	-0.132172086496
AAAGAGCC	-0.315022740606
AAAGAGCG	0.022055393666
AAAGAGCT	-0.184496732026
AAAGAGGA	-0.0224730056134
AAAGAGGC	-0.125004115226
AAAGAGGG	-0.175330484769
AAAGAGGT	0.0151453517935
AAAGAGTA	0.174087246701
AAAGAGTC	-0.168152314764
AAAGAGTG	-0.0620208077304
AAAGAGTT	0.181045428791
AAAGATAA	0.171311725481
AAAGATAC	0.162304417939
AAAGATAG	0.0352203121913
AAAGATAT	0.320603353466
AAAGATCA	0.203944329028
AAAGATCC	0.309658280094
AAAGATCG	0.312799093458
AAAGATCT	0.396754433631
AAAGATGA	-0.0749538738686
AAAGATGC	-0.0247739959492
AAAGATGG	0.0439454912615
AAAGATGT	-0.0912467015897
AAAGATTA	0.342872510922
AAAGATTC	0.394951549402
AAAGATTG	0.347112450328
AAAGATTT	0.432915651277
AAAGCAAA	0.10283074795
AAAGCAAC	-0.0772376621679
AAAGCAAG	-0.0567519510027
AAAGCAAT	0.328716166156
AAAGCACA	0.0298011128383
AAAGCACC	-0.247472222222
AAAGCACG	0.058264043248
AAAGCACT	-0.182251965998
AAAGCAGA	0.184691935554
AAAGCAGC	-0.100805268525
AAAGCAGG	-0.155050180805
AAAGCAGT	-0.32060769405
AAAGCATA	-0.0511513372946
AAAGCATC	-0.0538651518894
AAAGCATG	-0.0459380865356
AAAGCATT	-0.0242107709965
AAAGCCAA	-0.132269413915
AAAGCCAC	-0.0266537880541

AAAGCCAG -0.201192447349
AAAGCCAT -0.289772290809
AAAGCCCA -0.189159383076
AAAGCCCC -0.254421047434
AAAGCCCG -0.352368566898
AAAGCCCT -0.216013620701
AAAGCCGA 0.146320885452
AAAGCCGC -0.1954745719
AAAGCCGG -0.0508121755147
AAAGCCGT -0.272545953361
AAAGCCTA -0.0766905778996
AAAGCCTC -0.197193041579
AAAGCCTG 0.00115206369038
AAAGCCTT -0.166283244041
AAAGCGAA 0.0620208007464
AAAGCGAC -0.21187248172
AAAGCGAG -0.0641661050038
AAAGCGAT 0.163052427057
AAAGCGCA -0.26469426289
AAAGCGCC -0.201769547325
AAAGCGCG -0.0136473907221
AAAGCGCT -0.0557723550462
AAAGCGGA 0.195260792255
AAAGCGGC -0.258637917663
AAAGCGGG 0.0541992939433
AAAGCGGT 0.0801577731611
AAAGCGTA -0.0203850098344
AAAGCGTC -0.073878323866
AAAGCGTG -0.0345050002615
AAAGCGTT -0.0789611310295
AAAGCTAA -0.0779099727781
AAAGCTAC -0.165582614101
AAAGCTAG -0.0392881644302
AAAGCTAT 0.0134141254473
AAAGCTCA -0.122278746371
AAAGCTCC -0.243018536863
AAAGCTCG -0.1305708061
AAAGCTCT -0.192762688615
AAAGCTGA -0.196399934925
AAAGCTGC -0.00536806473639
AAAGCTGG 0.0263638080572
AAAGCTGT 0.0293236935691
AAAGCTTA -0.00496031746032
AAAGCTTC -0.317534657175
AAAGCTTG 0.0198732066174
AAAGCTTT -0.200870353663
AAAGGAAA -0.0464231117212
AAAGGAAC -0.0985662476765
AAAGGAAG -0.109608240116
AAAGGAAT 0.230254780566
AAAGGACA -0.0310267048635
AAAGGACC -0.0723819245503
AAAGGACG 0.0664681184897
AAAGGACT -0.0743785684192

AAAGGAGA -0.0983999209838
AAAGGAGC -0.293235286857
AAAGGAGG -0.084809473638
AAAGGAGT 0.0365035923986
AAAGGATA 0.0972935739533
AAAGGATC 0.145993874728
AAAGGATG -0.108309183517
AAAGGATT 0.329050019613
AAAGGCAA -0.0183803957081
AAAGGCAC -0.219931526015
AAAGGCAG -0.0383859649123
AAAGGCAT -0.313451021054
AAAGGCCA -0.263549802915
AAAGGCCC -0.494610964481
AAAGGCCG -0.261397242386
AAAGGCCT -0.305770887166
AAAGGCGA -0.0740216049383
AAAGGCGC -0.221460411933
AAAGGCGG -0.171825708061
AAAGGCGT -0.172098333604
AAAGGCTA -0.123938800261
AAAGGCTC -0.284838460146
AAAGGCTG -0.273627867262
AAAGGCTT -0.123252227929
AAAGGGAA -0.015451611841
AAAGGGAC -0.225286825354
AAAGGGAG -0.178174039576
AAAGGGAT 0.165945113536
AAAGGGCA 0.0482526096822
AAAGGGCC -0.338101508916
AAAGGGCG -0.268353853169
AAAGGGCT -0.279604757829
AAAGGGGA 0.0279769363149
AAAGGGGC -0.212894386017
AAAGGGGG -0.0359570734655
AAAGGGGT -0.160756034114
AAAGGGTA -0.0881219931671
AAAGGGTC -0.268133763701
AAAGGGTG -0.266161148179
AAAGGGTT -0.162925027744
AAAGGTAA 0.0132857786236
AAAGGTAC -0.0165268305873
AAAGGTAG -0.0438547047783
AAAGGTAT 0.0371550418231
AAAGGTCA -0.246565493236
AAAGGTCC -0.294627934843
AAAGGTCG -0.133590941348
AAAGGTCT -0.0515337815458
AAAGGTGA 0.0491755408017
AAAGGTGC -0.137345602801
AAAGGTGG -0.161408151231
AAAGGTGT -0.144952483883
AAAGGTTA -0.0837518180072
AAAGGTTC -0.202087848871

AAAGGTTG 0.0760585969051
AAAGTAAA 0.123247766142
AAAGTAAC 0.0421135211309
AAAGTAAG 0.0367788699002
AAAGTAAT 0.20100969012
AAAGTACA 0.120147189568
AAAGTACC -0.162990680271
AAAGTACG 0.139680513342
AAAGTACT -0.170814106147
AAAGTAGA -0.0911407336816
AAAGTAGC -0.0576873209523
AAAGTAGG 0.0968968372728
AAAGTAGT -0.0104250706622
AAAGTATA 0.110806494105
AAAGTATC 0.234480998485
AAAGTATG -0.0345052518414
AAAGTATT -0.0678351953278
AAAGTCAA -0.00602922127818
AAAGTCAC -0.168730497237
AAAGTCAG -0.173305268173
AAAGTCAT -0.172552796493
AAAGTCCA -0.13580426244
AAAGTCCC -0.339445717555
AAAGTCCG -0.0232540270574
AAAGTCCT -0.274705893245
AAAGTCGA -0.0590811147881
AAAGTCGC 0.00150380066409
AAAGTCGG -0.16188430133
AAAGTCGT -0.0310270258403
AAAGTCTA -0.160728593851
AAAGTCTC -0.185571428156
AAAGTCTG -0.137430196814
AAAGTCTT -0.102854021893
AAAGTGAA 0.0764357789697
AAAGTGAC -0.186592592593
AAAGTGAG -0.0912069277753
AAAGTGAT 0.0981014879738
AAAGTGCA -0.150631369256
AAAGTGCC -0.118765810109
AAAGTGCG -0.106246113975
AAAGTGCT -0.0730560768433
AAAGTGGA 0.238759025467
AAAGTGGC -0.138743809388
AAAGTGGG -0.180805213508
AAAGTGGT 0.0432539624889
AAAGTGTA 0.0600205273442
AAAGTGTC 0.0993737513447
AAAGTGTG -0.184495690199
AAAGTGTT -0.0159236331207
AAAGTTAA -0.0501653195112
AAAGTTAC 0.0318859909109
AAAGTTAG 0.00985339963556
AAAGTTAT 0.126426278093
AAAGTTCA 0.0342505927097

AAAGTTCC -0.162521423384
AAAGTTCG 0.0583571833278
AAAGTTCT 0.0237436606731
AAAGTTGA 0.0795330727994
AAAGTTGC 0.0438292679383
AAAGTTGG -0.0214521723858
AAAGTTGT -0.143317309803
AAAGTTTA -0.081911787306
AAAGTTTC -0.201330217275
AAAGTTTG 0.111882220576
AAATAAAA 0.284606220853
AAATAAAC 0.12002237299
AAATAAAG 0.145051065623
AAATAAAT 0.167836648119
AAATAACA 0.135584754879
AAATAACC 0.186126479176
AAATAACG 0.0881858992053
AAATAACT 0.121508569594
AAATAAGA 0.150293668767
AAATAAGC -0.0148555124905
AAATAAGG 0.0940926602786
AAATAAGT 0.0327641660629
AAATAATA 0.235307775911
AAATAATC 0.20982231058
AAATAATG 0.116390394142
AAATAATT 0.15080737228
AAATACAA 0.212886122637
AAATACAC 0.102935849305
AAATACAG 0.169277817326
AAATACAT 0.173864958616
AAATACCA 0.121826740081
AAATACCC 0.104468786774
AAATACCG 0.19519288019
AAATACCT 0.163010474627
AAATACGA 0.139298011128
AAATACGC 0.205100751093
AAATACGG 0.161503929801
AAATACGT 0.228542995366
AAATACTA 0.107147734372
AAATACTC 0.082194449933
AAATACTG 0.103018178226
AAATACTT 0.0742661748886
AAATAGAA 0.0486234800169
AAATAGAC 0.0309255734895
AAATAGAG 0.012547353987
AAATAGAT 0.23106794706
AAATAGCA -0.0542794572878
AAATAGCC -0.0180480292883
AAATAGCG 0.0907676348548
AAATAGCT 0.0476995045981
AAATAGGA 0.204031496521
AAATAGGC -0.0856086470152
AAATAGGG -0.201862217407
AAATAGGT 0.158230790464

AAATAGTA −0.0333404685064
AAATAGTC 0.0920773132753
AAATAGTG −0.0309306639446
AAATAGTT 0.0533685666304
AAATATAA 0.155967341246
AAATATAC 0.241712222003
AAATATAG 0.241542574056
AAATATAT 0.342609661039
AAATATCA 0.387818612922
AAATATCC 0.427748139366
AAATATCG 0.366613397644
AAATATCT 0.389536543064
AAATATGA 0.264404816791
AAATATGC 0.176856242728
AAATATGG 0.230924530117
AAATATGT 0.230946343498
AAATATTA 0.326554123749
AAATATTC 0.325347428045
AAATATTG 0.253795363235
AAATATTT 0.304647133026
AAATCAAA 0.290237240858
AAATCAAC 0.34219778304
AAATCAAG 0.300173988452
AAATCAAT 0.289187685412
AAATCACA 0.345725160897
AAATCACC 0.33888484145
AAATCACG 0.36809233943
AAATCACT 0.287816890154
AAATCAGA 0.33548588613
AAATCAGC 0.365456975579
AAATCAGG 0.373276581264
AAATCAGT 0.326538453094
AAATCATA 0.352329551233
AAATCATC 0.191162897116
AAATCATG 0.32276128618
AAATCATT 0.226622404951
AAATCCAA 0.455704213178
AAATCCAC 0.453146611342
AAATCCAG 0.425585111792
AAATCCAT 0.414079002804
AAATCCCA 0.402492370853
AAATCCCC 0.378367252849
AAATCCCG 0.439604550143
AAATCCCT 0.349426832407
AAATCCGA 0.434680234473
AAATCCGC 0.418085468397
AAATCCGG 0.459702468293
AAATCCGT 0.366706376302
AAATCCTA 0.410598114277
AAATCCTC 0.377529673267
AAATCCTG 0.424649514041
AAATCCTT 0.379056356811
AAATCGAA 0.293230916156
AAATCGAC 0.337142068485

AAATCGAG 0.336924149754
AAATCGAT 0.289534347626
AAATCGCA 0.3801853762
AAATCGCC 0.339173071056
AAATCGCG 0.425728336517
AAATCGCT 0.330912498366
AAATCGGA 0.310660584313
AAATCGGC 0.310654460032
AAATCGGG 0.340399227115
AAATCGGT 0.340547411852
AAATCGTA 0.356897127831
AAATCGTC 0.374173724812
AAATCGTG 0.385171762323
AAATCGTT 0.188269775407
AAATCTAA 0.450345129902
AAATCTAC 0.454923989319
AAATCTAG 0.463121754889
AAATCTAT 0.435757558148
AAATCTCA 0.455484752684
AAATCTCC 0.446174073482
AAATCTCG 0.464273807172
AAATCTCT 0.407956266877
AAATCTGA 0.468756265164
AAATCTGC 0.453580404748
AAATCTGG 0.46083274011
AAATCTGT 0.450247700957
AAATCTTA 0.412701756425
AAATCTTC 0.430906825616
AAATCTTG 0.423536921145
AAATGAAA 0.208465059606
AAATGAAC 0.0973704473145
AAATGAAG -0.0223802536574
AAATGAAT 0.267386150351
AAATGACA -0.0944619586536
AAATGACC -0.207700709043
AAATGACG -0.0424433028167
AAATGACT -0.0243851059811
AAATGAGA 0.132944740829
AAATGAGC -0.136898097841
AAATGAGG 0.103276142177
AAATGAGT 0.101757877762
AAATGATA 0.213788996465
AAATGATC -0.0120941184219
AAATGATG 0.00863081516608
AAATGATT 0.181510322084
AAATGCAA 0.12713088199
AAATGCAC -0.0675649761016
AAATGCAG -0.15412654321
AAATGCAT 0.0851587423333
AAATGCCA -0.151506392473
AAATGCCC -0.0449653509218
AAATGCCG -0.0683988784449
AAATGCCT -0.0883892824145
AAATGCGA 0.220750221551

AAATGCGC 0.0823863553819
AAATGCGG 0.00337274144328
AAATGCGT 0.0756026476981
AAATGCTA 0.0278974859231
AAATGCTC -0.0985239637964
AAATGCTG 0.0149056411895
AAATGCTT -0.0578225560414
AAATGGAA -0.0193112219698
AAATGGAC -0.112030844146
AAATGGAG -0.141101618187
AAATGGAT 0.187112286258
AAATGGCA -0.0427460979947
AAATGGCC -0.10847458612
AAATGGCG -0.0027530399663
AAATGGCT -0.301396147377
AAATGGGA -0.0598334924869
AAATGGGC -0.121382716049
AAATGGGG 0.0496394349501
AAATGGGT 0.0287260470995
AAATGGTA 0.143743550901
AAATGGTC -0.191927293905
AAATGGTG -0.129578520464
AAATGGTT -0.0709993752996
AAATGTAA 0.127431447452
AAATGTAC -0.0625906178722
AAATGTAG 0.0593042657358
AAATGTAT 0.0209216509523
AAATGTCA 0.216670782909
AAATGTCC -0.181129000395
AAATGTCG -0.217869012481
AAATGTCT 0.0020295497799
AAATGTGA 0.0680503413906
AAATGTGC -0.0990032712024
AAATGTGG 0.0423503261517
AAATGTGT -0.0626029111506
AAATGTTA 0.0373701567562
AAATGTTC -0.0334634001778
AAATGTTG 0.0279973215658
AAATTAAA 0.104576293348
AAATTAAC 0.208904147182
AAATTAAG 0.19388229483
AAATTAAT 0.0113476695427
AAATTACA 0.319918131977
AAATTACC 0.216798628565
AAATTACG 0.31194194645
AAATTACT 0.211173419726
AAATTAGA 0.201137535775
AAATTAGC 0.0183338684185
AAATTAGG -0.00814049688827
AAATTAGT 0.115178216251
AAATTATA 0.260274868159
AAATTATC 0.0376955820609
AAATTATG 0.101130101649
AAATTATT 0.235981508878

AAATTCAA 0.175885361281
AAATTCAC 0.118863751125
AAATTCAG 0.208418926968
AAATTCAT -0.00266368881251
AAATTCCA 0.143749364404
AAATTCCC 0.335399789238
AAATTCCG 0.119615591359
AAATTCCT 0.23785538913
AAATTCGA 0.178012000058
AAATTCGC 0.265914604913
AAATTCGG 0.176378734989
AAATTCGT 0.23285197507
AAATTCTA 0.306620342927
AAATTCTC 0.278902939691
AAATTCTG 0.280799907021
AAATTCTT 0.11193877803
AAATTGAA 0.109977716306
AAATTGAC -0.0565208548225
AAATTGAG 0.00405332718611
AAATTGAT 0.0953801229062
AAATTGCA 0.0699795156393
AAATTGCC 0.204922171727
AAATTGCG 0.301720105182
AAATTGCT 0.211615068354
AAATTGGA -0.0411013414125
AAATTGGC -0.0794517164732
AAATTGGG -0.0586215913879
AAATTGGT 0.11760347723
AAATTGTA 0.172694783026
AAATTGTC 0.0809883598489
AAATTGTG 0.149155305276
AAATTGTT 0.114309996659
AAATTTAA 0.210449616112
AAATTTAC 0.172471575619
AAATTTAG 0.144243013019
AAATTTAT 0.181691532457
AAATTTCA 0.262596324837
AAATTTCC 0.27794533263
AAATTTCG 0.281899712398
AAATTTCT 0.15777344136
AAATTTGA 0.103081297065
AAATTTGC 0.249120640253
AAATTTGG 0.189079343125
AAATTTGT 0.18795711058
AAATTTTA 0.255343146941
AAATTTTC 0.26803428515
AAATTTTG 0.275967922363
AACAAAAA 0.114211020314
AACAAAAC -0.124047651388
AACAAAAG 0.0380322038921
AACAAAAT 0.311267851176
AACAAACA 0.103730323388
AACAAACC -0.144115613251
AACAAACG 0.0993020226218

AACAAACT -0.0389740386152
AACAAAGA 0.0308579472488
AACAAAGC 0.104074732705
AACAAAGG -0.116028728986
AACAAAGT -0.00965121703576
AACAAATA 0.193202388389
AACAAATC 0.271644414743
AACAAATG 0.0164878307511
AACAAATT 0.0859224499877
AACAACAA 0.0636144176627
AACAACAC -0.0845771650226
AACAACAG -0.0487030450723
AACAACAT -0.00660787224694
AACAACCA -0.172788670486
AACAACCC -0.0819282078695
AACAACCG -0.169561706466
AACAACCT -0.126159865233
AACAACGA 0.0806517222844
AACAACGC 0.029231082683
AACAACGG -0.150667379468
AACAACGT 0.112950183778
AACAACTA -0.00979326776401
AACAACTC -0.177820509655
AACAACTG -0.0862542594696
AACAACTT -0.0609423451607
AACAAGAA 0.15274214403
AACAAGAC -0.195973366877
AACAAGAG -0.136546114742
AACAAGAT 0.251369404378
AACAAGCA -0.0363793529266
AACAAGCC -0.187163237311
AACAAGCG -0.124895035811
AACAAGCT -0.263248285322
AACAAGGA 0.0979719030116
AACAAGGC -0.0531271337876
AACAAGGG -0.0117321649592
AACAAGGT -0.0737625848067
AACAAGTA 0.0146792465257
AACAAGTC -0.110705963705
AACAAGTG -0.14411882716
AACAAGTT -0.0495154940218
AACAATAA 0.0320209783098
AACAATAC 0.205929328855
AACAATAG 0.154038034082
AACAATAT 0.209957957365
AACAATCA 0.128675199396
AACAATCC 0.275400079028
AACAATCG 0.10061555369
AACAATCT 0.269089332367
AACAATGA -0.0764572238734
AACAATGC -0.096712948033
AACAATGG -0.084909854285
AACAATGT 0.0392736393774
AACAATTA 0.171744657359

AACAATTC 0.0989072207952
AACAATTG 0.0328171080732
AACACAAA 0.110332253425
AACACAAC -0.147372580008
AACACAAG 0.110732398077
AACACAAT 0.179528714425
AACACACA -0.0935998877082
AACACACC 0.0233013234931
AACACACG -0.0491697296355
AACACACT 0.00223111374564
AACACAGA 0.11925820464
AACACAGC -0.112219642965
AACACAGG -0.0403247052625
AACACAGT -0.0610167072823
AACACATA -0.0316058834829
AACACATC -0.152150829712
AACACATG -0.137374186396
AACACATT 0.0496580605502
AACACCAA -0.127119776641
AACACCAC -0.113694403528
AACACCAG -0.220239616985
AACACCAT -0.146039215686
AACACCCA -0.0703842734367
AACACCCC -0.18238722367
AACACCCG -0.203496092893
AACACCCT -0.203261905315
AACACCGA 0.136758531518
AACACCGC -0.0533504963698
AACACCGG -0.10371083782
AACACCGT -0.0902009210698
AACACCTA -0.0771156276786
AACACCTC -0.046463104855
AACACCTG -0.000306539014717
AACACCTT -0.0207743309403
AACACGAA 0.0757155819946
AACACGAC -0.155861435843
AACACGAG 0.0740697889069
AACACGAT 0.252981854634
AACACGCA -0.0230950270946
AACACGCC -0.234884796939
AACACGCG -0.137491972534
AACACGCT -0.0175976639112
AACACGGA 0.151013322098
AACACGGC -0.31246057215
AACACGGG 0.136624843575
AACACGGT -0.278562091503
AACACGTA 0.104511075984
AACACGTC -0.094846768337
AACACGTG -0.00515292380328
AACACGTT -0.282431670811
AACACTAA 0.0580172301077
AACACTAC 0.00330500938642
AACACTAG -0.251440258094
AACACTAT 0.0999280994094

AACACTCA -0.0825987413636
AACACTCC -0.185429953656
AACACTCG -0.116663546834
AACACTCT -0.0403469266195
AACACTGA -0.082084678039
AACACTGC -0.0642090276466
AACACTGG -0.210013275892
AACACTGT 0.0146574384388
AACACTTA -0.00965379977627
AACACTTC -0.258082461589
AACACTTG -0.000852704394841
AACAGAAA 0.180670608574
AACAGAAC -0.0940429050198
AACAGAAG -0.0149390553949
AACAGAAT 0.226006608268
AACAGACA -0.127080657908
AACAGACC -0.17099695398
AACAGACG 0.00076565896068
AACAGACT -0.280664426158
AACAGAGA -0.0397841839492
AACAGAGC -0.166807096246
AACAGAGG -0.248393451979
AACAGAGT -0.0558217523985
AACAGATA 0.274288495344
AACAGATC 0.285928340908
AACAGATG -0.077750080622
AACAGATT 0.422095506516
AACAGCAA 0.0264847887639
AACAGCAC -0.238851851852
AACAGCAG -0.0635409463598
AACAGCAT 0.0657097613064
AACAGCCA -0.226372263389
AACAGCCC -0.315150223737
AACAGCCG -0.260037993766
AACAGCCT -0.180323702243
AACAGCGA -0.184690311504
AACAGCGC -0.157047060889
AACAGCGG -0.12197794735
AACAGCGT -0.141867059463
AACAGCTA -0.121042846768
AACAGCTC -0.100005865356
AACAGCTG -0.0940295476747
AACAGCTT -0.022923597693
AACAGGAA 0.156265843683
AACAGGAC -0.100178966238
AACAGGAG -0.263249345711
AACAGGAT 0.254050582889
AACAGGCA -0.0835807799362
AACAGGCC -0.215400657597
AACAGGCG -0.0777558244834
AACAGGCT -0.258137340421
AACAGGGA -0.0510234857014
AACAGGGC -0.265557050021
AACAGGGG -0.197084456412

AACAGGGT -0.0348014598941
AACAGGTA 0.0853050135836
AACAGGTC -0.0986306632639
AACAGGTG -0.0906132349874
AACAGGTT -0.14527523602
AACAGTAA 0.168626966717
AACAGTAC 0.0645170194529
AACAGTAG -0.0267415338573
AACAGTAT 0.119777602143
AACAGTCA -0.0577893032573
AACAGTCC -0.24415308292
AACAGTCG -0.0971976898227
AACAGTCT -0.218781213299
AACAGTGA -0.262054144847
AACAGTGC -0.114057638169
AACAGTGG -0.0198814522104
AACAGTGT -0.0453052454806
AACAGTTA 0.028515993765
AACAGTTC -0.138198983297
AACAGTTG -0.0191812066887
AACATAAA 0.028845309447
AACATAAC 0.0478440800018
AACATAAG 0.0672598898784
AACATAAT 0.173848492832
AACATACA 0.239353694714
AACATACC 0.0137884444961
AACATACG -0.190288088562
AACATACT -0.139301299894
AACATAGA 0.14000581791
AACATAGC -0.12961542596
AACATAGG 0.0188267255532
AACATAGT -0.0606845553731
AACATATA 0.129599442359
AACATATC 0.255941403763
AACATATG 0.00519867816555
AACATATT 0.194934115904
AACATCAA 0.0855534698896
AACATCAC -0.00225032382709
AACATCAG 0.167325265498
AACATCAT -0.0459313047487
AACATCCA 0.132528002557
AACATCCC -0.0467987738439
AACATCCG 0.0403948687403
AACATCCT -0.00602647698083
AACATCGA 0.11964464719
AACATCGC -0.00926445164383
AACATCGG 0.0668181518804
AACATCGT -0.0455726177851
AACATCTA 0.058775587291
AACATCTC -0.152140688333
AACATCTG 0.071886869086
AACATCTT 0.0462161058924
AACATGAA -0.0151512656699
AACATGAC 0.01766778634

AACATGAG -0.0116455769762
AACATGAT 0.0733326920027
AACATGCA -0.0522717631951
AACATGCC -0.113668255864
AACATGCG -0.0805176842521
AACATGCT -0.180920474999
AACATGGA 0.20945912571
AACATGGC -0.25218115102
AACATGGG -0.118209205847
AACATGGT -0.148542791606
AACATGTA -0.0491290514724
AACATGTC -0.222202694105
AACATGTG -0.184241871383
AACATGTT 0.00861685842674
AACATTAA 0.101650371188
AACATTAC 0.121942963404
AACATTAG 0.0704735559507
AACATTAT -0.00730027748318
AACATTCA -0.0142703462206
AACATTCC -0.11054035365
AACATTCG -0.0858358852778
AACATTCT 0.114094162331
AACATTGA 0.0685296296834
AACATTGC 0.0357123011953
AACATTGG -0.0168297293151
AACATTGT -0.0896534501306
AACATTTA 0.0436142547906
AACATTTC 0.0886275478332
AACATTTG 0.0426541208527
AACCAAAA 0.192608509517
AACCAAAC -0.0347074738096
AACCAAAG -0.0487290782367
AACCAAAT 0.271007596215
AACCAACA 0.04657794953
AACCAACC -0.1337396307
AACCAACG -0.0154586269731
AACCAACT -0.0994212364389
AACCAAGA 0.12416161716
AACCAAGC -0.149444115194
AACCAAGG -0.190858710562
AACCAAGT 0.0302394125008
AACCAATA -0.0894585445934
AACCAATC 0.0782284154895
AACCAATG -0.0347725654846
AACCAATT -0.045459300045
AACCACAA 0.0227318292098
AACCACAC -0.123265946705
AACCACAG -0.131282823793
AACCACAT -0.153511155018
AACCACCA -0.0790898738002
AACCACCC -0.0905711521533
AACCACCG -0.117380591773
AACCACCT -0.299551159267
AACCACGA 0.0328464949834

AACCACGC -0.0120828672294
AACCACGG 0.0560879229439
AACCACGT -0.0918440283004
AACCACTA -0.0327648075778
AACCACTC -0.0848920942282
AACCACTG -0.0268565604884
AACCACTT -0.146329702251
AACCAGAA 0.146506762745
AACCAGAC -0.042723831272
AACCAGAG -0.112732265047
AACCAGAT 0.29876753606
AACCAGCA 0.00800052300495
AACCAGCC -0.0857907732294
AACCAGCG -0.132640551282
AACCAGCT -0.216720177822
AACCAGGA -0.0468496202643
AACCAGGC -0.224959547748
AACCAGGG -0.0280580304619
AACCAGGT -0.196717954378
AACCAGTA 0.0276680058838
AACCAGTC -0.221065335917
AACCAGTG -0.178647892561
AACCAGTT -0.226035477169
AACCATAA 0.0821971942304
AACCATAC 0.0255439197348
AACCATAG -0.187535668818
AACCATAT 0.222763790624
AACCATCA -0.139067268217
AACCATCC -0.17575450349
AACCATCG -0.0432616319273
AACCATCT -0.0141735956752
AACCATGA 0.096805089025
AACCATGC -0.201972403776
AACCATGG -0.248356400035
AACCATGT -0.159867828613
AACCATTA -0.0462844856392
AACCATTC -0.0969938965759
AACCATTG -0.206632037055
AACCCAAA -0.00167652143594
AACCCAAC 0.0925907631514
AACCCAAG -0.0306639473096
AACCCAAT 0.0167786339986
AACCCACA -0.158288740704
AACCCACC -0.136825988516
AACCCACG -0.0874682580728
AACCCACT -0.164280214432
AACCCAGA 0.102337592483
AACCCAGC -0.113885781529
AACCCAGG -0.0481692825308
AACCCAGT -0.171304300102
AACCCATA -0.0336107817954
AACCCATC -0.300971339106
AACCCATG -0.139377373817
AACCCATT 0.00163729606439

AACCCCAA -0.112065985084
AACCCCAC -0.0484670568479
AACCCCAG -0.23965969955
AACCCCAT -0.141755568726
AACCCCCA -0.332134030826
AACCCCCC -0.339296784564
AACCCCCG -0.136959838432
AACCCCCT -0.318948633124
AACCCCGA -0.0811703590845
AACCCCGC -0.139823898348
AACCCCGG -0.363041350446
AACCCCGT -0.175133895254
AACCCCTA -0.128157699757
AACCCCTC -0.146587105624
AACCCCTG -0.114427430641
AACCCCTT -0.16111220883
AACCCGAA -0.0321309604166
AACCCGAC -0.17500308642
AACCCGAG -0.0767698632923
AACCCGAT 0.134785640608
AACCCGCA -0.0580624711849
AACCCGCC -0.104185462421
AACCCGCG 0.0586099690555
AACCCGCT -0.219334785766
AACCCGGA 0.183641213079
AACCCGGC -0.245170707021
AACCCGGG -0.144962491014
AACCCGGT -0.17044649185
AACCCGTA -0.0100496168961
AACCCGTC -0.282932784636
AACCCGTG -0.17501042627
AACCCGTT -0.111825723127
AACCCTAA -0.0981229933317
AACCCTAC -0.0879302530969
AACCCTAG -0.0618152978111
AACCCTAT -0.0940607916749
AACCCTCA -0.211714677641
AACCCTCC -0.149940592564
AACCCTCG -0.216091517811
AACCCTCT -0.19871534153
AACCCTGA -0.0389039817788
AACCCTGC -0.18686223193
AACCCTGG -0.228233493012
AACCCTGT -0.15066003941
AACCCTTA -0.242544213432
AACCCTTC -0.243580246914
AACCCTTG -0.169979986464
AACCGAAA 0.0091772841515
AACCGAAC -0.0222059186727
AACCGAAG -0.0491104498988
AACCGAAT 0.263903941423
AACCGACA 0.0860015558629
AACCGACC -0.0521050322633
AACCGACG -0.00656887440115

AACCGACT -0.057566988864
AACCGAGA -0.0519267600687
AACCGAGC -0.131223395755
AACCGAGG -0.101962818021
AACCGAGT -0.255980096843
AACCGATA 0.0746859863189
AACCGATC -0.0594671160635
AACCGATG -0.257870073145
AACCGATT 0.213974401813
AACCGCAA 0.0840919326486
AACCGCAC -0.0721442995742
AACCGCAG 0.00719806976811
AACCGCAT -0.207253033072
AACCGCCA -0.2512389252
AACCGCCC -0.0827214759208
AACCGCCG -0.122595603984
AACCGCCT -0.10898184459
AACCGCGA 0.0020295497799
AACCGCGC 0.043070910755
AACCGCGG 0.000996179938089
AACCGCGT 0.0129661644851
AACCGCTA -0.29643718228
AACCGCTC -0.0803987422005
AACCGCTG -0.279277989036
AACCGCTT -0.020387023666
AACCGGAA -0.101073163363
AACCGGAC -0.147323205471
AACCGGAG -0.173421781177
AACCGGAT 0.293157351852
AACCGGCA -0.133378884169
AACCGGCC -0.11473066052
AACCGGCG -0.0435849305144
AACCGGCT -0.132018105805
AACCGGGA -0.171072702332
AACCGGGC -0.282566942763
AACCGGGG -0.135287888016
AACCGGGT -0.0128139696965
AACCGGTA -0.17155676533
AACCGGTC -0.229905591866
AACCGGTG -0.293407407407
AACCGGTT 0.0405120481928
AACCGTAA 0.00792827504446
AACCGTAC -0.108336900567
AACCGTAG -0.175495634901
AACCGTAT 0.246602646986
AACCGTCA -0.186228829837
AACCGTCC -0.179211735072
AACCGTCG 0.0461348481106
AACCGTCT -0.150617459686
AACCGTGA -0.0332340186566
AACCGTGC -0.177899051758
AACCGTGG -0.117679552266
AACCGTGT -0.100816888561
AACCGTTA 0.0566082622453

AACCGTTC -0.168641975309
AACCGTTG -0.154138680363
AACCTAAA 0.0139626345945
AACCTAAC -0.0956113196777
AACCTAAG -0.244358303294
AACCTAAT 0.0483351447485
AACCTACA -0.178710460873
AACCTACC -0.162837274557
AACCTACG -0.090919787681
AACCTACT -0.123802536574
AACCTAGA -0.0607496455714
AACCTAGC -0.233382716049
AACCTAGG 0.0765450971828
AACCTAGT -0.119724242917
AACCTATA -0.0406485323498
AACCTATC -0.0136967880744
AACCTATG -0.0114956887536
AACCTATT 0.106813592318
AACCTCAA -0.0444626850493
AACCTCAC -0.143622334178
AACCTCAG -0.150980585824
AACCTCAT -0.129929742678
AACCTCCA -0.332180443267
AACCTCCC -0.301087947769
AACCTCCG -0.278144249513
AACCTCCT -0.196992592593
AACCTCGA -0.0423939054644
AACCTCGC -0.103577398081
AACCTCGG -0.170200649656
AACCTCGT -0.329583888807
AACCTCTA -0.156848218618
AACCTCTC -0.317959881958
AACCTCTG -0.173866761932
AACCTCTT -0.166949273454
AACCTGAA -0.0871573760394
AACCTGAC -0.250921614139
AACCTGAG -0.196942756952
AACCTGAT 0.241070056423
AACCTGCA -0.2310048717
AACCTGCC -0.263396987432
AACCTGCG -0.23073018695
AACCTGCT -0.071704514435
AACCTGGA -0.188672229065
AACCTGGC -0.176381985458
AACCTGGG -0.180230452675
AACCTGGT -0.184442326911
AACCTGTA -0.0954384289447
AACCTGTC -0.16226213956
AACCTGTG -0.018870720743
AACCTTAA -0.183254789617
AACCTTAC -0.0965423561373
AACCTTAG 0.0126116833496
AACCTTAT -0.025182688536
AACCTTCA -0.0388723432075

AACCTTCC -0.285898491084
AACCTTCG -0.148511094013
AACCTTCT -0.249756753538
AACCTTGA -0.0510082548464
AACCTTGC -0.139493432578
AACCTTGG -0.171489711934
AACCTTGT -0.0915113394367
AACCTTTA 0.0762817253352
AACCTTTC -0.258423328504
AACCTTTG 0.070528670841
AACGAAAA -0.00554896924192
AACGAAAC 0.0448421910501
AACGAAAG 0.113405343696
AACGAAAT 0.379243967283
AACGAACA -0.0992271149013
AACGAACC -0.088769921404
AACGAACG -0.0687998233667
AACGAACT -0.0830649145341
AACGAAGA 0.106941437973
AACGAAGC -0.0527645344699
AACGAAGG -0.166496264712
AACGAAGT -0.144536901988
AACGAATA 0.0998692487615
AACGAATC 0.40752400738
AACGAATG -0.101781122427
AACGAATT 0.200863456373
AACGACAA 0.123495581319
AACGACAC 0.0698313309023
AACGACAG -0.0643785199091
AACGACAT 0.0211914641375
AACGACCA -0.142310117112
AACGACCC -0.235172125338
AACGACCG -0.0923218352954
AACGACCT -0.18593946542
AACGACGA -0.0410498059358
AACGACGC -0.276653458747
AACGACGG 0.0536634559974
AACGACGT -0.0235702010052
AACGACTA 0.07634635228
AACGACTC -0.00755467742378
AACGACTG -0.0978372976098
AACGACTT -0.0734922830359
AACGAGAA 0.139373556288
AACGAGAC -0.119910768674
AACGAGAG 0.04187671611
AACGAGAT 0.335435465104
AACGAGCA -0.140031606499
AACGAGCC -0.226419177191
AACGAGCG -0.293590155031
AACGAGCT -0.20956330527
AACGAGGA 0.0131962445758
AACGAGGC -0.114998628258
AACGAGGG -0.164912724669
AACGAGGT -0.108968344116

AACGAGTA -0.118552542764
AACGAGTC -0.0845096199377
AACGAGTG 0.158322435046
AACGAGTT -0.113664957215
AACGATAA 0.09306146761
AACGATAC 0.0804308979705
AACGATAG -0.133436508721
AACGATAT 0.300540438452
AACGATCA 0.0955864193047
AACGATCC 0.189836769194
AACGATCG 0.0505320877652
AACGATCT 0.161551296406
AACGATGA 0.0501794770467
AACGATGC -0.286572631126
AACGATGG 0.114195701333
AACGATGT 0.0305536588555
AACGATTA 0.0150378452196
AACGATTC 0.168013888687
AACGATTG 0.0318321154098
AACGCAAA 0.150019612686
AACGCAAC 0.0869713654788
AACGCAAG 0.0302077330479
AACGCAAT 0.356144581814
AACGCACA 0.00574187493826
AACGCACC -0.213859256333
AACGCACG -0.187406495019
AACGCACT -0.0699768381304
AACGCAGA -0.0137544183187
AACGCAGC -0.0367488858153
AACGCAGG -0.161822091148
AACGCAGT -0.233568712656
AACGCATA -0.121796026521
AACGCATC -0.0698286460735
AACGCATG -0.134062623469
AACGCATT 0.052769747069
AACGCCAA -0.137387570595
AACGCCAC -0.185620527679
AACGCCAG -0.221196159122
AACGCCAT 0.160168170542
AACGCCCA -0.163139701105
AACGCCCC -0.294945309738
AACGCCCG -0.410820904191
AACGCCCT -0.112249882007
AACGCCGA -0.0273591836231
AACGCCGC -0.301085048011
AACGCCGG -0.148570166631
AACGCCGT -0.0939681285394
AACGCCTA -0.108208218096
AACGCCTC -0.116061578991
AACGCCTG -0.0830295177975
AACGCCTT -0.267311546841
AACGCGAA -0.0325583557536
AACGCGAC -0.0779948302545
AACGCGAG -0.0645589470045

AACGCGAT 0.304213594151
AACGCGCA 0.0762613862537
AACGCGCC -0.209479480468
AACGCGCG 0.103163666713
AACGCGCT -0.0544550923182
AACGCGGA 0.0694471887418
AACGCGGC -0.198794830756
AACGCGGG -0.219185980649
AACGCGGT -0.191219315241
AACGCGTA 0.0282931152209
AACGCGTC -0.183944444444
AACGCGTG -0.0533191741579
AACGCGTT -0.0579004542761
AACGCTAA 0.00233253752641
AACGCTAC -0.0384146743068
AACGCTAG -0.252765748575
AACGCTAT 0.0198819229361
AACGCTCA -0.0148424380124
AACGCTCC -0.125141303393
AACGCTCG -0.282993110339
AACGCTCT -0.121990637845
AACGCTGA -0.0831040344021
AACGCTGC -0.0574707912832
AACGCTGG -0.113418818068
AACGCTGT -0.167006909117
AACGCTTA -0.0547789577673
AACGCTTC -0.165785437051
AACGCTTG 0.158403587345
AACGGAAA 0.0454419472572
AACGGAAC -0.232691100937
AACGGAAG -0.182769766013
AACGGAAT 0.330142713401
AACGGACA -0.0655833684425
AACGGACC -0.196735834369
AACGGACG -0.307653768069
AACGGACT -0.197775273262
AACGGAGA 0.201927854372
AACGGAGC -0.077385362641
AACGGAGG 0.0293007123492
AACGGAGT -0.173150480455
AACGGATA 0.2344093676
AACGGATC 0.239261564469
AACGGATG -0.112924500071
AACGGATT 0.378979559223
AACGGCAA 0.0516685310825
AACGGCAC 0.040096475198
AACGGCAG -0.171392183003
AACGGCAT -0.184065407255
AACGGCCA -0.0858177819716
AACGGCCC -0.245320078127
AACGGCCG -0.127293779358
AACGGCCT -0.274749419839
AACGGCGA -0.0707068212255
AACGGCGC -0.142901139513

AACGGCGG -0.141355524825
AACGGCGT -0.10507180762
AACGGCTA -0.0982751902888
AACGGCTC -0.157582990398
AACGGCTG -0.140045324598
AACGGCTT -0.107257293752
AACGGGAA 0.187766042451
AACGGGAC -0.171333563797
AACGGGAG -0.0866771198578
AACGGGAT 0.166200136117
AACGGGCA -0.236577011142
AACGGGCC -0.360497083241
AACGGGCG 0.0171035156464
AACGGGCT -0.254106995885
AACGGGGA 0.0932764807578
AACGGGGC -0.191082860492
AACGGGGG -0.160539558673
AACGGGGT -0.222330514506
AACGGGTA -0.114416210028
AACGGGTC -0.450907311634
AACGGGTG -0.205435025721
AACGGTAA 0.134079583921
AACGGTAC 0.189884212514
AACGGTAG -0.165015005854
AACGGTAT 0.071760638066
AACGGTCA -0.275599054924
AACGGTCC -0.159245908295
AACGGTCG -0.218112279893
AACGGTCT -0.182701449597
AACGGTGA 0.162517289073
AACGGTGC -0.326069304541
AACGGTGG 0.00713721228885
AACGGTGT 0.0179724841283
AACGGTTA -0.260626361387
AACGGTTC -0.107754835452
AACGGTTG 0.011397149623
AACGTAAA 0.184802347711
AACGTAAC -0.0395900222277
AACGTAAG -0.0413099080112
AACGTAAT 0.371303008731
AACGTACA -0.0453269765455
AACGTACC -0.265777566087
AACGTACG -0.138385506346
AACGTACT -0.151492086364
AACGTAGA 0.273529339129
AACGTAGC -0.259550068587
AACGTAGG -0.0347702474258
AACGTAGT 0.0731945267734
AACGTATA 0.199400645459
AACGTATC 0.302600496855
AACGTATG 0.0629491627976
AACGTATT 0.118229818437
AACGTCAA -0.072951927128
AACGTCAC -0.259171566691

AACGTCAG -0.164363170401
AACGTCAT -0.0313234099541
AACGTCCA -0.185694447672
AACGTCCC -0.0135937704299
AACGTCCG -0.0866520641542
AACGTCCT -0.202843563344
AACGTCGA 0.0820133263079
AACGTCGC -0.0670913660599
AACGTCGG -0.0215533269807
AACGTCGT -0.0744599247454
AACGTCTA -0.204196097802
AACGTCTC -0.19962962963
AACGTCTG -0.379963354289
AACGTCTT -0.130600148185
AACGTGAA -0.0399111323732
AACGTGAC -0.298349438381
AACGTGAG -0.0316777837172
AACGTGAT 0.21526542844
AACGTGCA -0.00366829863583
AACGTGCC -0.241238044618
AACGTGCG -0.138551406998
AACGTGCT -0.0533712027661
AACGTGGA -0.00825040314965
AACGTGGC -0.290819334993
AACGTGGG -0.11096022693
AACGTGGT -0.0438166112373
AACGTGTA -0.0968737653102
AACGTGTC -0.155947137844
AACGTGTG 0.0150320340534
AACGTTAA -0.0364077155985
AACGTTAC 0.0183456277855
AACGTTAG -0.0581544051461
AACGTTAT 0.0820215592
AACGTTCA -0.202044835911
AACGTTCC 0.0947059022352
AACGTTCG -0.0203046147206
AACGTTCT -0.190338211971
AACGTTGA -0.0993796732495
AACGTTGC -0.2352187488
AACGTTGG -0.00113328284325
AACGTTGT 0.0133290522295
AACGTTTA -0.0103695287027
AACGTTTC 0.0024036899045
AACGTTTG -0.132613589689
AACTAAAA 0.167804686706
AACTAAAC -0.0546252387539
AACTAAAG -0.228176171035
AACTAAAT 0.121716327924
AACTAACA 0.00941293991087
AACTAACC -0.169630215022
AACTAACG 0.16399097675
AACTAACT -0.0200092978658
AACTAAGA 0.049971670565
AACTAAGC 0.0573460404167

AACTAAGG -0.0139453459823
AACTAAGT -0.151345254561
AACTAATA 0.0749916464487
AACTAATC -0.123160402714
AACTAATG -0.0860011387952
AACTAATT 0.10864701524
AACTACAA 0.104357395332
AACTACAC 0.0668675492327
AACTACAG -0.0919311957927
AACTACAT 0.105322015851
AACTACCA -0.147079316549
AACTACCC -0.130195889823
AACTACCG -0.0452266890557
AACTACCT -0.12907480029
AACTACGA -0.127678550677
AACTACGC -0.213111133062
AACTACGG -0.166378572369
AACTACGT 0.045651386419
AACTACTA 0.0959872884147
AACTACTC -0.0205863831679
AACTACTG 0.175501576279
AACTACTT -0.0605098949587
AACTAGAA 0.174040593646
AACTAGAC -0.0110564535629
AACTAGAG -0.111589815697
AACTAGAT 0.266580435642
AACTAGCA 0.0626269376607
AACTAGCC -0.122246505655
AACTAGCG -0.230429978766
AACTAGCT -0.245636130629
AACTAGGA -0.0298098295876
AACTAGGC -0.0374367501948
AACTAGGG -0.198138268678
AACTAGGT -0.0299812589891
AACTAGTA -0.124940320377
AACTAGTC -0.04665384526
AACTAGTG -0.125194149447
AACTAGTT -0.196438511123
AACTATAA -0.106249019414
AACTATAC -0.0536869659703
AACTATAG -0.0831851412764
AACTATAT -0.0211134194354
AACTATCA 0.0260906832479
AACTATCC -0.143652989089
AACTATCG -0.144756699209
AACTATCT 0.125437773161
AACTATGA 0.0218721490374
AACTATGC -0.0588788996465
AACTATGG -0.237496440837
AACTATGT 0.043996344957
AACTATTA 0.0202330277628
AACTATTC -0.0126658468034
AACTATTG 0.119100977923
AACTCAAA 0.01505795956

AACTCAAC 0.000729983095128
AACTCAAG 0.0268617602097
AACTCAAT 0.119280884322
AACTCACA -0.0466999840193
AACTCACC -0.177078703475
AACTCACG -0.136561157474
AACTCACT -0.129389972833
AACTCAGA 0.123866459402
AACTCAGC -0.0376737140223
AACTCAGG 0.0999994800022
AACTCAGT -0.1814037717
AACTCATA 0.0524490109552
AACTCATC -0.161133058985
AACTCATG 0.0267328171081
AACTCATT 0.0350035127006
AACTCCAA -0.0824444670434
AACTCCAC -0.271858215792
AACTCCAG 0.00712594249851
AACTCCAT -0.0142969215347
AACTCCCA -0.155411549714
AACTCCCC -0.219248564483
AACTCCCG -0.0799499489611
AACTCCCT -0.15805274279
AACTCCGA -0.124297459878
AACTCCGC -0.160830699328
AACTCCGG -0.157003160806
AACTCCGT -0.0955544578908
AACTCCTA -0.0058128766176
AACTCCTC -0.0151764596127
AACTCCTG -0.236279509087
AACTCCTT -0.170788060447
AACTCGAA -0.144813530418
AACTCGAC -0.133598340823
AACTCGAG -0.118111730333
AACTCGAT 0.0709785066632
AACTCGCA 0.0367708401941
AACTCGCC -0.0886457681627
AACTCGCG -0.152142338417
AACTCGCT -0.22322085048
AACTCGGA -0.048708533667
AACTCGGC -0.0426599147586
AACTCGGG -0.0856689549585
AACTCGGT -0.0951115005317
AACTCGTA -0.0431689186905
AACTCGTC -0.153246297119
AACTCGTG -0.200605242509
AACTCTAA 0.0645239192957
AACTCTAC -0.075939473883
AACTCTAG -0.144854889953
AACTCTAT -0.00342059767844
AACTCTCA 0.0395123932468
AACTCTCC 0.0942858240576
AACTCTCG -0.200424110385
AACTCTCT -0.107817956779

AACTCTGA 0.0496055670972
AACTCTGC -0.0964836601307
AACTCTGG -0.0617722592791
AACTCTGT -0.052810969332
AACTCTTA -0.0549000694197
AACTCTTC -0.211625555946
AACTCTTG -0.188465315842
AACTGAAA 0.0576535708797
AACTGAAC -0.0915399051758
AACTGAAG 0.0338924972834
AACTGAAT 0.167060857438
AACTGACA -0.172612926652
AACTGACC -0.12043028457
AACTGACG -0.169963828947
AACTGACT -0.222518577058
AACTGAGA -0.0580062130892
AACTGAGC -0.226988125027
AACTGAGG -0.0155523726423
AACTGAGT -0.233899919834
AACTGATA 0.255323754594
AACTGATC 0.0330146877225
AACTGATG 0.0337274144328
AACTGATT 0.308896895384
AACTGCAA 0.194989321982
AACTGCAC 0.065824730811
AACTGCAG -0.0725262903686
AACTGCAT -0.0849030714176
AACTGCCA -0.217239070543
AACTGCCC -0.15452432825
AACTGCCG -0.143689179375
AACTGCCT -0.0805204285495
AACTGCGA 0.125839350312
AACTGCGC -0.186716049383
AACTGCGG -0.011447431882
AACTGCGT -0.00877077433094
AACTGCTA -0.100592592593
AACTGCTC -0.115585954862
AACTGCTG -0.143084574802
AACTGCTT -0.0203094721368
AACTGGAA -0.0731730742845
AACTGGAC 0.0274998910406
AACTGGAG -0.0598997782608
AACTGGAT 0.168379108213
AACTGGCA -0.123740917318
AACTGGCC -0.0487881182902
AACTGGCG -0.17607986451
AACTGGCT -0.22119742965
AACTGGGA -0.190535189828
AACTGGGC -0.286538148738
AACTGGGG -0.151280864198
AACTGGGT -0.117517766763
AACTGGTA 0.0566457948949
AACTGGTC -0.0285197947597
AACTGGTG -0.136059734295

AACTGTAA 0.0169740287091
AACTGTAC -0.228244764915
AACTGTAG 0.0451919864019
AACTGTAT 0.00277174032361
AACTGTCA 0.0789970858441
AACTGTCC -0.162646668203
AACTGTCG -0.172280389833
AACTGTCT -0.120545683795
AACTGTGA 0.147421095525
AACTGTGC -0.177475308642
AACTGTGG -0.092186496765
AACTGTGT -0.0654899119629
AACTGTTA 0.188515682885
AACTGTTC -0.165908411434
AACTGTTG -0.177344834587
AACTTAAA 0.100129479456
AACTTAAC 0.007685267241
AACTTAAG 0.0025851281038
AACTTAAT -0.0663271977104
AACTTACA 0.0809718654636
AACTTACC -0.0320451601572
AACTTACG 0.0118273373398
AACTTACT -0.0344172772427
AACTTAGA 0.203524472273
AACTTAGC -0.0344587625122
AACTTAGG -0.124520505095
AACTTAGT -0.206939776256
AACTTATA 0.123896792465
AACTTATC 0.0489837723185
AACTTATG -0.0206508375596
AACTTATT -0.0747665859803
AACTTCAA 0.0620869832488
AACTTCAC -0.18485776373
AACTTCAG -0.0574957737821
AACTTCAT -0.0302638728917
AACTTCCA -0.160309699793
AACTTCCC -0.0549555097855
AACTTCCG 0.015103866524
AACTTCCT -0.0016381027642
AACTTCGA -0.0616201531242
AACTTCGC -0.0606932721224
AACTTCGG -0.177143097844
AACTTCGT 0.122878561155
AACTTCTA -0.0876249903959
AACTTCTC 0.0642438946436
AACTTCTG -0.00291444378581
AACTTCTT -0.100601455697
AACTTGAA 0.0648214462116
AACTTGAC -0.0052800281016
AACTTGAG -0.122715823785
AACTTGAT 0.100040615601
AACTTGCA -0.0725865966262
AACTTGCC 0.0286650298549
AACTTGCG -0.0110457968342

AACTTGCT -0.104601635181
AACTTGGA 0.140638891633
AACTTGGC -0.18246887778
AACTTGGG -0.231539554765
AACTTGGT -0.236909386179
AACTTGTA 0.0113065050824
AACTTGTC -0.193794610046
AACTTGTG -0.172662364033
AACTTTAA 0.0240480583441
AACTTTAC -0.0343192945244
AACTTTAG -0.0441496037372
AACTTTAT 0.115512964061
AACTTTCA 0.0033574012465
AACTTTCC 0.0658448709195
AACTTTCG 0.130015203407
AACTTTCT -0.0926959509149
AACTTTGA -0.0291386533869
AACTTTGC -0.130505716735
AACTTTGG 0.0923113021142
AACTTTGT -0.0692612445863
AACTTTTA -0.107985496762
AACTTTTC -0.00723718328641
AACTTTTG -0.223635802469
AAGAAAAA 0.190039847198
AAGAAAAC -0.172952100923
AAGAAAAG -0.0339701573253
AAGAAAAT 0.355906324002
AAGAAACA 0.160313618301
AAGAAACC -0.0284171990604
AAGAAACG 0.0471053021577
AAGAAACT 0.0131325868291
AAGAAAGA 0.0821479549786
AAGAAAGC -0.2310781893
AAGAAAGG -0.111194714946
AAGAAAGT 0.0941016663519
AAGAAATA 0.160441939186
AAGAAATC 0.447369233429
AAGAAATG -0.0560358075918
AAGAAATT 0.151295163657
AAGAACAA -0.179040576468
AAGAACAC -0.0212480243011
AAGAACAG -0.169287564917
AAGAACAT -0.0851815263028
AAGAACCA -0.0732344435115
AAGAACCC -0.170993141289
AAGAACCG -0.109118562227
AAGAACCT -0.066051167318
AAGAACGA -0.0585431406447
AAGAACGC -0.129155252402
AAGAACGG -0.16382441701
AAGAACGT 0.062120295367
AAGAACTA -0.04888482773
AAGAACTC -0.121183933971
AAGAACTG -0.0594079781367

AAGAACTT -0.108594714744
AAGAAGAA -0.297246086999
AAGAAGAC 0.0383611605085
AAGAAGAG -0.12253562098
AAGAAGAT 0.338269434719
AAGAAGCA -0.100344568113
AAGAAGCC -0.301335908345
AAGAAGCG -0.127484877187
AAGAAGCT -0.181634377452
AAGAAGGA -0.212407003396
AAGAAGGC -0.350773304039
AAGAAGGG -0.129617741785
AAGAAGGT -0.200287581699
AAGAAGTA 0.0435209424084
AAGAAGTC -0.118929747036
AAGAAGTG 0.166805166127
AAGAATAA 0.193588223671
AAGAATAC 0.151835602109
AAGAATAG -0.059994421556
AAGAATAT 0.33628556258
AAGAATCA 0.32641432906
AAGAATCC 0.443728182836
AAGAATCG 0.351470423824
AAGAATCT 0.459592056136
AAGAATGA 0.0255618671277
AAGAATGC 0.0221804828021
AAGAATGG -0.0670788601287
AAGAATGT 0.108734549606
AAGAATTA 0.106948012031
AAGAATTC 0.234203636004
AAGAATTG 0.0222836944829
AAGACAAA -0.153838977517
AAGACAAC -0.198343663572
AAGACAAG -0.133836263664
AAGACAAT 0.196963709412
AAGACACA 0.0065695233391
AAGACACC -0.0801737431609
AAGACACG -0.00398500719132
AAGACACT -0.178220683365
AAGACAGA 0.00943878662851
AAGACAGC -0.166661178343
AAGACAGG -0.123122085048
AAGACAGT -0.0395460831769
AAGACATA -0.0936388779139
AAGACATC -0.136049569247
AAGACATG -0.0499818876375
AAGACATT 0.0690300555446
AAGACCAA -0.231220244752
AAGACCAC -0.214935477968
AAGACCAG -0.258696308861
AAGACCAT -0.0295586508172
AAGACCCA -0.240000987557
AAGACCCC -0.286256792748
AAGACCCG -0.443928771272

AAGACCCT -0.209185185185
AAGACCGA -0.21530527178
AAGACCGC -0.249084105416
AAGACCGG -0.0754928758041
AAGACCGT -0.0335888274166
AAGACCTA -0.213684822077
AAGACCTC -0.19712920892
AAGACCTG -0.130826939752
AAGACCTT -0.311436456064
AAGACGAA 0.241394389319
AAGACGAC -0.150794826631
AAGACGAG -0.0498069430765
AAGACGAT 0.228361215394
AAGACGCA -0.0671045916267
AAGACGCC -0.169375103491
AAGACGCG -0.0690321544997
AAGACGCT -0.170342935528
AAGACGGA -0.0924553980562
AAGACGGC -0.236532194351
AAGACGGG -0.0708417707973
AAGACGGT -0.135657933725
AAGACGTA -0.152331415059
AAGACGTC -0.0439489154146
AAGACGTG -0.0765079051492
AAGACTAA -0.0491098937384
AAGACTAC 0.0369604695422
AAGACTAG -0.101899782135
AAGACTAT -0.0654090334578
AAGACTCA -0.186768031189
AAGACTCC 0.0140857824977
AAGACTCG -0.0902517687737
AAGACTCT -0.127880016968
AAGACTGA 0.186455794858
AAGACTGC -0.0228734039317
AAGACTGG -0.17778600823
AAGACTGT -0.0165981433324
AAGACTTA -0.0443101419377
AAGACTTC -0.0236209376317
AAGACTTG 0.0247168062661
AAGAGAAA 0.0112049660805
AAGAGAAC -0.00952246000567
AAGAGAAG -0.0407939801093
AAGAGAAT 0.357873403745
AAGAGACA -0.107344807151
AAGAGACC -0.327314077912
AAGAGACG -0.236059673322
AAGAGACT -0.0496388504687
AAGAGAGA -0.249932465209
AAGAGAGC -0.122747951686
AAGAGAGG -0.278908571279
AAGAGAGT -0.22147780647
AAGAGATA 0.299909926925
AAGAGATC 0.389435004062
AAGAGATG -0.0475872968835

AAGAGATT 0.425160266965
AAGAGCAA -0.13543240291
AAGAGCAC -0.100529100529
AAGAGCAG -0.179088549811
AAGAGCAT -0.077299759732
AAGAGCCA -0.0469199435183
AAGAGCCC -0.350633716506
AAGAGCCG -0.237739772978
AAGAGCCT -0.217103638967
AAGAGCGA 0.0809842148016
AAGAGCGC -0.0685451342586
AAGAGCGG -0.179189811049
AAGAGCGT 0.0254868005137
AAGAGCTA 0.0350616710008
AAGAGCTC -0.317998482281
AAGAGCTG -0.120526349431
AAGAGCTT -0.158626308513
AAGAGGAA 0.0356700815266
AAGAGGAC -0.212352934721
AAGAGGAG -0.192034560918
AAGAGGAT 0.27008411663
AAGAGGCA -0.0912640116537
AAGAGGCC -0.233159041394
AAGAGGCG -0.270612280962
AAGAGGCT -0.247448927722
AAGAGGGA -0.159368562953
AAGAGGGC -0.294833590376
AAGAGGGG -0.179766959613
AAGAGGGT -0.110597342947
AAGAGGTA -0.0205235860706
AAGAGGTC -0.210758246764
AAGAGGTG -0.100048022831
AAGAGTAA 0.167155151595
AAGAGTAC -0.191651509957
AAGAGTAG -0.0613002943065
AAGAGTAT 0.168676361629
AAGAGTCA -0.229623259814
AAGAGTCC -0.313059986064
AAGAGTCG -0.134927505498
AAGAGTCT -0.199711199307
AAGAGTGA -0.0686322933797
AAGAGTGC -0.0105391188383
AAGAGTGG -0.200006390073
AAGAGTGT -0.137957983608
AAGAGTTA 0.0164191310457
AAGAGTTC -0.0111685096838
AAGAGTTG -0.209761316872
AAGATAAA 0.146986183952
AAGATAAC 0.15129806924
AAGATAAG 0.107233418955
AAGATAAT 0.257350217327
AAGATACA 0.0942440399227
AAGATACC 0.17530623333
AAGATACG 0.302355704845

AAGATACT 0.28237473305
AAGATAGA 0.0747795859257
AAGATAGC 0.0968654635667
AAGATAGG -0.0598225163818
AAGATAGT -0.0437358668686
AAGATATA 0.308903402606
AAGATATC 0.472408834442
AAGATATG 0.330803091177
AAGATATT 0.355929877714
AAGATCAA 0.257596215065
AAGATCAC 0.29087690161
AAGATCAG 0.281965045836
AAGATCAT 0.198731712987
AAGATCCA 0.173769848764
AAGATCCC 0.388977670594
AAGATCCG 0.341395842111
AAGATCCT 0.344408931762
AAGATCGA 0.231972325716
AAGATCGC 0.393243066552
AAGATCGG 0.11461661861
AAGATCGT 0.279434282975
AAGATCTA 0.36144317109
AAGATCTC 0.409059474412
AAGATCTG 0.373081225575
AAGATCTT 0.387828362631
AAGATGAA -0.0441923819773
AAGATGAC -0.00983281740543
AAGATGAG -0.0197773271118
AAGATGAT 0.131551726643
AAGATGCA -0.0876062043074
AAGATGCC -0.133284814889
AAGATGCG 0.0246355573119
AAGATGCT 0.028670841021
AAGATGGA -0.0880704267088
AAGATGGC -0.0536821001554
AAGATGGG 0.0377391658071
AAGATGGT -0.171465494534
AAGATGTA -0.170269751105
AAGATGTC -0.0365485521087
AAGATGTG 0.000123493380755
AAGATTAA 0.12184131375
AAGATTAC 0.47308558395
AAGATTAG 0.160819664986
AAGATTAT 0.316036027136
AAGATTCA 0.22713088199
AAGATTCC 0.452557639504
AAGATTCG 0.399844551305
AAGATTCT 0.45272398955
AAGATTGA -0.00542822015851
AAGATTGC 0.42363649837
AAGATTGG 0.215060999704
AAGATTGT 0.326342016184
AAGATTTA 0.285315183124
AAGATTTC 0.473934663769

AAGATTTG 0.392874057501
AAGCAAAA -0.109031447369
AAGCAAAC 0.0131342071176
AAGCAAAG -0.134061812784
AAGCAAAT 0.176203099958
AAGCAACA -0.0143027327009
AAGCAACC -0.250871099259
AAGCAACG -0.162380074206
AAGCAACT -0.0846527101826
AAGCAAGA 0.123354613257
AAGCAAGC -0.303867347146
AAGCAAGG -0.0754194002011
AAGCAAGT -0.0367488858153
AAGCAATA 0.141120002635
AAGCAATC 0.403293478419
AAGCAATG -0.129281155507
AAGCAATT 0.179032470526
AAGCACAA -0.087110124417
AAGCACAC -0.0485768073942
AAGCACAG -0.115133021753
AAGCACAT 0.0277119836097
AAGCACCA -0.338128566657
AAGCACCC -0.203668949471
AAGCACCG -0.0910742313982
AAGCACCT -0.266534587469
AAGCACGA 0.0292432325627
AAGCACGC 0.189201464357
AAGCACGG -0.214948438635
AAGCACGT 0.131943072296
AAGCACTA -0.055007046039
AAGCACTC -0.219385179332
AAGCACTG -0.152860090434
AAGCACTT -0.15687678875
AAGCAGAA 0.201175308355
AAGCAGAC 0.0767698632923
AAGCAGAG -0.0710193599368
AAGCAGAT 0.266362064707
AAGCAGCA -0.0671295694536
AAGCAGCC -0.24778167391
AAGCAGCG -0.119625481577
AAGCAGCT -0.151476687851
AAGCAGGA -0.139176954733
AAGCAGGC -0.235883747139
AAGCAGGG -0.189326706039
AAGCAGGT -0.149190672154
AAGCAGTA 0.0247715485305
AAGCAGTC -0.264934156379
AAGCAGTG -0.419675377392
AAGCATAA -0.046008106766
AAGCATAC -0.160872824041
AAGCATAG -0.119638836023
AAGCATAT 0.232578123172
AAGCATCA -0.072773308464
AAGCATCC -0.101423084298

AAGCATCG -0.00994258929944
AAGCATCT -0.0186200575205
AAGCATGA -0.167989026063
AAGCATGC 0.021675038218
AAGCATGG -0.226621977434
AAGCATGT -0.0421224349867
AAGCATTA 0.16144547071
AAGCATTC -0.24201016703
AAGCATTG -0.0144350040616
AAGCCAAA -0.170888694063
AAGCCAAC -0.115340481592
AAGCCAAG -0.162178649237
AAGCCAAT -0.0230877736064
AAGCCACA -0.228357721704
AAGCCACC -0.270664625716
AAGCCACG -0.118608883285
AAGCCACT -0.218389006876
AAGCCAGA -0.13159801113
AAGCCAGC -0.205498768236
AAGCCAGG -0.200724002568
AAGCCAGT -0.0931842215353
AAGCCATA -0.112038683615
AAGCCATC -0.136203991812
AAGCCATG -0.16443884746
AAGCCATT -0.243535712718
AAGCCCAA -0.259096471156
AAGCCCAC -0.237654320988
AAGCCCAG -0.325678774612
AAGCCCAT -0.14440767994
AAGCCCCA -0.303201646091
AAGCCCCC -0.437479090226
AAGCCCCG -0.159109658678
AAGCCCCT -0.22816417123
AAGCCCGA -0.186543707315
AAGCCCGC -0.408063627671
AAGCCCGG -0.407775463911
AAGCCCGT -0.350225206691
AAGCCCTA -0.189111165994
AAGCCCTC -0.297860916362
AAGCCCTG -0.328535216368
AAGCCCTT -0.285183162738
AAGCCGAA 0.0140376445015
AAGCCGAC -0.0739441427779
AAGCCGAG -0.0351574718888
AAGCCGAT -0.00961475961801
AAGCCGCA -0.158298213267
AAGCCGCC -0.241923491364
AAGCCGCG -0.126888888889
AAGCCGCT -0.182039765728
AAGCCGGA -0.159394997091
AAGCCGGC -0.264537433419
AAGCCGGG -0.18876103621
AAGCCGGT -0.244140496412
AAGCCGTA -0.0948856838662

AAGCCGTC	-0.370228672667
AAGCCGTG	-0.197136234376
AAGCCTAA	-0.193618492542
AAGCCTAC	-0.0517556985748
AAGCCTAG	-0.234312160168
AAGCCTAT	0.0528283364615
AAGCCTCA	-0.123159313604
AAGCCTCC	-0.214117969822
AAGCCTCG	-0.116120630498
AAGCCTCT	-0.20748696845
AAGCCTGA	-0.0779758152829
AAGCCTGC	-0.158022104847
AAGCCTGG	-0.268159597057
AAGCCTGT	-0.201937197678
AAGCCTTA	-0.137080486265
AAGCCTTC	-0.238123844478
AAGCCTTG	-0.211602315873
AAGCGAAA	0.126643066875
AAGCGAAC	-0.0896192765266
AAGCGAAG	-0.0981297551368
AAGCGAAT	0.325342495605
AAGCGACA	0.0704124475179
AAGCGACC	-0.333064511705
AAGCGACG	0.0301816681629
AAGCGACT	-0.08615015908
AAGCGAGA	-0.0257260334864
AAGCGAGC	-0.182602759622
AAGCGAGG	-0.0938021163222
AAGCGAGT	-0.0983163486497
AAGCGATA	0.0923292606744
AAGCGATC	0.262732991443
AAGCGATG	-0.0714660781454
AAGCGATT	0.320493077448
AAGCGCAA	-0.00998794183023
AAGCGCAC	-0.323089669097
AAGCGCAG	-0.0243971870897
AAGCGCAT	-0.191774770622
AAGCGCCA	-0.329137599545
AAGCGCCC	-0.235271604938
AAGCGCCG	-0.238375857339
AAGCGCCT	-0.0855450184947
AAGCGCGA	0.205176296253
AAGCGCGC	-0.0609069353883
AAGCGCGG	-0.212023781517
AAGCGCGT	-0.225391430646
AAGCGCTA	-0.219428410408
AAGCGCTC	-0.0558874377116
AAGCGCTG	-0.130190674581
AAGCGCTT	-0.156581617079
AAGCGGAA	0.0392762192553
AAGCGGAC	-0.15447531178
AAGCGGAG	-0.083060672659
AAGCGGAT	0.252821137676
AAGCGGCA	-0.209205355146

AAGCGGCC -0.207714677641
AAGCGGCG -0.156420761476
AAGCGGCT -0.243576348278
AAGCGGGA -0.104372448308
AAGCGGGC -0.275101883131
AAGCGGGG 0.0531620007845
AAGCGGGT -0.0667544798441
AAGCGGTA 0.00494522382489
AAGCGGTC -0.0748179012346
AAGCGGTG -0.163111903856
AAGCGTAA 0.153959583339
AAGCGTAC -0.160609783199
AAGCGTAG -0.163383362332
AAGCGTAT 0.176142818334
AAGCGTCA -0.0806359555248
AAGCGTCC -0.265246080658
AAGCGTCG -0.181361501249
AAGCGTCT -0.276367557246
AAGCGTGA -0.038063404246
AAGCGTGC -0.17844574399
AAGCGTGG -0.157162767983
AAGCGTGT -0.202459929434
AAGCGTTA -0.0104612614986
AAGCGTTC -0.124477513093
AAGCGTTG 0.124154112126
AAGCTAAA 0.118089514558
AAGCTAAC -0.312154519603
AAGCTAAG -0.236089506173
AAGCTAAT -0.0356965409033
AAGCTACA -0.0448986032216
AAGCTACC -0.236611796982
AAGCTACG -0.17975617284
AAGCTACT -0.163234398407
AAGCTAGA -0.0278642188743
AAGCTAGC -0.0594297379165
AAGCTAGG -0.16491679643
AAGCTAGT -0.231126147632
AAGCTATA 0.0434559485389
AAGCTATC -0.0735361305937
AAGCTATG -0.318205381387
AAGCTATT 0.0624327647149
AAGCTCAA -0.132213282032
AAGCTCAC -0.175279475678
AAGCTCAG -0.17973275236
AAGCTCAT -0.31600188761
AAGCTCCA -0.150044446639
AAGCTCCC -0.20651303155
AAGCTCCG 0.00343860457968
AAGCTCCT -0.339761499544
AAGCTCGA -0.227255332807
AAGCTCGC -0.13619629209
AAGCTCGG -0.12287654321
AAGCTCGT -0.19639153622
AAGCTCTA -0.181589467079

AAGCTCTC -0.231539211813
AAGCTCTG -0.299146354175
AAGCTGAA -0.244627381253
AAGCTGAC -0.257077328437
AAGCTGAG -0.159796303613
AAGCTGAT 0.0589746122691
AAGCTGCA -0.156423647924
AAGCTGCC -0.190745862417
AAGCTGCG -0.0516634272616
AAGCTGCT -0.208752360203
AAGCTGGA 0.0800401765132
AAGCTGGC -0.30247210703
AAGCTGGG -0.128349640923
AAGCTGGT -0.237841649245
AAGCTGTA -0.155637739094
AAGCTGTC -0.133633450417
AAGCTGTG -0.132878396251
AAGCTTAA -0.176168940871
AAGCTTAC -0.0561961639761
AAGCTTAG 0.0955646666228
AAGCTTAT -0.0954274517553
AAGCTTCA -0.240219324244
AAGCTTCC -0.0451323217092
AAGCTTCG -0.213239611284
AAGCTTCT 0.020541668725
AAGCTTGA -0.278389251997
AAGCTTGC 0.0877769313369
AAGCTTGG -0.18774841237
AAGCTTGT -0.147102407276
AAGCTTTA -0.153947280498
AAGCTTTC -0.240089828997
AAGCTTTG -0.0153545537751
AAGGAAAA 0.0870079474851
AAGGAAAC -0.0945591447419
AAGGAAAG -0.325899314006
AAGGAAAT 0.348649435672
AAGGAACA 0.072756811346
AAGGAACC -0.149255399911
AAGGAACG -0.141798641292
AAGGAACT -0.108853161968
AAGGAAGA -0.0941010868713
AAGGAAGC -0.136351488744
AAGGAAGG -0.282538082146
AAGGAAGT -0.108150160533
AAGGAATA 0.148471975235
AAGGAATC 0.349029034078
AAGGAATG -0.0583340353772
AAGGAATT 0.149175673462
AAGGACAA -0.0404980293056
AAGGACAC -0.32626647904
AAGGACAG -0.170071169208
AAGGACAT -0.0481498699752
AAGGACCA -0.198437782936
AAGGACCC -0.254694777728

AAGGACCG -0.24997531108
AAGGACCT -0.286417608718
AAGGACGA -0.120014992068
AAGGACGC -0.0629850066405
AAGGACGG -0.157234102096
AAGGACGT -0.127269017112
AAGGACTA -0.144633140925
AAGGACTC -0.343868312757
AAGGACTG -0.30755829904
AAGGACTT -0.178042353406
AAGGAGAA -0.0166626958993
AAGGAGAC -0.22945467562
AAGGAGAG -0.168932834108
AAGGAGAT 0.30908197765
AAGGAGCA -0.22231824417
AAGGAGCC -0.151305946844
AAGGAGCG -0.115970785786
AAGGAGCT -0.240718792867
AAGGAGGA -0.152888888889
AAGGAGGC -0.352425741159
AAGGAGGG -0.284384403574
AAGGAGGT -0.0387536284371
AAGGAGTA -0.0859892883796
AAGGAGTC -0.132753852232
AAGGAGTG -0.178685501663
AAGGATAA 0.0543014116666
AAGGATAC 0.164748013308
AAGGATAG -0.0523560209424
AAGGATAT 0.392548632197
AAGGATCA 0.0685690135898
AAGGATCC 0.262470036175
AAGGATCG 0.0484084668691
AAGGATCT 0.337545829766
AAGGATGA -0.101777755823
AAGGATGC -0.0583517183092
AAGGATGG -0.164427651833
AAGGATGT 0.0355029748183
AAGGATTA 0.254481437573
AAGGATTC 0.372165140837
AAGGATTG 0.265051646739
AAGGCAAA 0.0159363360083
AAGGCAAC -0.19608248568
AAGGCAAG 0.0121891696373
AAGGCAAT 0.176945651069
AAGGCACA -0.175228658118
AAGGCACC -0.22316425212
AAGGCACG -0.300464654444
AAGGCACT -0.330474481593
AAGGCAGA 0.136186712769
AAGGCAGC -0.217867236767
AAGGCAGG -0.178572154752
AAGGCAGT 0.104111400055
AAGGCATA -0.144182115534
AAGGCATC -0.0926247628786

AAGGCATG -0.15941504769
AAGGCATT -0.122372056136
AAGGCCAA -0.319628369841
AAGGCCAC -0.183922304599
AAGGCCAG -0.29711277882
AAGGCCAT -0.203354401211
AAGGCCCA -0.206672476398
AAGGCCCC -0.441575751592
AAGGCCCG -0.24919405898
AAGGCCCT -0.483075060322
AAGGCCGA -0.120196204315
AAGGCCGC -0.280320306304
AAGGCCGG -0.224480831709
AAGGCCGT -0.353475513428
AAGGCCTA -0.179579101494
AAGGCCTC -0.236086989353
AAGGCCTG -0.34717045174
AAGGCCTT -0.138028851764
AAGGCGAA -0.0272066449932
AAGGCGAC -0.302957296087
AAGGCGAG -0.19355165692
AAGGCGAT 0.154646644273
AAGGCGCA -0.0949170124481
AAGGCGCC -0.337684069612
AAGGCGCG -0.133587267878
AAGGCGCT -0.168043413839
AAGGCGGA 0.0487421874188
AAGGCGGC -0.198399419027
AAGGCGGG -0.266015912068
AAGGCGGT -0.0697539319681
AAGGCGTA -0.0818322026828
AAGGCGTC -0.21472628298
AAGGCGTG -0.00361954940863
AAGGCTAA -0.209809925254
AAGGCTAC -0.16030801396
AAGGCTAG -0.0855218816092
AAGGCTAT -0.284228520305
AAGGCTCA -0.229888162672
AAGGCTCC -0.334897684637
AAGGCTCG -0.212716489351
AAGGCTCT -0.238168902054
AAGGCTGA -0.26689030713
AAGGCTGC -0.099040623856
AAGGCTGG -0.172536336281
AAGGCTGT -0.333301389276
AAGGCTTA -0.239144525656
AAGGCTTC -0.24833358021
AAGGCTTG -0.142277091907
AAGGGAAA 0.102738259896
AAGGGAAC -0.289073596947
AAGGGAAG -0.338555476498
AAGGGAAT 0.255993545413
AAGGGACA -0.0499978208127
AAGGGACC -0.256976798225

AAGGGACG -0.236494607722
AAGGGACT -0.207087931721
AAGGGAGA -0.185055675277
AAGGGAGC -0.0387426539582
AAGGGAGG -0.148775429587
AAGGGAGT -0.126581414333
AAGGGATA 0.197278921448
AAGGGATC 0.165302750884
AAGGGATG -0.161623644585
AAGGGATT 0.319534235033
AAGGGCAA -0.151433119169
AAGGGCAC -0.299260891712
AAGGGCAG -0.208303839421
AAGGGCAT -0.0393251244981
AAGGGCCA -0.304729001658
AAGGGCCC -0.327591230063
AAGGGCCG -0.325541838134
AAGGGCCT -0.238972282573
AAGGGCGA -0.127917718522
AAGGGCGC -0.153318956766
AAGGGCGG -0.036722762742
AAGGGCGT -0.266213358739
AAGGGCTA -0.116803153678
AAGGGCTC -0.258966434688
AAGGGCTG -0.348602164278
AAGGGGAA 0.0486662056019
AAGGGGAC -0.228416397832
AAGGGGAG -0.117732756592
AAGGGGAT 0.185517245165
AAGGGGCA -0.258738428784
AAGGGGCC -0.375407266624
AAGGGGCG -0.207754172427
AAGGGGCT -0.308257220439
AAGGGGGA 0.0178940333851
AAGGGGGC -0.184137919017
AAGGGGGG -0.165544637028
AAGGGGGT -0.231622730072
AAGGGGTA -0.020727350554
AAGGGGTC -0.388948262593
AAGGGGTG -0.171602682295
AAGGGTAA 0.047877390851
AAGGGTAC -0.16178350589
AAGGGTAG -0.198286883852
AAGGGTAT 0.0475998375376
AAGGGTCA -0.201645815602
AAGGGTCC -0.295207294393
AAGGGTCG -0.286277897977
AAGGGTCT -0.198449168814
AAGGGTGA -0.207032566137
AAGGGTGC -0.389317089462
AAGGGTGG 0.00866289088654
AAGGGTGT -0.28467776603
AAGGGTTA -0.132803481733
AAGGGTTC -0.260854981341

AAGGGTTG -0.226002370136
AAGGTAAA 0.123720482166
AAGGTAAC -0.224581590774
AAGGTAAG -0.0707474533822
AAGGTAAT 0.257473886072
AAGGTACA -0.0299346405229
AAGGTACC -0.0309455587393
AAGGTACG -0.122980596163
AAGGTACT -0.122838310098
AAGGTAGA -0.0636874754841
AAGGTAGC -0.293718287196
AAGGTAGG -0.0648340248963
AAGGTAGT -0.00916185113844
AAGGTATA -0.00117455926585
AAGGTATC 0.204691063879
AAGGTATG -0.0332438780774
AAGGTATT -0.0296006276059
AAGGTCAA -0.269887127485
AAGGTCAC -0.263682411112
AAGGTCAG -0.0754324746791
AAGGTCAT -0.195801473493
AAGGTCCA -0.252496543722
AAGGTCCC -0.279371824776
AAGGTCCG -0.283776478233
AAGGTCCT -0.393598359656
AAGGTCGA 0.0343942786889
AAGGTCGC -0.186440910488
AAGGTCGG -0.192536445388
AAGGTCGT -0.155678836952
AAGGTCTA -0.0778806736801
AAGGTCTC -0.272451323846
AAGGTCTG -0.168203321369
AAGGTGAA -0.085541011498
AAGGTGAC -0.244525865449
AAGGTGAG -0.163064099547
AAGGTGAT 0.110669301062
AAGGTGCA -0.0827859271332
AAGGTGCC -0.198081871345
AAGGTGCG -0.290689905592
AAGGTGCT -0.212695608719
AAGGTGGA -0.144284402817
AAGGTGGC -0.285435861091
AAGGTGGG -0.296763899862
AAGGTGGT -0.214466775303
AAGGTGTA -0.126413943355
AAGGTGTC -0.205250162443
AAGGTGTG -0.0861651684283
AAGGTTAA 0.135324046631
AAGGTTAC -0.110528915522
AAGGTTAG -0.0622536632037
AAGGTTAT -0.0753293315746
AAGGTTCA -0.0682021443651
AAGGTTCC -0.247776296223
AAGGTTCG 0.0467580956809

AAGGTTCT -0.231092229484
AAGGTTGA 0.00466393334651
AAGGTTGC -0.247534857688
AAGGTTGG -0.221783178021
AAGGTTGT -0.083319533177
AAGGTTTA 0.0776328214664
AAGGTTTC -0.0394327343825
AAGGTTTG -0.136742117205
AAGTAAAA 0.108048475268
AAGTAAAC 0.0522987803451
AAGTAAAG 0.10382374733
AAGTAAAT 0.174333531882
AAGTAACA 0.0327998419285
AAGTAACC -0.10727318686
AAGTAACG -0.073673406661
AAGTAACT -0.131888674865
AAGTAAGA 0.0884648351808
AAGTAAGC 0.0320238838929
AAGTAAGG 0.0179449603723
AAGTAAGT 0.0561350642327
AAGTAATA 0.0765286998969
AAGTAATC 0.363399822759
AAGTAATG 0.0440843926321
AAGTAATT 0.118970551916
AAGTACAA -0.0458325100441
AAGTACAC -0.0351764415324
AAGTACAG 0.124322635945
AAGTACAT 0.0302863452123
AAGTACCA -0.00324408350646
AAGTACCC -0.202706707498
AAGTACCG -0.314448998853
AAGTACCT -0.133202782249
AAGTACGA 0.229452442869
AAGTACGC -0.197625671501
AAGTACGG -0.00378288231226
AAGTACGT 0.0501024218035
AAGTACTA 0.0411425058728
AAGTACTC -0.132777942419
AAGTACTG -0.0947583281275
AAGTACTT 0.106596307253
AAGTAGAA 0.0924484447634
AAGTAGAC -0.16183079484
AAGTAGAG -0.152092955701
AAGTAGAT 0.265453138378
AAGTAGCA 0.0068905902692
AAGTAGCC -0.258765432099
AAGTAGCG 0.0440113151525
AAGTAGCT -0.136165953936
AAGTAGGA 0.0333734000746
AAGTAGGC -0.179307771247
AAGTAGGG 0.107263957695
AAGTAGGT -0.0507667566796
AAGTAGTA -0.046453170879
AAGTAGTC 0.00235949009014

AAGTAGTG -0.0435312325068
AAGTATAA 0.0844580361164
AAGTATAC 0.00713047005203
AAGTATAG 0.0397758457924
AAGTATAT 0.141869452152
AAGTATCA 0.1196189976
AAGTATCC 0.112369067163
AAGTATCG 0.0911670274432
AAGTATCT 0.229667500933
AAGTATGA 0.102960789156
AAGTATGC 0.0977133061177
AAGTATGG -0.118032048581
AAGTATGT -0.00735746119564
AAGTATTA 0.000751093225633
AAGTATTC 0.094441619572
AAGTATTG 0.0409876076882
AAGTCAAA -0.0271812950706
AAGTCAAC -0.224621641249
AAGTCAAG -0.195374577462
AAGTCAAT -0.00826307932117
AAGTCACA -0.0731495067773
AAGTCACC -0.20899896597
AAGTCACG -0.12915171502
AAGTCACT -0.0865428755716
AAGTCAGA 0.0275725306176
AAGTCAGC -0.272436647173
AAGTCAGG -0.0265936275236
AAGTCAGT -0.145390537967
AAGTCATA -0.101553829079
AAGTCATC -0.17423362075
AAGTCATG -0.20526390878
AAGTCATT -0.136312133388
AAGTCCAA -0.18288453159
AAGTCCAC -0.265687581506
AAGTCCAG -0.0469659048497
AAGTCCAT -0.124358615542
AAGTCCCA -0.250857020011
AAGTCCCC -0.21360486527
AAGTCCCG -0.260200274348
AAGTCCCT -0.164548705836
AAGTCCGA -0.167738562092
AAGTCCGC -0.0518975878818
AAGTCCGG -0.210578300163
AAGTCCGT 0.00217590534675
AAGTCCTA -0.180168482208
AAGTCCTC -0.180282524943
AAGTCCTG -0.152046296296
AAGTCGAA -0.0216218964741
AAGTCGAC -0.129919153
AAGTCGAG -0.198618108378
AAGTCGAT 0.0721740242081
AAGTCGCA -0.021317973033
AAGTCGCC -0.257902417081
AAGTCGCG -0.0615040168576

AAGTCGCT -0.106624628184
AAGTCGGA -0.113944624716
AAGTCGGC -0.27659450062
AAGTCGGG -0.272031953522
AAGTCGGT -0.108861009582
AAGTCGTA -0.0882785571582
AAGTCGTC -0.0982323668873
AAGTCGTG -0.0472781950518
AAGTCTAA -0.18042271646
AAGTCTAC -0.271000702401
AAGTCTAG -0.07370000878
AAGTCTAT -0.0132371826534
AAGTCTCA -0.0628986096785
AAGTCTCC -0.415223034618
AAGTCTCG -0.253555906756
AAGTCTCT -0.195192447349
AAGTCTGA -0.115952051637
AAGTCTGC -0.170423214478
AAGTCTGG -0.216272544275
AAGTCTGT -0.23608282762
AAGTCTTA 0.0915999593218
AAGTCTTC 0.0405552262399
AAGTCTTG -0.155502044401
AAGTGAAA 0.00718506982271
AAGTGAAC -0.162139246967
AAGTGAAG -0.137557007988
AAGTGAAT 0.174232694461
AAGTGACA -0.0331199608962
AAGTGACC -0.215086419753
AAGTGACG -0.0560055758613
AAGTGACT -0.179390266577
AAGTGAGA 0.0543514717238
AAGTGAGC -0.172881803949
AAGTGAGG -0.157565203752
AAGTGAGT -0.0992116894661
AAGTGATA 0.110791335551
AAGTGATC 0.100294502618
AAGTGATG 0.0365507823282
AAGTGATT 0.244509174379
AAGTGCAA -0.0119716497191
AAGTGCAC -0.167267578736
AAGTGCAG -0.126426581694
AAGTGCAT -0.136871849258
AAGTGCCA -0.262167352538
AAGTGCCC -0.0403316939399
AAGTGCCG -0.0384807552651
AAGTGCCT -0.294797318493
AAGTGCGA 0.0288727524205
AAGTGCGC 0.0799601935118
AAGTGCGG 0.086422210277
AAGTGCGT -0.152008078413
AAGTGCTA -0.202909732853
AAGTGCTC -0.230703657555
AAGTGCTG -0.197163237311

AAGTGGAA 0.0282470526246
AAGTGGAC -0.0599050520019
AAGTGGAG -0.187173037207
AAGTGGAT 0.276458965903
AAGTGGCA -0.146324074074
AAGTGGCC -0.123478864606
AAGTGGCG -0.230655505339
AAGTGGCT -0.200583354638
AAGTGGGA -0.0703025115095
AAGTGGGC -0.188626598844
AAGTGGGG -0.235826488678
AAGTGGGT -0.141365005679
AAGTGGTA 0.139257332965
AAGTGGTC -0.211244457614
AAGTGGTG -0.0243298999027
AAGTGTAA 0.0820260630802
AAGTGTAC -0.0957510978454
AAGTGTAG 0.0182339866053
AAGTGTAT 0.0384530944697
AAGTGTCA -0.221141342814
AAGTGTCC -0.0353932029243
AAGTGTCG 0.19402456695
AAGTGTCT 0.0563582625305
AAGTGTGA -0.00351544490549
AAGTGTGC -0.0798966825691
AAGTGTGG -0.0315677073497
AAGTGTGT -0.083566587086
AAGTGTTA -0.0453385365211
AAGTGTTC -0.172433755302
AAGTGTTG 0.0191920626297
AAGTTAAA 0.0176595534479
AAGTTAAC 0.0440692989967
AAGTTAAG -0.142849049532
AAGTTAAT -6.53756192523E-5
AAGTTACA -0.059916028649
AAGTTACC 0.0731931546247
AAGTTACG -0.045596500472
AAGTTACT -0.0557279364554
AAGTTAGA -0.0550057818247
AAGTTAGC -0.0294117647059
AAGTTAGG -0.0268860132914
AAGTTAGT 0.0620979604382
AAGTTATA 0.0355948454956
AAGTTATC -0.0272383885636
AAGTTATG 0.0794953002194
AAGTTATT 0.164926782147
AAGTTCAA -0.0135359559441
AAGTTCAC -0.124739389717
AAGTTCAG 0.0510247206305
AAGTTCAT 0.1174394608
AAGTTCCA -0.246538852578
AAGTTCCC -0.112958605664
AAGTTCCG -0.000466530549518
AAGTTCCT -0.229903219435

AAGTTCGA 0.029449054864
AAGTTCGC -0.000751937473929
AAGTTCGG -0.241887035816
AAGTTCGT -0.0709730180685
AAGTTCTA -0.00576031845191
AAGTTCTC -0.138478395062
AAGTTCTG 0.023178335419
AAGTTGAA 0.0135668247148
AAGTTGAC 0.0220943379657
AAGTTGAG -0.0939381086295
AAGTTGAT 0.194374814778
AAGTTGCA 0.125785597025
AAGTTGCC -0.166430727023
AAGTTGCG 0.0307965048629
AAGTTGCT -0.0752833347054
AAGTTGGA 0.0456394461959
AAGTTGGC -0.0949461648639
AAGTTGGG -0.198154647082
AAGTTGGT -0.194325096987
AAGTTGTA 0.0763889791663
AAGTTGTC -0.098293131363
AAGTTGTG -0.0713603661448
AAGTTTAA -0.0858318926484
AAGTTTAC -0.161738556926
AAGTTTAG 0.0279040154559
AAGTTTAT 0.00988334083942
AAGTTTCA 0.0155560495291
AAGTTTCC -0.116116462227
AAGTTTCG 0.0176121918266
AAGTTTCT -0.0745585112409
AAGTTTGA 0.157636556236
AAGTTTGC 0.0693017409822
AAGTTTGG -0.00720148765854
AAGTTTGT -0.16249320887
AAGTTTTA 0.0332526509912
AAGTTTTC 0.0965224263979
AAGTTTTG -0.0692928444818
AATAAAAA 0.227526041288
AATAAAAC 0.0650535686843
AATAAAAG 0.0949609212057
AATAAAAT 0.304606028672
AATAAACA 0.213965685064
AATAAACC -0.178247176779
AATAAACG 0.0689029971089
AATAAACT -0.0207304221827
AATAAAGA 0.187986866764
AATAAAGC 0.0234417879646
AATAAAGG -0.0164712726953
AATAAAGT 0.131272790667
AATAAATA 0.167822120204
AATAAATC 0.237886671497
AATAAATG 0.0889268228902
AATAAATT 0.115411212645
AATAACAA 0.110797146717

AATAACAC 0.0843447183764
AATAACAG 0.0934246654947
AATAACAT 0.0304232804233
AATAACCA 0.0582148097569
AATAACCC 0.0143948395417
AATAACCG 0.0133407967161
AATAACCT -0.0326143629428
AATAACGA 0.158361541702
AATAACGC -0.092864022523
AATAACGG 0.0309254868383
AATAACGT 0.143148038376
AATAACTA -0.050275527454
AATAACTC -0.0267328171081
AATAACTG 0.148414344991
AATAAGAA 0.204977057674
AATAAGAC -0.121570818312
AATAAGAG -0.143493849184
AATAAGAT 0.309979362884
AATAAGCA -0.0876890984576
AATAAGCC -0.033606377432
AATAAGCG -0.136803634927
AATAAGCT -0.0150442380733
AATAAGGA 0.121414147284
AATAAGGC 0.0741199715253
AATAAGGG -0.0264277308849
AATAAGGT -0.0673225213359
AATAAGTA 0.158988122681
AATAAGTC -0.00639632520004
AATAAGTG 0.0526164130936
AATAATAA 0.282100046104
AATAATAC 0.120510510947
AATAATAG 0.199422108071
AATAATAT 0.310005680134
AATAATCA 0.147930498453
AATAATCC 0.305856712437
AATAATCG 0.097573492283
AATAATCT 0.254242683703
AATAATGA 0.156775093342
AATAATGC 0.205391446574
AATAATGG 0.0886740371624
AATAATGT 0.178769017981
AATAATTA 0.162259325122
AATAATTC 0.112691826385
AATAATTG 0.0364171255067
AATACAAA 0.228505291005
AATACAAC 0.125128981975
AATACAAG 0.234253221805
AATACAAT 0.165953830285
AATACACA 0.210560341697
AATACACC 0.116364243895
AATACACG -0.00208566598608
AATACACT -0.0115325727268
AATACAGA 0.104653291299
AATACAGC -0.0589420184856

AATACAGG 0.113554545058
AATACAGT -0.114127292642
AATACATA 0.0939128034518
AATACATC 0.14009912402
AATACATG -0.0143554194384
AATACATT 0.162327932556
AATACCAA 0.0201129552789
AATACCAC 0.211321604463
AATACCAG -0.0732119768134
AATACCAT -0.000455553360118
AATACCCA 0.0459230718567
AATACCCC -0.107623110738
AATACCCG -0.0578864788691
AATACCCT -0.0596190496
AATACCGA 0.0706107708182
AATACCGC 0.0744363213243
AATACCGG -0.0762492796575
AATACCGT 0.0265328885619
AATACCTA 0.0352587323542
AATACCTC 0.0122252408002
AATACCTG 0.0159635776856
AATACGAA 0.158902232611
AATACGAC 0.164825007735
AATACGAG 0.138048610405
AATACGAT 0.274322707414
AATACGCA 0.169777577615
AATACGCC 0.0331512501271
AATACGCG 0.185375321424
AATACGCT 0.089326878746
AATACGGA 0.194019078355
AATACGGC 0.106796158819
AATACGGG -0.054697532846
AATACGGT 0.045235570148
AATACGTA 0.125791371676
AATACGTC 0.0983380064795
AATACGTG 0.17723871382
AATACTAA 0.183457062746
AATACTAC -0.0272674443944
AATACTAG 0.0158922259545
AATACTAT 0.0341033831698
AATACTCA 0.0557531449648
AATACTCC 0.150577067576
AATACTCG -0.00661241726761
AATACTCT -0.0919681137339
AATACTGA 0.117867571187
AATACTGC 0.0521334243749
AATACTGG 0.0200034866997
AATACTGT -0.0936338674764
AATACTTA 0.16867274803
AATACTTC 0.0682973281521
AATACTTG -0.0145546253766
AATAGAAA 0.159082126306
AATAGAAC -0.0938195489596
AATAGAAG -0.0672846824299

AATAGAAT 0.254135371116
AATAGACA -0.00086441096567
AATAGACC -0.18399811239
AATAGACG -0.0980459940409
AATAGACT -0.0466593058562
AATAGAGA 0.263476820711
AATAGAGC -0.0466233089103
AATAGAGG 0.0792745175525
AATAGAGT -0.0531517885563
AATAGATA 0.193711717052
AATAGATC 0.207820149729
AATAGATG -0.0273057586336
AATAGATT 0.389846439934
AATAGCAA 0.12282626066
AATAGCAC -0.215138175442
AATAGCAG -0.0559270922909
AATAGCAT 0.0901007705987
AATAGCCA -0.175244492739
AATAGCCC -0.106700274578
AATAGCCG -0.037508189561
AATAGCCT -0.0439389625256
AATAGCGA 0.0693402873622
AATAGCGC -0.153113920994
AATAGCGG 0.0773644865969
AATAGCGT 0.00154778370546
AATAGCTA -0.0447608931924
AATAGCTC -0.0239020321255
AATAGCTG -0.0243117302246
AATAGGAA 0.121791916398
AATAGGAC -0.0331654072208
AATAGGAG -0.0130823878082
AATAGGAT 0.323514883849
AATAGGCA -0.189070534455
AATAGGCC -0.292310013717
AATAGGCG -0.103938226481
AATAGGCT -0.0186146179885
AATAGGGA 0.058513908099
AATAGGGC -0.177121278141
AATAGGGG -0.0399445033632
AATAGGGT -0.0974372757253
AATAGGTA 0.0312277541296
AATAGGTC -0.0131317827205
AATAGGTG -0.0837641880173
AATAGTAA 0.19059693956
AATAGTAC -0.0354421494586
AATAGTAG 0.158306335624
AATAGTAT -0.079742274781
AATAGTCA 0.0602922127818
AATAGTCC -0.0509653799776
AATAGTCG -0.0238467660053
AATAGTCT -0.08921631072
AATAGTGA -0.0968394305597
AATAGTGC -0.074374665301
AATAGTGG 0.0894905060073

AATAGTGT -0.00721530277543
AATAGTTA 0.134596807833
AATAGTTC -0.082650763442
AATAGTTG -0.019640288815
AATATAAA 0.17912774396
AATATAAC 0.210715383433
AATATAAG 0.0912193279386
AATATAAT 0.18002316187
AATATACA 0.216865456976
AATATACC 0.251904542361
AATATACG 0.219266613976
AATATACT 0.262930571092
AATATAGA 0.274828933796
AATATAGC 0.115716298868
AATATAGG 0.224632080543
AATATAGT 0.122818283607
AATATATA 0.279105952087
AATATATC 0.314771984368
AATATATG 0.248010401987
AATATATT 0.215798546424
AATATCAA 0.356080658986
AATATCAC 0.335247628318
AATATCAG 0.358737293903
AATATCAT 0.305514796682
AATATCCA 0.416842301697
AATATCCC 0.321310550496
AATATCCG 0.416778289483
AATATCCT 0.392286329008
AATATCGA 0.361495981717
AATATCGC 0.321760348607
AATATCGG 0.355172009286
AATATCGT 0.348556504989
AATATCTA 0.414796681824
AATATCTC 0.415752618657
AATATCTG 0.447684976683
AATATGAA 0.218629145904
AATATGAC 0.15168829173
AATATGAG 0.285324878988
AATATGAT 0.218138102364
AATATGCA 0.168225427562
AATATGCC -0.0277587834131
AATATGCG 0.163145414789
AATATGCT 0.0863740147973
AATATGGA 0.131092340117
AATATGGC 0.217164482206
AATATGGG 0.0154300989351
AATATGGT 0.188105995671
AATATGTA 0.18864352854
AATATGTC 0.102043952666
AATATGTG 0.158383005115
AATATTAA 0.27466848888
AATATTAC 0.294897261432
AATATTAG 0.23843329858
AATATTAT 0.270914962157

AATATTCA 0.211917385673
AATATTCC 0.354851368856
AATATTCG 0.292416871849
AATATTCT 0.356054204572
AATATTGA 0.289289084986
AATATTGC 0.209477705328
AATATTGG 0.276704757514
AATATTGT 0.205487496981
AATATTTA 0.230798151441
AATATTTC 0.357042200423
AATATTTG 0.252010441556
AATCAAAA 0.262732796522
AATCAAAC 0.284167477809
AATCAAAG 0.239296558337
AATCAAAT 0.307740016246
AATCAACA 0.186672594349
AATCAACC 0.135099443581
AATCAACG 0.240156610928
AATCAACT 0.267347057371
AATCAAGA 0.284314694066
AATCAAGC 0.249422725477
AATCAAGG 0.238265384531
AATCAAGT 0.161642621101
AATCAATA 0.252014314255
AATCAATC 0.251766042583
AATCAATG 0.193106045639
AATCAATT 0.131084949443
AATCACAA 0.383099676027
AATCACAC 0.267094271643
AATCACAG 0.310683828977
AATCACAT 0.281317100809
AATCACCA 0.264569319948
AATCACCC 0.150069156293
AATCACCG 0.292012552119
AATCACCT 0.193418077236
AATCACGA 0.320272253134
AATCACGC 0.255665160606
AATCACGG 0.258663746734
AATCACGT 0.344214472327
AATCACTA 0.232999321403
AATCACTC 0.0768869788579
AATCACTG 0.27315241236
AATCAGAA 0.359699389263
AATCAGAC 0.214898377232
AATCAGAG 0.295618454851
AATCAGAT 0.376469135444
AATCAGCA 0.346108697863
AATCAGCC 0.206321763376
AATCAGCG 0.27668829173
AATCAGCT 0.120751674342
AATCAGGA 0.4035026804
AATCAGGC 0.288650792498
AATCAGGG 0.257190919798
AATCAGGT 0.347022251309

AATCAGTA 0.317110978746
AATCAGTC 0.219030604404
AATCAGTG 0.280088039167
AATCATAA 0.282546162451
AATCATAC 0.290118882961
AATCATAG 0.179816367149
AATCATAT 0.321741421326
AATCATCA 0.191862060638
AATCATCC 0.14884287486
AATCATCG 0.194059535397
AATCATCT 0.191461393225
AATCATGA 0.223451667435
AATCATGC 0.0486234604492
AATCATGG 0.287849349053
AATCATGT 0.257730417532
AATCATTA 0.312259211696
AATCATTC 0.15853878227
AATCATTG 0.0437624395276
AATCCAAA 0.413146310636
AATCCAAC 0.338788996465
AATCCAAG 0.398609190103
AATCCAAT 0.415784580071
AATCCACA 0.415105161474
AATCCACC 0.39266778634
AATCCACG 0.422635944968
AATCCACT 0.394872829261
AATCCAGA 0.335054007772
AATCCAGC 0.36949864164
AATCCAGG 0.411487222698
AATCCAGT 0.390598586138
AATCCATA 0.37935437944
AATCCATC 0.327172385782
AATCCATG 0.38978901842
AATCCATT 0.351994555314
AATCCCAA 0.373249749393
AATCCCAC 0.344637094118
AATCCCAG 0.353663088103
AATCCCAT 0.33614349382
AATCCCCA 0.342451061706
AATCCCCC 0.286698240669
AATCCCCG 0.390822715849
AATCCCCT 0.260469542225
AATCCCGA 0.401718479001
AATCCCGC 0.385741179828
AATCCCGG 0.344278180524
AATCCCGT 0.346692572834
AATCCCTA 0.290411575843
AATCCCTC 0.277458486482
AATCCCTG 0.254264638082
AATCCGAA 0.401927045599
AATCCGAC 0.408753069022
AATCCGAG 0.397676986329
AATCCGAT 0.426145889326
AATCCGCA 0.375839754989

AATCCGCC 0.417033980794
AATCCGCG 0.369258929944
AATCCGCT 0.363201373111
AATCCGGA 0.396411007926
AATCCGGC 0.387292551615
AATCCGGG 0.40888338269
AATCCGGT 0.374165008063
AATCCGTA 0.305930295062
AATCCGTC 0.413168033192
AATCCGTG 0.279816717716
AATCCTAA 0.379241578583
AATCCTAC 0.34113806722
AATCCTAG 0.358132000639
AATCCTAT 0.385639640826
AATCCTCA 0.358428370113
AATCCTCC 0.369181933084
AATCCTCG 0.311184109421
AATCCTCT 0.386272572749
AATCCTGA 0.408596142448
AATCCTGC 0.343182616523
AATCCTGG 0.364983365537
AATCCTGT 0.353602713561
AATCCTTA 0.359044655206
AATCCTTC 0.319315462469
AATCCTTG 0.324573536791
AATCGAAA 0.280004523981
AATCGAAC 0.185267241004
AATCGAAG 0.273886364135
AATCGAAT 0.325839350312
AATCGACA 0.0452181366496
AATCGACC 0.282740836517
AATCGACG 0.258229337672
AATCGACT 0.293888298923
AATCGAGA 0.272211003443
AATCGAGC 0.243829267938
AATCGAGG 0.245329324311
AATCGAGT 0.222644844014
AATCGATA 0.288107313004
AATCGATC 0.22284514695
AATCGATG 0.212545070631
AATCGATT 0.364235660641
AATCGCAA 0.395228215768
AATCGCAC 0.310347098729
AATCGCAG 0.279470902085
AATCGCAT 0.245078668662
AATCGCCA 0.284775736021
AATCGCCC 0.268201298796
AATCGCCG 0.329145903854
AATCGCCT 0.134236485407
AATCGCGA 0.362505214165
AATCGCGC 0.352483547136
AATCGCGG 0.333898658587
AATCGCGT 0.301941315945
AATCGCTA 0.251746981826

AATCGCTC 0.130046634608
AATCGCTG 0.229946391992
AATCGGAA 0.312174393096
AATCGGAC 0.171689451281
AATCGGAG 0.308335528771
AATCGGAT 0.326661328022
AATCGGCA 0.289387357808
AATCGGCC 0.168016860963
AATCGGCG 0.289756635711
AATCGGCT 0.197496103098
AATCGGGA 0.303543985598
AATCGGGC 0.233035026804
AATCGGGG 0.342944517891
AATCGGGT 0.231471823108
AATCGGTA 0.313141952261
AATCGGTC 0.181628575819
AATCGGTG 0.17350819996
AATCGTAA 0.375255219654
AATCGTAC 0.258328127497
AATCGTAG 0.303905103657
AATCGTAT 0.348319941162
AATCGTCA 0.295911844609
AATCGTCC 0.291492452748
AATCGTCG 0.291788822222
AATCGTCT 0.223391396569
AATCGTGA 0.344315953104
AATCGTGC 0.317607833452
AATCGTGG 0.336848778239
AATCGTGT 0.295874072029
AATCGTTA 0.0624816585068
AATCGTTC 0.137150723967
AATCGTTG 0.143418127933
AATCTAAA 0.377793761731
AATCTAAC 0.432245875729
AATCTAAG 0.419837868464
AATCTAAT 0.404168058925
AATCTACA 0.422190937232
AATCTACC 0.410231289381
AATCTACG 0.368185318089
AATCTACT 0.398589980022
AATCTAGA 0.464535445345
AATCTAGC 0.399859079221
AATCTAGG 0.430604952908
AATCTAGT 0.445064830728
AATCTATA 0.379045094294
AATCTATC 0.390062061739
AATCTATG 0.40387275242
AATCTCAA 0.411194537751
AATCTCAC 0.418406551187
AATCTCAG 0.423016970735
AATCTCAT 0.421315506378
AATCTCCA 0.353202046148
AATCTCCC 0.371378553891
AATCTCCG 0.430109104645

AATCTCCT 0.398853432567
AATCTCGA 0.432790957825
AATCTCGC 0.430421441272
AATCTCGG 0.435263609025
AATCTCGT 0.413971766669
AATCTCTA 0.379360688489
AATCTCTC 0.349731959961
AATCTCTG 0.376129182224
AATCTGAA 0.427205505737
AATCTGAC 0.439206485261
AATCTGAG 0.384427527494
AATCTGAT 0.42951546686
AATCTGCA 0.420067409527
AATCTGCC 0.434066508797
AATCTGCG 0.447778765725
AATCTGCT 0.340613577991
AATCTGGA 0.446568530593
AATCTGGC 0.399951151507
AATCTGGG 0.459716996208
AATCTGGT 0.435473336407
AATCTGTA 0.420590414481
AATCTGTC 0.38359429023
AATCTGTG 0.40377580521
AATCTTAA 0.418658201735
AATCTTAC 0.405222785582
AATCTTAG 0.376303517208
AATCTTAT 0.350408960818
AATCTTCA 0.380932111051
AATCTTCC 0.330302899338
AATCTTCG 0.393577208606
AATCTTCT 0.390802376767
AATCTTGA 0.379165507505
AATCTTGC 0.348694666802
AATCTTGG 0.398699751573
AATCTTGT 0.376314518431
AATCTTTA 0.360874871065
AATCTTTC 0.406719160868
AATCTTTG 0.389237380223
AATGAAAA 0.0350374578085
AATGAAAC -0.00769834236485
AATGAAAG -0.107054755713
AATGAAAT 0.241034097017
AATGAACA -0.0435547432435
AATGAACC -0.210202424262
AATGAACG -0.0077614984355
AATGAACT 0.01613325004
AATGAAGA 0.155182833815
AATGAAGC -0.11745529034
AATGAAGG -0.169385593802
AATGAAGT -0.208235294118
AATGAATA 0.197569479755
AATGAATC 0.300299275057
AATGAATG -0.0225026478546
AATGAATT 0.136517815978

AATGACAA 0.0835979835253
AATGACAC -0.202917160543
AATGACAG -0.18802438004
AATGACAT 0.1447808953
AATGACCA -0.221895121564
AATGACCC -0.297481481481
AATGACCG -0.134899468809
AATGACCT -0.207270810156
AATGACGA -0.0658309188287
AATGACGC -0.241578583424
AATGACGG -0.0538622463063
AATGACGT 0.01239284795
AATGACTA -0.0882761292403
AATGACTC -0.119775293304
AATGACTG -0.0582801044822
AATGAGAA 0.190119431821
AATGAGAC -0.196511557728
AATGAGAG -0.107189510198
AATGAGAT 0.354434209227
AATGAGCA -0.0953090444126
AATGAGCC -0.173009269884
AATGAGCG -0.251967078189
AATGAGCT -0.266865649964
AATGAGGA -0.0398355439978
AATGAGGC -0.134725445975
AATGAGGG -0.0324976473585
AATGAGGT 0.155639010358
AATGAGTA 0.110257634635
AATGAGTC -0.0181555358622
AATGAGTG -0.0868787218591
AATGATAA 0.098882522119
AATGATAC 0.175871159735
AATGATAG 0.0498474170673
AATGATAT 0.25077663615
AATGATCA 0.0843048145953
AATGATCC 0.0965743175512
AATGATCG 0.0861055017216
AATGATCT 0.133684424622
AATGATGA 0.00774844413711
AATGATGC -0.113794901347
AATGATGG -0.0349437216356
AATGATGT 0.121876989616
AATGATTA 0.0940882346484
AATGATTC 0.233726555577
AATGATTG 0.136130925573
AATGCAAA 0.0787166039545
AATGCAAC 0.0545827021449
AATGCAAG -0.059856464196
AATGCAAT 0.319499330391
AATGCACA 0.0860721420887
AATGCACC -0.0621996501851
AATGCACG -0.0545363415803
AATGCACT -0.00584037141305
AATGCAGA 0.135491448769

AATGCAGC -0.167220987349
AATGCAGG -0.113882851435
AATGCAGT 0.0123136622099
AATGCATA 0.104660555257
AATGCATC -0.182268106531
AATGCATG -0.0867822336362
AATGCATT 0.065323373481
AATGCCAA -0.143959849408
AATGCCAC -0.203531968425
AATGCCAG 0.102644080601
AATGCCAT -0.088932599613
AATGCCCA -0.163892730146
AATGCCCC -0.0486932623701
AATGCCCG -0.23665775005
AATGCCCT -0.0984890967995
AATGCCGA -0.0735403077012
AATGCCGC -0.176151053014
AATGCCGG -0.0392055177383
AATGCCGT -0.129276655093
AATGCCTA -0.0513249530772
AATGCCTC -0.28068239244
AATGCCTG -0.051518893554
AATGCGAA 0.14079148083
AATGCGAC -0.0583397498293
AATGCGAG 0.0592331963903
AATGCGAT 0.348926948533
AATGCGCA -0.0705763552746
AATGCGCC -0.0070097191754
AATGCGCG 0.0484491450322
AATGCGCT 0.0716206722431
AATGCGGA 0.0275274101146
AATGCGGC -0.0299787042526
AATGCGGG -0.0567445847195
AATGCGGT -0.0948057641016
AATGCGTA 0.100069679707
AATGCGTC 0.0354729510136
AATGCGTG -0.0767341116037
AATGCTAA 0.094951316165
AATGCTAC -0.197730415885
AATGCTAG -0.175614485641
AATGCTAT 0.176573736435
AATGCTCA -0.153277901265
AATGCTCC -0.334206237605
AATGCTCG -0.190374284601
AATGCTCT -0.178700182725
AATGCTGA 0.104470239565
AATGCTGC -0.116028888837
AATGCTGG -0.191701925663
AATGCTGT 0.0846207487688
AATGCTTA -0.132377957768
AATGCTTC 0.0525111501751
AATGCTTG -0.061621191345
AATGGAAA 0.174008106577
AATGGAAC -0.0764002085563

AATGGAAG -0.214864479237
AATGGAAT 0.196349875331
AATGGACA -0.0744912072713
AATGGACC -0.22454335015
AATGGACG -0.154028058971
AATGGACT -0.102341899961
AATGGAGA 0.12917407627
AATGGAGC -0.102928830182
AATGGAGG -0.139455390406
AATGGAGT -0.0443670552592
AATGGATA 0.231207051746
AATGGATC 0.102556913109
AATGGATG 0.0424484720293
AATGGCAA -0.119200092979
AATGGCAC 0.0294466282587
AATGGCAG -0.00857582845438
AATGGCAT -0.240091740188
AATGGCCA -0.197604938272
AATGGCCC -0.198660016132
AATGGCCG -0.220260727865
AATGGCCT -0.139059336264
AATGGCGA -0.0945478789076
AATGGCGC -0.0419836016991
AATGGCGG -0.15597139147
AATGGCGT -0.0464906648227
AATGGCTA -0.092425144916
AATGGCTC -0.107455783578
AATGGCTG -0.210300435418
AATGGGAA -0.0659259259259
AATGGGAC -0.217031550069
AATGGGAG 0.0202737059259
AATGGGAT 0.181997007249
AATGGGCA -0.292936025939
AATGGGCC -0.28091829178
AATGGGCG -0.195975308642
AATGGGCT -0.0582497901131
AATGGGGA 0.0750817195241
AATGGGGC -0.100671189691
AATGGGGG -0.206899005714
AATGGGGT 0.064645485528
AATGGGTA -0.0287525931048
AATGGGTC -0.356660027763
AATGGGTG 0.0516830589979
AATGGTAA 0.104234862214
AATGGTAC 0.137535522351
AATGGTAG 0.151106300757
AATGGTAT 0.0839700103186
AATGGTCA -0.0397033399677
AATGGTCC -0.102664998299
AATGGTCG -0.246975159267
AATGGTCT -0.269603002173
AATGGTGA 0.181495794168
AATGGTGC -0.252758850541
AATGGTGG -0.218078736853

AATGGTGT -0.0100663925733
AATGGTTA -0.00118233231256
AATGGTTC -0.147631786936
AATGGTTG -0.184351488744
AATGTAAA 0.0704708116534
AATGTAAC -0.0600928104405
AATGTAAG 0.122093789106
AATGTAAT 0.37260446508
AATGTACA 0.00716536038113
AATGTACC -0.0533770139323
AATGTACG -0.0880522604656
AATGTACT -0.071760638066
AATGTAGA 0.0298505077506
AATGTAGC -0.0776361720345
AATGTAGG 0.0541469934479
AATGTAGT 0.0457337153395
AATGTATA 0.0583476549398
AATGTATC -0.080556104415
AATGTATG 0.0901587901152
AATGTCAA -0.0361666614669
AATGTCAC 0.117791786032
AATGTCAG 0.0193037404629
AATGTCAT 0.160206586957
AATGTCCA -0.130705719664
AATGTCCC -0.133695121668
AATGTCCG -0.151114416529
AATGTCCT -0.0109963994819
AATGTCGA -0.0398434413275
AATGTCGC -0.0613268128828
AATGTCGG -0.0286630692482
AATGTCGT -0.15743602854
AATGTCTA 0.0605035236778
AATGTCTC -0.010615547775
AATGTCTG -0.184802565831
AATGTGAA 0.138725611262
AATGTGAC -0.0222057572514
AATGTGAG -0.0538796798047
AATGTGAT 0.150426394317
AATGTGCA 0.118458967266
AATGTGCC -0.103564293398
AATGTGCG 0.0388400403961
AATGTGCT 0.0139395348161
AATGTGGA 0.157298098296
AATGTGGC -0.0443573458228
AATGTGGG -0.000326845807222
AATGTGGT -0.0441924658231
AATGTGTA 0.115982238908
AATGTGTC -0.050385299348
AATGTGTG -0.0333213968191
AATGTTAA 0.15800996615
AATGTTAC -0.0857277759214
AATGTTAG -0.0241155129641
AATGTTAT 0.211853326166
AATGTTCA 0.0458254035128

AATGTTCC -0.115958915055
AATGTTCG -0.0446079642032
AATGTTCT 0.0365101319458
AATGTTGA -0.0261106171376
AATGTTGC -0.0475586730773
AATGTTGG -0.0315237436607
AATGTTGT -0.0200005811166
AATGTTTA 0.151262925641
AATGTTTC 0.0673238127061
AATGTTTG -0.00341824964699
AATTAAAA 0.0961519462557
AATTAAAC 0.0223248589431
AATTAAAG 0.208915781674
AATTAAAT 0.204938637511
AATTAACA 0.0543535533162
AATTAACC -0.0113390379614
AATTAACG 0.0812639586914
AATTAACT 0.0311532635184
AATTAAGA 0.22193860503
AATTAAGC -0.0806683061501
AATTAAGG -0.0238165802256
AATTAAGT 0.134034226877
AATTAATA -0.0158833698953
AATTAATC 0.192057588657
AATTAATG -0.0064024457178
AATTAATT 0.184644080427
AATTACAA 0.200312350181
AATTACAC 0.253630234753
AATTACAG 0.137505266369
AATTACAT 0.257665417805
AATTACCA 0.107040227798
AATTACCC 0.0504840940526
AATTACCG 0.182471075106
AATTACCT 0.245045317594
AATTACGA 0.297852044848
AATTACGC 0.300645039443
AATTACGG 0.202670475751
AATTACGT 0.31329998414
AATTACTA 0.0655228435311
AATTACTC 0.0981113709994
AATTACTG 0.112500227499
AATTAGAA 0.0940553030802
AATTAGAC -0.0158412389406
AATTAGAG 0.0650622345155
AATTAGAT 0.219454599348
AATTAGCA 0.172784130199
AATTAGCC -0.139766530408
AATTAGCG -0.0458071601653
AATTAGCT -0.10271931991
AATTAGGA 0.169078904037
AATTAGGC 0.134050692661
AATTAGGG 0.0797904454543
AATTAGGT -0.0216168302268
AATTAGTA 0.090158400843

AATTAGTC -0.0090973457156
AATTAGTG 0.12889602371
AATTATAA 0.176655909021
AATTATAC 0.238709630555
AATTATAG 0.128181250272
AATTATAT 0.247249139221
AATTATCA 0.0807170979036
AATTATCC 0.210717243183
AATTATCG 0.146515291225
AATTATCT 0.203537547397
AATTATGA 0.12904711403
AATTATGC 0.0457624077979
AATTATGG 0.206283323406
AATTATGT 0.0848317196865
AATTATTA 0.217799624543
AATTATTC 0.125419573238
AATTATTG 0.0860909738062
AATTCAAA 0.151393953482
AATTCAAC -0.00281564908121
AATTCAAG 0.15699027421
AATTCAAT -0.0375282423814
AATTCACA 0.089182514201
AATTCACC 0.0255535247755
AATTCACG 0.0806219675514
AATTCACT 0.152196730218
AATTCAGA 0.244857844348
AATTCAGC -0.029949274213
AATTCAGG -0.0238797169811
AATTCAGT 0.0817616550201
AATTCATA 0.0689131666497
AATTCATC -0.0515987970886
AATTCATG 0.0829793189752
AATTCCAA 0.199475542254
AATTCCAC 0.0227297984787
AATTCCAG 0.0672221172984
AATTCCAT 0.159903228406
AATTCCCA 0.196067293304
AATTCCCC 0.25362979966
AATTCCCG 0.346418143099
AATTCCCT 0.257132428813
AATTCCGA 0.11176425388
AATTCCGC 0.122306161289
AATTCCGG 0.110368236453
AATTCCGT 0.215279008615
AATTCCTA 0.142098503186
AATTCCTC 0.0220751674342
AATTCCTG 0.208272958792
AATTCGAA 0.122330786211
AATTCGAC -0.0395288590309
AATTCGAG 0.087799456656
AATTCGAT 0.22175059271
AATTCGCA 0.313382291598
AATTCGCC 0.185957316985
AATTCGCG 0.163262029444

AATTCGCT 0.218992343789
AATTCGGA 0.19623291154
AATTCGGC 0.0664695712812
AATTCGGG 0.0797974808595
AATTCGGT 0.169902517688
AATTCGTA 0.192021229884
AATTCGTC 0.163193526361
AATTCGTG -0.0585838188078
AATTCTAA 0.135514941961
AATTCTAC 0.166447122879
AATTCTAG -0.00311477749237
AATTCTAT 0.300671189691
AATTCTCA 0.304861040489
AATTCTCC 0.186484680313
AATTCTCG 0.298932911768
AATTCTCT 0.258328942458
AATTCTGA 0.171688730817
AATTCTGC 0.21227610844
AATTCTGG 0.0724637891709
AATTCTGT 0.23149216219
AATTCTTA 0.179581329996
AATTCTTC 0.122859448067
AATTCTTG 0.159937649564
AATTGAAA 0.189029971089
AATTGAAC 0.0541724296911
AATTGAAG 0.123759577598
AATTGAAT 0.0546773804035
AATTGACA 0.109794720556
AATTGACC -0.245442622591
AATTGACG 0.0814362297154
AATTGACT -0.124409803437
AATTGAGA 0.189537640782
AATTGAGC -0.139801322686
AATTGAGG -0.0323744758392
AATTGAGT -0.034191200685
AATTGATA 0.0232025336684
AATTGATC -0.051208588553
AATTGATG 0.125798672149
AATTGCAA 0.0670562578077
AATTGCAC 0.0582312454719
AATTGCAG 0.0228160881688
AATTGCAT 0.159150036225
AATTGCCA 0.0123950202549
AATTGCCC 0.0666322839336
AATTGCCG 0.0313038685238
AATTGCCT 0.00218198511253
AATTGCGA 0.355717461102
AATTGCGC 0.236719905776
AATTGCGG 0.239421498409
AATTGCGT 0.224546642078
AATTGCTA 0.197487870206
AATTGCTC 0.0795269929087
AATTGCTG 0.0486060465184
AATTGGAA -0.036563211767

AATTGGAC 0.0365333488298
AATTGGAG -0.00339541800559
AATTGGAT 0.241660903073
AATTGGCA -0.165920438957
AATTGGCC -0.125583964852
AATTGGCG 0.052956705965
AATTGGCT -0.0312767568992
AATTGGGA 0.104272322115
AATTGGGC -0.247403252303
AATTGGGG 0.0301323493092
AATTGGGT 0.0414388899866
AATTGGTA 0.0502694888681
AATTGGTC -0.113641712551
AATTGGTG 0.00846832188049
AATTGTAA 0.237791466303
AATTGTAC -0.0125901590752
AATTGTAG 0.0861142184708
AATTGTAT 0.197374805689
AATTGTCA -0.218167552768
AATTGTCC 0.0681903005554
AATTGTCG -0.00464609541373
AATTGTCT 0.105055714556
AATTGTGA 0.0974488980576
AATTGTGC -0.001363915783
AATTGTGG 0.0792251202002
AATTGTGT 0.135116877079
AATTGTTA 0.117151607963
AATTGTTC 0.183358272921
AATTGTTG -0.0197245507242
AATTTAAA 0.174720566174
AATTTAAC 0.108728371566
AATTTAAG 0.164899103628
AATTTAAT 0.200583437617
AATTTACA 0.170884651694
AATTTACC 0.153296449977
AATTTACG 0.26042140623
AATTTACT 0.181532525412
AATTTAGA 0.289165080703
AATTTAGC 0.105608794924
AATTTAGG 0.156894222248
AATTTAGT 0.11404704139
AATTTATA 0.19054479791
AATTTATC 0.0239870410995
AATTTATG 0.155932474249
AATTTCAA 0.27801345285
AATTTCAC 0.168414859152
AATTTCAG 0.190816483348
AATTTCAT 0.296660054028
AATTTCCA 0.269684844892
AATTTCCC 0.106561066105
AATTTCCG 0.261262596325
AATTTCCT 0.258002371073
AATTTCGA 0.27372771781
AATTTCGC 0.124687150102

AATTTCGG 0.177218489978
AATTTCGT 0.295505938659
AATTTCTA 0.165914729193
AATTTCTC 0.118270306394
AATTTCTG 0.219596377695
AATTTGAA 0.132851434719
AATTTGAC 0.150257870498
AATTTGAG 0.0903779446311
AATTTGAT 0.0688405270728
AATTTGCA 0.201760453206
AATTTGCC 0.174996004823
AATTTGCG 0.268843432656
AATTTGCT 0.186041405958
AATTTGGA 0.145667662748
AATTTGGC 0.0682744132475
AATTTGGG 0.158826209137
AATTTGGT 0.194021822653
AATTTGTA 0.25462962963
AATTTGTC 0.122280011041
AATTTGTG 0.0705620850464
AATTTTAA 0.224395957537
AATTTTAC 0.182556148324
AATTTTAG 0.230568186771
AATTTTAT 0.212704662008
AATTTTCA 0.205269274912
AATTTTCC 0.156286955385
AATTTTCG 0.206909043009
AATTTTCT 0.281164119111
AATTTTGA 0.173813432511
AATTTTGC 0.0867785355302
AATTTTGG 0.262555750875
AATTTTGT 0.188248369241
AATTTTTA 0.117645605888
AATTTTTC 0.0886653204132
AATTTTTG 0.0543329507649
ACAAAAAA 0.210144569584
ACAAAAAC 0.0148823245296
ACAAAAAG -0.00470098136073
ACAAAAAT 0.205929328855
ACAAAACA 0.122133693075
ACAAAACC -0.00789592201415
ACAAAACG -0.058099085889
ACAAAACT -0.102291810728
ACAAAAGA -0.0131026476981
ACAAAAGC 0.00447895631456
ACAAAAGG -0.206614342937
ACAAAAGT -0.057767349963
ACAAAATA 0.220741504801
ACAAAATC 0.460086005259
ACAAAATG 0.0511222814638
ACAAACAA 0.179987486004
ACAAACAC 0.153782190608
ACAAACAG -0.0369561111676
ACAAACAT 0.0512956262966

ACAAACCA 0.0814681911293
ACAAACCC -0.161799655316
ACAAACCG 0.0914399939567
ACAAACCT -0.190755223464
ACAAACGA 0.13799699225
ACAAACGC -0.182034583623
ACAAACGG -0.0415030097381
ACAAACGT -0.0200369009051
ACAAACTA 0.0702595007574
ACAAACTC -0.0541641967991
ACAAACTG -0.132436460188
ACAAAGAA 0.0329864541483
ACAAAGAC -0.0693693431929
ACAAAGAG -0.0619581183138
ACAAAGAT 0.156973808426
ACAAAGCA 0.157963476821
ACAAAGCC -0.0668993600931
ACAAAGCG 0.0881655635681
ACAAAGCT -0.0891731980944
ACAAAGGA 0.146994900702
ACAAAGGC -0.399633823021
ACAAAGGG -0.0471264462447
ACAAAGGT -0.204766451556
ACAAAGTA -0.0649115773143
ACAAAGTC -0.241299068027
ACAAAGTG 0.0403878241015
ACAAATAA 0.288798977235
ACAAATAC 0.132344950823
ACAAATAG 0.0567735602094
ACAAATAT 0.335898478927
ACAAATCA 0.233454631276
ACAAATCC 0.323750236079
ACAAATCG 0.271350950852
ACAAATCT 0.34971162088
ACAAATGA 0.0607746284486
ACAAATGC -0.218366484445
ACAAATGG 0.0375474763442
ACAAATGT 0.0530930770091
ACAAATTA 0.203499774817
ACAAATTC 0.142370030077
ACAAATTG 0.0668008077521
ACAACAAA 0.0958421106155
ACAACAAC 0.0832180728446
ACAACAAG -0.0185898702496
ACAACAAT 0.128589540934
ACAACACA 0.113972949022
ACAACACC -0.0483067714614
ACAACACG -0.0128121685819
ACAACACT -0.0590646928072
ACAACAGA -0.00908240407691
ACAACAGC -0.1067294793
ACAACAGG -0.109179316628
ACAACAGT -0.0521663483282
ACAACATA 0.0460026564798

ACAACATC 0.222439000296
ACAACATG -0.142791592466
ACAACCAA 0.124880507896
ACAACCAC -0.0327733582814
ACAACCAG -0.257496940864
ACAACCAT -0.261181889509
ACAACCCA -0.205167039817
ACAACCCC -0.0432713959874
ACAACCCG -0.108866608496
ACAACCCT -0.312444444444
ACAACCGA -0.0979457527639
ACAACCGC -0.125185221772
ACAACCGG -0.0864999396314
ACAACCGT -0.0528644728661
ACAACCTA -0.198632579935
ACAACCTC -0.0790663725071
ACAACCTG -0.150964155582
ACAACGAA -0.06707021229
ACAACGAC -0.059859369779
ACAACGAG 0.0415309517237
ACAACGAT 0.207858149434
ACAACGCA 0.019416558918
ACAACGCC -0.144641617204
ACAACGCG -0.0187206717708
ACAACGCT -0.171587026751
ACAACGGA 0.04984009187
ACAACGGC -0.111612460849
ACAACGGG -0.167405507316
ACAACGGT -0.202936819172
ACAACGTA 0.0740618598637
ACAACGTC -0.0525096864127
ACAACGTG -0.233745824256
ACAACTAA -0.142418055281
ACAACTAC 0.0508869292345
ACAACTAG -0.150866563491
ACAACTAT 0.120031089739
ACAACTCA -0.0310782636726
ACAACTCC -0.324743465747
ACAACTCG -0.212651024161
ACAACTCT -0.0151250127119
ACAACTGA 0.00985331160172
ACAACTGC 0.0693519096945
ACAACTGG -0.142985315422
ACAACTGT -0.077167928174
ACAACTTA 0.127069200202
ACAACTTC -0.0561927838256
ACAACTTG -0.0737216485196
ACAAGAAA -0.0332089866618
ACAAGAAC -0.00679079415251
ACAAGAAG -0.205127125167
ACAAGAAT 0.288694592637
ACAAGACA -0.223845175785
ACAAGACC -0.0738918474487
ACAAGACG -0.137317365826

ACAAGACT -0.176316930355
ACAAGAGA 0.308876556303
ACAAGAGC -0.140095860566
ACAAGAGG -0.198476753274
ACAAGAGT -0.143514712419
ACAAGATA 0.327594322491
ACAAGATC 0.296469187029
ACAAGATG -0.0287363793072
ACAAGCAA -0.0142403086587
ACAAGCAC -0.192793674677
ACAAGCAG -0.014445981251
ACAAGCAT -0.163963906384
ACAAGCCA -0.294899336593
ACAAGCCC -0.301849941134
ACAAGCCG -0.267488149904
ACAAGCCT -0.0421427720592
ACAAGCGA 0.0971158859526
ACAAGCGC -0.141306376019
ACAAGCGG 0.00417366393362
ACAAGCGT -0.202925391808
ACAAGCTA -0.195313929809
ACAAGCTC -0.18766504083
ACAAGCTG -0.196403308224
ACAAGGAA -0.0335171921737
ACAAGGAC -0.098529193889
ACAAGGAG -0.103015980455
ACAAGGAT 0.228903287667
ACAAGGCA -0.221566561871
ACAAGGCC -0.241889289418
ACAAGGCG -0.100403920665
ACAAGGCT -0.270355647732
ACAAGGGA -0.168428244514
ACAAGGGC -0.16070203165
ACAAGGGG 0.107669932219
ACAAGGGT -0.182207296194
ACAAGGTA -0.0429125776636
ACAAGGTC -0.143932416138
ACAAGGTG -0.190056458117
ACAAGTAA -0.0853681293049
ACAAGTAC -0.216249818446
ACAAGTAG -0.0072497173653
ACAAGTAT -0.0205410656655
ACAAGTCA -0.0897228791744
ACAAGTCC -0.348666877743
ACAAGTCG -0.0558364197531
ACAAGTCT -0.199455838047
ACAAGTGA -0.0478878988708
ACAAGTGC 0.00119096621937
ACAAGTGG -0.21651303155
ACAAGTGT -0.111878878379
ACAAGTTA 0.00169323146502
ACAAGTTC -0.0992459415852
ACAAGTTG -0.146613322796
ACAATAAA 0.0185596829788

ACAATAAC -0.0978314562339
ACAATAAG -0.115229231389
ACAATAAT 0.096121046591
ACAATACA 0.153985733587
ACAATACC -0.0233271020262
ACAATACG 0.125079584623
ACAATACT 0.131432597737
ACAATAGA 0.148293595908
ACAATAGC 0.0150436563857
ACAATAGG 0.00964071659092
ACAATAGT 0.121451919864
ACAATATA 0.178870556983
ACAATATC 0.389190761597
ACAATATG 0.162014419539
ACAATCAA 0.218890645239
ACAATCAC 0.21855243011
ACAATCAG 0.186359744451
ACAATCAT 0.20991239667
ACAATCCA 0.332510044128
ACAATCCC 0.277344178796
ACAATCCG 0.320448857275
ACAATCCT 0.27743573477
ACAATCGA 0.176374618543
ACAATCGC 0.188375708822
ACAATCGG 0.169001786934
ACAATCGT 0.127774484621
ACAATCTA 0.378073613032
ACAATCTC 0.340131913472
ACAATCTG 0.411033951738
ACAATGAA 0.00485191771499
ACAATGAC -0.381577495222
ACAATGAG -0.00220668134882
ACAATGAT 0.113525489228
ACAATGCA -0.0355454505833
ACAATGCC -0.0969018413326
ACAATGCG -0.177219643474
ACAATGCT -0.159252673922
ACAATGGA -0.0983621558654
ACAATGGC -0.100186679216
ACAATGGG -0.148311850791
ACAATGGT -0.108883071841
ACAATGTA 0.0665077206676
ACAATGTC -0.102762276326
ACAATGTG -0.0836393073736
ACAATTAA 0.00363583478767
ACAATTAC 0.144222974445
ACAATTAG 0.172918512923
ACAATTAT 0.180106925457
ACAATTCA -0.0728712164869
ACAATTCC 0.0175713721229
ACAATTCG 0.158321258425
ACAATTCT 0.0984159915695
ACAATTGA 0.00621068382898
ACAATTGC -0.0464810607308

ACAATTGG 0.025696982418
ACAATTGT -0.178601730105
ACAATTTA 0.0863822476893
ACAATTTC 0.241969694768
ACAATTTG 0.0943581448187
ACACAAAA 0.190473446179
ACACAAAC -0.00273270088475
ACACAAAG 0.167162552845
ACACAAAT 0.209354211948
ACACAACA 0.0535455377508
ACACAACC -0.182151894689
ACACAACG -0.0397389882226
ACACAACT 0.0195095375765
ACACAAGA 0.0736615906213
ACACAAGC -0.120112482853
ACACAAGG -0.0742302452118
ACACAAGT -0.0169160863338
ACACAATA 0.254713582148
ACACAATC 0.376051065885
ACACAATG -0.193386965326
ACACACAA -0.0948411016674
ACACACAC -0.0389503227943
ACACACAG 0.100178879249
ACACACAT -0.204074074074
ACACACCA -0.140787942655
ACACACCC -0.241273055542
ACACACCG 0.139399328196
ACACACCT -0.124887414217
ACACACGA 0.0555794459053
ACACACGC -0.290150384238
ACACACGG -0.268521719092
ACACACGT -0.187754200851
ACACACTA 0.131327996746
ACACACTC -0.110382366015
ACACACTG -0.118778783432
ACACAGAA 0.171549983293
ACACAGAC 0.0121676134025
ACACAGAG 0.047157313066
ACACAGAT 0.338333357547
ACACAGCA -0.000351270060814
ACACAGCC -0.202204429777
ACACAGCG -0.101687659504
ACACAGCT -0.11969168121
ACACAGGA -0.0296529281013
ACACAGGC -0.153652392947
ACACAGGG -0.13099706938
ACACAGGT -0.196478838461
ACACAGTA 0.0344732904276
ACACAGTC -0.119765342465
ACACAGTG 0.0431755117458
ACACATAA -0.0461724177002
ACACATAC 0.0553353769268
ACACATAG -0.0432983131018
ACACATAT 0.163611930324

ACACATCA -0.0821258424993
ACACATCC -0.122902479838
ACACATCG 0.0417430592884
ACACATCT -0.0711048224486
ACACATGA -0.0624814778228
ACACATGC -0.151007120881
ACACATGG -0.158074696084
ACACATGT -0.163645060493
ACACATTA 0.0939784627544
ACACATTC 0.0912367614371
ACACATTG -0.0243041835424
ACACCAAA 0.122184350919
ACACCAAC -0.0845802511462
ACACCAAG -0.110424849513
ACACCAAT 0.0644763412898
ACACCACA -0.160506110687
ACACCACC -0.110696804074
ACACCACG 0.0612801999041
ACACCACT -0.127042140674
ACACCAGA 0.144702395653
ACACCAGC -0.109565614659
ACACCAGG -0.137668916724
ACACCAGT -0.278677596168
ACACCATA -0.00311477749237
ACACCATC -0.0820847823497
ACACCATG -0.00171574680749
ACACCCAA -0.154245073344
ACACCCAC -0.123283054826
ACACCCAG -0.230003166147
ACACCCAT -0.187570079884
ACACCCCA -0.0966556738774
ACACCCCC -0.26812149834
ACACCCCG -0.16426724764
ACACCCCT -0.175201161946
ACACCCGA -0.191619550122
ACACCCGC -0.0950464243382
ACACCCGG -0.165879247809
ACACCCGT -0.167934281575
ACACCCTA -0.0600260159389
ACACCCTC -0.128812461327
ACACCCTG -0.225852318114
ACACCGAA 0.168882686777
ACACCGAC -0.147488764877
ACACCGAG 0.0385116014202
ACACCGAT 0.120946348408
ACACCGCA -0.0203215422874
ACACCGCC -0.0796813951447
ACACCGCG -0.131312118861
ACACCGCT -0.104736108367
ACACCGGA -0.0287157990913
ACACCGGC -0.443740580598
ACACCGGG -0.125469862019
ACACCGGT -0.066523414345
ACACCGTA -0.12037037037

ACACCGTC -0.162626937661
ACACCGTG -0.227410312273
ACACCTAA 0.0729516564579
ACACCTAC -0.211923578497
ACACCTAG -0.0100489590749
ACACCTAT 0.0800291993238
ACACCTCA -0.122298506152
ACACCTCC 0.00638017617519
ACACCTCG -0.106111001339
ACACCTCT -0.236232296592
ACACCTGA 0.100183051734
ACACCTGC -0.161793998058
ACACCTGG -0.173592592593
ACACCTGT -0.070919729575
ACACCTTA -0.0585633054513
ACACCTTC -0.11168278058
ACACCTTG -0.137549382716
ACACGAAA 0.17908997138
ACACGAAC -0.136465674329
ACACGAAG -0.254608364476
ACACGAAT 0.268294173164
ACACGACA -0.0559479900766
ACACGACC -0.118519250187
ACACGACG -0.14634541194
ACACGACT -0.0719735488782
ACACGAGA 0.159264863403
ACACGAGC -0.145357197743
ACACGAGG -0.0816876215435
ACACGAGT -0.0553426408845
ACACGATA 0.1589629974
ACACGATC 0.273059433702
ACACGATG -0.149850443182
ACACGCAA 0.0851865800652
ACACGCAC -0.0564556406246
ACACGCAG -0.114468095614
ACACGCAT -0.174251904542
ACACGCCA -0.137945691594
ACACGCCC -0.170515613653
ACACGCCG -0.183944946576
ACACGCCT -0.11253980059
ACACGCGA 0.0289207211657
ACACGCGC -0.278645873944
ACACGCGG -0.276675006035
ACACGCGT 0.0817616550201
ACACGCTA -0.0546220562811
ACACGCTC -0.188326474623
ACACGCTG -0.126047376825
ACACGGAA 0.0742625065174
ACACGGAC 0.00866620738064
ACACGGAG -0.170393009041
ACACGGAT 0.219008780163
ACACGGCA -0.0162485183722
ACACGGCC -0.303865781159
ACACGGCG -0.170595336077

ACACGGCT -0.300709908546
ACACGGGA 0.0616359949097
ACACGGGC -0.0960003834612
ACACGGGG -0.0588537122098
ACACGGGT -0.0812861691496
ACACGGTA -0.0823645635088
ACACGGTC -0.368208467045
ACACGGTG -0.161549008956
ACACGTAA 0.145623394586
ACACGTAC -0.0954353591899
ACACGTAG 0.035682012988
ACACGTAT 0.0231334028397
ACACGTCA -0.226650643136
ACACGTCC -0.2940845704
ACACGTCG -0.0422189292281
ACACGTCT -0.0997914203461
ACACGTGA -0.0236597441723
ACACGTGC -0.0983093681917
ACACGTGG -0.0972267413485
ACACGTGT 0.108305352558
ACACGTTA 0.0575731008594
ACACGTTC -0.155935654594
ACACGTTG -0.156043451608
ACACTAAA 0.149139221013
ACACTAAC -0.0260049616896
ACACTAAG 0.0299278285448
ACACTAAT 0.169048716767
ACACTACA 0.176919361567
ACACTACC -0.114162769764
ACACTACG 0.00207448384866
ACACTACT -0.0624502825769
ACACTAGA 0.222960438209
ACACTAGC -0.238370593529
ACACTAGG -0.063530483655
ACACTAGT -0.17758186398
ACACTATA 0.0665654555228
ACACTATC 0.136993883748
ACACTATG -0.0107017023939
ACACTCAA 0.0869481208141
ACACTCAC -0.102879085681
ACACTCAG -0.0786211946005
ACACTCAT -0.0987427120584
ACACTCCA -0.307453952891
ACACTCCC -0.0876317868951
ACACTCCG -0.158201184257
ACACTCCT -0.130425475861
ACACTCGA -0.106694463411
ACACTCGC -0.0719228919421
ACACTCGG -0.132083703777
ACACTCGT -0.0353013816048
ACACTCTA -0.0675453906782
ACACTCTC -0.0302108000523
ACACTCTG -0.0538360960586
ACACTGAA -0.0372277831854

ACACTGAC -0.133871797938
ACACTGAG -0.165925889344
ACACTGAT 0.156479834903
ACACTGCA -0.126150170169
ACACTGCC -0.288942884447
ACACTGCG -0.159532932636
ACACTGCT -0.164002217173
ACACTGGA -0.0494350651803
ACACTGGC -0.234766910567
ACACTGGG -0.148087196753
ACACTGGT -0.232386347131
ACACTGTA 0.120818502753
ACACTGTC -0.0586971615156
ACACTGTG -0.00959568811471
ACACTTAA 0.0284151497102
ACACTTAC -0.100875387613
ACACTTAG -0.0210266661243
ACACTTAT 0.0203462205537
ACACTTCA -0.0795799896566
ACACTTCC -0.0336269587668
ACACTTCG -0.0613726038974
ACACTTCT 0.097873113187
ACACTTGA 0.0382609936552
ACACTTGC -0.162078881371
ACACTTGG 0.0579823631107
ACACTTTA 0.0889762178025
ACACTTTC -0.0407562909909
ACACTTTG 0.0527494079874
ACAGAAAA 0.182306417265
ACAGAAAC 0.0595594853894
ACAGAAAG -0.0859147365302
ACAGAAAT 0.264894917515
ACAGAACA -0.160972921914
ACAGAACC -0.127475193584
ACAGAACG -0.046954808793
ACAGAACT 0.0313962779481
ACAGAAGA 0.100618889196
ACAGAAGC -0.00695451309692
ACAGAAGG -0.160816980552
ACAGAAGT -0.0450633676369
ACAGAATA 0.277839117865
ACAGAATC 0.454167489956
ACAGAATG -0.161816998629
ACAGACAA -0.0597741529191
ACAGACAC -0.169729968447
ACAGACAG 0.00684634550583
ACAGACAT -0.0409257063821
ACAGACCA -0.130579156513
ACAGACCC -0.235340590908
ACAGACCG -0.202559186638
ACAGACCT -0.163206962209
ACAGACGA 0.120449954994
ACAGACGC -0.0348172294387
ACAGACGG -0.0192255975412

ACAGACGT	-0.15125137282
ACAGACTA	0.100438743045
ACAGACTC	-0.262149348084
ACAGACTG	-0.28972329197
ACAGAGAA	0.0974266521782
ACAGAGAC	-0.100080591699
ACAGAGAG	-0.285234567901
ACAGAGAT	0.269662807084
ACAGAGCA	-0.0529305629218
ACAGAGCC	-0.228175876998
ACAGAGCG	0.082241076228
ACAGAGCT	-0.037342553717
ACAGAGGA	-0.0201198160223
ACAGAGGC	-0.2199505051
ACAGAGGG	-0.265981666922
ACAGAGGT	-0.127510051828
ACAGAGTA	0.179609552504
ACAGAGTC	-0.252290809328
ACAGAGTG	-0.0204920652446
ACAGATAA	0.155888890503
ACAGATAC	0.271155788272
ACAGATAG	0.0685993636773
ACAGATAT	0.405061398742
ACAGATCA	0.0383043391218
ACAGATCC	0.198539217623
ACAGATCG	0.200338500429
ACAGATCT	0.353042876902
ACAGATGA	0.0752833893115
ACAGATGC	-0.0832647258996
ACAGATGG	-0.154580263555
ACAGATGT	0.0718539375178
ACAGATTA	0.371082559518
ACAGATTC	0.448033646652
ACAGATTG	0.316556012378
ACAGCAAA	0.155854023506
ACAGCAAC	-0.040348379411
ACAGCAAG	-0.118846886838
ACAGCAAT	0.203367245858
ACAGCACA	-0.220504631459
ACAGCACC	-0.12280456212
ACAGCACG	-0.195827123263
ACAGCACT	-0.112148548699
ACAGCAGA	0.0547570700203
ACAGCAGC	-0.0802807548992
ACAGCAGG	-0.164373919486
ACAGCAGT	-0.211943099872
ACAGCATA	-0.0342173617447
ACAGCATC	-0.105708017957
ACAGCATG	-0.0853202046148
ACAGCCAA	-0.100730282977
ACAGCCAC	-0.24831243973
ACAGCCAG	-0.111744949869
ACAGCCAT	-0.0101797103134
ACAGCCCA	-0.17776022121 3

ACAGCCCC -0.261046238132
ACAGCCCG -0.290579870107
ACAGCCCT -0.282918559484
ACAGCCGA -0.157328756976
ACAGCCGC -0.27101929607
ACAGCCGG -0.281508040929
ACAGCCGT -0.226406106586
ACAGCCTA -0.0524105907923
ACAGCCTC -0.220897603486
ACAGCCTG -0.158313384619
ACAGCGAA -0.0132712507082
ACAGCGAC -0.167582638988
ACAGCGAG -0.167558460421
ACAGCGAT 0.0340704516016
ACAGCGCA 0.0431546884004
ACAGCGCC -0.215431589467
ACAGCGCG -0.0225033338272
ACAGCGCT -0.15148696845
ACAGCGGA 0.19750947046
ACAGCGGC -0.221093731843
ACAGCGGG -0.221991746784
ACAGCGGT -0.188778875011
ACAGCGTA -0.0224735750272
ACAGCGTC -0.287041609059
ACAGCGTG -0.202526901713
ACAGCTAA -0.170094916022
ACAGCTAC -0.225871664091
ACAGCTAG -0.188845546119
ACAGCTAT 0.0601934180772
ACAGCTCA -0.130117426267
ACAGCTCC -0.195250544662
ACAGCTCG -0.183425724727
ACAGCTCT -0.145247281961
ACAGCTGA 0.206521581218
ACAGCTGC -0.276095298126
ACAGCTGG -0.0972703186371
ACAGCTGT -0.028356346399
ACAGCTTA -0.128975604402
ACAGCTTC -0.108863400662
ACAGCTTG -0.21080053336
ACAGGAAA 0.0941074447299
ACAGGAAC 0.0707191705636
ACAGGAAG 0.00503682826551
ACAGGAAT 0.13309842148
ACAGGACA -0.104456864972
ACAGGACC -0.298269947454
ACAGGACG -0.287399813498
ACAGGACT -0.144722965494
ACAGGAGA -0.0403988012909
ACAGGAGC -0.227416122004
ACAGGAGG -0.224233096857
ACAGGAGT -0.122900767794
ACAGGATA 0.218743825941
ACAGGATC 0.30246393445

ACAGGATG -0.154835916982
ACAGGCAA -0.0884386447552
ACAGGCAC -0.233754121474
ACAGGCAG -0.16652189323
ACAGGCAT 0.0582806428242
ACAGGCCA -0.321092831109
ACAGGCCC -0.229933982184
ACAGGCCG -0.235004746462
ACAGGCCT -0.271078231354
ACAGGCGA 0.00674756056066
ACAGGCGC -0.198587875073
ACAGGCGG -0.145324618736
ACAGGCGT -0.251963059904
ACAGGCTA -0.063838952544
ACAGGCTC -0.238905579176
ACAGGCTG -0.223689913804
ACAGGGAA 0.202154489852
ACAGGGAC -0.208388669565
ACAGGGAG -0.156261319108
ACAGGGAT 0.108606337077
ACAGGGCA -0.138613544983
ACAGGGCC -0.246269229925
ACAGGGCG -0.156433470508
ACAGGGCT -0.267915283429
ACAGGGGA -0.108608122986
ACAGGGGC -0.159001778129
ACAGGGGG -0.337527292277
ACAGGGGT -0.0551652178208
ACAGGGTA 0.066460854532
ACAGGGTC -0.266131254061
ACAGGGTG -0.254993773213
ACAGGTAA -0.0441442776241
ACAGGTAC 0.00139821949988
ACAGGTAG -0.120012955061
ACAGGTAT 0.176980518065
ACAGGTCA 0.0186173132231
ACAGGTCC -0.243703703704
ACAGGTCG -0.360085734699
ACAGGTCT -0.0299333168684
ACAGGTGA -0.126122417616
ACAGGTGC -0.0249481952176
ACAGGTGG -0.0954403292181
ACAGGTTA -0.0826373415681
ACAGGTTC -0.181203081786
ACAGGTTG 0.034754868064
ACAGTAAA -0.00577484636729
ACAGTAAC -0.0254198879137
ACAGTAAG 0.105421818023
ACAGTAAT 0.238301060396
ACAGTACA -0.0185404728973
ACAGTACC -0.00938364282892
ACAGTACG -0.156925208963
ACAGTACT -0.0445252134486
ACAGTAGA 0.0250773891853

ACAGTAGC -0.081475811054
ACAGTAGG -0.0382467700371
ACAGTAGT -0.0746890070323
ACAGTATA 0.117757405419
ACAGTATC 0.281281118619
ACAGTATG -0.10306773843
ACAGTCAA 0.00695673142572
ACAGTCAC -0.18716872428
ACAGTCAG -0.0163866116845
ACAGTCAT -0.0804298667369
ACAGTCCA -0.107799071422
ACAGTCCC -0.256470994896
ACAGTCCG -0.237305555556
ACAGTCCT -0.288855967078
ACAGTCGA 0.021133758517
ACAGTCGC -0.246448250922
ACAGTCGG 0.0213512682889
ACAGTCGT -0.225276225442
ACAGTCTA -0.0838652971685
ACAGTCTC -0.314850101811
ACAGTCTG -0.237594544039
ACAGTGAA -0.202133080293
ACAGTGAC -0.125469182141
ACAGTGAG -0.0620514727673
ACAGTGAT -0.0227282706536
ACAGTGCA -0.0689954188006
ACAGTGCC -0.19974691358
ACAGTGCG -0.0136120924071
ACAGTGCT -0.159536703263
ACAGTGGA 0.050385299348
ACAGTGGC -0.0997304151549
ACAGTGGG -0.306786472738
ACAGTGGT -0.197295994098
ACAGTGTA 0.255329565761
ACAGTGTC -0.0307196645371
ACAGTGTG -0.269001966497
ACAGTTAA 0.0621257245798
ACAGTTAC -0.0777528046719
ACAGTTAG 0.00331511119893
ACAGTTAT 0.127634525456
ACAGTTCA 0.0453906781708
ACAGTTCC -0.0277067836315
ACAGTTCG 0.0523618318215
ACAGTTCT -0.169229080933
ACAGTTGA 0.00984485865159
ACAGTTGC -0.113767409127
ACAGTTGG -0.293636810141
ACAGTTTA -0.120867579511
ACAGTTTC -0.0411452501701
ACAGTTTG -0.129686011109
ACATAAAA 0.0740531431145
ACATAAAC 0.0260214274737
ACATAAAG -0.0622716049383
ACATAAAT 0.165446806038

ACATAACA 0.00320921650952
ACATAACC -0.0740960284529
ACATAACG 0.197970866539
ACATAACT -0.099720315124
ACATAAGA 0.111892551538
ACATAAGC -0.0515132055588
ACATAAGG -0.0628488171087
ACATAAGT -0.144319399941
ACATAATA 0.149031811895
ACATAATC 0.311575842982
ACATAATG -0.120130244352
ACATACAA 0.252855438007
ACATACAC 0.0398252431447
ACATACAG 0.144363762102
ACATACAT 0.107367849055
ACATACCA 0.019716312881
ACATACCC -0.11533732903
ACATACCG -0.0183968606789
ACATACCT -0.0441198262461
ACATACGA -0.0910157997803
ACATACGC -0.233190579403
ACATACGG 0.0531542953742
ACATACGT -0.0347100954484
ACATACTA -0.0571594599303
ACATACTC -0.14527360727
ACATACTG -0.00853622076926
ACATAGAA 0.0701451338747
ACATAGAC 0.066306650422
ACATAGAG -0.0463780790396
ACATAGAT 0.212475120945
ACATAGCA -0.0969571532317
ACATAGCC -0.164588822048
ACATAGCG 0.0109854222925
ACATAGCT -0.155953826754
ACATAGGA 0.0559687940377
ACATAGGC -0.258230297255
ACATAGGG -0.0460191222639
ACATAGGT 0.0642828503524
ACATAGTA 0.0538273793093
ACATAGTC 0.0424268370326
ACATAGTG -0.102909944538
ACATATAA 0.0344994406753
ACATATAC 0.0617421876135
ACATATAG -0.205551132831
ACATATAT 0.20290704779
ACATATCA 0.176563958431
ACATATCC 0.233615518815
ACATATCG 0.182692894397
ACATATCT 0.334532854881
ACATATGA 0.0947468660124
ACATATGC 0.0137659322201
ACATATGG -0.0557750776559
ACATATGT 0.0306673968962
ACATATTA 0.183560561154

ACATATTC 0.133594351546
ACATATTG 0.0766194098663
ACATCAAA -0.166500324886
ACATCAAC 0.106390919669
ACATCAAG 0.111369075062
ACATCAAT 0.0748725175425
ACATCACA -0.142736013698
ACATCACC -0.138386463529
ACATCACG -0.0236256558871
ACATCACT -0.0809392886738
ACATCAGA 0.108702943448
ACATCAGC -0.0293362195459
ACATCAGG 0.0167923554853
ACATCAGT -0.116923604372
ACATCATA -0.0999884739511
ACATCATC 0.00868624061133
ACATCATG -0.132595775506
ACATCCAA 0.0214068833263
ACATCCAC 0.121335696541
ACATCCAG 0.20437864161
ACATCCAT -0.0175902261211
ACATCCCA -0.00213506333838
ACATCCCC -0.204535748215
ACATCCCG 0.0648065819228
ACATCCCT -0.0673527071156
ACATCCGA 0.0352155520947
ACATCCGC -0.0667344296729
ACATCCGG 0.209953074833
ACATCCGT 0.0879679804745
ACATCCTA -0.053725109223
ACATCCTC -0.0290834338181
ACATCCTG 0.093657112141
ACATCGAA 0.0775318887352
ACATCGAC -0.120845954607
ACATCGAG 0.0716920239742
ACATCGAT 0.205174647083
ACATCGCA 0.00277174032361
ACATCGCC -0.123197078723
ACATCGCG -0.0124141037003
ACATCGCT -0.0720511963738
ACATCGGA 0.160828064803
ACATCGGC -0.11704569224
ACATCGGG 0.0026862115555
ACATCGGT 0.0127994028409
ACATCGTA -0.0425599354541
ACATCGTC -0.0227177933638
ACATCGTG -0.113495905508
ACATCTAA 0.154814046412
ACATCTAC 0.0369601967112
ACATCTAG 0.120992837738
ACATCTAT -0.0964580942281
ACATCTCA -0.168267805986
ACATCTCC -0.13605247483
ACATCTCG -0.13853529557

ACATCTCT 0.0629322268326
ACATCTGA 0.18041201168
ACATCTGC 0.0796805653416
ACATCTGG 0.0698313309023
ACATCTTA 0.135799689103
ACATCTTC -0.0892900207749
ACATCTTG -0.185169874895
ACATGAAA 0.08302257236
ACATGAAC 0.0413538476736
ACATGAAG -0.121774872912
ACATGAAT 0.104666366423
ACATGACA 0.00957759775187
ACATGACC -0.0952686685105
ACATGACG -0.0606452535389
ACATGACT -0.138952684348
ACATGAGA 0.204412127904
ACATGAGC -0.127154170637
ACATGAGG -0.0775089410486
ACATGAGT 0.0683406598834
ACATGATA 0.240147972513
ACATGATC 0.146794415469
ACATGATG -0.0963228231937
ACATGCAA 0.0934653436578
ACATGCAC -0.159295686662
ACATGCAG -0.0928021939712
ACATGCAT 0.0502672945619
ACATGCCA -0.148776987498
ACATGCCC -0.13612654321
ACATGCCG -0.223089163237
ACATGCCT -0.164595233784
ACATGCGA 0.0572185997497
ACATGCGC -0.0903069316168
ACATGCGG -0.190569879373
ACATGCGT -0.194940623148
ACATGCTA 0.0266773145404
ACATGCTC -0.222137178411
ACATGCTG -0.0920590414481
ACATGGAA -0.0941336277658
ACATGGAC -0.164196380068
ACATGGAG 0.00580332888823
ACATGGAT 0.341525060923
ACATGGCA -0.183655030562
ACATGGCC -0.343035670205
ACATGGCG 0.102974044256
ACATGGCT -0.0786904537068
ACATGGGA 0.176132087807
ACATGGGC -0.129088115035
ACATGGGG -0.0730248895475
ACATGGGT -0.0929217681343
ACATGGTA -0.0691058800286
ACATGGTC -0.389378834913
ACATGGTG -0.0766022369302
ACATGTAA 0.234615469055
ACATGTAC -0.0954475842308

ACATGTAG -0.152062543227
ACATGTAT -0.158840317225
ACATGTCA -0.143653816542
ACATGTCC -0.0936097116577
ACATGTCG -0.164785946196
ACATGTCT -0.196213125406
ACATGTGA 0.213406166985
ACATGTGC -0.135829299615
ACATGTGG -0.281223764137
ACATGTTA 0.0694028085082
ACATGTTC -0.135702759665
ACATGTTG -0.112903137281
ACATTAAA 0.0208467104613
ACATTAAC 0.0392379635118
ACATTAAG -0.0188935539639
ACATTAAT -0.00324608636644
ACATTACA 0.148052532942
ACATTACC 0.112982145192
ACATTACG 0.121864512661
ACATTACT 0.117744395857
ACATTAGA 0.0839178686689
ACATTAGC 0.0469247403895
ACATTAGG 0.0867486170108
ACATTAGT -0.0425976235559
ACATTATA -0.0491668313245
ACATTATC 0.193370052032
ACATTATG 0.0741932883882
ACATTCAA 0.129901315067
ACATTCAC -0.0733907440339
ACATTCAG 0.0381292673824
ACATTCAT 0.0872648293696
ACATTCCA -0.111367881638
ACATTCCC 0.0743418453028
ACATTCCG 0.0884096291023
ACATTCCT -0.0126727005942
ACATTCGA 0.0896369843465
ACATTCGC -0.0139235270878
ACATTCGG 0.0450197860434
ACATTCGT -0.235693977914
ACATTCTA 0.0925181235744
ACATTCTC 0.110713187995
ACATTCTG -0.0210274116849
ACATTGAA 0.0184060023272
ACATTGAC -0.0441692211585
ACATTGAG 0.129648839711
ACATTGAT 0.0865297168509
ACATTGCA 0.0255539926732
ACATTGCC -0.0409977772289
ACATTGCG 0.00857318492173
ACATTGCT -0.0451629014907
ACATTGGA 0.120148082285
ACATTGGC -0.108963804506
ACATTGGG -0.0497962136111
ACATTGGT 0.00923830139613

ACATTGTA -0.140423782729
ACATTGTC -0.0623888559573
ACATTGTG -0.138494918321
ACATTTAA 0.0417188083163
ACATTTAC 0.0372277831854
ACATTTAG -0.0850188136504
ACATTTAT 0.0110005159279
ACATTTCA -0.0358157166848
ACATTTCC 0.105953539727
ACATTTCG 0.139540113932
ACATTTCT -0.146147127178
ACATTTGA -0.143062965158
ACATTTGC -0.0517179259948
ACATTTGG -0.00798002250213
ACATTTTA 0.193953215219
ACATTTTC 0.109162659993
ACATTTTG -0.034229221449
ACCAAAAA 0.177076402307
ACCAAAAC -0.0159059474412
ACCAAAAG -0.108317692384
ACCAAAAT 0.281136797734
ACCAAACA 0.0132799674575
ACCAAACC -0.125042532407
ACCAAACG -0.157611380026
ACCAAACT 0.103829558497
ACCAAAGA 0.303118426903
ACCAAAGC -0.181480201062
ACCAAAGG -0.182534195842
ACCAAAGT 0.0343512559383
ACCAAATA 0.0596559789636
ACCAAATC 0.403520113899
ACCAAATG 0.00630333964491
ACCAACAA 0.00989652788067
ACCAACAC -0.0436047309349
ACCAACAG 0.0496038916637
ACCAACAT 0.0758749437043
ACCAACCA -0.249196159122
ACCAACCC -0.305378914863
ACCAACCG -0.139568627451
ACCAACCT -0.0804018421397
ACCAACGA -0.208601548652
ACCAACGC -0.148888979267
ACCAACGG -0.159988696287
ACCAACGT -0.0232634086369
ACCAACTA -0.0708259002504
ACCAACTC -0.1224086039
ACCAACTG -0.188949891068
ACCAAGAA -0.0438387780193
ACCAAGAC -0.27815632252
ACCAAGAG -0.00101980928695
ACCAAGAT 0.243015763244
ACCAAGCA 0.00407808287205
ACCAAGCC -0.260032645853
ACCAAGCG -0.13222003701

ACCAAGCT -0.242065574378
ACCAAGGA -0.0753848686919
ACCAAGGC -0.20825
ACCAAGGG -0.220424110385
ACCAAGGT -0.149492860545
ACCAAGTA -0.084159285236
ACCAAGTC -0.192689104677
ACCAAGTG -0.16627741467
ACCAATAA -0.140542244457
ACCAATAC -0.132968833523
ACCAATAG -0.207100640424
ACCAATAT 0.226453928736
ACCAATCA -0.142151293711
ACCAATCC 0.0967893306989
ACCAATCG 0.0140854408411
ACCAATCT 0.192571989529
ACCAATGA -0.00607218709869
ACCAATGC -0.181622295762
ACCAATGG -0.135197671345
ACCAATTA -0.0734566071703
ACCAATTC 0.00997003227294
ACCAATTG -0.175227182932
ACCACAAA 0.0868028416603
ACCACAAC 0.0438234567722
ACCACAAG 0.0431623087225
ACCACAAT 0.0466302500254
ACCACACA -0.0193033875606
ACCACACC -0.027975367187
ACCACACG -0.216893611089
ACCACACT -0.122851639668
ACCACAGA -0.188727063587
ACCACAGC -0.284362139918
ACCACAGG 0.0739703339968
ACCACAGT -0.0700123771725
ACCACATA -0.0248151322767
ACCACATC -0.0970767744627
ACCACATG -0.118033605514
ACCACCAA -0.0551261749452
ACCACCAC -0.294004566774
ACCACCAG -0.0519673693262
ACCACCAT -0.115542713513
ACCACCCA -0.0642097869094
ACCACCCC -0.0961711563371
ACCACCCG -0.208403651817
ACCACCCT -0.11046090535
ACCACCGA -0.115491732657
ACCACCGC -0.0470444268844
ACCACCGG -0.0354193235396
ACCACCGT -0.201242433721
ACCACCTA -0.0132597897972
ACCACCTC -0.216029999971
ACCACCTG -0.0787947139078
ACCACGAA -0.0871371464429
ACCACGAC -0.202753967124

ACCACGAG -0.00454779175733
ACCACGAT -0.0544415359203
ACCACGCA -0.27743853727
ACCACGCC -0.25948696845
ACCACGCG 0.107708518555
ACCACGCT -0.147021056546
ACCACGGA 0.187365071986
ACCACGGC -0.246968449931
ACCACGGG -0.199494237914
ACCACGGT -0.00782909360336
ACCACGTA -0.0427151153838
ACCACGTC -0.204203703704
ACCACGTG -0.0954282264482
ACCACTAA 0.00444118373455
ACCACTAC -0.0855115320032
ACCACTAG -0.164087664137
ACCACTAT -0.0863410410838
ACCACTCA 0.0917655775573
ACCACTCC -0.197333941479
ACCACTCG -0.0955370792126
ACCACTCT -0.210951844191
ACCACTGA 0.0835631165284
ACCACTGC -0.14913147108
ACCACTGG -0.182481729297
ACCACTTA -9.05618125535E-5
ACCACTTC -0.0798746590716
ACCACTTG -0.108443616078
ACCAGAAA 0.131320116797
ACCAGAAC -0.0189494024101
ACCAGAAG -0.18866500952
ACCAGAAT 0.0521101797103
ACCAGACA -0.185135802469
ACCAGACC -0.0998082788671
ACCAGACG -0.130833546378
ACCAGACT -0.147028319427
ACCAGAGA 0.0627562361077
ACCAGAGC -0.0955878722038
ACCAGAGG -0.302779423706
ACCAGAGT -0.221849108368
ACCAGATA 0.270601440207
ACCAGATC 0.112358897622
ACCAGATG 0.122298897331
ACCAGCAA -0.0314426669611
ACCAGCAC -0.0905365159153
ACCAGCAG -0.128282379283
ACCAGCAT 0.00906578629608
ACCAGCCA -0.0464949055634
ACCAGCCC -0.115551301751
ACCAGCCG 0.0107201487659
ACCAGCCT -0.223434722497
ACCAGCGA 0.0624179378447
ACCAGCGC -0.236492315557
ACCAGCGG -0.17652546605
ACCAGCGT -0.153242135414

ACCAGCTA -0.0820023555901
ACCAGCTC -0.330994179224
ACCAGCTG -0.187231441695
ACCAGGAA -0.0167338986307
ACCAGGAC -0.131324211358
ACCAGGAG -0.0897488038625
ACCAGGAT 0.296275042494
ACCAGGCA -0.275996148719
ACCAGGCC -0.334049796099
ACCAGGCG 0.150662867216
ACCAGGCT -0.201463302224
ACCAGGGA -0.0427088992049
ACCAGGGC -0.350503384451
ACCAGGGG -0.147625272331
ACCAGGGT -0.223108323869
ACCAGGTA -0.139514514878
ACCAGGTC -0.399409847873
ACCAGGTG -0.244442249874
ACCAGTAA -0.0930672115975
ACCAGTAC -0.152944662654
ACCAGTAG -0.130858710562
ACCAGTAT 0.100581116616
ACCAGTCA -0.237261775452
ACCAGTCC -0.112728635508
ACCAGTCG -0.208322681107
ACCAGTCT -0.116654998213
ACCAGTGA -0.198831895838
ACCAGTGC -0.20422093388
ACCAGTGG -0.239825079005
ACCAGTTA -0.0917394273096
ACCAGTTC -0.190328934181
ACCAGTTG 0.0860866154316
ACCATAAA 0.120931820493
ACCATAAC 0.0073313139295
ACCATAAG -0.0832376912237
ACCATAAT 0.246668423017
ACCATACA 0.0328083913239
ACCATACC 0.0625782124745
ACCATACG 0.0494504923079
ACCATACT -0.101664899104
ACCATAGA 0.00746686424632
ACCATAGC -0.305047960841
ACCATAGG -0.052435605015
ACCATAGT -0.24730014487
ACCATATA 0.0919689683727
ACCATATC 0.33461711679
ACCATATG 0.0394899341423
ACCATCAA -0.182104951221
ACCATCAC -0.128664647063
ACCATCAG -0.0734840501438
ACCATCAT 0.0624660426751
ACCATCCA -0.096416203779
ACCATCCC 0.0689887118097
ACCATCCG -0.152078927185

ACCATCCT -0.197437881382
ACCATCGA -0.104985153204
ACCATCGC 0.0759717968982
ACCATCGG -0.274318756618
ACCATCGT -0.202799681564
ACCATCTA 0.0701716832422
ACCATCTC -0.0302576180363
ACCATCTG -0.0221307331586
ACCATGAA 0.135050048669
ACCATGAC -0.0829272174476
ACCATGAG -0.0605507279219
ACCATGAT 0.0490535063124
ACCATGCA -0.0878750196117
ACCATGCC -0.139636891794
ACCATGCG -0.245953921762
ACCATGCT -0.139728560923
ACCATGGA -0.0403716142061
ACCATGGC -0.312663221191
ACCATGGG -0.244049382716
ACCATGGT -0.149229781772
ACCATGTA 0.0330016125986
ACCATGTC -0.218326797386
ACCATGTG -0.224403292181
ACCATTAA -0.0378321444656
ACCATTAC -0.029937675243
ACCATTAG -0.0593072612065
ACCATTAT 0.167849458825
ACCATTCA 0.080463424874
ACCATTCC 0.0291967515581
ACCATTCG -0.113590577421
ACCATTCT 0.00617101024569
ACCATTGA -0.166773188928
ACCATTGC -0.0333924135226
ACCATTGG -0.264127403303
ACCATTTA 0.0450097417744
ACCATTTC -0.0874662451426
ACCATTTG -0.103112960585
ACCCAAAA 0.193780599422
ACCCAAAC 0.0671996092121
ACCCAAAG -0.0674153385731
ACCCAAAT 0.00706027034686
ACCCAACA -0.0502851324947
ACCCAACC -0.156277783266
ACCCAACG 0.0615652698883
ACCCAACT -0.0951909164337
ACCCAAGA 0.117065970911
ACCCAAGC -0.267607699838
ACCCAAGG -0.295895866245
ACCCAAGT -0.262814721424
ACCCAATA 0.118531691146
ACCCAATC 0.160684098443
ACCCAATG -0.0210779067534
ACCCACAA -0.0606101982476
ACCCACAC -0.09574876083

ACCCACAG -0.145835754294
ACCCACAT -0.151039275732
ACCCACCA -0.161991076867
ACCCACCC -0.056685926877
ACCCACCG -0.219449415483
ACCCACCT -0.0749159080558
ACCCACGA 0.0348645535689
ACCCACGC -0.277670781893
ACCCACGG -0.169797136758
ACCCACGT -0.138524059668
ACCCACTA -0.0207647494661
ACCCACTC -0.285405103274
ACCCACTG -0.254974582426
ACCCAGAA 0.0677841445476
ACCCAGAC -0.114862332546
ACCCAGAG 0.0187261600944
ACCCAGAT 0.302368050208
ACCCAGCA -0.200765767639
ACCCAGCC -0.143996893762
ACCCAGCG -0.112749132973
ACCCAGCT -0.141172984405
ACCCAGGA -0.0950773975121
ACCCAGGC -0.240660061981
ACCCAGGG -0.263597408084
ACCCAGGT -0.263866541976
ACCCAGTA -0.128443515428
ACCCAGTC -0.093041698223
ACCCAGTG -0.0846039430064
ACCCATAA 0.0774413269227
ACCCATAC -0.063034779172
ACCCATAG -0.121034801355
ACCCATAT 0.0508070256999
ACCCATCA -0.249982169994
ACCCATCC -0.195529653227
ACCCATCG -0.165363285914
ACCCATCT -0.132128507056
ACCCATGA -0.0938837476211
ACCCATGC -0.195467380947
ACCCATGG -0.194839781332
ACCCATTA 0.0866220487998
ACCCATTC -0.221216505568
ACCCATTG -0.193387291701
ACCCCAAA -0.0823156425961
ACCCCAAC -0.0908706579693
ACCCCAAG -0.000939863276767
ACCCCAAT -0.361803185851
ACCCCACA -0.163353824343
ACCCCACC -0.156600823045
ACCCCACG -0.19863033591
ACCCCACT -0.0991998814639
ACCCCAGA -0.137509115366
ACCCCAGC -0.157869752995
ACCCCAGG -0.320268342312
ACCCCAGT -0.19735160507

ACCCCATA -0.0758229334738
ACCCCATC -0.297464052288
ACCCCATG -0.123605962451
ACCCCCAA -0.154800061348
ACCCCCAC -0.235665294925
ACCCCCAG -0.167123721433
ACCCCCAT -0.0926249444417
ACCCCCCA -0.361301412136
ACCCCCCC -0.320450862284
ACCCCCCG -0.201275690999
ACCCCCCT -0.296181995743
ACCCCCGA -0.112450262067
ACCCCCGC -0.248364722148
ACCCCCGG -0.302545902334
ACCCCCGT -0.209873244182
ACCCCCTA -0.172332402762
ACCCCCTC -0.212223048414
ACCCCCTG -0.405404306484
ACCCCGAA -0.0870471894327
ACCCCGAC -0.195576187576
ACCCCGAG -0.295860146919
ACCCCGAT 0.244433629219
ACCCCGCA -0.121343585015
ACCCCGCC -0.248848010105
ACCCCGCG -0.196223681828
ACCCCGCT -0.0961580364847
ACCCCGGA -0.0611737978479
ACCCCGGC -0.32776550311
ACCCCGGG -0.169588910291
ACCCCGGT -0.251647986026
ACCCCGTA 0.00159508632061
ACCCCGTC -0.256567252617
ACCCCGTG -0.0915592811587
ACCCCTAA -0.158868336561
ACCCCTAC -0.245398193844
ACCCCTAG -0.219334661956
ACCCCTAT -0.0506040408853
ACCCCTCA -0.133068299669
ACCCCTCC -0.294167024898
ACCCCTCG -0.192005486968
ACCCCTCT -0.204302668181
ACCCCTGA -0.133675446573
ACCCCTGC -0.249833688229
ACCCCTGG -0.213724715642
ACCCCTTA -0.124260312056
ACCCCTTC -0.337728338682
ACCCCTTG -0.0829708976493
ACCCGAAA -0.104608732393
ACCCGAAC -0.247239092151
ACCCGAAG -0.211532595655
ACCCGAAT 0.128701349643
ACCCGACA -0.201585632277
ACCCGACC -0.303602080737
ACCCGACG -0.235760279523

ACCCGACT	−0.203359544736
ACCCGAGA	0.0860324261583
ACCCGAGC	−0.0748700072622
ACCCGAGG	−0.200504801097
ACCCGAGT	−0.160770466515
ACCCGATA	0.193045486903
ACCCGATC	−0.005181233397
ACCCGATG	−0.178433115727
ACCCGCAA	0.0810104597561
ACCCGCAC	−0.214804113641
ACCCGCAG	−0.188751147309
ACCCGCAT	−0.0820904736036
ACCCGCCA	−0.160719066892
ACCCGCCC	0.101539001954
ACCCGCCG	−0.143142677853
ACCCGCCT	−0.194240064962
ACCCGCGA	−0.068846305808
ACCCGCGC	−0.125725091281
ACCCGCGG	−0.104923331906
ACCCGCGT	−0.178134877519
ACCCGCTA	−0.180535664313
ACCCGCTC	−0.270733478577
ACCCGCTG	−0.147032828775
ACCCGGAA	−0.0447720649973
ACCCGGAC	−0.165901756486
ACCCGGAG	−0.0973840714238
ACCCGGAT	0.234264088446
ACCCGGCA	−0.341482212897
ACCCGGCC	−0.0548156734912
ACCCGGCG	−0.265286418046
ACCCGGCT	−0.246621081893
ACCCGGGA	−0.23348166049
ACCCGGGC	−0.243961069418
ACCCGGGG	−0.110935528121
ACCCGGGT	−0.192483795091
ACCCGGTA	−0.0107733699031
ACCCGGTC	−0.288562914427
ACCCGGTG	−0.213571152623
ACCCGTAA	−0.0203265459844
ACCCGTAC	−0.264039087233
ACCCGTAG	−0.262254377445
ACCCGTAT	0.0859596258974
ACCCGTCA	−0.238063798374
ACCCGTCC	−0.349539779709
ACCCGTCG	−0.209510171835
ACCCGTCT	−0.213497942387
ACCCGTGA	−0.157005427416
ACCCGTGC	−0.181762183236
ACCCGTGG	−0.185638344227
ACCCGTTA	−0.133628590558
ACCCGTTC	−0.0531544139842
ACCCGTTG	−0.338452400661
ACCCTAAA	−0.0186804320622
ACCCTAAC	−0.188972294928

ACCCTAAG -0.0203709192298
ACCCTAAT -0.0417126268363
ACCCTACA -0.0880980995813
ACCCTACC -0.168390374276
ACCCTACG -0.191800259682
ACCCTACT 0.0414466898145
ACCCTAGA 0.119792831927
ACCCTAGC -0.160324405359
ACCCTAGG 0.00939280187033
ACCCTAGT -0.0382849771342
ACCCTATA 0.106786098487
ACCCTATC -0.048214560144
ACCCTATG -0.181527256035
ACCCTCAA -0.0925608526783
ACCCTCAC -0.190342691798
ACCCTCAG -0.0777070211728
ACCCTCAT -0.0472369005877
ACCCTCCA -0.333311550068
ACCCTCCC -0.0777844280061
ACCCTCCG -0.157779643531
ACCCTCCT -0.225552154307
ACCCTCGA -0.170076985969
ACCCTCGC -0.287299927378
ACCCTCGG 0.108147845779
ACCCTCGT 0.0441735740081
ACCCTCTA -0.171426039921
ACCCTCTC -0.322937581603
ACCCTCTG -0.133835222073
ACCCTGAA -0.122423601467
ACCCTGAC -0.0999495676859
ACCCTGAG -0.146381956407
ACCCTGAT -0.0131721925579
ACCCTGCA -0.163149576415
ACCCTGCC -0.071803751133
ACCCTGCG -0.191266274916
ACCCTGCT -0.348273979287
ACCCTGGA -0.0652937617307
ACCCTGGC -0.0860070420899
ACCCTGGG -0.220474881636
ACCCTGTA -0.173472551473
ACCCTGTC -0.260478220554
ACCCTGTG -0.139825350852
ACCCTTAA -0.18601016703
ACCCTTAC -0.238455907769
ACCCTTAG -0.168072982872
ACCCTTAT -0.140833696442
ACCCTTCA -0.324978939724
ACCCTTCC 0.00473595539531
ACCCTTCG -0.250821551814
ACCCTTCT -0.19878420787
ACCCTTGA -0.193932784636
ACCCTTGC -0.0968298652267
ACCCTTGG -0.127439198384
ACCCTTTA -0.174056883271

ACCCTTTC -0.330141831614
ACCCTTTG -0.159845501457
ACCGAAAA 0.147961478562
ACCGAAAC -0.0265510884854
ACCGAAAG 0.0108556387439
ACCGAAAT 0.315786032862
ACCGAACA -0.25109475833
ACCGAACC -0.0984035255249
ACCGAACG -0.160083747681
ACCGAACT -0.112614837499
ACCGAAGA 0.0190747213095
ACCGAAGC -0.194314606676
ACCGAAGG -0.132384891543
ACCGAAGT -0.0488438374313
ACCGAATA 0.140782764081
ACCGAATC 0.374956091242
ACCGAATG -0.0688124306326
ACCGACAA 0.15474409077
ACCGACAC -0.051452429238
ACCGACAG -0.0740842959072
ACCGACAT -0.05675060067
ACCGACCA -0.176791560039
ACCGACCC -0.33269671505
ACCGACCG -0.083724337762
ACCGACCT -0.173340440922
ACCGACGA -0.105790181389
ACCGACGC -0.2158155538
ACCGACGG -0.390537315889
ACCGACGT 0.049721686383
ACCGACTA -0.0771571403486
ACCGACTC -0.170374302504
ACCGACTG -0.197068990559
ACCGAGAA 0.0604535615185
ACCGAGAC -0.107201646091
ACCGAGAG 0.0499913563173
ACCGAGAT 0.248457861781
ACCGAGCA -0.0373110831234
ACCGAGCC -0.377734443567
ACCGAGCG 0.00252349890314
ACCGAGCT -0.30034215938
ACCGAGGA -0.0958613196365
ACCGAGGC -0.013190534207
ACCGAGGG -0.133276260406
ACCGAGGT -0.222827271063
ACCGAGTA -0.0510097356471
ACCGAGTC -0.151213837306
ACCGAGTG -0.211675227183
ACCGATAA 0.0545758414016
ACCGATAC 0.104074732705
ACCGATAG -0.0427419214253
ACCGATAT 0.356923313355
ACCGATCA -0.0570512112778
ACCGATCC 0.0237848251334
ACCGATCG -0.0770633271832

ACCGATCT 0.0580538561738
ACCGATGA 0.105246257966
ACCGATGC -0.0446312088678
ACCGATGG -0.215297668038
ACCGATTA 0.149057864687
ACCGATTC 0.276487958023
ACCGATTG 0.108278006189
ACCGCAAA 0.114578188284
ACCGCAAC 0.0341885432859
ACCGCAAG -0.0323038255325
ACCGCAAT 0.299291037729
ACCGCACA 0.000965895943237
ACCGCACC -0.118809708024
ACCGCACG -0.0965971491827
ACCGCACT -0.0805656913101
ACCGCAGA 0.0525332527558
ACCGCAGC -0.0451107598411
ACCGCAGG -0.256839506173
ACCGCAGT -0.147273783588
ACCGCATA -0.125621702652
ACCGCATC -0.176708810747
ACCGCATG -0.245389347073
ACCGCCAA -0.125939183339
ACCGCCAC -0.189188188238
ACCGCCAG -0.152497777558
ACCGCCAT -0.0746567223155
ACCGCCCA -0.275890838207
ACCGCCCC -0.279023697852
ACCGCCCG -0.00312786018334
ACCGCCCT -0.0130550479107
ACCGCCGA -0.0562718171639
ACCGCCGC -0.139047454447
ACCGCCGG -0.0678546422273
ACCGCCGT -0.117396743872
ACCGCCTA -0.0490980508369
ACCGCCTC -0.282261460496
ACCGCCTG -0.188274796389
ACCGCGAA 0.0169439077187
ACCGCGAC -0.0732403205281
ACCGCGAG -0.250566392862
ACCGCGAT 0.279419755059
ACCGCGCA -0.175656096678
ACCGCGCC 0.0725575422306
ACCGCGCG 0.0357407498889
ACCGCGCT -0.184320599999
ACCGCGGA 0.0405951244142
ACCGCGGC -0.166373231764
ACCGCGGG -0.000867069469825
ACCGCGGT -0.0519306735137
ACCGCGTA 0.0290340389058
ACCGCGTC -0.122266128811
ACCGCGTG -0.281760348584
ACCGCTAA -0.131645673829
ACCGCTAC -0.257863809195

ACCGCTAG -0.128817370488
ACCGCTAT -0.191680247584
ACCGCTCA 0.0143979057592
ACCGCTCC -0.0885308641975
ACCGCTCG -0.0825270228817
ACCGCTCT -0.105098074898
ACCGCTGA -0.194534997311
ACCGCTGC -0.210428565942
ACCGCTGG -0.241757938548
ACCGCTTA -0.138990019322
ACCGCTTC -0.139778511167
ACCGCTTG -0.0172991959606
ACCGGAAA -0.123225502786
ACCGGAAC -0.220705075446
ACCGGAAG -0.229373426159
ACCGGAAT 0.206908023768
ACCGGACA -0.0553266601775
ACCGGACC -0.192694957527
ACCGGACG -0.238194482771
ACCGGACT -0.241514403292
ACCGGAGA 0.0803669751427
ACCGGAGC -0.276469426404
ACCGGAGG -0.225539578794
ACCGGAGT -0.136121464241
ACCGGATA 0.207597428091
ACCGGATC 0.249774967617
ACCGGATG 0.101956135151
ACCGGCAA -0.176119360299
ACCGGCAC -0.139278440669
ACCGGCAG -0.279754505002
ACCGGCAT -0.112533309069
ACCGGCCA -0.16217350516
ACCGGCCC -0.394423873392
ACCGGCCG -0.0944655734466
ACCGGCCT -0.23075804469
ACCGGCGA 0.0746421436255
ACCGGCGC -0.154708468066
ACCGGCGG -0.0463745587146
ACCGGCGT -0.108156086747
ACCGGCTA -0.279105689719
ACCGGCTC -0.31523814226
ACCGGCTG -0.1560229991
ACCGGGAA -0.188504588462
ACCGGGAC -0.264797920728
ACCGGGAG -0.341378906057
ACCGGGAT 0.159734229116
ACCGGGCA -0.123124453495
ACCGGGCC -0.268088598402
ACCGGGCG -0.29311631173
ACCGGGCT -0.326196197185
ACCGGGGA -0.0769776801146
ACCGGGGC -0.177194975387
ACCGGGGG -0.154590396301
ACCGGGTA -0.0137153206553

ACCGGGTC -0.246297027748
ACCGGGTG -0.0282360754352
ACCGGTAA 0.0552674043338
ACCGGTAC -0.292743781297
ACCGGTAG -0.1699128179
ACCGGTAT 0.0610698435067
ACCGGTCA -0.0121709587477
ACCGGTCC -0.127893575686
ACCGGTCG -0.255875962868
ACCGGTCT -0.139836996789
ACCGGTGA -0.171449504429
ACCGGTGC -0.19375308642
ACCGGTGG -0.272077943451
ACCGGTTA -0.135725315462
ACCGGTTC -0.0911754537346
ACCGGTTG -0.286368231347
ACCGTAAA -0.0795567461401
ACCGTAAC -0.00693237879959
ACCGTAAG 0.112671247803
ACCGTAAT 0.317009283338
ACCGTACA 0.0712858679664
ACCGTACC -0.0257654380531
ACCGTACG -0.0521779032644
ACCGTACT -0.0913964123632
ACCGTAGA -0.0645106464574
ACCGTAGC -0.281833113299
ACCGTAGG -0.0535415397617
ACCGTAGT -0.145383999034
ACCGTATA 0.0388880655997
ACCGTATC 0.311343396336
ACCGTATG -0.0523490771327
ACCGTCAA -0.180150145701
ACCGTCAC -0.211885985852
ACCGTCAG -0.0417383270969
ACCGTCAT -0.14445277296
ACCGTCCA -0.157364877101
ACCGTCCC -0.128240174533
ACCGTCCG 0.0759098107013
ACCGTCCT -0.175288307916
ACCGTCGA -0.121436371918
ACCGTCGC -0.183961894137
ACCGTCGG -0.111528161904
ACCGTCGT -0.162976820932
ACCGTCTA -0.24110906772
ACCGTCTC -0.22636051557
ACCGTCTG -0.228134138781
ACCGTGAA -0.157714768711
ACCGTGAC -0.278798303513
ACCGTGAG -0.104469523606
ACCGTGAT 0.0976057995438
ACCGTGCA 0.0323717315419
ACCGTGCC -0.169335813198
ACCGTGCG -0.121642163722
ACCGTGCT -0.0820231574971

ACCGTGGA -0.113040066292
ACCGTGGC -0.343215851537
ACCGTGGG -0.241627370136
ACCGTGTA 0.0704051835602
ACCGTGTC -0.163654469738
ACCGTGTG -0.269220323919
ACCGTTAA 0.139315532062
ACCGTTAC -0.0413275609083
ACCGTTAG 0.117631078117
ACCGTTAT 0.0336725284858
ACCGTTCA -0.125551198257
ACCGTTCC -0.0593828541543
ACCGTTCG -0.352207520051
ACCGTTCT -0.151827860841
ACCGTTGA -0.0518742625065
ACCGTTGC -0.134898455407
ACCGTTGG -0.0943495105141
ACCGTTTA 0.123294059535
ACCGTTTC -0.209295753006
ACCGTTTG -0.0923058149467
ACCTAAAA 0.178378103526
ACCTAAAC -0.0781224579915
ACCTAAAG -0.0714078193138
ACCTAAAT 0.19240044746
ACCTAACA -0.0699631166436
ACCTAACC 0.0152473160772
ACCTAACG -0.160103775682
ACCTAACT -0.0943887111816
ACCTAAGA -0.157481399835
ACCTAAGC -0.170788194101
ACCTAAGG -0.0886446584891
ACCTAAGT -0.0588119076535
ACCTAATA -0.00276625172891
ACCTAATC 0.0674320167855
ACCTAATG 0.0725482447474
ACCTACAA -0.106039827766
ACCTACAC -0.307430634427
ACCTACAG -0.258729845236
ACCTACAT 0.108486207878
ACCTACCA -0.114068264343
ACCTACCC -0.0947089332152
ACCTACCG -0.164867118748
ACCTACCT -0.288401073304
ACCTACGA -0.0114760846299
ACCTACGC -0.198462959681
ACCTACGG -0.0685219023162
ACCTACGT -0.197719278027
ACCTACTA -0.00145714991356
ACCTACTC -0.0616610139726
ACCTACTG 0.0244269907133
ACCTAGAA -0.0220766167102
ACCTAGAC -0.234656594085
ACCTAGAG -0.232880174292
ACCTAGAT 0.306116252379

ACCTAGCA -0.167247085556
ACCTAGCC -0.0665160606105
ACCTAGCG -0.251700785263
ACCTAGCT -0.145713282185
ACCTAGGA -0.133185712012
ACCTAGGC -0.0525038969022
ACCTAGGG 0.0360765138771
ACCTAGGT 0.0295810307908
ACCTAGTA -0.070735831624
ACCTAGTC -0.179952795858
ACCTAGTG -0.217159041394
ACCTATAA 0.00349623482404
ACCTATAC -0.0313806842504
ACCTATAG 0.0815714944346
ACCTATAT 0.0691165574652
ACCTATCA -0.0331953605795
ACCTATCC -0.168702432493
ACCTATCG -0.0744344561889
ACCTATCT 0.0806640817939
ACCTATGA -0.12971130073
ACCTATGC -0.187577503429
ACCTATGG -0.23298986607
ACCTATTA -0.0267486662715
ACCTATTC -0.0782097301807
ACCTATTG -0.123781531977
ACCTCAAA 0.114780701117
ACCTCAAC -0.0940002085357
ACCTCAAG -0.000856220773233
ACCTCAAT -0.0261965326484
ACCTCACA -0.132804010669
ACCTCACC -0.129665675041
ACCTCACG -0.0829830376518
ACCTCACT -0.152790406498
ACCTCAGA -0.0867192679154
ACCTCAGC -0.198265795207
ACCTCAGG -0.144729006225
ACCTCAGT -0.150635802469
ACCTCATA -0.0233507184054
ACCTCATC -0.184999694974
ACCTCATG -0.184148527306
ACCTCCAA -0.188070596369
ACCTCCAC -0.208617283951
ACCTCCAG -0.317889276773
ACCTCCAT -0.180628698074
ACCTCCCA -0.34261162996
ACCTCCCC -0.0167544466223
ACCTCCCG -0.15317707486
ACCTCCCT -0.250360876094
ACCTCCGA -0.257454046639
ACCTCCGC -0.229053016225
ACCTCCGG -0.125467580642
ACCTCCGT -0.243677559913
ACCTCCTA -0.262273177505
ACCTCCTC -0.22722285313

ACCTCCTG -0.216375308642
ACCTCGAA 0.00688269775407
ACCTCGAC -0.202826434277
ACCTCGAG -0.190451105953
ACCTCGAT 0.137703352434
ACCTCGCA -0.0172700482376
ACCTCGCC -0.260657099457
ACCTCGCG -0.171341794657
ACCTCGCT -0.255648834019
ACCTCGGA -0.0461248031258
ACCTCGGC -0.276746278647
ACCTCGGG -0.0790174063831
ACCTCGTA -0.0302166112185
ACCTCGTC -0.238609053498
ACCTCGTG -0.124911751632
ACCTCTAA -0.186020160118
ACCTCTAC -0.158253066601
ACCTCTAG -0.197693211066
ACCTCTAT -0.0786149085468
ACCTCTCA -0.206720282543
ACCTCTCC -0.202143975461
ACCTCTCG -0.13182646297
ACCTCTCT -0.368283314448
ACCTCTGA -0.0199109773318
ACCTCTGC -0.201615192811
ACCTCTGG -0.391489326684
ACCTCTTA -0.141713445383
ACCTCTTC -0.243185858187
ACCTCTTG -0.137566268234
ACCTGAAA 0.052993476966
ACCTGAAC -0.21815311141
ACCTGAAG -0.140346544948
ACCTGAAT 0.0578254616245
ACCTGACA -0.13698032436
ACCTGACC -0.0689121699947
ACCTGACG -0.247182098765
ACCTGACT -0.337225184308
ACCTGAGA 0.00577674592197
ACCTGAGC -0.329712954653
ACCTGAGG -0.209397587561
ACCTGAGT -0.135462563655
ACCTGATA -0.00220367077214
ACCTGATC 0.0274504961283
ACCTGATG 0.141312103449
ACCTGCAA -0.0710342422966
ACCTGCAC -0.167568321411
ACCTGCAG -0.217141973496
ACCTGCAT 0.0645112082867
ACCTGCCA -0.248562414266
ACCTGCCC -0.346836172139
ACCTGCCG -0.247116882832
ACCTGCCT -0.330738381694
ACCTGCGA -0.108536500337
ACCTGCGC -0.127484690602

ACCTGCGG -0.275609846427
ACCTGCGT -0.0821364752372
ACCTGCTA -0.167395895979
ACCTGCTC -0.12545136579
ACCTGCTG -0.237296923609
ACCTGGAA -0.0921124117153
ACCTGGAC -0.315837554481
ACCTGGAG -0.0814404778159
ACCTGGAT 0.237134804527
ACCTGGCA -0.115072897976
ACCTGGCC -0.134079086
ACCTGGCG -0.177843205038
ACCTGGCT -0.379794512592
ACCTGGGA -0.276535163515
ACCTGGGC -0.237818445897
ACCTGGGG -0.253918276772
ACCTGGTA -0.225163382554
ACCTGGTC -0.275872185911
ACCTGGTG -0.293493646718
ACCTGTAA -0.0743683160032
ACCTGTAC -0.0852544603558
ACCTGTAG -0.169158319195
ACCTGTAT 0.180359711185
ACCTGTCA -0.148575301568
ACCTGTCC -0.208148148148
ACCTGTCG -0.0464344122845
ACCTGTCT 0.00931093331577
ACCTGTGA -0.132075422654
ACCTGTGC -0.138064026915
ACCTGTGG -0.0950425253176
ACCTGTTA 0.0746516932285
ACCTGTTC -0.162997821351
ACCTGTTG -0.145437748391
ACCTTAAA -0.0397527348801
ACCTTAAC -0.0922111917815
ACCTTAAG -0.184090372785
ACCTTAAT 0.000507024247091
ACCTTACA -0.177156305842
ACCTTACC -0.0106852817689
ACCTTACG -0.183921219992
ACCTTACT -0.0377944631057
ACCTTAGA -0.0637175006319
ACCTTAGC -0.111562481136
ACCTTAGG 0.020066302224
ACCTTAGT -0.185883224616
ACCTTATA 0.041968241977
ACCTTATC -0.165997513189
ACCTTATG -0.185336206045
ACCTTCAA -0.171480164219
ACCTTCAC -0.0816003951593
ACCTTCAG -0.226549608385
ACCTTCAT -0.0705225788458
ACCTTCCA -0.19844516234
ACCTTCCC -0.265580849231

ACCTTCCG -0.106681929909
ACCTTCCT -0.157552812071
ACCTTCGA -0.0654339603021
ACCTTCGC -0.0203530515777
ACCTTCGG -0.208331154684
ACCTTCGT -0.289126919469
ACCTTCTA -0.105078144289
ACCTTCTC -0.230483816986
ACCTTCTG -0.162400176625
ACCTTGAA -0.0374980024116
ACCTTGAC -0.0667454307721
ACCTTGAG -0.073918579253
ACCTTGAT 0.163649702904
ACCTTGCA -0.117516314043
ACCTTGCC -0.175784972227
ACCTTGCG -0.00855397484028
ACCTTGCT -0.236400762161
ACCTTGGA 0.00936614705156
ACCTTGGC -0.245559890047
ACCTTGGG -0.120186556927
ACCTTGTA -0.0424867875136
ACCTTGTC -0.201963334266
ACCTTGTG -0.12899490759
ACCTTTAA 0.179444115129
ACCTTTAC 0.0306030537678
ACCTTTAG -0.157630075881
ACCTTTAT 0.0355441392786
ACCTTTCA -0.0286156349425
ACCTTTCC -0.26715177923
ACCTTTCG -0.177833104795
ACCTTTCT -0.131667949966
ACCTTTGA 0.15341719906
ACCTTTGC -0.167525805018
ACCTTTGG -0.204743701836
ACCTTTTA 0.0097483577814
ACCTTTTC 0.107061271362
ACCTTTTG -0.134770639937
ACGAAAAA 0.0945739752794
ACGAAAAC -0.0895848426969
ACGAAAAG 0.04638908663
ACGAAAAT 0.253070868735
ACGAAACA 0.0518980721456
ACGAAACC -0.0950017286512
ACGAAACG -0.0821148653099
ACGAAACT 0.00179710313367
ACGAAAGA 0.252846241233
ACGAAAGC -0.231839981124
ACGAAAGG -0.0451367803234
ACGAAAGT 0.184468923577
ACGAAATA 0.210248852884
ACGAAATC 0.484003490746
ACGAAATG 0.113932270858
ACGAACAA 0.0585742826407
ACGAACAC -0.025906166985

ACGAACAG -0.180529086388
ACGAACAT -0.0785015908067
ACGAACCA 0.0107860546986
ACGAACCC -0.186963897208
ACGAACCG -0.0649767283585
ACGAACCT -0.0720726654044
ACGAACGA -0.0142388098732
ACGAACGC -0.235440196692
ACGAACGG -0.17207133059
ACGAACGT -0.265196828892
ACGAACTA -0.0846527101826
ACGAACTC -0.128880174292
ACGAACTG -0.0073380500632
ACGAAGAA 0.017554080165
ACGAAGAC -0.162140885984
ACGAAGAG 0.100616918044
ACGAAGAT 0.305462496186
ACGAAGCA -0.168588287577
ACGAAGCC -0.00129530834925
ACGAAGCG -0.108553825955
ACGAAGCT -0.214850116055
ACGAAGGA -0.0357491740481
ACGAAGGC -0.25221161833
ACGAAGGG -0.252858105518
ACGAAGTA 0.0136265115549
ACGAAGTC -0.244887879087
ACGAAGTG -0.106824730386
ACGAATAA 0.0971438118344
ACGAATAC 0.149677471355
ACGAATAG -0.00372641029739
ACGAATAT 0.281485323498
ACGAATCA 0.234678914349
ACGAATCC 0.366097499396
ACGAATCG 0.251281117258
ACGAATCT 0.423092121511
ACGAATGA 0.160682974415
ACGAATGC -0.208104465429
ACGAATGG -0.17158720557
ACGAATTA 0.112428373839
ACGAATTC 0.124105359064
ACGAATTG 0.127148784825
ACGACAAA -0.0486865792882
ACGACAAC 0.136095194186
ACGACAAG -0.0925706447188
ACGACAAT 0.14488835297
ACGACACA -0.16204847048
ACGACACC 0.0777548559557
ACGACACG -0.194190548386
ACGACACT -0.0495348645756
ACGACAGA -0.0177667629336
ACGACAGC -0.102636647424
ACGACAGG -0.0158127454854
ACGACAGT -0.0852268985049
ACGACATA 0.00458297657468

ACGACATC 0.0997058113241
ACGACATG -0.104957328284
ACGACCAA -0.0458544593436
ACGACCAC -0.239543726236
ACGACCAG -0.184317307804
ACGACCAT -0.118185185185
ACGACCCA -0.105140976515
ACGACCCC -0.37420317523
ACGACCCG -0.204659404503
ACGACCCT -0.227371487828
ACGACCGA -0.0286240096488
ACGACCGC -0.182335478844
ACGACCGG -0.181233821133
ACGACCGT -0.112957840781
ACGACCTA -0.0239268366375
ACGACCTC -0.240516678464
ACGACCTG -0.123047204067
ACGACGAA -0.195023810019
ACGACGAC -0.146703317806
ACGACGAG -0.2590015713
ACGACGAT 0.211414583121
ACGACGCA -0.109544194726
ACGACGCC -0.0715273319836
ACGACGCG -0.149714909753
ACGACGCT -0.0467202274137
ACGACGGA 0.0166853278886
ACGACGGC 0.00797179001674
ACGACGGG -0.246831104553
ACGACGTA -0.0534557347726
ACGACGTC 0.0871921897927
ACGACGTG -0.00645025426637
ACGACTAA 0.0262072231471
ACGACTAC 0.0208621484555
ACGACTAG -0.144810457516
ACGACTAT -0.0421801391445
ACGACTCA -0.0118718303366
ACGACTCC -0.0564397339911
ACGACTCG -0.0579478737997
ACGACTCT -0.184855019975
ACGACTGA -0.147881062926
ACGACTGC -0.00610606160399
ACGACTGG -0.161148429868
ACGACTTA 0.0117307122395
ACGACTTC -0.188304443579
ACGACTTG -0.100883297256
ACGAGAAA 0.0639475251696
ACGAGAAC 0.0331144250668
ACGAGAAG -0.111315198064
ACGAGAAT 0.340707260313
ACGAGACA -0.0265653884254
ACGAGACC -0.157130490104
ACGAGACG -0.144896637636
ACGAGACT -0.256243106655
ACGAGAGA 0.0543939680095

ACGAGAGC -0.129413798212
ACGAGAGG -0.0837517766692
ACGAGAGT -0.141818731789
ACGAGATA 0.273255175745
ACGAGATC 0.405469933478
ACGAGATG -0.1161843145
ACGAGCAA -0.0754463702003
ACGAGCAC -0.203186770733
ACGAGCAG -0.0154024270843
ACGAGCAT -0.00193366553833
ACGAGCCA -0.145913580247
ACGAGCCC -0.276943882113
ACGAGCCG -0.334628345018
ACGAGCCT -0.0999163030652
ACGAGCGA -0.0739986170791
ACGAGCGC -0.0507046795604
ACGAGCGG -0.371619208039
ACGAGCGT -0.25027017919
ACGAGCTA -0.164548592249
ACGAGCTC -0.222161718188
ACGAGCTG -0.13387879721
ACGAGGAA 0.116138663857
ACGAGGAC -0.306223560976
ACGAGGAG -0.10478803601
ACGAGGAT 0.249896125405
ACGAGGCA -0.14888879852
ACGAGGCC -0.175483789552
ACGAGGCG -0.126693666551
ACGAGGCT -0.0648811189579
ACGAGGGA -0.180514802388
ACGAGGGC -0.215163857039
ACGAGGGG -0.184835390947
ACGAGGTA -0.0994863943898
ACGAGGTC -0.0976674624736
ACGAGGTG -0.0220482509577
ACGAGTAA -0.0272467562405
ACGAGTAC -0.0382505484288
ACGAGTAG -0.143723096758
ACGAGTAT -0.00487992677931
ACGAGTCA -0.0644929853156
ACGAGTCC -0.151484224966
ACGAGTCG -0.130978468545
ACGAGTCT -0.166475904402
ACGAGTGA 0.131104266849
ACGAGTGC -0.0959550470391
ACGAGTGG -0.0855846956751
ACGAGTTA -0.0792168873082
ACGAGTTC -0.220271604938
ACGAGTTG -0.0955023188167
ACGATAAA 0.0704735559507
ACGATAAC 0.0822497929772
ACGATAAG 0.0684096126796
ACGATAAT 0.0742195218336
ACGATACA 0.0717286766522

ACGATACC 0.00732178533008
ACGATACG 0.215555039007
ACGATACT 0.246797463451
ACGATAGA -0.0251789573988
ACGATAGC -0.112711954728
ACGATAGG -0.18140281551
ACGATAGT 0.0627097467784
ACGATATA 0.151608966629
ACGATATC 0.452776398339
ACGATATG 0.12187322941
ACGATCAA 0.0339444743074
ACGATCAC 0.147822991879
ACGATCAG 0.094406573141
ACGATCAT 0.154627434192
ACGATCCA 0.214875012351
ACGATCCC 0.0741262157237
ACGATCCG 0.224670135459
ACGATCCT 0.254504380166
ACGATCGA 0.188151770621
ACGATCGC 0.193388345706
ACGATCGG 0.168623350677
ACGATCGT 0.160070658688
ACGATCTA 0.233521436333
ACGATCTC 0.311607804396
ACGATCTG 0.242211082713
ACGATGAA -0.169666720896
ACGATGAC -0.0242787273985
ACGATGAG 0.0218078537911
ACGATGAT 0.184186364099
ACGATGCA -0.141993907961
ACGATGCC -0.230265360446
ACGATGCG -0.128807753654
ACGATGCT 0.0236412767132
ACGATGGA 0.131159472927
ACGATGGC -0.0726574074074
ACGATGGG -0.100184186673
ACGATGTA -0.125301817442
ACGATGTC -0.0308863481179
ACGATGTG 0.070191623204
ACGATTAA -0.0552763508841
ACGATTAC 0.376147116292
ACGATTAG -0.00429208105557
ACGATTAT 0.127143957114
ACGATTCA 0.0509392297299
ACGATTCC 0.388523897341
ACGATTCG 0.341997297808
ACGATTCT 0.378055583804
ACGATTGA -0.0353420597678
ACGATTGC 0.393934595325
ACGATTGG -0.00642569697674
ACGATTTA 0.239651039472
ACGATTTC 0.394588794485
ACGATTTG 0.285440123197
ACGCAAAA 0.17902614809

ACGCAAAC 0.0716647538245
ACGCAAAG -0.0822413187585
ACGCAAAT 0.150554239972
ACGCAACA 0.0127133787573
ACGCAACC -0.0357355499107
ACGCAACG -0.0852778487926
ACGCAACT 0.0604822459746
ACGCAAGA 0.0376160837779
ACGCAAGC 0.15838769195
ACGCAAGG 0.0012700946656
ACGCAAGT -0.142470749403
ACGCAATA 0.281518255066
ACGCAATC 0.480648281277
ACGCAATG 0.0808936529891
ACGCACAA -0.0349410893031
ACGCACAC 0.028325238056
ACGCACAG -0.203895130433
ACGCACAT 0.162721957424
ACGCACCA -0.327923605504
ACGCACCC -0.101077884235
ACGCACCG -0.0576796417045
ACGCACCT -0.20378804823
ACGCACGA 0.0485363125245
ACGCACGC -0.176254165057
ACGCACGG -0.0612448545026
ACGCACGT -0.0674509093703
ACGCACTA 0.0752357154272
ACGCACTC -0.141295637665
ACGCACTG -0.0718323153499
ACGCAGAA -0.0999983536656
ACGCAGAC 0.0120017780412
ACGCAGAG 0.030126538143
ACGCAGAT 0.257380907414
ACGCAGCA -0.185372665866
ACGCAGCC -0.222586980268
ACGCAGCG -0.0991935296442
ACGCAGCT -0.27175797884
ACGCAGGA -0.0715571359586
ACGCAGGC -0.296072428576
ACGCAGGG -0.222336324524
ACGCAGTA -0.0504319524029
ACGCAGTC -0.158782282356
ACGCAGTG -0.146638820371
ACGCATAA -0.0328403527378
ACGCATAC 0.128155941953
ACGCATAG -0.236142338417
ACGCATAT 0.101945157962
ACGCATCA -0.0303958374498
ACGCATCC -0.149670555698
ACGCATCG -0.200847945995
ACGCATCT -0.139751970405
ACGCATGA -0.121561042524
ACGCATGC -0.10456902939
ACGCATGG -0.141699346405

ACGCATTA	0.034752226403
ACGCATTC	-0.0934816131882
ACGCATTG	-0.0307903276883
ACGCCAAA	-0.196112482853
ACGCCAAC	-0.165582991235
ACGCCAAG	0.0475865097006
ACGCCAAT	0.0552859820144
ACGCCACA	-0.0492830473755
ACGCCACC	-0.119475762057
ACGCCACG	-0.129456790123
ACGCCACT	-0.351044673819
ACGCCAGA	0.0398922028678
ACGCCAGC	-0.240293486552
ACGCCAGG	-0.380328588277
ACGCCAGT	-0.293363290702
ACGCCATA	-0.0244736437682
ACGCCATC	0.0189923437886
ACGCCATG	-0.0854941472811
ACGCCCAA	-0.15843580753
ACGCCCAC	-0.0598584012513
ACGCCCAG	-0.180301727643
ACGCCCAT	-0.23756840283
ACGCCCCA	-0.0905426024721
ACGCCCCC	-0.375212578037
ACGCCCCG	-0.262407553712
ACGCCCCT	-0.196862945221
ACGCCCGA	-0.0159949290395
ACGCCCGC	-0.330192509203
ACGCCCGG	-0.39291290235
ACGCCCGT	-0.142501314387
ACGCCCTA	-0.100285816584
ACGCCCTC	-0.110576615832
ACGCCCTG	-0.201308641975
ACGCCGAA	-0.0301392456475
ACGCCGAC	-0.279399420856
ACGCCGAG	-0.17229961311
ACGCCGAT	0.0908125463077
ACGCCGCA	-0.272912127814
ACGCCGCC	-0.212334378168
ACGCCGCG	-0.248852464731
ACGCCGCT	-0.190879778272
ACGCCGGA	-0.192412693709
ACGCCGGC	-0.125395806237
ACGCCGGG	-0.247626584576
ACGCCGTA	-0.0391211477677
ACGCCGTC	-0.0999719256654
ACGCCGTG	-0.139395031027
ACGCCTAA	-0.0778540541727
ACGCCTAC	-0.293140304559
ACGCCTAG	-0.113638234471
ACGCCTAT	-0.10730552263
ACGCCTCA	-0.121346953361
ACGCCTCC	-0.177361246855
ACGCCTCG	-0.182491865168

ACGCCTCT -0.292056950169
ACGCCTGA -0.0408353088371
ACGCCTGC -0.189398054417
ACGCCTGG -0.0847366948162
ACGCCTTA -0.0752531489257
ACGCCTTC -0.139417087854
ACGCCTTG -0.131749637824
ACGCGAAA 0.0153261936998
ACGCGAAC 0.00108216933553
ACGCGAAG -0.0121496956402
ACGCGAAT 0.222548793607
ACGCGACA 0.0639531054469
ACGCGACC -0.0842710940932
ACGCGACG -0.0396267661298
ACGCGACT -0.136213583148
ACGCGAGA -0.0623262098122
ACGCGAGC -0.0858120378307
ACGCGAGG -0.0742505802756
ACGCGAGT -0.25621247462
ACGCGATA 0.249517003666
ACGCGATC 0.428563334447
ACGCGATG -0.00312301038442
ACGCGCAA 0.187007685267
ACGCGCAC 0.0210262519431
ACGCGCAG -0.149359359281
ACGCGCAT 0.0490707809173
ACGCGCCA -0.0648957595913
ACGCGCCC -0.197135802469
ACGCGCCG -0.152671301125
ACGCGCCT -0.251708871607
ACGCGCGA -0.00376907690028
ACGCGCGC -0.0526548332565
ACGCGCGG -0.00794946661224
ACGCGCGT 0.00659675616468
ACGCGCTA -0.0535774991646
ACGCGCTC -0.0880473302012
ACGCGCTG -0.118502668514
ACGCGGAA 0.00336093414764
ACGCGGAC -0.344182851633
ACGCGGAG -0.138948038102
ACGCGGAT 0.250930512981
ACGCGGCA -0.063213033386
ACGCGGCC -0.0379732493551
ACGCGGCG -0.281907342485
ACGCGGCT -0.175231601851
ACGCGGGA 0.00841085590406
ACGCGGGC -0.235398672158
ACGCGGGG -0.184221231096
ACGCGGTA -0.213074074074
ACGCGGTC -0.179497910125
ACGCGGTG -0.180797703912
ACGCGTAA 0.0732013875167
ACGCGTAC -0.13758343904
ACGCGTAG -0.0113010164877

ACGCGTAT 0.180434806472
ACGCGTCA 0.00167127708621
ACGCGTCC -0.137812018152
ACGCGTCG -0.0505286038929
ACGCGTCT -0.188255051107
ACGCGTGA 0.125411644603
ACGCGTGC -0.0421894036682
ACGCGTGG -0.0732357567171
ACGCGTTA 0.017621133285
ACGCGTTC -0.195915444263
ACGCGTTG -0.0957294689005
ACGCTAAA 0.0433847137274
ACGCTAAC -0.113575368404
ACGCTAAG -0.179016774415
ACGCTAAT -0.0965656008019
ACGCTACA 0.0366555797054
ACGCTACC -0.0774207139018
ACGCTACG -0.102003492166
ACGCTACT 0.102895413537
ACGCTAGA 0.026963544754
ACGCTAGC -0.111223616188
ACGCTAGG -0.158408362604
ACGCTAGT -0.22658430811
ACGCTATA -0.00183849759074
ACGCTATC 0.210200049395
ACGCTATG -0.089987268347
ACGCTCAA -0.0604923026262
ACGCTCAC -0.00178684533183
ACGCTCAG -0.164582703884
ACGCTCAT -0.153560395301
ACGCTCCA -0.112666897153
ACGCTCCC -0.203451303155
ACGCTCCG -0.16812345679
ACGCTCCT -0.149913267322
ACGCTCGA -0.0678081574903
ACGCTCGC -0.216473877833
ACGCTCGG -0.15949103801
ACGCTCTA -0.0668995975767
ACGCTCTC -0.274627923459
ACGCTCTG -0.125232740867
ACGCTGAA 0.00210226711449
ACGCTGAC -0.280258533043
ACGCTGAG -0.0986991369218
ACGCTGAT 0.159018203478
ACGCTGCA -0.020238838929
ACGCTGCC -0.284107510579
ACGCTGCG -0.0270151519879
ACGCTGCT 0.0716182644952
ACGCTGGA -0.146210868062
ACGCTGGC 0.110831192781
ACGCTGGG -0.264874339604
ACGCTGTA -0.080513017179
ACGCTGTC -0.116265726993
ACGCTGTG -0.32826926875

ACGCTTAA -0.180979507612
ACGCTTAC -0.155436869795
ACGCTTAG -0.0503965313795
ACGCTTAT 0.154656923278
ACGCTTCA -0.132779998606
ACGCTTCC -0.210374284021
ACGCTTCG -0.0237353769104
ACGCTTCT -0.119846476573
ACGCTTGA -0.00772862215798
ACGCTTGC 0.0282873040547
ACGCTTGG -0.10763880819
ACGCTTTA 0.0782284659974
ACGCTTTC -0.0457445325028
ACGCTTTG -0.0640025027992
ACGGAAAA 0.108596167536
ACGGAAAC -0.279144899285
ACGGAAAG -0.169432371922
ACGGAAAT 0.298370985093
ACGGAACA -0.0579321170607
ACGGAACC -0.0880856639801
ACGGAACG -0.141783587509
ACGGAACT -0.0833465499445
ACGGAAGA 0.021095985937
ACGGAAGC -0.165786354468
ACGGAAGG -0.18614266118
ACGGAAGT -0.0684429737917
ACGGAATA 0.251870238644
ACGGAATC 0.460466636642
ACGGAATG 0.102285450833
ACGGACAA -0.0073380500632
ACGGACAC -0.0760023007672
ACGGACAG -0.200372764776
ACGGACAT -0.245895747599
ACGGACCA -0.250780825638
ACGGACCC -0.0861930727153
ACGGACCG -0.196414071587
ACGGACCT -0.320334848331
ACGGACGA -0.0365653102436
ACGGACGC -0.254807077878
ACGGACGG -0.180124548338
ACGGACGT -0.154603132181
ACGGACTA -0.127959407203
ACGGACTC -0.236198212434
ACGGACTG -0.210869514703
ACGGAGAA 0.164936901116
ACGGAGAC -0.223500685871
ACGGAGAG -0.121692207244
ACGGAGAT 0.335189667095
ACGGAGCA -0.128640156181
ACGGAGCC 0.035461188674
ACGGAGCG -0.0554393609296
ACGGAGCT -0.216456586091
ACGGAGGA -0.149790123457
ACGGAGGC -0.136325874975

ACGGAGGG -0.266477924273
ACGGAGTA -0.105722452709
ACGGAGTC -0.314324801617
ACGGAGTG -0.275793944782
ACGGATAA 0.0232743858263
ACGGATAC 0.261602719626
ACGGATAG 0.0667252625921
ACGGATAT 0.359583374494
ACGGATCA 0.136026324583
ACGGATCC 0.209641019508
ACGGATCG 0.159026542844
ACGGATCT 0.302327372045
ACGGATGA -0.135171422407
ACGGATGC -0.00765766420176
ACGGATGG -0.226257973069
ACGGATTA 0.315971990179
ACGGATTC 0.398591079719
ACGGATTG 0.342996818387
ACGGCAAA -0.0288974765011
ACGGCAAC -0.0668535192152
ACGGCAAG -0.0186287609768
ACGGCAAT 0.141336802125
ACGGCACA 0.0530254383798
ACGGCACC -0.187625177649
ACGGCACG -0.095386720191
ACGGCACT -0.156014524328
ACGGCAGA -0.0106900368682
ACGGCAGC -0.281815066305
ACGGCAGG -0.243468892296
ACGGCAGT -0.22175313275
ACGGCATA -0.116210883906
ACGGCATC 0.0648709031694
ACGGCATG -0.345934226157
ACGGCCAA -0.0257045635411
ACGGCCAC -0.300054019831
ACGGCCAG -0.152125659865
ACGGCCAT -0.212720597919
ACGGCCCA -0.240393267057
ACGGCCCC -0.105140376973
ACGGCCCG -0.271724103881
ACGGCCCT -0.0498001255026
ACGGCCGA -0.0475931074756
ACGGCCGC -0.228121726609
ACGGCCGG -0.0811328416524
ACGGCCGT 0.0488840608335
ACGGCCTA -0.0124518762803
ACGGCCTC -0.235914275838
ACGGCCTG -0.0815208232854
ACGGCGAA 0.0230840298781
ACGGCGAC -0.235702271427
ACGGCGAG -0.0468647401665
ACGGCGAT 0.0711795214505
ACGGCGCA -0.111948039119
ACGGCGCC -0.219648961094

ACGGCGCG -0.0963145234388
ACGGCGCT -0.188964064069
ACGGCGGA -0.0399487531085
ACGGCGGC -0.235125668713
ACGGCGGG -0.0727101582911
ACGGCGTA 0.0834556099545
ACGGCGTC -0.190480109739
ACGGCGTG 0.0168774287032
ACGGCTAA -0.103289051311
ACGGCTAC -0.172939898393
ACGGCTAG -0.0536758047254
ACGGCTAT 0.063972513089
ACGGCTCA -0.284067965852
ACGGCTCC -0.0848945336444
ACGGCTCG -0.217273512713
ACGGCTCT -0.139094402013
ACGGCTGA -0.137517831255
ACGGCTGC -0.188751993169
ACGGCTGG -0.00994267590438
ACGGCTTA -0.0487194087433
ACGGCTTC -0.330442849496
ACGGCTTG -0.0927626886145
ACGGGAAA 0.21731291677
ACGGGAAC -0.176519491637
ACGGGAAG -0.146340192044
ACGGGAAT 0.329686342307
ACGGGACA -0.0030303734652
ACGGGACC -0.324075445613
ACGGGACG -0.28247116337
ACGGGACT -0.21895069815
ACGGGAGA -0.103876635601
ACGGGAGC -0.27128884223
ACGGGAGG -0.125350566669
ACGGGAGT -0.269013372681
ACGGGATA 0.105497363183
ACGGGATC 0.204670724798
ACGGGATG 0.0369924309561
ACGGGCAA -0.0257335069783
ACGGGCAC -0.201528174353
ACGGGCAG -0.0632768640622
ACGGGCAT -0.132379812511
ACGGGCCA -0.235988277699
ACGGGCCC -0.232704762103
ACGGGCCG -0.333180373957
ACGGGCCT -0.242752430487
ACGGGCGA 0.00409998024106
ACGGGCGC -0.1311282984
ACGGGCGG 0.0218545953361
ACGGGCTA -0.0530719277091
ACGGGCTC -0.259206700113
ACGGGCTG -0.215262002743
ACGGGGAA 0.139569474632
ACGGGGAC -0.21871434549
ACGGGGAG -0.163064633261

ACGGGGAT 0.295440659149
ACGGGGCA -0.0366071169208
ACGGGGCC -0.155453949968
ACGGGGCG -0.217056819271
ACGGGGCT -0.0392048822048
ACGGGGGA -0.134406743386
ACGGGGGC -0.242779217158
ACGGGGGG -0.0603051572269
ACGGGGTA -0.113442887774
ACGGGGTC -0.119873799726
ACGGGGTG -0.212610847711
ACGGGTAA -0.157752205979
ACGGGTAC -0.311188595409
ACGGGTAG -0.0522699867796
ACGGGTAT 0.065933115985
ACGGGTCA -0.314404287768
ACGGGTCC -0.419061018957
ACGGGTCG -0.286561012238
ACGGGTCT -0.388413300849
ACGGGTGA -0.093361872616
ACGGGTGC -0.222145089519
ACGGGTGG -0.0202368397352
ACGGGTTA -0.193224785016
ACGGGTTC -0.187988388428
ACGGGTTG -0.137728280434
ACGGTAAA 0.100905734466
ACGGTAAC -0.0622049880349
ACGGTAAG -0.166996885676
ACGGTAAT 0.222820479045
ACGGTACA -0.0736884767242
ACGGTACC 0.0117442205098
ACGGTACG 0.0718264945443
ACGGTACT -0.133728395062
ACGGTAGA 0.00457584130111
ACGGTAGC -0.163714823411
ACGGTAGG -0.157584191322
ACGGTAGT 0.0202529144438
ACGGTATA 0.0196273337943
ACGGTATC 0.313746313541
ACGGTATG -0.148708787219
ACGGTCAA -0.00738380994715
ACGGTCAC -0.180171387073
ACGGTCAG -0.121567045835
ACGGTCAT -0.0272704557473
ACGGTCCA -0.175977599982
ACGGTCCC -0.12739869281
ACGGTCCG -0.35470979608
ACGGTCCT -0.2245944407
ACGGTCGA -0.0515617418993
ACGGTCGC -0.21799382716
ACGGTCGG -0.226571289025
ACGGTCTA -0.196740740741
ACGGTCTC -0.249814805669
ACGGTCTG -0.170842592593

ACGGTGAA 0.0816889756001
ACGGTGAC -0.15751763124
ACGGTGAG -0.078157279212
ACGGTGAT 0.199644066073
ACGGTGCA -0.0813095140089
ACGGTGCC -0.304657322591
ACGGTGCG -0.273262771433
ACGGTGCT -0.248328249818
ACGGTGGA 0.160877776648
ACGGTGGC -0.230813966248
ACGGTGGG -0.109437337767
ACGGTGTA -0.114192832408
ACGGTGTC -0.253522633745
ACGGTGTG -0.0240745988867
ACGGTTAA -0.00831247667347
ACGGTTAC -0.108769926699
ACGGTTAG -0.211328707079
ACGGTTAT 0.0373527232577
ACGGTTCA -0.0751487115326
ACGGTTCC -0.299079241225
ACGGTTCG -0.0505847485944
ACGGTTCT -0.0353798323479
ACGGTTGA -0.0137564830822
ACGGTTGC -0.0742318364738
ACGGTTGG 0.00513347656443
ACGGTTTA -0.0926365013502
ACGGTTTC -0.22640034237
ACGGTTTG -3.63944534853E-5
ACGTAAAA 0.0594365771598
ACGTAAAC 0.00577629915883
ACGTAAAG 0.0958359240169
ACGTAAAT 0.161421120683
ACGTAACA 0.0690991239667
ACGTAACC 0.0090863685262
ACGTAACG 0.0398279327227
ACGTAACT -0.0275023461424
ACGTAAGA 0.140474772275
ACGTAAGC 0.00342213879558
ACGTAAGG -0.17173228438
ACGTAAGT -0.0350049529792
ACGTAATA 0.293369459416
ACGTAATC 0.474883927689
ACGTAATG 0.0458370258452
ACGTACAA 0.0617567155289
ACGTACAC -0.00154770725282
ACGTACAG -0.085681665001
ACGTACAT 0.0227421044832
ACGTACCA -0.149684822077
ACGTACCC -0.145777714513
ACGTACCG -0.193744855967
ACGTACCT -0.00712523859744
ACGTACGA -0.0842114217349
ACGTACGC -0.21684822077
ACGTACGG -0.155262002743

ACGTACGT -0.11310141063
ACGTACTA -0.0535896749234
ACGTACTC -0.101711005751
ACGTACTG -0.146646618027
ACGTAGAA 0.230789918549
ACGTAGAC -0.193800316096
ACGTAGAG 0.109993949422
ACGTAGAT 0.287596873696
ACGTAGCA -0.0938832633572
ACGTAGCC -0.166309978489
ACGTAGCG -0.0521311799279
ACGTAGCT -0.0883658774847
ACGTAGGA -0.00511527900862
ACGTAGGC -0.208449437501
ACGTAGGG -0.154504715817
ACGTAGTA 0.0355795991634
ACGTAGTC -0.257115577724
ACGTAGTG 0.034358519896
ACGTATAA 0.221409910207
ACGTATAC -0.0171299040594
ACGTATAG -0.00763076011261
ACGTATAT 0.154456251284
ACGTATCA 0.166212427179
ACGTATCC 0.28984288266
ACGTATCG 0.222581725175
ACGTATCT 0.303036334316
ACGTATGA 0.0133206456206
ACGTATGC -0.0812009089562
ACGTATGG -0.248007890005
ACGTATTA 0.0516318831461
ACGTATTC 0.0540598259556
ACGTATTG 0.176883705423
ACGTCAAA -0.0629548355897
ACGTCAAC -0.0314012644473
ACGTCAAG -0.00317984654226
ACGTCAAT 0.0414334013919
ACGTCACA -0.02437369357
ACGTCACC -0.187056951907
ACGTCACG -0.191965584034
ACGTCACT -0.107252335362
ACGTCAGA -0.0589557203827
ACGTCAGC -0.208364889635
ACGTCAGG -0.107398810308
ACGTCAGT -0.206294521826
ACGTCATA 0.0110018880766
ACGTCATC -0.0171381369514
ACGTCATG -0.0824828296168
ACGTCCAA -0.12970925233
ACGTCCAC -0.169216422246
ACGTCCAG -0.0582077291421
ACGTCCAT -0.0472677775582
ACGTCCCA -0.295420402859
ACGTCCCC -0.151500801822
ACGTCCCG -0.288021947874

ACGTCCCT -0.156100286462
ACGTCCGA -0.185968868478
ACGTCCGC -0.153127087872
ACGTCCGG -0.0229258600628
ACGTCCTA 0.104195481789
ACGTCCTC -0.302336964415
ACGTCCTG -0.162723695692
ACGTCGAA 0.0376055089855
ACGTCGAC -0.0960261793035
ACGTCGAG -0.105176075468
ACGTCGAT 0.174409077042
ACGTCGCA -0.0516303716271
ACGTCGCC -0.0839175976639
ACGTCGCG -0.0604139907124
ACGTCGCT -0.0535250764174
ACGTCGGA 0.21908822803
ACGTCGGC -0.175807667316
ACGTCGGG 0.0121587803352
ACGTCGTA 0.00641982017587
ACGTCGTC -0.0422596184577
ACGTCGTG 0.0511600540438
ACGTCTAA 0.0347464152369
ACGTCTAC -0.203685412002
ACGTCTAG -0.0351215174867
ACGTCTAT -0.00608864933971
ACGTCTCA -0.19922941525
ACGTCTCC -0.3137148406
ACGTCTCG -0.118052540588
ACGTCTCT -0.244734877208
ACGTCTGA -0.149258024031
ACGTCTGC -0.109094251814
ACGTCTGG -0.112451450035
ACGTCTTA -0.0413922369317
ACGTCTTC -0.13646954238
ACGTCTTG -0.148498932915
ACGTGAAA 0.16463314233
ACGTGAAC -0.0260022173923
ACGTGAAG -0.0993661896145
ACGTGAAT 0.106470852822
ACGTGACA -0.0927731424454
ACGTGACC -0.2613281908
ACGTGACG -0.267209026715
ACGTGACT -0.280377100719
ACGTGAGA -0.103934738652
ACGTGAGC -0.0754037583586
ACGTGAGG -0.0649593335309
ACGTGAGT -0.0360416503244
ACGTGATA 0.1604739006
ACGTGATC 0.221621769189
ACGTGATG -0.107179794083
ACGTGCAA 0.133356093734
ACGTGCAC 0.0687281828361
ACGTGCAG -0.0927098920576
ACGTGCAT -0.121021893568

ACGTGCCA -0.0255548969242
ACGTGCCC -0.181105072516
ACGTGCCG -0.137496296296
ACGTGCCT -0.30329537101
ACGTGCGA -0.0148411600694
ACGTGCGC -0.0693770686163
ACGTGCGG -0.0973964678894
ACGTGCTA -0.214411615604
ACGTGCTC -0.222026886205
ACGTGCTG -0.129201519243
ACGTGGAA -0.063428944653
ACGTGGAC -0.152408381489
ACGTGGAG -0.124069433758
ACGTGGAT 0.252980306922
ACGTGGCA -0.151057443259
ACGTGGCC -0.345041798315
ACGTGGCG -0.0680629108772
ACGTGGCT -0.141336318539
ACGTGGGA 0.0724711054035
ACGTGGGC -0.0872090789856
ACGTGGGG -0.232948654653
ACGTGGTA -0.0321724955542
ACGTGGTC -0.167735657226
ACGTGGTG -0.214055434053
ACGTGTAA 0.21439548618
ACGTGTAC -0.214243382062
ACGTGTAG -0.13903017135
ACGTGTAT -0.0521003422608
ACGTGTCA -0.116680659127
ACGTGTCC -0.147981668999
ACGTGTCG -0.0291298606092
ACGTGTCT 0.0347929045661
ACGTGTGA 0.00547266572719
ACGTGTGC -0.00846890162243
ACGTGTGG -0.220768175583
ACGTGTTA -0.162835267967
ACGTGTTC -0.255206618825
ACGTGTTG 0.0373294785931
ACGTTAAA 0.0987012043642
ACGTTAAC -0.139019013502
ACGTTAAG 0.042661223541
ACGTTAAT -0.0624025774441
ACGTTACA 0.0643250298349
ACGTTACC 0.0140063632269
ACGTTACG 0.0898153636743
ACGTTACT -0.194977021772
ACGTTAGA 0.0174756742073
ACGTTAGC -0.22881100573
ACGTTAGG 0.0102445799025
ACGTTAGT -0.0334831719686
ACGTTATA 0.107679577117
ACGTTATC 0.162319100069
ACGTTATG 0.0301817822565
ACGTTCAA 0.00811409115207

ACGTTCAC -0.10301235974
ACGTTCAG -0.195264827253
ACGTTCAT -0.133833769569
ACGTTCCA 0.0273371783883
ACGTTCCC -0.389171208791
ACGTTCCG 0.0721272601522
ACGTTCCT -0.0703820968928
ACGTTCGA 0.0496492441458
ACGTTCGC -0.178714707771
ACGTTCGG 0.00693758370107
ACGTTCTA 0.026164990107
ACGTTCTC -0.311166303558
ACGTTCTG -0.198062068425
ACGTTGAA -0.0652869989685
ACGTTGAC -0.302237797988
ACGTTGAG -0.131616924841
ACGTTGAT 0.0162311466772
ACGTTGCA 0.0637993404326
ACGTTGCC -0.143290741054
ACGTTGCG -0.2103190281
ACGTTGCT -0.0943612318483
ACGTTGGA -0.0899461754975
ACGTTGGC -0.135124281641
ACGTTGGG -0.0956210829025
ACGTTGTA -0.022967499753
ACGTTGTC -0.20687300997
ACGTTGTG -0.0346769413159
ACGTTTAA -0.110634708901
ACGTTTAC -0.0150799761742
ACGTTTAG -0.176348159338
ACGTTTAT -0.0165866303362
ACGTTTCA -0.0787419692837
ACGTTTCC 0.0408917234466
ACGTTTCG 0.00214889097466
ACGTTTCT -0.0349980241059
ACGTTTGA -0.0394292255352
ACGTTTGC -0.133777887544
ACGTTTGG -0.0169378032449
ACGTTTTA 0.13667149663
ACGTTTTC 0.0737200597159
ACGTTTTG -0.0664880789815
ACTAAAAA 0.044785204771
ACTAAAAC 0.0143317885317
ACTAAAAG 0.0647434630837
ACTAAAAT 0.303480888527
ACTAAACA -0.0768101385321
ACTAAACC -0.0980852184794
ACTAAACG 0.0184751500007
ACTAAACT -0.0462932023884
ACTAAAGA 0.166456496157
ACTAAAGC -0.248890231715
ACTAAAGG -0.24395095716
ACTAAAGT -0.153967940381
ACTAAATA 0.0981872876134

ACTAAATC 0.215095956881
ACTAAATG 0.0785306466375
ACTAACAA -0.0769540295004
ACTAACAC -0.330808286182
ACTAACAG -0.00692255168306
ACTAACAT 0.0755907825187
ACTAACCA -0.240704697109
ACTAACCC -0.273187701999
ACTAACCG -0.093129608253
ACTAACCT 0.0263549298867
ACTAACGA 0.0661540319217
ACTAACGC -0.064142461964
ACTAACGG 0.152503348043
ACTAACTA 0.0328380413848
ACTAACTC 0.00262629256405
ACTAACTG -0.118561878417
ACTAAGAA 0.119548797895
ACTAAGAC -0.0907743974714
ACTAAGAG -0.200571734027
ACTAAGAT 0.251160837779
ACTAAGCA 0.0744207874327
ACTAAGCC -0.0910121115337
ACTAAGCG -0.119231897827
ACTAAGCT -0.110891460145
ACTAAGGA 0.0910420873709
ACTAAGGC -0.151476616129
ACTAAGGG -0.26011335517
ACTAAGTA 0.0152555489692
ACTAAGTC -0.0890881248765
ACTAAGTG -0.158284494491
ACTAATAA 0.110340970174
ACTAATAC 0.0132066445321
ACTAATAG -0.0383707655492
ACTAATAT 0.208578923969
ACTAATCA -0.0231069836879
ACTAATCC -0.0160541394981
ACTAATCG -0.063439048131
ACTAATCT -0.0282524370578
ACTAATGA -0.0667583080908
ACTAATGC -0.0744116226481
ACTAATGG 0.0252771199861
ACTAATTA 0.0465707260313
ACTAATTC 0.164814841718
ACTAATTG 0.00814446579324
ACTACAAA 0.0148460767364
ACTACAAC -0.00983104034402
ACTACAAG -0.0753924328659
ACTACAAT 0.148685950053
ACTACACA -0.101715565215
ACTACACC -0.153687422481
ACTACACG 0.0465241962431
ACTACACT -0.078635560385
ACTACAGA 0.197389091987
ACTACAGC -0.219305322313

ACTACAGG -0.152259105579
ACTACAGT -0.151949442905
ACTACATA -0.0091138114997
ACTACATC 0.141997297808
ACTACATG 0.0381196623417
ACTACCAA -0.0428823903928
ACTACCAC -0.103402760627
ACTACCAG -0.275234819058
ACTACCAT 0.0426515208636
ACTACCCA -0.243360112384
ACTACCCC -0.066574172272
ACTACCCG -0.200031274176
ACTACCCT -0.0528356780902
ACTACCGA -0.0442563886508
ACTACCGC -0.134514994205
ACTACCGG -0.171306815064
ACTACCTA -0.0841933689306
ACTACCTC -0.171583003151
ACTACCTG -0.145426994865
ACTACGAA -0.0571862333474
ACTACGAC -0.215808278867
ACTACGAG -0.0336588069991
ACTACGAT 0.240121743931
ACTACGCA -0.0908732903028
ACTACGCC -0.321232343648
ACTACGCG 0.0305381717397
ACTACGCT -0.200748694796
ACTACGGA 0.13768541252
ACTACGGC -0.149004987408
ACTACGGG -0.114920686087
ACTACGTA -0.00282113767591
ACTACGTC -0.0494610732435
ACTACGTG -0.0191357325268
ACTACTAA 0.138993172188
ACTACTAC -0.0111937588075
ACTACTAG -0.209791495199
ACTACTAT 0.237587688964
ACTACTCA 0.0538598310861
ACTACTCC -0.0303448143392
ACTACTCG 0.075695953808
ACTACTCT -0.0195037264103
ACTACTGA 0.0975186320515
ACTACTGC -0.173009288979
ACTACTGG 0.1373454593
ACTACTTA 0.117881292674
ACTACTTC 0.00537157743937
ACTACTTG -0.219575495606
ACTAGAAA 0.00571092353958
ACTAGAAC -0.0273109503112
ACTAGAAG 0.0828135521831
ACTAGAAT 0.113438321735
ACTAGACA -0.0982964871548
ACTAGACC -0.14283473168
ACTAGACG -0.0974513484145

ACTAGACT -0.14126623537
ACTAGAGA -0.0427272328142
ACTAGAGC -0.188151952634
ACTAGAGG -0.215401583617
ACTAGAGT -0.0247583960147
ACTAGATA 0.355368791132
ACTAGATC 0.279867214853
ACTAGATG -0.0126000610172
ACTAGCAA -0.124154112126
ACTAGCAC -0.113393495302
ACTAGCAG -0.10800969111
ACTAGCAT 0.00885035895409
ACTAGCCA -0.182785249231
ACTAGCCC -0.209836002612
ACTAGCCG -0.0404379078511
ACTAGCCT -0.322714536118
ACTAGCGA -0.191243289098
ACTAGCGC -0.235201171126
ACTAGCGG -0.12715754549
ACTAGCTA -0.118200064477
ACTAGCTC -0.0728254187798
ACTAGCTG -0.236772694263
ACTAGGAA 0.139868603043
ACTAGGAC -0.096603373382
ACTAGGAG -0.135559177491
ACTAGGAT 0.172218267401
ACTAGGCA -0.0469954849469
ACTAGGCC -0.244852106069
ACTAGGCG -0.0177678699094
ACTAGGCT -0.0823496970894
ACTAGGGA -0.0474476439791
ACTAGGGC -0.176972211247
ACTAGGGG -0.140476464906
ACTAGGTA -0.180754447282
ACTAGGTC -0.231934806415
ACTAGGTG -0.0152829919427
ACTAGTAA 0.0405552262399
ACTAGTAC -0.0713192077839
ACTAGTAG -0.25714951989
ACTAGTAT 0.185226349645
ACTAGTCA -0.138697365821
ACTAGTCC -0.235575308642
ACTAGTCG -0.134207063239
ACTAGTCT -0.0287739577159
ACTAGTGA -0.0193325637746
ACTAGTGC -0.068179809397
ACTAGTGG -0.168639170165
ACTAGTTA -0.218658774659
ACTAGTTC -0.206995132819
ACTAGTTG -0.135868081821
ACTATAAA 0.160140727568
ACTATAAC -0.0962150650947
ACTATAAG -0.0274301570468
ACTATAAT 0.187914227188

ACTATACA 0.0943913035565
ACTATACC -0.129614996268
ACTATACG -0.211495198903
ACTATACT -0.0754768973208
ACTATAGA -0.0511586851335
ACTATAGC -0.173123703448
ACTATAGG -0.134572229629
ACTATAGT 0.0531354231665
ACTATATA 0.186538123779
ACTATATC 0.129648569727
ACTATATG -0.121940057821
ACTATCAA 0.0269972251682
ACTATCAC 0.110499132802
ACTATCAG -0.0842333739449
ACTATCAT 0.0759868493271
ACTATCCA 0.153119856897
ACTATCCC -0.0936848229379
ACTATCCG 0.0741490273561
ACTATCCT -0.13112134929
ACTATCGA 0.0545886420757
ACTATCGC -0.00985477178423
ACTATCGG 0.0543864848844
ACTATCTA 0.0951941655892
ACTATCTC 0.201189927331
ACTATCTG 0.0935612278994
ACTATGAA 0.17911194773
ACTATGAC -0.126006008449
ACTATGAG -0.168908808275
ACTATGAT 0.0862159138785
ACTATGCA 0.12429939128
ACTATGCC 0.0270230960065
ACTATGCG 0.0340567301148
ACTATGCT -0.267849108368
ACTATGGA 0.0510494855922
ACTATGGC -0.242533757873
ACTATGGG -0.119473217788
ACTATGTA 0.0621696388875
ACTATGTC 0.0443842343062
ACTATGTG 0.0373440065085
ACTATTAA -0.0393372364999
ACTATTAC 0.109971961123
ACTATTAG -0.0381429888691
ACTATTAT 0.0218857713671
ACTATTCA -0.088412525931
ACTATTCC 0.00241724834277
ACTATTCG 0.165500559325
ACTATTCT 0.0453425095615
ACTATTGA -0.00851481120974
ACTATTGC 0.101978702076
ACTATTGG -0.232799382716
ACTATTTA -0.129621236552
ACTATTTC -0.0285633763532
ACTATTTG 0.0859376715186
ACTCAAAA 0.231439861694

ACTCAAAC -0.182253008863
ACTCAAAG 0.164158179943
ACTCAAAT 0.122700278821
ACTCAACA -0.0531814766378
ACTCAACC 0.00668138828759
ACTCAACG -0.14784543544
ACTCAACT -0.112814576174
ACTCAAGA 0.195429647193
ACTCAAGC -0.0836077088546
ACTCAAGG 0.104622518028
ACTCAAGT 0.0934086847878
ACTCAATA 0.109518040024
ACTCAATC 0.125513925007
ACTCAATG 0.205418889548
ACTCACAA -0.0954785549506
ACTCACAC -0.106421338602
ACTCACAG -0.122279553398
ACTCACAT -0.00127494319866
ACTCACCA -0.0566051167318
ACTCACCC -0.185478945029
ACTCACCG -0.0955218639055
ACTCACCT -0.0136144591539
ACTCACGA -0.10397909349
ACTCACGC -0.0427935583776
ACTCACGG -0.221177806563
ACTCACTA -0.0422444574214
ACTCACTC -0.112191914452
ACTCACTG -0.197632127077
ACTCAGAA 0.174265568085
ACTCAGAC -0.0862377416682
ACTCAGAG -0.151426922537
ACTCAGAT 0.263200794557
ACTCAGCA -0.0173988451997
ACTCAGCC -0.15300550946
ACTCAGCG -0.10033995322
ACTCAGCT 0.0861187951437
ACTCAGGA 0.0517080506707
ACTCAGGC -0.100602528645
ACTCAGGG -0.119681551735
ACTCAGTA 0.139950484046
ACTCAGTC -0.30210233412
ACTCAGTG -0.0162481819928
ACTCATAA -0.00965718237502
ACTCATAC 0.0579376056554
ACTCATAG 0.0275290398684
ACTCATAT 0.144420293435
ACTCATCA 0.0563598982515
ACTCATCC -0.0380878357764
ACTCATCG -0.127479062147
ACTCATCT -0.113924657315
ACTCATGA -0.0656549375414
ACTCATGC -0.0587249058799
ACTCATGG -0.133107796655
ACTCATTA 0.0145119346825

ACTCATTC -0.03016140514
ACTCATTG 0.0519468045402
ACTCCAAA 0.00744954704197
ACTCCAAC -0.207808564232
ACTCCAAG -0.124690949131
ACTCCAAT 0.0201486311445
ACTCCACA -0.117271145794
ACTCCACC -0.200731449015
ACTCCACG -0.0939437421528
ACTCCACT -0.15430543894
ACTCCAGA -0.161683268846
ACTCCAGC -0.13332771615
ACTCCAGG -0.188097887187
ACTCCAGT -0.0102906067342
ACTCCATA 0.00986590734096
ACTCCATC -0.201045665673
ACTCCATG -0.15128561178
ACTCCCAA -0.155610438791
ACTCCCAC -0.04683891434
ACTCCCAG -0.171286694102
ACTCCCAT 0.0976132845946
ACTCCCCA -0.10011795502
ACTCCCCC -0.26443470748
ACTCCCCG -0.125999587529
ACTCCCCT -0.185634986798
ACTCCCGA 0.114568593553
ACTCCCGC 0.086176425388
ACTCCCGG -0.169247814923
ACTCCCTA -0.142831617756
ACTCCCTC -0.205251965965
ACTCCCTG -0.153679339285
ACTCCGAA 0.0322359914576
ACTCCGAC -0.124970580971
ACTCCGAG -0.202415123457
ACTCCGAT 0.0458050670706
ACTCCGCA 0.0255531503785
ACTCCGCC -0.176617298441
ACTCCGCG -0.0595310388912
ACTCCGCT -0.306487951212
ACTCCGGA 0.00485160480628
ACTCCGGC -0.0440843926321
ACTCCGGG -0.121983213199
ACTCCGTA -0.115559397382
ACTCCGTC -0.234453719253
ACTCCGTG -0.251245399646
ACTCCTAA -0.053160547993
ACTCCTAC -0.114524174033
ACTCCTAG -0.242934854171
ACTCCTAT 0.0280769061889
ACTCCTCA -0.353325976157
ACTCCTCC -0.228148440801
ACTCCTCG -0.0467842459286
ACTCCTCT -0.0358789435553
ACTCCTGA -0.0275239553492

ACTCCTGC -0.375314799269
ACTCCTGG -0.26188277118
ACTCCTTA -0.0613734659378
ACTCCTTC -0.252200291315
ACTCCTTG -0.192856292104
ACTCGAAA 0.0505197699181
ACTCGAAC -0.0832153402044
ACTCGAAG -0.120855148342
ACTCGAAT 0.0801403396627
ACTCGACA -0.0761353336277
ACTCGACC -0.271169050637
ACTCGACG -0.172007041742
ACTCGACT -0.0260483552307
ACTCGAGA 0.238405343696
ACTCGAGC -0.119726927995
ACTCGAGG -0.164276812508
ACTCGAGT -0.180848676356
ACTCGATA 0.0706942890765
ACTCGATC 0.0456815736899
ACTCGATG -0.0506748216698
ACTCGCAA 0.100652598044
ACTCGCAC -0.27199200502
ACTCGCAG -0.0718210059496
ACTCGCAT -0.141312999274
ACTCGCCA -0.0978293080456
ACTCGCCC -0.141574111628
ACTCGCCG -0.0563280033467
ACTCGCCT -0.018311176703
ACTCGCGA 0.209063821932
ACTCGCGC 0.00995631078619
ACTCGCGG -0.239432711891
ACTCGCTA -0.085847258698
ACTCGCTC -0.138607640089
ACTCGCTG -0.034638908663
ACTCGGAA 0.0347246233638
ACTCGGAC -0.219320261438
ACTCGGAG -0.114995925524
ACTCGGAT 0.224684381038
ACTCGGCA -0.172234295938
ACTCGGCC -0.24177536516
ACTCGGCG -0.264264429975
ACTCGGCT -0.132836601307
ACTCGGGA 0.00794868128583
ACTCGGGC -0.357751295391
ACTCGGGG -0.108326337103
ACTCGGTA -0.0576933631913
ACTCGGTC -0.197721376297
ACTCGGTG -0.0888249530772
ACTCGTAA 0.202888647391
ACTCGTAC -0.204479164246
ACTCGTAG -0.068962561562
ACTCGTAT -0.0200063922828
ACTCGTCA -0.138490682255
ACTCGTCC -0.166421762323

ACTCGTCG -0.231433932451
ACTCGTCT -0.14336873302
ACTCGTGA 0.00367992096814
ACTCGTGC -0.0173705441673
ACTCGTGG 0.00995305273433
ACTCGTTA -0.0484201780277
ACTCGTTC -0.0702642627809
ACTCGTTG -0.163450980392
ACTCTAAA 0.161726931436
ACTCTAAC -0.103804775726
ACTCTAAG -0.227182441701
ACTCTAAT 0.187671518584
ACTCTACA 0.0320958460931
ACTCTACC -0.165132983247
ACTCTACG -0.213718906484
ACTCTACT -0.0719946891188
ACTCTAGA 0.128968558332
ACTCTAGC -0.236966855438
ACTCTAGG -0.254554379524
ACTCTATA 0.0320229960758
ACTCTATC -0.0461321039488
ACTCTATG -0.158666857314
ACTCTCAA -0.132786174498
ACTCTCAC -0.0841878168902
ACTCTCAG -0.0104365628225
ACTCTCAT 0.0947029572548
ACTCTCCA -0.000515690051395
ACTCTCCC -0.153848475371
ACTCTCCG -0.0839726914507
ACTCTCCT -0.0357975906259
ACTCTCGA -0.161239518855
ACTCTCGC -0.19164565273
ACTCTCGG -0.048254470966
ACTCTCTA -0.0995824210798
ACTCTCTC -0.251687250689
ACTCTCTG -0.0702378738931
ACTCTGAA -0.029190940392
ACTCTGAC -0.0589150462378
ACTCTGAG -0.11068050294
ACTCTGAT 0.118797887989
ACTCTGCA -0.234605596486
ACTCTGCC -0.17584220077
ACTCTGCG 0.063328635974
ACTCTGCT -0.259844097607
ACTCTGGA 0.0298716766559
ACTCTGGC -0.168653243553
ACTCTGGG 0.126693567887
ACTCTGTA 0.164563370542
ACTCTGTC -0.102276219325
ACTCTGTG -0.157049804645
ACTCTTAA -0.0190737001148
ACTCTTAC 0.03574270739
ACTCTTAG -0.102677494806
ACTCTTAT -0.0925011114953

ACTCTTCA -0.138067376299
ACTCTTCC -0.272079690635
ACTCTTCG -0.101416945172
ACTCTTCT -0.18242267517
ACTCTTGA -0.18278462553
ACTCTTGC -0.217548293391
ACTCTTGG -0.148999369613
ACTCTTTA -0.0691165574652
ACTCTTTC 0.0615875211311
ACTCTTTG 0.0603580759182
ACTGAAAA 0.056163468104
ACTGAAAC -0.0255822062092
ACTGAAAG 0.0725498246567
ACTGAAAT 0.264807577761
ACTGAACA -0.0694487218971
ACTGAACC -0.134568338844
ACTGAACG -0.0577208606337
ACTGAACT -0.123552526151
ACTGAAGA 0.032991942743
ACTGAAGC -0.181523367409
ACTGAAGG -0.147410493827
ACTGAAGT -0.0566408179393
ACTGAATA 0.199463764298
ACTGAATC 0.167743669461
ACTGAATG -0.116300321067
ACTGACAA 0.0968983951349
ACTGACAC -0.170339506173
ACTGACAG 0.0297517011349
ACTGACAT 0.0136494607722
ACTGACCA -0.0161227589272
ACTGACCC -0.214971909496
ACTGACCG -0.129388131463
ACTGACCT -0.117764734938
ACTGACGA -0.0957745374042
ACTGACGC -0.196836419753
ACTGACGG -0.0272773854208
ACTGACTA -0.0616361338312
ACTGACTC -0.241665625808
ACTGACTG -0.246398999128
ACTGAGAA -0.0658169224422
ACTGAGAC -0.133578794481
ACTGAGAG -0.103501870918
ACTGAGAT 0.261166882164
ACTGAGCA -0.220213125406
ACTGAGCC -0.323898275056
ACTGAGCG -0.066295581844
ACTGAGCT -0.10572980983
ACTGAGGA -0.000857104671366
ACTGAGGC -0.202779956427
ACTGAGGG -0.102402323893
ACTGAGTA -0.0203764078245
ACTGAGTC -0.141816475701
ACTGAGTG -0.11884537508
ACTGATAA 0.162981075325

ACTGATAC 0.17182045709
ACTGATAG -0.0996773184505
ACTGATAT 0.325083898711
ACTGATCA -0.0598998484206
ACTGATCC 0.0543649121788
ACTGATCG 0.122840788575
ACTGATCT 0.0647516959758
ACTGATGA -0.087055627388
ACTGATGC 0.0187293961044
ACTGATGG -0.0430135075069
ACTGATTA 0.255320849011
ACTGATTC 0.337721464796
ACTGATTG 0.232782241076
ACTGCAAA 0.150153269356
ACTGCAAC 0.0836851510177
ACTGCAAG -0.0447466747244
ACTGCAAT 0.255801444598
ACTGCACA 0.0460493095348
ACTGCACC -0.117046146754
ACTGCACG -0.0699019704691
ACTGCACT -0.178454421231
ACTGCAGA 0.147733759248
ACTGCAGC -0.123047084974
ACTGCAGG -0.278477297167
ACTGCAGT 0.0670202593811
ACTGCATA 0.0163453576046
ACTGCATC -0.031189802924
ACTGCATG 0.00408351445696
ACTGCCAA -0.0333047926409
ACTGCCAC -0.101880662704
ACTGCCAG -0.189698547205
ACTGCCAT -0.177914430285
ACTGCCCA -0.199169768554
ACTGCCCC -0.12841355428
ACTGCCCG -0.179921093497
ACTGCCCT -0.132217864924
ACTGCCGA -0.0457813340358
ACTGCCGC -0.0239471727055
ACTGCCGG -0.238104436382
ACTGCCTA -0.123395394542
ACTGCCTC -0.281055593957
ACTGCCTG -0.190451800102
ACTGCGAA -0.119326319949
ACTGCGAC -0.106153145441
ACTGCGAG -0.132689521175
ACTGCGAT 0.243468572307
ACTGCGCA -0.138205693841
ACTGCGCC -0.106248456409
ACTGCGCG -0.0417854206433
ACTGCGCT 0.0336974997458
ACTGCGGA 0.00828228940262
ACTGCGGC -0.087937809389
ACTGCGGG -0.16812085693
ACTGCGTA 0.168086528264

ACTGCGTC -0.0953901701593
ACTGCGTG -0.103979871115
ACTGCTAA -0.0697296354946
ACTGCTAC -0.067240196482
ACTGCTAG -0.0724893671416
ACTGCTAT -0.07036825207
ACTGCTCA -0.140682328539
ACTGCTCC -0.0874277813441
ACTGCTCG -0.0284706786312
ACTGCTCT -0.10932947306
ACTGCTGA -0.0371660190125
ACTGCTGC -0.25079471111
ACTGCTGG -0.111714737258
ACTGCTTA -0.148642131685
ACTGCTTC -0.116725988988
ACTGCTTG -0.0839039255388
ACTGGAAA 0.0987232045836
ACTGGAAC -0.206964953002
ACTGGAAG -0.116465906846
ACTGGAAT 0.244706250412
ACTGGACA -0.0673707490273
ACTGGACC -0.102209078021
ACTGGACG -0.00903813852386
ACTGGACT -0.179953241793
ACTGGAGA -0.12249058799
ACTGGAGC -0.188030916634
ACTGGAGG -0.176614735075
ACTGGATA 0.309713166041
ACTGGATC 0.0820517553826
ACTGGATG -0.0218550896429
ACTGGCAA -0.0162122319086
ACTGGCAC -0.374171954637
ACTGGCAG -0.297110963293
ACTGGCAT -0.00759347076774
ACTGGCCA -0.0607080733754
ACTGGCCC -0.154833434847
ACTGGCCG -0.0912743839015
ACTGGCCT -0.245330589849
ACTGGCGA -0.033215602977
ACTGGCGC -0.20875728539
ACTGGCGG -0.139202455742
ACTGGCTA -0.252059462663
ACTGGCTC -0.270531967415
ACTGGCTG -0.169585878041
ACTGGGAA 0.0741751776038
ACTGGGAC -0.229681296308
ACTGGGAG -0.265215983847
ACTGGGAT 0.161978992634
ACTGGGCA -0.162781317397
ACTGGGCC -0.348243252271
ACTGGGCG -0.191632489456
ACTGGGCT -0.158577902874
ACTGGGGA -0.0140152164377
ACTGGGGC -0.190783950345

ACTGGGGG -0.179000726216
ACTGGGTA 0.0404989681442
ACTGGGTC -0.204993464052
ACTGGGTG -0.193379193744
ACTGGTAA 0.249367309285
ACTGGTAC -0.188764340856
ACTGGTAG -0.144733430462
ACTGGTAT -0.0473464762541
ACTGGTCA -0.238545164955
ACTGGTCC -0.125377669835
ACTGGTCG -0.279971594622
ACTGGTCT -0.0811550876092
ACTGGTGA -0.0746769470333
ACTGGTGC -0.183831002904
ACTGGTGG -0.169953843964
ACTGGTTA -0.0791499846012
ACTGGTTC -0.180426148827
ACTGGTTG -0.281703477362
ACTGTAAA 0.202232940595
ACTGTAAC -0.0685887117862
ACTGTAAG -0.10166066723
ACTGTAAT 0.355255473392
ACTGTACA -0.123979464353
ACTGTACC -0.128969331819
ACTGTACG -0.250571178304
ACTGTACT -0.1997557696
ACTGTAGA 0.10857538036
ACTGTAGC 0.0455141715515
ACTGTAGG -0.185574333452
ACTGTATA 0.0824277152078
ACTGTATC 0.105416006857
ACTGTATG 0.020239192957
ACTGTCAA 0.228369184036
ACTGTCAC -0.134714220553
ACTGTCAG -0.139212393931
ACTGTCAT -0.0690913528308
ACTGTCCA -0.194545963387
ACTGTCCC -0.112338880932
ACTGTCCG -0.142351507968
ACTGTCCT -0.0388868711229
ACTGTCGA -0.0891523284213
ACTGTCGC 0.035782893148
ACTGTCGG -0.271835684383
ACTGTCTA -0.216420044135
ACTGTCTC -0.293706628867
ACTGTCTG -0.163261363999
ACTGTGAA 0.0582038024984
ACTGTGAC -0.158219317357
ACTGTGAG -0.182876772466
ACTGTGAT 0.165674894309
ACTGTGCA -0.208078862605
ACTGTGCC -0.222304841352
ACTGTGCG -0.112903694449
ACTGTGCT -0.240291692204

ACTGTGGA -0.133415553212
ACTGTGGC 0.00619570526223
ACTGTGGG -0.161397984704
ACTGTGTA 0.0441941645261
ACTGTGTC -0.187624142661
ACTGTGTG 0.0487280810077
ACTGTTAA -0.0523234119145
ACTGTTAC 0.150737291706
ACTGTTAG -0.0330757049671
ACTGTTAT 0.188812052359
ACTGTTCA -0.0785509679968
ACTGTTCC -0.151044925198
ACTGTTCG -0.0790724428847
ACTGTTCT -0.157506202112
ACTGTTGA -0.17795221605
ACTGTTGC -0.233684822077
ACTGTTGG -0.0719959902954
ACTGTTTA -0.183011055744
ACTGTTTC -0.064583278859
ACTGTTTG -0.0621916552795
ACTTAAAA 0.179050800158
ACTTAAAC 0.0106475091889
ACTTAAAG -0.0149125118005
ACTTAAAT 0.151774584865
ACTTAACA 0.0593701288722
ACTTAACC -0.0268149873771
ACTTAACG 0.0691276708253
ACTTAACT -0.0253352316476
ACTTAAGA 0.0421056301608
ACTTAAGC -0.154977114821
ACTTAAGG -0.136725887698
ACTTAAGT 0.0094706695635
ACTTAATA 0.0587465314602
ACTTAATC 0.126821437392
ACTTAATG -0.0184169795166
ACTTACAA -0.0315347208501
ACTTACAC 0.0128883483157
ACTTACAG -0.198216412491
ACTTACAT 0.0962216958689
ACTTACCA 0.124882692878
ACTTACCC -0.0706898893609
ACTTACCG -0.0808238838579
ACTTACCT -0.0846958096649
ACTTACGA 0.102943355658
ACTTACGC -0.0933868929147
ACTTACGG 0.0134812395767
ACTTACTA 0.0870207603911
ACTTACTC -0.0380405818201
ACTTACTG -0.0303025313399
ACTTAGAA 0.0783025583659
ACTTAGAC -0.0966398314154
ACTTAGAG -0.12051476762
ACTTAGAT 0.341111094969
ACTTAGCA -0.119572007613

ACTTAGCC -0.132752320758
ACTTAGCG -0.103180875235
ACTTAGCT -0.20004967075
ACTTAGGA -0.00823482279078
ACTTAGGC -0.0399928106512
ACTTAGGG -0.037753281694
ACTTAGTA -0.0309446969198
ACTTAGTC -0.212589687727
ACTTAGTG -0.0204509464937
ACTTATAA -0.155723489962
ACTTATAC 0.174618279023
ACTTATAG -0.0603114228633
ACTTATAT 0.0895689567504
ACTTATCA -0.0641550110116
ACTTATCC 0.183601725614
ACTTATCG -0.00381938895588
ACTTATCT 0.192629290957
ACTTATGA 0.116210247992
ACTTATGC -0.124767587794
ACTTATGG -0.0599523484375
ACTTATTA 0.134152936558
ACTTATTC 0.00817485798963
ACTTATTG -0.11689277443
ACTTCAAA 0.0335812764226
ACTTCAAC -0.0866273741515
ACTTCAAG -0.0519560721873
ACTTCAAT 0.111174872518
ACTTCACA 0.188085656589
ACTTCACC -0.140067389956
ACTTCACG -0.28982095924
ACTTCACT -0.268131687243
ACTTCAGA 0.0402569309209
ACTTCAGC -0.0562826865983
ACTTCAGG 0.0211585325693
ACTTCATA -0.00239565324772
ACTTCATC -0.151820845887
ACTTCATG -0.193903978052
ACTTCCAA -0.0588162654541
ACTTCCAC -0.177265414017
ACTTCCAG -0.224139233409
ACTTCCAT -0.00997727946261
ACTTCCCA -0.235143064633
ACTTCCCC -0.208020591576
ACTTCCCG -0.173426277989
ACTTCCCT -0.168881251943
ACTTCCGA -0.104909222948
ACTTCCGC -0.178148230995
ACTTCCGG -0.163379970716
ACTTCCTA 0.00448418187007
ACTTCCTC -0.0795513641818
ACTTCCTG 0.0463882480781
ACTTCGAA 0.140197911169
ACTTCGAC -0.0605042431849
ACTTCGAG -0.180217337614

ACTTCGAT 0.155855045409
ACTTCGCA -0.214085693537
ACTTCGCC -0.147111118859
ACTTCGCG -0.125118673274
ACTTCGCT 0.025259891569
ACTTCGGA -0.102522308933
ACTTCGGC -0.152025988849
ACTTCGGG -0.201086419753
ACTTCGTA 0.145604184505
ACTTCGTC -0.107996141957
ACTTCGTG 0.0315237436607
ACTTCTAA 0.0279647843331
ACTTCTAC -0.067949615522
ACTTCTAG -0.163555555556
ACTTCTAT -0.00874435227289
ACTTCTCA -0.00053608007787
ACTTCTCC -0.0625531681348
ACTTCTCG -0.0647535315618
ACTTCTCT -0.0346824299106
ACTTCTGA 0.107310649295
ACTTCTGC -0.143682702756
ACTTCTGG -0.164740645629
ACTTCTTA 0.0942809690803
ACTTCTTC -0.111198754209
ACTTCTTG -0.00400118553646
ACTTGAAA 0.0760181023412
ACTTGAAC 0.0502700388593
ACTTGAAG -0.0889289457367
ACTTGAAT -0.035225800786
ACTTGACA -0.0733953513899
ACTTGACC 0.0530856879405
ACTTGACG -0.0679015394748
ACTTGACT -0.201109368827
ACTTGAGA -0.0814840529525
ACTTGAGC -0.223993074116
ACTTGAGG 0.0136228262607
ACTTGATA 0.158310281236
ACTTGATC 0.0312669795011
ACTTGATG -0.103608572761
ACTTGCAA -0.0359987215434
ACTTGCAC -0.0417517398845
ACTTGCAG 0.0174262817347
ACTTGCAT 0.0607862507809
ACTTGCCA 0.0757616259643
ACTTGCCC -0.185978169605
ACTTGCCG -0.15593696981
ACTTGCCT 0.0530196272137
ACTTGCGA 0.030876089486
ACTTGCGC 0.0588859994479
ACTTGCGG -0.0915271571436
ACTTGCTA -0.184985590443
ACTTGCTC -0.0792502222661
ACTTGCTG -0.155841483091
ACTTGGAA 0.126623368182

ACTTGGAC 0.0722560399808
ACTTGGAG -0.125
ACTTGGAT 0.294007552306
ACTTGGCA -0.318894375857
ACTTGGCC -0.222271604938
ACTTGGCG 0.160678609849
ACTTGGCT -0.129275584615
ACTTGGGA 0.0136180700714
ACTTGGGC -0.353006519814
ACTTGGGG -0.0290136998242
ACTTGGTA -0.0610545841703
ACTTGGTC -0.23922085048
ACTTGGTG -0.28412220357
ACTTGTAA -0.00382482226851
ACTTGTAC -0.127121746027
ACTTGTAG -0.174024449764
ACTTGTAT 0.0331069609508
ACTTGTCA -0.0122862192364
ACTTGTCC -0.286010967689
ACTTGTCG -0.267157750343
ACTTGTCT -0.144548895661
ACTTGTGA -0.0816920626297
ACTTGTGC -0.188826202152
ACTTGTGG -0.169665739232
ACTTGTTA -0.126231604027
ACTTGTTC -0.167792302106
ACTTGTTG -0.0691062265031
ACTTTAAA 0.109774638302
ACTTTAAC -0.00375546612817
ACTTTAAG -0.0412577663615
ACTTTAAT 0.125072276379
ACTTTACA -0.0481453126544
ACTTTACC -0.0785472787547
ACTTTACG 0.0981462379963
ACTTTACT -0.0503988442161
ACTTTAGA 0.0159471119015
ACTTTAGC -0.0924645317088
ACTTTAGG -0.138427188923
ACTTTATA 0.105916156227
ACTTTATC -0.0940745131617
ACTTTATG -0.0393333507651
ACTTTCAA 0.0217439309633
ACTTTCAC -0.0368504248172
ACTTTCAG -0.0514912905147
ACTTTCAT -0.140583408798
ACTTTCCA -0.0337875728212
ACTTTCCC 0.0241642816672
ACTTTCCG -0.0403137280731
ACTTTCCT -0.212351488744
ACTTTCGA 0.0478289863663
ACTTTCGC -0.0730082485429
ACTTTCGG -0.0257647006834
ACTTTCTA 0.0494065346563
ACTTTCTC 0.0637250059562

ACTTTCTG -0.0162588397999
ACTTTGAA -0.062276339766
ACTTTGAC -0.266169790057
ACTTTGAG -0.0852170143176
ACTTTGAT 0.135927534758
ACTTTGCA 0.0225874051329
ACTTTGCC -0.298829339143
ACTTTGCG 0.0024973105886
ACTTTGCT -0.101790574652
ACTTTGGA -0.027021910547
ACTTTGGC -0.117276633575
ACTTTGGG 0.00192059041448
ACTTTGTA -0.0333898658587
ACTTTGTC -0.173554853015
ACTTTGTG -0.0217274545634
ACTTTTAA 0.000158354277745
ACTTTTAC 0.0581218310985
ACTTTTAG -0.134277780522
ACTTTTAT 0.130683439812
ACTTTTCA -0.0594002876527
ACTTTTCC -0.151594759459
ACTTTTCG 0.0952856914561
ACTTTTCT -0.107318221174
ACTTTTGA -0.00731335260703
ACTTTTGC -0.11929323366
ACTTTTGG -0.176309344304
ACTTTTTA 0.0678816846571
ACTTTTTC 0.0996317152956
ACTTTTTG -0.0631318706858
AGAAAAAA 0.0222495024189
AGAAAAAC -0.12201016703
AGAAAAAG -0.00218935684919
AGAAAAAT 0.227074139937
AGAAAACA 0.0788175920437
AGAAAACC 0.0192910382226
AGAAAACG -0.11031772551
AGAAAACT -0.163471314452
AGAAAAGA 0.183593492722
AGAAAAGC 0.0313371314409
AGAAAAGG -0.140137420835
AGAAAATA 0.146863816988
AGAAAATC 0.478374936881
AGAAAATG 0.18401784028
AGAAACAA 0.0111997431591
AGAAACAC -0.0443624424331
AGAAACAG -0.0139629849173
AGAAACAT 0.0991076837477
AGAAACCA 0.0688386108778
AGAAACCC -0.141631730481
AGAAACCG -0.0776615831518
AGAAACCT -0.00537287743391
AGAAACGA 0.0809471667874
AGAAACGC 0.00683633357252
AGAAACGG -0.0543803179823

AGAAACTA 0.12244853486
AGAAACTC -0.171962403044
AGAAACTG -0.0669969896588
AGAAAGAA 0.211775610542
AGAAAGAC -0.0490999837311
AGAAAGAG -0.0207044758423
AGAAAGAT 0.203278337614
AGAAAGCA -0.15917312821
AGAAAGCC -0.135163398693
AGAAAGCG -0.0879899458878
AGAAAGCT -0.132456815772
AGAAAGGA 0.0279764066654
AGAAAGGC -0.138482853224
AGAAAGGG -0.181217388442
AGAAAGTA 0.25347870934
AGAAAGTC 0.131439861694
AGAAAGTG -0.0977447978105
AGAAATAA 0.229153167812
AGAAATAC 0.130162857932
AGAAATAG 0.14675248364
AGAAATAT 0.363448335474
AGAAATCA 0.377451222524
AGAAATCC 0.457439019075
AGAAATCG 0.293865065767
AGAAATCT 0.460943152267
AGAAATGA -0.0948774570337
AGAAATGC 0.00814289657577
AGAAATGG -0.0405198088126
AGAAATTA 0.231202728949
AGAAATTC 0.139870410995
AGAAATTG 0.108509088215
AGAACAAA -0.0158538057916
AGAACAAC -0.217160712262
AGAACAAG -0.104100810037
AGAACAAT 0.148738250548
AGAACACA -0.0780737720902
AGAACACC -0.186464341232
AGAACACG -0.0511288527857
AGAACACT -0.0878518189203
AGAACAGA 0.129835377794
AGAACAGC -0.193969498911
AGAACAGG -0.237687726943
AGAACATA 0.0775510988167
AGAACATC 0.0280082987552
AGAACATG 0.0566422973062
AGAACCAA 0.00541175437441
AGAACCAC 0.0209307558893
AGAACCAG -0.0848929560255
AGAACCAT -0.166559232295
AGAACCCA -0.0635332469449
AGAACCCC -0.15820134567
AGAACCCG -0.211628460955
AGAACCCT -0.292615085831
AGAACCGA 0.177824979692

AGAACCGC -0.229240377633
AGAACCGG -0.277837172008
AGAACCTA -0.076421193323
AGAACCTC -0.168950972161
AGAACCTG -0.300361347443
AGAACGAA 0.0727823875142
AGAACGAC -0.158414504129
AGAACGAG -0.16625512371
AGAACGAT -0.0326543941689
AGAACGCA -0.125011661536
AGAACGCC -0.205299575836
AGAACGCG -0.128170938483
AGAACGCT -0.125708061002
AGAACGGA 0.0239492685195
AGAACGGC -0.217387725537
AGAACGGG -0.0365442665435
AGAACGTA -0.104220180465
AGAACGTC 0.0169822942513
AGAACGTG -0.168680682461
AGAACTAA 0.0506899163538
AGAACTAC 0.0109344045483
AGAACTAG 0.0438072185997
AGAACTAT -0.0480718566818
AGAACTCA 0.0654130716371
AGAACTCC -0.202588235294
AGAACTCG -0.115800910811
AGAACTCT -0.150440421131
AGAACTGA 0.0469556753301
AGAACTGC -0.0287120725704
AGAACTGG -0.0212260914904
AGAACTTA -0.0462029901864
AGAACTTC -0.00880601520266
AGAACTTG -0.151956104252
AGAAGAAA -0.0897227984677
AGAAGAAC -0.230230452675
AGAAGAAG -0.117281045752
AGAAGAAT 0.251200732207
AGAAGACA 0.0228893750418
AGAAGACC -0.252248213125
AGAAGACG -0.10057904094
AGAAGACT -0.101958605019
AGAAGAGA 0.0741426815078
AGAAGAGC -0.177221458926
AGAAGAGG -0.260711620352
AGAAGATA 0.271276537355
AGAAGATC 0.402659772992
AGAAGATG -0.0353822257349
AGAAGCAA -0.102698572267
AGAAGCAC -0.187970234273
AGAAGCAG -0.152304751835
AGAAGCAT -0.043869776746
AGAAGCCA -0.266716148655
AGAAGCCC -0.375117260788
AGAAGCCG -0.252411560679

AGAAGCCT -0.324313609313
AGAAGCGA 0.0306785989278
AGAAGCGC -0.211678491668
AGAAGCGG -0.276378208946
AGAAGCTA -0.24389336928
AGAAGCTC -0.213695473251
AGAAGCTG -0.0320838889163
AGAAGGAA -0.0187662341436
AGAAGGAC -0.338742949942
AGAAGGAG -0.236700901969
AGAAGGAT 0.165977917202
AGAAGGCA -0.106369038107
AGAAGGCC -0.309076941397
AGAAGGCG -0.18024691358
AGAAGGCT -0.231253848567
AGAAGGGA -0.141758228991
AGAAGGGC -0.249973938187
AGAAGGGG -0.133111111111
AGAAGGTA -0.127491168336
AGAAGGTC -0.193981118373
AGAAGGTG -0.234264928567
AGAAGTAA 0.0224064039051
AGAAGTAC -0.236641367994
AGAAGTAG -0.14509513435
AGAAGTAT 0.244811355019
AGAAGTCA -0.219838487577
AGAAGTCC -0.0458819073964
AGAAGTCG -0.136333948056
AGAAGTCT 0.0285255618671
AGAAGTGA 0.00487242214875
AGAAGTGC -0.267731940947
AGAAGTGG -0.122267219808
AGAAGTTA -0.00528967778335
AGAAGTTC -0.0121320260792
AGAAGTTG -0.105878511494
AGAATAAA 0.0527784783304
AGAATAAC 0.148563296454
AGAATAAG 0.14083797016
AGAATAAT 0.247191027559
AGAATACA 0.128518297909
AGAATACC -0.134975520868
AGAATACG 0.120182180059
AGAATACT 0.0538979999561
AGAATAGA -0.0246151313081
AGAATAGC -0.235150333898
AGAATAGG -0.0106780609893
AGAATATA 0.290401406302
AGAATATC 0.432139015127
AGAATATG 0.288674306036
AGAATCAA 0.371608094954
AGAATCAC 0.334880072058
AGAATCAG 0.400944587148
AGAATCAT 0.382162616084
AGAATCCA 0.437592462112

AGAATCCC 0.426947830256
AGAATCCG 0.480637194369
AGAATCCT 0.43471154824
AGAATCGA 0.334079782213
AGAATCGC 0.384020745863
AGAATCGG 0.372404224718
AGAATCTA 0.473523019166
AGAATCTC 0.460646782793
AGAATCTG 0.482180058983
AGAATGAA -0.141433892534
AGAATGAC -0.0525227725074
AGAATGAG -0.0935984706522
AGAATGAT 0.15637702846
AGAATGCA 0.0958479217817
AGAATGCC -0.114144724418
AGAATGCG 0.186865311696
AGAATGCT -0.268460552953
AGAATGGA -0.0424765232584
AGAATGGC -0.175877907838
AGAATGGG -0.229723762684
AGAATGTA 0.0187737381721
AGAATGTC -0.107821186129
AGAATGTG 0.130565435026
AGAATTAA 0.137560472448
AGAATTAC 0.21571000461
AGAATTAG 0.115155521334
AGAATTAT 0.17950714607
AGAATTCA 0.147718390888
AGAATTCC 0.22083448346
AGAATTCG 0.262959626923
AGAATTCT 0.245417734702
AGAATTGA -0.101646029551
AGAATTGC 0.131041796813
AGAATTGG 0.0550533491405
AGAATTTA 0.224661136374
AGAATTTC 0.255312427688
AGAATTTG 0.343783617643
AGACAAAA -0.116884418607
AGACAAAC -0.211778458296
AGACAAAG -0.183541031227
AGACAAAT 0.0966091845481
AGACAACA -0.170677713994
AGACAACC -0.297940450254
AGACAACG -0.248677509987
AGACAACT -0.193863143124
AGACAAGA -0.0290950717934
AGACAAGC -0.106738210891
AGACAAGG -0.1965817242
AGACAATA 0.149807505121
AGACAATC 0.318572487034
AGACAATG -0.145172088327
AGACACAA -0.0800660050666
AGACACAC -0.0769796212469
AGACACAG -0.0998588095847

AGACACAT -0.135170630366
AGACACCA -0.0764595502909
AGACACCC -0.287020264107
AGACACCG -0.253239596249
AGACACCT -0.149280348549
AGACACGA -0.0457527639359
AGACACGC -0.10720001976
AGACACGG 0.132475465982
AGACACTA -0.122736383442
AGACACTC -0.23296151053
AGACACTG -0.10236955947
AGACAGAA -0.0502253057098
AGACAGAC -0.030835500414
AGACAGAG -0.0889413508056
AGACAGAT 0.156276756899
AGACAGCA -0.164482207698
AGACAGCC -0.202127265544
AGACAGCG -0.138246850169
AGACAGCT -0.231922355033
AGACAGGA -0.194486564996
AGACAGGC -0.276538552023
AGACAGGG -0.140967621062
AGACAGTA 0.0511339037961
AGACAGTC -0.355176139483
AGACAGTG -0.338603358607
AGACATAA -0.166648267367
AGACATAC -0.0840246454615
AGACATAG -0.0313525983346
AGACATAT 0.0134772442864
AGACATCA 0.0347742317953
AGACATCC -0.116968605175
AGACATCG -0.0336919584938
AGACATCT -0.0876440079613
AGACATGA 0.032835517794
AGACATGC 0.0222562515094
AGACATGG 0.0278281967699
AGACATTA 0.119769587262
AGACATTC -0.0966004677989
AGACATTG -0.0715916749948
AGACCAAA 0.0185487057894
AGACCAAC -0.258292733777
AGACCAAG -0.232425596515
AGACCAAT -0.24337149547
AGACCACA 0.00940101404849
AGACCACC -0.383081882469
AGACCACG -0.254322714134
AGACCACT -0.180256486237
AGACCAGA -0.20328843601
AGACCAGC -0.117873660725
AGACCAGG -0.243830674676
AGACCATA 0.0171352546056
AGACCATC -0.191629907609
AGACCATG -0.274633744856
AGACCCAA -0.143384309788

AGACCCAC -0.331172237583
AGACCCAG -0.195946873028
AGACCCAT -0.223855644287
AGACCCCA -0.28203374373
AGACCCCC -0.411396363038
AGACCCCG -0.129371992732
AGACCCCT -0.248244821606
AGACCCGA -0.274840083071
AGACCCGC -0.18421603
AGACCCGG -0.295138717447
AGACCCTA -0.0578008146853
AGACCCTC -0.265070636298
AGACCCTG -0.310155924243
AGACCGAA -0.100021400234
AGACCGAC -0.210255734971
AGACCGAG -0.0405895428065
AGACCGAT -0.0210734593515
AGACCGCA -0.088749531521
AGACCGCC -0.294623018812
AGACCGCG -0.144199294363
AGACCGCT -0.28624691358
AGACCGGA -0.0265333004034
AGACCGGC -0.106336741246
AGACCGGG -0.269619058592
AGACCGTA 0.0479784405736
AGACCGTC -0.195202216775
AGACCGTG -0.349821631363
AGACCTAA 0.0690053568684
AGACCTAC -0.14507437953
AGACCTAG -0.221382107444
AGACCTAT -0.109206944993
AGACCTCA -0.235468481666
AGACCTCC -0.20645751634
AGACCTCG -0.193846372796
AGACCTCT -0.266652142338
AGACCTGA -0.0982629455833
AGACCTGC -0.18079974977
AGACCTGG -0.0733667232203
AGACCTTA -0.204441244359
AGACCTTC -0.347839445213
AGACCTTG -0.141527686189
AGACGAAA 0.180089491959
AGACGAAC 0.0842546453009
AGACGAAG -0.0364578036698
AGACGAAT 0.112398745431
AGACGACA -0.090634032731
AGACGACC -0.341264171545
AGACGACG -0.200270520904
AGACGACT -0.0813594771242
AGACGAGA 0.0749685122987
AGACGAGC -0.290336168164
AGACGAGG -0.182030178326
AGACGATA 0.0466331556085
AGACGATC 0.268454084523

AGACGATG -0.0455041873841
AGACGCAA 0.0560440404839
AGACGCAC -0.129737109659
AGACGCAG -0.22510681061
AGACGCAT -0.0965136124419
AGACGCCA -0.339659052957
AGACGCCC -0.30549118344
AGACGCCG -0.270510288066
AGACGCCT -0.299764232459
AGACGCGA 0.0715388224835
AGACGCGC -0.160631696377
AGACGCGG -0.240267970306
AGACGCTA -0.0815473025779
AGACGCTC -0.220875104943
AGACGCTG -0.0420624546115
AGACGGAA -0.148893772532
AGACGGAC -0.328050947867
AGACGGAG -0.225523122767
AGACGGAT 0.280901602429
AGACGGCA -0.151654320988
AGACGGCC -0.253611975351
AGACGGCG -0.195241387257
AGACGGCT -0.31119560208
AGACGGGA -0.128435185185
AGACGGGC -0.249630374099
AGACGGGG -0.18679542332
AGACGGTA -0.249468462084
AGACGGTC -0.25874904678
AGACGGTG -0.194656843377
AGACGTAA 0.0657446283033
AGACGTAC -0.217718812548
AGACGTAG -0.0739753967378
AGACGTAT -0.0816134702831
AGACGTCA 0.132159871786
AGACGTCC -0.288654618474
AGACGTCG 0.00667687545281
AGACGTCT 0.0186006527175
AGACGTGA -0.101784925744
AGACGTGC -0.18769699593
AGACGTGG -0.127314929132
AGACGTTA -0.297094217024
AGACGTTC -0.218594045025
AGACGTTG -0.143158172001
AGACTAAA 0.0296732671829
AGACTAAC -0.358831662232
AGACTAAG 0.145250913761
AGACTAAT -0.0847137149004
AGACTACA -0.229535701568
AGACTACC -0.104483601699
AGACTACG -0.0684366510249
AGACTACT 0.0450873854111
AGACTAGA 0.138551276277
AGACTAGC -0.301587140214
AGACTAGG -0.144292981676

AGACTATA 0.0957985268694
AGACTATC -0.0198673957383
AGACTATG -0.0828613141891
AGACTCAA -0.0893509278063
AGACTCAC -0.373419339099
AGACTCAG -0.0611259288529
AGACTCAT -0.21087298844
AGACTCCA -0.209528265107
AGACTCCC -0.0835185276639
AGACTCCG -0.0404050382811
AGACTCCT -0.159946896434
AGACTCGA -0.363931412894
AGACTCGC -0.0295202344496
AGACTCGG -0.19759122085
AGACTCTA -0.117134324716
AGACTCTC -0.197155274595
AGACTCTG -0.202803043288
AGACTGAA 0.148973602778
AGACTGAC -0.267940614919
AGACTGAG -0.134560401267
AGACTGAT 0.102201670753
AGACTGCA 0.0738352243837
AGACTGCC -0.291340853987
AGACTGCG -0.0498496360757
AGACTGCT -0.23251983395
AGACTGGA -0.0865709644848
AGACTGGC -0.186100684841
AGACTGGG -0.227939361019
AGACTGTA -0.146747987555
AGACTGTC -0.155707025805
AGACTGTG -0.153273736027
AGACTTAA 0.138330451963
AGACTTAC -0.062002237299
AGACTTAG -0.236267507676
AGACTTAT -0.19485493837
AGACTTCA -0.200822132867
AGACTTCC -0.250292650762
AGACTTCG -0.252684631889
AGACTTGA -0.0887160731249
AGACTTGC -0.174496634547
AGACTTGG -0.205493062496
AGACTTTA -0.0934014208301
AGACTTTC -0.222614521094
AGACTTTG -0.180259171442
AGAGAAAA 0.111888193163
AGAGAAAC -0.185494540341
AGAGAAAG -0.0800066828411
AGAGAAAT 0.234917341764
AGAGAACA -0.0873026019496
AGAGAACC 0.0108654279197
AGAGAACG -0.178629116601
AGAGAACT 0.0644501910421
AGAGAAGA -0.0268927855986
AGAGAAGC -0.0597438490758

AGAGAAGG -0.270712254684
AGAGAATA 0.282804759345
AGAGAATC 0.477595556434
AGAGAATG 0.116387488559
AGAGACAA -0.0051019518743
AGAGACAC -0.185590864327
AGAGACAG -0.0525303473162
AGAGACAT -0.0564938627258
AGAGACCA -0.224577145065
AGAGACCC -0.327591787917
AGAGACCG -0.256306209909
AGAGACCT -0.140480908357
AGAGACGA -0.190863843891
AGAGACGC -0.174822158383
AGAGACGG -0.331249019801
AGAGACTA -0.103451777207
AGAGACTC -0.145824255628
AGAGACTG -0.128515779336
AGAGAGAA -0.0323783650284
AGAGAGAC -0.308168482208
AGAGAGAG -0.248365206125
AGAGAGAT 0.203156916014
AGAGAGCA -0.107903667246
AGAGAGCC -0.345265890716
AGAGAGCG -0.117750670815
AGAGAGCT -0.155736670234
AGAGAGGA 0.00399081835747
AGAGAGGC -0.387846622233
AGAGAGGG -0.137376906318
AGAGAGTA -0.143521033255
AGAGAGTC -0.244218392507
AGAGAGTG -0.152285451149
AGAGATAA 0.113702729795
AGAGATAC 0.237428268418
AGAGATAG 0.11739863147
AGAGATAT 0.365054666403
AGAGATCA 0.102891940548
AGAGATCC 0.332420495983
AGAGATCG 0.330744344562
AGAGATCT 0.400022619905
AGAGATGA 0.0855292403438
AGAGATGC -0.07589298685
AGAGATGG -0.182027596224
AGAGATTA 0.28054421571
AGAGATTC 0.453528104252
AGAGATTG 0.290732078925
AGAGCAAA -0.0779302089779
AGAGCAAC -0.1239872412
AGAGCAAG -0.048916745088
AGAGCAAT 0.123057037476
AGAGCACA 0.0166061302543
AGAGCACC -0.238065061259
AGAGCACG -0.0915037521899
AGAGCACT -0.0461814988612

AGAGCAGA -0.101808641975
AGAGCAGC -0.057904210018
AGAGCAGG -0.221981118373
AGAGCATA 0.0685804773873
AGAGCATC -0.0708918687258
AGAGCATG -0.208621776529
AGAGCCAA -0.23133697704
AGAGCCAC -0.288022689449
AGAGCCAG -0.178097312999
AGAGCCAT -0.174015491804
AGAGCCCA -0.311811864323
AGAGCCCC -0.249753110804
AGAGCCCG -0.295009264691
AGAGCCCT -0.215254439142
AGAGCCGA -0.151512202491
AGAGCCGC -0.320727185083
AGAGCCGG -0.273380623995
AGAGCCTA -0.00235764775609
AGAGCCTC -0.316660906203
AGAGCCTG -0.231644951225
AGAGCGAA -0.0741975308642
AGAGCGAC -0.15198265373
AGAGCGAG -0.0651811242269
AGAGCGAT 0.0958226305737
AGAGCGCA -0.00309008760333
AGAGCGCC -0.285411602058
AGAGCGCG 0.0259830073108
AGAGCGCT -0.0888773645478
AGAGCGGA -0.0419791761449
AGAGCGGC -0.280137174211
AGAGCGGG -0.278750214223
AGAGCGTA -0.0352165462421
AGAGCGTC -0.137476064282
AGAGCGTG -0.21136571902
AGAGCTAA -0.00164601281362
AGAGCTAC -0.192179524054
AGAGCTAG -0.00394336050054
AGAGCTAT -0.0781277691103
AGAGCTCA -0.276946502058
AGAGCTCC -0.331259566484
AGAGCTCG 0.0278108675061
AGAGCTCT -0.279289064429
AGAGCTGA -0.178687219653
AGAGCTGC -0.0985508200679
AGAGCTGG -0.119249491008
AGAGCTTA -0.246750907771
AGAGCTTC -0.265771862764
AGAGCTTG -0.254567450128
AGAGGAAA 0.0799809403165
AGAGGAAC -0.31428337522
AGAGGAAG -0.257682264057
AGAGGAAT 0.184213703923
AGAGGACA -0.0732590177611
AGAGGACC -0.184912638834

AGAGGACG -0.161474903666
AGAGGACT -0.184254717398
AGAGGAGA -0.057520504127
AGAGGAGC -0.220547325103
AGAGGAGG -0.129136687513
AGAGGATA 0.238290083207
AGAGGATC 0.362827422893
AGAGGATG 0.0432638477244
AGAGGCAA 0.0270320921651
AGAGGCAC -0.246487141924
AGAGGCAG -0.26859782574
AGAGGCAT -0.0160239522273
AGAGGCCA -0.278245023015
AGAGGCCC -0.194658639906
AGAGGCCG -0.119629923387
AGAGGCCT -0.301328977903
AGAGGCGA -0.125320955955
AGAGGCGC -0.318603489389
AGAGGCGG -0.222705653021
AGAGGCTA -0.0540181697216
AGAGGCTC -0.246029578803
AGAGGCTG -0.267282498184
AGAGGGAA -0.0344585132285
AGAGGGAC -0.284498318493
AGAGGGAG -0.237715203607
AGAGGGAT 0.0892308283387
AGAGGGCA -0.221509168662
AGAGGGCC -0.243065701912
AGAGGGCG -0.211026361957
AGAGGGCT -0.356917152167
AGAGGGGA -0.0500954826814
AGAGGGGC -0.0532473644305
AGAGGGGG -0.306311068769
AGAGGGTA 0.00575160170267
AGAGGGTC -0.31827025834
AGAGGGTG -0.305400556351
AGAGGTAA 0.0813107861863
AGAGGTAC -0.264371789806
AGAGGTAG -0.0934127874206
AGAGGTAT -0.109534019564
AGAGGTCA -0.366024849831
AGAGGTCC -0.351654448072
AGAGGTCG -0.131561328702
AGAGGTGA -0.00375382791663
AGAGGTGC -0.103952664329
AGAGGTGG -0.188036310821
AGAGGTTA -0.16805644732
AGAGGTTC -0.230800250181
AGAGGTTG -0.179272331155
AGAGTAAA 0.0154119739182
AGAGTAAC -0.229237166992
AGAGTAAG 0.189975797688
AGAGTAAT 0.204436431096
AGAGTACA 0.0143637499455

AGAGTACC -0.236024655282
AGAGTACG -0.167522802858
AGAGTACT -0.158651761852
AGAGTAGA -0.0302310986629
AGAGTAGC -0.119130065973
AGAGTAGG -0.228850627794
AGAGTATA 0.190142809408
AGAGTATC 0.370207603911
AGAGTATG -0.0874624853646
AGAGTCAA -0.214602437673
AGAGTCAC -0.294553333876
AGAGTCAG -0.160214701961
AGAGTCAT 0.0553411880929
AGAGTCCA -0.19582441701
AGAGTCCC -0.216225127088
AGAGTCCG -0.325858815268
AGAGTCCT -0.344804884873
AGAGTCGA 0.0261478261824
AGAGTCGC -0.303818940177
AGAGTCGG -0.276119099492
AGAGTCTA -0.308633097966
AGAGTCTC -0.0211698029338
AGAGTCTG -0.291055918664
AGAGTGAA -0.0930384287467
AGAGTGAC -0.123052362151
AGAGTGAG -0.14319186506
AGAGTGAT 0.0857977123537
AGAGTGCA 0.0951011869307
AGAGTGCC -0.085838629304
AGAGTGCG -0.077569952266
AGAGTGCT 0.0159923202292
AGAGTGGA 0.102066457572
AGAGTGGC -0.269835368475
AGAGTGGG -0.28935001641
AGAGTGTA 0.0187163133962
AGAGTGTC -0.163878873688
AGAGTGTG -0.103584731334
AGAGTTAA -0.0219763331797
AGAGTTAC 0.0585578632816
AGAGTTAG 0.0799304046192
AGAGTTAT -0.0168538346433
AGAGTTCA 0.0361178504496
AGAGTTCC -0.202493430475
AGAGTTCG -0.178448871772
AGAGTTGA -0.0980159767611
AGAGTTGC -0.113525615699
AGAGTTGG -0.0434451042369
AGAGTTTA -0.023499418209
AGAGTTTC -0.0845546475788
AGAGTTTG -0.0740066537852
AGATAAAA 0.137599069134
AGATAAAC -0.092913282873
AGATAAAG -0.171356439176
AGATAAAT -0.0160942284896

AGATAACA 0.172188192935
AGATAACC 0.18106286229
AGATAACG 0.126946377464
AGATAACT 0.0472404224718
AGATAAGA -0.0267322004874
AGATAAGC 0.157797858585
AGATAAGG -0.00155578604238
AGATAATA 0.110410704168
AGATAATC 0.357527058772
AGATAATG 0.138800111569
AGATACAA 0.0295423609739
AGATACAC 0.221681595644
AGATACAG 0.213670991679
AGATACAT 0.123092583011
AGATACCA 0.111067202354
AGATACCC 0.358726993347
AGATACCG 0.318508564206
AGATACCT 0.169850051593
AGATACGA 0.298932505186
AGATACGC 0.439012056149
AGATACGG 0.385080482662
AGATACTA 0.109774638302
AGATACTC 0.379075281565
AGATACTG 0.333097323761
AGATAGAA 0.096226895847
AGATAGAC -0.351269690386
AGATAGAG -0.0561087208018
AGATAGAT 0.310651715735
AGATAGCA 0.11690758793
AGATAGCC -0.0280082987552
AGATAGCG 0.308978251711
AGATAGCT 0.0667817099674
AGATAGGA 0.164001278457
AGATAGGC -0.0600162712652
AGATAGGG -0.091829500385
AGATAGTA 0.0873713330404
AGATAGTC -0.00519902730688
AGATAGTG 0.183151660849
AGATATAA 0.223937646187
AGATATAC 0.238180311313
AGATATAG 0.190331672308
AGATATAT 0.328667219598
AGATATCA 0.418672341576
AGATATCC 0.47929772057
AGATATCG 0.460023820501
AGATATCT 0.476239108282
AGATATGA 0.320507605364
AGATATGC 0.40662139219
AGATATGG 0.351754667589
AGATATTA 0.379793122485
AGATATTC 0.458728512152
AGATATTG 0.409830621968
AGATCAAA 0.177748073694
AGATCAAC 0.106025928121

AGATCAAG 0.175154359101
AGATCAAT 0.171413420888
AGATCACA 0.183113240686
AGATCACC 0.297538971133
AGATCACG 0.258296010889
AGATCACT 0.0816323565732
AGATCAGA 0.322680981506
AGATCAGC 0.0174611015065
AGATCAGG 0.230310925615
AGATCATA 0.15870723343
AGATCATC 0.0783795563175
AGATCATG 0.122692045929
AGATCCAA 0.171945142591
AGATCCAC 0.278783432365
AGATCCAG 0.207622797205
AGATCCAT 0.191925179477
AGATCCCA 0.210650414772
AGATCCCC 0.368890768547
AGATCCCG 0.345998285706
AGATCCCT 0.304576293348
AGATCCGA 0.293302267887
AGATCCGC 0.377436694609
AGATCCGG 0.298489096799
AGATCCTA 0.308167594032
AGATCCTC 0.376451733298
AGATCCTG 0.399719611233
AGATCGAA 0.177679531933
AGATCGAC 0.143637696722
AGATCGAG 0.0347675031285
AGATCGAT 0.174374210045
AGATCGCA 0.407672166782
AGATCGCC 0.333890425695
AGATCGCG 0.460364941235
AGATCGCT 0.350726689938
AGATCGGA 0.301830517339
AGATCGGC 0.056467862835
AGATCGGG 0.265486069947
AGATCGTA 0.261915739094
AGATCGTC 0.333745441867
AGATCGTG 0.325351303961
AGATCTAA 0.281024281543
AGATCTAC 0.37747535621
AGATCTAG 0.356665349404
AGATCTAT 0.346025590357
AGATCTCA 0.401998611606
AGATCTCC 0.430291692775
AGATCTCG 0.415245555969
AGATCTGA 0.284616904609
AGATCTGC 0.390709398108
AGATCTGG 0.350713827002
AGATCTTA 0.336689608993
AGATCTTC 0.439136448967
AGATCTTG 0.340040871828
AGATGAAA 0.155632775683

AGATGAAC 0.0547426379789
AGATGAAG -0.125688918329
AGATGAAT 0.031393372365
AGATGACA -0.0193088761553
AGATGACC -0.041019569102
AGATGACG -0.0138449801313
AGATGACT -0.155472976507
AGATGAGA 0.114132756091
AGATGAGC -0.27360260715
AGATGAGG -0.259506429603
AGATGATA 0.131391468528
AGATGATC 0.0878202595008
AGATGATG -0.00454592856045
AGATGCAA 0.116737565445
AGATGCAC -0.166865205037
AGATGCAG 0.0381812394703
AGATGCAT -0.0944816806557
AGATGCCA 0.13120886784
AGATGCCC -0.0641927817259
AGATGCCG -0.061143408319
AGATGCCT -0.269591142634
AGATGCGA -0.0185201865384
AGATGCGC -0.10291720541
AGATGCGG 0.0662230218633
AGATGCTA -0.0655891796086
AGATGCTC 0.201128819026
AGATGCTG 0.0496286303467
AGATGGAA -0.168409844305
AGATGGAC -0.325612097587
AGATGGAG -0.216763979666
AGATGGAT 0.100024149817
AGATGGCA -0.179480326307
AGATGGCC -0.224119302587
AGATGGCG 0.0591387851757
AGATGGCT -0.161275187673
AGATGGGA 0.00598985951506
AGATGGGC -0.233301576605
AGATGGGG -0.324952655366
AGATGGTA 0.0574477358244
AGATGGTC -0.0413510788817
AGATGGTG -0.10589281149
AGATGTAA 0.00349686923423
AGATGTAC -0.0213533776812
AGATGTAG 0.0335930554184
AGATGTAT 0.0443435561431
AGATGTCA 0.0639887813124
AGATGTCC -0.0375648308224
AGATGTCG 0.0402031002571
AGATGTGA -0.0613993288103
AGATGTGC 0.136619063531
AGATGTGG -0.16277544524
AGATGTTA 0.0682187322941
AGATGTTC 0.031965575534
AGATGTTG 0.0405991349975

AGATTAAA 0.0984309851379
AGATTAAC 0.0760957680182
AGATTAAG 0.131251348744
AGATTAAT 0.191557443632
AGATTACA 0.485663790643
AGATTACC 0.446596224296
AGATTACG 0.487249994511
AGATTACT 0.429086339401
AGATTAGA 0.23434258619
AGATTAGC -0.00211090610608
AGATTAGG 0.164284616567
AGATTATA 0.305508902501
AGATTATC 0.390009152587
AGATTATG 0.331901980638
AGATTCAA 0.255044818619
AGATTCAC 0.312206732194
AGATTCAG 0.191537489286
AGATTCAT 0.225817251751
AGATTCCA 0.425015617509
AGATTCCC 0.496484244476
AGATTCCG 0.462486016881
AGATTCCT 0.457351851583
AGATTCGA 0.38307849107
AGATTCGC 0.487508898348
AGATTCGG 0.406719160868
AGATTCTA 0.450529542516
AGATTCTC 0.487743968034
AGATTCTG 0.472565484579
AGATTGAA 0.0238737233594
AGATTGAC -0.0677553294255
AGATTGAG 0.0565133153307
AGATTGAT 0.149804599538
AGATTGCA 0.462452459745
AGATTGCC 0.446723228684
AGATTGCG 0.493753979231
AGATTGCT 0.421252387539
AGATTGGA 0.150665563568
AGATTGGC 0.150376517596
AGATTGGG 0.229174898745
AGATTGTA 0.334313047487
AGATTGTC 0.395962692313
AGATTGTG 0.401199806801
AGATTTAA 0.259504888644
AGATTTAC 0.35527388126
AGATTTAG 0.227380745001
AGATTTAT 0.190594195262
AGATTTCA 0.474387472831
AGATTTCC 0.496454367824
AGATTTCG 0.488081298215
AGATTTGA 0.388768139805
AGATTTGC 0.466280708381
AGATTTGG 0.425823369605
AGATTTTA 0.396881833096
AGATTTTC 0.486135809787

AGATTTTG 0.467027443232
AGCAAAAA -0.0579811549585
AGCAAAAC -0.11169275849
AGCAAAAG -0.0731337877441
AGCAAAAT 0.262250664652
AGCAAACA -0.0357636830666
AGCAAACC -0.0499812608404
AGCAAACG -0.024565252132
AGCAAACT 0.0123642480702
AGCAAAGA 0.0939318096994
AGCAAAGC -0.200241426612
AGCAAAGG -0.134317341148
AGCAAATA 0.173839582758
AGCAAATC 0.419358447256
AGCAAATG -0.0828657765897
AGCAACAA 0.0853936338675
AGCAACAC -0.0193618492542
AGCAACAG -0.0887033927141
AGCAACAT -0.174835148874
AGCAACCA -0.00874058706009
AGCAACCC -0.201101180189
AGCAACCG -0.193005854886
AGCAACCT -0.226177748855
AGCAACGA -0.0908838945144
AGCAACGC -0.082486843044
AGCAACGG -0.172240686081
AGCAACTA -0.00109976319498
AGCAACTC -0.0156834098483
AGCAACTG -0.163162545139
AGCAAGAA -0.010464457455
AGCAAGAC -0.0712839506173
AGCAAGAG -0.0691445936798
AGCAAGAT 0.324218034954
AGCAAGCA -0.147564320078
AGCAAGCC -0.243775487679
AGCAAGCG 0.00714738132732
AGCAAGCT -0.250942340296
AGCAAGGA -0.113553294478
AGCAAGGC -0.114999012248
AGCAAGGG -0.122701305543
AGCAAGTA -0.144738069757
AGCAAGTC -0.189001345019
AGCAAGTG -0.0937724656053
AGCAATAA 0.036094605785
AGCAATAC 0.150507750643
AGCAATAG -0.0877384639522
AGCAATAT 0.299647083361
AGCAATCA 0.31395036115
AGCAATCC 0.421911018903
AGCAATCG 0.32820740052
AGCAATGA -0.073465498408
AGCAATGC -0.137066085694
AGCAATGG -0.243779230211
AGCAATTA 0.077389185273

AGCAATTC 0.132937689771
AGCAATTG 0.11213102905
AGCACAAA 0.128835155547
AGCACAAC -0.0259099664164
AGCACAAG -0.0411672045489
AGCACAAT 0.0178928187227
AGCACACA -0.0680037191463
AGCACACC -0.283234190378
AGCACACG -0.142355794823
AGCACACT -0.251584604212
AGCACAGA -0.148485183001
AGCACAGC -0.119838996444
AGCACAGG -0.18278109723
AGCACATA 0.00173318030596
AGCACATC 0.00425640519002
AGCACATG -0.111893197223
AGCACCAA -0.044663439373
AGCACCAC -0.33568677792
AGCACCAG -0.239432098765
AGCACCAT -0.128587105624
AGCACCCA -0.0630411782902
AGCACCCC -0.183839144607
AGCACCCG -0.201653495052
AGCACCCT -0.227601929123
AGCACCGA -0.0544580712761
AGCACCGC -0.0900211859755
AGCACCGG -0.191936092956
AGCACCTA -0.0892113771777
AGCACCTC -0.325489905635
AGCACCTG -0.251930136468
AGCACGAA 0.0471827043404
AGCACGAC -0.15495676321
AGCACGAG -0.169031666859
AGCACGAT 0.165601879295
AGCACGCA -0.033223324889
AGCACGCC -0.248509137192
AGCACGCG -0.0247310961294
AGCACGCT -0.213237472767
AGCACGGA -0.0568216815522
AGCACGGC -0.349532807708
AGCACGGG -0.207136547441
AGCACGTA -0.109942594185
AGCACGTC -0.222321002288
AGCACGTG -0.202834847886
AGCACTAA -0.0481789258059
AGCACTAC -0.016621387997
AGCACTAG -0.103753933045
AGCACTAT -0.106110441213
AGCACTCA 0.00147596329849
AGCACTCC -0.239004686797
AGCACTCG -0.127421975537
AGCACTGA 0.0345232606643
AGCACTGC -0.123642137252
AGCACTGG -0.133641716189

AGCACTTA -0.149511135647
AGCACTTC -0.263660337246
AGCACTTG -0.263724263576
AGCAGAAA -0.00232579200422
AGCAGAAC 0.0801801356781
AGCAGAAG -0.155619885368
AGCAGAAT 0.287498078992
AGCAGACA -0.0987218325705
AGCAGACC -0.192274401366
AGCAGACG -0.06589827475
AGCAGACT -0.0420645504736
AGCAGAGA 0.052257073077
AGCAGAGC -0.139826088978
AGCAGAGG -0.163665294925
AGCAGATA 0.227764299101
AGCAGATC 0.325880028475
AGCAGATG -0.110733770225
AGCAGCAA 0.111699330263
AGCAGCAC -0.133249914935
AGCAGCAG -0.0787441606433
AGCAGCAT -0.129891188042
AGCAGCCA -0.194382713001
AGCAGCCC -0.242988679825
AGCAGCCG -0.260139917695
AGCAGCCT -0.228620767222
AGCAGCGA 0.0157380907414
AGCAGCGC -0.0972229082627
AGCAGCGG -0.352211141829
AGCAGCTA -0.27808776088
AGCAGCTC -0.185985328571
AGCAGCTG -0.233822111449
AGCAGGAA -0.0900887814628
AGCAGGAC -0.278336397513
AGCAGGAG -0.370670704971
AGCAGGAT 0.22155216248
AGCAGGCA -0.236547505279
AGCAGGCC -0.293534426014
AGCAGGCG -0.124421205519
AGCAGGCT -0.163432098765
AGCAGGGA -0.224063009234
AGCAGGGC -0.183649445158
AGCAGGGG -0.0970280919533
AGCAGGTA -0.170126361656
AGCAGGTC -0.272154674629
AGCAGGTG -0.17678140886
AGCAGTAA -0.0803228391403
AGCAGTAC -0.0252660888033
AGCAGTAG -0.164910750051
AGCAGTAT 0.150379261894
AGCAGTCA -0.0706332718318
AGCAGTCC -0.145679012346
AGCAGTCG -0.285330428468
AGCAGTGA -0.0728639950085
AGCAGTGC -0.324478240832

AGCAGTGG -0.184678927862
AGCAGTTA -0.0409533658888
AGCAGTTC -0.131741093718
AGCAGTTG 0.0255384311401
AGCATAAA 0.0382679819273
AGCATAAC -0.0552234955762
AGCATAAG -0.29668592878
AGCATAAT 0.193867766914
AGCATACA -0.131387656559
AGCATACC -0.0184722444176
AGCATACG -0.130204793028
AGCATACT -0.141142188095
AGCATAGA -0.070331407122
AGCATAGC -0.222801125982
AGCATAGG 0.0640572309596
AGCATATA 0.205324211289
AGCATATC 0.362533959002
AGCATATG 0.130320552654
AGCATCAA -0.000695887147153
AGCATCAC -0.093357837084
AGCATCAG -0.00278271751301
AGCATCAT 0.106822309067
AGCATCCA -0.0968421745859
AGCATCCC -0.202876004214
AGCATCCG -0.0886188310839
AGCATCCT -0.0293784253164
AGCATCGA 0.0636482501126
AGCATCGC -0.130609053498
AGCATCGG -0.0127033524336
AGCATCTA 0.136006803813
AGCATCTC 0.129528090628
AGCATCTG -0.13758260335
AGCATGAA 0.0538273793093
AGCATGAC -0.341955252705
AGCATGAG -0.223528249704
AGCATGAT 0.18316581053
AGCATGCA -0.0671761568473
AGCATGCC -0.195886216603
AGCATGCG -0.0879273479443
AGCATGCT -0.101598117151
AGCATGGA 0.0159021380074
AGCATGGC -0.3068659602
AGCATGGG -0.24296846368
AGCATGTA 0.0692531198698
AGCATGTC -0.0811915150517
AGCATGTG -0.140322571794
AGCATTAA 0.0331639583128
AGCATTAC 0.0745151308239
AGCATTAG -0.0656364220093
AGCATTAT -0.00510027662517
AGCATTCA -0.0573417552341
AGCATTCC -0.180185009253
AGCATTCG -0.11772630493
AGCATTGA -0.131728706036

AGCATTGC -0.0166039544986
AGCATTGG -0.117662874984
AGCATTTA 0.0603073064173
AGCATTTC -0.109013118708
AGCATTTG -0.0608114782437
AGCCAAAA 0.0235715427193
AGCCAAAC -0.192661003556
AGCCAAAG -0.210808275317
AGCCAAAT 0.0527764054935
AGCCAACA -0.120923747277
AGCCAACC -0.114192652595
AGCCAACG -0.289674897119
AGCCAACT -0.093328026588
AGCCAAGA 0.0345768031959
AGCCAAGC -0.264237976776
AGCCAAGG -0.262135583966
AGCCAATA -0.128393842101
AGCCAATC 0.0737248122267
AGCCAATG -0.0789264271681
AGCCACAA -0.0839916900324
AGCCACAC -0.137635802469
AGCCACAG -0.221140858694
AGCCACAT -0.0313988890697
AGCCACCA -0.0212847702474
AGCCACCC -0.303754112594
AGCCACCG -0.129006205406
AGCCACCT -0.259990298707
AGCCACGA -0.019308818903
AGCCACGC -0.197795775923
AGCCACGG -0.363411409039
AGCCACTA -0.103964960811
AGCCACTC -0.269236889613
AGCCACTG -0.265595504631
AGCCAGAA 0.0119492104078
AGCCAGAC -0.12366422416
AGCCAGAG -0.191773794435
AGCCAGAT 0.0924338616652
AGCCAGCA -0.205661299209
AGCCAGCC -0.35136522008
AGCCAGCG -0.0483027359293
AGCCAGCT -0.329558671846
AGCCAGGA -0.0787528761011
AGCCAGGC -0.205150290646
AGCCAGGG -0.235945808023
AGCCAGTA -0.147660493827
AGCCAGTC -0.241298527443
AGCCAGTG -0.22991483775
AGCCATAA -0.0139931721882
AGCCATAC -0.203447955309
AGCCATAG -0.292702331962
AGCCATAT 0.105273633286
AGCCATCA -0.0402902677495
AGCCATCC -0.286267603702
AGCCATCG -0.174137061512

AGCCATGA -0.0494167041971
AGCCATGC -0.286278188937
AGCCATGG -0.217597726487
AGCCATTA -0.131070189609
AGCCATTC -0.216808631789
AGCCATTG -0.060406681191
AGCCCAAA -0.148287158214
AGCCCAAC -0.150173954081
AGCCCAAG -0.264476431959
AGCCCAAT -0.103049398749
AGCCCACA -0.275004501653
AGCCCACC -0.238054828676
AGCCCACG -0.166085693537
AGCCCACT -0.38206249764
AGCCCAGA -0.10440000416
AGCCCAGC -0.399404039289
AGCCCAGG -0.337319670869
AGCCCATA 0.0541504753123
AGCCCATC -0.266123015481
AGCCCATG -0.126422657952
AGCCCCAA -0.300699155296
AGCCCCAC -0.224106995885
AGCCCCAG -0.204508419337
AGCCCCAT -0.253419246531
AGCCCCCA -0.401501812816
AGCCCCCC -0.287422132516
AGCCCCCG -0.247941320079
AGCCCCCT -0.358984064291
AGCCCCGA -0.0364429284506
AGCCCCGC -0.256594033552
AGCCCCGG -0.227742938969
AGCCCCTA -0.242345301003
AGCCCCTC -0.298797983289
AGCCCCTG -0.149482365601
AGCCCGAA -0.178690124805
AGCCCGAC -0.274154943692
AGCCCGAG -0.0970699588477
AGCCCGAT 0.149869109948
AGCCCGCA -0.184073049739
AGCCCGCC -0.339960936643
AGCCCGCG -0.2609890562
AGCCCGCT -0.337000466277
AGCCCGGA -0.321348234468
AGCCCGGC -0.371978298059
AGCCCGGG -0.348253670419
AGCCCGTA -0.262892367455
AGCCCGTC -0.28023331742
AGCCCGTG -0.192190368838
AGCCCTAA -0.136607569098
AGCCCTAC -0.18355265069
AGCCCTAG -0.30355171893
AGCCCTAT -0.285327160494
AGCCCTCA -0.193559068978
AGCCCTCC -0.266714398578

AGCCCTCG -0.245253918277
AGCCCTGA -0.220171387073
AGCCCTGC -0.284691594177
AGCCCTGG -0.380741218288
AGCCCTTA -0.337743718432
AGCCCTTC -0.195451495496
AGCCCTTG -0.268031625054
AGCCGAAA 0.0340741568885
AGCCGAAC -0.147724653429
AGCCGAAG -0.17945805347
AGCCGAAT 0.121308920064
AGCCGACA -0.177033950617
AGCCGACC -0.222631041749
AGCCGACG -0.221368998628
AGCCGACT -0.190594045025
AGCCGAGA -0.0387643746622
AGCCGAGC -0.225761290098
AGCCGAGG -0.106360541056
AGCCGATA 0.145327689624
AGCCGATC -0.0568042108499
AGCCGATG -0.0703767734146
AGCCGCAA -0.0495252148573
AGCCGCAC -0.136533455737
AGCCGCAG -0.283884170562
AGCCGCAT -0.114335501221
AGCCGCCA -0.23606927091
AGCCGCCC -0.312306673521
AGCCGCCG -0.359900161826
AGCCGCCT -0.205975299462
AGCCGCGA -0.0862033012863
AGCCGCGC -0.369975919853
AGCCGCGG 0.0947476743229
AGCCGCTA -0.295268505717
AGCCGCTC -0.259156845963
AGCCGCTG -0.255785920926
AGCCGGAA -0.0538767742217
AGCCGGAC -0.196996714425
AGCCGGAG -0.30368212589
AGCCGGAT 0.241908677524
AGCCGGCA -0.205246155947
AGCCGGCC -0.403257130631
AGCCGGCG -0.242856893918
AGCCGGCT -0.166392898176
AGCCGGGA -0.00999180666043
AGCCGGGC -0.354616870199
AGCCGGGG -0.189049076726
AGCCGGTA -0.339432529368
AGCCGGTC -0.301062494917
AGCCGGTG -0.202758402029
AGCCGTAA -0.00153697752933
AGCCGTAC -0.0691433024371
AGCCGTAG -0.102113067312
AGCCGTAT 0.0577470108814
AGCCGTCA -0.25495311617

AGCCGTCC -0.258345916641
AGCCGTCG -0.31039249568
AGCCGTGA -0.0991633986928
AGCCGTGC -0.0858331412515
AGCCGTGG -0.244619728292
AGCCGTTA -0.104147116445
AGCCGTTC -0.214302908355
AGCCGTTG -0.0080831028579
AGCCTAAA -0.0689909717077
AGCCTAAC -0.165047768157
AGCCTAAG 0.0685858063466
AGCCTAAT -0.141299635026
AGCCTACA -0.141764195137
AGCCTACC -0.00201685929731
AGCCTACG -0.178847003045
AGCCTACT -0.142822692645
AGCCTAGA -0.107637173872
AGCCTAGC -0.262230012563
AGCCTAGG -0.360055078284
AGCCTATA 0.0896415963273
AGCCTATC -0.0826521288156
AGCCTATG -0.310594683777
AGCCTCAA -0.0126987262859
AGCCTCAC -0.175276145324
AGCCTCAG -0.208659815979
AGCCTCAT -0.10857415901
AGCCTCCA -0.33132549949
AGCCTCCC -0.364908527387
AGCCTCCG -0.225176808827
AGCCTCCT -0.120073100859
AGCCTCGA -0.0793604912951
AGCCTCGC -0.158230036051
AGCCTCGG -0.191975387547
AGCCTCTA -0.126408999001
AGCCTCTC -0.269296342445
AGCCTCTG -0.152849650604
AGCCTGAA -0.18207697894
AGCCTGAC -0.192583877996
AGCCTGAG -0.186484618044
AGCCTGAT 0.0176476399114
AGCCTGCA -0.283519606714
AGCCTGCC -0.305288454557
AGCCTGCG -0.170161220044
AGCCTGGA -0.129199968623
AGCCTGGC -0.0832057676164
AGCCTGGG -0.303624377423
AGCCTGTA -0.149485849155
AGCCTGTC -0.155264746228
AGCCTGTG -0.0601490555062
AGCCTTAA -0.157426611797
AGCCTTAC -0.00493650323246
AGCCTTAG -0.123710965868
AGCCTTAT -0.112140978891
AGCCTTCA -0.273480181585

AGCCTTCC -0.393642598712
AGCCTTCG -0.20202643752
AGCCTTGA 0.00178653757492
AGCCTTGC -0.42568549692
AGCCTTGG -0.247219273122
AGCCTTTA -0.194237301156
AGCCTTTC -0.198074845186
AGCCTTTG -0.314823957956
AGCGAAAA 0.230050992983
AGCGAAAC -0.125801822112
AGCGAAAG -0.10646867729
AGCGAAAT 0.388368844541
AGCGAACA 0.143027289293
AGCGAACC -0.245009355743
AGCGAACG -0.173268896403
AGCGAACT -0.283069436215
AGCGAAGA 0.0613470283149
AGCGAAGC -0.368935774754
AGCGAAGG -0.301617434194
AGCGAATA 0.113586506472
AGCGAATC 0.462329030275
AGCGAATG -0.0451962382227
AGCGACAA -0.174068501586
AGCGACAC -0.143380718712
AGCGACAG 0.049515357088
AGCGACAT 0.0841907224732
AGCGACCA -0.238123583356
AGCGACCC -0.3050214783
AGCGACCG -0.223606092644
AGCGACCT -0.277120665065
AGCGACGA 0.0826623857743
AGCGACGC -0.0781393902946
AGCGACGG -0.295028806584
AGCGACTA -0.100602528645
AGCGACTC -0.170727713198
AGCGACTG -0.215293652892
AGCGAGAA 0.109096796856
AGCGAGAC -0.220305778955
AGCGAGAG -0.0226670652923
AGCGAGAT 0.330113463019
AGCGAGCA -0.0571290991703
AGCGAGCC -0.289024216669
AGCGAGCG -0.202713301013
AGCGAGCT -0.258652004763
AGCGAGGA -0.0537663620647
AGCGAGGC -0.197461147422
AGCGAGGG -0.228225476157
AGCGAGTA 0.00136707683814
AGCGAGTC 0.00442344438523
AGCGAGTG -0.131922978595
AGCGATAA -0.118666767379
AGCGATAC 0.126807967407
AGCGATAG -0.16677916063
AGCGATAT 0.35793442099

AGCGATCA 0.159715543417
AGCGATCC 0.264392079381
AGCGATCG 0.206052822235
AGCGATGA -0.0915408717029
AGCGATGC -0.0663493357041
AGCGATGG -0.26237037037
AGCGATTA 0.160441939186
AGCGATTC 0.294610143391
AGCGATTG 0.104541426351
AGCGCAAA 0.0160547992968
AGCGCAAC -0.0886284550704
AGCGCAAG -0.0282181722395
AGCGCAAT 0.321338602124
AGCGCACA -0.162522950705
AGCGCACC -0.280545816667
AGCGCACG -0.318326474623
AGCGCACT -0.134915285751
AGCGCAGA 0.000713068602756
AGCGCAGC -0.33660515922
AGCGCAGG -0.182589849108
AGCGCATA 0.141852018654
AGCGCATC 0.0222585724994
AGCGCATG -0.203404624899
AGCGCCAA -0.13065795207
AGCGCCAC -0.271041081486
AGCGCCAG -0.115750181554
AGCGCCAT -0.238011485272
AGCGCCCA -0.249253523254
AGCGCCCC -0.269463653208
AGCGCCCG -0.311485312996
AGCGCCCT -0.268357298475
AGCGCCGA -0.0641014032257
AGCGCCGC -0.262326474623
AGCGCCGG -0.201969498911
AGCGCCTA -0.220572983996
AGCGCCTC -0.335311092458
AGCGCCTG -0.188158934476
AGCGCGAA 0.0667514128398
AGCGCGAC 0.0156813355065
AGCGCGAG -0.0963563988203
AGCGCGAT 0.365230340098
AGCGCGCA 0.00573878790872
AGCGCGCC -0.240912682734
AGCGCGCG 0.0178296998836
AGCGCGCT 0.170292376134
AGCGCGGA -0.0634818729046
AGCGCGGC -0.312207922719
AGCGCGGG -0.220129357164
AGCGCGTA -0.0265584821234
AGCGCGTC -0.240321881465
AGCGCGTG -0.0934904555926
AGCGCTAA -0.254689077659
AGCGCTAC -0.18465932474
AGCGCTAG -0.176951267057

AGCGCTAT -0.241147710998
AGCGCTCA -0.111266646391
AGCGCTCC -0.148651244477
AGCGCTCG -0.204161772197
AGCGCTGA -0.125461173063
AGCGCTGC -0.176474219676
AGCGCTGG -0.120629345233
AGCGCTTA -0.136622323202
AGCGCTTC -0.238763117603
AGCGCTTG -0.128807336429
AGCGGAAA 0.244752049788
AGCGGAAC -0.102738673472
AGCGGAAG 0.0240916420903
AGCGGAAT 0.246122307844
AGCGGACA -0.280923182442
AGCGGACC -0.185832743397
AGCGGACG -0.184135137805
AGCGGACT -0.181076025294
AGCGGAGA 0.198975781965
AGCGGAGC 0.0184663768689
AGCGGAGG -0.240897672325
AGCGGATA 0.285271599378
AGCGGATC 0.325665015327
AGCGGATG -0.149798059616
AGCGGCAA -0.171209876543
AGCGGCAC -0.168594719595
AGCGGCAG -0.271967882887
AGCGGCAT -0.15594123106
AGCGGCCA -0.268769065332
AGCGGCCC -0.287303658118
AGCGGCCG -0.222555388323
AGCGGCCT -0.241242694984
AGCGGCGA -0.0290543779873
AGCGGCGC -0.186342296697
AGCGGCGG -0.104334035735
AGCGGCTA -0.289140728304
AGCGGCTC -0.32151125155
AGCGGCTG -0.351909447955
AGCGGGAA -0.0311315300973
AGCGGGAC -0.264460469682
AGCGGGAG -0.179598139448
AGCGGGAT 0.235353685042
AGCGGGCA -0.0891300165855
AGCGGGCC -0.36906276136
AGCGGGCG -0.25955461189
AGCGGGGA -0.0993782343612
AGCGGGGC -0.280288178077
AGCGGGGG -0.261109190767
AGCGGGTA -0.170321348611
AGCGGGTC -0.195761801017
AGCGGGTG -0.212108094917
AGCGGTAA 0.0749538738686
AGCGGTAC -0.127245193745
AGCGGTAG -0.0861027014777

AGCGGTAT 0.000780149056412
AGCGGTCA -0.142671342256
AGCGGTCC -0.276025037578
AGCGGTCG -0.140319256489
AGCGGTGA -0.0821919311647
AGCGGTGC -0.195650910866
AGCGGTGG -0.118859900126
AGCGGTTA -0.00336568586412
AGCGGTTC -0.180384765228
AGCGGTTG -0.255866812677
AGCGTAAA 0.236963375125
AGCGTAAC -0.146215001728
AGCGTAAG -0.0875001815989
AGCGTAAT 0.376734395925
AGCGTACA -0.0897406346384
AGCGTACC -0.25653223594
AGCGTACG -0.177828262874
AGCGTACT -0.0261504094492
AGCGTAGA 0.0478912730812
AGCGTAGC -0.179959504173
AGCGTAGG -0.187767845874
AGCGTATA -0.128712971976
AGCGTATC 0.388521153044
AGCGTATG -0.0387585412036
AGCGTCAA -0.095522496477
AGCGTCAC -0.208020792723
AGCGTCAG -0.216295782053
AGCGTCAT -0.179883040936
AGCGTCCA -0.0853214260813
AGCGTCCC -0.281231123966
AGCGTCCG -0.168274167761
AGCGTCCT -0.392410504243
AGCGTCGA -0.0766233992609
AGCGTCGC -0.146923753947
AGCGTCGG -0.228696844993
AGCGTCTA -0.0338079119027
AGCGTCTC -0.397487588798
AGCGTCTG -0.0311693942288
AGCGTGAA -0.0874470350611
AGCGTGAC -0.251702416032
AGCGTGAG -0.265440849657
AGCGTGAT 0.154074353871
AGCGTGCA -0.115394112999
AGCGTGCC -0.208821312541
AGCGTGCG -0.077019743437
AGCGTGGA 0.0067081604426
AGCGTGGC -0.288409586057
AGCGTGGG -0.0503251186872
AGCGTGTA -0.103624035475
AGCGTGTC -0.305266146553
AGCGTGTG -0.128319943808
AGCGTTAA 0.12062673427
AGCGTTAC 0.0367687010591
AGCGTTAG -0.0466082121099

AGCGTTAT 0.0135829299615
AGCGTTCA -0.350219962261
AGCGTTCC -0.254717188177
AGCGTTCG -0.209682641598
AGCGTTGA -0.0568641011708
AGCGTTGC -0.0196388314314
AGCGTTGG -0.0248441881074
AGCGTTTA -0.0343076721921
AGCGTTTC -0.180691358025
AGCGTTTG -0.0266073927719
AGCTAAAA -0.0191769121714
AGCTAAAC 0.0203393598103
AGCTAAAG -0.126052702675
AGCTAAAT 0.141786504497
AGCTAACA -0.151612938633
AGCTAACC -0.18353121287
AGCTAACG -0.04583067944
AGCTAACT -0.25944216724
AGCTAAGA -0.200189867861
AGCTAAGC -0.0965648944274
AGCTAAGG -0.264667314097
AGCTAATA 0.100961475962
AGCTAATC -0.0955728995148
AGCTAATG -0.10895715689
AGCTACAA 0.00979036218093
AGCTACAC 0.0215976201453
AGCTACAG -0.163018844111
AGCTACAT -0.138512920152
AGCTACCA -0.305911976719
AGCTACCC -0.0399308471227
AGCTACCG -0.165412770534
AGCTACCT -0.250589506173
AGCTACGA -0.0497217904203
AGCTACGC -0.0543561954295
AGCTACGG -0.129295822565
AGCTACTA -0.157270893552
AGCTACTC -0.29207797271
AGCTACTG -0.0472108694974
AGCTAGAA 0.0839094475803
AGCTAGAC -0.140015144781
AGCTAGAG -0.193670900783
AGCTAGAT 0.210744985673
AGCTAGCA -0.0752504047639
AGCTAGCC -0.237430874438
AGCTAGCG -0.194561048282
AGCTAGCT -0.230746445498
AGCTAGGA -0.0297781729176
AGCTAGGC -0.237598515253
AGCTAGGG -0.235838134431
AGCTAGTA -0.129766844497
AGCTAGTC -0.152145996938
AGCTAGTG -0.17291831292
AGCTATAA -0.0541668724991
AGCTATAC -0.0429330125886

AGCTATAG -0.0817059650047
AGCTATAT 0.0729588560703
AGCTATCA -0.177114111314
AGCTATCC -0.0301461063909
AGCTATCG -0.126772816088
AGCTATGA 0.0303007853015
AGCTATGC -0.14766349066
AGCTATGG -0.156609003376
AGCTATTA 0.0311959689769
AGCTATTC -0.0316895804177
AGCTATTG -0.106767262417
AGCTCAAA -0.181652868555
AGCTCAAC 0.0231325955933
AGCTCAAG -0.273754092027
AGCTCAAT -0.201985739278
AGCTCACA -0.349492500036
AGCTCACC -0.231385473757
AGCTCACG -0.045785184126
AGCTCACT -0.141065951131
AGCTCAGA -0.102260752157
AGCTCAGC -0.379496187888
AGCTCAGG -0.19729189984
AGCTCATA -0.0107288549388
AGCTCATC -0.163447266221
AGCTCATG -0.25251121729
AGCTCCAA -0.183871056241
AGCTCCAC -0.223914951989
AGCTCCAG -0.275259507977
AGCTCCAT -0.188260709068
AGCTCCCA -0.209651577503
AGCTCCCC -0.246071025062
AGCTCCCG -0.192798933175
AGCTCCCT -0.122344044954
AGCTCCGA 0.0162087952
AGCTCCGC -0.240957432902
AGCTCCGG -0.172919916004
AGCTCCTA -0.105246257966
AGCTCCTC -0.287755600666
AGCTCCTG -0.338838690564
AGCTCGAA -0.0618362886496
AGCTCGAC -0.176608187135
AGCTCGAG 0.061351511559
AGCTCGAT 0.0545538870228
AGCTCGCA -0.138692810458
AGCTCGCC -0.216036258971
AGCTCGCG -0.158501654403
AGCTCGGA -0.0124082925341
AGCTCGGC -0.12730573711
AGCTCGGG -0.16816349293
AGCTCGTA -0.0722650248035
AGCTCGTC -0.169185558354
AGCTCGTG -0.300954350339
AGCTCTAA -0.200155731561
AGCTCTAC -0.137576873803

AGCTCTAG -0.121868266399
AGCTCTAT -0.168706184504
AGCTCTCA -0.0843075588926
AGCTCTCC -0.301458244994
AGCTCTCG -0.248573247289
AGCTCTGA -0.212569122149
AGCTCTGC -0.270059747185
AGCTCTGG -0.155349719521
AGCTCTTA -0.0218555478708
AGCTCTTC -0.338459892696
AGCTCTTG -0.0799302832244
AGCTGAAA 0.0743819766868
AGCTGAAC -0.286084637796
AGCTGAAG -0.0351736591363
AGCTGAAT -0.0478387358438
AGCTGACA -0.191023950078
AGCTGACC -0.280723988852
AGCTGACG -0.201301427372
AGCTGACT -0.218946016694
AGCTGAGA 0.0555857398444
AGCTGAGC -0.0853200083418
AGCTGAGG -0.300425251331
AGCTGATA 0.103033428733
AGCTGATC 0.0361204417221
AGCTGATG -0.220855832126
AGCTGCAA -0.0525946049779
AGCTGCAC -0.0547152378683
AGCTGCAG -0.0858014630425
AGCTGCAT -0.213713484011
AGCTGCCA -0.147196838726
AGCTGCCC -0.229283388663
AGCTGCCG -0.340156426613
AGCTGCCT -0.342078208721
AGCTGCGA -0.16116194626
AGCTGCGC -0.118760233918
AGCTGCGG -0.315383602456
AGCTGCTA -0.331276024951
AGCTGCTC -0.316032323749
AGCTGCTG -0.133154151677
AGCTGGAA 0.049240927353
AGCTGGAC -0.285598249166
AGCTGGAG -0.23139688654
AGCTGGAT 0.209834463984
AGCTGGCA -0.112989336765
AGCTGGCC -0.30483141876
AGCTGGCG -0.137084958928
AGCTGGGA -0.311201623742
AGCTGGGC -0.247075445816
AGCTGGGG -0.101729727845
AGCTGGTA -0.186921645283
AGCTGGTC -0.265263533943
AGCTGGTG -0.230965198209
AGCTGTAA -0.0208065790773
AGCTGTAC -0.128166919142

AGCTGTAG 0.00475723945641
AGCTGTAT −0.101695530783
AGCTGTCA −0.102938997283
AGCTGTCC −0.292528999355
AGCTGTCG 0.0562167867802
AGCTGTGA −0.0453029493109
AGCTGTGC −0.157251028807
AGCTGTGG −0.257372749768
AGCTGTTA −0.200078508035
AGCTGTTC −0.0591184460942
AGCTGTTG −0.165953002763
AGCTTAAA 0.0224325541528
AGCTTAAC −0.167901417494
AGCTTAAG −0.189015182036
AGCTTAAT −0.15448586155
AGCTTACA −0.112516052554
AGCTTACC −0.18799492929
AGCTTACG −0.163878735992
AGCTTACT −0.0258408031032
AGCTTAGA −0.159524527439
AGCTTAGC −0.109081822989
AGCTTAGG −0.129257806826
AGCTTATA −0.091416916049
AGCTTATC 0.00467067568902
AGCTTATG −0.153351384015
AGCTTCAA −0.254145876777
AGCTTCAC −0.159053931124
AGCTTCAG −0.180177562926
AGCTTCAT −0.244639809464
AGCTTCCA −0.0299815929866
AGCTTCCC −0.124234567901
AGCTTCCG −0.0650649414786
AGCTTCCT −0.272743645606
AGCTTCGA −0.189442578691
AGCTTCGC −0.273492667029
AGCTTCGG −0.165957879448
AGCTTCTA −0.0272550149557
AGCTTCTC −0.0783751979428
AGCTTCTG −0.0985003248863
AGCTTGAA −0.0955352487583
AGCTTGAC −0.332169253779
AGCTTGAG −0.177280965714
AGCTTGAT 0.0132563283497
AGCTTGCA −0.0102089714455
AGCTTGCC −0.259607339577
AGCTTGCG 0.0958471698058
AGCTTGGA −0.207638441224
AGCTTGGC −0.255587654321
AGCTTGGG −0.150784574157
AGCTTGTA −0.0910667632659
AGCTTGTC −0.10320992963
AGCTTGTG −0.205689561525
AGCTTTAA −0.0620269347551
AGCTTTAC 0.0250032931568

AGCTTTAG -0.255794194622
AGCTTTAT -0.177672981855
AGCTTTCA -0.294717956315
AGCTTTCC -0.102783402754
AGCTTTCG -0.210063700558
AGCTTTGA 0.0733358580869
AGCTTTGC -0.05362077706
AGCTTTGG -0.32413362382
AGCTTTTA 0.168538277459
AGCTTTTC -0.128631718748
AGCTTTTG -0.0517319827257
AGGAAAAA 0.219875847988
AGGAAAAC -0.18018173737
AGGAAAAG -0.117005020104
AGGAAAAT 0.241513518225
AGGAAACA -0.0555068063284
AGGAAACC 0.0187814877013
AGGAAACG -0.202457347204
AGGAAACT -0.285029164678
AGGAAAGA -0.0784826969112
AGGAAAGC -0.0512556069445
AGGAAAGG -0.235002193065
AGGAAATA 0.183438329559
AGGAAATC 0.4885041384
AGGAAATG -0.130922012191
AGGAACAA 0.168228901835
AGGAACAC -0.0508719654067
AGGAACAG -0.175201161946
AGGAACAT 0.0181302004435
AGGAACCA -0.0425379810753
AGGAACCC -0.112262092569
AGGAACCG -0.10971861956
AGGAACCT -0.302444444444
AGGAACGA -0.0538249675333
AGGAACGC -0.217862879684
AGGAACGG 0.000921947284371
AGGAACTA 0.10725830795
AGGAACTC -0.336342141419
AGGAACTG -0.162146025991
AGGAAGAA -0.0589876786394
AGGAAGAC -0.278477366255
AGGAAGAG -0.249927225471
AGGAAGAT 0.273628400184
AGGAAGCA -0.0741207202164
AGGAAGCC -0.223475808797
AGGAAGCG -0.181279185255
AGGAAGGA -0.254025864292
AGGAAGGC -0.259656052004
AGGAAGGG -0.122669980959
AGGAAGTA 0.0542712243957
AGGAAGTC -0.292993892703
AGGAAGTG 0.0420316582142
AGGAATAA 0.226477125797
AGGAATAC 0.061845485082

AGGAATAG -0.197208852431
AGGAATAT 0.358814704139
AGGAATCA 0.294862929118
AGGAATCC 0.412838371863
AGGAATCG 0.283532569321
AGGAATGA -0.0154940217628
AGGAATGC -0.220905576195
AGGAATGG -0.189954974582
AGGAATTA 0.160741728688
AGGAATTC 0.0273057586336
AGGAATTG 0.0116797295221
AGGACAAA -0.0195000137208
AGGACAAC -0.207614601857
AGGACAAG -0.22427154693
AGGACAAT 0.155135582337
AGGACACA -0.200193783247
AGGACACC -0.163472124985
AGGACACG -0.144643631236
AGGACACT -0.119117388823
AGGACAGA -0.070415353101
AGGACAGC -0.119102540265
AGGACAGG -0.218025197637
AGGACATA 0.105331620892
AGGACATC -0.0430415596391
AGGACATG -0.19244734931
AGGACCAA -0.198556521167
AGGACCAC -0.132803239826
AGGACCAG -0.24074773359
AGGACCAT -0.240516463778
AGGACCCA -0.147561985965
AGGACCCC -0.353697707175
AGGACCCG -0.288978248684
AGGACCCT -0.376213571086
AGGACCGA -0.0584864984223
AGGACCGC -0.217197521752
AGGACCGG -0.238296148048
AGGACCTA -0.0300771432307
AGGACCTC -0.198939865343
AGGACCTG -0.296805436566
AGGACGAA -0.214382539212
AGGACGAC -0.233445444538
AGGACGAG -0.294486421826
AGGACGAT 0.171526738628
AGGACGCA -0.00806411776329
AGGACGCC -0.193289760349
AGGACGCG -0.119714483304
AGGACGGA -0.116669426698
AGGACGGC -0.301839396896
AGGACGGG -0.271128223152
AGGACGTA 0.0346934071
AGGACGTC -0.127825242399
AGGACGTG -0.22576808117
AGGACTAA -0.239694554496
AGGACTAC -0.337856284017

AGGACTAG -0.129717371664
AGGACTAT -0.0929597204386
AGGACTCA -0.135557641016
AGGACTCC -0.401715891965
AGGACTCG -0.156853060299
AGGACTGA -0.115151532758
AGGACTGC -0.148508351489
AGGACTGG -0.195427225164
AGGACTTA -0.19881702187
AGGACTTC -0.218902936675
AGGACTTG -0.0286621242718
AGGAGAAA 0.0176924776748
AGGAGAAC -0.106357635903
AGGAGAAG -0.0163955568727
AGGAGAAT 0.308266383857
AGGAGACA -0.121968686719
AGGAGACC -0.292756919091
AGGAGACG -0.233195075384
AGGAGACT -0.310818580987
AGGAGAGA -0.0387386863859
AGGAGAGC -0.37954803515
AGGAGAGG -0.280706013375
AGGAGATA 0.264643788496
AGGAGATC 0.375187773306
AGGAGATG -0.130874559108
AGGAGCAA -0.126972676914
AGGAGCAC -0.313625292123
AGGAGCAG -0.134928958002
AGGAGCAT -0.244919363508
AGGAGCCA -0.133668975295
AGGAGCCC -0.35026003094
AGGAGCCG -0.159162800966
AGGAGCCT -0.409744870548
AGGAGCGA -0.194609247433
AGGAGCGC -0.107782442853
AGGAGCGG -0.181020835572
AGGAGCTA -0.206799574169
AGGAGCTC -0.289548487642
AGGAGCTG -0.184594446204
AGGAGGAA 0.0284616859685
AGGAGGAC -0.379193616076
AGGAGGAG -0.2292198138
AGGAGGAT 0.283170862813
AGGAGGCA -0.432801239017
AGGAGGCC -0.267930128321
AGGAGGCG -0.263392818236
AGGAGGGA -0.265850286391
AGGAGGGC -0.41236583496
AGGAGGGG -0.317756249419
AGGAGGTA -0.0497020428992
AGGAGGTC -0.274387090907
AGGAGGTG -0.13081321031
AGGAGTAA 0.0316490636759
AGGAGTAC -0.19586297653

AGGAGTAG -0.107571666886
AGGAGTAT -0.0229862346045
AGGAGTCA -0.262446008476
AGGAGTCC -0.351798506834
AGGAGTCG -0.0523810353894
AGGAGTGA -0.141917902144
AGGAGTGC -0.376180406487
AGGAGTGG -0.21177280683
AGGAGTTA -0.0805736499875
AGGAGTTC -0.143409951891
AGGAGTTG -0.100874648166
AGGATAAA 0.0158281638168
AGGATAAC 0.0924718435092
AGGATAAG 0.144166172693
AGGATAAT 0.186728242616
AGGATACA 0.0614835907196
AGGATACC 0.21869305583
AGGATACG 0.348327037026
AGGATACT 0.234187358669
AGGATAGA -0.101149335123
AGGATAGC 0.0503331576983
AGGATAGG 0.0395973278672
AGGATATA 0.243633230148
AGGATATC 0.474305143911
AGGATATG 0.283115656734
AGGATCAA 0.0267239675953
AGGATCAC 0.171828919268
AGGATCAG 0.138994116225
AGGATCAT 0.0473055013169
AGGATCCA 0.218061867439
AGGATCCC 0.349874585611
AGGATCCG 0.223318756992
AGGATCCT 0.323637860082
AGGATCGA -0.100531844826
AGGATCGC 0.367557712144
AGGATCGG 0.0340663351555
AGGATCTA 0.341975263704
AGGATCTC 0.410217482893
AGGATCTG 0.318873630596
AGGATGAA -0.214381344307
AGGATGAC -0.0430744912073
AGGATGAG -0.0462125952271
AGGATGAT 0.0188094140376
AGGATGCA -0.096942466422
AGGATGCC -0.104787379973
AGGATGCG -0.11767030234
AGGATGGA -0.0705051479304
AGGATGGC -0.162469135802
AGGATGGG -0.14379310946
AGGATGTA -0.0440061151743
AGGATGTC -0.055082591199
AGGATGTG 0.0984861912164
AGGATTAA -0.0594826450636
AGGATTAC 0.442957593015

AGGATTAG 0.121694990614
AGGATTAT 0.260693272122
AGGATTCA 0.138883400294
AGGATTCC 0.443977452675
AGGATTCG 0.369699126872
AGGATTGA -0.100395159299
AGGATTGC 0.456913108538
AGGATTGG 0.0721180247846
AGGATTTA 0.129064547528
AGGATTTC 0.460701988872
AGGATTTG 0.370977583427
AGGCAAAA -0.0867447356721
AGGCAAAC -0.194938440492
AGGCAAAG -0.349721605091
AGGCAAAT 0.232968890645
AGGCAACA 0.000265458316871
AGGCAACC -0.23404272829
AGGCAACG -0.0230927457663
AGGCAACT -0.187576793593
AGGCAAGA 0.126135719786
AGGCAAGC -0.2721292227
AGGCAAGG -0.267408497046
AGGCAATA 0.0444768271532
AGGCAATC 0.422025772522
AGGCAATG -0.21703723718
AGGCACAA -0.10882309366
AGGCACAC -0.1630260631
AGGCACAG -0.336361840806
AGGCACAT -0.081762611048
AGGCACCA -0.0169148518879
AGGCACCC -0.191416836351
AGGCACCG -0.323676804582
AGGCACCT -0.29751891539
AGGCACGA -0.234878721859
AGGCACGC -0.0802980252703
AGGCACGG -0.185498825905
AGGCACTA -0.169763221569
AGGCACTC -0.424172680674
AGGCACTG -0.327026997372
AGGCAGAA -0.0737850651916
AGGCAGAC -0.392122496987
AGGCAGAG 0.0912643526751
AGGCAGAT 0.252226403033
AGGCAGCA -0.162380285942
AGGCAGCC -0.0962359396433
AGGCAGCG -0.246432274949
AGGCAGGA 0.0245194877635
AGGCAGGC -0.193650627099
AGGCAGGG -0.20721865713
AGGCAGTA 0.0822727544544
AGGCAGTC -0.193608929718
AGGCAGTG -0.225857518341
AGGCATAA -0.169371875703
AGGCATAC -0.0516895749261

AGGCATAG -0.175106874544
AGGCATAT 0.0171989277645
AGGCATCA 0.0495968503479
AGGCATCC -0.0795222573423
AGGCATCG 0.051424015895
AGGCATGA -0.284832669279
AGGCATGC -0.310611121115
AGGCATGG -0.0672146482179
AGGCATTA -0.113637948265
AGGCATTC -0.152914965538
AGGCATTG -0.121871587623
AGGCCAAA -0.18024564757
AGGCCAAC -0.379055440352
AGGCCAAG -0.145240467428
AGGCCAAT -0.111380644711
AGGCCACA -0.105681144748
AGGCCACC -0.136284676834
AGGCCACG -0.285964494429
AGGCCACT -0.152590234039
AGGCCAGA -0.0487418018166
AGGCCAGC -0.16179375167
AGGCCAGG -0.198465507876
AGGCCATA -0.156870008338
AGGCCATC -0.194133331784
AGGCCATG -0.318118982967
AGGCCCAA -0.316896845379
AGGCCCAC -0.232361800579
AGGCCCAG -0.226985159774
AGGCCCAT -0.19215660456
AGGCCCCA -0.339692594561
AGGCCCCC -0.406796188407
AGGCCCCG -0.366346854682
AGGCCCCT -0.407726885986
AGGCCCGA -0.140961898333
AGGCCCGC -0.126848856734
AGGCCCGG -0.321823153269
AGGCCCTA -0.382419736594
AGGCCCTC -0.273308641975
AGGCCCTG -0.35360477442
AGGCCGAA -0.107374301063
AGGCCGAC -0.245737786777
AGGCCGAG -0.141937545389
AGGCCGAT -0.000581791038223
AGGCCGCA -0.293434069099
AGGCCGCC -0.309593565519
AGGCCGCG -0.0839191540036
AGGCCGGA -0.171657064472
AGGCCGGC -0.19168627451
AGGCCGGG -0.418382280348
AGGCCGTA -0.230235148243
AGGCCGTC -0.130832824219
AGGCCGTG -0.180142548192
AGGCCTAA -0.115145028951
AGGCCTAC -0.146980078604

AGGCCTAG -0.0763289200672
AGGCCTAT -0.233905133203
AGGCCTCA -0.276037763253
AGGCCTCC -0.191893004115
AGGCCTCG -0.166393762183
AGGCCTGA -0.263770010735
AGGCCTGC -0.41843352457
AGGCCTGG -0.375230384413
AGGCCTTA -0.128428838736
AGGCCTTC -0.250109728532
AGGCCTTG -0.1909735941
AGGCGAAA -0.0535771604938
AGGCGAAC -0.249629556456
AGGCGAAG -0.15523271182
AGGCGAAT 0.230347362457
AGGCGACA -0.124290018274
AGGCGACC -0.293644200718
AGGCGACG -0.130804014861
AGGCGACT -0.142135076253
AGGCGAGA -0.0154054742666
AGGCGAGC -0.168148970036
AGGCGAGG -0.263570723278
AGGCGATA 0.119763880656
AGGCGATC 0.319458689873
AGGCGATG -0.184056270279
AGGCGCAA -0.131134270693
AGGCGCAC -0.193972565158
AGGCGCAG -0.127183235386
AGGCGCAT -0.101980671681
AGGCGCCA -0.213128021113
AGGCGCCC -0.347482471733
AGGCGCCG -0.0835342929032
AGGCGCCT -0.23694037387
AGGCGCGA 0.0503029704275
AGGCGCGC -0.0299814485499
AGGCGCGG -0.0303677015385
AGGCGCTA -0.342347977329
AGGCGCTC -0.31969200722
AGGCGCTG -0.262380583952
AGGCGGAA -0.0801901018105
AGGCGGAC -0.104285469435
AGGCGGAG -0.290536478256
AGGCGGAT 0.245845742594
AGGCGGCA -0.0888511560484
AGGCGGCC -0.243626531288
AGGCGGCG -0.17645315732
AGGCGGGA -0.168726521576
AGGCGGGC -0.177541242715
AGGCGGGG -0.129995786905
AGGCGGTA -0.074695046669
AGGCGGTC -0.399881516588
AGGCGGTG -0.106889783221
AGGCGTAA -0.144518518519
AGGCGTAC -0.186833267483

AGGCGTAG -0.346406519555
AGGCGTAT 0.0694042101899
AGGCGTCA -0.241036107956
AGGCGTCC -0.191375402666
AGGCGTCG -0.12667373724
AGGCGTGA 0.0268345125158
AGGCGTGC -0.0841935430763
AGGCGTGG -0.259396574509
AGGCGTTA -0.165870859746
AGGCGTTC -0.174663923182
AGGCGTTG -0.212089744297
AGGCTAAA 0.019605421818
AGGCTAAC -0.108444079005
AGGCTAAG -0.114955112165
AGGCTAAT -0.202437352404
AGGCTACA -0.251808374068
AGGCTACC -0.309185857723
AGGCTACG -0.068463777885
AGGCTACT -0.320919571296
AGGCTAGA 0.0984890967995
AGGCTAGC -0.236646254136
AGGCTAGG -0.160909222948
AGGCTATA -0.108087835836
AGGCTATC 0.00836461832312
AGGCTATG -0.209493941788
AGGCTCAA -0.254103285921
AGGCTCAC -0.174384894699
AGGCTCAG -0.160551018452
AGGCTCAT -0.205110057717
AGGCTCCA -0.0495382031906
AGGCTCCC -0.381082860199
AGGCTCCG -0.225670441328
AGGCTCGA -0.210068057641
AGGCTCGC -0.313005603209
AGGCTCGG -0.153234262941
AGGCTCTA -0.129703442025
AGGCTCTC -0.334016292274
AGGCTCTG -0.115870996599
AGGCTGAA -0.344825783365
AGGCTGAC -0.142647462277
AGGCTGAG -0.144257936064
AGGCTGAT 0.08145947438
AGGCTGCA -0.288798173691
AGGCTGCC -0.258983095485
AGGCTGCG -0.209343671292
AGGCTGGA -0.0405419539278
AGGCTGGC -0.273249887464
AGGCTGGG -0.0991420701159
AGGCTGTA -0.0564482152456
AGGCTGTC -0.186328728608
AGGCTGTG -0.341914487614
AGGCTTAA -0.129197945476
AGGCTTAC -0.317313614357
AGGCTTAG -0.0883901674157

AGGCTTAT -0.0596108257606
AGGCTTCA -0.239587790615
AGGCTTCC -0.373121108633
AGGCTTCG -0.100302701949
AGGCTTGA -0.131838134431
AGGCTTGC -0.143371742112
AGGCTTGG -0.314289490425
AGGCTTTA -0.138338711625
AGGCTTTC -0.119230283773
AGGCTTTG -0.179178808383
AGGGAAAA 0.0588729218844
AGGGAAAC -0.114306133358
AGGGAAAG 0.00865783046568
AGGGAAAT 0.299145758575
AGGGAACA -0.124106177596
AGGGAACC -0.23671888389
AGGGAACG -0.292398136422
AGGGAACT -0.137989385097
AGGGAAGA -0.161144517066
AGGGAAGC -0.00269175680348
AGGGAAGG -0.305179772445
AGGGAATA 0.209508520622
AGGGAATC 0.485567460617
AGGGAATG 0.0516888405893
AGGGACAA 0.109307440909
AGGGACAC -0.295118323859
AGGGACAG -0.248330659097
AGGGACAT -0.172571873475
AGGGACCA -0.278358595833
AGGGACCC -0.390117545101
AGGGACCG -0.226566720299
AGGGACCT -0.26353231663
AGGGACGA -0.222236283273
AGGGACGC -0.205512670565
AGGGACGG -0.280234527271
AGGGACTA -0.234373366174
AGGGACTC -0.185855282267
AGGGACTG -0.156168482208
AGGGAGAA -0.264854015871
AGGGAGAC -0.270578360701
AGGGAGAG -0.255357760111
AGGGAGAT 0.286157398139
AGGGAGCA -0.180849736569
AGGGAGCC -0.258862158402
AGGGAGCG -0.2510307115
AGGGAGGA -0.177412363724
AGGGAGGC -0.313397882109
AGGGAGGG 6.39196704069E-5
AGGGAGTA -0.0957898101201
AGGGAGTC -0.20828087197
AGGGAGTG -0.217184627048
AGGGATAA 0.0181452940789
AGGGATAC 0.252590616699
AGGGATAG -0.00376273008586

AGGGATAT 0.349601208723
AGGGATCA 0.106848266324
AGGGATCC 0.123498181308
AGGGATCG 0.109854222925
AGGGATGA 0.0218386082778
AGGGATGC -0.268545033628
AGGGATGG -0.344409397591
AGGGATTA 0.137255386225
AGGGATTC 0.351100489591
AGGGATTG 0.216627199163
AGGGCAAA 0.088239652277
AGGGCAAC -0.183438413968
AGGGCAAG -0.226193173566
AGGGCAAT 0.147522448352
AGGGCACA -0.251513285543
AGGGCACC -0.37661269089
AGGGCACG -0.211204389575
AGGGCACT -0.18939256772
AGGGCAGA -0.0975156180552
AGGGCAGC -0.256046905454
AGGGCAGG -0.157738691861
AGGGCATA -0.0291607670102
AGGGCATC -0.118180180651
AGGGCATG -0.256661935325
AGGGCCAA -0.311312991672
AGGGCCAC -0.235483632055
AGGGCCAG -0.116297386097
AGGGCCAT -0.26261479214
AGGGCCCA -0.241212489144
AGGGCCCC -0.138465382202
AGGGCCCG -0.197998452751
AGGGCCCT -0.413600911873
AGGGCCGA -0.191230210603
AGGGCCGC -0.344184413504
AGGGCCGG -0.229397142105
AGGGCCTA -0.246988093474
AGGGCCTC -0.23524005487
AGGGCCTG -0.233676268861
AGGGCGAA -0.252366372002
AGGGCGAC -0.224566448802
AGGGCGAG -0.193340381778
AGGGCGAT 0.192661949937
AGGGCGCA -0.23955829904
AGGGCGCC -0.260202729045
AGGGCGCG 0.0531670973344
AGGGCGGA 0.154052561998
AGGGCGGC -0.195107674163
AGGGCGGG -0.0488540305011
AGGGCGTA -0.0598423301067
AGGGCGTC -0.223708897008
AGGGCGTG -0.264201615204
AGGGCTAA -0.201643347051
AGGGCTAC -0.252651285194
AGGGCTAG -0.224866941015

AGGGCTAT -0.136409783617
AGGGCTCA -0.311135422243
AGGGCTCC -0.253101049502
AGGGCTCG -0.246003165522
AGGGCTGA -0.272472342514
AGGGCTGC -0.222169934641
AGGGCTGG -0.351254690432
AGGGCTTA -0.256863858575
AGGGCTTC -0.224989615618
AGGGCTTG -0.366115362462
AGGGGAAA -0.0133788828919
AGGGGAAC -0.0706242591861
AGGGGAAG -0.119699245063
AGGGGAAT 0.285389086478
AGGGGACA -0.189283950617
AGGGGACC -0.280048136263
AGGGGACG -0.2705327551
AGGGGACT -0.331119888093
AGGGGAGA -0.0407528156491
AGGGGAGC -0.196243992363
AGGGGAGG -0.0726527386773
AGGGGATA 0.104999560912
AGGGGATC 0.351280635742
AGGGGATG -0.383155977904
AGGGGCAA -0.0869106753813
AGGGGCAC -0.213261560242
AGGGGCAG -0.185251946598
AGGGGCAT -0.108639633254
AGGGGCCA -0.245848970421
AGGGGCCC -0.348763146201
AGGGGCCG -0.299145472924
AGGGGCGA -0.18203490637
AGGGGCGC -0.19019869294
AGGGGCGG -0.280603490841
AGGGGCTA -0.144240964695
AGGGGCTC -0.328958069501
AGGGGCTG -0.226987419003
AGGGGGAA 0.11463251638
AGGGGGAC -0.350309403904
AGGGGGAG -0.153520539452
AGGGGGAT 0.112653814304
AGGGGGCA -0.0818158868144
AGGGGGCC -0.162408891652
AGGGGGCG -0.367176334239
AGGGGGGA -0.226754537586
AGGGGGGC -0.281953495261
AGGGGGGG -0.289764436106
AGGGGGTA -0.25308009481
AGGGGGTC -0.290211789411
AGGGGGTG -0.102909950707
AGGGGTAA 0.140538695103
AGGGGTAC -0.224488340192
AGGGGTAG -0.127373203851
AGGGGTAT -0.063600643168

AGGGGTCA -0.333624232216
AGGGGTCC -0.337202818325
AGGGGTCG -0.198027434842
AGGGGTGA -0.181002787655
AGGGGTGC -0.335618317177
AGGGGTGG -0.224031953522
AGGGGTTA -0.0296827753323
AGGGGTTC -0.191681482945
AGGGGTTG -0.0835548621582
AGGGTAAA 0.0527481393664
AGGGTAAC 0.014673033122
AGGGTAAG -0.217629049546
AGGGTAAT 0.215449296362
AGGGTACA -0.0693606264437
AGGGTACC -0.250805093347
AGGGTACG -0.246512708787
AGGGTACT -0.0981564708723
AGGGTAGA 0.0252544913659
AGGGTAGC -0.265600404277
AGGGTAGG -0.161418122157
AGGGTATA -0.00920300116358
AGGGTATC 0.348784739878
AGGGTATG -0.00930512980692
AGGGTCAA -0.0685814481873
AGGGTCAC -0.000423756353096
AGGGTCAG -0.116265432099
AGGGTCAT -0.204293552812
AGGGTCCA -0.313204628121
AGGGTCCC -0.100808173534
AGGGTCCG -0.223100093823
AGGGTCGA -0.156968928832
AGGGTCGC -0.303445013878
AGGGTCGG -0.143067341016
AGGGTCTA -0.310694711895
AGGGTCTC -0.188527828535
AGGGTCTG -0.183376403909
AGGGTGAA -0.28126405758
AGGGTGAC -0.213833061742
AGGGTGAG -0.233079324325
AGGGTGAT -0.0811866977355
AGGGTGCA -0.206258332922
AGGGTGCC -0.234617350792
AGGGTGCG -0.144728891161
AGGGTGGA 0.00458478363192
AGGGTGGC -0.17257481112
AGGGTGGG -0.0152622740294
AGGGTGTA 0.0527446672993
AGGGTGTC -0.208081444835
AGGGTGTG -0.331685871722
AGGGTTAA -0.139973990485
AGGGTTAC -0.209430528656
AGGGTTAG -0.170708018503
AGGGTTAT -0.133670865234
AGGGTTCA -0.233543696765

AGGGTTCC -0.11900805559
AGGGTTCG -0.293194334738
AGGGTTGA -0.108616668039
AGGGTTGC -0.0625375835553
AGGGTTGG -0.0475117591858
AGGGTTTA -0.0429155808123
AGGGTTTC -0.157720733493
AGGGTTTG -0.189391077976
AGGTAAAA 0.086552394125
AGGTAAAC -0.043137061206
AGGTAAAG -0.0747797810159
AGGTAAAT 0.256918919704
AGGTAACA -0.164122847025
AGGTAACC -0.182319790158
AGGTAACG -0.204911870583
AGGTAACT 0.110340970174
AGGTAAGA 0.00277678833749
AGGTAAGC -0.118185938013
AGGTAAGG -0.13535396379
AGGTAATA 0.11836154471
AGGTAATC 0.416729785506
AGGTAATG 0.017157710165
AGGTACAA -0.0610864197531
AGGTACAC -0.245410811863
AGGTACAG -0.220612199252
AGGTACAT -0.0987966850526
AGGTACCA -0.0377468251616
AGGTACCC -0.104666837102
AGGTACCG -0.163588477366
AGGTACCT -0.163740051979
AGGTACGA -0.00827364781428
AGGTACGC -0.201770557284
AGGTACGG -0.0599869390801
AGGTACTA -0.24208426621
AGGTACTC -0.21775308642
AGGTACTG -0.104864335342
AGGTAGAA 0.142049664131
AGGTAGAC -0.220540305011
AGGTAGAG -0.175375453885
AGGTAGAT 0.280106314079
AGGTAGCA -0.172032748497
AGGTAGCC -0.335113590853
AGGTAGCG -0.0770068794013
AGGTAGGA -0.103257096211
AGGTAGGC -0.2435764489
AGGTAGGG 0.00485740630969
AGGTAGTA -0.0213447259676
AGGTAGTC -0.0998460040969
AGGTAGTG -0.0776658270361
AGGTATAA -0.00790593013364
AGGTATAC -0.122120134361
AGGTATAG -0.159702672001
AGGTATAT 0.102155942644
AGGTATCA 0.10407626288

AGGTATCC 0.21736756021
AGGTATCG 0.0739625531074
AGGTATGA 0.122192583811
AGGTATGC -0.0861725339121
AGGTATGG -0.153496641644
AGGTATTA -0.0164092804324
AGGTATTC -0.167007571576
AGGTATTG 0.0636018353861
AGGTCAAA 0.0189807214563
AGGTCAAC -0.317062145149
AGGTCAAG -0.178049460685
AGGTCAAT -0.0321082789962
AGGTCACA -0.138122132836
AGGTCACC -0.402736153703
AGGTCACG -0.268009336393
AGGTCACT -0.144469686992
AGGTCAGA -0.156227709191
AGGTCAGC -0.249970951344
AGGTCAGG -0.259927688898
AGGTCATA -0.0184307010033
AGGTCATC -0.199340090869
AGGTCATG -0.0870645754474
AGGTCCAA -0.236765650104
AGGTCCAC -0.203429012346
AGGTCCAG -0.242205761317
AGGTCCAT -0.17009958863
AGGTCCCA -0.30115600158
AGGTCCCC -0.282548971205
AGGTCCCG -0.302322845282
AGGTCCGA -0.128666302554
AGGTCCGC -0.332419888412
AGGTCCGG -0.330872186915
AGGTCCTA -0.0980372789179
AGGTCCTC -0.150785811493
AGGTCCTG -0.326303010589
AGGTCGAA -0.165258981198
AGGTCGAC -0.128493370038
AGGTCGAG -0.0357811256552
AGGTCGAT -0.0931595277384
AGGTCGCA -0.295435185185
AGGTCGCC -0.317801299573
AGGTCGCG -0.132958554881
AGGTCGGA -0.0369880284575
AGGTCGGC -0.286138769156
AGGTCGGG -0.315399090395
AGGTCGTA 0.0243684935491
AGGTCGTC -0.237757200451
AGGTCGTG -0.116245253473
AGGTCTAA -0.0307416189159
AGGTCTAC -0.200566601689
AGGTCTAG -0.0536072969146
AGGTCTAT 0.0104600018593
AGGTCTCA -0.274348591839
AGGTCTCC -0.174929633105

AGGTCTCG -0.329970868513
AGGTCTGA -0.0645372382458
AGGTCTGC -0.263209876543
AGGTCTGG 0.0491695756219
AGGTCTTA -0.262820810726
AGGTCTTC -0.343793207743
AGGTCTTG -0.135251518744
AGGTGAAA -0.0735403077012
AGGTGAAC -0.216605800714
AGGTGAAG -0.209298496897
AGGTGAAT -0.0201138988567
AGGTGACA -0.0644745219434
AGGTGACC -0.33269780882
AGGTGACG -0.119691806194
AGGTGACT -0.179864574589
AGGTGAGA -0.221291541158
AGGTGAGC -0.169243664276
AGGTGAGG -0.193066272341
AGGTGATA 0.167851176035
AGGTGATC 0.157071462816
AGGTGATG -0.178899295791
AGGTGCAA 0.0874769369343
AGGTGCAC -0.249555511655
AGGTGCAG -0.107353231634
AGGTGCAT -0.160755742159
AGGTGCCA -0.143462728829
AGGTGCCC -0.32682287885
AGGTGCCG -0.208817510479
AGGTGCGA -0.0604757435036
AGGTGCGC -0.240661121973
AGGTGCGG -0.160013388864
AGGTGCTA -0.144429270844
AGGTGCTC -0.293135899432
AGGTGCTG -0.211573546843
AGGTGGAA -0.111211646774
AGGTGGAC -0.300292171771
AGGTGGAG -0.378497596945
AGGTGGAT 0.220331817587
AGGTGGCA -0.181283211166
AGGTGGCC -0.211314451707
AGGTGGCG -0.0534950971516
AGGTGGGA -0.0590167316
AGGTGGGC -0.208205761317
AGGTGGGG -0.220134430727
AGGTGGTA -0.0750087740963
AGGTGGTC -0.252583367701
AGGTGGTG 0.0363473915128
AGGTGTAA 0.147854953293
AGGTGTAC -0.102190957869
AGGTGTAG -0.185525054466
AGGTGTAT 0.118807839263
AGGTGTCA -0.144871243638
AGGTGTCC -0.195402209227
AGGTGTCG -0.262234085813

AGGTGTGA 0.00640826347827
AGGTGTGC -0.262937081217
AGGTGTGG 0.00324924806253
AGGTGTTA -0.0266311217003
AGGTGTTC -0.0652405096393
AGGTGTTG 0.0250499462118
AGGTTAAA 0.0287712134185
AGGTTAAC -0.0397830676276
AGGTTAAG -0.112222441744
AGGTTAAT 0.0678745619235
AGGTTACA 0.0642028365057
AGGTTACC -0.15656523649
AGGTTACG -0.151977732711
AGGTTACT -0.075200039514
AGGTTAGA 0.0637749431987
AGGTTAGC -0.23338580939
AGGTTAGG -0.128628710066
AGGTTATA -0.0934999506075
AGGTTATC 0.0665470957226
AGGTTATG -0.283314144331
AGGTTCAA -0.087187623469
AGGTTCAC -0.116436476973
AGGTTCAG -0.0801000633386
AGGTTCAT -0.241753900849
AGGTTCCA -0.158766010231
AGGTTCCC -0.134738071444
AGGTTCCG -0.280742898522
AGGTTCGA -0.0135322746701
AGGTTCGC -0.3203296854
AGGTTCGG -0.140623415788
AGGTTCTA -0.231915016216
AGGTTCTC -0.290469135802
AGGTTCTG -0.160828929824
AGGTTGAA -0.175019536186
AGGTTGAC -0.141996038213
AGGTTGAG -0.068057016346
AGGTTGAT -0.0480145989408
AGGTTGCA -0.18042072843
AGGTTGCC -0.239548025536
AGGTTGCG -0.213085499346
AGGTTGGA -0.121718811734
AGGTTGGC -0.263680566044
AGGTTGGG -0.224886276963
AGGTTGTA 0.0488704545785
AGGTTGTC -0.287615105301
AGGTTGTG -0.120084241104
AGGTTTAA -0.145746900775
AGGTTTAC -0.0843679334343
AGGTTTAG -0.0392239187599
AGGTTTAT -0.105718566818
AGGTTTCA -0.200924142397
AGGTTTCC -0.302148544736
AGGTTTCG -0.0869456654758
AGGTTTGA -0.0907594977154

AGGTTTGC 0.0583920503247
AGGTTTGG -0.16831308242
AGGTTTTA -0.16522875817
AGGTTTTC -0.167593456111
AGGTTTTG -0.202580648701
AGTAAAAA 0.130960613844
AGTAAAAC 0.0779801724833
AGTAAAAG 0.14028573624
AGTAAAAT 0.282942545391
AGTAAACA -0.131581546972
AGTAAACC -0.109535872824
AGTAAACG 0.133989510845
AGTAAACT -0.14473445937
AGTAAAGA -0.00605378234277
AGTAAAGC -0.0696588310832
AGTAAAGG -0.0674138229048
AGTAAATA 0.146399256171
AGTAAATC 0.287958115183
AGTAAATG 0.145609009187
AGTAACAA -0.166837603361
AGTAACAC -0.161156832727
AGTAACAG 0.038632309761
AGTAACAT -0.0186035917364
AGTAACCA 0.0505624567816
AGTAACCC -0.29226897395
AGTAACCG -0.290334619998
AGTAACGA 0.0994886173783
AGTAACGC -0.225577342048
AGTAACGG -0.1432599613
AGTAACTA 0.0323812706115
AGTAACTC -0.10195855714
AGTAACTG -0.0565440994872
AGTAAGAA 0.19862130083
AGTAAGAC -0.116752183438
AGTAAGAG 0.0643562297038
AGTAAGAT 0.259153313091
AGTAAGCA 0.0329262570384
AGTAAGCC -0.299479193738
AGTAAGCG -0.0348724502395
AGTAAGGA 0.0854314064475
AGTAAGGC -0.0317593598736
AGTAAGGG -0.176290809328
AGTAAGTA 0.213372946116
AGTAAGTC -0.145392873609
AGTAAGTG 0.0113339480559
AGTAATAA 0.00642569697674
AGTAATAC -0.127041966424
AGTAATAG -0.0272245134841
AGTAATAT 0.281017825752
AGTAATCA 0.326546685987
AGTAATCC 0.376297706042
AGTAATCG 0.289045862697
AGTAATGA 0.0320122615606
AGTAATGC 0.0405113174823

AGTAATGG -0.0373759679224
AGTAATTA 0.149909987047
AGTAATTC -0.0357334957957
AGTAATTG 0.125978818299
AGTACAAA 0.159186727296
AGTACAAC -0.0775355743506
AGTACAAG -0.0643149200077
AGTACAAT 0.155511097938
AGTACACA 0.0563794632063
AGTACACC -0.0889558075416
AGTACACG -0.151624496294
AGTACACT -0.110099806779
AGTACAGA 0.0128268458144
AGTACAGC -0.0474321909549
AGTACAGG 0.0531895189173
AGTACATA -0.0559863676776
AGTACATC 0.0450829793539
AGTACATG -0.0557395576396
AGTACCAA -0.188870780999
AGTACCAC -0.0959518635585
AGTACCAG -0.145385372996
AGTACCAT 0.0334505251841
AGTACCCA -0.0475338863626
AGTACCCC -0.303189083821
AGTACCCG -0.286130758813
AGTACCGA -0.147817105802
AGTACCGC -0.292477769418
AGTACCGG -0.252147094494
AGTACCTA -0.0725701991262
AGTACCTC -0.119502757883
AGTACCTG -0.299986832576
AGTACGAA 0.206604426003
AGTACGAC -0.0435735765398
AGTACGAG -0.0486618806121
AGTACGAT 0.203442995455
AGTACGCA -0.0970802399021
AGTACGCC -0.189148248307
AGTACGCG -0.226422796115
AGTACGGA -0.125883489179
AGTACGGC -0.147924601757
AGTACGGG 0.0559777919169
AGTACGTA -0.0796395091001
AGTACGTC -0.18904658303
AGTACGTG 0.152367323813
AGTACTAA -0.132061751567
AGTACTAC 0.0246081143384
AGTACTAG -0.0531163571711
AGTACTAT 0.0509584859744
AGTACTCA -0.137322323246
AGTACTCC -0.153211781862
AGTACTCG -0.137531900881
AGTACTGA 0.0918091613034
AGTACTGC 0.0553140573887
AGTACTGG -0.101654907792

AGTACTTA 0.0771449428088
AGTACTTC 0.00496467914835
AGTACTTG -0.0313468830358
AGTAGAAA 0.21647320326
AGTAGAAC -0.181285192137
AGTAGAAG -0.0713027444395
AGTAGAAT 0.307612627664
AGTAGACA -0.134632716049
AGTAGACC -0.226589095049
AGTAGACG -0.259410835241
AGTAGACT -0.125804483315
AGTAGAGA -0.0603514274425
AGTAGAGC -0.186763085113
AGTAGAGG -0.210096021948
AGTAGATA 0.302844189774
AGTAGATC 0.200980634289
AGTAGATG 0.0952388597965
AGTAGCAA 0.0204916683132
AGTAGCAC -0.0397265846324
AGTAGCAG -0.165168428332
AGTAGCAT -0.0819708570017
AGTAGCCA -0.192696975289
AGTAGCCC -0.215711934156
AGTAGCCG -0.214254357083
AGTAGCGA 0.0168930600148
AGTAGCGC -0.271348326029
AGTAGCGG -0.192927297668
AGTAGCTA -0.100609997036
AGTAGCTC -0.183200374184
AGTAGCTG -0.177475308642
AGTAGGAA 0.0168060769721
AGTAGGAC -0.233038712226
AGTAGGAG 0.133581942112
AGTAGGAT 0.223658710212
AGTAGGCA -0.241658219623
AGTAGGCC -0.146600823045
AGTAGGCG -0.168902495899
AGTAGGGA -0.0876413255361
AGTAGGGC -0.260389678289
AGTAGGGG -0.122620252852
AGTAGGTA 0.108568564497
AGTAGGTC -0.0844787463066
AGTAGGTG -0.0908931256654
AGTAGTAA 0.238917563963
AGTAGTAC 0.00256973436878
AGTAGTAG -0.15595283169
AGTAGTAT 0.179563581422
AGTAGTCA -0.0418825272762
AGTAGTCC -0.15792463081
AGTAGTCG -0.189637385311
AGTAGTGA -0.00554896924192
AGTAGTGC -0.0346029171313
AGTAGTGG -0.0092818851423
AGTAGTTA 0.184491450322

AGTAGTTC -0.0448562456243
AGTAGTTG -0.132518946565
AGTATAAA 0.111651737689
AGTATAAC 0.0870730608865
AGTATAAG 0.0149506777273
AGTATAAT 0.273951447707
AGTATACA 0.132357461196
AGTATACC -0.1501652067
AGTATACG -0.05663166214
AGTATACT 0.171410231088
AGTATAGA 0.150368282655
AGTATAGC -0.149684726955
AGTATAGG 0.129788798876
AGTATATA 0.21610719235
AGTATATC 0.194257832225
AGTATATG 0.0630816614124
AGTATCAA 0.118857234175
AGTATCAC 0.14174570243
AGTATCAG 0.206815736899
AGTATCAT 0.0972331994116
AGTATCCA 0.270743683989
AGTATCCC 0.150208475586
AGTATCCG 0.356771090586
AGTATCGA 0.0712949471501
AGTATCGC 0.153590574288
AGTATCGG 0.172317174911
AGTATCTA 0.288174276873
AGTATCTC 0.282651649872
AGTATCTG 0.309891545369
AGTATGAA -0.0760237316304
AGTATGAC -0.128530189777
AGTATGAG -0.0485373286965
AGTATGAT 0.175954847239
AGTATGCA 0.031576317181
AGTATGCC -0.130706527265
AGTATGCG -0.0230199792552
AGTATGGA -0.097876672484
AGTATGGC -0.223575093988
AGTATGGG -0.106762367119
AGTATGTA -0.116297721335
AGTATGTC -0.222967928962
AGTATGTG 0.180130189466
AGTATTAA 0.0134688303575
AGTATTAC -0.149615610346
AGTATTAG 0.0390495837752
AGTATTAT 0.100537677742
AGTATTCA 0.0865645838479
AGTATTCC -0.0843784773136
AGTATTCG 0.0397603330066
AGTATTGA 0.0874188252728
AGTATTGC 0.149615071617
AGTATTGG -0.199919878391
AGTATTTA 0.0236997519155
AGTATTTC 0.0563930091671

AGTATTTG 0.169696370941
AGTCAAAA 0.047286911801
AGTCAAAC -0.0750894640936
AGTCAAAG 0.0452968340989
AGTCAAAT 0.0756802696381
AGTCAACA -0.266141178213
AGTCAACC -0.170711161599
AGTCAACG -0.125279118674
AGTCAACT -0.181013054143
AGTCAAGA -0.0956056662096
AGTCAAGC -0.228776242869
AGTCAAGG -0.241655145171
AGTCAATA 0.122840237985
AGTCAATC -0.0256461290369
AGTCAATG -0.332030694073
AGTCACAA 0.0267541548662
AGTCACAC -0.11520782183
AGTCACAG -0.188863128597
AGTCACAT -0.00144949391213
AGTCACCA -0.194062454611
AGTCACCC -0.188296903461
AGTCACCG -0.005383779512
AGTCACGA -0.016633186239
AGTCACGC -0.305436274408
AGTCACGG -0.100750528251
AGTCACTA -0.160412490922
AGTCACTC -0.158501089325
AGTCACTG -0.286173382053
AGTCAGAA 0.00838341428611
AGTCAGAC -0.218923091719
AGTCAGAG -0.106630116237
AGTCAGAT 0.242271875409
AGTCAGCA -0.216072663078
AGTCAGCC -0.213844035137
AGTCAGCG -0.0948974670378
AGTCAGGA 0.149447212819
AGTCAGGC -0.0182659480191
AGTCAGGG -0.220476616274
AGTCAGTA -0.109844115468
AGTCAGTC -0.226242798354
AGTCAGTG -0.151028034306
AGTCATAA 0.0228435311423
AGTCATAC -0.0510631407934
AGTCATAG -0.0947086134661
AGTCATAT 0.0231755911216
AGTCATCA -0.102652854317
AGTCATCC -0.0851674529946
AGTCATCG -0.106537561925
AGTCATGA -0.0746494587166
AGTCATGC -0.127783813145
AGTCATGG 0.0156087922944
AGTCATTA -0.0377226780645
AGTCATTC -0.191623864085
AGTCATTG -0.169487291213

AGTCCAAA 0.046343285425
AGTCCAAC -0.211305359387
AGTCCAAG 0.0431987564104
AGTCCAAT 0.0128993360743
AGTCCACA -0.261176993387
AGTCCACC -0.282605033719
AGTCCACG -0.207212650021
AGTCCACT -0.211773420479
AGTCCAGA 0.0742798963753
AGTCCAGC -0.144540572017
AGTCCAGG -0.122129709252
AGTCCATA -0.161802472866
AGTCCATC -0.300585686778
AGTCCATG -0.149530864198
AGTCCCAA 0.00209938747283
AGTCCCAC -0.218694275728
AGTCCCAG -0.241251892817
AGTCCCAT -0.164033560527
AGTCCCCA -0.213704769844
AGTCCCCC -0.26034777709
AGTCCCCG -0.31837796085
AGTCCCGA -0.0982038572616
AGTCCCGC -0.220901929802
AGTCCCGG -0.296663384655
AGTCCCTA -0.0329478662452
AGTCCCTC -0.357222920762
AGTCCCTG -0.121150511436
AGTCCGAA -0.19749268664
AGTCCGAC -0.156009921419
AGTCCGAG -0.277541215973
AGTCCGAT 0.00492381211103
AGTCCGCA -0.0648715246907
AGTCCGCC -0.240782590257
AGTCCGCG -0.200391240171
AGTCCGGA -0.103639625119
AGTCCGGC -0.257568274977
AGTCCGGG -0.363824298973
AGTCCGTA -0.0340561098489
AGTCCGTC -0.266694907326
AGTCCGTG 0.0222910063778
AGTCCTAA -0.107718118734
AGTCCTAC -0.27263381656
AGTCCTAG -0.136043209877
AGTCCTAT -0.119203244537
AGTCCTCA -0.0400367955747
AGTCCTCC -0.333048053238
AGTCCTCG -0.242735285404
AGTCCTGA -0.126194621425
AGTCCTGC -0.349575102086
AGTCCTGG -0.284130746062
AGTCCTTA -0.212837086976
AGTCCTTC -0.245828738224
AGTCCTTG -0.337929587201
AGTCGAAA -0.149156654512

AGTCGAAC -0.17436838755
AGTCGAAG -0.116094178077
AGTCGAAT 0.0351706303662
AGTCGACA 0.0683305972426
AGTCGACC -0.168110095771
AGTCGACG -0.158084489176
AGTCGACT -0.24411198341
AGTCGAGA -0.0816614124039
AGTCGAGC -0.29868577388
AGTCGAGG -0.232774022247
AGTCGATA 0.0569111714827
AGTCGATC 0.0285807679456
AGTCGATG -0.139171495676
AGTCGCAA 0.196514753098
AGTCGCAC -0.141126531777
AGTCGCAG -0.138508959491
AGTCGCAT -0.267736106148
AGTCGCCA -0.321506756868
AGTCGCCC -0.339657645298
AGTCGCCG -0.22633147265
AGTCGCGA -0.125208932837
AGTCGCGC -0.129899458878
AGTCGCGG -0.0978025837751
AGTCGCTA -0.028458296289
AGTCGCTC -0.327056861325
AGTCGCTG -0.204213489718
AGTCGGAA -0.11164086802
AGTCGGAC -0.0680805717999
AGTCGGAG -0.0844706760398
AGTCGGAT 0.0603814024457
AGTCGGCA -0.071050712744
AGTCGGCC -0.137603503054
AGTCGGCG -0.174548307628
AGTCGGGA -0.025088257086
AGTCGGGC -0.285606908508
AGTCGGGG -0.254505891649
AGTCGGTA -0.0712198154647
AGTCGGTC -0.254982816871
AGTCGGTG -0.138338847464
AGTCGTAA 0.00792788342801
AGTCGTAC -0.133790488107
AGTCGTAG -0.20659121017
AGTCGTAT 0.118336846034
AGTCGTCA -0.156064272896
AGTCGTCC -0.240721591091
AGTCGTCG -0.253567725051
AGTCGTGA -0.0786158588491
AGTCGTGC -0.0659552229881
AGTCGTGG -0.129319961884
AGTCGTTA 0.0556462662857
AGTCGTTC -0.108568717422
AGTCGTTG -0.254829009175
AGTCTAAA -0.0222611247512
AGTCTAAC -0.147915885868

AGTCTAAG -0.0328833618899
AGTCTAAT -0.174364420734
AGTCTACA -0.0766013394738
AGTCTACC -0.25931320113
AGTCTACG -0.148883619838
AGTCTAGA -0.0714683082298
AGTCTAGC -0.107617514881
AGTCTAGG -0.297634918284
AGTCTATA -0.0508607789868
AGTCTATC -0.209020382665
AGTCTATG -0.106507317234
AGTCTCAA -0.018150280956
AGTCTCAC -0.228550068587
AGTCTCAG -0.210709993909
AGTCTCAT -0.104030608688
AGTCTCCA -0.151114216243
AGTCTCCC -0.314331518787
AGTCTCCG -0.251450039355
AGTCTCGA 0.0381711202213
AGTCTCGC -0.284483430799
AGTCTCGG -0.210420884982
AGTCTCTA -0.180087791495
AGTCTCTC -0.238408133624
AGTCTCTG -0.109322182712
AGTCTGAA -0.156254115633
AGTCTGAC -0.289888162672
AGTCTGAG -0.0244943881891
AGTCTGAT 0.0966890880828
AGTCTGCA -0.215085421793
AGTCTGCC -0.280293055967
AGTCTGCG -0.271496787536
AGTCTGGA -0.193694444444
AGTCTGGC -0.331876131026
AGTCTGGG -0.129505950323
AGTCTGTA 0.0170397919603
AGTCTGTC -0.247387073348
AGTCTGTG -0.14355213118
AGTCTTAA -0.0603930349078
AGTCTTAC 0.00581843011346
AGTCTTAG 0.0388529368177
AGTCTTAT 0.0504384881583
AGTCTTCA -0.0439177069415
AGTCTTCC 0.134580342049
AGTCTTCG -0.009108322905
AGTCTTGA -0.0412839771621
AGTCTTGC 0.0123397330348
AGTCTTGG -0.189310094408
AGTCTTTA -0.0776475816735
AGTCTTTC -0.126536044565
AGTCTTTG 0.00713721228885
AGTGAAAA 0.117686447562
AGTGAAAC -0.199481353421
AGTGAAAG -0.104769039935
AGTGAAAT 0.230071332065

AGTGAACA -0.152168375304
AGTGAACC -0.0295693614499
AGTGAACG 0.158326674705
AGTGAACT -0.303896394368
AGTGAAGA -0.0133083659227
AGTGAAGC -0.265824034759
AGTGAAGG -0.207518598986
AGTGAATA 0.0998198538492
AGTGAATC 0.1306429513
AGTGAATG -0.0904480157224
AGTGACAA -0.0864687656595
AGTGACAC -0.0777227735644
AGTGACAG -0.103793922983
AGTGACAT -0.0409196424162
AGTGACCA -0.242787560884
AGTGACCC -0.246819087123
AGTGACCG -0.258615582107
AGTGACGA -0.000820215517802
AGTGACGC -0.0314395612058
AGTGACGG -0.116907508065
AGTGACTA -0.0922861611921
AGTGACTC -0.311317467002
AGTGACTG -0.29783066693
AGTGAGAA -0.0763093283745
AGTGAGAC -0.0473175818446
AGTGAGAG 0.0100715712749
AGTGAGAT 0.289499222757
AGTGAGCA -0.0428261791726
AGTGAGCC -0.295527750428
AGTGAGCG -0.109638344227
AGTGAGGA -0.22957560722
AGTGAGGC -0.186516605852
AGTGAGGG -0.0372320723034
AGTGAGTA -0.0472388227611
AGTGAGTC -0.236172709344
AGTGAGTG -0.0418202841961
AGTGATAA 0.029119739182
AGTGATAC 0.0952929554138
AGTGATAG 0.0408043564161
AGTGATAT 0.28378136422
AGTGATCA -0.116829209317
AGTGATCC 0.0253613818953
AGTGATCG 0.232066016817
AGTGATGA 0.00955564337307
AGTGATGC 0.00341390590353
AGTGATGG -0.0961535668536
AGTGATTA 0.267501053274
AGTGATTC 0.227461344662
AGTGATTG 0.046521328679
AGTGCAAA 0.0147471254905
AGTGCAAC -0.117708067028
AGTGCAAG 0.0408955191113
AGTGCAAT 0.17131276208
AGTGCACA -0.139688904529

AGTGCACC -0.206607511304
AGTGCACG -0.0302543837985
AGTGCACT -0.116627916371
AGTGCAGA 0.177878343237
AGTGCAGC -0.103247897015
AGTGCAGG -0.157937545389
AGTGCATA -0.0904578456068
AGTGCATC -0.167030815092
AGTGCATG -0.137455205984
AGTGCCAA -0.0855614139082
AGTGCCAC -0.292159960962
AGTGCCAG -0.303835407966
AGTGCCAT -0.102543877234
AGTGCCCA -0.222326474623
AGTGCCCC -0.240575311833
AGTGCCCG -0.166164633845
AGTGCCGA 0.0658005979099
AGTGCCGC -0.219968046478
AGTGCCGG -0.193647663006
AGTGCCTA -0.360222397771
AGTGCCTC -0.284424255392
AGTGCCTG -0.197168455242
AGTGCGAA 0.0652616558272
AGTGCGAC -0.194935432219
AGTGCGAG -0.143036869138
AGTGCGAT 0.319175503304
AGTGCGCA 0.232140112846
AGTGCGCC -0.117807576431
AGTGCGCG -0.035833103308
AGTGCGGA 0.081979573751
AGTGCGGC -0.257969034608
AGTGCGGG -0.177982720152
AGTGCGTA 0.0058532971104
AGTGCGTC -0.213593538227
AGTGCGTG -0.0438699461014
AGTGCTAA 0.113955515523
AGTGCTAC -0.0954807446487
AGTGCTAG -0.0443763925933
AGTGCTAT -0.00939510197809
AGTGCTCA -0.190987672968
AGTGCTCC -0.280621402308
AGTGCTCG -0.000706929763904
AGTGCTGA -0.239824031929
AGTGCTGC -0.300392060058
AGTGCTGG 0.0212703209217
AGTGCTTA 0.0587471733737
AGTGCTTC -0.248228993476
AGTGCTTG -0.198949974142
AGTGGAAA 0.117305652812
AGTGGAAC -0.206150891632
AGTGGAAG -0.152688773056
AGTGGAAT 0.206546795758
AGTGGACA 0.00436617708402
AGTGGACC -0.0872020281839

AGTGGACG -0.193952658837
AGTGGAGA 0.0818340659883
AGTGGAGC -0.00724184297906
AGTGGAGG -0.0641147616411
AGTGGATA 0.310218116753
AGTGGATC 0.199813060785
AGTGGATG 0.105529210301
AGTGGCAA -0.188542626981
AGTGGCAC -0.433641061085
AGTGGCAG -0.0775176569368
AGTGGCAT -0.142930397822
AGTGGCCA -0.134020335709
AGTGGCCC -0.328093795949
AGTGGCCG -0.159769547325
AGTGGCGA 0.0391556345913
AGTGGCGC -0.211581297869
AGTGGCGG -0.0989398129856
AGTGGCTA -0.284142338417
AGTGGCTC -0.221511589622
AGTGGCTG -0.0904173870091
AGTGGGAA -0.086532790624
AGTGGGAC -0.198295763172
AGTGGGAG 0.0555775099344
AGTGGGAT 0.0413044194165
AGTGGGCA 0.0331522186548
AGTGGGCC -0.205021620791
AGTGGGCG -0.097541417041
AGTGGGGA -0.110551329755
AGTGGGGC -0.320400359874
AGTGGGGG -0.19929557008
AGTGGGTA -0.105376379491
AGTGGGTC -0.416322767073
AGTGGGTG -0.153050754458
AGTGGTAA 0.10390800924
AGTGGTAC -0.0686302350849
AGTGGTAG -0.065561879599
AGTGGTAT 0.151085918461
AGTGGTCA -0.266573526507
AGTGGTCC -0.248660196882
AGTGGTCG -0.197626571861
AGTGGTGA -0.00586181913983
AGTGGTGC -0.200649592907
AGTGGTGG -0.0304781663703
AGTGGTTA -0.0872823772201
AGTGGTTC -0.0765013828526
AGTGGTTG -0.0577043403807
AGTGTAAA 0.0740960284529
AGTGTAAC -0.0133454774709
AGTGTAAG 0.00487411561315
AGTGTAAT 0.352410907559
AGTGTACA 0.0424570243035
AGTGTACC -0.0104150625427
AGTGTACG -0.177413263691
AGTGTAGA 0.0187493281503

AGTGTAGC -0.135989645525
AGTGTAGG -0.143884256275
AGTGTATA 0.0754945829228
AGTGTATC 0.145173100112
AGTGTATG 0.010612642192
AGTGTCAA 0.0887582732392
AGTGTCAC -0.23252391785
AGTGTCAG 0.0375639421283
AGTGTCAT -0.127480844804
AGTGTCCA -0.0823167878202
AGTGTCCC -0.26028892949
AGTGTCCG -0.0196262442545
AGTGTCGA 0.0510670753854
AGTGTCGC -0.0132741562913
AGTGTCGG -0.00747106862151
AGTGTCTA -0.0647637006003
AGTGTCTC -0.0343037168763
AGTGTCTG -0.0779684849048
AGTGTGAA -0.0244794582195
AGTGTGAC -0.112631301656
AGTGTGAG -0.123150960636
AGTGTGAT 0.0884761465674
AGTGTGCA -0.0472067030991
AGTGTGCC -0.255206502572
AGTGTGCG -0.0166405971296
AGTGTGGA -0.0861403687185
AGTGTGGC -0.166021947874
AGTGTGGG -0.12421383191
AGTGTGTA 0.07455871457
AGTGTGTC -0.120225509081
AGTGTGTG -0.0498263914111
AGTGTTAA -0.121985689925
AGTGTTAC -0.155956203178
AGTGTTAG -0.110192785437
AGTGTTAT 0.0589906004387
AGTGTTCA -0.184869850835
AGTGTTCC -0.211906202163
AGTGTTCG -0.128654681944
AGTGTTGA -0.0632647131463
AGTGTTGC 0.158587455268
AGTGTTGG -0.188212342279
AGTGTTTA -0.11862319009
AGTGTTTC -0.0622768148998
AGTGTTTG -0.0370727129026
AGTTAAAA 0.151661267125
AGTTAAAC -0.0684765369904
AGTTAAAG -0.00434147840787
AGTTAAAT 0.156342161463
AGTTAACA 0.00669882178606
AGTTAACC -0.244934414611
AGTTAACG -0.141237543351
AGTTAACT -0.115861842962
AGTTAAGA 0.0889701200905
AGTTAAGC -0.188364282892

AGTTAAGG -0.0268112678512
AGTTAATA 0.105950459944
AGTTAATC -0.0610523831478
AGTTAATG -0.0644066072959
AGTTACAA -0.0193410137579
AGTTACAC 0.0463193526361
AGTTACAG -0.0671349498061
AGTTACAT 0.164989176703
AGTTACCA 0.031094097308
AGTTACCC -0.102410233615
AGTTACCG -0.0587801049419
AGTTACGA 0.0583163186898
AGTTACGC -0.00279952929554
AGTTACGG -0.112013362624
AGTTACTA -0.05116586521
AGTTACTC 0.0315676524183
AGTTACTG 0.0199865187604
AGTTAGAA -0.103385914071
AGTTAGAC -0.213604087098
AGTTAGAG -0.032257705987
AGTTAGAT 0.212792816527
AGTTAGCA -0.0411459619659
AGTTAGCC -0.186390548779
AGTTAGCG 0.00359193036759
AGTTAGGA -0.0387774491403
AGTTAGGC -0.119953977837
AGTTAGGG -0.000581704834187
AGTTAGTA 0.141035107653
AGTTAGTC -0.184432824982
AGTTAGTG -0.0991120769606
AGTTATAA 0.0557788657288
AGTTATAC 0.0623339700103
AGTTATAG 0.00521142066785
AGTTATAT 0.109283409076
AGTTATCA 0.00394432902823
AGTTATCC -0.102365035456
AGTTATCG 0.0172606162742
AGTTATGA -0.000502641044564
AGTTATGC 0.102243110136
AGTTATGG -0.0848426875169
AGTTATTA 0.0528017084828
AGTTATTC 0.0759100563074
AGTTATTG 0.0301643107231
AGTTCAAA -0.0526977126604
AGTTCAAC -0.119109947644
AGTTCAAG -0.0948251565383
AGTTCAAT 0.0837694129269
AGTTCACA 0.0390787942655
AGTTCACC -0.312521058196
AGTTCACG -0.11109739098
AGTTCAGA 0.126562840498
AGTTCAGC 0.0216909262552
AGTTCAGG -0.102672423311
AGTTCATA 0.0324655325207

AGTTCATC -0.170911853309
AGTTCATG -0.00996760274868
AGTTCCAA -0.0565572240883
AGTTCCAC -0.329098021144
AGTTCCAG -0.120553305454
AGTTCCAT -0.144173722042
AGTTCCCA -0.16331138546
AGTTCCCC -0.200491902434
AGTTCCCG -0.154506343562
AGTTCCGA -0.0413250353979
AGTTCCGC -0.12167234652
AGTTCCGG -0.00940200242895
AGTTCCTA -0.0382119064843
AGTTCCTC -0.290820079484
AGTTCCTG -0.0905158388919
AGTTCGAA 0.0221449014281
AGTTCGAC -0.180768820852
AGTTCGAG -0.0206563261543
AGTTCGAT -0.00346055895848
AGTTCGCA -0.0654170252485
AGTTCGCC -0.169913658865
AGTTCGCG -0.0351070719045
AGTTCGGA -0.139182441701
AGTTCGGC -0.23820943826
AGTTCGGG -0.1067231964
AGTTCGTA 0.0082049613286
AGTTCGTC -0.0693470315124
AGTTCGTG -0.130390316751
AGTTCTAA 0.00357888496868
AGTTCTAC -0.0777947204662
AGTTCTAG 0.0564714599102
AGTTCTAT -0.152424466947
AGTTCTCA -0.237899782135
AGTTCTCC -0.231464564164
AGTTCTCG 0.018265718631
AGTTCTGA 0.0508335706109
AGTTCTGC 0.0077663615008
AGTTCTGG 0.1156904141
AGTTCTTA -0.0475863001375
AGTTCTTC -0.186273541611
AGTTCTTG -0.0650723086633
AGTTGAAA 0.181864803219
AGTTGAAC -0.180191187367
AGTTGAAG -0.0742494202311
AGTTGAAT 0.0788848273288
AGTTGACA -0.00152199286184
AGTTGACC -0.336290486565
AGTTGACG -0.00765002237188
AGTTGAGA 0.127757035143
AGTTGAGC -0.161715318289
AGTTGAGG -0.217076046831
AGTTGATA 0.203793238708
AGTTGATC -0.0163807108828
AGTTGATG 0.0115249952784

AGTTGCAA 0.153866053763
AGTTGCAC -0.0580942640812
AGTTGCAG -0.121372733004
AGTTGCAT -0.0605693456087
AGTTGCCA -0.292964206553
AGTTGCCC -0.105290602586
AGTTGCCG -0.18920905317
AGTTGCGA 0.00983845469815
AGTTGCGC -0.168524646844
AGTTGCGG -0.000774299643793
AGTTGCTA -0.120708787219
AGTTGCTC -0.224054869684
AGTTGCTG 0.19260576522
AGTTGGAA 0.00663812738399
AGTTGGAC -0.282375594654
AGTTGGAG -0.223799647754
AGTTGGAT 0.324960482118
AGTTGGCA -0.143880586608
AGTTGGCC -0.210871056241
AGTTGGCG -0.0226308036921
AGTTGGGA 0.0200625668889
AGTTGGGC -0.288426147352
AGTTGGGG -0.21070442992
AGTTGGTA -0.0278747112577
AGTTGGTC -0.325730756385
AGTTGGTG -0.179315219002
AGTTGTAA 0.0117538255505
AGTTGTAC 0.00278820610771
AGTTGTAG 0.00232990845024
AGTTGTAT 0.139298011128
AGTTGTCA 0.0709819418012
AGTTGTCC -0.24751839597
AGTTGTCG -0.0103220838842
AGTTGTGA -0.214175744372
AGTTGTGC -0.128955558387
AGTTGTGG -0.09645106561
AGTTGTTA 0.0482021704706
AGTTGTTC -0.337781386445
AGTTGTTG -0.0441634099923
AGTTTAAA 0.0574052119695
AGTTTAAC -0.103085700674
AGTTTAAG -0.0548821059666
AGTTTAAT -0.0757069309974
AGTTTACA -0.0232414542581
AGTTTACC -0.0312945825404
AGTTTACG -0.0238644097565
AGTTTAGA 0.157179630727
AGTTTAGC -0.161630322665
AGTTTAGG -0.0795304886079
AGTTTATA 0.0843901264447
AGTTTATC -0.101445527581
AGTTTATG -0.0260845463128
AGTTTCAA -0.0165414844624
AGTTTCAC -0.13052523706

AGTTTCAG -0.00810512399576
AGTTTCAT 0.0102814057211
AGTTTCCA 0.044004808009
AGTTTCCC -0.0974397266562
AGTTTCCG -0.212294434293
AGTTTCGA 0.0534150036225
AGTTTCGC 0.0526186567489
AGTTTCGG -0.272735004746
AGTTTCTA -0.160093599393
AGTTTCTC -0.0890625771719
AGTTTCTG 0.0500477507739
AGTTTGAA 0.101006608268
AGTTTGAC -0.00234916391847
AGTTTGAG -0.00309498121693
AGTTTGAT 0.126573010039
AGTTTGCA -0.0331338166287
AGTTTGCC -0.0483590719568
AGTTTGCG -0.0667275900678
AGTTTGGA 0.1544128543
AGTTTGGC -0.294263374486
AGTTTGGG -0.0956630355846
AGTTTGTA -0.0442847098338
AGTTTGTC -0.259320536459
AGTTTGTG 0.0165110650069
AGTTTTAA -0.061676389244
AGTTTTAC 0.088039530234
AGTTTTAG 0.00915985065303
AGTTTTAT -0.00319468859413
AGTTTTCA 0.0045254456438
AGTTTTCC 0.0517469818256
AGTTTTCG 0.0386720679775
AGTTTTGA 0.0468767151858
AGTTTTGC -0.0869046685202
AGTTTTGG -0.0882001042781
AGTTTTTA 0.0770313657693
AGTTTTTC -0.0941601914044
AGTTTTTG -0.0202058151356
ATAAAAAA 0.184545763903
ATAAAAAC 0.0495910391818
ATAAAAAG 0.0901391907616
ATAAAAAT 0.312672873654
ATAAAACA 0.08591518603
ATAAAACC 0.0707814565688
ATAAAACG 0.114911962941
ATAAAAGA 0.179188895475
ATAAAAGC 0.132807525961
ATAAAAGG -0.170626585202
ATAAAATA 0.222178313465
ATAAAATC 0.413067859893
ATAAAATG 0.0834979911743
ATAAACAA 0.0180073511252
ATAAACAC 0.122916333735
ATAAACAG -0.024437897844
ATAAACAT 0.0242825743357

ATAAACCA -0.117920170143
ATAAACCC -0.0864191432727
ATAAACCG -0.111128383188
ATAAACGA 0.0903752560545
ATAAACGC -0.0704763002481
ATAAACGG -0.00295228760039
ATAAACTA -0.058271468627
ATAAACTC -0.0494295169416
ATAAACTG -0.0299347696599
ATAAAGAA 0.00970610027167
ATAAAGAC 0.0334721947792
ATAAAGAG -0.0418679993608
ATAAAGAT 0.33480597969
ATAAAGCA -0.108907875675
ATAAAGCC -0.260207697894
ATAAAGCG 0.150309268701
ATAAAGGA 0.0887909270781
ATAAAGGC -0.287343834872
ATAAAGGG 0.0200155731561
ATAAAGTA 0.177953888397
ATAAAGTC -0.208160330074
ATAAAGTG -0.0678700623248
ATAAATAA 0.116521145381
ATAAATAC -0.000920396134336
ATAAATAG 0.0131040198468
ATAAATAT 0.336283887608
ATAAATCA 0.168688591508
ATAAATCC 0.230961893278
ATAAATCG 0.253500235299
ATAAATGA 0.199774817311
ATAAATGC 0.0179594090044
ATAAATGG -0.0851445170661
ATAAATTA 0.0596269231328
ATAAATTC 0.125993346215
ATAAATTG 0.147515000073
ATAACAAA 0.104387430448
ATAACAAC 0.151768773699
ATAACAAG 0.00703293929116
ATAACAAT 0.17214153988
ATAACACA 0.0790536515915
ATAACACC 0.0605770487993
ATAACACG 0.0737044731451
ATAACAGA 0.128521203493
ATAACAGC -0.0596938901511
ATAACAGG -0.0826058233725
ATAACATA 0.0598448923138
ATAACATC 0.112834529847
ATAACATG 0.0221217809392
ATAACCAA 0.0607310447024
ATAACCAC -0.0302790812546
ATAACCAG -0.0962287865815
ATAACCAT 0.0602043952666
ATAACCCA -0.0661046345694
ATAACCCC -0.198086959385

ATAACCCG 0.0891347779315
ATAACCGA -0.0269613896435
ATAACCGC 0.0387853371016
ATAACCGG -0.190666510296
ATAACCTA -0.141321348019
ATAACCTC -0.0828575588348
ATAACCTG -0.0471176748219
ATAACGAA 0.187037585897
ATAACGAC -0.00992983016867
ATAACGAG 0.0787983819623
ATAACGAT 0.202654250142
ATAACGCA 0.173231025928
ATAACGCC -0.127144940758
ATAACGCG 0.15593371973
ATAACGGA 0.167827504446
ATAACGGC 0.117538099458
ATAACGGG 0.0624739291752
ATAACGTA 0.241646358866
ATAACGTC 0.037890940858
ATAACGTG 0.0994846821643
ATAACTAA 0.0419383521043
ATAACTAC -0.16969013559
ATAACTAG -0.00632286109465
ATAACTAT 0.0485300961736
ATAACTCA 0.0149798370847
ATAACTCC 0.0388290632067
ATAACTCG 0.0899635557312
ATAACTGA 0.0139629849173
ATAACTGC 0.0897839698982
ATAACTGG 0.000408900305166
ATAACTTA 0.0933591078917
ATAACTTC -0.100778696265
ATAACTTG 0.0850623973966
ATAAGAAA 0.14720410986
ATAAGAAC -0.0206518644206
ATAAGAAG 0.0214446559063
ATAAGAAT 0.312183109846
ATAAGACA 0.0306640091579
ATAAGACC -0.207133058985
ATAAGACG -0.0719044639819
ATAAGAGA -0.0799414931166
ATAAGAGC 0.109277526768
ATAAGAGG -0.0192393183502
ATAAGATA 0.288159454653
ATAAGATC 0.330438099629
ATAAGATG -0.00400079027956
ATAAGCAA 0.064371740299
ATAAGCAC -0.0786783227145
ATAAGCAG -0.00561040379799
ATAAGCAT 0.126208359363
ATAAGCCA -0.319323997003
ATAAGCCC -0.256533042847
ATAAGCCG -0.0719382014764
ATAAGCGA 0.0172664274403

ATAAGCGC -0.108682325049
ATAAGCGG -0.147249111023
ATAAGCTA -0.0880974335331
ATAAGCTC -0.0747008566872
ATAAGCTG -0.00525474699635
ATAAGGAA 0.199329293728
ATAAGGAC -0.23025828436
ATAAGGAG -0.00574107005642
ATAAGGAT 0.273187279357
ATAAGGCA -0.0555390897715
ATAAGGCC -0.0588785713502
ATAAGGCG 0.0159457330279
ATAAGGGA 0.0318931326544
ATAAGGGC -0.20679210496
ATAAGGGG -0.10769754245
ATAAGGTA 0.0269509789395
ATAAGGTC -0.279260696772
ATAAGGTG -0.196446257145
ATAAGTAA 0.227258727645
ATAAGTAC 0.0477452852972
ATAAGTAG 0.0250766347537
ATAAGTAT 0.0925130079694
ATAAGTCA -0.195241544701
ATAAGTCC -0.0422660642424
ATAAGTCG -0.0717523985159
ATAAGTGA 0.0179812008775
ATAAGTGC 0.0183390229761
ATAAGTGG 0.187778546181
ATAAGTTA 0.0533334301861
ATAAGTTC -0.0215899350602
ATAAGTTG 0.028595295861
ATAATAAA 0.203512498765
ATAATAAC 0.175019249488
ATAATAAG 0.131159472927
ATAATAAT 0.252978949051
ATAATACA 0.161180816264
ATAATACC -0.0344994406753
ATAATACG 0.118511402252
ATAATAGA 0.0193686167972
ATAATAGC 0.0313316428462
ATAATAGG 0.0713443396226
ATAATATA 0.269876945707
ATAATATC 0.366877805704
ATAATATG 0.0826856304389
ATAATCAA 0.221549256897
ATAATCAC 0.240812092472
ATAATCAG 0.20258951898
ATAATCAT 0.238108959582
ATAATCCA 0.329609321829
ATAATCCC 0.246487299392
ATAATCCG 0.312764226461
ATAATCGA 0.214131200649
ATAATCGC 0.265540784729
ATAATCGG 0.110840730464

ATAATCTA 0.355441430709
ATAATCTC 0.301192122769
ATAATCTG 0.348124237878
ATAATGAA 0.0442738221493
ATAATGAC 0.132253424956
ATAATGAG 0.0257420132785
ATAATGAT 0.150597823718
ATAATGCA 0.233961907806
ATAATGCC 0.0539419884196
ATAATGCG 0.069362985548
ATAATGGA 0.218229269578
ATAATGGC -0.124756669437
ATAATGGG 0.0954288239039
ATAATGTA 0.147396399289
ATAATGTC 0.154479651185
ATAATGTG 0.110982129136
ATAATTAA 0.126312960353
ATAATTAC 0.186162160592
ATAATTAG 0.118926968169
ATAATTAT 0.22158052801
ATAATTCA 0.132324611741
ATAATTCC 0.216891607223
ATAATTCG 0.107083811544
ATAATTGA 0.126901798064
ATAATTGC 0.140966207942
ATAATTGG 0.066477859009
ATAATTTA 0.202395653248
ATAATTTC 0.205718017959
ATAATTTG 0.070601310418
ATACAAAA 0.243201678553
ATACAAAC 0.0812012249333
ATACAAAG -0.000126392863888
ATACAAAT 0.293218369096
ATACAACA 0.137917407627
ATACAACC -0.0446991152385
ATACAACG -0.0924627086832
ATACAAGA 0.129975411096
ATACAAGC -0.0390132639868
ATACAAGG 0.139045313838
ATACAATA 0.17443028387
ATACAATC 0.266597416937
ATACAATG -0.190731800672
ATACACAA 0.181205954028
ATACACAC 0.0495823224326
ATACACAG 0.143576148763
ATACACAT 0.170239565325
ATACACCA 0.125987535049
ATACACCC 0.110513835842
ATACACCG 0.110169540773
ATACACGA 0.141029738643
ATACACGC -0.0886037278643
ATACACGG 0.0664216291604
ATACACTA 0.189500675548
ATACACTC 0.00245667049235

ATACACTG -0.094915868155
ATACAGAA 0.260745949817
ATACAGAC 0.00493924725872
ATACAGAG 0.120092195613
ATACAGAT 0.256555721819
ATACAGCA 0.0172616303322
ATACAGCC -0.0787935482372
ATACAGCG 0.052407846495
ATACAGGA 0.189537640782
ATACAGGC 0.0977133061177
ATACAGGG -0.213595152121
ATACAGTA 0.118073393488
ATACAGTC -0.145902515696
ATACAGTG 0.136786725496
ATACATAA 0.061463251638
ATACATAC 0.0862807086873
ATACATAG 0.215103514896
ATACATAT 0.185489720943
ATACATCA 0.0550396276537
ATACATCC -0.0854314064475
ATACATCG 0.126813980548
ATACATGA 0.147844018572
ATACATGC 0.0223646512547
ATACATGG -0.106734389235
ATACATTA 0.0989566181475
ATACATTC 0.124058706009
ATACATTG 0.0444658499638
ATACCAAA 0.0563552365871
ATACCAAC 0.0900541891244
ATACCAAG 0.053903410527
ATACCAAT 0.169866386561
ATACCACA -0.0475147643197
ATACCACC 0.153647720038
ATACCACG -0.0403686140201
ATACCAGA -0.165188052551
ATACCAGC 0.0817498737623
ATACCAGG 0.0143143550332
ATACCATA 0.180569277042
ATACCATC 0.0162352631232
ATACCATG 0.09250203078
ATACCCAA 0.220262793914
ATACCCAC 0.0601806472413
ATACCCAG 0.20847703863
ATACCCAT 0.145013293043
ATACCCCA 0.000842499286483
ATACCCCC -0.0110746299014
ATACCCCG 0.0249170497004
ATACCCGA 0.0226706404092
ATACCCGC 0.0917130452919
ATACCCGG 0.0539614733618
ATACCCTA 0.189235768074
ATACCCTC 0.0580980559882
ATACCCTG 0.0690584458036
ATACCGAA 0.100759621507

ATACCGAC 0.0522212342752
ATACCGAG 0.0473857016257
ATACCGAT 0.293267764009
ATACCGCA 0.140077169641
ATACCGCC 0.131938169192
ATACCGCG -0.0128762431667
ATACCGGA -0.152706719078
ATACCGGC -0.148346613109
ATACCGGG 0.141081582472
ATACCGTA 0.270357241439
ATACCGTC 0.0102145773103
ATACCGTG 0.0701882470611
ATACCTAA 0.0670419711476
ATACCTAC 0.0665480220243
ATACCTAG 0.054979253112
ATACCTAT 0.108053963863
ATACCTCA 0.0420394287624
ATACCTCC -0.141403993959
ATACCTCG 0.12809408278
ATACCTGA -0.0153109700289
ATACCTGC -0.0588312529087
ATACCTGG -0.0891495842595
ATACCTTA 0.127184965823
ATACCTTC 0.106148213793
ATACCTTG -0.0701256893155
ATACGAAA 0.236324146848
ATACGAAC 0.140990907838
ATACGAAG 0.119050363345
ATACGAAT 0.312726404531
ATACGACA 0.106467827931
ATACGACC -0.0467076664691
ATACGACG 0.191535489253
ATACGAGA 0.22242964857
ATACGAGC 0.00837451590504
ATACGAGG -0.0440457374296
ATACGATA 0.314265955345
ATACGATC 0.214881226811
ATACGATG 0.0655756245824
ATACGCAA 0.384802347711
ATACGCAC 0.251319861113
ATACGCAG 0.164653408456
ATACGCAT 0.166633736725
ATACGCCA 0.0124954600264
ATACGCCC 0.149075298185
ATACGCCG 0.0800706056688
ATACGCGA 0.218754085976
ATACGCGC 0.184787262069
ATACGCGG 0.224652419624
ATACGCTA 0.199431958508
ATACGCTC 0.0997326863568
ATACGCTG 0.105973786472
ATACGGAA 0.179169685394
ATACGGAC 0.182273485697
ATACGGAG 0.0228422413668

ATACGGAT 0.37560100112
ATACGGCA 0.0380002854069
ATACGGCC 0.11162978331
ATACGGCG 0.229101937772
ATACGGGA 0.142848909965
ATACGGGC -0.0809241165721
ATACGGGG 0.126540427739
ATACGGTA 0.164413871254
ATACGGTC 0.0427259654438
ATACGGTG 0.0589121496956
ATACGTAA 0.294589804309
ATACGTAC 0.177926518694
ATACGTAG 0.0207938052969
ATACGTAT 0.204194181208
ATACGTCA -0.0624046605553
ATACGTCC 0.0325967639246
ATACGTCG 0.222775412956
ATACGTGA 0.199155928116
ATACGTGC 0.0788996556884
ATACGTGG 0.0928929437915
ATACGTTA 0.270099233792
ATACGTTC 0.0545236997519
ATACGTTG 0.0861517267119
ATACTAAA 0.167485072567
ATACTAAC -0.0185843816549
ATACTAAG -0.00231893126084
ATACTAAT 0.147266744048
ATACTACA 0.0585386067751
ATACTACC 0.00490898261009
ATACTACG 0.0859541373027
ATACTAGA 0.0622892383852
ATACTAGC -0.105512744517
ATACTAGG 0.225924279348
ATACTATA 0.146399256171
ATACTATC 0.219198006542
ATACTATG 0.0657344587625
ATACTCAA 0.147934092955
ATACTCAC 0.0843708686241
ATACTCAG 0.222565259391
ATACTCAT 0.234647625412
ATACTCCA 0.179772783403
ATACTCCC 0.140869931574
ATACTCCG 0.00559727299674
ATACTCGA 0.183841327866
ATACTCGC 0.038863336774
ATACTCGG 0.0352909216635
ATACTCTA 0.209008760333
ATACTCTC 0.167975696503
ATACTCTG 0.114775217944
ATACTGAA 0.0161971728677
ATACTGAC 0.000126392863888
ATACTGAG 0.054187671611
ATACTGAT 0.191546466443
ATACTGCA 0.213624338624

ATACTGCC 0.0708934334453
ATACTGCG 0.0782481968185
ATACTGGA -0.033135753684
ATACTGGC -0.0190257802182
ATACTGGG -0.0380994581088
ATACTGTA 0.208451669466
ATACTGTC 0.0587746163472
ATACTGTG 0.0650270268865
ATACTTAA 0.0539610361309
ATACTTAC 0.187578212474
ATACTTAG 0.0979631825068
ATACTTCA -0.0195158375511
ATACTTCC 0.00696228237722
ATACTTCG 0.0148526070509
ATACTTGA 0.0970333996775
ATACTTGC 0.0542328042328
ATACTTGG 0.0181180239043
ATACTTTA 0.0811443591937
ATACTTTC 0.0629706469955
ATACTTTG 0.136301016488
ATAGAAAA 0.133603372193
ATAGAAAC -0.154723100224
ATAGAAAG 0.109861548966
ATAGAAAT 0.263543649122
ATAGAACA 0.00116078043961
ATAGAACC -0.17140513192
ATAGAACG -0.0176238141589
ATAGAAGA 0.0882648356715
ATAGAAGC -0.116967441656
ATAGAAGG -0.0221146670179
ATAGAATA 0.251059298777
ATAGAATC 0.419372975172
ATAGAATG -0.0371852290939
ATAGACAA 0.0132770618744
ATAGACAC 0.0104969373642
ATAGACAG -0.112105722376
ATAGACAT -0.0557668664515
ATAGACCA -0.239694610417
ATAGACCC -0.248664422063
ATAGACCG -0.309108016767
ATAGACGA -0.126631686902
ATAGACGC -0.226935368942
ATAGACGG -0.132914752803
ATAGACTA -0.0236530988606
ATAGACTC -0.151494945504
ATAGACTG -0.218222024682
ATAGAGAA 0.279320965235
ATAGAGAC 0.0428659246087
ATAGAGAG -0.178421687272
ATAGAGAT 0.292827670124
ATAGAGCA -0.0056938544788
ATAGAGCC -0.234915696893
ATAGAGCG -0.169039268955
ATAGAGGA 0.12726426549

ATAGAGGC -0.156779464057
ATAGAGGG -0.00762570278791
ATAGAGTA -0.0150756177996
ATAGAGTC -0.0898133513341
ATAGAGTG -0.0489369642289
ATAGATAA 0.00764286812005
ATAGATAC 0.251973617306
ATAGATAG 0.121136029331
ATAGATAT 0.383515554677
ATAGATCA 0.100621794779
ATAGATCC 0.210727814943
ATAGATCG 0.162371841314
ATAGATGA 0.0200141605743
ATAGATGC -0.0736085732629
ATAGATGG -0.0716089020609
ATAGATTA 0.342378537399
ATAGATTC 0.422984614938
ATAGATTG 0.345466564003
ATAGCAAA 0.0656820127774
ATAGCAAC -0.0356268069095
ATAGCAAG 0.0243880115642
ATAGCAAT 0.310332905265
ATAGCACA -0.0850856420612
ATAGCACC -0.162873131076
ATAGCACG -0.122979024663
ATAGCAGA 0.181385382012
ATAGCAGC -0.24213795071
ATAGCAGG -0.216162938087
ATAGCATA 0.0595100880371
ATAGCATC 0.158521348772
ATAGCATG -0.00556817932337
ATAGCCAA -0.112145948967
ATAGCCAC 0.0988552002673
ATAGCCAG -0.0585882768857
ATAGCCAT -0.0991219761638
ATAGCCCA 0.0609867360132
ATAGCCCC -0.0439593006026
ATAGCCCG -0.254434966175
ATAGCCGA 0.130120353495
ATAGCCGC -0.093591516828
ATAGCCGG -0.246135229911
ATAGCCTA -0.0050330413401
ATAGCCTC 0.0847327867233
ATAGCCTG -0.136645758219
ATAGCGAA 0.0966324292127
ATAGCGAC -0.149695702518
ATAGCGAG -0.14292535151
ATAGCGAT 0.253938066697
ATAGCGCA 0.129868383499
ATAGCGCC -0.0625837010692
ATAGCGCG 0.0820179555246
ATAGCGGA 0.125617466904
ATAGCGGC -0.0724521943402
ATAGCGGG -0.0663692289118

ATAGCGTA 0.257541613158
ATAGCGTC 0.0486351023492
ATAGCGTG 0.0398210160824
ATAGCTAA -0.0957265801664
ATAGCTAC -0.00723344907239
ATAGCTAG 0.17064040923
ATAGCTAT -0.046186271385
ATAGCTCA -0.0719785567969
ATAGCTCC -0.176733517529
ATAGCTCG 0.0636100682781
ATAGCTGA -0.101308480977
ATAGCTGC -0.105522081742
ATAGCTGG -0.1262533959
ATAGCTTA -0.0891213656541
ATAGCTTC -0.177955121203
ATAGCTTG -0.0295839757473
ATAGGAAA 0.197647039452
ATAGGAAC -0.136148843336
ATAGGAAG -0.0519066436157
ATAGGAAT 0.310526927491
ATAGGACA -0.015070133847
ATAGGACC -0.119102540265
ATAGGACG -0.128579147977
ATAGGAGA 0.103300840853
ATAGGAGC -0.10634311014
ATAGGAGG -0.285363246622
ATAGGATA 0.340373076867
ATAGGATC 0.268694153549
ATAGGATG -0.0208237282926
ATAGGCAA -0.121111282184
ATAGGCAC -0.0747098048901
ATAGGCAG -0.157189268095
ATAGGCAT -0.16019108455
ATAGGCCA -0.134238210729
ATAGGCCC -0.256392156863
ATAGGCCG -0.172718067766
ATAGGCGA 0.0169729635495
ATAGGCGC -0.109706492495
ATAGGCGG -0.00783458192695
ATAGGCTA -0.0335380579157
ATAGGCTC -0.0457831080276
ATAGGCTG -0.242982983681
ATAGGGAA -0.0321488239652
ATAGGGAC -0.212799382716
ATAGGGAG -0.0755204625688
ATAGGGAT 0.164030334287
ATAGGGCA -0.0115438815684
ATAGGGCC -0.309604138552
ATAGGGCG -0.0658778689462
ATAGGGGA -0.0443950495321
ATAGGGGC -0.324996296479
ATAGGGGG -0.1949112258
ATAGGGTA -0.0253057764169
ATAGGGTC -0.0546336786135

ATAGGGTG -0.0451464357066
ATAGGTAA 0.0984845990033
ATAGGTAC -0.0918609245358
ATAGGTAG -0.025806114207
ATAGGTCA 0.052635623175
ATAGGTCC -0.157191495295
ATAGGTCG -0.198871712133
ATAGGTGA -0.137732338947
ATAGGTGC -0.112332142485
ATAGGTGG -0.134682576917
ATAGGTTA 0.0293628261525
ATAGGTTC 0.0464036145453
ATAGGTTG -0.0164712726953
ATAGTAAA 0.151828250895
ATAGTAAC -0.0551755698575
ATAGTAAG 0.013200070254
ATAGTAAT 0.274738860721
ATAGTACA 0.0116536586972
ATAGTACC -0.234970737851
ATAGTACG -0.133991618049
ATAGTAGA 0.122815539309
ATAGTAGC -0.0920503246989
ATAGTAGG 0.15088257086
ATAGTATA 0.142508680429
ATAGTATC 0.197570199126
ATAGTATG -0.000924296117956
ATAGTCAA 0.131880057531
ATAGTCAC -0.11592550085
ATAGTCAG -0.0362989304314
ATAGTCAT -0.130364752468
ATAGTCCA -0.0202282157676
ATAGTCCC -0.332082366303
ATAGTCCG -0.255931412894
ATAGTCGA -0.115992656137
ATAGTCGC -0.11702448769
ATAGTCGG -0.0810857538036
ATAGTCTA 0.0900815605172
ATAGTCTC -0.225967448088
ATAGTCTG 0.111463375482
ATAGTGAA 0.00579518544884
ATAGTGAC -0.0759958863039
ATAGTGAG 0.134152223497
ATAGTGAT 0.12657446283
ATAGTGCA 0.0345052518414
ATAGTGCC -0.14268861454
ATAGTGCG 0.112105029159
ATAGTGGA 0.153404616972
ATAGTGGC -0.246044244034
ATAGTGGG -0.0136974836036
ATAGTGTA 0.102211148722
ATAGTGTC 0.0809820458362
ATAGTGTG -0.0234911853169
ATAGTTAA 0.0432074731597
ATAGTTAC 0.0150739652277

ATAGTTAG 0.0238781734664
ATAGTTCA -0.122571296845
ATAGTTCC 0.0716869999012
ATAGTTCG -0.121031746032
ATAGTTGA -0.0863989656124
ATAGTTGC -0.048219603969
ATAGTTGG -0.124950612406
ATAGTTTA -0.142421741985
ATAGTTTC -0.0997907980184
ATAGTTTG 0.0932320138752
ATATAAAA 0.199766100562
ATATAAAC 0.0489386792453
ATATAAAG 0.102655702933
ATATAAAT 0.292390871958
ATATAACA 0.166137017278
ATATAACC -0.101009352565
ATATAACG 0.184686324317
ATATAAGA 0.158187206718
ATATAAGC -0.0219768363859
ATATAAGG 0.130510075109
ATATAATA 0.24555698259
ATATAATC 0.292565071227
ATATAATG 0.0854876067532
ATATACAA 0.184968385695
ATATACAC 0.0705009989242
ATATACAG 0.322202463281
ATATACAT 0.0581552557479
ATATACCA 0.227218049482
ATATACCC 0.0214960811434
ATATACCG 0.119047619048
ATATACGA 0.227902917737
ATATACGC 0.0556488662748
ATATACGG 0.0341418033327
ATATACTA 0.188646107686
ATATACTC 0.220144789128
ATATACTG 0.210951393005
ATATAGAA 0.284681904336
ATATAGAC 0.0555637884476
ATATAGAG 0.229554138277
ATATAGAT 0.386424509868
ATATAGCA 0.110245085933
ATATAGCC 0.114684816876
ATATAGCG 0.10798567735
ATATAGGA 0.106508924455
ATATAGGC -0.119365541446
ATATAGGG 0.0346505309953
ATATAGTA 0.153887955736
ATATAGTC 0.0400897825171
ATATAGTG 0.00843636046664
ATATATAA 0.222894541862
ATATATAC 0.225970932402
ATATATAG 0.205759235811
ATATATAT 0.276904395294
ATATATCA 0.303340907594

ATATATCC 0.213220926914
ATATATCG 0.316895563679
ATATATGA 0.230734730914
ATATATGC 0.134906913434
ATATATGG 0.283428286022
ATATATTA 0.206510632655
ATATATTC 0.244479573186
ATATATTG 0.286488896747
ATATCAAA 0.223574448302
ATATCAAC 0.348649256844
ATATCAAG 0.29780047361
ATATCAAT 0.275884212606
ATATCACA 0.347442360496
ATATCACC 0.291688621045
ATATCACG 0.354485722755
ATATCAGA 0.383457062746
ATATCAGC 0.236342752692
ATATCAGG 0.299073118998
ATATCATA 0.320036900905
ATATCATC 0.300305714725
ATATCATG 0.368162353718
ATATCCAA 0.412248347933
ATATCCAC 0.392847907254
ATATCCAG 0.397241148868
ATATCCAT 0.434610274637
ATATCCCA 0.371391248985
ATATCCCC 0.352403455619
ATATCCCG 0.268323288213
ATATCCGA 0.402953525199
ATATCCGC 0.408895584313
ATATCCGG 0.445235154629
ATATCCTA 0.447932446376
ATATCCTC 0.397293449363
ATATCCTG 0.341846207488
ATATCGAA 0.367527278316
ATATCGAC 0.313784086121
ATATCGAG 0.245222178313
ATATCGAT 0.370304507234
ATATCGCA 0.36675613076
ATATCGCC 0.344458326675
ATATCGCG 0.409411125383
ATATCGGA 0.35787624716
ATATCGGC 0.34240872467
ATATCGGG 0.35517794024
ATATCGTA 0.399020834705
ATATCGTC 0.31797393692
ATATCGTG 0.336105073657
ATATCTAA 0.453802498408
ATATCTAC 0.376083997453
ATATCTAG 0.406792684801
ATATCTCA 0.388315330743
ATATCTCC 0.416400563683
ATATCTCG 0.427525304394
ATATCTGA 0.405452326646

ATATCTGC 0.429108697743
ATATCTGG 0.394129269391
ATATCTTA 0.399684621325
ATATCTTC 0.395431293772
ATATCTTG 0.42342587104
ATATGAAA 0.29138400404
ATATGAAC 0.138894135081
ATATGAAG 0.17271802769
ATATGAAT 0.119494939516
ATATGACA 0.05439047156
ATATGACC 0.111692902149
ATATGACG 0.167374695383
ATATGAGA 0.16396641146
ATATGAGC 0.136548003249
ATATGAGG 0.119955544579
ATATGATA 0.219198640187
ATATGATC 0.238428098602
ATATGATG 0.169959823487
ATATGCAA 0.298993940591
ATATGCAC 0.216182644952
ATATGCAG 0.183335028257
ATATGCAT 0.161814544017
ATATGCCA 0.0730655447979
ATATGCCC 0.135774111397
ATATGCCG 0.0936738457485
ATATGCGA 0.187324393823
ATATGCGC 0.187008114566
ATATGCGG 0.0976640540956
ATATGCTA 0.261900800017
ATATGCTC 0.154514918
ATATGCTG 0.206527585677
ATATGGAA 0.206895321522
ATATGGAC 0.0411123186019
ATATGGAG -0.0459177704696
ATATGGAT 0.363092493798
ATATGGCA 0.151002049788
ATATGGCC -0.0646586642956
ATATGGCG 0.130096160179
ATATGGGA 0.223794704604
ATATGGGC 0.0942041043428
ATATGGGG 0.0117714505599
ATATGGTA 0.203146246786
ATATGGTC 0.165887286219
ATATGGTG 0.152495169468
ATATGTAA 0.132353744114
ATATGTAC 0.0882209269139
ATATGTAG 0.187333300405
ATATGTCA 0.161523853433
ATATGTCC 0.0449421062572
ATATGTCG 0.200490131507
ATATGTGA 0.11425002015
ATATGTGC 0.0170928560451
ATATGTGG 0.100262903686
ATATGTTA 0.170509784551

ATATGTTC 0.139986607829
ATATGTTG 0.171387270641
ATATTAAA 0.126975433295
ATATTAAC 0.113591955916
ATATTAAG 0.186380079322
ATATTAAT 0.249665195723
ATATTACA 0.320638213792
ATATTACC 0.170928560451
ATATTACG 0.356256792807
ATATTAGA 0.201535708797
ATATTAGC 0.232546923303
ATATTAGG 0.0873665247774
ATATTATA 0.280946819978
ATATTATC 0.193769756989
ATATTATG 0.222548777476
ATATTCAA 0.291128784386
ATATTCAC 0.30117016839
ATATTCAG 0.281632117145
ATATTCAT 0.147709279621
ATATTCCA 0.334195807244
ATATTCCC 0.377478884718
ATATTCCG 0.354626336473
ATATTCGA 0.309899198423
ATATTCGC 0.337128696569
ATATTCGG 0.217707853081
ATATTCTA 0.254854662012
ATATTCTC 0.426411389904
ATATTCTG 0.37032920591
ATATTGAA 0.198773843941
ATATTGAC 0.222784309851
ATATTGAG 0.21882381997
ATATTGAT 0.303782739467
ATATTGCA 0.146037940915
ATATTGCC 0.306796158819
ATATTGCG 0.308105007794
ATATTGGA 0.27478535005
ATATTGGC 0.176498958704
ATATTGGG 0.193501663446
ATATTGTA 0.229429536396
ATATTGTC 0.272294527314
ATATTGTG 0.301574033389
ATATTTAA 0.298867702913
ATATTTAC 0.250293267806
ATATTTAG 0.277923225537
ATATTTCA 0.307196645371
ATATTTCC 0.362173666701
ATATTTCG 0.34323105843
ATATTTGA 0.235849056604
ATATTTGC 0.290423499967
ATATTTGG 0.136460185734
ATATTTTA 0.294078264871
ATATTTTC 0.352445774556
ATATTTTG 0.219093723243
ATCAAAAA 0.141299957869

ATCAAAAC -0.0869818766603
ATCAAAAG 0.185300445674
ATCAAAAT 0.260782344289
ATCAAACA 0.122104766296
ATCAAACC -0.0526415436032
ATCAAACG 0.198775296733
ATCAAAGA 0.178535005012
ATCAAAGC 0.154064853235
ATCAAAGG 0.0043162436622
ATCAAATA 0.134085395087
ATCAAATC 0.365896349896
ATCAAATG 0.0660689587038
ATCAACAA -0.000530268911714
ATCAACAC 0.0826198160223
ATCAACAG -0.0203434399198
ATCAACAT 0.15166906299
ATCAACCA 0.0866197899263
ATCAACCC -0.118298425871
ATCAACCG -0.0572651683543
ATCAACGA 0.156934900411
ATCAACGC 0.181999055962
ATCAACGG 0.16146470443
ATCAACTA 0.0666809370129
ATCAACTC 0.101249400723
ATCAACTG 0.172336384992
ATCAAGAA 0.0726991811017
ATCAAGAC 0.154339986167
ATCAAGAG 0.192218492682
ATCAAGAT 0.301741702065
ATCAAGCA 0.138167739311
ATCAAGCC 0.0235433564971
ATCAAGCG 0.112247566088
ATCAAGGA 0.0244983424444
ATCAAGGC 0.13063404246
ATCAAGGG -0.0647886914707
ATCAAGTA 0.10197289091
ATCAAGTC 0.123254834164
ATCAAGTG 0.00971481261938
ATCAATAA -0.0223523019166
ATCAATAC 0.18954038508
ATCAATAG 0.248845450953
ATCAATCA 0.169234046231
ATCAATCC 0.0043848815835
ATCAATCG 0.0454592856045
ATCAATGA 0.154758618686
ATCAATGC 0.0901090034907
ATCAATGG -0.0305769035201
ATCAATTA 0.167935437944
ATCAATTC -0.0323652899046
ATCAATTG 0.0585946539025
ATCACAAA 0.280137123424
ATCACAAC 0.0603416101341
ATCACAAG 0.198455682594
ATCACAAT 0.265385788793

ATCACACA 0.260646782793
ATCACACC -0.00284309205471
ATCACACG 0.161309798239
ATCACAGA 0.116710008281
ATCACAGC 0.182265252805
ATCACAGG 0.0822207371464
ATCACATA 0.190093414496
ATCACATC 0.18561300539
ATCACATG 0.24532920591
ATCACCAA 0.0395987389769
ATCACCAC 0.0702512678654
ATCACCAG 0.16987636744
ATCACCAT 0.208819897433
ATCACCCA 0.039003094446
ATCACCCC -0.0457091801897
ATCACCCG 0.0779918223978
ATCACCGA 0.199828755845
ATCACCGC 0.187367977569
ATCACCGG 0.0686705525917
ATCACCTA 0.0921891808821
ATCACCTC 0.179371812939
ATCACCTG 0.0466912672701
ATCACGAA 0.241815698865
ATCACGAC 0.13491929743
ATCACGAG 0.0583165051647
ATCACGAT 0.267859887154
ATCACGCA 0.234458762512
ATCACGCC 0.170020198028
ATCACGCG 0.16656180044
ATCACGGA 0.0427831423491
ATCACGGC 0.128712971976
ATCACGGG 0.0420783112692
ATCACGTA 0.265033260884
ATCACGTC 0.0807301730275
ATCACGTG 0.206929230897
ATCACTAA 0.0734067916915
ATCACTAC 0.182486597998
ATCACTAG 0.0492659367196
ATCACTCA 0.189099705083
ATCACTCC 0.0673697890941
ATCACTCG 0.045862697315
ATCACTGA 0.106610201502
ATCACTGC 0.0810265425014
ATCACTGG 0.131708628129
ATCACTTA 0.200329783679
ATCACTTC 0.00926726266917
ATCACTTG 0.123497450351
ATCAGAAA 0.348168756265
ATCAGAAC 0.104395815495
ATCAGAAG 0.208218441736
ATCAGAAT 0.328974409643
ATCAGACA 0.179787311319
ATCAGACC -0.0166010489155
ATCAGACG 0.0957520375401

ATCAGAGA 0.228089529957
ATCAGAGC 0.174025116072
ATCAGAGG 0.0299732156579
ATCAGATA 0.331689499221
ATCAGATC 0.335436291168
ATCAGATG 0.192859529586
ATCAGCAA 0.152064260467
ATCAGCAC 0.0258485367407
ATCAGCAG 0.181628575819
ATCAGCAT 0.236579838159
ATCAGCCA 0.0415321960965
ATCAGCCC -0.0232611105623
ATCAGCCG 0.101754972179
ATCAGCGA 0.195001536807
ATCAGCGC 0.0592932885464
ATCAGCGG 0.0722371536908
ATCAGCTA -0.0820682122549
ATCAGCTC -0.0519742475137
ATCAGCTG 0.0783524336429
ATCAGGAA 0.333077157759
ATCAGGAC -0.0213109990847
ATCAGGAG 0.0771647623124
ATCAGGAT 0.377913210233
ATCAGGCA 0.0912425726033
ATCAGGCC -0.125887852411
ATCAGGCG -0.00196949172813
ATCAGGGA 0.0160946824645
ATCAGGGC 0.0619744670575
ATCAGGGG 0.0906239193795
ATCAGGTA 0.188815890579
ATCAGGTC 0.034741430406
ATCAGGTG 0.138323188006
ATCAGTAA 0.2793571267
ATCAGTAC 0.161038112802
ATCAGTAG 0.0944587147907
ATCAGTCA 0.0396912098831
ATCAGTCC -0.138494433728
ATCAGTCG 0.110814726997
ATCAGTGA 0.15186392676
ATCAGTGC 0.0716371380272
ATCAGTGG 0.0943704929369
ATCAGTTA 0.167256690597
ATCAGTTC 0.127400764917
ATCAGTTG 0.133496344596
ATCATAAA 0.225187046911
ATCATAAC 0.118770066683
ATCATAAG 0.0416558917961
ATCATAAT 0.213230572545
ATCATACA -0.0445893073013
ATCATACC 0.0273112472283
ATCATACG 0.276199806801
ATCATAGA 0.213207205427
ATCATAGC 0.146917909711
ATCATAGG 0.00630887350273

ATCATATA 0.258887412673
ATCATATC 0.308072076226
ATCATATG 0.141751191025
ATCATCAA 0.0261778524227
ATCATCAC 0.115436123735
ATCATCAG 0.0435648598783
ATCATCAT 0.0432393044166
ATCATCCA 0.170505426176
ATCATCCC 0.0436123734879
ATCATCCG 0.0357115414169
ATCATCGA 0.144355293708
ATCATCGC −0.00669301061991
ATCATCGG 0.169109091308
ATCATCTA 0.193239697908
ATCATCTC 0.0293812560836
ATCATCTG 0.134783420053
ATCATGAA 0.203629607675
ATCATGAC 0.0212238315924
ATCATGAG 0.0409628239323
ATCATGAT 0.201047310795
ATCATGCA 0.0264249728341
ATCATGCC 0.0321342960499
ATCATGCG 0.0201022765243
ATCATGGA 0.193511383345
ATCATGGC 0.155592446512
ATCATGGG 0.00535051321249
ATCATGTA 0.16566617756
ATCATGTC 0.176298759545
ATCATGTG 0.186343278667
ATCATTAA 0.23132780083
ATCATTAC 0.217248993942
ATCATTAG 0.147721296471
ATCATTCA 0.00108232969651
ATCATTCC 0.0650539368214
ATCATTCG 0.0424157858342
ATCATTGA 0.093433382244
ATCATTGC −0.00215929093098
ATCATTGG 0.21954149899
ATCATTTA 0.237797277469
ATCATTTC −0.0220610026091
ATCATTTG 0.0899030988044
ATCCAAAA 0.239308180669
ATCCAAAC 0.147305798091
ATCCAAAG 0.295653247715
ATCCAAAT 0.343860457968
ATCCAACA 0.181280050056
ATCCAACC −0.00575160170267
ATCCAACG 0.279416123296
ATCCAAGA 0.229089244984
ATCCAAGC 0.174487527785
ATCCAAGG 0.0984324573536
ATCCAATA 0.338761553492
ATCCAATC 0.207634419537
ATCCAATG 0.237422457252

ATCCACAA 0.292913282873
ATCCACAC 0.138892716651
ATCCACAG 0.17677712725
ATCCACAT 0.320312931297
ATCCACCA 0.172871522975
ATCCACCC 0.222327953162
ATCCACCG 0.276559309754
ATCCACGA 0.228746386181
ATCCACGC 0.278399895399
ATCCACGG 0.281046235922
ATCCACTA 0.178248973633
ATCCACTC 0.29829140047
ATCCACTG 0.339232275224
ATCCAGAA 0.238358055003
ATCCAGAC 0.121902285241
ATCCAGAG 0.143098513794
ATCCAGAT 0.337930031395
ATCCAGCA 0.223379774236
ATCCAGCC 0.247411851873
ATCCAGCG 0.0462084787811
ATCCAGGA 0.357867592579
ATCCAGGC 0.0714352127613
ATCCAGGG 0.21686981535
ATCCAGTA 0.26588673736
ATCCAGTC 0.0854662734444
ATCCAGTG 0.308153066117
ATCCATAA 0.300184965641
ATCCATAC 0.234865544143
ATCCATAG 0.057459758669
ATCCATCA 0.0733807918007
ATCCATCC 0.140710124504
ATCCATCG 0.138193889559
ATCCATGA 0.166342839579
ATCCATGC 0.252502433426
ATCCATGG 0.162651416864
ATCCATTA 0.261251619135
ATCCATTC 0.181060456386
ATCCATTG 0.212183582516
ATCCCAAA 0.190619325033
ATCCCAAC 0.188044743024
ATCCCAAG 0.152624097126
ATCCCAAT 0.257015420535
ATCCCACA 0.180566532745
ATCCCACC 0.0973370911842
ATCCCACG 0.184156747012
ATCCCAGA 0.280965525257
ATCCCAGC 0.161685528743
ATCCCAGG -0.00614997036159
ATCCCATA 0.254034117105
ATCCCATC 0.199437769674
ATCCCATG 0.113756613757
ATCCCCAA 0.241815884373
ATCCCCAC 0.168985597928
ATCCCCAG 0.266180377176

ATCCCCAT 0.281726788023
ATCCCCCA 0.216464486511
ATCCCCCC 0.101495093196
ATCCCCCG 0.269375154359
ATCCCCGA 0.296947545194
ATCCCCGC 0.22675315619
ATCCCCGG 0.245941184219
ATCCCCTA 0.273448781834
ATCCCCTC 0.138802109059
ATCCCCTG 0.173290427557
ATCCCGAA 0.244387911919
ATCCCGAC 0.289183665909
ATCCCGAG 0.229615155521
ATCCCGAT 0.33746898944
ATCCCGCA 0.283201002667
ATCCCGCC 0.234017212233
ATCCCGCG 0.283735647325
ATCCCGGA 0.295106998097
ATCCCGGC 0.036103153435
ATCCCGGG 0.0148987903137
ATCCCGTA 0.276633405783
ATCCCGTC 0.0866256010925
ATCCCGTG 0.20821263057
ATCCCTAA 0.28964450191
ATCCCTAC 0.135317362312
ATCCCTAG 0.146029550594
ATCCCTCA 0.107362747519
ATCCCTCC 0.0504394694405
ATCCCTCG 0.120869973323
ATCCCTGA 0.152056270374
ATCCCTGC 0.00271949143186
ATCCCTGG 0.0479493847428
ATCCCTTA 0.162242859338
ATCCCTTC 0.0737335289759
ATCCCTTG 0.133002371073
ATCCGAAA 0.205091038618
ATCCGAAC 0.164457455
ATCCGAAG 0.203543358563
ATCCGAAT 0.306082460647
ATCCGACA 0.275170695295
ATCCGACC 0.129152599497
ATCCGACG 0.235545785857
ATCCGAGA 0.293261952842
ATCCGAGC 0.183568794046
ATCCGAGG 0.248937956926
ATCCGATA 0.356808052866
ATCCGATC 0.322008647564
ATCCGATG 0.253481614923
ATCCGCAA 0.346410878503
ATCCGCAC 0.259970032273
ATCCGCAG 0.219027210786
ATCCGCAT 0.301127366234
ATCCGCCA 0.225550244795
ATCCGCCC 0.189425130388

ATCCGCCG	0.260745572618
ATCCGCGA	0.290737001148
ATCCGCGC	0.188881753716
ATCCGCGG	0.332531370418
ATCCGCTA	0.329677625558
ATCCGCTC	0.253394695822
ATCCGCTG	0.244802638269
ATCCGGAA	0.350975549518
ATCCGGAC	0.236399692008
ATCCGGAG	0.200030252889
ATCCGGAT	0.405637860082
ATCCGGCA	0.217661586739
ATCCGGCC	0.253121322621
ATCCGGCG	-0.0354976509099
ATCCGGGA	0.21926382331
ATCCGGGC	0.12014259369
ATCCGGGG	0.270011477053
ATCCGGTA	0.222258219168
ATCCGGTC	0.184232853428
ATCCGGTG	0.364865567565
ATCCGTAA	0.265567889954
ATCCGTAC	0.183041888955
ATCCGTAG	0.0650151485214
ATCCGTCA	0.159528829979
ATCCGTCC	0.0492412081399
ATCCGTCG	0.211791147994
ATCCGTGA	0.154081319019
ATCCGTGC	0.197092964131
ATCCGTGG	0.0461217729868
ATCCGTTA	0.292689552976
ATCCGTTC	0.14560603199
ATCCGTTG	0.244415563568
ATCCTAAA	0.239549344065
ATCCTAAC	0.205533682972
ATCCTAAG	0.155467189181
ATCCTAAT	0.252290325861
ATCCTACA	0.285670376957
ATCCTACC	0.0628492147662
ATCCTACG	0.197177764605
ATCCTAGA	0.204827626284
ATCCTAGC	0.250090799471
ATCCTAGG	0.159361347121
ATCCTATA	0.26711892687
ATCCTATC	0.330956082112
ATCCTATG	0.194115128762
ATCCTCAA	0.30703904475
ATCCTCAC	0.0704967094272
ATCCTCAG	0.277871079279
ATCCTCAT	0.269440529978
ATCCTCCA	0.221755553296
ATCCTCCC	0.0994505916705
ATCCTCCG	0.237527058242
ATCCTCGA	0.163514613239
ATCCTCGC	0.268180959714

ATCCTCGG 0.233648104833
ATCCTCTA 0.329812514787
ATCCTCTC 0.242011350414
ATCCTCTG 0.295286723303
ATCCTGAA 0.152791538942
ATCCTGAC 0.23973871382
ATCCTGAG 0.223831759907
ATCCTGCA 0.172394015236
ATCCTGCC 0.175003842016
ATCCTGCG 0.240078287069
ATCCTGGA 0.272911248965
ATCCTGGC 0.327707523766
ATCCTGGG 0.120214141473
ATCCTGTA 0.315322509825
ATCCTGTC 0.165971263783
ATCCTGTG 0.234877166475
ATCCTTAA 0.177156755942
ATCCTTAC 0.291641967991
ATCCTTAG 0.191406738047
ATCCTTCA 0.238221475773
ATCCTTCC 0.0701635213912
ATCCTTCG 0.0986766998178
ATCCTTGA 0.230635015182
ATCCTTGC 0.188796680498
ATCCTTGG 0.258271312213
ATCCTTTA 0.281925915911
ATCCTTTC 0.119205904145
ATCCTTTG 0.150033669859
ATCGAAAA 0.176454607528
ATCGAAAC 0.142732900522
ATCGAAAG 0.149326472829
ATCGAAAT 0.4160199323
ATCGAACA 0.147183763602
ATCGAACC 0.0331303608525
ATCGAACG 0.103570961603
ATCGAAGA 0.0922508099313
ATCGAAGC 0.14712565194
ATCGAAGG 0.168089433847
ATCGAATA 0.244440053569
ATCGAATC 0.358207545799
ATCGAATG 0.0338115579915
ATCGACAA -0.0591169933026
ATCGACAC 0.0893077644967
ATCGACAG 0.0440529978353
ATCGACAT 0.000276016758575
ATCGACCA 0.105209858119
ATCGACCC 0.0795187600167
ATCGACCG -0.00624845640899
ATCGACGA -0.0182541642655
ATCGACGC 0.136548003249
ATCGACGG -0.0258183494698
ATCGACTA -0.0250130347685
ATCGACTC 0.137102351314
ATCGACTG 0.124444670434

ATCGAGAA 0.223176383421
ATCGAGAC −0.0200039513978
ATCGAGAG 0.0362863742682
ATCGAGAT 0.334307558893
ATCGAGCA 0.154967727063
ATCGAGCC 0.00648751893565
ATCGAGCG 0.124894375444
ATCGAGGA −0.100169597576
ATCGAGGC −0.131201161946
ATCGAGGG 0.0897955922305
ATCGAGTA 0.156987200907
ATCGAGTC −0.0333255851118
ATCGAGTG 0.0760225251926
ATCGATAA 0.110238612785
ATCGATAC 0.29096961514
ATCGATAG 0.0867811355192
ATCGATCA 0.160506425873
ATCGATCC 0.0503396885733
ATCGATCG 0.189677599947
ATCGATGA 0.0568051575931
ATCGATGC 0.0787195095376
ATCGATGG −0.00496207915927
ATCGATTA 0.139909593914
ATCGATTC 0.334833093701
ATCGATTG 0.267758348153
ATCGCAAA 0.285706052822
ATCGCAAC 0.113412171488
ATCGCAAG 0.284012821357
ATCGCAAT 0.416364793958
ATCGCACA 0.289932154635
ATCGCACC 0.158063294474
ATCGCACG 0.172227176646
ATCGCAGA 0.237849577964
ATCGCAGC 0.032130233375
ATCGCAGG 0.0463456936486
ATCGCATA 0.233209361789
ATCGCATC 0.242774541281
ATCGCATG 0.340712748908
ATCGCCAA 0.212585533102
ATCGCCAC 0.176137898973
ATCGCCAG 0.114577158664
ATCGCCAT 0.128048914356
ATCGCCCA 0.189205580803
ATCGCCCC 0.223885345692
ATCGCCCG 0.197903289539
ATCGCCGA 0.23873191508
ATCGCCGC 0.208154954006
ATCGCCGG 0.163600412878
ATCGCCTA 0.155453053041
ATCGCCTC 0.111296907007
ATCGCCTG 0.136159981404
ATCGCGAA 0.341439425857
ATCGCGAC 0.197910491998
ATCGCGAG 0.325407802586

ATCGCGAT 0.423209179345
ATCGCGCA 0.384381038165
ATCGCGCC 0.299683291445
ATCGCGCG 0.320400554318
ATCGCGGA 0.297262288964
ATCGCGGC 0.270299567499
ATCGCGGG 0.30350007684
ATCGCGTA 0.326224529857
ATCGCGTC 0.256625455813
ATCGCGTG 0.291091482283
ATCGCTAA 0.157803669751
ATCGCTAC 0.172857495678
ATCGCTAG 0.0440651825507
ATCGCTCA -0.0242313659634
ATCGCTCC 0.240272834251
ATCGCTCG 0.0790165536016
ATCGCTGA 0.139846648664
ATCGCTGC 0.205562738803
ATCGCTGG 0.17726788733
ATCGCTTA 0.212888866935
ATCGCTTC 0.223164761088
ATCGCTTG 0.187317504226
ATCGGAAA 0.19860205493
ATCGGAAC 0.0546444488353
ATCGGAAG 0.221979283193
ATCGGAAT 0.257950401697
ATCGGACA 0.145818239698
ATCGGACC -0.0971117806204
ATCGGACG -0.0996543209877
ATCGGAGA 0.264938328999
ATCGGAGC 0.103196141386
ATCGGAGG -0.0657254191707
ATCGGATA 0.342765483326
ATCGGATC 0.304517834206
ATCGGATG -0.0116961953062
ATCGGCAA 0.219483413467
ATCGGCAC 0.0392942216075
ATCGGCAG 0.0582036716885
ATCGGCAT 0.21201718858
ATCGGCCA -0.0550067231964
ATCGGCCC 0.0578972389608
ATCGGCCG 0.164877384794
ATCGGCGA 0.106347010911
ATCGGCGC 0.136169290215
ATCGGCGG 0.0313497886188
ATCGGCTA 0.106045138203
ATCGGCTC -0.0853723462645
ATCGGCTG 0.138438824123
ATCGGGAA 0.297088605756
ATCGGGAC 0.174452238249
ATCGGGAG 0.1190970164
ATCGGGCA 0.211426205454
ATCGGGCC -0.0730219933076
ATCGGGCG 0.0738831315605

ATCGGGGA 0.315292083739
ATCGGGGC -0.0398103329053
ATCGGGGG -0.0932556457166
ATCGGGTA 0.0964086993157
ATCGGGTC 0.0210515049727
ATCGGGTG 0.104576293348
ATCGGTAA 0.2342205047
ATCGGTAC 0.0318530593427
ATCGGTAG 0.139164354307
ATCGGTCA -0.0149170042486
ATCGGTCC -0.125225523607
ATCGGTCG 0.17535236778
ATCGGTGA 0.200289105516
ATCGGTGC -0.0274853631252
ATCGGTGG 0.00445399459922
ATCGGTTA 0.0610166979547
ATCGGTTC 0.00945746027867
ATCGGTTG -0.104059660213
ATCGTAAA 0.219774893806
ATCGTAAC 0.237343300621
ATCGTAAG 0.120141595405
ATCGTAAT 0.42030566734
ATCGTACA 0.13574941272
ATCGTACC -0.0106588509078
ATCGTACG 0.13086181914
ATCGTAGA 0.265780948092
ATCGTAGC 0.132350761989
ATCGTAGG 0.0499270734669
ATCGTATA 0.197493870526
ATCGTATC 0.378559353663
ATCGTATG 0.100275980441
ATCGTCAA 0.293458692836
ATCGTCAC 0.110149837371
ATCGTCAG 0.154659828861
ATCGTCAT 0.257969439505
ATCGTCCA 0.0295367047782
ATCGTCCC 0.021281943254
ATCGTCCG 0.144135806953
ATCGTCGA 0.178823903928
ATCGTCGC 0.162946551799
ATCGTCGG 0.0612918222364
ATCGTCTA 0.173941798942
ATCGTCTC 0.112215071259
ATCGTCTG 0.151685547432
ATCGTGAA 0.186598587375
ATCGTGAC 0.100171079281
ATCGTGAG 0.119981694827
ATCGTGCA 0.137088324229
ATCGTGCC 0.10550317435
ATCGTGCG 0.228615634943
ATCGTGGA 0.213884328738
ATCGTGGC 0.10101484116
ATCGTGGG 0.20862697315
ATCGTGTA 0.180711286737

ATCGTGTC 0.145870381348
ATCGTGTG 0.15630694417
ATCGTTAA 0.163350183319
ATCGTTAC 0.00799413818086
ATCGTTAG -0.0150801392868
ATCGTTCA 0.154392515218
ATCGTTCC 0.144301425189
ATCGTTCG -0.00463081321031
ATCGTTGA 0.164611450903
ATCGTTGC 0.141114000552
ATCGTTGG -0.182521464995
ATCGTTTA 0.162166019012
ATCGTTTC 0.103486699693
ATCGTTTG 0.032250519373
ATCTAAAA 0.201313254484
ATCTAAAC 0.193414495954
ATCTAAAG 0.148703383551
ATCTAAAT 0.363577063327
ATCTAACA 0.223838613361
ATCTAACC 0.253545537751
ATCTAACG 0.304136753826
ATCTAAGA 0.267629366177
ATCTAAGC 0.261517815978
ATCTAAGG 0.274702410353
ATCTAATA 0.22815074165
ATCTAATC 0.380220166947
ATCTAATG 0.203633431639
ATCTACAA 0.0993782052213
ATCTACAC 0.316634283958
ATCTACAG 0.316375866227
ATCTACAT 0.334245036057
ATCTACCA 0.219121166217
ATCTACCC 0.197392470952
ATCTACCG 0.333045196345
ATCTACGA 0.3172972439
ATCTACGC 0.251796376738
ATCTACGG 0.23831737684
ATCTACTA 0.322674482426
ATCTACTC 0.242848080038
ATCTACTG 0.182023157497
ATCTAGAA 0.243482293793
ATCTAGAC 0.280909021054
ATCTAGAG 0.175760173173
ATCTAGAT 0.459658305353
ATCTAGCA 0.33908549837
ATCTAGCC 0.169924147621
ATCTAGCG 0.126732567723
ATCTAGGA 0.23922369526
ATCTAGGC 0.147983490307
ATCTAGGG 0.232985631892
ATCTAGTA 0.359130068426
ATCTAGTC 0.247225894556
ATCTAGTG 0.33847340227
ATCTATAA 0.236451992504

ATCTATAC 0.144783751979
ATCTATAG 0.277668005884
ATCTATCA 0.298571905917
ATCTATCC 0.219047549867
ATCTATCG 0.111056661164
ATCTATGA 0.239418592826
ATCTATGC 0.114437199499
ATCTATGG 0.212372939033
ATCTATTA 0.166903955951
ATCTATTC 0.201132297087
ATCTATTG 0.255941403763
ATCTCAAA 0.342286403324
ATCTCAAC 0.274413435416
ATCTCAAG 0.297050978068
ATCTCAAT 0.296609146194
ATCTCACA 0.230150497267
ATCTCACC 0.321321168626
ATCTCACG 0.160217567894
ATCTCAGA 0.300552060785
ATCTCAGC 0.239061230762
ATCTCAGG 0.351876280273
ATCTCATA 0.261470515596
ATCTCATC 0.347581828483
ATCTCATG 0.31765236586
ATCTCCAA 0.384256098092
ATCTCCAC 0.260948963433
ATCTCCAG 0.275030744871
ATCTCCAT 0.363537069969
ATCTCCCA 0.264534452951
ATCTCCCC 0.284306461174
ATCTCCCG 0.123994305057
ATCTCCGA 0.292994639199
ATCTCCGC 0.228890864783
ATCTCCGG 0.362161578921
ATCTCCTA 0.324656736442
ATCTCCTC 0.284775736021
ATCTCCTG 0.31312158335
ATCTCGAA 0.324281957782
ATCTCGAC 0.271156276786
ATCTCGAG 0.335545236998
ATCTCGCA 0.309812154054
ATCTCGCC 0.295560269057
ATCTCGCG 0.338920722738
ATCTCGGA 0.357257420133
ATCTCGGC 0.310576322403
ATCTCGGG 0.325525547339
ATCTCGTA 0.355833684425
ATCTCGTC 0.321299589453
ATCTCGTG 0.301580637194
ATCTCTAA 0.200317240774
ATCTCTAC 0.280950185515
ATCTCTAG 0.311664361457
ATCTCTCA 0.303681200465
ATCTCTCC 0.194140891723

ATCTCTCG 0.274072656327
ATCTCTGA 0.167668335199
ATCTCTGC 0.289701749764
ATCTCTGG 0.192295659619
ATCTCTTA 0.326057269042
ATCTCTTC 0.291147994467
ATCTCTTG 0.362175898483
ATCTGAAA 0.257058332784
ATCTGAAC 0.305356613652
ATCTGAAG 0.195381575698
ATCTGAAT 0.289889682463
ATCTGACA 0.200538985661
ATCTGACC 0.322556477639
ATCTGACG 0.230344456874
ATCTGAGA 0.313092709671
ATCTGAGC 0.162838616078
ATCTGAGG 0.243008440719
ATCTGATA 0.34136314738
ATCTGATC 0.382574260686
ATCTGATG 0.276261386254
ATCTGCAA 0.326924216975
ATCTGCAC 0.339049704714
ATCTGCAG 0.288878460559
ATCTGCAT 0.346776981971
ATCTGCCA 0.293378176166
ATCTGCCC 0.230620487266
ATCTGCCG 0.2370475044
ATCTGCGA 0.326891171386
ATCTGCGC 0.361462381172
ATCTGCGG 0.219878592285
ATCTGCTA 0.291905420536
ATCTGCTC 0.0652264594173
ATCTGCTG 0.280041823092
ATCTGGAA 0.310352237921
ATCTGGAC 0.275423170651
ATCTGGAG 0.177525954391
ATCTGGCA 0.0969491377682
ATCTGGCC 0.112483259786
ATCTGGCG 0.342708460624
ATCTGGGA 0.275245727551
ATCTGGGC 0.155592521029
ATCTGGGG 0.37340556324
ATCTGGTA 0.176046675009
ATCTGGTC 0.285003512701
ATCTGGTG 0.332452457397
ATCTGTAA 0.313511273574
ATCTGTAC 0.344757843202
ATCTGTAG 0.338517311028
ATCTGTCA 0.0631290348581
ATCTGTCC 0.259014812138
ATCTGTCG 0.334884430433
ATCTGTGA 0.275349033167
ATCTGTGC 0.268191947134
ATCTGTGG -0.00242894747546

ATCTGTTA 0.222275652667
ATCTGTTC 0.178391677095
ATCTGTTG 0.251429778919
ATCTTAAA 0.28647445608
ATCTTAAC 0.312526070825
ATCTTAAG 0.212212408708
ATCTTAAT 0.316044629756
ATCTTACA 0.307162262287
ATCTTACC 0.302416429851
ATCTTACG 0.248701947353
ATCTTAGA 0.133103308006
ATCTTAGC 0.302118319668
ATCTTAGG 0.198453862873
ATCTTATA 0.34002667457
ATCTTATC 0.247823644177
ATCTTATG 0.141174888582
ATCTTCAA 0.290308239478
ATCTTCAC 0.345620559906
ATCTTCAG 0.281796522017
ATCTTCAT 0.326280499852
ATCTTCCA 0.282208522671
ATCTTCCC 0.193461985993
ATCTTCCG 0.27090880151
ATCTTCGA 0.246726053261
ATCTTCGC 0.251094974643
ATCTTCGG 0.257572483454
ATCTTCTA 0.244832312556
ATCTTCTC 0.320176368893
ATCTTCTG 0.343662656617
ATCTTGAA 0.299869248761
ATCTTGAC 0.234319294524
ATCTTGAG 0.255518428661
ATCTTGCA 0.310923594121
ATCTTGCC 0.248332921709
ATCTTGCG 0.14042247178
ATCTTGGA 0.258562207732
ATCTTGGC 0.246824924092
ATCTTGGG 0.262625484869
ATCTTGTA 0.277104236162
ATCTTGTC 0.321653603811
ATCTTGTG 0.266029582676
ATCTTTAA 0.219442249007
ATCTTTAC 0.230239855883
ATCTTTAG 0.287541987749
ATCTTTCA 0.316942454927
ATCTTTCC 0.280663344617
ATCTTTCG 0.25150581843
ATCTTTGA 0.36381532114
ATCTTTGC 0.226205549937
ATCTTTGG 0.294635996399
ATCTTTTA 0.204778231372
ATCTTTTC 0.310471196021
ATCTTTTG 0.178020716807
ATGAAAAA 0.0882004271207

ATGAAAAC 0.139083734001
ATGAAAAG 0.0985297749626
ATGAAAAT 0.270469713934
ATGAAACA 0.0433033574012
ATGAAACC -0.0678225647105
ATGAAACG 0.118008803917
ATGAAAGA 0.209598593698
ATGAAAGC -0.205532777888
ATGAAAGG -0.287494553377
ATGAAATA 0.182265773684
ATGAAATC 0.450545607708
ATGAAATG -0.0500597038341
ATGAACAA 0.0795022942326
ATGAACAC -0.102489380618
ATGAACAG -0.101364327032
ATGAACAT -0.0836708819074
ATGAACCA -0.142879483761
ATGAACCC -0.207564270153
ATGAACCG -0.145574290562
ATGAACGA 0.0554265735801
ATGAACGC -0.0226190084187
ATGAACGG -0.0154024270843
ATGAACTA -0.206631993375
ATGAACTC -0.149374458862
ATGAACTG 0.119193066807
ATGAAGAA 0.12100229675
ATGAAGAC -0.0804191774507
ATGAAGAG -0.142898850488
ATGAAGCA -0.0594671160635
ATGAAGCC -0.174127674054
ATGAAGCG -0.0382341319418
ATGAAGGA 0.0347208500735
ATGAAGGC -0.11946414202
ATGAAGGG -0.202265616603
ATGAAGTA -0.255200266121
ATGAAGTC -0.130744453773
ATGAAGTG -0.15936280804
ATGAATAA 0.158236185207
ATGAATAC 0.0439338689291
ATGAATAG -0.0416669087866
ATGAATCA 0.0706546795758
ATGAATCC 0.0840890270655
ATGAATCG 0.117459648715
ATGAATGA -0.0323754594453
ATGAATGC -0.00459395376408
ATGAATGG 0.0940031614305
ATGAATTA 0.199609199076
ATGAATTC -0.0338399306242
ATGAATTG 0.100133656822
ATGACAAA 0.202250374094
ATGACAAC 0.0189022707132
ATGACAAG -0.0665432823332
ATGACAAT 0.0780410558889
ATGACACA -0.0751572646841

ATGACACC -0.0986343759534
ATGACACG -0.112203179621
ATGACAGA -0.187992423362
ATGACAGC -0.0895339614583
ATGACAGG -0.252030178326
ATGACATA -0.00754956201014
ATGACATC 0.0360074382927
ATGACATG -0.0243705780658
ATGACCAA -0.111810906935
ATGACCAC -0.374502721875
ATGACCAG -0.122768148998
ATGACCAT -0.133101781237
ATGACCCA -0.226864690243
ATGACCCC -0.279571750687
ATGACCCG -0.235254903897
ATGACCGA -0.0671861108367
ATGACCGC -0.0960634199348
ATGACCGG -0.101820297108
ATGACCTA -0.154733485894
ATGACCTC -0.0685189973071
ATGACCTG -0.0323125051453
ATGACGAA -0.160517730723
ATGACGAC -0.218094720727
ATGACGAG -0.15615240021
ATGACGCA -0.186852720836
ATGACGCC -0.21174982578
ATGACGCG -0.00296065809475
ATGACGGA 0.0399111323732
ATGACGGC -0.141726704748
ATGACGGG -0.196290012869
ATGACGTA 0.0758429822905
ATGACGTC 0.0107576456124
ATGACGTG -0.0309342902387
ATGACTAA 0.134614211207
ATGACTAC 0.0380181017826
ATGACTAG -0.0802588677008
ATGACTCA -0.1216387229
ATGACTCC -0.142225127088
ATGACTCG -0.0310213770204
ATGACTGA -0.0864891249397
ATGACTGC -0.116726034482
ATGACTGG -0.229885791962
ATGACTTA -0.307046026964
ATGACTTC -0.262214842978
ATGACTTG -0.0119041738701
ATGAGAAA 0.0874333135744
ATGAGAAC -0.0978987861074
ATGAGAAG -0.031935263609
ATGAGAAT 0.3847355193
ATGAGACA -0.0363991652672
ATGAGACC -0.20070761216
ATGAGACG -0.218778682715
ATGAGAGA 0.0521087180838
ATGAGAGC -0.0677335440745

ATGAGAGG -0.210957403851
ATGAGATA 0.209124983656
ATGAGATC 0.366517376891
ATGAGATG 0.0442447663185
ATGAGCAA 0.03095963338
ATGAGCAC 0.0339207841975
ATGAGCAG -0.253926990508
ATGAGCAT -0.11055415631
ATGAGCCA -0.274654555912
ATGAGCCC -0.265029379814
ATGAGCCG -0.230067586211
ATGAGCGA -0.0510762527233
ATGAGCGC 0.0834156622538
ATGAGCGG -0.0789412520083
ATGAGCTA -0.0774889951041
ATGAGCTC -0.218856183327
ATGAGCTG -0.123717917058
ATGAGGAA -0.114880031172
ATGAGGAC -0.0620006395973
ATGAGGAG -0.114171558703
ATGAGGCA -0.17465831646
ATGAGGCC -0.203458691177
ATGAGGCG -0.244829903978
ATGAGGGA -0.133355895571
ATGAGGGC -0.165214233842
ATGAGGGG -0.0963315544994
ATGAGGTA 0.15490389784
ATGAGGTC -0.120919632959
ATGAGGTG -0.130873120069
ATGAGTAA 0.0800291993238
ATGAGTAC 0.0204528140982
ATGAGTAG 0.0952871442477
ATGAGTCA -0.108271043937
ATGAGTCC -0.200681000044
ATGAGTCG -0.0250913760743
ATGAGTGA -0.0638032837262
ATGAGTGC -0.047289731937
ATGAGTGG -0.00391033537081
ATGAGTTA 0.0469576719577
ATGAGTTC -0.074629282053
ATGAGTTG -0.0835583434862
ATGATAAA 0.191864804935
ATGATAAC 0.0680311313091
ATGATAAG -0.00979165294518
ATGATAAT 0.193644037017
ATGATACA 0.0623029651475
ATGATACC 0.176618220883
ATGATACG 0.198874086557
ATGATAGA 0.138349318933
ATGATAGC 0.0578991854925
ATGATAGG -0.0367379086259
ATGATATA 0.160401640154
ATGATATC 0.421956127784
ATGATATG 0.235504621397

ATGATCAA -0.082791388148
ATGATCAC 0.0783795563175
ATGATCAG 0.104736100417
ATGATCAT 0.165300411523
ATGATCCA 0.147573111734
ATGATCCC 0.0888570581843
ATGATCCG 0.0803815030581
ATGATCGA 0.0292157895892
ATGATCGC 0.243890768547
ATGATCGG -0.00407756321612
ATGATCTA 0.0657606090102
ATGATCTC 0.219077257459
ATGATCTG 0.0209971961123
ATGATGAA 0.0316938900964
ATGATGAC -0.138037433683
ATGATGAG -0.182093788768
ATGATGCA -0.135255455737
ATGATGCC -0.0759069051182
ATGATGCG 0.00613899317219
ATGATGGA -0.0174748223155
ATGATGGC -0.113200942385
ATGATGGG -0.00877409626808
ATGATGTA 0.0731073758226
ATGATGTC -0.119955544579
ATGATGTG -0.0479188761205
ATGATTAA -0.00304891435597
ATGATTAC 0.314449464646
ATGATTAG 0.0560181889501
ATGATTCA 0.0299677270632
ATGATTCC 0.341384219778
ATGATTCG 0.193623697936
ATGATTGA 0.0345573477262
ATGATTGC 0.343437836176
ATGATTGG -0.00956953786701
ATGATTTA 0.0374980789919
ATGATTTC 0.395971409063
ATGATTTG 0.218319783057
ATGCAAAA 0.0708852005533
ATGCAAAC -0.0924951844718
ATGCAAAG 0.182111777781
ATGCAAAT 0.309002393027
ATGCAACA -0.0202998561736
ATGCAACC -0.0516010230741
ATGCAACG 0.0857887931661
ATGCAAGA 0.137249171222
ATGCAAGC -0.110831234257
ATGCAAGG -0.0496811122572
ATGCAATA 0.0670075083975
ATGCAATC 0.453263696192
ATGCAATG -0.0571174169061
ATGCACAA 0.00958308634657
ATGCACAC -0.319681034908
ATGCACAG 0.00449790335682
ATGCACAT 0.0726305736679

ATGCACCA 0.00594918135197
ATGCACCC -0.18044028318
ATGCACCG 0.0326763485477
ATGCACGA -0.0178364934831
ATGCACGC -0.0271930753037
ATGCACGG 0.0195763659872
ATGCACTA -0.0706751402197
ATGCACTC -0.19150475283
ATGCACTG -0.106641431821
ATGCAGAA 0.222749262708
ATGCAGAC -0.25724982863
ATGCAGAG -0.0446716161566
ATGCAGCA -0.177651401906
ATGCAGCC -0.180603371887
ATGCAGCG -0.107962979813
ATGCAGGA 0.0286024391315
ATGCAGGC -0.0578115454108
ATGCAGGG -0.160281119015
ATGCAGTA 0.217769093313
ATGCAGTC -0.183722378609
ATGCAGTG -0.0730916156227
ATGCATAA 0.0472145016991
ATGCATAC -0.0301693147828
ATGCATAG 0.0362905881578
ATGCATCA -0.045102526949
ATGCATCC -0.147607312844
ATGCATCG -0.0567634451368
ATGCATGA -0.0244187578212
ATGCATGC -0.197178028285
ATGCATGG -0.185943451107
ATGCATTA -0.117963621594
ATGCATTC 0.107071505412
ATGCATTG -0.0741567718189
ATGCCAAA -0.182510877378
ATGCCAAC -0.0210843636047
ATGCCAAG -0.0104857313863
ATGCCAAT 0.0295991748144
ATGCCACA -0.0750499363449
ATGCCACC -0.0272316626051
ATGCCACG -0.0958471698058
ATGCCAGA -0.235918602084
ATGCCAGC 0.0767295777358
ATGCCAGG 0.0478313417529
ATGCCATA 0.0370113172461
ATGCCATC -0.149017186524
ATGCCATG -0.245365569984
ATGCCCAA -0.0626186791355
ATGCCCAC -0.0672347081584
ATGCCCAG -0.163583331397
ATGCCCAT -0.0859788359788
ATGCCCCA -0.226063366291
ATGCCCCC -0.18522085048
ATGCCCCG 0.0443835210433
ATGCCCGA -0.0515110606235

ATGCCCGC -0.025618442425
ATGCCCGG -0.0779257745787
ATGCCCTA -0.0831179379112
ATGCCCTC -0.272469821426
ATGCCCTG -0.032722513089
ATGCCGAA -0.132104283974
ATGCCGAC -0.229162551034
ATGCCGAG -0.108772969769
ATGCCGCA -0.0805375135829
ATGCCGCC -0.0502705609887
ATGCCGCG -0.00976598944099
ATGCCGGA -0.0427964636736
ATGCCGGC -0.167206437745
ATGCCGGG -0.17162262287
ATGCCGTA -0.0533450525184
ATGCCGTC -0.112535190399
ATGCCGTG -0.180400564005
ATGCCTAA -0.0122789940871
ATGCCTAC -0.0414745658522
ATGCCTAG -0.228763011616
ATGCCTCA 0.00409190740698
ATGCCTCC -0.324247099482
ATGCCTCG -0.159785668428
ATGCCTGA 0.0862889415794
ATGCCTGC -0.181502312501
ATGCCTGG -0.152148399485
ATGCCTTA -0.0781161479261
ATGCCTTC 0.0422951107598
ATGCCTTG -0.167855434715
ATGCGAAA 0.097053738759
ATGCGAAC 0.0117455926585
ATGCGAAG -0.15600749668
ATGCGAAT 0.36653933127
ATGCGACA -0.134870504559
ATGCGACC -0.103713947056
ATGCGACG 0.0874449755205
ATGCGAGA 0.102466840033
ATGCGAGC -0.242365637413
ATGCGAGG -0.036460361088
ATGCGATA 0.264147856367
ATGCGATC 0.377753403164
ATGCGATG 0.0497876123679
ATGCGCAA 0.109428617088
ATGCGCAC -0.0231618696349
ATGCGCAG -0.097854720274
ATGCGCAT 0.156326735829
ATGCGCCA -0.090170030183
ATGCGCCC -0.204307604698
ATGCGCCG -0.0342728051952
ATGCGCGA 0.128875684628
ATGCGCGC 0.0120704493041
ATGCGCGG -0.0102389734132
ATGCGCTA 0.230854330412
ATGCGCTC -0.0491610128863

ATGCGCTG 0.0599087646914
ATGCGGAA 0.095010318558
ATGCGGAC -0.0964074883213
ATGCGGAG -0.0960286887045
ATGCGGCA -0.0102649179794
ATGCGGCC -0.338991552956
ATGCGGCG -0.0874421091167
ATGCGGGA 0.016111207152
ATGCGGGC -0.129436167988
ATGCGGGG -0.0113448335474
ATGCGGTA -0.0876948823588
ATGCGGTC -0.240562872059
ATGCGGTG -0.163509077705
ATGCGTAA 0.147891278857
ATGCGTAC -0.0755945717547
ATGCGTAG -0.0063969571231
ATGCGTCA 0.0975709325469
ATGCGTCC -0.167256515775
ATGCGTCG -0.0432642810345
ATGCGTGA 0.00403440210364
ATGCGTGC -0.105294350059
ATGCGTGG -0.316532281489
ATGCGTTA 0.0076699322192
ATGCGTTC -0.136306505082
ATGCGTTG -0.170708850954
ATGCTAAA 0.0940406491073
ATGCTAAC -0.103561633152
ATGCTAAG -0.00371577861204
ATGCTAAT 0.156843454302
ATGCTACA 0.0254012162726
ATGCTACC -0.071397029772
ATGCTACG -0.055939738207
ATGCTAGA -0.0855532505735
ATGCTAGC -0.0144032210256
ATGCTAGG -0.0795892944359
ATGCTATA 0.205267953193
ATGCTATC 0.0837984687577
ATGCTATG -0.0501605334651
ATGCTCAA -0.108765170187
ATGCTCAC -0.130542215404
ATGCTCAG -0.0519444170042
ATGCTCCA -0.153092527445
ATGCTCCC -0.266978201245
ATGCTCCG -0.0876600739071
ATGCTCGA 0.0625480252036
ATGCTCGC -0.123188363696
ATGCTCGG -0.20377072417
ATGCTCTA -0.234347037906
ATGCTCTC -0.091999476995
ATGCTCTG 0.06508070257
ATGCTGAA 0.080273441788
ATGCTGAC -0.0718187497276
ATGCTGAG -0.0868551170818
ATGCTGCA -0.00725965813655

ATGCTGCC -0.160401530359
ATGCTGCG -0.195693614499
ATGCTGGA -0.0709413107665
ATGCTGGC -0.0272182661774
ATGCTGGG -0.148709429175
ATGCTGTA 0.018710619333
ATGCTGTC -0.137738173408
ATGCTGTG 0.00738453939244
ATGCTTAA -0.0356360732398
ATGCTTAC -0.217295312252
ATGCTTAG -0.0344056458296
ATGCTTCA -0.066661178614
ATGCTTCC -0.103465945572
ATGCTTCG -0.0175017563503
ATGCTTGA 0.0738061685529
ATGCTTGC -0.0855917358301
ATGCTTGG -0.0311038661661
ATGCTTTA 0.0320238838929
ATGCTTTC 0.0261894730725
ATGCTTTG 0.0265410486249
ATGGAAAA 0.0673093811061
ATGGAAAC -0.0991011013977
ATGGAAAG -0.00823207876453
ATGGAAAT 0.363814244001
ATGGAACA -0.0617814436158
ATGGAACC -0.075733187674
ATGGAACG -0.0885722918168
ATGGAAGA 0.133123647088
ATGGAAGC -0.274439606468
ATGGAAGG -0.241332033788
ATGGAATA 0.118264495228
ATGGAATC 0.410246538724
ATGGAATG 0.0163024213933
ATGGACAA -0.113635901385
ATGGACAC -0.115744183885
ATGGACAG -0.107395699562
ATGGACAT 0.0386342180948
ATGGACCA -0.243290423023
ATGGACCC -0.279493670228
ATGGACCG -0.298947961567
ATGGACGA -0.106979338555
ATGGACGC -0.0726629072136
ATGGACGG -0.120109217639
ATGGACTA -0.10618832801
ATGGACTC -0.325649440225
ATGGACTG -0.194222200272
ATGGAGAA -0.100004648474
ATGGAGAC -0.131872081475
ATGGAGAG -0.0525029049138
ATGGAGCA -0.19298066953
ATGGAGCC -0.38474026917
ATGGAGCG -0.236304498099
ATGGAGGA -0.099950617284
ATGGAGGC -0.156529362375

ATGGAGGG -0.205995631514
ATGGAGTA -0.159907678811
ATGGAGTC -0.0912204439175
ATGGAGTG -0.113640457665
ATGGATAA 0.147384248834
ATGGATAC 0.218027690207
ATGGATAG 0.0064518430701
ATGGATCA 0.0147211366641
ATGGATCC 0.0749989022811
ATGGATCG 0.133088816439
ATGGATGA -0.0805896877269
ATGGATGC -0.122674884688
ATGGATGG -0.260665613273
ATGGATTA 0.31756656622
ATGGATTC 0.417028169628
ATGGATTG 0.253230037981
ATGGCAAA 0.0772580012494
ATGGCAAC -0.17032631241
ATGGCAAG -0.116742148487
ATGGCAAT 0.18982319527
ATGGCACA -0.195232063588
ATGGCACC -0.0905739419434
ATGGCACG -0.086605503521
ATGGCAGA -0.107771480364
ATGGCAGC -0.11477836233
ATGGCAGG -0.0967731158837
ATGGCATA -0.149993172554
ATGGCATC -0.201112799077
ATGGCATG -0.170357883817
ATGGCCAA -0.168387400395
ATGGCCAC -0.285599065067
ATGGCCAG -0.351767464824
ATGGCCAT 0.198701362828
ATGGCCCA -0.242942628903
ATGGCCCC -0.165951030748
ATGGCCCG -0.330093598437
ATGGCCGA -0.129152347393
ATGGCCGC -0.223810542717
ATGGCCGG -0.306046590735
ATGGCCTA -0.155391624199
ATGGCCTC -0.315644285362
ATGGCCTG -0.12891347415
ATGGCGAA -0.0361259303168
ATGGCGAC -0.137976868033
ATGGCGAG -0.0336041069265
ATGGCGCA 0.0153819084309
ATGGCGCC -0.228030465153
ATGGCGCG -0.10937148439
ATGGCGGA -0.153397866456
ATGGCGGC -0.132261437908
ATGGCGGG -0.230005548567
ATGGCGTA -0.0799112097669
ATGGCGTC -0.284759255602
ATGGCGTG 0.066158130001

ATGGCTAA -0.136288359066
ATGGCTAC -0.351064551962
ATGGCTAG -0.105285255619
ATGGCTCA -0.0777014976156
ATGGCTCC -0.0871737338703
ATGGCTCG -0.10102857641
ATGGCTGA -0.0735134318471
ATGGCTGC -0.257396687074
ATGGCTGG -0.214364495366
ATGGCTTA -0.124772694263
ATGGCTTC -0.185368129305
ATGGCTTG -0.0961566961419
ATGGGAAA 0.0407144828789
ATGGGAAC -0.101500788863
ATGGGAAG -0.276819086064
ATGGGAAT 0.327644404927
ATGGGACA -0.084856434795
ATGGGACC -0.302805242184
ATGGGACG -0.0440991257964
ATGGGAGA 0.00603690705836
ATGGGAGC -0.258715753574
ATGGGAGG -0.251530713016
ATGGGATA 0.255966102439
ATGGGATC 0.194322323212
ATGGGATG -0.116950687413
ATGGGCAA -0.0916021996729
ATGGGCAC -0.192977834851
ATGGGCAG -0.287459588137
ATGGGCCA -0.182265347665
ATGGGCCC -0.330047450561
ATGGGCCG -0.0631036261469
ATGGGCGA 0.00558601725614
ATGGGCGC -0.131942837983
ATGGGCGG -0.166617283951
ATGGGCTA -0.00900105176912
ATGGGCTC -0.136276494697
ATGGGCTG -0.121376722137
ATGGGGAA -0.10666282484
ATGGGGAC -0.104777868326
ATGGGGAG -0.0979264044993
ATGGGGCA -0.0431661156016
ATGGGGCC -0.208704407899
ATGGGGCG -0.118009584033
ATGGGGGA -0.144039857757
ATGGGGGC -0.279930180821
ATGGGGGG -0.271626582383
ATGGGGTA 0.0999593218369
ATGGGGTC -0.390097540719
ATGGGGTG -0.0729864712823
ATGGGTAA 0.0519547071026
ATGGGTAC -0.282203673521
ATGGGTAG -0.110200094395
ATGGGTCA -0.279563697815
ATGGGTCC -0.321738343291

ATGGGTCG -0.116315112148
ATGGGTGA -0.0530198248041
ATGGGTGC -0.107701586278
ATGGGTGG 0.0761981602231
ATGGGTTA -0.0315778768904
ATGGGTTC -0.197568216876
ATGGGTTG -0.159129198839
ATGGTAAA 0.0957210915717
ATGGTAAC -0.0545264440493
ATGGTAAG -0.0714587586994
ATGGTAAT 0.311660104892
ATGGTACA -0.017608129779
ATGGTACC 0.0982071360975
ATGGTACG -0.0448896532291
ATGGTAGA 0.088470646347
ATGGTAGC -0.246031423285
ATGGTAGG -0.204357373783
ATGGTATA 0.0884529284977
ATGGTATC 0.243855418186
ATGGTATG -0.00712013133236
ATGGTCAA -0.140367265701
ATGGTCAC -0.142709810119
ATGGTCAG -0.029350750475
ATGGTCCA -0.0328432502442
ATGGTCCC -0.0203680247287
ATGGTCCG -0.203982939957
ATGGTCGA -0.0958144311136
ATGGTCGC -0.151043381853
ATGGTCGG -0.0746915409778
ATGGTCTA -0.254390729676
ATGGTCTC -0.307327671341
ATGGTCTG -0.189563623583
ATGGTGAA -0.0193787863379
ATGGTGAC -0.218907127317
ATGGTGAG 0.0145991021748
ATGGTGCA 0.00940371504792
ATGGTGCC -0.271073347858
ATGGTGCG -0.0429030421661
ATGGTGGA -0.153841255319
ATGGTGGC -0.0199208544644
ATGGTGGG -0.16424691358
ATGGTGTA 0.139090315821
ATGGTGTC -0.0670749777734
ATGGTGTG -0.129692153473
ATGGTTAA 0.196968672732
ATGGTTAC -0.189732609757
ATGGTTAG -0.0422515363271
ATGGTTCA -0.225659807956
ATGGTTCC -0.050872794045
ATGGTTCG -0.0859255837466
ATGGTTGA -0.0442476719016
ATGGTTGC -0.224408671177
ATGGTTGG -0.141948407752
ATGGTTTA 0.0726305736679

ATGGTTTC -0.0384248834135
ATGGTTTG -0.111790271225
ATGTAAAA 0.236882258666
ATGTAAAC -0.0697812903049
ATGTAAAG 0.0733509517223
ATGTAAAT 0.190596080368
ATGTAACA 0.0159808328805
ATGTAACC 0.0431711533712
ATGTAACG -0.106134823169
ATGTAAGA 0.130510549079
ATGTAAGC 0.12422384612
ATGTAAGG -0.0724302399614
ATGTAATA 0.213900276598
ATGTAATC 0.46892083251
ATGTAATG 0.159701299022
ATGTACAA -0.0609663969317
ATGTACAC -0.0660813174425
ATGTACAG 0.0517135392654
ATGTACAT 0.189736625514
ATGTACCA -0.0233193588593
ATGTACCC -0.254326047359
ATGTACCG 0.0371115598623
ATGTACGA 0.01852163933
ATGTACGC -0.0902023096006
ATGTACGG -0.0796983801669
ATGTACTA 0.0334212713041
ATGTACTC -0.107147734372
ATGTACTG -0.122886760275
ATGTAGAA 0.0141770401107
ATGTAGAC -0.0209335001866
ATGTAGAG -0.0260790577181
ATGTAGCA -0.0909518801099
ATGTAGCC -0.136811866193
ATGTAGCG -0.152128565161
ATGTAGGA 0.0519568817811
ATGTAGGC -0.256957475995
ATGTAGGG -0.0469963534932
ATGTAGTA 0.169943195851
ATGTAGTC -0.0943573576627
ATGTAGTG 0.00765185303561
ATGTATAA 0.186243516918
ATGTATAC 0.0652676238776
ATGTATAG -0.113578191686
ATGTATCA 0.0929041098027
ATGTATCC 0.0815059637093
ATGTATCG -0.194448271828
ATGTATGA -0.0567317617266
ATGTATGC 0.0792221754101
ATGTATGG 0.0108952542399
ATGTATTA 0.0183385875961
ATGTATTC 0.210046053492
ATGTATTG -0.094809654403
ATGTCAAA 0.0585193966937
ATGTCAAC -0.0145924387118

ATGTCAAG -0.00708346165691
ATGTCAAT 0.142563503041
ATGTCACA 0.103071201313
ATGTCACC -0.00844217163279
ATGTCACG -0.0781356341522
ATGTCAGA 0.0073380500632
ATGTCAGC 0.00437277733873
ATGTCAGG -0.109448718247
ATGTCATA 0.0882242171293
ATGTCATC -0.0754229384745
ATGTCATG 0.0272097082263
ATGTCCAA 0.12755768513
ATGTCCAC -0.0959815786033
ATGTCCAG -0.265051476034
ATGTCCCA -0.226647462277
ATGTCCCC -0.139347406041
ATGTCCCG 0.0224895167841
ATGTCCGA 0.0113008525364
ATGTCCGC -0.133249269282
ATGTCCGG 0.0039051351292
ATGTCCTA -0.00623229928209
ATGTCCTC -0.105811872928
ATGTCCTG 0.0630206441678
ATGTCGAA 0.0501779669635
ATGTCGAC 0.0580693319283
ATGTCGAG -0.1428320081
ATGTCGCA 0.0026862115555
ATGTCGCC -0.0681332480579
ATGTCGCG 0.0743238051329
ATGTCGGA -0.01177196984
ATGTCGGC -0.113648992394
ATGTCGGG -0.154683504012
ATGTCGTA 0.0910585303739
ATGTCGTC -0.214893871388
ATGTCGTG -0.044507721587
ATGTCTAA -0.0326747345023
ATGTCTAC -0.146355741882
ATGTCTAG 0.000779888063125
ATGTCTCA -0.0243103576015
ATGTCTCC 0.0310517245165
ATGTCTCG -0.0389478883675
ATGTCTGA 0.0435430680052
ATGTCTGC -0.0922852768311
ATGTCTGG -0.0444714017986
ATGTCTTA -0.129270843927
ATGTCTTC -0.114124039342
ATGTCTTG -0.025759446777
ATGTGAAA 0.241509143999
ATGTGAAC -0.03817317614
ATGTGAAG 0.162173666701
ATGTGAAT 0.199208228611
ATGTGACA 0.0221361846789
ATGTGACC -0.230081528299
ATGTGACG -0.129756930926

ATGTGAGA 0.0144712565194
ATGTGAGC -0.22771714977
ATGTGAGG 0.0264888925045
ATGTGATA 0.247449624453
ATGTGATC 0.222445965727
ATGTGATG -0.0679954554436
ATGTGCAA 0.0708206819403
ATGTGCAC -0.00404783859141
ATGTGCAG -0.136126734376
ATGTGCCA -0.102426263606
ATGTGCCC -0.115457219369
ATGTGCCG -0.00573009286702
ATGTGCGA 0.0992914224242
ATGTGCGC 0.059684049327
ATGTGCGG -0.0792486485003
ATGTGCTA -0.130749785713
ATGTGCTC 0.0802871632747
ATGTGCTG -0.0471634942589
ATGTGGAA -0.0162204175323
ATGTGGAC -0.174999862805
ATGTGGAG -0.0374660409978
ATGTGGCA -0.122043790107
ATGTGGCC -0.114209252573
ATGTGGCG -0.0219821887757
ATGTGGGA -0.0412735266512
ATGTGGGC -0.368387272798
ATGTGGGG -0.109463484085
ATGTGGTA -0.0386553376476
ATGTGGTC -0.053568880547
ATGTGGTG -0.0803887022767
ATGTGTAA 0.0960174756855
ATGTGTAC -0.152021947874
ATGTGTAG 0.0199279415397
ATGTGTCA -0.0768814902632
ATGTGTCC -0.0503745111598
ATGTGTCG 0.122933767234
ATGTGTGA 0.0990614966658
ATGTGTGC -0.214786645595
ATGTGTGG 0.0171734487818
ATGTGTTA -0.0854343411758
ATGTGTTC -0.122310588597
ATGTGTTG -0.014492332545
ATGTTAAA 0.116506617465
ATGTTAAC 0.067286417397
ATGTTAAG -0.0426153200316
ATGTTAAT 0.189047404588
ATGTTACA 0.00339517382651
ATGTTACC -0.0379457670651
ATGTTACG 0.0354699054233
ATGTTAGA 0.165423499967
ATGTTAGC -0.0548488989879
ATGTTAGG -0.112877544201
ATGTTATA 0.120739592894
ATGTTATC 0.155034649078

ATGTTATG 0.113519423218
ATGTTCAA 0.0166304419416
ATGTTCAC -0.0759344319872
ATGTTCAG 0.0891534583703
ATGTTCCA -0.0813271604938
ATGTTCCC 0.0261998068015
ATGTTCCG -0.143582999951
ATGTTCGA 0.104860973896
ATGTTCGC -0.235012977321
ATGTTCGG -0.0703456191071
ATGTTCTA 0.134160736525
ATGTTCTC -0.0801548675781
ATGTTCTG 0.0661179957288
ATGTTGAA 0.150127269465
ATGTTGAC -0.13786668862
ATGTTGAG 0.0194703122049
ATGTTGCA -0.0160760938769
ATGTTGCC -0.0815594079781
ATGTTGCG 0.00883733093138
ATGTTGGA 0.0874042973574
ATGTTGGC -0.210144569584
ATGTTGGG 0.0774289747983
ATGTTGTA 0.0977114896117
ATGTTGTC 0.0702032455363
ATGTTGTG 0.0602126281587
ATGTTTAA 0.121078399087
ATGTTTAC 0.0776800807921
ATGTTTAG -0.0885421811716
ATGTTTCA 0.00024973105886
ATGTTTCC -0.0329476846552
ATGTTTCG 0.0396355335308
ATGTTTGA 0.105517702265
ATGTTTGC -0.196157834955
ATGTTTGG -0.138654995308
ATGTTTTA 0.0191608676071
ATGTTTTC 0.187038958045
ATGTTTTG -0.0506611012316
ATTAAAAA 0.212971195855
ATTAAAAC 0.0243880115642
ATTAAAAG 0.0504873390629
ATTAAAAT 0.13848735345
ATTAAACA -0.00194664450785
ATTAAACC -0.126215200098
ATTAAACG -0.148253018174
ATTAAAGA 0.0440581149559
ATTAAAGC 0.0805180654628
ATTAAAGG -0.0384366286856
ATTAAATA 0.099067307832
ATTAAATC 0.169327212238
ATTAAATG -0.00724096958793
ATTAACAA 0.045699010649
ATTAACAC 0.147459793994
ATTAACAG 0.0683509363241
ATTAACCA 0.0209636874575

ATTAACCC -0.0863525692802
ATTAACCG -0.13435559854
ATTAACGA 0.20481514413
ATTAACGC 0.00643887295673
ATTAACGG -0.0170333876624
ATTAACTA 0.0651664172708
ATTAACTC -0.166016816684
ATTAACTG 0.0200394158345
ATTAAGAA 0.218094799008
ATTAAGAC -0.0773103017448
ATTAAGAG -0.0443011921228
ATTAAGCA -0.156694488345
ATTAAGCC -0.122481710258
ATTAAGCG -0.0249017541549
ATTAAGGA -0.0699356736701
ATTAAGGC -0.0603653584442
ATTAAGGG -0.0149125118005
ATTAAGTA 0.162237589528
ATTAAGTC 0.144981229006
ATTAAGTG 0.0529004983075
ATTAATAA 0.0673313354849
ATTAATAC 0.101543873516
ATTAATAG -0.136098214188
ATTAATCA 0.150563129816
ATTAATCC 0.164364809668
ATTAATCG 0.0412330676853
ATTAATGA 0.129808008957
ATTAATGC 0.0350153529355
ATTAATGG 0.00520647813279
ATTAATTA 0.11535241552
ATTAATTC 0.0903146922241
ATTAATTG 0.171093581407
ATTACAAA 0.344425783222
ATTACAAC 0.241164921466
ATTACAAG 0.167918066258
ATTACAAT 0.359442418607
ATTACACA 0.338849371007
ATTACACC 0.366878025289
ATTACACG 0.280607153443
ATTACAGA 0.347945026368
ATTACAGC 0.218388817544
ATTACAGG 0.213382840458
ATTACATA 0.332306966124
ATTACATC 0.242361948484
ATTACATG 0.334145645349
ATTACCAA 0.295045980852
ATTACCAC 0.318758444351
ATTACCAG 0.232282480787
ATTACCCA 0.259112911192
ATTACCCC 0.186722068557
ATTACCCG 0.159053070475
ATTACCGA 0.155421091628
ATTACCGC 0.278487108565
ATTACCGG 0.181304748732

ATTACCTA 0.279243232563
ATTACCTC 0.299265904376
ATTACCTG 0.144516438336
ATTACGAA 0.391098859034
ATTACGAC 0.37279357285
ATTACGAG 0.355622516411
ATTACGCA 0.370483634303
ATTACGCC 0.237957084538
ATTACGCG 0.303160026728
ATTACGGA 0.346932430666
ATTACGGC 0.312502723984
ATTACGGG 0.251633664086
ATTACGTA 0.375738215987
ATTACGTC 0.345481091918
ATTACGTG 0.350628027362
ATTACTAA 0.117479996049
ATTACTAC 0.0708453793965
ATTACTAG 0.160346549869
ATTACTCA 0.145771650226
ATTACTCC 0.118555053535
ATTACTCG 0.209331280055
ATTACTGA 0.173846969843
ATTACTGC 0.152662517289
ATTACTGG 0.110041695117
ATTACTTA 0.225523611934
ATTACTTC 0.201675068644
ATTACTTG 0.044122812795
ATTAGAAA 0.270071790819
ATTAGAAC -0.125953710537
ATTAGAAG 0.0800647945026
ATTAGAAT 0.184042526919
ATTAGACA 0.0224920972044
ATTAGACC -0.0491201782696
ATTAGACG -0.0134445435329
ATTAGAGA 0.0965830343004
ATTAGAGC -0.00127409817965
ATTAGAGG 0.0198436796304
ATTAGATA 0.321812773058
ATTAGATC 0.0678322897447
ATTAGATG 0.0935757558148
ATTAGCAA 0.129180770851
ATTAGCAC -0.0428936701873
ATTAGCAG 0.0264335420511
ATTAGCCA -0.180548171261
ATTAGCCC -0.133639261009
ATTAGCCG -0.00743102872169
ATTAGCGA 0.148822512458
ATTAGCGC -0.0716896330321
ATTAGCGG 0.14692419153
ATTAGCTA -0.161182455794
ATTAGCTC 0.0728803047268
ATTAGCTG 0.0153400258597
ATTAGGAA 0.0512277986344
ATTAGGAC 0.0114107883817

ATTAGGAG -0.0661470515596
ATTAGGCA 0.062333300405
ATTAGGCC 0.0839289474984
ATTAGGCG -0.0153912432634
ATTAGGGA 0.0464442927084
ATTAGGGC -0.0838868596535
ATTAGGGG -0.0803175802304
ATTAGGTA 0.122031787466
ATTAGGTC -0.0786032862145
ATTAGGTG -0.0544840410849
ATTAGTAA 0.0626152604887
ATTAGTAC -0.0303025313399
ATTAGTAG 0.0409339392742
ATTAGTCA 0.0632240349832
ATTAGTCC -0.213261560242
ATTAGTCG -0.0446277635074
ATTAGTGA -0.0142998271178
ATTAGTGC 0.0915650923249
ATTAGTGG 0.190947098022
ATTAGTTA -0.0807218274053
ATTAGTTC -0.161496921203
ATTAGTTG 0.0644966803713
ATTATAAA 0.133655368791
ATTATAAC 0.0430580254232
ATTATAAG 0.237606753167
ATTATAAT 0.235995079929
ATTATACA 0.228487453073
ATTATACC 0.0778605729856
ATTATACG 0.165587726817
ATTATAGA 0.145268984353
ATTATAGC 0.104739006
ATTATAGG 0.108853311638
ATTATATA 0.296784853504
ATTATATC 0.278059942179
ATTATATG 0.139835671475
ATTATCAA 0.187553934886
ATTATCAC 0.135615184577
ATTATCAG 0.210352116521
ATTATCCA 0.103693275374
ATTATCCC 0.21514244621
ATTATCCG 0.271804221812
ATTATCGA 0.145288689788
ATTATCGC 0.211829568157
ATTATCGG 0.191697402797
ATTATCTA 0.21213080935
ATTATCTC 0.0387561198844
ATTATCTG 0.303172896721
ATTATGAA 0.154049656415
ATTATGAC 0.182558892621
ATTATGAG 0.0752321675558
ATTATGCA 0.0848220437474
ATTATGCC 0.154360553804
ATTATGCG 0.153227842543
ATTATGGA 0.299171908823

ATTATGGC 0.103974837651
ATTATGGG -0.03035026804
ATTATGTA 0.193241613761
ATTATGTC 0.162687954905
ATTATGTG 0.0572487870206
ATTATTAA 0.151314099397
ATTATTAC 0.291553469993
ATTATTAG 0.0702235846178
ATTATTCA 0.194685882319
ATTATTCC 0.28229047114
ATTATTCG 0.174065292323
ATTATTGA 0.148755684047
ATTATTGC 0.221322621417
ATTATTGG 0.0500588897527
ATTATTTA 0.207227637906
ATTATTTC 0.261542001524
ATTATTTG 0.0981250960504
ATTCAAAA 0.213842510264
ATTCAAAC 0.259295686662
ATTCAAAG -0.0155462807468
ATTCAAAT 0.206916740517
ATTCAACA 0.110167072823
ATTCAACC -0.00906396641146
ATTCAACG 0.00471470284748
ATTCAAGA 0.0382472721684
ATTCAAGC 0.0504722880164
ATTCAAGG -0.0145059517929
ATTCAATA 0.0267122878084
ATTCAATC 0.129154620603
ATTCAATG 0.0456376649323
ATTCACAA 0.20176514172
ATTCACAC 0.0677945171647
ATTCACAG 0.0902544512503
ATTCACCA 0.166545917583
ATTCACCC 0.00596047496601
ATTCACCG 0.112722013656
ATTCACGA 0.0618990890997
ATTCACGC 0.100252785728
ATTCACGG 0.0734044655206
ATTCACTA 0.135477169381
ATTCACTC 0.00536705026259
ATTCACTG -0.0696166405971
ATTCAGAA 0.05582932605
ATTCAGAC -0.0351475883115
ATTCAGAG -0.0736259273893
ATTCAGCA 0.168706164181
ATTCAGCC -0.205728995148
ATTCAGCG -0.154334403556
ATTCAGGA 0.14845640899
ATTCAGGC -0.103778710793
ATTCAGGG 0.0415643659291
ATTCAGTA 0.0781543267806
ATTCAGTC 0.0443337137984
ATTCAGTG 0.0554254500022

ATTCATAA 0.149514808229
ATTCATAC 0.039538674306
ATTCATAG 0.127170181764
ATTCATCA 0.0714697358888
ATTCATCC -0.0824112494237
ATTCATCG 0.00401987418825
ATTCATGA 0.0315374651474
ATTCATGC 0.0336510104165
ATTCATGG 0.00648671422138
ATTCATTA 0.223761773036
ATTCATTC 0.182716139061
ATTCATTG -0.00681067139518
ATTCCAAA 0.243850029638
ATTCCAAC 0.214574404719
ATTCCAAG 0.255675330147
ATTCCAAT 0.200882566028
ATTCCACA 0.151328788777
ATTCCACC 0.21780396031
ATTCCACG 0.179183406881
ATTCCAGA 0.166839999272
ATTCCAGC -0.0192969457479
ATTCCAGG 0.154827767898
ATTCCATA 0.254410231477
ATTCCATC 0.163861994775
ATTCCATG 0.25931901086
ATTCCCAA 0.35495016356
ATTCCCAC 0.313596346791
ATTCCCAG 0.327071317537
ATTCCCCA 0.30868478782
ATTCCCCC 0.38629291183
ATTCCCCG 0.363951883544
ATTCCCGA 0.381083201371
ATTCCCGC 0.192467275871
ATTCCCGG 0.30663101215
ATTCCCTA 0.364733583613
ATTCCCTC 0.379544556412
ATTCCCTG 0.361807563233
ATTCCGAA 0.18327515423
ATTCCGAC 0.190245669499
ATTCCGAG 0.304067816309
ATTCCGCA 0.28437667979
ATTCCGCC 0.16980065424
ATTCCGCG 0.249055961712
ATTCCGGA 0.213064501965
ATTCCGGC 0.10334142054
ATTCCGGG 0.223254562331
ATTCCGTA 0.326735722691
ATTCCGTC 0.254786221725
ATTCCGTG 0.325345401188
ATTCCTAA 0.243973523019
ATTCCTAC 0.183925552701
ATTCCTAG 0.183204330542
ATTCCTCA 0.218572306747
ATTCCTCC 0.220122615606

ATTCCTCG 0.235606160399
ATTCCTGA 0.237284525338
ATTCCTGC 0.199789345227
ATTCCTGG 0.0359231763834
ATTCCTTA 0.211989921944
ATTCCTTC 0.194479392152
ATTCCTTG 0.146358578008
ATTCGAAA 0.228723462645
ATTCGAAC 0.0867966365892
ATTCGAAG 0.0784429954554
ATTCGAAT 0.36395733544
ATTCGACA 0.227315638104
ATTCGACC -0.0382331149303
ATTCGACG 0.194454694696
ATTCGAGA 0.163855999303
ATTCGAGC 0.210310461552
ATTCGAGG 0.085442141143
ATTCGATA 0.273056528119
ATTCGATC 0.210191222639
ATTCGATG 0.124191884706
ATTCGCAA 0.333467803903
ATTCGCAC 0.302260430506
ATTCGCAG 0.253644426881
ATTCGCCA 0.271632792411
ATTCGCCC 0.280452095545
ATTCGCCG 0.291650200883
ATTCGCGA 0.277160310874
ATTCGCGC 0.212872805934
ATTCGCGG 0.22429601818
ATTCGCTA 0.267323377023
ATTCGCTC 0.269962018925
ATTCGCTG 0.268679615727
ATTCGGAA 0.249573989924
ATTCGGAC -0.0349469004693
ATTCGGAG 0.236123802233
ATTCGGCA 0.173967428414
ATTCGGCC 0.0207734662153
ATTCGGCG 0.0631266669959
ATTCGGGA 0.248817667687
ATTCGGGC 0.179416672155
ATTCGGGG 0.187751711443
ATTCGGTA 0.202770473465
ATTCGGTC 0.210454675185
ATTCGGTG 0.160835034798
ATTCGTAA 0.205553360118
ATTCGTAC 0.15624764376
ATTCGTAG 0.0511701683901
ATTCGTCA 0.244785204771
ATTCGTCC -0.072147875252
ATTCGTCG 0.244593436288
ATTCGTGA 0.0991459746646
ATTCGTGC 0.0493353478709
ATTCGTGG 0.131368541934
ATTCGTTA 0.236826812721

ATTCGTTC 0.138196795142
ATTCGTTG 0.188063953105
ATTCTAAA 0.232076184388
ATTCTAAC 0.0813656721333
ATTCTAAG 0.218733746895
ATTCTACA 0.171140296079
ATTCTACC 0.100740923685
ATTCTACG 0.134761465674
ATTCTAGA 0.168948549913
ATTCTAGC 0.10538113986
ATTCTAGG 0.180193308305
ATTCTATA 0.340108471544
ATTCTATC 0.325218446069
ATTCTATG 0.2268199846
ATTCTCAA 0.339146475585
ATTCTCAC 0.310820391382
ATTCTCAG 0.34058651123
ATTCTCCA 0.270022393466
ATTCTCCC 0.239383725829
ATTCTCCG 0.376466229861
ATTCTCGA 0.319154480783
ATTCTCGC 0.230266006131
ATTCTCGG 0.283234785641
ATTCTCTA 0.372659916029
ATTCTCTC 0.235446991152
ATTCTCTG 0.341796812575
ATTCTGAA 0.273521421411
ATTCTGAC 0.170964236317
ATTCTGAG 0.263617203451
ATTCTGCA 0.321412105644
ATTCTGCC 0.130023389944
ATTCTGCG 0.205373875903
ATTCTGGA 0.209552104369
ATTCTGGC 0.102285636669
ATTCTGGG 0.144992206196
ATTCTGTA 0.344638472826
ATTCTGTC 0.320166194648
ATTCTGTG 0.317114535994
ATTCTTAA 0.299339722058
ATTCTTAC 0.139876222161
ATTCTTAG 0.244102392748
ATTCTTCA 0.272328484639
ATTCTTCC 0.290827074223
ATTCTTCG 0.22713088199
ATTCTTGA 0.252369240049
ATTCTTGC 0.142818722694
ATTCTTGG 0.257808028126
ATTCTTTA 0.203935612279
ATTCTTTC 0.222864387802
ATTCTTTG 0.331476507799
ATTGAAAA 0.201817442215
ATTGAAAC -0.169108850033
ATTGAAAG 0.104837795825
ATTGAAAT 0.239531882841

ATTGAACA -0.0255631298162
ATTGAACC -0.0459560034249
ATTGAACG -0.0623381184424
ATTGAAGA 0.165423005223
ATTGAAGC -0.0910551269521
ATTGAAGG 0.0257071462816
ATTGAATA 0.12096668749
ATTGAATC 0.136360466637
ATTGAATG 0.0174167572374
ATTGACAA 0.138902610376
ATTGACAC 0.0386369909782
ATTGACAG 0.125786171698
ATTGACCA -0.0948149869975
ATTGACCC -0.238896684767
ATTGACCG -0.0503166919142
ATTGACGA -0.0508325125872
ATTGACGC 0.00300774989572
ATTGACGG -0.0108102218413
ATTGACTA -0.166488313769
ATTGACTC -0.100178370517
ATTGACTG -0.0906815534037
ATTGAGAA 0.194683198314
ATTGAGAC -0.131982497632
ATTGAGAG -0.00776542279957
ATTGAGCA 0.00492052514874
ATTGAGCC -0.269694522084
ATTGAGCG -0.0783950563482
ATTGAGGA 0.00464457455
ATTGAGGC -0.198498913688
ATTGAGGG -0.114885332807
ATTGAGTA -0.0153810268653
ATTGAGTC 0.180031394762
ATTGAGTG 0.12702773379
ATTGATAA 0.159792201805
ATTGATAC 0.128185814482
ATTGATAG 0.145695446014
ATTGATCA -0.133965501553
ATTGATCC -0.0441173242003
ATTGATCG -0.0417132429018
ATTGATGA 0.00476079787311
ATTGATGC 0.065189526204
ATTGATGG -0.13639295489
ATTGATTA 0.096606278965
ATTGATTC 0.256285502593
ATTGATTG 0.0839837318053
ATTGCAAA 0.224254173664
ATTGCAAC 0.115709181321
ATTGCAAG 0.117100291416
ATTGCAAT 0.347262312523
ATTGCACA 0.286852958609
ATTGCACC 0.218484489231
ATTGCACG 0.218492583499
ATTGCAGA 0.224055358967
ATTGCAGC 0.186881160948

ATTGCAGG 0.210119870908
ATTGCATA 0.247231003974
ATTGCATC 0.218472139893
ATTGCATG 0.191379064304
ATTGCCAA 0.0287361401776
ATTGCCAC 0.0246777963533
ATTGCCAG 0.188374058706
ATTGCCCA 0.111605084634
ATTGCCCC 0.183547135821
ATTGCCCG 0.264124898461
ATTGCCGA 0.202656754265
ATTGCCGC 0.139504307527
ATTGCCGG 0.0378960021076
ATTGCCTA 0.115516129407
ATTGCCTC 0.155856879405
ATTGCCTG 0.148540582669
ATTGCGAA 0.326343607983
ATTGCGAC 0.367272058662
ATTGCGAG 0.277275434748
ATTGCGCA 0.336840545347
ATTGCGCC 0.220593320065
ATTGCGCG 0.37225570265
ATTGCGGA 0.380908472195
ATTGCGGC 0.170538870228
ATTGCGGG 0.327375463786
ATTGCGTA 0.341536038113
ATTGCGTC 0.270560275747
ATTGCGTG 0.391907615974
ATTGCTAA 0.27816454317
ATTGCTAC 0.160104891549
ATTGCTAG 0.192287426727
ATTGCTCA 0.0971990179129
ATTGCTCC 0.212001809592
ATTGCTCG 0.051639443237
ATTGCTGA 0.11820720353
ATTGCTGC 0.0238136402555
ATTGCTGG 0.208017956503
ATTGCTTA 0.236675722401
ATTGCTTC 0.19996871501
ATTGCTTG 0.0823755735581
ATTGGAAA -0.0487064660235
ATTGGAAC -0.0298305121957
ATTGGAAG -0.100597477491
ATTGGACA -0.121142302619
ATTGGACC -0.0728298963176
ATTGGACG -0.121780939208
ATTGGAGA 0.172627280511
ATTGGAGC -0.0228189967022
ATTGGAGG -0.070455892522
ATTGGATA 0.282378314011
ATTGGATC 0.114777817933
ATTGGATG 0.0780366975143
ATTGGCAA -0.0438906999462
ATTGGCAC -0.0871777141164

ATTGGCAG -0.227672967417
ATTGGCCA -0.20709370849
ATTGGCCC -0.174906570521
ATTGGCCG -0.14047760162
ATTGGCGA 0.0312791686275
ATTGGCGC -0.0230736030909
ATTGGCGG 0.0135019429625
ATTGGCTA -0.0350953463097
ATTGGCTC -0.16269725986
ATTGGCTG 0.105389856609
ATTGGGAA 0.112874625927
ATTGGGAC -0.110246904254
ATTGGGAG 0.0744599247454
ATTGGGCA -0.182358977736
ATTGGGCC -0.271257559731
ATTGGGCG -0.173410816981
ATTGGGGA -0.0153698354467
ATTGGGGC -0.0173361614342
ATTGGGGG -0.166625943563
ATTGGGTA 0.250424941525
ATTGGGTC 0.00795403367571
ATTGGGTG -0.0281666831341
ATTGGTAA 0.0145213869406
ATTGGTAC 0.0734895387385
ATTGGTAG -0.00990026934556
ATTGGTCA -0.00771287028024
ATTGGTCC -0.149765374166
ATTGGTCG 0.0601034387576
ATTGGTGA 0.0105161474456
ATTGGTGC -0.0578312727907
ATTGGTGG -0.104978212432
ATTGGTTA -0.0639476168522
ATTGGTTC -0.0859212057345
ATTGGTTG 0.143732573712
ATTGTAAA 0.224388011564
ATTGTAAC -0.0365998221871
ATTGTAAG -0.00121165909315
ATTGTACA 0.0682692132693
ATTGTACC 0.0656589136025
ATTGTACG 0.1115364772
ATTGTAGA 0.122294538957
ATTGTAGC 0.0749604821182
ATTGTAGG 0.0979439724253
ATTGTATA 0.160104891549
ATTGTATC 0.136183226069
ATTGTATG 0.183418946952
ATTGTCAA 0.113429604986
ATTGTCAC 0.0322550989045
ATTGTCAG 0.113563261808
ATTGTCCA 0.171953859341
ATTGTCCC -0.00367701538506
ATTGTCCG 0.0951571384663
ATTGTCGA 0.115214616099
ATTGTCGC 0.111585874553

ATTGTCGG 0.0287609140964
ATTGTCTA 0.143441372597
ATTGTCTC 0.186882594093
ATTGTCTG 0.00461248031258
ATTGTGAA 0.164987156688
ATTGTGAC 0.15095282004
ATTGTGAG 0.0763436079826
ATTGTGCA 0.133961565063
ATTGTGCC 0.121077099647
ATTGTGCG 0.141965903743
ATTGTGGA 0.0892464370288
ATTGTGGC 0.0395947221673
ATTGTGGG 0.230781685657
ATTGTGTA 0.253742034283
ATTGTGTC 0.19456244923
ATTGTGTG 0.172183400404
ATTGTTAA 0.21022861704
ATTGTTAC 0.0103308006334
ATTGTTAG 0.10961115776
ATTGTTCA 0.10083971351
ATTGTTCC 0.182155480911
ATTGTTCG 0.141460295506
ATTGTTGA -0.0943131734839
ATTGTTGC 0.134681881051
ATTGTTGG 0.0451919864019
ATTGTTTA 0.0457062746067
ATTGTTTC 0.119656269522
ATTGTTTG 0.159248830929
ATTTAAAA 0.158606665349
ATTTAAAC 0.024221168412
ATTTAAAG 0.206937079598
ATTTAAAT 0.247796966255
ATTTAACA 0.187309271334
ATTTAACC 0.0545741141604
ATTTAACG 0.171425043221
ATTTAAGA 0.250491769936
ATTTAAGC 0.0187773306408
ATTTAAGG 0.0979236639336
ATTTAATA -0.00907849432685
ATTTAATC 0.0578603286214
ATTTAATG 0.213344420295
ATTTACAA 0.283112751151
ATTTACAC 0.163250016466
ATTTACAG 0.135831741641
ATTTACCA 0.0600287602362
ATTTACCC 0.0502798888245
ATTTACCG 0.113419065183
ATTTACGA 0.201146018573
ATTTACGC 0.151142176557
ATTTACGG 0.0606758386239
ATTTACTA 0.237808899801
ATTTACTC 0.184000406782
ATTTACTG 0.139012027067
ATTTAGAA 0.303027617567

ATTTAGAC 0.144467043424
ATTTAGAG -0.0340406747012
ATTTAGCA 0.160654046751
ATTTAGCC -0.0778607340241
ATTTAGCG 0.205792279866
ATTTAGGA 0.168048755684
ATTTAGGC -0.0447711893707
ATTTAGGG 0.0795846231531
ATTTAGTA 0.234132472722
ATTTAGTC 0.0380112625963
ATTTAGTG 0.0973762584807
ATTTATAA 0.19474906145
ATTTATAC 0.17412978331
ATTTATAG 0.151547949385
ATTTATCA 0.0796928798687
ATTTATCC -0.0337759504889
ATTTATCG 0.1841689306
ATTTATGA 0.0818267140881
ATTTATGC 0.121347318873
ATTTATGG 0.0481036979645
ATTTATTA 0.244090770415
ATTTATTC 0.0371879733913
ATTTATTG 0.182335507678
ATTTCAAA 0.357428849534
ATTTCAAC 0.128936701873
ATTTCAAG 0.235063123792
ATTTCACA 0.261779960194
ATTTCACC 0.107201859536
ATTTCACG 0.183875127724
ATTTCAGA 0.285454651112
ATTTCAGC 0.222606423851
ATTTCAGG 0.201264023359
ATTTCATA 0.345623048383
ATTTCATC 0.261136358647
ATTTCATG 0.345477448995
ATTTCCAA 0.336125114407
ATTTCCAC 0.332016619935
ATTTCCAG 0.34821384471
ATTTCCCA 0.325854489776
ATTTCCCC 0.302084019408
ATTTCCCG 0.309411293332
ATTTCCGA 0.332641770401
ATTTCCGC 0.316747780861
ATTTCCGG 0.328305780588
ATTTCCTA 0.334250696147
ATTTCCTC 0.233659718629
ATTTCCTG 0.361174146122
ATTTCGAA 0.359741693664
ATTTCGAC 0.160099080383
ATTTCGAG 0.225323278228
ATTTCGCA 0.210909011666
ATTTCGCC 0.211656011045
ATTTCGCG 0.318225976421
ATTTCGGA 0.296054798129

ATTTCGGC 0.31283031216
ATTTCGGG 0.226250850732
ATTTCGTA 0.413451396859
ATTTCGTC 0.310329347842
ATTTCGTG 0.35192594788
ATTTCTAA 0.326615140993
ATTTCTAC 0.245835467491
ATTTCTAG 0.222903563805
ATTTCTCA 0.171488966048
ATTTCTCC 0.265963999826
ATTTCTCG 0.234159915695
ATTTCTGA 0.197380616855
ATTTCTGC 0.184147840787
ATTTCTGG 0.206370490898
ATTTCTTA 0.314946319353
ATTTCTTC 0.169609053797
ATTTCTTG 0.183050281115
ATTTGAAA 0.205800346879
ATTTGAAC 0.133389316999
ATTTGAAG 0.206248456409
ATTTGACA 0.218193308442
ATTTGACC 0.0918062557204
ATTTGACG 0.203561000241
ATTTGAGA 0.128149081209
ATTTGAGC 0.0692327807883
ATTTGAGG 0.156780904508
ATTTGATA 0.197421295018
ATTTGATC 0.172115377115
ATTTGATG 0.110064939782
ATTTGCAA 0.302203339591
ATTTGCAC 0.247396760934
ATTTGCAG 0.273006619899
ATTTGCCA 0.322445629277
ATTTGCCC 0.220229862346
ATTTGCCG 0.221918265948
ATTTGCGA 0.330517339067
ATTTGCGC 0.275159718106
ATTTGCGG 0.136583679115
ATTTGCTA 0.156811894882
ATTTGCTC 0.309210698357
ATTTGCTG 0.260631729724
ATTTGGAA 0.214341958072
ATTTGGAC 0.0924542007467
ATTTGGAG 0.0761597400602
ATTTGGCA 0.163974510966
ATTTGGCC 0.0786405848647
ATTTGGCG 0.133615899437
ATTTGGGA 0.206745311115
ATTTGGGC 0.0944329028228
ATTTGGGG 0.17473096613
ATTTGGTA 0.118808865178
ATTTGGTC 0.18254470966
ATTTGGTG 0.254684515577
ATTTGTAA 0.301148214011

ATTTGTAC 0.246314408659
ATTTGTAG 0.121444189934
ATTTGTCA 0.159796899743
ATTTGTCC 0.138382752459
ATTTGTCG 0.181350515015
ATTTGTGA 0.262285531649
ATTTGTGC 0.074127235483
ATTTGTGG 0.118659654526
ATTTGTTA 0.179499658594
ATTTGTTC 0.107737567736
ATTTGTTG 0.192089837318
ATTTTAAA 0.237074359481
ATTTTAAC 0.202962743419
ATTTTAAG 0.224974752464
ATTTTACA 0.277069749061
ATTTTACC 0.100113879522
ATTTTACG 0.271046016378
ATTTTAGA 0.237596102096
ATTTTAGC 0.0987854662734
ATTTTAGG 0.202199828756
ATTTTATA 0.331328308421
ATTTTATC 0.220546830735
ATTTTATG 0.12225095521
ATTTTCAA 0.205289613993
ATTTTCAC 0.325205933201
ATTTTCAG 0.238503902005
ATTTTCCA 0.289103403141
ATTTTCCC 0.289807214563
ATTTTCCG 0.31969895288
ATTTTCGA 0.322249116336
ATTTTCGC 0.234101375794
ATTTTCGG 0.280500631964
ATTTTCTA 0.380220243197
ATTTTCTC 0.354314811878
ATTTTCTG 0.277602142747
ATTTTGAA 0.281713100178
ATTTTGAC 0.181861897636
ATTTTGAG 0.151965465762
ATTTTGCA 0.223572416518
ATTTTGCC 0.26549620095
ATTTTGCG 0.310255729527
ATTTTGGA 0.244558569291
ATTTTGGC 0.211434922203
ATTTTGGG 0.26030483655
ATTTTGTA 0.249747940668
ATTTTGTC 0.316721630613
ATTTTGTG 0.35687678875
ATTTTTAA 0.201370454244
ATTTTTAC 0.278888033356
ATTTTTAG 0.232986321457
ATTTTTCA 0.156039980823
ATTTTTCC 0.274981345078
ATTTTTCG 0.179762453621
ATTTTTGA 0.203923989947

ATTTTTGC 0.112798853981
ATTTTTGG 0.272693330234
ATTTTTTA 0.303683944763
ATTTTTTC 0.313461876221
ATTTTTTG 0.329856714602
CAAAAAAA 0.217952145047
CAAAAAAC 0.102565669224
CAAAAAAG -0.023224988474
CAAAAACA -0.00336725284858
CAAAAACC 0.186597998053
CAAAAACG -0.00673078319992
CAAAAAGA -0.0455086949574
CAAAAAGC -0.319964706096
CAAAAAGG -0.000354463626802
CAAAAATA 0.142171632792
CAAAAATC 0.394666802261
CAAAAATG -0.0246370846025
CAAAACAA 0.0623641707004
CAAAACAC -0.00998018490217
CAAAACAG 0.0594716678742
CAAAACCA -0.143327570594
CAAAACCC -0.0172923221741
CAAAACCG -0.107940958552
CAAAACGA 0.0938988781312
CAAAACGC -0.0954473353941
CAAAACGG 0.0173390670173
CAAAACTA -0.00585084195043
CAAAACTC -0.052023012218
CAAAACTG -0.0660377358491
CAAAAGAA 0.0504627141052
CAAAAGAC -0.10642971072
CAAAAGAG -0.0200413778722
CAAAAGCA -0.0924489885519
CAAAAGCC 0.0136257318437
CAAAAGCG -0.112176656518
CAAAAGGA -0.0105254746996
CAAAAGGC -0.204325191159
CAAAAGGG -0.0184896779161
CAAAAGTA 0.0499311181365
CAAAAGTC -0.170471259069
CAAAAGTG -0.131617941426
CAAAATAA 0.152495115151
CAAAATAC 0.241344486158
CAAAATAG 0.17140606819
CAAAATCA 0.317224296486
CAAAATCC 0.416699598235
CAAAATCG 0.407930789011
CAAAATGA 0.0718285983199
CAAAATGC 0.0431485872357
CAAAATGG -0.0469353362486
CAAAATTA 0.131066494269
CAAAATTC 0.192940885912
CAAAATTG 0.0295615710553
CAAACAAA 0.116399110892

CAAACAAC 0.0385710992557
CAAACAAG 0.112825243706
CAAACACA 0.0624511498845
CAAACACC -0.0687220438517
CAAACACG -0.0113913384568
CAAACAGA 0.00665492107401
CAAACAGC 0.0293972367905
CAAACAGG -0.167039780521
CAAACATA 0.0825749530772
CAAACATC -0.164673633567
CAAACATG -0.129202476209
CAAACCAA 0.1437970647
CAAACCAC -0.104192509144
CAAACCAG 0.0315825581076
CAAACCCA -0.0983446398384
CAAACCCC -0.264463338049
CAAACCCG -0.116140662814
CAAACCGA 0.0325381720977
CAAACCGC -0.129619569691
CAAACCGG 0.0324974939346
CAAACCTA 0.0555955860562
CAAACCTC -0.0924187138309
CAAACCTG -0.180730784091
CAAACGAA 0.0654954005576
CAAACGAC 0.0309721398933
CAAACGAG 0.106659596415
CAAACGCA -0.137312517975
CAAACGCC -0.233345402876
CAAACGCG -0.1833314128
CAAACGGA 0.0261023055802
CAAACGGC -0.0941992778147
CAAACGGG 0.00832594830968
CAAACGTA 0.0594716240245
CAAACGTC -0.101981607659
CAAACGTG -0.0308362971745
CAAACTAA 0.117049961501
CAAACTAC 0.0539583744978
CAAACTAG -0.116384582976
CAAACTCA 0.087799456656
CAAACTCC -0.139754548466
CAAACTCG -0.126096382911
CAAACTGA -0.0662279185394
CAAACTGC -0.143508685272
CAAACTGG -0.0819160437727
CAAACTTA 0.0782851248674
CAAACTTC -0.0664142037815
CAAACTTG -0.12600787413
CAAAGAAA 0.0104552564419
CAAAGAAC 0.00279643899976
CAAAGAAG -0.0716170006304
CAAAGACA 0.00414114470131
CAAAGACC -0.103550622521
CAAAGACG -0.049169707393
CAAAGAGA -0.153853852293

CAAAGAGC -0.164671893746
CAAAGAGG -0.134786852954
CAAAGATA 0.169789677051
CAAAGATC 0.356083558366
CAAAGATG -0.0216887248849
CAAAGCAA 0.164875160526
CAAAGCAC -0.0705042722379
CAAAGCAG -0.0642674086147
CAAAGCCA -0.192723387885
CAAAGCCC -0.305708131727
CAAAGCCG -0.070191468695
CAAAGCGA -0.00109814767472
CAAAGCGC -0.219750464998
CAAAGCGG 0.017876988047
CAAAGCTA 0.0809883598489
CAAAGCTC -0.280681854491
CAAAGCTG -0.194477850399
CAAAGGAA 0.0939866956464
CAAAGGAC 0.0612220882426
CAAAGGAG -0.0874166666667
CAAAGGCA -0.214930256855
CAAAGGCC -0.34257430631
CAAAGGCG -0.208310700706
CAAAGGGA -0.0907774281738
CAAAGGGC -0.251170428169
CAAAGGGG -0.0105283802827
CAAAGGTA -0.137168218732
CAAAGGTC -0.122231618192
CAAAGGTG -0.224187734545
CAAAGTAA 0.0788531663592
CAAAGTAC -0.119436632812
CAAAGTAG -0.246585110122
CAAAGTCA -0.207747460897
CAAAGTCC -0.253169521463
CAAAGTCG -0.263494613193
CAAAGTGA 0.0195763659872
CAAAGTGC -0.160542302563
CAAAGTGG -0.0218372837627
CAAAGTTA 0.0378603262421
CAAAGTTC -0.0675152034073
CAAAGTTG -0.0796429825717
CAAATAAA 0.199800967559
CAAATAAC 0.0826304243604
CAAATAAG 0.040606976305
CAAATACA 0.169841818701
CAAATACC 0.138716894513
CAAATACG 0.266309764212
CAAATAGA 0.12737640376
CAAATAGC -0.025236441823
CAAATAGG -0.0678445190893
CAAATATA 0.238189024993
CAAATATC 0.362354277811
CAAATATG 0.168551421557
CAAATCAA 0.254294825353

CAAATCAC 0.224645436782
CAAATCAG 0.280706928363
CAAATCCA 0.380133075705
CAAATCCC 0.32536574027
CAAATCCG 0.383134097346
CAAATCGA 0.246215613954
CAAATCGC 0.347108688136
CAAATCGG 0.248661252597
CAAATCTA 0.422368631325
CAAATCTC 0.456573155318
CAAATCTG 0.425140557581
CAAATGAA 0.181074939485
CAAATGAC -0.0463237392698
CAAATGAG 0.0380878357764
CAAATGCA -0.0449478354092
CAAATGCC -0.0542812720187
CAAATGCG 0.127727979312
CAAATGGA -0.0923272015246
CAAATGGC -0.10078808434
CAAATGGG 0.0190389945391
CAAATGTA -0.1465162866
CAAATGTC 0.0482914004698
CAAATGTG -0.00898482952425
CAAATTAA -0.0382198291949
CAAATTAC 0.284588787355
CAAATTAG 0.0934460690685
CAAATTCA 0.169805180655
CAAATTCC 0.146435284972
CAAATTCG 0.142341214955
CAAATTGA 0.00243968034424
CAAATTGC 0.117148565281
CAAATTGG -0.120586056107
CAAATTTA -0.0171491141408
CAAATTTC 0.234888788808
CAAATTTG 0.207498170685
CAACAAAA 0.179720345175
CAACAAAC 0.0611895980153
CAACAAAG 0.0607971634943
CAACAACA -0.0107795999912
CAACAACC -0.0919500965802
CAACAACG 0.0292374297212
CAACAAGA -0.0700724942978
CAACAAGC -0.179972922641
CAACAAGG 0.0183400424775
CAACAATA 0.0954178954862
CAACAATC 0.158608433845
CAACAATG -0.0318873214882
CAACACAA 0.0628724594308
CAACACAC -0.115601851852
CAACACAG 0.0103065377877
CAACACCA -0.106649060573
CAACACCC -0.244458161866
CAACACCG -0.0138386086431
CAACACGA 0.1145380569

CAACACGC -0.136210454675
CAACACGG 0.00603745417023
CAACACTA 0.00827405651057
CAACACTC 0.0178548080136
CAACACTG -0.0871834730435
CAACAGAA -0.0132557048306
CAACAGAC -0.162927798904
CAACAGAG -0.0454263540363
CAACAGCA -0.0866264901535
CAACAGCC -0.225651408161
CAACAGCG -0.115554128521
CAACAGGA 0.0016029440822
CAACAGGC -0.259161596024
CAACAGGG -0.032869237787
CAACAGTA 0.0517731320733
CAACAGTC -0.169771306389
CAACAGTG -0.28719383166
CAACATAA 0.00945410437112
CAACATAC 0.0969999341369
CAACATAG -0.105398573359
CAACATCA 0.0840309154039
CAACATCC 0.123971060393
CAACATCG 0.0348795318987
CAACATGA 0.0316275671642
CAACATGC -0.106242053783
CAACATGG -0.124282437134
CAACATTA -0.0917295391682
CAACATTC -0.136682473819
CAACATTG 0.00385716153589
CAACCAAA 0.0503406796159
CAACCAAC -0.0175569857481
CAACCAAG -0.127564286939
CAACCACA -0.0476239594381
CAACCACC -0.184443840759
CAACCACG 0.032773524327
CAACCAGA 0.042704011065
CAACCAGC -0.358527857892
CAACCAGG -0.0421777668011
CAACCATA -0.0852919384598
CAACCATC -0.131717376272
CAACCATG -0.22636882716
CAACCCAA 0.0512007322069
CAACCCAC -0.0711606351605
CAACCCAG -0.286668486959
CAACCCCA -0.0728618540526
CAACCCCC -0.271723691205
CAACCCCG -0.19700462963
CAACCCGA 0.0310254932631
CAACCCGC -0.108432002092
CAACCCGG -0.129392303067
CAACCCTA -0.0144624470351
CAACCCTC -0.176926790995
CAACCCTG -0.126456934018
CAACCGAA -0.0245914023797

CAACCGAC -0.080000878045
CAACCGAG -0.111391647442
CAACCGCA 0.0154256694528
CAACCGCC -0.200046766028
CAACCGCG -0.0616637368704
CAACCGGA 0.0248331467211
CAACCGGC -0.137017128412
CAACCGGG -0.182936331561
CAACCGTA -0.0537467752806
CAACCGTC -0.0137216160853
CAACCGTG 0.0256658243118
CAACCTAA -0.201247249521
CAACCTAC -0.296802976426
CAACCTAG -0.275127702884
CAACCTCA -0.0680821698894
CAACCTCC -0.355004777796
CAACCTCG -0.100025792573
CAACCTGA 0.0455224044436
CAACCTGC -0.236726619593
CAACCTGG -0.238935186933
CAACCTTA -0.110002469746
CAACCTTC -0.237325583813
CAACCTTG -0.266714410365
CAACGAAA -0.018187497276
CAACGAAC -0.198387799564
CAACGAAG -0.164463918993
CAACGACA 0.0161419667892
CAACGACC -0.165670683524
CAACGACG 0.0817635952491
CAACGAGA 0.125204480409
CAACGAGC -0.201196159122
CAACGAGG -0.169523945078
CAACGATA 0.130026295527
CAACGATC 0.177007179082
CAACGATG -0.0903852132052
CAACGCAA 0.149057864687
CAACGCAC -0.108220922677
CAACGCAG -0.0854231064215
CAACGCCA 0.132147371174
CAACGCCC -0.217853304285
CAACGCCG -0.230054096166
CAACGCGA 0.0065445026178
CAACGCGC 0.0220156029811
CAACGCGG -0.157738959696
CAACGCTA -0.0582787863997
CAACGCTC -0.216392156863
CAACGCTG -0.156541709883
CAACGGAA 0.0679053887188
CAACGGAC -0.103644615672
CAACGGAG -0.0792917819044
CAACGGCA -0.167905811154
CAACGGCC -0.277255360624
CAACGGCG -0.120176119047
CAACGGGA 0.106912336165

CAACGGGC -0.208649382716
CAACGGGG 0.0123026850205
CAACGGTA 0.129277079423
CAACGGTC -0.253959681003
CAACGGTG -0.177335023153
CAACGTAA 0.152275571363
CAACGTAC -0.158799124925
CAACGTAG 0.122284369416
CAACGTCA -0.0550157627882
CAACGTCC -0.138674139088
CAACGTCG -0.142006264128
CAACGTGA -0.106795397304
CAACGTGC -0.254798394643
CAACGTGG -0.125200788923
CAACGTTA -0.0793458692836
CAACGTTC -0.220764714597
CAACGTTG 0.126016223666
CAACTAAA 0.0547978440574
CAACTAAC 0.0932881030901
CAACTAAG -0.301927764044
CAACTACA -0.0618482293793
CAACTACC -0.0711201950035
CAACTACG 0.0171385817849
CAACTAGA 0.0135568289095
CAACTAGC -0.192883093505
CAACTAGG 0.0221794111836
CAACTATA -0.0789291711943
CAACTATC -0.122939610437
CAACTATG 0.0484200582831
CAACTCAA -0.0988248918747
CAACTCAC -0.0427327208667
CAACTCAG 0.104936442073
CAACTCCA -0.0654697224143
CAACTCCC -0.115333750384
CAACTCCG -0.157799725652
CAACTCGA -0.0706297851321
CAACTCGC -0.210178861411
CAACTCGG -0.189863341623
CAACTCTA 0.0845744402671
CAACTCTC -0.032212746793
CAACTCTG -0.086822007187
CAACTGAA -0.0106199061497
CAACTGAC -0.179528120713
CAACTGAG -0.0566640326617
CAACTGCA 0.123089128717
CAACTGCC -0.194445977947
CAACTGCG 0.0109311764415
CAACTGGA -0.133000790202
CAACTGGC -0.112033058395
CAACTGGG -0.177499675114
CAACTGTA -0.128800108138
CAACTGTC -0.12960757199
CAACTGTG -0.190241011458
CAACTTAA -0.153249703674

CAACTTAC 0.0213654102653
CAACTTAG 0.124309560821
CAACTTCA -0.0438538716547
CAACTTCC -0.0647224417226
CAACTTCG 0.0744033897561
CAACTTGA -0.0770214494281
CAACTTGC 0.0505992765098
CAACTTGG -0.162740740741
CAACTTTA 0.0122717301294
CAACTTTC 0.101270060814
CAAGAAAA -0.01859718449
CAAGAAAC 0.107546268853
CAAGAAAG -0.0967324135357
CAAGAACA -0.0503401906549
CAAGAACC -0.230189068368
CAAGAACG -0.171164386944
CAAGAAGA -0.0659276273903
CAAGAAGC -0.160516078767
CAAGAAGG -0.269646893585
CAAGAATA 0.163181904029
CAAGAATC 0.387831271109
CAAGAATG -0.0622943063477
CAAGACAA -0.104966803059
CAAGACAC -0.215796879629
CAAGACAG -0.212946637348
CAAGACCA -0.235631758258
CAAGACCC -0.33620374189
CAAGACCG -0.147373360056
CAAGACGA 0.212224746976
CAAGACGC -0.152526590722
CAAGACGG -0.101701402608
CAAGACTA -0.108197099159
CAAGACTC 0.000450064765417
CAAGACTG -0.0468396671716
CAAGAGAA 0.196277075636
CAAGAGAC -0.178416218085
CAAGAGAG -0.0465986114541
CAAGAGCA -0.10984840608
CAAGAGCC -0.0725795206972
CAAGAGCG -0.0414350674822
CAAGAGGA 0.0347807518325
CAAGAGGC -0.26014859825
CAAGAGGG -0.240287516666
CAAGAGTA -0.0907253788154
CAAGAGTC -0.312139267452
CAAGAGTG -0.128011702533
CAAGATAA 0.10098208708
CAAGATAC 0.279004256679
CAAGATAG 0.0031859718449
CAAGATCA 0.165220907834
CAAGATCC 0.339466837911
CAAGATCG 0.30103569782
CAAGATGA 0.0707143562889
CAAGATGC -0.0755582351488

CAAGATGG -0.0682055165904
CAAGATTA 0.312180204262
CAAGATTC 0.402154610951
CAAGATTG 0.298204349658
CAAGCAAA 0.0812215630103
CAAGCAAC -0.137365798382
CAAGCAAG -0.0874509416587
CAAGCACA -0.112617432723
CAAGCACC -0.18342594556
CAAGCACG -0.149853718343
CAAGCAGA 0.210696904101
CAAGCAGC -0.0138079401144
CAAGCAGG -0.170395614165
CAAGCATA 0.114016021133
CAAGCATC -0.0638515664449
CAAGCATG -0.111240067606
CAAGCCAA -0.0537901039805
CAAGCCAC -0.211056556399
CAAGCCAG -0.149069189114
CAAGCCCA -0.232063100137
CAAGCCCC -0.257303444757
CAAGCCCG -0.365758114425
CAAGCCGA -0.158478896736
CAAGCCGC -0.170445004198
CAAGCCGG -0.176987654321
CAAGCCTA -0.113027409917
CAAGCCTC -0.260038454724
CAAGCCTG -0.18800259909
CAAGCGAA 0.0609149035427
CAAGCGAC -0.236550732748
CAAGCGAG -0.0972277097326
CAAGCGCA -0.111532863256
CAAGCGCC -0.315531637098
CAAGCGCG -0.137074238419
CAAGCGGA 0.0118388987684
CAAGCGGC -0.112724359502
CAAGCGGG -0.197041280502
CAAGCGTA -0.213706447188
CAAGCGTC -0.235701160956
CAAGCGTG -0.143172313873
CAAGCTAA -0.105201776528
CAAGCTAC -0.248239012162
CAAGCTAG -0.159858925791
CAAGCTCA -0.135096644605
CAAGCTCC -0.218351093956
CAAGCTCG -0.109961098086
CAAGCTGA -0.0979987049475
CAAGCTGC -0.125727341655
CAAGCTGG -0.271720164609
CAAGCTTA -0.223599383295
CAAGCTTC -0.0892043060741
CAAGCTTG -0.235319968398
CAAGGAAA 0.120536793745
CAAGGAAC -0.224940235341

CAAGGAAG -0.147676198943
CAAGGACA -0.242095040574
CAAGGACC -0.206654970896
CAAGGACG -0.300293928859
CAAGGAGA 0.104336261788
CAAGGAGC -0.155492991757
CAAGGAGG -0.290047482829
CAAGGATA 0.261246130541
CAAGGATC 0.215865936397
CAAGGATG 0.0425454410748
CAAGGCAA -0.0510540825545
CAAGGCAC -0.258852867106
CAAGGCAG -0.0924529086557
CAAGGCCA -0.281867908794
CAAGGCCC -0.257883026803
CAAGGCCG -0.265562648482
CAAGGCGA -0.17636536235
CAAGGCGC -0.151015836769
CAAGGCGG -0.224510062106
CAAGGCTA -0.303259372651
CAAGGCTC -0.177370058399
CAAGGCTG -0.266717700514
CAAGGGAA -0.0072450714047
CAAGGGAC -0.141554103123
CAAGGGAG -0.273745652436
CAAGGGCA -0.194430727023
CAAGGGCC -0.179770841565
CAAGGGCG -0.118424951845
CAAGGGGA -0.00698354681858
CAAGGGGC -0.321183041224
CAAGGGGG -0.0775720588961
CAAGGGTA -0.069541907924
CAAGGGTC -0.0945509126631
CAAGGGTG -0.178685277545
CAAGGTAA 0.198300182221
CAAGGTAC 0.0455004500648
CAAGGTAG -0.0693683014768
CAAGGTCA -0.12331225713
CAAGGTCC -0.132094314671
CAAGGTCG -0.109983260613
CAAGGTGA -0.233480369566
CAAGGTGC -0.235823261858
CAAGGTGG -0.311002582043
CAAGGTTA 0.0643698032436
CAAGGTTC -0.109725446294
CAAGTAAA 0.0359638545465
CAAGTAAC -0.157610485411
CAAGTAAG -0.11419554897
CAAGTACA -0.164043209877
CAAGTACC -0.215280864198
CAAGTACG 0.0337614225735
CAAGTAGA 0.1451237052
CAAGTAGC -0.110017429194
CAAGTAGG 0.0711630011154

CAAGTATA 0.115806371944
CAAGTATC 0.0256316011216
CAAGTATG 0.0544736712106
CAAGTCAA -0.0264249728341
CAAGTCAC -0.0839548552801
CAAGTCAG -0.15474691358
CAAGTCCA -0.196496732026
CAAGTCCC -0.199623538192
CAAGTCCG -0.142248841073
CAAGTCGA -0.0285081283687
CAAGTCGC -0.109136643004
CAAGTCGG -0.179271155321
CAAGTCTA -0.200019136747
CAAGTCTC -0.242080330873
CAAGTCTG -0.278343342078
CAAGTGAA -0.0717935629761
CAAGTGAC -0.0839553702439
CAAGTGAG 0.00893915448439
CAAGTGCA -0.0683810292213
CAAGTGCC -0.0558025179185
CAAGTGCG 0.173585040286
CAAGTGGA -0.147000717608
CAAGTGGC -0.17641104143
CAAGTGGG -0.242877643921
CAAGTGTA -0.140091966133
CAAGTGTC -0.0552701627288
CAAGTGTG -0.0334403556434
CAAGTTAA -0.0888257731676
CAAGTTAC 0.0082445919835
CAAGTTAG -0.151088071161
CAAGTTCA 0.0574189334563
CAAGTTCC -0.248070054154
CAAGTTCG -0.0900278617893
CAAGTTGA 0.0105719640289
CAAGTTGC -0.11973918198
CAAGTTGG -0.158237654321
CAAGTTTA -0.0358140555967
CAAGTTTC -0.0758668378939
CAATAAAA 0.133173042
CAATAAAC -0.0142911103686
CAATAAAG -0.0655941952622
CAATAACA -0.105028205804
CAATAACC -0.0788628090146
CAATAACG 0.0606700274578
CAATAAGA -0.043996344957
CAATAAGC -0.1919476272
CAATAAGG -0.0731182920664
CAATAATA 0.223792367033
CAATAATC 0.154363824953
CAATAATG 0.0329562668774
CAATACAA 0.051107049551
CAATACAC 0.145669954818
CAATACAG -0.0575553050421
CAATACCA -0.0113682517728

CAATACCC -0.07903331251
CAATACCG -0.128146867539
CAATACGA 0.252151529122
CAATACGC 0.0421880831632
CAATACGG 0.0381009109003
CAATACTA 0.147395871166
CAATACTC 0.0120102276524
CAATACTG -0.0237522479728
CAATAGAA 0.0432830183197
CAATAGAC -0.130654237101
CAATAGAG 0.0958188659509
CAATAGCA 0.0398021297924
CAATAGCC -0.0340512415201
CAATAGCG -0.00162567373208
CAATAGGA 0.0179147731015
CAATAGGC -0.309092176418
CAATAGGG 0.0321662574637
CAATAGTA 0.0301378734989
CAATAGTC -0.146094312873
CAATAGTG -0.0236528990455
CAATATAA 0.165859843246
CAATATAC 0.23989275286
CAATATAG 0.0966905408743
CAATATCA 0.292536608927
CAATATCC 0.313048973603
CAATATCG 0.340018441678
CAATATGA 0.186932140107
CAATATGC 0.0755698574811
CAATATGG 0.178317086281
CAATATTA 0.238808755406
CAATATTC 0.340550469688
CAATATTG 0.108813930625
CAATCAAA 0.238959691761
CAATCAAC 0.141860735403
CAATCAAG 0.234555094514
CAATCACA 0.289715471251
CAATCACC 0.175270038859
CAATCACG 0.227038674306
CAATCAGA 0.278068658928
CAATCAGC 0.176262839045
CAATCAGG 0.324100144899
CAATCATA 0.256995213945
CAATCATC 0.129436462162
CAATCATG 0.244258929944
CAATCCAA 0.343805572021
CAATCCAC 0.37112286258
CAATCCAG 0.285192869218
CAATCCCA 0.275996978194
CAATCCCC 0.251476431974
CAATCCCG 0.319558157856
CAATCCGA 0.345545014746
CAATCCGC 0.38087399939
CAATCCGG 0.324354597359
CAATCCTA 0.284001859573

CAATCCTC 0.202209695931
CAATCCTG 0.330046634608
CAATCGAA 0.28145947438
CAATCGAC 0.0504374409976
CAATCGAG 0.161839524647
CAATCGCA 0.246166216602
CAATCGCC 0.0642824211289
CAATCGCG 0.258775587291
CAATCGGA 0.218077085119
CAATCGGC 0.204373441632
CAATCGGG 0.102641175018
CAATCGTA 0.266481980776
CAATCGTC 0.0602290939428
CAATCGTG 0.231538651519
CAATCTAA 0.342425664329
CAATCTAC 0.354417769874
CAATCTAG 0.373614530819
CAATCTCA 0.30823151686
CAATCTCC 0.310739035056
CAATCTCG 0.408205997936
CAATCTGA 0.339420045618
CAATCTGC 0.34430485392
CAATCTGG 0.41322531779
CAATCTTA 0.31302827724
CAATCTTC 0.264261328142
CAATGAAA 0.0441953714062
CAATGAAC -0.067347801269
CAATGAAG -0.00381126422021
CAATGACA -0.161519958865
CAATGACC -0.213803549672
CAATGACG -0.132089863471
CAATGAGA 0.0195532178765
CAATGAGC -0.211935495114
CAATGAGG -0.122688958035
CAATGATA 0.114222829166
CAATGATC 0.119505916705
CAATGATG 0.106072581176
CAATGCAA 0.0465140267023
CAATGCAC -0.00536806473639
CAATGCAG -0.216558398515
CAATGCCA -0.0839412665348
CAATGCCC -0.247340263316
CAATGCCG -0.16668573137
CAATGCGA -0.0624197411962
CAATGCGC 0.0296296834367
CAATGCGG -0.143700798838
CAATGCTA -0.0682418155374
CAATGCTC -0.245530154655
CAATGCTG -0.0580419661691
CAATGGAA -0.0488927946503
CAATGGAC -0.176356652949
CAATGGAG -0.0664976513455
CAATGGCA -0.133036459019
CAATGGCC -0.253476780797

CAATGGCG -0.118904530611
CAATGGGA 0.0422951107598
CAATGGGC -0.320425030202
CAATGGGG -0.123821117897
CAATGGTA -0.0758825660278
CAATGGTC -0.293982577476
CAATGGTG -0.0993545707256
CAATGTAA 0.159231131229
CAATGTAC -0.0995176001844
CAATGTAG -0.04188072186
CAATGTCA 0.0389847152374
CAATGTCC -0.0794376944733
CAATGTCG 0.0489053215754
CAATGTGA 0.00946784245929
CAATGTGC -0.0519181690952
CAATGTGG -0.148610098393
CAATGTTA 0.0144812555567
CAATGTTC -0.0905228294153
CAATTAAA 0.0648504906804
CAATTAAC -0.00728062086983
CAATTAAG -0.0687268815788
CAATTACA 0.059658280094
CAATTACC 0.0556760157656
CAATTACG 0.258585029142
CAATTAGA 0.0503261517005
CAATTAGC -0.0405360161584
CAATTAGG 0.0144747384943
CAATTATA 0.250751937474
CAATTATC 0.173813432511
CAATTATG -0.0297604346752
CAATTCAA -0.0280228959947
CAATTCAC -0.0771846629021
CAATTCAG -0.0663881342444
CAATTCCA 0.083028746419
CAATTCCC 0.180560141647
CAATTCCG 0.152195894411
CAATTCGA -0.0860416872469
CAATTCGC 0.236046626494
CAATTCGG 0.0101648773848
CAATTCTA -0.0681601132846
CAATTCTC 0.179403774352
CAATTCTG 0.0169163046794
CAATTGAA 0.0576357329469
CAATTGAC -0.000430298192748
CAATTGAG 0.0215845105206
CAATTGCA 0.0243995975217
CAATTGCC -0.027137386023
CAATTGCG 0.165930003094
CAATTGGA 0.0498612905826
CAATTGGC -0.0402478649367
CAATTGGG 0.0420659224867
CAATTGTA 0.0499418902441
CAATTGTC -0.149164398624
CAATTGTG -0.137706213573

CAATTTAA 0.020761843883
CAATTTAC 0.12233803157
CAATTTAG 0.21935263609
CAATTTCA 0.224350238984
CAATTTCC 0.10781141954
CAATTTCG 0.089938857055
CAATTTGA -0.0499106533204
CAATTTGC 0.0322043294035
CAATTTGG 0.0952600496169
CAATTTTA 0.183799315023
CAATTTTC 0.0944724362774
CACAAAAA 0.188706118685
CACAAAAC 0.0311028140572
CACAAAAG -0.110602472651
CACAAACA 0.0942446595974
CACAAACC 0.10287943283
CACAAACG -0.193577417797
CACAAAGA 0.118261589645
CACAAAGC 0.0339357575581
CACAAAGG -0.128746728557
CACAAATA 0.140204553049
CACAAATC 0.286483227522
CACAAATG 0.0296209666875
CACAACAA 0.235696540903
CACAACAC 0.196690086931
CACAACAG -0.110881408627
CACAACCA -0.183667506214
CACAACCC -0.1011331774
CACAACCG -0.185360899842
CACAACGA 0.238661340512
CACAACGC -0.0476829886522
CACAACGG -0.0095356923944
CACAACTA -0.0840880151046
CACAACTC -0.151933428811
CACAACTG -0.0977201488527
CACAAGAA -0.0189123617147
CACAAGAC -0.166683128385
CACAAGAG 0.103582583935
CACAAGCA -0.0273346394253
CACAAGCC -0.304182012525
CACAAGCG 0.0994725460493
CACAAGGA -0.119619075839
CACAAGGC -0.162652751027
CACAAGGG -0.192702977487
CACAAGTA 0.00205263302324
CACAAGTC -0.117060160032
CACAAGTG -0.166273502684
CACAATAA 0.155362979966
CACAATAC 0.100915976153
CACAATAG 0.0349541153483
CACAATCA 0.192813040257
CACAATCC 0.325591670509
CACAATCG 0.263628180641
CACAATGA -0.0970072494298

CACAATGC -0.163845668341
CACAATGG -0.0449078485602
CACAATTA 0.100682812023
CACAATTC 0.0837984687577
CACACAAA 0.107562848236
CACACAAC -0.0900949570285
CACACAAG -0.0229219743604
CACACACA -0.0626865021138
CACACACC -0.169775494861
CACACACG -0.301618633518
CACACAGA 0.191273081226
CACACAGC -0.0530670771183
CACACAGG -0.178726105029
CACACATA 0.0378263332994
CACACATC -0.131912106044
CACACATG -0.295026780216
CACACCAA 0.0191250082329
CACACCAC -0.108950055055
CACACCAG 0.0691525486516
CACACCCA -0.265112583628
CACACCCC -0.191115474434
CACACCCG -0.214671023965
CACACCGA 0.168707707695
CACACCGC -0.0783333744937
CACACCGG -0.136198603479
CACACCTA 0.0534180756441
CACACCTC -0.124017735634
CACACCTG -0.173728923712
CACACGAA -0.0380579156513
CACACGAC -0.176209876543
CACACGAG -0.0639034611125
CACACGCA -0.214145404664
CACACGCC -0.141556464546
CACACGCG -0.19880098988
CACACGGA -0.104106649985
CACACGGC -0.24517856457
CACACGGG -0.128885984023
CACACGTA 0.00988043525635
CACACGTC -0.260279260054
CACACGTG -0.210564582064
CACACTAA 0.0981258989148
CACACTAC 0.0276800371915
CACACTAG 0.0231795968412
CACACTCA 0.0175168499857
CACACTCC -0.252919067215
CACACTCG -0.0635780464208
CACACTGA -0.0257307545925
CACACTGC -0.161443157687
CACACTGG -0.273440329218
CACACTTA -0.141189545712
CACACTTC 0.14545494167
CACAGAAA -0.0195758833021
CACAGAAC -0.0724767866844
CACAGAAG 0.00537043977467

CACAGACA 0.0127889239173
CACAGACC -0.167445854665
CACAGACG -0.0359493266311
CACAGAGA 0.120818502753
CACAGAGC -0.157397348959
CACAGAGG -0.102998397489
CACAGATA 0.233391512437
CACAGATC 0.136528990455
CACAGATG 0.0778478346142
CACAGCAA 0.0882672555315
CACAGCAC -0.160610301583
CACAGCAG -0.126168326885
CACAGCCA -0.00986687275918
CACAGCCC -0.306718692747
CACAGCCG -0.141167755991
CACAGCGA 0.0343454447721
CACAGCGC -0.169332598621
CACAGCGG 0.0654032222916
CACAGCTA -0.242599130907
CACAGCTC -0.261885954085
CACAGCTG 0.0507502908955
CACAGGAA -0.0300738417465
CACAGGAC -0.213443718228
CACAGGAG -0.0402521211323
CACAGGCA -0.073842426496
CACAGGCC -0.336627769823
CACAGGCG -0.143407227082
CACAGGGA 0.0124660097148
CACAGGGC -0.201771968046
CACAGGGG -0.18815192859
CACAGGTA -0.0334592419334
CACAGGTC -0.158081456605
CACAGGTG -0.292470750892
CACAGTAA 0.0453056049529
CACAGTAC -0.178510288066
CACAGTAG -0.237548554875
CACAGTCA -0.0154614035954
CACAGTCC -0.249275720165
CACAGTCG -0.206318596353
CACAGTGA -0.0927155502983
CACAGTGC -0.201562062779
CACAGTGG -0.0923462880485
CACAGTTA -0.0203347231706
CACAGTTC -0.0134216953432
CACATAAA 0.0330506846684
CACATAAC -0.121660725336
CACATAAG -0.0057341682042
CACATACA 0.11190970164
CACATACC -0.105806384333
CACATACG 0.0355423835789
CACATAGA -0.0155744902719
CACATAGC -0.160953886172
CACATAGG -0.193278463649
CACATATA -0.133161823221

CACATATC 0.18492173264
CACATATG 0.0857437398763
CACATCAA 0.0455663132012
CACATCAC -0.112514346316
CACATCAG -0.146847928951
CACATCCA 0.012659443676
CACATCCC 0.0242093182049
CACATCCG 0.194732595666
CACATCGA -0.0726851394514
CACATCGC -0.0624097945923
CACATCGG 0.0545554386923
CACATCTA 0.0235049068037
CACATCTC -0.0375411846058
CACATCTG 0.00638501881365
CACATGAA 0.235850536806
CACATGAC -0.129870610894
CACATGAG 0.0482254151352
CACATGCA -0.0745213945421
CACATGCC -0.208674897119
CACATGCG -0.122981581156
CACATGGA -0.0317536646667
CACATGGC -0.196720406681
CACATGGG 0.068177047318
CACATGTA -0.135359196279
CACATGTC -0.0278573427494
CACATGTG -0.114949056014
CACATTAA -0.0576742577681
CACATTAC 0.178354858861
CACATTAG -0.0521004851918
CACATTCA 0.168610466265
CACATTCC 0.177731673582
CACATTCG 0.0719727456307
CACATTGA 0.064522830619
CACATTGC -0.0177632821466
CACATTGG -0.0220842461256
CACATTTA -0.11140200663
CACATTTC 0.0599683856945
CACCAAAA 0.143136286374
CACCAAAC -0.0794923946363
CACCAAAG 0.0999061450306
CACCAACA 0.102452312118
CACCAACC -0.260270152505
CACCAACG -0.249085002826
CACCAAGA -0.0200525807372
CACCAAGC -0.145806684762
CACCAAGG -0.129704299123
CACCAATA 0.0576302443522
CACCAATC 0.0215812093589
CACCAATG -0.209946422399
CACCACAA 0.0828948324205
CACCACAC -0.221877333085
CACCACAG -0.119416458141
CACCACCA -0.29891693409
CACCACCC -0.0635187197471

CACCACCG -0.0328766725263
CACCACGA -0.0838546468238
CACCACGC -0.158607428751
CACCACGG -0.174518518519
CACCACTA -0.249910832359
CACCACTC 0.0147380381002
CACCACTG -0.156263888203
CACCAGAA -0.16983409493
CACCAGAC -0.14420043573
CACCAGAG -0.0507506726807
CACCAGCA 0.0205898335227
CACCAGCC -0.0735395808032
CACCAGCG -0.201435878018
CACCAGGA 0.176639443237
CACCAGGC -0.0777826883831
CACCAGGG -0.213870643818
CACCAGTA -0.0862756515907
CACCAGTC -0.265482274231
CACCAGTG -0.256860227672
CACCATAA 0.0564336873302
CACCATAC -0.0381429888691
CACCATAG -0.0477654264314
CACCATCA -0.148865182844
CACCATCC 0.0450750839755
CACCATCG -0.100861710813
CACCATGA -0.0489527761312
CACCATGC -0.111741461218
CACCATGG -0.224739698329
CACCATTA -0.036913980213
CACCATTC -0.00617344922955
CACCCAAA -0.00842100064207
CACCCAAC -0.0368297183037
CACCCAAG -0.274824435551
CACCCACA -0.119372889925
CACCCACC -0.0791978107851
CACCCACG -0.0254475217977
CACCCAGA -0.147900103699
CACCCAGC -0.165123520972
CACCCAGG -0.340643202685
CACCCATA 0.0189370204645
CACCCATC -0.172540123457
CACCCATG -0.208852906316
CACCCCAA -0.0703499283845
CACCCCAC -0.132766582704
CACCCCAG -0.240444444444
CACCCCCA -0.059001882309
CACCCCCC -0.201136505558
CACCCCCG -0.0847521977479
CACCCCGA 0.125406156008
CACCCCGC -0.0872184907151
CACCCCGG -0.315005020328
CACCCCTA -0.29723360712
CACCCCTC -0.211221496006
CACCCCTG -0.221166246471

CACCCGAA -0.175659161606
CACCCGAC -0.273638473313
CACCCGAG -0.155162090761
CACCCGCA -0.0510404833638
CACCCGCC -0.155917695473
CACCCGCG -0.269052799136
CACCCGGA -0.0463176825034
CACCCGGC -0.0608716619797
CACCCGGG -0.264524659962
CACCCGTA -0.176787879786
CACCCGTC -0.123931668365
CACCCGTG -0.13535396379
CACCCTAA 0.0787064480011
CACCCTAC -0.162663330096
CACCCTAG -0.117282516187
CACCCTCA -0.0475050549655
CACCCTCC -0.138554150753
CACCCTCG 0.067136490373
CACCCTGA -0.166353863124
CACCCTGC -0.198660500301
CACCCTGG -0.211688032575
CACCCTTA -0.251071277687
CACCCTTC -0.176108950321
CACCGAAA 0.196777573534
CACCGAAC -0.280297202615
CACCGAAG 0.0163932997254
CACCGACA -0.0939291119678
CACCGACC -0.078731601304
CACCGACG -0.342597029924
CACCGAGA 0.156815771505
CACCGAGC -0.227494938522
CACCGAGG -0.154200889942
CACCGATA 0.202508970988
CACCGATC -0.0303574172869
CACCGATG -0.0102998567406
CACCGCAA 0.1632143406
CACCGCAC -0.0232951894844
CACCGCAG -0.0688308217049
CACCGCCA -0.119000296282
CACCGCCC -0.0920667061767
CACCGCCG 0.072721009736
CACCGCGA -0.0329852788648
CACCGCGC 0.106124722826
CACCGCGG -0.0532659449454
CACCGCTA -0.0748861180189
CACCGCTC -0.150502406392
CACCGCTG -0.164853050136
CACCGGAA -0.00759772445973
CACCGGAC -0.221598252692
CACCGGAG -0.202873383482
CACCGGCA -0.286677201126
CACCGGCC -0.255128000348
CACCGGCG -0.209350762527
CACCGGGA -0.133974865439

CACCGGGC -0.250303140037
CACCGGGG 0.031544462685
CACCGGTA -0.0221547291773
CACCGGTC -0.129764080275
CACCGGTG -0.28056378357
CACCGTAA 0.140361454535
CACCGTAC 0.0119230601601
CACCGTAG -0.322350152781
CACCGTCA -0.0908053168253
CACCGTCC -0.0351758720972
CACCGTCG -0.222701525054
CACCGTGA -0.216715131902
CACCGTGC -0.198850915317
CACCGTGG -0.197877221289
CACCGTTA 0.0538665737604
CACCGTTC -0.116329991274
CACCTAAA 0.00066957547817
CACCTAAC 0.0104165153342
CACCTAAG -0.0512984409411
CACCTACA -0.230413214019
CACCTACC -0.314890710383
CACCTACG -0.169058085548
CACCTAGA 0.106331265527
CACCTAGC -0.185776065505
CACCTAGG -0.00953094469692
CACCTATA 0.000119123022122
CACCTATC -0.162902234514
CACCTATG -0.047927216523
CACCTCAA -0.0152486882259
CACCTCAC -0.195718729939
CACCTCAG -0.174988476228
CACCTCCA -0.258536879759
CACCTCCC -0.20995437132
CACCTCCG -0.0218264954132
CACCTCGA -0.201756552087
CACCTCGC -0.243092614595
CACCTCGG -0.162084470435
CACCTCTA -0.198983297023
CACCTCTC -0.142771453552
CACCTCTG -0.0754665179155
CACCTGAA -0.0592666308311
CACCTGAC -0.209692729767
CACCTGAG -0.227462535873
CACCTGCA -0.0335692433737
CACCTGCC -0.299001371742
CACCTGCG -0.0789940037651
CACCTGGA -0.0681141975309
CACCTGGC -0.11768773096
CACCTGGG -0.292195368816
CACCTGTA 0.12233521712
CACCTGTC -0.133650542768
CACCTTAA -0.0770769534723
CACCTTAC -0.0805093487136
CACCTTAG -0.107060566879

CACCTTCA -0.0973968909331
CACCTTCC -0.113115020801
CACCTTCG -0.267352084283
CACCTTGA -0.185800670541
CACCTTGC -0.220683127572
CACCTTGG -0.23694702281
CACCTTTA 0.0524385105981
CACCTTTC -0.0945885129387
CACGAAAA 0.125618525998
CACGAAAC -0.107616524162
CACGAAAG 0.00899132683451
CACGAACA -0.102741437366
CACGAACC -0.0319279125028
CACGAACG -0.23154367941
CACGAAGA 0.0411973918198
CACGAAGC -0.105179375454
CACGAAGG -0.141257778546
CACGAATA 0.145047092143
CACGAATC 0.319586757736
CACGAATG 0.00194238228757
CACGACAA 0.0525196335079
CACGACAC -0.16702062857
CACGACAG -0.115620986069
CACGACCA -0.133792957211
CACGACCC -0.18682156582
CACGACCG -0.189461151438
CACGACGA -0.243782200056
CACGACGC -0.109710181979
CACGACGG -0.211665294925
CACGACTA -0.217599223007
CACGACTC -0.0452902977586
CACGACTG -0.0423472524095
CACGAGAA 0.0507425335046
CACGAGAC -0.194101041818
CACGAGAG -0.150185743055
CACGAGCA -0.0313380711368
CACGAGCC -0.278325452243
CACGAGCG -0.178348862141
CACGAGGA -0.0879910878429
CACGAGGC -0.271275715145
CACGAGGG -0.0707364200311
CACGAGTA 0.0105370970319
CACGAGTC -0.0166697654961
CACGAGTG -0.00267645460832
CACGATAA 0.029524350489
CACGATAC 0.00630639531054
CACGATAG 0.0121804537491
CACGATCA 0.0677364055032
CACGATCC 0.104258600628
CACGATCG 0.1684285926
CACGATGA -0.0164099226572
CACGATGC -0.146120686293
CACGATGG 0.0412694492467
CACGATTA 0.178575683175

CACGATTC 0.370765475862
CACGCAAA -0.0807098339459
CACGCAAC -0.0636178328051
CACGCAAG -0.0800074956638
CACGCACA -0.0639993414012
CACGCACC -0.178118210547
CACGCACG -0.113535861841
CACGCAGA 0.115739543533
CACGCAGC -0.280371822803
CACGCAGG -0.155946815049
CACGCATA -0.0971612453329
CACGCATC -0.174934987579
CACGCATG -0.208350480957
CACGCCAA -0.0300198687128
CACGCCAC -0.123629011462
CACGCCAG -0.216021335441
CACGCCCA -0.275824513467
CACGCCCC -0.372801196509
CACGCCCG -0.127629136688
CACGCCGA -0.0928053248266
CACGCCGC -0.348270129699
CACGCCGG -0.155992296677
CACGCCTA -0.0693539005224
CACGCCTC -0.23950617284
CACGCCTG -0.16162962963
CACGCGAA -0.0331826714088
CACGCGAC 0.0103532110595
CACGCGAG -0.0313179213594
CACGCGCA 0.0928348321299
CACGCGCC -0.136027434842
CACGCGCG -0.172843469766
CACGCGGA -0.147424711357
CACGCGGC -0.0508644708439
CACGCGGG -0.155595614382
CACGCGTA 0.0344272102571
CACGCGTC -0.0649762630005
CACGCGTG 0.125790045428
CACGCTAA -0.192181883044
CACGCTAC 0.147119036642
CACGCTAG -0.0942655339326
CACGCTCA -0.127852859832
CACGCTCC -0.161703213486
CACGCTCG -0.214583607709
CACGCTGA -0.0921682879858
CACGCTGC -0.129590250446
CACGCTGG -0.128327324806
CACGCTTA -0.0886812466852
CACGCTTC -0.254665249302
CACGGAAA 0.0119870908253
CACGGAAC 0.00984508196245
CACGGAAG -0.202497778107
CACGGACA -0.155272097643
CACGGACC -0.184395937248
CACGGACG -0.120770862359

CACGGAGA -0.0674877604338
CACGGAGC -0.116431072305
CACGGAGG -0.331205455422
CACGGATA 0.220828772519
CACGGATC 0.179065402094
CACGGATG -0.0828570598405
CACGGCAA 0.0390897714549
CACGGCAC -0.191680464779
CACGGCAG -0.191663737738
CACGGCCA -0.183143064633
CACGGCCC -0.206117612206
CACGGCCG -0.19495742533
CACGGCGA -0.0529905713829
CACGGCGC -0.354441317336
CACGGCGG -0.0719783950617
CACGGCTA -0.0591227896869
CACGGCTC -0.271860666711
CACGGCTG -0.239472560542
CACGGGAA -0.0786587981438
CACGGGAC -0.33975989685
CACGGGAG -0.210873311483
CACGGGCA -0.0577036484009
CACGGGCC -0.295171634381
CACGGGCG -0.0207565081874
CACGGGGA -0.0510663286026
CACGGGGC -0.0388273642132
CACGGGGG -0.331260255275
CACGGGTA -0.0489016205419
CACGGGTC -0.216996142852
CACGGTAA -0.0141894149608
CACGGTAC 0.0589008540253
CACGGTAG -0.148702977487
CACGGTCA -0.0932527404205
CACGGTCC -0.232945417784
CACGGTCG -0.10757457895
CACGGTGA -0.15942446596
CACGGTGC -0.256317873024
CACGGTGG -0.125878175152
CACGGTTA -0.0342234954504
CACGGTTC -0.275525488453
CACGTAAA -0.138916329151
CACGTAAC -0.0393076918607
CACGTAAG 0.090225618526
CACGTACA -0.0493910198674
CACGTACC -0.230342299851
CACGTACG -0.149629704726
CACGTAGA 0.0310795693926
CACGTAGC -0.0828219912094
CACGTAGG -0.224951719446
CACGTATA 0.0862973659285
CACGTATC 0.184099196606
CACGTATG -0.164902315062
CACGTCAA -0.0720574434456
CACGTCAC -0.104464751242

CACGTCAG -0.183640374706
CACGTCCA -0.0741358923181
CACGTCCC -0.237196373495
CACGTCCG -0.180199606055
CACGTCGA 0.00629141862702
CACGTCGC -0.188068571296
CACGTCGG -0.218481793547
CACGTCTA -0.206917079541
CACGTCTC -0.220987654321
CACGTCTG -0.0545162917171
CACGTGAA -0.000661310327417
CACGTGAC -0.275169110749
CACGTGAG -0.0174689007032
CACGTGCA 0.000902873828185
CACGTGCC -0.0902264922939
CACGTGCG -0.0507889465163
CACGTGGA -0.00763854588561
CACGTGGC -0.127437983015
CACGTGGG -0.219950729456
CACGTGTA 0.0818129926014
CACGTGTC 0.053709274421
CACGTTAA -0.00713791740763
CACGTTAC -0.00392983380535
CACGTTAG 0.0166390892028
CACGTTCA -0.186218513215
CACGTTCC -0.0851456731587
CACGTTCG -0.169651752855
CACGTTGA -0.198095476029
CACGTTGC 0.0348771664754
CACGTTGG -0.22959049312
CACGTTTA -0.102201670753
CACGTTTC -0.054379624141
CACTAAAA 0.180417822847
CACTAAAC -0.140609563327
CACTAAAG 0.0646576520562
CACTAACA 8.02588037734E-5
CACTAACC -0.171387772771
CACTAACG -0.172434324975
CACTAAGA 0.0264976088232
CACTAAGC -0.0968474089789
CACTAAGG -0.293574759945
CACTAATA 0.202273924784
CACTAATC 0.0820899859079
CACTAATG -0.00547761751081
CACTACAA 0.0780423280423
CACTACAC -0.110617694996
CACTACAG -0.199685016902
CACTACCA -0.00425914948737
CACTACCC -0.15253530661
CACTACCG -0.194331950481
CACTACGA 0.0488762657446
CACTACGC -0.069358702094
CACTACGG 0.162201694878
CACTACTA 0.054759709324

CACTACTC -0.131141492551
CACTACTG -0.0619899793111
CACTAGAA -0.0627006569199
CACTAGAC -0.0320059045612
CACTAGAG -0.0436903860296
CACTAGCA -0.15138357032
CACTAGCC -0.11589049665
CACTAGCG -0.130330128792
CACTAGGA -0.117761818018
CACTAGGC -0.12153069345
CACTAGGG -0.0232773524327
CACTAGTA -0.0680831653021
CACTAGTC -0.109337185756
CACTAGTG -0.121654981732
CACTATAA 0.0241904319149
CACTATAC 0.0222910063778
CACTATAG -0.0892532160276
CACTATCA 0.0447334189554
CACTATCC 0.198089579126
CACTATCG 0.165767872968
CACTATGA -0.0922852312894
CACTATGC 0.0864810423939
CACTATGG 0.0315429537421
CACTATTA -0.107586515444
CACTATTC 0.0892377202795
CACTCAAA 0.179533072799
CACTCAAC -0.1355247127
CACTCAAG 0.160025467079
CACTCACA 0.000773496751314
CACTCACC 0.0151221071288
CACTCACG -0.0163545996418
CACTCAGA -0.0472084610579
CACTCAGC -0.0225080993128
CACTCAGG -0.188568498662
CACTCATA 0.115390873564
CACTCATC -0.0360298799095
CACTCATG -0.0932519970951
CACTCCAA -0.160536512013
CACTCCAC -0.157765897171
CACTCCAG -0.161693522613
CACTCCCA -0.0478119134644
CACTCCCC -0.126981314059
CACTCCCG -0.0950994916485
CACTCCGA -0.31248354072
CACTCCGC -0.126898523165
CACTCCGG -0.0263610531637
CACTCCTA -0.170310212192
CACTCCTC -0.237582038298
CACTCCTG -0.244614995309
CACTCGAA 0.0188848372147
CACTCGAC -0.185072745021
CACTCGAG -0.0140430598377
CACTCGCA -0.111801891835
CACTCGCC -0.287141193316

CACTCGCG -0.175013438322
CACTCGGA 0.0849723243212
CACTCGGC -0.273965679497
CACTCGGG -0.0737281373219
CACTCGTA 0.0511862042915
CACTCGTC -0.245132897603
CACTCTAA 0.0553305231728
CACTCTAC -0.074990533577
CACTCTAG -0.02691889756
CACTCTCA -0.148438975491
CACTCTCC -0.00297095869714
CACTCTCG -0.178668661834
CACTCTGA 0.00866331878928
CACTCTGC -0.0873520404902
CACTCTGG -0.0712231716084
CACTCTTA 0.0716197172868
CACTCTTC -0.281221775306
CACTGAAA 0.0743175511746
CACTGAAC -0.0742924528302
CACTGAAG -0.382213542045
CACTGACA -0.101161271911
CACTGACC -0.0477899612543
CACTGACG -0.0124206898066
CACTGAGA 0.000354481135502
CACTGAGC -0.213783587509
CACTGAGG -0.21181670041
CACTGATA 0.0799427600134
CACTGATC 0.0703833916871
CACTGATG -0.130906175192
CACTGCAA 0.0481614923075
CACTGCAC -0.0879734817303
CACTGCAG -0.0460552674186
CACTGCCA -0.144461455727
CACTGCCC -0.256195887516
CACTGCCG -0.145083676269
CACTGCGA -0.015170186878
CACTGCGC 0.0492330067781
CACTGCGG -0.0925288975781
CACTGCTA -0.10431815523
CACTGCTC -0.185999529435
CACTGCTG -0.128823367319
CACTGGAA -0.186793860877
CACTGGAC -0.0535535826342
CACTGGAG -0.238274509804
CACTGGCA -0.293941928719
CACTGGCC -0.10129621398
CACTGGCG -0.159131964931
CACTGGGA -0.0830627445373
CACTGGGC -0.149385391131
CACTGGGG -0.232998592732
CACTGGTA -0.170368616606
CACTGGTC -0.238765089287
CACTGTAA 0.144142670157
CACTGTAC -0.189655456237

CACTGTAG -0.127901301282
CACTGTCA -0.077037037037
CACTGTCC -0.131874340926
CACTGTCG -0.12995365595
CACTGTGA -0.112644386083
CACTGTGC -0.366477125066
CACTGTGG 0.12865540407
CACTGTTA -0.0435684647303
CACTGTTC -0.0928113614309
CACTTAAA 0.0116824738194
CACTTAAC -0.0426352961972
CACTTAAG -0.131095854325
CACTTACA -0.121390741198
CACTTACC -0.186046296296
CACTTACG -0.00622406639004
CACTTAGA 0.122082757088
CACTTAGC -0.224662309368
CACTTAGG -0.0276748717104
CACTTATA 0.0178461656677
CACTTATC 0.16418801041
CACTTATG 0.0701334054397
CACTTCAA -0.122693645933
CACTTCAC 0.0323630456496
CACTTCAG -0.0120048649183
CACTTCCA -0.15893910705
CACTTCCC -0.288996874673
CACTTCCG -0.0509067158445
CACTTCGA -0.0665454205092
CACTTCGC -0.19411387092
CACTTCGG -0.135200092186
CACTTCTA -0.139604938272
CACTTCTC -0.185913266818
CACTTCTG -0.124392156863
CACTTGAA -0.0944997348142
CACTTGAC -0.0889500322504
CACTTGAG -0.0169845858818
CACTTGCA -0.0512751974246
CACTTGCC -0.00471674816022
CACTTGCG -0.145543538501
CACTTGGA 0.223617697425
CACTTGGC -0.0402366679318
CACTTGGG -0.0926587889564
CACTTGTA 0.0167813782959
CACTTGTC -0.0749697122885
CACTTTAA 0.0296587392675
CACTTTAC 0.0882033327038
CACTTTAG -0.130179268112
CACTTTCA 0.00273057586336
CACTTTCC 0.0509381664352
CACTTTCG 0.0145179258381
CACTTTGA -0.119286190547
CACTTTGC -0.0957356572259
CACTTTGG -0.0424342700976
CACTTTTA 0.0724376389232

CACTTTTC -0.0570868734769
CAGAAAAA -0.10437456773
CAGAAAAC 0.124470820682
CAGAAAAG -0.0464936876208
CAGAAACA 0.0566022111487
CAGAAACC -0.133456899156
CAGAAACG 0.050548428806
CAGAAAGA 0.0553515291328
CAGAAAGC -0.234567972953
CAGAAAGG -0.153254901961
CAGAAATA 0.200624602077
CAGAAATC 0.379116446025
CAGAAATG -0.0817616550201
CAGAACAA -0.153720009412
CAGAACAC -0.11468555841
CAGAACAG -0.0725292635946
CAGAACCA -0.0285230711634
CAGAACCC -0.0593654206558
CAGAACCG 0.0483325649301
CAGAACGA -0.112245653892
CAGAACGC 0.00476492088488
CAGAACGG 0.0159169246306
CAGAACTA 0.150012350558
CAGAACTC -0.0282250982458
CAGAACTG 0.104045183346
CAGAAGAA 0.126429778919
CAGAAGAC -0.201327160494
CAGAAGAG -0.196150157627
CAGAAGCA -0.196452227671
CAGAAGCC -0.247304393633
CAGAAGCG -0.0161482515065
CAGAAGGA -0.0904543982968
CAGAAGGC -0.207283212888
CAGAAGGG -0.185956942524
CAGAAGTA 0.000480252036269
CAGAAGTC -0.0826569642691
CAGAATAA 0.169419268004
CAGAATAC 0.00916869744671
CAGAATAG -0.0377609576802
CAGAATCA 0.367206136591
CAGAATCC 0.43056104415
CAGAATCG 0.345017250928
CAGAATGA -0.0569712201996
CAGAATGC -0.0290136998242
CAGAATGG -0.239894970431
CAGAATTA 0.11702090567
CAGAATTC 0.224467057455
CAGACAAA -0.0824743682627
CAGACAAC -0.190154845402
CAGACAAG -0.148332894332
CAGACACA -0.103003314456
CAGACACC -0.168496732026
CAGACACG -0.0363154300989
CAGACAGA 0.0115258787502

CAGACAGC -0.100983733247
CAGACAGG -0.245072868685
CAGACATA -0.0520898406288
CAGACATC 0.0394726009353
CAGACATG 0.00199490708727
CAGACCAA -0.0869890452107
CAGACCAC -0.254185805304
CAGACCAG -0.263315557311
CAGACCCA -0.224744577074
CAGACCCC -0.248144990891
CAGACCCG -0.253741638857
CAGACCGA -0.0611418411695
CAGACCGC -0.041703465266
CAGACCGG -0.0892264207898
CAGACCTA -0.111129670892
CAGACCTC -0.176986176427
CAGACCTG -0.125880261231
CAGACGAA 0.0682942774541
CAGACGAC -0.0983450485895
CAGACGAG -0.200132455333
CAGACGCA -0.171711435908
CAGACGCC -0.371418281937
CAGACGCG -0.0307859018756
CAGACGGA 0.0573694287316
CAGACGGC -0.159986752504
CAGACGGG -0.184017429194
CAGACGTA -0.00691092935075
CAGACGTC -0.0146262385698
CAGACTAA -0.130729767338
CAGACTAC -0.213216048432
CAGACTAG -0.029770137654
CAGACTCA -0.134148817657
CAGACTCC -0.244425650649
CAGACTCG -0.30703818913
CAGACTGA -0.0861995466739
CAGACTGC -0.139354103906
CAGACTGG -0.214145803499
CAGACTTA -0.180048621744
CAGACTTC -0.171108367627
CAGAGAAA 0.0793848880624
CAGAGAAC -0.0168027017683
CAGAGAAG -0.0233102020972
CAGAGACA -0.144058278476
CAGAGACC -0.191851595768
CAGAGACG -0.1614291939
CAGAGAGA -0.0868841979864
CAGAGAGC -0.310002120447
CAGAGAGG -0.237020974999
CAGAGATA 0.206277854618
CAGAGATC 0.268743550901
CAGAGATG -0.135718228032
CAGAGCAA -0.022945547543
CAGAGCAC -0.00157023826546
CAGAGCAG -0.277549929709

CAGAGCCA -0.209039920094
CAGAGCCC -0.170689905592
CAGAGCCG -0.177026897479
CAGAGCGA -0.0662995703492
CAGAGCGC -0.079118925326
CAGAGCGG -0.0746832178155
CAGAGCTA -0.0845
CAGAGCTC -0.1465753211
CAGAGCTG -0.224041144085
CAGAGGAA -0.206568162741
CAGAGGAC -0.183752558425
CAGAGGAG -0.177052985038
CAGAGGCA -0.0603463887654
CAGAGGCC -0.147490740741
CAGAGGCG -0.243860749751
CAGAGGGA -0.0256784592634
CAGAGGGC -0.195895424837
CAGAGGGG -0.177516339869
CAGAGGTA -0.0510965128914
CAGAGGTC -0.412472229079
CAGAGTAA 0.0512634745
CAGAGTAC -0.252422959158
CAGAGTAG -0.0188412534685
CAGAGTCA -0.0886013433426
CAGAGTCC -0.104792629603
CAGAGTCG -0.214383746803
CAGAGTGA -0.0127460019537
CAGAGTGC 0.0681325577747
CAGAGTGG -0.144633685614
CAGAGTTA -0.0626122226032
CAGAGTTC -0.0453926862461
CAGATAAA -0.0503348684497
CAGATAAC 0.148168756265
CAGATAAG 0.0630787558293
CAGATACA 0.174531822872
CAGATACC 0.271223105197
CAGATACG 0.405785527673
CAGATAGA -0.0107330715662
CAGATAGC 0.177308848953
CAGATAGG -0.102762958572
CAGATATA 0.153141397177
CAGATATC 0.46540714529
CAGATATG 0.357649673848
CAGATCAA -0.00316288671588
CAGATCAC 0.144092076665
CAGATCAG 0.0216964148499
CAGATCCA 0.184909854285
CAGATCCC 0.248876265745
CAGATCCG 0.27672046838
CAGATCGA -0.0672627280511
CAGATCGC 0.413989761243
CAGATCGG 0.274661546422
CAGATCTA 0.235208237283
CAGATCTC 0.402654250142

CAGATCTG	0.345978425308
CAGATGAA	0.131962866648
CAGATGAC	-0.132794524368
CAGATGAG	-0.159046296296
CAGATGCA	-0.0483689747136
CAGATGCC	-0.102687782607
CAGATGCG	0.0273620167292
CAGATGGA	-0.0807970014383
CAGATGGC	-0.272701184893
CAGATGGG	-0.198794830756
CAGATGTA	0.0574710751059
CAGATGTC	0.132215944693
CAGATTAA	0.0891854197841
CAGATTAC	0.476630395305
CAGATTAG	0.05571600831
CAGATTCA	0.303714132034
CAGATTCC	0.484216872721
CAGATTCG	0.446486342981
CAGATTGA	0.0862930580254
CAGATTGC	0.472062818706
CAGATTGG	0.0373178562608
CAGATTTA	0.268117036886
CAGATTTC	0.48725611262
CAGCAAAA	0.041364152714
CAGCAAAC	0.0801911128675
CAGCAAAG	-0.117740023706
CAGCAACA	-0.111184866065
CAGCAACC	-0.0642321665734
CAGCAACG	-0.131916318175
CAGCAAGA	0.105694942833
CAGCAAGC	-0.108334979261
CAGCAAGG	-0.195740333811
CAGCAATA	0.230835500414
CAGCAATC	0.347965638544
CAGCAATG	-0.212946043975
CAGCACAA	0.0241846207488
CAGCACAC	-0.274527777778
CAGCACAG	-0.248251115171
CAGCACCA	-0.158668960027
CAGCACCC	-0.123680464779
CAGCACCG	-0.116336717318
CAGCACGA	-0.0532892137495
CAGCACGC	-0.298402273687
CAGCACGG	-0.0525547339212
CAGCACTA	-0.106591861283
CAGCACTC	-0.176234567901
CAGCACTG	-0.127782135076
CAGCAGAA	0.0200074630142
CAGCAGAC	-0.192699416129
CAGCAGAG	-0.0783000670698
CAGCAGCA	-0.0416282465038
CAGCAGCC	-0.187554524056
CAGCAGCG	-0.144384087791
CAGCAGGA	-0.100376180102

CAGCAGGC -0.129351595768
CAGCAGGG -0.198926589947
CAGCAGTA -0.138588432854
CAGCAGTC -0.180894442798
CAGCATAA -0.0550170689283
CAGCATAC -0.0838354653364
CAGCATAG -0.0479734365138
CAGCATCA 0.0783301614051
CAGCATCC -0.0767553353769
CAGCATCG 0.0217846091264
CAGCATGA -0.103900522411
CAGCATGC -0.195606974574
CAGCATGG -0.185497942387
CAGCATTA 0.0451019133265
CAGCATTC -0.0478170192915
CAGCCAAA -0.0194688662781
CAGCCAAC -0.143875910286
CAGCCAAG 0.0293565668069
CAGCCACA -0.0686574708904
CAGCCACC -0.170461338532
CAGCCACG -0.23982615567
CAGCCAGA -0.0134429846225
CAGCCAGC -0.173387225613
CAGCCAGG -0.238889711975
CAGCCATA 0.0463155063777
CAGCCATC -0.232996416833
CAGCCATG -0.167772255609
CAGCCCAA -0.0103996888428
CAGCCCAC -0.233302680952
CAGCCCAG -0.190610235896
CAGCCCCA -0.176114293239
CAGCCCCC -0.295752279385
CAGCCCCG -0.0989651066445
CAGCCCGA -0.141410649017
CAGCCCGC -0.111452126902
CAGCCCGG -0.364086028141
CAGCCCTA -0.218094168189
CAGCCCTC -0.171564027853
CAGCCCTG -0.427139202425
CAGCCGAA 0.0581625092615
CAGCCGAC -0.308170757564
CAGCCGAG -0.0956868240407
CAGCCGCA -0.152419624308
CAGCCGCC -0.361687019908
CAGCCGCG -0.0806132498932
CAGCCGGA -0.00887966896067
CAGCCGGC -0.424603958224
CAGCCGGG -0.101053013798
CAGCCGTA 0.0236605697415
CAGCCGTC -0.248646157391
CAGCCTAA -0.149951926847
CAGCCTAC -0.0262377975069
CAGCCTAG -0.285244804354
CAGCCTCA 0.100197579649

CAGCCTCC -0.240643163591
CAGCCTCG -0.239334178368
CAGCCTGA -0.137435855218
CAGCCTGC -0.276562654291
CAGCCTGG -0.0774770770573
CAGCCTTA -0.0843581259054
CAGCCTTC -0.233117727415
CAGCGAAA 0.053050135836
CAGCGAAC -0.0602391457044
CAGCGAAG -0.102583098731
CAGCGACA -0.0971999833642
CAGCGACC -0.304834619641
CAGCGACG -0.227781344836
CAGCGAGA -0.0116410259077
CAGCGAGC -0.252166196586
CAGCGAGG -0.214993566676
CAGCGATA 0.0253011717571
CAGCGATC 0.225019756989
CAGCGATG -0.0797746541074
CAGCGCAA 0.133995806714
CAGCGCAC -0.199274586948
CAGCGCAG -0.0765661953128
CAGCGCCA -0.179590413943
CAGCGCCC -0.139378625008
CAGCGCCG -0.184145549058
CAGCGCGA 0.0426496012087
CAGCGCGC -0.195756088994
CAGCGCGG -0.157621805402
CAGCGCTA -0.217313886557
CAGCGCTC -0.177362352722
CAGCGCTG -0.0143737757806
CAGCGGAA 0.123352171197
CAGCGGAC -0.160129206692
CAGCGGAG -0.113523398396
CAGCGGCA -0.193170660857
CAGCGGCC -0.319765263534
CAGCGGCG -0.206263578906
CAGCGGGA -0.0322708584545
CAGCGGGC -0.407092426187
CAGCGGGG -0.304067288858
CAGCGGTA -0.141556464546
CAGCGGTC -0.279259289378
CAGCGTAA 0.122471779524
CAGCGTAC -0.240091740188
CAGCGTAG -0.0981258989148
CAGCGTCA -0.183880136742
CAGCGTCC -0.19429779321
CAGCGTCG -0.11110331384
CAGCGTGA -0.108087487945
CAGCGTGC -0.149043042741
CAGCGTGG -0.19803669826
CAGCGTTA 0.135510658851
CAGCGTTC -0.382459431882
CAGCTAAA -0.102302042903

CAGCTAAC -0.116589288948
CAGCTAAG -0.278208462264
CAGCTACA -0.245671939904
CAGCTACC -0.252581571861
CAGCTACG -0.263923866585
CAGCTAGA 0.0673343645605
CAGCTAGC -0.301079502192
CAGCTAGG -0.322812268576
CAGCTATA -0.0210291575262
CAGCTATC -0.173271211647
CAGCTATG -0.0120857728125
CAGCTCAA -0.129028374574
CAGCTCAC -0.156510789591
CAGCTCAG -0.250850357426
CAGCTCCA -0.264537408465
CAGCTCCC -0.23038126523
CAGCTCCG -0.213826832881
CAGCTCGA -0.17813295277
CAGCTCGC -0.338498871627
CAGCTCGG -0.173948952657
CAGCTCTA -0.145542184077
CAGCTCTC -0.081003655043
CAGCTGAA 0.0183845119508
CAGCTGAC -0.176817253051
CAGCTGAG -0.0603238066519
CAGCTGCA -0.0446579402322
CAGCTGCC -0.364449642625
CAGCTGCG -0.279501959521
CAGCTGGA -0.0663120456744
CAGCTGGC -0.16472702332
CAGCTGGG -0.14514739453
CAGCTGTA 0.0533381632967
CAGCTGTC -0.140266394242
CAGCTTAA -0.204485712561
CAGCTTAC -0.263614513368
CAGCTTAG -0.167688271605
CAGCTTCA -0.198352569335
CAGCTTCC -0.209852462704
CAGCTTCG -0.248962287992
CAGCTTGA -0.114563621618
CAGCTTGC -0.0988213396278
CAGCTTGG -0.231336076818
CAGCTTTA -0.0483503552075
CAGCTTTC -0.0874463128776
CAGGAAAA 0.0407143954862
CAGGAAAC -0.0632090738207
CAGGAAAG -0.126904859699
CAGGAACA 0.0652177702373
CAGGAACC -0.135078206015
CAGGAACG 0.0892696816934
CAGGAAGA -0.0294241935307
CAGGAAGC -0.156627266342
CAGGAAGG -0.181227070849
CAGGAATA 0.153439483968

CAGGAATC 0.322792487212
CAGGAATG -0.194452676523
CAGGACAA -0.0167714458363
CAGGACAC -0.181859114016
CAGGACAG -0.0631318706858
CAGGACCA -0.217139701105
CAGGACCC -0.3117728379
CAGGACCG -0.23111155009
CAGGACGA -0.130036310821
CAGGACGC -0.29914086802
CAGGACGG -0.245954048566
CAGGACTA -0.24702512376
CAGGACTC -0.30953874662
CAGGACTG -0.198408502703
CAGGAGAA 0.0277270107846
CAGGAGAC -0.136084771061
CAGGAGAG -0.182138744401
CAGGAGCA -0.3288493298
CAGGAGCC -0.222243137158
CAGGAGCG -0.184618736383
CAGGAGGA -0.15749691358
CAGGAGGC -0.216213429528
CAGGAGGG -0.304846539949
CAGGAGTA 0.00951726002751
CAGGAGTC -0.281047849489
CAGGATAA 0.142217721575
CAGGATAC 0.310945331454
CAGGATAG -0.0225785340314
CAGGATCA 0.090001888629
CAGGATCC 0.278500027055
CAGGATCG 0.212112230784
CAGGATGA 0.0486577641661
CAGGATGC -0.0965382201873
CAGGATGG -0.22895886826
CAGGATTA 0.222397857253
CAGGATTC 0.408471227464
CAGGCAAA -0.15373388203
CAGGCAAC 0.0252887000812
CAGGCAAG -0.173835875091
CAGGCACA -0.187129304743
CAGGCACC -0.0973841037798
CAGGCACG -0.0459566087128
CAGGCAGA -0.0563855005185
CAGGCAGC -0.279294861183
CAGGCAGG -0.148118994783
CAGGCATA 0.0134360798261
CAGGCATC 0.00125449473112
CAGGCATG -0.173045751634
CAGGCCAA -0.321476178721
CAGGCCAC -0.205443718228
CAGGCCAG -0.0619872405699
CAGGCCCA -0.0656823535221
CAGGCCCC -0.348251208066
CAGGCCCG -0.286115532525

CAGGCCGA -0.136325355071
CAGGCCGC -0.274006231965
CAGGCCGG -0.273106085544
CAGGCCTA -0.21313253957
CAGGCCTC -0.399758096367
CAGGCCTG -0.334370063191
CAGGCGAA -0.0689102191647
CAGGCGAC -0.0421523229602
CAGGCGAG -0.0679731783737
CAGGCGCA -0.131235238922
CAGGCGCC -0.172908093278
CAGGCGCG -0.072094013928
CAGGCGGA 0.0289236508591
CAGGCGGC -0.228870958841
CAGGCGGG -0.148306648189
CAGGCGTA -0.125721531637
CAGGCGTC -0.148495673312
CAGGCTAA -0.0960145243282
CAGGCTAC -0.215423894227
CAGGCTAG -0.214520143711
CAGGCTCA -0.238242410687
CAGGCTCC -0.258385045457
CAGGCTCG -0.167344340736
CAGGCTGA -0.159381416504
CAGGCTGC -0.259487403165
CAGGCTGG -0.204402470054
CAGGCTTA -0.24919405898
CAGGCTTC -0.346948627225
CAGGGAAA 0.145832667471
CAGGGAAC -0.0698852578068
CAGGGAAG -0.153266069743
CAGGGACA -0.203077322937
CAGGGACC -0.28216374269
CAGGGACG -0.15131798756
CAGGGAGA -0.0497554831061
CAGGGAGC -0.149194110675
CAGGGAGG -0.20451203722
CAGGGATA 0.093603366097
CAGGGATC 0.181257536356
CAGGGATG -0.282469827541
CAGGGCAA -0.127724266623
CAGGGCAC -0.214079479009
CAGGGCAG -0.102268700073
CAGGGCCA -0.197234567901
CAGGGCCC -0.0880503144654
CAGGGCCG -0.212884324182
CAGGGCGA -0.220846054852
CAGGGCGC -0.20495583806
CAGGGCGG -0.124047109954
CAGGGCTA -0.115816883708
CAGGGCTC -0.308992559907
CAGGGGAA -0.0408840491437
CAGGGGAC -0.382304112768
CAGGGGAG -0.138095943632

CAGGGGCA -0.187212475634
CAGGGGCC -0.303489733259
CAGGGGCG -0.167198257081
CAGGGGGA -0.00249694056184
CAGGGGGC -0.135466119669
CAGGGGGG -0.359703687536
CAGGGGTA -0.0974143040601
CAGGGGTC -0.210669098767
CAGGGTAA -0.00805451272256
CAGGGTAC -0.134525590923
CAGGGTAG -0.0395484265121
CAGGGTCA -0.120887596483
CAGGGTCC -0.283858742213
CAGGGTCG -0.262161435617
CAGGGTGA -0.322649229782
CAGGGTGC -0.255181331161
CAGGGTGG -0.0923996246728
CAGGGTTA -0.184484055858
CAGGGTTC -0.170781689793
CAGGTAAA 0.03960164456
CAGGTAAC -0.240755373379
CAGGTAAG -0.148439900786
CAGGTACA -0.24497864303
CAGGTACC -0.127575557485
CAGGTACG 0.0715163889438
CAGGTAGA 0.0592186691925
CAGGTAGC -0.0323112501779
CAGGTAGG -0.227097152953
CAGGTATA -0.224708187221
CAGGTATC 0.187248848663
CAGGTATG -0.0413208852006
CAGGTCAA -0.0798006001749
CAGGTCAC -0.282897259239
CAGGTCAG -0.219805143322
CAGGTCCA -0.27543010571
CAGGTCCC -0.368927725118
CAGGTCCG -0.306532656019
CAGGTCGA -0.196968121608
CAGGTCGC -0.32700892533
CAGGTCGG -0.194730573711
CAGGTCTA 0.0644319853345
CAGGTCTC -0.188599162647
CAGGTGAA -0.161525225581
CAGGTGAC -0.0754715264328
CAGGTGAG -0.168632470191
CAGGTGCA -0.10304721454
CAGGTGCC -0.37406781433
CAGGTGCG -0.135767404846
CAGGTGGA -0.00294451756106
CAGGTGGC -0.163264242165
CAGGTGGG -0.0393489155524
CAGGTGTA 0.0703939713276
CAGGTGTC -0.240145976921
CAGGTTAA -0.00975162623947

CAGGTTAC -0.13234640426
CAGGTTAG -0.0891737510968
CAGGTTCA -0.0673673964523
CAGGTTCC -0.272339492313
CAGGTTCG -0.171347744247
CAGGTTGA -0.111693830661
CAGGTTGC -0.140495435554
CAGGTTGG 0.0445142395593
CAGGTTTA -0.138609387939
CAGGTTTC -0.108279921669
CAGTAAAA 0.225085351503
CAGTAAAC -0.0939812070517
CAGTAAAG 0.086942309648
CAGTAACA -0.109787556684
CAGTAACC -0.194249666683
CAGTAACG -0.0573926004265
CAGTAAGA 0.178180523877
CAGTAAGC -0.200427852523
CAGTAAGG 0.00729737190011
CAGTAATA 0.0533741083492
CAGTAATC 0.377634274258
CAGTAATG -0.016358756504
CAGTACAA 0.238572745834
CAGTACAC -0.192046745358
CAGTACAG -0.049546824064
CAGTACCA -0.161278197552
CAGTACCC -0.128610021786
CAGTACCG -0.165659596843
CAGTACGA 0.0412879536324
CAGTACGC -0.170702730585
CAGTACGG -0.104504474844
CAGTACTA 0.000257144102393
CAGTACTC 0.096091571714
CAGTACTG -0.025320955955
CAGTAGAA 0.189721508705
CAGTAGAC -0.260941362373
CAGTAGAG -0.106427359565
CAGTAGCA -0.0205305537717
CAGTAGCC -0.197601398845
CAGTAGCG -0.181892252234
CAGTAGGA 0.0739231377198
CAGTAGGC -0.200229763527
CAGTAGGG -0.227533607682
CAGTAGTA 0.0854749901937
CAGTAGTC -0.210563430522
CAGTATAA 0.0805931524293
CAGTATAC -0.00945486028959
CAGTATAG 0.0427911063967
CAGTATCA 0.134550288379
CAGTATCC 0.259498410107
CAGTATCG 0.156229443836
CAGTATGA -0.0964964272778
CAGTATGC -0.185551933799
CAGTATGG -0.122507379088

CAGTATTA 0.0630749302948
CAGTATTC -0.0327970976311
CAGTCAAA 0.130127990935
CAGTCAAC -0.255072201374
CAGTCAAG -0.246374180505
CAGTCACA -0.101274262854
CAGTCACC 0.0777322224418
CAGTCACG -0.095708785967
CAGTCAGA -0.0864956673965
CAGTCAGC -0.110794918056
CAGTCAGG -0.216302106028
CAGTCATA -0.09548762948
CAGTCATC -0.223661008677
CAGTCATG -0.035508463413
CAGTCCAA -0.0462197325833
CAGTCCAC -0.179360141337
CAGTCCAG -0.254141614222
CAGTCCCA -0.130297405983
CAGTCCCC -0.261429679263
CAGTCCCG -0.197533455138
CAGTCCGA -0.0722125863236
CAGTCCGC -0.232852173722
CAGTCCGG -0.37735093379
CAGTCCTA -0.0908886062821
CAGTCCTC -0.141670297749
CAGTCGAA -0.168881251943
CAGTCGAC -0.0867231675137
CAGTCGAG -0.167108476716
CAGTCGCA -0.0243763892319
CAGTCGCC -0.185673291684
CAGTCGCG -0.117220082093
CAGTCGGA -0.0161409372033
CAGTCGGC -0.0405053149163
CAGTCGGG -0.21014266118
CAGTCGTA -0.129147673294
CAGTCGTC -0.254018881627
CAGTCTAA -0.185373868024
CAGTCTAC -0.218506994177
CAGTCTAG -0.0404339135891
CAGTCTCA -0.139200385804
CAGTCTCC -0.274876264944
CAGTCTCG -0.243889615105
CAGTCTGA -0.153122431136
CAGTCTGC -0.179491648511
CAGTCTGG -0.0857800770446
CAGTCTTA 0.0911957452414
CAGTCTTC 0.0231289380667
CAGTGAAA -0.0211420667852
CAGTGAAC -0.244081486839
CAGTGAAG -0.344633449311
CAGTGACA -0.174588049364
CAGTGACC -0.213988467531
CAGTGACG -0.0817664943418
CAGTGAGA -0.0903445390603

CAGTGAGC -0.085182129546
CAGTGAGG -0.237942033448
CAGTGATA 0.0819708570017
CAGTGATC 0.0363415803466
CAGTGATG -0.14688967909
CAGTGCAA 0.0277283853052
CAGTGCAC -0.00187101714173
CAGTGCAG -0.170696882635
CAGTGCCA -0.11899156528
CAGTGCCC -0.298540883718
CAGTGCCG -0.129632716049
CAGTGCGA -0.149147789631
CAGTGCGC -0.0376361790291
CAGTGCGG -0.0657700923013
CAGTGCTA -0.0805306053476
CAGTGCTC -0.0769976295126
CAGTGGAA 0.000613047747994
CAGTGGAC -0.149922539458
CAGTGGAG 0.0250683470899
CAGTGGCA -0.115882972768
CAGTGGCC -0.134709397067
CAGTGGCG -0.117097689323
CAGTGGGA -0.0862834529847
CAGTGGGC -0.119915982987
CAGTGGGG -0.182610324983
CAGTGGTA -0.0652445508481
CAGTGGTC -0.23317533205
CAGTGTAA 0.180832729588
CAGTGTAC 0.0536547524275
CAGTGTAG 0.0412721934085
CAGTGTCA -0.198592965116
CAGTGTCC -0.168485112564
CAGTGTCG -0.127102344864
CAGTGTGA -0.0761075984105
CAGTGTGC -0.245250030495
CAGTGTGG -0.303252646405
CAGTGTTA -0.0591533293815
CAGTGTTC -0.108263478274
CAGTTAAA 0.246795868261
CAGTTAAC -0.28651317414
CAGTTAAG -0.0525721674197
CAGTTACA -0.0190562666163
CAGTTACC -0.0376026034024
CAGTTACG -0.0332558511179
CAGTTAGA 0.0447677226723
CAGTTAGC -0.102632488764
CAGTTAGG -0.0601701341375
CAGTTATA -0.0240539472384
CAGTTATC 0.0293283057806
CAGTTATG 0.0198698298781
CAGTTCAA -0.0256619245208
CAGTTCAC -0.127372537398
CAGTTCAG -0.199675752189
CAGTTCCA -0.115951185016

CAGTTCCC -0.270988176044
CAGTTCCG -0.023745877704
CAGTTCGA 0.0103714787965
CAGTTCGC -0.29699673009
CAGTTCGG -0.189147421932
CAGTTCTA -0.00116368602269
CAGTTCTC -0.135821370561
CAGTTGAA 0.00415761048541
CAGTTGAC -0.140711355589
CAGTTGAG -0.0809023662145
CAGTTGCA -0.0933198133546
CAGTTGCC -0.30248907731
CAGTTGCG 0.123944817669
CAGTTGGA 0.0972167336275
CAGTTGGC -0.0438656126253
CAGTTGGG -0.0423403251122
CAGTTGTA 0.0741426815078
CAGTTGTC -0.0659087937472
CAGTTTAA 0.0236151264655
CAGTTTAC -0.0995072861227
CAGTTTAG 0.00665523803989
CAGTTTCA -0.0645048358653
CAGTTTCC -0.120787838992
CAGTTTCG 0.00108523527959
CAGTTTGA -0.0620527659871
CAGTTTGC -0.133532285064
CAGTTTGG -0.0691436507429
CAGTTTTA 0.0834323652899
CAGTTTTC 0.0642275351819
CATAAAAA 0.101833422922
CATAAAAC 0.080007244945
CATAAAAG 0.122272168434
CATAAACA -0.0780163584327
CATAAACC -0.0773002936253
CATAAACG -0.0543343432348
CATAAAGA 0.106165647291
CATAAAGC -0.085784343983
CATAAAGG -0.119103670047
CATAAATA 0.0454374091629
CATAAATC 0.208102218413
CATAAATG -0.102598610562
CATAACAA 0.149273310062
CATAACAC -0.0783337709982
CATAACAG 0.00939098553206
CATAACCA -0.10840776101
CATAACCC -0.119032723641
CATAACCG -0.0227118210714
CATAACGA -0.002798076504
CATAACGC 0.0789566114569
CATAACGG 0.132938674487
CATAACTA -0.132825663401
CATAACTC -0.030099453336
CATAAGAA 0.201808725466
CATAAGAC -0.0669690882346

CATAAGAG 0.0142319260577
CATAAGCA 0.107851447024
CATAAGCC -0.34928467289
CATAAGCG -0.144775373603
CATAAGGA 0.0231934290544
CATAAGGC -0.103358854038
CATAAGGG -0.253879286694
CATAAGTA -0.0466302500254
CATAAGTC -0.272104209814
CATAATAA 0.177500617436
CATAATAC 0.157799837241
CATAATAG -0.0995874072029
CATAATCA 0.233555719066
CATAATCC 0.264496680371
CATAATCG 0.218778129048
CATAATGA -0.00726826947327
CATAATGC 0.104211462312
CATAATGG 0.0337713231904
CATAATTA 0.163356013905
CATAATTC 0.13594787384
CATACAAA 0.130825224736
CATACAAC -0.00300774989572
CATACAAG 0.0480423634013
CATACACA 0.0860996905554
CATACACC 0.126287075457
CATACACG -0.0490817581067
CATACAGA 0.196508474664
CATACAGC -0.196538323012
CATACAGG 0.0700783771323
CATACATA 0.0994533359679
CATACATC -0.082947778601
CATACATG 0.0592381511998
CATACCAA 0.117132099497
CATACCAC -0.0422271617135
CATACCAG -0.0308063068167
CATACCCA 0.135611599644
CATACCCC -0.179237336137
CATACCCG -0.0507114870118
CATACCGA -0.0937413740502
CATACCGC -0.144277777778
CATACCGG -0.151764060357
CATACCTA 0.0408801011143
CATACCTC -0.100547653541
CATACGAA 0.245555184287
CATACGAC 0.0212463500845
CATACGAG -0.0121447373773
CATACGCA 0.0731657116512
CATACGCC -0.200536608183
CATACGCG -0.172603448176
CATACGGA 0.060603199047
CATACGGC -0.0183948820305
CATACGGG -0.0605837670339
CATACGTA -0.0603690531076
CATACGTC -0.20555111647

CATACTAA -0.208758699423
CATACTAC -0.151297441142
CATACTAG -0.0675041253167
CATACTCA 0.0705009989242
CATACTCC -0.239400873612
CATACTCG -0.0891970278176
CATACTGA -0.195728268264
CATACTGC 0.00335078706448
CATACTGG -0.0977373877063
CATACTTA -0.054297762521
CATACTTC 0.108463963506
CATAGAAA -0.0776513005558
CATAGAAC -0.0600133656822
CATAGAAG 0.0128844760588
CATAGACA -0.0340029088115
CATAGACC -0.0867863492691
CATAGACG -0.194526757101
CATAGAGA -0.0485711795215
CATAGAGC -0.169780682643
CATAGAGG 0.00871314086733
CATAGATA 0.23550477242
CATAGATC 0.126597181058
CATAGATG -0.168885234853
CATAGCAA 0.0075211017971
CATAGCAC -0.145182752509
CATAGCAG -0.133289161056
CATAGCCA -0.203810699588
CATAGCCC -0.103047204067
CATAGCCG -0.0270619863397
CATAGCGA -0.046858873536
CATAGCGC -0.174602575127
CATAGCGG -0.193770259437
CATAGCTA -0.00764698456607
CATAGCTC -0.11820544799
CATAGGAA 0.04195226127
CATAGGAC 0.0957898101201
CATAGGAG -0.129090454831
CATAGGCA -0.0980498689647
CATAGGCC -0.232247214509
CATAGGCG -0.0796463905394
CATAGGGA -0.203145751699
CATAGGGC -0.205077665418
CATAGGGG -0.132967320261
CATAGGTA -0.201740014524
CATAGGTC -0.218274509804
CATAGTAA 0.154604613257
CATAGTAC -0.0193088761553
CATAGTAG -0.0344766076094
CATAGTCA 0.0501550451415
CATAGTCC -0.157024481234
CATAGTCG -0.222543225729
CATAGTGA -0.063363502971
CATAGTGC -0.191325102881
CATAGTGG 0.138202814551

CATAGTTA -0.0874308505384
CATAGTTC -0.107802158451
CATATAAA -0.0385848207425
CATATAAC 0.0564035434367
CATATAAG 0.0337109486487
CATATACA 0.10067990644
CATATACC -0.0319875299128
CATATACG 0.0509334185638
CATATAGA 0.128243759468
CATATAGC -0.146913558726
CATATAGG -0.0951011869307
CATATATA 0.209804276713
CATATATC 0.25770461481
CATATATG 0.11080709819
CATATCAA 0.121197776021
CATATCAC 0.161458021876
CATATCAG 0.181797974809
CATATCCA 0.367960877297
CATATCCC 0.211705141429
CATATCCG 0.332812749699
CATATCGA 0.189244984237
CATATCGC 0.203851895761
CATATCGG 0.264641959525
CATATCTA 0.375013438322
CATATCTC 0.325792004215
CATATGAA 0.067187617812
CATATGAC -0.104973259112
CATATGAG 0.0825539528859
CATATGCA -0.00352916639224
CATATGCC 0.00502343629937
CATATGCG -0.0194338783777
CATATGGA 0.0971457859895
CATATGGC -0.0175880572801
CATATGGG 0.0111389684814
CATATGTA 0.0231289380667
CATATGTC -0.0180842240463
CATATTAA -0.0547600714773
CATATTAC 0.206088498101
CATATTAG 0.0401051614745
CATATTCA 0.126036929961
CATATTCC 0.230963346069
CATATTCG 0.156869380496
CATATTGA 0.0629775077389
CATATTGC 0.186922725477
CATATTGG -0.016955530051
CATATTTA 0.240342817625
CATATTTC 0.308711497508
CATCAAAA -0.0457172495554
CATCAAAC -0.0184444224901
CATCAAAG 0.158184301135
CATCAACA 0.0208054276292
CATCAACC -0.194083821626
CATCAACG 0.0996152495115
CATCAAGA 0.123150343586

CATCAAGC -0.199236348532
CATCAAGG -0.24915076582
CATCAATA 0.0291416935608
CATCAATC -0.105433159894
CATCAATG 0.00537451842339
CATCACAA 0.0145662483944
CATCACAC -0.149446976894
CATCACAG -0.123733008457
CATCACCA 0.10494665613
CATCACCC -0.105266912507
CATCACCG 0.0666962067613
CATCACGA -0.169495897478
CATCACGC -0.00598907484589
CATCACGG -0.169389781432
CATCACTA -0.1126078228
CATCACTC 0.0311477749237
CATCAGAA 0.109492539915
CATCAGAC -0.0714200626413
CATCAGAG -0.0932241802624
CATCAGCA 0.0597021888516
CATCAGCC -0.190935528121
CATCAGCG -0.0289966910312
CATCAGGA -0.0242083794549
CATCAGGC -0.373975785264
CATCAGGG -0.197277443906
CATCAGTA -0.0793296819839
CATCAGTC -0.0437114570659
CATCATAA 0.0237284442055
CATCATAC -0.178909825229
CATCATAG -0.154165500513
CATCATCA 0.0251899524937
CATCATCC -0.0207089503456
CATCATCG 0.117079957848
CATCATGA -0.0877626292564
CATCATGC -0.0985925925926
CATCATGG -0.135564665273
CATCATTA 0.026282451731
CATCATTC -0.159741428509
CATCCAAA 0.031468917525
CATCCAAC 0.0490158949703
CATCCAAG 0.0125245158572
CATCCACA 0.0259860822571
CATCCACC 0.00913370040533
CATCCACG 0.0261952842387
CATCCAGA 0.129470460383
CATCCAGC -0.0449979082901
CATCCAGG -0.129104414742
CATCCATA -0.173830065359
CATCCATC -0.239546469584
CATCCATG -0.00215669811165
CATCCCAA 0.0744424912469
CATCCCAC -0.188181957892
CATCCCAG -0.0580085133584
CATCCCCA -0.0637697959611

CATCCCCC -0.168555109108
CATCCCCG 0.127059695204
CATCCCGA 0.144336292185
CATCCCGC 0.143084919537
CATCCCGG -0.343607381078
CATCCCTA -0.144386347131
CATCCCTC -0.109212244943
CATCCGAA -0.132240349832
CATCCGAC -0.0980208883123
CATCCGAG -0.0158891810614
CATCCGCA 0.0465065267476
CATCCGCC -0.258960219479
CATCCGCG -0.0610490961178
CATCCGGA 0.153879053708
CATCCGGC -0.169213517094
CATCCGGG 0.0159772991723
CATCCGTA 0.049131155459
CATCCGTC -0.156534479069
CATCCTAA -0.164580917868
CATCCTAC -0.125440416214
CATCCTAG -0.114503373215
CATCCTCA -0.00775153116665
CATCCTCC -0.197500685871
CATCCTCG -0.11586892981
CATCCTGA -0.0339554233388
CATCCTGC -0.00442309480094
CATCCTGG -0.0475742121901
CATCCTTA 0.0631956793783
CATCCTTC -0.150346810659
CATCGAAA 0.127437421004
CATCGAAC -0.0152520510377
CATCGAAG 0.023371057487
CATCGACA -0.07796172941
CATCGACC -0.223368942
CATCGACG -0.194836782063
CATCGAGA 0.122384612032
CATCGAGC 0.00569716129882
CATCGAGG -0.107073233798
CATCGATA 0.0738642802144
CATCGATC 0.0753170717534
CATCGATG -0.0858247337843
CATCGCAA 0.117927945729
CATCGCAC -0.0136851348756
CATCGCAG 0.00419130358985
CATCGCCA -0.170460124392
CATCGCCC -0.19896945545
CATCGCCG -0.18225617284
CATCGCGA 0.146293003139
CATCGCGC 0.0658070983395
CATCGCGG 0.0566340111413
CATCGCTA -0.0899727469769
CATCGCTC -0.199715627696
CATCGGAA 0.0037953632352
CATCGGAC -0.178625998548

CATCGGAG -0.1926920094
CATCGGCA 0.0693606264437
CATCGGCC -0.192809166337
CATCGGCG -0.18479006492
CATCGGGA -0.0754526227717
CATCGGGC -0.0213553787199
CATCGGGG -0.0911647404554
CATCGGTA 0.0566377309098
CATCGGTC 0.0363427298075
CATCGTAA 0.117297861306
CATCGTAC -0.0768760016685
CATCGTAG -0.108112387953
CATCGTCA 0.0323689872445
CATCGTCC -0.230891031656
CATCGTCG -0.039634715225
CATCGTGA -0.0150349396365
CATCGTGC -0.00501939476704
CATCGTGG -0.0297536718699
CATCGTTA 0.0713916290152
CATCGTTC 0.0299976950179
CATCTAAA -0.00780646721284
CATCTAAC -0.00501882402011
CATCTAAG 0.0836502840207
CATCTACA -0.190209667418
CATCTACC -0.0303937062775
CATCTACG 0.16584757483
CATCTAGA -0.0471666529459
CATCTAGC 0.0538944511915
CATCTAGG -0.0439745470922
CATCTATA 0.0590956991372
CATCTATC -0.070548725219
CATCTCAA 0.00297095869714
CATCTCAC -0.21485048011
CATCTCAG -0.0515496729444
CATCTCCA -0.0125085073218
CATCTCCC -0.318864197531
CATCTCCG -0.0600447825085
CATCTCGA 0.042138218587
CATCTCGC -0.129506477358
CATCTCGG -0.073226897812
CATCTCTA 0.0170338536521
CATCTCTC 0.0297342844275
CATCTGAA -0.0750592768228
CATCTGAC 0.00310928889767
CATCTGAG 0.165971263783
CATCTGCA -0.0272975257415
CATCTGCC -0.156095499915
CATCTGCG 0.042995365595
CATCTGGA 0.0209320099617
CATCTGGC 0.0217439309633
CATCTGGG -0.0945607484782
CATCTGTA 0.01624656778
CATCTGTC -0.104671199574
CATCTTAA 0.187229686711

CATCTTAC -0.0571048770212
CATCTTAG -0.0151605363257
CATCTTCA 0.0111559862275
CATCTTCC -0.0730094444719
CATCTTCG -0.251938463058
CATCTTGA -0.125873629359
CATCTTGC 0.10753384053
CATCTTGG -0.159255051752
CATCTTTA 0.000440698149068
CATCTTTC 0.0501520340732
CATGAAAA 0.214046960416
CATGAAAC 0.00784652710183
CATGAAAG -0.124644464394
CATGAACA 0.023112460593
CATGAACC -0.21430355846
CATGAACG 0.0656588242329
CATGAAGA 0.154534128082
CATGAAGC -0.170120883885
CATGAAGG 0.0651916238641
CATGAATA 0.0654679575841
CATGAATC 0.0596559789636
CATGAATG -0.0496230005956
CATGACAA 0.0446646230732
CATGACAC -0.0474873213462
CATGACAG -0.181781872791
CATGACCA -0.141516066153
CATGACCC -0.244418401341
CATGACCG -0.107601848194
CATGACGA -0.0476582950561
CATGACGC -0.172159122085
CATGACGG -0.329379777328
CATGACTA -0.0516755024312
CATGACTC -0.094327399075
CATGAGAA 0.140898987404
CATGAGAC -0.0259179465653
CATGAGAG 0.0541063152848
CATGAGCA -0.0339764357212
CATGAGCC -0.217439101252
CATGAGCG -0.0758867102397
CATGAGGA -0.153223557301
CATGAGGC -0.216855702065
CATGAGGG -0.0364618736383
CATGAGTA 0.0884919283339
CATGAGTC -0.168430814142
CATGATAA 0.114263020095
CATGATAC 0.201044479571
CATGATAG 0.0859898131682
CATGATCA 0.0667001470943
CATGATCC 0.173019715032
CATGATCG 0.0258901980155
CATGATGA -0.16169133991
CATGATGC -0.0587855935366
CATGATGG -0.0689644153958
CATGATTA 0.159633032558

CATGATTC 0.243887681517
CATGCAAA 0.0374139019237
CATGCAAC 0.0767785800415
CATGCAAG 0.10962095765
CATGCACA -0.126040873724
CATGCACC -0.222731874753
CATGCACG -0.101248466385
CATGCAGA 0.111966924476
CATGCAGC -0.214705446645
CATGCAGG -0.165041071987
CATGCATA -0.0731382686777
CATGCATC -0.193383151937
CATGCATG -0.0580452727542
CATGCCAA 0.0305234472766
CATGCCAC -0.0776299158834
CATGCCAG -0.267435352987
CATGCCCA -0.0968394305597
CATGCCCC -0.195318399926
CATGCCCG -0.0452357825302
CATGCCGA 0.061845485082
CATGCCGC -0.141949698995
CATGCCGG -0.0593749512574
CATGCCTA -0.27603557926
CATGCCTC -0.105124183007
CATGCGAA 0.105289316408
CATGCGAC -0.0775641416739
CATGCGAG -0.0519475308642
CATGCGCA -0.0796311655491
CATGCGCC -0.328022996829
CATGCGCG -0.11352640678
CATGCGGA 0.024706909043
CATGCGGC -0.296864402155
CATGCGGG -0.132159031819
CATGCGTA -0.149070827778
CATGCGTC -0.0620586419753
CATGCTAA 0.0311434922203
CATGCTAC -0.120776406036
CATGCTAG -0.0708930001352
CATGCTCA -0.166533042847
CATGCTCC -0.222075438568
CATGCTCG -0.0480724980955
CATGCTGA -0.141832938233
CATGCTGC -0.205844285792
CATGCTGG -0.115650935329
CATGCTTA -0.0747572209861
CATGCTTC -0.110949229554
CATGGAAA 0.134308935902
CATGGAAC -0.327740197611
CATGGAAG -0.0960671012775
CATGGACA -0.0550177264096
CATGGACC -0.280281055594
CATGGACG -0.0773480208128
CATGGAGA 0.0849826560407
CATGGAGC -0.339395236058

CATGGAGG -0.0267239071155
CATGGATA 0.304933680066
CATGGATC 0.103772859997
CATGGCAA 0.0117272275631
CATGGCAC -0.153224345994
CATGGCAG -0.202108746111
CATGGCCA -0.0967324457562
CATGGCCC -0.297801923317
CATGGCCG -0.110143673007
CATGGCGA -0.16017481463
CATGGCGC -0.213721004236
CATGGCGG -0.205827160494
CATGGCTA -0.213055788763
CATGGCTC -0.102321126699
CATGGGAA 0.134278469341
CATGGGAC -0.171180827887
CATGGGAG -0.0552556496272
CATGGGCA -0.0426492740024
CATGGGCC -0.262104977286
CATGGGCG -0.06683960989
CATGGGGA -0.0864051190025
CATGGGGC -0.218034346185
CATGGGGG -0.0492137976455
CATGGGTA -0.0689607859279
CATGGGTC -0.21369932349
CATGGTAA 0.129095957797
CATGGTAC -0.0483426563247
CATGGTAG -0.272213477076
CATGGTCA -0.281755633467
CATGGTCC -0.3138702136
CATGGTCG -0.180720902909
CATGGTGA -0.144287760545
CATGGTGC -0.0738762102351
CATGGTGG -0.257531885915
CATGGTTA -0.000886320480314
CATGGTTC -0.1477046401
CATGTAAA 0.195041622478
CATGTAAC 0.0887670420865
CATGTAAG 0.0363344969154
CATGTACA -0.0219790534532
CATGTACC -0.0228054477421
CATGTACG -0.238328858345
CATGTAGA 0.121690377396
CATGTAGC -0.150948595434
CATGTAGG 0.0127033524336
CATGTATA -0.0283211486531
CATGTATC -0.0608355836572
CATGTCAA -0.0951796376738
CATGTCAC -0.133208193374
CATGTCAG -0.130462940095
CATGTCCA -0.018820914387
CATGTCCC -0.0811269182638
CATGTCCG -0.0779391105843
CATGTCGA -0.249787348469

CATGTCGC -0.0328505222786
CATGTCGG -0.215895919334
CATGTCTA -0.0855230662847
CATGTCTC 0.00983684663119
CATGTGAA 0.152108461144
CATGTGAC -0.169317003309
CATGTGAG -0.206164110995
CATGTGCA -0.00686103224867
CATGTGCC -0.103777557689
CATGTGCG 0.0881748246567
CATGTGGA 0.0525576395043
CATGTGGC -0.0769102023794
CATGTGGG -0.207061844888
CATGTGTA -0.0422793007876
CATGTGTC -0.11247947932
CATGTTAA 0.0631681652011
CATGTTAC -0.0543481243654
CATGTTAG -0.0877921926983
CATGTTCA -0.115214113696
CATGTTCC -0.0106397572445
CATGTTCG -0.270691329975
CATGTTGA 0.0472760104503
CATGTTGC -0.15766190782
CATGTTGG -0.0310380030297
CATGTTTA 0.0140261037564
CATGTTTC -0.0362453585603
CATTAAAA -0.0722889706609
CATTAAAC -0.0833454393315
CATTAAAG -0.0599519199104
CATTAACA 0.113444132901
CATTAACC 0.0332936135533
CATTAACG 0.0345699137193
CATTAAGA -0.00585575202784
CATTAAGC -0.0970588923527
CATTAAGG -0.180796296296
CATTAATA 0.0377275434748
CATTAATC 0.135177894324
CATTAATG 0.0818092040292
CATTACAA 0.102934638909
CATTACAC 0.077539842808
CATTACAG 0.000210654773147
CATTACCA 0.235103801955
CATTACCC -0.118921031148
CATTACCG -0.151929311827
CATTACGA 0.262663257449
CATTACGC 0.0273925839413
CATTACGG -0.0570045445564
CATTACTA 0.0466418723577
CATTACTC -0.062785707622
CATTAGAA 0.139574041521
CATTAGAC -0.217043949148
CATTAGAG 0.0867213357704
CATTAGCA -0.0292129035091
CATTAGCC -0.197977432775

CATTAGCG 0.00672061365915
CATTAGGA 0.0176238141589
CATTAGGC -0.196897603486
CATTAGGG -0.243867405241
CATTAGTA -0.00893422665915
CATTAGTC -0.160151619913
CATTATAA -0.0373397431731
CATTATAC -0.0273167585961
CATTATAG 0.0959263725248
CATTATCA 0.149820580245
CATTATCC 0.0805064431305
CATTATCG 0.0271076373251
CATTATGA 0.172848778929
CATTATGC 0.0526302790813
CATTATGG -0.00706287033594
CATTATTA 0.189904551596
CATTATTC 0.271287514545
CATTCAAA 0.162876792026
CATTCAAC 0.0570450604107
CATTCAAG -0.0296493885706
CATTCACA 0.00301323849042
CATTCACC -0.0503496234824
CATTCACG -0.0949225010428
CATTCAGA 0.0383687924718
CATTCAGC -0.278322639661
CATTCAGG -0.123456198304
CATTCATA 0.142824211289
CATTCATC -0.013027179521
CATTCCAA 0.00633676368898
CATTCCAC -0.201705362408
CATTCCAG -0.186255952773
CATTCCCA -0.0249577258543
CATTCCCC -0.189099286495
CATTCCCG -0.115016313237
CATTCCGA 0.0467683154405
CATTCCGC -0.0150434949645
CATTCCGG -0.0329362369794
CATTCCTA -0.0301378734989
CATTCCTC -0.0345664987323
CATTCGAA 0.143453097497
CATTCGAC -0.219070114507
CATTCGAG 0.0569247308704
CATTCGCA -0.0122053957639
CATTCGCC -0.127029774873
CATTCGCG -0.0602075728741
CATTCGGA -0.0652610259037
CATTCGGC -0.115308641975
CATTCGGG 0.133916337551
CATTCGTA -0.0385357381499
CATTCGTC -0.178778208484
CATTCTAA -0.00643731930905
CATTCTAC 0.128492147662
CATTCTAG 0.0269129632589
CATTCTCA -0.0472850604235

CATTCTCC -0.0248386353158
CATTCTCG 0.0153080644458
CATTCTGA 0.00690332440395
CATTCTGC -0.134552169594
CATTCTGG -0.0846394898306
CATTCTTA -0.0161142246201
CATTCTTC 0.0721470806154
CATTGAAA 0.014994840721
CATTGAAC 0.00314529368181
CATTGAAG 0.0104854422217
CATTGACA 0.0983720828119
CATTGACC -0.0437138817544
CATTGACG -0.175296629238
CATTGAGA -0.112400288946
CATTGAGC -0.189170660857
CATTGAGG -0.0779011560173
CATTGATA 0.160170527512
CATTGATC -0.0349204781191
CATTGCAA -0.0556625725795
CATTGCAC -0.0287985555446
CATTGCAG 0.138984208156
CATTGCCA -0.0555055174297
CATTGCCC -0.146367499644
CATTGCCG -0.17705503696
CATTGCGA 0.0918701785481
CATTGCGC -0.0685443149136
CATTGCGG -0.14136678628
CATTGCTA -0.078160843843
CATTGCTC -0.0764101550924
CATTGGAA 0.0613558423055
CATTGGAC -0.124479977688
CATTGGAG 0.0830616478957
CATTGGCA 0.0328011273662
CATTGGCC -0.322954305167
CATTGGCG -0.187474762733
CATTGGGA 0.156109844339
CATTGGGC -0.333827062967
CATTGGGG -0.385570420102
CATTGGTA -0.0136053927622
CATTGGTC -0.106431517337
CATTGTAA -0.0470739318151
CATTGTAC 0.0310764231926
CATTGTAG -0.101575095938
CATTGTCA -0.1860358031
CATTGTCC -0.18922085048
CATTGTCG -0.0375425423213
CATTGTGA -0.03730139129
CATTGTGC -0.105293972368
CATTGTGG -0.215825860949
CATTGTTA -0.0420614237694
CATTGTTC -0.140385688056
CATTTAAA 0.0533374759826
CATTTAAC 0.109003490746
CATTTAAG 0.124066944634

CATTTACA 0.144876730638
CATTTACC -0.116157624654
CATTTACG -0.100505449636
CATTTAGA 0.139635607033
CATTTAGC 0.0060558546101
CATTTAGG 0.104025335353
CATTTATA 0.055643707399
CATTTATC 0.0879607165168
CATTTCAA 0.0173623116819
CATTTCAC -0.186203810775
CATTTCAG -0.096867781442
CATTTCCA -0.133897832359
CATTTCCC 0.0543617862083
CATTTCCG 0.0578042572221
CATTTCGA -0.0828147123712
CATTTCGC 0.0975172341874
CATTTCGG -0.1319063692
CATTTCTA -0.120302630585
CATTTCTC 0.0437852642209
CATTTGAA 0.146500951578
CATTTGAC -0.254179688862
CATTTGAG 0.00164020164747
CATTTGCA -0.00335594845496
CATTTGCC -0.0644196359964
CATTTGCG 0.017588947162
CATTTGGA 0.0885639640826
CATTTGGC -0.0927563194925
CATTTGGG 0.165889907457
CATTTGTA 0.0487716525061
CATTTGTC -0.121761161246
CATTTTAA -0.0383954642253
CATTTTAC 0.170399384323
CATTTTAG 0.198269725277
CATTTTCA -0.0995461703401
CATTTTCC -0.121365103958
CATTTTCG 0.0285489253332
CATTTTGA 0.121410390076
CATTTTGC -0.171751400941
CATTTTGG -0.0332005605576
CATTTTTA 0.0138407449915
CATTTTTC -0.0367053458375
CCAAAAAA 0.0831999186437
CCAAAAAC 0.154386704052
CCAAAAAG -0.0625970747235
CCAAAACA 0.0652608487208
CCAAAACC -0.182424110385
CCAAAACG 0.030711431645
CCAAAAGA 0.0283431030319
CCAAAAGC -0.134059244839
CCAAAAGG -0.115838512024
CCAAAATA 0.224562346549
CCAAAATC 0.362236978605
CCAAACAA 0.124456292767
CCAAACAC 0.0646622986068

CCAAACAG 0.107362747519
CCAAACCA -0.0131246775769
CCAAACCC -0.192599542409
CCAAACCG -0.104292542654
CCAAACGA 0.0438583237691
CCAAACGC -0.184891632373
CCAAACGG -0.136177790949
CCAAACTA 0.124788689104
CCAAACTC -0.281291846154
CCAAAGAA 0.067764331385
CCAAAGAC -0.104320602037
CCAAAGAG 0.072185176038
CCAAAGCA -0.0303588912228
CCAAAGCC -0.301690406833
CCAAAGCG -0.090033494755
CCAAAGGA -0.0187518520348
CCAAAGGC -0.260452498121
CCAAAGGG -0.14405152832
CCAAAGTA -0.0420096360683
CCAAAGTC -0.212708683197
CCAAATAA 0.0655688405271
CCAAATAC 0.125225032383
CCAAATAG -0.258167029775
CCAAATCA 0.165807701596
CCAAATCC 0.375016465784
CCAAATCG 0.183072076226
CCAAATGA 0.0807737567736
CCAAATGC 0.152327713012
CCAAATGG -0.036342832683
CCAAATTA 0.158750889835
CCAAATTC 0.144791872489
CCAACAAA 0.218157917847
CCAACAAC -0.0361849327098
CCAACAAG -0.204116369044
CCAACACA -0.171898329702
CCAACACC -0.176139433551
CCAACACG -0.0857151481225
CCAACAGA -0.001175308355
CCAACAGC -0.10907768722
CCAACAGG -0.142944159094
CCAACATA 0.0475806274561
CCAACATC 0.0303217414213
CCAACCAA -0.126069096149
CCAACCAC -0.0965676832298
CCAACCAG -0.109649173775
CCAACCCA -0.0360641836264
CCAACCCC -0.319435003631
CCAACCCG -0.189794412505
CCAACCGA -0.130057676583
CCAACCGC -0.225621870316
CCAACCGG -0.17111544603
CCAACCTA -0.306262890341
CCAACCTC -0.218596322041
CCAACGAA -0.0329672440668

CCAACGAC -0.227103362746
CCAACGAG -0.209937996428
CCAACGCA -0.18628220591
CCAACGCC -0.146103949556
CCAACGCG -0.0911580219582
CCAACGGA -0.0334140077052
CCAACGGC -0.318706808501
CCAACGGG -0.119157738164
CCAACGTA 0.0563733561659
CCAACGTC -0.113048297965
CCAACTAA 0.0205991312307
CCAACTAC -0.21090065874
CCAACTAG -0.137811266157
CCAACTCA -0.0147915928201
CCAACTCC -0.0160225777308
CCAACTCG -0.214067781729
CCAACTGA -0.0657052073558
CCAACTGC 0.0460218665613
CCAACTGG 0.103938463458
CCAACTTA -0.13231590231
CCAACTTC -0.154342760516
CCAAGAAA 0.0763049699999
CCAAGAAC -0.238949710544
CCAAGAAG -0.181683869162
CCAAGACA -0.0726107933668
CCAAGACC -0.328096797731
CCAAGACG -0.0780735722606
CCAAGAGA 0.0096886667732
CCAAGAGC -0.0922171183334
CCAAGAGG -0.146472098616
CCAAGATA 0.250354014358
CCAAGATC 0.285193148635
CCAAGCAA -0.0659058881641
CCAAGCAC -0.101713749506
CCAAGCAG -0.00398726708927
CCAAGCCA -0.191238841429
CCAAGCCC -0.27407462269
CCAAGCCG -0.121039978924
CCAAGCGA -0.102438100882
CCAAGCGC -0.404952480869
CCAAGCGG -0.132465123426
CCAAGCTA -0.220964262508
CCAAGCTC -0.15097622831
CCAAGGAA 0.024709154957
CCAAGGAC -0.368517032831
CCAAGGAG -0.25651206562
CCAAGGCA -0.17536523227
CCAAGGCC -0.205882352941
CCAAGGCG -0.250184458969
CCAAGGGA -0.0769760348584
CCAAGGGC -0.29425706684
CCAAGGGG -0.111558460421
CCAAGGTA 0.0268201126148
CCAAGGTC -0.129556143659

CCAAGTAA -0.0797355595073
CCAAGTAC -0.113751249216
CCAAGTAG -0.0703038068094
CCAAGTCA -0.0189603823747
CCAAGTCC -0.132945592303
CCAAGTCG -0.120916465011
CCAAGTGA 0.107749678949
CCAAGTGC -0.074154288552
CCAAGTGG -0.205001371742
CCAAGTTA -0.0322509824585
CCAAGTTC -0.153971938258
CCAATAAA 0.0952408395354
CCAATAAC -0.113155249258
CCAATAAG -0.0531997733645
CCAATACA -0.0787649809035
CCAATACC -0.0488484928319
CCAATACG -0.0434029795245
CCAATAGA -0.100196398014
CCAATAGC -0.0239714373532
CCAATAGG -0.201360426646
CCAATATA 0.196991152385
CCAATATC 0.308016503712
CCAATCAA -0.161244470787
CCAATCAC 0.0639678642512
CCAATCAG -0.0474516454807
CCAATCCA 0.0064134229072
CCAATCCC -0.0775414274758
CCAATCCG 0.207753548443
CCAATCGA -0.00366539305275
CCAATCGC -0.0908299798062
CCAATCGG -0.11887160067
CCAATCTA 0.217658681156
CCAATCTC 0.183294350094
CCAATGAA -0.0839497707608
CCAATGAC -0.0908265830287
CCAATGAG -0.091834422658
CCAATGCA -0.173834063244
CCAATGCC -0.263037037037
CCAATGCG 0.0485768073942
CCAATGGA -0.055075350961
CCAATGGC -0.153945224066
CCAATGGG -0.21030355846
CCAATGTA -0.0340142083013
CCAATGTC 0.103358854038
CCAATTAA -0.0492539915448
CCAATTAC 0.0572211794922
CCAATTAG -0.153831556071
CCAATTCA -0.113571491705
CCAATTCC 0.12308448045
CCAATTCG -0.0956056662096
CCAATTGA -0.0141378689182
CCAATTGC 0.00296805311406
CCAATTGG -0.146180363852
CCAATTTA -0.0201458602705

CCAATTTC -0.0146630250025
CCACAAAA 0.0389943776967
CCACAAAC -0.133592395003
CCACAAAG -0.145157495069
CCACAACA 0.148253018174
CCACAACC -0.148819172113
CCACAACG 0.0816565570425
CCACAAGA 0.0527133900088
CCACAAGC -0.200357298475
CCACAAGG -0.25466071938
CCACAATA 0.122881466738
CCACAATC 0.284353435126
CCACACAA 0.165596443566
CCACACAC -0.300232087304
CCACACAG 0.0226322202463
CCACACCA 0.0691370831412
CCACACCC -0.158868914351
CCACACCG -0.0210789479462
CCACACGA -0.0747627708596
CCACACGC -0.218044424821
CCACACGG -0.281078729077
CCACACTA -0.00823797371093
CCACACTC -0.0917303652829
CCACAGAA -0.00960731044702
CCACAGAC -0.115216778666
CCACAGAG -0.220204506525
CCACAGCA -0.13852512603
CCACAGCC -0.177546250576
CCACAGCG -0.227184533699
CCACAGGA 0.0623145874798
CCACAGGC -0.10975223024
CCACAGGG -0.201605238471
CCACAGTA -0.247626115776
CCACAGTC -0.208242323198
CCACATAA -0.129067453111
CCACATAC 0.0693072295769
CCACATAG -0.0659356941769
CCACATCA -0.17578002799
CCACATCC 0.108369532056
CCACATCG -0.283268031959
CCACATGA 0.1638908663
CCACATGC -0.266183534696
CCACATGG 0.0507640123823
CCACATTA -0.0804757729922
CCACATTC 0.231500878939
CCACCAAA 0.107702693802
CCACCAAC -0.081099683935
CCACCAAG -0.124508723409
CCACCACA -0.106720452238
CCACCACC -0.12527491364
CCACCACG -0.298957733344
CCACCAGA -0.0615545077121
CCACCAGC -0.0848764400796
CCACCAGG -0.186409115207

CCACCATA 0.122066346133
CCACCATC -0.125047332115
CCACCCAA -0.168983551332
CCACCCAC 0.0338016670589
CCACCCAG -0.234987729972
CCACCCCA -0.106998222047
CCACCCCC 0.0794065354312
CCACCCCG -0.113379389686
CCACCCGA -0.18104788941
CCACCCGC -0.25256824697
CCACCCGG -0.00957534903317
CCACCCTA 0.0407691287697
CCACCCTC -0.205430547577
CCACCGAA -0.0170692153183
CCACCGAC -0.241359324237
CCACCGAG -0.112112581764
CCACCGCA -0.0527651329187
CCACCGCC 0.00166892773209
CCACCGCG -0.156855481858
CCACCGGA 0.125208838784
CCACCGGC -0.104949891068
CCACCGGG -0.131524691358
CCACCGTA 0.000461041954818
CCACCGTC -0.169327129459
CCACCTAA -0.115095240447
CCACCTAC -0.19696934483
CCACCTAG 0.0468888469194
CCACCTCA 0.0509467358195
CCACCTCC -0.338501555095
CCACCTCG -0.172334145324
CCACCTGA -0.0465227434515
CCACCTGC -0.187377444928
CCACCTGG -0.0656361122086
CCACCTTA -0.161998593767
CCACCTTC -0.255605837042
CCACGAAA 0.0453700731009
CCACGAAC -0.204313648023
CCACGAAG -0.0137485400166
CCACGACA -0.0958502090948
CCACGACC -0.055892519971
CCACGACG -0.0130560769205
CCACGAGA 0.0137167097458
CCACGAGC -0.384668334046
CCACGAGG -0.127868596075
CCACGATA 0.0687882265774
CCACGATC 0.0956445309663
CCACGCAA 0.115674877604
CCACGCAC -0.249175925926
CCACGCAG -0.231707852466
CCACGCCA -0.128279835391
CCACGCCC -0.291296332872
CCACGCCG -0.29454028414
CCACGCGA 0.0768793835819
CCACGCGC -0.0854996350625

CCACGCGG -0.0905
CCACGCTA 0.0868405453468
CCACGCTC -0.175090411199
CCACGGAA 0.0903418418491
CCACGGAC 0.0322427495664
CCACGGAG -0.13457412072
CCACGGCA -0.0762636988745
CCACGGCC -0.12202679264
CCACGGCG -0.129436680767
CCACGGGA -0.0577674592197
CCACGGGC -0.163643149454
CCACGGGG -0.112233306993
CCACGGTA -0.138264926674
CCACGGTC -0.241128828439
CCACGTAA 0.0814565687969
CCACGTAC -0.0636636377105
CCACGTAG -0.219034935094
CCACGTCA -0.0375502206415
CCACGTCC -0.221227368861
CCACGTCG -0.187333366249
CCACGTGA -0.116135175033
CCACGTGC -0.326573260025
CCACGTGG 0.0478930041152
CCACGTTA 0.0828256383236
CCACGTTC -0.308892169194
CCACTAAA 0.019769337153
CCACTAAC -0.143005690267
CCACTAAG -0.146657994687
CCACTACA -0.243031208335
CCACTACC -0.203378235091
CCACTACG -0.108758744509
CCACTAGA 0.149883050281
CCACTAGC -0.16295581844
CCACTAGG -0.217365853942
CCACTATA -0.22319355954
CCACTATC -0.0741193549804
CCACTCAA 0.0865276954489
CCACTCAC 0.13627911031
CCACTCAG -0.147340489754
CCACTCCA -0.105804487161
CCACTCCC -0.146320951216
CCACTCCG -0.210453836298
CCACTCGA -0.119382161201
CCACTCGC -0.163626090468
CCACTCGG 0.0516278035767
CCACTCTA -0.0285866198697
CCACTCTC -0.241445474432
CCACTGAA -0.146410942507
CCACTGAC 0.175160266965
CCACTGAG -0.0574650548256
CCACTGCA -0.047535307375
CCACTGCC -0.0466837498075
CCACTGCG -0.10988196948
CCACTGGA -0.0189749102901

CCACTGGC -0.17126394924
CCACTGGG -0.0805354989613
CCACTGTA -0.10634109919
CCACTGTC -0.278040803523
CCACTTAA -0.0557119496027
CCACTTAC 0.0760883883291
CCACTTAG -0.137045701447
CCACTTCA 0.0210257676793
CCACTTCC -0.217864332045
CCACTTCG -0.267986766086
CCACTTGA -0.106459111182
CCACTTGC -0.140511739949
CCACTTTA 0.0063585369602
CCACTTTC -0.0816622213441
CCAGAAAA 0.0499736547454
CCAGAAAC -0.0800049968624
CCAGAAAG -0.125457471158
CCAGAACA -0.132376907502
CCAGAACC -0.0794199653121
CCAGAACG -0.0293739995875
CCAGAAGA 0.00267239272735
CCAGAAGC -0.116314980578
CCAGAAGG -0.231348442206
CCAGAATA -0.0483188434433
CCAGAATC 0.403482341319
CCAGACAA -0.175485095922
CCAGACAC -0.137558167611
CCAGACAG -0.201330877996
CCAGACCA -0.32894538147
CCAGACCC -0.22261018949
CCAGACCG -0.0571333706521
CCAGACGA -0.162022525441
CCAGACGC -0.147692203239
CCAGACGG -0.140938271605
CCAGACTA -0.254988614051
CCAGACTC -0.263720147943
CCAGAGAA 0.183518079991
CCAGAGAC -0.170088129417
CCAGAGAG -0.158356961908
CCAGAGCA 0.0865037376634
CCAGAGCC -0.0532502846566
CCAGAGCG -0.226855051951
CCAGAGGA -0.216097756503
CCAGAGGC -0.298104695369
CCAGAGGG -0.167430003457
CCAGAGTA -0.000623966555393
CCAGAGTC -0.110337372141
CCAGATAA 0.029449054864
CCAGATAC 0.344135878692
CCAGATAG -0.0703171310018
CCAGATCA -0.0236470878794
CCAGATCC 0.192908924498
CCAGATCG 0.311212645098
CCAGATGA 0.109201981608

CCAGATGC -0.0277187812133
CCAGATGG -0.182326797386
CCAGATTA 0.298398428066
CCAGATTC 0.475787776212
CCAGCAAA 0.00668429387067
CCAGCAAC -0.00761225165755
CCAGCAAG 0.102875474763
CCAGCACA -0.112916858767
CCAGCACC -0.379695907588
CCAGCACG -0.174092603407
CCAGCAGA 0.106703180161
CCAGCAGC -0.203713647361
CCAGCAGG -0.195176604192
CCAGCATA 0.0384188815334
CCAGCATC -0.0506215526467
CCAGCCAA 0.0488259405693
CCAGCCAC -0.0512417332126
CCAGCCAG -0.311697326309
CCAGCCCA -0.144040966179
CCAGCCCC -0.038118506984
CCAGCCCG -0.256854256854
CCAGCCGA 0.0425722847922
CCAGCCGC -0.232322733557
CCAGCCGG -0.051440125987
CCAGCCTA -0.206399393494
CCAGCCTC -0.144166241406
CCAGCGAA 0.0300484595037
CCAGCGAC -0.333134379511
CCAGCGAG -0.0444394180472
CCAGCGCA 0.0503786884095
CCAGCGCC -0.219937238379
CCAGCGCG -0.231228557402
CCAGCGGA 0.0387096305551
CCAGCGGC -0.176746227709
CCAGCGGG -0.250343517327
CCAGCGTA -0.0891737510968
CCAGCGTC -0.135177336281
CCAGCTAA -0.0804175285286
CCAGCTAC -0.202346660522
CCAGCTAG -0.311163844179
CCAGCTCA -0.238913580247
CCAGCTCC -0.298765736855
CCAGCTCG -0.13939518674
CCAGCTGA -0.0814054644449
CCAGCTGC -0.162570962174
CCAGCTGG -0.18321978673
CCAGCTTA -0.107981616194
CCAGCTTC -0.281883921181
CCAGGAAA -0.0187243408198
CCAGGAAC -0.00330154285034
CCAGGAAG -0.127628330084
CCAGGACA -0.017017771696
CCAGGACC -0.365369529201
CCAGGACG -0.144628824795

CCAGGAGA 0.0013968473512
CCAGGAGC -0.324644770015
CCAGGAGG -0.153634473033
CCAGGATA 0.256070489445
CCAGGATC 0.346964258271
CCAGGCAA -0.0492927056732
CCAGGCAC -0.325274568535
CCAGGCAG -0.26595727171
CCAGGCCA -0.196490296775
CCAGGCCC -0.282786637919
CCAGGCCG -0.149549223281
CCAGGCGA 0.0713572196975
CCAGGCGC -0.216257508336
CCAGGCGG -0.0175650164908
CCAGGCTA -0.05522630493
CCAGGCTC -0.306630336766
CCAGGGAA 0.0701730684865
CCAGGGAC -0.136377353107
CCAGGGAG -0.154885984023
CCAGGGCA -0.25815315263
CCAGGGCC -0.300230031281
CCAGGGCG -0.172525391034
CCAGGGGA -0.129643518519
CCAGGGGC -0.132232538742
CCAGGGGG -0.225691610043
CCAGGGTA -0.130521878332
CCAGGGTC -0.125873638344
CCAGGTAA -0.135147697957
CCAGGTAC -0.119219421263
CCAGGTAG -0.133481669405
CCAGGTCA -0.283258343088
CCAGGTCC -0.353908855538
CCAGGTCG -0.389473913485
CCAGGTGA -0.119061728395
CCAGGTGC -0.347829441098
CCAGGTTA -0.0717516650462
CCAGGTTC -0.28569644154
CCAGTAAA 0.0345691746691
CCAGTAAC -0.194581632205
CCAGTAAG -0.16833903727
CCAGTACA 0.041205526419
CCAGTACC -0.185903152806
CCAGTACG -0.174705969495
CCAGTAGA -0.0268231459127
CCAGTAGC -0.0274539826797
CCAGTAGG -0.0223078316129
CCAGTATA -0.174915916361
CCAGTATC 0.188219313411
CCAGTCAA -0.103801025168
CCAGTCAC -0.176134430727
CCAGTCAG -0.393461599653
CCAGTCCA -0.0684618491047
CCAGTCCC -0.237909819994
CCAGTCCG -0.117533924858

CCAGTCGA -0.106580499986
CCAGTCGC -0.109116302552
CCAGTCGG -0.256008386767
CCAGTCTA -0.139768219007
CCAGTCTC -0.173566870243
CCAGTGAA -0.131933125895
CCAGTGAC -0.356021330173
CCAGTGAG -0.249392186878
CCAGTGCA -0.0903939290933
CCAGTGCC -0.245266819182
CCAGTGCG -0.0912064945294
CCAGTGGA -0.0017663327678
CCAGTGGC -0.180360587538
CCAGTGGG -0.176472040883
CCAGTGTA -0.0845228099636
CCAGTGTC -0.0655464467381
CCAGTTAA -0.175955195539
CCAGTTAC -0.0464397288353
CCAGTTAG 0.00294066791819
CCAGTTCA -0.0924334469304
CCAGTTCC -0.182539290575
CCAGTTCG -0.273369047342
CCAGTTGA -0.0235143049713
CCAGTTGC -0.108155217145
CCAGTTTA -0.0272500108959
CCAGTTTC -0.229179698217
CCATAAAA 0.115251405576
CCATAAAC -0.10590995598
CCATAAAG -0.00197569890349
CCATAACA -0.0415606192562
CCATAACC -0.0444559693547
CCATAACG 0.0820086295817
CCATAAGA -0.0143471865464
CCATAAGC -0.225304284677
CCATAAGG -0.257840667753
CCATAATA 0.143383260936
CCATAATC 0.280105472666
CCATACAA 0.0888977670594
CCATACAC -0.00292267667786
CCATACAG -0.0646922604153
CCATACCA 0.0361533732903
CCATACCC -0.000362229357043
CCATACCG -0.181347775747
CCATACGA 0.111468336408
CCATACGC -0.161928201414
CCATACGG -0.110585583829
CCATACTA -0.187940051475
CCATACTC -0.148871817817
CCATAGAA -0.020396004149
CCATAGAC -0.182433708407
CCATAGAG -0.112016595051
CCATAGCA -0.126647787766
CCATAGCC -0.0412961865244
CCATAGCG -0.0692811425948

CCATAGGA 0.0102499506026
CCATAGGC -0.181526111029
CCATAGGG -0.311770919067
CCATAGTA -0.0862330830781
CCATAGTC -0.263643996641
CCATATAA -0.00819996048212
CCATATAC 0.0432803135085
CCATATAG -0.17784802138
CCATATCA 0.199804057169
CCATATCC 0.290757340229
CCATATCG 0.255013335968
CCATATGA 0.135568289095
CCATATGC -0.0575975550236
CCATATGG 0.0683724279835
CCATATTA 0.00652727258546
CCATATTC 0.186316156495
CCATCAAA 0.0566283613964
CCATCAAC -0.0983699678933
CCATCAAG -0.208395309922
CCATCACA -0.201184812016
CCATCACC -0.037490994027
CCATCACG -0.181098496293
CCATCAGA -0.00489294181567
CCATCAGC -0.223299067294
CCATCAGG -0.0911548282704
CCATCATA 0.0816890154432
CCATCATC -0.000229137509948
CCATCCAA -0.0812241221507
CCATCCAC -0.113619274992
CCATCCAG -0.0110746299014
CCATCCCA -0.186911514685
CCATCCCC -0.00380303254803
CCATCCCG -0.0514234602046
CCATCCGA -0.184349419536
CCATCCGC -0.0564170946925
CCATCCGG -0.0707446414461
CCATCCTA -0.220677564324
CCATCCTC 0.019791379135
CCATCGAA -0.01276921557
CCATCGAC -0.179182146415
CCATCGAG -0.141213308744
CCATCGCA 0.106058140717
CCATCGCC -0.135805374001
CCATCGCG 0.0683213962295
CCATCGGA -0.177368103078
CCATCGGC -0.200769284403
CCATCGGG -0.2481315027
CCATCGTA -0.180871093807
CCATCGTC -0.0350604234581
CCATCTAA -0.0587993150234
CCATCTAC -0.182490064378
CCATCTAG -0.163763517851
CCATCTCA 0.0216264352675
CCATCTCC -0.148505518065

CCATCTCG -0.144686157341
CCATCTGA -0.0685244642024
CCATCTGC -0.0926388529833
CCATCTTA 0.0053273865733
CCATCTTC -0.160803100379
CCATGAAA 0.188504060552
CCATGAAC -0.122811404159
CCATGAAG 0.0313728395492
CCATGACA -0.181105019674
CCATGACC -0.255620808537
CCATGACG -0.09751580403
CCATGAGA -0.0858933941569
CCATGAGC 0.0223838535266
CCATGAGG -0.131308018961
CCATGATA 0.220589869604
CCATGATC 0.0497156907945
CCATGCAA 0.091977685122
CCATGCAC -0.124783918605
CCATGCAG -0.0958959481977
CCATGCCA -0.239128729669
CCATGCCC -0.0528348531373
CCATGCCG -0.222079568087
CCATGCGA 0.0163316376089
CCATGCGC -0.341970755224
CCATGCGG -0.197072655903
CCATGCTA -0.0572236763407
CCATGCTC -0.227234815437
CCATGGAA 0.0125185258374
CCATGGAC -0.157906433326
CCATGGAG -0.0768553272751
CCATGGCA -0.0871243928542
CCATGGCC -0.109179602834
CCATGGCG -0.245287383809
CCATGGGA 0.0329041252278
CCATGGGC -0.16261768385
CCATGGGG -0.360539179381
CCATGGTA -0.119236638046
CCATGGTC -0.204357455924
CCATGTAA 0.169437624395
CCATGTAC -0.172704243928
CCATGTAG 0.0656771228272
CCATGTCA -0.0866343178417
CCATGTCC -0.117177946187
CCATGTCG -0.157847801869
CCATGTGA -0.205586071276
CCATGTGC -0.100031378747
CCATGTTA 0.000629058736362
CCATGTTC -0.099262890162
CCATTAAA -0.0876019082927
CCATTAAC 0.128760559206
CCATTAAG -0.0792051879529
CCATTACA -0.00515595717171
CCATTACC 0.0743665415391
CCATTACG 0.0256564359261

CCATTAGA -0.0320695653078
CCATTAGC -0.08374828596
CCATTAGG -0.182069440289
CCATTATA 0.0333624228852
CCATTATC 0.14427320029
CCATTCAA -0.0462094021316
CCATTCAC -0.0261489356448
CCATTCAG -0.0422072932446
CCATTCCA 0.0197690870688
CCATTCCC -0.142832035278
CCATTCCG -0.118093021769
CCATTCGA 0.114950383104
CCATTCGC -0.162131427061
CCATTCGG -0.282498395141
CCATTCTA -0.000991895834038
CCATTCTC -0.0424726845678
CCATTGAA 0.0348031789941
CCATTGAC -0.139774872912
CCATTGAG -0.154153206126
CCATTGCA 0.0454207367033
CCATTGCC -0.0830989843587
CCATTGCG -0.0911683022679
CCATTGGA -0.0609364235484
CCATTGGC -0.197680059727
CCATTGGG -0.249617302849
CCATTGTA -0.121814342866
CCATTGTC -0.250801130029
CCATTTAA -0.00320808360227
CCATTTAC -0.0703711020023
CCATTTAG 0.134422442724
CCATTTCA -0.0788189641924
CCATTTCC -0.334398199172
CCATTTCG -0.0725180574766
CCATTTGA -0.284969676198
CCATTTGC 0.0160083099676
CCATTTTA -0.098920124573
CCATTTTC -0.153645894259
CCCAAAAA 0.226434348976
CCCAAAAC -0.101873427431
CCCAAAAG -0.165519663692
CCCAAACA 0.110910777405
CCCAAACC -0.205585455104
CCCAAACG -0.00770873125645
CCCAAAGA 0.0127627736696
CCCAAAGC -0.178216049383
CCCAAAGG -0.0699130100711
CCCAAATA -0.0539309315243
CCCAAATC 0.201414959714
CCCAACAA 0.0182528728953
CCCAACAC -0.168581256773
CCCAACAG -0.214617715327
CCCAACCA -0.160087789375
CCCAACCC -0.0124562346549
CCCAACCG -0.0791267410847

CCCAACGA -0.198809005084
CCCAACGC -0.0957927157032
CCCAACGG 0.0271950238089
CCCAACTA -0.108827855716
CCCAACTC -0.131637143861
CCCAAGAA 0.00482900917512
CCCAAGAC -0.279448796257
CCCAAGAG -0.216156378601
CCCAAGCA -0.211146229443
CCCAAGCC -0.248950669103
CCCAAGCG -0.272497092194
CCCAAGGA -0.128127713832
CCCAAGGC -0.230150108631
CCCAAGGG -0.0895946369857
CCCAAGTA -0.147587509078
CCCAAGTC -0.162451082078
CCCAATAA 0.0756427823003
CCCAATAC -0.0742121900622
CCCAATAG -0.0386350539228
CCCAATCA 0.0179065402094
CCCAATCC 0.0749015733732
CCCAATCG -0.0811231293525
CCCAATGA -0.0664727961513
CCCAATGC -0.248366335819
CCCAATTA -0.0636191942818
CCCAATTC -0.0590984434345
CCCACAAA -0.168690889544
CCCACAAC -0.11902527373
CCCACAAG -0.264447846197
CCCACACA 0.102666908165
CCCACACC -0.0561839996487
CCCACACG -0.199227962961
CCCACAGA -0.123680180543
CCCACAGC -0.273935907565
CCCACAGG 0.0331401347999
CCCACATA -0.229777777778
CCCACATC -0.193506021821
CCCACCAA -0.170415694795
CCCACCAC -0.0428755716452
CCCACCAG -0.183489090238
CCCACCCA -0.0738728653584
CCCACCCC -0.127455822074
CCCACCCG -0.167176929534
CCCACCGA -0.136610862857
CCCACCGC 0.0242458931612
CCCACCGG -0.145382798816
CCCACCTA -0.133618923936
CCCACCTC -0.118852578068
CCCACGAA -0.0545210576575
CCCACGAC -0.160318225651
CCCACGAG 0.0218728937644
CCCACGCA -0.112239480443
CCCACGCC -0.284205282528
CCCACGCG -0.293756044856

CCCACGGA -0.113100495038
CCCACGGC -0.0661230857731
CCCACGGG -0.166308417594
CCCACGTA -0.166834172314
CCCACGTC 0.0348364883123
CCCACTAA -0.0387895551385
CCCACTAC -0.145221475921
CCCACTAG -0.108958770944
CCCACTCA -0.0493100928976
CCCACTCC -0.266522338014
CCCACTCG 0.0197855679689
CCCACTGA -0.0473857823443
CCCACTGC -0.0563641962934
CCCACTTA -0.00501673979496
CCCACTTC -0.257355119395
CCCAGAAA -0.106669397645
CCCAGAAC -0.180689697241
CCCAGAAG -0.00853908687845
CCCAGACA -0.0827907692933
CCCAGACC -0.0396562973592
CCCAGACG -0.143712583547
CCCAGAGA -0.134339742746
CCCAGAGC -0.141030284074
CCCAGAGG -0.2678686563
CCCAGATA 0.260739761452
CCCAGATC 0.304898813069
CCCAGCAA 0.0375038135778
CCCAGCAC -0.15893450076
CCCAGCAG -0.0542491407587
CCCAGCCA -0.263326139305
CCCAGCCC -0.259841193315
CCCAGCCG -0.0978704023793
CCCAGCGA -0.0745715414629
CCCAGCGC -0.21975308642
CCCAGCGG -0.0466132156753
CCCAGCTA -0.215630280686
CCCAGCTC -0.279481474597
CCCAGGAA -0.134861454047
CCCAGGAC -0.32553657481
CCCAGGAG -0.285605358632
CCCAGGCA -0.201353891021
CCCAGGCC -0.35110310251
CCCAGGCG -0.0941060275962
CCCAGGGA -0.0159757208557
CCCAGGGC -0.34047556112
CCCAGGGG -0.209392883079
CCCAGGTA -0.18421572501
CCCAGGTC -0.301800572117
CCCAGTAA -0.0825225136234
CCCAGTAC -0.157911401598
CCCAGTAG 0.114344863656
CCCAGTCA -0.269608085197
CCCAGTCC -0.15602315935
CCCAGTCG -0.0769050323473

CCCAGTGA -0.153456137752
CCCAGTGC -0.0803386994152
CCCAGTTA -0.286504370155
CCCAGTTC -0.231270878722
CCCATAAA -0.104552730273
CCCATAAC 0.00799101682457
CCCATAAG -0.169555840906
CCCATACA -0.00164108981536
CCCATACC -0.0486841744189
CCCATACG -0.122585818207
CCCATAGA 0.0307383708988
CCCATAGC -0.133109808183
CCCATAGG -0.167552768313
CCCATATA -0.033311380039
CCCATATC 0.291800444554
CCCATCAA -0.186357799071
CCCATCAC -0.109848561875
CCCATCAG -0.235586042229
CCCATCCA -0.180241476282
CCCATCCC -0.122263346271
CCCATCCG -0.173073424301
CCCATCGA -0.0802064809326
CCCATCGC -0.104888451158
CCCATCGG -0.243399176955
CCCATCTA -0.276947430805
CCCATCTC -0.187009259259
CCCATGAA 0.0456376649323
CCCATGAC -0.25244916512
CCCATGAG 0.0778536457804
CCCATGCA -0.0360393997065
CCCATGCC -0.192222222222
CCCATGCG -0.142455066166
CCCATGGA -0.0209187453692
CCCATGGC -0.232614776024
CCCATGGG -0.200222167366
CCCATGTA -0.007854179016
CCCATGTC -0.0894612685058
CCCATTAA -0.0160498095646
CCCATTAC -0.0197332674735
CCCATTAG 0.110534808668
CCCATTCA -0.0922685185185
CCCATTCC -0.125028806584
CCCATTCG -0.206269751136
CCCATTGA -0.0432713959874
CCCATTGC -0.188824139464
CCCATTTA 0.0327605256659
CCCATTTC -0.285043851112
CCCCAAAA 0.0624135546335
CCCCAAAC 0.0152855175459
CCCCAAAG -0.181480749922
CCCCAACA -0.1657794953
CCCCAACC -0.223884932207
CCCCAACG -0.122801997484
CCCCAAGA -0.0419712234953

CCCCAAGC -0.153595416831
CCCCAAGG -0.0168766504083
CCCCAATA -0.104893147618
CCCCAATC 0.0264934466179
CCCCACAA -0.269859623027
CCCCACAC 0.0151539800077
CCCCACAG -0.117740023706
CCCCACCA -0.0673495873392
CCCCACCC -0.118466230937
CCCCACCG -0.0811764705882
CCCCACGA 0.124308573686
CCCCACGC -0.142342935528
CCCCACGG -0.126849673203
CCCCACTA -0.178156696216
CCCCACTC -0.100554320683
CCCCAGAA -0.137923472189
CCCCAGAC -0.0479969431679
CCCCAGAG -0.221508916324
CCCCAGCA 0.010876782843
CCCCAGCC -0.180512347813
CCCCAGCG -0.226990969219
CCCCAGGA -0.254577616154
CCCCAGGC -0.135882178658
CCCCAGGG -0.25324577432
CCCCAGTA -0.0826995258791
CCCCAGTC -0.245870296307
CCCCATAA -0.255373191447
CCCCATAC 0.0619019946828
CCCCATAG -0.103893965588
CCCCATCA -0.141501266647
CCCCATCC -0.311066479621
CCCCATCG -0.0840823468541
CCCCATGA 0.00200189269855
CCCCATGC -0.0641486740884
CCCCATTA 0.0919873061345
CCCCATTC -0.173056268826
CCCCCAAA -0.0154379725247
CCCCCAAC -0.106306463326
CCCCCAAG -0.261815019641
CCCCCACA -0.151903900171
CCCCCACC -0.22464407902
CCCCCACG -0.00935666738008
CCCCCAGA 0.0510798276666
CCCCCAGC -0.116843820917
CCCCCAGG -0.187573048875
CCCCCATA -0.2293141564
CCCCCATC -0.288076123201
CCCCCCAA -0.238684245177
CCCCCCAC -0.221344039961
CCCCCCAG -0.116139433551
CCCCCCCA -0.0962560242626
CCCCCCCC -0.158541910331
CCCCCCCG -0.360258116157
CCCCCCGA -0.185771124051

CCCCCCGC -0.237621374738
CCCCCCGG -0.22275048066
CCCCCCTA -0.231533607682
CCCCCCTC -0.305572663699
CCCCCGAA 0.0366986071323
CCCCCGAC -0.327956900761
CCCCCGAG -0.104674579266
CCCCCGCA -0.109577200435
CCCCCGCC -0.222366651465
CCCCCGCG -0.0128400460718
CCCCCGGA -0.0799197530864
CCCCCGGC -0.320224490685
CCCCCGGG -0.161218973359
CCCCCGTA 0.0143581637358
CCCCCGTC -0.141690631808
CCCCCTAA -0.261702399591
CCCCCTAC -0.203534989658
CCCCCTAG -0.107445170661
CCCCCTCA -0.349032385466
CCCCCTCC -0.164178410847
CCCCCTCG -0.252066403731
CCCCCTGA -0.149028127941
CCCCCTGC -0.21022437322
CCCCCTTA -0.387540262735
CCCCCTTC -0.0403311546841
CCCCGAAA 0.0695852702295
CCCCGAAC -0.0849987927477
CCCCGAAG -0.235327521952
CCCCGACA -0.163729259435
CCCCGACC -0.277185624917
CCCCGACG -0.129716515211
CCCCGAGA -0.0727000726216
CCCCGAGC -0.227750926561
CCCCGAGG -0.150592592593
CCCCGATA 0.148749872881
CCCCGATC 0.108259786164
CCCCGCAA -0.00952848927297
CCCCGCAC -0.167728826754
CCCCGCAG -0.160918499495
CCCCGCCA 0.00321019359937
CCCCGCCC -0.297219430875
CCCCGCCG -0.212942050945
CCCCGCGA 0.092989215778
CCCCGCGC -0.185485393604
CCCCGCGG -0.0596061384443
CCCCGCTA -0.279021137437
CCCCGCTC -0.196885095405
CCCCGGAA 0.0293545638645
CCCCGGAC -0.141983772619
CCCCGGAG -0.225923946509
CCCCGGCA -0.210568132631
CCCCGGCC -0.320935879042
CCCCGGCG -0.28665725769
CCCCGGGA -0.223997095134

CCCCGGGC -0.236604889503
CCCCGGGG -0.0937260112856
CCCCGGTA -0.197446623094
CCCCGGTC -0.345838407777
CCCCGTAA -0.0597299370163
CCCCGTAC -0.10936238199
CCCCGTAG -0.00393532240005
CCCCGTCA 0.00702424709078
CCCCGTCC -0.171451539445
CCCCGTCG -0.293265153187
CCCCGTGA -0.139432798011
CCCCGTGC -0.169438271605
CCCCGTTA -0.0809698663405
CCCCGTTC -0.248466392318
CCCCTAAA 0.0119008418673
CCCCTAAC -0.0761210165566
CCCCTAAG -0.0623649104051
CCCCTACA -0.0732250519373
CCCCTACC -0.181487951212
CCCCTACG -0.105469950393
CCCCTAGA 0.0570174355428
CCCCTAGC -0.223271501306
CCCCTAGG -0.323384204336
CCCCTATA -0.163747166933
CCCCTATC -0.277668032989
CCCCTCAA -0.31331211264
CCCCTCAC -0.234554938847
CCCCTCAG -0.240950139355
CCCCTCCA -0.296196202959
CCCCTCCC -0.246751714678
CCCCTCCG -0.304003045683
CCCCTCGA -0.121931735657
CCCCTCGC -0.148894607964
CCCCTCGG -0.277723561651
CCCCTCTA -0.166803854427
CCCCTCTC -0.1244155007
CCCCTGAA -0.363606662117
CCCCTGAC -0.231168367214
CCCCTGAG -0.178946863917
CCCCTGCA -0.232446037523
CCCCTGCC -0.283102575944
CCCCTGCG -0.0578854640058
CCCCTGGA -0.184791164997
CCCCTGGC -0.203686166056
CCCCTGTA -0.00794531692647
CCCCTGTC -0.24236566386
CCCCTTAA -0.105945978925
CCCCTTAC -0.291867717505
CCCCTTAG -0.243507455317
CCCCTTCA -0.128897955657
CCCCTTCC -0.309781172405
CCCCTTCG -0.282915816271
CCCCTTGA -0.193886117833
CCCCTTGC -0.269905578869

CCCCTTTA -0.0691048879669
CCCCTTTC -0.0849442438572
CCCGAAAA 0.0247167961895
CCCGAAAC -0.110652903991
CCCGAAAG -0.190483036645
CCCGAACA -0.141653021442
CCCGAACC 0.0453522580079
CCCGAACG -0.135608891523
CCCGAAGA 0.0676090338832
CCCGAAGC -0.276638481179
CCCGAAGG -0.297184882248
CCCGAATA 0.100854241425
CCCGAATC 0.32795675689
CCCGACAA -0.124879437418
CCCGACAC -0.232275278051
CCCGACAG -0.0396484353562
CCCGACCA -0.290591488422
CCCGACCC -0.118519927847
CCCGACCG -0.329523010073
CCCGACGA -0.175399176955
CCCGACGC -0.28195241954
CCCGACGG -0.0537743742301
CCCGACTA -0.191814944667
CCCGACTC -0.101626819469
CCCGAGAA 0.187205264917
CCCGAGAC -0.2768243834
CCCGAGAG -0.0622648525998
CCCGAGCA -0.030547735467
CCCGAGCC -0.250913399822
CCCGAGCG -0.175336504958
CCCGAGGA 0.0951208803271
CCCGAGGC -0.18441143063
CCCGAGGG -0.23022632926
CCCGAGTA -0.119320707195
CCCGAGTC -0.201202684092
CCCGATAA 0.0840047651562
CCCGATAC 0.139972106402
CCCGATAG -0.00739616172475
CCCGATCA 0.103501174559
CCCGATCC 0.118755538768
CCCGATCG 0.14723088163
CCCGATGA -0.0191688206209
CCCGATGC -0.0853694008144
CCCGATTA 0.288665320413
CCCGATTC 0.309829587552
CCCGCAAA 0.0554704907417
CCCGCAAC -0.109832879116
CCCGCAAG -0.216040391072
CCCGCACA -0.12320579375
CCCGCACC -0.178053250801
CCCGCACG -0.188140544952
CCCGCAGA -0.0790521068166
CCCGCAGC -0.250258507511
CCCGCAGG -0.0957576169078

CCCGCATA -0.00780323982616
CCCGCATC -0.0251839869604
CCCGCCAA -0.108817331753
CCCGCCAC -0.0817568123817
CCCGCCAG -0.0687407575269
CCCGCCCA -0.0299717351335
CCCGCCCC -0.294034854299
CCCGCCCG 0.20532448099
CCCGCCGA -0.118178649237
CCCGCCGC -0.10226427592
CCCGCCGG -0.267766081871
CCCGCCTA -0.092250611595
CCCGCCTC -0.250095870035
CCCGCGAA -0.137759284868
CCCGCGAC -0.215574759945
CCCGCGAG -0.146869997937
CCCGCGCA -0.141702470904
CCCGCGCC -0.263506591291
CCCGCGCG -0.0144334358916
CCCGCGGA -0.0260161999309
CCCGCGGC -0.0890313038515
CCCGCGGG -0.113763168644
CCCGCGTA 0.0790914241715
CCCGCGTC -0.2050781893
CCCGCTAA -0.191251267185
CCCGCTAC -0.24303627238
CCCGCTAG -0.0822426367692
CCCGCTCA -0.105616704014
CCCGCTCC -0.176754458162
CCCGCTCG -0.271911786489
CCCGCTGA 0.0152390831851
CCCGCTGC -0.43047430391
CCCGCTTA -0.179802469136
CCCGCTTC -0.117001953554
CCCGGAAA 0.0420539566778
CCCGGAAC -0.1862696686
CCCGGAAG -0.097
CCCGGACA -0.130455876543
CCCGGACC -0.239584019137
CCCGGACG 0.0708905462807
CCCGGAGA 0.0776967442942
CCCGGAGC -0.235541006843
CCCGGAGG -0.281110617357
CCCGGATA 0.254425929423
CCCGGATC 0.0418477902918
CCCGGCAA -0.182673316216
CCCGGCAC -0.264648399429
CCCGGCAG -0.263576348278
CCCGGCCA -0.158977060118
CCCGGCCC -0.125356110545
CCCGGCCG -0.0470746513109
CCCGGCGA -0.234562477906
CCCGGCGC -0.221103046462
CCCGGCGG -0.284487014911

CCCGGCTA −0.237521599605
CCCGGCTC −0.302633031093
CCCGGGAA 0.0530651057103
CCCGGGAC −0.185965287723
CCCGGGAG −0.252504553734
CCCGGGCA −0.0456594415011
CCCGGGCC −0.237174739545
CCCGGGCG −0.181537625913
CCCGGGGA −0.125160525536
CCCGGGGC −0.175174211248
CCCGGGTA −0.0600255743247
CCCGGGTC −0.285814599037
CCCGGTAA −0.17321149255
CCCGGTAC −0.249761086598
CCCGGTAG −0.144003357527
CCCGGTCA −0.146771901525
CCCGGTCC −0.176573716059
CCCGGTCG −0.439640327672
CCCGGTGA −0.16262531483
CCCGGTGC −0.195000027436
CCCGGTTA −0.19402608376
CCCGGTTC −0.199575048733
CCCGTAAA 0.0113637354176
CCCGTAAC −0.0677897159061
CCCGTAAG −0.200278867102
CCCGTACA −0.154367097985
CCCGTACC −0.145466721286
CCCGTACG −0.144068587106
CCCGTAGA −0.143113821162
CCCGTAGC −0.199244734931
CCCGTAGG 0.0130823878082
CCCGTATA 0.107371003326
CCCGTATC 0.242938922918
CCCGTCAA −0.133379338399
CCCGTCAC −0.165428388714
CCCGTCAG −0.118975336014
CCCGTCCA −0.191463501627
CCCGTCCC −0.250801663743
CCCGTCCG −0.204769899269
CCCGTCGA −0.25422813178
CCCGTCGC −0.297406920108
CCCGTCGG −0.186524787675
CCCGTCTA −0.270953631304
CCCGTCTC −0.237696204296
CCCGTGAA −0.0736339972575
CCCGTGAC −0.21102712407
CCCGTGAG −0.29983736768
CCCGTGCA −0.0524141297337
CCCGTGCC −0.235988380537
CCCGTGCG −0.17175186701
CCCGTGGA −0.0888163820304
CCCGTGGC −0.146349425817
CCCGTGTA −0.0702154396593
CCCGTGTC −0.157805555556

CCCGTTAA 0.00560922813186
CCCGTTAC -0.326096932626
CCCGTTAG -0.195196084886
CCCGTTCA -0.0985044862812
CCCGTTCC -0.198476967981
CCCGTTCG -0.146748971193
CCCGTTGA 0.0199603698043
CCCGTTGC -0.126405073809
CCCGTTTA -0.248942334601
CCCGTTTC 0.0861694245493
CCCTAAAA 0.0958101492017
CCCTAAAC -0.032462476248
CCCTAAAG 0.0143632389984
CCCTAACA -0.0564940588897
CCCTAACC -0.283577971526
CCCTAACG -0.0929677876682
CCCTAAGA 0.0800008716749
CCCTAAGC -0.131911647359
CCCTAAGG -0.0304662309368
CCCTAATA -0.0505005598367
CCCTAATC 0.00996953980407
CCCTACAA -0.0793663013134
CCCTACAC -0.0663362539801
CCCTACAG -0.193966594045
CCCTACCA -0.299825144358
CCCTACCC -0.120026378785
CCCTACCG -0.110857301219
CCCTACGA -0.113997256516
CCCTACGC -0.244498727794
CCCTACGG -0.173319615912
CCCTACTA -0.164175459968
CCCTACTC -0.0978580246914
CCCTAGAA 0.163861810469
CCCTAGAC -0.0500460443943
CCCTAGAG -0.076893018283
CCCTAGCA -0.129095538326
CCCTAGCC -0.326078250661
CCCTAGCG -0.0836560951869
CCCTAGGA -0.000924691175099
CCCTAGGC -0.206631771271
CCCTAGGG -0.319092928794
CCCTAGTA -0.0181826039712
CCCTAGTC -0.118743808088
CCCTATAA 0.0544580712761
CCCTATAC -0.0396975288016
CCCTATAG -0.0533737291815
CCCTATCA -0.0117208939823
CCCTATCC -0.253357693067
CCCTATCG -0.0646003128344
CCCTATGA -0.144359936784
CCCTATGC -0.219979313107
CCCTATTA -0.0929479170904
CCCTATTC -0.0473665722628
CCCTCAAA -0.106207536194

CCCTCAAC -0.0836859232918
CCCTCAAG -0.10821879611
CCCTCACA -0.110090622189
CCCTCACC -0.256029905543
CCCTCACG -0.138375751308
CCCTCAGA -0.0563539883226
CCCTCAGC -0.265042813491
CCCTCAGG -0.151444304393
CCCTCATA -0.205827160494
CCCTCATC -0.179764705882
CCCTCCAA -0.292851125635
CCCTCCAC -0.203111247956
CCCTCCAG -0.150196231319
CCCTCCCA -0.0915124680781
CCCTCCCC -0.0484262232101
CCCTCCCG -0.072956342116
CCCTCCGA -0.151510162869
CCCTCCGC -0.220233392587
CCCTCCGG -0.173680602556
CCCTCCTA -0.326534250483
CCCTCCTC -0.230883732548
CCCTCGAA -0.00404469469964
CCCTCGAC -0.303010589048
CCCTCGAG -0.311485285404
CCCTCGCA -0.132298784336
CCCTCGCC -0.361924273091
CCCTCGCG -0.241592385732
CCCTCGGA 0.148359459044
CCCTCGGC -0.0977688884499
CCCTCGTA -0.138601689409
CCCTCGTC -0.103898491084
CCCTCTAA -0.177587564237
CCCTCTAC -0.262449458791
CCCTCTAG -0.163151916239
CCCTCTCA -0.248611659862
CCCTCTCC -0.29818946439
CCCTCTCG -0.0850775385223
CCCTCTGA -0.0619937521608
CCCTCTGC -0.0342445811258
CCCTCTTA -0.153312999274
CCCTCTTC -0.189686927169
CCCTGAAA -0.143360564297
CCCTGAAC -0.265246779468
CCCTGAAG -0.0724027969879
CCCTGACA -0.0808598402324
CCCTGACC -0.326465216091
CCCTGACG -0.0904316979422
CCCTGAGA 0.0928522656284
CCCTGAGC -0.293820455855
CCCTGAGG -0.0887813818925
CCCTGATA 0.1514083734
CCCTGATC -0.0947396095087
CCCTGCAA 0.0384132610812
CCCTGCAC -0.0838519542955

CCCTGCAG -0.223415461084
CCCTGCCA -0.0977254901961
CCCTGCCC -0.0869889836614
CCCTGCCG -0.324963990335
CCCTGCGA -0.167442265795
CCCTGCGC -0.158059506563
CCCTGCGG -0.00804313822343
CCCTGCTA -0.392670871666
CCCTGCTC -0.0637177423572
CCCTGGAA -0.0299405547065
CCCTGGAC -0.270048619342
CCCTGGAG -0.220315688985
CCCTGGCA -0.320694822809
CCCTGGCC -0.203997002031
CCCTGGCG -0.211314128944
CCCTGGGA -0.0328387306716
CCCTGGGC -0.284949796835
CCCTGGTA -0.334178924594
CCCTGGTC -0.188157310028
CCCTGTAA -0.0671563300812
CCCTGTAC 0.0170851513405
CCCTGTAG -0.125729847495
CCCTGTCA -0.343327244001
CCCTGTCC -0.164148521207
CCCTGTCG -0.0661376122534
CCCTGTGA -0.101088173318
CCCTGTGC -0.110049330282
CCCTGTTA -0.024974939346
CCCTGTTC -0.276465649232
CCCTTAAA -0.0809457946387
CCCTTAAC -0.121704676629
CCCTTAAG -0.217121833177
CCCTTACA -0.104455818433
CCCTTACC -0.0790603691469
CCCTTACG -0.211073860599
CCCTTAGA 0.0506748216698
CCCTTAGC -0.255142407234
CCCTTAGG -0.333766696838
CCCTTATA 0.00376870462739
CCCTTATC -0.186948505202
CCCTTCAA -0.218375126017
CCCTTCAC -0.376055086628
CCCTTCAG -0.0841184895947
CCCTTCCA -0.0681231958027
CCCTTCCC -0.256416214521
CCCTTCCG -0.12923051899
CCCTTCGA -0.113577625233
CCCTTCGC -0.379374091445
CCCTTCGG -0.246785516162
CCCTTCTA -0.240190269274
CCCTTCTC -0.139688815424
CCCTTGAA -0.0828694773049
CCCTTGAC -0.252438634713
CCCTTGAG -0.0188399144723

CCCTTGCA 0.0848796771635
CCCTTGCC -0.30512001394
CCCTTGCG -0.0914395191629
CCCTTGGA -0.17835739165
CCCTTGGC -0.176554183813
CCCTTGTA -0.00051592790182
CCCTTGTC -0.22074144426
CCCTTTAA -0.0460168491534
CCCTTTAC -0.076287369946
CCCTTTAG -0.240166307969
CCCTTTCA -0.263718130476
CCCTTTCC -0.230832318445
CCCTTTCG -0.200392808747
CCCTTTGA -0.215560409429
CCCTTTGC -0.126369826765
CCCTTTTA -0.11452303009
CCCTTTTC -0.236655114414
CCGAAAAA -0.0927993194479
CCGAAAAC 0.0575349033167
CCGAAAAG 0.0447485125908
CCGAAACA -0.0596527914993
CCGAAACC -0.209466966226
CCGAAACG 0.0560167361585
CCGAAAGA 0.0865239902145
CCGAAAGC -0.258130889976
CCGAAAGG -0.281742459006
CCGAAATA 0.17662603693
CCGAAATC 0.460969302515
CCGAACAA -0.00386652397025
CCGAACAC -0.271986942303
CCGAACAG -0.146296296296
CCGAACCA -0.129073023915
CCGAACCC -0.17343660589
CCGAACCG -0.0330650277145
CCGAACGA -0.176929602343
CCGAACGC -0.155268784717
CCGAACGG -0.153341070723
CCGAACTA -0.152641625652
CCGAACTC -0.0457403350553
CCGAAGAA 0.0437973065245
CCGAAGAC 0.0228792070078
CCGAAGAG -0.304855967078
CCGAAGCA -0.0622928055501
CCGAAGCC -0.228371438447
CCGAAGCG -0.102575288643
CCGAAGGA -0.166663923318
CCGAAGGC -0.138471616609
CCGAAGTA -0.0966247400164
CCGAAGTC -0.147764937003
CCGAATAA 0.0422072932446
CCGAATAC 0.0985878866241
CCGAATAG -0.0762263053333
CCGAATCA 0.209370677732
CCGAATCC 0.362667615824

CCGAATCG 0.253077738875
CCGAATGA -0.0125774471747
CCGAATGC 0.0486184958023
CCGAATTA 0.0863670041986
CCGAATTC 0.189411403104
CCGACAAA 0.0429111036857
CCGACAAC -0.0102801378735
CCGACAAG -0.0734843774515
CCGACACA -0.207067655193
CCGACACC -0.20911450189
CCGACACG -0.0220543044032
CCGACAGA 0.121647485923
CCGACAGC -0.170930462268
CCGACAGG 0.0412605106589
CCGACATA 0.00289250795403
CCGACATC -0.167734794431
CCGACCAA -0.179685286603
CCGACCAC -0.253064330611
CCGACCAG -0.229051033834
CCGACCCA -0.139526843483
CCGACCCC -0.346537858091
CCGACCCG -0.133023898874
CCGACCGA -0.15510043877
CCGACCGC -0.319697493375
CCGACCGG -0.194746227709
CCGACCTA -0.130482841564
CCGACCTC -0.261627180868
CCGACGAA 0.0728528617533
CCGACGAC -0.264777689995
CCGACGAG -0.277348329092
CCGACGCA -0.294877156906
CCGACGCC -0.374714062371
CCGACGCG -0.211791433286
CCGACGGA 0.0234625833539
CCGACGGC -0.119352383387
CCGACGTA 0.254414307091
CCGACGTC 0.0582645852887
CCGACTAA 0.0984776142338
CCGACTAC -0.214259067244
CCGACTAG -0.209050340707
CCGACTCA 0.072693692507
CCGACTCC -0.215695150927
CCGACTCG -0.0178627286347
CCGACTGA -0.0222013655624
CCGACTGC -0.225325111033
CCGACTTA -0.117908735647
CCGACTTC -0.269628900294
CCGAGAAA 0.107672192117
CCGAGAAC 0.0162443126021
CCGAGAAG -0.0473653625441
CCGAGACA -0.144171728645
CCGAGACC -0.257224757529
CCGAGACG -0.276966109375
CCGAGAGA 0.0276830281653

CCGAGAGC -0.125694444444
CCGAGAGG -0.151937627223
CCGAGATA 0.324362225296
CCGAGATC 0.309475400203
CCGAGCAA -0.0681819502473
CCGAGCAC -0.138866293258
CCGAGCAG 0.0172763430457
CCGAGCCA -0.217621626367
CCGAGCCC -0.129477408186
CCGAGCCG -0.23975220581
CCGAGCGA 0.0270961293734
CCGAGCGC -0.14400245207
CCGAGCGG -0.0799761830285
CCGAGCTA -0.134537722908
CCGAGCTC -0.127503171203
CCGAGGAA 0.12053230282
CCGAGGAC -0.243220474968
CCGAGGAG -0.151944869643
CCGAGGCA -0.067360929557
CCGAGGCC -0.1191040648
CCGAGGCG -0.0716978647042
CCGAGGGA -0.00774299643793
CCGAGGGC -0.104323234553
CCGAGGTA -0.0461101506545
CCGAGGTC -0.248781165144
CCGAGTAA 0.0500819253027
CCGAGTAC -0.102445799025
CCGAGTAG -0.197331273019
CCGAGTCA -0.142259748484
CCGAGTCC -0.297412728978
CCGAGTCG -0.305883696243
CCGAGTGA -0.183915758896
CCGAGTGC -0.23583851775
CCGAGTTA 0.0376781485625
CCGAGTTC -0.193526794287
CCGATAAA 0.0984426074703
CCGATAAC 0.102784912951
CCGATAAG -0.173181628576
CCGATACA -0.0876339572325
CCGATACC 0.173853981427
CCGATACG 0.202285241091
CCGATAGA 0.137483808646
CCGATAGC 0.0262882628972
CCGATAGG -0.164049382716
CCGATATA 0.23444714018
CCGATATC 0.435579488193
CCGATCAA -0.0536278771115
CCGATCAC 0.011451952842
CCGATCAG 0.0529897642765
CCGATCCA 0.131025816106
CCGATCCC 0.123556499594
CCGATCCG -0.0149855447242
CCGATCGA -0.00835350192631
CCGATCGC 0.258159603678

CCGATCGG -0.180493957948
CCGATCTA 0.0527152077982
CCGATCTC 0.282066741243
CCGATGAA 0.0629028358081
CCGATGAC -0.133331299626
CCGATGAG -0.162811132057
CCGATGCA -0.00844969154098
CCGATGCC -0.0326193699845
CCGATGCG 0.0315735185158
CCGATGGA -0.139467878429
CCGATGGC -0.255990101998
CCGATGTA 0.0775648203034
CCGATGTC -0.155323574439
CCGATTAA 0.0131782720497
CCGATTAC 0.42932169163
CCGATTAG 0.0140877195531
CCGATTCA -0.00992788699002
CCGATTCC 0.366732934901
CCGATTCG 0.244365348016
CCGATTGA -0.0180108235087
CCGATTGC 0.375746448879
CCGATTTA 0.176636758126
CCGATTTC 0.401219016883
CCGCAAAA 0.14078888495
CCGCAAAC -0.0231572939989
CCGCAAAG -0.262904320988
CCGCAACA 0.0280403294931
CCGCAACC -0.0239275285956
CCGCAACG -0.133310962554
CCGCAAGA -0.0430330567628
CCGCAAGC -0.0144712565194
CCGCAAGG -0.29181202826
CCGCAATA 0.230486830445
CCGCAATC 0.477580520971
CCGCACAA -0.0416713249804
CCGCACAC -0.0306175816832
CCGCACAG -0.0847521881981
CCGCACCA -0.0474908623926
CCGCACCC -0.213535540123
CCGCACCG -0.0651206291979
CCGCACGA 0.0700042811039
CCGCACGC -0.0876616708479
CCGCACGG -0.229208843571
CCGCACTA -0.132927786284
CCGCACTC -0.381305027565
CCGCAGAA -0.101169033398
CCGCAGAC -0.164454018063
CCGCAGAG -0.197452849097
CCGCAGCA -0.099023432394
CCGCAGCC -0.252260407751
CCGCAGCG 0.131944726918
CCGCAGGA -0.150562091503
CCGCAGGC -0.07475986542
CCGCAGTA -0.183101760393

CCGCAGTC −0.118058158217
CCGCATAA −0.00295323344925
CCGCATAC 0.094554937312
CCGCATAG −0.210329805387
CCGCATCA −0.0923786555867
CCGCATCC −0.0711753956287
CCGCATCG −0.12903033701
CCGCATGA −0.200392156863
CCGCATGC −0.0855711589554
CCGCATTA 0.0464471982915
CCGCATTC −0.0190901906549
CCGCCAAA −0.15611507865
CCGCCAAC −0.00124062269381
CCGCCAAG −0.148069666857
CCGCCACA −0.124193982099
CCGCCACC −0.209330428468
CCGCCACG −0.105037451116
CCGCCAGA 0.00540901007706
CCGCCAGC −0.0438457866232
CCGCCAGG −0.243188770405
CCGCCATA −0.0852920838394
CCGCCATC −0.227393091699
CCGCCCAA −0.0490084941643
CCGCCCAC −0.13124204311
CCGCCCAG −0.311752474378
CCGCCCCA −0.248807726194
CCGCCCCC −0.148972940944
CCGCCCCG −0.251611394644
CCGCCCGA −0.082201590202
CCGCCCGC 0.0946081942112
CCGCCCGG −0.187805555556
CCGCCCTA 0.00750839754989
CCGCCCTC −0.00175077522571
CCGCCGAA −0.135962236747
CCGCCGAC −0.246909796562
CCGCCGAG −0.0692153635117
CCGCCGCA −0.186016957768
CCGCCGCC 0.078795861804
CCGCCGCG −0.135102986926
CCGCCGGA −0.208193401817
CCGCCGGC −0.292726075866
CCGCCGTA 0.0408283666458
CCGCCGTC −0.233603485839
CCGCCTAA −0.23988671024
CCGCCTAC −0.0434998572965
CCGCCTAG −0.0529144490669
CCGCCTCA −0.0949770336346
CCGCCTCC −0.345673285569
CCGCCTCG −0.170463648834
CCGCCTGA 0.00257263966484
CCGCCTGC −0.351014064888
CCGCCTTA 0.053856919404
CCGCCTTC −0.242724030161
CCGCGAAA 0.0848564183626

CCGCGAAC -0.230784636488
CCGCGAAG -0.261568472517
CCGCGACA -0.0925488065949
CCGCGACC -0.26402330634
CCGCGACG -0.0950914542032
CCGCGAGA -0.046477473787
CCGCGAGC 0.0531230569873
CCGCGAGG -0.313831502087
CCGCGATA 0.251514852137
CCGCGATC 0.371552888876
CCGCGCAA 0.0600095501548
CCGCGCAC -0.2361652976
CCGCGCAG -0.0837991261301
CCGCGCCA -0.132381986964
CCGCGCCC -0.0988222803812
CCGCGCCG -0.185066085694
CCGCGCGA -0.0247002003238
CCGCGCGC -0.0519388460779
CCGCGCGG -0.179901423877
CCGCGCTA -0.148516994077
CCGCGCTC -0.211142383743
CCGCGGAA 0.0582230125799
CCGCGGAC -0.0981578427499
CCGCGGAG -0.124965015393
CCGCGGCA -0.00549133899756
CCGCGGCC -0.234371059781
CCGCGGCG -0.163075268463
CCGCGGGA -0.0355877344634
CCGCGGGC -0.109125214608
CCGCGGTA -0.0297699408388
CCGCGGTC -0.142096655581
CCGCGTAA 0.0237720279517
CCGCGTAC -0.0633924945394
CCGCGTAG -0.0502794756781
CCGCGTCA -0.108114782437
CCGCGTCC -0.373857887186
CCGCGTCG -0.0188216190853
CCGCGTGA -0.142281191346
CCGCGTGC -0.145420720765
CCGCGTTA -0.205385547314
CCGCGTTC -0.0117734536196
CCGCTAAA -0.0490467252791
CCGCTAAC -0.0882003446837
CCGCTAAG -0.0708822222158
CCGCTACA -0.254820148482
CCGCTACC -0.10544166306
CCGCTACG -0.0701350133347
CCGCTAGA -0.0745844377494
CCGCTAGC -0.144684272339
CCGCTAGG -0.193125827968
CCGCTATA -0.127710545814
CCGCTATC -0.0285179802245
CCGCTCAA 0.0683686798832
CCGCTCAC -0.255123203792

CCGCTCAG -0.00338097433533
CCGCTCCA -0.134379084967
CCGCTCCC -0.249385893448
CCGCTCCG 0.00891868725757
CCGCTCGA -0.0234291691485
CCGCTCGC -0.101092592593
CCGCTCTA -0.17303960304
CCGCTCTC -0.20722085048
CCGCTGAA 0.026940767086
CCGCTGAC -0.243512623299
CCGCTGAG -0.221399258235
CCGCTGCA -0.199509707102
CCGCTGCC -0.295394272469
CCGCTGCG -0.180910133382
CCGCTGGA -0.0960102503908
CCGCTGGC -0.269652624271
CCGCTGTA -0.158032502235
CCGCTGTC -0.177278300434
CCGCTTAA -0.0624046605553
CCGCTTAC -0.0606481454579
CCGCTTAG -0.145637840774
CCGCTTCA -0.122348816064
CCGCTTCC -0.240873262507
CCGCTTCG -0.118313112856
CCGCTTGA -0.0433238533203
CCGCTTGC -0.112510241674
CCGCTTTA 0.051277185357
CCGCTTTC 0.0419813171008
CCGGAAAA 0.0902662966891
CCGGAAAC -0.163618354625
CCGGAAAG -0.190351407256
CCGGAACA -0.0459059451104
CCGGAACC -0.167826733637
CCGGAACG -0.113026491254
CCGGAAGA 0.0211922761795
CCGGAAGC -0.19937238379
CCGGAAGG -0.148467291053
CCGGAATA 0.143298974731
CCGGAATC 0.38305725702
CCGGACAA -0.0761004717968
CCGGACAC -0.154633517192
CCGGACAG 0.00327023375416
CCGGACCA -0.275461537568
CCGGACCC -0.18457152891
CCGGACCG -0.155748725463
CCGGACGA -0.122442820141
CCGGACGC -0.208452241715
CCGGACGG -0.082540351285
CCGGACTA -0.168994700193
CCGGACTC -0.251400479537
CCGGAGAA 0.0742710618453
CCGGAGAC 0.0440117801047
CCGGAGAG -0.0714609431287
CCGGAGCA -0.293673913878

CCGGAGCC -0.348514598413
CCGGAGCG -0.187268121058
CCGGAGGA -0.109045369502
CCGGAGGC -0.261205977908
CCGGAGTA 0.00345114453211
CCGGAGTC -0.0932147256374
CCGGATAA 0.134256514962
CCGGATAC 0.307362747519
CCGGATAG -0.0792149567584
CCGGATCA -0.120836420515
CCGGATCC 0.281034119657
CCGGATCG 0.0984489231377
CCGGATGA -0.0186154741129
CCGGATGC 0.0289770357198
CCGGATTA 0.227769443368
CCGGATTC 0.453585698918
CCGGCAAA -0.0401221228885
CCGGCAAC -0.0612414427859
CCGGCAAG -0.291929155013
CCGGCACA -0.104901777265
CCGGCACC -0.367138757537
CCGGCACG -0.174593892138
CCGGCAGA -0.0575148870777
CCGGCAGC -0.274171751718
CCGGCAGG -0.2046403851
CCGGCATA -0.0979150888516
CCGGCATC -0.222316953981
CCGGCCAA -0.103634378155
CCGGCCAC -0.359427592316
CCGGCCAG -0.00386966323471
CCGGCCCA -0.155970951344
CCGGCCCC -0.272018528468
CCGGCCCG -0.273500231273
CCGGCCGA 0.0784704384289
CCGGCCGC -0.0341175373032
CCGGCCGG -0.22178811068
CCGGCCTA -0.299274472046
CCGGCCTC -0.247478986986
CCGGCGAA 0.0410142421997
CCGGCGAC -0.256311235175
CCGGCGAG -0.042227025645
CCGGCGCA -0.0316110738265
CCGGCGCC -0.0928540230244
CCGGCGCG -0.116126136334
CCGGCGGA -0.240187122205
CCGGCGGC -0.379601744425
CCGGCGTA -0.101578037462
CCGGCGTC -0.265867713864
CCGGCTAA -0.0773969257448
CCGGCTAC -0.236135034726
CCGGCTAG 0.0490073732048
CCGGCTCA -0.260366013072
CCGGCTCC -0.323948914803
CCGGCTCG -0.189049076726

CCGGCTGA -0.148863318353
CCGGCTGC -0.23083750013
CCGGCTTA -0.289348799656
CCGGCTTC -0.289208576998
CCGGGAAA 0.0419932380513
CCGGGAAC -0.349337460077
CCGGGAAG -0.210821770689
CCGGGACA -0.16320421859
CCGGGACC -0.0697014951392
CCGGGACG -0.23945698031
CCGGGAGA -0.00314788828556
CCGGGAGC -0.261867131403
CCGGGAGG -0.192794967194
CCGGGATA 0.175588377352
CCGGGATC 0.239797346734
CCGGGCAA -0.0574938977314
CCGGGCAC -0.286031699008
CCGGGCAG -0.217761493577
CCGGGCCA -0.23573275236
CCGGGCCC -0.284216835083
CCGGGCCG -0.31756291494
CCGGGCGA -0.030838194622
CCGGGCGC -0.188310013717
CCGGGCTA -0.289598434385
CCGGGCTC -0.305324753294
CCGGGGAA 0.189330608515
CCGGGGAC -0.297757997299
CCGGGGAG -0.186286128119
CCGGGGCA -0.187667316439
CCGGGGCC -0.240744398358
CCGGGGCG -0.209472621419
CCGGGGGA -0.135762916662
CCGGGGGC -0.0626512569763
CCGGGGTA 0.046082241103
CCGGGGTC -0.220664000132
CCGGGTAA -0.141363756091
CCGGGTAC -0.061652835408
CCGGGTAG -0.215679497159
CCGGGTCA -0.274166095433
CCGGGTCC -0.264178269313
CCGGGTCG -0.0934847984696
CCGGGTGA 0.0373910513952
CCGGGTGC -0.196498418532
CCGGGTTA -0.0534530537546
CCGGGTTC -0.22910331367
CCGGTAAA 0.0405924483896
CCGGTAAC -0.202193355738
CCGGTAAG -0.249045751634
CCGGTACA -0.179014594034
CCGGTACC -0.227775034294
CCGGTACG -0.139701040432
CCGGTAGA -0.0585199259731
CCGGTAGC -0.216589849108
CCGGTAGG -0.0660595036243

CCGGTATA	-0.0843442825389
CCGGTATC	0.141589277481
CCGGTCAA	-0.0799731072144
CCGGTCAC	-0.07370000878
CCGGTCAG	-0.0370232644618
CCGGTCCA	-0.127098972738
CCGGTCCC	-0.283185725033
CCGGTCCG	0.0406759753233
CCGGTCGA	-0.253534455801
CCGGTCGC	-0.263157665417
CCGGTCTA	-0.193544581619
CCGGTCTC	-0.234631873686
CCGGTGAA	-0.182094963627
CCGGTGAC	-0.180043578095
CCGGTGAG	-0.240790123457
CCGGTGCA	-0.233410150892
CCGGTGCC	-0.0519197752685
CCGGTGCG	-0.0918146236665
CCGGTGGA	-0.0985185185185
CCGGTGGC	-0.161039946765
CCGGTGTA	-0.14266571006
CCGGTGTC	-0.345179072962
CCGGTTAA	-0.100997208025
CCGGTTAC	-0.108004857415
CCGGTTAG	-0.121366922985
CCGGTTCA	-0.186928927742
CCGGTTCC	-0.0732436718411
CCGGTTCG	-0.247307137252
CCGGTTGA	-0.249746215177
CCGGTTGC	-0.104253211811
CCGGTTTA	-0.0076785439856
CCGGTTTC	-0.0413147626809
CCGTAAAA	-0.0115191841123
CCGTAAAC	-0.0724675358438
CCGTAAAG	-0.165390946502
CCGTAACA	0.0603608202156
CCGTAACC	-0.0936409141803
CCGTAACG	-0.0912016013992
CCGTAAGA	0.0391222233522
CCGTAAGC	-0.0604753533915
CCGTAAGG	-0.222524005487
CCGTAATA	0.184512832334
CCGTAATC	0.467553353769
CCGTACAA	-0.0230209347261
CCGTACAC	-0.202113240265
CCGTACAG	-0.0272881853896
CCGTACCA	-0.150106328584
CCGTACCC	-0.142196649161
CCGTACCG	-0.0566038771433
CCGTACGA	-0.0283568245187
CCGTACGC	-0.0648742599086
CCGTACGG	0.0405145170847
CCGTACTA	-0.114102772678
CCGTACTC	-0.0756221579766

CCGTAGAA 0.0413827669868
CCGTAGAC -0.298744156956
CCGTAGAG -0.241536351166
CCGTAGCA -0.0727979758063
CCGTAGCC -0.289428436165
CCGTAGCG -0.288315311312
CCGTAGGA -0.179985624853
CCGTAGGC 0.0229700870222
CCGTAGTA -0.0892899686794
CCGTAGTC -0.120186009274
CCGTATAA 0.0480105549128
CCGTATAC 0.09653065929
CCGTATAG -0.0248924341708
CCGTATCA 0.226571933522
CCGTATCC 0.336762285598
CCGTATCG 0.2387706478
CCGTATGA 0.103614545349
CCGTATGC -0.0238280614498
CCGTATTA 0.0494254209463
CCGTATTC 0.109268810018
CCGTCAAA -0.000444576170717
CCGTCAAC -0.0388631596074
CCGTCAAG -0.238290338603
CCGTCACA -0.113651218797
CCGTCACC -0.21902912565
CCGTCACG -0.154879909548
CCGTCAGA -0.170895355548
CCGTCAGC -0.383675245878
CCGTCAGG -0.089465162866
CCGTCATA -0.00221728273099
CCGTCATC -0.0780430121273
CCGTCCAA -0.25436950482
CCGTCCAC -0.211299872147
CCGTCCAG -0.0748450693793
CCGTCCCA 0.0454286619078
CCGTCCCC -0.19387541715
CCGTCCCG -0.15798800753
CCGTCCGA -0.0966187363834
CCGTCCGC -0.0626599728645
CCGTCCTA -0.199869952426
CCGTCCTC -0.267585507008
CCGTCGAA 0.0587032646161
CCGTCGAC -0.215411064821
CCGTCGAG -0.184298521858
CCGTCGCA -0.167319357613
CCGTCGCC -0.307245539073
CCGTCGCG -0.224007819512
CCGTCGGA 0.0187206717708
CCGTCGGC -0.056883795473
CCGTCGTA -0.0628521203493
CCGTCGTC -0.0718169934641
CCGTCTAA -0.118643624594
CCGTCTAC -0.184572503964
CCGTCTAG -0.274579033134

CCGTCTCA -0.149451288762
CCGTCTCC -0.302392942958
CCGTCTCG -0.251225732244
CCGTCTGA -0.0501141514922
CCGTCTGC -0.3210956151
CCGTCTTA -0.101493755683
CCGTCTTC -0.201493751107
CCGTGAAA -0.0856357988101
CCGTGAAC -0.0795230646799
CCGTGAAG -0.318362421253
CCGTGACA -0.174235222952
CCGTGACC -0.160168812495
CCGTGACG -0.0957422083808
CCGTGAGA -0.0847258578774
CCGTGAGC -0.280458161866
CCGTGAGG -0.200039780521
CCGTGATA 0.0862807086873
CCGTGATC 0.142829100118
CCGTGCAA 0.0277497711853
CCGTGCAC -0.15140872531
CCGTGCAG -0.191358741907
CCGTGCCA -0.176122085048
CCGTGCCC -0.188455203307
CCGTGCCG -0.248201250773
CCGTGCGA -0.0899535473741
CCGTGCGC -0.0407508026673
CCGTGCTA -0.198925886768
CCGTGCTC -0.199957104418
CCGTGGAA -0.0217944084635
CCGTGGAC -0.256401281563
CCGTGGAG -0.0674432644549
CCGTGGCA -0.196574170629
CCGTGGCC -0.209807020245
CCGTGGCG -0.131330841881
CCGTGGGA -0.253215936056
CCGTGGGC -0.0317740037482
CCGTGGTA 0.117639794866
CCGTGGTC -0.192811425948
CCGTGTAA 0.0833525434148
CCGTGTAC 0.0429713195205
CCGTGTAG -0.134265417434
CCGTGTCA -0.219947712418
CCGTGTCC -0.189061106641
CCGTGTCG -0.142295314334
CCGTGTGA -0.095244734931
CCGTGTGC -0.131267194023
CCGTGTTA 0.135805500269
CCGTGTTC -0.0875871712963
CCGTTAAA 0.0642618388988
CCGTTAAC -0.20443964408
CCGTTAAG 0.198557378002
CCGTTACA -0.0583208635393
CCGTTACC -0.237750060273
CCGTTACG -0.106510519557

CCGTTAGA 0.0623930382229
CCGTTAGC -0.160214310404
CCGTTAGG -0.212375944305
CCGTTATA 0.051017680473
CCGTTATC 0.179072537882
CCGTTCAA -0.141001281236
CCGTTCAC -0.0318032642936
CCGTTCAG -0.139173850011
CCGTTCCA -0.0369368531765
CCGTTCCC -0.335502676702
CCGTTCCG -0.244996626014
CCGTTCGA -0.127173095301
CCGTTCGC -0.211381043299
CCGTTCTA -0.177455337691
CCGTTCTC -0.103059578981
CCGTTGAA -0.16081885788
CCGTTGAC -0.142357167547
CCGTTGAG 0.0481157654394
CCGTTGCA 0.0407379289007
CCGTTGCC -0.0369241182571
CCGTTGCG -0.0855777250977
CCGTTGGA -0.0792541368239
CCGTTGGC -0.317665067212
CCGTTGTA -0.124816845294
CCGTTGTC -0.163925817705
CCGTTTAA -0.134914685981
CCGTTTAC -0.113659933824
CCGTTTAG 0.0554850144553
CCGTTTCA -0.118339971935
CCGTTTCC -0.0262160968694
CCGTTTCG 0.0371056444708
CCGTTTGA 0.0968532535267
CCGTTTGC -0.0844668725756
CCGTTTTA -0.0999000578154
CCGTTTTC -0.111909985036
CCTAAAAA 0.0685827350765
CCTAAAAC 0.0363317526181
CCTAAAAG 0.00160796324655
CCTAAACA 0.0186785408162
CCTAAACC -0.144527305852
CCTAAACG 0.0160809495445
CCTAAAGA 0.151132451005
CCTAAAGC -0.191801078393
CCTAAAGG -0.0622009306818
CCTAAATA 0.130496999985
CCTAAATC 0.0372675580144
CCTAACAA 0.0165464783167
CCTAACAC -0.183918547418
CCTAACAG 0.00173971264557
CCTAACCA -0.131301275943
CCTAACCC -0.0704246832814
CCTAACCG -0.158485596708
CCTAACGA -0.128167978311
CCTAACGC 0.0162218703238

CCTAACTA -0.174088668926
CCTAACTC -0.0312548025204
CCTAAGAA 0.0432336604536
CCTAAGAC -0.109654105135
CCTAAGAG -0.168800227786
CCTAAGCA -0.178243713436
CCTAAGCC -0.0531422229582
CCTAAGCG -0.123075604519
CCTAAGGA -0.0115233045731
CCTAAGGC -0.0383281183269
CCTAAGTA 0.108678976654
CCTAAGTC -0.141279022474
CCTAATAA 0.0589097846488
CCTAATAC -0.0112983597984
CCTAATAG -0.129482650429
CCTAATCA -0.129290285049
CCTAATCC 0.13358854038
CCTAATCG 0.0204889240159
CCTAATGA -0.0300742376476
CCTAATGC -0.226452942919
CCTAATTA 0.194899081424
CCTAATTC 0.104780017124
CCTACAAA -0.186046917038
CCTACAAC -0.0726584632371
CCTACAAG 0.0970280727752
CCTACACA -0.0523514887436
CCTACACC -0.153092527445
CCTACACG -0.22286458651
CCTACAGA -0.0459254675348
CCTACAGC 0.0154006980113
CCTACAGG -0.264023349074
CCTACATA -0.0151554769189
CCTACATC -0.157606786953
CCTACCAA -0.194251270879
CCTACCAC -0.178927297668
CCTACCAG -0.0974533432496
CCTACCCA -0.146129359787
CCTACCCC -0.183583104288
CCTACCCG -0.0797662031802
CCTACCGA 0.0974513484145
CCTACCGC -0.249838134431
CCTACCTA -0.171466215765
CCTACCTC -0.142286129267
CCTACGAA -0.16752461269
CCTACGAC -0.17221744486
CCTACGAG 0.160648422578
CCTACGCA 0.0675765984339
CCTACGCC -0.273242860919
CCTACGCG -0.124330213838
CCTACGGA -0.140743667413
CCTACGGC -0.213407842685
CCTACGTA -0.166506373599
CCTACGTC -0.123383565902
CCTACTAA -0.0968091991547

CCTACTAC -0.181786492375
CCTACTAG -0.177351377159
CCTACTCA 0.0721674196969
CCTACTCC -0.0414841889207
CCTACTCG 0.0552338502568
CCTACTGA 0.00276756788168
CCTACTGC -0.119235771555
CCTACTTA 0.01341138115
CCTACTTC -0.150643136222
CCTAGAAA 0.122848470878
CCTAGAAC 0.0404311078181
CCTAGAAG -0.238279835391
CCTAGACA -0.2014324331
CCTAGACC -0.230465016978
CCTAGACG -0.0817417981055
CCTAGAGA -0.0831071932873
CCTAGAGC -0.185240972119
CCTAGAGG 0.00962844512496
CCTAGATA 0.18753331236
CCTAGATC 0.290786678696
CCTAGCAA -0.00282374248786
CCTAGCAC -0.090242791443
CCTAGCAG -0.199249894371
CCTAGCCA -0.242603290596
CCTAGCCC -0.135745041867
CCTAGCCG -0.187260324912
CCTAGCGA -0.138342935337
CCTAGCGC -0.10272935289
CCTAGCTA -0.0547726699548
CCTAGCTC -0.174764965651
CCTAGGAA -0.105847198859
CCTAGGAC 0.0150178444976
CCTAGGAG -0.150155563238
CCTAGGCA -0.143662714988
CCTAGGCC -0.142231597664
CCTAGGCG -0.194906744366
CCTAGGGA -0.0786146026959
CCTAGGGC -0.166315483343
CCTAGGTA -0.128426950261
CCTAGGTC -0.112936267564
CCTAGTAA -0.0610781505134
CCTAGTAC -0.207575483241
CCTAGTAG -0.124370245975
CCTAGTCA 0.0185042058315
CCTAGTCC -0.130060856491
CCTAGTCG -0.0882309363888
CCTAGTGA -0.119188166155
CCTAGTGC -0.162870897359
CCTAGTTA -0.22949555808
CCTAGTTC -0.158585110178
CCTATAAA 0.151177852423
CCTATAAC 0.119046097076
CCTATAAG -0.0186523905685
CCTATACA -0.0125423473221

CCTATACC −0.0959720975366
CCTATACG −0.0710570069304
CCTATAGA 0.0886740371624
CCTATAGC 0.00941943506633
CCTATAGG 0.0337340419801
CCTATATA −0.0702183362972
CCTATATC 0.0183678257434
CCTATCAA 0.00237918827694
CCTATCAC −0.121632066015
CCTATCAG 0.0954027530791
CCTATCCA −0.123397040183
CCTATCCC −0.139624538081
CCTATCCG −0.0402556208602
CCTATCGA 0.06893126084
CCTATCGC −0.0748007955181
CCTATCTA 0.0590574288495
CCTATCTC 0.121219473218
CCTATGAA −0.000367556259352
CCTATGAC −0.172608494465
CCTATGAG −0.215191527925
CCTATGCA −0.0825348216932
CCTATGCC −0.128341460625
CCTATGCG −0.245551198257
CCTATGGA −0.146652344264
CCTATGGC −0.28472751091
CCTATGTA 0.0124460651141
CCTATGTC 0.0342553716967
CCTATTAA −0.11773856661
CCTATTAC −0.000530268911714
CCTATTAG −0.138274691358
CCTATTCA −0.0274759050693
CCTATTCC 0.0355101007606
CCTATTCG −0.0329042755655
CCTATTGA −0.0734356306321
CCTATTGC −0.145458005398
CCTATTTA −0.079276246031
CCTATTTC 0.166927200616
CCTCAAAA 0.104032063065
CCTCAAAC −0.0128523950078
CCTCAAAG 0.000107027596654
CCTCAACA 0.00288960237096
CCTCAACC −0.0632061685246
CCTCAACG −0.0904012467624
CCTCAAGA −0.0100624929183
CCTCAAGC −0.0534756717502
CCTCAAGG −0.1205182972
CCTCAATA −0.200941335923
CCTCAATC −0.0550012074908
CCTCACAA −0.0184344718376
CCTCACAC −0.166888932793
CCTCACAG −0.0580556104415
CCTCACCA −0.0530713273061
CCTCACCC −0.145241740333
CCTCACCG −0.106783995434

CCTCACGA -0.109939354024
CCTCACGC -0.233599379613
CCTCACTA -0.129787509878
CCTCACTC -0.140777573439
CCTCAGAA 0.0316017672402
CCTCAGAC -0.277032098765
CCTCAGAG -0.233458423859
CCTCAGCA -0.158718954248
CCTCAGCC -0.252379855041
CCTCAGCG -0.107496049188
CCTCAGGA -0.0178399704822
CCTCAGGC -0.170609148261
CCTCAGTA -0.107417006119
CCTCAGTC -0.0986967485532
CCTCATAA -0.169509345491
CCTCATAC -0.0402541093823
CCTCATAG 0.0123821422864
CCTCATCA -0.20288423548
CCTCATCC -0.0895339138833
CCTCATCG -0.0851075884271
CCTCATGA -0.187094217024
CCTCATGC -0.186817800168
CCTCATTA 0.178880769398
CCTCATTC -0.165904015714
CCTCCAAA -0.200058672491
CCTCCAAC -0.159326676989
CCTCCAAG -0.290323731139
CCTCCACA -0.195796848597
CCTCCACC -0.331200933195
CCTCCACG -0.185716559111
CCTCCAGA -0.10053449528
CCTCCAGC -0.261657860517
CCTCCAGG -0.206630567762
CCTCCATA -0.210826525546
CCTCCATC -0.202358141143
CCTCCCAA -0.100375327177
CCTCCCAC -0.0847161859608
CCTCCCAG -0.242234265948
CCTCCCCA -0.124504332821
CCTCCCCC -0.207113955157
CCTCCCCG -0.12511665146
CCTCCCGA 0.0365275376636
CCTCCCGC -0.246322177776
CCTCCCTA -0.152275962237
CCTCCCTC -0.161537037037
CCTCCGAA -0.100157499328
CCTCCGAC -0.236078353917
CCTCCGAG -0.422738941548
CCTCCGCA -0.0905112923055
CCTCCGCC -0.210209226771
CCTCCGCG -0.0906369199528
CCTCCGGA 0.180322290281
CCTCCGGC -0.385394725294
CCTCCGTA -0.14567694701

CCTCCGTC -0.369935935417
CCTCCTAA -0.281943706234
CCTCCTAC -0.21289209312
CCTCCTAG -0.0832427938828
CCTCCTCA -0.181302432253
CCTCCTCC -0.189100664955
CCTCCTCG -0.155497874986
CCTCCTGA -0.103473251029
CCTCCTGC -0.443569618587
CCTCCTTA -0.115784856021
CCTCCTTC -0.268200090623
CCTCGAAA -0.029211309178
CCTCGAAC -0.0353798323479
CCTCGAAG -0.00188136504293
CCTCGACA -0.079139710603
CCTCGACC -0.282813920591
CCTCGACG -0.158114381667
CCTCGAGA 0.0655129408278
CCTCGAGC -0.0921545385082
CCTCGAGG -0.379879906834
CCTCGATA 0.0436549329537
CCTCGATC -0.203901234568
CCTCGCAA 0.122639904279
CCTCGCAC -0.0278038473148
CCTCGCAG -0.230219950152
CCTCGCCA -0.169799382716
CCTCGCCC -0.169193540422
CCTCGCCG -0.245437858153
CCTCGCGA -0.184189152851
CCTCGCGC -0.229094982172
CCTCGCTA -0.192765589049
CCTCGCTC -0.117002114951
CCTCGGAA -0.0459915559725
CCTCGGAC -0.104086750849
CCTCGGAG -0.150069139217
CCTCGGCA -0.0589019607843
CCTCGGCC -0.202377270518
CCTCGGCG -0.0512951983104
CCTCGGGA -0.0724376389232
CCTCGGGC -0.0304999259186
CCTCGGTA -0.104741860084
CCTCGGTC -0.176331154684
CCTCGTAA 0.163106414953
CCTCGTAC -0.0281838936635
CCTCGTAG -0.0528666754467
CCTCGTCA -0.052789527506
CCTCGTCC -0.31868529194
CCTCGTCG -0.156829826539
CCTCGTGA -0.197540835953
CCTCGTGC -0.158575478384
CCTCGTTA -0.0207136432408
CCTCGTTC -0.220152548477
CCTCTAAA -0.0426031118795
CCTCTAAC -0.135718892911

CCTCTAAG -0.116391908932
CCTCTACA -0.174148033454
CCTCTACC -0.100884953181
CCTCTACG -0.222285319243
CCTCTAGA -0.14674708505
CCTCTAGC -0.125055555556
CCTCTATA 0.104207284297
CCTCTATC -0.226386625626
CCTCTCAA -0.164193293496
CCTCTCAC -0.150489808725
CCTCTCAG -0.21321625299
CCTCTCCA -0.132048641472
CCTCTCCC -0.308088053785
CCTCTCCG -0.199005604661
CCTCTCGA -0.039592424938
CCTCTCGC -0.176511260371
CCTCTCTA -0.171212262068
CCTCTCTC -0.216656499637
CCTCTGAA -0.0310995749972
CCTCTGAC -0.19186637618
CCTCTGAG -0.0367164005637
CCTCTGCA -0.0464673405995
CCTCTGCC 0.0125965801835
CCTCTGCG -0.0813909465021
CCTCTGGA -0.0891171693226
CCTCTGGC -0.318338641189
CCTCTGTA -0.172469135802
CCTCTGTC -0.111193581092
CCTCTTAA -0.158513274439
CCTCTTAC -0.0253410824635
CCTCTTAG -0.256243553449
CCTCTTCA -0.0607692054398
CCTCTTCC -0.219367283951
CCTCTTCG -0.198655760942
CCTCTTGA -0.147898454092
CCTCTTGC -0.101533134695
CCTCTTTA 0.0778129676173
CCTCTTTC -0.0746275594394
CCTGAAAA -0.0107143375997
CCTGAAAC -0.163061194251
CCTGAAAG -0.0894398621469
CCTGAACA -0.157815541031
CCTGAACC -0.228660529351
CCTGAACG -0.228068289452
CCTGAAGA 0.0333157698303
CCTGAAGC -0.279333102915
CCTGAAGG -0.256207891676
CCTGAATA 0.187394127817
CCTGAATC 0.104738852664
CCTGACAA -0.213896877269
CCTGACAC -0.214392704887
CCTGACAG -0.037550302907
CCTGACCA -0.165057371097
CCTGACCC -0.259625117267

CCTGACCG -0.0254233841685
CCTGACGA -0.0668422930216
CCTGACGC -0.0902833255382
CCTGACTA -0.119234646301
CCTGACTC -0.142784921931
CCTGAGAA 0.0731657116512
CCTGAGAC -0.20120589665
CCTGAGAG -0.160898970414
CCTGAGCA -0.197669569416
CCTGAGCC -0.170653882016
CCTGAGCG -0.189835513319
CCTGAGGA -0.0279271584171
CCTGAGGC -0.149027488211
CCTGAGTA -0.185741335309
CCTGAGTC -0.0821780509912
CCTGATAA 0.0512472215362
CCTGATAC 0.219378786338
CCTGATAG -0.0610029857955
CCTGATCA 0.0448942792214
CCTGATCC -0.0534177479198
CCTGATCG 0.00873854110674
CCTGATGA -0.154069464338
CCTGATGC -0.130970883094
CCTGATTA 0.147293299524
CCTGATTC 0.39492368863
CCTGCAAA -0.0890517629625
CCTGCAAC -0.132925954223
CCTGCAAG -0.252945824715
CCTGCACA -0.0235970472819
CCTGCACC -0.161093748185
CCTGCACG -0.122715347808
CCTGCAGA -0.0491208139879
CCTGCAGC -0.286245175007
CCTGCAGG -0.143971561173
CCTGCATA 0.196112977233
CCTGCATC 0.0581886595092
CCTGCCAA -0.160864197531
CCTGCCAC -0.181700540569
CCTGCCAG -0.0981797927916
CCTGCCCA -0.284771935599
CCTGCCCC -0.236785941581
CCTGCCCG -0.25482450535
CCTGCCGA -0.0825266693007
CCTGCCGC -0.317110770969
CCTGCCTA -0.153888888889
CCTGCCTC -0.198741165108
CCTGCGAA -0.0667157661702
CCTGCGAC -0.263076333195
CCTGCGAG -0.176396503186
CCTGCGCA 0.0102691606841
CCTGCGCC -0.222129764363
CCTGCGCG -0.0195163263112
CCTGCGGA 0.0817361522756
CCTGCGGC -0.249320954121

CCTGCGTA -0.0834993223259
CCTGCGTC -0.236192039899
CCTGCTAA -0.245330394045
CCTGCTAC -0.0777847054176
CCTGCTAG -0.202988604129
CCTGCTCA -0.134416201669
CCTGCTCC -0.339365845583
CCTGCTCG -0.127565555532
CCTGCTGA -0.0820714040827
CCTGCTGC -0.26256531349
CCTGCTTA -0.0566158315178
CCTGCTTC -0.284689651389
CCTGGAAA 0.0886947274821
CCTGGAAC -0.179996598248
CCTGGAAG -0.0645675391799
CCTGGACA -0.256579272207
CCTGGACC -0.284461235397
CCTGGACG -0.143797874359
CCTGGAGA -0.0131587357851
CCTGGAGC -0.191522115463
CCTGGATA 0.248228320718
CCTGGATC 0.213317740037
CCTGGCAA -0.268497945435
CCTGGCAC -0.157523504143
CCTGGCAG -0.237359273158
CCTGGCCA -0.170414266118
CCTGGCCC 0.0152528583674
CCTGGCCG -0.0492678650798
CCTGGCGA -0.00114705963064
CCTGGCGC -0.139424836601
CCTGGCTA -0.213688408407
CCTGGCTC -0.191583898116
CCTGGGAA -0.192079677821
CCTGGGAC -0.273741580768
CCTGGGAG -0.117983878491
CCTGGGCA -0.300342508023
CCTGGGCC -0.173712282088
CCTGGGCG -0.335807124519
CCTGGGGA -0.0903065656111
CCTGGGGC -0.279647035587
CCTGGGTA -0.105091609462
CCTGGGTC -0.31657587079
CCTGGTAA -0.185840028096
CCTGGTAC -0.111520697168
CCTGGTAG -0.294456013779
CCTGGTCA -0.0327762637209
CCTGGTCC -0.159073347858
CCTGGTCG -0.348855644427
CCTGGTGA -0.00551742973194
CCTGGTGC -0.0913029639508
CCTGGTTA 0.0216053955867
CCTGGTTC -0.287894964206
CCTGTAAA -0.0438291729786
CCTGTAAC -0.0672725476637

CCTGTAAG -0.143549919103
CCTGTACA -0.0196481984578
CCTGTACC -0.15708882931
CCTGTACG -0.112749086844
CCTGTAGA 0.0182612384115
CCTGTAGC -0.282047669947
CCTGTATA 0.0850827364781
CCTGTATC 0.0432226832642
CCTGTCAA -0.154748771988
CCTGTCAC -0.0802926069677
CCTGTCAG -0.0933586332554
CCTGTCCA -0.269293019071
CCTGTCCC -0.0511615678915
CCTGTCCG -0.226621212958
CCTGTCGA -0.208697167756
CCTGTCGC -0.0843046226788
CCTGTCTA -0.0584191546396
CCTGTCTC -0.166466260627
CCTGTGAA -0.0827214759208
CCTGTGAC -0.105261169962
CCTGTGAG -0.172249932781
CCTGTGCA 0.0779778370546
CCTGTGCC -0.175565767268
CCTGTGCG -0.0931139318308
CCTGTGGA -0.179009746589
CCTGTGGC -0.377458562324
CCTGTGTA -0.0340287362166
CCTGTGTC -0.239877415071
CCTGTTAA 0.0409778480318
CCTGTTAC -0.0537968084639
CCTGTTAG 0.0924396728313
CCTGTTCA 0.00459640539017
CCTGTTCC -0.231880240572
CCTGTTCG -0.0481133887654
CCTGTTGA -0.0860364662232
CCTGTTGC -0.214014294997
CCTGTTTA -0.129613382295
CCTGTTTC -0.182895126783
CCTTAAAA -0.0852602709479
CCTTAAAC -0.134627434565
CCTTAAAG 0.00568767887496
CCTTAACA -0.160740740741
CCTTAACC -0.0769380642683
CCTTAACG 0.00656984785615
CCTTAAGA -0.0723441095126
CCTTAAGC -0.318161369783
CCTTAAGG -0.0234594114162
CCTTAATA 0.0201603881859
CCTTAATC -0.00362417267608
CCTTACAA -0.179854387583
CCTTACAC -0.125644909877
CCTTACAG 0.110058777042
CCTTACCA -0.0683659743346
CCTTACCC -0.106962553229

CCTTACCG -0.0055239976949
CCTTACGA 0.0442215216539
CCTTACGC -0.242914332861
CCTTACTA 0.00428966963878
CCTTACTC -0.202086068478
CCTTAGAA 0.0614385541819
CCTTAGAC -0.171626485549
CCTTAGAG -0.178087225803
CCTTAGCA -0.154162756622
CCTTAGCC -0.135875469089
CCTTAGCG -0.234688333498
CCTTAGGA -0.0609547745994
CCTTAGGC -0.143348642731
CCTTAGTA -0.100999520579
CCTTAGTC -0.0144953786033
CCTTATAA 0.0700893543217
CCTTATAC -0.154436817935
CCTTATAG -0.0498292969942
CCTTATCA 0.050389415794
CCTTATCC -0.232238531431
CCTTATCG -0.223640813728
CCTTATGA -0.186884990253
CCTTATGC -0.105818430113
CCTTATTA 0.0264771257972
CCTTATTC 0.080785379106
CCTTCAAA -0.0999774967617
CCTTCAAC -0.0918855135699
CCTTCAAG -0.185476923415
CCTTCACA -0.0619762315706
CCTTCACC -0.331342342892
CCTTCACG -0.0718892911309
CCTTCAGA -0.278707334786
CCTTCAGC -0.0643693678861
CCTTCATA -0.0568643914325
CCTTCATC -0.137708841028
CCTTCCAA -0.260223231924
CCTTCCAC -0.137666465243
CCTTCCAG -0.0805930801146
CCTTCCCA -0.324528053457
CCTTCCCC -0.211979364853
CCTTCCCG -0.157296296296
CCTTCCGA -0.156809975978
CCTTCCGC -0.177864197531
CCTTCCTA -0.0851723797293
CCTTCCTC -0.220009526548
CCTTCGAA -0.140497212368
CCTTCGAC -0.219264892269
CCTTCGAG -0.0339024443786
CCTTCGCA 0.0930124700872
CCTTCGCC -0.269506676967
CCTTCGCG -0.312726315096
CCTTCGGA -0.116976034858
CCTTCGGC -0.245530800085
CCTTCGTA -0.259390584111

CCTTCGTC -0.253523806204
CCTTCTAA -0.12492129241
CCTTCTAC -0.125515216377
CCTTCTAG -0.0844556400716
CCTTCTCA -0.14350261923
CCTTCTCC -0.236897649847
CCTTCTCG -0.151706257099
CCTTCTGA -0.122295209716
CCTTCTGC -0.0498823529412
CCTTCTTA -0.0388486324096
CCTTCTTC -0.258197042704
CCTTGAAA 0.0931631630177
CCTTGAAC -0.340135494665
CCTTGAAG -0.0971056011064
CCTTGACA -0.103681220834
CCTTGACC -0.0894442798029
CCTTGACG -0.215139602054
CCTTGAGA 0.0601685749644
CCTTGAGC -0.266041988591
CCTTGATA 0.0669900844611
CCTTGATC 0.0674749030262
CCTTGCAA 0.00209528067922
CCTTGCAC -0.126832828482
CCTTGCAG -0.171119825708
CCTTGCCA -0.197998547567
CCTTGCCC -0.285443451254
CCTTGCCG -0.160816454557
CCTTGCGA 0.0258704911195
CCTTGCGC 0.0148954716488
CCTTGCTA 0.0259252372291
CCTTGCTC -0.331310394103
CCTTGGAA 0.0326104854113
CCTTGGAC -0.24375308642
CCTTGGAG -0.108571362168
CCTTGGCA -0.125504850959
CCTTGGCC -0.222068602278
CCTTGGCG -0.126784214945
CCTTGGGA -0.164469728438
CCTTGGGC -0.146219441709
CCTTGGTA -0.100766454957
CCTTGGTC -0.206268861454
CCTTGTAA -0.0408510452835
CCTTGTAC -0.173656046761
CCTTGTAG -0.202134891193
CCTTGTCA -0.147212620027
CCTTGTCC -0.146006189912
CCTTGTCG -0.21564658252
CCTTGTGA -0.0415678866005
CCTTGTGC -0.185579244291
CCTTGTTA -0.0142689365252
CCTTGTTC -0.112473668153
CCTTTAAA -0.0726163950111
CCTTTAAC 0.0507229823175
CCTTTAAG -0.0536539453609

CCTTTACA 0.19388229483
CCTTTACC -0.134543941974
CCTTTACG -0.0703889418272
CCTTTAGA -0.0484464857983
CCTTTAGC -0.200993160832
CCTTTATA 0.0822759432249
CCTTTATC -0.10216175381
CCTTTCAA -0.163412735075
CCTTTCAC -0.0714028400772
CCTTTCAG -0.138677609303
CCTTTCCA -0.0559041394336
CCTTTCCC -0.163253435182
CCTTTCCG -0.213216776514
CCTTTCGA -0.164438957476
CCTTTCGC -0.193576734363
CCTTTCTA -0.0286215632045
CCTTTCTC -0.199229224954
CCTTTGAA -0.100519692904
CCTTTGAC 0.0116807428261
CCTTTGAG 0.0131325868291
CCTTTGCA -0.0683582002818
CCTTTGCC -0.31166188887
CCTTTGCG -0.142358971959
CCTTTGGA -0.177777777778
CCTTTGGC -0.231655039818
CCTTTGTA -0.0392820304215
CCTTTGTC -0.146210674219
CCTTTTAA 0.144719829152
CCTTTTAC -0.16469165062
CCTTTTAG -0.0677582403108
CCTTTTCA -0.00484663637262
CCTTTTCC -0.0918903402637
CCTTTTCG -0.043343347415
CCTTTTGA 0.00305231502332
CCTTTTGC -0.0742023509656
CCTTTTTA 0.033343559957
CCTTTTTC -0.291361473986
CGAAAAAA 0.0493369777602
CGAAAAAC -0.09508810063
CGAAAAAG 0.10378597475
CGAAAACA 0.160502956431
CGAAAACC 0.0346185695815
CGAAAACG -0.133754815766
CGAAAAGA 0.0820050934159
CGAAAAGC -0.0467518496564
CGAAAATA 0.250562580957
CGAAAATC 0.421623329182
CGAAACAA 0.0806411776329
CGAAACAC -0.0977617599121
CGAAACAG -0.14883968206
CGAAACCA -0.107525200147
CGAAACCC -0.151777336033
CGAAACCG -0.227854039107
CGAAACGA -0.0107109925575

CGAAACGC -0.179622678783
CGAAACTA -0.0493482615735
CGAAACTC -0.134471419068
CGAAAGAA 0.0977408944214
CGAAAGAC -0.117263578683
CGAAAGAG -0.0534884801864
CGAAAGCA -0.0618579612801
CGAAAGCC -0.310694711895
CGAAAGCG -0.154692670519
CGAAAGGA -0.185135802469
CGAAAGGC -0.211413217139
CGAAAGTA 0.112340556324
CGAAAGTC -0.180378891965
CGAAATAA 0.183453533557
CGAAATAC 0.12800059271
CGAAATAG 0.0637413901924
CGAAATCA 0.426375430389
CGAAATCC 0.478975200848
CGAAATCG 0.450786515621
CGAAATGA -0.0547561149219
CGAAATGC -0.0884299681839
CGAAATTA 0.099105080412
CGAAATTC 0.32913181409
CGAACAAA 0.110561794488
CGAACAAC -0.236649304396
CGAACAAG -0.0758369431637
CGAACACA -0.116061578991
CGAACACC 0.0541005041187
CGAACACG -0.147923182442
CGAACAGA 0.190915694507
CGAACAGC -0.151456529136
CGAACATA 0.0765839059753
CGAACATC -0.00359565905888
CGAACCAA 0.0254357513693
CGAACCAC -0.13954582678
CGAACCAG -0.171391879878
CGAACCCA -0.212770129534
CGAACCCC -0.0985277777778
CGAACCCG -0.115307073988
CGAACCGA -0.0348983534269
CGAACCGC -0.0179999231711
CGAACCTA -0.0664495549306
CGAACCTC -0.290452914651
CGAACGAA -0.0296732671829
CGAACGAC -0.197666581449
CGAACGAG -0.134173199063
CGAACGCA -0.108861009582
CGAACGCC -0.279023965142
CGAACGCG 0.0435184270329
CGAACGGA -0.0816515775034
CGAACGGC -0.0563063390204
CGAACGTA -0.0150963797229
CGAACGTC -0.242802855325
CGAACTAA -0.0702083127531

CGAACTAC 0.0222291633374
CGAACTAG -0.136486256559
CGAACTCA -0.138109739369
CGAACTCC -0.118215044204
CGAACTCG -0.104205456811
CGAACTGA -0.208084241104
CGAACTGC -0.127341563786
CGAACTTA -0.179629345557
CGAACTTC -0.115855063875
CGAAGAAA 0.143371638604
CGAAGAAC -0.282779396513
CGAAGAAG -0.0632352214852
CGAAGACA 0.0504830531867
CGAAGACC -0.185112391378
CGAAGACG -0.0976584793011
CGAAGAGA -0.0108031872717
CGAAGAGC -0.210210655122
CGAAGATA 0.256934839404
CGAAGATC 0.312625286974
CGAAGCAA 0.0376926764779
CGAAGCAC -0.179095523229
CGAAGCAG -0.299251236563
CGAAGCCA -0.0579128090567
CGAAGCCC -0.256264342774
CGAAGCCG -0.151454319381
CGAAGCGA -0.0607641713343
CGAAGCGC -0.173266734773
CGAAGCTA -0.233262063777
CGAAGCTC -0.228587419955
CGAAGGAA -0.0108213096559
CGAAGGAC -0.251629468694
CGAAGGAG -0.183641993238
CGAAGGCA -0.0840106463997
CGAAGGCC -0.347220165117
CGAAGGCG -0.0548695489918
CGAAGGGA -0.138255081435
CGAAGGGC -0.314154820161
CGAAGGTA -0.0217189206777
CGAAGGTC -0.270216140379
CGAAGTAA 0.058031758023
CGAAGTAC -0.0967790619821
CGAAGTAG 0.0198249167353
CGAAGTCA -0.062778546181
CGAAGTCC -0.0572240059274
CGAAGTCG -0.177724223487
CGAAGTGA -0.0926111022329
CGAAGTGC -0.0895371082784
CGAAGTTA -0.117566559492
CGAAGTTC 0.0150414937759
CGAATAAA -0.0458042547257
CGAATAAC 0.0065067290171
CGAATAAG 0.0818322026828
CGAATACA 0.204112604009
CGAATACC 0.122277105458

CGAATACG 0.0804627983051
CGAATAGA 0.120147313062
CGAATAGC -0.00211026042095
CGAATATA 0.103948863415
CGAATATC 0.408285582559
CGAATCAA 0.122399139947
CGAATCAC 0.258307615108
CGAATCAG 0.345268282509
CGAATCCA 0.395763353751
CGAATCCC 0.39828322721
CGAATCCG 0.425810665437
CGAATCGA 0.273171752679
CGAATCGC 0.325786575126
CGAATCTA 0.426993457595
CGAATCTC 0.464631495752
CGAATGAA 0.00404602443595
CGAATGAC -0.00706547032502
CGAATGAG 0.0978063568112
CGAATGCA 0.0739341149092
CGAATGCC -0.125228428903
CGAATGCG -0.0833548788175
CGAATGGA 0.0444191013032
CGAATGGC -0.230733928966
CGAATGTA 0.0308384059971
CGAATGTC -0.118969033825
CGAATTAA -0.00770246764964
CGAATTAC 0.150873466331
CGAATTAG 0.091977685122
CGAATTCA 0.0305508902501
CGAATTCC 0.160914619421
CGAATTCG 0.185690044487
CGAATTGA -0.0210872691877
CGAATTGC 0.12494785835
CGAATTTA 0.179659465663
CGAATTTC 0.178442446596
CGACAAAA 0.10659674197
CGACAAAC -0.154469867757
CGACAAAG -0.189232485251
CGACAACA -0.0553924945731
CGACAACC 0.101785988715
CGACAACG -0.135825032244
CGACAAGA -0.0834047410876
CGACAAGC -0.214008636062
CGACAATA 0.118196186901
CGACAATC 0.279243430801
CGACACAA 0.0513705020966
CGACACAC -0.179388023034
CGACACAG -0.130534979424
CGACACCA -0.1367265962
CGACACCC 0.0189401422363
CGACACCG -0.24845137963
CGACACGA 0.0114145854255
CGACACGC -0.219325407315
CGACACTA -0.103298096555

CGACACTC -0.0409957522474
CGACAGAA 0.0191762026289
CGACAGAC -0.22244961988
CGACAGAG -0.118508479107
CGACAGCA -0.0600484494361
CGACAGCC -0.268231319038
CGACAGCG -0.0562690728666
CGACAGGA 0.104834890242
CGACAGGC -0.305164941382
CGACAGTA 0.138748855927
CGACAGTC -0.225424358959
CGACATAA 0.120230411206
CGACATAC -0.0470361611008
CGACATAG -0.0539348858832
CGACATCA -0.0815030678214
CGACATCC 0.0324650376518
CGACATCG 0.00436321725529
CGACATGA -0.0162291342815
CGACATGC -0.281335056412
CGACATTA 0.02292119431
CGACATTC -0.19291375183
CGACCAAA 0.0489386792453
CGACCAAC -0.0271893229832
CGACCAAG -0.240561831369
CGACCACA -0.0593114134729
CGACCACC -0.196194625999
CGACCACG -0.200555857354
CGACCAGA 0.0687097758343
CGACCAGC -0.107960375071
CGACCATA 0.12660541395
CGACCATC -0.0608053963863
CGACCCAA -0.0901585051247
CGACCCAC -0.0186822215347
CGACCCAG -0.236680961663
CGACCCCA -0.345410508761
CGACCCCC -0.365514113663
CGACCCCG -0.320660313698
CGACCCGA -0.0877559439422
CGACCCGC -0.223717609884
CGACCCTA -0.130794976162
CGACCCTC -0.13394270741
CGACCGAA -0.0304532033762
CGACCGAC -0.193917211329
CGACCGAG -0.291396486839
CGACCGCA -0.299272535756
CGACCGCC -0.243217070213
CGACCGCG -0.197606461812
CGACCGGA 0.0767233739631
CGACCGGC -0.284928357013
CGACCGTA -0.114199737427
CGACCGTC -0.192029939857
CGACCTAA -0.108320597823
CGACCTAC -0.208601038677
CGACCTAG 0.0390947358021

CGACCTCA -0.174968772694
CGACCTCC -0.370026605228
CGACCTCG -0.212683598615
CGACCTGA 0.0485746968062
CGACCTGC -0.170169372555
CGACCTTA -0.12591019584
CGACCTTC -0.116659258162
CGACGAAA -0.0818000633355
CGACGAAC -0.191394609901
CGACGAAG -0.173128705336
CGACGACA -0.178010413752
CGACGACC -0.282942483337
CGACGACG -0.00597619400405
CGACGAGA -0.123177190527
CGACGAGC -0.240453159041
CGACGATA 0.184858613801
CGACGATC 0.0755093415882
CGACGCAA 0.147462073811
CGACGCAC -0.201792504074
CGACGCAG -0.184307140152
CGACGCCA -0.346570119861
CGACGCCC -0.172233632452
CGACGCCG -0.172312478396
CGACGCGA -0.131466688068
CGACGCGC -0.273659807956
CGACGCTA -0.116280090649
CGACGCTC -0.0652209106425
CGACGGAA 0.166026469862
CGACGGAC -0.264755260192
CGACGGAG -0.132084927615
CGACGGCA -0.122475422409
CGACGGCC -0.103802036211
CGACGGCG -0.0404728973194
CGACGGGA -0.200880668285
CGACGGGC -0.254847257884
CGACGGTA -0.107699816394
CGACGGTC -0.193802405725
CGACGTAA 0.229316230872
CGACGTAC -0.043456546198
CGACGTAG -0.0969125563209
CGACGTCA -0.0852397859272
CGACGTCC -0.155523602033
CGACGTCG 0.181376003879
CGACGTGA -0.0489342202026
CGACGTGC -0.109064209416
CGACGTTA 0.112656150519
CGACGTTC 0.0302531339876
CGACTAAA -0.00173745285924
CGACTAAC -0.0810163464862
CGACTAAG 0.0161794335183
CGACTACA -0.043817645606
CGACTACC -0.0608951078209
CGACTACG -0.179777941104
CGACTAGA -0.213714257494

CGACTAGC -0.235085120208
CGACTATA -0.0701239996048
CGACTATC -0.0378404448809
CGACTCAA 0.0166263254956
CGACTCAC -0.176787249866
CGACTCAG 0.0155539090585
CGACTCCA -0.164750328992
CGACTCCC -0.0434785134915
CGACTCCG -0.177835942516
CGACTCGA -0.0446610748809
CGACTCGC 0.0780143011585
CGACTCTA -0.218894047149
CGACTCTC -0.186614379085
CGACTGAA -0.0707374140464
CGACTGAC -0.307229720506
CGACTGAG -0.0777843640914
CGACTGCA -0.0400868715804
CGACTGCC -0.153847553495
CGACTGCG -0.0571978556797
CGACTGGA -0.0232664564962
CGACTGGC -0.30319065515
CGACTGTA -0.00295384957282
CGACTGTC -0.142174535757
CGACTTAA 0.0587662531817
CGACTTAC -0.130502658226
CGACTTAG -0.026244679151
CGACTTCA -0.214820301783
CGACTTCC -0.311151710668
CGACTTGA -0.11255186722
CGACTTGC -0.125511760897
CGACTTTA -0.12458768969
CGACTTTC 0.070738380645
CGAGAAAA 0.150985751608
CGAGAAAC 0.0550201211628
CGAGAAAG 0.042919820435
CGAGAACA 0.211748725175
CGAGAACC -0.262510288066
CGAGAACG -0.094584267969
CGAGAAGA 0.0542051051095
CGAGAAGC -0.110356578427
CGAGAATA 0.175103511397
CGAGAATC 0.437147357791
CGAGACAA -0.0187569915593
CGAGACAC -0.0785866368146
CGAGACAG -0.179451569228
CGAGACCA -0.197425435781
CGAGACCC -0.189784406363
CGAGACCG -0.257958847737
CGAGACGA -0.168024274256
CGAGACGC -0.244668119099
CGAGACTA -0.109564852317
CGAGACTC -0.124979423868
CGAGAGAA 0.041289325781
CGAGAGAC -0.273551198257

CGAGAGAG -0.142556059413
CGAGAGCA 0.0082968924789
CGAGAGCC -0.222354582263
CGAGAGCG -0.0520452637298
CGAGAGGA 0.0259619184036
CGAGAGGC -0.253853840188
CGAGAGTA 0.0182029243233
CGAGAGTC -0.20293856235
CGAGATAA -0.0203338712156
CGAGATAC 0.321312451876
CGAGATAG -0.00305361698171
CGAGATCA 0.283384377264
CGAGATCC 0.405494632154
CGAGATCG 0.320706821225
CGAGATGA -0.0238405998695
CGAGATGC 0.0250746448879
CGAGATTA 0.367578051225
CGAGATTC 0.444441700147
CGAGCAAA 0.133684424622
CGAGCAAC -0.190062883764
CGAGCAAG -0.0509346153424
CGAGCACA -0.149165116522
CGAGCACC -0.133485329865
CGAGCACG -0.313876021897
CGAGCAGA -0.039524691358
CGAGCAGC -0.0947015250545
CGAGCATA 0.00742231197246
CGAGCATC 0.03948832682
CGAGCCAA -0.109801016703
CGAGCCAC -0.280154537375
CGAGCCAG -0.138192735734
CGAGCCCA -0.194513831604
CGAGCCCC -0.378310565933
CGAGCCCG -0.190286078047
CGAGCCGA -0.220278094939
CGAGCCGC -0.191055894846
CGAGCCTA -0.18310943185
CGAGCCTC -0.257452791002
CGAGCGAA -0.0713808392072
CGAGCGAC -0.141899527355
CGAGCGAG 0.0298469779798
CGAGCGCA -0.157920116195
CGAGCGCC -0.279625251246
CGAGCGCG 0.180703747612
CGAGCGGA -0.0192974300118
CGAGCGGC -0.174040788109
CGAGCGTA -0.00145173329821
CGAGCGTC -0.205694444444
CGAGCTAA -0.0284994566828
CGAGCTAC -0.0157946151229
CGAGCTAG -0.202288985007
CGAGCTCA -0.283118550338
CGAGCTCC -0.337420640222
CGAGCTCG 0.0960019931676

CGAGCTGA -0.139787226625
CGAGCTGC -0.350647640679
CGAGCTTA -0.228910483913
CGAGCTTC -0.245890021029
CGAGGAAA 0.00137674717242
CGAGGAAC -0.08174340571
CGAGGAAG -0.048476828503
CGAGGACA -0.142268463512
CGAGGACC -0.213718519551
CGAGGACG -0.269806787137
CGAGGAGA -0.0205297904216
CGAGGAGC -0.324804521848
CGAGGATA 0.228856798338
CGAGGATC 0.328036722246
CGAGGCAA 0.0703747612461
CGAGGCAC -0.321676564973
CGAGGCAG -0.0199854756718
CGAGGCCA -0.129419028664
CGAGGCCC -0.308809006518
CGAGGCCG -0.275552921941
CGAGGCGA 0.0237562086661
CGAGGCGC -0.143346339375
CGAGGCTA 0.0536908169047
CGAGGCTC -0.303059079082
CGAGGGAA 0.167912193279
CGAGGGAC -0.314052239174
CGAGGGAG -0.326925450506
CGAGGGCA -0.231178967121
CGAGGGCC -0.204392618989
CGAGGGCG -0.229863739481
CGAGGGGA 0.0479936442073
CGAGGGGC -0.345006650829
CGAGGGTA -0.0562928915516
CGAGGGTC -0.113574034908
CGAGGTAA 0.0825142010373
CGAGGTAC -0.270744526188
CGAGGTAG -0.1392105783
CGAGGTCA -0.0655707567102
CGAGGTCC -0.186421696932
CGAGGTGA -0.020572980983
CGAGGTGC -0.370970130832
CGAGGTTA -0.139986983631
CGAGGTTC -0.356535415233
CGAGTAAA 0.146290258842
CGAGTAAC -0.022271294292
CGAGTAAG -0.0628364197531
CGAGTACA -0.0254311158892
CGAGTACC -0.18974793236
CGAGTACG -0.0785868058885
CGAGTAGA 0.102063162748
CGAGTAGC -0.312581949451
CGAGTATA 0.0954576390261
CGAGTATC 0.177593596095
CGAGTCAA -0.0486783463962

CGAGTCAC -0.199401277623
CGAGTCAG -0.142984550369
CGAGTCCA -0.287699161821
CGAGTCCC -0.230067424853
CGAGTCCG -0.238364883402
CGAGTCGA -0.0908676851486
CGAGTCGC -0.20353824212
CGAGTCTA -0.0509092563466
CGAGTCTC -0.334791699943
CGAGTGAA 0.0545512915494
CGAGTGAC -0.173872314221
CGAGTGAG 0.00695970809049
CGAGTGCA -0.245172601116
CGAGTGCC -0.158205761317
CGAGTGCG -0.150998015879
CGAGTGGA -0.10328979478
CGAGTGGC -0.142431144022
CGAGTGTA -0.115329856319
CGAGTGTC -0.0444082197194
CGAGTTAA -0.126422021369
CGAGTTAC -0.0672241929814
CGAGTTAG -0.162143865745
CGAGTTCA -0.113996554319
CGAGTTCC -0.183481135282
CGAGTTGA -0.076383420743
CGAGTTGC -0.121210874051
CGAGTTTA -0.0490273560647
CGAGTTTC -0.0077978366097
CGATAAAA 0.16658143623
CGATAAAC 0.0116194267285
CGATAAAG -0.0216159470408
CGATAACA 0.113598128804
CGATAACC -0.187770972593
CGATAACG 0.194244110738
CGATAAGA 0.0157962024029
CGATAAGC -0.0276158642341
CGATAATA 0.011295527893
CGATAATC 0.333948832682
CGATACAA 0.0706167086018
CGATACAC 0.199466508595
CGATACAG -0.0299231473276
CGATACCA 0.00389690223715
CGATACCC 0.212768584836
CGATACCG 0.187742797786
CGATACGA 0.175803756919
CGATACGC 0.271884758084
CGATACTA 0.137014718957
CGATACTC 0.273242485436
CGATAGAA 0.125101539002
CGATAGAC -0.131646227909
CGATAGAG -0.19871371603
CGATAGCA -0.11210531515
CGATAGCC -0.0946582692537
CGATAGCG -0.155603299254

CGATAGGA -0.1156250343
CGATAGGC -0.0789693896823
CGATAGTA 0.167569540495
CGATAGTC -0.201102495294
CGATATAA 0.286860953322
CGATATAC 0.154360553804
CGATATAG 0.077842373062
CGATATCA 0.396028845204
CGATATCC 0.472829892639
CGATATCG 0.441057905109
CGATATGA 0.0903395244682
CGATATGC 0.262225844695
CGATATTA 0.234990484215
CGATATTC 0.395384640717
CGATCAAA 0.165972359437
CGATCAAC -0.108718320217
CGATCAAG 0.0907660569785
CGATCACA 0.114341149092
CGATCACC 0.0575755814798
CGATCACG 0.111912445937
CGATCAGA 0.180380050267
CGATCAGC -0.00249338952776
CGATCATA 0.228147160201
CGATCATC 0.0891594766076
CGATCCAA 0.214697891999
CGATCCAC 0.204861797185
CGATCCAG 0.255343627557
CGATCCCA 0.177779553412
CGATCCCC 0.266388943775
CGATCCCG 0.185040605524
CGATCCGA 0.228556060506
CGATCCGC 0.2507040166
CGATCCTA 0.0821539087356
CGATCCTC 0.326660574327
CGATCGAA 0.286194537751
CGATCGAC 0.1444046935
CGATCGAG 0.106344266614
CGATCGCA 0.324211288942
CGATCGCC 0.326063689653
CGATCGCG 0.399032024065
CGATCGGA 0.190062740836
CGATCGGC 0.043404801897
CGATCGTA 0.225847248296
CGATCGTC 0.166765461371
CGATCTAA 0.18936992431
CGATCTAC 0.192804323508
CGATCTAG 0.297015302202
CGATCTCA 0.272836354087
CGATCTCC 0.379028573545
CGATCTGA 0.273405198088
CGATCTGC 0.235326311629
CGATCTTA 0.224928450017
CGATCTTC 0.338896024062
CGATGAAA -0.134166214014

CGATGAAC -0.115877995643
CGATGAAG -0.0390245360969
CGATGACA 0.0783442007508
CGATGACC -0.246712096522
CGATGACG -0.156018314082
CGATGAGA 0.113092557349
CGATGAGC -0.0331725333948
CGATGATA 0.185470631819
CGATGATC 0.0803539625134
CGATGCAA 0.000211310895958
CGATGCAC -0.0972318253655
CGATGCAG -0.0312742434588
CGATGCCA -0.214443144899
CGATGCCC -0.0976610056223
CGATGCCG -0.311316507246
CGATGCGA 0.164891106281
CGATGCGC 0.174530678787
CGATGCTA 0.0493644037017
CGATGCTC -0.0761429676825
CGATGGAA 0.0932764807578
CGATGGAC -0.108615053826
CGATGGAG -0.124339408307
CGATGGCA -0.154988667731
CGATGGCC -0.124866261748
CGATGGCG -0.0824720946313
CGATGGGA -0.115075998791
CGATGGGC -0.144586782861
CGATGGTA -0.208049489735
CGATGGTC -0.194986201888
CGATGTAA 0.144404926563
CGATGTAC 0.0955196988173
CGATGTAG 0.0100698903487
CGATGTCA -0.123525844707
CGATGTCC -0.114307091076
CGATGTGA 0.0930002150025
CGATGTGC -0.0437135080557
CGATGTTA 0.15677347472
CGATGTTC -0.0130417096451
CGATTAAA 0.0775430949323
CGATTAAC -0.0694663988232
CGATTAAG -0.0985118338553
CGATTACA 0.400107576456
CGATTACC 0.419207356936
CGATTACG 0.441295599494
CGATTAGA -0.0195841188962
CGATTAGC -0.0702210805945
CGATTATA 0.184769523968
CGATTATC 0.276694737882
CGATTCAA 0.0274272514637
CGATTCAC 0.163548007496
CGATTCAG -0.0587114975082
CGATTCCA 0.265403741026
CGATTCCC 0.457562185778
CGATTCCG 0.356205003939

CGATTCGA 0.200890561213
CGATTCGC 0.381899670216
CGATTCTA 0.262465425269
CGATTCTC 0.426162910116
CGATTGAA 0.1138392922
CGATTGAC 0.0780203736635
CGATTGAG -0.115316015673
CGATTGCA 0.309917498271
CGATTGCC 0.305357895196
CGATTGCG 0.456549910653
CGATTGGA -0.187844260581
CGATTGGC 0.0111240248137
CGATTGTA 0.0956771828141
CGATTGTC 0.24255662255
CGATTTAA 0.145702979209
CGATTTAC 0.258483157167
CGATTTAG 0.135686930115
CGATTTCA 0.307018266043
CGATTTCC 0.468323989924
CGATTTGA 0.273887524879
CGATTTGC 0.359355251115
CGATTTTA 0.286860953322
CGATTTTC 0.420097038354
CGCAAAAA 0.248660782893
CGCAAAAC 0.0332149850951
CGCAAAAG 0.0282891811854
CGCAAACA -0.0154096208092
CGCAAACC -0.100606459757
CGCAAACG -0.0439593006026
CGCAAAGA 0.0500588897527
CGCAAAGC -0.212278275697
CGCAAATA 0.121391248985
CGCAAATC 0.423115366176
CGCAACAA 0.107490368468
CGCAACAC 0.0446405125098
CGCAACAG -0.10679142008
CGCAACCA -0.0398050353755
CGCAACCC 0.0890110847994
CGCAACCG -0.0321399724564
CGCAACGA -0.0125743704582
CGCAACGC -0.0590908298516
CGCAACTA -0.215220504716
CGCAACTC -0.0127527970738
CGCAAGAA 0.00276756788168
CGCAAGAC 0.065565934944
CGCAAGAG -0.066524890118
CGCAAGCA -0.0200855123054
CGCAAGCC -0.0278457327809
CGCAAGCG -0.0564206886691
CGCAAGGA -0.016518060773
CGCAAGGC -0.162024232846
CGCAAGTA -1.37214867505E-5
CGCAAGTC -0.0187783811109
CGCAATAA 0.245986861602

CGCAATAC 0.085133592395
CGCAATAG 0.129870201147
CGCAATCA 0.434847636611
CGCAATCC 0.48350791045
CGCAATGA 0.0694361716037
CGCAATGC 0.0614504099575
CGCAATTA 0.229619335599
CGCAATTC 0.283098223236
CGCACAAA 0.163162198951
CGCACAAC -0.0639444581653
CGCACAAG -0.120802054524
CGCACACA -0.0229206921099
CGCACACC -0.119197034274
CGCACACG 0.0491493905539
CGCACAGA 0.0151805562417
CGCACAGC -0.113631741346
CGCACATA -0.00143519397219
CGCACATC 0.0881578080748
CGCACCAA -0.0399091178504
CGCACCAC -0.204488086282
CGCACCAG -0.236491193412
CGCACCCA -0.11626911617
CGCACCCC -0.133644459809
CGCACCCG -0.240844767692
CGCACCGA -0.0677045599245
CGCACCGC 0.00371303431469
CGCACCTA -0.113775751818
CGCACCTC -0.189845381637
CGCACGAA 0.0761844387363
CGCACGAC -0.156315177062
CGCACGAG -0.0778353615139
CGCACGCA 0.0367512675606
CGCACGCC -0.254175438596
CGCACGCG 0.0274359682129
CGCACGGA -0.0135153196868
CGCACGGC -0.0592664995483
CGCACGTA -0.0303612008977
CGCACGTC -0.123706053765
CGCACTAA 0.0530603053768
CGCACTAC -0.170565205863
CGCACTAG 0.0318115392549
CGCACTCA -0.133487961379
CGCACTCC -0.262530030532
CGCACTGA -0.0960761579935
CGCACTGC -0.100315045336
CGCACTTA -0.064266601588
CGCACTTC -0.0568683617264
CGCAGAAA -0.0895589139119
CGCAGAAC -0.170118785822
CGCAGAAG -0.136007077301
CGCAGACA -0.0619326725718
CGCAGACC -0.102803363295
CGCAGACG 0.0532578850261
CGCAGAGA -0.0560995452762

CGCAGAGC	-0.261202787371
CGCAGATA	0.325869698702
CGCAGATC	0.316365342818
CGCAGCAA	-0.0831666666667
CGCAGCAC	0.0769180480293
CGCAGCAG	-0.177632419746
CGCAGCCA	-0.196599770611
CGCAGCCC	-0.284702919829
CGCAGCCG	-0.14662962963
CGCAGCGA	0.0543462470437
CGCAGCGC	-0.138936418066
CGCAGCTA	-0.204128050219
CGCAGCTC	-0.330159435556
CGCAGGAA	-0.101697286975
CGCAGGAC	-0.336813643202
CGCAGGAG	-0.173051561365
CGCAGGCA	-0.0537518341493
CGCAGGCC	-0.231636384428
CGCAGGCG	-0.0962693842534
CGCAGGGA	-0.0237834001097
CGCAGGGC	-0.283366879562
CGCAGGTA	-0.111028467497
CGCAGGTC	-0.146530007409
CGCAGTAA	-0.0160780439615
CGCAGTAC	-0.121703703704
CGCAGTAG	-0.231572617194
CGCAGTCA	-0.296453786906
CGCAGTCC	-0.260015219241
CGCAGTGA	0.0692056048972
CGCAGTGC	-0.028455369097
CGCAGTTA	0.0462935519989
CGCAGTTC	0.0354318354824
CGCATAAA	-0.0728887623409
CGCATAAC	0.0619689784628
CGCATAAG	0.0961849694187
CGCATACA	0.0949413798614
CGCATACC	0.0655388161686
CGCATACG	0.0590490460822
CGCATAGA	-0.10601058091
CGCATAGC	-0.0571039764176
CGCATATA	0.0665922203127
CGCATATC	0.2816191172
CGCATCAA	0.0913713802718
CGCATCAC	-0.00230601106391
CGCATCAG	0.0355276734945
CGCATCCA	-0.0939343590876
CGCATCCC	-0.118347269758
CGCATCCG	0.0148576990688
CGCATCGA	0.0508636845699
CGCATCGC	0.116145412189
CGCATCTA	-0.140869931574
CGCATCTC	-0.0672600925001
CGCATGAA	-0.0701921915989
CGCATGAC	-0.067410920108

CGCATGAG −0.0298330118298
CGCATGCA 0.0585838188078
CGCATGCC −0.14991055358
CGCATGCG −0.0397450457568
CGCATGGA −0.0985816186557
CGCATGGC −0.236718792867
CGCATGTA −0.0414862380286
CGCATGTC −0.0996526586175
CGCATTAA 0.0333633576918
CGCATTAC −0.0800328330888
CGCATTAG 0.00283730187555
CGCATTCA −0.00658259846295
CGCATTCC −0.0146790083643
CGCATTGA −0.000353028343963
CGCATTGC −0.114409745924
CGCATTTA 0.0505554457837
CGCATTTC 0.123552656429
CGCCAAAA −0.0560101614974
CGCCAAAC −0.146773458894
CGCCAAAG −0.247853541588
CGCCAACA −0.133018836857
CGCCAACC −0.0140913818852
CGCCAACG −0.138664590861
CGCCAAGA 0.0569262773765
CGCCAAGC −0.216529211319
CGCCAATA 0.0892329252217
CGCCAATC 0.0778362122819
CGCCACAA 0.00356860614442
CGCCACAC −0.0446538822483
CGCCACAG −0.129242137305
CGCCACCA −0.233125366959
CGCCACCC −0.0835611344642
CGCCACCG −0.158561523469
CGCCACGA −0.0140281551
CGCCACGC −0.206272392584
CGCCACTA −0.266510071209
CGCCACTC −0.00790201501383
CGCCAGAA −0.00271368055273
CGCCAGAC −0.243523602033
CGCCAGAG −0.09864177409
CGCCAGCA −0.158400140473
CGCCAGCC −0.17350102337
CGCCAGCG 0.029668598652
CGCCAGGA −0.095519986692
CGCCAGGC −0.273687646762
CGCCAGTA −0.102371307183
CGCCAGTC −0.299576426953
CGCCATAA −0.0966212211025
CGCCATAC −0.205095679012
CGCCATAG −0.181557761644
CGCCATCA −0.0870469106388
CGCCATCC −0.0150564720603
CGCCATGA −0.154534150303
CGCCATGC −0.291033763954

CGCCATTA	-0.0758092992247
CGCCATTC	-0.0762076566367
CGCCCAAA	0.00128572051196
CGCCCAAC	-0.13998988907
CGCCCAAG	-0.10393858075
CGCCCACA	-0.128438248077
CGCCCACC	-0.238328099992
CGCCCACG	-0.0624375734027
CGCCCAGA	-0.080339869281
CGCCCAGC	-0.207163461957
CGCCCATA	-0.196729766804
CGCCCATC	-0.12757211301
CGCCCCAA	-0.0248180378597
CGCCCCAC	-0.202021595838
CGCCCCAG	-0.109812927986
CGCCCCCA	0.011531433651
CGCCCCCC	-0.131228087502
CGCCCCCG	-0.257180118739
CGCCCCGA	-0.195740804759
CGCCCCGC	-0.251239506173
CGCCCCTA	-0.197827558261
CGCCCCTC	-0.269300541364
CGCCCGAA	0.0180608241445
CGCCCGAC	-0.333610706911
CGCCCGAG	-0.0728283476839
CGCCCGCA	-0.168301864015
CGCCCGCC	-0.113745246209
CGCCCGCG	-0.122458722241
CGCCCGGA	0.0300460584807
CGCCCGGC	-0.323990859162
CGCCCGTA	0.0482255373334
CGCCCGTC	-0.31726547254
CGCCCTAA	0.000680484900369
CGCCCTAC	-0.144203542737
CGCCCTAG	-0.033415460425
CGCCCTCA	-0.0277695448857
CGCCCTCC	-0.0367544162921
CGCCCTGA	-0.205725490196
CGCCCTGC	-0.11855487417
CGCCCTTA	0.0258409794818
CGCCCTTC	-0.301819667994
CGCCGAAA	0.0335270807263
CGCCGAAC	-0.213245541838
CGCCGAAG	-0.177223022753
CGCCGACA	-0.189953946508
CGCCGACC	-0.266178503793
CGCCGACG	-0.153420181893
CGCCGAGA	-0.0973918828236
CGCCGAGC	-0.0927318040939
CGCCGATA	0.210896507058
CGCCGATC	-0.0628542310132
CGCCGCAA	-0.0369208732589
CGCCGCAC	-0.109292091242
CGCCGCAG	-0.185570206485

CGCCGCCA -0.153743394736
CGCCGCCC -0.131713807609
CGCCGCCG -0.0335920728111
CGCCGCGA 0.0457501811236
CGCCGCGC -0.0254081940671
CGCCGCTA 0.0471868207864
CGCCGCTC -0.225179990342
CGCCGGAA 0.0539290747171
CGCCGGAC -0.117926935678
CGCCGGAG -0.220913709483
CGCCGGCA -0.155721897336
CGCCGGCC -0.155767932698
CGCCGGCG -0.00221751387068
CGCCGGGA -0.0677180217404
CGCCGGGC -0.361451936097
CGCCGGTA 0.0511376732853
CGCCGGTC -0.0561620452699
CGCCGTAA 0.0484084668691
CGCCGTAC -0.125246239869
CGCCGTAG -0.136871459695
CGCCGTCA -0.0997820812692
CGCCGTCC -0.218957959589
CGCCGTGA -0.0639039414234
CGCCGTGC -0.3420858493
CGCCGTTA -0.042723831272
CGCCGTTC -0.160149781774
CGCCTAAA -0.157454970529
CGCCTAAC -0.0512120296558
CGCCTAAG -0.0378778524153
CGCCTACA 0.0310269696867
CGCCTACC -0.150038688859
CGCCTACG -0.133081444165
CGCCTAGA -0.0626877640318
CGCCTAGC -0.119837937645
CGCCTATA -0.0278251098928
CGCCTATC 0.0692124417067
CGCCTCAA -0.0588320847397
CGCCTCAC -0.0509143781488
CGCCTCAG -0.139347096009
CGCCTCCA -0.115747360102
CGCCTCCC -0.224329010029
CGCCTCCG -0.313560863791
CGCCTCGA -0.0692104758529
CGCCTCGC -0.250474913398
CGCCTCTA -0.199249894371
CGCCTCTC -0.244616178076
CGCCTGAA -0.090509270691
CGCCTGAC -0.155448005663
CGCCTGAG 0.0020056850608
CGCCTGCA -0.214645899839
CGCCTGCC -0.152636165577
CGCCTGGA -0.0278367714284
CGCCTGGC -0.232459528691
CGCCTGTA 0.0459532491683

CGCCTGTC -0.410122652273
CGCCTTAA 0.0229410311914
CGCCTTAC -0.139771049758
CGCCTTAG -0.17951503778
CGCCTTCA -0.103609361564
CGCCTTCC -0.208596681809
CGCCTTGA -0.0281180538405
CGCCTTGC -0.250942340296
CGCCTTTA -0.266776949286
CGCCTTTC -0.138071633167
CGCGAAAA 0.188184446414
CGCGAAAC -0.117404974731
CGCGAAAG -0.131768727821
CGCGAACA -0.0896939861921
CGCGAACC -0.0408203896681
CGCGAACG -0.132499108147
CGCGAAGA 0.113232452963
CGCGAAGC -0.119996568184
CGCGAATA 0.235379832348
CGCGAATC 0.421531823399
CGCGACAA -0.0255027552745
CGCGACAC 0.000912551087262
CGCGACAG 0.0868158466706
CGCGACCA -0.0194717649964
CGCGACCC -0.185732294992
CGCGACCG -0.0755677390321
CGCGACGA -0.0465661255447
CGCGACGC -0.171214233842
CGCGACTA -0.0838094454049
CGCGACTC -0.246486564996
CGCGAGAA 0.0549847417067
CGCGAGAC -0.15216448791
CGCGAGAG -0.280957475995
CGCGAGCA -0.0110688187352
CGCGAGCC -0.0822245334325
CGCGAGCG 2.7442973501E-5
CGCGAGGA -0.144446090616
CGCGAGGC -0.207987688459
CGCGAGTA 0.186894882434
CGCGAGTC -0.129687726943
CGCGATAA 0.198445629981
CGCGATAC 0.139890750076
CGCGATAG 0.00364717117829
CGCGATCA 0.318947307251
CGCGATCC 0.443379657073
CGCGATGA 0.00783597050398
CGCGATGC 0.128188561608
CGCGATTA 0.285365759951
CGCGATTC 0.37438873796
CGCGCAAA 0.0968901622429
CGCGCAAC -0.0491648726463
CGCGCAAG 0.186636918483
CGCGCACA 0.0463721052372
CGCGCACC -0.0432007288854

CGCGCACG -0.0308706008913
CGCGCAGA 0.0546612816527
CGCGCAGC -0.194824360726
CGCGCATA 0.190286833959
CGCGCATC -0.108977242296
CGCGCCAA -0.0555465173031
CGCGCCAC -0.0173422459306
CGCGCCAG 0.0044503709557
CGCGCCCA -0.204508555014
CGCGCCCC -0.188649838917
CGCGCCCG -0.178798953035
CGCGCCGA -0.13945819363
CGCGCCGC -0.156008230453
CGCGCCTA 0.0873976377088
CGCGCCTC -0.0829674719974
CGCGCGAA 0.00404234999671
CGCGCGAC 0.000315594195262
CGCGCGAG 0.0166048302597
CGCGCGCA 0.153911172583
CGCGCGCC -0.156211410225
CGCGCGCG 0.179009480545
CGCGCGGA -0.0249154756416
CGCGCGGC -0.161745859686
CGCGCGTA 0.0822805544217
CGCGCGTC -0.150670667183
CGCGCTAA 0.0677451222524
CGCGCTAC -0.112198714243
CGCGCTAG 0.106366220993
CGCGCTCA -0.118868090197
CGCGCTCC -0.0963820501023
CGCGCTGA -0.000702540121627
CGCGCTGC -0.0846614420407
CGCGCTTA 0.0499691901294
CGCGCTTC -0.0875786972071
CGCGGAAA 0.201999777734
CGCGGAAC -0.0691047936631
CGCGGAAG -0.218205761317
CGCGGACA -0.166047930283
CGCGGACC -0.224348752981
CGCGGACG -0.103894322061
CGCGGAGA -0.019957931313
CGCGGAGC -0.0754452402514
CGCGGATA 0.337758052097
CGCGGATC 0.292815619405
CGCGGCAA 0.0304374355324
CGCGGCAC -0.237450063655
CGCGGCAG -0.0371481860227
CGCGGCCA -0.14252432825
CGCGGCCC -0.276332032986
CGCGGCCG -0.11395796233
CGCGGCGA 0.0730681504511
CGCGGCGC -0.108609533218
CGCGGCTA -0.0669526224505
CGCGGCTC -0.304203151403

CGCGGGAA −0.0635653681934
CGCGGGAC −0.17435719411
CGCGGGAG −0.130410945372
CGCGGGCA −0.191643404683
CGCGGGCC −0.203571115061
CGCGGGGA −0.0159839529345
CGCGGGGC −0.169698241802
CGCGGGTA −0.00639077315875
CGCGGGTC −0.236480397966
CGCGGTAA 0.0555445789084
CGCGGTAC −0.157999143948
CGCGGTAG −0.151831914882
CGCGGTCA −0.164693534845
CGCGGTCC −0.14546106852
CGCGGTGA 0.207449669587
CGCGGTGC −0.130118042179
CGCGGTTA 0.164242610181
CGCGGTTC −0.0254311158892
CGCGTAAA 0.0474815858672
CGCGTAAC 0.022888730696
CGCGTAAG −0.0642479850777
CGCGTACA 0.0111006827812
CGCGTACC −0.0935424836601
CGCGTACG −0.0720785365904
CGCGTAGA 0.0970304940944
CGCGTAGC −0.0451635213912
CGCGTATA 0.0992896829833
CGCGTATC 0.356931994828
CGCGTCAA −0.138351221156
CGCGTCAC −0.0187151834472
CGCGTCAG −0.0336187980324
CGCGTCCA −0.0835648148148
CGCGTCCC −0.221807956104
CGCGTCCG −0.2748080396
CGCGTCGA −0.0347281074364
CGCGTCGC −0.128713878123
CGCGTCTA −0.228217623461
CGCGTCTC −0.203782472343
CGCGTGAA 0.0436607441198
CGCGTGAC −0.120968140282
CGCGTGAG −0.0031209868616
CGCGTGCA 0.126758719494
CGCGTGCC −0.106773448346
CGCGTGGA 0.108347740183
CGCGTGGC −0.21371539961
CGCGTGTA −0.0292377441678
CGCGTGTC −0.169254155623
CGCGTTAA 0.0196234175816
CGCGTTAC −0.180991812634
CGCGTTAG −0.272240731758
CGCGTTCA 0.074070576613
CGCGTTCC −0.0451542138219
CGCGTTGA 0.135824029246
CGCGTTGC −0.285453288564

CGCGTTTA -0.00668785264221
CGCGTTTC -0.0833388211148
CGCTAAAA -0.0902462183583
CGCTAAAC -0.102913394577
CGCTAAAG 0.0165481130211
CGCTAACA 0.0250794946661
CGCTAACC -0.0551130998213
CGCTAACG -0.0712848182537
CGCTAAGA 0.117311463978
CGCTAAGC -0.220994816095
CGCTAATA 0.149630671551
CGCTAATC 0.0486546725279
CGCTACAA -0.0589106925987
CGCTACAC -0.127283151998
CGCTACAG 0.0142847342279
CGCTACCA 0.0266071075768
CGCTACCC -0.310874786887
CGCTACCG -0.207789464443
CGCTACGA -0.0359585281784
CGCTACGC -0.0893904927792
CGCTACTA -0.112644139001
CGCTACTC -0.024620931496
CGCTAGAA 0.00350849156655
CGCTAGAC -0.145315139701
CGCTAGAG 0.00830973237612
CGCTAGCA -0.0610971481702
CGCTAGCC -0.206631808279
CGCTAGCG 0.0502518269801
CGCTAGGA 0.00643781811421
CGCTAGGC -0.21462962963
CGCTAGTA -0.0850990270163
CGCTAGTC -0.326410146693
CGCTATAA 0.0386475376803
CGCTATAC -0.126491083676
CGCTATAG -0.252261891045
CGCTATCA 0.0819968605238
CGCTATCC 0.176277959633
CGCTATGA -0.140175744372
CGCTATGC -0.241258375665
CGCTATTA -0.0773063773508
CGCTATTC 0.0579342833152
CGCTCAAA 0.0263833891898
CGCTCAAC 0.00616824819427
CGCTCAAG -0.171424573188
CGCTCACA -0.126510220633
CGCTCACC -0.0490254184353
CGCTCACG -0.167118902085
CGCTCAGA 0.050769500977
CGCTCAGC -0.100844486147
CGCTCATA 0.185952518999
CGCTCATC -0.133535822468
CGCTCCAA -0.141410649017
CGCTCCAC -0.267854290287
CGCTCCAG 0.0277132867901

CGCTCCCA -0.00127990934581
CGCTCCCC -0.102411038489
CGCTCCCG -0.188983808756
CGCTCCGA -0.0475163527811
CGCTCCGC -0.0952130895252
CGCTCCTA -0.0838798250839
CGCTCCTC -0.174747998529
CGCTCGAA 0.0759484291642
CGCTCGAC -0.261719827924
CGCTCGAG -0.0364955491894
CGCTCGCA -0.14549946911
CGCTCGCC -0.204333333333
CGCTCGGA -0.196690450182
CGCTCGGC -0.0881307137898
CGCTCGTA -0.106562806887
CGCTCGTC -0.233982635571
CGCTCTAA -0.0596261174495
CGCTCTAC -0.0697645024915
CGCTCTAG -0.240172839506
CGCTCTCA -0.317498910675
CGCTCTCC -0.238207410161
CGCTCTGA -0.0829631612737
CGCTCTGC -0.157998715859
CGCTCTTA -0.123691527285
CGCTCTTC -0.0233876333168
CGCTGAAA 0.0934296032844
CGCTGAAC 0.000463440500923
CGCTGAAG -0.0751085208783
CGCTGACA -0.219337690632
CGCTGACC -0.217248920279
CGCTGACG -0.238832593488
CGCTGAGA -0.114081512315
CGCTGAGC -0.161657957341
CGCTGATA 0.141572811697
CGCTGATC -0.0847808786806
CGCTGCAA 0.0321110513852
CGCTGCAC -0.269706826201
CGCTGCAG -0.179300411523
CGCTGCCA -0.251707892321
CGCTGCCC -0.184552977776
CGCTGCCG -0.234065602147
CGCTGCGA -0.0681509824259
CGCTGCGC -0.196359733088
CGCTGCTA -0.0137609348081
CGCTGCTC -0.092869725556
CGCTGGAA -0.180849779709
CGCTGGAC -0.18288453159
CGCTGGAG 0.0342960498598
CGCTGGCA -0.211591402894
CGCTGGCC -0.231104055864
CGCTGGGA -0.0253928845329
CGCTGGGC -0.294618612688
CGCTGGTA -0.042649125118
CGCTGGTC -0.209511543442

CGCTGTAA 0.0402184352789
CGCTGTAC -0.00326151700492
CGCTGTAG -0.12712092278
CGCTGTCA -0.155906190155
CGCTGTCC -0.25147356587
CGCTGTGA -0.00433948832682
CGCTGTGC -0.0346176010538
CGCTGTTA -0.122001075552
CGCTGTTC -0.17979956427
CGCTTAAA 0.0927418534714
CGCTTAAC 0.0169430314105
CGCTTAAG -0.269165555501
CGCTTACA 0.130254124271
CGCTTACC -0.136871459695
CGCTTACG 0.152421019122
CGCTTAGA -0.187060798936
CGCTTAGC -0.0690370977254
CGCTTATA 0.105243803377
CGCTTATC 0.0282525412193
CGCTTCAA 0.0765635668938
CGCTTCAC -0.1289218107
CGCTTCAG -0.105375694331
CGCTTCCA -0.148999369613
CGCTTCCC -0.0675149938817
CGCTTCCG -0.0891503094675
CGCTTCGA -0.000586506642959
CGCTTCGC -0.175934614055
CGCTTCTA -0.168697346396
CGCTTCTC -0.218027596224
CGCTTGAA -0.12066581053
CGCTTGAC -0.114395223907
CGCTTGAG -0.103198121076
CGCTTGCA -0.0815030581262
CGCTTGCC -0.224576131687
CGCTTGGA -0.161801988674
CGCTTGGC -0.297924285511
CGCTTGTA 0.000439044200775
CGCTTGTC -0.177416666667
CGCTTTAA 0.14652077982
CGCTTTAC -0.144161274921
CGCTTTAG -0.180211248285
CGCTTTCA -0.111942510001
CGCTTTCC -0.0192824981845
CGCTTTGA 0.120042712071
CGCTTTGC -0.317443712981
CGCTTTTA 0.0658768323333
CGCTTTTC -0.0280796504863
CGGAAAAA -0.0310419582339
CGGAAAAC 0.0232083448346
CGGAAAAG -0.0192102625194
CGGAAACA -0.0807425355681
CGGAAACC -0.189513916326
CGGAAACG -0.118185938013
CGGAAAGA 0.0467628268458

CGGAAAGC -0.232637749189
CGGAAATA 0.118122790841
CGGAAATC 0.47428593383
CGGAACAA -0.12775412956
CGGAACAC -0.029237743968
CGGAACAG -0.0412674699985
CGGAACCA -0.153987766036
CGGAACCC -0.235915628207
CGGAACCG -0.117591860925
CGGAACGA 0.0604788250016
CGGAACGC -0.225113854595
CGGAACTA -0.0763717387248
CGGAACTC -0.132924453226
CGGAAGAA -0.0103045045935
CGGAAGAC -0.0572952682011
CGGAAGAG -0.0938638658413
CGGAAGCA -0.00164003962824
CGGAAGCC -0.380909508071
CGGAAGGA -0.068276861273
CGGAAGGC -0.346712085308
CGGAAGTA -0.0121098721026
CGGAAGTC -0.407189671544
CGGAATAA 0.110983104034
CGGAATAC 0.00119514149597
CGGAATAG 0.123849179833
CGGAATCA 0.318954978761
CGGAATCC 0.456544099487
CGGAATGA -0.130263468388
CGGAATGC 0.0811805384045
CGGAATTA 0.0950240400448
CGGAATTC 0.170626687743
CGGACAAA -0.0246175526274
CGGACAAC -0.0119099616261
CGGACAAG -0.336124756335
CGGACACA -0.10030513572
CGGACACC -0.198527500858
CGGACACG -0.281177472717
CGGACAGA 0.164617262069
CGGACAGC -0.244438696999
CGGACATA -0.0523368151904
CGGACATC -0.149525785418
CGGACCAA -0.0808434907675
CGGACCAC -0.277303947174
CGGACCAG -0.30906961178
CGGACCCA -0.153156136529
CGGACCCC -0.107416666667
CGGACCCG -0.269276786544
CGGACCGA -0.0635477741428
CGGACCGC -0.243662118644
CGGACCTA -0.214742193174
CGGACCTC -0.312738304888
CGGACGAA -0.131661514863
CGGACGAC -0.0590216031087
CGGACGAG -0.304967165358

CGGACGCA -0.330813376007
CGGACGCC -0.222521423384
CGGACGGA -0.140939058719
CGGACGGC -0.0987519464522
CGGACGTA -0.0604077621439
CGGACGTC -0.183944093244
CGGACTAA -0.0543636796811
CGGACTAC -0.161498143699
CGGACTAG -0.153988399976
CGGACTCA -0.0465493183839
CGGACTCC -0.276248108486
CGGACTGA -0.252012873093
CGGACTGC -0.237598691971
CGGACTTA -0.0263302324305
CGGACTTC -0.164797366861
CGGAGAAA 0.0953670772136
CGGAGAAC -0.206811909949
CGGAGAAG -0.089353132271
CGGAGACA 0.053826648225
CGGAGACC -0.134959384722
CGGAGACG -0.245874967762
CGGAGAGA 0.0108235087488
CGGAGAGC -0.228277348265
CGGAGATA 0.224849999274
CGGAGATC 0.379495706118
CGGAGCAA 0.0444740828558
CGGAGCAC -0.255147857504
CGGAGCAG -0.246326185835
CGGAGCCA -0.108070579841
CGGAGCCC -0.264313802938
CGGAGCCG -0.0687762740514
CGGAGCGA -0.0714808564283
CGGAGCGC -0.103800664087
CGGAGCTA -0.129277533398
CGGAGCTC -0.310986014408
CGGAGGAA -0.151305392086
CGGAGGAC -0.304441385559
CGGAGGAG -0.180930041152
CGGAGGCA -0.239537707242
CGGAGGCC -0.208969614437
CGGAGGGA -0.158225308642
CGGAGGGC -0.189431840923
CGGAGGTA -0.233142377401
CGGAGGTC -0.290976680384
CGGAGTAA -0.15679414783
CGGAGTAC -0.0810144020725
CGGAGTAG 0.0328560620045
CGGAGTCA -0.0554751756978
CGGAGTCC -0.11696397469
CGGAGTGA -0.19289051061
CGGAGTGC -0.217056241427
CGGAGTTA -0.056201240684
CGGAGTTC -0.22412090083
CGGATAAA -0.122374208994

CGGATAAC 0.0206601484753
CGGATAAG -0.0133054441171
CGGATACA 0.105369517528
CGGATACC 0.351849107555
CGGATACG 0.341012305144
CGGATAGA 0.10291720541
CGGATAGC 0.101220344893
CGGATATA 0.246034605494
CGGATATC 0.476736591363
CGGATCAA -0.143865426306
CGGATCAC 0.187435509012
CGGATCAG 0.0174669126727
CGGATCCA 0.120213945421
CGGATCCC 0.346277221681
CGGATCCG 0.355347210688
CGGATCGA 0.0335541590725
CGGATCGC 0.364972337483
CGGATCTA 0.225007684033
CGGATCTC 0.369776946937
CGGATGAA -0.106015706031
CGGATGAC -0.114600554966
CGGATGAG -0.1468438847
CGGATGCA -0.0290743778876
CGGATGCC -0.0836127397714
CGGATGGA -0.00508493180758
CGGATGGC -0.182496968899
CGGATGTA -0.115949068357
CGGATGTC 0.122611585031
CGGATTAA 0.0782961755472
CGGATTAC 0.466164485058
CGGATTAG 0.124567773167
CGGATTCA 0.13776921557
CGGATTCC 0.442073371534
CGGATTGA 0.116506617465
CGGATTGC 0.452362965438
CGGATTTA 0.144545527512
CGGATTTC 0.486325166304
CGGCAAAA 0.0291589789781
CGGCAAAC -0.0339415687243
CGGCAAAG -0.224640122689
CGGCAACA -0.0883515002208
CGGCAACC -0.170663321678
CGGCAACG -0.032215652376
CGGCAAGA 0.0974939076599
CGGCAAGC -0.237966066253
CGGCAATA 0.0290660173978
CGGCAATC 0.357842652967
CGGCACAA -0.245905147579
CGGCACAC -0.105584642234
CGGCACAG -0.188178326475
CGGCACCA -0.244220565076
CGGCACCC -0.263297161646
CGGCACCG -0.328796282581
CGGCACGA -0.239574932657

CGGCACGC -0.169386723527
CGGCACTA -0.173147093685
CGGCACTC -0.0876107171596
CGGCAGAA 0.052198582436
CGGCAGAC -0.18506215267
CGGCAGAG -0.206320599966
CGGCAGCA -0.242972912174
CGGCAGCC -0.179807949251
CGGCAGGA -0.107450980392
CGGCAGGC -0.207581699346
CGGCAGTA -0.106066356131
CGGCAGTC -0.129108772256
CGGCATAA 0.082092891491
CGGCATAC -0.0791059520869
CGGCATAG -0.0226249930999
CGGCATCA -0.142628996674
CGGCATCC 0.079458385475
CGGCATGA 0.0804163700551
CGGCATGC -0.144859890019
CGGCATTA 0.0737044731451
CGGCATTC -0.273684701441
CGGCCAAA 0.204035763683
CGGCCAAC -0.309183626057
CGGCCAAG -0.158487532376
CGGCCACA -0.392577438736
CGGCCACC -0.176142338417
CGGCCACG -0.320294190251
CGGCCAGA -0.050824386924
CGGCCAGC -0.189421124829
CGGCCATA -0.320940107638
CGGCCATC -0.272230726603
CGGCCCAA -0.25243482447
CGGCCCAC -0.199556180363
CGGCCCAG -0.221126335083
CGGCCCCA -0.21238025874
CGGCCCCC -0.130975898854
CGGCCCCG -0.210712190475
CGGCCCGA -0.203820102009
CGGCCCGC -0.198621945734
CGGCCCTA -0.176092812975
CGGCCCTC -0.210350477607
CGGCCGAA -0.104948636902
CGGCCGAC 0.132157127489
CGGCCGAG 0.049584554425
CGGCCGCA -0.206434654726
CGGCCGCC -0.211366791215
CGGCCGGA 0.0825258233696
CGGCCGGC -0.196051136881
CGGCCGTA 0.0230034033857
CGGCCGTC -0.12472706856
CGGCCTAA -0.196096528052
CGGCCTAC -0.197383975068
CGGCCTAG -0.0218416633742
CGGCCTCA -0.190315500686

CGGCCTCC -0.27000899774
CGGCCTGA -0.22110008176
CGGCCTGC -0.144289170905
CGGCCTTA -0.245388525781
CGGCCTTC -0.323319384783
CGGCGAAA 0.0924685195623
CGGCGAAC -0.0973669624581
CGGCGAAG -0.162142437761
CGGCGACA -0.153857552109
CGGCGACC -0.331418286774
CGGCGACG -0.213071895425
CGGCGAGA 0.0656188939384
CGGCGAGC -0.156772685368
CGGCGATA 0.155994094272
CGGCGATC 0.286438780215
CGGCGCAA -0.0944642488793
CGGCGCAC 0.0824008832973
CGGCGCAG -0.103344992679
CGGCGCCA -0.130537220815
CGGCGCCC -0.271083676269
CGGCGCCG -0.13149656841
CGGCGCGA 0.0298185463368
CGGCGCGC -0.215993772272
CGGCGCTA -0.0954137302685
CGGCGCTC -0.219462711018
CGGCGGAA -0.244298311859
CGGCGGAC -0.232183620955
CGGCGGAG -0.0950867892637
CGGCGGCA -0.205327551
CGGCGGCC -0.340571958351
CGGCGGGA 0.0563029360917
CGGCGGGC -0.303498447426
CGGCGGTA -0.0650277891569
CGGCGGTC -0.317476269591
CGGCGTAA -0.0576793269785
CGGCGTAC -0.00732640730457
CGGCGTAG -0.167355281207
CGGCGTCA -0.213902745537
CGGCGTCC -0.26140644378
CGGCGTGA -0.0151126037139
CGGCGTGC -0.117284432002
CGGCGTTA 0.101496465345
CGGCGTTC -0.175416069929
CGGCTAAA -0.00751663044194
CGGCTAAC -0.165306408893
CGGCTAAG -0.0906255144033
CGGCTACA -0.0552482094344
CGGCTACC -0.399917231766
CGGCTACG -0.224254357809
CGGCTAGA -0.0733798420195
CGGCTAGC -0.0116268155321
CGGCTATA -0.0109003490746
CGGCTATC -0.0856769632703
CGGCTCAA -0.377222948439

CGGCTCAC -0.380253241756
CGGCTCAG -0.105916052711
CGGCTCCA -0.307540172375
CGGCTCCC -0.360724394418
CGGCTCGA -0.0377827495533
CGGCTCGC -0.217214233842
CGGCTCTA -0.223408581288
CGGCTCTC -0.307759400971
CGGCTGAA -0.170879099453
CGGCTGAC -0.174721536351
CGGCTGAG -0.12715031195
CGGCTGCA -0.224855100715
CGGCTGCC -0.306516909405
CGGCTGGA 0.0605973878808
CGGCTGGC -0.212158383318
CGGCTGTA -0.172116476571
CGGCTGTC -0.293477124183
CGGCTTAA -0.164595497458
CGGCTTAC -0.173283950617
CGGCTTAG -0.0880871532851
CGGCTTCA -0.273973128809
CGGCTTCC -0.214045428532
CGGCTTGA -0.100325344953
CGGCTTGC -0.212678111918
CGGCTTTA -0.0488498296044
CGGCTTTC -0.177782303856
CGGGAAAA 0.0525634506705
CGGGAAAC -0.103786307054
CGGGAAAG -0.111229075107
CGGGAACA -0.209339655258
CGGGAACC -0.141881841634
CGGGAACG -0.124203988193
CGGGAAGA 0.0281372807306
CGGGAAGC -0.111459694989
CGGGAATA 0.0723012579859
CGGGAATC 0.470773233221
CGGGACAA -0.111468946743
CGGGACAC -0.182643427741
CGGGACAG -0.103410312273
CGGGACCA 0.0213026828829
CGGGACCC -0.0917329250827
CGGGACCG -0.228487803864
CGGGACGA -0.0659362068776
CGGGACGC -0.435011571889
CGGGACTA -0.278491083676
CGGGACTC -0.132787379973
CGGGAGAA -0.0302310986629
CGGGAGAC -0.196888888889
CGGGAGAG -0.168505446623
CGGGAGCA -0.391177487241
CGGGAGCC -0.256808332656
CGGGAGGA -0.141193610142
CGGGAGGC -0.179401734284
CGGGAGTA 0.0182804759345

CGGGAGTC -0.102692047333
CGGGATAA 0.0757703427135
CGGGATAC 0.213143405053
CGGGATAG -0.018931326544
CGGGATCA 0.120513073833
CGGGATCC 0.348575160816
CGGGATGA -0.106641431821
CGGGATGC 0.0555329565761
CGGGATTA 0.134490935138
CGGGATTC 0.431908885983
CGGGCAAA -0.0942995455444
CGGGCAAC -0.0935224971601
CGGGCAAG -0.141558562711
CGGGCACA -0.166579520697
CGGGCACC -0.205745851219
CGGGCACG -0.132972434369
CGGGCAGA -0.041203196672
CGGGCAGC -0.133624142661
CGGGCATA 0.0231101775426
CGGGCATC -0.0394582784959
CGGGCCAA -0.195593242365
CGGGCCAC -0.223684282713
CGGGCCAG -0.246321714229
CGGGCCCA -0.336617501851
CGGGCCCC -0.271474184894
CGGGCCCG -0.154320572036
CGGGCCGA -0.294858241509
CGGGCCGC -0.164371669916
CGGGCCTA -0.165733743819
CGGGCCTC -0.199671136728
CGGGCGAA 0.104427308808
CGGGCGAC -0.211228538035
CGGGCGAG -0.124250470635
CGGGCGCA -0.0962684377351
CGGGCGCC -0.319300762667
CGGGCGGA -0.0675737969411
CGGGCGGC -0.267796201579
CGGGCGTA -0.149089324619
CGGGCGTC -0.217851260795
CGGGCTAA -0.160543209877
CGGGCTAC -0.231844760026
CGGGCTAG 0.0657780631847
CGGGCTCA -0.277463880926
CGGGCTCC -0.304442949541
CGGGCTGA -0.0713976016026
CGGGCTGC -0.236207927225
CGGGCTTA -0.28005716292
CGGGCTTC -0.347048368078
CGGGGAAA 0.234413587869
CGGGGAAC -0.201664979257
CGGGGAAG -0.111226337449
CGGGGACA -0.073605966316
CGGGGACC -0.166787397859
CGGGGACG -0.0843311546841

CGGGGAGA -0.103474219317
CGGGGAGC -0.281297655599
CGGGGATA 0.12086955448
CGGGGATC 0.336999694914
CGGGGCAA -0.26326704575
CGGGGCAC -0.17219919423
CGGGGCAG -0.113353091298
CGGGGCCA -0.0578882083031
CGGGGCCC -0.17276513094
CGGGGCGA -0.153493418702
CGGGGCGC -0.106334584609
CGGGGCTA 0.0701480394578
CGGGGCTC -0.32857682199
CGGGGGAA -0.0461964890668
CGGGGGAC -0.192731376713
CGGGGGAG -0.173489729917
CGGGGGCA -0.210924950692
CGGGGGCC -0.0847314869214
CGGGGGGA 0.0371320442535
CGGGGGGC -0.238969680599
CGGGGGTA -0.305319501159
CGGGGGTC -0.324020188644
CGGGGTAA -0.0638743866194
CGGGGTAC -0.114997855221
CGGGGTAG -0.0662638038157
CGGGGTCA -0.220489436519
CGGGGTCC -0.140920842411
CGGGGTGA -0.0435208508256
CGGGGTGC -0.132252703512
CGGGGTTA -0.27821998472
CGGGGTTC -0.125519992975
CGGGTAAA -0.184432552099
CGGGTAAC -0.0744038626909
CGGGTAAG -0.0396537334543
CGGGTACA -0.177793689201
CGGGTACC 0.0551274451689
CGGGTACG 0.0409953278196
CGGGTAGA 0.0694013046068
CGGGTAGC -0.180902179628
CGGGTATA 0.0492764587818
CGGGTATC 0.234542414922
CGGGTCAA -0.313117010987
CGGGTCAC -0.158043205429
CGGGTCAG -0.316675282733
CGGGTCCA -0.319236868244
CGGGTCCC -0.359206877024
CGGGTCGA -0.161280409169
CGGGTCGC -0.207246281391
CGGGTCTA -0.141832712146
CGGGTCTC -0.239415077618
CGGGTGAA -0.0568201298796
CGGGTGAC -0.238242701891
CGGGTGAG -0.0468009186092
CGGGTGCA -0.232381846017

CGGGTGCC -0.0844181121508
CGGGTGGA 0.0608218621704
CGGGTGGC -0.0861261533456
CGGGTGTA -0.123335196475
CGGGTGTC -0.158260538647
CGGGTTAA -0.0762696442266
CGGGTTAC -0.120780165199
CGGGTTAG -0.228551825941
CGGGTTCA -0.170133936741
CGGGTTCC -0.251297022513
CGGGTTGA -0.117208337449
CGGGTTGC 0.150532821298
CGGGTTTA -0.201341823707
CGGGTTTC 0.0705723506553
CGGTAAAA 0.0124777036854
CGGTAAAC 0.0181718394117
CGGTAAAG -0.0637221810598
CGGTAACA -0.0264041847357
CGGTAACC -0.143917276521
CGGTAACG -0.25468218099
CGGTAAGA -0.00676469296801
CGGTAAGC -0.13913027057
CGGTAATA 0.079405227144
CGGTAATC 0.402264902009
CGGTACAA -0.255326534347
CGGTACAC -0.0710620890822
CGGTACAG -0.131025286448
CGGTACCA -0.0764098089892
CGGTACCC -0.141937039533
CGGTACCG -0.18977878728
CGGTACGA -0.0768597834423
CGGTACGC -0.138161865569
CGGTACTA -0.0650088269629
CGGTACTC -0.273807025773
CGGTAGAA -0.0102160301018
CGGTAGAC -0.191798745865
CGGTAGAG -0.149441892626
CGGTAGCA -0.0796004543659
CGGTAGCC -0.0893802711794
CGGTAGGA 0.0403795912095
CGGTAGGC -0.102298169367
CGGTAGTA -0.194350663312
CGGTAGTC 0.031765286999
CGGTATAA 0.138323563635
CGGTATAC -0.113158934681
CGGTATAG 0.0178864465849
CGGTATCA 0.201643107231
CGGTATCC 0.290466880039
CGGTATGA -0.0938805191954
CGGTATGC -0.213591647052
CGGTATTA 0.00056368311711
CGGTATTC 0.0698615776417
CGGTCAAA 0.00177050510093
CGGTCAAC -0.155354722593

CGGTCAAG -0.104621489018
CGGTCACA -0.0251235477151
CGGTCACC -0.178047229903
CGGTCACG -0.123212962963
CGGTCAGA 0.102631232299
CGGTCAGC -0.303230313011
CGGTCATA 0.0457013080545
CGGTCATC -0.0658713692946
CGGTCCAA -0.321377225161
CGGTCCAC -0.168063076673
CGGTCCAG -0.0366080542659
CGGTCCCA -0.215327003974
CGGTCCCC -0.375275181663
CGGTCCGA -0.101817553218
CGGTCCGC -0.102517729569
CGGTCCTA -0.0324705262465
CGGTCCTC -0.184991590829
CGGTCGAA -0.0818568500593
CGGTCGAC -0.222189302037
CGGTCGAG -0.286788788492
CGGTCGCA -0.112889064489
CGGTCGCC -0.293006590815
CGGTCGGA -0.189737109659
CGGTCGGC -0.325134041464
CGGTCGTA 0.077577615388
CGGTCGTC -0.28418460551
CGGTCTAA -0.0128378230038
CGGTCTAC 0.000499735605
CGGTCTAG -0.229317222798
CGGTCTCA -0.213737044536
CGGTCTCC -0.182031134265
CGGTCTGA -0.106391385953
CGGTCTGC 0.0073024691358
CGGTCTTA -0.00906969277882
CGGTCTTC -0.35843784976
CGGTGAAA 0.0950751006846
CGGTGAAC -0.131122730574
CGGTGAAG -0.173300083946
CGGTGACA -0.0859401102269
CGGTGACC -0.267441725382
CGGTGACG -0.0419465849964
CGGTGAGA 0.0920009297866
CGGTGAGC -0.199703703704
CGGTGATA 0.143871398893
CGGTGATC 0.214982639141
CGGTGCAA -0.182719571432
CGGTGCAC -0.252870919708
CGGTGCAG -0.108424444005
CGGTGCCA -0.290823855999
CGGTGCCC -0.122107538918
CGGTGCGA -0.0891475120273
CGGTGCGC -0.158804688069
CGGTGCTA -0.145358250322
CGGTGCTC -0.270857780205

CGGTGGAA 0.0949239463629
CGGTGGAC -0.119938207581
CGGTGGAG -0.236399255303
CGGTGGCA -0.154975525703
CGGTGGCC -0.290741852409
CGGTGGGA 0.0599532006322
CGGTGGGC -0.154998213419
CGGTGGTA 0.0872494456519
CGGTGGTC -0.278501914574
CGGTGTAA -0.015471465678
CGGTGTAC -0.140111292208
CGGTGTAG -0.0867369616752
CGGTGTCA -0.275750443051
CGGTGTCC -0.195041122798
CGGTGTGA 0.07330155437
CGGTGTGC -0.276514733473
CGGTGTTA -0.0999574654922
CGGTGTTC -0.0889904228753
CGGTTAAA 0.116108552584
CGGTTAAC 0.07863509627
CGGTTAAG -0.102098682223
CGGTTACA -0.157100137174
CGGTTACC -0.119772492845
CGGTTACG 0.216317420725
CGGTTAGA 0.0458983731805
CGGTTAGC -0.208028836658
CGGTTATA -0.1690938301
CGGTTATC -0.0307425217556
CGGTTCAA -0.0157143339998
CGGTTCAC 0.0423405389245
CGGTTCAG -0.150159122085
CGGTTCCA -0.107066216624
CGGTTCCC -0.175112362325
CGGTTCGA -0.116483896463
CGGTTCGC -0.106214154139
CGGTTCTA -0.114272890746
CGGTTCTC -0.0808202461029
CGGTTGAA -0.0937962962963
CGGTTGAC -0.213932928335
CGGTTGAG 0.0322409364813
CGGTTGCA 0.0132092445212
CGGTTGCC -0.233577342048
CGGTTGGA -0.104687985729
CGGTTGGC -0.130627441942
CGGTTGTA 0.041175437441
CGGTTGTC -0.0803357941191
CGGTTTAA -0.156962732839
CGGTTTAC 0.0139849392961
CGGTTTAG 0.012608800641
CGGTTTCA 0.0177830468287
CGGTTTCC -0.1541902687
CGGTTTGA 0.0466393334651
CGGTTTGC -0.0642328840129
CGGTTTTA -0.00176514171981

CGGTTTTC -0.0100426518528
CGTAAAAA 0.125771147555
CGTAAAAC -0.0761230482149
CGTAAAAG -0.00972009581193
CGTAAACA -0.0210611100134
CGTAAACC 0.0387933873411
CGTAAACG -0.136032316917
CGTAAAGA 0.0678778845958
CGTAAAGC -0.0130271817297
CGTAAATA 0.117259163483
CGTAAATC 0.178956314558
CGTAACAA -0.00219516801534
CGTAACAC -0.0746586094096
CGTAACAG 0.0958116533843
CGTAACCA -0.151541434145
CGTAACCC -0.0529786603438
CGTAACGA 0.153169264742
CGTAACGC -0.0644510026672
CGTAACTA 0.00215427341983
CGTAACTC -0.00841311580201
CGTAAGAA 0.1172562579
CGTAAGAC -0.0607484782009
CGTAAGAG -0.054001714294
CGTAAGCA -0.205378408523
CGTAAGCC -0.121203928521
CGTAAGGA -0.0475280751965
CGTAAGGC -0.208848074314
CGTAAGTA -0.00879272870974
CGTAAGTC 0.0216278074162
CGTAATAA 0.266416386748
CGTAATAC 0.113290624179
CGTAATAG 0.190334577891
CGTAATCA 0.421584447518
CGTAATCC 0.482450237084
CGTAATGA 0.108704362335
CGTAATGC 0.12929756965
CGTAATTA 0.259303410926
CGTAATTC 0.266944634114
CGTACAAA 0.0288837296099
CGTACAAC -0.0517944238189
CGTACAAG -0.14562665404
CGTACACA 0.090115206369
CGTACACC -0.0446084089893
CGTACACG 0.0455553360118
CGTACAGA 0.0648340248963
CGTACAGC -0.0762684887901
CGTACATA 0.0562745614613
CGTACATC 0.1091544271
CGTACCAA 0.0138668952392
CGTACCAC -0.170994375625
CGTACCAG -0.0929034132171
CGTACCCA -0.0273865733006
CGTACCCC -0.106602575835
CGTACCGA -0.0966407307762

CGTACCGC -0.262873899971
CGTACCTA -0.0324884732379
CGTACCTC -0.108556675008
CGTACGAA -0.0150323942637
CGTACGAC -0.0303743724276
CGTACGAG -0.103299640019
CGTACGCA 0.115491305207
CGTACGCC -0.0539289094105
CGTACGGA 0.0290224165734
CGTACGGC 0.0535357527059
CGTACGTA -0.119439996673
CGTACGTC -0.130983638094
CGTACTAA 0.0470921425278
CGTACTAC 0.114630667786
CGTACTAG -0.14488399403
CGTACTCA -0.0128661036203
CGTACTCC -0.222520161759
CGTACTGA -0.0542584452785
CGTACTGC 0.0747070607283
CGTACTTA 0.160796420322
CGTACTTC -0.13101902159
CGTAGAAA 0.193968285333
CGTAGAAC -0.244814386701
CGTAGAAG 0.00383101128819
CGTAGACA -0.165165832565
CGTAGACC -0.297639423763
CGTAGACG -0.256273057371
CGTAGAGA 0.118043670914
CGTAGAGC -0.00450591366867
CGTAGATA 0.212508026277
CGTAGATC 0.246487299392
CGTAGCAA 0.0551465140267
CGTAGCAC -0.105632301637
CGTAGCAG -0.180254736359
CGTAGCCA -0.252776736713
CGTAGCCC -0.142239014004
CGTAGCGA -0.112892573206
CGTAGCGC -0.141515123942
CGTAGCTA 0.00730318306626
CGTAGCTC -0.0204542945302
CGTAGGAA -0.0357938703374
CGTAGGAC -0.104092172645
CGTAGGAG -0.210194193403
CGTAGGCA 0.0664811936135
CGTAGGCC -0.0479467465688
CGTAGGGA -0.165037037037
CGTAGGGC -0.303254549573
CGTAGGTA 0.00896227100373
CGTAGGTC -0.197041796277
CGTAGTAA -0.0385793321478
CGTAGTAC -0.197815314203
CGTAGTAG 0.00396137118353
CGTAGTCA -0.148106562703
CGTAGTCC -0.186870095497

CGTAGTGA 0.249986278513
CGTAGTGC -0.0543892291818
CGTAGTTA -0.379043260648
CGTAGTTC -0.0631749637824
CGTATAAA -0.0976145792177
CGTATAAC 0.106238286868
CGTATAAG 0.0948222509552
CGTATACA 0.0130817112934
CGTATACC -0.128429920116
CGTATACG 0.199431228985
CGTATAGA 0.00467218037684
CGTATAGC 0.0455031943621
CGTATATA 0.0753831039101
CGTATATC 0.179952929554
CGTATCAA 0.226012096863
CGTATCAC 0.24468704033
CGTATCAG 0.323659685174
CGTATCCA 0.350603196558
CGTATCCC 0.327737711036
CGTATCGA 0.341130267253
CGTATCGC 0.230152688391
CGTATCTA 0.391877428703
CGTATCTC 0.361299086194
CGTATGAA -0.0363328635974
CGTATGAC 0.0883993062416
CGTATGAG -0.0234416761568
CGTATGCA -0.0750765658961
CGTATGCC -0.0386079351474
CGTATGGA 0.00232301367077
CGTATGGC -0.246294741578
CGTATGTA 0.00571382912266
CGTATGTC -0.0988402021652
CGTATTAA -0.0839081475858
CGTATTAC 0.136970639083
CGTATTAG 0.0311173419726
CGTATTCA 0.15379977627
CGTATTCC 0.220401551581
CGTATTGA 0.177291415455
CGTATTGC 0.161583833336
CGTATTTA 0.141005041187
CGTATTTC 0.0714003457644
CGTCAAAA -0.0807679456075
CGTCAAAC 0.0393038266482
CGTCAAAG 0.0231211652233
CGTCAACA -0.0725102785001
CGTCAACC 0.0161378405673
CGTCAACG -0.0789034831595
CGTCAAGA 0.0349376495642
CGTCAAGC -0.0438830444016
CGTCAATA 0.0690497290544
CGTCAATC -0.0315100221739
CGTCACAA 0.110326442259
CGTCACAC -0.0305292142046
CGTCACAG -0.0889558431295

CGTCACCA -0.196168940871
CGTCACCC -0.298698167792
CGTCACGA -0.110167374106
CGTCACGC -0.0661293332455
CGTCACTA -0.0251085961675
CGTCACTC -0.126906775353
CGTCAGAA -0.0934493911126
CGTCAGAC -0.29337320397
CGTCAGAG -0.171248155592
CGTCAGCA -0.181622243076
CGTCAGCC -0.288981660302
CGTCAGGA 0.101258117473
CGTCAGGC -0.0165555281139
CGTCAGTA -0.0737734686881
CGTCAGTC -0.252271380604
CGTCATAA 0.0544026847588
CGTCATAC 0.0583300401765
CGTCATAG -0.174153265408
CGTCATCA -0.230868221904
CGTCATCC -0.0169059457561
CGTCATGA -0.229967843189
CGTCATGC -0.229484583601
CGTCATTA 0.00554073634986
CGTCATTC -0.166578849151
CGTCCAAA -0.0730907337253
CGTCCAAC -0.291138967067
CGTCCAAG -0.259390946502
CGTCCACA -0.238690544774
CGTCCACC -0.180045555042
CGTCCACG -0.164320387644
CGTCCAGA -0.101174211248
CGTCCAGC -0.0537038625605
CGTCCATA 0.00371928437901
CGTCCATC -0.176506900849
CGTCCCAA -0.151739024334
CGTCCCAC -0.0387585637831
CGTCCCAG -0.40183010408
CGTCCCCA -0.0778295514956
CGTCCCCC -0.149191180909
CGTCCCGA -0.116964408749
CGTCCCGC -0.271055291562
CGTCCCTA -0.272725820366
CGTCCCTC -0.217506477211
CGTCCGAA -0.190064544975
CGTCCGAC -0.244393425716
CGTCCGAG -0.19601920439
CGTCCGCA -0.0527648808544
CGTCCGCC -0.245729238669
CGTCCGGA -0.0786041698625
CGTCCGGC -0.181269653117
CGTCCGTA 0.0342401282915
CGTCCGTC -0.18971307225
CGTCCTAA -0.0296987859229
CGTCCTAC -0.00707073642003

CGTCCTAG -0.00786241190805
CGTCCTCA -0.228110611723
CGTCCTCC -0.371902685208
CGTCCTGA -0.202849673203
CGTCCTGC -0.227547606046
CGTCCTTA -0.0427687301149
CGTCCTTC -0.199528120713
CGTCGAAA 0.180214432031
CGTCGAAC -0.208708508092
CGTCGAAG -0.00745171636414
CGTCGACA -0.101007606441
CGTCGACC -0.202256827136
CGTCGACG -0.209536119489
CGTCGAGA -0.102442216515
CGTCGAGC -0.236868237979
CGTCGATA 0.166458100068
CGTCGATC 0.0470655073609
CGTCGCAA -0.02550423973
CGTCGCAC -0.0926355299549
CGTCGCAG -0.172872734456
CGTCGCCA -0.0465263030822
CGTCGCCC -0.271265496123
CGTCGCGA -0.0435903769204
CGTCGCGC -0.152641975309
CGTCGCTA 0.0622419479029
CGTCGCTC -0.173878323866
CGTCGGAA 0.0427658245318
CGTCGGAC -0.249551418177
CGTCGGAG -0.122548051224
CGTCGGCA -0.302402323893
CGTCGGCC -0.158933227275
CGTCGGGA 0.163736870396
CGTCGGGC -0.274704512615
CGTCGGTA -0.0950170378784
CGTCGGTC -0.216441415488
CGTCGTAA 0.17397323958
CGTCGTAC -0.0326562231916
CGTCGTAG -0.0989016895966
CGTCGTCA -0.0834626572358
CGTCGTCC -0.046812769258
CGTCGTGA -0.135560797483
CGTCGTGC -0.210756476804
CGTCGTTA -0.115037424821
CGTCGTTC -0.0998535730784
CGTCTAAA -0.0473857016257
CGTCTAAC -0.0845214696492
CGTCTAAG -0.174439423124
CGTCTACA 0.0274233848218
CGTCTACC -0.00811303925927
CGTCTAGA -0.0263148720571
CGTCTAGC -0.0807202106615
CGTCTATA -0.113033373714
CGTCTATC -0.13146455915
CGTCTCAA -0.11894414539

CGTCTCAC -0.14665216242
CGTCTCAG -0.184788891116
CGTCTCCA -0.219040094805
CGTCTCCC -0.20118174801
CGTCTCGA -0.297514403292
CGTCTCGC -0.0754185805304
CGTCTCTA -0.188685820916
CGTCTCTC -0.314900584795
CGTCTGAA -0.0388810599567
CGTCTGAC -0.0866259985476
CGTCTGAG -0.345372591616
CGTCTGCA -0.116705627991
CGTCTGCC -0.338259980599
CGTCTGGA -0.217178259809
CGTCTGGC -0.0437049338206
CGTCTGTA 0.171109787457
CGTCTGTC -0.138192297859
CGTCTTAA -0.0723901959612
CGTCTTAC -0.0817882939252
CGTCTTAG -0.264508855093
CGTCTTCA -0.0655615772061
CGTCTTCC -0.180557734205
CGTCTTGA -0.0447677226723
CGTCTTGC 0.151293690658
CGTCTTTA -0.00169323146502
CGTCTTTC -0.135764822106
CGTGAAAA 0.0740274210191
CGTGAAAC 0.0170969724912
CGTGAAAG -0.0411323079069
CGTGAACA -0.0884553113252
CGTGAACC -0.139203208792
CGTGAACG -0.0549572987332
CGTGAAGA -0.0473688759384
CGTGAAGC -0.232385105565
CGTGAATA 0.0651232264824
CGTGAATC 0.222804468787
CGTGACAA -0.0518277923233
CGTGACAC -0.338843904633
CGTGACAG -0.177625329355
CGTGACCA -0.0807189219657
CGTGACCC -0.143817153547
CGTGACGA -0.065871565674
CGTGACGC -0.127235223932
CGTGACTA -0.227542626342
CGTGACTC -0.26784635229
CGTGAGAA 0.0937067815865
CGTGAGAC -0.121359843819
CGTGAGAG -0.13665971056
CGTGAGCA -0.0970304940944
CGTGAGCC -0.230906206794
CGTGAGGA -0.089115555349
CGTGAGGC -0.297554183813
CGTGAGTA -0.0518331000796
CGTGAGTC -0.236226090437

CGTGATAA 0.0491813934381
CGTGATAC 0.102000043909
CGTGATAG 0.119354980351
CGTGATCA 0.148372276836
CGTGATCC 0.217713887234
CGTGATGA -0.0727756041128
CGTGATGC -0.133411812484
CGTGATTA 0.153979922421
CGTGATTC 0.356090419109
CGTGCAAA 0.136064097163
CGTGCAAC -0.196375470482
CGTGCAAG -0.0733057150162
CGTGCACA -0.0931137681054
CGTGCACC -0.161063180828
CGTGCACG 0.13982025579
CGTGCAGA -0.0342115508655
CGTGCAGC -0.213313733237
CGTGCATA 0.180252204611
CGTGCATC -0.111927418535
CGTGCCAA -0.132246752605
CGTGCCAC -0.205705631554
CGTGCCAG -0.144322440087
CGTGCCCA -0.254772343926
CGTGCCCC -0.133031260803
CGTGCCGA -0.25670885937
CGTGCCGC -0.258225447109
CGTGCCTA -0.163370449666
CGTGCCTC -0.255465582602
CGTGCGAA -0.12011526074
CGTGCGAC -0.0721653078894
CGTGCGAG -0.0919366413034
CGTGCGCA -0.00294188675931
CGTGCGCC -0.0635786920422
CGTGCGGA 0.105284967837
CGTGCGGC -0.204137120755
CGTGCGTA 0.076439792097
CGTGCGTC -0.0312160426751
CGTGCTAA -0.261803921569
CGTGCTAC -0.0750700971918
CGTGCTAG 0.0600161183398
CGTGCTCA -0.14856336977
CGTGCTCC -0.306272457134
CGTGCTGA 0.0171170281085
CGTGCTGC -0.106864972602
CGTGCTTA 0.240188572342
CGTGCTTC 0.0917363718194
CGTGGAAA 0.139547520253
CGTGGAAC -0.323204477336
CGTGGAAG -0.200743391831
CGTGGACA -0.119807359842
CGTGGACC -0.195725308642
CGTGGAGA 0.0539215686275
CGTGGAGC -0.315887517147
CGTGGATA 0.271109135217

CGTGGATC 0.221499569702
CGTGGCAA -0.0166427971752
CGTGGCAC -0.0952784274984
CGTGGCAG -0.211974157039
CGTGGCCA -0.233296027475
CGTGGCCC -0.290276474687
CGTGGCGA 0.0210623561949
CGTGGCGC -0.226773662551
CGTGGCTA -0.0231534478851
CGTGGCTC -0.339809740121
CGTGGGAA 0.0254719886572
CGTGGGAC -0.225952992297
CGTGGGAG -0.229733140211
CGTGGGCA -0.236829263333
CGTGGGCC -0.17420823889
CGTGGGGA 0.0680607008589
CGTGGGGC -0.205472827555
CGTGGGTA -0.0820097017215
CGTGGGTC -0.339549147726
CGTGGTAA 0.0711807270572
CGTGGTAC 0.0101052267562
CGTGGTAG -0.209052055215
CGTGGTCA -0.238892483765
CGTGGTCC -0.237436834395
CGTGGTGA -0.037933418947
CGTGGTGC -0.293394549763
CGTGGTTA 0.0548036552235
CGTGGTTC -0.199883805374
CGTGTAAA 0.12657385453
CGTGTAAC 0.0477178423237
CGTGTAAG 0.080637194369
CGTGTACA -0.166246438895
CGTGTACC -0.201033653287
CGTGTAGA -0.0414420015339
CGTGTAGC -0.187024052494
CGTGTATA -0.130137324639
CGTGTATC 0.186457167007
CGTGTCAA -0.151080246914
CGTGTCAC -0.138067879008
CGTGTCAG -0.266746227709
CGTGTCCA -0.0260761553325
CGTGTCCC -0.22851458522
CGTGTCGA -0.0529516032554
CGTGTCGC -0.0678728810304
CGTGTCTA 0.0569166598063
CGTGTCTC -0.0534002585969
CGTGTGAA 0.0169764833722
CGTGTGAC -0.266381824308
CGTGTGAG -0.278835262751
CGTGTGCA 0.112683593493
CGTGTGCC -0.268827479639
CGTGTGGA -0.150042562587
CGTGTGGC -0.235183732752
CGTGTGTA -0.0894642343528

CGTGTGTC -0.280379763381
CGTGTTAA 0.127963841138
CGTGTTAC 0.0344747432191
CGTGTTAG -0.284700960219
CGTGTTCA -0.249193899782
CGTGTTCC -0.00588409594664
CGTGTTGA -0.00974085908839
CGTGTTGC 0.0243095608211
CGTGTTTA 0.0435954200913
CGTGTTTC -0.132933763639
CGTTAAAA 0.105198088126
CGTTAAAC -0.0981909591868
CGTTAAAG 0.0239405517702
CGTTAACA -0.0700125139959
CGTTAACC -0.0480995519812
CGTTAACG 0.110358186577
CGTTAAGA 0.0951157148461
CGTTAAGC 0.0267376890821
CGTTAATA 0.101640073112
CGTTAATC -0.0444801185478
CGTTACAA 0.173284228984
CGTTACAC -0.0113413833379
CGTTACAG 0.0179449026968
CGTTACCA 0.111404413581
CGTTACCC 0.0607339502855
CGTTACGA 0.177262359624
CGTTACGC -0.075244734931
CGTTACTA -0.0262819896156
CGTTACTC -0.241381864876
CGTTAGAA -0.159273894789
CGTTAGAC -0.0547256093132
CGTTAGAG -0.186351976713
CGTTAGCA -0.213389267204
CGTTAGCC -0.130644054773
CGTTAGGA 0.0881800880392
CGTTAGGC -0.112948670654
CGTTAGTA -0.0262217611803
CGTTAGTC 0.020450503853
CGTTATAA 0.0777349667391
CGTTATAC 0.070742368863
CGTTATAG 0.0329665615404
CGTTATCA 0.228450016707
CGTTATCC 0.170780003777
CGTTATGA 0.0482258974512
CGTTATGC -0.000470704458617
CGTTATTA 0.0821829645664
CGTTATTC -0.0869946101434
CGTTCAAA 0.151937297517
CGTTCAAC -0.139830803914
CGTTCAAG -0.101843028796
CGTTCACA -0.29804563835
CGTTCACC 0.0207551208589
CGTTCAGA -0.0598578658965
CGTTCAGC -0.149386496334

CGTTCATA 0.00527976729508
CGTTCATC -0.107014684578
CGTTCCAA 0.0720105182107
CGTTCCAC -0.360923173161
CGTTCCAG 0.0890779132102
CGTTCCCA -0.132213662129
CGTTCCCC -0.22137037037
CGTTCCGA 0.0233251553272
CGTTCCGC -0.101162746034
CGTTCCTA -0.272806829841
CGTTCCTC -0.0372794020809
CGTTCGAA -0.0115738513898
CGTTCGAC -0.158422404292
CGTTCGAG -0.0941119590759
CGTTCGCA -0.0263728250227
CGTTCGCC -0.339185091378
CGTTCGGA 0.0012760982678
CGTTCGGC -0.140330661048
CGTTCGTA 0.0232112504177
CGTTCGTC -0.331090777052
CGTTCTAA -0.0364422339461
CGTTCTAC -0.168420012841
CGTTCTAG -0.010196801563
CGTTCTCA -0.143842978448
CGTTCTCC -0.242649479784
CGTTCTGA -0.0733459564776
CGTTCTGC -0.0697746069467
CGTTCTTA 0.104478144416
CGTTCTTC -0.341263157895
CGTTGAAA -0.0576249763921
CGTTGAAC -0.125656757354
CGTTGAAG -0.138410706208
CGTTGACA -0.236434311886
CGTTGACC -0.221893614146
CGTTGAGA 0.174383630815
CGTTGAGC -0.0263303754191
CGTTGATA 0.0999709441692
CGTTGATC 0.0792177672854
CGTTGCAA -0.0552344727656
CGTTGCAC -0.209626354986
CGTTGCAG -0.0218583283936
CGTTGCCA -0.142979989309
CGTTGCCC -0.275272218204
CGTTGCGA 0.0287841587611
CGTTGCGC -0.0984004765156
CGTTGCTA -0.0514331788532
CGTTGCTC -0.199349309808
CGTTGGAA -0.06798599449
CGTTGGAC -0.324583669747
CGTTGGAG -0.0579207893625
CGTTGGCA -0.163053803674
CGTTGGCC -0.334961422219
CGTTGGGA -0.00793660017724
CGTTGGGC -0.26976250432

CGTTGGTA 0.0210988915201
CGTTGGTC -0.28828873309
CGTTGTAA 0.00112785049807
CGTTGTAC -0.127274523363
CGTTGTAG -0.0324623255733
CGTTGTCA -0.158392383476
CGTTGTCC -0.260852501624
CGTTGTGA 0.0516615899673
CGTTGTGC 0.0347804866712
CGTTGTTA -0.108054265863
CGTTGTTC 0.0030552206064
CGTTTAAA -0.0800983104436
CGTTTAAC -0.106344502072
CGTTTAAG 0.109887699214
CGTTTACA -0.0494021195993
CGTTTACC -0.212936411785
CGTTTAGA -0.117620909867
CGTTTAGC 0.0602182092891
CGTTTATA -0.214930624163
CGTTTATC 0.128974474453
CGTTTCAA -0.09988668226
CGTTTCAC -0.0552414762823
CGTTTCAG -0.185439477512
CGTTTCCA 0.0682385044698
CGTTTCCC -0.101916993026
CGTTTCGA 0.0575104584561
CGTTTCGC -0.189872183793
CGTTTCTA -0.0672107368733
CGTTTCTC -0.068103987619
CGTTTGAA 0.0445964559907
CGTTTGAC -0.0549490957987
CGTTTGAG -0.178451902294
CGTTTGCA -0.070844036093
CGTTTGCC -0.225668456149
CGTTTGGA 0.0383929219996
CGTTTGGC -0.132431575308
CGTTTGTA 0.0847768337395
CGTTTGTC -0.117956442293
CGTTTTAA 0.233935317472
CGTTTTAC -0.105733892312
CGTTTTAG -0.126242377229
CGTTTTCA 0.0107411798283
CGTTTTCC 0.0148424380124
CGTTTTGA 0.0379396510395
CGTTTTGC -0.0181583041138
CGTTTTTA -0.0961279877062
CGTTTTTC -0.150868042945
CTAAAAAA 0.186818822367
CTAAAAAC 0.140803103163
CTAAAAAG -0.0170009220839
CTAAAACA 0.0196548167303
CTAAAACC -0.0414157801142
CTAAAAGA 0.0835309227425
CTAAAAGC 0.0559348650736

CTAAAATA -0.00465619688231
CTAAAATC 0.347468510743
CTAAACAA 0.158248223962
CTAAACAC -0.0389851880691
CTAAACAG -0.0939244257841
CTAAACCA 0.0244984237212
CTAAACCC -0.216606587329
CTAAACGA 0.066051167318
CTAAACGC 0.0322737640376
CTAAACTA -0.0407210066186
CTAAACTC -0.0774939745253
CTAAAGAA 0.0761446705522
CTAAAGAC 0.102587744573
CTAAAGAG -0.0637841408604
CTAAAGCA -0.10835871683
CTAAAGCC -0.108050391944
CTAAAGGA 0.0173816892752
CTAAAGGC -0.111484243392
CTAAAGTA -0.104604060663
CTAAAGTC -0.135697993695
CTAAATAA 0.171573470329
CTAAATAC 0.136613252364
CTAAATAG 0.0127366234219
CTAAATCA -0.0015443174059
CTAAATCC 0.248526312323
CTAAATGA 0.201400894004
CTAAATGC 0.0793790768963
CTAAATTA 0.156394461959
CTAAATTC 0.194751805748
CTAACAAA 0.0814246073831
CTAACAAC -0.158187206718
CTAACAAG -0.0866925527187
CTAACACA -0.183117410462
CTAACACC -0.147928916727
CTAACAGA 0.0763921374922
CTAACAGC -0.229976297467
CTAACATA 0.174717068848
CTAACATC 0.0673878169712
CTAACCAA -0.159963329712
CTAACCAC -0.210413260944
CTAACCAG 0.0550464579786
CTAACCCA -0.118867867571
CTAACCCC -0.120627394163
CTAACCGA -0.0013669417982
CTAACCGC -0.0638935214509
CTAACCTA 0.131609838304
CTAACCTC -0.221042407481
CTAACGAA 0.189489053216
CTAACGAC -0.0710411921921
CTAACGAG -0.144637576197
CTAACGCA 0.00257528918379
CTAACGCC -0.0652256654909
CTAACGGA 0.0378288231227
CTAACGGC -0.165072623614

CTAACGTA 0.00994433727659
CTAACGTC -0.264384087791
CTAACTAA 0.0559088651828
CTAACTAC -0.179890260631
CTAACTAG -0.275037443013
CTAACTCA -0.0677102552555
CTAACTCC -0.00100426400619
CTAACTGA -0.0414170409841
CTAACTGC -0.240770919067
CTAACTTA 0.065751892261
CTAACTTC -0.0898767053246
CTAAGAAA 0.089829085161
CTAAGAAC 0.0480022983903
CTAAGAAG -0.106625071338
CTAAGACA -0.128637353834
CTAAGACC -0.238713144517
CTAAGAGA 0.0384018001701
CTAAGAGC -0.344359961818
CTAAGATA 0.164387721006
CTAAGATC 0.23171879767
CTAAGCAA -0.0642944721107
CTAAGCAC 0.225604952459
CTAAGCAG -0.190549714873
CTAAGCCA -0.297082722938
CTAAGCCC -0.0904104938272
CTAAGCGA -0.113621523148
CTAAGCGC -0.133938506504
CTAAGCTA -0.221323856193
CTAAGCTC -0.208694679531
CTAAGGAA 0.0557658657834
CTAAGGAC -0.243783715557
CTAAGGAG 0.0355688379547
CTAAGGCA -0.156561577614
CTAAGGCC -0.0421262910644
CTAAGGGA -0.0525249846608
CTAAGGGC -0.223832567939
CTAAGGTA 0.00280810733856
CTAAGGTC -0.267392617723
CTAAGTAA 0.111416035913
CTAAGTAC 0.0109671233275
CTAAGTAG -0.0219575113368
CTAAGTCA -0.124406563969
CTAAGTCC -0.241301353702
CTAAGTGA 0.0964764947987
CTAAGTGC -0.211909949165
CTAAGTTA 0.0922914880944
CTAAGTTC -0.231417000049
CTAATAAA 0.209575348217
CTAATAAC 0.00685937963054
CTAATAAG -0.0107608269289
CTAATACA 0.122204465881
CTAATACC -0.0578690453707
CTAATAGA 0.0819364859821
CTAATAGC 0.0840425377363

CTAATATA 0.0997056812361
CTAATATC 0.274868822587
CTAATCAA -0.139164354307
CTAATCAC -0.129487592933
CTAATCAG 0.0650637971506
CTAATCCA 0.136030128673
CTAATCCC -0.0649081209247
CTAATCGA -0.0863078577936
CTAATCGC 0.0349822187099
CTAATCTA 0.0632038427344
CTAATCTC 0.143598274084
CTAATGAA 0.0292141850566
CTAATGAC -0.138637202128
CTAATGAG -0.0502415276515
CTAATGCA -0.159632716049
CTAATGCC -0.159571315686
CTAATGGA -0.0531763672786
CTAATGGC -0.229163398693
CTAATGTA 0.158804254759
CTAATGTC 0.020689271955
CTAATTAA 0.0864425493586
CTAATTAC 0.0899786439644
CTAATTAG 0.0302192376062
CTAATTCA 0.0469760144117
CTAATTCC 0.122897732886
CTAATTGA -0.0222041098597
CTAATTGC 0.0338021007366
CTAATTTA 0.188829485857
CTAATTTC 0.0523135705258
CTACAAAA 0.0810884981009
CTACAAAC 0.00990270163481
CTACAAAG -0.087211025489
CTACAACA -0.0453762471599
CTACAACC 0.101678961119
CTACAAGA 0.0895862148456
CTACAAGC -0.151884281962
CTACAATA -0.0141084326769
CTACAATC 0.292045073411
CTACACAA 0.177538155685
CTACACAC -0.330227039712
CTACACAG -0.0276190199468
CTACACCA -0.147226200864
CTACACCC -0.0174988740866
CTACACGA -0.0949850443182
CTACACGC -0.0945580359103
CTACACTA -0.0560955911404
CTACACTC -0.264074750767
CTACAGAA 0.0450438016649
CTACAGAC 0.0706861163248
CTACAGAG -0.0491752349508
CTACAGCA -0.196932027112
CTACAGCC 0.00461964890668
CTACAGGA -0.15977057852
CTACAGGC -0.266282011183

CTACAGTA -0.0358066371861
CTACAGTC -0.0640056368312
CTACATAA 0.0856879404597
CTACATAC 0.0211256010011
CTACATAG -0.0799048418696
CTACATCA 0.0687068702512
CTACATCC -0.202564186983
CTACATGA 0.185339079324
CTACATGC -0.160500535112
CTACATTA 0.0704066363517
CTACATTC -0.117755488306
CTACCAAA -0.087689044499
CTACCAAC -0.0590471574632
CTACCAAG -0.154363060645
CTACCACA -0.0591387851757
CTACCACC -0.0815319690709
CTACCAGA 0.0340073327625
CTACCAGC -0.100119084858
CTACCATA -0.0604945588647
CTACCATC -0.189143288829
CTACCCAA -0.162385017229
CTACCCAC -0.28762959763
CTACCCAG -0.00642860255982
CTACCCCA -0.108919118373
CTACCCCC -0.14625708061
CTACCCGA -0.00543290364005
CTACCCGC -0.138699002272
CTACCCTA -0.0442985017388
CTACCCTC -0.240556694683
CTACCGAA 0.0457584140157
CTACCGAC -0.111851888433
CTACCGAG 0.127292141851
CTACCGCA -0.0688424428891
CTACCGCC -0.195476064092
CTACCGGA 0.0169903970479
CTACCGGC -0.187216679289
CTACCGTA 0.146945505789
CTACCGTC -0.152589141523
CTACCTAA -0.107870576162
CTACCTAC 0.0206343717755
CTACCTAG -0.132827183913
CTACCTCA -0.0538678737549
CTACCTCC -0.222631630939
CTACCTGA -0.00694856028054
CTACCTGC -0.170524915477
CTACCTTA -0.149581618656
CTACCTTC -0.218485527268
CTACGAAA 0.121908096407
CTACGAAC -0.253079713127
CTACGAAG -0.0671108864349
CTACGACA -0.176926607103
CTACGACC -0.235685164151
CTACGAGA 0.0672715122107
CTACGAGC -0.158238954971

CTACGATA 0.158036116398
CTACGATC 0.255735581462
CTACGCAA 0.0766372477991
CTACGCAC -0.162631296035
CTACGCAG 0.0552921030099
CTACGCCA -0.170833625774
CTACGCCC -0.0537425297575
CTACGCGA 0.0281391193178
CTACGCGC -0.0696658659096
CTACGCTA -0.0577266717999
CTACGCTC -0.155121233546
CTACGGAA 0.153866604681
CTACGGAC -0.267898167303
CTACGGAG -0.0593841684822
CTACGGCA -0.158043572985
CTACGGCC -0.24888970315
CTACGGGA 0.0408621020274
CTACGGGC -0.199526193015
CTACGGTA 0.0573431348336
CTACGGTC -0.0122581707417
CTACGTAA -2.90543956395E-5
CTACGTAC -0.122844545906
CTACGTAG -0.0282746979947
CTACGTCA 0.105801763311
CTACGTCC -0.0339967748028
CTACGTGA -0.0353528017291
CTACGTGC -0.00786027850168
CTACGTTA 0.00715438319173
CTACGTTC -0.00369444888353
CTACTAAA 0.137148123978
CTACTAAC 0.0224935713974
CTACTAAG -0.202527233115
CTACTACA 0.0144712565194
CTACTACC -0.289628459192
CTACTAGA -0.0079612976334
CTACTAGC -0.172636783161
CTACTATA 0.166650200883
CTACTATC 0.131126549268
CTACTCAA -0.0425564611289
CTACTCAC 0.0237354277811
CTACTCAG -0.137010397028
CTACTCCA 0.0255473392123
CTACTCCC -0.0405864340995
CTACTCGA 0.243599726875
CTACTCGC -0.05007244945
CTACTCTA -0.115555555556
CTACTCTC -0.253169939482
CTACTGAA 0.156722792846
CTACTGAC -0.120475364542
CTACTGAG -0.0220735106897
CTACTGCA 0.0204882720318
CTACTGCC -0.183944069431
CTACTGGA 0.153888422404
CTACTGGC -0.14090006146

CTACTGTA 0.0457033690236
CTACTGTC 0.0505946887471
CTACTTAA 0.0991719088228
CTACTTAC 0.0249581515326
CTACTTCA -0.0119047619048
CTACTTCC -0.0113613910294
CTACTTGA -0.1200687427
CTACTTGC 0.0155002387185
CTACTTTA 0.0312685240071
CTACTTTC -0.0483922750173
CTAGAAAA 0.198338602384
CTAGAAAC -0.19456515775
CTAGAAAG 0.0322214635422
CTAGAACA -0.0478084041362
CTAGAACC -0.103734204793
CTAGAAGA 0.0501602669652
CTAGAAGC -0.166723855126
CTAGAATA 0.0279808557817
CTAGAATC 0.358177037177
CTAGACAA -0.0993557026354
CTAGACAC -0.101954199626
CTAGACAG -0.0647549124511
CTAGACCA -0.0832532034988
CTAGACCC -0.247561365287
CTAGACGA 0.130241308675
CTAGACGC -0.0728292008298
CTAGACTA -0.178484730344
CTAGACTC -0.31144523449
CTAGAGAA 0.0994505916705
CTAGAGAC -0.0764478912819
CTAGAGAG -0.130203340595
CTAGAGCA -0.104613898766
CTAGAGCC -0.156253168111
CTAGAGGA 0.00517048508709
CTAGAGGC -0.0493000220977
CTAGAGTA -0.056562712683
CTAGAGTC -0.151916238641
CTAGATAA 0.12388169883
CTAGATAC 0.335520538321
CTAGATAG -0.0156189618352
CTAGATCA 0.101967079744
CTAGATCC 0.292871869938
CTAGATGA 0.0810526927491
CTAGATGC -0.10190536565
CTAGATTA 0.323186552962
CTAGATTC 0.481701025269
CTAGCAAA 0.0632792410617
CTAGCAAC -0.0132502483221
CTAGCAAG -0.109017729271
CTAGCACA 0.00624264524283
CTAGCACC -0.166589199757
CTAGCAGA 0.13584472564
CTAGCAGC -0.114618828913
CTAGCATA 0.116843948275

CTAGCATC -0.0578799754111
CTAGCCAA 0.0374253628347
CTAGCCAC -0.213259259259
CTAGCCAG -0.274683433186
CTAGCCCA -0.160504192458
CTAGCCCC -0.18580804838
CTAGCCGA -0.0697243627742
CTAGCCGC -0.247830516492
CTAGCCTA -0.13323741269
CTAGCCTC -0.229855349225
CTAGCGAA -0.0461508288176
CTAGCGAC -0.187148165523
CTAGCGAG -0.0465762538531
CTAGCGCA -0.0546691612243
CTAGCGCC -0.318861523178
CTAGCGGA 0.0111879476414
CTAGCGGC -0.383242798354
CTAGCGTA 0.0814478520477
CTAGCGTC -0.180405034465
CTAGCTAA -0.204958468793
CTAGCTAC -0.183767960135
CTAGCTAG -0.0555550069129
CTAGCTCA -0.231106501602
CTAGCTCC -0.0460337844512
CTAGCTGA 0.0175779093619
CTAGCTGC -0.295417510246
CTAGCTTA -0.0954372331434
CTAGCTTC -0.248939695708
CTAGGAAA 0.161271378076
CTAGGAAC -0.155116522931
CTAGGAAG 0.0127548796857
CTAGGACA 0.00896227100373
CTAGGACC -0.309557124906
CTAGGAGA 0.00861069545131
CTAGGAGC -0.0805619616398
CTAGGATA 0.219543788009
CTAGGATC 0.183518079991
CTAGGCAA -0.0969368998628
CTAGGCAC -0.233369549114
CTAGGCAG -0.131398464122
CTAGGCCA -0.202934575644
CTAGGCCC -0.444713226259
CTAGGCGA -0.0715493547019
CTAGGCGC -0.135987721836
CTAGGCTA -0.180156064797
CTAGGCTC -0.121175715174
CTAGGGAA -0.0014955681787
CTAGGGAC -0.0382646390572
CTAGGGAG -0.183236209196
CTAGGGCA -0.055664019942
CTAGGGCC -0.115787876864
CTAGGGGA -0.0667396792289
CTAGGGGC -0.284553849193
CTAGGGTA -0.0472219478196

CTAGGGTC 0.0611188266673
CTAGGTAA -0.0180921721647
CTAGGTAC -0.313845546119
CTAGGTCA -0.171156605461
CTAGGTCC -0.217496579352
CTAGGTGA -0.0702530049374
CTAGGTGC -0.081094747241
CTAGGTTA -0.00649965161867
CTAGGTTC -0.0560274581461
CTAGTAAA -0.025200882566
CTAGTAAC -0.12686456584
CTAGTAAG -0.113564068523
CTAGTACA -0.051246143029
CTAGTACC -0.188634829704
CTAGTAGA 0.0720158510615
CTAGTAGC -0.232783024386
CTAGTATA 0.180430142193
CTAGTATC 0.129130167512
CTAGTCAA -0.230120551924
CTAGTCAC -0.151297650487
CTAGTCAG -0.0356235944936
CTAGTCCA 0.00452086396151
CTAGTCCC -0.280291852076
CTAGTCGA -0.0856604974862
CTAGTCGC -0.216757201646
CTAGTCTA -0.105987761038
CTAGTCTC -0.180276338113
CTAGTGAA -0.10853232418
CTAGTGAC -0.176906275052
CTAGTGAG -0.162292904847
CTAGTGCA 0.0897165231906
CTAGTGCC -0.278625616929
CTAGTGGA -0.0385788793166
CTAGTGGC -0.174604119884
CTAGTGTA 0.104071062902
CTAGTGTC -0.106130211421
CTAGTTAA -0.0673258468902
CTAGTTAC 0.0475949036073
CTAGTTCA -0.186598902488
CTAGTTCC -0.0315386515189
CTAGTTGA 0.0531542953742
CTAGTTGC -0.0468999030204
CTAGTTTA 0.0290327780623
CTAGTTTC 0.0854930953479
CTATAAAA 0.193336487987
CTATAAAC 0.0300829875519
CTATAAAG -0.091440959359
CTATAACA 0.138117741334
CTATAACC -0.089891476472
CTATAAGA 0.167876901798
CTATAAGC -0.0849030714176
CTATAATA 0.113635901385
CTATAATC 0.203764078245
CTATACAA 0.137958572087

CTATACAC -0.0573281965162
CTATACAG -0.0606333053401
CTATACCA 0.033854401232
CTATACCC 0.0183193443328
CTATACGA -0.0585373294786
CTATACGC -0.0159662554757
CTATACTA 0.0627315386515
CTATACTC -0.0461611650384
CTATAGAA 0.17095051483
CTATAGAC -0.307435172285
CTATAGAG -0.206713808478
CTATAGCA 0.0851535423551
CTATAGCC -0.208209876543
CTATAGGA 0.152676929832
CTATAGGC -0.18308835229
CTATAGTA -0.0131884446085
CTATAGTC -0.227416150841
CTATATAA 0.0899931718798
CTATATAC 0.204304704823
CTATATAG 0.171336164329
CTATATCA 0.193896822745
CTATATCC 0.187015336363
CTATATGA -0.0135184087466
CTATATGC 0.134300408234
CTATATTA 0.200248427353
CTATATTC 0.204611404722
CTATCAAA -0.225162898386
CTATCAAC -0.0169896286436
CTATCAAG 0.0164558838059
CTATCACA 0.0601731727514
CTATCACC -0.119519222853
CTATCAGA 0.101987418825
CTATCAGC -0.0442215216539
CTATCATA 0.0307004544556
CTATCATC 0.015055236768
CTATCCAA 0.0642796768315
CTATCCAC 0.00470372565808
CTATCCAG 0.0155327230016
CTATCCCA -0.00361149531274
CTATCCCC -0.172314151642
CTATCCGA 0.146488812605
CTATCCGC 0.141343541615
CTATCCTA 0.0525605450874
CTATCCTC -0.0535440849592
CTATCGAA 0.047274770325
CTATCGAC -0.0877233416743
CTATCGAG -0.141805788798
CTATCGCA -0.087090494385
CTATCGCC -0.178864197531
CTATCGGA 0.108298206327
CTATCGGC 0.0106396408264
CTATCGTA -0.0178765998867
CTATCGTC -0.102142747371
CTATCTAA 0.104338861777

CTATCTAC 0.0662225967196
CTATCTCA 0.156847570748
CTATCTCC 0.0790961382248
CTATCTGA 0.230280534046
CTATCTGC -0.10670824703
CTATCTTA 0.130356868427
CTATCTTC -0.0133915095634
CTATGAAA 0.0716892796768
CTATGAAC -0.0286767611035
CTATGAAG 0.00172446355672
CTATGACA -0.111753389401
CTATGACC -0.242193173566
CTATGAGA -0.0314965205643
CTATGAGC -0.255526506899
CTATGATA 0.122376451733
CTATGATC 0.155725153132
CTATGCAA 0.103736579838
CTATGCAC -0.14136238199
CTATGCAG -0.0101709391777
CTATGCCA -0.146596927081
CTATGCCC -0.249384087791
CTATGCGA 0.0281180706492
CTATGCGC 0.0572269115105
CTATGCTA -0.134420462839
CTATGCTC -0.222112477434
CTATGGAA 0.193115220897
CTATGGAC -0.210829583451
CTATGGAG -0.0170451443501
CTATGGCA -0.128209262665
CTATGGCC -0.212226721981
CTATGGGA 0.029414670289
CTATGGGC -0.172347106407
CTATGGTA -0.0970479275929
CTATGGTC -0.276569213294
CTATGTAA 0.138548596017
CTATGTAC -0.118512115474
CTATGTCA 0.105139976465
CTATGTCC 0.0149317218819
CTATGTGA 0.0215234962898
CTATGTGC -0.215321677861
CTATGTTA 0.0901181119521
CTATGTTC -0.181307055668
CTATTAAA -0.0295657453973
CTATTAAC 0.0641886885651
CTATTAAG -0.228377204829
CTATTACA 0.18798711278
CTATTACC -0.0308416344228
CTATTAGA 0.221125041768
CTATTAGC -0.0531424263301
CTATTATA 0.0901196532648
CTATTATC -0.132242200707
CTATTCAA 0.029080528235
CTATTCAC -0.180141744402
CTATTCAG 0.0360248717911

CTATTCCA 0.0981665770779
CTATTCCC 0.00350721201344
CTATTCGA 0.145933147288
CTATTCGC 0.163754967178
CTATTCTA 0.0377444685895
CTATTCTC -0.0590490460822
CTATTGAA 0.038892682289
CTATTGAC -0.037256580825
CTATTGAG -0.130536404812
CTATTGCA 0.11887224637
CTATTGCC -0.11225800079
CTATTGGA 0.0133668378939
CTATTGGC -0.302252321599
CTATTGTA 0.165114116775
CTATTGTC -0.0298113021142
CTATTTAA -0.051718228032
CTATTTAC -0.102655702933
CTATTTCA -0.138566481728
CTATTTCC 0.0615910972934
CTATTTGA 0.0786061917975
CTATTTGC 0.136961922334
CTATTTTA -0.00453997355919
CTATTTTC 0.171683640114
CTCAAAAA 0.248443256538
CTCAAAAC 0.0724027719263
CTCAAAAG -0.13491436317
CTCAAACA -0.0506682033973
CTCAAACC -0.112156862745
CTCAAAGA 0.0710616015413
CTCAAAGC 0.0516830589979
CTCAAATA -0.014255198301
CTCAAATC 0.20026675888
CTCAACAA -0.0292696705522
CTCAACAC -0.023859195444
CTCAACAG -0.113950069582
CTCAACCA -0.129311246427
CTCAACCC -0.0341181636069
CTCAACGA 0.0562780636321
CTCAACGC -0.106730573711
CTCAACTA 0.0124120378172
CTCAACTC -0.195249234643
CTCAAGAA 0.18042657358
CTCAAGAC -0.182891292537
CTCAAGAG -0.11367610748
CTCAAGCA -0.165333135773
CTCAAGCC -0.170927473345
CTCAAGGA -0.0562392685627
CTCAAGGC -0.0717398224066
CTCAAGTA 0.0468089433847
CTCAAGTC -0.121711934156
CTCAATAA 0.0265526709572
CTCAATAC 0.0662006849766
CTCAATCA -0.0798700300775
CTCAATCC 0.074762892709

CTCAATGA -0.0636889282757
CTCAATGC -0.0592724198244
CTCAATTA -0.0084014934697
CTCAATTC -0.065511851288
CTCACAAA -0.132094314671
CTCACAAC 0.108187933113
CTCACAAG -0.191848946986
CTCACACA 0.0468888469194
CTCACACC -0.0569610506588
CTCACAGA 0.00372350471431
CTCACAGC -0.248765554019
CTCACATA -0.00426933256634
CTCACATC -0.00997502812766
CTCACCAA -0.0851870146264
CTCACCAC -0.0065441793846
CTCACCAG -0.172013453382
CTCACCCA -0.0764231531115
CTCACCCC -0.211117146478
CTCACCGA 0.121061933303
CTCACCGC -0.12724713552
CTCACCTA -0.115655667523
CTCACCTC -0.153165152673
CTCACGAA -0.086503585911
CTCACGAC -0.101016867529
CTCACGAG 0.069205690575
CTCACGCA -0.0647896599984
CTCACGCC -0.0977942386831
CTCACGGA -0.179321573081
CTCACGGC -0.178626211449
CTCACGTA -0.0959259926434
CTCACGTC -0.151615682659
CTCACTAA -0.177575837394
CTCACTAC -0.0286037296805
CTCACTCA -0.0999345582062
CTCACTCC -0.0235921692593
CTCACTGA -0.127196295349
CTCACTGC -0.0936275236128
CTCACTTA -0.189313622196
CTCACTTC -0.100072983889
CTCAGAAA -0.0342638487777
CTCAGAAC 0.0612362510703
CTCAGAAG -0.0729773782476
CTCAGACA -0.0622070751973
CTCAGACC -0.177634574973
CTCAGAGA -0.059821597199
CTCAGAGC -0.196204128881
CTCAGATA 0.225526356232
CTCAGATC 0.141731980944
CTCAGCAA 0.156051724516
CTCAGCAC -0.0755595826498
CTCAGCAG -0.0707860859561
CTCAGCCA -0.185345679012
CTCAGCCC -0.211647789631
CTCAGCGA 0.0137370423044

CTCAGCGC -0.149382945592
CTCAGCTA -0.0218781213299
CTCAGCTC -0.0739230988256
CTCAGGAA -0.0275170952836
CTCAGGAC -0.208374604595
CTCAGGAG -0.166745098039
CTCAGGCA 0.047781043169
CTCAGGCC -0.155941327539
CTCAGGGA 0.00940688404955
CTCAGGGC -0.125231172794
CTCAGGTA -0.0110813464969
CTCAGGTC -0.173645466879
CTCAGTAA 0.078696264873
CTCAGTAC -0.194186786276
CTCAGTCA -0.173884188544
CTCAGTCC -0.188312555913
CTCAGTGA -0.203478339182
CTCAGTGC -0.0431045251327
CTCAGTTA -0.0673170330123
CTCAGTTC -0.244058691513
CTCATAAA -0.0102968047805
CTCATAAC -0.114145070345
CTCATAAG -0.232620540932
CTCATACA 0.0852584579244
CTCATACC 0.0720366684584
CTCATAGA 0.137589464094
CTCATAGC -0.0617693541265
CTCATATA 0.0834787810929
CTCATATC 0.101528273581
CTCATCAA -0.00861898646646
CTCATCAC 0.0778710792788
CTCATCAG 0.0630886517816
CTCATCCA -0.022777530852
CTCATCCC -0.103165588424
CTCATCGA -0.181917562491
CTCATCGC -0.0260983507588
CTCATCTA -0.141728896294
CTCATCTC -0.168235468418
CTCATGAA 0.0458215328547
CTCATGAC -0.0265264399989
CTCATGAG -0.195519855644
CTCATGCA -0.034090721989
CTCATGCC -0.141267996455
CTCATGGA -0.138769541431
CTCATGGC -0.153544886672
CTCATGTA 0.156827393837
CTCATGTC -0.0883318240443
CTCATTAA -0.0622245144044
CTCATTAC 0.12908077016
CTCATTCA -0.232265107212
CTCATTCC -0.0442244272369
CTCATTGA 0.0204971569079
CTCATTGC -0.076793672748
CTCATTTA 0.125246611364

CTCATTTC -0.147039938172
CTCCAAAA -0.0467774155663
CTCCAAAC -0.0254346108258
CTCCAAAG -0.274284237246
CTCCAACA 0.0618756723529
CTCCAACC -0.225170660857
CTCCAAGA -0.00714738132732
CTCCAAGC -0.308272574159
CTCCAATA 0.0233987458272
CTCCAATC -0.0980104826981
CTCCACAA -0.118384010107
CTCCACAC -0.164024952268
CTCCACAG -0.314279993856
CTCCACCA -0.190014409557
CTCCACCC -0.0439288121412
CTCCACGA -0.0751369256025
CTCCACGC -0.265205469903
CTCCACTA 0.0922130373513
CTCCACTC -0.123481769615
CTCCAGAA -0.12921530213
CTCCAGAC -0.273300106593
CTCCAGAG -0.232807552651
CTCCAGCA -0.215511991177
CTCCAGCC -0.235209876543
CTCCAGGA -0.0684408664109
CTCCAGGC -0.3133096308
CTCCAGTA -0.0879564270153
CTCCAGTC -0.0565790773486
CTCCATAA -0.0819563290863
CTCCATAC -0.228989729409
CTCCATCA -0.0647528304585
CTCCATCC -0.320224327511
CTCCATGA -0.135596382633
CTCCATGC -0.217950828217
CTCCATTA 0.0522695841154
CTCCATTC -0.0339531910566
CTCCCAAA -0.0988715830087
CTCCCAAC -0.184502057613
CTCCCAAG -0.333720126407
CTCCCACA -0.0995735446631
CTCCCACC 0.020739639279
CTCCCAGA -0.00483266350208
CTCCCAGC -0.262635019939
CTCCCATA -0.259833986507
CTCCCATC -0.108439632458
CTCCCCAA -0.12638635079
CTCCCCAC -0.0343810281916
CTCCCCAG -0.151244321548
CTCCCCCA -0.278493917812
CTCCCCCC -0.150141650422
CTCCCCGA -0.105404382109
CTCCCCGC -0.19567364678
CTCCCCTA -0.107615056047
CTCCCCTC -0.13948746075

CTCCCGAA 0.0525854405373
CTCCCGAC -0.11571284314
CTCCCGAG -0.165337140832
CTCCCGCA -0.0791262911685
CTCCCGCC -0.193739299355
CTCCCGGA 0.0857181277306
CTCCCGGC -0.148502470466
CTCCCGTA -0.148081437237
CTCCCGTC -0.369212317628
CTCCCTAA -0.207307189542
CTCCCTAC -0.251693850568
CTCCCTCA -0.0670984478536
CTCCCTCC -0.0427347261323
CTCCCTGA -0.0242432430077
CTCCCTGC -0.222422657952
CTCCCTTA -0.0356933124777
CTCCCTTC -0.0929758007593
CTCCGAAA 0.104541426351
CTCCGAAC -0.270018094632
CTCCGAAG -0.187608040697
CTCCGACA -0.222254175744
CTCCGACC -0.236110425206
CTCCGAGA -0.0337092834994
CTCCGAGC -0.241176470588
CTCCGATA -0.101035389707
CTCCGATC 0.176288173176
CTCCGCAA -9.05483940008E-5
CTCCGCAC -0.0384298036891
CTCCGCAG -0.153748068455
CTCCGCCA -0.136230250275
CTCCGCCC -0.187621465693
CTCCGCGA 0.0306620342927
CTCCGCGC -0.0490883733093
CTCCGCTA -0.0985858415666
CTCCGCTC -0.116592696727
CTCCGGAA -0.136335905362
CTCCGGAC -0.176141321348
CTCCGGAG 0.303866026818
CTCCGGCA -0.230618505489
CTCCGGCC -0.138979423868
CTCCGGGA 0.0509643460888
CTCCGGGC -0.312683714608
CTCCGGTA -0.155739172305
CTCCGGTC 0.00683125895194
CTCCGTAA 0.0643467700228
CTCCGTAC -0.169060275962
CTCCGTCA -0.15156578377
CTCCGTCC -0.233511659808
CTCCGTGA -0.159932826237
CTCCGTGC -0.282806336038
CTCCGTTA 0.0711678631803
CTCCGTTC 0.0901337021669
CTCCTAAA -0.092614007816
CTCCTAAC -0.0761835308064

CTCCTAAG -0.14196616234
CTCCTACA -0.0952029584613
CTCCTACC -0.16932932869
CTCCTAGA -0.0681477639465
CTCCTAGC -0.0787896747004
CTCCTATA 0.112235410341
CTCCTATC -0.143954361355
CTCCTCAA 0.00715367644638
CTCCTCAC -0.312864724182
CTCCTCAG -0.404674227133
CTCCTCCA -0.120713305898
CTCCTCCC -0.234361839927
CTCCTCGA -0.187356202311
CTCCTCGC -0.110576052419
CTCCTCTA -0.174254521709
CTCCTCTC -0.0776465359219
CTCCTGAA -0.0926459692299
CTCCTGAC -0.0143369279755
CTCCTGCA -0.336631808279
CTCCTGCC -0.194275728496
CTCCTGGA -0.121233823965
CTCCTGGC -0.202611635736
CTCCTGTA -0.0280590543618
CTCCTGTC -0.124774498668
CTCCTTAA 0.00806880420728
CTCCTTAC -0.167374520552
CTCCTTCA -0.188184754447
CTCCTTCC -0.352238407211
CTCCTTGA -0.0413682390714
CTCCTTGC -0.0683522140991
CTCCTTTA -0.18030072217
CTCCTTTC -0.0642654320988
CTCGAAAA 0.0785807699608
CTCGAAAC -0.0279037670885
CTCGAAAG -0.0278573624009
CTCGAACA -0.0191797538971
CTCGAACC -0.151680008365
CTCGAAGA -0.0538789555138
CTCGAAGC -0.253425070916
CTCGAATA 0.185598477474
CTCGAATC 0.225001089594
CTCGACAA 0.0835216328345
CTCGACAC -0.0739862244468
CTCGACAG -0.138398960364
CTCGACCA -0.11796953076
CTCGACCC -0.30526141532
CTCGACGA -0.232057127494
CTCGACGC -0.116507278104
CTCGACTA -0.192880194955
CTCGACTC -0.0604518674321
CTCGAGAA 0.19810486796
CTCGAGAC -0.106391385953
CTCGAGAG -0.167085120208
CTCGAGCA 0.0475993000829

CTCGAGCC -0.287804985821
CTCGAGGA 0.113962436058
CTCGAGGC -0.242668686071
CTCGAGTA 0.0578450745974
CTCGAGTC -0.191656789148
CTCGATAA -0.0358130804189
CTCGATAC 0.129479279858
CTCGATCA 0.00490405936464
CTCGATCC -0.0133665232481
CTCGATGA 0.0808057181875
CTCGATGC 0.0126959452588
CTCGATTA 0.160922852313
CTCGATTC 0.281877878343
CTCGCAAA 0.0590325802981
CTCGCAAC 0.125606903665
CTCGCAAG -0.231556823431
CTCGCACA -0.106465015697
CTCGCACC -0.187438945416
CTCGCAGA -0.20214379085
CTCGCAGC -0.0487767735138
CTCGCATA -0.109247433044
CTCGCATC -0.109413630396
CTCGCCAA -0.234897119342
CTCGCCAC -0.0556653111846
CTCGCCAG 0.0332732846164
CTCGCCCA -0.21078140886
CTCGCCCC -0.209885257807
CTCGCCGA -0.129515312142
CTCGCCGC -0.0899858964017
CTCGCCTA 0.0697257071356
CTCGCCTC -0.278155534384
CTCGCGAA 0.0856493251783
CTCGCGAC -0.217687718836
CTCGCGAG 0.0210732784545
CTCGCGCA -0.0180724457593
CTCGCGCC -0.274496732672
CTCGCGGA -0.0779819535006
CTCGCGGC -0.0996525437516
CTCGCGTA -0.0602167564976
CTCGCGTC -0.276784832626
CTCGCTAA 0.0490898261009
CTCGCTAC -0.246479650521
CTCGCTCA -0.0942918916546
CTCGCTCC -0.202007453517
CTCGCTGA -0.21595734176
CTCGCTGC -0.0244571779841
CTCGCTTA -0.096573260013
CTCGCTTC -0.1625171404
CTCGGAAA 0.041888954752
CTCGGAAC -0.129968333697
CTCGGAAG -0.325958975118
CTCGGACA -0.00773978514752
CTCGGACC -0.104092094215
CTCGGAGA 0.163992561707

CTCGGAGC -0.108372817249
CTCGGATA 0.244764865689
CTCGGATC 0.177889965569
CTCGGCAA -0.00211005196929
CTCGGCAC -0.105982779342
CTCGGCAG 0.0068905902692
CTCGGCCA -0.186495591439
CTCGGCCC -0.12176325345
CTCGGCGA -0.0428114419916
CTCGGCGC -0.180212586154
CTCGGCTA -0.105639793001
CTCGGCTC -0.240713421559
CTCGGGAA -0.00260011155786
CTCGGGAC -0.170697785657
CTCGGGCA -0.192151053014
CTCGGGCC -0.184548408057
CTCGGGGA -0.0595555555556
CTCGGGGC -0.0793455272271
CTCGGGTA 0.0189962783157
CTCGGGTC -0.215685144951
CTCGGTAA -0.103765635669
CTCGGTAC -0.0944858771944
CTCGGTCA -0.0528406845797
CTCGGTCC -0.265632488737
CTCGGTGA 0.0631702284127
CTCGGTGC -0.140764338201
CTCGGTTA -0.106014524328
CTCGGTTC -0.0160518937138
CTCGTAAA 0.125220461116
CTCGTAAC 0.000686074337527
CTCGTAAG 0.0321112812407
CTCGTACA -0.183570781137
CTCGTACC 0.0904108761993
CTCGTAGA -0.175042776259
CTCGTAGC -0.256457746827
CTCGTATA 0.0591943140968
CTCGTATC 0.0848097653077
CTCGTCAA -0.0627832523764
CTCGTCAC -0.0643392339896
CTCGTCAG -0.162282574906
CTCGTCCA -0.246246372728
CTCGTCCC -0.107173048488
CTCGTCGA -0.226128172213
CTCGTCGC 0.0137818612922
CTCGTCTA -0.130654066098
CTCGTCTC -0.130609037214
CTCGTGAA -0.284349553936
CTCGTGAC -0.210649291323
CTCGTGCA -0.0771797251315
CTCGTGCC -0.12339100418
CTCGTGGA -0.242288343344
CTCGTGGC -0.209244236874
CTCGTGTA -0.0479332696656
CTCGTGTC -0.152836601307

CTCGTTAA -0.0107906563287
CTCGTTAC 0.09479077477
CTCGTTCA -0.0884114047364
CTCGTTCC -0.0907746482965
CTCGTTGA -0.0411713699277
CTCGTTGC -0.191927173358
CTCGTTTA 0.106139497044
CTCGTTTC -0.0393813891575
CTCTAAAA 0.120762804891
CTCTAAAC -0.0543300451818
CTCTAAAG -0.00455278930383
CTCTAACA -0.02546017172
CTCTAACC -0.162061772405
CTCTAAGA -0.0858473766315
CTCTAAGC -0.213242354904
CTCTAATA 0.118265494303
CTCTAATC 0.0740502375314
CTCTACAA 0.101569014862
CTCTACAC 0.154956198335
CTCTACAG -0.205037037037
CTCTACCA -0.133104938272
CTCTACCC -0.0321282165259
CTCTACGA -0.0822412108839
CTCTACGC -0.149190994916
CTCTACTA -0.235793754539
CTCTACTC -0.0279987155419
CTCTAGAA 0.113749753013
CTCTAGAC -0.113549341111
CTCTAGAG 0.0126723646724
CTCTAGCA -0.0344059686402
CTCTAGCC -0.099079157589
CTCTAGGA 0.0819476123371
CTCTAGGC -0.312164863242
CTCTAGTA -0.139289556302
CTCTAGTC -0.143012145843
CTCTATAA -0.0836359832879
CTCTATAC 0.19313404246
CTCTATCA -0.097793036071
CTCTATCC -0.149580371434
CTCTATGA -0.0428508309732
CTCTATGC -0.183862383909
CTCTATTA 0.103598564642
CTCTATTC 0.022363279106
CTCTCAAA -0.0190882280302
CTCTCAAC -0.113967501358
CTCTCAAG -0.251659158701
CTCTCACA -0.139156808516
CTCTCACC -0.229411764706
CTCTCAGA 0.0102773935761
CTCTCAGC -0.0892346666047
CTCTCATA 0.00475861160508
CTCTCATC -0.0881063312535
CTCTCCAA -0.10808910684
CTCTCCAC -0.125935683641

CTCTCCAG -0.194599854757
CTCTCCCA -0.386590530104
CTCTCCCC -0.121176360498
CTCTCCGA -0.140059273895
CTCTCCGC -0.196935528121
CTCTCCTA -0.117392807746
CTCTCCTC -0.250065346569
CTCTCGAA -0.0929890472192
CTCTCGAC -0.179612343118
CTCTCGCA -0.0846120320195
CTCTCGCC -0.251168839721
CTCTCGGA -0.0936003160338
CTCTCGGC -0.168288225998
CTCTCGTA -0.0112024755568
CTCTCGTC -0.257180715356
CTCTCTAA 0.0408655731989
CTCTCTAC -0.0430653075097
CTCTCTCA -0.123567184345
CTCTCTCC -0.204046568506
CTCTCTGA -0.196998835091
CTCTCTGC -0.0551208225553
CTCTCTTA -0.22802383186
CTCTCTTC -0.324949750787
CTCTGAAA 0.0698720090654
CTCTGAAC -0.168696399935
CTCTGAAG -0.114678169057
CTCTGACA -0.169154322799
CTCTGACC -0.260884236169
CTCTGAGA 0.128423515756
CTCTGAGC -0.139844617092
CTCTGATA 0.143139805484
CTCTGATC 0.156568796943
CTCTGCAA 0.00463237392698
CTCTGCAC -0.0996202642785
CTCTGCAG -0.0784747948739
CTCTGCCA -0.112517066086
CTCTGCCC -0.108329702251
CTCTGCGA 0.0216223188215
CTCTGCGC -0.0802301461873
CTCTGCTA -0.0782221912324
CTCTGCTC -0.250605544874
CTCTGGAA 0.0610915717208
CTCTGGAC -0.114595852335
CTCTGGCA -0.0181904028591
CTCTGGCC -0.117610739035
CTCTGGGA -0.0454031598646
CTCTGGGC -0.146587577201
CTCTGGTA -0.205574452387
CTCTGGTC -0.233593570742
CTCTGTAA 0.284873534497
CTCTGTAC -0.116534385386
CTCTGTCA -0.0617128463476
CTCTGTCC -0.164067651852
CTCTGTGA -0.028144141474

CTCTGTGC -0.145069088818
CTCTGTTA -0.0927158451052
CTCTGTTC -0.152503079462
CTCTTAAA -0.00795846231531
CTCTTAAC -0.0752095651795
CTCTTAAG -0.102907688171
CTCTTACA 0.013049944299
CTCTTACC -0.114381990407
CTCTTAGA -0.0775876261504
CTCTTAGC -0.168389863548
CTCTTATA 0.083362145609
CTCTTATC 0.0420618454851
CTCTTCAA 0.0777083666265
CTCTTCAC -0.160583761359
CTCTTCAG -0.0252771199861
CTCTTCCA -0.229772663671
CTCTTCCC -0.337176679697
CTCTTCGA -0.201179607688
CTCTTCGC -0.0374332871391
CTCTTCTA 0.0119152076518
CTCTTCTC -0.215166633735
CTCTTGAA -0.130750851978
CTCTTGAC -0.24875994513
CTCTTGCA -0.0912918165389
CTCTTGCC -0.140139775233
CTCTTGGA -0.11268917109
CTCTTGGC 0.0447372626502
CTCTTGTA -0.00977873984862
CTCTTGTC -0.138360796709
CTCTTTAA 0.0665073438612
CTCTTTAC 0.137848800193
CTCTTTCA 0.144824430143
CTCTTTCC -0.158968772694
CTCTTTGA -0.163399560407
CTCTTTGC -0.146251111331
CTCTTTTA 0.21217860885
CTCTTTTC -0.00086586375721
CTGAAAAA -0.0507941996531
CTGAAAAC -0.0262437155591
CTGAAAAG -0.0801637816081
CTGAAACA -0.103378445125
CTGAAACC 0.0990005269051
CTGAAAGA 0.142098993215
CTGAAAGC -0.163632350037
CTGAAATA 0.178758734909
CTGAAATC 0.397368994523
CTGAACAA -0.0751766332768
CTGAACAC -0.260635964594
CTGAACAG 0.0869307517534
CTGAACCA -0.158111291527
CTGAACCC -0.226839288516
CTGAACGA 0.0283831882963
CTGAACGC -0.173597392335
CTGAACTA -0.0967806139497

CTGAACTC 0.0303683944763
CTGAAGAA 0.0288974765011
CTGAAGAC -0.225698216735
CTGAAGCA -0.203052874071
CTGAAGCC -0.260405177759
CTGAAGGA -0.122119057026
CTGAAGGC -0.25891415227
CTGAAGTA 0.0432556148324
CTGAAGTC -0.0588454698152
CTGAATAA 0.125500098785
CTGAATAC -0.0074949515494
CTGAATCA 0.0748891303871
CTGAATCC 0.0916667877326
CTGAATGA -0.283809508136
CTGAATGC -0.0754679705635
CTGAATTA 0.0625233265275
CTGAATTC -0.0443181138639
CTGACAAA -0.0903796144291
CTGACAAC -0.0279706666822
CTGACAAG -0.136918939015
CTGACACA -0.0512828956395
CTGACACC -0.2677789267
CTGACAGA 0.115928406433
CTGACAGC -0.0889848605022
CTGACATA -0.066205522114
CTGACATC 0.0450074818764
CTGACCAA -0.1077191902
CTGACCAC -0.179268518519
CTGACCAG -0.0519325074953
CTGACCCA -0.242713305898
CTGACCCC -0.200639231824
CTGACCGA -0.0930188523915
CTGACCGC -0.185363538082
CTGACCTA -0.126995839822
CTGACCTC -0.238673761935
CTGACGAA -0.0655446897483
CTGACGAC -0.165988925657
CTGACGCA 0.0371551436084
CTGACGCC -0.242985442251
CTGACGGA -0.162232818622
CTGACGGC -0.293821409279
CTGACGTA -0.124290669213
CTGACGTC 0.014324524574
CTGACTAA -0.151482725317
CTGACTAC -0.12830718436
CTGACTCA -0.141129407116
CTGACTCC -0.154184458969
CTGACTGA -0.0961095195759
CTGACTGC -0.217580824616
CTGACTTA 0.0428413200665
CTGACTTC -0.205382716049
CTGAGAAA 0.0744628303285
CTGAGAAC -0.17731739839
CTGAGAAG -0.0823455814566

CTGAGACA	-0.0931550901917
CTGAGACC	-0.236652236652
CTGAGAGA	0.0251075764561
CTGAGAGC	-0.198739288308
CTGAGATA	0.250441831873
CTGAGATC	0.281271268304
CTGAGCAA	-0.0708711971553
CTGAGCAC	-0.158992600942
CTGAGCAG	-0.142220674167
CTGAGCCA	-0.11825483062
CTGAGCCC	-0.286206423654
CTGAGCGA	-0.0690912967065
CTGAGCGC	-0.0368547656123
CTGAGCTA	-0.0698177127218
CTGAGCTC	-0.297330589849
CTGAGGAA	0.13748929724
CTGAGGAC	-0.149794271842
CTGAGGCA	-0.116220487998
CTGAGGCC	-0.401428409642
CTGAGGGA	-0.0555552126031
CTGAGGGC	-0.276051830685
CTGAGGTA	-0.0794158488661
CTGAGGTC	-0.214602671077
CTGAGTAA	-0.0268751906789
CTGAGTAC	-0.111091902927
CTGAGTCA	-0.109110518343
CTGAGTCC	-0.163070442992
CTGAGTGA	-0.0167149938114
CTGAGTGC	-0.101423543052
CTGAGTTA	-0.08384786367
CTGAGTTC	0.0615475135473
CTGATAAA	-0.034024757096
CTGATAAC	0.0742158557669
CTGATAAG	0.0363811499704
CTGATACA	0.0624286482689
CTGATACC	0.1974933588
CTGATAGA	-0.0265121360211
CTGATAGC	-0.111489786734
CTGATATA	0.179008615054
CTGATATC	0.407934312499
CTGATCAA	-0.0680037191463
CTGATCAC	0.0344506471693
CTGATCAG	0.118181069959
CTGATCCA	-0.132714974238
CTGATCCC	0.176117559891
CTGATCGA	-0.0138628180641
CTGATCGC	0.214277481394
CTGATCTA	0.080375691892
CTGATCTC	0.235339154185
CTGATGAA	-0.250512746528
CTGATGAC	-0.168795715563
CTGATGCA	-0.0265142160979
CTGATGCC	-0.0643401945543
CTGATGGA	-0.0651364259651

CTGATGGC -0.0472511636285
CTGATGTA 0.0132000639228
CTGATGTC 0.0178242251383
CTGATTAA 0.0861970076182
CTGATTAC 0.375560715344
CTGATTCA 0.166136882019
CTGATTCC 0.386169290215
CTGATTGA 0.141915941482
CTGATTGC 0.39901902412
CTGATTTA 0.168455948539
CTGATTTC 0.429763340258
CTGCAAAA 0.122266435345
CTGCAAAC -0.0537373062339
CTGCAAAG -0.151524043043
CTGCAACA 0.0624931392566
CTGCAACC -0.115852788479
CTGCAAGA -0.0481353420598
CTGCAAGC -0.137376428175
CTGCAATA 0.15322385646
CTGCAATC 0.397363279106
CTGCACAA 0.156202693476
CTGCACAC -0.1209112271
CTGCACAG -0.0261431451964
CTGCACCA -0.0715365907856
CTGCACCC -0.183269090561
CTGCACGA 0.029229511076
CTGCACGC -0.192258533043
CTGCACTA 0.0800735112519
CTGCACTC -0.141757060008
CTGCAGAA 0.102596869093
CTGCAGAC -0.0413073265092
CTGCAGCA -0.143328054857
CTGCAGCC -0.178237037037
CTGCAGGA -0.062040009879
CTGCAGGC -0.301247126342
CTGCAGTA -0.00262629256405
CTGCAGTC 0.0589996487299
CTGCATAA 0.173607136112
CTGCATAC 0.0327415629131
CTGCATCA -0.0308911831215
CTGCATCC -0.0231008382607
CTGCATGA -0.13218664583
CTGCATGC 0.000106589021331
CTGCATTA 0.0877558729098
CTGCATTC 0.0854711409691
CTGCCAAA 0.0389113921272
CTGCCAAC -0.106774903975
CTGCCAAG -0.279986230257
CTGCCACA -0.0932672669008
CTGCCACC -0.0975632783609
CTGCCAGA 0.0921491145236
CTGCCAGC -0.176998628258
CTGCCATA -0.162287633347
CTGCCATC -0.188039525508

CTGCCCAA -0.0370132578252
CTGCCCAC -0.205383224009
CTGCCCAG -0.092956711774
CTGCCCCA 0.093090283629
CTGCCCCC -0.21734091622
CTGCCCGA -0.141159234201
CTGCCCGC -0.266506632278
CTGCCCTA 0.0270277337905
CTGCCCTC -0.371956089274
CTGCCGAA -0.234150302591
CTGCCGAC -0.102528220916
CTGCCGCA -0.133447785596
CTGCCGCC -0.199913443552
CTGCCGGA -0.137773803272
CTGCCGGC -0.120380407234
CTGCCGTA -0.167271787245
CTGCCGTC -0.20003862899
CTGCCTAA -0.0390694458165
CTGCCTAC -0.120849999177
CTGCCTCA -0.254966539905
CTGCCTCC -0.322371506863
CTGCCTGA -0.116418057888
CTGCCTGC -0.0847440361535
CTGCCTTA -0.0453393843955
CTGCCTTC -0.0712317442822
CTGCGAAA 0.0215345013063
CTGCGAAC -0.159981806368
CTGCGAAG -0.0105322184193
CTGCGACA -0.201016136377
CTGCGACC -0.234342821124
CTGCGAGA -0.0881557212866
CTGCGAGC -0.118034461896
CTGCGATA 0.207991806256
CTGCGATC 0.180359711185
CTGCGCAA 0.137011631927
CTGCGCAC -0.121246542868
CTGCGCAG 0.0701895753745
CTGCGCCA -0.101614036243
CTGCGCCC -0.128059891545
CTGCGCGA 0.205037432216
CTGCGCGC -0.124318612504
CTGCGCTA -0.0899057568503
CTGCGCTC -0.26328540305
CTGCGGAA -0.024818199281
CTGCGGAC -0.111550133627
CTGCGGCA -0.171034724966
CTGCGGCC -0.300870792718
CTGCGGGA -0.169627400634
CTGCGGGC -0.103681990378
CTGCGGTA -0.0400710153995
CTGCGGTC -0.336091094711
CTGCGTAA 0.116335211392
CTGCGTAC -0.140611111111
CTGCGTCA -0.157508464067

CTGCGTCC -0.265861064481
CTGCGTGA 0.0937177545061
CTGCGTGC -0.207565556343
CTGCGTTA -0.092716994594
CTGCGTTC -0.125624265973
CTGCTAAA -0.0144363895225
CTGCTAAC -0.225539422275
CTGCTAAG -0.191497360247
CTGCTACA -0.0311446979925
CTGCTACC -0.0788150709366
CTGCTAGA 0.196009181643
CTGCTAGC -0.10201511335
CTGCTATA -0.000937050542618
CTGCTATC -0.0340155656546
CTGCTCAA -0.172367908429
CTGCTCAC -0.0589499222757
CTGCTCCA -0.152128815569
CTGCTCCC -0.273639086361
CTGCTCGA -0.0806923385083
CTGCTCGC -0.126874820253
CTGCTCTA -0.0353248492232
CTGCTCTC -0.0505369794153
CTGCTGAA -0.0436515286215
CTGCTGAC -0.119284274018
CTGCTGCA -0.291391565268
CTGCTGCC -0.169978213508
CTGCTGGA -0.0757298474946
CTGCTGGC -0.193023600276
CTGCTGTA -0.0641092307412
CTGCTGTC -0.132311963235
CTGCTTAA -0.214126361656
CTGCTTAC 0.0259603748889
CTGCTTCA -0.126993925626
CTGCTTCC -0.216539778718
CTGCTTGA -0.069289858483
CTGCTTGC -0.047929207242
CTGCTTTA -0.0454772584656
CTGCTTTC -0.176063100137
CTGGAAAA 0.176321104744
CTGGAAAC -0.102221097338
CTGGAAAG 0.0584698553827
CTGGAACA -0.133680595905
CTGGAACC -0.201180072088
CTGGAAGA 0.121014629611
CTGGAAGC -0.251124071987
CTGGAATA 0.266934464574
CTGGAATC 0.342871878314
CTGGACAA 0.0184032580298
CTGGACAC -0.0747290140227
CTGGACAG -0.222487009913
CTGGACCA -0.107028846796
CTGGACCC -0.359104084015
CTGGACGA -0.0311666502025
CTGGACGC -0.132262575641

CTGGACTA 0.0988219895288
CTGGACTC -0.10834384134
CTGGAGAA 0.0577564820303
CTGGAGAC -0.207665670831
CTGGAGCA -0.242281816585
CTGGAGCC -0.301781482944
CTGGAGGA -0.080067686997
CTGGAGGC -0.209160493827
CTGGAGTA -0.0489308217524
CTGGAGTC -0.341884719822
CTGGATAA -0.057956212863
CTGGATAC 0.357815292084
CTGGATCA 0.0193468249241
CTGGATCC 0.128929558496
CTGGATGA -0.0608409586057
CTGGATGC -0.232466049383
CTGGATTA 0.338504786948
CTGGATTC 0.424037849349
CTGGCAAA -0.195822383043
CTGGCAAC -0.306976399724
CTGGCAAG 0.0426244264419
CTGGCACA -0.145851849109
CTGGCACC -0.316564575764
CTGGCAGA -0.114230914868
CTGGCAGC -0.207130315501
CTGGCATA 0.00254920039075
CTGGCATC -0.0115534918439
CTGGCCAA -0.136944035282
CTGGCCAC -0.150021125872
CTGGCCAG 0.0278737902429
CTGGCCCA -0.189056540192
CTGGCCCC -0.0643003353034
CTGGCCGA -0.0108421832551
CTGGCCGC -0.224285424246
CTGGCCTA -0.244111401906
CTGGCCTC -0.0783974169013
CTGGCGAA -0.076458168708
CTGGCGAC -0.210283224401
CTGGCGCA -0.0530540771729
CTGGCGCC -0.114774024321
CTGGCGGA -0.034301861026
CTGGCGGC -0.0930605142447
CTGGCGTA -0.130781747127
CTGGCGTC -0.344377265124
CTGGCTAA -0.313923314516
CTGGCTAC -0.181629735721
CTGGCTCA -0.217992737836
CTGGCTCC -0.288255044278
CTGGCTGA -0.0852344197099
CTGGCTGC -0.0391646771494
CTGGCTTA -0.133509491118
CTGGCTTC -0.196108927944
CTGGGAAA 0.113642818334
CTGGGAAC -0.290265777997

CTGGGAAG -0.234444279819
CTGGGACA -0.203743654657
CTGGGACC -0.266654566048
CTGGGAGA -0.115421711948
CTGGGAGC -0.132618736383
CTGGGATA 0.183608153066
CTGGGATC 0.090449348423
CTGGGCAA -0.0940302149204
CTGGGCAC -0.291059104923
CTGGGCCA -0.290591114623
CTGGGCCC -0.213175551496
CTGGGCGA -0.123131445171
CTGGGCGC -0.235519233618
CTGGGCTA -0.0902966438901
CTGGGCTC -0.353439101193
CTGGGGAA 0.0274857436678
CTGGGGAC -0.274772158296
CTGGGGCA -0.0482681417095
CTGGGGCC -0.252324758472
CTGGGGGA 0.0255370927442
CTGGGGGC -0.296313074993
CTGGGGTA -0.0841157877612
CTGGGGTC -0.220521604938
CTGGGTAA 0.164334013919
CTGGGTAC -0.0217891890557
CTGGGTCA -0.219337026203
CTGGGTCC -0.121710237499
CTGGGTGA -0.206901960784
CTGGGTGC -0.195673525377
CTGGGTTA -0.0713538564351
CTGGGTTC -0.0101506544826
CTGGTAAA -0.11272382656
CTGGTAAC 0.00927416308813
CTGGTAAG -0.0610092160507
CTGGTACA -0.193990420226
CTGGTACC -0.168339697087
CTGGTAGA 0.0904435372568
CTGGTAGC -0.292329613953
CTGGTATA -0.0373389097455
CTGGTATC 0.0138350953463
CTGGTCAA -0.161647767716
CTGGTCAC -0.238602432875
CTGGTCCA -0.190103901913
CTGGTCCC 0.00471698113208
CTGGTCGA -0.10365370033
CTGGTCGC -0.193909517853
CTGGTCTA -0.112856053248
CTGGTCTC 0.016060610463
CTGGTGAA 0.0522363279106
CTGGTGAC -0.161539578794
CTGGTGCA -0.19977692835
CTGGTGCC -0.0433854762887
CTGGTGGA -0.119782135076
CTGGTGGC -0.197730578195

CTGGTGTA -0.0474751964529
CTGGTGTC -0.146721842473
CTGGTTAA -0.0282729448951
CTGGTTAC 0.117371950015
CTGGTTCA -0.0765254041951
CTGGTTCC -0.173647462277
CTGGTTGA -0.0467107518697
CTGGTTGC -0.223693843401
CTGGTTTA -0.00646637513983
CTGGTTTC -0.0446535392451
CTGTAAAA 0.011489237929
CTGTAAAC 0.173406660959
CTGTAAAG -0.0411411856389
CTGTAACA -0.102645790115
CTGTAACC 0.00545523222873
CTGTAAGA 0.23250784869
CTGTAAGC 0.000630355846042
CTGTAATA 0.184265126086
CTGTAATC 0.451462634594
CTGTACAA -0.0280025569131
CTGTACAC -0.20063161245
CTGTACAG -0.0498422231499
CTGTACCA -0.199814687874
CTGTACCC 0.0494709194554
CTGTACGA -0.0936939161933
CTGTACGC -0.146259635258
CTGTACTA -0.0790071681699
CTGTACTC -0.14236831418
CTGTAGAA 0.0756136248875
CTGTAGAC 0.0480022983903
CTGTAGCA 0.00375837171124
CTGTAGCC -0.203349435177
CTGTAGGA -0.030819380649
CTGTAGGC -0.114575778608
CTGTAGTA -0.0233311494504
CTGTAGTC -0.0310475358521
CTGTATAA 0.0212600715713
CTGTATAC 0.111702030851
CTGTATCA 0.032326064533
CTGTATCC 0.0105923031104
CTGTATGA 0.014232998707
CTGTATGC -0.0332953809921
CTGTATTA 0.00597533159967
CTGTATTC -0.11141298382
CTGTCAAA -0.0154417212674
CTGTCAAC -0.0855628177197
CTGTCAAG 0.0117298507346
CTGTCACA -0.0380458781695
CTGTCACC -0.156401332818
CTGTCAGA -0.0412975951354
CTGTCAGC -0.159689984691
CTGTCATA -0.219777777778
CTGTCATC -0.014764037991
CTGTCCAA -0.263602142417

CTGTCCAC -0.172483140457
CTGTCCCA -0.0439949729439
CTGTCCCC -0.0674005688008
CTGTCCGA -0.0625531681348
CTGTCCGC -0.195075383501
CTGTCCTA -0.0404093966557
CTGTCCTC -0.218619559818
CTGTCGAA 0.027401101216
CTGTCGAC -0.193962794784
CTGTCGCA 0.0118504205832
CTGTCGCC -0.175304227079
CTGTCGGA 0.00821658308686
CTGTCGGC -0.182743432254
CTGTCGTA 0.0432264474911
CTGTCGTC -0.116755603266
CTGTCTAA -0.10450004939
CTGTCTAC -0.080985702917
CTGTCTCA -0.132735713933
CTGTCTCC -0.155741038206
CTGTCTGA -0.103059555592
CTGTCTGC -0.185571532317
CTGTCTTA 0.0588075479154
CTGTCTTC -0.057484254073
CTGTGAAA 0.0275253024216
CTGTGAAC -0.0132522119037
CTGTGAAG 0.153934499111
CTGTGACA -0.121343079633
CTGTGACC 0.0122456413296
CTGTGAGA -0.0904890469292
CTGTGAGC -0.260769855378
CTGTGATA 0.128701348415
CTGTGATC 0.144152451206
CTGTGCAA 0.152198799994
CTGTGCAC 0.0292914769758
CTGTGCCA -0.0214462499245
CTGTGCCC -0.121111111111
CTGTGCGA 0.0863040847575
CTGTGCGC -0.102217282731
CTGTGCTA -0.0827240993216
CTGTGCTC -0.19233405955
CTGTGGAA -0.14667224715
CTGTGGAC -0.157760348584
CTGTGGCA -0.238690544774
CTGTGGCC -0.164144773146
CTGTGGGA -0.0153617529394
CTGTGGGC -0.248355038593
CTGTGGTA -0.154745477627
CTGTGGTC -0.175331880901
CTGTGTAA 0.163343322576
CTGTGTAC -0.0118039312539
CTGTGTCA -0.1398684095
CTGTGTCC -0.191701925663
CTGTGTGA 0.0742797785946
CTGTGTGC -0.15408929277

CTGTGTTA -0.0231508924455
CTGTGTTC -0.102079628532
CTGTTAAA 0.0936399067124
CTGTTAAC -0.190264405581
CTGTTAAG -0.0784649498342
CTGTTACA -0.172650205761
CTGTTACC -0.168883262191
CTGTTAGA -0.0196586858184
CTGTTAGC 0.000538985660948
CTGTTATA 0.217493578979
CTGTTATC -0.121398465541
CTGTTCAA -0.0732476119646
CTGTTCAC -0.206925148213
CTGTTCCA -0.1816621388
CTGTTCCC -0.197445789853
CTGTTCGA 0.145632182238
CTGTTCGC 0.0988158154697
CTGTTCTA -0.118839052449
CTGTTCTC -0.100977922655
CTGTTGAA 0.048379217985
CTGTTGAC -0.262699016607
CTGTTGCA -0.0923548004741
CTGTTGCC -0.187244114138
CTGTTGGA -0.0718158441445
CTGTTGGC -0.137060275962
CTGTTGTA -0.0736594237636
CTGTTGTC -0.0336972880627
CTGTTTAA -0.0077114174887
CTGTTTAC -0.154352569299
CTGTTTCA -0.0889294984717
CTGTTTCC -0.272222222222
CTGTTTGA -0.176577110497
CTGTTTGC -0.033422239784
CTGTTTTA 0.0605668792585
CTGTTTTC 0.037843860458
CTTAAAAA 0.0747462461721
CTTAAAAC -0.0709354524719
CTTAAAAG 0.039190991258
CTTAAACA -0.0515749522492
CTTAAACC -0.0810006805857
CTTAAAGA 0.116213153575
CTTAAAGC -0.0717276998397
CTTAAATA 0.118482413958
CTTAAATC 0.054155963907
CTTAACAA -0.0150716810468
CTTAACAC 0.0459893840482
CTTAACCA 0.0369958725768
CTTAACCC 0.0313206656568
CTTAACGA -0.0728684895531
CTTAACGC -0.055396732704
CTTAACTA 0.0723214156001
CTTAACTC -0.022665000799
CTTAAGAA 0.16538385535
CTTAAGAC -0.175233516634

CTTAAGCA 0.0380894750708
CTTAAGCC -0.345405022876
CTTAAGGA -0.00654724664406
CTTAAGGC -0.12166960263
CTTAAGTA -0.100907085473
CTTAAGTC -0.0165788303689
CTTAATAA 0.109489231377
CTTAATAC 0.0178998445513
CTTAATCA 0.0300422762338
CTTAATCC -0.0321069068476
CTTAATGA -0.115438440515
CTTAATGC -0.0263815409109
CTTAATTA 0.0395514277245
CTTAATTC 0.052816466637
CTTACAAA 0.00397629044208
CTTACAAC -0.14488380645
CTTACAAG -0.0786090973806
CTTACACA 0.0246146470443
CTTACACC 0.0961123298418
CTTACAGA 0.32691441605
CTTACAGC -0.0702854884916
CTTACATA 0.121476322202
CTTACATC 0.0664674208368
CTTACCAA -0.0533327910382
CTTACCAC 0.0882411052838
CTTACCCA -0.118119169434
CTTACCCC -0.123823841747
CTTACCGA 0.0965278282219
CTTACCGC 0.0728893938645
CTTACCTA -0.00216677366784
CTTACCTC -0.0855607048945
CTTACGAA 0.100360292302
CTTACGAC -0.0804412725068
CTTACGCA -0.0660903926896
CTTACGCC -0.251478249062
CTTACGGA 0.034583635206
CTTACGGC -0.137497804996
CTTACGTA 0.0442106303102
CTTACGTC -0.106753812636
CTTACTAA 0.0506196155914
CTTACTAC -0.0574654921654
CTTACTCA -0.0022009791815
CTTACTCC -0.219821673525
CTTACTGA 0.11465399899
CTTACTGC -0.00231618696349
CTTACTTA 0.169934479102
CTTACTTC 0.0991206836931
CTTAGAAA 0.0117025325466
CTTAGAAC -0.00289768782971
CTTAGAAG -0.0884096118815
CTTAGACA -0.170062454611
CTTAGACC -0.180346061775
CTTAGAGA -0.0543026654503
CTTAGAGC -0.194259268404

CTTAGATA 0.286044484477
CTTAGATC 0.206129662561
CTTAGCAA 0.0456714076097
CTTAGCAC -0.0239268366375
CTTAGCCA -0.311669108247
CTTAGCCC -0.14973116796
CTTAGCGA 0.0862536864585
CTTAGCGC -0.221426289034
CTTAGCTA -0.0138592417912
CTTAGCTC -0.198067878443
CTTAGGAA 0.0683631912885
CTTAGGAC -0.0470294800392
CTTAGGCA -0.12556021285
CTTAGGCC -0.247283414008
CTTAGGGA 0.0234169823599
CTTAGGGC 0.00108923400074
CTTAGGTA -0.114244789094
CTTAGGTC -0.174582791415
CTTAGTAA -0.050381357779
CTTAGTAC -0.00591474859231
CTTAGTCA -0.0404846189731
CTTAGTCC -0.225890079502
CTTAGTGA -0.139592338543
CTTAGTGC -0.192475702848
CTTAGTTA 0.109216509523
CTTAGTTC 0.0128964304912
CTTATAAA 0.0462391262589
CTTATAAC 0.0659604229662
CTTATAAG -0.0718609588703
CTTATACA 0.158073888978
CTTATACC 0.0401143468055
CTTATAGA 0.0212298843004
CTTATAGC 0.0516229774529
CTTATATA 0.0676816157372
CTTATATC 0.146596858639
CTTATCAA 0.0241174987065
CTTATCAC -0.0900072621641
CTTATCCA 0.0625198941903
CTTATCCC -0.083471913504
CTTATCGA -0.0279424356188
CTTATCGC -0.044080604534
CTTATCTA 0.18738611166
CTTATCTC 0.0164795055874
CTTATGAA 0.192781078843
CTTATGAC -0.0870350247511
CTTATGCA 0.0716531314922
CTTATGCC -0.115860301744
CTTATGGA -0.120456341367
CTTATGGC -0.137752749786
CTTATGTA -0.079194459293
CTTATGTC -0.103817849558
CTTATTAA 0.0204606140654
CTTATTAC 0.201277744846
CTTATTCA 0.000745282059477

CTTATTCC 0.137920764749
CTTATTGA -0.207470121981
CTTATTGC -0.0819915044947
CTTATTTA 0.15863685262
CTTATTTC 0.100293090957
CTTCAAAA 0.0988493891012
CTTCAAAC -0.0535137983271
CTTCAAAG -0.0270262809989
CTTCAACA -0.100264936831
CTTCAACC -0.0243679216229
CTTCAAGA -0.0495812202244
CTTCAAGC -0.199046176474
CTTCAATA 0.214264960121
CTTCAATC -0.070509207338
CTTCACAA 0.0828863798804
CTTCACAC -0.0838802553174
CTTCACCA -0.0450641067861
CTTCACCC -0.114337127869
CTTCACGA -0.127511982571
CTTCACGC -0.0746908038664
CTTCACTA -0.13862962963
CTTCACTC -0.153104954428
CTTCAGAA -0.0603415466053
CTTCAGAC -0.244861303212
CTTCAGCA 0.0217268770592
CTTCAGCC -0.251099491649
CTTCAGGA -0.024600356788
CTTCAGGC -0.228375523314
CTTCAGTA -0.0202725229675
CTTCAGTC -0.111408551766
CTTCATAA 0.0639421282575
CTTCATAC -0.126736383442
CTTCATCA -0.204145903357
CTTCATCC -0.218037582184
CTTCATGA -0.107470003535
CTTCATGC 0.05099498392
CTTCATTA -0.0706655161694
CTTCATTC -0.164714460622
CTTCCAAA -0.0711546840959
CTTCCAAC -0.0948439457827
CTTCCAAG -0.197486047316
CTTCCACA -0.0837173355957
CTTCCACC -0.243016629976
CTTCCAGA -0.052021773366
CTTCCAGC -0.135093900578
CTTCCATA 0.0991137971612
CTTCCATC -0.178232302254
CTTCCCAA -0.144316572821
CTTCCCAC -0.169128177717
CTTCCCCA -0.268409584712
CTTCCCCC -0.282708461619
CTTCCCGA 0.0653305827147
CTTCCCGC -0.253235460316
CTTCCCTA -0.0457810112092

CTTCCCTC -0.113694793933
CTTCCGAA -0.0768350700446
CTTCCGAC -0.130601785548
CTTCCGCA -0.0350282113768
CTTCCGCC -0.270329146867
CTTCCGGA -0.024319103516
CTTCCGGC -0.17300308642
CTTCCGTA -0.118374548608
CTTCCGTC 0.0505450877106
CTTCCTAA -0.0176019232036
CTTCCTAC -0.0516586533184
CTTCCTCA -0.104413580247
CTTCCTCC -0.187971835813
CTTCCTGA 0.0344639279932
CTTCCTGC -0.209886298784
CTTCCTTA -0.136869950232
CTTCCTTC -0.216496570645
CTTCGAAA -0.0493963651156
CTTCGAAC -0.145316926474
CTTCGAAG 0.1415755002
CTTCGACA -0.0784398459107
CTTCGACC -0.286265506054
CTTCGAGA -0.0334359972688
CTTCGAGC -0.244202460759
CTTCGATA 0.120527944445
CTTCGATC 0.156029770138
CTTCGCAA 0.122686792672
CTTCGCAC -0.203849047396
CTTCGCCA -0.0932553977735
CTTCGCCC -0.0596574695992
CTTCGCGA -0.0167809136116
CTTCGCGC -0.0616753592027
CTTCGCTA -0.0643740975424
CTTCGCTC -0.29722394068
CTTCGGAA -0.0189996077463
CTTCGGAC -0.193913501589
CTTCGGCA -0.198753154802
CTTCGGCC -0.146213271226
CTTCGGGA -0.0289381546642
CTTCGGGC -0.199587819919
CTTCGGTA -0.0328048338767
CTTCGGTC -0.252705940436
CTTCGTAA -0.0122725767181
CTTCGTAC 0.0768156271268
CTTCGTCA -0.196445842446
CTTCGTCC -0.248403292181
CTTCGTGA 0.0652539023908
CTTCGTGC 0.0286915385206
CTTCGTTA -0.0142367113965
CTTCGTTC -0.14222101503
CTTCTAAA 0.0197130846148
CTTCTAAC -0.188414850283
CTTCTACA -0.0682246031562
CTTCTACC -0.110153587831

CTTCTAGA -0.0829044208447
CTTCTAGC -0.0624653549573
CTTCTATA 0.122412127599
CTTCTATC -0.0844103104438
CTTCTCAA -0.0328054857409
CTTCTCAC -0.160865472337
CTTCTCCA -0.153776356723
CTTCTCCC -0.283576930674
CTTCTCGA 0.0462414103493
CTTCTCGC -0.184147389946
CTTCTCTA -0.0522378639445
CTTCTCTC -0.2058097313
CTTCTGAA -0.105746804739
CTTCTGAC -0.00872874751666
CTTCTGCA -0.0103985681994
CTTCTGCC -0.170140885984
CTTCTGGA -0.100456790123
CTTCTGGC -0.129343364502
CTTCTGTA 0.0652230761408
CTTCTGTC -0.176692821432
CTTCTTAA -0.0037404772882
CTTCTTAC 0.110340970174
CTTCTTCA -0.013596676013
CTTCTTCC -0.272106383658
CTTCTTGA 0.0257739746924
CTTCTTGC -0.113867871318
CTTCTTTA -0.124870363969
CTTCTTTC -0.0570791761336
CTTGAAAA 0.0497376451733
CTTGAAAC -0.148558837411
CTTGAAAG -0.11840247311
CTTGAACA -0.112167509041
CTTGAACC -0.214875534267
CTTGAAGA 0.00818062827225
CTTGAAGC -0.178826989771
CTTGAATA -0.198230181049
CTTGAATC 0.00496999985472
CTTGACAA -0.0495968503479
CTTGACAC -0.111725772992
CTTGACCA -0.115954556681
CTTGACCC -0.219831517792
CTTGACGA 0.0620509865262
CTTGACGC -0.111078508843
CTTGACTA -0.102110982569
CTTGACTC -0.264788662848
CTTGAGAA 0.12229744454
CTTGAGAC -0.239566529492
CTTGAGCA -0.11391824997
CTTGAGCC -0.357261201358
CTTGAGGA 0.131002571441
CTTGAGGC -0.206169934641
CTTGAGTA 0.15874113592
CTTGAGTC -0.0389629255169
CTTGATAA 0.186917612192

CTTGATAC 0.18210078534
CTTGATCA -0.0772707067714
CTTGATCC -0.158157020938
CTTGATGA -0.17664489739
CTTGATGC -0.0756602779424
CTTGATTA 0.157928823904
CTTGATTC 0.230661165429
CTTGCAAA 0.0203148334311
CTTGCAAC 0.09067307832
CTTGCAAG -0.0432773241277
CTTGCACA 0.0715688695829
CTTGCACC -0.283230669266
CTTGCAGA 0.115817994276
CTTGCAGC -0.202224965706
CTTGCATA 0.086286197282
CTTGCATC -0.157539578794
CTTGCCAA -0.0899523240916
CTTGCCAC -0.209565904489
CTTGCCCA -0.114943758573
CTTGCCCC -0.265908479951
CTTGCCGA 0.0433860571937
CTTGCCGC -0.13826966666
CTTGCCTA -0.235448075527
CTTGCCTC -0.0122630533329
CTTGCGAA 0.0631978847355
CTTGCGAC 0.0513105469393
CTTGCGCA 0.0957217666239
CTTGCGCC -0.320057613169
CTTGCGGA 0.0618146794429
CTTGCGGC -0.227913051591
CTTGCGTA 0.147363279106
CTTGCGTC -0.081966673253
CTTGCTAA -0.162603221472
CTTGCTAC -0.0451848558695
CTTGCTCA -0.0885781529208
CTTGCTCC -0.131952030121
CTTGCTGA -0.123069105427
CTTGCTGC -0.247632768296
CTTGCTTA -0.0867622362587
CTTGCTTC -0.138293435192
CTTGGAAA 0.0472229889733
CTTGGAAC -0.0327916090364
CTTGGACA -0.0362542477322
CTTGGACC -0.212220456797
CTTGGAGA 0.14962732442
CTTGGAGC -0.218456895375
CTTGGATA 0.246914997167
CTTGGATC 0.108757427397
CTTGGCAA 0.0297619047619
CTTGGCAC -0.262445958698
CTTGGCCA -0.164526053408
CTTGGCCC -0.225001234507
CTTGGCGA 0.0768156271268
CTTGGCGC -0.126545541172

CTTGGCTA -0.139313150745
CTTGGCTC -0.338593974175
CTTGGGAA -0.0256141676231
CTTGGGAC -0.142681190995
CTTGGGCA -0.214955809016
CTTGGGCC -0.41167178689
CTTGGGGA 0.0769116775341
CTTGGGGC -0.11289756044
CTTGGGTA -0.244035721756
CTTGGGTC -0.143590756468
CTTGGTAA 0.11165003107
CTTGGTAC -0.130171978577
CTTGGTCA -0.23816309751
CTTGGTCC -0.230078144289
CTTGGTGA -0.203322359396
CTTGGTGC -0.183915041718
CTTGGTTA 0.0380194954884
CTTGGTTC -0.00843334815034
CTTGTAAA 0.057955892522
CTTGTAAC 0.0635372419243
CTTGTACA -0.134393729829
CTTGTACC -0.161396750136
CTTGTAGA -0.145511269159
CTTGTAGC -0.256257281398
CTTGTATA 0.0205836022745
CTTGTATC -0.159296061731
CTTGTCAA 0.0487666951799
CTTGTCAC -0.186939822603
CTTGTCCA -0.0645583006019
CTTGTCCC -0.12959990121
CTTGTCGA -0.272013080931
CTTGTCGC -0.279538101406
CTTGTCTA -0.104611155011
CTTGTCTC -0.0166039544986
CTTGTGAA 0.0752129574744
CTTGTGAC 0.126780759229
CTTGTGCA -0.178422075142
CTTGTGCC -0.287402311517
CTTGTGGA -0.0227938271318
CTTGTGGC -0.249358024691
CTTGTGTA -0.156128423351
CTTGTGTC -0.101025066948
CTTGTTAA -0.00299420336176
CTTGTTAC -0.0950808478491
CTTGTTCA -0.125907223504
CTTGTTCC -0.0291706370419
CTTGTTGA -0.187256499874
CTTGTTGC 0.0918004445542
CTTGTTTA 0.0276103031976
CTTGTTTC 0.0310387305018
CTTTAAAA 0.147788124882
CTTTAAAC -0.00728865515087
CTTTAAAG 0.0583427978865
CTTTAACA -0.0381160317142

CTTTAACC -0.0245131936011
CTTTAAGA -0.196092141654
CTTTAAGC 0.158716437243
CTTTAATA 0.0603031899712
CTTTAATC 0.0453256432235
CTTTACAA 0.111758894716
CTTTACAC 0.113163845529
CTTTACCA -0.0245202968232
CTTTACCC -0.0014586027051
CTTTACGA -0.00861650661747
CTTTACGC 0.101868289919
CTTTACTA -0.154020256725
CTTTACTC -0.278082788671
CTTTAGAA -0.0686350366566
CTTTAGAC -0.165351665877
CTTTAGCA -0.037083690092
CTTTAGCC -0.00679454460635
CTTTAGGA -0.0170833757064
CTTTAGGC -0.152708494537
CTTTAGTA -0.0123858888611
CTTTAGTC -0.326692029564
CTTTATAA 0.050967668133
CTTTATAC 0.0720378054403
CTTTATCA 0.0865210001017
CTTTATCC -0.0045295802802
CTTTATGA 0.131297488123
CTTTATGC -0.135167446739
CTTTATTA 0.236998104608
CTTTATTC 0.0175964346089
CTTTCAAA 0.0114542518921
CTTTCAAC 0.00984507992954
CTTTCACA -0.0498973734181
CTTTCACC 0.0486124832598
CTTTCAGA -0.115970541589
CTTTCAGC -0.0203167703909
CTTTCATA 0.0189233023777
CTTTCATC -0.183524691358
CTTTCCAA -0.260534127886
CTTTCCAC -0.158386013433
CTTTCCCA -0.21942260039
CTTTCCCC 0.0553121322621
CTTTCCGA -0.0300160662872
CTTTCCGC 0.00927343672824
CTTTCCTA 0.0323405924484
CTTTCCTC -0.131391659671
CTTTCGAA -0.0832297926761
CTTTCGAC -0.201565509408
CTTTCGCA -0.064560603199
CTTTCGCC -0.267221247575
CTTTCGGA -0.0593689986283
CTTTCGGC -0.283462525921
CTTTCGTA 0.104291546206
CTTTCGTC 0.0259267492151
CTTTCTAA -0.036813371995

CTTTCTAC 0.0883046279831
CTTTCTCA 0.068994379679
CTTTCTCC -0.0933372459919
CTTTCTGA 0.0741015170476
CTTTCTGC -0.167048191196
CTTTCTTA -0.0591313750027
CTTTCTTC 0.0345932529882
CTTTGAAA 0.0825665532205
CTTTGAAC 0.0533990737459
CTTTGACA -0.0809639836903
CTTTGACC -0.167624237236
CTTTGAGA 0.154377987303
CTTTGAGC -0.273008123056
CTTTGATA 0.0725335231648
CTTTGATC 0.0215015697381
CTTTGCAA 0.0330783610709
CTTTGCAC -0.0285105051703
CTTTGCCA -0.138781893004
CTTTGCCC -0.202915752619
CTTTGCGA 0.0233375046653
CTTTGCGC 0.0615902654284
CTTTGCTA 0.0751950372641
CTTTGCTC -0.135587338475
CTTTGGAA 0.0216464397508
CTTTGGAC -0.197932128261
CTTTGGCA -0.0994254576584
CTTTGGCC -0.0851183732752
CTTTGGGA 0.0635610826203
CTTTGGGC -0.155498479809
CTTTGGTA 0.0521305187919
CTTTGGTC -0.0702202029181
CTTTGTAA 0.212373425537
CTTTGTAC -0.169620915033
CTTTGTCA -0.134858280185
CTTTGTCC -0.0118676501559
CTTTGTGA 0.105349178446
CTTTGTGC -0.168781684114
CTTTGTTA -0.106261531533
CTTTGTTC -0.0451338747403
CTTTTAAA 0.12034136943
CTTTTAAC 0.0367873059782
CTTTTACA 0.0752925420975
CTTTTACC -0.136509908711
CTTTTAGA 0.0607093459791
CTTTTAGC -0.219352112521
CTTTTATA -0.0341156713599
CTTTTATC 0.0526070344166
CTTTTCAA 0.0711097253779
CTTTTCAC -0.137978052126
CTTTTCCA -0.0540384520399
CTTTTCCC -0.025907631514
CTTTTCGA -0.00429070890689
CTTTTCGC 0.0195531186195
CTTTTCTA 0.132286144398

CTTTTCTC -0.0742623450961
CTTTTGAA 0.056672484577
CTTTTGAC -0.0760746668423
CTTTTGCA 0.0969134887703
CTTTTGCC -0.198465132089
CTTTTGGA -0.0126029666003
CTTTTGGC -0.138320556135
CTTTTGTA 0.0438168236405
CTTTTGTC -0.201472226338
CTTTTTAA 0.0970653610913
CTTTTTAC 0.154776049941
CTTTTTCA -0.0893191309549
CTTTTTCC -0.127043310781
CTTTTTGA -0.116160618235
CTTTTTGC -0.194020638918
CTTTTTTA -0.102760626767
CTTTTTTC 0.0776095768018
GAAAAAAA 0.049787331399
GAAAAAAC -0.106923727046
GAAAAACA 0.000551576520952
GAAAAACC 0.0255933170871
GAAAAAGA -0.0387140632626
GAAAAAGC -0.0441605044092
GAAAAATA 0.249618542668
GAAAAATC 0.405357417287
GAAAACAA 0.0944532419043
GAAAACAC 0.00710560341697
GAAAACCA 0.0198117182166
GAAAACCC -0.0491970185954
GAAAACGA -0.0915717139784
GAAAACGC -0.0787783691308
GAAAACTA 0.0103569781993
GAAAACTC -0.261500685871
GAAAAGAA 0.131259148712
GAAAAGAC 0.0175366466666
GAAAAGCA 0.0464936876208
GAAAAGCC -0.113254407342
GAAAAGGA 0.0366582889021
GAAAAGGC -0.186940758129
GAAAAGTA 0.0944154693243
GAAAAGTC -0.223716049383
GAAAATAA 0.0828393598103
GAAAATAC 0.123397330348
GAAAATCA 0.421897297416
GAAAATCC 0.464297006225
GAAAATGA 0.0328519835781
GAAAATGC -0.00637162896375
GAAAATTA 0.20418395574
GAAAATTC 0.227003036334
GAAACAAA 0.119452878706
GAAACAAC -0.0350940745132
GAAACACA -0.108378248805
GAAACACC -0.101370786386
GAAACAGA -0.06508070257

GAAACAGC -0.201292891854
GAAACATA 0.184290965089
GAAACATC -0.11173406661
GAAACCAA 0.0561518457717
GAAACCAC -0.014078245406
GAAACCCA -0.0988028997719
GAAACCCC -0.249237386705
GAAACCGA -0.130771088172
GAAACCGC -0.0619543087316
GAAACCTA -0.124285988227
GAAACCTC -0.288240522101
GAAACGAA 0.12517687737
GAAACGAC -0.156457920519
GAAACGCA -0.0654652132868
GAAACGCC -0.161669217855
GAAACGGA -0.0467856476451
GAAACGGC -0.0617574437182
GAAACGTA 0.0908139990993
GAAACGTC -0.223761231491
GAAACTAA 0.0851640928043
GAAACTAC 0.0316984585882
GAAACTCA -0.131580935006
GAAACTCC -0.292274348422
GAAACTGA -0.041131581547
GAAACTGC -0.00881973601164
GAAACTTA 0.0404833053443
GAAACTTC -0.0813558928115
GAAAGAAA 0.213181177633
GAAAGAAC 0.0186612219807
GAAAGACA 0.104372468636
GAAAGACC -0.0716780118478
GAAAGAGA -0.0283308878009
GAAAGAGC -0.144110384895
GAAAGATA 0.212280446879
GAAAGATC 0.304268301541
GAAAGCAA 0.0480087378428
GAAAGCAC -0.203410312273
GAAAGCCA -0.198052287582
GAAAGCCC -0.366751084753
GAAAGCGA -0.00608106253945
GAAAGCGC -0.283708332467
GAAAGCTA -0.0976004471539
GAAAGCTC -0.264850385147
GAAAGGAA -0.0936371585525
GAAAGGAC -0.0107288549388
GAAAGGCA -0.307084670452
GAAAGGCC -0.367440874454
GAAAGGGA -0.204924554184
GAAAGGGC -0.228674897119
GAAAGGTA -0.00305440295067
GAAAGGTC -0.319448559671
GAAAGTAA 0.182297735098
GAAAGTAC 0.10693853239
GAAAGTCA -0.0995845884154

GAAAGTCC -0.25534815839
GAAAGTGA 0.140790686953
GAAAGTGC -0.0634211604334
GAAAGTTA 0.0911801025671
GAAAGTTC 0.023101149973
GAAATAAA 0.212556477271
GAAATAAC 0.0799907791609
GAAATACA 0.134346897564
GAAATACC 0.143139191957
GAAATAGA -0.00432226832642
GAAATAGC 0.0403640944571
GAAATATA 0.264322345387
GAAATATC 0.39839513491
GAAATCAA 0.392486662715
GAAATCAC 0.439996803859
GAAATCCA 0.476819258205
GAAATCCC 0.467317618295
GAAATCGA 0.428275681722
GAAATCGC 0.451612549123
GAAATCTA 0.487023665974
GAAATCTC 0.484140705614
GAAATGAA 0.112488196069
GAAATGAC -0.176254122296
GAAATGCA -0.0838391469208
GAAATGCC -0.157975906259
GAAATGGA -0.00676743726536
GAAATGGC -0.21317197662
GAAATGTA 0.00321906079167
GAAATGTC -0.0472183802059
GAAATTAA 0.246537271367
GAAATTAC 0.301875452809
GAAATTCA 0.205586291686
GAAATTCC 0.293994159778
GAAATTGA -0.0226624075172
GAAATTGC 0.26367440036
GAAATTTA 0.131941074775
GAAATTTC 0.308202513007
GAACAAAA 0.113560356224
GAACAAAC -0.184392939317
GAACAACA -0.146818144398
GAACAACC -0.134772933274
GAACAAGA 0.0624783907932
GAACAAGC -0.116845236712
GAACAATA 0.19049265626
GAACAATC 0.25586535612
GAACACAA -0.0913852806726
GAACACAC -0.0775950488864
GAACACCA -0.183771236665
GAACACCC -0.204776564978
GAACACGA 0.025255768513
GAACACGC -0.131326848057
GAACACTA -0.254763979666
GAACACTC -0.0221129400142
GAACAGAA 0.217839579354

GAACAGAC -0.0261697783924
GAACAGCA -0.215286009043
GAACAGCC -0.263207193706
GAACAGGA 0.115758013816
GAACAGGC -0.118209845253
GAACAGTA -0.0589223143569
GAACAGTC -0.128298274715
GAACATAA 0.064463544754
GAACATAC -0.126150247701
GAACATCA -0.0556695189807
GAACATCC 0.0457748797998
GAACATGA 0.00500834266394
GAACATGC -0.099633333527
GAACATTA 0.0270999363323
GAACATTC 0.0196722502288
GAACCAAA 0.161408592944
GAACCAAC -0.288633802326
GAACCACA -0.28776325345
GAACCACC -0.13611090894
GAACCAGA 0.0337556582262
GAACCAGC -0.0963468807289
GAACCATA -0.0105970673942
GAACCATC -0.118746981869
GAACCCAA -0.0977123698791
GAACCCAC -0.179295988658
GAACCCCA -0.246279409586
GAACCCCC -0.251473396549
GAACCCGA -0.0747895839326
GAACCCGC -0.222696868432
GAACCCTA -0.243421124829
GAACCCTC -0.213216120111
GAACCGAA -0.0132392892944
GAACCGAC 0.05590487121
GAACCGCA -0.0652671441508
GAACCGCC -0.0767755991285
GAACCGGA -0.203715109118
GAACCGGC -0.294842249657
GAACCGTA 0.0888396553978
GAACCGTC -0.203070442992
GAACCTAA -0.127009213985
GAACCTAC -0.217770824436
GAACCTCA -0.11881771968
GAACCTCC -0.225982981945
GAACCTGA -0.112514346316
GAACCTGC -0.235744188216
GAACCTTA -0.0869976379943
GAACCTTC -0.185424759146
GAACGAAA 0.132498946726
GAACGAAC -0.0223511978266
GAACGACA -0.0673296238723
GAACGACC -0.0616771757682
GAACGAGA 0.0543956715382
GAACGAGC -0.335703235575
GAACGATA 0.162647276742

GAACGATC -0.0493808865178
GAACGCAA 0.0278518738062
GAACGCAC -0.179098428525
GAACGCCA -0.266583256508
GAACGCCC -0.353938339258
GAACGCGA 0.150264001405
GAACGCGC 0.0347071285868
GAACGCTA -0.137245379516
GAACGCTC -0.239462599855
GAACGGAA 0.0123591294571
GAACGGAC -0.312182903277
GAACGGCA -0.164947462007
GAACGGCC -0.227342166253
GAACGGGA 0.0378661762527
GAACGGGC -0.308762371892
GAACGGTA -0.037305064262
GAACGGTC -0.421680765391
GAACGTAA 0.118226722648
GAACGTAC -0.206768175583
GAACGTCA -0.177439408773
GAACGTCC -0.296431029826
GAACGTGA -0.0252502799183
GAACGTGC -0.0728997510407
GAACGTTA 0.112093129507
GAACGTTC -0.0453692522479
GAACTAAA 0.00382006191135
GAACTAAC -0.0711157215307
GAACTACA 0.099038947068
GAACTACC -0.163868866557
GAACTAGA 0.128114421862
GAACTAGC -0.0957244345009
GAACTATA -0.0412142431682
GAACTATC 0.0703833613279
GAACTCAA -0.00567707615628
GAACTCAC -0.226139917695
GAACTCCA -0.125826597082
GAACTCCC -0.268549019608
GAACTCGA -0.0950682129825
GAACTCGC -0.0644292981676
GAACTCTA -0.26573877448
GAACTCTC -0.0929706928208
GAACTGAA 0.0333322356144
GAACTGAC -0.295881917319
GAACTGCA -0.0435227876897
GAACTGCC -0.313535743121
GAACTGGA -0.0594859041997
GAACTGGC -0.10126780275
GAACTGTA -0.0119986053201
GAACTGTC -0.0454767624455
GAACTTAA 0.0379755867308
GAACTTAC -0.0495933754774
GAACTTCA -0.0418239814821
GAACTTCC -0.280528949014
GAACTTGA -0.0161942672846

GAACTTGC -0.0856148464917
GAACTTTA 0.0286185405256
GAAGAAAA 0.0129771458124
GAAGAAAC 0.00657969287987
GAAGAACA -0.184417240648
GAAGAACC -0.180107571142
GAAGAAGA 0.207811660105
GAAGAAGC -0.350120830477
GAAGAATA 0.21431144945
GAAGAATC 0.414740169927
GAAGACAA -0.0235892638197
GAAGACAC -0.00710415062543
GAAGACCA -0.246765432099
GAAGACCC -0.313448614362
GAAGACGA 0.0908909970508
GAAGACGC -0.0800635034991
GAAGACTA 0.017327444685
GAAGACTC -0.0542959230719
GAAGAGAA -0.053900989447
GAAGAGAC -0.0889475503843
GAAGAGCA -0.121277593955
GAAGAGCC -0.259979432536
GAAGAGGA 0.00360051812334
GAAGAGGC -0.218233787038
GAAGAGTA -0.0213284325832
GAAGAGTC -0.203851873803
GAAGATAA 0.135828997344
GAAGATAC 0.248718413138
GAAGATCA 0.326922626925
GAAGATCC 0.359985949198
GAAGATGA -0.056104333701
GAAGATGC -0.00871050781273
GAAGATTA 0.32718754086
GAAGATTC 0.412181657054
GAAGCAAA -0.0608631479857
GAAGCAAC -0.253279917261
GAAGCACA -0.200255144033
GAAGCACC -0.244346684405
GAAGCAGA 0.03649162154
GAAGCAGC -0.349929425705
GAAGCATA -0.0139533798766
GAAGCATC -0.214112243742
GAAGCCAA -0.0642469998518
GAAGCCAC -0.223639796659
GAAGCCCA -0.412268625418
GAAGCCCC -0.336256647526
GAAGCCGA -0.0109725309404
GAAGCCGC -0.234348630569
GAAGCCTA -0.185658678286
GAAGCCTC -0.216642250863
GAAGCGAA 0.162624032078
GAAGCGAC -0.167187928669
GAAGCGCA 0.0558235148838
GAAGCGCC -0.165235292894

GAAGCGGA	-0.141751633987
GAAGCGGC	-0.292041782562
GAAGCGTA	0.134305912314
GAAGCGTC	-0.188063027078
GAAGCTAA	-0.0505312434829
GAAGCTAC	-0.158499314384
GAAGCTCA	-0.409118461834
GAAGCTCC	-0.124720406681
GAAGCTGA	-0.0458602705098
GAAGCTGC	-0.274481729099
GAAGCTTA	-0.0289207211657
GAAGCTTC	-0.0794489433142
GAAGGAAA	-0.17427741467
GAAGGAAC	-0.0557778436409
GAAGGACA	-0.119208809728
GAAGGACC	-0.434976267858
GAAGGAGA	-0.13805708941
GAAGGAGC	-0.284067840911
GAAGGATA	0.0965813654777
GAAGGATC	0.216210247992
GAAGGCAA	-0.0736789538093
GAAGGCAC	-0.359142021329
GAAGGCCA	-0.264445102848
GAAGGCCC	-0.371350387482
GAAGGCGA	-0.0693236848298
GAAGGCGC	-0.141561306466
GAAGGCTA	-0.285005083515
GAAGGCTC	-0.352520116503
GAAGGGAA	-0.173276846169
GAAGGGAC	-0.217634436926
GAAGGGCA	-0.31141231593
GAAGGGCC	-0.35115448218
GAAGGGGA	-0.126968772694
GAAGGGGC	-0.3396328116
GAAGGGTA	-0.0163931330422
GAAGGGTC	-0.302528868044
GAAGGTAA	-0.108007617417
GAAGGTAC	-0.185543209877
GAAGGTCA	-0.183481283026
GAAGGTCC	-0.383451222111
GAAGGTGA	-0.116459651549
GAAGGTGC	-0.212308550445
GAAGGTTA	-0.0669808009775
GAAGTAAA	-0.0292403522381
GAAGTAAC	-0.191893004115
GAAGTACA	0.00142947626546
GAAGTACC	-0.240836601307
GAAGTAGA	0.0661423316734
GAAGTAGC	-0.085148342377
GAAGTATA	-0.130684095861
GAAGTATC	0.177791175744
GAAGTCAA	-0.15378655043
GAAGTCAC	-0.223015250545
GAAGTCCA	-0.144560727645

GAAGTCCC -0.146144391218
GAAGTCGA -0.0299100968188
GAAGTCGC -0.413122785074
GAAGTCTA -0.197794807587
GAAGTCTC -0.258499314129
GAAGTGAA -0.170493531997
GAAGTGAC -0.0271686545698
GAAGTGCA -0.180953238664
GAAGTGCC -0.258737267681
GAAGTGGA -0.00152308502931
GAAGTGGC -0.211921568627
GAAGTGTA 0.0709180189735
GAAGTGTC -0.101880202589
GAAGTTAA 0.101703659795
GAAGTTAC -0.109253313575
GAAGTTCA -0.0595780993618
GAAGTTCC -0.182983573915
GAAGTTGA 0.0454039663474
GAAGTTGC -0.00483987967251
GAAGTTTA -0.0201836328505
GAATAAAA 0.142360495692
GAATAAAC 0.0190010605378
GAATAACA 0.0906265193686
GAATAACC -0.100174334985
GAATAAGA 0.184215419929
GAATAAGC 0.0342941118356
GAATAATA 0.228708613601
GAATAATC 0.281302572894
GAATACAA 0.247686429474
GAATACAC 0.0830372059913
GAATACCA 0.0774846367295
GAATACCC -0.145903312916
GAATACGA 0.214318645854
GAATACGC 0.172825534264
GAATACTA -0.017702264902
GAATACTC 0.0726854596149
GAATAGAA 0.130192210586
GAATAGAC -0.159458014735
GAATAGCA -0.10127607464
GAATAGCC -0.178126766205
GAATAGGA 0.186700037322
GAATAGGC -0.105478946328
GAATAGTA 0.0533079760258
GAATAGTC -0.0400698011326
GAATATAA 0.17723039821
GAATATAC 0.237332323432
GAATATCA 0.343298999027
GAATATCC 0.447019598158
GAATATGA 0.318621881946
GAATATGC 0.172749989104
GAATATTA 0.230976530769
GAATATTC 0.351698597669
GAATCAAA 0.344508112143
GAATCAAC 0.0415716298868

GAATCACA 0.34757623658
GAATCACC 0.269967507519
GAATCAGA 0.367955398597
GAATCAGC 0.230932330084
GAATCATA 0.348325429757
GAATCATC 0.235472811006
GAATCCAA 0.403130765766
GAATCCAC 0.437073787282
GAATCCCA 0.382725197041
GAATCCCC 0.382541804077
GAATCCGA 0.46129472782
GAATCCGC 0.417156475353
GAATCCTA 0.388334540824
GAATCCTC 0.411814748952
GAATCGAA 0.230869677961
GAATCGAC 0.225452260203
GAATCGCA 0.36159998128
GAATCGCC 0.323294059535
GAATCGGA 0.319019078355
GAATCGGC 0.255048134976
GAATCGTA 0.407788864739
GAATCGTC 0.343489977826
GAATCTAA 0.437695582061
GAATCTAC 0.443617160374
GAATCTCA 0.459260905838
GAATCTCC 0.433575465256
GAATCTGA 0.443042039223
GAATCTGC 0.468290032599
GAATCTTA 0.429833074252
GAATGAAA -0.0164353452534
GAATGAAC -0.209253131842
GAATGACA -0.0283686603809
GAATGACC -0.246176765931
GAATGAGA 0.147593969179
GAATGAGC -0.164222222222
GAATGATA 0.170355002305
GAATGATC -0.0429352499593
GAATGCAA 0.138946519133
GAATGCAC -0.226209598945
GAATGCCA 0.0455929568666
GAATGCCC -0.144365880087
GAATGCGA 0.209026193831
GAATGCGC -0.0356526488893
GAATGCTA -0.0773771301556
GAATGCTC -0.270722238681
GAATGGAA -0.0227666962068
GAATGGAC -0.176968449931
GAATGGCA -0.183893972404
GAATGGCC -0.155245139194
GAATGGGA -0.109592661872
GAATGGGC -0.159170890188
GAATGGTA 0.193522360535
GAATGGTC -0.176833215057
GAATGTAA 0.0915552481943

GAATGTAC 0.0142566247338
GAATGTCA 0.101173855563
GAATGTCC −0.0283948608585
GAATGTGA 0.0876483663359
GAATGTGC 0.178113695466
GAATGTTA 0.155339326287
GAATTAAA 0.180446878677
GAATTAAC −0.0570210103405
GAATTACA 0.278777621199
GAATTACC 0.185078378104
GAATTAGA 0.168961590361
GAATTAGC −0.0997437402305
GAATTATA 0.18648177473
GAATTATC 0.215575378089
GAATTCAA 0.047496926387
GAATTCAC 0.0879574743683
GAATTCCA 0.076181694439
GAATTCCC 0.240226184988
GAATTCGA 0.231382128195
GAATTCGC 0.176112538146
GAATTCTA 0.223067363117
GAATTCTC 0.263921820457
GAATTGAA 0.0892231923641
GAATTGAC −0.0132509116267
GAATTGCA −0.0222073714643
GAATTGCC −0.0632066014848
GAATTGGA 0.19113482184
GAATTGGC −0.138552536779
GAATTGTA 0.103714931344
GAATTGTC −0.121377527498
GAATTTAA 0.113664957215
GAATTTAC 0.237474757747
GAATTTCA 0.149841379613
GAATTTCC 0.304221827043
GAATTTGA 0.256770734967
GAATTTGC 0.308132727035
GAATTTTA 0.240526685547
GACAAAAA 0.132164804672
GACAAAAC −0.0348388548596
GACAAACA −0.0675359202708
GACAAACC −0.00979326776401
GACAAAGA −0.0957582842153
GACAAAGC −0.107000726216
GACAAATA 0.226204000988
GACAAATC 0.258377522409
GACAACAA −0.0593399416014
GACAACAC −0.148190436346
GACAACCA −0.100173133614
GACAACCC −0.184436516606
GACAACGA −0.167769710739
GACAACGC −0.155187440444
GACAACTA −0.147933404036
GACAACTC −0.115078903944
GACAAGAA −0.276364285901

GACAAGAC -0.185055494718
GACAAGCA -0.250138700485
GACAAGCC -0.234784636488
GACAAGGA 0.0372611973908
GACAAGGC -0.407632170007
GACAAGTA -0.186855569017
GACAAGTC -0.315635183174
GACAATAA -0.0983292897302
GACAATAC 0.0520757865156
GACAATCA 0.233912512894
GACAATCC 0.344612395442
GACAATGA -0.011502154167
GACAATGC -0.190346848404
GACAATTA 0.164195952523
GACACAAA 0.139632153182
GACACAAC -0.187017333852
GACACACA 0.0352311520292
GACACACC -0.156469361434
GACACAGA 0.0404083103089
GACACAGC -0.169108850033
GACACATA -0.00294188675931
GACACATC -0.0251042377929
GACACCAA -0.0683241403913
GACACCAC -0.13344143195
GACACCCA -0.242189587666
GACACCCC -0.306422287475
GACACCGA -0.11207615362
GACACCGC -0.298824284565
GACACCTA -0.0682894515111
GACACCTC -0.277906721536
GACACGAA -0.0761742187613
GACACGAC -0.0852117120825
GACACGCA -0.129603804776
GACACGCC -0.203090501313
GACACGGA 0.0336198096743
GACACGGC -0.133090248627
GACACGTA 0.104217436168
GACACGTC -0.263401662082
GACACTAA 0.0883253671931
GACACTAC -0.225632373114
GACACTCA -0.0607949675301
GACACTCC -0.109297309757
GACACTGA -0.0701015501286
GACACTGC -0.399208857287
GACACTTA 0.00354545704015
GACAGAAA -0.0357783814934
GACAGAAC -0.135752528084
GACAGACA -0.149992042717
GACAGACC -0.164195472253
GACAGAGA -0.038331904755
GACAGAGC -0.0926748057714
GACAGATA 0.202419372544
GACAGATC 0.154229511076
GACAGCAA 0.107116040113

GACAGCAC -0.134964430392
GACAGCCA -0.128381597602
GACAGCCC -0.31481390041
GACAGCGA -0.206366060846
GACAGCGC -0.157749462224
GACAGCTA -0.164758169935
GACAGCTC -0.154789663446
GACAGGAA -0.0499561278626
GACAGGAC -0.178720740037
GACAGGCA -0.206636969195
GACAGGCC -0.303126760727
GACAGGGA -0.00152175523508
GACAGGGC -0.19771257965
GACAGGTA 0.0849414915805
GACAGGTC -0.199382716049
GACAGTAA 0.029234541646
GACAGTAC -0.036579838159
GACAGTCA -0.148002858247
GACAGTCC -0.290071169208
GACAGTGA -0.102864182322
GACAGTGC -0.15807643486
GACAGTTA 0.0170650283269
GACATAAA 0.0208301250854
GACATAAC 0.0540859762033
GACATACA -0.0683330040177
GACATACC -0.0202124973932
GACATAGA 0.120666754484
GACATAGC -0.178573710966
GACATATA 0.127870535028
GACATATC 0.225363106412
GACATCAA 0.0639504307527
GACATCAC -0.207643347051
GACATCCA 0.142931238886
GACATCCC -0.108221812878
GACATCGA -0.0133345408242
GACATCGC -0.164328987225
GACATCTA 0.0320151671437
GACATCTC -0.145575523368
GACATGAA -0.151056905845
GACATGAC -0.10055193362
GACATGCA -0.000748187642555
GACATGCC -0.19234038571
GACATGGA 0.0256286955385
GACATGGC -0.112586283032
GACATGTA -0.115230301434
GACATGTC -0.19195036018
GACATTAA -0.183318470838
GACATTAC 0.144467043424
GACATTCA -0.0374340795839
GACATTCC 0.0343956830979
GACATTGA 0.0029735705217
GACATTGC -0.0885889964441
GACATTTA -0.116574467385
GACCAAAA -0.120373948542

GACCAAAC -0.00529100529101
GACCAACA -0.138658413451
GACCAACC -0.157673726807
GACCAAGA -0.127335974792
GACCAAGC -0.253718595301
GACCAATA -0.135681439045
GACCAATC -0.0243766379416
GACCACAA 0.0327531852454
GACCACAC 0.0421019163333
GACCACCA -0.292947101811
GACCACCC -0.180085515022
GACCACGA -0.202929203151
GACCACGC -0.293340748672
GACCACTA -0.168136528686
GACCACTC -0.215033938576
GACCAGAA -0.0634020498757
GACCAGAC -0.21823437097
GACCAGCA -0.241554412273
GACCAGCC -0.148737835875
GACCAGGA -0.0734328106706
GACCAGGC -0.275010012234
GACCAGTA -0.252525780683
GACCAGTC -0.163587052839
GACCATAA 0.0662342190519
GACCATAC 0.104326413203
GACCATCA -0.227964334705
GACCATCC -0.167185720594
GACCATGA -0.139822088456
GACCATGC -0.268600537875
GACCATTA 0.0585053680647
GACCCAAA -0.0155535246752
GACCCAAC -0.376657905818
GACCCACA -0.177514835869
GACCCACC -0.0374557349237
GACCCAGA 0.026345254561
GACCCAGC -0.316709551657
GACCCATA -0.100187091826
GACCCATC -0.238131891113
GACCCCAA -0.128854030501
GACCCCAC -0.369123863616
GACCCCCA -0.241315904139
GACCCCCC -0.407919423324
GACCCCGA -0.0966302862693
GACCCCGC -0.277506993119
GACCCCTA -0.275108235798
GACCCCTC -0.263811360428
GACCCGAA -0.342787846862
GACCCGAC -0.300685220845
GACCCGCA -0.244060290124
GACCCGCC -0.179534444828
GACCCGGA -0.0412798991281
GACCCGGC -0.356948703652
GACCCGTA -0.284953701014
GACCCGTC -0.400923137681

GACCCTAA	−0.200381930726
GACCCTAC	−0.135103530225
GACCCTCA	−0.220913580247
GACCCTCC	−0.254069840053
GACCCTGA	−0.256709237133
GACCCTGC	−0.22326539859
GACCCTTA	−0.313221570661
GACCGAAA	0.122303255706
GACCGAAC	−0.17320810189
GACCGACA	−0.0609880818869
GACCGACC	−0.415377288356
GACCGAGA	−0.0961547400469
GACCGAGC	−0.124100536555
GACCGATA	0.018334217286
GACCGATC	−0.0216393910457
GACCGCAA	0.114173444412
GACCGCAC	−0.279570048618
GACCGCCA	−0.140015804811
GACCGCCC	−0.31929184057
GACCGCGA	−0.169506937504
GACCGCGC	−0.192877450658
GACCGCTA	−0.328913798496
GACCGCTC	−0.115803830545
GACCGGAA	−0.00740813907546
GACCGGAC	−0.29613362382
GACCGGCA	−0.0846817660134
GACCGGCC	−0.291043302227
GACCGGGA	−0.235111025626
GACCGGGC	−0.262963221135
GACCGGTA	−0.106375963248
GACCGGTC	−0.0107835876166
GACCGTAA	−0.0129001516639
GACCGTAC	−0.00362384465082
GACCGTCA	−0.314220696426
GACCGTCC	−0.214159520243
GACCGTGA	−0.101290078887
GACCGTGC	−0.0963937684132
GACCGTTA	−0.0559658280094
GACCTAAA	0.0574796972382
GACCTAAC	−0.159178588872
GACCTACA	−0.315020218926
GACCTACC	−0.20353789481
GACCTAGA	−0.0913236238339
GACCTAGC	−0.149476388066
GACCTATA	−0.04176339837
GACCTATC	−0.0417482974158
GACCTCAA	−0.0605073148054
GACCTCAC	−0.0706814952978
GACCTCCA	−0.157798642199
GACCTCCC	−0.238502677966
GACCTCGA	0.0442023974182
GACCTCGC	−0.29633835267
GACCTCTA	−0.227160015699
GACCTCTC	−0.267229836684

GACCTGAA -0.0750115237719
GACCTGAC -0.18765155197
GACCTGCA -0.155952640938
GACCTGCC -0.311413848432
GACCTGGA 0.0349403938616
GACCTGGC -0.435106108725
GACCTGTA -0.126045854928
GACCTTAA -0.14772416919
GACCTTAC -0.242105624143
GACCTTCA -0.334613831706
GACCTTCC -0.292035971756
GACCTTGA -0.0192328800202
GACCTTGC -0.103949264415
GACCTTTA -0.154661958615
GACGAAAA 0.0507722452743
GACGAAAC -0.119367886112
GACGAACA -0.100145440575
GACGAACC -0.161228543834
GACGAAGA 0.0495338452465
GACGAAGC -0.277515074764
GACGAATA 0.155139250069
GACGAATC 0.266475382447
GACGACAA -0.0268117373907
GACGACAC -0.298220927553
GACGACCA -0.0832441612889
GACGACCC -0.286776686678
GACGACGA -0.138730984962
GACGACGC -0.0661027487006
GACGACTA -0.0942460807392
GACGACTC -0.235888074679
GACGAGAA -0.0393148038376
GACGAGAC -0.165521068167
GACGAGCA -0.216234352895
GACGAGCC -0.364644317443
GACGAGGA -0.0164176589694
GACGAGGC -0.146715301908
GACGAGTA -0.119728909099
GACGAGTC -0.196365673407
GACGATAA -0.0416936643761
GACGATAC 0.19869536104
GACGATCA 0.0636656836111
GACGATCC 0.246907176886
GACGATGA -0.152419992098
GACGATGC -0.0842226838871
GACGATTA 0.18239361934
GACGCAAA 0.0849305143911
GACGCAAC -0.0184548109192
GACGCACA -0.131049681466
GACGCACC -0.202725097678
GACGCAGA -0.198940066867
GACGCAGC -0.319914196525
GACGCATA 0.0635918329143
GACGCATC -0.215859768109
GACGCCAA -0.166013396373

GACGCCAC -0.3640480927
GACGCCCA -0.198942971589
GACGCCCC -0.380431562315
GACGCCGA -0.229509276178
GACGCCGC -0.266498200097
GACGCCTA -0.287445829606
GACGCCTC -0.161173565723
GACGCGAA 0.0202682148735
GACGCGAC -0.147863712191
GACGCGCA -0.105156574138
GACGCGCC -0.326517928194
GACGCGGA -0.114241581203
GACGCGGC -0.266927885118
GACGCGTA -0.0544633791608
GACGCGTC -0.112850807103
GACGCTAA -0.205012840774
GACGCTAC -0.159257806826
GACGCTCA -0.192327950883
GACGCTCC -0.237928525016
GACGCTGA -0.0176964537359
GACGCTGC -0.115655675081
GACGCTTA -0.253827160494
GACGGAAA -0.0350864195605
GACGGAAC -0.186126528968
GACGGACA -0.224335141918
GACGGACC -0.384418554903
GACGGAGA -0.0296209356915
GACGGAGC -0.129036147028
GACGGATA 0.219799007662
GACGGATC 0.0812135730515
GACGGCAA -0.194301842829
GACGGCAC -0.191339127
GACGGCCA -0.328473004845
GACGGCCC -0.202814971939
GACGGCGA -0.0725256815488
GACGGCGC -0.0873492663399
GACGGCTA -0.207849108368
GACGGCTC -0.195294142112
GACGGGAA 0.0157232704403
GACGGGAC -0.358567345564
GACGGGCA -0.14617904649
GACGGGCC -0.233557179538
GACGGGGA 0.0190097477442
GACGGGGC -0.178896955529
GACGGGTA -0.240561036207
GACGGTAA -0.0179114668836
GACGGTAC -0.267589844272
GACGGTCA -0.229733576144
GACGGTCC -0.379825759695
GACGGTGA -0.00551878197106
GACGGTGC -0.229339143065
GACGGTTA -0.0786483844466
GACGTAAA 0.117075595179
GACGTAAC -0.0676296159556

GACGTACA -0.00911917248994
GACGTACC -0.103728167656
GACGTAGA -0.011451952842
GACGTAGC 0.0677276887539
GACGTATA -0.0371173710284
GACGTATC 0.216078043961
GACGTCAA 0.0198110825704
GACGTCAC -0.0985962255039
GACGTCCA -0.237634749524
GACGTCCC -0.354072720889
GACGTCGA -0.0469855382673
GACGTCGC -0.128899754696
GACGTCTA 0.0343421370392
GACGTCTC -0.151216581062
GACGTGAA 0.127309575349
GACGTGAC -0.289378310566
GACGTGCA -0.04430287798
GACGTGCC -0.363399531828
GACGTGGA -0.060007554516
GACGTGGC -0.370077833177
GACGTGTA -0.0469983903865
GACGTTAA -0.0882034988366
GACGTTAC 0.048515973443
GACGTTCA -0.190629475088
GACGTTCC -0.259756968849
GACGTTGA -0.118180180651
GACGTTGC -0.258092133426
GACGTTTA -0.221220241035
GACTAAAA 0.116687523327
GACTAAAC -0.0379392298261
GACTAACA -0.204463648834
GACTAACC -0.184327346755
GACTAAGA 0.187564490988
GACTAAGC -0.105294924554
GACTAATA -0.00444301740982
GACTAATC -0.0731233565296
GACTACAA -0.221740128292
GACTACAC -0.273891607692
GACTACCA -0.056930448528
GACTACCC -0.170337811142
GACTACGA 0.0107329469363
GACTACGC -0.159941722639
GACTACTA -0.0407987447881
GACTACTC 0.0747770368541
GACTAGAA 0.00839277672047
GACTAGAC -0.338957475995
GACTAGCA -0.22550617284
GACTAGCC -0.208401927214
GACTAGGA 0.121636589126
GACTAGGC -0.143089128584
GACTAGTA -0.013379549192
GACTAGTC -0.249107128162
GACTATAA -0.0175936903115
GACTATAC -0.156210054152

GACTATCA -0.0349369422767
GACTATCC -0.162177217969
GACTATGA -0.0127225625151
GACTATGC -0.263396530726
GACTATTA -0.0595826497529
GACTCAAA 0.00785814943414
GACTCAAC -0.116804122014
GACTCACA -0.22862745098
GACTCACC -0.232931098819
GACTCAGA 0.0212024413269
GACTCAGC -0.155918315826
GACTCATA -0.0753499832623
GACTCATC -0.207343500363
GACTCCAA -0.125188834918
GACTCCAC -0.271611338787
GACTCCCA -0.0317126455481
GACTCCCC -0.233427695211
GACTCCGA 0.154880653175
GACTCCGC -0.102714805486
GACTCCTA -0.0932248936714
GACTCCTC -0.318696669939
GACTCGAA -0.20291953629
GACTCGAC -0.267178045103
GACTCGCA -0.146838021029
GACTCGCC -0.0242764002766
GACTCGGA -0.172682269273
GACTCGGC -0.38105485085
GACTCGTA -0.210628830401
GACTCTAA -0.225558134303
GACTCTAC -0.222864197531
GACTCTCA -0.0899031811895
GACTCTCC -0.320226907399
GACTCTGA -0.0662418494369
GACTCTGC -0.218046728863
GACTCTTA -0.228359699519
GACTGAAA 0.0938488806241
GACTGAAC -0.1202616437
GACTGACA -0.0158171580975
GACTGACC -0.347984112093
GACTGAGA 0.0377136013433
GACTGAGC -0.169182441701
GACTGATA 0.0621967551428
GACTGATC -0.0298301480031
GACTGCAA 0.0949911084766
GACTGCAC -0.337647981743
GACTGCCA -0.0771666627931
GACTGCCC -0.282897985705
GACTGCGA -0.0283134543024
GACTGCGC -0.255387530508
GACTGCTA -0.157174564808
GACTGCTC -0.183521801393
GACTGGAA 0.0439542080107
GACTGGAC -0.327809079285
GACTGGCA -0.305452679977

GACTGGCC -0.192247586902
GACTGGGA -0.0850412366043
GACTGGGC -0.291670660265
GACTGGTA -0.0797966594045
GACTGTAA 0.0422954800963
GACTGTAC -0.328782117756
GACTGTCA -0.147506283323
GACTGTCC -0.0911455760809
GACTGTGA -0.186077643909
GACTGTGC -0.179137650022
GACTGTTA -0.0653113759581
GACTTAAA -0.0607630061163
GACTTAAC -0.0628965509671
GACTTACA -0.163317582623
GACTTACC -0.00466507505283
GACTTAGA -0.0944764865689
GACTTAGC -0.251198902606
GACTTATA -0.0519686144823
GACTTATC -0.169383818518
GACTTCAA -0.267468782233
GACTTCAC -0.269220323919
GACTTCCA -0.192833052032
GACTTCCC -0.346698092657
GACTTCGA 0.113641712551
GACTTCGC -0.200868307222
GACTTCTA -0.133299341369
GACTTCTC -0.0496713947853
GACTTGAA -0.0186090803311
GACTTGAC -0.243083676269
GACTTGCA -0.0748946189818
GACTTGCC -0.17535221496
GACTTGGA 0.111558409484
GACTTGGC -0.20327384872
GACTTGTA -0.191567175018
GACTTTAA -0.0857864928052
GACTTTAC -0.0198378684643
GACTTTCA -0.203808278867
GACTTTCC -0.187271250706
GACTTTGA -0.26393605494
GACTTTGC -0.167061786931
GACTTTTA 0.0824545589252
GAGAAAAA 0.0915360364941
GAGAAAAC -0.174107093857
GAGAAACA -0.0941989043646
GAGAAACC -0.00872643451058
GAGAAAGA 0.0626321509732
GAGAAAGC -0.0232867955777
GAGAAATA 0.19732831636
GAGAAATC 0.454191568705
GAGAACAA 0.0863582874493
GAGAACAC -0.00588525852425
GAGAACCA 0.0166097656647
GAGAACCC -0.271185510729
GAGAACGA -0.0672457714506

GAGAACGC -0.289435515726
GAGAACTA 0.0411773270552
GAGAACTC -0.0925872126804
GAGAAGAA 0.112964883971
GAGAAGAC -0.254180636777
GAGAAGCA -0.0690479326506
GAGAAGCC -0.347862699275
GAGAAGGA -0.159667316439
GAGAAGGC -0.182114771461
GAGAAGTA -0.0418040765331
GAGAATAA 0.106735141575
GAGAATAC 0.11113306549
GAGAATCA 0.458752342626
GAGAATCC 0.487875001816
GAGAATGA -0.0161160323127
GAGAATGC 0.0396539450554
GAGAATTA 0.316398274386
GAGACAAA -0.0303327186107
GAGACAAC -0.313393126526
GAGACACA -0.114102772678
GAGACACC -0.186053372356
GAGACAGA 0.0614777795534
GAGACAGC -0.256427424063
GAGACATA -0.0231620027196
GAGACATC 0.0281201818952
GAGACCAA -0.208929707495
GAGACCAC -0.277128614831
GAGACCCA -0.113410605113
GAGACCCC -0.323521828336
GAGACCGA -0.220935528121
GAGACCGC -0.255335538986
GAGACCTA -0.126990952461
GAGACCTC -0.289467416435
GAGACGAA -0.0801789081316
GAGACGAC -0.338610522158
GAGACGCA -0.0545703860367
GAGACGCC -0.309516894884
GAGACGGA -0.2190865746
GAGACGGC -0.34391887073
GAGACGTA -0.213224521376
GAGACTAA -0.0231838240137
GAGACTAC -0.202994773889
GAGACTCA -0.0930651194589
GAGACTCC -0.233454571388
GAGACTGA -0.142678014838
GAGACTGC -0.10602209078
GAGACTTA -0.218737825188
GAGAGAAA 0.0235897009233
GAGAGAAC -0.105217524913
GAGAGACA -0.158112929091
GAGAGACC -0.245960784314
GAGAGAGA -0.0574885458703
GAGAGAGC -0.167062716242
GAGAGATA 0.165157700522

GAGAGATC 0.308574375663
GAGAGCAA -0.0321486887751
GAGAGCAC -0.127754730234
GAGAGCCA -0.328073315231
GAGAGCCC -0.377564472993
GAGAGCGA -0.022005007579
GAGAGCGC -0.181047300819
GAGAGCTA -0.182406401264
GAGAGCTC -0.312227596298
GAGAGGAA -0.267671457285
GAGAGGAC -0.167003798584
GAGAGGCA -0.298483551693
GAGAGGCC -0.341946436403
GAGAGGGA -0.0597788205593
GAGAGGGC -0.270741299038
GAGAGGTA -0.215572698847
GAGAGTAA -0.00716810467848
GAGAGTAC -0.164307063994
GAGAGTCA -0.13683900149
GAGAGTCC -0.333443701877
GAGAGTGA -0.118221585545
GAGAGTGC -0.117523583741
GAGAGTTA 0.0193618492542
GAGATAAA -0.144089464295
GAGATAAC 0.16707247977
GAGATACA 0.194431450031
GAGATACC 0.25794336868
GAGATAGA -0.0110122537485
GAGATAGC 0.0741659885028
GAGATATA 0.310112881903
GAGATATC 0.456626982167
GAGATCAA 0.209626196737
GAGATCAC 0.286447013107
GAGATCCA 0.359715543417
GAGATCCC 0.341451048189
GAGATCGA 0.298445513053
GAGATCGC 0.412725855672
GAGATCTA 0.425047578923
GAGATCTC 0.424300155373
GAGATGAA -0.071908822803
GAGATGAC -0.0440580855478
GAGATGCA -0.0571972690009
GAGATGCC 0.0292774621992
GAGATGGA -0.152761919703
GAGATGGC -0.0423282546424
GAGATGTA -0.0289441041516
GAGATTAA 0.140712980342
GAGATTAC 0.459572769794
GAGATTCA 0.206340141262
GAGATTCC 0.461344122732
GAGATTGA 0.0107400454501
GAGATTGC 0.472457978005
GAGATTTA 0.292505309691
GAGCAAAA -0.0363592960274

GAGCAAAC −0.0623623548007
GAGCAACA −0.0648767137615
GAGCAACC −0.216909767512
GAGCAAGA 0.0999857296538
GAGCAAGC −0.0616637368704
GAGCAATA −0.0193966936706
GAGCAATC 0.378924345211
GAGCACAA −0.013350943926
GAGCACAC −0.237536096649
GAGCACCA −0.124972039287
GAGCACCC −0.259463117659
GAGCACGA −0.119573270114
GAGCACGC −0.328623288367
GAGCACTA −0.0706129327503
GAGCACTC −0.0968888230323
GAGCAGAA −0.0476864294742
GAGCAGAC −0.149154580602
GAGCAGCA −0.0230526997702
GAGCAGCC −0.260803583957
GAGCAGGA −0.248861566485
GAGCAGGC −0.371837019182
GAGCAGTA 0.0531910566153
GAGCATAA 0.0185013002484
GAGCATAC −0.08490308411
GAGCATCA 0.0699246964807
GAGCATCC −0.268268861454
GAGCATGA −0.14501797426
GAGCATGC −0.0726952689836
GAGCATTA −0.195832921729
GAGCCAAA −0.100807131111
GAGCCAAC −0.229246784631
GAGCCACA −0.134294843863
GAGCCACC −0.252080932785
GAGCCAGA −0.0752253783422
GAGCCAGC −0.312836043628
GAGCCATA −0.126192987196
GAGCCATC −0.101691049351
GAGCCCAA −0.29074704982
GAGCCCAC −0.344455072822
GAGCCCCA −0.305102925363
GAGCCCCC −0.267439627838
GAGCCCGA −0.0728640182633
GAGCCCGC −0.379705971091
GAGCCCTA −0.211771966254
GAGCCCTC −0.226523742027
GAGCCGAA −0.145967777339
GAGCCGAC −0.289731768133
GAGCCGCA −0.244929581965
GAGCCGCC −0.309553591512
GAGCCGGA −0.183707703614
GAGCCGGC −0.272631255521
GAGCCGTA −0.163654889892
GAGCCTAA −0.0577616437003
GAGCCTAC −0.200513630976

GAGCCTCA -0.145327160494
GAGCCTCC -0.273073560634
GAGCCTGA -0.170006128964
GAGCCTGC -0.180411508407
GAGCCTTA -0.131047204067
GAGCGAAA 0.0595833393866
GAGCGAAC -0.150075178893
GAGCGACA -0.105595628542
GAGCGACC -0.150679662118
GAGCGAGA 0.0794297631788
GAGCGAGC -0.0901999198784
GAGCGATA -0.00135836008891
GAGCGATC 0.298276989235
GAGCGCAA 0.149944742171
GAGCGCAC -0.173131592523
GAGCGCCA -0.0893478576616
GAGCGCCC -0.386880508807
GAGCGCGA 0.160566879258
GAGCGCGC 0.0714567998814
GAGCGCTA -0.188000636425
GAGCGCTC -0.1663584749
GAGCGGAA -0.0137896608742
GAGCGGAC -0.275261754247
GAGCGGCA -0.161391430646
GAGCGGCC -0.390753813497
GAGCGGGA -0.124865649964
GAGCGGGC -0.296747353595
GAGCGGTA 0.0637063617741
GAGCGTAA 0.165224528932
GAGCGTAC -0.292117283951
GAGCGTCA -0.317383910694
GAGCGTCC -0.20998703118
GAGCGTGA -0.203662400927
GAGCGTGC -0.250050835149
GAGCGTTA 0.0488617378292
GAGCTAAA -0.0619612491611
GAGCTAAC -0.159132041379
GAGCTACA -0.0831837765155
GAGCTACC -0.240518551435
GAGCTAGA 0.0375401333663
GAGCTAGC -0.214698473848
GAGCTATA -0.112538075245
GAGCTATC 0.0203517091484
GAGCTCAA -0.221961183821
GAGCTCAC -0.28539331391
GAGCTCCA -0.297890024523
GAGCTCCC -0.236822739171
GAGCTCGA 0.138943774836
GAGCTCGC -0.188240813908
GAGCTCTA -0.159775034294
GAGCTGAA -0.227196404761
GAGCTGAC -0.170463648834
GAGCTGCA -0.116930615918
GAGCTGCC -0.189082269289

GAGCTGGA -0.0217320907155
GAGCTGGC -0.193814845906
GAGCTGTA -0.0955650837161
GAGCTTAA -0.0951992887835
GAGCTTAC -0.147779265788
GAGCTTCA -0.280677910975
GAGCTTCC -0.265267353567
GAGCTTGA -0.15060435973
GAGCTTGC -0.263359334184
GAGCTTTA -0.156345169309
GAGGAAAA 0.142543547426
GAGGAAAC -0.16899199921
GAGGAACA -0.207097979185
GAGGAACC -0.154431372549
GAGGAAGA -0.0876894750129
GAGGAAGC -0.261130698947
GAGGAATA 0.26744000966
GAGGAATC 0.398946738677
GAGGACAA -0.0944136457922
GAGGACAC -0.133268028734
GAGGACCA -0.150515442956
GAGGACCC -0.366921057523
GAGGACGA -0.239407813698
GAGGACGC -0.200397153972
GAGGACTA -0.208551440329
GAGGACTC -0.258196399887
GAGGAGAA 0.117806805211
GAGGAGAC -0.277089299233
GAGGAGCA -0.303984013988
GAGGAGCC -0.41811376876
GAGGAGGA 0.0331692567716
GAGGAGGC -0.241956104252
GAGGAGTA -0.164657070968
GAGGATAA 0.23152993477
GAGGATAC 0.113940987608
GAGGATCA 0.0671996092121
GAGGATCC 0.343792656071
GAGGATGA -0.0503720899602
GAGGATGC -0.190365650878
GAGGATTA 0.329942033618
GAGGCAAA -0.0867115633713
GAGGCAAC -0.180017226529
GAGGCACA -0.179816662988
GAGGCACC -0.293383918186
GAGGCAGA -0.085837133622
GAGGCAGC -0.26114777335
GAGGCATA -0.0957593014978
GAGGCATC -0.108970225127
GAGGCCAA -0.179614950024
GAGGCCAC -0.194570632256
GAGGCCCA -0.377424357716
GAGGCCCC -0.362069366109
GAGGCCGA -0.0659773672714
GAGGCCGC -0.249470507545

GAGGCCTA -0.14273102188
GAGGCCTC -0.138648370836
GAGGCGAA 0.0354699693767
GAGGCGAC -0.155395376263
GAGGCGCA -0.208482207698
GAGGCGCC -0.326289109432
GAGGCGGA -0.155307189542
GAGGCGGC -0.152309304712
GAGGCGTA -0.268094234863
GAGGCTAA -0.151149359059
GAGGCTAC -0.264456113244
GAGGCTCA -0.145375537011
GAGGCTCC -0.214625889531
GAGGCTGA -0.0905480910668
GAGGCTGC -0.253224649362
GAGGCTTA -0.00448501086527
GAGGGAAA -0.0332903349516
GAGGGAAC -0.213407407407
GAGGGACA -0.245876950425
GAGGGACC -0.382140948797
GAGGGAGA -0.0628409586057
GAGGGAGC -0.220854730397
GAGGGATA 0.198040184214
GAGGGATC 0.0913941310147
GAGGGCAA -0.01392559024
GAGGGCAC -0.292888551618
GAGGGCCA -0.211871729041
GAGGGCCC -0.393246994032
GAGGGCGA -0.125354428615
GAGGGCGC -0.220683524299
GAGGGCTA -0.32056979213
GAGGGGAA -0.0569411764706
GAGGGGAC -0.256633089736
GAGGGGCA -0.182314600737
GAGGGGCC -0.32209164527
GAGGGGGA -0.131238305321
GAGGGGGC -0.351673578199
GAGGGGTA -0.155588772774
GAGGGTAA 0.0849054959104
GAGGGTAC -0.105683640505
GAGGGTCA -0.104301783265
GAGGGTCC -0.327619502186
GAGGGTGA -0.0454904672528
GAGGGTGC -0.101515251298
GAGGGTTA -0.272043572985
GAGGTAAA 0.0693086017256
GAGGTAAC -0.0371724156856
GAGGTACA -0.199034908313
GAGGTACC -0.0711592914527
GAGGTAGA 0.082578123865
GAGGTAGC -0.28150056941
GAGGTATA -0.0420423465302
GAGGTATC -0.0431663187015
GAGGTCAA -0.0785740548668

GAGGTCAC -0.178227858396
GAGGTCCA -0.0759853558613
GAGGTCCC -0.316892444269
GAGGTCGA -0.139148160952
GAGGTCGC -0.216092451498
GAGGTCTA -0.237886887977
GAGGTGAA -0.0241584705011
GAGGTGAC -0.154852962964
GAGGTGCA -0.256595191683
GAGGTGCC -0.177005486968
GAGGTGGA -0.145873799726
GAGGTGGC -0.124243682955
GAGGTGTA 0.0378553891302
GAGGTTAA -0.140354776406
GAGGTTAC -0.0501396847351
GAGGTTCA -0.340938386182
GAGGTTCC -0.232780080577
GAGGTTGA -0.210514403292
GAGGTTGC -0.266119561877
GAGGTTTA -0.283366807147
GAGTAAAA 0.21332936237
GAGTAAAC -0.0477761411277
GAGTAACA -0.07295343682
GAGTAACC -0.213850399419
GAGTAAGA 0.118775877849
GAGTAAGC -0.000429097105601
GAGTAATA 0.111819911961
GAGTAATC 0.365637121729
GAGTACAA 0.0816338093647
GAGTACAC 0.0147211366641
GAGTACCA -0.203735551211
GAGTACCC -0.166358205833
GAGTACGA -0.150505150283
GAGTACGC -0.149438143491
GAGTACTA -0.208864962162
GAGTACTC -0.291870517159
GAGTAGAA 0.0631368674909
GAGTAGAC -0.147309743691
GAGTAGCA -0.167330622637
GAGTAGCC -0.240768066646
GAGTAGGA 0.1786455246
GAGTAGGC -0.217029575865
GAGTAGTA 0.212422820449
GAGTATAA 0.10767559024
GAGTATAC 0.0521828141123
GAGTATCA 0.249407987448
GAGTATCC 0.305064431305
GAGTATGA 0.0358418200572
GAGTATGC -0.134915538872
GAGTATTA -0.0326818371424
GAGTCAAA -0.0906672671538
GAGTCAAC -0.137785790334
GAGTCACA -0.0633901374825
GAGTCACC -0.189438175893

GAGTCAGA 0.049570243035
GAGTCAGC -0.225276172602
GAGTCATA 0.0270553368297
GAGTCATC -0.0387829799377
GAGTCCAA 0.00801701570681
GAGTCCAC -0.341841520612
GAGTCCCA -0.163956427015
GAGTCCCC -0.320356472795
GAGTCCGA -0.201281882436
GAGTCCGC -0.238880933963
GAGTCCTA -0.139135622431
GAGTCGAA -0.0798703721997
GAGTCGAC -0.199367318929
GAGTCGCA -0.112074976178
GAGTCGCC -0.350475877013
GAGTCGGA -0.181338673334
GAGTCGGC 0.0669635996399
GAGTCGTA -0.103123250683
GAGTCTAA -0.146524196243
GAGTCTAC -0.0977733810964
GAGTCTCA -0.226497600818
GAGTCTCC -0.185978257397
GAGTCTGA -0.0745396668588
GAGTCTGC -0.38603757807
GAGTCTTA -0.132534351815
GAGTGAAA 0.0578166331895
GAGTGAAC 0.108391323929
GAGTGACA -0.187056034445
GAGTGACC -0.283753169465
GAGTGAGA 0.12639842438
GAGTGAGC -0.155171136465
GAGTGATA 0.186525358476
GAGTGATC 0.0935928889767
GAGTGCAA -0.000852788633359
GAGTGCAC -0.150099295057
GAGTGCCA -0.17758186398
GAGTGCCC -0.214471192388
GAGTGCGA 0.00593116191505
GAGTGCGC -0.0988832230344
GAGTGCTA 0.195927825316
GAGTGGAA 0.184360699083
GAGTGGAC -0.265589079277
GAGTGGCA -0.359307384453
GAGTGGCC -0.196278102664
GAGTGGGA 0.0326434169795
GAGTGGGC -0.234782367174
GAGTGGTA -0.0425561275478
GAGTGTAA 0.0562622579286
GAGTGTAC -0.101077971322
GAGTGTCA 0.0413455838767
GAGTGTCC -0.281069958848
GAGTGTGA -0.109413580247
GAGTGTGC -0.125047213393
GAGTGTTA -0.227375249008

GAGTTAAA 0.0900513732464
GAGTTAAC -0.133562151217
GAGTTACA 0.118150233814
GAGTTACC -0.0707706968987
GAGTTAGA 0.0553305231728
GAGTTAGC -0.19697985705
GAGTTATA 0.110335159008
GAGTTATC 0.0146455915041
GAGTTCAA -0.0531463005038
GAGTTCAC -0.191772723707
GAGTTCCA -0.140030178326
GAGTTCCC -0.150913770615
GAGTTCGA -0.258604266194
GAGTTCGC -0.125410150892
GAGTTCTA -0.109856507427
GAGTTGAA -0.108994659802
GAGTTGAC -0.193511659808
GAGTTGCA -0.0875422538303
GAGTTGCC -0.142903002766
GAGTTGGA 0.0938253598387
GAGTTGGC -0.124427469136
GAGTTGTA -0.0013467377566
GAGTTTAA -0.0553469992591
GAGTTTAC 0.0405401478942
GAGTTTCA -0.0891907466864
GAGTTTCC -0.171416122004
GAGTTTGA 0.111400432118
GAGTTTGC 0.0169148518879
GAGTTTTA -0.0545157236846
GATAAAAA 0.123973965976
GATAAAAC 0.0877132319041
GATAAACA -0.0586815330666
GATAAACC -0.219969437795
GATAAAGA 0.170789382685
GATAAAGC -0.115075837394
GATAAATA 0.0359502952864
GATAAATC 0.149002658609
GATAACAA 0.102760303924
GATAACAC 0.145521855584
GATAACCA -0.0123795253463
GATAACCC -0.000754681771279
GATAACGA 0.22689552976
GATAACGC 0.138551340315
GATAACTA -0.0414618937074
GATAAGAA -0.122840958606
GATAAGAC -0.0235021006328
GATAAGCA 0.0502389842082
GATAAGCC -0.0356915494967
GATAAGGA -0.0415413757739
GATAAGGC -0.0217359087092
GATAAGTA 0.159166388215
GATAATAA 0.0410791020448
GATAATAC 0.0353565876832
GATAATCA 0.27893587126

GATAATCC 0.345937268461
GATAATGA 0.0533454139089
GATAATGC 0.0651678700623
GATAATTA 0.220987288415
GATACAAA 0.121265962547
GATACAAC −0.015127918295
GATACACA 0.250238984208
GATACACC 0.176400689367
GATACAGA 0.060584312757
GATACAGC 0.00202373861375
GATACATA 0.194463654526
GATACATC −0.00882566027794
GATACCAA 0.0996619025665
GATACCAC 0.0703935612279
GATACCCA 0.284960701989
GATACCCC 0.325808469999
GATACCGA 0.290431732859
GATACCGC 0.244077806318
GATACCTA 0.197285871399
GATACGAA 0.272908343382
GATACGAC 0.178758040791
GATACGCA 0.431187075966
GATACGCC 0.372947265582
GATACGGA 0.371144262223
GATACGGC 0.305317217033
GATACGTA 0.332833871216
GATACTAA −0.0380587799457
GATACTAC 0.164731937035
GATACTCA 0.325002542385
GATACTCC 0.31539668473
GATACTGA 0.13328635974
GATACTGC 0.278746514742
GATACTTA 0.177121406698
GATAGAAA 0.171249643241
GATAGAAC −0.1081559717
GATAGACA −0.222743432747
GATAGACC −0.28293639037
GATAGAGA 0.0710778260427
GATAGAGC −0.290608569354
GATAGATA 0.216601901249
GATAGATC 0.171593567039
GATAGCAA 0.065278601067
GATAGCAC 0.0032905728357
GATAGCCA 0.0902052794445
GATAGCCC −0.1395515385
GATAGCGA 0.14784627544
GATAGCGC 0.137398735428
GATAGCTA 0.0694314083881
GATAGGAA 0.152913573431
GATAGGAC −0.173725308642
GATAGGCA −0.125084249425
GATAGGCC −0.275761416446
GATAGGGA −0.0231389206873
GATAGGGC −0.098409138448

GATAGGTA -0.0390678170761
GATAGTAA 0.0724055412852
GATAGTAC -0.0814315352697
GATAGTCA -0.0444164526115
GATAGTCC -0.239961591221
GATAGTGA 0.216471772202
GATAGTGC 0.183008957387
GATAGTTA 0.0792594833547
GATATAAA 0.282618802028
GATATAAC 0.0861752357154
GATATACA 0.206480903055
GATATACC 0.211758216426
GATATAGA 0.253060305377
GATATAGC 0.184067526916
GATATATA 0.272396955933
GATATATC 0.346904009481
GATATCAA 0.360861080921
GATATCAC 0.427036506447
GATATCCA 0.478586247777
GATATCCC 0.469585327742
GATATCGA 0.436711014512
GATATCGC 0.458001273354
GATATCTA 0.472227710817
GATATGAA 0.247118487782
GATATGAC 0.262727596544
GATATGCA 0.373592608197
GATATGCC 0.281573141013
GATATGGA 0.262994247953
GATATGGC 0.183094030604
GATATGTA 0.260899568521
GATATTAA 0.239485421237
GATATTAC 0.355310679471
GATATTCA 0.405126896309
GATATTCC 0.430019031569
GATATTGA 0.281197172276
GATATTGC 0.379854504611
GATATTTA 0.40783490477
GATCAAAA 0.172127818393
GATCAAAC 0.00619760870513
GATCAACA -0.284029560866
GATCAACC -0.193866641876
GATCAAGA 0.0892966914751
GATCAAGC -0.0854732510288
GATCAATA -0.0562826865983
GATCAATC 0.0461014511844
GATCACAA 0.0726799710202
GATCACAC 0.184601862479
GATCACCA 0.148268897232
GATCACCC -0.026134266994
GATCACGA 0.180705475571
GATCACGC 0.227171560153
GATCACTA 0.0390650727788
GATCAGAA 0.26724245638
GATCAGAC 0.0418285802103

GATCAGCA 0.0321125041768
GATCAGCC 0.0346642546676
GATCAGGA 0.209999564163
GATCAGGC -0.0105326132297
GATCAGTA 0.0868367750222
GATCATAA 0.177207512788
GATCATAC 0.0472694783026
GATCATCA -0.0357334957957
GATCATCC 0.0462496432413
GATCATGA 0.105968297877
GATCATGC 0.116769852247
GATCATTA 0.226514303278
GATCCAAA 0.152758567696
GATCCAAC 0.113317526181
GATCCACA 0.1705185013
GATCCACC 0.175600366103
GATCCAGA 0.230565281188
GATCCAGC 0.0881279298627
GATCCATA 0.207494950493
GATCCATC -0.0299289584937
GATCCCAA 0.102697095436
GATCCCAC 0.119461595456
GATCCCCA 0.362949457382
GATCCCCC 0.32187322941
GATCCCGA 0.325284383944
GATCCCGC 0.302333183212
GATCCCTA 0.202932556148
GATCCGAA 0.238257151639
GATCCGAC 0.178626314518
GATCCGCA 0.256726272805
GATCCGCC 0.314202490085
GATCCGGA 0.243844481771
GATCCGGC 0.167675096315
GATCCGTA 0.285483764737
GATCCTAA 0.209165661819
GATCCTAC 0.223469847312
GATCCTCA 0.321569902742
GATCCTCC 0.287931677973
GATCCTGA 0.233854835523
GATCCTGC 0.206832202683
GATCCTTA 0.191030297673
GATCGAAA 0.155887066675
GATCGAAC -0.0170194528787
GATCGACA -0.0645564062457
GATCGACC -0.0386075714921
GATCGAGA 0.158477683374
GATCGAGC 0.114450219309
GATCGATA 0.223978572526
GATCGATC 0.0784070709066
GATCGCAA 0.30545668502
GATCGCAC 0.377765025496
GATCGCCA 0.281075937414
GATCGCCC 0.360268611825
GATCGCGA 0.443117438907

GATCGCGC 0.435421194757
GATCGCTA 0.2235916266
GATCGGAA 0.172200833902
GATCGGAC -0.073153231663
GATCGGCA 0.175237107291
GATCGGCC -0.0513108497385
GATCGGGA 0.333366264902
GATCGGGC 0.146290258842
GATCGGTA 0.0987041427913
GATCGTAA 0.281775116545
GATCGTAC 0.133846137846
GATCGTCA 0.28824691645
GATCGTCC 0.192273705241
GATCGTGA 0.131328349689
GATCGTGC 0.194737882101
GATCGTTA 0.161172583372
GATCTAAA 0.197317071268
GATCTAAC 0.173540171131
GATCTACA 0.325040314965
GATCTACC 0.258409068458
GATCTAGA 0.321384662671
GATCTAGC 0.200764561242
GATCTATA 0.260603964961
GATCTCAA 0.313632022657
GATCTCAC 0.351262424698
GATCTCCA 0.429518211157
GATCTCCC 0.337320797383
GATCTCGA 0.341980463682
GATCTCGC 0.404817618295
GATCTCTA 0.354019846758
GATCTGAA 0.257201036247
GATCTGAC 0.103727863089
GATCTGCA 0.322847144042
GATCTGCC 0.293313868082
GATCTGGA 0.272796878087
GATCTGGC 0.170168390085
GATCTGTA 0.326403739699
GATCTTAA 0.225531844826
GATCTTAC 0.328059215783
GATCTTCA 0.420772135502
GATCTTCC 0.351165777514
GATCTTGA 0.291939912542
GATCTTGC 0.304894179894
GATCTTTA 0.313614582819
GATGAAAA 0.260353318902
GATGAAAC -0.161268626579
GATGAACA -0.112658894817
GATGAACC -0.214593184696
GATGAAGA -0.0868146043423
GATGAAGC -0.168730956021
GATGAATA 0.0822294538957
GATGAATC -0.0266253105342
GATGACAA 0.0854662734444
GATGACAC -0.0582065467958

GATGACCA -0.27623311988
GATGACCC -0.158999546886
GATGACGA -0.163670485974
GATGACGC -0.152401135278
GATGACTA -0.000900129530835
GATGAGAA -0.014866415818
GATGAGAC -0.269519482444
GATGAGCA 0.0449315524314
GATGAGCC -0.279184492639
GATGAGGA -0.00629267382379
GATGAGGC -0.244700842927
GATGAGTA 0.143720308573
GATGATAA 0.0536181773277
GATGATAC -0.0543974620738
GATGATCA -0.00731355243803
GATGATCC 0.0406679935496
GATGATGA 0.00466507505283
GATGATGC 0.0744424912469
GATGATTA 0.170286394871
GATGCAAA 0.189641924082
GATGCAAC -0.0478389725858
GATGCACA -0.157883956665
GATGCACC 0.0515871747563
GATGCAGA 0.0985036247149
GATGCAGC -0.236943918393
GATGCATA 0.188254180408
GATGCATC -0.0721109170815
GATGCCAA -0.11476853681
GATGCCAC 0.0142497733981
GATGCCCA -0.0209461869872
GATGCCCC -0.130893773497
GATGCCGA -0.137425609937
GATGCCGC -0.0851699039705
GATGCCTA -0.0276995653033
GATGCGAA 0.140756613833
GATGCGAC -0.0875495765113
GATGCGCA 0.231989176491
GATGCGCC -0.15031872672
GATGCGGA 0.0907660569785
GATGCGGC -0.116786318031
GATGCGTA 0.0702306856353
GATGCTAA 0.0287144247672
GATGCTAC -0.100677000857
GATGCTCA 0.053471709735
GATGCTCC 0.0120886298272
GATGCTGA 0.0669228422414
GATGCTGC 0.112267371755
GATGCTTA 0.0971975235461
GATGGAAA 0.05359202708
GATGGAAC 0.0310534191449
GATGGACA -0.222298897331
GATGGACC -0.25726676561
GATGGAGA -0.016537761998
GATGGAGC -0.254543209877

GATGGATA 0.214020891142
GATGGCAA 0.00530942855732
GATGGCAC -0.105920989562
GATGGCCA -0.137406395637
GATGGCCC -0.430095880785
GATGGCGA 0.225721674197
GATGGCGC -0.265514403292
GATGGCTA -0.166714115149
GATGGGAA -0.0148596361241
GATGGGAC -0.0675963768342
GATGGGCA -0.224316630356
GATGGGCC -0.250950476473
GATGGGGA -0.0670810182597
GATGGGGC -0.154594143729
GATGGGTA -0.120594465848
GATGGTAA 0.150562956721
GATGGTAC -0.173384016059
GATGGTCA -0.0827107823768
GATGGTCC -0.216712074915
GATGGTGA 0.0163056900128
GATGGTGC -0.0630377760218
GATGGTTA 0.0574974585107
GATGTAAA 0.0701607060528
GATGTAAC -0.105241059079
GATGTACA 0.140349750531
GATGTACC -0.110420873709
GATGTAGA 0.0644263064309
GATGTAGC -0.102620141255
GATGTATA 0.0955806085153
GATGTCAA 0.0998169482661
GATGTCAC -0.0219967166911
GATGTCCA -0.0827553644328
GATGTCCC -0.136069908329
GATGTCGA 0.0929026981932
GATGTCGC 0.0521553711388
GATGTCTA -0.0795928560451
GATGTGAA 0.14599247454
GATGTGAC -0.0616741296808
GATGTGCA 0.0204727383668
GATGTGCC -0.0169434656178
GATGTGGA -0.0707279800348
GATGTGGC -0.149527777778
GATGTGTA 0.0918265948019
GATGTTAA 0.120719712928
GATGTTAC 0.0408568564497
GATGTTCA 0.0633605973879
GATGTTCC -0.14326450687
GATGTTGA 0.138770884103
GATGTTGC -0.102144320311
GATGTTTA -0.0224990055042
GATTAAAA 0.173615227557
GATTAAAC -0.205034490706
GATTAACA 0.0839088809147
GATTAACC -0.00757506818471

GATTAAGA 0.106063951884
GATTAAGC -0.154105619133
GATTAATA 0.0934450045763
GATTAATC 0.179127987359
GATTACAA 0.426845265538
GATTACAC 0.453574721728
GATTACCA 0.439000188863
GATTACCC 0.438610068278
GATTACGA 0.464712165366
GATTACGC 0.458328127497
GATTACTA 0.399844551305
GATTAGAA 0.00718805748122
GATTAGAC -0.0950953757645
GATTAGCA 0.00116495422512
GATTAGCC -0.0710728626134
GATTAGGA 0.067849026776
GATTAGGC 0.0286230213616
GATTAGTA 0.00894774308834
GATTATAA 0.243919275539
GATTATAC 0.206848668467
GATTATCA 0.338994818767
GATTATCC 0.341931739603
GATTATGA 0.203868783868
GATTATGC 0.309928377377
GATTATTA 0.181678845902
GATTCAAA 0.132403062485
GATTCAAC -0.0131172548051
GATTCACA 0.293660017724
GATTCACC 0.163063074187
GATTCAGA 0.0920981131879
GATTCAGC -0.025463077303
GATTCATA 0.117354387583
GATTCCAA 0.34582249793
GATTCCAC 0.376986329232
GATTCCCA 0.468173533671
GATTCCCC 0.486175235715
GATTCCGA 0.425675184868
GATTCCGC 0.411276866671
GATTCCTA 0.424388011564
GATTCGAA 0.323750246987
GATTCGAC 0.292181171589
GATTCGCA 0.46338009616
GATTCGCC 0.45615491949
GATTCGGA 0.366742195989
GATTCGGC 0.322771054581
GATTCGTA 0.381844277591
GATTCTAA 0.308859122804
GATTCTAC 0.359036392047
GATTCTCA 0.482400883297
GATTCTCC 0.472768337395
GATTCTGA 0.398343026959
GATTCTGC 0.405055544578
GATTCTTA 0.406486714221
GATTGAAA 0.126064787372

GATTGAAC -0.176463325713
GATTGACA 0.200833168675
GATTGACC -0.0384040608636
GATTGAGA 0.13869129948
GATTGAGC -0.249562731346
GATTGATA 0.0797935394646
GATTGCAA 0.380422731564
GATTGCAC 0.457300207448
GATTGCCA 0.398356892769
GATTGCCC 0.403570879712
GATTGCGA 0.467957229817
GATTGCGC 0.482575218282
GATTGCTA 0.422466166156
GATTGGAA 0.0491800934436
GATTGGAC -0.0493731204509
GATTGGCA -0.0201981607659
GATTGGCC -0.107205846033
GATTGGGA 0.136866341742
GATTGGGC -0.133164705699
GATTGGTA 0.160038063138
GATTGTAA 0.208791630991
GATTGTAC 0.262137346912
GATTGTCA 0.353628346869
GATTGTCC 0.33283308878
GATTGTGA 0.306639257333
GATTGTGC 0.269586050188
GATTGTTA 0.251694779216
GATTTAAA 0.226327691058
GATTTAAC 0.206045065594
GATTTACA 0.298093937501
GATTTACC 0.268852149405
GATTTAGA 0.202082851252
GATTTAGC 0.129194757382
GATTTATA 0.267789907572
GATTTCAA 0.370870076853
GATTTCAC 0.442169421941
GATTTCCA 0.48044688138
GATTTCCC 0.485335522206
GATTTCGA 0.463276335479
GATTTCGC 0.459077037915
GATTTCTA 0.421889064524
GATTTGAA 0.301343059123
GATTTGAC 0.333533334302
GATTTGCA 0.404117602706
GATTTGCC 0.408253308733
GATTTGGA 0.330380776662
GATTTGGC 0.279899119629
GATTTGTA 0.339678208041
GATTTTAA 0.322794299246
GATTTTAC 0.368961108771
GATTTTCA 0.44703780544
GATTTTCC 0.45042426837
GATTTTGA 0.320554094693
GATTTTGC 0.462445338718

GATTTTTA 0.407970786523
GCAAAAAA 0.165576404579
GCAAAAAC -0.10083971351
GCAAAACA -0.108758497875
GCAAAACC -0.0585931926197
GCAAAAGA 0.104541426351
GCAAAAGC -0.0649812857409
GCAAAATA 0.196181584667
GCAAACAA 0.0641886051566
GCAAACAC -0.161524869397
GCAAACCA -0.212178326475
GCAAACCC -0.184311756442
GCAAACGA 0.067878779074
GCAAACGC -0.230335511983
GCAAACTA -0.134184184911
GCAAAGAA 0.14474529207
GCAAAGAC 0.0843011346302
GCAAAGCA -0.236406736135
GCAAAGCC -0.360665522319
GCAAAGGA -0.0699214039777
GCAAAGGC -0.316593962558
GCAAAGTA -0.0213400549155
GCAAATAA -0.00126538143042
GCAAATAC 0.243545412633
GCAAATCA 0.360186247875
GCAAATCC 0.424175904
GCAAATGA 0.00670172736914
GCAAATGC -0.0774512225241
GCAAATTA 0.0901064896198
GCAACAAA 0.0549547455436
GCAACAAC -0.0721449698125
GCAACACA -0.0788582685807
GCAACACC -0.101690949365
GCAACAGA 0.0210377834859
GCAACAGC -0.174033649414
GCAACATA 0.0914895471649
GCAACCAA 0.023279874421
GCAACCAC -0.0151540099673
GCAACCCA -0.114969593185
GCAACCCC -0.254394627691
GCAACCGA -0.0507038775006
GCAACCGC -0.137277174797
GCAACCTA -0.262640480267
GCAACGAA -0.0743347823223
GCAACGAC -0.111874025774
GCAACGCA -0.196438634713
GCAACGCC -0.119292746483
GCAACGGA 0.146341144509
GCAACGGC -0.136130179268
GCAACGTA 0.112318219459
GCAACTAA -0.140865038232
GCAACTAC -0.0893619590152
GCAACTCA -0.0962770919067
GCAACTCC 0.0330451963448

GCAACTGA -0.0173480131546
GCAACTGC -0.188824981845
GCAACTTA -0.0152444378588
GCAAGAAA 0.104137550303
GCAAGAAC -0.14832135625
GCAAGACA -0.0261081167463
GCAAGACC -0.275863471314
GCAAGAGA 0.0997065361091
GCAAGAGC -0.06627492572
GCAAGATA 0.203063937356
GCAAGCAA 0.0155535024797
GCAAGCAC -0.200892906143
GCAAGCCA -0.0460987068871
GCAAGCCC -0.300590829795
GCAAGCGA -0.0801614433713
GCAAGCGC -0.0691700689231
GCAAGCTA -0.152888888889
GCAAGGAA -0.0191935156374
GCAAGGAC -0.296701397462
GCAAGGCA -0.128345679012
GCAAGGCC -0.191737491878
GCAAGGGA 0.0198698298781
GCAAGGGC -0.0860012317847
GCAAGGTA 0.00943297546235
GCAAGTAA -0.172488486627
GCAAGTAC -0.0161160323127
GCAAGTCA -0.0351732513612
GCAAGTCC -0.152630630997
GCAAGTGA 0.0445737560137
GCAAGTGC -0.0645509179221
GCAAGTTA -0.136421765571
GCAATAAA 0.00415934217599
GCAATAAC 0.135320267895
GCAATACA -0.0658804643122
GCAATACC -0.0632676187294
GCAATAGA 0.118865950925
GCAATAGC -0.109333245538
GCAATATA 0.217043188419
GCAATCAA 0.378356275659
GCAATCAC 0.347406090145
GCAATCCA 0.468716042675
GCAATCCC 0.440127555097
GCAATCGA 0.339879672505
GCAATCGC 0.348229279858
GCAATCTA 0.466577202573
GCAATGAA 0.113745636567
GCAATGAC -0.226963785712
GCAATGCA -0.0676665730717
GCAATGCC -0.294942384537
GCAATGGA 0.0678637291708
GCAATGGC -0.229755085917
GCAATGTA 0.0230165763515
GCAATTAA 0.126893767046
GCAATTAC 0.170149180004

GCAATTCA 0.147538244737
GCAATTCC 0.157989198446
GCAATTGA 0.000548859470021
GCAATTGC 0.205361351404
GCAATTTA 0.287363619194
GCACAAAA 0.260593005509
GCACAAAC −0.000137994747484
GCACAACA 0.0169874914648
GCACAACC −0.225122514721
GCACAAGA −0.0465306146167
GCACAAGC −0.255382716049
GCACAATA 0.173182920983
GCACACAA 0.0164528641785
GCACACAC −0.245679885213
GCACACCA −0.190676033341
GCACACCC −0.379296173886
GCACACGA −0.0435147683493
GCACACGC −0.0182485145207
GCACACTA −0.0595347867132
GCACAGAA −0.0163734399331
GCACAGAC −0.068474423605
GCACAGCA 0.0217933258757
GCACAGCC −0.330447757919
GCACAGGA −0.0372062519894
GCACAGGC −0.184067036141
GCACAGTA −0.112650357786
GCACATAA 0.0654899119629
GCACATAC −0.0298826538453
GCACATCA −0.00991384946174
GCACATCC −0.00973806168553
GCACATGA 0.110149201691
GCACATGC −0.00334738136002
GCACATTA 0.0700548364786
GCACCAAA 0.185156922341
GCACCAAC −0.19254413871
GCACCACA −0.120806256228
GCACCACC −0.222104240748
GCACCAGA −0.076690912305
GCACCAGC −0.271417637764
GCACCATA −0.0222507629147
GCACCCAA −0.114974318451
GCACCCAC −0.113387818338
GCACCCCA −0.188076405813
GCACCCCC −0.214415422681
GCACCCGA −0.0311518651611
GCACCCGC −0.261307828644
GCACCCTA −0.0709607121691
GCACCGAA −0.166975107283
GCACCGAC −0.312099814001
GCACCGCA −0.0614802008726
GCACCGCC −0.0963983697089
GCACCGGA −0.0100198712436
GCACCGGC −0.214588235294
GCACCGTA −0.115324504042

GCACCTAA -0.0646172620691
GCACCTAC -0.19306545276
GCACCTCA -0.262120806486
GCACCTCC -0.196547221262
GCACCTGA -0.219602710954
GCACCTGC -0.279005829777
GCACCTTA -0.134390985532
GCACGAAA 0.111240093087
GCACGAAC -0.222317271188
GCACGACA -0.041571029281
GCACGACC -0.292341584834
GCACGAGA -0.136307915759
GCACGAGC -0.104942001493
GCACGATA 0.0764753342554
GCACGCAA -0.0154234550399
GCACGCAC -0.0349843977895
GCACGCCA -0.106149349649
GCACGCCC -0.410491501733
GCACGCGA 0.0775908911282
GCACGCGC 0.0450174537311
GCACGCTA -0.0361094645327
GCACGGAA 0.0461590814288
GCACGGAC -0.330781242285
GCACGGCA -0.190544724704
GCACGGCC -0.253276317739
GCACGGGA -0.0790997190395
GCACGGGC -0.181882887489
GCACGGTA 0.139698981593
GCACGTAA 0.117047055918
GCACGTAC -0.189642893346
GCACGTCA -0.231754014393
GCACGTCC -0.181644664978
GCACGTGA 0.090479483633
GCACGTGC 0.00982443066785
GCACGTTA -0.00520825766711
GCACTAAA -0.079504448228
GCACTAAC -0.251459804454
GCACTACA -0.0800456651079
GCACTACC -0.108746684331
GCACTAGA 0.0902297525742
GCACTAGC -0.14578943325
GCACTATA -0.0186978214691
GCACTCAA -0.120944081336
GCACTCAC -0.224255953928
GCACTCCA -0.2475986542
GCACTCCC -0.24275017137
GCACTCGA -0.00195649178273
GCACTCGC -0.290569879263
GCACTCTA 0.0292622959816
GCACTGAA -0.155061728395
GCACTGAC -0.165641556227
GCACTGCA -0.0504374409976
GCACTGCC -0.209791495199
GCACTGGA -0.113789894758

GCACTGGC -0.247941561327
GCACTGTA -0.0337679870921
GCACTTAA -0.114906099422
GCACTTAC -0.147434736559
GCACTTCA -0.256229902953
GCACTTCC -0.125230585098
GCACTTGA -0.0933198313904
GCACTTTA -0.141721098638
GCAGAAAA 0.00067264248253
GCAGAAAC -0.0744504361065
GCAGAACA -0.0214163899372
GCAGAACC -0.0425604852767
GCAGAAGA 0.0560110639139
GCAGAAGC -0.294747295192
GCAGAATA 0.162196206283
GCAGACAA -0.0600589475071
GCAGACAC -0.0536099756246
GCAGACCA -0.220849647384
GCAGACCC -0.298682418007
GCAGACGA 0.0547745993927
GCAGACGC -0.13025210084
GCAGACTA -0.126974511838
GCAGAGAA 0.127576895212
GCAGAGAC -0.293944833155
GCAGAGCA -0.224823302164
GCAGAGCC -0.213282488862
GCAGAGGA 0.00765782079986
GCAGAGGC -0.0503586265181
GCAGAGTA -0.180960723303
GCAGATAA 0.214859272432
GCAGATAC 0.316016817054
GCAGATCA 0.211681896765
GCAGATCC 0.313033765835
GCAGATGA -0.170669878146
GCAGATGC -0.137874729068
GCAGATTA 0.293230668431
GCAGCAAA 0.217420423343
GCAGCAAC -0.0553662270954
GCAGCACA -0.240541371042
GCAGCACC -0.0386534281762
GCAGCAGA -0.0255578605376
GCAGCAGC -0.13048655352
GCAGCATA -0.11182733734
GCAGCCAA -0.0495019346341
GCAGCCAC -0.218686368429
GCAGCCCA -0.173870733479
GCAGCCCC -0.225415007187
GCAGCCGA -0.226057613169
GCAGCCGC -0.271021830838
GCAGCCTA -0.36542069919
GCAGCGAA 0.0514763985405
GCAGCGAC -0.290303289661
GCAGCGCA -0.0562391937954
GCAGCGCC -0.321269872618

GCAGCGGA -0.0450107834906
GCAGCGGC -0.400009292817
GCAGCGTA -0.0297429848162
GCAGCTAA -0.195660130719
GCAGCTAC -0.34951486273
GCAGCTCA -0.222189463411
GCAGCTCC -0.229239015345
GCAGCTGA -0.226516939198
GCAGCTGC -0.299754057159
GCAGCTTA -0.239013981809
GCAGGAAA 0.0256753694266
GCAGGAAC -0.304309756487
GCAGGACA -0.124694958979
GCAGGACC -0.25714708932
GCAGGAGA 0.110468333169
GCAGGAGC -0.393514632875
GCAGGATA 0.080526782212
GCAGGCAA 0.0256809960339
GCAGGCAC -0.00529279337829
GCAGGCCA -0.138385751298
GCAGGCCC -0.223300251815
GCAGGCGA 0.0379728424334
GCAGGCGC -0.078613608175
GCAGGCTA -0.337600011617
GCAGGGAA 0.0373132690134
GCAGGGAC -0.207262300416
GCAGGGCA -0.131527877844
GCAGGGCC -0.224403076788
GCAGGGGA -0.0265878169315
GCAGGGGC -0.172171387073
GCAGGGTA -0.0834321133995
GCAGGTAA -0.0521196952732
GCAGGTAC -0.309885503931
GCAGGTCA -0.107864516448
GCAGGTCC -0.385397095167
GCAGGTGA -0.0425378923239
GCAGGTTA -0.19403009648
GCAGTAAA 0.131761948671
GCAGTAAC -0.0760945920731
GCAGTACA -0.119779345628
GCAGTACC -0.195873799726
GCAGTAGA -0.0609242429232
GCAGTAGC -0.271116287716
GCAGTATA 0.154514918
GCAGTCAA -0.147254185392
GCAGTCAC -0.0730149322533
GCAGTCCA -0.334677717982
GCAGTCCC -0.232303706135
GCAGTCGA -0.0630001976089
GCAGTCGC -0.170307611414
GCAGTCTA -0.27655888307
GCAGTGAA -0.0655098994688
GCAGTGAC -0.114192196048
GCAGTGCA -0.140885069393

GCAGTGCC -0.16540976606
GCAGTGGA 3.21051071817E-5
GCAGTGGC -0.206897119342
GCAGTGTA -0.0451135356588
GCAGTTAA -0.0144305783563
GCAGTTAC 0.108912928934
GCAGTTCA -0.16782441701
GCAGTTCC -0.174521604938
GCAGTTGA 0.00234335275231
GCAGTTTA -0.114264153014
GCATAAAA 0.084153878241
GCATAAAC -0.0453895660512
GCATAACA 0.0838547498299
GCATAACC -0.213415041556
GCATAAGA 0.133358999317
GCATAAGC -0.158943377176
GCATAATA 0.0523494801322
GCATACAA -0.0868580477387
GCATACAC 0.0107494127204
GCATACCA 0.000349698531266
GCATACCC 0.0787078872053
GCATACGA 0.101728821931
GCATACGC -0.148993141289
GCATACTA -0.0561675338646
GCATAGAA 0.0657423873192
GCATAGAC -0.250702331962
GCATAGCA -0.0330724377468
GCATAGCC -0.267790545015
GCATAGGA 0.0639688313309
GCATAGGC -0.0742797785946
GCATAGTA 0.200059564453
GCATATAA 0.107957913456
GCATATAC -0.0384030915404
GCATATCA 0.178522031219
GCATATCC 0.362291158972
GCATATGA -0.00837326483229
GCATATGC 0.102544879939
GCATATTA 0.0257158630308
GCATCAAA 0.161136907506
GCATCAAC 0.0629015152616
GCATCACA -0.00730608864934
GCATCACC -0.0729228712972
GCATCAGA 0.155859623702
GCATCAGC -0.0331103892708
GCATCATA 0.0665437221454
GCATCCAA 0.0620414626705
GCATCCAC 0.0510109991438
GCATCCCA -0.077130155594
GCATCCCC -0.10113059466
GCATCCGA -0.0253759098107
GCATCCGC -0.217324377274
GCATCCTA -0.0451040757753
GCATCGAA 0.185163118641
GCATCGAC -0.115135026863

GCATCGCA -0.172360203341
GCATCGCC -0.2311015652
GCATCGGA 0.0760928624352
GCATCGGC -0.020442309592
GCATCGTA 0.0469643920794
GCATCTAA 0.0260180436709
GCATCTAC 0.209959683394
GCATCTCA 0.106188891956
GCATCTCC 0.0499113723295
GCATCTGA 0.0547761751081
GCATCTTA -0.0523131682364
GCATGAAA 0.15039766833
GCATGAAC -0.0476791655165
GCATGACA -0.19669426289
GCATGACC -0.255947712418
GCATGAGA 0.0879931502338
GCATGAGC -0.298179499914
GCATGATA 0.1262619697
GCATGCAA 0.232443108166
GCATGCAC -0.164638409694
GCATGCCA -0.0772211462219
GCATGCCC -0.115144431274
GCATGCGA 0.169948846297
GCATGCGC -0.209220079388
GCATGCTA -0.128967470038
GCATGGAA -0.100343998947
GCATGGAC -0.0873603907686
GCATGGCA -0.242750700926
GCATGGCC -0.225066934404
GCATGGGA -0.259541031227
GCATGGGC -0.159820472572
GCATGGTA 0.0298156407537
GCATGTAA 0.122367178534
GCATGTAC -0.110128076154
GCATGTCA -0.113835774915
GCATGTCC -0.141356540276
GCATGTGA -0.0823323156118
GCATGTTA -0.199226809781
GCATTAAA 0.00427556549911
GCATTAAC -0.0494573002074
GCATTACA -0.087761684076
GCATTACC -0.178417378706
GCATTAGA 0.0275198138269
GCATTAGC -0.195092616264
GCATTATA -0.087781592728
GCATTCAA 0.0126813980548
GCATTCAC -0.0519632703243
GCATTCCA -0.15716194626
GCATTCCC -0.140362116101
GCATTCGA -0.020395617906
GCATTCGC -0.11188755549
GCATTCTA -0.0333333333333
GCATTGAA -0.00340567301148
GCATTGAC -0.0460026440806

GCATTGCA -0.0996543209877
GCATTGCC -0.274345679012
GCATTGGA 0.0746814185518
GCATTGGC -0.0980387920391
GCATTGTA 0.0099385469179
GCATTTAA 0.00753346835943
GCATTTAC 0.104162550221
GCATTTCA -0.101080876905
GCATTTCC 0.00620311346904
GCATTTGA 0.13850468726
GCATTTTA 0.0711925618565
GCCAAAAA -0.0535440849592
GCCAAAAC -0.0976242220686
GCCAAACA -0.187744371823
GCCAAACC -0.0870036497352
GCCAAAGA -0.0171991064192
GCCAAAGC -0.338964316081
GCCAAATA 0.0392006740953
GCCAACAA -0.0380753575693
GCCAACAC -0.256279933837
GCCAACCA -0.115195110047
GCCAACCC -0.146985710523
GCCAACGA -0.219712861944
GCCAACGC -0.20353254871
GCCAACTA -0.128580600553
GCCAAGAA 0.01382040591
GCCAAGAC -0.0541671468475
GCCAAGCA -0.25901049212
GCCAAGCC -0.298402000359
GCCAAGGA -0.156641975309
GCCAAGGC -0.2421091937
GCCAAGTA -0.0941333364325
GCCAATAA 0.122324310084
GCCAATAC -0.277907192899
GCCAATCA -0.0699124063679
GCCAATCC -0.0897670206635
GCCAATGA -0.153083217387
GCCAATTA -0.104743185765
GCCACAAA -0.0524558716986
GCCACAAC 0.0170300044158
GCCACACA -0.122695817762
GCCACACC 0.0183922808404
GCCACAGA -0.104202519536
GCCACAGC -0.190203436698
GCCACATA -0.0694341946993
GCCACCAA -0.055711957327
GCCACCAC -0.249787965749
GCCACCCA -0.0683950617284
GCCACCCC -0.315575341511
GCCACCGA -0.113867455319
GCCACCGC -0.220050188643
GCCACCTA -0.147424711357
GCCACGAA 0.0365814836769
GCCACGAC -0.115854720517

GCCACGCA −0.074826011548
GCCACGCC −0.23181223462
GCCACGGA −0.213993592276
GCCACGGC −0.227917695473
GCCACGTA 0.0495074229577
GCCACTAA −0.166336415036
GCCACTAC −0.24096324292
GCCACTCA −0.112515844404
GCCACTCC −0.266244739629
GCCACTGA −0.0985292664927
GCCACTTA −0.103648096931
GCCAGAAA 0.102019380239
GCCAGAAC −0.0218388294387
GCCAGACA −0.268107816022
GCCAGACC −0.208261995698
GCCAGAGA 0.118597554282
GCCAGAGC −0.130434424745
GCCAGATA 0.0403027508837
GCCAGCAA −0.0206570296465
GCCAGCAC −0.254310013717
GCCAGCCA −0.318428920157
GCCAGCCC −0.329619205934
GCCAGCGA −0.150088748028
GCCAGCGC −0.130618393757
GCCAGCTA −0.204270232116
GCCAGGAA −0.0338661705957
GCCAGGAC −0.226439912656
GCCAGGCA −0.297347861391
GCCAGGCC −0.27609627106
GCCAGGGA −0.183696798389
GCCAGGGC −0.406187640309
GCCAGGTA −0.0534182260843
GCCAGTAA −0.146235656311
GCCAGTAC −0.173693423768
GCCAGTCA −0.356727167015
GCCAGTCC −0.14975189997
GCCAGTGA −0.19818010167
GCCAGTTA −0.009762837823
GCCATAAA −0.0337094279289
GCCATAAC −0.14638382641
GCCATACA −0.0423884168697
GCCATACC −0.280419361741
GCCATAGA −0.0443987622216
GCCATAGC −0.189076875256
GCCATATA −0.114051625722
GCCATCAA −0.144645855972
GCCATCAC −0.101287982307
GCCATCCA −0.217217365536
GCCATCCC −0.246168913469
GCCATCGA −0.132276831042
GCCATCGC −0.093665453867
GCCATCTA −0.0986082257057
GCCATGAA −0.128787255176
GCCATGAC −0.22321483035

GCCATGCA -0.255837557882
GCCATGCC -0.247779478486
GCCATGGA -0.0509728053566
GCCATGGC -0.119913099294
GCCATGTA 0.0411532259236
GCCATTAA -0.109969596189
GCCATTAC -0.115564064425
GCCATTCA -0.0946879566762
GCCATTCC -0.0757815200751
GCCATTGA -0.121697041537
GCCATTTA -0.0944952114377
GCCCAAAA 0.0585979946656
GCCCAAAC -0.288064504886
GCCCAACA -0.146991198033
GCCCAACC -0.0987746671586
GCCCAAGA -0.032133892672
GCCCAAGC -0.389592983577
GCCCAATA 0.00750839754989
GCCCACAA -0.145301678246
GCCCACAC -0.154271248769
GCCCACCA -0.355759652664
GCCCACCC -0.189924597135
GCCCACGA -0.195170795322
GCCCACGC -0.266896151053
GCCCACTA -0.0418615117785
GCCCAGAA -0.0367745122252
GCCCAGAC -0.269821098159
GCCCAGCA -0.141893462245
GCCCAGCC -0.283261403702
GCCCAGGA -0.257095510171
GCCCAGGC -0.343726767958
GCCCAGTA -0.148136581908
GCCCATAA -0.00358113114349
GCCCATAC -0.171383382155
GCCCATCA -0.374448668576
GCCCATCC -0.0466722815336
GCCCATGA -0.145210502925
GCCCATTA -0.185836864991
GCCCCAAA -0.1331775458
GCCCCAAC -0.256542240656
GCCCCACA -0.188321876217
GCCCCACC -0.0586216312788
GCCCCAGA 0.0861690678341
GCCCCAGC -0.279846160599
GCCCCATA -0.159696861409
GCCCCCAA -0.148232285819
GCCCCCAC -0.124391975309
GCCCCCCA -0.251314192113
GCCCCCCC -0.280881515923
GCCCCCGA -0.355165489886
GCCCCCGC -0.191925162737
GCCCCCTA -0.321754538853
GCCCCGAA -0.100407883422
GCCCCGAC -0.21948944203

GCCCCGCA -0.185315951476
GCCCCGCC -0.334304334946
GCCCCGGA -0.156339869281
GCCCCGGC -0.331033361212
GCCCCGTA -0.126614540466
GCCCCTAA -0.166866124258
GCCCCTAC -0.0683466195645
GCCCCTCA -0.274005884386
GCCCCTCC -0.3956644883
GCCCCTGA -0.23756840283
GCCCCTTA -0.32549000244
GCCCGAAA -0.133306443204
GCCCGAAC -0.0681079716349
GCCCGACA -0.206155437284
GCCCGACC -0.351512607808
GCCCGAGA 0.118845611843
GCCCGAGC -0.113231929149
GCCCGATA -0.0420442759779
GCCCGCAA -0.123708360326
GCCCGCAC -0.287009743418
GCCCGCCA -0.207697893972
GCCCGCCC -0.016794687057
GCCCGCGA -0.106135076253
GCCCGCGC -0.332892386775
GCCCGCTA -0.130493284614
GCCCGGAA -0.206963016329
GCCCGGAC -0.398449842022
GCCCGGCA -0.214855967078
GCCCGGCC -0.345535046146
GCCCGGGA -0.103754376722
GCCCGGGC -0.190129972713
GCCCGGTA -0.244854849418
GCCCGTAA 0.0135269420191
GCCCGTAC -0.165544470784
GCCCGTCA -0.34293779731
GCCCGTCC -0.130144626119
GCCCGTGA -0.12237037037
GCCCGTTA -0.311206016918
GCCCTAAA -0.0851177574655
GCCCTAAC -0.184363865072
GCCCTACA -0.10978581189
GCCCTACC -0.0266705631839
GCCCTAGA 0.0557973646361
GCCCTAGC -0.285226731419
GCCCTATA -0.0456760695583
GCCCTCAA -0.10803947715
GCCCTCAC -0.0834647979802
GCCCTCCA -0.142762527233
GCCCTCCC -0.120067050922
GCCCTCGA -0.269517554689
GCCCTCGC -0.273333772247
GCCCTCTA -0.225839765508
GCCCTGAA -0.034093778129
GCCCTGAC -0.298550429502

GCCCTGCA -0.0504546626935
GCCCTGCC -0.30847356223
GCCCTGGA -0.0444452515509
GCCCTGTA -0.171503190515
GCCCTTAA -0.0524352894685
GCCCTTAC -0.204133796517
GCCCTTCA -0.422169565527
GCCCTTCC -0.102574074074
GCCCTTGA -0.133112713622
GCCCTTTA -0.172337728612
GCCGAAAA 0.0239796702452
GCCGAAAC -0.269256094951
GCCGAACA -0.101159316509
GCCGAACC -0.281160660883
GCCGAAGA -0.040165341302
GCCGAAGC -0.192376061624
GCCGAATA -0.0687625539201
GCCGACAA -0.229874823158
GCCGACAC -0.182831105676
GCCGACCA -0.220238198983
GCCGACCC -0.0803371526406
GCCGACGA -0.226002556801
GCCGACGC -0.360146824025
GCCGACTA -0.239137065127
GCCGAGAA 0.159703921084
GCCGAGAC -0.297590242457
GCCGAGCA -0.142115042718
GCCGAGCC -0.0729905591866
GCCGAGGA 0.00198712436377
GCCGAGGC -0.136901136407
GCCGAGTA -0.165628712598
GCCGATAA -0.00679767144978
GCCGATAC 0.0437123164083
GCCGATCA -0.00513561809016
GCCGATCC -0.0244780846396
GCCGATGA -0.00978890864783
GCCGATTA 0.11000682812
GCCGCAAA -0.0583436239401
GCCGCAAC -0.12686067629
GCCGCACA -0.0995685964566
GCCGCACC -0.259887899865
GCCGCAGA -0.280655834856
GCCGCAGC -0.217084207041
GCCGCATA -0.0680734531402
GCCGCCAA -0.168062378168
GCCGCCAC -0.197488646791
GCCGCCCA -0.203907239372
GCCGCCCC -0.119471786781
GCCGCCGA -0.168362223649
GCCGCCGC -0.120399554953
GCCGCCTA -0.110537049034
GCCGCGAA -0.137343849481
GCCGCGAC -0.265370692321
GCCGCGCA -0.0328779541057

GCCGCGCC	-0.266829024603
GCCGCGGA	-0.0143107816022
GCCGCGGC	-0.0555352557772
GCCGCGTA	-0.0426076194295
GCCGCTAA	0.0591140877196
GCCGCTAC	-0.164601851852
GCCGCTCA	-0.224424110385
GCCGCTCC	-0.335988824285
GCCGCTGA	-0.197436254905
GCCGCTTA	-0.128341703027
GCCGGAAA	-0.0898867154054
GCCGGAAC	-0.104559346457
GCCGGACA	-0.124455310788
GCCGGACC	-0.354456732826
GCCGGAGA	-0.0519278092034
GCCGGAGC	-0.34895284799
GCCGGATA	0.212585218852
GCCGGCAA	-0.0894256734506
GCCGGCAC	-0.253838937441
GCCGGCCA	-0.244584115381
GCCGGCCC	-0.179833442067
GCCGGCGA	-0.0348368699197
GCCGGCGC	-0.092696673027
GCCGGCTA	-0.0433117104064
GCCGGGAA	-0.0249032682967
GCCGGGAC	-0.222002754322
GCCGGGCA	-0.312206136466
GCCGGGCC	-0.310935331812
GCCGGGGA	-0.0585286709874
GCCGGGTA	-0.28124005487
GCCGGTAA	-0.122225582659
GCCGGTAC	-0.230274204029
GCCGGTCA	-0.111574137285
GCCGGTCC	-0.183046076349
GCCGGTGA	-0.166919067215
GCCGGTTA	-0.0765439034026
GCCGTAAA	-0.140171387073
GCCGTAAC	-0.170744578526
GCCGTACA	-0.13136825585
GCCGTACC	-0.2258686729
GCCGTAGA	-0.0137543508957
GCCGTAGC	-0.211144257219
GCCGTATA	-0.122529004358
GCCGTCAA	-0.0457577676927
GCCGTCAC	-0.179739368999
GCCGTCCA	-0.369446913093
GCCGTCCC	-0.207689000941
GCCGTCGA	-0.164302127306
GCCGTCGC	-0.325269426289
GCCGTCTA	-0.177530864198
GCCGTGAA	-0.140528040533
GCCGTGAC	-0.240306512937
GCCGTGCA	-0.189683441158
GCCGTGCC	-0.25642306698

GCCGTGGA -0.0451402607665
GCCGTGTA -0.0437683831599
GCCGTTAA -0.0965033583564
GCCGTTAC -0.131837437991
GCCGTTCA -0.170824703035
GCCGTTCC -0.312650471198
GCCGTTGA -0.0394495626077
GCCGTTTA -0.146899024353
GCCTAAAA 0.0298152019052
GCCTAAAC -0.188602989596
GCCTAACA -0.117440641788
GCCTAACC -0.227354059495
GCCTAAGA -0.0147763427426
GCCTAAGC -0.272598079561
GCCTAATA 0.047586186858
GCCTACAA -0.133334204922
GCCTACAC -0.17621352032
GCCTACCA -0.152604212055
GCCTACCC -0.161059285579
GCCTACGA 0.00130024842735
GCCTACGC -0.148607193384
GCCTACTA -0.0564637833863
GCCTAGAA -0.088432844493
GCCTAGAC -0.299843741377
GCCTAGCA -0.0631455321765
GCCTAGCC -0.24087844609
GCCTAGGA -0.211128540305
GCCTAGGC -0.375574940879
GCCTAGTA -0.0814643866534
GCCTATAA 0.170585329711
GCCTATAC 0.0302048713598
GCCTATCA -0.0690235788067
GCCTATCC -0.165379972565
GCCTATGA -0.156697530864
GCCTATTA -0.132027564088
GCCTCAAA -0.0437469940596
GCCTCAAC -0.0913049204076
GCCTCACA 0.0461906781877
GCCTCACC -0.104775144611
GCCTCAGA -0.201518169199
GCCTCAGC -0.259889377253
GCCTCATA -0.0934545858646
GCCTCCAA -0.241925925926
GCCTCCAC -0.233112226465
GCCTCCCA -0.190300616156
GCCTCCCC -0.319781282161
GCCTCCGA -0.279012284716
GCCTCCGC -0.269356112976
GCCTCCTA -0.271179868778
GCCTCGAA -0.0275555336055
GCCTCGAC -0.163962562355
GCCTCGCA -0.207537076358
GCCTCGCC -0.24705185254
GCCTCGGA -0.139285341937

GCCTCGTA 0.0604521087269
GCCTCTAA −0.196747650952
GCCTCTAC −0.153302495048
GCCTCTCA −0.303280172485
GCCTCTCC −0.274610223682
GCCTCTGA −0.0827588110366
GCCTCTTA −0.198003579148
GCCTGAAA 0.0203891221426
GCCTGAAC −0.252324733538
GCCTGACA −0.333369334452
GCCTGACC −0.36473298774
GCCTGAGA −0.00407782442853
GCCTGAGC −0.087341563786
GCCTGATA 0.0100029638411
GCCTGCAA 0.00502641044564
GCCTGCAC −0.263075606465
GCCTGCCA −0.134184458969
GCCTGCCC −0.279121885921
GCCTGCGA −0.000344498553106
GCCTGCGC −0.160788681001
GCCTGCTA −0.171838013831
GCCTGGAA 0.0477906384218
GCCTGGAC −0.0435649690762
GCCTGGCA −0.307233302068
GCCTGGCC −0.150105888033
GCCTGGGA −0.236004125793
GCCTGGTA −0.0648767779534
GCCTGTAA −0.0755839794558
GCCTGTAC −0.158828532236
GCCTGTCA −0.156353400168
GCCTGTCC −0.182441834455
GCCTGTGA −0.217761521835
GCCTGTTA −0.0810804877781
GCCTTAAA 0.0176412476574
GCCTTAAC −0.176989081149
GCCTTACA −0.00298548661253
GCCTTACC −0.270323060345
GCCTTAGA −0.0883500533122
GCCTTAGC −0.28170798583
GCCTTATA −0.134946112648
GCCTTCAA −0.138596722247
GCCTTCAC −0.043053154414
GCCTTCCA −0.272178994543
GCCTTCCC −0.270604790576
GCCTTCGA −0.00677259131198
GCCTTCGC −0.210328784925
GCCTTCTA −0.0827296475573
GCCTTGAA −0.24352117003
GCCTTGAC −0.263047449761
GCCTTGCA −0.315856127185
GCCTTGCC −0.376297283658
GCCTTGGA −0.194139827439
GCCTTGTA −0.246421119561
GCCTTTAA −0.191562271937

GCCTTTAC -0.0556393205675
GCCTTTCA -0.283041484261
GCCTTTCC -0.307985641181
GCCTTTGA -0.122490616821
GCCTTTTA -0.0472076110041
GCGAAAAA 0.0605710649944
GCGAAAAC 0.0415152281093
GCGAAACA -0.0283484788621
GCGAAACC -0.121498530283
GCGAAAGA -0.142226831959
GCGAAAGC -0.112520988691
GCGAAATA 0.194745682068
GCGAACAA -0.0409934188543
GCGAACAC -0.00460195933152
GCGAACCA -0.138630559647
GCGAACCC -0.132800496071
GCGAACGA -0.271200199825
GCGAACGC -0.153567909996
GCGAACTA -0.0881335987099
GCGAAGAA 0.0267592293993
GCGAAGAC -0.0915924860487
GCGAAGCA -0.1313651893
GCGAAGCC -0.0991209008297
GCGAAGGA -0.141700682511
GCGAAGTA 0.0246756642889
GCGAATAA 0.0773041120551
GCGAATAC 0.0980329202563
GCGAATCA 0.346567779989
GCGAATCC 0.471615358912
GCGAATGA 0.0944198854095
GCGAATTA 0.207282267448
GCGACAAA -0.102022896155
GCGACAAC -0.185785241898
GCGACACA 0.0403384267492
GCGACACC -0.216235294118
GCGACAGA -0.0219516345035
GCGACAGC 0.0275829326659
GCGACATA 0.119580012734
GCGACCAA -0.0713986928105
GCGACCAC -0.0985934816132
GCGACCCA -0.236197530864
GCGACCCC -0.266286051233
GCGACCGA -0.0406589727971
GCGACCGC -0.166984942271
GCGACCTA -0.305112460972
GCGACGAA 0.0311110836708
GCGACGAC -0.147887478529
GCGACGCA -0.0536634687462
GCGACGCC -0.326273832169
GCGACGGA -0.107324108961
GCGACGTA 0.0626168370093
GCGACTAA -0.194986362863
GCGACTAC -0.185271604938
GCGACTCA -0.283807335585

GCGACTCC -0.0493265099511
GCGACTGA -0.160315975682
GCGACTTA -0.00788186689616
GCGAGAAA 0.144465301104
GCGAGAAC -0.0419755059347
GCGAGACA -0.0497799020819
GCGAGACC -0.298473278255
GCGAGAGA -0.144227128299
GCGAGAGC -0.0343462906253
GCGAGATA 0.200339640352
GCGAGCAA 0.101785537884
GCGAGCAC -0.25169853631
GCGAGCCA -0.0524844864852
GCGAGCCC -0.290374613207
GCGAGCGA -0.0490070169831
GCGAGCGC -0.197530407018
GCGAGCTA -0.0931457031971
GCGAGGAA -0.0102968778146
GCGAGGAC -0.189156136529
GCGAGGCA -0.175265188042
GCGAGGCC -0.25955265069
GCGAGGGA -0.164647318783
GCGAGGTA -0.0693182379377
GCGAGTAA 0.13278831588
GCGAGTAC -0.110844308089
GCGAGTCA -0.178308187996
GCGAGTCC -0.323122444896
GCGAGTGA -0.116054799297
GCGAGTTA 0.00305812618947
GCGATAAA 0.071283123669
GCGATAAC -0.0574159134687
GCGATACA 0.0174647083361
GCGATACC 0.124557988174
GCGATAGA -0.0817619747692
GCGATAGC -0.277341628401
GCGATATA 0.335230708063
GCGATCAA 0.134233305344
GCGATCAC 0.292855171211
GCGATCCA 0.36273831868
GCGATCCC 0.354273386312
GCGATCGA 0.286441524512
GCGATCGC 0.426950424649
GCGATCTA 0.389209265321
GCGATGAA -0.0576379213542
GCGATGAC -0.0735613684431
GCGATGCA -0.138698207319
GCGATGCC -0.215209876543
GCGATGGA 0.0155644397441
GCGATGTA 0.0133671349498
GCGATTAA -0.139873671488
GCGATTAC 0.414099341885
GCGATTCA 0.172432435399
GCGATTCC 0.404159202531
GCGATTGA 0.000941293991086

GCGATTTA 0.259612395217
GCGCAAAA 0.0191442183143
GCGCAAAC -0.120296274342
GCGCAACA 0.0130097482312
GCGCAACC -0.0980900583674
GCGCAAGA 0.00996179938089
GCGCAAGC -0.130619351015
GCGCAATA 0.13063404246
GCGCACAA 0.128799031716
GCGCACAC -0.170650402489
GCGCACCA -0.0122191489128
GCGCACCC -0.219639117313
GCGCACGA -0.0818437562562
GCGCACGC -0.05116586521
GCGCACTA -0.0405923295122
GCGCAGAA 0.023594166749
GCGCAGAC -0.151709972808
GCGCAGCA -0.182986848386
GCGCAGCC -0.24694641078
GCGCAGGA 0.0317307428446
GCGCAGTA 0.0215125469275
GCGCATAA 0.120328794619
GCGCATAC 0.0301672163061
GCGCATCA 0.0205204138143
GCGCATCC -0.0358465869801
GCGCATGA 0.00339226824343
GCGCATTA -0.133208979475
GCGCCAAA 0.0796908183928
GCGCCAAC -0.183485145984
GCGCCACA -0.0934471378437
GCGCCACC -0.126609454529
GCGCCAGA 0.0857112034791
GCGCCAGC -0.101276795416
GCGCCATA -0.0943780390091
GCGCCCAA -0.147933360838
GCGCCCAC -0.150263365088
GCGCCCCA -0.359326386488
GCGCCCCC -0.251966570255
GCGCCCGA -0.249318277835
GCGCCCGC -0.369638077058
GCGCCCTA -0.177855700426
GCGCCGAA -0.169708570017
GCGCCGAC -0.298454183174
GCGCCGCA -0.0436716620858
GCGCCGCC -0.16093177679
GCGCCGGA 0.00848262310918
GCGCCGTA 0.0106972710707
GCGCCTAA -0.0779051131748
GCGCCTAC 0.0239741816505
GCGCCTCA -0.220079132448
GCGCCTCC -0.291724962828
GCGCCTGA -0.150794826631
GCGCCTTA -0.212135679909
GCGCGAAA 0.13781038003

GCGCGAAC -0.129993032373
GCGCGACA 0.171987676578
GCGCGACC -0.0809055941618
GCGCGAGA -0.0337576017037
GCGCGAGC -0.111791663236
GCGCGATA 0.254865229566
GCGCGCAA 0.257111620131
GCGCGCAC -0.0787837378028
GCGCGCCA -0.11059650856
GCGCGCCC -0.157392641933
GCGCGCGA 0.143584381655
GCGCGCGC 0.087946869445
GCGCGCTA 0.176280559622
GCGCGGAA 0.115294379137
GCGCGGAC -0.283616816431
GCGCGGCA -0.237354814537
GCGCGGCC -0.234910713248
GCGCGGGA 0.0650771118668
GCGCGGTA -0.153508410856
GCGCGTAA -0.0293739921259
GCGCGTAC -0.175724161959
GCGCGTCA -0.206108966034
GCGCGTCC -0.231096586783
GCGCGTGA 0.0201981607659
GCGCGTTA -0.0137653955081
GCGCTAAA 0.0241196294101
GCGCTAAC -0.0662981418796
GCGCTACA -0.0598476462744
GCGCTACC -0.215314470067
GCGCTAGA -0.00578008651972
GCGCTAGC -0.186981430265
GCGCTATA -0.0301002766252
GCGCTCAA -0.300238198983
GCGCTCAC -0.0637029741516
GCGCTCCA -0.18842383852
GCGCTCCC -0.211139380949
GCGCTCGA -0.0244862675361
GCGCTCTA -0.0696008693934
GCGCTGAA -0.00234088563964
GCGCTGAC -0.176658653318
GCGCTGCA -0.173036649215
GCGCTGCC -0.360668734318
GCGCTGGA 0.0922358339371
GCGCTGTA 0.141438341127
GCGCTTAA -0.0579332147797
GCGCTTAC 0.0187810211197
GCGCTTCA -0.0751382989232
GCGCTTCC -0.099287101979
GCGCTTGA -0.13053785746
GCGCTTTA -0.0934908482204
GCGGAAAA 0.172160155739
GCGGAAAC -0.084493272787
GCGGAACA -0.11367690239
GCGGAACC -0.277697421733

GCGGAAGA 0.0419813171008
GCGGAAGC -0.126821437392
GCGGAATA 0.196230275833
GCGGACAA -0.107018395952
GCGGACAC -0.333524997677
GCGGACCA -0.170051183671
GCGGACCC -0.297824792716
GCGGACGA -0.078926725528
GCGGACGC -0.301331319805
GCGGACTA -0.136500726697
GCGGAGAA -0.101184087076
GCGGAGAC -0.170635877443
GCGGAGCA -0.0589521567595
GCGGAGCC -0.0844665042364
GCGGAGGA 0.0341239221884
GCGGAGTA -0.0883688702286
GCGGATAA 0.0847631122527
GCGGATAC 0.307522554589
GCGGATCA 0.108571643999
GCGGATCC 0.357282816308
GCGGATGA -0.127002941306
GCGGATTA 0.334699993414
GCGGCAAA -0.0464659434485
GCGGCAAC -0.14534794215
GCGGCACA -0.246430727023
GCGGCACC -0.361939397929
GCGGCAGA -0.0112686026603
GCGGCAGC -0.279662179213
GCGGCATA 0.0935775069745
GCGGCCAA -0.205400007681
GCGGCCAC -0.149864923747
GCGGCCCA -0.237351790584
GCGGCCCC -0.394944707741
GCGGCCGA -0.174576615832
GCGGCCGC -0.310205446556
GCGGCCTA -0.125733593242
GCGGCGAA -0.10498245614
GCGGCGAC -0.264141276974
GCGGCGCA 0.0232484194409
GCGGCGCC -0.177108367627
GCGGCGGA 0.0160386869224
GCGGCGTA -0.0656182354394
GCGGCTAA -0.113654823449
GCGGCTAC -0.174199018736
GCGGCTCA -0.397654144596
GCGGCTCC -0.29969333705
GCGGCTGA -0.133140721717
GCGGCTTA -0.136452572585
GCGGGAAA -0.00325667604063
GCGGGAAC -0.197123932046
GCGGGACA -0.124224812583
GCGGGACC -0.192496732026
GCGGGAGA -0.173957383012
GCGGGAGC -0.217267083632

GCGGGATA 0.116106412353
GCGGGCAA -0.0513979450701
GCGGGCAC -0.12933972048
GCGGGCCA -0.378184770148
GCGGGCCC -0.284625871613
GCGGGCGA -0.0462834908404
GCGGGCTA -0.207909849042
GCGGGGAA 0.0134340890008
GCGGGGAC -0.09136995375
GCGGGGCA -0.214775464194
GCGGGGCC -0.184214135629
GCGGGGGA 0.0955872985333
GCGGGGTA -0.12976437463
GCGGGTAA -0.0229203714681
GCGGGTAC -0.129026010321
GCGGGTCA -0.20046768337
GCGGGTCC -0.290239176581
GCGGGTGA -0.083977162117
GCGGGTTA 0.0356552833213
GCGGTAAA -0.109306582598
GCGGTAAC -0.149735789422
GCGGTACA -0.0797410599625
GCGGTACC -0.157870039386
GCGGTAGA 0.105511891099
GCGGTAGC -0.228189814815
GCGGTATA 0.0383213681969
GCGGTCAA -0.211419573756
GCGGTCAC -0.188571584509
GCGGTCCA -0.307551323907
GCGGTCCC -0.348881866263
GCGGTCGA -0.168576320295
GCGGTCTA -0.219046096498
GCGGTGAA 0.11034278376
GCGGTGAC -0.0605249871031
GCGGTGCA -0.0782013926614
GCGGTGCC -0.288307270233
GCGGTGGA -0.0318855850813
GCGGTGTA 0.0788037714468
GCGGTTAA 0.137412456915
GCGGTTAC -0.142356078276
GCGGTTCA 0.0501657555599
GCGGTTCC -0.0827290689059
GCGGTTGA -0.0778290375811
GCGGTTTA -0.153931869814
GCGTAAAA 0.249367309285
GCGTAAAC -0.109506017165
GCGTAACA -0.0876963350785
GCGTAACC -0.0925367973921
GCGTAAGA 0.0253817209769
GCGTAAGC -0.173122895892
GCGTAATA 0.200887637304
GCGTACAA -0.109887335847
GCGTACAC 0.0435968212921
GCGTACCA -0.130322640611

GCGTACCC -0.121004128426
GCGTACGA 0.0675039588569
GCGTACGC 0.00544006057314
GCGTACTA -0.0234991951932
GCGTAGAA 0.0115315374651
GCGTAGAC -0.198498142569
GCGTAGCA 0.000592709671046
GCGTAGCC -0.0983670623102
GCGTAGGA -0.0137593886653
GCGTAGTA -0.163403858291
GCGTATAA -0.0405325297634
GCGTATAC -0.163532404663
GCGTATCA 0.319458165931
GCGTATCC 0.425692618366
GCGTATGA 0.158701101452
GCGTATTA 0.0193253419394
GCGTCAAA -0.0242321456014
GCGTCAAC -0.190894670788
GCGTCACA -0.0109244527605
GCGTCACC -0.14758933667
GCGTCAGA -0.0100576758241
GCGTCAGC -0.176929557008
GCGTCATA 0.0128920721166
GCGTCCAA -0.192087558716
GCGTCCAC -0.278609414437
GCGTCCCA -0.260908093278
GCGTCCCC -0.176450819987
GCGTCCGA -0.253959471509
GCGTCCTA -0.142642373802
GCGTCGAA -0.0260011155786
GCGTCGAC -0.221749187784
GCGTCGCA -0.0127737843002
GCGTCGCC -0.302230781048
GCGTCGGA -0.232166861444
GCGTCGTA 0.0322728844539
GCGTCTAA -0.0747697534523
GCGTCTAC -0.155934503006
GCGTCTCA -0.288233551542
GCGTCTCC -0.185675330162
GCGTCTGA -0.265838724014
GCGTCTTA -0.121307551073
GCGTGAAA 0.0141690758793
GCGTGAAC -0.0885716794538
GCGTGACA -0.248981068056
GCGTGACC -0.00682203433896
GCGTGAGA -0.123389943777
GCGTGAGC -0.0698040958864
GCGTGATA 0.185827719524
GCGTGCAA -0.0315821822712
GCGTGCAC -0.0193608929718
GCGTGCCA -0.233405483405
GCGTGCCC -0.21818675621
GCGTGCGA -0.00516467392094
GCGTGCTA -0.0701004360849

GCGTGGAA -0.115130948161
GCGTGGAC -0.231697280927
GCGTGGCA -0.0389478883675
GCGTGGCC -0.389854356949
GCGTGGGA -0.109873939204
GCGTGGTA -0.138439979617
GCGTGTAA 0.122681068739
GCGTGTAC -0.0641715439324
GCGTGTCA -0.163465840747
GCGTGTCC -0.269413307436
GCGTGTGA -0.1124473199
GCGTGTTA -0.123630780751
GCGTTAAA -0.193868953716
GCGTTAAC 0.178656949641
GCGTTACA 0.0462368423941
GCGTTACC -0.141914028416
GCGTTAGA -0.115978532859
GCGTTAGC -0.164259382453
GCGTTATA 0.116882368438
GCGTTCAA -0.118702331962
GCGTTCAC -0.18762243403
GCGTTCCA -0.182603184011
GCGTTCCC -0.0264349948426
GCGTTCGA -0.0200703151105
GCGTTCTA -0.112058721844
GCGTTGAA 0.0630302830737
GCGTTGAC -0.0929959445295
GCGTTGCA -0.261957666733
GCGTTGCC -0.190987654321
GCGTTGGA 0.0360163487313
GCGTTGTA -0.0700684778282
GCGTTTAA -0.147021050949
GCGTTTAC 0.0595920561359
GCGTTTCA 0.00669059693956
GCGTTTCC -0.282287909186
GCGTTTGA -0.0506083205742
GCGTTTTA -0.0139688086536
GCTAAAAA 0.183069331928
GCTAAAAC -0.128439700653
GCTAAACA -0.0113408231866
GCTAAACC -0.134035185917
GCTAAAGA 0.132886741876
GCTAAAGC -0.129909506484
GCTAAATA 0.238831096909
GCTAACAA -0.0397331571668
GCTAACAC -0.082194449933
GCTAACCA 0.00100094634928
GCTAACCC -0.227130235707
GCTAACGA 0.0358781398457
GCTAACTA -0.101800630028
GCTAAGAA -0.00388048370197
GCTAAGAC -0.210326185835
GCTAAGCA -0.158972222222
GCTAAGCC -0.268603827073

GCTAAGGA -0.113756613757
GCTAAGTA -0.0294233864641
GCTAATAA 0.0132247689302
GCTAATAC 0.0292679312389
GCTAATCA -0.0594321889372
GCTAATCC 0.0516929570353
GCTAATGA -0.0778293603758
GCTAATTA 0.0495921974138
GCTACAAA 0.165933491203
GCTACAAC -0.103912854031
GCTACACA -0.124464336038
GCTACACC -0.168760151894
GCTACAGA 0.0112280528422
GCTACAGC -0.0473816658983
GCTACATA -0.00242870315484
GCTACCAA -0.0636622666206
GCTACCAC -0.105453283096
GCTACCCA -0.220276734601
GCTACCCC -0.227227595385
GCTACCGA 0.0878107311949
GCTACCTA -0.203927281812
GCTACGAA 0.0178653757492
GCTACGAC -0.21168627451
GCTACGCA 0.00718731475993
GCTACGCC -0.104304486649
GCTACGGA -0.0937669778239
GCTACGTA -0.0875576815588
GCTACTAA -0.137848580112
GCTACTAC -0.129489711934
GCTACTCA -0.193729499505
GCTACTCC -0.124216736919
GCTACTGA -0.0633443933733
GCTACTTA -0.0838686341332
GCTAGAAA 0.0711606351605
GCTAGAAC 0.0989456409581
GCTAGACA -0.0925869977602
GCTAGACC -0.0808602044343
GCTAGAGA 0.164899856762
GCTAGAGC -0.208351165981
GCTAGATA 0.110652813454
GCTAGCAA -0.0683924555771
GCTAGCAC -0.0510094261635
GCTAGCCA -0.278263786629
GCTAGCCC -0.0902582446435
GCTAGCGA 0.0827734966739
GCTAGCTA -0.13083694155
GCTAGGAA -0.0618829572726
GCTAGGAC -0.146899862826
GCTAGGCA -0.251651659067
GCTAGGCC -0.236244862572
GCTAGGGA -0.0705289672544
GCTAGGTA -0.162253836528
GCTAGTAA -0.0830081501605
GCTAGTAC -0.159161689172

GCTAGTCA -0.117142989956
GCTAGTCC -0.217597097602
GCTAGTGA -0.139027441684
GCTAGTTA -0.0329243087962
GCTATAAA 0.0367948513068
GCTATAAC -0.0963996597239
GCTATACA -0.0962360843191
GCTATACC -0.0336856663045
GCTATAGA -0.0317385840426
GCTATAGC -0.257385774614
GCTATATA 0.17408605869
GCTATCAA -0.0281125820545
GCTATCAC 0.000103148199265
GCTATCCA 0.0738655074755
GCTATCCC 0.0642851654262
GCTATCGA -0.153231250515
GCTATCTA 0.0803019627318
GCTATGAA -0.0381517586042
GCTATGAC -0.165811565952
GCTATGCA -0.128925433903
GCTATGCC -0.29727492419
GCTATGGA 0.248567335244
GCTATGTA 0.0215578094572
GCTATTAA -0.223349849924
GCTATTAC 0.076572283643
GCTATTCA -0.0695378670115
GCTATTCC -0.0245367626073
GCTATTGA 0.00929060189153
GCTATTTA 0.0178940333851
GCTCAAAA -0.162624631046
GCTCAAAC -0.159204937168
GCTCAACA -0.170389803573
GCTCAACC -0.152947890601
GCTCAAGA -0.126676336715
GCTCAAGC -0.355109551598
GCTCAATA 0.017448242552
GCTCACAA -0.180717176417
GCTCACAC -0.167026870007
GCTCACCA -0.161552775821
GCTCACCC -0.252532792694
GCTCACGA -0.144899317344
GCTCACTA -0.120409697528
GCTCAGAA -0.0535257220387
GCTCAGAC -0.174637275561
GCTCAGCA -0.109278373143
GCTCAGCC -0.221163289604
GCTCAGGA -0.0465430825331
GCTCAGTA -0.07860263207
GCTCATAA -0.14192116828
GCTCATAC 0.0260874576048
GCTCATCA -0.0361197535132
GCTCATCC -0.17825971557
GCTCATGA -0.196646416753
GCTCATTA -0.195914774444

GCTCCAAA -0.110293391287
GCTCCAAC -0.207934861927
GCTCCACA -0.081178007167
GCTCCACC -0.23981170584
GCTCCAGA -0.282049122236
GCTCCAGC -0.307136783785
GCTCCATA -0.214520996784
GCTCCCAA -0.133047578448
GCTCCCAC -0.181554282453
GCTCCCCA -0.160749555599
GCTCCCCC -0.226800096427
GCTCCCGA -0.208767026985
GCTCCCTA -0.378395457912
GCTCCGAA -0.140206606307
GCTCCGAC -0.162954549196
GCTCCGCA -0.18645346413
GCTCCGCC -0.182041911535
GCTCCGGA -0.176315139541
GCTCCGTA -0.0695589905674
GCTCCTAA -0.00139273090518
GCTCCTAC -0.17157564789
GCTCCTCA -0.28152152757
GCTCCTCC -0.340191771036
GCTCCTGA -0.166032984397
GCTCCTTA -0.0680887615535
GCTCGAAA 0.0748667063763
GCTCGAAC -0.29387656702
GCTCGACA -0.148944444444
GCTCGACC -0.31916399243
GCTCGAGA -0.171352909218
GCTCGAGC 0.0759401331472
GCTCGATA 0.0350858416211
GCTCGCAA -0.23976672727
GCTCGCAC -0.18256045566
GCTCGCCA -0.0810610551274
GCTCGCCC -0.246612143426
GCTCGCGA -0.0524730031143
GCTCGCTA -0.324109739369
GCTCGGAA -0.0145903854256
GCTCGGAC -0.285088441196
GCTCGGCA -0.143339506173
GCTCGGCC -0.0205327413984
GCTCGGGA -0.185142636384
GCTCGGTA -0.178663761801
GCTCGTAA 0.0737667127709
GCTCGTAC -0.0397265331486
GCTCGTCA -0.216408204926
GCTCGTCC -0.319685125316
GCTCGTGA -0.149775275349
GCTCGTTA -0.116662277309
GCTCTAAA -0.130164600415
GCTCTAAC -0.170499124872
GCTCTACA -0.139908889915
GCTCTACC -0.220385802469

GCTCTAGA 0.00969830107499
GCTCTATA -0.0696433863479
GCTCTCAA -0.222675381264
GCTCTCAC -0.202721546936
GCTCTCCA -0.304812291557
GCTCTCCC -0.374617340641
GCTCTCGA -0.217598766347
GCTCTCTA -0.140914337564
GCTCTGAA -0.301386283094
GCTCTGAC -0.21805604371
GCTCTGCA -0.184878525515
GCTCTGCC -0.331056964874
GCTCTGGA 0.0124394942211
GCTCTGTA 0.143089797045
GCTCTTAA -0.163286575126
GCTCTTAC -0.0175779093619
GCTCTTCA -0.113072896089
GCTCTTCC -0.233057188009
GCTCTTGA -0.0800443470508
GCTCTTTA -0.0928377013916
GCTGAAAA -0.0291497909052
GCTGAAAC -0.132923161856
GCTGAACA -0.0798142934756
GCTGAACC -0.275162938824
GCTGAAGA 0.0573420048846
GCTGAAGC -0.23170462436
GCTGAATA 0.106698280972
GCTGACAA -0.0651381904073
GCTGACAC -0.174789096723
GCTGACCA -0.147526553095
GCTGACCC -0.254484125025
GCTGACGA -0.0954131825604
GCTGACTA -0.181925925926
GCTGAGAA -0.0960675854696
GCTGAGAC -0.0940046422046
GCTGAGCA -0.00426897118314
GCTGAGCC -0.0715895061728
GCTGAGGA -0.12704621579
GCTGAGTA 0.0530544942106
GCTGATAA 0.067732002898
GCTGATAC 0.195092098619
GCTGATCA -0.0153865151889
GCTGATCC -0.00689951101452
GCTGATGA -0.0372602288631
GCTGATTA 0.196958220817
GCTGCAAA 0.02102334636
GCTGCAAC -0.136041940854
GCTGCACA -0.0943934105975
GCTGCACC -0.31337945591
GCTGCAGA -0.0481682720013
GCTGCAGC -0.18261704725
GCTGCATA -0.0656385745209
GCTGCCAA -0.142375254952
GCTGCCAC -0.226885568786

GCTGCCCA -0.115802469136
GCTGCCCC -0.264441565684
GCTGCCGA -0.203695473251
GCTGCCTA -0.0860855916669
GCTGCGAA -0.0181552300113
GCTGCGAC -0.395207184546
GCTGCGCA -0.0786204682406
GCTGCGCC -0.105777067075
GCTGCGGA -0.0360669034039
GCTGCGTA -0.120500393898
GCTGCTAA -0.127855764985
GCTGCTAC -0.307015233329
GCTGCTCA -0.298201484821
GCTGCTCC -0.213306456298
GCTGCTGA -0.0227968780873
GCTGCTTA -0.232814324979
GCTGGAAA 0.11023514578
GCTGGAAC -0.0321446798703
GCTGGACA -0.155026870007
GCTGGACC -0.219083663487
GCTGGAGA 0.0489390546444
GCTGGATA 0.192301148214
GCTGGCAA -0.077601062487
GCTGGCAC -0.372639821305
GCTGGCCA -0.281749950622
GCTGGCCC -0.355932006528
GCTGGCGA -0.142854914845
GCTGGCTA -0.213537637771
GCTGGGAA -0.0279562113777
GCTGGGAC -0.343269541942
GCTGGGCA -0.162519132968
GCTGGGCC -0.242247679242
GCTGGGGA 0.103504670333
GCTGGGTA 0.115948745515
GCTGGTAA -0.0489942457769
GCTGGTAC -0.274805568841
GCTGGTCA -0.327338766832
GCTGGTCC -0.285604165295
GCTGGTGA -0.102400199765
GCTGGTTA 0.0043162436622
GCTGTAAA -0.0933401514315
GCTGTAAC -0.0893539436026
GCTGTACA -0.127850563028
GCTGTACC -0.153886254884
GCTGTAGA -0.15272152524
GCTGTATA 0.025906166985
GCTGTCAA -0.133174736449
GCTGTCAC -0.110057722531
GCTGTCCA -0.294842465039
GCTGTCCC -0.230540539755
GCTGTCGA -0.0736990275362
GCTGTCTA -0.286986906159
GCTGTGAA 0.0815193527849
GCTGTGAC -0.183117577335

GCTGTGCA 0.0178866248762
GCTGTGCC -0.209629444588
GCTGTGGA -0.0776391560279
GCTGTGTA -0.0784492903113
GCTGTTAA -0.0943097260635
GCTGTTAC -0.068495393957
GCTGTTCA -0.0935905034739
GCTGTTCC -0.275890862937
GCTGTTGA -0.116694168778
GCTGTTTA -0.0393062391409
GCTTAAAA 0.0634399218424
GCTTAAAC -0.119864122531
GCTTAACA -0.0109524907243
GCTTAACC -0.167350210872
GCTTAAGA -0.033035026804
GCTTAAGC -0.277897713247
GCTTAATA -0.0523908862172
GCTTACAA -0.0823206876111
GCTTACAC 0.103919631572
GCTTACCA -0.320204892044
GCTTACCC -0.270587472842
GCTTACGA -0.0598924890513
GCTTACTA -0.223490272566
GCTTAGAA 0.0564904614419
GCTTAGAC -0.251663498099
GCTTAGCA -0.136391103261
GCTTAGCC -0.19472421296
GCTTAGGA 0.0471968387256
GCTTAGTA -0.121201337466
GCTTATAA 0.0219348078738
GCTTATAC 0.0290830945559
GCTTATCA 0.0231153661761
GCTTATCC -0.110064281502
GCTTATGA -0.0496781372518
GCTTATTA -0.121752970555
GCTTCAAA 0.164421367184
GCTTCAAC -0.247198902606
GCTTCACA -0.129413830184
GCTTCACC -0.126467150641
GCTTCAGA -0.070248523568
GCTTCATA -0.0216306132233
GCTTCCAA 0.0312295260974
GCTTCCAC -0.2558066924
GCTTCCCA -0.323870619088
GCTTCCCC -0.189706447188
GCTTCCGA -0.088306144062
GCTTCCTA -0.224496682964
GCTTCGAA -0.0797053722365
GCTTCGAC -0.158012530972
GCTTCGCA -0.0663624003533
GCTTCGCC -0.123732385092
GCTTCGGA 0.000383518022442
GCTTCGTA -0.0712560982971
GCTTCTAA -0.134215327937

GCTTCTAC -0.192645315818
GCTTCTCA -0.167109826907
GCTTCTCC -0.241911401598
GCTTCTGA -0.127266341806
GCTTCTTA -0.161039941903
GCTTGAAA -0.113752509449
GCTTGAAC -0.246811026253
GCTTGACA -0.0147385701626
GCTTGACC -0.36916810664
GCTTGAGA -0.171619636597
GCTTGATA 0.149016718259
GCTTGCAA -0.105654417595
GCTTGCAC -0.178829805161
GCTTGCCA -0.108594590441
GCTTGCCC -0.142034138232
GCTTGCGA 0.00288382915439
GCTTGCTA -0.13683900149
GCTTGGAA 0.0583165051647
GCTTGGAC -0.34576922022
GCTTGGCA -0.269740263136
GCTTGGCC -0.339653384684
GCTTGGGA -0.12265900648
GCTTGGTA -0.111115145846
GCTTGTAA 0.070081211047
GCTTGTAC -0.250887473321
GCTTGTCA -0.193828874893
GCTTGTCC -0.235679012346
GCTTGTGA 0.119853849171
GCTTGTTA -0.212811606609
GCTTTAAA 0.0855342598081
GCTTTAAC -0.0946469535821
GCTTTACA 0.061520941612
GCTTTACC -0.0121366550308
GCTTTAGA -0.0877094874692
GCTTTATA -0.169921915615
GCTTTCAA -0.15441031213
GCTTTCAC -0.0494632154383
GCTTTCCA -0.233222948439
GCTTTCCC -0.0609467682903
GCTTTCGA -0.187698850049
GCTTTCTA -0.107647029771
GCTTTGAA 0.0480859471474
GCTTTGAC -0.0277498532753
GCTTTGCA -0.162717478343
GCTTTGCC -0.360740813886
GCTTTGGA -0.0350065578988
GCTTTGTA 0.0468848708583
GCTTTTAA 0.0419054205361
GCTTTTAC 0.0494247952754
GCTTTTCA -0.0677345125544
GCTTTTCC -0.0896733446441
GCTTTTGA -0.0110302835688
GCTTTTTA -0.0206508375596
GGAAAAAA 0.106400999521

GGAAAAAC	0.0192596574318
GGAAAACA	-0.0881045557948
GGAAAACC	-0.20601598996
GGAAAAGA	0.108934883312
GGAAAATA	0.262212892072
GGAAACAA	0.080823151686
GGAAACAC	-0.159309568892
GGAAACCA	-0.0134484435165
GGAAACCC	-0.100181114679
GGAAACGA	-0.0843650872052
GGAAACTA	-0.0241352258364
GGAAAGAA	-0.0142736768701
GGAAAGAC	-0.0647175046853
GGAAAGCA	-0.109868128612
GGAAAGCC	-0.316323863405
GGAAAGGA	0.118560864702
GGAAAGTA	-0.115225255328
GGAAATAA	0.0674465959736
GGAAATAC	0.129264638082
GGAAATCA	0.448565368355
GGAAATCC	0.489656743287
GGAAATGA	0.0544552010274
GGAAATTA	0.186925069705
GGAACAAA	0.0365967351576
GGAACAAC	-0.143555218057
GGAACACA	-0.190403700002
GGAACACC	-0.216090534979
GGAACAGA	0.0096196591751
GGAACATA	-0.0751717930141
GGAACCAA	-0.0947786668423
GGAACCAC	-0.350492858346
GGAACCCA	-0.202589684068
GGAACCCC	-0.329083240624
GGAACCGA	-0.0441169206631
GGAACCTA	-0.159135802469
GGAACGAA	-0.0314918539522
GGAACGAC	-0.0476497678783
GGAACGCA	-0.0837360751843
GGAACGCC	-0.268469595466
GGAACGGA	-0.0706299359978
GGAACGTA	-0.107670764869
GGAACTAA	-0.122916495157
GGAACTAC	-0.140016572281
GGAACTCA	-0.0986949328329
GGAACTCC	-0.323110587131
GGAACTGA	-0.128516469646
GGAACTTA	0.0124808604169
GGAAGAAA	0.0197683607448
GGAAGAAC	-0.220378509776
GGAAGACA	-0.230582444357
GGAAGACC	-0.401252207044
GGAAGAGA	-0.112808766155
GGAAGATA	0.212866912556
GGAAGCAA	-0.149327299555

GGAAGCAC -0.0526358024691
GGAAGCCA -0.28127940886
GGAAGCCC -0.354417936022
GGAAGCGA 0.0280941820044
GGAAGCTA 0.017440762425
GGAAGGAA -0.158725269241
GGAAGGAC -0.271076252723
GGAAGGCA -0.240256106393
GGAAGGCC -0.293768342522
GGAAGGGA -0.186268053909
GGAAGGTA -0.0408074127227
GGAAGTAA 0.00172616303322
GGAAGTAC -0.101478941787
GGAAGTCA -0.312219121932
GGAAGTCC -0.392621664911
GGAAGTGA -0.190223385517
GGAAGTTA 0.050756177996
GGAATAAA 0.135341548948
GGAATAAC -0.141398747694
GGAATACA 0.142196330249
GGAATACC -0.0528743480598
GGAATAGA 0.0858759606584
GGAATATA 0.329067453111
GGAATCAA 0.378122412215
GGAATCAC 0.372950009879
GGAATCCA 0.469211728029
GGAATCCC 0.43843069458
GGAATCGA 0.336006278952
GGAATCTA 0.482694112417
GGAATGAA -0.046318706951
GGAATGAC -0.269185185185
GGAATGCA -0.0132168445764
GGAATGCC -0.22260080435
GGAATGGA -0.082427033545
GGAATGTA -0.0433132450767
GGAATTAA 0.00927846934071
GGAATTAC 0.262761780105
GGAATTCA -0.0487827842537
GGAATTCC 0.171731187043
GGAATTGA 0.174301570468
GGAATTTA 0.242066334091
GGACAAAA -0.00778841544027
GGACAAAC -0.0126347449999
GGACAACA -0.0878353531362
GGACAACC -0.194788734713
GGACAAGA -0.0464600420106
GGACAATA -0.0525844611281
GGACACAA 0.0100523611934
GGACACAC -0.327325696228
GGACACCA -0.134848467241
GGACACCC -0.298711914245
GGACACGA -0.0298766579983
GGACACTA -0.012084578979
GGACAGAA 0.0374139019237

GGACAGAC -0.248192078253
GGACAGCA -0.0999354992417
GGACAGCC -0.215754458162
GGACAGGA -0.148854119158
GGACAGTA -0.135508916324
GGACATAA 0.0885665305885
GGACATAC -0.198251592829
GGACATCA -0.158477683374
GGACATCC 0.0132534443355
GGACATGA -0.117461846199
GGACATTA -0.0838409781905
GGACCAAA -0.0411384416126
GGACCAAC -0.265809729148
GGACCACA -0.000704492635322
GGACCACC -0.151804905253
GGACCAGA -0.113746698857
GGACCATA 0.133925588017
GGACCCAA -0.239203680682
GGACCCAC -0.265526263823
GGACCCCA -0.187388899862
GGACCCCC -0.211731656599
GGACCCGA -0.243808278867
GGACCCTA -0.0952239838001
GGACCGAA -0.0397693599368
GGACCGAC -0.159178588872
GGACCGCA -0.30263526081
GGACCGCC -0.370540087568
GGACCGGA -0.0733313166733
GGACCGTA -0.0702424709078
GGACCTAA -0.13856415739
GGACCTAC -0.217593022986
GGACCTCA -0.0959089390263
GGACCTCC -0.244804956075
GGACCTGA -0.252624027788
GGACCTTA -0.339875897031
GGACGAAA -0.0225142154603
GGACGAAC -0.279592428882
GGACGACA -0.143408574533
GGACGACC -0.137585588831
GGACGAGA -0.104623259814
GGACGATA -0.0136446464247
GGACGCAA -0.0941655891796
GGACGCAC -0.203002216954
GGACGCCA -0.25464682935
GGACGCCC -0.334327943718
GGACGCGA 0.0243328229697
GGACGCTA -0.28105970837
GGACGGAA 0.178781979574
GGACGGAC -0.214773707616
GGACGGCA -0.290802234092
GGACGGCC -0.281926298636
GGACGGGA -0.227328976035
GGACGGTA 0.0298446538194
GGACGTAA -0.0173961009023

GGACGTAC -0.0760661387421
GGACGTCA -0.243110759997
GGACGTCC -0.221495926932
GGACGTGA -0.0700510460518
GGACGTTA -0.0531076423193
GGACTAAA -0.0798978124623
GGACTAAC -0.090488340192
GGACTACA -0.166093193505
GGACTACC -0.0879389685546
GGACTAGA 0.0541681072805
GGACTATA -0.22511641824
GGACTCAA 0.0471261020704
GGACTCAC -0.116415395788
GGACTCCA -0.388033455792
GGACTCCC -0.235221496006
GGACTCGA -0.220732510288
GGACTCTA -0.341345483691
GGACTGAA 0.0227143957114
GGACTGAC -0.172002904866
GGACTGCA -0.267009602195
GGACTGCC -0.243820606938
GGACTGGA -0.196587105624
GGACTGTA -0.172827167022
GGACTTAA -0.129920239036
GGACTTAC -0.0856872236201
GGACTTCA -0.165366126696
GGACTTGA 0.0199458426065
GGACTTTA -0.153878655187
GGAGAAAA -0.0641553338382
GGAGAAAC -0.192973129993
GGAGAACA -0.205867825743
GGAGAACC -0.117225770087
GGAGAAGA 0.0262178758993
GGAGAATA 0.198025656299
GGAGACAA -0.151615168925
GGAGACAC -0.134558815459
GGAGACCA -0.220573469623
GGAGACCC -0.2098296755
GGAGACGA -0.0807883746838
GGAGACTA -0.181310088239
GGAGAGAA -0.0208054276292
GGAGAGAC -0.179997095134
GGAGAGCA -0.2632072677
GGAGAGCC -0.464867103257
GGAGAGGA -0.0995061728395
GGAGAGTA -0.158326797386
GGAGATAA 0.113888340029
GGAGATAC 0.249728341401
GGAGATCA 0.238413576588
GGAGATCC 0.401199619678
GGAGATGA -0.195443171342
GGAGATTA 0.29783824619
GGAGCAAA 0.0227812241222
GGAGCAAC -0.188252723312

GGAGCACA	-0.190458739441
GGAGCACC	-0.392182837458
GGAGCAGA	-0.124285181161
GGAGCATA	-0.223548076448
GGAGCCAA	-0.260395487446
GGAGCCAC	-0.123211669218
GGAGCCCA	-0.352332757344
GGAGCCCC	-0.307552614831
GGAGCCGA	-0.205368191721
GGAGCCTA	-0.246997404792
GGAGCGAA	-0.0177632821466
GGAGCGAC	-0.202896298512
GGAGCGCA	-0.111013688555
GGAGCGCC	-0.266288721473
GGAGCGGA	0.0602896868517
GGAGCGTA	-0.138398991381
GGAGCTAA	-0.131877562108
GGAGCTAC	-0.228083144768
GGAGCTCA	-0.253215368707
GGAGCTCC	-0.299235130149
GGAGCTGA	-0.147491691264
GGAGCTTA	-0.063673941584
GGAGGAAA	-0.0156828846629
GGAGGAAC	-0.28826666202
GGAGGACA	-0.428251711427
GGAGGACC	-0.242464393091
GGAGGAGA	0.0712160697736
GGAGGATA	0.18576409571
GGAGGCAA	-0.0559040641036
GGAGGCAC	-0.290041272027
GGAGGCCA	-0.0759047483494
GGAGGCCC	-0.355358073452
GGAGGCGA	-0.0682207095314
GGAGGCTA	-0.289929612623
GGAGGGAA	0.0705880173862
GGAGGGAC	-0.258849666749
GGAGGGCA	-0.229796982167
GGAGGGCC	-0.327900155372
GGAGGGGA	-0.22832312858
GGAGGGTA	-0.217867236767
GGAGGTAA	0.0860433269666
GGAGGTAC	-0.0215348819072
GGAGGTCA	-0.162380264652
GGAGGTGA	-0.0394964318675
GGAGGTTA	-0.180929922465
GGAGTAAA	-0.0895310227491
GGAGTAAC	-0.141575290592
GGAGTACA	-0.111267851176
GGAGTACC	-0.267403285933
GGAGTAGA	0.0794197550593
GGAGTATA	0.0104789853704
GGAGTCAA	-0.160040497827
GGAGTCAC	-0.146693115161
GGAGTCCA	-0.31866690679

GGAGTCCC	-0.148508279911
GGAGTCGA	-0.163548469566
GGAGTCTA	-0.0435968212921
GGAGTGAA	-0.0854058152472
GGAGTGAC	-0.370502259762
GGAGTGCA	-0.280875171468
GGAGTGCC	-0.319823674956
GGAGTGGA	0.0518755138742
GGAGTGTA	-0.0670546396964
GGAGTTAA	-0.102741417634
GGAGTTAC	-0.0652805499144
GGAGTTCA	-0.109009187
GGAGTTGA	-0.171828612927
GGAGTTTA	-0.0932820426402
GGATAAAA	0.0450582831177
GGATAAAC	-0.0570594305034
GGATAACA	0.126468199082
GGATAACC	0.0465369702657
GGATAAGA	0.182407956267
GGATAATA	0.125426010076
GGATACAA	0.0657867592579
GGATACAC	0.208775165207
GGATACCA	0.21810614095
GGATACCC	0.355584096248
GGATACGA	0.279037328043
GGATACTA	0.224536356947
GGATAGAA	-0.0250664119959
GGATAGAC	-0.1770521262
GGATAGCA	0.00454578472535
GGATAGCC	0.0182543256868
GGATAGGA	0.200434653759
GGATAGTA	0.0625260708248
GGATATAA	0.198693940406
GGATATAC	0.284705010678
GGATATCA	0.422289731937
GGATATCC	0.486788531206
GGATATGA	0.326244813278
GGATATTA	0.340166780469
GGATCAAA	0.0168131564802
GGATCAAC	0.00711321873148
GGATCACA	0.177539572768
GGATCACC	0.131726061627
GGATCAGA	0.165494851698
GGATCATA	0.100240949307
GGATCCAA	0.153803654025
GGATCCAC	0.22148949483
GGATCCCA	0.179947693693
GGATCCCC	0.389128760914
GGATCCGA	0.280533052317
GGATCCTA	0.265107356912
GGATCGAA	0.122303255706
GGATCGAC	-0.194929379229
GGATCGCA	0.333556578966
GGATCGCC	0.325447096596

GGATCGGA 0.182764081182
GGATCGTA 0.236994774858
GGATCTAA 0.102532437595
GGATCTAC 0.250474336437
GGATCTCA 0.337746429765
GGATCTGA 0.254149377593
GGATCTTA 0.265804408439
GGATGAAA 0.170387750062
GGATGAAC -0.224567382302
GGATGACA -0.174853790976
GGATGACC -0.177174062183
GGATGAGA 0.00757082761987
GGATGATA 0.0250039951767
GGATGCAA -0.0478389725858
GGATGCAC -0.0655310764232
GGATGCCA -0.00836612282752
GGATGCCC -0.0547633781621
GGATGCGA -0.0423252980307
GGATGCTA 0.0418115683079
GGATGGAA 0.0402129469554
GGATGGAC -0.226406035665
GGATGGCA -0.180021961869
GGATGGCC -0.343995150478
GGATGGGA 0.0320177171837
GGATGGTA -0.0597489576221
GGATGTAA 0.161961559136
GGATGTAC 0.0325583437617
GGATGTCA 0.224274693824
GGATGTGA 0.162669616788
GGATGTTA -0.012815868625
GGATTAAA -0.176029731331
GGATTAAC -0.0256742738589
GGATTACA 0.466854563864
GGATTACC 0.452168291372
GGATTAGA 0.208912380074
GGATTATA 0.247179405227
GGATTCAA 0.0240208347055
GGATTCAC 0.238257151639
GGATTCCA 0.379338734111
GGATTCCC 0.479565961931
GGATTCGA 0.330456321822
GGATTCTA 0.386550747547
GGATTGAA -0.140296713429
GGATTGAC 0.129843243793
GGATTGCA 0.460405619398
GGATTGCC 0.434011656624
GGATTGGA 0.163271977104
GGATTGTA 0.3009443145
GGATTTAA 0.131118794764
GGATTTAC 0.173557597313
GGATTTCA 0.445191986402
GGATTTGA 0.30601659751
GGATTTTA 0.39783529825
GGCAAAAA -0.25007771667

GGCAAAAC -0.128952124167
GGCAAACA -0.168499636892
GGCAAACC -0.120267022257
GGCAAAGA 0.0380849301934
GGCAAATA 0.236618506202
GGCAACAA -0.124976392137
GGCAACAC -0.0682759622367
GGCAACCA -0.240895297985
GGCAACCC -0.186181554103
GGCAACGA -0.108435817652
GGCAACTA -0.12972702047
GGCAAGAA -0.0393603125995
GGCAAGAC -0.0582092910931
GGCAAGCA -0.023648426182
GGCAAGCC -0.244867173612
GGCAAGGA -0.0547597486348
GGCAAGTA -0.102567119602
GGCAATAA 0.06915903752
GGCAATAC -0.151713262804
GGCAATCA 0.316798941799
GGCAATGA -0.215683853669
GGCAATTA -0.0876793564161
GGCACAAA -0.188732792345
GGCACAAC -0.172414833838
GGCACACA -0.213106074323
GGCACACC -0.288324219907
GGCACAGA -0.0297372060858
GGCACATA -0.0940455992749
GGCACCAA -0.088656562047
GGCACCAC -0.146159122085
GGCACCCA -0.368158426054
GGCACCCC -0.411661628964
GGCACCGA -0.30927654065
GGCACCTA -0.181830778771
GGCACGAA -0.292704778776
GGCACGAC -0.342202026715
GGCACGCA -0.135457096707
GGCACGCC -0.203301688805
GGCACGGA -0.183014281874
GGCACGTA 0.032540768567
GGCACTAA -0.247086707486
GGCACTAC -0.0930509157557
GGCACTCA -0.28270442992
GGCACTGA -0.202595336077
GGCACTTA -0.233022252946
GGCAGAAA 0.0730594337019
GGCAGAAC -0.0722241430406
GGCAGACA -0.19191345526
GGCAGACC -0.242263491828
GGCAGAGA 0.0863553818663
GGCAGATA 0.227504815766
GGCAGCAA 0.0639558497442
GGCAGCAC -0.222224965842
GGCAGCCA -0.123528733929

GGCAGCCC -0.194308178019
GGCAGCGA 0.0432123157981
GGCAGCTA -0.323105406073
GGCAGGAA -0.0568864740265
GGCAGGAC -0.379771407995
GGCAGGCA -0.0808006414894
GGCAGGCC -0.243952666984
GGCAGGGA -0.100527601765
GGCAGGTA -0.250454024218
GGCAGTAA 0.0897491029012
GGCAGTAC -0.0212727422409
GGCAGTCA -0.0508896819125
GGCAGTGA -0.244049382716
GGCAGTTA -0.0540910358612
GGCATAAA -0.0327793354932
GGCATAAC -0.141960784314
GGCATACA 0.0917500933061
GGCATACC -0.157261827095
GGCATAGA -0.0924269800211
GGCATATA -0.141253772291
GGCATCAA -0.164938356954
GGCATCAC -0.0372593251224
GGCATCCA 0.0839757093255
GGCATCCC -0.00328992498203
GGCATCGA -0.08257041867
GGCATCTA 0.0454912614589
GGCATGAA 0.164434210335
GGCATGAC -0.374444554162
GGCATGCA -0.0964505029552
GGCATGCC -0.233935624013
GGCATGGA 0.00631874464862
GGCATGTA -0.124793199664
GGCATTAA -0.043252870535
GGCATTAC -0.147855923786
GGCATTCA -0.0636308166141
GGCATTGA 0.0897451230705
GGCATTTA 0.02329841791
GGCCAAAA 0.00654014445846
GGCCAAAC -0.147064633261
GGCCAACA -0.2334255053
GGCCAACC -0.347631586165
GGCCAAGA 0.0487556840469
GGCCAATA -0.0836162830897
GGCCACAA -0.109047807191
GGCCACAC -0.320973508645
GGCCACCA -0.303665035503
GGCCACCC -0.268423376065
GGCCACGA -0.165146528712
GGCCACTA -0.315141289438
GGCCAGAA 0.0497678324442
GGCCAGAC -0.328769998025
GGCCAGCA -0.194670678858
GGCCAGCC -0.354044966623
GGCCAGGA 0.0160322155139

GGCCAGTA -0.145618655693
GGCCATAA -0.069792198243
GGCCATAC -0.347209191316
GGCCATCA -0.360222397771
GGCCATGA -0.0930983581369
GGCCATTA -0.249731138546
GGCCCAAA -0.240494296578
GGCCCAAC -0.3725136858
GGCCCACA -0.27678468553
GGCCCACC -0.265800854485
GGCCCAGA -0.182517340533
GGCCCATA -0.000872210162353
GGCCCCAA -0.127618502584
GGCCCCAC -0.332022206862
GGCCCCCA -0.237173380116
GGCCCCCC -0.335442229224
GGCCCCGA -0.14940928843
GGCCCCTA -0.398820102686
GGCCCGAA -0.125965552402
GGCCCGAC -0.311814950133
GGCCCGCA -0.195387330898
GGCCCGCC -0.234979878531
GGCCCGGA -0.302368284614
GGCCCGTA -0.0891770987128
GGCCCTAA -0.152927231738
GGCCCTAC -0.280811472082
GGCCCTCA -0.276136812481
GGCCCTGA -0.0327788365445
GGCCCTTA -0.236990275362
GGCCGAAA 0.0817965220171
GGCCGAAC -0.275942658701
GGCCGACA -0.219856136844
GGCCGACC -0.203452230068
GGCCGAGA 0.00671122525133
GGCCGATA 0.1325605392
GGCCGCAA 0.00700001097611
GGCCGCAC -0.351387380271
GGCCGCCA -0.169549404037
GGCCGCCC -0.226548125201
GGCCGCGA -0.0790342214823
GGCCGCTA -0.210877262305
GGCCGGAA -0.12671508862
GGCCGGAC -0.387309032063
GGCCGGCA -0.122774814522
GGCCGGCC -0.215706559548
GGCCGGGA -0.0569576837172
GGCCGGTA -0.31682386055
GGCCGTAA -0.104153411253
GGCCGTAC -0.123848107452
GGCCGTCA -0.164310820625
GGCCGTGA -0.0638714813233
GGCCGTTA -0.0590871173455
GGCCTAAA -0.109748231226
GGCCTAAC -0.314421102466

GGCCTACA -0.387810105774
GGCCTACC -0.194103959907
GGCCTAGA -0.0811667087935
GGCCTATA -0.0719507978066
GGCCTCAA -0.292277505541
GGCCTCAC -0.202332957674
GGCCTCCA -0.225112410656
GGCCTCCC -0.216312695333
GGCCTCGA -0.288139433551
GGCCTCTA -0.144688101941
GGCCTGAA -0.212354396765
GGCCTGAC -0.272702582589
GGCCTGCA -0.239531898084
GGCCTGGA -0.141054466231
GGCCTGTA -0.311365681304
GGCCTTAA -0.260380903867
GGCCTTAC -0.3380871606
GGCCTTCA -0.210675144583
GGCCTTGA -0.232222373913
GGCCTTTA -0.311573091041
GGCGAAAA 0.111055743611
GGCGAAAC -0.0215525756902
GGCGAACA -0.264927400237
GGCGAACC -0.192116713029
GGCGAAGA 0.0331417963995
GGCGAATA 0.211675887506
GGCGACAA -0.218904320988
GGCGACAC -0.0896038237473
GGCGACCA -0.0524851418317
GGCGACCC -0.339991440752
GGCGACGA -0.0596990352004
GGCGACTA -0.285390409188
GGCGAGAA 0.0335897867988
GGCGAGAC -0.199795346183
GGCGAGCA -0.1065708061
GGCGAGCC -0.179723552878
GGCGAGGA -0.0576773901144
GGCGAGTA 0.00965961094243
GGCGATAA -0.102810021679
GGCGATAC -0.0449537285895
GGCGATCA 0.207744831694
GGCGATGA -0.210247693988
GGCGATTA 0.190709455751
GGCGCAAA -0.0158373400075
GGCGCAAC -0.166578543705
GGCGCACA -0.120818517055
GGCGCACC -0.104935185185
GGCGCAGA -0.034254319524
GGCGCATA 0.00175642497058
GGCGCCAA -0.0910230870966
GGCGCCAC -0.258736842105
GGCGCCCA -0.131202112011
GGCGCCCC -0.179808470208
GGCGCCGA -0.155313508529

GGCGCCTA −0.0697475670602
GGCGCGAA 0.0383725829181
GGCGCGAC −0.110122297445
GGCGCGCA −0.0237239844648
GGCGCGCC −0.0369566486055
GGCGCGGA 0.00728393698683
GGCGCGTA −0.0270395617906
GGCGCTAA −0.169155252645
GGCGCTAC −0.385835692497
GGCGCTCA −0.16790891953
GGCGCTGA −0.0899063235706
GGCGCTTA −0.201177952289
GGCGGAAA 0.00367120421891
GGCGGAAC −0.290413018193
GGCGGACA −0.163276688453
GGCGGACC −0.242265892021
GGCGGAGA −0.204365863295
GGCGGATA 0.154026433072
GGCGGCAA −0.0539231824417
GGCGGCAC −0.268032584547
GGCGGCCA −0.275771234731
GGCGGCCC −0.212336008318
GGCGGCGA −0.158076393347
GGCGGCTA −0.233590329806
GGCGGGAA 0.00927343672824
GGCGGGAC −0.138683268645
GGCGGGCA −0.108361732602
GGCGGGGA 0.0544953324113
GGCGGGTA −0.0628143477692
GGCGGTAA 0.0356732962387
GGCGGTAC −0.342047177319
GGCGGTCA −0.200661631852
GGCGGTGA 0.0147985335924
GGCGGTTA −0.13979981562
GGCGTAAA 0.109228523129
GGCGTAAC −0.149710965868
GGCGTACA −0.0816236360842
GGCGTACC −0.158908819318
GGCGTAGA −0.155257569023
GGCGTATA −0.154612754702
GGCGTCAA −0.139752364794
GGCGTCAC −0.170673642562
GGCGTCCA −0.280564882481
GGCGTCCC −0.366634298348
GGCGTCGA −0.107494484507
GGCGTCTA −0.287374435612
GGCGTGAA −0.080165577342
GGCGTGAC −0.185162969439
GGCGTGCA −0.0493409499978
GGCGTGGA −0.234851976211
GGCGTGTA −0.0383304519634
GGCGTTAA −0.00495547193933
GGCGTTAC −0.185651961753
GGCGTTCA −0.263605155699

GGCGTTGA -0.0945704932509
GGCGTTTA 0.00173439592527
GGCTAAAA -0.00233754158616
GGCTAAAC 0.00449057864687
GGCTAACA 0.0357691804803
GGCTAACC -0.34957623632
GGCTAAGA -0.15503848947
GGCTAATA -0.196595038825
GGCTACAA -0.127965999385
GGCTACAC -0.304777451174
GGCTACCA -0.2754913095
GGCTACCC -0.386859211717
GGCTACGA -0.117169921082
GGCTACTA -0.184487356776
GGCTAGAA -0.0961757938932
GGCTAGAC -0.0853602742966
GGCTAGCA -0.113034673516
GGCTAGCC -0.0646490582299
GGCTAGGA 0.00209918008496
GGCTAGTA -0.272907100183
GGCTATAA 0.0763638496493
GGCTATAC -0.0929016605407
GGCTATCA -0.0141907615974
GGCTATGA -0.0406575011663
GGCTATTA -0.0800859386576
GGCTCAAA -0.212143192256
GGCTCAAC -0.342761130522
GGCTCACA -0.243349794239
GGCTCACC -0.276672756901
GGCTCAGA -0.173557607884
GGCTCATA -0.261371590556
GGCTCCAA -0.112419494935
GGCTCCAC -0.15163909923
GGCTCCCA -0.306948241629
GGCTCCCC -0.365980159836
GGCTCCGA -0.168147106751
GGCTCCTA -0.283533361992
GGCTCGAA -0.226272033773
GGCTCGAC -0.269358440461
GGCTCGCA -0.321885904273
GGCTCGGA -0.0336068050294
GGCTCGTA -0.0451454970726
GGCTCTAA -0.220631084178
GGCTCTAC -0.205332452718
GGCTCTCA -0.299461016634
GGCTCTGA -0.175564225394
GGCTCTTA -0.226043034239
GGCTGAAA -0.0627754452403
GGCTGAAC -0.0423542194436
GGCTGACA -0.0376965505886
GGCTGACC -0.277002654523
GGCTGAGA -0.0365704142081
GGCTGATA 0.105589584843
GGCTGCAA -0.0111548842385

GGCTGCAC -0.308329848527
GGCTGCCA -0.336836243739
GGCTGCCC -0.271239631678
GGCTGCGA -0.18428582402
GGCTGCTA -0.277935293827
GGCTGGAA 0.00392434521065
GGCTGGAC -0.0737458242556
GGCTGGCA -0.232646432725
GGCTGGGA -0.0587843117888
GGCTGGTA -0.116003468278
GGCTGTAA -0.0985657660772
GGCTGTAC -0.226990969219
GGCTGTCA -0.146802859949
GGCTGTGA -0.0388205014883
GGCTGTTA -0.230023499203
GGCTTAAA -0.0735353834672
GGCTTAAC -0.196277272927
GGCTTACA -0.0677268092005
GGCTTACC -0.34265911223
GGCTTAGA -0.0347717712134
GGCTTATA -0.252231934636
GGCTTCAA -0.213596533266
GGCTTCAC -0.243328122
GGCTTCCA -0.235339140841
GGCTTCCC -0.411339248116
GGCTTCGA -0.127644647666
GGCTTCTA -0.299575617284
GGCTTGAA -0.198964843012
GGCTTGAC -0.208859693203
GGCTTGCA -0.173305898491
GGCTTGGA -0.109188559664
GGCTTGTA -0.243738979122
GGCTTTAA -0.13415704933
GGCTTTAC -0.107175788711
GGCTTTCA -0.210677070473
GGCTTTGA -0.0233731805309
GGCTTTTA -0.113069312684
GGGAAAAA 0.0222782058882
GGGAAAAC -0.0758691325382
GGGAAACA -0.111706623636
GGGAAACC -0.0790240529461
GGGAAAGA 0.0521169509759
GGGAAATA 0.197200470704
GGGAACAA -0.030794822251
GGGAACAC -0.305750214181
GGGAACCA -0.287938460824
GGGAACCC -0.286803917696
GGGAACGA 0.00166021788645
GGGAACTA -0.112303294571
GGGAAGAA -0.017045049198
GGGAAGAC -0.16059473638
GGGAAGCA -0.10946324401
GGGAAGGA -0.195958847737
GGGAAGTA -0.0956034888518

GGGAATAA 0.0320035448114
GGGAATAC 0.128600518123
GGGAATCA 0.454348613581
GGGAATGA -0.0631592523077
GGGAATTA 0.242571187073
GGGACAAA 0.0856115525983
GGGACAAC -0.0591881080547
GGGACACA -0.126788154148
GGGACACC -0.312950506048
GGGACAGA -0.0627870228631
GGGACATA -0.178658168127
GGGACCAA -0.280622188144
GGGACCAC -0.179050142928
GGGACCCA -0.273454687097
GGGACCCC -0.233390854185
GGGACCGA -0.297815197055
GGGACCTA -0.193828874893
GGGACGAA -0.203354995018
GGGACGAC -0.120256047236
GGGACGCA -0.231493288639
GGGACGGA 0.017877739331
GGGACGTA -0.233000839465
GGGACTAA -0.285406911175
GGGACTAC -0.112739618482
GGGACTCA -0.215002084842
GGGACTGA -0.0777906589865
GGGACTTA -0.15071967738
GGGAGAAA -0.00435692182529
GGGAGAAC -0.224024691358
GGGAGACA -0.24985510787
GGGAGACC -0.218032513702
GGGAGAGA -0.0590327003974
GGGAGATA 0.229976900615
GGGAGCAA 0.145408352267
GGGAGCAC -0.379175645769
GGGAGCCA -0.217719942274
GGGAGCCC -0.320285477805
GGGAGCGA 0.0928435488792
GGGAGCTA -0.0858097312999
GGGAGGAA 0.0677828948324
GGGAGGAC -0.324001382511
GGGAGGCA -0.266848276354
GGGAGGGA 0.211051385237
GGGAGGTA 0.0171269594526
GGGAGTAA -0.0889657467123
GGGAGTAC -0.19428700909
GGGAGTCA -0.151726712116
GGGAGTGA -0.0346241232836
GGGAGTTA -0.0830520845338
GGGATAAA -0.148900872213
GGGATAAC 0.0273458951375
GGGATACA 0.0397456585216
GGGATACC 0.200986849327
GGGATAGA -0.0589571862333

GGGATATA 0.104227623378
GGGATCAA 0.108289973435
GGGATCAC 0.120131616501
GGGATCCA 0.205394214984
GGGATCCC 0.265044150457
GGGATCGA 0.113998111923
GGGATCTA 0.265975622158
GGGATGAA 0.031617102262
GGGATGAC -0.203307707925
GGGATGCA -0.0434869850835
GGGATGGA -0.174287689497
GGGATGTA 0.00110847994421
GGGATTAA -0.125746301418
GGGATTAC 0.429995786905
GGGATTCA 0.0710051864658
GGGATTGA 0.0272398954972
GGGATTTA 0.202633427737
GGGCAAAA -0.0808947004494
GGGCAAAC -0.0793402908926
GGGCAACA -0.163476651588
GGGCAACC -0.301092648584
GGGCAAGA -0.114271938284
GGGCAATA 0.0519742475137
GGGCACAA -0.0711428289214
GGGCACAC -0.306740946428
GGGCACCA -0.193826303421
GGGCACCC -0.366465128706
GGGCACGA -0.144680384088
GGGCACTA -0.261436456207
GGGCAGAA 0.155192759446
GGGCAGAC -0.334332844468
GGGCAGCA -0.082779417575
GGGCAGGA -0.253327523602
GGGCAGTA -0.037842101135
GGGCATAA -0.0326807675211
GGGCATAC -0.0433249972704
GGGCATCA -0.109470408871
GGGCATGA -0.111177923021
GGGCATTA -0.114358750908
GGGCCAAA -0.18995945208
GGGCCAAC -0.254046439408
GGGCCACA -0.146428498811
GGGCCACC -0.43758638989
GGGCCAGA -0.122986670179
GGGCCATA -0.0938887516808
GGGCCCAA -0.345557091755
GGGCCCAC -0.223448499114
GGGCCCCA -0.212431693989
GGGCCCCC -0.0986617900508
GGGCCCGA -0.20471259069
GGGCCCTA -0.210611796982
GGGCCGAA -0.314177569324
GGGCCGAC -0.325521854424
GGGCCGCA -0.265562690785

GGGCCGGA -0.19093539055
GGGCCGTA 0.0189849067002
GGGCCTAA -0.120572224793
GGGCCTAC -0.292363442842
GGGCCTCA -0.341704470867
GGGCCTGA -0.266710487335
GGGCCTTA -0.152492760103
GGGCGAAA -0.098796045311
GGGCGAAC -0.339705503163
GGGCGACA -0.161420606699
GGGCGACC -0.297481149284
GGGCGAGA -0.110334789783
GGGCGATA 0.0682149992316
GGGCGCAA -0.077467203231
GGGCGCAC -0.233508616008
GGGCGCCA -0.156412240552
GGGCGCCC -0.109867000624
GGGCGCGA -0.0403821626443
GGGCGCTA -0.368587369772
GGGCGGAA 0.0197221237601
GGGCGGAC -0.292616128114
GGGCGGCA -0.177856233678
GGGCGGGA 0.0732018072727
GGGCGGTA -0.0629376318343
GGGCGTAA 0.00395846588747
GGGCGTAC -0.203050388633
GGGCGTCA -0.149138692701
GGGCGTGA -0.17479157589
GGGCGTTA -0.179256259124
GGGCTAAA 0.0103666083642
GGGCTAAC -0.182644037465
GGGCTACA -0.299351181176
GGGCTACC -0.245276186714
GGGCTAGA -0.0517838914683
GGGCTATA -0.0788116247421
GGGCTCAA -0.33464795339
GGGCTCAC -0.209647736489
GGGCTCCA -0.235401920439
GGGCTCGA -0.274400887595
GGGCTCTA -0.187968046478
GGGCTGAA -0.169947390285
GGGCTGAC -0.143736146773
GGGCTGCA -0.107854273673
GGGCTGGA -0.0414251684999
GGGCTGTA -0.0835116950399
GGGCTTAA -0.262208067941
GGGCTTAC -0.27650878146
GGGCTTCA -0.316067180415
GGGCTTGA -0.304223659737
GGGCTTTA -0.297067748804
GGGGAAAA 0.000979714153988
GGGGAAAC -0.0532956576061
GGGGAACA -0.000288151221761
GGGGAACC -0.136858832826

GGGGAAGA -0.135811919381
GGGGAATA 0.27239072208
GGGGACAA -0.111390424099
GGGGACAC -0.25017065243
GGGGACCA -0.0444379559499
GGGGACCC -0.251869914921
GGGGACGA -0.0254645636449
GGGGACTA -0.325750626876
GGGGAGAA -0.0382421219006
GGGGAGAC -0.318898289578
GGGGAGCA -0.0927361824487
GGGGAGGA -0.0887959608753
GGGGAGTA -0.0970185891533
GGGGATAA -0.0151395406273
GGGGATAC 0.148395391745
GGGGATCA 0.241959208764
GGGGATGA -0.134111111111
GGGGATTA 0.206167100083
GGGGCAAA -0.0417163012592
GGGGCAAC -0.16072643663
GGGGCACA -0.0699624049834
GGGGCACC -0.276905435951
GGGGCAGA 0.165096683277
GGGGCATA -0.0973304237246
GGGGCCAA -0.020183310008
GGGGCCAC -0.367189632839
GGGGCCCA -0.301560262677
GGGGCCCC -0.154473642872
GGGGCCGA -0.0104156376568
GGGGCCTA -0.388193183963
GGGGCGAA 0.0116110157206
GGGGCGAC -0.265786600387
GGGGCGCA -0.211336132378
GGGGCGGA -0.189761940723
GGGGCGTA 0.0383204919247
GGGGCTAA 0.0526506181628
GGGGCTAC -0.179312537033
GGGGCTCA -0.259471572936
GGGGCTGA -0.165720742178
GGGGCTTA -0.324084060826
GGGGGAAA -0.0611610709979
GGGGGAAC -0.206210554137
GGGGGACA -0.238113267596
GGGGGACC -0.305532641656
GGGGGAGA -0.0162682053115
GGGGGATA 0.115779042774
GGGGGCAA 0.00784281441234
GGGGGCAC -0.348799656132
GGGGGCCA -0.157387505664
GGGGGCGA 0.0741564029946
GGGGGCTA -0.234283324232
GGGGGGAA -0.100748459458
GGGGGGAC -0.328179488912
GGGGGGCA -0.255989671828

GGGGGGGA -0.00900633901375
GGGGGGTA -0.243698216735
GGGGGTAA -0.285776737242
GGGGGTAC -0.38256671878
GGGGGTCA -0.327305364176
GGGGGTGA -0.00715209274621
GGGGGTTA -0.323302701747
GGGGTAAA 0.068204548822
GGGGTAAC -0.210328908567
GGGGTACA -0.109368998628
GGGGTACC -0.259298765432
GGGGTAGA 0.0446180843059
GGGGTATA -0.148403689851
GGGGTCAA -0.29695060235
GGGGTCAC -0.207859100336
GGGGTCCA -0.16556480871
GGGGTCGA -0.241250250532
GGGGTCTA -0.193916245479
GGGGTGAA 0.097071285868
GGGGTGAC -0.208552056311
GGGGTGCA -0.093816367857
GGGGTGGA 0.094205165355
GGGGTGTA -0.0801527604671
GGGGTTAA -0.0902603854937
GGGGTTAC -0.184853820029
GGGGTTCA -0.231052175884
GGGGTTGA -0.141050967997
GGGGTTTA -0.270437587228
GGGTAAAA 0.0860968407649
GGGTAAAC -0.119910768674
GGGTAACA -0.105772608032
GGGTAACC -0.0999668796249
GGGTAAGA -0.0415629131376
GGGTAATA 0.143046499374
GGGTACAA -0.14739453005
GGGTACAC -0.0750592621858
GGGTACCA -0.13971052184
GGGTACCC -0.28888971169
GGGTACGA 0.0763759792209
GGGTACTA -0.170281849046
GGGTAGAA 0.133457789142
GGGTAGAC -0.0475991285403
GGGTAGCA -0.156911641255
GGGTAGGA -0.115174529755
GGGTAGTA -0.160836043286
GGGTATAA 0.120238838929
GGGTATAC -0.204590576847
GGGTATCA 0.205318669824
GGGTATGA -0.0895483582341
GGGTATTA 0.0322563305391
GGGTCAAA -0.107315393934
GGGTCAAC -0.34202470727
GGGTCACA -0.0548779424839
GGGTCACC -0.139782135076

GGGTCAGA -0.074504816004
GGGTCATA -0.121261068631
GGGTCCAA -0.328379823912
GGGTCCAC -0.174852476421
GGGTCCCA -0.264811248814
GGGTCCGA -0.175907099952
GGGTCCTA -0.35265946015
GGGTCGAA -0.20931573459
GGGTCGAC -0.154210017999
GGGTCGCA -0.128708551483
GGGTCGGA -0.238697961244
GGGTCGTA -0.193922166507
GGGTCTAA -0.137461147422
GGGTCTAC -0.217917805672
GGGTCTCA -0.152281724114
GGGTCTGA -0.115101851852
GGGTCTTA -0.246297850861
GGGTGAAA 0.094375703622
GGGTGAAC -0.104641165142
GGGTGACA -0.153445433666
GGGTGACC -0.133026936575
GGGTGAGA -0.0776586782861
GGGTGATA 0.0117428483611
GGGTGCAA -0.0415575862353
GGGTGCAC -0.235598028886
GGGTGCCA -0.247830568311
GGGTGCGA -0.0222905323346
GGGTGCTA -0.114307270233
GGGTGGAA -0.0314845679012
GGGTGGAC -0.133442771249
GGGTGGCA 0.0169378032449
GGGTGGGA 0.0603031899712
GGGTGGTA -0.0760487809129
GGGTGTAA 0.017943994747
GGGTGTAC -0.189477292003
GGGTGTCA -0.251586213308
GGGTGTGA -0.183783226391
GGGTGTTA -0.0108229741226
GGGTTAAA -0.187197334364
GGGTTAAC -0.180058274483
GGGTTACA -0.0979588278878
GGGTTACC -0.177181255934
GGGTTAGA 0.0714400375383
GGGTTATA -0.11304606802
GGGTTCAA -0.232951989026
GGGTTCAC -0.0488628506519
GGGTTCCA -0.162937903332
GGGTTCGA -0.216357086979
GGGTTCTA -0.262048323996
GGGTTGAA -0.0651312387051
GGGTTGAC -0.198753299203
GGGTTGCA 0.0246822103669
GGGTTGGA -0.0401723967595
GGGTTGTA -0.237031857597

GGGTTTAA -0.249406006332
GGGTTTAC 0.0266879151147
GGGTTTCA 0.0680124358956
GGGTTTGA -0.0910124828241
GGGTTTTA 0.0165892774814
GGTAAAAA 0.207474368263
GGTAAAAC -0.115420369343
GGTAAACA 0.144671123406
GGTAAACC -0.191157425699
GGTAAAGA 0.191217875147
GGTAAATA 0.0479697238243
GGTAACAA -0.0113800389671
GGTAACAC -0.101700553777
GGTAACCA -0.0871922413142
GGTAACGA 0.0996920081937
GGTAACTA -0.0136402597591
GGTAAGAA 0.00689367494347
GGTAAGAC -0.170439193606
GGTAAGCA -0.194611111111
GGTAAGGA 0.111506108988
GGTAAGTA 0.0891512437156
GGTAATAA 0.209008760333
GGTAATAC 0.0666013523897
GGTAATCA 0.334073772754
GGTAATGA -0.0194470913612
GGTAATTA 0.102391469956
GGTACAAA -0.0288655960211
GGTACAAC -0.330085368798
GGTACACA -0.0976228104833
GGTACACC -0.124872427984
GGTACAGA -0.0645465994962
GGTACATA -0.170513411475
GGTACCAA 0.0833803498025
GGTACCAC -0.0516314690048
GGTACCCA -0.264879707045
GGTACCGA -0.137074319742
GGTACCTA -0.173868235208
GGTACGAA 0.1217379887
GGTACGAC -0.226927762413
GGTACGCA -0.0570050835149
GGTACGGA 0.0452347885712
GGTACGTA 0.11703604909
GGTACTAA -0.0230273990647
GGTACTAC -0.178909907092
GGTACTCA -0.180923218754
GGTACTGA -0.0928615337329
GGTACTTA -0.0483648831229
GGTAGAAA 0.277298679412
GGTAGAAC -0.0158592943863
GGTAGACA -0.202695557951
GGTAGACC -0.228941176471
GGTAGAGA 0.0480772303982
GGTAGATA 0.268552822235
GGTAGCAA -0.0825375657194

GGTAGCAC -0.245837848202
GGTAGCCA -0.128346009482
GGTAGCGA -0.141210105799
GGTAGCTA -0.0576586578336
GGTAGGAA 0.00307086873477
GGTAGGAC -0.0336315256581
GGTAGGCA -0.198684806729
GGTAGGGA -0.209667766751
GGTAGGTA -0.00469822689781
GGTAGTAA -0.1435089024
GGTAGTAC -0.123342891757
GGTAGTCA 0.000555665316606
GGTAGTGA -0.062273568488
GGTAGTTA -0.104898426357
GGTATAAA 0.169503688085
GGTATAAC 0.0183850769253
GGTATACA -0.0475951353903
GGTATACC -0.119333333333
GGTATAGA 0.189009557443
GGTATATA 0.0373279325561
GGTATCAA 0.0865616782648
GGTATCAC 0.135911326264
GGTATCCA 0.339573865507
GGTATCGA 0.172802289599
GGTATCTA 0.356336033722
GGTATGAA -0.0086775238621
GGTATGAC -0.143055523276
GGTATGCA 0.0137971612453
GGTATGGA -0.128518353885
GGTATGTA -0.0507168761618
GGTATTAA 0.0672933040838
GGTATTAC 0.0921397130132
GGTATTCA -0.0293216960794
GGTATTGA 0.0653182256635
GGTATTTA 0.12227618644
GGTCAAAA 0.00269215570045
GGTCAAAC -0.07364966468
GGTCAACA -0.215301095091
GGTCAACC -0.305487188417
GGTCAAGA -0.192932336605
GGTCAATA -0.0919962242199
GGTCACAA -0.00626298432438
GGTCACAC -0.167739474955
GGTCACCA -0.306704848425
GGTCACGA -0.146000702401
GGTCACTA -0.217695858157
GGTCAGAA 0.0241251180048
GGTCAGAC -0.289391969365
GGTCAGCA -0.162482233308
GGTCAGGA 0.0421114410399
GGTCAGTA -0.0544695131696
GGTCATAA 0.00358403672657
GGTCATAC -0.0481243654126
GGTCATCA -0.214525181863

GGTCATGA -0.0036795574653
GGTCATTA -0.151024160961
GGTCCAAA -0.0611152273538
GGTCCAAC -0.356387289029
GGTCCACA -0.10400819915
GGTCCACC -0.226976445608
GGTCCAGA -0.190858450618
GGTCCATA -0.207012835921
GGTCCCAA -0.172557653517
GGTCCCAC -0.286106027596
GGTCCCCA -0.230345204143
GGTCCCGA -0.00532448099022
GGTCCCTA -0.168966238414
GGTCCGAA 0.0240483232708
GGTCCGAC -0.24244170096
GGTCCGCA -0.192611615962
GGTCCGGA -0.19870549629
GGTCCGTA -0.121627117856
GGTCCTAA -0.176435726404
GGTCCTAC -0.148404320988
GGTCCTCA -0.175761871013
GGTCCTGA -0.109229794935
GGTCCTTA -0.245841684822
GGTCGAAA -0.180310820625
GGTCGAAC -0.148203025963
GGTCGACA -0.0909571532317
GGTCGACC -0.195581337941
GGTCGAGA 0.026721223298
GGTCGATA 0.102867241871
GGTCGCAA -0.0664397630441
GGTCGCAC -0.160117322973
GGTCGCCA -0.0838632554113
GGTCGCGA -0.0360216397139
GGTCGCTA -0.309848684355
GGTCGGAA -0.0297807190834
GGTCGGAC -0.315199491143
GGTCGGCA -0.168108366625
GGTCGGGA -0.132200617284
GGTCGGTA -0.204353027036
GGTCGTAA 0.105090122725
GGTCGTAC -0.145953336522
GGTCGTCA -0.138421101728
GGTCGTGA -0.134057623642
GGTCGTTA -0.196492899807
GGTCTAAA -0.0681286592187
GGTCTAAC 0.136509583086
GGTCTACA -0.00669882178606
GGTCTAGA -0.100372820017
GGTCTATA -0.307032218901
GGTCTCAA -0.157656296752
GGTCTCAC -0.126527510275
GGTCTCCA -0.164839415938
GGTCTCGA -0.182639984428
GGTCTCTA -0.200390161991

GGTCTGAA -0.0424949590624
GGTCTGAC -0.30869790638
GGTCTGCA -0.229423384168
GGTCTGGA -0.132401600079
GGTCTGTA -0.180919170295
GGTCTTAA -0.120557734205
GGTCTTAC -0.197588787919
GGTCTTCA -0.346393689986
GGTCTTGA -0.149194722286
GGTCTTTA -0.16554721454
GGTGAAAA 0.205925635112
GGTGAAAC -0.126195892492
GGTGAACA -0.207827160494
GGTGAACC -0.16327605504
GGTGAAGA 0.104126874355
GGTGAATA 0.0655749851808
GGTGACAA -0.0441648627838
GGTGACAC -0.185152041652
GGTGACCA -0.320517330766
GGTGACGA -0.0737182466823
GGTGACTA -0.248361272961
GGTGAGAA -0.0857569541696
GGTGAGAC -0.208071514987
GGTGAGCA -0.148534141175
GGTGAGGA -0.0670538296483
GGTGAGTA 0.0803262969796
GGTGATAA -0.0877152974875
GGTGATAC 0.105756986981
GGTGATCA -0.00262078845964
GGTGATGA 0.087575726759
GGTGATTA 0.167149397973
GGTGCAAA 0.0147643197436
GGTGCAAC -0.259610748003
GGTGCACA -0.241383266386
GGTGCACC -0.218477920966
GGTGCAGA 0.0880946892358
GGTGCATA -0.0375299638255
GGTGCCAA -0.172916921173
GGTGCCAC -0.306028736483
GGTGCCCA -0.214305426848
GGTGCCGA -0.0969957817185
GGTGCCTA -0.238558864194
GGTGCGAA -0.0298111012326
GGTGCGAC -0.247493348515
GGTGCGCA -0.0631394348144
GGTGCGGA 0.113647523717
GGTGCGTA -0.0632352214852
GGTGCTAA -0.0261531537465
GGTGCTAC -0.185905878221
GGTGCTCA -0.166267692379
GGTGCTGA -0.039479112795
GGTGCTTA -0.22386677859
GGTGGAAA 0.0734233632206
GGTGGAAC -0.288047676857

GGTGGACA -0.0581431723139
GGTGGAGA -0.108068770772
GGTGGATA 0.285862022128
GGTGGCAA -0.0686129223474
GGTGGCAC -0.216847471206
GGTGGCCA -0.340431367386
GGTGGCGA -0.0547625272331
GGTGGCTA -0.0398899096667
GGTGGGAA 0.107233418955
GGTGGGAC -0.181687984592
GGTGGGCA -0.167195352215
GGTGGGGA -0.0444081910152
GGTGGGTA -0.154858538459
GGTGGTAA 0.130584436432
GGTGGTAC -0.237593205274
GGTGGTCA -0.260845697157
GGTGGTGA 0.0351129222579
GGTGGTTA -0.187569951939
GGTGTAAA -0.0570339554233
GGTGTAAC 0.0354176049279
GGTGTACA -0.254486746882
GGTGTAGA 0.117782497969
GGTGTATA 0.0986218138708
GGTGTCAA -0.190058335117
GGTGTCAC -0.258889821627
GGTGTCCA -0.246528436542
GGTGTCGA -0.289845768933
GGTGTCTA -0.348513801966
GGTGTGAA 0.0708163235657
GGTGTGAC -0.0670556858097
GGTGTGCA -0.339930761297
GGTGTGGA 0.00103180483521
GGTGTGTA -0.00343875757268
GGTGTTAA 0.0957814661134
GGTGTTAC -0.187800756522
GGTGTTCA -0.219994182952
GGTGTTGA 0.0689567503959
GGTGTTTA -0.0852192988828
GGTTAAAA 0.198817050282
GGTTAAAC -0.184887852123
GGTTAACA 0.0199885162501
GGTTAACC -0.0784703643187
GGTTAAGA 0.122220026784
GGTTAATA -0.051326593339
GGTTACAA -0.0159184331426
GGTTACAC -0.146364492063
GGTTACCA -0.170151868855
GGTTACGA 0.0459422819381
GGTTACTA -0.0943570552789
GGTTAGAA -0.0148671342487
GGTTAGAC 0.000948672874929
GGTTAGCA -0.110851906942
GGTTAGGA 0.0142852992024
GGTTAGTA -0.0578342761019

GGTTATAA -0.0733514448012
GGTTATAC -0.0650481262293
GGTTATCA 0.0309845154598
GGTTATGA -0.172401398287
GGTTATTA 0.0238847996838
GGTTCAAA 0.0198677314014
GGTTCAAC -0.219069647482
GGTTCACA -0.000906497143953
GGTTCAGA -0.0437864596649
GGTTCATA -0.0573019978942
GGTTCCAA -0.0568343981207
GGTTCCAC -0.166105911695
GGTTCCCA -0.285198536149
GGTTCCGA -0.231556112373
GGTTCCTA -0.170499692699
GGTTCGAA -0.137360466265
GGTTCGAC -0.198163053676
GGTTCGCA -0.178287850923
GGTTCGGA -0.165703171979
GGTTCGTA -0.0373608641243
GGTTCTAA -0.136160519725
GGTTCTAC -0.266906423508
GGTTCTCA -0.197999506051
GGTTCTGA -0.0488878880769
GGTTCTTA -0.0216716737075
GGTTGAAA -0.0803694921952
GGTTGAAC -0.308300212304
GGTTGACA -0.184304107431
GGTTGAGA -0.03941278166
GGTTGATA 0.00348251333729
GGTTGCAA 0.111152275571
GGTTGCAC -0.0698224527318
GGTTGCCA -0.260599126388
GGTTGCGA 0.0432161899089
GGTTGCTA -0.0776328214664
GGTTGGAA 0.056605249292
GGTTGGAC -0.209400666475
GGTTGGCA -0.113384081592
GGTTGGGA -0.0439818110499
GGTTGGTA -0.161381970932
GGTTGTAA -0.0234692309381
GGTTGTAC -0.205407659845
GGTTGTCA -0.16750686444
GGTTGTGA -0.0299986634978
GGTTGTTA -0.229025417574
GGTTTAAA -0.160478581817
GGTTTAAC -0.181762062324
GGTTTACA 0.216221816066
GGTTTAGA 0.0177371318989
GGTTTATA 0.0432281718589
GGTTTCAA -0.0593070100332
GGTTTCAC -0.142187534304
GGTTTCCA -0.294379059149
GGTTTCGA -0.12437427186

GGTTTCTA 0.035994204044
GGTTTGAA 0.0823238853457
GGTTTGAC 0.0602196620807
GGTTTGCA -0.0601817553218
GGTTTGGA -0.108592343825
GGTTTGTA -0.0855554800948
GGTTTTAA -0.130526055816
GGTTTTAC -0.000457575473955
GGTTTTCA -0.045459300045
GGTTTTGA -0.219629245485
GGTTTTTA 0.0566806618918
GTAAAAAA 0.099973451546
GTAAAAAC -0.0198138268678
GTAAAACA 0.0310853805587
GTAAAAGA 0.144708206819
GTAAAATA 0.166526707502
GTAAACAA -0.0498274907245
GTAAACAC -0.0086320609445
GTAAACCA 0.0517084307463
GTAAACGA -0.13708833988
GTAAACTA -0.0984940783913
GTAAAGAA 0.0202591458395
GTAAAGAC -0.191710213294
GTAAAGCA 0.169797916697
GTAAAGGA 0.00738599218398
GTAAAGTA -0.0081254630773
GTAAATAA 0.162865195473
GTAAATAC 0.0707370084963
GTAAATCA 0.212199090552
GTAAATGA 0.0280394892818
GTAAATTA 0.244506873071
GTAACAAA 0.0689088082751
GTAACAAC -0.109221284088
GTAACACA 0.0390663587869
GTAACAGA -0.179862688926
GTAACATA 0.0623240393164
GTAACCAA -0.0670150116648
GTAACCAC -0.0228625407488
GTAACCCA 0.00920437331226
GTAACCGA 0.0400173439593
GTAACCTA -0.0143724666948
GTAACGAA 0.210317460317
GTAACGAC 0.0313947616852
GTAACGCA 0.00821642626622
GTAACGGA -0.0660686008165
GTAACGTA 0.0587064534329
GTAACTAA 0.0854952830189
GTAACTAC 0.010934258619
GTAACTCA 0.0854574194823
GTAACTGA -0.0504917699359
GTAACTTA 0.00841127137808
GTAAGAAA 0.219582177153
GTAAGAAC -0.0934137076624
GTAAGACA -0.106916740517

GTAAGAGA 0.00115787485654
GTAAGATA 0.230433159894
GTAAGCAA -0.0511975038929
GTAAGCAC -0.112073798851
GTAAGCCA -0.0139083391926
GTAAGCGA 0.296917176354
GTAAGCTA -0.0727333945197
GTAAGGAA -0.0282931704738
GTAAGGAC -0.0865218918415
GTAAGGCA 0.0751333728512
GTAAGGGA 0.142011899273
GTAAGGTA 0.0017012188921
GTAAGTAA 0.0120035566094
GTAAGTAC 0.0319454331498
GTAAGTCA -0.01173419726
GTAAGTGA 0.00110889534264
GTAAGTTA 0.063266969667
GTAATAAA 0.214012174393
GTAATAAC 0.0341198489539
GTAATACA -0.00853202046148
GTAATAGA 0.14295323464
GTAATATA 0.277067004764
GTAATCAA 0.414171760479
GTAATCAC 0.383987573822
GTAATCCA 0.468166150739
GTAATCGA 0.408205997936
GTAATCTA 0.464482152456
GTAATGAA -0.0870797460506
GTAATGAC 0.0270546911446
GTAATGCA 0.0574913195706
GTAATGGA 0.114862195001
GTAATGTA 0.0584683394251
GTAATTAA 0.101267316516
GTAATTAC 0.274195141221
GTAATTCA 0.110862752201
GTAATTGA 0.133187569916
GTAATTTA 0.258120375859
GTACAAAA 0.0330553658855
GTACAAAC 0.0280069266065
GTACAACA -0.070687611144
GTACAAGA 0.109346527915
GTACAATA 0.0525505499572
GTACACAA 0.112228040133
GTACACAC -0.145699010649
GTACACCA -0.143520347689
GTACACGA -0.190070898635
GTACACTA -0.0232304770687
GTACAGAA -0.0513540363125
GTACAGAC -0.238768438035
GTACAGCA 0.00857318492173
GTACAGGA -0.0215583615645
GTACAGTA -0.0213313381663
GTACATAA -0.0173233770225
GTACATAC -0.134477362468

GTACATCA 0.029280280577
GTACATGA -0.0235985543961
GTACATTA 0.0126731651628
GTACCAAA 0.140768236166
GTACCAAC -0.190854938272
GTACCACA -0.149298696168
GTACCAGA -0.00876990791048
GTACCATA 0.172561417375
GTACCCAA -0.0079055396402
GTACCCAC -0.0386704859739
GTACCCCA -0.0956411996059
GTACCCGA 0.0161390612061
GTACCCTA -0.289255948952
GTACCGAA -0.147384394174
GTACCGAC -0.226660965714
GTACCGCA -0.171101451184
GTACCGGA -0.0443883977082
GTACCGTA -0.0677801925209
GTACCTAA 0.0563568903818
GTACCTAC 0.011730499023
GTACCTCA 0.0364461813374
GTACCTGA -0.0614196678919
GTACCTTA -0.0980655659298
GTACGAAA 0.171694408772
GTACGAAC 0.0578442995455
GTACGACA 0.0145228215768
GTACGAGA 0.038668952392
GTACGATA 0.145217238578
GTACGCAA 0.107661529342
GTACGCAC -0.0800219478738
GTACGCCA -0.110710463489
GTACGCGA 0.087651271919
GTACGCTA 0.0116681759557
GTACGGAA -0.0917857412648
GTACGGAC -0.156182105471
GTACGGCA -0.11030900876
GTACGGGA 0.0823156425961
GTACGGTA -0.00178836083391
GTACGTAA 0.101261827922
GTACGTAC -0.174714857058
GTACGTCA -0.0801627675979
GTACGTGA 0.0466612878439
GTACGTTA -0.196049382716
GTACTAAA 0.104042156475
GTACTAAC 0.0166543179386
GTACTACA 0.0660332828383
GTACTAGA -0.0165409902642
GTACTATA -0.0261051903582
GTACTCAA 0.0731705899786
GTACTCAC -0.25767354989
GTACTCCA -0.199037813466
GTACTCGA -0.149134939101
GTACTCTA 0.0256241923784
GTACTGAA -0.120845133812

GTACTGAC 0.00720378054403
GTACTGCA 0.0080586661536
GTACTGGA 0.118646579402
GTACTGTA -0.118105634035
GTACTTAA 0.141449318317
GTACTTCA -0.00388005312435
GTACTTGA -0.0437646361518
GTACTTTA 0.061275242644
GTAGAAAA 0.187187831418
GTAGAAAC 0.162022576381
GTAGAACA -0.0478415723181
GTAGAAGA 0.151095523502
GTAGAATA 0.00375837171124
GTAGACAA -0.184529739998
GTAGACAC -0.230453398089
GTAGACCA -0.281023805928
GTAGACGA 0.126177303563
GTAGACTA -0.012254296631
GTAGAGAA -0.00887219792832
GTAGAGAC -0.09783682339
GTAGAGCA -0.1987598153
GTAGAGGA -0.121466488507
GTAGAGTA -0.0162785291938
GTAGATAA 0.241989396035
GTAGATAC 0.326972527712
GTAGATCA 0.117047055918
GTAGATGA -0.00627840347757
GTAGATTA 0.310901747708
GTAGCAAA 0.0352229308617
GTAGCAAC -0.161421897878
GTAGCACA -0.146402775033
GTAGCAGA -0.00993063101628
GTAGCATA 0.0875559322651
GTAGCCAA -0.100306812444
GTAGCCAC -0.219877842755
GTAGCCCA -0.206640448002
GTAGCCGA -0.256318868256
GTAGCCTA -0.256205825705
GTAGCGAA -0.0742089927768
GTAGCGAC -0.103888256211
GTAGCGCA -0.0372757555303
GTAGCGGA 0.0547036161101
GTAGCGTA 0.021319715834
GTAGCTAA 0.114618319668
GTAGCTAC -0.0140617283951
GTAGCTCA -0.0944087433472
GTAGCTGA -0.0286285099563
GTAGCTTA -0.163091568792
GTAGGAAA 0.0288041449867
GTAGGAAC -0.115961623944
GTAGGACA -0.149141817928
GTAGGAGA 0.166057492838
GTAGGATA 0.288650683879
GTAGGCAA -0.136809445232

GTAGGCAC -0.273440382029
GTAGGCCA -0.102372250757
GTAGGCGA -0.0506547246644
GTAGGCTA -0.227438358495
GTAGGGAA 0.00322407160457
GTAGGGAC -0.363118394416
GTAGGGCA -0.0875801498552
GTAGGGGA 0.0176011528784
GTAGGGTA -0.0539145468017
GTAGGTAA 0.161479232345
GTAGGTCA -0.285609103662
GTAGGTGA -0.1706960702
GTAGGTTA -0.109374228281
GTAGTAAA 0.0283952446816
GTAGTAAC -0.0142593690312
GTAGTACA -0.260528640646
GTAGTAGA -0.104430067167
GTAGTATA 0.294819864322
GTAGTCAA -0.00250554348065
GTAGTCAC -0.199657225853
GTAGTCCA -0.0523337504665
GTAGTCGA -0.146543728218
GTAGTCTA -0.0281739863147
GTAGTGAA 0.0401624220941
GTAGTGAC -0.121528463852
GTAGTGCA -0.00184068687984
GTAGTGGA -0.181047204067
GTAGTGTA 0.104524797471
GTAGTTAA 0.155835137216
GTAGTTCA -0.180285295667
GTAGTTGA -0.0375403816208
GTAGTTTA -0.0535196494878
GTATAAAA 0.146708700769
GTATAAAC -0.102515529574
GTATAACA 0.0545464709048
GTATAAGA 0.136313963252
GTATAATA 0.250050121308
GTATACAA -0.0500432898182
GTATACAC -0.0358447494521
GTATACCA 0.0131468447833
GTATACGA 0.222419965836
GTATACTA 0.192655162572
GTATAGAA 0.0238067795122
GTATAGAC -0.0742433882725
GTATAGCA -0.0143608443624
GTATAGGA -0.083195352215
GTATAGTA -0.106674020657
GTATATAA 0.0441111094969
GTATATAC -0.00284834418951
GTATATCA -0.00809350166345
GTATATGA 0.102016474656
GTATATTA 0.219403828844
GTATCAAA 0.251508724013
GTATCAAC 0.0633149757977

GTATCACA 0.218333552877
GTATCAGA 0.245791620014
GTATCATA 0.160677291415
GTATCCAA 0.336700419857
GTATCCAC 0.322550230267
GTATCCCA 0.20623973966
GTATCCGA 0.318790405765
GTATCCTA 0.304968275923
GTATCGAA 0.187441346439
GTATCGAC 0.00592303110427
GTATCGCA 0.298217304441
GTATCGGA 0.0968407648906
GTATCGTA 0.273096006498
GTATCTAA 0.355935366309
GTATCTCA 0.271674158969
GTATCTGA 0.267220465872
GTATCTTA 0.126004968547
GTATGAAA 0.0385411067366
GTATGAAC -0.0344615552615
GTATGACA -0.0874395031853
GTATGAGA 0.12299623727
GTATGATA 0.215007396969
GTATGCAA 0.01975195081
GTATGCAC -0.0397375439983
GTATGCCA -0.070304940944
GTATGCGA -0.0871694820376
GTATGCTA -0.0605489973328
GTATGGAA -0.0130875540627
GTATGGAC -0.185877491893
GTATGGCA -0.113726361785
GTATGGGA -0.155901671252
GTATGGTA 0.0194257689246
GTATGTAA 0.136145453489
GTATGTCA -0.0619463927031
GTATGTGA 0.147445266079
GTATGTTA -0.0405543070236
GTATTAAA -0.0670843487234
GTATTAAC 0.0705214068833
GTATTACA 0.071766198582
GTATTAGA 0.119412476539
GTATTATA 0.0542893670187
GTATTCAA 0.0425613076028
GTATTCAC 0.176293559567
GTATTCCA 0.0330867825743
GTATTCGA 0.148653988639
GTATTCTA -0.00443752881512
GTATTGAA 0.100938503334
GTATTGAC 0.0454128107158
GTATTGCA 0.0660063234858
GTATTGGA 0.114965017147
GTATTGTA 0.106866051163
GTATTTAA 0.137999215493
GTATTTCA 0.031131869888
GTATTTGA 0.117925201431

GTATTTTA 0.105360800779
GTCAAAAA 0.0808057181875
GTCAAAAC -0.118971808399
GTCAAACA -0.138795345256
GTCAAAGA 0.109268441073
GTCAAATA 0.108847500472
GTCAACAA -0.0230230662847
GTCAACAC -0.0895631455842
GTCAACCA -0.0266456496157
GTCAACGA -0.136073935064
GTCAACTA -0.112587251416
GTCAAGAA 0.111256558871
GTCAAGAC -0.156501211535
GTCAAGCA 0.0714729853413
GTCAAGGA -0.017378028408
GTCAAGTA -0.0326543941689
GTCAATAA 0.027646051505
GTCAATCA 0.0605145008672
GTCAATGA -0.273397240378
GTCAATTA -0.0965232898506
GTCACAAA 0.09761161071
GTCACAAC -0.0697245760865
GTCACACA 0.0503639242805
GTCACAGA 0.0719059172199
GTCACATA -0.106349755209
GTCACCAA -0.19257179987
GTCACCAC -0.147190530284
GTCACCCA -0.153624468357
GTCACCGA -0.0330496430551
GTCACCTA -0.136031848773
GTCACGAA -0.023288107313
GTCACGAC -0.246640522876
GTCACGCA -0.0815852159213
GTCACGGA 0.0855740521197
GTCACGTA 0.0060959132974
GTCACTAA -0.0849987943021
GTCACTCA 0.0190485736449
GTCACTGA -0.147673021204
GTCACTTA -0.11478261824
GTCAGAAA -0.103570961603
GTCAGAAC -0.0995483555863
GTCAGACA -0.235441114348
GTCAGAGA -0.219967532468
GTCAGATA 0.27901820018
GTCAGCAA -0.181656285588
GTCAGCAC -0.142838329465
GTCAGCCA -0.265344889684
GTCAGCGA -0.0813875112943
GTCAGCTA -0.211443992877
GTCAGGAA 0.00234637423434
GTCAGGAC -0.0475969109367
GTCAGGCA -0.0705237345358
GTCAGGGA -0.116676737862
GTCAGGTA -0.164345335111

GTCAGTAA -0.0165728116973
GTCAGTCA -0.0952945437782
GTCAGTGA -0.294185746657
GTCAGTTA -0.155325207386
GTCATAAA 0.093912599618
GTCATAAC -0.282885640924
GTCATACA -0.042477526425
GTCATAGA -0.163044909785
GTCATATA 0.0230575863356
GTCATCAA -0.0404902057632
GTCATCAC -0.176177461666
GTCATCCA -0.00806469316298
GTCATCGA -0.0682832668235
GTCATCTA -0.0192020500782
GTCATGAA -0.0377263113859
GTCATGAC -0.0671702957587
GTCATGCA -0.0701949346622
GTCATGGA 0.0172341873587
GTCATGTA -0.0291677077825
GTCATTAA -0.08963332354
GTCATTCA -0.180801097394
GTCATTGA -0.0475600366103
GTCATTTA 0.0592524449274
GTCCAAAA -0.0295548958339
GTCCAAAC -0.02305466294
GTCCAACA -0.130853586248
GTCCAAGA -0.0510997998251
GTCCAATA -0.104365628225
GTCCACAA -0.0992115026482
GTCCACAC -0.285741574815
GTCCACCA -0.184892532416
GTCCACGA -0.00774128042589
GTCCACTA -0.0721851188003
GTCCAGAA -0.136932784636
GTCCAGAC -0.38209159361
GTCCAGCA -0.0913697989443
GTCCAGGA -0.121179375454
GTCCAGTA 0.0299265626029
GTCCATAA -0.0100825484643
GTCCATCA -0.320998740927
GTCCATGA -0.100395159299
GTCCATTA -0.080513017179
GTCCCAAA -0.0196491690268
GTCCCAAC -0.0244846209576
GTCCCACA -0.133091685674
GTCCCAGA -0.192423568924
GTCCCATA -0.183229759529
GTCCCCAA -0.132652333289
GTCCCCAC -0.258670167652
GTCCCCCA -0.128171493816
GTCCCCGA -0.192693538201
GTCCCCTA -0.118862685067
GTCCCGAA -0.178532422272
GTCCCGAC -0.0923987890128

GTCCCGCA -0.216893849598
GTCCCGGA -0.0148801915077
GTCCCGTA -0.0363464229851
GTCCCTAA 0.0156232848142
GTCCCTCA -0.301234965916
GTCCCTGA -0.177817287853
GTCCCTTA -0.195684763756
GTCCGAAA 0.0633053913094
GTCCGAAC -0.234853506562
GTCCGACA -0.0121293565586
GTCCGAGA 0.00931698009223
GTCCGATA 0.0254027057435
GTCCGCAA -0.114365851037
GTCCGCAC -0.172304805176
GTCCGCCA -0.239003264747
GTCCGCGA 0.0130610451436
GTCCGCTA -0.176811909949
GTCCGGAA -0.0618888285263
GTCCGGAC -0.223591833803
GTCCGGCA -0.1898995299
GTCCGGGA -0.0116346713205
GTCCGGTA -0.0870436233507
GTCCGTAA 0.0485466201234
GTCCGTCA -0.26988790677
GTCCGTGA -0.155077044646
GTCCGTTA -0.293655496302
GTCCTAAA -0.167781442041
GTCCTAAC -0.185494437811
GTCCTACA -0.136017607833
GTCCTAGA -0.0997059695161
GTCCTATA -0.255523148565
GTCCTCAA 0.0408475748296
GTCCTCAC -0.249086555078
GTCCTCCA -0.354816332576
GTCCTCGA -0.0582745098039
GTCCTCTA -0.135974445103
GTCCTGAA -0.0427985774968
GTCCTGCA -0.0185267514106
GTCCTGGA 0.00974291035128
GTCCTGTA -0.243260318299
GTCCTTAA -0.11988145221
GTCCTTCA -0.128553001604
GTCCTTGA -0.308713886977
GTCCTTTA -0.123092767332
GTCGAAAA 0.083723023557
GTCGAAAC -0.196441539579
GTCGAACA -0.077353885491
GTCGAAGA -0.135775637332
GTCGAATA 0.0564598375779
GTCGACAA -0.139517239865
GTCGACAC -0.0155606043145
GTCGACCA -0.0976291613158
GTCGACGA -0.0954074074074
GTCGACTA -0.107541132954

GTCGAGAA 0.137333836968
GTCGAGAC -0.210793995655
GTCGAGCA -0.179921568627
GTCGAGGA -0.0274043042741
GTCGAGTA 0.00488735215828
GTCGATAA 0.12221453819
GTCGATCA -0.146564923198
GTCGATGA -0.229427996444
GTCGATTA 0.15914314355
GTCGCAAA 0.0346487544752
GTCGCAAC -0.167111176964
GTCGCACA 0.0737886671497
GTCGCAGA -0.0227760684953
GTCGCATA -0.110304367392
GTCGCCAA -0.098867621635
GTCGCCAC -0.171388710554
GTCGCCCA -0.239987315186
GTCGCCGA -0.169319908318
GTCGCCTA -0.146750877356
GTCGCGAA -0.0160742092175
GTCGCGAC -0.169143910589
GTCGCGCA 0.0600685717606
GTCGCGGA 0.0776158722318
GTCGCGTA 0.0376461871486
GTCGCTAA 0.0610274141763
GTCGCTCA -0.275725310121
GTCGCTGA -0.0580850560428
GTCGCTTA 0.121385088413
GTCGGAAA 0.0279940502475
GTCGGAAC -0.148681272255
GTCGGACA -0.0856218512299
GTCGGAGA -0.13876751807
GTCGGATA 0.193864861331
GTCGGCAA -0.138625488388
GTCGGCAC -0.282327961799
GTCGGCCA -0.226543209877
GTCGGCGA 0.00679454460635
GTCGGCTA -0.1485634313
GTCGGGAA -0.0421298945772
GTCGGGCA -0.229419092146
GTCGGGGA -0.108869352387
GTCGGGTA -0.145240682207
GTCGGTAA -0.0675989761737
GTCGGTCA -0.342715586062
GTCGGTGA 0.0679069375565
GTCGGTTA 0.0807069283628
GTCGTAAA 0.0676521435939
GTCGTAAC -0.0584966513155
GTCGTACA -0.039191957346
GTCGTAGA -0.135285474391
GTCGTATA 0.0900102087861
GTCGTCAA -0.069494154833
GTCGTCAC -0.211551606288
GTCGTCCA -0.149730994152

GTCGTCGA -0.135214715636
GTCGTCTA -0.148417950744
GTCGTGAA -0.113632622152
GTCGTGCA 0.0188917429581
GTCGTGGA 0.222946842358
GTCGTGTA -0.0242047026279
GTCGTTAA 0.0237604056194
GTCGTTCA 0.0459011174779
GTCGTTGA 0.007854179016
GTCGTTTA 0.0324647461227
GTCTAAAA -0.0218136649572
GTCTAAAC -0.211533712028
GTCTAACA 0.0925471794052
GTCTAAGA -0.0171385817849
GTCTAATA 0.0924023119103
GTCTACAA -0.0764478912819
GTCTACAC -0.149330652846
GTCTACCA -0.168800985428
GTCTACGA -0.169915272839
GTCTACTA -0.203112910926
GTCTAGAA -0.128430985195
GTCTAGAC -0.141431347993
GTCTAGCA 0.0174216661987
GTCTAGGA -0.0854420133573
GTCTAGTA -0.121140332275
GTCTATAA -0.00611770517049
GTCTATCA -0.187366901124
GTCTATGA 0.00367992096814
GTCTATTA -0.087918648125
GTCTCAAA 0.10957979319
GTCTCAAC -0.0824706172911
GTCTCACA -0.248813596733
GTCTCAGA -0.170167029775
GTCTCATA -0.0114542518921
GTCTCCAA -0.135734538778
GTCTCCAC -0.110603864243
GTCTCCCA -0.235925832339
GTCTCCGA -0.106408133624
GTCTCCTA -0.200854278801
GTCTCGAA -0.177232211465
GTCTCGCA -0.165454249657
GTCTCGGA -0.139839519372
GTCTCGTA -0.103640695597
GTCTCTAA -0.329080061809
GTCTCTCA -0.259918663762
GTCTCTGA 0.0549984631935
GTCTCTTA -0.255491648511
GTCTGAAA 0.0503348684497
GTCTGAAC -0.0372394055177
GTCTGACA -0.0418906276331
GTCTGAGA 0.0153646582355
GTCTGATA 0.144185888571
GTCTGCAA -0.141185412002
GTCTGCAC -0.228410239882

GTCTGCCA -0.228402376603
GTCTGCGA -0.130022006004
GTCTGCTA -0.118813212383
GTCTGGAA -0.18440877915
GTCTGGCA -0.128364696004
GTCTGGGA -0.0393141289438
GTCTGGTA -0.146396481453
GTCTGTAA 0.0671087995584
GTCTGTCA -0.179641718889
GTCTGTGA -0.132051486932
GTCTGTTA -0.270646053541
GTCTTAAA -0.0409550760935
GTCTTAAC -0.213643770678
GTCTTACA 0.0239135182354
GTCTTAGA -0.0713206604319
GTCTTATA -0.0155022169542
GTCTTCAA 0.021214790665
GTCTTCAC -0.155692510596
GTCTTCCA -0.0678478246974
GTCTTCGA -0.0655106248554
GTCTTCTA -0.00188350599888
GTCTTGAA -0.176056260735
GTCTTGCA -0.0942033405955
GTCTTGGA -0.0251975308642
GTCTTGTA 0.0211482864324
GTCTTTAA -0.0432850799197
GTCTTTCA -0.0587581537925
GTCTTTGA -0.038647700894
GTCTTTTA 0.0605254780566
GTGAAAAA 0.0117272275631
GTGAAAAC 0.12558337255
GTGAAACA -0.0333102812356
GTGAAAGA -0.0497217904203
GTGAAATA 0.0795650342132
GTGAACAA 0.0707230343738
GTGAACAC -0.248374406564
GTGAACCA -0.202905159694
GTGAACGA 0.0837806538014
GTGAACTA -0.194770967441
GTGAAGAA 0.0101826158964
GTGAAGCA -0.1829218107
GTGAAGGA -0.203587574341
GTGAAGTA 0.0333629673532
GTGAATAA 0.00210751625916
GTGAATCA 0.0133866824738
GTGAATGA -0.0562855386507
GTGAATTA 0.117754565397
GTGACAAA 0.0161905545951
GTGACAAC -0.118974200308
GTGACACA 0.0339590022228
GTGACAGA -0.13173704883
GTGACATA -0.047991898434
GTGACCAA -0.146069619076
GTGACCAC -0.289618898809

GTGACCCA -0.2931272835
GTGACCGA -0.107581699346
GTGACCTA -0.204769844336
GTGACGAA -0.0538764602916
GTGACGCA 0.0422243367755
GTGACGGA -0.190462473732
GTGACGTA -0.0246136029331
GTGACTAA -0.131321612011
GTGACTCA -0.183912871678
GTGACTGA 0.0725262903686
GTGACTTA 0.00494966077318
GTGAGAAA 0.00669591620298
GTGAGAAC -0.147378390313
GTGAGACA -0.109878680828
GTGAGAGA -0.046248786979
GTGAGATA 0.0763834002942
GTGAGCAA -0.0348221133223
GTGAGCAC -0.181191046471
GTGAGCCA -0.280340065511
GTGAGCGA -0.065633207056
GTGAGCTA -0.128863320879
GTGAGGAA 0.14802191047
GTGAGGCA -0.0647699957193
GTGAGGGA -0.0370526933547
GTGAGGTA -0.0579605712376
GTGAGTAA 0.0662211439281
GTGAGTCA -0.253185185185
GTGAGTGA 0.133268926242
GTGAGTTA 0.0353536821002
GTGATAAA 0.0226184987596
GTGATAAC -0.062207080906
GTGATACA -0.0383754885012
GTGATAGA 0.119058596237
GTGATATA 0.146277221681
GTGATCAA -0.0974993962546
GTGATCAC 0.18656991125
GTGATCCA 0.0609053796871
GTGATCGA -0.0790880347792
GTGATCTA 0.151809040813
GTGATGAA -0.0327641660629
GTGATGCA -0.097986430927
GTGATGGA -0.0752015243324
GTGATGTA -0.0549771073977
GTGATTAA 0.0532666017753
GTGATTCA 0.101537053448
GTGATTGA 0.0128323344091
GTGATTTA 0.114931791437
GTGCAAAA -0.00554320682747
GTGCAAAC -0.0434845840605
GTGCAACA -0.194501746169
GTGCAAGA 0.103109135315
GTGCAATA 0.0217658085836
GTGCACAA -0.173866191782
GTGCACAC -0.197111374512

GTGCACCA -0.165991736217
GTGCACGA 0.080993559827
GTGCACTA -0.197517898272
GTGCAGAA -0.0331291576105
GTGCAGCA -0.114872242848
GTGCAGGA 0.0784701117432
GTGCAGTA 0.054748449145
GTGCATAA 0.0298947940334
GTGCATCA -0.103008724604
GTGCATGA -0.0815505380343
GTGCATTA 0.1362381278
GTGCCAAA -0.155959331881
GTGCCAAC -0.153449853397
GTGCCACA -0.23316817593
GTGCCAGA -0.0551748407171
GTGCCATA -0.114050306233
GTGCCCAA -0.153385459534
GTGCCCAC -0.144280045088
GTGCCCCA -0.0427493201943
GTGCCCGA -0.0687794872987
GTGCCCTA -0.225071957386
GTGCCGAA -0.132748041927
GTGCCGCA -0.337900557942
GTGCCGGA -0.1406728107
GTGCCGTA -0.0199829861969
GTGCCTAA -0.273750740668
GTGCCTCA -0.154181202381
GTGCCTGA -0.012455247688
GTGCCTTA -0.15875845078
GTGCGAAA -0.0439251521799
GTGCGAAC -0.103340412571
GTGCGACA -0.0435270872982
GTGCGAGA -0.0490378493491
GTGCGATA 0.0879098688129
GTGCGCAA 0.126693231465
GTGCGCAC 0.116531700573
GTGCGCCA -0.0780318943402
GTGCGCGA 0.187981055598
GTGCGCTA -0.28109770619
GTGCGGAA 0.15909240598
GTGCGGCA -0.211602194787
GTGCGGGA -0.0405936464028
GTGCGGTA -0.075706312358
GTGCGTAA 0.0708831519765
GTGCGTCA -0.0582235265062
GTGCGTGA -0.0208228611277
GTGCGTTA 0.0462331774572
GTGCTAAA 0.0593512298726
GTGCTAAC -0.156138322167
GTGCTACA -0.147813343871
GTGCTAGA 0.252994418651
GTGCTATA 0.0206920020198
GTGCTCAA -0.244451577151
GTGCTCCA -0.234278816964

GTGCTCGA -0.0178016282083
GTGCTCTA -0.180604620355
GTGCTGAA 0.0978983088272
GTGCTGCA -0.303612669326
GTGCTGGA -0.142608569354
GTGCTGTA -0.123357418238
GTGCTTAA 0.13012363256
GTGCTTCA -0.176409260265
GTGCTTGA -0.0853820115352
GTGCTTTA -0.0199043886803
GTGGAAAA 0.0796669520736
GTGGAAAC -0.0443112219698
GTGGAACA -0.151336549173
GTGGAAGA -0.146181498861
GTGGAATA 0.192205492737
GTGGACAA 0.00840029418868
GTGGACAC -0.0666867132095
GTGGACCA -0.112963237352
GTGGACGA -0.201065847841
GTGGACTA -0.1408853743
GTGGAGAA -0.189827160494
GTGGAGCA -0.0663895063253
GTGGAGGA -0.110118727722
GTGGAGTA 0.0926077110476
GTGGATAA 0.179282201585
GTGGATCA 0.0199867176008
GTGGATGA 0.0022749101287
GTGGATTA 0.299782081269
GTGGCAAA 0.0836533486559
GTGGCAAC -0.312199618145
GTGGCACA -0.191145969499
GTGGCAGA -0.0202368397352
GTGGCATA -0.0237723065123
GTGGCCAA -0.270333407396
GTGGCCAC -0.127871846769
GTGGCCCA -0.31322875817
GTGGCCGA -0.181039949271
GTGGCCTA -0.116920505442
GTGGCGAA 0.076572283643
GTGGCGCA -0.100212915405
GTGGCGGA -0.0930539779742
GTGGCGTA -0.0102000493949
GTGGCTAA -0.0761542514655
GTGGCTCA -0.312517902119
GTGGCTGA -0.0789662155488
GTGGCTTA -0.366815341486
GTGGGAAA -0.0581247366815
GTGGGAAC -0.179144032922
GTGGGACA -0.221558233461
GTGGGAGA 0.0903844570676
GTGGGATA 0.0766345035017
GTGGGCAA -0.057767349963
GTGGGCCA -0.284782584837
GTGGGCGA -0.115812636166

GTGGGCTA -0.230921490208
GTGGGGAA -0.0263315330743
GTGGGGCA -0.053733702094
GTGGGGGA -0.0436891370535
GTGGGGTA -0.0551275508804
GTGGGTAA -0.110970358706
GTGGGTCA -0.400895014404
GTGGGTGA -0.185534539429
GTGGGTTA -0.174473970167
GTGGTAAA -0.0549347906298
GTGGTAAC -0.0418476602792
GTGGTACA -0.089217606998
GTGGTAGA -0.00444365033019
GTGGTATA 0.0342321270321
GTGGTCAA 0.0186888768152
GTGGTCCA -0.0750289480472
GTGGTCGA -0.0719546664471
GTGGTCTA -0.112392830891
GTGGTGAA 0.180058983336
GTGGTGCA -0.179397095859
GTGGTGGA -0.0732715412665
GTGGTGTA -0.019399307442
GTGGTTAA -0.0839294326839
GTGGTTCA -0.242817250668
GTGGTTGA -0.119131556851
GTGGTTTA -0.118556022063
GTGTAAAA 0.0910595208694
GTGTAAAC -0.0883469169342
GTGTAACA -0.0136583679115
GTGTAAGA 0.113852664164
GTGTAATA 0.287410261477
GTGTACAA -0.0779756802696
GTGTACAC -0.259919361475
GTGTACCA 0.020779819535
GTGTACGA 0.0872851215175
GTGTACTA -0.0620194286655
GTGTAGAA -0.0601659751037
GTGTAGCA -0.107139017622
GTGTAGGA 0.0371129438491
GTGTAGTA -0.0390379614429
GTGTATAA 0.0667687545281
GTGTATCA 0.153481614923
GTGTATGA 0.118295681574
GTGTATTA 0.0873235416804
GTGTCAAA -0.0590835534095
GTGTCAAC -0.218912293339
GTGTCACA -0.106126107281
GTGTCAGA 0.0269270455992
GTGTCATA -0.132969941526
GTGTCCAA -0.140060761745
GTGTCCCA -0.227040349496
GTGTCCGA 0.0117428483611
GTGTCCTA -0.0204630108489
GTGTCGAA -0.163066148543

GTGTCGCA 0.00959567901235
GTGTCGGA 0.0517179259948
GTGTCGTA 0.0547005070242
GTGTCTAA −0.153618658052
GTGTCTCA −0.122985685745
GTGTCTGA −0.0366016622462
GTGTCTTA −0.208832035473
GTGTGAAA −0.0844774792572
GTGTGAAC −0.131299927378
GTGTGACA −0.0915644345545
GTGTGAGA −0.0352700048807
GTGTGATA 0.144476278294
GTGTGCAA 0.0502418897912
GTGTGCCA −0.402752579483
GTGTGCGA 0.117680958967
GTGTGCTA −0.124582866803
GTGTGGAA 0.00974266521782
GTGTGGCA −0.107245257907
GTGTGGGA −0.0741584394303
GTGTGGTA −0.0281565528162
GTGTGTAA 0.195535537884
GTGTGTCA 0.0476094315227
GTGTGTGA −0.0120625281478
GTGTGTTA −0.110349315546
GTGTTAAA 0.00027438907273
GTGTTAAC −0.0837833980988
GTGTTACA 0.0539900732417
GTGTTAGA −0.0616972930251
GTGTTATA 0.0870463344054
GTGTTCAA −0.131378489029
GTGTTCCA −0.27614556587
GTGTTCGA 0.0328336461523
GTGTTCTA −0.139584313746
GTGTTGAA 0.0277311247228
GTGTTGCA 0.0148628375761
GTGTTGGA −0.150469472657
GTGTTGTA −0.0307855276735
GTGTTTAA −0.0158230238653
GTGTTTCA −0.137564210214
GTGTTTGA −0.136446464247
GTGTTTTA 0.153901471678
GTTAAAAA 0.188006322861
GTTAAAAC 0.14416891699
GTTAAACA 0.0771127220955
GTTAAAGA 0.0975192630643
GTTAAATA 0.201375793587
GTTAACAA 0.0668531082475
GTTAACCA −0.184729699324
GTTAACGA −0.0532503457815
GTTAACTA −0.0582956551582
GTTAAGAA 0.124766734725
GTTAAGCA 0.0450915497596
GTTAAGGA 0.126133394806
GTTAAGTA 0.161008527886

GTTAATAA 0.142447663185
GTTAATCA -0.0567187617812
GTTAATGA 0.0781935990005
GTTAATTA -0.0461875060125
GTTACAAA 0.173231025928
GTTACAAC -0.199293792286
GTTACACA -0.0675009878495
GTTACAGA 0.155200267314
GTTACATA 0.158993611276
GTTACCAA 0.0116275878724
GTTACCCA -0.0482215804429
GTTACCGA -0.112145337266
GTTACCTA -0.053661990384
GTTACGAA 0.113899317219
GTTACGCA -0.0148373599872
GTTACGGA -0.0760129944247
GTTACGTA 0.206471632819
GTTACTAA -0.00459082126306
GTTACTCA -0.121610090434
GTTACTGA 0.0161063042228
GTTACTTA 0.015705234038
GTTAGAAA -0.105575863356
GTTAGAAC -0.0733499920096
GTTAGACA -0.0719553121323
GTTAGAGA -0.024198098368
GTTAGATA 0.26386326326
GTTAGCAA 0.0286940856856
GTTAGCCA -0.103871597535
GTTAGCGA 0.147388607209
GTTAGCTA -0.0813369556269
GTTAGGAA 0.0620850464167
GTTAGGCA -0.138700286753
GTTAGGGA -0.0358031003788
GTTAGGTA -0.043025093855
GTTAGTAA -0.0883660324405
GTTAGTCA -0.0568317522119
GTTAGTGA -0.0613366477896
GTTAGTTA -0.000933015010565
GTTATAAA 0.0856522645942
GTTATAAC 0.0358433478033
GTTATACA -0.105268790648
GTTATAGA 0.0810802652089
GTTATATA 0.0680118143504
GTTATCAA 0.212631507458
GTTATCCA 0.185220861051
GTTATCGA 0.0867447299987
GTTATCTA 0.00631188390524
GTTATGAA 0.129209826682
GTTATGCA 0.062859638941
GTTATGGA -0.00850648139001
GTTATGTA 0.00963927186088
GTTATTAA 0.0554806560807
GTTATTCA 0.00509616017915
GTTATTGA -0.0480018360564

GTTATTTA 0.0862478752924
GTTCAAAA -0.078073944678
GTTCAAAC -0.0195192316073
GTTCAACA -0.0539458734273
GTTCAAGA 0.0113806011109
GTTCAATA 0.0517469818256
GTTCACAA -0.0659418563434
GTTCACCA -0.136546790269
GTTCACGA -0.252288517897
GTTCACTA -0.0357772683825
GTTCAGAA -0.00215739543533
GTTCAGCA -0.0909110717928
GTTCAGGA 0.0331309110456
GTTCAGTA -0.0488265384531
GTTCATAA -0.0663367986962
GTTCATCA -0.270840805718
GTTCATGA 0.0580550026877
GTTCATTA -0.119688513525
GTTCCAAA 0.0492567931215
GTTCCAAC -0.182130471048
GTTCCACA -0.184684768114
GTTCCAGA 0.0131442085873
GTTCCATA 0.0295047433644
GTTCCCAA -0.167398353197
GTTCCCCA -0.157617190905
GTTCCCGA 0.0520898406288
GTTCCCTA -0.21253676632
GTTCCGAA -0.198852578068
GTTCCGCA -0.0736505584369
GTTCCGGA 0.0565709455751
GTTCCGTA -0.00570827602524
GTTCCTAA 0.00352246317661
GTTCCTCA -0.028065093468
GTTCCTGA 0.122003227294
GTTCCTTA -0.0499135589034
GTTCGAAA 0.102208243139
GTTCGAAC -0.0228570017776
GTTCGACA -0.0594579463874
GTTCGAGA 0.179004256679
GTTCGATA 0.0550196689174
GTTCGCAA 0.0406567652418
GTTCGCCA -0.178038970374
GTTCGCGA 0.0578607653296
GTTCGCTA -0.214689614302
GTTCGGAA -0.0815279424031
GTTCGGCA -0.116622760078
GTTCGGGA 0.0395947221673
GTTCGGTA -0.242093953764
GTTCGTAA 0.0657547978441
GTTCGTCA -0.287534744308
GTTCGTGA 0.0818430113463
GTTCGTTA 0.0121243056928
GTTCTAAA 0.0389667113843
GTTCTACA -0.119032639638

GTTCTAGA -0.117158944977
GTTCTATA -0.102027935567
GTTCTCAA -0.105326132297
GTTCTCCA -0.208091835167
GTTCTCGA 0.0320570367723
GTTCTCTA -0.0477891940548
GTTCTGAA -0.0787739085096
GTTCTGCA 0.110402036311
GTTCTGGA 0.0750950805097
GTTCTGTA 0.0304272659916
GTTCTTAA 0.167061845485
GTTCTTCA -0.155639514465
GTTCTTGA -0.164243691484
GTTCTTTA 0.0254109744263
GTTGAAAA 0.112711925966
GTTGAAAC 0.0529594502624
GTTGAACA -0.192987222552
GTTGAAGA 0.13956124174
GTTGAATA 0.111398602415
GTTGACAA -0.0460244359537
GTTGACCA -0.265687775431
GTTGACGA 0.168373619618
GTTGACTA -0.0934647786834
GTTGAGAA 0.199657237261
GTTGAGCA -0.146287059443
GTTGAGGA -0.00214846643947
GTTGAGTA 0.107334957957
GTTGATAA 0.00746008455247
GTTGATCA -0.284449146374
GTTGATGA -0.100446771609
GTTGATTA 0.214276582453
GTTGCAAA 0.119047619048
GTTGCAAC -0.00752375864716
GTTGCACA 0.0631833568201
GTTGCAGA 0.00821053209297
GTTGCATA -0.0663241783574
GTTGCCAA -0.0916842301697
GTTGCCCA -0.288572806172
GTTGCCGA -0.175004526973
GTTGCCTA -0.0974074074074
GTTGCGAA -0.0370302035361
GTTGCGCA -0.114780417922
GTTGCGGA 0.0213662051632
GTTGCGTA 0.0259855908605
GTTGCTAA -0.0599872154345
GTTGCTCA -0.211944807553
GTTGCTGA 0.0618207864058
GTTGCTTA -0.0340934661508
GTTGGAAA 0.0523368151904
GTTGGACA -0.070267168364
GTTGGAGA 0.115213632996
GTTGGATA 0.287045269929
GTTGGCAA -0.156088483697
GTTGGCCA -0.191493177388

GTTGGCGA 0.0733906701727
GTTGGCTA -0.136887884352
GTTGGGAA 0.0456496157366
GTTGGGCA -0.145968799557
GTTGGGGA -0.122042554675
GTTGGGTA 0.0044059693956
GTTGGTAA 0.0437711562768
GTTGGTCA -0.267381131505
GTTGGTGA -0.191647600393
GTTGGTTA -0.00375968736965
GTTGTAAA 0.127280511098
GTTGTACA -0.00985202748688
GTTGTAGA 0.086011767547
GTTGTATA 0.0594903607281
GTTGTCAA 0.00171793014117
GTTGTCCA -0.374631118495
GTTGTCGA -0.0962208947592
GTTGTCTA -0.161332543093
GTTGTGAA -0.107122573954
GTTGTGCA -0.0129051472404
GTTGTGGA 0.117576675668
GTTGTGTA 0.19740386152
GTTGTTAA -0.0595832643326
GTTGTTCA 0.0190070034468
GTTGTTGA -0.0820710996179
GTTGTTTA 0.000639421282575
GTTTAAAA 0.0523145403851
GTTTAAAC -0.000352395616827
GTTTAACA 0.0859737725971
GTTTAAGA 0.127892489407
GTTTAATA -0.0863350427847
GTTTACAA -0.0243970923159
GTTTACCA -0.116949026968
GTTTACGA 0.00469205314368
GTTTACTA -0.178922294844
GTTTAGAA 0.122082431392
GTTTAGCA -0.135408010878
GTTTAGGA 0.122047564395
GTTTAGTA 0.067057618358
GTTTATAA 0.0178086252038
GTTTATCA -0.0734126984127
GTTTATGA -0.00901776109245
GTTTATTA 0.0548420382445
GTTTCAAA 0.0783592943863
GTTTCACA -0.0250390781621
GTTTCAGA 0.0961052932007
GTTTCATA 0.176299150366
GTTTCCAA -0.0117632530908
GTTTCCCA -0.0881179927891
GTTTCCGA 0.0327230016027
GTTTCCTA -0.0633734338316
GTTTCGAA 0.0896764633243
GTTTCGCA -0.0983041080102
GTTTCGGA -0.0716374991082

GTTTCGTA 0.0214185056586
GTTTCTAA -0.12173749321
GTTTCTCA -0.0264824694285
GTTTCTGA 0.0346086881359
GTTTCTTA 0.0734119916696
GTTTGAAA 0.0228696019671
GTTTGACA -0.104510762669
GTTTGAGA -0.012887713742
GTTTGATA 0.155796504863
GTTTGCAA -0.00117389506964
GTTTGCCA -0.262448578799
GTTTGCGA -0.0605419438407
GTTTGCTA -0.154873683966
GTTTGGAA 0.170155303416
GTTTGGCA -0.223414541589
GTTTGGGA -0.0472366364307
GTTTGGTA -0.0530620427564
GTTTGTAA 0.0300800488138
GTTTGTCA -0.0965781574947
GTTTGTGA 0.0474022481284
GTTTGTTA 0.101045028431
GTTTTAAA 0.180972789214
GTTTTACA 0.0696134121715
GTTTTAGA -0.08130838406
GTTTTATA -0.08618588952
GTTTTCAA -0.0675071619085
GTTTTCCA 0.0183431295071
GTTTTCGA 0.0499964900147
GTTTTCTA 0.0498365609519
GTTTTGAA 0.0466516828031
GTTTTGCA -0.0853851986787
GTTTTGGA -0.00369913010071
GTTTTGTA 0.0619854442469
GTTTTTAA -0.0128731858266
GTTTTTCA -0.111024436445
GTTTTTGA 0.0574792585871
GTTTTTTA 0.00788537734377
TAAAAAAA 0.231805965162
TAAAAACA 0.109678587894
TAAAAAGA 0.187086983249
TAAAAATA 0.24166810728
TAAAACAA 0.0271860880682
TAAAACCA 0.1052191047
TAAAACGA 0.130889253701
TAAAACTA 0.0162709389888
TAAAAGAA -0.0918669141979
TAAAAGCA 0.125974225559
TAAAAGGA -0.115577239835
TAAAAGTA 0.0472924762344
TAAAATAA 0.107356912336
TAAAATCA 0.293235802595
TAAAATGA 0.177981404641
TAAAATTA 0.117814129851
TAAACAAA 0.115696831983

TAAACACA 0.158894816571
TAAACAGA 0.0200325425305
TAAACATA 0.0226969622129
TAAACCAA -0.0397691725114
TAAACCCA -0.0951107020664
TAAACCGA -0.00607153443336
TAAACCTA -0.172027699585
TAAACGAA -0.0868789655097
TAAACGCA -0.0894265831796
TAAACGGA 0.119939515686
TAAACGTA -0.0536729675734
TAAACTAA -0.0964066007035
TAAACTCA 0.124300204176
TAAACTGA 0.1041724173
TAAACTTA -0.0852788633359
TAAAGAAA 0.152481393664
TAAAGACA 0.0790125209918
TAAAGAGA 0.139429515467
TAAAGATA 0.238340621504
TAAAGCAA 0.102565629858
TAAAGCCA -0.112013133236
TAAAGCGA 0.0217952095545
TAAAGCTA -0.103735814191
TAAAGGAA -0.0631399583564
TAAAGGCA 0.0516400234877
TAAAGGGA 0.0928406432961
TAAAGGTA 0.125768403258
TAAAGTAA 0.112107564686
TAAAGTCA -0.0977801345285
TAAAGTGA -0.072989699708
TAAAGTTA 0.024606712437
TAAATAAA 0.191113274156
TAAATACA 0.0884938910116
TAAATAGA 0.0580299812309
TAAATATA 0.227708343625
TAAATCAA 0.181443493673
TAAATCCA 0.275270582424
TAAATCGA 0.0723329962014
TAAATCTA 0.245099007743
TAAATGAA 0.154860314094
TAAATGCA 0.0447445266079
TAAATGGA 0.0541636646757
TAAATGTA 0.14458617233
TAAATTAA 0.100435794419
TAAATTCA 0.0317507590836
TAAATTGA 0.0444568738832
TAAATTTA 0.240843325358
TAACAAAA 0.116433977889
TAACAACA 0.0476602120793
TAACAAGA 0.140126102306
TAACAATA 0.145796285319
TAACACAA 0.179540336757
TAACACCA -0.141031227306
TAACACGA 0.151144920854

TAACACTA 0.013149216219
TAACAGAA 0.0685772464818
TAACAGCA 0.140447169236
TAACAGGA 0.021995085449
TAACAGTA 0.20266878057
TAACATAA 0.0919660627897
TAACATCA 0.140480583441
TAACATGA 0.088402050539
TAACATTA 0.158240960005
TAACCAAA 0.00476410019978
TAACCACA -0.144101524258
TAACCAGA 0.0728655188243
TAACCATA 0.0961202962948
TAACCCAA 0.0449900107576
TAACCCCA -0.0518922609795
TAACCCGA -0.0187802362239
TAACCCTA -0.0750813756383
TAACCGAA 0.0622132209269
TAACCGCA -0.0807301730275
TAACCGGA -0.0537581457238
TAACCGTA 0.0150407508027
TAACCTAA 0.0583629944939
TAACCTCA -0.034034775736
TAACCTGA 0.0712434442782
TAACCTTA -0.0395508134097
TAACGAAA 0.272773016892
TAACGACA 0.141367906255
TAACGAGA 0.213775274979
TAACGATA 0.112102390596
TAACGCAA 0.214070286055
TAACGCCA -0.078325818672
TAACGCGA 0.0621634971598
TAACGCTA -0.151892274516
TAACGGAA 0.0380268185318
TAACGGCA -0.028153672242
TAACGGGA 0.0360518342883
TAACGGTA 0.150943588105
TAACGTAA 0.19731772377
TAACGTCA -0.111986541966
TAACGTGA 0.0539144657402
TAACGTTA 0.0543923756852
TAACTAAA -0.00724226958247
TAACTACA 0.0707945316926
TAACTAGA 0.114882396525
TAACTATA -0.0666469096776
TAACTCAA 0.0252447913236
TAACTCCA 0.0299627086832
TAACTCGA -0.0782819355778
TAACTCTA 0.10803612593
TAACTGAA 0.136408044084
TAACTGCA 0.0226102658675
TAACTGGA 0.119991657336
TAACTGTA 0.0933047377549
TAACTTAA -0.0395835337492

TAACTTCA -0.188848372147
TAACTTGA 0.0434704923605
TAAGAAAA 0.189241256669
TAAGAACA 0.0125661375661
TAAGAAGA 0.0988581058504
TAAGAATA 0.241856377028
TAAGACAA -0.0934025376755
TAAGACCA -0.245115465828
TAAGACGA -0.131477273975
TAAGACTA -0.0219963442348
TAAGAGAA 0.131955602691
TAAGAGCA -0.0337905332719
TAAGAGGA -0.142994715788
TAAGAGTA 0.0634913486264
TAAGATAA 0.219044644284
TAAGATCA 0.237683959729
TAAGATGA 0.0959932009356
TAAGATTA 0.355628889657
TAAGCAAA -0.0106620371043
TAAGCACA 0.027496184516
TAAGCAGA 0.0657012496296
TAAGCATA -0.0984295937744
TAAGCCAA -0.230198480024
TAAGCCCA -0.073091438383
TAAGCCGA -0.108421832551
TAAGCCTA -0.142037192509
TAAGCGAA 0.118098876992
TAAGCGCA -0.126969622129
TAAGCGGA -0.0280200591547
TAAGCGTA 0.124294715581
TAAGCTAA -0.218180471181
TAAGCTCA -0.135166271992
TAAGCTGA -0.238290634541
TAAGCTTA 0.0245217969034
TAAGGAAA 0.087743419175
TAAGGACA -0.260483707252
TAAGGAGA 0.0945369272651
TAAGGATA 0.222871297198
TAAGGCAA 0.113550623087
TAAGGCCA -0.25800534997
TAAGGCGA 0.00696228237722
TAAGGCTA -0.0422583494085
TAAGGGAA 0.0691837361962
TAAGGGCA -0.187967161777
TAAGGGGA -0.0238538202334
TAAGGGTA -0.00878911609426
TAAGGTAA 0.103504265282
TAAGGTCA -0.185949471997
TAAGGTGA -0.0624602076884
TAAGTAAA 0.238818779018
TAAGTACA 0.0606903665393
TAAGTAGA 0.115390873564
TAAGTATA 0.0758853103251
TAAGTCAA -0.199552025472

TAAGTCCA -0.182832872382
TAAGTCGA -0.069202609349
TAAGTCTA -0.0184092226813
TAAGTGAA 0.0938686908604
TAAGTGCA -0.130258283397
TAAGTGGA 0.109068324786
TAAGTGTA -0.00917437856842
TAAGTTAA -0.0642686996421
TAAGTTCA -0.00237656150519
TAAGTTGA 0.0481624184944
TAATAAAA 0.148655675646
TAATAACA 0.106366132524
TAATAAGA 0.20725959932
TAATAATA 0.241699475542
TAATACAA 0.189737974489
TAATACCA 0.0191442183143
TAATACGA 0.143990527799
TAATACTA -0.0376549038978
TAATAGAA 0.0845475649511
TAATAGCA 0.0505609343784
TAATAGGA 0.0781666748987
TAATAGTA 0.18140281551
TAATATAA 0.181680717469
TAATATCA 0.292704080891
TAATATGA 0.0962488922465
TAATATTA 0.289783669493
TAATCAAA 0.2963941714
TAATCACA 0.259821840216
TAATCAGA 0.304693846188
TAATCATA 0.209204904673
TAATCCAA 0.324044066792
TAATCCCA 0.325918241893
TAATCCGA 0.404838745088
TAATCCTA 0.335875234263
TAATCGAA 0.318738105269
TAATCGCA 0.211667368849
TAATCGGA 0.300229541063
TAATCGTA 0.256778414455
TAATCTAA 0.368536893641
TAATCTCA 0.340248962656
TAATCTGA 0.388776372698
TAATGAAA -0.0220606395188
TAATGACA 0.163804364531
TAATGAGA 0.0556735428568
TAATGATA 0.069967893307
TAATGCAA 0.148383769413
TAATGCCA -0.173268793836
TAATGCGA 0.114739072208
TAATGCTA 0.0217918730841
TAATGGAA 0.187783475949
TAATGGCA -0.0805272892928
TAATGGGA 0.0551837032646
TAATGGTA 0.163425973007
TAATGTAA 0.269957645955

TAATGTCA -0.0487571368384
TAATGTGA 0.109126436448
TAATTAAA 0.15584041362
TAATTACA 0.226161350122
TAATTAGA 0.0663324112494
TAATTATA 0.0784539726448
TAATTCAA 0.0754972179042
TAATTCCA 0.078062847762
TAATTCGA 0.000672097177192
TAATTCTA 0.101993229991
TAATTGAA 0.142209405372
TAATTGCA 0.175064216558
TAATTGGA 0.129215557295
TAATTGTA 0.0964290383973
TAATTTAA 0.239116116709
TAATTTCA 0.325842255895
TAATTTGA 0.0926502228369
TACAAAAA 0.0630282772399
TACAAACA 0.230443667885
TACAAAGA 0.00946006026775
TACAAATA 0.280703595772
TACAACAA 0.0848831434908
TACAACCA -0.0110688187352
TACAACGA -0.00634080866011
TACAACTA 0.0522246806563
TACAAGAA 0.173229410312
TACAAGCA -0.0535397265846
TACAAGGA 0.0789287360864
TACAAGTA -0.100582282371
TACAATAA -0.0582339897693
TACAATCA 0.255062828736
TACAATGA -0.0271370159985
TACACAAA 0.188887597519
TACACACA -0.131426878056
TACACAGA 0.295385934072
TACACATA 0.116736158529
TACACCAA 0.158299304046
TACACCCA -0.0650260301671
TACACCGA 0.0629421934247
TACACCTA -0.0363721665267
TACACGAA 0.117242607581
TACACGCA 0.0203219154401
TACACGGA 0.145148631144
TACACGTA -0.0291430657095
TACACTAA 0.158413842198
TACACTCA -0.0773117283951
TACACTGA 0.0079026668088
TACAGAAA 0.170645897824
TACAGACA -0.12366352719
TACAGAGA 0.0652480259583
TACAGATA 0.30574765637
TACAGCAA -0.0560193418077
TACAGCCA -0.0961322788525
TACAGCGA 0.105991312307

TACAGCTA -0.0643438645833
TACAGGAA 0.146686908895
TACAGGCA -0.0483881589779
TACAGGGA 0.00532448099022
TACAGGTA -0.00922038363382
TACAGTAA 0.103476475883
TACAGTCA -0.0784368603305
TACAGTGA -0.0339648133889
TACATAAA 0.187539792312
TACATACA 0.212410007878
TACATAGA 0.155407847287
TACATATA 0.0696186076018
TACATCAA 0.114363103471
TACATCCA 0.125394796101
TACATCGA 0.266317592044
TACATCTA 0.182687320952
TACATGAA 0.0810730318307
TACATGCA -0.00055119768497
TACATGGA 0.0248294671913
TACATGTA 0.0794156378601
TACATTAA 0.0899973220112
TACATTCA 0.0770857762397
TACATTGA 0.0517527929917
TACCAAAA 0.142218122122
TACCAACA 0.0420700783771
TACCAAGA 0.0848961434362
TACCAATA 0.0320039956969
TACCACAA 0.148656894222
TACCACCA 0.0882154383192
TACCACGA -0.137994424689
TACCACTA 0.0768977089477
TACCAGAA 0.0423307866254
TACCAGCA -0.0250691562932
TACCAGGA 0.0284659804429
TACCAGTA 0.0577993113768
TACCATAA 0.0984873433006
TACCATCA 0.068812610987
TACCATGA 0.181873519969
TACCCAAA 0.152788754967
TACCCACA -0.00756759112635
TACCCAGA 0.254600264408
TACCCATA 0.0234117356501
TACCCCAA -0.122937864269
TACCCCCA -0.00187073989924
TACCCCGA -0.0725833333333
TACCCCTA 0.101895892958
TACCCGAA 0.157517179301
TACCCGCA -8.28091177197E-5
TACCCGGA 0.0181343168895
TACCCGTA -0.0932562223869
TACCCTAA -0.00122944521285
TACCCTCA 0.00949433084176
TACCCTGA 0.0736904820705
TACCGAAA 0.237623218951

TACCGACA 0.108048465126
TACCGAGA 0.225829180771
TACCGATA 0.236120756033
TACCGCAA 0.174059803728
TACCGCCA -0.00265484540156
TACCGCGA 0.136970639083
TACCGCTA -0.0783826209137
TACCGGAA -0.181720394701
TACCGGCA -0.125454136345
TACCGGGA -0.0357060528222
TACCGGTA -0.0715435512542
TACCGTAA 0.223620937632
TACCGTCA -0.0394939765649
TACCGTGA 0.0101099763195
TACCTAAA 0.190285182979
TACCTACA 0.162893215848
TACCTAGA 0.0116593675568
TACCTATA 0.195010880954
TACCTCAA 0.106511411678
TACCTCCA -0.145338973203
TACCTCGA 0.0268981870276
TACCTCTA -0.132730685635
TACCTGAA -0.124552015587
TACCTGCA -0.0658082504556
TACCTGGA -0.037290927632
TACCTTAA 0.0167197038239
TACCTTCA -0.141006248148
TACCTTGA 0.114060116514
TACGAAAA 0.209438786629
TACGAACA 0.143659291328
TACGAAGA 0.26852217722
TACGAATA 0.184410093996
TACGACAA 0.0540627315387
TACGACCA -0.145056850608
TACGACGA 0.154067597532
TACGACTA 0.109768570308
TACGAGAA 0.194053724231
TACGAGCA 0.0773015849956
TACGAGGA 0.134300408234
TACGAGTA 0.0245202968232
TACGATAA 0.11378405673
TACGATCA 0.205210322949
TACGATGA 0.140082518559
TACGCAAA 0.211559862275
TACGCACA 0.0979151573031
TACGCAGA 0.235644580562
TACGCATA 0.174966948992
TACGCCAA 0.0883361874026
TACGCCCA 0.0447248839277
TACGCCGA -0.0304831204179
TACGCCTA -0.0727907402404
TACGCGAA 0.224812838921
TACGCGCA 0.0291416935608
TACGCGGA 0.0524365893988

TACGCGTA 0.120930055081
TACGCTAA 0.0774090915695
TACGCTCA -0.0446471372665
TACGCTGA 0.0176441532405
TACGGAAA -0.00387295093875
TACGGACA -0.0724304835284
TACGGAGA 0.157245959222
TACGGATA 0.210833388219
TACGGCAA 0.0519343919341
TACGGCCA -0.12779893671
TACGGCGA 0.0254370799808
TACGGCTA 0.0319013632322
TACGGGAA 0.203584036727
TACGGGCA -0.108406764691
TACGGGGA 0.0445679457085
TACGGTAA 0.0794023215609
TACGGTCA 0.196884124789
TACGGTGA 0.0546264146558
TACGTAAA 0.10139047416
TACGTACA 0.108053963863
TACGTAGA 0.128844622903
TACGTATA 0.09907123802
TACGTCAA 0.132368438385
TACGTCCA 0.0385352955704
TACGTCGA 0.0957956212863
TACGTCTA 0.0282727761394
TACGTGAA 0.084739877675
TACGTGCA 0.0826013685296
TACGTGGA 0.0189778158732
TACGTTAA 0.0517601923204
TACGTTCA 0.156693737016
TACGTTGA -0.00645447289121
TACTAAAA 0.16257841055
TACTAACA -0.0770168378539
TACTAAGA 0.0161886100683
TACTAATA 0.124002447913
TACTACAA 0.00740136995324
TACTACCA -0.139574793817
TACTACGA 0.0933636482501
TACTACTA 0.0470877383658
TACTAGAA 0.0385672569843
TACTAGCA -0.0483493866798
TACTAGGA 0.0844151454564
TACTAGTA 0.059987654321
TACTATAA 0.212126450976
TACTATCA 0.106385431074
TACTATGA 0.0366391139213
TACTCAAA 0.190778063185
TACTCACA -0.0418505658623
TACTCAGA 0.175264550265
TACTCATA 0.0718419943922
TACTCCAA 0.0703780993777
TACTCCCA 0.0682492409164
TACTCCGA -0.00986590734096

TACTCCTA -0.110525826452
TACTCGAA 0.204439175394
TACTCGCA -0.163741245691
TACTCGGA 0.110854519309
TACTCTAA 0.192523436299
TACTCTCA 0.0743347823223
TACTCTGA -0.0779093619074
TACTGAAA 0.187542312554
TACTGACA 0.16033557268
TACTGAGA 0.0836069288923
TACTGATA 0.195619278399
TACTGCAA 0.119623921491
TACTGCCA -0.076235236006
TACTGCGA -0.0344849551632
TACTGCTA 0.0144679356298
TACTGGAA 0.185585402351
TACTGGCA -0.0805126357567
TACTGGGA 0.117143076687
TACTGTAA 0.160380921942
TACTGTCA 0.0628097924935
TACTGTGA 0.0405524819425
TACTTAAA 0.240487289953
TACTTACA 0.0271192596574
TACTTAGA 0.0993545412633
TACTTATA 0.122346386145
TACTTCAA 0.198305067119
TACTTCCA -0.0127424345881
TACTTCGA 0.0947413774177
TACTTCTA 0.109984688333
TACTTGAA 0.031353597225
TACTTGCA 0.0949856198819
TACTTGGA 0.0380035738672
TACTTTAA 0.185939519054
TACTTTCA 0.0277969878592
TACTTTGA 0.104146267052
TAGAAAAA -0.0883718430327
TAGAAACA -0.0617329688906
TAGAAAGA 0.187273595469
TAGAAATA 0.279823631107
TAGAACAA 0.0425832619816
TAGAACCA -0.127666112455
TAGAACGA -0.0238042663475
TAGAACTA -0.124114066281
TAGAAGAA -0.00988043525635
TAGAAGCA -0.0723047689885
TAGAAGGA -0.0111898724451
TAGAATAA 0.171389602406
TAGAATCA 0.23872231004
TAGAATGA -0.0676347100954
TAGACAAA -0.0592023664482
TAGACACA -0.244823018105
TAGACAGA 0.0737987431739
TAGACATA -0.0132218557959
TAGACCAA -0.202030927609

TAGACCCA -0.237487291213
TAGACCGA 0.0247483038659
TAGACCTA -0.0289303826648
TAGACGAA 0.0295948164398
TAGACGCA -0.0519045668349
TAGACGGA 0.00994822506697
TAGACTAA -0.0636918338588
TAGACTCA -0.307650918089
TAGACTGA -0.0935234553194
TAGAGAAA 0.0829471329159
TAGAGACA 0.0888861447271
TAGAGAGA 0.0206921098892
TAGAGATA 0.208749223253
TAGAGCAA 0.0842032755933
TAGAGCCA -0.311442265795
TAGAGCGA -0.15143107816
TAGAGCTA -0.0655328350182
TAGAGGAA 0.0681957891501
TAGAGGCA -0.0286260248938
TAGAGGGA -0.169618512675
TAGAGTAA -0.00882581744542
TAGAGTCA -0.172173867572
TAGAGTGA 0.00720439324161
TAGATAAA -0.0850565862304
TAGATACA 0.141331919283
TAGATAGA -0.0107356912336
TAGATATA 0.271205185624
TAGATCAA -0.0140953768646
TAGATCCA -0.0270495256636
TAGATCGA -0.0432936468728
TAGATCTA 0.164156626506
TAGATGAA 0.0915644466398
TAGATGCA 0.0590394410415
TAGATGGA -0.193879230871
TAGATTAA 0.139410631361
TAGATTCA 0.265594990775
TAGATTGA 0.0382884366287
TAGCAAAA 0.020294078904
TAGCAACA -0.00750076271556
TAGCAAGA 0.00697291512308
TAGCAATA 0.14576583906
TAGCACAA 0.04896633882
TAGCACCA -0.0943761215653
TAGCACGA 0.0614365847768
TAGCACTA -0.0204354102176
TAGCAGAA 0.0125551603767
TAGCAGCA -0.147728461199
TAGCAGGA -0.0358992908172
TAGCATAA 0.0414937759336
TAGCATCA 0.0160199322999
TAGCATGA -0.00477758568645
TAGCCAAA -0.0111363586467
TAGCCACA -0.0386457077274
TAGCCAGA -0.0268255065973

TAGCCATA -0.117830083436
TAGCCCAA -0.136101670298
TAGCCCCA -0.146393510731
TAGCCCGA 0.0192179474516
TAGCCCTA -0.218493458888
TAGCCGAA -0.0597041504725
TAGCCGCA -0.0754252110802
TAGCCGGA 0.0665601879295
TAGCCTAA 0.103448927113
TAGCCTCA -0.295661668285
TAGCCTGA -0.169765432099
TAGCGAAA 0.204381545149
TAGCGACA -0.118448999753
TAGCGAGA 0.22920508683
TAGCGATA 0.156570396716
TAGCGCAA 0.139286812005
TAGCGCCA -0.145829883294
TAGCGCGA 0.0376781485625
TAGCGCTA -0.23922996131
TAGCGGAA 0.233764736877
TAGCGGCA -0.137721201979
TAGCGGGA 0.0053608007787
TAGCGTAA 0.250861281241
TAGCGTCA -0.112683438155
TAGCGTGA -0.0437784034776
TAGCTAAA 0.0111061713759
TAGCTACA 0.0328547278755
TAGCTAGA -0.00543634593872
TAGCTATA 0.0179175173988
TAGCTCAA -0.133010603965
TAGCTCCA -0.21644084446
TAGCTCGA 0.129953456717
TAGCTGAA 0.100008716749
TAGCTGCA -0.222762123074
TAGCTGGA -0.222967928962
TAGCTTAA -0.0717010716579
TAGCTTCA -0.210081915236
TAGCTTGA -0.0269339480256
TAGGAAAA 0.071376693704
TAGGAACA 0.0316461580928
TAGGAAGA 0.0719790534532
TAGGAATA 0.173165487484
TAGGACAA -0.132908579049
TAGGACCA -0.240168963674
TAGGACGA 0.00167797422748
TAGGACTA -0.202987940189
TAGGAGAA 0.146762454055
TAGGAGCA 0.0512878996993
TAGGAGGA -0.0604548531518
TAGGATAA 0.000326878096262
TAGGATCA 0.138917379745
TAGGATGA -0.124726511993
TAGGCAAA -0.00248608778689
TAGGCACA -0.11660708947

TAGGCAGA -0.115474004695
TAGGCATA -0.0747537114788
TAGGCCAA 0.00862334462581
TAGGCCCA -0.332228195678
TAGGCCGA -0.182840449856
TAGGCCTA -0.0256695142627
TAGGCGAA -0.178205297068
TAGGCGCA -0.125984846724
TAGGCGGA -0.0788453772382
TAGGCTAA 0.0605810455596
TAGGCTCA -0.100493949123
TAGGCTGA -0.0836077088546
TAGGGAAA 0.121178795055
TAGGGACA -0.191058091467
TAGGGAGA -0.0814894387646
TAGGGATA 0.185220751674
TAGGGCAA 0.0294819864322
TAGGGCCA -0.139350611206
TAGGGCGA 0.0373059781773
TAGGGGAA 0.125661375661
TAGGGGCA -0.0158950332409
TAGGGGGA -0.0773752974297
TAGGGTAA -0.0115741278732
TAGGGTCA -0.063571252161
TAGGGTGA -0.186192858867
TAGGTAAA -0.0136112039283
TAGGTACA -0.0751457221893
TAGGTAGA 0.0635296958521
TAGGTATA 0.0485246657446
TAGGTCAA -0.0931432271944
TAGGTCCA -0.29218455443
TAGGTCGA -0.00780752596105
TAGGTGAA -0.238887613913
TAGGTGCA -0.11177523107
TAGGTGGA -0.0950333511516
TAGGTTAA -0.0140535467299
TAGGTTCA -0.0798362446014
TAGGTTGA -0.0710169268261
TAGTAAAA 0.138728917339
TAGTAACA -0.0405648453503
TAGTAAGA 0.0344064620168
TAGTAATA -0.0421324074209
TAGTACAA -0.0398565326026
TAGTACCA -0.172589687727
TAGTACGA 0.131402499259
TAGTACTA -0.0134022562298
TAGTAGAA 0.153369749975
TAGTAGCA -0.158181395552
TAGTAGGA -0.107310447024
TAGTATAA 0.240438452486
TAGTATCA -0.026430636468
TAGTATGA -0.17201285176
TAGTCAAA 0.134018566676
TAGTCACA -0.0571692023974

TAGTCAGA -0.0306779246843
TAGTCATA 0.0378905135129
TAGTCCAA 0.0443533337724
TAGTCCCA -0.124975953665
TAGTCCGA -0.159951253947
TAGTCGAA -0.0803730182249
TAGTCGCA -0.188941571591
TAGTCGGA 0.0858178489968
TAGTCTAA 0.0696228237722
TAGTCTCA -0.224984910837
TAGTCTGA 0.0354321328433
TAGTGAAA -0.0159808328805
TAGTGACA 0.011075984105
TAGTGAGA 0.00685799907792
TAGTGATA 0.116236398239
TAGTGCAA 0.111300467628
TAGTGCCA -0.186932880994
TAGTGCGA 0.220352156669
TAGTGGAA -0.069186291459
TAGTGGCA -0.141936786568
TAGTGGGA -0.113398483004
TAGTGTAA 0.255919399125
TAGTGTCA 0.0671872503015
TAGTGTGA -0.0482859118751
TAGTTAAA -0.0317855088296
TAGTTACA 0.0906178722415
TAGTTAGA -0.104371308458
TAGTTATA 0.112538145733
TAGTTCAA -0.0387585412036
TAGTTCCA -0.188352970232
TAGTTCGA -0.015222617401
TAGTTGAA 0.0991553887188
TAGTTGCA -0.00883620098241
TAGTTGGA -0.17646413266
TAGTTTAA -0.028644728661
TAGTTTCA -0.0320819955545
TAGTTTGA 0.0794342829747
TATAAAAA 0.234889513464
TATAAACA 0.0515958915055
TATAAAGA 0.0332271447622
TATAAATA 0.253177836653
TATAACAA 0.135186831764
TATAACCA 0.00176458319612
TATAACGA 0.1012837823
TATAAGAA 0.180495529981
TATAAGCA 0.105246547674
TATAAGGA 0.136199743159
TATAATAA 0.230394275412
TATAATCA 0.210842214463
TATAATGA 0.212739529005
TATACAAA 0.192699722517
TATACACA -0.0571211100007
TATACAGA 0.264747853959
TATACATA 0.144495693118

TATACCAA 0.166738337716
TATACCCA 0.108199555446
TATACCGA 0.193524908111
TATACGAA 0.210639415314
TATACGCA 0.00320259500757
TATACGGA 0.106993450628
TATACTAA 0.183725218995
TATACTCA 0.0934631074549
TATACTGA 0.0839096357769
TATAGAAA 0.241563685433
TATAGACA 0.0334072596895
TATAGAGA 0.194309973874
TATAGATA 0.324068585479
TATAGCAA 0.134416631558
TATAGCCA 0.0721173900634
TATAGCGA 0.0612918222364
TATAGGAA 0.0765490389784
TATAGGCA -0.0499870363969
TATAGGGA -0.0221594293435
TATAGTAA 0.0854683966717
TATAGTCA -0.159645809423
TATAGTGA 0.139907265633
TATATAAA 0.127886962875
TATATACA 0.0632070345305
TATATAGA 0.326445528929
TATATATA 0.190327301458
TATATCAA 0.1470123342
TATATCCA 0.256701000973
TATATCGA 0.160738540946
TATATGAA 0.206640710125
TATATGCA 0.190887298273
TATATGGA 0.16218238345
TATATTAA 0.0672540787122
TATATTCA 0.100825185594
TATATTGA 0.215910338317
TATCAAAA 0.150608267445
TATCAACA 0.138821495504
TATCAAGA 0.266613976158
TATCAATA 0.339440274351
TATCACAA 0.254495159059
TATCACCA -0.0844406026179
TATCACGA 0.252630279081
TATCAGAA 0.214280940828
TATCAGCA 0.167838395079
TATCAGGA 0.319455077546
TATCATAA 0.15329370568
TATCATCA 0.123574745578
TATCATGA 0.272227956643
TATCCAAA 0.317575872038
TATCCACA 0.240831302553
TATCCAGA 0.122167885135
TATCCATA 0.336686864695
TATCCCAA 0.196310566643
TATCCCCA 0.164718957477

TATCCCGA	0.204645362047
TATCCGAA	0.221009705621
TATCCGCA	0.269020724934
TATCCGGA	0.393634446735
TATCCTAA	0.264097455487
TATCCTCA	0.229664756636
TATCCTGA	0.274248611386
TATCGAAA	0.300972578981
TATCGACA	0.166976772267
TATCGAGA	0.128576409571
TATCGATA	0.3194387707
TATCGCAA	0.331115889181
TATCGCCA	-0.0419918337779
TATCGCGA	0.235071022415
TATCGGAA	0.256430781747
TATCGGCA	0.10740701867
TATCGGGA	0.280791016268
TATCGTAA	0.251293710865
TATCGTCA	0.215269995866
TATCGTGA	0.161743397221
TATCTAAA	0.321745383755
TATCTACA	0.302574346607
TATCTAGA	0.29995553799
TATCTCAA	0.348594370897
TATCTCCA	0.287610593756
TATCTCGA	0.244712565194
TATCTGAA	0.29657401462
TATCTGCA	0.344589077913
TATCTGGA	0.297742361948
TATCTTAA	0.263537837956
TATCTTCA	0.226285357314
TATCTTGA	0.299918768798
TATGAAAA	0.0890808187933
TATGAACA	-0.057204878263
TATGAAGA	0.201547222989
TATGAATA	-0.020340731959
TATGACAA	0.0731163142989
TATGACCA	-0.0681373759679
TATGACGA	0.0990032712024
TATGAGAA	0.128670841021
TATGAGCA	-0.138426415401
TATGAGGA	0.221142341215
TATGATAA	0.243785684192
TATGATCA	0.174331489166
TATGATGA	-0.0103249894673
TATGCAAA	0.186853689364
TATGCACA	0.0772391755955
TATGCAGA	0.221029157526
TATGCATA	0.0698416688738
TATGCCAA	0.013354059826
TATGCCCA	-0.0869142875959
TATGCCGA	0.0675998430985
TATGCGAA	0.194569297639
TATGCGCA	0.0930673560342

TATGCGGA 0.236969186291
TATGCTAA 0.0306922215636
TATGCTCA -0.107935133753
TATGCTGA 0.200166304419
TATGGAAA 0.131012755494
TATGGACA 0.0111443638952
TATGGAGA 0.0203472409856
TATGGCAA 0.136863132509
TATGGCCA -0.0932236874599
TATGGCGA -0.067059404646
TATGGGAA 0.0834101736591
TATGGGCA -0.151825506597
TATGGGGA 0.0835144569584
TATGGTAA -0.0225581242179
TATGGTCA -0.0714439295106
TATGGTGA -0.00696228237722
TATGTAAA 0.121209303677
TATGTACA -0.00319436211552
TATGTAGA 0.208828614182
TATGTCAA 0.169803398538
TATGTCCA 0.0951622041753
TATGTCGA 0.129584646899
TATGTGAA 0.201162484358
TATGTGCA -0.107970492356
TATGTGGA 0.142680109831
TATGTTAA 0.0444743073816
TATGTTCA -0.0819581557757
TATGTTGA 0.0539203579678
TATTAAAA 0.162732016526
TATTAACA 0.172901079424
TATTAAGA 0.197465270646
TATTAATA 0.160492635605
TATTACAA 0.231428239362
TATTACCA 0.170223276032
TATTACGA 0.340301104305
TATTAGAA 0.203794265516
TATTAGCA 0.0242807584359
TATTAGGA 0.0180712739529
TATTATAA 0.210574415587
TATTATCA -0.0270670047641
TATTATGA 0.127413764862
TATTCAAA 0.244525072995
TATTCACA 0.134015016795
TATTCAGA 0.0058271468627
TATTCCAA 0.202267807592
TATTCCCA 0.226910057676
TATTCCGA 0.18420670318
TATTCGAA 0.229576939121
TATTCGCA 0.204943849607
TATTCGGA 0.157355156574
TATTCTAA 0.198467304926
TATTCTCA 0.204142042603
TATTCTGA 0.205115738514
TATTGAAA 0.150455450147

TATTGACA 0.118468572307
TATTGAGA 0.114658666628
TATTGCAA 0.100057081385
TATTGCCA -0.0602835127927
TATTGCGA 0.277908851859
TATTGGAA 0.0512210712885
TATTGGCA -0.0722197201923
TATTGGGA 0.0733934883312
TATTGTAA 0.241016663519
TATTGTCA 0.0915186029957
TATTGTGA 0.099514767626
TATTTAAA 0.241082896947
TATTTACA 0.0692303892511
TATTTAGA 0.251632856923
TATTTCAA 0.159007332763
TATTTCCA 0.228144415904
TATTTCGA 0.136915739094
TATTTGAA 0.158861301992
TATTTGCA 0.276863737172
TATTTGGA 0.234645656326
TATTTTAA 0.160622085337
TATTTTCA 0.136987090825
TATTTTGA 0.182707422312
TCAAAAAA 0.260705503963
TCAAAACA -0.0484656373663
TCAAAAGA -0.0147051560289
TCAAACAA 0.017103714788
TCAAACCA 0.0739499949152
TCAAACGA -0.00777878305723
TCAAAGAA 0.128172533523
TCAAAGCA 0.0908822803016
TCAAAGGA 0.111934400316
TCAAATAA -0.0358460119871
TCAAATCA 0.198415004431
TCAAATGA 0.18312970647
TCAACAAA 0.168103961763
TCAACACA 0.0174814405881
TCAACAGA 0.095897316694
TCAACCAA 0.0823456772188
TCAACCCA -0.0670836940921
TCAACCGA -0.0442200587451
TCAACGAA -0.000248402813489
TCAACGCA 0.0739631893546
TCAACGGA 0.0335405982596
TCAACTAA 0.147343570671
TCAACTCA -0.026843229265
TCAACTGA -0.00788084340391
TCAAGAAA 0.094343454302
TCAAGACA -0.125662109744
TCAAGAGA 0.102019380239
TCAAGCAA 0.0494960972236
TCAAGCCA 0.00985525100092
TCAAGCGA -0.079638684469
TCAAGGAA -0.076802954105

TCAAGGCA	-0.125078622482
TCAAGGGA	0.0446602646986
TCAAGTAA	0.0979980532593
TCAAGTCA	-0.131176192985
TCAAGTGA	-0.0302195168015
TCAATAAA	0.127713498963
TCAATACA	0.0886687493826
TCAATAGA	0.123108102218
TCAATCAA	-0.0204073627475
TCAATCCA	-0.0149245274796
TCAATCGA	0.0978287119366
TCAATGAA	-0.0853621183678
TCAATGCA	0.0668156672923
TCAATGGA	0.0406520128427
TCAATTAA	0.144267711695
TCAATTCA	-0.13549138725
TCAATTGA	0.0881639963908
TCACAAAA	0.133199192248
TCACAACA	0.171079278834
TCACAAGA	0.152393576149
TCACACAA	0.145307800391
TCACACCA	0.0781905200992
TCACACGA	-0.168963333692
TCACAGAA	0.191723446603
TCACAGCA	0.00179202616962
TCACAGGA	0.0506158203254
TCACATAA	0.10906251362
TCACATCA	0.0880013831259
TCACATGA	0.16601484753
TCACCAAA	0.0728967705109
TCACCACA	0.0414663329601
TCACCAGA	0.1021125601
TCACCCAA	0.162240495111
TCACCCCA	-0.0890749246802
TCACCCGA	-0.107111111111
TCACCGAA	-0.0148459153152
TCACCGCA	0.0235163366409
TCACCGGA	-0.0435656680689
TCACCTAA	0.0129668049793
TCACCTCA	-0.0892033853652
TCACCTGA	-0.101134630192
TCACGAAA	0.0918049792531
TCACGACA	-0.0473575010985
TCACGAGA	0.00448967046477
TCACGCAA	0.135469494391
TCACGCCA	0.134791451775
TCACGCGA	0.120876614415
TCACGGAA	0.08584299326
TCACGGCA	-0.185963689179
TCACGGGA	-0.224576131687
TCACGTAA	0.107572174751
TCACGTCA	0.0449570791894
TCACGTGA	0.0372439520383
TCACTAAA	0.0907282843985

TCACTACA −0.125603029555
TCACTAGA −0.162745872708
TCACTCAA 0.194820798164
TCACTCCA 0.0146978919995
TCACTCGA 0.0677347471953
TCACTGAA −0.00107361294728
TCACTGCA 0.0743960019177
TCACTGGA 0.0833196118466
TCACTTAA 0.0777128815569
TCACTTCA 0.0106588509078
TCAGAAAA 5.4885947002E−6
TCAGAACA −0.0272398954972
TCAGAAGA 0.171388696769
TCAGACAA −0.0550796856159
TCAGACCA −0.168462930631
TCAGACGA 0.0517986124833
TCAGAGAA 0.0584480449626
TCAGAGCA −0.00578852338459
TCAGAGGA −0.00190151627402
TCAGATAA 0.237178388273
TCAGATCA −0.017977624494
TCAGATGA 0.0789112780207
TCAGCAAA 0.0565876832333
TCAGCACA 0.068959655979
TCAGCAGA 0.10344602153
TCAGCCAA −0.0139250069008
TCAGCCCA −0.0528010322517
TCAGCCGA 0.0567358679703
TCAGCGAA 0.118870200745
TCAGCGCA −0.0590100219677
TCAGCGGA 0.0223134252466
TCAGCTAA 0.00467530623333
TCAGCTCA −0.0630648985035
TCAGCTGA −0.00567845151096
TCAGGAAA 0.225396248892
TCAGGACA 0.0696599015007
TCAGGAGA 0.0700658057236
TCAGGCAA 0.0112643891894
TCAGGCCA −0.104135451826
TCAGGCGA 0.00216030101841
TCAGGGAA 0.0447073930323
TCAGGGCA 0.115496927346
TCAGGGGA −0.0334897505557
TCAGGTAA 0.0283948106286
TCAGGTCA −0.132334057685
TCAGTAAA 0.16669475397
TCAGTACA 0.00445149659192
TCAGTAGA 0.100040678163
TCAGTCAA −0.242142453519
TCAGTCCA −0.176558881315
TCAGTCGA −0.071768063445
TCAGTGAA −0.166451706609
TCAGTGCA −0.110925916781
TCAGTGGA 0.16137755606

TCAGTTAA 0.178566966426
TCAGTTCA -0.0551014864932
TCATAAAA 0.109917341764
TCATAACA 0.0834759490361
TCATAAGA 0.0338234648401
TCATACAA -0.0529249084196
TCATACCA -0.0352142141124
TCATACGA 0.204877021196
TCATAGAA 0.0374776633301
TCATAGCA -0.0109467132562
TCATAGGA 0.0971097060309
TCATATAA 0.230062615315
TCATATCA 0.171826778328
TCATATGA 0.00848805056291
TCATCAAA 0.0819098397571
TCATCACA 0.0419417238448
TCATCAGA 0.044561474874
TCATCCAA 0.129642758561
TCATCCCA 0.0204364185783
TCATCCGA -0.0692879868668
TCATCGAA 0.0125853476476
TCATCGCA 0.0997036305261
TCATCGGA -0.117210227347
TCATCTAA 0.0594879394089
TCATCTCA -0.0318100866069
TCATGAAA 0.190343900556
TCATGACA 0.15479294987
TCATGAGA 0.299825462689
TCATGCAA 0.0617712434443
TCATGCCA 0.0207012568932
TCATGCGA 0.155958418407
TCATGGAA 0.224510046053
TCATGGCA -0.076107480029
TCATGGGA 0.0600754132912
TCATGTAA 0.14667238098
TCATGTCA -0.0312575468177
TCATTAAA 0.0174930629204
TCATTACA 0.230097482312
TCATTAGA 0.176579547601
TCATTCAA -0.00287862572377
TCATTCCA -0.123946397998
TCATTCGA -0.141795359784
TCATTGAA 0.0519968619703
TCATTGCA -0.0289339784773
TCATTGGA 0.0949762468583
TCATTTAA 0.154410634701
TCATTTCA -0.0839517682505
TCCAAAAA 0.128503769994
TCCAAACA 0.197055191551
TCCAAAGA 0.0963178954648
TCCAACAA 0.0985559507344
TCCAACCA -0.0212441706739
TCCAACGA 0.0642567013964
TCCAAGAA 0.0320093559775

TCCAAGCA 0.177654833256
TCCAAGGA 0.158656062702
TCCAATAA 0.0263699532372
TCCAATCA 0.0123877582384
TCCACAAA 0.0826972527712
TCCACACA 0.0335260703442
TCCACAGA 0.0695747856915
TCCACCAA -0.00748666855599
TCCACCCA -0.15404601599
TCCACCGA 0.0101806602321
TCCACGAA 0.0308949916033
TCCACGCA -0.186465272069
TCCACGGA 0.127530242157
TCCACTAA 0.1762634745
TCCACTCA 0.0780231179609
TCCAGAAA -0.0531213226214
TCCAGACA -0.0652464989683
TCCAGAGA -0.00800448599869
TCCAGCAA 0.259702468293
TCCAGCCA 0.0893103067563
TCCAGCGA -0.0421085356004
TCCAGGAA 0.0755536767974
TCCAGGCA 0.0230227031048
TCCAGGGA 0.0910958406578
TCCAGTAA 0.137214708062
TCCAGTCA -0.0385672569843
TCCATAAA 0.0959757674371
TCCATACA 0.10155739253
TCCATAGA -0.0602592812136
TCCATCAA 0.0563348975056
TCCATCCA 0.0262025510988
TCCATCGA -0.0407611380026
TCCATGAA 0.0788822221899
TCCATGCA 0.0569712201996
TCCATGGA 0.023466854611
TCCATTAA 0.146371505483
TCCATTCA 0.0423506489943
TCCCAAAA 0.0732013875167
TCCCAACA 0.118770066683
TCCCAAGA -0.0119569035544
TCCCACAA 0.0907893016431
TCCCACCA -0.024166282465
TCCCACGA 0.109478254188
TCCCAGAA 0.0115754462227
TCCCAGCA -0.0430476660904
TCCCAGGA -0.0330321212209
TCCCATAA -0.192897544137
TCCCATCA 0.0955534986753
TCCCCAAA 0.190325700632
TCCCCACA 0.0307541440879
TCCCCAGA -0.0352302106028
TCCCCCAA -0.0088357645406
TCCCCCCA -0.135689986283
TCCCCCGA 0.0577921578959

TCCCCGAA 0.0644252641978
TCCCCGCA 0.108218621704
TCCCCGGA 0.227575436201
TCCCCTAA 0.303684279343
TCCCCTCA −0.129755393343
TCCCGAAA 0.1246393445
TCCCGACA 0.0207276778854
TCCCGAGA 0.182518559412
TCCCGCAA 0.151907835518
TCCCGCCA −0.0471389410254
TCCCGCGA 0.039377914663
TCCCGGAA 0.0997588366045
TCCCGGCA −0.159159896243
TCCCGGGA 0.129751168292
TCCCGTAA 0.107008266384
TCCCGTCA 0.00098935103802
TCCCTAAA 0.181022939704
TCCCTACA −0.0884861056236
TCCCTAGA 0.0999848917143
TCCCTCAA 0.0461074064927
TCCCTCCA −0.0773985397982
TCCCTCGA 0.0421825945685
TCCCTGAA 0.0243473334011
TCCCTGCA −0.0193373746852
TCCCTTAA −0.0845242407279
TCCCTTCA −0.00806444583267
TCCGAAAA 0.133600627895
TCCGAACA 0.0367774178083
TCCGAAGA 0.0288393648395
TCCGACAA 0.100180146151
TCCGACCA −0.0191526576056
TCCGACGA −0.0221464796154
TCCGAGAA 0.0495757316297
TCCGAGCA −0.103044869157
TCCGAGGA 0.0195816954727
TCCGATAA 0.0877762119913
TCCGATCA 0.0478088264349
TCCGCAAA −0.0531945182874
TCCGCACA −0.0108044106751
TCCGCAGA 0.108781547884
TCCGCCAA 0.0521479522903
TCCGCCCA −0.125165319416
TCCGCCGA −0.101437908497
TCCGCGAA 0.22464818108
TCCGCGCA 0.125002542385
TCCGCGGA 0.295049402723
TCCGCTAA 0.226533784667
TCCGCTCA 0.00239675087736
TCCGGAAA 0.276084546313
TCCGGACA 0.0543826715928
TCCGGAGA 0.240861688381
TCCGGCAA 0.0046707247977
TCCGGCCA −0.132176623263
TCCGGCGA 0.127164296195

TCCGGGAA -3.01842888033E-5
TCCGGGCA -0.156016986815
TCCGGTAA 0.0879957769436
TCCGGTCA -0.0223879777822
TCCGTAAA 0.0625237894615
TCCGTACA -0.0825416817588
TCCGTAGA 0.0291880458595
TCCGTCAA 0.077114755538
TCCGTCCA -0.0144665064155
TCCGTCGA 0.136069908329
TCCGTGAA 0.0759940726105
TCCGTGCA -0.0691034823413
TCCGTTAA 0.0199657141197
TCCGTTCA 0.0819941016664
TCCTAAAA 0.0173336708486
TCCTAACA -0.012002849588
TCCTAAGA 0.078847355193
TCCTACAA 0.185519989462
TCCTACCA -0.0575367425116
TCCTACGA 0.0420509233861
TCCTAGAA 0.0770589688086
TCCTAGCA -0.00699629847
TCCTAGGA 0.151037037037
TCCTATAA 0.124420953259
TCCTATCA 0.0335749589852
TCCTCAAA 0.202235846178
TCCTCACA -0.0162160530418
TCCTCAGA 0.0782720497436
TCCTCCAA 0.0125246986762
TCCTCCCA 0.0308937734969
TCCTCCGA -0.18251773575
TCCTCGAA -0.0660184951147
TCCTCGCA 0.185160031611
TCCTCTAA 0.205405168061
TCCTCTCA -0.0549875421208
TCCTGAAA 0.0682449619678
TCCTGACA -0.0896950439846
TCCTGAGA 0.132357461196
TCCTGCAA -0.140559676234
TCCTGCCA -0.0729781862866
TCCTGCGA -0.0751514535179
TCCTGGAA 0.164666656981
TCCTGGCA 0.0543694905602
TCCTGTAA 0.155801993458
TCCTGTCA 0.100769979516
TCCTTAAA -0.0504031496521
TCCTTACA -0.0193739668041
TCCTTAGA 0.155057893743
TCCTTCAA 0.210682451865
TCCTTCCA -0.197184219336
TCCTTCGA -0.0215158426917
TCCTTGAA 0.00334868449726
TCCTTGCA 0.0249871950538
TCCTTTAA 0.00486289490439

TCCTTTCA -0.041596327343
TCGAAAAA 0.150810116578
TCGAAACA -0.0824923308254
TCGAAAGA 0.0946304824721
TCGAACAA -0.0636224176162
TCGAACCA 0.00756322236491
TCGAACGA -0.0188396013085
TCGAAGAA 0.0645042671716
TCGAAGCA -0.00129734284428
TCGAATAA 0.0652978111484
TCGAATCA 0.184346171168
TCGACAAA 0.200065375619
TCGACACA 0.093034424466
TCGACAGA 0.0385326790928
TCGACCAA 0.0140720616888
TCGACCCA -0.0870616704383
TCGACCGA -0.163218106996
TCGACGAA -0.147654350396
TCGACGCA 0.0814110479954
TCGACTAA -0.0499920096465
TCGACTCA 0.0883787240115
TCGAGAAA 0.230417096451
TCGAGACA -0.107752091155
TCGAGAGA -0.017773441788
TCGAGCAA 0.073357391899
TCGAGCCA -0.263053013798
TCGAGCGA 0.10009275725
TCGAGGAA -0.0303184951051
TCGAGGCA -0.0710475626019
TCGAGTAA 0.148064155274
TCGAGTCA -0.0820347798294
TCGATAAA 0.150403149652
TCGATACA 0.0927331367222
TCGATAGA -0.00208374493727
TCGATCAA 0.101671272981
TCGATCCA -0.0346398933017
TCGATCGA 0.19931002285
TCGATGAA -0.100087167492
TCGATGCA 0.0618829675727
TCGATTAA 0.0687060114348
TCGATTCA 0.00363052605582
TCGCAAAA 0.257480954576
TCGCAACA 0.045793442099
TCGCAAGA 0.110117240277
TCGCACAA 0.148160039516
TCGCACCA -0.169328623345
TCGCACGA 0.0862856478724
TCGCAGAA 0.0708687347691
TCGCAGCA -0.00694238642475
TCGCATAA 0.053222902808
TCGCATCA 0.181510322084
TCGCCAAA 0.00989253309859
TCGCCACA 0.0127225625151
TCGCCAGA -0.082855872765

TCGCCCAA	-0.113928096182
TCGCCCCA	0.038331904755
TCGCCCGA	-0.0622561367116
TCGCCGAA	-0.0236967650836
TCGCCGCA	0.0530226942181
TCGCCTAA	0.0948551565741
TCGCCTCA	-0.0236944952089
TCGCGAAA	0.19375562581
TCGCGACA	0.011342180948
TCGCGAGA	0.28673329572
TCGCGCAA	0.203078003908
TCGCGCCA	0.0678546422273
TCGCGCGA	0.197877994802
TCGCGGAA	0.131112318483
TCGCGGCA	-0.0184005137325
TCGCGTAA	0.280206480933
TCGCGTCA	0.0902198073598
TCGCTAAA	0.116686763616
TCGCTACA	-0.0446500425626
TCGCTAGA	-0.0227035719774
TCGCTCAA	-0.0435037922443
TCGCTCCA	-0.106013717421
TCGCTGAA	-0.113838807936
TCGCTGCA	-0.0698649456984
TCGCTTAA	0.0907253788154
TCGCTTCA	0.14731451484
TCGGAAAA	0.0252338141342
TCGGAACA	-0.0936755682878
TCGGAAGA	0.117971031337
TCGGACAA	0.0187984368482
TCGGACCA	-0.175973024731
TCGGACGA	-0.156919398371
TCGGAGAA	0.185261429837
TCGGAGCA	-0.077733871189
TCGGATAA	-0.0482050760536
TCGGATCA	0.102581834947
TCGGCAAA	0.11940929496
TCGGCACA	-0.214226129716
TCGGCAGA	0.00839987925688
TCGGCCAA	-0.000293449395959
TCGGCCCA	-0.137411764706
TCGGCCGA	0.248075291456
TCGGCGAA	0.142649700871
TCGGCGCA	-0.0121688325649
TCGGCTAA	0.0292075566972
TCGGCTCA	-0.179645920004
TCGGGAAA	0.0664932442074
TCGGGACA	-0.0069428907646
TCGGGAGA	0.0317391367513
TCGGGCAA	0.112609497464
TCGGGCCA	-0.21548455769
TCGGGGAA	0.165347476045
TCGGGGCA	-0.00133359675985
TCGGGTAA	0.102395222727

TCGGGTCA -0.227660076492
TCGGTAAA 0.161575116587
TCGGTACA -0.0709641620378
TCGGTAGA 0.0680566531661
TCGGTCAA -0.105615369419
TCGGTCCA -0.14607683346
TCGGTGAA -0.0361869151276
TCGGTGCA -0.194662583757
TCGGTTAA 0.0430580254232
TCGGTTCA -0.137126227927
TCGTAAAA 0.102455217701
TCGTAACA 0.00400392983381
TCGTAAGA 0.141341906364
TCGTACAA 0.0718457046258
TCGTACCA 0.0280604404048
TCGTACGA -0.043191282735
TCGTAGAA -0.125766960281
TCGTAGCA -0.0602051341653
TCGTATAA 0.0876116929021
TCGTATCA 0.273444532262
TCGTCAAA 0.218660124262
TCGTCACA 0.0111253814573
TCGTCAGA 0.0243938227304
TCGTCCAA -0.0798729645335
TCGTCCCA -0.125114384734
TCGTCGAA 0.144301425189
TCGTCGCA 0.0368151903883
TCGTCTAA -0.155428850143
TCGTCTCA -0.0324155441227
TCGTGAAA 0.194343944483
TCGTGACA -0.086873476915
TCGTGAGA 0.209118467846
TCGTGCAA 0.00224143591353
TCGTGCCA -0.154379084967
TCGTGGAA 0.0989452733427
TCGTGGCA -0.0123661615795
TCGTGTAA 0.180728720236
TCGTGTCA -0.0811373052629
TCGTTAAA 0.132083448346
TCGTTACA 0.0777816197941
TCGTTAGA 0.0635661595205
TCGTTCAA 0.0855185739398
TCGTTCCA 0.00963734303363
TCGTTGAA -0.0310337257485
TCGTTGCA -0.0113201373111
TCGTTTAA 0.11296625612
TCGTTTCA 0.079795934049
TCTAAAAA 0.171296296296
TCTAAACA 0.0229962372699
TCTAAAGA 0.0301207269769
TCTAACAA 0.0769775406705
TCTAACCA -0.032674593767
TCTAAGAA 0.187295337992
TCTAAGCA 0.208997138001

TCTAATAA 0.123658038596
TCTAATCA 0.0293885200413
TCTACAAA 0.119626665788
TCTACACA 0.109681402816
TCTACAGA 0.299041157584
TCTACCAA 0.022574590002
TCTACCCA 0.115512908053
TCTACGAA 0.0368568370791
TCTACGCA −0.0250419836017
TCTACTAA −0.00623717167726
TCTACTCA 0.00779132102335
TCTAGAAA 0.176891557055
TCTAGACA 0.142836560627
TCTAGAGA 0.160788524688
TCTAGCAA 0.22968122242
TCTAGCCA 0.0675594162505
TCTAGGAA 0.257332762519
TCTAGGCA −0.19722875817
TCTAGTAA 0.160465183851
TCTAGTCA −0.0825566971833
TCTATAAA 0.0447128367253
TCTATACA −0.0722088894829
TCTATAGA −0.0336840829409
TCTATCAA −0.0223820059956
TCTATCCA −0.00433706228083
TCTATGAA −0.0406128564842
TCTATGCA 0.0665810530475
TCTATTAA 0.072728197231
TCTATTCA −0.0287363793072
TCTCAAAA 0.265433928297
TCTCAACA 0.0692807229091
TCTCAAGA 0.161566399837
TCTCACAA 0.239653945055
TCTCACCA −0.0226830990212
TCTCAGAA 0.0978120959424
TCTCAGCA 0.0905206480933
TCTCATAA 0.121046391605
TCTCATCA 0.0438408902707
TCTCCAAA −0.0130300873128
TCTCCACA −0.149414436918
TCTCCAGA 0.0775369372249
TCTCCCAA −0.139945158845
TCTCCCCA −0.0323628191831
TCTCCGAA 0.0043081212201
TCTCCGCA −0.063638582007
TCTCCTAA −0.0167347057371
TCTCCTCA 0.0185258362833
TCTCGAAA 0.108073173945
TCTCGACA 0.0909636366278
TCTCGAGA 0.223
TCTCGCAA 0.199579024786
TCTCGCCA 0.0334052592583
TCTCGGAA 0.0803887022767
TCTCGGCA 0.155114048521

TCTCGTAA 0.230523721706
TCTCGTCA -0.00943349632044
TCTCTAAA 0.0492052514874
TCTCTACA 0.251424290325
TCTCTCAA 0.0415948745515
TCTCTCCA -0.137714012385
TCTCTGAA 0.0787979602807
TCTCTGCA 0.0897867988448
TCTCTTAA 0.0850347976904
TCTCTTCA -0.0394500699761
TCTGAAAA 0.17825897462
TCTGAACA 0.141035549809
TCTGAAGA 0.101115743902
TCTGACAA 0.0161642602745
TCTGACCA -0.042702837753
TCTGAGAA 0.315966179013
TCTGAGCA 0.0200330057934
TCTGATAA 0.057524758396
TCTGATCA 0.259034226877
TCTGCAAA 0.135037267558
TCTGCACA -0.0461753229963
TCTGCAGA 0.187400397736
TCTGCCAA -0.07177969721
TCTGCCCA -0.0563238449592
TCTGCGAA 0.206083009506
TCTGCGCA 0.265133003065
TCTGCTAA 0.00879665276829
TCTGCTCA -0.0716008309968
TCTGGAAA 0.257938779365
TCTGGACA -0.013747182002
TCTGGCAA 0.100837819816
TCTGGCCA -0.0832736389685
TCTGGGAA 0.152498075051
TCTGGGCA 0.0218659654526
TCTGGTAA 0.036167245362
TCTGGTCA 0.0836880566008
TCTGTAAA 0.251680153415
TCTGTACA -0.0286667827651
TCTGTCAA 0.0486444956931
TCTGTCCA -0.0725841414092
TCTGTGAA 0.143835484862
TCTGTGCA 0.203066842939
TCTGTTAA -0.0391047898537
TCTGTTCA -0.105144117211
TCTTAAAA 0.245150826582
TCTTAACA 0.0435437660541
TCTTAAGA 0.219780392439
TCTTACAA 0.0327943533338
TCTTACCA 0.144418648049
TCTTAGAA 0.110374267273
TCTTAGCA 0.193847427833
TCTTATAA 0.23662630617
TCTTATCA -0.0158809797387
TCTTCAAA 0.0989703396342

TCTTCACA 0.105234407915
TCTTCCAA -0.107870250673
TCTTCCCA -0.0451941124173
TCTTCGAA 0.175834897737
TCTTCGCA -0.0305013583601
TCTTCTAA 0.0297662458414
TCTTCTCA 0.0204383146126
TCTTGAAA -0.0307827833761
TCTTGACA 0.119722716196
TCTTGCAA 0.213105666425
TCTTGCCA -0.136156623593
TCTTGGAA 0.0276015864484
TCTTGGCA 0.137290253222
TCTTGTAA 0.0916293442227
TCTTGTCA -0.0476284552444
TCTTTAAA 0.215495010374
TCTTTACA 0.0227747792718
TCTTTCAA 0.218350018661
TCTTTCCA 0.041205526419
TCTTTGAA 0.176838144495
TCTTTGCA 0.190218226525
TCTTTTAA 0.106582652353
TCTTTTCA 0.187116644633
TGAAAAAA 0.230181744222
TGAAAACA 0.125699882324
TGAAACAA 0.109678587894
TGAAACCA -0.0213458660817
TGAAAGAA 0.110338064591
TGAAAGCA 0.0230027003886
TGAAATAA 0.164027428704
TGAAATCA 0.375266196843
TGAACAAA -0.0855068168346
TGAACACA -0.104313822343
TGAACCAA -0.15366739647
TGAACCCA -0.105335866752
TGAACGAA 0.106593997673
TGAACGCA -0.0303015727334
TGAACTAA -0.0942899039824
TGAACTCA -0.17690640417
TGAAGAAA 0.10071767902
TGAAGACA -0.0334621475165
TGAAGCAA -0.0464965932038
TGAAGCCA -0.0474710850409
TGAAGGAA -0.0249691281798
TGAAGGCA -0.0276639063126
TGAAGTAA 0.0854110673659
TGAAGTCA -0.197349251815
TGAATAAA -0.028093371973
TGAATACA 0.00916275623611
TGAATCAA -0.143227622999
TGAATCCA 0.124255807534
TGAATGAA -0.0353425056417
TGAATGCA 0.0209728119142
TGAATTAA 0.118989988803

TGAATTCA -0.0295427612088
TGACAAAA 0.19524210771
TGACAACA 0.0480828338712
TGACACAA 0.0753721267207
TGACACCA -0.109356796504
TGACAGAA 0.0236439087047
TGACAGCA -0.0108372302356
TGACATAA 0.0200034866997
TGACATCA -0.0474875005525
TGACCAAA -0.0120460932183
TGACCACA -0.0831977595463
TGACCCAA -0.0826692789797
TGACCCCA -0.245931612165
TGACCGAA -0.00376544967488
TGACCGCA -0.116829653776
TGACCTAA -0.204171948135
TGACCTCA 0.0203297547696
TGACGAAA 0.146692720062
TGACGACA -0.31863259769
TGACGCAA 0.0979110408571
TGACGCCA -0.183203401843
TGACGGAA 0.0229817093545
TGACGGCA -0.187701999001
TGACGTAA 0.114398779337
TGACGTCA 0.0328805311522
TGACTAAA -0.10798703722
TGACTACA -0.14501038746
TGACTCAA -0.0907045518588
TGACTCCA -0.110422119611
TGACTGAA -0.0911820237546
TGACTGCA -0.0683838782969
TGACTTAA -0.108121104703
TGAGAAAA 0.15084479828
TGAGAACA -0.0568782415411
TGAGACAA 0.0089075491891
TGAGACCA -0.129657072603
TGAGAGAA 0.00981166940746
TGAGAGCA -0.0138885458856
TGAGATAA -0.0161762607302
TGAGATCA 0.252152140961
TGAGCAAA 0.162397396598
TGAGCACA -0.0202489134729
TGAGCCAA -0.273353240363
TGAGCCCA -0.276803452963
TGAGCGAA 0.0945931853608
TGAGCGCA -0.0215957462264
TGAGCTAA -0.127111319637
TGAGCTCA -0.269614685487
TGAGGAAA 0.0216393299725
TGAGGACA 0.0210088184446
TGAGGCAA -0.112974304938
TGAGGCCA -0.260238860764
TGAGGGAA 0.068110308281
TGAGGGCA -0.259495582572

TGAGGTAA 0.0988180311313
TGAGTAAA 0.066574172272
TGAGTACA 0.0081343228292
TGAGTCAA -0.0982125735803
TGAGTCCA -0.0234718228942
TGAGTGAA 0.0950202499136
TGAGTGCA -0.0399051758198
TGAGTTAA -0.0539638630925
TGATAAAA 0.134489780237
TGATAACA -0.0943399241643
TGATACAA -0.0252974590676
TGATACCA 0.0328711936596
TGATAGAA 0.219628861226
TGATAGCA 0.114769078785
TGATATAA 0.098650628993
TGATATCA 0.388939266748
TGATCAAA 0.0499242573931
TGATCACA 0.10789447683
TGATCCAA -0.0158949922276
TGATCCCA -0.0372771780977
TGATCGAA -0.0176732749347
TGATCGCA 0.169980968431
TGATCTAA -0.0276625838171
TGATGAAA 0.0246163472305
TGATGACA 0.0670628183362
TGATGCAA 0.122821027904
TGATGCCA -0.234873921917
TGATGGAA 0.161171240539
TGATGGCA 0.0867347218792
TGATGTAA 0.00545703528069
TGATTAAA -0.00749186853415
TGATTACA 0.365312630354
TGATTCAA -0.0381633809365
TGATTCCA 0.269848764401
TGATTGAA 0.000513132909656
TGATTGCA 0.339855155986
TGATTTAA 0.0633809364694
TGCAAAAA 0.0520318828411
TGCAAACA -0.023371057487
TGCAACAA 0.018263042436
TGCAACCA -0.0640848317197
TGCAAGAA 0.0988435987356
TGCAAGCA -0.196871459695
TGCAATAA -0.0214878482513
TGCACAAA 0.174262881732
TGCACACA -0.242172839506
TGCACCAA -0.0380965525257
TGCACCCA 0.00145072224129
TGCACGAA 0.106064348284
TGCACGCA -0.208742072114
TGCACTAA -0.151697264071
TGCAGAAA -0.0286092998749
TGCAGACA -0.11677111206
TGCAGCAA -0.0912947165622

TGCAGCCA -0.0498088409603
TGCAGGAA -0.00816965852517
TGCAGGCA 0.136595818866
TGCAGTAA 0.145429089198
TGCATAAA 0.0474031351241
TGCATACA -0.0195308527916
TGCATCAA 0.1418294035
TGCATCCA -0.0936280563102
TGCATGAA 0.0252829311522
TGCATGCA 0.0340452674897
TGCATTAA 0.0170494283924
TGCCAAAA -0.0462953815757
TGCCAACA -0.074855084044
TGCCACAA 0.0416210247992
TGCCACCA -0.035892665042
TGCCAGAA -0.0141207591074
TGCCAGCA 0.0525670157413
TGCCATAA -0.00937426062811
TGCCCAAA 0.0420783112692
TGCCCACA -0.113530089734
TGCCCCAA -0.135473251029
TGCCCCCA -0.161373294565
TGCCCGAA 0.0797273266153
TGCCCGCA -0.131584604212
TGCCCTAA -0.0491781235422
TGCCGAAA -0.291181117914
TGCCGACA -0.13766890665
TGCCGCAA -0.101650643136
TGCCGCCA -0.0648587105624
TGCCGGAA -0.119734514046
TGCCGGCA -0.112398163235
TGCCGTAA 0.0541474553964
TGCCTAAA 0.0381488530211
TGCCTACA -0.212087145969
TGCCTCAA -0.186534953701
TGCCTCCA -0.162254926169
TGCCTGAA -0.0526328788777
TGCCTTAA -0.0152354248689
TGCGAAAA 0.00695852775566
TGCGAACA -0.00420131170934
TGCGACAA -0.109453719253
TGCGACCA -0.156379108851
TGCGAGAA 0.0160093351773
TGCGAGCA -0.192637473714
TGCGATAA 0.104168038815
TGCGCAAA 0.170382445279
TGCGCACA 0.0914023796725
TGCGCCAA -0.0106010198597
TGCGCCCA -0.112377759668
TGCGCGAA 0.0981055598332
TGCGCGCA 0.00574382409847
TGCGCTAA 0.117099167929
TGCGGAAA 0.0299579873439
TGCGGACA -0.139235956783

TGCGGCAA 0.0490460822411
TGCGGCCA -0.123588198492
TGCGGGAA -0.139787226625
TGCGGTAA 0.0369918749383
TGCGTAAA 0.0788683615447
TGCGTACA -0.176340939398
TGCGTCAA 0.0309446969198
TGCGTCCA -0.235071906398
TGCGTGAA 0.0917061845485
TGCGTTAA 0.00800237107291
TGCTAAAA 0.0845408241674
TGCTAACA -0.0728473731586
TGCTACAA 0.100049394912
TGCTACCA -0.173075749784
TGCTAGAA 0.199504598085
TGCTAGCA 0.0486463070286
TGCTATAA 0.192951694162
TGCTCAAA -0.148217656614
TGCTCACA -0.140098421709
TGCTCCAA -0.134343976885
TGCTCCCA -0.187836436367
TGCTCGAA 0.0362306761496
TGCTCTAA 0.0141303870557
TGCTGAAA 0.0997058113241
TGCTGACA -0.00387750061744
TGCTGCAA -0.189896877269
TGCTGCCA -0.0806292290216
TGCTGGAA 0.0615488317228
TGCTGTAA 0.00605087675969
TGCTTAAA 0.0955922304708
TGCTTACA 0.133477134514
TGCTTCAA 0.00311623785103
TGCTTCCA -0.358681566866
TGCTTGAA -0.0559150585084
TGCTTTAA 0.0457995784759
TGGAAAAA 0.116201531242
TGGAAACA -0.115235382572
TGGAACAA 0.0967021632066
TGGAACCA -0.205385620915
TGGAAGAA -0.0400955936833
TGGAATAA 0.169369799557
TGGACAAA 0.0803059578981
TGGACACA -0.137366755859
TGGACCAA -0.189452432825
TGGACCCA -0.413316973296
TGGACGAA -0.101007107379
TGGACTAA -0.0616973723205
TGGAGAAA -0.132562869554
TGGAGACA -0.0911373982157
TGGAGCAA -0.0562164326157
TGGAGCCA -0.333189086833
TGGAGGAA -0.107256325415
TGGAGTAA -0.124517744672
TGGATAAA 0.11012014586

TGGATACA 0.147555678236
TGGATCAA -0.159522543645
TGGATCCA -0.0406018109094
TGGATGAA -0.0905316252827
TGGATTAA -0.00548173395684
TGGCAAAA -0.0760379791993
TGGCAACA -0.22046768337
TGGCACAA -0.161736382044
TGGCACCA -0.13732704302
TGGCAGAA -0.272347886211
TGGCATAA -0.0121688325649
TGGCCAAA -0.0822330874929
TGGCCACA -0.0854087428995
TGGCCCAA -0.340102119947
TGGCCCCA -0.196509421702
TGGCCGAA 0.105586840545
TGGCCTAA -0.101757877762
TGGCGAAA 0.0535571600831
TGGCGACA -0.113986024145
TGGCGCAA 0.0750468525271
TGGCGCCA -0.217125789889
TGGCGGAA -0.0488706947431
TGGCGTAA 0.161985700879
TGGCTAAA -0.061910711432
TGGCTACA -0.242632736929
TGGCTCAA -0.229561334193
TGGCTGAA 0.13446174553
TGGCTTAA -0.165266313964
TGGGAAAA 0.141295637665
TGGGAACA -0.195041122798
TGGGACAA -0.052690509122
TGGGACCA -0.226526536412
TGGGAGAA 0.108965968586
TGGGATAA 0.0889122949748
TGGGCAAA 0.0181787805268
TGGGCACA -0.120775599129
TGGGCCAA -0.290880658436
TGGGCCCA -0.25479632181
TGGGCGAA 0.0329768491076
TGGGCTAA -0.186189296203
TGGGGAAA 0.0372538365277
TGGGGACA -0.150773609602
TGGGGCAA -0.0624388132457
TGGGGGAA -0.0574246293569
TGGGGTAA 0.0307529882446
TGGGTAAA 0.0504525445644
TGGGTACA -0.0737312999274
TGGGTCAA -0.243949592282
TGGGTGAA 0.00259884959055
TGGGTTAA -0.0662083916468
TGGTAAAA 0.0155429324113
TGGTAACA -0.0898071307822
TGGTACAA -0.0110540297262
TGGTACCA 0.0655392060043

TGGTAGAA 0.12980526466
TGGTATAA -0.00645184722444
TGGTCAAA -0.0220284748293
TGGTCACA -0.219854066756
TGGTCCAA 0.0589935060218
TGGTCGAA -0.164965454416
TGGTCTAA -0.0257250433599
TGGTGAAA 0.144882541804
TGGTGACA 0.0133061177052
TGGTGCAA -0.146003446157
TGGTGGAA -0.128364883402
TGGTGTAA 0.281715165691
TGGTTAAA 0.101192828213
TGGTTACA 0.0616592384262
TGGTTCAA -0.132906911653
TGGTTGAA -0.0508216426266
TGGTTTAA -0.0684263101276
TGTAAAAA 0.067923910896
TGTAAACA -0.202375660592
TGTAACAA -0.0100907813563
TGTAAGAA 0.108221366001
TGTAATAA 0.244870908253
TGTACAAA 0.101655673046
TGTACACA -0.162652011833
TGTACCAA 0.0224151206543
TGTACGAA 0.107258146529
TGTACTAA 0.130429752195
TGTAGAAA 0.0164566308822
TGTAGACA -0.118580350577
TGTAGCAA 0.0578283672076
TGTAGGAA 0.0987590287383
TGTAGTAA -0.0554046192013
TGTATAAA 0.0305495181014
TGTATACA 0.00238596522731
TGTATCAA 0.161386817149
TGTATGAA 0.0768238600189
TGTATTAA 0.134780675756
TGTCAAAA 0.0883834788546
TGTCAACA 0.0449624453387
TGTCACAA 0.182686932715
TGTCAGAA 0.0550477242021
TGTCATAA -0.0157497130737
TGTCCAAA 0.0299139966098
TGTCCACA -0.164144025844
TGTCCCAA -0.0597171491336
TGTCCGAA -0.0208298125148
TGTCCTAA 0.0240619991657
TGTCGAAA 0.0311820853502
TGTCGACA -0.123719669291
TGTCGCAA 0.221275439636
TGTCGGAA 0.112880449784
TGTCGTAA 0.204823267909
TGTCTAAA 0.0377391658071
TGTCTCAA -0.0807491608535

TGTCTGAA -0.0666163032267
TGTCTTAA -0.179605557525
TGTGAAAA 0.135773538855
TGTGAACA -0.0441837490738
TGTGACAA 0.0188299501161
TGTGAGAA 0.138857815292
TGTGATAA 0.0285662400302
TGTGCAAA 0.020071915698
TGTGCACA 0.102742938969
TGTGCCAA -0.183795935913
TGTGCGAA 0.103605457859
TGTGCTAA -0.0675475426031
TGTGGAAA -0.0357517469818
TGTGGCAA -0.102575835491
TGTGGGAA -0.00473464762541
TGTGGTAA 0.156063225488
TGTGTAAA 0.167278644976
TGTGTCAA -0.0636002458405
TGTGTGAA -0.170156895672
TGTGTTAA -0.0418360379469
TGTTAAAA 0.147311137456
TGTTAACA 0.0183761969106
TGTTACAA -0.0659948626754
TGTTAGAA 0.0192422239333
TGTTATAA 0.14257448023
TGTTCAAA 0.0320655305837
TGTTCCAA -0.128445640781
TGTTCGAA 0.122287274999
TGTTCTAA 0.00935530966651
TGTTGAAA 0.129322268326
TGTTGCAA 0.110554018749
TGTTGGAA -0.0168480234771
TGTTGTAA 0.0789722952654
TGTTTAAA 0.170253784934
TGTTTCAA 0.0888143269798
TGTTTGAA -0.116967152383
TGTTTTAA 0.136755625935
TTAAAAAA 0.181204938939
TTAAACAA 0.0454157162989
TTAAAGAA 0.206373396481
TTAAATAA 0.169739805035
TTAACAAA 0.0910599025226
TTAACCAA -0.0140528525562
TTAACGAA -0.00734922830359
TTAACTAA -0.0589066525921
TTAAGAAA 0.0999883776677
TTAAGCAA 0.141271706659
TTAAGGAA -0.0136824425337
TTAAGTAA 0.247837493688
TTAATAAA 0.13956124174
TTAATCAA 0.00682376185841
TTAATGAA 0.0124489706972
TTAATTAA -0.0148118386971
TTACAAAA 0.0413346263946

TTACACAA 0.0804134009528
TTACAGAA 0.322372609717
TTACATAA 0.267021504314
TTACCAAA 0.186981379038
TTACCCAA 0.125226272282
TTACCGAA 0.176063140793
TTACCTAA 0.121319897254
TTACGAAA 0.305662981419
TTACGCAA 0.208230064068
TTACGGAA 0.0985486612526
TTACGTAA 0.326028793191
TTACTAAA 0.158146528555
TTACTCAA 0.153085139081
TTACTGAA 0.117732773524
TTAGAAAA 0.140719335221
TTAGACAA 0.0897810643151
TTAGAGAA 0.084098992294
TTAGATAA 0.00189589295832
TTAGCAAA 0.0213371493324
TTAGCCAA -0.0976987782023
TTAGCGAA 0.197854226897
TTAGCTAA -0.0243702081051
TTAGGAAA 0.0456772187759
TTAGGCAA 0.121950014818
TTAGGGAA 0.211725480511
TTAGTAAA 0.0120139925969
TTAGTCAA -0.0717330350268
TTAGTGAA 0.111188266673
TTATAAAA 0.182736332507
TTATACAA 0.184516147778
TTATAGAA 0.154638411381
TTATATAA 0.184944851208
TTATCAAA 0.11228189967
TTATCCAA 0.162327662604
TTATCGAA 0.138281027026
TTATGAAA 0.231631630177
TTATGCAA 0.0159086917386
TTATGGAA 0.191592695364
TTATTAAA 0.0545828707519
TTATTCAA -0.0829487516152
TTATTGAA 0.0694115128762
TTCAAAAA 0.00993710070474
TTCAACAA -0.0589282969988
TTCAAGAA -0.0975259726079
TTCACAAA 0.198740429736
TTCACCAA 0.0101738991472
TTCACGAA 0.0836764342684
TTCAGAAA -0.0243998814581
TTCAGCAA 0.0982513337285
TTCAGGAA 0.255367338341
TTCATAAA -0.0392777558234
TTCATCAA -0.0312081494654
TTCATGAA -0.0604287580291
TTCCAAAA 0.158809743353

TTCCACAA -0.00564227535182
TTCCAGAA 0.141645722255
TTCCCAAA 0.22735131397
TTCCCCAA 0.0739752793695
TTCCCGAA 0.212324030625
TTCCGAAA 0.0255748657888
TTCCGCAA 0.191813519678
TTCCGGAA 0.234168009661
TTCCTAAA 0.147309962462
TTCCTCAA -0.132805441038
TTCGAAAA 0.182930031694
TTCGACAA 0.237419551669
TTCGAGAA 0.247118387983
TTCGCAAA 0.189169904938
TTCGCCAA 0.233700405329
TTCGCGAA 0.121863791238
TTCGGAAA 0.154635667084
TTCGGCAA 0.102953525199
TTCGTAAA 0.0605218427208
TTCGTCAA 0.175282204756
TTCTAAAA 0.0864069463655
TTCTACAA 0.0513016845329
TTCTAGAA -0.137799940752
TTCTCAAA -0.0138320282423
TTCTCCAA 0.0977902917737
TTCTGAAA 0.102771926256
TTCTGCAA 0.207551610419
TTCTTAAA 0.143478958807
TTCTTCAA 0.0579794575276
TTGAAAAA 0.130834047623
TTGAACAA -0.0914081908387
TTGACAAA 0.142996818387
TTGACCAA -0.156554269028
TTGAGAAA 0.180313221856
TTGAGCAA 0.0182136475237
TTGATAAA 0.0583788155685
TTGATCAA -0.124886666607
TTGCAAAA 0.198999978046
TTGCACAA 0.170309299319
TTGCCAAA 0.103416481436
TTGCCCAA -0.0632066014848
TTGCGAAA 0.0661528627257
TTGCGCAA 0.248296464547
TTGCTAAA -0.0356264681991
TTGGAAAA 0.149575058475
TTGGACAA 0.0476826214038
TTGGCAAA 0.0087355317755
TTGGCCAA -0.0661087246334
TTGGGAAA 0.144680292343
TTGGTAAA 0.00287216887249
TTGTAAAA 0.126764583196
TTGTACAA 0.107197848816
TTGTCAAA 0.189016224286
TTGTGAAA 0.0582914755769

TTGTTAAA 0.191232403062
TTTAAAAA 0.0822081714198
TTTACAAA 0.0608904696042
TTTAGAAA 0.166276976443
TTTATAAA 0.182399522358
TTTCAAAA 0.0967104938159
TTTCCAAA 0.23698661979
TTTCGAAA 0.202315486104
TTTGAAAA -0.00259323289701
TTTGCAAA 0.223757749337
TTTTAAAA 0.1756793155
