# Supplementary material for: The intracellular immune receptor Rx1 regulates the DNA-binding activity of a Golden2-like transcription factor
Source: J Biol Chem. 2017 Dec 7;293(9):3218–33. doi: 10.1074/jbc.RA117.000485 (PMC5836133; doi:10.1074/jbc.RA117.000485)
Supplement: Supporting Information [file supp_RA117.000485_133217_1_supp_23416_1z1jtx.pdf]

#8mers	all
AAAAAAAA	0.150851800382
AAAAAAAC	-0.00289993649774
AAAAAAAG	0.0282372136884
AAAAAAAT	0.25173400005
AAAAAACA	-0.0589735741251
AAAAAACC	0.0281534978505
AAAAAACG	0.159940293278
AAAAAACT	0.151590984924
AAAAAAGA	0.202885338277
AAAAAAGC	0.169347386554
AAAAAAGG	-0.00791858634485
AAAAAAGT	-0.0383675686706
AAAAAATA	0.15169243368
AAAAAATC	0.427073129785
AAAAAATG	-0.109036445194
AAAAAATT	0.164074509317
AAAAACAA	0.12225586468
AAAAACAC	-0.0918647002545
AAAAACAG	-0.0908856514917
AAAAACAT	-0.0538100083555
AAAAACCA	-0.188923630929
AAAAACCC	-0.111125303825
AAAAACCG	0.0375663306643
AAAAACCT	0.0648541103087
AAAAACGA	0.0726896491318
AAAAACGC	-0.0342914654137
AAAAACGG	0.133829916133
AAAAACGT	-0.0125306130446
AAAAACTA	0.1694232973
AAAAACTC	0.0576627404057
AAAAACTG	0.0208075735568
AAAAACTT	0.308529740225
AAAAAGAA	0.167215742317
AAAAAGAC	0.0658102740836
AAAAAGAG	-0.057731197737
AAAAAGAT	0.27914609028
AAAAAGCA	-0.0563644591724
AAAAAGCC	-0.277542017328
AAAAAGCG	0.250227367284
AAAAAGCT	-0.013323240596
AAAAAGGA	0.082429581834
AAAAAGGC	-0.0563413874206
AAAAAGGG	-0.111859719362
AAAAAGGT	-0.0885800554246
AAAAAGTA	-0.0230825440157
AAAAAGTC	0.111801201434
AAAAAGTG	-0.00903321203416
AAAAAGTT	0.0035393520242
AAAAATAA	0.254484208375
AAAAATAC	-0.0771902805048
AAAAATAG	-0.0186455771042
AAAAATAT	0.267123347664
AAAAATCA	0.320406852405

AAAAATCC 0.433608539669
AAAAATCG 0.397994413146
AAAAATCT 0.452252140464
AAAAATGA 0.165817536842
AAAAATGC 0.0308686671095
AAAAATGG 0.043262270535
AAAAATGT -0.0701422596585
AAAAATTA 0.22501624052
AAAAATTC 0.165298907723
AAAAATTG 0.048961461151
AAAAATTT 0.281835434261
AAAACAAA 0.174525765435
AAAACAAC 0.0864294942447
AAAACAAG -0.0480230359919
AAAACAAT 0.166884515369
AAAACACA 0.0255038210842
AAAACACC -0.0399779945234
AAAACACG 0.0178908491055
AAAACACT -0.0640460427874
AAAACAGA 0.113993854149
AAAACAGC 0.00481376320227
AAAACAGG -0.0454845885468
AAAACAGT 0.032256774681
AAAACATA 0.0932067890309
AAAACATC -0.0816795577064
AAAACATG 0.0208834843033
AAAACATT -0.0399083030815
AAAACCAA -0.0321781274863
AAAACCAC -0.0842445367934
AAAACCAG -0.31016208612
AAAACCAT -0.107507864791
AAAACCCA -0.205609433948
AAAACCCC -0.279085226767
AAAACCCG 0.124756988393
AAAACCCT -0.187782356298
AAAACCGA 0.0451181208757
AAAACCGC -0.174829099072
AAAACCGG 0.115547104808
AAAACCGT 0.0479661029321
AAAACCTA 0.131786646881
AAAACCTC -0.0689372969541
AAAACCTG -0.0587877637716
AAAACCTT 0.21916268886
AAAACGAA 0.0701670766334
AAAACGAC -0.0652685063404
AAAACGAG -0.0659359446306
AAAACGAT 0.135534988285
AAAACGCA -0.0833057197368
AAAACGCC -0.123473790128
AAAACGCG 0.156027928755
AAAACGCT -0.0647388929747
AAAACGGA 0.0240092314564
AAAACGGC -0.00736115267549
AAAACGGG 0.0462183947032

AAAACGGT -0.144819789904
AAAACGTA 0.100846025088
AAAACGTC -0.0266501925948
AAAACGTG -0.150465202828
AAAACGTT 0.00187101702253
AAAACTAA 0.0702429873798
AAAACTAC 0.0900776809868
AAAACTAG 0.136644453041
AAAACTAT 0.14872010102
AAAACTCA -0.0411839394719
AAAACTCC -0.130926481982
AAAACTCG 0.0931799568163
AAAACTCT -0.26559535205
AAAACTGA 0.03904205717
AAAACTGC -0.095945808067
AAAACTGG -0.0675404241156
AAAACTGT -0.0338285944347
AAAACTTA 0.259203538725
AAAACTTC 0.0441559673876
AAAACTTG -0.0999019145635
AAAACTTT 0.115656979293
AAAAGAAA 0.15577104151
AAAAGAAC 0.0281341948009
AAAAGAAG -0.0483086392177
AAAAGAAT 0.286395790414
AAAAGACA 0.0142256247363
AAAAGACC -0.332349931787
AAAAGACG 0.194452027785
AAAAGACT 0.081712038117
AAAAGAGA 0.00153857607266
AAAAGAGC -0.105631130036
AAAAGAGG -0.0624157298724
AAAAGAGT -0.129707641264
AAAAGATA 0.233772339833
AAAAGATC 0.293427035851
AAAAGATG 0.0812704831281
AAAAGATT 0.39476296783
AAAAGCAA 0.122206228267
AAAAGCAC 0.0392665392665
AAAAGCAG -0.0988423611162
AAAAGCAT -0.0413008350813
AAAAGCCA -0.179377327853
AAAAGCCC -0.38003775821
AAAAGCCG -0.147049580284
AAAAGCCT -0.11758664141
AAAAGCGA 0.183934623329
AAAAGCGC 0.182894535156
AAAAGCGG -0.106438024454
AAAAGCGT 0.236224231871
AAAAGCTA 0.081664635081
AAAAGCTC -0.146844272285
AAAAGCTG -0.208105330914
AAAAGCTT 0.202876579345
AAAAGGAA 0.201142608575

AAAAGGAC -0.133673715174
AAAAGGAG 0.000246803277106
AAAAGGAT 0.287607570637
AAAAGGCA -0.272120911384
AAAAGGCC -0.20281341344
AAAAGGCG -0.121494605601
AAAAGGCT -0.168997424053
AAAAGGGA -0.199576837723
AAAAGGGC -0.107612542598
AAAAGGGG -0.0735823303139
AAAAGGGT -0.217738801095
AAAAGGTA 0.0243140661153
AAAAGGTC -0.118310449164
AAAAGGTG -0.0875818615489
AAAAGGTT 0.0679812043448
AAAAGTAA -0.0132846344968
AAAAGTAC -0.164375573807
AAAAGTAG -0.203406418601
AAAAGTAT 0.140169094715
AAAAGTCA -0.111891467097
AAAAGTCC -0.214366193072
AAAAGTCG 0.12368999219
AAAAGTCT -0.036864050872
AAAAGTGA 0.122788077334
AAAAGTGC -0.163273875684
AAAAGTGG 0.0209204013144
AAAAGTGT -0.0137293019536
AAAAGTTA 0.0525167040319
AAAAGTTC -0.156602848847
AAAAGTTG -0.0791775478834
AAAAGTTT 0.170204978038
AAAATAAA 0.323624300198
AAAATAAC 0.194923468829
AAAATAAG 0.123383714293
AAAATAAT 0.240029050459
AAAATACA 0.14445742064
AAAATACC 0.091214790917
AAAATACG 0.33372042948
AAAATACT 0.191986306869
AAAATAGA 0.243506591991
AAAATAGC 0.09166880379
AAAATAGG -0.088827952453
AAAATAGT -0.0591293317744
AAAATATA 0.300162040247
AAAATATC 0.400743779333
AAAATATG 0.245145420255
AAAATATT 0.316208404196
AAAATCAA 0.361074575009
AAAATCAC 0.338400618965
AAAATCAG 0.36789778326
AAAATCAT 0.24412239148
AAAATCCA 0.442976431173
AAAATCCC 0.396326078144
AAAATCCG 0.451905153169

AAAATCCT 0.453320730203
AAAATCGA 0.368287019802
AAAATCGC 0.411159472731
AAAATCGG 0.37364364284
AAAATCGT 0.385620022919
AAAATCTA 0.455476539752
AAAATCTC 0.453691525003
AAAATCTG 0.476058918418
AAAATCTT 0.461271225519
AAAATGAA 0.124034502822
AAAATGAC 0.0139230527799
AAAATGAG -0.112030950994
AAAATGAT 0.177020211236
AAAATGCA 0.13563133654
AAAATGCC -0.0679666709973
AAAATGCG 0.0958599446727
AAAATGCT -0.132324435511
AAAATGGA -0.0859771617347
AAAATGGC -0.0583635258208
AAAATGGG -1.39581627336E-5
AAAATGGT -0.0948541272819
AAAATGTA -0.163825264081
AAAATGTC -0.0675758924987
AAAATGTG -0.0686375080314
AAAATGTT 0.140579973913
AAAATTAA 0.207183054386
AAAATTAC 0.172932709503
AAAATTAG 0.0146055146055
AAAATTAT 0.0312475009445
AAAATTCA 0.112682085515
AAAATTCC 0.190210872029
AAAATTCG 0.269199214616
AAAATTCT 0.185148500398
AAAATTGA 0.161954847704
AAAATTGC 0.0690254957921
AAAATTGG 0.121871053919
AAAATTGT 0.0424267242449
AAAATTTA 0.229829002556
AAAATTTC 0.271633892846
AAAATTTG 0.110431888353
AAAATTTT 0.282408441038
AAACAAAA 0.23710201752
AAACAAAC -0.130971288185
AAACAAAG -0.0726414750042
AAACAAAT 0.181590681591
AAACAACA -0.0303212338416
AAACAACC -0.116415760041
AAACAACG -0.112597055101
AAACAACT 0.191212601184
AAACAAGA -0.0376524601651
AAACAAGC 0.0175609293227
AAACAAGG -0.167250537526
AAACAAGT -0.0400331257289
AAACAATA 0.105429684592

AAACAATC 0.0715384806294
AAACAATG -0.125364025055
AAACAATT 0.0617001087567
AAACACAA -0.0504598033944
AAACACAC 0.158604703414
AAACACAG -0.00754300181191
AAACACAT -0.0418832462636
AAACACCA -0.0794241628991
AAACACCC -0.263266997737
AAACACCG 0.102431632735
AAACACCT -0.0774920926436
AAACACGA 0.187101742298
AAACACGC -0.141506390371
AAACACGG -0.0664284723692
AAACACGT -0.0129968949576
AAACACTA 0.215649432848
AAACACTC -0.132841774822
AAACACTG -0.0194338810099
AAACACTT 0.137606397499
AAACAGAA 0.11941271359
AAACAGAC -0.0421399531397
AAACAGAG 0.040201503834
AAACAGAT 0.160153266214
AAACAGCA -0.160922149064
AAACAGCC -0.233191070528
AAACAGCG 0.0597856809978
AAACAGCT -0.0589249870441
AAACAGGA 0.200815310614
AAACAGGC -0.124006603272
AAACAGGG -0.057452769574
AAACAGGT -0.151491423059
AAACAGTA 0.038842215134
AAACAGTC -0.0438184119735
AAACAGTG -0.173992678776
AAACAGTT 0.0213243505617
AAACATAA 0.100788474375
AAACATAC -0.0645742129395
AAACATAG -0.0515092677601
AAACATAT 0.0686016595108
AAACATCA 0.0186587155026
AAACATCC 0.0435136456866
AAACATCG 0.167317045934
AAACATCT 0.0571715767789
AAACATGA 0.180479989489
AAACATGC -0.0382355130692
AAACATGG 0.058175255145
AAACATGT -0.0179421908959
AAACATTA 0.0748273416161
AAACATTC 0.039349266622
AAACATTG -0.0811022059006
AAACATTT 0.255354262315
AAACCAAA 0.00356999481763
AAACCAAC 0.140997642387
AAACCAAG 0.0795220487878

AAACCAAT 0.028833675175
AAACCACA -0.0428183003941
AAACCACC -0.301196432756
AAACCACG -0.0301113785962
AAACCACT 0.0824368809442
AAACCAGA -0.158330369378
AAACCAGC 0.146286577666
AAACCAGG -0.245444383315
AAACCAGT -0.134030391889
AAACCATA 0.0890542542864
AAACCATC -0.148608769894
AAACCATG 0.0515329925305
AAACCATT -0.119072575053
AAACCCAA -0.091076534509
AAACCCAC -0.319979250599
AAACCCAG -0.132610913507
AAACCCAT -0.308397774253
AAACCCCA -0.348930172314
AAACCCCC -0.389934352486
AAACCCCG -0.240608843115
AAACCCCT -0.236916537716
AAACCCGA -0.161432183319
AAACCCGC -0.0171186845828
AAACCCGG -0.156246811866
AAACCCGT -0.0689766901888
AAACCCTA 0.0244018122806
AAACCCTC -0.252498559451
AAACCCTG -0.149442094425
AAACCCTT -0.180448346067
AAACCGAA 0.0986537501689
AAACCGAC -0.0667234643264
AAACCGAG -0.134435328855
AAACCGAT 0.123973197667
AAACCGCA -0.00335540097662
AAACCGCC -0.316648741414
AAACCGCG 0.0528419085713
AAACCGCT -0.265936329123
AAACCGGA 0.00352585015246
AAACCGGC -0.0649009891434
AAACCGGG -0.166726020979
AAACCGGT -0.0994278917373
AAACCGTA -0.0246746421611
AAACCGTC -0.102461016669
AAACCGTG -0.0749164011402
AAACCGTT 0.0307852354165
AAACCTAA -0.0943899038707
AAACCTAC -0.0429341186917
AAACCTAG 0.123157592855
AAACCTAT 0.0230907352394
AAACCTCA -0.0684576250155
AAACCTCC -0.152025118977
AAACCTCG -0.0325490780036
AAACCTCT -0.260003499805
AAACCTGA 0.0935534258374

AAACCTGC -0.0971314496763
AAACCTGG -0.305963900261
AAACCTGT -0.121396215699
AAACCTTA 0.0174640792119
AAACCTTC -0.155803030916
AAACCTTG 0.0706367524549
AAACCTTT 0.0274668608002
AAACGAAA 0.127479690226
AAACGAAC -0.0413612748869
AAACGAAG -0.130703140294
AAACGAAT 0.0984511288074
AAACGACA -0.0891199462785
AAACGACC -0.236246462459
AAACGACG 0.0394611796822
AAACGACT 0.0288980440496
AAACGAGA 0.074502748115
AAACGAGC -0.0110860682958
AAACGAGG -0.222298033054
AAACGAGT -0.19840880594
AAACGATA 0.164264286184
AAACGATC 0.157009626707
AAACGATG -0.250984949367
AAACGATT 0.176403676404
AAACGCAA 0.249897447501
AAACGCAC -0.114835220896
AAACGCAG 0.0467947286129
AAACGCAT -0.0382069384818
AAACGCCA -0.203718188757
AAACGCCC -0.177750488587
AAACGCCG 0.00271732387026
AAACGCCT -0.1828830433
AAACGCGA 0.191393619118
AAACGCGC 0.138465642604
AAACGCGG -0.0954246257277
AAACGCGT 0.214823762983
AAACGCTA -0.00245615059524
AAACGCTC 0.0247823581157
AAACGCTG 0.0773085260907
AAACGCTT -0.0496005593483
AAACGGAA -0.0283154383481
AAACGGAC -0.0698914502979
AAACGGAG -0.0906148040554
AAACGGAT 0.242949561131
AAACGGCA -0.189274578402
AAACGGCC -0.17648445104
AAACGGCG -0.153879217866
AAACGGCT -0.14263038284
AAACGGGA -0.0696687390245
AAACGGGC -0.0552384340263
AAACGGGG -0.0514722302906
AAACGGGT -0.207189964084
AAACGGTA 0.0142304839275
AAACGGTC -0.210086373499
AAACGGTG -0.0969535130496

AAACGGTT -0.0907005664726
AAACGTAA 0.0257971938359
AAACGTAC -0.163538402484
AAACGTAG -0.0280906257527
AAACGTAT 0.147923038182
AAACGTCA 0.0966161324934
AAACGTCC -0.303397544531
AAACGTCG 0.193738093326
AAACGTCT -0.129473382358
AAACGTGA 0.0649727245788
AAACGTGC -0.165051046431
AAACGTGG -0.0412541254125
AAACGTGT 0.065375292648
AAACGTTA 0.0226164478745
AAACGTTC -0.192108433901
AAACGTTG 0.0345386597374
AAACGTTT 0.128245943346
AAACTAAA 0.201589016299
AAACTAAC 0.0208396896418
AAACTAAG 0.040173586123
AAACTAAT 0.107036788855
AAACTACA -0.122666236442
AAACTACC 0.0480273662092
AAACTACG 0.164021955724
AAACTACT 0.140971365591
AAACTAGA 0.178868345949
AAACTAGC -0.0391700240185
AAACTAGG 0.0203404305015
AAACTAGT -0.124728763603
AAACTATA 0.225074937196
AAACTATC 0.219945841158
AAACTATG 0.0517059759484
AAACTATT 0.136311613614
AAACTCAA 0.0552163733982
AAACTCAC -0.0922506666745
AAACTCAG -0.0255806770958
AAACTCAT -0.208102191379
AAACTCCA -0.240125659493
AAACTCCC -0.283590913977
AAACTCCG -0.228573476347
AAACTCCT -0.102707969898
AAACTCGA 0.214009182281
AAACTCGC 0.0312120008934
AAACTCGG -0.082836329145
AAACTCGT 0.114372658014
AAACTCTA -0.0395423356577
AAACTCTC -0.122684897966
AAACTCTG -0.0659426717614
AAACTCTT 0.0768026980148
AAACTGAA 0.0665941423517
AAACTGAC -0.160996550783
AAACTGAG 0.0340744363262
AAACTGAT 0.0762980611465
AAACTGCA -0.201098480924

AAACTGCC -0.22045066755
AAACTGCG -0.0770127584977
AAACTGCT -0.261902792597
AAACTGGA -0.0519619764426
AAACTGGC -0.128460926611
AAACTGGG -0.181922671061
AAACTGGT -0.085767464946
AAACTGTA -0.081289278259
AAACTGTC -0.0787045890148
AAACTGTG -0.0540433421166
AAACTGTT -0.0147626965809
AAACTTAA 0.0493967285388
AAACTTAC -0.0509154721829
AAACTTAG -0.0539966278111
AAACTTAT 0.120221455005
AAACTTCA -0.1175811268
AAACTTCC -0.238981077062
AAACTTCG 0.0801586710678
AAACTTCT -0.00337583848529
AAACTTGA -0.0590245899605
AAACTTGC -0.0428679368073
AAACTTGG -0.142457176414
AAACTTGT -0.0136541500178
AAACTTTA 0.0294263629264
AAACTTTC 0.00636993350511
AAACTTTG 0.0102761163367
AAAGAAAA 0.093858321131
AAAGAAAC -0.0512178845512
AAAGAAAG -0.0254919721071
AAAGAAAT 0.274534865444
AAAGAACA 0.262463595688
AAAGAACC -0.186166509775
AAAGAACG -0.191954113589
AAAGAACT 0.0434470282955
AAAGAAGA -0.0244834878803
AAAGAAGC -0.151433910206
AAAGAAGG -0.010780753205
AAAGAAGT 0.0873842178638
AAAGAATA 0.0976406432241
AAAGAATC 0.427494988101
AAAGAATG -0.0493598916931
AAAGAATT 0.206812259586
AAAGACAA 0.095194265819
AAAGACAC -0.154966961847
AAAGACAG 0.0442805916073
AAAGACAT -0.0919549206952
AAAGACCA -0.0828721969941
AAAGACCC -0.229159603877
AAAGACCG -0.18991417788
AAAGACCT -0.130785846158
AAAGACGA -0.0643480946511
AAAGACGC -0.201694118815
AAAGACGG -0.05585950786
AAAGACGT 0.158829628527

AAAGACTA 0.0643949402568
AAAGACTC -0.0831864922774
AAAGACTG 0.0806982328854
AAAGACTT 0.0387546258111
AAAGAGAA 0.0183017233834
AAAGAGAC -0.279834643199
AAAGAGAG -0.0853857214806
AAAGAGAT 0.27649540521
AAAGAGCA 0.00515218619398
AAAGAGCC -0.150482748894
AAAGAGCG -0.0358522562766
AAAGAGCT -0.297560900864
AAAGAGGA -0.138404921598
AAAGAGGC -0.0911429256714
AAAGAGGG -0.212998326083
AAAGAGGT -0.152532884509
AAAGAGTA -0.100261159773
AAAGAGTC -0.0625395064342
AAAGAGTG -0.157868805793
AAAGAGTT 0.0434607963905
AAAGATAA 0.0734061021855
AAAGATAC 0.257553273619
AAAGATAG 0.146244242827
AAAGATAT 0.364307834005
AAAGATCA 0.109968094817
AAAGATCC 0.274933395619
AAAGATCG 0.242839257991
AAAGATCT 0.364238894542
AAAGATGA 0.00700838579626
AAAGATGC -0.0681935378905
AAAGATGG -0.0221403910863
AAAGATGT -0.02188638205
AAAGATTA 0.29389306662
AAAGATTC 0.407042028254
AAAGATTG 0.323244732336
AAAGATTT 0.421284921285
AAAGCAAA 0.0319489354248
AAAGCAAC -0.0675145799727
AAAGCAAG 0.115141274279
AAAGCAAT 0.211813609921
AAAGCACA 0.00540071752193
AAAGCACC -0.238379070117
AAAGCACG -0.0375651166151
AAAGCACT -0.144451614149
AAAGCAGA -0.0301418769697
AAAGCAGC 0.0107377210718
AAAGCAGG -0.0831727283004
AAAGCAGT 0.00413498898347
AAAGCATA -0.0120812951849
AAAGCATC -0.105030952282
AAAGCATG -0.0745708731439
AAAGCATT -0.227859989988
AAAGCCAA -0.200743594796
AAAGCCAC -0.346019945867

AAAGCCAG -0.0760623530158
AAAGCCAT -0.084088557137
AAAGCCCA -0.321620020119
AAAGCCCC -0.466840013129
AAAGCCCG -0.234747549932
AAAGCCCT -0.209789209919
AAAGCCGA -0.0258099468169
AAAGCCGC -0.234058156814
AAAGCCGG -0.238920145654
AAAGCCGT -0.242270741154
AAAGCCTA 0.0100511058333
AAAGCCTC -0.113999101208
AAAGCCTG -0.120510576603
AAAGCCTT -0.109671726572
AAAGCGAA 0.0284574534084
AAAGCGAC -0.277660864629
AAAGCGAG -0.000365667273436
AAAGCGAT 0.266759932501
AAAGCGCA -0.00686774929199
AAAGCGCC -0.222958892555
AAAGCGCG 0.120501740838
AAAGCGCT -0.191152019783
AAAGCGGA 0.0256983460398
AAAGCGGC -0.189377321453
AAAGCGGG -0.22048655319
AAAGCGGT -0.0780467476204
AAAGCGTA 0.113339853872
AAAGCGTC 0.0436625475355
AAAGCGTG -0.146138532507
AAAGCGTT -0.0678689112242
AAAGCTAA -0.126799964384
AAAGCTAC -0.185366375568
AAAGCTAG 0.0962570162697
AAAGCTAT -0.0997492274659
AAAGCTCA -0.108273703658
AAAGCTCC -0.283552611631
AAAGCTCG -0.127989521078
AAAGCTCT -0.247431881363
AAAGCTGA 0.00383713427799
AAAGCTGC -0.274013612193
AAAGCTGG -0.071821394116
AAAGCTGT 0.203108066744
AAAGCTTA 0.123743703005
AAAGCTTC -0.0335477827134
AAAGCTTG 0.0934805328745
AAAGCTTT -0.183425523999
AAAGGAAA 0.027538569715
AAAGGAAC -0.0604573136472
AAAGGAAG -0.187022681409
AAAGGAAT 0.309120415181
AAAGGACA 0.0985285991778
AAAGGACC -0.185510285105
AAAGGACG -0.210314408193
AAAGGACT -0.0587877673216

AAAGGAGA -0.121006681613
AAAGGAGC -0.0959733838522
AAAGGAGG -0.330934343619
AAAGGAGT -0.0255446961015
AAAGGATA 0.248277202823
AAAGGATC 0.218931052264
AAAGGATG 0.0101106616258
AAAGGATT 0.415258598956
AAAGGCAA -0.0368652461357
AAAGGCAC -0.386894206206
AAAGGCAG -0.032979260252
AAAGGCAT -0.241536736788
AAAGGCCA -0.157280354223
AAAGGCCC -0.327638638994
AAAGGCCG -0.236151426689
AAAGGCCT -0.265394610701
AAAGGCGA -0.144311847465
AAAGGCGC -0.0418786239526
AAAGGCGG -0.203975028201
AAAGGCGT -0.0887754282753
AAAGGCTA -0.118913896402
AAAGGCTC -0.252379284427
AAAGGCTG -0.159766142133
AAAGGCTT -0.143294689784
AAAGGGAA -0.073791895879
AAAGGGAC -0.111113215161
AAAGGGAG -0.127865083246
AAAGGGAT 0.181709159653
AAAGGGCA -0.192815937708
AAAGGGCC -0.387644521615
AAAGGGCG -0.156937656603
AAAGGGCT -0.257087176614
AAAGGGGA -0.142834727499
AAAGGGGC -0.268508479968
AAAGGGGG -0.306886314205
AAAGGGGT -0.182115535707
AAAGGGTA -0.207344804284
AAAGGGTC -0.1994064112
AAAGGGTG -0.213574080871
AAAGGGTT -0.19150154914
AAAGGTAA 0.0760764362824
AAAGGTAC -0.276741339817
AAAGGTAG 0.0255586164677
AAAGGTAT 0.0682929250316
AAAGGTCA -0.189704605009
AAAGGTCC -0.246250840889
AAAGGTCG 0.0823200238231
AAAGGTCT -0.0431824517515
AAAGGTGA 0.0536776445867
AAAGGTGC -0.210200466105
AAAGGTGG -0.200804892109
AAAGGTGT 0.0175602402164
AAAGGTTA -0.0722196025226
AAAGGTTC 0.159574174726

AAAGGTTG -0.0523460527803
AAAGTAAA 0.0721115596009
AAAGTAAC -0.149167847672
AAAGTAAG 0.0449121552083
AAAGTAAT 0.228646001373
AAAGTACA 0.0120865966439
AAAGTACC -0.119898722573
AAAGTACG -0.0268526966563
AAAGTACT 0.00379410838779
AAAGTAGA 0.0107043884684
AAAGTAGC -0.190956283927
AAAGTAGG 0.053941073472
AAAGTAGT 0.0159813385612
AAAGTATA 0.132916067531
AAAGTATC 0.251214206988
AAAGTATG -0.0896144077962
AAAGTATT 0.145292438852
AAAGTCAA -0.0634648032477
AAAGTCAC -0.272541333501
AAAGTCAG 0.0437220536387
AAAGTCAT -0.0138267917902
AAAGTCCA -0.390730240738
AAAGTCCC -0.34101527292
AAAGTCCG -0.160754506833
AAAGTCCT -0.253538095551
AAAGTCGA 0.0579574064423
AAAGTCGC 0.0596698627002
AAAGTCGG -0.134980086355
AAAGTCGT 0.0556973204966
AAAGTCTA -0.0319007612972
AAAGTCTC -0.0956088542571
AAAGTCTG -0.0247147974421
AAAGTCTT 0.032262289838
AAAGTGAA -0.145891255646
AAAGTGAC 0.0592330094961
AAAGTGAG 0.0221505287394
AAAGTGAT 0.336779534152
AAAGTGCA -0.050347493391
AAAGTGCC -0.145821100829
AAAGTGCG -0.179698824464
AAAGTGCT -0.249705371542
AAAGTGGA -0.0342749996553
AAAGTGGC -0.210408132084
AAAGTGGG -0.0960340505795
AAAGTGGT -0.0609334927301
AAAGTGTA -0.0253419828363
AAAGTGTC -0.100320008359
AAAGTGTG -0.044405014986
AAAGTGTT 0.0768092669504
AAAGTTAA -0.104850988664
AAAGTTAC -0.23367184752
AAAGTTAG -0.104880362456
AAAGTTAT -0.18759606977
AAAGTTCA -0.162811763246

AAAGTTCC -0.028274437303
AAAGTTCG 0.0738750246345
AAAGTTCT 0.0621438946592
AAAGTTGA 0.0308073545835
AAAGTTGC -0.252139508347
AAAGTTGG -0.0488508361942
AAAGTTGT -0.00897863550433
AAAGTTTA -0.0453398830683
AAAGTTTC 0.0535771893689
AAAGTTTG 0.0963515018634
AAATAAAA 0.261888425801
AAATAAAC 0.0436800654
AAATAAAG 0.258282665343
AAATAAAT 0.312065509035
AAATAACA 0.162902571993
AAATAACC 0.13964134605
AAATAACG 0.108990743728
AAATAACT 0.131948753161
AAATAAGA 0.174361615009
AAATAAGC 0.196147938572
AAATAAGG 0.0658255658635
AAATAAGT 0.0352239291633
AAATAATA 0.219463264918
AAATAATC 0.211568317629
AAATAATG 0.143696037635
AAATAATT 0.167604243362
AAATACAA 0.0364977409254
AAATACAC -0.0518304367847
AAATACAG 0.157014809895
AAATACAT 0.121357196558
AAATACCA -0.00934933121572
AAATACCC -0.0491221248797
AAATACCG 0.105707636011
AAATACCT 0.0693379329743
AAATACGA 0.171053974084
AAATACGC 0.202739356072
AAATACGG 0.252387903915
AAATACGT 0.304535667102
AAATACTA 0.191682663883
AAATACTC 0.158737399911
AAATACTG 0.263453234732
AAATACTT 0.223762329823
AAATAGAA 0.215436888243
AAATAGAC -0.113630574413
AAATAGAG 0.0653670199125
AAATAGAT 0.297873631207
AAATAGCA 0.0986073297665
AAATAGCC -0.0528900826989
AAATAGCG 0.0340948738349
AAATAGCT -0.0332949304241
AAATAGGA 0.00551487925343
AAATAGGC -0.00462813608954
AAATAGGG -0.0431363303939
AAATAGGT -0.0837600686086

AAATAGTA 0.0558892225559
AAATAGTC -0.058341058225
AAATAGTG -0.102010511101
AAATAGTT 0.174920099163
AAATATAA 0.260451911967
AAATATAC 0.209265739569
AAATATAG 0.231908216757
AAATATAT 0.308786748181
AAATATCA 0.345924541798
AAATATCC 0.412151821243
AAATATCG 0.383690138172
AAATATCT 0.425426450516
AAATATGA 0.186734271334
AAATATGC 0.291023554229
AAATATGG 0.312520893703
AAATATGT 0.287625088502
AAATATTA 0.300183103213
AAATATTC 0.286474748728
AAATATTG 0.203624552434
AAATATTT 0.340770967182
AAATCAAA 0.327752676949
AAATCAAC 0.356775938655
AAATCAAG 0.335689642334
AAATCAAT 0.33042053273
AAATCACA 0.351454347715
AAATCACC 0.326964373043
AAATCACG 0.393152105273
AAATCACT 0.232454217303
AAATCAGA 0.294422521695
AAATCAGC 0.359664386911
AAATCAGG 0.356520432278
AAATCAGT 0.218059657454
AAATCATA 0.3333642329
AAATCATC 0.279812120902
AAATCATG 0.340902753954
AAATCATT 0.220277658153
AAATCCAA 0.396351411899
AAATCCAC 0.407530119651
AAATCCAG 0.401263475982
AAATCCAT 0.430070566434
AAATCCCA 0.396665260302
AAATCCCC 0.356969494297
AAATCCCG 0.430889104618
AAATCCCT 0.358030198725
AAATCCGA 0.419310698876
AAATCCGC 0.445969066371
AAATCCGG 0.421996376542
AAATCCGT 0.435053510811
AAATCCTA 0.423438172887
AAATCCTC 0.44967143452
AAATCCTG 0.419950253284
AAATCCTT 0.405196236579
AAATCGAA 0.364702167732
AAATCGAC 0.344423902524

AAATCGAG 0.238289253441
AAATCGAT 0.381582155135
AAATCGCA 0.37702027096
AAATCGCC 0.305741480114
AAATCGCG 0.423477196204
AAATCGCT 0.259026726555
AAATCGGA 0.295983311135
AAATCGGC 0.292788545053
AAATCGGG 0.367708006394
AAATCGGT 0.309242133384
AAATCGTA 0.389036006511
AAATCGTC 0.333458803156
AAATCGTG 0.344718728787
AAATCGTT 0.30849762414
AAATCTAA 0.449518574584
AAATCTAC 0.426877290514
AAATCTAG 0.440833395379
AAATCTAT 0.402204075554
AAATCTCA 0.432101853751
AAATCTCC 0.43271508423
AAATCTCG 0.460973117377
AAATCTCT 0.375953088074
AAATCTGA 0.468886241614
AAATCTGC 0.457996564057
AAATCTGG 0.47066341613
AAATCTGT 0.442059662927
AAATCTTA 0.46724159325
AAATCTTC 0.460260536018
AAATCTTG 0.419100919101
AAATGAAA 0.155076166215
AAATGAAC -0.245358322472
AAATGAAG 0.0452333160588
AAATGAAT 0.246242053094
AAATGACA 0.00623163851402
AAATGACC -0.0190336749634
AAATGACG 0.118554338226
AAATGACT -0.0358189236732
AAATGAGA 0.0920906841456
AAATGAGC -0.274588051069
AAATGAGG -0.138738586957
AAATGAGT -0.0201345955928
AAATGATA 0.0864732889061
AAATGATC -0.0342189587089
AAATGATG 0.0932891980468
AAATGATT 0.212785851405
AAATGCAA 0.0669496288402
AAATGCAC -0.0774899151577
AAATGCAG 0.0742712409379
AAATGCAT 0.0416071180923
AAATGCCA -0.261024962439
AAATGCCC -0.0108910023868
AAATGCCG 0.187370349554
AAATGCCT -0.231330097061
AAATGCGA 0.0332406878924

AAATGCGC 0.130192694245
AAATGCGG -0.123609353417
AAATGCGT 0.0979474902009
AAATGCTA 0.0714692378999
AAATGCTC -0.0734122610454
AAATGCTG -0.0895451600006
AAATGCTT 0.174652614047
AAATGGAA 0.0089688950098
AAATGGAC -0.160659354439
AAATGGAG -0.116823137987
AAATGGAT 0.282279220163
AAATGGCA -0.232086636297
AAATGGCC -0.340315286062
AAATGGCG -0.0278225884286
AAATGGCT -0.293979755472
AAATGGGA -0.121425245615
AAATGGGC -0.103328278988
AAATGGGG -0.0542754538222
AAATGGGT -0.120501093109
AAATGGTA 0.00784393208636
AAATGGTC -0.159626937063
AAATGGTG -0.083297143708
AAATGGTT -0.118475010547
AAATGTAA 0.155375429735
AAATGTAC -0.265513744511
AAATGTAG -0.0202624590316
AAATGTAT -0.00719865871381
AAATGTCA 0.00904072116193
AAATGTCC -0.122274965223
AAATGTCG 0.210482252213
AAATGTCT -0.0784048511321
AAATGTGA -0.0146767790639
AAATGTGC -0.03398577641
AAATGTGG 0.0879798252593
AAATGTGT -0.00527674327165
AAATGTTA 0.164156259352
AAATGTTC 0.0377181521573
AAATGTTG 0.101245873973
AAATTAAA 0.0653091107637
AAATTAAC 0.0277154514865
AAATTAAG 0.101510915819
AAATTAAT 0.201781671479
AAATTACA 0.241602738626
AAATTACC 0.288135242681
AAATTACG 0.323008255294
AAATTACT 0.157049845624
AAATTAGA 0.188911921637
AAATTAGC 0.0368189017759
AAATTAGG 0.162528787283
AAATTAGT 0.118326605987
AAATTATA 0.250927235776
AAATTATC 0.178182314546
AAATTATG 0.0618022963001
AAATTATT 0.151202166354

AAATTCAA 0.100526145981
AAATTCAC -0.0680542559729
AAATTCAG 0.206822525004
AAATTCAT 0.115535426232
AAATTCCA 0.0378553754297
AAATTCCC 0.251830251892
AAATTCCG 0.204387583175
AAATTCCT 0.113578554235
AAATTCGA 0.260095764326
AAATTCGC 0.301472960446
AAATTCGG 0.278569124252
AAATTCGT 0.15052242325
AAATTCTA 0.184020108263
AAATTCTC 0.286392998693
AAATTCTG 0.188871046619
AAATTCTT 0.26968679518
AAATTGAA 0.181950651118
AAATTGAC -0.0882936869996
AAATTGAG 0.0859010386634
AAATTGAT 0.261105961183
AAATTGCA 0.172968831427
AAATTGCC 0.0908264782523
AAATTGCG 0.223039641468
AAATTGCT 0.0774948502221
AAATTGGA 0.0641058954913
AAATTGGC 0.0702824036157
AAATTGGG 0.0647161011073
AAATTGGT 0.0159084107647
AAATTGTA 0.118636088261
AAATTGTC 0.177866908024
AAATTGTG 0.036710263983
AAATTGTT 0.113839113012
AAATTTAA 0.151020780567
AAATTTAC -0.0475179375634
AAATTTAG 0.167940667941
AAATTTAT 0.258587852322
AAATTTCA 0.195495719072
AAATTTCC 0.331927768005
AAATTTCG 0.38289015569
AAATTTCT 0.196776666474
AAATTTGA 0.161671642227
AAATTTGC 0.198877508233
AAATTTGG 0.104604561134
AAATTTGT 0.126866564966
AAATTTTA 0.225615422585
AAATTTTC 0.277599030678
AAATTTTG 0.109357459326
AACAAAAA 0.12535126968
AACAAAAC 0.213924512602
AACAAAAG -0.0501619227238
AACAAAAT 0.39530237983
AACAAACA 0.169609400203
AACAAACC 0.0889084676963
AACAAACG 0.0396042422429

AACAAACT -0.158567489333
AACAAAGA 0.0919884966023
AACAAAGC -0.129039674534
AACAAAGG -0.0447099992555
AACAAAGT -0.165673938401
AACAAATA 0.199932987516
AACAAATC 0.332450384298
AACAAATG -0.0956838381081
AACAAATT 0.172472865558
AACAACAA -0.00178983547162
AACAACAC -0.207081969414
AACAACAG -0.201250257217
AACAACAT 0.104078742801
AACAACCA -0.208712987401
AACAACCC -0.152211228155
AACAACCG -0.179648791947
AACAACCT -0.25088908768
AACAACGA 0.0453501018226
AACAACGC -0.148126549261
AACAACGG -0.010101010101
AACAACGT -0.0216177747933
AACAACTA 0.29073563922
AACAACTC 0.146466135778
AACAACTG -0.0436731497338
AACAACTT 0.106198484986
AACAAGAA 0.084388663022
AACAAGAC -0.0605757272424
AACAAGAG -0.0626618353891
AACAAGAT 0.205419589352
AACAAGCA 0.0233299004889
AACAAGCC -0.00927798595429
AACAAGCG 0.0461759001051
AACAAGCT -0.0308614399523
AACAAGGA -0.0910737772043
AACAAGGC -0.177716299035
AACAAGGG -0.103006990115
AACAAGGT -0.126779075691
AACAAGTA 0.00520207586695
AACAAGTC -0.119519512067
AACAAGTG -0.153745199081
AACAAGTT -0.120749270004
AACAATAA 0.242437756837
AACAATAC 0.0399657542745
AACAATAG 0.0532860684376
AACAATAT 0.145951736861
AACAATCA 0.13057547906
AACAATCC 0.212879280016
AACAATCG 0.122881835003
AACAATCT 0.218524053302
AACAATGA 0.0677550829066
AACAATGC -0.116982504908
AACAATGG 0.224861499383
AACAATGT 0.00798449668985
AACAATTA 0.0849346364678

AACAATTC 0.195858392828
AACAATTG 0.0498200769326
AACACAAA 0.0653058692145
AACACAAC -0.0632559434698
AACACAAG 0.00679912118713
AACACAAT 0.247325838235
AACACACA 0.0147726692116
AACACACC -0.139510967578
AACACACG 0.104986103184
AACACACT -0.232183433326
AACACAGA -0.109314001434
AACACAGC -0.0143554326485
AACACAGG -0.025772428341
AACACAGT -0.18633484612
AACACATA 0.0473091830106
AACACATC 0.0184966752553
AACACATG -0.13156074072
AACACATT 0.017237635556
AACACCAA -0.172791106764
AACACCAC -0.0956213415828
AACACCAG -0.318079364603
AACACCAT 0.0548937367119
AACACCCA -0.0895592562259
AACACCCC -0.270867721856
AACACCCG -0.271107666957
AACACCCT -0.210666713684
AACACCGA 0.00988012806195
AACACCGC -0.00280524970315
AACACCGG 0.0229608110771
AACACCGT -0.153178020612
AACACCTA -0.0276323299501
AACACCTC 0.0210995379663
AACACCTG -0.136766639955
AACACCTT 0.098621927987
AACACGAA 0.159359083602
AACACGAC -0.221826548985
AACACGAG -0.0886273415209
AACACGAT 0.221208812118
AACACGCA 0.126039262403
AACACGCC -0.173071422694
AACACGCG -0.0717416664598
AACACGCT -0.201889072628
AACACGGA -0.0199369513343
AACACGGC -0.0415545644986
AACACGGG -0.163754413261
AACACGGT -0.175818874732
AACACGTA 0.130665021934
AACACGTC -0.112964111897
AACACGTG -0.0700397367064
AACACGTT -0.106406915664
AACACTAA 0.216275504154
AACACTAC -0.0991916307951
AACACTAG 0.0672518120041
AACACTAT 0.00912607753115

AACACTCA -0.274188463565
AACACTCC -0.162077532941
AACACTCG -0.0405675360449
AACACTCT -0.278082495683
AACACTGA 0.0855938583211
AACACTGC 0.0217564578878
AACACTGG -0.0569454683474
AACACTGT 0.0157250914827
AACACTTA 0.196380371233
AACACTTC -0.026694738816
AACACTTG -0.0783805246048
AACAGAAA 0.18465800019
AACAGAAC -0.16776931892
AACAGAAG 0.0730769180527
AACAGAAT 0.37583175462
AACAGACA 0.258295485568
AACAGACC 0.108208180226
AACAGACG -0.0324496543871
AACAGACT -0.25317818099
AACAGAGA -0.0248004301609
AACAGAGC -0.101559376801
AACAGAGG -0.215973596067
AACAGAGT -0.133002830174
AACAGATA 0.268082131718
AACAGATC 0.323550823551
AACAGATG -0.102588264491
AACAGATT 0.429201898295
AACAGCAA -0.0152314849285
AACAGCAC -0.277046227766
AACAGCAG -0.225479007441
AACAGCAT 0.0443924585593
AACAGCCA -0.218943059174
AACAGCCC -0.204778946539
AACAGCCG -0.197116402078
AACAGCCT -0.294107188634
AACAGCGA -0.124447904518
AACAGCGC 0.0632650379919
AACAGCGG -0.0535000868508
AACAGCGT -0.0185999360661
AACAGCTA 0.0483775786806
AACAGCTC -0.0762156870855
AACAGCTG -0.239095497611
AACAGCTT -0.0943519276853
AACAGGAA 0.258474728172
AACAGGAC -0.0301941059517
AACAGGAG -0.00886184974052
AACAGGAT 0.213521601717
AACAGGCA -0.0162724031078
AACAGGCC -0.14628381189
AACAGGCG -0.252949931058
AACAGGCT -0.234296626334
AACAGGGA -0.259846956431
AACAGGGC -0.147265854405
AACAGGGG -0.132265278019

AACAGGGT -0.0883887367141
AACAGGTA -0.13397784612
AACAGGTC -0.162502377821
AACAGGTG -0.31352679985
AACAGGTT -0.131878272414
AACAGTAA 0.0809390657876
AACAGTAC -0.16136698174
AACAGTAG -0.118931429879
AACAGTAT 0.204605008649
AACAGTCA 0.0148677320892
AACAGTCC 0.00347364656248
AACAGTCG 0.0389064473041
AACAGTCT -0.179521962221
AACAGTGA -0.0653843714934
AACAGTGC 0.0755654985657
AACAGTGG 0.0371809376437
AACAGTGT -0.129186850782
AACAGTTA 0.102578045736
AACAGTTC -0.149448315022
AACAGTTG 0.0258729584379
AACATAAA 0.140675021715
AACATAAC -0.0572345131129
AACATAAG 0.0554520703926
AACATAAT 0.291336824081
AACATACA 0.0972032638699
AACATACC -0.0565746735473
AACATACG -0.00562408138166
AACATACT -0.0169246381368
AACATAGA 0.0471561158399
AACATAGC 0.038645682348
AACATAGG -0.00247950774801
AACATAGT -0.141169026235
AACATATA 0.096693503062
AACATATC 0.30178244272
AACATATG -0.18378266048
AACATATT 0.148571199171
AACATCAA -0.109869856864
AACATCAC 0.00686335335723
AACATCAG 0.0189944745735
AACATCAT 0.0462046068107
AACATCCA -0.0178660321307
AACATCCC -0.0399721173989
AACATCCG 0.127468011649
AACATCCT -0.165583045884
AACATCGA 0.135127697934
AACATCGC 0.135309700874
AACATCGG 0.106956819078
AACATCGT 0.0533849623731
AACATCTA 0.140920271819
AACATCTC 0.11163478172
AACATCTG 0.102674393991
AACATCTT 0.138299456481
AACATGAA 0.0717991576827
AACATGAC -0.178628639806

AACATGAG 0.222823587805
AACATGAT 0.178126756348
AACATGCA -0.0493193579703
AACATGCC 0.0330481814267
AACATGCG -0.105864817986
AACATGCT -0.282758250027
AACATGGA 0.0121016333138
AACATGGC 0.0730429260673
AACATGGG -0.11357026083
AACATGGT -0.124644786332
AACATGTA -0.062054364437
AACATGTC -0.170195551411
AACATGTG -0.198727452051
AACATGTT 0.11762298132
AACATTAA 0.100744509244
AACATTAC -0.0452918483222
AACATTAG 0.00462032280214
AACATTAT 0.10188836739
AACATTCA -0.0998251306776
AACATTCC 0.135861757074
AACATTCG -0.0648458375731
AACATTCT 0.0245024638964
AACATTGA -0.105746171822
AACATTGC 0.0375312949351
AACATTGG 0.0107793384123
AACATTGT -0.161925716381
AACATTTA 0.181918833434
AACATTTC 0.264307006731
AACATTTG 0.00317259408168
AACCAAAA 0.0560214009912
AACCAAAC -0.111272922405
AACCAAAG -0.0662502929782
AACCAAAT 0.114408443611
AACCAACA 0.0299376511498
AACCAACC -0.0718797754072
AACCAACG -0.0979147191268
AACCAACT -0.0164908797618
AACCAAGA 0.00840007367454
AACCAAGC 0.051184793609
AACCAAGG -0.0955278411521
AACCAAGT -0.131384032383
AACCAATA -0.0606736212797
AACCAATC 0.166348912068
AACCAATG -0.145368601275
AACCAATT 0.0668707984497
AACCACAA -0.113425983373
AACCACAC -0.153489339649
AACCACAG -0.266393499108
AACCACAT -0.0639165336135
AACCACCA -0.0308321715583
AACCACCC -0.138819625357
AACCACCG -0.145675642139
AACCACCT -0.388338934497
AACCACGA -0.000409439440185

AACCACGC -0.164988037946
AACCACGG -0.304526697925
AACCACGT -0.074555271525
AACCACTA -0.179310531071
AACCACTC 0.108973987762
AACCACTG -0.109736338517
AACCACTT 0.0956148986452
AACCAGAA 0.0673497188649
AACCAGAC -0.255511102841
AACCAGAG -0.100752242936
AACCAGAT 0.303073045497
AACCAGCA 0.0804456787398
AACCAGCC -0.140274952242
AACCAGCG -0.0669274947545
AACCAGCT -0.132764205213
AACCAGGA -0.214667277724
AACCAGGC -0.172746695337
AACCAGGG -0.235537093599
AACCAGGT -0.231470628364
AACCAGTA -0.0516141982292
AACCAGTC -0.368565150593
AACCAGTG -0.169622347559
AACCAGTT -0.128274481583
AACCATAA 0.146884703257
AACCATAC -0.233644116406
AACCATAG 0.0851944847923
AACCATAT 0.0334494186373
AACCATCA -0.0161776748817
AACCATCC -0.0585191565148
AACCATCG -0.0541274134361
AACCATCT -0.135166847288
AACCATGA 0.00360795019087
AACCATGC -0.00407495992967
AACCATGG -0.269035884878
AACCATGT -0.0173171390408
AACCATTA 0.0162733662767
AACCATTC -0.0401867112399
AACCATTG -0.149782753203
AACCCAAA -0.102738646266
AACCCAAC -0.189207071189
AACCCAAG -0.0765450391597
AACCCAAT -0.01606427364
AACCCACA 0.0756829591041
AACCCACC -0.0559347037818
AACCCACG -0.273302848134
AACCCACT -0.388373477538
AACCCAGA 0.155051745961
AACCCAGC -0.335901484561
AACCCAGG -0.169064559207
AACCCAGT -0.158812436966
AACCCATA -0.0361546827442
AACCCATC -0.111225527903
AACCCATG -0.275466351309
AACCCATT -0.161474623842

AACCCCAA -0.29400738223
AACCCCAC -0.260678485473
AACCCCAG -0.33396249574
AACCCCAT -0.167370682672
AACCCCCA -0.310827758621
AACCCCCC -0.351386661959
AACCCCCG -0.332211500032
AACCCCCT -0.422833458722
AACCCCGA -0.117073190905
AACCCCGC -0.203412377416
AACCCCGG -0.211490478593
AACCCCGT -0.160044926633
AACCCCTA -0.19060615835
AACCCCTC -0.051366398608
AACCCCTG -0.0215968965318
AACCCCTT -0.111036667577
AACCCGAA 0.0925760471215
AACCCGAC -0.292336641436
AACCCGAG -0.317612476489
AACCCGAT 0.0224260072745
AACCCGCA 0.0836061984044
AACCCGCC -0.0560990742809
AACCCGCG -0.102705791848
AACCCGCT -0.210051741771
AACCCGGA -0.15452547761
AACCCGGC -0.110507756684
AACCCGGG -0.106134930865
AACCCGGT -0.372494787114
AACCCGTA 0.0221778252081
AACCCGTC -0.0989531715021
AACCCGTG -0.0909009291767
AACCCGTT -0.210724357225
AACCCTAA -0.129494062144
AACCCTAC -0.078907957138
AACCCTAG -0.0664311549479
AACCCTAT -0.244629552603
AACCCTCA -0.0904831969265
AACCCTCC -0.334129231721
AACCCTCG -0.245110200174
AACCCTCT -0.259808421123
AACCCTGA 0.0379594470504
AACCCTGC -0.0670372416784
AACCCTGG -0.287562975186
AACCCTGT -0.144350985728
AACCCTTA -0.18656690277
AACCCTTC -0.222144461586
AACCCTTG -0.147924412079
AACCGAAA 0.102990872978
AACCGAAC -0.247160844836
AACCGAAG 0.0315664620483
AACCGAAT 0.204914490924
AACCGACA 0.146968314562
AACCGACC -0.148841089328
AACCGACG 0.080657792779

AACCGACT -0.176473426356
AACCGAGA 0.0321685321685
AACCGAGC -0.374380826071
AACCGAGG -0.335692802512
AACCGAGT -0.0827011898361
AACCGATA 0.245243534813
AACCGATC 0.12302723298
AACCGATG -0.0941627488444
AACCGATT 0.177662532937
AACCGCAA 0.0329870158517
AACCGCAC -0.0488994217932
AACCGCAG -0.19345420953
AACCGCAT -0.0750378477651
AACCGCCA -0.127829157976
AACCGCCC -0.279858613419
AACCGCCG -0.106940659796
AACCGCCT -0.386461964539
AACCGCGA 0.0699305854616
AACCGCGC 0.147821177755
AACCGCGG -0.143995599778
AACCGCGT 0.0342233381751
AACCGCTA -0.0585352288668
AACCGCTC -0.204276947102
AACCGCTG -0.12225407246
AACCGCTT -0.164070285145
AACCGGAA 0.0727132090768
AACCGGAC -0.297730181407
AACCGGAG -0.125449140601
AACCGGAT 0.17271403635
AACCGGCA -0.0314919078781
AACCGGCC -0.191718348837
AACCGGCG -0.370337284355
AACCGGCT -0.327367295051
AACCGGGA -0.285218758528
AACCGGGC -0.234182738968
AACCGGGG -0.225086492315
AACCGGGT -0.262063193961
AACCGGTA -0.0284594537219
AACCGGTC -0.106681500966
AACCGGTG -0.268515586813
AACCGGTT -0.211382281858
AACCGTAA 0.210126104065
AACCGTAC -0.179225728853
AACCGTAG -0.193771106425
AACCGTAT -0.0780661737334
AACCGTCA -0.167175095641
AACCGTCC -0.0127676998288
AACCGTCG -0.190387958275
AACCGTCT -0.0156028984788
AACCGTGA -0.0388374677067
AACCGTGC -0.0487149916425
AACCGTGG -0.156585663471
AACCGTGT -0.0752805146745
AACCGTTA -0.00534728272868

AACCGTTC -0.121667017135
AACCGTTG -0.0801320486843
AACCTAAA 0.0694196477449
AACCTAAC 0.0216192346153
AACCTAAG -0.0955665204412
AACCTAAT 0.0611865308835
AACCTACA -0.0220259454573
AACCTACC -0.171591617366
AACCTACG -0.157527108508
AACCTACT -0.118917644723
AACCTAGA -0.0118229412203
AACCTAGC -0.0580784012074
AACCTAGG -0.112121563016
AACCTAGT 0.0186149208411
AACCTATA -0.107122695352
AACCTATC -0.0452004163206
AACCTATG -0.0409078633315
AACCTATT -0.0557952788779
AACCTCAA -0.0301251664888
AACCTCAC -0.151657809738
AACCTCAG -0.157123677035
AACCTCAT -0.159395357941
AACCTCCA -0.17254567276
AACCTCCC -0.0578211458773
AACCTCCG -0.345455926486
AACCTCCT -0.22734351528
AACCTCGA 0.0576016788138
AACCTCGC -0.0473332726726
AACCTCGG -0.162429530386
AACCTCGT 0.00386198871047
AACCTCTA -0.200807930402
AACCTCTC -0.1923093763
AACCTCTG -0.195885539429
AACCTCTT -0.0100610252125
AACCTGAA 0.183796705182
AACCTGAC -0.180891346416
AACCTGAG 0.101413107742
AACCTGAT 0.0252308343613
AACCTGCA 0.0621468143033
AACCTGCC -0.157096988405
AACCTGCG -0.246645881746
AACCTGCT -0.129504770737
AACCTGGA -0.281017455733
AACCTGGC -0.061462157763
AACCTGGG -0.286720989299
AACCTGGT -0.238386290225
AACCTGTA -0.0665023794004
AACCTGTC -0.174768420269
AACCTGTG -0.129895575647
AACCTTAA 0.0125483610332
AACCTTAC -0.2957187556
AACCTTAG -0.183882753581
AACCTTAT -0.242134215669
AACCTTCA 0.0422961540988

AACCTTCC 0.029966497084
AACCTTCG -0.305066920483
AACCTTCT -0.218490210189
AACCTTGA -0.0315620825821
AACCTTGC -0.168792337659
AACCTTGG -0.248485445537
AACCTTGT -0.169107246859
AACCTTTA 0.174760159609
AACCTTTC 0.0511669603196
AACCTTTG -0.19035648711
AACGAAAA 0.154755005365
AACGAAAC 0.177093202339
AACGAAAG -0.0325876841028
AACGAAAT 0.30823717978
AACGAACA -0.139190382692
AACGAACC -0.21768873451
AACGAACG 0.0155551338292
AACGAACT -0.118745411368
AACGAAGA -0.00530254040222
AACGAAGC 0.0319093197881
AACGAAGG -0.146979456944
AACGAAGT -0.169873942281
AACGAATA -0.121478227539
AACGAATC 0.327457792895
AACGAATG -0.127901578798
AACGAATT 0.0808177323329
AACGACAA -0.0442991200567
AACGACAC -0.284434686491
AACGACAG -0.0726312562499
AACGACAT -0.0853560425295
AACGACCA -0.0112734757633
AACGACCC -0.0830579012881
AACGACCG -0.175807197895
AACGACCT -0.30871452242
AACGACGA -0.129834383189
AACGACGC -0.144384827701
AACGACGG -0.175044190196
AACGACGT -0.0483379520481
AACGACTA -0.0300686846273
AACGACTC 0.164521214864
AACGACTG -0.134394036047
AACGACTT 0.0867878898182
AACGAGAA 0.104069983869
AACGAGAC 0.0636533506566
AACGAGAG -0.0618415616985
AACGAGAT 0.282060297212
AACGAGCA 0.0817551440479
AACGAGCC -0.151151866551
AACGAGCG 0.0663773785975
AACGAGCT 0.0571880420365
AACGAGGA -0.0290044743546
AACGAGGC -0.119551396685
AACGAGGG -0.307013790709
AACGAGGT -0.120465064028

AACGAGTA 0.0496857733042
AACGAGTC -0.183886559082
AACGAGTG -0.183103833271
AACGAGTT -0.0567089771757
AACGATAA 0.145331281695
AACGATAC -0.146707313374
AACGATAG -0.0351649233958
AACGATAT 0.279264112597
AACGATCA 0.0464638191911
AACGATCC 0.182126668759
AACGATCG 0.146901162748
AACGATCT 0.140447610145
AACGATGA -0.0774751838271
AACGATGC -0.0717315111255
AACGATGG -0.0842958210058
AACGATGT -0.201595848808
AACGATTA 0.185084268228
AACGATTC 0.259751487024
AACGATTG 0.0768063473063
AACGCAAA 0.0802248529521
AACGCAAC 0.169902685054
AACGCAAG -0.0803697729247
AACGCAAT 0.33088873998
AACGCACA 0.00733633570068
AACGCACC -0.272945174994
AACGCACG -0.196842123702
AACGCACT -0.0302278052305
AACGCAGA 0.195650460209
AACGCAGC -0.0594555696171
AACGCAGG -0.286853528463
AACGCAGT -0.118582028439
AACGCATA 0.0333267151449
AACGCATC 0.000784531087561
AACGCATG -0.202609676148
AACGCATT -0.106874302023
AACGCCAA -0.156196048271
AACGCCAC -0.266452595774
AACGCCAG -0.359063976807
AACGCCAT -0.0579308557422
AACGCCCA -0.0814924067595
AACGCCCC -0.330846054281
AACGCCCG 0.0456719154396
AACGCCCT -0.354742513815
AACGCCGA -0.231781323496
AACGCCGC -0.0904333497287
AACGCCGG -0.107681194185
AACGCCGT -0.298867740076
AACGCCTA -0.144093297339
AACGCCTC -0.111683251855
AACGCCTG -0.182669491027
AACGCCTT -0.111749490537
AACGCGAA 0.156072050011
AACGCGAC -0.129268864348
AACGCGAG -0.18126075653

AACGCGAT 0.377574544241
AACGCGCA -0.0246017367229
AACGCGCC -0.113318815852
AACGCGCG -0.071734268704
AACGCGCT -0.130969463498
AACGCGGA -0.0705737837731
AACGCGGC -0.0752362239781
AACGCGGG -0.11659236099
AACGCGGT -0.169494423074
AACGCGTA -0.0889476165662
AACGCGTC 0.0750552150284
AACGCGTG -0.211342196191
AACGCGTT 0.076288173857
AACGCTAA 0.0791622205127
AACGCTAC -0.117093111346
AACGCTAG -0.0799687321637
AACGCTAT -0.0582745279715
AACGCTCA -0.0714161641728
AACGCTCC 0.0211666897805
AACGCTCG -0.0801238305581
AACGCTCT -0.173193371133
AACGCTGA 0.125289201047
AACGCTGC -0.118395217623
AACGCTGG -0.167287060244
AACGCTGT -0.057530870324
AACGCTTA -0.0853014342788
AACGCTTC 0.0937921392467
AACGCTTG -0.121322708466
AACGGAAA 0.156546353516
AACGGAAC -0.016190812628
AACGGAAG -0.149306391704
AACGGAAT 0.240331195814
AACGGACA 0.106243525719
AACGGACC -0.170392064421
AACGGACG -0.0471453823843
AACGGACT -0.245011205663
AACGGAGA -0.00681517922965
AACGGAGC -0.355051970926
AACGGAGG -0.291813775358
AACGGAGT -0.05554728282
AACGGATA 0.19550155836
AACGGATC 0.260460719838
AACGGATG -0.0506737474254
AACGGATT 0.404527290327
AACGGCAA 0.00987528710436
AACGGCAC -0.0868764021328
AACGGCAG -0.18610497763
AACGGCAT -0.110667612537
AACGGCCA -0.325041187123
AACGGCCC -0.261128351929
AACGGCCG 0.00340581857138
AACGGCCT -0.185858625656
AACGGCGA 0.0795782733582
AACGGCGC -0.0464541092981

AACGGCGG -0.205057625983
AACGGCGT -0.16199510927
AACGGCTA -0.0414947117946
AACGGCTC -0.0356043298322
AACGGCTG -0.244753188652
AACGGCTT -0.206046212775
AACGGGAA -0.0836531401403
AACGGGAC -0.270627458439
AACGGGAG -0.0165676104921
AACGGGAT 0.0758662219075
AACGGGCA -0.096018292674
AACGGGCC -0.323067827583
AACGGGCG -0.137401372106
AACGGGCT -0.19040478119
AACGGGGA -0.261769279683
AACGGGGC 0.059374801799
AACGGGGG -0.0495527732758
AACGGGGT -0.169333180341
AACGGGTA -0.0881199738997
AACGGGTC -0.377461740705
AACGGGTG -0.276492222581
AACGGTAA 0.0780463659252
AACGGTAC -0.0207118214986
AACGGTAG -0.0848380577406
AACGGTAT -0.098378137705
AACGGTCA -0.0289826409212
AACGGTCC -0.131558268609
AACGGTCG -0.129333963914
AACGGTCT -0.132860096197
AACGGTGA -0.0607315304285
AACGGTGC -0.110766499323
AACGGTGG -0.135087145699
AACGGTGT -0.107111316125
AACGGTTA -0.0298687116869
AACGGTTC -0.308500387141
AACGGTTG 0.0328348775051
AACGTAAA 0.111421647701
AACGTAAC 0.202975702976
AACGTAAG -0.0313908950273
AACGTAAT 0.349789183123
AACGTACA -0.0507948325544
AACGTACC -0.228005669019
AACGTACG -0.0689948395291
AACGTACT 0.0757289986351
AACGTAGA 0.0856514090932
AACGTAGC -0.02134016042
AACGTAGG -0.11621074632
AACGTAGT -0.11676021693
AACGTATA 0.250168901684
AACGTATC 0.342330459917
AACGTATG -0.162260655807
AACGTATT 0.183975987006
AACGTCAA 0.0440625387765
AACGTCAC -0.0440143646489

AACGTCAG -0.0637235031174
AACGTCAT -0.0804675198615
AACGTCCA -0.27697802675
AACGTCCC -0.396630879398
AACGTCCG -0.344431488327
AACGTCCT -0.303206108975
AACGTCGA 0.0755246235484
AACGTCGC 0.0206473691322
AACGTCGG -0.147638200342
AACGTCGT 0.111728210331
AACGTCTA -0.0811816185774
AACGTCTC -0.131455560113
AACGTCTG -0.0843997738898
AACGTCTT -0.0661646857819
AACGTGAA -0.0479763216864
AACGTGAC -0.201598548887
AACGTGAG 0.0896498616819
AACGTGAT 0.0968916574977
AACGTGCA -0.124579023817
AACGTGCC -0.229419758906
AACGTGCG -0.0893542988384
AACGTGCT -0.0849008611063
AACGTGGA 0.0177985733116
AACGTGGC -0.113038356952
AACGTGGG 0.0367040350398
AACGTGGT -0.317868691129
AACGTGTA -0.112967787289
AACGTGTC -0.143966074632
AACGTGTG -0.109798034496
AACGTTAA 0.161082570173
AACGTTAC 0.0369620223401
AACGTTAG -0.107131230703
AACGTTAT 0.0103064497004
AACGTTCA -0.136421790428
AACGTTCC -0.144309060779
AACGTTCG -0.168438322924
AACGTTCT -0.0886484032529
AACGTTGA 0.0797581074867
AACGTTGC -0.0772165391514
AACGTTGG -0.0865755588286
AACGTTGT -0.00749837594797
AACGTTTA -0.0877302959494
AACGTTTC 0.00896257746181
AACGTTTG 0.0592125719875
AACTAAAA 0.222074691772
AACTAAAC 0.092683371897
AACTAAAG 0.0341766238696
AACTAAAT 0.103655997595
AACTAACA 0.0368260822806
AACTAACC -0.227239769854
AACTAACG 0.103579483661
AACTAACT -0.0432260607432
AACTAAGA -0.0278687328015
AACTAAGC -0.138340901759

AACTAAGG -0.179459817182
AACTAAGT -0.0810689906313
AACTAATA 0.00143959889796
AACTAATC -0.0380035683066
AACTAATG 0.0552467067619
AACTAATT 0.12614249323
AACTACAA 0.0495909132273
AACTACAC -0.177333858739
AACTACAG -0.288850573762
AACTACAT -0.0433486857952
AACTACCA 0.0332108968473
AACTACCC -0.0456960796479
AACTACCG 0.0923802590469
AACTACCT -0.0550447800413
AACTACGA -0.0502556402174
AACTACGC 0.0148485799581
AACTACGG 0.000396341685948
AACTACGT -0.138994361032
AACTACTA 0.118005445136
AACTACTC 0.145692430093
AACTACTG 0.0612125281928
AACTACTT 0.184751428801
AACTAGAA 0.240023211171
AACTAGAC -0.19229410743
AACTAGAG -0.188884640028
AACTAGAT 0.148587981921
AACTAGCA 0.00898301497048
AACTAGCC 0.00588053618357
AACTAGCG -0.038005310569
AACTAGCT -0.0768098340411
AACTAGGA 0.0447155144125
AACTAGGC 0.0703977285169
AACTAGGG -0.131701641342
AACTAGGT -0.102288303464
AACTAGTA 0.00851502664641
AACTAGTC -0.153878773635
AACTAGTG -0.203111000841
AACTAGTT 0.212791159179
AACTATAA 0.203411400381
AACTATAC -0.131990116839
AACTATAG 0.120297365751
AACTATAT 0.109310685813
AACTATCA 0.0749413325171
AACTATCC 0.15062370897
AACTATCG 0.0564076473167
AACTATCT -0.0956913249927
AACTATGA 0.0876280081458
AACTATGC 0.0858916768008
AACTATGG 0.0432484826424
AACTATGT -0.107659999504
AACTATTA 0.0639275639276
AACTATTC 0.130332812151
AACTATTG 0.141169901389
AACTCAAA 0.0562111778574

AACTCAAC 0.0402978736312
AACTCAAG -0.124842504597
AACTCAAT -0.0755314299001
AACTCACA -0.0517418244691
AACTCACC -0.25767601307
AACTCACG -0.143481332386
AACTCACT -0.0692337336453
AACTCAGA -0.0166602191193
AACTCAGC -0.190875979829
AACTCAGG -0.0249315708415
AACTCAGT -0.0310186219277
AACTCATA -0.0758338089684
AACTCATC -0.196039838683
AACTCATG -0.1961475159
AACTCATT -0.107323927213
AACTCCAA 0.0274916790068
AACTCCAC -0.123447850249
AACTCCAG -0.369661564921
AACTCCAT -0.161203150838
AACTCCCA -0.327631016853
AACTCCCC -0.34120920464
AACTCCCG -0.0558850861881
AACTCCCT -0.271630818593
AACTCCGA -0.156219757314
AACTCCGC -0.0351794405151
AACTCCGG -0.127566277721
AACTCCGT -0.0974424728737
AACTCCTA -0.0486478398283
AACTCCTC 0.0308316210098
AACTCCTG -0.149078715967
AACTCCTT -0.0362166574288
AACTCGAA 0.088277628957
AACTCGAC -0.176309309919
AACTCGAG -0.132564948761
AACTCGAT 0.0481921731146
AACTCGCA 0.0601439384539
AACTCGCC -0.228825207374
AACTCGCG 0.0233593424961
AACTCGCT -0.116877900196
AACTCGGA -0.156674303264
AACTCGGC 0.0144325578069
AACTCGGG -0.0990184643726
AACTCGGT -0.127971193109
AACTCGTA -0.0322760777306
AACTCGTC -0.0803075803076
AACTCGTG -0.104264140201
AACTCTAA 0.0470434953979
AACTCTAC -0.0763584905936
AACTCTAG -0.0681014284359
AACTCTAT -0.158197265753
AACTCTCA -0.178981557304
AACTCTCC -0.24068835455
AACTCTCG -0.0778355218499
AACTCTCT -0.222999666416

AACTCTGA -0.0231114854558
AACTCTGC 0.0665665665666
AACTCTGG -0.163222269085
AACTCTGT -0.00646190229411
AACTCTTA 0.0569410888813
AACTCTTC -0.199149196415
AACTCTTG 0.0168310182989
AACTGAAA 0.184812741327
AACTGAAC -0.0256760800858
AACTGAAG -0.238644154114
AACTGAAT 0.0549488882822
AACTGACA -0.0385605991667
AACTGACC -0.318715609182
AACTGACG 0.0877783698167
AACTGACT -0.125429752226
AACTGAGA -0.0321688211796
AACTGAGC -0.118436906347
AACTGAGG -0.0748484640612
AACTGAGT -0.144051252781
AACTGATA 0.172760450501
AACTGATC 0.0923213360291
AACTGATG -0.121545126649
AACTGATT 0.283879185127
AACTGCAA -0.0264024354933
AACTGCAC -0.237478090946
AACTGCAG -0.246223825113
AACTGCAT -0.151234338171
AACTGCCA -0.180979751616
AACTGCCC -0.158230637979
AACTGCCG -0.0691962949716
AACTGCCT -0.176916453221
AACTGCGA -0.140425320728
AACTGCGC 0.115658051284
AACTGCGG 0.0652594743504
AACTGCGT -0.154651540894
AACTGCTA -0.0164089709544
AACTGCTC -0.0809144877066
AACTGCTG -0.0652424596728
AACTGCTT 0.0137945884397
AACTGGAA 0.170707940702
AACTGGAC -0.204740885411
AACTGGAG -0.253727270472
AACTGGAT 0.177299889421
AACTGGCA -0.0884469368006
AACTGGCC -0.162574057073
AACTGGCG -0.130940548903
AACTGGCT -0.283199479473
AACTGGGA -0.210845648536
AACTGGGC -0.281882510578
AACTGGGG -0.09093064481
AACTGGGT -0.220583765477
AACTGGTA -0.170746383464
AACTGGTC -0.211495992655
AACTGGTG -0.208712245598

AACTGTAA 0.0617384668834
AACTGTAC -0.106205623299
AACTGTAG -0.128192931223
AACTGTAT -0.0973090975974
AACTGTCA -0.112521472511
AACTGTCC -0.102361290944
AACTGTCG -0.147973094047
AACTGTCT -0.0698311383311
AACTGTGA 0.0440123318911
AACTGTGC -0.04735727463
AACTGTGG -0.0655101610382
AACTGTGT -0.173868713718
AACTGTTA 0.1104451559
AACTGTTC -0.0612285862353
AACTGTTG -0.0807267322419
AACTTAAA 0.224071178617
AACTTAAC 0.00580060290651
AACTTAAG -0.110123865901
AACTTAAT -0.0415647832529
AACTTACA -0.127762748975
AACTTACC -0.0323526086945
AACTTACG 0.183452187178
AACTTACT -0.0246323073217
AACTTAGA -0.0353438221208
AACTTAGC 0.104446617957
AACTTAGG 0.0791128661416
AACTTAGT -0.0840426851967
AACTTATA 0.0877520930199
AACTTATC 0.147035465217
AACTTATG -0.00428396572827
AACTTATT 0.165753304672
AACTTCAA 0.0396139941594
AACTTCAC -0.183142123736
AACTTCAG 0.0748365445335
AACTTCAT -0.134358172659
AACTTCCA -0.373530651875
AACTTCCC -0.11678423654
AACTTCCG -0.143405614237
AACTTCCT -0.218774554808
AACTTCGA -0.0200820419991
AACTTCGC 0.140016641971
AACTTCGG -0.266835339947
AACTTCGT -0.0893622767385
AACTTCTA 0.0445712642453
AACTTCTC 0.165394188448
AACTTCTG -0.283509563111
AACTTCTT 0.121511937695
AACTTGAA 0.246148624483
AACTTGAC -0.0977424137161
AACTTGAG 0.0341369741435
AACTTGAT -0.0617601072147
AACTTGCA 0.06173806413
AACTTGCC -0.124993248658
AACTTGCG -0.0405837828369

AACTTGCT -0.0923461530039
AACTTGGA -0.244514741761
AACTTGGC 0.12517317139
AACTTGGG -0.22932846993
AACTTGGT -0.0084699024093
AACTTGTA 0.120107711017
AACTTGTC -0.144400850005
AACTTGTG -0.209998924758
AACTTTAA 0.0467450921996
AACTTTAC -0.0622446223805
AACTTTAG -0.123949017888
AACTTTAT 0.0259913889371
AACTTTCA -0.07801879014
AACTTTCC 0.00109062230274
AACTTTCG -0.111473047913
AACTTTCT -0.108520981292
AACTTTGA -0.163810098905
AACTTTGC 0.106639270673
AACTTTGG -0.000832828478209
AACTTTGT -0.125471452321
AACTTTTA 0.0495539370176
AACTTTTC 0.092441041437
AACTTTTG -0.0975834477138
AAGAAAAA 0.138731998569
AAGAAAAC -0.244152889517
AAGAAAAG 0.0505996219061
AAGAAAAT 0.323352277898
AAGAAACA -0.156678825777
AAGAAACC -0.021915578491
AAGAAACG -0.00940287601164
AAGAAACT -0.239877096488
AAGAAAGA -0.150590133063
AAGAAAGC -0.103896808854
AAGAAAGG -0.107573596918
AAGAAAGT 0.0546565849596
AAGAAATA 0.282699283763
AAGAAATC 0.439413242444
AAGAAATG -0.0820544075677
AAGAAATT 0.128335145946
AAGAACAA 0.263478050475
AAGAACAC -0.162494521216
AAGAACAG -0.0373307191489
AAGAACAT 0.093603059787
AAGAACCA -0.285353450579
AAGAACCC -0.153557250961
AAGAACCG -0.00557433048911
AAGAACCT -0.242869283641
AAGAACGA -0.0691325243401
AAGAACGC -0.145515710524
AAGAACGG -0.0815543590041
AAGAACGT -0.130977684048
AAGAACTA 0.00811968993787
AAGAACTC -0.0595248928045
AAGAACTG -0.0239483806924

AAGAACTT 0.00818035666521
AAGAAGAA 0.0376831975293
AAGAAGAC -0.317850602972
AAGAAGAG -0.0360172938156
AAGAAGAT 0.247058353119
AAGAAGCA -0.159765709941
AAGAAGCC -0.115539805698
AAGAAGCG -0.00859032283965
AAGAAGCT -0.182671182383
AAGAAGGA 0.0283273704326
AAGAAGGC -0.0914423639769
AAGAAGGG -0.141174864943
AAGAAGGT -0.140122093456
AAGAAGTA 0.0170908666234
AAGAAGTC -0.0183077266748
AAGAAGTG -0.330221589641
AAGAATAA 0.135464916827
AAGAATAC -0.0342919732629
AAGAATAG -0.0939242206375
AAGAATAT 0.326358038479
AAGAATCA 0.324421363036
AAGAATCC 0.46630022086
AAGAATCG 0.397152617096
AAGAATCT 0.438790039306
AAGAATGA 0.0185390100947
AAGAATGC -0.259033243386
AAGAATGG 0.147007889432
AAGAATGT -0.0989921888799
AAGAATTA 0.153210513638
AAGAATTC 0.131910250141
AAGAATTG -0.106212759152
AAGACAAA 0.213607712733
AAGACAAC -0.251056231657
AAGACAAG 0.0259023525032
AAGACAAT 0.101838753354
AAGACACA -0.0765461995116
AAGACACC -0.155699196877
AAGACACG -0.0983393862182
AAGACACT -0.262219244776
AAGACAGA 0.0369356875397
AAGACAGC 0.0562504653414
AAGACAGG 0.214477562962
AAGACAGT 0.214832521916
AAGACATA 0.0583439778691
AAGACATC 0.0740718912078
AAGACATG -0.073480304973
AAGACATT -0.00885309080823
AAGACCAA 0.0495964283843
AAGACCAC -0.254161918284
AAGACCAG -0.138264268709
AAGACCAT -0.117111006334
AAGACCCA -0.322681549734
AAGACCCC -0.23826002828
AAGACCCG -0.262713347007

AAGACCCT -0.318838179481
AAGACCGA -0.253408234613
AAGACCGC -0.30708537911
AAGACCGG -0.333421139811
AAGACCGT -0.12183801454
AAGACCTA -0.0805229504264
AAGACCTC -0.279353765567
AAGACCTG -0.112498047682
AAGACCTT -0.0226567141087
AAGACGAA -0.159001309265
AAGACGAC -0.273296082373
AAGACGAG -0.0288344623936
AAGACGAT 0.212980007737
AAGACGCA -0.141406622611
AAGACGCC -0.367395779084
AAGACGCG -0.0570638761357
AAGACGCT -0.0440713023094
AAGACGGA -0.178524574135
AAGACGGC -0.258608864171
AAGACGGG -0.0241238411608
AAGACGGT -0.113761935278
AAGACGTA -0.0382578472489
AAGACGTC 0.0461530039488
AAGACGTG -0.0670020185331
AAGACTAA 0.0415837609395
AAGACTAC -0.155946797687
AAGACTAG 0.081544531851
AAGACTAT 0.00652759428626
AAGACTCA -0.174172326565
AAGACTCC -0.288040167484
AAGACTCG -0.100791031484
AAGACTCT -0.347700566596
AAGACTGA -0.116138707065
AAGACTGC -0.216772301994
AAGACTGG 0.131538948309
AAGACTGT -0.0256288193125
AAGACTTA 0.0140456778319
AAGACTTC 0.00325807901565
AAGACTTG -0.120059175312
AAGAGAAA 0.0492828624191
AAGAGAAC -0.226216962068
AAGAGAAG -0.115084595589
AAGAGAAT 0.283182609673
AAGAGACA -0.0328993077095
AAGAGACC -0.119428144307
AAGAGACG -0.255121101257
AAGAGACT -0.265842590065
AAGAGAGA -0.22766771018
AAGAGAGC -0.24730852472
AAGAGAGG -0.115819886581
AAGAGAGT -0.0984998249198
AAGAGATA 0.318951339531
AAGAGATC 0.365524112611
AAGAGATG 0.0697466478836

AAGAGATT 0.375628486968
AAGAGCAA 0.0120865966439
AAGAGCAC -0.182523055784
AAGAGCAG 0.0762379339438
AAGAGCAT -0.156996709628
AAGAGCCA -0.125067743423
AAGAGCCC -0.160444047432
AAGAGCCG -0.0824900048607
AAGAGCCT -0.186097807776
AAGAGCGA -0.0622008277191
AAGAGCGC -0.0254746246433
AAGAGCGG -0.126974872155
AAGAGCGT 0.020813412845
AAGAGCTA 0.0126961720986
AAGAGCTC -0.405556974123
AAGAGCTG -0.234982692049
AAGAGCTT -0.121758443557
AAGAGGAA -0.191104990502
AAGAGGAC -0.290342566821
AAGAGGAG -0.2223297411
AAGAGGAT 0.296836565623
AAGAGGCA -0.159955788899
AAGAGGCC -0.224785381891
AAGAGGCG -0.21727299799
AAGAGGCT -0.303586047781
AAGAGGGA -0.290521522031
AAGAGGGC -0.190812925423
AAGAGGGG -0.101754823868
AAGAGGGT -0.359520498269
AAGAGGTA -0.102114675304
AAGAGGTC -0.188287304699
AAGAGGTG -0.281908287888
AAGAGTAA -0.0486478398283
AAGAGTAC -0.292024155781
AAGAGTAG 0.0267389631983
AAGAGTAT 0.136974372824
AAGAGTCA 0.0833498771621
AAGAGTCC -0.186749510011
AAGAGTCG 0.04190492179
AAGAGTCT -0.373486003604
AAGAGTGA -0.0918423353658
AAGAGTGC -0.182040782562
AAGAGTGG -0.0725598541937
AAGAGTGT -0.138533885897
AAGAGTTA 0.0307483792332
AAGAGTTC -0.103996911665
AAGAGTTG -0.204151347659
AAGATAAA 0.0325620606848
AAGATAAC -0.0576695400831
AAGATAAG 0.0913520141895
AAGATAAT 0.184735653385
AAGATACA -0.0120657847931
AAGATACC 0.280925347048
AAGATACG 0.334756903134

AAGATACT 0.284480631811
AAGATAGA 0.0384309929764
AAGATAGC 0.159972409363
AAGATAGG 0.112612612613
AAGATAGT 0.0401572371269
AAGATATA 0.310435780133
AAGATATC 0.477331883243
AAGATATG 0.37345472194
AAGATATT 0.429201929202
AAGATCAA 0.233290511887
AAGATCAC 0.159636650292
AAGATCAG 0.162199389472
AAGATCAT 0.228716319625
AAGATCCA 0.306853685642
AAGATCCC 0.27626938233
AAGATCCG 0.322240568287
AAGATCCT 0.333317518594
AAGATCGA 0.218015536197
AAGATCGC 0.403517441224
AAGATCGG 0.186474353141
AAGATCGT 0.301616745663
AAGATCTA 0.351095231491
AAGATCTC 0.402315914877
AAGATCTG 0.350271111993
AAGATCTT 0.409469349277
AAGATGAA -0.0169521835288
AAGATGAC -0.0199607320819
AAGATGAG -0.0527553735644
AAGATGAT 0.104378483166
AAGATGCA -0.117743149467
AAGATGCC -0.0794646832551
AAGATGCG -0.0339328335876
AAGATGCT -0.142904376183
AAGATGGA -0.139049501893
AAGATGGC -0.0794130054315
AAGATGGG 0.0108123867894
AAGATGGT -0.104059765115
AAGATGTA 0.175356518172
AAGATGTC 0.0199404392605
AAGATGTG -0.273337127929
AAGATTAA 0.112155777247
AAGATTAC 0.434973541034
AAGATTAG 0.11060122771
AAGATTAT 0.260857276009
AAGATTCA 0.183586490807
AAGATTCC 0.466802739658
AAGATTCG 0.420352109078
AAGATTCT 0.451732443815
AAGATTGA 0.101138559088
AAGATTGC 0.462797894937
AAGATTGG 0.197624139617
AAGATTGT 0.268076616561
AAGATTTA 0.379727399062
AAGATTTC 0.480832536514

AAGATTTG 0.392306007898
AAGCAAAA 0.0142996868682
AAGCAAAC -0.165691067354
AAGCAAAG -0.17096368289
AAGCAAAT 0.238173435143
AAGCAACA -0.193178403562
AAGCAACC -0.281849915287
AAGCAACG -0.173912377071
AAGCAACT -0.191798166168
AAGCAAGA 0.0901462011781
AAGCAAGC -0.180641408694
AAGCAAGG -0.0357744973967
AAGCAAGT 0.0792321246867
AAGCAATA 0.0814544207061
AAGCAATC 0.367798928405
AAGCAATG 0.0214443093231
AAGCAATT 0.0371984555083
AAGCACAA -0.00605899140895
AAGCACAC -0.209568582699
AAGCACAG -0.176364701942
AAGCACAT -0.0888586071945
AAGCACCA -0.155051294564
AAGCACCC -0.314452021908
AAGCACCG -0.137976712311
AAGCACCT -0.109362979874
AAGCACGA -0.0492025722064
AAGCACGC -0.132845849283
AAGCACGG 0.0369506230323
AAGCACGT -0.0852017839025
AAGCACTA -0.092807456771
AAGCACTC -0.252112828162
AAGCACTG -0.0045552935986
AAGCACTT -0.0471375040326
AAGCAGAA 0.0352218564557
AAGCAGAC -0.149687183772
AAGCAGAG -0.111788644588
AAGCAGAT 0.221104024134
AAGCAGCA -0.0693762653576
AAGCAGCC -0.312194911952
AAGCAGCG -0.199316154472
AAGCAGCT -0.347572159616
AAGCAGGA -0.105644595133
AAGCAGGC -0.262914021313
AAGCAGGG -0.140480227157
AAGCAGGT 0.0892779832174
AAGCAGTA 0.0717810821014
AAGCAGTC -0.15991887204
AAGCAGTG -0.132289245649
AAGCATAA 0.0353573248045
AAGCATAC -0.0464858798192
AAGCATAG -0.0279880431396
AAGCATAT 0.192453449924
AAGCATCA 0.0866309496872
AAGCATCC -0.136436712194

AAGCATCG -0.0324495670083
AAGCATCT -0.110675678635
AAGCATGA -0.0536119467714
AAGCATGC -0.260039421632
AAGCATGG -0.17405835203
AAGCATGT -0.0588841060134
AAGCATTA -0.0126632263527
AAGCATTC -0.192349569657
AAGCATTG -0.396080555487
AAGCCAAA -0.239774860839
AAGCCAAC -0.262496479402
AAGCCAAG -0.237740780685
AAGCCAAT -0.0103522464046
AAGCCACA -0.236742703433
AAGCCACC -0.390511811541
AAGCCACG -0.164366557937
AAGCCACT -0.250483398103
AAGCCAGA -0.0609305733758
AAGCCAGC -0.205439915317
AAGCCAGG -0.134661785506
AAGCCAGT 0.0280680129165
AAGCCATA -0.153511415732
AAGCCATC 0.00594337047423
AAGCCATG -0.164045703143
AAGCCATT -0.0634794007436
AAGCCCAA -0.0856773251524
AAGCCCAC -0.157390773919
AAGCCCAG -0.26804098137
AAGCCCAT -0.112153444882
AAGCCCCA -0.352661258982
AAGCCCCC -0.245255637419
AAGCCCCG -0.336046822503
AAGCCCCT -0.316674087412
AAGCCCGA -0.243737606173
AAGCCCGC -0.313842685619
AAGCCCGG -0.308175260876
AAGCCCGT -0.056213205388
AAGCCCTA -0.275671615046
AAGCCCTC -0.174834128579
AAGCCCTG -0.231352113007
AAGCCCTT -0.309107445533
AAGCCGAA -0.0350422983438
AAGCCGAC -0.185815532455
AAGCCGAG -0.253175143072
AAGCCGAT 0.175726808902
AAGCCGCA -0.121516317161
AAGCCGCC -0.480328580285
AAGCCGCG -0.143634193209
AAGCCGCT -0.477433353999
AAGCCGGA -0.186933666463
AAGCCGGC -0.363824397618
AAGCCGGG -0.233117549285
AAGCCGGT -0.189153865879
AAGCCGTA -0.142079146508

AAGCCGTC -0.273285621093
AAGCCGTG -0.213878733481
AAGCCTAA -0.240466916417
AAGCCTAC -0.226520574735
AAGCCTAG -0.248493547318
AAGCCTAT 0.00849751052062
AAGCCTCA -0.209083590426
AAGCCTCC -0.182879673042
AAGCCTCG -0.249245300184
AAGCCTCT -0.33592007062
AAGCCTGA -0.119445064838
AAGCCTGC -0.304180353826
AAGCCTGG -0.163721670897
AAGCCTGT -0.0724721356467
AAGCCTTA -0.0829044336108
AAGCCTTC -0.332450335957
AAGCCTTG -0.23967836475
AAGCGAAA 0.0511256482791
AAGCGAAC -0.316177838696
AAGCGAAG -0.239874632399
AAGCGAAT 0.257614363675
AAGCGACA -0.0946800885776
AAGCGACC -0.303701435312
AAGCGACG -0.129593330521
AAGCGACT -0.373229654984
AAGCGAGA -0.0264367950322
AAGCGAGC -0.0338581619709
AAGCGAGG -0.109711245868
AAGCGAGT -0.0223535251053
AAGCGATA 0.222560819836
AAGCGATC 0.325571702809
AAGCGATG 0.0336383215171
AAGCGATT 0.226195776735
AAGCGCAA 0.101505086354
AAGCGCAC -0.146818024602
AAGCGCAG -0.0830359241143
AAGCGCAT 0.0159726429348
AAGCGCCA -0.137130417759
AAGCGCCC -0.284808944294
AAGCGCCG -0.159669321688
AAGCGCCT -0.253697317532
AAGCGCGA 0.0673221430797
AAGCGCGC 0.0210002700671
AAGCGCGG 0.0102899042293
AAGCGCGT 0.0854251366758
AAGCGCTA -0.233100927144
AAGCGCTC -0.103165590695
AAGCGCTG -0.275416326751
AAGCGCTT 0.0198002199565
AAGCGGAA -0.20628185936
AAGCGGAC -0.0863750279149
AAGCGGAG -0.17801373731
AAGCGGAT 0.200964212463
AAGCGGCA -0.183779196119

AAGCGGCC -0.436103746232
AAGCGGCG -0.10150324365
AAGCGGCT -0.37597530558
AAGCGGGA -0.0927686092843
AAGCGGGC -0.179975481074
AAGCGGGG 0.058049413964
AAGCGGGT -0.0264341710978
AAGCGGTA -0.0545659584097
AAGCGGTC -0.267223075879
AAGCGGTG -0.139049462045
AAGCGTAA 0.0874484500339
AAGCGTAC -0.225301730273
AAGCGTAG 0.047075611483
AAGCGTAT 0.0248416460538
AAGCGTCA 0.0293254687194
AAGCGTCC -0.333384328286
AAGCGTCG -0.0601745947169
AAGCGTCT 0.0499851105541
AAGCGTGA -0.105845424942
AAGCGTGC -0.280235549049
AAGCGTGG -0.194103927455
AAGCGTGT -0.145624670896
AAGCGTTA 0.0206611570248
AAGCGTTC -0.0635492341804
AAGCGTTG -0.0860705118131
AAGCTAAA -0.0858515230297
AAGCTAAC -0.326972619275
AAGCTAAG -0.223232006847
AAGCTAAT -0.0636285336817
AAGCTACA -0.0994572920361
AAGCTACC -0.163115800842
AAGCTACG -0.189748591916
AAGCTACT -0.303531808046
AAGCTAGA 0.12110902681
AAGCTAGC -0.182812985751
AAGCTAGG -0.034631829256
AAGCTAGT -0.0627142293809
AAGCTATA 0.106317060863
AAGCTATC 0.0656182711327
AAGCTATG -0.00380502616731
AAGCTATT -0.0594382261049
AAGCTCAA 0.0355796567918
AAGCTCAC -0.253275554563
AAGCTCAG -0.0824495218339
AAGCTCAT -0.146762509068
AAGCTCCA -0.256874650167
AAGCTCCC -0.235782185874
AAGCTCCG -0.210926652778
AAGCTCCT -0.26936870629
AAGCTCGA -0.178676671274
AAGCTCGC -0.375001468697
AAGCTCGG 0.0177127508157
AAGCTCGT -0.0220951293948
AAGCTCTA -0.212599120486

AAGCTCTC -0.20763890612
AAGCTCTG -0.26224896277
AAGCTGAA -0.192764889936
AAGCTGAC -0.138834434822
AAGCTGAG -0.139006184345
AAGCTGAT 0.0213244697458
AAGCTGCA -0.165963623731
AAGCTGCC -0.344354872049
AAGCTGCG -0.215302161176
AAGCTGCT -0.406950817865
AAGCTGGA -0.204234622766
AAGCTGGC -0.235942750993
AAGCTGGG 0.0544885878411
AAGCTGGT -0.258754263059
AAGCTGTA 0.0903853073277
AAGCTGTC -0.0892807407959
AAGCTGTG -0.144983677561
AAGCTTAA 0.0872732236369
AAGCTTAC -0.23960616613
AAGCTTAG -0.0943501851692
AAGCTTAT 0.00935526959264
AAGCTTCA -0.0105232537299
AAGCTTCC -0.0909078303414
AAGCTTCG 0.0190470281673
AAGCTTCT -0.126824230126
AAGCTTGA 0.0314146405553
AAGCTTGC -0.287426753359
AAGCTTGG 0.0126077531149
AAGCTTGT -0.079571306844
AAGCTTTA -0.158812873769
AAGCTTTC 0.0640295943326
AAGCTTTG -0.121510286126
AAGGAAAA 0.0852637913492
AAGGAAAC -0.163140423866
AAGGAAAG -0.297721604334
AAGGAAAT 0.348061721276
AAGGAACA -0.0470659763024
AAGGAACC -0.132777419435
AAGGAACG -0.100373969285
AAGGAACT -0.167098897224
AAGGAAGA -0.0305801669438
AAGGAAGC -0.0142585489257
AAGGAAGG -0.0811390481806
AAGGAAGT -0.169727730759
AAGGAATA 0.187210626605
AAGGAATC 0.443221458373
AAGGAATG -0.0477047947855
AAGGAATT 0.141545126394
AAGGACAA 0.117780645802
AAGGACAC -0.172716070823
AAGGACAG -0.00319742279403
AAGGACAT -0.0270036644581
AAGGACCA -0.329097995761
AAGGACCC -0.245672579912

AAGGACCG -0.244743813454
AAGGACCT -0.237289280348
AAGGACGA -0.0652482983814
AAGGACGC -0.338389616682
AAGGACGG -0.189372738349
AAGGACGT -0.238332492606
AAGGACTA -0.130276288576
AAGGACTC -0.168118000283
AAGGACTG -0.138771025809
AAGGACTT -0.0455455455455
AAGGAGAA 0.0128516946699
AAGGAGAC -0.345611321993
AAGGAGAG -0.244067852683
AAGGAGAT 0.212187324365
AAGGAGCA -0.27272952792
AAGGAGCC -0.226626516046
AAGGAGCG -0.0637394801574
AAGGAGCT -0.194599268811
AAGGAGGA -0.257981817147
AAGGAGGC -0.245270229845
AAGGAGGG -0.202772746196
AAGGAGGT -0.228255111616
AAGGAGTA -0.0700292796705
AAGGAGTC -0.188875313205
AAGGAGTG -0.216727444122
AAGGATAA 0.205694675392
AAGGATAC 0.0269125493602
AAGGATAG 0.0047261738794
AAGGATAT 0.381953578923
AAGGATCA 0.0172965729459
AAGGATCC 0.182302652803
AAGGATCG 0.210006350226
AAGGATCT 0.331836547015
AAGGATGA -0.0114895294264
AAGGATGC -0.045335098096
AAGGATGG -0.0750769576583
AAGGATGT -0.057543769665
AAGGATTA 0.30174940781
AAGGATTC 0.37551463309
AAGGATTG 0.22922928695
AAGGCAAA 0.0397457183203
AAGGCAAC -0.0376937351278
AAGGCAAG -0.319160439349
AAGGCAAT 0.101021875433
AAGGCACA -0.281188757411
AAGGCACC -0.356948588688
AAGGCACG -0.279215871103
AAGGCACT -0.276997692823
AAGGCAGA -0.0963905914401
AAGGCAGC -0.0867783917141
AAGGCAGG -0.183453138226
AAGGCAGT 0.0193097501055
AAGGCATA -0.0905038575798
AAGGCATC -0.287106645164

AAGGCATG −0.128572012442
AAGGCATT −0.106070778558
AAGGCCAA 0.042577012274
AAGGCCAC −0.220617637188
AAGGCCAG −0.251235593926
AAGGCCAT 0.0665511700623
AAGGCCCA −0.278464142279
AAGGCCCC −0.4043349873
AAGGCCCG −0.237287154423
AAGGCCCT −0.238461991714
AAGGCCGA −0.202209296099
AAGGCCGC −0.280405694048
AAGGCCGG −0.115421729265
AAGGCCGT −0.250835945231
AAGGCCTA −0.250692600954
AAGGCCTC −0.259952089932
AAGGCCTG −0.279228985499
AAGGCCTT 0.113124622725
AAGGCGAA −0.0104841311199
AAGGCGAC −0.263619747083
AAGGCGAG −0.238265754777
AAGGCGAT 0.144243392478
AAGGCGCA −0.141249128262
AAGGCGCC −0.336567097754
AAGGCGCG −0.0201783011142
AAGGCGCT −0.126200223297
AAGGCGGA −0.213458998812
AAGGCGGC −0.111278705751
AAGGCGGG 0.0690925084864
AAGGCGGT −0.249168272239
AAGGCGTA −0.157413150937
AAGGCGTC −0.291127322454
AAGGCGTG −0.19433621486
AAGGCTAA −0.0300974755622
AAGGCTAC −0.287593961646
AAGGCTAG −0.198076774317
AAGGCTAT −0.134994591896
AAGGCTCA −0.343421440638
AAGGCTCC −0.350128765384
AAGGCTCG −0.283064065389
AAGGCTCT −0.261141852008
AAGGCTGA −0.126215243063
AAGGCTGC −0.267407208498
AAGGCTGG −0.308233630581
AAGGCTGT −0.256041044642
AAGGCTTA −0.167357443387
AAGGCTTC −0.334093280125
AAGGCTTG −0.199607418251
AAGGGAAA 0.161712517244
AAGGGAAC −0.181965432344
AAGGGAAG −0.167339477659
AAGGGAAT 0.261660693561
AAGGGACA −0.287962761033
AAGGGACC −0.268790707788

AAGGGACG -0.222766222336
AAGGGACT -0.300314857694
AAGGGAGA -0.0364394126258
AAGGGAGC -0.169210505192
AAGGGAGG -0.173238454005
AAGGGAGT -0.179395247666
AAGGGATA 0.16347014299
AAGGGATC 0.112251369827
AAGGGATG 0.00829948963397
AAGGGATT 0.316176787555
AAGGGCAA -0.073217421258
AAGGGCAC -0.191985983949
AAGGGCAG -0.232353808787
AAGGGCAT -0.268017962141
AAGGGCCA -0.309672443808
AAGGGCCC -0.275122473014
AAGGGCCG -0.250747659172
AAGGGCCT -0.1751272221
AAGGGCGA -0.135863862502
AAGGGCGC -0.175386939294
AAGGGCGG -0.148589720951
AAGGGCGT -0.235385329362
AAGGGCTA -0.0879070126533
AAGGGCTC -0.271891598378
AAGGGCTG -0.256363332476
AAGGGGAA 0.0827011087348
AAGGGGAC -0.276071260204
AAGGGGAG -0.0550978883899
AAGGGGAT 0.219699568623
AAGGGGCA -0.316034741648
AAGGGGCC -0.396965824497
AAGGGGCG -0.2391798069
AAGGGGCT -0.305267896358
AAGGGGGA -0.196697972081
AAGGGGGC -0.212414571108
AAGGGGGG -0.268656551876
AAGGGGGT -0.318794404374
AAGGGGTA -0.0539976783494
AAGGGGTC -0.2882734271
AAGGGGTG -0.273183408172
AAGGGTAA -0.0766709956352
AAGGGTAC -0.209288894402
AAGGGTAG -0.224826108892
AAGGGTAT 0.0815493091392
AAGGGTCA -0.0999457027048
AAGGGTCC -0.229089377706
AAGGGTCG -0.10806986252
AAGGGTCT -0.308864025404
AAGGGTGA -0.0859631127765
AAGGGTGC -0.175933048256
AAGGGTGG -0.315090490565
AAGGGTGT -0.204025389263
AAGGGTTA -0.177197423609
AAGGGTTC -0.127355633746

AAGGGTTG -0.303674664417
AAGGTAAA 0.0969964454813
AAGGTAAC -0.0131254222333
AAGGTAAG 0.0460860309345
AAGGTAAT 0.176343009676
AAGGTACA -0.109871825623
AAGGTACC -0.199786086233
AAGGTACG -0.258172853126
AAGGTACT -0.0169480775541
AAGGTAGA 0.14246667922
AAGGTAGC -0.171795359505
AAGGTAGG -0.0795589731463
AAGGTAGT 0.00697138018875
AAGGTATA -0.0929797157726
AAGGTATC 0.147295314701
AAGGTATG 0.130504441509
AAGGTATT 0.0872236374386
AAGGTCAA 0.0217090368606
AAGGTCAC -0.273500587433
AAGGTCAG -0.235408696617
AAGGTCAT -0.281545753985
AAGGTCCA -0.34024712507
AAGGTCCC -0.277099459884
AAGGTCCG -0.131808819515
AAGGTCCT -0.21418230113
AAGGTCGA -0.158798099854
AAGGTCGC -0.170176042924
AAGGTCGG -0.0246792565203
AAGGTCGT -0.0765003818678
AAGGTCTA -0.24045841634
AAGGTCTC -0.237997767026
AAGGTCTG -0.0462480935806
AAGGTGAA -0.0296282025326
AAGGTGAC 0.0282058918423
AAGGTGAG -0.113202734415
AAGGTGAT 0.0363284015678
AAGGTGCA -0.214145323531
AAGGTGCC -0.230790172235
AAGGTGCG -0.18164309639
AAGGTGCT -0.157243945195
AAGGTGGA -0.0208472908034
AAGGTGGC -0.277829345359
AAGGTGGG -0.145529309603
AAGGTGGT -0.193958565935
AAGGTGTA -0.0895825092648
AAGGTGTC -0.0651847532074
AAGGTGTG -0.171446896256
AAGGTTAA -0.14881679175
AAGGTTAC -0.205241705338
AAGGTTAG -0.218248737744
AAGGTTAT -0.0975941137057
AAGGTTCA -0.118219382057
AAGGTTCC -0.125030102853
AAGGTTCG -0.0312916222007

AAGGTTCT -0.120073772083
AAGGTTGA -0.118690049909
AAGGTTGC -0.187835807523
AAGGTTGG -0.221337259151
AAGGTTGT -0.213769584288
AAGGTTTA -0.0847317212032
AAGGTTTC -0.128714688177
AAGGTTTG -0.110571912454
AAGTAAAA 0.311139747808
AAGTAAAC -0.0702320387145
AAGTAAAG -0.166710787923
AAGTAAAT 0.113906264826
AAGTAACA -0.216574886412
AAGTAACC -0.0507022173689
AAGTAACG 0.097214294184
AAGTAACT -0.094410881645
AAGTAAGA 0.0988525798705
AAGTAAGC 0.0832250983766
AAGTAAGG -0.100124954565
AAGTAAGT -0.0377209165088
AAGTAATA 0.168056486238
AAGTAATC 0.310685160196
AAGTAATG -0.137904733822
AAGTAATT 0.249912446882
AAGTACAA 0.108253833858
AAGTACAC -0.306242109795
AAGTACAG -0.0376789657825
AAGTACAT 0.0573327266962
AAGTACCA -0.297569332462
AAGTACCC -0.161411243737
AAGTACCG -0.0193788830152
AAGTACCT -0.165449295925
AAGTACGA 0.0246234210983
AAGTACGC -0.0530068684627
AAGTACGG -0.231415542487
AAGTACGT -0.13206181388
AAGTACTA 0.0753659996084
AAGTACTC -0.0540219826151
AAGTACTG -0.059687014153
AAGTACTT 0.142404435294
AAGTAGAA -0.0446450077736
AAGTAGAC -0.0676758831556
AAGTAGAG -0.0744501031262
AAGTAGAT 0.166865689073
AAGTAGCA -0.0571768507719
AAGTAGCC -0.154751271554
AAGTAGCG -0.0972940505019
AAGTAGCT -0.271588012019
AAGTAGGA -0.142069211447
AAGTAGGC 0.041811784236
AAGTAGGG -0.153860581477
AAGTAGGT -0.177450615527
AAGTAGTA 0.0780325978263
AAGTAGTC -0.00605899140895

AAGTAGTG -0.167090438567
AAGTATAA 0.245039805646
AAGTATAC -0.097263601988
AAGTATAG -0.0608445911476
AAGTATAT 0.0888496662283
AAGTATCA 0.181612811398
AAGTATCC 0.143362370635
AAGTATCG 0.279750808376
AAGTATCT 0.268244490997
AAGTATGA 0.0781511539087
AAGTATGC -0.0256410443567
AAGTATGG 0.0384001323572
AAGTATGT 0.0779526082556
AAGTATTA 0.139096954081
AAGTATTC -0.0807157019278
AAGTATTG 0.100164903195
AAGTCAAA -0.143696853354
AAGTCAAC -0.0927054123136
AAGTCAAG -0.113128179675
AAGTCAAT -0.215449575279
AAGTCACA -0.189701321541
AAGTCACC -0.284520937943
AAGTCACG -0.032962457432
AAGTCACT -0.230041052569
AAGTCAGA -0.0202718188653
AAGTCAGC -0.0284236764248
AAGTCAGG -0.0456834790786
AAGTCAGT 0.125228644628
AAGTCATA 0.137488728398
AAGTCATC -0.287624514418
AAGTCATG -0.109615320066
AAGTCATT 0.0756555453525
AAGTCCAA -0.101032094188
AAGTCCAC -0.230201656811
AAGTCCAG -0.140275349051
AAGTCCAT -0.32929566126
AAGTCCCA -0.272312433484
AAGTCCCC -0.261336491134
AAGTCCCG -0.0590278095278
AAGTCCCT -0.260319232519
AAGTCCGA 0.0540250994796
AAGTCCGC -0.109096899962
AAGTCCGG -0.224251324572
AAGTCCGT -0.253251606421
AAGTCCTA -0.101830599737
AAGTCCTC -0.269809360219
AAGTCCTG -0.199185848711
AAGTCGAA 0.0320014890185
AAGTCGAC -0.217458781629
AAGTCGAG -0.25299683847
AAGTCGAT 0.0661970510455
AAGTCGCA -0.136201362911
AAGTCGCC -0.313023907318
AAGTCGCG -0.0423473425037

AAGTCGCT -0.18400905404
AAGTCGGA -0.199891919016
AAGTCGGC -0.0449441892442
AAGTCGGG -0.0629860053806
AAGTCGGT -0.166528017981
AAGTCGTA 0.0164418895583
AAGTCGTC -0.197179910194
AAGTCGTG -0.11974086178
AAGTCTAA 0.107223199492
AAGTCTAC -0.171445150836
AAGTCTAG -0.0743887966859
AAGTCTAT -0.0770541671593
AAGTCTCA -0.132915436342
AAGTCTCC -0.291041258342
AAGTCTCG -0.102167835741
AAGTCTCT -0.183096135013
AAGTCTGA -0.217826298282
AAGTCTGC -0.260260831452
AAGTCTGG -0.171516451761
AAGTCTGT 0.0493344584254
AAGTCTTA 0.165196223788
AAGTCTTC -0.193709501072
AAGTCTTG -0.130745312146
AAGTGAAA 0.149546360299
AAGTGAAC -0.275699270853
AAGTGAAG -0.253482473914
AAGTGAAT 0.0747333999985
AAGTGACA 0.0378688239819
AAGTGACC 0.0753136056166
AAGTGACG -0.013711378583
AAGTGACT -0.204386109607
AAGTGAGA 0.0635161273841
AAGTGAGC 0.11064921671
AAGTGAGG 0.0284915750552
AAGTGAGT -0.0452105260853
AAGTGATA 0.233772339833
AAGTGATC 0.00583271899155
AAGTGATG 0.272069224762
AAGTGATT 0.31898427353
AAGTGCAA 0.229454099545
AAGTGCAC -0.08459029938
AAGTGCAG -0.155315154372
AAGTGCAT -0.0460556975708
AAGTGCCA -0.197482757337
AAGTGCCC -0.240348956851
AAGTGCCG -0.0627714221902
AAGTGCCT -0.230110464684
AAGTGCGA -0.0510085342819
AAGTGCGC -0.0151500111671
AAGTGCGG -0.257608207299
AAGTGCGT 0.00827411433472
AAGTGCTA 0.00675334772504
AAGTGCTC -0.180748994797
AAGTGCTG -0.177190631608

AAGTGGAA 0.0495135143026
AAGTGGAC -0.0257870265615
AAGTGGAG -0.252273065568
AAGTGGAT 0.0505340050795
AAGTGGCA -0.148829411911
AAGTGGCC -0.421475249822
AAGTGGCG -0.134808969118
AAGTGGCT -0.233203765731
AAGTGGGA 0.0312277833332
AAGTGGGC -0.204923568898
AAGTGGGG -0.170237021965
AAGTGGGT -0.236077117859
AAGTGGTA -0.09867029564
AAGTGGTC -0.261331946011
AAGTGGTG -0.0696096551094
AAGTGTAA 0.112863951884
AAGTGTAC -0.225730022081
AAGTGTAG -0.0693324178173
AAGTGTAT 0.0433275551939
AAGTGTCA -0.111680551074
AAGTGTCC -0.199313228609
AAGTGTCG -0.0290469532894
AAGTGTCT -0.116091618432
AAGTGTGA 0.0414903323285
AAGTGTGC -0.21556780914
AAGTGTGG 0.024135597737
AAGTGTGT -0.168448128302
AAGTGTTA 0.0271083755932
AAGTGTTC -0.00790497282811
AAGTGTTG -0.0214387941661
AAGTTAAA 0.246207017365
AAGTTAAC -0.00443832262014
AAGTTAAG -0.289312613923
AAGTTAAT 0.0709845769801
AAGTTACA -0.085577353941
AAGTTACC -0.123549930375
AAGTTACG 0.0258729804184
AAGTTACT -0.0276543822641
AAGTTAGA 0.0661091872418
AAGTTAGC -0.0619376542444
AAGTTAGG 0.0797391865399
AAGTTAGT -0.0480549419943
AAGTTATA 0.109532637971
AAGTTATC -0.0627383345328
AAGTTATG 0.0557527937344
AAGTTATT 0.145249232883
AAGTTCAA 0.111593294786
AAGTTCAC -0.0219275390143
AAGTTCAG -0.140108185901
AAGTTCAT -0.176855642393
AAGTTCCA -0.089941558925
AAGTTCCC -0.206051658685
AAGTTCCG -0.115493305191
AAGTTCCT -0.121938813488

AAGTTCGA 0.0488069604315
AAGTTCGC -0.12412422857
AAGTTCGG -0.000530436508921
AAGTTCGT 0.0328496540618
AAGTTCTA -0.0660178084421
AAGTTCTC -0.123529995607
AAGTTCTG -0.0442191502798
AAGTTGAA -0.0315825200908
AAGTTGAC 0.0336672035237
AAGTTGAG -0.0406071399896
AAGTTGAT -0.105315212076
AAGTTGCA -0.252310851976
AAGTTGCC -0.169271445273
AAGTTGCG -0.0831755509003
AAGTTGCT -0.273684602218
AAGTTGGA 0.0499602198492
AAGTTGGC -0.141555687029
AAGTTGGG -0.0208927936201
AAGTTGGT -0.000459091247487
AAGTTGTA 0.0530033253096
AAGTTGTC -0.0114701478338
AAGTTGTG -0.113157821753
AAGTTTAA 0.0235428265731
AAGTTTAC -0.223692141538
AAGTTTAG -0.109737905172
AAGTTTAT -0.0367181740546
AAGTTTCA 0.0328569447384
AAGTTTCC 0.0196601560238
AAGTTTCG -0.0679392942658
AAGTTTCT -0.222285222239
AAGTTTGA 0.0997402361039
AAGTTTGC -0.197707056547
AAGTTTGG -0.0560437436246
AAGTTTGT -0.112740354795
AAGTTTTA 0.0687072545857
AAGTTTTC -0.138583517383
AAGTTTTG 0.0549930095385
AATAAAAA 0.265620897445
AATAAAAC -0.0389962965721
AATAAAAG 0.0407877757649
AATAAAAT 0.266005123975
AATAAACA -0.0320775320775
AATAAACC -0.145331281695
AATAAACG 0.0867696327818
AATAAACT 0.0534988284928
AATAAAGA 0.195491614295
AATAAAGC 0.13593789917
AATAAAGG 0.221807551659
AATAAAGT -0.0441204709423
AATAAATA 0.250058598543
AATAAATC 0.268816741239
AATAAATG 0.0769736678828
AATAAATT 0.337162689868
AATAACAA 0.104963089812

AATAACAC 0.00909240538242
AATAACAG 0.0239123420942
AATAACAT -0.0268789734531
AATAACCA 0.0469821828719
AATAACCC -0.0406784194663
AATAACCG 0.143447223783
AATAACCT -0.0213232962457
AATAACGA 0.0915295174971
AATAACGC 0.0438297114513
AATAACGG 0.00732688611476
AATAACGT 0.0645671992584
AATAACTA 0.0196747516478
AATAACTC 0.0553936775107
AATAACTG -0.0243259788714
AATAAGAA 0.138911638912
AATAAGAC -0.212830416169
AATAAGAG 0.005172879426
AATAAGAT 0.290713578592
AATAAGCA 0.149848195303
AATAAGCC -0.000321874785599
AATAAGCG 0.0588476164756
AATAAGCT 0.0709373285131
AATAAGGA 0.0235316014978
AATAAGGC 0.1786759211
AATAAGGG -0.143053668206
AATAAGGT -0.158756377598
AATAAGTA 0.0973548024496
AATAAGTC -0.0105822075519
AATAAGTG 0.0182850010584
AATAATAA 0.277552316373
AATAATAC 0.0853236156266
AATAATAG 0.161796783009
AATAATAT 0.207883768969
AATAATCA 0.220817792311
AATAATCC 0.308226959742
AATAATCG 0.227165098574
AATAATCT 0.297240766938
AATAATGA 0.187349554557
AATAATGC 0.14186776308
AATAATGG 0.242847530726
AATAATGT 0.18018802508
AATAATTA 0.129907313199
AATAATTC -0.0098349037743
AATAATTG 0.0145507762604
AATACAAA 0.162162142435
AATACAAC -0.112821856539
AATACAAG -0.102090588492
AATACAAT 0.182955847682
AATACACA -0.0340571617653
AATACACC 0.0203178896692
AATACACG 0.161242454545
AATACACT -0.091461500843
AATACAGA 0.191986752593
AATACAGC 0.135779029718

AATACAGG 0.18711050123
AATACAGT 0.106811529675
AATACATA 0.228274563331
AATACATC -0.12150171894
AATACATG 0.0819186441173
AATACATT 0.122300241601
AATACCAA -0.000246803277106
AATACCAC 0.0919943358905
AATACCAG 0.0463948797282
AATACCAT 0.00417083750417
AATACCCA 0.249556418592
AATACCCC -0.149183618881
AATACCCG -0.0547382174113
AATACCCT -0.0947299187882
AATACCGA 0.032706259979
AATACCGC 0.09644971278
AATACCGG -0.0107421471058
AATACCGT -0.135867272231
AATACCTA 0.0689049931474
AATACCTC -0.0256056031214
AATACCTG 0.0434318956519
AATACGAA 0.251976234097
AATACGAC -0.102797061961
AATACGAG 0.128314708437
AATACGAT 0.312935483165
AATACGCA 0.145893885535
AATACGCC 0.0895184776976
AATACGCG 0.112168642472
AATACGCT 0.120306124683
AATACGGA 0.162967375089
AATACGGC 0.0887863163904
AATACGGG 0.117222980519
AATACGGT 0.0547404273271
AATACGTA 0.204497151643
AATACGTC 0.18155483307
AATACGTG 0.254283208829
AATACTAA 0.143858893601
AATACTAC 0.0878863966482
AATACTAG 0.0582578483683
AATACTAT 0.0693627511809
AATACTCA 0.238646086562
AATACTCC -0.0556288674241
AATACTCG 0.0704740553225
AATACTCT 0.0773955773956
AATACTGA 0.223919511798
AATACTGC 0.120322182726
AATACTGG 0.126139573586
AATACTGT 0.260658361665
AATACTTA 0.0951592300899
AATACTTC 0.0912395219515
AATACTTG 0.103325969868
AATAGAAA 0.104911655955
AATAGAAC -0.0499427019846
AATAGAAG -0.141915176532

AATAGAAT 0.257027218382
AATAGACA 0.0790311161069
AATAGACC -0.254834004704
AATAGACG 0.184809821683
AATAGACT -0.0821245998461
AATAGAGA 0.249680810287
AATAGAGC 0.0476250603971
AATAGAGG -0.0490752460449
AATAGAGT 0.0257914060276
AATAGATA 0.295362855969
AATAGATC 0.286778514051
AATAGATG 0.250739764823
AATAGATT 0.42244586184
AATAGCAA 0.218461639526
AATAGCAC 0.00529317195984
AATAGCAG -0.0763125890724
AATAGCAT 0.0328306679416
AATAGCCA -0.0737099405336
AATAGCCC -0.000424057257218
AATAGCCG -0.000329530632561
AATAGCCT -0.222511696124
AATAGCGA 0.0831396134426
AATAGCGC 0.0583541966234
AATAGCGG -0.0775590618025
AATAGCGT 0.0133566418254
AATAGCTA -0.164772586288
AATAGCTC -0.108563487928
AATAGCTG -0.236862634833
AATAGGAA 0.12098786158
AATAGGAC -0.207370928127
AATAGGAG -0.107851629396
AATAGGAT 0.111716531755
AATAGGCA -0.198442395789
AATAGGCC -0.329979904235
AATAGGCG -0.0772284150006
AATAGGCT -0.131870176919
AATAGGGA 0.0868265658416
AATAGGGC -0.0122205404332
AATAGGGG -0.0618128117942
AATAGGGT -0.167948686137
AATAGGTA 0.0415442991201
AATAGGTC -0.204797791837
AATAGGTG -0.00221847191544
AATAGTAA 0.224501360865
AATAGTAC -0.105811834316
AATAGTAG -0.0283987990372
AATAGTAT -0.0163244600483
AATAGTCA -0.175605858272
AATAGTCC -0.0622154259396
AATAGTCG 0.0366854457764
AATAGTCT 0.0561787683
AATAGTGA -0.175453822179
AATAGTGC 0.0521685004748
AATAGTGG 0.00905726663302

AATAGTGT -0.151458710702
AATAGTTA 0.16168372229
AATAGTTC 0.052447764569
AATAGTTG 0.127990579064
AATATAAA 0.329802254045
AATATAAC 0.146053767266
AATATAAG 0.195549544034
AATATAAT 0.200415319373
AATATACA 0.202824036157
AATATACC 0.0288251653432
AATATACG 0.287674722451
AATATACT 0.180733998526
AATATAGA 0.266410954505
AATATAGC 0.270191893608
AATATAGG 0.0658430837281
AATATAGT 0.139308730218
AATATATA 0.259954169045
AATATATC 0.30673707875
AATATATG 0.25635570024
AATATATT 0.353599375963
AATATCAA 0.28698533244
AATATCAC 0.345737684576
AATATCAG 0.305726881893
AATATCAT 0.258393611819
AATATCCA 0.375567688299
AATATCCC 0.356887805377
AATATCCG 0.435155541216
AATATCCT 0.385220031678
AATATCGA 0.289971789972
AATATCGC 0.340190052311
AATATCGG 0.387040203572
AATATCGT 0.295986949191
AATATCTA 0.389354919658
AATATCTC 0.367001988214
AATATCTG 0.399086775289
AATATGAA 0.33888236024
AATATGAC -0.0121844047211
AATATGAG 0.143616563141
AATATGAT 0.226817660927
AATATGCA 0.152095209594
AATATGCC -0.00173589567529
AATATGCG 0.181503636228
AATATGCT 0.144431143844
AATATGGA 0.181879958833
AATATGGC 0.134535073132
AATATGGG 0.271906893119
AATATGGT 0.152393013292
AATATGTA 0.0726056635148
AATATGTC 0.127302233363
AATATGTG 0.235033539412
AATATTAA 0.2635701408
AATATTAC 0.176056728685
AATATTAG 0.180140195292
AATATTAT 0.150109851609

AATATTCA 0.0503076574966
AATATTCC 0.327778191415
AATATTCG 0.223090830001
AATATTCT 0.270489497762
AATATTGA 0.154456109002
AATATTGC 0.23994438078
AATATTGG 0.128028583316
AATATTGT 0.0440391816238
AATATTTA 0.201110194667
AATATTTC 0.32355422874
AATATTTG 0.253872158991
AATCAAAA 0.329279650811
AATCAAAC 0.215965208328
AATCAAAG 0.203444491323
AATCAAAT 0.28629081115
AATCAACA 0.331142383743
AATCAACC 0.00716407669905
AATCAACG 0.253748458063
AATCAACT 0.280915746371
AATCAAGA 0.330854575691
AATCAAGC 0.191369055005
AATCAAGG 0.278460555376
AATCAAGT 0.303392924605
AATCAATA 0.295487690051
AATCAATC 0.251993751962
AATCAATG 0.114983745688
AATCAATT 0.295536583415
AATCACAA 0.309755990745
AATCACAC 0.304255381269
AATCACAG 0.311616023737
AATCACAT 0.248980679255
AATCACCA 0.254587810507
AATCACCC 0.0412823291611
AATCACCG 0.270207749013
AATCACCT 0.159039204494
AATCACGA 0.357821301834
AATCACGC 0.255652066013
AATCACGG 0.252725176968
AATCACGT 0.263055066085
AATCACTA 0.23544894757
AATCACTC 0.190075750682
AATCACTG 0.264548221572
AATCAGAA 0.305399881754
AATCAGAC 0.116211252575
AATCAGAG 0.161490691794
AATCAGAT 0.305462627804
AATCAGCA 0.298401494858
AATCAGCC 0.215048381715
AATCAGCG 0.346782917162
AATCAGCT 0.262737944556
AATCAGGA 0.384611884612
AATCAGGC 0.29190820639
AATCAGGG 0.368196019711
AATCAGGT 0.293173338628

AATCAGTA 0.21793556642
AATCAGTC 0.0989589795766
AATCAGTG 0.252022948403
AATCATAA 0.287190061531
AATCATAC 0.234907994715
AATCATAG 0.312007036342
AATCATAT 0.237310313068
AATCATCA 0.232368732369
AATCATCC 0.125260760713
AATCATCG 0.255291957218
AATCATCT 0.218955546179
AATCATGA 0.284083560214
AATCATGC 0.0514346401174
AATCATGG 0.227457062984
AATCATGT 0.349745061866
AATCATTA 0.0905795602765
AATCATTC 0.19170164157
AATCATTG 0.196301833883
AATCCAAA 0.412524094342
AATCCAAC 0.358165620269
AATCCAAG 0.390192185573
AATCCAAT 0.3997219039
AATCCACA 0.42318416385
AATCCACC 0.371313766852
AATCCACG 0.388510470574
AATCCACT 0.332758378213
AATCCAGA 0.363267227725
AATCCAGC 0.35926439567
AATCCAGG 0.418711269096
AATCCAGT 0.404641504201
AATCCATA 0.378075662577
AATCCATC 0.407076487376
AATCCATG 0.372245133318
AATCCATT 0.407182664758
AATCCCAA 0.360256951166
AATCCCAC 0.291245008354
AATCCCAG 0.330654345806
AATCCCAT 0.32558630103
AATCCCCA 0.348993087743
AATCCCCC 0.144042224396
AATCCCCG 0.334076626059
AATCCCCT 0.263774515887
AATCCCGA 0.315178815179
AATCCCGC 0.284418026842
AATCCCGG 0.307326846857
AATCCCGT 0.385234915986
AATCCCTA 0.272463376714
AATCCCTC 0.309332748727
AATCCCTG 0.31201495959
AATCCGAA 0.409569863434
AATCCGAC 0.341273548754
AATCCGAG 0.369509426801
AATCCGAT 0.438567768589
AATCCGCA 0.414384356547

AATCCGCC 0.327405239301
AATCCGCG 0.422246072909
AATCCGCT 0.412944972008
AATCCGGA 0.443472398018
AATCCGGC 0.389680313923
AATCCGGG 0.327753096225
AATCCGGT 0.411026729209
AATCCGTA 0.368005810092
AATCCGTC 0.366011693175
AATCCGTG 0.363681863682
AATCCTAA 0.410098319015
AATCCTAC 0.34412896068
AATCCTAG 0.417760627342
AATCCTAT 0.36563797873
AATCCTCA 0.393011468769
AATCCTCC 0.380414297497
AATCCTCG 0.378117787209
AATCCTCT 0.346686409006
AATCCTGA 0.389111917257
AATCCTGC 0.368797033642
AATCCTGG 0.360585103009
AATCCTGT 0.388530908082
AATCCTTA 0.315829603708
AATCCTTC 0.21036546645
AATCCTTG 0.347756847757
AATCGAAA 0.359693583352
AATCGAAC 0.240095467368
AATCGAAG 0.296019065276
AATCGAAT 0.309388115589
AATCGACA 0.266979100312
AATCGACC 0.116448404327
AATCGACG 0.22824536689
AATCGACT 0.231871564856
AATCGAGA 0.313881447852
AATCGAGC 0.17032838697
AATCGAGG 0.149198451498
AATCGAGT 0.110738450983
AATCGATA 0.316234680992
AATCGATC 0.296949539577
AATCGATG 0.323497728909
AATCGATT 0.34227078906
AATCGCAA 0.321981923611
AATCGCAC 0.282644903857
AATCGCAG 0.168111637809
AATCGCAT 0.303577682366
AATCGCCA 0.234158249994
AATCGCCC 0.058089770211
AATCGCCG 0.272305715933
AATCGCCT 0.316502452866
AATCGCGA 0.38909019212
AATCGCGC 0.269794822011
AATCGCGG 0.301072969205
AATCGCGT 0.402478235812
AATCGCTA 0.196400808741

AATCGCTC 0.135566696173
AATCGCTG 0.238157558594
AATCGGAA 0.27996453754
AATCGGAC 0.21643268613
AATCGGAG 0.0548595793878
AATCGGAT 0.386385007597
AATCGGCA 0.201169489048
AATCGGCC 0.135343751597
AATCGGCG 0.149059527847
AATCGGCT 0.137167069334
AATCGGGA 0.297666711516
AATCGGGC 0.150632467902
AATCGGGG 0.311362326514
AATCGGGT 0.312887267433
AATCGGTA 0.200762463707
AATCGGTC 0.162366517522
AATCGGTG 0.193606709342
AATCGTAA 0.362780135507
AATCGTAC 0.25520975521
AATCGTAG 0.278588790026
AATCGTAT 0.329824314673
AATCGTCA 0.160760713269
AATCGTCC 0.205126614218
AATCGTCG 0.351877696109
AATCGTCT 0.330712254955
AATCGTGA 0.278749370452
AATCGTGC 0.279399233945
AATCGTGG 0.234960856173
AATCGTGT 0.332946621337
AATCGTTA 0.268497339748
AATCGTTC 0.0495192161859
AATCGTTG 0.262989131625
AATCTAAA 0.427496478179
AATCTAAC 0.393902166629
AATCTAAG 0.383313504084
AATCTAAT 0.420582760961
AATCTACA 0.379380758169
AATCTACC 0.398253364667
AATCTACG 0.391056400225
AATCTACT 0.413899695627
AATCTAGA 0.439032696608
AATCTAGC 0.408862030074
AATCTAGG 0.435745663018
AATCTAGT 0.397531139955
AATCTATA 0.424445106263
AATCTATC 0.342765630644
AATCTATG 0.357016796411
AATCTCAA 0.431175229739
AATCTCAC 0.370032043094
AATCTCAG 0.433116793063
AATCTCAT 0.419440101258
AATCTCCA 0.423905315942
AATCTCCC 0.40217440494
AATCTCCG 0.443411458143

AATCTCCT 0.402718145142
AATCTCGA 0.439732706583
AATCTCGC 0.390734381062
AATCTCGG 0.422906377452
AATCTCGT 0.458663678898
AATCTCTA 0.375426390578
AATCTCTC 0.386808318066
AATCTCTG 0.371418874039
AATCTGAA 0.460980264011
AATCTGAC 0.394431171125
AATCTGAG 0.446116915814
AATCTGAT 0.39765247341
AATCTGCA 0.407929023452
AATCTGCC 0.245488784917
AATCTGCG 0.440339788825
AATCTGCT 0.398962796435
AATCTGGA 0.457116896511
AATCTGGC 0.431353450629
AATCTGGG 0.397274685153
AATCTGGT 0.449992335934
AATCTGTA 0.407648695527
AATCTGTC 0.397429983285
AATCTGTG 0.417771143698
AATCTTAA 0.402597753334
AATCTTAC 0.345252966465
AATCTTAG 0.408928211959
AATCTTAT 0.404160358706
AATCTTCA 0.395121016333
AATCTTCC 0.391570257586
AATCTTCG 0.414950767501
AATCTTCT 0.428550469698
AATCTTGA 0.37548301862
AATCTTGC 0.34701648869
AATCTTGG 0.372379436947
AATCTTGT 0.417484978091
AATCTTTA 0.344085166018
AATCTTTC 0.366287775379
AATCTTTG 0.366958723225
AATGAAAA 0.148204678508
AATGAAAC 0.106090377583
AATGAAAG -0.0719738380452
AATGAAAT 0.230516849996
AATGAACA 0.0986336065634
AATGAACC -0.133852482293
AATGAACG -0.214626587312
AATGAACT -0.2576406372
AATGAAGA 0.105205756721
AATGAAGC -0.121704649778
AATGAAGG 0.138809608507
AATGAAGT 0.149842135308
AATGAATA -0.0687320715605
AATGAATC 0.223643753947
AATGAATG -0.012648000362
AATGAATT 0.0593060005985

AATGACAA 0.118822945483
AATGACAC -0.211141435444
AATGACAG -0.101774425228
AATGACAT -0.0828403273764
AATGACCA -0.14838719566
AATGACCC -0.183262329211
AATGACCG 0.144486617081
AATGACCT -0.270516478518
AATGACGA 0.0427224221367
AATGACGC -0.0833069348846
AATGACGG -0.0569865257202
AATGACGT 0.0980553556311
AATGACTA 0.0542843118601
AATGACTC 0.137434535481
AATGACTG -0.0653504744414
AATGAGAA 0.0987006290037
AATGAGAC -0.168903267617
AATGAGAG -0.134222237142
AATGAGAT 0.268825500172
AATGAGCA -0.0284838201355
AATGAGCC -0.26932945117
AATGAGCG -0.133396754491
AATGAGCT -0.219888024684
AATGAGGA -0.0625395064342
AATGAGGC -0.209146703752
AATGAGGG 0.0120077662533
AATGAGGT -0.071563298836
AATGAGTA 0.240431961344
AATGAGTC 0.0337293216081
AATGAGTG -0.15124288731
AATGATAA 0.0559425706006
AATGATAC 0.0196050044535
AATGATAG 0.162941687408
AATGATAT 0.196509181358
AATGATCA 0.0722444207293
AATGATCC 0.0645948979282
AATGATCG 0.0578461785508
AATGATCT 0.0826239462603
AATGATGA 0.108830593679
AATGATGC -0.0541975155451
AATGATGG 0.0720569053902
AATGATGT 0.0266147456625
AATGATTA 0.151251802767
AATGATTC 0.206476500515
AATGATTG 0.00894068013109
AATGCAAA 0.0329562126377
AATGCAAC 0.01823724551
AATGCAAG 0.0881970124394
AATGCAAT 0.287251374058
AATGCACA 0.0360925663956
AATGCACC -0.166522180141
AATGCACG -0.283905441575
AATGCACT -0.0964774493989
AATGCAGA 0.0601994116917

AATGCAGC 0.00447337723326
AATGCAGG 0.287918455468
AATGCAGT -0.0717568228433
AATGCATA 0.208868648263
AATGCATC 0.0382407684503
AATGCATG -0.102628262926
AATGCATT 0.00820702372388
AATGCCAA -0.156150144004
AATGCCAC -0.0879533179642
AATGCCAG -0.110701347361
AATGCCAT -0.203974838912
AATGCCCA 0.0459661467267
AATGCCCC -0.316339009864
AATGCCCG -0.0793526259644
AATGCCCT -0.280676652138
AATGCCGA 0.00433491089168
AATGCCGC -0.275899140761
AATGCCGG -0.105241607381
AATGCCGT 0.00113198598047
AATGCCTA 0.079615044926
AATGCCTC -0.128032447314
AATGCCTG -0.164438985523
AATGCGAA -0.0378670681701
AATGCGAC -0.307023999515
AATGCGAG -0.0554978200804
AATGCGAT 0.143890984441
AATGCGCA 0.0201856893645
AATGCGCC -0.233283811453
AATGCGCG -0.064057461583
AATGCGCT -0.271459138205
AATGCGGA -0.125559631501
AATGCGGC -0.244822201303
AATGCGGG -0.122716039291
AATGCGGT -0.0591975936632
AATGCGTA 0.157379765407
AATGCGTC 0.0109304175821
AATGCGTG -0.126388769271
AATGCTAA 0.165209286421
AATGCTAC -0.147291630481
AATGCTAG 0.0311482281179
AATGCTAT -0.0224381947841
AATGCTCA -0.0845306335145
AATGCTCC -0.148731696565
AATGCTCG -0.149734677343
AATGCTCT -0.269764864485
AATGCTGA 0.0108924622089
AATGCTGC -0.256897411937
AATGCTGG -0.163045848748
AATGCTGT -0.0736673312431
AATGCTTA 0.0198666543005
AATGCTTC -0.102103443427
AATGCTTG -0.176163310946
AATGGAAA -0.0404903542258
AATGGAAC -0.203770891533

AATGGAAG -0.18235873008
AATGGAAT 0.108011503398
AATGGACA 0.0562987671803
AATGGACC -0.319701843904
AATGGACG -0.208849434824
AATGGACT -0.231352130771
AATGGAGA -0.118339744385
AATGGAGC -0.117606913717
AATGGAGG -0.106699666739
AATGGAGT 0.012284522713
AATGGATA 0.36888170332
AATGGATC 0.201577337723
AATGGATG 0.159582447461
AATGGCAA 0.0459702126369
AATGGCAC -0.221083624875
AATGGCAG 0.0128917785443
AATGGCAT -0.18211186393
AATGGCCA -0.247782165354
AATGGCCC -0.310101178919
AATGGCCG -0.167645964229
AATGGCCT -0.0897722578771
AATGGCGA 0.0199215183226
AATGGCGC -0.0735305065226
AATGGCGG -0.106968260665
AATGGCGT -0.0590271442688
AATGGCTA -0.0600343917004
AATGGCTC -0.177582187552
AATGGCTG -0.247578751676
AATGGGAA 0.0582552249219
AATGGGAC -0.128744207076
AATGGGAG -0.164108233543
AATGGGAT 0.165058813311
AATGGGCA -0.0636592743325
AATGGGCC -0.278236709583
AATGGGCG -0.260028545618
AATGGGCT -0.0666084933101
AATGGGGA -0.0462432208133
AATGGGGC -0.173423116052
AATGGGGG -0.101695100788
AATGGGGT 0.0895213973417
AATGGGTA -0.0615934042674
AATGGGTC -0.0711520805935
AATGGGTG -0.00585296039841
AATGGTAA 0.0994351618293
AATGGTAC -0.0309579552004
AATGGTAG -0.124672103747
AATGGTAT 0.107526614884
AATGGTCA -0.0417078458136
AATGGTCC -0.0568543750362
AATGGTCG 0.0694241083123
AATGGTCT -0.131311752524
AATGGTGA 0.00393767453131
AATGGTGC -0.18069124471
AATGGTGG -0.053841886674

AATGGTGT 0.0361146270237
AATGGTTA -0.0929943139931
AATGGTTC -0.0801084647781
AATGGTTG 0.0362327832237
AATGTAAA 0.221198805866
AATGTAAC 0.0360831514638
AATGTAAG -0.0313561511658
AATGTAAT 0.33359943966
AATGTACA 0.111319460158
AATGTACC -0.00913247008027
AATGTACG -0.062805543837
AATGTACT -0.0160733706561
AATGTAGA 0.209483733933
AATGTAGC -0.170275296044
AATGTAGG 0.0249680663927
AATGTAGT 0.0642226248287
AATGTATA 0.159925695058
AATGTATC 0.197216914411
AATGTATG 0.0312540601301
AATGTCAA 0.06620803924
AATGTCAC -0.0703203579484
AATGTCAG -0.0880002744329
AATGTCAT 0.0759070969249
AATGTCCA -0.0380545835091
AATGTCCC -0.11042733346
AATGTCCG -0.227265209589
AATGTCCT -0.231713140986
AATGTCGA 0.0676006585097
AATGTCGC -0.00110143573498
AATGTCGG 0.0331956234535
AATGTCGT 0.0428154909873
AATGTCTA 0.00198462807384
AATGTCTC 0.0309674747789
AATGTCTG -0.185024787696
AATGTGAA 0.224426472413
AATGTGAC -0.230025412979
AATGTGAG 0.0382410601285
AATGTGAT 0.0418393600212
AATGTGCA -0.016377013642
AATGTGCC -0.140964973866
AATGTGCG -0.0354851339912
AATGTGCT -0.225322333206
AATGTGGA 0.0123210164528
AATGTGGC -0.0899315293255
AATGTGGG -0.0127366084265
AATGTGGT 0.0746848777152
AATGTGTA 0.147193127158
AATGTGTC 0.0140771936791
AATGTGTG 0.0783000631485
AATGTTAA 0.218760082396
AATGTTAC 0.0377531878864
AATGTTAG 0.0706781161327
AATGTTAT 0.00597286190813
AATGTTCA -0.122389290745

AATGTTCC -0.00233704779159
AATGTTCG -0.243685492356
AATGTTCT -0.0701422596585
AATGTTGA 0.125438110287
AATGTTGC 0.129511053753
AATGTTGG -0.0477485917066
AATGTTGT 0.142121705364
AATGTTTA 0.1097392158
AATGTTTC 0.107180182938
AATGTTTG 0.0459729702154
AATTAAAA 0.105578579051
AATTAAAC -0.0625393220047
AATTAAAG -0.0665306599126
AATTAAAT 0.0948001138661
AATTAACA 0.10489805089
AATTAACC -0.186142178877
AATTAACG -0.0821561949428
AATTAACT 0.056553798978
AATTAAGA 0.152728772363
AATTAAGC 0.0981606242199
AATTAAGG 0.195815573283
AATTAAGT 0.120001021875
AATTAATA 0.243729057559
AATTAATC 0.0581867136511
AATTAATG -0.0259115865176
AATTAATT 0.0991334044471
AATTACAA 0.191408217338
AATTACAC 0.196398878217
AATTACAG 0.0753961592082
AATTACAT 0.0891272453888
AATTACCA 0.196073808603
AATTACCC 0.165308564404
AATTACCG 0.187414144216
AATTACCT 0.180260149957
AATTACGA 0.180520864507
AATTACGC 0.0902486508547
AATTACGG 0.299973527246
AATTACGT 0.206439221591
AATTACTA 0.239968618756
AATTACTC 0.205256089283
AATTACTG -0.0429895695715
AATTAGAA 0.168359819875
AATTAGAC -0.0944087373474
AATTAGAG -0.0151244863251
AATTAGAT 0.228470179485
AATTAGCA 0.0688503171463
AATTAGCC -0.0750881367561
AATTAGCG -0.0131566462048
AATTAGCT -0.0455617760195
AATTAGGA 0.0301720453236
AATTAGGC 0.117310569842
AATTAGGG 0.00843120223645
AATTAGGT 0.0250401917069
AATTAGTA 0.127707422465

AATTAGTC -0.0132454028836
AATTAGTG 0.137187506843
AATTATAA 0.310316562411
AATTATAC -0.0835142296154
AATTATAG 0.119765771281
AATTATAT 0.137581658796
AATTATCA 0.0437180207733
AATTATCC 0.198210988081
AATTATCG 0.116422998037
AATTATCT 0.113509193229
AATTATGA 0.0209456467358
AATTATGC 0.111652975289
AATTATGG 0.0673291825726
AATTATGT 0.0907891362437
AATTATTA 0.0881528911832
AATTATTC 0.0125443581408
AATTATTG -0.0468443650262
AATTCAAA 0.0611479247843
AATTCAAC 0.0574491069539
AATTCAAG -0.0366931781522
AATTCAAT 0.107138529813
AATTCACA 0.0963446055926
AATTCACC 0.00102406516646
AATTCACG 0.0953418983722
AATTCACT -0.132689048521
AATTCAGA 0.126650246917
AATTCAGC -0.00960050960051
AATTCAGG 0.241885135825
AATTCAGT 0.169196838474
AATTCATA 0.226828757132
AATTCATC -0.0109848140151
AATTCATG 0.0283548010821
AATTCCAA 0.0826211886818
AATTCCAC 0.0484011299023
AATTCCAG 0.0153098837252
AATTCCAT 0.149668985351
AATTCCCA 0.10564659168
AATTCCCC 0.196809558915
AATTCCCG 0.193947390917
AATTCCCT 0.235526959264
AATTCCGA -0.00753049203302
AATTCCGC 0.126265118282
AATTCCGG 0.0282643445764
AATTCCGT 0.153925826442
AATTCCTA 0.191878280038
AATTCCTC 0.122233089786
AATTCCTG -0.061103803528
AATTCGAA 0.0297008094713
AATTCGAC -0.0278293961454
AATTCGAG 0.0189412268294
AATTCGAT 0.14138518684
AATTCGCA 0.215172472748
AATTCGCC 0.0894338080188
AATTCGCG 0.192408864183

AATTCGCT 0.064917983969
AATTCGGA 0.143055991475
AATTCGGC 0.164103705758
AATTCGGG 0.228266336089
AATTCGGT 0.0831478142814
AATTCGTA 0.25366054062
AATTCGTC -0.0424757122107
AATTCGTG -0.0436990430866
AATTCTAA 0.243973489632
AATTCTAC 0.0986277672752
AATTCTAG 0.115652211995
AATTCTAT 0.139324887709
AATTCTCA 0.180278074217
AATTCTCC 0.114551506171
AATTCTCG 0.0698103347352
AATTCTCT 0.191475171849
AATTCTGA 0.161470186784
AATTCTGC 0.195863594228
AATTCTGG 0.186388868207
AATTCTGT 0.214943468391
AATTCTTA 0.190741078663
AATTCTTC 0.133884573279
AATTCTTG 0.105232002219
AATTGAAA 0.26289788411
AATTGAAC -0.00878301934994
AATTGAAG -0.0019042433461
AATTGAAT 0.0648403224161
AATTGACA 0.0638999881424
AATTGACC 0.00295103026941
AATTGACG 0.0114033999255
AATTGACT -0.196269000334
AATTGAGA 0.0803332773735
AATTGAGC -0.0489945488084
AATTGAGG 0.150849196304
AATTGAGT -0.0164089709544
AATTGATA 0.235959099595
AATTGATC 0.202952453036
AATTGATG -0.134157808385
AATTGCAA 0.196383290877
AATTGCAC 0.188748421567
AATTGCAG 0.0527893549776
AATTGCAT 0.205984221136
AATTGCCA 0.107419183336
AATTGCCC 0.0803710046134
AATTGCCG 0.0478026028627
AATTGCCT -0.0904659022065
AATTGCGA 0.271302983424
AATTGCGC 0.217528663186
AATTGCGG 0.157949096005
AATTGCGT 0.242147105783
AATTGCTA 0.189558462188
AATTGCTC 0.0545955803111
AATTGCTG -0.210841762538
AATTGGAA 0.0841516447577

AATTGGAC 0.0248312529469
AATTGGAG 0.0372783251571
AATTGGAT 0.158918212112
AATTGGCA -0.0594591872629
AATTGGCC -0.223956075395
AATTGGCG 0.168109598729
AATTGGCT -0.150886986726
AATTGGGA -0.0664590725256
AATTGGGC -0.129814254163
AATTGGGG 0.0896640442095
AATTGGGT -0.0272352780604
AATTGGTA 0.1041303011
AATTGGTC 0.0851914166544
AATTGGTG -0.011959703682
AATTGTAA 0.1395575353
AATTGTAC -0.0323095114706
AATTGTAG 0.0304015240542
AATTGTAT 0.0255490755677
AATTGTCA 0.0507572826872
AATTGTCC 0.166194170931
AATTGTCG 0.118836467321
AATTGTCT 0.0794194287716
AATTGTGA 0.0873752540419
AATTGTGC 0.0626921687528
AATTGTGG 0.220198827763
AATTGTGT 0.11120624757
AATTGTTA 0.190552761618
AATTGTTC 0.0801503983322
AATTGTTG 0.0642664759166
AATTTAAA 0.277295387692
AATTTAAC -0.0175507105684
AATTTAAG 0.0702400677357
AATTTAAT 0.113085844836
AATTTACA 0.0972307175755
AATTTACC 0.156595989929
AATTTACG 0.0910086358786
AATTTACT 0.0694926388473
AATTTAGA 0.213266985994
AATTTAGC 0.0976672043678
AATTTAGG 0.187376081315
AATTTAGT 0.165292000905
AATTTATA 0.337221058902
AATTTATC 0.201834266403
AATTTATG 0.116740841954
AATTTCAA 0.213965387619
AATTTCAC 0.218537550273
AATTTCAG 0.0781346084376
AATTTCAT 0.180269775114
AATTTCCA 0.262020974142
AATTTCCC 0.278279307752
AATTTCCG 0.252951298406
AATTTCCT 0.251677297132
AATTTCGA 0.243702380066
AATTTCGC 0.266308766961

AATTTCGG 0.311931125596
AATTTCGT 0.272596287748
AATTTCTA 0.139019184474
AATTTCTC 0.215556593651
AATTTCTG 0.0594928578206
AATTTGAA 0.208301278074
AATTTGAC 0.0651920030948
AATTTGAG 0.143809705446
AATTTGAT 0.168205805712
AATTTGCA 0.156609777822
AATTTGCC 0.149598913892
AATTTGCG 0.177798595429
AATTTGCT -0.0018517842675
AATTTGGA 0.168313832544
AATTTGGC 0.118930972314
AATTTGGG 0.122506789423
AATTTGGT 0.00655095143902
AATTTGTA 0.201619672562
AATTTGTC 0.12296300081
AATTTGTG 0.0628576234637
AATTTTAA 0.124466617519
AATTTTAC 0.0269183886484
AATTTTAG 0.213439851682
AATTTTAT 0.146310222068
AATTTTCA 0.2508375729
AATTTTCC 0.193119398481
AATTTTCG 0.188295876733
AATTTTCT 0.205612285862
AATTTTGA 0.108919398466
AATTTTGC 0.0125041057495
AATTTTGG 0.236747767051
AATTTTGT 0.270857632082
AATTTTTA 0.243749258901
AATTTTTC 0.238330617118
AATTTTTG 0.0330963555543
ACAAAAAA 0.238317988932
ACAAAAAC -0.0210455245506
ACAAAAAG 0.118850255214
ACAAAAAT 0.269612587794
ACAAAACA 0.219156514821
ACAAAACC 0.180576337744
ACAAAACG -0.0461559035274
ACAAAACT 0.0927550437998
ACAAAAGA 0.163597481779
ACAAAAGC 0.0282058918423
ACAAAAGG -0.0325839580155
ACAAAAGT -0.169343743883
ACAAAATA 0.365162076743
ACAAAATC 0.455459591823
ACAAAATG -0.0492230097151
ACAAACAA 0.0310963993489
ACAAACAC -0.0110896019987
ACAAACAG 0.215874834858
ACAAACAT 0.206117384291

ACAAACCA 0.119476205343
ACAAACCC -0.181105927893
ACAAACCG -0.128589528568
ACAAACCT 0.0589556433071
ACAAACGA 0.00210265361781
ACAAACGC 0.10561739524
ACAAACGG -0.192161223634
ACAAACGT -0.0669861243914
ACAAACTA 0.0999332665999
ACAAACTC -0.035350777775
ACAAACTG -0.123931199731
ACAAAGAA 0.169680745465
ACAAAGAC 0.0640896687127
ACAAAGAG 0.0378601742238
ACAAAGAT 0.251988903504
ACAAAGCA 0.00518011124072
ACAAAGCC -0.132836435237
ACAAAGCG -0.351816591828
ACAAAGCT -0.16017844295
ACAAAGGA 0.0545738576042
ACAAAGGC -0.217444976551
ACAAAGGG -0.150526172098
ACAAAGGT -0.151116524623
ACAAAGTA 0.00291338170126
ACAAAGTC -0.0767296221842
ACAAAGTG -0.0948237015171
ACAAATAA 0.170557609952
ACAAATAC 0.103849028091
ACAAATAG 0.121894621895
ACAAATAT 0.291087786399
ACAAATCA 0.290832154469
ACAAATCC 0.350917133201
ACAAATCG 0.3235701266
ACAAATCT 0.397448941609
ACAAATGA 0.0657834330099
ACAAATGC -0.114716935291
ACAAATGG -0.237802978293
ACAAATGT -0.0758540910056
ACAAATTA 0.132221192237
ACAAATTC 0.218133179565
ACAAATTG 0.130089781053
ACAACAAA 0.0366554017065
ACAACAAC -0.235075604074
ACAACAAG -0.0238338456153
ACAACAAT 0.123278322373
ACAACACA 0.08573461895
ACAACACC 0.0442260388459
ACAACACG -0.0644709093165
ACAACACT -0.0586960128225
ACAACAGA -0.0104034218229
ACAACAGC -0.0769665865304
ACAACAGG 0.0168748129603
ACAACAGT -0.00970854652818
ACAACATA 0.194129157746

ACAACATC 0.0742216045246
ACAACATG 0.0338341095917
ACAACCAA 0.0244491064349
ACAACCAC -0.196974584191
ACAACCAG 0.0962453376933
ACAACCAT -0.0616224603442
ACAACCCA 0.0252525252525
ACAACCCC -0.233952011116
ACAACCCG -0.235116929317
ACAACCCT -0.0786792620822
ACAACCGA -0.0357009902464
ACAACCGC -0.0497045547918
ACAACCGG -0.12853773757
ACAACCGT -0.0583002091821
ACAACCTA -0.175938562319
ACAACCTC -0.0350605090536
ACAACCTG -0.192163803632
ACAACGAA 0.154541871346
ACAACGAC 0.0115639803508
ACAACGAG 0.101218298188
ACAACGAT 0.127522928589
ACAACGCA -0.0182979122373
ACAACGCC -0.109620639924
ACAACGCG -0.143755246235
ACAACGCT -0.129218392896
ACAACGGA 0.0856876159906
ACAACGGC -0.0468484191778
ACAACGGG -0.101615804491
ACAACGGT 0.00797202674712
ACAACGTA 0.117182105501
ACAACGTC -0.177942599585
ACAACGTG -0.178894706513
ACAACTAA 0.0997927052692
ACAACTAC 0.150589331038
ACAACTAG -0.196193960479
ACAACTAT 0.0262121625758
ACAACTCA -0.109466133876
ACAACTCC 0.0305581063157
ACAACTCG 0.154854273264
ACAACTCT -0.101529233675
ACAACTGA -0.0258234410114
ACAACTGC -0.188530834738
ACAACTGG -0.181095652106
ACAACTGT -0.0958244746124
ACAACTTA -0.0485281344204
ACAACTTC 0.0879827449034
ACAACTTG 0.0349715859741
ACAAGAAA 0.0502346663942
ACAAGAAC -0.00576747546445
ACAAGAAG -0.074142090968
ACAAGAAT 0.25648416458
ACAAGACA -0.0784267497792
ACAAGACC -0.096438035832
ACAAGACG -0.263265466953

ACAAGACT -0.0850090873922
ACAAGAGA 0.244837704284
ACAAGAGC -0.0579031116107
ACAAGAGG -0.0660255614841
ACAAGAGT 0.0617322248418
ACAAGATA 0.316894520558
ACAAGATC 0.293029349722
ACAAGATG -0.0781381396285
ACAAGCAA 0.0561552860832
ACAAGCAC -0.0274687415604
ACAAGCAG 0.0493470945892
ACAAGCAT -0.112958413636
ACAAGCCA -0.162403552733
ACAAGCCC -0.144065025698
ACAAGCCG -0.28319010867
ACAAGCCT -0.134735992504
ACAAGCGA 0.127424359006
ACAAGCGC -0.117508166894
ACAAGCGG -0.235339525318
ACAAGCGT -0.276539439415
ACAAGCTA 0.0378794772734
ACAAGCTC -0.169387153328
ACAAGCTG -0.333041160532
ACAAGGAA 0.0325766537888
ACAAGGAC -0.18220253702
ACAAGGAG -0.299898842059
ACAAGGAT 0.225452725453
ACAAGGCA -0.0789233689853
ACAAGGCC -0.203745915742
ACAAGGCG -0.184435985644
ACAAGGCT -0.264750031014
ACAAGGGA -0.110605607176
ACAAGGGC -0.263205142517
ACAAGGGG -0.0363068108186
ACAAGGGT -0.106853487853
ACAAGGTA -0.103175112954
ACAAGGTC -0.189909853186
ACAAGGTG -0.164178287616
ACAAGTAA 0.180044962519
ACAAGTAC -0.00398312445713
ACAAGTAG -0.0698729032062
ACAAGTAT 0.226304817214
ACAAGTCA -0.115312281979
ACAAGTCC -0.113655977127
ACAAGTCG -0.0931022597234
ACAAGTCT -0.102795559221
ACAAGTGA 0.0546086431735
ACAAGTGC -0.139019152145
ACAAGTGG -0.177081632946
ACAAGTGT -0.281452555714
ACAAGTTA 0.0275633760482
ACAAGTTC -0.239415790794
ACAAGTTG -0.101155537779
ACAATAAA 0.170802106857

ACAATAAC −0.0830055587265
ACAATAAG 0.172255352073
ACAATAAT 0.203974365525
ACAATACA 0.120706115924
ACAATACC 0.11922804347
ACAATACG 0.117240498383
ACAATACT 0.0878771333317
ACAATAGA 0.144112902637
ACAATAGC 0.0941607118092
ACAATAGG 0.0126809452285
ACAATAGT 0.115503310146
ACAATATA 0.232958854171
ACAATATC 0.385047772677
ACAATATG 0.00394195848741
ACAATCAA 0.226750509113
ACAATCAC 0.152556513361
ACAATCAG 0.288562667351
ACAATCAT 0.23236597479
ACAATCCA 0.333775902717
ACAATCCC 0.274046774047
ACAATCCG 0.34734321098
ACAATCCT 0.326007826008
ACAATCGA 0.225745028775
ACAATCGC 0.200716042714
ACAATCGG 0.0761932220462
ACAATCGT 0.0701524784129
ACAATCTA 0.382560235907
ACAATCTC 0.406615256719
ACAATCTG 0.396017229351
ACAATGAA −0.0792797303551
ACAATGAC 0.0615920819252
ACAATGAG −0.0545924452119
ACAATGAT 0.0619190820639
ACAATGCA 0.0702755096694
ACAATGCC −0.232842711537
ACAATGCG 0.00436997729977
ACAATGCT −0.00506429294308
ACAATGGA 0.135608730178
ACAATGGC −0.0596005216821
ACAATGGG 0.0124515555138
ACAATGGT 0.152691258752
ACAATGTA 0.123249125932
ACAATGTC −0.0241257490712
ACAATGTG −0.098579295549
ACAATTAA 0.0748969000679
ACAATTAC 0.223833784662
ACAATTAG 0.0897802565948
ACAATTAT 0.0325737392612
ACAATTCA 0.181210135756
ACAATTCC 0.147984350708
ACAATTCG 0.0010378444187
ACAATTCT 0.148860243936
ACAATTGA 0.152036816712
ACAATTGC −0.023477588082

ACAATTGG -0.0269760118245
ACAATTGT -0.0856299456751
ACAATTTA 0.228753384962
ACAATTTC 0.133035239096
ACAATTTG 0.0104360558906
ACACAAAA 0.147258829077
ACACAAAC -0.0426391423447
ACACAAAG -0.025464797696
ACACAAAT 0.197664606756
ACACAACA 0.0491800340285
ACACAACC 0.0233523441389
ACACAACG 0.138234475768
ACACAACT 0.0072688297896
ACACAAGA 0.0334700699985
ACACAAGC -0.112041701478
ACACAAGG -0.17113697078
ACACAAGT 0.00838594775297
ACACAATA 0.197936982139
ACACAATC 0.404698086516
ACACAATG 0.032834330099
ACACACAA 0.0798398575214
ACACACAC -0.044718101549
ACACACAG 0.0532474623384
ACACACAT 0.145625278279
ACACACCA -0.0700231337872
ACACACCC -0.081340463635
ACACACCG -0.245322703804
ACACACCT -0.226923872142
ACACACGA -0.0893151047482
ACACACGC 0.109753003692
ACACACGG -0.0663711260463
ACACACGT -0.0744487346992
ACACACTA -0.0479363661182
ACACACTC -0.0156529419064
ACACACTG -0.229150209369
ACACAGAA 0.00665313898236
ACACAGAC -0.0326759266153
ACACAGAG -0.0552208527624
ACACAGAT 0.249705628494
ACACAGCA 0.00506485259447
ACACAGCC -0.123540431233
ACACAGCG -0.117436145943
ACACAGCT -0.118450650975
ACACAGGA -0.0323036535158
ACACAGGC -0.00561520550645
ACACAGGG -0.0746297261449
ACACAGGT -0.160932963597
ACACAGTA 0.0433808464111
ACACAGTC -0.0761675442195
ACACAGTG -0.271409237622
ACACATAA 0.166401465661
ACACATAC 0.107842001781
ACACATAG 0.0643246552337
ACACATAT 0.172880155909

ACACATCA -0.00420944360338
ACACATCC 0.0479267145934
ACACATCG 0.136872185281
ACACATCT 0.0316905469223
ACACATGA 0.228350474077
ACACATGC -0.0151726141212
ACACATGG -0.292753781825
ACACATGT -0.276483155962
ACACATTA 0.193130807355
ACACATTC 0.134845952278
ACACATTG -0.128639157591
ACACCAAA 0.0198236534966
ACACCAAC -0.234222200166
ACACCAAG -0.213454738615
ACACCAAT -0.0674211513616
ACACCACA 0.179650810566
ACACCACC -0.0346861017642
ACACCACG -0.0899877869582
ACACCACT -0.0794448442536
ACACCAGA 0.0378033574034
ACACCAGC -0.116383708251
ACACCAGG -0.259630521025
ACACCAGT -0.197279869693
ACACCATA 0.178789515558
ACACCATC -0.148281890706
ACACCATG -0.0995459395082
ACACCCAA -0.238298564842
ACACCCAC -0.0969949507481
ACACCCAG -0.0485268931967
ACACCCAT 0.167983912761
ACACCCCA -0.123357478862
ACACCCCC -0.151941018833
ACACCCCG -0.167108883002
ACACCCCT -0.23846187526
ACACCCGA -0.158557589434
ACACCCGC -0.122137025733
ACACCCGG -0.350096319997
ACACCCGT -0.147441202011
ACACCCTA 0.0653321460114
ACACCCTC -0.0542944315088
ACACCCTG -0.150360121719
ACACCGAA 0.116177747933
ACACCGAC -0.288622365908
ACACCGAG -0.193616769075
ACACCGAT 0.169835124381
ACACCGCA -0.000499989051335
ACACCGCC -0.0157960044671
ACACCGCG -0.148483570288
ACACCGCT -0.044450105029
ACACCGGA 0.0909286657956
ACACCGGC -0.129301526644
ACACCGGG -0.0305619332172
ACACCGGT -0.199337325046
ACACCGTA 0.130270869981

ACACCGTC -0.257684769395
ACACCGTG -0.224179811465
ACACCTAA -0.123546881795
ACACCTAC 0.0287314876317
ACACCTAG -0.150003170586
ACACCTAT -0.10513405968
ACACCTCA 0.112037692605
ACACCTCC -0.0149281512918
ACACCTCG -0.0641745071276
ACACCTCT -0.0843639271484
ACACCTGA 0.0344600799146
ACACCTGC -0.210389944675
ACACCTGG -0.238358902259
ACACCTGT -0.162310179003
ACACCTTA -0.0432083447072
ACACCTTC 0.00171917953639
ACACCTTG -0.195868005027
ACACGAAA 0.14114251993
ACACGAAC -0.238164003041
ACACGAAG 0.0843945023102
ACACGAAT 0.232035064926
ACACGACA -0.097578294548
ACACGACC -0.176981743885
ACACGACG -0.205891433124
ACACGACT -0.24855549961
ACACGAGA 0.291660036642
ACACGAGC -0.00579827970989
ACACGAGG -0.0130340211528
ACACGAGT -0.0862429336887
ACACGATA 0.218975496887
ACACGATC 0.341750841751
ACACGATG -0.00521521426538
ACACGCAA 0.135527850676
ACACGCAC -0.101092557212
ACACGCAG -0.0359730527778
ACACGCAT 0.152080179666
ACACGCCA -0.28425827427
ACACGCCC -0.138021156407
ACACGCCG -0.0766344857254
ACACGCCT -0.197914857365
ACACGCGA 0.098124295094
ACACGCGC -0.107345193493
ACACGCGG -0.168264286916
ACACGCGT -0.215512321016
ACACGCTA 0.0204074598014
ACACGCTC -0.0282885684901
ACACGCTG -0.156759090072
ACACGGAA 0.0264633998189
ACACGGAC -0.212406950426
ACACGGAG -0.169990102749
ACACGGAT 0.157092354062
ACACGGCA -0.159710677154
ACACGGCC -0.0685539732707
ACACGGCG -0.106510097774

ACACGGCT -0.169823148474
ACACGGGA 0.015259682067
ACACGGGC -0.203621873965
ACACGGGG 0.0775820415766
ACACGGGT -0.251337782961
ACACGGTA -0.0252001780983
ACACGGTC -0.213938171592
ACACGGTG -0.251321044928
ACACGTAA 0.170167949222
ACACGTAC -0.0354638384941
ACACGTAG 0.0154940646069
ACACGTAT 0.23663642402
ACACGTCA -0.034276297828
ACACGTCC -0.12790106985
ACACGTCG -0.165128884999
ACACGTCT -0.157708099492
ACACGTGA -0.0609935548857
ACACGTGC -0.232213745193
ACACGTGG -0.084197662589
ACACGTGT -0.193435082527
ACACGTTA -0.106901645494
ACACGTTC -0.109634720947
ACACGTTG -0.27275254541
ACACTAAA 0.0953336256367
ACACTAAC -0.0463767502485
ACACTAAG -0.074320270359
ACACTAAT 0.202675126918
ACACTACA -0.00417083750417
ACACTACC -0.0528673636139
ACACTACG -0.177265746278
ACACTACT -0.0614595311565
ACACTAGA -0.00192011812294
ACACTAGC -0.0424457776013
ACACTAGG -0.0691583395984
ACACTAGT 0.0834602161996
ACACTATA 0.103529849711
ACACTATC -0.0320555024343
ACACTATG 0.000945187862474
ACACTCAA -0.0346145043115
ACACTCAC -0.112401448655
ACACTCAG -0.153117943373
ACACTCAT -0.0422333817508
ACACTCCA -0.114818307401
ACACTCCC -0.0402480333524
ACACTCCG -0.0476892449535
ACACTCCT -0.0748101595652
ACACTCGA -0.141119397212
ACACTCGC -0.122821398072
ACACTCGG -0.0836606659339
ACACTCGT -0.210267594596
ACACTCTA 0.209250162405
ACACTCTC -0.180113574155
ACACTCTG -0.0413708995844
ACACTGAA 0.0761830032919

ACACTGAC -0.00835675131202
ACACTGAG -0.198307380417
ACACTGAT 0.217011777618
ACACTGCA 0.0212646555285
ACACTGCC -0.183773720401
ACACTGCG -0.148875967929
ACACTGCT -0.0072381683646
ACACTGGA -0.164130362383
ACACTGGC -0.136115793545
ACACTGGG -0.0115158062232
ACACTGGT -0.182299519644
ACACTGTA 0.022068859806
ACACTGTC -0.0341165376491
ACACTGTG -0.172339382122
ACACTTAA 0.0630120478605
ACACTTAC 0.0904254659156
ACACTTAG -0.117892702241
ACACTTAT 0.0861867377019
ACACTTCA 0.0507978261409
ACACTTCC 0.0262147544214
ACACTTCG 0.031705258978
ACACTTCT -0.107294730772
ACACTTGA -0.0190074805679
ACACTTGC -0.214983996229
ACACTTGG -0.141492473937
ACACTTTA 0.0732823368831
ACACTTTC -0.0762300453791
ACACTTTG -0.0801263851199
ACAGAAAA 0.179455194412
ACAGAAAC -0.0261625261625
ACAGAAAG 0.0163521966672
ACAGAAAT 0.23179565411
ACAGAACA -0.0344844926561
ACAGAACC -0.223190687941
ACAGAACG -0.093058989318
ACAGAACT 0.000941713062925
ACAGAAGA 0.16224075315
ACAGAAGC -0.182934764926
ACAGAAGG -0.305063069504
ACAGAAGT -0.0759723867372
ACAGAATA 0.235510707993
ACAGAATC 0.453799529557
ACAGAATG -0.143799364457
ACAGACAA 0.281597024021
ACAGACAC 0.0103853884905
ACAGACAG -0.120011195769
ACAGACAT 0.0243039182433
ACAGACCA -0.204859818753
ACAGACCC -0.134268850954
ACAGACCG 0.0459398792732
ACAGACCT -0.180325751439
ACAGACGA -0.285574087235
ACAGACGC -0.0156003883127
ACAGACGG -0.0858277572379

ACAGACGT -0.00174968356787
ACAGACTA 0.0804938577987
ACAGACTC -0.415265290164
ACAGACTG -0.237586199348
ACAGAGAA 0.279753728021
ACAGAGAC 0.00424151295957
ACAGAGAG -0.0337563038881
ACAGAGAT 0.263405278557
ACAGAGCA -0.170385163501
ACAGAGCC -0.216191465794
ACAGAGCG -0.220334641561
ACAGAGCT -0.177614131775
ACAGAGGA -0.0613062001075
ACAGAGGC -0.281453609947
ACAGAGGG -0.239438366699
ACAGAGGT -0.269299790772
ACAGAGTA 0.0203813055189
ACAGAGTC -0.257474617604
ACAGAGTG -0.287457651414
ACAGATAA 0.223026056359
ACAGATAC 0.281315751013
ACAGATAG -0.0436172119508
ACAGATAT 0.424136120251
ACAGATCA 0.116385042663
ACAGATCC 0.269243009277
ACAGATCG 0.343162558484
ACAGATCT 0.359503806486
ACAGATGA 0.0498328606754
ACAGATGC -0.243121555263
ACAGATGG -0.0873431438238
ACAGATGT -0.193892431147
ACAGATTA 0.353591527193
ACAGATTC 0.409139954202
ACAGATTG 0.401154598124
ACAGCAAA 0.147694526482
ACAGCAAC -0.0250815553846
ACAGCAAG 0.0575161938798
ACAGCAAT 0.132632862054
ACAGCACA -0.244722958033
ACAGCACC -0.237998722179
ACAGCACG -0.0927386216168
ACAGCACT -0.0250512220209
ACAGCAGA 0.0807507864791
ACAGCAGC -0.0585592763207
ACAGCAGG -0.203363325233
ACAGCAGT -0.288794065841
ACAGCATA 0.277143534719
ACAGCATC 0.165335795567
ACAGCATG -0.188030900805
ACAGCCAA 0.0416876932028
ACAGCCAC -0.208433109048
ACAGCCAG -0.251313878733
ACAGCCAT -0.290246621883
ACAGCCCA -0.30063813162

ACAGCCCC -0.251592057276
ACAGCCCG -0.24923714307
ACAGCCCT -0.125187969925
ACAGCCGA -0.00839178704116
ACAGCCGC -0.311244523665
ACAGCCGG -0.214437282095
ACAGCCGT -0.217044653581
ACAGCCTA -0.21507448723
ACAGCCTC -0.176147516256
ACAGCCTG -0.323898356941
ACAGCGAA -0.00487653555032
ACAGCGAC -0.201912819113
ACAGCGAG -0.195181696953
ACAGCGAT 0.0669029145347
ACAGCGCA -0.103689551646
ACAGCGCC 0.0339755096189
ACAGCGCG 0.0247169770005
ACAGCGCT -0.00636096085191
ACAGCGGA -0.0565193146768
ACAGCGGC -0.189671571182
ACAGCGGG -0.033875678966
ACAGCGGT -0.241785558459
ACAGCGTA -0.10671881089
ACAGCGTC -0.286587599096
ACAGCGTG -0.234279214402
ACAGCTAA -0.103963416859
ACAGCTAC -0.133022907035
ACAGCTAG -0.0272165192223
ACAGCTAT -0.0180528893528
ACAGCTCA -0.246160525989
ACAGCTCC -0.0573689611332
ACAGCTCG 0.144258884842
ACAGCTCT -0.0409720955016
ACAGCTGA -0.211676054824
ACAGCTGC -0.263251017457
ACAGCTGG -0.218167806756
ACAGCTGT -0.195077620965
ACAGCTTA -0.132931366953
ACAGCTTC -0.194782967903
ACAGCTTG -0.21928961645
ACAGGAAA 0.179464588555
ACAGGAAC -0.279968211387
ACAGGAAG -0.109298543054
ACAGGAAT 0.300500718962
ACAGGACA -0.104379674528
ACAGGACC -0.0832609468973
ACAGGACG -0.0670441574233
ACAGGACT -0.302515066022
ACAGGAGA 0.133263505179
ACAGGAGC 0.0226411100487
ACAGGAGG 0.00315925327453
ACAGGAGT -0.125717284457
ACAGGATA 0.303887246597
ACAGGATC 0.286463070152

ACAGGATG -0.205549076532
ACAGGCAA 0.29841861677
ACAGGCAC -0.0792026970818
ACAGGCAG -0.0517229299224
ACAGGCAT -0.01606427364
ACAGGCCA -0.133634429721
ACAGGCCC -0.0406731739074
ACAGGCCG -0.239424913787
ACAGGCCT 0.0147351207957
ACAGGCGA 0.00932906350302
ACAGGCGC -0.174560250081
ACAGGCGG -0.138969536051
ACAGGCGT -0.247818067467
ACAGGCTA -0.0599937343358
ACAGGCTC -0.281379383817
ACAGGCTG -0.309827628507
ACAGGGAA 0.0822709762104
ACAGGGAC -0.0990705586849
ACAGGGAG -0.285481548063
ACAGGGAT 0.0349649277753
ACAGGGCA -0.178382886049
ACAGGGCC -0.304722692828
ACAGGGCG -0.0619844602771
ACAGGGCT -0.14567395829
ACAGGGGA -0.0207360422764
ACAGGGGC -0.270097127053
ACAGGGGG -0.15804818739
ACAGGGGT -0.124589668626
ACAGGGTA -0.04895895518
ACAGGGTC -0.263583724775
ACAGGGTG -0.272534425009
ACAGGTAA 0.067309601211
ACAGGTAC -0.0421750699701
ACAGGTAG -0.0770901059632
ACAGGTAT 0.194500120435
ACAGGTCA -0.180585126953
ACAGGTCC -0.265972549571
ACAGGTCG -0.195171503158
ACAGGTCT -0.253986545364
ACAGGTGA -0.154417056036
ACAGGTGC -0.376208680173
ACAGGTGG -0.265358163806
ACAGGTTA -0.110181439102
ACAGGTTC -0.0734370871851
ACAGGTTG -0.239687603525
ACAGTAAA 0.128703811351
ACAGTAAC -0.0468140316625
ACAGTAAG -0.0751127104273
ACAGTAAT 0.196550545035
ACAGTACA 0.0288585196095
ACAGTACC -0.157930118319
ACAGTACG -0.0694027402095
ACAGTACT 0.0206469931315
ACAGTAGA 0.00946475624621

ACAGTAGC -0.0467986472771
ACAGTAGG -0.00232578659012
ACAGTAGT -0.0740253863054
ACAGTATA 0.287984204725
ACAGTATC 0.32732319096
ACAGTATG -0.0144651606928
ACAGTCAA 0.107523922834
ACAGTCAC -0.161345596608
ACAGTCAG -0.312064346489
ACAGTCAT -0.00888228724918
ACAGTCCA -0.153820836575
ACAGTCCC -0.0935088459343
ACAGTCCG -0.046827384148
ACAGTCCT -0.208653192014
ACAGTCGA -0.0290249118632
ACAGTCGC -0.204641750505
ACAGTCGG -0.144900502657
ACAGTCGT -0.228946287428
ACAGTCTA 0.102344474209
ACAGTCTC -0.232028090027
ACAGTCTG -0.0776818283332
ACAGTGAA -0.158905624068
ACAGTGAC -0.121938600524
ACAGTGAG -0.189216148414
ACAGTGAT 0.186920724364
ACAGTGCA 0.101711065105
ACAGTGCC -0.185585644753
ACAGTGCG 0.17144077137
ACAGTGCT -0.118584785744
ACAGTGGA -0.0410760448004
ACAGTGGC 0.00297774138316
ACAGTGGG -0.124209845713
ACAGTGGT -0.124524020261
ACAGTGTA -0.0447712499398
ACAGTGTC -0.278078564164
ACAGTGTG -0.0882674849129
ACAGTTAA 0.152451406174
ACAGTTAC 0.0449004766319
ACAGTTAG -0.0923853248956
ACAGTTAT 0.0730756117665
ACAGTTCA -0.225400571228
ACAGTTCC -0.025009596901
ACAGTTCG -0.0227728235498
ACAGTTCT -0.0778053832646
ACAGTTGA -0.043384487825
ACAGTTGC -0.205282455405
ACAGTTGG -0.0555169532885
ACAGTTTA -0.0770764143851
ACAGTTTC -0.106243089505
ACAGTTTG -0.0616563951965
ACATAAAA 0.171414494573
ACATAAAC -0.0909432640161
ACATAAAG -0.0534841303323
ACATAAAT 0.197091030424

ACATAACA -0.192011812294
ACATAACC -0.106030272697
ACATAACG -0.0324776037918
ACATAACT 0.0945957387794
ACATAAGA -0.0915228133691
ACATAAGC -0.136782186384
ACATAAGG -0.146953894169
ACATAAGT -0.0399881754414
ACATAATA 0.110177670784
ACATAATC 0.342332690818
ACATAATG 0.0419020021459
ACATACAA 0.109435882163
ACATACAC 0.227016196726
ACATACAG -0.00750129559207
ACATACAT 0.0170412326737
ACATACCA -0.269588750432
ACATACCC -0.21751711512
ACATACCG -0.028096595595
ACATACCT 0.119140382659
ACATACGA 0.0656033364037
ACATACGC 0.0676585676586
ACATACGG 0.0427907246089
ACATACGT -0.0800221469515
ACATACTA -0.0214420355075
ACATACTC -0.15121703099
ACATACTG 0.0657146193879
ACATAGAA 0.00661649750602
ACATAGAC 0.119074034875
ACATAGAG 0.0347033569861
ACATAGAT 0.233579556652
ACATAGCA -0.0378101209463
ACATAGCC -0.199455713404
ACATAGCG -0.0265391311617
ACATAGCT 0.0309416582119
ACATAGGA 0.131248950753
ACATAGGC -0.15018513464
ACATAGGG -0.00608028516253
ACATAGGT -0.0447348174621
ACATAGTA 0.0154943296027
ACATAGTC -0.0824339613001
ACATAGTG -0.0497845166307
ACATATAA 0.160550498894
ACATATAC 0.0289008016281
ACATATAG -0.0635351050707
ACATATAT 0.271707571283
ACATATCA 0.255879798253
ACATATCC 0.194673879108
ACATATCG 0.268572980694
ACATATCT 0.352587169624
ACATATGA 0.0506054611943
ACATATGC -0.0821922307224
ACATATGG -0.0491938132742
ACATATGT -0.177224661226
ACATATTA 0.160145296176

ACATATTC 0.132552130618
ACATATTG -0.0727849061182
ACATCAAA 0.027610254883
ACATCAAC -0.155539126894
ACATCAAG -0.105190833064
ACATCAAT -0.0961388385631
ACATCACA -0.0468738824347
ACATCACC -0.0476860156732
ACATCACG 0.000798318980137
ACATCACT 0.0831069392641
ACATCAGA 0.0382845631118
ACATCAGC 0.00101892526135
ACATCAGG 0.0111961051948
ACATCAGT -0.0464610264009
ACATCATA 0.192905026238
ACATCATC -0.194655418809
ACATCATG -0.0332903193786
ACATCCAA 0.0658385658386
ACATCCAC -0.0055830894214
ACATCCAG 0.0743319076652
ACATCCAT 0.0615044926024
ACATCCCA 0.000998094796948
ACATCCCC -0.179554003185
ACATCCCG -0.0702967962876
ACATCCCT 0.0293168762728
ACATCCGA 0.045239155347
ACATCCGC 0.0769819406183
ACATCCGG 0.0263886476008
ACATCCGT 0.0305329080385
ACATCCTA -0.00523612179567
ACATCCTC -0.233628306386
ACATCCTG -0.263057906158
ACATCGAA 0.0586023663715
ACATCGAC -0.168491821267
ACATCGAG -0.104991749175
ACATCGAT 0.276831928347
ACATCGCA -0.158044276925
ACATCGCC -0.171683333747
ACATCGCG 0.153152445161
ACATCGCT 0.00163386527023
ACATCGGA 0.0788734553258
ACATCGGC -0.123332940476
ACATCGGG -0.0930962602675
ACATCGGT -0.268591805959
ACATCGTA 0.0892060757794
ACATCGTC -0.118707619541
ACATCGTG -0.179260031266
ACATCTAA 0.15427410882
ACATCTAC 0.045173272446
ACATCTAG 0.12858769516
ACATCTAT 0.146234024072
ACATCTCA 0.142548884973
ACATCTCC 0.13151232677
ACATCTCG 0.0933261084776

ACATCTCT 0.146384385743
ACATCTGA -0.00800111406172
ACATCTGC -0.13225165139
ACATCTGG -0.00384882082874
ACATCTTA -0.00742634465983
ACATCTTC 0.210120588908
ACATCTTG 0.0633953512741
ACATGAAA 0.175457826973
ACATGAAC -0.187268549942
ACATGAAG 0.01578575821
ACATGAAT 0.0423329665754
ACATGACA 0.0308113518119
ACATGACC -0.327164628348
ACATGACG -0.236431584171
ACATGACT 0.0476974956753
ACATGAGA 0.191621351357
ACATGAGC -0.00801214437578
ACATGAGG -0.0546512493487
ACATGAGT 0.0072152041849
ACATGATA 0.155694261755
ACATGATC 0.105664109545
ACATGATG -0.060830803255
ACATGCAA 0.189233082487
ACATGCAC 0.0890951293037
ACATGCAG -0.0399808763312
ACATGCAT 0.00285880743904
ACATGCCA -0.167250908104
ACATGCCC -0.063942395422
ACATGCCG 0.0190047232631
ACATGCCT 0.0593858321131
ACATGCGA 0.0543256755378
ACATGCGC -0.223407686773
ACATGCGG -0.0916801321884
ACATGCGT -0.0953410798219
ACATGCTA 0.0289531813948
ACATGCTC -0.229842575877
ACATGCTG -0.0755355181193
ACATGGAA 0.078207776472
ACATGGAC -0.0643292482646
ACATGGAG -0.152491586757
ACATGGAT 0.211267636475
ACATGGCA -0.106351569284
ACATGGCC -0.23061243554
ACATGGCG -0.146411505681
ACATGGCT -0.0523564298049
ACATGGGA -0.0709170842352
ACATGGGC -0.10313443328
ACATGGGG -0.126396659257
ACATGGGT -0.248625826438
ACATGGTA 0.0239228338066
ACATGGTC -0.198309112815
ACATGGTG -0.159099367324
ACATGTAA 0.060911804851
ACATGTAC 0.0684914509765

ACATGTAG 0.00229388263904
ACATGTAT 0.155713564804
ACATGTCA -0.165286841068
ACATGTCC -0.0245851912519
ACATGTCG -0.0118853622961
ACATGTCT -0.0600829189346
ACATGTGA 0.18939187121
ACATGTGC -0.170487507573
ACATGTGG -0.168222226358
ACATGTTA -0.16763720747
ACATGTTC 0.0249242717313
ACATGTTG -0.124956225614
ACATTAAA 0.0742602106238
ACATTAAC -0.00661080414297
ACATTAAG 0.0579794670704
ACATTAAT 0.175904907192
ACATTACA 0.0522133703952
ACATTACC -0.000660327694966
ACATTACG 0.0599579373154
ACATTACT 0.00940052407612
ACATTAGA 0.198502604082
ACATTAGC 0.00950753157803
ACATTAGG -0.0736116969802
ACATTAGT 0.0294026809178
ACATTATA 0.272583403111
ACATTATC 0.0739071407195
ACATTATG 0.118309088122
ACATTCAA -0.0117514208423
ACATTCAC 0.0504234116674
ACATTCAG 0.074839302112
ACATTCAT -0.0269227681146
ACATTCCA -0.0313399982192
ACATTCCC 0.0143668386824
ACATTCCG -0.0441888169161
ACATTCCT 0.139016426895
ACATTCGA -0.0510868946472
ACATTCGC -0.00945161784778
ACATTCGG -0.131444657071
ACATTCGT -0.084909819493
ACATTCTA 0.119109467594
ACATTCTC 0.048807760929
ACATTCTG 0.0524725827756
ACATTGAA -0.00762056822663
ACATTGAC -0.0633717387768
ACATTGAG -0.130923035385
ACATTGAT -0.0100005109377
ACATTGCA -0.108716597447
ACATTGCC -0.0669790707281
ACATTGCG 0.0461883220623
ACATTGCT 0.0173873230145
ACATTGGA 0.0690483872302
ACATTGGC -0.180161703371
ACATTGGG -0.163915006574
ACATTGGT -0.191024686763

ACATTGTA -0.013440365115
ACATTGTC -0.0173686082777
ACATTGTG -0.099908031211
ACATTTAA 0.0705402372069
ACATTTAC 0.116029252393
ACATTTAG 0.0729845331854
ACATTTAT 0.217974058962
ACATTTCA 0.116187309229
ACATTTCC 0.0414588141861
ACATTTCG 0.243749258901
ACATTTCT 0.120720714145
ACATTTGA 0.169051972082
ACATTTGC -0.155069007357
ACATTTGG -0.0638353309015
ACATTTTA 0.0712909769958
ACATTTTC 0.0746166142347
ACATTTTG -0.199970353189
ACCAAAAA 0.056366283639
ACCAAAAC -0.0754968869295
ACCAAAAG -0.173098673563
ACCAAAAT 0.20847645672
ACCAAACA -0.162788881835
ACCAAACC -0.173856906586
ACCAAACG -0.0611867916153
ACCAAACT -0.0968611298823
ACCAAAGA 0.113665844142
ACCAAAGC -0.123562400468
ACCAAAGG -0.109955857741
ACCAAAGT -0.251932247625
ACCAAATA 0.0533908016613
ACCAAATC 0.409372906172
ACCAAATG 0.0743626051984
ACCAACAA 0.128035749248
ACCAACAC -0.156290928743
ACCAACAG -0.226182304374
ACCAACAT -0.0690130615561
ACCAACCA -0.105384999324
ACCAACCC -0.319204645768
ACCAACCG -0.0741326532847
ACCAACCT -0.0221633677168
ACCAACGA 0.0672482688442
ACCAACGC -0.190221571595
ACCAACGG -0.202368496703
ACCAACGT -0.252852737592
ACCAACTA -0.0643870888637
ACCAACTC -0.154395524992
ACCAACTG 0.0745497563679
ACCAAGAA 0.0877053787143
ACCAAGAC -0.0137561804228
ACCAAGAG -0.282854094822
ACCAAGAT 0.334745224557
ACCAAGCA -0.0106492396355
ACCAAGCC -0.106357407124
ACCAAGCG -0.358466872819

ACCAAGCT -0.0242708273011
ACCAAGGA -0.25863105531
ACCAAGGC 0.0339430523419
ACCAAGGG -0.224283102983
ACCAAGGT -0.131553482371
ACCAAGTA -0.1606090065
ACCAAGTC -0.185754020332
ACCAAGTG -0.024339766764
ACCAATAA 0.141396217154
ACCAATAC 0.0598311755662
ACCAATAG -0.0307787125969
ACCAATAT 0.181872659723
ACCAATCA 0.00874841783933
ACCAATCC 0.161951207406
ACCAATCG 0.137643152795
ACCAATCT 0.1502131206
ACCAATGA -0.0138913017701
ACCAATGC -0.203206687903
ACCAATGG -0.138817898861
ACCAATTA 0.101252527317
ACCAATTC -0.0178275414779
ACCAATTG 0.109964745298
ACCACAAA -0.14251776792
ACCACAAC -0.0932732211055
ACCACAAG -0.261257783235
ACCACAAT 0.262837217383
ACCACACA -0.0383619336803
ACCACACC -0.0889128484163
ACCACACG -0.175278939265
ACCACACT -0.0878796652466
ACCACAGA -0.00449114252973
ACCACAGC -0.214611192431
ACCACAGG 0.0573556783428
ACCACAGT -0.0734863086031
ACCACATA 0.094023775842
ACCACATC 0.115187988584
ACCACATG -0.152296307325
ACCACCAA -0.0778083027637
ACCACCAC -0.0251801606521
ACCACCAG -0.193544079336
ACCACCAT -0.0656675959706
ACCACCCA -0.0703390807701
ACCACCCC -0.178214556715
ACCACCCG -0.128784572562
ACCACCCT -0.191103143646
ACCACCGA -0.0616547137504
ACCACCGC -0.230603555214
ACCACCGG -0.225705720345
ACCACCGT -0.181117009243
ACCACCTA -0.274334442899
ACCACCTC -0.27346271627
ACCACCTG -0.286774372885
ACCACGAA 0.0270366342343
ACCACGAC -0.131874508247

ACCACGAG -0.110128036877
ACCACGAT 0.150354199514
ACCACGCA -0.0929938416221
ACCACGCC -0.197641911284
ACCACGCG -0.075666226287
ACCACGCT -0.103799746333
ACCACGGA -0.174385587672
ACCACGGC -0.342187771355
ACCACGGG -0.128691157861
ACCACGGT -0.224723044749
ACCACGTA -0.0527415863562
ACCACGTC -0.204233893754
ACCACGTG -0.0145227872501
ACCACTAA 0.162558627193
ACCACTAC -0.208241191213
ACCACTAG -0.142084660023
ACCACTAT -0.00207831848525
ACCACTCA -0.0197850066287
ACCACTCC -0.149600112481
ACCACTCG 0.166604380926
ACCACTCT 0.00472786836423
ACCACTGA 0.0510197891765
ACCACTGC -0.179221359271
ACCACTGG -0.231809830142
ACCACTTA -0.0649113227585
ACCACTTC -0.240743038153
ACCACTTG -0.0566515298146
ACCAGAAA 0.157756780902
ACCAGAAC -0.312754445299
ACCAGAAG -0.31709959395
ACCAGAAT 0.24523559372
ACCAGACA -0.197710837219
ACCAGACC -0.374361462308
ACCAGACG -0.237743174401
ACCAGACT -0.267169206118
ACCAGAGA 0.112706291103
ACCAGAGC -0.0877025383986
ACCAGAGG 0.0259639805094
ACCAGAGT -0.198653532822
ACCAGATA 0.264519025131
ACCAGATC 0.287721436757
ACCAGATG -0.10825529368
ACCAGCAA 0.167165049507
ACCAGCAC -0.125049811483
ACCAGCAG -0.275522325331
ACCAGCAT 0.0379896790581
ACCAGCCA -0.172670884506
ACCAGCCC -0.240841347413
ACCAGCCG -0.0929836897327
ACCAGCCT -0.019611005928
ACCAGCGA 0.0770453649242
ACCAGCGC -0.227939169713
ACCAGCGG -0.287480601244
ACCAGCGT -0.186034683802

ACCAGCTA -0.167212504652
ACCAGCTC -0.16678781987
ACCAGCTG -0.221711979127
ACCAGGAA -0.1597674035
ACCAGGAC -0.277176348
ACCAGGAG -0.296285730823
ACCAGGAT 0.10402764903
ACCAGGCA -0.333505732956
ACCAGGCC -0.141372038209
ACCAGGCG -0.274979390243
ACCAGGCT -0.16828614005
ACCAGGGA -0.241803392324
ACCAGGGC -0.324761012539
ACCAGGGG -0.16273342236
ACCAGGGT -0.421813046773
ACCAGGTA -0.159465463563
ACCAGGTC -0.333187659506
ACCAGGTG -0.253570335822
ACCAGTAA 0.0512950967496
ACCAGTAC -0.225325568763
ACCAGTAG -0.0363848697182
ACCAGTAT 0.102994178752
ACCAGTCA -0.302797835179
ACCAGTCC -0.319009913127
ACCAGTCG -0.302200058992
ACCAGTCT -0.199428799984
ACCAGTGA -0.233254218319
ACCAGTGC -0.172135262739
ACCAGTGG -0.160012172499
ACCAGTTA -0.0476865523488
ACCAGTTC -0.337531250086
ACCAGTTG -0.190735364263
ACCATAAA 0.211653802563
ACCATAAC -0.0044852014549
ACCATAAG -0.201844919193
ACCATAAT 0.114305765821
ACCATACA -0.0583168592548
ACCATACC -0.183443503695
ACCATACG -0.168720380617
ACCATACT 0.126672292685
ACCATAGA 0.00798136337693
ACCATAGC 0.065778851558
ACCATAGG -0.0308553854059
ACCATAGT 0.0668037183189
ACCATATA 0.144147938366
ACCATATC 0.30316128801
ACCATATG -0.0633190514076
ACCATCAA -0.0552024408225
ACCATCAC -0.0760675065
ACCATCAG -0.183383644158
ACCATCAT 0.0662722714101
ACCATCCA -0.00351452157982
ACCATCCC -0.245495923932
ACCATCCG -0.0216068709923

ACCATCCT -0.159262292144
ACCATCGA -0.0735532095245
ACCATCGC -0.325867118448
ACCATCGG -0.285425023088
ACCATCGT -0.0419454991135
ACCATCTA -0.0572914512308
ACCATCTC -0.167135361957
ACCATCTG -0.0983722984168
ACCATGAA 0.00854159945069
ACCATGAC -0.234597156398
ACCATGAG -0.25207294535
ACCATGAT 0.191803980445
ACCATGCA -0.127841606216
ACCATGCC -0.101932124563
ACCATGCG -0.0174733962613
ACCATGCT 0.167433273494
ACCATGGA -0.257206827259
ACCATGGC -0.278092310027
ACCATGGG -0.282420208924
ACCATGGT -0.32541217838
ACCATGTA 0.0588943307811
ACCATGTC -0.275451352049
ACCATGTG -0.0797948463416
ACCATTAA 0.114169032795
ACCATTAC 0.067462582839
ACCATTAG 0.047453789878
ACCATTAT 0.130971584564
ACCATTCA -0.0548141281578
ACCATTCC -0.090102163213
ACCATTCG -0.147595253656
ACCATTCT 0.0279053157841
ACCATTGA -0.0495071400108
ACCATTGC -0.221246063766
ACCATTGG -0.102472831375
ACCATTTA 0.054531135729
ACCATTTC -0.105477657829
ACCATTTG -0.156979293343
ACCCAAAA -0.0431822660818
ACCCAAAC 0.0221184937556
ACCCAAAG -0.314527323942
ACCCAAAT 0.0630655402996
ACCCAACA -0.167894863079
ACCCAACC -0.166236919825
ACCCAACG -0.0649570528464
ACCCAACT -0.327711484339
ACCCAAGA -0.0695608614269
ACCCAAGC -0.246576626499
ACCCAAGG -0.254937232814
ACCCAAGT -0.187447370841
ACCCAATA -0.1853038245
ACCCAATC -0.0120768151071
ACCCAATG -0.175545928652
ACCCACAA -0.0015450682618
ACCCACAC -0.0367196338766

ACCCACAG -0.367039009964
ACCCACAT 0.0511505425266
ACCCACCA -0.0972418699691
ACCCACCC 0.00318760254236
ACCCACCG 0.0261763140551
ACCCACCT -0.140370030353
ACCCACGA 0.0387108311497
ACCCACGC -0.165384774428
ACCCACGG -0.0594819575761
ACCCACGT -0.275101011059
ACCCACTA -0.163258202583
ACCCACTC -0.258696423073
ACCCACTG -0.18907604722
ACCCAGAA 0.154833897258
ACCCAGAC 0.00169833436241
ACCCAGAG -0.00299581165402
ACCCAGAT 0.190977478856
ACCCAGCA -0.312334094123
ACCCAGCC -0.218129815711
ACCCAGCG -0.293711247988
ACCCAGCT -0.0381173337303
ACCCAGGA -0.066433933923
ACCCAGGC -0.1779205616
ACCCAGGG -0.212052174728
ACCCAGGT -0.338183115839
ACCCAGTA -0.0942707595929
ACCCAGTC -0.23975407145
ACCCAGTG -0.184394875485
ACCCATAA 0.130556176011
ACCCATAC -0.10908842727
ACCCATAG -0.222894181863
ACCCATAT 0.20729407093
ACCCATCA 0.15961329314
ACCCATCC -0.190585754212
ACCCATCG 0.0730925600169
ACCCATCT -0.023156765581
ACCCATGA -0.186696738285
ACCCATGC -0.305761673576
ACCCATGG -0.238575224687
ACCCATTA 0.0769552491551
ACCCATTC -0.122297764875
ACCCATTG -0.00669255417765
ACCCCAAA -0.115783583221
ACCCCAAC -0.125279521773
ACCCCAAG -0.380889466043
ACCCCAAT -0.0658270256856
ACCCCACA -0.280270164179
ACCCCACC -0.197574208702
ACCCCACG -0.145259584654
ACCCCACT -0.210937435388
ACCCCAGA -0.240435064982
ACCCCAGC -0.0930737183994
ACCCCAGG -0.1237139058
ACCCCAGT -0.0938795855306

ACCCCATA -0.0284181618152
ACCCCATC -0.0278401617986
ACCCCATG -0.17120360464
ACCCCCAA -0.228094407089
ACCCCCAC -0.21700505865
ACCCCCAG -0.423697620711
ACCCCCAT -0.193121855308
ACCCCCCA -0.321914267251
ACCCCCCC -0.384741203738
ACCCCCCG -0.206855716064
ACCCCCCT -0.351104496534
ACCCCCGA -0.0894583821669
ACCCCCGC -0.198846296985
ACCCCCGG -0.341863381427
ACCCCCGT -0.348593794457
ACCCCCTA -0.253680960454
ACCCCCTC -0.258896304199
ACCCCCTG -0.311602425776
ACCCCGAA -0.0750906508941
ACCCCGAC -0.215058470722
ACCCCGAG -0.0517171335004
ACCCCGAT 0.153338977979
ACCCCGCA -0.0767584222841
ACCCCGCC -0.193831846284
ACCCCGCG -0.178793293132
ACCCCGCT 0.00292441201532
ACCCCGGA -0.108615874119
ACCCCGGC -0.113154902399
ACCCCGGG -0.245117499646
ACCCCGGT -0.280947734308
ACCCCGTA -0.147153693128
ACCCCGTC -0.209967827043
ACCCCGTG -0.1984049256
ACCCCTAA -0.0407288383751
ACCCCTAC -0.270043527048
ACCCCTAG -0.242089080925
ACCCCTAT -0.0156587339008
ACCCCTCA -0.105228544746
ACCCCTCC -0.105742661838
ACCCCTCG -0.014296807832
ACCCCTCT -0.184645752607
ACCCCTGA -0.158256910863
ACCCCTGC -0.14433812035
ACCCCTGG -0.112350212122
ACCCCTTA -0.0613696212654
ACCCCTTC -0.211984905594
ACCCCTTG -0.277268673458
ACCCGAAA 0.116037605016
ACCCGAAC -0.193856536734
ACCCGAAG -0.239341894548
ACCCGAAT 0.21202674394
ACCCGACA -0.294519708298
ACCCGACC -0.263393893047
ACCCGACG -0.299079345588

ACCCGACT -0.13096035665
ACCCGAGA -0.0577476128634
ACCCGAGC -0.162035084237
ACCCGAGG 0.0191492157106
ACCCGAGT -0.184580566375
ACCCGATA 0.0145696660848
ACCCGATC 0.0456365456365
ACCCGATG -0.192668138994
ACCCGCAA 0.0937866240897
ACCCGCAC -0.101340601589
ACCCGCAG -0.0696145813113
ACCCGCAT -0.128562446744
ACCCGCCA -0.269820243879
ACCCGCCC -0.100525775709
ACCCGCCG -0.151737343159
ACCCGCCT 0.0378489906979
ACCCGCGA -0.0661379754244
ACCCGCGC -0.162048177826
ACCCGCGG -0.136978955581
ACCCGCGT -0.209031864649
ACCCGCTA -0.107150832444
ACCCGCTC -0.195277510642
ACCCGCTG -0.216543579551
ACCCGGAA -0.0881739876774
ACCCGGAC -0.29106828385
ACCCGGAG -0.391359058567
ACCCGGAT 0.160037447916
ACCCGGCA -0.135506625547
ACCCGGCC -0.315622045247
ACCCGGCG -0.221072825449
ACCCGGCT -0.135602954677
ACCCGGGA -0.37036850606
ACCCGGGC -0.145691377501
ACCCGGGG -0.111151084095
ACCCGGGT -0.299463170107
ACCCGGTA -0.271200154378
ACCCGGTC -0.257425720902
ACCCGGTG -0.148694705634
ACCCGTAA 0.166131696435
ACCCGTAC -0.199164356317
ACCCGTAG -0.0943160791646
ACCCGTAT 0.0400509477895
ACCCGTCA -0.350820110414
ACCCGTCC -0.0865468639304
ACCCGTCG -0.175649560586
ACCCGTCT -0.0781460481476
ACCCGTGA -0.167536948041
ACCCGTGC -0.175893529203
ACCCGTGG -0.208245218445
ACCCGTTA -0.155357019757
ACCCGTTC -0.248255840254
ACCCGTTG -0.294823576832
ACCCTAAA -0.0900454734568
ACCCTAAC -0.0122970501385

ACCCTAAG -0.284014013269
ACCCTAAT -0.0266437236134
ACCCTACA -0.0692342503449
ACCCTACC -0.0982241081251
ACCCTACG -0.0236831005136
ACCCTACT -0.104086544426
ACCCTAGA -0.056268354221
ACCCTAGC -0.00420192395179
ACCCTAGG 0.0436567082473
ACCCTAGT -0.088683510455
ACCCTATA -0.0451925754956
ACCCTATC -0.177193786479
ACCCTATG -0.105081701779
ACCCTCAA -0.0633361213167
ACCCTCAC -0.197274031564
ACCCTCAG -0.222359012831
ACCCTCAT -0.1810381945
ACCCTCCA -0.298106882617
ACCCTCCC -0.25651807199
ACCCTCCG -0.00832463522697
ACCCTCCT -0.137071244149
ACCCTCGA -0.325335395183
ACCCTCGC -0.283820501483
ACCCTCGG -0.172531959297
ACCCTCGT -0.169743078074
ACCCTCTA -0.0743529937222
ACCCTCTC -0.0362222802821
ACCCTCTG -0.297528626692
ACCCTGAA -0.0829631467924
ACCCTGAC -0.162588388537
ACCCTGAG -0.318483608252
ACCCTGAT 0.152898077141
ACCCTGCA -0.0821735557576
ACCCTGCC -0.223552489737
ACCCTGCG -0.127798383554
ACCCTGCT -0.0844342948227
ACCCTGGA -0.184247521232
ACCCTGGC -0.166978149352
ACCCTGGG -0.0426145996146
ACCCTGTA -0.151216368174
ACCCTGTC -0.313733848426
ACCCTGTG -0.129488568538
ACCCTTAA -0.130081318614
ACCCTTAC -0.0681564384445
ACCCTTAG -0.117005351132
ACCCTTAT -0.158063253419
ACCCTTCA -0.144617594592
ACCCTTCC -0.34798121367
ACCCTTCG -0.175153294985
ACCCTTCT -0.204646190445
ACCCTTGA -0.293095346159
ACCCTTGC -0.171690475994
ACCCTTGG -0.160029225982
ACCCTTTA 0.0168198501532

ACCCTTTC	-0.346399878733
ACCCTTTG	-0.215337151065
ACCGAAAA	-0.00529041438132
ACCGAAAC	-0.213908719241
ACCGAAAG	-0.126678247922
ACCGAAAT	0.360268753239
ACCGAACA	-0.108388515816
ACCGAACC	-0.0190010947587
ACCGAACG	-0.0554538799414
ACCGAACT	-0.276228384648
ACCGAAGA	0.0278474066353
ACCGAAGC	-0.0937051098594
ACCGAAGG	-0.0175368205671
ACCGAAGT	-0.140161849002
ACCGAATA	0.16251541937
ACCGAATC	0.350712971925
ACCGAATG	-0.0972949425268
ACCGACAA	0.0356348083621
ACCGACAC	-0.0742454695086
ACCGACAG	-0.170413400679
ACCGACAT	0.178652292286
ACCGACCA	-0.149651959365
ACCGACCC	-0.169475523801
ACCGACCG	-0.336179151681
ACCGACCT	-0.352232714177
ACCGACGA	-0.0790557901791
ACCGACGC	-0.250866268059
ACCGACGG	-0.205024147962
ACCGACGT	-0.101245564846
ACCGACTA	-0.0283520916865
ACCGACTC	-0.235795117782
ACCGACTG	-0.145733888158
ACCGAGAA	-0.0334552710828
ACCGAGAC	-0.166003607425
ACCGAGAG	-0.203796991768
ACCGAGAT	0.300111406172
ACCGAGCA	-0.227312758937
ACCGAGCC	-0.350316912211
ACCGAGCG	-0.244211349031
ACCGAGCT	-0.214970602099
ACCGAGGA	-0.257441177687
ACCGAGGC	-0.239182543226
ACCGAGGG	-0.31936680182
ACCGAGGT	-0.322121312494
ACCGAGTA	0.164015341026
ACCGAGTC	-0.280576570617
ACCGAGTG	-0.113176434904
ACCGATAA	0.193422771764
ACCGATAC	0.139885257987
ACCGATAG	0.0825030399285
ACCGATAT	0.370490325036
ACCGATCA	-0.0396024094891
ACCGATCC	0.117904405783
ACCGATCG	0.154042472224

ACCGATCT 0.0800763486931
ACCGATGA -0.0528176970207
ACCGATGC -0.145946715121
ACCGATGG -0.235711706049
ACCGATTA 0.179776990813
ACCGATTC 0.143478188933
ACCGATTG 0.119681320847
ACCGCAAA -0.142002420793
ACCGCAAC -0.198261864395
ACCGCAAG -0.31947442567
ACCGCAAT 0.304763399342
ACCGCACA -0.0309153067883
ACCGCACC -0.0465416855807
ACCGCACG -0.0773043144456
ACCGCACT 0.00271745874178
ACCGCAGA 0.0162675269885
ACCGCAGC -0.149744385159
ACCGCAGG -0.128711043259
ACCGCAGT -0.0980015092313
ACCGCATA 0.053574087401
ACCGCATC 0.12773517319
ACCGCATG -0.22267635748
ACCGCCAA -0.0112630397436
ACCGCCAC -0.176364766894
ACCGCCAG -0.0832912865221
ACCGCCAT -0.0416181908155
ACCGCCCA -0.306100812401
ACCGCCCC -0.173419738799
ACCGCCCG -0.102762036663
ACCGCCCT -0.285705425587
ACCGCCGA -0.0118486456501
ACCGCCGC -0.292323054405
ACCGCCGG -0.213077586753
ACCGCCGT -0.12202014187
ACCGCCTA -0.108202298927
ACCGCCTC -0.347055824273
ACCGCCTG -0.24858915934
ACCGCGAA -0.0621740520232
ACCGCGAC -0.239605877829
ACCGCGAG -0.169541014252
ACCGCGAT 0.309906325058
ACCGCGCA -0.0621333473462
ACCGCGCC -0.112283023811
ACCGCGCG -0.0530103651674
ACCGCGCT -0.033854003197
ACCGCGGA -0.177903119166
ACCGCGGC -0.216815378975
ACCGCGGG -0.092268181931
ACCGCGGT -0.370481985818
ACCGCGTA -0.0381031137919
ACCGCGTC -0.375694323842
ACCGCGTG -0.106143184303
ACCGCTAA -0.057549284822
ACCGCTAC -0.258028056791

ACCGCTAG -0.114003517298
ACCGCTAT 0.134072246593
ACCGCTCA -0.127511777682
ACCGCTCC -0.0942146459007
ACCGCTCG -0.111047702542
ACCGCTCT -0.0998677663756
ACCGCTGA -0.16426661787
ACCGCTGC -0.213175954187
ACCGCTGG -0.124787820495
ACCGCTTA -0.205291596025
ACCGCTTC -0.178819472315
ACCGCTTG -0.189120456519
ACCGGAAA 0.0771508653095
ACCGGAAC -0.129618872336
ACCGGAAG -0.301364594963
ACCGGAAT 0.278953745538
ACCGGACA -0.170652740219
ACCGGACC -0.219430518765
ACCGGACG -0.369002830628
ACCGGACT -0.196289476949
ACCGGAGA -0.038969256346
ACCGGAGC -0.0933173855106
ACCGGAGG -0.213658585006
ACCGGAGT -0.104083795466
ACCGGATA 0.315779216514
ACCGGATC 0.170027663628
ACCGGATG -0.0751149148278
ACCGGCAA -0.000431109346699
ACCGGCAC -0.227656974869
ACCGGCAG -0.149804748801
ACCGGCAT -0.00415538345876
ACCGGCCA -0.265776233994
ACCGGCCC -0.240236087985
ACCGGCCG -0.144927176637
ACCGGCCT -0.17779128723
ACCGGCGA -0.150618976095
ACCGGCGC -0.178881245046
ACCGGCGG -0.240541784869
ACCGGCGT -0.195571269073
ACCGGCTA -0.276955089779
ACCGGCTC -0.309000300442
ACCGGCTG -0.297665619469
ACCGGGAA -0.135616781825
ACCGGGAC -0.273565870318
ACCGGGAG -0.17455531567
ACCGGGAT 0.196917585746
ACCGGGCA -0.298010744241
ACCGGGCC -0.293088526723
ACCGGGCG -0.33062757062
ACCGGGCT -0.232191368745
ACCGGGGA 0.00722830886915
ACCGGGGC -0.0897678030098
ACCGGGGG -0.404851456692
ACCGGGTA -0.194586119118

ACCGGGTC -0.238989649592
ACCGGGTG -0.212587905489
ACCGGTAA 0.126891354164
ACCGGTAC -0.133535375698
ACCGGTAG 0.124373188908
ACCGGTAT 0.0420187879098
ACCGGTCA 0.00777912899125
ACCGGTCC -0.178927128017
ACCGGTCG -0.041717453788
ACCGGTCT -0.110334080276
ACCGGTGA -0.181084441616
ACCGGTGC -0.207436523445
ACCGGTGG -0.238366857895
ACCGGTTA 0.0239593660646
ACCGGTTC -0.282726069515
ACCGGTTG -0.294382473317
ACCGTAAA 0.0270848083619
ACCGTAAC 0.112447157902
ACCGTAAG 0.0148369013817
ACCGTAAT 0.397657698141
ACCGTACA -0.178273929854
ACCGTACC -0.0983839256653
ACCGTACG -0.125059260972
ACCGTACT -0.0995264572315
ACCGTAGA -0.0430030581546
ACCGTAGC -0.0909627539026
ACCGTAGG -0.0698977214129
ACCGTAGT -0.125592140318
ACCGTATA 0.129415414261
ACCGTATC 0.237439919258
ACCGTATG -0.114308523399
ACCGTCAA -0.354590932108
ACCGTCAC -0.317836221037
ACCGTCAG -0.2062471998
ACCGTCAT 0.0133566418254
ACCGTCCA -0.043129712488
ACCGTCCC 0.0508857470274
ACCGTCCG 0.0371382193099
ACCGTCCT -0.146186066537
ACCGTCGA -0.254586991958
ACCGTCGC -0.354853419055
ACCGTCGG -0.172034437318
ACCGTCGT -0.117206133349
ACCGTCTA -0.132489785909
ACCGTCTC -0.182501276388
ACCGTCTG -0.258768120957
ACCGTGAA 0.0369794822011
ACCGTGAC -0.116068731311
ACCGTGAG -0.25256710304
ACCGTGAT 0.188380546411
ACCGTGCA -0.0606562450277
ACCGTGCC -0.0800279368341
ACCGTGCG -0.0147799683219
ACCGTGCT -0.00669806101461

ACCGTGGA -0.17026579218
ACCGTGGC -0.289122177782
ACCGTGGG -0.21629654169
ACCGTGTA -0.126038231203
ACCGTGTC -0.0957061997359
ACCGTGTG -0.150877744416
ACCGTTAA 0.0265155588645
ACCGTTAC -0.13404271744
ACCGTTAG -0.0555555555556
ACCGTTAT 0.0482314270193
ACCGTTCA -0.0735239371603
ACCGTTCC -0.0279709203448
ACCGTTCG -0.062533298885
ACCGTTCT 0.0162343726937
ACCGTTGA -0.139658269838
ACCGTTGC -0.114955827977
ACCGTTGG -0.270740474281
ACCGTTTA -0.00486485697393
ACCGTTTC -0.322653547756
ACCGTTTG 0.0434966647088
ACCTAAAA -0.113738579284
ACCTAAAC -0.0504428963501
ACCTAAAG -0.124120052931
ACCTAAAT 0.251955796588
ACCTAACA -0.0775046748481
ACCTAACC -0.123956595959
ACCTAACG -0.0704368746269
ACCTAACT -0.0268907030154
ACCTAAGA -0.0552958694335
ACCTAAGC -0.236593637678
ACCTAAGG -0.130467933498
ACCTAAGT -0.234950056381
ACCTAATA 0.0539834894126
ACCTAATC 0.0376754164633
ACCTAATG 0.104031028273
ACCTACAA -0.0890932936571
ACCTACAC 0.170740056787
ACCTACAG -0.0698783942967
ACCTACAT 0.0950468374711
ACCTACCA -0.0518052487749
ACCTACCC -0.0189532104249
ACCTACCG 0.00906553936857
ACCTACCT -0.268289960059
ACCTACGA 0.0714061168607
ACCTACGC -0.217398950648
ACCTACGG -0.0744756005179
ACCTACGT -0.0977980399454
ACCTACTA -0.271111769522
ACCTACTC -0.215378571724
ACCTACTG -0.279283703458
ACCTAGAA 0.115290176128
ACCTAGAC -0.170424098836
ACCTAGAG -0.203895830931
ACCTAGAT 0.276608680547

ACCTAGCA -0.124606030525
ACCTAGCC -0.145462174449
ACCTAGCG -0.130420347363
ACCTAGCT -0.201609634648
ACCTAGGA -0.221561330152
ACCTAGGC -0.194341745035
ACCTAGGG -0.219327483204
ACCTAGGT -0.180471154986
ACCTAGTA -0.12264913907
ACCTAGTC -0.175198542731
ACCTAGTG -0.146276397747
ACCTATAA -0.0310190287804
ACCTATAC -0.0606416475318
ACCTATAG -0.0801855741428
ACCTATAT 0.156728353698
ACCTATCA -0.0459133626348
ACCTATCC -0.117570738783
ACCTATCG 0.0198212098697
ACCTATCT -0.0542494144572
ACCTATGA -0.0485573308614
ACCTATGC -0.231476197958
ACCTATGG -0.111365961664
ACCTATTA 0.0399090550606
ACCTATTC -0.061170076885
ACCTATTG -0.0616805638465
ACCTCAAA -0.165379231422
ACCTCAAC -0.169913314076
ACCTCAAG -0.154236447091
ACCTCAAT 0.0841580111384
ACCTCACA -0.00343974094936
ACCTCACC -0.133815087415
ACCTCACG -0.229161588459
ACCTCACT -0.205504352839
ACCTCAGA -0.0955694400853
ACCTCAGC -0.264392145189
ACCTCAGG -0.10533825817
ACCTCAGT -0.265759507802
ACCTCATA -0.0266178039819
ACCTCATC 0.0667419410874
ACCTCATG -0.234095052378
ACCTCCAA -0.143999371272
ACCTCCAC -0.312150805267
ACCTCCAG -0.293098933569
ACCTCCAT 0.0669269224288
ACCTCCCA -0.0611497558448
ACCTCCCC -0.290876287515
ACCTCCCG -0.198362486044
ACCTCCCT -0.259181463869
ACCTCCGA -0.288930130734
ACCTCCGC -0.293963395645
ACCTCCGG -0.311861701688
ACCTCCGT -0.265453271144
ACCTCCTA -0.352183175143
ACCTCCTC -0.338005341801

ACCTCCTG -0.102859791391
ACCTCGAA -0.0527704012552
ACCTCGAC -0.221612948581
ACCTCGAG -0.00105764107355
ACCTCGAT 0.107348395227
ACCTCGCA -0.170605748812
ACCTCGCC -0.177295021629
ACCTCGCG -0.198445666944
ACCTCGCT -0.227217795785
ACCTCGGA -0.1263511433
ACCTCGGC -0.238761964839
ACCTCGGG -0.150917907958
ACCTCGTA -0.0252443782658
ACCTCGTC -0.0993755501158
ACCTCGTG -0.203258113905
ACCTCTAA -0.16280441368
ACCTCTAC -0.294907866146
ACCTCTAG -0.126450515682
ACCTCTAT -0.105085366156
ACCTCTCA -0.251739215038
ACCTCTCC -0.0423046064005
ACCTCTCG -0.109598299115
ACCTCTCT -0.0408930153536
ACCTCTGA 0.0382144916535
ACCTCTGC -0.258827761425
ACCTCTGG -0.161106286785
ACCTCTTA 0.0742238373726
ACCTCTTC -0.0764405248541
ACCTCTTG -0.271851661167
ACCTGAAA 0.0811040634147
ACCTGAAC -0.180985932964
ACCTGAAG -0.080101243207
ACCTGAAT 0.077079379096
ACCTGACA -0.125663937692
ACCTGACC -0.383575483218
ACCTGACG -0.235140144632
ACCTGACT -0.272904483431
ACCTGAGA 0.000861743285986
ACCTGAGC 0.0692297193339
ACCTGAGG 0.0700970051751
ACCTGAGT -0.175156408114
ACCTGATA 0.19560134234
ACCTGATC 0.0385444114362
ACCTGATG -0.102796113855
ACCTGCAA -0.0451851419312
ACCTGCAC -0.073710127905
ACCTGCAG 0.0489106225891
ACCTGCAT 0.0564827047583
ACCTGCCA -0.249301214245
ACCTGCCC -0.173616614491
ACCTGCCG -0.246395218089
ACCTGCCT -0.147179147886
ACCTGCGA 0.0173641269132
ACCTGCGC -0.182275877872

ACCTGCGG -0.176405635826
ACCTGCGT -0.260335260211
ACCTGCTA -0.151501290098
ACCTGCTC -0.324496995223
ACCTGCTG -0.201223631135
ACCTGGAA -0.117700742505
ACCTGGAC -0.281941653366
ACCTGGAG -0.3762582895
ACCTGGAT 0.210707236434
ACCTGGCA -0.11849683095
ACCTGGCC -0.237495494365
ACCTGGCG -0.299713876967
ACCTGGCT -0.187047180111
ACCTGGGA -0.314690307446
ACCTGGGC -0.0497961175488
ACCTGGGG -0.38455235934
ACCTGGTA -0.202704793488
ACCTGGTC -0.233508822873
ACCTGGTG -0.311578398864
ACCTGTAA 0.163394232243
ACCTGTAC -0.179844129688
ACCTGTAG -0.033798261071
ACCTGTAT 0.0956380517215
ACCTGTCA -0.255558979569
ACCTGTCC -0.186315863218
ACCTGTCG -0.202077918667
ACCTGTCT -0.0861292935485
ACCTGTGA -0.108899862417
ACCTGTGC -0.224169678125
ACCTGTGG -0.228087677023
ACCTGTTA -0.163369257449
ACCTGTTC -0.300377378866
ACCTGTTG -0.0896424228889
ACCTTAAA 0.00561966607382
ACCTTAAC -0.229642977566
ACCTTAAG -0.0191331576681
ACCTTAAT 0.118886103735
ACCTTACA -0.12518577341
ACCTTACC -0.272129786244
ACCTTACG -0.181035501965
ACCTTACT -0.140023528828
ACCTTAGA -0.0993057106539
ACCTTAGC 0.0105998560544
ACCTTAGG -0.113417825539
ACCTTAGT 0.049819792244
ACCTTATA -0.071196251177
ACCTTATC -0.0344156291792
ACCTTATG 0.0268541564783
ACCTTCAA -0.0879036840146
ACCTTCAC -0.112074464746
ACCTTCAG -0.178782014704
ACCTTCAT 0.109166994327
ACCTTCCA -0.0281723757874
ACCTTCCC -0.167235630825

ACCTTCCG -0.0851497078636
ACCTTCCT -0.187981770578
ACCTTCGA -0.342918635362
ACCTTCGC -0.36415695936
ACCTTCGG -0.223338653611
ACCTTCGT -0.223752810727
ACCTTCTA -0.0188804081883
ACCTTCTC 0.00471840638288
ACCTTCTG -0.191612565301
ACCTTGAA -0.098638929478
ACCTTGAC -0.0925500997689
ACCTTGAG -0.24587913224
ACCTTGAT 0.140618088655
ACCTTGCA -0.00722392940301
ACCTTGCC -0.123429682008
ACCTTGCG -0.0848366045508
ACCTTGCT 0.0857792454629
ACCTTGGA -0.273267532435
ACCTTGGC -0.048477121426
ACCTTGGG -0.142640819323
ACCTTGTA -0.152043227624
ACCTTGTC -0.157431532627
ACCTTGTG -0.124435961256
ACCTTTAA 0.173240334462
ACCTTTAC 0.0132794128595
ACCTTTAG -0.110430090628
ACCTTTAT 0.0492131249707
ACCTTTCA -0.0187818681583
ACCTTTCC -0.0439734967864
ACCTTTCG -0.0935093694028
ACCTTTCT -0.168867859807
ACCTTTGA -0.158338640882
ACCTTTGC -0.0539191763788
ACCTTTGG -0.175828281074
ACCTTTTA 0.00405465573747
ACCTTTTC 0.0211754487128
ACCTTTTG -0.0505466628062
ACGAAAAA 0.252212725269
ACGAAAAC -0.134738043829
ACGAAAAG -0.126999408772
ACGAAAAT 0.291447094477
ACGAAACA -0.151918877336
ACGAAACC 0.00843078378335
ACGAAACG 0.101791076259
ACGAAACT 0.15988482004
ACGAAAGA 0.0468712363963
ACGAAAGC -0.238461474847
ACGAAAGG -0.211083440747
ACGAAAGT -0.181809092462
ACGAAATA 0.282515711335
ACGAAATC 0.46946809068
ACGAAATG 0.0597718931052
ACGAACAA 0.0979167183661
ACGAACAC -0.0198898181006

ACGAACAG -0.240420619031
ACGAACAT -0.0640737794063
ACGAACCA -0.240211790111
ACGAACCC -0.178110292232
ACGAACCG -0.153073799304
ACGAACCT -0.133126973399
ACGAACGA 0.075470787592
ACGAACGC -0.0057575381561
ACGAACGG -0.0568859041594
ACGAACGT -0.126248206422
ACGAACTA -0.0692455805071
ACGAACTC -0.176262604886
ACGAACTG -0.0487637031837
ACGAAGAA 0.0343873986396
ACGAAGAC -0.0515510489449
ACGAAGAG -0.190021477272
ACGAAGAT 0.313922252277
ACGAAGCA -0.080225250542
ACGAAGCC -0.27486141777
ACGAAGCG -0.136347469333
ACGAAGCT -0.112531193559
ACGAAGGA -0.119504405817
ACGAAGGC -0.145034085615
ACGAAGGG -0.192147961365
ACGAAGTA -0.00871903048352
ACGAAGTC -0.292254646555
ACGAAGTG -0.254110810639
ACGAATAA 0.0457559323518
ACGAATAC 0.0390962241703
ACGAATAG -0.0621219973285
ACGAATAT 0.179558346225
ACGAATCA 0.259733975234
ACGAATCC 0.355702396643
ACGAATCG 0.3151760576
ACGAATCT 0.382338342956
ACGAATGA 0.126338109384
ACGAATGC -0.186887748639
ACGAATGG -0.130258357531
ACGAATTA 0.0848674846536
ACGAATTC 0.104567560471
ACGAATTG 0.0704382198454
ACGACAAA 0.0288073983781
ACGACAAC -0.0769047284199
ACGACAAG -0.137115083993
ACGACAAT 0.154039714646
ACGACACA -0.190862016196
ACGACACC -0.0796058019207
ACGACACG -0.238540525
ACGACACT -0.135266479721
ACGACAGA 0.0971577264731
ACGACAGC -0.213907126273
ACGACAGG -0.174291101243
ACGACAGT -0.331518368764
ACGACATA -0.154675206811

ACGACATC 0.117818920849
ACGACATG -0.0615351488654
ACGACCAA -0.0306156568988
ACGACCAC -0.232343965944
ACGACCAG -0.169842766515
ACGACCAT -0.13729601212
ACGACCCA -0.237166050654
ACGACCCC -0.280492914458
ACGACCCG -0.0833932898339
ACGACCCT -0.136154603277
ACGACCGA -0.0170367130698
ACGACCGC -0.352629581792
ACGACCGG -0.000318939456971
ACGACCGT -0.268937476015
ACGACCTA -0.277933469756
ACGACCTC -0.329711972959
ACGACCTG -0.274350997562
ACGACGAA 0.134689786205
ACGACGAC -0.138973671803
ACGACGAG -0.280422755353
ACGACGAT 0.140860361814
ACGACGCA -0.257462491145
ACGACGCC -0.273012958874
ACGACGCG -0.0809923761063
ACGACGCT -0.152953292392
ACGACGGA 0.00936060026969
ACGACGGC -0.344936501057
ACGACGGG -0.162521875826
ACGACGTA 0.0417262993021
ACGACGTC -0.235743446823
ACGACGTG -0.103067809748
ACGACTAA -0.0967021514181
ACGACTAC -0.229956846038
ACGACTAG -0.191895052232
ACGACTAT -0.0417458011868
ACGACTCA 0.0803361970176
ACGACTCC -0.0217050567852
ACGACTCG -0.0460658544453
ACGACTCT 0.0987912673445
ACGACTGA 0.0531397122691
ACGACTGC -0.217783119819
ACGACTGG -0.18278671417
ACGACTTA -0.0592401447921
ACGACTTC -0.0638793721325
ACGACTTG -0.187673784186
ACGAGAAA 0.202768884587
ACGAGAAC -0.114614121725
ACGAGAAG -0.0700375801991
ACGAGAAT 0.279420505298
ACGAGACA -0.196813719409
ACGAGACC -0.133904618532
ACGAGACG -0.0186738466883
ACGAGACT 0.116373949707
ACGAGAGA 0.0729503608291

ACGAGAGC 0.0875214411363
ACGAGAGG 0.0753125151017
ACGAGAGT -0.153868528214
ACGAGATA 0.276106685198
ACGAGATC 0.414680520741
ACGAGATG -0.11469310891
ACGAGCAA -0.0520183384382
ACGAGCAC -0.18288760267
ACGAGCAG -0.163977502083
ACGAGCAT -0.126951040969
ACGAGCCA -0.197102384083
ACGAGCCC -0.220252974301
ACGAGCCG -0.361340577014
ACGAGCCT -0.277229723912
ACGAGCGA 0.0290991505166
ACGAGCGC -0.169557856637
ACGAGCGG -0.227222163469
ACGAGCGT 0.0680695833437
ACGAGCTA -0.0605136817258
ACGAGCTC -0.151285419266
ACGAGCTG -0.130050893891
ACGAGGAA -0.125279549522
ACGAGGAC -0.190630023963
ACGAGGAG -0.208233776129
ACGAGGAT 0.185560170215
ACGAGGCA -0.0989387093713
ACGAGGCC -0.226802366808
ACGAGGCG -0.240300049468
ACGAGGCT -0.263060989475
ACGAGGGA 0.0105697275064
ACGAGGGC -0.279353033104
ACGAGGGG -0.255130841381
ACGAGGTA -0.0287245986651
ACGAGGTC -0.275252875358
ACGAGGTG -0.178056222025
ACGAGTAA 0.134682452209
ACGAGTAC 0.0555602591931
ACGAGTAG -0.140538445707
ACGAGTAT 0.190969206121
ACGAGTCA 0.0947760268023
ACGAGTCC -0.346273647187
ACGAGTCG -0.132413312049
ACGAGTCT -0.185806834002
ACGAGTGA -0.00131109842751
ACGAGTGC -0.195383996052
ACGAGTGG -0.0634237019456
ACGAGTTA -0.0501865501866
ACGAGTTC -0.139095494259
ACGAGTTG -0.144488332071
ACGATAAA 0.0505178718714
ACGATAAC 0.0656124444003
ACGATAAG 0.0931800134005
ACGATAAT 0.216911308511
ACGATACA -0.0516786431344

ACGATACC 0.0991694173512
ACGATACG 0.198524971252
ACGATACT 0.14047518593
ACGATAGA 0.0477240325725
ACGATAGC -0.143014915742
ACGATAGG 0.0222148420108
ACGATAGT 0.0962518992822
ACGATATA 0.239589225229
ACGATATC 0.435963511721
ACGATATG 0.265105873594
ACGATCAA 0.0205145722762
ACGATCAC 0.164451143406
ACGATCAG 0.0985212002657
ACGATCAT 0.0767888294417
ACGATCCA 0.0249301110195
ACGATCCC 0.238854557036
ACGATCCG 0.221439684192
ACGATCCT 0.288959758657
ACGATCGA 0.079780779043
ACGATCGC 0.311058992877
ACGATCGG 0.140060436633
ACGATCGT 0.197711404938
ACGATCTA 0.270861770862
ACGATCTC 0.278976367976
ACGATCTG 0.353425107479
ACGATGAA -0.0235727663277
ACGATGAC -0.104058604059
ACGATGAG -0.273329970256
ACGATGAT 0.0732653795568
ACGATGCA 0.0833959840295
ACGATGCC -0.180720860127
ACGATGCG 0.0738808639227
ACGATGCT -0.00818887177653
ACGATGGA -0.0690132495071
ACGATGGC -0.145467201478
ACGATGGG 0.170340379006
ACGATGTA -0.0942380823778
ACGATGTC -0.17645509867
ACGATGTG -0.208512195727
ACGATTAA 0.0363793545612
ACGATTAC 0.437336785822
ACGATTAG -0.0120139713441
ACGATTAT 0.201016766056
ACGATTCA 0.166457800829
ACGATTCC 0.412250096713
ACGATTCG 0.306634891207
ACGATTCT 0.346164106509
ACGATTGA 0.0644735644736
ACGATTGC 0.295122946638
ACGATTGG 0.137902679062
ACGATTTA 0.104937118165
ACGATTTC 0.427895612054
ACGATTTG 0.242218802825
ACGCAAAA 0.165014634716

ACGCAAAC -0.185527197489
ACGCAAAG -0.15647304998
ACGCAAAT 0.200518700519
ACGCAACA 0.115287463772
ACGCAACC 0.0903796358342
ACGCAACG 0.117639678246
ACGCAACT -0.0217170426925
ACGCAAGA -0.0295028165868
ACGCAAGC -0.13956232716
ACGCAAGG -0.121229041037
ACGCAAGT -0.158292843475
ACGCAATA 0.282080684365
ACGCAATC 0.478902651767
ACGCAATG -0.0124463306281
ACGCACAA 0.0203521090779
ACGCACAC -0.133521893681
ACGCACAG -0.272027181629
ACGCACAT 0.0820485682795
ACGCACCA -0.269141759847
ACGCACCC -0.175381155514
ACGCACCG -0.109747623893
ACGCACCT -0.129851295309
ACGCACGA -0.0575878909212
ACGCACGC 0.0185390100947
ACGCACGG -0.169335472789
ACGCACGT 0.0414581351422
ACGCACTA -0.0277453762302
ACGCACTC -0.213287284795
ACGCACTG -0.0832835777319
ACGCAGAA 0.133829421708
ACGCAGAC -0.149296286512
ACGCAGAG -0.0920792488729
ACGCAGAT 0.328552659431
ACGCAGCA -0.0638698453457
ACGCAGCC -0.232825808749
ACGCAGCG -0.0928729185795
ACGCAGCT -0.20605040302
ACGCAGGA -0.129244526061
ACGCAGGC -0.318071711435
ACGCAGGG -0.222065094842
ACGCAGTA -0.131311929779
ACGCAGTC -0.13391087403
ACGCAGTG -0.203564730693
ACGCATAA 0.0472741472814
ACGCATAC -0.00616180919211
ACGCATAG 0.00152356212962
ACGCATAT 0.167767859098
ACGCATCA 0.0864183742972
ACGCATCC 0.100490500208
ACGCATCG 0.0510726042495
ACGCATCT -0.147536486036
ACGCATGA -0.118899337518
ACGCATGC -0.187852745174
ACGCATGG -0.145569878943

ACGCATTA 0.0530112479289
ACGCATTC −0.111344573491
ACGCATTG −0.0701323919895
ACGCCAAA 0.0807653846996
ACGCCAAC −0.340321584471
ACGCCAAG −0.222683979104
ACGCCAAT −0.0459054677398
ACGCCACA −0.144720536784
ACGCCACC −0.184078861405
ACGCCACG −0.134034446347
ACGCCACT −0.0601627831956
ACGCCAGA −0.129764224261
ACGCCAGC −0.326384582096
ACGCCAGG −0.152927683906
ACGCCAGT −0.230791177711
ACGCCATA −0.0454609149548
ACGCCATC −0.25440323092
ACGCCATG −0.0575889669167
ACGCCCAA −0.0146772116469
ACGCCCAC −0.274697316599
ACGCCCAG −0.250581114543
ACGCCCAT −0.194469943186
ACGCCCCA −0.259201832511
ACGCCCCC −0.244970355486
ACGCCCCG −0.296431161456
ACGCCCCT −0.305196559147
ACGCCCGA −0.134833985371
ACGCCCGC −0.226996770376
ACGCCCGG −0.23266781964
ACGCCCGT −0.0725255686255
ACGCCCTA −0.13721614327
ACGCCCTC −0.2983143017
ACGCCCTG −0.332927927205
ACGCCGAA 0.109959822081
ACGCCGAC −0.234795736837
ACGCCGAG −0.0800678839104
ACGCCGAT 0.0825660371115
ACGCCGCA −0.143411452366
ACGCCGCC −0.186765910056
ACGCCGCG −0.00831295665058
ACGCCGCT −0.122404168217
ACGCCGGA −0.103437151011
ACGCCGGC −0.250957960671
ACGCCGGG −0.0578990320064
ACGCCGTA −0.333245138975
ACGCCGTC −0.398432370268
ACGCCGTG −0.133140075043
ACGCCTAA 0.0152673334492
ACGCCTAC −0.222009983489
ACGCCTAG −0.062499483031
ACGCCTAT −0.215802301568
ACGCCTCA −0.0973790361492
ACGCCTCC −0.197698437465
ACGCCTCG −0.1536695206

ACGCCTCT -0.235325857749
ACGCCTGA 0.0094088160117
ACGCCTGC -0.358312557366
ACGCCTGG -0.2519689808
ACGCCTTA -0.108399032641
ACGCCTTC -0.374407365089
ACGCCTTG -0.213806402405
ACGCGAAA 0.191760631155
ACGCGAAC 0.000823137186774
ACGCGAAG -0.0684673889405
ACGCGAAT 0.265278132596
ACGCGACA 0.00637965789481
ACGCGACC -0.205220455053
ACGCGACG -0.133741434697
ACGCGACT -0.222919241877
ACGCGAGA 0.0995941694707
ACGCGAGC -0.140648898583
ACGCGAGG -0.102995549444
ACGCGAGT -0.12693547542
ACGCGATA 0.254421087754
ACGCGATC 0.444731137274
ACGCGATG 0.111858134493
ACGCGCAA 0.111833317519
ACGCGCAC -0.0488298215571
ACGCGCAG -0.0676601235824
ACGCGCAT -0.0302190462983
ACGCGCCA -0.163819218757
ACGCGCCC -0.156480104386
ACGCGCCG -0.255834266125
ACGCGCCT -0.13458212247
ACGCGCGA 0.227085453327
ACGCGCGC 0.0668171943845
ACGCGCGG -0.0698283991364
ACGCGCGT 0.0833450478088
ACGCGCTA -0.0842178638424
ACGCGCTC -0.165327332953
ACGCGCTG -0.0163405991921
ACGCGGAA 0.0310555243316
ACGCGGAC -0.189136733687
ACGCGGAG -0.273110355722
ACGCGGAT 0.218350693051
ACGCGGCA -0.0557719717584
ACGCGGCC -0.153287433321
ACGCGGCG -0.12538154205
ACGCGGCT -0.272249374936
ACGCGGGA -0.11333049211
ACGCGGGC -0.190921429979
ACGCGGGG -0.151335434295
ACGCGGTA -0.123931942066
ACGCGGTC -0.320612241026
ACGCGGTG -0.245408675912
ACGCGTAA 0.122396589856
ACGCGTAC -0.0131478872725
ACGCGTAG 0.00878447917199

ACGCGTAT 0.242101997766
ACGCGTCA -0.265731729918
ACGCGTCC 0.0995357765888
ACGCGTCG 0.168227456106
ACGCGTCT -0.142256247363
ACGCGTGA 0.093267300716
ACGCGTGC -0.0529085429574
ACGCGTGG 0.072103784225
ACGCGTTA -0.0480341045212
ACGCGTTC -0.120525928316
ACGCGTTG -0.152280606994
ACGCTAAA 0.0801614286463
ACGCTAAC -0.117460949987
ACGCTAAG -0.0165606377728
ACGCTAAT -0.101651238015
ACGCTACA -0.00754812186184
ACGCTACC -0.140789078288
ACGCTACG -0.107886921051
ACGCTACT -0.168202657356
ACGCTAGA 0.0474738952169
ACGCTAGC -0.239390091727
ACGCTAGG -0.00135239087819
ACGCTAGT -0.18354788761
ACGCTATA 0.0683231440807
ACGCTATC 0.0407195462873
ACGCTATG 0.0831864922774
ACGCTCAA 0.00809661335977
ACGCTCAC -0.28551368374
ACGCTCAG -0.215097927335
ACGCTCAT -0.0963271765773
ACGCTCCA -0.241695720638
ACGCTCCC -0.0534080119528
ACGCTCCG 0.00264718906795
ACGCTCCT 0.0457238162668
ACGCTCGA -0.0125458178904
ACGCTCGC 0.0126968022598
ACGCTCGG -0.142979822189
ACGCTCTA -0.080785193181
ACGCTCTC -0.332639463466
ACGCTCTG -0.131211108727
ACGCTGAA 0.00552315561622
ACGCTGAC -0.11179740265
ACGCTGAG 0.146169585564
ACGCTGAT 0.311277125318
ACGCTGCA 0.0214586541901
ACGCTGCC -0.0570593344671
ACGCTGCG -0.195403750283
ACGCTGCT -0.144685270689
ACGCTGGA -0.0781946380736
ACGCTGGC -0.0598329462564
ACGCTGGG -0.153883935153
ACGCTGTA -0.217128492636
ACGCTGTC -0.341314922761
ACGCTGTG -0.0288045691466

ACGCTTAA -0.0822050685425
ACGCTTAC 0.04952196455
ACGCTTAG -0.109578693385
ACGCTTAT -0.052891534129
ACGCTTCA -0.100190595999
ACGCTTCC -0.112259462773
ACGCTTCG 0.00912517169452
ACGCTTCT -0.107812972091
ACGCTTGA 0.0612328461176
ACGCTTGC -0.283838208417
ACGCTTGG -0.343521252574
ACGCTTTA -0.0320499562924
ACGCTTTC -0.299915947625
ACGCTTTG -0.190313722571
ACGGAAAA 0.209247242761
ACGGAAAC -0.123410837324
ACGGAAAG -0.0931165325105
ACGGAAAT 0.390828667985
ACGGAACA -0.0395027490794
ACGGAACC 0.0420304664861
ACGGAACG -0.171738054823
ACGGAACT -0.0790282775641
ACGGAAGA -0.025947778918
ACGGAAGC -0.251189706576
ACGGAAGG -0.196265971964
ACGGAAGT -0.218149479698
ACGGAATA 0.276382443049
ACGGAATC 0.445157405312
ACGGAATG -0.065187777309
ACGGACAA 0.0972994267563
ACGGACAC -0.187779018627
ACGGACAG -0.0281507913921
ACGGACAT -0.064979717786
ACGGACCA -0.228587502682
ACGGACCC 0.0199491981927
ACGGACCG -0.147644095973
ACGGACCT -0.325939841396
ACGGACGA -0.111111111111
ACGGACGC -0.168046274257
ACGGACGG -0.0446540100095
ACGGACGT 0.109340833575
ACGGACTA -0.0405262981188
ACGGACTC -0.279029221486
ACGGACTG -0.276176260609
ACGGAGAA 0.220307083943
ACGGAGAC -0.026476062568
ACGGAGAG -0.248387456895
ACGGAGAT 0.313382188711
ACGGAGCA -0.138342897526
ACGGAGCC -0.295828681305
ACGGAGCG -0.190187223496
ACGGAGCT -0.349756776496
ACGGAGGA -0.105637565083
ACGGAGGC -0.179336522083

ACGGAGGG -0.249064260667
ACGGAGTA 0.14881410336
ACGGAGTC -0.266156585448
ACGGAGTG -0.188790138551
ACGGATAA 0.0285446304096
ACGGATAC 0.259828699223
ACGGATAG -0.109392448569
ACGGATAT 0.401990144414
ACGGATCA 0.0671058297993
ACGGATCC 0.185387911214
ACGGATCG 0.194490999035
ACGGATCT 0.227386991526
ACGGATGA -0.16909009702
ACGGATGC -0.192490479728
ACGGATGG -0.004254651358
ACGGATTA 0.307732677387
ACGGATTC 0.410742698621
ACGGATTG 0.211662075298
ACGGCAAA 0.141586222299
ACGGCAAC -0.158753001569
ACGGCAAG -0.140885767775
ACGGCAAT 0.200707283782
ACGGCACA 0.032824367657
ACGGCACC -0.0237724721356
ACGGCACG -0.257273222138
ACGGCACT -0.143761493944
ACGGCAGA -0.111922934885
ACGGCAGC -0.192613449988
ACGGCAGG -0.242970082633
ACGGCAGT -0.236691063971
ACGGCATA 0.108031940906
ACGGCATC -0.131682517901
ACGGCATG 0.100906691816
ACGGCCAA -0.12814329481
ACGGCCAC -0.298395416811
ACGGCCAG -0.236511795203
ACGGCCAT -0.236436096154
ACGGCCCA -0.389185137418
ACGGCCCC -0.110641338349
ACGGCCCG -0.2602833568
ACGGCCCT -0.267993229786
ACGGCCGA -0.0630379288653
ACGGCCGC -0.266650859773
ACGGCCGG -0.0115343784037
ACGGCCGT -0.133216762982
ACGGCCTA -0.162390935781
ACGGCCTC -0.05582839715
ACGGCCTG -0.441522353103
ACGGCGAA -0.0717095777265
ACGGCGAC -0.126611346154
ACGGCGAG -0.206708284266
ACGGCGAT 0.0687229929654
ACGGCGCA -0.0388184502394
ACGGCGCC -0.190149620235

ACGGCGCG -0.146966701683
ACGGCGCT -0.193803602465
ACGGCGGA -0.11776164348
ACGGCGGC -0.244581928135
ACGGCGGG -0.188401810224
ACGGCGTA -0.0864459075445
ACGGCGTC -0.310798296746
ACGGCGTG -0.323812910179
ACGGCTAA -0.0212878550105
ACGGCTAC -0.236950199176
ACGGCTAG -0.139603162671
ACGGCTAT -0.145729057647
ACGGCTCA -0.127510673505
ACGGCTCC 0.0911972730524
ACGGCTCG -0.126038012912
ACGGCTCT -0.0567967676909
ACGGCTGA -0.0889137058637
ACGGCTGC -0.353407809834
ACGGCTGG -0.18642238706
ACGGCTTA 0.0556631011176
ACGGCTTC -0.342236902203
ACGGCTTG -0.347965328593
ACGGGAAA 0.0673846558105
ACGGGAAC -0.345269501024
ACGGGAAG -0.275077935716
ACGGGAAT 0.318690101677
ACGGGACA -0.0763373617556
ACGGGACC -0.204244608544
ACGGGACG -0.142099578946
ACGGGACT -0.182521898806
ACGGGAGA -0.0897051160605
ACGGGAGC -0.0911514396097
ACGGGAGG 0.0100086312208
ACGGGAGT -0.171777462194
ACGGGATA 0.183259490668
ACGGGATC 0.166427742458
ACGGGATG -0.367247610974
ACGGGCAA -0.0540820658172
ACGGGCAC -0.23060192299
ACGGGCAG -0.296149762511
ACGGGCAT -0.0964124055042
ACGGGCCA -0.234598221054
ACGGGCCC -0.293095396883
ACGGGCCG -0.248337348768
ACGGGCCT -0.277203365659
ACGGGCGA 0.0991387049919
ACGGGCGC -0.335075690533
ACGGGCGG -0.0995910187603
ACGGGCTA 0.0048996003872
ACGGGCTC -0.171305520645
ACGGGCTG -0.312352018611
ACGGGGAA -0.111038057037
ACGGGGAC -0.339718728379
ACGGGGAG -0.162606365824

ACGGGGAT 0.132916067531
ACGGGGCA -0.212666870646
ACGGGGCC -0.213820632815
ACGGGGCG -0.0755849000974
ACGGGGCT -0.243551651726
ACGGGGGA -0.0421302385344
ACGGGGGC -0.273381079752
ACGGGGGG -0.14065232182
ACGGGGTA -0.115591590964
ACGGGGTC -0.281425441316
ACGGGGTG -0.183683596755
ACGGGTAA -0.153366609018
ACGGGTAC -0.114631189674
ACGGGTAG -0.120419856548
ACGGGTAT -0.053015627395
ACGGGTCA -0.166360636213
ACGGGTCC -0.233695449922
ACGGGTCG -0.283645975443
ACGGGTCT -0.251336139295
ACGGGTGA -0.181193139274
ACGGGTGC -0.468003682762
ACGGGTGG -0.0401918532673
ACGGGTTA 0.0607011970648
ACGGGTTC -0.326819840629
ACGGGTTG -0.202491529022
ACGGTAAA 0.0237661904329
ACGGTAAC -0.12623693447
ACGGTAAG -0.0103760866149
ACGGTAAT 0.21736677299
ACGGTACA -0.0143317589904
ACGGTACC 0.0602903178661
ACGGTACG -0.095485292455
ACGGTACT -0.0119086028177
ACGGTAGA -0.0260585534623
ACGGTAGC -0.113686224881
ACGGTAGG 0.00997819080154
ACGGTAGT -0.123205250169
ACGGTATA -0.0271056180147
ACGGTATC 0.280384371145
ACGGTATG -0.303042283744
ACGGTCAA -0.0773588595799
ACGGTCAC -0.124413126125
ACGGTCAG -0.226655702346
ACGGTCAT -0.133289214267
ACGGTCCA -0.178466795806
ACGGTCCC -0.277648784144
ACGGTCCG -0.123780134526
ACGGTCCT -0.375472987684
ACGGTCGA -0.210746080191
ACGGTCGC -0.225831162387
ACGGTCGG -0.0921647450017
ACGGTCTA 0.095752777571
ACGGTCTC -0.29383675679
ACGGTCTG -0.0988755711068

ACGGTGAA -0.089746134948
ACGGTGAC -0.197996108694
ACGGTGAG -0.165816249176
ACGGTGAT 0.245836745837
ACGGTGCA 0.0311123729835
ACGGTGCC -0.279186687698
ACGGTGCG -0.116345821225
ACGGTGCT -0.230335602793
ACGGTGGA -0.0739757523558
ACGGTGGC -0.0865568319976
ACGGTGGG 9.78746047106E-5
ACGGTGTA 0.0425793595761
ACGGTGTC -0.184106049347
ACGGTGTG -0.247312983959
ACGGTTAA -0.0210922388561
ACGGTTAC 0.116091148224
ACGGTTAG -0.157164168429
ACGGTTAT -0.033608753093
ACGGTTCA -0.182296311324
ACGGTTCC -0.25630476789
ACGGTTCG -0.124321101359
ACGGTTCT -0.195015754087
ACGGTTGA -0.216714079493
ACGGTTGC -0.024833811702
ACGGTTGG -0.0090950179549
ACGGTTTA 0.0800402598938
ACGGTTTC -0.120418393429
ACGGTTTG -0.333927296681
ACGTAAAA 0.144090371363
ACGTAAAC -0.129759588508
ACGTAAAG 0.00569257607498
ACGTAAAT 0.196505292419
ACGTAACA 0.108758932286
ACGTAACC 0.183554374722
ACGTAACG -0.0119858150161
ACGTAACT 0.034526261799
ACGTAAGA 0.0195112515784
ACGTAAGC -0.0653504744414
ACGTAAGG -0.119679659253
ACGTAAGT 0.091830657369
ACGTAATA 0.276051533627
ACGTAATC 0.477013642037
ACGTAATG -0.0339637157819
ACGTACAA 0.118393757801
ACGTACAC -0.00884493308736
ACGTACAG -0.172839046713
ACGTACAT -0.0886239118986
ACGTACCA -0.200219448787
ACGTACCC -0.0238047965321
ACGTACCG -0.291486840127
ACGTACCT -0.233514218034
ACGTACGA 0.0432953614772
ACGTACGC -0.203120562683
ACGTACGG 0.0177469056958

ACGTACGT 0.0229005929197
ACGTACTA 0.138613820432
ACGTACTC -0.099522967944
ACGTACTG -0.0863480252278
ACGTAGAA 0.150332150332
ACGTAGAC -0.0797919131252
ACGTAGAG -0.0480732931084
ACGTAGAT 0.155310249514
ACGTAGCA 0.0506801567408
ACGTAGCC -0.254801987784
ACGTAGCG -0.134403219144
ACGTAGCT -0.164566244275
ACGTAGGA -0.0556796465887
ACGTAGGC -0.168760320099
ACGTAGGG -0.0808155996107
ACGTAGTA -0.0413545688781
ACGTAGTC -0.225550526406
ACGTAGTG -0.240702800433
ACGTATAA 0.260460719838
ACGTATAC 0.0575851333427
ACGTATAG 0.15712575637
ACGTATAT 0.111865308835
ACGTATCA 0.159350810866
ACGTATCC 0.294634855241
ACGTATCG 0.224311087947
ACGTATCT 0.333358393612
ACGTATGA -0.0860900856186
ACGTATGC -0.198647810445
ACGTATGG -0.186904666321
ACGTATTA 0.174058069649
ACGTATTC 0.121205728326
ACGTATTG 0.180334007285
ACGTCAAA 0.0526257006593
ACGTCAAC -0.174768043833
ACGTCAAG 0.13428993732
ACGTCAAT 0.0660619296983
ACGTCACA 0.0106539045933
ACGTCACC 0.0727130062845
ACGTCACG 0.0286789340379
ACGTCACT -0.147934673286
ACGTCAGA -0.00683465834981
ACGTCAGC -0.185625456701
ACGTCAGG -0.0814134645876
ACGTCAGT -0.192640819013
ACGTCATA -0.0545104332983
ACGTCATC -0.143915895349
ACGTCATG -0.0524119160483
ACGTCCAA -0.176086309155
ACGTCCAC -0.171730205602
ACGTCCAG -0.142675013028
ACGTCCAT -0.0349853463796
ACGTCCCA -0.290850808282
ACGTCCCC -0.132631402232
ACGTCCCG -0.226503254191

ACGTCCCT -0.114775144034
ACGTCCGA -0.285142575301
ACGTCCGC -0.209204899796
ACGTCCGG -0.176016673697
ACGTCCTA -0.0892440634227
ACGTCCTC -0.194200144947
ACGTCCTG -0.24683270332
ACGTCGAA 0.0188356400478
ACGTCGAC -0.025772428341
ACGTCGAG -0.0342094161929
ACGTCGAT 0.0464800040875
ACGTCGCA -0.0620220943996
ACGTCGCC -0.0653183628845
ACGTCGCG -0.104315759206
ACGTCGCT -0.127130840426
ACGTCGGA -0.0657677406902
ACGTCGGC -0.195430625505
ACGTCGGG -0.260906963088
ACGTCGTA 0.120055317025
ACGTCGTC -0.149214980694
ACGTCGTG -0.0774896251258
ACGTCTAA 0.074691487847
ACGTCTAC -0.227788503211
ACGTCTAG -0.218264107674
ACGTCTAT -0.0252147763188
ACGTCTCA -0.0686902170978
ACGTCTCC -0.186082338054
ACGTCTCG -0.0114676320956
ACGTCTCT -0.0778268529317
ACGTCTGA -0.143133280942
ACGTCTGC -0.235102227242
ACGTCTGG -0.0624665153318
ACGTCTTA -0.0504288227265
ACGTCTTC 0.0276630714818
ACGTCTTG -0.149524502789
ACGTGAAA 0.16813281461
ACGTGAAC 0.0507339255345
ACGTGAAG -0.197013215409
ACGTGAAT -0.00540061166544
ACGTGACA 0.0250498164274
ACGTGACC -0.136510260291
ACGTGACG -0.24003308487
ACGTGACT -0.00385028065079
ACGTGAGA -0.0432169797276
ACGTGAGC -0.109965564681
ACGTGAGG 0.0474259687744
ACGTGAGT -0.119257568112
ACGTGATA 0.211139172135
ACGTGATC 0.241727953849
ACGTGATG -0.163198122363
ACGTGCAA 0.108344342825
ACGTGCAC -0.047415076549
ACGTGCAG -0.0796347311499
ACGTGCAT -0.135377306859

ACGTGCCA -0.123681301566
ACGTGCCC 0.0278117997416
ACGTGCCG -0.157047385094
ACGTGCCT -0.281523467754
ACGTGCGA 0.0881666824814
ACGTGCGC -0.149856469246
ACGTGCGG 0.013038400619
ACGTGCTA 0.128053280803
ACGTGCTC -0.109843967366
ACGTGCTG -0.218610302327
ACGTGGAA 0.0284906627117
ACGTGGAC 0.0175643963523
ACGTGGAG -0.242294787281
ACGTGGAT 0.313913563937
ACGTGGCA -0.215766633858
ACGTGGCC -0.171375332276
ACGTGGCG -0.0521720411276
ACGTGGCT -0.16241097794
ACGTGGGA -0.078397070136
ACGTGGGC -0.12143774348
ACGTGGGG -0.00594512528923
ACGTGGTA 0.0363269417458
ACGTGGTC -0.234921225654
ACGTGGTG -0.327867496878
ACGTGTAA -0.0149198785562
ACGTGTAC -0.109935237339
ACGTGTAG 0.084479796601
ACGTGTAT 0.0182860479485
ACGTGTCA -0.215419923086
ACGTGTCC -0.206947028905
ACGTGTCG -0.215574642449
ACGTGTCT -0.0715660564145
ACGTGTGA -0.123450970013
ACGTGTGC -0.136167091232
ACGTGTGG -0.0902584066456
ACGTGTTA 0.0949296403842
ACGTGTTC -0.0926061493411
ACGTGTTG -0.124528365228
ACGTTAAA 0.089133084677
ACGTTAAC 0.0189561257662
ACGTTAAG 0.0345386597374
ACGTTAAT 0.153375138224
ACGTTACA 0.0523343988275
ACGTTACC -0.0961443537201
ACGTTACG 0.0244647818698
ACGTTACT 0.0650530913689
ACGTTAGA 0.0283906496028
ACGTTAGC -0.122682944986
ACGTTAGG -0.090144551828
ACGTTAGT 0.0792490756136
ACGTTATA 0.201270624627
ACGTTATC 0.00256855689291
ACGTTATG -0.123800871298
ACGTTCAA -0.0368623266365

ACGTTCAC -0.136864375233
ACGTTCAG -0.230625502502
ACGTTCAT -0.15997915581
ACGTTCCA -0.113224375464
ACGTTCCC -0.150425857919
ACGTTCCG -0.140976952526
ACGTTCCT -0.0724309501948
ACGTTCGA -0.176651113152
ACGTTCGC -0.17468668569З
ACGTTCGG -0.0765969837306
ACGTTCTA 0.126507448742
ACGTTCTC -0.215023955845
ACGTTCTG -0.000839682657864
ACGTTGAA 0.0268366386138
ACGTTGAC -0.133448037213
ACGTTGAG -0.0378714334723
ACGTTGAT 0.0979299723364
ACGTTGCA 0.107489031731
ACGTTGCC -0.27414127819
ACGTTGCG 0.0815760664246
ACGTTGCT -0.0972990859081
ACGTTGGA -0.0923147780562
ACGTTGGC -0.372795157409
ACGTTGGG -0.110601009725
ACGTTGTA -0.0529718327336
ACGTTGTC -0.0519160855647
ACGTTGTG -0.0762411269875
ACGTTTAA 0.0020514744197
ACGTTTAC 0.12910844729
ACGTTTAG -0.0872150304255
ACGTTTAT -0.0408949659886
ACGTTTCA 0.0265761629398
ACGTTTCC 0.0595775274994
ACGTTTCG 0.0133976952159
ACGTTTCT -0.0482624468077
ACGTTTGA 0.0872677084798
ACGTTTGC -0.0513284451185
ACGTTTGG 0.0116136143004
ACGTTTTA -0.0938247094346
ACGTTTTC -0.0882048015706
ACGTTTTG 0.0505781263357
ACTAAAAA 0.150728816157
ACTAAAAC 0.071320336051
ACTAAAAG 0.0657029408115
ACTAAAAT 0.257664962652
ACTAAACA 0.143237009409
ACTAAACC -0.123864278722
ACTAAACG 0.0489594277473
ACTAAACT -0.0186017824427
ACTAAAGA 0.0401103582922
ACTAAAGC -0.245038201362
ACTAAAGG 0.0335169880624
ACTAAAGT 0.0376914698543
ACTAAATA 0.01753757217

ACTAAATC 0.211296832916
ACTAAATG -0.0372495492799
ACTAACAA -0.0154410608956
ACTAACAC -0.0541018041119
ACTAACAG 0.0880784365633
ACTAACAT 0.166670072918
ACTAACCA -0.00270380573411
ACTAACCC -0.332438854295
ACTAACCG -0.0794196400257
ACTAACCT -0.269942295339
ACTAACGA -0.110745750093
ACTAACGC -0.21017587078
ACTAACGG 0.109794011723
ACTAACTA 0.235038068371
ACTAACTC 0.0259281319887
ACTAACTG -0.0146110297625
ACTAAGAA 0.0948620797106
ACTAAGAC -0.0775174167332
ACTAAGAG 0.0384695990757
ACTAAGAT 0.210611437884
ACTAAGCA 0.00710317994157
ACTAAGCC -0.157881822969
ACTAAGCG -0.191646828206
ACTAAGCT -0.0661787513338
ACTAAGGA -0.195723052898
ACTAAGGC -0.181174945885
ACTAAGGG -0.0561939032965
ACTAAGTA -0.0227789773244
ACTAAGTC -0.0752403561158
ACTAAGTG -0.0993297495172
ACTAATAA 0.0341925947987
ACTAATAC -0.0673993552781
ACTAATAG -0.125853279058
ACTAATAT 0.210020948446
ACTAATCA 0.122615563163
ACTAATCC 0.00751698053716
ACTAATCG 0.000813163185196
ACTAATCT 0.142504763717
ACTAATGA 0.081144938432
ACTAATGC -0.151492610419
ACTAATGG 0.0216411427437
ACTAATTA 0.177297577425
ACTAATTC 0.100826259279
ACTAATTG 0.0295898629957
ACTACAAA 0.0966635233392
ACTACAAC 0.0338613023072
ACTACAAG -0.104171380441
ACTACAAT 0.164988357919
ACTACACA -0.0503128817968
ACTACACC -0.0286029831484
ACTACACG 0.0704449021116
ACTACACT 0.109966441506
ACTACAGA -0.0435203038313
ACTACAGC -0.0909603761109

ACTACAGG -0.0548910451012
ACTACAGT -0.143604618506
ACTACATA -0.00775530462837
ACTACATC -0.0157223339042
ACTACATG 0.058336702922
ACTACCAA -0.00282876285549
ACTACCAC 0.00270104815559
ACTACCAG -0.248315334868
ACTACCAT 0.0201331357707
ACTACCCA -0.0323270293351
ACTACCCC -0.337398204136
ACTACCCG 0.020435764449
ACTACCCT -0.239483912967
ACTACCGA 0.0846231906838
ACTACCGC -0.203994972173
ACTACCGG 0.0925227914717
ACTACCTA 0.00482976400228
ACTACCTC -0.238418246489
ACTACCTG -0.157140178618
ACTACGAA -0.0354014145676
ACTACGAC -0.336364087425
ACTACGAG -0.0281590130075
ACTACGAT 0.140153865244
ACTACGCA 0.10154920761
ACTACGCC -0.193015937424
ACTACGCG 0.0458819701244
ACTACGCT -0.0286663192463
ACTACGGA -0.00915118826328
ACTACGGC -0.194540237014
ACTACGGG -0.155317096032
ACTACGTA -0.200710283865
ACTACGTC -0.149429303213
ACTACGTG -0.171579939949
ACTACTAA 0.0542098572402
ACTACTAC 0.0322992927162
ACTACTAG -0.0390073268861
ACTACTAT 0.0743042123165
ACTACTCA -0.10147588009
ACTACTCC -0.126875711004
ACTACTCG -0.0422046857638
ACTACTCT 0.0699501154047
ACTACTGA 0.127964351146
ACTACTGC -0.0358550137183
ACTACTGG -0.0710206670251
ACTACTTA 0.0157186338985
ACTACTTC -0.0120763778895
ACTACTTG 0.163296905721
ACTAGAAA 0.234630628526
ACTAGAAC 0.0180221543858
ACTAGAAG 0.0676620219995
ACTAGAAT 0.240611134551
ACTAGACA 0.091219318492
ACTAGACC -0.209203607658
ACTAGACG -0.209643267744

ACTAGACT -0.00842098348211
ACTAGAGA -0.053819274664
ACTAGAGC -0.111123031808
ACTAGAGG 0.0159153644002
ACTAGAGT -0.0848910077965
ACTAGATA 0.17872151153
ACTAGATC 0.210994741298
ACTAGATG -0.220603926045
ACTAGCAA 0.0336656861529
ACTAGCAC -0.214496689544
ACTAGCAG -0.0521306430397
ACTAGCAT -0.094870474678
ACTAGCCA -0.170789358842
ACTAGCCC -0.190719191017
ACTAGCCG -0.216021151362
ACTAGCCT -0.0275110763998
ACTAGCGA -0.0376213964257
ACTAGCGC -0.209123491616
ACTAGCGG 0.0260791563815
ACTAGCTA -0.127530609938
ACTAGCTC 0.0272119220669
ACTAGCTG -0.204058859403
ACTAGGAA -0.0150865309519
ACTAGGAC -0.0472732607449
ACTAGGAG -0.149488131771
ACTAGGAT 0.223842543594
ACTAGGCA -0.0246792565203
ACTAGGCC -0.161300985524
ACTAGGCG -0.0103798738809
ACTAGGCT -0.196745990389
ACTAGGGA -0.307101484036
ACTAGGGC -0.103166013348
ACTAGGGG -0.108012208134
ACTAGGTA -0.0318944338288
ACTAGGTC -0.31664309522
ACTAGGTG -0.248319713465
ACTAGTAA 0.168055314291
ACTAGTAC 0.00677399162248
ACTAGTAG 0.00716991598724
ACTAGTAT 0.0772327964861
ACTAGTCA -0.246731174792
ACTAGTCC -0.156120167509
ACTAGTCG -0.166160801665
ACTAGTCT -0.0718181353693
ACTAGTGA -0.0645379217162
ACTAGTGC -0.188938578424
ACTAGTGG 0.0613322336007
ACTAGTTA 0.0821492960008
ACTAGTTC -0.0732086636297
ACTAGTTG 0.039913724517
ACTATAAA 0.129310307074
ACTATAAC -0.0983378966728
ACTATAAG -0.0438888199528
ACTATAAT 0.193633026966

ACTATACA 0.0535094322973
ACTATACC 0.0269126304615
ACTATACG 0.173378612773
ACTATACT -0.0798734334285
ACTATAGA -0.0529455559367
ACTATAGC -0.0749394082272
ACTATAGG 0.150921512668
ACTATAGT -0.043700506341
ACTATATA 0.0634197791289
ACTATATC 0.154395442274
ACTATATG -0.105661189901
ACTATCAA -0.00100800712393
ACTATCAC 0.000139413005555
ACTATCAG 0.077558274528
ACTATCAT 0.229499094225
ACTATCCA 0.136209211967
ACTATCCC -0.0551467851205
ACTATCCG 0.148575578637
ACTATCCT 0.114391024845
ACTATCGA 0.158051467851
ACTATCGC -0.0487724235785
ACTATCGG 0.0229444320353
ACTATCTA 0.06345877558
ACTATCTC 0.140690277054
ACTATCTG 0.167430515915
ACTATGAA -0.121361576024
ACTATGAC -0.0380111789374
ACTATGAG -0.133538180745
ACTATGAT 0.173702035722
ACTATGCA 0.1055421813
ACTATGCC -0.0478930256071
ACTATGCG 0.0415084505994
ACTATGCT 0.125498777014
ACTATGGA -0.0735655387741
ACTATGGC -0.205200649881
ACTATGGG -0.0658606264667
ACTATGTA 0.0567413143171
ACTATGTC -0.185121965815
ACTATGTG 0.0184368225513
ACTATTAA -0.036382031249
ACTATTAC 0.0665720817236
ACTATTAG 0.0486395486395
ACTATTAT 0.188269536754
ACTATTCA -0.0488149894528
ACTATTCC 0.0414778538762
ACTATTCG -0.0472524866464
ACTATTCT -0.0339402763645
ACTATTGA 0.120933848164
ACTATTGC -0.0230466972743
ACTATTGG -0.0695495719072
ACTATTTA 0.0739982406649
ACTATTTC -0.0105430414467
ACTATTTG 0.139286730947
ACTCAAAA 0.116324313294

ACTCAAAC 0.0206031984701
ACTCAAAG -0.148652870087
ACTCAAAT 0.18234856171
ACTCAACA 0.0592330094961
ACTCAACC -0.112066260215
ACTCAACG -0.0243513299939
ACTCAACT -0.0160500135034
ACTCAAGA -0.0646089597903
ACTCAAGC -0.149830168056
ACTCAAGG 0.0384042685197
ACTCAAGT -0.153374219698
ACTCAATA 0.114112735325
ACTCAATC -0.140344128047
ACTCAATG -0.158539914142
ACTCACAA -0.0517517725009
ACTCACAC -0.242860277322
ACTCACAG -0.114578421884
ACTCACAT 0.157952718559
ACTCACCA -0.0573221769538
ACTCACCC -0.230794359674
ACTCACCG -0.179056277979
ACTCACCT -0.10102581019
ACTCACGA -0.0525240493974
ACTCACGC -0.189777213453
ACTCACGG 0.262018159991
ACTCACTA 0.0863740210068
ACTCACTC -0.0545140465059
ACTCACTG -0.173159474951
ACTCAGAA 0.0690091426974
ACTCAGAC -0.131100840962
ACTCAGAG -0.068472223236
ACTCAGAT 0.2465730193
ACTCAGCA -0.301491929659
ACTCAGCC -0.374333076071
ACTCAGCG -0.102114675304
ACTCAGCT -0.143208965459
ACTCAGGA -0.0536489739199
ACTCAGGC -0.215878193481
ACTCAGGG -0.267955450799
ACTCAGTA -0.00958008218798
ACTCAGTC 0.0289828994068
ACTCAGTG -0.146467528386
ACTCATAA 0.0210207075757
ACTCATAC -0.11135004343
ACTCATAG -0.213901819196
ACTCATAT 0.122711911418
ACTCATCA -0.122192440374
ACTCATCC -0.146764831589
ACTCATCG -0.0683922924438
ACTCATCT -0.129587947738
ACTCATGA 0.155861550477
ACTCATGC -0.142571998651
ACTCATGG -0.145537915422
ACTCATTA 0.222157908951

ACTCATTC -0.0311375592446
ACTCATTG -0.0292964387641
ACTCCAAA 0.00483265634781
ACTCCAAC -0.144914983385
ACTCCAAG -0.172230955957
ACTCCAAT 0.157437051376
ACTCCACA 0.0375371342233
ACTCCACC -0.177252128987
ACTCCACG -0.177199696971
ACTCCACT -0.200366415823
ACTCCAGA -0.0810248944694
ACTCCAGC -0.259807935083
ACTCCAGG -0.270146748688
ACTCCAGT -0.272758220524
ACTCCATA -0.0159332277395
ACTCCATC -0.0202876134863
ACTCCATG -0.244141653982
ACTCCCAA -0.0929947194992
ACTCCCAC -0.21510738416
ACTCCCAG -0.306591150247
ACTCCCAT -0.114365786347
ACTCCCCA -0.295550593392
ACTCCCCC -0.167943270899
ACTCCCCG -0.168206806875
ACTCCCCT -0.180719383936
ACTCCCGA -0.0166936850204
ACTCCCGC -0.11861414293
ACTCCCGG -0.142094954251
ACTCCCTA -0.00870437920985
ACTCCCTC -0.242618780386
ACTCCCTG -0.347942540862
ACTCCGAA -0.10033189764
ACTCCGAC -0.230783757482
ACTCCGAG -0.0266979555192
ACTCCGAT 0.0187804884775
ACTCCGCA -0.144370283988
ACTCCGCC -0.265178794279
ACTCCGCG 0.0517004607914
ACTCCGCT -0.15148098687
ACTCCGGA -0.157269144169
ACTCCGGC -0.31169876433
ACTCCGGG -0.158381256016
ACTCCGTA 0.0522630068085
ACTCCGTC -0.234249666812
ACTCCGTG -0.135873236446
ACTCCTAA -0.175217641951
ACTCCTAC -0.255717636811
ACTCCTAG -0.230631710982
ACTCCTAT -0.132519571914
ACTCCTCA -0.117097720852
ACTCCTCC -0.156420728138
ACTCCTCG -0.122838142974
ACTCCTCT 0.0846397361549
ACTCCTGA -0.0534241589531

ACTCCTGC -0.169140087368
ACTCCTGG -0.206425298949
ACTCCTTA 0.0247971313001
ACTCCTTC -0.194016844873
ACTCCTTG -0.146158292114
ACTCGAAA 0.0353357225754
ACTCGAAC -0.11023566684
ACTCGAAG -0.079469276439
ACTCGAAT 0.103984149439
ACTCGACA -0.115172544641
ACTCGACC -0.10355196003
ACTCGACG -0.07525129497
ACTCGACT -0.133683528422
ACTCGAGA -0.0815955522925
ACTCGAGC -0.239287929537
ACTCGAGG 0.0639307168456
ACTCGAGT -0.234301913732
ACTCGATA 0.115367433549
ACTCGATC -0.0305093874477
ACTCGATG -0.26965361342
ACTCGCAA 0.131062093531
ACTCGCAC -0.0532754908205
ACTCGCAG -0.0348262446808
ACTCGCAT 0.0554403918162
ACTCGCCA -0.226363083869
ACTCGCCC -0.150090839667
ACTCGCCG -0.128624016044
ACTCGCCT -0.300984113226
ACTCGCGA 0.12980664657
ACTCGCGC -0.166725945778
ACTCGCGG -0.0540058649828
ACTCGCTA 0.0852090830128
ACTCGCTC -0.0848981409166
ACTCGCTG -0.129064006815
ACTCGGAA -0.0834879528186
ACTCGGAC -0.364396960689
ACTCGGAG -0.241977382414
ACTCGGAT 0.280185835347
ACTCGGCA -0.182912233946
ACTCGGCC -0.0142807091816
ACTCGGCG -0.0328885431248
ACTCGGCT -0.0876974574893
ACTCGGGA -0.120197718054
ACTCGGGC -0.184528196155
ACTCGGGG -0.00361932180114
ACTCGGTA 0.0414712426897
ACTCGGTC -0.184116669318
ACTCGGTG -0.1434857112
ACTCGTAA 0.290524295088
ACTCGTAC -0.111112489764
ACTCGTAG 0.0255490755677
ACTCGTAT 0.0275082512346
ACTCGTCA -0.184763332394
ACTCGTCC -0.0644019289147

ACTCGTCG -0.112110534892
ACTCGTCT -0.0583137310731
ACTCGTGA -0.0774521238627
ACTCGTGC -0.237052124276
ACTCGTGG -0.10546327974
ACTCGTTA 0.00452741336751
ACTCGTTC -0.133799774345
ACTCGTTG -0.0655233826996
ACTCTAAA 0.0962540966256
ACTCTAAC 0.112361672968
ACTCTAAG -0.24650236392
ACTCTAAT 0.0881812807019
ACTCTACA 0.0687340232795
ACTCTACC 0.00659739866158
ACTCTACG -0.0600760527034
ACTCTACT 0.00553199564973
ACTCTAGA -0.0539382349292
ACTCTAGC -0.10387550429
ACTCTAGG 0.0071959011353
ACTCTATA 0.0753604844514
ACTCTATC -0.157173884169
ACTCTATG -0.229807822804
ACTCTCAA -0.234247437902
ACTCTCAC -0.086736131284
ACTCTCAG -0.297286235209
ACTCTCAT 0.0322760777306
ACTCTCCA -0.0657394363627
ACTCTCCC -0.23895097577
ACTCTCCG -0.15200006421
ACTCTCCT -0.180801320454
ACTCTCGA -0.139504426633
ACTCTCGC -0.0987137963297
ACTCTCGG -0.160729481242
ACTCTCTA -0.0070332040029
ACTCTCTC -0.0952447918821
ACTCTCTG -0.12975606837
ACTCTGAA -0.152065267684
ACTCTGAC -0.141985741077
ACTCTGAG -0.0987369465978
ACTCTGAT 0.153245549368
ACTCTGCA -0.0481788338055
ACTCTGCC 0.160570936403
ACTCTGCG 0.0437011530589
ACTCTGCT 0.0903975600945
ACTCTGGA -0.238855399271
ACTCTGGC -0.280157657099
ACTCTGGG 0.0084460437606
ACTCTGTA -0.24356537821
ACTCTGTC -0.184113672266
ACTCTGTG -0.0405082303896
ACTCTTAA -0.0497698818975
ACTCTTAC -0.00427779618684
ACTCTTAG -0.167534346948
ACTCTTAT 0.24812020746

ACTCTTCA -0.224350188225
ACTCTTCC -0.253161721586
ACTCTTCG -0.00780532598714
ACTCTTCT -0.0445361887094
ACTCTTGA -0.129304770371
ACTCTTGC -0.101257882445
ACTCTTGG 0.00106785982789
ACTCTTTA 0.16398400035
ACTCTTTC -0.177126536029
ACTCTTTG -0.125451898179
ACTGAAAA 0.289079071261
ACTGAAAC 0.0950336853938
ACTGAAAG -0.0763180606221
ACTGAAAT 0.29056808975
ACTGAACA -0.0415911779548
ACTGAACC -0.0269638861277
ACTGAACG -0.0446931819011
ACTGAACT -0.0737801362014
ACTGAAGA -0.0322510374874
ACTGAAGC -0.159864733341
ACTGAAGG -0.088258648952
ACTGAAGT -0.316128133176
ACTGAATA 0.209351224503
ACTGAATC 0.146153040092
ACTGAATG -0.0793644884554
ACTGACAA 0.0465465465465
ACTGACAC -0.127388289145
ACTGACAG -0.340991894711
ACTGACAT -0.0583664882779
ACTGACCA -0.06812632003
ACTGACCC -0.286599272458
ACTGACCG -0.309219420534
ACTGACCT -0.32426921107
ACTGACGA 0.0904766507866
ACTGACGC -0.109146726439
ACTGACGG 0.0650372619578
ACTGACTA -0.216334702131
ACTGACTC -0.224725285488
ACTGACTG -0.0867699237102
ACTGAGAA 0.14763691306
ACTGAGAC -0.0144668483568
ACTGAGAG -0.102187044251
ACTGAGAT 0.232741618797
ACTGAGCA -0.295758808596
ACTGAGCC -0.253559634179
ACTGAGCG -0.013253706323
ACTGAGCT -0.144994737603
ACTGAGGA 0.0079081934285
ACTGAGGC -0.150066919555
ACTGAGGG -0.171008017609
ACTGAGTA -0.00596556279789
ACTGAGTC -0.270381696134
ACTGAGTG 0.0265521152501
ACTGATAA 0.0429618329526

ACTGATAC 0.236064173777
ACTGATAG 0.0202207250936
ACTGATAT 0.335114896891
ACTGATCA 0.111360335175
ACTGATCC -0.0567328452662
ACTGATCG 0.166352302716
ACTGATCT 0.0286971492915
ACTGATGA 0.0517710992683
ACTGATGC -0.152498228256
ACTGATGG -0.125433706573
ACTGATTA 0.272294037357
ACTGATTC 0.231497337558
ACTGATTG 0.181099089262
ACTGCAAA 0.018702510164
ACTGCAAC -0.038517203589
ACTGCAAG -0.054639572511
ACTGCAAT 0.166442420862
ACTGCACA -0.0813474194919
ACTGCACC -0.304597717923
ACTGCACG -0.0179890634436
ACTGCACT -0.118441853031
ACTGCAGA 0.000875163317592
ACTGCAGC -0.225052097546
ACTGCAGG -0.132652864284
ACTGCAGT -0.270764613973
ACTGCATA 0.0505670960216
ACTGCATC -0.0640778980774
ACTGCATG -0.202618838767
ACTGCCAA -0.183876054909
ACTGCCAC -0.0864531167669
ACTGCCAG -0.259053431579
ACTGCCAT -0.147387591577
ACTGCCCA -0.240650231694
ACTGCCCC -0.263270838039
ACTGCCCG -0.26633018057
ACTGCCCT -0.210164346625
ACTGCCGA 0.0437393316181
ACTGCCGC -0.222253921788
ACTGCCGG -0.0554593695029
ACTGCCTA -0.112262114483
ACTGCCTC -0.281982968104
ACTGCCTG -0.260747596558
ACTGCGAA 0.0473617366043
ACTGCGAC -0.170395285127
ACTGCGAG -0.243955119988
ACTGCGAT 0.173137084589
ACTGCGCA -0.199605429685
ACTGCGCC 0.119441910031
ACTGCGCG -0.0014105014105
ACTGCGCT -0.0568497654912
ACTGCGGA -0.145717646475
ACTGCGGC -0.141511691882
ACTGCGGG 0.113908674515
ACTGCGTA 0.0172648639408

ACTGCGTC -0.14225282223
ACTGCGTG -0.367474520504
ACTGCTAA 0.0687860849762
ACTGCTAC -0.164538449875
ACTGCTAG -0.215663317744
ACTGCTAT 0.0137009832417
ACTGCTCA 0.0019027835965
ACTGCTCC 0.00033773631203
ACTGCTCG 0.0460989094318
ACTGCTCT 0.059561919415
ACTGCTGA -0.0319863230894
ACTGCTGC -0.209682597826
ACTGCTGG -0.111585391074
ACTGCTTA 0.0481514572424
ACTGCTTC -0.195463269044
ACTGCTTG -0.0369253551072
ACTGGAAA 0.143464741648
ACTGGAAC -0.0310186219277
ACTGGAAG -0.0247457328051
ACTGGAAT 0.267237213784
ACTGGACA -0.0804485031971
ACTGGACC -0.244063282179
ACTGGACG -0.116874083049
ACTGGACT -0.20879685919
ACTGGAGA 0.135292657825
ACTGGAGC -0.152787084143
ACTGGAGG -0.0342797879965
ACTGGATA 0.268837708232
ACTGGATC 0.132276905004
ACTGGATG -0.18248808155
ACTGGCAA 0.133505835639
ACTGGCAC -0.40655577228
ACTGGCAG -0.174144000353
ACTGGCAT -0.179894179894
ACTGGCCA -0.247741962789
ACTGGCCC -0.187335538559
ACTGGCCG -0.111461718808
ACTGGCCT -0.237725824832
ACTGGCGA 0.0844879309212
ACTGGCGC -0.16082659151
ACTGGCGG -0.0258679392491
ACTGGCTA -0.169850842578
ACTGGCTC -0.305640330055
ACTGGCTG -0.238442409856
ACTGGGAA -0.0438793665899
ACTGGGAC -0.258932244629
ACTGGGAG -0.180588979582
ACTGGGAT 0.209858150377
ACTGGGCA -0.320307723734
ACTGGGCC -0.121709664701
ACTGGGCG -0.324397156962
ACTGGGCT -0.354197852486
ACTGGGGA -0.162445976381
ACTGGGGC -0.123727898305

ACTGGGGG -0.0842478519334
ACTGGGTA 0.0312943797792
ACTGGGTC -0.371291726261
ACTGGGTG -0.24291652497
ACTGGTAA 0.0951154354284
ACTGGTAC -0.33517532351
ACTGGTAG -0.237035658658
ACTGGTAT -0.0108525083607
ACTGGTCA -0.149173685801
ACTGGTCC -0.327547703531
ACTGGTCG -0.240821597603
ACTGGTCT -0.328337054956
ACTGGTGA -0.0848854509142
ACTGGTGC -0.210950352001
ACTGGTGG -0.290513126881
ACTGGTTA -0.138362933
ACTGGTTC -0.202633602887
ACTGGTTG -0.0875655269595
ACTGTAAA 0.200491230119
ACTGTAAC -0.0743991007496
ACTGTAAG -0.101922007482
ACTGTAAT 0.269146661022
ACTGTACA -0.0250546284173
ACTGTACC -0.196137539497
ACTGTACG 0.069083888674
ACTGTACT -0.0712737530919
ACTGTAGA -0.00295155376217
ACTGTAGC 0.0257654348563
ACTGTAGG 0.00278015425709
ACTGTATA 0.0932737144858
ACTGTATC -0.137665628644
ACTGTATG -0.182459552346
ACTGTCAA -0.0264658438136
ACTGTCAC -0.18956478207
ACTGTCAG -0.157755735597
ACTGTCAT -0.0911384478855
ACTGTCCA -0.0632714900563
ACTGTCCC -0.0801781307303
ACTGTCCG 0.0224910918781
ACTGTCCT -0.00602608961792
ACTGTCGA -0.162406630767
ACTGTCGC 0.0170999400415
ACTGTCGG -0.0712418625042
ACTGTCTA 0.155744764713
ACTGTCTC -0.150154248236
ACTGTCTG 0.0193535907973
ACTGTGAA 0.129587673263
ACTGTGAC -0.00387144807048
ACTGTGAG -0.262569233127
ACTGTGAT 0.250662508238
ACTGTGCA -0.0885374023074
ACTGTGCC -0.103526171121
ACTGTGCG -0.0376983518785
ACTGTGCT -0.0807599164439

ACTGTGGA -0.148283715634
ACTGTGGC -0.133699815518
ACTGTGGG -0.183217670115
ACTGTGTA -0.0257069202349
ACTGTGTC -0.200765870762
ACTGTGTG -0.0295796442414
ACTGTTAA 0.174611504858
ACTGTTAC -0.00686518734104
ACTGTTAG -0.0275709291037
ACTGTTAT 0.188351490185
ACTGTTCA -0.129961625088
ACTGTTCC -0.0150193791377
ACTGTTCG 0.0195296324502
ACTGTTCT 0.025835200689
ACTGTTGA -0.0150191513828
ACTGTTGC -0.213057395965
ACTGTTGG -0.0969337535257
ACTGTTTA 0.0438961190631
ACTGTTTC -0.176604142066
ACTGTTTG -0.0416656325747
ACTTAAAA 0.100597843022
ACTTAAAC 0.118256534528
ACTTAAAG -0.0850750013095
ACTTAAAT 0.126011686618
ACTTAACA 0.157489445368
ACTTAACC -0.0448143471311
ACTTAACG 0.0288687109041
ACTTAACT -0.063185775307
ACTTAAGA -0.0370510134815
ACTTAAGC -0.0318763739816
ACTTAAGG -0.107689376964
ACTTAAGT -0.2302468064
ACTTAATA 0.198412443523
ACTTAATC 0.0170777282249
ACTTAATG 0.0894048318291
ACTTACAA 0.119854013793
ACTTACAC 0.0433764224141
ACTTACAG -0.126139800867
ACTTACAT 0.0677875666956
ACTTACCA -0.107830663502
ACTTACCC -0.24797811903
ACTTACCG -0.15029760674
ACTTACCT -0.038156686482
ACTTACGA 0.199704692657
ACTTACGC -0.0310210325148
ACTTACGG 0.0166240620786
ACTTACTA 0.0616592337394
ACTTACTC -0.0461384484313
ACTTACTG -0.00271759362668
ACTTAGAA 0.000694110932211
ACTTAGAC -0.0103683860937
ACTTAGAG -0.195789186373
ACTTAGAT 0.17377940629
ACTTAGCA -0.0477151204625

ACTTAGCC -0.228312791436
ACTTAGCG 0.00297129085008
ACTTAGCT -0.0465481301338
ACTTAGGA 0.010877268453
ACTTAGGC -0.0661482676373
ACTTAGGG -0.107530812846
ACTTAGTA -0.0207448012087
ACTTAGTC -0.128719800453
ACTTAGTG -0.0226071419709
ACTTATAA 0.135486726396
ACTTATAC 0.106585772714
ACTTATAG 0.121666678832
ACTTATAT 0.0685685685686
ACTTATCA -0.0827094311943
ACTTATCC 0.0857510402965
ACTTATCG 0.0658385658386
ACTTATCT 0.111823980542
ACTTATGA 0.221471792588
ACTTATGC -0.0884286490347
ACTTATGG -0.141476186931
ACTTATTA 0.115640533419
ACTTATTC -0.110640642906
ACTTATTG 0.216995232147
ACTTCAAA 0.0739655985505
ACTTCAAC 0.0191800665029
ACTTCAAG -0.220605223471
ACTTCAAT 0.144539170675
ACTTCACA 0.0153215623016
ACTTCACC -0.03963781815
ACTTCACG -0.211041543525
ACTTCACT -0.0441734013836
ACTTCAGA 0.0103932030685
ACTTCAGC -0.0872471417291
ACTTCAGG -0.121478062593
ACTTCATA 0.0868044352893
ACTTCATC -0.20990568447
ACTTCATG -0.208430443406
ACTTCCAA -0.015273388174
ACTTCCAC -0.145990985403
ACTTCCAG -0.245292380976
ACTTCCAT -0.125415804892
ACTTCCCA -0.0863659803054
ACTTCCCC -0.203805703731
ACTTCCCG -0.218940951725
ACTTCCCT -0.0606576948422
ACTTCCGA 0.146994591359
ACTTCCGC -0.150933253943
ACTTCCGG -0.0337179964429
ACTTCCTA -0.0913359561469
ACTTCCTC -0.243617845494
ACTTCCTG -0.0576716819552
ACTTCGAA 0.0656095122005
ACTTCGAC -0.131205351112
ACTTCGAG -0.147367448836

ACTTCGAT 0.147713829532
ACTTCGCA 0.131605147333
ACTTCGCC -0.149010668652
ACTTCGCG 0.0257158621656
ACTTCGCT 0.0942140487595
ACTTCGGA -0.169773840737
ACTTCGGC -0.0213043154577
ACTTCGGG -0.106286067203
ACTTCGTA -0.0281369345521
ACTTCGTC -0.257088583393
ACTTCGTG -0.124455749955
ACTTCTAA 0.0980029616393
ACTTCTAC -0.0572261506017
ACTTCTAG -0.116226274656
ACTTCTAT -0.0329491110069
ACTTCTCA 0.0465383969694
ACTTCTCC -0.12834032206
ACTTCTCG 0.161140267001
ACTTCTCT 0.153893710357
ACTTCTGA 0.136253220732
ACTTCTGC -0.189223804056
ACTTCTGG -0.0728633679554
ACTTCTTA -0.0167970272247
ACTTCTTC -0.130960981341
ACTTCTTG -0.0613186043808
ACTTGAAA 0.170573637074
ACTTGAAC 0.0971949911344
ACTTGAAG -0.000584600698018
ACTTGAAT 0.107675744327
ACTTGACA 0.0202116717268
ACTTGACC -0.251732571765
ACTTGACG -0.0835118776799
ACTTGACT -0.0324495732859
ACTTGAGA 0.0730952060601
ACTTGAGC -0.192801536824
ACTTGAGG 0.0605440150895
ACTTGATA 0.10529951439
ACTTGATC -0.087855778059
ACTTGATG -0.24389191919
ACTTGCAA -0.0769788279679
ACTTGCAC -0.101762469222
ACTTGCAG -0.18457237759
ACTTGCAT 0.109140821262
ACTTGCCA -0.219418843086
ACTTGCCC -0.264904997781
ACTTGCCG -0.14199414929
ACTTGCCT -0.101656898024
ACTTGCGA 0.292464640949
ACTTGCGC -0.0219820371336
ACTTGCGG -0.0150910737772
ACTTGCTA -0.0282804026189
ACTTGCTC -0.0988091953822
ACTTGCTG 0.0778223834515
ACTTGGAA 0.0132542333471

ACTTGGAC -0.175255467314
ACTTGGAG -0.182902245379
ACTTGGAT 0.231524127209
ACTTGGCA -0.168635089199
ACTTGGCC -0.0102788739152
ACTTGGCG -0.215963625438
ACTTGGCT 0.0773756779049
ACTTGGGA 0.0526375334847
ACTTGGGC -0.186085681285
ACTTGGGG -0.154234952536
ACTTGGTA -0.0926656575432
ACTTGGTC -0.0710250949838
ACTTGGTG -0.0711117351637
ACTTGTAA 0.249275563309
ACTTGTAC -0.0205729145123
ACTTGTAG -0.10773900845
ACTTGTAT 0.0630368660672
ACTTGTCA -0.259799665233
ACTTGTCC -0.0599649236013
ACTTGTCG -0.123461232007
ACTTGTCT -0.0367739171214
ACTTGTGA 0.0263858900223
ACTTGTGC -0.247324950996
ACTTGTGG -0.140129048269
ACTTGTTA -0.0774617592799
ACTTGTTC -0.179419947007
ACTTGTTG -0.12195383625
ACTTTAAA 0.0858660029342
ACTTTAAC 0.080163938016
ACTTTAAG 0.0724237749321
ACTTTAAT 0.0218761632957
ACTTTACA 0.134523070813
ACTTTACC -0.10298217314
ACTTTACG 0.20983986237
ACTTTACT 0.0805363922873
ACTTTAGA 0.128146052388
ACTTTAGC -0.0209939793267
ACTTTAGG -0.081879228922
ACTTTATA 0.0743217301811
ACTTTATC -0.131468018648
ACTTTATG 0.0385706882331
ACTTTCAA -0.168528032164
ACTTTCAC -0.100733130957
ACTTTCAG -0.10888078362
ACTTTCAT 0.00552031707335
ACTTTCCA -0.00742753773057
ACTTTCCC 0.0932176667664
ACTTTCCG -0.0898726464125
ACTTTCCT -0.164722539265
ACTTTCGA -0.00901075158938
ACTTTCGC -0.0752068667918
ACTTTCGG -0.167267699451
ACTTTCTA 0.0580534732816
ACTTTCTC -0.135538163555

ACTTTCTG 0.120121498909
ACTTTGAA 0.106201324059
ACTTTGAC -0.0677799011132
ACTTTGAG -0.0776796079826
ACTTTGAT -0.0638963855809
ACTTTGCA -0.101004210404
ACTTTGCC 0.180322328708
ACTTTGCG -0.0428554096941
ACTTTGCT 0.0652620745531
ACTTTGGA 0.0379594470504
ACTTTGGC 0.00398166463508
ACTTTGGG -0.14295899736
ACTTTGTA -0.0112605718666
ACTTTGTC 0.0146434780578
ACTTTGTG -0.110742830449
ACTTTTAA 0.104924063826
ACTTTTAC -0.0413292079959
ACTTTTAG -0.161697925653
ACTTTTAT 0.17862076953
ACTTTTCA -0.0340082880468
ACTTTTCC -0.148105308173
ACTTTTCG 0.0731075428045
ACTTTTCT 0.00653683987017
ACTTTTGA -0.0362200517551
ACTTTTGC -0.0816422596432
ACTTTTGG -0.230941048192
ACTTTTTA 0.0449793070225
ACTTTTTC -0.0853950443041
ACTTTTTG 0.135053786569
AGAAAAAA 0.0292862200098
AGAAAAAC 0.0840575601969
AGAAAAAG -0.166254530698
AGAAAAAT 0.276878472272
AGAAAACA -0.0462245352292
AGAAAACC -0.145638790325
AGAAAACG -0.0884063366009
AGAAAACT -0.246892907643
AGAAAAGA 0.143781522569
AGAAAAGC -0.0807264419705
AGAAAAGG 0.105142332415
AGAAAATA 0.204102829865
AGAAAATC 0.480499697087
AGAAAATG 0.18541418801
AGAAACAA -0.0528067955303
AGAAACAC -0.01741640694
AGAAACAG 0.0117017844291
AGAAACAT 0.0627044663256
AGAAACCA -0.0779172718846
AGAAACCC -0.218179838525
AGAAACCG 0.0202178054495
AGAAACCT -0.132106608432
AGAAACGA 0.00455391487778
AGAAACGC -0.058180770302
AGAAACGG -0.055716606601

AGAAACTA 0.0384539024693
AGAAACTC -0.165261553866
AGAAACTG -0.0944144141514
AGAAAGAA -0.0379093077443
AGAAAGAC -0.00102444041838
AGAAAGAG -0.237198668309
AGAAAGAT 0.166813135479
AGAAAGCA -0.0581904360126
AGAAAGCC -0.239325475408
AGAAAGCG -0.115210251574
AGAAAGCT -0.0229724896535
AGAAAGGA 0.0218386430508
AGAAAGGC -0.18024986973
AGAAAGGG -0.264576009759
AGAAAGTA 0.0653263067232
AGAAAGTC -0.280216250517
AGAAAGTG 0.229875680738
AGAAATAA 0.141716096262
AGAAATAC 0.104361937695
AGAAATAG -0.0125125125125
AGAAATAT 0.360677503412
AGAAATCA 0.381427413998
AGAAATCC 0.469968540835
AGAAATCG 0.381459972369
AGAAATCT 0.469928606292
AGAAATGA -0.0541426100158
AGAAATGC -0.00948037239608
AGAAATGG -0.131928419996
AGAAATTA 0.10972722083
AGAAATTC 0.0459165127771
AGAAATTG 0.16861075952
AGAACAAA 0.0468420399553
AGAACAAC 0.0595687685671
AGAACAAG -0.0623440399478
AGAACAAT 0.260335175142
AGAACACA -0.187781233626
AGAACACC -0.288524463631
AGAACACG -0.16916827961
AGAACACT 0.00128089522029
AGAACAGA 0.149477300992
AGAACAGC -0.10633764884
AGAACAGG -0.0612956153501
AGAACATA 0.0590955902424
AGAACATC -0.0588082028017
AGAACATG 0.0947462614129
AGAACCAA -0.143051006228
AGAACCAC -0.157407637183
AGAACCAG -0.232504344942
AGAACCAT -0.165452762083
AGAACCCA -0.129297197107
AGAACCCC -0.21698687994
AGAACCCG -0.133836411702
AGAACCCT -0.239903592866
AGAACCGA 0.0861054137501

AGAACCGC -0.0455989279599
AGAACCGG -0.174283587644
AGAACCTA -0.100318162248
AGAACCTC -0.263411891762
AGAACCTG -0.0450456828636
AGAACGAA -0.0393478878327
AGAACGAC -0.101649727113
AGAACGAG -0.20904599095
AGAACGAT -0.00482738080465
AGAACGCA -0.281965292423
AGAACGCC -0.25354685231
AGAACGCG -0.124306916907
AGAACGCT -0.150401318315
AGAACGGA 0.14078954988
AGAACGGC -0.114095287024
AGAACGGG -0.0906830512161
AGAACGTA 0.0778165441633
AGAACGTC -0.188461419179
AGAACGTG 0.140613064855
AGAACTAA 0.146968305577
AGAACTAC -0.00711599429937
AGAACTAG -0.161212717793
AGAACTAT -0.069911607775
AGAACTCA -0.152137770168
AGAACTCC -0.309509832293
AGAACTCG -0.171039573254
AGAACTCT -0.029138777983
AGAACTGA 0.0822985519955
AGAACTGC -0.120286055353
AGAACTGG -0.020683328541
AGAACTTA 0.00306258809268
AGAACTTC -0.258771821016
AGAACTTG -0.0295095727831
AGAAGAAA -0.0938567540965
AGAAGAAC -0.157350934419
AGAAGAAG -0.050756684251
AGAAGAAT 0.170845163975
AGAAGACA -0.140069470289
AGAAGACC -0.25842450404
AGAAGACG -0.0664585314753
AGAAGACT -0.242375795184
AGAAGAGA -0.0336204316694
AGAAGAGC 0.0420421450625
AGAAGAGG -0.120665951531
AGAAGATA 0.249307679394
AGAAGATC 0.363416129574
AGAAGATG -0.0681558770254
AGAAGCAA -0.118888861313
AGAAGCAC -0.0483875859977
AGAAGCAG -0.0719408392684
AGAAGCAT -0.0710509988832
AGAAGCCA -0.117078571001
AGAAGCCC -0.33442703568
AGAAGCCG -0.301963346095

AGAAGCCT -0.190551301796
AGAAGCGA -0.00262840959687
AGAAGCGC -0.181981052352
AGAAGCGG -0.0761623178817
AGAAGCTA -0.217344792123
AGAAGCTC -0.352306981418
AGAAGCTG -0.016285044853
AGAAGGAA -0.0308672072874
AGAAGGAC -0.104909622949
AGAAGGAG -0.274554310822
AGAAGGAT 0.0568593388466
AGAAGGCA -0.251293415913
AGAAGGCC -0.20913581472
AGAAGGCG -0.23928739796
AGAAGGCT -0.355857328172
AGAAGGGA 0.0325925590724
AGAAGGGC -0.347782024174
AGAAGGGG -0.249854588257
AGAAGGTA 0.0532096106091
AGAAGGTC -0.373184425195
AGAAGGTG 0.147709506911
AGAAGTAA 0.161407797106
AGAAGTAC -0.143390654855
AGAAGTAG -0.0901007615136
AGAAGTAT 0.182605470484
AGAAGTCA -0.17502268884
AGAAGTCC -0.174715500671
AGAAGTCG -0.111295446085
AGAAGTCT 0.103859769494
AGAAGTGA -0.147331783156
AGAAGTGC -0.084686995068
AGAAGTGG -0.27175486037
AGAAGTTA 0.130335102151
AGAAGTTC -0.170923911061
AGAAGTTG 0.0900148171938
AGAATAAA 0.169005093248
AGAATAAC 0.123729407385
AGAATAAG -0.242531926788
AGAATAAT 0.165548924238
AGAATACA 0.0541243723062
AGAATACC -0.0325193428335
AGAATACG 0.200204695054
AGAATACT -0.0714943593731
AGAATAGA 0.18716374777
AGAATAGC -0.0617496574434
AGAATAGG -0.185507282336
AGAATATA 0.244291123079
AGAATATC 0.436970027879
AGAATATG 0.217225604778
AGAATCAA 0.31233575173
AGAATCAC 0.411523105333
AGAATCAG 0.379444182474
AGAATCAT 0.335956876857
AGAATCCA 0.474435596301

AGAATCCC 0.439375139589
AGAATCCG 0.475123884215
AGAATCCT 0.443323488778
AGAATCGA 0.34971132019
AGAATCGC 0.370172186011
AGAATCGG 0.378519448479
AGAATCTA 0.485947752969
AGAATCTC 0.478339160456
AGAATCTG 0.476342733918
AGAATGAA 0.00498432316614
AGAATGAC -0.131919116735
AGAATGAG -0.0596533172291
AGAATGAT 0.158307067398
AGAATGCA -0.0479860025315
AGAATGCC -0.348074355626
AGAATGCG -0.247069769478
AGAATGCT -0.24380901184
AGAATGGA -0.0455426568752
AGAATGGC 0.254948431786
AGAATGGG -0.0961324313871
AGAATGTA 0.197758443246
AGAATGTC -0.199039498025
AGAATGTG 0.105310837343
AGAATTAA -0.128688786614
AGAATTAC 0.243354925173
AGAATTAG 0.0237464765294
AGAATTAT 0.0741774832684
AGAATTCA 0.0491692672649
AGAATTCC 0.0681339309157
AGAATTCG 0.179833288322
AGAATTCT 0.352429737306
AGAATTGA -0.0165917946936
AGAATTGC 0.0843501904108
AGAATTGG -0.0888415044311
AGAATTTA 0.220267637789
AGAATTTC 0.278647182908
AGAATTTG 0.242500075833
AGACAAAA 0.0455055039858
AGACAAAC 0.145695349737
AGACAAAG -0.138492565666
AGACAAAT 0.192514331092
AGACAACA -0.0726181178514
AGACAACC -0.145865213088
AGACAACG -0.212368825821
AGACAACT -0.155053475272
AGACAAGA 0.119809892537
AGACAAGC -0.21431281585
AGACAAGG -0.128816040896
AGACAATA -0.0792137979132
AGACAATC 0.319144213084
AGACAATG 0.0673189071147
AGACACAA 0.076674963322
AGACACAC -0.119405495581
AGACACAG -0.133016227544

AGACACAT 0.0135639365561
AGACACCA -0.075388848908
AGACACCC -0.230430467684
AGACACCG 0.0411512823159
AGACACCT 0.0311240509803
AGACACGA 0.0346586255677
AGACACGC -0.295258158341
AGACACGG -0.228385565288
AGACACTA -0.106964502841
AGACACTC -0.114169003251
AGACACTG 0.0350364590556
AGACAGAA -0.137262768389
AGACAGAC -0.041491414179
AGACAGAG -0.0848893819843
AGACAGAT 0.288039677963
AGACAGCA -0.096298768564
AGACAGCC -0.294716371585
AGACAGCG -0.162364938532
AGACAGCT -0.0880584299031
AGACAGGA 0.00678539031848
AGACAGGC -0.115437837459
AGACAGGG -0.136598371617
AGACAGTA 0.186377670562
AGACAGTC -0.22498187342
AGACAGTG 0.214958066612
AGACATAA -0.220263838147
AGACATAC -0.022252797384
AGACATAG -0.029934202362
AGACATAT 0.11336029138
AGACATCA 0.0174929092663
AGACATCC -0.0931999024887
AGACATCG -0.0178153359972
AGACATCT 0.176091764414
AGACATGA -0.0644165942838
AGACATGC -0.160658635523
AGACATGG -0.0533384624294
AGACATTA -0.0205900600717
AGACATTC -0.0752588548345
AGACATTG 0.0386928516098
AGACCAAA -0.0712637403538
AGACCAAC 0.00674948723751
AGACCAAG -0.0990262942358
AGACCAAT 0.135596300811
AGACCACA -0.127238364639
AGACCACC -0.358763392402
AGACCACG -0.303756349935
AGACCACT -0.164932579949
AGACCAGA -0.098402810524
AGACCAGC -0.150443478883
AGACCAGG -0.240698466264
AGACCATA -0.107206812759
AGACCATC -0.190356345367
AGACCATG -0.0899269759764
AGACCCAA -0.191611797693

AGACCCAC -0.203217063561
AGACCCAG -0.355128357445
AGACCCAT -0.234670322374
AGACCCCA -0.245841456297
AGACCCCC -0.368818233412
AGACCCCG -0.257044159596
AGACCCCT -0.200850222013
AGACCCGA -0.155896015107
AGACCCGC -0.412983782128
AGACCCGG -0.197178051759
AGACCCTA -0.352773436316
AGACCCTC -0.28791129403
AGACCCTG -0.103166530214
AGACCGAA -0.0825660371115
AGACCGAC -0.207007819879
AGACCGAG -0.251248108457
AGACCGAT -0.0539357193865
AGACCGCA -0.159196896297
AGACCGCC -0.376334759703
AGACCGCG -0.275189439359
AGACCGCT -0.0977015772192
AGACCGGA 0.0584768216754
AGACCGGC -0.389659740673
AGACCGGG -0.182429701147
AGACCGTA -0.0153927516812
AGACCGTC -0.282463975346
AGACCGTG -0.0735699968846
AGACCTAA -0.168185291371
AGACCTAC -0.120745223571
AGACCTAG -0.266344774444
AGACCTAT -0.0374013707729
AGACCTCA -0.353526709057
AGACCTCC -0.334065034601
AGACCTCG -0.0900466635626
AGACCTCT -0.00983701086838
AGACCTGA -0.124299369467
AGACCTGC -0.188162204412
AGACCTGG -0.256617300489
AGACCTTA -0.179689586675
AGACCTTC -0.183792701382
AGACCTTG -0.157852504446
AGACGAAA -0.120032936364
AGACGAAC -0.132621156853
AGACGAAG -0.149912953306
AGACGAAT -0.098478519217
AGACGACA -0.217011691989
AGACGACC -0.354092758441
AGACGACG -0.238610740165
AGACGACT -0.168193078159
AGACGAGA 0.189971752443
AGACGAGC -0.194115895881
AGACGAGG -0.264853265858
AGACGATA 0.0395698729032
AGACGATC 0.250015693087

AGACGATG 0.0908332574999
AGACGCAA 0.0187804084087
AGACGCAC -0.192917049205
AGACGCAG -0.0825202218197
AGACGCAT -0.0849553995647
AGACGCCA -0.111239300335
AGACGCCC -0.403311877253
AGACGCCG -0.255426558508
AGACGCCT -0.219653132794
AGACGCGA 0.168460471491
AGACGCGC -0.149391271856
AGACGCGG -0.152156463846
AGACGCTA -0.171092140854
AGACGCTC -0.143407254975
AGACGCTG -0.131559355992
AGACGGAA -0.0846312701446
AGACGGAC -0.261251581454
AGACGGAG -0.109314905337
AGACGGAT 0.041545689919
AGACGGCA -0.138274922386
AGACGGCC -0.415712518153
AGACGGCG -0.40284977306
AGACGGCT -0.273073594716
AGACGGGA -0.0227476770582
AGACGGGC -0.132495705711
AGACGGGG -0.274780993297
AGACGGTA -0.0932115767968
AGACGGTC -0.395180737048
AGACGGTG -0.11989266067
AGACGTAA -0.0442158200915
AGACGTAC 0.0491662461359
AGACGTAG -0.295438135143
AGACGTAT 0.154659718966
AGACGTCA -0.034893396495
AGACGTCC -0.16446804346
AGACGTCG -0.103728427537
AGACGTCT 0.134067348587
AGACGTGA -0.0776727140364
AGACGTGC -0.207338821603
AGACGTGG -0.344450508107
AGACGTTA -0.0869912243699
AGACGTTC -0.0799825186876
AGACGTTG -0.0830181490667
AGACTAAA -0.115347565719
AGACTAAC -0.0196408529742
AGACTAAG -0.0349845354881
AGACTAAT -0.108841736637
AGACTACA -0.16892042776
AGACTACC -0.181899049162
AGACTACG -0.0574506349181
AGACTACT -0.04588293687
AGACTAGA 0.117146071692
AGACTAGC -0.0458756192981
AGACTAGG -0.111412357305

AGACTATA 0.00438749516434
AGACTATC -0.0871404275819
AGACTATG -0.012964755389
AGACTCAA -0.142535097081
AGACTCAC -0.181298710019
AGACTCAG -0.278446258301
AGACTCAT 0.0635044524767
AGACTCCA -0.141437327841
AGACTCCC -0.447592309746
AGACTCCG -0.283919833492
AGACTCCT -0.413392410272
AGACTCGA 0.0161566525203
AGACTCGC -0.290678771198
AGACTCGG -0.363511200417
AGACTCTA -0.170631729356
AGACTCTC -0.141015038112
AGACTCTG -0.00669782575726
AGACTGAA -0.0608498349835
AGACTGAC -0.209979770647
AGACTGAG -0.0294440584601
AGACTGAT 0.00844872010102
AGACTGCA -0.330010724369
AGACTGCC -0.374550437781
AGACTGCG -0.171589424754
AGACTGCT -0.185884023212
AGACTGGA 0.132010977862
AGACTGGC 0.0541285795039
AGACTGGG -0.102015883111
AGACTGTA -0.107548739809
AGACTGTC -0.0763107617691
AGACTGTG 0.0791273367031
AGACTTAA -0.100481133466
AGACTTAC -0.144891202964
AGACTTAG -0.0761009514514
AGACTTAT 0.0118617840485
AGACTTCA -0.194826944548
AGACTTCC -0.293797295621
AGACTTCG -0.0594212968707
AGACTTGA -0.126626912251
AGACTTGC -0.197481702022
AGACTTGG -0.213448150575
AGACTTTA -0.07073878286
AGACTTTC -0.13947691332
AGACTTTG 0.0700025093964
AGAGAAAA -0.0225666591243
AGAGAAAC -0.0138844082686
AGAGAAAG -0.0498380003033
AGAGAAAT 0.247469232318
AGAGAACA -0.0214046407743
AGAGAACC -0.0982978089874
AGAGAACG -0.246161446195
AGAGAACT -0.0997411046687
AGAGAAGA 0.0211082968986
AGAGAAGC -0.119161062445

AGAGAAGG -0.185126822234
AGAGAATA 0.203416915538
AGAGAATC 0.480298590909
AGAGAATG -0.118049868614
AGAGACAA -0.0780774819337
AGAGACAC -0.0834015766269
AGAGACAG -0.179872845725
AGAGACAT -0.0432027035904
AGAGACCA -0.0554493887827
AGAGACCC -0.333093554922
AGAGACCG -0.0975003247943
AGAGACCT -0.270477883913
AGAGACGA -0.0347305642686
AGAGACGC -0.357338548083
AGAGACGG -0.396908179151
AGAGACTA -0.135805271216
AGAGACTC -0.156951717558
AGAGACTG -0.0486033565763
AGAGAGAA -0.108995089728
AGAGAGAC -0.331725570937
AGAGAGAG -0.0557471787279
AGAGAGAT 0.278514051241
AGAGAGCA -0.230394953606
AGAGAGCC -0.280310248774
AGAGAGCG -0.11553041268
AGAGAGCT -0.226143125919
AGAGAGGA 0.00743962671564
AGAGAGGC -0.418112710451
AGAGAGGG -0.23175186003
AGAGAGTA 0.0761629397993
AGAGAGTC -0.119732000683
AGAGAGTG -0.0744559986984
AGAGATAA 0.0354934059056
AGAGATAC 0.205873917995
AGAGATAG 0.0938629081115
AGAGATAT 0.416668734645
AGAGATCA 0.187056202208
AGAGATCC 0.359676065488
AGAGATCG 0.276101253257
AGAGATCT 0.421568951872
AGAGATGA -0.111073893038
AGAGATGC -0.0430779404101
AGAGATGG -0.0718453928307
AGAGATTA 0.315858046904
AGAGATTC 0.41392305278
AGAGATTG 0.358610395393
AGAGCAAA -0.0660146288844
AGAGCAAC -0.234607719089
AGAGCAAG -0.120268978451
AGAGCAAT 0.223664445304
AGAGCACA -0.151300013865
AGAGCACC -0.412523393336
AGAGCACG -0.306726714622
AGAGCACT -0.203979379454

AGAGCAGA 0.00865266572172
AGAGCAGC -0.437124024151
AGAGCAGG -0.310555929265
AGAGCATA -0.0404221094378
AGAGCATC -0.0858499448917
AGAGCATG -0.249217770059
AGAGCCAA -0.318912124896
AGAGCCAC -0.120567229265
AGAGCCAG -0.160461708441
AGAGCCAT -0.1124001511
AGAGCCCA -0.187382998492
AGAGCCCC -0.432190616329
AGAGCCCG -0.105201345956
AGAGCCCT -0.32894015186
AGAGCCGA -0.196938620794
AGAGCCGC -0.432653266669
AGAGCCGG -0.295170490612
AGAGCCTA -0.228712030208
AGAGCCTC 0.0166637714966
AGAGCCTG -0.116736833109
AGAGCGAA -0.0642910215182
AGAGCGAC -0.224066320403
AGAGCGAG -0.113459189217
AGAGCGAT 0.147970284334
AGAGCGCA -0.243712176379
AGAGCGCC -0.191698618261
AGAGCGCG -0.180888778619
AGAGCGCT -0.118158726451
AGAGCGGA -0.0869770775614
AGAGCGGC -0.262028194634
AGAGCGGG -0.0637311256124
AGAGCGTA 0.0707451661642
AGAGCGTC -0.31206065497
AGAGCGTG -0.162740998983
AGAGCTAA -0.116050743414
AGAGCTAC -0.0356761720398
AGAGCTAG -0.047567571513
AGAGCTAT -0.0152938256826
AGAGCTCA -0.157814116888
AGAGCTCC -0.330853713884
AGAGCTCG -0.359921117257
AGAGCTCT -0.178664450673
AGAGCTGA -0.119844007179
AGAGCTGC -0.165661025936
AGAGCTGG -0.211384149163
AGAGCTTA -0.107066704613
AGAGCTTC -0.232875062939
AGAGCTTG -0.206888706889
AGAGGAAA -0.124346238996
AGAGGAAC -0.210591686448
AGAGGAAG -0.370625008208
AGAGGAAT 0.23630783419
AGAGGACA -0.119843928803
AGAGGACC -0.430203140115

AGAGGACG	-0.288944294216
AGAGGACT	-0.153515616446
AGAGGAGA	-0.0113639847303
AGAGGAGC	-0.336449545957
AGAGGAGG	-0.365386682542
AGAGGATA	0.262550562077
AGAGGATC	0.383324095445
AGAGGATG	-0.138964014259
AGAGGCAA	-0.143994839606
AGAGGCAC	-0.104493514728
AGAGGCAG	-0.212923334574
AGAGGCAT	-0.0917562101397
AGAGGCCA	-0.258148855646
AGAGGCCC	-0.459502021179
AGAGGCCG	-0.0893341052254
AGAGGCCT	-0.174024232437
AGAGGCGA	0.114587038829
AGAGGCGC	-0.331778599251
AGAGGCGG	-0.149433175276
AGAGGCTA	-0.231406348655
AGAGGCTC	-0.151772878858
AGAGGCTG	-0.242022632979
AGAGGGAA	-0.227312334102
AGAGGGAC	-0.229111038282
AGAGGGAG	-0.0988672991924
AGAGGGAT	4.30626130394E-5
AGAGGGCA	-0.200812123242
AGAGGGCC	-0.481059252377
AGAGGGCG	-0.216893218084
AGAGGGCT	-0.207668359545
AGAGGGGA	-0.124133628144
AGAGGGGC	-0.298491292834
AGAGGGGG	-0.243138081888
AGAGGGTA	-0.0133502167459
AGAGGGTC	-0.39336613061
AGAGGGTG	-0.137844887477
AGAGGTAA	-0.0422984341266
AGAGGTAC	0.0258180944158
AGAGGTAG	-0.272725481927
AGAGGTAT	-0.0525320207911
AGAGGTCA	-0.25144311254
AGAGGTCC	-0.245545128638
AGAGGTCG	-0.274226453587
AGAGGTGA	-0.222696021781
AGAGGTGC	-0.42344968452
AGAGGTGG	-0.117735360526
AGAGGTTA	-0.13488577298
AGAGGTTC	-0.169712677487
AGAGGTTG	0.0419195200105
AGAGTAAA	-0.160678395077
AGAGTAAC	0.110344661477
AGAGTAAG	-0.0485306968561
AGAGTAAT	0.230376707079
AGAGTACA	-0.138925593687

AGAGTACC -0.266223569981
AGAGTACG -0.131897053268
AGAGTACT -0.0338200217838
AGAGTAGA 0.144588927215
AGAGTAGC -0.219348302527
AGAGTAGG -0.0701332892252
AGAGTATA 0.128573477058
AGAGTATC 0.267183215789
AGAGTATG -0.0396670145909
AGAGTCAA -0.0803734729952
AGAGTCAC -0.187178617611
AGAGTCAG -0.0840143267688
AGAGTCAT 0.0249381613018
AGAGTCCA -0.242856642545
AGAGTCCC -0.122269369794
AGAGTCCG -0.2586923435
AGAGTCCT -0.0946011894423
AGAGTCGA 0.10838585999
AGAGTCGC -0.306610184761
AGAGTCGG -0.107751668693
AGAGTCTA -0.20780785251
AGAGTCTC -0.0936449639207
AGAGTCTG -0.068262026447
AGAGTGAA -0.166065796061
AGAGTGAC -0.13234746793
AGAGTGAG -0.0441537722754
AGAGTGAT -0.0300176209267
AGAGTGCA -0.135644474939
AGAGTGCC -0.332579374922
AGAGTGCG -0.230857765097
AGAGTGCT -0.264768642241
AGAGTGGA 0.0176164025605
AGAGTGGC -0.302231514117
AGAGTGGG -0.30363920518
AGAGTGTA 0.0825551265301
AGAGTGTC -0.105849912451
AGAGTGTG 0.150140498625
AGAGTTAA -0.115858313252
AGAGTTAC -0.0692083267841
AGAGTTAG 0.0809806630215
AGAGTTAT 0.0753343921384
AGAGTTCA -0.177999372432
AGAGTTCC -0.244443356251
AGAGTTCG -0.218734172411
AGAGTTGA -0.224397438686
AGAGTTGC 0.0170211533848
AGAGTTGG -0.214089540651
AGAGTTTA 0.0881725217696
AGAGTTTC -0.176255243106
AGAGTTTG 0.0740151675511
AGATAAAA 0.108903124506
AGATAAAC -0.146143560534
AGATAAAG -0.176043759768
AGATAAAT 0.206271009301

AGATAACA 0.0239870659767
AGATAACC -0.0620052115647
AGATAACG 0.110864307834
AGATAACT -0.0263527990801
AGATAAGA 0.145102402678
AGATAAGC -0.114415740902
AGATAAGG 0.117717944062
AGATAATA 0.120939687452
AGATAATC 0.320649182865
AGATAATG 0.226511635603
AGATACAA 0.0477150135398
AGATACAC 0.144384197335
AGATACAG -0.0737335131275
AGATACAT 0.115307693992
AGATACCA 0.296074311226
AGATACCC 0.344957905564
AGATACCG 0.40956694379
AGATACCT 0.261497193492
AGATACGA 0.365243826778
AGATACGC 0.405321299261
AGATACGG 0.358174926173
AGATACTA 0.278332051059
AGATACTC 0.357507645386
AGATACTG 0.327463632183
AGATAGAA -0.0820477399751
AGATAGAC -0.0585168971907
AGATAGAG -0.0868619423951
AGATAGAT 0.22259863169
AGATAGCA 0.0757815522288
AGATAGCC -0.0131654051371
AGATAGCG 0.193069583344
AGATAGCT 0.0999240892535
AGATAGGA -0.085767464946
AGATAGGC 0.0597877418743
AGATAGGG -0.186955658669
AGATAGTA 0.266807005352
AGATAGTC 0.0114985846389
AGATAGTG 0.118592293599
AGATATAA 0.192171510353
AGATATAC 0.244444168687
AGATATAG 0.259529353372
AGATATAT 0.302561987694
AGATATCA 0.449677014372
AGATATCC 0.479778544995
AGATATCG 0.464030145848
AGATATCT 0.483235896461
AGATATGA 0.31582684613
AGATATGC 0.423575396159
AGATATGG 0.393932499993
AGATATTA 0.378166171544
AGATATTC 0.442907094422
AGATATTG 0.339872819972
AGATCAAA 0.0879189423262
AGATCAAC 0.105773817895

AGATCAAG 0.0507718809077
AGATCAAT 0.179132441621
AGATCACA 0.187519251403
AGATCACC 0.0155974686686
AGATCACG 0.262542156482
AGATCACT 0.0626898681051
AGATCAGA 0.172413012854
AGATCAGC -0.0506349009389
AGATCAGG 0.168073926099
AGATCATA 0.0951226708802
AGATCATC 0.215233139476
AGATCATG 0.119171402338
AGATCCAA 0.351501062021
AGATCCAC 0.295617931982
AGATCCAG 0.313560287001
AGATCCAT 0.198634538992
AGATCCCA 0.226428908247
AGATCCCC 0.386560148318
AGATCCCG 0.293011831858
AGATCCCT 0.291096882006
AGATCCGA 0.319767425828
AGATCCGC 0.302756002712
AGATCCGG 0.282571267712
AGATCCTA 0.320529477457
AGATCCTC 0.396899654475
AGATCCTG 0.338498427042
AGATCGAA 0.114525380489
AGATCGAC 0.0819565994905
AGATCGAG 0.170370094613
AGATCGAT 0.293256689165
AGATCGCA 0.385059451253
AGATCGCC 0.371196265136
AGATCGCG 0.466967146705
AGATCGCT 0.341758209674
AGATCGGA 0.253050571232
AGATCGGC 0.13490434516
AGATCGGG 0.213331824851
AGATCGTA 0.303569264907
AGATCGTC 0.337483850719
AGATCGTG 0.35270945877
AGATCTAA 0.324041809303
AGATCTAC 0.31644588252
AGATCTAG 0.341661861419
AGATCTAT 0.327819828763
AGATCTCA 0.423482711361
AGATCTCC 0.451681709257
AGATCTCG 0.447688735568
AGATCTGA 0.379576546243
AGATCTGC 0.431686167456
AGATCTGG 0.37645234046
AGATCTTA 0.356922841106
AGATCTTC 0.41710301015
AGATCTTG 0.405172879426
AGATGAAA 0.090298022671

AGATGAAC -0.193360808734
AGATGAAG -0.119861417861
AGATGAAT 0.0939621760107
AGATGACA -0.0644493933153
AGATGACC -0.17703240936
AGATGACG -0.106785949375
AGATGACT -0.13643715831
AGATGAGA 0.0316887135069
AGATGAGC -0.187604239893
AGATGAGG -0.0495246561366
AGATGATA 0.232666550848
AGATGATC 0.141280398856
AGATGATG -0.0351549897004
AGATGCAA 0.0331476288557
AGATGCAC -0.130543856704
AGATGCAG -0.00151602519653
AGATGCAT 0.065869360525
AGATGCCA -0.10027100271
AGATGCCC -0.158407719014
AGATGCCG 0.149059527847
AGATGCCT -0.115465187199
AGATGCGA 0.13397005905
AGATGCGC -0.042862517292
AGATGCGG -0.0116822259367
AGATGCTA 0.227452683518
AGATGCTC -0.110316967917
AGATGCTG 0.0584709823872
AGATGGAA -0.228131107533
AGATGGAC -0.258133127473
AGATGGAG -0.0175206445086
AGATGGAT 0.164227485917
AGATGGCA -0.0433451512661
AGATGGCC -0.189433424165
AGATGGCG -0.0778434242963
AGATGGCT -0.0982311632313
AGATGGGA -0.111853157928
AGATGGGC -0.116253752822
AGATGGGG -0.132945964449
AGATGGTA -0.0236464408322
AGATGGTC -0.147554926952
AGATGGTG -0.212213286933
AGATGTAA 0.0867685867686
AGATGTAC -0.0152694137637
AGATGTAG 0.0315687272005
AGATGTAT 0.1979540594
AGATGTCA 0.00359174601599
AGATGTCC -0.0727435424405
AGATGTCG 0.0854173732962
AGATGTGA -0.10870491887
AGATGTGC -0.23347936937
AGATGTGG 0.000475172076524
AGATGTTA 0.16966966967
AGATGTTC 0.0779742049444
AGATGTTG 0.180468310913

AGATTAAA	-0.0488210562886
AGATTAAC	-0.0911854480482
AGATTAAG	0.174731210265
AGATTAAT	0.111366174463
AGATTACA	0.467780452629
AGATTACC	0.471179616349
AGATTACG	0.479950803997
AGATTACT	0.429182626152
AGATTAGA	0.023508244345
AGATTAGC	0.170766333584
AGATTAGG	0.173329161926
AGATTATA	0.314839091115
AGATTATC	0.406796201543
AGATTATG	0.367109533776
AGATTCAA	0.11699992003
AGATTCAC	0.264863543134
AGATTCAG	0.284539024693
AGATTCAT	0.254196117979
AGATTCCA	0.44067867127
AGATTCCC	0.489248579299
AGATTCCG	0.463793493573
AGATTCCT	0.452952952953
AGATTCGA	0.394917109607
AGATTCGC	0.475974248739
AGATTCGG	0.418679009588
AGATTCTA	0.452415225142
AGATTCTC	0.492455639658
AGATTCTG	0.466321693594
AGATTGAA	0.0558988740807
AGATTGAC	0.0882893075334
AGATTGAG	0.130080170766
AGATTGAT	0.110020218535
AGATTGCA	0.465715026321
AGATTGCC	0.4486989336
AGATTGCG	0.494782596002
AGATTGCT	0.430147515018
AGATTGGA	0.156417980246
AGATTGGC	0.194739531251
AGATTGGG	0.143406491891
AGATTGTA	0.330408921318
AGATTGTC	0.391257855667
AGATTGTG	0.440710787355
AGATTTAA	0.278691597575
AGATTTAC	0.342134843763
AGATTTAG	0.324163005981
AGATTTAT	0.331773922683
AGATTTCA	0.460068027707
AGATTTCC	0.497392757823
AGATTTCG	0.484207645088
AGATTTGA	0.264910916426
AGATTTGC	0.47263133732
AGATTTGG	0.361786968169
AGATTTTA	0.430125718005
AGATTTTC	0.489603147376

AGATTTTG 0.462778205202
AGCAAAAA 0.0281256614819
AGCAAAAC 0.114082401961
AGCAAAAG 0.0204718144858
AGCAAAAT 0.220680569039
AGCAAACA -0.089386306673
AGCAAACC -0.12280542113
AGCAAACG -0.19560235697
AGCAAACT -0.163500626931
AGCAAAGA -0.108969377159
AGCAAAGC -0.0486116280801
AGCAAAGG -0.164101376517
AGCAAATA 0.267558374634
AGCAAATC 0.418091645364
AGCAAATG -0.0866003744792
AGCAACAA -0.0773468324276
AGCAACAC -0.223484887192
AGCAACAG -0.160532881225
AGCAACAT -0.0893570560064
AGCAACCA -0.0998709581358
AGCAACCC -0.0830422782715
AGCAACCG -0.271476455492
AGCAACCT -0.17811604757
AGCAACGA -0.140184767618
AGCAACGC -0.322415195247
AGCAACGG -0.179102724082
AGCAACTA 0.0455400303885
AGCAACTC -0.0785437660412
AGCAACTG -0.187743171885
AGCAAGAA -0.0448361731352
AGCAAGAC -0.0110953774735
AGCAAGAG -0.0868316637853
AGCAAGAT 0.294032434176
AGCAAGCA -0.174435758503
AGCAAGCC -0.107884326455
AGCAAGCG -0.25034253956
AGCAAGCT -0.341392092825
AGCAAGGA -0.0367064954782
AGCAAGGC -0.246753388894
AGCAAGGG -0.0788826112784
AGCAAGTA 0.214558075371
AGCAAGTC -0.141175270409
AGCAAGTG 0.00299886663523
AGCAATAA -0.125074447229
AGCAATAC 0.182863878893
AGCAATAG -0.18752308229
AGCAATAT 0.298451128807
AGCAATCA 0.327985009803
AGCAATCC 0.401675780464
AGCAATCG 0.28705978706
AGCAATGA -0.059780442764
AGCAATGC 0.00425006094455
AGCAATGG -0.153805320472
AGCAATTA 0.0102588994402

AGCAATTC 0.055092282365
AGCAATTG 0.0401158734492
AGCACAAA -0.0713426925548
AGCACAAC -0.0308033806235
AGCACAAG -0.0621627136779
AGCACAAT 0.0518863426467
AGCACACA -0.185720941645
AGCACACC -0.292368858528
AGCACACG -0.296276596521
AGCACACT -0.146581556474
AGCACAGA -0.0557046001538
AGCACAGC -0.230579643343
AGCACAGG -0.289670037679
AGCACATA 0.0729261403035
AGCACATC -0.309267850301
AGCACATG -0.0731130579615
AGCACCAA -0.257372624591
AGCACCAC -0.128854633588
AGCACCAG -0.294203008517
AGCACCAT -0.260854558907
AGCACCCA -0.188315696102
AGCACCCC -0.128036739347
AGCACCCG -0.385592788264
AGCACCCT -0.223758704286
AGCACCGA -0.21984073826
AGCACCGC -0.183638725308
AGCACCGG -0.250332375489
AGCACCTA -0.0463500799253
AGCACCTC -0.112133037199
AGCACCTG -0.203202651678
AGCACGAA -0.00570571447341
AGCACGAC -0.0826457304514
AGCACGAG -0.122736728393
AGCACGAT 0.0829590106299
AGCACGCA -0.23656749637
AGCACGCC -0.272221257549
AGCACGCG 0.0114384356547
AGCACGCT -0.245630742062
AGCACGGA -0.0635342129572
AGCACGGC -0.157781870346
AGCACGGG -0.0757246191689
AGCACGTA 0.154074745018
AGCACGTC -0.326582147334
AGCACGTG -0.0266340720886
AGCACTAA -0.230393981188
AGCACTAC -0.123936702116
AGCACTAG -0.176305669272
AGCACTAT 0.122066670073
AGCACTCA 0.0276461099392
AGCACTCC -0.161693539558
AGCACTCG -0.175419371735
AGCACTGA -0.0555878338431
AGCACTGC -0.0885872846521
AGCACTGG -0.229484602596

AGCACTTA 0.0753027305971
AGCACTTC 0.0362933658387
AGCACTTG -0.211951644137
AGCAGAAA -0.156434308592
AGCAGAAC -0.0372264883224
AGCAGAAG -0.434983521942
AGCAGAAT 0.221571060488
AGCAGACA -0.235948750359
AGCAGACC -0.112066537158
AGCAGACG -0.121189317022
AGCAGACT -0.307923407547
AGCAGAGA 0.02334428092
AGCAGAGC -0.150329635365
AGCAGAGG -0.122300546568
AGCAGATA 0.290955012808
AGCAGATC 0.329214889821
AGCAGATG -0.0259184105458
AGCAGCAA -0.189404079884
AGCAGCAC -0.215156374672
AGCAGCAG -0.143100375556
AGCAGCAT -0.0126298565283
AGCAGCCA -0.13633948654
AGCAGCCC -0.371632949347
AGCAGCCG -0.251242241916
AGCAGCCT -0.367072202793
AGCAGCGA -0.11727412982
AGCAGCGC -0.140154929772
AGCAGCGG -0.172163626587
AGCAGCTA -0.165129698093
AGCAGCTC -0.318269779443
AGCAGCTG -0.182170508695
AGCAGGAA -0.16016136457
AGCAGGAC -0.0520239608709
AGCAGGAG -0.173601808439
AGCAGGAT 0.0380320138975
AGCAGGCA -0.171319552976
AGCAGGCC -0.310394613011
AGCAGGCG -0.371939876479
AGCAGGCT -0.256558870194
AGCAGGGA -0.0596191292106
AGCAGGGC -0.220870977581
AGCAGGGG -0.199807341906
AGCAGGTA 0.02566923359
AGCAGGTC -0.263898883127
AGCAGGTG -0.222376349751
AGCAGTAA -0.017586920125
AGCAGTAC -0.178851815147
AGCAGTAG -0.162890370152
AGCAGTAT 0.272726144683
AGCAGTCA 0.111526754889
AGCAGTCC -0.243148880778
AGCAGTCG -0.241998114284
AGCAGTGA -0.227956319118
AGCAGTGC -0.294898299525

AGCAGTGG -0.0359700130414
AGCAGTTA -0.0421482088149
AGCAGTTC -0.000771477660722
AGCAGTTG 0.0646147957817
AGCATAAA -0.222477234945
AGCATAAC -0.158098361804
AGCATAAG -0.167100920618
AGCATAAT 0.198637256118
AGCATACA -0.0419948929278
AGCATACC -0.157610704179
AGCATACG -0.0563532710308
AGCATACT -0.28273200052
AGCATAGA 0.133630587009
AGCATAGC -0.232244527711
AGCATAGG -0.119072038466
AGCATATA 0.30757358637
AGCATATC 0.340878660377
AGCATATG -0.0482083522907
AGCATCAA 0.042892755014
AGCATCAC 0.186176215119
AGCATCAG -0.0582797996739
AGCATCAT 0.0772063385473
AGCATCCA -0.0923486860064
AGCATCCC -0.225638183778
AGCATCCG -0.230218594149
AGCATCCT -0.151142225305
AGCATCGA 0.00949545843995
AGCATCGC 0.0232058217733
AGCATCGG -0.162993206214
AGCATCTA 0.185526594308
AGCATCTC -0.0889815205375
AGCATCTG 0.134203538725
AGCATGAA -0.185179482764
AGCATGAC -0.156220980274
AGCATGAG -0.205856731313
AGCATGAT -0.187589095411
AGCATGCA -0.246338672059
AGCATGCC -0.22804420517
AGCATGCG -0.256743464498
AGCATGCT -0.327917648518
AGCATGGA 0.0259931367721
AGCATGGC -0.206358160464
AGCATGGG -0.167976019155
AGCATGTA 0.12702923309
AGCATGTC -0.16133525982
AGCATGTG -0.0400993279781
AGCATTAA -0.0897326660325
AGCATTAC -0.12998072644
AGCATTAG -0.156767136245
AGCATTAT 0.0952881255912
AGCATTCA -0.117136131305
AGCATTCC -0.191504905893
AGCATTCG -0.190633159698
AGCATTGA -0.282202478332

AGCATTGC -0.247963344333
AGCATTGG -0.257118857071
AGCATTTA 0.142921687846
AGCATTTC 0.000654924897349
AGCATTTG -0.0278015809873
AGCCAAAA -0.0235070969503
AGCCAAAC -0.189685119476
AGCCAAAG -0.179335974438
AGCCAAAT 0.0380363933636
AGCCAACA 0.00775092516222
AGCCAACC -0.205731867696
AGCCAACG -0.434107492242
AGCCAACT -0.22627092242
AGCCAAGA -0.139882109349
AGCCAAGC -0.347198327124
AGCCAAGG -0.173798398324
AGCCAATA 0.0188981263184
AGCCAATC -0.00918301059101
AGCCAATG -0.191125754358
AGCCACAA -0.138652508663
AGCCACAC -0.174385542907
AGCCACAG -0.16973125884
AGCCACAT -0.236996577567
AGCCACCA -0.252745015332
AGCCACCC -0.200132937003
AGCCACCG -0.267926430824
AGCCACCT -0.18023125977
AGCCACGA -0.0229147417174
AGCCACGC -0.282510677478
AGCCACGG -0.225090201461
AGCCACTA -0.0873439253212
AGCCACTC -0.326043811556
AGCCACTG -0.0415594518581
AGCCAGAA -0.00575688507781
AGCCAGAC -0.119305734947
AGCCAGAG -0.293244247015
AGCCAGAT 0.177907783041
AGCCAGCA -0.211518063171
AGCCAGCC -0.278853996886
AGCCAGCG -0.211646178298
AGCCAGCT -0.323751367769
AGCCAGGA -0.00536066432826
AGCCAGGC -0.275601857594
AGCCAGGG -0.266883072131
AGCCAGTA -0.0503012504998
AGCCAGTC -0.178612882504
AGCCAGTG -0.206168396961
AGCCATAA -0.206469055802
AGCCATAC -0.104868882878
AGCCATAG -0.277238886027
AGCCATAT 0.154827996467
AGCCATCA -0.106702399563
AGCCATCC -0.174155155177
AGCCATCG -0.240650799234

AGCCATGA -0.107310355096
AGCCATGC -0.256794543058
AGCCATGG -0.251987769481
AGCCATTA -0.115080645384
AGCCATTC -0.0108150916403
AGCCATTG -0.198989648376
AGCCCAAA 0.0422485627586
AGCCCAAC -0.162807854813
AGCCCAAG -0.120289542013
AGCCCAAT -0.3190400211
AGCCCACA -0.212016427207
AGCCCACC -0.247405359266
AGCCCACG -0.259598806777
AGCCCACT -0.293266461033
AGCCCAGA -0.26445927035
AGCCCAGC -0.357931448824
AGCCCAGG -0.326475541553
AGCCCATA -0.0507486607965
AGCCCATC -0.0963823199363
AGCCCATG -0.202795518083
AGCCCCAA -0.348260887963
AGCCCCAC -0.20361154609
AGCCCCAG -0.2528700465
AGCCCCAT -0.185008545986
AGCCCCCA -0.348374929781
AGCCCCCC -0.289340068195
AGCCCCCG -0.385319596381
AGCCCCCT -0.288381809346
AGCCCCGA -0.198835320677
AGCCCCGC -0.165160322934
AGCCCCGG -0.373778430935
AGCCCCTA -0.140505439054
AGCCCCTC -0.224945493235
AGCCCCTG -0.323758501491
AGCCCGAA -0.118806599677
AGCCCGAC -0.235254231682
AGCCCGAG -0.186043915147
AGCCCGAT -0.113324439475
AGCCCGCA -0.295325290163
AGCCCGCC -0.195334011361
AGCCCGCG -0.225655617946
AGCCCGCT -0.349509767441
AGCCCGGA -0.0836665046425
AGCCCGGC -0.254341910397
AGCCCGGG -0.092747720072
AGCCCGTA -0.0497673982522
AGCCCGTC -0.0873677593323
AGCCCGTG -0.171922181601
AGCCCTAA -0.306511271523
AGCCCTAC -0.202524469065
AGCCCTAG -0.133450765376
AGCCCTAT -0.219599037293
AGCCCTCA -0.238940578092
AGCCCTCC -0.359950716565

AGCCCTCG -0.366792519443
AGCCCTGA -0.149518460618
AGCCCTGC -0.24333487519
AGCCCTGG -0.315929348151
AGCCCTTA -0.260673794934
AGCCCTTC -0.317577456404
AGCCCTTG -0.154441218878
AGCCGAAA 0.00544759635669
AGCCGAAC -0.131916176142
AGCCGAAG -0.172844560228
AGCCGAAT 0.016422758847
AGCCGACA -0.106604419391
AGCCGACC -0.238861236241
AGCCGACG -0.113548689526
AGCCGACT -0.18372931706
AGCCGAGA 0.0053796401312
AGCCGAGC -0.241814419812
AGCCGAGG -0.275958102226
AGCCGATA 0.248405509368
AGCCGATC 0.0970949541251
AGCCGATG -0.198529959062
AGCCGCAA -0.138352281435
AGCCGCAC -0.175089003814
AGCCGCAG -0.169026982614
AGCCGCAT -0.23700258496
AGCCGCCA -0.282989771703
AGCCGCCC -0.306189493036
AGCCGCCG -0.393936137085
AGCCGCCT -0.266321424245
AGCCGCGA 0.0828646088042
AGCCGCGC -0.245190624647
AGCCGCGG -0.19110824084
AGCCGCTA -0.288982884991
AGCCGCTC -0.182963810447
AGCCGCTG -0.348451269499
AGCCGGAA -0.221210442013
AGCCGGAC -0.309858445896
AGCCGGAG -0.308407685798
AGCCGGAT 0.0158828638789
AGCCGGCA -0.220941094733
AGCCGGCC -0.384884512461
AGCCGGCG -0.327446018967
AGCCGGCT -0.446821086394
AGCCGGGA -0.177160784833
AGCCGGGC -0.403688651638
AGCCGGGG -0.247751519663
AGCCGGTA -0.223970093211
AGCCGGTC -0.278073026365
AGCCGGTG -0.287049211508
AGCCGTAA -0.110431144839
AGCCGTAC -0.105431997839
AGCCGTAG -0.287921638675
AGCCGTAT -0.0202912492391
AGCCGTCA -0.0906639558287

AGCCGTCC -0.00662182538466
AGCCGTCG -0.261845624901
AGCCGTGA -0.0435516010598
AGCCGTGC -0.361122824424
AGCCGTGG -0.240519259241
AGCCGTTA -0.183279698184
AGCCGTTC 0.0220980562469
AGCCGTTG -0.161439562797
AGCCTAAA -0.150996055471
AGCCTAAC -0.268211874895
AGCCTAAG -0.152514825935
AGCCTAAT -0.14210079238
AGCCTACA -0.00457581220849
AGCCTACC -0.155479744724
AGCCTACG -0.303548993829
AGCCTACT -0.176446508343
AGCCTAGA -0.101888332076
AGCCTAGC -0.292489780843
AGCCTAGG -0.273205970637
AGCCTATA -0.0386765626
AGCCTATC 0.221533240688
AGCCTATG -0.164352220132
AGCCTCAA -0.132923066743
AGCCTCAC -0.0908348980261
AGCCTCAG -0.189624464791
AGCCTCAT -0.205971943114
AGCCTCCA -0.168903886394
AGCCTCCC -0.381392902425
AGCCTCCG -0.272778012056
AGCCTCCT -0.278690569821
AGCCTCGA -0.0799336981734
AGCCTCGC -0.225912971094
AGCCTCGG -0.111044952214
AGCCTCTA -0.283822268538
AGCCTCTC -0.233216885772
AGCCTCTG -0.245212201409
AGCCTGAA -0.128099093809
AGCCTGAC -0.126888221687
AGCCTGAG -0.130472607498
AGCCTGAT -0.137661898618
AGCCTGCA -0.182225627156
AGCCTGCC -0.299792808107
AGCCTGCG -0.425996264376
AGCCTGGA -0.187862967433
AGCCTGGC -0.194428077009
AGCCTGGG -0.314044396755
AGCCTGTA -0.0766238695503
AGCCTGTC -0.208583813131
AGCCTGTG -0.16672456432
AGCCTTAA -0.29263862359
AGCCTTAC -0.0826917476077
AGCCTTAG -0.303802889449
AGCCTTAT 0.0323918960283
AGCCTTCA -0.291186758249

AGCCTTCC -0.360021800003
AGCCTTCG -0.277420263335
AGCCTTGA -0.112331908273
AGCCTTGC -0.363060294446
AGCCTTGG -0.387816829405
AGCCTTTA -0.100703856321
AGCCTTTC -0.113457228163
AGCCTTTG -0.315747610343
AGCGAAAA -0.19095569442
AGCGAAAC 0.0852019161558
AGCGAAAG -0.0642262451299
AGCGAAAT 0.297927403988
AGCGAACA -0.102559330306
AGCGAACC -0.251539942182
AGCGAACG -0.201095562149
AGCGAACT -0.28776160046
AGCGAAGA -0.111233798996
AGCGAAGC -0.280147428489
AGCGAAGG -0.315652368075
AGCGAATA 0.281429603731
AGCGAATC 0.456005592369
AGCGAATG 0.131535075874
AGCGACAA -0.115575252755
AGCGACAC -0.0457697186625
AGCGACAG -0.129102117676
AGCGACAT -0.094593647777
AGCGACCA -0.182685017466
AGCGACCC -0.299629389149
AGCGACCG -0.252217904057
AGCGACCT -0.20958537009
AGCGACGA 0.0597083846595
AGCGACGC -0.183114298081
AGCGACGG -0.200378170833
AGCGACTA -0.149709728793
AGCGACTC -0.214363015058
AGCGACTG -0.139791216881
AGCGAGAA -0.205899219484
AGCGAGAC 0.109658182668
AGCGAGAG -0.0461283304795
AGCGAGAT 0.207668814637
AGCGAGCA -0.0323806372448
AGCGAGCC -0.329511089057
AGCGAGCG -0.0255533221387
AGCGAGCT -0.169292947276
AGCGAGGA -0.0316802470668
AGCGAGGC -0.148162175179
AGCGAGGG -0.267683901044
AGCGAGTA 0.0825274310123
AGCGAGTC -0.284164882743
AGCGAGTG 0.145207769173
AGCGATAA -0.0288372721432
AGCGATAC 0.19085890298
AGCGATAG -0.0270483128107
AGCGATAT 0.327174587418

AGCGATCA 0.209898323394
AGCGATCC 0.284462770189
AGCGATCG 0.202349732653
AGCGATGA -0.0127799976285
AGCGATGC -0.188838154481
AGCGATGG -0.265202049462
AGCGATTA 0.123722108275
AGCGATTC 0.21042969862
AGCGATTG 0.231351868207
AGCGCAAA -0.00382782837432
AGCGCAAC -0.0321823200611
AGCGCAAG 0.0208517737243
AGCGCAAT 0.284470607906
AGCGCACA -0.0730356269571
AGCGCACC -0.238571739676
AGCGCACG -0.278051453261
AGCGCACT -0.0944393439369
AGCGCAGA -0.0119479135493
AGCGCAGC -0.277735459742
AGCGCAGG -0.179532230851
AGCGCATA 0.0160041913113
AGCGCATC -0.0732040580525
AGCGCATG -0.217843839522
AGCGCCAA -0.0975565122454
AGCGCCAC -0.25092700571
AGCGCCAG -0.187757167388
AGCGCCAT -0.238117022503
AGCGCCCA -0.199099918676
AGCGCCCC -0.235579427891
AGCGCCCG -0.359480123588
AGCGCCCT -0.30239830344
AGCGCCGA -0.0976531310805
AGCGCCGC -0.187945110301
AGCGCCGG -0.316847620859
AGCGCCTA -0.0366202658329
AGCGCCTC -0.165138049861
AGCGCCTG -0.0674027818527
AGCGCGAA -0.041825475258
AGCGCGAC -0.126420286139
AGCGCGAG -0.0607087024492
AGCGCGAT 0.285636810873
AGCGCGCA 0.0858089494453
AGCGCGCC -0.142714923707
AGCGCGCG -0.0931690028799
AGCGCGCT 0.00806920636496
AGCGCGGA 0.162888784101
AGCGCGGC -0.068743380076
AGCGCGGG -0.341324965478
AGCGCGTA -0.0521692491389
AGCGCGTC -0.0772243107769
AGCGCGTG 0.115450756553
AGCGCTAA -0.124237425458
AGCGCTAC -0.0798617548521
AGCGCTAG -0.29208184146

AGCGCTAT 0.0530579622344
AGCGCTCA -0.0394582600381
AGCGCTCC -0.17546409089
AGCGCTCG -0.176523853234
AGCGCTGA 0.0297504434209
AGCGCTGC -0.12335538561
AGCGCTGG -0.127676998288
AGCGCTTA -0.284433170757
AGCGCTTC 0.0155171784559
AGCGCTTG -0.132211724421
AGCGGAAA -0.0964017824693
AGCGGAAC -0.132415336181
AGCGGAAG -0.318269265222
AGCGGAAT 0.0787187141887
AGCGGACA -0.177386353852
AGCGGACC -0.306127080071
AGCGGACG -0.117644322608
AGCGGACT -0.183393574463
AGCGGAGA 0.119236316206
AGCGGAGC -0.266911718089
AGCGGAGG -0.268645527581
AGCGGATA 0.254198730551
AGCGGATC 0.321617761012
AGCGGATG -0.0181171215229
AGCGGCAA -0.131895005884
AGCGGCAC -0.156837850729
AGCGGCAG -0.218424328572
AGCGGCAT -0.280201175799
AGCGGCCA -0.137727037224
AGCGGCCC -0.468712543914
AGCGGCCG -0.217963149016
AGCGGCCT -0.383750826255
AGCGGCGA -0.00382216461363
AGCGGCGC -0.255690919066
AGCGGCGG -0.304738906669
AGCGGCTA -0.229275386291
AGCGGCTC -0.384014288904
AGCGGCTG -0.140843902356
AGCGGGAA -0.285501121484
AGCGGGAC -0.0790236183135
AGCGGGAG -0.16644479574
AGCGGGAT 0.0841378568651
AGCGGGCA -0.295302993966
AGCGGGCC -0.266749085536
AGCGGGCG -0.14525201223
AGCGGGGA 0.117744466229
AGCGGGGC -0.220177843667
AGCGGGGG -0.297444155662
AGCGGGTA -0.0553314251434
AGCGGGTC -0.25478060646
AGCGGGTG -0.0422950561263
AGCGGTAA 0.134080361353
AGCGGTAC -0.0324918363504
AGCGGTAG -0.162579267377

AGCGGTAT 0.12942556882
AGCGGTCA -0.116561842722
AGCGGTCC -0.330397823588
AGCGGTCG -0.324283802773
AGCGGTGA -0.168211733618
AGCGGTGC -0.30818255049
AGCGGTGG -0.185389154876
AGCGGTTA -0.0505854325749
AGCGGTTC -0.0506900497718
AGCGGTTG -0.164931373927
AGCGTAAA -0.0135764011042
AGCGTAAC 0.123960048202
AGCGTAAG -0.211703922396
AGCGTAAT 0.297593736988
AGCGTACA 0.0706655136513
AGCGTACC -0.0960906932691
AGCGTACG -0.26814847891
AGCGTACT -0.160375458156
AGCGTAGA 0.0878396823427
AGCGTAGC -0.210348521713
AGCGTAGG -0.254606773784
AGCGTATA 0.131228513244
AGCGTATC 0.331669134699
AGCGTATG 0.0848824030642
AGCGTCAA 0.0916407209054
AGCGTCAC -0.0703662001539
AGCGTCAG -0.0919997132118
AGCGTCAT -0.16613053181
AGCGTCCA -0.0990680953955
AGCGTCCC -0.277952823193
AGCGTCCG -0.26776883262
AGCGTCCT -0.322859806227
AGCGTCGA -0.0621215062537
AGCGTCGC -0.08445143537
AGCGTCGG -0.279257862779
AGCGTCTA -0.0526758598346
AGCGTCTC -0.277463538609
AGCGTCTG -0.0771552447757
AGCGTGAA -0.206619489444
AGCGTGAC -0.158000189777
AGCGTGAG -0.189688438345
AGCGTGAT 0.15722502427
AGCGTGCA -0.198554384572
AGCGTGCC -0.444852343352
AGCGTGCG -0.187829965787
AGCGTGGA 0.121557119325
AGCGTGGC -0.137222064114
AGCGTGGG -0.200592515009
AGCGTGTA -0.093594313899
AGCGTGTC -0.217353546882
AGCGTGTG 0.0859830261166
AGCGTTAA -0.105359136584
AGCGTTAC 0.0621584928797
AGCGTTAG -0.136940647199

AGCGTTAT 0.107883365459
AGCGTTCA -0.0413370510135
AGCGTTCC -0.165888671352
AGCGTTCG -0.0022284183558
AGCGTTGA -0.0233462040977
AGCGTTGC -0.28880766587
AGCGTTGG -0.254096662109
AGCGTTTA -0.174891706854
AGCGTTTC 0.0709845769801
AGCGTTTG 0.189075421706
AGCTAAAA -0.135539452896
AGCTAAAC -0.248571695418
AGCTAAAG -0.152720965833
AGCTAAAT -0.122453284364
AGCTAACA -0.0309469248863
AGCTAACC -0.215787311591
AGCTAACG -0.337471842422
AGCTAACT -0.168169397777
AGCTAAGA 0.00334477671616
AGCTAAGC -0.238931279887
AGCTAAGG -0.23598533405
AGCTAATA 0.112887309037
AGCTAATC -0.0697762168963
AGCTAATG -0.129078715503
AGCTACAA -0.18998817175
AGCTACAC 0.0317313068116
AGCTACAG -0.0884328985711
AGCTACAT -0.00854422319603
AGCTACCA -0.151736068037
AGCTACCC -0.228216818273
AGCTACCG -0.259891840937
AGCTACCT -0.195340674416
AGCTACGA -0.0715943408206
AGCTACGC -0.270986774228
AGCTACGG -0.086815098735
AGCTACTA -0.105240013344
AGCTACTC -0.266459979015
AGCTACTG -0.225187114915
AGCTAGAA -0.017916077322
AGCTAGAC -0.212230354657
AGCTAGAG -0.259041956442
AGCTAGAT 0.220301568786
AGCTAGCA -0.125114083486
AGCTAGCC -0.223743535776
AGCTAGCG -0.0921808350773
AGCTAGCT -0.312696523007
AGCTAGGA 0.142220973263
AGCTAGGC -0.119425587162
AGCTAGGG -0.186095017741
AGCTAGTA -0.00941575183999
AGCTAGTC -0.262184185987
AGCTAGTG -0.0279218541038
AGCTATAA 0.101310988872
AGCTATAC -0.0221257928658

AGCTATAG -0.195631337368
AGCTATAT 0.103708092414
AGCTATCA -0.0842749934676
AGCTATCC 0.0380597505164
AGCTATCG 0.0113239961725
AGCTATGA 0.00828238707027
AGCTATGC -0.134466188768
AGCTATGG -0.137146100593
AGCTATTA -0.16366947636
AGCTATTC -0.265053199329
AGCTATTG -0.0472230535098
AGCTCAAA -0.111207693082
AGCTCAAC -0.0487563663329
AGCTCAAG -0.132491259315
AGCTCAAT -0.222256198576
AGCTCACA -0.337105207308
AGCTCACC -0.165678418141
AGCTCACG -0.265524598705
AGCTCACT -0.260154330943
AGCTCAGA -0.0658270256856
AGCTCAGC -0.108148209779
AGCTCAGG -0.146241526011
AGCTCATA -0.229743062093
AGCTCATC -0.219390443055
AGCTCATG -0.133548631474
AGCTCCAA -0.143447855569
AGCTCCAC -0.232173726045
AGCTCCAG -0.251969209476
AGCTCCAT -0.211123273277
AGCTCCCA -0.308157849046
AGCTCCCC -0.125888716818
AGCTCCCG -0.11968111893
AGCTCCCT -0.283210538067
AGCTCCGA -0.298068627532
AGCTCCGC -0.362714695643
AGCTCCGG -0.329041700776
AGCTCCTA -0.203359993824
AGCTCCTC -0.284030723624
AGCTCCTG -0.129819845897
AGCTCGAA -0.187237505748
AGCTCGAC -0.141939157313
AGCTCGAG -0.0627698578128
AGCTCGAT -0.140353634361
AGCTCGCA -0.25705833864
AGCTCGCC -0.273458853883
AGCTCGCG -0.294569732856
AGCTCGGA 0.195545353021
AGCTCGGC -0.178302940082
AGCTCGGG -0.0746768723252
AGCTCGTA -0.066637819797
AGCTCGTC -0.246098886105
AGCTCGTG -0.145879332159
AGCTCTAA -0.028980771405
AGCTCTAC -0.133953512741

AGCTCTAG −0.168146011661
AGCTCTAT −0.192730661832
AGCTCTCA −0.159562502584
AGCTCTCC −0.323439821592
AGCTCTCG −0.09293177475
AGCTCTGA −0.0448493786649
AGCTCTGC −0.120526129114
AGCTCTGG −0.176807158319
AGCTCTTA −0.124113195706
AGCTCTTC −0.225734505565
AGCTCTTG −0.178162345449
AGCTGAAA −0.0777694089621
AGCTGAAC −0.224875338014
AGCTGAAG −0.350209932883
AGCTGAAT −0.152566185411
AGCTGACA −0.237267645381
AGCTGACC −0.200310717407
AGCTGACG −0.140285134226
AGCTGACT −0.235814415085
AGCTGAGA 0.105599509906
AGCTGAGC −0.143471991309
AGCTGAGG −0.260095935161
AGCTGATA 0.204282795192
AGCTGATC −0.0774621209647
AGCTGATG −0.166294467466
AGCTGCAA −0.0207811220565
AGCTGCAC −0.133347483363
AGCTGCAG −0.117612754708
AGCTGCAT −0.213205351949
AGCTGCCA −0.179728913919
AGCTGCCC −0.267449666878
AGCTGCCG −0.202702590948
AGCTGCCT −0.0653822631755
AGCTGCGA −0.0818188085095
AGCTGCGC −0.335498336627
AGCTGCGG −0.173554933109
AGCTGCTA −0.264141099415
AGCTGCTC −0.287290403852
AGCTGCTG −0.119474431622
AGCTGGAA −0.191348587094
AGCTGGAC −0.234212550557
AGCTGGAG −0.123477449837
AGCTGGAT −0.0974893493816
AGCTGGCA −0.11032458236
AGCTGGCC −0.386738899689
AGCTGGCG −0.126191037443
AGCTGGGA 0.155481996745
AGCTGGGC −0.202468676343
AGCTGGGG −0.27378361454
AGCTGGTA −0.0968820397438
AGCTGGTC −0.41762050456
AGCTGGTG −0.164830210531
AGCTGTAA −0.0670115883769
AGCTGTAC −0.161532982135

AGCTGTAG -0.103957375819
AGCTGTAT 0.0828470909396
AGCTGTCA -0.147454192001
AGCTGTCC -0.0206046582922
AGCTGTCG -0.204708772514
AGCTGTGA 0.0511950944468
AGCTGTGC -0.175400074432
AGCTGTGG -0.281465036093
AGCTGTTA -0.154193371351
AGCTGTTC 0.0516903997105
AGCTGTTG -0.037438264711
AGCTTAAA -0.053991028379
AGCTTAAC -0.0899867328516
AGCTTAAG 0.0853784223703
AGCTTAAT 0.00526559617469
AGCTTACA -0.228551139059
AGCTTACC -0.0695361157291
AGCTTACG -0.222413816869
AGCTTACT -0.253533366508
AGCTTAGA 0.0312875429349
AGCTTAGC -0.125576966027
AGCTTAGG -0.208058407583
AGCTTATA 0.156072050011
AGCTTATC -0.139892547832
AGCTTATG -0.181562166588
AGCTTCAA -0.247941271326
AGCTTCAC -0.096629480804
AGCTTCAG -0.255829563655
AGCTTCAT -0.0843321174223
AGCTTCCA -0.219402712214
AGCTTCCC -0.177288586576
AGCTTCCG -0.196264856214
AGCTTCCT -0.237577216006
AGCTTCGA -0.135435018155
AGCTTCGC -0.0493813372601
AGCTTCGG -0.0917994719412
AGCTTCTA -0.0199375196164
AGCTTCTC -0.210647975851
AGCTTCTG -0.238685272036
AGCTTGAA -0.134663474522
AGCTTGAC -0.0978185830569
AGCTTGAG -0.182181061199
AGCTTGAT -0.0203771264377
AGCTTGCA -0.27568502898
AGCTTGCC -0.363250301071
AGCTTGCG -0.155072547674
AGCTTGGA 0.0514521579819
AGCTTGGC -0.239041490561
AGCTTGGG -0.260598716878
AGCTTGTA -0.0174456033809
AGCTTGTC -0.242198092486
AGCTTGTG -0.223707030851
AGCTTTAA -0.242355166853
AGCTTTAC -0.0980472439106

AGCTTTAG -0.26384355553
AGCTTTAT 0.0539280161748
AGCTTTCA 0.0341319280713
AGCTTTCC -0.00138756085633
AGCTTTCG -0.0982839061918
AGCTTTGA -0.12442035448
AGCTTTGC -0.160038763858
AGCTTTGG -0.117895921427
AGCTTTTA 0.014801131014
AGCTTTTC -0.0479734020423
AGCTTTTG -0.033706023341
AGGAAAAA -0.021511113317
AGGAAAAC -0.0120267439399
AGGAAAAG -0.20369359459
AGGAAAAT 0.211072020321
AGGAAACA 0.0437267797056
AGGAAACC -0.240685824532
AGGAAACG -0.191497948526
AGGAAACT -0.168310668105
AGGAAAGA -0.168555045555
AGGAAAGC -0.146352120636
AGGAAAGG -0.30349101893
AGGAAATA 0.295405940016
AGGAAATC 0.489766647446
AGGAAATG -0.0280498859262
AGGAACAA -0.140050913539
AGGAACAC -0.159103815245
AGGAACAG -0.148494932026
AGGAACAT -0.041622851649
AGGAACCA -0.0218055521086
AGGAACCC -0.312953115555
AGGAACCG -0.379309596448
AGGAACCT -0.168296477711
AGGAACGA -0.207390960387
AGGAACGC -0.201693910998
AGGAACGG -0.112005856515
AGGAACTA -0.0727426893328
AGGAACTC -0.228062123204
AGGAACTG -0.0485360161483
AGGAAGAA -0.120861519152
AGGAAGAC -0.139032058889
AGGAAGAG -0.148754235489
AGGAAGAT 0.278276388108
AGGAAGCA -0.0581922817014
AGGAAGCC -0.249458432844
AGGAAGCG -0.165477575363
AGGAAGGA 0.0775537685318
AGGAAGGC -0.282550960401
AGGAAGGG -0.305714710916
AGGAAGTA -0.0606239848664
AGGAAGTC -0.336711069217
AGGAAGTG 0.00909980073429
AGGAATAA 0.105585157453
AGGAATAC 0.13992321336

AGGAATAG -0.0771446377507
AGGAATAT 0.355413385108
AGGAATCA 0.345871988205
AGGAATCC 0.430192063266
AGGAATCG 0.305833613096
AGGAATGA -0.254887935145
AGGAATGC -0.0334298243437
AGGAATGG 0.00545636151575
AGGAATTA 0.192719137537
AGGAATTC 0.121684196696
AGGAATTG 0.0907119240453
AGGACAAA 0.116790873193
AGGACAAC -0.0866362229999
AGGACAAG 0.0355634548149
AGGACAAT 0.134924180379
AGGACACA -0.131065512079
AGGACACC -0.150891530256
AGGACACG -0.277821397247
AGGACACT -0.165788471166
AGGACAGA -0.110720504673
AGGACAGC -0.161526472704
AGGACAGG -0.0917282924287
AGGACATA -0.0924541798355
AGGACATC -0.237352787926
AGGACATG -0.295305750586
AGGACCAA -0.125292059911
AGGACCAC -0.336263039674
AGGACCAG -0.348704866021
AGGACCAT -0.0956495245028
AGGACCCA -0.267276431169
AGGACCCC -0.345211222186
AGGACCCG -0.287797596897
AGGACCCT -0.341610790044
AGGACCGA -0.118835410237
AGGACCGC -0.335544486416
AGGACCGG -0.277579310934
AGGACCTA -0.176784842997
AGGACCTC -0.37930166485
AGGACCTG -0.211998949189
AGGACGAA -0.141626349088
AGGACGAC -0.152326680947
AGGACGAG -0.273617227967
AGGACGAT 0.181282891616
AGGACGCA -0.00274811500478
AGGACGCC -0.354006994568
AGGACGCG -0.237842182025
AGGACGGA -0.176160453016
AGGACGGC -0.27472454514
AGGACGGG -0.127560039199
AGGACGTA -0.0811062309238
AGGACGTC -0.275031176751
AGGACGTG -0.133071669603
AGGACTAA -0.228543873577
AGGACTAC -0.23351321176

AGGACTAG -0.0924249875593
AGGACTAT -0.125043247228
AGGACTCA -0.228379583479
AGGACTCC -0.217340326024
AGGACTCG -0.291374809449
AGGACTGA -0.0603835962423
AGGACTGC -0.213841578386
AGGACTGG -0.12093214334
AGGACTTA -0.076648273618
AGGACTTC -0.105161142681
AGGACTTG -0.171740478849
AGGAGAAA 0.043680252479
AGGAGAAC -0.11227909611
AGGAGAAG -0.226742412633
AGGAGAAT 0.225830821223
AGGAGACA -0.139340397548
AGGAGACC -0.307285852497
AGGAGACG -0.278814215739
AGGAGACT -0.288246903779
AGGAGAGA -0.0037213522062
AGGAGAGC -0.273650467953
AGGAGAGG -0.271716269595
AGGAGATA 0.192279055915
AGGAGATC 0.388251606819
AGGAGATG 0.0187017643993
AGGAGCAA -0.183963840878
AGGAGCAC -0.165459522357
AGGAGCAG -0.373181825897
AGGAGCAT -0.217277706106
AGGAGCCA -0.176121397014
AGGAGCCC -0.395226915117
AGGAGCCG -0.0789186344027
AGGAGCCT -0.373221987956
AGGAGCGA -0.086628109814
AGGAGCGC -0.215162688863
AGGAGCGG -0.181294114729
AGGAGCTA -0.130232500434
AGGAGCTC -0.296104043631
AGGAGCTG -0.175614615031
AGGAGGAA -0.100102469336
AGGAGGAC -0.33160829013
AGGAGGAG -0.226445582784
AGGAGGAT 0.204146624526
AGGAGGCA -0.0869655010355
AGGAGGCC -0.334305786354
AGGAGGCG -0.254885178662
AGGAGGGA -0.0841488062249
AGGAGGGC -0.28549041984
AGGAGGGG -0.131731614981
AGGAGGTA -0.0991836521444
AGGAGGTC -0.304226550336
AGGAGGTG -0.377695267767
AGGAGTAA -0.106693689595
AGGAGTAC -0.0969729215292

AGGAGTAG	-0.240284427795
AGGAGTAT	0.0067593781555
AGGAGTCA	-0.17166304171
AGGAGTCC	-0.178417278401
AGGAGTCG	-0.280087482119
AGGAGTGA	-0.0831585369948
AGGAGTGC	-0.0365893882674
AGGAGTGG	-0.264177286021
AGGAGTTA	2.93914404288E-5
AGGAGTTC	-0.0190484068881
AGGAGTTG	-0.0258074640701
AGGATAAA	0.111842076451
AGGATAAC	0.099664240929
AGGATAAG	-0.0321074356238
AGGATAAT	0.28875018269
AGGATACA	0.0125318423684
AGGATACC	0.181002605782
AGGATACG	0.3649606213
AGGATACT	0.164078591458
AGGATAGA	0.0253738587072
AGGATAGC	-0.0128910396934
AGGATAGG	-0.149347345956
AGGATATA	0.179230381816
AGGATATC	0.464460328097
AGGATATG	0.318441931928
AGGATCAA	-0.0367794865806
AGGATCAC	0.250945599731
AGGATCAG	0.1594362958
AGGATCAT	0.161169463442
AGGATCCA	0.256196968318
AGGATCCC	0.294016189427
AGGATCCG	0.296948463615
AGGATCCT	0.29019236547
AGGATCGA	0.00524232573144
AGGATCGC	0.346169791902
AGGATCGG	0.137866516654
AGGATCTA	0.285438275074
AGGATCTC	0.394224803316
AGGATCTG	0.354216331029
AGGATGAA	-0.210767498697
AGGATGAC	-0.0897504389979
AGGATGAG	0.144010715094
AGGATGAT	0.118166025561
AGGATGCA	-0.162531325212
AGGATGCC	-0.272984987281
AGGATGCG	-0.00981559898582
AGGATGGA	-0.173241212815
AGGATGGC	-0.220730883338
AGGATGGG	-0.214760247232
AGGATGTA	-0.0756837441516
AGGATGTC	-0.206444516124
AGGATGTG	-0.0324613329635
AGGATTAA	-0.174963546372
AGGATTAC	0.454795150471

AGGATTAG -0.152816678367
AGGATTAT 0.358613315037
AGGATTCA 0.14065020474
AGGATTCC 0.451378375621
AGGATTCG 0.33400756128
AGGATTGA -0.0588578301224
AGGATTGC 0.442226082641
AGGATTGG 0.010644897149
AGGATTTA 0.256811908327
AGGATTTC 0.477825303096
AGGATTTG 0.39553465311
AGGCAAAA -0.0621278366167
AGGCAAAC 0.0135282293675
AGGCAAAG 0.0531206137267
AGGCAAAT 0.243748677036
AGGCAACA -0.144704130566
AGGCAACC -0.196370824339
AGGCAACG -0.281627398605
AGGCAACT -0.0660066175193
AGGCAAGA 0.014178573983
AGGCAAGC -0.443635444828
AGGCAAGG -0.163714472368
AGGCAATA 0.00535071213884
AGGCAATC 0.411845730028
AGGCAATG 0.0303139347314
AGGCACAA -0.205253450573
AGGCACAC -0.170103135416
AGGCACAG -0.0454148707447
AGGCACAT -0.154372539776
AGGCACCA -0.0400788037709
AGGCACCC -0.35653970065
AGGCACCG -0.281713506731
AGGCACCT -0.210707087807
AGGCACGA -0.0335470300679
AGGCACGC -0.138702137102
AGGCACGG -0.206302656523
AGGCACTA -0.185081806779
AGGCACTC -0.212957220003
AGGCACTG -0.278311183886
AGGCAGAA -0.113279300886
AGGCAGAC -0.378308157849
AGGCAGAG -0.00649101763384
AGGCAGAT 0.17462779584
AGGCAGCA 0.0223637438596
AGGCAGCC -0.171121335846
AGGCAGCG -0.146778551607
AGGCAGGA -0.0981533081211
AGGCAGGC -0.305348130139
AGGCAGGG -0.305235114298
AGGCAGTA -0.139388625579
AGGCAGTC -0.357760086149
AGGCAGTG -0.0358802361992
AGGCATAA -0.0714557532739
AGGCATAC -0.105533360354

AGGCATAG -0.195052470699
AGGCATAT 0.110509856583
AGGCATCA 0.0804344289193
AGGCATCC -0.288749556733
AGGCATCG -0.277553090892
AGGCATGA -0.14577320156
AGGCATGC -0.299752792034
AGGCATGG -0.125400824052
AGGCATTA 0.0549498916082
AGGCATTC -0.238210414031
AGGCATTG -0.0531794076044
AGGCCAAA -0.221532823813
AGGCCAAC -0.0701378801924
AGGCCAAG -0.13604190963
AGGCCAAT -0.0987481214929
AGGCCACA -0.13827638199
AGGCCACC -0.250898617814
AGGCCACG -0.28535710643
AGGCCACT -0.376168230738
AGGCCAGA -0.0736297645687
AGGCCAGC -0.453590038219
AGGCCAGG -0.325963592266
AGGCCATA -0.0874894731234
AGGCCATC -0.189024755127
AGGCCATG -0.0595264337277
AGGCCCAA -0.418877126031
AGGCCCAC -0.303509555352
AGGCCCAG -0.336141020048
AGGCCCAT -0.229492448612
AGGCCCCA -0.328988167589
AGGCCCCC -0.351796397415
AGGCCCCG -0.268321706179
AGGCCCCT -0.366932430204
AGGCCCGA -0.268995619947
AGGCCCGC -0.255097537697
AGGCCCGG -0.32710994667
AGGCCCTA -0.174088374652
AGGCCCTC -0.325341611282
AGGCCCTG -0.188759086511
AGGCCGAA -0.202146321103
AGGCCGAC -0.243346027029
AGGCCGAG -0.0343912177106
AGGCCGAT -0.141977299732
AGGCCGCA -0.224098485887
AGGCCGCC -0.239526569809
AGGCCGCG -0.290546865514
AGGCCGGA 0.051336115478
AGGCCGGC -0.309273274009
AGGCCGGG -0.239027404676
AGGCCGTA -0.323558138654
AGGCCGTC -0.344647015861
AGGCCGTG 0.0178052956796
AGGCCTAA -0.331376326837
AGGCCTAC -0.328018508873

AGGCCTAG	-0.32043904254
AGGCCTAT	-0.261101016514
AGGCCTCA	-0.194402811364
AGGCCTCC	-0.176336356336
AGGCCTCG	-0.396644865042
AGGCCTGA	-0.126286586799
AGGCCTGC	-0.186478337583
AGGCCTGG	-0.0713764504487
AGGCCTTA	-0.0659580555551
AGGCCTTC	-0.184046998113
AGGCCTTG	-0.0676917979281
AGGCGAAA	-0.0771860014284
AGGCGAAC	-0.0655720117878
AGGCGAAG	-0.0718058124309
AGGCGAAT	0.280678750376
AGGCGACA	0.00759326438107
AGGCGACC	-0.184134941658
AGGCGACG	-0.116985160935
AGGCGACT	-0.267046785802
AGGCGAGA	-0.0217801938256
AGGCGAGC	-0.356796651245
AGGCGAGG	-0.19425077262
AGGCGATA	0.0128558310376
AGGCGATC	0.315358057782
AGGCGATG	-0.113102354021
AGGCGCAA	0.181292863111
AGGCGCAC	-0.225447376757
AGGCGCAG	-0.129012606027
AGGCGCAT	-0.141665195191
AGGCGCCA	-0.163947071521
AGGCGCCC	-0.229222081987
AGGCGCCG	-0.259665401378
AGGCGCCT	-0.274217460436
AGGCGCGA	0.0613754250118
AGGCGCGC	-0.119797052295
AGGCGCGG	-0.126042450658
AGGCGCTA	-0.065739093116
AGGCGCTC	-0.0390997783342
AGGCGCTG	0.0722234630094
AGGCGGAA	-0.144160760021
AGGCGGAC	-0.27791722551
AGGCGGAG	-0.137021620052
AGGCGGAT	0.166062786946
AGGCGGCA	-0.109702094867
AGGCGGCC	-0.152900114962
AGGCGGCG	-0.262157665448
AGGCGGGA	-0.0717365633069
AGGCGGGC	-0.325709520245
AGGCGGGG	-0.244748136436
AGGCGGTA	-0.179521771492
AGGCGGTC	-0.289316485617
AGGCGGTG	-0.0829990101276
AGGCGTAA	0.0665496375992
AGGCGTAC	-0.232035791963

AGGCGTAG -0.303603178935
AGGCGTAT 0.131714358987
AGGCGTCA -0.228628184026
AGGCGTCC -0.307363629598
AGGCGTCG -0.310231993475
AGGCGTGA -0.0766988930719
AGGCGTGC -0.359246030762
AGGCGTGG -0.15551805302
AGGCGTTA -0.116930549406
AGGCGTTC -0.206928211618
AGGCGTTG -0.0493620342105
AGGCTAAA -0.191786109112
AGGCTAAC -0.067535260785
AGGCTAAG -0.0642898301393
AGGCTAAT -0.0935461267272
AGGCTACA -0.153121372781
AGGCTACC -0.0747340701176
AGGCTACG -0.282144048338
AGGCTACT -0.314603849875
AGGCTAGA -0.107448088516
AGGCTAGC -0.33326546845
AGGCTAGG -0.342147500201
AGGCTATA -0.175208885192
AGGCTATC -0.183601428
AGGCTATG 0.0755866058896
AGGCTCAA -0.181323542788
AGGCTCAC -0.196600648844
AGGCTCAG -0.204727481259
AGGCTCAT -0.254306803473
AGGCTCCA -0.176447329248
AGGCTCCC -0.372009784469
AGGCTCCG -0.336648237237
AGGCTCGA -0.188289737373
AGGCTCGC -0.367413249994
AGGCTCGG -0.164080274001
AGGCTCTA -0.246039924046
AGGCTCTC -0.100278492579
AGGCTCTG -0.00845559952646
AGGCTGAA -0.207833650766
AGGCTGAC -0.184188617348
AGGCTGAG -0.232758557788
AGGCTGAT -0.020218170885
AGGCTGCA -0.132688091988
AGGCTGCC -0.327669488495
AGGCTGCG -0.255357069015
AGGCTGGA -0.230295963681
AGGCTGGC -0.178652885579
AGGCTGGG -0.260223327427
AGGCTGTA -0.224613460183
AGGCTGTC -0.253242475115
AGGCTGTG -0.167753531703
AGGCTTAA -0.153582609076
AGGCTTAC -0.356250086127
AGGCTTAG -0.0599750764387

AGGCTTAT -0.0628256315555
AGGCTTCA -0.241812795085
AGGCTTCC -0.333025855552
AGGCTTCG -0.389412516915
AGGCTTGA -0.196307508223
AGGCTTGC -0.447175685671
AGGCTTGG -0.215952086686
AGGCTTTA -0.0866555260495
AGGCTTTC -0.065182262152
AGGCTTTG -0.0535883973183
AGGGAAAA -0.156566688609
AGGGAAAC 0.0958541053846
AGGGAAAG -0.171775952442
AGGGAAAT 0.367979533295
AGGGAACA -0.105651778052
AGGGAACC -0.320238656379
AGGGAACG -0.303896152224
AGGGAACT -0.207595229296
AGGGAAGA -0.100741807927
AGGGAAGC -0.212798046853
AGGGAAGG -0.339424860598
AGGGAATA 0.183084312022
AGGGAATC 0.467496985744
AGGGAATG -0.0328767236602
AGGGACAA -0.22410775079
AGGGACAC -0.228939796096
AGGGACAG -0.314670106588
AGGGACAT -0.116082705834
AGGGACCA -0.0717351036298
AGGGACCC -0.445379992229
AGGGACCG -0.262613733775
AGGGACCT -0.336912272578
AGGGACGA 0.0119335905284
AGGGACGC -0.336470442289
AGGGACGG -0.057755448042
AGGGACTA -0.102967245339
AGGGACTC -0.101433614058
AGGGACTG -0.166169958072
AGGGAGAA -0.137170492344
AGGGAGAC -0.251398249871
AGGGAGAG -0.177870418621
AGGGAGAT 0.275623891448
AGGGAGCA -0.264374326451
AGGGAGCC -0.264228710015
AGGGAGCG 0.0219517250338
AGGGAGGA -0.0210801942186
AGGGAGGC -0.40737522779
AGGGAGGG -0.274304304114
AGGGAGTA -0.0266023599357
AGGGAGTC -0.329959974961
AGGGAGTG -0.188154873889
AGGGATAA 0.00600324842749
AGGGATAC 0.083439778691
AGGGATAG -0.156038311704

AGGGATAT 0.356283280526
AGGGATCA 0.146527448304
AGGGATCC 0.0831624125019
AGGGATCG 0.00248454913252
AGGGATGA -0.22030895997
AGGGATGC -0.168289340535
AGGGATGG -0.282927444997
AGGGATTA 0.197936541536
AGGGATTC 0.366245264702
AGGGATTG 0.27933305206
AGGGCAAA -0.116500263323
AGGGCAAC -0.0127937855211
AGGGCAAG -0.189441537687
AGGGCAAT 0.254243492071
AGGGCACA -0.165758269994
AGGGCACC -0.310179463346
AGGGCACG -0.23420210495
AGGGCACT -0.26293710478
AGGGCAGA -0.149563629516
AGGGCAGC -0.308828270774
AGGGCAGG -0.357430313678
AGGGCATA -0.213483924915
AGGGCATC -0.324784098395
AGGGCATG -0.272681872128
AGGGCCAA -0.184694893151
AGGGCCAC -0.314258304091
AGGGCCAG -0.125230765638
AGGGCCAT -0.351456795023
AGGGCCCA -0.157750279122
AGGGCCCC -0.305812124258
AGGGCCCG -0.439629980319
AGGGCCCT -0.209810244326
AGGGCCGA -0.0507700223439
AGGGCCGC -0.381543222215
AGGGCCGG -0.363473520893
AGGGCCTA -0.289634205166
AGGGCCTC -0.1955820703
AGGGCCTG -0.11068616505
AGGGCGAA -0.166378571212
AGGGCGAC -0.139341663841
AGGGCGAG -0.116703748735
AGGGCGAT 0.0750574804931
AGGGCGCA -0.141573814428
AGGGCGCC -0.260648862089
AGGGCGCG -0.136972954984
AGGGCGGA -0.016963781004
AGGGCGGC -0.232046250907
AGGGCGGG 0.0116282125209
AGGGCGTA -0.0490063065821
AGGGCGTC -0.376817398587
AGGGCGTG -0.244289618802
AGGGCTAA -0.165086071645
AGGGCTAC -0.0976864603754
AGGGCTAG -0.299248969917

AGGGCTAT -0.0896862132034
AGGGCTCA -0.323516371078
AGGGCTCC -0.337940950333
AGGGCTCG -0.422893777436
AGGGCTGA -0.0774269212412
AGGGCTGC -0.266820754119
AGGGCTGG -0.0338102523811
AGGGCTTA -0.148405971822
AGGGCTTC -0.415844708463
AGGGCTTG -0.227739872362
AGGGGAAA -0.048825642595
AGGGGAAC -0.213341303171
AGGGGAAG -0.0528285811185
AGGGGAAT 0.355516673698
AGGGGACA -0.23808637533
AGGGGACC -0.214586303353
AGGGGACG -0.264463612017
AGGGGACT -0.350913458241
AGGGGAGA 0.0913268640541
AGGGGAGC -0.319725550016
AGGGGAGG -0.229681035372
AGGGGATA 0.194570603662
AGGGGATC 0.280256528492
AGGGGATG -0.130614906541
AGGGGCAA -0.187155493353
AGGGGCAC -0.26566319301
AGGGGCAG -0.231278650205
AGGGGCAT -0.20645157053
AGGGGCCA -0.219469727931
AGGGGCCC -0.415847586296
AGGGGCCG -0.256060746523
AGGGGCGA -0.119028506125
AGGGGCGC -0.215460648744
AGGGGCGG -0.400378152071
AGGGGCTA -0.239480785987
AGGGGCTC -0.265184490188
AGGGGCTG -0.115316508299
AGGGGGAA -0.0588312336457
AGGGGGAC -0.315679934282
AGGGGGAG -0.0606650035253
AGGGGGAT -0.0457523639342
AGGGGGCA -0.174281831389
AGGGGGCC -0.221468323865
AGGGGGCG -0.179575280955
AGGGGGGA -0.0464833013923
AGGGGGGC -0.249493054694
AGGGGGGG -0.415993629222
AGGGGGTA -0.164656990448
AGGGGGTC -0.317147317813
AGGGGGTG -0.237983172428
AGGGGTAA 0.0303051757991
AGGGGTAC -0.315120340277
AGGGGTAG -0.21003046217
AGGGGTAT 0.0158616964592

AGGGGTCA -0.219457574878
AGGGGTCC -0.307787396952
AGGGGTCG -0.297997134777
AGGGGTGA -0.0118544849383
AGGGGTGC -0.183383095276
AGGGGTGG -0.0286673663039
AGGGGTTA -0.0582659861638
AGGGGTTC -0.172085205885
AGGGGTTG 0.134332094918
AGGGTAAA -0.0459332106171
AGGGTAAC -0.0955180931257
AGGGTAAG -0.149075291582
AGGGTAAT 0.308363320511
AGGGTACA -0.0963878342721
AGGGTACC -0.413559474689
AGGGTACG 0.0079553002489
AGGGTACT -0.0780113904261
AGGGTAGA -0.0455332109272
AGGGTAGC -0.290786595235
AGGGTAGG -0.21430697859
AGGGTATA -0.0719028050481
AGGGTATC 0.265529221988
AGGGTATG -0.0228272349484
AGGGTCAA -0.21594141142
AGGGTCAC -0.088719906931
AGGGTCAG -0.213835225241
AGGGTCAT -0.301644811255
AGGGTCCA -0.279507557276
AGGGTCCC -0.442103508235
AGGGTCCG -0.237819337008
AGGGTCGA -0.238823295933
AGGGTCGC -0.410127284676
AGGGTCGG -0.233456272517
AGGGTCTA -0.225034844604
AGGGTCTC -0.337325559079
AGGGTCTG -0.221800011968
AGGGTGAA 0.0554987846981
AGGGTGAC 0.0281066837952
AGGGTGAG -0.246146161832
AGGGTGAT -0.0770649791124
AGGGTGCA -0.146870270203
AGGGTGCC -0.426257935275
AGGGTGCG 0.0542812931104
AGGGTGGA -0.0552991389364
AGGGTGGC -0.250126589298
AGGGTGGG -0.220673970052
AGGGTGTA 0.0401158734492
AGGGTGTC -0.117292068139
AGGGTGTG -0.117008630633
AGGGTTAA -0.0542072335134
AGGGTTAC 0.0576857206682
AGGGTTAG -0.220950104884
AGGGTTAT -0.162869152127
AGGGTTCA -0.164932513977

AGGGTTCC -0.408051773407
AGGGTTCG -0.33027807407
AGGGTTGA -0.0297723407517
AGGGTTGC -0.302969156171
AGGGTTGG -0.33340086719
AGGGTTTA -0.182493049322
AGGGTTTC -0.319099590723
AGGGTTTG 0.0816946423007
AGGTAAAA 0.124958291625
AGGTAAAC 0.140256052787
AGGTAAAG -0.0097552608337
AGGTAAAT 0.152355430036
AGGTAACA -0.162787523883
AGGTAACC -0.137745410232
AGGTAACG -0.0271650985745
AGGTAACT -0.0907699322564
AGGTAAGA 0.0207383692232
AGGTAAGC 0.147739100604
AGGTAAGG -0.0357208024535
AGGTAATA -0.0540637796253
AGGTAATC 0.415242731911
AGGTAATG -0.154715149923
AGGTACAA -0.0677250909131
AGGTACAC -0.0448605010877
AGGTACAG -0.29662097778
AGGTACAT -0.0709871343288
AGGTACCA -0.0183390144741
AGGTACCC -0.375629751864
AGGTACCG -0.0599506993904
AGGTACCT -0.347308681167
AGGTACGA -0.218003511983
AGGTACGC -0.108107172664
AGGTACGG -0.111640353732
AGGTACTA 0.0107035410066
AGGTACTC -0.186049277676
AGGTACTG -0.00819965971481
AGGTAGAA 0.147011564136
AGGTAGAC -0.160089841908
AGGTAGAG -0.127776537175
AGGTAGAT 0.194884967612
AGGTAGCA -0.0923168347411
AGGTAGCC -0.231868375622
AGGTAGCG -0.0463846655701
AGGTAGGA 0.080257366627
AGGTAGGC -0.288179715214
AGGTAGGG -0.171749782905
AGGTAGTA -0.0210791478734
AGGTAGTC -0.18267123231
AGGTAGTG -0.0368407991066
AGGTATAA -0.0471926501284
AGGTATAC -0.0897681072677
AGGTATAG -0.164387054449
AGGTATAT -0.0733420941597
AGGTATCA 0.0808120990051

AGGTATCC 0.144071068313
AGGTATCG -0.0177711740328
AGGTATGA -0.0235073706398
AGGTATGC -0.0703319519236
AGGTATGG -0.143840235417
AGGTATTA 0.000760911735545
AGGTATTC -0.0607464394535
AGGTATTG 0.0987299548185
AGGTCAAA 0.0162903950783
AGGTCAAC 0.046097530711
AGGTCAAG -0.112184113021
AGGTCAAT 0.0234264943103
AGGTCACA -0.308382473329
AGGTCACC -0.356413555748
AGGTCACG -0.307705384751
AGGTCACT -0.155922563055
AGGTCAGA -0.102413190669
AGGTCAGC -0.248531491392
AGGTCAGG -0.20996429999
AGGTCATA -0.204284891238
AGGTCATC -0.394687542403
AGGTCATG -0.289483767639
AGGTCCAA -0.328673497234
AGGTCCAC -0.149558969099
AGGTCCAG -0.303606645829
AGGTCCAT -0.353747328027
AGGTCCCA -0.212509683591
AGGTCCCC -0.427936082798
AGGTCCCG -0.323403199802
AGGTCCGA -0.0575410757429
AGGTCCGC -0.141296705297
AGGTCCGG -0.1312883036
AGGTCCTA -0.328555754469
AGGTCCTC -0.320263302681
AGGTCCTG -0.149923885392
AGGTCGAA -0.117857961238
AGGTCGAC -0.259630013177
AGGTCGAG -0.321571205318
AGGTCGAT -0.144887391595
AGGTCGCA -0.22960643733
AGGTCGCC -0.116473996366
AGGTCGCG -0.256861383206
AGGTCGGA 0.0888148434706
AGGTCGGC -0.356035112379
AGGTCGGG -0.164955070603
AGGTCGTA -0.137737715971
AGGTCGTC -0.370580062724
AGGTCGTG -0.144674116534
AGGTCTAA -0.202430874337
AGGTCTAC -0.0845381153876
AGGTCTAG -0.286726946106
AGGTCTAT -0.218105329218
AGGTCTCA -0.342505920583
AGGTCTCC -0.317455441253

AGGTCTCG -0.283151809859
AGGTCTGA 0.000460573856047
AGGTCTGC -0.0637239090361
AGGTCTGG -0.137733871803
AGGTCTTA -0.130191008989
AGGTCTTC -0.236279347305
AGGTCTTG -0.0587292133685
AGGTGAAA 0.0194314567279
AGGTGAAC -0.309965147719
AGGTGAAG -0.0666029792478
AGGTGAAT 0.00272042583815
AGGTGACA -0.0198367918951
AGGTGACC -0.186878837252
AGGTGACG 0.0150026059117
AGGTGACT -0.187813095346
AGGTGAGA 0.0378881792852
AGGTGAGC -0.127167330544
AGGTGAGG -0.241399605784
AGGTGATA -0.0750193700661
AGGTGATC 0.0477226537833
AGGTGATG -0.218040762483
AGGTGCAA -0.128107981415
AGGTGCAC -0.143170822479
AGGTGCAG -0.111629458685
AGGTGCAT -0.298084794959
AGGTGCCA -0.326174141791
AGGTGCCC -0.383003877411
AGGTGCCG -0.288561986114
AGGTGCGA -0.027298047293
AGGTGCGC -0.317321051979
AGGTGCGG -0.105810718786
AGGTGCTA -0.168841964219
AGGTGCTC -0.265539192579
AGGTGCTG -0.0221418467876
AGGTGGAA -0.0449165346744
AGGTGGAC -0.0856429075017
AGGTGGAG -0.101820372622
AGGTGGAT 0.165905126165
AGGTGGCA -0.284399901874
AGGTGGCC -0.336494139243
AGGTGGCG -0.183975313237
AGGTGGGA -0.237939651184
AGGTGGGC -0.217803497394
AGGTGGGG -0.236689628178
AGGTGGTA -0.0430699637854
AGGTGGTC -0.286990868222
AGGTGGTG -0.316460139021
AGGTGTAA 0.0642985920016
AGGTGTAC -0.136755741922
AGGTGTAG -0.266179296124
AGGTGTAT 0.019238404844
AGGTGTCA -0.138792442917
AGGTGTCC -0.194871461749
AGGTGTCG -0.289919719097

AGGTGTGA 0.145708555154
AGGTGTGC -0.20148556209
AGGTGTGG -0.00349247318944
AGGTGTTA 0.0482230316124
AGGTGTTC -0.0309079337832
AGGTGTTG 0.0364388167854
AGGTTAAA -0.0896085811584
AGGTTAAC -0.0665773742181
AGGTTAAG -0.078646199031
AGGTTAAT -0.091629211925
AGGTTACA 0.0417414217207
AGGTTACC -0.254441136335
AGGTTACG -0.0210805602797
AGGTTACT -0.0332771816323
AGGTTAGA 0.0789435267841
AGGTTAGC -0.386727533521
AGGTTAGG -0.242452312621
AGGTTATA 0.00891878280038
AGGTTATC -0.197987351066
AGGTTATG -0.0263863289307
AGGTTCAA -0.0830548999172
AGGTTCAC -0.12647839712
AGGTTCAG -0.227773537657
AGGTTCAT -0.0956329806741
AGGTTCCA -0.158704360796
AGGTTCCC -0.327901302666
AGGTTCCG -0.257991339557
AGGTTCGA 0.00885596340142
AGGTTCGC -0.074233989815
AGGTTCGG 0.0486230031685
AGGTTCTA -0.000295613964658
AGGTTCTC -0.203336172512
AGGTTCTG -0.0466166719561
AGGTTGAA 0.0495350274124
AGGTTGAC -0.139184595151
AGGTTGAG -0.231409186916
AGGTTGAT -0.0349167536477
AGGTTGCA -0.118616918022
AGGTTGCC -0.302483838509
AGGTTGCG -0.0126515477763
AGGTTGGA 0.0595934314703
AGGTTGGC -0.147462826608
AGGTTGGG -0.126055807874
AGGTTGTA -0.12576059596
AGGTTGTC -0.25855851385
AGGTTGTG -0.074970153657
AGGTTTAA -0.140373008192
AGGTTTAC -0.241556740961
AGGTTTAG -0.227096822207
AGGTTTAT -0.0776740928256
AGGTTTCA -0.156768099969
AGGTTTCC -0.112127278794
AGGTTTCG -0.115042832604
AGGTTTGA 0.0432479580739

AGGTTTGC -0.107993978046
AGGTTTGG -0.043748776446
AGGTTTTA 0.0567799204163
AGGTTTTC -0.037124001834
AGGTTTTG 0.127202960536
AGTAAAAA 0.166982474836
AGTAAAAC 0.102351662958
AGTAAAAG 0.184798143106
AGTAAAAT 0.301423590746
AGTAAACA -0.154078320745
AGTAAACC -0.209162813907
AGTAAACG 0.0470890373469
AGTAAACT -0.0910437129516
AGTAAAGA 0.0505174596084
AGTAAAGC -0.167127460955
AGTAAAGG -0.131145543099
AGTAAATA 0.175177915812
AGTAAATC 0.193073238528
AGTAAATG 0.0130006039097
AGTAACAA 0.0299635774399
AGTAACAC -0.0947892445149
AGTAACAG -0.197467337865
AGTAACAT 0.0476741385225
AGTAACCA 0.076921273891
AGTAACCC 0.0584125895053
AGTAACCG -0.000809349294198
AGTAACGA -0.0325656234747
AGTAACGC -0.0887650736136
AGTAACGG -0.240685557013
AGTAACTA 0.0828531642825
AGTAACTC -0.210788570483
AGTAACTG 0.0633497076706
AGTAAGAA 0.229200090509
AGTAAGAC 0.0834660554878
AGTAAGAG -0.0512215555403
AGTAAGAT 0.202902856142
AGTAAGCA -0.0024831994529
AGTAAGCC -0.128144223823
AGTAAGCG 0.127459415338
AGTAAGGA -0.0197511561148
AGTAAGGC -0.339659879729
AGTAAGGG -0.234935929243
AGTAAGTA 0.172495751304
AGTAAGTC -0.196148383816
AGTAAGTG -0.021947694576
AGTAATAA 0.106493545887
AGTAATAC 0.229401545952
AGTAATAG -0.101136667521
AGTAATAT 0.299361344816
AGTAATCA 0.316984072452
AGTAATCC 0.362234134961
AGTAATCG 0.302209975063
AGTAATGA -0.026893284469
AGTAATGC -0.0825837046811

AGTAATGG -0.149505728585
AGTAATTA 0.291733875971
AGTAATTC 0.168176609271
AGTAATTG 0.10672977964
AGTACAAA 0.121862294986
AGTACAAC 0.0311460332985
AGTACAAG -0.166625795707
AGTACAAT 0.173271388218
AGTACACA -0.264306030036
AGTACACC -0.0348513049593
AGTACACG -0.168566208393
AGTACACT -0.175785201784
AGTACAGA -0.0196119792997
AGTACAGC 0.0304613767582
AGTACAGG -0.206294467738
AGTACATA 0.191279752998
AGTACATC -0.0905180026009
AGTACATG -0.112369820748
AGTACCAA -0.0323194810386
AGTACCAC -0.209355695041
AGTACCAG -0.298098577379
AGTACCAT -0.227447656067
AGTACCCA -0.126594253244
AGTACCCC -0.282155508691
AGTACCCG -0.168653403372
AGTACCGA -0.0887242616954
AGTACCGC -0.148290852187
AGTACCGG -0.219398204426
AGTACCTA -0.141828281575
AGTACCTC -0.194005409027
AGTACCTG 0.0426613643696
AGTACGAA 0.16643650139
AGTACGAC 0.0296336356325
AGTACGAG 0.075726078991
AGTACGAT 0.165064268666
AGTACGCA -0.00946814583178
AGTACGCC -0.220722127448
AGTACGCG 0.0559609195973
AGTACGGA 0.145811100357
AGTACGGC -0.282713507166
AGTACGGG -0.266395690851
AGTACGTA 0.0916853492611
AGTACGTC -0.140876551588
AGTACGTG -0.0544636454008
AGTACTAA 0.0137712312869
AGTACTAC -0.0721743853583
AGTACTAG -0.115850682813
AGTACTAT -0.0700891469077
AGTACTCA -0.112541584617
AGTACTCC -0.0969145344433
AGTACTCG -0.225649064483
AGTACTGA 0.0669691730298
AGTACTGC -0.13587752165
AGTACTGG -0.0577746472705

AGTACTTA 0.0813637328789
AGTACTTC -0.0275329737305
AGTACTTG 0.104618876959
AGTAGAAA 0.0646197161349
AGTAGAAC 0.0387984204725
AGTAGAAG -0.0147449248414
AGTAGAAT 0.225492142508
AGTAGACA 0.00492762932199
AGTAGACC 0.0210971759398
AGTAGACG -0.0107911988777
AGTAGACT 0.0338148065421
AGTAGAGA 0.0502058532362
AGTAGAGC -0.127380588023
AGTAGAGG -0.13317153639
AGTAGATA 0.302906505697
AGTAGATC 0.1434019693
AGTAGATG 0.0512886579126
AGTAGCAA 0.111459603074
AGTAGCAC -0.182148002505
AGTAGCAG -0.050929172854
AGTAGCAT -0.05666054545
AGTAGCCA -0.241552390139
AGTAGCCC -0.247533513616
AGTAGCCG -0.236646374303
AGTAGCGA 0.071991854193
AGTAGCGC -0.219856681042
AGTAGCGG -0.0747813929632
AGTAGCTA -0.0759631792762
AGTAGCTC -0.161100983579
AGTAGCTG -0.247322380988
AGTAGGAA -0.0613580155008
AGTAGGAC 0.196971858947
AGTAGGAG -0.137588908172
AGTAGGAT 0.0332191695828
AGTAGGCA 0.0263822368911
AGTAGGCC -0.366724375872
AGTAGGCG 0.0241138271911
AGTAGGGA -0.114326128496
AGTAGGGC -0.24567576582
AGTAGGGG -0.125823682655
AGTAGGTA -0.117428815427
AGTAGGTC -0.445740984158
AGTAGGTG -0.259356033465
AGTAGTAA 0.0954163529921
AGTAGTAC -0.0456470996965
AGTAGTAG -0.0318045318045
AGTAGTAT 0.118706861131
AGTAGTCA -0.14408959693
AGTAGTCC -0.0629679266043
AGTAGTCG -0.109339306332
AGTAGTGA 0.0587512682204
AGTAGTGC -0.199524778519
AGTAGTGG -0.109250892316
AGTAGTTA 0.157415564434

AGTAGTTC 0.0888527988438
AGTAGTTG 0.125657617192
AGTATAAA 0.143290673594
AGTATAAC 0.20442691036
AGTATAAG 0.00994649752195
AGTATAAT 0.191137057349
AGTATACA 0.138167092713
AGTATACC -0.0719098195895
AGTATACG 0.122001042468
AGTATACT 0.0942381318581
AGTATAGA 0.169718181354
AGTATAGC -0.199858001602
AGTATAGG -0.161912512865
AGTATATA 0.238125442509
AGTATATC 0.181955232399
AGTATATG 0.164643839916
AGTATCAA 0.16916636862
AGTATCAC 0.0637030079853
AGTATCAG 0.336610715399
AGTATCAT 0.122008277191
AGTATCCA 0.259724969526
AGTATCCC 0.0273884513478
AGTATCCG 0.298553316351
AGTATCGA 0.247783625176
AGTATCGC 0.159251257272
AGTATCGG 0.23173448931
AGTATCTA 0.322715277261
AGTATCTC 0.187875447983
AGTATCTG 0.367820989033
AGTATGAA 0.0536031899668
AGTATGAC 0.0990535990536
AGTATGAG 0.0565576222455
AGTATGAT 0.147146412852
AGTATGCA 0.0850397436552
AGTATGCC -0.140122059386
AGTATGCG -0.0587556510556
AGTATGGA 0.00176898661747
AGTATGGC -0.185415374437
AGTATGGG 0.103864578421
AGTATGTA 0.120437508668
AGTATGTC -0.142996542025
AGTATGTG -0.0504769968541
AGTATTAA -0.0669356851652
AGTATTAC 0.264761355591
AGTATTAG 0.091889228703
AGTATTAT 0.165563527806
AGTATTCA 0.0978219455048
AGTATTCC 0.10307139095
AGTATTCG 0.100774328047
AGTATTGA 0.081296759925
AGTATTGC 0.110518017854
AGTATTGG -0.0372590221075
AGTATTTA 0.14374291647
AGTATTTC 0.239185273315

AGTATTTG 0.246017240498
AGTCAAAA -0.0271913753713
AGTCAAAC -0.0719907235059
AGTCAAAG 0.00775293199536
AGTCAAAT 0.0469997007365
AGTCAACA 0.0407370641519
AGTCAACC -0.200423162277
AGTCAACG -0.227897203696
AGTCAACT -0.132172783747
AGTCAAGA 0.0857014038832
AGTCAAGC -0.155456555529
AGTCAAGG -0.206328969818
AGTCAATA -0.0351579432855
AGTCAATC -0.179976894933
AGTCAATG -0.111822023106
AGTCACAA -0.0662462475501
AGTCACAC -0.145308583766
AGTCACAG -0.119931291375
AGTCACAT -0.258351112655
AGTCACCA -0.177453485674
AGTCACCC 0.0388976883718
AGTCACCG -0.306391583753
AGTCACGA 0.0701416978931
AGTCACGC -0.252719933335
AGTCACGG -0.144862189585
AGTCACTA -0.14212341513
AGTCACTC -0.226348883431
AGTCACTG -0.0389468490575
AGTCAGAA 0.00694767819528
AGTCAGAC -0.0343883828732
AGTCAGAG 0.00110278608577
AGTCAGAT 0.17334552183
AGTCAGCA -0.0883931242317
AGTCAGCC -0.344824290985
AGTCAGCG -0.15884279715
AGTCAGGA 0.13798513739
AGTCAGGC -0.279425991616
AGTCAGGG -0.285313892347
AGTCAGTA 0.0226217953491
AGTCAGTC -0.219805677611
AGTCAGTG -0.00368824040349
AGTCATAA 0.0105466908207
AGTCATAC 0.0946628905936
AGTCATAG -0.161698351484
AGTCATAT 0.074350926622
AGTCATCA -0.145034050349
AGTCATCC -0.304807896383
AGTCATCG -0.286618699086
AGTCATGA 0.137326031265
AGTCATGC -0.310153127379
AGTCATGG -0.2979407929
AGTCATTA 0.0122946638098
AGTCATTC 0.0655600504085
AGTCATTG 0.100586812708

AGTCCAAA 0.035535414255
AGTCCAAC -0.231283256571
AGTCCAAG -0.0912863221973
AGTCCAAT -0.155160433779
AGTCCACA -0.362098295505
AGTCCACC -0.205921758357
AGTCCACG -0.350738559011
AGTCCACT -0.147092392772
AGTCCAGA -0.0216031765728
AGTCCAGC -0.154940688963
AGTCCAGG -0.347862696621
AGTCCATA -0.0743815440785
AGTCCATC -0.243282471157
AGTCCATG -0.237622233801
AGTCCCAA -0.161910515213
AGTCCCAC -0.254141544467
AGTCCCAG -0.0950255576785
AGTCCCAT -0.191720457255
AGTCCCCA -0.280788195311
AGTCCCCC -0.0698550394624
AGTCCCCG -0.340064651993
AGTCCCGA -0.0742866192855
AGTCCCGC -0.290382425992
AGTCCCGG -0.336756280428
AGTCCCTA -0.120569140412
AGTCCCTC -0.0770925861598
AGTCCCTG -0.0879871490024
AGTCCGAA 0.0653934585374
AGTCCGAC 0.00747063932907
AGTCCGAG -0.145376872369
AGTCCGAT -0.0315562432939
AGTCCGCA -0.0825040327584
AGTCCGCC -0.213306625218
AGTCCGCG -0.16533928793
AGTCCGGA 0.0311489529426
AGTCCGGC -0.386997249601
AGTCCGGG -0.269330992121
AGTCCGTA -0.0720751273122
AGTCCGTC -0.358399202709
AGTCCGTG -0.0930288738737
AGTCCTAA -0.22188448591
AGTCCTAC -0.0847625498322
AGTCCTAG -0.165532123576
AGTCCTAT -0.229863815643
AGTCCTCA -0.239926943065
AGTCCTCC -0.350983893818
AGTCCTCG -0.357126689818
AGTCCTGA 0.156671399777
AGTCCTGC -0.300586878266
AGTCCTGG -0.0738895871011
AGTCCTTA -0.123329666664
AGTCCTTC -0.1627358525
AGTCCTTG -0.115420542333
AGTCGAAA 0.152706777179

AGTCGAAC -0.129729276001
AGTCGAAG -0.130895160973
AGTCGAAT 0.0324651959203
AGTCGACA 0.042983755105
AGTCGACC -0.298396449924
AGTCGACG -0.145664970204
AGTCGACT -0.0615917040859
AGTCGAGA 0.166220352201
AGTCGAGC -0.337524313424
AGTCGAGG -0.335557484807
AGTCGATA 0.146156653504
AGTCGATC -0.0636314533258
AGTCGATG 0.0294991107251
AGTCGCAA 0.186697717001
AGTCGCAC -0.112707352913
AGTCGCAG -0.119428506372
AGTCGCAT -0.180399438668
AGTCGCCA -0.276570727969
AGTCGCCC -0.308056864691
AGTCGCCG -0.322616198318
AGTCGCGA 0.156538080781
AGTCGCGC 0.0208075735568
AGTCGCGG -0.0608490325029
AGTCGCTA -0.0784522145293
AGTCGCTC -0.130547043644
AGTCGCTG -0.116456692109
AGTCGGAA -0.0804675198615
AGTCGGAC -0.0195250346765
AGTCGGAG -0.250592205222
AGTCGGAT 0.0140939430783
AGTCGGCA 0.0674567899072
AGTCGGCC -0.259432441148
AGTCGGCG -0.213005976488
AGTCGGGA 0.0579512857383
AGTCGGGC -0.162340069379
AGTCGGGG -0.0717856246778
AGTCGGTA -0.0767624802061
AGTCGGTC -0.190059205139
AGTCGGTG -0.129099047123
AGTCGTAA -0.0011592714942
AGTCGTAC -0.184530749957
AGTCGTAG -0.271087791257
AGTCGTAT 0.118417624141
AGTCGTCA -0.142082065428
AGTCGTCC -0.290308894678
AGTCGTCG -0.188346353502
AGTCGTGA -0.0787240378173
AGTCGTGC 0.010559622782
AGTCGTGG -0.0956190740349
AGTCGTTA 0.133330656993
AGTCGTTC -0.0161142456735
AGTCGTTG -0.0044108523171
AGTCTAAA -0.0547172516869
AGTCTAAC -0.0730099749386

AGTCTAAG 0.110367656183
AGTCTAAT 0.000280198398754
AGTCTACA 0.0208338503536
AGTCTACC −0.207216233058
AGTCTACG −0.282296551781
AGTCTAGA 0.0890684072502
AGTCTAGC −0.219327022282
AGTCTAGG −0.304909475884
AGTCTATA −0.139504457183
AGTCTATC −0.0736441095293
AGTCTATG −0.129216041427
AGTCTCAA 0.121370334956
AGTCTCAC −0.116618482765
AGTCTCAG −0.217901004838
AGTCTCAT −0.14898407105
AGTCTCCA −0.0868671579696
AGTCTCCC −0.43953231202
AGTCTCCG −0.288917834843
AGTCTCGA 0.0490930855529
AGTCTCGC −0.00813646177135
AGTCTCGG −0.260431336551
AGTCTCTA −0.0987782988825
AGTCTCTC −0.192858619692
AGTCTCTG 0.0157711874922
AGTCTGAA −0.0134962981346
AGTCTGAC 0.0115239206148
AGTCTGAG 0.0505000372236
AGTCTGAT 0.0137871981336
AGTCTGCA −0.0863745433123
AGTCTGCC −0.45295255574
AGTCTGCG −0.180848160004
AGTCTGGA −0.0492410031983
AGTCTGGC −0.223633612028
AGTCTGGG −0.169972343236
AGTCTGTA 0.0605234921863
AGTCTGTC −0.197115377599
AGTCTGTG −0.0452361246739
AGTCTTAA −0.261126931604
AGTCTTAC 0.0631637146789
AGTCTTAG −0.110581083941
AGTCTTAT −0.0695537080697
AGTCTTCA 0.0558170259045
AGTCTTCC −0.14951971707
AGTCTTCG −0.301064108396
AGTCTTGA −0.0232790522835
AGTCTTGC −0.0727144661066
AGTCTTGG −0.10872742112
AGTCTTTA 0.149677225435
AGTCTTTC −0.00271015963154
AGTCTTTG −0.0476405626154
AGTGAAAA 0.138337846616
AGTGAAAC −0.124449884175
AGTGAAAG −0.0968560811384
AGTGAAAT 0.185262366518

AGTGAACA 0.0272572812345
AGTGAACC -0.208881245321
AGTGAACG -0.22118187059
AGTGAACT -0.10016669513
AGTGAAGA -0.0146502874633
AGTGAAGC -0.386974362566
AGTGAAGG -0.366968166172
AGTGAATA 0.0493573724836
AGTGAATC 0.155990014817
AGTGAATG -0.0781240952594
AGTGACAA 0.018569282846
AGTGACAC -0.165458967169
AGTGACAG -0.0382073426875
AGTGACAT -0.0452464896823
AGTGACCA 0.10880991184
AGTGACCC -0.144204881014
AGTGACCG -0.125511748505
AGTGACGA 0.00451887914863
AGTGACGC -0.245253788286
AGTGACGG -0.308206472454
AGTGACTA -0.158763095779
AGTGACTC -0.148938956574
AGTGACTG -0.119367117613
AGTGAGAA 0.0321546341941
AGTGAGAC -0.228880121965
AGTGAGAG 0.102321117056
AGTGAGAT 0.333545250834
AGTGAGCA 0.215908746212
AGTGAGCC -0.265930186914
AGTGAGCG -0.17067736554
AGTGAGGA -0.00112479288775
AGTGAGGC -0.187403589217
AGTGAGGG -0.188493421331
AGTGAGTA -0.0126895031496
AGTGAGTC -0.0926581158779
AGTGAGTG 0.0221443928555
AGTGATAA -0.207455572598
AGTGATAC 0.0961746870838
AGTGATAG 0.0193565104414
AGTGATAT 0.313008600887
AGTGATCA 0.0564379806804
AGTGATCC 0.023548589225
AGTGATCG -0.0228040424095
AGTGATGA 0.0431960886506
AGTGATGC 0.139587788314
AGTGATGG -0.131922014271
AGTGATTA 0.221731640913
AGTGATTC 0.335831332161
AGTGATTG 0.258647620855
AGTGCAAA 0.188217046342
AGTGCAAC -0.0858696161726
AGTGCAAG 0.208269161989
AGTGCAAT 0.304188277043
AGTGCACA -0.0407804498714

AGTGCACC -0.326169312648
AGTGCACG -0.329564117195
AGTGCACT 0.149740778409
AGTGCAGA 0.081583454056
AGTGCAGC -0.178452974699
AGTGCAGG -0.183792333457
AGTGCATA -0.00970343715101
AGTGCATC -0.00281526681897
AGTGCATG -0.0641288671592
AGTGCCAA -0.313554054705
AGTGCCAC -0.290232950458
AGTGCCAG -0.284997009215
AGTGCCAT -0.103440361281
AGTGCCCA -0.169632937739
AGTGCCCC -0.218316333902
AGTGCCCG -0.332977835098
AGTGCCGA -0.102055517361
AGTGCCGC -0.165032314928
AGTGCCGG -0.167438272731
AGTGCCTA -0.0897596025267
AGTGCCTC -0.312109460059
AGTGCCTG -0.236827204149
AGTGCGAA 0.0588867104019
AGTGCGAC 0.023333250606
AGTGCGAG -0.0684006919557
AGTGCGAT 0.306923935972
AGTGCGCA 0.0880867092988
AGTGCGCC -0.157812265083
AGTGCGCG -0.0327357795085
AGTGCGGA -0.082668166566
AGTGCGGC -0.259416127841
AGTGCGGG -0.132051942518
AGTGCGTA 0.0200922607534
AGTGCGTC -0.200303017145
AGTGCGTG -0.0466314509377
AGTGCTAA -0.14856375067
AGTGCTAC -0.0619730954797
AGTGCTAG -0.177981783926
AGTGCTAT 0.00172210778271
AGTGCTCA -0.00761478375999
AGTGCTCC -0.116296367889
AGTGCTCG -0.266820087835
AGTGCTGA -0.0776658200901
AGTGCTGC -0.137842879391
AGTGCTGG -0.159828213744
AGTGCTTA 0.0445845844604
AGTGCTTC -0.191176835417
AGTGCTTG -0.110880876161
AGTGGAAA -0.0207383692232
AGTGGAAC -0.0113225477302
AGTGGAAG -0.0303459117202
AGTGGAAT 0.1706155191
AGTGGACA 0.0905464693343
AGTGGACC -0.0788961406005

AGTGGACG -0.0801866343202
AGTGGAGA 0.138487356379
AGTGGAGC -0.211356462529
AGTGGAGG -0.340804041707
AGTGGATA 0.203303206018
AGTGGATC 0.205089669569
AGTGGATG -0.111198294928
AGTGGCAA 0.0377691741328
AGTGGCAC -0.101910330644
AGTGGCAG -0.128580770505
AGTGGCAT -0.0772315556989
AGTGGCCA -0.0979888568246
AGTGGCCC -0.339255568282
AGTGGCCG -0.149200362807
AGTGGCGA 0.0790048393101
AGTGGCGC -0.371342042634
AGTGGCGG -0.119174383561
AGTGGCTA -0.0566184682087
AGTGGCTC -0.223544514177
AGTGGCTG -0.154645425931
AGTGGGAA -0.159714427914
AGTGGGAC -0.0533168973686
AGTGGGAG -0.0858362767562
AGTGGGAT 0.167855183007
AGTGGGCA -0.0826584212823
AGTGGGCC -0.205632810072
AGTGGGCG -0.297418936982
AGTGGGGA -0.197754870578
AGTGGGGC -0.228868661547
AGTGGGGG -0.211211507004
AGTGGGTA -0.0159752275877
AGTGGGTC -0.279912568667
AGTGGGTG -0.230102346841
AGTGGTAA -0.222275819491
AGTGGTAC -0.184878848952
AGTGGTAG -0.00139395593941
AGTGGTAT 0.0673736723233
AGTGGTCA -0.0169797897071
AGTGGTCC -0.264537912047
AGTGGTCG -0.232430839846
AGTGGTGA 0.110720913751
AGTGGTGC -0.19832737132
AGTGGTGG -0.0817817411601
AGTGGTTA 0.0126318401787
AGTGGTTC -0.0630120478605
AGTGGTTG -0.158886286185
AGTGTAAA 0.0989007539981
AGTGTAAC 0.0644487462669
AGTGTAAG -0.00459985866684
AGTGTAAT 0.370648087998
AGTGTACA 0.00470850875669
AGTGTACC -0.287185858889
AGTGTACG -0.222403258359
AGTGTAGA 0.0564975330784

AGTGTAGC -0.00585987391309
AGTGTAGG -0.170010095588
AGTGTATA 0.106758273425
AGTGTATC 0.0883506200594
AGTGTATG 0.0363780355175
AGTGTCAA -0.12630799646
AGTGTCAC -0.180568996082
AGTGTCAG -0.0280015766078
AGTGTCAT -0.187276808489
AGTGTCCA -0.166434065252
AGTGTCCC -0.164505841932
AGTGTCCG -0.317257735994
AGTGTCGA -0.265029039558
AGTGTCGC -0.074560786682
AGTGTCGG -0.35362079182
AGTGTCTA -0.0646970918149
AGTGTCTC 0.0195482366955
AGTGTCTG -0.0201256730268
AGTGTGAA -0.0729582785401
AGTGTGAC -0.0625526448326
AGTGTGAG -0.0638181568825
AGTGTGAT 0.246788110424
AGTGTGCA 0.0771198195441
AGTGTGCC -0.246811190056
AGTGTGCG -0.207064519626
AGTGTGGA 0.203659582447
AGTGTGGC -0.196465674745
AGTGTGGG 0.0665525572433
AGTGTGTA 0.0671649611044
AGTGTGTC -0.0152039091433
AGTGTGTG -0.275353044788
AGTGTTAA -0.0750028262374
AGTGTTAC 0.0485182151849
AGTGTTAG -0.180432190429
AGTGTTAT -0.0590738888929
AGTGTTCA 0.0890776114392
AGTGTTCC -0.103093362919
AGTGTTCG -0.168696501675
AGTGTTGA 0.0976169006472
AGTGTTGC -0.0898653474411
AGTGTTGG -0.00065473018839
AGTGTTTA 0.101535732794
AGTGTTTC 0.140910883335
AGTGTTTG 0.0617351444859
AGTTAAAA 0.0709730252872
AGTTAAAC -0.106426290319
AGTTAAAG -0.00874754944537
AGTTAAAT 0.0605440150895
AGTTAACA -0.016038334927
AGTTAACC -0.063100290373
AGTTAACG -0.037529264802
AGTTAACT 0.0138802748766
AGTTAAGA 0.00703269271476
AGTTAAGC -0.0960576056117

AGTTAAGG -0.315114583805
AGTTAATA 0.0942802306439
AGTTAATC -0.194959320006
AGTTAATG -0.0117493777509
AGTTACAA 0.192306888439
AGTTACAC -0.175921128865
AGTTACAG -0.152928767983
AGTTACAT 0.124119179872
AGTTACCA -0.113208249572
AGTTACCC -0.12331477483
AGTTACCG -0.0433282482865
AGTTACGA 0.0423412393109
AGTTACGC 0.0518273094031
AGTTACGG -0.10418545267
AGTTACTA 0.0430932169369
AGTTACTC -0.122964844796
AGTTACTG 0.00927205973592
AGTTAGAA 0.013778241051
AGTTAGAC -0.140986867936
AGTTAGAG 0.125098928129
AGTTAGAT 0.307650927352
AGTTAGCA 0.234467249619
AGTTAGCC -0.103843216402
AGTTAGCG -0.0653365254775
AGTTAGGA 0.138217485173
AGTTAGGC -0.0545138326305
AGTTAGGG -0.339207514516
AGTTAGTA 0.173632220334
AGTTAGTC -0.112667764183
AGTTAGTG 0.149076297599
AGTTATAA 0.0151735757796
AGTTATAC 0.0763771596242
AGTTATAG -0.209585186331
AGTTATAT 0.121984920038
AGTTATCA 0.0995270176565
AGTTATCC -0.0239812266885
AGTTATCG -0.122072776303
AGTTATGA 0.105380053106
AGTTATGC 0.0973245973246
AGTTATGG -0.0605278716524
AGTTATTA 0.0713086574747
AGTTATTC 0.119434404183
AGTTATTG -0.0578797544579
AGTTCAAA 0.133637219623
AGTTCAAC -0.099777209773
AGTTCAAG 0.156959990293
AGTTCAAT 0.0275330426846
AGTTCACA -0.00655135344131
AGTTCACC -0.0830845112749
AGTTCACG -0.0480392643518
AGTTCAGA -0.00488213251899
AGTTCAGC -0.233067608282
AGTTCAGG -0.220061005422
AGTTCATA -0.117712001564

AGTTCATC -0.215689436987
AGTTCATG -0.16100327111
AGTTCCAA -0.0947215949976
AGTTCCAC -0.237833910931
AGTTCCAG 0.029253771678
AGTTCCAT -0.0733421895871
AGTTCCCA -0.181677469638
AGTTCCCC -0.160794289176
AGTTCCCG -0.218413491076
AGTTCCGA -0.106836883201
AGTTCCGC -0.244059654651
AGTTCCGG -0.327853715143
AGTTCCTA -0.0554569819093
AGTTCCTC -0.135450181826
AGTTCCTG -0.0737224444876
AGTTCGAA -0.0204047022229
AGTTCGAC -0.120567287887
AGTTCGAG 0.102686072568
AGTTCGAT 0.0646884383453
AGTTCGCA -0.0702970007956
AGTTCGCC -0.106030410634
AGTTCGCG -0.190746294796
AGTTCGGA 0.0838014322863
AGTTCGGC -0.208384677942
AGTTCGGG -0.221335188004
AGTTCGTA 0.12101219677
AGTTCGTC -0.111089051578
AGTTCGTG -0.148334697996
AGTTCTAA 0.0364313190222
AGTTCTAC -0.0797449690883
AGTTCTAG -0.127965136638
AGTTCTAT 0.00422983438319
AGTTCTCA -0.067738102131
AGTTCTCC -0.176574949972
AGTTCTCG -0.43124619374
AGTTCTGA 0.0218674043634
AGTTCTGC -0.0447155144125
AGTTCTGG -0.0446990307493
AGTTCTTA -0.0238045882207
AGTTCTTC -0.0153288614118
AGTTCTTG -0.0866827693266
AGTTGAAA 0.123491729249
AGTTGAAC -0.136302604406
AGTTGAAG -0.094037798756
AGTTGAAT -0.201293127986
AGTTGACA 0.0154383480654
AGTTGACC -0.147829299019
AGTTGACG 0.0499552608954
AGTTGAGA 0.0321795873083
AGTTGAGC -0.11501479236
AGTTGAGG -0.145715309653
AGTTGATA 0.112885612886
AGTTGATC -0.191137422287
AGTTGATG -0.316592375738

AGTTGCAA 0.032336744458
AGTTGCAC -0.204158474229
AGTTGCAG 0.0325077143259
AGTTGCAT -0.0759669496288
AGTTGCCA -0.110830758092
AGTTGCCC -0.224279069511
AGTTGCCG -0.191619819926
AGTTGCGA 0.0033215033215
AGTTGCGC -0.0704651845105
AGTTGCGG -0.0712692422794
AGTTGCTA -0.160987754161
AGTTGCTC -0.231607603137
AGTTGCTG -0.00866017532684
AGTTGGAA 0.102155874883
AGTTGGAC -0.319203787402
AGTTGGAG 0.0152105156382
AGTTGGAT 0.213120834333
AGTTGGCA -0.127752313494
AGTTGGCC -0.153721120679
AGTTGGCG -0.342240351602
AGTTGGGA 0.107833405108
AGTTGGGC -0.241228831987
AGTTGGGG -0.0332605332605
AGTTGGTA 0.183212776363
AGTTGGTC 0.00335686079867
AGTTGGTG -0.338499039136
AGTTGTAA 0.023937432027
AGTTGTAC -0.196688201217
AGTTGTAG -0.0164470851003
AGTTGTAT 0.0520068903601
AGTTGTCA -0.00518123285216
AGTTGTCC -0.224204770864
AGTTGTCG -0.193269026602
AGTTGTGA 0.0374051737688
AGTTGTGC -0.0626979741812
AGTTGTGG -0.120866865724
AGTTGTTA 0.168136456015
AGTTGTTC 0.188418445994
AGTTGTTG 0.0690208114451
AGTTTAAA 0.122303008769
AGTTTAAC 0.0572081527937
AGTTTAAG 0.0526738860072
AGTTTAAT 0.0712061924183
AGTTTACA -0.0293805407343
AGTTTACC -0.11916308402
AGTTTACG -0.216822002244
AGTTTAGA 0.173135945863
AGTTTAGC -0.138562042847
AGTTTAGG 0.0421712534432
AGTTTATA 0.23210784922
AGTTTATC -0.171767478856
AGTTTATG -0.0331902150084
AGTTTCAA 0.0362061691937
AGTTTCAC -0.0760428603753

AGTTTCAG 0.0319138996956
AGTTTCAT -0.0065782035479
AGTTTCCA 0.0658218042687
AGTTTCCC -0.113513572695
AGTTTCCG -0.0271939474649
AGTTTCGA -0.0835263756362
AGTTTCGC -0.000930682748865
AGTTTCGG -0.18824536489
AGTTTCTA 0.0415029354423
AGTTTCTC -0.146778565517
AGTTTCTG -0.0485806880141
AGTTTGAA -0.178343897914
AGTTTGAC -0.0503621319088
AGTTTGAG 0.0656538080781
AGTTTGAT 0.119092922123
AGTTTGCA -0.00544395364419
AGTTTGCC -0.149333209279
AGTTTGCG -0.219866339131
AGTTTGGA 0.126195046824
AGTTTGGC -0.153618938207
AGTTTGGG 0.0900063877397
AGTTTGTA 0.0735404826314
AGTTTGTC 0.0448297843713
AGTTTGTG -0.173711205531
AGTTTTAA -0.221931103319
AGTTTTAC -0.0539837358019
AGTTTTAG -0.112946279613
AGTTTTAT 0.142318286721
AGTTTTCA -0.00728138606926
AGTTTTCC 0.00525732343914
AGTTTTCG -0.191245440334
AGTTTTGA 0.122038015977
AGTTTTGC 0.117173422026
AGTTTTGG -0.135384626614
AGTTTTTA 0.176172039808
AGTTTTTC 0.229059947592
AGTTTTTG 0.0689904780814
ATAAAAAA 0.250227367284
ATAAAAAC 0.189291475369
ATAAAAAG 0.0885058612331
ATAAAAAT 0.271418144128
ATAAAACA 0.0738217556399
ATAAAACC -0.047030501576
ATAAAACG 0.0684795223462
ATAAAAGA 0.167179550813
ATAAAAGC -0.062582927908
ATAAAAGG -0.0884005702106
ATAAAATA 0.217919020949
ATAAAATC 0.405420572087
ATAAAATG -0.0676475696852
ATAAACAA 0.0791800179558
ATAAACAC -0.0616305010244
ATAAACAG -0.0716377534559
ATAAACAT 0.190128917785

ATAAACCA 0.148048582878
ATAAACCC -0.178602446711
ATAAACCG -0.144780719741
ATAAACGA 0.027561997259
ATAAACGC -0.0198264000904
ATAAACGG -0.0493748312081
ATAAACTA 0.050633277906
ATAAACTC 0.159622052072
ATAAACTG -0.0245658882023
ATAAAGAA 0.188217142763
ATAAAGAC 0.208209586997
ATAAAGAG 0.193930845446
ATAAAGAT 0.222826507449
ATAAAGCA 0.0892283468041
ATAAAGCC -0.06078158913
ATAAAGCG -0.184970596263
ATAAAGGA 0.158599478269
ATAAAGGC -0.110624170762
ATAAAGGG 0.0680695833437
ATAAAGTA 0.0517165254354
ATAAAGTC -0.130377778329
ATAAAGTG -0.0498170346655
ATAAATAA 0.142495419808
ATAAATAC 0.130648683525
ATAAATAG 0.0947212834755
ATAAATAT 0.248621563031
ATAAATCA 0.309808544338
ATAAATCC 0.257435121071
ATAAATCG 0.109816427998
ATAAATGA 0.149450012044
ATAAATGC -0.0404909041273
ATAAATGG -0.0687734186953
ATAAATTA 0.314690233812
ATAAATTC 0.301954701722
ATAAATTG 0.178620176201
ATAACAAA 0.113831813902
ATAACAAC 0.141902256199
ATAACAAG 0.0366481025963
ATAACAAT 0.0998890535244
ATAACACA 0.176844888966
ATAACACC -0.0114836901382
ATAACACG 0.197559818772
ATAACAGA 0.272797634054
ATAACAGC -0.059375281668
ATAACAGG -0.0862326170698
ATAACATA 0.0862484763107
ATAACATC 0.0395546082932
ATAACATG -0.205831569021
ATAACCAA -0.0785840937356
ATAACCAC -0.0108524502464
ATAACCAG -0.172417794632
ATAACCAT -0.0721616933738
ATAACCCA -0.00233352554324
ATAACCCC -0.151617395265

ATAACCCG -0.0704517419327
ATAACCGA 0.0958575655545
ATAACCGC 0.0476945760312
ATAACCGG -0.255519991619
ATAACCTA -0.0304708797532
ATAACCTC 0.0837683413441
ATAACCTG -0.0845983724772
ATAACGAA 0.180713561017
ATAACGAC 0.0733198763502
ATAACGAG 0.0983035376975
ATAACGAT 0.228563608096
ATAACGCA 0.227269969694
ATAACGCC -0.126716422049
ATAACGCG -0.0348544136423
ATAACGGA 0.0974730480354
ATAACGGC 0.000574382957977
ATAACGGG -0.0970433517215
ATAACGTA 0.167521516006
ATAACGTC 0.000539404246622
ATAACGTG 0.0115289446217
ATAACTAA -0.158920748735
ATAACTAC 0.0562790033998
ATAACTAG 0.0540610963595
ATAACTAT 0.0653890805406
ATAACTCA 0.0523514083633
ATAACTCC -0.147082178364
ATAACTCG 0.0848548272791
ATAACTGA 0.0742298772602
ATAACTGC 0.0406005707904
ATAACTGG -0.0798442369875
ATAACTTA 0.081512813588
ATAACTTC 0.184368955424
ATAACTTG 0.125388473873
ATAAGAAA 0.0774340707868
ATAAGAAC 0.151219316365
ATAAGAAG 0.163655390928
ATAAGAAT 0.2863404451
ATAAGACA 0.213182923002
ATAAGACC -0.331611993055
ATAAGACG -0.105884542674
ATAAGAGA 0.17754255633
ATAAGAGC -0.117301567606
ATAAGAGG -0.275181465356
ATAAGATA 0.314237644431
ATAAGATC 0.329615860405
ATAAGATG 0.0828252494919
ATAAGCAA 0.117678444998
ATAAGCAC -0.0612665416086
ATAAGCAG -0.0844302870897
ATAAGCAT 0.0945169083889
ATAAGCCA 0.133495616884
ATAAGCCC -0.200317984744
ATAAGCCG -0.138556819924
ATAAGCGA 0.137048043279

ATAAGCGC -0.0198627354418
ATAAGCGG 0.0309993188781
ATAAGCTA 0.0489521552473
ATAAGCTC 0.075476302749
ATAAGCTG -0.04126578369
ATAAGGAA 0.0282086494208
ATAAGGAC -0.0184164881135
ATAAGGAG -0.00182689576629
ATAAGGAT 0.167732823369
ATAAGGCA 0.0715422290023
ATAAGGCC -0.105025437399
ATAAGGCG -0.148869040235
ATAAGGGA -0.0968609544719
ATAAGGGC -0.210296780897
ATAAGGGG -0.220041001911
ATAAGGTA 0.0733290511887
ATAAGGTC -0.0607468449596
ATAAGGTG 0.00951147055174
ATAAGTAA 0.133035239096
ATAAGTAC 0.0795778842097
ATAAGTAG -0.0151500111671
ATAAGTAT 0.09575154478
ATAAGTCA 0.0110413640577
ATAAGTCC 0.00248972650234
ATAAGTCG -0.104233355666
ATAAGTGA -0.0236531297137
ATAAGTGC 0.0859909496273
ATAAGTGG -0.0397601458208
ATAAGTTA 0.103176178934
ATAAGTTC 0.154491957522
ATAAGTTG -0.0289927957782
ATAATAAA 0.170853922907
ATAATAAC 0.0836572921761
ATAATAAG 0.0547922308271
ATAATAAT 0.141820884245
ATAATACA 0.237335131275
ATAATACC 0.071006267976
ATAATACG 0.12799438557
ATAATAGA 0.257441807844
ATAATAGC -0.00765895637322
ATAATAGG -0.00709622974013
ATAATATA 0.195390611884
ATAATATC 0.326243220951
ATAATATG 0.0691510404882
ATAATCAA 0.210916242232
ATAATCAC 0.187992233747
ATAATCAG 0.241956015562
ATAATCAT 0.211114797741
ATAATCCA 0.378735484796
ATAATCCC 0.177828952651
ATAATCCG 0.361886236068
ATAATCGA 0.217574323635
ATAATCGC 0.147532694742
ATAATCGG 0.0831901861327

ATAATCTA 0.312164781862
ATAATCTC 0.391727918367
ATAATCTG 0.364010015525
ATAATGAA 0.156985613454
ATAATGAC 0.108612303645
ATAATGAG 0.167332832128
ATAATGAT 0.129141538232
ATAATGCA 0.150909863031
ATAATGCC 0.061451258421
ATAATGCG -0.0225418255721
ATAATGGA 0.14942628579
ATAATGGC 0.0204513769771
ATAATGGG 0.0780617942673
ATAATGTA 0.252971685237
ATAATGTC 0.192670632065
ATAATGTG 0.0482396997549
ATAATTAA -0.00600643781523
ATAATTAC 0.194348298942
ATAATTAG 0.0375721699525
ATAATTAT 0.105484028346
ATAATTCA 0.176613252371
ATAATTCC 0.0883514368363
ATAATTCG -0.208957740065
ATAATTGA -0.00944577855959
ATAATTGC 0.129102932133
ATAATTGG 0.189203008265
ATAATTTA 0.144219977553
ATAATTTC 0.248972112608
ATAATTTG 0.130051539142
ATACAAAA 0.259161365222
ATACAAAC 0.105459453944
ATACAAAG -0.0849652927308
ATACAAAT 0.268048874842
ATACAACA 0.124075385211
ATACAACC -0.117536847729
ATACAACG -0.0119809377249
ATACAAGA 0.123257884864
ATACAAGC -0.0439216756758
ATACAAGG -0.173547862293
ATACAATA 0.172763370145
ATACAATC 0.280773817844
ATACAATG -0.014601870032
ATACACAA 0.169107975738
ATACACAC -0.0667016879138
ATACACAG -0.0134661284789
ATACACAT 0.170068671468
ATACACCA 0.12056013372
ATACACCC -0.0263334960305
ATACACCG 0.0670849913274
ATACACGA 0.229829002556
ATACACGC -0.0591556389276
ATACACGG 0.0223124550215
ATACACTA 0.00906705213788
ATACACTC 0.0342379363956

ATACACTG -0.0204820332401
ATACAGAA 0.181224498734
ATACAGAC -0.0378347023352
ATACAGAG 0.159856125633
ATACAGAT 0.207183054386
ATACAGCA 0.0408769651194
ATACAGCC -0.0381078435428
ATACAGCG -0.0345752234106
ATACAGGA 0.159885781098
ATACAGGC 0.0890916197236
ATACAGGG 0.0187694581634
ATACAGTA 0.158033765684
ATACAGTC -0.113878715245
ATACAGTG -0.0465511298617
ATACATAA 0.186809777888
ATACATAC 0.191165886878
ATACATAG 0.1105176943
ATACATAT 0.222009007102
ATACATCA 0.130168682438
ATACATCC 0.102621840398
ATACATCG -0.10270450604
ATACATGA 0.126568717478
ATACATGC 0.15373896922
ATACATGG -0.0260301623938
ATACATTA 0.187151376247
ATACATTC 0.156165193463
ATACATTG 0.142863294964
ATACCAAA 0.155244186912
ATACCAAC -0.0736457514718
ATACCAAG -0.211792716855
ATACCAAT 0.0513171573778
ATACCACA 0.223025043247
ATACCACC 0.0287782019372
ATACCACG 0.16634575568
ATACCAGA 0.0316611377217
ATACCAGC -0.113928162157
ATACCAGG -0.0472276243628
ATACCATA 0.178262284323
ATACCATC 0.0248804770698
ATACCATG -0.141158840057
ATACCCAA 0.0378787325825
ATACCCAC 0.235891914776
ATACCCAG 0.161680964711
ATACCCAT 0.157770718377
ATACCCCA 0.0238673605687
ATACCCCC -0.0138479145287
ATACCCCG 0.0422903148106
ATACCCGA 0.0988826291857
ATACCCGC 0.0722925775348
ATACCCGG -0.210093312529
ATACCCTA 0.103746680235
ATACCCTC 0.0917019472809
ATACCCTG -0.0470088651749
ATACCGAA 0.285750676992

ATACCGAC 0.143587636784
ATACCGAG 0.104033785852
ATACCGAT 0.259264508595
ATACCGCA 0.28266169354
ATACCGCC 0.0135931329971
ATACCGCG 0.143164018306
ATACCGGA 0.154332017968
ATACCGGC 0.0461014467578
ATACCGGG -0.111254085474
ATACCGTA 0.170747882869
ATACCGTC -0.0306307161157
ATACCGTG -0.208892207222
ATACCTAA 0.1513758938
ATACCTAC -0.0952008734708
ATACCTAG 0.0590811880032
ATACCTAT 0.0468858346168
ATACCTCA 0.101416843841
ATACCTCC -0.161473879644
ATACCTCG 0.00614499838698
ATACCTGA 0.0581256187317
ATACCTGC 0.123298229359
ATACCTGG -0.0972911311476
ATACCTTA 0.139330525609
ATACCTTC 0.0697880735539
ATACCTTG 0.0909578622366
ATACGAAA 0.315274710276
ATACGAAC 0.179461830977
ATACGAAG 0.0970460818946
ATACGAAT 0.391124029084
ATACGACA 0.143298321935
ATACGACC 0.0340218827325
ATACGACG 0.0718166755473
ATACGAGA 0.240081604052
ATACGAGC 0.0828646088042
ATACGAGG -0.025067767492
ATACGATA 0.275677904863
ATACGATC 0.278898272301
ATACGATG 0.269117602451
ATACGCAA 0.236490441815
ATACGCAC 0.170317700621
ATACGCAG 0.162302285351
ATACGCAT 0.241891783392
ATACGCCA 0.164220694524
ATACGCCC -0.0562391710202
ATACGCCG 0.176118267027
ATACGCGA 0.176620219995
ATACGCGC 0.260831514638
ATACGCGG 0.108186699096
ATACGCTA 0.279167597349
ATACGCTC 0.221842587389
ATACGCTG 0.190947145493
ATACGGAA 0.166521171069
ATACGGAC 0.0637552152704
ATACGGAG 0.0983666196839

ATACGGAT 0.286807588155
ATACGGCA 0.187323687324
ATACGGCC -0.0233650353268
ATACGGCG -0.027623317097
ATACGGGA 0.0916381393108
ATACGGGC -0.0753825450795
ATACGGGG -0.153584392346
ATACGGTA 0.228890608235
ATACGGTC 0.0931385931386
ATACGGTG 0.155534550338
ATACGTAA 0.192179003379
ATACGTAC 0.149096735108
ATACGTAG 0.0123756414093
ATACGTAT 0.183279171111
ATACGTCA 0.143400509478
ATACGTCC 0.0220664403308
ATACGTCG -0.0386568177339
ATACGTGA 0.133346845468
ATACGTGC 0.321621336576
ATACGTGG 0.0675510220965
ATACGTTA 0.283262601444
ATACGTTC 0.128821659125
ATACGTTG 0.167033424609
ATACTAAA 0.102492299462
ATACTAAC 0.0960947173068
ATACTAAG -6.78817252177E-5
ATACTAAT 0.104353189346
ATACTACA 0.23288715713
ATACTACC 0.000375904177281
ATACTACG 0.0452829500084
ATACTAGA 0.214934709459
ATACTAGC 0.0588797325606
ATACTAGG -0.0948658058583
ATACTATA 0.120278680885
ATACTATC 0.087022283992
ATACTATG -0.0596461391356
ATACTCAA 0.244639168485
ATACTCAC 0.201244498296
ATACTCAG 0.132256909928
ATACTCAT 0.29381181434
ATACTCCA 0.23542477172
ATACTCCC 0.039364831427
ATACTCCG 0.196394969453
ATACTCGA 0.115909140676
ATACTCGC 0.165406453285
ATACTCGG 0.248729445699
ATACTCTA 0.247863566045
ATACTCTC 0.158236330694
ATACTCTG 0.0541820252111
ATACTGAA 0.216402352766
ATACTGAC 0.111077129698
ATACTGAG 0.152815349785
ATACTGAT 0.175434844492
ATACTGCA 0.147056147056

ATACTGCC -0.0106340737064
ATACTGCG 0.0388626526426
ATACTGGA 0.0927738808639
ATACTGGC -0.110784386709
ATACTGGG -0.0166593922479
ATACTGTA 0.339399137245
ATACTGTC 0.217924425013
ATACTGTG 0.193845360512
ATACTTAA 0.205422508996
ATACTTAC 0.0909187424339
ATACTTAG -0.151074395365
ATACTTCA 0.0808033400728
ATACTTCC 0.0291198002963
ATACTTCG -0.0603526930067
ATACTTGA -0.0212705818766
ATACTTGC 0.299344799345
ATACTTGG 0.0567337941505
ATACTTTA 0.118985376561
ATACTTTC 0.240680131092
ATACTTTG 0.00616555841843
ATAGAAAA 0.2196119793
ATAGAAAC -0.0235826952694
ATAGAAAG -0.0987518521492
ATAGAAAT 0.234293522172
ATAGAACA -0.00358897250425
ATAGAACC -0.0257571621208
ATAGAACG -0.00806315957831
ATAGAAGA 0.125216966052
ATAGAAGC -0.14069974881
ATAGAAGG -0.299375442716
ATAGAATA 0.135205453387
ATAGAATC 0.374138601411
ATAGAATG -0.0267364420398
ATAGACAA 0.155073246571
ATAGACAC 0.0134914528854
ATAGACAG 0.11876477028
ATAGACAT 0.142121705364
ATAGACCA -0.219302086298
ATAGACCC -0.247237288902
ATAGACCG -0.213321606096
ATAGACGA 0.0828307646489
ATAGACGC -0.0390913642674
ATAGACGG 0.0253155040401
ATAGACTA 0.00102995557541
ATAGACTC -0.176202332013
ATAGACTG -0.243899174281
ATAGAGAA 0.183501821128
ATAGAGAC 0.0530579622344
ATAGAGAG 0.151928183239
ATAGAGAT 0.25874396911
ATAGAGCA -0.0146894626148
ATAGAGCC -0.203426082007
ATAGAGCG -0.0895459541035
ATAGAGGA 0.153245549368

ATAGAGGC -0.078725969433
ATAGAGGG -0.0929638543588
ATAGAGTA 0.224073752389
ATAGAGTC -0.21658603151
ATAGAGTG -0.00263716852916
ATAGATAA 0.19589148377
ATAGATAC 0.268374435041
ATAGATAG 0.101047119051
ATAGATAT 0.404816662392
ATAGATCA -0.0127643760067
ATAGATCC 0.20918852737
ATAGATCG 0.250193719891
ATAGATGA 0.0940297493629
ATAGATGC 0.187157215535
ATAGATGG 0.0140815746876
ATAGATTA 0.335454698072
ATAGATTC 0.450498796436
ATAGATTG 0.308523900936
ATAGCAAA 0.145826611402
ATAGCAAC -0.00265468639373
ATAGCAAG -0.00735458331326
ATAGCAAT 0.331597848222
ATAGCACA 0.0174092183971
ATAGCACC -0.113648273275
ATAGCACG 0.0949006858098
ATAGCAGA -0.0141814412823
ATAGCAGC -0.0585863201026
ATAGCAGG -0.050596702262
ATAGCATA 0.00883484303263
ATAGCATC 0.00877875120299
ATAGCATG -0.164335943853
ATAGCCAA 0.036795748917
ATAGCCAC -0.0987447502599
ATAGCCAG -0.208605840172
ATAGCCAT 0.00263809275664
ATAGCCCA 0.0176718757983
ATAGCCCC -0.133564108291
ATAGCCCG -0.0688366817556
ATAGCCGA 0.0470948847137
ATAGCCGC -0.0819648849952
ATAGCCGG -0.196100746731
ATAGCCTA -0.121708159494
ATAGCCTC 0.0257874954845
ATAGCCTG -0.152472766074
ATAGCGAA 0.238293389277
ATAGCGAC 0.155299518989
ATAGCGAG 0.0207812967599
ATAGCGAT 0.239254405921
ATAGCGCA 0.0358451476038
ATAGCGCC -0.167550020049
ATAGCGCG 0.15191419166
ATAGCGGA 0.0633503107002
ATAGCGGC -0.010646558204
ATAGCGGG -0.257680634464

ATAGCGTA 0.27489835989
ATAGCGTC -0.0956493623084
ATAGCGTG -0.0378393173872
ATAGCTAA 0.108580833996
ATAGCTAC -0.0848516928709
ATAGCTAG -0.192828296244
ATAGCTAT -0.0630131719297
ATAGCTCA -0.0926311986918
ATAGCTCC -0.282238925686
ATAGCTCG -0.0129376728977
ATAGCTGA 0.0438330892876
ATAGCTGC 0.0275578608912
ATAGCTGG -0.172348343878
ATAGCTTA 0.0884065884066
ATAGCTTC -0.0619393498181
ATAGCTTG 0.13274380853
ATAGGAAA 0.186948656646
ATAGGAAC -0.023813347153
ATAGGAAG 0.059406513952
ATAGGAAT 0.239681612811
ATAGGACA -0.13726390509
ATAGGACC -0.257928823369
ATAGGACG -0.136639617507
ATAGGAGA 0.0641757459939
ATAGGAGC -0.0804223265184
ATAGGAGG -0.109951988836
ATAGGATA 0.152874754567
ATAGGATC 0.241372226221
ATAGGATG -0.114729057798
ATAGGCAA 0.034059919207
ATAGGCAC -0.111717800164
ATAGGCAG 0.0122745186662
ATAGGCAT -0.210513678686
ATAGGCCA -0.211270751135
ATAGGCCC -0.253777614343
ATAGGCCG -0.00676846492413
ATAGGCGA 0.0761656973778
ATAGGCGC 0.0628325327004
ATAGGCGG -0.172062176566
ATAGGCTA 0.0041570496116
ATAGGCTC -0.0424335445486
ATAGGCTG -0.0277067771794
ATAGGGAA 0.0858776815106
ATAGGGAC -0.0414730769848
ATAGGGAG -0.00192176034123
ATAGGGAT 0.188640394736
ATAGGGCA -0.0944540592081
ATAGGGCC -0.107555433104
ATAGGGCG -0.0165632024325
ATAGGGGA -0.18811179751
ATAGGGGC -0.0878791328934
ATAGGGGG -0.26373971993
ATAGGGTA 0.0810777866178
ATAGGGTC -0.320687089173

ATAGGGTG -0.0489310453056
ATAGGTAA 0.208739042072
ATAGGTAC -0.182718683748
ATAGGTAG -0.139490397514
ATAGGTCA -0.267950239519
ATAGGTCC -0.191682513182
ATAGGTCG -0.237612584671
ATAGGTGA 0.0557306617913
ATAGGTGC 0.181583614957
ATAGGTGG -0.172374614155
ATAGGTTA -0.0577543159004
ATAGGTTC -0.14364797768
ATAGGTTG -0.0658153471092
ATAGTAAA 0.127108895426
ATAGTAAC 0.00731723458996
ATAGTAAG 0.115781070327
ATAGTAAT 0.354963038631
ATAGTACA 0.0690196565039
ATAGTACC -0.264352137819
ATAGTACG 0.11842972449
ATAGTAGA 0.278767748465
ATAGTAGC -0.0471302052846
ATAGTAGG -0.021130605256
ATAGTATA 0.0211212993523
ATAGTATC 0.118599315569
ATAGTATG -0.120141164792
ATAGTCAA -0.037422140606
ATAGTCAC -0.196786941424
ATAGTCAG -0.109838903428
ATAGTCAT -0.16109869513
ATAGTCCA 0.00494295948841
ATAGTCCC -0.0746172361597
ATAGTCCG -0.0836894896162
ATAGTCGA 0.0740340891856
ATAGTCGC -0.269497058612
ATAGTCGG -0.0586354650057
ATAGTCTA -0.0456814814274
ATAGTCTC 0.0498434340854
ATAGTCTG -0.00566544505938
ATAGTGAA 0.113922462407
ATAGTGAC -0.120492822426
ATAGTGAG 0.0449085449085
ATAGTGAT -0.0335299227024
ATAGTGCA 0.0286691650328
ATAGTGCC 0.0877337392489
ATAGTGCG -0.0573542185208
ATAGTGGA 0.0850147668329
ATAGTGGC 0.0238095238095
ATAGTGGG -0.228122596571
ATAGTGTA 0.137257578301
ATAGTGTC -0.134374399925
ATAGTGTG -0.0159347327142
ATAGTTAA 0.0818952869645
ATAGTTAC -0.112687067233

ATAGTTAG 0.206975759655
ATAGTTCA 0.129593781109
ATAGTTCC -0.111779886525
ATAGTTCG -0.0342627533704
ATAGTTGA 0.195597687173
ATAGTTGC 0.178950095983
ATAGTTGG -0.113037279704
ATAGTTTA 0.245208868639
ATAGTTTC 0.16040144828
ATAGTTTG 0.0325435628466
ATATAAAA 0.223706780144
ATATAAAC 0.26174536324
ATATAAAG 0.110390004329
ATATAAAT 0.259029001453
ATATAACA 0.261931945099
ATATAACC 0.0702948127191
ATATAACG 0.147741405317
ATATAAGA 0.262565783231
ATATAAGC 0.103306994248
ATATAAGG -0.0109493952687
ATATAATA 0.156245777458
ATATAATC 0.30099121917
ATATAATG 0.207773889592
ATATACAA 0.238140040729
ATATACAC 0.114380220441
ATATACAG 0.202240096932
ATATACAT 0.339761644787
ATATACCA 0.109025002964
ATATACCC 0.0811155508125
ATATACCG 0.0253896325286
ATATACGA 0.243823127961
ATATACGC 0.0921782734685
ATATACGG 0.0662301163858
ATATACTA 0.120784946315
ATATACTC 0.268010919469
ATATACTG 0.294325671701
ATATAGAA 0.22298416823
ATATAGAC 0.0853171098443
ATATAGAG 0.233172747211
ATATAGAT 0.318317740775
ATATAGCA 0.178874466753
ATATAGCC 0.0605088939658
ATATAGCG 0.076375273345
ATATAGGA 0.243626051984
ATATAGGC 0.0554754275454
ATATAGGG -0.0177050328565
ATATAGTA 0.146866127019
ATATAGTC -0.0305197696401
ATATAGTG 0.0897081654657
ATATATAA 0.135351605049
ATATATAC 0.228023473939
ATATATAG 0.0841521718503
ATATATAT 0.211925887337
ATATATCA 0.249377750852

ATATATCC	0.310269848106
ATATATCG	0.235699887215
ATATATGA	0.339624748716
ATATATGC	0.248122303891
ATATATGG	0.164016552101
ATATATTA	0.250831733612
ATATATTC	0.317107654577
ATATATTG	0.346216506208
ATATCAAA	0.233160157403
ATATCAAC	0.22597092055
ATATCAAG	0.297884717853
ATATCAAT	0.285820748451
ATATCACA	0.391295811041
ATATCACC	0.261891367952
ATATCACG	0.338527232467
ATATCAGA	0.302138226381
ATATCAGC	0.299156885128
ATATCAGG	0.310027517646
ATATCATA	0.294474573549
ATATCATC	0.322500186137
ATATCATG	0.37804057501
ATATCCAA	0.402020477778
ATATCCAC	0.392826711009
ATATCCAG	0.391812588046
ATATCCAT	0.426182638336
ATATCCCA	0.370113346457
ATATCCCC	0.367423897727
ATATCCCG	0.365398567915
ATATCCGA	0.401220780009
ATATCCGC	0.413563701442
ATATCCGG	0.426200156201
ATATCCTA	0.38472953147
ATATCCTC	0.406419567455
ATATCCTG	0.403228297168
ATATCGAA	0.176491918916
ATATCGAC	0.326558542514
ATATCGAG	0.346816513483
ATATCGAT	0.324546907732
ATATCGCA	0.343645298191
ATATCGCC	0.321055214995
ATATCGCG	0.420965042177
ATATCGGA	0.379146363995
ATATCGGC	0.365310978592
ATATCGGG	0.331316164649
ATATCGTA	0.329726356357
ATATCGTC	0.337045904104
ATATCGTG	0.35904657915
ATATCTAA	0.380624426079
ATATCTAC	0.407879389502
ATATCTAG	0.377760346114
ATATCTCA	0.390673042188
ATATCTCC	0.359331547484
ATATCTCG	0.442918124736
ATATCTGA	0.393330802975

ATATCTGC 0.447288745502
ATATCTGG 0.407204725387
ATATCTTA 0.415676610553
ATATCTTC 0.428551140672
ATATCTTG 0.44787073575
ATATGAAA 0.240478675649
ATATGAAC 0.151819863941
ATATGAAG 0.242265497836
ATATGAAT 0.298392735925
ATATGACA 0.129727816179
ATATGACC -0.0941395941396
ATATGACG 0.102525492143
ATATGAGA 0.297344583695
ATATGAGC 0.0399393884242
ATATGAGG -0.0839448263691
ATATGATA 0.218867470055
ATATGATC 0.104395028637
ATATGATG 0.197929144422
ATATGCAA 0.281276738047
ATATGCAC 0.315697466479
ATATGCAG 0.153782763881
ATATGCAT 0.276899372852
ATATGCCA 0.239268193814
ATATGCCC 0.121684196696
ATATGCCG 0.210000510938
ATATGCGA 0.271133425383
ATATGCGC 0.166795617614
ATATGCGG 0.131219754312
ATATGCTA 0.187028626423
ATATGCTC 0.220205053538
ATATGCTG 0.310002730274
ATATGGAA 0.23027148423
ATATGGAC 0.143875280239
ATATGGAG 0.150219338263
ATATGGAT 0.268670759034
ATATGGCA 0.201299971533
ATATGGCC -0.0868117371229
ATATGGCG 0.116598176682
ATATGGGA 0.192105328469
ATATGGGC 0.102426117578
ATATGGGG 0.0953577658883
ATATGGTA 0.253074020277
ATATGGTC 0.0390063603077
ATATGGTG 0.226046874886
ATATGTAA 0.131915662219
ATATGTAC 0.152550674073
ATATGTAG 0.138768244829
ATATGTCA 0.1177996178
ATATGTCC -0.107794478935
ATATGTCG -0.0108209309285
ATATGTGA 0.214271950249
ATATGTGC 0.279911388802
ATATGTGG 0.197883987942
ATATGTTA 0.12564323409

ATATGTTC 0.0983292800953
ATATGTTG 0.174379393152
ATATTAAA 0.213697168243
ATATTAAC 0.151353619994
ATATTAAG 0.315538377547
ATATTAAT 0.307352807353
ATATTACA 0.241223316981
ATATTACC 0.103605727474
ATATTACG 0.306392560747
ATATTAGA 0.222043188436
ATATTAGC 0.184278446457
ATATTAGG 0.0493631526317
ATATTATA 0.276670583856
ATATTATC 0.219670372182
ATATTATG 0.110420337693
ATATTCAA 0.135599220455
ATATTCAC 0.114540159995
ATATTCAG 0.27248046945
ATATTCAT -0.000686846273439
ATATTCCA 0.31800836478
ATATTCCC 0.409301256177
ATATTCCG 0.364975219521
ATATTCGA 0.27617424436
ATATTCGC 0.304710206929
ATATTCGG 0.238528353394
ATATTCTA 0.24538367773
ATATTCTC 0.377352784806
ATATTCTG 0.313750389508
ATATTGAA 0.207083786486
ATATTGAC 0.0833657348809
ATATTGAG 0.209729012759
ATATTGAT 0.191369055005
ATATTGCA 0.303406712498
ATATTGCC 0.190494902616
ATATTGCG 0.348407636286
ATATTGGA 0.207577206339
ATATTGGC 0.0933054266388
ATATTGGG 0.0275023174675
ATATTGTA 0.234993947115
ATATTGTC 0.249181080477
ATATTGTG 0.168983032619
ATATTTAA 0.196932183967
ATATTTAC 0.252570752571
ATATTTAG 0.317972242215
ATATTTCA 0.297000857607
ATATTTCC 0.375291269231
ATATTTCG 0.350602668784
ATATTTGA 0.290418517691
ATATTTGC 0.385582067546
ATATTTGG 0.170325973356
ATATTTTA 0.328244038018
ATATTTTC 0.374072419527
ATATTTTG 0.328111793172
ATCAAAAA 0.232650005377

ATCAAAAC 0.208369526551
ATCAAAAG 0.00639912994606
ATCAAAAT 0.275064779603
ATCAAACA -0.00997278270006
ATCAAACC 0.155957898732
ATCAAACG 0.0847922922302
ATCAAAGA 0.152801763465
ATCAAAGC 0.152340744969
ATCAAAGG -0.0878054362903
ATCAAATA 0.232420457946
ATCAAATC 0.353492259294
ATCAAATG -0.0439807887419
ATCAACAA 0.239091844704
ATCAACAC 0.128946780592
ATCAACAG 0.228916885032
ATCAACAT -0.00611446464676
ATCAACCA 0.0037159770224
ATCAACCC 0.0609672780888
ATCAACCG -0.176157487487
ATCAACGA 0.265832444103
ATCAACGC 0.0140882126635
ATCAACGG 0.101721162752
ATCAACTA 0.109369137902
ATCAACTC 0.0775141532717
ATCAACTG -0.0745507194764
ATCAAGAA 0.118417624141
ATCAAGAC 0.227327716703
ATCAAGAG 0.235233856446
ATCAAGAT 0.285455547732
ATCAAGCA 0.044900272173
ATCAAGCC -0.145437444087
ATCAAGCG 0.070829835843
ATCAAGGA 0.304702086816
ATCAAGGC -0.0325520510212
ATCAAGGG -0.112842293033
ATCAAGTA 0.106463212524
ATCAAGTC -0.0285414802672
ATCAAGTG 0.0613437128589
ATCAATAA 0.214794684492
ATCAATAC 0.159598694919
ATCAATAG 0.0656328693532
ATCAATCA 0.168704517189
ATCAATCC 0.115787252998
ATCAATCG 0.0420765117735
ATCAATGA 0.130081023401
ATCAATGC -0.0589970135425
ATCAATGG -0.026325854063
ATCAATTA 0.205597687642
ATCAATTC 0.119903466759
ATCAATTG 0.142215217973
ATCACAAA 0.169645434726
ATCACAAC 0.209685189375
ATCACAAG 0.0699743801231
ATCACAAT 0.215409624501

ATCACACA 0.287994606176
ATCACACC 0.280080728159
ATCACACG 0.0962896245603
ATCACAGA 0.316593797216
ATCACAGC 0.160424954198
ATCACAGG -0.0112052116313
ATCACATA 0.2341985212
ATCACATC 0.196135121129
ATCACATG 0.147010647011
ATCACCAA 0.163236571462
ATCACCAC 0.155677612899
ATCACCAG 0.0248050236249
ATCACCAT 0.0370986199005
ATCACCCA 0.0805419744814
ATCACCCC -0.0554011311587
ATCACCCG -0.0458020739417
ATCACCGA 0.0880783092476
ATCACCGC 0.179544558332
ATCACCGG 0.0988274776154
ATCACCTA 0.0410684437567
ATCACCTC 0.0258674652614
ATCACCTG -0.114296860569
ATCACGAA 0.190180538665
ATCACGAC 0.205306846664
ATCACGAG 0.19919782041
ATCACGAT 0.312342795413
ATCACGCA 0.0913315766808
ATCACGCC 0.0993045332051
ATCACGCG 0.193575204109
ATCACGGA 0.275608762046
ATCACGGC 0.0908673532696
ATCACGGG 0.021009990707
ATCACGTA 0.249161697189
ATCACGTC 0.0571521935158
ATCACGTG 0.185255503437
ATCACTAA 0.0658547623045
ATCACTAC 0.0922065316005
ATCACTAG 0.161414713546
ATCACTCA 0.162316883572
ATCACTCC -0.0695146754728
ATCACTCG 0.0978861776749
ATCACTGA 0.214596030744
ATCACTGC 0.033089056444
ATCACTGG 0.0530544318423
ATCACTTA 0.146682495167
ATCACTTC 0.191865419138
ATCACTTG 0.127415458056
ATCAGAAA 0.195594986971
ATCAGAAC 0.219507386174
ATCAGAAG 0.271409385196
ATCAGAAT 0.220191265646
ATCAGACA 0.0922512645708
ATCAGACC 0.0528834619744
ATCAGACG -0.10499885421

ATCAGAGA 0.248743679207
ATCAGAGC -0.0757831434929
ATCAGAGG -0.0723708505263
ATCAGATA 0.290474299471
ATCAGATC 0.364289103158
ATCAGATG -0.0071793556642
ATCAGCAA 0.096844778663
ATCAGCAC 0.0901972949497
ATCAGCAG 0.0998219017102
ATCAGCAT 0.184482821544
ATCAGCCA 0.236160402827
ATCAGCCC 0.09595260271
ATCAGCCG -0.230364305504
ATCAGCGA 0.263978854888
ATCAGCGC 0.0502171485296
ATCAGCGG 0.0047904060495
ATCAGCTA 0.300701527974
ATCAGCTC 0.0434663313451
ATCAGCTG 0.0637730560645
ATCAGGAA 0.242562332714
ATCAGGAC 0.262657974779
ATCAGGAG 0.0750075144015
ATCAGGAT 0.311012114042
ATCAGGCA 0.182316445625
ATCAGGCC 0.00793951981924
ATCAGGCG 0.126406020345
ATCAGGGA 0.176734086115
ATCAGGGC -0.0457928334092
ATCAGGGG 0.131475750755
ATCAGGTA 0.253900279556
ATCAGGTC 0.128082628083
ATCAGGTG 0.0135442288927
ATCAGTAA 0.149607672825
ATCAGTAC 0.217844566329
ATCAGTAG 0.0612446442779
ATCAGTCA 0.0872649509013
ATCAGTCC -0.11691494964
ATCAGTCG -0.0923471681047
ATCAGTGA 0.283656935172
ATCAGTGC 0.14329540229
ATCAGTGG 0.174092825608
ATCAGTTA 0.0752311685326
ATCAGTTC 0.217339929461
ATCAGTTG 0.0100296961726
ATCATAAA 0.23860167283
ATCATAAC 0.188217046342
ATCATAAG 0.252427358488
ATCATAAT 0.102073147528
ATCATACA 0.167108019532
ATCATACC 0.161496463581
ATCATACG 0.000361305956804
ATCATAGA 0.214777048678
ATCATAGC 0.204845341209
ATCATAGG 0.0871623249126

ATCATATA 0.162960481142
ATCATATC 0.350055838193
ATCATATG -0.0455559367313
ATCATCAA 0.136050365172
ATCATCAC 0.119719174584
ATCATCAG 0.0697466478836
ATCATCAT -0.0785484827177
ATCATCCA 0.171402815997
ATCATCCC 0.0806077239185
ATCATCCG 0.0933473371978
ATCATCGA 0.232892672287
ATCATCGC 0.0739155133095
ATCATCGG 0.106732699284
ATCATCTA 0.164762093872
ATCATCTC 0.222223601011
ATCATCTG 0.184398151865
ATCATGAA 0.226119865988
ATCATGAC 0.177650101893
ATCATGAG 0.233509485194
ATCATGAT 0.208035426332
ATCATGCA 0.0659013077897
ATCATGCC 0.0618138799957
ATCATGCG -0.0184256892573
ATCATGGA 0.221105274914
ATCATGGC -0.0768866375189
ATCATGGG -0.11428153956
ATCATGTA 0.141376914104
ATCATGTC 0.266078115078
ATCATGTG 0.0751976234097
ATCATTAA 0.044167645964
ATCATTAC 0.117733435915
ATCATTAG 0.0498858475817
ATCATTCA 0.0980376951138
ATCATTCC -0.258864104409
ATCATTCG 0.308249454392
ATCATTGA 0.206228330766
ATCATTGC 0.220715205564
ATCATTGG 0.00225615497471
ATCATTTA 0.0647659875425
ATCATTTC 0.0850838772117
ATCATTTG 0.0549075246045
ATCCAAAA 0.282691782692
ATCCAAAC 0.334845865149
ATCCAAAG 0.197237182086
ATCCAAAT 0.259672415932
ATCCAACA 0.293631096661
ATCCAACC 0.314771223559
ATCCAACG 0.0645116261756
ATCCAAGA 0.205302803588
ATCCAAGC 0.221827989168
ATCCAAGG -0.107193580924
ATCCAATA 0.309169142282
ATCCAATC 0.300916619098
ATCCAATG 0.188228724918

ATCCACAA 0.286833864952
ATCCACAC 0.268068343826
ATCCACAG 0.208037187357
ATCCACAT 0.192205280176
ATCCACCA 0.202619650665
ATCCACCC 0.287470347365
ATCCACCG -0.0270862681839
ATCCACGA 0.331198967563
ATCCACGC 0.273674500947
ATCCACGG 0.243266682661
ATCCACTA 0.315046385846
ATCCACTC 0.157280129166
ATCCACTG 0.226106271561
ATCCAGAA 0.338127621553
ATCCAGAC 0.257195351189
ATCCAGAG 0.193008182303
ATCCAGAT 0.389445919749
ATCCAGCA 0.277064735809
ATCCAGCC 0.183063228518
ATCCAGCG 0.0851477704868
ATCCAGGA 0.298255512653
ATCCAGGC 0.114563184748
ATCCAGGG 0.142241410772
ATCCAGTA 0.386037532025
ATCCAGTC 0.0926008653281
ATCCAGTG 0.134212844629
ATCCATAA 0.246106988531
ATCCATAC 0.141000562031
ATCCATAG 0.190489387459
ATCCATCA 0.303963416859
ATCCATCC 0.198301497048
ATCCATCG 0.135006532704
ATCCATGA 0.297615797616
ATCCATGC 0.160311088078
ATCCATGG 0.218539476115
ATCCATTA 0.379912118713
ATCCATTC 0.297951869667
ATCCATTG 0.294552127885
ATCCCAAA 0.268344101677
ATCCCAAC 0.16224075315
ATCCCAAG 0.260180434005
ATCCCAAT 0.145447099993
ATCCCACA 0.286949483919
ATCCCACC 0.212006772613
ATCCCACG -0.0836525072038
ATCCCAGA 0.271953773142
ATCCCAGC 0.0598548936885
ATCCCAGG -0.0057699466435
ATCCCATA 0.303916864523
ATCCCATC 0.153482683786
ATCCCATG 0.14600964601
ATCCCCAA 0.310801672581
ATCCCCAC 0.221825069524
ATCCCCAG 0.119054316024

ATCCCCAT 0.271322286474
ATCCCCCA 0.12028695362
ATCCCCCC 0.053425108449
ATCCCCCG -0.0234396327088
ATCCCCGA 0.270616701824
ATCCCCGC 0.173571643269
ATCCCCGG 0.266054757925
ATCCCCTA 0.278552657341
ATCCCCTC 0.176918023693
ATCCCCTG 0.0136734530674
ATCCCGAA 0.102486646489
ATCCCGAC 0.18289901493
ATCCCGAG 0.191438873601
ATCCCGAT 0.250099604326
ATCCCGCA 0.295258067985
ATCCCGCC 0.115818297636
ATCCCGCG 0.0182645635497
ATCCCGGA 0.321571790399
ATCCCGGC -0.030488763162
ATCCCGGG 0.0880031824121
ATCCCGTA 0.343752843753
ATCCCGTC 0.20222659255
ATCCCGTG 0.00898832717015
ATCCCTAA 0.0882396735838
ATCCCTAC 0.105871404276
ATCCCTAG 0.0160981876309
ATCCCTCA 0.305887462318
ATCCCTCC 0.139015612202
ATCCCTCG 0.157740385013
ATCCCTGA 0.0343868382444
ATCCCTGC 0.187715263473
ATCCCTGG 0.0904877995372
ATCCCTTA 0.26865324117
ATCCCTTC 0.0517569911509
ATCCCTTG 0.146690767903
ATCCGAAA 0.293744157381
ATCCGAAC 0.313601480268
ATCCGAAG 0.181116471902
ATCCGAAT 0.359551011066
ATCCGACA 0.121674015613
ATCCGACC 0.18731265701
ATCCGACG 0.156025050546
ATCCGAGA 0.321933826267
ATCCGAGC 0.163589209044
ATCCGAGG -0.0261935187712
ATCCGATA 0.394632924936
ATCCGATC 0.250716171432
ATCCGATG 0.161563615395
ATCCGCAA 0.303142663439
ATCCGCAC 0.307126685915
ATCCGCAG 0.226718393028
ATCCGCAT 0.242796873061
ATCCGCCA 0.282970298122
ATCCGCCC 0.20436000739

ATCCGCCG 0.170823266644
ATCCGCGA 0.341419530959
ATCCGCGC 0.240244376608
ATCCGCGG 0.226858535944
ATCCGCTA 0.375340905644
ATCCGCTC 0.289265849872
ATCCGCTG 0.276626789194
ATCCGGAA 0.312385388143
ATCCGGAC 0.307379400451
ATCCGGAG 0.266834302898
ATCCGGAT 0.437811294959
ATCCGGCA 0.288819122152
ATCCGGCC 0.262227792531
ATCCGGCG 0.201584133325
ATCCGGGA 0.263286935323
ATCCGGGC 0.116415313385
ATCCGGGG 0.287884303036
ATCCGGTA 0.349190788585
ATCCGGTC 0.0947931402477
ATCCGGTG 0.225596818622
ATCCGTAA 0.27008413372
ATCCGTAC 0.26888922562
ATCCGTAG 0.117342685927
ATCCGTCA 0.236324022102
ATCCGTCC 0.00586123313396
ATCCGTCG 0.150792862992
ATCCGTGA 0.318546005113
ATCCGTGC 0.111360671967
ATCCGTGG 0.0897432883079
ATCCGTTA 0.338207353359
ATCCGTTC 0.176205604229
ATCCGTTG 0.221484569969
ATCCTAAA 0.265482507682
ATCCTAAC 0.303083144165
ATCCTAAG 0.134146543237
ATCCTAAT 0.243521146815
ATCCTACA 0.310992810993
ATCCTACC 0.28687309229
ATCCTACG 0.0341415881404
ATCCTAGA 0.321747367202
ATCCTAGC 0.224644754948
ATCCTAGG 0.0116136143004
ATCCTATA 0.384361656314
ATCCTATC 0.184226926651
ATCCTATG -0.00269277542005
ATCCTCAA 0.337717422246
ATCCTCAC 0.21446464676
ATCCTCAG 0.184654351321
ATCCTCAT 0.155122880521
ATCCTCCA 0.347617935374
ATCCTCCC 0.35122312395
ATCCTCCG 0.277441369897
ATCCTCGA 0.283987281997
ATCCTCGC 0.142727746482

ATCCTCGG 0.154426179733
ATCCTCTA 0.354728006243
ATCCTCTC 0.132544292901
ATCCTCTG 0.305504988942
ATCCTGAA 0.178003054097
ATCCTGAC 0.362633983846
ATCCTGAG 0.23637949534
ATCCTGCA 0.350528214165
ATCCTGCC 0.256895834398
ATCCTGCG 0.174424647635
ATCCTGGA 0.3200790322
ATCCTGGC 0.159485932213
ATCCTGGG 0.117858905738
ATCCTGTA 0.292597242396
ATCCTGTC 0.350125909652
ATCCTGTG 0.175819069758
ATCCTTAA 0.275464169404
ATCCTTAC 0.240478770782
ATCCTTAG 0.0251166339926
ATCCTTCA 0.145747903331
ATCCTTCC 0.16484091393
ATCCTTCG 0.113821595148
ATCCTTGA 0.205945125289
ATCCTTGC 0.0802717317869
ATCCTTGG 0.128942992579
ATCCTTTA 0.266215251064
ATCCTTTC 0.183330713634
ATCCTTTG 0.251484266636
ATCGAAAA 0.175563308833
ATCGAAAC 0.228218576703
ATCGAAAG 0.0403867068604
ATCGAAAT 0.3583269226
ATCGAACA 0.0750350901866
ATCGAACC -0.0225754180565
ATCGAACG 0.0588315588316
ATCGAAGA 0.270460500865
ATCGAAGC 0.0429246667027
ATCGAAGG -0.141964803092
ATCGAATA 0.225226454895
ATCGAATC 0.349606213003
ATCGAATG -0.0369088096361
ATCGACAA 0.137345334315
ATCGACAC -0.0242205063427
ATCGACAG 0.177589541835
ATCGACAT 0.0145082681388
ATCGACCA 0.0156316743063
ATCGACCC -0.16858979131
ATCGACCG -0.0758892017964
ATCGACGA 0.0670499510554
ATCGACGC 0.117341241283
ATCGACGG -0.0876572045868
ATCGACTA 0.148626588021
ATCGACTC 0.00692466588323
ATCGACTG 0.0439194762158

ATCGAGAA 0.249161697189
ATCGAGAC 0.00142405640752
ATCGAGAG 0.152433666758
ATCGAGAT 0.320027298672
ATCGAGCA 0.0167674561614
ATCGAGCC 0.0280424516251
ATCGAGCG -0.107682102194
ATCGAGGA 0.217596384263
ATCGAGGC 0.0153186426575
ATCGAGGG -0.00352370713585
ATCGAGTA 0.0769102435769
ATCGAGTC -0.126203142217
ATCGAGTG -0.135695236927
ATCGATAA 0.177831872295
ATCGATAC 0.198704407933
ATCGATAG 0.0984438296972
ATCGATCA 0.181411355956
ATCGATCC 0.078756669562
ATCGATCG 0.0661588261189
ATCGATGA 0.189379064692
ATCGATGC -0.0204350355866
ATCGATGG 0.0604137792298
ATCGATTA 0.274587259436
ATCGATTC 0.35885973839
ATCGATTG 0.275826806712
ATCGCAAA 0.181954681955
ATCGCAAC 0.259866572265
ATCGCAAG 0.112058339331
ATCGCAAT 0.37720502872
ATCGCACA 0.221344020647
ATCGCACC 0.336149573367
ATCGCACG 0.184923687802
ATCGCAGA 0.222020685678
ATCGCAGC 0.0298101051691
ATCGCAGG 0.132693606872
ATCGCATA 0.310672931885
ATCGCATC 0.136139166687
ATCGCATG 0.239725078998
ATCGCCAA 0.199930374943
ATCGCCAC 0.263850426633
ATCGCCAG 0.168869500698
ATCGCCAT 0.0587454289322
ATCGCCCA 0.16159391518
ATCGCCCC 0.15887926494
ATCGCCCG 0.177962999727
ATCGCCGA 0.165946938674
ATCGCCGC 0.10138099532
ATCGCCGG 0.0408509302716
ATCGCCTA 0.30201017496
ATCGCCTC 0.185382071925
ATCGCCTG 0.162007401298
ATCGCGAA 0.305207411268
ATCGCGAC 0.37545598928
ATCGCGAG 0.335224046189

ATCGCGAT	0.424596284077
ATCGCGCA	0.331823559096
ATCGCGCC	0.265809507821
ATCGCGCG	0.391474551455
ATCGCGGA	0.343264746027
ATCGCGGC	0.145019326505
ATCGCGGG	0.21832714257
ATCGCGTA	0.387389327241
ATCGCGTC	0.312816400725
ATCGCGTG	0.316301149634
ATCGCTAA	0.15568776832
ATCGCTAC	0.108263911294
ATCGCTAG	0.122259366583
ATCGCTCA	0.118387918513
ATCGCTCC	0.136617333587
ATCGCTCG	0.206653100727
ATCGCTGA	0.108680546939
ATCGCTGC	0.089562272359
ATCGCTGG	0.0737728370912
ATCGCTTA	0.249301840106
ATCGCTTC	0.18898374959
ATCGCTTG	0.196175514357
ATCGGAAA	0.301677010277
ATCGGAAC	0.103617439034
ATCGGAAG	0.0789844018014
ATCGGAAT	0.320117807639
ATCGGACA	−0.0558960995988
ATCGGACC	−0.0171215228864
ATCGGACG	−0.0811703365668
ATCGGAGA	0.232837967052
ATCGGAGC	−0.0594244382123
ATCGGAGG	−0.141947287836
ATCGGATA	0.317542681547
ATCGGATC	0.244794381158
ATCGGATG	0.173364816829
ATCGGCAA	0.212869894688
ATCGGCAC	−0.0149960219849
ATCGGCAG	0.0724838640992
ATCGGCAT	0.100924616076
ATCGGCCA	0.0427355730386
ATCGGCCC	0.125183390145
ATCGGCCG	−0.0199260527557
ATCGGCGA	0.261334337092
ATCGGCGC	0.00591638470426
ATCGGCGG	0.00916120747413
ATCGGCTA	0.059034340959
ATCGGCTC	0.0387546258111
ATCGGCTG	−0.00295198780047
ATCGGGAA	0.255885637541
ATCGGGAC	0.0153244819457
ATCGGGAG	0.118183543426
ATCGGGCA	0.0111406622087
ATCGGGCC	−0.283742093514
ATCGGGCG	0.0497756709878

ATCGGGGA 0.230306635621
ATCGGGGC 0.109129790948
ATCGGGGG 0.0238799672367
ATCGGGTA 0.222535207384
ATCGGGTC -0.0482040539258
ATCGGGTG -0.12810131538
ATCGGTAA 0.168649591615
ATCGGTAC 0.0657846908462
ATCGGTAG 0.0511893900134
ATCGGTCA -0.0545119674875
ATCGGTCC -0.157935957607
ATCGGTCG 0.00334753415781
ATCGGTGA 0.256952544831
ATCGGTGC 0.0150162818848
ATCGGTGG 0.130979361984
ATCGGTTA 0.212282530464
ATCGGTTC 0.142404910841
ATCGGTTG 0.0333275429933
ATCGTAAA 0.257330333088
ATCGTAAC 0.251418084751
ATCGTAAG 0.224302815212
ATCGTAAT 0.329130748962
ATCGTACA 0.163029276731
ATCGTACC 0.147733261315
ATCGTACG 0.112656657153
ATCGTAGA 0.110738450983
ATCGTAGC 0.1347612826
ATCGTAGG 0.148254314921
ATCGTATA 0.275570336176
ATCGTATC 0.341507120282
ATCGTATG 0.161521025157
ATCGTCAA 0.168710904141
ATCGTCAC -0.0208204084671
ATCGTCAG 0.165497453376
ATCGTCAT 0.0924264432166
ATCGTCCA 0.133854239915
ATCGTCCC 0.125882644906
ATCGTCCG 0.149595994248
ATCGTCGA 0.262222277374
ATCGTCGC 0.130582250966
ATCGTCGG 0.11373218949
ATCGTCTA 0.241874265527
ATCGTCTC 0.234691941052
ATCGTCTG -0.0259651248513
ATCGTGAA 0.145968282332
ATCGTGAC 0.157695086969
ATCGTGAG 0.234840842901
ATCGTGCA 0.119859419137
ATCGTGCC 0.203739505623
ATCGTGCG 0.238563044395
ATCGTGGA 0.332506141896
ATCGTGGC 0.0227549761684
ATCGTGGG 0.0653881172366
ATCGTGTA 0.342934826245

ATCGTGTC 0.303271461209
ATCGTGTG 0.0596592775341
ATCGTTAA 0.193147693148
ATCGTTAC 0.0641837213221
ATCGTTAG −0.0970668648738
ATCGTTCA 0.108465214526
ATCGTTCC −0.189401956162
ATCGTTCG 0.100067151814
ATCGTTGA 0.200452266918
ATCGTTGC 0.110464458949
ATCGTTGG 0.0330178663512
ATCGTTTA 0.145484364407
ATCGTTTC 0.0617468230623
ATCGTTTG −0.01447924498
ATCTAAAA 0.286179864674
ATCTAAAC 0.283367389428
ATCTAAAG 0.203367279125
ATCTAAAT 0.307259695043
ATCTAACA 0.153593789393
ATCTAACC 0.278924549097
ATCTAACG 0.132445117294
ATCTAAGA 0.253269636431
ATCTAAGC 0.262507390349
ATCTAAGG 0.0106106354524
ATCTAATA 0.291749085786
ATCTAATC 0.281186144823
ATCTAATG 0.282501509774
ATCTACAA 0.309855572375
ATCTACAC 0.362548498912
ATCTACAG 0.201307367974
ATCTACAT 0.257490272642
ATCTACCA 0.286369948101
ATCTACCC 0.103043815165
ATCTACCG 0.116163149712
ATCTACGA 0.351171142238
ATCTACGC 0.229646796056
ATCTACGG 0.150997423414
ATCTACTA 0.360257074663
ATCTACTC 0.212712712713
ATCTACTG 0.195896998927
ATCTAGAA 0.273888163033
ATCTAGAC 0.294036460703
ATCTAGAG 0.300425588057
ATCTAGAT 0.337012874259
ATCTAGCA 0.30546119428
ATCTAGCC 0.211846833059
ATCTAGCG 0.232500748159
ATCTAGGA 0.375012407872
ATCTAGGC 0.0171357262359
ATCTAGGG 0.18386677664
ATCTAGTA 0.272217120702
ATCTAGTC 0.253891520624
ATCTAGTG 0.145371269242
ATCTATAA 0.212015065364

ATCTATAC 0.316228841704
ATCTATAG 0.297354641785
ATCTATCA 0.254120511696
ATCTATCC 0.162575813588
ATCTATCG 0.216534716535
ATCTATGA 0.255377619468
ATCTATGC 0.205474352929
ATCTATGG 0.196748246389
ATCTATTA 0.380974390153
ATCTATTC 0.364139621715
ATCTATTG 0.189927957782
ATCTCAAA 0.33099056225
ATCTCAAC 0.384568951045
ATCTCAAG 0.214601870032
ATCTCAAT 0.193092851981
ATCTCACA 0.348409158924
ATCTCACC 0.311907768443
ATCTCACG 0.240139996934
ATCTCAGA 0.305712048136
ATCTCAGC 0.199991970979
ATCTCAGG 0.201594855587
ATCTCATA 0.319665262804
ATCTCATC 0.275513805817
ATCTCATG 0.318558717692
ATCTCCAA 0.299675779755
ATCTCCAC 0.4058424816
ATCTCCAG 0.310581432861
ATCTCCAT 0.306052559744
ATCTCCCA 0.317014225966
ATCTCCCC 0.316701824048
ATCTCCCG 0.243983653075
ATCTCCGA 0.329031481062
ATCTCCGC 0.358056403511
ATCTCCGG 0.27404290417
ATCTCCTA 0.349541001056
ATCTCCTC 0.307639248776
ATCTCCTG 0.310173810174
ATCTCGAA 0.409137756108
ATCTCGAC 0.409362568703
ATCTCGAG 0.249276643994
ATCTCGCA 0.338713179641
ATCTCGCC 0.331468174295
ATCTCGCG 0.327510706299
ATCTCGGA 0.363009761877
ATCTCGGC 0.359198575576
ATCTCGGG 0.157569542273
ATCTCGTA 0.396504456107
ATCTCGTC 0.317571877988
ATCTCGTG 0.411689525047
ATCTCTAA 0.231211725291
ATCTCTAC 0.314034478119
ATCTCTAG 0.277225316234
ATCTCTCA 0.285400319701
ATCTCTCC 0.200220882039

ATCTCTCG 0.188038948052
ATCTCTGA 0.370251016401
ATCTCTGC 0.332289803873
ATCTCTGG 0.217957627049
ATCTCTTA 0.347208089632
ATCTCTTC 0.280671927517
ATCTCTTG 0.396802259805
ATCTGAAA 0.310377874937
ATCTGAAC 0.378998270111
ATCTGAAG 0.247917928804
ATCTGAAT 0.348111355226
ATCTGACA 0.259454798124
ATCTGACC 0.145272001343
ATCTGACG 0.131761237822
ATCTGAGA 0.340704218156
ATCTGAGC 0.275406377963
ATCTGAGG 0.286890798012
ATCTGATA 0.348137632023
ATCTGATC 0.275822654611
ATCTGATG 0.205215214265
ATCTGCAA 0.3076547622
ATCTGCAC 0.411152310533
ATCTGCAG 0.279750808376
ATCTGCAT 0.272902378963
ATCTGCCA 0.218779385446
ATCTGCCC 0.206226674626
ATCTGCCG 0.134994854127
ATCTGCGA 0.373606388758
ATCTGCGC 0.272889644752
ATCTGCGG 0.175179311543
ATCTGCTA 0.370790629575
ATCTGCTC 0.229258483391
ATCTGCTG 0.302881406191
ATCTGGAA 0.391719159434
ATCTGGAC 0.41642882552
ATCTGGAG 0.27285840137
ATCTGGCA 0.226605393272
ATCTGGCC 0.277368378795
ATCTGGCG 0.270092625709
ATCTGGGA 0.392672286612
ATCTGGGC 0.180862462866
ATCTGGGG 0.16593724225
ATCTGGTA 0.299128486238
ATCTGGTC 0.238537112326
ATCTGGTG 0.332923366642
ATCTGTAA 0.348029605191
ATCTGTAC 0.295514522787
ATCTGTAG 0.260692483311
ATCTGTCA 0.226721312672
ATCTGTCC -0.0186076217309
ATCTGTCG 0.201827171524
ATCTGTGA 0.413461299849
ATCTGTGC 0.193148325219
ATCTGTGG 0.118864043106

ATCTGTTA 0.366791238148
ATCTGTTC 0.250370429845
ATCTGTTG 0.350493784808
ATCTTAAA 0.257012620162
ATCTTAAC 0.321376281113
ATCTTAAG 0.210420939687
ATCTTAAT 0.326196506646
ATCTTACA 0.207895223047
ATCTTACC 0.200637276395
ATCTTACG 0.29878104859
ATCTTAGA 0.352532973745
ATCTTAGC 0.190719181332
ATCTTAGG 0.0280321643958
ATCTTATA 0.344911026729
ATCTTATC 0.328170186054
ATCTTATG 0.249555849142
ATCTTCAA 0.316240482907
ATCTTCAC 0.357935070056
ATCTTCAG 0.318755394513
ATCTTCAT 0.268171499894
ATCTTCCA 0.355179813581
ATCTTCCC 0.287263052634
ATCTTCCG 0.361803952713
ATCTTCGA 0.388373221707
ATCTTCGC 0.223158756886
ATCTTCGG 0.311148660978
ATCTTCTA 0.333545250834
ATCTTCTC 0.301802863737
ATCTTCTG 0.350651445589
ATCTTGAA 0.271254644059
ATCTTGAC 0.310439578383
ATCTTGAG 0.238744407057
ATCTTGCA 0.286774012248
ATCTTGCC 0.27465344132
ATCTTGCG 0.242577899754
ATCTTGGA 0.255260833704
ATCTTGGC 0.0901812369072
ATCTTGGG 0.168801413108
ATCTTGTA 0.282223746925
ATCTTGTC 0.297541308037
ATCTTGTG 0.370437005236
ATCTTTAA 0.298727415944
ATCTTTAC 0.228853884905
ATCTTTAG 0.232186732187
ATCTTTCA 0.17799245272
ATCTTTCC 0.156109720225
ATCTTTCG 0.136113077816
ATCTTTGA 0.272279166219
ATCTTTGC 0.172107369077
ATCTTTGG 0.312763224163
ATCTTTTA 0.313236206506
ATCTTTTC 0.267122494395
ATCTTTTG 0.301300799839
ATGAAAAA 0.0929811755947

ATGAAAAC 0.134722877147
ATGAAAAG 0.151038693505
ATGAAAAT 0.371415954395
ATGAAACA 0.16784966785
ATGAAACC -0.0676622800125
ATGAAACG 0.102593761961
ATGAAAGA 0.0340492007159
ATGAAAGC 0.114086468311
ATGAAAGG -0.0826654756883
ATGAAATA 0.18564192025
ATGAAATC 0.453957212616
ATGAAATG -0.0415877348996
ATGAACAA 0.0198145804206
ATGAACAC 0.0330757755
ATGAACAG 0.00586783582192
ATGAACAT -0.0922005763053
ATGAACCA 0.091276103443
ATGAACCC -0.309449198672
ATGAACCG -0.289169512878
ATGAACGA -0.0153335153335
ATGAACGC -0.214342030903
ATGAACGG -0.165103683861
ATGAACTA -0.176183790143
ATGAACTC -0.246652441092
ATGAACTG -0.149188313031
ATGAAGAA 0.159389416965
ATGAAGAC -0.0288876885908
ATGAAGAG -0.0390983269771
ATGAAGCA -0.124492361645
ATGAAGCC -0.418669345172
ATGAAGCG -0.142410306885
ATGAAGGA 0.129377855052
ATGAAGGC -0.0421578115823
ATGAAGGG -0.109585094387
ATGAAGTA -0.0944517505769
ATGAAGTC -0.227717707769
ATGAAGTG 0.167297796399
ATGAATAA 0.218406166288
ATGAATAC 0.0743870592355
ATGAATAG 0.0210665210665
ATGAATCA 0.27874017268
ATGAATCC 0.219617818588
ATGAATCG 0.0894489530853
ATGAATGA 0.0991264858426
ATGAATGC -0.0654937674219
ATGAATGG -0.0418166025561
ATGAATTA -0.0755873958964
ATGAATTC 0.119335126429
ATGAATTG 0.223610663005
ATGACAAA 0.0978540615899
ATGACAAC -0.129804583322
ATGACAAG -0.00204729880636
ATGACAAT 0.198999291986
ATGACACA 0.142588869862

ATGACACC -0.199370080911
ATGACACG -0.0455213787627
ATGACAGA 0.0278191778982
ATGACAGC -0.110174141019
ATGACAGG -0.0934523453381
ATGACATA -0.182218067008
ATGACATC 0.00927006031124
ATGACATG 0.0508185952132
ATGACCAA 0.00309701247418
ATGACCAC -0.0984482091633
ATGACCAG -0.142185445252
ATGACCAT -0.181566996629
ATGACCCA -0.102657754173
ATGACCCC -0.255891295299
ATGACCCG -0.0722041618758
ATGACCGA -0.193214302451
ATGACCGC -0.136973955939
ATGACCGG 0.0829249075614
ATGACCTA -0.253793581629
ATGACCTC -0.249604597216
ATGACCTG -0.306651838255
ATGACGAA -0.0198917926191
ATGACGAC -0.0523057090576
ATGACGAG -0.0483941241517
ATGACGCA -0.00894943906338
ATGACGCC -0.306483152938
ATGACGCG -0.263038689418
ATGACGGA 0.147499689788
ATGACGGC -0.0997822269773
ATGACGGG -0.250445455912
ATGACGTA 0.017878760303
ATGACGTC -0.227123493851
ATGACGTG 0.072018299291
ATGACTAA -0.0647496770144
ATGACTAC 0.0080166127749
ATGACTAG 0.00460923100235
ATGACTCA -0.00702539360452
ATGACTCC -0.127014628155
ATGACTCG -0.0153865243827
ATGACTGA -0.183250208845
ATGACTGC -0.132618237498
ATGACTGG -0.247562458134
ATGACTTA -0.0461740693002
ATGACTTC -0.165650192825
ATGACTTG 0.0257008970606
ATGAGAAA 0.129867959096
ATGAGAAC 0.0827976737068
ATGAGAAG 0.124139617381
ATGAGAAT 0.317258029379
ATGAGACA 0.114220280887
ATGAGACC -0.344184856753
ATGAGACG -0.0378316003673
ATGAGAGA 0.14542025461
ATGAGAGC -0.187653524552

ATGAGAGG -0.167133161037
ATGAGATA 0.209288117778
ATGAGATC 0.372031999299
ATGAGATG 0.0499076662555
ATGAGCAA -0.083451713357
ATGAGCAC -0.0185378215681
ATGAGCAG 0.0398732065399
ATGAGCAT -0.228483301491
ATGAGCCA 0.0161271310519
ATGAGCCC -0.308564797588
ATGAGCCG -0.259316097112
ATGAGCGA 0.0291957110428
ATGAGCGC -0.134776418649
ATGAGCGG -0.341930380183
ATGAGCTA -0.264816974489
ATGAGCTC -0.29601420211
ATGAGCTG -0.0793788652048
ATGAGGAA 0.0435749582126
ATGAGGAC -0.18399632727
ATGAGGAG -0.124707137738
ATGAGGCA -0.213299695979
ATGAGGCC -0.210101232683
ATGAGGCG -0.0608633269938
ATGAGGGA 0.106096607688
ATGAGGGC -0.193986478104
ATGAGGGG -0.248991765974
ATGAGGTA -0.079989168658
ATGAGGTC -0.241473587128
ATGAGGTG 0.0811974920257
ATGAGTAA 0.245864321622
ATGAGTAC 0.0221447705525
ATGAGTAG 0.000159850514222
ATGAGTCA -0.0753963329721
ATGAGTCC -0.308664026086
ATGAGTCG -0.0381546389495
ATGAGTGA -0.0976287827586
ATGAGTGC -0.05255578345
ATGAGTGG -0.16074790831
ATGAGTTA -0.252581270385
ATGAGTTC -0.0888309015131
ATGAGTTG -0.0623174400663
ATGATAAA 0.150526559617
ATGATAAC -0.0497952599578
ATGATAAG 0.0719772559725
ATGATAAT 0.0448304051736
ATGATACA 0.0897495291435
ATGATACC 0.0435807975008
ATGATACG 0.126040305687
ATGATAGA 0.193572360239
ATGATAGC -0.0729651779978
ATGATAGG -0.0771870890097
ATGATATA 0.0549966059137
ATGATATC 0.422914650187
ATGATATG 0.266264887477

ATGATCAA 0.0689463568251
ATGATCAC -0.0613503864648
ATGATCAG 0.075664766465
ATGATCAT 0.0943933116674
ATGATCCA 0.14947628884
ATGATCCC 0.0496252822791
ATGATCCG 0.121131846043
ATGATCGA 0.0948526674598
ATGATCGC 0.220949176295
ATGATCGG -0.112688211177
ATGATCTA -0.0812398496717
ATGATCTC 0.254697342394
ATGATCTG -0.00378750830274
ATGATGAA 0.0341757137875
ATGATGAC -0.0177223706891
ATGATGAG 0.121893243105
ATGATGCA 0.0644332219942
ATGATGCC -0.0880542941512
ATGATGCG -0.0523154008002
ATGATGGA 0.184596687664
ATGATGGC -0.0935980897392
ATGATGGG -0.124941065535
ATGATGTA 0.0733115333241
ATGATGTC -0.059106004978
ATGATGTG 0.0353882761691
ATGATTAA 0.271479456654
ATGATTAC 0.289494728889
ATGATTAG 0.0784238301351
ATGATTCA 0.11974922581
ATGATTCC 0.269964161369
ATGATTCG 0.204393098332
ATGATTGA -0.149683583571
ATGATTGC 0.31660724085
ATGATTGG -0.101941662543
ATGATTTA 0.195461301522
ATGATTTC 0.377754501726
ATGATTTG 0.295201564929
ATGCAAAA 0.0955154266695
ATGCAAAC -0.0846342209979
ATGCAAAG -0.136281102352
ATGCAAAT 0.289944214187
ATGCAACA 0.0237388962285
ATGCAACC -0.138757234119
ATGCAACG 0.0462551914922
ATGCAAGA 0.0757187798807
ATGCAAGC -0.256412588935
ATGCAAGG 0.0756560075327
ATGCAATA 0.0888411202674
ATGCAATC 0.449869710882
ATGCAATG -0.141512325632
ATGCACAA -0.0487364304763
ATGCACAC 0.123452653756
ATGCACAG 0.0518784260199
ATGCACAT -0.0373108618059

ATGCACCA 0.031467194149
ATGCACCC -0.157270684259
ATGCACCG -0.0890628920932
ATGCACGA -0.132263441359
ATGCACGC -0.227422130304
ATGCACGG -0.102670811533
ATGCACTA -0.0199944526762
ATGCACTC -0.172934020454
ATGCACTG 0.0651219316365
ATGCAGAA 0.0972418699691
ATGCAGAC -0.0305621044795
ATGCAGAG -0.00490281234717
ATGCAGCA -0.246686825672
ATGCAGCC -0.312073950951
ATGCAGCG -0.0648892178028
ATGCAGGA 0.158187112733
ATGCAGGC -0.149883018488
ATGCAGGG 0.0392684831719
ATGCAGTA -0.042812785237
ATGCAGTC -0.316421489569
ATGCAGTG 0.138734918213
ATGCATAA 0.215711334788
ATGCATAC 0.156196141045
ATGCATAG 0.0496916125924
ATGCATCA 0.0382115720094
ATGCATCC -0.194797564675
ATGCATCG -0.0527954934611
ATGCATGA -0.131124440844
ATGCATGC -0.250058586911
ATGCATGG -0.043945530553
ATGCATTA 0.0602765299735
ATGCATTC 0.0038415217185
ATGCATTG 0.0421414129618
ATGCCAAA 0.00968372955337
ATGCCAAC -0.0935274117092
ATGCCAAG -0.0992525317695
ATGCCAAT -0.00889250600352
ATGCCACA 0.0768764187646
ATGCCACC -0.223034087307
ATGCCACG -0.0969651898873
ATGCCAGA -0.13421968862
ATGCCAGC -0.237466956649
ATGCCAGG -0.120458680012
ATGCCATA -0.113076306675
ATGCCATC -0.0426368451381
ATGCCATG -0.265470487553
ATGCCCAA 0.106572118859
ATGCCCAC 0.0631292745414
ATGCCCAG -0.244940919631
ATGCCCAT 0.0375955271052
ATGCCCCA -0.0514591870458
ATGCCCCC -0.162984022248
ATGCCCCG -0.0943341279237
ATGCCCGA -0.124609757557

ATGCCCGC -0.0271974344714
ATGCCCGG -0.185518060649
ATGCCCTA -0.241780038319
ATGCCCTC -0.332438943048
ATGCCCTG -0.248635078578
ATGCCGAA 0.185048685049
ATGCCGAC -0.0782401548426
ATGCCGAG -0.100281745655
ATGCCGCA 0.10589515135
ATGCCGCC -0.237975849165
ATGCCGCG -0.0630008102012
ATGCCGGA 0.0557651315227
ATGCCGGC 0.0639511543543
ATGCCGGG -0.0577192288435
ATGCCGTA 0.0944380779983
ATGCCGTC 0.0523280512106
ATGCCGTG -0.00851678124405
ATGCCTAA 0.115166130318
ATGCCTAC 0.115745640606
ATGCCTAG -0.100684029007
ATGCCTCA -0.115496224545
ATGCCTCC -0.308910719159
ATGCCTCG -0.0918468938636
ATGCCTGA -0.0595508901959
ATGCCTGC -0.112617828617
ATGCCTGG -0.23724449218
ATGCCTTA -0.181902507163
ATGCCTTC -0.246671102764
ATGCCTTG 0.0557127375309
ATGCGAAA 0.223836704306
ATGCGAAC -0.0741158952724
ATGCGAAG 0.0128351491988
ATGCGAAT 0.307578928791
ATGCGACA 0.0204822939673
ATGCGACC -0.344265842009
ATGCGACG -0.157539987813
ATGCGAGA -0.0752024836634
ATGCGAGC -0.052549794974
ATGCGAGG -0.159525956069
ATGCGATA 0.117424587122
ATGCGATC 0.347457122291
ATGCGATG 0.0148729997215
ATGCGCAA 0.236708067598
ATGCGCAC -0.0100665403696
ATGCGCAG -0.12218499676
ATGCGCAT 0.16639930099
ATGCGCCA -0.0181667554725
ATGCGCCC -0.239153452283
ATGCGCCG -0.0174223810587
ATGCGCGA 0.127302233363
ATGCGCGC 0.0099714039108
ATGCGCGG -0.110969124009
ATGCGCTA -0.163957505879
ATGCGCTC -0.264328247116

ATGCGCTG -0.0759395759396
ATGCGGAA 0.0590656918288
ATGCGGAC -0.218671342359
ATGCGGAG -0.143659910189
ATGCGGCA -0.14873217924
ATGCGGCC -0.329196547775
ATGCGGCG -0.172655377745
ATGCGGGA -0.0699286240065
ATGCGGGC -0.195720541583
ATGCGGGG -0.227472918761
ATGCGGTA 0.0226289816192
ATGCGGTC -0.162834773739
ATGCGGTG 0.107918074787
ATGCGTAA 0.272902378963
ATGCGTAC 0.0765990525755
ATGCGTAG -0.139854539038
ATGCGTCA -0.0668751779158
ATGCGTCC -0.217386386359
ATGCGTCG 0.109558914768
ATGCGTGA -0.165388539476
ATGCGTGC -0.255671057963
ATGCGTGG -0.303634097831
ATGCGTTA -0.19305635643
ATGCGTTC -0.093847474227
ATGCGTTG 0.0428187703919
ATGCTAAA 0.0116273298091
ATGCTAAC -0.000638379426258
ATGCTAAG 0.0727290643271
ATGCTAAT 0.0853930205908
ATGCTACA -0.0319130456362
ATGCTACC -0.211833734908
ATGCTACG 0.000630106690713
ATGCTAGA 0.0170294261203
ATGCTAGC -0.124900382476
ATGCTAGG 0.0253228031503
ATGCTATA 0.021422564145
ATGCTATC -0.134356911892
ATGCTATG -0.147575523597
ATGCTCAA -0.067411175911
ATGCTCAC 0.00253145707691
ATGCTCAG -0.126676903361
ATGCTCCA 0.0452566732115
ATGCTCCC -0.158064243042
ATGCTCCG -0.109229250592
ATGCTCGA -0.202544899475
ATGCTCGC -0.226600604923
ATGCTCGG -0.181656182536
ATGCTCTA -0.122347391435
ATGCTCTC -0.221956168719
ATGCTCTG -0.214733055191
ATGCTGAA -0.0523032342357
ATGCTGAC -0.114541202452
ATGCTGAG -0.109054062535
ATGCTGCA -0.121802442282

ATGCTGCC −0.18697400814
ATGCTGCG 0.0424742523886
ATGCTGGA 0.0860378284621
ATGCTGGC −0.206129650305
ATGCTGGG −0.189464889045
ATGCTGTA 0.102859057405
ATGCTGTC 0.0696211031875
ATGCTGTG 0.176912184405
ATGCTTAA 0.226228529553
ATGCTTAC 0.049072602083
ATGCTTAG −0.0507153502888
ATGCTTCA −0.253879761326
ATGCTTCC −0.0738433335815
ATGCTTCG −0.084648001578
ATGCTTGA −0.20923506496
ATGCTTGC −0.23349425981
ATGCTTGG −0.0796430255354
ATGCTTTA −0.179924195429
ATGCTTTC −0.0659308068399
ATGCTTTG −0.113674547464
ATGGAAAA −0.0423412393109
ATGGAAAC −0.0521168551472
ATGGAAAG −0.127448385024
ATGGAAAT 0.306302169939
ATGGAACA −0.0163916118625
ATGGAACC −0.248953017433
ATGGAACG −0.118605837504
ATGGAAGA −0.0113824029732
ATGGAAGC −0.189222456547
ATGGAAGG −0.133155237961
ATGGAATA 0.103177655905
ATGGAATC 0.360146648025
ATGGAATG −0.187631807468
ATGGACAA 0.0202475202475
ATGGACAC 0.0482843441384
ATGGACAG −0.0864647868296
ATGGACAT −0.0672476884598
ATGGACCA −0.219623462458
ATGGACCC −0.225167201019
ATGGACCG −0.325460277151
ATGGACGA −0.110912504069
ATGGACGC −0.17972153506
ATGGACGG −0.0833366176069
ATGGACTA −0.0545576980101
ATGGACTC −0.193367484694
ATGGACTG −0.297441326397
ATGGAGAA 0.03388465946
ATGGAGAC −0.204490097327
ATGGAGAG −0.247570732305
ATGGAGCA −0.14728005669
ATGGAGCC −0.281022848495
ATGGAGCG −0.131747570123
ATGGAGGA −0.133694815991
ATGGAGGC −0.277843939958

ATGGAGGG	-0.113703841542
ATGGAGTA	0.00115535282999
ATGGAGTC	-0.0940880185536
ATGGAGTG	-0.0570887031206
ATGGATAA	0.197736303797
ATGGATAC	0.282393086283
ATGGATAG	0.194791209943
ATGGATCA	-0.00230395684941
ATGGATCC	0.253654118173
ATGGATCG	-0.0223622840376
ATGGATGA	0.0187249379241
ATGGATGC	-0.0845623409459
ATGGATGG	-0.0767067639979
ATGGATTA	0.334983744075
ATGGATTC	0.432687605381
ATGGATTG	0.16764815369
ATGGCAAA	0.0618052159442
ATGGCAAC	-0.225738264369
ATGGCAAG	-0.111304112867
ATGGCAAT	0.28408064057
ATGGCACA	0.0926891078406
ATGGCACC	-0.315866548559
ATGGCACG	-0.136217955735
ATGGCAGA	0.158002354972
ATGGCAGC	-0.247742904795
ATGGCAGG	-0.19550112906
ATGGCATA	0.0250650099135
ATGGCATC	-0.14209908099
ATGGCATG	-0.128906816285
ATGGCCAA	-0.0930328377473
ATGGCCAC	-0.00174959672416
ATGGCCAG	-0.276199455789
ATGGCCAT	-0.45383068127
ATGGCCCA	-0.239997927258
ATGGCCCC	-0.246877592818
ATGGCCCG	-0.375763157242
ATGGCCGA	-0.244380349833
ATGGCCGC	-0.208359176653
ATGGCCGG	-0.243598761856
ATGGCCTA	-0.0927719706955
ATGGCCTC	-0.311132063508
ATGGCCTG	-0.0349261512997
ATGGCGAA	-0.0327941723904
ATGGCGAC	-0.265464991295
ATGGCGAG	-0.0194650309686
ATGGCGCA	-0.118567476625
ATGGCGCC	-0.104685850089
ATGGCGCG	-0.15489923673
ATGGCGGA	-0.0986326525548
ATGGCGGC	-0.164551317885
ATGGCGGG	-0.212554902107
ATGGCGTA	-0.0407596281696
ATGGCGTC	-0.264396538327
ATGGCGTG	-0.160080119324

ATGGCTAA 0.0546039137829
ATGGCTAC -0.29663399909
ATGGCTAG -0.00917133201463
ATGGCTCA -0.0439676503434
ATGGCTCC -0.195226709756
ATGGCTCG -0.106538121117
ATGGCTGA -0.160852665095
ATGGCTGC -0.118033324786
ATGGCTGG -0.306952415557
ATGGCTTA -0.138361635445
ATGGCTTC -0.189880287805
ATGGCTTG 0.0158806969364
ATGGGAAA 0.154068889002
ATGGGAAC -0.161333454637
ATGGGAAG -0.0233609201678
ATGGGAAT 0.265319038046
ATGGGACA -0.123441166172
ATGGGACC -0.225820974151
ATGGGACG -0.272317183094
ATGGGAGA -0.080774165111
ATGGGAGC -0.28421692426
ATGGGAGG -0.106432833784
ATGGGATA 0.229060041202
ATGGGATC 0.187974715882
ATGGGATG -0.179144510328
ATGGGCAA -0.0344284260936
ATGGGCAC -0.141667578829
ATGGGCAG -0.233349595782
ATGGGCCA -0.0127829317606
ATGGGCCC -0.353429244698
ATGGGCCG -0.215191323497
ATGGGCGA -0.0349004231814
ATGGGCGC -0.100892154673
ATGGGCGG -0.123220000496
ATGGGCTA -0.063481821566
ATGGGCTC -0.139873838256
ATGGGCTG -0.11728594151
ATGGGGAA 0.0587162378166
ATGGGGAC -0.17172807344
ATGGGGAG 0.0106578327006
ATGGGGCA -0.0668251468143
ATGGGGCC -0.222940286288
ATGGGGCG -0.15299357628
ATGGGGGA -0.0404378006321
ATGGGGGC -0.0968795021917
ATGGGGGG -0.352573617241
ATGGGGTA 0.0555125932525
ATGGGGTC -0.191334893968
ATGGGGTG 0.165000036496
ATGGGTAA 0.0444356927611
ATGGGTAC -0.0994634660712
ATGGGTAG 0.0124919693498
ATGGGTCA -0.163455044177
ATGGGTCC -0.163189189636

ATGGGTCG -0.251763859522
ATGGGTGA -0.0234489835538
ATGGGTGC -0.0710722882597
ATGGGTGG -0.0282086280349
ATGGGTTA -0.0145034842005
ATGGGTTC -0.082669821359
ATGGGTTG -0.217857697919
ATGGTAAA 0.115331585029
ATGGTAAC -0.174641774954
ATGGTAAG 0.0357036503958
ATGGTAAT 0.298608789589
ATGGTACA -0.0675774044387
ATGGTACC -0.195834677386
ATGGTACG 0.176894525379
ATGGTAGA 0.183779189518
ATGGTAGC 0.0551088278361
ATGGTAGG 0.0249330306636
ATGGTATA 0.158236749146
ATGGTATC 0.156303686607
ATGGTATG -0.116745842364
ATGGTCAA -0.0743808107157
ATGGTCAC -0.215110140781
ATGGTCAG -0.0689062493158
ATGGTCCA 0.0510614934114
ATGGTCCC -0.221415315255
ATGGTCCG -0.0421261836745
ATGGTCGA -0.200685054239
ATGGTCGC -0.159562746606
ATGGTCGG -0.314147412655
ATGGTCTA -0.202835151973
ATGGTCTC 0.0047775047775
ATGGTCTG -0.168771555467
ATGGTGAA 0.044718271991
ATGGTGAC -0.301494767075
ATGGTGAG -0.0774083313748
ATGGTGCA -0.00530255093178
ATGGTGCC -0.262871432345
ATGGTGCG -0.149351311853
ATGGTGGA -0.0403093362919
ATGGTGGC -0.141825524818
ATGGTGGG -0.182646905565
ATGGTGTA -0.052003794428
ATGGTGTC -0.0968307263345
ATGGTGTG 0.16995759217
ATGGTTAA -0.048876833172
ATGGTTAC -0.0585425276148
ATGGTTAG -0.0339995643026
ATGGTTCA 0.103246644234
ATGGTTCC -0.00641080852244
ATGGTTCG -0.106563910027
ATGGTTGA -0.0605890965464
ATGGTTGC -0.0409072984831
ATGGTTGG -0.123642956401
ATGGTTTA 0.0123814806975

ATGGTTTC -0.00466048188726
ATGGTTTG -0.10668882779
ATGTAAAA 0.021090779034
ATGTAAAC 0.0777403599341
ATGTAAAG 0.212467610198
ATGTAAAT 0.201912656458
ATGTAACA 0.166559126442
ATGTAACC -0.143139165381
ATGTAACG -0.0194545770367
ATGTAAGA 0.0593877506332
ATGTAAGC -0.252160198993
ATGTAAGG -0.233822489392
ATGTAATA 0.0700438730742
ATGTAATC 0.454162220513
ATGTAATG -0.0342891100467
ATGTACAA 0.0690196565039
ATGTACAC 0.13687103081
ATGTACAG 0.0665362332029
ATGTACAT 0.0570329576432
ATGTACCA 0.0370219867484
ATGTACCC -0.0791406027605
ATGTACCG -0.128253276448
ATGTACGA 0.0757698736524
ATGTACGC -0.0360992095064
ATGTACGG -0.0488977127022
ATGTACTA -0.0693218396677
ATGTACTC 0.0118774350448
ATGTACTG -0.0255738925425
ATGTAGAA 0.0604855368131
ATGTAGAC -0.0663200734546
ATGTAGAG 0.124717025743
ATGTAGCA 0.0115990160799
ATGTAGCC -0.403878620384
ATGTAGCG -0.313325434358
ATGTAGGA 0.172514830351
ATGTAGGC 0.0154576063667
ATGTAGGG -0.0836385426444
ATGTAGTA 0.275669145931
ATGTAGTC -0.163692478803
ATGTAGTG -0.013505240778
ATGTATAA 0.161986963789
ATGTATAC 0.201104355379
ATGTATAG 0.0904031298585
ATGTATCA 0.236044584529
ATGTATCC 0.167579425155
ATGTATCG -0.0715524477566
ATGTATGA -0.0272293307446
ATGTATGC 0.134270782392
ATGTATGG -0.0728437624523
ATGTATTA 0.16374166989
ATGTATTC 0.0901548931852
ATGTATTG 0.111465442363
ATGTCAAA -0.0424056407524
ATGTCAAC 0.00293586689496

ATGTCAAG -0.0583006701557
ATGTCAAT -0.112693398574
ATGTCACA 0.19732633592
ATGTCACC -0.245038684903
ATGTCACG 0.0813863314887
ATGTCAGA -0.0420035647085
ATGTCAGC -0.127473359887
ATGTCAGG -0.20974444858
ATGTCATA 0.0441374526635
ATGTCATC -0.00287074005679
ATGTCATG 0.021240248513
ATGTCCAA -0.11357616921
ATGTCCAC 0.130252842374
ATGTCCAG 0.0568184638293
ATGTCCCA -0.0243645849706
ATGTCCCC -0.317403998048
ATGTCCCG -0.141750504633
ATGTCCGA -0.173792884809
ATGTCCGC -0.298713303693
ATGTCCGG -0.141091092187
ATGTCCTA -0.103453695661
ATGTCCTC -0.285073655296
ATGTCCTG -0.349944621742
ATGTCGAA 0.0240921731641
ATGTCGAC -0.151030571689
ATGTCGAG 0.0380393130077
ATGTCGCA 0.0471146077207
ATGTCGCC -0.0273316335393
ATGTCGCG -0.137142252359
ATGTCGGA 0.0134473316291
ATGTCGGC -0.121234210983
ATGTCGGG -0.180819183573
ATGTCGTA 0.104383998323
ATGTCGTC -0.119685396801
ATGTCGTG 0.0106103099948
ATGTCTAA -0.0578774366653
ATGTCTAC -0.0454625081203
ATGTCTAG -0.183185039744
ATGTCTCA 0.158389962264
ATGTCTCC -0.085277694649
ATGTCTCG -0.0875871331285
ATGTCTGA -0.0179176963984
ATGTCTGC -0.128785678105
ATGTCTGG -0.327965509341
ATGTCTTA 0.0657087800997
ATGTCTTC -0.0891388791167
ATGTCTTG 0.138600578207
ATGTGAAA 0.0914512820887
ATGTGAAC 0.206179648322
ATGTGAAG 0.0524973905681
ATGTGAAT 0.0521119975475
ATGTGACA 0.0588257190362
ATGTGACC -0.233973696325
ATGTGACG -0.111760456389

ATGTGAGA 0.0753273935092
ATGTGAGC -0.134414472541
ATGTGAGG -0.0782413806693
ATGTGATA 0.213647146413
ATGTGATC 0.228742516621
ATGTGATG -0.0875637759757
ATGTGCAA -0.0205067326279
ATGTGCAC -0.053240683294
ATGTGCAG -0.0393597220499
ATGTGCCA 0.00203718166755
ATGTGCCC -0.148236841452
ATGTGCCG -0.00992314036919
ATGTGCGA 0.0725027919097
ATGTGCGC -0.0295475281563
ATGTGCGG -0.106353445495
ATGTGCTA -0.0972715090453
ATGTGCTC -0.195778006768
ATGTGCTG -0.0627263636563
ATGTGGAA 0.0532535783888
ATGTGGAC -0.369097730474
ATGTGGAG 0.0630909248827
ATGTGGCA -0.0481490577104
ATGTGGCC -0.200335240473
ATGTGGCG -0.191637317905
ATGTGGGA 0.0592067326993
ATGTGGGC -0.0868345948629
ATGTGGGG -0.258090150835
ATGTGGTA 0.0999211696094
ATGTGGTC -0.0851378592083
ATGTGGTG 0.116981413008
ATGTGTAA 0.255330905163
ATGTGTAC 0.147978511419
ATGTGTAG 0.0700152551404
ATGTGTCA 0.136175850164
ATGTGTCC -0.205804993892
ATGTGTCG -0.265529099409
ATGTGTGA 0.107015904761
ATGTGTGC 0.142536294826
ATGTGTGG -0.153024175206
ATGTGTTA 0.0800646701167
ATGTGTTC -0.0410421669598
ATGTGTTG 0.023740637531
ATGTTAAA 0.185133902177
ATGTTAAC 0.15438017371
ATGTTAAG -0.0234647448577
ATGTTAAT 0.184290350957
ATGTTACA 0.194225906347
ATGTTACC -0.053699797328
ATGTTACG 0.0121374599173
ATGTTAGA 0.0772990621475
ATGTTAGC 0.0247607716619
ATGTTAGG -0.0269432056232
ATGTTATA 0.0847086756178
ATGTTATC 0.0754165967169

ATGTTATG -0.156182258495
ATGTTCAA -0.00080245534791
ATGTTCAC 0.0183025189229
ATGTTCAG -0.203663267247
ATGTTCCA 0.160822025795
ATGTTCCC -0.139075849204
ATGTTCCG 0.0107712969789
ATGTTCGA -0.182566075195
ATGTTCGC -0.179866021585
ATGTTCGG 0.0304396108891
ATGTTCTA -0.0666704133573
ATGTTCTC -0.00872419345186
ATGTTCTG 0.0251397264202
ATGTTGAA -0.0459530083283
ATGTTGAC 0.118133909476
ATGTTGAG 0.0466405845127
ATGTTGCA 0.0733474521353
ATGTTGCC -0.0194809134203
ATGTTGCG 0.100652654044
ATGTTGGA 0.115246100095
ATGTTGGC -0.0162504101898
ATGTTGGG -0.146921607068
ATGTTGTA 0.109202866779
ATGTTGTC 0.0521162490252
ATGTTGTG 0.171899155493
ATGTTTAA 0.0771832438499
ATGTTTAC -0.052731795156
ATGTTTAG 0.198710303594
ATGTTTCA 0.295327007448
ATGTTTCC 0.0440564531474
ATGTTTCG -0.0437837127654
ATGTTTGA -0.0526987042139
ATGTTTGC -0.183872773242
ATGTTTGG -0.0767947498311
ATGTTTTA -0.0046351266447
ATGTTTTC 0.00530718305439
ATGTTTTG 0.0397765012445
ATTAAAAA -0.0652024651528
ATTAAAAC -0.127630051897
ATTAAAAG 0.221947694576
ATTAAAAT 0.154387169539
ATTAAACA 0.0581973157731
ATTAAACC 0.0209126807442
ATTAAACG -0.0547352977672
ATTAAAGA 0.225551998279
ATTAAAGC -0.0757871673943
ATTAAAGG -0.0740988885856
ATTAAATA 0.122671036401
ATTAAATC 0.2644393705
ATTAAATG 0.10233863492
ATTAACAA 0.230716942838
ATTAACAC 0.0961195355135
ATTAACAG 0.231331882847
ATTAACCA -0.0557127375309

ATTAACCC -0.130003666851
ATTAACCG -0.191357412048
ATTAACGA -0.0300838028111
ATTAACGC -0.23738914939
ATTAACGG -0.10190234249
ATTAACTA -0.0774893350651
ATTAACTC -0.00598308066247
ATTAACTG 0.0253592792077
ATTAAGAA 0.183942896064
ATTAAGAC 0.0496240041695
ATTAAGAG 0.250612871825
ATTAAGCA 0.203507915629
ATTAAGCC -0.18086819602
ATTAAGCG 0.0774924636687
ATTAAGGA 0.250960197952
ATTAAGGC 0.00908759999669
ATTAAGGG 0.0606350151805
ATTAAGTA 0.236721093699
ATTAAGTC 0.089071772151
ATTAAGTG 0.1500032846
ATTAATAA 0.0533631535846
ATTAATAC 0.146958253019
ATTAATAG 0.211507650902
ATTAATCA 0.0880878520908
ATTAATCC 0.129505538596
ATTAATCG 0.141194913922
ATTAATGA 0.107734137209
ATTAATGC -0.0357167361299
ATTAATGG -0.0505935482553
ATTAATTA 0.078479305752
ATTAATTC 0.052147599895
ATTAATTG 0.158145749055
ATTACAAA 0.259946862477
ATTACAAC 0.271890347648
ATTACAAG 0.269864893469
ATTACAAT 0.333930349082
ATTACACA 0.280505022929
ATTACACC 0.301011861925
ATTACACG 0.256169392533
ATTACAGA 0.24161489313
ATTACAGC 0.13657654416
ATTACAGG 0.0617847784355
ATTACATA 0.201341164084
ATTACATC 0.215010620205
ATTACATG 0.252215644913
ATTACCAA 0.33323510435
ATTACCAC 0.191551279899
ATTACCAG 0.204981140035
ATTACCCA 0.209500227947
ATTACCCC 0.30799749361
ATTACCCG 0.101044570742
ATTACCGA 0.148816860938
ATTACCGC 0.29892564741
ATTACCGG 0.178513223968

ATTACCTA 0.170345276406
ATTACCTC 0.160194302315
ATTACCTG 0.280705531996
ATTACGAA 0.290134487104
ATTACGAC 0.330062115428
ATTACGAG 0.365567907272
ATTACGCA 0.339507164077
ATTACGCC 0.0865368914829
ATTACGCG 0.360516922987
ATTACGGA 0.38595206777
ATTACGGC 0.244355963008
ATTACGGG 0.276267672971
ATTACGTA 0.332059151989
ATTACGTC 0.207842829055
ATTACGTG 0.334548397252
ATTACTAA 0.164386911236
ATTACTAC 0.261902398266
ATTACTAG 0.254294431509
ATTACTCA 0.270056557935
ATTACTCC 0.19004766319
ATTACTCG 0.228386788993
ATTACTGA 0.124876392664
ATTACTGC 0.0426984182965
ATTACTGG 0.103440235445
ATTACTTA 0.214678866194
ATTACTTC 0.155919943359
ATTACTTG 0.195105573893
ATTAGAAA -0.00756844740626
ATTAGAAC 0.130410285686
ATTAGAAG 0.112317978438
ATTAGAAT 0.148147850777
ATTAGACA 0.0898746742772
ATTAGACC -0.0571907184514
ATTAGACG -0.0161872367758
ATTAGAGA 0.0485938264126
ATTAGAGC 0.0113836314496
ATTAGAGG -0.0809638839942
ATTAGATA 0.286356503142
ATTAGATC 0.228524667919
ATTAGATG 0.0598081793829
ATTAGCAA 0.208558206755
ATTAGCAC 0.00126159217068
ATTAGCAG 0.10363984912
ATTAGCCA 0.0868616015708
ATTAGCCC -0.237116434127
ATTAGCCG -0.0550178277451
ATTAGCGA 0.211518725867
ATTAGCGC -0.164170618743
ATTAGCGG -0.128422055868
ATTAGCTA -0.0122267437533
ATTAGCTC 0.0317245620276
ATTAGCTG 0.0688444778581
ATTAGGAA -0.00791484339887
ATTAGGAC 0.00354663766487

ATTAGGAG 0.0260386182214
ATTAGGCA 0.153295168447
ATTAGGCC -0.255127749248
ATTAGGCG -0.0305746517868
ATTAGGGA -0.0803479816858
ATTAGGGC -0.119535219694
ATTAGGGG -0.0679603785235
ATTAGGTA 0.150769226527
ATTAGGTC -0.02046368216
ATTAGGTG 0.0885610128034
ATTAGTAA 0.00900547816834
ATTAGTAC 0.174255308278
ATTAGTAG 0.123962805781
ATTAGTCA -0.00304574546999
ATTAGTCC -0.0483675539952
ATTAGTCG -0.0457220305705
ATTAGTGA -0.0415305112275
ATTAGTGC -0.0799697816836
ATTAGTGG 0.143625322073
ATTAGTTA -0.050504733473
ATTAGTTC -0.0282015722283
ATTAGTTG 0.176086554874
ATTATAAA 0.227778223835
ATTATAAC 0.123792179733
ATTATAAG 0.268638642949
ATTATAAT 0.378456114818
ATTATACA 0.0425177243359
ATTATACC 0.0209010663556
ATTATACG 0.109932629212
ATTATAGA 0.209080823048
ATTATAGC 0.0255861922529
ATTATAGG 0.133242057484
ATTATATA 0.133644663948
ATTATATC 0.267795343553
ATTATATG 0.148565921293
ATTATCAA 0.107345824544
ATTATCAC 0.129042265406
ATTATCAG 0.184401071509
ATTATCCA 0.260181669273
ATTATCCC 0.306462632205
ATTATCCG 0.285970543546
ATTATCGA 0.0708325405295
ATTATCGC 0.0101129172354
ATTATCGG 0.0454172536368
ATTATCTA 0.189195314613
ATTATCTC 0.183970471849
ATTATCTG 0.26873016267
ATTATGAA 0.00646514282878
ATTATGAC 0.056503142267
ATTATGAG 0.0843423305975
ATTATGCA 0.185183536127
ATTATGCC 0.0802602862711
ATTATGCG 0.170673428249
ATTATGGA 0.255518604003

ATTATGGC 0.078482223017
ATTATGGG 0.00929541688868
ATTATGTA 0.236190268429
ATTATGTC 0.0590257147654
ATTATGTG 0.144032462214
ATTATTAA 0.258052708638
ATTATTAC 0.214715736152
ATTATTAG 0.250443420947
ATTATTCA 0.198596668294
ATTATTCC 0.221614855149
ATTATTCG 0.21049393079
ATTATTGA 0.122777047019
ATTATTGC -0.00350138318139
ATTATTGG 0.172159003817
ATTATTTA 0.0880207002766
ATTATTTC 0.176007048721
ATTATTTG 0.148428733595
ATTCAAAA 0.00724553754857
ATTCAAAC -0.116353007679
ATTCAAAG 0.105921038225
ATTCAAAT 0.213992229144
ATTCAACA 0.0263583142371
ATTCAACC 0.181633248907
ATTCAACG 0.0557888924734
ATTCAAGA -0.0179973361792
ATTCAAGC -0.192144649507
ATTCAAGG -0.182701559653
ATTCAATA 0.0351935957997
ATTCAATC 0.0532364320243
ATTCAATG 0.0751742662569
ATTCACAA 0.23353165248
ATTCACAC 0.195621241076
ATTCACAG 0.243030444589
ATTCACCA 0.104872089721
ATTCACCC 0.0306555330905
ATTCACCG 0.0291227199404
ATTCACGA -0.0083386782212
ATTCACGC 0.0279285855054
ATTCACGG 0.0376060214571
ATTCACTA 0.0434143777873
ATTCACTC -0.0400414188293
ATTCACTG -0.0357699297093
ATTCAGAA 0.142218053619
ATTCAGAC 0.0808500543784
ATTCAGAG 0.191029872848
ATTCAGCA 0.0449186118357
ATTCAGCC -0.0432818351754
ATTCAGCG -0.0648109895404
ATTCAGGA 0.262767238674
ATTCAGGC -0.0580929247397
ATTCAGGG 0.0628408590661
ATTCAGTA 0.203636228251
ATTCAGTC 0.108642146522
ATTCAGTG 0.155070326927

ATTCATAA 0.159759275344
ATTCATAC 0.128512810331
ATTCATAG 0.0665196877318
ATTCATCA 0.177846299748
ATTCATCC 0.042149539668
ATTCATCG 0.122603319573
ATTCATGA 0.0296376753853
ATTCATGC 0.0951626557687
ATTCATGG 0.0111144202053
ATTCATTA -0.0895525062791
ATTCATTC 0.0137186776932
ATTCATTG 0.0314663622602
ATTCCAAA 0.0963825609658
ATTCCAAC 0.21380313671
ATTCCAAG 0.154103924732
ATTCCAAT 0.131894192098
ATTCCACA 0.13445814961
ATTCCACC 0.251288657913
ATTCCACG 0.264095676737
ATTCCAGA 0.0560318968651
ATTCCAGC 0.0355888775839
ATTCCAGG 0.104537126924
ATTCCATA 0.260112415316
ATTCCATC 0.138968489741
ATTCCATG 0.19800369335
ATTCCCAA 0.332456223586
ATTCCCAC 0.35038867762
ATTCCCAG 0.255722664814
ATTCCCCA 0.202668350905
ATTCCCCC 0.353652550622
ATTCCCCG 0.275138347768
ATTCCCGA 0.2855988555
ATTCCCGC 0.163255542043
ATTCCCGG 0.259774300816
ATTCCCTA 0.369150310577
ATTCCCTC 0.363911099384
ATTCCCTG 0.284136753834
ATTCCGAA 0.182740591832
ATTCCGAC 0.0954745516522
ATTCCGAG 0.246264170507
ATTCCGCA 0.192527237982
ATTCCGCC 0.161282590209
ATTCCGCG 0.262434610919
ATTCCGGA 0.250195251199
ATTCCGGC 0.0378962504471
ATTCCGGG 0.141407247468
ATTCCGTA 0.28848638351
ATTCCGTC 0.269704313044
ATTCCGTG 0.287455749144
ATTCCTAA 0.228233688313
ATTCCTAC 0.168009607404
ATTCCTAG 0.221053023465
ATTCCTCA 0.354400268607
ATTCCTCC 0.15336817442

ATTCCTCG 0.229980669375
ATTCCTGA 0.100547027459
ATTCCTGC 0.152874754567
ATTCCTGG 0.20200635283
ATTCCTTA 0.243310803917
ATTCCTTC 0.0784986088016
ATTCCTTG 0.30939337916
ATTCGAAA 0.186859411838
ATTCGAAC 0.130411012898
ATTCGAAG 0.191282672642
ATTCGAAT 0.0774905664018
ATTCGACA 0.13200221893
ATTCGACC -0.13967139748
ATTCGACG 0.111677793496
ATTCGAGA 0.243480069779
ATTCGAGC -0.0158514777049
ATTCGAGG 0.0759009698404
ATTCGATA 0.204237133493
ATTCGATC 0.204003574177
ATTCGATG 0.0915713186568
ATTCGCAA 0.370218900316
ATTCGCAC 0.291622081268
ATTCGCAG 0.329726356357
ATTCGCCA 0.284939209182
ATTCGCCC 0.269383152194
ATTCGCCG 0.23392870437
ATTCGCGA 0.359168047415
ATTCGCGC 0.186462340241
ATTCGCGG 0.254341145814
ATTCGCTA 0.335107835108
ATTCGCTC 0.191779012138
ATTCGCTG 0.327224221367
ATTCGGAA -0.00446683375934
ATTCGGAC 0.100324842749
ATTCGGAG 0.034343043583
ATTCGGCA 0.219231580515
ATTCGGCC 0.000667676040607
ATTCGGCG 0.180506953234
ATTCGGGA 0.323992175354
ATTCGGGC 0.195942005608
ATTCGGGG 0.0765379704774
ATTCGGTA 0.279732900945
ATTCGGTC 0.0730248154491
ATTCGGTG 0.121471062677
ATTCGTAA 0.211098297118
ATTCGTAC 0.131324861499
ATTCGTAG 0.172077035713
ATTCGTCA 0.153020736772
ATTCGTCC 0.0167521879083
ATTCGTCG 0.17392185574
ATTCGTGA 0.167438788651
ATTCGTGC 0.0338699581124
ATTCGTGG 0.178802769712
ATTCGTTA 0.16673502898

ATTCGTTC 0.135022590746
ATTCGTTG 0.0478159547189
ATTCTAAA 0.210459771066
ATTCTAAC 0.194598114506
ATTCTAAG 0.16630803705
ATTCTACA 0.122338196974
ATTCTACC 0.234817485748
ATTCTACG 0.241903863811
ATTCTAGA 0.231565002226
ATTCTAGC 0.208903117994
ATTCTAGG 0.0812004116698
ATTCTATA 0.228175295431
ATTCTATC 0.197535000565
ATTCTATG 0.147788284152
ATTCTCAA 0.317860922754
ATTCTCAC 0.287236272085
ATTCTCAG 0.298448209163
ATTCTCCA 0.350174950989
ATTCTCCC 0.269727670197
ATTCTCCG 0.299014620118
ATTCTCGA 0.297502736897
ATTCTCGC 0.234536189082
ATTCTCGG 0.173899795112
ATTCTCTA 0.239679073012
ATTCTCTC 0.302154771852
ATTCTCTG 0.373718093764
ATTCTGAA 0.100730206791
ATTCTGAC 0.313744482573
ATTCTGAG 0.127639774673
ATTCTGCA 0.210003430582
ATTCTGCC 0.219530229265
ATTCTGCG 0.228876010014
ATTCTGGA 0.287415514688
ATTCTGGC 0.21528001831
ATTCTGGG 0.121570330577
ATTCTGTA 0.198184711284
ATTCTGTC 0.349693802325
ATTCTGTG 0.182371918863
ATTCTTAA 0.253078147018
ATTCTTAC 0.256346941308
ATTCTTAG 0.26160230068
ATTCTTCA 0.376568028083
ATTCTTCC 0.150390059481
ATTCTTCG 0.0665663874262
ATTCTTGA 0.189378083317
ATTCTTGC 0.0748239089655
ATTCTTGG 0.226832259148
ATTCTTTA 0.312412963928
ATTCTTTC 0.181859071395
ATTCTTTG 0.263660649767
ATTGAAAA 0.225620606848
ATTGAAAC -0.0241783026649
ATTGAAAG -0.00730421961563
ATTGAAAT 0.247869081202

ATTGAACA -0.135800631297
ATTGAACC 0.128612512135
ATTGAACG 0.099392781211
ATTGAAGA 0.0579775625351
ATTGAAGC -0.120677928004
ATTGAAGG -0.124057736232
ATTGAATA 0.0846455917024
ATTGAATC 0.075542141413
ATTGAATG -0.00977423704696
ATTGACAA 0.225492142508
ATTGACAC -0.073171975857
ATTGACAG 0.0555125932525
ATTGACCA -0.0408815865346
ATTGACCC -0.284698965543
ATTGACCG -0.0645245814533
ATTGACGA 0.00336873712683
ATTGACGC -0.224627493143
ATTGACGG -0.158534232036
ATTGACTA -0.00980489478333
ATTGACTC -0.142945372231
ATTGACTG 0.00673747425367
ATTGAGAA 0.107985226601
ATTGAGAC 0.123758603826
ATTGAGAG -0.0265458295761
ATTGAGCA -0.0964580635838
ATTGAGCC -0.298912399364
ATTGAGCG 0.0388718128249
ATTGAGGA 0.140167779405
ATTGAGGC 0.0713057378306
ATTGAGGG 0.0435727955227
ATTGAGTA 0.068564192025
ATTGAGTC -0.134587175257
ATTGAGTG -0.023109712926
ATTGATAA 0.0913806008655
ATTGATAC 0.134107282322
ATTGATAG 0.124737685344
ATTGATCA 0.0924409257743
ATTGATCC -0.0121318511273
ATTGATCG 0.113671225397
ATTGATGA -0.0336493518312
ATTGATGC -0.118836757593
ATTGATGG -0.240519454364
ATTGATTA 0.193980481859
ATTGATTC 0.321452306301
ATTGATTG 0.181066741673
ATTGCAAA 0.20040656044
ATTGCAAC 0.0837609395415
ATTGCAAG 0.169586797369
ATTGCAAT 0.262744660266
ATTGCACA 0.262466515332
ATTGCACC 0.226018029048
ATTGCACG 0.283514229615
ATTGCAGA 0.281432523375
ATTGCAGC 0.0535921596528

ATTGCAGG -0.00487677760405
ATTGCATA 0.332900009489
ATTGCATC 0.173449486508
ATTGCATG 0.250894144834
ATTGCCAA 0.239713728896
ATTGCCAC 0.160678963234
ATTGCCAG 0.213931562416
ATTGCCCA 0.21165594914
ATTGCCCC 0.225883374817
ATTGCCCG -0.0187184286944
ATTGCCGA 0.166284679898
ATTGCCGC -0.0203605809666
ATTGCCGG -0.0278907056284
ATTGCCTA 0.150028101574
ATTGCCTC 0.180292973878
ATTGCCTG 0.215158684856
ATTGCGAA 0.298534071261
ATTGCGAC 0.325395643577
ATTGCGAG 0.203651309712
ATTGCGCA 0.264273775027
ATTGCGCC 0.25433092706
ATTGCGCG 0.383188974098
ATTGCGGA 0.33666310939
ATTGCGGC 0.175863191015
ATTGCGGG 0.192791965519
ATTGCGTA 0.392520619793
ATTGCGTC 0.271181652957
ATTGCGTG 0.384464104028
ATTGCTAA 0.12486660876
ATTGCTAC 0.298950465617
ATTGCTAG 0.128288412327
ATTGCTCA 0.256017021525
ATTGCTCC 0.00593568775387
ATTGCTCG 0.0637786546877
ATTGCTGA 0.181564384445
ATTGCTGC -0.0610796843865
ATTGCTGG 0.0492595052663
ATTGCTTA 0.16120114605
ATTGCTTC 0.120411231871
ATTGCTTG 0.207396188405
ATTGGAAA 0.238948314706
ATTGGAAC 0.100560571666
ATTGGAAG 0.102140099122
ATTGGACA -0.0795220487878
ATTGGACC -0.253585163131
ATTGGACG -0.100167879535
ATTGGAGA 0.110210761726
ATTGGAGC -0.0241969931903
ATTGGAGG -0.185988360392
ATTGGATA 0.354022066143
ATTGGATC 0.200902003932
ATTGGATG 0.0292013776862
ATTGGCAA 0.096204462676
ATTGGCAC -0.139846986876

ATTGGCAG -0.0357993598174
ATTGGCCA -0.0804544426034
ATTGGCCC -0.185146123367
ATTGGCCG -0.120838421902
ATTGGCGA 0.213942030466
ATTGGCGC -0.0475869447133
ATTGGCGG -0.0796961200301
ATTGGCTA -0.089257169551
ATTGGCTC -0.305941160419
ATTGGCTG 0.095275946437
ATTGGGAA 0.0247616762768
ATTGGGAC 0.0373969010333
ATTGGGAG -0.0375473529777
ATTGGGCA -0.0795639511544
ATTGGGCC -0.379187990729
ATTGGGCG -0.0385574608532
ATTGGGGA 0.090935287905
ATTGGGGC -0.0565317383499
ATTGGGGG -0.176309309919
ATTGGGTA -0.172323963709
ATTGGGTC -0.100369334978
ATTGGGTG -0.117151523025
ATTGGTAA 0.149520043459
ATTGGTAC 0.194800843777
ATTGGTAG 0.00969540812975
ATTGGTCA 0.0644790796306
ATTGGTCC -0.195875829803
ATTGGTCG 0.118141180804
ATTGGTGA 0.120300285395
ATTGGTGC -0.0509693467183
ATTGGTGG 0.118090114815
ATTGGTTA 0.215813522332
ATTGGTTC -0.0635555425794
ATTGGTTG -0.102689759559
ATTGTAAA 0.0151755800968
ATTGTAAC 0.201048882141
ATTGTAAG 0.127742139613
ATTGTACA 0.0806106435625
ATTGTACC 0.185302382272
ATTGTACG 0.163960643198
ATTGTAGA 0.177120004598
ATTGTAGC -0.0974336328402
ATTGTAGG -0.0489746306149
ATTGTATA 0.222957891433
ATTGTATC 0.041078662511
ATTGTATG 0.116574819529
ATTGTCAA 0.209691028664
ATTGTCAC 0.081712038117
ATTGTCAG 0.16158113326
ATTGTCCA 0.213057410027
ATTGTCCC 0.153587471769
ATTGTCCG 0.143806340007
ATTGTCGA 0.177570132116
ATTGTCGC -0.0416318539659

ATTGTCGG 0.101623322117
ATTGTCTA 0.170780973811
ATTGTCTC 0.0239870659767
ATTGTCTG 0.255740589074
ATTGTGAA 0.173577950848
ATTGTGAC 0.233553366256
ATTGTGAG 0.249342715123
ATTGTGCA 0.244884418589
ATTGTGCC 0.155217200672
ATTGTGCG 0.258323982501
ATTGTGGA 0.315148573389
ATTGTGGC 0.0899811657387
ATTGTGGG 0.151152164551
ATTGTGTA 0.243672046702
ATTGTGTC 0.256300096781
ATTGTGTG 0.234137208674
ATTGTTAA 0.123894367277
ATTGTTAC 0.159452841271
ATTGTTAG 0.17553995168
ATTGTTCA 0.0129829273812
ATTGTTCC 0.0608475726809
ATTGTTCG 0.194111844554
ATTGTTGA 0.052587899535
ATTGTTGC 0.137783789299
ATTGTTGG 0.149867498352
ATTGTTTA -0.0171480019965
ATTGTTTC 0.0293942477107
ATTGTTTG -0.0360696826066
ATTTAAAA 0.218140478676
ATTTAAAC 0.167698587984
ATTTAAAG 0.237257919076
ATTTAAAT 0.121648103589
ATTTAACA 0.192786289351
ATTTAACC 0.096389778208
ATTTAACG -0.00422323149596
ATTTAAGA 0.221057203127
ATTTAAGC 0.00898301497048
ATTTAAGG -0.0995001817388
ATTTAATA 0.201293580081
ATTTAATC 0.175149514852
ATTTAATG 0.164677271261
ATTTACAA 0.206172857529
ATTTACAC 0.223505875021
ATTTACAG 0.266551675643
ATTTACCA 0.200167149624
ATTTACCC 0.0748861809468
ATTTACCG 0.0385385721481
ATTTACGA 0.18063765027
ATTTACGC 0.185714911352
ATTTACGG -0.0496209948284
ATTTACTA 0.132274114698
ATTTACTC 0.0191775797836
ATTTACTG 0.218161687859
ATTTAGAA 0.255885361946

ATTTAGAC 0.143702589974
ATTTAGAG 0.163443866193
ATTTAGCA 0.111891083704
ATTTAGCC -0.103531280244
ATTTAGCG 0.0581498215368
ATTTAGGA -0.000546703356861
ATTTAGGC 0.13754112239
ATTTAGGG 0.0922103895535
ATTTAGTA 0.129689860806
ATTTAGTC 0.0912110457565
ATTTAGTG 0.0945490244739
ATTTATAA 0.263565218111
ATTTATAC 0.284213966032
ATTTATAG 0.220789660184
ATTTATCA 0.230067224805
ATTTATCC 0.104539419808
ATTTATCG 0.184778442354
ATTTATGA 0.281225228645
ATTTATGC -0.109817691873
ATTTATGG -0.00551653581957
ATTTATTA 0.254597572779
ATTTATTC 0.25476565878
ATTTATTG 0.250335394115
ATTTCAAA 0.104921726134
ATTTCAAC 0.25169302862
ATTTCAAG 0.225654028684
ATTTCACA 0.346079282935
ATTTCACC 0.32022794144
ATTTCACG 0.299543344998
ATTTCAGA 0.244808507843
ATTTCAGC 0.154080567579
ATTTCAGG 0.117491587775
ATTTCATA 0.360499405123
ATTTCATC 0.276183003292
ATTTCATG 0.318948726959
ATTTCCAA 0.383514901854
ATTTCCAC 0.321314427375
ATTTCCAG 0.338261063814
ATTTCCCA 0.331718771113
ATTTCCCC 0.363906629782
ATTTCCCG 0.308243615103
ATTTCCGA 0.349157697643
ATTTCCGC 0.348447114297
ATTTCCGG 0.261075124711
ATTTCCTA 0.381413093534
ATTTCCTC 0.384212035727
ATTTCCTG 0.361845316391
ATTTCGAA 0.273078863988
ATTTCGAC 0.371757552754
ATTTCGAG 0.31212674175
ATTTCGCA 0.347524506763
ATTTCGCC 0.127514725955
ATTTCGCG 0.291573943089
ATTTCGGA 0.321396611753

ATTTCGGC 0.253102965224
ATTTCGGG 0.313621599527
ATTTCGTA 0.385279218613
ATTTCGTC 0.295630752611
ATTTCGTG 0.382747761536
ATTTCTAA 0.0734721137493
ATTTCTAC 0.324354211222
ATTTCTAG 0.298848435212
ATTTCTCA 0.324458066882
ATTTCTCC 0.218915939174
ATTTCTCG 0.336342269877
ATTTCTGA 0.202731052645
ATTTCTGC 0.254560119121
ATTTCTGG 0.227547571951
ATTTCTTA 0.312941322453
ATTTCTTC 0.239728327117
ATTTCTTG 0.332052438113
ATTTGAAA 0.184161660694
ATTTGAAC 0.232654029474
ATTTGAAG 0.149995985489
ATTTGACA 0.0948426681887
ATTTGACC 0.152460165106
ATTTGACG 0.134800089346
ATTTGAGA 0.194700004833
ATTTGAGC 0.105710823851
ATTTGAGG 0.0224133778092
ATTTGATA 0.260082396446
ATTTGATC 0.156171032751
ATTTGATG 0.121690561085
ATTTGCAA 0.305834908725
ATTTGCAC 0.363515397473
ATTTGCAG 0.264854784202
ATTTGCCA 0.394551214207
ATTTGCCC 0.108330207036
ATTTGCCG 0.253891520624
ATTTGCGA 0.258341191652
ATTTGCGC 0.220737266192
ATTTGCGG 0.253864056894
ATTTGCTA 0.17607552456
ATTTGCTC 0.19672780888
ATTTGCTG 0.188716305482
ATTTGGAA 0.0829410677896
ATTTGGAC 0.14541214426
ATTTGGAG 0.0536214535448
ATTTGGCA 0.158769515996
ATTTGGCC -0.0730714835124
ATTTGGCG 0.100602981729
ATTTGGGA 0.232407338468
ATTTGGGC 0.0804267059845
ATTTGGGG 0.0779654460121
ATTTGGTA 0.23547440567
ATTTGGTC 0.177771435347
ATTTGGTG 0.17231082531
ATTTGTAA 0.230918301059

ATTTGTAC 0.210393727736
ATTTGTAG 0.298646744962
ATTTGTCA 0.212890958592
ATTTGTCC 0.0404772158274
ATTTGTCG 0.162708115881
ATTTGTGA 0.228662875995
ATTTGTGC 0.0305533455472
ATTTGTGG 0.126339433255
ATTTGTTA 0.294679194481
ATTTGTTC 0.11591206032
ATTTGTTG 0.177859362611
ATTTTAAA 0.251685435823
ATTTTAAC 0.20026349788
ATTTTAAG 0.23383300656
ATTTTACA 0.263173069203
ATTTTACC 0.19642124625
ATTTTACG 0.155048429596
ATTTTAGA 0.20439861349
ATTTTAGC 0.00500881133808
ATTTTAGG 0.129857740341
ATTTTATA 0.230430142222
ATTTTATC 0.246788110424
ATTTTATG 0.25324043999
ATTTTCAA 0.319256512631
ATTTTCAC 0.355223608242
ATTTTCAG 0.325285340437
ATTTTCCA 0.298378137705
ATTTTCCC 0.318549003369
ATTTTCCG 0.344698693184
ATTTTCGA 0.208759662197
ATTTTCGC 0.137530092076
ATTTTCGG 0.193273540258
ATTTTCTA 0.365874010472
ATTTTCTC 0.278001941563
ATTTTCTG 0.371094793545
ATTTTGAA 0.0126846925459
ATTTTGAC 0.172747127293
ATTTTGAG 0.236326941746
ATTTTGCA 0.252286150334
ATTTTGCC 0.15025291417
ATTTTGCG 0.276401976599
ATTTTGGA 0.356426501609
ATTTTGGC 0.161476026072
ATTTTGGG 0.260790250925
ATTTTGTA 0.34529097903
ATTTTGTC 0.365740625853
ATTTTGTG 0.28815454573
ATTTTTAA 0.240316073649
ATTTTTAC 0.347489362641
ATTTTTAG 0.237649540521
ATTTTTCA 0.349433455494
ATTTTTCC 0.0518686730808
ATTTTTCG 0.299597364568
ATTTTTGA 0.27653113135

ATTTTTGC 0.0218644847193
ATTTTTGG 0.117728078947
ATTTTTTA 0.224998722656
ATTTTTTC 0.285660168026
ATTTTTTG 0.300208754553
CAAAAAAA 0.0780150799618
CAAAAAAC 0.262073823201
CAAAAAAG 0.126516323486
CAAAAACA -0.0910790274666
CAAAAACC -0.151621318288
CAAAAACG -0.13036165772
CAAAAAGA 0.0976847222324
CAAAAAGC 0.0670372181631
CAAAAAGG 0.0477763260659
CAAAAATA 0.143923125771
CAAAAATC 0.402660247661
CAAAAATG 0.055647686547
CAAAACAA 0.108669275336
CAAAACAC 0.0621818500325
CAAAACAG 0.123196198954
CAAAACCA -0.0776161836768
CAAAACCC -0.106425536725
CAAAACCG 0.0471194061444
CAAAACGA -0.0369474157353
CAAAACGC -0.0603406205282
CAAAACGG -0.195765832697
CAAAACTA 0.201406640801
CAAAACTC -0.179912758876
CAAAACTG -0.0120878454212
CAAAAGAA -0.0380389075016
CAAAAGAC -0.106340007153
CAAAAGAG -0.0801221567898
CAAAAGCA 0.0633346845468
CAAAAGCC -0.199305220116
CAAAAGCG -0.0110136274388
CAAAAGGA 0.155613380729
CAAAAGGC -0.400190412268
CAAAAGGG -0.185001392232
CAAAAGTA -0.100471284682
CAAAAGTC -0.0747120564809
CAAAAGTG -0.0977703907367
CAAAATAA 0.258370254666
CAAAATAC 0.246534017503
CAAAATAG 0.211863243871
CAAAATCA 0.311009356464
CAAAATCC 0.423656438808
CAAAATCG 0.364876268957
CAAAATGA 0.109935548857
CAAAATGC -0.177887783319
CAAAATGG -0.0937197573141
CAAAATTA 0.058707473559
CAAAATTC 0.193539557528
CAAAATTG 0.186081128213
CAAACAAA -0.0742122435275

CAAACAAC -0.0736438196392
CAAACAAG 0.042630785055
CAAACACA -0.0012325764156
CAAACACC -0.13848717391
CAAACACG -0.0275506763414
CAAACAGA 0.0215024488515
CAAACAGC -0.0866169199503
CAAACAGG 0.106035787854
CAAACATA -0.157361686641
CAAACATC -0.130996401539
CAAACATG 0.0969082029688
CAAACCAA 0.0444012174916
CAAACCAC 0.0189362916636
CAAACCAG 0.143286537226
CAAACCCA -0.153871811624
CAAACCCC -0.350237584434
CAAACCCG -0.134878787127
CAAACCGA -0.0637485420751
CAAACCGC -0.10727470231
CAAACCGG -0.131669447352
CAAACCTA 0.129567235754
CAAACCTC -0.0915121066913
CAAACCTG -0.0238308650176
CAAACGAA -0.0870294811063
CAAACGAC 0.0293364990335
CAAACGAG -0.103050292753
CAAACGCA 0.0530024889966
CAAACGCC -0.207532397655
CAAACGCG 0.132603665613
CAAACGGA 0.0673193855012
CAAACGGC -0.237711592936
CAAACGGG -0.0485102064539
CAAACGTA -0.105227622753
CAAACGTC -0.0385692284111
CAAACGTG 0.0713261470837
CAAACTAA 0.160489186368
CAAACTAC 0.0656649854383
CAAACTAG 0.0959788990092
CAAACTCA 0.0172996688148
CAAACTCC -0.210559180872
CAAACTCG -0.0184849966789
CAAACTGA -0.0840689174023
CAAACTGC -0.281866049153
CAAACTGG -0.227356173559
CAAACTTA -0.0687816244089
CAAACTTC -0.216723143922
CAAACTTG -0.0406217382101
CAAAGAAA 0.0903242994679
CAAAGAAC 0.1200754728
CAAAGAAG -0.0100968591929
CAAAGACA 0.0291709751692
CAAAGACC -0.130672487859
CAAAGACG -0.0445931604892
CAAAGAGA 0.101281723758

CAAAGAGC -0.186128563819
CAAAGAGG -0.101656898024
CAAAGATA 0.280797326252
CAAAGATC 0.260857791435
CAAAGATG -0.0158133339952
CAAAGCAA 0.00656846930359
CAAAGCAC -0.0286994983965
CAAAGCAG 0.0514083633205
CAAAGCCA -0.196944134309
CAAAGCCC -0.285471935183
CAAAGCCG -0.00832609504901
CAAAGCGA 0.108776707944
CAAAGCGC -0.176084484468
CAAAGCGG -0.0900129304759
CAAAGCTA -0.272662067879
CAAAGCTC -0.210951057014
CAAAGCTG 0.0646720161623
CAAAGGAA -0.154194476838
CAAAGGAC 0.0341429583854
CAAAGGAG -0.141610902945
CAAAGGCA -0.275462101476
CAAAGGCC -0.293229093455
CAAAGGCG -0.250634711919
CAAAGGGA -0.110093716139
CAAAGGGC -0.304649085812
CAAAGGGG -0.209043699856
CAAAGGTA -0.176382290914
CAAAGGTC -0.0636633291925
CAAAGGTG -0.0858415423935
CAAAGTAA 0.0647194580242
CAAAGTAC -0.0490311247887
CAAAGTAG 0.0186178404852
CAAAGTCA 0.0878054362903
CAAAGTCC -0.229834007394
CAAAGTCG 0.0143463022251
CAAAGTGA 0.107039546433
CAAAGTGC -0.23012011622
CAAAGTGG -0.206097228081
CAAAGTTA -0.269084696482
CAAAGTTC -0.0175185944833
CAAAGTTG 0.129017048416
CAAATAAA 0.121941500729
CAAATAAC 0.176031403304
CAAATAAG 0.0654635299957
CAAATACA 0.116224462238
CAAATACC -0.0855741706068
CAAATACG 0.183822981978
CAAATAGA 0.147849095466
CAAATAGC -0.164461041772
CAAATAGG -0.0830882170472
CAAATATA 0.252303267455
CAAATATC 0.386662335861
CAAATATG 0.254817047802
CAAATCAA 0.202032611124

CAAATCAC 0.319901753976
CAAATCAG 0.254859542738
CAAATCCA 0.378262519799
CAAATCCC 0.304871426173
CAAATCCG 0.395604216169
CAAATCGA 0.314552965993
CAAATCGC 0.29334175164
CAAATCGG 0.255138058168
CAAATCTA 0.401508726086
CAAATCTC 0.396273804223
CAAATCTG 0.435507981577
CAAATGAA 0.0107141970866
CAAATGAC 0.085430975964
CAAATGAG -0.0320926987594
CAAATGCA 0.0158188491522
CAAATGCC -0.13248806954
CAAATGCG -0.13141452493
CAAATGGA -0.0255779195173
CAAATGGC -0.344784739824
CAAATGGG -0.0356391528877
CAAATGTA -0.0998309693862
CAAATGTC -0.0213579264688
CAAATGTG -0.0100399612798
CAAATTAA -0.108436392715
CAAATTAC 0.266008432675
CAAATTAG -0.0172791836675
CAAATTCA 0.236960504514
CAAATTCC 0.168663153512
CAAATTCG 0.284420779107
CAAATTGA 0.034173291749
CAAATTGC 0.0332452574031
CAAATTGG 0.00998299307314
CAAATTTA 0.0838069474433
CAAATTTC 0.229285759589
CAAATTTG 0.152386031935
CAACAAAA 0.189787814865
CAACAAAC 0.199090274848
CAACAAAG 0.0429005204266
CAACAACA -0.198940696075
CAACAACC -0.248288742781
CAACAACG -0.0317673044946
CAACAAGA 0.0487250335735
CAACAAGC 0.0348569009438
CAACAAGG -0.19524065572
CAACAATA 0.124043269125
CAACAATC 0.110367656183
CAACAATG 0.120794348067
CAACACAA 0.104718144858
CAACACAC -0.161846812722
CAACACAG -0.170989691461
CAACACCA -0.137447803477
CAACACCC -0.28277635149
CAACACCG -0.0893811975494
CAACACGA -0.0143559073876

CAACACGC -0.280456165797
CAACACGG -0.175718479924
CAACACTA 0.00607908183666
CAACACTC -0.195882601074
CAACACTG 0.0992496514675
CAACAGAA 0.088545959722
CAACAGAC 0.0145834539774
CAACAGAG -0.294863625859
CAACAGCA -0.148278931967
CAACAGCC -0.31906121085
CAACAGCG -0.0135676426066
CAACAGGA -0.0877346000067
CAACAGGC -0.22450999038
CAACAGGG -0.190957894783
CAACAGTA -0.135765241826
CAACAGTC -0.150364911178
CAACAGTG 0.0372568483902
CAACATAA 0.116456677063
CAACATAC 0.00650334605221
CAACATAG 0.0627161449019
CAACATCA 0.0483436943604
CAACATCC -0.0310832609505
CAACATCG -0.00903362250156
CAACATGA 0.0290685410825
CAACATGC -0.0840439933718
CAACATGG -0.0122997262132
CAACATTA 0.149797721576
CAACATTC -0.0147708316733
CAACATTG 0.0463778165442
CAACCAAA 0.07330861368
CAACCAAC -0.155688204357
CAACCAAG -0.0744866900725
CAACCACA -0.154768606256
CAACCACC -0.229297360084
CAACCACG -0.170915429957
CAACCAGA 0.0508704296583
CAACCAGC -0.0533057676337
CAACCAGG -0.117481144714
CAACCATA -0.0581191652738
CAACCATC -0.15399778432
CAACCATG -0.116237600636
CAACCCAA -0.0548854639764
CAACCCAC 0.00209968759737
CAACCCAG 0.0106413728167
CAACCCCA -0.09212222146
CAACCCCC -0.365405976015
CAACCCCG -0.0699882215745
CAACCCGA -0.204603414859
CAACCCGC 0.00750502568684
CAACCCGG -0.22728554881
CAACCCTA -0.198948967578
CAACCCTC -0.166227369251
CAACCCTG -0.0570975468424
CAACCGAA -0.0531210176206

CAACCGAC 0.0158295803742
CAACCGAG -0.296177308073
CAACCGCA -0.158951727865
CAACCGCC -0.147266066829
CAACCGCG 0.13118532397
CAACCGGA -0.0456749981384
CAACCGGC -0.210948721978
CAACCGGG -0.302895255419
CAACCGTA 0.0157664551604
CAACCGTC -0.12039449559
CAACCGTG -0.16816656603
CAACCTAA 0.0421371785008
CAACCTAC -0.100916969185
CAACCTAG -0.0812802776752
CAACCTCA -0.120824203816
CAACCTCC -0.268715284313
CAACCTCG 0.0149171209777
CAACCTGA -0.0108530470136
CAACCTGC -0.208127113585
CAACCTGG -0.247794902185
CAACCTTA -0.310854430334
CAACCTTC -0.150340096666
CAACCTTG -0.223387392226
CAACGAAA 0.149379940585
CAACGAAC -0.0886425642545
CAACGAAG 0.0679890044142
CAACGACA -0.149157271617
CAACGACC -0.118345479677
CAACGACG -0.200935752579
CAACGAGA 0.15118720028
CAACGAGC -0.193566345196
CAACGAGG 0.0183640941217
CAACGATA 0.0827729111348
CAACGATC 0.0971591426137
CAACGATG -0.0382269321663
CAACGCAA 0.161271650986
CAACGCAC -0.103802836434
CAACGCAG -0.142328009891
CAACGCCA -0.234928121356
CAACGCCC -0.184903454446
CAACGCCG -0.269991921899
CAACGCGA -0.0113992637631
CAACGCGC -0.0558602377348
CAACGCGG -0.163600067152
CAACGCTA -0.237200712729
CAACGCTC -0.28642202347
CAACGCTG -0.115195895999
CAACGGAA 0.0799794284643
CAACGGAC 0.0401210192478
CAACGGAG -0.291355513104
CAACGGCA -0.10500657454
CAACGGCC -0.248560964755
CAACGGCG -0.232518046288
CAACGGGA -0.0240643775522

CAACGGGC -0.231229378055
CAACGGGG -0.0989716135567
CAACGGTA -0.0944176654508
CAACGGTC -0.169329826564
CAACGGTG -0.232473372651
CAACGTAA 0.143855977189
CAACGTAC -0.0642793435214
CAACGTAG -0.0806748757326
CAACGTCA -0.0678678569082
CAACGTCC -0.375787157099
CAACGTCG -0.0881462582303
CAACGTGA -0.12690766777
CAACGTGC -0.237828396869
CAACGTGG -0.160084908131
CAACGTTA -0.0180400786461
CAACGTTC -0.0634358371714
CAACGTTG 0.0777478728946
CAACTAAA 0.136131999768
CAACTAAC 0.026250768675
CAACTAAG -0.145753936919
CAACTACA -0.158520530511
CAACTACC -0.0411609957065
CAACTACG 0.0557242116236
CAACTAGA 0.0320744801209
CAACTAGC -0.153588446916
CAACTAGG -0.0722614084717
CAACTATA 0.141167338137
CAACTATC -0.0278074202755
CAACTATG 0.154203790567
CAACTCAA -0.0654730258423
CAACTCAC -0.152616167065
CAACTCAG -0.131577205913
CAACTCCA -0.111293084196
CAACTCCC -0.223999242617
CAACTCCG 0.0316617658006
CAACTCGA -0.00116496127365
CAACTCGC -0.0394363627074
CAACTCGG 0.0634000876823
CAACTCTA -0.0706759662448
CAACTCTC -0.135819309485
CAACTCTG -0.02470237878
CAACTGAA -0.0487394990915
CAACTGAC -0.0273360007824
CAACTGAG -0.0444347304463
CAACTGCA -0.242803669338
CAACTGCC -0.115852107784
CAACTGCG -0.148473711637
CAACTGGA -0.113198251133
CAACTGGC -0.267542509277
CAACTGGG -0.191747378753
CAACTGTA -0.127900560954
CAACTGTC -0.202223487087
CAACTGTG -0.0973163963454
CAACTTAA -0.0698977214129

CAACTTAC -0.0332508817357
CAACTTAG -0.215175656129
CAACTTCA 0.136702845923
CAACTTCC -0.0574648943591
CAACTTCG -0.0477427501588
CAACTTGA -0.0597873290816
CAACTTGC -0.203431919846
CAACTTGG -0.128951265315
CAACTTTA -0.0802666732839
CAACTTTC -0.199915918896
CAAGAAAA -0.0576053683751
CAAGAAAC -0.149021699632
CAAGAAAG -0.0937528737394
CAAGAACA 0.0157022137633
CAAGAACC -0.149238337847
CAAGAACG 0.0568216815522
CAAGAAGA -0.0995154159129
CAAGAAGC -0.0686415625935
CAAGAAGG -0.0460697940921
CAAGAATA 0.129042265406
CAAGAATC 0.382452209076
CAAGAATG -0.172893355993
CAAGACAA 0.0234253316186
CAAGACAC -0.205374435708
CAAGACAG 0.107477208528
CAAGACCA 0.0379608258396
CAAGACCC -0.222258951962
CAAGACCG -0.288493052663
CAAGACGA -0.0770184160387
CAAGACGC -0.135785469131
CAAGACGG -0.13342319633
CAAGACTA 0.0569410888813
CAAGACTC -0.254299779967
CAAGACTG -0.0746966727043
CAAGAGAA 0.0505494202479
CAAGAGAC -0.0701971683777
CAAGAGAG -0.20130571394
CAAGAGCA -0.129091657507
CAAGAGCC -0.196927840225
CAAGAGCG -0.0716434122632
CAAGAGGA 0.0298164628308
CAAGAGGC -0.304806798566
CAAGAGGG -0.203179486331
CAAGAGTA 0.0542520966694
CAAGAGTC -0.215848039337
CAAGAGTG -0.147816844909
CAAGATAA 0.0762760005184
CAAGATAC 0.247766107312
CAAGATAG 0.173444794657
CAAGATCA 0.205781539115
CAAGATCC 0.320865236528
CAAGATCG 0.266051838281
CAAGATGA 0.0222440952596
CAAGATGC -0.275404651652

CAAGATGG -0.259472437928
CAAGATTA 0.316733265494
CAAGATTC 0.408892363438
CAAGATTG 0.318605431998
CAAGCAAA 0.0793026430078
CAAGCAAC -0.352278145732
CAAGCAAG -0.0057115537616
CAAGCACA -0.262175474574
CAAGCACC -0.152832133176
CAAGCACG -0.0780331634924
CAAGCAGA 0.0153114547054
CAAGCAGC -0.321458600394
CAAGCAGG 0.0245826733721
CAAGCATA -0.0412527468286
CAAGCATC -0.159714020861
CAAGCATG -0.204030428105
CAAGCCAA -0.221256236748
CAAGCCAC -0.276236468021
CAAGCCAG -0.22357654764
CAAGCCCA 0.028710389836
CAAGCCCC -0.121883366281
CAAGCCCG -0.322025935849
CAAGCCGA -0.145965742339
CAAGCCGC -0.326106908099
CAAGCCGG -0.255987772237
CAAGCCTA -0.172794938593
CAAGCCTC -0.315598207187
CAAGCCTG -0.196065509713
CAAGCGAA -0.0808978612412
CAAGCGAC -0.0434525636588
CAAGCGAG -0.129218960492
CAAGCGCA -0.0132578107551
CAAGCGCC -0.190317094081
CAAGCGCG 0.122413046655
CAAGCGGA -0.207071812722
CAAGCGGC -0.426449798154
CAAGCGGG -0.0617885692351
CAAGCGTA -0.148147688643
CAAGCGTC -0.240397878797
CAAGCGTG -0.370155132083
CAAGCTAA -0.185624953473
CAAGCTAC -0.21703006043
CAAGCTAG -0.107279455764
CAAGCTCA -0.105701198016
CAAGCTCC -0.252688661169
CAAGCTCG -0.194435194798
CAAGCTGA -0.275136124363
CAAGCTGC -0.266018989348
CAAGCTGG -0.299396270107
CAAGCTTA -0.108534119691
CAAGCTTC -0.104165541443
CAAGCTTG -0.188679830328
CAAGGAAA -0.0779768940903
CAAGGAAC -0.110915361902

CAAGGAAG -0.0414687098725
CAAGGACA -0.0384840188691
CAAGGACC -0.340431102817
CAAGGACG -0.189977364775
CAAGGAGA 0.0671208398481
CAAGGAGC -0.371179237873
CAAGGAGG -0.223456883808
CAAGGATA 0.197486916345
CAAGGATC 0.0836387351539
CAAGGATG -0.0939703258859
CAAGGCAA 0.080566792688
CAAGGCAC -0.230499538127
CAAGGCAG -0.0508580104085
CAAGGCCA -0.0336824768616
CAAGGCCC -0.236590719048
CAAGGCCG -0.240843374595
CAAGGCGA -0.0824833519461
CAAGGCGC -0.100157621664
CAAGGCGG -0.204142990662
CAAGGCTA -0.378334386368
CAAGGCTC -0.263478659306
CAAGGCTG -0.278148180307
CAAGGGAA 0.0330992751984
CAAGGGAC -0.334322117384
CAAGGGAG -0.113718532747
CAAGGGCA -0.235969722574
CAAGGGCC -0.383021174836
CAAGGGCG -0.207912087231
CAAGGGGA 0.0772770015194
CAAGGGGC -0.445774953503
CAAGGGGG -0.137345717303
CAAGGGTA -0.0784715156629
CAAGGGTC -0.234659333692
CAAGGGTG -0.29127082935
CAAGGTAA 0.148909446931
CAAGGTAC -0.226076360458
CAAGGTAG -0.108764634362
CAAGGTCA -0.220014312634
CAAGGTCC -0.229551062041
CAAGGTCG 0.0214608547942
CAAGGTGA -0.158664716538
CAAGGTGC -0.327541157112
CAAGGTGG -0.0511691190458
CAAGGTTA -0.304703839109
CAAGGTTC -0.184829868261
CAAGTAAA 0.156212686516
CAAGTAAC 0.0369581034814
CAAGTAAG -0.0119251482888
CAAGTACA 0.151488055972
CAAGTACC -0.298627134764
CAAGTACG -0.0528900826989
CAAGTAGA 0.0674470226524
CAAGTAGC -0.236830122924
CAAGTAGG -0.153000107546

CAAGTATA 0.183774683775
CAAGTATC 0.267482463888
CAAGTATG 0.147923038182
CAAGTCAA -0.198411910533
CAAGTCAC -0.0475889112253
CAAGTCAG -0.123254454673
CAAGTCCA 0.0312920155033
CAAGTCCC -0.0372934536561
CAAGTCCG -0.122095668213
CAAGTCGA -0.281373156977
CAAGTCGC -0.303131917052
CAAGTCGG -0.00464250317115
CAAGTCTA -0.16456746396
CAAGTCTC -0.145319517158
CAAGTCTG -0.197878277927
CAAGTGAA -0.167014975085
CAAGTGAC -0.0639964088378
CAAGTGAG 0.0268960420476
CAAGTGCA -0.000780506029164
CAAGTGCC -0.181990083446
CAAGTGCG -0.143263474584
CAAGTGGA -0.0673146654534
CAAGTGGC -0.31082982229
CAAGTGGG -0.110690705592
CAAGTGTA -0.1369033456
CAAGTGTC -0.222071484972
CAAGTGTG -0.295871338356
CAAGTTAA -0.0389824304539
CAAGTTAC -0.0836456135997
CAAGTTAG 0.0228037955311
CAAGTTCA 0.104183849989
CAAGTTCC -0.1396274286
CAAGTTCG -0.0333316789505
CAAGTTGA -0.0474445988535
CAAGTTGC -0.232182970208
CAAGTTGG -0.199709684623
CAAGTTTA -0.120033306589
CAAGTTTC -0.0593251653858
CAATAAAA 0.0695530240985
CAATAAAC 0.0285409376318
CAATAAAG 0.14216841967
CAATAACA -0.106170909201
CAATAACC -0.0582710141902
CAATAACG -0.139109027237
CAATAAGA 0.0415808412954
CAATAAGC 0.191749815697
CAATAAGG 0.0170515174827
CAATAATA 0.0909656212687
CAATAATC 0.26001897214
CAATAATG 0.156895104487
CAATACAA -0.0427410881956
CAATACAC 0.0369678036247
CAATACAG 0.193837361226
CAATACCA 0.162450329117

CAATACCC 0.0332108968473
CAATACCG -0.113328094194
CAATACGA 0.0750071345261
CAATACGC -0.196750612699
CAATACGG -0.0467602899207
CAATACTA -0.0724195820529
CAATACTC 0.0370108400411
CAATACTG 0.188992211849
CAATAGAA 0.0311766019657
CAATAGAC -0.006091107494
CAATAGAG 0.0527280424516
CAATAGCA 0.0920989860384
CAATAGCC -0.147593038853
CAATAGCG 0.044261074575
CAATAGGA 0.0131581060269
CAATAGGC -0.191320349005
CAATAGGG -0.045728605811
CAATAGTA 0.133151057393
CAATAGTC 0.0156782126479
CAATAGTG -0.0596986927294
CAATATAA 0.216784127851
CAATATAC 0.131681638832
CAATATAG 0.271634197791
CAATATCA 0.304890289739
CAATATCC 0.361642926272
CAATATCG 0.271220256069
CAATATGA 0.143075166886
CAATATGC -0.151765507857
CAATATGG 0.105455835115
CAATATTA 0.0851915651482
CAATATTC 0.0935494723374
CAATATTG 0.249609307171
CAATCAAA 0.188041867696
CAATCAAC 0.208638360176
CAATCAAG 0.180066986358
CAATCACA 0.120641883754
CAATCACC 0.0877392544059
CAATCACG 0.0902513083655
CAATCAGA 0.134755968089
CAATCAGC 0.263138033474
CAATCAGG 0.264080885293
CAATCATA 0.252008350182
CAATCATC 0.0816918847222
CAATCATG 0.103978634282
CAATCCAA 0.251368448338
CAATCCAC 0.284279176368
CAATCCAG 0.295975270615
CAATCCCA 0.282466077385
CAATCCCC 0.135972060214
CAATCCCG 0.248870082203
CAATCCGA 0.354134580995
CAATCCGC 0.229468697766
CAATCCGG 0.33127755855
CAATCCTA 0.292200170799

CAATCCTC 0.336207966249
CAATCCTG 0.326167765562
CAATCGAA 0.122332357686
CAATCGAC 0.0780331042013
CAATCGAG 0.0898805135654
CAATCGCA 0.213198046531
CAATCGCC 0.183043925468
CAATCGCG 0.255372452342
CAATCGGA 0.236524403191
CAATCGGC −0.0410121272537
CAATCGGG 0.177986613432
CAATCGTA 0.203306612398
CAATCGTC 0.188310474953
CAATCGTG 0.184462384035
CAATCTAA 0.359643949403
CAATCTAC 0.326757706109
CAATCTAG 0.374767705817
CAATCTCA 0.349411394866
CAATCTCC 0.387299302678
CAATCTCG 0.398848930315
CAATCTGA 0.410406274043
CAATCTGC 0.266349641979
CAATCTGG 0.414612129935
CAATCTTA 0.295164310316
CAATCTTC 0.323904776958
CAATGAAA 0.0193083363138
CAATGAAC −0.230528724427
CAATGAAG 0.103167813843
CAATGACA −0.0194484792304
CAATGACC −0.161079786372
CAATGACG −0.058595089938
CAATGAGA 0.0893352965343
CAATGAGC −0.306871963562
CAATGAGG −0.0235599190171
CAATGATA 0.139374912102
CAATGATC 0.110125325723
CAATGATG 0.0175142150172
CAATGCAA 0.149864601505
CAATGCAC −0.170823266644
CAATGCAG 0.217359232511
CAATGCCA −0.181278273525
CAATGCCC −0.200187002258
CAATGCCG −0.234394610811
CAATGCGA −0.0468747491055
CAATGCGC −0.261226875329
CAATGCGG −0.175257476114
CAATGCTA −0.0409397361128
CAATGCTC −0.238412198208
CAATGCTG −0.128424292061
CAATGGAA −0.130552615636
CAATGGAC −0.0828076757443
CAATGGAG −0.0609576518667
CAATGGCA −0.155437786202
CAATGGCC −0.130728766036

CAATGGCG −0.275640155578
CAATGGGA −0.0572053166719
CAATGGGC −0.366351616184
CAATGGGG 0.0140843322662
CAATGGTA −0.106730333513
CAATGGTC 0.0428337789947
CAATGGTG −0.0276555987825
CAATGTAA 0.158541578778
CAATGTAC 0.00297146777808
CAATGTAG −0.0370393349051
CAATGTCA 0.132801449239
CAATGTCC −0.165070911376
CAATGTCG −0.0948796634049
CAATGTGA −0.0407969953424
CAATGTGC −0.284738735562
CAATGTGG −0.1234920241
CAATGTTA 0.0423399487602
CAATGTTC −0.247905348416
CAATTAAA −0.0460943036701
CAATTAAC −0.0114720115618
CAATTAAG 0.226861455589
CAATTACA 0.197980336197
CAATTACC 0.0810852174489
CAATTACG 0.102324614736
CAATTAGA 0.164836536426
CAATTAGC −0.0688478622298
CAATTAGG 0.140428311789
CAATTATA 0.12180923257
CAATTATC 0.148885800401
CAATTATG 0.0896061350607
CAATTCAA 0.127870922535
CAATTCAC 0.0582028309301
CAATTCAG 0.193323944711
CAATTCCA 0.0957519178412
CAATTCCC 0.252863805902
CAATTCCG −0.0638384991439
CAATTCGA −0.119195127533
CAATTCGC −0.0964248958052
CAATTCGG 0.0181513564497
CAATTCTA 0.0182813667662
CAATTCTC 0.000272973174182
CAATTCTG 0.203480945673
CAATTGAA 0.0368564156443
CAATTGAC 0.12858844846
CAATTGAG 0.088580315853
CAATTGCA 0.097795668708
CAATTGCC −0.0756253512697
CAATTGCG 0.0841172528482
CAATTGGA 0.00401089795029
CAATTGGC −0.19531055035
CAATTGGG −0.0321452505647
CAATTGTA −0.0386629277614
CAATTGTC −0.00804030076681
CAATTGTG 0.106496208112

CAATTTAA 0.0713962467975
CAATTTAC 0.0980656128248
CAATTTAG 0.113922462407
CAATTTCA 0.1400516777
CAATTTCC 0.103327845752
CAATTTCG 0.321251003069
CAATTTGA 0.0961443537201
CAATTTGC -0.117685744108
CAATTTGG -0.0926027027102
CAATTTTA 0.0382904023999
CAATTTTC 0.108332664248
CACAAAAA 0.0754321814928
CACAAAAC 0.0990453263181
CACAAAAG -0.0335804123683
CACAAACA 0.0406627312617
CACAAACC -0.0350529592954
CACAAACG 0.028451355464
CACAAAGA 0.102855019209
CACAAAGC -0.225359830603
CACAAAGG -0.0566089505483
CACAAATA 0.274507127581
CACAAATC 0.261178952286
CACAAATG -0.224591655295
CACAACAA 0.0461938789662
CACAACAC 0.0163510618056
CACAACAG 0.0285174982145
CACAACCA 0.0950473980693
CACAACCC -0.0661675697288
CACAACCG -0.000598364837104
CACAACGA 0.112957836999
CACAACGC -0.143783308382
CACAACGG -0.152851132266
CACAACTA -0.0324728834173
CACAACTC 0.0717436844449
CACAACTG 0.0401268585359
CACAAGAA 0.0183678473995
CACAAGAC -0.113754443333
CACAAGAG -0.0566446909475
CACAAGCA -0.0939689145431
CACAAGCC -0.252244568604
CACAAGCG -0.0666817722951
CACAAGGA 0.00853041169318
CACAAGGC -0.430591649752
CACAAGGG -0.29458389821
CACAAGTA 0.160278680795
CACAAGTC -0.0675785978816
CACAAGTG -0.244389193039
CACAATAA 0.123800938666
CACAATAC 0.191777176626
CACAATAG 0.169195565061
CACAATCA 0.347166725955
CACAATCC 0.406299862047
CACAATCG 0.27171886747
CACAATGA 0.00195925953502

CACAATGC 0.0320744801209
CACAATGG -0.145547147445
CACAATTA 0.0964559600923
CACAATTC 0.170547360277
CACACAAA 0.115279191037
CACACAAC 0.084758312031
CACACAAG -0.029592863741
CACACACA 0.0951533908017
CACACACC -0.164968504046
CACACACG -0.0812810068991
CACACAGA 0.209837010868
CACACAGC -0.0721065418035
CACACAGG 0.00224728450082
CACACATA 0.231816264238
CACACATC -0.0994408881557
CACACATG 0.0307460420575
CACACCAA -0.298914496625
CACACCAC -0.0396811610986
CACACCAG -0.138385016447
CACACCCA 0.0564816665295
CACACCCC -0.236795029755
CACACCCG -0.204247271364
CACACCGA -0.297144833936
CACACCGC -0.285653310219
CACACCGG -0.129601601204
CACACCTA -0.191128673278
CACACCTC 0.0845845845846
CACACCTG -0.0579832419332
CACACGAA -0.0680185732051
CACACGAC -0.225482899865
CACACGAG -0.0637781575994
CACACGCA 0.0927709612198
CACACGCC -0.138518064464
CACACGCG 0.0296486885531
CACACGGA 0.139447311373
CACACGGC -0.190728347433
CACACGGG -0.103897440088
CACACGTA 0.0888620740998
CACACGTC -0.0716312781472
CACACGTG -0.24817330356
CACACTAA -0.157261521751
CACACTAC -0.0845331854047
CACACTAG 0.00100237979026
CACACTCA 0.102657754173
CACACTCC 0.140264811719
CACACTCG -0.115208059353
CACACTGA 0.0512068542372
CACACTGC -0.234059652344
CACACTGG -0.186801297998
CACACTTA -0.018437367254
CACACTTC 0.0376772771399
CACAGAAA 0.100297266964
CACAGAAC -0.0976941128456
CACAGAAG -0.0630762987842

CACAGACA -0.0823235205726
CACAGACC -0.153115331319
CACAGACG -0.208321272519
CACAGAGA 0.258676031403
CACAGAGC -0.335939654556
CACAGAGG -0.278165268538
CACAGATA 0.326272553545
CACAGATC 0.291683393794
CACAGATG -0.224160826048
CACAGCAA 0.158442515857
CACAGCAC -0.0306686714889
CACAGCAG 0.0915142566444
CACAGCCA 0.0254206112275
CACAGCCC -0.331604536606
CACAGCCG -0.298471571035
CACAGCGA -0.00501465652981
CACAGCGC 0.0516935668451
CACAGCGG -0.108642474433
CACAGCTA -0.0609334116369
CACAGCTC -0.209552793422
CACAGCTG -0.0757767265902
CACAGGAA -0.106300258509
CACAGGAC -0.161430544871
CACAGGAG 0.0675372342039
CACAGGCA -0.108140845402
CACAGGCC -0.102130973549
CACAGGCG -0.199366835639
CACAGGGA 0.0242130950904
CACAGGGC -0.286391686987
CACAGGGG -0.176014416487
CACAGGTA -0.00869251038298
CACAGGTC -0.272655165721
CACAGGTG -0.315237079126
CACAGTAA 0.0428210579726
CACAGTAC -0.0484050898137
CACAGTAG -0.0634497191685
CACAGTCA -0.0766591691638
CACAGTCC -0.0713770733452
CACAGTCG -0.23356684691
CACAGTGA -0.105904974569
CACAGTGC -0.181218234112
CACAGTGG -0.239547583408
CACAGTTA -0.00385757976103
CACAGTTC -0.0338464839741
CACATAAA 0.039991782416
CACATAAC 0.00427654868871
CACATAAG -0.00285906148041
CACATACA 0.257286211832
CACATACC -0.104136346451
CACATACG 0.114860988446
CACATAGA 0.228770902827
CACATAGC -0.282414643896
CACATAGG -0.0452747723156
CACATATA 0.307537061232

CACATATC 0.228330036569
CACATATG 0.0550141237783
CACATCAA -0.184256019587
CACATCAC 0.00441074683499
CACATCAG 0.118274052393
CACATCCA -0.002703917725
CACATCCC -0.0833614321184
CACATCCG -0.0512580016496
CACATCGA 0.0984165984166
CACATCGC -0.110011894598
CACATCGG -0.328532916553
CACATCTA 0.073920279118
CACATCTC -0.0542713003011
CACATCTG -0.14617125873
CACATGAA 0.0681714772624
CACATGAC 0.168594118377
CACATGAG -0.053277795702
CACATGCA 0.0128696189302
CACATGCC -0.063548413112
CACATGCG -0.218032158737
CACATGGA -0.0237238504107
CACATGGC -0.243382772515
CACATGGG -0.317629659945
CACATGTA 0.135128241189
CACATGTC -0.218845102369
CACATGTG -0.117304353218
CACATTAA -0.0288876885908
CACATTAC 0.0132810755143
CACATTAG 0.0460115763146
CACATTCA 0.169683457562
CACATTCC 0.145368349598
CACATTCG 0.0323051320044
CACATTGA -0.0939658666931
CACATTGC -0.107552456037
CACATTGG -0.225143851979
CACATTTA 0.0748503324261
CACATTTC 0.0157887053568
CACCAAAA -0.0651555075436
CACCAAAC -0.0480443970259
CACCAAAG -0.058799442348
CACCAACA -0.146329081907
CACCAACC -0.128500332027
CACCAACG -0.30245466498
CACCAAGA 0.159648629346
CACCAAGC -0.284600766838
CACCAAGG -0.113756060996
CACCAATA 0.0795654109764
CACCAATC 0.0942636851728
CACCAATG -0.177761787791
CACCACAA 0.14187645526
CACCACAC 0.0182636570523
CACCACAG 0.0550942149278
CACCACCA -0.00897399310122
CACCACCC 0.0411602588802

CACCACCG -0.0160075494502
CACCACGA 0.0566929191332
CACCACGC -0.173335924943
CACCACGG -0.30023381875
CACCACTA 0.086652768471
CACCACTC -0.0665442484098
CACCACTG -0.135506533109
CACCAGAA -0.089240861957
CACCAGAC -0.243431661994
CACCAGAG -0.0697138295062
CACCAGCA -0.140836837427
CACCAGCC -0.128152853248
CACCAGCG -0.196424363077
CACCAGGA -0.00999467164953
CACCAGGC -0.297648109426
CACCAGGG -0.171823399533
CACCAGTA 0.0710421164967
CACCAGTC -0.26205072962
CACCAGTG -0.341189702368
CACCATAA -0.0190424584364
CACCATAC 0.0887184952154
CACCATAG -0.051330992752
CACCATCA -0.0405334143981
CACCATCC -0.267145857078
CACCATCG -0.186246287978
CACCATGA -0.0480527670283
CACCATGC -0.122835223765
CACCATGG -0.371487959657
CACCATTA 0.0471421835058
CACCATTC -0.12831218991
CACCCAAA -0.0966905834179
CACCCAAC -0.187931647685
CACCCAAG -0.203870044581
CACCCACA -0.125861799667
CACCCACC -0.0841277626215
CACCCACG -0.0636227452051
CACCCAGA 0.115208426093
CACCCAGC -0.259502760756
CACCCAGG -0.206168421268
CACCCATA 0.136276577885
CACCCATC 0.101658357846
CACCCATG -0.17768625797
CACCCCAA -0.249858811271
CACCCCAC -0.151381066201
CACCCCAG 0.0453538724967
CACCCCCA -0.31264146235
CACCCCCC -0.325186379484
CACCCCCG -0.201147191696
CACCCCGA 0.000860565097115
CACCCCGC -0.0720773258555
CACCCCGG -0.373490820541
CACCCCTA -0.0290731364132
CACCCCTC -0.0964580635838
CACCCCTG -0.104023473363

CACCCGAA -0.101353570235
CACCCGAC -0.278746826125
CACCCGAG -0.155610004218
CACCCGCA -0.135881657196
CACCCGCC -0.19022020697
CACCCGCG -0.120386221726
CACCCGGA 0.0261716030481
CACCCGGC -0.302942854683
CACCCGGG -0.288528295499
CACCCGTA -0.0592256704662
CACCCGTC -0.222997570602
CACCCGTG -0.301769565401
CACCCTAA -0.098430633016
CACCCTAC -0.175918237498
CACCCTAG 0.101932804391
CACCCTCA -0.0982089691105
CACCCTCC -0.0860574558119
CACCCTCG -0.211200990326
CACCCTGA -0.122894077364
CACCCTGC -0.167078822365
CACCCTGG -0.207121188465
CACCCTTA -0.0552908280181
CACCCTTC -0.194373623615
CACCGAAA 0.0192689211185
CACCGAAC -0.0924965607744
CACCGAAG -0.110072985858
CACCGACA -0.153285343579
CACCGACC -0.233131113441
CACCGACG -0.272744849968
CACCGAGA -0.000838667766399
CACCGAGC -0.393802481925
CACCGAGG -0.214274884704
CACCGATA 0.308692990511
CACCGATC 0.180704802085
CACCGATG -0.231239527816
CACCGCAA -0.154902447392
CACCGCAC 0.105554363486
CACCGCAG -0.00454077647935
CACCGCCA -0.0937289736131
CACCGCCC -0.178913906463
CACCGCCG -0.300990291177
CACCGCGA 0.0491485587907
CACCGCGC -0.117530435262
CACCGCGG -0.336248170475
CACCGCTA -0.075218706983
CACCGCTC -0.256616975966
CACCGCTG -0.312829316481
CACCGGAA 0.0259373882324
CACCGGAC -0.191330078738
CACCGGAG 0.010653051393
CACCGGCA -0.236043918481
CACCGGCC -0.131628278818
CACCGGCG -0.25215160869
CACCGGGA -0.0603406205282

CACCGGGC -0.29946331719
CACCGGGG -0.285156346903
CACCGGTA -0.0404798738132
CACCGGTC -0.229592047701
CACCGGTG -0.245184413523
CACCGTAA 0.104557564433
CACCGTAC -0.0968261011076
CACCGTAG -0.086317087947
CACCGTCA -0.0778296825617
CACCGTCC -0.0775845585499
CACCGTCG -0.202066189748
CACCGTGA 0.0366302942061
CACCGTGC -0.151907149164
CACCGTGG -0.274098095148
CACCGTTA 0.0684582164751
CACCGTTC -0.126288069929
CACCTAAA -0.0601840544484
CACCTAAC -0.0479136853309
CACCTAAG -0.212386513707
CACCTACA 0.0657000211674
CACCTACC 0.0110238461931
CACCTACG -0.0768939883772
CACCTAGA 0.0404275818778
CACCTAGC -0.367719376572
CACCTAGG -0.196373028256
CACCTATA 0.159257053196
CACCTATC 0.00640309731219
CACCTATG -0.08968791616
CACCTCAA -0.120319138085
CACCTCAC 0.0449559498697
CACCTCAG -0.0604372825885
CACCTCCA -0.0684080939422
CACCTCCC -0.10381812104
CACCTCCG -0.24074958446
CACCTCGA -0.00841514419392
CACCTCGC -0.23325020472
CACCTCGG -0.282182639113
CACCTCTA -0.20853338978
CACCTCTC -0.142435120165
CACCTCTG -0.209509435003
CACCTGAA -0.237504031758
CACCTGAC -0.223069925154
CACCTGAG 0.0182185041812
CACCTGCA -0.133683801817
CACCTGCC -0.225179138097
CACCTGCG -0.122493435754
CACCTGGA -0.190534673117
CACCTGGC -0.303061630973
CACCTGGG -0.326989140469
CACCTGTA 0.0111377123129
CACCTGTC -0.278739695218
CACCTTAA -0.174715587761
CACCTTAC -0.018376057771
CACCTTAG -0.195943289901

CACCTTCA -0.0768673284843
CACCTTCC -0.21607710482
CACCTTCG -0.27229540324
CACCTTGA -0.118295981932
CACCTTGC -0.190514174951
CACCTTGG -0.21034708958
CACCTTTA 0.0131960614001
CACCTTTC -0.186360139947
CACGAAAA -0.0984737560491
CACGAAAC 0.0672339005672
CACGAAAG -0.195596446793
CACGAACA -0.206956568698
CACGAACC -0.0675407477238
CACGAACG -0.137546579635
CACGAAGA 0.191856713899
CACGAAGC -0.203020878997
CACGAAGG -0.15979958373
CACGAATA 0.037618884258
CACGAATC 0.344050662232
CACGAATG -0.0339471703108
CACGACAA -0.127390641081
CACGACAC 0.00747574261111
CACGACAG -0.34246971764
CACGACCA -0.243772864992
CACGACCC -0.218599455065
CACGACCG 0.00338313759553
CACGACGA -0.043080284492
CACGACGC -0.268845753677
CACGACGG -0.264244148383
CACGACTA -0.0520365655645
CACGACTC -0.0490257202412
CACGACTG -0.160517413923
CACGAGAA 0.0398617555991
CACGAGAC 0.0510609256732
CACGAGAG -0.073041807765
CACGAGCA -0.203415743391
CACGAGCC -0.265914195195
CACGAGCG -0.140709218685
CACGAGGA -0.0261636606498
CACGAGGC -0.325822287272
CACGAGGG -0.123211249517
CACGAGTA -0.0155916293804
CACGAGTC -0.102888383652
CACGAGTG -0.169250653538
CACGATAA 0.125862777378
CACGATAC 0.225302365642
CACGATAG 0.0320102479508
CACGATCA 0.0811775963291
CACGATCC 0.283201827697
CACGATCG 0.290930466288
CACGATGA -0.157000664054
CACGATGC -0.046770508675
CACGATGG -0.100448660222
CACGATTA 0.226078990971

CACGATTC 0.371413034751
CACGCAAA -0.0773436918266
CACGCAAC 0.110227698755
CACGCAAG -0.0921238740415
CACGCACA -0.299255988069
CACGCACC -0.0796730392278
CACGCACG 0.0728984663172
CACGCAGA 0.00399986763623
CACGCAGC -0.153222048512
CACGCAGG -0.274784871156
CACGCATA -0.0118328728052
CACGCATC -0.173159803557
CACGCATG -0.223149871122
CACGCCAA -0.129556564679
CACGCCAC -0.0729619341098
CACGCCAG -0.24398370321
CACGCCCA -0.285730233943
CACGCCCC -0.306472466824
CACGCCCG -0.159962548402
CACGCCGA 0.0807763686552
CACGCCGC -0.208986372671
CACGCCGG -0.23659923247
CACGCCTA -0.11390384504
CACGCCTC -0.151431579934
CACGCCTG -0.295380558891
CACGCGAA -0.00161914179607
CACGCGAC -0.152676644255
CACGCGAG -0.0334890476851
CACGCGCA -0.0882367537224
CACGCGCC -0.0895270442859
CACGCGCG 0.243101223416
CACGCGGA 0.0285241929009
CACGCGGC -0.156521858341
CACGCGGG -0.372447141104
CACGCGTA 0.155712584063
CACGCGTC 0.0217200671746
CACGCGTG -0.0539982193997
CACGCTAA -0.170361967013
CACGCTAC -0.142884768347
CACGCTAG -0.0941843476839
CACGCTCA -0.211511489598
CACGCTCC -0.1070998299
CACGCTCG 0.0661000124085
CACGCTGA 0.0280548040408
CACGCTGC -0.0763465033612
CACGCTGG -0.136962508994
CACGCTTA -0.0820782257169
CACGCTTC -0.145216336545
CACGGAAA 0.0757289986351
CACGGAAC 0.0119940877517
CACGGAAG -0.202290655131
CACGGACA -0.0629782200495
CACGGACC -0.28267053677
CACGGACG 0.0778515798924

CACGGAGA 0.165797099334
CACGGAGC -0.246738148375
CACGGAGG -0.09240788984
CACGGATA 0.240335376699
CACGGATC 0.181041923466
CACGGATG -0.0856708384688
CACGGCAA 0.0477266921162
CACGGCAC 0.0403392373089
CACGGCAG -0.102392000199
CACGGCCA -0.305673401091
CACGGCCC -0.222350080777
CACGGCCG -0.166359272952
CACGGCGA -0.156020121266
CACGGCGC -0.174116778723
CACGGCGG -0.14756528123
CACGGCTA -0.213844891787
CACGGCTC -0.121895238332
CACGGCTG -0.218415572248
CACGGGAA 0.097299779118
CACGGGAC -0.147473694947
CACGGGAG 0.00331598816447
CACGGGCA -0.288740314405
CACGGGCC -0.194906844625
CACGGGCG -0.240213227085
CACGGGGA -0.169479848658
CACGGGGC -0.231375321581
CACGGGGG -0.301109955009
CACGGGTA -0.245372782174
CACGGGTC -0.275796126295
CACGGTAA -0.0846589734045
CACGGTAC -0.0550233429021
CACGGTAG -0.159715157681
CACGGTCA -0.198435540118
CACGGTCC -0.123716222626
CACGGTCG -0.22523703575
CACGGTGA -0.0406302865058
CACGGTGC -0.272045508601
CACGGTGG -0.148716443254
CACGGTTA -0.0104822522135
CACGGTTC -0.0222938432141
CACGTAAA 0.092311319584
CACGTAAC 0.0480828886959
CACGTAAG 0.117418596673
CACGTACA 0.0312482208419
CACGTACC -0.274244951385
CACGTACG -0.0856094117524
CACGTAGA 0.0954196379236
CACGTAGC -0.263087421686
CACGTAGG -0.0149091323088
CACGTATA 0.254338360399
CACGTATC 0.248782143457
CACGTATG -0.0479259316559
CACGTCAA -0.0411122384181
CACGTCAC 0.236739494315

CACGTCAG -0.154356847079
CACGTCCA 0.0112282212798
CACGTCCC -0.187390078975
CACGTCCG -0.1331797667
CACGTCGA 0.0809309122996
CACGTCGC -0.127992656362
CACGTCGG -0.336029827477
CACGTCTA -0.23650360446
CACGTCTC -0.246004363785
CACGTCTG -0.189804145364
CACGTGAA 0.00278315073393
CACGTGAC -0.131459342539
CACGTGAG -0.0674503478026
CACGTGCA -0.0044059543799
CACGTGCC -0.0891879970778
CACGTGCG -0.0621773041053
CACGTGGA 0.0321575018545
CACGTGGC -0.115037626913
CACGTGGG -0.0590533702831
CACGTGTA 0.0530200879624
CACGTGTC -0.310712503756
CACGTTAA -0.0644597929989
CACGTTAC -0.188764271082
CACGTTAG -0.157280801162
CACGTTCA -0.172278497823
CACGTTCC -0.0298278139895
CACGTTCG -0.10551130301
CACGTTGA -0.104790952337
CACGTTGC -0.0732018033668
CACGTTGG -0.345852662218
CACGTTTA 0.0651096290522
CACGTTTC -0.0734028036224
CACTAAAA 0.0032663517512
CACTAAAC -0.00179940929736
CACTAAAG -0.00972087803614
CACTAACA 0.0746779837689
CACTAACC -0.183722048004
CACTAACG -0.0141092689278
CACTAAGA 0.137954930512
CACTAAGC -0.0918117770333
CACTAAGG -0.177275480345
CACTAATA 0.0995609935004
CACTAATC 0.0743217301811
CACTAATG -0.0326869569294
CACTACAA 0.0360597943111
CACTACAC 0.186175180823
CACTACAG -0.0974203740003
CACTACCA -0.191889958614
CACTACCC -0.304587436642
CACTACCG -0.00114025871602
CACTACGA -0.129808872233
CACTACGC -0.263471947453
CACTACGG -0.204049251604
CACTACTA -0.0110896019987

CACTACTC -0.0521426538105
CACTACTG -0.06810434808
CACTAGAA 0.0606402779501
CACTAGAC -0.0723409359773
CACTAGAG -0.0381759188029
CACTAGCA -0.212220209689
CACTAGCC -0.257605610164
CACTAGCG -0.206129650305
CACTAGGA 0.146060305249
CACTAGGC -0.223083171293
CACTAGGG -0.240156802433
CACTAGTA 0.0785260176784
CACTAGTC -0.146105748952
CACTAGTG 0.139342997087
CACTATAA 0.0212329701963
CACTATAC 0.145693370205
CACTATAG -0.0120004671431
CACTATCA 0.118358686751
CACTATCC 0.0739756429867
CACTATCG 0.101557480345
CACTATGA -0.00181966818245
CACTATGC 0.081198278168
CACTATGG -0.212879240064
CACTATTA 0.131393155818
CACTATTC -0.158610708483
CACTCAAA 0.0631334400897
CACTCAAC 0.0775099815333
CACTCAAG -0.0592282855355
CACTCACA -0.185892131322
CACTCACC -0.205357758205
CACTCACG -0.118142918979
CACTCAGA 0.132256909928
CACTCAGC -0.286225660424
CACTCAGG -0.38335091459
CACTCATA -0.103284599607
CACTCATC -0.112841491629
CACTCATG -0.0605860895687
CACTCCAA -0.0855783068258
CACTCCAC -0.0833395369612
CACTCCAG -0.142275044317
CACTCCCA -0.0996187512498
CACTCCCC -0.125643562514
CACTCCCG -0.209643007765
CACTCCGA 0.0185653973533
CACTCCGC -0.27989222034
CACTCCGG -0.392422000094
CACTCCTA -0.24923714307
CACTCCTC -0.139858950801
CACTCCTG -0.0151128077487
CACTCGAA -0.179253763032
CACTCGAC -0.0673026821825
CACTCGAG -0.284166246282
CACTCGCA 0.128934719844
CACTCGCC -0.252894186527

CACTCGCG 0.0467865667175
CACTCGGA 0.0374787413414
CACTCGGC -0.13422889813
CACTCGGG -0.103574915465
CACTCGTA 0.0575483748531
CACTCGTC -0.174528939062
CACTCTAA -0.0706094027138
CACTCTAC -0.0231016140107
CACTCTAG 0.0438961190631
CACTCTCA -0.284859019525
CACTCTCC -0.153701154928
CACTCTCG -0.340894398989
CACTCTGA 0.0516204910144
CACTCTGC 0.0356013290869
CACTCTGG -0.203080050967
CACTCTTA -0.0163549526116
CACTCTTC -0.0695974356261
CACTGAAA 0.143823857872
CACTGAAC -0.0153431668583
CACTGAAG 0.0020379929027
CACTGACA -0.169556035279
CACTGACC -0.218207116143
CACTGACG -0.0810467409216
CACTGAGA 0.207346464922
CACTGAGC -0.289409167125
CACTGAGG -0.202551369688
CACTGATA 0.336191279748
CACTGATC 0.145904858026
CACTGATG -0.0486303219638
CACTGCAA 0.194070059222
CACTGCAC -0.0799297311421
CACTGCAG -0.170680769517
CACTGCCA -0.309962265607
CACTGCCC -0.359934839953
CACTGCCG -0.0964105400975
CACTGCGA 0.0786348859717
CACTGCGC 0.0593906702773
CACTGCGG -0.175763585184
CACTGCTA -0.11157151246
CACTGCTC -0.270430491166
CACTGCTG -0.151067295916
CACTGGAA -0.0844549783944
CACTGGAC -0.108339963358
CACTGGAG 0.0291052020759
CACTGGCA -0.192300507988
CACTGGCC -0.345620879369
CACTGGCG -0.186372770349
CACTGGGA -0.184108671381
CACTGGGC -0.331609323709
CACTGGGG -0.199552026906
CACTGGTA -0.00321210065869
CACTGGTC -0.31692222717
CACTGTAA 0.115745221806
CACTGTAC -0.0859802048409

CACTGTAG -0.0506349009389
CACTGTCA -0.0529213248614
CACTGTCC -0.00352125298148
CACTGTCG -0.0881360262184
CACTGTGA -0.0745266866827
CACTGTGC -0.207216233058
CACTGTGG -0.257928823369
CACTGTTA 0.11591206032
CACTGTTC 0.031675836327
CACTTAAA -0.234776764801
CACTTAAC 0.00896350896351
CACTTAAG 0.00471683805017
CACTTACA 0.168824770261
CACTTACC -0.124694060728
CACTTACG 0.0973168470763
CACTTAGA 0.00278461055597
CACTTAGC -0.182686056034
CACTTAGG -0.1877106183
CACTTATA 0.127685536776
CACTTATC -0.104324679013
CACTTATG 0.0544477128238
CACTTCAA 0.0267694867996
CACTTCAC 0.203110150873
CACTTCAG -0.124277313218
CACTTCCA 0.0322126534248
CACTTCCC -0.131164376952
CACTTCCG -0.0464172317395
CACTTCGA 0.155057261118
CACTTCGC 0.0760738676752
CACTTCGG -0.00953044823836
CACTTCTA -0.194603087495
CACTTCTC -0.0975179194075
CACTTCTG -0.00758471970593
CACTTGAA -0.0650153646271
CACTTGAC -0.0680489035578
CACTTGAG -0.0388413230205
CACTTGCA -0.318186578909
CACTTGCC -0.342426522675
CACTTGCG -0.0643749534751
CACTTGGA 0.0880849324467
CACTTGGC -0.190909065844
CACTTGGG -0.207980106251
CACTTGTA 0.117377708287
CACTTGTC -0.275005801641
CACTTTAA -0.175293358615
CACTTTAC 0.0385909325303
CACTTTAG -0.00302256154975
CACTTTCA 0.0140236655388
CACTTTCC -0.0594651212017
CACTTTCG -0.116234891267
CACTTTGA -0.198403120882
CACTTTGC -0.106065515153
CACTTTGG -0.0836214836298
CACTTTTA 0.103424742524

CACTTTTC -0.0543617550739
CAGAAAAA 0.109471730684
CAGAAAAC 0.0192973422339
CAGAAAAG 0.047811361795
CAGAAACA 0.0907284695163
CAGAAACC -0.143435164524
CAGAAACG -0.164626625817
CAGAAAGA 0.127152690087
CAGAAAGC -0.10106586823
CAGAAAGG -0.251240417773
CAGAAATA 0.148458375731
CAGAAATC 0.431683749866
CAGAAATG 0.0714778139021
CAGAACAA 0.00754160290083
CAGAACAC -0.206644728681
CAGAACAG -0.156744997808
CAGAACCA -0.208792827549
CAGAACCC -0.296008189676
CAGAACCG -0.129674866151
CAGAACGA -0.177630436117
CAGAACGC -0.272390635284
CAGAACGG 0.0224600921148
CAGAACTA 0.13645792648
CAGAACTC -0.227262752986
CAGAACTG 0.134732086159
CAGAAGAA -0.0930714445244
CAGAAGAC 0.0765447398344
CAGAAGAG -0.0629970619615
CAGAAGCA -0.174462559038
CAGAAGCC -0.364596601346
CAGAAGCG -0.127787612428
CAGAAGGA -0.168299396775
CAGAAGGC -0.363952804598
CAGAAGGG -0.237335334541
CAGAAGTA -0.0607243799203
CAGAAGTC -0.0152327303108
CAGAATAA 0.12912928914
CAGAATAC 0.025435209448
CAGAATAG 0.00515536156143
CAGAATCA 0.378717984278
CAGAATCC 0.464184725882
CAGAATCG 0.314363189127
CAGAATGA -0.0717549563766
CAGAATGC -0.164949847014
CAGAATGG -0.0037032441301
CAGAATTA 0.11872782093
CAGAATTC 0.242864024875
CAGACAAA 0.102484617125
CAGACAAC -0.00238668420487
CAGACAAG 0.0801872951687
CAGACACA -0.143899601018
CAGACACC -0.0562117940955
CAGACACG -0.259195863274
CAGACAGA -0.117793770939

CAGACAGC -0.259531124932
CAGACAGG -0.178763741036
CAGACATA -0.112690968345
CAGACATC -0.0155332364985
CAGACATG -0.119086815969
CAGACCAA -0.0922137556157
CAGACCAC -0.34325468188
CAGACCAG -0.165808086415
CAGACCCA -0.260759211016
CAGACCCC -0.156906851029
CAGACCCG -0.172804645399
CAGACCGA -0.102828433197
CAGACCGC -0.262735644301
CAGACCGG -0.0798215080599
CAGACCTA -0.162276662234
CAGACCTC -0.098523098661
CAGACCTG -0.0597454070349
CAGACGAA -0.148585094212
CAGACGAC -0.355252096787
CAGACGAG -0.0719970380779
CAGACGCA 0.0998058436677
CAGACGCC -0.1467116853
CAGACGCG 0.0138734188302
CAGACGGA -0.012978547915
CAGACGGC -0.351228925156
CAGACGGG -0.191786829098
CAGACGTA -0.07474872813
CAGACGTC -0.0377774468684
CAGACTAA -0.180094976934
CAGACTAC -0.133650699115
CAGACTAG -0.0850690972471
CAGACTCA -0.164685641496
CAGACTCC -0.311742865884
CAGACTCG -0.298539881831
CAGACTGA -0.0152523761074
CAGACTGC -0.283353009764
CAGACTGG -0.099287058298
CAGACTTA -0.105719307004
CAGACTTC -0.260660012514
CAGAGAAA 0.120212696072
CAGAGAAC -0.0304379177414
CAGAGAAG 0.104712150167
CAGAGACA -0.0238049937268
CAGAGACC -0.246607457392
CAGAGACG -0.185769536694
CAGAGAGA -0.0850006479023
CAGAGAGC -0.235861981514
CAGAGAGG -0.13099461632
CAGAGATA 0.116277434459
CAGAGATC 0.354471176514
CAGAGATG 0.204094070933
CAGAGCAA -0.121667140991
CAGAGCAC -0.349460498574
CAGAGCAG -0.174937907118

CAGAGCCA -0.235003401862
CAGAGCCC -0.361500973948
CAGAGCCG -0.364757904716
CAGAGCGA -0.00665459880441
CAGAGCGC -0.225041965386
CAGAGCGG -0.135132117603
CAGAGCTA -0.0648165313722
CAGAGCTC -0.16059362177
CAGAGCTG -0.0886746787456
CAGAGGAA -0.21183181928
CAGAGGAC -0.140636693603
CAGAGGAG -0.116678659157
CAGAGGCA -0.173819479411
CAGAGGCC -0.319710763277
CAGAGGCG -0.156518167509
CAGAGGGA -0.0653951122689
CAGAGGGC -0.3188985424
CAGAGGGG -0.24652642514
CAGAGGTA -0.134135483428
CAGAGGTC -0.192527632595
CAGAGTAA -0.0416697689425
CAGAGTAC -0.276478700162
CAGAGTAG -0.0382413517778
CAGAGTCA -0.00151579949118
CAGAGTCC -0.0238045199472
CAGAGTCG -0.0682489268256
CAGAGTGA -0.011508753933
CAGAGTGC -0.296005083749
CAGAGTGG -0.108385515326
CAGAGTTA -0.00262383595717
CAGAGTTC -0.0638584358584
CAGATAAA -0.0423164211043
CAGATAAC 0.18252666
CAGATAAG 0.17366264336
CAGATACA 0.205988919951
CAGATACC 0.364440197774
CAGATACG 0.376683846381
CAGATAGA 0.101471500624
CAGATAGC 0.0618511073057
CAGATAGG -0.240314404558
CAGATATA 0.192951905073
CAGATATC 0.47135537178
CAGATATG 0.428150478457
CAGATCAA 0.0185189780921
CAGATCAC 0.197665014635
CAGATCAG 0.127782465196
CAGATCCA 0.27539761903
CAGATCCC 0.283166086196
CAGATCCG 0.255158646161
CAGATCGA 0.19427554276
CAGATCGC 0.420223253557
CAGATCGG 0.155291655292
CAGATCTA 0.2626938233
CAGATCTC 0.44419212601

CAGATCTG 0.413948354129
CAGATGAA -0.0397445759614
CAGATGAC -0.101488540882
CAGATGAG -0.142972943911
CAGATGCA -0.191905250573
CAGATGCC -0.0818987443857
CAGATGCG -0.00142989569571
CAGATGGA -0.0504413333859
CAGATGGC -0.102969275256
CAGATGGG -0.189731312179
CAGATGTA -0.157795675775
CAGATGTC -0.176801643435
CAGATTAA -0.143142021998
CAGATTAC 0.476464067373
CAGATTAG 0.116426343699
CAGATTCA 0.282407752105
CAGATTCC 0.461942658912
CAGATTCG 0.4292791414
CAGATTGA 0.0789122455789
CAGATTGC 0.478677839171
CAGATTGG 0.040441267714
CAGATTTA 0.283226752924
CAGATTTC 0.485633463918
CAGCAAAA 0.171496244608
CAGCAAAC -0.0492379431773
CAGCAAAG 0.0773100924616
CAGCAACA -0.119924885014
CAGCAACC -0.137615127426
CAGCAACG -0.201476243642
CAGCAAGA 0.00176857441078
CAGCAAGC -0.318976852656
CAGCAAGG 0.0416925176821
CAGCAATA 0.137412319438
CAGCAATC 0.330064893168
CAGCAATG -0.158205271539
CAGCACAA -0.124038131215
CAGCACAC -0.307776812913
CAGCACAG -0.15490565845
CAGCACCA -0.293522976185
CAGCACCC -0.272443981355
CAGCACCG -0.239307764484
CAGCACGA -0.0173105318014
CAGCACGC -0.137767978479
CAGCACGG -0.202808577963
CAGCACTA -0.212499397853
CAGCACTC 0.0337458670792
CAGCACTG -0.123887082854
CAGCAGAA -0.109258397054
CAGCAGAC -0.253616715055
CAGCAGAG -0.0301065365478
CAGCAGCA -0.0576593791101
CAGCAGCC -0.181473773664
CAGCAGCG -0.164872414986
CAGCAGGA -0.164575942144

CAGCAGGC -0.27267577915
CAGCAGGG -0.223452202507
CAGCAGTA -0.101591516325
CAGCAGTC -0.333447391806
CAGCATAA -0.0619635318869
CAGCATAC 0.0596125632285
CAGCATAG 0.0616939845245
CAGCATCA 0.239786719999
CAGCATCC -0.0930935818924
CAGCATCG 0.0129316644468
CAGCATGA -0.140488656195
CAGCATGC -0.221763640318
CAGCATGG 0.160646872768
CAGCATTA 0.013421437986
CAGCATTC -0.341563296616
CAGCCAAA 0.0372397190579
CAGCCAAC -0.316631518009
CAGCCAAG -0.0216849266074
CAGCCACA -0.0769257155702
CAGCCACC -0.129727054399
CAGCCACG -0.230517049907
CAGCCAGA -0.104507693877
CAGCCAGC -0.290976235954
CAGCCAGG -0.11001151571
CAGCCATA -0.00301112627704
CAGCCATC -0.396227663252
CAGCCATG -0.320293054832
CAGCCCAA -0.21595016731
CAGCCCAC -0.410354557777
CAGCCCAG -0.38637773202
CAGCCCCA -0.251340340316
CAGCCCCC -0.368473272975
CAGCCCCG -0.181618471936
CAGCCCGA -0.116412618857
CAGCCCGC -0.176180236805
CAGCCCGG -0.0507941628137
CAGCCCTA -0.0596758923733
CAGCCCTC -0.242099186895
CAGCCCTG -0.0564832504887
CAGCCGAA -0.157905765514
CAGCCGAC -0.275013333829
CAGCCGAG -0.0703915860522
CAGCCGCA -0.0386976927513
CAGCCGCC -0.0289399882701
CAGCCGCG -0.19773633936
CAGCCGGA -0.209087651105
CAGCCGGC -0.360995726514
CAGCCGGG -0.254759630888
CAGCCGTA -0.181806438052
CAGCCGTC -0.2129434369
CAGCCTAA -0.146928925157
CAGCCTAC -0.140602697723
CAGCCTAG -0.231183062482
CAGCCTCA -0.309545259293

CAGCCTCC -0.291134373527
CAGCCTCG -0.185904168079
CAGCCTGA -0.0622778646329
CAGCCTGC -0.265629953057
CAGCCTGG -0.181198977693
CAGCCTTA -0.201308178781
CAGCCTTC -0.318817093169
CAGCGAAA -0.0175974638311
CAGCGAAC -0.203417569861
CAGCGAAG 0.0246060305249
CAGCGACA -0.17198934534
CAGCGACC -0.156958196502
CAGCGACG -0.0565677103327
CAGCGAGA 0.019496653358
CAGCGAGC -0.2578984075
CAGCGAGG -0.058385507558
CAGCGATA 0.0841988861558
CAGCGATC 0.163327239085
CAGCGATG -0.208283402689
CAGCGCAA 0.215784325891
CAGCGCAC -0.192316673599
CAGCGCAG -0.238554957074
CAGCGCCA -0.241656812237
CAGCGCCC -0.363759271663
CAGCGCCG -0.257439848692
CAGCGCGA 0.0333046545168
CAGCGCGC -0.0222133821887
CAGCGCGG 0.0968061725637
CAGCGCTA -0.0918715303577
CAGCGCTC 0.0413655132595
CAGCGCTG 0.0440862748989
CAGCGGAA -0.222537515341
CAGCGGAC 0.0144369101407
CAGCGGAG -0.153279852238
CAGCGGCA -0.403252444111
CAGCGGCC -0.358713810697
CAGCGGCG -0.299414029662
CAGCGGGA -0.0717271481472
CAGCGGGC -0.266292393939
CAGCGGGG -0.141700880536
CAGCGGTA -0.0313054100933
CAGCGGTC -0.285165843856
CAGCGTAA 0.0305771314655
CAGCGTAC -0.260995874466
CAGCGTAG -0.255951937946
CAGCGTCA 0.070289297562
CAGCGTCC -0.125973373891
CAGCGTCG -0.131373035627
CAGCGTGA -0.113960461222
CAGCGTGC -0.190102293119
CAGCGTGG 0.00417293139777
CAGCGTTA 0.0469471471428
CAGCGTTC -0.1833442555
CAGCTAAA -0.0581251432166

CAGCTAAC -0.0190017736838
CAGCTAAG -0.107158907003
CAGCTACA -0.0113796459421
CAGCTACC -0.15246639062
CAGCTACG -0.103820651159
CAGCTAGA -0.101296365215
CAGCTAGC -0.215144572101
CAGCTAGG -0.0325122894662
CAGCTATA -0.0238234819723
CAGCTATC -0.0111537703554
CAGCTATG 0.0887798077414
CAGCTCAA -0.224169740351
CAGCTCAC -0.269962335079
CAGCTCAG -0.19670016127
CAGCTCCA -0.220381920181
CAGCTCCC -0.162558365943
CAGCTCCG -0.434074991502
CAGCTCGA -0.084474281444
CAGCTCGC -0.226637169361
CAGCTCGG 0.0410638209869
CAGCTCTA -0.0865769362715
CAGCTCTC -0.185105308408
CAGCTGAA -0.289586978762
CAGCTGAC -0.181403322352
CAGCTGAG -0.106726053117
CAGCTGCA -0.226599816993
CAGCTGCC -0.0886900872136
CAGCTGCG -0.304590001241
CAGCTGGA -0.165679389358
CAGCTGGC -0.185260540296
CAGCTGGG -0.0860312910058
CAGCTGTA -0.187728348244
CAGCTGTC -0.346644336931
CAGCTTAA -0.20012707642
CAGCTTAC -0.233456110371
CAGCTTAG -0.334337485645
CAGCTTCA -0.0779095379684
CAGCTTCC -0.219417940987
CAGCTTCG -0.128473859209
CAGCTTGA -0.324578659269
CAGCTTGC -0.368891810182
CAGCTTGG -0.0823730066154
CAGCTTTA 0.0525090691445
CAGCTTTC 0.0526931890568
CAGGAAAA 0.074464271434
CAGGAAAC 0.0235899943797
CAGGAAAG -0.136309253057
CAGGAACA -0.288165240045
CAGGAACC -0.248578272867
CAGGAACG -0.188461294582
CAGGAAGA -0.0288125077553
CAGGAAGC -0.163887579299
CAGGAAGG -0.0394009565949
CAGGAATA 0.0945587460739

CAGGAATC 0.369442274987
CAGGAATG 0.0348754249696
CAGGACAA 0.0499047466114
CAGGACAC -0.165293869551
CAGGACAG -0.121717735599
CAGGACCA -0.165471369046
CAGGACCC -0.31436095883
CAGGACCG -0.336642482088
CAGGACGA 0.0257096559929
CAGGACGC -0.159100118253
CAGGACGG -0.250918273981
CAGGACTA -0.154122609222
CAGGACTC -0.186201536811
CAGGACTG 0.0775444866354
CAGGAGAA -0.0702433135839
CAGGAGAC -0.362991535678
CAGGAGAG -0.0394382870397
CAGGAGCA -0.162693425468
CAGGAGCC -0.326085497569
CAGGAGCG -0.114903867906
CAGGAGGA 0.0426010019548
CAGGAGGC -0.169406423348
CAGGAGGG -0.139410024876
CAGGAGTA -0.0932290974806
CAGGAGTC -0.284128716774
CAGGATAA 0.181028135574
CAGGATAC 0.297381403442
CAGGATAG -0.0114282169004
CAGGATCA 0.26797588374
CAGGATCC 0.282857309694
CAGGATCG 0.271558287045
CAGGATGA 0.0291635949578
CAGGATGC -0.244799321609
CAGGATGG -0.207830372429
CAGGATTA 0.296617592315
CAGGATTC 0.405881087699
CAGGCAAA 0.107398378138
CAGGCAAC -0.206542127012
CAGGCAAG 0.0847581705834
CAGGCACA -0.155480112712
CAGGCACC -0.217118592209
CAGGCACG -0.195659364741
CAGGCAGA -0.192508607548
CAGGCAGC -0.192307268191
CAGGCAGG -0.265549282745
CAGGCATA 0.0783741961855
CAGGCATC -0.127350277662
CAGGCATG -0.196518988727
CAGGCCAA -0.0918504917165
CAGGCCAC -0.242314500911
CAGGCCAG -0.226992168633
CAGGCCCA -0.298157197706
CAGGCCCC -0.332651691459
CAGGCCCG -0.269609180843

CAGGCCGA -0.118206330695
CAGGCCGC -0.318160055568
CAGGCCGG -0.242453810512
CAGGCCTA -0.249434206744
CAGGCCTC -0.167963656713
CAGGCCTG 0.205682760978
CAGGCGAA -0.0402380379022
CAGGCGAC -0.24788646594
CAGGCGAG -0.165523366382
CAGGCGCA -0.13920508034
CAGGCGCC -0.185029428386
CAGGCGCG -0.189642289545
CAGGCGGA 0.0309071577014
CAGGCGGC -0.147648302622
CAGGCGGG -0.368334692794
CAGGCGTA -0.0901081528475
CAGGCGTC -0.238020847774
CAGGCTAA -0.134239751529
CAGGCTAC -0.204754742404
CAGGCTAG -0.311882061417
CAGGCTCA -0.227871399509
CAGGCTCC -0.359160596715
CAGGCTCG -0.3459171121
CAGGCTGA -0.121965003984
CAGGCTGC -0.24630373254
CAGGCTGG -0.205926292545
CAGGCTTA -0.123440399478
CAGGCTTC -0.190821404122
CAGGGAAA 0.0803719879892
CAGGGAAC -0.216348238097
CAGGGAAG -0.0958440512918
CAGGGACA -0.218263795954
CAGGGACC -0.261749989662
CAGGGACG -0.131410494976
CAGGGAGA -0.133040028889
CAGGGAGC -0.239723505673
CAGGGAGG -0.305313478915
CAGGGATA 0.0804650470258
CAGGGATC 0.0866951818573
CAGGGATG -0.214384504701
CAGGGCAA -0.00268726026302
CAGGGCAC -0.285218561612
CAGGGCAG -0.36707013584
CAGGGCCA -0.158927680317
CAGGGCCC -0.292692696637
CAGGGCCG -0.327848184982
CAGGGCGA 0.0237335009775
CAGGGCGC -0.204276303998
CAGGGCGG -0.0287600676091
CAGGGCTA -0.163215206274
CAGGGCTC -0.394385059894
CAGGGGAA -0.0535324043999
CAGGGGAC -0.164656591475
CAGGGGAG -0.232720291305

CAGGGGCA -0.326968675993
CAGGGGCC -0.350175319303
CAGGGGCG -0.284003864266
CAGGGGGA -0.0101023888903
CAGGGGGC -0.288943568826
CAGGGGGG -0.263855143903
CAGGGGTA -0.254014808414
CAGGGGTC -0.108494510514
CAGGGTAA 0.00683965672529
CAGGGTAC -0.197071075492
CAGGGTAG -0.131535319365
CAGGGTCA -0.285017080435
CAGGGTCC -0.347201530378
CAGGGTCG -0.329549052191
CAGGGTGA -0.115532449341
CAGGGTGC -0.251359500795
CAGGGTGG -0.241259639165
CAGGGTTA 0.0362082951789
CAGGGTTC -0.310255340198
CAGGTAAA 0.0422932344547
CAGGTAAC -0.0161755581995
CAGGTAAG 0.0145303387517
CAGGTACA -0.115064142329
CAGGTACC -0.16034428658
CAGGTACG -0.0168308804672
CAGGTAGA -0.0423136635258
CAGGTAGC -0.073866597186
CAGGTAGG -0.129548224202
CAGGTATA 0.0022295022295
CAGGTATC 0.189311901433
CAGGTATG 0.0832441625366
CAGGTCAA 0.0262673141461
CAGGTCAC -0.26016157536
CAGGTCAG -0.2312529457
CAGGTCCA -0.202084649953
CAGGTCCC -0.488356369206
CAGGTCCG -0.261812842648
CAGGTCGA -0.319187518699
CAGGTCGC -0.0402956576829
CAGGTCGG -0.260361717062
CAGGTCTA -0.241526122469
CAGGTCTC -0.405606277934
CAGGTGAA -0.121866823274
CAGGTGAC -0.024054720227
CAGGTGAG -0.0467512695542
CAGGTGCA -0.351544020977
CAGGTGCC -0.381115687588
CAGGTGCG -0.154835597424
CAGGTGGA -0.128962567309
CAGGTGGC -0.33571665142
CAGGTGGG -0.236990655542
CAGGTGTA -0.203582130071
CAGGTGTC -0.26355734444
CAGGTTAA -0.0452256664378

CAGGTTAC -0.00140774383199
CAGGTTAG -0.275311019325
CAGGTTCA 0.0680853703933
CAGGTTCC -0.160621575942
CAGGTTCG -0.00139631978862
CAGGTTGA -0.180996379659
CAGGTTGC -0.153702371205
CAGGTTGG 0.0391700240185
CAGGTTTA -0.10823814211
CAGGTTTC -0.271823673595
CAGTAAAA 0.162059954891
CAGTAAAC -0.153559012701
CAGTAAAG 0.0725670240798
CAGTAACA -0.0214338372152
CAGTAACC -0.0862458528981
CAGTAACG -0.136315671937
CAGTAAGA 0.120711955213
CAGTAAGC -0.110295029809
CAGTAAGG -0.26839211211
CAGTAATA 0.0823847700365
CAGTAATC 0.323758472899
CAGTAATG -0.0931716840808
CAGTACAA 0.0405735640825
CAGTACAC -0.121839981433
CAGTACAG -0.0950953062401
CAGTACCA -0.0179812769712
CAGTACCC -0.113902257099
CAGTACCG -0.189628348548
CAGTACGA 0.0869637891141
CAGTACGC -0.133585313473
CAGTACGG -0.231172081511
CAGTACTA 0.0810865455501
CAGTACTC -0.156039325638
CAGTACTG 0.06212015975
CAGTAGAA -0.0386973940712
CAGTAGAC -0.137515966804
CAGTAGAG -0.124239289311
CAGTAGCA -0.0805719890875
CAGTAGCC -0.236415656892
CAGTAGCG -0.00619114364054
CAGTAGGA 0.0934998359241
CAGTAGGC -0.242716555253
CAGTAGGG -0.159786329084
CAGTAGTA 0.154112683664
CAGTAGTC -0.104801354715
CAGTATAA 0.094887703189
CAGTATAC 0.0777733656522
CAGTATAG -0.0806468092311
CAGTATCA 0.166294393567
CAGTATCC 0.243436275118
CAGTATCG 0.151590984924
CAGTATGA 0.143689554243
CAGTATGC -0.164607344365
CAGTATGG -0.144658995196

CAGTATTA 0.169315270469
CAGTATTC 0.13490434516
CAGTCAAA 0.0808967686839
CAGTCAAC -0.078909950877
CAGTCAAG 0.0856382706948
CAGTCACA -0.211006516488
CAGTCACC -0.280517305545
CAGTCACG -0.145889796186
CAGTCAGA 0.11817186485
CAGTCAGC -0.274923297029
CAGTCAGG -0.0489047685087
CAGTCATA -0.145682211338
CAGTCATC -0.25498540152
CAGTCATG -0.0219878396823
CAGTCCAA -0.105403409099
CAGTCCAC -0.282732471353
CAGTCCAG -0.26455119496
CAGTCCCA -0.204863573149
CAGTCCCC -0.181843242006
CAGTCCCG -0.2898332188
CAGTCCGA -0.0422474816414
CAGTCCGC -0.0645140388173
CAGTCCGG -0.271935047114
CAGTCCTA -0.296612098564
CAGTCCTC -0.286041068043
CAGTCGAA -0.0450741954556
CAGTCGAC -0.254073228501
CAGTCGAG -0.145163958152
CAGTCGCA 0.052378005077
CAGTCGCC -0.135457001449
CAGTCGCG 0.00975472072653
CAGTCGGA -0.0707699831391
CAGTCGGC -0.261695008807
CAGTCGGG -0.161384554318
CAGTCGTA 0.00252706983977
CAGTCGTC -0.268792775541
CAGTCTAA -0.0974679426207
CAGTCTAC -0.122700638348
CAGTCTAG -0.08130655056
CAGTCTCA -0.0977017702383
CAGTCTCC -0.275033199144
CAGTCTCG 0.0703996007026
CAGTCTGA 0.206820606993
CAGTCTGC -0.299763781047
CAGTCTGG -0.198064096307
CAGTCTTA -0.0993472647738
CAGTCTTC -0.146708553164
CAGTGAAA -0.0308131243167
CAGTGAAC 0.0883039057539
CAGTGAAG -0.230145735306
CAGTGACA -0.104883700577
CAGTGACC -0.0423921824895
CAGTGACG -0.35124635025
CAGTGAGA 0.150289407865

CAGTGAGC -0.307049312811
CAGTGAGG -0.150300929947
CAGTGATA 0.0390183572002
CAGTGATC 0.0982175572798
CAGTGATG 0.0386132962752
CAGTGCAA 0.108594820716
CAGTGCAC 0.00583917294166
CAGTGCAG -0.120913735253
CAGTGCCA -0.248619328669
CAGTGCCC -0.222998658889
CAGTGCCG -0.14427257641
CAGTGCGA 0.195444587991
CAGTGCGC 0.112802639358
CAGTGCGG 0.0215689808991
CAGTGCTA -0.191470931225
CAGTGCTC -0.176256756145
CAGTGGAA -0.239459894537
CAGTGGAC -0.00281962403175
CAGTGGAG -0.156706833874
CAGTGGCA -0.192574766445
CAGTGGCC -0.208605921258
CAGTGGCG -0.136959943544
CAGTGGGA 0.0556506061911
CAGTGGGC -0.336944988742
CAGTGGGG -0.15710279051
CAGTGGTA -0.149261014482
CAGTGGTC -0.144637699772
CAGTGTAA 0.0488854629377
CAGTGTAC -0.191794116755
CAGTGTAG 0.0367090529291
CAGTGTCA -0.0620649060266
CAGTGTCC -0.0914461840447
CAGTGTCG -0.349014192432
CAGTGTGA -0.00706007891406
CAGTGTGC -0.237007929534
CAGTGTGG 0.00930360811239
CAGTGTTA 0.0373693252481
CAGTGTTC -0.0364729239506
CAGTTAAA -0.0844134799968
CAGTTAAC 0.0375663306643
CAGTTAAG -0.0549924166009
CAGTTACA 0.161259972409
CAGTTACC -0.0538214491653
CAGTTACG -0.00588089311913
CAGTTAGA 0.125505620766
CAGTTAGC -0.172286008336
CAGTTAGG -0.0864124287601
CAGTTATA 0.0285709072064
CAGTTATC -0.0590587112909
CAGTTATG 0.147115434994
CAGTTCAA -0.0859748395634
CAGTTCAC -0.110481906466
CAGTTCAG -0.187535505845
CAGTTCCA -0.0489242375363

CAGTTCCC -0.223562072854
CAGTTCCG -0.274120631062
CAGTTCGA 0.0250925856986
CAGTTCGC -0.202018971001
CAGTTCGG -0.218064464342
CAGTTCTA -0.0662963465741
CAGTTCTC -0.187399585867
CAGTTGAA -0.102284289153
CAGTTGAC 0.0992817675525
CAGTTGAG -0.137579161382
CAGTTGCA -0.0817525461682
CAGTTGCC -0.282891055485
CAGTTGCG -0.106043355331
CAGTTGGA -0.00739421764487
CAGTTGGC -0.308537153047
CAGTTGGG -0.102618712667
CAGTTGTA -0.15427346716
CAGTTGTC -0.279633075577
CAGTTTAA -0.10175530032
CAGTTTAC -0.197363523452
CAGTTTAG -0.0344380474973
CAGTTTCA -0.0312175645789
CAGTTTCC -0.147752101362
CAGTTTCG 0.0191560345852
CAGTTTGA -0.185111147116
CAGTTTGC -0.281361238206
CAGTTTGG -0.0551091965684
CAGTTTTA -0.161603800433
CAGTTTTC 0.00010754022418
CATAAAAA 0.0995766516062
CATAAAAC 0.177826033007
CATAAAAG 0.0320336051035
CATAAACA -0.0419939709349
CATAAACC -0.0878834944654
CATAAACG -0.248986820272
CATAAAGA 0.151394495011
CATAAAGC -0.248487055146
CATAAAGG -0.00147806982329
CATAAATA 0.149613801129
CATAAATC 0.17789552638
CATAAATG -0.0826885209529
CATAACAA -0.117781362452
CATAACAC 0.112138309108
CATAACAG -0.00031298516147
CATAACCA -0.199534570212
CATAACCC -0.238285482684
CATAACCG -0.159091804757
CATAACGA 0.0154821427268
CATAACGC -0.0115736246604
CATAACGG -0.194485110653
CATAACTA 0.0901680025808
CATAACTC 0.0172059111453
CATAAGAA 0.0133866649018
CATAAGAC 0.0469383882105

CATAAGAG 0.127470445652
CATAAGCA -0.108374214435
CATAAGCC -0.169397667023
CATAAGCG -0.150520662303
CATAAGGA 0.0988771140286
CATAAGGC -0.261317415154
CATAAGGG -0.251338556708
CATAAGTA 0.137076560367
CATAAGTC -0.111186936993
CATAATAA -0.0747041807648
CATAATAC 0.225789150032
CATAATAG 0.0821895708719
CATAATCA 0.264684794988
CATAATCC 0.338179777574
CATAATCG 0.14266767881
CATAATGA 0.00325330588391
CATAATGC -0.215099369058
CATAATGG -0.101358392259
CATAATTA 0.0936994080422
CATAATTC 0.0139843653059
CATACAAA 0.126487011233
CATACAAC 0.100934267601
CATACAAG 0.146434019693
CATACACA 0.0492489734914
CATACACC 0.00250724436691
CATACACG -0.118474775796
CATACAGA 0.0493896099957
CATACAGC -0.178233216717
CATACAGG -0.0501589349566
CATACATA 0.00822098786158
CATACATC 0.0603716706933
CATACATG 0.0229243155259
CATACCAA -0.222487640184
CATACCAC -0.0965138692411
CATACCAG -0.146257233039
CATACCCA -0.0229666503653
CATACCCC -0.188135981055
CATACCCG -0.259389333921
CATACCGA 0.282859994981
CATACCGC -0.0355202104166
CATACCGG -0.290295672126
CATACCTA -0.105457775001
CATACCTC -0.0772515930308
CATACGAA 0.0485916366795
CATACGAC 0.00805456814814
CATACGAG -0.0113026722043
CATACGCA -0.0759300155221
CATACGCC -0.130215527818
CATACGCG -0.058797824151
CATACGGA 0.0900717020518
CATACGGC -0.279236746794
CATACGGG -0.183387354992
CATACGTA -0.0371045977107
CATACGTC -0.23988963746

CATACTAA -0.0130486193733
CATACTAC 0.0205343084131
CATACTAG 0.115336584244
CATACTCA 0.0551776238477
CATACTCC -0.121305372875
CATACTCG 0.00265141174232
CATACTGA 0.0856178331861
CATACTGC -0.082567086442
CATACTGG -0.101192205127
CATACTTA 0.0799070093356
CATACTTC -0.0462999727027
CATAGAAA 0.0432333598534
CATAGAAC -0.0594933776752
CATAGAAG -0.00217297186994
CATAGACA 0.243754774058
CATAGACC -0.173548567175
CATAGACG -0.0599345902376
CATAGAGA 0.13855390028
CATAGAGC -0.204978353636
CATAGAGG -0.126386166861
CATAGATA 0.276047674223
CATAGATC 0.161978204857
CATAGATG 0.124117230178
CATAGCAA 0.0926278631164
CATAGCAC 0.0520828011065
CATAGCAG 0.0706353736657
CATAGCCA -0.291087078672
CATAGCCC -0.125247622315
CATAGCCG -0.252468066539
CATAGCGA 0.170369261987
CATAGCGC -0.0319203501022
CATAGCGG -0.0464286040792
CATAGCTA -0.2355662799
CATAGCTC -0.0507587425093
CATAGGAA 0.192430092772
CATAGGAC -0.145529309603
CATAGGAG 0.107450931731
CATAGGCA -0.179376334398
CATAGGCC -0.260154504609
CATAGGCG -0.259093913655
CATAGGGA 0.0110372080069
CATAGGGC -0.112883706185
CATAGGGG -0.283266527656
CATAGGTA -0.067484523773
CATAGGTC -0.175458836895
CATAGTAA 0.07046026743
CATAGTAC -0.232825441304
CATAGTAG 0.0438727619103
CATAGTCA -0.0262612601434
CATAGTCC -0.0252648219267
CATAGTCG -0.198139918168
CATAGTGA 0.0639044400488
CATAGTGC -0.16239425414
CATAGTGG -0.0888366811029

CATAGTTA -0.102098836549
CATAGTTC 0.00950132764226
CATATAAA 0.0545367619687
CATATAAC 0.145073465545
CATATAAG 0.188149582089
CATATACA 0.143400509478
CATATACC -0.0201141580841
CATATACG 0.0396555574857
CATATAGA 0.131181798939
CATATAGC -0.261570651436
CATATAGG -0.0359718793838
CATATATA 0.0913359561469
CATATATC 0.191422815559
CATATATG 0.360346913856
CATATCAA 0.147617314284
CATATCAC 0.281391648358
CATATCAG 0.235416012788
CATATCCA 0.364382288625
CATATCCC 0.273051288203
CATATCCG 0.342663600239
CATATCGA 0.211662075298
CATATCGC 0.325634299942
CATATCGG 0.189031627045
CATATCTA 0.374181080477
CATATCTC 0.33943125333
CATATGAA 0.0321718706933
CATATGAC -0.0241081301687
CATATGAG -0.060116201835
CATATGCA 0.0662861397196
CATATGCC 0.0486140407475
CATATGCG 0.0133070078757
CATATGGA 0.0586550738066
CATATGGC -0.287748969662
CATATGGG -0.0444392060853
CATATGTA 0.0566960885871
CATATGTC -0.174479317427
CATATTAA 0.176941380846
CATATTAC 0.259056371028
CATATTAG 0.07092034481
CATATTCA -0.0407852382795
CATATTCC 0.361957767348
CATATTCG 0.107014234601
CATATTGA -0.0448950986834
CATATTGC 0.173428249186
CATATTGG -0.171514254477
CATATTTA 0.128991194324
CATATTTC 0.345010693196
CATCAAAA 0.0968497040211
CATCAAAC 0.12507962508
CATCAAAG -0.101560025514
CATCAACA 0.0754349390713
CATCAACC -0.227469611905
CATCAACG 0.0587220717795
CATCAAGA 0.0775088716282

CATCAAGC -0.147157413609
CATCAAGG -0.0903144862197
CATCAATA 0.0178053346118
CATCAATC -0.209284117256
CATCAATG -0.158854061227
CATCACAA -0.0721622478664
CATCACAC 0.152515638344
CATCACAG 0.0863002183677
CATCACCA 0.046220155763
CATCACCC -0.185114887869
CATCACCG -0.0116162994951
CATCACGA 0.170750640448
CATCACGC -0.130051304003
CATCACGG -0.116238934515
CATCACTA -0.014602757027
CATCACTC -0.127244324214
CATCAGAA 0.129981825216
CATCAGAC 0.0238534399089
CATCAGAG -0.066858308861
CATCAGCA -0.0216440515901
CATCAGCC -0.0769801989898
CATCAGCG -0.0957227419226
CATCAGGA 0.0171866080957
CATCAGGC -0.103177754067
CATCAGGG -0.123430455476
CATCAGTA 0.104443698313
CATCAGTC -0.204796407037
CATCATAA 0.0330729984015
CATCATAC 0.0583021037566
CATCATAG 0.0796957589895
CATCATCA -0.032808290384
CATCATCC -0.180521438989
CATCATCG -0.0079983564832
CATCATGA 0.0770924419584
CATCATGC -0.144868200127
CATCATGG -0.0410915215941
CATCATTA 0.0781688218213
CATCATTC -0.0103124010107
CATCCAAA 0.0580732247399
CATCCAAC 0.0864680107104
CATCCAAG -0.186570868389
CATCCACA 0.0560389188558
CATCCACC -0.0904688218506
CATCCACG -0.0335007262615
CATCCAGA 0.152580955611
CATCCAGC -0.190394078485
CATCCAGG -0.0660136720743
CATCCATA -0.0654036772917
CATCCATC -0.138746787205
CATCCATG -0.0891373745955
CATCCCAA -0.170450128596
CATCCCAC -0.225187114915
CATCCCAG -0.115496438011
CATCCCCA -0.168355432034

CATCCCCC -0.295592208087
CATCCCCG -0.0937052473304
CATCCCGA 0.0188273673122
CATCCCGC -0.093696863903
CATCCCGG -0.0513435634418
CATCCCTA -0.151003066934
CATCCCTC -0.181457315107
CATCCGAA 0.095103756852
CATCCGAC -0.194351936869
CATCCGAG -0.139409884795
CATCCGCA 0.0730283278468
CATCCGCC -0.0961934539319
CATCCGCG -0.0582943439195
CATCCGGA 0.113575007514
CATCCGGC -0.0852872821709
CATCCGGG -0.238911568399
CATCCGTA 0.0641288671592
CATCCGTC -0.29749640261
CATCCTAA -0.0193570094986
CATCCTAC -0.168335729875
CATCCTAG -0.142128250373
CATCCTCA -0.168241015484
CATCCTCC -0.049977977121
CATCCTCG -0.300799254146
CATCCTGA -0.0268684662624
CATCCTGC -0.207470481679
CATCCTGG -0.249629868572
CATCCTTA 0.0105800602907
CATCCTTC -0.106085424267
CATCGAAA 0.172154247912
CATCGAAC -0.048368513799
CATCGAAG -0.0818993325228
CATCGACA -0.175588257188
CATCGACC -0.10696033988
CATCGACG -0.205211030322
CATCGAGA 0.210774216623
CATCGAGC -0.377782497086
CATCGAGG -0.116690430359
CATCGATA 0.156976535764
CATCGATC -0.0208748693597
CATCGATG 0.157016695195
CATCGCAA 0.0166670803445
CATCGCAC 0.0677817274074
CATCGCAG -0.123109898138
CATCGCCA -0.0588217451848
CATCGCCC -0.196473215263
CATCGCCG -0.134822351822
CATCGCGA 0.229569790176
CATCGCGC 0.0145069004977
CATCGCGG 0.0337926007782
CATCGCTA -0.118404544264
CATCGCTC -0.177719280065
CATCGGAA 0.0449223328011
CATCGGAC -0.130495229282

CATCGGAG -0.0333898608871
CATCGGCA -0.293652959352
CATCGGCC -0.118602227901
CATCGGCG -0.0680737742605
CATCGGGA -0.120456068088
CATCGGGC -0.309882848354
CATCGGGG -0.0190706042933
CATCGGTA 0.0597001960638
CATCGGTC -0.362441575573
CATCGTAA 0.114504311474
CATCGTAC -0.126674896405
CATCGTAG -0.069634241586
CATCGTCA -0.0125989941826
CATCGTCC 0.103655997595
CATCGTCG -0.238126845488
CATCGTGA 0.0160798716588
CATCGTGC -0.25025889414
CATCGTGG -0.240692952201
CATCGTTA -0.0682319023838
CATCGTTC -0.171662407054
CATCTAAA 0.0992190537645
CATCTAAC 0.112265424845
CATCTAAG -0.0530798595651
CATCTACA 0.24227571659
CATCTACC 0.0662687480869
CATCTACG 0.0882571914484
CATCTAGA 0.0702809427531
CATCTAGC -0.159916210289
CATCTAGG 0.00144279315349
CATCTATA 0.147566841602
CATCTATC -0.105779435487
CATCTCAA -0.0564958431567
CATCTCAC 0.151931285207
CATCTCAG -0.0921251406694
CATCTCCA -0.16500012406
CATCTCCC -0.0892945286885
CATCTCCG -0.048449275722
CATCTCGA 0.185601045233
CATCTCGC 0.0107669175128
CATCTCGG -0.0984384373864
CATCTCTA -0.088526543072
CATCTCTC -0.247996725297
CATCTGAA 0.0886426249038
CATCTGAC -0.196211099807
CATCTGAG -0.0661603221083
CATCTGCA -0.109420231674
CATCTGCC -0.109822071122
CATCTGCG -0.0844222389291
CATCTGGA 0.201600694875
CATCTGGC -0.244269189405
CATCTGGG -0.0884534672413
CATCTGTA -0.0329752367317
CATCTGTC -0.114172642235
CATCTTAA -0.0691938868072

CATCTTAC 0.000444790465155
CATCTTAG -0.119256273016
CATCTTCA 0.112811158266
CATCTTCC 0.290294426658
CATCTTCG -0.0112909936279
CATCTTGA -0.0536583415371
CATCTTGC -0.0828865061349
CATCTTGG -0.159722353992
CATCTTTA 0.0867594140274
CATCTTTC -0.212881852247
CATGAAAA 0.236438918257
CATGAAAC 0.132737969242
CATGAAAG 0.0620358650662
CATGAACA -0.0445473021231
CATGAACC -0.0283657146638
CATGAACG -0.0230513200441
CATGAAGA 0.124154215601
CATGAAGC -0.229536104515
CATGAAGG 0.0962161412524
CATGAATA 0.163420996754
CATGAATC 0.160724084967
CATGAATG -0.0914206258257
CATGACAA -0.0818188836776
CATGACAC -0.00293638386395
CATGACAG -0.246317692441
CATGACCA -0.114266840872
CATGACCC -0.208987686175
CATGACCG -0.180331397699
CATGACGA -0.119878175345
CATGACGC -0.316687200434
CATGACGG -0.1250037552
CATGACTA -0.120017647627
CATGACTC -0.190221571595
CATGAGAA 0.142915642916
CATGAGAC -0.0553004922033
CATGAGAG 0.0977816381961
CATGAGCA -0.104855269362
CATGAGCC -0.167689474453
CATGAGCG -0.2018098113
CATGAGGA -0.133849400905
CATGAGGC -0.215240392964
CATGAGGG -0.167432434602
CATGAGTA 0.201825507471
CATGAGTC -0.122677336821
CATGATAA 0.0864183742972
CATGATAC 0.132974460413
CATGATAG 0.0291840324664
CATGATCA -0.0758268067123
CATGATCC 0.104528367992
CATGATCG 0.114904160359
CATGATGA 0.0648270475829
CATGATGC 0.0229173387597
CATGATGG -0.119918379727
CATGATTA 0.26634380269

CATGATTC 0.298633344088
CATGCAAA 0.0822158246401
CATGCAAC -0.13416033113
CATGCAAG 0.0247899681029
CATGCACA -0.0610445384199
CATGCACC -0.192490479728
CATGCACG -0.234699387757
CATGCAGA 0.102586731022
CATGCAGC -0.286558040646
CATGCAGG -0.0530185470391
CATGCATA 0.144848409646
CATGCATC -0.210551628408
CATGCATG -0.22499813965
CATGCCAA -0.0973114943955
CATGCCAC 0.00997918131558
CATGCCAG -0.294210774399
CATGCCCA -0.0786651757757
CATGCCCC -0.108249373402
CATGCCCG -0.160951341158
CATGCCGA 0.130488383466
CATGCCGC -0.158749096959
CATGCCGG 0.142136303585
CATGCCTA -0.0521916437121
CATGCCTC -0.136601128785
CATGCGAA 0.110392290821
CATGCGAC -0.182442619743
CATGCGAG -0.134290393826
CATGCGCA 0.121195278036
CATGCGCC -0.13379925987
CATGCGCG -0.00287213716781
CATGCGGA 0.0445133804856
CATGCGGC -0.215274504453
CATGCGGG -0.171005911979
CATGCGTA 0.0712709955134
CATGCGTC -0.237143344498
CATGCTAA -0.0130809985855
CATGCTAC -0.0936424749823
CATGCTAG -0.207318379068
CATGCTCA -0.153061313862
CATGCTCC -0.311627828924
CATGCTCG -0.259868013
CATGCTGA -0.0957475383751
CATGCTGC -0.0833045047096
CATGCTGG 0.00865836676731
CATGCTTA -0.0655104139953
CATGCTTC -0.256336295403
CATGGAAA 0.111997672604
CATGGAAC -0.0405515708546
CATGGAAG -0.151464714201
CATGGACA -0.24559776016
CATGGACC -0.206698191
CATGGACG -0.106563000396
CATGGAGA 0.198257486136
CATGGAGC -0.21063703721

CATGGAGG -0.222850794123
CATGGATA 0.310164740918
CATGGATC 0.169945427521
CATGGCAA -0.104038975326
CATGGCAC -0.0690863750279
CATGGCAG -0.191466457153
CATGGCCA -0.283307384423
CATGGCCC -0.338331219956
CATGGCCG -0.381556354817
CATGGCGA -0.149188111605
CATGGCGC -0.246923246437
CATGGCGG -0.186780506103
CATGGCTA -0.11279606257
CATGGCTC -0.0712575370338
CATGGGAA 0.0549033718756
CATGGGAC -0.31154113882
CATGGGAG -0.314901176075
CATGGGCA -0.337248628151
CATGGGCC -0.275682849984
CATGGGCG -0.267695573827
CATGGGGA 0.103197010284
CATGGGGC -0.154837744551
CATGGGGG -0.390136012237
CATGGGTA -0.0227191449795
CATGGGTC -0.38457627539
CATGGTAA 0.014723035262
CATGGTAC -0.26581345022
CATGGTAG 0.184535375138
CATGGTCA -0.227196297491
CATGGTCC 0.0461442450165
CATGGTCG -0.353570060174
CATGGTGA -0.144925898885
CATGGTGC -0.297119889803
CATGGTGG -0.213041003525
CATGGTTA -0.0567770415774
CATGGTTC -0.191125016969
CATGTAAA -0.0265023393648
CATGTAAC 0.0896998927302
CATGTAAG -0.0764194944636
CATGTACA 0.0980811226642
CATGTACC -0.180652877979
CATGTACG 0.0026293511142
CATGTAGA 0.0903097012474
CATGTAGC -0.184137773257
CATGTAGG 0.101547828821
CATGTATA 0.274337788224
CATGTATC 0.0243724589972
CATGTCAA -0.16989579882
CATGTCAC 0.0542229002285
CATGTCAG -0.12087043586
CATGTCCA 0.0180503511428
CATGTCCC -0.152703675212
CATGTCCG -0.0158338227549
CATGTCGA -0.025588490763

CATGTCGC -0.108226164385
CATGTCGG -0.00665323871065
CATGTCTA -0.0996890579038
CATGTCTC -0.0674674366359
CATGTGAA -0.00643562549725
CATGTGAC 0.0410570689975
CATGTGAG 0.0306102826233
CATGTGCA -0.0526915469004
CATGTGCC -0.157868656011
CATGTGCG -0.168331506485
CATGTGGA -0.104763399342
CATGTGGC -0.151189299877
CATGTGGG -0.135965635789
CATGTGTA 0.0258067985341
CATGTGTC -0.254835075512
CATGTTAA 0.00165041074132
CATGTTAC -0.125843651522
CATGTTAG -0.216967586275
CATGTTCA -0.0341181401787
CATGTTCC 0.0495441705656
CATGTTCG -0.0802938621782
CATGTTGA -0.0348702550231
CATGTTGC -0.114298047523
CATGTTGG -0.129761048113
CATGTTTA 0.0853463062853
CATGTTTC -0.0528702361612
CATTAAAA -0.0800566406627
CATTAAAC 0.078746790868
CATTAAAG 0.148851970337
CATTAACA 0.203063945488
CATTAACC -0.0589862834138
CATTAACG -0.108360426542
CATTAAGA 0.194616402514
CATTAAGC -0.199629716661
CATTAAGG 0.0716093808165
CATTAATA 0.128620355893
CATTAATC 0.145820894433
CATTAATG 0.153387244809
CATTACAA 0.161225964256
CATTACAC -0.041442268715
CATTACAG -0.179888252663
CATTACCA 0.0732261186807
CATTACCC -0.132632963405
CATTACCG -0.0535650424839
CATTACGA 0.136396727306
CATTACGC -0.102575874135
CATTACGG 0.0291052020759
CATTACTA 0.0994313873102
CATTACTC -0.164502585794
CATTAGAA -0.14904748906
CATTAGAC -0.176677831535
CATTAGAG -0.0155872125569
CATTAGCA -0.0134544499026
CATTAGCC -0.0416611315081

CATTAGCG -0.0807015264602
CATTAGGA 0.0388714115749
CATTAGGC -0.233121900525
CATTAGGG -0.204310514679
CATTAGTA 0.0716123004606
CATTAGTC -0.164700513185
CATTATAA 0.275237038605
CATTATAC 0.141928653258
CATTATAG 0.134457639614
CATTATCA 0.0478259600155
CATTATCC 0.206383071904
CATTATCG -0.0594014073928
CATTATGA 0.0073420527966
CATTATGC -0.0555514193931
CATTATGG -0.114485971077
CATTATTA 0.220345186987
CATTATTC 0.17340861149
CATTCAAA -0.0460033035791
CATTCAAC 0.00725166602191
CATTCAAG -0.00422648913366
CATTCACA 0.155368130625
CATTCACC -0.200167433278
CATTCACG -0.116839937474
CATTCAGA 0.187201010197
CATTCAGC -0.213742015345
CATTCAGG 0.00705021057933
CATTCATA 0.108283030299
CATTCATC -0.0439985439985
CATTCCAA -0.101728640909
CATTCCAC 0.0220425830091
CATTCCAG -0.0266286710067
CATTCCCA 0.0283713465532
CATTCCCC -0.147469458988
CATTCCCG -0.258654156228
CATTCCGA -0.0900004963149
CATTCCGC -0.218217942862
CATTCCGG -0.250682628474
CATTCCTA -0.029711459472
CATTCCTC 0.0715246927368
CATTCGAA -0.0750106191513
CATTCGAC -0.0840209970382
CATTCGAG 0.0617614212827
CATTCGCA 0.120551374787
CATTCGCC 0.0780408507681
CATTCGCG 0.0201331357707
CATTCGGA 0.0403833585652
CATTCGGC -0.310788308402
CATTCGGG -0.140692586987
CATTCGTA -0.0364244017581
CATTCGTC -0.107726838098
CATTCTAA 0.0894159463587
CATTCTAC -0.00194219683555
CATTCTAG 0.0899659990569
CATTCTCA 0.000972046426592

CATTCTCC -0.0386764174643
CATTCTCG 0.00910875637282
CATTCTGA 0.0502251562858
CATTCTGC -0.161030755617
CATTCTGG -0.0612775309745
CATTCTTA 0.0181798938709
CATTCTTC -0.0371156280247
CATTGAAA 0.0892732275936
CATTGAAC 0.0596359203813
CATTGAAG 0.152407611512
CATTGACA 0.121295884032
CATTGACC -0.0408080256565
CATTGACG -0.186117002803
CATTGAGA -0.00608788568409
CATTGAGC -0.340008874998
CATTGAGG -0.110919009105
CATTGATA 0.0716298183251
CATTGATC 0.0958823837612
CATTGCAA -0.090497793698
CATTGCAC -0.0186053822417
CATTGCAG -0.327873575101
CATTGCCA 0.0991363264091
CATTGCCC -0.161234776231
CATTGCCG -0.258151773375
CATTGCGA 0.0774242386637
CATTGCGC -0.075106787228
CATTGCGG 0.0448459877386
CATTGCTA -0.0360760209245
CATTGCTC -0.0274920987132
CATTGGAA 0.104347350058
CATTGGAC -0.162086328753
CATTGGAG -0.0288401349007
CATTGGCA -0.184914712231
CATTGGCC -0.240525127718
CATTGGCG -0.254498540245
CATTGGGA -0.0501543761815
CATTGGGC -0.299766264122
CATTGGGG -0.284259557938
CATTGGTA -0.169973972665
CATTGGTC -0.0927483725816
CATTGTAA -0.0341839229798
CATTGTAC 0.0244473123261
CATTGTAG -0.0953017548214
CATTGTCA 0.141341166836
CATTGTCC 0.0457220305705
CATTGTCG -0.0860018474859
CATTGTGA 0.073950935381
CATTGTGC -0.282381105956
CATTGTGG -0.0199097829975
CATTGTTA 0.101877359453
CATTGTTC 0.0560038831266
CATTTAAA 0.121640402035
CATTTAAC 0.18359819875
CATTTAAG 0.0850781911388

CATTTACA 0.1744421655
CATTTACC 0.106834886827
CATTTACG 0.225518907337
CATTTAGA 0.116859484829
CATTTAGC -0.214510251467
CATTTAGG -0.130105332374
CATTTATA 0.171499164252
CATTTATC 0.104995511047
CATTTCAA -0.0100735020401
CATTTCAC 0.0320219265272
CATTTCAG -0.0706018096179
CATTTCCA 0.0768468192711
CATTTCCC -0.211887491074
CATTTCCG -0.0952347474146
CATTTCGA 0.158559385832
CATTTCGC -0.102929134732
CATTTCGG 0.116139792559
CATTTCTA 0.00883951366983
CATTTCTC 0.0973880216304
CATTTGAA 0.120373276498
CATTTGAC 0.082032510237
CATTTGAG 0.0841580111384
CATTTGCA 0.0484186477668
CATTTGCC -0.00432180317219
CATTTGCG -0.110261987666
CATTTGGA -0.0334976850128
CATTTGGC -0.101905067772
CATTTGGG -0.254158889348
CATTTGTA 0.113774880842
CATTTGTC -0.107952775227
CATTTTAA -0.0751656697526
CATTTTAC 0.0458226821863
CATTTTAG -0.038690393641
CATTTTCA 0.156208988124
CATTTTCC 0.104026189208
CATTTTCG 0.0144235144235
CATTTTGA -0.105707636011
CATTTTGC 0.0556184901061
CATTTTGG -0.136135304059
CATTTTTA 0.142995612693
CATTTTTC 0.018183735762
CCAAAAAA 0.0860295030036
CCAAAAAC -0.127508878046
CCAAAAAG 0.138373911101
CCAAAACA 0.0420363057743
CCAAAACC -0.113296275157
CCAAAACG -0.0623407960598
CCAAAAGA -0.0381429603731
CCAAAAGC -0.0630491661359
CCAAAAGG -0.166954762121
CCAAAATA 0.234497582982
CCAAAATC 0.394842500903
CCAAACAA -0.0434147513644
CCAAACAC -0.138422652276

CCAAACAG -0.22987652591
CCAAACCA 0.0496033223306
CCAAACCC -0.0993722551671
CCAAACCG -0.307948305224
CCAAACGA -0.161565652806
CCAAACGC 0.25990598746
CCAAACGG -0.0313932936833
CCAAACTA -0.0254400405916
CCAAACTC 0.133565808741
CCAAAGAA 0.159330998564
CCAAAGAC 0.0534656427124
CCAAAGAG -0.086317087947
CCAAAGCA -0.206651283196
CCAAAGCC -0.261393237242
CCAAAGCG -0.110231749103
CCAAAGGA 0.0829801955773
CCAAAGGC -0.320764009789
CCAAAGGG -0.33097080636
CCAAAGTA -0.189574189518
CCAAAGTC -0.0970672175062
CCAAATAA -0.00545643536785
CCAAATAC -0.0466382696712
CCAAATAG -0.116624686701
CCAAATCA 0.259307516883
CCAAATCC 0.3137588228
CCAAATCG 0.231059903798
CCAAATGA 0.0728097243249
CCAAATGC -0.123279223101
CCAAATGG -0.20020899333
CCAAATTA 0.191777176626
CCAAATTC 0.213109931899
CCAACAAA 0.219909782997
CCAACAAC -0.179951361144
CCAACAAG 0.0685131647517
CCAACACA -0.053750989134
CCAACACC -0.193240887775
CCAACACG -0.301092789313
CCAACAGA 0.0276295800006
CCAACAGC -0.247218634383
CCAACAGG -0.307631752128
CCAACATA 0.0655686371831
CCAACATC 0.0428100276585
CCAACCAA -0.199346243034
CCAACCAC -0.276305815354
CCAACCAG -0.09499241889
CCAACCCA -0.101851622065
CCAACCCC -0.188695999872
CCAACCCG -0.325069763115
CCAACCGA -0.188896316286
CCAACCGC 0.00897453927757
CCAACCGG -0.293167227611
CCAACCTA 0.0032663517512
CCAACCTC -0.0595615217709
CCAACGAA -0.0658883382116

CCAACGAC -0.238107843543
CCAACGAG -0.0559764291334
CCAACGCA -0.027024344244
CCAACGCC -0.285975402989
CCAACGCG -0.155953519266
CCAACGGA -0.131507282698
CCAACGGC -0.20598741841
CCAACGGG -0.28795338243
CCAACGTA -0.00807930402173
CCAACGTC -0.36134214025
CCAACTAA -0.0539092824017
CCAACTAC -0.172269324572
CCAACTAG -0.083613875172
CCAACTCA -0.108909621763
CCAACTCC -0.177488561483
CCAACTCG -0.00754800989759
CCAACTGA -0.0214551636842
CCAACTGC -0.0155948563554
CCAACTGG -0.288923348358
CCAACTTA 0.0350422983438
CCAACTTC 0.121861530952
CCAAGAAA 0.0485999090044
CCAAGAAC -0.0744634660712
CCAAGAAG 0.0520857207506
CCAAGACA 0.0913403356131
CCAAGACC -0.207029236575
CCAAGACG 0.0471131727211
CCAAGAGA -0.117655978187
CCAAGAGC -0.153181318181
CCAAGAGG -0.332618583117
CCAAGATA 0.233226279717
CCAAGATC 0.259745407035
CCAAGCAA 0.00343569118924
CCAAGCAC -0.200221055504
CCAAGCAG -0.181458292286
CCAAGCCA -0.123383750443
CCAAGCCC -0.13749450851
CCAAGCCG -0.295379442446
CCAAGCGA -0.126539239591
CCAAGCGC -0.0627853385604
CCAAGCGG -0.225588578233
CCAAGCTA -0.166963091644
CCAAGCTC -0.227465527786
CCAAGGAA -0.0931835996514
CCAAGGAC -0.214020304261
CCAAGGAG -0.322840649246
CCAAGGCA 0.0796821967402
CCAAGGCC -0.22492397864
CCAAGGCG -0.00722347692045
CCAAGGGA -0.110124239288
CCAAGGGC -0.334201668858
CCAAGGGG -0.342541375556
CCAAGGTA -0.105735086134
CCAAGGTC -0.0635561152329

CCAAGTAA -0.0368862577381
CCAAGTAC -0.0403608029976
CCAAGTAG -0.223083794447
CCAAGTCA 0.0318263103728
CCAAGTCC -0.0643495427237
CCAAGTCG -0.256293741955
CCAAGTGA -0.182395853555
CCAAGTGC -0.0883225847747
CCAAGTGG -0.0610453936432
CCAAGTTA 0.0903534959088
CCAAGTTC -0.158196108919
CCAATAAA 0.18478671509
CCAATAAC -0.0957183419341
CCAATAAG 0.205319644714
CCAATACA 0.0844470559039
CCAATACC -0.128721998788
CCAATACG -0.0148615281535
CCAATAGA 0.182497463559
CCAATAGC -0.207375528559
CCAATAGG -0.074077922239
CCAATATA 0.203962686948
CCAATATC 0.243534167777
CCAATCAA -0.0139433456585
CCAATCAC -0.0334728668062
CCAATCAG -0.157900767314
CCAATCCA -0.0178777107071
CCAATCCC 0.0599765491228
CCAATCCG 0.0200190263474
CCAATCGA 0.0378904111589
CCAATCGC 0.00381762154014
CCAATCGG 0.153467442319
CCAATCTA 0.145954494439
CCAATCTC 0.245637686766
CCAATGAA 0.0354682657737
CCAATGAC -0.0642511477851
CCAATGAG -0.205624608904
CCAATGCA -0.0669471124017
CCAATGCC -0.0911384478855
CCAATGCG -0.164062133759
CCAATGGA -0.280902312045
CCAATGGC -0.300546659409
CCAATGGG -0.239740546858
CCAATGTA -0.107950174934
CCAATGTC -0.0681165893319
CCAATTAA 0.123622840376
CCAATTAC 0.0832062071633
CCAATTAG -0.0346218695941
CCAATTCA 0.0431107348014
CCAATTCC -0.00282513918878
CCAATTCG -0.297142293713
CCAATTGA 0.00550398371764
CCAATTGC 0.026293584812
CCAATTGG 0.055315190156
CCAATTTA -0.13341386685

CCAATTTC 0.155041130486
CCACAAAA 0.0966379602743
CCACAAAC -0.0356740904665
CCACAAAG -0.0370204915659
CCACAACA 0.0803420363058
CCACAACC 0.00708197515798
CCACAACG -0.208979173082
CCACAAGA -0.168568342856
CCACAAGC -0.259316364549
CCACAAGG -0.127707480461
CCACAATA 0.049906922485
CCACAATC 0.376490295833
CCACACAA 0.146265523579
CCACACAC -0.162680881806
CCACACAG 0.030456997292
CCACACCA -0.0394818476891
CCACACCC -0.155639373461
CCACACCG -0.243952963358
CCACACGA -0.148434592194
CCACACGC -0.0357855421652
CCACACGG 0.00536873888406
CCACACTA -0.0553349711724
CCACACTC 0.0825357037478
CCACAGAA 0.0331693466566
CCACAGAC -0.326027272652
CCACAGAG -0.00960927862894
CCACAGCA 0.0338021440802
CCACAGCC -0.336921011738
CCACAGCG -0.0592172563163
CCACAGGA -0.000505828339525
CCACAGGC -0.183348907468
CCACAGGG -0.106850542392
CCACAGTA -0.0996306650219
CCACAGTC -0.0947859196947
CCACATAA -0.106010664931
CCACATAC -0.120042239007
CCACATAG -0.0306944616785
CCACATCA 0.131178879295
CCACATCC -0.104852167548
CCACATCG -0.234987731095
CCACATGA -0.0531035006255
CCACATGC -0.15765810481
CCACATGG -0.137269740539
CCACATTA -0.119692370468
CCACATTC 0.147851708458
CCACCAAA -0.0831976982668
CCACCAAC -0.24897670611
CCACCAAG 0.05433092706
CCACCACA 0.136422924302
CCACCACC -0.172004278912
CCACCACG -0.277357312042
CCACCAGA -0.159766971076
CCACCAGC -0.315644475316
CCACCAGG -0.219505437704

CCACCATA -0.0161951918768
CCACCATC -0.115174972885
CCACCCAA -0.0397662824902
CCACCCAC -0.150751115853
CCACCCAG -0.222455748601
CCACCCCA 0.143987357941
CCACCCCC -0.314120743589
CCACCCCG -0.308836296518
CCACCCGA -0.182911094018
CCACCCGC -0.000263445576231
CCACCCGG -0.140706551858
CCACCCTA -0.146435665094
CCACCCTC -0.0815392843654
CCACCGAA -0.102669065641
CCACCGAC -0.157163180401
CCACCGAG -0.0382765685796
CCACCGCA -0.0768614897757
CCACCGCC -0.376655164112
CCACCGCG -0.295256131414
CCACCGGA -0.0724927789641
CCACCGGC -0.3410788521
CCACCGGG -0.243504138936
CCACCGTA -0.0416377743553
CCACCGTC -0.0843390103421
CCACCTAA 0.026689331712
CCACCTAC -0.183769168618
CCACCTAG -0.319027446044
CCACCTCA -0.0360051263861
CCACCTCC -0.274933960798
CCACCTCG -0.231646559738
CCACCTGA -0.240498617555
CCACCTGC -0.152018068113
CCACCTGG -0.400625223424
CCACCTTA -0.144939477491
CCACCTTC -0.186706509921
CCACGAAA 0.0399860749887
CCACGAAC -0.184655815199
CCACGAAG -0.110430056828
CCACGACA 0.00212471424593
CCACGACC -0.289485051507
CCACGACG -0.21722559925
CCACGAGA 0.154379467746
CCACGAGC -0.2205873839
CCACGAGG -0.19414887795
CCACGATA 0.223168105808
CCACGATC 0.236292766596
CCACGCAA 0.114504791866
CCACGCAC -0.13935871334
CCACGCAG -0.24695301269
CCACGCCA 0.0265789205183
CCACGCCC -0.283616207516
CCACGCCG -0.169592883674
CCACGCGA 0.0864623402407
CCACGCGC 0.0442961103042

CCACGCGG -0.19433745262
CCACGCTA -0.0669481690182
CCACGCTC -0.101047068456
CCACGGAA -0.0685612822335
CCACGGAC -0.0956005018544
CCACGGAG -0.237919619404
CCACGGCA 0.0221998858362
CCACGGCC -0.218185837931
CCACGGCG -0.0610409411869
CCACGGGA -0.0450346892274
CCACGGGC -0.253814473913
CCACGGGG -0.32633463138
CCACGGTA -0.31437561504
CCACGGTC -0.235208662405
CCACGTAA 0.0154514864638
CCACGTAC -0.198110242732
CCACGTAG -0.151762795327
CCACGTCA 0.0534345963227
CCACGTCC -0.167748126418
CCACGTCG -0.141846833764
CCACGTGA -0.00178828966708
CCACGTGC 0.0174706386828
CCACGTGG 0.00341081040855
CCACGTTA -0.0383347711153
CCACGTTC -0.19512560005
CCACTAAA 0.0416333948892
CCACTAAC 0.111884611885
CCACTAAG 0.190532173611
CCACTACA 0.136749697356
CCACTACC -0.2292488525
CCACTACG -0.220772501244
CCACTAGA 0.0493446454195
CCACTAGC -0.15046895224
CCACTAGG 0.00177180138583
CCACTATA 0.000554002467099
CCACTATC 0.130670861222
CCACTCAA 0.0775886078916
CCACTCAC -0.18940139554
CCACTCAG -0.118677783016
CCACTCCA -0.025643065345
CCACTCCC -0.186180140766
CCACTCCG -0.284150784815
CCACTCGA -0.17293522217
CCACTCGC -0.0422595712478
CCACTCGG -0.10139809048
CCACTCTA -0.0418779661204
CCACTCTC -0.148492013106
CCACTGAA 0.0654489317752
CCACTGAC -0.313415902913
CCACTGAG -0.227600956774
CCACTGCA 0.0520393431711
CCACTGCC -0.226678009435
CCACTGCG -0.123545465823
CCACTGGA 0.0254731648212

CCACTGGC -0.300275532348
CCACTGGG -0.344776794508
CCACTGTA -0.207361279954
CCACTGTC 0.129262871687
CCACTTAA -0.101870921317
CCACTTAC -0.10797888723
CCACTTAG -0.175286857105
CCACTTCA 0.0558870973628
CCACTTCC -0.186484175132
CCACTTCG -0.210908622957
CCACTTGA 0.0531068258341
CCACTTGC -0.314251141817
CCACTTTA -0.234449048458
CCACTTTC 0.061740804165
CCAGAAAA 0.245558856375
CCAGAAAC -0.0516707365311
CCAGAAAG -0.00387420551213
CCAGAACA 0.0053626562922
CCAGAACC -0.255123036042
CCAGAACG -0.281212356766
CCAGAAGA -0.126994169458
CCAGAAGC -0.352629689987
CCAGAAGG -0.265136542432
CCAGAATA 0.163373794734
CCAGAATC 0.417676874209
CCAGACAA 0.0772742439409
CCAGACAC -0.268548149251
CCAGACAG 0.000332076551308
CCAGACCA -0.32712435326
CCAGACCC -0.367174560657
CCAGACCG -0.259847327576
CCAGACGA -0.0798946988905
CCAGACGC -0.0903000074444
CCAGACGG -0.248735138701
CCAGACTA -0.173840042381
CCAGACTC -0.23042973236
CCAGAGAA 0.0937593481109
CCAGAGAC -0.196255278034
CCAGAGAG -0.158295549235
CCAGAGCA -0.196949399224
CCAGAGCC -0.318673052312
CCAGAGCG -0.157344237984
CCAGAGGA -0.0434124637995
CCAGAGGC -0.296035171268
CCAGAGGG -0.295047775789
CCAGAGTA -0.181921941295
CCAGAGTC -0.10813243463
CCAGATAA 0.182859499427
CCAGATAC 0.384551217885
CCAGATAG 0.107605672868
CCAGATCA 0.0995620533857
CCAGATCC 0.313311934524
CCAGATCG 0.281572666292
CCAGATGA -0.080156386566

CCAGATGC 0.106918629178
CCAGATGG -0.200639712998
CCAGATTA 0.386081326686
CCAGATTC 0.448950022992
CCAGCAAA 0.196094246111
CCAGCAAC -0.172812724837
CCAGCAAG -0.0235509044441
CCAGCACA -0.0194426399422
CCAGCACC -0.296188939198
CCAGCACG -0.172814540963
CCAGCAGA -0.224132749974
CCAGCAGC -0.140819997519
CCAGCAGG -0.30476886014
CCAGCATA 0.0371225447618
CCAGCATC -0.0788836740801
CCAGCCAA -0.115920089341
CCAGCCAC -0.193600103664
CCAGCCAG -0.18757890988
CCAGCCCA -0.1935059022
CCAGCCCC -0.316414291738
CCAGCCCG -0.229738327003
CCAGCCGA -0.142283544681
CCAGCCGC 0.209050648445
CCAGCCGG -0.454771293701
CCAGCCTA -0.0180735553197
CCAGCCTC -0.178795891356
CCAGCGAA -0.0415415415415
CCAGCGAC 0.0293577539709
CCAGCGAG -0.0658898549371
CCAGCGCA -0.0301077003251
CCAGCGCC -0.423785153554
CCAGCGCG -0.12227719521
CCAGCGGA -0.178736013596
CCAGCGGC -0.416732116923
CCAGCGGG -0.424383920586
CCAGCGTA -0.27473018236
CCAGCGTC 0.068372780494
CCAGCTAA -0.00484125953034
CCAGCTAC -0.0955964443484
CCAGCTAG -0.326664020442
CCAGCTCA -0.170191475354
CCAGCTCC -0.364660593357
CCAGCTCG -0.136617847735
CCAGCTGA -0.155824318289
CCAGCTGC -0.187777192401
CCAGCTGG -0.116528442714
CCAGCTTA -0.159059262752
CCAGCTTC -0.0744551660519
CCAGGAAA 0.223795829288
CCAGGAAC -0.20699313809
CCAGGAAG 0.0146606661758
CCAGGACA -0.228093872458
CCAGGACC -0.321410801701
CCAGGACG -0.15047395717

CCAGGAGA	-0.09838882655
CCAGGAGC	-0.282464653355
CCAGGAGG	-0.103205690443
CCAGGATA	0.247044200808
CCAGGATC	0.159617672606
CCAGGCAA	-0.0172850614912
CCAGGCAC	-0.406333753026
CCAGGCAG	-0.252045695702
CCAGGCCA	-0.120603048077
CCAGGCCC	-0.363507184775
CCAGGCCG	-0.313238905206
CCAGGCGA	-0.0980834925598
CCAGGCGC	-0.140100809482
CCAGGCGG	-0.202901988029
CCAGGCTA	-0.293834878266
CCAGGCTC	-0.147359425792
CCAGGGAA	-0.145930605078
CCAGGGAC	-0.0812538426121
CCAGGGAG	-0.296446494862
CCAGGGCA	-0.301792152874
CCAGGGCC	-0.340794662566
CCAGGGCG	-0.264314785926
CCAGGGGA	-0.197134784645
CCAGGGGC	-0.306990994567
CCAGGGGG	-0.339936679814
CCAGGGTA	-0.0428862889091
CCAGGGTC	-0.216860605088
CCAGGTAA	0.0343985168208
CCAGGTAC	-0.0879210268713
CCAGGTAG	-0.156162989801
CCAGGTCA	-0.232165920676
CCAGGTCC	-0.38750496044
CCAGGTCG	-0.303281415769
CCAGGTGA	-0.0898991666656
CCAGGTGC	-0.324048850596
CCAGGTTA	-0.147712726439
CCAGGTTC	-0.163523651191
CCAGTAAA	0.177463582897
CCAGTAAC	-0.0647058823529
CCAGTAAG	0.0375455662757
CCAGTACA	-0.0510861266858
CCAGTACC	-0.164953256857
CCAGTACG	-0.104810982351
CCAGTAGA	0.0256744347653
CCAGTAGC	-0.155632541595
CCAGTAGG	-0.108184232077
CCAGTATA	-0.175891697885
CCAGTATC	0.146555519125
CCAGTCAA	0.090045576327
CCAGTCAC	-0.231446251742
CCAGTCAG	-0.0572317514812
CCAGTCCA	-0.283762995311
CCAGTCCC	-0.255698058705
CCAGTCCG	-0.259885525649

CCAGTCGA -0.093969475121
CCAGTCGC 0.0200828294359
CCAGTCGG -0.222067615902
CCAGTCTA -0.220140973583
CCAGTCTC 0.0314842847414
CCAGTGAA -0.112360108191
CCAGTGAC -0.205891111004
CCAGTGAG -0.129809773543
CCAGTGCA -0.0203778228331
CCAGTGCC -0.377110402638
CCAGTGCG -0.102544469829
CCAGTGGA -0.0696838755356
CCAGTGGC -0.227756208291
CCAGTGGG -0.253986169336
CCAGTGTA -0.0854724448525
CCAGTGTC -0.130293581674
CCAGTTAA -0.251031524438
CCAGTTAC -0.00407234735719
CCAGTTAG -5.10152025303E-5
CCAGTTCA -0.144665271678
CCAGTTCC -0.246662296914
CCAGTTCG -0.283047830509
CCAGTTGA -0.0157517929374
CCAGTTGC -0.316096382729
CCAGTTTA -0.0968261846659
CCAGTTTC -0.0119771099903
CCATAAAA 0.154428369358
CCATAAAC 0.0175037296249
CCATAAAG -0.00151238167716
CCATAACA 0.103531906562
CCATAACC -0.315822259709
CCATAACG -0.0346417879293
CCATAAGA 0.114969015277
CCATAAGC -0.198334026577
CCATAAGG -0.160861449553
CCATAATA 0.161151509636
CCATAATC 0.294970913046
CCATACAA 0.0751784842694
CCATACAC -0.221532998646
CCATACAG -0.102379565688
CCATACCA -0.135462418422
CCATACCC -0.0946619336551
CCATACCG -0.0353669519221
CCATACGA -0.0462128795462
CCATACGC -0.0694316931755
CCATACGG -0.195931283774
CCATACTA 0.07319027016
CCATACTC -0.00972184305518
CCATAGAA -0.00102168283986
CCATAGAC -0.0927249563613
CCATAGAG -0.151616034436
CCATAGCA 0.0427173822602
CCATAGCC -0.142732688771
CCATAGCG -0.0129773185912

CCATAGGA 0.070891148369
CCATAGGC -0.260626224791
CCATAGGG -0.195017752567
CCATAGTA -0.144490220248
CCATAGTC -0.083946762655
CCATATAA 0.0661088085331
CCATATAC -0.0438275074268
CCATATAG -0.173906792562
CCATATCA 0.239566012293
CCATATCC 0.33894659241
CCATATCG 0.256081150021
CCATATGA -0.071472298745
CCATATGC 0.0526754888579
CCATATGG -0.123226034061
CCATATTA 0.178819315183
CCATATTC 0.215896732188
CCATCAAA 0.122280682887
CCATCAAC -0.0527832231845
CCATCAAG -0.127510818246
CCATCACA 0.00722856474885
CCATCACC -0.246049530227
CCATCACG -0.144259857308
CCATCAGA -0.106521452387
CCATCAGC -0.212275478424
CCATCAGG -0.127329920298
CCATCATA 0.0241412211109
CCATCATC 0.101705072152
CCATCCAA 0.0584510129965
CCATCCAC -0.0788015160162
CCATCCAG -0.170917375807
CCATCCCA -0.301304919497
CCATCCCC -0.223490267751
CCATCCCG -0.0747996327635
CCATCCGA -0.176427764611
CCATCCGC 0.0112260217882
CCATCCGG -0.157366476995
CCATCCTA -0.273314587382
CCATCCTC -0.192722403438
CCATCGAA 0.0885306794398
CCATCGAC 0.0150963635812
CCATCGAG 0.0626575617219
CCATCGCA 0.0736385334628
CCATCGCC -0.13441954351
CCATCGCG 0.0419158704554
CCATCGGA -0.14053293187
CCATCGGC -0.138687361807
CCATCGGG -0.223776802031
CCATCGTA 0.078204856828
CCATCGTC 0.0136858823237
CCATCTAA -0.042423158617
CCATCTAC -0.0330248242739
CCATCTAG -0.275198357395
CCATCTCA -0.0158850310365
CCATCTCC -0.181668307019

CCATCTCG -0.131473164438
CCATCTGA -0.144002107878
CCATCTGC -0.0120866455457
CCATCTTA -0.0452215279025
CCATCTTC 0.0384116899268
CCATGAAA 0.196383290877
CCATGAAC 0.0229805186748
CCATGAAG -0.158950100469
CCATGACA -0.157321258735
CCATGACC -0.257187199356
CCATGACG -0.21601752369
CCATGAGA 0.0197905551282
CCATGAGC -0.166480819557
CCATGAGG -0.295857074729
CCATGATA 0.171037428613
CCATGATC 0.0155346551852
CCATGCAA -0.13245646689
CCATGCAC -0.264520521754
CCATGCAG -0.284203867231
CCATGCCA -0.226639821003
CCATGCCC -0.291595221035
CCATGCCG 0.182243715413
CCATGCGA -0.00870429658308
CCATGCGC -0.0957907653279
CCATGCGG -0.212927576329
CCATGCTA -0.0932506693357
CCATGCTC -0.0793178089369
CCATGGAA -0.0696284022978
CCATGGAC -0.217838977794
CCATGGAG -0.242054140171
CCATGGCA -0.177054991827
CCATGGCC -0.369953801315
CCATGGCG -0.0668568625991
CCATGGGA -0.234626447895
CCATGGGC -0.312586177966
CCATGGGG -0.140048564765
CCATGGTA -0.228713442314
CCATGGTC -0.287901160712
CCATGTAA 0.137812310679
CCATGTAC -0.229103818677
CCATGTAG -0.0789710208459
CCATGTCA -0.0845027144044
CCATGTCC -0.0761171355231
CCATGTCG -0.170916419235
CCATGTGA -0.0683316083959
CCATGTGC -0.170537103036
CCATGTTA -0.170335442776
CCATGTTC 0.02579576822
CCATTAAA 0.148323029423
CCATTAAC 0.128465931496
CCATTAAG 0.121696076242
CCATTACA 0.0152162752815
CCATTACC -0.173354030717
CCATTACG 0.0274439245856

CCATTAGA -0.0158748348576
CCATTAGC -0.081755309028
CCATTAGG -0.0380738109077
CCATTATA 0.0639578972912
CCATTATC 0.15265578126
CCATTCAA 0.0388749631174
CCATTCAC -0.189465194193
CCATTCAG -0.252131588663
CCATTCCA 0.0325376308409
CCATTCCC -0.174631889705
CCATTCCG -0.134927446747
CCATTCGA -0.129761392086
CCATTCGC 0.0220834580265
CCATTCGG -0.0797290255341
CCATTCTA 0.00685587921495
CCATTCTC 0.0232853558212
CCATTGAA 0.190529404466
CCATTGAC -0.0279140616219
CCATTGAG -0.233428707678
CCATTGCA -0.229934634036
CCATTGCC -0.159239497594
CCATTGCG -0.120836953014
CCATTGGA -0.193520960653
CCATTGGC -0.252742739164
CCATTGGG -0.270884082487
CCATTGTA -0.0766124250973
CCATTGTC 0.164970840055
CCATTTAA 0.157715566806
CCATTTAC 0.0780956697917
CCATTTAG -0.255225701564
CCATTTCA -0.108009515678
CCATTTCC -0.233864284828
CCATTTCG -0.138224418983
CCATTTGA -0.0951110559623
CCATTTGC 0.0194285194285
CCATTTTA -0.242529007434
CCATTTTC 0.0653339289703
CCCAAAAA 0.110592468778
CCCAAAAC -0.119679558382
CCCAAAAG -0.0849380470781
CCCAAACA -0.123212077953
CCCAAACC -0.0888569931657
CCCAAACG 0.114981372557
CCCAAAGA 0.0767789332605
CCCAAAGC -0.293551570075
CCCAAAGG -0.295796429074
CCCAAATA 0.122025795056
CCCAAATC 0.340180410452
CCCAACAA -0.0214083549001
CCCAACAC -0.200755779252
CCCAACAG -0.194823843196
CCCAACCA -0.393226733691
CCCAACCC -0.183504105987
CCCAACCG -0.086441172821

CCCAACGA -0.0167233349052
CCCAACGC -0.109542856726
CCCAACGG -0.232376072467
CCCAACTA -0.266784078487
CCCAACTC -0.118734923982
CCCAAGAA -0.104029676683
CCCAAGAC -0.05278295532
CCCAAGAG 0.0313657206354
CCCAAGCA -0.249341516766
CCCAAGCC -0.167784877452
CCCAAGCG -0.190537655435
CCCAAGGA -0.201211085541
CCCAAGGC -0.201363559922
CCCAAGGG -0.324613116155
CCCAAGTA -0.0875788933833
CCCAAGTC -0.125747388791
CCCAATAA 0.0634256184171
CCCAATAC -0.264967125667
CCCAATAG -0.194876566183
CCCAATCA -0.0495850455829
CCCAATCC -0.0294605900667
CCCAATCG 0.124522090757
CCCAATGA -0.11569756324
CCCAATGC -0.261589869565
CCCAATTA -0.00806511650991
CCCAATTC -0.114504311474
CCCACAAA 0.0890548621622
CCCACAAC -0.0745740719929
CCCACAAG -0.223082087319
CCCACACA -0.102463683008
CCCACACC -0.070525910173
CCCACACG 0.121491805013
CCCACAGA -0.228166491766
CCCACAGC -0.189872207783
CCCACAGG -0.300647515498
CCCACATA -0.0490659166425
CCCACATC -0.0860077162364
CCCACCAA 0.0769923027404
CCCACCAC -0.146319777011
CCCACCAG -0.350502415417
CCCACCCA -0.0726982472683
CCCACCCC -0.158426566777
CCCACCCG 0.065833283101
CCCACCGA 0.0383532171871
CCCACCGC -0.161314568339
CCCACCGG -0.115450462745
CCCACCTA -0.313334099346
CCCACCTC -0.244693302433
CCCACGAA -0.100354331251
CCCACGAC -0.268636792914
CCCACGAG -0.10927208009
CCCACGCA -0.29125972038
CCCACGCC -0.227706785624
CCCACGCG 0.0751203563541

CCCACGGA -0.0428674055159
CCCACGGC -0.0291130026222
CCCACGGG -0.374046673657
CCCACGTA -0.233953971838
CCCACGTC -0.194154281319
CCCACTAA 0.0195282017344
CCCACTAC -0.16855705986
CCCACTAG -0.166317905552
CCCACTCA -0.198311050919
CCCACTCC -0.278775529391
CCCACTCG -0.13441323139
CCCACTGA -0.149796393252
CCCACTGC -0.145460860935
CCCACTTA -0.11944644349
CCCACTTC -0.17351022082
CCCAGAAA 0.0899681028883
CCCAGAAC -0.0852474832766
CCCAGAAG -0.128203649888
CCCAGACA -0.101926984678
CCCAGACC -0.325995651131
CCCAGACG -0.00767647423779
CCCAGAGA -0.146528932227
CCCAGAGC -0.181053017222
CCCAGAGG -0.265286632104
CCCAGATA 0.319130425191
CCCAGATC 0.251640475026
CCCAGCAA -0.0314246594938
CCCAGCAC -0.23596318039
CCCAGCAG -0.18669584903
CCCAGCCA -0.215326877648
CCCAGCCC -0.06269893275
CCCAGCCG -0.160577917937
CCCAGCGA -0.192081214076
CCCAGCGC -0.189107891538
CCCAGCGG -0.346668096512
CCCAGCTA -0.175663689369
CCCAGCTC -0.178571330096
CCCAGGAA -0.0481243469607
CCCAGGAC -0.326771523316
CCCAGGAG -0.294179355902
CCCAGGCA -0.352614462164
CCCAGGCC -0.141201133012
CCCAGGCG -0.16629108036
CCCAGGGA -0.266631381922
CCCAGGGC -0.386001326918
CCCAGGGG -0.260989010989
CCCAGGTA -0.178559908692
CCCAGGTC -0.298078087896
CCCAGTAA -0.0992990235414
CCCAGTAC -0.167488788256
CCCAGTAG 0.03088326533
CCCAGTCA -0.178945102418
CCCAGTCC -0.210884241226
CCCAGTCG -0.0786068581796

CCCAGTGA -0.227633443414
CCCAGTGC -0.304577287513
CCCAGTTA -0.214392855272
CCCAGTTC -0.229325328901
CCCATAAA 0.148036466218
CCCATAAC -0.0643698479963
CCCATAAG 0.00265906585987
CCCATACA -0.189626398988
CCCATACC 0.0215825910027
CCCATACG -0.132171521076
CCCATAGA 0.0227348560682
CCCATAGC -0.0388513932657
CCCATAGG -0.147525850683
CCCATATA -0.0193511705002
CCCATATC 0.266612409947
CCCATCAA -0.031812096316
CCCATCAC -0.106613461247
CCCATCAG -0.0216355981906
CCCATCCA -0.120158184108
CCCATCCC -0.217640656397
CCCATCCG -0.0721561523336
CCCATCGA 0.0186260640806
CCCATCGC 0.0631752683439
CCCATCGG -0.00387669023694
CCCATCTA -0.0500624591534
CCCATCTC -0.136963195333
CCCATGAA -0.101965795623
CCCATGAC -0.266407106038
CCCATGAG -0.285129320444
CCCATGCA -0.290459873944
CCCATGCC -0.177155872647
CCCATGCG -0.206982731068
CCCATGGA -0.243534139945
CCCATGGC -0.305315700916
CCCATGGG -0.16074617426
CCCATGTA -0.112076665426
CCCATGTC -0.0155360338376
CCCATTAA 0.0927415018324
CCCATTAC -0.127300626184
CCCATTAG 0.209792437065
CCCATTCA -0.17658079386
CCCATTCC -0.250457791947
CCCATTCG -0.010096939502
CCCATTGA 0.0746487303198
CCCATTGC -0.202031342379
CCCATTTA -0.0786365031576
CCCATTTC -0.243944693906
CCCCAAAA -0.22752431908
CCCCAAAC -0.0381073308462
CCCCAAAG -0.1141408759
CCCCAACA -0.129923712949
CCCCAACC -0.230124669583
CCCCAACG 0.0170470719619
CCCCAAGA -0.127032175713

CCCCAAGC -0.316178476848
CCCCAAGG -0.38179599894
CCCCAATA -0.0647111371907
CCCCAATC 0.117587936031
CCCCACAA -0.257644074524
CCCCACAC 0.0711625047217
CCCCACAG -0.228417419876
CCCCACCA -0.193246144125
CCCCACCC -0.153953131935
CCCCACCG -0.0601680073033
CCCCACGA -0.0714640260095
CCCCACGC -0.161094382419
CCCCACGG -0.19745994723
CCCCACTA -0.256448425005
CCCCACTC -0.215168029816
CCCCAGAA -0.305704453613
CCCCAGAC -0.217701345343
CCCCAGAG -0.212880462941
CCCCAGCA -0.15698493432
CCCCAGCC -0.305461164714
CCCCAGCG -0.0873172027551
CCCCAGGA -0.204222944564
CCCCAGGC -0.276130292855
CCCCAGGG -0.157976592155
CCCCAGTA -0.00669547382174
CCCCAGTC -0.0683423415271
CCCCATAA -0.0907449686081
CCCCATAC -0.0750969833825
CCCCATAG -0.13543745083
CCCCATCA -0.126434251995
CCCCATCC -0.138365742234
CCCCATCG 0.151623909741
CCCCATGA -0.136458240592
CCCCATGC -0.233605438801
CCCCATTA -0.0701499698075
CCCCATTC -0.156217388576
CCCCCAAA -0.449125911618
CCCCCAAC -0.148815382791
CCCCCAAG -0.314264059421
CCCCCACA -0.159611014873
CCCCCACC -0.245518301091
CCCCCACG 0.0354203922542
CCCCCAGA -0.264576563391
CCCCCAGC -0.339976519417
CCCCCAGG -0.312961690369
CCCCCATA -0.178655427892
CCCCCATC -0.0395505698536
CCCCCCAA -0.232997626903
CCCCCCAC -0.044781077944
CCCCCCAG -0.208623812241
CCCCCCCA -0.167029162291
CCCCCCCC 0.0501004987717
CCCCCCCG -0.350040102862
CCCCCCGA -0.139737292662

CCCCCCGC -0.207986789625
CCCCCCGG -0.234264673051
CCCCCCTA -0.2683466927
CCCCCCTC -0.24154593249
CCCCCGAA -0.125999232286
CCCCCGAC -0.104720534603
CCCCCGAG -0.0005377011209
CCCCCGCA -0.24146252472
CCCCCGCC -0.147892254556
CCCCCGCG 0.0594119909671
CCCCCGGA -0.141958153782
CCCCCGGC -0.170993539726
CCCCCGGG -0.278873204495
CCCCCGTA -0.161155046781
CCCCCGTC -0.331438250625
CCCCCTAA -0.249142372698
CCCCCTAC -0.115843896174
CCCCCTAG -0.247679614514
CCCCCTCA -0.132435769633
CCCCCTCC -0.234708888393
CCCCCTCG -0.14625612061
CCCCCTGA -0.0832251252319
CCCCCTGC -0.224268911547
CCCCCTTA -0.381702215822
CCCCCTTC -0.219882330053
CCCCGAAA -0.196588406205
CCCCGAAC 0.0795354583233
CCCCGAAG -0.0251643150002
CCCCGACA -0.177322384036
CCCCGACC -0.321505847591
CCCCGACG 0.0519720822751
CCCCGAGA -0.182976130534
CCCCGAGC -0.160682827581
CCCCGAGG -0.326403529804
CCCCGATA 0.193078889247
CCCCGATC 0.0325124267352
CCCCGCAA -0.073613716488
CCCCGCAC 0.0412782313544
CCCCGCAG -0.057622944957
CCCCGCCA -0.157104094496
CCCCGCCC -0.0510222927917
CCCCGCCG 0.00667291408486
CCCCGCGA -0.0272214363123
CCCCGCGC -0.0528757796888
CCCCGCGG -0.172707184256
CCCCGCTA -0.150081664884
CCCCGCTC -0.0972308379445
CCCCGGAA -0.272947043208
CCCCGGAC -0.280418227371
CCCCGGAG -0.297583510429
CCCCGGCA -0.159568474687
CCCCGGCC -0.282554452585
CCCCGGCG 0.00202524036804
CCCCGGGA -0.290493952752

CCCCGGGC -0.233914348808
CCCCGGGG -0.187063356235
CCCCGGTA -0.153394653149
CCCCGGTC -0.184786833726
CCCCGTAA -0.157689406044
CCCCGTAC -0.0576903150672
CCCCGTAG -0.252044439839
CCCCGTCA 0.0102729877717
CCCCGTCC -0.291190828627
CCCCGTCG -0.277074944118
CCCCGTGA -0.285424805792
CCCCGTGC -0.248200437243
CCCCGTTA -0.133977035719
CCCCGTTC -0.160581624925
CCCCTAAA -0.109976486935
CCCCTAAC -0.0857572542501
CCCCTAAG -0.068543766977
CCCCTACA -0.242974906262
CCCCTACC -0.210378056414
CCCCTACG 0.155429443151
CCCCTAGA -0.0501736113645
CCCCTAGC -0.234785561869
CCCCTAGG -0.244962336384
CCCCTATA -0.071157683611
CCCCTATC -0.106475974177
CCCCTCAA -0.237285353001
CCCCTCAC 0.039529242459
CCCCTCAG -0.0912506878046
CCCCTCCA -0.165603989381
CCCCTCCC -0.294610068151
CCCCTCCG -0.237982419145
CCCCTCGA -0.0299329211769
CCCCTCGC -0.0784398856424
CCCCTCGG -0.217872085201
CCCCTCTA -0.274344577031
CCCCTCTC -0.151209939324
CCCCTGAA -0.27954200211
CCCCTGAC -0.126355492692
CCCCTGAG -0.258739039264
CCCCTGCA -0.114106625421
CCCCTGCC -0.301957833401
CCCCTGCG -0.245390388659
CCCCTGGA -0.240990735118
CCCCTGGC -0.264487247873
CCCCTGTA 0.00335394115457
CCCCTGTC -0.266570736266
CCCCTTAA -0.0575900425919
CCCCTTAC -0.0171986460035
CCCCTTAG -0.0342284433194
CCCCTTCA -0.308667041168
CCCCTTCC -0.251571728998
CCCCTTCG -0.159956052924
CCCCTTGA -0.237463497072
CCCCTTGC -0.0554949008709

CCCCTTTA -0.0610845070811
CCCCTTTC -0.40301230749
CCCGAAAA -0.0153514395939
CCCGAAAC -0.333501875856
CCCGAAAG -0.190833862388
CCCGAACA -0.145285139741
CCCGAACC 0.0463424857364
CCCGAACG -0.0683215269335
CCCGAAGA -0.00284815448963
CCCGAAGC -0.221790832486
CCCGAAGG -0.295616944468
CCCGAATA 0.15685520231
CCCGAATC 0.363794924401
CCCGACAA -0.162814474474
CCCGACAC -0.177872807562
CCCGACAG 0.0292122655946
CCCGACCA -0.357152653457
CCCGACCC -0.351479817487
CCCGACCG -0.157434638496
CCCGACGA -0.0467354406748
CCCGACGC -0.162149638419
CCCGACGG -0.272910151433
CCCGACTA -0.131008008381
CCCGACTC 0.066890346414
CCCGAGAA -0.153543198542
CCCGAGAC -0.098126138942
CCCGAGAG -0.0823499062833
CCCGAGCA -0.14932121083
CCCGAGCC -0.23847421703
CCCGAGCG -0.104889577242
CCCGAGGA -0.250127763031
CCCGAGGC -0.048128113722
CCCGAGGG -0.359923342802
CCCGAGTA -0.169367044637
CCCGAGTC -0.059725772076
CCCGATAA -0.0797166946402
CCCGATAC 0.0200489745944
CCCGATAG 0.0705574168287
CCCGATCA -0.0059412029109
CCCGATCC -0.069076417318
CCCGATCG 0.0798194200127
CCCGATGA -0.111722434607
CCCGATGC -0.216213617528
CCCGATTA 0.213845682211
CCCGATTC 0.305138471805
CCCGCAAA -0.0322025289111
CCCGCAAC -0.0876433272426
CCCGCAAG 0.0631394932958
CCCGCACA -0.0392290679766
CCCGCACC -0.118035051154
CCCGCACG -0.0378949872795
CCCGCAGA 0.00201956356407
CCCGCAGC -0.330864901966
CCCGCAGG -0.260970950857

CCCGCATA -0.20432756664
CCCGCATC -0.0403226918378
CCCGCCAA -0.0770155235042
CCCGCCAC -0.207997420187
CCCGCCAG -0.0351287119978
CCCGCCCA -0.259550378163
CCCGCCCC -0.260514253742
CCCGCCCG -0.0534008288047
CCCGCCGA -0.138379767072
CCCGCCGC -0.153289566825
CCCGCCGG -0.137803297194
CCCGCCTA -0.205083138273
CCCGCCTC -0.0624426192898
CCCGCGAA -0.149218824113
CCCGCGAC -0.19129314195
CCCGCGAG -0.0830858406616
CCCGCGCA -0.150660512362
CCCGCGCC -0.113941498413
CCCGCGCG -0.0204119617819
CCCGCGGA -0.139701685573
CCCGCGGC -0.0817969777425
CCCGCGGG -0.279549757352
CCCGCGTA 0.0466847108339
CCCGCGTC -0.193040426347
CCCGCTAA -0.231056575961
CCCGCTAC -0.288421090354
CCCGCTAG -0.0331070267722
CCCGCTCA -0.0133480176854
CCCGCTCC -0.326307809721
CCCGCTCG -0.341526490796
CCCGCTGA -0.157386986456
CCCGCTGC -0.242944956284
CCCGCTTA 0.118425588123
CCCGCTTC -0.103958936362
CCCGGAAA -0.0755899064586
CCCGGAAC -0.222843871039
CCCGGAAG 0.0922425056386
CCCGGACA -0.0911530439326
CCCGGACC -0.202651589772
CCCGGACG -0.192814166977
CCCGGAGA -0.0680470436444
CCCGGAGC -0.333003257244
CCCGGAGG -0.328141391239
CCCGGATA 0.24629450387
CCCGGATC 0.250747993172
CCCGGCAA -0.0340129048269
CCCGGCAC -0.28156513517
CCCGGCAG -0.153904146027
CCCGGCCA -0.333263042993
CCCGGCCC -0.377831645568
CCCGGCCG -0.123485280859
CCCGGCGA -0.183018129395
CCCGGCGC -0.0136018130237
CCCGGCGG -0.155283724757

CCCGGCTA -0.164540825634
CCCGGCTC -0.304594799293
CCCGGGAA -0.0589639321075
CCCGGGAC -0.323785346707
CCCGGGAG -0.188731753711
CCCGGGCA -0.234942532538
CCCGGGCC -0.334457811402
CCCGGGCG -0.169475199277
CCCGGGGA -0.0935855340242
CCCGGGGC -0.192276836298
CCCGGGTA -0.208043598496
CCCGGGTC -0.285180568991
CCCGGTAA -0.115464111648
CCCGGTAC -0.327673455213
CCCGGTAG -0.0492331966657
CCCGGTCA -0.0464885276675
CCCGGTCC -0.282992203653
CCCGGTCG -0.177578992993
CCCGGTGA -0.151495350036
CCCGGTGC -0.329449265381
CCCGGTTA -0.218125070652
CCCGGTTC -0.342681127068
CCCGTAAA -0.145656352203
CCCGTAAC -0.0735378040753
CCCGTAAG 0.0131743313561
CCCGTACA 0.0517123592619
CCCGTACC 0.0429857578139
CCCGTACG 0.00750163840629
CCCGTAGA -0.0827731822276
CCCGTAGC -0.201467060042
CCCGTAGG -0.285311847445
CCCGTATA -0.141374033172
CCCGTATC 0.192748333978
CCCGTCAA -0.067110435152
CCCGTCAC -0.189625429628
CCCGTCAG -0.0284439987564
CCCGTCCA -0.163213485191
CCCGTCCC -0.237142065061
CCCGTCCG -0.308078869695
CCCGTCGA -0.049824437331
CCCGTCGC -0.283311010244
CCCGTCGG -0.0457834267796
CCCGTCTA -0.262089070835
CCCGTCTC -0.01993408098
CCCGTGAA -0.251464454754
CCCGTGAC -0.159405082021
CCCGTGAG -0.0823484328434
CCCGTGCA -0.225350581347
CCCGTGCC -0.18808021153
CCCGTGCG -0.0975100016267
CCCGTGGA -0.334506749392
CCCGTGGC -0.0754042095687
CCCGTGTA 0.0315728952093
CCCGTGTC -0.104391000798

CCCGTTAA -0.128424195091
CCCGTTAC -0.261969800322
CCCGTTAG 0.179318436894
CCCGTTCA -0.222830026931
CCCGTTCC -0.365191883733
CCCGTTCG -0.228050448105
CCCGTTGA -0.0295051886799
CCCGTTGC -0.156543382481
CCCGTTTA -0.200053650731
CCCGTTTC -0.104786756494
CCCTAAAA 0.0881446184476
CCCTAAAC -0.200744104732
CCCTAAAG -0.0792358180897
CCCTAACA -0.0326228950676
CCCTAACC -0.0134198489698
CCCTAACG -0.0053400507946
CCCTAAGA -0.342161556409
CCCTAAGC -0.28654344822
CCCTAAGG -0.136513017322
CCCTAATA -0.282624241301
CCCTAATC 0.189857886324
CCCTACAA -0.165543799834
CCCTACAC -0.103250247768
CCCTACAG -0.0962504718256
CCCTACCA -0.185519688361
CCCTACCC -0.247974673426
CCCTACCG -0.0717367412439
CCCTACGA 0.163007359977
CCCTACGC 0.00236012901625
CCCTACGG -0.126086776689
CCCTACTA -0.127135771221
CCCTACTC -0.0999068725979
CCCTAGAA -0.0919457934294
CCCTAGAC -0.270745057996
CCCTAGAG 0.0290059341766
CCCTAGCA -0.0787807856987
CCCTAGCC -0.263030588702
CCCTAGCG -0.115978698203
CCCTAGGA -0.143939957822
CCCTAGGC -0.0393633496177
CCCTAGGG -0.235997222016
CCCTAGTA -0.0687716076553
CCCTAGTC -0.104774713895
CCCTATAA 0.0336714124593
CCCTATAC -0.165891864021
CCCTATAG -0.137865229708
CCCTATCA -0.0213663548155
CCCTATCC -0.0526055007042
CCCTATCG 0.103135697758
CCCTATGA -0.120117317205
CCCTATGC -0.127060740667
CCCTATTA -0.0170047371225
CCCTATTC -0.0118106902769
CCCTCAAA -0.0230980620828

CCCTCAAC -0.119950587593
CCCTCAAG -0.178903142032
CCCTCACA -0.00740337727245
CCCTCACC -0.151702351122
CCCTCACG -0.0328107981415
CCCTCAGA -0.0762498850504
CCCTCAGC -0.171881549891
CCCTCAGG -0.0968862090546
CCCTCATA -0.20653486101
CCCTCATC -0.0519229867849
CCCTCCAA -0.312828388208
CCCTCCAC -0.227151659009
CCCTCCAG -0.264940820415
CCCTCCCA -0.182464076123
CCCTCCCC -0.212926902543
CCCTCCCG -0.159199467615
CCCTCCGA -0.0512693252599
CCCTCCGC -0.196833745448
CCCTCCGG -0.310503399137
CCCTCCTA -0.0954813289106
CCCTCCTC -0.294051488162
CCCTCGAA -0.211898544992
CCCTCGAC -0.316533802626
CCCTCGAG 0.0178140541771
CCCTCGCA -0.157236142106
CCCTCGCC -0.235580739179
CCCTCGCG -0.112119146224
CCCTCGGA -0.0506373363966
CCCTCGGC -0.222795599331
CCCTCGTA -0.0428727838077
CCCTCGTC -0.276104026488
CCCTCTAA -0.148499524875
CCCTCTAC -0.29414472143
CCCTCTAG -0.0703959613639
CCCTCTCA -0.200878858228
CCCTCTCC -0.204008499472
CCCTCTCG -0.0880791164137
CCCTCTGA -0.240094281107
CCCTCTGC -0.170574628532
CCCTCTTA -0.282558568866
CCCTCTTC -0.18893718319
CCCTGAAA -0.206002034588
CCCTGAAC -0.29238616978
CCCTGAAG -0.112209788012
CCCTGACA 0.0390670277293
CCCTGACC -0.160730778668
CCCTGACG -0.261607602825
CCCTGAGA -0.298880121649
CCCTGAGC -0.248310310354
CCCTGAGG -0.205718901548
CCCTGATA 0.208187526369
CCCTGATC -0.0788982723006
CCCTGCAA -0.115102364296
CCCTGCAC -0.202306853721

CCCTGCAG -0.115619788005
CCCTGCCA -0.211949161507
CCCTGCCC -0.285142575301
CCCTGCCG -0.135895887949
CCCTGCGA -0.0273334938739
CCCTGCGC -0.0499987590281
CCCTGCGG -0.0737384793888
CCCTGCTA -0.100862334463
CCCTGCTC 0.00892754173266
CCCTGGAA -0.176055197667
CCCTGGAC -0.18324856304
CCCTGGAG -0.2911929665
CCCTGGCA -0.119519094767
CCCTGGCC -0.258178365402
CCCTGGCG -0.021435420805
CCCTGGGA -0.291936430763
CCCTGGGC -0.275073801196
CCCTGGTA -0.238134614484
CCCTGGTC -0.467119169071
CCCTGTAA -0.0487149916425
CCCTGTAC -0.286424959003
CCCTGTAG 0.0139023320842
CCCTGTCA -0.243759699859
CCCTGTCC -0.273017315973
CCCTGTCG -0.164140500628
CCCTGTGA -0.321804586983
CCCTGTGC -0.0871072069613
CCCTGTTA -0.0970109542283
CCCTGTTC -0.332860596338
CCCTTAAA -0.0856213205388
CCCTTAAC -0.224937143124
CCCTTAAG -0.0235676447798
CCCTTACA -0.0365006880158
CCCTTACC -0.00899388184432
CCCTTACG 0.0272660392974
CCCTTAGA 0.00460332034643
CCCTTAGC -0.148890354762
CCCTTAGG -0.0904882050434
CCCTTATA -0.145710282624
CCCTTATC -0.155655042069
CCCTTCAA -0.19922754623
CCCTTCAC -0.312293132481
CCCTTCAG -0.127684375086
CCCTTCCA -0.313230531784
CCCTTCCC -0.297071250796
CCCTTCCG -0.28930653765
CCCTTCGA -0.0618432770595
CCCTTCGC -0.116629308895
CCCTTCGG -0.257997645846
CCCTTCTA -0.182375868637
CCCTTCTC -0.182899492957
CCCTTGAA -0.219499789098
CCCTTGAC -0.15475017696
CCCTTGAG -0.0604866664429

CCCTTGCA -0.0157492901615
CCCTTGCC -0.2768226087
CCCTTGCG -0.241820750492
CCCTTGGA -0.223835737188
CCCTTGGC -0.215595407715
CCCTTGTA -0.138274050469
CCCTTGTC -0.0580261006471
CCCTTTAA -0.122786918243
CCCTTTAC -0.0487195076557
CCCTTTAG 0.0819731577307
CCCTTTCA -0.240182093329
CCCTTTCC -0.255392104168
CCCTTTCG -0.271020965141
CCCTTTGA -0.297648825033
CCCTTTGC -0.0932607131337
CCCTTTTA -0.127541074909
CCCTTTTC -0.183541861343
CCGAAAAA 0.0490638891119
CCGAAAAC -0.13767384802
CCGAAAAG -0.0109699371059
CCGAAACA -0.176159124808
CCGAAACC -0.181878799156
CCGAAACG -0.181403322352
CCGAAAGA 0.00216272636366
CCGAAAGC -0.179940392987
CCGAAAGG -0.0868238563632
CCGAAATA 0.183283834799
CCGAAATC 0.449919616586
CCGAACAA 0.0782106961161
CCGAACAC -0.238421165553
CCGAACAG -0.0153424053163
CCGAACCA 0.0841054575447
CCGAACCC -0.143300527913
CCGAACCG -0.171767478856
CCGAACGA -0.152565852045
CCGAACGC 0.0518135215105
CCGAACGG -0.192075700561
CCGAACTA -0.226770332938
CCGAACTC -0.122257677918
CCGAAGAA -0.0891815767923
CCGAAGAC -0.0966656465652
CCGAAGAG -0.0377952582226
CCGAAGCA -0.109786357659
CCGAAGCC -0.247373858382
CCGAAGCG -0.160387041842
CCGAAGGA -0.0226162930739
CCGAAGGC -0.216595549381
CCGAAGTA -0.0444523112633
CCGAAGTC -0.0816151842203
CCGAATAA 0.0823045240611
CCGAATAC -0.108599158788
CCGAATAG -0.0263972321774
CCGAATCA 0.305141229384
CCGAATCC 0.321879730971

CCGAATCG 0.195610210762
CCGAATGA -0.124476158363
CCGAATGC -0.210504624461
CCGAATTA 0.00214953245256
CCGAATTC 0.173709945643
CCGACAAA 0.107554532595
CCGACAAC -0.126028232089
CCGACAAG -0.157797529134
CCGACACA -0.0656373290985
CCGACACC -0.309068317362
CCGACACG -0.0593440609414
CCGACAGA 0.0019114472261
CCGACAGC -0.109237533784
CCGACAGG -0.112244868174
CCGACATA 0.0577493361789
CCGACATC -0.0173879113273
CCGACCAA -0.190632456928
CCGACCAC -0.268146332737
CCGACCAG -0.233117224992
CCGACCCA -0.213721647524
CCGACCCC -0.259175078402
CCGACCCG -0.346491418438
CCGACCGA -0.122472585497
CCGACCGC -0.159114904221
CCGACCGG -0.249899672092
CCGACCTA -0.21293332782
CCGACCTC -0.300461136883
CCGACGAA -0.122146940329
CCGACGAC -0.245071765027
CCGACGAG -0.116779050283
CCGACGCA -0.0796319570038
CCGACGCC -0.382734404068
CCGACGCG -0.0618357227001
CCGACGGA -0.0895868210073
CCGACGGC -0.223750519933
CCGACGTA -0.0431793464377
CCGACGTC -0.155743377797
CCGACTAA -0.0600166970493
CCGACTAC -0.0168418672996
CCGACTAG -0.123609977946
CCGACTCA 0.170477288818
CCGACTCC -0.289879066564
CCGACTCG -0.179736129658
CCGACTGA -0.124817722816
CCGACTGC -0.157417206652
CCGACTTA -0.171524226734
CCGACTTC -0.0849571846206
CCGAGAAA -0.109957064503
CCGAGAAC -0.321361733326
CCGAGAAG -0.00241783520187
CCGAGACA 0.0121654270344
CCGAGACC -0.343843153743
CCGAGACG -0.189921824494
CCGAGAGA 0.165467120013

CCGAGAGC -0.114170837694
CCGAGAGG -0.0984983196729
CCGAGATA 0.368878783676
CCGAGATC 0.336333510945
CCGAGCAA 0.0532947278224
CCGAGCAC -0.180682729927
CCGAGCAG -0.145601040148
CCGAGCCA -0.173217775793
CCGAGCCC -0.298572781296
CCGAGCCG -0.162927818467
CCGAGCGA -0.022203521208
CCGAGCGC -0.112070997841
CCGAGCGG -0.226185486788
CCGAGCTA -0.0576228853702
CCGAGCTC -0.104450454474
CCGAGGAA -0.22954486084
CCGAGGAC -0.331370814142
CCGAGGAG -0.224796995839
CCGAGGCA 0.0220368371424
CCGAGGCC -0.173412061529
CCGAGGCG -0.176758860165
CCGAGGGA -0.250237758537
CCGAGGGC -0.331529393606
CCGAGGTA -0.0428112928175
CCGAGGTC -0.264656187035
CCGAGTAA 0.0658547623045
CCGAGTAC -0.097514841193
CCGAGTAG -0.208599705027
CCGAGTCA 0.0460115763146
CCGAGTCC -0.313883858805
CCGAGTCG -0.108960733554
CCGAGTGA -0.0669878283563
CCGAGTGC -0.140104691118
CCGAGTTA -0.177447728394
CCGAGTTC -0.162108773543
CCGATAAA 0.0351908382211
CCGATAAC -0.193804123967
CCGATAAG 0.0785347766107
CCGATACA -0.0691904556835
CCGATACC 0.192248722552
CCGATACG 0.210896841675
CCGATAGA 0.171718550506
CCGATAGC 0.201501426976
CCGATAGG 0.177147215754
CCGATATA 0.239188192959
CCGATATC 0.450204109487
CCGATCAA -0.0234221148442
CCGATCAC 0.098275179647
CCGATCAG -0.106805844841
CCGATCCA 0.00835821113406
CCGATCCC 0.0218220975797
CCGATCCG -0.00578827207981
CCGATCGA -0.0857787180187
CCGATCGC 0.269897009555

CCGATCGG	-0.118264500393
CCGATCTA	0.0250439771392
CCGATCTC	0.249454688849
CCGATGAA	-0.163231746819
CCGATGAC	-0.221072089449
CCGATGAG	-0.171734051126
CCGATGCA	0.0903727418879
CCGATGCC	-0.232953873771
CCGATGCG	-0.129494082326
CCGATGGA	-0.138713849271
CCGATGGC	-0.306439653913
CCGATGTA	-0.23804403863
CCGATGTC	-0.0151515151515
CCGATTAA	0.0598461347562
CCGATTAC	0.408128888651
CCGATTAG	0.0253986769138
CCGATTCA	-0.117281193039
CCGATTCC	0.315501850324
CCGATTCG	0.224484865295
CCGATTGA	-0.282768720657
CCGATTGC	0.35620362558
CCGATTTA	0.04576177164
CCGATTTC	0.44240418093
CCGCAAAA	0.0694079691686
CCGCAAAC	-0.318723887382
CCGCAAAG	-0.17063961848
CCGCAACA	0.0391341795435
CCGCAACC	-0.263764670861
CCGCAACG	-0.0835040108611
CCGCAAGA	0.0341999810223
CCGCAAGC	-0.0468416074477
CCGCAAGG	-0.40982347251
CCGCAATA	0.0825660371115
CCGCAATC	0.468880726456
CCGCACAA	0.137692789208
CCGCACAC	-0.064173976
CCGCACAG	-0.0529113002622
CCGCACCA	-0.147158091429
CCGCACCC	-0.112747165505
CCGCACCG	-0.0993919588529
CCGCACGA	-0.076798399185
CCGCACGC	-0.0771957973903
CCGCACGG	-0.136065257969
CCGCACTA	-0.192515956337
CCGCACTC	-0.118838826069
CCGCAGAA	-0.0650267353138
CCGCAGAC	-0.1575794921
CCGCAGAG	0.091117288087
CCGCAGCA	-0.0641020073944
CCGCAGCC	-0.270312570686
CCGCAGCG	-0.115720113328
CCGCAGGA	0.0474694145966
CCGCAGGC	-0.317322409554
CCGCAGTA	-0.0997924264603

CCGCAGTC -0.0212267662605
CCGCATAA -0.0901297376294
CCGCATAC -0.295608263399
CCGCATAG 0.00760786260155
CCGCATCA 0.141434823253
CCGCATCC -0.075273361038
CCGCATCG -0.0971699604124
CCGCATGA -0.0892715620352
CCGCATGC -0.141223173304
CCGCATTA -0.00235849980863
CCGCATTC -0.111143790039
CCGCCAAA 0.142323160807
CCGCCAAC -0.324136336248
CCGCCAAG -0.079741659236
CCGCCACA -0.0657796208635
CCGCCACC -0.265873311225
CCGCCACG -0.107490451958
CCGCCAGA 0.0991388937142
CCGCCAGC 0.0447690926476
CCGCCAGG -0.252257925175
CCGCCATA -0.222296897918
CCGCCATC -0.0756054528663
CCGCCCAA -0.223844574012
CCGCCCAC -0.193230721727
CCGCCCAG -0.205634232454
CCGCCCCA -0.115913864243
CCGCCCCC -0.25088908768
CCGCCCCG -0.0787765861947
CCGCCCGA -0.177325144384
CCGCCCGC -0.0250338064164
CCGCCCGG -0.155339457937
CCGCCCTA -0.204247757844
CCGCCCTC -0.168054195353
CCGCCGAA 0.100954723619
CCGCCGAC -0.171987420349
CCGCCGAG 0.0161884022646
CCGCCGCA -0.16213796274
CCGCCGCC -0.207059567817
CCGCCGCG -0.329821747028
CCGCCGGA -0.13110826326
CCGCCGGC -0.262530286867
CCGCCGTA -0.0854682955376
CCGCCGTC -0.249906964051
CCGCCTAA -0.108807154262
CCGCCTAC -0.169658912684
CCGCCTAG -0.0995233167579
CCGCCTCA -0.136338818157
CCGCCTCC -0.290968184372
CCGCCTCG -0.25852714253
CCGCCTGA -0.0640479252019
CCGCCTGC -0.202901906944
CCGCCTTA -0.1625854897
CCGCCTTC 0.0704269249578
CCGCGAAA 0.0694951149497

CCGCGAAC -0.325138713172
CCGCGAAG -0.0734349023597
CCGCGACA -0.107315681068
CCGCGACC -0.229025318913
CCGCGACG -0.148970715938
CCGCGAGA 0.0914426823518
CCGCGAGC -0.142838896893
CCGCGAGG -0.336491019168
CCGCGATA 0.177305404578
CCGCGATC 0.399571748057
CCGCGCAA 0.164628816144
CCGCGCAC -0.0757642180258
CCGCGCAG -0.0749251587685
CCGCGCCA -0.0301689316801
CCGCGCCC -0.159947781831
CCGCGCCG -0.0965318620355
CCGCGCGA 0.0332582048875
CCGCGCGC -0.0764927742269
CCGCGCGG -0.0149951628507
CCGCGCTA -0.179581118215
CCGCGCTC -0.183065368037
CCGCGGAA -0.184260068679
CCGCGGAC -0.211921597817
CCGCGGAG 0.00299466120813
CCGCGGCA 0.123252045576
CCGCGGCC -0.226684461464
CCGCGGCG 0.133245987314
CCGCGGGA -0.162755698327
CCGCGGGC -0.104793233083
CCGCGGTA -0.00797629585508
CCGCGGTC -0.313868009559
CCGCGTAA 0.0335108140906
CCGCGTAC -0.0301554998525
CCGCGTAG -0.128819603075
CCGCGTCA -0.190791499846
CCGCGTCC -0.295965360661
CCGCGTCG -0.189210650388
CCGCGTGA -0.0934581825983
CCGCGTGC -0.129513845815
CCGCGTTA -0.258937653509
CCGCGTTC 0.0174745249846
CCGCTAAA -0.0756215978711
CCGCTAAC -0.17503829924
CCGCTAAG -0.0152410947201
CCGCTACA -0.234677557048
CCGCTACC -0.344296123952
CCGCTACG -0.140794821836
CCGCTAGA 0.232362065064
CCGCTAGC -0.13516422572
CCGCTAGG -0.108926597589
CCGCTATA -0.137086804152
CCGCTATC 0.157560783341
CCGCTCAA 0.00141601656753
CCGCTCAC -0.171012938112

CCGCTCAG 0.0182643869271
CCGCTCCA -0.137436127219
CCGCTCCC -0.146225891503
CCGCTCCG -0.191532763815
CCGCTCGA -0.187201393886
CCGCTCGC -0.0716780951278
CCGCTCTA -0.269332681603
CCGCTCTC 0.0465548220231
CCGCTGAA -0.177351222727
CCGCTGAC -0.31320870074
CCGCTGAG -0.153084826881
CCGCTGCA -0.148935875186
CCGCTGCC -0.31953297892
CCGCTGCG -0.133301353359
CCGCTGGA -0.293933034548
CCGCTGGC -0.283599740281
CCGCTGTA -0.272294320492
CCGCTGTC -0.0442771326175
CCGCTTAA -0.0282812306165
CCGCTTAC 0.0458610040527
CCGCTTAG -0.173585822687
CCGCTTCA -0.124615595686
CCGCTTCC -0.259869881046
CCGCTTCG -0.272011213377
CCGCTTGA -0.160547941607
CCGCTTGC -0.231473729255
CCGCTTTA -0.154395569559
CCGCTTTC -0.0842314807922
CCGGAAAA 0.131264873689
CCGGAAAC -0.00573766649848
CCGGAAAG -0.120105762957
CCGGAACA -0.0517167385107
CCGGAACC -0.189026457915
CCGGAACG -0.122749806996
CCGGAAGA -0.100700906878
CCGGAAGC -0.183015294011
CCGGAAGG -0.130233272136
CCGGAATA 0.210435537908
CCGGAATC 0.401418217119
CCGGACAA 0.0407954570338
CCGGACAC -0.307207741251
CCGGACAG -0.0172235180268
CCGGACCA -0.09965467463
CCGGACCC -0.195457103572
CCGGACCG -0.189598337454
CCGGACGA -0.128986322369
CCGGACGC -0.166583477455
CCGGACGG -0.277568264208
CCGGACTA -0.202238804881
CCGGACTC -0.172851759036
CCGGAGAA -0.129263020123
CCGGAGAC -0.281457156573
CCGGAGAG -0.067172369506
CCGGAGCA -0.13584661975

CCGGAGCC -0.365291097464
CCGGAGCG -0.155798014676
CCGGAGGA -0.0893123220459
CCGGAGGC -0.261762881739
CCGGAGTA -0.117821724854
CCGGAGTC -0.123883219946
CCGGATAA 0.0888808919112
CCGGATAC 0.2774027471
CCGGATAG 0.0967834007595
CCGGATCA 0.17891214061
CCGGATCC 0.286433873711
CCGGATCG 0.239302059079
CCGGATGA 0.0354817047802
CCGGATGC -0.137999057095
CCGGATTA 0.397710382559
CCGGATTC 0.447300667543
CCGGCAAA 0.091052723019
CCGGCAAC -0.276726126975
CCGGCAAG -0.17862110233
CCGGCACA 0.077685160179
CCGGCACC -0.410870817247
CCGGCACG -0.271393591298
CCGGCAGA 0.121768661804
CCGGCAGC -0.34805271569
CCGGCAGG -0.104279187999
CCGGCATA -0.062612121451
CCGGCATC 0.113494595009
CCGGCCAA -0.201605143517
CCGGCCAC -0.32341559084
CCGGCCAG -0.201340739238
CCGGCCCA -0.240730633633
CCGGCCCC -0.244082406349
CCGGCCCG -0.338107326646
CCGGCCGA -0.225163828444
CCGGCCGC -0.0398743093217
CCGGCCGG -0.0221668223926
CCGGCCTA -0.203227798036
CCGGCCTC -0.0874308982799
CCGGCGAA 0.0688065224849
CCGGCGAC -0.0817316182856
CCGGCGAG -0.25767601307
CCGGCGCA -0.0624102152628
CCGGCGCC -0.314155237149
CCGGCGCG -0.0720163651704
CCGGCGGA -0.183617590093
CCGGCGGC -0.235875712399
CCGGCGTA 0.0307911217002
CCGGCGTC -0.305937955603
CCGGCTAA -0.302489636243
CCGGCTAC -0.33663260139
CCGGCTAG -0.210888629741
CCGGCTCA -0.285295504203
CCGGCTCC -0.288837377624
CCGGCTCG -0.241408770489

CCGGCTGA -0.230458437375
CCGGCTGC -0.216018755117
CCGGCTTA -0.179337839396
CCGGCTTC -0.228113487699
CCGGGAAA 0.0442120036507
CCGGGAAC -0.251070042743
CCGGGAAG -0.227770619027
CCGGGACA -0.11967667844
CCGGGACC -0.385452235478
CCGGGACG -0.312768584904
CCGGGAGA 0.0703899491778
CCGGGAGC -0.200343371519
CCGGGAGG -0.176028599858
CCGGGATA 0.168056903863
CCGGGATC 0.194463058099
CCGGGCAA -0.0317624623664
CCGGGCAC -0.27966457849
CCGGGCAG -0.209114735725
CCGGGCCA -0.249355046759
CCGGGCCC -0.250613353946
CCGGGCCG -0.409943383648
CCGGGCGA -0.154752747934
CCGGGCGC -0.160154020792
CCGGGCTA -0.101250111278
CCGGGCTC -0.183078620625
CCGGGGAA -0.141508222275
CCGGGGAC -0.205041745778
CCGGGGAG -0.271504642836
CCGGGGCA 0.0208612496588
CCGGGGCC -0.355974478758
CCGGGGCG -0.178319480627
CCGGGGGA -0.123957902051
CCGGGGGC -0.313190602595
CCGGGGTA -0.0681998042216
CCGGGGTC -0.16711881977
CCGGGTAA -0.227440034443
CCGGGTAC -0.149047993272
CCGGGTAG -0.124728114791
CCGGGTCA -0.157340556038
CCGGGTCC -0.282155233419
CCGGGTCG -0.31910834531
CCGGGTGA -0.0155550495341
CCGGGTGC -0.31041454762
CCGGGTTA -0.309085747204
CCGGGTTC -0.0181713278904
CCGGTAAA 0.0357656897536
CCGGTAAC -0.123461988065
CCGGTAAG -0.161410404422
CCGGTACA -0.181122847372
CCGGTACC -0.277741947042
CCGGTACG -0.31105704343
CCGGTAGA 0.0804004291877
CCGGTAGC -0.231640537792
CCGGTAGG 0.169122920229

CCGGTATA -0.0303926516048
CCGGTATC 0.326460068884
CCGGTCAA -0.184503124129
CCGGTCAC -0.260919147744
CCGGTCAG -0.0219098350938
CCGGTCCA -0.129923064236
CCGGTCCC -0.161816881572
CCGGTCCG -0.285846621465
CCGGTCGA -0.0683467203412
CCGGTCGC -0.212289132047
CCGGTCTA -0.237501769771
CCGGTCTC 0.0641022459362
CCGGTGAA -0.19350631805
CCGGTGAC -0.324346668729
CCGGTGAG -0.155644218433
CCGGTGCA 0.0068892882115
CCGGTGCC -0.276556655393
CCGGTGCG -0.131159919129
CCGGTGGA -0.056698969322
CCGGTGGC -0.439158482752
CCGGTGTA -0.0504423875972
CCGGTGTC -0.0188053436396
CCGGTTAA -0.135153059395
CCGGTTAC -0.0749362341619
CCGGTTAG -0.151156340641
CCGGTTCA -0.107710120774
CCGGTTCC -0.362065745918
CCGGTTCG -0.420260497741
CCGGTTGA -0.182306701848
CCGGTTGC -0.305264057379
CCGGTTTA -0.256005446164
CCGGTTTC -0.0630811004139
CCGTAAAA 0.00679330967566
CCGTAAAC -0.301949354714
CCGTAAAG -0.074338762197
CCGTAACA 0.0223460374976
CCGTAACC 0.0175022762038
CCGTAACG 0.0383633935023
CCGTAAGA 0.142419509062
CCGTAAGC -0.151547980362
CCGTAAGG -0.00905912488661
CCGTAATA 0.291125741772
CCGTAATC 0.455652622319
CCGTACAA 0.0772549408913
CCGTACAC -0.143055658949
CCGTACAG -0.0797149095601
CCGTACCA 0.128148288724
CCGTACCC -0.178224946034
CCGTACCG -0.165609397701
CCGTACGA -0.100145077508
CCGTACGC 0.0287766114379
CCGTACGG -0.150446368279
CCGTACTA -0.0356311403746
CCGTACTC -0.029784019328

CCGTAGAA 0.0144924538864
CCGTAGAC -0.227147948529
CCGTAGAG -0.192570619614
CCGTAGCA -0.0685717756187
CCGTAGCC -0.230566666618
CCGTAGCG -0.226536333639
CCGTAGGA -0.117381150287
CCGTAGGC -0.35224294391
CCGTAGTA -0.16491270834
CCGTAGTC -0.0695601846784
CCGTATAA 0.0840996182565
CCGTATAC -0.140993610691
CCGTATAG 0.0113653598502
CCGTATCA 0.198784183633
CCGTATCC 0.285965028389
CCGTATCG 0.153680576338
CCGTATGA -0.18181493088
CCGTATGC -0.073388641965
CCGTATTA -0.0882265351854
CCGTATTC 0.2035244611
CCGTCAAA -0.0577191740327
CCGTCAAC -0.239291749017
CCGTCAAG -0.0802772469439
CCGTCACA -0.0795301319436
CCGTCACC -0.160076870414
CCGTCACG -0.382926309739
CCGTCAGA -0.0660820890378
CCGTCAGC -0.228076000765
CCGTCAGG -0.117732514066
CCGTCATA 0.0131058143286
CCGTCATC -0.0593316543014
CCGTCCAA -0.0467807274293
CCGTCCAC -0.298048588197
CCGTCCAG -0.0481794848467
CCGTCCCA -0.12933370829
CCGTCCCC -0.131184058534
CCGTCCCG -0.161668751158
CCGTCCGA -0.0704028823765
CCGTCCGC -0.128436990912
CCGTCCTA -0.0499917528241
CCGTCCTC -0.198716093484
CCGTCGAA -0.105433820549
CCGTCGAC -0.205787478635
CCGTCGAG -0.173542182038
CCGTCGCA -0.171828545824
CCGTCGCC -0.293994359408
CCGTCGCG -0.161820538533
CCGTCGGA 0.0165190763038
CCGTCGGC -0.0727862278851
CCGTCGTA -0.128125594514
CCGTCGTC -0.110060386844
CCGTCTAA -0.0606474865302
CCGTCTAC -0.266449245555
CCGTCTAG -0.266990585673

CCGTCTCA 0.0476414077756
CCGTCTCC -0.3208810178
CCGTCTCG -0.221488178532
CCGTCTGA -0.207371474102
CCGTCTGC -0.214581868806
CCGTCTTA -0.0873855586774
CCGTCTTC -0.232516311494
CCGTGAAA 0.0950882011488
CCGTGAAC -0.103444425143
CCGTGAAG -0.157208914682
CCGTGACA -0.154952795077
CCGTGACC -0.350175593714
CCGTGACG -0.0656748641338
CCGTGAGA -0.021119245564
CCGTGAGC -0.135143965524
CCGTGAGG -0.233239573771
CCGTGATA 0.210156711897
CCGTGATC 0.174214159063
CCGTGCAA 0.0120244211153
CCGTGCAC -0.257330869037
CCGTGCAG -0.144903094354
CCGTGCCA -0.0313999436537
CCGTGCCC -0.132935688357
CCGTGCCG -0.193500897369
CCGTGCGA -0.0745339847063
CCGTGCGC 0.00688379086589
CCGTGCTA -0.0378403588648
CCGTGCTC -0.131815415066
CCGTGGAA -0.225846773075
CCGTGGAC -0.27025633816
CCGTGGAG -0.313629924029
CCGTGGCA -0.19310687532
CCGTGGCC -0.255135074793
CCGTGGCG 0.0541243723062
CCGTGGGA -0.1839051943
CCGTGGGC -0.245304319778
CCGTGGTA -0.0525167040319
CCGTGGTC -0.30835896609
CCGTGTAA -0.0800514513074
CCGTGTAC -0.0938597579692
CCGTGTAG 0.149260490855
CCGTGTCA -0.000876623139639
CCGTGTCC -0.273566843195
CCGTGTCG -0.174266292887
CCGTGTGA 0.0594665810238
CCGTGTGC -0.194008165921
CCGTGTTA -0.0462024309463
CCGTGTTC -0.15041185559
CCGTTAAA 0.0509237024007
CCGTTAAC -0.178948636161
CCGTTAAG -0.0036448122689
CCGTTACA -0.19151839996
CCGTTACC -0.0954281221553
CCGTTACG -0.201106177005

CCGTTAGA 0.0691067354306
CCGTTAGC -0.111605515912
CCGTTAGG 0.0572873148631
CCGTTATA 0.0878951555805
CCGTTATC 0.115248857673
CCGTTCAA -0.0216762487764
CCGTTCAC -0.178238902811
CCGTTCAG 0.0720998810245
CCGTTCCA -0.0165046587908
CCGTTCCC -0.222091918424
CCGTTCCG -0.216263490882
CCGTTCGA 0.0112429102801
CCGTTCGC -0.144982036491
CCGTTCTA 0.00533054020715
CCGTTCTC 0.0709125103064
CCGTTGAA 0.0876212869444
CCGTTGAC -0.144485412716
CCGTTGAG -0.0331657058413
CCGTTGCA -0.171880212512
CCGTTGCC -0.361768281382
CCGTTGCG -0.0101713283531
CCGTTGGA -0.128955995026
CCGTTGGC -0.370129673267
CCGTTGTA -0.0494452099973
CCGTTGTC 0.126085688761
CCGTTTAA 0.111652975289
CCGTTTAC -0.0520936879181
CCGTTTAG -0.0320934578075
CCGTTTCA -0.139711214229
CCGTTTCC -0.181759681941
CCGTTTCG -0.187253061146
CCGTTTGA -0.0537796828863
CCGTTTGC 0.00628849080139
CCGTTTTA -0.0108501273695
CCGTTTTC -0.0129233917113
CCTAAAAA -0.0502653226572
CCTAAAAC -0.109563695444
CCTAAAAG 0.125871050113
CCTAAACA -0.0802582491666
CCTAAACC -0.180632444527
CCTAAACG -0.0951991068106
CCTAAAGA 0.0839255233195
CCTAAAGC -0.259203718704
CCTAAAGG -0.095496322769
CCTAAATA 0.180920855748
CCTAAATC 0.228430910249
CCTAACAA -0.0268163299268
CCTAACAC -0.18004826183
CCTAACAG 0.0801961780677
CCTAACCA -0.224008504888
CCTAACCC -0.16176377345
CCTAACCG -0.030973707499
CCTAACGA 0.0142617314949
CCTAACGC -0.0839046996777

CCTAACTA -0.130011785138
CCTAACTC -0.179132045345
CCTAAGAA 0.00274373553864
CCTAAGAC -0.233616148586
CCTAAGAG -0.0257452376747
CCTAAGCA -0.243576806751
CCTAAGCC -0.148260957567
CCTAAGCG -0.202750507502
CCTAAGGA -0.163978027614
CCTAAGGC -0.225054768949
CCTAAGTA -0.252093275877
CCTAAGTC -0.0842809374922
CCTAATAA -0.0205646615082
CCTAATAC -0.150217094653
CCTAATAG 0.108242155281
CCTAATCA 0.06891878104
CCTAATCC 0.0117314467906
CCTAATCG 0.080099014815
CCTAATGA -0.00219922191485
CCTAATGC -0.146850089697
CCTAATTA -0.00412395866941
CCTAATTC -0.0414450262935
CCTACAAA -0.0499496526621
CCTACAAC -0.175382415701
CCTACAAG -0.0179614888862
CCTACACA 0.0890656496717
CCTACACC -0.258952659571
CCTACACG 0.0441917293507
CCTACAGA 0.067615307694
CCTACAGC -0.155276557852
CCTACAGG -0.0212148639081
CCTACATA 0.074257498011
CCTACATC 0.0621789463235
CCTACCAA -0.107534643507
CCTACCAC 0.0879736485797
CCTACCAG -0.227681438664
CCTACCCA -0.146322902967
CCTACCCC -0.194893106728
CCTACCCG -0.203004726487
CCTACCGA -0.0369849456024
CCTACCGC -0.0953670849083
CCTACCTA -0.124572497055
CCTACCTC -0.317603133441
CCTACGAA 0.040611600557
CCTACGAC -0.19020425019
CCTACGAG 0.0700001021825
CCTACGCA -0.176408570424
CCTACGCC -0.288795444083
CCTACGCG 0.0957484428121
CCTACGGA -0.0278454506622
CCTACGGC -0.293013754146
CCTACGTA -0.0686751314009
CCTACGTC -0.18704461812
CCTACTAA -0.0516634266727

CCTACTAC -0.183102454893
CCTACTAG -0.139055016502
CCTACTCA -0.0805475561109
CCTACTCC -0.207921218357
CCTACTCG -0.142745477555
CCTACTGA -0.345533698807
CCTACTGC -0.166651587078
CCTACTTA -0.0649482128129
CCTACTTC -0.256852139163
CCTAGAAA 0.0358526570648
CCTAGAAC -0.121980946078
CCTAGAAG 0.0126926226457
CCTAGACA -0.12485618806
CCTAGACC -0.326120688467
CCTAGACG -0.173848798705
CCTAGAGA 0.000615314993102
CCTAGAGC -0.17733892376
CCTAGAGG -0.20334325766
CCTAGATA 0.301647377405
CCTAGATC 0.105296756812
CCTAGCAA -0.0832554356068
CCTAGCAC -0.180051195311
CCTAGCAG -0.108357481551
CCTAGCCA 0.00736687100323
CCTAGCCC -0.224511709327
CCTAGCCG -0.17052218335
CCTAGCGA 0.0376987573444
CCTAGCGC -0.202908917501
CCTAGCTA -0.20016159428
CCTAGCTC 0.000828652343804
CCTAGGAA -0.210345521563
CCTAGGAC -0.203710134861
CCTAGGAG -0.15182956106
CCTAGGCA -0.109375387746
CCTAGGCC -0.224278661275
CCTAGGCG 0.0973431238732
CCTAGGGA -0.19344310197
CCTAGGGC -0.227697977568
CCTAGGTA -0.182352016806
CCTAGGTC -0.33036158573
CCTAGTAA 0.0675957409843
CCTAGTAC -0.232031657032
CCTAGTAG -0.118714918651
CCTAGTCA -0.0403765926882
CCTAGTCC -0.163100277694
CCTAGTCG -0.095758843528
CCTAGTGA -0.19025162969
CCTAGTGC -0.12686035633
CCTAGTTA 0.07890419269
CCTAGTTC -0.135594271958
CCTATAAA -0.00910418020043
CCTATAAC -0.0191678315701
CCTATAAG 0.0663887324974
CCTATACA -0.0580578527478

CCTATACC -0.213151111662
CCTATACG 0.130898115747
CCTATAGA -0.0433224089983
CCTATAGC -0.167579270277
CCTATAGG 0.042495982624
CCTATATA 0.0627424216988
CCTATATC 0.308908081635
CCTATCAA 0.165767696071
CCTATCAC -0.148099573336
CCTATCAG 0.0410348678496
CCTATCCA 0.0646004130853
CCTATCCC -0.112306956805
CCTATCCG -0.132394335134
CCTATCGA -0.119977317743
CCTATCGC -0.0799742467733
CCTATCTA -0.0881361073196
CCTATCTC -0.0439209360379
CCTATGAA -0.182523535817
CCTATGAC -0.232335062793
CCTATGAG -0.136634326692
CCTATGCA -0.101940757281
CCTATGCC -0.0367707276483
CCTATGCG -0.129502364562
CCTATGGA -0.0589833402658
CCTATGGC -0.200924567817
CCTATGTA 0.00986133838601
CCTATGTC 0.000605288484076
CCTATTAA 0.124101662007
CCTATTAC -0.134800698115
CCTATTAG 0.0299927738809
CCTATTCA -0.203167622904
CCTATTCC -0.15339350797
CCTATTCG -0.0561425662212
CCTATTGA -0.170781331415
CCTATTGC -0.212878539597
CCTATTTA 0.0117333197083
CCTATTTC -0.0555776139939
CCTCAAAA -0.102283357583
CCTCAAAC -0.188125267061
CCTCAAAG 0.0381993563812
CCTCAACA -0.0627983455251
CCTCAACC -0.245530990822
CCTCAACG -0.0259365875044
CCTCAAGA 0.0310434401343
CCTCAAGC -0.239836235668
CCTCAAGG -0.189258551727
CCTCAATA 0.215191638139
CCTCAATC 0.0708561126399
CCTCACAA -0.00984868944476
CCTCACAC -0.110542785459
CCTCACAG -0.0931551332852
CCTCACCA 0.0488429869082
CCTCACCC -0.281183474258
CCTCACCG -0.20480521812

CCTCACGA -0.0723243905062
CCTCACGC -0.127128887345
CCTCACTA -0.27866200441
CCTCACTC -0.154904101113
CCTCAGAA -0.0504165188744
CCTCAGAC -0.274029593972
CCTCAGAG 0.0232688335292
CCTCAGCA -0.149955202691
CCTCAGCC -0.402494949745
CCTCAGCG -0.105470055532
CCTCAGGA -0.00753887678108
CCTCAGGC -0.152861635909
CCTCAGTA -0.123150468534
CCTCAGTC -0.153982501076
CCTCATAA -0.00233936483143
CCTCATAC -0.154778348483
CCTCATAG -0.0762760005184
CCTCATCA 0.211831127786
CCTCATCC -0.117919248086
CCTCATCG -0.184117807607
CCTCATGA -0.0750047568229
CCTCATGC -0.340804041707
CCTCATTA -0.0378794772734
CCTCATTC -0.100092737116
CCTCCAAA -0.155331863025
CCTCCAAC -0.169923371368
CCTCCAAG -0.102817468832
CCTCCACA -0.168037517932
CCTCCACC -0.330177061523
CCTCCACG -0.15285748797
CCTCCAGA -0.0916648055783
CCTCCAGC -0.190131170875
CCTCCAGG -0.378635713108
CCTCCATA 0.107894395773
CCTCCATC -0.181928238359
CCTCCCAA -0.0859979421616
CCTCCCAC -0.289971252725
CCTCCCAG 0.00756265907781
CCTCCCCA -0.242405335785
CCTCCCCC -0.264279275927
CCTCCCCG -0.274373422704
CCTCCCGA -0.20476154339
CCTCCCGC -0.165249348716
CCTCCCTA -0.310736866374
CCTCCCTC -0.220929367107
CCTCCGAA -0.118459328845
CCTCCGAC -0.162691873762
CCTCCGAG -0.155018565841
CCTCCGCA -0.249953671345
CCTCCGCC -0.329949945803
CCTCCGCG -0.132315583111
CCTCCGGA -0.138619415843
CCTCCGGC -0.208122179242
CCTCCGTA -0.197166996389

CCTCCGTC -0.196623080168
CCTCCTAA -0.207631428784
CCTCCTAC -0.192493932597
CCTCCTAG -0.0853109120436
CCTCCTCA -0.134269481728
CCTCCTCC -0.237171526429
CCTCCTCG -0.290491978875
CCTCCTGA -0.142438022303
CCTCCTGC -0.114702144143
CCTCCTTA -0.28271561695
CCTCCTTC -0.155089035689
CCTCGAAA 0.0636270738597
CCTCGAAC -0.200368787205
CCTCGAAG -0.200529842412
CCTCGACA -0.0931385930058
CCTCGACC -0.36364637885
CCTCGACG -0.172284127644
CCTCGAGA 0.0287215590246
CCTCGAGC -0.258755959773
CCTCGAGG 0.0615744300846
CCTCGATA 0.184915920886
CCTCGATC -0.0138673086947
CCTCGCAA -0.208721002357
CCTCGCAC -0.171238635153
CCTCGCAG -0.116529731418
CCTCGCCA -0.00437727641001
CCTCGCCC -0.364060798986
CCTCGCCG -0.233943655601
CCTCGCGA 0.0525090691445
CCTCGCGC -0.130968141208
CCTCGCTA -0.211315111166
CCTCGCTC -0.189394481271
CCTCGGAA -0.00996644983342
CCTCGGAC -0.294347443569
CCTCGGAG -0.0843629628224
CCTCGGCA -0.35735691781
CCTCGGCC -0.0112440263955
CCTCGGCG 0.00792902345204
CCTCGGGA -0.209693246123
CCTCGGGC -0.307809770896
CCTCGGTA -0.279077524444
CCTCGGTC -0.292692696637
CCTCGTAA -0.0392782122155
CCTCGTAC -0.158750244674
CCTCGTAG 0.0498473680292
CCTCGTCA -0.0750631675286
CCTCGTCC -0.204308977145
CCTCGTCG -0.139438281868
CCTCGTGA -0.268624872857
CCTCGTGC -0.20582397165
CCTCGTTA -0.220323160898
CCTCGTTC -0.235198518283
CCTCTAAA -0.0945422006028
CCTCTAAC -0.0334377540877

CCTCTAAG -0.0778501200704
CCTCTACA -0.253999751953
CCTCTACC -0.284657279014
CCTCTACG -0.302574811507
CCTCTAGA 0.0296674084553
CCTCTAGC -0.214600409695
CCTCTATA -0.0198132016314
CCTCTATC 0.102467099261
CCTCTCAA -0.044651550473
CCTCTCAC -0.165918095569
CCTCTCAG -0.0958113502193
CCTCTCCA -0.119938622395
CCTCTCCC -0.151714271162
CCTCTCCG -0.104690061023
CCTCTCGA -0.229443284347
CCTCTCGC -0.295263825273
CCTCTCTA -0.102963679827
CCTCTCTC -0.231261100466
CCTCTGAA -0.0822186375481
CCTCTGAC -0.244725302287
CCTCTGAG -0.054087136778
CCTCTGCA -0.248448004267
CCTCTGCC -0.0502654037584
CCTCTGCG -0.0355076790127
CCTCTGGA -0.157670434967
CCTCTGGC -0.26632458701
CCTCTGTA -0.114561535556
CCTCTGTC -0.260263727412
CCTCTTAA 0.0816150011314
CCTCTTAC -0.0457607164225
CCTCTTAG 0.0562045561704
CCTCTTCA -0.257760576465
CCTCTTCC -0.357404048614
CCTCTTCG -0.162260930939
CCTCTTGA -0.125418724101
CCTCTTGC -0.166613992862
CCTCTTTA -0.171183957246
CCTCTTTC -0.264148368689
CCTGAAAA -0.117828761575
CCTGAAAC 0.0581118661635
CCTGAAAG -0.0812342308806
CCTGAACA -0.171341062043
CCTGAACC -0.270118213245
CCTGAACG -0.179742129837
CCTGAAGA 0.225052876568
CCTGAAGC -0.241766254531
CCTGAAGG -0.32076296226
CCTGAATA -0.0932452799762
CCTGAATC 0.102227688445
CCTGACAA 0.119990803121
CCTGACAC -0.0704686818023
CCTGACAG -0.0274078268878
CCTGACCA -0.137998672687
CCTGACCC -0.353852337869

CCTGACCG -0.177092080125
CCTGACGA 0.0512923391711
CCTGACGC -0.154455366069
CCTGACTA -0.293311415509
CCTGACTC -0.26892938652
CCTGAGAA -0.0298336532777
CCTGAGAC -0.342707670087
CCTGAGAG -0.226912845112
CCTGAGCA -0.174492111147
CCTGAGCC -0.207086872496
CCTGAGCG -0.122332887203
CCTGAGGA 0.0178812026585
CCTGAGGC -0.124043776464
CCTGAGTA -0.0781819986599
CCTGAGTC -0.224401581416
CCTGATAA 0.031619015642
CCTGATAC 0.21394421374
CCTGATAG -0.0282804026189
CCTGATCA -0.100414147179
CCTGATCC -0.00163281096034
CCTGATCG 0.0977685674655
CCTGATGA 0.0246017367229
CCTGATGC -0.142324164134
CCTGATTA 0.249733204279
CCTGATTC 0.399888835041
CCTGCAAA 0.0749109439355
CCTGCAAC -0.0312335415202
CCTGCAAG -0.309627287043
CCTGCACA -0.0759202353233
CCTGCACC -0.165347237727
CCTGCACG -0.113216428727
CCTGCAGA -0.0408226286288
CCTGCAGC 0.0867157702059
CCTGCAGG -0.194137876871
CCTGCATA -0.071621883982
CCTGCATC -0.182599549501
CCTGCCAA -0.322983709173
CCTGCCAC -0.183706810267
CCTGCCAG -0.143338984686
CCTGCCCA -0.178386034246
CCTGCCCC -0.37230804919
CCTGCCCG -0.10527861178
CCTGCCGA -0.0433741942647
CCTGCCGC -0.322409929465
CCTGCCTA -0.219788126132
CCTGCCTC -0.18216438832
CCTGCGAA 0.0043228338756
CCTGCGAC -0.190703020981
CCTGCGAG -0.0349750326051
CCTGCGCA 0.0150500354007
CCTGCGCC -0.291015856479
CCTGCGCG -0.0677890265177
CCTGCGGA 0.134693649651
CCTGCGGC -0.311613145295

CCTGCGTA -0.135062836513
CCTGCGTC -0.20919094843
CCTGCTAA -0.16343688201
CCTGCTAC -0.129594334074
CCTGCTAG -0.218553585318
CCTGCTCA 0.0269374474363
CCTGCTCC -0.251193086042
CCTGCTCG -0.100868378613
CCTGCTGA -0.116063476307
CCTGCTGC -0.249091896168
CCTGCTTA -0.181466553229
CCTGCTTC -0.248564256369
CCTGGAAA 0.0949751404297
CCTGGAAC -0.188643249606
CCTGGAAG -0.332937674636
CCTGGACA -0.137632696313
CCTGGACC -0.18515115646
CCTGGACG -0.130911073514
CCTGGAGA 0.073156792187
CCTGGAGC -0.227157302994
CCTGGATA 0.250916205462
CCTGGATC 0.144451581352
CCTGGCAA 0.134016356504
CCTGGCAC -0.162000623058
CCTGGCAG -0.136043733425
CCTGGCCA -0.0696096551094
CCTGGCCC -0.222713881524
CCTGGCCG -0.284466444397
CCTGGCGA 0.0663686196653
CCTGGCGC 0.0558078614661
CCTGGCTA -0.302101163454
CCTGGCTC -0.133693090187
CCTGGGAA -0.214289258864
CCTGGGAC -0.175263089694
CCTGGGAG -0.181653669845
CCTGGGCA -0.320243361307
CCTGGGCC -0.170524557356
CCTGGGCG -0.263526755811
CCTGGGGA -0.200074585801
CCTGGGGC -0.382212684788
CCTGGGTA -0.267336040604
CCTGGGTC -0.293510917635
CCTGGTAA -0.0906381612081
CCTGGTAC -0.293938907573
CCTGGTAG -0.2975380307
CCTGGTCA -0.143396876594
CCTGGTCC -0.426222067905
CCTGGTCG -0.257023513231
CCTGGTGA -0.0571385830104
CCTGGTGC -0.274862714939
CCTGGTTA -0.243039816467
CCTGGTTC -0.225062130337
CCTGTAAA 0.117284293045
CCTGTAAC 0.0469004328372

CCTGTAAG 0.0188940864084
CCTGTACA -0.211888626322
CCTGTACC -0.276310087473
CCTGTACG -0.11632052141
CCTGTAGA 0.100054600055
CCTGTAGC -0.14865595178
CCTGTATA 0.0437779377173
CCTGTATC 0.0891787103908
CCTGTCAA 0.0230326745478
CCTGTCAC -0.209703650412
CCTGTCAG -0.0721207380414
CCTGTCCA -0.139213222994
CCTGTCCC -0.209743007399
CCTGTCCG -0.199628039055
CCTGTCGA 0.0520522259448
CCTGTCGC 0.0709991752005
CCTGTCTA -0.147788625884
CCTGTCTC -0.2142506942
CCTGTGAA -0.235105189302
CCTGTGAC -0.196365119927
CCTGTGAG -0.0999776669396
CCTGTGCA -0.108732965434
CCTGTGCC -0.193225207939
CCTGTGCG -0.0480968062608
CCTGTGGA 0.037158962039
CCTGTGGC -0.340618838649
CCTGTGTA -0.0809858178288
CCTGTGTC -0.1702554704
CCTGTTAA 0.101538282678
CCTGTTAC -0.284901239162
CCTGTTAG 0.097657553322
CCTGTTCA -0.0621955354854
CCTGTTCC -0.274113214204
CCTGTTCG -0.217125688021
CCTGTTGA 0.0159181219787
CCTGTTGC -0.24754847231
CCTGTTTA -0.169063391639
CCTGTTTC -0.0165086731022
CCTTAAAA -0.0288599793591
CCTTAAAC 0.138305730531
CCTTAAAG -0.0948002746276
CCTTAACA -0.104963823697
CCTTAACC -0.258062421098
CCTTAACG -0.10088943897
CCTTAAGA 0.00347437647351
CCTTAAGC 0.0952260800746
CCTTAAGG -0.136407487341
CCTTAATA 0.0439145593965
CCTTAATC -0.00022198507047
CCTTACAA -0.0810163929906
CCTTACAC -0.0577132536433
CCTTACAG 0.0590025286995
CCTTACCA -0.0989921888799
CCTTACCC -0.246304244158

CCTTACCG -0.118371031992
CCTTACGA 0.0435552785275
CCTTACGC 0.0669617129228
CCTTACTA -0.182135757435
CCTTACTC -0.160925221916
CCTTAGAA -0.0176636999689
CCTTAGAC -0.11548006055
CCTTAGAG -0.150719512142
CCTTAGCA -0.0817218707356
CCTTAGCC -0.093828848615
CCTTAGCG -0.0580024202648
CCTTAGGA -0.242551281771
CCTTAGGC -0.147435314102
CCTTAGTA -0.0587819224183
CCTTAGTC 0.00735093392115
CCTTATAA 0.0519864528514
CCTTATAC -0.191093629799
CCTTATAG -0.112661531522
CCTTATCA -0.0191697343205
CCTTATCC -0.0873332136398
CCTTATCG 0.0642431187638
CCTTATGA 0.0933269673651
CCTTATGC -0.0716264521242
CCTTATTA 0.0281943939639
CCTTATTC -0.0867900702904
CCTTCAAA -0.0603964876682
CCTTCAAC -0.199946140586
CCTTCAAG -0.0410749682268
CCTTCACA -0.132109702198
CCTTCACC -0.269710564834
CCTTCACG -0.12471846477
CCTTCAGA -0.0595453397618
CCTTCAGC -0.30022494025
CCTTCATA -0.129229521535
CCTTCATC -0.0631346858702
CCTTCCAA -0.0852533216954
CCTTCCAC -0.217897945766
CCTTCCAG -0.206597381827
CCTTCCCA -0.21905025792
CCTTCCCC -0.396042297968
CCTTCCCG -0.167650829095
CCTTCCGA -0.101061116213
CCTTCCGC -0.222478506872
CCTTCCTA -0.225095694583
CCTTCCTC -0.422622043832
CCTTCGAA -0.149387008581
CCTTCGAC -0.151677784767
CCTTCGAG -0.214490895808
CCTTCGCA -0.220433175379
CCTTCGCC -0.246119969866
CCTTCGCG -0.206673262443
CCTTCGGA -0.243324397018
CCTTCGGC -0.180503805542
CCTTCGTA -0.0507507849414

CCTTCGTC -0.342795842945
CCTTCTAA -0.00784117450784
CCTTCTAC -0.174770962897
CCTTCTAG 0.0261957741547
CCTTCTCA -0.0909339693002
CCTTCTCC -0.202258514124
CCTTCTCG -0.235259823744
CCTTCTGA -0.168394203858
CCTTCTGC -0.226196013495
CCTTCTTA -0.070962879333
CCTTCTTC -0.208614326223
CCTTGAAA -0.0418386620893
CCTTGAAC -0.159971548851
CCTTGAAG -0.0879207991772
CCTTGACA -0.0520443953369
CCTTGACC -0.223089136421
CCTTGACG 0.106686576384
CCTTGAGA 0.0492710341195
CCTTGAGC -0.158698593449
CCTTGATA 0.12994970913
CCTTGATC -0.113381181608
CCTTGCAA 0.121103196861
CCTTGCAC -0.0991974695037
CCTTGCAG -0.11590145268
CCTTGCCA -0.0136310734397
CCTTGCCC -0.377009621931
CCTTGCCG -0.161197958102
CCTTGCGA -0.19811579628
CCTTGCGC 0.156349186652
CCTTGCTA 0.0382076291167
CCTTGCTC -0.248974466365
CCTTGGAA -0.185827146952
CCTTGGAC -0.0645099754336
CCTTGGAG -0.16483796347
CCTTGGCA -0.11670125633
CCTTGGCC -0.0302409847864
CCTTGGCG -0.115776668209
CCTTGGGA -0.0175746948656
CCTTGGGC -0.22795485687
CCTTGGTA -0.185073071484
CCTTGGTC -0.129226184076
CCTTGTAA 0.14658876083
CCTTGTAC -0.257452204717
CCTTGTAG -0.125702257281
CCTTGTCA -0.0801589861942
CCTTGTCC -0.0897605810394
CCTTGTCG -0.108057051393
CCTTGTGA -0.222575852008
CCTTGTGC -0.253236421606
CCTTGTTA 0.0904141055656
CCTTGTTC -0.288204359709
CCTTTAAA 0.116142313112
CCTTTAAC 0.0529878907761
CCTTTAAG -0.134541524328

CCTTTACA 0.102302026544
CCTTTACC -0.202024115444
CCTTTACG -0.0554593695029
CCTTTAGA 0.159331507816
CCTTTAGC -0.0815944222514
CCTTTATA -0.0831947787677
CCTTTATC 0.058732286005
CCTTTCAA -0.0362554104655
CCTTTCAC -0.108442172538
CCTTTCAG -0.0165907840169
CCTTTCCA -0.238002990356
CCTTTCCC -0.23394352159
CCTTTCCG -0.0776225082723
CCTTTCGA -0.0774182442994
CCTTTCGC -0.0264721208202
CCTTTCTA -0.0674678015733
CCTTTCTC -0.108008559122
CCTTTGAA -0.00802975117333
CCTTTGAC -0.202010789859
CCTTTGAG -0.0683283083947
CCTTTGCA -0.284768221048
CCTTTGCC 0.0316369704941
CCTTTGCG -0.284138052612
CCTTTGGA -0.1267781694
CCTTTGGC -0.196180498882
CCTTTGTA -0.150444869048
CCTTTGTC -0.203306150628
CCTTTTAA 0.105139574837
CCTTTTAC -0.209546262977
CCTTTTAG 0.0741144354503
CCTTTTCA 0.0692331449907
CCTTTTCC -0.176770522118
CCTTTTCG -0.0480994790154
CCTTTTGA -0.136228556582
CCTTTTGC -0.0801948311564
CCTTTTTA -0.0104267789756
CCTTTTTC -0.149777634405
CGAAAAAA 0.0813317956541
CGAAAAAC 0.14073439831
CGAAAAAG 0.0883360218389
CGAAAACA -0.0674918221588
CGAAAACC -0.0798970422392
CGAAAACG -0.134197608173
CGAAAAGA 0.00215104778727
CGAAAAGC -0.0602651036839
CGAAAATA 0.078359597965
CGAAAATC 0.405798360344
CGAAACAA -0.0398539034903
CGAAACAC -0.0977328963599
CGAAACAG -0.13296627918
CGAAACCA 0.038061145991
CGAAACCC -0.34650436635
CGAAACCG 0.0534184322063
CGAAACGA 0.031773756779

CGAAACGC 0.0324803106501
CGAAACTA 0.0695072370678
CGAAACTC -0.103909694819
CGAAAGAA 0.101772223966
CGAAAGAC -0.103861229316
CGAAAGAG -0.235592591998
CGAAAGCA -0.182300689921
CGAAAGCC -0.16184964682
CGAAAGCG -0.0997822269773
CGAAAGGA -0.0999582573768
CGAAAGGC -0.0163952089708
CGAAAGTA -0.0239946340425
CGAAAGTC -0.2592555834
CGAAATAA 0.220695167259
CGAAATAC 0.157959398011
CGAAATAG 0.122624322095
CGAAATCA 0.405806442195
CGAAATCC 0.482114260272
CGAAATCG 0.445183682109
CGAAATGA 0.0191871710838
CGAAATGC 0.129102932133
CGAAATTA 0.211667590455
CGAAATTC 0.185524650275
CGAACAAA 0.0162558484121
CGAACAAC 0.0131770889347
CGAACAAG -0.0841932474903
CGAACACA -0.069645383031
CGAACACC -0.247300096188
CGAACACG -0.00326359417269
CGAACAGA 0.0375214035784
CGAACAGC -0.191253870182
CGAACATA -0.140036316767
CGAACATC -0.0110758141061
CGAACCAA -0.11164297485
CGAACCAC -0.209810417132
CGAACCAG -0.188129785641
CGAACCCA 0.0445238425436
CGAACCCC -0.231967113819
CGAACCCG 0.0846314634193
CGAACCGA -0.0238360747142
CGAACCGC -0.194263593044
CGAACCTA 0.0378045769657
CGAACCTC -0.176640615308
CGAACGAA -0.129021184373
CGAACGAC -0.171866722126
CGAACGAG -0.0806701629065
CGAACGCA 0.0919914404763
CGAACGCC -0.211732252104
CGAACGCG -0.0198973077761
CGAACGGA -0.064922568781
CGAACGGC -0.0421286422001
CGAACGTA -0.065576886151
CGAACGTC -0.204383338904
CGAACTAA -0.108752074669

CGAACTAC -0.172972124698
CGAACTAG -0.160169400399
CGAACTCA -0.0788719955037
CGAACTCC -0.170874011714
CGAACTCG -0.0236435767637
CGAACTGA -0.0875069152205
CGAACTGC -0.051082763204
CGAACTTA 0.149455851332
CGAACTTC -0.348496832557
CGAAGAAA 0.169954672525
CGAAGAAC -0.124761148473
CGAAGAAG -0.191757642798
CGAAGACA -0.149171567776
CGAAGACC -0.21757176564
CGAAGACG -0.0730415631007
CGAAGAGA -0.0321558215604
CGAAGAGC 0.0615146827268
CGAAGATA 0.286103804253
CGAAGATC 0.324598703387
CGAAGCAA -0.078168212136
CGAAGCAC -0.133542633543
CGAAGCAG -0.0526810878186
CGAAGCCA -0.189078306327
CGAAGCCC -0.296366957966
CGAAGCCG -0.154225420061
CGAAGCGA 0.0158133339952
CGAAGCGC -0.153059556287
CGAAGCTA -0.171176014845
CGAAGCTC -0.152190933025
CGAAGGAA -0.0174222462282
CGAAGGAC -0.246118850324
CGAAGGAG -0.163146636676
CGAAGGCA -0.165080073995
CGAAGGCC -0.431381221255
CGAAGGCG -0.0846620044579
CGAAGGGA -0.108161526947
CGAAGGGC -0.0931259440881
CGAAGGTA -0.137441937503
CGAAGGTC -0.405520578648
CGAAGTAA 0.0278323940311
CGAAGTAC -0.0675083096246
CGAAGTAG -0.154900079143
CGAAGTCA -0.189787822904
CGAAGTCC -0.259953881069
CGAAGTCG -0.0599146922337
CGAAGTGA 0.110359670966
CGAAGTGC 0.0221892821746
CGAAGTTA -0.00131748939804
CGAAGTTC -0.333020325791
CGAATAAA 0.0932544606298
CGAATAAC -0.0241286687153
CGAATAAG -0.175880236724
CGAATACA 0.107447668054
CGAATACC -0.0488551345591

CGAATACG 0.259835916002
CGAATAGA 0.0382346858585
CGAATAGC 0.100642321701
CGAATATA 0.180336926929
CGAATATC 0.377024590702
CGAATCAA 0.326335977851
CGAATCAC 0.29351984993
CGAATCAG 0.271508653095
CGAATCCA 0.41109975694
CGAATCCC 0.305078579649
CGAATCCG 0.437166339423
CGAATCGA 0.314418662365
CGAATCGC 0.288617818921
CGAATCTA 0.427084108902
CGAATCTC 0.441180850054
CGAATGAA 0.0844246450307
CGAATGAC -0.0344041083193
CGAATGAG 0.00835529148997
CGAATGCA -0.164566575375
CGAATGCC -0.197636813924
CGAATGCG 0.059738802163
CGAATGGA -0.169725744504
CGAATGGC -0.0716129352493
CGAATGTA 0.0639540739984
CGAATGTC -0.247908125362
CGAATTAA -0.0571702809428
CGAATTAC 0.0190265906586
CGAATTAG 0.0641234133559
CGAATTCA 0.0321988655322
CGAATTCC 0.0813744482573
CGAATTCG 0.184210816458
CGAATTGA -0.0359602026269
CGAATTGC 0.194149763144
CGAATTTA 0.270139340014
CGAATTTC 0.237630192176
CGACAAAA 0.0694634424064
CGACAAAC 0.0660357802384
CGACAAAG -0.0997649686503
CGACAACA 0.0594078927412
CGACAACC -0.142526102192
CGACAACG -0.0361973543792
CGACAAGA -0.102521946628
CGACAAGC -0.147411561334
CGACAATA 0.019344831865
CGACAATC 0.288533097815
CGACACAA 0.1015824471
CGACACAC -0.265632279924
CGACACAG -0.149989709787
CGACACCA -0.209816222989
CGACACCC -0.0224701285307
CGACACCG -0.0551668141678
CGACACGA 0.0836412341336
CGACACGC -0.328183167385
CGACACTA -0.231015191503

CGACACTC -0.208013188982
CGACAGAA 0.123406786713
CGACAGAC 0.0509091041802
CGACAGAG -0.0127881442148
CGACAGCA -0.170352655094
CGACAGCC -0.255396679268
CGACAGCG 0.0263390111875
CGACAGGA 0.144099911101
CGACAGGC 0.0563290179372
CGACAGTA -0.0213911705586
CGACAGTC -0.234757064203
CGACATAA 0.00652759428626
CGACATAC -0.186083606996
CGACATAG -0.0616690287936
CGACATCA -0.111107785961
CGACATCC 0.117965072511
CGACATCG -0.0160112634279
CGACATGA 0.101643759626
CGACATGC -0.116184300134
CGACATTA 0.0689518719822
CGACATTC -0.107344364722
CGACCAAA -0.160396555575
CGACCAAC -0.202747588727
CGACCAAG -0.244231368236
CGACCACA -0.174654200315
CGACCACC -0.234798680195
CGACCACG -0.338520182971
CGACCAGA -0.0761062347365
CGACCAGC -0.141946780449
CGACCATA -0.273141763962
CGACCATC -0.217436728663
CGACCCAA -0.193269074992
CGACCCAC -0.375556800591
CGACCCAG -0.161664382952
CGACCCCA -0.256850533072
CGACCCCC -0.299615343644
CGACCCCG -0.00179362676571
CGACCCGA 0.0124363366708
CGACCCGC -0.104556433664
CGACCCTA -0.216695746129
CGACCCTC -0.349115961717
CGACCGAA 0.0166266432122
CGACCGAC -0.130238963746
CGACCGAG -0.217427880581
CGACCGCA -0.187140742211
CGACCGCC -0.343007382308
CGACCGCG -0.032258704986
CGACCGGA 0.00278915222457
CGACCGGC -0.317644205194
CGACCGTA -0.207863650774
CGACCGTC -0.187234300275
CGACCTAA -0.0997813403631
CGACCTAC -0.238414707998
CGACCTAG -0.313574273939

CGACCTCA -0.308933975838
CGACCTCC -0.270629505576
CGACCTCG -0.318442491978
CGACCTGA -0.138550172581
CGACCTGC -0.209160787298
CGACCTTA -0.22631714126
CGACCTTC -0.371338211584
CGACGAAA 0.126863645322
CGACGAAC -0.19723316466
CGACGAAG -0.240008930565
CGACGACA -0.160577835995
CGACGACC -0.384064632774
CGACGACG -0.093306805428
CGACGAGA 0.0111961051948
CGACGAGC -0.208822854378
CGACGATA 0.125920600279
CGACGATC 0.173922468851
CGACGCAA -0.0271994043926
CGACGCAC -0.176172113884
CGACGCAG -0.0952869944226
CGACGCCA -0.15403119305
CGACGCCC -0.323062454125
CGACGCCG -0.161518422032
CGACGCGA 0.0822894389174
CGACGCGC -0.151928548177
CGACGCTA -0.100213351857
CGACGCTC -0.282775428238
CGACGGAA 0.116362919393
CGACGGAC -0.26954343486
CGACGGAG -0.174825359208
CGACGGCA -0.212345161869
CGACGGCC -0.388067618821
CGACGGCG -0.169735910739
CGACGGGA -0.0749820112647
CGACGGGC -0.0423915291393
CGACGGTA -0.170508832798
CGACGGTC -0.284501509325
CGACGTAA 0.143277884426
CGACGTAC -0.18267317845
CGACGTAG -0.0686790287994
CGACGTCA -0.268877144519
CGACGTCC -0.196654565102
CGACGTCG -0.136840437113
CGACGTGA -0.0784249835775
CGACGTGC -0.159754864262
CGACGTTA 0.118469668547
CGACGTTC -0.231987066577
CGACTAAA -0.0245770740487
CGACTAAC -0.148799766993
CGACTAAG -0.0644364393235
CGACTACA 0.00978981065588
CGACTACC -0.0412795289096
CGACTACG -0.00895819799566
CGACTAGA -0.0355835859917

CGACTAGC -0.22320820059
CGACTATA -0.0989022138201
CGACTATC 0.0571863389853
CGACTCAA 0.120180579987
CGACTCAC -0.0458952631448
CGACTCAG -0.0475231007471
CGACTCCA -0.100265645876
CGACTCCC -0.0864673884574
CGACTCCG -0.0256322854244
CGACTCGA -0.113278460245
CGACTCGC -0.261691236661
CGACTCTA -0.209329426067
CGACTCTC -0.0238077511685
CGACTGAA 0.164547491661
CGACTGAC -0.204813975313
CGACTGAG 0.080293792415
CGACTGCA -0.202422999192
CGACTGCC -0.226329227707
CGACTGCG 0.0221360116202
CGACTGGA 0.0337866342408
CGACTGGC -0.174072052251
CGACTGTA -0.237662208769
CGACTGTC -0.307401710674
CGACTTAA -0.0239277576586
CGACTTAC -0.264620354521
CGACTTAG -0.183938215636
CGACTTCA -0.0850959959719
CGACTTCC -0.175068791554
CGACTTGA -0.213080051779
CGACTTGC -0.195466410865
CGACTTTA 0.0471135668562
CGACTTTC -0.28181658968
CGAGAAAA 0.101361692271
CGAGAAAC -0.0562216169938
CGAGAAAG -0.162182133
CGAGAACA -0.165430076842
CGAGAACC -0.149065680412
CGAGAACG -0.307432125106
CGAGAAGA -0.0254093168148
CGAGAAGC -0.154601312054
CGAGAATA 0.100320706381
CGAGAATC 0.437166339423
CGAGACAA -0.0513303679992
CGAGACAC -0.139956496208
CGAGACAG -0.191913096397
CGAGACCA -0.122702307326
CGAGACCC -0.311880997075
CGAGACCG -0.0326069979842
CGAGACGA -0.105378351435
CGAGACGC -0.216276191343
CGAGACTA 0.0993284818581
CGAGACTC -0.177357342103
CGAGAGAA 0.0999415393355
CGAGAGAC -0.10938391683

CGAGAGAG -0.0223443587008
CGAGAGCA -0.169688072889
CGAGAGCC -0.0826926249071
CGAGAGCG 0.0137706635783
CGAGAGGA -0.23827824631
CGAGAGGC -0.158465155637
CGAGAGTA 0.122615563163
CGAGAGTC -0.235821335737
CGAGATAA 0.0711597556258
CGAGATAC 0.320736772187
CGAGATAG 0.0903510186863
CGAGATCA 0.219638256097
CGAGATCC 0.425332530958
CGAGATCG 0.322789731881
CGAGATGA -0.0495630673301
CGAGATGC 0.00764873761888
CGAGATTA 0.39527544073
CGAGATTC 0.45752777571
CGAGCAAA 0.118687495347
CGAGCAAC -0.282464773883
CGAGCAAG -0.0816750220743
CGAGCACA -0.133069718397
CGAGCACC -0.183678082966
CGAGCACG -0.172756476541
CGAGCAGA -0.307406948062
CGAGCAGC -0.323150046326
CGAGCATA -0.132578107551
CGAGCATC -0.192924932673
CGAGCCAA -0.163841572947
CGAGCCAC -0.385352332763
CGAGCCAG -0.228612975294
CGAGCCCA -0.263588746916
CGAGCCCC -0.249208647162
CGAGCCCG -0.0432426545798
CGAGCCGA -0.160572479259
CGAGCCGC -0.361722756819
CGAGCCTA -0.117670815587
CGAGCCTC -0.280627678622
CGAGCGAA -0.113558382043
CGAGCGAC -0.233666170961
CGAGCGAG -0.2119185301
CGAGCGCA -0.234176845688
CGAGCGCC -0.176116634093
CGAGCGCG 0.0505737545781
CGAGCGGA -0.0735385342941
CGAGCGGC -0.27382468951
CGAGCGTA 0.0783046755903
CGAGCGTC -0.141511384595
CGAGCTAA -0.0523705523706
CGAGCTAC -0.248754901231
CGAGCTAG -0.0740207635356
CGAGCTCA -0.192380889833
CGAGCTCC -0.22666429609
CGAGCTCG -0.120629211625

CGAGCTGA -0.221570354564
CGAGCTGC -0.207583765498
CGAGCTTA -0.0662164233017
CGAGCTTC -0.221662815934
CGAGGAAA -0.0873799233086
CGAGGAAC -0.184330837849
CGAGGAAG -0.264045005776
CGAGGACA -0.160641611038
CGAGGACC -0.333436701481
CGAGGACG -0.0212644248666
CGAGGAGA -0.0751851599137
CGAGGAGC -0.204997964155
CGAGGATA 0.270828376021
CGAGGATC 0.186024867843
CGAGGCAA -0.121508391441
CGAGGCAC -0.231474171031
CGAGGCAG -0.149383858449
CGAGGCCA -0.279400049068
CGAGGCCC -0.250926775757
CGAGGCCG -0.157461788917
CGAGGCGA -0.020381753714
CGAGGCGC -0.131828471068
CGAGGCTA -0.0128020984319
CGAGGCTC -0.257594802374
CGAGGGAA 0.0381475429322
CGAGGGAC -0.172719418959
CGAGGGAG -0.145508723328
CGAGGGCA -0.272163919694
CGAGGGCC -0.377793260427
CGAGGGCG -0.294777014232
CGAGGGGA -0.24756075668
CGAGGGGC -0.18192094106
CGAGGGTA -0.11390664899
CGAGGGTC -0.427628632246
CGAGGTAA 0.0171988934549
CGAGGTAC -0.309854951483
CGAGGTAG -0.105443785255
CGAGGTCA -0.206401639162
CGAGGTCC -0.338590209035
CGAGGTGA -0.332879936717
CGAGGTGC -0.148432379726
CGAGGTTA 0.0416790219574
CGAGGTTC -0.166252625464
CGAGTAAA -0.0169880624426
CGAGTAAC -0.137177299644
CGAGTAAG 0.0523681112186
CGAGTACA -0.0349328116903
CGAGTACC -0.181664281716
CGAGTACG 0.0954599534317
CGAGTAGA -0.117945452089
CGAGTAGC -0.225838016751
CGAGTATA 0.1482184664
CGAGTATC 0.328806668467
CGAGTCAA 0.0390508885856

CGAGTCAC -0.136496593857
CGAGTCAG -0.0915356257565
CGAGTCCA -0.226608441059
CGAGTCCC -0.248583504967
CGAGTCCG -0.264049125419
CGAGTCGA -0.216374051335
CGAGTCGC -0.178738507767
CGAGTCTA -0.150778167878
CGAGTCTC -0.208334367169
CGAGTGAA -0.0793128617622
CGAGTGAC -0.192449511732
CGAGTGAG -0.0472802455639
CGAGTGCA 0.057361769483
CGAGTGCC -0.238861737522
CGAGTGCG -0.00785165288351
CGAGTGGA -0.248794851026
CGAGTGGC -0.1601899863
CGAGTGTA -0.0298044178589
CGAGTGTC -0.359427102107
CGAGTTAA -0.11846816797
CGAGTTAC -0.29408456761
CGAGTTAG -0.266599126108
CGAGTTCA -0.124499918664
CGAGTTCC -0.149425762777
CGAGTTGA -0.0943705589016
CGAGTTGC -0.220825924548
CGAGTTTA 0.221944774932
CGAGTTTC 0.0523601672956
CGATAAAA 0.171858280476
CGATAAAC -0.152190381157
CGATAAAG -0.0468305771336
CGATAACA 0.0741835620518
CGATAACC -0.175713708071
CGATAACG -0.0427238008576
CGATAAGA 0.0995591337416
CGATAAGC 0.0852557973183
CGATAATA 0.0166032860594
CGATAATC 0.25449475147
CGATACAA 0.0300628453392
CGATACAC -0.0471468099362
CGATACAG -0.0192820595169
CGATACCA 0.119596651168
CGATACCC 0.212194287952
CGATACCG 0.171572155354
CGATACGA 0.186888608279
CGATACGC 0.188884476763
CGATACTA 0.0833375911476
CGATACTC 0.211819257274
CGATAGAA 0.226211059544
CGATAGAC -0.0672776691886
CGATAGAG -0.00723852762348
CGATAGCA 0.0333357663701
CGATAGCC -0.159473880134
CGATAGCG 0.144765443092

CGATAGGA -0.0821856621752
CGATAGGC -0.0688177679081
CGATAGTA 0.148340547287
CGATAGTC -0.0598803881182
CGATATAA 0.187446260301
CGATATAC 0.141666240885
CGATATAG 0.278403748101
CGATATCA 0.367203291446
CGATATCC 0.466823572884
CGATATCG 0.457420683154
CGATATGA 0.246782595267
CGATATGC 0.225670240798
CGATATTA 0.266350372411
CGATATTC 0.353329913936
CGATCAAA 0.111261067276
CGATCAAC -0.0640197659905
CGATCAAG 0.00283519865002
CGATCACA 0.193276789559
CGATCACC 0.0560769089087
CGATCACG 0.171786343667
CGATCAGA 0.0427632971541
CGATCAGC -0.0479700999733
CGATCATA 0.0802276105306
CGATCATC -0.000583169963025
CGATCCAA 0.244912957034
CGATCCAC 0.137678007051
CGATCCAG 0.0618168945206
CGATCCCA 0.259529353372
CGATCCCC 0.160103793348
CGATCCCG 0.238275465548
CGATCCGA 0.216829777436
CGATCCGC 0.226403071466
CGATCCTA 0.11578359598
CGATCCTC 0.326517978033
CGATCGAA 0.0740120285575
CGATCGAC -0.0226484091589
CGATCGAG 0.0684417199569
CGATCGCA 0.35657282627
CGATCGCC 0.286869514142
CGATCGCG 0.382878477114
CGATCGGA 0.0899885403969
CGATCGGC 0.0136530902095
CGATCGTA 0.268047977281
CGATCGTC 0.12409262092
CGATCTAA 0.210587359401
CGATCTAC 0.219361234513
CGATCTAG 0.263540399904
CGATCTCA 0.31822318186
CGATCTCC 0.350045637924
CGATCTGA 0.293727611909
CGATCTGC 0.339514897541
CGATCTTA 0.271616679927
CGATCTTC 0.394063633643
CGATGAAA 0.0192989132383

CGATGAAC	-0.203506234392
CGATGAAG	-0.0079442928813
CGATGACA	0.0111639891097
CGATGACC	-0.212749806535
CGATGACG	-0.0864922665927
CGATGAGA	0.0165314208667
CGATGAGC	-0.189773808047
CGATGATA	0.012213481566
CGATGATC	0.17089403453
CGATGCAA	0.0319372568484
CGATGCAC	-0.093946036867
CGATGCAG	0.0754165967169
CGATGCCA	-0.0987377058673
CGATGCCC	-0.115574966018
CGATGCCG	-0.0833390509697
CGATGCGA	-0.0417933607669
CGATGCGC	-0.106741727954
CGATGCTA	0.0679867195019
CGATGCTC	-0.188185524067
CGATGGAA	0.0626204717114
CGATGGAC	-0.175655358548
CGATGGAG	0.0345093821952
CGATGGCA	0.0935356844448
CGATGGCC	-0.39442577795
CGATGGCG	-0.133769517303
CGATGGGA	0.0579919245385
CGATGGGC	0.0308423903126
CGATGGTA	-0.120175823983
CGATGGTC	-0.356947372773
CGATGTAA	-0.0710476316537
CGATGTAC	-0.0985218822634
CGATGTAG	-0.167335198801
CGATGTCA	-0.0668634993394
CGATGTCC	-0.183910184841
CGATGTGA	-0.126851599137
CGATGTGC	-0.28083747495
CGATGTTA	0.159353444815
CGATGTTC	-0.0370542549667
CGATTAAA	-0.0556841820982
CGATTAAC	-0.113094991907
CGATTAAG	0.0416567520419
CGATTACA	0.399870805749
CGATTACC	0.408679371985
CGATTACG	0.447195316891
CGATTAGA	0.213848835061
CGATTAGC	-0.0648489449136
CGATTATA	0.0769990438166
CGATTATC	0.212458851266
CGATTCAA	0.0117376329498
CGATTCAC	0.122142580819
CGATTCAG	0.0706505403475
CGATTCCA	0.197639788549
CGATTCCC	0.443364743838
CGATTCCG	0.403476065216

CGATTCGA 0.20548966081
CGATTCGC 0.401456172493
CGATTCTA 0.20768523317
CGATTCTC 0.41165545711
CGATTGAA -0.127226178955
CGATTGAC -0.194543013199
CGATTGAG -0.0434856343947
CGATTGCA 0.297340039764
CGATTGCC 0.30074888871
CGATTGCG 0.426778017687
CGATTGGA 0.0151763333582
CGATTGGC 0.0299541966209
CGATTGTA -0.0285256527229
CGATTGTC 0.141964044583
CGATTTAA 0.0643467158619
CGATTTAC -0.0592782639796
CGATTTAG 0.174220272549
CGATTTCA 0.371147347138
CGATTTCC 0.468425726001
CGATTTGA 0.10890366951
CGATTTGC 0.305779435487
CGATTTTA 0.338651708357
CGATTTTC 0.414302606512
CGCAAAAA 0.117179938953
CGCAAAAC -0.00342255279081
CGCAAAAG 0.129342423159
CGCAAACA -0.0597057112209
CGCAAACC -0.278572898529
CGCAAACG -0.163821410552
CGCAAAGA -0.200576689821
CGCAAAGC 0.0219900294154
CGCAAATA 0.159662417238
CGCAAATC 0.419543367663
CGCAACAA 0.121815580681
CGCAACAC -0.0574666921636
CGCAACAG -0.0299758666898
CGCAACCA -0.136009615876
CGCAACCC -0.0200616044904
CGCAACCG 0.0107866733027
CGCAACGA 0.147415020109
CGCAACGC -0.065932132873
CGCAACTA 0.0787041159683
CGCAACTC 0.022223600943
CGCAAGAA 0.021853363774
CGCAAGAC -0.149554303801
CGCAAGAG 0.00843926226178
CGCAAGCA -0.0208403996283
CGCAAGCC -0.284750130237
CGCAAGCG -0.028900745888
CGCAAGGA -0.174519290411
CGCAAGGC -0.0757851515427
CGCAAGTA 0.230730730731
CGCAAGTC -0.0612295255977
CGCAATAA 0.105871404276

CGCAATAC 0.131316102567
CGCAATAG -0.000993408903455
CGCAATCA 0.430600021509
CGCAATCC 0.478275796458
CGCAATGA 0.0708719068775
CGCAATGC 0.0444596103735
CGCAATTA 0.190068100699
CGCAATTC 0.309799785406
CGCACAAA -0.00700787573995
CGCACAAC 0.0358990743728
CGCACAAG 0.0336496281103
CGCACACA 0.0718604702087
CGCACACC -0.0693072414473
CGCACACG 0.0198835198835
CGCACAGA -0.185699940597
CGCACAGC -0.0449297541741
CGCACATA 0.169907189121
CGCACATC -0.0420643763985
CGCACCAA -0.179749588574
CGCACCAC -0.169403153893
CGCACCAG 0.0396898616011
CGCACCCA -0.126054277312
CGCACCCC -0.22391478734
CGCACCCG -0.0563153231915
CGCACCGA -0.217008935094
CGCACCGC -0.0529055621157
CGCACCTA -0.0158684855655
CGCACCTC -0.209570774043
CGCACGAA 0.0134500704364
CGCACGAC -0.185776298063
CGCACGAG -0.14898482405
CGCACGCA 0.0785758210001
CGCACGCC -0.351002020302
CGCACGCG 0.0955446231104
CGCACGGA -0.0925281103595
CGCACGGC -0.174085337824
CGCACGTA 0.162191765433
CGCACGTC 0.101635000693
CGCACTAA 0.0460770932023
CGCACTAC -0.017690853485
CGCACTAG -0.130269720354
CGCACTCA -0.139592656007
CGCACTCC -0.284582516806
CGCACTGA 0.0675539398246
CGCACTGC 0.127292833004
CGCACTTA -0.0258220622906
CGCACTTC -0.0385611832146
CGCAGAAA 0.0492048522352
CGCAGAAC -0.115073866018
CGCAGAAG -0.251208208928
CGCAGACA -0.148249860973
CGCAGACC -0.132992549457
CGCAGACG -0.137389608296
CGCAGAGA -0.111171762786

CGCAGAGC -0.106913531233
CGCAGATA 0.292506733429
CGCAGATC 0.332794226734
CGCAGCAA -0.00642038993815
CGCAGCAC -0.0585279301189
CGCAGCAG -0.168731187851
CGCAGCCA -0.253801691846
CGCAGCCC -0.272306157158
CGCAGCCG -0.0938623702823
CGCAGCGA 0.0299194754475
CGCAGCGC 0.0427203017594
CGCAGCTA -0.13564965971
CGCAGCTC -0.16363929626
CGCAGGAA -0.0516839153203
CGCAGGAC -0.278300296926
CGCAGGAG -0.0697842485298
CGCAGGCA -0.20564777367
CGCAGGCC -0.256864747275
CGCAGGCG -0.187068933436
CGCAGGGA 0.116306278821
CGCAGGGC -0.148580061589
CGCAGGTA 0.00406029349725
CGCAGGTC -0.23038262936
CGCAGTAA -0.0187906266559
CGCAGTAC -0.143454315413
CGCAGTAG -0.273201171422
CGCAGTCA -0.207625673027
CGCAGTCC -0.154572771506
CGCAGTGA -0.131455243735
CGCAGTGC 0.0933432114625
CGCAGTTA 0.0976063610915
CGCAGTTC 0.00875236308694
CGCATAAA 0.115982131778
CGCATAAC -0.0826184665867
CGCATAAG -0.0233856445978
CGCATACA 0.112317978438
CGCATACC 0.0168343797305
CGCATACG 0.0340251081878
CGCATAGA -0.012274913688
CGCATAGC 0.149718589113
CGCATATA 0.155630837449
CGCATATC 0.288419231696
CGCATCAA 0.0329060035609
CGCATCAC 0.0117041232674
CGCATCAG 0.0664093845912
CGCATCCA -0.0107779956265
CGCATCCC -0.0913516822608
CGCATCCG -0.0585429470265
CGCATCGA -0.0620430071426
CGCATCGC -0.134721867404
CGCATCTA 0.0974365524843
CGCATCTC -0.147440404792
CGCATGAA -0.0215965897654
CGCATGAC -0.231917992333

CGCATGAG -0.144196964766
CGCATGCA 0.141315883594
CGCATGCC -0.0522602492299
CGCATGCG 0.0463989702287
CGCATGGA -0.108924325121
CGCATGGC -0.224792574268
CGCATGTA 0.0234772850719
CGCATGTC -0.168211824839
CGCATTAA -0.00982938861727
CGCATTAC -0.183060854896
CGCATTAG -0.18005756681
CGCATTCA -0.112988499204
CGCATTCC -0.112018994262
CGCATTGA -0.279576302475
CGCATTGC -0.157406393894
CGCATTTA -0.000135033539412
CGCATTTC 0.195952150498
CGCCAAAA 0.0954072263444
CGCCAAAC -0.0163432751944
CGCCAAAG 0.0933876460192
CGCCAACA -0.25940632065
CGCCAACC -0.271394921599
CGCCAACG -0.344189803027
CGCCAAGA -0.230882097574
CGCCAAGC -0.0626006314877
CGCCAATA 0.0109245782939
CGCCAATC 0.0838014322863
CGCCACAA 0.0639799579194
CGCCACAC -0.18822757558
CGCCACAG -0.289071395292
CGCCACCA -0.248620035228
CGCCACCC -0.201916583283
CGCCACCG -0.253556729876
CGCCACGA -0.113879463211
CGCCACGC 0.0393589708035
CGCCACTA -0.00286374528799
CGCCACTC -0.213611268806
CGCCAGAA 0.126311832588
CGCCAGAC -0.243715512851
CGCCAGAG -0.18452266675
CGCCAGCA -0.179149744548
CGCCAGCC -0.222014107685
CGCCAGCG -0.165319031863
CGCCAGGA 0.0925317021118
CGCCAGGC 0.00827646109939
CGCCAGTA -0.0706730467456
CGCCAGTC -0.0103395406426
CGCCATAA -0.113054549289
CGCCATAC -0.222115924896
CGCCATAG -0.257947459134
CGCCATCA -0.156294285485
CGCCATCC -0.208055948374
CGCCATGA -0.277123080358
CGCCATGC -0.0787590700689

CGCCATTA -0.00457827737661
CGCCATTC 0.0278060429576
CGCCCAAA -0.0870966329204
CGCCCAAC -0.197371431722
CGCCCAAG -0.113885674342
CGCCCACA 0.0135723943732
CGCCCACC -0.209732599091
CGCCCACG -0.223131105406
CGCCCAGA -0.0893722133438
CGCCCAGC -0.100954920791
CGCCCATA 0.0611959024417
CGCCCATC 0.0148134476512
CGCCCCAA 0.0357364437275
CGCCCCAC -0.271407543394
CGCCCCAG -0.255176903378
CGCCCCCA -0.234089453093
CGCCCCCC -0.248339829073
CGCCCCCG -0.168052529504
CGCCCCGA -0.0997846651833
CGCCCCGC -0.171707245086
CGCCCCTA -0.223890143276
CGCCCCTC -0.30058752023
CGCCCGAA -0.0522325283091
CGCCCGAC -0.461117911116
CGCCCGAG -0.186316430814
CGCCCGCA -0.242754865721
CGCCCGCC -0.288518984959
CGCCCGCG -0.145003904055
CGCCCGGA -0.147417850653
CGCCCGGC -0.127974491948
CGCCCGTA 0.056504602089
CGCCCGTC 0.00283037336664
CGCCCTAA -0.198396040384
CGCCCTAC -0.0889334193612
CGCCCTAG -0.227715165056
CGCCCTCA -0.0784108549575
CGCCCTCC -0.287970587506
CGCCCTGA -0.0486105942191
CGCCCTGC -0.149284778471
CGCCCTTA -0.00103393798026
CGCCCTTC -0.294591785822
CGCCGAAA 0.0622151076697
CGCCGAAC -0.149287750535
CGCCGAAG -0.0459656494969
CGCCGACA -0.16051634068
CGCCGACC -0.391042974209
CGCCGACG -0.190193524377
CGCCGAGA 0.00986229996278
CGCCGAGC 0.088518485539
CGCCGATA 0.161686240447
CGCCGATC 0.153060774273
CGCCGCAA 0.0942278636234
CGCCGCAC -0.178113859871
CGCCGCAG -0.0188732565224

CGCCGCCA -0.180229427089
CGCCGCCC -0.0738962367415
CGCCGCCG -0.0392102527621
CGCCGCGA -0.103662252222
CGCCGCGC -0.109737123641
CGCCGCTA -0.00652580955611
CGCCGCTC -0.259494490894
CGCCGGAA -0.00397144588075
CGCCGGAC -0.270259664793
CGCCGGAG -0.238093952444
CGCCGGCA -0.171406489694
CGCCGGCC -0.276039728418
CGCCGGCG -0.178008036312
CGCCGGGA -0.173859046989
CGCCGGGC -0.0339073472122
CGCCGGTA -0.0449406312812
CGCCGGTC -0.183763153248
CGCCGTAA -0.12984252115
CGCCGTAC -0.232425833962
CGCCGTAG -0.295906563312
CGCCGTCA -0.203483119995
CGCCGTCC -0.163221103303
CGCCGTGA -0.145110541301
CGCCGTGC -0.386613718243
CGCCGTTA -0.102382862799
CGCCGTTC 0.138848784333
CGCCTAAA 0.1026948315
CGCCTAAC -0.144267511972
CGCCTAAG -0.0309721595777
CGCCTACA 0.0954614390833
CGCCTACC -0.142763014609
CGCCTACG -0.154562621397
CGCCTAGA -0.111291678727
CGCCTAGC 0.0393794296475
CGCCTATA 0.0641292526441
CGCCTATC 0.00225323533061
CGCCTCAA 0.049482290331
CGCCTCAC -0.368713805716
CGCCTCAG 0.0207339472313
CGCCTCCA -0.233926145558
CGCCTCCC -0.234763682953
CGCCTCCG -0.161027439369
CGCCTCGA 0.0529677439132
CGCCTCGC -0.234150744324
CGCCTCTA -0.26353539555
CGCCTCTC -0.00388950279469
CGCCTGAA 0.0405326890652
CGCCTGAC -0.187631253658
CGCCTGAG -0.234903426585
CGCCTGCA -0.142180340384
CGCCTGCC -0.255794862664
CGCCTGGA -0.197193113039
CGCCTGGC -0.0535329721085
CGCCTGTA -0.179958313689

CGCCTGTC 0.0945723816267
CGCCTTAA -0.0561815258785
CGCCTTAC -0.286225724879
CGCCTTAG -0.168410229268
CGCCTTCA -0.170052295042
CGCCTTCC -0.0732100756918
CGCCTTGA -0.293244333677
CGCCTTGC -0.183050736115
CGCCTTTA -0.019927300862
CGCCTTTC -0.0109193120052
CGCGAAAA 0.215325941768
CGCGAAAC -0.0731489064822
CGCGAAAG -0.106676855213
CGCGAACA -0.103283724496
CGCGAACC -0.215197330886
CGCGAACG -0.0951014049165
CGCGAAGA -0.0667364264778
CGCGAAGC -0.000815817554657
CGCGAATA 0.207062560488
CGCGAATC 0.425578471033
CGCGACAA -0.0847197059318
CGCGACAC -0.149956570294
CGCGACAG 0.0373999109509
CGCGACCA -0.0339551364244
CGCGACCC -0.0827982665734
CGCGACCG -0.116764964582
CGCGACGA -0.156996754201
CGCGACGC 0.0342358029546
CGCGACTA -0.0130061190667
CGCGACTC -0.0492597357645
CGCGAGAA 0.180319437895
CGCGAGAC -0.178816902806
CGCGAGAG -0.183742330841
CGCGAGCA -0.267246108341
CGCGAGCC -0.246508780405
CGCGAGCG 0.0624358590688
CGCGAGGA -0.129432052205
CGCGAGGC -0.159607822463
CGCGAGTA -0.0943017468393
CGCGAGTC -0.244059928286
CGCGATAA 0.0177653044094
CGCGATAC 0.094404321677
CGCGATAG 0.0570584358463
CGCGATCA 0.350847061743
CGCGATCC 0.449381888776
CGCGATGA 0.0501071807861
CGCGATGC 0.199410961804
CGCGATTA 0.194786245557
CGCGATTC 0.389429374278
CGCGCAAA 0.158664173816
CGCGCAAC -0.112716999254
CGCGCAAG 0.151969664898
CGCGCACA 0.0668285365255
CGCGCACC -0.277482833996

CGCGCACG -0.166594663074
CGCGCAGA -0.18608722175
CGCGCAGC -0.0235850227042
CGCGCATA 0.0608650905455
CGCGCATC -0.02617095976
CGCGCCAA -0.151838036989
CGCGCCAC -0.257150653668
CGCGCCAG -0.0909692413303
CGCGCCCA -0.178575859075
CGCGCCCC -0.213260492571
CGCGCCCG -0.11756076712
CGCGCCGA -0.216902734298
CGCGCCGC -0.049483321631
CGCGCCTA 0.0697670853923
CGCGCCTC -0.156408772002
CGCGCGAA 0.173802763443
CGCGCGAC 0.026882254155
CGCGCGAG -0.18036631673
CGCGCGCA 0.0371026379135
CGCGCGCC -0.126323030308
CGCGCGCG 0.2830313755
CGCGCGGA -0.0803323985142
CGCGCGGC 0.00108818931429
CGCGCGTA 0.235961857174
CGCGCGTC -0.0919140456778
CGCGCTAA -0.165189473392
CGCGCTAC -0.325443938087
CGCGCTAG -0.252797142077
CGCGCTCA -0.0921130989305
CGCGCTCC -0.0601366523584
CGCGCTGA 0.0923885317825
CGCGCTGC 0.0763975971329
CGCGCTTA 0.109504821626
CGCGCTTC -0.242533768252
CGCGGAAA 0.211197565017
CGCGGAAC -0.333691577397
CGCGGAAG -0.194826906591
CGCGGACA -0.153086990981
CGCGGACC -0.190534622533
CGCGGACG -0.160723642823
CGCGGAGA -0.12570125019
CGCGGAGC -0.157472174219
CGCGGATA 0.235595099231
CGCGGATC 0.218301059101
CGCGGCAA 0.0878881636457
CGCGGCAC -0.133232711685
CGCGGCAG 0.024904281671
CGCGGCCA -0.242026181084
CGCGGCCC -0.0809883567332
CGCGGCCG 0.082306956782
CGCGGCGA -0.0376574809184
CGCGGCGC 0.173365883316
CGCGGCTA -0.0251217735661
CGCGGCTC -0.380501346018

CGCGGGAA -0.0150011167084
CGCGGGAC -0.250411405228
CGCGGGAG -0.191777706095
CGCGGGCA -0.0957865787703
CGCGGGCC -0.300522357027
CGCGGGGA -0.0695647318659
CGCGGGGC -0.175026606337
CGCGGGTA -0.121823555522
CGCGGGTC -0.159421608108
CGCGGTAA -0.109223391319
CGCGGTAC -0.0393993714318
CGCGGTAG -0.227610475611
CGCGGTCA -0.235720946115
CGCGGTCC -0.436513193892
CGCGGTGA -0.0687836596928
CGCGGTGC -0.208911032714
CGCGGTTA -0.0869925936035
CGCGGTTC 0.0155522141851
CGCGTAAA 0.0798529959198
CGCGTAAC -0.00157595612141
CGCGTAAG -0.100659609067
CGCGTACA -0.0292468048145
CGCGTACC -0.161061506416
CGCGTACG 0.177454313818
CGCGTAGA -0.132896981833
CGCGTAGC 0.0467369327679
CGCGTATA 0.213942725265
CGCGTATC 0.324015532507
CGCGTCAA -0.022908825192
CGCGTCAC -0.133673792885
CGCGTCAG -0.0751934842607
CGCGTCCA -0.0735046341107
CGCGTCCC -0.236999820811
CGCGTCCG 0.0515956728078
CGCGTCGA -0.10010134266
CGCGTCGC 0.129362144514
CGCGTCTA -0.10165397838
CGCGTCTC -0.207881614528
CGCGTGAA 0.0818766424827
CGCGTGAC -0.286197040234
CGCGTGAG -0.143512336084
CGCGTGCA 0.0261054318742
CGCGTGCC -0.125926661098
CGCGTGGA -0.0625278278578
CGCGTGGC 0.0732000518348
CGCGTGTA -0.131701469019
CGCGTGTC -0.159756511095
CGCGTTAA -0.08622657898
CGCGTTAC -0.0824806791456
CGCGTTAG -0.213942301065
CGCGTTCA -0.0873714942643
CGCGTTCC -0.159343706716
CGCGTTGA -0.0360985457237
CGCGTTGC 0.0438873601308

CGCGTTTA -0.0120023604872
CGCGTTTC 0.154781282162
CGCTAAAA 0.0529692036628
CGCTAAAC -0.0515715276626
CGCTAAAG 0.180091676825
CGCTAACA -0.0772603519631
CGCTAACC -0.242529772198
CGCTAACG -0.235499516237
CGCTAAGA -0.0469794863734
CGCTAAGC 0.0528944621651
CGCTAATA -0.0223279648609
CGCTAATC -0.0513120634842
CGCTACAA -0.0870998343513
CGCTACAC -0.127778742739
CGCTACAG 0.00031416184102 8
CGCTACCA -0.164508968579
CGCTACCC -0.110530402885
CGCTACCG -0.0443029947087
CGCTACGA -0.0703727825332
CGCTACGC -0.0765331303957
CGCTACTA -0.0746131806738
CGCTACTC -0.242984393221
CGCTAGAA 0.143981447012
CGCTAGAC -0.241922727266
CGCTAGAG -0.00259045422363
CGCTAGCA -0.21088684078
CGCTAGCC -0.324672888503
CGCTAGCG -0.133805655463
CGCTAGGA 0.0532336957712
CGCTAGGC -0.181403476856
CGCTAGTA -0.179824670223
CGCTAGTC 0.0588067406249
CGCTATAA 0.224083607545
CGCTATAC -0.104744242668
CGCTATAG -0.0899096269026
CGCTATCA -0.0296890121343
CGCTATCC 0.162089151332
CGCTATGA 0.11322170682
CGCTATGC 0.0550702217369
CGCTATTA -0.104919403926
CGCTATTC 0.129307387429
CGCTCAAA 0.0580346186407
CGCTCAAC 0.0254951921619
CGCTCAAG -0.142449258699
CGCTCACA -0.102105917675
CGCTCACC -0.10451491416
CGCTCACG -0.307762295214
CGCTCAGA -0.159002900686
CGCTCAGC -0.0339869929727
CGCTCATA -0.134604939191
CGCTCATC -0.136288013038
CGCTCCAA -0.175710626377
CGCTCCAC -0.308316863342
CGCTCCAG -0.064441998917

CGCTCCCA	-0.165253142476
CGCTCCCC	-0.094736972695
CGCTCCCG	-0.10385119023
CGCTCCGA	-0.225257261628
CGCTCCGC	-0.069814920501
CGCTCCTA	-0.0938872416445
CGCTCCTC	-0.204028930897
CGCTCGAA	-0.212418706079
CGCTCGAC	-0.237107316568
CGCTCGAG	-0.147739220498
CGCTCGCA	-0.0586741023796
CGCTCGCC	-0.19031137576
CGCTCGGA	-0.0704918756014
CGCTCGGC	-0.115668859153
CGCTCGTA	-0.132248839441
CGCTCGTC	-0.0991144876418
CGCTCTAA	-0.299729164569
CGCTCTAC	-0.0874688064414
CGCTCTAG	-0.0808511538905
CGCTCTCA	-0.301410492832
CGCTCTCC	-0.325743106371
CGCTCTGA	-0.0818050971536
CGCTCTGC	-0.0911821370028
CGCTCTTA	-0.035072262345
CGCTCTTC	-0.0197492025722
CGCTGAAA	0.140688160113
CGCTGAAC	-0.195998861258
CGCTGAAG	-0.153149652071
CGCTGACA	-0.0468767349114
CGCTGACC	-0.175156292639
CGCTGACG	-0.0899636485447
CGCTGAGA	0.0404219646644
CGCTGAGC	0.0870820919379
CGCTGATA	0.209728984037
CGCTGATC	0.106973364549
CGCTGCAA	0.045945709218
CGCTGCAC	-0.331454951675
CGCTGCAG	-0.0375648708422
CGCTGCCA	-0.277531084705
CGCTGCCC	-0.258203819816
CGCTGCCG	-0.155250933852
CGCTGCGA	-0.0879785861393
CGCTGCGC	0.0810894651942
CGCTGCTA	-0.0386568177339
CGCTGCTC	-0.184124699843
CGCTGGAA	-0.0243701001277
CGCTGGAC	-0.086760314033
CGCTGGAG	-0.209072890502
CGCTGGCA	-0.157908522682
CGCTGGCC	-0.218336765326
CGCTGGGA	-0.163062557372
CGCTGGGC	-0.189346215527
CGCTGGTA	-0.158533096901
CGCTGGTC	-0.238126480587

CGCTGTAA -0.113397208805
CGCTGTAC -0.2585242239
CGCTGTAG -0.20955170831
CGCTGTCA -0.280197970677
CGCTGTCC -0.16476303115
CGCTGTGA 0.150241235593
CGCTGTGC -0.0196032210557
CGCTGTTA -0.0696667631771
CGCTGTTC -0.100948190057
CGCTTAAA 0.0296745326745
CGCTTAAC -0.179934788098
CGCTTAAG -0.0617421538704
CGCTTACA -0.0987970298
CGCTTACC 0.0360015663046
CGCTTACG -0.000990390945331
CGCTTAGA -0.098384425477
CGCTTAGC 0.0198255891184
CGCTTATA 0.0778428209601
CGCTTATC 0.102370751005
CGCTTCAA 0.158311203766
CGCTTCAC -0.284800650271
CGCTTCAG 0.0674352037988
CGCTTCCA -0.147205681677
CGCTTCCC -0.163647717592
CGCTTCCG -0.120929142892
CGCTTCGA 0.0294686949401
CGCTTCGC -0.113831918906
CGCTTCTA -0.235162721333
CGCTTCTC 0.0774428297191
CGCTTGAA -0.0576462997914
CGCTTGAC -0.0558492230908
CGCTTGAG -0.124919701469
CGCTTGCA -0.132210286055
CGCTTGCC -0.229273414777
CGCTTGGA -0.1214725225
CGCTTGGC -0.0443282054875
CGCTTGTA -0.212789290963
CGCTTGTC -0.148157395231
CGCTTTAA -0.0162792823976
CGCTTTAC -0.163512309987
CGCTTTAG -0.0815870967386
CGCTTTCA -0.0650067593602
CGCTTTCC -0.140772349992
CGCTTTGA 0.0545236235703
CGCTTTGC 0.00166930651154
CGCTTTTA 0.0550523224552
CGCTTTTC 0.184261212738
CGGAAAAA -0.052700158975
CGGAAAAC 0.158787033861
CGGAAAAG -0.0646279888704
CGGAAACA -0.0720734508613
CGGAAACC -0.205924032765
CGGAAACG -0.0667112863459
CGGAAAGA -0.0886674014438

CGGAAAGC -0.0512208009832
CGGAAATA 0.266809563987
CGGAAATC 0.468877968878
CGGAACAA -0.159350423003
CGGAACAC -0.232075362463
CGGAACAG -0.0092172061869
CGGAACCA -0.143411288142
CGGAACCC -0.298068546463
CGGAACCG -0.119118075316
CGGAACGA -0.0757871673943
CGGAACGC -0.14455730934
CGGAACTA -0.193880023412
CGGAACTC -0.207573388918
CGGAAGAA -0.265287806976
CGGAAGAC -0.224963817558
CGGAAGAG -0.173857166002
CGGAAGCA -0.0940191090706
CGGAAGCC -0.277514494372
CGGAAGGA -0.0831940696284
CGGAAGGC -0.152015803302
CGGAAGTA 0.143505764718
CGGAAGTC -0.209587982014
CGGAATAA 0.0531894433829
CGGAATAC 0.0912615052225
CGGAATAG 0.0929957738152
CGGAATCA 0.3239423997
CGGAATCC 0.435925751297
CGGAATGA -0.262932902565
CGGAATGC -0.150000364956
CGGAATTA 0.0970957183078
CGGAATTC 0.0274006789158
CGGACAAA -0.0520200287585
CGGACAAC 0.0256118479157
CGGACAAG -0.118287095489
CGGACACA -0.155439042011
CGGACACC -0.277707476973
CGGACACG -0.125219842049
CGGACAGA -0.0114875018957
CGGACAGC -0.276858201538
CGGACATA -0.182097584482
CGGACATC -0.198521404211
CGGACCAA -0.0668880782413
CGGACCAC -0.295521762739
CGGACCAG -0.297385458785
CGGACCCA -0.0605887656435
CGGACCCC -0.232396360107
CGGACCCG -0.0578967397149
CGGACCGA -0.191127864322
CGGACCGC -0.392992071956
CGGACCTA -0.0771408387181
CGGACCTC -0.241037092172
CGGACGAA -0.164730795501
CGGACGAC -0.339596997191
CGGACGAG -0.160491692025

CGGACGCA -0.0971909841772
CGGACGCC -0.335261108043
CGGACGGA -0.108493310064
CGGACGGC -0.151825568155
CGGACGTA -0.0372300387934
CGGACGTC -0.0521852522696
CGGACTAA -0.111788029437
CGGACTAC -0.173180884643
CGGACTAG -0.249525381337
CGGACTCA -0.044502350719
CGGACTCC -0.0980972855495
CGGACTGA -0.244443844563
CGGACTGC -0.159920038886
CGGACTTA 0.0588738932724
CGGACTTC -0.259748099999
CGGAGAAA 0.0149113523062
CGGAGAAC -0.0925855638198
CGGAGAAG -0.248781836781
CGGAGACA -0.192933052144
CGGAGACC -0.180056854913
CGGAGACG -0.250918735027
CGGAGAGA -0.0659011799733
CGGAGAGC -0.250638148821
CGGAGATA 0.241545953667
CGGAGATC 0.317860922754
CGGAGCAA -0.106007167726
CGGAGCAC -0.161288810585
CGGAGCAG 0.0576572657912
CGGAGCCA -0.187273316393
CGGAGCCC -0.376680250194
CGGAGCCG -0.27715131409
CGGAGCGA -0.178359671537
CGGAGCGC -0.194078529653
CGGAGCTA -0.29821155224
CGGAGCTC -0.454914588207
CGGAGGAA -0.0447069624604
CGGAGGAC -0.408695099571
CGGAGGAG -0.179293414882
CGGAGGCA -0.315864116368
CGGAGGCC -0.208842198684
CGGAGGGA -0.222814785196
CGGAGGGC -0.334819004463
CGGAGGTA -0.159746175119
CGGAGGTC -0.412767736128
CGGAGTAA -0.0332870152105
CGGAGTAC -0.271059739979
CGGAGTAG -0.0421730270215
CGGAGTCA 0.00130847100544
CGGAGTCC -0.274226453587
CGGAGTGA -0.138569497326
CGGAGTGC -0.345750758441
CGGAGTTA -0.00286782041269
CGGAGTTC -0.225137006794
CGGATAAA -0.0193842470603

CGGATAAC -0.0444940688073
CGGATAAG -0.173226696662
CGGATACA 0.136176121025
CGGATACC 0.28843674956
CGGATACG 0.389065202952
CGGATAGA 0.0235637175828
CGGATAGC 0.0352294443204
CGGATATA 0.283436328891
CGGATATC 0.460292840303
CGGATCAA -0.0115832085529
CGGATCAC 0.239617713477
CGGATCAG 0.106543182301
CGGATCCA 0.144702670744
CGGATCCC 0.196533999564
CGGATCCG 0.325539761686
CGGATCGA 0.014873936025
CGGATCGC 0.402974599944
CGGATCTA 0.184882812785
CGGATCTC 0.359158830038
CGGATGAA 0.0371376886528
CGGATGAC -0.238639763346
CGGATGAG -0.0859440707926
CGGATGCA -0.131959020065
CGGATGCC -0.147873282958
CGGATGGA -0.145283133311
CGGATGGC -0.178151239654
CGGATGTA -0.00661785994954
CGGATGTC 0.0436676345767
CGGATTAA -0.125999232286
CGGATTAC 0.457246502701
CGGATTAG -0.0296065601796
CGGATTCA 0.285017846325
CGGATTCC 0.462453376933
CGGATTGA -0.134157573552
CGGATTGC 0.431540185251
CGGATTTA 0.266186141909
CGGATTTC 0.480073737649
CGGCAAAA 0.0756693332451
CGGCAAAC -0.0452826852932
CGGCAAAG 0.131923388539
CGGCAACA -0.188571099167
CGGCAACC -0.335335918915
CGGCAACG -0.214970409451
CGGCAAGA -0.0816863787462
CGGCAAGC -0.203719323779
CGGCAATA 0.108349396228
CGGCAATC 0.326841747991
CGGCACAA 0.0956562623229
CGGCACAC -0.134308982246
CGGCACAG 0.00119253432155
CGGCACCA -0.304300603976
CGGCACCC -0.320661147912
CGGCACCG -0.16842027689
CGGCACGA -0.106444974715

CGGCACGC -0.291485772408
CGGCACTA -0.196938868349
CGGCACTC -0.182572684875
CGGCAGAA 0.0592505273607
CGGCAGAC -0.209793123173
CGGCAGAG 0.0651252567868
CGGCAGCA -0.313038498297
CGGCAGCC -0.23347550202
CGGCAGGA -0.0910657326309
CGGCAGGC -0.215167513757
CGGCAGTA -0.0408557146441
CGGCAGTC -0.208516416564
CGGCATAA 0.0784324269173
CGGCATAC -0.282753562906
CGGCATAG 0.0829872338562
CGGCATCA -0.126978971263
CGGCATCC 0.00460646847149
CGGCATGA -0.0624791094076
CGGCATGC 0.122371682978
CGGCATTA -0.197495637215
CGGCATTC -0.137475221183
CGGCCAAA -0.160504572599
CGGCCAAC -0.0390459329853
CGGCCAAG -0.221801591924
CGGCCACA -0.198479331563
CGGCCACC -0.353330006755
CGGCCACG -0.175068916152
CGGCCAGA -0.18985403196
CGGCCAGC -0.141754417892
CGGCCATA -0.115294150088
CGGCCATC -0.302158541124
CGGCCCAA -0.165672831237
CGGCCCAC -0.252641725027
CGGCCCAG -0.151475917184
CGGCCCCA -0.113107055116
CGGCCCCC -0.192315101138
CGGCCCCG -0.0829787102057
CGGCCCGA -0.288746661374
CGGCCCGC -0.143240605173
CGGCCCTA -0.0928637006526
CGGCCCTC -0.399375161389
CGGCCGAA 0.106148848573
CGGCCGAC -0.24490005881
CGGCCGAG -0.122621874594
CGGCCGCA -0.235856468273
CGGCCGCC -0.177476785946
CGGCCGGA 0.0617594913324
CGGCCGGC -0.146949336876
CGGCCGTA 0.0375721699525
CGGCCGTC -0.320596916065
CGGCCTAA -0.258013926857
CGGCCTAC -0.298677780258
CGGCCTAG -0.347275623799
CGGCCTCA -0.0898092315497

CGGCCTCC -0.116930118374
CGGCCTGA -0.185925797284
CGGCCTGC -0.409421338205
CGGCCTTA -0.199371702533
CGGCCTTC -0.264085504611
CGGCGAAA -0.0155449128873
CGGCGAAC -0.187494071239
CGGCGAAG 0.0538934698423
CGGCGACA -0.117431777812
CGGCGACC -0.186050261728
CGGCGACG -0.0513522530198
CGGCGAGA -0.0500982832819
CGGCGAGC -0.110366919045
CGGCGATA 0.10397071597
CGGCGATC 0.150516179008
CGGCGCAA 0.224041079392
CGGCGCAC -0.0925228859926
CGGCGCAG 0.170515244192
CGGCGCCA -0.0896365841803
CGGCGCCC -0.110671364648
CGGCGCCG -0.355010155628
CGGCGCGA -0.0377254512675
CGGCGCGC -0.133331241233
CGGCGCTA -0.216314147802
CGGCGCTC -0.283735350301
CGGCGGAA -0.0892922052802
CGGCGGAC -0.334775236714
CGGCGGAG -0.136377922798
CGGCGGCA -0.2196807711
CGGCGGCC -0.342352550907
CGGCGGGA -0.20672432828
CGGCGGGC -0.269569124989
CGGCGGTA 0.0115084636616
CGGCGGTC -0.365099109221
CGGCGTAA -0.0985648663933
CGGCGTAC -0.353038459312
CGGCGTAG -0.130224366975
CGGCGTCA -0.211706135454
CGGCGTCC 0.0200056051603
CGGCGTGA -0.159352989014
CGGCGTGC -0.184463857821
CGGCGTTA -0.130144303956
CGGCGTTC -0.274176886431
CGGCTAAA -0.162514577802
CGGCTAAC -0.187152855304
CGGCTAAG 0.024869366202
CGGCTACA -0.212741473894
CGGCTACC -0.207493516671
CGGCTACG -0.0886220893658
CGGCTAGA -0.095644615324
CGGCTAGC -0.270792383337
CGGCTATA -0.0399933273224
CGGCTATC -0.0393989030353
CGGCTCAA -0.207398581254

CGGCTCAC	-0.288335111265
CGGCTCAG	-0.135419935573
CGGCTCCA	-0.108869725901
CGGCTCCC	-0.276290809544
CGGCTCGA	-0.0637676950045
CGGCTCGC	-0.322434979797
CGGCTCTA	-0.238691964097
CGGCTCTC	-0.204290404479
CGGCTGAA	-0.0708910672678
CGGCTGAC	-0.214790935056
CGGCTGAG	-0.211175288543
CGGCTGCA	-0.305152399776
CGGCTGCC	-0.245190147349
CGGCTGGA	-0.146489581352
CGGCTGGC	-0.189936417644
CGGCTGTA	0.00987642606366
CGGCTGTC	-0.289782966845
CGGCTTAA	-0.0591964769232
CGGCTTAC	-0.378971649676
CGGCTTAG	-0.0282806271902
CGGCTTCA	-0.243012471084
CGGCTTCC	-0.227529243756
CGGCTTGA	-0.170876235372
CGGCTTGC	-0.340753693645
CGGCTTTA	-0.2391680518
CGGCTTTC	-0.309830385127
CGGGAAAA	-0.01585635222
CGGGAAAC	-0.0735545924452
CGGGAAAG	-0.0789799400711
CGGGAACA	-0.211341003662
CGGGAACC	-0.309686349421
CGGGAACG	-0.0889710079133
CGGGAAGA	-0.197489972295
CGGGAAGC	-0.276374588209
CGGGAATA	0.279496612651
CGGGAATC	0.457919169653
CGGGACAA	-0.0221585221585
CGGGACAC	-0.0524005388861
CGGGACAG	-0.280614664801
CGGGACCA	-0.233733302783
CGGGACCC	-0.386092193054
CGGGACCG	-0.23721145655
CGGGACGA	-0.251256585676
CGGGACGC	-0.155451355293
CGGGACTA	-0.176443586888
CGGGACTC	-0.294569282651
CGGGAGAA	0.0349620081312
CGGGAGAC	-0.190289395826
CGGGAGAG	-0.31633716171
CGGGAGCA	-0.148701123333
CGGGAGCC	-0.270306733425
CGGGAGGA	0.0608475726809
CGGGAGGC	-0.20268921323
CGGGAGTA	-0.0235885345576

CGGGAGTC -0.231511116625
CGGGATAA 0.129447629448
CGGGATAC 0.132720451377
CGGGATAG -0.230755765069
CGGGATCA -0.159414144098
CGGGATCC 0.19158414613
CGGGATGA -0.249956578915
CGGGATGC -0.142239869849
CGGGATTA 0.319802486077
CGGGATTC 0.373062633665
CGGGCAAA 0.0164809253146
CGGGCAAC -0.146257540309
CGGGCAAG -0.384620491711
CGGGCACA -0.193417106329
CGGGCACC -0.418810370308
CGGGCACG -0.0737421808282
CGGGCAGA -0.136192780052
CGGGCAGC -0.0893555050994
CGGGCATA 0.0352983837832
CGGGCATC -0.239124279975
CGGGCCAA -0.130968627743
CGGGCCAC -0.238884584123
CGGGCCAG -0.233915726157
CGGGCCCA -0.230280143319
CGGGCCCC -0.386194538826
CGGGCCCG -0.120100045475
CGGGCCGA -0.133677836799
CGGGCCGC -0.338805789144
CGGGCCTA -0.315643679159
CGGGCCTC -0.33754779658
CGGGCGAA 0.02525127187
CGGGCGAC -0.0684134780513
CGGGCGAG -0.398404233329
CGGGCGCA -0.208212228929
CGGGCGCC -0.248856861724
CGGGCGGA -0.122418256863
CGGGCGGC -0.222329476555
CGGGCGTA -0.145175921101
CGGGCGTC -0.401847947414
CGGGCTAA -0.0182786091877
CGGGCTAC -0.184486347158
CGGGCTAG -0.175884556944
CGGGCTCA -0.35880806144
CGGGCTCC -0.382282907954
CGGGCTGA -0.0849266564182
CGGGCTGC -0.112187731763
CGGGCTTA -0.131564146465
CGGGCTTC -0.261946697758
CGGGGAAA 0.220163792034
CGGGGAAC -0.142846310588
CGGGGAAG -0.232167122842
CGGGGACA -0.193207745784
CGGGGACC -0.309639265239
CGGGGACG -0.0198163543864

CGGGGAGA -0.0805507996769
CGGGGAGC -0.0806584860688
CGGGGATA 0.252893389257
CGGGGATC 0.300737376495
CGGGGCAA 0.141139762352
CGGGGCAC -0.110824369936
CGGGGCAG -0.138033523793
CGGGGCCA -0.328275621581
CGGGGCCC -0.0509033775036
CGGGGCGA -0.114023768258
CGGGGCGC -0.00404516689416
CGGGGCTA -0.0533427459012
CGGGGCTC -0.405199092791
CGGGGGAA 0.0903727418879
CGGGGGAC -0.0539495052262
CGGGGGAG -0.0456325022006
CGGGGGCA -0.191157133705
CGGGGGCC -0.35326328089
CGGGGGGA -0.0174847427246
CGGGGGGC -0.193142694904
CGGGGGTA -0.0120173885372
CGGGGGTC -0.39214105742
CGGGGTAA 0.0924206039284
CGGGGTAC -0.156526809419
CGGGGTAG -0.226171865307
CGGGGTCA -0.167883714712
CGGGGTCC -0.201503460937
CGGGGTGA -0.054798195985
CGGGGTGC -0.0703313066137
CGGGGTTA -0.14116148457
CGGGGTTC -0.196978593778
CGGGTAAA -0.00247768429587
CGGGTAAC -0.0212722009666
CGGGTAAG -0.235490395518
CGGGTACA -0.135436950897
CGGGTACC -0.125152685753
CGGGTACG -0.123171380747
CGGGTAGA 0.00114973903405
CGGGTAGC -0.120893035089
CGGGTATA -0.107714211904
CGGGTATC 0.186453581308
CGGGTCAA -0.16408718662
CGGGTCAC -0.291799295081
CGGGTCAG -0.276997692823
CGGGTCCA -0.184387709091
CGGGTCCC -0.369834340628
CGGGTCGA -0.140125273818
CGGGTCGC -0.192773643042
CGGGTCTA -0.236906740765
CGGGTCTC -0.358325271418
CGGGTGAA -0.0199681758794
CGGGTGAC -0.0847279094456
CGGGTGAG -0.154001129566
CGGGTGCA -0.306628463695

CGGGTGCC -0.401526945889
CGGGTGGA -0.132387853521
CGGGTGGC -0.212301144273
CGGGTGTA -0.0373002515149
CGGGTGTC -0.212295631032
CGGGTTAA -0.0324054542196
CGGGTTAC 0.042554697752
CGGGTTAG -0.27771024057
CGGGTTCA -0.263180822482
CGGGTTCC -0.311891374427
CGGGTTGA 0.0139989635263
CGGGTTGC -0.114624091768
CGGGTTTA -0.0373838529083
CGGGTTTC -0.317590869619
CGGTAAAA 0.136076848198
CGGTAAAC 0.0897109230443
CGGTAAAG -0.0687729465778
CGGTAACA -0.114496749622
CGGTAACC -0.118832869955
CGGTAACG -0.137537633636
CGGTAAGA 0.108823699733
CGGTAAGC -0.204012935348
CGGTAATA -0.0131219373644
CGGTAATC 0.368919658694
CGGTACAA 0.139794064036
CGGTACAC -0.133530175587
CGGTACAG -0.167346221031
CGGTACCA -0.279157740335
CGGTACCC -0.257672745317
CGGTACCG 0.164301838117
CGGTACGA -0.0208545269583
CGGTACGC -0.242690493714
CGGTACTA -0.0787415539431
CGGTACTC -0.165041316162
CGGTAGAA 0.193828602293
CGGTAGAC -0.0811542650729
CGGTAGAG -0.171411370652
CGGTAGCA -0.174103117685
CGGTAGCC -0.194671338652
CGGTAGGA 0.143673977007
CGGTAGGC -0.0568553501345
CGGTAGTA -0.0255835435883
CGGTAGTC -0.26947846332
CGGTATAA 0.141649108002
CGGTATAC 0.0250236462358
CGGTATAG -0.0423432739105
CGGTATCA 0.203781669015
CGGTATCC 0.308430472325
CGGTATGA -0.109484868966
CGGTATGC -0.113306941997
CGGTATTA 0.0643053521841
CGGTATTC -0.0591176835544
CGGTCAAA 0.137946486431
CGGTCAAC -0.161656340988

CGGTCAAG -0.197256517177
CGGTCACA -0.100258306486
CGGTCACC -0.295937871614
CGGTCACG -0.129318787886
CGGTCAGA -0.197002026217
CGGTCAGC -0.151154067879
CGGTCATA -0.0223000471499
CGGTCATC -0.309690002619
CGGTCCAA -0.161882568963
CGGTCCAC -0.239107659308
CGGTCCAG -0.145346792167
CGGTCCCA -0.160926527878
CGGTCCCC -0.147806066975
CGGTCCGA -0.278220389106
CGGTCCGC -0.363049416097
CGGTCCTA -0.164127693007
CGGTCCTC -0.325111539672
CGGTCGAA -0.150625463549
CGGTCGAC -0.343089099895
CGGTCGAG -0.201159549744
CGGTCGCA -0.10135065117
CGGTCGCC -0.158580687342
CGGTCGGA 0.00658433579917
CGGTCGGC -0.232043332132
CGGTCGTA -0.129296619037
CGGTCGTC -0.340899707188
CGGTCTAA 0.109700609701
CGGTCTAC -0.0658383560884
CGGTCTAG -0.195972128772
CGGTCTCA -0.118021447432
CGGTCTCC -0.307917064879
CGGTCTGA -0.101151336195
CGGTCTGC -0.118742437798
CGGTCTTA -0.188543109475
CGGTCTTC -0.3235717319
CGGTGAAA 0.164477149326
CGGTGAAC -0.215895675826
CGGTGAAG -0.105975121127
CGGTGACA -0.128657766618
CGGTGACC -0.402621722846
CGGTGACG -0.15038364088
CGGTGAGA 0.0381191710374
CGGTGAGC -0.151265890838
CGGTGATA 0.119222785812
CGGTGATC 0.121423075969
CGGTGCAA 0.141313489798
CGGTGCAC 0.101404916518
CGGTGCAG -0.151344683885
CGGTGCCA -0.207544673497
CGGTGCCC -0.245262058148
CGGTGCGA -0.00762556964329
CGGTGCGC -0.336672482271
CGGTGCTA -0.188389615331
CGGTGCTC -0.256582278666

CGGTGGAA 0.236593342654
CGGTGGAC -0.369498948506
CGGTGGAG -0.239540170707
CGGTGGCA -0.235361969063
CGGTGGCC -0.313978978048
CGGTGGGA -0.0587402425877
CGGTGGGC -0.0963686151897
CGGTGGTA -0.141430587768
CGGTGGTC -0.370496696682
CGGTGTAA 0.199113158106
CGGTGTAC -0.0292012363755
CGGTGTAG -0.0599614042207
CGGTGTCA -0.0993708166974
CGGTGTCC -0.00223998575284
CGGTGTGA -0.252165773411
CGGTGTGC -0.127409281686
CGGTGTTA 0.0921373984511
CGGTGTTC -0.165705841322
CGGTTAAA 0.144416545623
CGGTTAAC -0.0835964411538
CGGTTAAG 0.00371305737831
CGGTTACA 0.13038630803
CGGTTACC -0.0727342131731
CGGTTACG 0.0689962993511
CGGTTAGA -0.176299789056
CGGTTAGC -0.080074888871
CGGTTATA -0.137363150605
CGGTTATC -0.0286250437766
CGGTTCAA 0.0906892549798
CGGTTCAC -0.0545587404007
CGGTTCAG -0.176013916802
CGGTTCCA -0.36216915139
CGGTTCCC -0.223334301346
CGGTTCGA -0.228570657257
CGGTTCGC -0.267741257609
CGGTTCTA -0.0946192883029
CGGTTCTC -0.117256838466
CGGTTGAA 0.0648182886506
CGGTTGAC -0.214647279224
CGGTTGAG -0.276775746148
CGGTTGCA -0.104555618974
CGGTTGCC -0.309293040726
CGGTTGGA -0.111865408612
CGGTTGGC -0.173445533364
CGGTTGTA -0.0296331794834
CGGTTGTC -0.0914225762602
CGGTTTAA 0.0340306416648
CGGTTTAC -0.0426808591585
CGGTTTAG -0.0540642520687
CGGTTTCA -0.0929917729281
CGGTTTCC -0.0651574439453
CGGTTTGA -0.0276130451684
CGGTTTGC -0.187858824697
CGGTTTTA -0.0324379758107

CGGTTTTC 0.0368771137344
CGTAAAAA 0.220207586695
CGTAAAAC -0.137954759167
CGTAAAAG 0.0974949453662
CGTAAACA 0.0671744336088
CGTAAACC -0.157841345451
CGTAAACG -0.144472843274
CGTAAAGA -0.0359611137306
CGTAAAGC -0.084742460047
CGTAAATA 0.0355382931141
CGTAAATC 0.239952726009
CGTAACAA 0.183959441535
CGTAACAC 0.0478580761005
CGTAACAG 0.0326839348999
CGTAACCA 0.122360652664
CGTAACCC 0.0873090721576
CGTAACGA -0.05256370396
CGTAACGC 0.115231783246
CGTAACTA -0.0137396349518
CGTAACTC 0.0119610519478
CGTAAGAA 0.147596038043
CGTAAGAC -0.134839298466
CGTAAGAG -0.0759717363196
CGTAAGCA -0.095253064207
CGTAAGCC -0.214889538581
CGTAAGGA 0.0377181521573
CGTAAGGC 0.0493395747104
CGTAAGTA 0.0576678606982
CGTAAGTC 0.00477198962047
CGTAATAA 0.272444936769
CGTAATAC 0.283213506274
CGTAATAG 0.0842743570016
CGTAATCA 0.396209572053
CGTAATCC 0.490296081205
CGTAATGA -0.0115590271645
CGTAATGC -0.0166456208988
CGTAATTA 0.0978871433417
CGTAATTC 0.234607886123
CGTACAAA 0.200526041469
CGTACAAC -0.054664611647
CGTACAAG -0.113918186706
CGTACACA 0.0261294352203
CGTACACC -0.175337819748
CGTACACG -0.0261549017175
CGTACAGA -0.202713708365
CGTACAGC -0.142903815455
CGTACATA 0.00146493142486
CGTACATC -0.0548979710001
CGTACCAA -0.0832995546732
CGTACCAC -0.165788379941
CGTACCAG -0.145326926311
CGTACCCA 0.023694696061
CGTACCCC -0.140998706952
CGTACCGA -0.25937462053

CGTACCGC -0.0449172248992
CGTACCTA -0.195217021326
CGTACCTC -0.205197243798
CGTACGAA 0.0457500930637
CGTACGAC -0.28111157969
CGTACGAG -0.0379631643244
CGTACGCA -0.0436012606397
CGTACGCC -0.325842196576
CGTACGGA -0.0208265512434
CGTACGGC -0.0393276703914
CGTACGTA 0.0798456968096
CGTACGTC 0.0658168069312
CGTACTAA 0.205256089283
CGTACTAC 0.115923738896
CGTACTAG -0.0730050227491
CGTACTCA -0.120354057936
CGTACTCC -0.137339810729
CGTACTGA -0.0305838854562
CGTACTGC -0.0524306158626
CGTACTTA -0.13788586949
CGTACTTC -0.226500922181
CGTAGAAA 0.173615906221
CGTAGAAC -0.212794354809
CGTAGAAG -0.046333319835
CGTAGACA 0.013516271092
CGTAGACC -0.282453492971
CGTAGACG -0.26984107293
CGTAGAGA 0.0678846890968
CGTAGAGC -0.177422067805
CGTAGATA 0.209025830238
CGTAGATC 0.162267530035
CGTAGCAA 0.204907386726
CGTAGCAC -0.0858953155176
CGTAGCAG 0.0100619045649
CGTAGCCA -0.282923415725
CGTAGCCC -0.147515657133
CGTAGCGA -0.108402652394
CGTAGCGC -0.162612759099
CGTAGCTA -0.201037919199
CGTAGCTC -0.0618801849416
CGTAGGAA -0.0252718283021
CGTAGGAC -0.222791436156
CGTAGGAG -0.215912756507
CGTAGGCA -0.112844974198
CGTAGGCC -0.435644669295
CGTAGGGA -0.1392312607
CGTAGGGC -0.0457534990374
CGTAGGTA -0.0867567145466
CGTAGGTC -0.137708528222
CGTAGTAA 0.0187473975353
CGTAGTAC 0.0888919222253
CGTAGTAG -0.0831480667806
CGTAGTCA 0.0369853214893
CGTAGTCC -0.246767909874

CGTAGTGA	-0.237847351432
CGTAGTGC	-0.159009838954
CGTAGTTA	-0.151336197157
CGTAGTTC	-0.195339628122
CGTATAAA	0.224222097326
CGTATAAC	-0.010657303789
CGTATAAG	0.048631275904
CGTATACA	0.200333942758
CGTATACC	0.00333642329
CGTATACG	0.23743581475
CGTATAGA	0.0705057553484
CGTATAGC	0.130556995102
CGTATATA	0.0427983328832
CGTATATC	0.120206856784
CGTATCAA	0.215493469222
CGTATCAC	0.178045814409
CGTATCAG	0.279169799201
CGTATCCA	0.338422444483
CGTATCCC	0.211256711257
CGTATCGA	0.0549351003896
CGTATCGC	0.229962117618
CGTATCTA	0.36549491617
CGTATCTC	0.368472953147
CGTATGAA	0.104805734181
CGTATGAC	-0.137805086705
CGTATGAG	-0.309004388468
CGTATGCA	-0.0678846890968
CGTATGCC	-0.195521593988
CGTATGGA	-0.0647438377262
CGTATGGC	-0.104536281764
CGTATGTA	0.152833957863
CGTATGTC	-0.139029936467
CGTATTAA	0.183559592651
CGTATTAC	0.228643243795
CGTATTAG	0.0356485962547
CGTATTCA	-0.0189640105078
CGTATTCC	0.188494412531
CGTATTGA	0.014420756845
CGTATTGC	-0.0239978270281
CGTATTTA	0.167618957249
CGTATTTC	0.226624696322
CGTCAAAA	0.1883863857
CGTCAAAC	-0.171201279453
CGTCAAAG	-0.0409925330102
CGTCAACA	-0.0254629460669
CGTCAACC	-0.08141815714
CGTCAACG	-0.163503373709
CGTCAAGA	-0.00337389731329
CGTCAAGC	-0.0211243549411
CGTCAATA	-0.13424375019
CGTCAATC	-0.0800446391426
CGTCACAA	0.0965304147122
CGTCACAC	-0.168399045513
CGTCACAG	0.0606798621153

CGTCACCA -0.114302823238
CGTCACCC -0.054361583323
CGTCACGA -0.209456311697
CGTCACGC -0.237890169466
CGTCACTA -0.153237512717
CGTCACTC -0.136711631613
CGTCAGAA 0.165712429655
CGTCAGAC -0.159424213281
CGTCAGAG -0.251959398033
CGTCAGCA -0.12829573075
CGTCAGCC -0.313405138758
CGTCAGGA 0.16740582323
CGTCAGGC 0.0058035225506
CGTCAGTA 0.0585888919222
CGTCAGTC -0.1263720357
CGTCATAA 0.129359941023
CGTCATAC -0.226117713873
CGTCATAG -0.219124834542
CGTCATCA -0.148902395768
CGTCATCC -0.0537922527244
CGTCATGA -0.0129159052106
CGTCATGC -0.111111111111
CGTCATTA -0.121110369431
CGTCATTC -0.0916460517962
CGTCCAAA -0.0204129749584
CGTCCAAC -0.266097615444
CGTCCAAG -0.196851663483
CGTCCACA -0.122809719495
CGTCCACC -0.19630588644
CGTCCACG 0.034531776956
CGTCCAGA -0.0803126728376
CGTCCAGC -0.143767734677
CGTCCATA 0.0861426164456
CGTCCATC -0.282085734682
CGTCCCAA 0.0547187135519
CGTCCCAC -0.302365297937
CGTCCCAG -0.147730175062
CGTCCCCA -0.193384395022
CGTCCCCC -0.115905020602
CGTCCCGA -0.167971651241
CGTCCCGC -0.201524459989
CGTCCCTA -0.0674324026457
CGTCCCTC -0.0666608769013
CGTCCGAA -0.133075044771
CGTCCGAC -0.167425943385
CGTCCGAG -0.247972118175
CGTCCGCA 0.0482810634326
CGTCCGCC -0.385669635105
CGTCCGGA -0.0144172168706
CGTCCGGC -0.30059919475
CGTCCGTA -0.0386229468419
CGTCCGTC -0.167543315552
CGTCCTAA 0.0956274660645
CGTCCTAC -0.00084442323047

CGTCCTAG -0.157322695372
CGTCCTCA -0.306059302995
CGTCCTCC -0.412163709759
CGTCCTGA -0.137042000678
CGTCCTGC -0.208342636928
CGTCCTTA -0.15254718672
CGTCCTTC -0.2330418884
CGTCGAAA 0.131920468895
CGTCGAAC -0.112787718848
CGTCGAAG -0.278089291375
CGTCGACA -0.133143640179
CGTCGACC -0.306947851709
CGTCGACG -0.0407560836455
CGTCGAGA -0.12030361002
CGTCGAGC -0.174969053861
CGTCGATA 0.0674196915396
CGTCGATC -0.0510197891765
CGTCGCAA 0.104452564707
CGTCGCAC -0.104928793184
CGTCGCAG 0.0343671226958
CGTCGCCA -0.102513104902
CGTCGCCC -0.121705855919
CGTCGCGA -0.0823258641654
CGTCGCGC -0.0176850141968
CGTCGCTA -0.00752956813563
CGTCGCTC -0.253467353255
CGTCGGAA 0.102415087264
CGTCGGAC -0.237842937805
CGTCGGAG -0.137588388537
CGTCGGCA -0.112095727803
CGTCGGCC -0.340873950969
CGTCGGGA -0.188292069923
CGTCGGGC -0.3555004694
CGTCGGTA -0.0798081597083
CGTCGGTC -0.321113233709
CGTCGTAA 0.227763625614
CGTCGTAC -0.237668046898
CGTCGTAG -0.293092113424
CGTCGTCA 0.0516156580513
CGTCGTCC -0.202685977252
CGTCGTGA -0.0474779150847
CGTCGTGC -0.215095922999
CGTCGTTA -0.132793782343
CGTCGTTC -0.245795382159
CGTCTAAA 0.156845470537
CGTCTAAC -0.0582356558112
CGTCTAAG -0.0657465540583
CGTCTACA -0.00513879340168
CGTCTACC -0.147368229018
CGTCTAGA -0.00746625986292
CGTCTAGC -0.166404002994
CGTCTATA -0.109106451035
CGTCTATC 0.0761684050714
CGTCTCAA 0.0673758968782

CGTCTCAC -0.314973304778
CGTCTCAG -0.0884746574113
CGTCTCCA -0.288424329621
CGTCTCCC -0.232979073114
CGTCTCGA -0.216623444475
CGTCTCGC -0.193078700426
CGTCTCTA -0.2432189279
CGTCTCTC -0.282609901054
CGTCTGAA 0.0808442150902
CGTCTGAC -0.240581010968
CGTCTGAG -0.229363075893
CGTCTGCA -0.0210465074599
CGTCTGCC -0.309346225282
CGTCTGGA -0.0922636032057
CGTCTGGC -0.0515988231823
CGTCTGTA -0.207413473177
CGTCTGTC 0.0537866567399
CGTCTTAA 0.0132640146909
CGTCTTAC 0.13264454063
CGTCTTAG -0.240659428499
CGTCTTCA -0.0677172510484
CGTCTTCC -0.237116162862
CGTCTTGA -0.178765908358
CGTCTTGC -0.0825980209697
CGTCTTTA -0.0618092990192
CGTCTTTC -0.240586308712
CGTGAAAA 0.269816648605
CGTGAAAC -0.195258822654
CGTGAAAG 0.0177302686802
CGTGAACA -0.057185284458
CGTGAACC -0.268366207667
CGTGAACG -0.0334894122773
CGTGAAGA -0.242284524942
CGTGAAGC -0.0946275375922
CGTGAATA -0.0519777750203
CGTGAATC 0.0920322912637
CGTGACAA 0.0101829886937
CGTGACAC -0.258402869091
CGTGACAG -0.215884964856
CGTGACCA -0.274151803065
CGTGACCC -0.177081402494
CGTGACGA -0.22955888556
CGTGACGC -0.181566292315
CGTGACTA 0.0519835332073
CGTGACTC -0.146743548912
CGTGAGAA 0.118315284982
CGTGAGAC -0.272113547843
CGTGAGAG -0.115694675346
CGTGAGCA 0.213391677555
CGTGAGCC -0.256175573199
CGTGAGGA 0.0260715260715
CGTGAGGC -0.151403038323
CGTGAGTA 0.203077733381
CGTGAGTC -0.104627468497

CGTGATAA 0.131399995036
CGTGATAC 0.205388933089
CGTGATAG -0.129109173633
CGTGATCA 0.0937451909951
CGTGATCC 0.337022607142
CGTGATGA -0.154378578971
CGTGATGC 0.0848324489245
CGTGATTA 0.293242483741
CGTGATTC 0.298605869944
CGTGCAAA 0.0292902032451
CGTGCAAC -0.159172620766
CGTGCAAG -0.112398789636
CGTGCACA 0.00826186287892
CGTGCACC -0.19276588355
CGTGCACG -0.0908846608621
CGTGCAGA -0.00870800071693
CGTGCAGC -0.145482293967
CGTGCATA 0.178459595775
CGTGCATC -0.20889321862
CGTGCCAA -0.0999845289579
CGTGCCAC -0.206142123905
CGTGCCAG -0.238218166257
CGTGCCCA -0.0245812135501
CGTGCCCC -0.113046039082
CGTGCCGA -0.114968353183
CGTGCCGC -0.0495679871075
CGTGCCTA -0.0899781486331
CGTGCCTC -0.156277132793
CGTGCGAA 0.0308697126879
CGTGCGAC -0.093334899898
CGTGCGAG -0.095777882545
CGTGCGCA 0.161137347357
CGTGCGCC -0.193078064994
CGTGCGGA 0.0272546051598
CGTGCGGC -0.169886559429
CGTGCGTA 0.129295708853
CGTGCGTC -0.137118915455
CGTGCTAA -0.0446799561632
CGTGCTAC -0.0313547878514
CGTGCTAG -0.104616437776
CGTGCTCA -0.0117143420217
CGTGCTCC -0.0998437990409
CGTGCTGA -0.208200320253
CGTGCTGC -0.10545425088
CGTGCTTA -0.052215972872
CGTGCTTC -0.136407559376
CGTGGAAA 0.0851214936899
CGTGGAAC -0.210441931779
CGTGGAAG -0.282520471173
CGTGGACA 0.10985963811
CGTGGACC -0.313859328532
CGTGGAGA -0.0412538219605
CGTGGAGC -0.181566545185
CGTGGATA 0.353492259294

CGTGGATC 0.329596432195
CGTGGCAA 0.0473062633665
CGTGGCAC -0.11155570086
CGTGGCAG -0.193677300536
CGTGGCCA -0.1121665058
CGTGGCCC -0.267186717898
CGTGGCGA 0.205438589064
CGTGGCGC 0.0271549918327
CGTGGCTA -0.117481393689
CGTGGCTC -0.187635983172
CGTGGGAA -0.0105298135601
CGTGGGAC -0.203268460343
CGTGGGAG -0.346764085766
CGTGGGCA 0.0759596505186
CGTGGGCC -0.33428574579
CGTGGGGA -0.185405115077
CGTGGGGC -0.125536275507
CGTGGGTA 0.0851085245025
CGTGGGTC -0.30024050705
CGTGGTAA -0.00428665580181
CGTGGTAC -0.0523703860499
CGTGGTAG -0.0248819368919
CGTGGTCA -0.14983978453
CGTGGTCC -0.259230313305
CGTGGTGA -0.1793201836
CGTGGTGC -0.147993746743
CGTGGTTA -0.112028589318
CGTGGTTC -0.215506806407
CGTGTAAA 0.154461624159
CGTGTAAC -0.190416456869
CGTGTAAG -0.239086712272
CGTGTACA 0.0784076087106
CGTGTACC -0.176672814541
CGTGTAGA 0.00593017259684
CGTGTAGC 0.193936629125
CGTGTATA 0.1086650371
CGTGTATC 0.0731516640608
CGTGTCAA 0.163125624986
CGTGTCAC -0.164944110543
CGTGTCAG 0.0872980418435
CGTGTCCA -0.106790171214
CGTGTCCC -0.211244142384
CGTGTCGA -0.159317151167
CGTGTCGC -0.150749536106
CGTGTCTA -0.138602790118
CGTGTCTC -0.115029387682
CGTGTGAA 0.0613350285892
CGTGTGAC -0.188955051069
CGTGTGAG -0.257397214659
CGTGTGCA 0.0375721699525
CGTGTGCC -0.252089477963
CGTGTGGA -0.180198811543
CGTGTGGC -0.0994182304339
CGTGTGTA 0.0105187832461

CGTGTGTC -0.120496492026
CGTGTTAA -0.10249483588
CGTGTTAC -0.0160600978276
CGTGTTAG -0.0505298687117
CGTGTTCA 0.0650609286973
CGTGTTCC -0.0907501619997
CGTGTTGA -0.157678907398
CGTGTTGC 0.0122001059673
CGTGTTTA -0.0104472164843
CGTGTTTC 0.0520857207506
CGTTAAAA 0.253266716787
CGTTAAAC -0.100280549948
CGTTAAAG 0.0126428913862
CGTTAACA 0.0248566677422
CGTTAACC -0.150631935859
CGTTAACG -0.0424745808493
CGTTAAGA 0.0216951453618
CGTTAAGC -0.0726117108547
CGTTAATA 0.0274668608002
CGTTAATC 0.0407556316647
CGTTACAA 0.091839773658
CGTTACAC -0.157982880536
CGTTACAG 0.0384006596128
CGTTACCA -0.100870843295
CGTTACCC 0.0169478040627
CGTTACGA -0.065739362362
CGTTACGC -0.0789763186845
CGTTACTA -0.117370503647
CGTTACTC -0.141301980375
CGTTAGAA 0.133975898338
CGTTAGAC -0.0513183037003
CGTTAGAG -0.145195221277
CGTTAGCA -0.163662015418
CGTTAGCC 0.186271846005
CGTTAGGA 0.130552039643
CGTTAGGC -0.14920710228
CGTTAGTA -0.0371123260075
CGTTAGTC -0.160301569242
CGTTATAA 0.169606245364
CGTTATAC 0.112380976017
CGTTATAG 0.23295767246
CGTTATCA 0.0789480940996
CGTTATCC 0.2532167407
CGTTATGA 0.0521278854612
CGTTATGC -0.179692186002
CGTTATTA -0.000998243422486
CGTTATTC 0.186728027853
CGTTCAAA 0.0311371978039
CGTTCAAC -0.086185907401
CGTTCAAG -0.199103834642
CGTTCACA -0.0195714746532
CGTTCACC -0.238383371015
CGTTCAGA 0.0977839901316
CGTTCAGC -0.000371524711138

CGTTCATA -0.169988988007
CGTTCATC -0.19480359319
CGTTCCAA -0.121378353093
CGTTCCAC -0.113993587146
CGTTCCAG -0.233471171208
CGTTCCCA -0.172364838726
CGTTCCCC -0.134507059729
CGTTCCGA -0.163188773835
CGTTCCGC -0.130972963285
CGTTCCTA -0.140003094362
CGTTCCTC -0.172032880343
CGTTCGAA -0.13292277065
CGTTCGAC -0.160451230995
CGTTCGAG -0.193346565769
CGTTCGCA 0.0598207252762
CGTTCGCC -0.103636163145
CGTTCGGA -0.00693162094974
CGTTCGGC -0.209035619804
CGTTCGTA 0.134551907279
CGTTCGTC -0.0594709283569
CGTTCTAA -0.0258191426465
CGTTCTAC -0.099909491033
CGTTCTAG 0.198803675832
CGTTCTCA 0.00537114863316
CGTTCTCC -0.0365371561207
CGTTCTGA 0.0480273662092
CGTTCTGC -0.0600945297915
CGTTCTTA 0.0318379889491
CGTTCTTC -0.240360192594
CGTTGAAA 0.126396502266
CGTTGAAC -0.0496214847725
CGTTGAAG -0.100984941258
CGTTGACA -0.0148982139077
CGTTGACC -0.133452442989
CGTTGAGA 0.0881871199901
CGTTGAGC -0.0543079370594
CGTTGATA 0.102222056768
CGTTGATC 0.0160802385683
CGTTGCAA -0.0439150967497
CGTTGCAC -0.114640555316
CGTTGCAG 0.10904420342
CGTTGCCA -0.170663181606
CGTTGCCC -0.285290947533
CGTTGCGA 0.0960285354225
CGTTGCGC 0.0201623322117
CGTTGCTA -0.0563962360122
CGTTGCTC -0.331660402868
CGTTGGAA -0.215541516008
CGTTGGAC -0.193115190544
CGTTGGAG -0.200778013445
CGTTGGCA -0.248459585308
CGTTGGCC -0.239607713407
CGTTGGGA -0.202413156959
CGTTGGGC -0.133616531246

CGTTGGTA 0.0124037355965
CGTTGGTC -0.137759066765
CGTTGTAA 0.0971650255834
CGTTGTAC 0.0910952274589
CGTTGTAG 0.0656007532682
CGTTGTCA 0.155255806771
CGTTGTCC 0.0105229196138
CGTTGTGA 0.0505806442195
CGTTGTGC -0.198681735328
CGTTGTTA 0.0681260931983
CGTTGTTC -0.0222279804092
CGTTTAAA 0.118595213244
CGTTTAAC -0.172339382122
CGTTTAAG 0.0713407735597
CGTTTACA 0.123085625862
CGTTTACC 0.123444742086
CGTTTAGA 0.0844494632373
CGTTTAGC -0.293350700906
CGTTTATA 0.0648568678872
CGTTTATC -0.0629644335255
CGTTTCAA 0.0132074222983
CGTTTCAC -0.266423751844
CGTTTCAG 0.0425177243359
CGTTTCCA 0.062993511091
CGTTTCCC -0.0198090652636
CGTTTCGA 0.0424405121375
CGTTTCGC -0.101097969151
CGTTTCTA 0.0130150434662
CGTTTCTC -0.035567834281
CGTTTGAA 0.0690371743684
CGTTTGAC -0.128209476694
CGTTTGAG 0.0672339005672
CGTTTGCA -0.0225870966329
CGTTTGCC 0.0319948047221
CGTTTGGA 0.0203996190869
CGTTTGGC -0.012056315193
CGTTTGTA -0.0792398706598
CGTTTGTC -0.15274600795
CGTTTTAA -0.13820580053
CGTTTTAC -0.118809633975
CGTTTTAG -0.0427764355525
CGTTTTCA 0.211477317538
CGTTTTCC -0.0274470333586
CGTTTTGA 0.0261570110055
CGTTTTGC -0.0703616384718
CGTTTTTA 0.0613001175157
CGTTTTTC 0.114189470304
CTAAAAAA 0.0890133792691
CTAAAAAC 0.000619076376652
CTAAAAAG -0.0301914534581
CTAAAACA -0.0759223873848
CTAAAACC -0.0547498959877
CTAAAAGA 0.034248865817
CTAAAAGC 0.027671656825

CTAAAATA 0.258133359803
CTAAAATC 0.20079445837
CTAAACAA -0.108526137871
CTAAACAC 0.0896282601683
CTAAACAG 0.0606607154588
CTAAACCA -0.179825250695
CTAAACCC -0.203880936914
CTAAACGA -0.0154310109459
CTAAACGC 0.054176766298
CTAAACTA 0.217413487296
CTAAACTC -0.0451448346224
CTAAAGAA 0.119620008321
CTAAAGAC -0.0716686658119
CTAAAGAG -0.11958906289
CTAAAGCA -0.200435378426
CTAAAGCC -0.073898345164
CTAAAGGA 0.0446077157103
CTAAAGGC 0.032336744458
CTAAAGTA 0.209855861371
CTAAAGTC -0.197661210502
CTAAATAA 0.0896031473763
CTAAATAC 0.123378199136
CTAAATAG -0.102892809193
CTAAATCA 0.0258398894763
CTAAATCC 0.222049882119
CTAAATGA -0.0567324381176
CTAAATGC -0.0240148025956
CTAAATTA 0.207399108049
CTAAATTC 0.182509142136
CTAACAAA 0.154033853273
CTAACAAC -0.0700141477823
CTAACAAG -0.0579637672168
CTAACACA -0.116957395598
CTAACACC -0.0487967416772
CTAACAGA 0.0961081144209
CTAACAGC -0.175088436162
CTAACATA 0.175850294372
CTAACATC 0.0770012436679
CTAACCAA -0.0163624154216
CTAACCAC -0.0462900115752
CTAACCAG -0.108826448331
CTAACCCA -0.254318938829
CTAACCCC -0.271561744348
CTAACCGA 0.129657400308
CTAACCGC -0.207127395759
CTAACCTA -0.11025160666
CTAACCTC -0.213761976692
CTAACGAA 0.193802325497
CTAACGAC -0.0175726690878
CTAACGAG -0.0721963616087
CTAACGCA -0.193825682649
CTAACGCC -0.211381273346
CTAACGGA 0.154299540886
CTAACGGC -0.0636638127146

CTAACGTA 0.260708889586
CTAACGTC -0.164219152492
CTAACTAA 0.00445172733444
CTAACTAC 0.0735538625704
CTAACTAG 0.186993715466
CTAACTCA -0.0389195857025
CTAACTCC 0.163219053597
CTAACTGA 0.070744298017
CTAACTGC -0.0543188429775
CTAACTTA 0.120140801959
CTAACTTC 0.00496226253802
CTAAGAAA -0.0232946445068
CTAAGAAC 0.0688678350109
CTAAGAAG -0.104597216364
CTAAGACA -0.181201427352
CTAAGACC 0.0179063360882
CTAAGAGA 0.127940993993
CTAAGAGC -0.131759541869
CTAAGATA 0.290611884411
CTAAGATC 0.141729884154
CTAAGCAA -0.0417691583396
CTAAGCAC -0.174068284021
CTAAGCAG -0.192266133011
CTAAGCCA -0.0555416082217
CTAAGCCC -0.118920500736
CTAAGCGA -0.0583323968001
CTAAGCGC -0.175833795136
CTAAGCTA 0.1486959327
CTAAGCTC -0.132778051048
CTAAGGAA 0.0829142427538
CTAAGGAC -0.20956732691
CTAAGGAG -0.283743076687
CTAAGGCA -0.160568924724
CTAAGGCC -0.0353401020416
CTAAGGGA -0.0887592922085
CTAAGGGC -0.123655223447
CTAAGGTA 0.236422372786
CTAAGGTC -0.153547257715
CTAAGTAA 0.0714336099612
CTAAGTAC -0.120535030062
CTAAGTAG -0.0990205081114
CTAAGTCA -0.126902006843
CTAAGTCC 0.0303372918841
CTAAGTGA -0.0898140517186
CTAAGTGC -0.137267795136
CTAAGTTA 0.130931206689
CTAAGTTC 0.113424523551
CTAATAAA -0.0159236784522
CTAATAAC 0.147445676372
CTAATAAG 0.01698867908
CTAATACA 0.000465295183264
CTAATACC -0.0859523590572
CTAATAGA 0.205110107078
CTAATAGC -0.143634930712

CTAATATA 0.219772113712
CTAATATC 0.222521419491
CTAATCAA 0.0630589266953
CTAATCAC 0.0402007417518
CTAATCAG -0.0509175805008
CTAATCCA 0.00231162821252
CTAATCCC 0.00490135252513
CTAATCGA 0.145482215718
CTAATCGC -0.0055220509766
CTAATCTA 0.225024999453
CTAATCTC 0.0948205513748
CTAATGAA 0.0460041021
CTAATGAC -0.193251560605
CTAATGAG -0.0762577250503
CTAATGCA -0.209091134655
CTAATGCC -0.103371437664
CTAATGGA -0.117577724159
CTAATGGC -0.272892318636
CTAATGTA 0.216312781472
CTAATGTC -0.124702570508
CTAATTAA 0.0654284942665
CTAATTAC -0.0166074701285
CTAATTAG -0.0704611415672
CTAATTCA -0.114961982709
CTAATTCC 0.088927770746
CTAATTGA -0.0651636371067
CTAATTGC 0.227187242339
CTAATTTA 0.280182386243
CTAATTTC 0.135690787206
CTACAAAA -0.0499704386035
CTACAAAC -0.211082408401
CTACAAAG -0.160078832198
CTACAACA 0.100754727999
CTACAACC -0.196092296241
CTACAAGA 0.0846455917024
CTACAAGC -0.0390490563394
CTACAATA 0.137823989256
CTACAATC 0.166833572987
CTACACAA 0.0717993701088
CTACACAC 0.128936592629
CTACACAG -0.139145982041
CTACACCA 0.0644956251017
CTACACCC -0.0149770173833
CTACACGA 0.223620650643
CTACACGC -0.0952713500733
CTACACTA 0.0927801079316
CTACACTC -0.141401233496
CTACAGAA -0.11621074632
CTACAGAC 0.0788901849508
CTACAGAG -0.154398811357
CTACAGCA -0.00259875976431
CTACAGCC -0.209984487481
CTACAGGA 0.0396889119216
CTACAGGC -0.227114816745

CTACAGTA 0.207559688474
CTACAGTC -0.163211830972
CTACATAA -0.142924812678
CTACATAC 0.0218615650752
CTACATAG -0.0496332965433
CTACATCA 0.0117712750816
CTACATCC 0.0997635088283
CTACATGA 0.0981869010168
CTACATGC -0.0426481170895
CTACATTA 0.220499551105
CTACATTC 0.0918454340416
CTACCAAA -0.108481034318
CTACCAAC 0.174325379736
CTACCAAG -0.150226697596
CTACCACA 0.0405901769538
CTACCACC -0.109435645024
CTACCAGA -0.149625990168
CTACCAGC -0.21785022177
CTACCATA 0.23255184193
CTACCATC 0.0334094425004
CTACCCAA -0.114936112145
CTACCCAC -0.00756817423484
CTACCCAG -0.154031009223
CTACCCCA -0.299023839647
CTACCCCC -0.218702785994
CTACCCGA 0.162188359158
CTACCCGC -0.19819138115
CTACCCTA -0.0865989148222
CTACCCTC -0.229675116128
CTACCGAA -0.0364096879248
CTACCGAC -0.0258519654791
CTACCGAG 0.00425155297485
CTACCGCA -0.0904847376968
CTACCGCC -0.0538875517288
CTACCGGA 0.066092263062
CTACCGGC -0.0460733465781
CTACCGTA 0.162956285629
CTACCGTC -0.239028794332
CTACCTAA -0.0182341118378
CTACCTAC 0.0487994881934
CTACCTAG -0.0648752217105
CTACCTCA -0.208192635419
CTACCTCC -0.158374802386
CTACCTGA 0.130229994964
CTACCTGC -0.227036421422
CTACCTTA 0.158951038539
CTACCTTC -0.178505765186
CTACGAAA 0.203524740334
CTACGAAC -0.0284600079702
CTACGAAG -0.164908199975
CTACGACA -0.163303088702
CTACGACC -0.206258837538
CTACGAGA 0.0822544031883
CTACGAGC -0.205629605279

CTACGATA 0.104477755993
CTACGATC 0.176091764414
CTACGCAA 0.0804646613578
CTACGCAC −0.072189063223
CTACGCAG −0.237656253421
CTACGCCA −0.0465446835028
CTACGCCC −0.0943342901181
CTACGCGA 0.192181923024
CTACGCGC −0.095596811323
CTACGCTA −0.0277619232752
CTACGCTC −0.022311542678
CTACGGAA 0.0372714312108
CTACGGAC −0.102506514456
CTACGGAG −0.231975219804
CTACGGCA −0.132985608175
CTACGGCC −0.23377117129
CTACGGGA −0.251293983397
CTACGGGC −0.158050848626
CTACGGTA 0.197437282395
CTACGGTC −0.216598386614
CTACGTAA −0.0459959798496
CTACGTAC −0.111714990832
CTACGTAG −0.294734216843
CTACGTCA 0.0330694723608
CTACGTCC 0.0345400496916
CTACGTGA 0.0277481338087
CTACGTGC −0.159024176406
CTACGTTA 0.0671622035258
CTACGTTC −0.248868497601
CTACTAAA 0.0287022911907
CTACTAAC 0.122946497258
CTACTAAG 0.0712963598224
CTACTACA 0.109915111348
CTACTACC −0.135498261606
CTACTAGA 0.0969109605473
CTACTAGC 0.0259171016747
CTACTATA 0.233582476296
CTACTATC 0.11724258694
CTACTCAA −0.218697046345
CTACTCAC 0.0370232768626
CTACTCAG −0.233920172549
CTACTCCA −0.119551857681
CTACTCCC −0.2008593666
CTACTCGA 0.065043101246
CTACTCGC −0.115396915985
CTACTCTA 0.0376643861492
CTACTCTC −0.149065481255
CTACTGAA −0.148132522645
CTACTGAC −0.0868723207763
CTACTGAG −0.181446933549
CTACTGCA −0.0786835973584
CTACTGCC −0.121650620789
CTACTGGA −0.203488957545
CTACTGGC −0.139932201077

CTACTGTA 0.14910549404
CTACTGTC -0.0808204899114
CTACTTAA -0.0556141106399
CTACTTAC 0.124974837096
CTACTTCA -0.145870705296
CTACTTCC -0.0500285381046
CTACTTGA -0.0421087901307
CTACTTGC 0.185323679044
CTACTTTA 0.256630146785
CTACTTTC -0.279795724269
CTAGAAAA -0.0852134624789
CTAGAAAC 0.0105129084765
CTAGAAAG -0.0972880561415
CTAGAACA 0.00040802026233
CTAGAACC -0.0951559669454
CTAGAAGA 0.0945008369251
CTAGAAGC -0.063201613739
CTAGAATA 0.171773610797
CTAGAATC 0.339428960641
CTAGACAA 0.0318155621186
CTAGACAC -0.00247950774801
CTAGACAG -0.0255479182003
CTAGACCA -0.318119543614
CTAGACCC -0.203974338828
CTAGACGA 0.0305197146481
CTAGACGC -0.234850803261
CTAGACTA -0.177342745332
CTAGACTC -0.174842236723
CTAGAGAA 0.122305621342
CTAGAGAC -0.159070178127
CTAGAGAG -0.12783112633
CTAGAGCA -0.205895486776
CTAGAGCC -0.34155049303
CTAGAGGA 0.110831879594
CTAGAGGC -0.317541161063
CTAGAGTA -0.000669328408867
CTAGAGTC -0.23773786191
CTAGATAA 0.100887388766
CTAGATAC 0.277654503916
CTAGATAG -0.0192588181124
CTAGATCA -0.166316488958
CTAGATCC 0.25096603724
CTAGATGA -0.290567863256
CTAGATGC 0.0770067588249
CTAGATTA 0.405330540207
CTAGATTC 0.443538579902
CTAGCAAA 0.00560753591057
CTAGCAAC -0.131300931082
CTAGCAAG -0.245570579884
CTAGCACA -0.062381845653
CTAGCACC -0.226001468144
CTAGCAGA 0.0362466515332
CTAGCAGC -0.231316359815
CTAGCATA -0.0507413013927

CTAGCATC 0.0709451617848
CTAGCCAA -0.0326811983841
CTAGCCAC -0.00946814583178
CTAGCCAG -0.215965899223
CTAGCCCA -0.2626841929
CTAGCCCC -0.130646258992
CTAGCCGA -0.0686113937349
CTAGCCGC -0.381840797835
CTAGCCTA -0.101901573016
CTAGCCTC -0.268279922185
CTAGCGAA 0.019803945899
CTAGCGAC -0.15963417828
CTAGCGAG -0.0323310032951
CTAGCGCA -0.179221990203
CTAGCGCC -0.179060285008
CTAGCGGA 0.254660997085
CTAGCGGC -0.0575324449738
CTAGCGTA -0.00432483256267
CTAGCGTC -0.299122758056
CTAGCTAA -0.239515395808
CTAGCTAC -0.0106664803032
CTAGCTAG -0.234872723365
CTAGCTCA -0.273816317248
CTAGCTCC -0.0268023812084
CTAGCTGA 0.084501166282
CTAGCTGC -0.304977239463
CTAGCTTA 0.0649303918406
CTAGCTTC -0.204249217365
CTAGGAAA 0.0263665759144
CTAGGAAC -0.0935181279003
CTAGGAAG -0.147564059084
CTAGGACA -0.0212323817727
CTAGGACC -0.280971098165
CTAGGAGA 0.198812434764
CTAGGAGC -0.0972619376046
CTAGGATA 0.164556250593
CTAGGATC 0.218733166427
CTAGGCAA -0.00282388559067
CTAGGCAC -0.0977420175156
CTAGGCAG -0.133684561668
CTAGGCCA -0.222596778511
CTAGGCCC -0.211626501892
CTAGGCGA 0.181938136484
CTAGGCGC -0.197952483621
CTAGGCTA -0.197264242504
CTAGGCTC -0.227741057968
CTAGGGAA 0.0179171259024
CTAGGGAC -0.207141448919
CTAGGGAG -0.286464320666
CTAGGGCA -0.204303698375
CTAGGGCC -0.185436981532
CTAGGGGA 0.0241457180435
CTAGGGGC -0.162184665455
CTAGGGTA 0.218608415578

CTAGGGTC -0.343432464386
CTAGGTAA -0.0461820979229
CTAGGTAC -0.043525324263
CTAGGTCA -0.334819910849
CTAGGTCC -0.0762470741007
CTAGGTGA -0.0801603601315
CTAGGTGC -0.264884422942
CTAGGTTA -0.195460897897
CTAGGTTC -0.04786938225
CTAGTAAA -0.0425603818894
CTAGTAAC 0.0692152726583
CTAGTAAG -0.0656461301155
CTAGTACA -0.084997730202
CTAGTACC -0.00991396422103
CTAGTAGA 0.153651326401
CTAGTAGC -0.0104415710476
CTAGTATA 0.167255006576
CTAGTATC 0.0492710341195
CTAGTCAA -0.100475294468
CTAGTCAC 0.0145121005943
CTAGTCAG -0.245370458204
CTAGTCCA -0.24823669513
CTAGTCCC -0.0812061866654
CTAGTCGA 0.104469589003
CTAGTCGC -0.224564898531
CTAGTCTA 0.0335822692975
CTAGTCTC -0.212434518484
CTAGTGAA -0.201632048705
CTAGTGAC -0.244018822206
CTAGTGAG -0.0749885791599
CTAGTGCA 0.0551281308857
CTAGTGCC -0.355501417654
CTAGTGGA 0.194950811792
CTAGTGGC -0.00248996980601
CTAGTGTA 0.15428786231
CTAGTGTC -0.281898319432
CTAGTTAA 0.00777775020199
CTAGTTAC 0.161686240447
CTAGTTCA -0.0680619321394
CTAGTTCC -0.0732254038233
CTAGTTGA -0.0433331954613
CTAGTTGC 0.117548678155
CTAGTTTA -0.0111491905762
CTAGTTTC -0.21563202856
CTATAAAA 0.119774749458
CTATAAAC 0.014632526295
CTATAAAG -0.186055590826
CTATAACA 0.0978000619149
CTATAACC -0.0710669347033
CTATAAGA 0.170240797647
CTATAAGC 0.113649336146
CTATAATA 0.20187514142
CTATAATC 0.111753441085
CTATACAA 0.0820944911854

CTATACAC 0.110028977468
CTATACAG -0.187173824943
CTATACCA -0.00777521361159
CTATACCC -0.255048523022
CTATACGA 0.198791997256
CTATACGC 0.143423037362
CTATACTA -0.0782267541587
CTATACTC 0.0192228950046
CTATAGAA -0.0350627358525
CTATAGAC -0.270868333438
CTATAGAG -0.21153934077
CTATAGCA -0.0934621385836
CTATAGCC -0.183684164827
CTATAGGA 0.161303767071
CTATAGGC -0.0412920770184
CTATAGTA 0.159850900488
CTATAGTC -0.28430788823
CTATATAA 0.0819105640618
CTATATAC 0.157853445732
CTATATAG 0.0541760722348
CTATATCA 0.205101348146
CTATATCC 0.262314812517
CTATATGA -0.0835532502199
CTATATGC 0.148579958103
CTATATTA 0.252686051084
CTATATTC -0.00831003700649
CTATCAAA 0.146884383453
CTATCAAC 0.00769215048851
CTATCAAG -0.27446878299
CTATCACA 0.0258819149946
CTATCACC -0.0374311397305
CTATCAGA 0.0152368926228
CTATCAGC -0.0385264411743
CTATCATA 0.128565797829
CTATCATC 0.0165957135523
CTATCCAA 0.123496954394
CTATCCAC 0.079615044926
CTATCCAG 0.0644515038454
CTATCCCA -0.178992055372
CTATCCCC 0.156207528302
CTATCCGA 0.154746246433
CTATCCGC -0.0323825025729
CTATCCTA 0.0384305453165
CTATCCTC 0.0209769129143
CTATCGAA 0.191329386948
CTATCGAC -0.0351328073108
CTATCGAG 0.0133712400458
CTATCGCA -0.0840971094013
CTATCGCC -0.0749728108144
CTATCGGA 0.201495587688
CTATCGGC 0.161493543937
CTATCGTA 0.134263365594
CTATCGTC -0.213733566675
CTATCTAA 0.000848886520733

CTATCTAC 0.00525170981657
CTATCTCA -0.0589133084677
CTATCTCC 0.0786515623745
CTATCTGA 0.0506404969234
CTATCTGC -0.0216679764514
CTATCTTA 0.153861594272
CTATCTTC -0.0620455617043
CTATGAAA 0.154693692839
CTATGAAC -0.179397889664
CTATGAAG -0.110285462239
CTATGACA -0.0198417375983
CTATGACC -0.0919470688948
CTATGAGA 0.0281183623716
CTATGAGC -0.174807451204
CTATGATA 0.199186149208
CTATGATC 0.003913003913
CTATGCAA 0.15534956444
CTATGCAC -0.0853870399325
CTATGCAG -0.111921436316
CTATGCCA -0.162764850376
CTATGCCC 0.0984386590447
CTATGCGA 0.0950920782757
CTATGCGC -0.0817224579601
CTATGCTA 0.0219464780577
CTATGCTC -0.145573285025
CTATGGAA 0.146451537558
CTATGGAC -0.116284517827
CTATGGAG -0.27813476016
CTATGGCA -0.205123433817
CTATGGCC -0.157347901486
CTATGGGA -0.0337590611929
CTATGGGC 0.0141406223771
CTATGGTA 0.25520975521
CTATGGTC -0.366661900753
CTATGTAA 0.075658302931
CTATGTAC 0.0835742342877
CTATGTCA -0.136174011032
CTATGTCC 0.00277439180164
CTATGTGA 0.0532506587447
CTATGTGC -0.0422888549886
CTATGTTA 0.100716418898
CTATGTTC -0.232723406677
CTATTAAA 0.167703626928
CTATTAAC 0.0417649054013
CTATTAAG -0.0456806668928
CTATTACA 0.0638075108846
CTATTACC -0.0671700619694
CTATTAGA 0.117079917958
CTATTAGC 0.0175813668314
CTATTATA 0.109094691357
CTATTATC 0.046526718393
CTATTCAA -0.0315007700561
CTATTCAC 0.0982621740197
CTATTCAG -0.123265605146

CTATTCCA -0.111229675217
CTATTCCC 0.121066704717
CTATTCGA -0.0283578254986
CTATTCGC 0.136873788389
CTATTCTA 0.14398558338
CTATTCTC -0.0461354092515
CTATTGAA 0.0647793878481
CTATTGAC -0.15425506298
CTATTGAG 0.0506813719408
CTATTGCA -0.106329452052
CTATTGCC -0.0338256093485
CTATTGGA 0.0624913323066
CTATTGGC 0.0354738951339
CTATTGTA 0.221098965184
CTATTGTC -0.097652253985
CTATTTAA -0.0772439105772
CTATTTAC 0.191285592286
CTATTTCA 0.0474084509098
CTATTTCC 0.0605337109406
CTATTTGA -0.0823149549639
CTATTTGC 0.171902075137
CTATTTTA -0.0157774854745
CTATTTTC 0.0674398838623
CTCAAAAA 0.089393801515
CTCAAAAC 0.0909073361735
CTCAAAAG -0.262549715146
CTCAAACA -0.114375192495
CTCAAACC -0.184868586112
CTCAAAGA -0.0658113507209
CTCAAAGC 0.141803249779
CTCAAATA 0.150479680783
CTCAAATC 0.15644839894
CTCAACAA -0.00725788441974
CTCAACAC -0.0961638669016
CTCAACAG -0.0250607985706
CTCAACCA -0.132819580309
CTCAACCC 0.0769961241725
CTCAACGA -0.0100739216416
CTCAACGC 0.0938860578763
CTCAACTA 0.0630478963812
CTCAACTC -0.249819427684
CTCAAGAA -0.0252938889303
CTCAAGAC -0.188916749738
CTCAAGAG -0.304689416428
CTCAAGCA -0.12322490468
CTCAAGCC -0.207680139854
CTCAAGGA 0.00422323149596
CTCAAGGC -0.051148198045
CTCAAGTA -0.0469684560594
CTCAAGTC -0.215528864845
CTCAATAA 0.184742669869
CTCAATAC 0.0291986306869
CTCAATCA -0.0937493967797
CTCAATCC -0.0517544077363

CTCAATGA 0.00459964096328
CTCAATGC -0.263693341107
CTCAATTA 0.055463748969
CTCAATTC 0.0752939716649
CTCACAAA -0.0399001806124
CTCACAAC -0.065038104872
CTCACAAG -0.159499651867
CTCACACA -0.195268025748
CTCACACC 0.0238047965321
CTCACAGA -0.0164046797846
CTCACAGC 0.00879536575211
CTCACATA 0.157517021153
CTCACATC -0.172248553176
CTCACCAA 0.137303970637
CTCACCAC -0.0605182115691
CTCACCAG 0.0103328643998
CTCACCCA -0.200080313066
CTCACCCC -0.043429044364
CTCACCGA 0.0471106472121
CTCACCGC -0.193018856221
CTCACCTA 0.0438709702702
CTCACCTC -0.216336836998
CTCACGAA 0.147377404953
CTCACGAC -0.143243578608
CTCACGAG -0.206098169419
CTCACGCA -0.0338377254766
CTCACGCC -0.237053409202
CTCACGGA 0.172217672218
CTCACGGC 0.005468951856
CTCACGTA -0.103308631956
CTCACGTC -0.156778177508
CTCACTAA 0.0208707329919
CTCACTAC -0.15275071696
CTCACTCA -0.155211366273
CTCACTCC -0.0398761901843
CTCACTGA 0.138942212944
CTCACTGC -0.19425354846
CTCACTTA 0.0583409863735
CTCACTTC 0.0729779366143
CTCAGAAA 0.149019174976
CTCAGAAC -0.191753436615
CTCAGAAG -0.177184190287
CTCAGACA -0.0861261384222
CTCAGACC -0.19392784888
CTCAGAGA 0.0841726093589
CTCAGAGC -0.157793514732
CTCAGATA 0.287307861622
CTCAGATC 0.233389779786
CTCAGCAA -0.0178079072626
CTCAGCAC -0.363414271187
CTCAGCAG -0.253728820917
CTCAGCCA -0.301356949716
CTCAGCCC -0.285873744264
CTCAGCGA 0.00314372677971

CTCAGCGC -0.0834363519866
CTCAGCTA -0.0177712147409
CTCAGCTC -0.187138347875
CTCAGGAA -0.115229145815
CTCAGGAC -0.297122957243
CTCAGGAG -0.241926632953
CTCAGGCA -0.321766032257
CTCAGGCC -0.169573523209
CTCAGGGA -0.25566509078
CTCAGGGC -0.198708395792
CTCAGGTA -0.0479170630686
CTCAGGTC -0.145215617197
CTCAGTAA -0.111908907671
CTCAGTAC -0.0471588213397
CTCAGTCA -0.153244475897
CTCAGTCC -0.15846946243
CTCAGTGA 0.181005525426
CTCAGTGC -0.100732100757
CTCAGTTA 0.0326379714313
CTCAGTTC -0.364056761926
CTCATAAA 0.121715179961
CTCATAAC 0.020384225163
CTCATAAG -0.0491422207236
CTCATACA -0.224045888979
CTCATACC -0.138091902187
CTCATAGA -0.0761456625806
CTCATAGC -0.123298759881
CTCATATA 0.074833786955
CTCATATC 0.200774435596
CTCATCAA 0.120604511502
CTCATCAC -0.200703243911
CTCATCAG -0.00605607176485
CTCATCCA -0.193269318248
CTCATCCC -0.267315699385
CTCATCGA 0.189098778859
CTCATCGC -0.200367439309
CTCATCTA 0.115481582017
CTCATCTC -0.297715231257
CTCATGAA 0.0575191784122
CTCATGAC -0.0967227806042
CTCATGAG -0.127569005195
CTCATGCA -0.081626029639
CTCATGCC -0.112732327765
CTCATGGA 0.0268366386138
CTCATGGC -0.101219766761
CTCATGTA 0.126040305687
CTCATGTC -0.116926743899
CTCATTAA 0.00931986252078
CTCATTAC 0.14345700816
CTCATTCA -0.168397738581
CTCATTCC -0.22169200658
CTCATTGA 0.122570228631
CTCATTGC -0.126732819235
CTCATTTA 0.00621227272395

CTCATTTC -0.199789373275
CTCCAAAA -0.0514605514606
CTCCAAAC -0.066374145665
CTCCAAAG -0.191432709724
CTCCAACA 0.0721512180998
CTCCAACC -0.283000993689
CTCCAAGA -0.0466391246907
CTCCAAGC -0.115861143832
CTCCAATA 0.146224737134
CTCCAATC -0.101853266925
CTCCACAA -0.0199422068916
CTCCACAC -0.153234411057
CTCCACAG -0.0772149127608
CTCCACCA -0.305164658318
CTCCACCC -0.215470032993
CTCCACGA 0.0758076722662
CTCCACGC -0.0100472252432
CTCCACTA 0.0274718527972
CTCCACTC -0.280380889385
CTCCAGAA -0.129669689658
CTCCAGAC -0.1500025465
CTCCAGAG -0.0641006219989
CTCCAGCA -0.201722604399
CTCCAGCC -0.304526697925
CTCCAGGA 0.111170558309
CTCCAGGC -0.209648520733
CTCCAGTA 0.0597293489924
CTCCAGTC -0.237910150704
CTCCATAA -0.0151653613425
CTCCATAC -0.124995347279
CTCCATCA -0.138778896915
CTCCATCC -0.199643162066
CTCCATGA -0.00480976844613
CTCCATGC -0.15634575045
CTCCATTA 0.0496624161515
CTCCATTC -0.224907264968
CTCCCAAA -0.0629151251122
CTCCCAAC -0.216353037506
CTCCCAAG 0.00164294816867
CTCCCACA -0.245832609828
CTCCCACC -0.131509738246
CTCCCAGA -0.152218270133
CTCCCAGC -0.129908621831
CTCCCATA 0.0517897192538
CTCCCATC -0.244637845711
CTCCCCAA -0.132867026806
CTCCCCAC -0.234521371999
CTCCCCAG -0.196131709588
CTCCCCCA -0.102357085631
CTCCCCCC -0.212653238716
CTCCCCGA -0.209916667356
CTCCCCGC -0.0136493885839
CTCCCCTA -0.174121207611
CTCCCCTC -0.274910083096

CTCCCGAA -0.0639083958521
CTCCCGAC -0.196833626899
CTCCCGAG -0.170537346269
CTCCCGCA -0.123486045287
CTCCCGCC -0.251934458586
CTCCCGGA 0.104233710294
CTCCCGGC -0.195494027781
CTCCCGTA -0.0592613731929
CTCCCGTC -0.337385692252
CTCCCTAA -0.109929550337
CTCCCTAC -0.220992941673
CTCCCTCA -0.152633406931
CTCCCTCC -0.295319171222
CTCCCTGA -0.11250203331
CTCCCTGC -0.22223819203
CTCCCTTA -0.129073007264
CTCCCTTC -0.21531825761
CTCCGAAA -0.0651017075068
CTCCGAAC -0.305364850785
CTCCGAAG -0.188360357415
CTCCGACA -0.222525480596
CTCCGACC -0.27313312801
CTCCGAGA 0.100112509203
CTCCGAGC -0.0850395003516
CTCCGATA 0.028404775497
CTCCGATC 0.0132894900112
CTCCGCAA -0.0172514470486
CTCCGCAC -0.264075458261
CTCCGCAG -0.18608334902
CTCCGCCA -0.158267266124
CTCCGCCC -0.196915238048
CTCCGCGA 0.0192327313539
CTCCGCGC -0.151297793627
CTCCGCTA 0.0195208257409
CTCCGCTC -0.216371004425
CTCCGGAA -0.230032296244
CTCCGGAC -0.272368983147
CTCCGGAG -0.392076875387
CTCCGGCA -0.00346291217413
CTCCGGCC -0.188543033119
CTCCGGGA -0.0843481738709
CTCCGGGC -0.156115700885
CTCCGGTA -0.175980353106
CTCCGGTC -0.160623156703
CTCCGTAA 0.139970549061
CTCCGTAC 0.0261680413196
CTCCGTCA -0.189604925405
CTCCGTCC -0.186231210346
CTCCGTGA 0.00067698552547
CTCCGTGC 0.0411527229709
CTCCGTTA -0.12124717384
CTCCGTTC -0.199512697171
CTCCTAAA -0.186980842341
CTCCTAAC -0.0415858562508

CTCCTAAG -0.21134519467
CTCCTACA -0.169028164287
CTCCTACC -0.23461185402
CTCCTAGA -0.014555907969
CTCCTAGC 0.016465501314
CTCCTATA -0.0184528805939
CTCCTATC -0.235478638946
CTCCTCAA -0.0541549081497
CTCCTCAC -0.211606070468
CTCCTCAG -0.0918049557879
CTCCTCCA -0.240838136507
CTCCTCCC -0.182813300502
CTCCTCGA -0.0289419341676
CTCCTCGC -0.180642057407
CTCCTCTA -0.0100267877346
CTCCTCTC -0.268201752572
CTCCTGAA -0.0324834607924
CTCCTGAC -0.145935039442
CTCCTGCA 0.0186577935097
CTCCTGCC -0.345185626201
CTCCTGGA -0.114296369929
CTCCTGGC -0.0532882532464
CTCCTGTA -0.0640049105976
CTCCTGTC -0.104133190306
CTCCTTAA -0.18412034706
CTCCTTAC -0.102020753536
CTCCTTCA -0.110930380466
CTCCTTCC -0.133040643383
CTCCTTGA 0.0371177828155
CTCCTTGC -0.11707562358
CTCCTTTA -0.184294116187
CTCCTTTC -0.279190686108
CTCGAAAA -0.0358977540638
CTCGAAAC -0.124760041751
CTCGAAAG -0.243524975667
CTCGAACA -0.244571556195
CTCGAACC -0.242559317508
CTCGAAGA -0.0662952341338
CTCGAAGC -0.067453355784
CTCGAATA 0.113970496996
CTCGAATC 0.183551455078
CTCGACAA -0.250271770455
CTCGACAC -0.191646734856
CTCGACAG -0.064426847475
CTCGACCA -0.152856004654
CTCGACCC -0.211746205011
CTCGACGA -0.140394842402
CTCGACGC -0.107259353313
CTCGACTA -0.0410093288881
CTCGACTC -0.121068841029
CTCGAGAA -0.00936402852492
CTCGAGAC -0.142542019413
CTCGAGAG -0.274641554833
CTCGAGCA -0.11073544372

CTCGAGCC -0.406746376272
CTCGAGGA -0.105658595539
CTCGAGGC -0.00949249286512
CTCGAGTA -0.0104994801964
CTCGAGTC -0.0473352140019
CTCGATAA -0.0780546386607
CTCGATAC 0.0341760493276
CTCGATCA -0.0662318397013
CTCGATCC 0.0605633181391
CTCGATGA -0.0672930330524
CTCGATGC -0.242163616466
CTCGATTA 0.149908802144
CTCGATTC 0.297023422845
CTCGCAAA -0.0562021756215
CTCGCAAC -0.237859929151
CTCGCAAG -0.111298823179
CTCGCACA -0.0436318912725
CTCGCACC -0.0943372957668
CTCGCAGA -0.109652470769
CTCGCAGC -0.149951885031
CTCGCATA 0.056548283821
CTCGCATC -0.119263764484
CTCGCCAA -0.287981652581
CTCGCCAC -0.156739582903
CTCGCCAG 0.100569330599
CTCGCCCA -0.14505060677
CTCGCCCC -0.268600600916
CTCGCCGA 0.00393211178668
CTCGCCGC -0.156485998602
CTCGCCTA -0.223804026604
CTCGCCTC -0.219311519602
CTCGCGAA 0.0438556220066
CTCGCGAC -0.296270866381
CTCGCGAG -0.0128122441892
CTCGCGCA -0.103749432579
CTCGCGCC -0.289689114503
CTCGCGGA -0.00522105355357
CTCGCGGC -0.215936495833
CTCGCGTA -0.0515120106859
CTCGCGTC 0.0112247233459
CTCGCTAA 0.168255439662
CTCGCTAC -0.173154130285
CTCGCTCA -0.0245062767186
CTCGCTCC -0.30414582783
CTCGCTGA -0.0817115902593
CTCGCTGC -0.151033094874
CTCGCTTA 0.0520230974776
CTCGCTTC -0.0964230295935
CTCGGAAA 0.154965389946
CTCGGAAC -0.272923693009
CTCGGAAG 0.0360043238831
CTCGGACA -0.250055080943
CTCGGACC -0.239311044404
CTCGGAGA -0.0111026765837

CTCGGAGC -0.17472726344
CTCGGATA 0.271456099501
CTCGGATC 0.24491361503
CTCGGCAA -0.126103955905
CTCGGCAC -0.288041234105
CTCGGCAG -0.075209333854
CTCGGCCA 0.00186191060785
CTCGGCCC -0.213379922535
CTCGGCGA 0.0967503550206
CTCGGCGC -0.111701145052
CTCGGCTA -0.148039710306
CTCGGCTC -0.248972520612
CTCGGGAA 0.0148770379929
CTCGGGAC -0.227433228035
CTCGGGCA -0.291960370903
CTCGGGCC -0.309535124493
CTCGGGGA -0.209758303076
CTCGGGGC -0.29640105647
CTCGGGTA 0.00289407865165
CTCGGGTC 0.123161536609
CTCGGTAA -0.00251454347715
CTCGGTAC -0.291811428513
CTCGGTCA -0.246723635499
CTCGGTCC -0.0310786247798
CTCGGTGA -0.205431335086
CTCGGTGC -0.280353584216
CTCGGTTA -0.0455705349518
CTCGGTTC -0.254281204257
CTCGTAAA -0.0362862428217
CTCGTAAC 0.0459638653585
CTCGTAAG -0.0223760645586
CTCGTACA -0.103151714002
CTCGTACC -0.306221935548
CTCGTAGA 0.0563911018456
CTCGTAGC -0.0904569227378
CTCGTATA 0.128797863961
CTCGTATC 0.0728911045744
CTCGTCAA -0.0104071022657
CTCGTCAC -0.226805394844
CTCGTCAG -0.1128162573
CTCGTCCA -0.175057101367
CTCGTCCC -0.108619569897
CTCGTCGA -0.0449697758846
CTCGTCGC -0.0897412564079
CTCGTCTA 0.0801872951687
CTCGTCTC -0.259745916674
CTCGTGAA -0.151460991334
CTCGTGAC -0.298620140175
CTCGTGCA -0.158027208601
CTCGTGCC -0.311357265127
CTCGTGGA -0.0882513497695
CTCGTGGC -0.0339916906443
CTCGTGTA 0.208929001555
CTCGTGTC 0.0915998643271

CTCGTTAA -0.0101051464688
CTCGTTAC 0.0726692116231
CTCGTTCA 0.0210444604384
CTCGTTCC -0.0226686741838
CTCGTTGA 0.018545007641
CTCGTTGC -0.131111607401
CTCGTTTA -0.0269548841996
CTCGTTTC -0.125442464636
CTCTAAAA -0.198937219342
CTCTAAAC -0.0608839659645
CTCTAAAG -0.255821709375
CTCTAACA -0.0523117474655
CTCTAACC -0.248975124543
CTCTAAGA -0.196078188141
CTCTAAGC -0.138765167117
CTCTAATA 0.166884515369
CTCTAATC -0.0286132420458
CTCTACAA -0.142561761325
CTCTACAC -0.219131184893
CTCTACAG -0.0189891526008
CTCTACCA -0.0780140670709
CTCTACCC -0.133829549684
CTCTACGA -0.0636801816332
CTCTACGC -0.153545955573
CTCTACTA 0.113135478785
CTCTACTC -0.209765337436
CTCTAGAA -0.0302904467412
CTCTAGAC -0.286206021907
CTCTAGAG -0.0998643944468
CTCTAGCA -0.194191499392
CTCTAGCC -0.104053461694
CTCTAGGA 0.00174229761392
CTCTAGGC -0.165091101572
CTCTAGTA 0.185297402247
CTCTAGTC -0.259599992412
CTCTATAA -0.0131688161991
CTCTATAC -0.0404490082192
CTCTATCA -0.0865405248384
CTCTATCC 0.044996787421
CTCTATGA -0.179138237605
CTCTATGC -0.0939770941004
CTCTATTA 0.166154755735
CTCTATTC -0.103256086187
CTCTCAAA -0.190366674501
CTCTCAAC -0.161224287239
CTCTCAAG -0.293568484748
CTCTCACA -0.304843415025
CTCTCACC -0.116527105818
CTCTCAGA -0.200304621704
CTCTCAGC -0.0851907908703
CTCTCATA -0.00394195848741
CTCTCATC -0.105790139758
CTCTCCAA 0.00179148697067
CTCTCCAC -0.246058754942

CTCTCCAG 0.0218358854722
CTCTCCCA -0.354174762633
CTCTCCCC 0.0495694468571
CTCTCCGA -0.0428674664417
CTCTCCGC -0.19125970034
CTCTCCTA -0.195069829393
CTCTCCTC -0.255031694247
CTCTCGAA -0.0410010261021
CTCTCGAC -0.374914217206
CTCTCGCA -0.180652381858
CTCTCGCC -0.28328744243
CTCTCGGA 0.102423304599
CTCTCGGC 0.0788240030664
CTCTCGTA 0.336428715217
CTCTCGTC -0.168602979489
CTCTCTAA -0.235928140411
CTCTCTAC -0.0778205181233
CTCTCTCA -0.208909122485
CTCTCTCC -0.194422105492
CTCTCTGA -0.123277825854
CTCTCTGC -0.215762570865
CTCTCTTA 0.032380408798
CTCTCTTC -0.354711335731
CTCTGAAA 0.056809704897
CTCTGAAC -0.0707682297052
CTCTGAAG -0.27717677482
CTCTGACA -0.155553074843
CTCTGACC -0.261212628826
CTCTGAGA -0.0541019693482
CTCTGAGC -0.00557168738987
CTCTGATA 0.192476807077
CTCTGATC 0.0775129011773
CTCTGCAA 0.152375495427
CTCTGCAC -0.1392090694
CTCTGCAG -0.209094467461
CTCTGCCA -0.160381259563
CTCTGCCC -0.109009847953
CTCTGCGA 0.0216841798377
CTCTGCGC -0.216831958885
CTCTGCTA 0.138848784333
CTCTGCTC -0.0872646569856
CTCTGGAA -0.167194690534
CTCTGGAC -0.272406618723
CTCTGGCA -0.207551726503
CTCTGGCC -0.278232357683
CTCTGGGA -0.179280836552
CTCTGGGC -0.307076445569
CTCTGGTA -0.0158657279869
CTCTGGTC -0.0698646304707
CTCTGTAA -0.0523557878295
CTCTGTAC -0.137864172778
CTCTGTCA -0.132524784047
CTCTGTCC -0.146870391046
CTCTGTGA 0.0158010594863

CTCTGTGC -0.248173483667
CTCTGTTA -0.0581513480848
CTCTGTTC -0.172809533192
CTCTTAAA 0.049990762112
CTCTTAAC -0.0184081722621
CTCTTAAG -0.018284645802
CTCTTACA -0.0594038086757
CTCTTACC -0.109261732233
CTCTTAGA -0.0268683211267
CTCTTAGC -0.0296481054057
CTCTTATA 0.0594300854726
CTCTTATC -0.0358829904284
CTCTTCAA -0.186420949543
CTCTTCAC -0.303044083399
CTCTTCAG -0.278313886031
CTCTTCCA -0.217405812603
CTCTTCCC -0.221338506037
CTCTTCGA 0.12929002159
CTCTTCGC -0.0322219221477
CTCTTCTA 0.127301591936
CTCTTCTC -0.187840665658
CTCTTGAA -0.234905718893
CTCTTGAC -0.0924312411976
CTCTTGCA -0.038207111442
CTCTTGCC -0.283082124716
CTCTTGGA -0.125695649572
CTCTTGGC -0.149468511321
CTCTTGTA 0.207276482997
CTCTTGTC 0.0129916863134
CTCTTTAA -0.0467367538759
CTCTTTAC 0.084847047145
CTCTTTCA -0.0858820609768
CTCTTTCC -0.284179604035
CTCTTTGA -0.0689325689326
CTCTTTGC -0.173244543712
CTCTTTTA -0.0915309248643
CTCTTTTC -0.0473379715804
CTGAAAAA 0.197501514565
CTGAAAAC 0.00120766163006
CTGAAAAG 0.0463268564244
CTGAAACA 0.050502183677
CTGAAACC -0.136387179614
CTGAAAGA -0.13616249626
CTGAAAGC -0.240178200048
CTGAAATA 0.161606510091
CTGAAATC 0.409762559944
CTGAACAA 5.27334541033E-5
CTGAACAC 0.115173298464
CTGAACAG -0.137182637183
CTGAACCA -0.27412231733
CTGAACCC -0.204731463793
CTGAACGA 0.116458731419
CTGAACGC -0.0840093190414
CTGAACTA 0.0277426186517

CTGAACTC −0.302425432753
CTGAAGAA 0.168509448698
CTGAAGAC −0.0103450557996
CTGAAGCA −0.322877097948
CTGAAGCC −0.366814965503
CTGAAGGA −0.195012676667
CTGAAGGC −0.318270562262
CTGAAGTA −0.0900847968539
CTGAAGTC −0.355674066725
CTGAATAA 0.00449623176896
CTGAATAC 0.00630278169091
CTGAATCA −0.105496959921
CTGAATCC 0.166818974767
CTGAATGA 0.0327724418634
CTGAATGC −0.0627979760378
CTGAATTA 0.170319628037
CTGAATTC 0.0596855543309
CTGACAAA 0.119357240352
CTGACAAC 0.105775875244
CTGACAAG −0.0381489584991
CTGACACA −0.0958847616476
CTGACACC 0.0812286115316
CTGACAGA −0.0233754883391
CTGACAGC −0.222445762823
CTGACATA −0.0200480184629
CTGACATC −0.123359992501
CTGACCAA −0.0477496887238
CTGACCAC −0.00507723498322
CTGACCAG −0.0111001148766
CTGACCCA −0.390101441008
CTGACCCC −0.18024023577
CTGACCGA −0.257083501771
CTGACCGC −0.321139253416
CTGACCTA −0.141102383797
CTGACCTC −0.253505833807
CTGACGAA 0.134457639614
CTGACGAC −0.0870268519401
CTGACGCA −0.224860308245
CTGACGCC −0.227190566306
CTGACGGA 0.0461308454676
CTGACGGC −0.118237682885
CTGACGTA −0.0167150621696
CTGACGTC −0.193554767615
CTGACTAA −0.121676297625
CTGACTAC −0.266519702321
CTGACTCA −0.27943475348
CTGACTCC −0.186136840922
CTGACTGA 0.111001218951
CTGACTGC −0.0966820815306
CTGACTTA 0.127940665558
CTGACTTC −0.0529467226932
CTGAGAAA 0.234105435588
CTGAGAAC 0.0348288954513
CTGAGAAG −0.0124480146676

CTGAGACA	-0.17540928926
CTGAGACC	-0.157292556025
CTGAGAGA	-0.0111861172467
CTGAGAGC	-0.23593851401
CTGAGATA	0.125917928948
CTGAGATC	0.2707542253
CTGAGCAA	0.0160623546637
CTGAGCAC	-0.263522312902
CTGAGCAG	0.0339737854377
CTGAGCCA	-0.2951529797
CTGAGCCC	-0.236966310253
CTGAGCGA	-0.0201218116799
CTGAGCGC	-0.0944561606638
CTGAGCTA	0.0279869783873
CTGAGCTC	-0.11990153018
CTGAGGAA	0.195417180266
CTGAGGAC	-0.236961467794
CTGAGGCA	-0.240529410305
CTGAGGCC	-0.282025519528
CTGAGGGA	0.0914123489881
CTGAGGGC	-0.175979917785
CTGAGGTA	-0.120726310129
CTGAGGTC	-0.484756911469
CTGAGTAA	-0.0353809770589
CTGAGTAC	-0.299653010345
CTGAGTCA	-0.279171249549
CTGAGTCC	-0.0864908933522
CTGAGTGA	-0.129327545514
CTGAGTGC	-0.170488721286
CTGAGTTA	0.119640797409
CTGAGTTC	-0.233213098281
CTGATAAA	0.125950011169
CTGATAAC	0.164089107538
CTGATAAG	0.136174390342
CTGATACA	0.107875092724
CTGATACC	0.118078436239
CTGATAGA	0.0040302009999
CTGATAGC	-0.11518677299
CTGATATA	0.243754516324
CTGATATC	0.405943665467
CTGATCAA	-0.0721584100029
CTGATCAC	0.0492158825492
CTGATCAG	-0.121621398156
CTGATCCA	-0.0706959161559
CTGATCCC	-0.093756978735
CTGATCGA	-0.140069123449
CTGATCGC	0.266386220932
CTGATCTA	-0.0952481407027
CTGATCTC	0.191776092494
CTGATGAA	0.18857562797
CTGATGAC	-0.216624510509
CTGATGCA	-0.0878762135391
CTGATGCC	-0.143246552337
CTGATGGA	-0.151743697781

CTGATGGC -0.113916111869
CTGATGTA 0.0155139753993
CTGATGTC -0.183093058071
CTGATTAA 0.135517059759
CTGATTAC 0.398705238603
CTGATTCA -0.110209476062
CTGATTCC 0.435991087506
CTGATTGA -0.0611438013923
CTGATTGC 0.401016719199
CTGATTTA 0.164816331483
CTGATTTC 0.403043539407
CTGCAAAA -0.0949510582339
CTGCAAAC 0.0142120975453
CTGCAAAG -0.111924962527
CTGCAACA 0.0266697930451
CTGCAACC -0.0728415389084
CTGCAAGA -0.0867213715937
CTGCAAGC -0.255120241945
CTGCAATA 0.162524783737
CTGCAATC 0.336742958137
CTGCACAA -0.0956179747401
CTGCACAC 0.0571714965654
CTGCACAG 0.0468819356564
CTGCACCA -0.104900233671
CTGCACCC -0.148963513153
CTGCACGA -0.0268330788252
CTGCACGC -0.0494355539015
CTGCACTA 0.0769552491551
CTGCACTC -0.269150933254
CTGCAGAA 0.117821678428
CTGCAGAC 0.0375485678516
CTGCAGCA -0.193414187409
CTGCAGCC 0.0029027888977
CTGCAGGA 0.10985749172
CTGCAGGC -0.231515408872
CTGCAGTA 0.101732808771
CTGCAGTC -0.317238928911
CTGCATAA -0.209195995324
CTGCATAC -0.0111596096436
CTGCATCA -0.173870074019
CTGCATCC -0.17682012887
CTGCATGA -0.145834194863
CTGCATGC -0.0513998847332
CTGCATTA 0.0475544331146
CTGCATTC -0.0454303920352
CTGCCAAA -0.176112283024
CTGCCAAC -0.330915776967
CTGCCAAG -0.138853740231
CTGCCACA -0.191452524902
CTGCCACC -0.162864080681
CTGCCAGA -0.103063418365
CTGCCAGC -0.226451398243
CTGCCATA -0.131223789672
CTGCCATC -0.138422944928

CTGCCCAA -0.205891879255
CTGCCCAC 0.0406512787502
CTGCCCAG -0.138907497002
CTGCCCCA -0.241452832425
CTGCCCCC -0.188707512246
CTGCCCGA 0.036351778776
CTGCCCGC -0.166963880494
CTGCCCTA -0.061063292839
CTGCCCTC -0.342782091461
CTGCCGAA 0.0932702203601
CTGCCGAC -0.00790926062973
CTGCCGCA -0.343806425249
CTGCCGCC -0.249879071267
CTGCCGGA 0.174331219024
CTGCCGGC -0.244868730478
CTGCCGTA -0.185161292101
CTGCCGTC -0.402000909504
CTGCCTAA -0.0796369848611
CTGCCTAC -0.0884279906279
CTGCCTCA -0.139058333078
CTGCCTCC -0.1219794654
CTGCCTGA -0.14483969683
CTGCCTGC -0.318801465663
CTGCCTTA -0.0319120773666
CTGCCTTC -0.251331392252
CTGCGAAA 0.119316838422
CTGCGAAC -0.0272230700514
CTGCGAAG -0.123950361763
CTGCGACA -0.326074132879
CTGCGACC -0.214684873197
CTGCGAGA -0.0222947321406
CTGCGAGC -0.346759019264
CTGCGATA 0.0786447604629
CTGCGATC 0.253982029591
CTGCGCAA -0.16353711886
CTGCGCAC -0.0858371635794
CTGCGCAG -0.280969469457
CTGCGCCA -0.0505012282574
CTGCGCCC 0.0938950241966
CTGCGCGA 0.0582841251651
CTGCGCGC -0.147998715675
CTGCGCTA -0.119449119698
CTGCGCTC -0.0745071217648
CTGCGGAA 0.00431742370605
CTGCGGAC 0.0399094890692
CTGCGGCA -0.163878068079
CTGCGGCC -0.338005098594
CTGCGGGA 0.0367488303176
CTGCGGGC -0.326827298611
CTGCGGTA 0.0783469419833
CTGCGGTC -0.247946480818
CTGCGTAA 0.107839244203
CTGCGTAC -0.252253446621
CTGCGTCA -0.258908907813

CTGCGTCC -0.0241116156478
CTGCGTGA -0.143565502924
CTGCGTGC -0.26505467595
CTGCGTTA -0.0588841030072
CTGCGTTC -0.140344181933
CTGCTAAA -0.091949081407
CTGCTAAC 0.142349277428
CTGCTAAG -0.125955284898
CTGCTACA 0.00977326682714
CTGCTACC -0.184987688845
CTGCTAGA -0.207122663376
CTGCTAGC -0.241821551844
CTGCTATA 0.09621880834
CTGCTATC -0.135813814296
CTGCTCAA -0.155612069084
CTGCTCAC 0.0778624959674
CTGCTCCA -0.147395942027
CTGCTCCC -0.143189906358
CTGCTCGA -0.0320240842286
CTGCTCGC -0.292118385472
CTGCTCTA -0.275627396013
CTGCTCTC -0.305757600076
CTGCTGAA 0.0486989336
CTGCTGAC 0.00103547073244
CTGCTGCA -0.34878265197
CTGCTGCC -0.186490012682
CTGCTGGA -0.0534998828087
CTGCTGGC -0.264149019154
CTGCTGTA 0.0195608831972
CTGCTGTC -0.348279625909
CTGCTTAA -0.132927989411
CTGCTTAC -0.119705933442
CTGCTTCA -0.201198481427
CTGCTTCC -0.221470843641
CTGCTTGA -0.021150631738
CTGCTTGC -0.115846368328
CTGCTTTA 0.0744850946208
CTGCTTTC -0.163973314587
CTGGAAAA -0.0775515178406
CTGGAAAC 0.129289869565
CTGGAAAG -0.0351801225192
CTGGAACA 0.145662191117
CTGGAACC -0.210448695154
CTGGAAGA -0.015730210792
CTGGAAGC -0.149850989863
CTGGAATA 0.127691364423
CTGGAATC 0.324362970154
CTGGACAA 0.149525677911
CTGGACAC -0.00117886481523
CTGGACAG -0.0827508526965
CTGGACCA -0.248321823871
CTGGACCC -0.0753100816868
CTGGACGA -0.0145722761646
CTGGACGC -0.196908257832

CTGGACTA -0.142562308195
CTGGACTC -0.259402205244
CTGGAGAA 0.0872265570827
CTGGAGAC -0.216425999616
CTGGAGCA -0.0922798587686
CTGGAGCC -0.292987407571
CTGGAGGA -0.00750377768287
CTGGAGGC -0.0824833941319
CTGGAGTA -0.126703625692
CTGGAGTC -0.243526200953
CTGGATAA 0.0623023654099
CTGGATAC 0.27753235329
CTGGATCA -0.041165181858
CTGGATCC 0.30568992161
CTGGATGA -0.138715885186
CTGGATGC -0.0772607574692
CTGGATTA 0.327273855317
CTGGATTC 0.43607657244
CTGGCAAA 0.165852572571
CTGGCAAC -0.0361681127295
CTGGCAAG -0.187731716931
CTGGCACA -0.252282944551
CTGGCACC -0.286871037637
CTGGCAGA -0.0387765231418
CTGGCAGC -0.149685590577
CTGGCATA -0.0542843118601
CTGGCATC -0.286073172974
CTGGCCAA -0.102548888237
CTGGCCAC -0.0419985278184
CTGGCCAG -0.157700225711
CTGGCCCA -0.22443442562
CTGGCCCC 0.0240231715345
CTGGCCGA 0.0202917018814
CTGGCCGC -0.322170127261
CTGGCCTA -0.315356437035
CTGGCCTC -0.325235012526
CTGGCGAA 0.1314303284
CTGGCGAC -0.0551413110595
CTGGCGCA -0.0319079365126
CTGGCGCC -0.130055173669
CTGGCGGA 0.0372672948431
CTGGCGGC -0.121331282408
CTGGCGTA 0.0309708546528
CTGGCGTC -0.25893165929
CTGGCTAA -0.139712633695
CTGGCTAC -0.175553363231
CTGGCTCA -0.297826312187
CTGGCTCC -0.0532112435494
CTGGCTGA -0.112149807315
CTGGCTGC -0.201920312644
CTGGCTTA 0.139009364758
CTGGCTTC -0.243293481259
CTGGGAAA 0.0625029297817
CTGGGAAC -0.299575756072

CTGGGAAG -0.0640776373082
CTGGGACA -0.144779026775
CTGGGACC -0.19707960771
CTGGGAGA -0.0273152858076
CTGGGAGC -0.281281268865
CTGGGATA 0.137905122754
CTGGGATC 0.0420421450625
CTGGGCAA -0.273027213416
CTGGGCAC -0.213903566438
CTGGGCCA -0.138518252533
CTGGGCCC -0.134293607272
CTGGGCGA -0.052817389705
CTGGGCGC -0.223739990923
CTGGGCTA -0.226816226999
CTGGGCTC -0.378438181672
CTGGGGAA -0.0376285376285
CTGGGGAC -0.261963829418
CTGGGGCA -0.213054102699
CTGGGGCC -0.0750581345276
CTGGGGGA -0.0804705823972
CTGGGGGC -0.260538905643
CTGGGGTA -0.0830184079885
CTGGGGTC -0.244360922981
CTGGGTAA -0.025800344209
CTGGGTAC -0.220798011829
CTGGGTCA -0.340135592825
CTGGGTCC -0.235640739362
CTGGGTGA -0.101129262286
CTGGGTGC -0.265620133566
CTGGGTTA -0.136921868819
CTGGGTTC -0.167739369659
CTGGTAAA 0.0106296942403
CTGGTAAC 0.0896965759874
CTGGTAAG -0.0462402913224
CTGGTACA -0.0512708048406
CTGGTACC -0.153297704821
CTGGTAGA -0.0395466439465
CTGGTAGC -0.34120835504
CTGGTATA -0.0522905825936
CTGGTATC 0.078359597965
CTGGTCAA -0.0671596480192
CTGGTCAC -0.186510444106
CTGGTCCA -0.147642132491
CTGGTCCC -0.206499581948
CTGGTCGA -0.0726634582963
CTGGTCGC -0.248978742967
CTGGTCTA -0.183257139667
CTGGTCTC -0.240356505354
CTGGTGAA 0.140799106589
CTGGTGAC -0.216414229489
CTGGTGCA -0.180389967673
CTGGTGCC -0.199494134998
CTGGTGGA -0.219371350257
CTGGTGGC -0.253364317923

CTGGTGTA 0.184920768158
CTGGTGTC -0.216911491057
CTGGTTAA -0.162512424198
CTGGTTAC 0.140273570652
CTGGTTCA -0.118042289977
CTGGTTCC -0.0419138146411
CTGGTTGA -0.111036763429
CTGGTTGC -0.202120951604
CTGGTTTA 0.0257461318067
CTGGTTTC -0.220447776223
CTGTAAAA 0.0293095771625
CTGTAAAC -0.0976910826863
CTGTAAAG 0.050199630665
CTGTAACA 0.136353984839
CTGTAACC 0.126052050282
CTGTAAGA -0.00301335392349
CTGTAAGC -0.249716686933
CTGTAATA 0.179148631782
CTGTAATC 0.405199156223
CTGTACAA -0.127763301209
CTGTACAC -0.045785610341
CTGTACAG -0.200186034975
CTGTACCA -0.222349026775
CTGTACCC -0.20851770064
CTGTACGA -0.137981091342
CTGTACGC -0.00530695985241
CTGTACTA 0.0836442503109
CTGTACTC -0.230108318377
CTGTAGAA -0.024493138447
CTGTAGAC -0.0713085856627
CTGTAGCA -0.0235882638019
CTGTAGCC -0.00565316087969
CTGTAGGA 0.0634168594848
CTGTAGGC -0.0259735764526
CTGTAGTA 0.169954672525
CTGTAGTC -0.250058584004
CTGTATAA 0.0632685026624
CTGTATAC 0.101570768523
CTGTATCA 0.0969956862258
CTGTATCC 0.183931185944
CTGTATGA -0.156034501631
CTGTATGC -0.0947870565689
CTGTATTA 0.238423246206
CTGTATTC 0.0826763402521
CTGTCAAA -0.111337187663
CTGTCAAC -0.0811321525905
CTGTCAAG -0.0272966925175
CTGTCACA -0.125149246529
CTGTCACC -0.054275540182
CTGTCAGA -0.0130239693585
CTGTCAGC -0.0451632738031
CTGTCATA 0.0826222783443
CTGTCATC -0.0601002958148
CTGTCCAA -0.157622706956

CTGTCCAC 0.107218789037
CTGTCCCA -0.0341804387206
CTGTCCCC -0.185032749482
CTGTCCGA -0.210935496944
CTGTCCGC -0.213348231032
CTGTCCTA -0.110881061191
CTGTCCTC -0.225973811772
CTGTCGAA 0.134486836055
CTGTCGAC -0.0919456988314
CTGTCGCA -0.098840495788
CTGTCGCC -0.0338469937219
CTGTCGGA -0.0874552238189
CTGTCGGC -0.087072742362
CTGTCGTA 0.0433270886578
CTGTCGTC -0.233909642007
CTGTCTAA 0.00894505959724
CTGTCTAC -0.0629219798107
CTGTCTCA -0.033491886228
CTGTCTCC -0.154233690333
CTGTCTGA -0.0967081012825
CTGTCTGC -0.285501217169
CTGTCTTA 0.239714921533
CTGTCTTC 0.116069721101
CTGTGAAA 0.168664189835
CTGTGAAC -0.0933178899491
CTGTGAAG -0.0653999365946
CTGTGACA -0.0822990817627
CTGTGACC -0.0725248886742
CTGTGAGA 0.0810894651942
CTGTGAGC -0.169944116407
CTGTGATA 0.29215053685
CTGTGATC -0.00360649036882
CTGTGCAA -0.0200243170145
CTGTGCAC -0.0714629763967
CTGTGCCA -0.069941251644
CTGTGCCC -0.201705632466
CTGTGCGA 0.158556381977
CTGTGCGC -0.168663117529
CTGTGCTA -0.0959531449781
CTGTGCTC -0.158494826431
CTGTGGAA -0.0945929179384
CTGTGGAC -0.0922247586909
CTGTGGCA -0.264747316853
CTGTGGCC -0.149662368028
CTGTGGGA -0.245941135614
CTGTGGGC -0.116573572833
CTGTGGTA 0.0757727932965
CTGTGGTC -0.0282190900929
CTGTGTAA 0.04613153098
CTGTGTAC -0.115663195392
CTGTGTCA -0.137785415618
CTGTGTCC -0.0304059035204
CTGTGTGA 0.0739808681317
CTGTGTGC 0.010556179193

CTGTGTTA 0.0572747350919
CTGTGTTC -0.112652277687
CTGTTAAA 0.108966227017
CTGTTAAC 0.18987444745
CTGTTAAG 0.0272891834485
CTGTTACA 0.0305708634118
CTGTTACC -0.312153234275
CTGTTAGA -0.00998921947333
CTGTTAGC -0.0885798121209
CTGTTATA 0.313606016232
CTGTTATC 0.122846215046
CTGTTCAA -0.17004887246
CTGTTCAC -0.223937894308
CTGTTCCA -0.001407998365
CTGTTCCC -0.0284498374649
CTGTTCGA 0.0197973766998
CTGTTCGC -0.0651491712098
CTGTTCTA 0.211587337504
CTGTTCTC -0.106923728136
CTGTTGAA 0.0427684976048
CTGTTGAC -0.0760857276009
CTGTTGCA -0.242420637643
CTGTTGCC -0.235359060388
CTGTTGGA -0.0186749124602
CTGTTGGC -0.162975330294
CTGTTGTA 0.18822288563
CTGTTGTC -0.100926987
CTGTTTAA -0.123637681999
CTGTTTAC 0.130878155953
CTGTTTCA -0.248291154944
CTGTTTCC 0.05764580007
CTGTTTGA -0.0537245234215
CTGTTTGC -0.0229722463498
CTGTTTTA 0.0658138872871
CTGTTTTC -0.0102576829218
CTTAAAAA 0.0650839762633
CTTAAAAC -0.0464207679177
CTTAAAAG -0.0050779909929
CTTAAACA -0.105611290895
CTTAAACC 0.18796595695
CTTAAAGA -0.101251478531
CTTAAAGC -0.192006539678
CTTAAATA 0.0468553953402
CTTAAATC 0.100352418534
CTTAACAA 0.0933426539487
CTTAACAC -0.00537552788195
CTTAACCA -0.0770135287517
CTTAACCC -0.0190776844303
CTTAACGA 0.0286939832394
CTTAACGC -0.149880195107
CTTAACTA 0.0604008671343
CTTAACTC -0.252377589007
CTTAAGAA 0.0144252315643
CTTAAGAC -0.139002865346

CTTAAGCA 0.119967074513
CTTAAGCC -0.0999854054015
CTTAAGGA 0.0757678677098
CTTAAGGC -0.125969489179
CTTAAGTA -0.113689408771
CTTAAGTC -0.162086913622
CTTAATAA 0.0860966548178
CTTAATAC 0.164894929308
CTTAATCA 0.192155646227
CTTAATCC -0.0735487534468
CTTAATGA 0.00941074283045
CTTAATGC 0.0588446968315
CTTAATTA 0.173534156186
CTTAATTC 0.0158877064096
CTTACAAA 0.178419759408
CTTACAAC 0.00130847100544
CTTACAAG 0.0195994892965
CTTACACA -0.0651934805866
CTTACACC 0.139179124028
CTTACAGA -0.0633439494837
CTTACAGC -0.235084685158
CTTACATA -0.202501405079
CTTACATC -0.0383181390189
CTTACCAA 0.158366605111
CTTACCAC -0.223928776205
CTTACCCA -0.27673184781
CTTACCCC -0.0908000741553
CTTACCGA -0.0449863709327
CTTACCGC -0.127662947432
CTTACCTA 0.108816909614
CTTACCTC -0.0842753865398
CTTACGAA 0.117021738591
CTTACGAC -0.0622468623705
CTTACGCA 0.017173092071
CTTACGCC 0.0328189893652
CTTACGGA 0.118778558172
CTTACGGC -0.119726265733
CTTACGTA -0.0142047984351
CTTACGTC -0.165122891419
CTTACTAA -0.0658227164523
CTTACTAC -0.163226591753
CTTACTCA 0.0278611945279
CTTACTCC -0.157880951002
CTTACTGA 0.0201302161267
CTTACTGC -0.264014678434
CTTACTTA -0.0512895815926
CTTACTTC -0.0413171694999
CTTAGAAA 0.149045739955
CTTAGAAC -0.0884466830426
CTTAGAAG -0.102232372006
CTTAGACA -0.103670376436
CTTAGACC -0.209202294627
CTTAGAGA -0.176262604886
CTTAGAGC -0.177491480403

CTTAGATA 0.263038765574
CTTAGATC 0.118317847054
CTTAGCAA 0.0488273979402
CTTAGCAC -0.313626023426
CTTAGCCA -0.0968034981211
CTTAGCCC -0.0367221906754
CTTAGCGA 0.153842097427
CTTAGCGC -0.212757868313
CTTAGCTA -0.00268361643772
CTTAGCTC -0.184243472122
CTTAGGAA 0.00105201620353
CTTAGGAC -0.267652336418
CTTAGGCA 0.0401898556423
CTTAGGCC -0.117583884105
CTTAGGGA -0.325624884173
CTTAGGGC -0.219324103652
CTTAGGTA -0.0948190057101
CTTAGGTC -0.217758286222
CTTAGTAA 0.00751114153878
CTTAGTAC 0.0515963471012
CTTAGTCA -0.0575323808954
CTTAGTCC -0.118927682242
CTTAGTGA -0.108857461853
CTTAGTGC -0.236706285204
CTTAGTTA -0.115406620191
CTTAGTTC -0.201584790969
CTTATAAA 0.154822157179
CTTATAAC 0.152207819691
CTTATAAG 0.0110570867064
CTTATACA -0.0159569395803
CTTATACC -0.044632787057
CTTATAGA -0.0101815288716
CTTATAGC 0.20521485673
CTTATATA 0.0373882323745
CTTATATC 0.0683507744318
CTTATCAA -0.0726608150851
CTTATCAC -0.0964970667788
CTTATCCA 0.0810852174489
CTTATCCC 0.0627859264223
CTTATCGA 0.188709376131
CTTATCGC -0.0437385393832
CTTATCTA 0.111460832966
CTTATCTC -0.0757646666738
CTTATGAA 0.0912910155334
CTTATGAC 0.0359605264118
CTTATGCA 0.0935615180322
CTTATGCC 0.0874864054072
CTTATGGA -0.0778012160318
CTTATGGC -0.108389056508
CTTATGTA -0.104729539965
CTTATGTC -0.271746472893
CTTATTAA -0.0988845323473
CTTATTAC 0.245908442878
CTTATTCA -0.0467781831418

CTTATTCC 0.0563100230786
CTTATTGA 0.153957942527
CTTATTGC 0.0547503426291
CTTATTTA 0.159670689974
CTTATTTC 0.121199712109
CTTCAAAA 0.00671056731663
CTTCAAAC -0.00254981760429
CTTCAAAG 0.0532156230156
CTTCAACA -0.0559790661518
CTTCAACC 0.0479501540108
CTTCAAGA -0.0215890598889
CTTCAAGC -0.21315848242
CTTCAATA 0.107608592513
CTTCAATC -0.281086772566
CTTCACAA -0.0718969657599
CTTCACAC -0.0651435581469
CTTCACCA -0.0165743558309
CTTCACCC -0.208063542468
CTTCACGA -0.0782433081429
CTTCACGC -0.197473570208
CTTCACTA -0.158808221048
CTTCACTC -0.174394085981
CTTCAGAA 0.0701320409042
CTTCAGAC -0.0731363936924
CTTCAGCA 0.0189964513487
CTTCAGCC -0.24214161366
CTTCAGGA -0.0142201265666
CTTCAGGC -0.210762949915
CTTCAGTA -0.107031677576
CTTCAGTC -0.316616087696
CTTCATAA -0.0979246310301
CTTCATAC -0.116588497076
CTTCATCA 0.119176222418
CTTCATCC -0.287727084978
CTTCATGA 0.0968695968696
CTTCATGC -0.150272622385
CTTCATTA 0.206505696956
CTTCATTC 0.0915612582279
CTTCCAAA -0.116693207146
CTTCCAAC -0.197358684436
CTTCCAAG -0.0704248893145
CTTCCACA -0.108292079208
CTTCCACC -0.123450888928
CTTCCAGA -0.156694152387
CTTCCAGC -0.14436784662
CTTCCATA 0.0504978475992
CTTCCATC -0.132364253674
CTTCCCAA -0.0395813354002
CTTCCCAC -0.257692829756
CTTCCCCA -0.322271971821
CTTCCCCC -0.231573593986
CTTCCCGA -0.0870150040214
CTTCCCGC -0.25818590841
CTTCCCTA -0.0755970768216

CTTCCCTC -0.284992516517
CTTCCGAA -0.0141808475142
CTTCCGAC -0.110916701826
CTTCCGCA 0.0441034137939
CTTCCGCC -0.257234420874
CTTCCGGA -0.0155265458296
CTTCCGGC -0.149662279505
CTTCCGTA -0.105156711418
CTTCCGTC -0.239879320949
CTTCCTAA -0.118628156456
CTTCCTAC -0.191694943701
CTTCCTCA -0.149511891864
CTTCCTCC -0.238305414205
CTTCCTGA 0.0249339444101
CTTCCTGC -0.20836041982
CTTCCTTA 0.0332569359795
CTTCCTTC -0.150338047389
CTTCGAAA 0.0213593862908
CTTCGAAC -0.0829250100257
CTTCGAAG -0.0865090192512
CTTCGACA 0.00791363966118
CTTCGACC -0.193901590191
CTTCGAGA -0.160962407639
CTTCGAGC -0.0894159971395
CTTCGATA 0.178896553886
CTTCGATC -0.0763001064052
CTTCGCAA 0.111495793314
CTTCGCAC -0.209213644146
CTTCGCCA -0.0287768051462
CTTCGCCC -0.188219614588
CTTCGCGA 0.0544745847776
CTTCGCGC -0.101044690935
CTTCGCTA -0.043649824544
CTTCGCTC -0.324849296718
CTTCGGAA -0.199176873055
CTTCGGAC -0.187566490041
CTTCGGCA 0.102461101323
CTTCGGCC -0.120950983798
CTTCGGGA -0.124023666355
CTTCGGGC -0.236193785361
CTTCGGTA -0.0252277070459
CTTCGGTC -0.10766894047
CTTCGTAA 0.0501762735123
CTTCGTAC -0.0339382235437
CTTCGTCA -0.142188057047
CTTCGTCC -0.305298189136
CTTCGTGA 0.0179559725014
CTTCGTGC -0.264787603994
CTTCGTTA 0.0671656825033
CTTCGTTC -0.205730480499
CTTCTAAA 0.193901593396
CTTCTAAC -0.00617577717276
CTTCTACA -0.0891812588046
CTTCTACC -0.100968253713

CTTCTAGA -0.0319971434322
CTTCTAGC -0.0699245363133
CTTCTATA -0.0489544024583
CTTCTATC -0.126805348575
CTTCTCAA -0.0245724546178
CTTCTCAC -0.247348789425
CTTCTCCA -0.178965534856
CTTCTCCC -0.205533640421
CTTCTCGA 0.0950610832218
CTTCTCGC -0.199035808175
CTTCTCTA 0.138548060991
CTTCTCTC -0.189326694722
CTTCTGAA -0.125544642036
CTTCTGAC -0.00367171579293
CTTCTGCA 0.147817930994
CTTCTGCC -0.0854291800371
CTTCTGGA -0.136378496319
CTTCTGGC -0.292201162279
CTTCTGTA -0.0969437279862
CTTCTGTC -0.243017159234
CTTCTTAA -0.182862609848
CTTCTTAC -0.0131474817664
CTTCTTCA 0.0299652269349
CTTCTTCC -0.314491759745
CTTCTTGA -0.0697672444084
CTTCTTGC -0.069298482515
CTTCTTTA -0.0894849017905
CTTCTTTC -0.0926992083778
CTTGAAAA 0.0522583056103
CTTGAAAC -0.0262966590711
CTTGAAAG -0.222238847148
CTTGAACA -0.205572962805
CTTGAACC 0.0325518355821
CTTGAAGA 0.0703836513884
CTTGAAGC -0.0524572243532
CTTGAATA -0.0382738110011
CTTGAATC -0.224235060604
CTTGACAA 0.141178660321
CTTGACAC 0.0156966382176
CTTGACCA -0.260844343195
CTTGACCC -0.245694331833
CTTGACGA 0.00582104041517
CTTGACGC -0.00331841631442
CTTGACTA -0.0829769340006
CTTGACTC -0.218025475425
CTTGAGAA 0.158468849645
CTTGAGAC 0.028927103786
CTTGAGCA -0.022803150296
CTTGAGCC -0.207601340752
CTTGAGGA -0.0399816924048
CTTGAGGC -0.0453542623154
CTTGAGTA 0.0521703904294
CTTGAGTC -0.265896941777
CTTGATAA 0.189231890627

CTTGATAC 0.128112961446
CTTGATCA -0.0677034753453
CTTGATCC -0.0247680707722
CTTGATGA 0.0508256382188
CTTGATGC -0.252617776215
CTTGATTA 0.271318876229
CTTGATTC 0.262200827719
CTTGCAAA 0.0859495859496
CTTGCAAC -0.0315873390079
CTTGCAAG 0.0151364805305
CTTGCACA -0.241217659789
CTTGCACC -0.111885439158
CTTGCAGA -0.167689312283
CTTGCAGC -0.131706701191
CTTGCATA 0.00192754738209
CTTGCATC -0.0577624494455
CTTGCCAA 0.118792962255
CTTGCCAC -0.16119147458
CTTGCCCA -0.384458628283
CTTGCCCC -0.161688456672
CTTGCCGA -0.0544174215163
CTTGCCGC -0.0996566985151
CTTGCCTA -0.0372721261511
CTTGCCTC -0.358127797047
CTTGCGAA 0.195332219003
CTTGCGAC 0.0690691063299
CTTGCGCA 0.0312803369269
CTTGCGCC -0.148772646688
CTTGCGGA 0.0220011260187
CTTGCGGC -0.167006720813
CTTGCGTA -0.109949833386
CTTGCGTC -0.151511266608
CTTGCTAA 0.121712621713
CTTGCTAC 0.0407473589292
CTTGCTCA -0.0304467785377
CTTGCTCC -0.32242383457
CTTGCTGA 0.070187514142
CTTGCTGC -0.227853168068
CTTGCTTA 0.0477062546076
CTTGCTTC -0.132382015966
CTTGGAAA 0.183032895154
CTTGGAAC -0.181272403189
CTTGGACA -0.165751053417
CTTGGACC -0.138219486704
CTTGGAGA 0.0741099983506
CTTGGAGC -0.263702453912
CTTGGATA 0.113427443195
CTTGGATC 0.174909068848
CTTGGCAA 0.0638834426713
CTTGGCAC -0.14954866204
CTTGGCCA -0.0459215326203
CTTGGCCC -0.000587520052315
CTTGGCGA 0.00720787136048
CTTGGCGC -0.305516275659

CTTGGCTA -0.0675869824448
CTTGGCTC -0.187616659783
CTTGGGAA 0.0910979850374
CTTGGGAC -0.0180627748755
CTTGGGCA -0.0866457845886
CTTGGGCC -0.245473513726
CTTGGGGA -0.0999537574671
CTTGGGGC -0.254082068958
CTTGGGTA -0.173661254615
CTTGGGTC -0.275688072796
CTTGGTAA 0.143792552883
CTTGGTAC -0.130094068451
CTTGGTCA -0.120773796407
CTTGGTCC -0.019301539014
CTTGGTGA 0.0520798814624
CTTGGTGC -0.202917505162
CTTGGTTA -0.0889875955014
CTTGGTTC -0.113595566034
CTTGTAAA 0.210225323533
CTTGTAAC 0.184246330372
CTTGTACA -0.0666357671
CTTGTACC -0.149061401191
CTTGTAGA 0.0360627139552
CTTGTAGC -0.0883549876438
CTTGTATA 0.0522092215301
CTTGTATC -0.0179035785096
CTTGTCAA -0.0546331102239
CTTGTCAC -0.0133256209215
CTTGTCCA -0.0114599274793
CTTGTCCC -0.0867124103531
CTTGTCGA -0.0658110401941
CTTGTCGC -0.20339263413
CTTGTCTA -0.092108009792
CTTGTCTC -0.199989683411
CTTGTGAA 0.0431669851354
CTTGTGAC 0.0499317533193
CTTGTGCA -0.231894137205
CTTGTGCC -0.254388525114
CTTGTGGA -0.0644197572316
CTTGTGGC -0.154215153545
CTTGTGTA -0.0132170738231
CTTGTGTC -0.214803693685
CTTGTTAA 0.139345123829
CTTGTTAC -0.0467287979946
CTTGTTCA -0.176475696598
CTTGTTCC -0.196234958442
CTTGTTGA -0.0436260519843
CTTGTTGC -0.103583863127
CTTGTTTA -0.0349693072414
CTTGTTTC -0.171186089383
CTTTAAAA 0.131850397437
CTTTAAAC -0.0231885433166
CTTTAAAG -0.161053578073
CTTTAACA 0.0193706102797

CTTTAACC 0.0958754898149
CTTTAAGA -0.165887724287
CTTTAAGC -0.0393851151427
CTTTAATA 0.151742651743
CTTTAATC -0.0709713435058
CTTTACAA 0.0735487553669
CTTTACAC 0.12896578907
CTTTACCA -0.0176371000874
CTTTACCC -0.249677801818
CTTTACGA 0.121227287894
CTTTACGC 0.0340161097737
CTTTACTA 0.127079698985
CTTTACTC -0.228653975547
CTTTAGAA 0.119754311949
CTTTAGAC 0.142766946709
CTTTAGCA -0.024181222309
CTTTAGCC -0.264344008136
CTTTAGGA 0.0108807836325
CTTTAGGC -0.171879092671
CTTTAGTA 0.0581669824094
CTTTAGTC -0.211646933316
CTTTATAA 0.142401991197
CTTTATAC 0.209471567922
CTTTATCA 0.0355189900644
CTTTATCC 0.0956921108436
CTTTATGA 0.0832289004144
CTTTATGC 0.0351225885565
CTTTATTA 0.166556206798
CTTTATTC 0.0566804131522
CTTTCAAA 0.0851010561813
CTTTCAAC -0.0485954273833
CTTTCACA -0.100762432615
CTTTCACC -0.0321958886545
CTTTCAGA -0.124672807344
CTTTCAGC -0.211001884069
CTTTCATA 0.0599814690724
CTTTCATC 0.0305095508857
CTTTCCAA -0.0666729432784
CTTTCCAC -0.0724142975251
CTTTCCCA -0.259689730168
CTTTCCCC -0.104786756494
CTTTCCGA 0.00560470856829
CTTTCCGC 0.0351220217112
CTTTCCTA -0.0767318963818
CTTTCCTC -0.287883651538
CTTTCGAA 0.0471871355188
CTTTCGAC -0.089313238096
CTTTCGCA 0.0686527533067
CTTTCGCC -0.0556754923613
CTTTCGGA -0.0595890920821
CTTTCGGC 0.000932061538122
CTTTCGTA -0.0801560711971
CTTTCGTC -0.282998558359
CTTTCTAA 0.0747620899136

CTTTCTAC 0.0704269249578
CTTTCTCA -0.125550480745
CTTTCTCC -0.150557113433
CTTTCTGA 0.133248906958
CTTTCTGC -0.112703612704
CTTTCTTA -0.0485984201774
CTTTCTTC -0.0307985268207
CTTTGAAA 0.0725700789911
CTTTGAAC -0.0616777864219
CTTTGACA -0.0915044843111
CTTTGACC -0.115394047145
CTTTGAGA 0.0738189980614
CTTTGAGC -0.0544677448264
CTTTGATA 0.0493802268159
CTTTGATC 0.0237447355167
CTTTGCAA 0.139526141763
CTTTGCAC -0.242640146659
CTTTGCCA -0.0157432566565
CTTTGCCC 0.00636409421691
CTTTGCGA 0.073348046879
CTTTGCGC -0.202200053492
CTTTGCTA 0.0771860014284
CTTTGCTC -0.18091291798
CTTTGGAA 0.0529626896376
CTTTGGAC 0.0214057032239
CTTTGGCA -0.0951205912276
CTTTGGCC -0.156166295991
CTTTGGGA 0.0421092140854
CTTTGGGC -0.161246284219
CTTTGGTA -0.111208513682
CTTTGGTC -0.0976934000634
CTTTGTAA -0.0071959011353
CTTTGTAC 0.155613380729
CTTTGTCA 0.102470238834
CTTTGTCC -0.0477368310051
CTTTGTGA -0.0188061575294
CTTTGTGC -0.149627589022
CTTTGTTA 0.00986182784319
CTTTGTTC -0.246050451672
CTTTTAAA 0.20791296541
CTTTTAAC 0.00491155799766
CTTTTACA -0.266460347708
CTTTTACC -0.135280087135
CTTTTAGA -0.100966598208
CTTTTAGC -0.0419055419055
CTTTTATA 0.203393476121
CTTTTATC -0.00576029559774
CTTTTCAA 0.0293859757964
CTTTTCAC 0.00920526204046
CTTTTCCA -0.0627183224017
CTTTTCCC -0.00381678205415
CTTTTCGA 0.113044969818
CTTTTCGC -0.14578732766
CTTTTCTA 0.074080145886

CTTTTCTC -0.0671469252697
CTTTTGAA 0.0655931413507
CTTTTGAC -0.0414888725064
CTTTTGCA -0.0493853199121
CTTTTGCC -0.337510487266
CTTTTGGA -0.0100730020772
CTTTTGGC -0.10460930532
CTTTTGTA 0.142416521204
CTTTTGTC -0.0834071613411
CTTTTTAA -0.00894514069846
CTTTTTAC 0.136402242463
CTTTTTCA -0.0268940715258
CTTTTTCC -0.357751808456
CTTTTTGA 0.0606431975942
CTTTTTGC 0.0938833456202
CTTTTTTA 0.232729975154
CTTTTTTC -0.017019335343
GAAAAAAA 0.134577345022
GAAAAAAC -0.109922653762
GAAAAACA -0.141398601013
GAAAAACC 0.0492633198742
GAAAAAGA -0.0413542504419
GAAAAAGC -0.179170127146
GAAAAATA 0.176626228399
GAAAAATC 0.396703866401
GAAAACAA -0.0392513000169
GAAAACAC -0.128901899478
GAAAACCA -0.252383426267
GAAAACCC -0.172062070989
GAAAACGA 0.0416830288388
GAAAACGC 0.0650636862758
GAAAACTA -0.0399421460028
GAAAACTC -0.24858402934
GAAAAGAA 0.087376961356
GAAAAGAC -0.120922816466
GAAAAGCA -0.171384848012
GAAAAGCC -0.117864303394
GAAAAGGA 0.0776618030262
GAAAAGGC -0.10549751717
GAAAAGTA 0.0355284190857
GAAAAGTC -0.196248961856
GAAAATAA 0.165327036634
GAAAATAC 0.248887250644
GAAAATCA 0.393114434594
GAAAATCC 0.478088071064
GAAAATGA 0.00288029075908
GAAAATGC 0.224439784599
GAAAATTA 0.167651122197
GAAAATTC 0.238315219375
GAAACAAA 0.0546754450632
GAAACAAC -0.0197589197221
GAAACACA -0.120587424789
GAAACACC 0.0469050317535
GAAACAGA 0.00703596158142

GAAACAGC -0.159389844153
GAAACATA 0.148743458172
GAAACATC 0.170814064753
GAAACCAA 0.0941699275033
GAAACCAC -0.240175281128
GAAACCCA -0.321613521255
GAAACCCC -0.364678159494
GAAACCGA 0.0928904110722
GAAACCGC -0.107443632621
GAAACCTA -0.0352459897914
GAAACCTC -0.151304602927
GAAACGAA 0.126519127318
GAAACGAC -0.166509515893
GAAACGCA -0.0822544842895
GAAACGCC -0.228599177781
GAAACGGA -0.0691379020897
GAAACGGC -0.133166265539
GAAACGTA 0.0650343423137
GAAACGTC -0.0535949172313
GAAACTAA 0.091308219528
GAAACTAC 0.0134721498358
GAAACTCA -0.26781714199
GAAACTCC -0.24758811709
GAAACTGA 0.0251169682416
GAAACTGC -0.120435633622
GAAACTTA -0.130689191576
GAAACTTC -0.034435012372
GAAAGAAA 0.125038867762
GAAAGAAC -0.201022110454
GAAAGACA -0.177120874405
GAAAGACC -0.0899633039158
GAAAGAGA -0.0931799568163
GAAAGAGC -0.257719610194
GAAAGATA 0.0807215900382
GAAAGATC 0.19598621928
GAAAGCAA 0.161959480141
GAAAGCAC -0.309318552912
GAAAGCCA -0.227035124253
GAAAGCCC -0.312809951721
GAAAGCGA -0.109461512972
GAAAGCGC 0.0477354510485
GAAAGCTA -0.029526771951
GAAAGCTC -0.209008210176
GAAAGGAA -0.0752096240966
GAAAGGAC -0.178901927705
GAAAGGCA -0.183749644257
GAAAGGCC -0.198769346196
GAAAGGGA -0.0631752683439
GAAAGGGC -0.265007915606
GAAAGGTA 0.00736261249754
GAAAGGTC -0.189355888265
GAAAGTAA 0.0428596454092
GAAAGTAC -0.0977416552922
GAAAGTCA -0.263741761559

GAAAGTCC -0.415613458303
GAAAGTGA 0.105426363002
GAAAGTGC 0.0212469799931
GAAAGTTA -0.238170554223
GAAAGTTC 0.0684999598549
GAAATAAA 0.24353682225
GAAATAAC 0.0684488660832
GAAATACA -0.10582069673
GAAATACC 0.0522216294017
GAAATAGA 0.10070614808
GAAATAGC 0.140915892353
GAAATATA 0.334021152821
GAAATATC 0.421718502515
GAAATCAA 0.423005650278
GAAATCAC 0.4382904024
GAAATCCA 0.471889244617
GAAATCCC 0.479469827955
GAAATCGA 0.409146060661
GAAATCGC 0.439058738864
GAAATCTA 0.481165738741
GAAATCTC 0.487962307395
GAAATGAA -0.0379969781684
GAAATGAC -0.203029576141
GAAATGCA -0.0462542432239
GAAATGCC -0.0192138533868
GAAATGGA 0.0158040866743
GAAATGGC -0.27013334423
GAAATGTA 0.118925133026
GAAATGTC 0.0652236258297
GAAATTAA 0.175278612028
GAAATTAC 0.28793749042
GAAATTCA -0.150994308436
GAAATTCC 0.175388130187
GAAATTGA 0.181541045177
GAAATTGC 0.265082516441
GAAATTTA 0.235534432504
GAAATTTC 0.402111086304
GAACAAAA 0.0310584439757
GAACAAAC -0.158693910081
GAACAACA 0.0705407910776
GAACAACC -0.25342335808
GAACAAGA -0.0370549612974
GAACAAGC -0.0371239007603
GAACAATA 0.189080264838
GAACAATC 0.267721874703
GAACACAA 0.0277650854361
GAACACAC -0.181385075428
GAACACCA -0.228424392159
GAACACCC -0.285564625738
GAACACGA -0.0528699643532
GAACACGC 0.0965363426558
GAACACTA 0.064371583104
GAACACTC -0.233268204937
GAACAGAA 0.136136434969

GAACAGAC -0.0508869751294
GAACAGCA -0.249653303337
GAACAGCC -0.208594191512
GAACAGGA 0.224231952751
GAACAGGC -0.279719624365
GAACAGTA 0.031335743457
GAACAGTC -0.084479796601
GAACATAA 0.0149901826967
GAACATAC -0.0705685276965
GAACATCA -0.11785653289
GAACATCC -0.0378146741783
GAACATGA 0.113060525597
GAACATGC -0.105793750538
GAACATTA -0.0627804112653
GAACATTC -0.145731845288
GAACCAAA 0.0448450033941
GAACCAAC -0.134502706295
GAACCACA -0.261706543521
GAACCACC -0.186172829055
GAACCAGA 0.150783014419
GAACCAGC -0.290126060265
GAACCATA 0.0682926651241
GAACCATC 0.0452917089407
GAACCCAA -0.0565192003095
GAACCCAC -0.244295902389
GAACCCCA -0.325222379342
GAACCCCC -0.327483566911
GAACCCGA -0.0340437800632
GAACCCGC -0.225495061427
GAACCCTA -0.28478021986
GAACCCTC -0.361778736844
GAACCGAA 0.0589832256499
GAACCGAC -0.173404840685
GAACCGCA -0.139129451792
GAACCGCC -0.240778953175
GAACCGGA -0.0064061110957
GAACCGGC -0.395586861447
GAACCGTA -0.172730931333
GAACCGTC -0.238366827937
GAACCTAA -0.079726413731
GAACCTAC -0.295254908122
GAACCTCA -0.292975522095
GAACCTCC -0.261343620531
GAACCTGA 0.080073429049
GAACCTGC 0.0738099996473
GAACCTTA -0.215684977019
GAACCTTC -0.0956066259097
GAACGAAA 0.0498697108822
GAACGAAC -0.299083459212
GAACGACA -0.199552675555
GAACGACC -0.188180713028
GAACGAGA 0.0909374247279
GAACGAGC -0.0253582412642
GAACGATA 0.0819759153092

GAACGATC 0.0732040580525
GAACGCAA 0.0527510982056
GAACGCAC -0.280063129747
GAACGCCA -0.23366049553
GAACGCCC -0.34491355146
GAACGCGA 0.0073420527966
GAACGCGC -0.204759916919
GAACGCTA 0.0087757202397
GAACGCTC -0.115831860203
GAACGGAA 0.123298759881
GAACGGAC -0.0867872040672
GAACGGCA -0.0974867278855
GAACGGCC -0.10374091993
GAACGGGA -0.232209243093
GAACGGGC -0.33318174928
GAACGGTA -0.043404159033
GAACGGTC 0.013820297528
GAACGTAA 0.221636236788
GAACGTAC -0.271911053219
GAACGTCA -0.27117452941
GAACGTCC -0.304784984247
GAACGTGA 0.0103985262368
GAACGTGC 0.0869202535869
GAACGTTA -0.0142663324482
GAACGTTC -0.221027464274
GAACTAAA 0.059565848923
GAACTAAC -0.012939418363
GAACTACA -0.232200413024
GAACTACC -0.0117687609436
GAACTAGA -0.168782816996
GAACTAGC -0.0345819688085
GAACTATA 0.0221564491289
GAACTATC -0.0957731581429
GAACTCAA -0.0937020563493
GAACTCAC -0.24513363382
GAACTCCA -0.269222605393
GAACTCCC -0.43848351036
GAACTCGA -0.206039982427
GAACTCGC -0.0264325636456
GAACTCTA -0.110144303409
GAACTCTC -0.33371238749
GAACTGAA -0.0419675874221
GAACTGAC -0.22896808891
GAACTGCA -0.170704992082
GAACTGCC -0.277607865511
GAACTGGA 0.0477827133534
GAACTGGC -0.165668031262
GAACTGTA -0.0482780918516
GAACTGTC -0.148316877923
GAACTTAA -0.0471510550221
GAACTTAC -0.0157522098056
GAACTTCA -0.258383502266
GAACTTCC -0.361156219315
GAACTTGA 0.168828608223

GAACTTGC 0.011724560776
GAACTTTA 0.0670693342482
GAAGAAAA 0.113433282483
GAAGAAAC -0.135454966086
GAAGAACA -0.0684342678627
GAAGAACC -0.22769614533
GAAGAAGA 0.116897596303
GAAGAAGC -0.181762385111
GAAGAATA 0.096233659117
GAAGAATC 0.437358860461
GAAGACAA -0.106051707928
GAAGACAC -0.177950376491
GAAGACCA -0.268288110971
GAAGACCC -0.340907267037
GAAGACGA 0.0208147224497
GAAGACGC -0.241313301739
GAAGACTA -0.041535586812
GAAGACTC -0.283378662986
GAAGAGAA -0.0490824612924
GAAGAGAC -0.203444568499
GAAGAGCA -0.0962518992822
GAAGAGCC -0.116240967191
GAAGAGGA -0.258748748997
GAAGAGGC -0.349962375861
GAAGAGTA -0.0859258556382
GAAGAGTC -0.123755846103
GAAGATAA -0.181163481396
GAAGATAC 0.200970051751
GAAGATCA 0.264279614315
GAAGATCC 0.363193910217
GAAGATGA 0.0196656711808
GAAGATGC 0.0444771282381
GAAGATTA 0.3503740794
GAAGATTC 0.454036043006
GAAGCAAA -0.0815405502069
GAAGCAAC -0.404504027802
GAAGCACA -0.178726295371
GAAGCACC -0.151043939923
GAAGCAGA 0.12061049758
GAAGCAGC -0.247860796719
GAAGCATA 0.0265563527806
GAAGCATC -0.10227394926
GAAGCCAA -0.277945923298
GAAGCCAC -0.27632318279
GAAGCCCA -0.306825605531
GAAGCCCC -0.415616110743
GAAGCCGA -0.0751073334161
GAAGCCGC -0.282537753192
GAAGCCTA -0.29187249089
GAAGCCTC -0.31303349732
GAAGCGAA -0.0625641377478
GAAGCGAC -0.230597940702
GAAGCGCA -0.00953760452594
GAAGCGCC -0.244243727168

GAAGCGGA -0.122233526611
GAAGCGGC -0.34399552458
GAAGCGTA -0.101836918674
GAAGCGTC -0.153176089707
GAAGCTAA -0.359146605729
GAAGCTAC -0.376337080783
GAAGCTCA -0.303707930522
GAAGCTCC -0.265606369083
GAAGCTGA -0.0627626636927
GAAGCTGC -0.170221071633
GAAGCTTA -0.0920246159954
GAAGCTTC 0.0280802868806
GAAGGAAA 0.0192708842725
GAAGGAAC -0.206400409776
GAAGGACA -0.0297359591298
GAAGGACC -0.270883086055
GAAGGAGA -0.0880526813506
GAAGGAGC -0.227169381236
GAAGGATA 0.160939399955
GAAGGATC 0.225350695048
GAAGGCAA -0.21708224786
GAAGGCAC -0.380859068171
GAAGGCCA -0.216663237447
GAAGGCCC -0.387150216884
GAAGGCGA 0.0252015273099
GAAGGCGC -0.332363050987
GAAGGCTA -0.126034677289
GAAGGCTC -0.421525340882
GAAGGGAA 0.0994996226735
GAAGGGAC -0.351249268591
GAAGGGCA -0.261343954147
GAAGGGCC -0.105604143407
GAAGGGGA -0.030971743093
GAAGGGGC -0.48277933465
GAAGGGTA -0.120144285216
GAAGGGTC -0.310685980215
GAAGGTAA -0.0243328469594
GAAGGTAC -0.137402960322
GAAGGTCA -0.24233766377
GAAGGTCC -0.327812988366
GAAGGTGA 0.0374989314383
GAAGGTGC -0.235706394598
GAAGGTTA -0.0518656329693
GAAGTAAA 0.0799380647865
GAAGTAAC -0.1047478022
GAAGTACA -0.145527622201
GAAGTACC -0.2636725049
GAAGTAGA -0.115849466415
GAAGTAGC -0.112623330594
GAAGTATA 0.00135690459333
GAAGTATC 0.142935578331
GAAGTCAA -0.239868340598
GAAGTCAC -0.294420424881
GAAGTCCA -0.243721768573

GAAGTCCC -0.428644494934
GAAGTCGA -0.132541947581
GAAGTCGC -0.173148457577
GAAGTCTA 0.0121420698817
GAAGTCTC -0.200320351438
GAAGTGAA 0.065688342591
GAAGTGAC -0.0116162994951
GAAGTGCA 0.0532915835946
GAAGTGCC -0.160017741495
GAAGTGGA 0.0662656021241
GAAGTGGC -0.255836165621
GAAGTGTA 0.0496653357956
GAAGTGTC -0.0167537741875
GAAGTTAA -0.0703996007026
GAAGTTAC -0.0951611184659
GAAGTTCA -0.241082490701
GAAGTTCC -0.283598696604
GAAGTTGA -0.0243480394996
GAAGTTGC -0.0801311665886
GAAGTTTA -0.0906379227043
GAATAAAA 0.167283198178
GAATAAAC -0.11139975037
GAATAACA -0.159838105735
GAATAACC 0.0901374422458
GAATAAGA 0.0106566621718
GAATAAGC -0.147960769719
GAATAATA 0.208923162267
GAATAATC 0.272991832296
GAATACAA 0.0328949001117
GAATACAC -0.0735407253856
GAATACCA -0.0209451876119
GAATACCC -0.0646086858208
GAATACGA 0.13777727495
GAATACGC 0.210210725313
GAATACTA 0.154296169448
GAATACTC 0.0117337161623
GAATAGAA -0.0392918403247
GAATAGAC -0.0257649452813
GAATAGCA -0.073064823398
GAATAGCC 0.145011402587
GAATAGGA -0.0925488541696
GAATAGGC -0.406645461111
GAATAGTA -0.0102078056685
GAATAGTC -0.242753173717
GAATATAA 0.178562860381
GAATATAC 0.241877185171
GAATATCA 0.305157551294
GAATATCC 0.445988535127
GAATATGA 0.21780596023
GAATATGC 0.207244434517
GAATATTA 0.204464865733
GAATATTC 0.264355354884
GAATCAAA 0.326498674984
GAATCAAC 0.298195275083

GAATCACA 0.358225002372
GAATCACC 0.249841974263
GAATCAGA 0.241504589989
GAATCAGC 0.291510518783
GAATCATA 0.293471157108
GAATCATC 0.223171025452
GAATCCAA 0.440028182452
GAATCCAC 0.411756676861
GAATCCCA 0.354540490904
GAATCCCC 0.327149463513
GAATCCGA 0.457428669445
GAATCCGC 0.445723816267
GAATCCTA 0.408606380882
GAATCCTC 0.379807011525
GAATCGAA 0.374156264734
GAATCGAC 0.207261884776
GAATCGCA 0.249376504454
GAATCGCC 0.315142208852
GAATCGGA 0.362316862317
GAATCGGC 0.14323117012
GAATCGTA 0.355174733963
GAATCGTC 0.319425143311
GAATCTAA 0.456307526113
GAATCTAC 0.445758851996
GAATCTCA 0.460765822646
GAATCTCC 0.459058231786
GAATCTGA 0.44403626198
GAATCTGC 0.431356247673
GAATCTTA 0.425134486106
GAATGAAA 0.0676309918734
GAATGAAC -0.0812566689
GAATGACA -0.134999986217
GAATGACC -0.00827208163325
GAATGAGA -0.190703876657
GAATGAGC -0.192154770645
GAATGATA 0.146874885951
GAATGATC 0.0665748393021
GAATGCAA 0.167513243271
GAATGCAC -0.241857807899
GAATGCCA -0.210258030711
GAATGCCC -0.339014894489
GAATGCGA -0.112708241191
GAATGCGC -0.105713878068
GAATGCTA 0.0809583688372
GAATGCTC -0.248108269035
GAATGGAA -0.0736303069403
GAATGGAC -0.346034844024
GAATGGCA 0.0622869572199
GAATGGCC -0.152409387776
GAATGGGA -0.018437367254
GAATGGGC -0.17405010296
GAATGGTA 0.0184532525874
GAATGGTC -0.223291653957
GAATGTAA 0.265834705229

GAATGTAC -0.088296285266
GAATGTCA -0.262014638382
GAATGTCC -0.288109945176
GAATGTGA 0.106566279571
GAATGTGC 0.0254135105664
GAATGTTA 0.181188075127
GAATTAAA 0.0208280110655
GAATTAAC -0.106120141733
GAATTACA 0.0374437056123
GAATTACC 0.125315304841
GAATTAGA 0.042812785237
GAATTAGC 0.0524780979326
GAATTATA 0.0813677196814
GAATTATC 0.10359992117
GAATTCAA 0.0167674561614
GAATTCAC 0.0470610132625
GAATTCCA -0.0197235803296
GAATTCCC 0.143540652394
GAATTCGA 0.0599428629732
GAATTCGC 0.188596600074
GAATTCTA 0.216408789736
GAATTCTC 0.13772588015
GAATTGAA 0.017611275187
GAATTGAC -0.0486167369501
GAATTGCA 0.142113187568
GAATTGCC 0.126008189602
GAATTGGA 0.020906841456
GAATTGGC -0.205570028532
GAATTGTA 0.129919175374
GAATTGTC 0.0597663779482
GAATTTAA 0.140212050525
GAATTTAC 0.159788471785
GAATTTCA 0.082674831938
GAATTTCC 0.294019109071
GAATTTGA 0.21962657752
GAATTTGC 0.248448172691
GAATTTTA 0.199813872689
GACAAAAA 0.195601938026
GACAAAAC 0.0450844142099
GACAAACA 0.0782194550484
GACAAACC 0.10916288189
GACAAAGA 0.226867294877
GACAAAGC -0.1979669166
GACAAATA 0.123462259951
GACAAATC 0.294476015584
GACAACAA -0.108488865207
GACAACAC 0.0420599663024
GACAACCA -0.00993141902233
GACAACCC -0.140237833307
GACAACGA 0.0154017679278
GACAACGC -0.142479410848
GACAACTA -0.0586515990903
GACAACTC -0.227522269919
GACAAGAA 0.121083893811

GACAAGAC -0.228274983665
GACAAGCA -0.0630796405918
GACAAGCC -0.0500497044737
GACAAGGA -0.0289796788259
GACAAGGC -0.155239584109
GACAAGTA 0.159086530335
GACAAGTC -0.30871946698
GACAATAA -0.0143843565469
GACAATAC -0.0380735538626
GACAATCA 0.0696619782049
GACAATCC 0.379968122392
GACAATGA -0.0368896907382
GACAATGC 0.056537253507
GACAATTA 0.175000620394
GACACAAA 0.16372708797
GACACAAC 0.0504974343628
GACACACA -0.179576051369
GACACACC -0.0609687379108
GACACAGA 0.0717874791063
GACACAGC -0.0966061584394
GACACATA 0.0592865592866
GACACATC -0.0543134091954
GACACCAA -0.167202288433
GACACCAC -0.133158158195
GACACCCA -0.120801004358
GACACCCC -0.0647169758993
GACACCGA 0.0445390293875
GACACCGC -0.0681973114332
GACACCTA -0.102422920435
GACACCTC -0.139987389641
GACACGAA -0.031860312182
GACACGAC -0.0926491145355
GACACGCA -0.232110147982
GACACGCC -0.1641414252
GACACGGA -0.197853067432
GACACGGC -0.248727049308
GACACGTA -0.0102516003299
GACACGTC -0.284548408702
GACACTAA 0.0458543841389
GACACTAC -0.200131510384
GACACTCA -0.210231327503
GACACTCC -0.140068080259
GACACTGA 0.0209837937111
GACACTGC -0.0614197607448
GACACTTA 0.0366744154623
GACAGAAA -0.0628752655051
GACAGAAC -0.0611963523777
GACAGACA -0.0559195559196
GACAGACC -0.0532760264085
GACAGAGA 0.213331824851
GACAGAGC -0.230324666891
GACAGATA 0.217981487456
GACAGATC 0.126860725678
GACAGCAA 0.129558476822

GACAGCAC -0.231119657059
GACAGCCA -0.271659863489
GACAGCCC -0.200936734316
GACAGCGA -0.0639876499055
GACAGCGC -0.134783619347
GACAGCTA -0.0590300942315
GACAGCTC -0.0359438199459
GACAGGAA -0.0700500790376
GACAGGAC -0.21226339031
GACAGGCA 0.0644005648382
GACAGGCC 0.0968584629534
GACAGGGA -0.122636965156
GACAGGGC -0.244873315852
GACAGGTA 0.0510346488763
GACAGGTC -0.348147548327
GACAGTAA 0.104023269563
GACAGTAC -0.254051825419
GACAGTCA -0.205348164127
GACAGTCC -0.0808262539533
GACAGTGA 0.0337706852858
GACAGTGC 0.100899250381
GACAGTTA -0.0220079147623
GACATAAA -0.145078238228
GACATAAC -0.121548433246
GACATACA -0.196211099807
GACATACC 0.0433348563387
GACATAGA 0.0537382393086
GACATAGC -0.140527335942
GACATATA -0.0582009153084
GACATATC 0.143823857872
GACATCAA -0.0749523628312
GACATCAC -0.146769850478
GACATCCA 0.0315051495223
GACATCCC 0.025073282649
GACATCGA -0.0640394268424
GACATCGC -0.0659576413425
GACATCTA 0.173312263235
GACATCTC 0.18174164532
GACATGAA -0.049637792062
GACATGAC -0.214557812803
GACATGCA -0.0588753530945
GACATGCC -0.0571038590396
GACATGGA 0.0313270512325
GACATGGC -0.218295610851
GACATGTA 0.162069783282
GACATGTC -0.0994765696708
GACATTAA -0.00997887839993
GACATTAC 0.0709320233863
GACATTCA -0.131256342708
GACATTCC -0.149253772495
GACATTGA -0.139394064701
GACATTGC 4.82576240153E-5
GACATTTA 0.168180577271
GACCAAAA -0.103005914409

GACCAAAC -0.148354496698
GACCAACA -0.0255241800115
GACCAACC -0.131542374984
GACCAAGA -0.0608824843056
GACCAAGC -0.23065130689
GACCAATA 0.118767472245
GACCAATC 0.0641454126303
GACCACAA -0.138981371065
GACCACAC -0.236897820783
GACCACCA -0.240769281289
GACCACCC -0.235522477788
GACCACGA -0.254787798591
GACCACGC -0.277831531882
GACCACTA 0.0695802281702
GACCACTC -0.258601264705
GACCAGAA -0.0358100973005
GACCAGAC -0.363685636461
GACCAGCA -0.157215708654
GACCAGCC -0.0248941831768
GACCAGGA -0.137743394442
GACCAGGC -0.279975964653
GACCAGTA -0.0935263611909
GACCAGTC -0.384049160939
GACCATAA -0.236473280128
GACCATAC -0.101790042269
GACCATCA -0.0735294523173
GACCATCC -0.159378124156
GACCATGA -0.225123421568
GACCATGC 0.00771005014489
GACCATTA 0.131482522281
GACCCAAA -0.150082589477
GACCCAAC -0.195175224148
GACCCACA -0.284791431644
GACCCACC 0.0218790829398
GACCCAGA -0.0105260468749
GACCCAGC -0.353327088704
GACCCATA -0.147784631177
GACCCATC -0.185462685004
GACCCCAA -0.166066982638
GACCCCAC -0.283897334269
GACCCCCA -0.364587847193
GACCCCCC -0.357033364627
GACCCCGA -0.0573226371142
GACCCCGC -0.150336770361
GACCCCTA -0.20614504268
GACCCCTC -0.373864153127
GACCCGAA -0.0841038186204
GACCCGAC -0.268631701025
GACCCGCA -0.203643208195
GACCCGCC -0.337024082205
GACCCGGA -0.123925761726
GACCCGGC -0.263477807825
GACCCGTA -0.0584814914958
GACCCGTC -0.234671881934

GACCCTAA -0.266853994072
GACCCTAC -0.0395501790205
GACCCTCA -0.384765598084
GACCCTCC -0.140514503362
GACCCTGA -0.262983084297
GACCCTGC -0.301255532354
GACCCTTA -0.0718336379702
GACCGAAA -0.0736241738426
GACCGAAC -0.119467952801
GACCGACA -0.0281519382787
GACCGACC -0.190887891042
GACCGAGA 0.0403748556816
GACCGAGC -0.390451660203
GACCGATA 0.138845457027
GACCGATC -0.236112144895
GACCGCAA -0.27677176097
GACCGCAC -0.188085392714
GACCGCCA -0.196960674854
GACCGCCC -0.289835499579
GACCGCGA -0.194460296587
GACCGCGC -0.0884717500806
GACCGCTA -0.141012581379
GACCGCTC -0.140275192933
GACCGGAA -0.110016515436
GACCGGAC -0.164429912189
GACCGGCA -0.180451462524
GACCGGCC -0.259100375209
GACCGGGA 0.0854105384554
GACCGGGC -0.359601689328
GACCGGTA 0.065472288928
GACCGGTC -0.194956359738
GACCGTAA -0.023077988044
GACCGTAC -0.280688300525
GACCGTCA -0.358004290753
GACCGTCC -0.0307167808434
GACCGTGA 0.0184660189923
GACCGTGC -0.164214652542
GACCGTTA -0.0710286198899
GACCTAAA -0.160405448996
GACCTAAC -0.111299949
GACCTACA 0.0328277482975
GACCTACC 0.000293652960104
GACCTAGA 0.0605556082714
GACCTAGC -0.305673389397
GACCTATA 0.0338963380364
GACCTATC -0.358435254327
GACCTCAA -0.256458810526
GACCTCAC -0.328235702726
GACCTCCA -0.33065967657
GACCTCCC -0.329386224158
GACCTCGA -0.181962675313
GACCTCGC -0.284461204698
GACCTCTA -0.148162394223
GACCTCTC -0.169936181064

GACCTGAA -0.287407977024
GACCTGAC -0.30396378845
GACCTGCA -0.0982709799802
GACCTGCC -0.196727161278
GACCTGGA -0.035212160817
GACCTGGC -0.480024239343
GACCTGTA -0.0210610059095
GACCTTAA -0.0563185718634
GACCTTAC -0.293702492532
GACCTTCA -0.131321152165
GACCTTCC -0.214040615195
GACCTTGA -0.25759075412
GACCTTGC -0.0556725035218
GACCTTTA -0.0353917635218
GACGAAAA 0.0383041443648
GACGAAAC -0.0222597215674
GACGAACA -0.14055660723
GACGAACC -0.237779217993
GACGAAGA -0.0124307281592
GACGAAGC -0.215130491128
GACGAATA 0.183788471667
GACGAATC 0.236931308074
GACGACAA -0.0683336504076
GACGACAC -0.210439452066
GACGACCA -0.285686250192
GACGACCC -0.248948876199
GACGACGA 0.0243432625563
GACGACGC -0.203575175884
GACGACTA -0.175775052074
GACGACTC -0.0644764228315
GACGAGAA 0.0363587130264
GACGAGAC -0.157126216823
GACGAGCA -0.28850601327
GACGAGCC -0.287295010792
GACGAGGA -0.179768728753
GACGAGGC -0.192008810174
GACGAGTA 0.183658865477
GACGAGTC -0.328136498565
GACGATAA 0.00742753773057
GACGATAC -0.00112042605527
GACGATCA -0.0159098705868
GACGATCC 0.189149204301
GACGATGA -0.0175244337715
GACGATGC 0.0714225235944
GACGATTA 0.270378391936
GACGCAAA 0.0239111552302
GACGCAAC -0.0418114939646
GACGCACA -0.193906296297
GACGCACC -0.33778037523
GACGCAGA 0.0781759721154
GACGCAGC -0.276913300483
GACGCATA -0.00395392801617
GACGCATC -0.0500120970744
GACGCCAA -0.333726132295

GACGCCAC 0.0105196398781
GACGCCCA -0.306693181411
GACGCCCC -0.300360269861
GACGCCGA -0.0177576731261
GACGCCGC 0.00375958508078
GACGCCTA -0.202981090717
GACGCCTC -0.352851446405
GACGCGAA 0.0339928337137
GACGCGAC -0.120888111082
GACGCGCA -0.175918182681
GACGCGCC -0.296698283537
GACGCGGA -0.040546911834
GACGCGGC -0.252041946551
GACGCGTA 0.065134432395
GACGCGTC -0.184151198203
GACGCTAA -0.169775114414
GACGCTAC -0.259704824507
GACGCTCA -0.276522261638
GACGCTCC -0.121276185833
GACGCTGA 0.152295340869
GACGCTGC -0.131938067861
GACGCTTA 0.056537253507
GACGGAAA 0.129447629448
GACGGAAC -0.032353289929
GACGGACA -0.338465142558
GACGGACC -0.271816066126
GACGGAGA 0.101316759487
GACGGAGC -0.323370346427
GACGGATA 0.172582352211
GACGGATC 0.0308686671095
GACGGCAA -0.0486950568499
GACGGCAC -0.234010650081
GACGGCCA -0.230793406612
GACGGCCC -0.359496173516
GACGGCGA -0.0602912347847
GACGGCGC -0.199050621365
GACGGCTA -0.102902861847
GACGGCTC -0.0313407280578
GACGGGAA -0.0374626021977
GACGGGAC -0.0546498258848
GACGGGCA -0.211711724861
GACGGGCC -0.399137570051
GACGGGGA -0.0341965169397
GACGGGGC -0.272083447027
GACGGGTA -0.105477657829
GACGGTAA -0.0871676743615
GACGGTAC -0.130331328872
GACGGTCA -0.251397728318
GACGGTCC -0.419581822559
GACGGTGA -0.014402436906
GACGGTGC -0.131192248132
GACGGTTA 0.0103243877019
GACGTAAA -0.0872096069267
GACGTAAC 0.0606227600855

GACGTACA -0.184360291502
GACGTACC -0.156756421392
GACGTAGA 0.105190659008
GACGTAGC -0.0877143186531
GACGTATA 0.105512288052
GACGTATC 0.213647531829
GACGTCAA -0.0499358440053
GACGTCAC -0.225532526716
GACGTCCA -0.218045238988
GACGTCCC -0.232735335706
GACGTCGA -0.137739393282
GACGTCGC -0.226462196341
GACGTCTA 0.0291848322151
GACGTCTC -0.121560600422
GACGTGAA -0.0858641010156
GACGTGAC -0.0523789827798
GACGTGCA -0.218347599553
GACGTGCC -0.0930926175398
GACGTGGA 0.0517849974088
GACGTGGC -0.226845957469
GACGTGTA -0.0972915461073
GACGTTAA -0.0473077231885
GACGTTAC -0.0311813190601
GACGTTCA -0.27306296486
GACGTTCC -0.207230231
GACGTTGA -0.00903410874214
GACGTTGC -0.0114603329854
GACGTTTA 0.0901521356067
GACTAAAA 0.107275753086
GACTAAAC -0.0275534112392
GACTAACA -0.0329815012422
GACTAACC -0.13330355592
GACTAAGA -0.0163950099034
GACTAAGC -0.063417635236
GACTAATA 0.0883955580925
GACTAATC -0.0866224351073
GACTACAA 0.0310409261111
GACTACAC 0.00482228278561
GACTACCA -0.15966438673
GACTACCC -0.0850094076192
GACTACGA -0.0268436480558
GACTACGC 0.0464683255111
GACTACTA 0.0443156655278
GACTACTC -0.300519270045
GACTAGAA 0.168894790107
GACTAGAC -0.127641730473
GACTAGCA -0.217168355991
GACTAGCC -0.128691320039
GACTAGGA -0.109698719698
GACTAGGC -0.0658373117751
GACTAGTA -0.0460814562981
GACTAGTC -0.264303960715
GACTATAA -0.0112026627178
GACTATAC -0.0761946818683

GACTATCA −0.123463989038
GACTATCC 0.0955983531741
GACTATGA −0.124329696809
GACTATGC 0.0905082370459
GACTATTA 0.108475726809
GACTCAAA 0.00666189791464
GACTCAAC −0.123124091858
GACTCACA −0.162965168697
GACTCACC −0.0909406714474
GACTCAGA 0.0937866240897
GACTCAGC −0.249010097502
GACTCATA −0.0904582268219
GACTCATC −0.198406002101
GACTCCAA −0.0793120944636
GACTCCAC −0.200445216337
GACTCCCA −0.242938360132
GACTCCCC −0.20579771267
GACTCCGA −0.0493251161572
GACTCCGC −0.153423331762
GACTCCTA −0.195334187928
GACTCCTC −0.297364569611
GACTCGAA −0.00542250899615
GACTCGAC −0.159729652016
GACTCGCA −0.269124742626
GACTCGCC −0.115592563237
GACTCGGA −0.177649300128
GACTCGGC −0.284189635069
GACTCGTA 0.0501122912376
GACTCTAA −0.0851001640678
GACTCTAC −0.0693189200236
GACTCTCA −0.173582682049
GACTCTCC −0.145930686163
GACTCTGA 0.0276601521276
GACTCTGC 0.136962694248
GACTCTTA −0.0799111684574
GACTGAAA 0.242735560535
GACTGAAC −0.0709013671233
GACTGACA −0.36623860595
GACTGACC −0.271087783614
GACTGAGA 0.0102740764635
GACTGAGC −0.0862799103413
GACTGATA 0.0965910814396
GACTGATC −0.18169949639
GACTGCAA −0.196801143106
GACTGCAC −0.251604961334
GACTGCCA −0.292482322293
GACTGCCC −0.184596128769
GACTGCGA −0.0544963165047
GACTGCGC −0.205669828824
GACTGCTA −0.0854197166323
GACTGCTC 0.134627370216
GACTGGAA 0.250190962312
GACTGGAC −0.0471255274647
GACTGGCA −0.155322285222

GACTGGCC -0.0594231532716
GACTGGGA -0.0185950202102
GACTGGGC -0.234893658079
GACTGGTA -0.0953358685576
GACTGTAA -0.0205701569338
GACTGTAC -0.260872960385
GACTGTCA -0.207856806172
GACTGTCC 0.0184091454285
GACTGTGA -0.0603642951088
GACTGTGC -0.0685549608772
GACTGTTA 0.0551396684744
GACTTAAA 0.013609299895
GACTTAAC 0.0749716658808
GACTTACA -0.231258391713
GACTTACC -0.117789833495
GACTTAGA -0.0310350057217
GACTTAGC -0.171273665666
GACTTATA 0.0266384421753
GACTTATC 0.0442756727955
GACTTCAA 0.0327286999293
GACTTCAC -0.227769586493
GACTTCCA -0.228377756276
GACTTCCC -0.262685159914
GACTTCGA -0.0692248722552
GACTTCGC -0.0806449729449
GACTTCTA -0.0380802691263
GACTTCTC -0.143496119864
GACTTGAA 0.143047232542
GACTTGAC -0.130359747138
GACTTGCA -0.0801818556045
GACTTGCC -0.316854082417
GACTTGGA 0.092408925352
GACTTGGC -0.283452772342
GACTTGTA 0.0294468021741
GACTTTAA 0.0215987337199
GACTTTAC 0.0972280820766
GACTTTCA -0.256508918626
GACTTTCC -0.153817626347
GACTTTGA 0.0102678436012
GACTTTGC 0.144446098992
GACTTTTA 0.00732274974699
GAGAAAAA -0.0096529035923
GAGAAAAC -0.0846872751185
GAGAAACA -0.017568228433
GAGAAACC -0.0468230622687
GAGAAAGA -0.0443851594491
GAGAAAGC -0.105074408902
GAGAAATA 0.243410076743
GAGAAATC 0.449167177207
GAGAACAA 0.00874068451056
GAGAACAC -0.135615278498
GAGAACCA -0.0930041361873
GAGAACCC -0.143067164598
GAGAACGA -0.232594712698

GAGAACGC -0.200830273181
GAGAACTA -0.0844229958068
GAGAACTC -0.308367573693
GAGAAGAA -0.0820532545847
GAGAAGAC -0.30337702794
GAGAAGCA -0.0962616461661
GAGAAGCC -0.333496038886
GAGAAGGA -0.16214458686
GAGAAGGC -0.343751427829
GAGAAGTA 0.181634802847
GAGAATAA 0.140267731364
GAGAATAC 0.131205156091
GAGAATCA 0.42298890516
GAGAATCC 0.484295234411
GAGAATGA -0.153603691048
GAGAATGC -0.212280942449
GAGAATTA 0.050379060476
GAGACAAA -0.0878003534987
GAGACAAC -0.3031918387
GAGACACA -0.0758568485841
GAGACACC -0.0247700983028
GAGACAGA 0.0618663695301
GAGACAGC -0.23956084853
GAGACATA -0.00856988533098
GAGACATC -0.00506923206061
GAGACCAA 0.0437676547229
GAGACCAC -0.173920001985
GAGACCCA -0.219444553888
GAGACCCC -0.349721512568
GAGACCGA -0.174326945956
GAGACCGC -0.144919255384
GAGACCTA -0.229742511133
GAGACCTC -0.311678672009
GAGACGAA -0.197236350604
GAGACGAC -0.254863850775
GAGACGCA -0.435890945749
GAGACGCC -0.355362345204
GAGACGGA -0.195805390864
GAGACGGC -0.461946555364
GAGACGTA 0.0873476782568
GAGACTAA -0.0205212408974
GAGACTAC -0.0357989949162
GAGACTCA -0.173188341272
GAGACTCC -0.385055592606
GAGACTGA -0.0213505516536
GAGACTGC -0.219692625134
GAGACTTA -0.22577041237
GAGAGAAA -0.0154657759536
GAGAGAAC -0.159468838517
GAGAGACA -0.267837261663
GAGAGACC -0.218852890433
GAGAGAGA 0.00943318197232
GAGAGAGC -0.194674266293
GAGAGATA 0.258331334089

GAGAGATC 0.259055821688
GAGAGCAA -0.0278184480234
GAGAGCAC -0.275978531623
GAGAGCCA -0.231240954389
GAGAGCCC -0.256858756106
GAGAGCGA -0.151350881981
GAGAGCGC -0.252155603001
GAGAGCTA -0.0870994961904
GAGAGCTC -0.262214955194
GAGAGGAA -0.117837782519
GAGAGGAC -0.368811054125
GAGAGGCA -0.206629995287
GAGAGGCC -0.217234031719
GAGAGGGA -0.181513433724
GAGAGGGC -0.310498673016
GAGAGGTA 0.00310930571248
GAGAGTAA 0.0421092968767
GAGAGTAC -0.0805711680191
GAGAGTCA -0.299113364182
GAGAGTCC -0.248473597019
GAGAGTGA -0.240617324534
GAGAGTGC -0.256252690879
GAGAGTTA -0.130929239287
GAGATAAA -0.0201204585365
GAGATAAC -0.11953651712
GAGATACA -0.00819909053086
GAGATACC 0.314034420095
GAGATAGA -0.0838482600028
GAGATAGC 0.0662781106983
GAGATATA 0.324038889659
GAGATATC 0.476435552506
GAGATCAA 0.194151451727
GAGATCAC 0.202076596863
GAGATCCA 0.348366824084
GAGATCCC 0.399635172362
GAGATCGA 0.118837543703
GAGATCGC 0.42357922661
GAGATCTA 0.40901921205
GAGATCTC 0.463577628384
GAGATGAA 0.0325792641184
GAGATGAC -0.297373946974
GAGATGCA -0.106915871874
GAGATGCC -0.146146804422
GAGATGGA -0.0336128067694
GAGATGGC -0.00602043839943
GAGATGTA 0.200170731612
GAGATTAA 0.085748282718
GAGATTAC 0.467830089042
GAGATTCA 0.217874791063
GAGATTCC 0.473626854886
GAGATTGA 0.138331168634
GAGATTGC 0.474023926483
GAGATTTA 0.325314602001
GAGCAAAA 0.083960935162

GAGCAAAC -0.170636200495
GAGCAACA -0.202047647012
GAGCAACC -0.164243110597
GAGCAAGA -0.00527600305364
GAGCAAGC -0.226564358531
GAGCAATA -0.0316379933286
GAGCAATC 0.353327156357
GAGCACAA 0.058459285732
GAGCACAC -0.3051776102
GAGCACCA -0.244106191436
GAGCACCC -0.168392806841
GAGCACGA -0.178336380004
GAGCACGC -0.170039159251
GAGCACTA 0.00809533262202
GAGCACTC -0.173554767084
GAGCAGAA -0.209034629804
GAGCAGAC -0.213285257868
GAGCAGCA -0.312164259117
GAGCAGCC -0.422066081101
GAGCAGGA 0.038019908065
GAGCAGGC -0.298671995028
GAGCAGTA 0.11310675363
GAGCATAA -0.0173425550581
GAGCATAC -0.363408055004
GAGCATCA -0.00455093155542
GAGCATCC -0.210300624406
GAGCATGA -0.322725856827
GAGCATGC -0.233537657713
GAGCATTA -0.105399881754
GAGCCAAA -0.121827889035
GAGCCAAC -0.178832150743
GAGCCACA -0.207838175338
GAGCCACC -0.254254154917
GAGCCAGA -0.0173554298025
GAGCCAGC -0.285828488572
GAGCCATA -0.0797329826042
GAGCCATC -0.163944233542
GAGCCCAA -0.184615289184
GAGCCCAC -0.329952505782
GAGCCCCA -0.325640621603
GAGCCCCC -0.429518392677
GAGCCCGA -0.0315924141424
GAGCCCGC -0.400643649081
GAGCCCTA -0.295654806633
GAGCCCTC -0.435449269611
GAGCCGAA -0.213921719423
GAGCCGAC -0.197015554839
GAGCCGCA -0.402673391486
GAGCCGCC -0.41586634902
GAGCCGGA -0.176490289966
GAGCCGGC -0.339909977701
GAGCCGTA -0.0472193957042
GAGCCTAA -0.0678923332229
GAGCCTAC -0.125868282352

GAGCCTCA -0.179397227718
GAGCCTCC -0.289403473376
GAGCCTGA -0.242094580496
GAGCCTGC -0.359816219617
GAGCCTTA -0.327581527489
GAGCGAAA -0.00897831280606
GAGCGAAC -0.186281108106
GAGCGACA -0.239007713245
GAGCGACC -0.207973343878
GAGCGAGA -0.107891957769
GAGCGAGC -0.19475802561
GAGCGATA 0.121037014976
GAGCGATC 0.32400385393
GAGCGCAA -0.0769988005129
GAGCGCAC -0.11497644694
GAGCGCCA -0.289610328961
GAGCGCCC -0.153758984921
GAGCGCGA 0.0365999284687
GAGCGCGC 0.0661438070699
GAGCGCTA -0.00754398590466
GAGCGCTC -0.287779073068
GAGCGGAA -0.12887395375
GAGCGGAC -0.402005369144
GAGCGGCA -0.239357781965
GAGCGGCC -0.174101051822
GAGCGGGA -0.0556994005701
GAGCGGGC -0.317437980475
GAGCGGTA 0.104563240927
GAGCGTAA -0.0400539880278
GAGCGTAC 0.00353383686717
GAGCGTCA -0.180526955616
GAGCGTCC -0.253689120213
GAGCGTGA -0.0840456048408
GAGCGTGC -0.205588772835
GAGCGTTA 0.0251147785085
GAGCTAAA -0.195589731315
GAGCTAAC -0.264615947602
GAGCTACA -0.209879606426
GAGCTACC -0.369669833552
GAGCTAGA -0.163734418555
GAGCTAGC -0.218719576364
GAGCTATA -0.0977354765234
GAGCTATC -0.0922114546705
GAGCTCAA -0.230079452701
GAGCTCAC -0.174560424134
GAGCTCCA -0.224649547203
GAGCTCCC -0.210506151199
GAGCTCGA -0.226439284375
GAGCTCGC -0.331879198059
GAGCTCTA -0.19584073548
GAGCTGAA -0.343181761802
GAGCTGAC -0.376793859468
GAGCTGCA -0.104282321411
GAGCTGCC -0.219839241857

GAGCTGGA -0.168119866498
GAGCTGGC -0.380051538578
GAGCTGTA 0.0909372906172
GAGCTTAA 0.026670218903
GAGCTTAC -0.183624130709
GAGCTTCA -0.321816005896
GAGCTTCC -0.22015051896
GAGCTTGA -0.162896239499
GAGCTTGC -0.214344787661
GAGCTTTA -0.197036424155
GAGGAAAA -0.0719245416215
GAGGAAAC -0.340915425034
GAGGAACA -0.222325631417
GAGGAACC -0.181050098012
GAGGAAGA -0.126871083098
GAGGAAGC -0.246033960954
GAGGAATA 0.195949392919
GAGGAATC 0.420106858974
GAGGACAA -0.0850918917428
GAGGACAC -0.183827361445
GAGGACCA -0.333140966058
GAGGACCC -0.410895045652
GAGGACGA -0.277542839715
GAGGACGC -0.32189643825
GAGGACTA -0.264793441255
GAGGACTC -0.302553055425
GAGGAGAA -0.170009968605
GAGGAGAC -0.231476900399
GAGGAGCA -0.326713163251
GAGGAGCC -0.285448311408
GAGGAGGA -0.102286908659
GAGGAGGC -0.273932759353
GAGGAGTA -0.0858061918668
GAGGATAA 0.18753092998
GAGGATAC 0.241074407741
GAGGATCA 0.20677564617
GAGGATCC 0.342691176025
GAGGATGA -0.247280174422
GAGGATGC -0.0159637591358
GAGGATTA 0.183595441171
GAGGCAAA -0.0203769260527
GAGGCAAC -0.291211891946
GAGGCACA -0.17831868044
GAGGCACC -0.304692129802
GAGGCAGA 0.0123896246838
GAGGCAGC -0.10860609971
GAGGCATA -0.153716341978
GAGGCATC -0.142479410848
GAGGCCAA -0.297420970522
GAGGCCAC -0.339410232653
GAGGCCCA -0.396249996555
GAGGCCCC -0.378918395247
GAGGCCGA -0.133307015713
GAGGCCGC -0.244443893175

GAGGCCTA -0.313286036762
GAGGCCTC -0.169612763295
GAGGCGAA 0.0697746264348
GAGGCGAC -0.17822987979
GAGGCGCA -0.112579789602
GAGGCGCC -0.18025953403
GAGGCGGA -0.0673327984428
GAGGCGGC -0.327588535766
GAGGCGTA -0.0628830169954
GAGGCTAA -0.105186453671
GAGGCTAC -0.130466848079
GAGGCTCA -0.359543438653
GAGGCTCC -0.360730183881
GAGGCTGA -0.342147257018
GAGGCTGC -0.29656721621
GAGGCTTA -0.131623576114
GAGGGAAA 0.101308000555
GAGGGAAC -0.404868215736
GAGGGACA -0.225448686673
GAGGGACC -0.271104267005
GAGGGAGA -0.0806912359495
GAGGGAGC -0.141887011075
GAGGGATA 0.144754947785
GAGGGATC 0.216203807113
GAGGGCAA 0.0235935677594
GAGGGCAC -0.215353826893
GAGGGCCA -0.42486326376
GAGGGCCC -0.230186737888
GAGGGCGA -0.0829115536079
GAGGGCGC -0.26955250803
GAGGGCTA -0.310662266246
GAGGGGAA -0.0762222608207
GAGGGGAC -0.415636037892
GAGGGGCA -0.244291314164
GAGGGGCC -0.268509052642
GAGGGGGA -0.134974785899
GAGGGGGC -0.129918318085
GAGGGGTA 0.00690911296972
GAGGGTAA 0.127646930677
GAGGGTAC -0.2397021974
GAGGGTCA -0.349769669979
GAGGGTCC -0.427770815675
GAGGGTGA -0.199584783672
GAGGGTGC -0.238917226734
GAGGGTTA -0.189975716581
GAGGTAAA 0.0403441943569
GAGGTAAC -0.0731867355474
GAGGTACA -0.181917751843
GAGGTACC -0.224416573387
GAGGTAGA -0.103953777238
GAGGTAGC -0.206622165976
GAGGTATA -0.182393228266
GAGGTATC 0.105865564988
GAGGTCAA -0.0646206372075

GAGGTCAC -0.385804689416
GAGGTCCA -0.392401679083
GAGGTCCC -0.340718254657
GAGGTCGA -0.307761512901
GAGGTCGC -0.27536333744
GAGGTCTA -0.195061299545
GAGGTGAA -0.227394155935
GAGGTGAC -0.254575317438
GAGGTGCA -0.122546708338
GAGGTGCC -0.469340629969
GAGGTGGA 0.0439213318001
GAGGTGGC -0.192465282692
GAGGTGTA -0.00110969016197
GAGGTTAA -0.0358940207425
GAGGTTAC -0.0708984474793
GAGGTTCA -0.320478135438
GAGGTTCC -0.314027457368
GAGGTTGA -0.123780297829
GAGGTTGC -0.0898934873585
GAGGTTTA -0.171469252697
GAGTAAAA 0.0658165052104
GAGTAAAC -0.0203234548908
GAGTAACA -0.0650471062144
GAGTAACC 0.0988730173792
GAGTAAGA 0.178502193654
GAGTAAGC -0.140485899164
GAGTAATA 0.240788157924
GAGTAATC 0.391310409261
GAGTACAA -0.0114094811065
GAGTACAC -0.0823776097203
GAGTACCA -0.277540720159
GAGTACCC -0.203117142652
GAGTACGA -0.0219765383139
GAGTACGC -0.13902728126
GAGTACTA -0.0117238450572
GAGTACTC -0.274112381598
GAGTAGAA 0.106088031659
GAGTAGAC 0.0272453887871
GAGTAGCA -0.265950616311
GAGTAGCC -0.300608272684
GAGTAGGA -0.00932023386349
GAGTAGGC -0.15598005893
GAGTAGTA -0.0528181073636
GAGTATAA 0.227439545119
GAGTATAC 0.103649241244
GAGTATCA 0.259017971139
GAGTATCC 0.316301832807
GAGTATGA 0.00100237979026
GAGTATGC -0.0711845726006
GAGTATTA 0.17118384269
GAGTCAAA 0.070432764246
GAGTCAAC -0.195318371463
GAGTCACA -0.27946052773
GAGTCACC -0.0659087757202

GAGTCAGA 0.0987480457602
GAGTCAGC -0.245978180856
GAGTCATA 0.0648533243798
GAGTCATC -0.234927081707
GAGTCCAA -0.112622189133
GAGTCCAC -0.342346211088
GAGTCCCA -0.219063237593
GAGTCCCC -0.367903776659
GAGTCCGA -0.0323806372448
GAGTCCGC -0.184584857936
GAGTCCTA -0.148004553515
GAGTCGAA -0.0361752937511
GAGTCGAC -0.0540983922954
GAGTCGCA -0.17872618344
GAGTCGCC -0.357570073174
GAGTCGGA -0.0316772960766
GAGTCGGC -0.213273783551
GAGTCGTA 0.0277619217013
GAGTCTAA -0.0258366605111
GAGTCTAC -0.208107462724
GAGTCTCA -0.151161772369
GAGTCTCC -0.346874977202
GAGTCTGA -0.0117284910173
GAGTCTGC -0.207951867014
GAGTCTTA -0.225549058038
GAGTGAAA 0.0286964519025
GAGTGAAC -0.356513600306
GAGTGACA -0.259501964968
GAGTGACC -0.207848440485
GAGTGAGA 0.159706721751
GAGTGAGC -0.21598619351
GAGTGATA 0.121906089648
GAGTGATC -0.0765379704774
GAGTGCAA 0.216269988997
GAGTGCAC -0.291545024742
GAGTGCCA -0.378931356742
GAGTGCCC -0.309414858147
GAGTGCGA -0.138844168222
GAGTGCGC -0.124070686914
GAGTGCTA -0.157931445308
GAGTGGAA 0.0588481043026
GAGTGGAC -0.296266092824
GAGTGGCA -0.162991544186
GAGTGGCC -0.17548336162
GAGTGGGA -0.236208469246
GAGTGGGC -0.207849411998
GAGTGGTA 0.111766165704
GAGTGTAA 0.206734282492
GAGTGTAC -0.220826480293
GAGTGTCA -0.221550818506
GAGTGTCC -0.298839405092
GAGTGTGA 0.0814398224856
GAGTGTGC 0.0644619946894
GAGTGTTA -0.184092832938

GAGTTAAA -0.16798022479
GAGTTAAC -0.138915767275
GAGTTACA -0.0703378758129
GAGTTACC -0.164463361433
GAGTTAGA 0.0269213082925
GAGTTAGC 0.054214598815
GAGTTATA 0.0441860593376
GAGTTATC -0.0472635169605
GAGTTCAA 0.0504788535092
GAGTTCAC -0.204122620909
GAGTTCCA -0.243590005531
GAGTTCCC -0.193769220222
GAGTTCGA -0.0503762691328
GAGTTCGC -0.168620104303
GAGTTCTA -0.135928982684
GAGTTGAA -0.13601660918
GAGTTGAC -0.204752787065
GAGTTGCA -0.217627857866
GAGTTGCC -0.130489390863
GAGTTGGA 0.0180213011438
GAGTTGGC -0.186898765064
GAGTTGTA -0.00918776427804
GAGTTTAA 0.164242755152
GAGTTTAC -0.0324292168785
GAGTTTCA -0.211489462845
GAGTTTCC -0.156615366653
GAGTTTGA -0.10843339197
GAGTTTGC -0.0687004348378
GAGTTTTA -0.0457082426779
GATAAAAA 0.149003306497
GATAAAAC 0.0697495675277
GATAAACA -0.0208128238431
GATAAACC -0.0375209920664
GATAAAGA 0.182646365408
GATAAAGC -0.186611191957
GATAAATA 0.116302252666
GATAAATC 0.264754647671
GATAACAA 0.133462040977
GATAACAC -0.0321381988049
GATAACCA -0.0835615229555
GATAACCC -0.0766834091636
GATAACGA 0.157003196482
GATAACGC 0.157296414872
GATAACTA 0.0722140873656
GATAAGAA 0.182360045996
GATAAGAC -0.103836145816
GATAAGCA -0.0713316119728
GATAAGCC -0.0622040773556
GATAAGGA 0.114320854288
GATAAGGC 0.0352744100494
GATAAGTA 0.150813347783
GATAATAA 0.108634805605
GATAATAC 0.133015335431
GATAATCA 0.211915797464

GATAATCC 0.420944796829
GATAATGA 0.119135273535
GATAATGC 0.075647272617
GATAATTA 0.0708830682185
GATACAAA 0.216847076341
GATACAAC -0.0243314940285
GATACACA 0.0247724502383
GATACACC 0.169747377795
GATACAGA 0.0971329094983
GATACAGC 0.0747363196426
GATACATA 0.17498076589
GATACATC 0.140533418976
GATACCAA 0.103435391314
GATACCAC 0.209575992549
GATACCCA 0.287546258111
GATACCCC 0.30599548915
GATACCGA 0.309553355008
GATACCGC 0.275789563668
GATACCTA 0.123085625862
GATACGAA 0.338350985015
GATACGAC 0.260565827026
GATACGCA 0.357824766916
GATACGCC 0.322473989141
GATACGGA 0.281583236129
GATACGGC 0.309785187186
GATACGTA 0.281006902219
GATACTAA 0.202631005661
GATACTAC 0.207431694667
GATACTCA 0.289952044846
GATACTCC 0.340617403129
GATACTGA 0.214207718079
GATACTGC 0.271759742487
GATACTTA 0.202085355795
GATAGAAA 0.0661000124085
GATAGAAC 0.00247220863777
GATAGACA -0.0941767551829
GATAGACC -0.238541640825
GATAGAGA 0.149917155099
GATAGAGC -0.182991048689
GATAGATA 0.31125668781
GATAGATC 0.143801037504
GATAGCAA 0.195885968613
GATAGCAC -0.0616986489347
GATAGCCA -0.0186729429154
GATAGCCC 0.041750180653
GATAGCGA 0.151294545234
GATAGCGC 0.131829959928
GATAGCTA 0.0878688787837
GATAGGAA -0.145466631836
GATAGGAC -0.253815852223
GATAGGCA -0.114150737919
GATAGGCC -0.0019046082835
GATAGGGA 0.161128588425
GATAGGGC -0.0477120127945

GATAGGTA 0.148405981739
GATAGTAA 0.24813380874
GATAGTAC 0.0886506341052
GATAGTCA -0.0990878835756
GATAGTCC -0.00747441656533
GATAGTGA 0.104772647411
GATAGTGC 0.169916495024
GATAGTTA 0.165668423244
GATATAAA 0.114727675334
GATATAAC 0.2103158325
GATATACA 0.212881852247
GATATACC 0.193983239438
GATATAGA 0.291046911382
GATATAGC 0.182318162642
GATATATA 0.238423246206
GATATATC 0.349542032916
GATATCAA 0.386392285596
GATATCAC 0.402969084787
GATATCCA 0.478604848069
GATATCCC 0.465728814214
GATATCGA 0.415987612957
GATATCGC 0.467714575593
GATATCTA 0.462587932285
GATATGAA 0.22738991117
GATATGAC 0.274238520324
GATATGCA 0.371218325764
GATATGCC 0.391757114808
GATATGGA 0.366519711247
GATATGGC 0.349650007664
GATATGTA 0.361068735721
GATATTAA 0.292775340686
GATATTAC 0.367313594586
GATATTCA 0.319334486001
GATATTCC 0.438100625534
GATATTGA 0.166316795983
GATATTGC 0.396926177846
GATATTTA 0.383034549701
GATCAAAA -0.0211010947985
GATCAAAC -0.14785726693
GATCAACA 0.0525966584673
GATCAACC -0.0587527280425
GATCAAGA 0.160591373912
GATCAAGC -0.039760443202
GATCAATA 0.0789581250046
GATCAATC -0.0539760740612
GATCACAA 0.111313793132
GATCACAC 0.30007831523
GATCACCA -0.0864163558462
GATCACCC 0.102814936148
GATCACGA 0.231842947818
GATCACGC 0.24198716623
GATCACTA 0.101444419626
GATCAGAA 0.177346768256
GATCAGAC -0.0739027612534

GATCAGCA -0.0544205379548
GATCAGCC -0.167175313666
GATCAGGA 0.22581121066
GATCAGGC 0.010871753296
GATCAGTA 0.0928358037383
GATCATAA 0.150253559344
GATCATAC 0.12038071129
GATCATCA 0.0858951993752
GATCATCC 0.243973489632
GATCATGA 0.120730923761
GATCATGC 0.0688036028408
GATCATTA 0.0882484325161
GATCCAAA 0.244915714613
GATCCAAC 0.222769483586
GATCCACA 0.0801814558805
GATCCACC 0.107278457453
GATCCAGA 0.348961909568
GATCCAGC 0.0779206274354
GATCCATA 0.174751647774
GATCCATC 0.0216075560389
GATCCCAA 0.221360846113
GATCCCAC 0.248598205879
GATCCCCA 0.338513444574
GATCCCCC 0.244022259174
GATCCCGA 0.243207638117
GATCCCGC 0.147048604775
GATCCCTA 0.160533812049
GATCCGAA 0.27047570987
GATCCGAC 0.0528798639446
GATCCGCA 0.194642528239
GATCCGCC 0.183989401692
GATCCGGA 0.380861577831
GATCCGGC 0.103842571607
GATCCGTA 0.217725990453
GATCCTAA 0.220050629142
GATCCTAC 0.224341802734
GATCCTCA 0.305681714773
GATCCTCC 0.38581271943
GATCCTGA 0.302531321478
GATCCTGC 0.324211148661
GATCCTTA 0.300146412884
GATCGAAA 0.0207053860134
GATCGAAC -0.128863022802
GATCGACA -0.16650535633
GATCGACC -0.152394186787
GATCGAGA 0.147438568624
GATCGAGC -0.123223349904
GATCGATA 0.137447355167
GATCGATC 0.0984122652509
GATCGCAA 0.336652079076
GATCGCAC 0.389514828142
GATCGCCA 0.210965772295
GATCGCCC 0.3413157307
GATCGCGA 0.415888340131

GATCGCGC 0.443028427877
GATCGCTA 0.253100297074
GATCGGAA 0.10295176018
GATCGGAC -0.239942314849
GATCGGCA 0.0601192396456
GATCGGCC 0.0958278285877
GATCGGGA 0.229064818721
GATCGGGC -0.14913436965
GATCGGTA 0.165621544409
GATCGTAA 0.303230227473
GATCGTAC 0.17637334304
GATCGTCA 0.150283892708
GATCGTCC 0.224043602831
GATCGTGA 0.241357488522
GATCGTGC 0.254136770728
GATCGTTA 0.198692729356
GATCTAAA 0.177605980636
GATCTAAC 0.247488741122
GATCTACA 0.30556046218
GATCTACC 0.128256355529
GATCTAGA 0.307186703941
GATCTAGC 0.199749640519
GATCTATA 0.249576286651
GATCTCAA 0.329846061765
GATCTCAC 0.390162989132
GATCTCCA 0.390641810763
GATCTCCC 0.43236644453
GATCTCGA 0.367810193937
GATCTCGC 0.405845239179
GATCTCTA 0.346312854463
GATCTGAA 0.202622570309
GATCTGAC 0.196478847994
GATCTGCA 0.382849280673
GATCTGCC 0.286769632782
GATCTGGA 0.35423164211
GATCTGGC 0.252198479471
GATCTGTA 0.330079633293
GATCTTAA 0.291476099064
GATCTTAC 0.301976573939
GATCTTCA 0.378551753232
GATCTTCC 0.38006463764
GATCTTGA 0.336508689591
GATCTTGC 0.35595643891
GATCTTTA 0.26375273345
GATGAAAA 0.0730037660232
GATGAAAC -0.124132655171
GATGAACA -0.101339707906
GATGAACC -0.273063794203
GATGAAGA 0.129861266225
GATGAAGC -0.167125689568
GATGAATA 0.16543798311
GATGAATC 0.222571055904
GATGACAA 0.0598870097735
GATGACAC -0.125943628524

GATGACCA -0.00593636635694
GATGACCC -0.237976057561
GATGACGA 0.0100895600826
GATGACGC -0.220924292601
GATGACTA -0.148866497351
GATGAGAA 0.1227703043
GATGAGAC -0.117247896553
GATGAGCA -0.273875213618
GATGAGCC -0.22472247721
GATGAGGA 0.113328175295
GATGAGGC -0.195518943999
GATGAGTA 0.106242199076
GATGATAA -0.0831301645202
GATGATAC 0.159053403479
GATGATCA 0.0815953694742
GATGATCC 0.226765107333
GATGATGA -0.110100659206
GATGATGC -0.0514364665885
GATGATTA 0.215335169881
GATGCAAA 0.0788530178171
GATGCAAC -0.142368607161
GATGCACA -0.140738107785
GATGCACC -0.220252841179
GATGCAGA 0.199434972162
GATGCAGC -0.254915987815
GATGCATA 0.113197219258
GATGCATC -0.0476707265646
GATGCCAA -0.0345669514963
GATGCCAC -0.155214769597
GATGCCCA -0.149338726086
GATGCCCC -0.107449471909
GATGCCGA 0.0502113683932
GATGCCGC -0.0709772778698
GATGCCTA -0.0184743972623
GATGCGAA 0.146668707275
GATGCGAC -0.177374903109
GATGCGCA -0.0896338036393
GATGCGCC -0.0981532496684
GATGCGGA 0.120565973008
GATGCGGC -0.281964876882
GATGCGTA 0.220782756582
GATGCTAA 0.0802894827121
GATGCTAC -0.0109172791837
GATGCTCA -0.107774772316
GATGCTCC -0.0692345446173
GATGCTGA 0.092027288997
GATGCTGC 0.0392597242396
GATGCTTA 0.227442489515
GATGGAAA -0.0869292912141
GATGGAAC -0.22947452148
GATGGACA -0.176335594897
GATGGACC -0.298493104998
GATGGAGA 0.0621468143033
GATGGAGC -0.253044149471

GATGGATA 0.253050663124
GATGGCAA 0.0200678390603
GATGGCAC -0.121032287591
GATGGCCA -0.188659872057
GATGGCCC -0.393646697003
GATGGCGA -0.120210854496
GATGGCGC -0.120505552791
GATGGCTA -0.0682143623473
GATGGGAA 0.10463218039
GATGGGAC -0.223674051413
GATGGGCA -0.229180013981
GATGGGCC -0.121705855919
GATGGGGA 0.0139967523785
GATGGGGC -0.246700523009
GATGGGTA 0.19743297016
GATGGTAA 0.00682539798399
GATGGTAC -0.216053778677
GATGGTCA -0.280509182315
GATGGTCC -0.107796666253
GATGGTGA -0.161980383885
GATGGTGC -0.197470443756
GATGGTTA 0.0677035511328
GATGTAAA 0.106621752808
GATGTAAC -0.0320681030035
GATGTACA -0.00432837946048
GATGTACC -0.0793273406288
GATGTAGA 0.021517047072
GATGTAGC -0.101118404631
GATGTATA 0.216526443799
GATGTCAA -0.111906593543
GATGTCAC -0.0123884214793
GATGTCCA 0.0503685503686
GATGTCCC -0.180639110092
GATGTCGA -0.0581836730162
GATGTCGC 0.00501521864485
GATGTCTA 0.116618614191
GATGTGAA -0.0164473684211
GATGTGAC -0.257565099625
GATGTGCA -0.268892449441
GATGTGCC -0.0590748358325
GATGTGGA -0.0088122157909
GATGTGGC -0.244308918764
GATGTGTA 0.226031816941
GATGTTAA 0.154439683803
GATGTTAC 0.144914823606
GATGTTCA -0.048488285361
GATGTTCC -0.0353897359912
GATGTTGA 0.0847682167544
GATGTTGC -0.0257782676292
GATGTTTA 0.219953577659
GATTAAAA -0.0813501418633
GATTAAAC -0.160246201367
GATTAACA 0.128976083522
GATTAACC -0.125762050111

GATTAAGA 0.1674700554
GATTAAGC 0.0375926074612
GATTAATA 0.235518200331
GATTAATC 0.030048318766
GATTACAA 0.405277178004
GATTACAC 0.453186426575
GATTACCA 0.44996525796
GATTACCC 0.456584857434
GATTACGA 0.476727516916
GATTACGC 0.477466186872
GATTACTA 0.399348032537
GATTAGAA -0.0286482777749
GATTAGAC 0.118326605987
GATTAGCA 0.043434815296
GATTAGCC -0.0798222464889
GATTAGGA 0.184695955563
GATTAGGC -0.0321988655322
GATTAGTA 0.223729238881
GATTATAA 0.226817660927
GATTATAC 0.202733516784
GATTATCA 0.278182959497
GATTATCC 0.423223498981
GATTATGA 0.140857499471
GATTATGC 0.317146051946
GATTATTA 0.286150668234
GATTCAAA 0.197052424325
GATTCAAC -0.0739430890946
GATTCACA 0.231494930768
GATTCACC 0.202111632592
GATTCAGA 0.283331540907
GATTCAGC 0.0179112866142
GATTCATA 0.253161539275
GATTCCAA 0.32106669197
GATTCCAC 0.407021014138
GATTCCCA 0.466191651984
GATTCCCC 0.488049896718
GATTCCGA 0.424261512522
GATTCCGC 0.443284882679
GATTCCTA 0.412743516565
GATTCGAA 0.339770900377
GATTCGAC 0.227010757314
GATTCGCA 0.448479415341
GATTCGCC 0.404287207318
GATTCGGA 0.327501587556
GATTCGGC 0.297327077093
GATTCGTA 0.379725455483
GATTCTAA 0.310201385959
GATTCTAC 0.39479286449
GATTCTCA 0.469363302697
GATTCTCC 0.484671868499
GATTCTGA 0.380740244377
GATTCTGC 0.449357272254
GATTCTTA 0.440666992694
GATTGAAA -0.0343941373547

GATTGAAC -0.00214391087949
GATTGACA -0.127987736117
GATTGACC -0.100051667443
GATTGAGA 0.0162311071402
GATTGAGC -0.101406432241
GATTGATA 0.234992664394
GATTGCAA 0.338485288643
GATTGCAC 0.43093289928
GATTGCCA 0.382612789501
GATTGCCC 0.450635283024
GATTGCGA 0.468593388466
GATTGCGC 0.480669374609
GATTGCTA 0.424266748876
GATTGGAA 0.154349174836
GATTGGAC -0.309316388608
GATTGGCA 0.00663562111779
GATTGGCC -0.0527510982056
GATTGGGA 0.179853407126
GATTGGGC -0.238532250696
GATTGGTA 0.128769265133
GATTGTAA 0.255027755028
GATTGTAC 0.195945107576
GATTGTCA 0.248326678978
GATTGTCC 0.324383407663
GATTGTGA 0.373188178361
GATTGTGC 0.373115539782
GATTGTTA 0.226513440393
GATTTAAA 0.142867385292
GATTTAAC 0.0551233199526
GATTTACA 0.249123779427
GATTTACC 0.170822337489
GATTTAGA 0.319974244217
GATTTAGC 0.0604125457107
GATTTATA 0.336014467578
GATTTCAA 0.397362086508
GATTTCAC 0.449338335657
GATTTCCA 0.476828067737
GATTTCCC 0.4922863003
GATTTCGA 0.461595001569
GATTTCGC 0.471965577396
GATTTCTA 0.440833412407
GATTTGAA 0.263625614038
GATTTGAC 0.266592426235
GATTTGCA 0.399675189594
GATTTGCC 0.445684542274
GATTTGGA 0.232338399005
GATTTGGC 0.214358987086
GATTTGTA 0.411967063482
GATTTTAA 0.349466070086
GATTTTAC 0.402423084241
GATTTTCA 0.470149558769
GATTTTCC 0.470386049941
GATTTTGA 0.34795350666
GATTTTGC 0.402733926474

GATTTTTA	0.423408976446
GCAAAAAA	0.250611087674
GCAAAAAC	-0.194645018458
GCAAAACA	-0.159855813091
GCAAAACC	-0.0339634087494
GCAAAAGA	0.0507171749758
GCAAAAGC	-0.0272532005415
GCAAAATA	0.157808953089
GCAAACAA	0.000122712243924
GCAAACAC	-0.017906907148
GCAAACCA	-0.0326708720375
GCAAACCC	-0.106160699559
GCAAACGA	-0.161250782463
GCAAACGC	-0.139793974186
GCAAACTA	0.0231209170603
GCAAAGAA	0.0222357343569
GCAAAGAC	-0.241026467889
GCAAAGCA	-0.0742487033774
GCAAAGCC	-0.0938333181689
GCAAAGGA	-0.140403338545
GCAAAGGC	-0.410987813365
GCAAAGTA	0.0023904586031
GCAAATAA	0.179012345679
GCAAATAC	0.273596198623
GCAAATCA	0.326821310482
GCAAATCC	0.43637439092
GCAAATGA	-0.0902118931702
GCAAATGC	0.0908256197732
GCAAATTA	0.0423136719634
GCAACAAA	0.113082925191
GCAACAAC	-0.0958146901893
GCAACACA	-0.22246200284
GCAACACC	-0.201143770574
GCAACAGA	0.00838496386552
GCAACAGC	-0.181741427498
GCAACATA	0.104262664869
GCAACCAA	-0.0582417903258
GCAACCAC	-0.206854061743
GCAACCCA	-0.0698151213083
GCAACCCC	-0.105607244759
GCAACCGA	-0.170287586564
GCAACCGC	-0.127506361534
GCAACCTA	-0.0776574235601
GCAACGAA	0.0349089088475
GCAACGAC	-0.167776371692
GCAACGCA	0.0201234292143
GCAACGCC	-0.246013652465
GCAACGGA	-0.134200533722
GCAACGGC	-0.269369390402
GCAACGTA	-0.00301541210632
GCAACTAA	0.163583693887
GCAACTAC	-0.0211798281789
GCAACTCA	-0.0217090368606
GCAACTCC	-0.19652758826

GCAACTGA -0.0485492440849
GCAACTGC -0.156338990275
GCAACTTA -0.238246299008
GCAAGAAA 0.0474969323462
GCAAGAAC -0.220908627273
GCAAGACA -0.0114749312059
GCAAGACC -0.095181588417
GCAAGAGA 0.159481909155
GCAAGAGC -0.192886109939
GCAAGATA 0.274854565228
GCAAGCAA 0.0201214571944
GCAAGCAC -0.182421358227
GCAAGCCA -0.136342334147
GCAAGCCC -0.230630645093
GCAAGCGA -0.108796745665
GCAAGCGC 0.0131648837152
GCAAGCTA -0.268264975113
GCAAGGAA -0.0444793000209
GCAAGGAC -0.0958425358381
GCAAGGCA -0.146571048082
GCAAGGCC -0.0971685182671
GCAAGGGA -0.146314262949
GCAAGGGC -0.205505849664
GCAAGGTA 0.135786077677
GCAAGTAA 0.162213177365
GCAAGTAC -0.00875674255308
GCAAGTCA -0.0495529294979
GCAAGTCC -0.222618608065
GCAAGTGA -0.084867651803
GCAAGTGC -0.10822166141
GCAAGTTA 0.0013911983609
GCAATAAA 0.0640764383278
GCAATAAC -0.137752383899
GCAATACA 0.180100435757
GCAATACC 0.142766946709
GCAATAGA 0.0559554044403
GCAATAGC -0.0865821277018
GCAATATA 0.306468471493
GCAATCAA 0.411437608407
GCAATCAC 0.288185660168
GCAATCCA 0.445992824781
GCAATCCC 0.419969635701
GCAATCGA 0.238843526722
GCAATCGC 0.379510769677
GCAATCTA 0.468025877117
GCAATGAA -0.0469783913268
GCAATGAC -0.0953724069585
GCAATGCA 0.177644586735
GCAATGCC -0.12878804466
GCAATGGA -0.0385341926819
GCAATGGC -0.27756550745
GCAATGTA 0.0251946161037
GCAATTAA 0.154971059028
GCAATTAC 0.00820346999701

GCAATTCA 0.22077862987
GCAATTCC 0.162152510637
GCAATTGA 0.0167394050389
GCAATTGC 0.203753317953
GCAATTTA 0.192249074838
GCACAAAA 0.0986775158093
GCACAAAC -0.0519045216015
GCACAACA -0.00082822542621
GCACAACC -0.143525295884
GCACAAGA 0.114949660404
GCACAAGC -0.150947210476
GCACAATA 0.206714979442
GCACACAA 0.114914653788
GCACACAC -0.248999175405
GCACACCA -0.156917964069
GCACACCC -0.272617998289
GCACACGA -0.103005841859
GCACACGC 0.0183984093898
GCACACTA -0.199038945686
GCACAGAA 0.0819799566433
GCACAGAC -0.228352459674
GCACAGCA -0.0465212511148
GCACAGCC -0.134654358058
GCACAGGA -0.127477742468
GCACAGGC -0.182504830978
GCACAGTA 0.00255291957218
GCACATAA 0.0433355473968
GCACATAC 0.0665715349299
GCACATCA -0.0709759346123
GCACATCC -0.134550850043
GCACATGA -0.0446070524003
GCACATGC -0.0418083232002
GCACATTA -0.047309017006
GCACCAAA 0.0233056748208
GCACCAAC -0.333727471425
GCACCACA -0.031083095041
GCACCACC -0.119317493516
GCACCAGA -0.0928815783308
GCACCAGC -0.107972386947
GCACCATA -0.00786047755745
GCACCCAA -0.19322508692
GCACCCAC -0.23190356741
GCACCCCA -0.0373217551742
GCACCCCC -0.390913653227
GCACCCGA -0.167922182806
GCACCCGC -0.244981299806
GCACCCTA -0.133043801961
GCACCGAA -0.126512037014
GCACCGAC -0.253176001036
GCACCGCA 0.133167156924
GCACCGCC -0.169391126919
GCACCGGA 0.092195791333
GCACCGGC -0.232567658076
GCACCGTA -0.0144967628446

GCACCTAA -0.0231812442063
GCACCTAC -0.260431741936
GCACCTCA -0.1421141437
GCACCTCC -0.29186957226
GCACCTGA -0.254989775976
GCACCTGC -0.125586242835
GCACCTTA -0.0964470364396
GCACGAAA 0.0533384624294
GCACGAAC -0.240303216476
GCACGACA -0.260305448731
GCACGACC -0.175112426053
GCACGAGA -0.0508479152771
GCACGAGC -0.321385353698
GCACGATA 0.0771304278009
GCACGCAA 0.186711504893
GCACGCAC -0.0910346770274
GCACGCCA -0.297948698493
GCACGCCC -0.395803029662
GCACGCGA -0.0942563962578
GCACGCGC 0.207025393605
GCACGCTA -0.344082242455
GCACGGAA 0.0556603435391
GCACGGAC -0.205505830983
GCACGGCA 0.0675951433527
GCACGGCC -0.251636743002
GCACGGGA -0.0146164682525
GCACGGGC -0.220876991314
GCACGGTA 0.0800617504726
GCACGTAA 0.179688765939
GCACGTAC -0.112900335514
GCACGTCA 0.0154325087772
GCACGTCC -0.110113016314
GCACGTGA -0.0930782654724
GCACGTGC 0.137248173231
GCACGTTA -0.202234800771
GCACTAAA -0.027188433129
GCACTAAC -0.162757454186
GCACTACA 0.127082618629
GCACTACC -0.133176535442
GCACTAGA -0.0814237644431
GCACTAGC -0.191529864441
GCACTATA 0.117135340399
GCACTCAA -0.0219593731524
GCACTCAC -0.319960458449
GCACTCCA -0.139743284724
GCACTCCC -0.213243503172
GCACTCGA -0.325852756564
GCACTCGC 0.178593899404
GCACTCTA -0.258768048215
GCACTGAA 0.0240033421852
GCACTGAC -0.163533145294
GCACTGCA 0.0482615112204
GCACTGCC -0.121740520232
GCACTGGA -0.125019890075

GCACTGGC -0.207813026722
GCACTGTA 0.0603220367437
GCACTTAA 0.0301899695839
GCACTTAC -0.121149843554
GCACTTCA -0.0718483120401
GCACTTCC -0.144818077787
GCACTTGA -0.229156054651
GCACTTTA -0.27763029857
GCAGAAAA -0.0369860218345
GCAGAAAC -0.147474120939
GCAGAACA -0.20439224443
GCAGAACC -0.18598134963
GCAGAAGA 0.0611829804252
GCAGAAGC -0.225429388269
GCAGAATA 0.00294519098122
GCAGACAA 0.185830237294
GCAGACAC -0.249901455699
GCAGACCA -0.122911789926
GCAGACCC -0.153319162897
GCAGACGA -0.0980496761867
GCAGACGC -0.0421461168328
GCAGACTA -0.298057382361
GCAGAGAA 0.119233558627
GCAGAGAC -0.274678816088
GCAGAGCA -0.203802999479
GCAGAGCC -0.163236052188
GCAGAGGA 0.0973494155312
GCAGAGGC -0.0777099771538
GCAGAGTA 0.00942217810316
GCAGATAA 0.225533017525
GCAGATAC 0.30522749628
GCAGATCA 0.112800127952
GCAGATCC 0.325825825826
GCAGATGA -0.148113097874
GCAGATGC 0.0221739669934
GCAGATTA 0.233728218577
GCAGCAAA 0.164153339708
GCAGCAAC -0.213354173386
GCAGCACA -0.293588969654
GCAGCACC -0.106158745916
GCAGCAGA -0.104658920178
GCAGCAGC -0.292108600878
GCAGCATA 0.0579629643147
GCAGCCAA 0.0207025207025
GCAGCCAC -0.167668878831
GCAGCCCA -0.267483096645
GCAGCCCC -0.221239422315
GCAGCCGA -0.196854142372
GCAGCCGC -0.0881168052281
GCAGCCTA -0.276155506488
GCAGCGAA -0.0425423686757
GCAGCGAC -0.356587094166
GCAGCGCA -0.198104589756
GCAGCGCC -0.261069630144

GCAGCGGA 0.100877557902
GCAGCGGC -0.396672427146
GCAGCGTA 0.0206914903885
GCAGCTAA 0.159712053651
GCAGCTAC -0.198151594095
GCAGCTCA -0.0787604525942
GCAGCTCC -0.335451424002
GCAGCTGA -0.229982940607
GCAGCTGC -0.0179160568551
GCAGCTTA -0.407380846423
GCAGGAAA 0.0425039364433
GCAGGAAC -0.349138645617
GCAGGACA -0.173558560421
GCAGGACC -0.198153982706
GCAGGAGA -0.0184840539192
GCAGGAGC -0.206955435924
GCAGGATA 0.186750110993
GCAGGCAA -0.098426130426
GCAGGCAC -0.220970792509
GCAGGCCA -0.296951453153
GCAGGCCC -0.43895819039
GCAGGCGA -0.191855416904
GCAGGCGC -0.143599560367
GCAGGCTA -0.263702490911
GCAGGGAA 0.138787471807
GCAGGGAC -0.291058638071
GCAGGGCA -0.18807532938
GCAGGGCC -0.212998908541
GCAGGGGA -0.127231114972
GCAGGGGC -0.346706517515
GCAGGGTA -0.0817195406102
GCAGGTAA 0.237176558178
GCAGGTAC -0.0932124650891
GCAGGTCA -0.22797419675
GCAGGTCC -0.27447687976
GCAGGTGA -0.117956120252
GCAGGTTA -0.152726749897
GCAGTAAA 0.0846950278827
GCAGTAAC -0.119402059298
GCAGTACA -0.199820057529
GCAGTACC -0.055169571199
GCAGTAGA -0.254896365911
GCAGTAGC -0.205755853866
GCAGTATA 0.120052115647
GCAGTCAA 0.0288824230092
GCAGTCAC -0.217350219419
GCAGTCCA -0.282272001852
GCAGTCCC -0.270793919382
GCAGTCGA -0.185173163707
GCAGTCGC -0.0982018784585
GCAGTCTA -0.29025867994
GCAGTGAA -0.0766809617924
GCAGTGAC -0.28815972653
GCAGTGCA -0.117212723779

GCAGTGCC -0.195877126972
GCAGTGGA -0.0522802025866
GCAGTGGC 0.0487665845801
GCAGTGTA -0.016015460208
GCAGTTAA 0.0825405283096
GCAGTTAC -0.0387095084065
GCAGTTCA -0.0575985156144
GCAGTTCC -0.23968468475
GCAGTTGA -0.120762739697
GCAGTTTA -0.203718868127
GCATAAAA 0.107243637001
GCATAAAC -0.301251354324
GCATAACA -0.0715612066889
GCATAACC -0.158243424923
GCATAAGA 0.227629321986
GCATAAGC -0.192914236179
GCATAATA 0.0734963613751
GCATACAA 0.194953907075
GCATACAC -0.0911461792807
GCATACCA -0.101130901729
GCATACCC -0.180691001431
GCATACGA 0.102544123084
GCATACGC -0.135447360932
GCATACTA -0.0733639976064
GCATAGAA 0.138235659073
GCATAGAC -0.101868891551
GCATAGCA -0.0256182326208
GCATAGCC -0.148686282133
GCATAGGA 0.100300723342
GCATAGGC -0.206737553979
GCATAGTA -0.0272738303041
GCATATAA 0.245019790555
GCATATAC 0.18759516215
GCATATCA 0.27557571732
GCATATCC 0.396151179171
GCATATGA 0.0352873534692
GCATATGC 0.136754892963
GCATATTA -0.0743576169166
GCATCAAA -0.00812244751639
GCATCAAC -0.119381794021
GCATCACA 0.0604945777601
GCATCACC -0.105942339718
GCATCAGA 0.179604096261
GCATCAGC -0.1012768622
GCATCATA 0.0496419786428
GCATCCAA -0.0750599083932
GCATCCAC -0.139068277128
GCATCCCA -0.212018226639
GCATCCCC -0.224069524747
GCATCCGA -0.0929827318571
GCATCCGC -0.115896089185
GCATCCTA -0.261176908426
GCATCGAA -0.0742528393346
GCATCGAC -0.179737828058

GCATCGCA 0.0800588308285
GCATCGCC -0.188483349737
GCATCGGA 0.0368943515259
GCATCGGC -0.0937038901902
GCATCGTA 0.0142485930965
GCATCTAA 0.128957030138
GCATCTAC 0.20779180018
GCATCTCA -0.034472009231
GCATCTCC -0.32928896939
GCATCTGA -0.0856735607218
GCATCTTA -0.0373936754889
GCATGAAA 0.218045952537
GCATGAAC -0.241775295658
GCATGACA -0.32877101127
GCATGACC -0.252809685663
GCATGAGA 0.116905587127
GCATGAGC -0.244189948944
GCATGATA 0.0317854353554
GCATGCAA 0.155330261391
GCATGCAC -0.219709961746
GCATGCCA -0.152765715767
GCATGCCC -0.272423816591
GCATGCGA 0.0827715867067
GCATGCGC 0.151587191521
GCATGCTA -0.326437826613
GCATGGAA 0.0549703291169
GCATGGAC -0.208868555983
GCATGGCA -0.234798631027
GCATGGCC -0.323103312919
GCATGGGA -0.0201868535202
GCATGGGC -0.269213382178
GCATGGTA 0.00714350714351
GCATGTAA 0.1620803924
GCATGTAC 0.106998386897
GCATGTCA 0.0244133340146
GCATGTCC -0.337246292429
GCATGTGA -0.0450353735201
GCATGTTA -0.284971893463
GCATTAAA 0.0315966449115
GCATTAAC -0.217469004721
GCATTACA -0.14333391231
GCATTACC 0.00420743828769
GCATTAGA -0.0105318861631
GCATTAGC -0.241053875961
GCATTATA 0.205264970097
GCATTCAA -0.0641619581014
GCATTCAC -0.14565621301
GCATTCCA -0.225359337672
GCATTCCC -0.124111085426
GCATTCGA -0.0405107586926
GCATTCGC 0.0249855842573
GCATTCTA -0.246588708183
GCATTGAA -0.0452046876665
GCATTGAC -0.200518837978

GCATTGCA -0.180227091622
GCATTGCC -0.165098311635
GCATTGGA 0.0747567571513
GCATTGGC -0.241696541544
GCATTGTA -0.118015663891
GCATTTAA 0.199872198923
GCATTTAC 0.114574863324
GCATTTCA -0.0962629295963
GCATTTCC 0.142282285789
GCATTTGA -0.0250541958935
GCATTTTA -0.0210853530185
GCCAAAAA -0.0339970657577
GCCAAAAC -0.0788710004686
GCCAAACA -0.247607942376
GCCAAACC -0.273160208962
GCCAAAGA 0.0420082447505
GCCAAAGC -0.195560242043
GCCAAATA -0.141777973689
GCCAACAA -0.0124159972645
GCCAACAC -0.157227384912
GCCAACCA -0.273733704034
GCCAACCC -0.299448951519
GCCAACGA -0.168491263102
GCCAACGC -0.259328630772
GCCAACTA -0.217185876178
GCCAAGAA 0.155376889557
GCCAAGAC -0.249093805905
GCCAAGCA -0.337396419977
GCCAAGCC -0.370485228097
GCCAAGGA -0.0151237356744
GCCAAGGC -0.325955647062
GCCAAGTA -0.00391305299884
GCCAATAA 0.0996438034204
GCCAATAC -0.0435873017843
GCCAATCA -0.156301040552
GCCAATCC -0.16683071816
GCCAATGA -0.20251150248
GCCAATTA 0.0131289067562
GCCACAAA -0.00330868667109
GCCACAAC -0.028146224618
GCCACACA -0.0192800319863
GCCACACC -0.191959916746
GCCACAGA -0.167147792473
GCCACAGC -0.259900696667
GCCACATA -0.0870712590594
GCCACCAA -0.306284686094
GCCACCAC -0.105886293874
GCCACCCA -0.206701109919
GCCACCCC -0.174712930495
GCCACCGA -0.224253255275
GCCACCGC -0.330711091332
GCCACCTA -0.184792709324
GCCACGAA -0.0109103593952
GCCACGAC -0.251085987442

GCCACGCA -0.214044301886
GCCACGCC -0.196825711899
GCCACGGA -0.0912331636804
GCCACGGC -0.181709660433
GCCACGTA -0.081352916362
GCCACTAA 0.0371167054736
GCCACTAC -0.0875930361723
GCCACTCA -0.185899291684
GCCACTCC -0.284769936748
GCCACTGA -0.302864805122
GCCACTTA -0.175833502396
GCCAGAAA 0.0239068269371
GCCAGAAC -0.231810003021
GCCAGACA -0.259941455186
GCCAGACC -0.354420053192
GCCAGAGA -0.0117723127997
GCCAGAGC -0.370288858121
GCCAGATA 0.249155857901
GCCAGCAA -0.184397644432
GCCAGCAC -0.296458007067
GCCAGCCA -0.188025068171
GCCAGCCC -0.414757302797
GCCAGCGA -0.126123257039
GCCAGCGC -0.255908258354
GCCAGCTA -0.116807181354
GCCAGGAA 0.193930789837
GCCAGGAC -0.249040230782
GCCAGGCA -0.127989213824
GCCAGGCC -0.295306985993
GCCAGGGA 0.0244880293234
GCCAGGGC -0.36778164057
GCCAGGTA -0.0748762376238
GCCAGTAA 0.0330090742901
GCCAGTAC -0.201875604734
GCCAGTCA -0.0806515643997
GCCAGTCC -0.23360178997
GCCAGTGA -0.207559105907
GCCAGTTA -0.0946111400657
GCCATAAA -0.134507786023
GCCATAAC -0.270041173698
GCCATACA -0.150955968105
GCCATACC -0.230107669696
GCCATAGA -0.118366021182
GCCATAGC -0.323902940812
GCCATATA -0.0748172414839
GCCATCAA -0.132469089857
GCCATCAC -0.232848274934
GCCATCCA -0.140734721223
GCCATCCC -0.310490994529
GCCATCGA -0.100046128087
GCCATCGC -0.127167263534
GCCATCTA -0.192105735539
GCCATGAA -0.0175538735162
GCCATGAC -0.390357154842

GCCATGCA -0.33999031111
GCCATGCC -0.0900847951765
GCCATGGA 0.0952367578498
GCCATGGC -0.324049502741
GCCATGTA -0.175517615042
GCCATTAA 0.0662983596794
GCCATTAC -0.183440990056
GCCATTCA -0.118011951345
GCCATTCC -0.0578534776611
GCCATTGA -0.215948286558
GCCATTTA -0.206718623437
GCCCAAAA 0.0979650080655
GCCCAAAC -0.226507642111
GCCCAACA -0.350090970237
GCCCAACC -0.170595436011
GCCCAAGA 0.0261188242884
GCCCAAGC -0.212437270808
GCCCAATA -0.168698052351
GCCCACAA 0.0411750107662
GCCCACAC -0.106255036116
GCCCACCA -0.3291790386
GCCCACCC -0.123841121454
GCCCACGA -0.0988535126684
GCCCACGC -0.221178251218
GCCCACTA -0.179176383755
GCCCAGAA -0.119863578574
GCCCAGAC -0.183567370432
GCCCAGCA -0.256070767967
GCCCAGCC -0.29423179226
GCCCAGGA -0.238048694494
GCCCAGGC -0.407897558905
GCCCAGTA -0.263390260376
GCCCATAA 0.0864156167186
GCCCATAC -0.180635156928
GCCCATCA -0.289618610044
GCCCATCC -0.153308850897
GCCCATGA -0.32391732065
GCCCATTA -0.0162468461761
GCCCCAAA -0.134423231039
GCCCCAAC -0.117922194311
GCCCCACA -0.236841234795
GCCCCACC -0.17091389376
GCCCCAGA -0.0803319631071
GCCCCAGC -0.35003480639
GCCCCATA -0.0311685773841
GCCCCCAA -0.401510328386
GCCCCCAC -0.425936949674
GCCCCCCA -0.319799598204
GCCCCCCC -0.346645886902
GCCCCCGA -0.311695438649
GCCCCCGC -0.180865315438
GCCCCCTA -0.225804951256
GCCCCGAA 0.0509984739075
GCCCCGAC -0.132578803797

GCCCCGCA -0.0973125758082
GCCCCGCC 0.0620855017773
GCCCCGGA -0.291093966791
GCCCCGGC -0.305082210512
GCCCCGTA -0.305521226226
GCCCCTAA -0.204100529294
GCCCCTAC -0.257879893143
GCCCCTCA -0.269302430702
GCCCCTCC -0.334752781705
GCCCCTGA -0.324540516407
GCCCCTTA -0.101427039307
GCCCGAAA -0.116503458533
GCCCGAAC -0.134359433176
GCCCGACA -0.231430256822
GCCCGACC -0.383212264543
GCCCGAGA -0.164550750289
GCCCGAGC -0.289313663064
GCCCGATA -0.0819815646602
GCCCGCAA -0.0421311942074
GCCCGCAC -0.241868086737
GCCCGCCA -0.134264373872
GCCCGCCC -0.234641106573
GCCCGCGA -0.137243468958
GCCCGCGC -0.310737690924
GCCCGCTA -0.327629476695
GCCCGGAA -0.119136511514
GCCCGGAC -0.20195316269
GCCCGGCA -0.267145168537
GCCCGGCC -0.348167164113
GCCCGGGA -0.108145924143
GCCCGGGC -0.385090895563
GCCCGGTA -0.269218963165
GCCCGTAA -0.00113320331431
GCCCGTAC -0.0964681526375
GCCCGTCA -0.265699319371
GCCCGTCC -0.264943738901
GCCCGTGA -0.142131685813
GCCCGTTA -0.135155156383
GCCCTAAA -0.127519815693
GCCCTAAC -0.27601849244
GCCCTACA -0.126866165438
GCCCTACC -0.235166421246
GCCCTAGA -0.135845815792
GCCCTAGC -0.290769820203
GCCCTATA -0.133025663929
GCCCTCAA -0.000406560440282
GCCCTCAC -0.308118396498
GCCCTCCA -0.400790820011
GCCCTCCC -0.31182073246
GCCCTCGA -0.285723333268
GCCCTCGC -0.361106288221
GCCCTCTA -0.286407472638
GCCCTGAA -0.0759252257868
GCCCTGAC -0.250686473309

GCCCTGCA -0.256999749148
GCCCTGCC -0.278932221516
GCCCTGGA -0.154955960799
GCCCTGTA -0.110008063516
GCCCTTAA 0.0410948138221
GCCCTTAC -0.283984763669
GCCCTTCA -0.280472107145
GCCCTTCC -0.288193496997
GCCCTTGA -0.211897467067
GCCCTTTA -0.219619470744
GCCGAAAA 0.160732357702
GCCGAAAC -0.153728957229
GCCGAACA -0.0526816952433
GCCGAACC -0.179257980086
GCCGAAGA 0.0819850112912
GCCGAAGC -0.124931216128
GCCGAATA 0.0028196188518
GCCGACAA -0.00476558907469
GCCGACAC -0.45403613781
GCCGACCA -0.298240941667
GCCGACCC -0.343858013478
GCCGACGA -0.23163792747
GCCGACGC -0.183657615303
GCCGACTA -0.113640509418
GCCGAGAA -0.119313843058
GCCGAGAC -0.322854934463
GCCGAGCA -0.14544482111
GCCGAGCC -0.160574210581
GCCGAGGA -0.127980764319
GCCGAGGC -0.0576404023654
GCCGAGTA -0.0084892054589
GCCGATAA -0.0266629197901
GCCGATAC 0.0620358650662
GCCGATCA -0.0796770022634
GCCGATCC 0.13960103354
GCCGATGA -0.317412672031
GCCGATTA 0.096294971643
GCCGCAAA -0.0708009102547
GCCGCAAC -0.175095846051
GCCGCACA -0.111287146921
GCCGCACC -0.406356291431
GCCGCAGA -0.0366572424856
GCCGCAGC -0.185664090605
GCCGCATA -0.0988193910217
GCCGCCAA -0.154156931819
GCCGCCAC -0.209208215832
GCCGCCCA -0.145492667676
GCCGCCCC -0.182669643965
GCCGCCGA -0.176866772608
GCCGCCGC -0.348858904295
GCCGCCTA -0.1657127339
GCCGCGAA 0.00398730601753
GCCGCGAC -0.245502816168
GCCGCGCA 0.0142734959155

GCCGCGCC	-0.211614826793
GCCGCGGA	0.00889826419048
GCCGCGGC	0.0765943988292
GCCGCGTA	-0.106066121218
GCCGCTAA	-0.121548433246
GCCGCTAC	-0.320858997763
GCCGCTCA	-0.206539142466
GCCGCTCC	-0.118000977838
GCCGCTGA	-0.442771027213
GCCGCTTA	-0.20204213295
GCCGGAAA	-0.0402249409247
GCCGGAAC	-0.146444208777
GCCGGACA	-0.148605850975
GCCGGACC	-0.299215267958
GCCGGAGA	-0.0578420913606
GCCGGAGC	-0.218643773416
GCCGGATA	0.215045624137
GCCGGCAA	-0.253480619601
GCCGGCAC	-0.249529712399
GCCGGCCA	-0.247632508731
GCCGGCCC	-0.184210449506
GCCGGCGA	-0.080563992463
GCCGGCGC	-0.315359679807
GCCGGCTA	-0.320712271212
GCCGGGAA	-0.110858842764
GCCGGGAC	-0.316622042591
GCCGGGCA	-0.267461616688
GCCGGGCC	-0.386099346859
GCCGGGGA	-0.140715322401
GCCGGGTA	-0.0865335707019
GCCGGTAA	-0.128461613695
GCCGGTAC	-0.310196234092
GCCGGTCA	-0.11134198338
GCCGGTCC	-0.193239948336
GCCGGTGA	-0.300537816701
GCCGGTTA	-0.160700327552
GCCGTAAA	-0.256143656262
GCCGTAAC	-0.241675289214
GCCGTACA	-0.208182766785
GCCGTACC	-0.11712233093
GCCGTAGA	-0.155983366441
GCCGTAGC	-0.275659524971
GCCGTATA	-0.0893243213652
GCCGTCAA	-0.040743957756
GCCGTCAC	-0.191694162594
GCCGTCCA	-0.253552890979
GCCGTCCC	-0.0701633654479
GCCGTCGA	-0.209439762703
GCCGTCGC	-0.238384865801
GCCGTCTA	-0.175962564192
GCCGTGAA	0.0152018568936
GCCGTGAC	-0.350885216421
GCCGTGCA	-0.3083084573
GCCGTGCC	-0.250282756316

GCCGTGGA 0.000441190663303
GCCGTGTA 0.00289847667569
GCCGTTAA -0.148848298904
GCCGTTAC -0.345370301462
GCCGTTCA 0.00059779712853
GCCGTTCC -0.00943024421858
GCCGTTGA -0.219820270761
GCCGTTTA -0.153951236758
GCCTAAAA 0.0363444596104
GCCTAAAC -0.158707778952
GCCTAACA -0.188373803731
GCCTAACC -0.302726854264
GCCTAAGA 0.0629100174555
GCCTAAGC -0.149221743033
GCCTAATA -0.0441753448528
GCCTACAA 0.0125731792398
GCCTACAC -0.281525322561
GCCTACCA -0.0755822449413
GCCTACCC -0.195607222319
GCCTACGA -0.0788316504999
GCCTACGC -0.317806830037
GCCTACTA -0.178648318599
GCCTAGAA -0.0646296834241
GCCTAGAC -0.371080518693
GCCTAGCA -0.265516937216
GCCTAGCC -0.214758301286
GCCTAGGA -0.0792348051986
GCCTAGGC -0.217566066699
GCCTAGTA -0.187609075044
GCCTATAA 0.151020405497
GCCTATAC -0.177206987357
GCCTATCA 0.0918149554513
GCCTATCC -0.0161263191566
GCCTATGA -0.24491125616
GCCTATTA -0.171881332242
GCCTCAAA -0.203006668927
GCCTCAAC -0.111218629989
GCCTCACA -0.276223684383
GCCTCACC -0.256601903917
GCCTCAGA -0.0635049877168
GCCTCAGC -0.342571692367
GCCTCATA -0.0456926763227
GCCTCCAA -0.125826601575
GCCTCCAC -0.31774380181
GCCTCCCA -0.189948354325
GCCTCCCC -0.293676188901
GCCTCCGA -0.290401973349
GCCTCCGC -0.230454461507
GCCTCCTA -0.148734366585
GCCTCGAA -0.0887284217144
GCCTCGAC -0.369911213896
GCCTCGCA -0.293305051808
GCCTCGCC -0.348730949654
GCCTCGGA -0.114276436291

GCCTCGTA -0.106630268437
GCCTCTAA -0.186492001699
GCCTCTAC -0.294913782409
GCCTCTCA 0.0772855299402
GCCTCTCC -0.200909973219
GCCTCTGA -0.275363652862
GCCTCTTA -0.172582931091
GCCTGAAA -0.0362061691937
GCCTGAAC -0.214243598444
GCCTGACA -0.0533049460696
GCCTGACC -0.289809233212
GCCTGAGA -0.119598645523
GCCTGAGC -0.399307434971
GCCTGATA 0.0970540791078
GCCTGCAA -0.127142293809
GCCTGCAC -0.346588678882
GCCTGCCA -0.41619335797
GCCTGCCC -0.36281221321
GCCTGCGA -0.216556312653
GCCTGCGC -0.248941989308
GCCTGCTA -0.279402805689
GCCTGGAA -0.210464439979
GCCTGGAC -0.310604258573
GCCTGGCA -0.176756094599
GCCTGGCC -0.178118884558
GCCTGGGA -0.0816440463489
GCCTGGTA -0.157330455564
GCCTGTAA 0.0409005642212
GCCTGTAC -0.134454511351
GCCTGTCA 0.0962891323548
GCCTGTCC 0.0209289820904
GCCTGTGA -0.257759806394
GCCTGTTA -0.109359017835
GCCTTAAA -0.152732025495
GCCTTAAC -0.257658500421
GCCTTACA -0.257215934383
GCCTTACC -0.170068667586
GCCTTAGA -0.175025136701
GCCTTAGC -0.176629483543
GCCTTATA -0.0935619203882
GCCTTCAA -0.173235330604
GCCTTCAC -0.35651121355
GCCTTCCA -0.283811122412
GCCTTCCC -0.304174516565
GCCTTCGA -0.301806744577
GCCTTCGC -0.330079304043
GCCTTCTA -0.234506856251
GCCTTGAA -0.171062534633
GCCTTGAC -0.240959870341
GCCTTGCA -0.130986358259
GCCTTGCC -0.266406153083
GCCTTGGA -0.00246786034745
GCCTTGTA -0.0142164770115
GCCTTTAA -0.111093513019

GCCTTTAC -0.216393569181
GCCTTTCA 0.00216126654161
GCCTTTCC -0.326581995882
GCCTTTGA -0.12985621236
GCCTTTTA -0.283239479712
GCGAAAAA 0.22395349007
GCGAAAAC -0.095550099336
GCGAAACA -0.0254236119728
GCGAAACC 0.0235952205649
GCGAAAGA -0.0364670846624
GCGAAAGC -0.0164696376818
GCGAAATA 0.139686518474
GCGAACAA 0.00564614200978
GCGAACAC -0.169226940385
GCGAACCA -0.230417497222
GCGAACCC -0.135256459846
GCGAACGA -0.0395890832824
GCGAACGC -0.000415015566531
GCGAACTA -0.169737827387
GCGAAGAA 0.0533439775864
GCGAAGAC -0.305069992572
GCGAAGCA -0.168916180788
GCGAAGCC -0.300357240319
GCGAAGGA -0.102906666214
GCGAAGTA -0.0394723589116
GCGAATAA 0.160235253245
GCGAATAC 0.284409754107
GCGAATCA 0.350093793567
GCGAATCC 0.448317463469
GCGAATGA 0.322097326336
GCGAATTA -0.103045178467
GCGACAAA 0.0498434340854
GCGACAAC -0.0715524477566
GCGACACA -0.0495843156719
GCGACACC -0.205358458847
GCGACAGA -0.128299351003
GCGACAGC -0.152237028214
GCGACATA -0.182402007418
GCGACCAA -0.194937239301
GCGACCAC -0.166844486349
GCGACCCA -0.244596059753
GCGACCCC -0.204627404285
GCGACCGA -0.0390712664974
GCGACCGC -0.26233303612
GCGACCTA -0.287523732111
GCGACGAA 0.0381969430039
GCGACGAC -0.162310125099
GCGACGCA 0.142026816931
GCGACGCC -0.160004232434
GCGACGGA -0.0444846646319
GCGACGTA -0.137484801112
GCGACTAA -0.184042287668
GCGACTAC -0.0201160792878
GCGACTCA -0.16000633437

GCGACTCC -0.185296233759
GCGACTGA 0.0655664474501
GCGACTTA -0.314175781928
GCGAGAAA 0.0673505121948
GCGAGAAC -0.311453742065
GCGAGACA -0.152612343848
GCGAGACC -0.171717621371
GCGAGAGA -0.140648648984
GCGAGAGC -0.233145037452
GCGAGATA 0.234638219487
GCGAGCAA 0.00988454018757
GCGAGCAC -0.177329804684
GCGAGCCA -0.272902208892
GCGAGCCC -0.238248048808
GCGAGCGA -0.0976531310805
GCGAGCGC -0.0436743292167
GCGAGCTA -0.132643023217
GCGAGGAA 0.134203630577
GCGAGGAC -0.284006362281
GCGAGGCA -0.245174410035
GCGAGGCC -0.342930934209
GCGAGGGA -0.0616127529231
GCGAGGTA -0.210119585414
GCGAGTAA -0.0181638358284
GCGAGTAC -0.18423088093
GCGAGTCA -0.103095043134
GCGAGTCC -0.275414470679
GCGAGTGA 0.0325582549569
GCGAGTTA -0.232707883704
GCGATAAA 0.210883864082
GCGATAAC -0.120719254323
GCGATACA -0.0196440953848
GCGATACC 0.11035889725
GCGATAGA 0.0562316153661
GCGATAGC -0.0565082989325
GCGATATA 0.170681700985
GCGATCAA 0.277216666253
GCGATCAC 0.229401577886
GCGATCCA 0.36013837529
GCGATCCC 0.288109749422
GCGATCGA 0.132665723575
GCGATCGC 0.397089021745
GCGATCTA 0.389287095903
GCGATGAA 0.0931799568163
GCGATGAC -0.113217587423
GCGATGCA 0.0608066976636
GCGATGCC -0.0685724752646
GCGATGGA -0.0154945120714
GCGATGTA -0.155303312244
GCGATTAA 0.0866572264841
GCGATTAC 0.346022330871
GCGATTCA 0.0714817480209
GCGATTCC 0.339555809253
GCGATTGA 0.0309007831945

GCGATTTA 0.037352779777
GCGCAAAA 0.262790338548
GCGCAAAC -0.105706525486
GCGCAACA -0.0130499789066
GCGCAACC -0.0459500886842
GCGCAAGA 0.0715685057991
GCGCAAGC -0.159909183395
GCGCAATA 0.187133414406
GCGCACAA 0.150721517731
GCGCACAC -0.117279508072
GCGCACCA -0.128593737458
GCGCACCC -0.107034840602
GCGCACGA -0.0171931740163
GCGCACGC -0.219711945389
GCGCACTA -0.186085734261
GCGCAGAA 0.0441968424049
GCGCAGAC -0.172103588018
GCGCAGCA -0.135134688071
GCGCAGCC -0.325833670192
GCGCAGGA -0.0978825967194
GCGCAGTA -0.130847717429
GCGCATAA 0.217987691391
GCGCATAC 0.105185287913
GCGCATCA -0.100265687613
GCGCATCC -0.0794403028105
GCGCATGA -0.0906635898199
GCGCATTA -0.288357031192
GCGCCAAA 0.0966083584031
GCGCCAAC -0.226843926174
GCGCCACA -0.162761887721
GCGCCACC -0.299379207388
GCGCCAGA -0.141500486616
GCGCCAGC -0.261606297236
GCGCCATA -0.222781816227
GCGCCCAA -0.163217617287
GCGCCCAC -0.0974782687807
GCGCCCCA -0.232833831813
GCGCCCCC -0.262150748519
GCGCCCGA -0.23643744228
GCGCCCGC -0.269528398592
GCGCCCTA -0.140775668335
GCGCCGAA 0.0831317562389
GCGCCGAC -0.21640453636
GCGCCGCA 0.182012802639
GCGCCGCC -0.165235280981
GCGCCGGA -0.238836240046
GCGCCGTA -0.132285833244
GCGCCTAA 0.0732647247799
GCGCCTAC -0.0248619510337
GCGCCTCA 0.00934704262111
GCGCCTCC -0.272693190871
GCGCCTGA -0.183762486524
GCGCCTTA -0.182946619986
GCGCGAAA 0.115036167091

GCGCGAAC -0.143770002729
GCGCGACA -0.151169682416
GCGCGACC -0.107544906159
GCGCGAGA 0.000129599757345
GCGCGAGC -0.343594164785
GCGCGATA 0.224297300055
GCGCGCAA 0.222006087458
GCGCGCAC -0.193615226184
GCGCGCCA -0.242786970652
GCGCGCCC -0.220758311347
GCGCGCGA 0.0391429384758
GCGCGCGC 0.140643216828
GCGCGCTA -0.233107279218
GCGCGGAA 0.108517608518
GCGCGGAC -0.0821608476637
GCGCGGCA -0.0869469325416
GCGCGGCC 0.108192990574
GCGCGGGA -0.187119048272
GCGCGGTA -0.100325540317
GCGCGTAA 0.119332831454
GCGCGTAC -0.109780579478
GCGCGTCA -0.125393241078
GCGCGTCC -0.259129219291
GCGCGTGA -0.0147566111691
GCGCGTTA -0.215376487475
GCGCTAAA 0.0856765856766
GCGCTAAC -0.272563614206
GCGCTACA -0.233588583746
GCGCTACC -0.157069786249
GCGCTAGA -0.0914852375636
GCGCTAGC -0.37406323298
GCGCTATA 0.00361670912316
GCGCTCAA 0.157373926118
GCGCTCAC -0.291205059177
GCGCTCCA -0.166093320848
GCGCTCCC -0.0372086742626
GCGCTCGA -0.206168674566
GCGCTCTA -0.347183977905
GCGCTGAA 0.129194974978
GCGCTGAC -0.196409713433
GCGCTGCA -0.135072549652
GCGCTGCC -0.258335656192
GCGCTGGA -0.0213284910255
GCGCTGTA -0.0307746234211
GCGCTTAA 0.0321211944264
GCGCTTAC -0.161552373942
GCGCTTCA -0.0709483588271
GCGCTTCC -0.0776996848713
GCGCTTGA -0.00899880884433
GCGCTTTA -0.185948499061
GCGGAAAA 0.0742105742106
GCGGAAAC -0.30886589639
GCGGAACA -0.239569118802
GCGGAACC -0.34259649703

GCGGAAGA -0.069258000215
GCGGAAGC -0.267951721068
GCGGAATA 0.053186795611
GCGGACAA 0.0166529200091
GCGGACAC -0.150280152461
GCGGACCA -0.299148635613
GCGGACCC -0.257512853382
GCGGACGA -0.175299885876
GCGGACGC -0.130030797566
GCGGACTA -0.316879626184
GCGGAGAA 0.00526630803705
GCGGAGAC -0.253592744214
GCGGAGCA -0.126563939222
GCGGAGCC -0.190819267789
GCGGAGGA -0.200552934464
GCGGAGTA -0.0272573881541
GCGGATAA 0.0915447127568
GCGGATAC 0.285887816191
GCGGATCA 0.107047819169
GCGGATCC 0.274467067871
GCGGATGA -0.176140020578
GCGGATTA 0.304535667102
GCGGCAAA 0.106110284522
GCGGCAAC -0.306720187269
GCGGCACA -0.0166022737722
GCGGCACC -0.238441286654
GCGGCAGA 0.00342058363754
GCGGCAGC -0.255945844666
GCGGCATA 0.0619338908604
GCGGCCAA -0.179694509344
GCGGCCAC -0.32420980397
GCGGCCCA -0.139898501225
GCGGCCCC -0.197354602363
GCGGCCGA -0.174403365931
GCGGCCGC -0.152055413632
GCGGCCTA -0.279569021505
GCGGCGAA 0.0338754732694
GCGGCGAC -0.261103255522
GCGGCGCA 0.21423691452
GCGGCGCC -0.146917248898
GCGGCGGA -0.327517323999
GCGGCGTA -0.130794293547
GCGGCTAA -0.0560643003504
GCGGCTAC -0.155630271099
GCGGCTCA -0.352762824594
GCGGCTCC -0.438712176589
GCGGCTGA -0.272097760797
GCGGCTTA -0.388277131575
GCGGGAAA -0.049416256599
GCGGGAAC -0.205447412322
GCGGGACA -0.0860383709778
GCGGGACC -0.23629490714
GCGGGAGA -0.0549940664124
GCGGGAGC -0.330691243477

GCGGGATA 0.158286385559
GCGGGCAA -0.222492424681
GCGGGCAC -0.329432341048
GCGGGCCA -0.298914826116
GCGGGCCC -0.293548813865
GCGGGCGA -0.237548141543
GCGGGCTA -0.160220088068
GCGGGGAA 0.12309146515
GCGGGGAC -0.184799544458
GCGGGGCA -0.0253405230867
GCGGGGCC -0.0658223691412
GCGGGGGA -0.112341522093
GCGGGGTA -0.0466825487423
GCGGGTAA 0.168045455924
GCGGGTAC -0.152146824905
GCGGGTCA -0.196049000393
GCGGGTCC -0.213685310389
GCGGGTGA -0.159173198642
GCGGGTTA -0.102372210828
GCGGTAAA 0.0199276411398
GCGGTAAC -0.111008518062
GCGGTACA -0.13114943955
GCGGTACC 0.00393257472937
GCGGTAGA -0.0850840075491
GCGGTAGC -0.153727371744
GCGGTATA 0.0549148558791
GCGGTCAA 0.007344730085
GCGGTCAC -0.278156775515
GCGGTCCA -0.394070078109
GCGGTCCC -0.297593005795
GCGGTCGA -0.190876511048
GCGGTCTA -0.196122787138
GCGGTGAA 0.0257378590712
GCGGTGAC -0.286805749117
GCGGTGCA -0.0570929055778
GCGGTGCC -0.244751208678
GCGGTGGA -0.23088837172
GCGGTGTA 0.0804529827814
GCGGTTAA 0.102674420472
GCGGTTAC -0.142760641979
GCGGTTCA -0.0123864278722
GCGGTTCC -0.223462824659
GCGGTTGA -0.240757739902
GCGGTTTA -0.0971557121472
GCGTAAAA 0.159338401763
GCGTAAAC -0.156068516283
GCGTAACA 0.0848851606427
GCGTAACC 0.0500711663248
GCGTAAGA 0.0105463590312
GCGTAAGC -0.251844868413
GCGTAATA 0.208493974584
GCGTACAA -0.0163535747078
GCGTACAC -0.186954097887
GCGTACCA -0.120384819188

GCGTACCC -0.0348433833282
GCGTACGA -0.018176629693
GCGTACGC -0.146040211115
GCGTACTA 0.0171667773698
GCGTAGAA 0.0126530075984
GCGTAGAC -0.185247050067
GCGTAGCA -0.0339595794141
GCGTAGCC -0.333592429968
GCGTAGGA -0.084781697984
GCGTAGTA 0.119836061984
GCGTATAA 0.123544009985
GCGTATAC 0.235789727232
GCGTATCA 0.190518582575
GCGTATCC 0.405073611527
GCGTATGA -0.109926862838
GCGTATTA 0.159768034277
GCGTCAAA 0.0457385760416
GCGTCAAC 0.0302407402812
GCGTCACA -0.0342725645756
GCGTCACC -0.180946000711
GCGTCAGA -0.0244413617619
GCGTCAGC -0.245035029819
GCGTCATA -0.10580631998
GCGTCCAA -0.23677634623
GCGTCCAC -0.00388677620198
GCGTCCCA -0.165313098733
GCGTCCCC -0.204074542793
GCGTCCGA -0.223286258072
GCGTCCTA -0.179872550748
GCGTCGAA -0.130621241822
GCGTCGAC -0.25399251918
GCGTCGCA 0.0913724516981
GCGTCGCC 0.0314218398976
GCGTCGGA -0.168781042427
GCGTCGTA -0.153486420005
GCGTCTAA 0.0649000386853
GCGTCTAC 0.00885747027437
GCGTCTCA -0.231422017345
GCGTCTCC -0.421734145784
GCGTCTGA -0.0824561454728
GCGTCTTA -0.0510464116669
GCGTGAAA 0.15202668233
GCGTGAAC -0.369970422619
GCGTGACA -0.191652666625
GCGTGACC -0.228447401488
GCGTGAGA -0.0988157862088
GCGTGAGC -0.256618285573
GCGTGATA 0.127692824245
GCGTGCAA -0.0472205934781
GCGTGCAC -0.193354944167
GCGTGCCA -0.318868590824
GCGTGCCC -0.333061475105
GCGTGCGA -0.096733302421
GCGTGCTA -0.195795983844

GCGTGGAA 0.0688158123337
GCGTGGAC -0.167293559589
GCGTGGCA 0.0335576593213
GCGTGGCC -0.207466677694
GCGTGGGA -0.0661554898117
GCGTGGTA 0.101649598914
GCGTGTAA 0.0792851251432
GCGTGTAC -0.129877290635
GCGTGTCA 0.090388531638
GCGTGTCC -0.283888434001
GCGTGTGA -0.146942466373
GCGTGTTA -0.0185736700888
GCGTTAAA 0.0524535959067
GCGTTAAC -0.117675276448
GCGTTACA 0.0605550454035
GCGTTACC -0.11010926768
GCGTTAGA -0.119410043652
GCGTTAGC -0.27924867816
GCGTTATA 0.0528973818092
GCGTTCAA -0.102311977793
GCGTTCAC -0.22306055711
GCGTTCCA -0.289504656849
GCGTTCCC -0.149349056976
GCGTTCGA -0.196028763937
GCGTTCTA -0.272510299116
GCGTTGAA -0.0468143033364
GCGTTGAC -0.194239734884
GCGTTGCA 0.0669305669306
GCGTTGCC -0.140020609619
GCGTTGGA -0.192189222134
GCGTTGTA -0.0533770685286
GCGTTTAA -0.0979119741799
GCGTTTAC 0.0294637830523
GCGTTTCA 0.143663277446
GCGTTTCC -0.0813987393603
GCGTTTGA -0.017321388689
GCGTTTTA -0.122192440374
GCTAAAAA 0.0607894395773
GCTAAAAC -0.237596758349
GCTAAACA -0.0407157411773
GCTAAACC -0.201367695264
GCTAAAGA 0.0242206375043
GCTAAAGC -0.184651941509
GCTAAATA 0.139156806785
GCTAACAA 0.175271344423
GCTAACAC -0.100012716175
GCTAACCA -0.0751424797682
GCTAACCC -0.342133445386
GCTAACGA -0.0737705560469
GCTAACTA 0.0209039218119
GCTAAGAA 0.210521667409
GCTAAGAC -0.221155600312
GCTAAGCA -0.146260459373
GCTAAGCC -0.0823107586004

GCTAAGGA -0.121636933562
GCTAAGTA -0.0248388884753
GCTAATAA 0.0449530302256
GCTAATAC 0.0481704780187
GCTAATCA -0.142742558201
GCTAATCC -0.190641809274
GCTAATGA -0.133747204782
GCTAATTA -0.104515881346
GCTACAAA -0.0552454049464
GCTACAAC 0.0043583528432
GCTACACA -0.123860482973
GCTACACC -0.107750123383
GCTACAGA -0.0188697604497
GCTACAGC -0.192079466565
GCTACATA -0.146228794984
GCTACCAA -0.141641231792
GCTACCAC -0.212280307464
GCTACCCA -0.165034666226
GCTACCCC -0.286259043625
GCTACCGA -0.0906193310269
GCTACCTA -0.0558217033557
GCTACGAA 0.143822741147
GCTACGAC -0.295651964554
GCTACGCA -0.339549875756
GCTACGCC -0.235509650929
GCTACGGA -0.108593038792
GCTACGTA -0.147580602912
GCTACTAA -0.0871302636746
GCTACTAC -0.237588857742
GCTACTCA -0.32502294659
GCTACTCC -0.302938703497
GCTACTGA -0.143791255059
GCTACTTA -0.0203311131644
GCTAGAAA -0.0161953743455
GCTAGAAC -0.159278274537
GCTAGACA -0.223837519029
GCTAGACC -0.368945291126
GCTAGAGA -0.0549019940735
GCTAGAGC -0.25800470775
GCTAGATA 0.201086837515
GCTAGCAA -0.169618813955
GCTAGCAC -0.180702922128
GCTAGCCA -0.15454184552
GCTAGCCC -0.358118407162
GCTAGCGA -0.0544181108767
GCTAGCTA -0.170783802915
GCTAGGAA 0.133245987314
GCTAGGAC -0.173839749686
GCTAGGCA -0.164080888737
GCTAGGCC -0.393772071255
GCTAGGGA -0.0806778359807
GCTAGGTA 0.0615570461961
GCTAGTAA -0.220110949035
GCTAGTAC -0.295830153243

GCTAGTCA -0.0252644102684
GCTAGTCC -0.372891259112
GCTAGTGA -0.203788235878
GCTAGTTA 0.0610461084794
GCTATAAA 0.0536721294297
GCTATAAC 0.0898026070861
GCTATACA -0.110635428817
GCTATACC -0.177718507491
GCTATAGA -0.0724159877615
GCTATAGC -0.224786494374
GCTATATA 0.0914746392415
GCTATCAA -0.0415410798448
GCTATCAC -0.0567191381768
GCTATCCA 0.147374647375
GCTATCCC -0.0397254074728
GCTATCGA 0.00711317377984
GCTATCTA 0.0833551090122
GCTATGAA 0.185895635567
GCTATGAC -0.216304254926
GCTATGCA -0.0964992181877
GCTATGCC -0.117895933777
GCTATGGA 0.0250499981059
GCTATGTA 0.0486643668462
GCTATTAA 0.0668952703541
GCTATTAC -0.143955250016
GCTATTCA -0.143625922526
GCTATTCC 0.05849433954
GCTATTGA 0.113687291519
GCTATTTA -0.0603262670363
GCTCAAAA 0.0658661416237
GCTCAAAC -0.0896322744796
GCTCAACA -0.132667303541
GCTCAACC -0.15983785401
GCTCAAGA -0.0545809777793
GCTCAAGC -0.0427204404503
GCTCAATA -0.062991844379
GCTCACAA 0.091839773658
GCTCACAC -0.247459554595
GCTCACCA -0.0808102849586
GCTCACCC -0.268876154565
GCTCACGA -0.0186010091206
GCTCACTA -0.0241939708773
GCTCAGAA -0.137189639027
GCTCAGAC -0.201664304657
GCTCAGCA -0.279789811393
GCTCAGCC -0.236211388165
GCTCAGGA -0.136471541891
GCTCAGTA -0.076759773326
GCTCATAA 0.0405974169496
GCTCATAC -0.345993985949
GCTCATCA -0.258266745477
GCTCATCC -0.212597787496
GCTCATGA -0.210261384387
GCTCATTA 0.0781698210988

GCTCCAAA -0.0436815252221
GCTCCAAC -0.207506570566
GCTCCACA -0.202703820418
GCTCCACC -0.393579796913
GCTCCAGA -0.0526314637211
GCTCCAGC -0.273988802607
GCTCCATA -0.181013590081
GCTCCCAA -0.184611129517
GCTCCCAC -0.339556547742
GCTCCCCA -0.185098002729
GCTCCCCC -0.164228516722
GCTCCCGA -0.0881557722324
GCTCCCTA -0.269398066602
GCTCCGAA -0.20589986559
GCTCCGAC -0.257460474989
GCTCCGCA -0.198520025696
GCTCCGCC -0.132697593507
GCTCCGGA -0.27776813152
GCTCCGTA -0.0326569491179
GCTCCTAA -0.18438786455
GCTCCTAC -0.371588675823
GCTCCTCA -0.224905555207
GCTCCTCC -0.380335008864
GCTCCTGA -0.21498208513
GCTCCTTA -0.0697061523932
GCTCGAAA -0.201262704034
GCTCGAAC -0.371911024067
GCTCGACA -0.241049814612
GCTCGACC -0.336301528773
GCTCGAGA -0.159309529543
GCTCGAGC -0.151107858783
GCTCGATA 0.0954687469839
GCTCGCAA -0.215811382652
GCTCGCAC -0.170566924008
GCTCGCCA -0.246328911437
GCTCGCCC -0.279961561642
GCTCGCGA -0.0108527083695
GCTCGCTA -0.0527534619893
GCTCGGAA 0.185367473705
GCTCGGAC -0.142992259793
GCTCGGCA -0.259347277575
GCTCGGCC -0.185826986529
GCTCGGGA -0.0130890052356
GCTCGGTA 0.0823882671167
GCTCGTAA -0.0988980693469
GCTCGTAC -0.359644336417
GCTCGTCA -0.149739718251
GCTCGTCC -0.395249856082
GCTCGTGA -0.143581812217
GCTCGTTA 0.210490104429
GCTCTAAA -0.225560668833
GCTCTAAC -0.289490657506
GCTCTACA -0.0690862135856
GCTCTACC -0.283975424409

GCTCTAGA -0.193772012664
GCTCTATA -0.0972039559846
GCTCTCAA -0.202999671881
GCTCTCAC -0.23913519271
GCTCTCCA -0.220076355151
GCTCTCCC -0.3429393236
GCTCTCGA -0.0998525579732
GCTCTCTA -0.176700441757
GCTCTGAA -0.0807565748652
GCTCTGAC -0.291009039111
GCTCTGCA -0.0949206154583
GCTCTGCC -0.133892382847
GCTCTGGA -0.10158045998
GCTCTGTA -0.0907860087411
GCTCTTAA 0.0278176390298
GCTCTTAC -0.238132037333
GCTCTTCA -0.246135177221
GCTCTTCC -0.194686409949
GCTCTTGA -0.137189082176
GCTCTTTA 0.0216930954331
GCTGAAAA 0.237728615033
GCTGAAAC -0.0796406238368
GCTGAACA -0.18771993327
GCTGAACC -0.378977556178
GCTGAAGA -0.0121172529069
GCTGAAGC -0.343710465607
GCTGAATA 0.0112936628088
GCTGACAA -0.0141362433457
GCTGACAC -0.115242360064
GCTGACCA -0.223730661292
GCTGACCC -0.323582271398
GCTGACGA -0.00482058378328
GCTGACTA -0.29150448419
GCTGAGAA 0.233682097923
GCTGAGAC -0.249747953352
GCTGAGCA -0.153152606115
GCTGAGCC -0.270448571916
GCTGAGGA -0.0289309632718
GCTGAGTA -0.152959406216
GCTGATAA 0.185627962379
GCTGATAC 0.159619132428
GCTGATCA -0.183271707571
GCTGATCC 0.0418056538908
GCTGATGA -0.156139475071
GCTGATTA 0.185545571995
GCTGCAAA -0.0906422590047
GCTGCAAC -0.118850622364
GCTGCACA -0.259368902497
GCTGCACC -0.238873077923
GCTGCAGA -0.0555652066013
GCTGCAGC -0.192630168928
GCTGCATA -0.286278869291
GCTGCCAA -0.194342145404
GCTGCCAC -0.263115388962

GCTGCCCA -0.166004609589
GCTGCCCC -0.328050145414
GCTGCCGA -0.106464596342
GCTGCCTA 0.034772231265
GCTGCGAA 0.164369393371
GCTGCGAC -0.308363723569
GCTGCGCA -0.328466485575
GCTGCGCC -0.0943846087603
GCTGCGGA 0.00402797909158
GCTGCGTA -0.061649804074
GCTGCTAA -0.107966114756
GCTGCTAC -0.212207338092
GCTGCTCA -0.159635207746
GCTGCTCC -0.26449152519
GCTGCTGA -0.0371794778217
GCTGCTTA -0.342557420398
GCTGGAAA 0.0107476622628
GCTGGAAC -0.133532400859
GCTGGACA -0.0997103856804
GCTGGACC -0.349988838455
GCTGGAGA -0.0861872654365
GCTGGATA 0.0994686247746
GCTGGCAA 0.0310324098203
GCTGGCAC -0.361952400197
GCTGGCCA -0.248486904996
GCTGGCCC -0.224189467417
GCTGGCGA -0.143593773697
GCTGGCTA -0.272625398007
GCTGGGAA 0.176564746757
GCTGGGAC -0.130713521545
GCTGGGCA -0.134470772109
GCTGGGCC -0.36456016164
GCTGGGGA -0.0690750630914
GCTGGGTA 0.00624408147805
GCTGGTAA 0.107739976497
GCTGGTAC -0.214472367627
GCTGGTCA -0.26511799715
GCTGGTCC -0.231080183361
GCTGGTGA -0.136068995371
GCTGGTTA -0.0319663264964
GCTGTAAA -0.055916798341
GCTGTAAC -0.0367327722751
GCTGTACA -0.173124444438
GCTGTACC -0.195871070197
GCTGTAGA -0.0741268142863
GCTGTATA 0.0204736416858
GCTGTCAA -0.0185395497256
GCTGTCAC -0.166361639557
GCTGTCCA -0.0143157449107
GCTGTCCC -0.315549877199
GCTGTCGA 0.015978482223
GCTGTCTA -0.174181198248
GCTGTGAA 0.22750377729
GCTGTGAC -0.0708341542795

GCTGTGCA 0.0612341269893
GCTGTGCC -0.135672117749
GCTGTGGA -0.0343405267845
GCTGTGTA -0.110073523938
GCTGTTAA 0.09467456917
GCTGTTAC -0.301279984124
GCTGTTCA -0.298961356852
GCTGTTCC 0.0213091879759
GCTGTTGA -0.0492865119742
GCTGTTTA -0.119547827359
GCTTAAAA 0.110166389558
GCTTAAAC -0.0548987978365
GCTTAACA -0.228054142717
GCTTAACC -0.123424924617
GCTTAAGA 0.0567835293534
GCTTAAGC -0.11882122728
GCTTAATA 0.0728676334737
GCTTACAA 0.131233161536
GCTTACAC -0.297525627138
GCTTACCA 0.033696677172
GCTTACCC -0.243639624704
GCTTACGA -0.160347864923
GCTTACTA -0.0328902564442
GCTTAGAA 0.0385414917922
GCTTAGAC -0.217844358215
GCTTAGCA -0.135635797107
GCTTAGCC -0.0732159305393
GCTTAGGA -0.0974175747976
GCTTAGTA -0.0949192885778
GCTTATAA 0.174458223543
GCTTATAC -0.0698646304707
GCTTATCA -0.0347916718366
GCTTATCC -0.00916549272644
GCTTATGA -0.0798398302772
GCTTATTA 0.106278454763
GCTTCAAA -0.049649277753
GCTTCAAC -0.13422973312
GCTTCACA -0.13854220943
GCTTCACC -0.227683060125
GCTTCAGA -0.0624136053588
GCTTCATA -0.196836383383
GCTTCCAA -0.25574479885
GCTTCCAC -0.261759379118
GCTTCCCA -0.169912988645
GCTTCCCC -0.259961884583
GCTTCCGA -0.0692167324803
GCTTCCTA -0.197657626362
GCTTCGAA -0.00536411611425
GCTTCGAC -0.209577521931
GCTTCGCA -0.184959097414
GCTTCGCC -0.058457880286
GCTTCGGA -0.176175091604
GCTTCGTA -0.188443264201
GCTTCTAA -0.0100334494274

GCTTCTAC -0.0753792794384
GCTTCTCA -0.0703994110982
GCTTCTCC -0.344820164565
GCTTCTGA -0.17776206114
GCTTCTTA -0.100972436916
GCTTGAAA -0.143030590399
GCTTGAAC -0.0468295520652
GCTTGACA -0.0999635044488
GCTTGACC -0.186173335649
GCTTGAGA -0.0812068675539
GCTTGATA -0.0136541500178
GCTTGCAA 0.0231530883192
GCTTGCAC -0.257402745244
GCTTGCCA -0.349180757093
GCTTGCCC -0.295191515282
GCTTGCGA 0.00185607160948
GCTTGCTA -0.184931806738
GCTTGGAA -0.0281198221937
GCTTGGAC -0.176589563732
GCTTGGCA -0.284508422437
GCTTGGCC -0.303252229469
GCTTGGGA -0.0663716351849
GCTTGGTA -0.13085794795
GCTTGTAA -0.00376415114997
GCTTGTAC -0.238090807592
GCTTGTCA 0.00988810464004
GCTTGTCC -0.245897416062
GCTTGTGA -0.0406983278139
GCTTGTTA -0.0913426144213
GCTTTAAA -0.0618377298558
GCTTTAAC -0.0783237512236
GCTTTACA -0.0772111235196
GCTTTACC -0.208326095767
GCTTTAGA -0.0106457522828
GCTTTATA 0.0984580597208
GCTTTCAA -0.0159974599096
GCTTTCAC -0.137544013478
GCTTTCCA -0.178609426651
GCTTTCCC -0.343034631687
GCTTTCGA -0.0575459757278
GCTTTCTA -0.136727881871
GCTTTGAA -0.161426425703
GCTTTGAC -0.0590271644802
GCTTTGCA -0.0650927351956
GCTTTGCC -0.0973025366965
GCTTTGGA -0.147041714568
GCTTTGTA -0.147002715406
GCTTTTAA 0.129549659853
GCTTTTAC -0.0942576741237
GCTTTTCA 0.127018202776
GCTTTTCC -0.0570156209069
GCTTTTGA -0.0589494323533
GCTTTTTA -0.0628751844039
GGAAAAAA -0.0214473811198

GGAAAAAC -0.0764421393376
GGAAAACA -0.0244781370368
GGAAAACC -0.20575856636
GGAAAAGA -0.00658744699021
GGAAAATA 0.254044966127
GGAAACAA -0.029997153347
GGAAACAC 0.106113000052
GGAAACCA -0.111911257468
GGAAACCC -0.370266894125
GGAAACGA 0.0209156003883
GGAAACTA -0.0670503565615
GGAAAGAA -0.0801121120127
GGAAAGAC -0.236993489996
GGAAAGCA -0.0883644755453
GGAAAGCC -0.347839625856
GGAAAGGA -0.2568399895
GGAAAGTA -0.0350125459251
GGAAATAA 0.176916223944
GGAAATAC 0.210371305738
GGAAATCA 0.444710699766
GGAAATCC 0.4905277178
GGAAATGA -0.190617862329
GGAAATTA 0.253552841909
GGAACAAA -0.223053549616
GGAACAAC -0.0959635920381
GGAACACA -0.150870418896
GGAACACC -0.257159053932
GGAACAGA 0.110497549891
GGAACATA -0.0488376166945
GGAACCAA -0.134353163058
GGAACCAC -0.174231256049
GGAACCCA -0.259891935106
GGAACCCC -0.27360921631
GGAACCGA -0.227513007436
GGAACCTA -0.270750365194
GGAACGAA -0.155554760921
GGAACGAC -0.289727135655
GGAACGCA -0.160118881701
GGAACGCC -0.32668689949
GGAACGGA -0.197842451207
GGAACGTA -0.138490000744
GGAACTAA -0.16083749199
GGAACTAC -0.146470161622
GGAACTCA -0.161922976967
GGAACTCC -0.314010920106
GGAACTGA -0.125629303311
GGAACTTA -0.0357254950622
GGAAGAAA -0.0816672319141
GGAAGAAC -0.313842959227
GGAAGACA -0.0713299340631
GGAAGACC -0.360312499139
GGAAGAGA 0.0110705604987
GGAAGATA 0.160769472201
GGAAGCAA -0.180124527294

GGAAGCAC -0.179943372958
GGAAGCCA -0.282039301948
GGAAGCCC -0.25884856353
GGAAGCGA -0.205588957834
GGAAGCTA -0.291399807672
GGAAGGAA -0.0505283587933
GGAAGGAC -0.345428184752
GGAAGGCA -0.331241126421
GGAAGGCC -0.236411897497
GGAAGGGA -0.333358382422
GGAAGGTA -0.124344455035
GGAAGTAA 0.055373240002
GGAAGTAC -0.111188319477
GGAAGTCA -0.180066146003
GGAAGTCC -0.3672250749
GGAAGTGA 0.105225059771
GGAAGTTA -0.205115163545
GGAATAAA 0.0408129748984
GGAATAAC 0.109339941461
GGAATACA 0.0613555907535
GGAATACC -0.040831952585
GGAATAGA 0.0719845550827
GGAATATA 0.249246366868
GGAATCAA 0.35828047561
GGAATCAC 0.328715537137
GGAATCCA 0.450843405389
GGAATCCC 0.405017965624
GGAATCGA 0.347754951151
GGAATCTA 0.468725959749
GGAATGAA -0.158184641293
GGAATGAC -0.125707558224
GGAATGCA 0.07501027198
GGAATGCC -0.263738394894
GGAATGGA 0.0923563717583
GGAATGTA -0.0568047386229
GGAATTAA 0.152857236703
GGAATTAC 0.209392138373
GGAATTCA -0.00278607037802
GGAATTCC 0.096338286735
GGAATTGA 0.140542177909
GGAATTTA 0.172027619833
GGACAAAA 0.220245542068
GGACAAAC -0.283857634839
GGACAACA 0.0370962679649
GGACAACC -0.210261627667
GGACAAGA 0.122368925399
GGACAATA -0.0260255105839
GGACACAA -0.0493086239186
GGACACAC 0.00293790825344
GGACACCA -0.266340477389
GGACACCC -0.089611701359
GGACACGA 0.0726896491318
GGACACTA -0.212840712239
GGACAGAA 0.0830216010323

GGACAGAC -0.232714104144
GGACAGCA -0.170550289338
GGACAGCC -0.34987488236
GGACAGGA -0.107809762922
GGACAGTA -0.0528230665089
GGACATAA -0.257976646882
GGACATAC -0.0179358659421
GGACATCA -0.161270191164
GGACATCC -0.164434658909
GGACATGA -0.0816101483396
GGACATTA 0.00781953679846
GGACCAAA -0.00247216913884
GGACCAAC -0.107239774089
GGACCACA -0.128859915551
GGACCACC -0.380153513305
GGACCAGA -0.113747918342
GGACCATA -0.126744045178
GGACCCAA -0.236439120719
GGACCCAC -0.196806413639
GGACCCCA -0.288656160571
GGACCCCC -0.379401105186
GGACCCGA -0.0378998436608
GGACCCTA -0.277177631973
GGACCGAA -0.188290456226
GGACCGAC -0.272157898654
GGACCGCA -0.282609330105
GGACCGCC -0.341384803212
GGACCGGA -0.139896543218
GGACCGTA -0.246227515197
GGACCTAA -0.218908032137
GGACCTAC -0.147174115137
GGACCTCA -0.384295000401
GGACCTCC -0.411358347134
GGACCTGA -0.271968199623
GGACCTTA -0.179761835833
GGACGAAA -0.094138521478
GGACGAAC -0.201339981221
GGACGACA -0.211376032437
GGACGACC -0.414042748123
GGACGAGA -0.118921349708
GGACGATA 0.0567133566418
GGACGCAA 0.0539110863207
GGACGCAC -0.220433152294
GGACGCCA -0.314304814677
GGACGCCC -0.395517074857
GGACGCGA -0.047199696357
GGACGCTA -0.301888560851
GGACGGAA 0.00500638379426
GGACGGAC -0.320679256121
GGACGGCA -0.138576442858
GGACGGCC -0.402561356876
GGACGGGA -0.12658317439
GGACGGTA 0.00964091619724
GGACGTAA -0.082874931412

GGACGTAC -0.139292124701
GGACGTCA -0.190922147089
GGACGTCC -0.237188422332
GGACGTGA -0.0679758837398
GGACGTTA -0.283334051648
GGACTAAA -0.00146412884334
GGACTAAC -0.22734252638
GGACTACA -0.312351157252
GGACTACC -0.2469492614
GGACTAGA 0.0924760771662
GGACTATA -0.174582103008
GGACTCAA -0.264612229032
GGACTCAC -0.0680799909565
GGACTCCA -0.244486900617
GGACTCCC -0.246988637441
GGACTCGA -0.131347601045
GGACTCTA -0.190509033212
GGACTGAA -0.194783327436
GGACTGAC -0.271659173456
GGACTGCA -0.245039141007
GGACTGCC -0.323970950983
GGACTGGA -0.171773813364
GGACTGTA -0.255067358027
GGACTTAA -0.121093933102
GGACTTAC -0.116109136296
GGACTTCA -0.260527752277
GGACTTGA 0.109427530784
GGACTTTA -0.328734139881
GGAGAAAA -0.0625708352981
GGAGAAAC -0.107923824964
GGAGAACA -0.057544122391
GGAGAACC -0.15390787199
GGAGAAGA -0.176080993535
GGAGAATA 0.220044086626
GGAGACAA -0.261308259813
GGAGACAC -0.0117440504256
GGAGACCA -0.106085373881
GGAGACCC -0.304807933476
GGAGACGA -0.0649225166878
GGAGACTA -0.242547642987
GGAGAGAA -0.101773127802
GGAGAGAC -0.372171923877
GGAGAGCA -0.187359969173
GGAGAGCC -0.204575341786
GGAGAGGA -0.279759254496
GGAGAGTA -0.133259540637
GGAGATAA 0.0537464942543
GGAGATAC 0.22712933319
GGAGATCA 0.310544209246
GGAGATCC 0.388092961468
GGAGATGA 0.0152310533346
GGAGATTA 0.364125603089
GGAGCAAA -0.178552154926
GGAGCAAC -0.269909210965

GGAGCACA -0.115008430472
GGAGCACC -0.341189165999
GGAGCAGA -0.0863739511902
GGAGCATA 0.0881473760262
GGAGCCAA -0.0356087092983
GGAGCCAC -0.236733731921
GGAGCCCA -0.383943114639
GGAGCCCC -0.428932975133
GGAGCCGA -0.100035785138
GGAGCCTA -0.162506719596
GGAGCGAA -0.0962851762708
GGAGCGAC -0.170197722593
GGAGCGCA -0.0391700240185
GGAGCGCC -0.316527965655
GGAGCGGA -0.0743226663799
GGAGCGTA 0.0705612285862
GGAGCTAA -0.284938922841
GGAGCTAC -0.26075571257
GGAGCTCA -0.273712809677
GGAGCTCC -0.398522210925
GGAGCTGA -0.220547733273
GGAGCTTA -0.103267179025
GGAGGAAA -0.0104136056661
GGAGGAAC -0.298658531068
GGAGGACA -0.335805778882
GGAGGACC -0.401236252001
GGAGGAGA -0.0520933539061
GGAGGATA 0.27472789594
GGAGGCAA -0.146333363987
GGAGGCAC -0.268586738111
GGAGGCCA -0.35826724991
GGAGGCCC -0.328657886251
GGAGGCGA -0.0937867873084
GGAGGCTA -0.290005131667
GGAGGGAA -0.155226135706
GGAGGGAC -0.0910657326309
GGAGGGCA -0.268784465487
GGAGGGCC -0.360537215705
GGAGGGGA -0.121324390856
GGAGGGTA -0.0199360597943
GGAGGTAA -0.00924386430752
GGAGGTAC -0.280667003008
GGAGGTCA -0.288936091965
GGAGGTGA -0.189571920483
GGAGGTTA -0.137233736642
GGAGTAAA -0.0675805837258
GGAGTAAC -0.241212663397
GGAGTACA -0.192299271511
GGAGTACC -0.241909119435
GGAGTAGA -0.00714509901243
GGAGTATA 0.132279585119
GGAGTCAA -0.174850231363
GGAGTCAC -0.00359424659288
GGAGTCCA -0.231608251786

GGAGTCCC	-0.342633248713
GGAGTCGA	0.0495633374421
GGAGTCTA	-0.220428690172
GGAGTGAA	-0.134701343883
GGAGTGAC	-0.276531723387
GGAGTGCA	-0.105059127467
GGAGTGCC	-0.197665765386
GGAGTGGA	-0.0511660933137
GGAGTGTA	-0.0766041523617
GGAGTTAA	-0.133502339341
GGAGTTAC	-0.0580967565824
GGAGTTCA	-0.180337810914
GGAGTTGA	0.0272241938909
GGAGTTTA	-0.141129909198
GGATAAAA	-5.27334541033E-5
GGATAAAC	-0.0178075581476
GGATAACA	0.104354635814
GGATAACC	-0.0653420412262
GGATAAGA	0.0385523264311
GGATAATA	0.231555246707
GGATACAA	-0.0158850310365
GGATACAC	0.161286630984
GGATACCA	0.158906739268
GGATACCC	0.274286683378
GGATACGA	0.323498755502
GGATACTA	0.100213134019
GGATAGAA	0.00232611097892
GGATAGAC	-0.123635384326
GGATAGCA	-0.0594154872521
GGATAGCC	0.0949778980082
GGATAGGA	-0.0944797929594
GGATAGTA	0.000599256950578
GGATATAA	0.194257789975
GGATATAC	0.177164768074
GGATATCA	0.4061761486
GGATATCC	0.48066698089
GGATATGA	0.293209187149
GGATATTA	0.291314730709
GGATCAAA	-0.126533846994
GGATCAAC	-0.133616656668
GGATCACA	0.213492405276
GGATCACC	0.105551194011
GGATCAGA	0.0564372037621
GGATCATA	0.128457658761
GGATCCAA	0.122799397541
GGATCCAC	0.221445515792
GGATCCCA	0.121024357131
GGATCCCC	0.376054336282
GGATCCGA	0.212496806639
GGATCCTA	0.286787150646
GGATCGAA	0.0914840460295
GGATCGAC	-0.193358727935
GGATCGCA	0.336669270016
GGATCGCC	0.194503040079

GGATCGGA -0.100051842476
GGATCGTA 0.226434423404
GGATCTAA 0.191212601184
GGATCTAC 0.204371437122
GGATCTCA 0.359426920033
GGATCTGA 0.218657255681
GGATCTTA 0.298310985891
GGATGAAA 0.00448945987805
GGATGAAC -0.293838209578
GGATGACA 0.00482252213455
GGATGACC -0.357339845124
GGATGAGA 0.045361780399
GGATGATA 0.202158349257
GGATGCAA -0.0992877835913
GGATGCAC -0.211419183153
GGATGCCA -0.201978297832
GGATGCCC -0.268318224742
GGATGCGA -0.0725392874609
GGATGCTA -0.170058319891
GGATGGAA 0.0152682508842
GGATGGAC -0.416467407875
GGATGGCA -0.0920984843509
GGATGGCC -0.355956850973
GGATGGGA -0.0774822240419
GGATGGTA 0.0193843981723
GGATGTAA 0.0766841221387
GGATGTAC -0.158911362038
GGATGTCA 0.0720899963324
GGATGTGA 0.00884871134209
GGATGTTA 0.00982971175814
GGATTAAA -0.12312341018
GGATTAAC -0.194692178201
GGATTACA 0.457980742983
GGATTACC 0.434529900805
GGATTAGA 0.0706196214681
GGATTATA 0.242588318346
GGATTCAA 0.132958026897
GGATTCAC 0.205260317145
GGATTCCA 0.386892402044
GGATTCCC 0.483731743101
GGATTCGA 0.265655462625
GGATTCTA 0.404545155945
GGATTGAA -0.166109584202
GGATTGAC -0.185229971203
GGATTGCA 0.420797453905
GGATTGCC 0.42163675248
GGATTGGA 0.0306601367207
GGATTGTA 0.250580644219
GGATTTAA 0.178400165115
GGATTTAC 0.283730283278
GGATTTCA 0.458246169792
GGATTTGA 0.333060589914
GGATTTTA 0.386489689059
GGCAAAAA 0.119605677842

GGCAAAAC -0.0908745982135
GGCAAACA -0.00715177987905
GGCAAACC -0.18351759014
GGCAAAGA 0.0352463838353
GGCAAATA 0.253893059571
GGCAACAA 0.0408852378549
GGCAACAC -0.139582557995
GGCAACCA -0.103504920813
GGCAACCC -0.160497253806
GGCAACGA -0.157054754678
GGCAACTA 0.0349590884871
GGCAAGAA -0.0180221543858
GGCAAGAC -0.195899152158
GGCAAGCA -0.240915318057
GGCAAGCC -0.466113340657
GGCAAGGA -0.127219065422
GGCAAGTA 0.115850747794
GGCAATAA 0.0163535747078
GGCAATAC -0.0405595346486
GGCAATCA 0.36324970986
GGCAATGA 0.0610734701644
GGCAATTA 0.0423896416726
GGCACAAA 0.00362546805544
GGCACAAC -0.230748559191
GGCACACA 0.0451705148675
GGCACACC -0.290751903947
GGCACAGA 0.022913927003
GGCACATA 0.110878593899
GGCACCAA -0.192549768834
GGCACCAC -0.177848075094
GGCACCCA -0.287091937009
GGCACCCC -0.195877785512
GGCACCGA -0.0814688910394
GGCACCTA -0.212567158172
GGCACGAA -0.118056457628
GGCACGAC -0.213123833402
GGCACGCA -0.194851775578
GGCACGCC -0.333443489755
GGCACGGA -0.0124017414273
GGCACGTA -0.13088631395
GGCACTAA -0.147543412293
GGCACTAC -0.28805492278
GGCACTCA -0.178297192882
GGCACTGA -0.156192123295
GGCACTTA -0.183980611573
GGCAGAAA 0.027455603162
GGCAGAAC -0.178789332495
GGCAGACA -0.104903347478
GGCAGACC -0.243147730061
GGCAGAGA -0.0875899226025
GGCAGATA 0.220338970679
GGCAGCAA -0.113797351401
GGCAGCAC -0.144642015431
GGCAGCCA 0.0245327972601

GGCAGCCC -0.189249523098
GGCAGCGA -0.18884838278
GGCAGCTA -0.0683544913536
GGCAGGAA -0.149122499763
GGCAGGAC -0.28858319482
GGCAGGCA -0.204948344982
GGCAGGCC -0.43060687979
GGCAGGGA -0.173895626871
GGCAGGTA -0.164528376802
GGCAGTAA -0.0890868582339
GGCAGTAC -0.00296095509595
GGCAGTCA -0.0597015687929
GGCAGTGA -0.160035997425
GGCAGTTA 0.015610607067
GGCATAAA -0.00369352434722
GGCATAAC -0.212841423841
GGCATACA -0.025007481588
GGCATACC -0.304288189525
GGCATAGA -0.0227990217568
GGCATATA 0.12675561849
GGCATCAA -0.183664833423
GGCATCAC -0.0295884031737
GGCATCCA -0.104123753981
GGCATCCC -0.1710216119
GGCATCGA -0.136692006298
GGCATCTA 0.145866117302
GGCATGAA 0.112756006695
GGCATGAC -0.271571618898
GGCATGCA -0.0650238158146
GGCATGCC -0.285218069437
GGCATGGA -0.00807062619067
GGCATGTA 0.0146952986431
GGCATTAA 0.0738399889054
GGCATTAC -0.209544868137
GGCATTCA -0.125074933295
GGCATTGA -0.175036597772
GGCATTTA -0.000172988912652
GGCCAAAA -0.0728245554535
GGCCAAAC -0.30841386737
GGCCAACA 0.0473228048986
GGCCAACC -0.197039628775
GGCCAAGA -0.0498060043515
GGCCAATA -0.0489938176536
GGCCACAA -0.0429786774377
GGCCACAC -0.175772236695
GGCCACCA -0.170083896021
GGCCACCC -0.315386837923
GGCCACGA -0.20104861852
GGCCACTA -0.214809347788
GGCCAGAA -0.0036179893701
GGCCAGAC -0.243623589748
GGCCAGCA -0.216171802469
GGCCAGCC -0.26452784592
GGCCAGGA -0.163169893291

GGCCAGTA -0.263179438835
GGCCATAA -0.0739370063799
GGCCATAC -0.112152011017
GGCCATCA -0.118393100881
GGCCATGA -0.124940373284
GGCCATTA -0.168193488964
GGCCCAAA -0.252114077751
GGCCCAAC -0.30962854552
GGCCCACA -0.0690293687021
GGCCCACC -0.33151757615
GGCCCAGA -0.0527105935762
GGCCCATA -0.167730430468
GGCCCCAA -0.158716383792
GGCCCCAC -0.281069664626
GGCCCCCA -0.251935224515
GGCCCCCC -0.173751059199
GGCCCCGA -0.106309741418
GGCCCCTA -0.292587707074
GGCCCGAA -0.0560318968651
GGCCCGAC -0.331426694854
GGCCCGCA -0.0868632656904
GGCCCGCC -0.327730779727
GGCCCGGA -0.239572910378
GGCCCGTA -0.20530781515
GGCCCTAA -0.218274626561
GGCCCTAC -0.085389488616
GGCCCTCA -0.245052311743
GGCCCTGA -0.136214170493
GGCCCTTA -0.170958208252
GGCCGAAA 0.138086757224
GGCCGAAC -0.074518336104
GGCCGACA -0.119171767275
GGCCGACC -0.373036601469
GGCCGAGA -0.082304220397
GGCCGATA 0.0444011504618
GGCCGCAA -0.222488151071
GGCCGCAC -0.299769305187
GGCCGCCA 0.0610603736296
GGCCGCCC -0.287575213824
GGCCGCGA -0.222100942946
GGCCGCTA -0.241135026041
GGCCGGAA 0.0333078363737
GGCCGGAC -0.418851922047
GGCCGGCA -0.10934824951
GGCCGGCC -0.0505792176221
GGCCGGGA -0.186055115226
GGCCGGTA -0.238599064486
GGCCGTAA -0.160416596738
GGCCGTAC -0.163047044067
GGCCGTCA -0.00061379424099
GGCCGTGA -0.0788906259909
GGCCGTTA -0.301472229802
GGCCTAAA -0.154432669584
GGCCTAAC -0.338212067839

GGCCTACA -0.270908815538
GGCCTACC -0.350589840302
GGCCTAGA -0.294531321819
GGCCTATA -0.189552296421
GGCCTCAA -0.189103915974
GGCCTCAC -0.273684602218
GGCCTCCA -0.308057878009
GGCCTCCC -0.186910316264
GGCCTCGA -0.265920963762
GGCCTCTA -0.311695398735
GGCCTGAA -0.124341535825
GGCCTGAC -0.272136714469
GGCCTGCA -0.374647549591
GGCCTGGA -0.128545710686
GGCCTGTA -0.162616185931
GGCCTTAA -0.117806056621
GGCCTTAC -0.138037454517
GGCCTTCA -0.215900774059
GGCCTTGA -0.0895056367501
GGCCTTTA -0.291469851318
GGCGAAAA -0.0457585538889
GGCGAAAC -0.0607536797647
GGCGAACA -0.0341868426239
GGCGAACC -0.331031555037
GGCGAAGA 0.147733261315
GGCGAATA 0.205200616045
GGCGACAA -0.150666026972
GGCGACAC -0.0859132252026
GGCGACCA -0.245149725854
GGCGACCC -0.340522065207
GGCGACGA 0.00769226526802
GGCGACTA -0.481883309224
GGCGAGAA -0.0993769728046
GGCGAGAC -0.225557814363
GGCGAGCA -0.174809962199
GGCGAGCC -0.359729965958
GGCGAGGA -0.015911027289
GGCGAGTA -0.18691667214
GGCGATAA 0.0439300692359
GGCGATAC -0.0157697276702
GGCGATCA 0.184386473289
GGCGATGA -0.00748470385168
GGCGATTA 0.105564241928
GGCGCAAA 0.0713399349763
GGCGCAAC 0.0244834054729
GGCGCACA -0.2304992404
GGCGCACC -0.274676887202
GGCGCAGA 0.243525895041
GGCGCATA 0.159201623322
GGCGCCAA -0.127089910049
GGCGCCAC -0.109491435708
GGCGCCCA -0.0469137334381
GGCGCCCC -0.139462898189
GGCGCCGA -0.112419225116

GGCGCCTA -0.251508261331
GGCGCGAA -0.157120839056
GGCGCGAC -0.105849506945
GGCGCGCA -0.199808326924
GGCGCGCC -0.185679557463
GGCGCGGA 0.0252719526823
GGCGCGTA -0.107675769248
GGCGCTAA -0.1703398229
GGCGCTAC -0.0950932959712
GGCGCTCA -0.0733870456306
GGCGCTGA -0.150701635325
GGCGCTTA -0.164753097143
GGCGGAAA -0.0160256675408
GGCGGAAC -0.376781829245
GGCGGACA -0.190741246171
GGCGGACC -0.336697621981
GGCGGAGA 0.0914555017232
GGCGGATA 0.17169770005
GGCGGCAA -0.143134141233
GGCGGCAC -0.0531037845049
GGCGGCCA -0.19088478132
GGCGGCCC -0.210510503061
GGCGGCGA 0.0355268382262
GGCGGCTA -0.371855508424
GGCGGGAA -0.0646758144959
GGCGGGAC -0.15897329972
GGCGGGCA -0.156045163767
GGCGGGGA -0.201361619292
GGCGGGTA 0.00190296285268
GGCGGTAA 0.0239344027223
GGCGGTAC -0.112086544076
GGCGGTCA -0.0647042928478
GGCGGTGA -0.0543011686475
GGCGGTTA -0.112433107532
GGCGTAAA 0.0115873375036
GGCGTAAC -0.0861478521974
GGCGTACA -0.19134980725
GGCGTACC -0.255078132537
GGCGTAGA 0.0448864842804
GGCGTATA -0.0290906197951
GGCGTCAA -0.194981322968
GGCGTCAC -0.269405636495
GGCGTCCA -0.231938256236
GGCGTCCC -0.219840501926
GGCGTCGA -0.228835598546
GGCGTCTA -0.195503728515
GGCGTGAA -0.0868473954598
GGCGTGAC -0.239996760321
GGCGTGCA -0.261123441711
GGCGTGGA 0.0998919029222
GGCGTGTA -0.186068310327
GGCGTTAA -0.0732123307881
GGCGTTAC -0.0345413247811
GGCGTTCA -0.213703893405

GGCGTTGA -0.0734816049655
GGCGTTTA -0.0299941180849
GGCTAAAA -0.102639481979
GGCTAAAC -0.210672213538
GGCTAACA 0.0259373882324
GGCTAACC -0.222097456978
GGCTAAGA 0.15352956262
GGCTAATA -0.0966146726714
GGCTACAA -0.0585031143758
GGCTACAC -0.0265110982971
GGCTACCA -0.129383010493
GGCTACCC -0.223741120236
GGCTACGA -0.0281169025496
GGCTACTA -0.318477513801
GGCTAGAA -0.0993054856127
GGCTAGAC -0.276267461662
GGCTAGCA -0.288412497553
GGCTAGCC -0.307723022742
GGCTAGGA -0.161228666053
GGCTAGTA -0.265600405775
GGCTATAA 0.0076150530696
GGCTATAC -0.193252836014
GGCTATCA -0.086340786829
GGCTATGA -0.0938848054422
GGCTATTA -0.164502287703
GGCTCAAA -0.0711914899207
GGCTCAAC -0.341683408153
GGCTCACA -0.150078595813
GGCTCACC -0.313307862158
GGCTCAGA -0.0181192461351
GGCTCATA -0.0995950914636
GGCTCCAA -0.165223432499
GGCTCCAC -0.126668265665
GGCTCCCA -0.282808348205
GGCTCCCC -0.397754413917
GGCTCCGA -0.300382905265
GGCTCCTA -0.258141041296
GGCTCGAA 0.0185351704769
GGCTCGAC -0.388113173687
GGCTCGCA -0.271991917989
GGCTCGGA -0.0867200099243
GGCTCGTA -0.291198925798
GGCTCTAA -0.0712988815604
GGCTCTAC -0.30541089504
GGCTCTCA -0.210232488897
GGCTCTGA -0.282469313443
GGCTCTTA -0.0790578484167
GGCTGAAA -0.111765581645
GGCTGAAC -0.181467697694
GGCTGACA 0.0775457928568
GGCTGACC -0.354147061012
GGCTGAGA -0.0211055665102
GGCTGATA 0.128586235338
GGCTGCAA -0.198841219821

GGCTGCAC -0.0924057987093
GGCTGCCA -0.145849358577
GGCTGCCC -0.193093106241
GGCTGCGA -0.134444919627
GGCTGCTA -0.199656606136
GGCTGGAA -0.207518082767
GGCTGGAC -0.34662338553
GGCTGGCA -0.125505130477
GGCTGGGA -0.0557760961557
GGCTGGTA -0.243050903527
GGCTGTAA -0.109434829894
GGCTGTAC -0.108104893033
GGCTGTCA -0.203979311724
GGCTGTGA -0.0958880016114
GGCTGTTA -0.208494656453
GGCTTAAA -0.026998678861
GGCTTAAC -0.244868904019
GGCTTACA -0.324529042558
GGCTTACC -0.260209977866
GGCTTAGA 0.119967074513
GGCTTATA 0.00293059276074
GGCTTCAA -0.235040901362
GGCTTCAC -0.287178645183
GGCTTCCA -0.273569761825
GGCTTCCC -0.334636956192
GGCTTCGA -0.218118381402
GGCTTCTA -0.17719371471
GGCTTGAA -0.201548624379
GGCTTGAC -0.0969759991827
GGCTTGCA -0.230056538291
GGCTTGGA -0.104504230664
GGCTTGTA -0.262911419271
GGCTTTAA -0.195841914593
GGCTTTAC -0.282481484238
GGCTTTCA -0.160157316196
GGCTTTGA -0.043097596403
GGCTTTTA -0.225324807042
GGGAAAAA -0.111452611419
GGGAAAAC -0.178141208587
GGGAAACA 0.17280779402
GGGAAACC -0.139193840166
GGGAAAGA -0.140151288448
GGGAAATA 0.20124118609
GGGAACAA -0.154172536368
GGGAACAC -0.186433120182
GGGAACCA -0.240474240958
GGGAACCC -0.305444618086
GGGAACGA -0.124275380831
GGGAACTA -0.139165565718
GGGAAGAA -0.101592927489
GGGAAGAC -0.317209745506
GGGAAGCA -0.241521212112
GGGAAGGA -0.264809038002
GGGAAGTA -0.120710495391

GGGAATAA 0.125330832172
GGGAATAC 0.0947066852551
GGGAATCA 0.447440553501
GGGAATGA -0.1413130085
GGGAATTA 0.0026651996349
GGGACAAA -0.0194864285773
GGGACAAC -0.278874318615
GGGACACA 0.0415829052193
GGGACACC -0.231013004601
GGGACAGA -0.225712509431
GGGACATA -0.0811332100515
GGGACCAA -0.0511236980212
GGGACCAC -0.320190995907
GGGACCCA -0.315574674896
GGGACCCC -0.346883731789
GGGACCGA -0.0881652365683
GGGACCTA -0.167049855959
GGGACGAA -0.0968595276623
GGGACGAC -0.378148157334
GGGACGCA -0.0663408830464
GGGACGGA -0.152004124314
GGGACGTA -0.0725687727049
GGGACTAA -0.128591555897
GGGACTAC -0.186202580158
GGGACTCA -0.220584951998
GGGACTGA -0.262947669456
GGGACTTA -0.0390509696868
GGGAGAAA -0.128208309553
GGGAGAAC -0.0739647388694
GGGAGACA -0.0561542447976
GGGAGACC -0.296874749205
GGGAGAGA -0.208278667112
GGGAGATA 0.0955037480931
GGGAGCAA -0.348534993368
GGGAGCAC -0.212443276112
GGGAGCCA -0.0743625224973
GGGAGCCC -0.406485484146
GGGAGCGA -0.00743229930603
GGGAGCTA -0.208677218561
GGGAGGAA -0.108454975258
GGGAGGAC -0.323780401496
GGGAGGCA -0.307263801719
GGGAGGGA -0.0619729365101
GGGAGGTA -0.303167140191
GGGAGTAA 0.0313167379268
GGGAGTAC -0.138363095195
GGGAGTCA -0.248731421057
GGGAGTGA -0.196536237997
GGGAGTTA -0.0995848472153
GGGATAAA 0.00274385424981
GGGATAAC 0.123338175076
GGGATACA 0.216219352861
GGGATACC 0.115574251938
GGGATAGA -0.127837737253

GGGATATA 0.157139489292
GGGATCAA -0.136832832932
GGGATCAC 0.127105975782
GGGATCCA 0.276462412826
GGGATCCC 0.196311180571
GGGATCGA 0.200298533609
GGGATCTA 0.20031188213
GGGATGAA -0.271310424678
GGGATGAC -0.256690675642
GGGATGCA -0.254286068726
GGGATGGA -0.109011840273
GGGATGTA -0.039699130676
GGGATTAA -0.0898627533691
GGGATTAC 0.441217708514
GGGATTCA 0.0598432151121
GGGATTGA 0.0747455444425
GGGATTTA 0.167670670273
GGGCAAAA -0.128782803596
GGGCAAAC -0.136297711713
GGGCAACA 0.0390872966631
GGGCAACC -0.085959770375
GGGCAAGA -0.13467715075
GGGCAATA -0.0656135546508
GGGCACAA -0.137001105515
GGGCACAC -0.216000529271
GGGCACCA -0.286342458498
GGGCACCC -0.348856634699
GGGCACGA -0.0991212614756
GGGCACTA -0.181777711622
GGGCAGAA 0.0392887273269
GGGCAGAC -0.313350240252
GGGCAGCA -0.160267232436
GGGCAGGA -0.226561143226
GGGCAGTA -0.29029772783
GGGCATAA -0.12111632592
GGGCATAC -0.0610997829568
GGGCATCA -0.25304215606
GGGCATGA -0.00586045561046
GGGCATTA -0.0772950138888
GGGCCAAA -0.10819816208
GGGCCAAC -0.226026817446
GGGCCACA 0.0138960862654
GGGCCACC -0.447299170311
GGGCCAGA 0.101138196254
GGGCCATA -0.147572553398
GGGCCCAA -0.315502029949
GGGCCCAC -0.175477608237
GGGCCCCA -0.417088454735
GGGCCCCC -0.222103711053
GGGCCCGA -0.345024448397
GGGCCCTA -0.262778480611
GGGCCGAA 0.00810865962381
GGGCCGAC -0.185443835261
GGGCCGCA -0.306208201819

GGGCCGGA -0.272175502138
GGGCCGTA -0.260270329119
GGGCCTAA -0.241406458636
GGGCCTAC -0.426953842473
GGGCCTCA -0.202237164867
GGGCCTGA -0.149883096425
GGGCCTTA -0.225881798374
GGGCGAAA -0.122832047242
GGGCGAAC -0.137499896601
GGGCGACA -0.0203502880005
GGGCGACC -0.212447245171
GGGCGAGA -0.159781131932
GGGCGATA 0.0809872776509
GGGCGCAA -0.149143242498
GGGCGCAC -0.264691287754
GGGCGCCA -0.160822050909
GGGCGCCC -0.221749625919
GGGCGCGA -0.0601425597331
GGGCGCTA -0.226346327561
GGGCGGAA -0.182246114016
GGGCGGAC -0.311924362868
GGGCGGCA -0.121234555647
GGGCGGGA -0.131537589849
GGGCGGTA -0.0835672465964
GGGCGTAA -0.186274509804
GGGCGTAC -0.202217956503
GGGCGTCA -0.275576700553
GGGCGTGA -0.140162533515
GGGCGTTA -0.0531677296077
GGGCTAAA -0.0849245820372
GGGCTAAC 0.0581251657351
GGGCTACA 0.0469234682483
GGGCTACC -0.00600262436927
GGGCTAGA -0.133218851671
GGGCTATA -0.147386439753
GGGCTCAA -0.372107988635
GGGCTCAC -0.24860039528
GGGCTCCA -0.379386889556
GGGCTCGA -0.264905664812
GGGCTCTA -0.254402069381
GGGCTGAA -0.200287427996
GGGCTGAC -0.242523576543
GGGCTGCA 0.0415962945716
GGGCTGGA -0.0631280238184
GGGCTGTA -0.199589474314
GGGCTTAA -0.158546163223
GGGCTTAC -0.0235172958431
GGGCTTCA -0.475388206119
GGGCTTGA -0.033826411702
GGGCTTTA -0.210232895328
GGGGAAAA 0.0366715127481
GGGGAAAC -0.0467395770426
GGGGAACA -0.0606942913659
GGGGAACC -0.122341411444

GGGGAAGA 0.0638869221842
GGGGAATA 0.197043130442
GGGGACAA -0.215429589133
GGGGACAC -0.256763501828
GGGGACCA -0.299250638067
GGGGACCC -0.32354830085
GGGGACGA -0.0275755819809
GGGGACTA -0.208548726389
GGGGAGAA 0.125193608899
GGGGAGAC -0.293010811529
GGGGAGCA -0.167368666856
GGGGAGGA -0.142313131063
GGGGAGTA -0.115268920376
GGGGATAA 0.171330992382
GGGGATAC 0.13582403305
GGGGATCA -0.0539272054424
GGGGATGA -0.0981834876045
GGGGATTA 0.229106516985
GGGGCAAA -0.179973535128
GGGGCAAC -0.20474672412
GGGGCACA -0.160815665903
GGGGCACC -0.278818195599
GGGGCAGA 0.147609041548
GGGGCATA 0.000492227764955
GGGGCCAA -0.260444682475
GGGGCCAC -0.225739321222
GGGGCCCA -0.296769557383
GGGGCCCC -0.12439234604
GGGGCCGA -0.133207947066
GGGGCCTA -0.243204334749
GGGGCGAA -0.00286745466527
GGGGCGAC -0.202667481937
GGGGCGCA -0.0147378783742
GGGGCGGA -0.248765539317
GGGGCGTA -0.18152492583
GGGGCTAA -0.17004271627
GGGGCTAC -0.127403786507
GGGGCTCA -0.261266447488
GGGGCTGA -0.202338717014
GGGGCTTA -0.321366058311
GGGGGAAA 0.0215987971066
GGGGGAAC -0.123363884376
GGGGGACA -0.208813496692
GGGGGACC -0.452551204579
GGGGGAGA 0.120209741422
GGGGGATA 0.0054713799216
GGGGGCAA -0.337252324477
GGGGGCAC -0.280390665267
GGGGGCCA -0.173895499103
GGGGGCGA -0.0698149058914
GGGGGCTA -0.215515265978
GGGGGGAA 0.0208386553371
GGGGGGAC -0.242265611432
GGGGGGCA -0.304612558411

GGGGGGGA -0.186057558303
GGGGGGTA -0.240064550448
GGGGGTAA -0.08230608554
GGGGGTAC -0.205996179513
GGGGGTCA -0.250830390787
GGGGGTGA -0.23006706218
GGGGGTTA -0.320489118159
GGGGTAAA -0.0943635255811
GGGGTAAC -0.0154748436166
GGGGTACA -0.161970396125
GGGGTACC -0.305130734357
GGGGTAGA -0.105381716116
GGGGTATA -0.231748183977
GGGGTCAA -0.314580193545
GGGGTCAC -0.128305420488
GGGGTCCA -0.271496860595
GGGGTCGA -0.0950044889528
GGGGTCTA -0.356553706414
GGGGTGAA 0.0789854083431
GGGGTGAC -0.172269741499
GGGGTGCA -0.200166824539
GGGGTGGA 0.019712707021
GGGGTGTA -0.139854634891
GGGGTTAA -0.233274611485
GGGGTTAC -0.171252790138
GGGGTTCA -0.263636313516
GGGGTTGA 0.0657475657476
GGGGTTTA -0.199593889316
GGGTAAAA 0.165403695707
GGGTAAAC -0.218042014328
GGGTAACA -0.0902207332037
GGGTAACC -0.121146321893
GGGTAAGA 0.0426146936953
GGGTAATA 0.0967913111392
GGGTACAA -0.0811580768304
GGGTACAC -0.167669752298
GGGTACCA -0.311324663804
GGGTACCC -0.351016738996
GGGTACGA 0.0951185345125
GGGTACTA -0.186839108508
GGGTAGAA 0.153780502265
GGGTAGAC -0.207904863218
GGGTAGCA -0.295549822638
GGGTAGGA -0.233446360391
GGGTAGTA -0.234626447895
GGGTATAA -0.028893527879
GGGTATAC -0.0860983267721
GGGTATCA 0.147972672131
GGGTATGA 0.0161507412246
GGGTATTA 0.0868926778018
GGGTCAAA -0.00476558907469
GGGTCAAC -0.191745210401
GGGTCACA -0.165308761453
GGGTCACC -0.211322418218

GGGTCAGA -0.0569079464337
GGGTCATA -0.192144162924
GGGTCCAA -0.207877974049
GGGTCCAC -0.324927834432
GGGTCCCA -0.371654144663
GGGTCCGA -0.075273361038
GGGTCCTA -0.302624869418
GGGTCGAA -0.077251768676
GGGTCGAC -0.262178665454
GGGTCGCA -0.37323111401
GGGTCGGA -0.119622599898
GGGTCGTA -0.187905618496
GGGTCTAA -0.226415934176
GGGTCTAC -0.318355342766
GGGTCTCA -0.343881682114
GGGTCTGA -0.146825505661
GGGTCTTA -0.304284920041
GGGTGAAA 0.0340652154556
GGGTGAAC 0.0149653657219
GGGTGACA -0.0231623942229
GGGTGACC -0.292531126051
GGGTGAGA 0.0705378714335
GGGTGATA -0.0786392453059
GGGTGCAA -0.147028173352
GGGTGCAC -0.297809530897
GGGTGCCA -0.291750143363
GGGTGCGA 0.000792725127043
GGGTGCTA -0.204212813714
GGGTGGAA -0.196876026183
GGGTGGAC -0.172997438464
GGGTGGCA -0.125441690956
GGGTGGGA -0.0174469863703
GGGTGGTA -0.18767766501
GGGTGTAA 0.109827458312
GGGTGTAC -0.230814083319
GGGTGTCA -0.0962435305902
GGGTGTGA 0.0721423903242
GGGTGTTA -0.15410589653
GGGTTAAA -0.0688657838215
GGGTTAAC -0.0648246956474
GGGTTACA -0.0951240496695
GGGTTACC -0.244936541686
GGGTTAGA -0.141678315123
GGGTTATA -0.106062486041
GGGTTCAA -0.206897264749
GGGTTCAC -0.155730902735
GGGTTCCA -0.328917666627
GGGTTCGA 0.0206452009003
GGGTTCTA -0.143438453713
GGGTTGAA -0.0366656204609
GGGTTGAC -0.147743404423
GGGTTGCA -0.206017705356
GGGTTGGA -0.0360641737772
GGGTTGTA -0.142225958015

GGGTTTAA -0.0559980438385
GGGTTTAC -0.197542350687
GGGTTTCA -0.331169248054
GGGTTTGA -0.0605919282773
GGGTTTTA -0.174242501646
GGTAAAAA 0.263576248425
GGTAAAAC 0.0111116626268
GGTAAACA 0.151519287883
GGTAAACC -0.0828193543207
GGTAAAGA -0.0215758288823
GGTAAATA 0.0666264765933
GGTAACAA 0.00567371779493
GGTAACAC -0.188435697844
GGTAACCA -0.102777984555
GGTAACGA 0.0645263242411
GGTAACTA -0.00675094705955
GGTAAGAA 0.109769520137
GGTAAGAC 0.0893986568051
GGTAAGCA -0.0385771446378
GGTAAGGA -0.0661325767289
GGTAAGTA -0.232994477393
GGTAATAA 0.122563009569
GGTAATAC -0.0128471639307
GGTAATCA 0.335720385685
GGTAATGA 0.0770165616811
GGTAATTA 0.138131279155
GGTACAAA 0.221136188798
GGTACAAC -0.00745750093064
GGTACACA 0.19414593657
GGTACACC -0.171417920095
GGTACAGA -0.0633419600253
GGTACATA 0.0645728373001
GGTACCAA -0.130488359591
GGTACCAC -0.288829305511
GGTACCCA -0.429287182472
GGTACCGA -0.134502923977
GGTACCTA -0.191663151278
GGTACGAA 0.162845339153
GGTACGAC -0.166077472899
GGTACGCA 0.0263100566131
GGTACGGA -0.0520872104083
GGTACGTA -0.0508200550353
GGTACTAA -0.0523657496973
GGTACTAC -0.122377142624
GGTACTCA -0.0995819277161
GGTACTGA 0.0585060181164
GGTACTTA 0.0397998583973
GGTAGAAA 0.269662224208
GGTAGAAC 0.0807446565022
GGTAGACA -0.00439695894241
GGTAGACC -0.212963300784
GGTAGAGA 0.0340709489735
GGTAGATA 0.15535061276
GGTAGCAA -0.248238622016

GGTAGCAC -0.246336024797
GGTAGCCA -0.342183964123
GGTAGCGA 0.106719667326
GGTAGCTA -0.110096543269
GGTAGGAA 0.0360484451394
GGTAGGAC -0.206451514041
GGTAGGCA -0.0120878454212
GGTAGGGA -0.086451568642
GGTAGGTA -0.112102263798
GGTAGTAA 0.0926424968796
GGTAGTAC -0.2174806804
GGTAGTCA -0.173852511669
GGTAGTGA -0.0528638059021
GGTAGTTA 0.041966234316
GGTATAAA 0.27589710923
GGTATAAC -0.0168733266607
GGTATACA 0.0648504047357
GGTATACC -0.274897680764
GGTATAGA -0.0983605719899
GGTATATA -0.0762180913696
GGTATCAA 0.0997021963023
GGTATCAC 0.140839186294
GGTATCCA 0.289330160653
GGTATCGA 0.132938723848
GGTATCTA 0.361235891539
GGTATGAA 0.00594152460359
GGTATGAC -0.196834219763
GGTATGCA -0.0906527594286
GGTATGGA -0.0379473442187
GGTATGTA -0.125593876666
GGTATTAA 0.14444282242
GGTATTAC 0.111682901628
GGTATTCA -0.0512200462764
GGTATTGA 0.100963482551
GGTATTTA 0.0274668608002
GGTCAAAA 0.190202404327
GGTCAAAC 0.0605164393043
GGTCAACA 0.0974657489252
GGTCAACC -0.243070888497
GGTCAAGA -0.101885627159
GGTCAATA -0.250798610148
GGTCACAA -0.0860565908207
GGTCACAC -0.39166177415
GGTCACCA -0.309023840277
GGTCACGA -0.111727368732
GGTCACTA -0.220822843466
GGTCAGAA 0.151441209317
GGTCAGAC -0.186277915379
GGTCAGCA -0.198944851755
GGTCAGGA -0.056055526446
GGTCAGTA -0.198998422402
GGTCATAA -0.214310720774
GGTCATAC -0.122652696355
GGTCATCA -0.145969958416

GGTCATGA -0.00440647285096
GGTCATTA -0.193514276415
GGTCCAAA 0.106609364185
GGTCCAAC -0.27727698395
GGTCCACA -0.0881491646168
GGTCCACC -0.316010210017
GGTCCAGA 0.00409990873774
GGTCCATA -0.28931400199
GGTCCCAA -0.209891400544
GGTCCCAC -0.214837154253
GGTCCCCA -0.22742330205
GGTCCCGA -0.260862427972
GGTCCCTA -0.321362921073
GGTCCGAA -0.126001736757
GGTCCGAC -0.105596096046
GGTCCGCA -0.188341694021
GGTCCGGA -0.0279887207346
GGTCCGTA 0.0139799858397
GGTCCTAA -0.245110200174
GGTCCTAC -0.146573042536
GGTCCTCA -0.207625775109
GGTCCTGA -0.296465335483
GGTCCTTA -0.219740008435
GGTCGAAA 0.111451672058
GGTCGAAC -0.232864156488
GGTCGACA -0.0897840884375
GGTCGACC -0.458155963884
GGTCGAGA -0.138004746665
GGTCGATA -0.125244702671
GGTCGCAA -0.10120259492
GGTCGCAC -0.236438788139
GGTCGCCA -0.16975017186
GGTCGCGA 0.0388159383371
GGTCGCTA -0.281744844605
GGTCGGAA 0.015733232119
GGTCGGAC -0.220025195513
GGTCGGCA -0.212555800558
GGTCGGGA -0.0399842062933
GGTCGGTA -0.254950842987
GGTCGTAA -0.0428292449402
GGTCGTAC -0.289649537347
GGTCGTCA -0.17723738219
GGTCGTGA 0.0684678437698
GGTCGTTA -0.160036664875
GGTCTAAA 0.212015065364
GGTCTAAC -0.202377842033
GGTCTACA -0.123874462318
GGTCTAGA -0.078289054027
GGTCTATA -0.23255695337
GGTCTCAA -0.183062165866
GGTCTCAC -0.281045022013
GGTCTCCA -0.321324386759
GGTCTCGA -0.0170164156989
GGTCTCTA -0.188400342975

GGTCTGAA -0.0244077430957
GGTCTGAC -0.254916950766
GGTCTGCA -0.0349751465296
GGTCTGGA -0.0128917785443
GGTCTGTA -0.304398740965
GGTCTTAA -0.217050288509
GGTCTTAC -0.100130793564
GGTCTTCA -0.357814881893
GGTCTTGA -0.0987080574878
GGTCTTTA -0.0651656122069
GGTGAAAA 0.105024707488
GGTGAAAC -0.245052529086
GGTGAACA 0.0834485376233
GGTGAACC -0.035496001016
GGTGAAGA 0.129108851631
GGTGAATA 0.0797560646045
GGTGACAA -0.0961448920403
GGTGACAC -0.173947786692
GGTGACCA -0.299760277371
GGTGACGA -0.0290551626201
GGTGACTA -0.0251135320247
GGTGAGAA 0.0202411626023
GGTGAGAC -0.131295960824
GGTGAGCA 0.0131164222073
GGTGAGGA -0.154093095701
GGTGAGTA 0.0163318437316
GGTGATAA -0.176572020695
GGTGATAC 0.0428416167121
GGTGATCA 0.127613993854
GGTGATGA -0.127153479063
GGTGATTA 0.0918775501266
GGTGCAAA -0.0637411793895
GGTGCAAC -0.208178653022
GGTGCACA 0.0403808675722
GGTGCACC -0.111594802045
GGTGCAGA 0.0241084944289
GGTGCATA 0.0353921414527
GGTGCCAA -0.239670898747
GGTGCCAC -0.298645046952
GGTGCCCA -0.297368271281
GGTGCCGA -0.271483034378
GGTGCCTA -0.0170914781278
GGTGCGAA -0.00243733646548
GGTGCGAC -0.243591227998
GGTGCGCA -0.0563177448669
GGTGCGGA 0.0511455953519
GGTGCGTA 0.0239958249089
GGTGCTAA -0.127242753787
GGTGCTAC -0.342578879095
GGTGCTCA 0.0170474532087
GGTGCTGA -0.160564583936
GGTGCTTA -0.143622572984
GGTGGAAA 0.0752151412743
GGTGGAAC -0.177488814114

GGTGGACA -0.174992738467
GGTGGAGA -0.0457142481779
GGTGGATA 0.222908257483
GGTGGCAA -0.175784189678
GGTGGCAC -0.28910612691
GGTGGCCA -0.0586036047214
GGTGGCGA -0.110061139807
GGTGGCTA -0.221489133401
GGTGGGAA -0.0264354482519
GGTGGGAC -0.330227537778
GGTGGGCA 0.0201758232061
GGTGGGGA -0.0380040312282
GGTGGGTA -0.0319313804162
GGTGGTAA -0.0593103317408
GGTGGTAC -0.257479354341
GGTGGTCA -0.141571347046
GGTGGTGA 0.0271001028577
GGTGGTTA -0.0581805097154
GGTGTAAA 0.11048589225
GGTGTAAC -0.0831727203293
GGTGTACA 0.115314377378
GGTGTAGA 0.123330875966
GGTGTATA -0.0203081869749
GGTGTCAA -0.243770001737
GGTGTCAC -0.159772368214
GGTGTCCA -0.0551791647709
GGTGTCGA -0.0248886682936
GGTGTCTA 0.0403283139785
GGTGTGAA -0.0266560310137
GGTGTGAC -0.278374608011
GGTGTGCA -0.0504540353025
GGTGTGGA -0.0666923096012
GGTGTGTA -0.250288593866
GGTGTTAA 0.0133370284885
GGTGTTAC -0.0367599003963
GGTGTTCA 0.00799790400764
GGTGTTGA -0.0784428061192
GGTGTTTA -0.172242213674
GGTTAAAA -0.00299336510879
GGTTAAAC -0.252982623074
GGTTAACA 0.104607362183
GGTTAACC -0.13674121846
GGTTAAGA 0.117859269921
GGTTAATA -0.0589832256499
GGTTACAA 0.0661215475097
GGTTACAC -0.128576234637
GGTTACCA -0.245546093391
GGTTACGA 0.0724898452171
GGTTACTA 0.0320278956643
GGTTAGAA -0.0297335903397
GGTTAGAC -0.187274762665
GGTTAGCA -0.217230341679
GGTTAGGA -0.136618122576
GGTTAGTA -0.0908084392933

GGTTATAA -0.100069484081
GGTTATAC -0.116515247707
GGTTATCA -0.0707203491894
GGTTATGA -0.0253435253435
GGTTATTA -0.0286218577065
GGTTCAAA -0.018058728641
GGTTCAAC -0.249015173574
GGTTCACA 0.0593417108569
GGTTCAGA -0.0734546033108
GGTTCATA -0.129937660283
GGTTCCAA -0.131990459252
GGTTCCAC -0.176012087424
GGTTCCCA -0.363854809798
GGTTCCGA -0.307277020312
GGTTCCTA -0.274269248226
GGTTCGAA 0.0810024900934
GGTTCGAC -0.290525186628
GGTTCGCA -0.000373651888803
GGTTCGGA -0.0735035037811
GGTTCGTA -0.128218770572
GGTTCTAA -0.171303727716
GGTTCTAC -0.0524728966763
GGTTCTCA -0.0440906822399
GGTTCTGA 0.0358821858809
GGTTCTTA -0.11267804412
GGTTGAAA 0.0324275137527
GGTTGAAC -0.275612237302
GGTTGACA -0.0962713692935
GGTTGAGA -0.0479115479115
GGTTGATA 0.0893956125766
GGTTGCAA -0.188520952936
GGTTGCAC -0.243030368488
GGTTGCCA -0.135931136973
GGTTGCGA -0.0108965715026
GGTTGCTA -0.0840275192676
GGTTGGAA 0.0101737247992
GGTTGGAC -0.337631702995
GGTTGGCA -0.15457974539
GGTTGGGA -0.149008399295
GGTTGGTA -0.140741027284
GGTTGTAA 0.0978546790084
GGTTGTAC -0.14768794438
GGTTGTCA -0.144797783626
GGTTGTGA -0.0138077268381
GGTTGTTA -0.171644405262
GGTTTAAA 0.112996795691
GGTTTAAC -0.253028443509
GGTTTACA -0.204011139703
GGTTTAGA -0.000809349294198
GGTTTATA 0.026609253882
GGTTTCAA -0.0980287541475
GGTTTCAC -0.33226633083
GGTTTCCA -0.0944677459829
GGTTTCGA -0.242183156042

GGTTTCTA -0.153126249466
GGTTTGAA -0.0155945419103
GGTTTGAC -0.0558564410998
GGTTTGCA 0.0681262454107
GGTTTGGA 0.0702579103554
GGTTTGTA -0.168657666297
GGTTTTAA 0.00786047755745
GGTTTTAC -0.0278807203907
GGTTTTCA 0.0147825882494
GGTTTTGA 0.124164109013
GGTTTTTA -0.0530273059714
GTAAAAAA 0.167460849279
GTAAAAAC 0.102664301418
GTAAAACA 0.124857309477
GTAAAAGA 0.109905929658
GTAAAATA 0.237754647708
GTAAACAA -0.07662298609
GTAAACAC 0.135191665495
GTAAACCA -0.150749923477
GTAAACGA 0.14650594337
GTAAACTA 0.017531813983
GTAAAGAA -0.0334838971203
GTAAAGAC -0.110478858036
GTAAAGCA -0.0672576512923
GTAAAGGA 0.0848392640016
GTAAAGTA 0.208248193097
GTAAATAA 0.1382788895
GTAAATAC 0.238575067699
GTAAATCA 0.185181279003
GTAAATGA 0.15636956855
GTAAATTA 0.129098795765
GTAACAAA 0.209078839616
GTAACAAC 0.0657087800997
GTAACACA 0.0573755573756
GTAACAGA 0.168021868134
GTAACATA 0.102677313635
GTAACCAA -0.0475741407122
GTAACCAC -0.071355506478
GTAACCCA -0.0682045682046
GTAACCGA 0.125203716113
GTAACCTA 0.0646489492931
GTAACGAA 0.0445969385363
GTAACGAC -0.209952503425
GTAACGCA -0.0319007612972
GTAACGGA 0.0357649102574
GTAACGTA 0.251310555243
GTAACTAA -0.0785261845868
GTAACTAC 0.0491368802143
GTAACTCA -0.024243251516
GTAACTGA 0.14291000927
GTAACTTA 0.108561729774
GTAAGAAA 0.0687740081679
GTAAGAAC -0.000594258170016
GTAAGACA 0.0178263663112

GTAAGAGA 0.151632348602
GTAAGATA 0.181668284636
GTAAGCAA -0.134331968738
GTAAGCAC -0.0456412606981
GTAAGCCA -0.162913049888
GTAAGCGA 0.0508302737896
GTAAGCTA -0.119749723756
GTAAGGAA 0.10537092651
GTAAGGAC -0.186007239964
GTAAGGCA -0.215136787875
GTAAGGGA 0.219752810662
GTAAGGTA -0.133336641935
GTAAGTAA -0.0105325711386
GTAAGTAC 0.0798924111151
GTAAGTCA -0.0391782967541
GTAAGTGA 0.222391813301
GTAAGTTA 0.121403910863
GTAATAAA 0.175137040795
GTAATAAC 0.0141537046634
GTAATACA 0.247827717525
GTAATAGA 0.183341240703
GTAATATA 0.193962950096
GTAATCAA 0.333095625643
GTAATCAC 0.386113442771
GTAATCCA 0.468984620775
GTAATCGA 0.380089455847
GTAATCTA 0.481924483405
GTAATGAA -0.0852636863991
GTAATGAC -0.139656108947
GTAATGCA 0.16920304241
GTAATGGA 0.0923242556732
GTAATGTA 0.0663045966076
GTAATTAA 0.111323839624
GTAATTAC 0.273150100464
GTAATTCA 0.0799245272001
GTAATTGA 0.126011109246
GTAATTTA 0.279628112961
GTACAAAA 0.193057816252
GTACAAAC -0.0103785821489
GTACAACA 0.0949139799858
GTACAAGA 0.054085766207
GTACAATA 0.259497237287
GTACACAA -0.0659350525171
GTACACAC 0.0675382643897
GTACACCA 0.123460926491
GTACACGA 0.0917375983324
GTACACTA 0.0225171849021
GTACAGAA -0.170781778879
GTACAGAC -0.154900069474
GTACAGCA -0.0158445989636
GTACAGGA -0.306152750599
GTACAGTA 0.222630891294
GTACATAA 0.0526648892465
GTACATAC 0.101781770355

GTACATCA -0.112460677844
GTACATGA -0.0856893644665
GTACATTA 0.208482296008
GTACCAAA -0.0995842839481
GTACCAAC -0.118534350467
GTACCACA -0.159506332495
GTACCAGA 0.0442727531514
GTACCATA 0.218834537016
GTACCCAA -0.229390147411
GTACCCAC -0.0898475086088
GTACCCCA -0.198834109766
GTACCCGA -0.0624467442649
GTACCCTA 0.00161522293734
GTACCGAA 0.0828004312853
GTACCGAC -0.260887423306
GTACCGCA -0.0722223601011
GTACCGGA -0.0995221053415
GTACCGTA 0.229404465596
GTACCTAA -0.0973543498348
GTACCTAC -0.255987772237
GTACCTCA -0.236824133538
GTACCTGA -0.0109903291721
GTACCTTA 0.000101457632315
GTACGAAA 0.191621351357
GTACGAAC 0.127313263677
GTACGACA 0.0320223805072
GTACGAGA 0.247882869095
GTACGATA 0.187717787202
GTACGCAA 0.128706851627
GTACGCAC 0.00738013036211
GTACGCCA -0.34249081042
GTACGCGA 0.135078604776
GTACGCTA -0.0291071846096
GTACGGAA 0.01669575912
GTACGGAC -0.0620853398808
GTACGGCA -0.190085537941
GTACGGGA 0.206707152398
GTACGGTA 0.0589832256499
GTACGTAA -0.00635483968817
GTACGTAC 0.106002221616
GTACGTCA -0.119270949651
GTACGTGA 0.0087281395101
GTACGTTA 0.140323519111
GTACTAAA 0.062275774397
GTACTAAC -0.0792194331511
GTACTACA 0.0865404407203
GTACTAGA 0.134088634089
GTACTATA 0.121638167093
GTACTCAA -0.0722918400217
GTACTCAC 0.0406977225159
GTACTCCA -0.187070559916
GTACTCGA -0.12687678372
GTACTCTA 0.060117661657
GTACTGAA 0.0479573439998

GTACTGAC -0.0766478903529
GTACTGCA -0.00908867976581
GTACTGGA 0.0927277139398
GTACTGTA 0.193635905783
GTACTTAA -0.181788394091
GTACTTCA -0.287782861563
GTACTTGA 0.0409852339
GTACTTTA 0.128601052843
GTAGAAAA 0.222176429632
GTAGAAAC 0.148223761523
GTAGAACA 0.0409954526543
GTAGAAGA 0.0431864762798
GTAGAATA 0.117261889989
GTAGACAA -0.0164324868798
GTAGACAC 0.0174403053191
GTAGACCA -0.0911212190661
GTAGACGA 0.10802026233
GTAGACTA 0.110405611556
GTAGAGAA 0.0170608233864
GTAGAGAC -0.0950691118446
GTAGAGCA -0.132191289955
GTAGAGGA -0.0727975053144
GTAGAGTA 0.179919417823
GTAGATAA -0.030058465873
GTAGATAC 0.36378400473
GTAGATCA -0.086441172821
GTAGATGA 0.152034955065
GTAGATTA 0.369900203234
GTAGCAAA -0.000520426560002
GTAGCAAC -0.16228966255
GTAGCACA -0.0930626860268
GTAGCAGA 0.169825702227
GTAGCATA -0.0353138070699
GTAGCCAA -0.281547830738
GTAGCCAC -0.314661320731
GTAGCCCA -0.0628599470125
GTAGCCGA 0.00123533385725
GTAGCCTA -0.273380749562
GTAGCGAA 0.102169516341
GTAGCGAC -0.0663438026905
GTAGCGCA -0.31856749448
GTAGCGGA -0.0225395880035
GTAGCGTA 0.0471642441339
GTAGCTAA -0.184166667883
GTAGCTAC -0.0738016686098
GTAGCTCA -0.296889947536
GTAGCTGA 0.0709358016726
GTAGCTTA -0.0114214457652
GTAGGAAA -0.019613277189
GTAGGAAC -0.15087223008
GTAGGACA 0.00235761260702
GTAGGAGA 0.0893228615432
GTAGGATA 0.184427500807
GTAGGCAA -0.131311723101

GTAGGCAC -0.028760116364
GTAGGCCA -0.395676761664
GTAGGCGA 0.158297415873
GTAGGCTA -0.21929650485
GTAGGGAA -0.0200566938605
GTAGGGAC -0.176592003077
GTAGGGCA -0.25064025693
GTAGGGGA 0.205043179393
GTAGGGTA 0.052984971132
GTAGGTAA 0.0821755727977
GTAGGTCA -0.0566999181121
GTAGGTGA 0.126938232999
GTAGGTTA -0.0261377079559
GTAGTAAA 0.106995425177
GTAGTAAC 0.0175257902531
GTAGTACA 0.0305825419881
GTAGTAGA 0.199151113479
GTAGTATA 0.0907064088882
GTAGTCAA -0.102270023284
GTAGTCAC 0.0542650088105
GTAGTCCA -0.171823949312
GTAGTCGA 0.0456710327107
GTAGTCTA -0.0684313482187
GTAGTGAA -0.0826902558249
GTAGTGAC -0.0591624682534
GTAGTGCA -0.0124539851925
GTAGTGGA 0.00591914228278
GTAGTGTA 0.0632124843982
GTAGTTAA -0.0327532973731
GTAGTTCA -0.0350436046111
GTAGTTGA 0.0415224484135
GTAGTTTA 0.129390917914
GTATAAAA 0.165163786376
GTATAAAC 0.207553849186
GTATAACA 0.240715922534
GTATAAGA 0.208639769246
GTATAATA 0.0381745381745
GTATACAA 0.00258247227944
GTATACAC 0.15209228995
GTATACCA -0.0240338206845
GTATACGA 0.317224938437
GTATACTA 0.18807982307
GTATAGAA -0.0214324706583
GTATAGAC 0.0562146168207
GTATAGCA 0.0112952990711
GTATAGGA 0.0613847871944
GTATAGTA 0.226052714174
GTATATAA 0.0650664583987
GTATATAC 0.217457639733
GTATATCA 0.109041548435
GTATATGA 0.145561046108
GTATATTA 0.191741056765
GTATCAAA 0.200850346343
GTATCAAC 0.195396451173

GTATCACA 0.182912077792
GTATCAGA 0.289039728434
GTATCATA 0.0905519844914
GTATCCAA 0.279794103163
GTATCCAC 0.283318613461
GTATCCCA 0.259547857161
GTATCCGA 0.330321963753
GTATCCTA 0.182748864567
GTATCGAA 0.0461242522773
GTATCGAC 0.085153609775
GTATCGCA 0.151023700211
GTATCGGA 0.302968257514
GTATCGTA 0.173146281852
GTATCTAA 0.270732164672
GTATCTCA 0.246914959036
GTATCTGA 0.341241807952
GTATCTTA 0.289698035809
GTATGAAA 0.257649452813
GTATGAAC -0.182012157301
GTATGACA -0.0722761645868
GTATGAGA 0.0360452771918
GTATGATA 0.035703747825
GTATGCAA -0.132085481886
GTATGCAC 0.0331915937977
GTATGCCA -0.23287676898
GTATGCGA 0.203921811931
GTATGCTA -0.260609869377
GTATGGAA 0.12483695817
GTATGGAC -0.04765808048
GTATGGCA -0.209101989926
GTATGGGA 0.212641015671
GTATGGTA 0.0740017687116
GTATGTAA -0.124100518598
GTATGTCA -0.253136169983
GTATGTGA 0.219479810389
GTATGTTA 0.144901099447
GTATTAAA 0.118359406238
GTATTAAC 0.0698594188153
GTATTACA 0.222532449805
GTATTAGA 0.172410477207
GTATTATA 0.179241010497
GTATTCAA -0.128097194952
GTATTCAC 0.0403999040363
GTATTCCA 0.0648358065152
GTATTCGA 0.0907670756156
GTATTCTA 0.0529762121997
GTATTGAA -0.0119452372089
GTATTGAC -0.0706240009343
GTATTGCA 0.171037860485
GTATTGGA 0.149674824639
GTATTGTA 0.173469612864
GTATTTAA -0.0217785718816
GTATTTCA 0.143631161361
GTATTTGA 0.133663966997

GTATTTTA 0.285645569805
GTCAAAAA 0.0764530703707
GTCAAAAC 0.129781296448
GTCAAACA 0.0552039006445
GTCAAAGA 0.123117741947
GTCAAATA −0.00700562821775
GTCAACAA 0.0326204535667
GTCAACAC −0.0830498332567
GTCAACCA −0.0665953355663
GTCAACGA −0.068302413404
GTCAACTA −0.0849130689274
GTCAAGAA 0.0642856116489
GTCAAGAC −0.156319474957
GTCAAGCA −0.20860597533
GTCAAGGA −0.0228703021102
GTCAAGTA −0.0705393312555
GTCAATAA −0.101292836051
GTCAATCA −0.143502549128
GTCAATGA 0.103366451851
GTCAATTA −0.132363037778
GTCACAAA 0.0692795048284
GTCACAAC 0.048471710412
GTCACACA −0.0964432836191
GTCACAGA 0.0993900236324
GTCACATA −0.0487081945963
GTCACCAA −0.229634781238
GTCACCAC −0.183706424942
GTCACCCA −0.143028703635
GTCACCGA −0.155527155316
GTCACCTA −0.14656040014
GTCACGAA −0.103505734626
GTCACGAC −0.10821209289
GTCACGCA −0.210728000105
GTCACGGA −0.0549137841692
GTCACGTA −0.0968844823988
GTCACTAA −0.167805214843
GTCACTCA −0.221148358602
GTCACTGA 0.107270266704
GTCACTTA −0.0956142890626
GTCAGAAA 0.11353838967
GTCAGAAC −0.0991022094407
GTCAGACA −0.02796874009
GTCAGAGA −0.0051326999622
GTCAGATA 0.176712906668
GTCAGCAA −0.147806215322
GTCAGCAC −0.223008985205
GTCAGCCA −0.254336638358
GTCAGCGA 0.115722014046
GTCAGCTA −0.173989023032
GTCAGGAA 0.0711248438521
GTCAGGAC −0.210733767182
GTCAGGCA −0.0789369186498
GTCAGGGA −0.170613052269
GTCAGGTA −0.222988071243

GTCAGTAA 0.0230469405779
GTCAGTCA -0.272258212338
GTCAGTGA -0.0704873816137
GTCAGTTA 0.0319889906196
GTCATAAA -0.0200407018589
GTCATAAC 0.0420684218594
GTCATACA -0.147027173154
GTCATAGA -0.0227841051642
GTCATATA 0.014402436906
GTCATCAA -0.215517455276
GTCATCAC -0.159954720561
GTCATCCA -0.120491945692
GTCATCGA -0.159467343861
GTCATCTA -0.166445790782
GTCATGAA -0.0584727840231
GTCATGAC 0.100891953947
GTCATGCA -0.344819358689
GTCATGGA -0.0854777207044
GTCATGTA -0.15056342727
GTCATTAA -0.103280933189
GTCATTCA 0.135529148997
GTCATTGA 0.0238352444837
GTCATTTA 0.0453968161281
GTCCAAAA 0.198511748085
GTCCAAAC -0.116893060736
GTCCAACA -0.211467632096
GTCCAAGA -0.0929652328059
GTCCAATA -0.0949233193538
GTCCACAA -0.133946618795
GTCCACAC -0.132485484951
GTCCACCA -0.283176619446
GTCCACGA -0.076972642425
GTCCACTA 0.0319617137799
GTCCAGAA 0.0929724166624
GTCCAGAC -0.165821639411
GTCCAGCA -0.029607351737
GTCCAGGA 0.019774019547
GTCCAGTA -0.247756952462
GTCCATAA -0.137541256173
GTCCATCA -0.180471138235
GTCCATGA -0.221548333871
GTCCATTA -0.159159270931
GTCCCAAA 0.151412012876
GTCCCAAC -0.254913962574
GTCCCACA -0.165431516726
GTCCCAGA 0.0499452012922
GTCCCATA -0.108932735482
GTCCCCAA -0.176011542554
GTCCCCAC -0.392979874391
GTCCCCCA -0.283086177759
GTCCCCGA -0.172095799798
GTCCCCTA -0.18851332122
GTCCCGAA -0.241957749089
GTCCCGAC -0.216364713764

GTCCCGCA -0.286912765553
GTCCCGGA -0.218530236278
GTCCCGTA -0.174362855796
GTCCCTAA -0.30022986795
GTCCCTCA -0.12179940454
GTCCCTGA -0.236270008686
GTCCCTTA -0.294677266605
GTCCGAAA -0.147665958829
GTCCGAAC -0.130040697819
GTCCGACA -0.0462362138055
GTCCGAGA -0.132095724141
GTCCGATA -0.020435764449
GTCCGCAA -0.01760576003
GTCCGCAC -0.107631981107
GTCCGCCA -0.181559310073
GTCCGCGA -0.166155267725
GTCCGCTA -0.215374776369
GTCCGGAA -0.088540351502
GTCCGGAC -0.0833540049116
GTCCGGCA -0.164994610272
GTCCGGGA -0.00527774330976
GTCCGGTA -0.294187690705
GTCCGTAA 0.0613326825448
GTCCGTCA -0.334836846124
GTCCGTGA 0.116431756969
GTCCGTTA -0.163207890039
GTCCTAAA -0.0721386353385
GTCCTAAC -0.0762906647808
GTCCTACA 0.0110880783632
GTCCTAGA -0.225262520341
GTCCTATA -0.0804942420177
GTCCTCAA -0.328685171754
GTCCTCAC -0.168110044087
GTCCTCCA -0.381335949982
GTCCTCGA -0.0292370813377
GTCCTCTA -0.220745832058
GTCCTGAA -0.052289284656
GTCCTGCA -0.206892148837
GTCCTGGA 0.000337674993371
GTCCTGTA -0.213688787425
GTCCTTAA -0.0518889889628
GTCCTTCA -0.0742643186624
GTCCTTGA -0.149488657827
GTCCTTTA 0.0348890170288
GTCGAAAA 0.162573812252
GTCGAAAC -0.0450018903757
GTCGAACA -0.0086808318066
GTCGAAGA -0.0689343936875
GTCGAATA -0.00987487084095
GTCGACAA -0.110640642906
GTCGACAC -0.224539276903
GTCGACCA -0.326497216771
GTCGACGA -0.104508744792
GTCGACTA 0.0850601811639

GTCGAGAA -0.0563743125336
GTCGAGAC -0.208472894182
GTCGAGCA -0.276986134881
GTCGAGGA -0.156022825952
GTCGAGTA 0.0307256045488
GTCGATAA -0.0456552046306
GTCGATCA -0.262021795222
GTCGATGA 0.0128311058882
GTCGATTA 0.207078373256
GTCGCAAA 0.0435019671102
GTCGCAAC -0.0122560577106
GTCGCACA -0.0995477920194
GTCGCAGA 0.0352288668402
GTCGCATA -0.124085603965
GTCGCCAA -0.127314981083
GTCGCCAC -0.27055189835
GTCGCCCA -0.268048045418
GTCGCCGA -0.221512427608
GTCGCCTA 0.0865093743882
GTCGCGAA 0.0328832215353
GTCGCGAC -0.253551537599
GTCGCGCA 0.00985233899988
GTCGCGGA -0.00614465372388
GTCGCGTA -0.0916136256483
GTCGCTAA -0.160297924783
GTCGCTCA -0.234823588703
GTCGCTGA 0.0486062858277
GTCGCTTA -0.30317350069
GTCGGAAA 0.179988528473
GTCGGAAC -0.134039149518
GTCGGACA -0.151956040363
GTCGGAGA -0.266333992068
GTCGGATA 0.281202690294
GTCGGCAA -0.0775949775429
GTCGGCAC -0.264877531048
GTCGGCCA -0.409715606275
GTCGGCGA -0.265947821062
GTCGGCTA -0.032871371245
GTCGGGAA 0.0365983643496
GTCGGGCA -0.0619107138426
GTCGGGGA 0.0511558390346
GTCGGGTA -0.200084640855
GTCGGTAA -0.158834914329
GTCGGTCA -0.226179154917
GTCGGTGA -0.0984871155202
GTCGGTTA 0.0974307131961
GTCGTAAA 0.0737812627765
GTCGTAAC -0.0972873635479
GTCGTACA -0.11329220328
GTCGTAGA -0.121170139147
GTCGTATA 0.00528765680281
GTCGTCAA -0.166008969395
GTCGTCAC -0.0362397955386
GTCGTCCA -0.151059717501

GTCGTCGA -0.303356383359
GTCGTCTA -0.0580841295446
GTCGTGAA -0.216535881229
GTCGTGCA -0.00814175056599
GTCGTGGA 0.00226810832871
GTCGTGTA -0.201789824433
GTCGTTAA -0.13156880746
GTCGTTCA -0.0208645066166
GTCGTTGA -0.0481029305938
GTCGTTTA -0.0254600264228
GTCTAAAA 0.0553049854828
GTCTAAAC 0.101855298825
GTCTAACA -0.0579089962908
GTCTAAGA 0.0830269075643
GTCTAATA 0.108512093361
GTCTACAA 0.0107064437208
GTCTACAC -0.0462858477552
GTCTACCA -0.0952603715862
GTCTACGA -0.278467311748
GTCTACTA 0.186380590206
GTCTAGAA -0.0382073426875
GTCTAGAC -0.0866719588532
GTCTAGCA -0.238637898573
GTCTAGGA -0.106370866587
GTCTAGTA -0.0158464249373
GTCTATAA -0.192725322358
GTCTATCA -0.00501229900075
GTCTATGA 0.030519228065
GTCTATTA 0.158751998131
GTCTCAAA 0.073377785499
GTCTCAAC -0.166188298028
GTCTCACA -0.098808260431
GTCTCAGA -0.0148536966719
GTCTCATA -0.0617384668834
GTCTCCAA -0.159103877738
GTCTCCAC -0.272523318697
GTCTCCCA -0.265125011372
GTCTCCGA -0.32952084152
GTCTCCTA -0.137213303227
GTCTCGAA -0.0451332678392
GTCTCGCA -0.261963285103
GTCTCGGA -0.241008592387
GTCTCGTA -0.164204557893
GTCTCTAA -0.288152050556
GTCTCTCA -0.0821166725949
GTCTCTGA 0.0466568496872
GTCTCTTA -0.113563490291
GTCTGAAA 0.0482507682314
GTCTGAAC -0.15610172342
GTCTGACA -0.117312289583
GTCTGAGA 0.144057429399
GTCTGATA 0.111196835106
GTCTGCAA 0.0222476692605
GTCTGCAC -0.00150142697605

GTCTGCCA -0.186639879384
GTCTGCGA -0.132964120318
GTCTGCTA -0.0725860017664
GTCTGGAA -0.218654911787
GTCTGGCA -0.208629192689
GTCTGGGA -0.19866752686
GTCTGGTA -0.287979038399
GTCTGTAA -0.160021102742
GTCTGTCA -0.101074139324
GTCTGTGA 0.0812621076406
GTCTGTTA 0.145834449119
GTCTTAAA -0.120500180586
GTCTTAAC -0.104290416998
GTCTTACA 0.0291241654878
GTCTTAGA -0.0650136459496
GTCTTATA 0.031651131727
GTCTTCAA -0.103002114967
GTCTTCAC -0.193728224522
GTCTTCCA -0.139022239997
GTCTTCGA -0.349177246867
GTCTTCTA -0.040839081458
GTCTTGAA -0.164049255299
GTCTTGCA -0.0227564359905
GTCTTGGA 0.180584165433
GTCTTGTA -0.0844522208159
GTCTTTAA 0.0320938633136
GTCTTTCA -0.0466917593856
GTCTTTGA 0.00376561097202
GTCTTTTA 0.168865835533
GTGAAAAA 0.161476903901
GTGAAAAC 0.0676182273381
GTGAAACA 0.0700714488593
GTGAAAGA 0.102955572653
GTGAAATA 0.200185033518
GTGAACAA 0.0078817557414
GTGAACAC 0.174772085283
GTGAACCA -0.12200324908
GTGAACGA 0.000647431078152
GTGAACTA 0.0382739044944
GTGAAGAA 0.0192575495606
GTGAAGCA -0.235906786739
GTGAAGGA -0.0780664532619
GTGAAGTA -0.0896250447071
GTGAATAA 0.153672166567
GTGAATCA -0.0229272829638
GTGAATGA 0.111176397597
GTGAATTA 0.0443763322551
GTGACAAA 0.180658087779
GTGACAAC -0.137618100266
GTGACACA 0.00563390356018
GTGACAGA 0.148015947142
GTGACATA 0.0306818098874
GTGACCAA -0.0750900904627
GTGACCAC -0.125738122523

GTGACCCA -0.180712017072
GTGACCGA -0.0169104691781
GTGACCTA -0.200472672137
GTGACGAA -0.0300219400364
GTGACGCA -0.155492958523
GTGACGGA -0.226063763801
GTGACGTA -0.0596023444742
GTGACTAA 0.0142208564776
GTGACTCA -0.281003184055
GTGACTGA -0.0738173473722
GTGACTTA -0.150158716259
GTGAGAAA -0.00082552936797
GTGAGAAC 0.0166616789413
GTGAGACA -0.094643912907
GTGAGAGA 0.0681533364767
GTGAGATA 0.280320265169
GTGAGCAA 0.139634124483
GTGAGCAC 0.262212447577
GTGAGCCA -0.235841280081
GTGAGCGA 0.0538550250724
GTGAGCTA -0.282037535963
GTGAGGAA 0.0352294443204
GTGAGGCA -0.16105445131
GTGAGGGA 0.148650029561
GTGAGGTA -0.0898927408367
GTGAGTAA 0.095854807976
GTGAGTCA -0.243888957808
GTGAGTGA 0.0547167277542
GTGAGTTA -0.0356495843157
GTGATAAA -0.0495172836533
GTGATAAC -0.00382546367598
GTGATACA 0.210567316628
GTGATAGA 0.0688941118078
GTGATATA 0.227565089816
GTGATCAA 0.0999707391882
GTGATCAC 0.270148208788
GTGATCCA 0.0847054781726
GTGATCGA -0.0241798272101
GTGATCTA 0.0113943144246
GTGATGAA -0.00745262496174
GTGATGCA 0.0823288853592
GTGATGGA -0.058504621609
GTGATGTA 0.079378276348
GTGATTAA -0.0265344554499
GTGATTCA 0.0642956723575
GTGATTGA 0.0743705137645
GTGATTTA 0.0475971839608
GTGCAAAA 0.0269561009504
GTGCAAAC -0.052525127187
GTGCAACA -0.252057638699
GTGCAAGA 0.201577337723
GTGCAATA 0.237211593907
GTGCACAA 0.0850529503128
GTGCACAC 0.125035948118

GTGCACCA -0.249702929897
GTGCACGA -0.077562911174
GTGCACTA -0.188902122638
GTGCAGAA 0.0194195634734
GTGCAGCA -0.230043189703
GTGCAGGA 0.0236418215378
GTGCAGTA 0.011332268908
GTGCATAA 0.0478315781346
GTGCATCA -0.155296525227
GTGCATGA -0.148762266762
GTGCATTA 0.0163930716846
GTGCCAAA -0.0305781046319
GTGCCAAC -0.357024056677
GTGCCACA -0.320612241026
GTGCCAGA -0.0346902192398
GTGCCATA -0.0773315058175
GTGCCCAA -0.186939488927
GTGCCCAC -0.172651722029
GTGCCCCA -0.104996180754
GTGCCCGA -0.155967885374
GTGCCCTA -0.279737540622
GTGCCGAA -0.109365991502
GTGCCGCA -0.139419856608
GTGCCGGA 0.0172602059809
GTGCCGTA -0.112584890521
GTGCCTAA -0.0100559841755
GTGCCTCA -0.361024979535
GTGCCTGA -0.22585305202
GTGCCTTA -0.253604785294
GTGCGAAA 0.086409056736
GTGCGAAC 0.0460019247898
GTGCGACA -0.06891878104
GTGCGAGA 0.0481516702829
GTGCGATA 0.185060302256
GTGCGCAA 0.13622986358
GTGCGCAC 0.172738899501
GTGCGCCA -0.289032460123
GTGCGCGA -0.0332113043237
GTGCGCTA -0.200178655616
GTGCGGAA 0.0429118108456
GTGCGGCA -0.324517859358
GTGCGGGA -0.00956280762708
GTGCGGTA 0.00738560307365
GTGCGTAA 0.109126807442
GTGCGTCA -0.226847493743
GTGCGTGA -0.00249994525667
GTGCGTTA 0.0082162051859
GTGCTAAA -0.19781852872
GTGCTAAC -0.0288566268216
GTGCTACA 0.00804340273469
GTGCTAGA -0.0100146120934
GTGCTATA 0.00394544174448
GTGCTCAA -0.155501650247
GTGCTCCA -0.237986316155

GTGCTCGA -0.217694327615
GTGCTCTA -0.340292155258
GTGCTGAA -0.026230812464
GTGCTGCA -0.206293474934
GTGCTGGA 0.014693109544
GTGCTGTA 0.0266395872456
GTGCTTAA 0.0664948695252
GTGCTTCA -0.269375690438
GTGCTTGA -0.170492777146
GTGCTTTA -0.187528434706
GTGGAAAA 0.0755013853166
GTGGAAAC -0.111028736631
GTGGAACA -0.121060513836
GTGGAAGA 0.0352076799181
GTGGAATA 0.164681547009
GTGGACAA -0.0401137201375
GTGGACAC -0.150321931312
GTGGACCA -0.0286132420458
GTGGACGA -0.0055536759548
GTGGACTA -0.169212658556
GTGGAGAA 0.0497967197799
GTGGAGCA -0.285788624893
GTGGAGGA -0.172404005143
GTGGAGTA -0.105739742484
GTGGATAA 0.169945913593
GTGGATCA 0.0447044840984
GTGGATGA -0.00796259935914
GTGGATTA 0.394255136679
GTGGCAAA 0.0637265211377
GTGGCAAC 0.0318188917873
GTGGCACA -0.0772677361721
GTGGCAGA -0.0907273684762
GTGGCATA -0.0384900645453
GTGGCCAA -0.249445375167
GTGGCCAC -0.246526658123
GTGGCCCA -0.274029908686
GTGGCCGA 0.0557615526667
GTGGCCTA -0.242637389902
GTGGCGAA -0.0290498186621
GTGGCGCA -0.249063391639
GTGGCGGA 0.0441742151632
GTGGCGTA -0.138167235994
GTGGCTAA -0.15802803076
GTGGCTCA -0.125043767869
GTGGCTGA 0.0259610879266
GTGGCTTA -0.169851208068
GTGGGAAA -0.107000173702
GTGGGAAC -0.270934466856
GTGGGACA 0.0299211056787
GTGGGAGA -0.0949469881844
GTGGGATA 0.132064571459
GTGGGCAA 0.107591074648
GTGGGCCA -0.168772667236
GTGGGCGA -0.153280192172

GTGGGCTA -0.0547200922043
GTGGGGAA -0.0238709784164
GTGGGGCA -0.0797538400457
GTGGGGGA 0.072972854609
GTGGGGTA -0.19196242718
GTGGGTAA -0.0803742532668
GTGGGTCA -0.10122560576
GTGGGTGA -0.0720254301001
GTGGGTTA -0.0441442888112
GTGGTAAA -0.0479215783596
GTGGTAAC -0.18440837062
GTGGTACA 0.0565235797756
GTGGTAGA 0.00120210379112
GTGGTATA 0.163489936217
GTGGTCAA -0.111019065789
GTGGTCCA -0.115270505386
GTGGTCGA -0.226044466499
GTGGTCTA -0.259184471744
GTGGTGAA 0.0881958789224
GTGGTGCA -0.108576904718
GTGGTGGA -0.094174063871
GTGGTGTA 0.171037860485
GTGGTTAA -0.048143801554
GTGGTTCA -0.181933242338
GTGGTTGA -0.111762954444
GTGGTTTA 0.00519665671181
GTGTAAAA 0.0492857820632
GTGTAAAC -0.113734005825
GTGTAACA 0.175322705626
GTGTAAGA 0.0598081793829
GTGTAATA 0.21045149833
GTGTACAA -0.00547462865998
GTGTACAC 0.015483780275
GTGTACCA -0.00821166122072
GTGTACGA -0.0835944428409
GTGTACTA -0.0671858671586
GTGTAGAA 0.105087180845
GTGTAGCA 0.050354762476
GTGTAGGA 0.000174422762827
GTGTAGTA 0.0312144100023
GTGTATAA 0.0836447737447
GTGTATCA 0.0097403233581
GTGTATGA 0.0369484518626
GTGTATTA 0.0765710614195
GTGTCAAA 0.0527309620957
GTGTCAAC -0.0310848038121
GTGTCACA 0.0570336176397
GTGTCAGA 0.159195784034
GTGTCATA -0.00568089749859
GTGTCCAA -0.204295605132
GTGTCCCA -0.033298587844
GTGTCCGA -0.198367594153
GTGTCCTA -0.0835835835836
GTGTCGAA -0.154525072145

GTGTCGCA -0.136068995371
GTGTCGGA -0.0651077639092
GTGTCGTA -0.147139105773
GTGTCTAA -0.0582563885462
GTGTCTCA 0.00215094102758
GTGTCTGA -0.00649693802326
GTGTCTTA -0.0673627584797
GTGTGAAA -0.00701920592542
GTGTGAAC -0.0965419045214
GTGTGACA 0.04917727645
GTGTGAGA 0.095255578345
GTGTGATA 0.151182384991
GTGTGCAA -0.129841326372
GTGTGCCA -0.202629424003
GTGTGCGA 0.0227403779479
GTGTGCTA -0.0199855502886
GTGTGGAA 0.081349107874
GTGTGGCA -0.00684111647488
GTGTGGGA 0.0739421764487
GTGTGGTA -0.0117137606215
GTGTGTAA 0.0606046818168
GTGTGTCA -0.0648821931065
GTGTGTGA 0.0128980316578
GTGTGTTA -0.00259045422363
GTGTTAAA -0.113374970702
GTGTTAAC 0.0964029984745
GTGTTACA 0.215613685311
GTGTTAGA -0.0887257943257
GTGTTATA 0.210893922031
GTGTTCAA -0.188793537512
GTGTTCCA -0.0201726302205
GTGTTCGA -0.0207880056365
GTGTTCTA -0.170992877882
GTGTTGAA -0.14197224497
GTGTTGCA -0.0475199717624
GTGTTGGA -0.0246761913429
GTGTTGTA 0.0850222257907
GTGTTTAA -0.173306738016
GTGTTTCA 0.0573424664334
GTGTTTGA 0.0266986376157
GTGTTTTA 0.0670664146041
GTTAAAAA 0.0702589641984
GTTAAAAC 0.039119581323
GTTAAACA -0.0483164602235
GTTAAAGA 0.0454233362286
GTTAAATA 0.090175397619
GTTAACAA 0.047630274903
GTTAACCA 0.0547034637944
GTTAACGA 0.114498952578
GTTAACTA 0.0802358832662
GTTAAGAA -0.119215404084
GTTAAGCA 0.00994649752195
GTTAAGGA 0.0320949176295
GTTAAGTA 0.0238907177215

GTTAATAA 0.0668386823646
GTTAATCA 0.128643722184
GTTAATGA 0.152222870189
GTTAATTA 0.013274982972
GTTACAAA 0.0050929947195
GTTACAAC 0.00497329285208
GTTACACA 0.0293571808723
GTTACAGA 0.222782010661
GTTACATA 0.0318045318045
GTTACCAA -0.280462888507
GTTACCCA -0.130422883067
GTTACCGA -0.0409283008401
GTTACCTA -0.213397575863
GTTACGAA -0.127518453262
GTTACGCA 0.0502930592761
GTTACGGA 0.0173740721006
GTTACGTA 0.0323781231068
GTTACTAA -0.0962109700576
GTTACTCA 0.0266588902953
GTTACTGA -0.0589270649828
GTTACTTA 0.00235542287395
GTTAGAAA 0.115801113844
GTTAGAAC -0.205356625888
GTTAGACA -0.142964429128
GTTAGAGA -0.0799948539985
GTTAGATA 0.138271880696
GTTAGCAA -0.0193843981723
GTTAGCCA 0.00382062503275
GTTAGCGA -0.0139216318833
GTTAGCTA -0.191055413416
GTTAGGAA -0.00646742057867
GTTAGGCA 0.0590928398457
GTTAGGGA -0.00425632243814
GTTAGGTA -0.110808080956
GTTAGTAA 0.00212185134632
GTTAGTCA -0.0453614428715
GTTAGTGA 0.0890546193576
GTTAGTTA -0.1776829312
GTTATAAA 0.186371831274
GTTATAAC -0.110662631174
GTTATACA 0.134002175135
GTTATAGA 0.137990607688
GTTATATA 0.0606753136793
GTTATCAA 0.0867594140274
GTTATCCA 0.22127325679
GTTATCGA 0.0278115581146
GTTATCTA -0.000824069545922
GTTATGAA -0.109312204842
GTTATGCA 0.108888378788
GTTATGGA 0.107220279848
GTTATGTA -0.0644625341595
GTTATTAA -0.0741535149756
GTTATTCA 0.115709373285
GTTATTGA 0.00211893170223

GTTATTTA 0.105182368269
GTTCAAAA -0.0453083937932
GTTCAAAC -0.0274743169806
GTTCAACA -0.117913449903
GTTCAAGA -0.112591710299
GTTCAATA 0.0642391702998
GTTCACAA -0.0471024700423
GTTCACCA 0.177498435074
GTTCACGA -0.12394930816
GTTCACTA 0.0161001221607
GTTCAGAA -0.127243300771
GTTCAGCA -0.122992138144
GTTCAGGA -0.17241215734
GTTCAGTA -0.00949541250922
GTTCATAA -0.239900902676
GTTCATCA -0.123369155121
GTTCATGA 0.132963542054
GTTCATTA 0.0594340645488
GTTCCAAA 0.045707496836
GTTCCAAC -0.206847638189
GTTCCACA -0.123658142512
GTTCCAGA 0.0438377261812
GTTCCATA 0.00368274610699
GTTCCCAA -0.152010940442
GTTCCCCA -0.091627583811
GTTCCCGA -0.0225561318323
GTTCCCTA -0.172569601056
GTTCCGAA -0.263420162021
GTTCCGCA -0.160380635716
GTTCCGGA -0.204720565248
GTTCCGTA -0.0867275224524
GTTCCTAA -0.150271732387
GTTCCTCA -0.00569111625293
GTTCCTGA 0.0568598901932
GTTCCTTA -0.0501644895584
GTTCGAAA -0.0208945485438
GTTCGAAC -0.0896322757229
GTTCGACA -0.181534938102
GTTCGAGA 0.136040999677
GTTCGATA 0.0579601640208
GTTCGCAA -0.0553646527475
GTTCGCCA -0.217687581148
GTTCGCGA -0.10525389955
GTTCGCTA -0.0844301046391
GTTCGGAA 0.0793522769574
GTTCGGCA -0.226754935768
GTTCGGGA -0.165157360495
GTTCGGTA 0.0636628363901
GTTCGTAA 0.0839399944352
GTTCGTCA -0.0629155326125
GTTCGTGA 0.0702219841208
GTTCGTTA -0.03519802963
GTTCTAAA -0.0391369330763
GTTCTACA -0.0327192046284

GTTCTAGA 0.174664058451
GTTCTATA 0.0675262038898
GTTCTCAA -0.190463194979
GTTCTCCA -0.170938686385
GTTCTCGA -0.268500101422
GTTCTCTA -0.0561539500933
GTTCTGAA -0.00920883031725
GTTCTGCA -0.128961409604
GTTCTGGA 0.0465915576842
GTTCTGTA -0.00475382939709
GTTCTTAA -0.295456568596
GTTCTTCA 0.0163171609381
GTTCTTGA 0.060056349131
GTTCTTTA 0.0804821792224
GTTGAAAA 0.244879684506
GTTGAAAC 0.00367218236097
GTTGAACA -0.13151494252
GTTGAAGA 0.12294840259
GTTGAATA 0.0951993944959
GTTGACAA 0.0884203762992
GTTGACCA -0.0697756733521
GTTGACGA 0.12325496522
GTTGACTA -0.131295960824
GTTGAGAA 0.0713144967628
GTTGAGCA -0.145779398912
GTTGAGGA 0.00844796259075
GTTGAGTA 0.0027670926914
GTTGATAA -0.128441712955
GTTGATCA -0.0269429623196
GTTGATGA 0.0688678350109
GTTGATTA 0.0969909303243
GTTGCAAA 0.147566841602
GTTGCAAC -0.315908921795
GTTGCACA -0.247918135467
GTTGCAGA 0.207895447545
GTTGCATA 0.0487806836347
GTTGCCAA -0.11310070401
GTTGCCCA -0.214730179948
GTTGCCGA -0.0908973948707
GTTGCCTA -0.103651709586
GTTGCGAA 0.0198132016314
GTTGCGCA 0.116133953271
GTTGCGGA 0.0536535696299
GTTGCGTA 0.0725670574155
GTTGCTAA -0.0966390725335
GTTGCTCA -0.187908131903
GTTGCTGA -0.095836538223
GTTGCTTA -0.0883170441523
GTTGGAAA 0.116958556352
GTTGGACA -0.240823555683
GTTGGAGA 0.0450354646164
GTTGGATA 0.2963087054
GTTGGCAA 0.0895196912922
GTTGGCCA -0.235384942003

GTTGGCGA -0.341435041611
GTTGGCTA -0.0786282149919
GTTGGGAA -0.0145244994635
GTTGGGCA -0.222839827842
GTTGGGGA -0.0480525845596
GTTGGGTA -0.120527686914
GTTGGTAA 0.0384188667401
GTTGGTCA -0.0427968730612
GTTGGTGA -0.0537720659398
GTTGGTTA -0.0469270923816
GTTGTAAA 0.143044312898
GTTGTACA -0.0858249654814
GTTGTAGA 0.0966876637738
GTTGTATA 0.0557060794289
GTTGTCAA 0.135610817429
GTTGTCCA -0.0906512573179
GTTGTCGA -0.0918653310311
GTTGTCTA 0.0285300321891
GTTGTGAA 0.0461033699992
GTTGTGCA -0.192398038097
GTTGTGGA -0.0267223669879
GTTGTGTA 0.0486702659207
GTTGTTAA 0.021487850631
GTTGTTCA 0.0500238530542
GTTGTTGA 0.112423085626
GTTGTTTA -0.00397728516894
GTTTAAAA 0.080930793052
GTTTAAAC 0.138261851016
GTTTAACA 0.0887410973522
GTTTAAGA 0.106151606152
GTTTAATA 0.178583393305
GTTTACAA 0.0463930317393
GTTTACCA -0.0188295146822
GTTTACGA 0.121383383255
GTTTACTA -0.102398487624
GTTTAGAA -0.0318955318955
GTTTAGCA -0.104433422666
GTTTAGGA 0.0988146244973
GTTTAGTA -0.0142856354978
GTTTATAA 0.0635166847288
GTTTATCA 0.0097080551626
GTTTATGA -0.00637138515926
GTTTATTA 0.0161829664739
GTTTCAAA -0.0656506807711
GTTTCACA -0.0700599519158
GTTTCAGA -0.030715288291
GTTTCATA 0.057422830157
GTTTCCAA 0.0769736678828
GTTTCCCA -0.0322209261603
GTTTCCGA 0.147305327427
GTTTCCTA -0.216700406188
GTTTCGAA -0.246805607245
GTTTCGCA -0.114579699526
GTTTCGGA -0.0282221278298

GTTTCGTA 0.0489490532794
GTTTCTAA 0.0242819500303
GTTTCTCA −0.0939607161887
GTTTCTGA 0.0107145713206
GTTTCTTA −0.103645870587
GTTTGAAA 0.0673690219145
GTTTGACA −0.00308679371985
GTTTGAGA −0.00111268293086
GTTTGATA 0.0660950206405
GTTTGCAA 0.0704356838901
GTTTGCCA −0.102793701213
GTTTGCGA 0.0104203719789
GTTTGCTA −0.160528055792
GTTTGGAA −0.17435645573
GTTTGGCA −0.148128847973
GTTTGGGA 0.155579344479
GTTTGGTA −0.172255147624
GTTTGTAA 0.0925622592289
GTTTGTCA 0.111041144502
GTTTGTGA 0.0452477959685
GTTTGTTA 0.104353189346
GTTTTAAA −0.170041272756
GTTTTACA 0.0624796537302
GTTTTAGA −0.10599548915
GTTTTATA −0.0589862995701
GTTTTCAA 0.0861343437101
GTTTTCCA −0.145095557765
GTTTTCGA −0.0607204740099
GTTTTCTA −0.20519288611
GTTTTGAA −0.0316369704941
GTTTTGCA −0.127787209382
GTTTTGGA 0.0143821507458
GTTTTGTA 0.127688294355
GTTTTTAA 0.0118562088259
GTTTTTCA 0.0997236906328
GTTTTTGA 0.0930395684766
GTTTTTTA 0.118952285619
TAAAAAAA 0.12933658387
TAAAAACA 0.140425549516
TAAAAAGA 0.204395855911
TAAAAATA 0.0966434754314
TAAAACAA 0.112345127497
TAAAACCA 0.0563676624283
TAAAACGA 0.0296803719627
TAAAACTA −0.118593800412
TAAAAGAA 0.156027928755
TAAAAGCA 0.158349087246
TAAAAGGA 0.156679050824
TAAAAGTA −0.0313711172274
TAAAATAA 0.228408866959
TAAAATCA 0.250817135391
TAAAATGA 0.0592162410344
TAAAATTA 0.0116879965365
TAAACAAA 0.127389181259

TAAACACA 0.0930643854514
TAAACAGA 0.165086574177
TAAACATA -0.0594095897869
TAAACCAA 0.0762866962801
TAAACCCA -0.192133748896
TAAACCGA -0.00799578704738
TAAACCTA -0.25685742565
TAAACGAA 0.02093002093
TAAACGCA 0.194986998017
TAAACGGA 0.128818901546
TAAACGTA -0.0616049442184
TAAACTAA 0.0577761070925
TAAACTCA 0.0417063859915
TAAACTGA 0.1011123844
TAAACTTA -0.0628955009058
TAAAGAAA 0.0417764574498
TAAAGACA 0.0603629117611
TAAAGAGA 0.0640737155889
TAAAGATA 0.227511302642
TAAAGCAA 0.0131376685182
TAAAGCCA -0.148343148201
TAAAGCGA -0.0913682277319
TAAAGCTA -0.166226942025
TAAAGGAA 0.168521127275
TAAAGGCA 0.0339196951892
TAAAGGGA 0.143627098173
TAAAGGTA 0.00939760443202
TAAAGTAA 0.211644270563
TAAAGTCA 0.0395229940684
TAAAGTGA 0.0963037305752
TAAAGTTA -0.213702235487
TAAATAAA 0.143070589695
TAAATACA 0.0953110515828
TAAATAGA 0.0654938685242
TAAATATA 0.0241710035547
TAAATCAA 0.208451438358
TAAATCCA 0.275493967285
TAAATCGA 0.0579250089414
TAAATCTA 0.284766756932
TAAATGAA 0.180415757319
TAAATGCA 0.0430526945678
TAAATGGA 0.158007488887
TAAATGTA -0.00626383959717
TAAATTAA 0.122883213792
TAAATTCA 0.104582543976
TAAATTGA 0.0810047894384
TAAATTTA -0.0455746285317
TAACAAAA 0.287730195689
TAACAACA -0.07276532938
TAACAAGA 0.152971997142
TAACAATA 0.127832096682
TAACACAA 0.23126135924
TAACACCA -0.0585420130875
TAACACGA 0.10715020839

TAACACTA -0.00524441070633
TAACAGAA 0.216096727809
TAACAGCA 0.0281154427275
TAACAGGA 0.186144099034
TAACAGTA 0.0341999810223
TAACATAA 0.175066969336
TAACATCA 0.030204603614
TAACATGA -0.0144091735217
TAACATTA 0.00203134237936
TAACCAAA -0.0237199185419
TAACCACA -0.0776039378205
TAACCAGA 0.0497839437233
TAACCATA -0.124462643559
TAACCCAA -0.12535856879
TAACCCCA -0.294824860637
TAACCCGA 0.00507982983604
TAACCCTA -0.120402720328
TAACCGAA 0.0723988451261
TAACCGCA 0.0937590483045
TAACCGGA -0.140409334102
TAACCGTA -0.0246250082115
TAACCTAA 0.117833186135
TAACCTCA -0.0319093197881
TAACCTGA 0.039087465238
TAACCTTA -0.247284878864
TAACGAAA 0.227272727273
TAACGACA -0.139750945412
TAACGAGA 0.0425811486418
TAACGATA 0.0204251001803
TAACGCAA 0.164362094261
TAACGCCA -0.152931689982
TAACGCGA 0.110014973651
TAACGCTA 0.0385998846741
TAACGGAA 0.182320061108
TAACGGCA 0.0378436968534
TAACGGGA 0.0770545170544
TAACGGTA 0.0363415363755
TAACGTAA 0.286941891783
TAACGTCA -0.0463150318277
TAACGTGA -0.0132543011331
TAACGTTA 0.0936482735619
TAACTAAA 0.0882438912742
TAACTACA 0.0631073772107
TAACTAGA 0.0898874080692
TAACTATA -0.0817437876707
TAACTCAA 0.0131833635258
TAACTCCA -0.0957178424609
TAACTCGA -0.206533239738
TAACTCTA 0.0397385458713
TAACTGAA 0.0890711648287
TAACTGCA -0.0918730707785
TAACTGGA 0.0870489556895
TAACTGTA 0.0275329737305
TAACTTAA 0.143599045276

TAACTTCA 0.0122179806282
TAACTTGA 0.0643131902221
TAAGAAAA 0.133076647957
TAAGAACA 0.0624963806782
TAAGAAGA 0.0767116979238
TAAGAATA 0.141257490712
TAAGACAA 0.108019827779
TAAGACCA -0.140985129855
TAAGACGA 0.0115785785713
TAAGACTA -0.187106929713
TAAGAGAA 0.0961224199214
TAAGAGCA -0.15210954236
TAAGAGGA 0.0346934008744
TAAGAGTA 0.103444425143
TAAGATAA 0.187300278096
TAAGATCA 0.159557819902
TAAGATGA 0.0308145611176
TAAGATTA 0.133589512377
TAAGCAAA -0.0372174331949
TAAGCACA -0.0317940760442
TAAGCAGA -0.120228497861
TAAGCATA 0.132131506448
TAAGCCAA -0.0571971858947
TAAGCCCA -0.0116734670044
TAAGCCGA -0.1068374197
TAAGCCTA -0.173151046798
TAAGCGAA 0.0726254169617
TAAGCGCA -0.0528589114712
TAAGCGGA 0.0825605603121
TAAGCGTA -0.0382004808177
TAAGCTAA -0.0746242109878
TAAGCTCA 0.120790785603
TAAGCTGA -0.100191889366
TAAGCTTA -0.17201344964
TAAGGAAA 0.128700647691
TAAGGACA -0.0885524810081
TAAGGAGA -0.222562337179
TAAGGATA 0.0749266193704
TAAGGCAA 0.028130040948
TAAGGCCA 0.0510000358485
TAAGGCGA -0.153401006636
TAAGGCTA -0.0516963020164
TAAGGGAA 0.0257397172413
TAAGGGCA -0.0780992959977
TAAGGGGA 0.131359034171
TAAGGGTA -0.0525969675162
TAAGGTAA 0.116622131774
TAAGGTCA -0.0847975838225
TAAGGTGA -0.0776632628483
TAAGTAAA 0.084279628858
TAAGTACA -0.155789312375
TAAGTAGA 0.0200547595192
TAAGTATA -0.121855794602
TAAGTCAA -0.0737252403919

TAAGTCCA −0.180895379913
TAAGTCGA −0.0872383841008
TAAGTCTA 0.00307332125514
TAAGTGAA −0.00431104603872
TAAGTGCA 0.0252111615748
TAAGTGGA −0.10414960415
TAAGTGTA −0.0869174960084
TAAGTTAA 0.217979687677
TAAGTTCA 0.103862689138
TAAGTTGA −0.123363884376
TAATAAAA 0.138997123846
TAATAACA 0.121134909014
TAATAAGA −0.040162752284
TAATAATA 0.217991576827
TAATACAA 0.233950418145
TAATACCA 0.0908718635991
TAATACGA 0.2473018839
TAATACTA 0.0722820557362
TAATAGAA 0.0445471996964
TAATAGCA 0.0945261381345
TAATAGGA 0.0955530188351
TAATAGTA 0.0298935059816
TAATATAA 0.22263133732
TAATATCA 0.269659466629
TAATATGA 0.279136784376
TAATATTA 0.146286557686
TAATCAAA 0.233289763593
TAATCACA 0.290667357649
TAATCAGA 0.26676125161
TAATCATA 0.2323445472
TAATCCAA 0.403301387561
TAATCCCA 0.190106056072
TAATCCGA 0.275000547433
TAATCCTA 0.31360895352
TAATCGAA 0.306249775947
TAATCGCA 0.259377531879
TAATCGGA 0.212947106887
TAATCGTA 0.291168579047
TAATCTAA 0.329862920772
TAATCTCA 0.388657252294
TAATCTGA 0.410587319357
TAATGAAA 0.193484117727
TAATGACA −0.0526635734313
TAATGAGA 0.0276045050108
TAATGATA 0.0948510493965
TAATGCAA 0.0706670858186
TAATGCCA 0.0922279074181
TAATGCGA −0.132737931024
TAATGCTA −0.0548041154102
TAATGGAA −0.0911192727333
TAATGGCA −0.0962574144392
TAATGGGA 0.184739836255
TAATGGTA 0.0463535160505
TAATGTAA −0.0254337496259

TAATGTCA 0.0894542455275
TAATGTGA 0.108426608427
TAATTAAA 0.0238615212806
TAATTACA 0.106909731702
TAATTAGA −0.0607264074509
TAATTATA 0.0949869710882
TAATTCAA −0.0775056020671
TAATTCCA 0.16629343883
TAATTCGA −0.186407408326
TAATTCTA 0.0550729793154
TAATTGAA 0.0460712539141
TAATTGCA 0.241567702897
TAATTGGA 0.215848079484
TAATTGTA −0.0991854232325
TAATTTAA 0.191235958337
TAATTTCA 0.240294738071
TAATTTGA 0.0301168937533
TACAAAAA 0.220435318935
TACAAACA 0.157255051194
TACAAAGA 0.153598826303
TACAAATA 0.196794960694
TACAACAA 0.11874546723
TACAACCA 0.00197002985336
TACAACGA 0.264646407909
TACAACTA −0.0249180631955
TACAAGAA 0.0788617767494
TACAAGCA 0.0837545534515
TACAAGGA −0.0371289517586
TACAAGTA 0.117784822692
TACAATAA 0.11224790698
TACAATCA 0.235059816208
TACAATGA 0.0595103756852
TACACAAA −0.0210572031269
TACACACA 0.250387947709
TACACAGA 0.0116661678941
TACACATA 0.135718362991
TACACCAA 0.132174874599
TACACCCA −0.0153347007
TACACCGA 0.0361382002862
TACACCTA −0.0742418101154
TACACGAA 0.18701483853
TACACGCA 0.174077209988
TACACGGA −0.0202678932456
TACACGTA 0.143634081006
TACACTAA 0.00661226396502
TACACTCA −0.00219365370881
TACACTGA 0.0353517806179
TACAGAAA 0.109550155836
TACAGACA 0.133410947206
TACAGAGA 0.150427351544
TACAGATA 0.24226841748
TACAGCAA 0.0148952942636
TACAGCCA −0.077407196014
TACAGCGA −0.101852514179

TACAGCTA -0.144791944918
TACAGGAA -0.00202686231204
TACAGGCA 0.175586665985
TACAGGGA 0.0498832165499
TACAGGTA 0.191084136844
TACAGTAA 0.0856937439326
TACAGTCA -0.0487526093587
TACAGTGA -0.0188070877756
TACATAAA 0.153736049576
TACATACA 0.0867710926038
TACATAGA 0.156153514887
TACATATA -0.0590631954268
TACATCAA 0.0254400405916
TACATCCA 0.115672649504
TACATCGA -0.0112732406327
TACATCTA -0.0485018576236
TACATGAA -0.011632591987
TACATGCA 0.197866470077
TACATGGA 0.119978104827
TACATGTA -0.0159551156425
TACATTAA 0.153479120895
TACATTCA 0.139086174397
TACATTGA 0.1347612826
TACCAAAA 0.131762808114
TACCAACA -0.0627351225886
TACCAAGA 0.107573144403
TACCAATA 0.195530240985
TACCACAA 0.0900904533836
TACCACCA -0.0283674963397
TACCACGA 0.0759625701627
TACCACTA -0.154592965118
TACCAGAA -0.0256147675598
TACCAGCA 0.00228442701828
TACCAGGA -0.224924112718
TACCAGTA -0.154182070814
TACCATAA 0.150581711188
TACCATCA -0.015731772297
TACCATGA 0.00797629585508
TACCCAAA 0.10377733105
TACCCACA 0.0777026780435
TACCCAGA 0.104673544067
TACCCATA 0.110969102866
TACCCCAA -0.0302575302575
TACCCCCA -0.137965835298
TACCCCGA -0.00957960671426
TACCCCTA -0.167195069794
TACCCGAA 0.0558111866164
TACCCGCA 0.0465821916308
TACCCGGA -0.290193889242
TACCCGTA -0.0161230046057
TACCCTAA 0.00992973486308
TACCCTCA -0.0505485281344
TACCCTGA -0.179681318833
TACCGAAA 0.0999737232031

TACCGACA 0.105474936829
TACCGAGA 0.0641380115764
TACCGATA 0.183247812092
TACCGCAA 0.163252784465
TACCGCCA 0.0591308219528
TACCGCGA 0.0662199611201
TACCGCTA -0.0064341707268
TACCGGAA 0.0176305782366
TACCGGCA 0.012413239686
TACCGGGA -0.000728254579513
TACCGGTA 0.189828252007
TACCGTAA 0.287414874127
TACCGTCA -0.175138698938
TACCGTGA -0.221058569571
TACCTAAA 0.109187894907
TACCTACA 0.0557790705313
TACCTAGA 0.0520944796829
TACCTATA -0.0541712511409
TACCTCAA 0.0457509243864
TACCTCCA -0.123662229723
TACCTCGA -0.107614675104
TACCTCTA -0.166609258397
TACCTGAA 0.175069888981
TACCTGCA 0.178176390298
TACCTGGA 0.00514702029854
TACCTTAA 0.142990097536
TACCTTCA -0.0408541738738
TACCTTGA 0.0732153843943
TACGAAAA 0.257941066984
TACGAACA 0.156609777822
TACGAAGA 0.194184542669
TACGAATA 0.0776968387554
TACGACAA 0.132820147972
TACGACCA -0.0507332688156
TACGACGA 0.024501410778
TACGACTA -0.321603402616
TACGAGAA 0.213038106978
TACGAGCA -0.151727867288
TACGAGGA -0.0268818930972
TACGAGTA 0.117620052116
TACGATAA 0.121497339474
TACGATCA 0.104805907836
TACGATGA -0.035441777866
TACGCAAA 0.128081136909
TACGCACA 0.104243702693
TACGCAGA 0.0667335751772
TACGCATA 0.139791829376
TACGCCAA 0.164512997846
TACGCCCA -0.0561576937055
TACGCCGA 0.101078469921
TACGCCTA -0.0248746804973
TACGCGAA 0.186819050455
TACGCGCA 0.117876980796
TACGCGGA 0.0979198697894

TACGCGTA 0.216791109568
TACGCTAA 0.128727901455
TACGCTCA 0.0397433595637
TACGCTGA 0.0945796902444
TACGGAAA 0.162149753059
TACGGACA 0.0728093902772
TACGGAGA 0.207498375948
TACGGATA 0.209083739387
TACGGCAA 0.112659576798
TACGGCCA −0.0908707412095
TACGGCGA −0.240455752103
TACGGCTA −0.178078183264
TACGGGAA −0.0056820752968
TACGGGCA −0.134377248927
TACGGGGA −0.0436304369402
TACGGTAA 0.212890958592
TACGGTCA 0.00368678058145
TACGGTGA 0.0331775378804
TACGTAAA 0.141896892769
TACGTACA 0.0626011686618
TACGTAGA −0.0630883995241
TACGTATA −0.0573396203003
TACGTCAA 0.159554871676
TACGTCCA −0.0941462567309
TACGTCGA 0.00389407531222
TACGTCTA −0.0852461720117
TACGTGAA 0.209183010591
TACGTGCA 0.148939074327
TACGTGGA 0.115812792421
TACGTTAA 0.174453844076
TACGTTCA −0.0197054079108
TACGTTGA 0.0596811748648
TACTAAAA 0.18071928678
TACTAACA 0.0289183448538
TACTAAGA 0.204931508549
TACTAATA −0.0607232992207
TACTACAA 0.124218304092
TACTACCA 0.0587279110676
TACTACGA 0.0146303328122
TACTACTA −0.0412283532041
TACTAGAA 0.163950451829
TACTAGCA 0.0107488661395
TACTAGGA 0.00172210778271
TACTAGTA 0.188538281528
TACTATAA 0.132662965996
TACTATCA 0.194344482223
TACTATGA 0.0256746202638
TACTCAAA 0.112400279067
TACTCACA 0.168574910999
TACTCAGA −0.0563224309502
TACTCATA 0.0990094777974
TACTCCAA 0.0106814803785
TACTCCCA −0.0923824427158
TACTCCGA −0.0725364337805

TACTCCTA -0.195133012966
TACTCGAA 0.0523776851602
TACTCGCA 0.100572250243
TACTCGGA -0.0462616542758
TACTCTAA 0.163373794734
TACTCTCA -0.00113063217594
TACTCTGA -0.189033654575
TACTGAAA 0.097392485793
TACTGACA 0.120165981767
TACTGAGA 0.0797312463979
TACTGATA 0.17750390848
TACTGCAA 0.0188529761773
TACTGCCA 0.0845173729539
TACTGCGA -0.0827439886846
TACTGCTA -0.114556499224
TACTGGAA 0.0119662973014
TACTGGCA -0.0269625480028
TACTGGGA 0.115877024591
TACTGTAA 0.242198346022
TACTGTCA -0.0882250758705
TACTGTGA 0.195154120713
TACTTAAA 0.0699189068853
TACTTACA 0.165287877409
TACTTAGA -0.0324568023601
TACTTATA -0.00347317013984
TACTTCAA -0.00250566017905
TACTTCCA 0.0203083144165
TACTTCGA -0.0850307434467
TACTTCTA 0.0217581567779
TACTTGAA 0.112731717001
TACTTGCA 0.109121518212
TACTTGGA 0.0384061747698
TACTTTAA 0.204084671315
TACTTTCA -0.0565980307001
TACTTTGA 0.162979642782
TAGAAAAA 0.0180957581732
TAGAAACA 0.151704899488
TAGAAAGA -0.00213632510283
TAGAAATA 0.142994515721
TAGAACAA 0.0821113406276
TAGAACCA -0.12610265835
TAGAACGA 0.121730911002
TAGAACTA 0.00388680691711
TAGAAGAA -0.0126311102677
TAGAAGCA 0.112210006149
TAGAAGGA 0.0271723976847
TAGAATAA -0.0934921133114
TAGAATCA 0.283099904312
TAGAATGA 0.167451197754
TAGACAAA -0.0339649496726
TAGACACA 0.145111420918
TAGACAGA 0.0415621172909
TAGACATA -0.0967455058364
TAGACCAA -0.00721827927637

TAGACCCA -0.212646755733
TAGACCGA -0.0623263724152
TAGACCTA -0.120552546188
TAGACGAA -0.0448258655126
TAGACGCA 0.0427165828486
TAGACGGA -0.0435132101799
TAGACTAA -0.0854853310587
TAGACTCA -0.0460067482747
TAGACTGA -0.109929606009
TAGAGAAA 0.114531887259
TAGAGACA -0.073372270342
TAGAGAGA 0.0490724884664
TAGAGATA 0.24436472194
TAGAGCAA 0.0520127296483
TAGAGCCA -0.120284802068
TAGAGCGA 0.0422144040641
TAGAGCTA -0.0754998065736
TAGAGGAA 0.101146601147
TAGAGGCA 0.026935906513
TAGAGGGA -0.0107892712098
TAGAGTAA 0.234686101764
TAGAGTCA -0.0670853922907
TAGAGTGA -0.00197421334349
TAGATAAA 0.128737774794
TAGATACA 0.013742034846
TAGATAGA 0.0527967351891
TAGATATA 0.172897486165
TAGATCAA -0.0668226243221
TAGATCCA 0.122376152347
TAGATCGA 0.114592381189
TAGATCTA 0.146294504671
TAGATGAA -0.0756089979272
TAGATGCA 0.217098165734
TAGATGGA -0.0568025060981
TAGATTAA 0.0718280263735
TAGATTCA 0.195627103056
TAGATTGA 0.0214498732249
TAGCAAAA 0.151201798501
TAGCAACA -0.0473690357145
TAGCAAGA 0.0805347328161
TAGCAATA 0.1541812953
TAGCACAA -0.0860938414624
TAGCACCA -0.25998768277
TAGCACGA 0.0277972015211
TAGCACTA -0.046954446253
TAGCAGAA -0.0857540892446
TAGCAGCA -0.122651313571
TAGCAGGA -0.0708951901646
TAGCATAA -0.274641136452
TAGCATCA -0.0930322693664
TAGCATGA -0.15360312301
TAGCCAAA -0.00757428669445
TAGCCACA -0.167075376268
TAGCCAGA -0.0214248077336

TAGCCATA -0.0829264483282
TAGCCCAA -0.0707328964789
TAGCCCCA -0.00233429021308
TAGCCCGA -0.223268515532
TAGCCCTA -0.173062081052
TAGCCGAA 0.128294961628
TAGCCGCA -0.105542734088
TAGCCGGA -0.317187543321
TAGCCTAA -0.323198180123
TAGCCTCA 0.0509704167062
TAGCCTGA -0.131017104259
TAGCGAAA 0.117022999782
TAGCGACA 0.0940513516271
TAGCGAGA 0.173414461293
TAGCGATA 0.213730259185
TAGCGCAA -0.0462680311165
TAGCGCCA -0.104080649752
TAGCGCGA 0.000809390978903
TAGCGCTA -0.110690768166
TAGCGGAA 0.0219903098691
TAGCGGCA 0.0153368904331
TAGCGGGA -0.21161539573
TAGCGTAA 0.21781398119
TAGCGTCA -0.199883377584
TAGCGTGA -0.065135070035
TAGCTAAA -0.305332656245
TAGCTACA 0.00559153659727
TAGCTAGA -0.123859736178
TAGCTATA 0.0565124353003
TAGCTCAA -0.199374298512
TAGCTCCA -0.198409006327
TAGCTCGA -0.0956342016948
TAGCTGAA -0.0347006999847
TAGCTGCA -0.0212003725528
TAGCTGGA -0.116993982946
TAGCTTAA 0.00025619876937
TAGCTTCA -0.33198926104
TAGCTTGA 0.0329007393999
TAGGAAAA 0.0415222676084
TAGGAACA -0.000850712971925
TAGGAAGA 0.122716380292
TAGGAATA 0.177916541974
TAGGACAA 0.0765260614731
TAGGACCA -0.0618908887095
TAGGACGA 0.0931496234527
TAGGACTA -0.142616752277
TAGGAGAA 0.203143915265
TAGGAGCA -0.164351017227
TAGGAGGA -0.0373129971113
TAGGATAA 0.0178704370052
TAGGATCA 0.132805121056
TAGGATGA 0.0972666881758
TAGGCAAA 0.119827303052
TAGGCACA 0.00456919869966

TAGGCAGA 0.0208071803942
TAGGCATA -0.0112384400342
TAGGCCAA -0.1489652565
TAGGCCCA -0.290775417651
TAGGCCGA -0.181637560425
TAGGCCTA -0.454635760677
TAGGCGAA 0.0966045859493
TAGGCGCA 0.119914892375
TAGGCGGA -0.141538781232
TAGGCTAA -0.150468460132
TAGGCTCA 0.0786574016627
TAGGCTGA -0.0846877248704
TAGGGAAA -0.0649046614551
TAGGGACA -0.2093744938
TAGGGAGA -0.0320263059933
TAGGGATA 0.0370558795549
TAGGGCAA 0.0709057465895
TAGGGCCA -0.0925057418023
TAGGGCGA -0.22955069839
TAGGGGAA 0.199968613826
TAGGGGCA -0.0902290152098
TAGGGGGA -0.0989172114912
TAGGGTAA 0.15412436224
TAGGGTCA -0.192522199891
TAGGGTGA 0.0596477405266
TAGGTAAA 0.209990982718
TAGGTACA -0.105508545842
TAGGTAGA 0.0508842175509
TAGGTATA -0.0813784114743
TAGGTCAA 0.00850669076227
TAGGTCCA -0.265702643972
TAGGTCGA -0.13901117773
TAGGTGAA 0.0423129420524
TAGGTGCA -0.0431209535558
TAGGTGGA 0.0687802456881
TAGGTTAA -0.0508070053525
TAGGTTCA -0.211933420132
TAGGTTGA 0.0643511455954
TAGTAAAA 0.133338089684
TAGTAACA 0.060698670832
TAGTAAGA 0.0862484763107
TAGTAATA 0.131844123475
TAGTACAA -0.011539163376
TAGTACCA -0.174041724909
TAGTACGA 0.268101428436
TAGTACTA -0.317089268638
TAGTAGAA 0.282001853974
TAGTAGCA 0.0792004554645
TAGTAGGA 0.11136939293
TAGTATAA -0.00422177829615
TAGTATCA 0.0750681811288
TAGTATGA 0.0319038046311
TAGTCAAA 0.0362662938421
TAGTCACA 0.0157146599385

TAGTCAGA 0.00570129358008
TAGTCATA -0.0552191309767
TAGTCCAA -0.124599846142
TAGTCCCA 0.0121391502376
TAGTCCGA -0.070932281198
TAGTCGAA 0.043312190244
TAGTCGCA 0.0215125749645
TAGTCGGA -0.0249593074604
TAGTCTAA -0.108014875553
TAGTCTCA 0.0523209159573
TAGTCTGA 0.0320041513218
TAGTGAAA 0.000938566664359
TAGTGACA 0.000431561037622
TAGTGAGA 0.0235993303001
TAGTGATA 0.00939369121187
TAGTGCAA 0.0971039910434
TAGTGCCA -0.172429472049
TAGTGCGA 0.193081173405
TAGTGGAA 0.150722347692
TAGTGGCA 0.131998389574
TAGTGGGA 0.0836497654679
TAGTGTAA 0.224382784989
TAGTGTCA -0.0899899131303
TAGTGTGA 0.0292263673058
TAGTTAAA 0.070981657336
TAGTTACA -0.182537319605
TAGTTAGA 0.206675036313
TAGTTATA -0.107674779468
TAGTTCAA 0.0751147245891
TAGTTCCA -0.0884863835099
TAGTTCGA 0.00659788569869
TAGTTGAA 0.0685870645749
TAGTTGCA 0.18259444017
TAGTTGGA 0.0669664154513
TAGTTTAA 0.131082925861
TAGTTTCA 0.0963387663044
TAGTTTGA 0.242913712611
TATAAAAA 0.284448515726
TATAAACA 0.260912427579
TATAAAGA 0.307774151922
TATAAATA -0.00679053709357
TATAACAA 0.209293315354
TATAACCA -0.00947497500055
TATAACGA 0.193425691408
TATAAGAA 0.29603018997
TATAAGCA 0.107433413867
TATAAGGA -0.0163886922184
TATAATAA 0.0281921039497
TATAATCA 0.18276316116
TATAATGA 0.179489406762
TATACAAA 0.18729335396
TATACACA 0.190232505888
TATACAGA 0.187526366727
TATACATA 0.18509302716

TATACCAA -0.0245014479464
TATACCCA 0.0964847485092
TATACCGA 0.133993416203
TATACGAA 0.27339047036
TATACGCA 0.235018941191
TATACGGA 0.0498473680292
TATACTAA -0.00761516171179
TATACTCA 0.218933809843
TATACTGA 0.14762558702
TATAGAAA 0.083746341321
TATAGACA 0.169804791017
TATAGAGA 0.0708269161989
TATAGATA 0.254936754937
TATAGCAA 0.103724937058
TATAGCCA 0.0603730452215
TATAGCGA 0.215687977636
TATAGGAA 0.178252301045
TATAGGCA 0.152337540054
TATAGGGA -0.0159916206214
TATAGTAA 0.20944013878
TATAGTCA -0.138071692682
TATAGTGA 0.0650197440932
TATATAAA 0.226911089538
TATATACA 0.277641343437
TATATAGA 0.145866937985
TATATATA 0.0123223277851
TATATCAA 0.124453155922
TATATCCA 0.269846981968
TATATCGA 0.135410284914
TATATGAA 0.274725138362
TATATGCA 0.231943096137
TATATGGA 0.0261806089783
TATATTAA 0.255634548149
TATATTCA 0.0995474551652
TATATTGA 0.297075464675
TATCAAAA 0.283135583368
TATCAACA 0.148811345781
TATCAAGA 0.265211492484
TATCAATA 0.180401159099
TATCACAA 0.244587562769
TATCACCA 0.145698039637
TATCACGA 0.173064093487
TATCAGAA 0.266410954505
TATCAGCA 0.163405910819
TATCAGGA 0.218619445892
TATCATAA 0.198678131136
TATCATCA 0.0925640962001
TATCATGA 0.236621825799
TATCCAAA 0.319839122869
TATCCACA 0.23439003742
TATCCAGA 0.248487259403
TATCCATA 0.266209499062
TATCCCAA 0.229185492288
TATCCCCA 0.271896296548

TATCCCGA 0.0830987460958
TATCCGAA 0.279071082101
TATCCGCA 0.289119946279
TATCCGGA 0.340060446121
TATCCTAA 0.129280715686
TATCCTCA 0.238491699134
TATCCTGA 0.277829682562
TATCGAAA 0.161620297984
TATCGACA 0.237415101051
TATCGAGA 0.160664365014
TATCGATA 0.161972607422
TATCGCAA 0.190743998307
TATCGCCA 0.179867195019
TATCGCGA 0.315297475238
TATCGGAA 0.345860309628
TATCGGCA 0.196277544762
TATCGGGA 0.148857008116
TATCGTAA 0.215112033639
TATCGTCA 0.0959212571987
TATCGTGA 0.232368732369
TATCTAAA 0.186146201298
TATCTACA 0.291870251016
TATCTAGA 0.292748671537
TATCTCAA 0.125447617935
TATCTCCA 0.296325627906
TATCTCGA 0.27702970008
TATCTGAA 0.25738925425
TATCTGCA 0.367572806967
TATCTGGA 0.373796661675
TATCTTAA 0.315282877017
TATCTTCA 0.21331722663
TATCTTGA 0.274590017014
TATGAAAA 0.290821771612
TATGAACA 0.0350352501342
TATGAAGA 0.150877718006
TATGAATA 0.0697212363879
TATGACAA 0.100801903832
TATGACCA -0.128711043259
TATGACGA 0.0337513822362
TATGAGAA 0.275180138817
TATGAGCA 0.0717315111255
TATGAGGA -0.00686773282337
TATGATAA 0.160343204163
TATGATCA 0.0353425050395
TATGATGA 0.0802323994342
TATGCAAA 0.141146544236
TATGCACA 0.164717058656
TATGCAGA 0.0566520441158
TATGCATA 0.190485995683
TATGCCAA 0.0731213306971
TATGCCCA 0.102324614736
TATGCCGA 0.0376285376285
TATGCGAA 0.264633345046
TATGCGCA 0.182298927761

TATGCGGA -0.0165418924322
TATGCTAA -0.0875652690537
TATGCTCA -0.106189566325
TATGCTGA -0.0916694031539
TATGGAAA 0.18296671327
TATGGACA 0.18489701823
TATGGAGA -0.144361206279
TATGGCAA 0.0697232907309
TATGGCCA -0.0873994667637
TATGGCGA 0.0307467057101
TATGGGAA 0.0964234359832
TATGGGCA 0.155444041371
TATGGGGA 0.0841874084919
TATGGTAA 0.170608672803
TATGGTCA -0.105565895962
TATGGTGA 0.141257490712
TATGTAAA 0.12127140915
TATGTACA 0.15517962089
TATGTAGA 0.0578053035335
TATGTCAA -0.0225892194897
TATGTCCA -0.0188825188825
TATGTCGA -0.0726053487196
TATGTGAA 0.149998905133
TATGTGCA 0.164121223623
TATGTGGA 0.00759161365222
TATGTTAA 0.17059991387
TATGTTCA -0.0075455368886
TATGTTGA -0.0634367149519
TATTAAAA 0.247332307616
TATTAACA 0.228621645947
TATTAAGA 0.280769750467
TATTAATA 0.220186659831
TATTACAA 0.221502385932
TATTACCA 0.0444011504618
TATTACGA 0.250455099523
TATTAGAA 0.297807529096
TATTAGCA 0.213404815953
TATTAGGA 0.193638825427
TATTATAA 0.23757071013
TATTATCA 0.101509455997
TATTATGA 0.0766730918246
TATTCAAA -0.00911199967604
TATTCACA 0.256395904009
TATTCAGA 0.143603312219
TATTCCAA 0.200970051751
TATTCCCA 0.28030262111
TATTCCGA 0.189218484267
TATTCGAA 0.0505762647533
TATTCGCA 0.291678731073
TATTCGGA -0.0742684833594
TATTCTAA -0.0463381809211
TATTCTCA 0.305312292432
TATTCTGA 0.0933607293271
TATTGAAA 0.153805320472

TATTGACA 0.104379466143
TATTGAGA 0.216567807477
TATTGCAA 0.0753659996084
TATTGCCA 0.0666217181369
TATTGCGA 0.18853702187
TATTGGAA 0.197939672929
TATTGGCA 0.168458354927
TATTGGGA -0.0197235803296
TATTGTAA 0.205454625081
TATTGTCA 0.195104486763
TATTGTGA 0.236528397188
TATTTAAA 0.0749640518821
TATTTACA 0.325759643941
TATTTAGA 0.176877979908
TATTTCAA 0.0751730898082
TATTTCCA 0.287657204587
TATTTCGA 0.303537148822
TATTTGAA 0.146883644884
TATTTGCA 0.369574658163
TATTTGGA 0.0304175282713
TATTTTAA 0.289940938531
TATTTTCA 0.317042938255
TATTTTGA 0.223012879371
TCAAAAAA 0.225827901579
TCAAAACA 0.159630811004
TCAAAAGA 0.0920400109211
TCAAACAA 0.00638161208149
TCAAACCA -0.0189706940619
TCAAACGA 0.0774953833128
TCAAAGAA 0.119090164545
TCAAAGCA 0.139366639367
TCAAAGGA 0.108495563199
TCAAATAA 0.0822434004252
TCAAATCA 0.221309899
TCAAATGA 0.0625292876798
TCAACAAA 0.26387670343
TCAACACA -0.0083359000597
TCAACAGA 0.21576517676
TCAACCAA -0.0644267434262
TCAACCCA -0.159905442114
TCAACCGA 0.0208652178349
TCAACGAA 0.0944655053872
TCAACGCA 0.0285913447151
TCAACGGA 0.271383108399
TCAACTAA -0.0154753731323
TCAACTCA -0.194926235632
TCAACTGA 0.010946848776
TCAAGAAA -0.0716814305683
TCAAGACA 0.0937402830595
TCAAGAGA -0.076541521504
TCAAGCAA -0.0236270641659
TCAAGCCA -0.219399988034
TCAAGCGA -0.0544637708663
TCAAGGAA 0.252661752662

TCAAGGCA 0.01460041021
TCAAGGGA 0.169438033074
TCAAGTAA 0.0737169676564
TCAAGTCA -0.0408184086804
TCAAGTGA 0.0418566768862
TCAATAAA 0.100029781848
TCAATACA 0.00130143135552
TCAATAGA -0.0348729589863
TCAATCAA -0.00130040795319
TCAATCCA -0.0851939170837
TCAATCGA -0.0993533799341
TCAATGAA 0.0155171784559
TCAATGCA 0.0878426019868
TCAATGGA 0.179084399612
TCAATTAA 0.0774203956022
TCAATTCA 0.0555531429139
TCAATTGA 0.154668287399
TCACAAAA 0.153819108365
TCACAACA 0.147517207652
TCACAAGA 0.218756979428
TCACACAA 0.101471500624
TCACACCA 0.057276284549
TCACACGA -0.00796349147261
TCACAGAA 0.00564889958829
TCACAGCA 0.283618329073
TCACAGGA 0.166554761479
TCACATAA 0.074838507186
TCACATCA -0.0215711579348
TCACATGA 0.075556272526
TCACCAAA 0.0710234244668
TCACCACA 0.0963241680839
TCACCAGA 0.248408429013
TCACCCAA -0.119771375634
TCACCCCA 0.00633781018046
TCACCCGA -0.053418577342
TCACCGAA 0.0638503517291
TCACCGCA 0.0327945024915
TCACCGGA -0.0240474634414
TCACCTAA -0.10278791037
TCACCTCA 0.00356417023084
TCACCTGA 0.0548330699846
TCACGAAA 0.145488055006
TCACGACA -0.108995251906
TCACGAGA 0.12860714617
TCACGCAA 0.0084209742544
TCACGCCA -0.0321836988504
TCACGCGA -0.0322788353091
TCACGGAA 0.127035904323
TCACGGCA 0.100543053802
TCACGGGA 0.121205728326
TCACGTAA 0.179598256972
TCACGTCA 0.0656912622351
TCACGTGA 0.103359958326
TCACTAAA 0.00758471970593

TCACTACA -0.0774730804759
TCACTAGA 0.0821587214929
TCACTCAA -0.0790791955135
TCACTCCA -0.0595176058959
TCACTCGA -0.1274826083
TCACTGAA 0.165709510011
TCACTGCA -0.0191516940283
TCACTGGA 0.118384224445
TCACTTAA -0.06775662925
TCACTTCA 0.256450665542
TCAGAAAA 0.17671656825
TCAGAACA -0.182383539666
TCAGAAGA 0.218613461019
TCAGACAA -0.104007941432
TCAGACCA -0.132439661827
TCAGACGA -0.113852706447
TCAGAGAA 0.119266731385
TCAGAGCA -0.21244448855
TCAGAGGA -0.0611435110504
TCAGATAA 0.0286963049359
TCAGATCA -0.0412933594752
TCAGATGA 0.0503748093107
TCAGCAAA -0.0392757822821
TCAGCACA -0.108570506935
TCAGCAGA 0.150973287337
TCAGCCAA -0.129627743146
TCAGCCCA -0.2806059303
TCAGCCGA -0.152515473155
TCAGCGAA 0.25716152201
TCAGCGCA 0.000951074064072
TCAGCGGA 0.0691393873212
TCAGCTAA -0.0609218153129
TCAGCTCA -0.293641227979
TCAGCTGA 0.0477054787913
TCAGGAAA 0.117900824136
TCAGGACA -0.0724629114765
TCAGGAGA 0.0547944638854
TCAGGCAA -0.0443432413129
TCAGGCCA -0.0873610776652
TCAGGCGA -0.0238384407539
TCAGGGAA 0.114350050729
TCAGGGCA -0.274568912046
TCAGGGGA -0.0667784348273
TCAGGTAA 0.0575804909382
TCAGGTCA -0.104304339076
TCAGTAAA 0.00512299120209
TCAGTACA -0.0093440547986
TCAGTAGA 0.0922541842149
TCAGTCAA -0.153554954493
TCAGTCCA -0.204155811934
TCAGTCGA -0.0947611051832
TCAGTGAA 0.134352433184
TCAGTGCA 0.0835036138066
TCAGTGGA 0.11961961962

TCAGTTAA -0.0458006941261
TCAGTTCA -0.104823065234
TCATAAAA 0.158195385468
TCATAACA -0.000372944721885
TCATAAGA 0.138218141209
TCATACAA -0.0247417939753
TCATACCA -0.0636927658518
TCATACGA 0.0819428243671
TCATAGAA 0.0423757090424
TCATAGCA 0.114246477883
TCATAGGA -0.0980952538513
TCATATAA 0.0667452537536
TCATATCA 0.113395764911
TCATATGA -0.0683312247277
TCATCAAA 0.197723407517
TCATCACA 0.00312036962694
TCATCAGA 0.173273824789
TCATCCAA -0.0210196422318
TCATCCCA -0.169289518733
TCATCCGA 0.0604723180481
TCATCGAA 0.13052032749
TCATCGCA -0.0566295790765
TCATCGGA 0.0936360026801
TCATCTAA 0.0426611096107
TCATCTCA -0.119340440607
TCATGAAA 0.209494618586
TCATGACA 0.0635525332495
TCATGAGA 0.111136873979
TCATGCAA 0.00985257988845
TCATGCCA -0.0262491721822
TCATGCGA -0.0539241230625
TCATGGAA 0.168654880776
TCATGGCA -0.0554464750986
TCATGGGA -0.104567947181
TCATGTAA -0.0391969518916
TCATGTCA -0.0251008584342
TCATTAAA -0.000169591078682
TCATTACA 0.0242031196397
TCATTAGA -0.0999277514429
TCATTCAA -0.0306049851504
TCATTCCA -0.137543188198
TCATTCGA 0.0886520128944
TCATTGAA 0.0902612347701
TCATTGCA -0.0317426377543
TCATTGGA 0.1349014815
TCATTTAA 0.018697761122
TCATTTCA -0.111327581025
TCCAAAAA 0.0841288146975
TCCAAACA 0.0373389918844
TCCAAAGA 0.259316219353
TCCAACAA 0.112288781997
TCCAACCA -0.0218392325869
TCCAACGA -0.134072075938
TCCAAGAA 0.142334487208

TCCAAGCA 0.12256185324
TCCAAGGA 0.00507808083566
TCCAATAA 0.180101589192
TCCAATCA -0.0635344036128
TCCACAAA 0.159935913812
TCCACACA 0.0636803573644
TCCACAGA 0.127005639238
TCCACCAA 0.0721816310592
TCCACCCA -0.0311929781023
TCCACCGA 0.0165507324657
TCCACGAA 0.193704517419
TCCACGCA 0.0749057187905
TCCACGGA 0.0346399252878
TCCACTAA 0.25813084385
TCCACTCA -0.0194021908457
TCCAGAAA 0.157661564748
TCCAGACA -0.0321569216502
TCCAGAGA 0.189164470851
TCCAGCAA 0.179294613987
TCCAGCCA -0.179701909795
TCCAGCGA 0.151676469858
TCCAGGAA 0.192438851704
TCCAGGCA -0.119219137257
TCCAGGGA 0.0638668972002
TCCAGTAA 0.235824762961
TCCAGTCA 0.135543747217
TCCATAAA -0.0808786427443
TCCATACA 0.118119496907
TCCATAGA 0.117453632402
TCCATCAA -0.0603605838812
TCCATCCA 0.0340243444955
TCCATCGA 0.219670972622
TCCATGAA 0.273710349468
TCCATGCA -0.0581922503748
TCCATGGA 0.0728268269758
TCCATTAA 0.144622215488
TCCATTCA -0.0977827332953
TCCCAAAA 0.304395524186
TCCCAACA 0.0309328237834
TCCCAAGA 0.227603045189
TCCCACAA 0.134918943381
TCCCACCA 0.047931607812
TCCCACGA -0.309371300447
TCCCAGAA 0.0852243428001
TCCCAGCA 0.0845410328312
TCCCAGGA -0.0101085377692
TCCCATAA -0.0106575119604
TCCCATCA 0.0170404564344
TCCCCAAA 0.233909492651
TCCCCACA 0.0373163087604
TCCCCAGA 0.0417122252797
TCCCCCAA -0.215815835683
TCCCCCCA -0.0619370207697
TCCCCCGA 0.157883779096

TCCCCGAA 0.12203250082
TCCCCGCA 0.134059584941
TCCCCGGA -0.0187847876575
TCCCCTAA 0.171227637351
TCCCCTCA -0.0958496221291
TCCCGAAA -0.0723130150435
TCCCGACA 0.175110936359
TCCCGAGA 0.145066554157
TCCCGCAA 0.145135183577
TCCCGCCA 0.0399088240403
TCCCGCGA 0.0908118800318
TCCCGGAA 0.313866849631
TCCCGGCA -0.0120208410867
TCCCGGGA 0.152661672413
TCCCGTAA 0.0103396230822
TCCCGTCA 0.0248994962404
TCCCTAAA 0.163493500142
TCCCTACA 0.0754991410996
TCCCTAGA 0.0753990788523
TCCCTCAA 0.0315514390258
TCCCTCCA -0.0153903624489
TCCCTCGA 0.137605015949
TCCCTGAA -0.0280857194733
TCCCTGCA -0.0395832451533
TCCCTTAA -0.116841074851
TCCCTTCA 0.0468601516096
TCCGAAAA 0.0539534024383
TCCGAACA 0.0944205601337
TCCGAAGA 0.0798242169538
TCCGACAA 0.0404363408101
TCCGACCA -0.196420350384
TCCGACGA 0.0374758216973
TCCGAGAA 0.0442433679927
TCCGAGCA 0.0621643321679
TCCGAGGA -0.0722995531074
TCCGATAA -0.0939035017933
TCCGATCA -0.0324613329635
TCCGCAAA 0.122940476455
TCCGCACA 0.083300764287
TCCGCAGA 0.109446912477
TCCGCCAA -0.0384829160567
TCCGCCCA -0.0240750849855
TCCGCCGA 0.221652810522
TCCGCGAA 0.0962073823201
TCCGCGCA -0.00687216141762
TCCGCGGA 0.110820316484
TCCGCTAA 0.00309979400888
TCCGCTCA -0.168792949465
TCCGGAAA 0.185658982058
TCCGGACA -0.0322451386689
TCCGGAGA -0.0618033124269
TCCGGCAA -0.115531466786
TCCGGCCA -0.0966893186511
TCCGGCGA 0.111246469055

TCCGGGAA 0.118110506318
TCCGGGCA 0.0941009880404
TCCGGTAA 0.037979988475
TCCGGTCA -0.213314644372
TCCGTAAA 0.011689593656
TCCGTACA 0.143337552428
TCCGTAGA -0.0204907921724
TCCGTCAA -0.0785586140553
TCCGTCCA -0.0259420110022
TCCGTCGA -0.0635897605595
TCCGTGAA 0.128132264496
TCCGTGCA 0.108335583892
TCCGTTAA -0.026330738452
TCCGTTCA 0.0805364593243
TCCTAAAA 0.132837237141
TCCTAACA 0.143213652256
TCCTAAGA 0.0229973005986
TCCTACAA -0.0991530627434
TCCTACCA 0.068367372261
TCCTACGA -0.125668957873
TCCTAGAA 0.0591425005292
TCCTAGCA 0.116973615627
TCCTAGGA -0.0907013518541
TCCTATAA 0.113100443056
TCCTATCA -0.0234577416167
TCCTCAAA 0.188033108764
TCCTCACA -0.00489490532794
TCCTCAGA 0.189642810855
TCCTCCAA -0.0493281477931
TCCTCCCA -0.0545560821746
TCCTCCGA 0.0738633460581
TCCTCGAA 0.103087691429
TCCTCGCA -0.0341021729451
TCCTCTAA 0.171777293871
TCCTCTCA -0.125748594766
TCCTGAAA 0.106120734812
TCCTGACA 0.0595981090404
TCCTGAGA 0.0111347926688
TCCTGCAA 0.0385756178448
TCCTGCCA -0.0840460207011
TCCTGCGA 0.0993897943841
TCCTGGAA 0.0814726098881
TCCTGGCA 0.196117605209
TCCTGTAA 0.0491485587907
TCCTGTCA 0.0227015012719
TCCTTAAA 0.0736502120392
TCCTTACA 0.156193299013
TCCTTAGA -0.0162135135727
TCCTTCAA 0.0311518725867
TCCTTCCA -0.0817609477732
TCCTTCGA -0.00593344671285
TCCTTGAA 0.00791734487566
TCCTTGCA 0.107059699423
TCCTTTAA -0.0123858601636

TCCTTTCA -0.0224891305909
TCGAAAAA 0.0416246359569
TCGAAACA 0.0948234710189
TCGAAAGA -0.0408416040312
TCGAACAA 0.019431277007
TCGAACCA -0.287016997485
TCGAACGA -0.172704977263
TCGAAGAA 0.00774216622994
TCGAAGCA 0.0498375947972
TCGAATAA -0.101980978519
TCGAATCA 0.182453668898
TCGACAAA -0.0398305146603
TCGACACA -0.269062586204
TCGACAGA 0.257628665066
TCGACCAA -0.154988681826
TCGACCCA -0.308640020547
TCGACCGA -0.0949078095144
TCGACGAA -0.0631497120501
TCGACGCA -0.0734852521478
TCGACTAA 0.0464420487143
TCGACTCA -0.216424967784
TCGAGAAA 0.0606625909656
TCGAGACA -0.184255824628
TCGAGAGA 0.116414239104
TCGAGCAA -0.147600363752
TCGAGCCA -0.254372038796
TCGAGCGA -0.258872375092
TCGAGGAA -0.0162046802492
TCGAGGCA -0.218660314782
TCGAGTAA 0.0486809123173
TCGAGTCA -0.140224047467
TCGATAAA -0.112160369736
TCGATACA -0.0177767298979
TCGATAGA 0.172272869937
TCGATCAA -0.23936236199
TCGATCCA -0.172903292652
TCGATCGA 0.00566015893098
TCGATGAA 0.0525674620264
TCGATGCA -0.311172859415
TCGATTAA -0.0395450546966
TCGATTCA 0.112130036372
TCGCAAAA -0.0156967365678
TCGCAACA 0.037099187609
TCGCAAGA 0.1351320774
TCGCACAA 0.0587446951083
TCGCACCA 0.107178826668
TCGCACGA 0.189349868251
TCGCAGAA -0.116108230014
TCGCAGCA 0.0422860877406
TCGCATAA 0.0450518530323
TCGCATCA -0.0632216238277
TCGCCAAA -0.032650866526
TCGCCACA -0.20549703642
TCGCCAGA 0.195631561965

TCGCCCAA -0.0599614606979
TCGCCCCA 0.0500001959332
TCGCCCGA -0.0113827273298
TCGCCGAA -0.120756010122
TCGCCGCA -0.18621953136
TCGCCTAA -0.0524495328071
TCGCCTCA -0.0917928117234
TCGCGAAA 0.0739596943133
TCGCGACA -0.152835339372
TCGCGAGA 0.0585819288629
TCGCGCAA 0.205890463466
TCGCGCCA -0.215856771149
TCGCGCGA 0.232096346092
TCGCGGAA 0.15720817236
TCGCGGCA -0.0179945786006
TCGCGTAA 0.105278716524
TCGCGTCA 0.0816383582841
TCGCTAAA 0.0629375932406
TCGCTACA -0.0408202740086
TCGCTAGA 0.0484603060361
TCGCTCAA -0.1759327239
TCGCTCCA -0.184564826154
TCGCTGAA -0.132119016304
TCGCTGCA -0.172061065756
TCGCTTAA 0.00262986941892
TCGCTTCA -0.0315737611585
TCGGAAAA 0.120320044562
TCGGAACA -0.0389867375167
TCGGAAGA 0.0400480281454
TCGGACAA -0.078059379694
TCGGACCA -0.0812702824167
TCGGACGA -0.0501660547579
TCGGAGAA -0.0687144842304
TCGGAGCA 0.0224508254811
TCGGATAA -0.0191724917621
TCGGATCA 0.15215652212
TCGGCAAA 0.109701977329
TCGGCACA -0.17575312131
TCGGCAGA 0.108945033187
TCGGCCAA -0.043169998701
TCGGCCCA -0.00676814051923
TCGGCCGA 0.181972662549
TCGGCGAA 0.119832121498
TCGGCGCA 0.124386094083
TCGGCTAA -0.0126252829602
TCGGCTCA -0.18965511895
TCGGGAAA 0.227229330745
TCGGGACA -0.143290124169
TCGGGAGA -0.021587212162
TCGGGCAA -0.0943811642995
TCGGGCCA -0.096243350336
TCGGGGAA 0.19910406274
TCGGGGCA 0.111870823992
TCGGGTAA 0.0255573959652

TCGGGTCA -0.159109897837
TCGGTAAA 0.0596560748076
TCGGTACA 0.098778542162
TCGGTAGA 0.0682852849127
TCGGTCAA -0.0358375824211
TCGGTCCA -0.229626229291
TCGGTGAA 0.151391542795
TCGGTGCA -0.117440504256
TCGGTTAA -0.0033215033215
TCGGTTCA -0.135014165464
TCGTAAAA 0.127332566727
TCGTAACA 0.115152397884
TCGTAAGA 0.25539451297
TCGTACAA 0.112107975744
TCGTACCA -0.0526478333402
TCGTACGA -0.0713431725164
TCGTAGAA 0.0549482591756
TCGTAGCA 0.111573469194
TCGTATAA 0.0479909970752
TCGTATCA 0.2343155828
TCGTCAAA 0.134819675482
TCGTCACA -0.140615222611
TCGTCAGA 0.0708999073013
TCGTCCAA -0.155991231336
TCGTCCCA -0.191132725162
TCGTCGAA 0.066370778492
TCGTCGCA 0.0370437005236
TCGTCTAA -0.00780493857799
TCGTCTCA -0.212041579156
TCGTGAAA 0.108373539266
TCGTGACA -0.0278623799717
TCGTGAGA 0.185779443355
TCGTGCAA 0.02262067254
TCGTGCCA -0.244415775589
TCGTGGAA 0.050932461333
TCGTGGCA 0.0188439127833
TCGTGTAA 0.162956285629
TCGTGTCA 0.112569067831
TCGTTAAA -0.0216483701332
TCGTTACA 0.0458493609629
TCGTTAGA 0.187361590622
TCGTTCAA 0.00342109296877
TCGTTCCA -0.198121817194
TCGTTGAA 0.0418238330397
TCGTTGCA 0.0375955271052
TCGTTTAA -0.0634104275753
TCGTTTCA -0.0681456874919
TCTAAAAA 0.152669553565
TCTAAACA 0.165775968806
TCTAAAGA 0.0564186776308
TCTAACAA 0.0903231054746
TCTAACCA 0.0578939821364
TCTAAGAA 0.0403545907754
TCTAAGCA -0.0867160248721

TCTAATAA 0.29198068592
TCTAATCA 0.0737721192267
TCTACAAA 0.0868097778881
TCTACACA 0.0676824595082
TCTACAGA 0.0928352595019
TCTACCAA 0.148922509794
TCTACCCA 0.0655416934882
TCTACGAA 0.113579466578
TCTACGCA 0.0425939577965
TCTACTAA 0.305833381591
TCTACTCA 0.0949636139355
TCTAGAAA 0.0403020371817
TCTAGACA 0.0412740732226
TCTAGAGA 0.156584959615
TCTAGCAA 0.0196280373422
TCTAGCCA 0.0886299522663
TCTAGGAA 0.128986226579
TCTAGGCA -0.0390784347783
TCTAGTAA 0.17683043437
TCTAGTCA -0.0715601211991
TCTATAAA -0.0094399392714
TCTATACA 0.243290292913
TCTATAGA 0.00514196566143
TCTATCAA -0.0651707943519
TCTATCCA 0.069106296379
TCTATGAA -0.0453807580856
TCTATGCA 0.116370444443
TCTATTAA 0.227256371442
TCTATTCA 0.167420421451
TCTCAAAA 0.243907807542
TCTCAACA 0.0553035874539
TCTCAAGA 0.0243169857594
TCTCACAA 0.0453941980793
TCTCACCA 0.0615453676197
TCTCAGAA -0.0371020402802
TCTCAGCA 0.0267012787597
TCTCATAA 0.122013197771
TCTCATCA 0.159690663708
TCTCCAAA -0.0419147386505
TCTCCACA 0.228488819398
TCTCCAGA 0.211031195422
TCTCCCAA 0.0334111888632
TCTCCCCA 0.108404547798
TCTCCGAA 0.000536640444698
TCTCCGCA -0.0529341696725
TCTCCTAA 0.0950732478017
TCTCCTCA -0.0200606088032
TCTCGAAA 0.200879943304
TCTCGACA 0.0220489995181
TCTCGAGA 0.213400533078
TCTCGCAA 0.0601750750676
TCTCGCCA 0.0949285782063
TCTCGGAA 0.202724757852
TCTCGGCA 0.13157923764

TCTCGTAA 0.284439756794
TCTCGTCA 0.0239783070444
TCTCTAAA -0.0767011828922
TCTCTACA -0.0321664489099
TCTCTCAA -0.140587825003
TCTCTCCA -0.133692574488
TCTCTGAA 0.227080428896
TCTCTGCA 0.139721757918
TCTCTTAA 0.141467705087
TCTCTTCA -0.114305625488
TCTGAAAA 0.213912834026
TCTGAACA 0.176757443268
TCTGAAGA 0.120233133581
TCTGACAA 0.176666934301
TCTGACCA 0.229787638879
TCTGAGAA 0.260717648519
TCTGAGCA 0.119583771099
TCTGATAA 0.264866462778
TCTGATCA 0.135544635545
TCTGCAAA 0.185710531886
TCTGCACA 0.351350596819
TCTGCAGA 0.24886920857
TCTGCCAA 0.0271373619556
TCTGCCCA 0.03153924415
TCTGCGAA 0.155029685333
TCTGCGCA -0.154638320324
TCTGCTAA 0.219030970125
TCTGCTCA 0.0668919608314
TCTGGAAA 0.184668139214
TCTGGACA 0.317472610089
TCTGGCAA -0.00458680780071
TCTGGCCA -0.0336126344464
TCTGGGAA 0.165969358335
TCTGGGCA 0.0115004811975
TCTGGTAA 0.140883776268
TCTGGTCA 0.0273754140215
TCTGTAAA 0.096633650358
TCTGTACA 0.152976942111
TCTGTCAA -0.0412500310151
TCTGTCCA -0.015330757755
TCTGTGAA 0.127529661022
TCTGTGCA 0.114700554267
TCTGTTAA 0.237961101597
TCTGTTCA 0.0129384905313
TCTTAAAA 0.130078173471
TCTTAACA 0.189565921914
TCTTAAGA 0.111099397217
TCTTACAA 0.0807303489705
TCTTACCA 0.161151945578
TCTTAGAA 0.274019198262
TCTTAGCA 0.126455512266
TCTTATAA 0.19462851281
TCTTATCA 0.207358233031
TCTTCAAA 0.203422552791

TCTTCACA 0.204059431332
TCTTCCAA 0.102008715138
TCTTCCCA 0.133405026816
TCTTCGAA 0.141168394666
TCTTCGCA 0.0982569724751
TCTTCTAA 0.116595257038
TCTTCTCA -0.0182983827605
TCTTGAAA 0.117129551908
TCTTGACA 0.202806660545
TCTTGCAA 0.16506718831
TCTTGCCA 0.124676831894
TCTTGGAA 0.32697022091
TCTTGGCA 0.143208813775
TCTTGTAA 0.236253950643
TCTTGTCA 0.138667766399
TCTTTAAA 0.158779992113
TCTTTACA 0.250802537171
TCTTTCAA -0.0268436480558
TCTTTCCA 0.00621082903883
TCTTTGAA 0.220070696211
TCTTTGCA 0.13227374583
TCTTTTAA 0.192730816114
TCTTTTCA 0.210349467925
TGAAAAAA 0.250196477469
TGAAAACA -0.0101172967015
TGAAACAA 0.0362670890418
TGAAACCA -0.0624809320209
TGAAAGAA -0.0513920253231
TGAAAGCA -0.0150536676938
TGAAATAA 0.0857754939673
TGAAATCA 0.34458590073
TGAACAAA 0.00678160332255
TGAACACA 0.00791734487566
TGAACCAA -0.0325138865572
TGAACCCA -0.125605553504
TGAACGAA -0.032749002446
TGAACGCA -0.158918394302
TGAACTAA -0.106660525075
TGAACTCA -0.17345300525
TGAAGAAA 0.14499171551
TGAAGACA 0.0364335087553
TGAAGCAA -0.170065748666
TGAAGCCA -0.354611688623
TGAAGGAA -0.116631903748
TGAAGGCA -0.290500960682
TGAAGTAA -0.000978810682978
TGAAGTCA -0.206977487796
TGAATAAA 0.133247572642
TGAATACA -0.0822517805296
TGAATCAA 0.0310467653993
TGAATCCA 0.194129391099
TGAATGAA 0.0513295329299
TGAATGCA 0.0386626295717
TGAATTAA -0.0461825461825

TGAATTCA	0.167705809553
TGACAAAA	0.305864105166
TGACAACA	−0.0699045696812
TGACACAA	0.0216221542594
TGACACCA	−0.104920961858
TGACAGAA	0.146246797762
TGACAGCA	0.0704409643804
TGACATAA	0.0414348590907
TGACATCA	−0.167173910047
TGACCAAA	0.0572564853846
TGACCACA	−0.111011843212
TGACCCAA	−0.115491524582
TGACCCCA	−0.240173739479
TGACCGAA	−0.0299591979738
TGACCGCA	−0.319951703862
TGACCTAA	0.0251111289534
TGACCTCA	−0.271337572595
TGACGAAA	0.177682970446
TGACGACA	−0.139177412003
TGACGCAA	0.0725615422585
TGACGCCA	−0.288796518831
TGACGGAA	0.112621621424
TGACGGCA	−0.15893595141
TGACGTAA	0.0500594877484
TGACGTCA	−0.209304690795
TGACTAAA	0.0380655898046
TGACTACA	−0.0774893350651
TGACTCAA	−0.109512759328
TGACTCCA	−0.085458315068
TGACTGAA	0.0124311146471
TGACTGCA	−0.288565407556
TGACTTAA	0.103053577189
TGAGAAAA	0.200775155321
TGAGAACA	−0.0219944088816
TGAGACAA	−0.183760789795
TGAGACCA	−0.171857278607
TGAGAGAA	0.0114516551543
TGAGAGCA	−0.245932005302
TGAGATAA	−0.0799463375221
TGAGATCA	0.133978817982
TGAGCAAA	0.143120223645
TGAGCACA	−0.0471033481019
TGAGCCAA	−0.257052854635
TGAGCCCA	−0.286229034588
TGAGCGAA	0.0131740576222
TGAGCGCA	−0.055465208791
TGAGCTAA	−0.117429788642
TGAGCTCA	−0.209494903147
TGAGGAAA	0.0781990175398
TGAGGACA	−0.17841424482
TGAGGCAA	−0.0650962617415
TGAGGCCA	−0.43527879712
TGAGGGAA	0.188374324738
TGAGGGCA	−0.167548582644

TGAGGTAA 0.118306168478
TGAGTAAA 0.0536418910535
TGAGTACA 0.0475135580973
TGAGTCAA -0.246303919866
TGAGTCCA -0.299335945766
TGAGTGAA -0.0747317014789
TGAGTGCA -0.251275806162
TGAGTTAA -0.153364941778
TGATAAAA 0.206213732546
TGATAACA 0.0202778536112
TGATACAA 0.0950904537908
TGATACCA -0.0173025408203
TGATAGAA -0.0222301317259
TGATAGCA -0.139176309383
TGATATAA 0.141955285651
TGATATCA 0.341824260817
TGATCAAA -0.0842316145346
TGATCACA 0.241918226767
TGATCCAA 0.0939957859206
TGATCCCA 0.0660883338321
TGATCGAA -0.155519164551
TGATCGCA 0.203504302825
TGATCTAA 0.0362448711936
TGATGAAA 0.207321342496
TGATGACA -0.068141402612
TGATGCAA 0.00573714210078
TGATGCCA -0.18072064918
TGATGGAA -0.0976599617292
TGATGGCA -0.155887534281
TGATGTAA 0.162316883572
TGATTAAA 0.0639594758915
TGATTACA 0.369118668884
TGATTCAA -0.122105030952
TGATTCCA 0.206579858095
TGATTGAA -0.0784642152849
TGATTGCA 0.312669795552
TGATTTAA 0.0487581245157
TGCAAAAA 0.0656345385513
TGCAAACA -0.0743456955578
TGCAACAA 0.0214028469202
TGCAACCA -0.198698925186
TGCAAGAA -0.0715541036634
TGCAAGCA -0.137116455298
TGCAATAA -0.0705731040586
TGCACAAA 0.0165440923017
TGCACACA 0.149106406682
TGCACCAA 0.025340767765
TGCACCCA -0.0863377138245
TGCACGAA -0.211707240407
TGCACGCA -0.0448301929748
TGCACTAA -0.167912252847
TGCAGAAA 0.16183263153
TGCAGACA 0.0815566082495
TGCAGCAA -0.112038944173

TGCAGCCA -0.216631432701
TGCAGGAA 0.0220309044327
TGCAGGCA -0.143474390128
TGCAGTAA -0.0183861547498
TGCATAAA -0.108757913667
TGCATACA 0.132046013591
TGCATCAA -0.297486272436
TGCATCCA -0.197184568475
TGCATGAA 0.0278529180626
TGCATGCA -0.145696377333
TGCATTAA -0.0959402348854
TGCCAAAA 0.0160368751049
TGCCAACA -0.157688597112
TGCCACAA 0.0513747921682
TGCCACCA -0.155315969551
TGCCAGAA -0.197410540421
TGCCAGCA -0.341339735537
TGCCATAA -0.0314129556554
TGCCCAAA 0.077310528152
TGCCCACA 0.0105298135601
TGCCCCAA -0.162476004175
TGCCCCCA -0.353303068641
TGCCCGAA -0.0641362880611
TGCCCGCA -0.226810389739
TGCCCTAA 0.0200800721311
TGCCGAAA 0.123739441921
TGCCGACA -0.293747637265
TGCCGCAA 0.0986190083429
TGCCGCCA -0.205098623
TGCCGGAA -0.0516736557406
TGCCGGCA -0.213939664509
TGCCGTAA 0.0588784376663
TGCCTAAA -0.0294613267427
TGCCTACA -0.0687904644424
TGCCTCAA -0.00813697026492
TGCCTCCA -0.14656239575
TGCCTGAA -0.265816896167
TGCCTTAA -0.097521734455
TGCGAAAA 0.189005810218
TGCGAACA -0.0545298890791
TGCGACAA 0.0596198228156
TGCGACCA -0.304945622958
TGCGAGAA -0.0851122050031
TGCGAGCA -0.165097537242
TGCGATAA 0.0525167040319
TGCGCAAA 0.153195915418
TGCGCACA 0.0602526242648
TGCGCCAA -0.0909971905853
TGCGCCCA -0.170785058013
TGCGCGAA 0.0537517193993
TGCGCGCA 0.0402578287151
TGCGCTAA -0.0777154565033
TGCGGAAA 0.140174609872
TGCGGACA -0.0736494642521

TGCGGCAA -0.111490278157
TGCGGCCA -0.39464052936
TGCGGGAA 0.0738633460581
TGCGGTAA 0.151660182624
TGCGTAAA 0.144277886702
TGCGTACA -0.0325956365919
TGCGTCAA -0.0355300203785
TGCGTCCA -0.12599317713
TGCGTGAA -0.196741755876
TGCGTTAA -0.153137207873
TGCTAAAA -0.0306004083451
TGCTAACA 0.117161667993
TGCTACAA -0.0155974686686
TGCTACCA -0.281897219705
TGCTAGAA -0.0666824814055
TGCTAGCA -0.153224628394
TGCTATAA 0.0849678879982
TGCTCAAA 0.106777576475
TGCTCACA 0.103290438895
TGCTCCAA -0.0599701627183
TGCTCCCA -0.299021999498
TGCTCGAA -0.282355146473
TGCTCTAA -0.294184045893
TGCTGAAA 0.143339196952
TGCTGACA -0.164271087687
TGCTGCAA -0.148607284971
TGCTGCCA -0.290524262097
TGCTGGAA 0.126720384296
TGCTGTAA 0.0750341233404
TGCTTAAA 0.0405618855062
TGCTTACA -0.0826505693471
TGCTTCAA -0.142566160232
TGCTTCCA -0.142819703248
TGCTTGAA -0.202874406182
TGCTTTAA -0.0745080767227
TGGAAAAA -0.192496318147
TGGAAACA 0.0277066925542
TGGAACAA -0.00108099822632
TGGAACCA 0.0112660656879
TGGAAGAA -0.0531002974984
TGGAATAA 0.156337452465
TGGACAAA -0.0789432326691
TGGACACA 0.0341236553358
TGGACCAA -0.111400370466
TGGACCCA -0.1710847189
TGGACGAA -0.110509071955
TGGACTAA -0.0704860163288
TGGAGAAA -0.0757154558316
TGGAGACA -0.189616912419
TGGAGCAA -0.169182582419
TGGAGCCA -0.14169278402
TGGAGGAA -0.220852045753
TGGAGTAA -0.141839277087
TGGATAAA 0.11065086166

TGGATACA 0.169878761779
TGGATCAA -0.153909768399
TGGATCCA 0.302232089945
TGGATGAA -0.0671437851726
TGGATTAA 0.158153832792
TGGCAAAA -0.023477588082
TGGCAACA -0.00167368597768
TGGCACAA -0.0475197895729
TGGCACCA -0.340614224405
TGGCAGAA 0.0558230406715
TGGCATAA 0.0718693900512
TGGCCAAA -0.0433406953371
TGGCCACA -0.295162921983
TGGCCCAA -0.284579909671
TGGCCCCA 0.066115343574
TGGCCGAA 0.0748764625592
TGGCCTAA -0.0919417822967
TGGCGAAA 0.0765406596936
TGGCGACA -0.210079045964
TGGCGCAA -0.173615013333
TGGCGCCA -0.0623799325831
TGGCGGAA -0.116595934778
TGGCGTAA 0.182850740495
TGGCTAAA -0.123642980283
TGGCTACA -0.160823937817
TGGCTCAA -0.190510057531
TGGCTGAA -0.033732194647
TGGCTTAA -0.123542550163
TGGGAAAA 0.142598521386
TGGGAACA -0.137385523618
TGGGACAA 0.110818140136
TGGGACCA -0.220599547231
TGGGAGAA -0.0299313839826
TGGGATAA 0.171941885938
TGGGCAAA 0.0715307937296
TGGGCACA -0.201441508498
TGGGCCAA 0.0411750107662
TGGGCCCA -0.291627742919
TGGGCGAA -0.0536600025917
TGGGCTAA -0.0539426143953
TGGGGAAA 0.128253964315
TGGGGACA -0.385513569497
TGGGGCAA -0.055187842602
TGGGGGAA 0.0135543588853
TGGGGTAA 0.112110683708
TGGGTAAA 0.0199112428195
TGGGTACA -0.135647998809
TGGGTCAA -0.05731600729
TGGGTGAA -0.102403677036
TGGGTTAA -0.0948054153251
TGGTAAAA 0.0392720544236
TGGTAACA -0.0819401359419
TGGTACAA 0.196511938936
TGGTACCA -0.15501180919

TGGTAGAA 0.122049152208
TGGTATAA 0.119497108966
TGGTCAAA 0.150405226163
TGGTCACA -0.239265840556
TGGTCCAA 0.111650217711
TGGTCGAA -0.150617846699
TGGTCTAA -0.00901121671588
TGGTGAAA 0.0322016231107
TGGTGACA -0.37356018721
TGGTGCAA 0.044077136997
TGGTGGAA -0.0653482040539
TGGTGTAA 0.142863294964
TGGTTAAA 0.0475544414938
TGGTTACA -0.158621812041
TGGTTCAA 0.0683838108081
TGGTTGAA -0.146860336989
TGGTTTAA 0.219432931554
TGTAAAAA 0.181932621327
TGTAAACA 0.0714231596713
TGTAACAA 0.173666183776
TGTAAGAA -0.0367502901396
TGTAATAA 0.0930979613585
TGTACAAA 0.043218761633
TGTACACA 0.00883376070665
TGTACCAA 0.0630010175465
TGTACGAA 0.051810763932
TGTACTAA -0.0332913580021
TGTAGAAA 0.232759136661
TGTAGACA -0.177585346555
TGTAGCAA 0.0243461822004
TGTAGGAA 0.0294584790114
TGTAGTAA 0.149588715777
TGTATAAA 0.262075283023
TGTATACA 0.0429017934661
TGTATCAA 0.0674389866354
TGTATGAA -0.0177609249432
TGTATTAA 0.155601702153
TGTCAAAA 0.0678307318163
TGTCAACA 0.00379580682611
TGTCACAA 0.258047303502
TGTCAGAA 0.167940667941
TGTCATAA 0.0969819562681
TGTCCAAA 0.133053163356
TGTCCACA -0.101324950711
TGTCCCAA 0.172894754129
TGTCCGAA -0.202709329768
TGTCCTAA -0.0429954088597
TGTCGAAA 0.180593855609
TGTCGACA -0.403848299978
TGTCGCAA 0.210685892504
TGTCGGAA 0.0343455309988
TGTCGTAA 0.176284460924
TGTCTAAA 0.0870526173556
TGTCTCAA 0.0057422100246

TGTCTGAA -0.192770403451
TGTCTTAA 0.177172032729
TGTGAAAA 0.0945052298125
TGTGAACA -0.00202671960955
TGTGACAA 0.187218528061
TGTGAGAA 0.086744815807
TGTGATAA 0.155730110276
TGTGCAAA -0.0275881942549
TGTGCACA 0.0292985923335
TGTGCCAA -0.0630240862528
TGTGCGAA 0.187536769268
TGTGCTAA -0.165642510695
TGTGGAAA 0.196233423506
TGTGGCAA 0.100727449212
TGTGGGAA 0.0728443902688
TGTGGTAA 0.043990271263
TGTGTAAA 0.145768340839
TGTGTCAA 0.0872791106764
TGTGTGAA 0.0837931595507
TGTGTTAA 0.250274081589
TGTTAAAA 0.0695061452637
TGTTAACA 0.268714908255
TGTTACAA 0.182465327897
TGTTAGAA -0.0283768617102
TGTTATAA 0.211100908071
TGTTCAAA 0.034801427706
TGTTCCAA 0.0265446742042
TGTTCGAA -0.0117439439688
TGTTCTAA 0.0767829901535
TGTTGAAA 0.213285110545
TGTTGCAA 0.0492069516726
TGTTGGAA 0.059010801435
TGTTGTAA 0.222965389632
TGTTTAAA 0.0870864141661
TGTTTCAA -0.0696888227103
TGTTTGAA 0.0226939970717
TGTTTTAA 0.0289446216506
TTAAAAAA 0.117620052116
TTAAACAA 0.112131121216
TTAAAGAA 0.0806357321509
TTAAATAA 0.147610243939
TTAACAAA 0.24846390225
TTAACCAA 0.1367055761
TTAACGAA 0.0772230705826
TTAACTAA 0.063977328096
TTAAGAAA 0.228276485852
TTAAGCAA 0.159157828661
TTAAGGAA 0.204779159325
TTAAGTAA 0.1800878013
TTAATAAA 0.228836274291
TTAATCAA 0.093659775478
TTAATGAA 0.244507784501
TTAATTAA 0.130931709374
TTACAAAA 0.310266928461

TTACACAA 0.075428233287
TTACAGAA 0.210534805807
TTACATAA 0.102594329867
TTACCAAA 0.174538513755
TTACCCAA -0.0447238381641
TTACCGAA 0.0796062859937
TTACCTAA 0.15289811172
TTACGAAA 0.149414976314
TTACGCAA 0.210807225959
TTACGGAA 0.196377451589
TTACGTAA 0.275076557543
TTACTAAA 0.112598264272
TTACTCAA 0.148036847722
TTACTGAA 0.106081618651
TTAGAAAA 0.151337287701
TTAGACAA 0.0224396546061
TTAGAGAA 0.0230984064614
TTAGATAA 0.201494883313
TTAGCAAA 0.174903469267
TTAGCCAA 0.059545771667
TTAGCGAA 0.139355269363
TTAGCTAA -0.146130142744
TTAGGAAA 0.116049283592
TTAGGCAA 0.233755794362
TTAGGGAA 0.0729834517713
TTAGTAAA 0.121158348431
TTAGTCAA 0.10411950429
TTAGTGAA 0.0186528762144
TTATAAAA 0.26019795187
TTATACAA 0.198427041744
TTATAGAA 0.169543002708
TTATATAA 0.179387208895
TTATCAAA 0.158253294617
TTATCCAA 0.243975380339
TTATCGAA 0.12736617424
TTATGAAA 0.213948107888
TTATGCAA 0.208942353029
TTATGGAA -0.0076343561192
TTATTAAA 0.237927140978
TTATTCAA 0.093750775569
TTATTGAA 0.0521616314971
TTCAAAAA 0.183156986187
TTCAACAA 0.124002394108
TTCAAGAA -0.0216521987831
TTCACAAA 0.0985401779523
TTCACCAA 0.0887768880973
TTCACGAA 0.0436410638918
TTCAGAAA 0.0788488212731
TTCAGCAA 0.151836409412
TTCAGGAA 0.0930576584179
TTCATAAA 0.171203811698
TTCATCAA 0.12736290009
TTCATGAA 0.144798928279
TTCCAAAA 0.126996142148

TTCCACAA 0.140357843016
TTCCAGAA -0.0838151006129
TTCCCAAA 0.253177419844
TTCCCCAA 0.0590238906691
TTCCCGAA 0.214228155588
TTCCGAAA 0.167218354889
TTCCGCAA 0.0642915642916
TTCCGGAA 0.121449656438
TTCCTAAA 0.130154084217
TTCCTCAA 0.110541671148
TTCGAAAA 0.0775041422451
TTCGACAA 0.199117850633
TTCGAGAA 0.0129307262195
TTCGCAAA 0.15094846913
TTCGCCAA 0.0100499948985
TTCGCGAA 0.101503863021
TTCGGAAA 0.13103289709
TTCGGCAA 0.0913666124099
TTCGTAAA 0.145176857298
TTCGTCAA 0.156149961536
TTCTAAAA 0.076126070232
TTCTACAA 0.125517689394
TTCTAGAA 0.0702027844619
TTCTCAAA 0.19728406092
TTCTCCAA -0.0913719846391
TTCTGAAA -0.0743024708208
TTCTGCAA 0.0973428543068
TTCTTAAA 0.0228600833558
TTCTTCAA 0.257663700795
TTGAAAAA 0.323076520046
TTGAACAA -0.0655352755788
TTGACAAA 0.282260371586
TTGACCAA 0.0860353422918
TTGAGAAA 0.216479201185
TTGAGCAA 0.17479836208
TTGATAAA 0.216356576133
TTGATCAA -0.107462353321
TTGCAAAA 0.17233292801
TTGCACAA -0.0441502108169
TTGCCAAA 0.164336512821
TTGCCCAA -0.0606242199076
TTGCGAAA 0.201097792007
TTGCGCAA 0.230208738172
TTGCTAAA 0.128063619045
TTGGAAAA 0.116032009971
TTGGACAA 0.0397531440917
TTGGCAAA 0.13592330095
TTGGCCAA -0.0303728695825
TTGGGAAA 0.130734039825
TTGGTAAA 0.123866290533
TTGTAAAA 0.184374794713
TTGTACAA 0.027689579044
TTGTCAAA 0.149610592469
TTGTGAAA 0.0803829113231

TTGTTAAA	0.153902469289
TTTAAAAA	0.216879413849
TTTACAAA	0.188984291635
TTTAGAAA	0.0293695899757
TTTATAAA	0.212044410266
TTTCAAAA	0.223806451079
TTTCCAAA	0.147207725378
TTTCGAAA	0.115263548135
TTTGAAAA	0.319989343299
TTTGCAAA	0.242287051508
TTTTAAAA	0.208521798572
