# Supplementary material for: The intracellular immune receptor Rx1 regulates the DNA-binding activity of a Golden2-like transcription factor
Source: J Biol Chem. 2017 Dec 7;293(9):3218–33. doi: 10.1074/jbc.RA117.000485 (PMC5836133; doi:10.1074/jbc.RA117.000485)
Supplement: Supporting Information [file supp_RA117.000485_133217_1_supp_23410_mzmjtw.pdf]

#8mers	all
AAAAAAAA	2.76945325626
AAAAAAAC	2.10934789419
AAAAAAAG	-0.693604288528
AAAAAAAT	3.59896447281
AAAAAACA	0.865741580653
AAAAAACC	1.25806254534
AAAAAACG	0.212310858958
AAAAAACT	0.276540942162
AAAAAAGA	0.493169563669
AAAAAAGC	-0.120084977379
AAAAAAGG	0.357562178957
AAAAAAGT	0.972469958586
AAAAAATA	3.59625054772
AAAAAATC	14.6460460797
AAAAAATG	1.25721776003
AAAAAATT	2.98736787521
AAAAACAA	1.41921998086
AAAAACAC	0.302169668822
AAAAACAG	0.664096397633
AAAAACAT	0.595039903298
AAAAACCA	2.06589268144
AAAAACCC	0.843569886954
AAAAACCG	1.38949932534
AAAAACCT	1.60383327784
AAAAACGA	0.403243055972
AAAAACGC	1.93711728883
AAAAACGG	-0.734373021249
AAAAACGT	0.628485507904
AAAAACTA	1.0723765383
AAAAACTC	0.880923930533
AAAAACTG	-0.345028408768
AAAAACTT	-0.220629589564
AAAAAGAA	1.84296502491
AAAAAGAC	-0.12517878188
AAAAAGAG	-0.328296850182
AAAAAGAT	3.13834188383
AAAAAGCA	-0.11130987451
AAAAAGCC	-0.117993663246
AAAAAGCG	-0.297841451009
AAAAAGCT	0.119446944908
AAAAAGGA	0.904787808671
AAAAAGGC	-0.455171998606
AAAAAGGG	-0.629323497294
AAAAAGGT	-0.279855522154
AAAAAGTA	0.197201957306
AAAAAGTC	-0.110475544462
AAAAAGTG	0.782269630035
AAAAAGTT	1.86721496366
AAAAATAA	2.18654851622
AAAAATAC	3.23949499236
AAAAATAG	0.130653157982
AAAAATAT	4.78573022218
AAAAATCA	4.92078373929

AAAAATCC 13.8412296734
AAAAATCG 5.46688961026
AAAAATCT 13.1795243946
AAAAATGA 1.22225326698
AAAAATGC 0.405497472219
AAAAATGG 0.58184536044
AAAAATGT 0.805255004636
AAAAATTA 1.3995682671
AAAAATTC 1.64715257215
AAAAATTG 1.15086158949
AAAAATTT 2.13249480261
AAAACAAA 0.978506566496
AAAACAAC 1.60353164348
AAAACAAG 0.990809798119
AAAACAAT 1.24332480556
AAAACACA 0.944545778769
AAAACACC 0.821674996362
AAAACACG 0.367606550846
AAAACACT 1.0986508775
AAAACAGA 0.76165655478
AAAACAGC 0.73015536785
AAAACAGG 0.255865919469
AAAACAGT 0.962275292281
AAAACATA 2.20077185703
AAAACATC 1.06527114105
AAAACATG -0.195885378218
AAAACATT -0.0729286012669
AAAACCAA 2.31040993487
AAAACCAC 0.840184995314
AAAACCAG 0.563475462023
AAAACCAT 1.10965373569
AAAACCCA 1.9867594039
AAAACCCC -1.1617591109
AAAACCCG 0.441468807166
AAAACCCT -0.328420222294
AAAACCGA 0.67421081979
AAAACCGC 0.258375444152
AAAACCGG 0.868659906149
AAAACCGT 0.634374173832
AAAACCTA 0.324643519644
AAAACCTC -0.340963924983
AAAACCTG -0.189976324525
AAAACCTT 0.215241469388
AAAACGAA 1.96657917656
AAAACGAC -0.327578573562
AAAACGAG -0.131081301033
AAAACGAT 1.41491868535
AAAACGCA 1.16482720805
AAAACGCC -0.882453274239
AAAACGCG 1.30719731874
AAAACGCT 0.618771783731
AAAACGGA -0.0776857462752
AAAACGGC -0.196603916219
AAAACGGG -0.839234089075

AAAACGGT 0.181986673346
AAAACGTA 1.80576310541
AAAACGTC 0.610419857663
AAAACGTG 0.218991249734
AAAACGTT -0.257271629681
AAAACTAA 0.594331559179
AAAACTAC 0.19765519299
AAAACTAG 0.539300278407
AAAACTAT 1.28280179044
AAAACTCA 0.451166848443
AAAACTCC 0.177581609344
AAAACTCG -0.239137236081
AAAACTCT -0.252957787856
AAAACTGA 1.25025037227
AAAACTGC 0.249871132614
AAAACTGG 0.569933155681
AAAACTGT -0.170588083419
AAAACTTA 1.10971568313
AAAACTTC 0.364722204958
AAAACTTG 0.142572158662
AAAACTTT -0.236907912466
AAAAGAAA 1.52901096692
AAAAGAAC -0.0988717699842
AAAAGAAG -0.659383164733
AAAAGAAT 3.24023052017
AAAAGACA 1.03649668691
AAAAGACC -0.265672434108
AAAAGACG 1.23224483979
AAAAGACT -0.108628883502
AAAAGAGA -0.0582342508231
AAAAGAGC -0.813216165222
AAAAGAGG 0.57436409646
AAAAGAGT 1.74025408207
AAAAGATA 1.71863730149
AAAAGATC 4.46836016814
AAAAGATG -0.477042319328
AAAAGATT 7.40169393336
AAAAGCAA 0.126300108918
AAAAGCAC -0.274484914545
AAAAGCAG 0.329272849053
AAAAGCAT 0.235153519208
AAAAGCCA 0.00365097810308
AAAAGCCC 0.211001859936
AAAAGCCG -0.310293931523
AAAAGCCT 0.430727069185
AAAAGCGA 0.123713738047
AAAAGCGC 0.950026689411
AAAAGCGG 0.160033494659
AAAAGCGT -0.431731297268
AAAAGCTA 0.108540797904
AAAAGCTC 0.102681145333
AAAAGCTG 2.5130533342
AAAAGCTT -0.0697601335861
AAAAGGAA 0.627545318311

AAAAGGAC -0.0183777398645
AAAAGGAG -0.822589831941
AAAAGGAT 0.97861216466
AAAAGGCA 0.029414839047
AAAAGGCC -1.5398436657
AAAAGGCG 0.422536415559
AAAAGGCT -0.336781819482
AAAAGGGA -0.0302078708028
AAAAGGGC -1.40040416544
AAAAGGGG -0.18084103782
AAAAGGGT -0.324750163334
AAAAGGTA 0.888294368717
AAAAGGTC -0.295586511999
AAAAGGTG -1.55108333562
AAAAGGTT -0.270582225967
AAAAGTAA 0.960692365348
AAAAGTAC 0.559207623359
AAAAGTAG 1.65434108875
AAAAGTAT 0.518674656835
AAAAGTCA -0.815525732985
AAAAGTCC -0.205572441468
AAAAGTCG 0.50865720725
AAAAGTCT -0.209391226554
AAAAGTGA 0.0820346132339
AAAAGTGC 0.0960786462722
AAAAGTGG 0.974445742054
AAAAGTGT -0.932476222291
AAAAGTTA 0.683495878148
AAAAGTTC -0.353030344882
AAAAGTTG 0.766696253291
AAAAGTTT -0.1763410923
AAAATAAA 1.58301920014
AAAATAAC 2.97774066835
AAAATAAG 1.35094632439
AAAATAAT 1.88711237611
AAAATACA 3.23949499236
AAAATACC 2.15776569787
AAAATACG 2.93421226876
AAAATACT 1.71697360765
AAAATAGA 0.626175678759
AAAATAGC 0.36346156153
AAAATAGG 1.59807295027
AAAATAGT 1.16415702564
AAAATATA 4.02684378953
AAAATATC 8.25900438933
AAAATATG 3.47243121151
AAAATATT 5.18755266919
AAAATCAA 4.93142014049
AAAATCAC 5.81346644359
AAAATCAG 6.82064604817
AAAATCAT 5.03520170233
AAAATCCA 15.8149926617
AAAATCCC 10.3171656646
AAAATCCG 10.9053973615

AAAATCCT	9.28057775552
AAAATCGA	4.51881727102
AAAATCGC	7.0619893449
AAAATCGG	6.026409846
AAAATCGT	8.45443705462
AAAATCTA	13.7203487891
AAAATCTC	12.9056121671
AAAATCTG	18.8565744774
AAAATCTT	10.8243026765
AAAATGAA	1.80775692421
AAAATGAC	-0.109410675849
AAAATGAG	1.77326134974
AAAATGAT	1.01266731114
AAAATGCA	0.682851572519
AAAATGCC	0.0228776853845
AAAATGCG	0.917408618931
AAAATGCT	0.59220809511
AAAATGGA	-0.590246164867
AAAATGGC	0.0944230552567
AAAATGGG	0.398234200488
AAAATGGT	0.38666806496
AAAATGTA	1.47332414111
AAAATGTC	-0.0486912088312
AAAATGTG	1.09381871597
AAAATGTT	1.38039122232
AAAATTAA	2.00089753431
AAAATTAC	2.78208765836
AAAATTAG	0.545323033092
AAAATTAT	2.40307911963
AAAATTCA	1.19208120546
AAAATTCC	2.90594855425
AAAATTCG	4.0060357236
AAAATTCT	3.50143594284
AAAATTGA	1.64531192297
AAAATTGC	1.88701252834
AAAATTGG	0.837940250185
AAAATTGT	1.28023005694
AAAATTTA	3.34202401361
AAAATTTC	4.00362578531
AAAATTTG	2.6120228609
AAAATTTT	5.22084954766
AAACAAAA	1.03742825091
AAACAAAC	0.811167456289
AAACAAAG	0.533015619364
AAACAAAT	2.0739578719
AAACAACA	1.06011251392
AAACAACC	0.0780571695201
AAACAACG	1.19178296906
AAACAACT	0.548315590961
AAACAAGA	0.0583670326728
AAACAAGC	-0.256632290303
AAACAAGG	1.048639953
AAACAAGT	0.354382733249
AAACAATA	2.10855799901

AAACAATC 2.72201432665
AAACAATG 0.139750544356
AAACAATT 0.454211159867
AAACACAA 0.62259265987
AAACACAC 0.718966405962
AAACACAG 0.0713132630169
AAACACAT 0.635771519833
AAACACCA 0.128319543112
AAACACCC 0.597477286661
AAACACCG 0.482229698439
AAACACCT 0.300524010307
AAACACGA 0.898711470759
AAACACGC 0.194712297624
AAACACGG -0.253570989075
AAACACGT 1.14378964909
AAACACTA 2.1078026062
AAACACTC 0.276958368568
AAACACTG 0.121948628143
AAACACTT 0.428108286996
AAACAGAA 0.645671609082
AAACAGAC 0.146103162614
AAACAGAG -0.514116684601
AAACAGAT 4.24193574864
AAACAGCA 1.34442302396
AAACAGCC -0.872589255649
AAACAGCG -0.284678796706
AAACAGCT 0.974787106416
AAACAGGA -0.556134821341
AAACAGGC -0.203905872425
AAACAGGG -0.252729601725
AAACAGGT -0.430288993633
AAACAGTA 0.67245616515
AAACAGTC -0.740938404124
AAACAGTG -0.0166962720712
AAACAGTT -0.197798952866
AAACATAA 2.01366262721
AAACATAC 1.67574615023
AAACATAG 1.03717340386
AAACATAT 2.30051350496
AAACATCA 1.22188262788
AAACATCC 0.2621124168
AAACATCG 0.385561898055
AAACATCT 0.131187944723
AAACATGA 0.840367439666
AAACATGC -0.0819023541474
AAACATGG -0.416223265923
AAACATGT -0.399202619294
AAACATTA 1.16912641251
AAACATTC 0.529570871338
AAACATTG 0.676609780058
AAACATTT 0.509988946471
AAACCAAA 2.23917012008
AAACCAAC 0.778943810635
AAACCAAG 0.0573168014287

AAACCAAT 1.2442699614
AAACCACA 0.827436630836
AAACCACC -0.532881269225
AAACCACG 0.913783256224
AAACCACT -0.14873788908
AAACCAGA 1.84067349247
AAACCAGC -0.0265453919095
AAACCAGG -0.032583829491
AAACCAGT 0.749908497662
AAACCATA 1.11348402157
AAACCATC -0.143501892992
AAACCATG -0.394758870816
AAACCATT 1.2354441505
AAACCCAA 0.694313678174
AAACCCAC 0.612760007072
AAACCCAG 1.21676895838
AAACCCAT 0.287615941676
AAACCCCA -0.517618936577
AAACCCCC -1.50390866279
AAACCCCG -0.738060070012
AAACCCCT -0.577044826087
AAACCCGA -0.00390870035463
AAACCCGC 0.98390095983
AAACCCGG -0.414454758059
AAACCCGT 0.0770511117653
AAACCCTA 0.61197899887
AAACCCTC -0.40585190566
AAACCCTG -0.0833227017285
AAACCCTT -0.333912110963
AAACCGAA 1.53060434912
AAACCGAC -0.294456297987
AAACCGAG -0.297505837043
AAACCGAT 1.56370858936
AAACCGCA -0.68070824345
AAACCGCC -0.557150550215
AAACCGCG -0.0532162469841
AAACCGCT -0.244257440123
AAACCGGA 0.811453146371
AAACCGGC 0.953807051403
AAACCGGG -0.0456445449731
AAACCGGT 0.402815174302
AAACCGTA 0.00834695981842
AAACCGTC -0.162058679248
AAACCGTG -0.191047727678
AAACCGTT 0.662113818244
AAACCTAA -0.465142660885
AAACCTAC 0.0601156755358
AAACCTAG -0.245879051531
AAACCTAT 0.0673546387778
AAACCTCA -0.709389907126
AAACCTCC -1.50702694521
AAACCTCG 0.77119436914
AAACCTCT -0.535604342669
AAACCTGA 0.112361935425

AAACCTGC -0.922050233275
AAACCTGG -0.178349025704
AAACCTGT 0.129248754678
AAACCTTA 0.39498418175
AAACCTTC -0.22060763351
AAACCTTG 0.488740713943
AAACCTTT 1.08332842708
AAACGAAA 2.23752942782
AAACGAAC -0.0391392798651
AAACGAAG 1.16794705875
AAACGAAT 2.06759950325
AAACGACA 1.35075865241
AAACGACC -0.327578573562
AAACGACG -0.809702151074
AAACGACT 0.712431604734
AAACGAGA 2.14865759485
AAACGAGC -0.728904395542
AAACGAGG 0.359778694873
AAACGAGT 1.07432566085
AAACGATA 1.43099286923
AAACGATC 2.07659181422
AAACGATG 0.391792451108
AAACGATT 1.96468677383
AAACGCAA 2.53091562956
AAACGCAC -0.258686226867
AAACGCAG 0.200548164471
AAACGCAT 0.609157645946
AAACGCCA -0.487454455119
AAACGCCC -0.744395959847
AAACGCCG -0.599617479151
AAACGCCT 0.0726802887528
AAACGCGA 0.512059872839
AAACGCGC 0.0497058921786
AAACGCGG -0.751738168819
AAACGCGT 0.713263843729
AAACGCTA 0.076128434739
AAACGCTC -0.220831898918
AAACGCTG 0.305342841372
AAACGCTT 0.54793501936
AAACGGAA 0.442046199107
AAACGGAC -0.221025321297
AAACGGAG -0.497624028792
AAACGGAT 2.35490440083
AAACGGCA -0.0598088135446
AAACGGCC -0.352912723165
AAACGGCG 0.486583270267
AAACGGCT -0.324521977203
AAACGGGA 0.0592275008798
AAACGGGC -0.630997646403
AAACGGGG 0.229289161769
AAACGGGT -0.382364155635
AAACGGTA 0.807783871557
AAACGGTC -0.578824311978
AAACGGTG 0.587711286169

AAACGGTT	0.148880342048
AAACGTAA	1.66353178835
AAACGTAC	-0.0897926803214
AAACGTAG	-0.10702478466
AAACGTAT	2.3733744084
AAACGTCA	0.306710912634
AAACGTCC	0.377503242134
AAACGTCG	-0.227892599913
AAACGTCT	-0.48296940835
AAACGTGA	1.04671827552
AAACGTGC	0.356032051106
AAACGTGG	0.418211595707
AAACGTGT	-0.0790093826666
AAACGTTA	0.992522893086
AAACGTTC	-0.535813970708
AAACGTTG	-0.212805392933
AAACGTTT	0.150701648995
AAACTAAA	1.66004286684
AAACTAAC	1.39053282817
AAACTAAG	1.34221382672
AAACTAAT	0.545596961003
AAACTACA	0.570190877933
AAACTACC	-0.452170292382
AAACTACG	-0.0406158244888
AAACTACT	2.14682949198
AAACTAGA	1.08147444744
AAACTAGC	0.203644490831
AAACTAGG	0.669751127035
AAACTAGT	0.500354420921
AAACTATA	0.122655926736
AAACTATC	0.233481199771
AAACTATG	0.12596266528
AAACTATT	1.0371713128
AAACTCAA	1.85320778542
AAACTCAC	-0.0619534495222
AAACTCAG	-0.112307568054
AAACTCAT	0.575069826764
AAACTCCA	-0.0401957842675
AAACTCCC	-0.084050910849
AAACTCCG	0.192458926904
AAACTCCT	-0.363586240551
AAACTCGA	0.133322909582
AAACTCGC	0.0559320017444
AAACTCGG	0.497109107052
AAACTCGT	0.590789838582
AAACTCTA	-0.232854406709
AAACTCTC	-0.0580199179162
AAACTCTG	-0.164931785728
AAACTCTT	0.49417013241
AAACTGAA	0.388077173132
AAACTGAC	0.53945005006
AAACTGAG	-0.402455513229
AAACTGAT	1.9784093075
AAACTGCA	0.714286891287

AAACTGCC -0.42867470091
AAACTGCG 0.13356364203
AAACTGCT -0.396477193414
AAACTGGA 1.15066712158
AAACTGGC -0.109290963079
AAACTGGG 0.262004466201
AAACTGGT -0.220402971722
AAACTGTA -0.0449053578257
AAACTGTC -0.268682765924
AAACTGTG 0.123397989081
AAACTGTT -0.1580888156
AAACTTAA 2.04176820586
AAACTTAC 1.11262825823
AAACTTAG -0.220629589564
AAACTTAT 1.43621292104
AAACTTCA 0.72380379512
AAACTTCC -0.514364474352
AAACTTCG 0.266355424213
AAACTTCT 0.268530380455
AAACTTGA 0.643048644788
AAACTTGC 0.392159953629
AAACTTGG -0.596479593117
AAACTTGT 0.291344288731
AAACTTTA 0.515683928638
AAACTTTC 0.353354980822
AAACTTTG 0.0335527710594
AAAGAAAA 1.08769402246
AAAGAAAC 0.203141854026
AAAGAAAG 0.445292819884
AAAGAAAT 2.22301752172
AAAGAACA -0.326062560317
AAAGAACC -0.0707165288381
AAAGAACG 1.25256412214
AAAGAACT 0.0617974047107
AAAGAAGA -0.414531342866
AAAGAAGC -0.250312083363
AAAGAAGG -0.685837073085
AAAGAAGT 0.646890692836
AAAGAATA 1.88898334556
AAAGAATC 9.93290989037
AAAGAATG 1.13606268641
AAAGAATT 0.780100946951
AAAGACAA 1.3056585653
AAAGACAC 0.962558629928
AAAGACAG 0.344913400867
AAAGACAT 0.747987342947
AAAGACCA -0.0481543310374
AAAGACCC -1.13829122588
AAAGACCG 0.445292819884
AAAGACCT 0.164223441608
AAAGACGA 2.76261107028
AAAGACGC -0.207775888304
AAAGACGG -0.0381496891507
AAAGACGT 0.563324383462

AAAGACTA -0.15386436628
AAAGACTC -0.697947405091
AAAGACTG -0.277338417405
AAAGACTT -0.949874826705
AAAGAGAA 0.816207416182
AAAGAGAC -0.539072353657
AAAGAGAG -0.626143267441
AAAGAGAT 1.7311906753
AAAGAGCA -0.598970559707
AAAGAGCC -1.33233490938
AAAGAGCG -0.425638753697
AAAGAGCT -0.765716072314
AAAGAGGA 0.359431057353
AAAGAGGC -0.643872780953
AAAGAGGG -0.666272138021
AAAGAGGT 1.32338206703
AAAGAGTA 1.0909369839
AAAGAGTC 0.133418575245
AAAGAGTG -0.14190929494
AAAGAGTT 1.90381413719
AAAGATAA 2.27619717529
AAAGATAC 2.31713894262
AAAGATAG 0.645790537707
AAAGATAT 5.04968903855
AAAGATCA 2.55147668988
AAAGATCC 4.68808014976
AAAGATCG 4.60567855677
AAAGATCT 8.34578516954
AAAGATGA 0.299934856195
AAAGATGC 0.443362255432
AAAGATGG 0.43682353348
AAAGATGT 0.0592836979225
AAAGATTA 5.30363484884
AAAGATTC 6.35909111104
AAAGATTG 4.60528177951
AAAGATTT 11.5162466691
AAAGCAAA 0.67049815563
AAAGCAAC -0.0616965114154
AAAGCAAG 0.124746979487
AAAGCAAT 4.39298033029
AAAGCACA 0.819808209019
AAAGCACC -0.504005660406
AAAGCACG 0.122140482233
AAAGCACT -0.527981409866
AAAGCAGA 2.89339125972
AAAGCAGC -0.751734770858
AAAGCAGG -1.19856974215
AAAGCAGT -0.743985590745
AAAGCATA 0.559374907579
AAAGCATC 0.180453147534
AAAGCATG 0.0350617270008
AAAGCATT 0.615991206336
AAAGCCAA -0.139980298777
AAAGCCAC 0.144427445215

AAAGCCAG -0.691072285028
AAAGCCAT -0.611962793211
AAAGCCCA -1.35417987609
AAAGCCCC -1.49535939362
AAAGCCCG -0.880644774991
AAAGCCCT -0.034302152089
AAAGCCGA 1.08829258631
AAAGCCGC -0.840417624932
AAAGCCGG -0.155255439123
AAAGCCGT -0.685619080835
AAAGCCTA 0.263253086075
AAAGCCTC -0.272101637172
AAAGCCTG 0.439113236242
AAAGCCTT -0.215547808616
AAAGCGAA -0.203602669776
AAAGCGAC -0.128069139545
AAAGCGAG -0.349167647688
AAAGCGAT 0.732940650115
AAAGCGCA -0.424732020948
AAAGCGCC -0.734812142327
AAAGCGCG 0.26288819737
AAAGCGCT 0.138263544469
AAAGCGGA 1.22582347818
AAAGCGGC -1.64272163137
AAAGCGGG 0.229487027635
AAAGCGGT 0.709178449417
AAAGCGTA -0.182990640048
AAAGCGTC 0.0655294111079
AAAGCGTG 0.0902121977795
AAAGCGTT -0.0415625486218
AAAGCTAA 0.218897675123
AAAGCTAC -0.720901413901
AAAGCTAG 0.115670765022
AAAGCTAT 0.953936958055
AAAGCTCA -0.2558413496
AAAGCTCC -0.861668732658
AAAGCTCG -0.689553396586
AAAGCTCT -0.350950270159
AAAGCTGA -0.518012315876
AAAGCTGC 0.238453200449
AAAGCTGG 0.3050981882
AAAGCTGT 0.679312988502
AAAGCTTA 1.10327550204
AAAGCTTC -1.06377708386
AAAGCTTG -0.479383775645
AAAGCTTT -0.213573332056
AAAGGAAA -0.479484668941
AAAGGAAC -0.28762639694
AAAGGAAG -1.27743667184
AAAGGAAT 2.65644361725
AAAGGACA -0.141986663892
AAAGGACC -0.155037969637
AAAGGACG 0.351068937402
AAAGGACT 0.17592131346

AAAGGAGA	−0.385907444522
AAAGGAGC	−0.872677341247
AAAGGAGG	−0.42862712946
AAAGGAGT	−0.747731711748
AAAGGATA	1.80598423424
AAAGGATC	2.45946435706
AAAGGATG	0.397946157972
AAAGGATT	4.17464619388
AAAGGCAA	0.141096920946
AAAGGCAC	−1.25841697879
AAAGGCAG	−0.857375017216
AAAGGCAT	−0.611825306493
AAAGGCCA	−1.00854663032
AAAGGCCC	−1.78011712757
AAAGGCCG	−1.53394872661
AAAGGCCT	−1.45768019134
AAAGGCGA	−0.999330053935
AAAGGCGC	−0.0514438183962
AAAGGCGG	−0.906773001876
AAAGGCGT	−0.152047502821
AAAGGCTA	−0.412466689656
AAAGGCTC	−1.31916519778
AAAGGCTG	−0.848050490236
AAAGGCTT	−1.18686742681
AAAGGGAA	−0.154899437392
AAAGGGAC	−0.593761747304
AAAGGGAG	−1.36632366494
AAAGGGAT	1.84853297562
AAAGGGCA	−0.0726763680288
AAAGGGCC	−1.45840369559
AAAGGGCG	−0.899977080436
AAAGGGCT	−0.974278980597
AAAGGGGA	−0.454530045411
AAAGGGGC	−1.28521983156
AAAGGGGG	−0.597287262242
AAAGGGGT	−0.242723391548
AAAGGGTA	−0.255357009506
AAAGGGTC	−1.06323158048
AAAGGGTG	−1.0465410588
AAAGGGTT	−0.5573481547
AAAGGTAA	0.193918743105
AAAGGTAC	0.493011427805
AAAGGTAG	−0.372438973753
AAAGGTAT	0.63849668433
AAAGGTCA	−1.42368673091
AAAGGTCC	−0.991622172113
AAAGGTCG	−0.248510118655
AAAGGTCT	0.0802112152351
AAAGGTGA	0.601933058691
AAAGGTGC	−0.963193264438
AAAGGTGG	−1.08433709865
AAAGGTGT	−0.103178031743
AAAGGTTA	−0.0342974472203
AAAGGTTC	0.00657662228319

AAAGGTTG -0.0709760807608
AAAGTAAA 1.57590726835
AAAGTAAC 0.189152449741
AAAGTAAG 1.22044372221
AAAGTAAT 2.15689268335
AAAGTACA 1.08174576153
AAAGTACC -0.446954684058
AAAGTACG 1.26951210468
AAAGTACT -0.0183777398645
AAAGTAGA -0.0469023132028
AAAGTAGC 0.0895422767545
AAAGTAGG 0.838875473528
AAAGTAGT -0.0817308878219
AAAGTATA 1.13101671474
AAAGTATC 2.87441234219
AAAGTATG 0.656817443008
AAAGTATT 0.3347054041
AAAGTCAA -0.192319871896
AAAGTCAC -0.191453391912
AAAGTCAG -0.15824616732
AAAGTCAT -0.0860355812912
AAAGTCCA -1.12364889037
AAAGTCCC -1.09319009324
AAAGTCCG -0.129535228905
AAAGTCCT -0.591421336513
AAAGTCGA 0.07879348147
AAAGTCGC -0.24491298516
AAAGTCGG -0.550095338233
AAAGTCGT 0.583366601316
AAAGTCTA -0.659186605774
AAAGTCTC -0.363089354141
AAAGTCTG 0.0951622424041
AAAGTCTT -0.786096517951
AAAGTGAA 1.33861094284
AAAGTGAC -0.210628084256
AAAGTGAG -0.145492836592
AAAGTGAT 1.1940758084
AAAGTGCA -0.368330055098
AAAGTGCC -0.612164057038
AAAGTGCG 0.10372196684
AAAGTGCT -0.440285794072
AAAGTGGA 2.40907887274
AAAGTGGC -0.103908070535
AAAGTGGG -0.521056365918
AAAGTGGT 1.53528647761
AAAGTGTA 1.33889611015
AAAGTGTC 0.574056188943
AAAGTGTG -1.2251159182
AAAGTGTT 0.615803011588
AAAGTTAA 0.10372196684
AAAGTTAC 0.407779856296
AAAGTTAG 0.79421895098
AAAGTTAT 2.14188990262
AAAGTTCA 0.872301474515

AAAGTTCC	-0.302502668973
AAAGTTCG	0.631634110584
AAAGTTCT	0.209719521836
AAAGTTGA	0.815708961483
AAAGTTGC	0.523056457874
AAAGTTGG	0.528450328445
AAAGTTGT	-0.516469118946
AAAGTTTA	0.471458162956
AAAGTTTC	0.0624566090904
AAAGTTTG	0.0809294918551
AAATAAAA	3.3088282898
AAATAAAC	1.98495639367
AAATAAAG	1.26827655389
AAATAAAT	2.3995975168
AAATAACA	2.01635355072
AAATAACC	2.33864176082
AAATAACG	1.13207870815
AAATAACT	1.88088365273
AAATAAGA	1.43314168731
AAATAAGC	0.0584318553081
AAATAAGG	1.48156210481
AAATAAGT	0.990765363248
AAATAATA	2.30375777331
AAATAATC	2.36323123427
AAATAATG	0.80521788845
AAATAATT	1.8079247312
AAATACAA	2.9095577113
AAATACAC	1.86779104869
AAATACAG	2.37680216662
AAATACAT	2.49000575078
AAATACCA	0.315772751112
AAATACCC	1.65832140766
AAATACCG	1.89571862647
AAATACCT	2.5817438943
AAATACGA	1.12277064822
AAATACGC	1.58143444353
AAATACGG	1.41833860212
AAATACGT	2.93421226876
AAATACTA	1.28797871429
AAATACTC	1.20948529889
AAATACTG	1.8261059121
AAATACTT	0.509806240737
AAATAGAA	0.91832685247
AAATAGAC	0.693649768925
AAATAGAG	-0.265817239511
AAATAGAT	2.03395603278
AAATAGCA	0.215279631101
AAATAGCC	0.595978524602
AAATAGCG	0.53155867836
AAATAGCT	0.866137051005
AAATAGGA	0.702192764939
AAATAGGC	0.681057972022
AAATAGGG	-0.789719528223
AAATAGGT	2.03056800456

AAATAGTA 0.170324872154
AAATAGTC 0.221884221214
AAATAGTG -0.1875057457
AAATAGTT 1.08903046655
AAATATAA 1.92062593993
AAATATAC 2.54860201511
AAATATAG 2.4461291909
AAATATAT 4.86374687755
AAATATCA 5.69407099085
AAATATCC 11.5516225768
AAATATCG 6.30154743039
AAATATCT 8.41548518536
AAATATGA 3.08855313368
AAATATGC 2.72441015034
AAATATGG 2.55174565154
AAATATGT 2.84708960142
AAATATTA 4.44823901305
AAATATTC 4.96559944323
AAATATTG 3.61282109524
AAATATTT 5.30178897202
AAATCAAA 3.80709453317
AAATCAAC 4.79172134969
AAATCAAG 3.14975014487
AAATCAAT 4.03510004993
AAATCACA 4.60786449104
AAATCACC 4.19095353014
AAATCACG 5.88678868553
AAATCACT 2.86301479779
AAATCAGA 3.8663455584
AAATCAGC 5.05115172995
AAATCAGG 4.55042693131
AAATCAGT 4.42030463935
AAATCATA 5.74640376805
AAATCATC 2.42024509443
AAATCATG 3.42310432264
AAATCATT 3.08682304891
AAATCCAA 10.4328390434
AAATCCAC 12.6406695104
AAATCCAG 6.93778630051
AAATCCAT 8.12032524812
AAATCCCA 6.71116218547
AAATCCCC 7.95805589531
AAATCCCG 11.1884041002
AAATCCCT 5.2154954071
AAATCCGA 8.60642391691
AAATCCGC 9.14983076154
AAATCCGG 11.1857840111
AAATCCGT 5.3814868338
AAATCCTA 7.72129984555
AAATCCTC 6.8162702589
AAATCCTG 10.4289708572
AAATCCTT 7.06150918691
AAATCGAA 2.05875147492
AAATCGAC 5.75394776361

AAATCGAG	3.74742634293
AAATCGAT	3.58713460325
AAATCGCA	7.43717099572
AAATCGCC	5.37918510749
AAATCGCG	9.07684805429
AAATCGCT	3.48682889384
AAATCGGA	4.70758392153
AAATCGGC	4.49749898823
AAATCGGG	4.7880502452
AAATCGGT	4.15874086252
AAATCGTA	6.29800283459
AAATCGTC	6.43624651406
AAATCGTG	8.17302735751
AAATCGTT	3.02521070237
AAATCTAA	8.53513914449
AAATCTAC	11.8438095598
AAATCTAG	15.2229042794
AAATCTAT	10.9442742143
AAATCTCA	10.3394230915
AAATCTCC	9.20305772517
AAATCTCG	14.9478788277
AAATCTCT	8.0419797747
AAATCTGA	14.0591879431
AAATCTGC	11.0335281871
AAATCTGG	11.2066563769
AAATCTGT	10.505689753
AAATCTTA	9.20994068668
AAATCTTC	8.76117619647
AAATCTTG	10.6001731874
AAATGAAA	2.82759837596
AAATGAAC	1.23065825352
AAATGAAG	-0.0322714784863
AAATGAAT	3.5576985923
AAATGACA	0.208644197959
AAATGACC	-0.61738254056
AAATGACG	0.164496062611
AAATGACT	0.728649287107
AAATGAGA	1.00365670346
AAATGAGC	-0.868138449869
AAATGAGG	0.909971789822
AAATGAGT	1.79409137172
AAATGATA	2.50053289447
AAATGATC	0.74221865117
AAATGATG	0.546546821715
AAATGATT	3.18753128598
AAATGCAA	1.52352090793
AAATGCAC	0.423750794444
AAATGCAG	-0.336771364218
AAATGCAT	1.93711937988
AAATGCCA	-0.527376572858
AAATGCCC	0.664284330999
AAATGCCG	0.359094136479
AAATGCCT	0.027699653028
AAATGCGA	1.99240890567

AAATGCGC	1.4716201945
AAATGCGG	0.411851397384
AAATGCGT	0.951751285167
AAATGCTA	1.44900336795
AAATGCTC	0.409869079376
AAATGCTG	0.327459906318
AAATGCTT	0.428168143381
AAATGGAA	-0.238866183368
AAATGGAC	-0.367506964459
AAATGGAG	-1.25717750727
AAATGGAT	1.56726494733
AAATGGCA	0.178423258076
AAATGGCC	0.0757980484044
AAATGGCG	0.660540301049
AAATGGCT	-0.531109102018
AAATGGGA	-0.236615687845
AAATGGGC	-0.675502044863
AAATGGGG	0.35998492495
AAATGGGT	0.599453592892
AAATGGTA	1.92049551051
AAATGGTC	0.0969534904669
AAATGGTG	-0.0929674211604
AAATGGTT	0.677283360425
AAATGTAA	1.60321380346
AAATGTAC	0.315179676275
AAATGTAG	1.32664855281
AAATGTAT	0.715694692552
AAATGTCA	1.05543430615
AAATGTCC	-0.545591210608
AAATGTCG	-0.184429022959
AAATGTCT	0.825566445532
AAATGTGA	1.17500383903
AAATGTGC	0.557451139048
AAATGTGG	0.821748705972
AAATGTGT	0.496837008812
AAATGTTA	0.621022017873
AAATGTTC	0.489083385212
AAATGTTG	1.03514482131
AAATTAAA	1.158343899
AAATTAAC	2.06210839873
AAATTAAG	2.7929621782
AAATTAAT	0.546699991329
AAATTACA	3.48215225437
AAATTACC	2.47842288683
AAATTACG	4.89480789787
AAATTACT	2.68639272027
AAATTAGA	1.21162444585
AAATTAGC	0.641789831032
AAATTAGG	0.469747420425
AAATTAGT	0.763471065804
AAATTATA	3.32976051199
AAATTATC	1.6863389007
AAATTATG	1.24332480556
AAATTATT	2.86860182935

AAATTCAA 1.59307637972
AAATTCAC 2.24979711154
AAATTCAG 2.36316144538
AAATTCAT 1.51995383331
AAATTCCA 1.75208264472
AAATTCCC 5.72963718433
AAATTCCG 2.07469235418
AAATTCCT 4.24716128947
AAATTCGA 2.47262073821
AAATTCGC 4.04077176914
AAATTCGG 2.29743599808
AAATTCGT 3.48916067904
AAATTCTA 2.76641704767
AAATTCTC 5.06286894404
AAATTCTG 4.03654183081
AAATTCTT 1.3043208143
AAATTGAA 2.23956715872
AAATTGAC 0.412331032609
AAATTGAG 0.753519223
AAATTGAT 1.4303825432
AAATTGCA 1.55617923117
AAATTGCC 2.76026177251
AAATTGCG 4.54084206827
AAATTGCT 3.06573817988
AAATTGGA 0.568408778226
AAATTGGC 0.481505671424
AAATTGGG 0.511052246795
AAATTGGT 0.865868350726
AAATTGTA 1.2003981064
AAATTGTC 0.920253496198
AAATTGTG 2.10752397342
AAATTGTT 1.81228614447
AAATTTAA 3.0152342897
AAATTTAC 2.06154747383
AAATTTAG 1.92183665947
AAATTTAT 2.2196551089
AAATTTCA 3.81845835935
AAATTTCC 4.00362578531
AAATTTCG 3.26021209749
AAATTTCT 2.02234964448
AAATTTGA 1.97608510237
AAATTTGC 3.2381311032
AAATTTGG 1.93271300897
AAATTTGT 2.31307341331
AAATTTTA 3.11334543924
AAATTTTC 4.68700796246
AAATTTTG 3.82073656132
AACAAAAA 0.894041888584
AACAAAAC 0.479485191704
AACAAAAG -0.147472279402
AACAAAAT 3.74586537205
AACAAACA 1.80897287139
AACAAACC 0.063335374009
AACAAACG 0.659625988234

AACAAACT 0.243477477447
AACAAAGA 0.801588605019
AACAAAGC 1.0617273294
AACAAAGG 0.0833122464647
AACAAAGT 0.122044555188
AACAAATA 2.80712540124
AACAAATC 4.03405373941
AACAAATG 1.05663666148
AACAAATT 1.32677192492
AACAACAA 1.44423158557
AACAACAC 0.288993422676
AACAACAG 0.595448442729
AACAACAT 0.563852635663
AACAACCA -0.0150328396078
AACAACCC -0.387767697325
AACAACCG -0.455797223378
AACAACCT -0.430288993633
AACAACGA 0.906902385765
AACAACGC 0.656721515963
AACAACGG 0.144839905371
AACAACGT 0.856721563231
AACAACTA 0.577677892307
AACAACTC -0.43416031642
AACAACTG -0.23244456037
AACAACTT 0.0659209607355
AACAAGAA 1.40911522982
AACAAGAC -0.256060910138
AACAAGAG -0.339383611867
AACAAGAT 3.58855730327
AACAAGCA -0.0508321854665
AACAAGCC -0.330717766504
AACAAGCG -0.0239940461718
AACAAGCT -0.567421801327
AACAAGGA 0.696467201125
AACAAGGC 0.118322481292
AACAAGGG -0.08112709634
AACAAGGT 0.456184852282
AACAAGTA 0.163250579316
AACAAGTC -0.0265111509208
AACAAGTG -0.509565508289
AACAAGTT 0.101907455815
AACAATAA 1.12400881283
AACAATAC 1.3593403329
AACAATAG 2.21164899068
AACAATAT 2.69768911
AACAATCA 1.34478477608
AACAATCC 3.76701166575
AACAATCG 2.34934272328
AACAATCT 2.99168119427
AACAATGA -0.0487021868581
AACAATGC -0.223988604427
AACAATGG -0.461147704605
AACAATGT 0.341084421898
AACAATTA 1.1525286813

AACAATTC 1.13606268641
AACAATTG 0.604884056886
AACACAAA 0.846679282395
AACACAAC -0.269184357203
AACACAAG 0.765817226991
AACACAAT 1.92025190287
AACACACA -0.218063867839
AACACACC 0.668416512617
AACACACG 0.211462414304
AACACACT 0.318698918055
AACACAGA 1.07786868835
AACACAGC 0.0386980677346
AACACAGG 0.677144044036
AACACAGT 0.222049414382
AACACATA 0.0750933636273
AACACATC 0.0653325907677
AACACATG -0.388047375631
AACACATT 1.01027070331
AACACCAA -0.146663826133
AACACCAC -0.0761514363193
AACACCAG -0.466048348108
AACACCAT -0.935553729177
AACACCCA 0.00901792636987
AACACCCC -0.170692636056
AACACCCG -0.586954325073
AACACCCT -0.425070771493
AACACCGA 1.56457114862
AACACCGC -0.00414577346026
AACACCGG -0.596951125512
AACACCGT 0.410343486969
AACACCTA 0.409069251699
AACACCTC 0.252072488398
AACACCTG 0.498729672934
AACACCTT 0.666201042227
AACACGAA 0.358647435335
AACACGAC -0.316919954927
AACACGAG 0.801988780239
AACACGAT 3.06421667762
AACACGCA 0.47147018651
AACACGCC -0.955401217744
AACACGCG 0.048735120939
AACACGCT 0.557733692551
AACACGGA 1.56404969234
AACACGGC -0.833687310272
AACACGGG 0.650628449628
AACACGGT -0.249307855279
AACACGTA 1.88379465954
AACACGTC -0.396875538963
AACACGTG 0.0909001541346
AACACGTT -0.789946668828
AACACTAA 1.1297646969
AACACTAC 0.829552514838
AACACTAG -1.17840336803
AACACTAT 2.03909061281

AACACTCA	0.112197526403
AACACTCC	0.0298785299945
AACACTCG	-0.049459147954
AACACTCT	0.344551125978
AACACTGA	0.642242282571
AACACTGC	0.281086629461
AACACTGG	-0.453480598312
AACACTGT	1.045964451
AACACTTA	0.549901393091
AACACTTC	-0.683509992754
AACACTTG	-0.43146259699
AACAGAAA	2.02872212774
AACAGAAC	0.358519358354
AACAGAAG	0.169994747201
AACAGAAT	1.66907987406
AACAGACA	0.200934225085
AACAGACC	0.0584253207682
AACAGACG	1.15646404257
AACAGACT	-0.501620553362
AACAGAGA	-0.507919588392
AACAGAGC	-0.0712656915668
AACAGAGG	-0.315996755138
AACAGAGT	0.534629912088
AACAGATA	2.9284177002
AACAGATC	4.24193574864
AACAGATG	-0.207664801127
AACAGATT	9.01405164912
AACAGCAA	0.467362836144
AACAGCAC	-0.656970874003
AACAGCAG	-0.369808952156
AACAGCAT	1.07541353104
AACAGCCA	-1.37059516295
AACAGCCC	-1.02074740035
AACAGCCG	-0.833851980676
AACAGCCT	-0.723320761934
AACAGCGA	-0.243313591187
AACAGCGC	-0.338566010241
AACAGCGG	-0.263006341851
AACAGCGT	-0.0409428128627
AACAGCTA	-0.632508170633
AACAGCTC	0.352494773997
AACAGCTG	0.0683314217941
AACAGCTT	0.478996930887
AACAGGAA	0.317512507
AACAGGAC	-0.16244761506
AACAGGAG	-0.783173226205
AACAGGAT	2.63785990869
AACAGGCA	0.0161000606556
AACAGGCC	-1.02722391349
AACAGGCG	-0.448698883434
AACAGGCT	-1.19796934863
AACAGGGA	0.344415207549
AACAGGGC	-0.306104507336
AACAGGGG	-0.486265953012

AACAGGGT 0.124142665242
AACAGGTA 0.209355678657
AACAGGTC -0.196583005692
AACAGGTG -0.374032355949
AACAGGTT -0.553985219113
AACAGTAA 1.9058262527
AACAGTAC 0.82102677001
AACAGTAG 0.386567694428
AACAGTAT 1.00276643775
AACAGTCA -0.0724685696617
AACAGTCC -1.33010872235
AACAGTCG -0.761206978438
AACAGTCT -0.0901123500107
AACAGTGA 0.0822181031128
AACAGTGC -0.494118901618
AACAGTGG 0.475618051023
AACAGTGT 0.395314829466
AACAGTTA 0.993124070751
AACAGTTC -0.606165610841
AACAGTTG -0.0229495653228
AACATAAA 0.69756997007
AACATAAC 1.06333744002
AACATAAG 0.812977785208
AACATAAT 1.45835847658
AACATACA 3.01311605326
AACATACC 0.625243069232
AACATACG 0.222381369006
AACATACT -0.110292315965
AACATAGA 1.10861605076
AACATAGC -0.164066089889
AACATAGG 0.679933508406
AACATAGT 0.480813271582
AACATATA 2.22220776155
AACATATC 4.1016553838
AACATATG 0.678905233215
AACATATT 2.35127537878
AACATCAA 0.950327016862
AACATCAC 1.06527114105
AACATCAG 1.86246618286
AACATCAT 0.92853040575
AACATCCA 2.0019386172
AACATCCC 0.563810553226
AACATCCG 0.682770021462
AACATCCT 0.704841606011
AACATCGA 0.864619208089
AACATCGC 0.289401700725
AACATCGG 1.17569388643
AACATCGT 0.212484939099
AACATCTA 1.35082112261
AACATCTC 0.240260915553
AACATCTG 1.56347909633
AACATCTT 0.670898069469
AACATGAA 0.621665277975
AACATGAC 0.847882944634

AACATGAG -0.0700212537984
AACATGAT 1.81811077191
AACATGCA -0.0386460527974
AACATGCC 0.0175648431076
AACATGCG 0.348653771475
AACATGCT -0.307554129656
AACATGGA 2.27822392816
AACATGGC -0.153595143239
AACATGGG -0.38465255149
AACATGGT -0.419403234394
AACATGTA 0.84060581968
AACATGTC -0.251654277847
AACATGTG -0.384040395797
AACATGTT 1.04884696722
AACATTAA 1.15080565383
AACATTAC 0.225127444031
AACATTAG 0.516653131588
AACATTAT 0.936131905262
AACATTCA 0.855303829466
AACATTCC -0.0151993396831
AACATTCG -0.18084103782
AACATTCT 1.25901711092
AACATTGA 0.909671985134
AACATTGC 0.89946764771
AACATTGG 0.293178926138
AACATTGT 0.264348797717
AACATTTA 0.690940548705
AACATTTC 0.85010756338
AACATTTG 0.946640752244
AACCAAAA 2.40307911963
AACCAAAC -0.0805201682791
AACCAAAG -0.527724471759
AACCAAAT 3.77769877498
AACCAACA 1.37676585962
AACCAACC -0.000341625743179
AACCAACG 0.285685115842
AACCAACT -0.312865142262
AACCAAGA 0.755137436447
AACCAAGC -0.360748943349
AACCAAGG -0.902819866651
AACCAAGT 0.880398030766
AACCAATA 0.342560443759
AACCAATC 1.55502340176
AACCAATG 0.715727626632
AACCAATT 0.251365973949
AACCACAA 0.793414679816
AACCACAC -0.847011759782
AACCACAG -0.273549168439
AACCACAT -0.147653416847
AACCACCA -0.622075124314
AACCACCC -0.650139927429
AACCACCG -0.806770756499
AACCACCT -0.53274273698
AACCACGA 0.592003171941

AACCACGC 0.244479614478
AACCACGG 0.454991906688
AACCACGT 0.583128482684
AACCACTA 0.322262594706
AACCACTC 0.246802251321
AACCACTG 0.0536109331909
AACCACTT -0.591845036076
AACCAGAA 1.8891503684
AACCAGAC -0.239490885377
AACCAGAG 0.116395576182
AACCAGAT 2.89354861144
AACCAGCA 0.287581439306
AACCAGCC -0.968820810154
AACCAGCG -0.253224919845
AACCAGCT -0.384595308921
AACCAGGA 0.0144883817478
AACCAGGC -0.463123488073
AACCAGGG -0.724295715279
AACCAGGT -0.935712649186
AACCAGTA 0.342362577892
AACCAGTC -1.44441638236
AACCAGTG -1.11663706773
AACCAGTT 0.187360417534
AACCATAA 0.695723570491
AACCATAC 0.701360525944
AACCATAG -0.707444443923
AACCATAT 3.22773438893
AACCATCA 0.34078461721
AACCATCC -0.320645165403
AACCATCG 0.196039593358
AACCATCT 0.0106952120577
AACCATGA 1.42669340339
AACCATGC -0.485622954291
AACCATGG -0.507569075675
AACCATGT -0.21981721557
AACCATTA 0.255352566019
AACCATTC 0.0178487035185
AACCATTG -0.286267212652
AACCCAAA 0.744548083935
AACCCAAC 0.943298465803
AACCCAAG -0.383216521013
AACCCAAT 0.976041476685
AACCCACA -0.267347628743
AACCCACC -0.50655151713
AACCCACG -0.0381452456636
AACCCACT 0.458773575588
AACCCAGA 1.03368761892
AACCCAGC 0.546346342032
AACCCAGG -0.163384929455
AACCCAGT -0.468867871361
AACCCATA 0.750258748998
AACCCATC -0.568394140856
AACCCATG 0.239728742627
AACCCATT 0.918134214235

AACCCCAA -0.134293942203
AACCCCAC -0.459025024681
AACCCCAG -0.786529888633
AACCCCAT 0.128175521854
AACCCCCA -1.63162389166
AACCCCCC -1.50823818751
AACCCCCG -0.00495605640124
AACCCCCT -1.70490849466
AACCCCGA 0.158235189293
AACCCCGC -0.739956654857
AACCCCGG -0.851459428983
AACCCCGT -0.625207259954
AACCCCTA -0.0781110141284
AACCCCTC -0.975583274751
AACCCCTG -0.202727041437
AACCCCTT 0.401589294627
AACCCGAA -0.29391889743
AACCCGAC -0.647378953653
AACCCGAG 0.278613959583
AACCCGAT 1.08969646685
AACCCGCA 0.438378753963
AACCCGCC 0.308920109865
AACCCGCG 0.995121548892
AACCCGCT -0.652318020251
AACCCGGA 1.05651172108
AACCCGGC -1.44595304475
AACCCGGG -0.538740399033
AACCCGGT -0.904019608167
AACCCGTA 0.465432794455
AACCCGTC -1.24876886139
AACCCGTG -0.236289483615
AACCCGTT 0.155705799609
AACCCTAA 0.319570887052
AACCCTAC -0.657596360157
AACCCTAG 0.16126852269
AACCCTAT 0.449276013993
AACCCTCA -0.435351955106
AACCCTCC -0.636767906469
AACCCTCG -0.634904778468
AACCCTCT -0.455961371019
AACCCTGA 0.0890741423199
AACCCTGC -0.87880464857
AACCCTGG -0.750792490212
AACCCTGT -0.140917613173
AACCCTTA -0.455381103881
AACCCTTC -0.810485511711
AACCCTTG -0.941122464034
AACCGAAA 0.825792279229
AACCGAAC 0.857961557512
AACCGAAG -0.771943488788
AACCGAAT 3.01818790171
AACCGACA 0.414957133482
AACCGACC -0.696740606273
AACCGACG -0.362160665338

AACCGACT -0.0606865329368
AACCGAGA -0.53307730542
AACCGAGC 0.135211391597
AACCGAGG -0.258390342903
AACCGAGT -0.0847702329953
AACCGATA 0.874558243196
AACCGATC 0.799244796267
AACCGATG -0.308546072805
AACCGATT 3.00489795457
AACCGCAA 0.864358349259
AACCGCAC -0.0703231495393
AACCGCAG 0.334164344201
AACCGCAT -0.285354729507
AACCGCCA -0.526024184491
AACCGCCC -0.39360983733
AACCGCCG -0.658598235807
AACCGCCT -1.14361818276
AACCGCGA 0.410071127348
AACCGCGC 0.478563560204
AACCGCGG 0.80275306002
AACCGCGT 0.40271637206
AACCGCTA -0.829573425366
AACCGCTC -0.708854859003
AACCGCTG -0.663061849285
AACCGCTT 1.02648263529
AACCGGAA -0.194874354213
AACCGGAC -0.497255480744
AACCGGAG -0.284370105043
AACCGGAT 3.9134509651
AACCGGCA -0.130654987653
AACCGGCC -0.146317495521
AACCGGCG 0.0364807676739
AACCGGCT -0.469639731208
AACCGGGA -0.87815694498
AACCGGGC -1.571632111
AACCGGGG -0.312651593499
AACCGGGT -0.421846368152
AACCGGTA -0.212637063187
AACCGGTC -0.591180342683
AACCGGTG -0.315170266538
AACCGGTT 0.994707781829
AACCGTAA 0.55742029602
AACCGTAC -0.055002267415
AACCGTAG -0.622075124314
AACCGTAT 2.46738631041
AACCGTCA -0.0477476212773
AACCGTCC -0.175624906733
AACCGTCG 0.931610526452
AACCGTCT -0.757614027049
AACCGTGA -0.0384523690364
AACCGTGC -0.635936713
AACCGTGG -0.15109947178
AACCGTGT -0.00263420370295
AACCGTTA 1.08370821454

AACCGTTC -0.821066500012
AACCGTTG -1.20068458062
AACCTAAA 0.512998755524
AACCTAAC 0.224193266215
AACCTAAG -0.527323773776
AACCTAAT 0.735977904235
AACCTACA -0.402733100482
AACCTACC -0.643510506064
AACCTACG -0.0236806496408
AACCTACT 0.446984220178
AACCTAGA -0.261260835567
AACCTAGC -0.251575340606
AACCTAGG 0.104731422555
AACCTAGT -0.78200145252
AACCTATA 0.421351834176
AACCTATC 0.390960734876
AACCTATG 0.0257617698911
AACCTATT 0.685292353843
AACCTCAA 0.248080668696
AACCTCAC -0.182038949664
AACCTCAG -0.430518748054
AACCTCAT -0.517454527555
AACCTCCA -1.15588795754
AACCTCCC -1.75082513787
AACCTCCG -0.85010756338
AACCTCCT -0.959782234638
AACCTCGA -0.0348424278435
AACCTCGC -0.642073691443
AACCTCGG -0.487772033756
AACCTCGT -0.995126776524
AACCTCTA -0.243544652516
AACCTCTC -0.892530318827
AACCTCTG -1.4041764246
AACCTCTT -0.535604342669
AACCTGAA 0.0745329614901
AACCTGAC -0.402028938468
AACCTGAG 0.0214847828708
AACCTGAT 2.81225893574
AACCTGCA -0.150251027127
AACCTGCC -0.970268341421
AACCTGCG -0.65076645911
AACCTGCT -0.23176888895
AACCTGGA -0.393579517065
AACCTGGC -0.771272783618
AACCTGGG -0.513684882208
AACCTGGT -0.58553685269
AACCTGTA 0.689026974056
AACCTGTC -0.757726421134
AACCTGTG -0.115052597552
AACCTTAA 0.0771804956543
AACCTTAC 0.102137733
AACCTTAG 0.716077877968
AACCTTAT 0.606250559859
AACCTTCA 0.114698164111

AACCTTCC -0.303503237714
AACCTTCG -0.685059201461
AACCTTCT -0.752851654409
AACCTTGA 0.608246208328
AACCTTGC -0.321501451504
AACCTTGG -0.978957972509
AACCTTGT 0.507078201042
AACCTTTA 0.693263185548
AACCTTTC -0.67224784402
AACCTTTG 1.5041656009
AACGAAAA -0.0286108292643
AACGAAAC 0.782381501357
AACGAAAG 1.09124698247
AACGAAAT 6.9612973135
AACGAACA 0.28311834859
AACGAACC 0.0192625165597
AACGAACG -0.401329219941
AACGAACT 0.147729217509
AACGAAGA 0.848165498137
AACGAAGC -0.577494925192
AACGAAGG -0.837205245143
AACGAAGT 0.136532675554
AACGAATA 1.56477764008
AACGAATC 5.89491713034
AACGAATG 0.376895529929
AACGAATT 3.05399456625
AACGACAA 1.30328156108
AACGACAC 0.266027913076
AACGACAG 0.71025481882
AACGACAT 1.02988373259
AACGACCA -0.510221053326
AACGACCC -0.897704628859
AACGACCG 0.00420928918757
AACGACCT -0.316454172927
AACGACGA -0.533721611049
AACGACGC -1.15992525764
AACGACGG 0.135789306301
AACGACGT 1.13204106921
AACGACTA 0.585726092964
AACGACTC 0.0486974819894
AACGACTG -0.156479227745
AACGACTT 0.616694845586
AACGAGAA 1.72135619283
AACGAGAC -0.222424758351
AACGAGAG 0.158849958802
AACGAGAT 5.49822978612
AACGAGCA -1.30397422231
AACGAGCC -0.87633825185
AACGAGCG -1.24503554809
AACGAGCT -1.16561814875
AACGAGGA 0.869123074333
AACGAGGC -0.892295075392
AACGAGGG -1.10782955355
AACGAGGT -0.903076543376

AACGAGTA -0.368330055098
AACGAGTC -0.594206618777
AACGAGTG 0.723737404195
AACGAGTT 0.28110936966
AACGATAA 1.40607666879
AACGATAC 1.32209999031
AACGATAG -0.188559113523
AACGATAT 4.57661893531
AACGATCA 1.27613603703
AACGATCC 2.00351657788
AACGATCG 1.23902664663
AACGATCT 2.5956933072
AACGATGA 0.835989297969
AACGATGC -1.13471291186
AACGATGG 0.428833359537
AACGATGT 0.228145878677
AACGATTA 0.279554410558
AACGATTC 2.64307211905
AACGATTG 0.9876972661
AACGCAAA 1.92839080294
AACGCAAC 1.28790029981
AACGCAAG -0.0550331104431
AACGCAAT 3.34609006568
AACGCACA 0.481796589138
AACGCACC -0.443520391296
AACGCACG -0.731751886626
AACGCACT 0.156555551171
AACGCAGA 0.347217218235
AACGCAGC 0.188000279676
AACGCAGG 0.353610612021
AACGCAGT -0.846970984253
AACGCATA 0.144908910111
AACGCATC 0.384429854372
AACGCATG -0.466901759012
AACGCATT 1.34999594092
AACGCCAA -0.182344504748
AACGCCAC -0.191222591965
AACGCCAG -0.822683929315
AACGCCAT 1.97007855334
AACGCCCA -1.10505028306
AACGCCCC -1.54907749327
AACGCCCG -0.706914884814
AACGCCCT -0.108669920412
AACGCCGA -0.0057966596071
AACGCCGC -0.7655202975
AACGCCGG -0.552986218661
AACGCCGT -0.88471161121
AACGCCTA -0.25584474756
AACGCCTC 0.0989899144646
AACGCCTG -0.957878069727
AACGCCTT -0.367211603258
AACGCGAA 0.11281203453
AACGCGAC -0.198188411441
AACGCGAG 0.0901865823833

AACGCGAT	3.83063926438
AACGCGCA	0.198211935785
AACGCGCC	-0.456838567648
AACGCGCG	1.71619286083
AACGCGCT	0.310037516179
AACGCGGA	0.451325507071
AACGCGGC	-1.34482450608
AACGCGGG	-0.800874771886
AACGCGGT	-0.556307333193
AACGCGTA	0.72816285596
AACGCGTC	-0.297316335387
AACGCGTG	-0.287273531788
AACGCGTT	1.01451658592
AACGCTAA	-0.0441316683078
AACGCTAC	1.42317102503
AACGCTAG	-1.10064443491
AACGCTAT	-0.03419080353
AACGCTCA	0.977967336268
AACGCTCC	-0.550320126404
AACGCTCG	-1.04899177262
AACGCTCT	-0.339294219362
AACGCTGA	0.164593557945
AACGCTGC	-0.131081301033
AACGCTGG	-0.400372824689
AACGCTGT	-0.359101716545
AACGCTTA	-0.0558371202258
AACGCTTC	-0.927794616563
AACGCTTG	1.1862171094
AACGGAAA	0.0567548310019
AACGGAAC	-0.658347570858
AACGGAAG	-0.685878371376
AACGGAAT	4.90939351358
AACGGACA	0.238325646232
AACGGACC	-0.668324506296
AACGGACG	-1.36656047667
AACGGACT	-0.761258209231
AACGGAGA	1.3919552668
AACGGAGC	-0.114823365894
AACGGAGG	-0.2910834299
AACGGAGT	-0.451390591088
AACGGATA	2.41722665978
AACGGATC	2.89199443648
AACGGATG	-0.122085592098
AACGGATT	5.45933881877
AACGGCAA	0.588756812545
AACGGCAC	-0.429070432643
AACGGCAG	-0.928365735346
AACGGCAT	-0.0179783487891
AACGGCCA	-0.334891507795
AACGGCCC	-0.973882203338
AACGGCCG	-0.308702117616
AACGGCCT	-1.12496076459
AACGGCGA	0.285174898971
AACGGCGC	0.0764211821241

AACGGCGG -0.619153139477
AACGGCGT -0.635453679815
AACGGCTA -0.332289715409
AACGGCTC -0.599311924068
AACGGCTG -0.722868049014
AACGGCTT -0.277052727323
AACGGGAA 0.733789878913
AACGGGAC -0.64891169532
AACGGGAG -0.817738066796
AACGGGAT 1.28493152767
AACGGGCA -0.882971593939
AACGGGCC -1.50968049115
AACGGGCG -0.261491896896
AACGGGCT -0.39360983733
AACGGGGA 1.13159306115
AACGGGGC -1.03641095375
AACGGGGG -0.107070787821
AACGGGGT -1.13504329819
AACGGGTA -0.272588591082
AACGGGTC -1.55787141561
AACGGGTG -0.701118486588
AACGGTAA 0.562434379135
AACGGTAC 1.5263074971
AACGGTAG -0.438590473054
AACGGTAT 0.807783871557
AACGGTCA -0.591670694553
AACGGTCC -1.21372151038
AACGGTCG -1.03158114466
AACGGTCT -0.20138118761
AACGGTGA 1.76519642066
AACGGTGC -1.25788898797
AACGGTGG -0.164118366208
AACGGTGT 0.29493724012
AACGGTTA -1.33567092267
AACGGTTC 0.040385285923
AACGGTTG 0.913024204075
AACGTAAA 2.52765933766
AACGTAAC 0.44488872394
AACGTAAG 0.21514005333
AACGTAAT 5.5258222727
AACGTACA -0.0721841864876
AACGTACC -0.822233046065
AACGTACG -0.242096337105
AACGTACT -0.262824681643
AACGTAGA 2.11756965222
AACGTAGC -0.42388619011
AACGTAGG 0.385942469655
AACGTAGT 1.26392010686
AACGTATA 1.44042377851
AACGTATC 4.29758545827
AACGTATG 0.502941314556
AACGTATT 1.43992244862
AACGTCAA 0.370483316668
AACGTCAC -0.323279630487

AACGTCAG -0.490589988719
AACGTCAT 0.56018205394
AACGTCCA -0.700110337781
AACGTCCC 0.421416918193
AACGTCCG -0.731602899117
AACGTCCT -0.266712210088
AACGTCGA 0.58205864782
AACGTCGC 0.422658742145
AACGTCGG -1.00238638891
AACGTCGT 0.684598647093
AACGTCTA -0.436907698353
AACGTCTC -0.633008977767
AACGTCTG -0.8529119265
AACGTCTT 0.010998153325
AACGTGAA 0.811773338824
AACGTGAC -0.582462221001
AACGTGAG -0.554282671367
AACGTGAT 3.24712289142
AACGTGCA 0.773826220408
AACGTGCC -1.11545849813
AACGTGCG -0.442924179881
AACGTGCT 0.460401198773
AACGTGGA 0.630926027846
AACGTGGC -0.432142189134
AACGTGGG -0.95602879495
AACGTGGT -0.194178817791
AACGTGTA -0.862689950545
AACGTGTC -0.195747630118
AACGTGTG 0.181704642606
AACGTTAA 0.796770035336
AACGTTAC 0.849531478347
AACGTTAG 0.221757973905
AACGTTAT 1.67317310982
AACGTTCA -0.688427103298
AACGTTCC 0.526954180202
AACGTTCG 0.471640084546
AACGTTCT -0.324037375728
AACGTTGA 0.10028793546
AACGTTGC -0.592120793658
AACGTTGG -0.0338815891045
AACGTTGT 0.17249878287
AACGTTTA 0.348316589219
AACGTTTC 0.830858377281
AACGTTTG -0.502511864597
AACTAAAA 1.83340211791
AACTAAAC 0.524060163195
AACTAAAG -0.577817470078
AACTAAAT 2.13480306347
AACTAACA 1.00457781219
AACTAACC -0.0905621877337
AACTAACG 0.722430496225
AACTAACT 0.783144735611
AACTAAGA 0.910166257728
AACTAAGC 0.814803535641

AACTAAGG 1.14595179763
AACTAAGT 0.615879857777
AACTAATA 0.382422182349
AACTAATC 0.133227766682
AACTAATG -0.00175230220526
AACTAATT 1.25156120096
AACTACAA 0.997496984817
AACTACAC 0.563475462023
AACTACAG 0.643888986612
AACTACAT 0.649189282572
AACTACCA -0.150476338061
AACTACCC -0.275305391368
AACTACCG -0.474453857404
AACTACCT -0.465998946987
AACTACGA -0.0122501711596
AACTACGC -0.42619523511
AACTACGG -0.701899494791
AACTACGT 0.760299984308
AACTACTA 0.404759853361
AACTACTC -0.142442774773
AACTACTG 2.0557581329
AACTACTT -0.132331750578
AACTAGAA 2.38477143003
AACTAGAC 0.692848372958
AACTAGAG -0.0583343599736
AACTAGAT 3.17777103588
AACTAGCA 1.00051254427
AACTAGCC 0.108311304865
AACTAGCG -1.02320464872
AACTAGCT -0.977459471831
AACTAGGA -0.218409937069
AACTAGGC -0.0285269257726
AACTAGGG 0.331335933973
AACTAGGT 0.604570660355
AACTAGTA -0.517185043132
AACTAGTC -0.356861153522
AACTAGTG 0.0119077612716
AACTAGTT -0.201586894925
AACTATAA -0.0850878116318
AACTATAC -0.160045518212
AACTATAG -0.532222587608
AACTATAT 0.749430169345
AACTATCA 1.15208041186
AACTATCC 0.192700443496
AACTATCG -0.131454553949
AACTATCT 1.60009630519
AACTATGA -0.140215019449
AACTATGC 0.392140350009
AACTATGG 0.0060907139002
AACTATGT 0.995739193598
AACTATTA 0.903020084952
AACTATTC 0.856976671666
AACTATTG 0.0730284490358
AACTCAAA 0.380757181596

AACTCAAC 0.390893559806
AACTCAAG -0.152224196779
AACTCAAT 1.72104122801
AACTCACA 0.228910681221
AACTCACC -0.649679373061
AACTCACG -0.476340248367
AACTCACT 0.502216503396
AACTCAGA 1.05563844517
AACTCAGC 0.271017164939
AACTCAGG 1.24312511002
AACTCAGT -0.829643475633
AACTCATA 1.36313533226
AACTCATC -0.65762302108
AACTCATG 0.263019149549
AACTCATT 0.718181999799
AACTCCAA 0.238100335298
AACTCCAC -0.810288429989
AACTCCAG 0.616618783543
AACTCCAT 0.472331700243
AACTCCCA -0.279237354685
AACTCCCC -0.83293923615
AACTCCCG -0.137036619267
AACTCCCT -0.084050910849
AACTCCGA -0.0546982806214
AACTCCGC -0.285971851451
AACTCCGG -0.524370684528
AACTCCGT 0.90513361652
AACTCCTA 0.104024646726
AACTCCTC 0.026975626013
AACTCCTG -0.35368092367
AACTCCTT -1.09167800072
AACTCGAA -0.449546543943
AACTCGAC -0.746926133676
AACTCGAG -0.270201131603
AACTCGAT 1.39841714256
AACTCGCA 0.772281193807
AACTCGCC -0.0713213658463
AACTCGCG -0.583053466167
AACTCGCT -0.626070080595
AACTCGGA 0.186795310528
AACTCGGC -0.0959045661307
AACTCGGG -0.416626054959
AACTCGGT 0.220880777276
AACTCGTA 0.561143938206
AACTCGTC -0.124050658921
AACTCGTG -0.467613762474
AACTCTAA 0.582088445322
AACTCTAC -0.103991712645
AACTCTAG -0.548010558641
AACTCTAT 0.0599797571069
AACTCTCA 0.547621622829
AACTCTCC 0.749255043677
AACTCTCG -0.758203965306
AACTCTCT -0.0230643118425

AACTCTGA 0.641435136209
AACTCTGC -0.460030559673
AACTCTGG -0.282426471511
AACTCTGT -0.0232336871153
AACTCTTA 0.590746710619
AACTCTTC -0.388968745749
AACTCTTG -0.514096558218
AACTGAAA 0.82102677001
AACTGAAC -0.198184490717
AACTGAAG 1.05826611434
AACTGAAT 2.80128587505
AACTGACA -0.451910740459
AACTGACC 0.0662853266773
AACTGACG -0.993149424766
AACTGACT 0.00483974159197
AACTGAGA 0.384797618274
AACTGAGC -0.326056809922
AACTGAGG -0.456838567648
AACTGAGT -0.758423787227
AACTGATA 2.94672329875
AACTGATC 1.40928695753
AACTGATG 0.834998661728
AACTGATT 2.56688827141
AACTGCAA 1.7706532842
AACTGCAC 0.794284296378
AACTGCAG 0.810945020553
AACTGCAT -0.0468994380053
AACTGCCA -0.558432626933
AACTGCCC -0.781928265673
AACTGCCG -0.953160916103
AACTGCCT -0.0295690541874
AACTGCGA 0.289413201515
AACTGCGC -0.44754697475
AACTGCGG -0.484318137374
AACTGCGT 0.185202451095
AACTGCTA -0.581225363299
AACTGCTC -0.495433651035
AACTGCTG -0.26595158965
AACTGCTT -0.146431719277
AACTGGAA 0.0508172867157
AACTGGAC 0.593297533593
AACTGGAG 0.695962473268
AACTGGAT 0.610022819021
AACTGGCA -0.145850406612
AACTGGCC 0.304082982089
AACTGGCG -0.174585130752
AACTGGCT -0.600753966321
AACTGGGA -0.39232906752
AACTGGGC -1.02871065199
AACTGGGG -0.843490949713
AACTGGGT -0.217185625683
AACTGGTA 0.902569463084
AACTGGTC -0.0970190972469
AACTGGTG -0.0974725943123

AACTGTAA 0.372505887441
AACTGTAC -0.263838058082
AACTGTAG 0.827069389697
AACTGTAT 0.86017023198
AACTGTCA 0.915003385504
AACTGTCC -0.261981203239
AACTGTCG -0.421577929255
AACTGTCT -0.349801759435
AACTGTGA 2.46352152216
AACTGTGC -0.482054050008
AACTGTGG -0.351149442933
AACTGTGT 0.191604731855
AACTGTTA 1.57724920145
AACTGTTC -0.1580888156
AACTGTTG -0.478423982433
AACTTAAA 1.33428769127
AACTTAAC 0.803946528377
AACTTAAG 1.59882834308
AACTTAAT 0.350193309063
AACTTACA 1.23626201351
AACTTACC 0.390560036893
AACTTACG 0.00504623305112
AACTTACT -0.524370684528
AACTTAGA 1.68959284017
AACTTAGC 0.478644588498
AACTTAGG -0.463879142261
AACTTAGT -0.383523383004
AACTTATA 1.40504342735
AACTTATC 0.751624729207
AACTTATG 0.369977543284
AACTTATT 0.439151397955
AACTTCAA 0.856572052959
AACTTCAC -0.641383382653
AACTTCAG 0.231994461265
AACTTCAT 0.900693788766
AACTTCCA -0.396227312611
AACTTCCC -0.593186185034
AACTTCCG 0.1294884416
AACTTCCT -0.549557937676
AACTTCGA 0.334073906169
AACTTCGC -0.132778713103
AACTTCGG -0.658347570858
AACTTCGT 1.10836120371
AACTTCTA -0.0515739864299
AACTTCTC 1.60235385802
AACTTCTG 0.726103953146
AACTTCTT 0.287010059141
AACTTGAA 0.925269670366
AACTTGAC 0.212812711618
AACTTGAG -0.333143387695
AACTTGAT 1.09502420788
AACTTGCA 0.0301493213257
AACTTGCC 0.695185647171
AACTTGCG 0.181347072585

AACTTGCT 0.0861586920219
AACTTGGA 1.39761679212
AACTTGGC -0.313262965047
AACTTGGG -0.694262970144
AACTTGGT -0.621205769133
AACTTGTA 0.687521416075
AACTTGTC -0.691957061723
AACTTGTG -0.0907072545183
AACTTTAA 0.367390911031
AACTTTAC 0.374608702364
AACTTTAG -0.0443841629275
AACTTTAT 0.702717096416
AACTTTCA 0.634056333814
AACTTTCC 0.884934046946
AACTTTCG 1.17169370252
AACTTTCT 0.0583670326728
AACTTTGA -0.00634765200696
AACTTTGC 0.188327790813
AACTTTGG 0.982393310797
AACTTTGT -0.679047424802
AACTTTTA -0.0986519480638
AACTTTTC -0.0393347932973
AACTTTTG -0.19843907639
AAGAAAAA 1.9150057129
AAGAAAAC 0.0869974655566
AAGAAAAG 0.196139179745
AAGAAAAT 5.2444706023
AAGAAACA 1.92754261967
AAGAAACC 0.982060049265
AAGAAACG 0.581369645939
AAGAAACT 0.110354263403
AAGAAAGA 1.48976791857
AAGAAAGC -0.757083422413
AAGAAAGG -0.400138626781
AAGAAAGT 1.15899264811
AAGAAATA 1.08307175036
AAGAAATC 12.742660347
AAGAAATG 0.340010666311
AAGAAATT 1.95767965606
AAGAACAA 0.197064993351
AAGAACAC 0.354609873854
AAGAACAG -1.02240272999
AAGAACAT 0.972699190244
AAGAACCA -0.198876890559
AAGAACCC -0.868451323637
AAGAACCG -0.0669429627675
AAGAACCT 0.141194416281
AAGAACGA 0.194555991431
AAGAACGC -1.25144514753
AAGAACGG -0.877275043483
AAGAACGT 0.658486103103
AAGAACTA -0.147117061816
AAGAACTC -0.679845161427
AAGAACTG -0.244889460817

AAGAACTT -0.131733709491
AAGAAGAA -1.01684000691
AAGAAGAC 0.405953060337
AAGAAGAG 0.265124316906
AAGAAGAT 4.64891447036
AAGAAGCA 0.0587962212499
AAGAAGCC -1.04953230976
AAGAAGCG -0.0237365853019
AAGAAGCT -0.924270408533
AAGAAGGA -0.66020913057
AAGAAGGC -1.325347918
AAGAAGGG -0.757083422413
AAGAAGGT -0.647900148551
AAGAAGTA 0.012241545567
AAGAAGTC -0.606200897356
AAGAAGTG 0.719148850314
AAGAATAA 2.1647534734
AAGAATAC 1.77312516993
AAGAATAG -0.0541749946704
AAGAATAT 3.78693286393
AAGAATCA 4.48094935123
AAGAATCC 11.8435236083
AAGAATCG 7.37999795417
AAGAATCT 15.7675168773
AAGAATGA 0.535200769489
AAGAATGC 0.0495349486162
AAGAATGG 0.201990990869
AAGAATGT 1.01004852895
AAGAATTA 2.34121584676
AAGAATTC 2.55876636115
AAGAATTG 0.493722647122
AAGACAAA -0.264346706664
AAGACAAC -0.404940729424
AAGACAAG -0.269280022866
AAGACAAT 2.53073370797
AAGACACA 0.324098539021
AAGACACC -0.647205919038
AAGACACG 0.1223482806
AAGACACT -1.18196286258
AAGACAGA 0.392374547917
AAGACAGC -0.158068427836
AAGACAGG -0.892886320558
AAGACAGT -0.566029160195
AAGACATA -0.0659721915279
AAGACATC 0.529250940267
AAGACATG 0.747987342947
AAGACATT 0.883735350957
AAGACCAA -0.83328190742
AAGACCAC -1.05803818959
AAGACCAG -0.919392243847
AAGACCAT -0.190750275425
AAGACCCA -1.01317047071
AAGACCCC -0.71228629002
AAGACCCG -1.36659131969

AAGACCCT −1.27637128047
AAGACCGA −0.867049795531
AAGACCGC −0.275934014101
AAGACCGG 0.250949331689
AAGACCGT 0.39567945679
AAGACCTA −0.843998552768
AAGACCTC −0.841034485494
AAGACCTG −0.312794830613
AAGACCTT −0.449650051054
AAGACGAA 3.12958821425
AAGACGAC −0.79894028671
AAGACGAG −0.53642926298
AAGACGAT 1.88810614893
AAGACGCA −0.359084726741
AAGACGCC −0.252748421199
AAGACGCG −0.0321549022954
AAGACGCT −0.741374388622
AAGACGGA −0.36377469668
AAGACGGC −0.72422043738
AAGACGGG −0.985184866219
AAGACGGT −0.45439203593
AAGACGTA −0.440313761903
AAGACGTC 0.297443889605
AAGACGTG −0.565550309115
AAGACTAA 0.209275173126
AAGACTAC 0.185226498202
AAGACTAG −0.742531524938
AAGACTAT 0.372505887441
AAGACTCA −0.697947405091
AAGACTCC −0.111854070988
AAGACTCG 0.263253086075
AAGACTCT −0.421528266752
AAGACTGA 1.44984606221
AAGACTGC 0.218419346806
AAGACTGG −1.17443768649
AAGACTGT 0.710547043442
AAGACTTA 0.853031900652
AAGACTTC 0.674787950349
AAGACTTG −0.381889486661
AAGAGAAA 0.227199677307
AAGAGAAC 0.440475034346
AAGAGAAG 0.670855202888
AAGAGAAT 5.31208897511
AAGAGACA −0.538009837478
AAGAGACC −0.929213134473
AAGAGACG −0.703443737247
AAGAGACT 0.302173328165
AAGAGAGA −0.903872188948
AAGAGAGC −1.10128560396
AAGAGAGG −0.97960593748
AAGAGAGT −0.927614786026
AAGAGATA 4.13444361369
AAGAGATC 6.55158035821
AAGAGATG −0.348423755672

AAGAGATT 6.61820443543
AAGAGCAA -0.207316902225
AAGAGCAC 0.152460224358
AAGAGCAG -0.50605463072
AAGAGCAT -0.560366327964
AAGAGCCA -0.0728078429706
AAGAGCCC -1.70014716755
AAGAGCCG -0.896762609595
AAGAGCCT -1.45769587424
AAGAGCGA 0.933840634211
AAGAGCGC -0.369808952156
AAGAGCGG -0.642324356391
AAGAGCGT -0.198266041775
AAGAGCTA 0.373243506299
AAGAGCTC -1.0626191634
AAGAGCTG -0.280813224314
AAGAGCTT -1.03929425411
AAGAGGAA -0.000345807848681
AAGAGGAC -0.740779484115
AAGAGGAG -0.645683371254
AAGAGGAT 2.1786234263
AAGAGGCA -0.555457843013
AAGAGGCC -0.542248140022
AAGAGGCG -1.44342391645
AAGAGGCT -0.763177795656
AAGAGGGA 0.0176309726508
AAGAGGGC -0.822976153937
AAGAGGGG -1.28808196002
AAGAGGGT -0.43090663834
AAGAGGTA 0.299340213069
AAGAGGTC -0.253658551909
AAGAGGTG -0.10111285577
AAGAGTAA 1.42138840256
AAGAGTAC -0.236036988996
AAGAGTAG -0.107068696768
AAGAGTAT 1.45793477701
AAGAGTCA -0.029576372872
AAGAGTCC -1.29836758712
AAGAGTCG -0.364590730016
AAGAGTCT 0.131343728153
AAGAGTGA -0.171928970995
AAGAGTGC 0.145946856421
AAGAGTGG -0.903805013878
AAGAGTGT -0.293195915942
AAGAGTTA 0.607319087815
AAGAGTTC 0.0775148027128
AAGAGTTG -0.67795641803
AAGATAAA 1.17557940129
AAGATAAC 2.11419024959
AAGATAAG 0.829471225163
AAGATAAT 2.50189521534
AAGATACA 1.01610055838
AAGATACC 2.38115103358
AAGATACG 4.01943675792

AAGATACT 3.67825510207
AAGATAGA 0.756260070393
AAGATAGC 1.51332990096
AAGATAGG -0.21321314822
AAGATAGT 0.293290013316
AAGATATA 5.27692844586
AAGATATC 19.8613595652
AAGATATG 5.52610142824
AAGATATT 6.32295432154
AAGATCAA 2.36302552695
AAGATCAC 3.51709452998
AAGATCAG 3.98936454417
AAGATCAT 2.75760874934
AAGATCCA 2.69270404024
AAGATCCC 7.62721135873
AAGATCCG 5.66819891793
AAGATCCT 5.66236488075
AAGATCGA 2.81166952025
AAGATCGC 8.27236098877
AAGATCGG 1.13528272373
AAGATCGT 3.46732982694
AAGATCTA 5.29220463174
AAGATCTC 9.67573366497
AAGATCTG 7.18364470289
AAGATCTT 5.73661973223
AAGATGAA -0.466141661337
AAGATGAC 0.97078483145
AAGATGAG -0.0690781890077
AAGATGAT 1.78065661918
AAGATGCA 0.2037344061
AAGATGCC -0.0573162786655
AAGATGCG 1.01651641649
AAGATGCT 0.71208658103
AAGATGGA -0.277625414395
AAGATGGC 0.147583627961
AAGATGGG 0.56696307663
AAGATGGT -0.697085368595
AAGATGTA 0.141492129916
AAGATGTC 0.427653221641
AAGATGTG 0.6259791198
AAGATTAA 1.25980439229
AAGATTAC 14.9713898407
AAGATTAG 1.6238697453
AAGATTAT 5.60571460239
AAGATTCA 3.79299561
AAGATTCC 12.5977464706
AAGATTCG 7.88032362311
AAGATTCT 13.6531661394
AAGATTGA 0.678619281752
AAGATTGC 9.17694674947
AAGATTGG 2.67087318814
AAGATTGT 4.49147518802
AAGATTTA 4.44850902024
AAGATTTC 18.5186130428

AAGATTTG 7.79575779052
AAGCAAAA -0.259157497881
AAGCAAAC 0.228496130013
AAGCAAAG -0.416161318485
AAGCAAAT 1.8079247312
AAGCAACA 0.209738341311
AAGCAACC -0.268871744817
AAGCAACG 0.0672584503513
AAGCAACT 0.21586983074
AAGCAAGA 1.48007327525
AAGCAAGC -0.407835530576
AAGCAAGG -0.302866773533
AAGCAAGT 0.218870491437
AAGCAATA 1.53092297328
AAGCAATC 7.56512199078
AAGCAATG -0.111523946035
AAGCAATT 2.5157429508
AAGCACAA -0.390209262794
AAGCACAC 0.226048030005
AAGCACAG -1.20430785228
AAGCACAT 0.095327696953
AAGCACCA -0.660011526085
AAGCACCC -0.786949406091
AAGCACCG -0.0983623372578
AAGCACCT -0.803931106863
AAGCACGA 0.604935287678
AAGCACGC 2.07834385505
AAGCACGG -0.74350595552
AAGCACGT 1.24629723704
AAGCACTA 0.37142821113
AAGCACTC -0.488075759168
AAGCACTG -0.582522861531
AAGCACTT 0.490345074166
AAGCAGAA 2.09365192947
AAGCAGAC 0.707953353886
AAGCAGAG -0.0215937789954
AAGCAGAT 3.6889364609
AAGCAGCA -0.355414667782
AAGCAGCC -0.742774087057
AAGCAGCG 0.279606164114
AAGCAGCT -0.756611890018
AAGCAGGA -0.366423537752
AAGCAGGC -0.802925571872
AAGCAGGG -0.116730406003
AAGCAGGT -0.920365890284
AAGCAGTA 0.532221019319
AAGCAGTC -0.903872188948
AAGCAGTG -0.823053522889
AAGCATAA 0.391701751695
AAGCATAC -0.223067234308
AAGCATAG 0.443630432947
AAGCATAT 2.00054283949
AAGCATCA -0.117411305055
AAGCATCC 0.141667255584

AAGCATCG 0.931524270526
AAGCATCT 0.702622214898
AAGCATGA -1.22632663774
AAGCATGC 0.0867091616586
AAGCATGG -0.523994817796
AAGCATGT -0.43830608988
AAGCATTA 1.58167569875
AAGCATTC -0.715266549501
AAGCATTG 0.301368011474
AAGCCAAA 0.0272312572118
AAGCCAAC -0.436645532615
AAGCCAAG -0.456632076189
AAGCCAAT 0.639563643997
AAGCCACA -1.29285975417
AAGCCACC -1.24822257386
AAGCCACG -0.269702415522
AAGCCACT -0.103908070535
AAGCCAGA -0.103908070535
AAGCCAGC -0.949574499253
AAGCCAGG -1.02762121351
AAGCCAGT -0.497506145693
AAGCCATA 0.397381050966
AAGCCATC -0.30297786071
AAGCCATG -0.646725499668
AAGCCATT -0.518787573684
AAGCCCAA -0.318753285426
AAGCCCAC -1.16971216866
AAGCCCAG -1.38653656498
AAGCCCAT -0.427330676754
AAGCCCCA -1.28743347228
AAGCCCCC -1.64670770068
AAGCCCCG -0.423332583894
AAGCCCCT -1.13236831896
AAGCCCGA -0.78927465675
AAGCCCGC -1.98749440894
AAGCCCGG -1.25366035654
AAGCCCGT -1.23742986647
AAGCCCTA -0.251306901709
AAGCCCTC -0.922328604672
AAGCCCTG -1.38489169061
AAGCCCTT -1.49798732417
AAGCCGAA 0.539302369459
AAGCCGAC -0.884076715318
AAGCCGAG -0.913426993111
AAGCCGAT 0.750753282973
AAGCCGCA -1.05997633411
AAGCCGCC -0.840417624932
AAGCCGCG -0.749917384636
AAGCCGCT -0.642324356391
AAGCCGGA 0.0180400348452
AAGCCGGC -0.655826284004
AAGCCGGG -0.464490252427
AAGCCGGT -1.01049026385
AAGCCGTA -0.375991933759

AAGCCGTC -1.65647526946
AAGCCGTG -1.41952083107
AAGCCTAA -0.575678845877
AAGCCTAC 0.0185463309926
AAGCCTAG -0.843808266968
AAGCCTAT 0.579786980388
AAGCCTCA -0.664572112134
AAGCCTCC -1.34379100326
AAGCCTCG -0.388089980831
AAGCCTCT -0.586281528851
AAGCCTGA -0.735011576483
AAGCCTGC -0.491818743592
AAGCCTGG -1.49977700394
AAGCCTGT -0.376040812117
AAGCCTTA -0.32301929442
AAGCCTTC -1.47343444415
AAGCCTTG -0.627151939012
AAGCGAAA -0.112430678784
AAGCGAAC -1.02237214834
AAGCGAAG -1.03006408589
AAGCGAAT 3.3716341047
AAGCGACA 0.810059721094
AAGCGACC -1.63410832371
AAGCGACG -0.145542760476
AAGCGACT -0.752176767133
AAGCGAGA -0.262101700154
AAGCGAGC -0.843808266968
AAGCGAGG -0.25801107821
AAGCGAGT -0.33611581918
AAGCGATA 1.54829491677
AAGCGATC 3.80747562754
AAGCGATG -0.611962793211
AAGCGATT 3.54095056667
AAGCGCAA 0.24818130061
AAGCGCAC -0.663061849285
AAGCGCAG -0.368461791421
AAGCGCAT -0.42124100838
AAGCGCCA -1.18175637112
AAGCGCCC -0.759832895399
AAGCGCCG -1.10088804256
AAGCGCCT -0.262927404609
AAGCGCGA 2.65828792577
AAGCGCGC 0.364130175647
AAGCGCGG -0.815097589935
AAGCGCGT -0.942480864177
AAGCGCTA -0.11808776062
AAGCGCTC 0.501892912983
AAGCGCTG 0.0425116251891
AAGCGCTT -0.25971005857
AAGCGGAA 0.769189049552
AAGCGGAC -0.804745049146
AAGCGGAG -0.291778966321
AAGCGGAT 2.88907950894
AAGCGGCA -0.820524133205

AAGCGGCC -0.768795931634
AAGCGGCG -1.01670434986
AAGCGGCT -0.799289231138
AAGCGGGA -0.759370772741
AAGCGGGC -0.753457536943
AAGCGGGG 0.135087758104
AAGCGGGT -0.175344967046
AAGCGGTA 0.935262550081
AAGCGGTC -0.433271619001
AAGCGGTG -0.855776930151
AAGCGTAA 1.42619886941
AAGCGTAC -0.185427762029
AAGCGTAG -0.900826047853
AAGCGTAT 1.14389707692
AAGCGTCA -0.704092486363
AAGCGTCC -1.1298783979
AAGCGTCG -0.825714910277
AAGCGTCT -0.764374400593
AAGCGTGA 0.558782616887
AAGCGTGC -0.615915405673
AAGCGTGG -0.441385687819
AAGCGTGT -0.587032216788
AAGCGTTA 0.167969562611
AAGCGTTC -1.02741707448
AAGCGTTG 1.13588703798
AAGCTAAA -0.134710845845
AAGCTAAC -0.927779717812
AAGCTAAG -0.464843640342
AAGCTAAT 0.613230493941
AAGCTACA -0.222154489782
AAGCTACC -0.210012791984
AAGCTACG -0.369504442599
AAGCTACT -0.714258662075
AAGCTAGA -0.106653622797
AAGCTAGC -0.0768665763601
AAGCTAGG -0.119681404198
AAGCTAGT -0.558987540056
AAGCTATA 0.595615465568
AAGCTATC 0.411142530501
AAGCTATG -1.17905656064
AAGCTATT 0.913160122504
AAGCTCAA -0.37216295479
AAGCTCAC -0.155602031116
AAGCTCAG -0.670356748188
AAGCTCAT -0.816032290514
AAGCTCCA -0.728465797228
AAGCTCCC -1.16697419646
AAGCTCCG 0.226869813736
AAGCTCCT -1.1598363879
AAGCTCGA -0.932950629883
AAGCTCGC -0.910681702231
AAGCTCGG -0.614981489239
AAGCTCGT -0.796308958204
AAGCTCTA -0.222143773137

AAGCTCTC -0.572360867925
AAGCTCTG -1.37774159711
AAGCTGAA -0.524784712973
AAGCTGAC -0.564823145521
AAGCTGAG -0.41468451248
AAGCTGAT 0.481540435176
AAGCTGCA -0.91456478719
AAGCTGCC -0.944836435101
AAGCTGCG -0.140471696174
AAGCTGCT -0.711379282437
AAGCTGGA 0.665373508101
AAGCTGGC -1.36819332748
AAGCTGGG -0.668338882283
AAGCTGGT -0.494267889126
AAGCTGTA -0.546965032265
AAGCTGTC -0.23404839783
AAGCTGTG -0.242738813062
AAGCTTAA -0.622075124314
AAGCTTAC 0.451138357849
AAGCTTAG 0.0473403887541
AAGCTTAT 0.677173057393
AAGCTTCA -0.370675170758
AAGCTTCC -0.263720959128
AAGCTTCG -1.20465000078
AAGCTTCT -0.0467954081309
AAGCTTGA -0.503273791943
AAGCTTGC 0.958003794274
AAGCTTGG -0.529264270729
AAGCTTGT -0.120942570388
AAGCTTTA -0.812358833594
AAGCTTTC -0.660643546779
AAGCTTTG 0.185194086884
AAGGAAAA 1.23629573173
AAGGAAAC -0.800422581729
AAGGAAAG -1.29729749087
AAGGAAAT 3.88854783374
AAGGAACA 0.129772040629
AAGGAACC 0.00707141764037
AAGGAACG -0.77577795677
AAGGAACT -0.275062567867
AAGGAAGA -0.0442168787074
AAGGAAGC -0.123857759305
AAGGAAGG -1.70490849466
AAGGAAGT 0.141931773757
AAGGAATA 0.843123447192
AAGGAATC 5.78341775417
AAGGAATG -0.66020913057
AAGGAATT 1.9446597161
AAGGACAA -0.122028088148
AAGGACAC -0.579138231272
AAGGACAG -0.548666103678
AAGGACAT 0.362509348384
AAGGACCA -0.784026114346
AAGGACCC -1.16818387048

AAGGACCG	-0.913435095941
AAGGACCT	-1.06238313582
AAGGACGA	-0.123746410746
AAGGACGC	-0.480045593841
AAGGACGG	-0.466701540711
AAGGACGT	-0.331933190915
AAGGACTA	-0.223632864077
AAGGACTC	-1.10449772237
AAGGACTG	-0.835740985455
AAGGACTT	-0.365307438346
AAGGAGAA	-0.0680224687501
AAGGAGAC	-1.1139976364
AAGGAGAG	-0.425408476513
AAGGAGAT	3.78488493914
AAGGAGCA	-0.838933761624
AAGGAGCC	-0.102089377405
AAGGAGCG	-0.238055377664
AAGGAGCT	-1.21159046624
AAGGAGGA	-0.46946957179
AAGGAGGC	-0.8193426884
AAGGAGGG	-0.58718982989
AAGGAGGT	-1.26565829446
AAGGAGTA	-0.52169779635
AAGGAGTC	-0.672873591555
AAGGAGTG	-0.633120064945
AAGGATAA	1.21965304289
AAGGATAC	2.2201208909
AAGGATAG	-0.100258660721
AAGGATAT	5.64461419533
AAGGATCA	1.86845495794
AAGGATCC	3.77312198327
AAGGATCG	1.23173148634
AAGGATCT	4.95959132591
AAGGATGA	0.574954296099
AAGGATGC	-0.0763114018547
AAGGATGG	-0.498079355528
AAGGATGT	0.76205176375
AAGGATTA	2.4037796223
AAGGATTC	5.06870664056
AAGGATTG	2.82641693116
AAGGCAAA	-0.177790191856
AAGGCAAC	-0.725581451339
AAGGCAAG	-0.330407767934
AAGGCAAT	0.690741637312
AAGGCACA	-1.45478565157
AAGGCACC	-0.386148438351
AAGGCACG	-1.25841697879
AAGGCACT	-0.994805277163
AAGGCAGA	-0.268626568882
AAGGCAGC	-0.669596911895
AAGGCAGG	-0.877530151918
AAGGCAGT	0.920510434305
AAGGCATA	0.0276285572345
AAGGCATC	-0.650399479352

AAGGCATG −0.440954146808
AAGGCATT −0.669543851431
AAGGCCAA −0.982241448091
AAGGCCAC −0.0376081064882
AAGGCCAG −1.7838486112
AAGGCCAT −1.02929039637
AAGGCCCA −0.363406148632
AAGGCCCC −1.9737486123
AAGGCCCG −1.68002496692
AAGGCCCT −1.63582011177
AAGGCCGA −0.617714756566
AAGGCCGC −1.11673822241
AAGGCCGG −1.16177374827
AAGGCCGT −1.49860104815
AAGGCCTA −0.576071441031
AAGGCCTC −1.57317818313
AAGGCCTG −1.22953405128
AAGGCCTT −0.872649112034
AAGGCGAA −0.501803781859
AAGGCGAC −1.02060416324
AAGGCGAG −1.01765630163
AAGGCGAT 1.66831899224
AAGGCGCA 0.427988312844
AAGGCGCC −0.814076110666
AAGGCGCG −0.618831901498
AAGGCGCT −0.183852676544
AAGGCGGA −0.218255721928
AAGGCGGC −1.13641581294
AAGGCGGG −0.537133947757
AAGGCGGT −1.34002658555
AAGGCGTA 0.159864642149
AAGGCGTC −0.768345309767
AAGGCGTG −0.0575499538104
AAGGCTAA −1.19207414816
AAGGCTAC −0.420290102142
AAGGCTAG −0.563188465033
AAGGCTAT −0.45289484216
AAGGCTCA −1.42875727245
AAGGCTCC −1.4489401136
AAGGCTCG −1.19517805458
AAGGCTCT −1.38784948472
AAGGCTGA −1.20107926683
AAGGCTGC −0.337579556106
AAGGCTGG −0.716694999911
AAGGCTGT −1.36824795624
AAGGCTTA −0.708148083174
AAGGCTTC −1.6339750191
AAGGCTTG −1.2403406119
AAGGGAAA 0.192700443496
AAGGGAAC −1.0490035348
AAGGGAAG −1.55502131071
AAGGGAAT 1.65907863014
AAGGGACA 0.292122160354
AAGGGACC −1.24770529969

AAGGGACG	−0.214400866182
AAGGGACT	−0.602577102939
AAGGGAGA	−1.45047128698
AAGGGAGC	−1.25652274637
AAGGGAGG	−1.03151632202
AAGGGAGT	−0.545667272652
AAGGGATA	2.2884763598
AAGGGATC	1.61341500431
AAGGGATG	−1.01559347809
AAGGGATT	3.12348495403
AAGGGCAA	−1.19037856576
AAGGGCAC	−1.39945012262
AAGGGCAG	−1.14875511522
AAGGGCAT	0.122712907924
AAGGGCCA	−1.27296077343
AAGGGCCC	−0.466583918994
AAGGGCCG	−0.870699205344
AAGGGCCT	−1.44288233379
AAGGGCGA	−0.714936947311
AAGGGCGC	−0.728000537991
AAGGGCGG	−0.223719381385
AAGGGCGT	−1.17573675301
AAGGGCTA	−0.805398764513
AAGGGCTC	−1.80084076724
AAGGGCTG	−1.34735049781
AAGGGGAA	−0.0534376371942
AAGGGGAC	−1.03603456425
AAGGGGAG	−0.589189137701
AAGGGGAT	1.16652932499
AAGGGGCA	−0.914927846223
AAGGGGCC	−1.5098459457
AAGGGGCG	−0.947813048692
AAGGGGCT	−1.8267421149
AAGGGGGA	0.241294418375
AAGGGGGC	−1.52525256099
AAGGGGGG	−1.49190654277
AAGGGGGT	−0.86804513664
AAGGGGTA	−0.529407507842
AAGGGGTC	−1.41317997499
AAGGGGTG	−0.499085413283
AAGGGTAA	0.113057471846
AAGGGTAC	−0.984193184452
AAGGGTAG	−0.449661551844
AAGGGTAT	0.897520093454
AAGGGTCA	−1.11786922057
AAGGGTCC	−1.49709130806
AAGGGTCG	−1.40325819106
AAGGGTCT	−0.504998387699
AAGGGTGA	−1.21089728226
AAGGGTGC	−0.822875783405
AAGGGTGG	−0.23396972197
AAGGGTGT	−0.85878281848
AAGGGTTA	0.0537722056343
AAGGGTTC	−1.0211258809

AAGGGTTG -0.614107167807
AAGGTAAA 1.39030856276
AAGGTAAC -0.811366106301
AAGGTAAG -0.152201195199
AAGGTAAT 2.57476814232
AAGGTACA -0.564210467065
AAGGTACC -0.0689490665004
AAGGTACG -0.833988683249
AAGGTACT -0.544293450994
AAGGTAGA 0.298478699335
AAGGTAGC -1.20093707524
AAGGTAGG 0.339057407638
AAGGTAGT -0.182321764549
AAGGTATA 0.223305091559
AAGGTATC 3.03690805146
AAGGTATG 0.116472160989
AAGGTATT 0.56773389095
AAGGTCAA -0.862380474738
AAGGTCAC -0.938117359849
AAGGTCAG -0.279461097329
AAGGTCAT -0.805878138356
AAGGTCCA -1.55782698074
AAGGTCCC -1.15522666211
AAGGTCCG -1.11585161604
AAGGTCCT -1.18610001045
AAGGTCGA 0.362168245404
AAGGTCGC -0.480489419788
AAGGTCGG -0.460951929791
AAGGTCGT -0.0151993396831
AAGGTCTA -0.499338692047
AAGGTCTC -1.24346647438
AAGGTCTG -0.542120063041
AAGGTGAA 0.00564113755875
AAGGTGAC -1.20609099751
AAGGTGAG -0.149183021934
AAGGTGAT 1.48730335152
AAGGTGCA -0.395039071885
AAGGTGCC -0.654685353346
AAGGTGCG -1.07083281861
AAGGTGCT 0.121455401076
AAGGTGGA -0.0949369314702
AAGGTGGC -1.45289899923
AAGGTGGG -1.20609517962
AAGGTGGT -0.964003024616
AAGGTGTA -0.11146408965
AAGGTGTC -0.555309378268
AAGGTGTG 0.315233782265
AAGGTTAA 0.87596761275
AAGGTTAC -0.029985696448
AAGGTTAG -0.365709443238
AAGGTTAT 0.0779706522125
AAGGTTCA -0.321501451504
AAGGTTCC -0.97851859005
AAGGTTCG 0.146301812625

AAGGTTCT -0.601857258029
AAGGTTGA 1.17723734474
AAGGTTGC -0.859393405883
AAGGTTGG -0.00522815464045
AAGGTTGT -0.303503237714
AAGGTTTA 1.16692793192
AAGGTTTC 0.171487758865
AAGGTTTG -0.494727397968
AAGTAAAA 1.06166825716
AAGTAAAC 1.02648263529
AAGTAAAG 0.833357708082
AAGTAAAT 2.14686242606
AAGTAACA 1.05374891763
AAGTAACC 0.0145014508275
AAGTAACG 0.179882290133
AAGTAACT -0.51605979537
AAGTAAGA 0.685989197172
AAGTAAGC 1.36785666799
AAGTAAGG 0.261662056313
AAGTAAGT 0.669741194535
AAGTAATA 1.47091943045
AAGTAATC 5.59935466545
AAGTAATG 1.41840499305
AAGTAATT 2.08912689132
AAGTACAA 0.8269486314
AAGTACAC 0.167378840209
AAGTACAG 0.802888717067
AAGTACAT 0.437533445889
AAGTACCA 0.37614954686
AAGTACCC -0.426825164751
AAGTACCG -0.73687235205
AAGTACCT -0.00705834856068
AAGTACGA 2.91582041429
AAGTACGC -0.544136360656
AAGTACGG -0.0239049150483
AAGTACGT 0.737973814086
AAGTACTA 0.242539117525
AAGTACTC 0.023943076761
AAGTACTG 0.32499873723
AAGTACTT 0.481571016823
AAGTAGAA 0.0845768106158
AAGTAGAC -0.747383028702
AAGTAGAG -0.29391889743
AAGTAGAT 3.65801083624
AAGTAGCA 1.1420739403
AAGTAGCC -0.836972092762
AAGTAGCG 0.44488872394
AAGTAGCT -0.306036809503
AAGTAGGA 0.0667775082185
AAGTAGGC -0.644404431115
AAGTAGGG 0.621895293778
AAGTAGGT 0.838875473528
AAGTAGTA 0.0924119852735
AAGTAGTC 0.390580424657

AAGTAGTG -0.450011280417
AAGTATAA 1.22321227605
AAGTATAC 0.876834615497
AAGTATAG 0.534842153942
AAGTATAT 2.18593949711
AAGTATCA 1.85983825232
AAGTATCC 1.05148273921
AAGTATCG 1.42350977557
AAGTATCT 2.87441234219
AAGTATGA 1.16949234674
AAGTATGC 0.799267797847
AAGTATGG 0.518957733101
AAGTATGT 0.841592796578
AAGTATTA 0.518653746307
AAGTATTC 1.65486385193
AAGTATTG 0.337794934539
AAGTCAAA 0.317393055612
AAGTCAAC -0.806591187344
AAGTCAAG -0.910509713142
AAGTCAAT 0.17149350926
AAGTCACA 0.711027985575
AAGTCACC -0.824766617855
AAGTCACG 0.147423923807
AAGTCACT 0.056994779305
AAGTCAGA 0.499193363881
AAGTCAGC -0.524472100587
AAGTCAGG -0.295206724543
AAGTCAGT -0.169810473177
AAGTCATA 0.0634603144108
AAGTCATC -0.331844843937
AAGTCATG -0.417593428238
AAGTCATT -0.0793465649226
AAGTCCAA -0.278815223411
AAGTCCAC -0.982776496214
AAGTCCAG -0.0702661683518
AAGTCCAT -0.261043104699
AAGTCCCA -0.318481448569
AAGTCCCC -1.28351849877
AAGTCCCG -0.796926602911
AAGTCCCT 0.0278881091572
AAGTCCGA -0.890278255014
AAGTCCGC -0.453310961658
AAGTCCGG -0.450206793849
AAGTCCGT -0.36377469668
AAGTCCTA -0.270537529715
AAGTCCTC -0.328526604603
AAGTCCTG -0.940389027282
AAGTCGAA 0.987383608187
AAGTCGAC 0.255529259976
AAGTCGAG -0.264326580281
AAGTCGAT -0.00715924185591
AAGTCGCA -0.274647232515
AAGTCGCC -1.36669116746
AAGTCGCG -0.631141929042

AAGTCGCT -0.272372951267
AAGTCGGA -0.410106152482
AAGTCGGC -0.966441192124
AAGTCGGG -1.15079101646
AAGTCGGT 0.10220177149
AAGTCGTA 0.183547905606
AAGTCGTC 0.284329852278
AAGTCGTG 0.143921933213
AAGTCTAA -0.209391226554
AAGTCTAC -0.541303506942
AAGTCTAG -0.649141711122
AAGTCTAT 1.02061305021
AAGTCTCA 0.160419816654
AAGTCTCC -1.16650945999
AAGTCTCG -0.749525835008
AAGTCTCT -0.579799003941
AAGTCTGA 0.0375968670797
AAGTCTGC -0.194084720418
AAGTCTGG -1.12101756187
AAGTCTGT -0.661705278813
AAGTCTTA 0.715239627197
AAGTCTTC 0.912170793171
AAGTCTTG -0.316650209122
AAGTGAAA 0.903353085102
AAGTGAAC -1.44540152959
AAGTGAAG -0.63358009655
AAGTGAAT 2.41874476408
AAGTGACA 0.758334394722
AAGTGACC -0.544082254666
AAGTGACG 0.0897077313034
AAGTGACT 0.056994779305
AAGTGAGA 0.61197899887
AAGTGAGC -0.368915549868
AAGTGAGG -0.559251796848
AAGTGAGT 0.0949680358799
AAGTGATA 1.17972569752
AAGTGATC 1.24317451114
AAGTGATG 1.2247878843
AAGTGATT 3.26068310712
AAGTGCAA 0.188272639296
AAGTGCAC -0.444644593531
AAGTGCAG -0.903857551579
AAGTGCAT 0.0243531844818
AAGTGCCA -1.17926801835
AAGTGCCC -0.659500002305
AAGTGCCG -0.647205919038
AAGTGCCT -0.994384191415
AAGTGCGA 0.166160279219
AAGTGCGC 1.2754350116
AAGTGCGG 0.646930422838
AAGTGCGT -0.699319135696
AAGTGCTA -0.81571837122
AAGTGCTC -1.08727633468
AAGTGCTG -0.790877971447

AAGTGGAA 0.426775502249
AAGTGGAC -0.0957785802024
AAGTGGAG -0.805454700174
AAGTGGAT 3.9705594454
AAGTGGCA -0.81256637058
AAGTGGCC -0.830558311212
AAGTGGCG -0.760465700238
AAGTGGCT -0.500131462422
AAGTGGGA -0.821157460807
AAGTGGGC -0.378730951481
AAGTGGGG -0.80044244673
AAGTGGGT -0.772917657988
AAGTGGTA 1.7398646235
AAGTGGTC -0.727376358745
AAGTGGTG 0.318461060805
AAGTGTAA 0.97697382483
AAGTGTAC 0.30266943043
AAGTGTAG 0.469210281249
AAGTGTAT 0.932335598993
AAGTGTCA -1.19198527842
AAGTGTCC 0.0629971462265
AAGTGTCG 1.66567485604
AAGTGTCT 0.681783828709
AAGTGTGA 0.309828149522
AAGTGTGC -0.041851375283
AAGTGTGG 0.389310632874
AAGTGTGT -0.0374946668765
AAGTGTTA 0.442352799717
AAGTGTTC -0.779338758223
AAGTGTTG 0.910007076337
AAGTTAAA 1.22703550463
AAGTTAAC 1.16832370963
AAGTTAAG -0.163776479083
AAGTTAAT 0.685745850908
AAGTTACA 0.0765241664721
AAGTTACC 1.18842264729
AAGTTACG 0.198486909221
AAGTTACT -0.345649974198
AAGTTAGA -0.321520793742
AAGTTAGC 0.40381835686
AAGTTAGG -0.0482588836749
AAGTTAGT 0.206322606642
AAGTTATA 1.30057782988
AAGTTATC 0.743647362962
AAGTTATG 1.95298210605
AAGTTATT 1.39414564456
AAGTTCAA -0.0100459401785
AAGTTCAC -0.363608196605
AAGTTCAG 1.76111703813
AAGTTCAT 1.44822549633
AAGTTCCA -0.251974993063
AAGTTCCC -0.581357883767
AAGTTCCG 0.268044733454
AAGTTCCT -0.836747827354

AAGTTCGA 1.28630691761
AAGTTCGC 0.570414620577
AAGTTCGG -0.413193591868
AAGTTCGT 0.0928168653624
AAGTTCTA 0.503908426453
AAGTTCTC -0.365866533576
AAGTTCTG 0.145649665548
AAGTTGAA 0.132995398445
AAGTTGAC 0.320364441571
AAGTTGAG -0.222898904562
AAGTTGAT 2.40903705168
AAGTTGCA 0.592410665845
AAGTTGCC -0.187838223087
AAGTTGCG 0.788067335168
AAGTTGCT 0.0836259043774
AAGTTGGA 0.741593165016
AAGTTGGC -0.280343260208
AAGTTGGG -0.391358557662
AAGTTGGT -0.371113246309
AAGTTGTA 1.03528753566
AAGTTGTC -0.427424251365
AAGTTGTG -0.373123270766
AAGTTTAA 0.126793074604
AAGTTTAC -0.315549792612
AAGTTTAG 0.414218730479
AAGTTTAT 1.55617923117
AAGTTTCA 0.181347072585
AAGTTTCC 0.00586592572948
AAGTTTCG 0.394465600668
AAGTTTCT -0.199799044823
AAGTTTGA 1.10821718245
AAGTTTGC 0.8097031966
AAGTTTGG 0.175952679251
AAGTTTGT -0.241202934817
AAGTTTTA 0.60243961622
AAGTTTTC 0.432353908225
AAGTTTTG -0.313340072618
AATAAAAA 2.08170208577
AATAAAAC 1.19759295913
AATAAAAG 1.08434154214
AATAAAAT 3.34509681562
AATAAACA 3.13937564803
AATAAACC -0.468867871361
AATAAACG 1.03097683041
AATAAACT 1.08807093472
AATAAAGA 1.75308138379
AATAAAGC 0.750753282973
AATAAAGG 0.166423751865
AATAAAGT 1.84889159116
AATAAATA 2.50918201141
AATAAATC 3.80192231419
AATAAATG 1.55207083528
AATAAATT 1.49140991774
AATAACAA 0.478870422195

AATAACAC 0.389593447758
AATAACAG 1.20930782079
AATAACAT 1.18749840198
AATAACCA 0.928197144217
AATAACCC 0.479697433558
AATAACCG -0.00263420370295
AATAACCT 0.110157181681
AATAACGA 1.50209885664
AATAACGC 0.24206601684
AATAACGG 0.910209908454
AATAACGT 1.32036232548
AATAACTA 0.559870487081
AATAACTC 0.85652787947
AATAACTG 2.3970940039
AATAAGAA 1.67063300349
AATAAGAC -0.615625794867
AATAAGAG -0.116017095634
AATAAGAT 4.97684460216
AATAAGCA -0.0319348189934
AATAAGCC 0.0988704630762
AATAAGCG -0.188339552984
AATAAGCT 0.32571858214
AATAAGGA 1.88333619622
AATAAGGC 1.23997023418
AATAAGGG 0.827534126171
AATAAGGT -0.0446298616257
AATAAGTA 1.79368361644
AATAAGTC 0.732486891668
AATAAGTG 0.593945759946
AATAATAA 4.27395786909
AATAATAC 2.22261656236
AATAATAG 2.52167761989
AATAATAT 4.03167046204
AATAATCA 1.94190893621
AATAATCC 3.67307896237
AATAATCG 2.02283685978
AATAATCT 2.87565338199
AATAATGA 1.11098808873
AATAATGC 1.79843082894
AATAATGG 0.661789443686
AATAATGT 1.92309416632
AATAATTA 2.99090802752
AATAATTC 1.76112853892
AATAATTG 1.13058804892
AATACAAA 2.97637782472
AATACAAC 1.88898334556
AATACAAG 2.81645201927
AATACAAT 1.52530718974
AATACACA 2.3087807434
AATACACC 1.640571245
AATACACG 0.343945243443
AATACACT 0.129054025391
AATACAGA 0.753967753815
AATACAGC 0.271035984414

AATACAGG 0.884668744628
AATACAGT 0.0631623393938
AATACATA 1.48770535641
AATACATC 1.59549990986
AATACATG 0.300410570696
AATACATT 2.49000575078
AATACCAA 0.750794842647
AATACCAC 1.35298144148
AATACCAG 0.267367755126
AATACCAT 0.717843772016
AATACCCA 1.41566675947
AATACCCC 0.279122085402
AATACCCG 0.239822317238
AATACCCT 1.1420739403
AATACCGA 1.2299739565
AATACCGC 1.17544060767
AATACCGG -0.292001924821
AATACCGT 0.917192717734
AATACCTA 1.70199435127
AATACCTC 0.211922445909
AATACCTG 0.758334394722
AATACGAA 1.74548537329
AATACGAC 2.19941267275
AATACGAG 1.4666500235
AATACGAT 4.31552669087
AATACGCA 1.8019788227
AATACGCC 1.27982125612
AATACGCG 2.31225633445
AATACGCT 1.43087838409
AATACGGA 1.79656979199
AATACGGC 1.26428708662
AATACGGG 0.537370236718
AATACGGT 1.0253492847
AATACGTA 1.63730109988
AATACGTC 1.24420147942
AATACGTG 0.892325657039
AATACTAA 1.85580147498
AATACTAC 0.612108121377
AATACTAG 1.01345799046
AATACTAT 1.02077249299
AATACTCA 0.629715046921
AATACTCC 1.15306451356
AATACTCG 0.840728146266
AATACTCT 0.70489231404
AATACTGA 1.80274728458
AATACTGC 1.61699201142
AATACTGG 0.947025244568
AATACTGT 0.057795391127
AATACTTA 1.88711237611
AATACTTC 1.65726699431
AATACTTG 0.137151365787
AATAGAAA 0.711129401633
AATAGAAC 0.0674944779305
AATAGAAG 0.22965666429

AATAGAAT 2.5600011278
AATAGACA 0.470173733804
AATAGACC -1.09277240545
AATAGACG -0.376585531358
AATAGACT -0.0344435595313
AATAGAGA 2.86686756248
AATAGAGC 0.102059579903
AATAGAGG 0.893132542019
AATAGAGT -0.25713205191
AATAGATA 0.989261112176
AATAGATC 1.7762980811
AATAGATG 0.260152054846
AATAGATT 6.49630625393
AATAGCAA 1.18087028752
AATAGCAC 0.0665469696528
AATAGCAG -0.799164813499
AATAGCAT 1.6184847617
AATAGCCA -0.51674356962
AATAGCCC 0.137742088189
AATAGCCG 0.126118710092
AATAGCCT 0.0436261563053
AATAGCGA 0.36346156153
AATAGCGC -0.145850406612
AATAGCGG 0.546699991329
AATAGCGT 0.351068937402
AATAGCTA 0.529070848349
AATAGCTC -0.316064191589
AATAGCTG 0.15110104007
AATAGGAA 1.61045930124
AATAGGAC -0.303000339528
AATAGGAG 0.0913536511999
AATAGGAT 5.02679802271
AATAGGCA -0.184278205779
AATAGGCC -0.842395760834
AATAGGCG -0.552278397305
AATAGGCT 0.0752964571257
AATAGGGA 1.27857368178
AATAGGGC -0.595347288052
AATAGGGG 0.31605295218
AATAGGGT 0.169096117281
AATAGGTA 0.642084669469
AATAGGTC 0.427856576521
AATAGGTG 0.332259395145
AATAGTAA 1.38511072838
AATAGTAC -0.00390268857797
AATAGTAG 1.75795902571
AATAGTAT 0.545292712828
AATAGTCA 1.01421678123
AATAGTCC 0.189510803907
AATAGTCG 0.0298513463087
AATAGTCT -0.0769925622883
AATAGTGA 0.0559320017444
AATAGTGC -0.208171620037
AATAGTGG 1.34637815828

AATAGTGT	-0.178825262968
AATAGTTA	1.60547789083
AATAGTTC	0.292672368609
AATAGTTG	0.916729026787
AATATAAA	2.91917550842
AATATAAC	2.22051766816
AATATAAG	1.49207173593
AATATAAT	1.88479731933
AATATACA	2.644972886
AATATACC	2.65596032268
AATATACG	2.85980581596
AATATACT	3.64513726998
AATATAGA	3.05346108642
AATATAGC	1.19491066121
AATATAGG	2.10755403231
AATATAGT	1.75109096295
AATATATA	2.99628647657
AATATATC	4.40267967848
AATATATG	2.67344962651
AATATATT	3.01933588967
AATATCAA	6.09846804656
AATATCAC	5.38708641169
AATATCAG	4.99076238789
AATATCAT	4.35265646904
AATATCCA	8.36826660043
AATATCCC	4.62971599229
AATATCCG	9.08213841775
AATATCCT	6.49135542517
AATATCGA	6.33368482012
AATATCGC	4.32977041945
AATATCGG	5.36644928933
AATATCGT	5.44751600652
AATATCTA	6.88717890321
AATATCTC	9.71195200553
AATATCTG	12.3092705835
AATATGAA	1.15628813276
AATATGAC	2.521627696
AATATGAG	2.62112103142
AATATGAT	2.51056132208
AATATGCA	2.30255672488
AATATGCC	0.997473983237
AATATGCG	2.54636275899
AATATGCT	1.28591850457
AATATGGA	1.83920112995
AATATGGC	2.55174565154
AATATGGG	0.804907367116
AATATGGT	2.44721052656
AATATGTA	2.26627120926
AATATGTC	1.3812861929
AATATGTG	1.68594552141
AATATTAA	3.80717477732
AATATTAC	5.16654543049
AATATTAG	1.3815687464
AATATTAT	3.5966640534

AATATTCA 2.56824222807
AATATTCC 7.82596357027
AATATTCG 4.07876201552
AATATTCT 6.26927464499
AATATTGA 3.97731772789
AATATTGC 2.45114797889
AATATTGG 3.92724119661
AATATTGT 3.05129527853
AATATTTA 2.52018199441
AATATTTC 6.01405669049
AATATTTG 4.14303888603
AATCAAAA 2.59945981597
AATCAAAC 2.78572321495
AATCAAAG 2.57460713126
AATCAAAT 3.58654806296
AATCAACA 1.9867594039
AATCAACC 1.37983813887
AATCAACG 2.23993884335
AATCAACT 3.03936111772
AATCAAGA 2.8649430098
AATCAAGC 1.79279465763
AATCAAGG 2.9028705246
AATCAAGT 2.12634083437
AATCAATA 2.56669615594
AATCAATC 3.11072430462
AATCAATG 1.41429398334
AATCAATT 2.35229528976
AATCACAA 6.89449549679
AATCACAC 2.79411565517
AATCACAG 3.96508140995
AATCACAT 3.95177734821
AATCACCA 2.79893004275
AATCACCC 1.25694200245
AATCACCG 2.72104198712
AATCACCT 1.82026612453
AATCACGA 2.87748122348
AATCACGC 2.98207489793
AATCACGG 2.9159537189
AATCACGT 5.03487968021
AATCACTA 2.34351077715
AATCACTC 1.11736475409
AATCACTG 3.70707059312
AATCAGAA 5.44388018855
AATCAGAC 2.86429216963
AATCAGAG 3.43687834849
AATCAGAT 4.17331863677
AATCAGCA 4.14051106463
AATCAGCC 2.46807766472
AATCAGCG 3.14256005999
AATCAGCT 1.79409137172
AATCAGGA 5.85207720987
AATCAGGC 3.40728733825
AATCAGGG 2.05941355449
AATCAGGT 3.63251645972

AATCAGTA 4.73754086601
AATCAGTC 1.94783968457
AATCAGTG 4.09549618792
AATCATAA 3.56973077121
AATCATAC 2.58924580743
AATCATAG 2.2077917825
AATCATAT 4.19850144643
AATCATCA 2.55674091518
AATCATCC 1.88242894071
AATCATCG 1.88214978517
AATCATCT 2.48174295583
AATCATGA 1.9959686616
AATCATGC 0.673088708607
AATCATGG 2.88757970136
AATCATGT 2.95993091068
AATCATTA 3.17773993147
AATCATTC 2.26545334625
AATCATTG 1.32628209582
AATCCAAA 7.82338948434
AATCCAAC 3.71936650606
AATCCAAG 5.27559566112
AATCCAAT 7.59956685722
AATCCACA 6.0675680373
AATCCACC 4.1136167283
AATCCACG 8.82544365724
AATCCACT 5.58446218776
AATCCAGA 3.01666927465
AATCCAGC 4.51137364599
AATCCAGG 6.82924445708
AATCCAGT 6.17850152231
AATCCATA 5.09109475822
AATCCATC 4.84186244222
AATCCATG 6.82495701479
AATCCATT 5.39409745018
AATCCCAA 6.75736347324
AATCCCAC 3.51381497512
AATCCCAG 5.64253595028
AATCCCAT 4.07523231847
AATCCCCA 4.11661425241
AATCCCCC 2.38983517565
AATCCCCG 6.36386550723
AATCCCCT 2.75475498509
AATCCCGA 6.39525482284
AATCCCGC 5.30644313268
AATCCCGG 3.86398658951
AATCCCGT 4.28901527857
AATCCCTA 3.76150148037
AATCCCTC 3.07558729972
AATCCCTG 3.43612400121
AATCCGAA 6.94690694985
AATCCGAC 6.88393228244
AATCCGAG 5.42454291685
AATCCGAT 9.39180660173
AATCCGCA 4.57790728519

AATCCGCC 8.28648212938
AATCCGCG 4.45993322556
AATCCGCT 4.17891952156
AATCCGGA 5.39965468425
AATCCGGC 4.452022773
AATCCGGG 6.92756784848
AATCCGGT 6.47114540033
AATCCGTA 4.09498597105
AATCCGTC 6.71539395348
AATCCGTG 3.18061408348
AATCCTAA 5.73298522117
AATCCTAC 3.51276892598
AATCCTAG 4.40963870203
AATCCTAT 6.28058175136
AATCCTCA 5.22265125099
AATCCTCC 4.94993667398
AATCCTCG 2.45741617089
AATCCTCT 6.06446596054
AATCCTGA 9.26779645696
AATCCTGC 4.98738716737
AATCCTGG 4.6285371613
AATCCTGT 4.41222036803
AATCCTTA 5.35761877356
AATCCTTC 3.14537043489
AATCCTTG 4.75238472672
AATCGAAA 3.12816943496
AATCGAAC 1.8964551998
AATCGAAG 2.46834662638
AATCGAAT 4.36924975677
AATCGACA 0.947738293556
AATCGACC 1.84535248438
AATCGACG 3.63258468032
AATCGACT 2.38221381114
AATCGAGA 3.18971695886
AATCGAGC 2.47450137877
AATCGAGG 3.35691805959
AATCGAGT 2.37691063998
AATCGATA 4.21633917192
AATCGATC 2.50494423163
AATCGATG 2.04866737303
AATCGATT 3.41608857928
AATCGCAA 6.82466139221
AATCGCAC 2.7355682692
AATCGCAG 2.98720581862
AATCGCAT 3.33403880591
AATCGCCA 2.98923649223
AATCGCCC 2.03209107511
AATCGCCG 3.38621945902
AATCGCCT 1.64817091484
AATCGCGA 6.82129296761
AATCGCGC 5.52556036834
AATCGCGG 4.96359830575
AATCGCGT 4.11449758427
AATCGCTA 2.92410751772

AATCGCTC 1.85389600316
AATCGCTG 2.5817438943
AATCGGAA 3.91418910672
AATCGGAC 2.05116539692
AATCGGAG 3.67139775596
AATCGGAT 4.67117712485
AATCGGCA 2.57772410677
AATCGGCC 1.83589177759
AATCGGCG 4.43627087263
AATCGGCT 2.0440908427
AATCGGGA 3.59147406047
AATCGGGC 2.52079388872
AATCGGGG 4.31272677124
AATCGGGT 2.90663703337
AATCGGTA 3.48235613201
AATCGGTC 1.42056452777
AATCGGTG 1.14733293797
AATCGTAA 5.59889960009
AATCGTAC 2.32332950429
AATCGTAG 3.58489116503
AATCGTAT 6.1655448367
AATCGTCA 3.45468157161
AATCGTCC 3.41388983731
AATCGTCG 4.12132121216
AATCGTCT 2.78938517108
AATCGTGA 4.73978299732
AATCGTGC 5.11641296355
AATCGTGG 3.62487862817
AATCGTGT 4.21177858587
AATCGTTA 1.42138840256
AATCGTTC 2.03926626124
AATCGTTG 0.990744975484
AATCTAAA 4.89786005075
AATCTAAC 6.78034179054
AATCTAAG 8.1184197763
AATCTAAT 7.70173569463
AATCTACA 8.83716688311
AATCTACC 5.23610822097
AATCTACG 7.28694715228
AATCTACT 5.51981415538
AATCTAGA 14.7840309914
AATCTAGC 6.61996196527
AATCTAGG 6.32750392957
AATCTAGT 8.48025240774
AATCTATA 6.08146360559
AATCTATC 7.67050268522
AATCTATG 5.97886348855
AATCTCAA 6.46240558397
AATCTCAC 8.60148485032
AATCTCAG 9.01313498387
AATCTCAT 7.89768589548
AATCTCCA 4.45318592109
AATCTCCC 4.70171642751
AATCTCCG 7.85782441827

AATCTCCT	7.02637035234
AATCTCGA	10.4063169145
AATCTCGC	9.13646161578
AATCTCGG	10.5360071427
AATCTCGT	6.88334835596
AATCTCTA	4.91180188358
AATCTCTC	2.98759057233
AATCTCTG	6.14964133501
AATCTGAA	7.17692170692
AATCTGAC	8.70018306292
AATCTGAG	6.67544046995
AATCTGAT	9.11786614505
AATCTGCA	7.65222531588
AATCTGCC	6.54170692988
AATCTGCG	10.3043550913
AATCTGCT	4.40593387932
AATCTGGA	7.91524524958
AATCTGGC	4.96294380624
AATCTGGG	10.3576646515
AATCTGGT	9.55930280228
AATCTGTA	8.63797006148
AATCTGTC	4.9465381905
AATCTGTG	6.59723065358
AATCTTAA	7.99625969045
AATCTTAC	7.75800174205
AATCTTAG	5.4432560093
AATCTTAT	5.9183348301
AATCTTCA	6.04419607932
AATCTTCC	4.05311185557
AATCTTCG	6.36321911055
AATCTTCT	6.78986444477
AATCTTGA	5.14221969107
AATCTTGC	5.21324647986
AATCTTGG	6.60899413221
AATCTTGT	6.09450445607
AATCTTTA	6.1286910775
AATCTTTC	5.65049554258
AATCTTTG	5.69364127951
AATGAAAA	0.0445336731992
AATGAAAC	0.955445391233
AATGAAAG	0.591817591009
AATGAAAT	1.65427417506
AATGAACA	0.327569425206
AATGAACC	-0.308932656182
AATGAACG	-0.0255746206699
AATGAACT	1.2754350116
AATGAAGA	0.891889411159
AATGAAGC	0.18009662304
AATGAAGG	-0.292907873425
AATGAAGT	-0.959179488683
AATGAATA	2.92920550433
AATGAATC	4.21014599644
AATGAATG	0.0681887074438
AATGAATT	1.62315460526

AATGACAA 0.685790808542
AATGACAC -0.195747630118
AATGACAG -0.396462033282
AATGACAT 2.00369928362
AATGACCA -0.376223779232
AATGACCC -0.545207502428
AATGACCG -0.579175086077
AATGACCT -0.705971820023
AATGACGA -0.426861235411
AATGACGC 0.071274578541
AATGACGG 0.232148415024
AATGACGT 0.705864130806
AATGACTA 0.490011028489
AATGACTC -0.379354607964
AATGACTG -0.315304616677
AATGAGAA 1.53639369004
AATGAGAC -1.06587597806
AATGAGAG 0.242673990427
AATGAGAT 4.67158174356
AATGAGCA -0.672369125079
AATGAGCC -0.392429438052
AATGAGCG -0.86043579568
AATGAGCT -0.814862869263
AATGAGGA 0.30266943043
AATGAGGC -0.893915641274
AATGAGGG -0.435351955106
AATGAGGT 1.5041784086
AATGAGTA 1.42507231474
AATGAGTC 0.752765398483
AATGAGTG -0.629418378812
AATGATAA 1.47007699757
AATGATAC 2.0773291717
AATGATAG 1.24346020122
AATGATAT 3.44179023141
AATGATCA 0.785729799574
AATGATCC 0.800888102347
AATGATCG 0.316719998008
AATGATCT 2.61935592151
AATGATGA 0.71229490216
AATGATGC -0.217523853466
AATGATGG -0.121636799902
AATGATGT 1.32766898655
AATGATTA 1.17201415635
AATGATTC 3.47642224707
AATGATTG 2.08966376911
AATGCAAA 0.640894599073
AATGCAAC 0.682851572519
AATGCAAG 0.439334887834
AATGCAAT 3.34501813976
AATGCACA 1.12400881283
AATGCACC -0.180105771396
AATGCACG 0.682851572519
AATGCACT 0.111574915446
AATGCAGA 0.598295672431

AATGCAGC 0.107558003111
AATGCAGG 0.258803325821
AATGCAGT 0.394759916343
AATGCATA 2.24241621809
AATGCATC -0.178784487439
AATGCATG -0.70163366971
AATGCATT 2.01332779739
AATGCCAA -0.0737545671035
AATGCCAC -0.696438187768
AATGCCAG 0.616968773497
AATGCCAT -0.0763155839602
AATGCCCA -0.583544602181
AATGCCCC -0.377962750976
AATGCCCG -1.59237770672
AATGCCCT 0.216594903281
AATGCCGA 0.532661970067
AATGCCGC -0.795652106259
AATGCCGG -0.323855454139
AATGCCGT 0.343099412605
AATGCCTA 0.281177590256
AATGCCTC -1.2877413798
AATGCCTG 0.0349906312073
AATGCGAA 1.90540647386
AATGCGAC 0.682522754474
AATGCGAG -0.0309690140041
AATGCGAT 4.91777602129
AATGCGCA -0.0186997619882
AATGCGCC 0.87170055823
AATGCGCG 0.898524582919
AATGCGCT 0.234543454568
AATGCGGA -0.443969706256
AATGCGGC 0.317646072995
AATGCGGG 0.275054465038
AATGCGGT 0.342568285207
AATGCGTA 0.811990285547
AATGCGTC 0.539802392449
AATGCGTG 0.126505032087
AATGCTAA 1.10137185989
AATGCTAC 0.12954908213
AATGCTAG -0.418835774953
AATGCTAT 2.12557367939
AATGCTCA -0.0790318614836
AATGCTCC -0.800578365159
AATGCTCG -0.647734694002
AATGCTCT -0.870161282024
AATGCTGA 0.993573647093
AATGCTGC -0.318946707806
AATGCTGG -0.5587355682
AATGCTGT 1.03072642684
AATGCTTA -0.56902616155
AATGCTTC 0.607370318607
AATGCTTG -0.53772153358
AATGGAAA 0.613314397433
AATGGAAC -0.444948580325

AATGGAAG −0.16231326492
AATGGAAT 2.00257142204
AATGGACA 0.275391647294
AATGGACC −0.908596138494
AATGGACG −1.17675927781
AATGGACT 0.225177106534
AATGGAGA 1.08403206633
AATGGAGC −1.08295700384
AATGGAGG −0.634274848826
AATGGAGT 0.716810791957
AATGGATA 3.03778472532
AATGGATC 0.967795410161
AATGGATG 0.105636325633
AATGGCAA −0.0685264124631
AATGGCAC −0.347551786675
AATGGCAG 0.132183285833
AATGGCAT −0.138998549511
AATGGCCA −0.649524635157
AATGGCCC −0.474148825084
AATGGCCG −1.07795625118
AATGGCCT −0.557475708918
AATGGCGA −0.459336591541
AATGGCGC 0.0907148345845
AATGGCGG −0.788534424077
AATGGCGT 0.0375555687878
AATGGCTA 0.31568727933
AATGGCTC −0.0835610817421
AATGGCTG −0.408255570797
AATGGGAA −0.560966721485
AATGGGAC −0.840073123991
AATGGGAG 0.313601715593
AATGGGAT 1.67160325197
AATGGGCA −0.967463194156
AATGGGCC −0.990892917466
AATGGGCG −1.07175706392
AATGGGCT −0.971809447298
AATGGGGA 0.326043740842
AATGGGGC −0.0449382919065
AATGGGGG −1.39110682215
AATGGGGT 0.432983053721
AATGGGTA 1.08403389601
AATGGGTC −0.647301584701
AATGGGTG 0.390610222159
AATGGTAA 1.77795497902
AATGGTAC 0.492615957453
AATGGTAG 1.57093474491
AATGGTAT 1.15450132818
AATGGTCA 0.293976139999
AATGGTCC 0.032010358274
AATGGTCG −0.829806577748
AATGGTCT −0.406398454573
AATGGTGA 1.64711231939
AATGGTGC −0.741345898029
AATGGTGG −0.350805987519

AATGGTGT	0.204846846163
AATGGTTA	0.710087011837
AATGGTTC	0.0681887074438
AATGGTTG	-0.440072506692
AATGTAAA	1.02232562242
AATGTAAC	0.0765241664721
AATGTAAG	0.922812944765
AATGTAAT	6.11669653754
AATGTACA	1.23425538701
AATGTACC	0.0831428711919
AATGTACG	-0.209260274376
AATGTACT	0.181150252245
AATGTAGA	0.650829974837
AATGTAGC	0.222233688405
AATGTAGG	0.894765915599
AATGTAGT	0.436528695042
AATGTATA	1.83873430243
AATGTATC	0.394759916343
AATGTATG	0.512930273547
AATGTCAA	0.072416293343
AATGTCAC	0.778572126008
AATGTCAG	-0.0278543909316
AATGTCAT	2.23241523555
AATGTCCA	-0.545591210608
AATGTCCC	-0.82009206943
AATGTCCG	-0.327986328848
AATGTCCT	0.393037934402
AATGTCGA	-0.0956800393416
AATGTCGC	0.23294641303
AATGTCGG	0.936154384079
AATGTCGT	-0.285162614036
AATGTCTA	0.937155998347
AATGTCTC	0.782215001282
AATGTCTG	-0.478555718756
AATGTGAA	1.74806102752
AATGTGAC	0.104507679911
AATGTGAG	0.682888427324
AATGTGAT	1.3392149957
AATGTGCA	0.877168399792
AATGTGCC	0.175896220827
AATGTGCG	0.790632272749
AATGTGCT	0.831662909827
AATGTGGA	0.821748705972
AATGTGGC	-0.319405693885
AATGTGGG	-0.173618018855
AATGTGGT	0.689069317874
AATGTGTA	1.37779727139
AATGTGTC	0.344526033345
AATGTGTG	0.350511933226
AATGTTAA	2.40088665082
AATGTTAC	0.374321182611
AATGTTAG	0.223232950239
AATGTTAT	2.31532626127
AATGTTCA	0.462005036233

AATGTTCC 0.187608730048
AATGTTCG 0.0342138051103
AATGTTCT 1.08941757269
AATGTTGA 0.804907367116
AATGTTGC 0.471070272671
AATGTTGG 0.693700999718
AATGTTGT 0.313049939048
AATGTTTA 1.38039122232
AATGTTTC 1.15250881629
AATGTTTG 0.0235645962131
AATTAAAA 1.31921381475
AATTAAAC 0.590195979601
AATTAAAG 1.72825614415
AATTAAAT 2.75120490029
AATTAACA 0.885201701698
AATTAACC 0.428868123289
AATTAACG 0.363321460996
AATTAACT 1.56394749214
AATTAAGA 1.48830653407
AATTAAGC -0.372065198074
AATTAAGG -0.0595296580023
AATTAAGT 1.42813727531
AATTAATA 0.518653746307
AATTAATC 1.96512040589
AATTAATG 1.35915971822
AATTAATT 2.22863121422
AATTACAA 1.97195840977
AATTACAC 3.10500475258
AATTACAG 1.95705730648
AATTACAT 2.83362949486
AATTACCA 1.86676800113
AATTACCC 1.67440134193
AATTACCG 1.05195061227
AATTACCT 3.08305758567
AATTACGA 4.17964537825
AATTACGC 4.59936410023
AATTACGG 2.16559930424
AATTACGT 4.85430106951
AATTACTA 1.41555828611
AATTACTC 1.62811353686
AATTACTG 1.10725608233
AATTAGAA 0.870394173024
AATTAGAC 0.406548226226
AATTAGAG 0.458981112573
AATTAGAT 2.89534508713
AATTAGCA 1.81053593332
AATTAGCC -0.0957785802024
AATTAGCG -0.581682781088
AATTAGCT -0.141172721609
AATTAGGA 2.23476322641
AATTAGGC 1.86612134307
AATTAGGG 0.420758236576
AATTAGGT 0.647618901956
AATTAGTA 1.31860009077

AATTAGTC 0.458580937353
AATTAGTG 1.79768850522
AATTATAA 1.75176637299
AATTATAC 3.32976051199
AATTATAG 1.41238929566
AATTATAT 3.25440158466
AATTATCA 1.79826615854
AATTATCC 1.60128062519
AATTATCG 1.68789464395
AATTATCT 2.6064021111
AATTATGA 2.14570555113
AATTATGC 1.74228135771
AATTATGG 2.35945975925
AATTATGT 1.40317637862
AATTATTA 2.8399070966
AATTATTC 2.0621131036
AATTATTG 2.3859037351
AATTCAAA 1.84109876033
AATTCAAC 0.710271024479
AATTCAAG 1.13590324364
AATTCAAT 1.2306059772
AATTCACA 1.82064878718
AATTCACC 1.19773384381
AATTCACG 0.507901553062
AATTCACT 1.45770894332
AATTCAGA 1.92674017817
AATTCAGC 0.660123658788
AATTCAGG 0.283319873799
AATTCAGT 1.37638999289
AATTCATA 0.934739786893
AATTCATC 0.752933205466
AATTCATG 1.80242029621
AATTCCAA 2.07646060066
AATTCCAC 1.22408999544
AATTCCAG 1.40543785217
AATTCCAT 1.72489164027
AATTCCCA 2.07833130873
AATTCCCC 3.32877588752
AATTCCCG 4.07400539327
AATTCCCT 3.50705381743
AATTCCGA 1.68837192674
AATTCCGC 2.44580560049
AATTCCGG 1.93941666271
AATTCCGT 3.50625320561
AATTCCTA 1.65572588843
AATTCCTC 0.899519662647
AATTCCTG 1.48688697064
AATTCGAA 0.647506507871
AATTCGAC 0.478870422195
AATTCGAG 1.28504000103
AATTCGAT 3.41928240098
AATTCGCA 4.28189472119
AATTCGCC 1.82120187064
AATTCGCG 2.61635865878

AATTCGCT 2.40209580208
AATTCGGA 2.66466276147
AATTCGGC 0.759373386557
AATTCGGG 0.796728214281
AATTCGGT 1.96759595096
AATTCGTA 2.04163542401
AATTCGTC 1.43853608064
AATTCGTG 0.500803213118
AATTCTAA 1.34229720745
AATTCTAC 2.31868475337
AATTCTAG 1.05052555982
AATTCTAT 4.25237088602
AATTCTCA 4.38014884647
AATTCTCC 1.1558884803
AATTCTCG 4.59410928466
AATTCTCT 4.63541750899
AATTCTGA 2.06935415789
AATTCTGC 2.37183696186
AATTCTGG 1.28657012888
AATTCTGT 2.71215945641
AATTCTTA 1.75799274394
AATTCTTC 1.87929445264
AATTCTTG 2.14008689239
AATTGAAA 2.09570900262
AATTGAAC 0.486195902745
AATTGAAG 1.54747417857
AATTGAAT 1.46197155435
AATTGACA 1.26865163647
AATTGACC -0.111517411495
AATTGACG 1.18271930091
AATTGACT 0.148671498155
AATTGAGA 0.927175403567
AATTGAGC -0.192088287804
AATTGAGG 0.17206750324
AATTGAGT 0.216495055512
AATTGATA 0.900444953489
AATTGATC 0.393091256248
AATTGATG 2.03797895689
AATTGCAA 0.554544575724
AATTGCAC 1.46727132755
AATTGCAG 0.988933339657
AATTGCAT 2.30139070159
AATTGCCA 0.647618901956
AATTGCCC 1.2484690567
AATTGCCG 1.2144724597
AATTGCCT 0.0906000880648
AATTGCGA 3.17273003046
AATTGCGC 2.7082771556
AATTGCGG 3.53429370024
AATTGCGT 3.33634732815
AATTGCTA 2.39858309484
AATTGCTC 1.39228878971
AATTGCTG 0.741039036037
AATTGGAA -0.0857841321979

AATTGGAC 0.975318756578
AATTGGAG 0.100329233752
AATTGGAT 3.6478791629
AATTGGCA -0.244621806065
AATTGGCC -0.05610346807
AATTGGCG 1.28560432389
AATTGGCT 0.458916028556
AATTGGGA 2.22958604118
AATTGGGC -0.787748188242
AATTGGGG 0.651203489135
AATTGGGT 0.582080865256
AATTGGTA 0.64712750456
AATTGGTC 0.011516211644
AATTGGTG 0.834708005396
AATTGTAA 1.7035843355
AATTGTAC 0.940895323429
AATTGTAG 1.44679835282
AATTGTAT 1.17279621008
AATTGTCA -0.85993237473
AATTGTCC 1.23471123651
AATTGTCG 0.511479867082
AATTGTCT 0.219417824495
AATTGTGA 1.22773835973
AATTGTGC 1.12553684962
AATTGTGG 1.73561508154
AATTGTGT 1.79335558254
AATTGTTA 1.79744855691
AATTGTTC 2.18918951585
AATTGTTG 0.415207798431
AATTTAAA 2.41766839467
AATTTAAC 1.90076329123
AATTTAAG 2.69216507139
AATTTAAT 2.80606994236
AATTTACA 2.85711619936
AATTTACC 2.29147911155
AATTTACG 2.95559354452
AATTTACT 2.17679872139
AATTTAGA 3.03671253803
AATTTAGC 2.14018151252
AATTTAGG 2.19397070796
AATTTAGT 0.750779421133
AATTTATA 2.01855516789
AATTTATC 0.999238570377
AATTTATG 2.40971141619
AATTTCAA 3.07649533938
AATTTCAC 1.2163554527
AATTTCAG 2.76923657092
AATTTCAT 4.17780342216
AATTTCCA 4.33570116781
AATTTCCC 2.03083748898
AATTTCCG 2.69165041103
AATTTCCT 3.49348994238
AATTTCGA 2.85490778627
AATTTCGC 0.596953216565

AATTTCGG	2.58138684704
AATTTCGT	3.25127389251
AATTTCTA	2.46628406423
AATTTCTC	1.97608510237
AATTTCTG	2.17798931455
AATTTGAA	2.16342042727
AATTTGAC	2.27458575776
AATTTGAG	1.46892273646
AATTTGAT	1.7633555101
AATTTGCA	2.10239828037
AATTTGCC	2.08811377626
AATTTGCG	2.24797057696
AATTTGCT	2.10703205326
AATTTGGA	1.15000974688
AATTTGGC	0.832838081473
AATTTGGG	1.35629889667
AATTTGGT	3.02037409736
AATTTGTA	3.28569706427
AATTTGTC	2.23400548117
AATTTGTG	1.24332480556
AATTTTAA	2.56190163337
AATTTTAC	2.46668711464
AATTTTAG	3.21777522744
AATTTTAT	2.22734573954
AATTTTCA	2.47517103842
AATTTTCC	2.64296024773
AATTTTCG	2.24952945679
AATTTTCT	4.34334605667
AATTTTGA	2.05671295987
AATTTTGC	1.78297794911
AATTTTGG	1.97753916818
AATTTTGT	2.32731400531
AATTTTTA	1.85804360629
AATTTTTC	0.956728774859
AATTTTTG	1.04503236424
ACAAAAAA	1.98546974712
ACAAAAAC	1.02831910237
ACAAAAAG	0.569250426958
ACAAAAAT	1.910584966
ACAAAACA	1.42099972813
ACAAAACC	0.463394279404
ACAAAACG	0.0815949693931
ACAAAACT	0.0493718465016
ACAAAAGA	0.339421250816
ACAAAAGC	0.218507693785
ACAAAAGG	-1.4043667104
ACAAAAGT	0.119618933997
ACAAAATA	3.04553782616
ACAAAATC	16.1522413087
ACAAAATG	0.319112685118
ACAAACAA	1.27243539643
ACAAACAC	1.699828282
ACAAACAG	0.335571622702
ACAAACAT	1.33519573093

ACAAACCA 0.175728936607
ACAAACCC 0.741058639657
ACAAACCG -0.236853545095
ACAAACCT -0.603880874329
ACAAACGA 2.05766020676
ACAAACGC -0.844462243715
ACAAACGG -0.476335543498
ACAAACGT 0.280028556769
ACAAACTA 0.632318407596
ACAAACTC 0.492429853758
ACAAACTG -0.44474600959
ACAAAGAA 0.432068479524
ACAAAGAC 0.342066432546
ACAAAGAG -0.0982128269861
ACAAAGAT 1.60239228111
ACAAAGCA 1.30057207948
ACAAAGCC -0.197798952866
ACAAAGCG -0.104824213021
ACAAAGCT 0.185194086884
ACAAAGGA 0.966599589369
ACAAAGGC -1.61820874274
ACAAAGGG 0.686320367652
ACAAAGGT -0.263144351332
ACAAAGTA -0.106084072304
ACAAAGTC -0.462001899654
ACAAAGTG 1.16264205793
ACAAATAA 2.63631279103
ACAAATAC 2.40172307192
ACAAATAG 0.509242179257
ACAAATAT 6.19294860577
ACAAATCA 3.78751077863
ACAAATCC 5.42111097653
ACAAATCG 3.5460454167
ACAAATCT 4.31847089314
ACAAATGA 0.393285985535
ACAAATGC -0.222333013411
ACAAATGG 1.3888552811
ACAAATGT 1.11027059625
ACAAATTA 2.17445386711
ACAAATTC 1.78595247165
ACAAATTG 0.926296638649
ACAACAAA 1.28542501612
ACAACAAC 0.574386575277
ACAACAAG 0.799386465091
ACAACAAT 1.77302349249
ACAACACA 0.306530036571
ACAACACC 0.651819042788
ACAACACG 0.677300088847
ACAACACT 0.62259265987
ACAACAGA 0.124360134728
ACAACAGC -0.390209262794
ACAACAGG -0.293291058842
ACAACAGT 0.57876889908
ACAACATA 0.66339563358

ACAACATC	2.77296491798
ACAACATG	-0.0927303480548
ACAACCAA	1.33861094284
ACAACCAC	0.141043076338
ACAACCAG	-0.55110662362
ACAACCAT	-0.857319081554
ACAACCCA	-0.637317853342
ACAACCCC	0.027820672706
ACAACCCG	-0.0792294659686
ACAACCCT	-0.389642326116
ACAACCGA	0.235105424995
ACAACCGC	-0.0667887476271
ACAACCGG	-0.32232062142
ACAACCGT	0.109796997845
ACAACCTA	-0.22067820654
ACAACCTC	-0.11130987451
ACAACCTG	-0.430288993633
ACAACGAA	-0.456086834184
ACAACGAC	0.202297330097
ACAACGAG	0.404795924021
ACAACGAT	2.38753266519
ACAACGCA	0.654215650622
ACAACGCC	-0.233721670837
ACAACGCG	0.317356984952
ACAACGCT	-0.554013971088
ACAACGGA	0.448191018997
ACAACGGC	0.00933550500641
ACAACGGG	-0.674900083053
ACAACGGT	-0.897144749485
ACAACGTA	1.61936979978
ACAACGTC	-0.142029530473
ACAACGTG	-0.890276686725
ACAACTAA	0.38666806496
ACAACTAC	1.1888202087
ACAACTAG	-0.0527624885372
ACAACTAT	0.845051397828
ACAACTCA	-0.333596100616
ACAACTCC	-1.12932897379
ACAACTCG	-0.282808611402
ACAACTCT	0.556748545323
ACAACTGA	-0.0200965852258
ACAACTGC	0.91601623918
ACAACTGG	-0.39232906752
ACAACTGT	0.105131597776
ACAACTTA	1.91256989782
ACAACTTC	-0.183852676544
ACAACTTG	-0.480191706152
ACAAGAAA	-0.276560023019
ACAAGAAC	-0.766969658438
ACAAGAAG	-0.66020913057
ACAAGAAT	2.95053947002
ACAAGACA	-0.528555403846
ACAAGACC	-0.231759217831
ACAAGACG	-0.27866466761 2

ACAAGACT -0.465658628152
ACAAGAGA 2.6009504752
ACAAGAGC -1.06621316032
ACAAGAGG -0.674216831566
ACAAGAGT -0.358472309667
ACAAGATA 4.20409631945
ACAAGATC 4.31643629882
ACAAGATG 0.500373763159
ACAAGCAA -0.0934015759878
ACAAGCAC -0.776166369818
ACAAGCAG 0.344480030184
ACAAGCAT -0.211772935637
ACAAGCCA -0.784931017424
ACAAGCCC -0.584782766792
ACAAGCCG -1.20671386985
ACAAGCCT -0.362770207215
ACAAGCGA 0.0181315184031
ACAAGCGC -0.442882358826
ACAAGCGG 0.136652649706
ACAAGCGT -0.47393501494
ACAAGCTA -0.307516490706
ACAAGCTC -0.488636161305
ACAAGCTG -0.622696689745
ACAAGGAA -0.598142764199
ACAAGGAC -0.548666103678
ACAAGGAG -0.805529978073
ACAAGGAT 3.44675386787
ACAAGGCA -1.11857155291
ACAAGGCC -1.29892432991
ACAAGGCG -0.153754324629
ACAAGGCT -0.877961431548
ACAAGGGA -0.654746255258
ACAAGGGC -1.06559943633
ACAAGGGG 0.0236341237171
ACAAGGGT -0.366326826562
ACAAGGTA 0.309839650313
ACAAGGTC 0.202389597799
ACAAGGTG -0.685641298271
ACAAGTAA -0.0797344552079
ACAAGTAC -0.810811454558
ACAAGTAG -0.0310270407179
ACAAGTAT 0.491350870539
ACAAGTCA -0.00956081594032
ACAAGTCC -0.858424725696
ACAAGTCG -0.387083400313
ACAAGTCT -0.121313993633
ACAAGTGA 0.542794950317
ACAAGTGC 0.0678787088735
ACAAGTGG -0.702410495807
ACAAGTGT -0.448793242189
ACAAGTTA 1.06528159632
ACAAGTTC 0.0722887391252
ACAAGTTG -0.222066926949
ACAATAAA 1.2007431301

ACAATAAC 0.170405377685
ACAATAAG 0.0328002534507
ACAATAAT 1.99086231078
ACAATACA 1.3056585653
ACAATACC 0.56171375008
ACAATACG 1.38721197502
ACAATACT 1.89723568524
ACAATAGA 1.223502671
ACAATAGC 0.421904394865
ACAATAGG 1.13725406371
ACAATAGT 1.68656368888
ACAATATA 2.9186475176
ACAATATC 7.91896732347
ACAATATG 1.9867594039
ACAATCAA 2.98829630263
ACAATCAC 2.99090619785
ACAATCAG 1.79409137172
ACAATCAT 2.31854752803
ACAATCCA 5.65761557719
ACAATCCC 3.41810304722
ACAATCCG 4.90677081066
ACAATCCT 3.48264469729
ACAATCGA 1.18317933252
ACAATCGC 2.76513235713
ACAATCGG 2.29001380634
ACAATCGT 1.26770255991
ACAATCTA 6.28711838226
ACAATCTC 4.59216199179
ACAATCTG 8.41358860052
ACAATGAA 0.723256462062
ACAATGAC -0.965799761692
ACAATGAG 0.0012609048088
ACAATGAT 1.92926512437
ACAATGCA 0.465553029988
ACAATGCC -0.298588218223
ACAATGCG -0.915155770973
ACAATGCT -0.306888129355
ACAATGGA -0.563347385042
ACAATGGC 0.160653491799
ACAATGGG -0.475578059639
ACAATGGT -0.397995559093
ACAATGTA 1.06073800007
ACAATGTC -1.09809282779
ACAATGTG -0.353821285586
ACAATTAA 0.134541470572
ACAATTAC 2.06318999576
ACAATTAG 2.80097927444
ACAATTAT 1.26019358948
ACAATTCA -0.292907873425
ACAATTCC 1.06903974087
ACAATTCG 1.926049608
ACAATTCT 1.29598796909
ACAATTGA 0.130782019108
ACAATTGC -0.0659081530374

ACAATTGG -0.294559020954
ACAATTGT 0.154263234592
ACAATTTA 1.56012504771
ACAATTTC 2.15931203138
ACAATTTG 1.11394718975
ACACAAAA 1.45876231114
ACACAAAC 0.344551125978
ACACAAAG 1.30057207948
ACACAAAT 2.1118456567
ACACAACA 1.03514482131
ACACAACC 0.0378407361067
ACACAACG -0.470302856311
ACACAACT 0.724370731797
ACACAAGA 0.497713421297
ACACAAGC -1.50003132823
ACACAAGG -0.843908114736
ACACAAGT 0.00669450538202
ACACAATA 2.94103694217
ACACAATC 5.22084954766
ACACAATG 0.462008434193
ACACACAA 0.242026809601
ACACACAC 0.0357052484849
ACACACAG 0.252291787555
ACACACAT -0.206179108147
ACACACCA 0.240900254931
ACACACCC -0.31878987885
ACACACCG 2.07349470372
ACACACCT -0.165061953761
ACACACGA 0.519042159356
ACACACGC -0.968146968405
ACACACGG -0.808404130079
ACACACGT -0.0280556547589
ACACACTA 2.28021774696
ACACACTC -0.459724220445
ACACACTG 0.252285514397
ACACAGAA 1.44844165891
ACACAGAC 0.0014438719245
ACACAGAG -0.15959907845
ACACAGAT 4.25454270568
ACACAGCA 0.340611582595
ACACAGCC -0.224078781077
ACACAGCG -0.0651728866138
ACACAGCT 0.0818568737501
ACACAGGA 0.249015107894
ACACAGGC -0.153905141809
ACACAGGG -0.449480675781
ACACAGGT -0.212380386462
ACACAGTA 0.274097285641
ACACAGTC 0.104167361076
ACACAGTG 0.415871969061
ACACATAA 0.0291118977797
ACACATAC 1.00601384267
ACACATAG -0.0210613446887
ACACATAT 2.03481937618

ACACATCA 0.44272239329
ACACATCC 0.0182297978824
ACACATCG 0.67421081979
ACACATCT 0.0698704366187
ACACATGA −0.136254565539
ACACATGC −0.104903934407
ACACATGG −0.110533571176
ACACATGT −0.578583840912
ACACATTA 1.225539095
ACACATTC 0.824219284797
ACACATTG −0.0693468892862
ACACCAAA 1.59131989541
ACACCAAC 0.00548692241837
ACACCAAG −0.345649974198
ACACCAAT 1.42619886941
ACACCACA −0.377912304329
ACACCACC −0.292246316611
ACACCACG 0.742160101693
ACACCACT −0.287550596277
ACACCAGA 1.99055649431
ACACCAGC −0.0479980248443
ACACCAGG −0.829960792888
ACACCAGT −0.842605911636
ACACCATA 0.477193397889
ACACCATC −0.757019122541
ACACCATG 0.592744450141
ACACCCAA −0.220167989669
ACACCCAC −0.519406525298
ACACCCAG −1.2160857069
ACACCCAT −0.373017149839
ACACCCCA 0.519178077785
ACACCCCC −1.19717893069
ACACCCCG −1.12089366699
ACACCCCT −0.75568241707
ACACCCGA −0.49818129435
ACACCCGC −0.255925775854
ACACCCGG −0.917827875007
ACACCCGT −0.607722660995
ACACCCTA 0.43562640578
ACACCCTC −1.12934622497
ACACCCTG −0.8523510016
ACACCGAA 0.908207202682
ACACCGAC −0.216626269072
ACACCGAG −0.0654708616308
ACACCGAT 0.882977605716
ACACCGCA −0.143343234364
ACACCGCC −0.69740268585
ACACCGCG −0.269717575655
ACACCGCT 0.358332209132
ACACCGGA −0.0376151637912
ACACCGGC −1.46146029195
ACACCGGG −1.56236116725
ACACCGGT −0.582892455105
ACACCGTA −0.338173415087

ACACCGTC 0.224846720199
ACACCGTG -0.416553129494
ACACCTAA 1.35933275283
ACACCTAC -0.835380017474
ACACCTAG 0.448147629652
ACACCTAT 1.56036395049
ACACCTCA -0.421064053041
ACACCTCC -0.157149410152
ACACCTCG 0.788919700546
ACACCTCT -0.806772324788
ACACCTGA 0.703627488508
ACACCTGC -1.00082698632
ACACCTGG -0.856455476768
ACACCTGT -0.340769457078
ACACCTTA 0.253312221297
ACACCTTC -0.15691782606
ACACCTTG -0.314921692642
ACACGAAA 2.79487549146
ACACGAAC 0.321826610207
ACACGAAG -0.52388059404
ACACGAAT 3.02520861132
ACACGACA 0.576906293842
ACACGACC -0.851976964539
ACACGACG -0.45824924411
ACACGACT 0.0953391977432
ACACGAGA 0.0652479031313
ACACGAGC 0.297253081042
ACACGAGG -0.282426471511
ACACGAGT 0.284543662422
ACACGATA 1.56982700971
ACACGATC 3.29349250893
ACACGATG 0.0592400471963
ACACGCAA 0.440071983928
ACACGCAC -0.178255451093
ACACGCAG -0.127024658696
ACACGCAT 0.328742767181
ACACGCCA -0.0698296610901
ACACGCCC -1.21756878606
ACACGCCG -0.679936122222
ACACGCCT -0.249717701619
ACACGCGA 0.196163226852
ACACGCGC -0.320358168413
ACACGCGG -0.785082618748
ACACGCGT 0.802349225457
ACACGCTA 0.608978860936
ACACGCTC -0.348724605887
ACACGCTG -0.667633936125
ACACGGAA 0.0172611176955
ACACGGAC -0.441038050299
ACACGGAG -0.97209017113
ACACGGAT 3.15898162
ACACGGCA -0.709930967025
ACACGGCC -1.0913345453
ACACGGCG -0.997823711809

ACACGGCT -0.996974221629
ACACGGGA 0.0623295776358
ACACGGGC -1.28478071049
ACACGGGG -0.521290563826
ACACGGGT -0.32727171157
ACACGGTA 0.284529547816
ACACGGTC -0.671555182796
ACACGGTG -0.808404130079
ACACGTAA 1.72807579085
ACACGTAC -0.277511974784
ACACGTAG 0.233943845192
ACACGTAT 0.882910430646
ACACGTCA -0.593576427754
ACACGTCC -0.877275043483
ACACGTCG 0.0511599579852
ACACGTCT -0.334917384573
ACACGTGA -0.484677537066
ACACGTGC -0.802185600579
ACACGTGG -0.384916024136
ACACGTGT 0.93459654978
ACACGTTA 0.481864286971
ACACGTTC -1.19200566618
ACACGTTG -0.60823993517
ACACTAAA 1.79118167182
ACACTAAC 0.837238701987
ACACTAAG -0.360794423747
ACACTAAT 1.72935446961
ACACTACA 0.84097985674
ACACTACC 0.151884400707
ACACTACG -0.0514035656307
ACACTACT -0.534395191416
ACACTAGA 1.5377377142
ACACTAGC -0.625337427987
ACACTAGG 0.0618522948454
ACACTAGT -0.283431222358
ACACTATA 1.31010414345
ACACTATC 1.63386184087
ACACTATG -0.0535680666095
ACACTCAA 0.809739790024
ACACTCAC 0.00254559534263
ACACTCAG 0.109336443476
ACACTCAT -0.589122746776
ACACTCCA -0.602841882493
ACACTCCC -1.04772746985
ACACTCCG 0.0298610174277
ACACTCCT 0.246127364045
ACACTCGA 0.183873587072
ACACTCGC 0.071341492229
ACACTCGG -0.234134392374
ACACTCGT 0.326918062274
ACACTCTA 0.170625199605
ACACTCTC 0.81701560807
ACACTCTG 0.354941567097
ACACTGAA 0.642242282571

ACACTGAC -0.252554214675
ACACTGAG -0.411593936514
ACACTGAT 1.83011498299
ACACTGCA 0.221566381196
ACACTGCC -0.877995411155
ACACTGCG -0.700542140174
ACACTGCT -0.100468550141
ACACTGGA 0.134745870979
ACACTGGC -0.815024403088
ACACTGGG -0.61847642253
ACACTGGT -0.790189753711
ACACTGTA 1.41472787678
ACACTGTC -0.314105659306
ACACTGTG 0.164257159834
ACACTTAA 1.31685981212
ACACTTAC 0.00882136741129
ACACTTAG 0.140482674201
ACACTTAT 0.751978639885
ACACTTCA -0.0667955435485
ACACTTCC 0.904952740457
ACACTTCG -0.450473403075
ACACTTCT 1.07367089995
ACACTTGA 0.703233063683
ACACTTGC -0.182869358988
ACACTTGG 0.316237226204
ACACTTTA 1.5327787826
ACACTTTC 0.00860729588591
ACACTTTG 0.41003374978
ACAGAAAA 1.39870570784
ACAGAAAC 1.15465266813
ACAGAAAG -0.0425147617682
ACAGAAAT 2.94435596565
ACAGAACA 0.0367520817683
ACAGAACC 0.186944298036
ACAGAACG -0.163972253897
ACAGAACT 0.949650038534
ACAGAAGA 0.265177115988
ACAGAAGC 0.05259076083
ACAGAAGG -0.783706183275
ACAGAAGT -0.13993481838
ACAGAATA 2.49838067843
ACAGAATC 11.9571785985
ACAGAATG -0.115540596988
ACAGACAA 0.574093566511
ACAGACAC 0.0698440370777
ACAGACAG -0.220103428415
ACAGACAT 0.275986029039
ACAGACCA 0.301847646699
ACAGACCC -1.46593331517
ACAGACCG -0.0359169675759
ACAGACCT -0.91657533441
ACAGACGA 1.64194951015
ACAGACGC -0.0847702329953
ACAGACGG -0.238030023649

ACAGACGT -0.139424078746
ACAGACTA 0.758567547103
ACAGACTC -0.557157868899
ACAGACTG -0.521908469914
ACAGAGAA 0.872994135738
ACAGAGAC -0.115077951567
ACAGAGAG -0.501722492183
ACAGAGAT 3.26012740985
ACAGAGCA -0.0419496547623
ACAGAGCC -0.867442913448
ACAGAGCG 0.00400907088667
ACAGAGCT -0.804075650885
ACAGAGGA 0.19663841859
ACAGAGGC -0.297076387084
ACAGAGGG -0.843014189685
ACAGAGGT -0.829960792888
ACAGAGTA 2.22785804746
ACAGAGTC -0.700391322994
ACAGAGTG -0.061580980751
ACAGATAA 1.0171340612
ACAGATAC 2.89202763194
ACAGATAG 0.670652370771
ACAGATAT 8.15879486834
ACAGATCA 0.885201701698
ACAGATCC 3.75546199727
ACAGATCG 1.45825261703
ACAGATCT 4.97034378054
ACAGATGA 0.876985694058
ACAGATGC 0.33756387321
ACAGATGG -0.308655068929
ACAGATGT 0.905888486563
ACAGATTA 5.34796333748
ACAGATTC 10.8732479482
ACAGATTG 2.33952758304
ACAGCAAA 0.736421207419
ACAGCAAC 0.262837227959
ACAGCAAG -0.438298509814
ACAGCAAT 1.53221367559
ACAGCACA -0.281257573024
ACAGCACC 0.52728665759
ACAGCACG -0.195405742993
ACAGCACT -0.67305969525
ACAGCAGA 0.150010033297
ACAGCAGC -0.760091924559
ACAGCAGG -0.0576142536825
ACAGCAGT -0.563889751849
ACAGCATA -0.0387127051039
ACAGCATC 0.884434808102
ACAGCATG 0.223843537642
ACAGCCAA -0.515823245028
ACAGCCAC -1.05061965719
ACAGCCAG -0.248974332366
ACAGCCAT 0.359198427734
ACAGCCCA -1.20507892798

ACAGCCCC -1.16417479959
ACAGCCCG -0.568980419771
ACAGCCCT -0.876853696353
ACAGCCGA -0.642190790397
ACAGCCGC -1.2656975017
ACAGCCGG -1.03934574628
ACAGCCGT -1.20152283139
ACAGCCTA 0.349431643098
ACAGCCTC -1.15016082544
ACAGCCTG -0.203758191825
ACAGCGAA 0.209738341311
ACAGCGAC 0.0986485501031
ACAGCGAG -0.586804292039
ACAGCGAT 0.404540292822
ACAGCGCA 0.0209377111948
ACAGCGCC -0.664775989777
ACAGCGCG -0.70897901526
ACAGCGCT -0.653299508136
ACAGCGGA 1.77962651432
ACAGCGGC -0.566993135513
ACAGCGGG -0.6498662609
ACAGCGGT -0.244257440123
ACAGCGTA 0.126118710092
ACAGCGTC -1.12611920781
ACAGCGTG -0.782453642677
ACAGCTAA -0.0286327853181
ACAGCTAC -0.956029317714
ACAGCTAG -0.875029252828
ACAGCTAT 0.915236015123
ACAGCTCA -0.86579673217
ACAGCTCC -0.429281106207
ACAGCTCG -0.280646201476
ACAGCTCT -0.0714423855242
ACAGCTGA 1.92975260104
ACAGCTGC -0.823082797627
ACAGCTGG -0.416270837373
ACAGCTGT -0.19741576745
ACAGCTTA -0.308333308187
ACAGCTTC -0.633983408349
ACAGCTTG -0.519363135953
ACAGGAAA 0.2882659977
ACAGGAAC 0.750779421133
ACAGGAAG 0.118282228526
ACAGGAAT 0.637435997823
ACAGGACA -0.56798141932
ACAGGACC -0.862525802904
ACAGGACG -0.249632752601
ACAGGACT -0.0810298623871
ACAGGAGA 0.326772472726
ACAGGAGC -0.637342684594
ACAGGAGG -0.913818804121
ACAGGAGT -0.194315258983
ACAGGATA 2.52697843861
ACAGGATC 3.7140761426

ACAGGATG -0.351750097836
ACAGGCAA -0.374967579292
ACAGGCAC -1.29068035444
ACAGGCAG -1.13449465823
ACAGGCAT 0.819808209019
ACAGGCCA -1.29793107986
ACAGGCCC -0.957519715562
ACAGGCCG -1.03470099536
ACAGGCCT -0.669714010849
ACAGGCGA -0.00933811882235
ACAGGCGC -0.970203518786
ACAGGCGG -0.415573732662
ACAGGCGT -0.559211544082
ACAGGCTA -0.333512981269
ACAGGCTC -1.34667979264
ACAGGCTG -0.421778408937
ACAGGGAA 1.62143889647
ACAGGGAC -0.822771230767
ACAGGGAG 0.0215545717563
ACAGGGAT 0.623548793741
ACAGGGCA -0.349539855078
ACAGGGCC -1.48145964322
ACAGGGCG -0.936120665854
ACAGGGCT -1.02454161557
ACAGGGGA -0.47233771202
ACAGGGGC -0.411368102817
ACAGGGGG -1.35105113841
ACAGGGGT 0.0800807858198
ACAGGGTA 1.2768493474
ACAGGGTC -0.700296180094
ACAGGGTG -1.17920946887
ACAGGTAA -0.431603220287
ACAGGTAC 0.328608939805
ACAGGTAG -1.52811939431
ACAGGTAT 2.76241294303
ACAGGTCA 0.438434166861
ACAGGTCC -0.613444042703
ACAGGTCG -1.4666215329
ACAGGTCT 0.145424615996
ACAGGTGA 0.227269466193
ACAGGTGC 0.16228869505
ACAGGTGG -0.732388612188
ACAGGTTA -0.510486617026
ACAGGTTC -0.639878347436
ACAGGTTG -0.196782178466
ACAGTAAA 0.40390043068
ACAGTAAC -0.269905247639
ACAGTAAG 0.504862207889
ACAGTAAT 2.21396613851
ACAGTACA 0.380296104465
ACAGTACC -0.102073694509
ACAGTACG -0.0598088135446
ACAGTACT -0.084168009803
ACAGTAGA 0.153436745993

ACAGTAGC -0.730530711819
ACAGTAGG 0.123062636496
ACAGTAGT -0.0764332056774
ACAGTATA 0.953595855075
ACAGTATC 4.49742161928
ACAGTATG -0.178272440897
ACAGTCAA -0.202280340293
ACAGTCAC -0.592931599362
ACAGTCAG -0.264970624528
ACAGTCAT 0.211572717337
ACAGTCCA -0.0930847814961
ACAGTCCC -0.622696689745
ACAGTCCG -1.19366726897
ACAGTCCT -1.01210717039
ACAGTCGA 0.12959665358
ACAGTCGC -0.884948684315
ACAGTCGG -0.186381543465
ACAGTCGT -0.652481122365
ACAGTCTA 0.155481011438
ACAGTCTC -1.05252852697
ACAGTCTG -0.863080454646
ACAGTGAA 0.0011286457223
ACAGTGAC -0.579989551123
ACAGTGAG 0.137708369964
ACAGTGAT 0.264093950663
ACAGTGCA -0.742736186726
ACAGTGCC -0.467469479834
ACAGTGCG -0.0655443098587
ACAGTGCT -1.02163871159
ACAGTGGA 0.653973088503
ACAGTGGC -0.782531534392
ACAGTGGG -0.539108424317
ACAGTGGT -0.457986555609
ACAGTGTA 2.3102348092
ACAGTGTC 0.328066311616
ACAGTGTG -0.809289690919
ACAGTTAA 1.16511237537
ACAGTTAC 0.256007849675
ACAGTTAG 1.1748253154
ACAGTTAT 1.56609996957
ACAGTTCA 0.430398773903
ACAGTTCC 0.902682379933
ACAGTTCG -0.310793170367
ACAGTTCT -0.320758082251
ACAGTTGA 0.642605602986
ACAGTTGC -0.425027382149
ACAGTTGG -1.04337050007
ACAGTTTA -0.278765560908
ACAGTTTC 0.635634555878
ACAGTTTG -0.239941768626
ACATAAAA 2.20416824946
ACATAAAC 0.732940650115
ACATAAAG -0.523354432891
ACATAAAT 0.844507724113

ACATAACA 1.36626616099
ACATAACC 0.317277263566
ACATAACG 0.0647899625788
ACATAACT 0.575554428239
ACATAAGA 1.23868554365
ACATAAGC 0.246969535541
ACATAAGG -0.0473236603321
ACATAAGT -0.251530644354
ACATAATA 2.47335940259
ACATAATC 4.2367729394
ACATAATG 0.202913145132
ACATACAA 4.06742537303
ACATACAC 0.358122581094
ACATACAG 0.941785850519
ACATACAT 0.714155154964
ACATACCA -0.346029500272
ACATACCC 0.529546040087
ACATACCG -0.0863955037459
ACATACCT 0.355520527327
ACATACGA 0.133682047892
ACATACGC -0.285623429786
ACATACGG 0.967767703712
ACATACGT 0.534417931615
ACATACTA 0.45997932888
ACATACTC -0.38140070308
ACATACTG -0.4990553544
ACATAGAA 0.896751108805
ACATAGAC -0.206681483571
ACATAGAG -0.118875826126
ACATAGAT 2.49764619615
ACATAGCA -1.35999457103
ACATAGCC -0.958929869261
ACATAGCG 0.528324342517
ACATAGCT 0.133814306978
ACATAGGA 1.27703309866
ACATAGGC -0.599559975201
ACATAGGG 0.233787016236
ACATAGGT -0.543090311518
ACATAGTA 0.728005242859
ACATAGTC 0.567470941067
ACATAGTG -0.330141942853
ACATATAA 1.14811420756
ACATATAC 0.902862733232
ACATATAG -0.307493750508
ACATATAT 2.86795830787
ACATATCA 2.90478174681
ACATATCC 3.11491895644
ACATATCG 2.57442520967
ACATATCT 5.97225105699
ACATATGA 1.49306106527
ACATATGC 0.57795547956
ACATATGG 0.0474606242873
ACATATGT 1.4457094371
ACATATTA 2.81217058877

ACATATTC 1.30153971414
ACATATTG 1.61448248674
ACATCAAA -0.516898830286
ACATCAAC 1.96800945665
ACATCAAG 1.02205430832
ACATCAAT 1.87631313418
ACATCACA -0.399213074557
ACATCACC -0.0830161011189
ACATCACG 0.481763393676
ACATCACT 0.0472243353264
ACATCAGA 1.80326011527
ACATCAGC 0.727234167157
ACATCAGG 0.418264917552
ACATCAGT 0.0182297978824
ACATCATA 0.196163226852
ACATCATC 1.08125567104
ACATCATG -0.298231693729
ACATCCAA 0.926461309053
ACATCCAC 1.46505481163
ACATCCAG 1.87866818234
ACATCCAT 0.469293923359
ACATCCCA 0.881411145824
ACATCCCC -0.337421943005
ACATCCCG 1.11895264727
ACATCCCT 0.0288643694103
ACATCCGA 0.744709879142
ACATCCGC -0.143030621978
ACATCCGG 0.79677395606
ACATCCGT 1.33602457196
ACATCCTA 0.7081491287
ACATCCTC 0.359266909712
ACATCCTG 0.703385971915
ACATCGAA 0.706383757415
ACATCGAC -0.708457036218
ACATCGAG 1.36620761152
ACATCGAT 1.47668446289
ACATCGCA 0.401132922364
ACATCGCC -0.389957290937
ACATCGCG 0.555129024968
ACATCGCT 0.437077596389
ACATCGGA 0.7742282253
ACATCGGC -0.782021317521
ACATCGGG 0.879090600034
ACATCGGT 0.8097031966
ACATCGTA 0.384457822202
ACATCGTC -0.229768535612
ACATCGTG 0.167689361543
ACATCTAA 1.90696744474
ACATCTAC 0.487260771358
ACATCTAG 1.58281114039
ACATCTAT 0.253018428386
ACATCTCA 0.378200085464
ACATCTCC 0.168188077624
ACATCTCG 0.14752429434

ACATCTCT 1.01687686171
ACATCTGA 1.93012167185
ACATCTGC 1.53308329216
ACATCTGG 1.60490076027
ACATCTTA 1.94990642884
ACATCTTC 0.774454843142
ACATCTTG -0.0623039622396
ACATGAAA 0.458945564677
ACATGAAC 0.739816292941
ACATGAAG -0.57331281969
ACATGAAT 1.03791285239
ACATGACA 0.732732328984
ACATGACC -0.548165819307
ACATGACG -0.031687290624
ACATGACT -0.37312222524
ACATGAGA 1.48302401206
ACATGAGC -0.340443252848
ACATGAGG -0.216626269072
ACATGAGT 0.334832696936
ACATGATA 2.43218944912
ACATGATC 2.35396708643
ACATGATG -0.0265694390162
ACATGCAA 1.51565933373
ACATGCAC 0.292796786248
ACATGCAG 0.0186584636963
ACATGCAT 1.03912095811
ACATGCCA 0.0233737876496
ACATGCCC -0.741583493897
ACATGCCG -0.805374717406
ACATGCCT -0.497289983115
ACATGCGA 0.44252714124
ACATGCGC -0.00640071247052
ACATGCGG -0.215826441395
ACATGCGT -1.64007409721
ACATGCTA 0.85570792541
ACATGCTC -0.757770071861
ACATGCTG 0.223843537642
ACATGGAA 0.155208390436
ACATGGAC -0.316731498798
ACATGGAG -0.154341126307
ACATGGAT 3.30422745099
ACATGGCA -0.340113127895
ACATGGCC -1.49816244983
ACATGGCG 1.22711365772
ACATGGCT 0.153250119535
ACATGGGA 0.700465032604
ACATGGGC 0.0344788460465
ACATGGGG 0.0400567292595
ACATGGGT -0.342853713907
ACATGGTA -0.0396782487116
ACATGGTC -1.41548901999
ACATGGTG -0.278777061698
ACATGTAA 1.53610381785
ACATGTAC 0.311925475432

ACATGTAG 0.218832852488
ACATGTAT 0.086437324801
ACATGTCA -0.273229760131
ACATGTCC -0.0337007130415
ACATGTCG -0.385753229381
ACATGTCT -0.701630794512
ACATGTGA 1.34349459653
ACATGTGC -0.217577175311
ACATGTGG -1.38792999025
ACATGTTA 1.42606138269
ACATGTTC 0.28799703604
ACATGTTG 0.737971723033
ACATTAAA 0.665600910088
ACATTAAC 0.635455248104
ACATTAAG 0.732521132657
ACATTAAT 0.259745083704
ACATTACA 0.804280835436
ACATTACC 0.929344348033
ACATTACG 0.915912470688
ACATTACT 2.15143294461
ACATTAGA 0.844873658344
ACATTAGC 0.364998223921
ACATTAGG 0.629840248705
ACATTAGT 0.0342862078118
ACATTATA 0.353722483343
ACATTATC 2.55611699731
ACATTATG 1.04884696722
ACATTCAA 1.28221368185
ACATTCAC 0.496973711386
ACATTCAG 0.529350788036
ACATTCAT 1.66651728892
ACATTCCA -0.311087486042
ACATTCCC 0.579531349189
ACATTCCG 1.59362319002
ACATTCCT 0.943977012421
ACATTCGA 0.36194136618
ACATTCGC -0.444599374516
ACATTCGG -0.18084103782
ACATTCGT -0.0052898406966
ACATTCTA 1.54144750316
ACATTCTC 1.41879183781
ACATTCTG 0.371012353014
ACATTGAA 1.28544174454
ACATTGAC 0.403506005855
ACATTGAG 0.86129025211
ACATTGAT 0.804613574205
ACATTGCA 0.629689170144
ACATTGCC -0.0106374467254
ACATTGCG 0.740520193574
ACATTGCT 0.393037934402
ACATTGGA 1.8878494722
ACATTGGC -0.0289767634957
ACATTGGG -0.542234809561
ACATTGGT 0.537422774418

ACATTGTA	-0.60565539397
ACATTGTC	0.686585669969
ACATTGTG	0.25042317054
ACATTTAA	0.804907367116
ACATTTAC	0.843448605894
ACATTTAG	0.338309333516
ACATTTAT	1.1852813633
ACATTTCA	0.348164726513
ACATTTCC	1.13270027358
ACATTTCG	1.43181700539
ACATTTCT	-0.604872033333
ACATTTGA	0.291914100605
ACATTTGC	0.592660023886
ACATTTGG	-0.230321619064
ACATTTTA	1.89810869976
ACATTTTC	1.00383548847
ACATTTTG	0.315179676275
ACCAAAAA	2.37684634011
ACCAAAAC	0.685114091596
ACCAAAAG	0.0760523726952
ACCAAAAT	2.69616917603
ACCAAACA	0.0869250628551
ACCAAACC	-0.284370105043
ACCAAACG	0.0935009009935
ACCAAACT	0.512656084255
ACCAAAGA	2.20001280489
ACCAAAGC	-0.39306537947
ACCAAAGG	-0.985016536473
ACCAAAGT	0.681007263993
ACCAAATA	0.681057972022
ACCAAATC	6.3010672724
ACCAAATG	-0.029985696448
ACCAACAA	-0.0795961843448
ACCAACAC	-0.0986976898427
ACCAACAG	0.826192715831
ACCAACAT	0.812977785208
ACCAACCA	-0.412445256365
ACCAACCC	-0.745582632283
ACCAACCG	-0.472624447628
ACCAACCT	-0.036876499407
ACCAACGA	-0.23240195517
ACCAACGC	-0.275334666107
ACCAACGG	-0.796526427691
ACCAACGT	0.927175403567
ACCAACTA	-0.375585223999
ACCAACTC	-0.603584990365
ACCAACTG	-0.632989112766
ACCAAGAA	0.438529832525
ACCAAGAC	-0.976202749128
ACCAAGAG	0.0536754944446
ACCAAGAT	2.08393480734
ACCAAGCA	1.25831817654
ACCAAGCC	-1.4887464393
ACCAAGCG	-0.345850192499

ACCAAGCT -0.124849441072
ACCAAGGA 0.121650130363
ACCAAGGC -1.31920832574
ACCAAGGG -0.903502333992
ACCAAGGT -0.902819866651
ACCAAGTA 0.0403128832215
ACCAAGTC -0.725884392607
ACCAAGTG -0.807925801762
ACCAATAA -0.135791135973
ACCAATAC -0.222984637725
ACCAATAG -0.0104782653348
ACCAATAT 2.02994853018
ACCAATCA -0.0540359396625
ACCAATCC 1.50686645691
ACCAATCG 0.947738293556
ACCAATCT 2.95704813309
ACCAATGA 0.764391651778
ACCAATGC 0.126649314727
ACCAATGG -0.518787573684
ACCAATTA 0.368671419459
ACCAATTC 0.913061320262
ACCAATTG -0.662069644755
ACCACAAA 0.940895323429
ACCACAAC 0.855100474586
ACCACAAG 0.427653221641
ACCACAAT 0.912796802089
ACCACACA 0.376637807677
ACCACACC 0.499908503922
ACCACACG -0.846569502125
ACCACACT -0.597287262242
ACCACAGA -0.642313116982
ACCACAGC -0.790308420954
ACCACAGG 0.420515413076
ACCACAGT -0.20043001999
ACCACATA 0.453638734176
ACCACATC 0.234158962244
ACCACATG -0.2241153745
ACCACCAA 0.426721134877
ACCACCAC -1.42639046212
ACCACCAG -0.526487875439
ACCACCAT -1.0035652199
ACCACCCA -0.407076217046
ACCACCCC 0.168988428064
ACCACCCG -1.23844167462
ACCACCCT -0.861637366867
ACCACCGA -0.247125318971
ACCACCGC -0.206273466903
ACCACCGG -0.329838478822
ACCACCGT -0.456398923808
ACCACCTA -0.148021703513
ACCACCTC -0.420461568467
ACCACCTG -0.084168009803
ACCACGAA -0.381783888497
ACCACGAC -0.470042520244

ACCACGAG -0.251462423758
ACCACGAT -0.299289243658
ACCACGCA -0.415676978392
ACCACGCC -1.09947684333
ACCACGCG -0.562003622268
ACCACGCT -0.501722492183
ACCACGGA 1.6053320399
ACCACGGC -0.934066990671
ACCACGGG -0.669028929691
ACCACGGT 0.641498651936
ACCACGTA -0.228095954793
ACCACGTC -0.398369857536
ACCACGTG 0.0404817357311
ACCACTAA 0.556290082008
ACCACTAC -0.053537484963
ACCACTAG -0.512812390448
ACCACTAT -0.125304506427
ACCACTCA 0.886102422671
ACCACTCC -0.863222646233
ACCACTCG -0.305301020317
ACCACTCT -0.156698788284
ACCACTGA 1.00873743888
ACCACTGC -1.07863584333
ACCACTGG -0.641008300066
ACCACTTA 0.495631778283
ACCACTTC -0.573411621932
ACCACTTG -0.550892552095
ACCAGAAA 1.84282074227
ACCAGAAC -0.171081310486
ACCAGAAG -0.21321314822
ACCAGAAT 0.0455878251673
ACCAGACA -1.01765630163
ACCAGACC -0.55644952478
ACCAGACG -0.374897529025
ACCAGACT -0.478731105806
ACCAGAGA 0.838895338529
ACCAGAGC -1.22377476924
ACCAGAGG -1.32271998745
ACCAGAGT -0.149275551018
ACCAGATA 2.89354861144
ACCAGATC 1.22284921702
ACCAGATG 0.924912361727
ACCAGCAA -0.294639787866
ACCAGCAC 0.742160101693
ACCAGCAG -0.825656622182
ACCAGCAT 0.625371407595
ACCAGCCA -0.716537909573
ACCAGCCC -0.882983094729
ACCAGCCG 0.217902856777
ACCAGCCT -1.00425866527
ACCAGCGA 0.284706241773
ACCAGCGC -0.599739544356
ACCAGCGG -0.253224919845
ACCAGCGT -0.896294736542

ACCAGCTA 0.448796901532
ACCAGCTC -1.34482450608
ACCAGCTG -1.22806273429
ACCAGGAA -0.20977754855
ACCAGGAC -0.829960792888
ACCAGGAG -0.391253743643
ACCAGGAT 3.0834987978
ACCAGGCA -1.69752368049
ACCAGGCC -0.667329949331
ACCAGGCG 1.02945558954
ACCAGGCT -1.35760554326
ACCAGGGA -0.147433594926
ACCAGGGC -1.67507544506
ACCAGGGG -1.25409869347
ACCAGGGT -0.829672750372
ACCAGGTA -0.245301920972
ACCAGGTC -1.27354365439
ACCAGGTG -1.00360599543
ACCAGTAA 0.0692703044792
ACCAGTAC -0.00894081879968
ACCAGTAG -0.987661718203
ACCAGTAT 1.39683447701
ACCAGTCA -1.16816217581
ACCAGTCC -0.495952232117
ACCAGTCG -1.15824614228
ACCAGTCT -0.911154541534
ACCAGTGA -0.694574014241
ACCAGTGC -1.20925005545
ACCAGTGG -1.23373837422
ACCAGTTA 0.866758877817
ACCAGTTC -0.69102340667
ACCAGTTG 0.835121511077
ACCATAAA 1.77019560503
ACCATAAC -0.2935728282
ACCATAAG 0.506860470174
ACCATAAT 1.68085720592
ACCATACA 0.190290505201
ACCATACC 1.38968699733
ACCATACG 0.50760932844
ACCATACT 0.134450248396
ACCATAGA 0.0250019335978
ACCATAGC -0.748147569864
ACCATAGG 0.529864402868
ACCATAGT -0.25883260056
ACCATATA 1.53775653367
ACCATATC 4.61245409045
ACCATATG 0.251621082385
ACCATCAA -0.194629962422
ACCATCAC -0.342735046663
ACCATCAG 0.145484472381
ACCATCAT 0.784027159872
ACCATCCA -0.136394404691
ACCATCCC 0.85681984271
ACCATCCG -0.406322915292

ACCATCCT -0.406573318859
ACCATCGA -0.136254565539
ACCATCGC 0.238257425636
ACCATCGG -1.10787686362
ACCATCGT -0.042139679181
ACCATCTA 1.24883107021
ACCATCTC -0.589176591384
ACCATCTG -0.0975073580642
ACCATGAA 1.94251534151
ACCATGAC -0.159876665702
ACCATGAG -0.256128085208
ACCATGAT 0.984665762374
ACCATGCA 0.0014438719245
ACCATGCC -0.212984700707
ACCATGCG -0.897365878313
ACCATGCT -0.420513844786
ACCATGGA -0.286062812245
ACCATGGC -0.720063947275
ACCATGGG -0.396399301699
ACCATGGT -0.282810441073
ACCATGTA 0.382572215384
ACCATGTC -0.697251607289
ACCATGTG -0.193733162174
ACCATTAA 0.723929258285
ACCATTAC 0.139913646471
ACCATTAG -0.28715747836
ACCATTAT 1.67875622067
ACCATTCA 1.01310094321
ACCATTCC 0.180986627367
ACCATTCG -0.0352358071423
ACCATTCT -0.373888596073
ACCATTGA -0.196617769444
ACCATTGC 0.494672769215
ACCATTGG -0.373910552127
ACCATTTA 0.0674048240438
ACCATTTC 0.511052246795
ACCATTTG 0.249908771564
ACCCAAAA 2.12853382594
ACCCAAAC 0.185226498202
ACCCAAAG 0.317423375877
ACCCAAAT 0.574024823151
ACCCAACA 0.355818763726
ACCCAACC -0.0271136354946
ACCCAACG 0.0701846172946
ACCCAACT -0.353690594789
ACCCAAGA -0.014913649601
ACCCAAGC -0.680261803687
ACCCAAGG -1.59114581527
ACCCAAGT -0.702906859453
ACCCAATA 1.25147389951
ACCCAATC 1.32968214759
ACCCAATG -0.187668847815
ACCCACAA -0.524088131025
ACCCACAC -0.148445925839

ACCCACAG -0.831426620866
ACCCACAT -0.44033624072
ACCCACCA -0.327701422911
ACCCACCC -0.137627341669
ACCCACCG -0.448583091388
ACCCACCT -0.854502433499
ACCCACGA 0.0303563355481
ACCCACGC -0.753053963762
ACCCACGG -0.319745489957
ACCCACGT -0.95602879495
ACCCACTA 0.331185116794
ACCCACTC 0.100021326234
ACCCACTG -1.05559662412
ACCCAGAA 0.21547462177
ACCCAGAC -0.296188735191
ACCCAGAG -0.305005136352
ACCCAGAT 3.6382425463
ACCCAGCA -0.331538504709
ACCCAGCC -0.4545216812
ACCCAGCG -0.808469998241
ACCCAGCT -0.923246838211
ACCCAGGA -0.41995527232
ACCCAGGC -1.24875108744
ACCCAGGG -0.549557937676
ACCCAGGT -1.44356506251
ACCCAGTA 0.057081035231
ACCCAGTC -0.329372696822
ACCCAGTG 0.245623420332
ACCCATAA 1.04314466637
ACCCATAC 0.455325690983
ACCCATAG 0.0138187221044
ACCCATAT 1.75000413828
ACCCATCA -0.568394140856
ACCCATCC -0.377116135994
ACCCATCG -0.776547986945
ACCCATCT -0.444404645228
ACCCATGA 0.287615941676
ACCCATGC -0.497450471413
ACCCATGG -0.522279109014
ACCCATTA 1.62856232905
ACCCATTC -0.0810042469909
ACCCATTG -0.440168172355
ACCCCAAA 0.136070814278
ACCCCAAC 0.668263604384
ACCCCAAG -0.204863574585
ACCCCAAT -1.29992646694
ACCCCACA -0.426791185144
ACCCCACC -0.731206906003
ACCCCACG -0.992091613456
ACCCCACT -0.280507407849
ACCCCAGA -0.974279241979
ACCCCAGC -0.596388109559
ACCCCAGG -1.14939001112
ACCCCAGT -0.468727248064

ACCCCATA 0.116655650867
ACCCCATC -0.52766879748
ACCCCATG -0.450206793849
ACCCCCAA -0.388207079785
ACCCCCAC -1.02588041209
ACCCCCAG -1.04015707475
ACCCCCAT -0.785628644898
ACCCCCCA -0.901482115654
ACCCCCCC -1.55147201005
ACCCCCCG -1.35714812547
ACCCCCCT -1.17758524365
ACCCCCGA 0.0757557045862
ACCCCCGC -0.884416250009
ACCCCCGG -1.14172473449
ACCCCCGT -0.881635672613
ACCCCCTA -0.44731408375
ACCCCCTC -0.854700560747
ACCCCCTG -1.51789257807
ACCCCGAA -0.261260835567
ACCCCGAC -0.949313379041
ACCCCGAG -1.2232073098
ACCCCGAT 3.14256005999
ACCCCGCA -0.327701422911
ACCCCGCC -0.790892608816
ACCCCGCG -0.728432863147
ACCCCGCT -0.839291593026
ACCCCGGA -0.80044244673
ACCCCGGC -0.762801144779
ACCCCGGG -0.398484342674
ACCCCGGT -0.640992355789
ACCCCGTA -0.336339561825
ACCCCGTC -0.724400006535
ACCCCGTG 0.262112155418
ACCCCTAA -0.105609141948
ACCCCTAC -0.498266243368
ACCCCTAG -0.205335629744
ACCCCTAT -0.172921436907
ACCCCTCA -0.29493070558
ACCCCTCC -0.883528859497
ACCCCTCG -0.919570506094
ACCCCTCT -0.658626987782
ACCCCTGA -0.369341601866
ACCCCTGC -0.368043580871
ACCCCTGG -1.08142060283
ACCCCTTA -0.31878987885
ACCCCTTC -1.54019182598
ACCCCTTG -0.137959296294
ACCCGAAA -0.388089980831
ACCCGAAC -0.640601328924
ACCCGAAG -0.74821579046
ACCCGAAT 2.25165292086
ACCCGACA -0.285705242225
ACCCGACC -0.974324460994
ACCCGACG -1.34905156922

ACCCGACT -0.776266217587
ACCCGAGA 1.13343083514
ACCCGAGC -0.773684812966
ACCCGAGG -0.588854569261
ACCCGAGT -0.858563519323
ACCCGATA 2.77254357085
ACCCGATC 0.877168399792
ACCCGATG -0.576513698688
ACCCGCAA 0.438378753963
ACCCGCAC -0.466701540711
ACCCGCAG -0.96819035775
ACCCGCAT -0.231232795301
ACCCGCCA -0.458038831927
ACCCGCCC 0.409708068314
ACCCGCCG -0.759799699937
ACCCGCCT -0.382297503329
ACCCGCGA -0.341959266093
ACCCGCGC -0.365101992413
ACCCGCGG -0.189961164393
ACCCGCGT -0.224383290633
ACCCGCTA -0.930502791257
ACCCGCTC -1.55465746753
ACCCGCTG -0.258240309868
ACCCGGAA -0.219324772647
ACCCGGAC -0.564619267878
ACCCGGAG -0.0182998481495
ACCCGGAT 2.09548891932
ACCCGGCA -1.45150897191
ACCCGGCC -0.0697619632573
ACCCGGCG -0.912026510532
ACCCGGCT -0.903917146582
ACCCGGGA -1.03856264703
ACCCGGGC -1.6962199091
ACCCGGGG -1.42510524882
ACCCGGGT -0.374527412688
ACCCGGTA -0.0389066502465
ACCCGGTC -1.79048691954
ACCCGGTG -0.450575341896
ACCCGTAA 0.215620211318
ACCCGTAC -0.882155821985
ACCCGTAG -0.913302314091
ACCCGTAT 0.75627104842
ACCCGTCA -0.850740106837
ACCCGTCC -0.701851923341
ACCCGTCG -1.01206796315
ACCCGTCT -1.32009728454
ACCCGTGA -0.204783591818
ACCCGTGC -0.521908469914
ACCCGTGG -0.449546543943
ACCCGTTA -0.137627341669
ACCCGTTC -0.014292606934
ACCCGTTG -1.430572829
ACCCTAAA 0.557499756024
ACCCTAAC -0.745389209904

ACCCTAAG 0.319570887052
ACCCTAAT -0.36712351766
ACCCTACA -0.409426560338
ACCCTACC 0.141492129916
ACCCTACG -0.86303026938
ACCCTACT 1.01292372649
ACCCTAGA 1.53479978508
ACCCTAGC -0.109887174494
ACCCTAGG -0.149069059559
ACCCTAGT -0.223714415135
ACCCTATA 0.768461885957
ACCCTATC 0.293864530059
ACCCTATG -0.468324459028
ACCCTCAA -0.359176210299
ACCCTCAC -0.552793319045
ACCCTCAG -0.728795138036
ACCCTCAT -0.559932957281
ACCCTCCA -0.553985219113
ACCCTCCC -0.364336667107
ACCCTCCG -1.09872537125
ACCCTCCT -1.33986191514
ACCCTCGA -0.856537550589
ACCCTCGC -0.817467275464
ACCCTCGG 0.306672750921
ACCCTCGT -0.456842488372
ACCCTCTA -0.619499470089
ACCCTCTC -1.74200586151
ACCCTCTG -0.590928370827
ACCCTGAA 0.0508167639525
ACCCTGAC -0.141795332565
ACCCTGAG -0.641383382653
ACCCTGAT -0.142957435132
ACCCTGCA -0.77227178407
ACCCTGCC -0.257709705232
ACCCTGCG -1.19627036827
ACCCTGCT -1.08170629291
ACCCTGGA 0.228363086782
ACCCTGGC -0.600770694743
ACCCTGGG -1.53234802573
ACCCTGTA -0.925925999548
ACCCTGTC -0.612622520354
ACCCTGTG -0.468488345287
ACCCTTAA -0.339403999631
ACCCTTAC -0.890910798471
ACCCTTAG -0.59163253284
ACCCTTAT -0.895807259869
ACCCTTCA -0.546870412128
ACCCTTCC -0.283740959547
ACCCTTCG -1.10945142634
ACCCTTCT -0.784837442813
ACCCTTGA -0.848271619064
ACCCTTGC -0.53642926298
ACCCTTGG -0.539067648788
ACCCTTTA -0.800925479915

ACCCTTTC -1.31755848512
ACCCTTTG -1.14429855905
ACCGAAAA 1.89862518979
ACCGAAAC 0.338456229972
ACCGAAAG -0.21453992119
ACCGAAAT 3.50184003878
ACCGAACA -0.916864161071
ACCGAACC -0.91924351772
ACCGAACG -0.249717701619
ACCGAACT -0.0574339003827
ACCGAAGA -0.864524326571
ACCGAAGC -1.1426050677
ACCGAAGG -1.09232988641
ACCGAAGT -0.303216763487
ACCGAATA 1.92975260104
ACCGAATC 6.77728153484
ACCGAATG -0.033648959486
ACCGACAA 2.21068606089
ACCGACAC -0.250630707526
ACCGACAG -0.494881613109
ACCGACAT -0.420697857428
ACCGACCA -1.07061221254
ACCGACCC -1.19226574087
ACCGACCG -0.385210601193
ACCGACCT -0.51420346329
ACCGACGA 0.0198367719215
ACCGACGC -0.587594709978
ACCGACGG -0.985276872541
ACCGACGT -0.888849543222
ACCGACTA -0.253622742631
ACCGACTC -1.02110941386
ACCGACTG -1.20038921942
ACCGAGAA 1.07370383403
ACCGAGAC -0.53307730542
ACCGAGAG 0.558575602665
ACCGAGAT 2.31424466423
ACCGAGCA -0.473449106557
ACCGAGCC -1.43802743206
ACCGAGCG 0.742304384333
ACCGAGCT -1.2685831545
ACCGAGGA -0.299735944802
ACCGAGGC -0.303000339528
ACCGAGGG -1.49729570847
ACCGAGGT -0.258132097888
ACCGAGTA -0.229265114662
ACCGAGTC -0.279366477192
ACCGAGTG -0.300234399501
ACCGATAA 0.781495940517
ACCGATAC 1.1420739403
ACCGATAG 0.105328940879
ACCGATAT 3.96882099641
ACCGATCA -0.027649467762
ACCGATCC 0.995607980038
ACCGATCG 0.87231715741

ACCGATCT	0.481026297581
ACCGATGA	0.578797128292
ACCGATGC	0.11941113563
ACCGATGG	-0.839001459456
ACCGATTA	1.11767161608
ACCGATTC	3.54685099477
ACCGATTG	0.795841346533
ACCGCAAA	0.805304144376
ACCGCAAC	0.718212058682
ACCGCAAG	-0.852083869611
ACCGCAAT	3.69693029419
ACCGCACA	0.326772472726
ACCGCACC	-1.08915985044
ACCGCACG	-0.161103590904
ACCGCACT	-0.54663778251
ACCGCAGA	-0.20660594429
ACCGCAGC	-0.038391467125
ACCGCAGG	-1.06710028945
ACCGCAGT	-0.937021648208
ACCGCATA	-0.199079461295
ACCGCATC	-0.448434626643
ACCGCATG	-0.596890484983
ACCGCCAA	-0.188083399022
ACCGCCAC	-1.25997690414
ACCGCCAG	-0.761923164005
ACCGCCAT	0.0943443793969
ACCGCCCA	-0.815113795593
ACCGCCCC	-0.936363227973
ACCGCCCG	0.173957814927
ACCGCCCT	0.145620129428
ACCGCCGA	0.164503642677
ACCGCCGC	-1.12748989289
ACCGCCGG	-0.512311060551
ACCGCCGT	-0.64858209313
ACCGCCTA	0.0819449593472
ACCGCCTC	-2.13650335074
ACCGCCTG	-0.678764348536
ACCGCGAA	0.399814513605
ACCGCGAC	-0.0123074137286
ACCGCGAG	-0.796951172781
ACCGCGAT	2.45521220129
ACCGCGCA	-0.0468521279368
ACCGCGCC	0.662260976081
ACCGCGCG	1.03662084317
ACCGCGCT	-0.636784634891
ACCGCGGA	-0.158338696404
ACCGCGGC	-0.742816430876
ACCGCGGG	-0.238616563946
ACCGCGGT	0.917460372486
ACCGCGTA	0.575635979296
ACCGCGTC	-0.0525873628693
ACCGCGTG	-0.7422693592
ACCGCTAA	-0.467833323013
ACCGCTAC	-1.36326706858

ACCGCTAG -0.511313628389
ACCGCTAT -0.42619523511
ACCGCTCA 0.263179376466
ACCGCTCC -0.706734792896
ACCGCTCG -0.148379012151
ACCGCTCT -0.709124604808
ACCGCTGA -0.55841250055
ACCGCTGC -0.92194777169
ACCGCTGG -0.911385602863
ACCGCTTA 0.185908181398
ACCGCTTC -0.595657286623
ACCGCTTG -0.392777075572
ACCGGAAA -0.0547254643071
ACCGGAAC -1.24142900486
ACCGGAAG -0.9581851931
ACCGGAAT 2.05957169036
ACCGGACA 0.39852642511
ACCGGACC -0.399390814041
ACCGGACG -1.4677321433
ACCGGACT -1.02468354578
ACCGGAGA 0.389232741159
ACCGGAGC -0.656398186931
ACCGGAGG -0.432339532237
ACCGGAGT -0.331844843937
ACCGGATA 1.75625743154
ACCGGATC 2.87869429546
ACCGGATG 0.712469243683
ACCGGCAA 0.17358926688
ACCGGCAC -0.227568748118
ACCGGCAG -0.560234330259
ACCGGCAT -0.345850192499
ACCGGCCA -1.18358970162
ACCGGCCC -1.1753734326
ACCGGCCG -0.605064932949
ACCGGCCT -1.18920130306
ACCGGCGA 0.0685980310198
ACCGGCGC -0.824206738481
ACCGGCGG 0.0726863005294
ACCGGCGT -0.573284851859
ACCGGCTA -0.552895257867
ACCGGCTC -1.24732655776
ACCGGCTG -1.78117337059
ACCGGGAA -0.631508386037
ACCGGGAC -1.03514848065
ACCGGGAG -1.24175024283
ACCGGGAT 0.700164443771
ACCGGGCA -0.997105173808
ACCGGGCC -0.56979200962
ACCGGGCG -1.31613996721
ACCGGGCT -1.87691561875
ACCGGGGA -0.646091910685
ACCGGGGC -0.395734869688
ACCGGGGG -0.781158758261
ACCGGGTA 0.00635470931

ACCGGGTC -0.975592684488
ACCGGGTG 0.18549519848
ACCGGTAA 1.32736421561
ACCGGTAC -0.656398186931
ACCGGTAG -0.271851494987
ACCGGTAT 1.44508996273
ACCGGTCA 0.302169668822
ACCGGTCC -0.236853545095
ACCGGTCG -1.29992542142
ACCGGTCT -0.138131808146
ACCGGTGA -0.346854943346
ACCGGTGC -0.832715232124
ACCGGTGG -0.77371826981
ACCGGTTA -0.110129997995
ACCGGTTC -0.231265990763
ACCGGTTG -0.939127861091
ACCGTAAA 0.0653359887284
ACCGTAAC 0.0662853266773
ACCGTAAG 1.36192330581
ACCGTAAT 2.5066458258
ACCGTACA 0.382572215384
ACCGTACC -0.221273111048
ACCGTACG 0.093112487945
ACCGTACT -0.0109641737178
ACCGTAGA -1.02609004013
ACCGTAGC -1.07427416867
ACCGTAGG -0.772817026074
ACCGTAGT -0.126472098007
ACCGTATA 0.957912833479
ACCGTATC 3.08461123787
ACCGTATG 0.1084712704
ACCGTCAA -0.120276308706
ACCGTCAC -0.00468291263565
ACCGTCAG -0.497552148853
ACCGTCAT -0.441157763069
ACCGTCCA -0.189191134217
ACCGTCCC -0.917348501164
ACCGTCCG 0.505556176021
ACCGTCCT -1.00943245254
ACCGTCGA -0.383174699958
ACCGTCGC -0.0236043262154
ACCGTCGG -0.764120860447
ACCGTCGT -0.809702151074
ACCGTCTA -0.900448090068
ACCGTCTC -0.616796000263
ACCGTCTG -0.63847368275
ACCGTGAA 0.164714839005
ACCGTGAC -1.70720865268
ACCGTGAG -0.362789810834
ACCGTGAT 1.00824943944
ACCGTGCA 1.02475255052
ACCGTGCC -0.869317019476
ACCGTGCG -0.0993124593515
ACCGTGCT 0.226846028011

ACCGTGGA -0.171220104113
ACCGTGGC -1.06885154613
ACCGTGGG -1.03613493478
ACCGTGTA 0.913845203662
ACCGTGTC -0.788746143168
ACCGTGTG -0.528256383303
ACCGTTAA 1.13001797567
ACCGTTAC 0.57436409646
ACCGTTAG 0.889586900698
ACCGTTAT 0.421092020872
ACCGTTCA -0.880295046418
ACCGTTCC 0.512064316326
ACCGTTCG -1.31653099408
ACCGTTCT -0.78750353507
ACCGTTGA -0.162634241518
ACCGTTGC -0.423083748617
ACCGTTGG -0.356860630759
ACCGTTTA 0.931935685155
ACCGTTTC -0.427824687966
ACCGTTTG -0.229792059955
ACCTAAAA 1.46153870643
ACCTAAAC -0.198830364636
ACCTAAAG -0.180301023447
ACCTAAAT 2.19251690354
ACCTAACA 0.224193266215
ACCTAACC 0.426813141198
ACCTAACG -0.364948038655
ACCTAACT -0.01981194067
ACCTAAGA -0.136678003721
ACCTAAGC -0.523994817796
ACCTAAGG -0.392393367392
ACCTAAGT -0.256357839629
ACCTAATA 0.795562190991
ACCTAATC 1.22759590676
ACCTAATG 0.356990798793
ACCTACAA 0.10136874835
ACCTACAC -0.991762795411
ACCTACAG -0.879321661363
ACCTACAT 0.0299535465119
ACCTACCA -0.348456951135
ACCTACCC 0.238325646232
ACCTACCG -1.04421659229
ACCTACCT -0.288817512863
ACCTACGA -0.365508702174
ACCTACGC -0.671306086137
ACCTACGG -0.52385602417
ACCTACGT -0.134293158058
ACCTACTA 0.427581603084
ACCTACTC -0.152454473963
ACCTACTG 0.459125917976
ACCTAGAA -0.719161396631
ACCTAGAC -0.901496753023
ACCTAGAG -0.81920650859
ACCTAGAT 3.76847095919

ACCTAGCA 0.0344788460465
ACCTAGCC 0.355640501479
ACCTAGCG -0.148530874857
ACCTAGCT -0.210821506636
ACCTAGGA 0.00967582384162
ACCTAGGC 0.409670690746
ACCTAGGG 0.0633824226959
ACCTAGGT 0.436809418874
ACCTAGTA -0.324497407333
ACCTAGTC -0.799952356241
ACCTAGTG -0.56534617009
ACCTATAA 0.184546906057
ACCTATAC -0.201153001479
ACCTATAG 0.421351834176
ACCTATAT 1.10716381463
ACCTATCA 0.484681980553
ACCTATCC -0.580255114823
ACCTATCG -0.363855724974
ACCTATCT 0.847813939894
ACCTATGA 0.00293426977271
ACCTATGC -0.413865342565
ACCTATGG -0.443663367028
ACCTATTA 0.354679139977
ACCTATTC 0.180453147534
ACCTATTG 0.199643261393
ACCTCAAA 1.54016124434
ACCTCAAC -0.334596669357
ACCTCAAG 0.270899543222
ACCTCAAT 0.598330436183
ACCTCACA 0.265256053229
ACCTCACC -0.332960681961
ACCTCACG -0.25308795589
ACCTCACT -0.635871890365
ACCTCAGA -0.368533932741
ACCTCAGC -0.520474791872
ACCTCAGG -0.909923695609
ACCTCAGT -0.608761652831
ACCTCATA 0.675726310271
ACCTCATC -0.0833227017285
ACCTCATG -0.82923624311
ACCTCCAA -0.546990647661
ACCTCCAC -1.19435653223
ACCTCCAG -0.709162243758
ACCTCCAT -0.390227820887
ACCTCCCA -1.18651665271
ACCTCCCC -1.15833971689
ACCTCCCG -0.103039238117
ACCTCCCT -1.53643603386
ACCTCCGA -0.716939391702
ACCTCCGC -1.19397752892
ACCTCCGG -0.647073137188
ACCTCCGT -0.677626293077
ACCTCCTA -0.91286267025
ACCTCCTC -1.20199619346

ACCTCCTG -0.653544945452
ACCTCGAA 0.661948886458
ACCTCGAC -0.725366857051
ACCTCGAG -1.0055007506
ACCTCGAT 1.89954891234
ACCTCGCA -0.606893035817
ACCTCGCC -1.4323266995
ACCTCGCG -0.920147898034
ACCTCGCT -0.696332066841
ACCTCGGA -0.346273107918
ACCTCGGC -1.37609410892
ACCTCGGG -0.962586336377
ACCTCGTA 0.818098250632
ACCTCGTC -0.958475849432
ACCTCGTG -0.376392631742
ACCTCTAA -0.383344336612
ACCTCTAC -0.059321336872
ACCTCTAG -0.652503339801
ACCTCTAT 0.349431643098
ACCTCTCA -0.744048322327
ACCTCTCC -1.33332371595
ACCTCTCG -0.438301907775
ACCTCTCT -1.21862398355
ACCTCTGA -0.111517411495
ACCTCTGC -0.734295913679
ACCTCTGG -1.48620319639
ACCTCTTA -0.0987619897148
ACCTCTTC -0.603874601171
ACCTCTTG -0.125892876395
ACCTGAAA 0.486550074804
ACCTGAAC -1.08920846742
ACCTGAAG -0.873184421539
ACCTGAAT 0.854087359528
ACCTGACA -0.127628972941
ACCTGACC -0.486202698666
ACCTGACG -0.595867698806
ACCTGACT -1.33672141529
ACCTGAGA 0.26128435991
ACCTGAGC -0.979376444441
ACCTGAGG -0.437121769879
ACCTGAGT -0.479725140007
ACCTGATA 0.904994561512
ACCTGATC 1.08652224878
ACCTGATG 1.7633356451
ACCTGCAA 0.277802108353
ACCTGCAC -0.0100532588632
ACCTGCAG -0.925451330574
ACCTGCAT 0.29681657378
ACCTGCCA -0.734827302459
ACCTGCCC -1.06306926251
ACCTGCCG -1.02760814443
ACCTGCCT -1.13102743138
ACCTGCGA -0.408865635437
ACCTGCGC -0.164093796338

ACCTGCGG -1.10551423539
ACCTGCGT 0.09900063111
ACCTGCTA -0.269184357203
ACCTGCTC -0.986371800037
ACCTGCTG -0.985016536473
ACCTGGAA -0.135903530058
ACCTGGAC -0.502009489173
ACCTGGAG -0.914112074269
ACCTGGAT 2.709313795
ACCTGGCA -1.13910255434
ACCTGGCC -0.415003398024
ACCTGGCG -0.735864464624
ACCTGGCT -1.02153520448
ACCTGGGA -0.545724515221
ACCTGGGC -1.09384328584
ACCTGGGG -0.718252050066
ACCTGGTA -0.295586511999
ACCTGGTC -1.00218878443
ACCTGGTG -0.681769452721
ACCTGTAA 0.0374915302973
ACCTGTAC -0.11857314624
ACCTGTAG -0.677800895981
ACCTGTAT 1.46295513329
ACCTGTCA -0.123717136007
ACCTGTCC -0.5306961191
ACCTGTCG -0.00479687501058
ACCTGTCT -1.35670848163
ACCTGTGA -0.298231693729
ACCTGTGC -0.623646027694
ACCTGTGG -0.568108189393
ACCTGTTA 0.550041232243
ACCTGTTC -0.722066130284
ACCTGTTG -0.455863614303
ACCTTAAA 0.479591835394
ACCTTAAC -0.0698416846434
ACCTTAAG -0.907890930953
ACCTTAAT 0.846139790785
ACCTTACA 0.0918659591239
ACCTTACC 0.504305987857
ACCTTACG -0.321803608627
ACCTTACT 0.265687071477
ACCTTAGA -0.0368866932891
ACCTTAGC -0.155364696629
ACCTTAGG 0.0138493037508
ACCTTAGT 0.358122581094
ACCTTATA 0.393947019586
ACCTTATC -0.145646528969
ACCTTATG -0.207225941431
ACCTTCAA 0.420947215469
ACCTTCAC -0.029985696448
ACCTTCAG -0.834404541365
ACCTTCAT -0.463731200279
ACCTTCCA -0.295916375571
ACCTTCCC -0.773451660584

ACCTTCCG 0.0429387227135
ACCTTCCT -1.10187972433
ACCTTCGA -0.507940237538
ACCTTCGC -0.372252608677
ACCTTCGG -0.785130712961
ACCTTCGT -1.0105775653
ACCTTCTA -0.988738871751
ACCTTCTC -0.76632875077
ACCTTCTG -0.724032504014
ACCTTGAA 0.35683292431
ACCTTGAC -0.275888272322
ACCTTGAG -0.245547096907
ACCTTGAT 1.2526001928
ACCTTGCA 0.0425116251891
ACCTTGCC -0.394975294776
ACCTTGCG 0.11891424922
ACCTTGCT -0.631310258789
ACCTTGGA 0.178291521753
ACCTTGGC -1.18612039821
ACCTTGGG -0.854403631256
ACCTTGTA 0.313255384981
ACCTTGTC -0.826890081923
ACCTTGTG -0.870161282024
ACCTTTAA 2.22301752172
ACCTTTAC 0.703032322618
ACCTTTAG -0.596702290235
ACCTTTAT 0.229708156464
ACCTTTCA 0.257567513645
ACCTTTCC -0.942421269174
ACCTTTCG -0.158752724849
ACCTTTCT 0.0715045943436
ACCTTTGA 1.92314252192
ACCTTTGC -0.292450978399
ACCTTTGG -1.03035578774
ACCTTTTA 0.0990795683513
ACCTTTTC 0.0641840800442
ACCTTTTG -0.664556952002
ACGAAAAA 1.65785458013
ACGAAAAC 0.283360649328
ACGAAAAG 0.694811871491
ACGAAAAT 2.9166395842
ACGAAACA 1.00452449035
ACGAAACC -0.58411990307
ACGAAACG 0.460838228798
ACGAAACT 0.717870432939
ACGAAAGA 2.42520481017
ACGAAAGC -0.615915405673
ACGAAAGG 0.0582794698389
ACGAAAGT 2.06210839873
ACGAAATA 2.388055167
ACGAAATC 25.9070264388
ACGAAATG 0.723386630096
ACGAACAA 0.413083027454
ACGAACAC 0.149860523025

ACGAACAG -0.0612589586273
ACGAACAT 0.0385861964124
ACGAACCA 0.803736900339
ACGAACCC -0.326983669054
ACGAACCG 0.128384365747
ACGAACCT -0.0819067976345
ACGAACGA 0.51221748594
ACGAACGC -0.0962945474687
ACGAACGG -0.808172023224
ACGAACGT -0.608450870116
ACGAACTA 0.449406182027
ACGAACTC -0.505211413698
ACGAACTG 0.613233107757
ACGAAGAA 0.848165498137
ACGAAGAC -0.605268810592
ACGAAGAG 0.213284244013
ACGAAGAT 3.6805921149
ACGAAGCA -0.448539963425
ACGAAGCC 0.010155720448
ACGAAGCG -0.918921756978
ACGAAGCT -0.612927291292
ACGAAGGA -0.0605636835876
ACGAAGGC -1.34051249393
ACGAAGGG -0.620156060652
ACGAAGTA -0.154899437392
ACGAAGTC -0.135563472604
ACGAAGTG 0.0998075160902
ACGAATAA 0.615267440702
ACGAATAC 1.43026492149
ACGAATAG 0.902819605269
ACGAATAT 3.06156679102
ACGAATCA 2.37111476452
ACGAATCC 5.86201598359
ACGAATCG 2.91004257415
ACGAATCT 10.5886765794
ACGAATGA 1.93174955642
ACGAATGC -0.884206883352
ACGAATGG -0.864799561389
ACGAATTA 1.70233022662
ACGAATTC 1.17349383756
ACGAATTG 1.37262818899
ACGACAAA 0.457464053803
ACGACAAC 1.47826033251
ACGACAAG -0.434419606961
ACGACAAT 1.18631277507
ACGACACA -0.677973146452
ACGACACC 0.427526712949
ACGACACG -1.18212883989
ACGACACT -0.0140923886331
ACGACAGA -0.177885857519
ACGACAGC -0.801353100203
ACGACAGG -0.440433213291
ACGACAGT 0.738832714004
ACGACATA 0.457416220971

ACGACATC 1.26653444556
ACGACATG -0.0562555921576
ACGACCAA 0.361419648519
ACGACCAC -0.531699301657
ACGACCAG -1.20370562908
ACGACCAT -1.14331759393
ACGACCCA -0.601985857773
ACGACCCC -1.14461718321
ACGACCCG -1.04267836161
ACGACCCT -0.906336233233
ACGACCGA -1.23158981752
ACGACCGC -1.31935731325
ACGACCGG -0.406458572339
ACGACCGT -0.242789521092
ACGACCTA -0.0810395335061
ACGACCTC -0.331440486611
ACGACCTG -0.519596026953
ACGACGAA -0.525037730356
ACGACGAC -0.525037730356
ACGACGAG -0.717232139087
ACGACGAT 2.07163811026
ACGACGCA 0.017411412112
ACGACGCC -0.648564580563
ACGACGCG -0.083761300043
ACGACGCT -0.1875057457
ACGACGGA 0.370394446926
ACGACGGC 0.135789306301
ACGACGGG -1.33598196676
ACGACGTA -0.240708400841
ACGACGTC 0.811093223917
ACGACGTG -0.023620009111
ACGACTAA -0.296376668557
ACGACTAC 0.697998374502
ACGACTAG -0.587850079796
ACGACTAT 0.0922954090826
ACGACTCA -0.185632423817
ACGACTCC -0.316209519755
ACGACTCG -0.982025808276
ACGACTCT -0.575324935199
ACGACTGA 0.338744272488
ACGACTGC 0.0380474889475
ACGACTGG -0.0278622323794
ACGACTTA 0.7252568154
ACGACTTC -0.0769366266272
ACGACTTG 0.135795056697
ACGAGAAA 0.734269514138
ACGAGAAC -0.237551433951
ACGAGAAG 0.277163814501
ACGAGAAT 3.6360584417
ACGAGACA 0.434003748845
ACGAGACC -1.31328280501
ACGAGACG -0.41997330765
ACGAGACT -0.577537007628
ACGAGAGA 0.523922415095

ACGAGAGC -0.596501026408
ACGAGAGG -0.346604278397
ACGAGAGT -0.478325964335
ACGAGATA 3.63535140449
ACGAGATC 8.68226718433
ACGAGATG -0.214900889171
ACGAGCAA 0.39320652553
ACGAGCAC -1.11112845064
ACGAGCAG -0.20363116037
ACGAGCAT 0.664565316213
ACGAGCCA -0.65252294342
ACGAGCCC -1.25330330928
ACGAGCCG -1.57296567989
ACGAGCCT -0.319233704796
ACGAGCGA -0.407208998895
ACGAGCGC -0.65403163798
ACGAGCGG -0.807238890933
ACGAGCGT -0.334898303716
ACGAGCTA -0.95255869291
ACGAGCTC -0.773327242946
ACGAGCTG -0.607115471553
ACGAGGAA 0.88432136849
ACGAGGAC -0.876078961309
ACGAGGAG -1.46512512328
ACGAGGAT 2.737215496
ACGAGGCA -0.738422344901
ACGAGGCC -1.36415106113
ACGAGGCG -0.407842587879
ACGAGGCT -1.01177051089
ACGAGGGA -0.905390293245
ACGAGGGC -1.18280424993
ACGAGGGG -0.91037327195
ACGAGGTA -0.890298120015
ACGAGGTC -0.290818388964
ACGAGGTG -0.00071252622487
ACGAGTAA 0.661948886458
ACGAGTAC 0.411884070083
ACGAGTAG -0.58995969064
ACGAGTAT 1.29885950728
ACGAGTCA -1.15014540392
ACGAGTCC -1.16788589546
ACGAGTCG -0.489928170523
ACGAGTCT -0.587082402054
ACGAGTGA 1.0412114881
ACGAGTGC -0.267021685895
ACGAGTGG -0.305301020317
ACGAGTTA 0.222381369006
ACGAGTTC -0.635124600388
ACGAGTTG 0.00162030450036
ACGATAAA 0.905715974711
ACGATAAC 0.976672190471
ACGATAAG 0.758919889492
ACGATAAT 1.66796063808
ACGATACA 0.629168498009

ACGATACC 0.68989998858
ACGATACG 2.0039266856
ACGATACT 2.84378521531
ACGATAGA −1.18098895476
ACGATAGC −0.370478089036
ACGATAGG 0.307819170592
ACGATAGT 0.894614052893
ACGATATA 1.47229978665
ACGATATC 15.0146037982
ACGATATG 2.02321690861
ACGATCAA 0.570482841173
ACGATCAC 1.24261280209
ACGATCAG 1.24538972015
ACGATCAT 0.849759403097
ACGATCCA 2.7480322505
ACGATCCC 1.45804194347
ACGATCCG 2.64883741287
ACGATCCT 2.65698938202
ACGATCGA 2.3216608442
ACGATCGC 2.59646490567
ACGATCGG 1.27908468279
ACGATCGT 1.55563947818
ACGATCTA 2.77184176127
ACGATCTC 4.02566260611
ACGATCTG 2.46766729562
ACGATGAA −0.308375390624
ACGATGAC 0.467763795509
ACGATGAG −0.203605806356
ACGATGAT 2.13006813589
ACGATGCA −0.122946321687
ACGATGCC −0.434673408489
ACGATGCG −0.200875414226
ACGATGCT 1.31389888142
ACGATGGA 1.34853717024
ACGATGGC −0.357899622595
ACGATGGG −0.452863999132
ACGATGTA 0.156026253443
ACGATGTC 0.788070733129
ACGATGTG 0.367190169967
ACGATTAA 0.476582810486
ACGATTAC 5.81730718473
ACGATTAG 0.354843810381
ACGATTAT 1.38152770949
ACGATTCA 0.69095936818
ACGATTCC 5.43865046562
ACGATTCG 4.53233749535
ACGATTCT 7.63473365962
ACGATTGA −0.0732223941784
ACGATTGC 6.10523992089
ACGATTGG 0.612369502971
ACGATTTA 3.80192231419
ACGATTTC 6.27327116956
ACGATTTG 3.65627421693
ACGCAAAA 1.31040264123

ACGCAAAC 0.272471492128
ACGCAAAG -0.457986555609
ACGCAAAT 1.80011517193
ACGCAACA 1.1940758084
ACGCAACC 0.44236482327
ACGCAACG -0.275710532839
ACGCAACT 1.1139905791
ACGCAAGA 0.364122856963
ACGCAAGC 2.06154224619
ACGCAAGG 0.422412520684
ACGCAAGT -0.648104026195
ACGCAATA 2.87605068202
ACGCAATC 17.2749988878
ACGCAATG 0.964340206872
ACGCACAA -0.0075983629336
ACGCACAC 0.0815949693931
ACGCACAG -0.335561167438
ACGCACAT 1.06932307852
ACGCACCA -1.00008309431
ACGCACCC -0.180441646744
ACGCACCG 0.350353274598
ACGCACCT -1.24072458146
ACGCACGA 1.30369924887
ACGCACGC -0.739927380119
ACGCACGG -0.368804724072
ACGCACGT -0.147785414552
ACGCACTA 0.773303195839
ACGCACTC 0.040964507535
ACGCACTG 0.0258344339742
ACGCAGAA -0.419001490884
ACGCAGAC -0.173055525665
ACGCAGAG 0.31000327519
ACGCAGAT 2.37580081373
ACGCAGCA -0.479974236666
ACGCAGCC -0.505861208341
ACGCAGCG -0.179102327457
ACGCAGCT -0.413214241014
ACGCAGGA -0.635988989319
ACGCAGGC -0.592368583409
ACGCAGGG -0.797517848076
ACGCAGTA 0.147049102602
ACGCAGTC -0.935265948042
ACGCAGTG -0.790218244304
ACGCATAA 0.23294641303
ACGCATAC 0.679888550771
ACGCATAG -0.49635946464
ACGCATAT 1.30688862708
ACGCATCA 0.366259912874
ACGCATCC -0.106901412548
ACGCATCG 0.113958192819
ACGCATCT 0.343232717218
ACGCATGA -0.698585437562
ACGCATGC -0.346038387247
ACGCATGG -1.12102095983

ACGCATTA 1.10201093789
ACGCATTC -0.081714682163
ACGCATTG -0.0192729718235
ACGCCAAA -0.935000122961
ACGCCAAC 0.10028793546
ACGCCAAG 0.122712907924
ACGCCAAT 1.15357002557
ACGCCACA 0.406530452278
ACGCCACC -0.803860272451
ACGCCACG -1.53237390251
ACGCCACT -0.757112174389
ACGCCAGA 0.404540292822
ACGCCAGC -0.288869005037
ACGCCAGG -1.24843011085
ACGCCAGT -0.693034999416
ACGCCATA 0.421650331956
ACGCCATC 0.294514586083
ACGCCATG -0.27597975588
ACGCCCAA -0.59293944081
ACGCCCAC -0.00257879080505
ACGCCCAG -1.02510384738
ACGCCCAT -1.09294413316
ACGCCCCA 0.168589036989
ACGCCCCC -1.50410888109
ACGCCCCG -1.14484301691
ACGCCCCT -0.77027116935
ACGCCCGA -0.606863238315
ACGCCCGC -0.613545720144
ACGCCCGG -1.38184528813
ACGCCCGT -0.739360704823
ACGCCCTA -0.802320996245
ACGCCCTC -0.875916381958
ACGCCCTG -1.00817912779
ACGCCGAA 0.214981917465
ACGCCGAC -0.723630237741
ACGCCGAG -0.901906860744
ACGCCGAT 0.995900727423
ACGCCGCA -0.871866796924
ACGCCGCC -0.774165232336
ACGCCGCG -0.86304333846
ACGCCGCT -0.296263490327
ACGCCGGA -0.133297294186
ACGCCGGC -0.189976324525
ACGCCGGG -0.960480646257
ACGCCGTA -0.258746344634
ACGCCGTC -0.418835774953
ACGCCGTG -1.40186345888
ACGCCTAA -0.361569158791
ACGCCTAC -0.98342524533
ACGCCTAG -0.721912699288
ACGCCTAT -0.475728092674
ACGCCTCA -0.210588877017
ACGCCTCC -1.14484719901
ACGCCTCG -1.28154349945

ACGCCTCT -1.13910255434
ACGCCTGA -0.851886003744
ACGCCTGC -0.475614653062
ACGCCTGG -0.865673360057
ACGCCTTA 0.187246455159
ACGCCTTC -0.229792059955
ACGCCTTG -0.453016645983
ACGCGAAA 0.0920682684776
ACGCGAAC -0.19814057861
ACGCGAAG 0.343821871331
ACGCGAAT 2.13746863296
ACGCGACA 0.23294641303
ACGCGACC -0.198341058292
ACGCGACG 0.228225338682
ACGCGACT -0.982645805417
ACGCGAGA 0.520177601
ACGCGAGC 0.641165390404
ACGCGAGG -0.428108025614
ACGCGAGT -0.57034300202
ACGCGATA 3.63160188553
ACGCGATC 9.01935037679
ACGCGATG 0.457596574271
ACGCGCAA 1.36785954319
ACGCGCAC 0.356663287656
ACGCGCAG -0.820956196979
ACGCGCAT 0.761637735305
ACGCGCCA -0.0468521279368
ACGCGCCC -0.917496704528
ACGCGCCG -0.705105601421
ACGCGCCT -0.751942046462
ACGCGCGA 0.015664076157
ACGCGCGC 0.247902406449
ACGCGCGG 0.349091585645
ACGCGCGT 0.396150989185
ACGCGCTA 0.336992493046
ACGCGCTC 0.167690407069
ACGCGCTG -0.620351574085
ACGCGGAA -0.430677406682
ACGCGGAC -1.1447792398
ACGCGGAG -0.401862699774
ACGCGGAT 2.37133354091
ACGCGGCA -0.72496981841
ACGCGGCC -0.796586545457
ACGCGGCG -0.864239943397
ACGCGGCT -1.70794365773
ACGCGGGA 0.0359326504715
ACGCGGGC -0.625147926332
ACGCGGGG 0.170716421781
ACGCGGTA -0.554684153495
ACGCGGTC -0.522571333636
ACGCGGTG -0.480307498198
ACGCGTAA 1.24332480556
ACGCGTAC -0.449650051054
ACGCGTAG 0.358252749128

ACGCGTAT 1.72046932509
ACGCGTCA 0.182366722183
ACGCGTCC -0.378396905804
ACGCGTCG 0.153568482316
ACGCGTCT 0.0540793290071
ACGCGTGA 1.39224958247
ACGCGTGC -0.400907872812
ACGCGTGG 0.101903796473
ACGCGTTA 1.21472181774
ACGCGTTC -0.19504451363
ACGCGTTG -0.0841322005247
ACGCTAAA 0.444002640337
ACGCTAAC -0.0969375461896
ACGCTAAG -0.262621065381
ACGCTAAT 0.61735300444
ACGCTACA 1.44001863704
ACGCTACC 0.505693401357
ACGCTACG -0.895298872669
ACGCTACT 1.32721758054
ACGCTAGA -0.0743928609557
ACGCTAGC -0.843020462844
ACGCTAGG -0.617943726842
ACGCTAGT -0.750792490212
ACGCTATA -0.0449053578257
ACGCTATC 1.87237306803
ACGCTATG -0.33023002845
ACGCTCAA -0.220831898918
ACGCTCAC -0.171784688356
ACGCTCAG 0.03800854309
ACGCTCAT -0.673090538278
ACGCTCCA -0.265174763553
ACGCTCCC -0.616110134961
ACGCTCCG -0.60460751516
ACGCTCCT -0.451448879183
ACGCTCGA -0.395859025945
ACGCTCGC -0.518938390863
ACGCTCGG -0.0790020639819
ACGCTCTA 0.0452639733725
ACGCTCTC -0.507351606189
ACGCTCTG -1.33792586168
ACGCTGAA 0.353931588618
ACGCTGAC -0.67058179774
ACGCTGAG -0.555063679569
ACGCTGAT 1.46946588741
ACGCTGCA -0.238170646946
ACGCTGCC -1.16445813724
ACGCTGCG 0.00497095515209
ACGCTGCT -0.215909560742
ACGCTGGA -1.30298201778
ACGCTGGC 0.945209688017
ACGCTGGG -1.04886944604
ACGCTGTA -0.278777061698
ACGCTGTC -0.915887116673
ACGCTGTG -0.969175243595

ACGCTTAA -0.332086883293
ACGCTTAC -0.154149794981
ACGCTTAG 0.0538163791237
ACGCTTAT 1.25005982508
ACGCTTCA -0.640417839045
ACGCTTCC -1.09064188408
ACGCTTCG -0.764947349046
ACGCTTCT 0.0139630047442
ACGCTTGA -0.440714982649
ACGCTTGC 0.529289886125
ACGCTTGG -0.549488932935
ACGCTTTA 0.700887948022
ACGCTTTC -0.456943120286
ACGCTTTG 0.140574157759
ACGGAAAA 0.243693901406
ACGGAAAC -0.271251885611
ACGGAAAG -0.143558612797
ACGGAAAT 3.04530519654
ACGGAACA 0.0688361496518
ACGGAACC 0.143164449354
ACGGAACG -0.733313903031
ACGGAACT 0.560980313328
ACGGAAGA 0.539840815543
ACGGAAGC -0.807660760826
ACGGAAGG -0.83553188018
ACGGAAGT -0.179857720264
ACGGAATA 2.7858092095
ACGGAATC 13.4950762783
ACGGAATG 1.559680699
ACGGACAA 0.653109483717
ACGGACAC -0.450712567233
ACGGACAG -0.58783936315
ACGGACAT -0.878519742633
ACGGACCA -0.683753861782
ACGGACCC -0.586040796403
ACGGACCG -0.727793001005
ACGGACCT -1.22647248867
ACGGACGA 0.123723931929
ACGGACGC -1.32539261425
ACGGACGG -1.00040537781
ACGGACGT -0.728480434597
ACGGACTA 0.10161967468
ACGGACTC -0.946532278882
ACGGACTG -1.0540461085
ACGGAGAA 0.782029943113
ACGGAGAC -0.795142412151
ACGGAGAG -0.758521543943
ACGGAGAT 3.92172892017
ACGGAGCA -0.15451050158
ACGGAGCC 0.39075319789
ACGGAGCG -1.01213435407
ACGGAGCT -0.593318182739
ACGGAGGA -1.20764203589
ACGGAGGC -0.791641467083

ACGGAGGG -1.37551541007
ACGGAGTA -0.0598514187444
ACGGAGTC -1.20131712408
ACGGAGTG -0.112806545516
ACGGATAA 0.495312631357
ACGGATAC 2.82440716808
ACGGATAG 0.936131905262
ACGGATAT 6.67361158293
ACGGATCA 0.819787821255
ACGGATCC 2.81875270006
ACGGATCG 1.8977830183
ACGGATCT 4.58012223281
ACGGATGA -0.530765646604
ACGGATGC 0.730819277099
ACGGATGG -0.590920006616
ACGGATTA 2.2972723732
ACGGATTC 5.76814967113
ACGGATTG 5.48928373969
ACGGCAAA 0.466659719656
ACGGCAAC 0.402496811521
ACGGCAAG -0.42388619011
ACGGCAAT 1.27192831614
ACGGCACA 0.202159320615
ACGGCACC -0.200589985526
ACGGCACG -0.966546790287
ACGGCACT -0.50728782908
ACGGCAGA 0.0265477443439
ACGGCAGC -0.795417124206
ACGGCAGG -1.54830537204
ACGGCAGT -1.08251762138
ACGGCATA -0.457602586047
ACGGCATC -0.100064192815
ACGGCATG -0.885529474217
ACGGCCAA 0.00911542170438
ACGGCCAC -1.06170537335
ACGGCCAG -0.289052494916
ACGGCCAT -0.624343916549
ACGGCCCA -0.932142699377
ACGGCCCC -0.287310125211
ACGGCCCG -1.65722804845
ACGGCCCT -0.357754033047
ACGGCCGA -0.273302946978
ACGGCCGC -0.365711011527
ACGGCCGG -0.226086975862
ACGGCCGT 0.168729660286
ACGGCCTA 0.341087035714
ACGGCCTC -1.2591566887
ACGGCCTG -0.404652164144
ACGGCGAA -0.572604998334
ACGGCGAC -0.466599079126
ACGGCGAG -0.192730240998
ACGGCGAT 1.05804864485
ACGGCGCA -0.0487257112016
ACGGCGCC -0.368804724072

ACGGCGCG -0.207124525372
ACGGCGCT -0.992716838228
ACGGCGGA -0.772359608285
ACGGCGGC -1.11043474389
ACGGCGGG 0.150720468469
ACGGCGTA 0.661498525972
ACGGCGTC -1.01356881626
ACGGCGTG 0.0784095119086
ACGGCTAA -0.357884462462
ACGGCTAC -0.907746909695
ACGGCTAG 0.00450857111254
ACGGCTAT 0.373681843232
ACGGCTCA -0.880006219757
ACGGCTCC -0.364527998433
ACGGCTCG -1.23730858541
ACGGCTCT -0.201287874381
ACGGCTGA -0.547378276565
ACGGCTGC -0.749699131005
ACGGCTGG -0.817010380438
ACGGCTTA -0.94432621823
ACGGCTTC -1.16492705582
ACGGCTTG -0.959113097758
ACGGGAAA 1.57953733593
ACGGGAAC -0.366001145096
ACGGGAAG -0.36423054618
ACGGGAAT 3.34196650966
ACGGGACA -0.534501312343
ACGGGACC -1.20906813386
ACGGGACG -0.79224944067
ACGGGACT -0.767428644517
ACGGGAGA -0.449626788092
ACGGGAGC -1.44637491464
ACGGGAGG -0.263474737667
ACGGGAGT -0.739648224576
ACGGGATA 1.72927370269
ACGGGATC 1.4799901559
ACGGGATG 0.298775890208
ACGGGCAA -0.608029522987
ACGGGCAC -0.699292474773
ACGGGCAG -0.961652419942
ACGGGCAT -0.910168871544
ACGGGCCA -1.07334547987
ACGGGCCC -1.0934263822
ACGGGCCG -1.71718140602
ACGGGCCT -0.901194334519
ACGGGCGA 0.0202126386534
ACGGGCGC -0.0504518752475
ACGGGCGG -0.959306520137
ACGGGCTA -0.045887891237
ACGGGCTC -0.537111730321
ACGGGCTG -0.39360983733
ACGGGGAA 1.08132284611
ACGGGGAC -0.893839840612
ACGGGGAG -0.711379282437

ACGGGGAT 2.37890027667
ACGGGGCA -1.00802961752
ACGGGGCC -0.679561823779
ACGGGGCG -1.10786118072
ACGGGGCT 0.0410212273409
ACGGGGGA -0.1292654831
ACGGGGGC -1.14631198147
ACGGGGGG -0.671024316779
ACGGGGTA -0.302865466625
ACGGGGTC -1.63497506508
ACGGGGTG -0.385846281229
ACGGGTAA -0.388546353093
ACGGGTAC -0.840513290595
ACGGGTAG 0.260814395804
ACGGGTAT 0.832678638701
ACGGGTCA -1.70481230623
ACGGGTCC -1.33405845961
ACGGGTCG -1.0755319369
ACGGGTCT -1.50331009894
ACGGGTGA -0.196643646221
ACGGGTGC -0.896136077914
ACGGGTGG -0.121714168854
ACGGGTTA -0.648919014004
ACGGGTTC -0.25654498885
ACGGGTTG -0.448642163628
ACGGTAAA 0.677511285175
ACGGTAAC 0.542448096942
ACGGTAAG -0.435729912891
ACGGTAAT 2.38052920677
ACGGTACA -0.296963731617
ACGGTACC 0.18009662304
ACGGTACG 0.592159478134
ACGGTACT -0.61269727549
ACGGTAGA -0.322710341376
ACGGTAGC -1.27744817263
ACGGTAGG -0.0305965452328
ACGGTAGT 0.304603131461
ACGGTATA 0.867076195072
ACGGTATC 4.11207353137
ACGGTATG -0.55318434591
ACGGTCAA -0.254539669262
ACGGTCAC -0.364305039934
ACGGTCAG -0.441890677059
ACGGTCAT 0.0334171140122
ACGGTCCA -0.583573615538
ACGGTCCC -1.29296064747
ACGGTCCG -1.15331439437
ACGGTCCT -0.465959739748
ACGGTCGA 0.0296422410336
ACGGTCGC -0.243171660982
ACGGTCGG -0.498725490828
ACGGTCTA -1.12385982532
ACGGTCTC -0.437653158659
ACGGTCTG -0.457032774173

ACGGTGAA 0.898966056431
ACGGTGAC -0.0530356323028
ACGGTGAG 0.310953135902
ACGGTGAT 2.44464271378
ACGGTGCA -0.122525758702
ACGGTGCC -1.46291906263
ACGGTGCG -1.16540982762
ACGGTGCT -0.761146337908
ACGGTGGA 0.583891716938
ACGGTGGC -0.803860272451
ACGGTGGG -0.910509713142
ACGGTGTA -0.285971851451
ACGGTGTC -0.740597301144
ACGGTGTG 0.466788842164
ACGGTTAA 0.192679010206
ACGGTTAC -0.355087156645
ACGGTTAG -0.520797075377
ACGGTTAT 0.751103011546
ACGGTTCA -0.159876665702
ACGGTTCC -0.658403506519
ACGGTTCG 0.588668988329
ACGGTTCT 0.957549513064
ACGGTTGA 0.303404958235
ACGGTTGC 0.192906150811
ACGGTTGG 0.0484434190802
ACGGTTTA 0.197422040608
ACGGTTTC -0.56824881269
ACGGTTTG -0.26273241394
ACGTAAAA -0.203921032558
ACGTAAAC 0.563932618431
ACGTAAAG -0.360392418855
ACGTAAAT 1.80831392839
ACGTAACA 1.55064682836
ACGTAACC 0.997457254815
ACGTAACG 0.602688974261
ACGTAACT 0.149357102076
ACGTAAGA 1.36941188847
ACGTAAGC 0.114984376956
ACGTAAGG -0.249717701619
ACGTAAGT 0.39202743316
ACGTAATA 3.88847726071
ACGTAATC 15.1871948488
ACGTAATG 1.21133797162
ACGTACAA 0.672996179523
ACGTACAC -0.0652207194455
ACGTACAG -0.311638478442
ACGTACAT 1.12765822264
ACGTACCA -0.316770967419
ACGTACCC -0.336614535262
ACGTACCG -1.2361200833
ACGTACCT -0.271179744291
ACGTACGA -0.0774201825759
ACGTACGC -0.871174658464
ACGTACGG -0.821568352672

ACGTACGT -0.242096337105
ACGTACTA 0.332508753185
ACGTACTC -0.351875822382
ACGTACTG -0.71708837921
ACGTAGAA 1.04637168353
ACGTAGAC -0.230659324084
ACGTAGAG 0.384196179227
ACGTAGAT 2.32720866853
ACGTAGCA -0.14058095368
ACGTAGCC -0.295831165171
ACGTAGCG -0.184016824185
ACGTAGCT 0.0172747095384
ACGTAGGA 0.531289455318
ACGTAGGC -0.576357915258
ACGTAGGG -0.646781696711
ACGTAGTA 0.911896603879
ACGTAGTC -0.261537900056
ACGTAGTG 0.506568245552
ACGTATAA 1.62171282439
ACGTATAC 0.496933197239
ACGTATAG -0.249876098864
ACGTATAT 1.33998999212
ACGTATCA 1.51759042094
ACGTATCC 4.54920497236
ACGTATCG 2.09176057226
ACGTATCT 3.77543233518
ACGTATGA 0.0169030249119
ACGTATGC -0.104790494796
ACGTATGG -0.199168069655
ACGTATTA 1.33239790235
ACGTATTC 0.422658742145
ACGTATTG 2.09348752045
ACGTCAAA 0.160928465236
ACGTCAAC 0.665936785436
ACGTCAAG -0.137959296294
ACGTCAAT 0.370483316668
ACGTCACA 0.103013099957
ACGTCACC -0.704727643636
ACGTCACG -0.509149388791
ACGTCACT 0.316443717663
ACGTCAGA -0.389746878754
ACGTCAGC -0.00738507555301
ACGTCAGG -0.488759010654
ACGTCAGT -0.65015482618
ACGTCATA 1.05259570204
ACGTCATC 0.447744579235
ACGTCATG -0.0897354377523
ACGTCCAA -0.288869005037
ACGTCCAC -0.665037371371
ACGTCCAG -0.236605755344
ACGTCCAT 0.257566729501
ACGTCCCA -0.651156963211
ACGTCCCC -0.872358978465
ACGTCCCG -0.759815382832

ACGTCCCT −0.238241481358
ACGTCCGA −0.424690461274
ACGTCCGC −0.753053963762
ACGTCCGG 0.285095177585
ACGTCCTA 1.83151102208
ACGTCCTC −0.657979284192
ACGTCCTG −0.266712210088
ACGTCGAA 0.355439237652
ACGTCGAC −0.0243403767837
ACGTCGAG −0.689033769978
ACGTCGAT 2.06603173645
ACGTCGCA −0.650099413282
ACGTCGCC 0.627019679926
ACGTCGCG 0.228225338682
ACGTCGCT −0.311731268907
ACGTCGGA 1.7976461614
ACGTCGGC −1.06959857472
ACGTCGGG −0.796228452674
ACGTCGTA 0.515022633206
ACGTCGTC −0.172875172365
ACGTCGTG 0.527677161691
ACGTCTAA 0.576737964096
ACGTCTAC −0.815020743746
ACGTCTAG −0.209241193519
ACGTCTAT 0.369560639642
ACGTCTCA −0.50476392841
ACGTCTCC −1.50150369075
ACGTCTCG −0.375711994072
ACGTCTCT −0.330548391231
ACGTCTGA −0.274560715207
ACGTCTGC −0.128703251292
ACGTCTGG −0.188339552984
ACGTCTTA 0.285289906872
ACGTCTTC −0.381282035837
ACGTCTTG −0.494479085454
ACGTGAAA 2.13154023703
ACGTGAAC 0.736842554548
ACGTGAAG −0.692434083132
ACGTGAAT 1.33681838786
ACGTGACA −0.153965259575
ACGTGACC −0.7618664442
ACGTGACG −0.874427552399
ACGTGACT −0.998494416979
ACGTGAGA −0.282608393101
ACGTGAGC 0.0644880668379
ACGTGAGG −0.262965566322
ACGTGAGT −0.434225139055
ACGTGATA 1.99476499936
ACGTGATC 3.30838367971
ACGTGATG −0.761386286212
ACGTGCAA 1.43202663343
ACGTGCAC 1.03286086894
ACGTGCAG 0.0865356042803
ACGTGCAT 0.220961021425

ACGTGCCA 0.185175790172
ACGTGCCC -1.33135812637
ACGTGCCG -0.644158732417
ACGTGCCT -1.11545849813
ACGTGCGA 0.151314327451
ACGTGCGC 0.114467102782
ACGTGCGG -0.438508660616
ACGTGCTA -0.0294718202344
ACGTGCTC -1.07694287475
ACGTGCTG 0.313960853903
ACGTGGAA 0.284537389263
ACGTGGAC -0.0371099131703
ACGTGGAG -0.87680560214
ACGTGGAT 3.24987759203
ACGTGGCA -0.513912284195
ACGTGGCC -1.10958943582
ACGTGGCG -0.432142189134
ACGTGGCT -0.521211365203
ACGTGGGA 0.240389515297
ACGTGGGC -0.550275952915
ACGTGGGG -0.922045005643
ACGTGGTA -0.220831898918
ACGTGGTC -0.526579097615
ACGTGGTG -0.61257599443
ACGTGTAA 1.54632932719
ACGTGTAC -0.737027612716
ACGTGTAG -0.492032030972
ACGTGTAT -0.561028407541
ACGTGTCA -0.394738483052
ACGTGTCC -0.149140939498
ACGTGTCG -0.520201125343
ACGTGTCT 0.874851513344
ACGTGTGA 0.309828149522
ACGTGTGC 0.181704642606
ACGTGTGG -0.747245803365
ACGTGTTA -0.38644014021
ACGTGTTC -0.856970921271
ACGTGTTG 0.79712002529
ACGTTAAA 1.01390390746
ACGTTAAC -0.146868487921
ACGTTAAG 0.163810720072
ACGTTAAT 0.363321460996
ACGTTACA 0.0606585651062
ACGTTACC 1.30428082292
ACGTTACG 0.286473965492
ACGTTACT -0.548666103678
ACGTTAGA 0.524189024321
ACGTTAGC -0.0410290687887
ACGTTAGG -0.309275588833
ACGTTAGT 0.241877822092
ACGTTATA 1.70039809388
ACGTTATC 1.70813551182
ACGTTATG -0.0942706697874
ACGTTCAA -0.109410675849

ACGTTCAC -0.284788838357
ACGTTCAG -0.50252545644
ACGTTCAT -0.350060527213
ACGTTCCA 0.284537389263
ACGTTCCC -1.22528424795
ACGTTCCG 0.441005638982
ACGTTCCT -0.465229439575
ACGTTCGA 0.403067930304
ACGTTCGC -0.55284820918
ACGTTCGG 0.547309533206
ACGTTCTA 0.85575549686
ACGTTCTC -0.280914640372
ACGTTCTG -0.5613201094
ACGTTGAA 0.49371088495
ACGTTGAC -0.257559672198
ACGTTGAG -1.22568311626
ACGTTGAT 0.835121511077
ACGTTGCA 1.00240886773
ACGTTGCC -0.464234621228
ACGTTGCG -0.894148270893
ACGTTGCT 0.0468348767516
ACGTTGGA -0.616930611784
ACGTTGGC -0.497428253978
ACGTTGGG -0.527261826338
ACGTTGTA -0.139808832452
ACGTTGTC -0.366326826562
ACGTTGTG -0.368491066159
ACGTTTAA 0.165016473364
ACGTTTAC 0.529938635241
ACGTTTAG 0.21586983074
ACGTTTAT 0.718497226001
ACGTTTCA 0.847070570641
ACGTTTCC 0.567385730667
ACGTTTCG 0.646697531838
ACGTTTCT 0.655327306541
ACGTTTGA -0.0648547852141
ACGTTTGC 0.0301456619834
ACGTTTGG 0.578397737217
ACGTTTTA 0.404339028995
ACGTTTTC 0.669239864637
ACGTTTTG 0.00420928918757
ACTAAAAA 1.23525464884
ACTAAAAC 0.158029481978
ACTAAAAG 0.841005733518
ACTAAAAT 4.37577514826
ACTAAACA 0.534629912088
ACTAAACC -0.368785120453
ACTAAACG 0.448488994014
ACTAAACT 0.298009780756
ACTAAAGA 0.996359190739
ACTAAAGC -0.560522111394
ACTAAAGG -1.42138029973
ACTAAAGT -0.066640805645
ACTAAATA 1.20256182323

ACTAAATC 3.58411642999
ACTAAATG 0.756051487881
ACTAACAA -0.116488889411
ACTAACAC -0.718854273258
ACTAACAG 0.33045638491
ACTAACAT 0.898249870864
ACTAACCA -0.52535164965
ACTAACCC -0.967694255485
ACTAACCG -0.181247486198
ACTAACCT -0.0696069639721
ACTAACGA 0.285792282296
ACTAACGC 0.172718343409
ACTAACGG 1.08322178339
ACTAACTA -0.436290053647
ACTAACTC 0.990447784612
ACTAACTG 0.167743206151
ACTAAGAA 0.802129142155
ACTAAGAC -0.466501845174
ACTAAGAG -0.0899811364506
ACTAAGAT 2.81489000287
ACTAAGCA 0.485416985595
ACTAAGCC -0.482108678761
ACTAAGCG -0.167366816656
ACTAAGCT -0.511313628389
ACTAAGGA 1.27481475308
ACTAAGGC -0.891899343659
ACTAAGGG -1.14304837089
ACTAAGTA 0.971062418703
ACTAAGTC 0.646091387922
ACTAAGTG -0.428747364993
ACTAATAA 0.561876852195
ACTAATAC 1.06638880875
ACTAATAG 0.438529832525
ACTAATAT 2.50848386117
ACTAATCA 0.149833862103
ACTAATCC 0.572618590176
ACTAATCG 0.625065329748
ACTAATCT 0.539560091711
ACTAATGA -0.412717354604
ACTAATGC 0.669751127035
ACTAATGG 0.2438502076
ACTAATTA 0.33611085293
ACTAATTC 2.24954148034
ACTAATTG 0.950628128458
ACTACAAA 0.304434278951
ACTACAAC 0.532511414269
ACTACAAG -0.548145692925
ACTACAAT 1.33749275238
ACTACACA -0.148448016892
ACTACACC 0.105398468383
ACTACACG 0.437079687442
ACTACACT -0.21595347285
ACTACAGA 1.61744498572
ACTACAGC -0.0536067510854

ACTACAGG 0.27430534539
ACTACAGT -0.0307196559635
ACTACATA 0.236977962734
ACTACATC 1.07367089995
ACTACATG 0.824921878521
ACTACCAA 0.649049704801
ACTACCAC -0.187834040982
ACTACCAG -0.768212005154
ACTACCAT 0.656572789836
ACTACCCA -0.371974237279
ACTACCCC 0.128162975537
ACTACCCG -0.558448048447
ACTACCCT 0.189104355528
ACTACCGA 0.0331891892624
ACTACCGC -0.817899077858
ACTACCGG -0.0775161096208
ACTACCTA -0.0884823743913
ACTACCTC -0.198478283629
ACTACCTG -1.15833658031
ACTACGAA 0.274904170622
ACTACGAC -0.105199818372
ACTACGAG 0.616584542554
ACTACGAT 2.71762154758
ACTACGCA 0.384430115753
ACTACGCC -1.31549513882
ACTACGCG 0.679789748529
ACTACGCT -0.554230395048
ACTACGGA 1.02720587816
ACTACGGC -1.45344737781
ACTACGGG -0.904203098046
ACTACGTA 0.707178880224
ACTACGTC -0.234078718095
ACTACGTG -0.0481012705738
ACTACTAA 1.5083477064
ACTACTAC 0.402497072903
ACTACTAG -0.934574070963
ACTACTAT 2.93407844138
ACTACTCA -0.306147112536
ACTACTCC 0.0116576190863
ACTACTCG 0.384927263545
ACTACTCT 0.0413646827552
ACTACTGA 1.46859391841
ACTACTGC -1.17187876069
ACTACTGG 1.2778334491
ACTACTTA 1.36567752964
ACTACTTC 0.672550262524
ACTACTTG -0.793332344614
ACTAGAAA 0.579700463081
ACTAGAAC 0.0818142685503
ACTAGAAG 0.919718186694
ACTAGAAT 1.06016113089
ACTAGACA 0.191895910951
ACTAGACC -1.00761611184
ACTAGACG -0.195304326934

ACTAGACT -0.841751455206
ACTAGAGA -0.0783240401274
ACTAGAGC -0.326666351799
ACTAGAGG -0.736932469816
ACTAGAGT 0.275070147934
ACTAGATA 6.53034310371
ACTAGATC 3.53386163646
ACTAGATG 0.43286647753
ACTAGCAA 0.263705537614
ACTAGCAC -0.00212346406854
ACTAGCAG -0.603458481673
ACTAGCAT 0.808574028115
ACTAGCCA -0.654735277231
ACTAGCCC -0.831066959793
ACTAGCCG 0.0912268811269
ACTAGCCT -0.9628458883
ACTAGCGA -0.183259601708
ACTAGCGC -1.08857749225
ACTAGCGG -0.597982014519
ACTAGCTA 0.129236469743
ACTAGCTC 0.373243506299
ACTAGCTG -0.373613884018
ACTAGGAA 1.62811353686
ACTAGGAC 0.233738399259
ACTAGGAG -0.28100612393
ACTAGGAT 1.48890535931
ACTAGGCA -0.0120457707532
ACTAGGCC -1.23220432564
ACTAGGCG -0.10353168104
ACTAGGCT -0.537996507017
ACTAGGGA 0.0627987575968
ACTAGGGC -0.797446229519
ACTAGGGG 0.292680994202
ACTAGGTA -0.113488751476
ACTAGGTC -0.804728059343
ACTAGGTG 0.558130731192
ACTAGTAA 0.479082402668
ACTAGTAC -0.12583641797
ACTAGTAG -0.686199609355
ACTAGTAT 1.94853365271
ACTAGTCA 0.0178356344388
ACTAGTCC -0.830955611234
ACTAGTCG -0.494479085454
ACTAGTCT 0.196902936763
ACTAGTGA -0.035438116496
ACTAGTGC -0.289536573628
ACTAGTGG -0.512812390448
ACTAGTTA -0.257798574974
ACTAGTTC -0.479829169881
ACTAGTTG -0.63398523802
ACTATAAA 0.671087309743
ACTATAAC 0.266069211367
ACTATAAG -0.0634610985556
ACTATAAT 2.31586104801

ACTATACA 1.46602506011
ACTATACC -0.0801929185236
ACTATACG -0.431991894717
ACTATACT -0.302048126381
ACTATAGA 0.235105424995
ACTATAGC 0.186930706193
ACTATAGG -0.994107911071
ACTATAGT 0.963022582257
ACTATATA 1.78924274316
ACTATATC 1.35656681281
ACTATATG 0.609996158099
ACTATCAA 1.874591675
ACTATCAC 0.908500211449
ACTATCAG -0.0322257367074
ACTATCAT 0.8034321294
ACTATCCA 1.75397713851
ACTATCCC 0.139681016852
ACTATCCG 0.913707455562
ACTATCCT 0.152345216457
ACTATCGA 0.358626002044
ACTATCGC 0.634947645049
ACTATCGG 0.536400511004
ACTATCTA 1.64273522322
ACTATCTC 2.1233822561
ACTATCTG 0.895089506012
ACTATGAA 1.73239747412
ACTATGAC -0.103510770512
ACTATGAG 0.165939673154
ACTATGAT 1.02945558954
ACTATGCA 1.12790392134
ACTATGCC 0.373999421869
ACTATGCG 0.488017993836
ACTATGCT -0.867223352909
ACTATGGA 0.779495848561
ACTATGGC -0.16244761506
ACTATGGG 0.194192932397
ACTATGTA 0.550380244171
ACTATGTC 0.733075784399
ACTATGTG 0.302173328165
ACTATTAA 0.315756806834
ACTATTAC 2.09114292755
ACTATTAG 0.806851000648
ACTATTAT 1.11658897352
ACTATTCA -0.0572995502435
ACTATTCC -0.82143687773
ACTATTCG 1.96533813676
ACTATTCT 0.845103935528
ACTATTGA 0.000203877643213
ACTATTGC 0.889410468123
ACTATTGG -0.62046893442
ACTATTTA 0.0951622424041
ACTATTTC -0.18275487385
ACTATTTG 0.436713230447
ACTCAAAA 1.67590637715

ACTCAAAC -0.469375735798
ACTCAAAG 0.915046252085
ACTCAAAT 1.45556117076
ACTCAACA 0.75908351437
ACTCAACC 0.303404958235
ACTCAACG -1.21582484807
ACTCAACT -0.289068700575
ACTCAAGA 2.08023155292
ACTCAAGC -0.585874296328
ACTCAAGG 0.370680921153
ACTCAAGT 0.356620421074
ACTCAATA 0.837675732012
ACTCAATC 1.47599441548
ACTCAATG 2.02881910031
ACTCACAA -0.116331537691
ACTCACAC 0.100021326234
ACTCACAG -0.152476691398
ACTCACAT 0.00569602769346
ACTCACCA 0.333415485934
ACTCACCC -0.759074627395
ACTCACCG -0.394504807907
ACTCACCT 0.368671419459
ACTCACGA -0.0741364456122
ACTCACGC -0.166290970016
ACTCACGG -1.40316383231
ACTCACTA 0.0368710103935
ACTCACTC 0.233039203496
ACTCACTG -0.602345518846
ACTCAGAA 1.59317361368
ACTCAGAC -0.241367082458
ACTCAGAG -0.522446393234
ACTCAGAT 2.93006832497
ACTCAGCA 1.21230456076
ACTCAGCC -0.330875118223
ACTCAGCG 0.151921255511
ACTCAGCT 0.781068058848
ACTCAGGA 0.27975802682
ACTCAGGC -0.57013468089
ACTCAGGG 0.237817259032
ACTCAGTA 1.28376576576
ACTCAGTC -0.984881402189
ACTCAGTG -0.0190541954294
ACTCATAA 0.730190131602
ACTCATAC 0.725677901148
ACTCATAG -0.0852778360506
ACTCATAT 1.630111015
ACTCATCA 0.880366403593
ACTCATCC 0.183813730687
ACTCATCG -0.486655672968
ACTCATCT -0.761658645832
ACTCATGA -0.153712242193
ACTCATGC -0.573746190372
ACTCATGG -0.381253283861
ACTCATTA 0.834359322349

ACTCATTC 0.709098205267
ACTCATTG 0.964340206872
ACTCCAAA 0.0333956807215
ACTCCAAC -0.128225184357
ACTCCAAG -0.0111270144508
ACTCCAAT 0.498352760675
ACTCCACA -0.254630368675
ACTCCACC -0.593087382792
ACTCCACG -0.42326462468
ACTCCACT -0.370972623012
ACTCCAGA -0.986005604424
ACTCCAGC -0.353395233588
ACTCCAGG -0.363530827653
ACTCCAGT 0.107791678256
ACTCCATA 0.673196659205
ACTCCATC -0.791570632671
ACTCCATG -0.510810730202
ACTCCCAA -0.223632864077
ACTCCCAC -0.71152251955
ACTCCCAG -1.0108530615
ACTCCCAT 0.309197174355
ACTCCCCA -0.26233720497
ACTCCCCC -1.5849908015
ACTCCCCG -1.02752633199
ACTCCCCT -1.31623354182
ACTCCCGA 0.240926915854
ACTCCCGC 0.609504237939
ACTCCCGG -0.696104142092
ACTCCCTA -0.864630970261
ACTCCCTC -0.892391263819
ACTCCCTG -0.448584398296
ACTCCGAA 0.55067037774
ACTCCGAC 0.0580507609442
ACTCCGAG -0.716724013268
ACTCCGAT 0.326900549707
ACTCCGCA 0.504032844092
ACTCCGCC -0.13975603337
ACTCCGCG 0.278467585891
ACTCCGCT -0.944197095723
ACTCCGGA -0.262082096535
ACTCCGGC 0.363779401549
ACTCCGGG -0.551840321754
ACTCCGTA 0.219041173618
ACTCCGTC 0.0915972588454
ACTCCGTG -0.916771893368
ACTCCTAA 0.398704425976
ACTCCTAC -0.663390405948
ACTCCTAG -0.834553267492
ACTCCTAT 0.518953289614
ACTCCTCA -0.584369783873
ACTCCTCC -0.318205429606
ACTCCTCG 0.116959637661
ACTCCTCT 0.250310515073
ACTCCTGA 0.284796157041

ACTCCTGC -0.74583460414
ACTCCTGG -1.12557919344
ACTCCTTA 0.323839771243
ACTCCTTC -1.1120861528
ACTCCTTG -0.414468349902
ACTCGAAA 1.01643016057
ACTCGAAC 0.0186584636963
ACTCGAAG -0.170872727975
ACTCGAAT 0.893132542019
ACTCGACA -0.59270001527
ACTCGACC -0.432038682023
ACTCGACG -0.676019580419
ACTCGACT 0.355037755523
ACTCGAGA 2.63845533596
ACTCGAGC -0.0226236224752
ACTCGAGG -0.674470894475
ACTCGAGT -0.1580888156
ACTCGATA 1.86915729028
ACTCGATC 0.0909508621638
ACTCGATG 0.5455472985
ACTCGCAA 0.745126260021
ACTCGCAC -0.692336849179
ACTCGCAG 0.378987628206
ACTCGCAT -0.333571530746
ACTCGCCA -0.279640666484
ACTCGCCC -0.146711658964
ACTCGCCG -0.590636407586
ACTCGCCT -0.189929014457
ACTCGCGA 0.88908374113
ACTCGCGC 0.390160907199
ACTCGCGG -0.302145621716
ACTCGCTA -0.744929701062
ACTCGCTC -1.02696410018
ACTCGCTG 0.18377818279
ACTCGGAA 0.277168519369
ACTCGGAC -0.525255199842
ACTCGGAG -0.454277812173
ACTCGGAT 2.5668103797
ACTCGGCA -0.127307996344
ACTCGGCC -1.18309987251
ACTCGGCG -0.970183392403
ACTCGGCT -0.550995536443
ACTCGGGA -0.228544224226
ACTCGGGC -0.896120133637
ACTCGGGG -0.607640064412
ACTCGGTA 0.265677138977
ACTCGGTC -0.307475715178
ACTCGGTG -0.65076645911
ACTCGTAA 0.641435136209
ACTCGTAC -0.36423054618
ACTCGTAG 0.715142654625
ACTCGTAT 0.222381369006
ACTCGTCA 0.0468471616865
ACTCGTCC 0.173152236855

ACTCGTCG -0.6335424576
ACTCGTCT 0.147933095152
ACTCGTGA 1.204118612
ACTCGTGC -0.134884664605
ACTCGTGG -0.3180109617
ACTCGTTA -0.0653608199798
ACTCGTTC 0.0240596529519
ACTCGTTG -0.583132142026
ACTCTAAA 1.45715742815
ACTCTAAC -0.244257440123
ACTCTAAG -0.38140070308
ACTCTAAT 1.38128410185
ACTCTACA 1.30928366662
ACTCTACC -0.869981974251
ACTCTACG -0.558225351329
ACTCTACT -0.0715145268441
ACTCTAGA 0.950243374752
ACTCTAGC -1.06578397174
ACTCTAGG -0.712814267387
ACTCTATA 0.115172571704
ACTCTATC -0.0434154827407
ACTCTATG -0.28052387489
ACTCTCAA -0.532604204735
ACTCTCAC 0.462974239183
ACTCTCAG 0.632193205813
ACTCTCAT 0.952413887507
ACTCTCCA -0.156481057416
ACTCTCCC -0.476250855861
ACTCTCCG -0.466701540711
ACTCTCCT 0.0845768106158
ACTCTCGA -0.582789209375
ACTCTCGC -0.698247471161
ACTCTCGG -0.00490848495115
ACTCTCTA -0.730914681381
ACTCTCTC -0.485647001398
ACTCTCTG -0.19236482953
ACTCTGAA 0.375496615638
ACTCTGAC -0.595868482951
ACTCTGAG -0.940647533678
ACTCTGAT 1.57474621131
ACTCTGCA -0.687805537868
ACTCTGCC -0.516695475406
ACTCTGCG 0.547655079673
ACTCTGCT -1.28098074488
ACTCTGGA 0.24174294919
ACTCTGGC -1.19988788952
ACTCTGGG 2.12880461727
ACTCTGTA 1.00875834941
ACTCTGTC -0.412749765922
ACTCTGTG -0.352101917461
ACTCTTAA 0.691341769451
ACTCTTAC -0.193225559119
ACTCTTAG 0.206475253493
ACTCTTAT 0.298954675218

ACTCTTCA	-0.698405345644
ACTCTTCC	-0.611018682894
ACTCTTCG	-0.33084009309
ACTCTTCT	0.246227734577
ACTCTTGA	-0.655711276102
ACTCTTGC	-0.532371575117
ACTCTTGG	-0.274236863412
ACTCTTTA	0.705025618653
ACTCTTTC	0.527656773926
ACTCTTTG	0.561427798617
ACTGAAAA	0.928820539319
ACTGAAAC	0.251161573543
ACTGAAAG	0.147246445705
ACTGAAAT	2.54261768352
ACTGAACA	-0.0418529435725
ACTGAACC	-0.512546826748
ACTGAACG	0.22219840189
ACTGAACT	-0.470357223683
ACTGAAGA	0.610971372826
ACTGAAGC	-0.792621909441
ACTGAAGG	-0.97540605803
ACTGAAGT	0.960142941238
ACTGAATA	1.64747511704
ACTGAATC	2.62911015983
ACTGAATG	0.164714839005
ACTGACAA	1.54417031522
ACTGACAC	-0.665691348119
ACTGACAG	-0.0440629249487
ACTGACAT	0.131215128409
ACTGACCA	-0.44868189363
ACTGACCC	-0.275034077274
ACTGACCG	0.248171629491
ACTGACCT	0.188598843526
ACTGACGA	0.0497058921786
ACTGACGC	-0.808459020214
ACTGACGG	-0.574817070762
ACTGACTA	0.867614379773
ACTGACTC	-0.25232053953
ACTGACTG	-0.609132553313
ACTGAGAA	0.119356245495
ACTGAGAC	-0.641083577965
ACTGAGAG	-0.761046228758
ACTGAGAT	2.49805944045
ACTGAGCA	-1.01989059149
ACTGAGCC	-1.22069543269
ACTGAGCG	-0.116525482834
ACTGAGCT	0.567221321645
ACTGAGGA	-0.353383210034
ACTGAGGC	-1.16920116764
ACTGAGGG	-0.836968433419
ACTGAGTA	-0.0150090538828
ACTGAGTC	-0.431501804229
ACTGAGTG	-0.324037375728
ACTGATAA	2.97316675183

ACTGATAC 1.98845760012
ACTGATAG -0.482722141362
ACTGATAT 4.81730773253
ACTGATCA 0.237065002804
ACTGATCC 0.830592813582
ACTGATCG 1.87591348172
ACTGATCT 1.12790392134
ACTGATGA 0.577093965827
ACTGATGC -0.106478497129
ACTGATGG -0.0483647432205
ACTGATTA 2.01074952935
ACTGATTC 4.95901314983
ACTGATTG 3.75837509513
ACTGCAAA 1.71179276308
ACTGCAAC 0.881859676639
ACTGCAAG -0.321624823617
ACTGCAAT 3.08461123787
ACTGCACA 0.593255712538
ACTGCACC -0.45790970942
ACTGCACG 0.134212652527
ACTGCACT -0.102905410741
ACTGCAGA 1.95609672912
ACTGCAGC 0.207235351168
ACTGCAGG -0.918854059145
ACTGCAGT 1.16155418773
ACTGCATA 0.692305222006
ACTGCATC 1.29476601014
ACTGCATG 0.246124750229
ACTGCCAA 0.128462518844
ACTGCCAC -0.523354432891
ACTGCCAG -0.800988211498
ACTGCCAT -0.0801524043765
ACTGCCCA -0.648361748446
ACTGCCCC -0.464660673226
ACTGCCCG -1.28293744749
ACTGCCCT -0.45491349221
ACTGCCGA -0.418119066623
ACTGCCGC -0.484546846269
ACTGCCGG -0.99061480745
ACTGCCTA -0.201520504
ACTGCCTC -1.55433492265
ACTGCCTG 0.281483406721
ACTGCGAA -0.414961054206
ACTGCGAC -0.714137903779
ACTGCGAG 0.215556172827
ACTGCGAT 3.12272354945
ACTGCGCA -0.00894081879968
ACTGCGCC 0.0167307744416
ACTGCGCG -0.363726079703
ACTGCGCT 0.826975815086
ACTGCGGA 0.105472962137
ACTGCGGC -0.607362738541
ACTGCGGG -0.748458091198
ACTGCGTA 1.01065415011

ACTGCGTC -0.84759359521
ACTGCGTG 0.100848337597
ACTGCTAA 0.263054697445
ACTGCTAC -0.136031084276
ACTGCTAG -0.75775857107
ACTGCTAT -0.120951718744
ACTGCTCA -0.630838465012
ACTGCTCC -0.950753591623
ACTGCTCG -0.0287274054551
ACTGCTCT -0.274526474218
ACTGCTGA 0.393206264149
ACTGCTGC -1.41031941482
ACTGCTGG -0.733585478507
ACTGCTTA -0.0935599732337
ACTGCTTC 0.255455550367
ACTGCTTG -0.621771921666
ACTGGAAA 0.431511213966
ACTGGAAC -0.560082206172
ACTGGAAG 0.557100103567
ACTGGAAT 2.43670063405
ACTGGACA -0.656398186931
ACTGGACC -0.560022088405
ACTGGACG 0.211001859936
ACTGGACT 0.0955830667702
ACTGGAGA 0.061636916412
ACTGGAGC -0.449439377489
ACTGGAGG -0.932266071489
ACTGGATA 2.71970423612
ACTGGATC 0.518953289614
ACTGGATG -0.180004616719
ACTGGCAA 0.348283132375
ACTGGCAC -1.41878295083
ACTGGCAG -0.983721390675
ACTGGCAT 0.52728665759
ACTGGCCA -0.756681417522
ACTGGCCC -0.68144795336
ACTGGCCG -0.22917467663
ACTGGCCT -0.893366217164
ACTGGCGA 0.398224267988
ACTGGCGC -0.166736887015
ACTGGCGG -0.914932289711
ACTGGCTA -1.08330803932
ACTGGCTC -0.519574593663
ACTGGCTG -0.611119053426
ACTGGGAA 1.13442434658
ACTGGGAC -0.820397885895
ACTGGGAG -1.08895231346
ACTGGGAT 1.92089803817
ACTGGGCA -0.100864020493
ACTGGGCC -1.22244146173
ACTGGGCG -1.15892677995
ACTGGGCT -1.2747533284
ACTGGGGA -0.375140875289
ACTGGGGC -0.612932518924

ACTGGGGG -1.28401407827
ACTGGGTA 1.15447858799
ACTGGGTC -0.798039304356
ACTGGGTG -0.533960775207
ACTGGTAA 1.92578953332
ACTGGTAC -0.821373362003
ACTGGTAG -0.259091891101
ACTGGTAT 0.738497100037
ACTGGTCA -0.0190006122027
ACTGGTCC -0.462622942321
ACTGGTCG -1.72126366375
ACTGGTCT -0.677081573835
ACTGGTGA -0.215124893197
ACTGGTGC -0.428223033515
ACTGGTGG -1.36763109568
ACTGGTTA -0.705069269379
ACTGGTTC -0.8908564311
ACTGGTTG -0.875286190935
ACTGTAAA 1.50421186544
ACTGTAAC 0.0780234512945
ACTGTAAG -0.147348384527
ACTGTAAT 4.16525161664
ACTGTACA -0.0153140862028
ACTGTACC -0.349810123646
ACTGTACG -0.787190399921
ACTGTACT 0.0649407797585
ACTGTAGA 1.05405682515
ACTGTAGC 0.161155083078
ACTGTAGG -0.179295227074
ACTGTATA 0.639882529541
ACTGTATC 1.40181431914
ACTGTATG 0.564766687097
ACTGTCAA 1.32483038244
ACTGTCAC -0.0126589719724
ACTGTCAG -0.410368840983
ACTGTCAT -0.0648192373173
ACTGTCCA -0.296334063358
ACTGTCCC -0.361386453057
ACTGTCCG -0.298063625364
ACTGTCCT 0.531449420853
ACTGTCGA -0.106653622797
ACTGTCGC 0.72041419861
ACTGTCGG -1.0221426553
ACTGTCTA -0.588131065009
ACTGTCTC -0.807283325804
ACTGTCTG -0.869383410401
ACTGTGAA 0.770083497366
ACTGTGAC -0.774381133532
ACTGTGAG -0.0789934383893
ACTGTGAT 2.35888942461
ACTGTGCA -0.307047572127
ACTGTGCC -0.785965565772
ACTGTGCG 0.155908108963
ACTGTGCT -0.734913558385

ACTGTGGA -0.524009193784
ACTGTGGC -0.303443642711
ACTGTGGG -0.936664600951
ACTGTGTA 0.248239327324
ACTGTGTC -0.161845391867
ACTGTGTG 0.546259563343
ACTGTTAA -0.667286821368
ACTGTTAC 1.5849027159
ACTGTTAG -0.100865327401
ACTGTTAT 1.76400425922
ACTGTTCA -0.345028408768
ACTGTTCC -0.263158204557
ACTGTTCG -0.353633090838
ACTGTTCT 0.150162680148
ACTGTTGA -0.262276825822
ACTGTTGC -0.777589069834
ACTGTTGG 0.485259633876
ACTGTTTA 0.144002438744
ACTGTTTC -0.116713677581
ACTGTTTG -0.217262471872
ACTTAAAA 2.40648910391
ACTTAAAC 0.44488872394
ACTTAAAG 0.585928140936
ACTTAAAT 2.16387523124
ACTTAACA 1.05922956689
ACTTAACC 0.47234267827
ACTTAACG -0.134580939193
ACTTAACT 0.473082649562
ACTTAAGA -0.123308335194
ACTTAAGC 0.0232527679717
ACTTAAGG -0.343844872911
ACTTAAGT 1.577647547
ACTTAATA 0.441787692711
ACTTAATC 1.30477300446
ACTTAATG 1.14802089433
ACTTACAA 0.636841354697
ACTTACAC 0.354793363733
ACTTACAG -0.742265961239
ACTTACAT 1.48651528601
ACTTACCA 1.11356374295
ACTTACCC 0.309041652306
ACTTACCG -0.104700579527
ACTTACCT -0.0781706091319
ACTTACGA 0.344864522509
ACTTACGC 0.304261244336
ACTTACGG -0.423418055675
ACTTACTA 0.811929122254
ACTTACTC -0.0109231368075
ACTTACTG 0.554805434555
ACTTAGAA 1.58124206668
ACTTAGAC -0.209391226554
ACTTAGAG 0.00163102114571
ACTTAGAT 2.84453564186
ACTTAGCA 0.386637744695

ACTTAGCC -0.523048355045
ACTTAGCG -0.519512123462
ACTTAGCT -1.10543085466
ACTTAGGA -0.0109231368075
ACTTAGGC -0.340938309587
ACTTAGGG 0.0492312232041
ACTTAGTA 0.802072422349
ACTTAGTC -0.656710537936
ACTTAGTG 0.378023130124
ACTTATAA -0.244903314041
ACTTATAC 2.55029446093
ACTTATAG 0.136981990514
ACTTATAT 1.82665324516
ACTTATCA 0.0894040058913
ACTTATCC 0.749908497662
ACTTATCG 0.558160528693
ACTTATCT 1.91767990798
ACTTATGA 0.943987990448
ACTTATGC -0.447080931368
ACTTATGG 0.402684483505
ACTTATTA 1.82064878718
ACTTATTC 0.624999461587
ACTTATTG 0.0373872390414
ACTTCAAA 0.623910545867
ACTTCAAC -0.248252396403
ACTTCAAG 0.15304598051
ACTTCAAT 1.38761476405
ACTTCACA 2.06154747383
ACTTCACC -0.230755251129
ACTTCACG -0.894293076296
ACTTCACT -1.01142705548
ACTTCAGA 0.735831530543
ACTTCAGC -0.593744496119
ACTTCAGG 0.873394049577
ACTTCATA 0.478682750211
ACTTCATC -1.02042903757
ACTTCATG -0.455997964443
ACTTCCAA 0.218656942675
ACTTCCAC -0.687392032186
ACTTCCAG -0.260052207077
ACTTCCAT 0.573481672199
ACTTCCCA -0.764034604521
ACTTCCCC -0.922084474263
ACTTCCCG -0.702758394708
ACTTCCCT -0.419308875638
ACTTCCGA -0.350667716656
ACTTCCGC -0.213650962389
ACTTCCGG 0.0659209607355
ACTTCCTA 0.55325335065
ACTTCCTC -0.449804527576
ACTTCCTG -0.279014396185
ACTTCGAA 1.30451449806
ACTTCGAC -0.732354632581
ACTTCGAG 0.257738979971

ACTTCGAT 1.43034438149
ACTTCGCA -0.451763582622
ACTTCGCC -0.359754125003
ACTTCGCG -0.368915549868
ACTTCGCT -0.188983597232
ACTTCGGA -0.299360339451
ACTTCGGC -0.0423495686009
ACTTCGGG -0.689538497835
ACTTCGTA 0.911320780228
ACTTCGTC -0.170122301419
ACTTCGTG 0.520876796763
ACTTCTAA 0.644825778244
ACTTCTAC -0.170701784412
ACTTCTAG -0.398833809865
ACTTCTAT 0.465373460833
ACTTCTCA 1.10870282945
ACTTCTCC -0.109177523467
ACTTCTCG 0.363253763163
ACTTCTCT 0.838895338529
ACTTCTGA 1.2754350116
ACTTCTGC -0.0774267171157
ACTTCTGG -0.348463485675
ACTTCTTA 0.914371626192
ACTTCTTC -0.698438541106
ACTTCTTG 0.0707565202219
ACTTGAAA 1.36637149777
ACTTGAAC 1.46194541619
ACTTGAAG -0.520797075377
ACTTGAAT 0.856976671666
ACTTGACA 0.128621700235
ACTTGACC 0.357802388642
ACTTGACG 0.0770511117653
ACTTGACT -0.130331920003
ACTTGAGA -0.268915656925
ACTTGAGC -0.358557520066
ACTTGAGG 0.655458258719
ACTTGATA 1.79829125117
ACTTGATC 0.765494682104
ACTTGATG 0.086732686002
ACTTGCAA 0.65646196404
ACTTGCAC 0.246451999985
ACTTGCAG 0.413988191914
ACTTGCAT 1.39102474833
ACTTGCCA 0.635413949812
ACTTGCCC -0.595322718183
ACTTGCCG -0.401042484333
ACTTGCCT 0.270430886024
ACTTGCGA 0.152736243321
ACTTGCGC 0.863585966649
ACTTGCGG 0.0777537054896
ACTTGCTA -0.353197106339
ACTTGCTC -0.0624503359321
ACTTGCTG 0.0571487330638
ACTTGGAA 0.437533445889

ACTTGGAC 1.3995682671
ACTTGGAG 0.176546538233
ACTTGGAT 2.7768981882
ACTTGGCA -0.453808109449
ACTTGGCC -0.942464135755
ACTTGGCG 1.50395884806
ACTTGGCT -0.261577630058
ACTTGGGA -0.159876665702
ACTTGGGC -1.53234802573
ACTTGGGG 0.863810493438
ACTTGGTA -0.0226236224752
ACTTGGTC -0.724306693306
ACTTGGTG -1.13876484933
ACTTGTAA -0.155949407255
ACTTGTAC -0.199799044823
ACTTGTAG 0.0236341237171
ACTTGTAT 0.834470148145
ACTTGTCA 0.679536208383
ACTTGTCC -0.791862857293
ACTTGTCG -0.673142291834
ACTTGTCT -0.689547123428
ACTTGTGA 0.493491324411
ACTTGTGC -0.33872493025
ACTTGTGG -0.625566136882
ACTTGTTA 0.127411503455
ACTTGTTC -0.77227178407
ACTTGTTG -0.0792527289304
ACTTTAAA 1.51217145774
ACTTTAAC 0.351730755598
ACTTTAAG 0.136717472341
ACTTTAAT 1.84091892979
ACTTTACA 0.496623198669
ACTTTACC 0.502765404743
ACTTTACG 1.02386254619
ACTTTACT 0.220546208835
ACTTTAGA 0.173538036087
ACTTTAGC -0.0193986963701
ACTTTAGG -0.222002627076
ACTTTATA 1.32036232548
ACTTTATC 0.430111776913
ACTTTATG 0.010995539509
ACTTTCAA 0.467583442209
ACTTTCAC 0.545839523122
ACTTTCAG 0.164024791597
ACTTTCAT 0.0345096890746
ACTTTCCA 0.146919718713
ACTTTCCC -0.088328159251
ACTTTCCG 0.443809217958
ACTTTCCT -0.516469118946
ACTTTCGA 0.351176103856
ACTTTCGC 0.143219862252
ACTTTCGG -0.449609275525
ACTTTCTA 0.93459654978
ACTTTCTC 0.0825646951063

ACTTTCTG -0.0798476334381
ACTTTGAA 0.220901949185
ACTTTGAC -0.900448090068
ACTTTGAG -0.0138239497362
ACTTTGAT 1.2825168845
ACTTTGCA 0.41114880366
ACTTTGCC -0.514235351845
ACTTTGCG 0.89135985205
ACTTTGCT -0.355443419757
ACTTTGGA -0.265574938773
ACTTTGGC -0.383442093328
ACTTTGGG 0.316139992251
ACTTTGTA 0.527677161691
ACTTTGTC 0.302169668822
ACTTTGTG -0.345315928521
ACTTTTAA 0.454495804423
ACTTTTAC 0.633847228539
ACTTTTAG -0.365890319301
ACTTTTAT 1.22853635774
ACTTTTCA 0.431522453375
ACTTTTCC 0.270580919059
ACTTTTCG 2.19025177064
ACTTTTCT -0.0229111422285
ACTTTTGA 0.0761945642822
ACTTTTGC 0.294595614377
ACTTTTGG -0.114285181193
ACTTTTTA 1.05384301501
ACTTTTTC 1.22374680141
ACTTTTTG -0.209829563487
AGAAAAAA 0.920315705018
AGAAAAAC -0.573143444417
AGAAAAAG 0.102738910665
AGAAAAAT 2.09172920647
AGAAAACA 0.352682184599
AGAAAACC 0.69037570308
AGAAAACG 0.610474486416
AGAAAACT -0.309516059899
AGAAAAGA 2.35137679484
AGAAAAGC 1.12400881283
AGAAAAGG -0.249751681226
AGAAAATA 2.05148062312
AGAAAATC 21.724022307
AGAAAATG 1.37182522473
AGAAACAA 0.615062778914
AGAAACAC 0.612983749717
AGAAACAG 0.831968203529
AGAAACAT 1.10245580936
AGAAACCA 0.974115878483
AGAAACCC -0.0219591904636
AGAAACCG -0.373910552127
AGAAACCT 0.171487758865
AGAAACGA 0.397539970975
AGAAACGC 0.0201671582561
AGAAACGG -0.246705017368

AGAAACTA 1.77628553478
AGAAACTC -0.390780120195
AGAAACTG 0.0202126386534
AGAAAGAA 1.46759648625
AGAAAGAC -0.731345699629
AGAAAGAG 0.228960343723
AGAAAGAT 2.186381232
AGAAAGCA -0.277786686839
AGAAAGCC -0.298147528856
AGAAAGCG -0.242421495807
AGAAAGCT 0.0806788269066
AGAAAGGA 0.625450606218
AGAAAGGC -0.596900417483
AGAAAGGG -0.488851278357
AGAAAGTA 2.62163882835
AGAAAGTC 0.746513673521
AGAAAGTG 0.0451505337607
AGAAATAA 2.37995442864
AGAAATAC 2.11990718781
AGAAATAG 1.8652347367
AGAAATAT 4.45356753822
AGAAATCA 4.66044584213
AGAAATCC 14.6315114334
AGAAATCG 3.33572314891
AGAAATCT 15.7564280245
AGAAATGA 0.52230786099
AGAAATGC 1.17246869895
AGAAATGG 0.568465498032
AGAAATTA 2.50843419867
AGAAATTC 2.71932549419
AGAAATTG 1.01860067332
AGAACAAA 0.816207416182
AGAACAAC -0.253392726828
AGAACAAG 0.146047488334
AGAACAAT 1.88223917767
AGAACACA 0.920200697116
AGAACACC 0.273087829926
AGAACACG 0.0674944779305
AGAACACT 0.063836703906
AGAACAGA -0.053085033424
AGAACAGC -0.799037520663
AGAACAGG -1.19688670606
AGAACATA 0.845044601907
AGAACATC 1.43478786859
AGAACATG 1.03285747098
AGAACCAA 0.595248224428
AGAACCAC 0.533786695066
AGAACCAG -0.346572389843
AGAACCAT -0.648430753187
AGAACCCA -0.0938945416739
AGAACCCC -0.556543622154
AGAACCCG -0.976140017545
AGAACCCT -0.665349722376
AGAACCGA 1.68401443419

AGAACCGC -0.817147344394
AGAACCGG -0.326683341603
AGAACCTA 0.392769495505
AGAACCTC -0.425320652297
AGAACCTG -1.08728888099
AGAACGAA -0.218726731561
AGAACGAC -0.742400311378
AGAACGAG -0.50728782908
AGAACGAT 0.321831315076
AGAACGCA -0.702872357083
AGAACGCC -0.767031605876
AGAACGCG -0.348023841834
AGAACGCT -0.845525021276
AGAACGGA 0.941286873057
AGAACGGC -0.816032290514
AGAACGGG -0.694858397415
AGAACGTA -0.00455771085219
AGAACGTC -0.179253144637
AGAACGTG 0.0886716146653
AGAACTAA 0.687250101981
AGAACTAC -0.109410675849
AGAACTAG 0.213961745104
AGAACTAT 0.323934652762
AGAACTCA 1.00276643775
AGAACTCC -0.605666633378
AGAACTCG -0.418625624152
AGAACTCT -0.282614666259
AGAACTGA 0.430057932304
AGAACTGC -0.226828515444
AGAACTGG 0.0512982288484
AGAACTTA 0.407008257831
AGAACTTC -0.386801108191
AGAACTTG -0.0875293771001
AGAAGAAA 0.0583670326728
AGAAGAAC -0.795694188696
AGAAGAAG -1.11280024732
AGAAGAAT 2.18859016785
AGAAGACA -0.0696838101607
AGAAGACC -1.31056783439
AGAAGACG -0.381282035837
AGAAGACT -0.163721066185
AGAAGAGA 0.360791809931
AGAAGAGC -0.777530520357
AGAAGAGG -0.95562208519
AGAAGATA 2.85431262038
AGAAGATC 8.49870699379
AGAAGATG 0.188868066567
AGAAGCAA 0.00381094363852
AGAAGCAC -0.31832096027
AGAAGCAG -0.673137586965
AGAAGCAT 0.397087780818
AGAAGCCA -0.354082144416
AGAAGCCC -1.55217486515
AGAAGCCG -0.790550721692

AGAAGCCT −1.15788909502
AGAAGCGA 0.473561239261
AGAAGCGC −1.04075825242
AGAAGCGG −0.83586644862
AGAAGCTA −0.319786526867
AGAAGCTC −0.544508045283
AGAAGCTG −0.564823145521
AGAAGGAA 0.0335331674399
AGAAGGAC −1.11263975902
AGAAGGAG −0.754710077541
AGAAGGAT 1.22494445188
AGAAGGCA 0.213545102844
AGAAGGCC −1.02946238546
AGAAGGCG −0.720901413901
AGAAGGCT −1.44163449806
AGAAGGGA −0.757083422413
AGAAGGGC −1.49933343938
AGAAGGGG −0.945917509373
AGAAGGTA −0.272543110684
AGAAGGTC −0.779201794268
AGAAGGTG −1.20516622943
AGAAGTAA 1.06810033542
AGAAGTAC −0.465998946987
AGAAGTAG −0.771810706938
AGAAGTAT 3.22631064339
AGAAGTCA −0.813780226701
AGAAGTCC −0.0738970200722
AGAAGTCG 0.501886117061
AGAAGTCT −0.00199172774524
AGAAGTGA −0.0683170458064
AGAAGTGC −0.675679784347
AGAAGTGG −0.662616977812
AGAAGTTA 0.76471994706
AGAAGTTC 1.42348128498
AGAAGTTG −0.394504807907
AGAATAAA 1.36695777669
AGAATAAC 1.26308342438
AGAATAAG 1.76339001247
AGAATAAT 2.43186925667
AGAATACA 1.12880072159
AGAATACC 0.26288819737
AGAATACG 2.0240052355
AGAATACT 1.43835259076
AGAATAGA −0.336749408164
AGAATAGC −0.557413500098
AGAATAGG 0.576737964096
AGAATATA 2.91298442399
AGAATATC 10.0135126552
AGAATATG 3.43094080421
AGAATCAA 4.99808708429
AGAATCAC 5.49705670553
AGAATCAG 5.90919745234
AGAATCAT 6.55147423728
AGAATCCA 10.5677697112

AGAATCCC 9.09626922947
AGAATCCG 18.6284168366
AGAATCCT 12.6952112235
AGAATCGA 5.12249766567
AGAATCGC 8.23077204061
AGAATCGG 6.91112746899
AGAATCTA 16.4513436645
AGAATCTC 15.5694241315
AGAATCTG 21.6105063719
AGAATGAA 0.0104646734919
AGAATGAC 0.268840901789
AGAATGAG -0.338394543916
AGAATGAT 1.14086766425
AGAATGCA 0.813898371182
AGAATGCC -0.546124951822
AGAATGCG 2.36079489643
AGAATGCT -1.15079101646
AGAATGGA -0.0271392508908
AGAATGGC -0.137110328877
AGAATGGG -0.288608930351
AGAATGTA 0.766117293061
AGAATGTC -0.405924831125
AGAATGTG 1.50977223609
AGAATTAA 1.13546542947
AGAATTAC 2.53318808114
AGAATTAG 0.729000583969
AGAATTAT 3.04021714244
AGAATTCA 1.00908716745
AGAATTCC 2.44580560049
AGAATTCG 2.59825693787
AGAATTCT 3.49478717923
AGAATTGA 0.246385870442
AGAATTGC 1.77688540554
AGAATTGG 1.00064009848
AGAATTTA 2.1943408243
AGAATTTC 3.83804681875
AGAATTTG 5.18581552711
AGACAAAA -0.201520504
AGACAAAC -0.264346706664
AGACAAAG -0.632749425845
AGACAAAT 1.40145021458
AGACAACA -0.559938446295
AGACAACC -0.610751028142
AGACAACG -0.406573318859
AGACAACT -0.326721764697
AGACAAGA -0.135769179919
AGACAAGC -0.905505301146
AGACAAGG -0.503941099152
AGACAATA 2.00933493216
AGACAATC 3.96618626995
AGACAATG -1.06909463101
AGACACAA -0.226769443204
AGACACAC 0.1157491795
AGACACAG -0.0726165116439

AGACACAT	0.152539684363
AGACACCA	-0.604189565991
AGACACCC	-0.74821579046
AGACACCG	-0.587244458643
AGACACCT	-1.20464608006
AGACACGA	1.35877548728
AGACACGC	0.0383797049533
AGACACGG	1.10460645711
AGACACTA	-1.01199373078
AGACACTC	-1.18081957949
AGACACTG	-0.00752099398182
AGACAGAA	-0.256632290303
AGACAGAC	0.33070234499
AGACAGAG	-0.0157796068215
AGACAGAT	1.97529154785
AGACAGCA	-0.800308619354
AGACAGCC	-0.802088366626
AGACAGCG	-0.498400332125
AGACAGCT	-0.184667664354
AGACAGGA	-0.862525802904
AGACAGGC	-0.529850288262
AGACAGGG	-0.812917144679
AGACAGTA	0.141561918802
AGACAGTC	-0.755858065501
AGACAGTG	-1.30904188865
AGACATAA	-0.0692896467172
AGACATAC	-0.0947056087596
AGACATAG	0.123219465453
AGACATAT	1.63006710289
AGACATCA	1.62811353686
AGACATCC	0.544095585128
AGACATCG	0.486666389614
AGACATCT	0.501390014796
AGACATGA	0.236807541934
AGACATGC	0.712551578885
AGACATGG	-0.441798147974
AGACATTA	1.12691537615
AGACATTC	0.574298751062
AGACATTG	-0.593378823269
AGACCAAA	0.150475292534
AGACCAAC	-0.834774657702
AGACCAAG	-1.48049723619
AGACCAAT	-0.173708456886
AGACCACA	0.34668347702
AGACCACC	-0.791226915875
AGACCACG	-0.244875346211
AGACCACT	-0.946427203481
AGACCAGA	-1.02467936367
AGACCAGC	-0.64208806743
AGACCAGG	-1.11069142062
AGACCATA	-0.139436625062
AGACCATC	-0.947303093203
AGACCATG	-0.685615944256
AGACCCAA	-1.02366023684

AGACCCAC -1.29402603884
AGACCCAG -0.128091095599
AGACCCAT -0.30110662988
AGACCCCA -1.05772217924
AGACCCCC -1.48100117991
AGACCCCG -0.722308953784
AGACCCCT -0.703057415251
AGACCCGA -0.460498955489
AGACCCGC -1.0820685678
AGACCCGG -1.34377741142
AGACCCTA -0.213180214139
AGACCCTC -1.66383185442
AGACCCTG -0.965381028379
AGACCGAA -0.177830444622
AGACCGAC -0.898725062601
AGACCGAG 0.215414504003
AGACCGAT 0.442251383658
AGACCGCA -0.443663367028
AGACCGCC -1.10786118072
AGACCGCG 0.19195655148
AGACCGCT -0.42619523511
AGACCGGA -0.0154894732523
AGACCGGC -0.364910661087
AGACCGGG -1.18700961839
AGACCGTA 0.51075949941
AGACCGTC -0.958728605433
AGACCGTG -0.983834046142
AGACCTAA 0.436438257011
AGACCTAC -0.890910798471
AGACCTAG -0.367997839092
AGACCTAT -0.357591453695
AGACCTCA -1.10709167331
AGACCTCC -1.12695327648
AGACCTCG -0.955460812747
AGACCTCT -1.21227633155
AGACCTGA -0.373076222079
AGACCTGC -0.747291545144
AGACCTGG -0.503506682943
AGACCTTA -0.972815243672
AGACCTTC -0.788653614083
AGACCTTG -1.06114131187
AGACGAAA 2.38769968803
AGACGAAC 0.65160941475
AGACGAAG 0.155210481489
AGACGAAT 0.937648964033
AGACGACA -0.674463314409
AGACGACC -0.917057060687
AGACGACG -0.691964380408
AGACGACT -0.469463821395
AGACGAGA 0.875076824278
AGACGAGC -1.21152329117
AGACGAGG -0.913318258368
AGACGATA 0.641926010842
AGACGATC 2.62738321164

AGACGATG -0.366079036811
AGACGCAA 0.856268327547
AGACGCAC -0.997684656801
AGACGCAG -0.675214002347
AGACGCAT -0.159667037664
AGACGCCA -1.20049220377
AGACGCCC -1.44767973156
AGACGCCG -1.00817965056
AGACGCCT -0.879628000591
AGACGCGA 0.692926526055
AGACGCGC -0.277942208887
AGACGCGG -0.881482241617
AGACGCTA -0.391234140023
AGACGCTC -0.306445871698
AGACGCTG -0.516529498094
AGACGGAA -0.650774300557
AGACGGAC -0.896245858184
AGACGGAG -0.431238592964
AGACGGAT 2.9522878515
AGACGGCA -0.918142578446
AGACGGCC -0.521089299999
AGACGGCG -0.701630794512
AGACGGCT -0.94432621823
AGACGGGA -1.11860474837
AGACGGGC -0.69114965398
AGACGGGG -1.26235913598
AGACGGTA -0.520926982029
AGACGGTC -0.948957900073
AGACGGTG -0.397053278447
AGACGTAA -0.169508316055
AGACGTAC -0.407167439222
AGACGTAG 0.0242661444111
AGACGTAT 0.496950709806
AGACGTCA 0.467528552074
AGACGTCC -0.862660153043
AGACGTCG 0.446102580062
AGACGTCT 1.33998999212
AGACGTGA -0.277250070427
AGACGTGC -0.48884683487
AGACGTGG -1.04010584396
AGACGTTA -0.528128829085
AGACGTTC -1.15989441461
AGACGTTG -0.340435411401
AGACTAAA 0.244585735405
AGACTAAC -0.880827219343
AGACTAAG 1.0182004981
AGACTAAT 0.438529832525
AGACTACA -0.73959464135
AGACTACC 0.256994042428
AGACTACG 0.00668797084217
AGACTACT 0.70794080757
AGACTAGA 1.67750995323
AGACTAGC -0.539979870551
AGACTAGG -0.732187871124

AGACTATA 0.98998174123
AGACTATC 0.0418869231797
AGACTATG 0.17149350926
AGACTCAA -0.157160910942
AGACTCAC -1.18501370854
AGACTCAG -0.469075408347
AGACTCAT -0.429974028813
AGACTCCA -0.816474025408
AGACTCCC -1.07632209346
AGACTCCG -0.0799359804168
AGACTCCT -0.58958094871
AGACTCGA -0.298526793549
AGACTCGC -0.408958164521
AGACTCGG -0.48721685925
AGACTCTA -0.94718468734
AGACTCTC -0.574220859347
AGACTCTG -0.415818385834
AGACTGAA 1.45101652899
AGACTGAC -1.22449017066
AGACTGAG -0.147371124726
AGACTGAT 0.957141757777
AGACTGCA 0.186730749274
AGACTGCC -0.351849422841
AGACTGCG 0.159633842202
AGACTGCT -0.570226948593
AGACTGGA -0.945988082404
AGACTGGC -0.94483957168
AGACTGGG -0.969750544483
AGACTGTA -0.467387406014
AGACTGTC -0.244681923831
AGACTGTG -0.564362329771
AGACTTAA 0.964107577254
AGACTTAC 0.737984792113
AGACTTAG 0.215980656535
AGACTTAT -0.426731590141
AGACTTCA -1.08794782399
AGACTTCC -1.12103219923
AGACTTCG -0.395622998366
AGACTTGA -0.346521943195
AGACTTGC -0.800615481345
AGACTTGG -0.704522197703
AGACTTTA 0.395855105221
AGACTTTC -0.677855001971
AGACTTTG 0.233481199771
AGAGAAAA 1.65009259232
AGAGAAAC -0.246705017368
AGAGAAAG 0.227199677307
AGAGAAAT 2.01413546652
AGAGAACA 0.135522697076
AGAGAACC 0.524658204282
AGAGAACG -0.8223830791
AGAGAACT 1.0145168473
AGAGAAGA -0.0821856917952
AGAGAAGC 0.158849958802

AGAGAAGG -0.746127874288
AGAGAATA 1.98932669392
AGAGAATC 18.6044125965
AGAGAATG 1.02674819899
AGAGACAA -0.128788984455
AGAGACAC -0.789335035899
AGAGACAG 0.040929743783
AGAGACAT 0.427988312844
AGAGACCA -1.02753234377
AGAGACCC -1.0117448955
AGAGACCG -0.456623189215
AGAGACCT -0.188083399022
AGAGACGA -0.703443737247
AGAGACGC -0.828059503174
AGAGACGG -0.760423617801
AGAGACTA 0.14362631063
AGAGACTC -0.237155179454
AGAGACTG 0.0313691892243
AGAGAGAA 0.444361778647
AGAGAGAC -0.484743143846
AGAGAGAG -0.54361046089
AGAGAGAT 1.36936640808
AGAGAGCA -0.13560372537
AGAGAGCC -1.29033402383
AGAGAGCG -0.501722492183
AGAGAGCT -0.143629708591
AGAGAGGA 0.534877701839
AGAGAGGC -1.13294597228
AGAGAGGG -1.4241143512
AGAGAGTA -0.704030800307
AGAGAGTC -0.84974685678
AGAGAGTG -0.494246194454
AGAGATAA 1.50350221442
AGAGATAC 3.37781760907
AGAGATAG 1.00841959886
AGAGATAT 5.55140133686
AGAGATCA 1.2754350116
AGAGATCC 6.47636100865
AGAGATCG 4.04984510841
AGAGATCT 8.57406487559
AGAGATGA 0.424482662907
AGAGATGC -0.204107659016
AGAGATGG -0.848071923527
AGAGATTA 2.73426109985
AGAGATTC 14.923611115
AGAGATTG 3.12559482626
AGAGCAAA -0.0810298623871
AGAGCAAC -0.39421990197
AGAGCAAG -0.263020456457
AGAGCAAT 1.46397530565
AGAGCACA 0.37745174996
AGAGCACC -1.03322523488
AGAGCACG -0.132124997737
AGAGCACT -0.0272014597101

AGAGCAGA -0.553043722612
AGAGCAGC -0.556950331914
AGAGCAGG -0.973780525898
AGAGCATA 0.844005871453
AGAGCATC 0.317442456733
AGAGCATG -0.788137646817
AGAGCCAA -0.38442959299
AGAGCCAC -1.12756726184
AGAGCCAG -0.394989932145
AGAGCCAT 0.138591316988
AGAGCCCA -1.59123311672
AGAGCCCC -1.70670680002
AGAGCCCG -1.09082537396
AGAGCCCT -1.57318210385
AGAGCCGA -0.763914630369
AGAGCCGC -1.26082796261
AGAGCCGG -1.27379771729
AGAGCCTA -0.100009825444
AGAGCCTC -1.16468083436
AGAGCCTG -1.46904244923
AGAGCGAA -0.808725629439
AGAGCGAC 0.00893088629911
AGAGCGAG -0.536005563416
AGAGCGAT 0.844321097655
AGAGCGCA 0.331185116794
AGAGCGCC -0.952356644938
AGAGCGCG 0.375075791272
AGAGCGCT -0.445889292681
AGAGCGGA -0.369580504643
AGAGCGGC -1.31383092221
AGAGCGGG -0.811025264702
AGAGCGTA -0.481697002751
AGAGCGTC -1.38616252792
AGAGCGTG -0.259890673252
AGAGCTAA 0.501562526648
AGAGCTAC -0.387538988431
AGAGCTAG -0.145410762772
AGAGCTAT -0.28795939709
AGAGCTCA -0.90133887854
AGAGCTCC -1.27452278984
AGAGCTCG -0.0485929293519
AGAGCTCT -1.04824578955
AGAGCTGA -0.0543158793495
AGAGCTGC -0.748386734022
AGAGCTGG -0.537286071844
AGAGCTTA -0.581682781088
AGAGCTTC -1.65490488884
AGAGCTTG -1.03858617137
AGAGGAAA -0.0674563162178
AGAGGAAC -1.61377623367
AGAGGAAG -0.66930102793
AGAGGAAT 1.25700604094
AGAGGACA 0.522024523342
AGAGGACC -0.808229004411

AGAGGACG 0.187005461329
AGAGGACT -0.667633936125
AGAGGAGA 0.0496423764513
AGAGGAGC -0.734812142327
AGAGGAGG -1.26902698044
AGAGGATA 2.25039515263
AGAGGATC 5.55110022526
AGAGGATG 0.545657078769
AGAGGCAA 0.324841385511
AGAGGCAC -1.35728169147
AGAGGCAG -1.16095457836
AGAGGCAT 0.251151118279
AGAGGCCA -0.838589522065
AGAGGCCC -0.173021284676
AGAGGCCG -0.719092914653
AGAGGCCT -1.53936716705
AGAGGCGA -0.766096382533
AGAGGCGC -1.18037261696
AGAGGCGG -0.787648863237
AGAGGCTA -0.217451189383
AGAGGCTC -1.65465579219
AGAGGCTG -1.43787949008
AGAGGGAA -0.0228860495955
AGAGGGAC -0.87415545416
AGAGGGAG -0.746595224578
AGAGGGAT 1.06348877996
AGAGGGCA -0.602602456953
AGAGGGCC -0.852014342107
AGAGGGCG -0.968892690092
AGAGGGCT -1.11090679905
AGAGGGGA -0.316734896759
AGAGGGGC -0.998548261587
AGAGGGGG -0.511211689567
AGAGGGTA 0.498153587901
AGAGGGTC -1.87981852273
AGAGGGTG -1.23133575461
AGAGGTAA 0.948025290546
AGAGGTAC -0.39616327412
AGAGGTAG -0.0463926190948
AGAGGTAT -0.20980055013
AGAGGTCA -0.992066520823
AGAGGTCC -0.699047298838
AGAGGTCG -0.0348727481084
AGAGGTGA 1.44375639384
AGAGGTGC -0.434284734059
AGAGGTGG -1.14846419751
AGAGGTTA -0.483738131618
AGAGGTTC -0.692509883794
AGAGGTTG -0.892530318827
AGAGTAAA 0.845930162747
AGAGTAAC -0.817074157547
AGAGTAAG 1.16046893135
AGAGTAAT 1.67179510606
AGAGTACA 0.861362654811

AGAGTACC -0.637531140723
AGAGTACG -0.276635300918
AGAGTACT -0.236036988996
AGAGTAGA -0.107068696768
AGAGTAGC -0.885608934221
AGAGTAGG -0.80551481794
AGAGTATA 2.49319042412
AGAGTATC 6.83368689865
AGAGTATG -0.0708422533847
AGAGTCAA -0.102089377405
AGAGTCAC -0.892655781992
AGAGTCAG -0.0425103182811
AGAGTCAT 0.582130004996
AGAGTCCA -0.652542024277
AGAGTCCC -1.07091358552
AGAGTCCG -1.76522804784
AGAGTCCT -1.30874312949
AGAGTCGA -0.410522010597
AGAGTCGC -0.952672655285
AGAGTCGG -0.959203013026
AGAGTCTA -0.441438748283
AGAGTCTC 0.122476618963
AGAGTCTG -0.578050883842
AGAGTGAA -0.617791602754
AGAGTGAC -0.45790970942
AGAGTGAG 0.136996627883
AGAGTGAT 1.5063525807
AGAGTGCA 0.530493809746
AGAGTGCC -0.0180497059642
AGAGTGCG -0.466701540711
AGAGTGCT -0.0546935757527
AGAGTGGA 0.50837517651
AGAGTGGC -1.30106138582
AGAGTGGG -0.556543622154
AGAGTGTA 0.808253574281
AGAGTGTC -0.378730951481
AGAGTGTG -0.0842919046785
AGAGTTAA 0.524919063112
AGAGTTAC 0.423885667347
AGAGTTAG 1.4146708956
AGAGTTAT 0.263270598642
AGAGTTCA 0.547621622829
AGAGTTCC -0.717078969473
AGAGTTCG -0.728473115912
AGAGTTGA -0.993963105668
AGAGTTGC -0.554418067033
AGAGTTGG -0.592691651059
AGAGTTTA 0.243489501
AGAGTTTC -0.365031680765
AGAGTTTG 0.225588521163
AGATAAAA 1.38862840187
AGATAAAC 0.356032051106
AGATAAAG -0.249435409497
AGATAAAT 0.596610806677

AGATAACA 1.38876301339
AGATAACC 1.71231996975
AGATAACG 2.16225518813
AGATAACT 1.18698191195
AGATAAGA 0.241016831122
AGATAAGC 1.30579134715
AGATAAGG -0.111618827554
AGATAATA 1.4216079631
AGATAATC 6.12385081314
AGATAATG -0.17905684706
AGATACAA 0.416049969926
AGATACAC 2.96030808432
AGATACAG 3.08317938949
AGATACAT 2.31713894262
AGATACCA 2.19115824201
AGATACCC 5.16357822663
AGATACCG 4.38881861255
AGATACCT 2.36194366853
AGATACGA 4.60672748111
AGATACGC 12.9061087921
AGATACGG 6.99571943974
AGATACTA 1.97068861798
AGATACTC 6.35873929141
AGATACTG 4.99153712293
AGATAGAA 1.25825283114
AGATAGAC -0.54517509111
AGATAGAG 0.0216612154466
AGATAGAT 3.52629751452
AGATAGCA 1.53162818082
AGATAGCC 0.953058454518
AGATAGCG 2.57911491823
AGATAGCT 0.651024181361
AGATAGGA 1.00323404942
AGATAGGC 0.438557800355
AGATAGGG 0.255612902087
AGATAGTA 0.915893651213
AGATAGTC -0.257725388128
AGATAGTG 1.67331739246
AGATATAA 2.43626987718
AGATATAC 3.17302669857
AGATATAG 1.49138587063
AGATATAT 5.29153601762
AGATATCA 9.55754762487
AGATATCC 23.3788921708
AGATATCG 14.5716751748
AGATATCT 22.7796390575
AGATATGA 5.92285620891
AGATATGC 7.92839274375
AGATATGG 5.62215524326
AGATATTA 6.18839847498
AGATATTC 14.0135564672
AGATATTG 8.33720087524
AGATCAAA 0.935050569609
AGATCAAC 1.42182700087

AGATCAAG 1.89763638322
AGATCAAT 1.40810995621
AGATCACA 2.31500136395
AGATCACC 4.60095852795
AGATCACG 4.44538524881
AGATCACT 1.92093489297
AGATCAGA 4.4326742619
AGATCAGC 1.27697088984
AGATCAGG 1.76540029831
AGATCATA 1.96408402787
AGATCATC 1.50457048099
AGATCATG 2.11059102505
AGATCCAA 2.16795748898
AGATCCAC 3.14066112271
AGATCCAG 2.48258957081
AGATCCAT 3.06501336872
AGATCCCA 2.47071631191
AGATCCCC 6.74679790645
AGATCCCG 5.25824593506
AGATCCCT 4.53846663234
AGATCCGA 4.27918602373
AGATCCGC 6.92080642941
AGATCCGG 3.84060286936
AGATCCTA 4.55119016557
AGATCCTC 7.21539707754
AGATCCTG 7.23920162205
AGATCGAA 2.49431384221
AGATCGAC 1.65797115632
AGATCGAG 0.266257928878
AGATCGAT 1.49483689182
AGATCGCA 9.29395683378
AGATCGCC 5.78684943312
AGATCGCG 16.8269137284
AGATCGCT 5.29657597751
AGATCGGA 3.62812864691
AGATCGGC 0.750947228116
AGATCGGG 3.05569067141
AGATCGTA 3.45943636419
AGATCGTC 3.20748986172
AGATCGTG 5.21418902189
AGATCTAA 3.21964306031
AGATCTAC 6.93491476232
AGATCTAG 5.36796164323
AGATCTAT 4.94317708458
AGATCTCA 8.55395600543
AGATCTCC 12.1177589035
AGATCTCG 9.56233404462
AGATCTGA 4.00222739378
AGATCTGC 7.44746315736
AGATCTGG 4.40448582529
AGATCTTA 5.21911136006
AGATCTTC 9.689346157
AGATCTTG 6.05204014095
AGATGAAA -0.29731999473

AGATGAAC 1.30360724255
AGATGAAG -0.463731200279
AGATGAAT 0.47404662488
AGATGACA 0.645947366664
AGATGACC 0.191728888112
AGATGACG 0.16640153443
AGATGACT 0.11077195119
AGATGAGA 0.815805934054
AGATGAGC -0.705211199585
AGATGAGG -0.772383132629
AGATGATA 1.18719467656
AGATGATC 1.50643517728
AGATGATG 0.245686674678
AGATGCAA 1.02853657185
AGATGCAC -0.234312393239
AGATGCAG -0.687055111312
AGATGCAT -0.100972755236
AGATGCCA 0.471080727935
AGATGCCC 0.0938934961475
AGATGCCG -0.160913566485
AGATGCCT -0.702211584414
AGATGCGA 0.21547462177
AGATGCGC -0.000698150237208
AGATGCGG 0.562450323412
AGATGCTA 0.427647732627
AGATGCTC 1.89125030812
AGATGCTG 1.37134585089
AGATGGAA -0.528165161127
AGATGGAC -1.27974362579
AGATGGAG -1.0927943615
AGATGGAT 1.30545390351
AGATGGCA -0.540948812119
AGATGGCC -0.898966579194
AGATGGCG 0.159178254083
AGATGGCT -0.251886646084
AGATGGGA 0.302334077845
AGATGGGC -1.26287405772
AGATGGGG -0.562342372814
AGATGGTA 0.761029238954
AGATGGTC -0.0975073580642
AGATGGTG -0.63378841768
AGATGTAA 0.966799023526
AGATGTAC 0.395026002805
AGATGTAG 0.706825230927
AGATGTAT 1.14277444298
AGATGTCA 1.19118126863
AGATGTCC 0.347290927844
AGATGTCG 0.905888486563
AGATGTGA 0.0541804836839
AGATGTGC 1.30028246867
AGATGTGG -0.0622783468434
AGATGTTA 1.56347909633
AGATGTTC 0.421904394865
AGATGTTG 1.54178076469

AGATTAAA 2.23474466831
AGATTAAC 0.575294876316
AGATTAAG 1.68610078207
AGATTAAT 2.07086050002
AGATTACA 25.9009346794
AGATTACC 11.5597102461
AGATTACG 22.0894588679
AGATTACT 10.3806048071
AGATTAGA 2.55750414943
AGATTAGC 0.95250615521
AGATTAGG 1.36626616099
AGATTATA 5.60571460239
AGATTATC 9.27114266412
AGATTATG 6.3371131011
AGATTCAA 2.85284470135
AGATTCAC 5.01866617994
AGATTCAG 3.01485476362
AGATTCAT 2.44557872127
AGATTCCA 11.2937623158
AGATTCCC 37.1991070254
AGATTCCG 17.1139839053
AGATTCCT 13.6638684087
AGATTCGA 6.8938945805
AGATTCGC 29.7793132008
AGATTCGG 9.63864623062
AGATTCTA 14.1926284744
AGATTCTC 29.3557678523
AGATTCTG 15.4424052232
AGATTGAA 0.818067930367
AGATTGAC 0.177453532363
AGATTGAG 1.15569087582
AGATTGAT 0.862405828753
AGATTGCA 16.8468333583
AGATTGCC 12.1994061492
AGATTGCG 32.8331383918
AGATTGCT 10.566943484
AGATTGGA 1.82176305692
AGATTGGC 2.23995766282
AGATTGGG 1.69167788114
AGATTGTA 4.08370212764
AGATTGTC 7.6795530229
AGATTGTG 7.02494007226
AGATTTAA 3.80202686683
AGATTTAC 5.70027436022
AGATTTAG 3.27177614197
AGATTTAT 3.70378293543
AGATTTCA 21.5380344043
AGATTTCC 37.2518987887
AGATTTCG 28.3656596024
AGATTTGA 7.61999984056
AGATTTGC 15.7091545495
AGATTTGG 10.0350541579
AGATTTTA 8.27867439979
AGATTTTC 24.2307421038

AGATTTTG 17.8285509976
AGCAAAAA -0.0747018139997
AGCAAAAC -0.382267444446
AGCAAAAG -0.14109744371
AGCAAAAT 1.79588079011
AGCAAACA 0.4959297533
AGCAAACC -0.0113941464397
AGCAAACG 0.0587457746023
AGCAAACT 0.739390502325
AGCAAAGA 1.52142227511
AGCAAAGC -0.820669461371
AGCAAAGG -0.280512635481
AGCAAATA 2.78728549274
AGCAAATC 9.89574142773
AGCAAATG 0.821748705972
AGCAACAA 0.728682743951
AGCAACAC 0.891753754112
AGCAACAG -0.675506749732
AGCAACAT -0.50493461059
AGCAACCA 0.333671639896
AGCAACCC -0.468488345287
AGCAACCG -0.300502054253
AGCAACCT -0.401303081782
AGCAACGA -0.246706847039
AGCAACGC -0.650774300557
AGCAACGG -0.337061759169
AGCAACTA 0.612108121377
AGCAACTC -0.119457661554
AGCAACTG -0.372158772685
AGCAAGAA 0.226736770505
AGCAAGAC -0.285594939192
AGCAAGAG -0.49582154132
AGCAAGAT 3.8997286928
AGCAAGCA -0.466982525925
AGCAAGCC -0.704841606011
AGCAAGCG 0.125221648462
AGCAAGCT -0.663021596519
AGCAAGGA -0.0901123500107
AGCAAGGC -0.283222117083
AGCAAGGG -0.925454467153
AGCAAGTA -0.148189771877
AGCAAGTC -0.3624267518
AGCAAGTG -0.12265984746
AGCAATAA 0.596888132548
AGCAATAC 1.39900577391
AGCAATAG 0.111556618734
AGCAATAT 3.25980617188
AGCAATCA 4.60222622868
AGCAATCC 11.3012681496
AGCAATCG 5.78344519924
AGCAATGA -0.611754210699
AGCAATGC -1.01895562953
AGCAATGG -0.518121573382
AGCAATTA 1.10335234823

AGCAATTC 1.56745627866
AGCAATTG 1.21358846715
AGCACAAA 0.773368541237
AGCACAAC -0.217027228437
AGCACAAG 0.0478508670069
AGCACAAT 0.990809798119
AGCACACA 0.819808209019
AGCACACC -0.927914067951
AGCACACG -0.472003666343
AGCACACT -0.519935300262
AGCACAGA -0.429291561471
AGCACAGC -0.901235894192
AGCACAGG -0.898586007594
AGCACATA 1.03668409751
AGCACATC 0.423844369055
AGCACATG -0.0265694390162
AGCACCAA 0.415398606994
AGCACCAC -1.46697335253
AGCACCAG -0.923685436526
AGCACCAT -0.183097545119
AGCACCCA -0.268063030165
AGCACCCC -0.230141004383
AGCACCCG -1.57653406141
AGCACCCT -1.33007735656
AGCACCGA 0.156253394048
AGCACCGC 0.0499126450193
AGCACCGG -1.01525342063
AGCACCTA -0.676965259026
AGCACCTC -0.730735634989
AGCACCTG -0.607640064412
AGCACGAA 0.534842153942
AGCACGAC -0.647770764662
AGCACGAG -0.907478209417
AGCACGAT 1.86126617996
AGCACGCA 0.0634603144108
AGCACGCC -0.759467222549
AGCACGCG 0.266355946976
AGCACGCT -0.393138304934
AGCACGGA 0.0400292841922
AGCACGGC -0.973245739157
AGCACGGG -0.82625649294
AGCACGTA -0.683293568795
AGCACGTC -0.433484122237
AGCACGTG -0.164342108852
AGCACTAA 0.46818226744
AGCACTAC 0.105398468383
AGCACTAG -0.116525482834
AGCACTAT 0.149602016629
AGCACTCA -0.596181095337
AGCACTCC -0.158781999587
AGCACTCG -0.155551323087
AGCACTGA 0.128034114412
AGCACTGC -0.394496182315
AGCACTGG -1.19666557723

AGCACTTA 0.46349700237
AGCACTTC -1.09574196174
AGCACTTG -0.609961655728
AGCAGAAA 0.258685181341
AGCAGAAC 0.597435204224
AGCAGAAG -0.4902948889
AGCAGAAT 1.74174265025
AGCAGACA 0.0958256288893
AGCAGACC -0.853238914874
AGCAGACG -0.0708430375295
AGCAGACT -0.145883602075
AGCAGAGA 0.0682409837626
AGCAGAGC -0.69268631637
AGCAGAGG -0.455569298629
AGCAGATA 3.17302669857
AGCAGATC 5.05930448325
AGCAGATG 0.317570010951
AGCAGCAA 0.519751026238
AGCAGCAC -0.621069327941
AGCAGCAG -0.74830100086
AGCAGCAT -0.196551117137
AGCAGCCA -0.872768302041
AGCAGCCC -0.551556984106
AGCAGCCG -0.853416392976
AGCAGCCT -1.01317047071
AGCAGCGA 0.379490526392
AGCAGCGC 0.0861022335976
AGCAGCGG -1.10237634935
AGCAGCTA -1.29641506661
AGCAGCTC -1.57540175635
AGCAGCTG -1.56599306449
AGCAGGAA -0.724196390274
AGCAGGAC -1.3459986322
AGCAGGAG -1.67694406208
AGCAGGAT 1.38765318715
AGCAGGCA -0.72988797448
AGCAGGCC -2.14788312119
AGCAGGCG -0.336782342245
AGCAGGCT -0.852497113911
AGCAGGGA -0.730074339556
AGCAGGGC -0.184538541846
AGCAGGGG -0.49583617869
AGCAGGTA -1.09151385308
AGCAGGTC -1.03067179809
AGCAGGTG -1.14107180327
AGCAGTAA 0.183846664768
AGCAGTAC -0.00510582805452
AGCAGTAG -0.831103030453
AGCAGTAT 1.75504828028
AGCAGTCA 0.206475253493
AGCAGTCC -0.903872188948
AGCAGTCG -0.923745554292
AGCAGTGA -0.0796777354021
AGCAGTGC -0.770652786477

AGCAGTGG -1.11519633239
AGCAGTTA -0.430020293355
AGCAGTTC -1.08374036448
AGCAGTTG 0.542684647284
AGCATAAA 1.21152459808
AGCATAAC 0.954755343825
AGCATAAG -0.373224686824
AGCATAAT 2.46032273421
AGCATACA -0.296061180974
AGCATACC 0.148262435961
AGCATACG -0.448836108771
AGCATACT 0.00926153401535
AGCATAGA 0.00919383618254
AGCATAGC -1.05143072428
AGCATAGG 0.70863948057
AGCATATA 1.95463351496
AGCATATC 5.72320196949
AGCATATG 1.32408596766
AGCATCAA 0.992276410243
AGCATCAC 0.163751125068
AGCATCAG 0.32499873723
AGCATCAT 1.41653977399
AGCATCCA 0.102059579903
AGCATCCC -0.2241153745
AGCATCCG 0.516654699878
AGCATCCT -0.282208479262
AGCATCGA 0.670126993767
AGCATCGC -0.905196086721
AGCATCGG 0.625337950751
AGCATCTA 1.3021850653
AGCATCTC 1.60476222802
AGCATCTG 0.270230406342
AGCATGAA 0.6290195105
AGCATGAC -1.38342952197
AGCATGAG -0.629514567239
AGCATGAT 1.94993073733
AGCATGCA -0.291461387685
AGCATGCC -1.10316572177
AGCATGCG -0.623741954739
AGCATGCT -0.154895255286
AGCATGGA -0.0154894732523
AGCATGGC -1.27655633864
AGCATGGG -1.02229007452
AGCATGTA 0.808574028115
AGCATGTC -0.0735452004468
AGCATGTG -0.437581017339
AGCATTAA 0.72338401628
AGCATTAC 1.16950254062
AGCATTAG 0.369822543999
AGCATTAT 0.612127986378
AGCATTCA 0.0997139414796
AGCATTCC -0.523775780021
AGCATTCG -0.896636100903
AGCATTGA -0.355326843566

AGCATTGC 0.475763117807
AGCATTGG -0.748659093643
AGCATTTA 1.55207083528
AGCATTTC 0.433070093792
AGCATTTG -0.0931511724209
AGCCAAAA 1.05695554703
AGCCAAAC -0.829695751952
AGCCAAAG -1.32827434632
AGCCAAAT 0.957184101595
AGCCAACA -0.512105614618
AGCCAACC -0.0486682072509
AGCCAACG -1.20933239066
AGCCAACT -0.21981721557
AGCCAAGA -0.347752527739
AGCCAAGC -0.174954201563
AGCCAAGG -1.49314549152
AGCCAATA 0.0468636287269
AGCCAATC 1.07260184923
AGCCAATG -0.250150026775
AGCCACAA 0.185194086884
AGCCACAC -1.04582513461
AGCCACAG -1.29782234511
AGCCACAT -0.415652669903
AGCCACCA 1.10175478392
AGCCACCC -1.85626438178
AGCCACCG -1.09510105407
AGCCACCT -1.4703407316
AGCCACGA 0.262880094541
AGCCACGC -0.922683299495
AGCCACGG -0.838999368404
AGCCACTA 0.159039721839
AGCCACTC -0.725044312164
AGCCACTG -0.596702290235
AGCCAGAA 0.256003144806
AGCCAGAC -0.0343554739342
AGCCAGAG -1.22283823899
AGCCAGAT 1.08335691768
AGCCAGCA -1.32546135761
AGCCAGCC -1.6692738192
AGCCAGCG -0.587813747754
AGCCAGCT -0.648138005802
AGCCAGGA -0.31624637456
AGCCAGGC -1.08770029562
AGCCAGGG -1.28397356413
AGCCAGTA -0.670817302556
AGCCAGTC -0.968211268277
AGCCAGTG -0.547698730399
AGCCATAA 0.776818255513
AGCCATAC -0.213627176664
AGCCATAG -0.532604204735
AGCCATAT 2.1169535758
AGCCATCA 0.887770560003
AGCCATCC -0.832891403318
AGCCATCG -0.236907912466

AGCCATGA 0.245277873865
AGCCATGC -1.15960872453
AGCCATGG -1.12640934138
AGCCATTA -0.150506135562
AGCCATTC -0.355687288784
AGCCATTG -0.83553188018
AGCCCAAA -0.578661732627
AGCCCAAC -0.450206793849
AGCCCAAG -1.10136375706
AGCCCAAT -0.318753285426
AGCCCACA -1.10590081877
AGCCCACC -1.48800542248
AGCCCACG -0.819984902977
AGCCCACT -1.33868883455
AGCCCAGA -0.361512177603
AGCCCAGC -1.39596930394
AGCCCAGG -1.59469903666
AGCCCATA 0.371566481993
AGCCCATC -1.78135947428
AGCCCATG -0.43144952791
AGCCCCAA -1.07565112691
AGCCCCAC -1.29840104396
AGCCCCAG -0.933621073672
AGCCCCAT -1.08802911367
AGCCCCCA -1.41788275262
AGCCCCCC -1.25727055911
AGCCCCCG -1.24786395831
AGCCCCCT -1.25263940003
AGCCCCGA -0.111449975044
AGCCCCGC -1.3673524629
AGCCCCGG -1.09601170754
AGCCCCTA -0.76205673
AGCCCCTC -1.7510392094
AGCCCCTG -1.09486842445
AGCCCGAA -0.634484738246
AGCCCGAC -0.761206978438
AGCCCGAG -0.896120133637
AGCCCGAT 1.48381782796
AGCCCGCA -0.332086883293
AGCCCGCC -1.39784105753
AGCCCGCG -1.89945533773
AGCCCGCT -1.28304827328
AGCCCGGA -1.1308653748
AGCCCGGC -1.5674471303
AGCCCGGG -0.922972387538
AGCCCGTA -0.845807836161
AGCCCGTC -0.69963958953
AGCCCGTG -0.39450533067
AGCCCTAA -0.575324935199
AGCCCTAC -0.507093361174
AGCCCTAG -1.12276411368
AGCCCTAT -0.746030378954
AGCCCTCA -0.987112294093
AGCCCTCC -1.59299822663

AGCCCTCG -0.88532507381
AGCCCTGA -0.901901894493
AGCCCTGC -1.27062062402
AGCCCTGG -1.68065751038
AGCCCTTA -1.57985203936
AGCCCTTC -0.839724440945
AGCCCTTG -1.67355629524
AGCCGAAA 0.325740538194
AGCCGAAC -0.357591453695
AGCCGAAG -0.480541696106
AGCCGAAT 0.776593467343
AGCCGACA -0.457843057114
AGCCGACC -1.06377708386
AGCCGACG -0.997873897075
AGCCGACT -1.06401912322
AGCCGAGA -0.840284581701
AGCCGAGC -1.6939179214
AGCCGAGG -0.516910069695
AGCCGATA 0.99132158328
AGCCGATC 0.778200964145
AGCCGATG -0.348220923555
AGCCGCAA 0.154101439386
AGCCGCAC -0.781928265673
AGCCGCAG -1.12584475714
AGCCGCAT 0.11358441714
AGCCGCCA -0.624917910529
AGCCGCCC -0.989569281075
AGCCGCCG -0.911172315483
AGCCGCCT -0.907746909695
AGCCGCGA -0.38564972227
AGCCGCGC -0.705953523311
AGCCGCGG -0.797778445525
AGCCGCTA -1.27744817263
AGCCGCTC -1.22518283189
AGCCGCTG -0.963429030636
AGCCGGAA 0.218642043924
AGCCGGAC -0.988639546746
AGCCGGAG -1.4840332064
AGCCGGAT 2.23097868231
AGCCGGCA -0.720204831954
AGCCGGCC -1.25419671157
AGCCGGCG -0.943917417417
AGCCGGCT -0.66851217828
AGCCGGGA 0.122140482233
AGCCGGGC -1.70538159534
AGCCGGGG -0.658791658186
AGCCGGTA -1.30483390637
AGCCGGTC -1.27873861356
AGCCGGTG -0.95491687765
AGCCGTAA -0.455307917035
AGCCGTAC -0.181262907712
AGCCGTAG -0.656211821855
AGCCGTAT 0.491889316622
AGCCGTCA -1.40297380789

AGCCGTCC −0.672213864412
AGCCGTCG −1.21858242388
AGCCGTGA −0.299832917373
AGCCGTGC −0.349936632338
AGCCGTGG −1.00046157485
AGCCGTTA −0.332289715409
AGCCGTTC −0.843804869007
AGCCGTTG −0.307400437279
AGCCTAAA 0.144839905371
AGCCTAAC −1.0923641274
AGCCTAAG −0.38083324364
AGCCTAAT 0.13501666231
AGCCTACA −0.541468438728
AGCCTACC −0.122606786997
AGCCTACG −0.192104754844
AGCCTACT −0.215184749582
AGCCTAGA −0.531033301356
AGCCTAGC −1.31881154848
AGCCTAGG −1.37655989092
AGCCTATA 0.550986649468
AGCCTATC 0.799951572097
AGCCTATG −0.83268177528
AGCCTCAA −0.368325350229
AGCCTCAC −0.664572112134
AGCCTCAG −0.0842919046785
AGCCTCAT −0.958638428784
AGCCTCCA −1.20459328098
AGCCTCCC −1.3565485161
AGCCTCCG −0.739292222846
AGCCTCCT −0.210550453923
AGCCTCGA −0.762806372411
AGCCTCGC −1.31325222336
AGCCTCGG −0.229703451595
AGCCTCTA −0.494000757137
AGCCTCTC −0.751508937161
AGCCTCTG 0.306263427345
AGCCTGAA −0.689835427326
AGCCTGAC −0.565468758058
AGCCTGAG −0.975105469197
AGCCTGAT −0.0243733108645
AGCCTGCA −0.585877432907
AGCCTGCC −1.89081824435
AGCCTGCG −0.533756636182
AGCCTGGA −0.0485929293519
AGCCTGGC −0.511664141106
AGCCTGGG −1.49150427649
AGCCTGTA −0.919859332755
AGCCTGTC −0.584926526668
AGCCTGTG 0.0377027266252
AGCCTTAA −0.827277449445
AGCCTTAC −0.430490780224
AGCCTTAG −1.05588257558
AGCCTTAT 0.164593557945
AGCCTTCA −1.29615211673

AGCCTTCC −1.46423328928
AGCCTTCG −1.13250188496
AGCCTTGA 0.516041237277
AGCCTTGC −1.49749854059
AGCCTTGG −1.31907998738
AGCCTTTA −1.21717514538
AGCCTTTC −1.16532278755
AGCCTTTG −1.47959834489
AGCGAAAA 1.73569558707
AGCGAAAC −0.616035641207
AGCGAAAG −0.0912556331022
AGCGAAAT 4.28449024042
AGCGAACA 0.742304384333
AGCGAACC −0.47705904775
AGCGAACG −1.03869751993
AGCGAACT −0.955041818052
AGCGAAGA 0.985699526578
AGCGAAGC −1.45731216606
AGCGAAGG −1.49178578447
AGCGAATA 1.2778334491
AGCGAATC 11.2533853941
AGCGAATG −0.501709945867
AGCGACAA −0.991169459193
AGCGACAC −0.517130414378
AGCGACAG 0.765236959852
AGCGACAT 0.334495776062
AGCGACCA −0.460030559673
AGCGACCC −1.13829122588
AGCGACCG −1.3144943087
AGCGACCT −0.55435376716
AGCGACGA 0.590400641389
AGCGACGC −0.107068696768
AGCGACGG −1.28994064453
AGCGACTA −0.614357571374
AGCGACTC −1.1012267931
AGCGACTG −0.995438604765
AGCGAGAA 0.466872484274
AGCGAGAC −0.749525835008
AGCGAGAG −0.324079458165
AGCGAGAT 3.31812615586
AGCGAGCA −0.345499941163
AGCGAGCC −0.760138973246
AGCGAGCG −1.41985147879
AGCGAGCT −0.825144052876
AGCGAGGA 0.449885294488
AGCGAGGC −0.509149388791
AGCGAGGG −1.20904382538
AGCGAGTA 0.809739790024
AGCGAGTC −0.33611581918
AGCGAGTG −0.360627139527
AGCGATAA −0.150115631461
AGCGATAC 1.66482405895
AGCGATAG −0.409214841247
AGCGATAT 5.04646751041

AGCGATCA 2.20739683491
AGCGATCC 4.15952840526
AGCGATCG 2.68029155111
AGCGATGA −0.294639787866
AGCGATGC −0.571745314271
AGCGATGG −0.981022625719
AGCGATTA 1.83890629152
AGCGATTC 2.86980470745
AGCGATTG 1.16082205789
AGCGCAAA 0.512921647954
AGCGCAAC 0.270592158468
AGCGCAAG −0.0810042469909
AGCGCAAT 2.96902986535
AGCGCACA −0.474751571039
AGCGCACC −0.769140955338
AGCGCACG −0.845460460022
AGCGCACT −0.669991598101
AGCGCAGA −0.134100519823
AGCGCAGC −1.18880138922
AGCGCAGG −0.949400680493
AGCGCATA 2.43227910301
AGCGCATC 0.0209377111948
AGCGCATG −0.778302902966
AGCGCCAA −0.646640812032
AGCGCCAC −0.742396652036
AGCGCCAG −0.821816665186
AGCGCCAT −0.1151129767
AGCGCCCA −0.788758166721
AGCGCCCC −1.32476242323
AGCGCCCG −1.44246098666
AGCGCCCT −0.590959736618
AGCGCCGA −0.814831503472
AGCGCCGC −1.62393430655
AGCGCCGG −1.25674727316
AGCGCCTA −0.796844790472
AGCGCCTC −1.42316788845
AGCGCCTG −0.932651870722
AGCGCGAA 0.562858601462
AGCGCGAC −0.217581618798
AGCGCGAG 0.182037904138
AGCGCGAT 6.25027847628
AGCGCGCA 0.462696129167
AGCGCGCC −0.364910661087
AGCGCGCG 1.06976324512
AGCGCGCT 1.90494173739
AGCGCGGA 0.0400567292595
AGCGCGGC −1.32202157584
AGCGCGGG −1.01732931325
AGCGCGTA 0.485644910345
AGCGCGTC −0.766851775339
AGCGCGTG −1.2031841728
AGCGCTAA −0.609716479793
AGCGCTAC −0.686879462881
AGCGCTAG −0.400781102739

AGCGCTAT -0.552757771148
AGCGCTCA -0.266060324393
AGCGCTCC -0.0725731222993
AGCGCTCG -1.52921562871
AGCGCTGA 0.292245793848
AGCGCTGC -0.228415624482
AGCGCTGG -0.323226308642
AGCGCTTA 0.0425116251891
AGCGCTTC -0.344501986238
AGCGCTTG -0.29370561005
AGCGGAAA 1.16690858968
AGCGGAAC -0.078384157894
AGCGGAAG 1.09315689778
AGCGGAAT 2.68518043244
AGCGGACA -0.774717793025
AGCGGACC -1.07000580724
AGCGGACG -0.156151455227
AGCGGACT -0.962000318844
AGCGGAGA -0.0900101498075
AGCGGAGC 0.241266189163
AGCGGAGG -1.42388276711
AGCGGATA 3.05666797719
AGCGGATC 3.96111991051
AGCGGATG -0.424077782818
AGCGGCAA -0.63301760336
AGCGGCAC -0.1457795722
AGCGGCAG -0.595189152188
AGCGGCAT -0.758884341595
AGCGGCCA -0.868001224532
AGCGGCCC -1.92792449817
AGCGGCCG -0.219593734307
AGCGGCCT -0.679401074099
AGCGGCGA -0.0229947843385
AGCGGCGC -0.626194236852
AGCGGCGG -1.16139160838
AGCGGCTA -1.22518283189
AGCGGCTC -1.28369440858
AGCGGCTG -1.1946793385
AGCGGGAA -1.10152738194
AGCGGGAC -0.637543164276
AGCGGGAG -0.12476057133
AGCGGGAT 2.16943638604
AGCGGGCA -0.207414136178
AGCGGGCC -1.762345793
AGCGGGCG -0.814281556598
AGCGGGGA -0.567935938922
AGCGGGGC -1.33484704788
AGCGGGGG -0.87180223567
AGCGGGTA -1.10786118072
AGCGGGTC -0.966686368059
AGCGGGTG -0.197933825769
AGCGGTAA 1.03845913992
AGCGGTAC -0.201791295331
AGCGGTAG -0.534375849178

AGCGGTAT 0.800580717593
AGCGGTCA 0.0490550520098
AGCGGTCC -0.989308945007
AGCGGTCG -1.23217635781
AGCGGTGA -0.596650275298
AGCGGTGC -0.949544701751
AGCGGTGG -0.682969717
AGCGGTTA 0.593557346898
AGCGGTTC -1.00893190679
AGCGGTTG -0.52038749042
AGCGTAAA 2.73721575738
AGCGTAAC -0.627429003502
AGCGTAAG 0.300336338323
AGCGTAAT 6.6628578214
AGCGTACA -0.137111635785
AGCGTACC -0.942421269174
AGCGTACG -0.306020342463
AGCGTACT 0.34916947736
AGCGTAGA 0.16226569347
AGCGTAGC -1.25263940003
AGCGTAGG -0.998508008822
AGCGTATA 0.578221566022
AGCGTATC 5.23008259109
AGCGTATG 0.0782937198626
AGCGTCAA 0.674787950349
AGCGTCAC -0.704092486363
AGCGTCAG -0.847866477594
AGCGTCAT -0.822622766022
AGCGTCCA -0.266973330301
AGCGTCCC -1.66093051873
AGCGTCCG -0.378270397112
AGCGTCCT -1.08265040323
AGCGTCGA -0.67058179774
AGCGTCGC -1.04695195067
AGCGTCGG -1.02098212103
AGCGTCTA 0.229540088099
AGCGTCTC -0.947992095084
AGCGTCTG -0.0511826981839
AGCGTGAA 0.0867839167944
AGCGTGAC -0.423973230181
AGCGTGAG -0.251806663317
AGCGTGAT 1.35317303419
AGCGTGCA -0.568422892832
AGCGTGCC -1.51304760884
AGCGTGCG 0.256879034527
AGCGTGGA -0.346197830019
AGCGTGGC -0.849303030834
AGCGTGGG -0.608031614039
AGCGTGTA -0.259840487986
AGCGTGTC -0.566517943776
AGCGTGTG -0.512980720194
AGCGTTAA 0.700441246879
AGCGTTAC 0.306429143276
AGCGTTAG -0.110596825522

AGCGTTAT 0.0385158847637
AGCGTTCA -1.46202958106
AGCGTTCC -0.836036346656
AGCGTTCG -0.861935080502
AGCGTTGA -0.0818898078309
AGCGTTGC -0.550057699284
AGCGTTGG 0.235979746427
AGCGTTTA 0.616584542554
AGCGTTTC -0.764947349046
AGCGTTTG 0.0495882704613
AGCTAAAA -0.471417910191
AGCTAAAC 0.299223375496
AGCTAAAG -0.460882663669
AGCTAAAT 1.09009141444
AGCTAACA -0.360281070296
AGCTAACC -0.755651574042
AGCTAACG -0.116488889411
AGCTAACT -0.761820702421
AGCTAAGA -0.693467063191
AGCTAAGC 0.269149593451
AGCTAAGG -1.08500074652
AGCTAATA 1.42012331564
AGCTAATC 0.207263057617
AGCTAATG -0.0846207227236
AGCTACAA 0.0155263280571
AGCTACAC 0.392076050137
AGCTACAG -0.249307855279
AGCTACAT 0.286177820147
AGCTACCA -1.05339448419
AGCTACCC -0.637220619389
AGCTACCG -0.511313628389
AGCTACCT -0.291431590183
AGCTACGA 0.337527279787
AGCTACGC -0.0282618848364
AGCTACGG -1.05489586007
AGCTACTA -0.158068427836
AGCTACTC -0.949277308381
AGCTACTG -0.369776018075
AGCTAGAA 0.915781257127
AGCTAGAC -0.359101716545
AGCTAGAG -0.676845546256
AGCTAGAT 2.10315524146
AGCTAGCA -0.15274094819
AGCTAGCC -1.49650685882
AGCTAGCG -1.10487254358
AGCTAGCT -0.950388180155
AGCTAGGA -0.774236066748
AGCTAGGC -0.732092728224
AGCTAGGG -0.742144941561
AGCTAGTA 0.32705842419
AGCTAGTC 0.0584318553081
AGCTAGTG -0.431950596426
AGCTATAA 0.276682872368
AGCTATAC -0.234149029743

AGCTATAG 0.591205173934
AGCTATAT 1.20429739701
AGCTATCA -0.0876496126333
AGCTATCC 0.295352575473
AGCTATCG 0.153281746708
AGCTATGA -0.654986987706
AGCTATGC 0.0776188325872
AGCTATGG -0.163582795322
AGCTATTA 0.696868944635
AGCTATTC -0.116091589388
AGCTATTG -0.0339981652954
AGCTCAAA -0.696244765389
AGCTCAAC 0.971704371898
AGCTCAAG -0.471695236062
AGCTCAAT -0.244861754368
AGCTCACA -0.942848889461
AGCTCACC -0.953940878779
AGCTCACG -0.344501986238
AGCTCACT -0.379923374312
AGCTCAGA 0.252630015337
AGCTCAGC -1.22806273429
AGCTCAGG -1.28640258328
AGCTCATA 0.649984405381
AGCTCATC -0.756942799116
AGCTCATG -0.111149908974
AGCTCCAA -0.722537924061
AGCTCCAC -1.12161769401
AGCTCCAG -1.12169767677
AGCTCCAT -0.653382888864
AGCTCCCA -1.05791664715
AGCTCCCC -1.10455940843
AGCTCCCG -1.08847555343
AGCTCCCT -0.959801054113
AGCTCCGA 0.266733643379
AGCTCCGC -0.478905970092
AGCTCCGG -1.06255747735
AGCTCCTA 0.143419819171
AGCTCCTC -0.77251120961
AGCTCCTG -0.894800940733
AGCTCGAA 0.184862655023
AGCTCGAC -0.773977560351
AGCTCGAG 0.603983858677
AGCTCGAT 0.568672250872
AGCTCGCA -0.686369768773
AGCTCGCC -1.28186447604
AGCTCGCG -0.416159227432
AGCTCGGA 0.689569602245
AGCTCGGC -0.81662693364
AGCTCGGG -0.983944871938
AGCTCGTA -0.577277717087
AGCTCGTC -0.53676879767
AGCTCGTG -1.45273432882
AGCTCTAA 0.128319543112
AGCTCTAC -1.03192878218

AGCTCTAG -0.749866937988
AGCTCTAT 0.00303464030475
AGCTCTCA 0.1868596104
AGCTCTCC -0.827971156195
AGCTCTCG -0.536784219184
AGCTCTGA -1.00853434538
AGCTCTGC -1.29273141581
AGCTCTGG -0.534774978872
AGCTCTTA 0.578033894038
AGCTCTTC -1.14331759393
AGCTCTTG -0.811079893455
AGCTGAAA 1.73296545633
AGCTGAAC -0.389648860656
AGCTGAAG 0.162625354543
AGCTGAAT -0.0243403767837
AGCTGACA -0.256720114518
AGCTGACC -1.16937472502
AGCTGACG -0.62388153251
AGCTGACT -0.274744466468
AGCTGAGA -0.347285438831
AGCTGAGC -0.68453094926
AGCTGAGG -0.316064191589
AGCTGATA 0.685336004569
AGCTGATC 0.740361012183
AGCTGATG -0.353947271514
AGCTGCAA -0.404527485124
AGCTGCAC -0.114615828909
AGCTGCAG -0.150251027127
AGCTGCAT -0.708121945014
AGCTGCCA -0.00795462604603
AGCTGCCC -1.33463428327
AGCTGCCG -1.16996753847
AGCTGCCT -0.992724156913
AGCTGCGA -0.836582634187
AGCTGCGC -0.950388180155
AGCTGCGG -1.39600459045
AGCTGCTA -1.57540175635
AGCTGCTC -1.12853123716
AGCTGCTG -1.07776282881
AGCTGGAA 0.643888986612
AGCTGGAC -0.763855558129
AGCTGGAG -1.0466304513
AGCTGGAT 2.36755971346
AGCTGGCA -0.924526562495
AGCTGGCC -1.25740334096
AGCTGGCG -0.686160663498
AGCTGGGA -1.11378042829
AGCTGGGC -0.943619965163
AGCTGGGG -0.871639133556
AGCTGGTA -0.81883586949
AGCTGGTC -1.41963427068
AGCTGGTG -0.852289576925
AGCTGTAA 0.154556766122
AGCTGTAC 0.0394215719864

AGCTGTAG 0.29444688825
AGCTGTAT -0.244788567521
AGCTGTCA 0.0335331674399
AGCTGTCC -0.936185749871
AGCTGTCG 0.0891488974557
AGCTGTGA -0.38143834203
AGCTGTGC -0.628771197986
AGCTGTGG -1.19475801436
AGCTGTTA -0.580302686273
AGCTGTTC 0.166165506851
AGCTGTTG -0.497699829454
AGCTTAAA 0.168621448307
AGCTTAAC -0.546965032265
AGCTTAAG -0.670630676098
AGCTTAAT -0.59163253284
AGCTTACA 0.0742647839748
AGCTTACC -0.25308795589
AGCTTACG 0.051093828442
AGCTTACT 0.357562178957
AGCTTAGA -0.692059000545
AGCTTAGC -0.445594977007
AGCTTAGG -0.935983440517
AGCTTATA 0.142942274999
AGCTTATC 0.450310039578
AGCTTATG -0.827585095581
AGCTTCAA -1.29292666786
AGCTTCAC -1.34170387124
AGCTTCAG -0.639416486159
AGCTTCAT -0.497547705366
AGCTTCCA -0.16231326492
AGCTTCCC -1.09843314663
AGCTTCCG -0.90597003762
AGCTTCCT -0.772888644631
AGCTTCGA -0.565747390837
AGCTTCGC -1.11448354478
AGCTTCGG -0.883235589349
AGCTTCTA 0.00178680457565
AGCTTCTC 0.355495434694
AGCTTCTG -0.324387365682
AGCTTGAA -0.0278622323794
AGCTTGAC -0.778780447138
AGCTTGAG -0.74584767322
AGCTTGAT 0.548379629451
AGCTTGCA 0.729834914016
AGCTTGCC -0.87955742756
AGCTTGCG 0.00235896888461
AGCTTGGA -0.496527271624
AGCTTGGC -0.389715774344
AGCTTGGG -0.431912173331
AGCTTGTA 0.500701012915
AGCTTGTC -0.133297294186
AGCTTGTG -0.614436247234
AGCTTTAA 0.535768751692
AGCTTTAC 0.661788659541

AGCTTTAG -0.840590920929
AGCTTTAT -0.923218608999
AGCTTTCA -0.524109302935
AGCTTTCC -0.692181849894
AGCTTTCG -0.769440237263
AGCTTTGA 1.23382463014
AGCTTTGC -0.299390136953
AGCTTTGG -0.494267889126
AGCTTTTA 1.91756725252
AGCTTTTC -0.0897435405817
AGCTTTTG 0.395549288756
AGGAAAAA 2.17031175299
AGGAAAAC -0.488139013514
AGGAAAAG -0.550461272465
AGGAAAAT 1.28022665898
AGGAAACA 0.269729860589
AGGAAACC 0.693312586669
AGGAAACG -0.91566363541
AGGAAACT -0.663427260753
AGGAAAGA -1.00501039873
AGGAAAGC -0.30430933855
AGGAAAGG -1.16826646706
AGGAAATA 2.47461429562
AGGAAATC 23.9715950612
AGGAAATG 0.0728465274465
AGGAACAA 1.12611110499
AGGAACAC -0.373017149839
AGGAACAG -0.602345518846
AGGAACAT 0.358122581094
AGGAACCA 0.343927469495
AGGAACCC -0.76298332775
AGGAACCG -1.2044892511
AGGAACCT -0.636664137976
AGGAACGA -0.561734137845
AGGAACGC -0.843274787135
AGGAACGG -0.723439951941
AGGAACTA -0.338316129438
AGGAACTC -0.578063430158
AGGAACTG -0.10521602403
AGGAAGAA 0.0060907139002
AGGAAGAC -0.859633354186
AGGAAGAG -1.18550719699
AGGAAGAT 3.32651049325
AGGAAGCA -0.356992889845
AGGAAGCC -1.24959430447
AGGAAGCG -0.67795328145
AGGAAGGA -0.670427321218
AGGAAGGC -1.43370261221
AGGAAGGG -1.23281099232
AGGAAGTA 0.79997666473
AGGAAGTC -0.724196390274
AGGAAGTG -0.0290928169233
AGGAATAA 2.53784093489
AGGAATAC 0.854436826719

AGGAATAG −1.14705116861
AGGAATAT 6.00968351505
AGGAATCA 4.03125747912
AGGAATCC 8.05071201099
AGGAATCG 4.63371434653
AGGAATGA 0.308188764166
AGGAATGC −0.989172503815
AGGAATGG −0.850675545583
AGGAATTA 1.9312343733
AGGAATTC 0.899519662647
AGGAATTG 0.811247177675
AGGACAAA −0.168252377496
AGGACAAC −0.440433213291
AGGACAAG −1.24428512153
AGGACAAT 1.08193970668
AGGACACA −0.0876621589498
AGGACACC −0.612977476558
AGGACACG −0.940383015505
AGGACACT −0.488075759168
AGGACAGA 0.155066198849
AGGACAGC −0.789376595572
AGGACAGG −1.20104345755
AGGACATA 1.22540500624
AGGACATC 0.916629701781
AGGACATG −0.567062663017
AGGACCAA −0.796526427691
AGGACCAC −0.862525802904
AGGACCAG −1.40587122286
AGGACCAT −0.693211693374
AGGACCCA −0.96713437611
AGGACCCC −1.52559444811
AGGACCCG −0.710243840793
AGGACCCT −1.59151932957
AGGACCGA 0.0107480111397
AGGACCGC −0.874177148832
AGGACCGG −1.14724354547
AGGACCTA 0.134881789408
AGGACCTC −1.56891165137
AGGACCTG −1.14012220394
AGGACGAA −0.137247815595
AGGACGAC −1.23182767477
AGGACGAG −1.19176833169
AGGACGAT 2.63021188325
AGGACGCA 0.703213721445
AGGACGCC −0.851525297145
AGGACGCG 0.189433696336
AGGACGGA −0.371915687802
AGGACGGC −1.56771295538
AGGACGGG −0.699466032152
AGGACGTA 0.687250101981
AGGACGTC −0.669895932438
AGGACGTG −0.382708656576
AGGACTAA −0.722659989265
AGGACTAC −0.960070277155

AGGACTAG -0.356778034175
AGGACTAT 0.232589888536
AGGACTCA -0.676874298231
AGGACTCC -1.85590759591
AGGACTCG -0.42329259251
AGGACTGA -0.421184288574
AGGACTGC -1.21048299243
AGGACTGG -0.425410828947
AGGACTTA -0.7327676155
AGGACTTC -1.02276212968
AGGACTTG 0.248104977184
AGGAGAAA -0.505622828327
AGGAGAAC -0.24957524865
AGGAGAAG -0.00156358469449
AGGAGAAT 3.45226745121
AGGAGACA -0.821856395189
AGGAGACC -1.27366023058
AGGAGACG -1.49773430678
AGGAGACT -1.61139164939
AGGAGAGA 0.331522821813
AGGAGAGC -1.03742955782
AGGAGAGG -1.70633642231
AGGAGATA 3.14691206353
AGGAGATC 6.14776670622
AGGAGATG -0.451308517267
AGGAGCAA -0.705655025531
AGGAGCAC -1.59903195934
AGGAGCAG -0.863028178328
AGGAGCAT -0.332990740844
AGGAGCCA -0.853506569626
AGGAGCCC -1.72540917583
AGGAGCCG -0.65263272369
AGGAGCCT -1.31155376576
AGGAGCGA 0.00748962819055
AGGAGCGC -0.13224026702
AGGAGCGG -0.587757550711
AGGAGCTA -0.480646771507
AGGAGCTC -0.265817239511
AGGAGCTG -1.08986558075
AGGAGGAA -0.10817434091
AGGAGGAC -1.37044878926
AGGAGGAG -1.223522536
AGGAGGAT 2.69053770959
AGGAGGCA -1.86278846637
AGGAGGCC -1.19913589468
AGGAGGCG -1.38310985228
AGGAGGGA -0.724148818823
AGGAGGGC -1.68908027086
AGGAGGGG -1.55450534344
AGGAGGTA -0.540374556757
AGGAGGTC -1.32479640284
AGGAGGTG -0.267429963945
AGGAGTAA 0.500724537258
AGGAGTAC -0.28100612393

AGGAGTAG -0.400720984972
AGGAGTAT 0.676845023493
AGGAGTCA -0.915846079763
AGGAGTCC -1.34906803626
AGGAGTCG -0.186579932095
AGGAGTGA -0.753032530472
AGGAGTGC -0.96190622147
AGGAGTGG -0.659724267713
AGGAGTTA -0.185283218007
AGGAGTTC 4.07755286431E-5
AGGAGTTG -0.502084244309
AGGATAAA 0.737125630814
AGGATAAC 1.70600472906
AGGATAAG 1.23839044383
AGGATAAT 2.20980651183
AGGATACA 1.60383327784
AGGATACC 2.41609696853
AGGATACG 5.58046984529
AGGATACT 2.67581251612
AGGATAGA -0.623391964784
AGGATAGC 0.16524204568
AGGATAGG 0.212805131552
AGGATATA 2.93407844138
AGGATATC 20.2620444795
AGGATATG 3.9328222164
AGGATCAA 0.825792279229
AGGATCAC 2.80811342367
AGGATCAG 1.73062530692
AGGATCAT 0.873617269458
AGGATCCA 3.20817572702
AGGATCCC 5.41332729404
AGGATCCG 3.46949537345
AGGATCCT 6.43539362592
AGGATCGA 0.127506384974
AGGATCGC 6.38079989794
AGGATCGG 1.18868794961
AGGATCTA 4.10746955597
AGGATCTC 9.53074006723
AGGATCTG 5.27824790015
AGGATGAA -0.874793748012
AGGATGAC 0.467784706036
AGGATGAG 0.448147629652
AGGATGAT 1.11523789206
AGGATGCA -0.218670273136
AGGATGCC -0.808549981008
AGGATGCG -0.254913967705
AGGATGGA -0.227957683929
AGGATGGC -1.02959778112
AGGATGGG 0.00319695827454
AGGATGTA 0.308817125517
AGGATGTC 0.53842674112
AGGATGTG 0.770051870193
AGGATTAA -0.108702331729
AGGATTAC 12.7307970206

AGGATTAG	1.38773918169
AGGATTAT	2.99455848286
AGGATTCA	1.74993199696
AGGATTCC	13.4950762783
AGGATTCG	6.38881751695
AGGATTGA	0.349777712328
AGGATTGC	12.170400111
AGGATTGG	0.897371105945
AGGATTTA	1.72443160867
AGGATTTC	14.7779387092
AGGATTTG	7.63242121666
AGGCAAAA	0.65405777614
AGGCAAAC	-0.974122674404
AGGCAAAG	-1.10878176669
AGGCAAAT	2.64975120292
AGGCAACA	-0.51975782216
AGGCAACC	-1.17913131577
AGGCAACG	-0.504593246229
AGGCAACT	-0.444644593531
AGGCAAGA	1.27857368178
AGGCAAGC	-0.805753197954
AGGCAAGG	-0.88423615809
AGGCAATA	0.63993323757
AGGCAATC	9.15729974059
AGGCAATG	-0.392339522784
AGGCACAA	-0.560155915781
AGGCACAC	-1.57185114878
AGGCACAG	-0.896120133637
AGGCACAT	-0.353383210034
AGGCACCA	0.152539684363
AGGCACCC	-0.73687235205
AGGCACCG	-1.49590437425
AGGCACCT	-0.677613485378
AGGCACGA	-0.958972213079
AGGCACGC	-0.297574057639
AGGCACGG	-1.05699945913
AGGCACTA	-0.820675734529
AGGCACTC	-1.54892092569
AGGCACTG	-0.900885642856
AGGCAGAA	-0.458059742455
AGGCAGAC	-1.69033255008
AGGCAGAG	0.41191961798
AGGCAGAT	2.63401759926
AGGCAGCA	-0.452092400667
AGGCAGCC	-1.46664505725
AGGCAGCG	-0.925454467153
AGGCAGGA	-0.478334589928
AGGCAGGC	-1.10830657496
AGGCAGGG	-0.572249519366
AGGCAGTA	0.689987290032
AGGCAGTC	-0.509764681063
AGGCAGTG	-0.507657422654
AGGCATAA	-0.559190372173
AGGCATAC	-0.262532718402

AGGCATAG -0.203905872425
AGGCATAT 1.55072890218
AGGCATCA 1.06019458774
AGGCATCC -0.788447645387
AGGCATCG 0.531090282543
AGGCATGA -0.640188346006
AGGCATGC -1.41344762974
AGGCATGG -0.411540614669
AGGCATTA -0.250911692739
AGGCATTC -0.695803553259
AGGCATTG -0.698034183781
AGGCCAAA -0.807156555731
AGGCCAAC -1.11242751716
AGGCCAAG -1.35617369489
AGGCCAAT -0.523199433606
AGGCCACA -0.167835996617
AGGCCACC -0.36566553113
AGGCCACG -0.664893088732
AGGCCACT -0.942065790206
AGGCCAGA 0.00826227218201
AGGCCAGC -1.67693883444
AGGCCAGG -0.967316036318
AGGCCATA -0.256508134046
AGGCCATC -1.26162779028
AGGCCATG -1.3124976147
AGGCCCAA -1.10294276327
AGGCCCAC -0.895914687704
AGGCCCAG -1.19102705349
AGGCCCAT -1.37304300158
AGGCCCCA -1.06710028945
AGGCCCCC -1.7336006136
AGGCCCCG -1.31289047124
AGGCCCCT -1.44051421654
AGGCCCGA -0.731619104776
AGGCCCGC -0.824571104422
AGGCCCGG -1.55618942505
AGGCCCTA -1.04653138768
AGGCCCTC -0.98102445539
AGGCCCTG -1.06727855169
AGGCCGAA -0.688515711659
AGGCCGAC -1.23995246023
AGGCCGAG -1.05374996316
AGGCCGAT 0.462017582549
AGGCCGCA -1.32931516783
AGGCCGCC -1.11673822241
AGGCCGCG -0.791445953651
AGGCCGGA -0.854009206431
AGGCCGGC -1.23616007469
AGGCCGGG -1.19815309989
AGGCCGTA -1.3933526128
AGGCCGTC -0.232016940082
AGGCCGTG -0.890844407546
AGGCCTAA -0.00193187136024
AGGCCTAC -0.482773894918

AGGCCTAG 0.149937630596
AGGCCTAT -0.747291545144
AGGCCTCA -0.960940155099
AGGCCTCC -1.31453142488
AGGCCTCG -0.897433314765
AGGCCTGA -1.11393150686
AGGCCTGC -1.31526433887
AGGCCTGG -1.61589394734
AGGCCTTA -0.643689552456
AGGCCTTC -1.26631122568
AGGCCTTG -1.11393150686
AGGCGAAA -0.707584544457
AGGCGAAC -0.336375893866
AGGCGAAG -0.862178165384
AGGCGAAT 1.6266317646
AGGCGACA -0.318683235159
AGGCGACC -1.3014210469
AGGCGACG -0.484148239338
AGGCGACT -0.828377081811
AGGCGAGA 0.0430788232478
AGGCGAGC -0.707170516013
AGGCGAGG -0.891983247151
AGGCGATA 1.2427845298
AGGCGATC 4.50186928848
AGGCGATG -1.13387230865
AGGCGCAA -0.144626095227
AGGCGCAC -0.934027783432
AGGCGCAG -1.21717514538
AGGCGCAT 0.349502738892
AGGCGCCA -0.766834001391
AGGCGCCC -0.983144521498
AGGCGCCG -0.335292989923
AGGCGCCT -1.05752352923
AGGCGCGA 0.77823128441
AGGCGCGC 0.308968726842
AGGCGCGG -0.27372638516
AGGCGCTA -1.2964720478
AGGCGCTC -1.39962054342
AGGCGCTG -0.50780771707
AGGCGGAA -0.15274094819
AGGCGGAC -0.678641499187
AGGCGGAG -0.76385581951
AGGCGGAT 1.66764070701
AGGCGGCA -0.718854273258
AGGCGGCC -1.25436608684
AGGCGGCG -0.627527544362
AGGCGGGA -0.611626656481
AGGCGGGC -0.0922399961847
AGGCGGGG 0.126488565047
AGGCGGTA -0.945029073336
AGGCGGTC -2.12378635205
AGGCGGTG -0.480783212699
AGGCGTAA -0.3653719996
AGGCGTAC -0.745389209904

AGGCGTAG −0.8436430738
AGGCGTAT 1.03111457851
AGGCGTCA −0.996602798384
AGGCGTCC −0.461612963842
AGGCGTCG −1.35670848163
AGGCGTGA −0.0212769845036
AGGCGTGC −0.476835305105
AGGCGTGG −1.938952449
AGGCGTTA −0.323130381597
AGGCGTTC −1.10806610389
AGGCGTTG −0.925758715328
AGGCTAAA 0.208909500277
AGGCTAAC −0.544420482449
AGGCTAAG −0.679936122222
AGGCTAAT −0.68901939399
AGGCTACA −1.62379838812
AGGCTACC −1.32125677329
AGGCTACG −0.0786518126462
AGGCTACT −1.01679897
AGGCTAGA 0.393162090659
AGGCTAGC −0.860902884588
AGGCTAGG −0.613670137782
AGGCTATA −0.296827029044
AGGCTATC 0.463180207879
AGGCTATG −1.02435028424
AGGCTCAA −0.926439352999
AGGCTCAC −0.919945327299
AGGCTCAG −0.785412743701
AGGCTCAT −1.24785663963
AGGCTCCA −0.559540100746
AGGCTCCC −1.21362192399
AGGCTCCG −0.70568430027
AGGCTCGA −1.22338870863
AGGCTCGC −1.48904284602
AGGCTCGG −1.0569346365
AGGCTCTA −0.512708883337
AGGCTCTC −1.17960127988
AGGCTCTG −1.53091094973
AGGCTGAA −0.836972092762
AGGCTGAC −1.28560406251
AGGCTGAG −1.12504754328
AGGCTGAT 0.964461749313
AGGCTGCA −1.35193277853
AGGCTGCC −1.37176772078
AGGCTGCG −1.12994923231
AGGCTGGA −0.435729912891
AGGCTGGC −1.74460660837
AGGCTGGG −0.668320846953
AGGCTGTA 0.373088768396
AGGCTGTC −1.23671551057
AGGCTGTG −1.01264770752
AGGCTTAA −0.368991089149
AGGCTTAC −1.46615313709
AGGCTTAG −0.889554227999

AGGCTTAT -0.277382329513
AGGCTTCA -1.10567498507
AGGCTTCC -1.55826191971
AGGCTTCG -0.587961166973
AGGCTTGA -0.927604853526
AGGCTTGC -1.02880109002
AGGCTTGG -1.28727350675
AGGCTTTA 0.082961995129
AGGCTTTC 0.0359326504715
AGGCTTTG -0.71486297632
AGGGAAAA 0.00742166897615
AGGGAAAC -0.124772594883
AGGGAAAG -0.0820994358692
AGGGAAAT 2.76726000331
AGGGAACA -0.350005114315
AGGGAACC -0.858047290675
AGGGAACG -1.17700001026
AGGGAACT -0.717154508753
AGGGAAGA -0.236088219788
AGGGAAGC -0.342596514419
AGGGAAGG -1.42207139266
AGGGAATA 2.13569489747
AGGGAATC 25.4860044682
AGGGAATG 0.215921584295
AGGGACAA 0.737971723033
AGGGACAC -1.00922151759
AGGGACAG -1.40985650802
AGGGACAT -0.89408867589
AGGGACCA -0.840688677645
AGGGACCC -2.16291543803
AGGGACCG -0.883404964622
AGGGACCT -0.671410900156
AGGGACGA -0.532150969051
AGGGACGC -1.06621316032
AGGGACGG -0.774708906051
AGGGACTA -1.30864092928
AGGGACTC -1.20220399183
AGGGACTG -0.64077279525
AGGGAGAA -1.29485017501
AGGGAGAC -1.28017621233
AGGGAGAG -1.36632366494
AGGGAGAT 3.63019669808
AGGGAGCA -1.00995652263
AGGGAGCC -1.48361917795
AGGGAGCG -0.974853235959
AGGGAGGA -0.808404130079
AGGGAGGC -1.68678429494
AGGGAGGG 0.059417263917
AGGGAGTA -0.193748583688
AGGGAGTC -0.949719566038
AGGGAGTG -1.38408611254
AGGGATAA 0.802416400527
AGGGATAC 2.58798594814
AGGGATAG 0.210601423334

AGGGATAT 5.32682201003
AGGGATCA 1.17228677736
AGGGATCC 1.59359705186
AGGGATCG 1.23188491734
AGGGATGA 0.606075434191
AGGGATGC -1.06820933155
AGGGATGG -1.0669220272
AGGGATTA 1.46273139064
AGGGATTC 4.31346648115
AGGGATTG 2.23284651518
AGGGCAAA 1.55890831639
AGGGCAAC -0.834672980262
AGGGCAAG -0.724961192817
AGGGCAAT 1.10593140041
AGGGCACA -1.63106401229
AGGGCACC -1.85507718658
AGGGCACG -1.10368560976
AGGGCACT -0.604720954772
AGGGCAGA -0.717874353663
AGGGCAGC -0.954791153103
AGGGCAGG -0.0298011610427
AGGGCATA -0.186366644714
AGGGCATC -0.268516527231
AGGGCATG -1.20801920953
AGGGCCAA -1.54457911604
AGGGCCAC -0.881846346178
AGGGCCAG -0.876347661588
AGGGCCAT -1.42987494015
AGGGCCCA -1.09512379427
AGGGCCCC -1.60798349479
AGGGCCCG -0.710871940763
AGGGCCCT -1.21333544977
AGGGCCGA -0.807925801762
AGGGCCGC -2.01340307529
AGGGCCGG -0.528383414757
AGGGCCTA -0.861271694017
AGGGCCTC -0.987112294093
AGGGCCTG -0.892635655609
AGGGCGAA -0.600588250391
AGGGCGAC -1.38369404014
AGGGCGAG -1.4850599133
AGGGCGAT 2.3595271957
AGGGCGCA -0.654491146822
AGGGCGCC -0.728000537991
AGGGCGCG -0.368915549868
AGGGCGGA 1.72938792645
AGGGCGGC -0.896762609595
AGGGCGGG -0.634439519231
AGGGCGTA -1.18287926645
AGGGCGTC -1.54464393867
AGGGCGTG -1.17584078289
AGGGCTAA -0.987112294093
AGGGCTAC -0.646893829415
AGGGCTAG -0.91698047588

AGGGCTAT -0.030094431191
AGGGCTCA -1.29776170458
AGGGCTCC -1.45678626629
AGGGCTCG -1.44001759152
AGGGCTGA -1.34047929847
AGGGCTGC -0.680465681331
AGGGCTGG -1.24868129856
AGGGCTTA -0.974523372387
AGGGCTTC -1.37615030596
AGGGCTTG -1.60476641013
AGGGGAAA -0.166290970016
AGGGGAAC -0.0549627987943
AGGGGAAG -0.827477406365
AGGGGAAT 2.64478390711
AGGGGACA -0.706024880487
AGGGGACC -1.40437376771
AGGGGACG -1.0878511128
AGGGGACT -1.19895266618
AGGGGAGA 0.243971488659
AGGGGAGC -1.52939598201
AGGGGAGG -0.157620942547
AGGGGATA 1.00680426061
AGGGGATC 5.29263329755
AGGGGATG -0.475557149111
AGGGGCAA -0.402928875296
AGGGGCAC -0.82910502955
AGGGGCAG -0.683959307714
AGGGGCAT -0.540016986737
AGGGGCCA -0.646920228956
AGGGGCCC -1.49659625132
AGGGGCCG -1.10222788461
AGGGGCGA -0.814992775915
AGGGGCGC -1.29643074951
AGGGGCGG -0.849755743754
AGGGGCTA -1.21525399066
AGGGGCTC -2.02605655825
AGGGGCTG -0.999641359413
AGGGGGAA 0.435508784063
AGGGGGAC -1.66229336236
AGGGGGAG -0.862698053375
AGGGGGAT 1.09259388182
AGGGGGCA -0.596304990212
AGGGGGCC -1.6166898543
AGGGGGCG -1.76345953997
AGGGGGGA -0.907209247757
AGGGGGGC -1.51019567427
AGGGGGGG -1.88872248673
AGGGGGTA -0.369659441884
AGGGGGTC -1.1898937029
AGGGGGTG -0.630533694073
AGGGGTAA 1.68026021036
AGGGGTAC -0.635446883893
AGGGGTAG -0.529407507842
AGGGGTAT -0.00372573323892

AGGGGTCA -1.50300375972
AGGGGTCC -1.03286243723
AGGGGTCG -0.953397205063
AGGGGTGA -0.70074157433
AGGGGTGC -1.15532964646
AGGGGTGG -0.329300032739
AGGGGTTA 1.01920420342
AGGGGTTC -0.701172331197
AGGGGTTG -0.20720921300 9
AGGGTAAA 1.3834107025
AGGGTAAC -0.039755879045
AGGGTAAG -1.01212808091
AGGGTAAT 1.77967539268
AGGGTACA 0.311925475432
AGGGTACC -0.962420359065
AGGGTACG -1.10117556231
AGGGTACT -0.473579274591
AGGGTAGA -0.472189508656
AGGGTAGC -1.66212869195
AGGGTAGG -0.28613416942
AGGGTATA 0.554394542689
AGGGTATC 4.78361930442
AGGGTATG 0.593549766831
AGGGTCAA -0.234006576775
AGGGTCAC -0.38619078217
AGGGTCAG -0.417722027982
AGGGTCAT -0.966899132676
AGGGTCCA -1.4592584134
AGGGTCCC -1.09911535259
AGGGTCCG -1.64183293395
AGGGTCGA -1.12479557142
AGGGTCGC -1.1770619577
AGGGTCGG -0.499164611906
AGGGTCTA -1.273543393
AGGGTCTC -0.624343916549
AGGGTCTG -1.20371294777
AGGGTGAA -1.16877668393
AGGGTGAC -1.17651932951
AGGGTGAG -1.03159003163
AGGGTGAT -0.0714308847341
AGGGTGCA -0.597197085592
AGGGTGCC -1.11142119803
AGGGTGCG -0.173708456886
AGGGTGGA -0.177684593692
AGGGTGGC -1.22421389032
AGGGTGGG 0.0781578014338
AGGGTGTA -0.305155692151
AGGGTGTC -0.829672750372
AGGGTGTG -1.12548195949
AGGGTTAA -0.390773847036
AGGGTTAC -0.465353595832
AGGGTTAG -0.728314195903
AGGGTTAT 0.0801871681285
AGGGTTCA -0.874750097286

AGGGTTCC -0.546817874428
AGGGTTCG -1.61126409517
AGGGTTGA 4.07755286431E-5
AGGGTTGC -0.276857998036
AGGGTTGG -0.0304274313416
AGGGTTTA -0.117982423838
AGGGTTTC -0.219232766326
AGGGTTTG -0.275697463759
AGGTAAAA 0.844601298723
AGGTAAAC -0.316642629056
AGGTAAAG -0.0374410836497
AGGTAAAT 2.25383833237
AGGTAACA -0.749852039238
AGGTAACC -0.540374556757
AGGTAACG -0.513466367196
AGGTAACT 1.1377086063
AGGTAAGA 0.171449074389
AGGTAAGC -0.31595127474
AGGTAAGG -0.865673360057
AGGTAATA 2.39778091472
AGGTAATC 8.24342238699
AGGTAATG 0.471804232187
AGGTACAA -0.596989287225
AGGTACAC -0.218255721928
AGGTACAG -0.474173394953
AGGTACAT 0.0344788460465
AGGTACCA -0.0401497811069
AGGTACCC -0.210693168273
AGGTACCG -0.731802594655
AGGTACCT -0.467933693545
AGGTACGA 0.395013456489
AGGTACGC -0.879359300312
AGGTACGG -0.701541140625
AGGTACTA -0.595760793734
AGGTACTC -0.894002419964
AGGTACTG -0.384412341805
AGGTAGAA 0.889090537052
AGGTAGAC -0.334390177898
AGGTAGAG -0.66968133815
AGGTAGAT 3.47697637604
AGGTAGCA -0.173109893036
AGGTAGCC -1.56003356415
AGGTAGCG -0.785498476864
AGGTAGGA -0.244656831198
AGGTAGGC -0.98342524533
AGGTAGGG 0.510433556562
AGGTAGTA -0.305508034539
AGGTAGTC 0.125148200234
AGGTAGTG -0.483843991163
AGGTATAA -0.213180214139
AGGTATAC -0.15381836312
AGGTATAG -0.54368103392
AGGTATAT 1.16739319116
AGGTATCA 2.19115249162

AGGTATCC 2.40620184553
AGGTATCG 0.850592687618
AGGTATGA 1.4492908877
AGGTATGC -0.22531746845
AGGTATGG -0.510221053326
AGGTATTA 1.2389730634
AGGTATTC 0.110157181681
AGGTATTG 1.8261059121
AGGTCAAA 0.225159332586
AGGTCAAC -1.72044658489
AGGTCAAG -0.493738330017
AGGTCAAT 0.381883213503
AGGTCACA -0.856351969657
AGGTCACC -1.28248499595
AGGTCACG -0.497532806615
AGGTCACT -0.764649896793
AGGTCAGA -1.03196746665
AGGTCAGC -0.561485563949
AGGTCAGG -1.15461293812
AGGTCATA 0.256909354792
AGGTCATC -1.01658097775
AGGTCATG -0.387096208011
AGGTCCAA -0.242738813062
AGGTCCAC -1.03897615271
AGGTCCAG -0.795322504069
AGGTCCAT -0.699047298838
AGGTCCCA -1.15357917392
AGGTCCCC -1.43522489861
AGGTCCCG -0.599880429035
AGGTCCGA -0.264642067865
AGGTCCGC -0.721473839592
AGGTCCGG -1.46153975196
AGGTCCTA -0.861725452464
AGGTCCTC -1.22309700677
AGGTCCTG -1.09085621699
AGGTCGAA -0.127077980541
AGGTCGAC -0.144967459588
AGGTCGAG -0.0485929293519
AGGTCGAT -0.106594550556
AGGTCGCA -0.819203633393
AGGTCGCC -1.36662137858
AGGTCGCG -0.352577631962
AGGTCGGA -0.932136948982
AGGTCGGC -1.14490261191
AGGTCGGG -1.04716523805
AGGTCGTA -0.136678003721
AGGTCGTC -0.402184460516
AGGTCGTG -0.129281427377
AGGTCTAA 1.04903228677
AGGTCTAC -0.900479455859
AGGTCTAG -0.369375320092
AGGTCTAT -0.960348648552
AGGTCTCA -0.577044826087
AGGTCTCC -1.103378225

AGGTCTCG −1.39457849248
AGGTCTGA −0.382085261475
AGGTCTGC −0.886921069823
AGGTCTGG 0.708556099842
AGGTCTTA −0.927235521334
AGGTCTTC −0.921854719842
AGGTCTTG −0.285896050788
AGGTGAAA 0.146393034801
AGGTGAAC −0.539186577413
AGGTGAAG −0.311251633683
AGGTGAAT 1.13216600961
AGGTGACA 0.389595016048
AGGTGACC −1.3453681798
AGGTGACG −1.21718873722
AGGTGACT −0.833349866634
AGGTGAGA −0.898027173746
AGGTGAGC −0.25308795589
AGGTGAGG −0.217845875589
AGGTGATA 1.59921989271
AGGTGATC 1.9592965626
AGGTGATG −0.247125318971
AGGTGCAA 0.99265645908
AGGTGCAC −0.74821579046
AGGTGCAG −0.790185310223
AGGTGCAT −0.108991419772
AGGTGCCA −0.772462854015
AGGTGCCC −1.33351138794
AGGTGCCG −0.510671936576
AGGTGCGA 0.259928312201
AGGTGCGC −0.124730773828
AGGTGCGG −0.763188773683
AGGTGCTA 0.187556453729
AGGTGCTC −1.05701252821
AGGTGCTG −0.486202698666
AGGTGGAA −0.613232584994
AGGTGGAC −1.10095757006
AGGTGGAG −1.44678920447
AGGTGGAT 2.51533336584
AGGTGGCA −0.192761606789
AGGTGGCC −1.0211614288
AGGTGGCG −1.13470768423
AGGTGGGA −1.06993366592
AGGTGGGC −0.998268060519
AGGTGGGG −0.56674011813
AGGTGGTA 0.0448818334822
AGGTGGTC −1.55018261465
AGGTGGTG 0.292855597107
AGGTGTAA 1.25809129732
AGGTGTAC −0.784837442813
AGGTGTAG −1.02902953754
AGGTGTAT 0.902487912027
AGGTGTCA −1.16808167027
AGGTGTCC −0.424097386438
AGGTGTCG −1.04309918597

AGGTGTGA	0.411916742782
AGGTGTGC	-0.407043805728
AGGTGTGG	0.508355311509
AGGTGTTA	0.380312832887
AGGTGTTC	0.499339476192
AGGTGTTG	0.952447867115
AGGTTAAA	0.153496863759
AGGTTAAC	-0.507093361174
AGGTTAAG	-0.564017044686
AGGTTAAT	-0.017724808643
AGGTTACA	0.63354559418
AGGTTACC	-0.565651463792
AGGTTACG	0.069150068946
AGGTTACT	0.10040764823
AGGTTAGA	1.04502217036
AGGTTAGC	-1.05940260151
AGGTTAGG	0.483572677069
AGGTTATA	0.0718637326535
AGGTTATC	0.585218489909
AGGTTATG	-0.844927502953
AGGTTCAA	-0.466823867297
AGGTTCAC	-0.629014805632
AGGTTCAG	-0.755898579648
AGGTTCAT	-0.802767436007
AGGTTCCA	-1.32055705477
AGGTTCCC	-0.724251803171
AGGTTCCG	-0.940410199191
AGGTTCGA	-0.633372559564
AGGTTCGC	-0.886921069823
AGGTTCGG	-0.292578271235
AGGTTCTA	-0.0368866932891
AGGTTCTC	-0.695565173245
AGGTTCTG	-0.537419115076
AGGTTGAA	-0.253550862693
AGGTTGAC	-0.714298653459
AGGTTGAG	-0.321803608627
AGGTTGAT	0.0533343914646
AGGTTGCA	0.270026528699
AGGTTGCC	-1.09790123509
AGGTTGCG	-0.551417406335
AGGTTGGA	-0.0957367591474
AGGTTGGC	-0.970412624061
AGGTTGGG	-0.927903351306
AGGTTGTA	0.977001269897
AGGTTGTC	-0.338975595199
AGGTTGTG	-0.303503237714
AGGTTTAA	-0.747412826204
AGGTTTAC	0.248639763925
AGGTTTAG	0.478210172289
AGGTTTAT	0.31418172135
AGGTTTCA	-0.533262102207
AGGTTTCC	-0.185315367943
AGGTTTCG	-0.0482588836749
AGGTTTGA	-0.59910072774

AGGTTTGC 0.676097994897
AGGTTTGG -0.306888129355
AGGTTTTA -0.54773663073
AGGTTTTC -0.289719802125
AGGTTTTG -0.757330166638
AGTAAAAA 1.71863730149
AGTAAAAC 0.793276670334
AGTAAAAG 0.628828440555
AGTAAAAT 3.15706543154
AGTAAACA 0.139793410938
AGTAAACC -0.125796426586
AGTAAACG 1.4502679321
AGTAAACT -0.512708883337
AGTAAAGA 0.705025618653
AGTAAAGC -0.303000339528
AGTAAAGG -0.464098180036
AGTAAATA 2.41664221054
AGTAAATC 4.06048987382
AGTAAATG 1.55390625683
AGTAACAA -0.146810722588
AGTAACAC -0.208957071727
AGTAACAG -0.0286327853181
AGTAACAT 0.370483316668
AGTAACCA 1.0831449372
AGTAACCC -0.82923624311
AGTAACCG -0.385028679603
AGTAACGA 1.18126262129
AGTAACGC -0.581905739588
AGTAACGG -0.15735903819
AGTAACTA 0.334164344201
AGTAACTC -0.51605979537
AGTAACTG 0.147449539204
AGTAAGAA 1.6006415472
AGTAAGAC 0.120737385837
AGTAAGAG 0.776770684063
AGTAAGAT 2.17968463557
AGTAAGCA 0.864128856219
AGTAAGCC -1.29792010183
AGTAAGCG 0.0898496615088
AGTAAGGA 0.699144532791
AGTAAGGC -0.254115708317
AGTAAGGG -1.26578192796
AGTAAGTA 2.50562983555
AGTAAGTC 0.522728162593
AGTAAGTG 0.73660678835
AGTAATAA 0.695329407047
AGTAATAC 0.233371942265
AGTAATAG 0.297363645456
AGTAATAT 3.69757852054
AGTAATCA 3.96465274414
AGTAATCC 7.06998657615
AGTAATCG 2.64669460656
AGTAATGA 0.438095939079
AGTAATGC 0.898861503793

AGTAATGG 0.838895338529
AGTAATTA 1.78297794911
AGTAATTC 0.847753560745
AGTAATTG 1.86675676172
AGTACAAA 1.77369681148
AGTACAAC 0.551319649619
AGTACAAG -0.00833676593626
AGTACAAT 1.91482928032
AGTACACA 0.751710985133
AGTACACC -0.382375133662
AGTACACG 0.0863113388727
AGTACACT 0.206322606642
AGTACAGA 0.391709593142
AGTACAGC 0.0445253089882
AGTACAGG 0.0914725798251
AGTACATA 0.797250454706
AGTACATC 1.27447757082
AGTACATG 0.0144381964818
AGTACCAA -0.322860635792
AGTACCAC -0.514116684601
AGTACCAG -0.120574806486
AGTACCAT 0.57588925806
AGTACCCA 0.175438280275
AGTACCCC -0.668694622633
AGTACCCG -0.340112866514
AGTACCGA -0.195090516791
AGTACCGC -0.431912173331
AGTACCGG -0.102073694509
AGTACCTA 0.367363727345
AGTACCTC -0.277727353217
AGTACCTG -1.14536212075
AGTACGAA 1.99847191312
AGTACGAC -0.394790236608
AGTACGAG 0.554544575724
AGTACGAT 2.34404216593
AGTACGCA -0.641152321324
AGTACGCC -0.691034384697
AGTACGCG -0.154899437392
AGTACGGA -0.51712414122
AGTACGGC -0.068633317535
AGTACGGG -0.360481288597
AGTACGTA 0.348534058705
AGTACGTC -1.17815610105
AGTACGTG 0.283625428882
AGTACTAA -0.0499897525895
AGTACTAC 0.65549929563
AGTACTAG -1.13575582442
AGTACTAT 1.31579180693
AGTACTCA 0.224877563227
AGTACTCC -0.423220189809
AGTACTCG 0.020138929044
AGTACTGA 0.778729216346
AGTACTGC 0.350713197053
AGTACTGG -0.088573335186

AGTACTTA 0.252596558493
AGTACTTC 0.507078201042
AGTACTTG 0.0663775943799
AGTAGAAA 1.75242557737
AGTAGAAC -0.061580980751
AGTAGAAG -0.385165382177
AGTAGAAT 3.56681715058
AGTAGACA -0.442726575396
AGTAGACC -1.17701412486
AGTAGACG -0.948250862862
AGTAGACT 0.280280005863
AGTAGAGA 0.129923380572
AGTAGAGC -0.829634065896
AGTAGAGG -0.757477063094
AGTAGATA 2.63243284265
AGTAGATC 2.27131691955
AGTAGATG 1.31240325594
AGTAGCAA 0.376173332585
AGTAGCAC 0.712469243683
AGTAGCAG -0.426514120655
AGTAGCAT 0.246000071209
AGTAGCCA -1.03353000582
AGTAGCCC -1.51272767777
AGTAGCCG -0.639776408614
AGTAGCGA -0.0423495686009
AGTAGCGC -0.607335293473
AGTAGCGG -1.26178174404
AGTAGCTA -0.171784688356
AGTAGCTC -0.264642067865
AGTAGCTG -0.7967804906
AGTAGGAA 0.753519223
AGTAGGAC -1.17452289689
AGTAGGAG -0.283398288277
AGTAGGAT 3.11852367
AGTAGGCA -0.804728059343
AGTAGGCC -1.12888906856
AGTAGGCG -0.997552397715
AGTAGGGA -1.08251762138
AGTAGGGC -0.562198874319
AGTAGGGG -0.234302983502
AGTAGGTA 1.82669271378
AGTAGGTC -0.558350814494
AGTAGGTG 0.0616149603582
AGTAGTAA 2.94769485413
AGTAGTAC -0.141417636162
AGTAGTAG -0.639049506402
AGTAGTAT 1.44582209257
AGTAGTCA 0.278467585891
AGTAGTCC -0.596599305887
AGTAGTCG -0.253622742631
AGTAGTGA 1.19148028918
AGTAGTGC -0.0630455018214
AGTAGTGG 0.0111210026741
AGTAGTTA 1.93778930091

AGTAGTTC -0.708056599616
AGTAGTTG 0.0659209607355
AGTATAAA 1.20102516084
AGTATAAC 0.130107654596
AGTATAAG 1.36127795465
AGTATAAT 3.54624197566
AGTATACA 1.57064252029
AGTATACC 0.397620215124
AGTATACG 0.505991376374
AGTATACT 3.03518502399
AGTATAGA 1.59549990986
AGTATAGC -0.214086685506
AGTATAGG 0.787480794872
AGTATATA 2.63364905121
AGTATATC 2.18943782836
AGTATATG 1.01974526332
AGTATCAA 1.85983825232
AGTATCAC 2.04263546998
AGTATCAG 2.30853582884
AGTATCAT 1.00256569669
AGTATCCA 2.26125712614
AGTATCCC 2.40659208825
AGTATCCG 4.01652366006
AGTATCGA 1.62400566373
AGTATCGC 1.57197269122
AGTATCGG 2.18919422072
AGTATCTA 4.39939594151
AGTATCTC 3.77214101815
AGTATCTG 4.06643630508
AGTATGAA -0.160389757771
AGTATGAC -0.0962945474687
AGTATGAG -0.0669089831603
AGTATGAT 1.09528506671
AGTATGCA -0.0511471502871
AGTATGCC -1.18193594028
AGTATGCG 0.673954665828
AGTATGGA -0.527981409866
AGTATGGC -0.72827760248
AGTATGGG 0.0637005240956
AGTATGTA -0.209391226554
AGTATGTC -0.437702298398
AGTATGTG 1.70130717906
AGTATTAA 0.727456341512
AGTATTAC 0.0885929388055
AGTATTAG 0.937930733391
AGTATTAT 2.14147535141
AGTATTCA 1.99904433881
AGTATTCC 0.0942975920916
AGTATTCG 1.11375847224
AGTATTGA 1.65448955349
AGTATTGC 0.957912833479
AGTATTGG -0.14109744371
AGTATTTA 1.42625480507
AGTATTTC 0.676460531168

AGTATTTG 1.77986750815
AGTCAAAA 0.770609919896
AGTCAAAC 0.898249870864
AGTCAAAG -0.45716032839
AGTCAAAT 1.08690517281
AGTCAACA -0.281325270856
AGTCAACC -0.773146366883
AGTCAACG -0.447254750128
AGTCAACT -0.591508637965
AGTCAAGA -0.582462221001
AGTCAAGC -1.3667390003
AGTCAAGG -0.964686798866
AGTCAATA 1.36107982741
AGTCAATC 0.184329959335
AGTCAATG -1.39353375025
AGTCACAA 0.283506500257
AGTCACAC 0.219634248454
AGTCACAG -0.179854060921
AGTCACAT 0.300435663329
AGTCACCA -0.772102147415
AGTCACCC -1.00651569533
AGTCACCG 0.0789053527922
AGTCACGA 0.729111671146
AGTCACGC -0.630783836259
AGTCACGG -0.0375694220123
AGTCACTA -0.216230014576
AGTCACTC -0.592931599362
AGTCACTG -0.766345740574
AGTCAGAA -0.0345454983529
AGTCAGAC -0.840689984553
AGTCAGAG -1.15416153211
AGTCAGAT 2.99329496423
AGTCAGCA -0.438206503493
AGTCAGCC -0.798496722145
AGTCAGCG -0.503197991281
AGTCAGGA 1.49349574286
AGTCAGGC 0.509763112774
AGTCAGGG -0.358190278927
AGTCAGTA 0.246451999985
AGTCAGTC -0.566638179309
AGTCAGTG -0.252554214675
AGTCATAA 1.09945305761
AGTCATAC 0.216065866935
AGTCATAG -0.0421835912888
AGTCATAT 1.08249279013
AGTCATCA -0.346880558742
AGTCATCC -0.399306387786
AGTCATCG 0.273989073661
AGTCATGA -0.334653650544
AGTCATGC -0.339628003657
AGTCATGG -0.0362915273999
AGTCATTA 0.0725062086112
AGTCATTC -0.394099143673
AGTCATTG -0.805398764513

AGTCCAAA 0.0713702442043
AGTCCAAC -0.958770165107
AGTCCAAG 0.640163253373
AGTCCAAT 0.598866791214
AGTCCACA -0.617140239822
AGTCCACC -0.707975048558
AGTCCACG -0.802775800218
AGTCCACT -0.714440322283
AGTCCAGA 0.446215235529
AGTCCAGC -0.694928709064
AGTCCAGG -0.600092148126
AGTCCATA -0.261551230518
AGTCCATC -1.11860239594
AGTCCATG -0.352273122405
AGTCCCAA 0.649043954406
AGTCCCAC -1.13609509773
AGTCCCAG -1.15536519435
AGTCCCAT -0.512512324378
AGTCCCCA -0.734151892421
AGTCCCCC -1.16347664935
AGTCCCCG -1.83089416152
AGTCCCGA -0.277252684242
AGTCCCGC -0.748491025278
AGTCCCGG -1.4587855741
AGTCCCTA -0.114610078514
AGTCCCTC -1.32546135761
AGTCCCTG -0.389249208199
AGTCCGAA -1.02872293693
AGTCCGAC -0.87680560214
AGTCCGAG -1.3453681798
AGTCCGAT 0.094911316074
AGTCCGCA -0.790877971447
AGTCCGCC -1.44306686919
AGTCCGCG -1.42628198876
AGTCCGGA -0.470357223683
AGTCCGGC -0.971645038276
AGTCCGGG -1.18728563736
AGTCCGTA -0.128160623103
AGTCCGTC -0.909452163214
AGTCCGTG 0.447209531112
AGTCCTAA 0.0501714127972
AGTCCTAC -1.472888418
AGTCCTAG -1.43318246284
AGTCCTAT -0.294637696813
AGTCCTCA -0.113536061545
AGTCCTCC -1.58844678893
AGTCCTCG -0.859652696424
AGTCCTGA -0.24294661143
AGTCCTGC -1.23686083874
AGTCCTGG -1.3100905516
AGTCCTTA -0.631066912525
AGTCCTTC -0.49527211721
AGTCCTTG -0.843772719071
AGTCGAAA 0.283649998752

AGTCGAAC -0.378767544904
AGTCGAAG -0.660643546779
AGTCGAAT 0.616584542554
AGTCGACA 0.927589432012
AGTCGACC 0.121545054962
AGTCGACG -0.70062186156
AGTCGACT -0.270073838767
AGTCGAGA 0.0662181516077
AGTCGAGC -0.501022512275
AGTCGAGG -0.596757703133
AGTCGATA -0.00715924185591
AGTCGATC 1.26044137923
AGTCGATG -0.588980555189
AGTCGCAA 1.21358846715
AGTCGCAC -0.199437292697
AGTCGCAG -0.465770238092
AGTCGCAT -0.473677031307
AGTCGCCA -0.998975881875
AGTCGCCC -1.78790917426
AGTCGCCG -1.09160455249
AGTCGCGA -0.301923970124
AGTCGCGC -0.327233027095
AGTCGCGG -0.47433022391
AGTCGCTA -0.0842166267795
AGTCGCTC -1.00395676953
AGTCGCTG -0.308375390624
AGTCGGAA -0.469726509897
AGTCGGAC -0.971408487933
AGTCGGAG -0.381783888497
AGTCGGAT 1.05933960854
AGTCGGCA -0.951966402219
AGTCGGCC -0.828221559762
AGTCGGCG -0.979616915507
AGTCGGGA 0.176537651258
AGTCGGGC -1.15079101646
AGTCGGGG -0.601343120434
AGTCGGTA -0.376792284199
AGTCGGTC -0.48484874201
AGTCGGTG -1.09295720224
AGTCGTAA 0.291169685826
AGTCGTAC -0.373632964874
AGTCGTAG -0.240193740483
AGTCGTAT 1.10233923317
AGTCGTCA -1.1461922687
AGTCGTCC -0.649141711122
AGTCGTCG -1.13366764686
AGTCGTGA -0.264855355246
AGTCGTGC 0.201770907567
AGTCGTGG -0.23795474575
AGTCGTTA 1.16047598866
AGTCGTTC -0.167904739976
AGTCGTTG -0.243537595213
AGTCTAAA 0.864674620987
AGTCTAAC -0.968944182266

AGTCTAAG 0.148696329406
AGTCTAAT 0.180063166196
AGTCTACA 0.124016940695
AGTCTACC -0.972558044183
AGTCTACG 0.27430534539
AGTCTAGA -0.105629006949
AGTCTAGC -0.17707086971
AGTCTAGG -0.863647129942
AGTCTATA 0.854147477294
AGTCTATC 0.124060852803
AGTCTATG -0.114546824168
AGTCTCAA 0.11680202456
AGTCTCAC -0.414680330374
AGTCTCAG -0.704522197703
AGTCTCAT -0.296982028329
AGTCTCCA -0.534927364341
AGTCTCCC -0.988828264256
AGTCTCCG -0.999038613457
AGTCTCGA 1.06203968041
AGTCTCGC -0.898623646543
AGTCTCGG -0.3963240238
AGTCTCTA -0.544508045283
AGTCTCTC -0.213028090051
AGTCTCTG -0.15959907845
AGTCTGAA -0.658060312486
AGTCTGAC -0.584183680178
AGTCTGAG -0.357477229939
AGTCTGAT 2.21495233126
AGTCTGCA -0.320535385133
AGTCTGCC -0.815354789423
AGTCTGCG -0.895338079908
AGTCTGGA -0.52484326245
AGTCTGGC -0.837616659772
AGTCTGGG -1.35260322232
AGTCTGTA 0.186247454707
AGTCTGTC -0.198422086586
AGTCTGTG -0.0566528921803
AGTCTTAA -0.204029767301
AGTCTTAC 0.207087931949
AGTCTTAG 0.452397694369
AGTCTTAT 0.747643364769
AGTCTTCA -0.198341058292
AGTCTTCC 0.933099878774
AGTCTTCG 0.637887665217
AGTCTTGA 0.348371740735
AGTCTTGC 0.689026974056
AGTCTTGG -0.991392679074
AGTCTTTA -0.863734431394
AGTCTTTC -0.68070824345
AGTCTTTG -0.100403204743
AGTGAAAA 0.656817443008
AGTGAAAC 0.027285885965
AGTGAAAG 0.629547239938
AGTGAAAT 0.903353085102

AGTGAACA -0.439624760021
AGTGAACC -0.536586353318
AGTGAACG 1.78749514582
AGTGAACT -0.624283537401
AGTGAAGA 0.0121152982571
AGTGAAGC -1.41896487242
AGTGAAGG -0.976993689831
AGTGAATA 0.520560263653
AGTGAATC 1.1439370683
AGTGAATG -0.197362445605
AGTGACAA 0.458852774211
AGTGACAC 0.356944272869
AGTGACAG -0.309650148657
AGTGACAT -0.28808721269
AGTGACCA -1.03514377578
AGTGACCC -1.1042530692
AGTGACCG -1.25583348311
AGTGACGA -0.433513396975
AGTGACGC -0.188920865649
AGTGACGG -0.545440132047
AGTGACTA 0.0820346132339
AGTGACTC -0.785082618748
AGTGACTG -0.63958873663
AGTGAGAA 0.181863039852
AGTGAGAC -0.307264257468
AGTGAGAG 0.422522823716
AGTGAGAT 1.59432264716
AGTGAGCA -0.27370834983
AGTGAGCC -0.868630108647
AGTGAGCG -0.511265534175
AGTGAGGA -0.676807384543
AGTGAGGC -0.394148283413
AGTGAGGG -0.888144858445
AGTGAGTA 0.0150357148054
AGTGAGTC -0.912654087738
AGTGAGTG -0.162058679248
AGTGATAA 0.409199158351
AGTGATAC 1.4351569394
AGTGATAG 0.224001412125
AGTGATAT 3.50599861994
AGTGATCA 0.16000578821
AGTGATCC 0.303404958235
AGTGATCG 1.84224988486
AGTGATGA 0.245855004424
AGTGATGC 0.807501318054
AGTGATGG -1.23776103695
AGTGATTA 3.76759951296
AGTGATTC 2.41250192609
AGTGATTG 1.0412114881
AGTGCAAA -0.050250088657
AGTGCAAC -0.713965391927
AGTGCAAG 0.512456650099
AGTGCAAT 1.9878357733
AGTGCACA 0.0092466352645

AGTGCACC -0.367128222529
AGTGCACG -0.313698165401
AGTGCACT -0.444644593531
AGTGCAGA 0.962205503395
AGTGCAGC -0.958552695621
AGTGCAGG -1.10796024434
AGTGCATA -0.287969852354
AGTGCATC -0.139715519223
AGTGCATG -0.505487432661
AGTGCCAA -0.0652601880662
AGTGCCAC -0.849943415739
AGTGCCAG -1.39399116803
AGTGCCAT -0.469216815789
AGTGCCCA -1.41605203594
AGTGCCCC -1.67711709669
AGTGCCCG -0.701975034071
AGTGCCGA -0.0774201825759
AGTGCCGC -0.60427634468
AGTGCCGG -0.928812697871
AGTGCCTA -1.15808460845
AGTGCCTC -1.33620100454
AGTGCCTG -0.527092712447
AGTGCGAA 0.122026519858
AGTGCGAC -0.521985838866
AGTGCGAG -0.387258787362
AGTGCGAT 2.82618011943
AGTGCGCA 3.27639527749
AGTGCGCC 0.0902359835046
AGTGCGCG 0.358647435335
AGTGCGGA 0.652629064347
AGTGCGGC -1.19419029354
AGTGCGGG -0.50917709524
AGTGCGTA 0.35482394538
AGTGCGTC -0.730481049316
AGTGCGTG 0.395670569816
AGTGCTAA 0.748450511131
AGTGCTAC -0.0864828051983
AGTGCTAG -0.0841586000657
AGTGCTAT 0.498901923404
AGTGCTCA -0.853502387521
AGTGCTCC -1.73109056615
AGTGCTCG 0.357245645847
AGTGCTGA -0.688515711659
AGTGCTGC -1.33314728338
AGTGCTGG 0.501156339651
AGTGCTTA 0.611246084881
AGTGCTTC -0.457989169425
AGTGCTTG 0.0918233539241
AGTGGAAA 1.49109364601
AGTGGAAC -1.12584475714
AGTGGAAG -0.0590889686351
AGTGGAAT 1.74089028487
AGTGGACA 0.33993355874
AGTGGACC -1.07957707845

AGTGGACG −0.859793842485
AGTGGAGA 0.479986521601
AGTGGAGC −0.477721127327
AGTGGAGG −1.23282510693
AGTGGATA 3.73739660841
AGTGGATC 2.7480322505
AGTGGATG 0.505427837658
AGTGGCAA −0.522928903657
AGTGGCAC −0.832954396282
AGTGGCAG −0.793332344614
AGTGGCAT −0.0215409799134
AGTGGCCA −0.446791581943
AGTGGCCC −1.1781283946
AGTGGCCG −1.07688275698
AGTGGCGA 0.239911448362
AGTGGCGC −0.554230395048
AGTGGCGG −0.887924775143
AGTGGCTA −0.854529094422
AGTGGCTC −0.495429730311
AGTGGCTG −0.180071791789
AGTGGGAA −0.00439487011921
AGTGGGAC −0.87229415583
AGTGGGAG 0.397639034599
AGTGGGAT 0.490248362976
AGTGGGCA 0.122046907622
AGTGGGCC −0.371626076996
AGTGGGCG −0.463731200279
AGTGGGGA −0.345953176847
AGTGGGGC −1.26716594349
AGTGGGGG −0.657909233925
AGTGGGTA −0.589398242976
AGTGGGTC −1.13829122588
AGTGGGTG −1.07839929299
AGTGGTAA 0.274859997132
AGTGGTAC 0.0416464521134
AGTGGTAG −0.402198836504
AGTGGTAT 2.08554910006
AGTGGTCA −0.762415084165
AGTGGTCC −0.594698538936
AGTGGTCG −0.732953196431
AGTGGTGA 0.402904044044
AGTGGTGC −1.3890398165
AGTGGTGG 0.385561898055
AGTGGTTA 0.756242819208
AGTGGTTC −0.358330640843
AGTGGTTG 0.245623420332
AGTGTAAA 0.65501391001
AGTGTAAC −0.411456188414
AGTGTAAG 0.221469408625
AGTGTAAT 5.32232311003
AGTGTACA 0.787928541542
AGTGTACC 0.710982505177
AGTGTACG −0.400810116096
AGTGTAGA −0.188375885026

AGTGTAGC -0.00187044668568
AGTGTAGG -0.385846281229
AGTGTATA 1.06499120137
AGTGTATC 2.12697180953
AGTGTATG 0.427458492353
AGTGTCAA 0.777720806157
AGTGTCAC -0.148448016892
AGTGTCAG 0.70696507008
AGTGTCAT 0.251621082385
AGTGTCCA -0.304700888177
AGTGTCCC -1.0525862923
AGTGTCCG 0.0134658569526
AGTGTCGA 0.360791809931
AGTGTCGC 0.0890213432379
AGTGTCGG 0.291344288731
AGTGTCTA 0.112074938435
AGTGTCTC 0.441147307806
AGTGTCTG -0.776999131576
AGTGTGAA -0.147021396153
AGTGTGAC -0.245525663616
AGTGTGAG 0.0210041021197
AGTGTGAT 1.14636530331
AGTGTGCA -0.180274362524
AGTGTGCC -0.527282998247
AGTGTGCG 0.0313273681693
AGTGTGGA 0.111826364539
AGTGTGGC -0.61539499492
AGTGTGGG -0.429848827029
AGTGTGTA 1.17569388643
AGTGTGTC -0.0532366347485
AGTGTGTG 0.076731442076
AGTGTTAA -0.420689754599
AGTGTTAC -0.0654483828138
AGTGTTAG 0.206322606642
AGTGTTAT 1.16892959217
AGTGTTCA 0.0764342512038
AGTGTTCC -0.732126185068
AGTGTTCG -0.589555071932
AGTGTTGA 0.73783998671
AGTGTTGC 1.97216908333
AGTGTTGG -0.466006265672
AGTGTTTA -0.351902744687
AGTGTTTC 0.315419101815
AGTGTTTG 0.572641068993
AGTTAAAA 1.83152539807
AGTTAAAC -0.31337980262
AGTTAAAG 0.803814269291
AGTTAAAT 2.32002930029
AGTTAACA 0.564771914729
AGTTAACC -1.12965570078
AGTTAACG -0.584507793355
AGTTAACT 0.500354420921
AGTTAAGA 1.07129938475
AGTTAAGC -0.668694622633

AGTTAAGG 0.181298194227
AGTTAATA 1.66280880686
AGTTAATC 0.641435136209
AGTTAATG 0.634941894654
AGTTACAA 0.306414244525
AGTTACAC 1.30720254637
AGTTACAG 0.155391357552
AGTTACAT 1.41265120002
AGTTACCA 1.15793745062
AGTTACCC -0.0226115989219
AGTTACCG 0.61197899887
AGTTACGA 1.46734190058
AGTTACGC 0.55126083876
AGTTACGG -0.412298359909
AGTTACTA 0.203091407379
AGTTACTC 0.334164344201
AGTTACTG -0.114970785113
AGTTAGAA 0.256909354792
AGTTAGAC -0.480235879641
AGTTAGAG -0.185802583234
AGTTAGAT 2.54527854814
AGTTAGCA 0.143977868874
AGTTAGCC -0.85810061252
AGTTAGCG -0.43207266163
AGTTAGGA 0.0913617540293
AGTTAGGC -0.123306244142
AGTTAGGG -0.766030514371
AGTTAGTA 1.53503790372
AGTTAGTC -1.02051006587
AGTTAGTG -0.346273107918
AGTTATAA 1.41760281293
AGTTATAC 0.781865011328
AGTTATAG 0.253498063611
AGTTATAT 1.34873477473
AGTTATCA 0.587138076334
AGTTATCC 0.813376653521
AGTTATCG 0.450045260024
AGTTATGA 0.141370848857
AGTTATGC 1.86916931384
AGTTATGG -0.617621704718
AGTTATTA 1.25055226801
AGTTATTC 0.955240729445
AGTTATTG 0.91834985405
AGTTCAAA -0.155352673076
AGTTCAAC -0.23997182751
AGTTCAAG 0.164063737454
AGTTCAAT 0.453638734176
AGTTCACA 1.22672498329
AGTTCACC -0.93306642193
AGTTCACG -0.75942461735
AGTTCAGA 0.628850919372
AGTTCAGC 0.834897768433
AGTTCAGG 0.0613459986981
AGTTCATA 0.732848905175

AGTTCATC -0.276300209714
AGTTCATG 0.519611448467
AGTTCCAA 0.736190668853
AGTTCCAC -1.19487406779
AGTTCCAG -0.0202440044447
AGTTCCAT -0.171437312217
AGTTCCCA -0.535723009913
AGTTCCCC -0.970231225235
AGTTCCCG -0.60178198013
AGTTCCGA -0.363685042793
AGTTCCGC -0.250160482039
AGTTCCGG -0.0100258137958
AGTTCCTA -0.306691047633
AGTTCCTC -0.387200237885
AGTTCCTG -0.67492909641
AGTTCGAA 0.872238742932
AGTTCGAC -0.736610970456
AGTTCGAG 0.458882571712
AGTTCGAT 0.20067101382
AGTTCGCA 0.152736243321
AGTTCGCC -0.698429131369
AGTTCGCG -0.510241441091
AGTTCGGA -0.817559543167
AGTTCGGC -0.366001406478
AGTTCGGG 0.130504170474
AGTTCGTA -0.251541099617
AGTTCGTC -0.200574564012
AGTTCGTG 0.0887118674308
AGTTCTAA 0.289159661369
AGTTCTAC -0.020112529503
AGTTCTAG 0.571238233979
AGTTCTAT 0.143341666075
AGTTCTCA -0.739731605305
AGTTCTCC -0.791570632671
AGTTCTCG -0.0621272682821
AGTTCTGA 0.0964320341871
AGTTCTGC 0.0982274643553
AGTTCTGG 0.145875237864
AGTTCTTA 0.139133422413
AGTTCTTC -1.16062366926
AGTTCTTG 0.0512982288484
AGTTGAAA 2.08611002496
AGTTGAAC 0.145309346713
AGTTGAAG -0.123398250463
AGTTGAAT 1.06109478595
AGTTGACA -0.381253283861
AGTTGACC -0.942691799124
AGTTGACG -0.0119341608126
AGTTGAGA 1.52487198939
AGTTGAGC -0.627682020884
AGTTGAGG -0.732773888658
AGTTGATA 2.10811260477
AGTTGATC 0.624085671534
AGTTGATG 0.609867296973

AGTTGCAA 1.43621292104
AGTTGCAC -0.18174960024
AGTTGCAG -0.578661732627
AGTTGCAT 0.139667947773
AGTTGCCA -0.745211993183
AGTTGCCC -0.356839720231
AGTTGCCG -0.788669035597
AGTTGCGA 0.196364490679
AGTTGCGC -0.730580374322
AGTTGCGG -0.128882559065
AGTTGCTA -0.586211739965
AGTTGCTC -0.35654435903
AGTTGCTG 1.41500128193
AGTTGGAA -0.0411456449796
AGTTGGAC -0.918072005416
AGTTGGAG -0.460143476521
AGTTGGAT 4.16304712427
AGTTGGCA -0.692041226597
AGTTGGCC -1.05949042572
AGTTGGCG -0.194787836905
AGTTGGGA -0.25629563081
AGTTGGGC -0.789674047826
AGTTGGGG -0.94645804651
AGTTGGTA 0.191604731855
AGTTGGTC -0.843236102659
AGTTGGTG -0.432157087885
AGTTGTAA 0.336468161569
AGTTGTAC 0.647618901956
AGTTGTAG 0.46416770754
AGTTGTAT 2.20000914554
AGTTGTCA 1.01531850465
AGTTGTCC -0.224351663461
AGTTGTCG 0.658607122781
AGTTGTGA -0.788758166721
AGTTGTGC 0.162286081235
AGTTGTGG -0.389305928005
AGTTGTTA 0.166468970881
AGTTGTTC -0.39232906752
AGTTGTTG 0.245425293084
AGTTTAAA 1.25917498541
AGTTTAAC -0.259448415595
AGTTTAAG 0.420600100712
AGTTTAAT 0.460219799947
AGTTTACA 0.274097285641
AGTTTACC 0.202584327087
AGTTTACG 0.665492698108
AGTTTAGA 2.05169025116
AGTTTAGC 0.515000938533
AGTTTAGG -0.198830364636
AGTTTATA 1.18655925791
AGTTTATC 0.821935593812
AGTTTATG 0.369560639642
AGTTTCAA 0.626923491499
AGTTTCAC -0.320505064869

AGTTTCAG 0.251161573543
AGTTTCAT 0.631518579919
AGTTTCCA 1.56021783818
AGTTTCCC -0.0243733108645
AGTTTCCG -0.216437551562
AGTTTCGA 0.187360417534
AGTTTCGC 0.404759853361
AGTTTCGG -0.29391889743
AGTTTCTA -0.411195852346
AGTTTCTC -0.0321614368353
AGTTTCTG 0.319881408386
AGTTTGAA 0.224904746913
AGTTTGAC 0.576906293842
AGTTTGAG 0.00929525224096
AGTTTGAT 1.58110196615
AGTTTGCA 0.465657844007
AGTTTGCC 0.364122856963
AGTTTGCG 0.258160849864
AGTTTGGA 1.00289399197
AGTTTGGC -1.08223977274
AGTTTGGG -0.705313922551
AGTTTGTA -0.149778710587
AGTTTGTC -0.482773894918
AGTTTGTG 0.646170325163
AGTTTTAA -0.400534881278
AGTTTTAC 0.625833007489
AGTTTTAG 0.158029481978
AGTTTTAT 1.56538509091
AGTTTTCA 0.142372201743
AGTTTTCC 0.594331559179
AGTTTTCG 0.713376237814
AGTTTTGA 0.343295971564
AGTTTTGC -0.119410612867
AGTTTTGG 0.0674944779305
AGTTTTTA 0.948883144937
AGTTTTTC -0.556752204666
AGTTTTTG 0.646590888148
ATAAAAAA 1.80143567175
ATAAAAAC 1.01698716474
ATAAAAAG 1.09490946136
ATAAAAAT 3.49047961057
ATAAAACA 1.19759295913
ATAAAACC 1.08764435996
ATAAAACG 1.32687490927
ATAAAAGA 2.49369593612
ATAAAAGC 1.08434154214
ATAAAAGG 0.0833362935714
ATAAAATA 2.61811252927
ATAAAATC 8.69307139252
ATAAAATG 1.12400881283
ATAAACAA 0.58353284001
ATAAACAC 1.68282828452
ATAAACAG -0.0558687473987
ATAAACAT 0.672604107132

ATAAACCA 0.140182608131
ATAAACCC −0.384551135431
ATAAACCG 0.168171871965
ATAAACGA 1.07054163951
ATAAACGC 0.134313545822
ATAAACGG 0.937871661151
ATAAACTA 0.987121181067
ATAAACTC 0.332110407636
ATAAACTG 0.789403256495
ATAAAGAA 0.388604379807
ATAAAGAC 0.260809168173
ATAAAGAG 0.266470954877
ATAAAGAT 4.65991706717
ATAAAGCA −0.40488766896
ATAAAGCC −0.437681126489
ATAAAGCG 1.34343447877
ATAAAGGA 0.58471533034
ATAAAGGC −1.20261775889
ATAAAGGG 0.12444900447
ATAAAGTA 2.05484251318
ATAAAGTC 0.127535921094
ATAAAGTG 0.228764830291
ATAAATAA 1.95636359973
ATAAATAC 1.25437497382
ATAAATAG 0.546374832626
ATAAATAT 3.47928385276
ATAAATCA 1.28092977546
ATAAATCC 3.18207625211
ATAAATCG 2.62364074998
ATAAATGA 3.01964797929
ATAAATGC 1.12886266902
ATAAATGG −0.668532566044
ATAAATTA 0.285514433661
ATAAATTC 2.06104980327
ATAAATTG 1.54581126887
ATAACAAA 0.570652216446
ATAACAAC 1.3310026474
ATAACAAG −0.0380059292741
ATAACAAT 2.04491628577
ATAACACA 2.10899032417
ATAACACC 0.534982777239
ATAACACG 1.65843118793
ATAACAGA 0.956553649191
ATAACAGC 0.60467155365
ATAACAGG 0.0304977429904
ATAACATA 1.22853635774
ATAACATC 1.87947898804
ATAACATG 1.28107196705
ATAACCAA 1.36785954319
ATAACCAC −0.435874979676
ATAACCAG 0.194791496247
ATAACCAT 0.755652619569
ATAACCCA 0.235388501261
ATAACCCC −0.475027590002

ATAACCCG 1.1856689922
ATAACCGA 0.0219654636219
ATAACCGC 0.893051513725
ATAACCGG -0.72892530607
ATAACCTA -0.340048043878
ATAACCTC 0.0759172384112
ATAACCTG -0.22550121971
ATAACGAA 2.42162048437
ATAACGAC -0.0112381016281
ATAACGAG 0.158849958802
ATAACGAT 2.76609162758
ATAACGCA 2.69896465217
ATAACGCC 0.086732686002
ATAACGCG 2.23239981404
ATAACGGA 1.01686797474
ATAACGGC 0.79546208184
ATAACGGG 0.271930432228
ATAACGTA 2.17608724069
ATAACGTC 0.941443702013
ATAACGTG 1.16918600751
ATAACTAA 1.13748904577
ATAACTAC -0.0606865329368
ATAACTAG 0.452750298139
ATAACTAT 1.02829610078
ATAACTCA 0.874145260278
ATAACTCC 1.18546067107
ATAACTCG 1.28515161097
ATAACTGA 0.70699303791
ATAACTGC 0.726785113579
ATAACTGG 0.20698311793
ATAACTTA 2.29507206294
ATAACTTC 0.164714839005
ATAACTTG 1.57925112308
ATAAGAAA 1.87947898804
ATAAGAAC 0.0170922651859
ATAAGAAG 0.381156572672
ATAAGAAT 3.56931595862
ATAAGACA -0.202363721022
ATAAGACC -0.69802268299
ATAAGACG -0.303713388516
ATAAGAGA -0.307974431259
ATAAGAGC 1.01956412588
ATAAGAGG 0.339421250816
ATAAGATA 3.52629751452
ATAAGATC 4.82165607673
ATAAGATG 0.0317931501695
ATAAGCAA 0.70696507008
ATAAGCAC -0.198905119772
ATAAGCAG 0.10040764823
ATAAGCAT 0.702554255683
ATAAGCCA -0.492626412717
ATAAGCCC -0.748608908377
ATAAGCCG -0.246705017368
ATAAGCGA 1.06334841805

ATAAGCGC	0.0199387107431
ATAAGCGG	-0.345953176847
ATAAGCTA	0.198211935785
ATAAGCTC	-0.224900042044
ATAAGCTG	0.254074932788
ATAAGGAA	2.72043035419
ATAAGGAC	-0.528615521613
ATAAGGAG	0.287731210959
ATAAGGAT	2.11059102505
ATAAGGCA	0.167690407069
ATAAGGCC	-0.504104985411
ATAAGGCG	0.486371289794
ATAAGGGA	0.476410560015
ATAAGGGC	-0.674621188892
ATAAGGGG	-0.00372573323892
ATAAGGTA	-0.145646528969
ATAAGGTC	-0.89055636503
ATAAGGTG	-0.194815543354
ATAAGTAA	2.09826583737
ATAAGTAC	1.12629799282
ATAAGTAG	0.967376676848
ATAAGTAT	1.23937271586
ATAAGTCA	-0.313320207616
ATAAGTCC	0.24260394016
ATAAGTCG	0.126535875115
ATAAGTGA	0.412660373417
ATAAGTGC	0.315920693094
ATAAGTGG	1.74186915894
ATAAGTTA	0.394752336277
ATAAGTTC	0.775229316804
ATAAGTTG	0.61369758285
ATAATAAA	2.48544359644
ATAATAAC	1.8525179994
ATAATAAG	2.02354677218
ATAATAAT	3.24794702758
ATAATACA	2.52678423209
ATAATACC	1.00990503046
ATAATACG	1.58175542013
ATAATAGA	0.819215918327
ATAATAGC	0.491350870539
ATAATAGG	1.40841289748
ATAATATA	2.90779024896
ATAATATC	6.4717549422
ATAATATG	1.54089964734
ATAATCAA	2.80601557499
ATAATCAC	3.32714721881
ATAATCAG	2.3176860143
ATAATCAT	2.8184965461
ATAATCCA	4.6131151245
ATAATCCC	2.91873351215
ATAATCCG	3.66877818962
ATAATCGA	3.14699518287
ATAATCGC	4.03170287336
ATAATCGG	0.978431549979

ATAATCTA 7.07554459436
ATAATCTC 5.32360231155
ATAATCTG 6.14395889916
ATAATGAA 0.359940751461
ATAATGAC 1.09170361611
ATAATGAG 0.653993999031
ATAATGAT 1.63579345085
ATAATGCA 2.68265339519
ATAATGCC 0.0772168276958
ATAATGCG 0.857883665797
ATAATGGA 1.72489164027
ATAATGGC -0.0478634133234
ATAATGGG 0.81570530214
ATAATGTA 1.8310379214
ATAATGTC 1.40200277527
ATAATGTG 1.09162520164
ATAATTAA 1.44240348271
ATAATTAC 2.3195988048
ATAATTAG 0.771011924787
ATAATTAT 4.00461694431
ATAATTCA 1.97600067612
ATAATTCC 2.34222425695
ATAATTCG 1.65143687786
ATAATTGA 1.35355177612
ATAATTGC 1.64232537688
ATAATTGG 1.08985277305
ATAATTTA 2.82496469502
ATAATTTC 2.44154037564
ATAATTTG 1.78836188718
ATACAAAA 1.98865311355
ATACAAAC 2.1516234918
ATACAAAG 0.603890545448
ATACAAAT 2.83642941449
ATACAACA 1.41454673934
ATACAACC 0.99804849998
ATACAACG -0.185579624735
ATACAAGA 1.53298736511
ATACAAGC 0.238093016613
ATACAAGG 1.09163931624
ATACAATA 2.15108217051
ATACAATC 4.31648282474
ATACAATG -0.192014578194
ATACACAA 1.89954891234
ATACACAC 0.638167866285
ATACACAG 0.734570887116
ATACACAT 2.31867612778
ATACACCA 1.99348736613
ATACACCC 0.973021996512
ATACACCG 0.616846969674
ATACACGA 1.00557655126
ATACACGC 0.645551112167
ATACACGG 0.805305189902
ATACACTA 2.10309773751
ATACACTC 0.561427798617

ATACACTG -0.217031671924
ATACAGAA 2.65383476756
ATACAGAC -0.283597983815
ATACAGAG 1.27715830045
ATACAGAT 3.2481441093
ATACAGCA 0.415398606994
ATACAGCC -0.438903085441
ATACAGCG 1.09294909941
ATACAGGA 1.83873430243
ATACAGGC 1.17262918724
ATACAGGG -0.580283866798
ATACAGTA 1.34754078361
ATACAGTC -0.592931599362
ATACAGTG 1.75090381373
ATACATAA 1.0547377242
ATACATAC 1.96489117423
ATACATAG 2.90437712811
ATACATAT 2.22220776155
ATACATCA 1.32319465643
ATACATCC 0.662561564914
ATACATCG 1.05560864767
ATACATGA 2.01193724731
ATACATGC 0.962319727152
ATACATGG -0.833051368854
ATACATTA 0.858372710759
ATACATTC 2.49000575078
ATACATTG 1.1439370683
ATACCAAA 1.41824084541
ATACCAAC 2.25808029425
ATACCAAG -0.221114713802
ATACCAAT 1.72853033344
ATACCACA 0.718317918228
ATACCACC 1.59278284819
ATACCACG 0.771992105764
ATACCAGA -0.00259133712156
ATACCAGC 1.47865318905
ATACCAGG 0.626946754461
ATACCATA 1.02593477947
ATACCATC 0.674943211016
ATACCATG 1.42035908184
ATACCCAA 1.50134032725
ATACCCAC 1.24515918158
ATACCCAG 1.57305637931
ATACCCAT 2.41672585265
ATACCCCA 0.282258141765
ATACCCCC 0.309565199639
ATACCCCG 0.0285282326806
ATACCCGA 0.732860667347
ATACCCGC 0.394700321339
ATACCCGG 0.512605376225
ATACCCTA 2.27269701436
ATACCCTC 0.626371192191
ATACCCTG 1.04599241883
ATACCGAA 1.35412550872

ATACCGAC 1.2299739565
ATACCGAG 0.901300716827
ATACCGAT 3.83821933061
ATACCGCA 1.43094399087
ATACCGCC 1.58476679747
ATACCGCG 0.179772509864
ATACCGGA -0.228306105594
ATACCGGC -0.824081013934
ATACCGGG 1.89309644632
ATACCGTA 2.53585678721
ATACCGTC 0.457687012302
ATACCGTG 1.25319771112
ATACCTAA 1.839490218
ATACCTAC 0.295688973584
ATACCTAG 1.43060680861
ATACCTAT 1.15347645095
ATACCTCA 0.809776644828
ATACCTCC -0.351756893757
ATACCTCG 1.08893244846
ATACCTGA 0.263560209448
ATACCTGC 0.451794164268
ATACCTGG 0.0944230552567
ATACCTTA 1.89091443277
ATACCTTC 1.43386205498
ATACCTTG -0.375756690324
ATACGAAA 2.1088747935
ATACGAAC 2.09820990171
ATACGAAG 1.34228126318
ATACGAAT 4.48653089379
ATACGACA 1.04681707776
ATACGACC -0.146645529421
ATACGACG 1.19935963732
ATACGAGA 2.38596228458
ATACGAGC -0.274938150229
ATACGAGG 0.17435511495
ATACGATA 3.67726760241
ATACGATC 2.39701454389
ATACGATG 0.525817170269
ATACGCAA 8.61376508036
ATACGCAC 2.39292052399
ATACGCAG 1.66680820663
ATACGCAT 1.10856220615
ATACGCCA 0.670094582449
ATACGCCC 1.04232549645
ATACGCCG 0.624946662505
ATACGCGA 2.8411410791
ATACGCGC 2.02466391712
ATACGCGG 1.39106186451
ATACGCTA 1.05259570204
ATACGCTC 1.61200249817
ATACGCTG 1.36100193569
ATACGGAA 1.559680699
ATACGGAC 1.48064465541
ATACGGAG 0.473580058735

ATACGGAT 6.2831500869
ATACGGCA 0.849829714745
ATACGGCC 1.17785472807
ATACGGCG 2.53506689203
ATACGGGA 1.87929445264
ATACGGGC 0.00098854518799
ATACGGGG 0.215743060667
ATACGGTA 1.69799155354
ATACGGTC 0.64890359249
ATACGGTG 1.17136958934
ATACGTAA 3.38741658672
ATACGTAC 2.40173666377
ATACGTAG 0.640523698591
ATACGTAT 1.97529154785
ATACGTCA 0.530282613418
ATACGTCC 1.30338402267
ATACGTCG 2.23624264623
ATACGTGA 1.41292643484
ATACGTGC 0.421461353064
ATACGTGG 0.982380764481
ATACGTTA 2.62012464478
ATACGTTC 1.16450936803
ATACGTTG 0.53348401518
ATACTAAA 2.03796118294
ATACTAAC 0.700678058603
ATACTAAG 0.538559000207
ATACTAAT 1.53984915471
ATACTACA 0.550905621174
ATACTACC 0.745822057823
ATACTACG 0.911896603879
ATACTAGA 0.919718186694
ATACTAGC 0.313195789978
ATACTAGG 2.32345810404
ATACTATA 1.19394773142
ATACTATC 2.25962767329
ATACTATG 0.728005242859
ATACTCAA 1.4874484183
ATACTCAC 1.14194324951
ATACTCAG 1.48416389719
ATACTCAT 3.10762850102
ATACTCCA 0.768595713334
ATACTCCC 1.41477963034
ATACTCCG 0.620577930545
ATACTCGA 2.30853582884
ATACTCGC 1.12905556864
ATACTCGG -0.0669089831603
ATACTCTA 1.71694485567
ATACTCTC 1.83920112995
ATACTCTG 1.79081050996
ATACTGAA 0.634374173832
ATACTGAC 0.826678885595
ATACTGAG 1.33681838786
ATACTGAT 1.89628869972
ATACTGCA 2.23761568374

ATACTGCC 0.980269846728
ATACTGCG 1.57556642675
ATACTGGA 0.135990047366
ATACTGGC -0.0101988484109
ATACTGGG 0.193118915428
ATACTGTA 2.57304825144
ATACTGTC 0.351379981499
ATACTGTG 1.08829258631
ATACTTAA 0.853031900652
ATACTTAC 2.38249165977
ATACTTAG 0.841592796578
ATACTTCA -0.32341946964
ATACTTCC 1.27142071308
ATACTTCG 0.0175133509336
ATACTTGA 1.21078358126
ATACTTGC 1.59308160736
ATACTTGG 0.668020780884
ATACTTTA 1.25917498541
ATACTTTC 1.01331736717
ATACTTTG 1.2662741095
ATAGAAAA 0.751924272514
ATAGAAAC -0.277158064105
ATAGAAAG 0.573671435237
ATAGAAAT 2.66042602721
ATAGAACA 0.323841339533
ATAGAACC -0.751601204864
ATAGAACG 0.681698095546
ATAGAAGA 1.06502596512
ATAGAAGC 0.547309533206
ATAGAAGG -0.545382628096
ATAGAATA 3.46037498549
ATAGAATC 7.17503740701
ATAGAATG 0.91149982662
ATAGACAA 0.475933277225
ATAGACAC 0.537859804443
ATAGACAG -1.00795956726
ATAGACAT 0.21798702165
ATAGACCA -0.407914206435
ATAGACCC -1.33860885178
ATAGACCG -1.21414259613
ATAGACGA -0.376585531358
ATAGACGC -0.846623869497
ATAGACGG -0.0607965745878
ATAGACTA 0.451868396641
ATAGACTC -0.127000611589
ATAGACTG -0.560334962173
ATAGAGAA 3.31399423563
ATAGAGAC 0.811906643437
ATAGAGAG 0.160084202688
ATAGAGAT 2.83268120244
ATAGAGCA 0.0128173692183
ATAGAGCC -0.693275993246
ATAGAGCG -0.444404645228
ATAGAGGA 0.273878770629

ATAGAGGC -1.16501906214
ATAGAGGG 0.294379974562
ATAGAGTA 0.151495464895
ATAGAGTC -0.433828623177
ATAGAGTG -0.146810722588
ATAGATAA 0.20283577618
ATAGATAC 3.76281544565
ATAGATAG 0.823805256352
ATAGATAT 7.33479723514
ATAGATCA 1.6127944844
ATAGATCC 2.85821635449
ATAGATCG 0.752298048193
ATAGATGA 0.200352128275
ATAGATGC -0.109985715355
ATAGATGG -0.143030621978
ATAGATTA 4.56433007967
ATAGATTC 8.55557291197
ATAGATTG 4.91171458213
ATAGCAAA 0.76900346862
ATAGCAAC 0.695946267609
ATAGCAAG 0.241499080163
ATAGCAAT 3.10450080887
ATAGCACA 0.284543662422
ATAGCACC -0.267458193157
ATAGCACG -0.154899437392
ATAGCAGA 0.788411836109
ATAGCAGC -1.00645296375
ATAGCAGG -0.615780532771
ATAGCATA 1.48604871987
ATAGCATC 1.10404840741
ATAGCATG 0.15019090936
ATAGCCAA -0.381889486661
ATAGCCAC 1.27013889774
ATAGCCAG -0.633733788927
ATAGCCAT -0.0280551319957
ATAGCCCA 0.210691338602
ATAGCCCC -0.320024645499
ATAGCCCG -0.554219678403
ATAGCCGA 1.45916536156
ATAGCCGC 0.116435044802
ATAGCCGG -0.346546513065
ATAGCCTA 0.57254697162
ATAGCCTC 0.558941014133
ATAGCCTG 0.00826227218201
ATAGCGAA 1.24825524656
ATAGCGAC -0.16456480597
ATAGCGAG -0.246084758845
ATAGCGAT 3.32502584579
ATAGCGCA 1.10973973024
ATAGCGCC 0.454778096544
ATAGCGCG 0.399093100406
ATAGCGGA 0.894484930386
ATAGCGGC 0.61181171465
ATAGCGGG -0.362114923559

ATAGCGTA 1.30130159551
ATAGCGTC 0.314941296262
ATAGCGTG 1.54508593495
ATAGCTAA 0.183570907186
ATAGCTAC 0.681949283257
ATAGCTAG 1.52363304063
ATAGCTAT 1.20256182323
ATAGCTCA 0.492660392324
ATAGCTCC 0.0298445503873
ATAGCTCG 0.0301582082999
ATAGCTGA 0.385175053296
ATAGCTGC -0.226636661354
ATAGCTGG 0.144409148504
ATAGCTTA -0.564556013532
ATAGCTTC -0.726123818147
ATAGCTTG 0.291337492809
ATAGGAAA 1.92975260104
ATAGGAAC -0.178810625599
ATAGGAAG -0.203921032558
ATAGGAAT 2.82771756597
ATAGGACA -0.153376366845
ATAGGACC -0.469681290881
ATAGGACG -0.758039294902
ATAGGAGA 0.883735350957
ATAGGAGC -0.28715747836
ATAGGAGG -0.570580597889
ATAGGATA 2.02171396445
ATAGGATC 3.87736723606
ATAGGATG 0.666730601336
ATAGGCAA -0.221535015405
ATAGGCAC -0.0722500546493
ATAGGCAG -0.45126643483
ATAGGCAT -0.592846388962
ATAGGCCA -0.861700359831
ATAGGCCC -0.159667037664
ATAGGCCG -0.556164618843
ATAGGCGA 0.643674130942
ATAGGCGC -0.295016961506
ATAGGCGG -0.0882330163508
ATAGGCTA 0.686613899182
ATAGGCTC -0.509825060212
ATAGGCTG -0.192132461293
ATAGGGAA 0.37572480177
ATAGGGAC -0.498155940335
ATAGGGAG 0.199800613112
ATAGGGAT 1.78711405146
ATAGGGCA -0.0906421705014
ATAGGGCC -0.570403119787
ATAGGGCG -0.434326032351
ATAGGGGA -0.125519100715
ATAGGGGC -0.946974013776
ATAGGGGG -0.756123629201
ATAGGGTA -0.810786361925
ATAGGGTC 0.59944836526

ATAGGGTG 0.768461885957
ATAGGTAA 1.25774235289
ATAGGTAC -0.435089266605
ATAGGTAG 0.0196483157923
ATAGGTCA 1.00964391025
ATAGGTCC -0.28811674881
ATAGGTCG -0.339403999631
ATAGGTGA -0.140910817252
ATAGGTGC -0.78750353507
ATAGGTGG -0.584453948747
ATAGGTTA 0.111514797679
ATAGGTTC 0.744501558011
ATAGGTTG 0.254546987947
ATAGTAAA 1.75072894944
ATAGTAAC 0.147449539204
ATAGTAAG 0.502765404743
ATAGTAAT 2.74160592263
ATAGTACA 0.740691137136
ATAGTACC -0.398729257227
ATAGTACG -0.164895976449
ATAGTAGA 1.58033481117
ATAGTAGC 0.817108659918
ATAGTAGG 2.32478278595
ATAGTATA 1.25785108763
ATAGTATC 2.33306596866
ATAGTATG 0.599453592892
ATAGTCAA 1.77954783846
ATAGTCAC 0.221115759328
ATAGTCAG -0.352050686669
ATAGTCAT 0.63849668433
ATAGTCCA 0.275045578064
ATAGTCCC -0.820104092983
ATAGTCCG -0.96190622147
ATAGTCGA -0.725839957736
ATAGTCGC -0.0218703207217
ATAGTCGG 0.227813662671
ATAGTCTA 0.291023834897
ATAGTCTC -0.180274362524
ATAGTCTG 1.30012799215
ATAGTGAA 0.327296020059
ATAGTGAC -0.0029517823395
ATAGTGAG 1.35000561204
ATAGTGAT 1.4006106569
ATAGTGCA 0.528665184116
ATAGTGCC -0.791013367113
ATAGTGCG 1.19705399028
ATAGTGGA 2.2911262464
ATAGTGGC 0.0199345286376
ATAGTGGG -0.126916446716
ATAGTGTA 1.47229978665
ATAGTGTC 0.469609410943
ATAGTGTG 1.14636530331
ATAGTTAA 1.50395884806
ATAGTTAC 0.703089826569

ATAGTTAG 0.197671398649
ATAGTTCA 0.55268589121
ATAGTTCC 0.12759107261
ATAGTTCG 0.522064776107
ATAGTTGA 0.186025280352
ATAGTTGC 0.508990207401
ATAGTTGG -0.454982758332
ATAGTTTA -0.129397480805
ATAGTTTC 0.201766202698
ATAGTTTG 1.28203306717
ATATAAAA 2.27739116641
ATATAAAC 1.00047020045
ATATAAAG 1.70214856641
ATATAAAT 4.8507794753
ATATAACA 2.40915101406
ATATAACC 0.100330279278
ATATAACG 1.96921913066
ATATAAGA 1.78896646281
ATATAAGC -0.106653622797
ATATAAGG 1.49068353829
ATATAATA 2.86317868404
ATATAATC 4.39946834421
ATATAATG 1.23214368511
ATATACAA 2.03369256013
ATATACAC 1.35000561204
ATATACAG 2.4655686628
ATATACAT 1.16618482405
ATATACCA 2.72331470008
ATATACCC 0.735493825524
ATATACCG 1.1401512173
ATATACGA 3.34262100917
ATATACGC 0.830107166581
ATATACGG 0.627805654378
ATATACTA 1.70459901885
ATATACTC 2.56711750307
ATATACTG 2.55660368984
ATATAGAA 2.55119622743
ATATAGAC 1.00519676381
ATATAGAG 2.18568883216
ATATAGAT 7.8888200932
ATATAGCA 0.89931839882
ATATAGCC 0.414865911306
ATATAGCG 1.19491066121
ATATAGGA 0.761085697379
ATATAGGC -0.345448710371
ATATAGGG 0.475811212021
ATATAGTA 2.50120072444
ATATAGTC 0.718317918228
ATATAGTG 0.660133591289
ATATATAA 2.97544443104
ATATATAC 2.9186475176
ATATATAG 2.07761303211
ATATATAT 4.13291244032
ATATATCA 3.6719424752

ATATATCC 2.68241972005
ATATATCG 4.31358567115
ATATATGA 2.90084899935
ATATATGC 2.66113803067
ATATATGG 1.91831794046
ATATATTA 2.76956329791
ATATATTC 3.15584556364
ATATATTG 3.66741691428
ATATCAAA 3.76752684888
ATATCAAC 5.38037413236
ATATCAAG 2.62365904669
ATATCAAT 2.87869429546
ATATCACA 4.73571014932
ATATCACC 3.26571313452
ATATCACG 6.23219662038
ATATCAGA 5.67279452911
ATATCAGC 2.38660606744
ATATCAGG 3.00235654133
ATATCATA 4.24206121181
ATATCATC 3.58740251939
ATATCATG 5.00616273002
ATATCCAA 7.72795958718
ATATCCAC 6.73473775971
ATATCCAG 8.02386367782
ATATCCAT 9.85185781055
ATATCCCA 4.69264857726
ATATCCCC 4.56239115101
ATATCCCG 3.29513607639
ATATCCGA 8.81499283697
ATATCCGC 8.79132943852
ATATCCGG 9.9849028715
ATATCCTA 13.1499197925
ATATCCTC 7.19312004705
ATATCCTG 3.6058009084
ATATCGAA 5.22319178813
ATATCGAC 4.00073934837
ATATCGAG 2.58888327116
ATATCGAT 5.57880719697
ATATCGCA 4.71685355838
ATATCGCC 4.27421611411
ATATCGCG 9.50372837055
ATATCGGA 5.24728620483
ATATCGGC 4.82634735357
ATATCGGG 3.77540044663
ATATCGTA 6.00906613172
ATATCGTC 3.99119552223
ATATCGTG 3.78802831419
ATATCTAA 10.9943857707
ATATCTAC 5.15131263396
ATATCTAG 8.58651657196
ATATCTCA 6.71236297251
ATATCTCC 7.59515395177
ATATCTCG 9.48993552523
ATATCTGA 6.79918243721

ATATCTGC 10.9236036351
ATATCTGG 6.47299807307
ATATCTTA 7.74797566687
ATATCTTC 7.44134264596
ATATCTTG 9.01620595622
ATATGAAA 2.25797521885
ATATGAAC 1.55201646791
ATATGAAG 0.94711411431
ATATGAAT 1.41592918659
ATATGACA 0.359516267753
ATATGACC 0.80990811977
ATATGACG 2.27066451109
ATATGAGA 2.27513936398
ATATGAGC 1.05541156595
ATATGAGG 1.03211410173
ATATGATA 2.65957287769
ATATGATC 3.48815854201
ATATGATG 1.96746003254
ATATGCAA 3.82826696504
ATATGCAC 2.59204807949
ATATGCAG 2.32794942396
ATATGCAT 1.39896525977
ATATGCCA 0.979641485377
ATATGCCC 1.46473697161
ATATGCCG 1.07576404376
ATATGCGA 2.55035928356
ATATGCGC 2.62747966145
ATATGCGG 1.39138806874
ATATGCTA 3.22344642388
ATATGCTC 0.738146325938
ATATGCTG 3.01431788583
ATATGGAA 2.79765031846
ATATGGAC 0.897463635029
ATATGGAG 0.0462316080329
ATATGGAT 4.65326255317
ATATGGCA 1.24896646588
ATATGGCC -0.0638597054863
ATATGGCG 1.15696092898
ATATGGGA 2.46738631041
ATATGGGC 0.848090743001
ATATGGGG 0.438452986336
ATATGGTA 2.1615923244
ATATGGTC 1.88223917767
ATATGGTG 1.87465963421
ATATGTAA 2.03369256013
ATATGTAC 1.83233306719
ATATGTAG 2.26078846895
ATATGTCA 1.32479248211
ATATGTCC 0.755516178377
ATATGTCG 1.53766923222
ATATGTGA 0.937727639893
ATATGTGC 1.27196229574
ATATGTGG 1.38671874795
ATATGTTA 2.66836653865

ATATGTTC 1.98711775807
ATATGTTG 2.10714706117
ATATTAAA 1.65888912848
ATATTAAC 1.17483995277
ATATTAAG 2.76956329791
ATATTAAT 2.17798931455
ATATTACA 4.97590127599
ATATTACC 2.9850347831
ATATTACG 5.82875360749
ATATTAGA 2.18332463564
ATATTAGC 1.50761191722
ATATTAGG 1.3815687464
ATATTATA 4.27836920625
ATATTATC 2.46684603465
ATATTATG 2.81277699406
ATATTCAA 4.85475587348
ATATTCAC 4.15442806622
ATATTCAG 2.41021562129
ATATTCAT 1.60143667
ATATTCCA 4.49664479318
ATATTCCC 7.85387311272
ATATTCCG 5.13105242386
ATATTCGA 3.93785825557
ATATTCGC 5.23869250079
ATATTCGG 2.72676206192
ATATTCTA 3.42603127373
ATATTCTC 11.4461266131
ATATTCTG 5.72075413086
ATATTGAA 2.72265235912
ATATTGAC 2.31078031259
ATATTGAG 1.69394615061
ATATTGAT 3.89040834793
ATATTGCA 2.22652003508
ATATTGCC 3.07073788701
ATATTGCG 3.90762921286
ATATTGGA 3.68098732387
ATATTGGC 1.87522996885
ATATTGGG 1.48127092571
ATATTGTA 2.85589920666
ATATTGTC 4.27861124561
ATATTGTG 3.37088681473
ATATTTAA 4.89867007231
ATATTTAC 2.84776840942
ATATTTAG 2.40505594863
ATATTTCA 4.6298009413
ATATTTCC 5.5287646453
ATATTTCG 3.78751077863
ATATTTGA 2.73211437282
ATATTTGC 4.96643168222
ATATTTGG 1.70143107393
ATATTTTA 4.4872747858
ATATTTTC 5.63635898045
ATATTTTG 3.27407786828
ATCAAAAA 1.79092290404

ATCAAAAC 0.605079047555
ATCAAAAG 1.93711937988
ATCAAAAT 3.01501002429
ATCAAACA 1.25467033502
ATCAAACC -0.0510415521232
ATCAAACG 1.08422601148
ATCAAAGA 1.59994261281
ATCAAAGC 1.82390403355
ATCAAAGG 0.537482369421
ATCAAATA 1.24230855392
ATCAAATC 5.40341073775
ATCAAATG 0.704463125463
ATCAACAA 0.740504510678
ATCAACAC 1.08217416596
ATCAACAG 0.707543507547
ATCAACAT 1.4021088962
ATCAACCA 0.675101608261
ATCAACCC 0.107339226717
ATCAACCG 0.0296422410336
ATCAACGA 1.40607666879
ATCAACGC 2.17983780518
ATCAACGG 1.41630975819
ATCAACTA 1.08504570415
ATCAACTC 1.24155865013
ATCAACTG 1.60406590746
ATCAAGAA 0.890759458528
ATCAAGAC 1.62259211207
ATCAAGAG 1.72540760754
ATCAAGAT 3.70594560674
ATCAAGCA 1.36084327706
ATCAAGCC -0.566055298355
ATCAAGCG -0.200295669851
ATCAAGGA 0.647459459184
ATCAAGGC 1.35870047076
ATCAAGGG 0.287883596428
ATCAAGTA 0.883479719758
ATCAAGTC 1.15375194715
ATCAAGTG 0.638940248895
ATCAATAA 0.121618241809
ATCAATAC 1.95021773432
ATCAATAG 1.71027465878
ATCAATCA 1.6700307803
ATCAATCC 0.373376288149
ATCAATCG 0.651201920845
ATCAATGA 1.49084899284
ATCAATGC 1.59798094395
ATCAATGG 0.557499756024
ATCAATTA 2.57142977661
ATCAATTC 0.823594321406
ATCAATTG 1.54078490082
ATCACAAA 2.53659283778
ATCACAAC 0.921878244186
ATCACAAG 1.05500302652
ATCACAAT 2.99672977976

ATCACACA	2.90823825701
ATCACACC	0.568722436138
ATCACACG	1.46749271776
ATCACAGA	2.12818305184
ATCACAGC	1.27206841667
ATCACAGG	0.94802424502
ATCACATA	2.59406855921
ATCACATC	2.04960390328
ATCACATG	2.08764956255
ATCACCAA	0.76205176375
ATCACCAC	1.27532366304
ATCACCAG	1.25058101998
ATCACCAT	1.59010865311
ATCACCCA	1.04427226656
ATCACCCC	0.524751778892
ATCACCCG	0.125513873083
ATCACCGA	1.93178562708
ATCACCGC	1.97618521152
ATCACCGG	0.201254417537
ATCACCTA	1.27757938619
ATCACCTC	2.41270266715
ATCACCTG	0.646570500383
ATCACGAA	2.99809549859
ATCACGAC	1.51395146639
ATCACGAG	1.26262992732
ATCACGAT	2.91564162928
ATCACGCA	2.35914557857
ATCACGCC	2.65903913647
ATCACGCG	2.03198521556
ATCACGGA	0.463338343743
ATCACGGC	1.59999358222
ATCACGGG	0.79997666473
ATCACGTA	2.31991586068
ATCACGTC	0.616687004139
ATCACGTG	2.44975507637
ATCACTAA	0.397323547015
ATCACTAC	1.94945711388
ATCACTAG	0.0863447957167
ATCACTCA	2.5410473029
ATCACTCC	1.03855193038
ATCACTCG	1.31179162302
ATCACTGA	0.835859129935
ATCACTGC	1.18576204405
ATCACTGG	0.201307216619
ATCACTTA	3.03512229241
ATCACTTC	0.43886858307
ATCACTTG	1.35964092173
ATCAGAAA	4.86184192849
ATCAGAAC	1.51016639953
ATCAGAAG	1.60785489504
ATCAGAAT	5.0647540281
ATCAGACA	2.37929391735
ATCAGACC	0.541234502201
ATCAGACG	0.861753681676

ATCAGAGA 2.13401186138
ATCAGAGC 1.94352139926
ATCAGAGG -0.0855026242213
ATCAGATA 5.35045273578
ATCAGATC 4.41054569616
ATCAGATG 2.49819718855
ATCAGCAA 1.59654674315
ATCAGCAC 0.612369502971
ATCAGCAG 2.30394701358
ATCAGCAT 2.9599604468
ATCAGCCA 1.46602506011
ATCAGCCC -0.272142151319
ATCAGCCG 0.459573664647
ATCAGCGA 0.991656674484
ATCAGCGC 0.382975527183
ATCAGCGG 0.460894687222
ATCAGCTA 0.62271394093
ATCAGCTC 0.342511304019
ATCAGCTG 1.04817678481
ATCAGGAA 5.14357312496
ATCAGGAC 0.394219640588
ATCAGGAG 0.0137434442053
ATCAGGAT 5.39605598246
ATCAGGCA 0.206169959791
ATCAGGCC 0.0814235030675
ATCAGGCG 0.95552851058
ATCAGGGA 0.0288643694103
ATCAGGGC 0.59448263774
ATCAGGGG 0.803736900339
ATCAGGTA 1.44568486723
ATCAGGTC 0.31726158067
ATCAGGTG 1.27460015879
ATCAGTAA 4.21948150144
ATCAGTAC 1.30206875049
ATCAGTAG 1.05118162762
ATCAGTCA 0.00483974159197
ATCAGTCC -0.689312664138
ATCAGTCG 1.24538972015
ATCAGTGA 1.90876679563
ATCAGTGC 0.881859676639
ATCAGTGG 0.247739827098
ATCAGTTA 1.52413802987
ATCAGTTC 1.318381053
ATCAGTTG 1.62129382969
ATCATAAA 1.90987766741
ATCATAAC 1.30153971414
ATCATAAG 0.522728162593
ATCATAAT 3.34772370064
ATCATACA -0.418462522037
ATCATACC 1.01920420342
ATCATACG 2.14682269606
ATCATAGA 2.69323673593
ATCATAGC 0.92460288592
ATCATAGG 0.517200987409

ATCATATA 3.95105776468
ATCATATC 3.52981780182
ATCATATG 1.87829937291
ATCATCAA 0.678619281752
ATCATCAC 1.53240396139
ATCATCAG 1.20998244668
ATCATCAT 1.48700459236
ATCATCCA 1.81082920347
ATCATCCC 0.623944002711
ATCATCCG 0.778200964145
ATCATCGA 1.84187244984
ATCATCGC 0.227272864154
ATCATCGG 1.26468595493
ATCATCTA 1.50643517728
ATCATCTC 1.459559525
ATCATCTG 1.65531055308
ATCATGAA 1.57984576621
ATCATGAC 0.778200964145
ATCATGAG -0.153712242193
ATCATGAT 2.4009906807
ATCATGCA -0.205617399102
ATCATGCC 0.803080832538
ATCATGCG 0.352677479731
ATCATGGA 1.54675328813
ATCATGGC 1.09889892863
ATCATGGG 0.107274142701
ATCATGTA 3.23776464621
ATCATGTC 1.68359596226
ATCATGTG 1.74338700185
ATCATTAA 1.7969289303
ATCATTAC 2.3695443839
ATCATTAG 1.85944983927
ATCATTCA 0.50195486042
ATCATTCC -0.193246992409
ATCATTCG 1.02444124504
ATCATTGA 1.59383752293
ATCATTGC 0.92853040575
ATCATTGG 1.70762529495
ATCATTTA 3.01964797929
ATCATTTC 0.0379045132156
ATCATTTG 0.876315511652
ATCCAAAA 1.75736490535
ATCCAAAC 1.28988653854
ATCCAAAG 3.80406146116
ATCCAAAT 4.49990474442
ATCCAACA 2.16795748898
ATCCAACC 0.787553197573
ATCCAACG 3.23254119644
ATCCAAGA 1.97313436556
ATCCAAGC 0.978116585158
ATCCAAGG 1.27083521831
ATCCAATA 4.23837886792
ATCCAATC 2.36530529721
ATCCAATG 2.65515500599

ATCCACAA 2.85185589478
ATCCACAC 2.27822392816
ATCCACAG 1.94251534151
ATCCACAT 4.26460119217
ATCCACCA 1.44035398963
ATCCACCC 2.20625250629
ATCCACCG 2.54795509566
ATCCACGA 1.97166017337
ATCCACGC 3.41019233329
ATCCACGG 1.92633477532
ATCCACTA 2.75095083738
ATCCACTC 2.56260657952
ATCCACTG 1.95235714266
ATCCAGAA 2.34634807435
ATCCAGAC 1.54957777764
ATCCAGAG 0.959980361886
ATCCAGAT 3.76187185809
ATCCAGCA 1.84973872891
ATCCAGCC 2.25944731999
ATCCAGCG 0.523090960245
ATCCAGGA 3.22322241985
ATCCAGGC 0.991711564618
ATCCAGGG 1.7431904429
ATCCAGTA 2.21350976625
ATCCAGTC 1.01702924718
ATCCAGTG 2.84820779187
ATCCATAA 2.55724485889
ATCCATAC 1.99914549349
ATCCATAG 0.817923386346
ATCCATCA 0.833550607698
ATCCATCC 1.39507459474
ATCCATCG 0.608360954848
ATCCATGA 2.16814568373
ATCCATGC 2.68394540441
ATCCATGG 1.73001471951
ATCCATTA 3.08520901757
ATCCATTC 0.947506448083
ATCCATTG 2.85247510778
ATCCCAAA 2.17914305291
ATCCCAAC 2.86356082394
ATCCCAAG 1.24212035917
ATCCCAAT 3.32257251815
ATCCCACA 1.64971202072
ATCCCACC 1.55426434962
ATCCCACG 1.49997983606
ATCCCAGA 3.46134941607
ATCCCAGC 1.18480773985
ATCCCAGG 0.392762960966
ATCCCATA 2.94189453518
ATCCCATC 1.83479815701
ATCCCATG 1.24287183125
ATCCCCAA 1.70546000982
ATCCCCAC 0.927237089623
ATCCCCAG 1.66379369271

ATCCCCAT 2.85704431942
ATCCCCCA 2.25931819748
ATCCCCCC 1.90430814841
ATCCCCCG 1.90369546995
ATCCCCGA 2.29240283411
ATCCCCGC 2.49140335816
ATCCCCGG 1.37361621141
ATCCCCTA 3.31212326618
ATCCCCTC 0.514627685617
ATCCCCTG 1.36204066615
ATCCCGAA 2.08088056342
ATCCCGAC 1.60960615172
ATCCCGAG 1.45953887585
ATCCCGAT 5.22852109744
ATCCCGCA 2.02378776601
ATCCCGCC 2.03096661149
ATCCCGCG 3.56936431421
ATCCCGGA 3.83989687768
ATCCCGGC 0.122140482233
ATCCCGGG 0.0979880388154
ATCCCGTA 3.31940065251
ATCCCGTC 1.02335598866
ATCCCGTG 1.74402425018
ATCCCTAA 2.38025580162
ATCCCTAC 1.52708589149
ATCCCTAG 1.572018433
ATCCCTCA 0.802416400527
ATCCCTCC 0.626946754461
ATCCCTCG 0.677735811964
ATCCCTGA 0.302058320263
ATCCCTGC -0.00189867589782
ATCCCTGG 0.923662957709
ATCCCTTA 1.84522074806
ATCCCTTC 0.714376806555
ATCCCTTG 1.03247297865
ATCCGAAA 1.79005041228
ATCCGAAC 1.6522045556
ATCCGAAG 2.3373782422
ATCCGAAT 2.96499361078
ATCCGACA 3.04553782616
ATCCGACC -0.180787715975
ATCCGACG 2.54882732604
ATCCGAGA 2.62758525961
ATCCGAGC 1.71237721232
ATCCGAGG 2.87555039765
ATCCGATA 4.39411263535
ATCCGATC 4.84237161357
ATCCGATG 2.93724481801
ATCCGCAA 3.68464169993
ATCCGCAC 2.92871933456
ATCCGCAG 2.88306224327
ATCCGCAT 3.57453444214
ATCCGCCA 0.853657125424
ATCCGCCC 1.3976654091

ATCCGCCG 1.14941092164
ATCCGCGA 3.92365112042
ATCCGCGC 1.04681707776
ATCCGCGG 4.09467544971
ATCCGCTA 3.95680763698
ATCCGCTC 2.81328668817
ATCCGCTG 2.47348747957
ATCCGGAA 4.18136422361
ATCCGGAC 2.08189420124
ATCCGGAG 1.80951210161
ATCCGGAT 6.47399289141
ATCCGGCA 1.39487594473
ATCCGGCC 1.04792219914
ATCCGGCG 0.840726055213
ATCCGGGA 1.89749811236
ATCCGGGC 0.515912898914
ATCCGGGG 1.03484580076
ATCCGGTA 2.71405891646
ATCCGGTC 2.11460846014
ATCCGGTG 3.94946804182
ATCCGTAA 2.84613817241
ATCCGTAC 1.49882034731
ATCCGTAG 0.373376288149
ATCCGTCA 0.85681984271
ATCCGTCC 0.146632460341
ATCCGTCG 2.34797491339
ATCCGTGA 2.21278965996
ATCCGTGC 1.08816660039
ATCCGTGG -0.0650150121311
ATCCGTTA 3.04984068996
ATCCGTTC 0.936837112803
ATCCGTTG 2.57913818119
ATCCTAAA 2.53992728277
ATCCTAAC 2.27933087921
ATCCTAAG 0.87911464714
ATCCTAAT 2.50340155746
ATCCTACA 2.20340762903
ATCCTACC 0.709384940876
ATCCTACG 1.2418885137
ATCCTAGA 1.16021068634
ATCCTAGC 2.42096807592
ATCCTAGG 1.6849415547
ATCCTATA 2.57272518379
ATCCTATC 3.85636966848
ATCCTATG 1.78338465887
ATCCTCAA 3.20412326679
ATCCTCAC 1.2558648489
ATCCTCAG 2.38839653136
ATCCTCAT 2.53371554919
ATCCTCCA 1.91357229624
ATCCTCCC 0.372136032486
ATCCTCCG 1.39083420114
ATCCTCGA 1.57089867425
ATCCTCGC 1.8748671712

ATCCTCGG 1.53663468387
ATCCTCTA 3.20584368044
ATCCTCTC 2.42146365542
ATCCTCTG 2.5768275679
ATCCTGAA 0.987231484099
ATCCTGAC 2.65876311751
ATCCTGAG 2.31256345782
ATCCTGCA 1.56106863527
ATCCTGCC 2.05979883096
ATCCTGCG 2.93080385277
ATCCTGGA 2.45812765159
ATCCTGGC 3.49382137424
ATCCTGGG 1.11001940854
ATCCTGTA 3.53604966178
ATCCTGTC 2.41373826103
ATCCTGTG 3.06350885627
ATCCTTAA 1.84726239969
ATCCTTAC 2.9723535937
ATCCTTAG 2.71791717016
ATCCTTCA 2.6839114248
ATCCTTCC 1.31946369556
ATCCTTCG 0.332401586732
ATCCTTGA 2.98496551698
ATCCTTGC 1.53649667439
ATCCTTGG 3.91337228924
ATCCTTTA 3.35118805229
ATCCTTTC 1.62991158084
ATCCTTTG 1.66597622902
ATCGAAAA 2.43118339137
ATCGAAAC 0.937244345326
ATCGAAAG 1.57093474491
ATCGAAAT 6.52829230372
ATCGAACA 0.898697356153
ATCGAACC 0.814482820426
ATCGAACG 1.11798710367
ATCGAAGA 0.0332749224251
ATCGAAGC 0.743647362962
ATCGAAGG 1.53240396139
ATCGAATA 2.89536573628
ATCGAATC 5.81030869255
ATCGAATG 1.07089816401
ATCGACAA 0.548040094761
ATCGACAC 0.651793688773
ATCGACAG 0.141332687144
ATCGACAT 0.136532675554
ATCGACCA 0.912796802089
ATCGACCC 0.181806581427
ATCGACCG 0.173729106032
ATCGACGA -0.0536093649013
ATCGACGC 1.19676464086
ATCGACGG 0.187005461329
ATCGACTA 0.0584318553081
ATCGACTC 1.61566706812
ATCGACTG 0.839014005773

ATCGAGAA 2.64293437095
ATCGAGAC 0.660778681063
ATCGAGAG 0.416244437832
ATCGAGAT 3.92452544185
ATCGAGCA 1.01338140566
ATCGAGCC 0.751381644325
ATCGAGCG 1.52941009662
ATCGAGGA 0.18754678261
ATCGAGGC -0.351824330208
ATCGAGGG 0.155877527316
ATCGAGTA 1.87653478577
ATCGAGTC 0.469828971482
ATCGAGTG 0.866753911566
ATCGATAA 0.911816621112
ATCGATAC 3.62822378981
ATCGATAG 1.50025663916
ATCGATCA 2.41664586988
ATCGATCC 1.41675253861
ATCGATCG 2.49202544635
ATCGATGA 0.926072896004
ATCGATGC 0.260501522037
ATCGATGG 0.636025582742
ATCGATTA 0.794179482359
ATCGATTC 4.25674824357
ATCGATTG 3.06766220979
ATCGCAAA 3.31183862162
ATCGCAAC 1.30151148493
ATCGCAAG 3.19492733956
ATCGCAAT 7.74756372948
ATCGCACA 3.1292452816
ATCGCACC 1.23839044383
ATCGCACG 1.73304570048
ATCGCAGA 2.93563836673
ATCGCAGC 0.452661167015
ATCGCAGG 1.58026894301
ATCGCATA 2.72974599419
ATCGCATC 3.23350151241
ATCGCATG 4.13128586266
ATCGCCAA 2.66891308756
ATCGCCAC 1.31475098542
ATCGCCAG 1.00709491694
ATCGCCAT 1.39942659828
ATCGCCCA 1.37700058029
ATCGCCCC 1.63860068916
ATCGCCCG 2.00110141196
ATCGCCGA 2.13106765911
ATCGCCGC 2.39701454389
ATCGCCGG 1.43100724521
ATCGCCTA 1.71158208951
ATCGCCTC 1.15647632751
ATCGCCTG 0.793568110811
ATCGCGAA 4.7035910563
ATCGCGAC 2.18902275439
ATCGCGAG 2.96147018689

ATCGCGAT 9.24270173289
ATCGCGCA 5.03574563743
ATCGCGCC 3.31892415387
ATCGCGCG 5.27595845877
ATCGCGGA 3.68331362006
ATCGCGGC 2.81536336493
ATCGCGGG 4.80248896445
ATCGCGTA 4.7429969454
ATCGCGTC 3.84172759436
ATCGCGTG 4.54055062779
ATCGCTAA 1.21637636323
ATCGCTAC 1.22039536662
ATCGCTAG 1.21998264508
ATCGCTCA -0.0696069639721
ATCGCTCC 1.31450005909
ATCGCTCG 1.02682739761
ATCGCTGA 1.23859170765
ATCGCTGC 1.80798511034
ATCGCTGG 1.14637157647
ATCGCTTA 1.6794742359
ATCGCTTC 2.58248726355
ATCGCTTG 2.37360468558
ATCGGAAA 2.00489902513
ATCGGAAC 0.883735350957
ATCGGAAG 3.06639294077
ATCGGAAT 2.32353704128
ATCGGACA 0.555448956039
ATCGGACC -0.505824092154
ATCGGACG -0.218409937069
ATCGGAGA 2.27040757298
ATCGGAGC 1.0290697903
ATCGGAGG 0.0359101716545
ATCGGATA 4.82929260137
ATCGGATC 4.24193574864
ATCGGATG 0.383253375818
ATCGGCAA 2.16523859764
ATCGGCAC 0.339421250816
ATCGGCAG -0.405230862993
ATCGGCAT 2.89579466347
ATCGGCCA -0.163776479083
ATCGGCCC 1.21504174881
ATCGGCCG 1.6216161132
ATCGGCGA 0.735238194325
ATCGGCGC 2.0609656384
ATCGGCGG 0.164503642677
ATCGGCTA 1.41980599839
ATCGGCTC 0.444276045484
ATCGGCTG 1.31914271896
ATCGGGAA 2.69019425417
ATCGGGAC 1.4727833426
ATCGGGAG 1.39138388664
ATCGGGCA 1.87166158733
ATCGGGCC 0.0141815197566
ATCGGGCG -0.233721670837

ATCGGGGA 2.80340175905
ATCGGGGC -0.172338294571
ATCGGGGG -0.355677617665
ATCGGGTA 1.87788717414
ATCGGGTC 1.14366052658
ATCGGGTG 1.2484776823
ATCGGTAA 1.52079861863
ATCGGTAC 0.902841822705
ATCGGTAG 0.562680339215
ATCGGTCA 0.227844244318
ATCGGTCC -0.421578452018
ATCGGTCG 1.08949755546
ATCGGTGA 1.24034740782
ATCGGTGC 0.779306608287
ATCGGTGG 0.511863052499
ATCGGTTA 0.926333232072
ATCGGTTC 0.987764179788
ATCGGTTG -0.35278882829
ATCGTAAA 2.02203677072
ATCGTAAC 2.41785998738
ATCGTAAG 1.00519676381
ATCGTAAT 8.79109210403
ATCGTACA 1.44418845761
ATCGTACC 0.426721134877
ATCGTACG 1.80524687677
ATCGTAGA 2.17594844707
ATCGTAGC 1.66796063808
ATCGTAGG -0.0804017624171
ATCGTATA 1.57251976289
ATCGTATC 6.83133629397
ATCGTATG 1.01374472607
ATCGTCAA 2.16069108067
ATCGTCAC 1.72659323445
ATCGTCAG 0.85538485776
ATCGTCAT 3.88751720612
ATCGTCCA 0.920357526073
ATCGTCCC 0.762060127961
ATCGTCCG 1.4634264043
ATCGTCGA 1.97575419327
ATCGTCGC 0.879982695414
ATCGTCGG 1.30777627897
ATCGTCTA 0.955784141779
ATCGTCTC 1.33763912607
ATCGTCTG 1.64310481679
ATCGTGAA 0.49883736215
ATCGTGAC 0.950964265188
ATCGTGAG 0.760547512677
ATCGTGCA 1.44824849791
ATCGTGCC 0.69120219168
ATCGTGCG 1.87763232708
ATCGTGGA 2.18164656581
ATCGTGGC 1.14145864803
ATCGTGGG 1.69845446034
ATCGTGTA 2.1135184989

ATCGTGTC 1.95374011267
ATCGTGTG 0.870037125767
ATCGTTAA 1.57358097216
ATCGTTAC 0.485259633876
ATCGTTAG 0.505575518259
ATCGTTCA 2.49747028633
ATCGTTCC 1.83273507208
ATCGTTCG -0.136678003721
ATCGTTGA 0.802370397366
ATCGTTGC 1.62246168265
ATCGTTGG 0.202360061679
ATCGTTTA 2.09426513069
ATCGTTTC 1.48716638756
ATCGTTTG 1.30747359908
ATCTAAAA 2.54317547184
ATCTAAAC 2.19736762316
ATCTAAAG 0.740199739739
ATCTAAAT 5.0266817079
ATCTAACA 1.47183295912
ATCTAACC 2.71943501308
ATCTAACG 2.93615276571
ATCTAAGA 3.54257400775
ATCTAAGC 2.05778828374
ATCTAAGG 2.31166613481
ATCTAATA 2.81754877644
ATCTAATC 4.21388140079
ATCTAATG 1.77190138131
ATCTACAA 0.823733637796
ATCTACAC 2.32946203925
ATCTACAG 2.23130384101
ATCTACAT 3.48398898283
ATCTACCA 2.30017370889
ATCTACCC 1.14877053674
ATCTACCG 3.77828662219
ATCTACGA 3.12015495253
ATCTACGC 2.65497674374
ATCTACGG 1.83273507208
ATCTACTA 3.84290172048
ATCTACTC 1.57527681595
ATCTACTG 2.12456605334
ATCTAGAA 1.61448248674
ATCTAGAC 2.82524750991
ATCTAGAG 1.57657170036
ATCTAGAT 12.2636851107
ATCTAGCA 3.794799143
ATCTAGCC 1.97317435694
ATCTAGCG 1.11560591735
ATCTAGGA 1.65017884825
ATCTAGGC 1.52363304063
ATCTAGGG 1.93178562708
ATCTAGTA 3.48949289505
ATCTAGTC 2.94687829803
ATCTAGTG 4.64430056246
ATCTATAA 3.19870613326

ATCTATAC 1.46074358362
ATCTATAG 3.82270659439
ATCTATCA 3.10545589721
ATCTATCC 1.64812778688
ATCTATCG −0.31595127474
ATCTATGA 1.34740538794
ATCTATGC 1.44508996273
ATCTATGG 1.53310498683
ATCTATTA 1.45430052733
ATCTATTC 1.44888417794
ATCTATTG 1.7762980811
ATCTCAAA 6.00551238757
ATCTCAAC 2.77211778023
ATCTCAAG 2.24202362294
ATCTCAAT 3.04546986695
ATCTCACA 1.34387412261
ATCTCACC 2.81329426824
ATCTCACG 1.10134964245
ATCTCAGA 4.02755213365
ATCTCAGC 1.17249692816
ATCTCAGG 3.49721436871
ATCTCATA 2.41211194475
ATCTCATC 5.0120278716
ATCTCATG 4.0213754252
ATCTCCAA 5.15498765917
ATCTCCAC 3.11345156017
ATCTCCAG 3.25339056065
ATCTCCAT 4.48292670299
ATCTCCCA 2.63545153868
ATCTCCCC 2.58417160654
ATCTCCCG 1.25917498541
ATCTCCGA 2.97322190335
ATCTCCGC 2.52067652838
ATCTCCGG 3.89411578445
ATCTCCTA 3.04965667732
ATCTCCTC 2.70854794693
ATCTCCTG 3.79255335234
ATCTCGAA 4.94380361626
ATCTCGAC 1.66998268609
ATCTCGAG 4.32908220171
ATCTCGCA 3.09910066514
ATCTCGCC 3.08671692799
ATCTCGCG 3.37080657058
ATCTCGGA 6.58316675554
ATCTCGGC 2.616060161
ATCTCGGG 4.1082685995
ATCTCGTA 4.37955838545
ATCTCGTC 2.76261107028
ATCTCGTG 3.16921575493
ATCTCTAA 2.19489495328
ATCTCTAC 3.65815825546
ATCTCTAG 2.60912309349
ATCTCTCA 2.8867370071
ATCTCTCC 1.12463429898

ATCTCTCG 2.7661794518
ATCTCTGA 1.71592102397
ATCTCTGC 2.52518980436
ATCTCTGG 2.20340762903
ATCTCTTA 2.31177094883
ATCTCTTC 3.43496346694
ATCTCTTG 5.21147771062
ATCTGAAA 1.75193993037
ATCTGAAC 4.24374006579
ATCTGAAG 2.15052202976
ATCTGAAT 3.23763761476
ATCTGACA 2.21618762068
ATCTGACC 3.41012751065
ATCTGACG 2.36722174706
ATCTGAGA 3.58702220917
ATCTGAGC 1.15763346382
ATCTGAGG 2.14407740518
ATCTGATA 6.52222720522
ATCTGATC 5.52104447854
ATCTGATG 2.94085083848
ATCTGCAA 4.64030090131
ATCTGCAC 3.47879245536
ATCTGCAG 3.57166656329
ATCTGCAT 4.7185175136
ATCTGCCA 1.99447172921
ATCTGCCC 2.11205110263
ATCTGCCG 2.23437193816
ATCTGCGA 4.41340364251
ATCTGCGC 4.25753291111
ATCTGCGG 0.866341974175
ATCTGCTA 3.41019233329
ATCTGCTC 1.17507022995
ATCTGCTG 3.76288157519
ATCTGGAA 3.01399847752
ATCTGGAC 3.08256096064
ATCTGGAG 1.05181364831
ATCTGGCA 1.10195657051
ATCTGGCC 1.0844698805
ATCTGGCG 4.43601550281
ATCTGGGA 2.29129928102
ATCTGGGC 1.76735673954
ATCTGGGG 3.57454228359
ATCTGGTA 1.37342801666
ATCTGGTC 3.45723004215
ATCTGGTG 3.18939859608
ATCTGTAA 3.31356635396
ATCTGTAC 3.02189664514
ATCTGTAG 3.90233754249
ATCTGTCA 1.00981014894
ATCTGTCC 3.10869023306
ATCTGTCG 4.0027752496
ATCTGTGA 2.62561026029
ATCTGTGC 2.91331350342
ATCTGTGG −0.172338294571

ATCTGTTA 0.914637451273
ATCTGTTC 0.0497443152729
ATCTGTTG 1.97068861798
ATCTTAAA 3.3438560372
ATCTTAAC 3.88482732813
ATCTTAAG 2.46799323847
ATCTTAAT 4.06422710785
ATCTTACA 3.48425167133
ATCTTACC 3.20140803479
ATCTTACG 2.60850989228
ATCTTAGA 1.76111703813
ATCTTAGC 4.13070428861
ATCTTAGG 1.16000628593
ATCTTATA 3.79474634392
ATCTTATC 2.08926098008
ATCTTATG 2.03699015032
ATCTTCAA 2.89726415079
ATCTTCAC 3.25991438386
ATCTTCAG 3.75047013159
ATCTTCAT 3.73824087095
ATCTTCCA 2.59002211076
ATCTTCCC 1.67651513488
ATCTTCCG 2.27918894901
ATCTTCGA 2.40786710767
ATCTTCGC 2.5828686193
ATCTTCGG 2.31440410701
ATCTTCTA 2.28689291011
ATCTTCTC 3.82122639043
ATCTTCTG 4.49664479318
ATCTTGAA 2.91479553706
ATCTTGAC 2.1271001479
ATCTTGAG 3.52151841346
ATCTTGCA 3.76638879342
ATCTTGCC 2.32334649409
ATCTTGCG 1.28266639477
ATCTTGGA 1.74217053192
ATCTTGGC 2.09214846255
ATCTTGGG 3.13406306713
ATCTTGTA 3.25639383517
ATCTTGTC 4.65147810103
ATCTTGTG 2.39468066764
ATCTTTAA 2.66798858087
ATCTTTAC 2.38245088424
ATCTTTAG 3.41181237641
ATCTTTCA 3.99418259109
ATCTTTCC 2.50098377772
ATCTTTCG 2.52329112847
ATCTTTGA 6.15792948397
ATCTTTGC 2.07143397123
ATCTTTGG 2.00899670438
ATCTTTTA 3.16167672562
ATCTTTTC 3.59623251239
ATCTTTTG 1.22370445759
ATGAAAAA 0.29364340123

ATGAAAAC 1.04940344863
ATGAAAAG 0.847364624934
ATGAAAAT 2.62815690116
ATGAAACA 0.794247180192
ATGAAACC 0.29866976928
ATGAAACG 1.46007706056
ATGAAAGA 1.64664627601
ATGAAAGC 0.455142723867
ATGAAAGG -0.879016106279
ATGAAATA 1.72731438627
ATGAAATC 11.6686012953
ATGAAATG 0.0520374159958
ATGAACAA 1.17548269011
ATGAACAC -0.108669920412
ATGAACAG -0.401074111506
ATGAACAT 0.462477614154
ATGAACCA -0.0884823743913
ATGAACCC -0.414781485051
ATGAACCG -0.263896084796
ATGAACGA 1.08279076514
ATGAACGC -0.664224997378
ATGAACGG -0.253909216858
ATGAACTA 0.495926616721
ATGAACTC -0.124050658921
ATGAACTG 1.17010293414
ATGAAGAA 0.797991210143
ATGAAGAC -0.298157461356
ATGAAGAG -0.717230048034
ATGAAGCA 0.659295863281
ATGAAGCC -0.333989741296
ATGAAGCG -0.185778013365
ATGAAGGA 0.167689361543
ATGAAGGC -0.442900655537
ATGAAGGG -0.468556304501
ATGAAGTA -0.879002253055
ATGAAGTC -0.961588642833
ATGAAGTG -0.534679051827
ATGAATAA 2.07465471523
ATGAATAC 0.991925897525
ATGAATAG -0.122781389901
ATGAATCA 0.727102953597
ATGAATCC 1.50716547745
ATGAATCG 1.09649395658
ATGAATGA 0.162387497293
ATGAATGC 0.895906323493
ATGAATGG 1.09896244436
ATGAATTA 2.02493575397
ATGAATTC 0.892724786733
ATGAATTG 0.991781876267
ATGACAAA 2.07385148959
ATGACAAC 0.950688246225
ATGACAAG -0.0914783302202
ATGACAAT 1.25963815359
ATGACACA 0.568996886812

ATGACACC 0.0914626473246
ATGACACG -0.240058867581
ATGACAGA -0.325975258865
ATGACAGC -0.17861563493
ATGACAGG -0.93320103345
ATGACATA 0.15019090936
ATGACATC 0.538472221517
ATGACATG 0.372337819076
ATGACCAA 0.212241592835
ATGACCAC -1.06354027214
ATGACCAG 0.0255147642849
ATGACCAT -0.2935728282
ATGACCCA -1.77166326268
ATGACCCC -0.868521896667
ATGACCCG -1.10149157266
ATGACCGA 0.602785424069
ATGACCGC -0.392777075572
ATGACCGG -0.194787836905
ATGACCTA -0.683010492529
ATGACCTC -0.534208564958
ATGACCTG -0.155993319362
ATGACGAA -0.419288749256
ATGACGAC -0.501620553362
ATGACGAG -0.224198232465
ATGACGCA 0.00497095515209
ATGACGCC -0.8578481179
ATGACGCG -0.30430933855
ATGACGGA 0.559207623359
ATGACGGC 0.168589036989
ATGACGGG -0.565550309115
ATGACGTA 1.05259570204
ATGACGTC 0.551395973044
ATGACGTG 0.422603852011
ATGACTAA 1.06044629821
ATGACTAC 0.508498548623
ATGACTAG 0.477473337576
ATGACTCA -0.398674889856
ATGACTCC -0.771765749304
ATGACTCG -0.196391151602
ATGACTGA 0.068281236528
ATGACTGC -0.161909430358
ATGACTGG -1.10298824367
ATGACTTA -0.0324994032362
ATGACTTC -0.609945972833
ATGACTTG -0.00161559963167
ATGAGAAA 1.36371952012
ATGAGAAC -0.0921328297312
ATGAGAAG 0.400940022748
ATGAGAAT 5.06052722634
ATGAGACA -0.0253030451939
ATGAGACC -0.522446393234
ATGAGACG -0.859612182277
ATGAGAGA 0.561143938206
ATGAGAGC -0.596260555341

ATGAGAGG	-0.300032351529
ATGAGATA	1.81575415546
ATGAGATC	6.4320453277
ATGAGATG	0.866769071699
ATGAGCAA	-0.145850406612
ATGAGCAC	0.00984363082488
ATGAGCAG	-0.858559598599
ATGAGCAT	-1.13350428337
ATGAGCCA	-0.852809987679
ATGAGCCC	-0.861309332966
ATGAGCCG	-0.769978944728
ATGAGCGA	-0.848617688295
ATGAGCGC	0.396577563946
ATGAGCGG	-0.48445510133
ATGAGCTA	0.189242103628
ATGAGCTC	-0.734474960071
ATGAGCTG	-0.385555624896
ATGAGGAA	-0.169704613632
ATGAGGAC	0.301672259649
ATGAGGAG	-0.586171748581
ATGAGGCA	-1.12664981245
ATGAGGCC	-1.13081832611
ATGAGGCG	-0.850013204624
ATGAGGGA	-0.159114476975
ATGAGGGC	-0.322810189145
ATGAGGGG	-0.287628487992
ATGAGGTA	0.964800238477
ATGAGGTC	-0.425423636645
ATGAGGTG	-0.118435136758
ATGAGTAA	0.964340206872
ATGAGTAC	0.95569422651
ATGAGTAG	0.988614715494
ATGAGTCA	-0.2539554814
ATGAGTCC	-0.451042430804
ATGAGTCG	0.0174129804016
ATGAGTGA	-0.366901866069
ATGAGTGC	0.235016555253
ATGAGTGG	-0.671281254886
ATGAGTTA	1.14666301695
ATGAGTTC	-0.208093989704
ATGAGTTG	-0.35397288691
ATGATAAA	1.58292065928
ATGATAAC	0.70787206421
ATGATAAG	0.304044820377
ATGATAAT	1.82905220543
ATGATACA	1.5666491323
ATGATACC	2.18858049673
ATGATACG	2.84634440249
ATGATAGA	0.806131155739
ATGATAGC	0.718686727657
ATGATAGG	0.723828887753
ATGATATA	1.59383752293
ATGATATC	11.5019971902
ATGATATG	2.99462565793

ATGATCAA -0.250622081933
ATGATCAC 0.64638622636
ATGATCAG 1.23025572586
ATGATCAT 2.55514883989
ATGATCCA 2.00460497084
ATGATCCC 0.962975533571
ATGATCCG 0.268279976888
ATGATCGA 0.953185224591
ATGATCGC 2.8671924598
ATGATCGG -0.130579971136
ATGATCTA 1.50049998543
ATGATCTC 1.84037421055
ATGATCTG 1.2672780762
ATGATGAA 0.847501066126
ATGATGAC -0.0795744896725
ATGATGAG -0.0333867937473
ATGATGCA -0.0907145732029
ATGATGCC 0.0823566353576
ATGATGCG 0.365692714816
ATGATGGA -0.366909184753
ATGATGGC -0.687888918596
ATGATGGG -0.0123913172203
ATGATGTA 1.4202346642
ATGATGTC -0.286438678977
ATGATGTG 0.258595527454
ATGATTAA 0.427906239024
ATGATTAC 4.45466377263
ATGATTAG 1.50613249739
ATGATTCA 1.09294204211
ATGATTCC 6.04166512135
ATGATTCG 2.44076041297
ATGATTGA 0.0497129494816
ATGATTGC 5.66715600537
ATGATTGG 0.791304546208
ATGATTTA 1.28092977546
ATGATTTC 8.45001683049
ATGATTTG 2.56986619191
ATGCAAAA 1.10189070235
ATGCAAAC -0.0830979135578
ATGCAAAG 1.83452449048
ATGCAAAT 3.01324778958
ATGCAACA 0.547309533206
ATGCAACC 0.164913227634
ATGCAACG 1.34435584889
ATGCAAGA 0.928765126421
ATGCAAGC -0.60623592249
ATGCAAGG 0.301401206936
ATGCAATA 1.4526394473
ATGCAATC 12.3337981095
ATGCAATG -0.145492836592
ATGCACAA 0.733284628292
ATGCACAC -1.23325272722
ATGCACAG 0.17592131346
ATGCACAT 1.21414886929

ATGCACCA 0.570760167044
ATGCACCC -0.208886237315
ATGCACCG 0.367606550846
ATGCACGA -0.290467876247
ATGCACGC -0.603324131534
ATGCACGG 0.295369042513
ATGCACTA 0.0185214997411
ATGCACTC -0.766011172133
ATGCACTG -0.53428436562
ATGCAGAA 1.71549680164
ATGCAGAC -0.918448917674
ATGCAGAG -0.694574014241
ATGCAGCA -0.359810583427
ATGCAGCC -0.82285199768
ATGCAGCG -0.0444189266795
ATGCAGGA 0.740520193574
ATGCAGGC -0.148893933891
ATGCAGGG -0.850068356141
ATGCAGTA 2.89774535431
ATGCAGTC -0.285611667614
ATGCAGTG 0.537346712374
ATGCATAA 1.58538182836
ATGCATAC -0.0774293309317
ATGCATAG 0.195984964605
ATGCATCA 0.792908906431
ATGCATCC -0.114681697071
ATGCATCG -0.595505685298
ATGCATGA 0.132873594622
ATGCATGC -0.379292399144
ATGCATGG -0.217642520709
ATGCATTA 0.228668119102
ATGCATTC 1.4544926428
ATGCATTG -0.12461132244
ATGCCAAA -0.661896087376
ATGCCAAC 0.256598310695
ATGCCAAG -0.0573162786655
ATGCCAAT 1.19663996184
ATGCCACA -0.654132531275
ATGCCACC 0.277709056505
ATGCCACG -0.477238878286
ATGCCAGA -1.29504542706
ATGCCAGC 0.529031379729
ATGCCAGG -0.238837170011
ATGCCATA 0.457754448753
ATGCCATC 0.390346226749
ATGCCATG -0.711897340756
ATGCCCAA -0.237956052658
ATGCCCAC 0.140083283125
ATGCCCAG -0.474751571039
ATGCCCAT 0.0600819573102
ATGCCCCA -0.809596291528
ATGCCCCC -0.89245373402
ATGCCCCG 0.604782902209
ATGCCCGA -0.680954464911

ATGCCCGC -0.704935964767
ATGCCCGG -0.513803549452
ATGCCCTA -0.240723822355
ATGCCCTC -1.38672188453
ATGCCCTG -0.184538541846
ATGCCGAA -0.419291885835
ATGCCGAC -0.149183021934
ATGCCGAG -0.130565333766
ATGCCGCA 0.10819891078
ATGCCGCC -0.0893527750989
ATGCCGCG -0.31802899703
ATGCCGGA -0.459430950296
ATGCCGGC -0.82539550197
ATGCCGGG -0.375750417166
ATGCCGTA 0.216528773738
ATGCCGTC -0.250517790677
ATGCCGTG -1.06422822849
ATGCCTAA 0.57436409646
ATGCCTAC 0.274883521476
ATGCCTAG -0.430172940206
ATGCCTCA -0.380961320621
ATGCCTCC -0.89665857972
ATGCCTCG -1.08370847592
ATGCCTGA 1.20998244668
ATGCCTGC -0.440405245461
ATGCCTGG -0.695803553259
ATGCCTTA 0.0276285572345
ATGCCTTC 0.415894186496
ATGCCTTG -0.811961533571
ATGCGAAA 1.23780834702
ATGCGAAC 1.10594734469
ATGCGAAG -0.310978228535
ATGCGAAT 4.85729963916
ATGCGACA 0.0135981160391
ATGCGACC -0.234758048857
ATGCGACG 1.07368971943
ATGCGAGA 1.48084330543
ATGCGAGC -0.939989374825
ATGCGAGG -0.0309690140041
ATGCGATA 1.87299541761
ATGCGATC 7.55850224054
ATGCGATG -0.167735364703
ATGCGCAA 1.82033251546
ATGCGCAC 0.365552614281
ATGCGCAG -0.31327002235
ATGCGCAT 1.85324673128
ATGCGCCA -0.2484682976
ATGCGCCC 0.133871288166
ATGCGCCG 0.382020177458
ATGCGCGA 0.988858845903
ATGCGCGC 0.462696129167
ATGCGCGG 1.00981014894
ATGCGCTA 2.69835563306
ATGCGCTC 0.151665362931

ATGCGCTG 0.359552077031
ATGCGGAA 0.452661167015
ATGCGGAC -0.393863900239
ATGCGGAG -0.797163937398
ATGCGGCA -0.0907995222209
ATGCGGCC -0.805574935707
ATGCGGCG -0.140380212616
ATGCGGGA -0.0288672446078
ATGCGGGC -0.30945228279
ATGCGGGG 0.534674346959
ATGCGGTA 0.00657244017769
ATGCGGTC -0.648564580563
ATGCGGTG -0.374817023494
ATGCGTAA 1.48612582744
ATGCGTAC -0.639980286257
ATGCGTAG 0.0401045620912
ATGCGTCA 0.672705523191
ATGCGTCC -1.10713663094
ATGCGTCG 0.0403541815133
ATGCGTGA 1.17596049566
ATGCGTGC -0.124815984228
ATGCGTGG -1.23342837565
ATGCGTTA 0.144908910111
ATGCGTTC 0.143989108283
ATGCGTTG -0.189702396615
ATGCTAAA 1.36200956174
ATGCTAAC -0.0286604917671
ATGCTAAG 0.341480937776
ATGCTAAT 1.19816538482
ATGCTACA 0.643996153065
ATGCTACC -0.510486617026
ATGCTACG 0.139488117236
ATGCTAGA -0.150258345811
ATGCTAGC 0.513814004715
ATGCTAGG -0.0210819938346
ATGCTATA 1.5148388569
ATGCTATC 1.92399776249
ATGCTATG -0.145011894459
ATGCTCAA -0.369258482519
ATGCTCAC -0.462456965008
ATGCTCAG 0.0145014508275
ATGCTCCA -0.806699399324
ATGCTCCC -0.303503237714
ATGCTCCG -0.800578365159
ATGCTCGA 1.00594588346
ATGCTCGC -0.551417406335
ATGCTCGG -1.06080308409
ATGCTCTA -0.207321607094
ATGCTCTC 0.274436820332
ATGCTCTG 1.02951675283
ATGCTGAA 0.625807653475
ATGCTGAC 0.153898868651
ATGCTGAG -0.291461387685
ATGCTGCA 0.361894840257

ATGCTGCC -0.586592050184
ATGCTGCG -0.446442898897
ATGCTGGA -0.309516059899
ATGCTGGC -0.595740667351
ATGCTGGG -0.207568089937
ATGCTGTA 0.457213911617
ATGCTGTC -0.105523408785
ATGCTGTG 0.838506141336
ATGCTTAA 0.152460224358
ATGCTTAC -0.572336298055
ATGCTTAG -0.322295267405
ATGCTTCA -0.303874138196
ATGCTTCC -0.0719941620689
ATGCTTCG 0.277017440808
ATGCTTGA 1.33934019748
ATGCTTGC -0.668084296611
ATGCTTGG 0.602996620397
ATGCTTTA 0.480476089326
ATGCTTTC 0.758983927982
ATGCTTTG 1.14305203023
ATGGAAAA 1.25935742976
ATGGAAAC -0.195088164356
ATGGAAAG -0.0943485615024
ATGGAAAT 5.10579512044
ATGGAACA -0.0039526124624
ATGGAACC -0.17241357247
ATGGAACG -0.779326473288
ATGGAAGA 1.01256092883
ATGGAAGC -0.743813078893
ATGGAAGG -1.18670537022
ATGGAATA 0.679078790594
ATGGAATC 6.35909111104
ATGGAATG -0.171091504369
ATGGACAA 0.462218323613
ATGGACAC -0.247230394371
ATGGACAG 0.0490827584588
ATGGACAT 0.692334496745
ATGGACCA -1.50355736593
ATGGACCC -0.993669312756
ATGGACCG -1.10941718535
ATGGACGA -0.298432434793
ATGGACGC -0.150795746368
ATGGACGG -0.191808086735
ATGGACTA -0.274707350281
ATGGACTC -1.02163871159
ATGGACTG -0.792528334831
ATGGAGAA -0.919967283353
ATGGAGAC -0.725347514813
ATGGAGAG -0.371138861705
ATGGAGCA -0.922296716118
ATGGAGCC -1.68297962446
ATGGAGCG -1.23763661931
ATGGAGGA -0.999641359413
ATGGAGGC -0.754730465305

ATGGAGGG -1.53007557416
ATGGAGTA -0.510810730202
ATGGAGTC 0.31740089706
ATGGAGTG -0.0880855971319
ATGGATAA 1.18004249201
ATGGATAC 3.23910710208
ATGGATAG 0.199587325732
ATGGATCA 1.26687842374
ATGGATCC 1.41323486512
ATGGATCG 1.6383246702
ATGGATGA -0.570481534265
ATGGATGC -0.605389307507
ATGGATGG -0.291499288016
ATGGATTA 3.86613802141
ATGGATTC 8.31705462751
ATGGATTG 3.04682905124
ATGGCAAA 0.521838681029
ATGGCAAC -0.405625287818
ATGGCAAG -0.368531318925
ATGGCAAT 2.25842662486
ATGGCACA -0.199168069655
ATGGCACC -0.394758870816
ATGGCACG -0.163579135979
ATGGCAGA -0.718915959314
ATGGCAGC -0.509091362078
ATGGCAGG -0.753695394194
ATGGCATA -0.0210195236337
ATGGCATC -0.534191313773
ATGGCATG -0.0922399961847
ATGGCCAA -0.189531453052
ATGGCCAC -0.878519742633
ATGGCCAG -1.19988788952
ATGGCCAT 1.24540853962
ATGGCCCA -0.254561625316
ATGGCCCC -0.454304473096
ATGGCCCG -1.10590081877
ATGGCCGA -0.51877058388
ATGGCCGC -1.22604539115
ATGGCCGG -1.18739959973
ATGGCCTA 0.0542309303315
ATGGCCTC -1.15654141152
ATGGCCTG -0.736138131152
ATGGCGAA 0.643627605018
ATGGCGAC -0.805394582407
ATGGCGAG -0.593761747304
ATGGCGCA 0.0847461858887
ATGGCGCC -0.411540614669
ATGGCGCG -0.316157766199
ATGGCGGA -0.512022495271
ATGGCGGC -0.844465903058
ATGGCGGG -0.812081507723
ATGGCGTA -0.05204630297
ATGGCGTC -1.05468204992
ATGGCGTG 0.574015152032

ATGGCTAA 0.128786893402
ATGGCTAC -1.49368994938
ATGGCTAG -0.181134307968
ATGGCTCA -1.05893603536
ATGGCTCC 0.405404681753
ATGGCTCG 0.285331989309
ATGGCTGA -0.00914600335086
ATGGCTGC -1.13705672061
ATGGCTGG -0.527642136557
ATGGCTTA -0.498079355528
ATGGCTTC -0.285348717731
ATGGCTTG 0.0879214494909
ATGGGAAA 0.228482799552
ATGGGAAC -0.624946923886
ATGGGAAG -1.29961150212
ATGGGAAT 3.45692579398
ATGGGACA -0.467856063211
ATGGGACC -0.333683140686
ATGGGACG -0.237790336727
ATGGGAGA -0.330090973442
ATGGGAGC -0.688457946326
ATGGGAGG -1.46707189339
ATGGGATA 2.94103694217
ATGGGATC 2.47071631191
ATGGGATG -0.0876903881619
ATGGGCAA -0.808850569841
ATGGGCAC -0.250911692739
ATGGGCAG -0.7723423571
ATGGGCCA -1.08472054545
ATGGGCCC -1.28598384996
ATGGGCCG -1.1028429155
ATGGGCGA 0.559415160344
ATGGGCGC -0.856251860507
ATGGGCGG -0.330505001887
ATGGGCTA 0.0567882878459
ATGGGCTC -1.07577920389
ATGGGCTG -1.12640934138
ATGGGGAA -0.231858020073
ATGGGGAC -0.462694038114
ATGGGGAG -1.10991877663
ATGGGGCA 0.1084712704
ATGGGGCC -0.576921192593
ATGGGGCG 0.19848507955
ATGGGGGA 0.0426736817773
ATGGGGGC -1.27070269784
ATGGGGGG -1.00323849291
ATGGGGTA 0.527780930184
ATGGGGTC -1.16856182826
ATGGGGTG -0.0511596966036
ATGGGTAA 0.969154071686
ATGGGTAC -0.259976145033
ATGGGTAG -0.117848073699
ATGGGTCA -0.395581700074
ATGGGTCC -1.13999438834

ATGGGTCG -0.644692735013
ATGGGTGA 0.348283132375
ATGGGTGC -0.593380130177
ATGGGTGG 1.00427774612
ATGGGTTA 0.835989297969
ATGGGTTC -0.30231525837
ATGGGTTG -0.596012242827
ATGGTAAA 1.4418739236
ATGGTAAC 1.09299640948
ATGGTAAG 0.557499756024
ATGGTAAT 2.69768911
ATGGTACA -0.349810123646
ATGGTACC -0.105954427033
ATGGTACG -0.161277148282
ATGGTAGA 0.131117110311
ATGGTAGC -1.09756483697
ATGGTAGG -0.706781318819
ATGGTATA 1.82446809503
ATGGTATC 2.44352792128
ATGGTATG 0.693690283072
ATGGTCAA 0.340441423177
ATGGTCAC -0.187569522809
ATGGTCAG -0.28715747836
ATGGTCCA -0.520777210376
ATGGTCCC -0.0722500546493
ATGGTCCG -0.818924216469
ATGGTCGA -0.167000359661
ATGGTCGC 0.188598843526
ATGGTCGG 0.185125604906
ATGGTCTA -0.710995574257
ATGGTCTC -0.7771408004
ATGGTCTG -0.865337746091
ATGGTGAA 0.647131686665
ATGGTGAC -0.697407390719
ATGGTGAG 0.713859270999
ATGGTGCA 0.509629808161
ATGGTGCC -0.30262185898
ATGGTGCG -0.346250629101
ATGGTGGA -0.39319005849
ATGGTGGC 0.512097250407
ATGGTGGG -0.542163191004
ATGGTGTA 1.9533151062
ATGGTGTC -0.20522192875
ATGGTGTG 0.275948128707
ATGGTTAA 2.1010715074
ATGGTTAC -0.42388619011
ATGGTTAG 0.824367749542
ATGGTTCA -0.507569075675
ATGGTTCC -0.0287083245988
ATGGTTCG -0.214418117367
ATGGTTGA 0.866769071699
ATGGTTGC -0.478036614911
ATGGTTGG -0.114021969928
ATGGTTTA 0.992253670044

ATGGTTTC 0.0813842958284
ATGGTTTG -0.313503697495
ATGTAAAA 2.28054630363
ATGTAAAC -0.00656799669059
ATGTAAAG 1.97948201756
ATGTAAAT 2.59548629298
ATGTAACA 0.438915893139
ATGTAACC 0.541513396362
ATGTAACG -0.101444287631
ATGTAAGA 0.303404958235
ATGTAAGC 0.928060964407
ATGTAAGG -0.0868186805464
ATGTAATA 2.37506162659
ATGTAATC 16.7083374456
ATGTAATG 1.57069009174
ATGTACAA 0.988052222304
ATGTACAC 0.09509924944
ATGTACAG 0.560638687585
ATGTACAT 1.47760792406
ATGTACCA -0.646725499668
ATGTACCC -0.81673514562
ATGTACCG 0.899352378427
ATGTACGA 1.08314755102
ATGTACGC 0.506318364748
ATGTACGG -0.885080420639
ATGTACTA 0.061636916412
ATGTACTC 0.592146931817
ATGTACTG 0.0661342481161
ATGTAGAA 0.708595045699
ATGTAGAC 0.520186487974
ATGTAGAG 0.172789961966
ATGTAGCA -0.0927638048988
ATGTAGCC -0.440003501951
ATGTAGCG -0.302509464894
ATGTAGGA 1.11989179134
ATGTAGGC -0.999103436093
ATGTAGGG 0.502641509867
ATGTAGTA 1.36635503073
ATGTAGTC 0.46286393615
ATGTAGTG 0.488740713943
ATGTATAA 2.17874862808
ATGTATAC 1.87273743397
ATGTATAG -0.126916446716
ATGTATCA 0.888257775294
ATGTATCC 1.50192817446
ATGTATCG -0.202938237765
ATGTATGA 0.186393305637
ATGTATGC 0.373689946061
ATGTATGG 0.890598186085
ATGTATTA 1.11229421255
ATGTATTC 3.01818790171
ATGTATTG -0.213018418932
ATGTCAAA 1.36322472477
ATGTCAAC 0.384979539864

ATGTCAAG -0.180275408051
ATGTCAAT 1.83920112995
ATGTCACA 0.27096488862
ATGTCACC 0.47574874182
ATGTCACG 0.19545122339
ATGTCAGA 0.525018910881
ATGTCAGC 0.0287679196022
ATGTCAGG -0.400795740108
ATGTCATA 0.846642166208
ATGTCATC -0.418547732437
ATGTCATG 0.5810549425
ATGTCCAA 1.3995682671
ATGTCCAC 0.175109984993
ATGTCCAG -0.722357832142
ATGTCCCA -0.795497106974
ATGTCCCC 0.14752429434
ATGTCCCG 0.880133773975
ATGTCCGA 0.361663778928
ATGTCCGC -0.192868773243
ATGTCCGG 0.941292623452
ATGTCCTA 0.459278042064
ATGTCCTC 0.157014798631
ATGTCCTG 1.17596049566
ATGTCGAA 1.12629799282
ATGTCGAC 0.544643963712
ATGTCGAG -0.538637937448
ATGTCGCA 1.12477570642
ATGTCGCC -0.267737348699
ATGTCGCG 1.54473934295
ATGTCGGA 0.337527279787
ATGTCGGC 0.397482728406
ATGTCGGG -0.233457152664
ATGTCGTA 1.21931533787
ATGTCGTC -0.674463314409
ATGTCGTG 0.816704302592
ATGTCTAA 1.22399171597
ATGTCTAC -0.0777845485177
ATGTCTAG 0.236807541934
ATGTCTCA 0.194755164206
ATGTCTCC 0.201893756916
ATGTCTCG 0.356944272869
ATGTCTGA 0.608603255585
ATGTCTGC -0.0253796300009
ATGTCTGG 0.0737378386815
ATGTCTTA 0.523468656648
ATGTCTTC 0.487266260372
ATGTCTTG 0.835782022365
ATGTGAAA 2.50344808339
ATGTGAAC 0.161089476298
ATGTGAAG 1.1575273429
ATGTGAAT 1.90960478502
ATGTGACA 0.425290332032
ATGTGACC -0.887328563727
ATGTGACG -0.0904194733835

ATGTGAGA 0.392180341393
ATGTGAGC -1.02060416324
ATGTGAGG -0.0253835507248
ATGTGATA 3.39969524848
ATGTGATC 2.81087988646
ATGTGATG 0.365692714816
ATGTGCAA 0.678783952156
ATGTGCAC 0.771645252389
ATGTGCAG -0.402230463677
ATGTGCCA -0.520196681856
ATGTGCCC -0.437581017339
ATGTGCCG 0.304619859883
ATGTGCGA 1.08794181221
ATGTGCGC 1.01205829203
ATGTGCGG 0.122007700383
ATGTGCTA 0.881418987272
ATGTGCTC 0.460467589698
ATGTGCTG 0.263253086075
ATGTGGAA 0.643566964488
ATGTGGAC -0.0286003740005
ATGTGGAG 0.200731654349
ATGTGGCA -0.607413707951
ATGTGGCC -0.595505685298
ATGTGGCG 0.406530452278
ATGTGGGA 0.00433475235263
ATGTGGGC -1.27564307135
ATGTGGGG 0.384430115753
ATGTGGTA 0.681861981805
ATGTGGTC -0.0513792571425
ATGTGGTG 0.396626180923
ATGTGTAA 0.99132158328
ATGTGTAC -0.501291735317
ATGTGTAG 0.552034005515
ATGTGTCA 0.584953710354
ATGTGTCC 0.0135981160391
ATGTGTCG 1.10811628916
ATGTGTGA 1.76585536366
ATGTGTGC -0.593134170097
ATGTGTGG 0.202046665148
ATGTGTTA 0.343927469495
ATGTGTTC 0.133263053197
ATGTGTTG -0.00367241139378
ATGTTAAA 1.37839008484
ATGTTAAC 1.99003033317
ATGTTAAG -0.233010712902
ATGTTAAT 2.81473421944
ATGTTACA 1.34967862366
ATGTTACC 0.389337555178
ATGTTACG 0.756184269731
ATGTTAGA 2.11792957468
ATGTTAGC 0.638333059453
ATGTTAGG 0.241877822092
ATGTTATA 1.54236390703
ATGTTATC 2.87460001417

ATGTTATG 1.34256146424
ATGTTCAA 0.321751855071
ATGTTCAC -0.252780571135
ATGTTCAG 1.21469986169
ATGTTCCA -0.0782035432127
ATGTTCCC 0.645332597155
ATGTTCCG -0.203605544974
ATGTTCGA 1.27280551276
ATGTTCGC -0.497976632562
ATGTTCGG 0.446644685488
ATGTTCTA 1.86560772824
ATGTTCTC 0.331745780313
ATGTTCTG 0.845044601907
ATGTTGAA 1.72711599764
ATGTTGAC 0.010279876705
ATGTTGAG 1.12225180575
ATGTTGCA 0.908512235002
ATGTTGCC -0.626669689971
ATGTTGCG 0.932655791446
ATGTTGGA 1.2342953784
ATGTTGGC 0.117189914845
ATGTTGGG 0.935366579956
ATGTTGTA 0.442867460075
ATGTTGTC 1.11588376598
ATGTTGTG 1.6044898684
ATGTTTAA 2.18657648405
ATGTTTAC 0.676609780058
ATGTTTAG 0.488476718533
ATGTTTCA 0.601730749338
ATGTTTCC 0.00587847204598
ATGTTTCG 0.247677356897
ATGTTTGA 1.90011794007
ATGTTTGC -0.410446732698
ATGTTTGG -0.00617853811574
ATGTTTTA 0.830265563826
ATGTTTTC 1.97261865967
ATGTTTTG 0.212886159846
ATTAAAAA 1.59307637972
ATTAAAAC 1.00427774612
ATTAAAAG -0.0183777398645
ATTAAAAT 1.50727447358
ATTAAACA 0.0969103625039
ATTAAACC -0.122817983324
ATTAAACG 0.165016473364
ATTAAAGA 0.649402308571
ATTAAAGC 0.563959017972
ATTAAAGG 0.152924438069
ATTAAATA 1.43872898026
ATTAAATC 1.82186734818
ATTAAATG 0.81002626425
ATTAACAA 0.786646726206
ATTAACAC 1.24127975597
ATTAACAG 0.792146194941
ATTAACCA 0.651203489135

ATTAACCC 0.127758095448
ATTAACCG -0.297115594323
ATTAACGA 1.93587363521
ATTAACGC 0.872873900205
ATTAACGG -0.0763459042251
ATTAACTA 1.31860636393
ATTAACTC 0.083512203384
ATTAACTG 1.56394749214
ATTAAGAA 2.47947520912
ATTAAGAC 0.289037334783
ATTAAGAG 0.289401700725
ATTAAGCA -0.631979918432
ATTAAGCC -0.0302967405447
ATTAAGCG 1.06226682101
ATTAAGGA 0.165565636093
ATTAAGGC -0.459108928173
ATTAAGGG 0.17746947664
ATTAAGTA 2.32730590248
ATTAAGTC 0.681101622749
ATTAAGTG 1.18789256542
ATTAATAA 1.68026021036
ATTAATAC 1.26428708662
ATTAATAG -0.051912475594
ATTAATCA 1.16204140302
ATTAATCC 1.80855047873
ATTAATCG 0.527107349816
ATTAATGA 1.42507231474
ATTAATGC 2.04778102804
ATTAATGG 0.448139265441
ATTAATTA 1.76938244689
ATTAATTC 1.42507231474
ATTAATTG 0.92558358966
ATTACAAA 4.2060140762
ATTACAAC 2.02603068147
ATTACAAG 1.95284827868
ATTACAAT 5.4405465277
ATTACACA 4.37800316496
ATTACACC 4.22741913768
ATTACACG 3.34166722773
ATTACAGA 4.73466253189
ATTACAGC 2.457034031
ATTACAGG 2.10636945092
ATTACATA 2.53720786867
ATTACATC 2.60030381714
ATTACATG 2.95245278328
ATTACCAA 3.77260862982
ATTACCAC 2.57172147847
ATTACCAG 1.88512796705
ATTACCCA 2.37576787965
ATTACCCC 1.61992941777
ATTACCCG 1.67468546372
ATTACCGA 1.22008432252
ATTACCGC 2.86350436551
ATTACCGG 2.48972973181

ATTACCTA 3.76080855777
ATTACCTC 3.85927048141
ATTACCTG 1.51783977898
ATTACGAA 5.24207294894
ATTACGAC 5.83393000857
ATTACGAG 4.70311612595
ATTACGCA 5.30925821245
ATTACGCC 1.65190605782
ATTACGCG 3.13178747898
ATTACGGA 5.16654543049
ATTACGGC 2.52950390757
ATTACGGG 1.87928086079
ATTACGTA 5.41709118899
ATTACGTC 3.15530241269
ATTACGTG 4.61877900225
ATTACTAA 1.02728272435
ATTACTAC 0.706239213394
ATTACTAG 2.12699716355
ATTACTCA 1.84638572582
ATTACTCC 0.937911652535
ATTACTCG 2.3695443839
ATTACTGA 2.36262901107
ATTACTGC 1.50737196891
ATTACTGG 0.900626352315
ATTACTTA 2.81328668817
ATTACTTC 2.19981284797
ATTACTTG 1.41291153609
ATTAGAAA 3.6061103842
ATTAGAAC 0.138842243318
ATTAGAAG 1.10305646426
ATTAGAAT 1.44498619423
ATTAGACA 1.18977921776
ATTAGACC 0.706239736157
ATTAGACG 0.758529908154
ATTAGAGA 0.213874182271
ATTAGAGC 0.807550457793
ATTAGAGG 0.244393881315
ATTAGATA 4.24105410853
ATTAGATC 1.55505973381
ATTAGATG 1.98336092042
ATTAGCAA 2.30202298367
ATTAGCAC 0.25097076498
ATTAGCAG 0.74446522597
ATTAGCCA -0.127611983137
ATTAGCCC -0.426015665955
ATTAGCCG 0.453310438894
ATTAGCGA 1.82663808503
ATTAGCGC -0.533212962467
ATTAGCGG 0.531090282543
ATTAGCTA -0.187600104455
ATTAGCTC 1.14801174597
ATTAGCTG 1.06967751196
ATTAGGAA 0.51568915627
ATTAGGAC 0.47230503932

ATTAGGAG 0.0985933985867
ATTAGGCA -0.0302188488297
ATTAGGCC 1.38832885857
ATTAGGCG -0.0236043262154
ATTAGGGA 0.439449634353
ATTAGGGC -0.451685690907
ATTAGGGG 0.153496863759
ATTAGGTA 1.35324308446
ATTAGGTC 0.243994490239
ATTAGGTG 0.347723514382
ATTAGTAA 1.49257829346
ATTAGTAC 0.345321156153
ATTAGTAG 1.21995206343
ATTAGTCA 1.04282525806
ATTAGTCC -0.410702102516
ATTAGTCG 0.63682227384
ATTAGTGA 0.272860950702
ATTAGTGC 0.538959959571
ATTAGTGG 1.65792123244
ATTAGTTA -0.316734896759
ATTAGTTC -0.153905141809
ATTAGTTG 0.629863511667
ATTATAAA 1.23937271586
ATTATAAC 1.13590324364
ATTATAAG 1.62332763987
ATTATAAT 2.95059828088
ATTATACA 2.94508469753
ATTATACC 0.786249426183
ATTATACG 2.37137013434
ATTATAGA 2.01332779739
ATTATAGC 1.88210587306
ATTATAGG 1.75133509336
ATTATATA 4.28584681089
ATTATATC 2.93407844138
ATTATATG 2.79765031846
ATTATCAA 2.28504755605
ATTATCAC 1.79727499954
ATTATCAG 2.94272964937
ATTATCCA 2.01332779739
ATTATCCC 1.71422831677
ATTATCCG 3.16034289534
ATTATCGA 1.40607666879
ATTATCGC 1.36417197166
ATTATCGG 1.72758753003
ATTATCTA 2.18763168155
ATTATCTC 0.578809151845
ATTATCTG 3.90387289797
ATTATGAA 2.37134530308
ATTATGAC 2.61252523632
ATTATGAG 1.69674502472
ATTATGCA 1.72495541738
ATTATGCC 1.74228135771
ATTATGCG 1.58700997431
ATTATGGA 2.74370298716

ATTATGGC 2.04820237517
ATTATGGG 0.575081850317
ATTATGTA 3.19395656832
ATTATGTC 1.9399394259
ATTATGTG 0.332441316734
ATTATTAA 1.24011530096
ATTATTAC 3.25216677203
ATTATTAG 1.42012331564
ATTATTCA 1.61566706812
ATTATTCC 3.68449715591
ATTATTCG 1.93700724718
ATTATTGA 2.20235922745
ATTATTGC 2.86860182935
ATTATTGG 0.647506507871
ATTATTTA 2.46471289946
ATTATTTC 3.66462379057
ATTATTTG 1.8079247312
ATTCAAAA 2.720373373
ATTCAAAC 1.84109876033
ATTCAAAG 1.08546522161
ATTCAAAT 2.70917369447
ATTCAACA 1.64371279038
ATTCAACC 0.432739707457
ATTCAACG 0.2883086029
ATTCAAGA 0.395855105221
ATTCAAGC 1.05282571784
ATTCAAGG 0.35181152251
ATTCAATA 0.960356751382
ATTCAATC 1.89842497149
ATTCAATG 0.722079722126
ATTCACAA 2.27112140611
ATTCACAC 0.761085697379
ATTCACAG 1.40433586738
ATTCACCA 1.86084143487
ATTCACCC 0.332882528864
ATTCACCG 1.44106364066
ATTCACGA 0.614177740837
ATTCACGC 1.00908716745
ATTCACGG 0.968582952904
ATTCACTA 1.61558551706
ATTCACTC 0.00411153247147
ATTCACTG 0.0215545717563
ATTCAGAA 0.583366601316
ATTCAGAC 0.588911027685
ATTCAGAG -0.488534483865
ATTCAGCA 2.24087615774
ATTCAGCC 0.192890990678
ATTCAGCG -0.159177208557
ATTCAGGA 1.21510369625
ATTCAGGC 0.068281236528
ATTCAGGG 0.24023425463
ATTCAGTA 1.07232739856
ATTCAGTC 1.32922211598
ATTCAGTG 1.19369262299

ATTCATAA	1.48064465541
ATTCATAC	1.01553701966
ATTCATAG	1.28668069329
ATTCATCA	1.56347909633
ATTCATCC	0.0168870806347
ATTCATCG	0.689730351925
ATTCATGA	0.480325272147
ATTCATGC	1.25981118821
ATTCATGG	1.11461057624
ATTCATTA	2.44782973955
ATTCATTC	1.37051700985
ATTCATTG	0.990846652924
ATTCCAAA	2.82157849647
ATTCCAAC	2.07646060066
ATTCCAAG	2.61238147644
ATTCCAAT	2.3486934514
ATTCCACA	1.53233678632
ATTCCACC	1.19175238742
ATTCCACG	1.74021330654
ATTCCAGA	1.70880517146
ATTCCAGC	-0.106369762386
ATTCCAGG	0.854056777881
ATTCCATA	2.9028705246
ATTCCATC	1.47002628954
ATTCCATG	2.35544912007
ATTCCCAA	3.26294039857
ATTCCCAC	3.41262527316
ATTCCCAG	3.2007362841
ATTCCCCA	2.89040732744
ATTCCCCC	4.36697652104
ATTCCCCG	4.55024004347
ATTCCCGA	3.23350151241
ATTCCCGC	0.641425726471
ATTCCCGG	2.78911751633
ATTCCCTA	4.389252506
ATTCCCTC	4.5659088245
ATTCCCTG	3.75340779932
ATTCCGAA	1.67869793257
ATTCCGAC	2.1314568563
ATTCCGAG	2.710711141
ATTCCGCA	3.85776806001
ATTCCGCC	1.71577334337
ATTCCGCG	3.17349143504
ATTCCGGA	2.59001191688
ATTCCGGC	1.5561248638
ATTCCGGG	1.19085035953
ATTCCGTA	3.63902460003
ATTCCGTC	2.7858092095
ATTCCGTG	3.87136460776
ATTCCTAA	2.5301424628
ATTCCTAC	2.01413442099
ATTCCTAG	2.0786768552
ATTCCTCA	2.32760779822
ATTCCTCC	1.90452927723

ATTCCTCG 3.29693673419
ATTCCTGA 2.3343548413
ATTCCTGC 1.9074920376
ATTCCTGG 0.684648832359
ATTCCTTA 3.248662429
ATTCCTTC 0.943397790809
ATTCCTTG 0.463001945632
ATTCGAAA 2.10651294942
ATTCGAAC 1.09413028283
ATTCGAAG 0.6479179225
ATTCGAAT 3.838244946
ATTCGACA 1.68481060253
ATTCGACC 0.47206718207
ATTCGACG 2.06154747383
ATTCGAGA 1.46957880426
ATTCGAGC 1.82390403355
ATTCGAGG 0.474031987511
ATTCGATA 3.52865909722
ATTCGATC 2.5898184945
ATTCGATG 1.46165502124
ATTCGCAA 3.50121455263
ATTCGCAC 3.04477563743
ATTCGCAG 2.11935462712
ATTCGCCA 3.56810393217
ATTCGCCC 1.63775119898
ATTCGCCG 1.64557800943
ATTCGCGA 3.92663113197
ATTCGCGC 2.09499281705
ATTCGCGG 2.84685775594
ATTCGCTA 2.04871991073
ATTCGCTC 2.95356313229
ATTCGCTG 2.34768242739
ATTCGGAA 2.25753557501
ATTCGGAC 0.305904289035
ATTCGGAG 2.16754189224
ATTCGGCA 1.10788575059
ATTCGGCC 0.0256245445544
ATTCGGCG 0.641405600089
ATTCGGGA 2.81824326734
ATTCGGGC 1.33032776013
ATTCGGGG 1.25863758485
ATTCGGTA 2.6064021111
ATTCGGTC 1.93295008208
ATTCGGTG 1.3159057693
ATTCGTAA 2.15776569787
ATTCGTAC 1.30153971414
ATTCGTAG 0.795448751379
ATTCGTCA 1.28437347796
ATTCGTCC 0.113609509773
ATTCGTCG 2.4684046531
ATTCGTGA 2.02892548262
ATTCGTGC 0.338247124697
ATTCGTGG 1.19516707656
ATTCGTTA 2.76237556546

ATTCGTTC 1.56477764008
ATTCGTTG 0.99649066568
ATTCTAAA 2.38786827916
ATTCTAAC 0.766117293061
ATTCTAAG 3.25015805448
ATTCTACA 2.51609581595
ATTCTACC 1.89776053948
ATTCTACG 1.04637168353
ATTCTAGA 1.29598796909
ATTCTAGC 1.44894429571
ATTCTAGG 2.56524339704
ATTCTATA 4.70482530019
ATTCTATC 4.2150955183
ATTCTATG 1.5544122916
ATTCTCAA 3.36091197034
ATTCTCAC 2.62382633091
ATTCTCAG 4.2416665256
ATTCTCCA 2.79057158214
ATTCTCCC 2.01080651054
ATTCTCCG 4.15600079927
ATTCTCGA 4.75851752306
ATTCTCGC 0.66293638612
ATTCTCGG 2.73050243253
ATTCTCTA 4.87905939546
ATTCTCTC 2.2852854133
ATTCTCTG 4.40635784026
ATTCTGAA 2.96382183709
ATTCTGAC 1.95574647779
ATTCTGAG 2.18467989921
ATTCTGCA 3.83602555489
ATTCTGCC 1.03610043241
ATTCTGCG 2.64545252123
ATTCTGGA 2.15689268335
ATTCTGGC 1.19577008389
ATTCTGGG 1.29295829503
ATTCTGTA 5.98760774839
ATTCTGTC 2.93499432249
ATTCTGTG 4.45031595119
ATTCTTAA 3.87622395297
ATTCTTAC 0.559791027076
ATTCTTAG 2.91002610711
ATTCTTCA 2.51224801751
ATTCTTCC 1.97658015911
ATTCTTCG 2.1647534734
ATTCTTGA 2.41294705894
ATTCTTGC 1.61911913483
ATTCTTGG 2.92742235909
ATTCTTTA 2.17555428362
ATTCTTTC 2.34121584676
ATTCTTTG 4.2784366427
ATTGAAAA 2.03730772896
ATTGAAAC -0.143629708591
ATTGAAAG 0.886175086754
ATTGAAAT 3.54294203303

ATTGAACA 1.05690170242
ATTGAACC 0.524407277952
ATTGAACG -0.266712210088
ATTGAAGA 1.60383327784
ATTGAAGC -0.726281169866
ATTGAAGG 0.609011795016
ATTGAATA 2.35292914012
ATTGAATC 1.83316582895
ATTGAATG 0.263287327064
ATTGACAA 1.39386518211
ATTGACAC 0.737811496116
ATTGACAG 1.25199509441
ATTGACCA 0.229461412239
ATTGACCC -0.234006576775
ATTGACCG 0.158760043533
ATTGACGA -0.734592059025
ATTGACGC 0.812939623496
ATTGACGG 0.93994598548
ATTGACTA -0.233101673697
ATTGACTC 0.130956360631
ATTGACTG -0.196665340894
ATTGAGAA 2.02892548262
ATTGAGAC -0.0957926948085
ATTGAGAG 0.58160436661
ATTGAGCA 0.460863844194
ATTGAGCC -0.955884250929
ATTGAGCG -0.471826188241
ATTGAGGA 0.586860489081
ATTGAGGC -0.990892917466
ATTGAGGG -0.588788439718
ATTGAGTA -0.0918460941228
ATTGAGTC 1.60931366572
ATTGAGTG 0.789736256645
ATTGATAA 1.71779983487
ATTGATAC 1.93163533266
ATTGATAG 1.28284021353
ATTGATCA -0.0653893105735
ATTGATCC 0.373376288149
ATTGATCG -0.141083851867
ATTGATGA 0.215620472699
ATTGATGC 0.912796802089
ATTGATGG -0.172712854395
ATTGATTA 1.69754746621
ATTGATTC 2.54708783153
ATTGATTG 1.726505933
ATTGCAAA 1.58447509562
ATTGCAAC 1.91609959487
ATTGCAAG 0.804625597758
ATTGCAAT 3.63936282781
ATTGCACA 2.55178851812
ATTGCACC 2.03497332994
ATTGCACG 2.33817179672
ATTGCAGA 1.91387053264
ATTGCAGC 1.08524017206

ATTGCAGG 2.37216081366
ATTGCATA 3.02269987078
ATTGCATC 1.97605739592
ATTGCATG 2.02604558022
ATTGCCAA -0.248631922478
ATTGCCAC 0.440980023586
ATTGCCAG 1.83340211791
ATTGCCCA 0.761670669386
ATTGCCCC 0.578383883992
ATTGCCCG 2.95464289966
ATTGCCGA 1.57927909091
ATTGCCGC 1.14410435253
ATTGCCGG 0.69054455559
ATTGCCTA -0.14620091933
ATTGCCTC 1.40122072154
ATTGCCTG 0.89812127112
ATTGCGAA 3.28001436704
ATTGCGAC 3.82740074644
ATTGCGAG 3.03246090502
ATTGCGCA 4.32540822203
ATTGCGCC 1.88701252834
ATTGCGCG 3.6592241696
ATTGCGGA 4.7837729968
ATTGCGGC 0.529069018678
ATTGCGGG 3.10002700151
ATTGCGTA 5.24010396139
ATTGCGTC 2.37543540226
ATTGCGTG 4.38417595268
ATTGCTAA 3.92083159716
ATTGCTAC 1.9461621375
ATTGCTAG 1.36551834825
ATTGCTCA 0.579421830301
ATTGCTCC 1.2345125865
ATTGCTCG 0.956221955948
ATTGCTGA 0.860201075008
ATTGCTGC 0.823733637796
ATTGCTGG 0.697690205603
ATTGCTTA 2.53814309201
ATTGCTTC 0.85634255992
ATTGCTTG 1.13204106921
ATTGGAAA -0.0289767634957
ATTGGAAC 0.795954002
ATTGGAAG 0.235016555253
ATTGGACA 0.144764627472
ATTGGACC -0.239705218284
ATTGGACG 0.173843852552
ATTGGAGA 0.520936391767
ATTGGAGC 0.550861709066
ATTGGAGG -0.352014093246
ATTGGATA 4.53483368957
ATTGGATC 2.0708976162
ATTGGATG 0.62271394093
ATTGGCAA -0.20980055013
ATTGGCAC -0.00788143919975

ATTGGCAG -0.561329519137
ATTGGCCA -0.593454885313
ATTGGCCC -0.147545204867
ATTGGCCG 0.06254181949
ATTGGCGA 0.695760163914
ATTGGCGC 0.369579720498
ATTGGCGG 0.941292623452
ATTGGCTA 0.306061117992
ATTGGCTC -0.681315171511
ATTGGCTG 0.89092203788
ATTGGGAA 1.88801179017
ATTGGGAC -0.252888521734
ATTGGGAG 0.542602312082
ATTGGGCA -0.98408131313
ATTGGGCC -1.17473644565
ATTGGGCG -0.659491899476
ATTGGGGA 0.514760728848
ATTGGGGC 0.649933697352
ATTGGGGG -0.690953356403
ATTGGGTA 2.22355152432
ATTGGGTC 0.438268450931
ATTGGGTG 0.653109483717
ATTGGTAA 0.874376321607
ATTGGTAC 0.880518527681
ATTGGTAG -0.200016252927
ATTGGTCA 0.252601786125
ATTGGTCC -0.0433728775409
ATTGGTCG 0.857174276151
ATTGGTGA 1.02335598866
ATTGGTGC 0.644009222145
ATTGGTGG -0.465728678419
ATTGGTTA 0.803955415351
ATTGGTTC 0.273682473052
ATTGGTTG 1.38418439201
ATTGTAAA 2.02321534032
ATTGTAAC 0.354374107657
ATTGTAAG 0.582059954728
ATTGTACA 1.11301510299
ATTGTACC 0.999179759518
ATTGTACG 0.226171663499
ATTGTAGA 2.07001310089
ATTGTAGC 1.41189972794
ATTGTAGG 0.637236825048
ATTGTATA 0.991887213049
ATTGTATC 1.81078450721
ATTGTATG 1.41051518964
ATTGTCAA 0.175795588913
ATTGTCAC 0.458852774211
ATTGTCAG 1.07999110689
ATTGTCCA 2.45602091594
ATTGTCCC 0.677771359861
ATTGTCCG 1.23902664663
ATTGTCGA 1.03482645853
ATTGTCGC 1.06684831759

ATTGTCGG 0.570701617567
ATTGTCTA 1.84840803522
ATTGTCTC 1.79851891454
ATTGTCTG -0.97905494508
ATTGTGAA 1.31622413208
ATTGTGAC 1.65700378304
ATTGTGAG 1.05663666148
ATTGTGCA 2.00400117936
ATTGTGCC 1.19491066121
ATTGTGCG 1.84535248438
ATTGTGGA 1.32972762799
ATTGTGGC 1.39306143371
ATTGTGGG 1.16557240697
ATTGTGTA 2.81111722094
ATTGTGTC 2.06620581659
ATTGTGTG 1.31475098542
ATTGTTAA 2.2431091407
ATTGTTAC 0.720017944114
ATTGTTAG 1.63758809687
ATTGTTCA 0.523010193332
ATTGTTCC 2.43894903852
ATTGTTCG 1.07552853894
ATTGTTGA -0.166419308378
ATTGTTGC 1.07368135522
ATTGTTGG 1.30078223028
ATTGTTTA 0.698886026395
ATTGTTTC 0.985241847407
ATTGTTTG 0.987479012469
ATTTAAAA 2.95464289966
ATTTAAAC 0.95984967109
ATTTAAAG 2.47003149214
ATTTAAAT 3.12195378065
ATTTAACA 2.84031981814
ATTTAACC 0.950649561749
ATTTAACG 1.07435389006
ATTTAAGA 2.21534492642
ATTTAAGC 0.725591122458
ATTTAAGG 0.839531018566
ATTTAATA 1.07388575562
ATTTAATC 1.64826605774
ATTTAATG 1.67592101452
ATTTACAA 2.91976335563
ATTTACAC 2.42764010248
ATTTACAG 1.03254590412
ATTTACCA 1.16928036626
ATTTACCC 1.21753323816
ATTTACCG 1.42051538804
ATTTACGA 2.94766244281
ATTTACGC 1.62037193681
ATTTACGG 0.615133874708
ATTTACTA 3.68463621092
ATTTACTC 1.57170817304
ATTTACTG 2.11280754096
ATTTAGAA 3.94647077909

ATTTAGAC 2.23834127905
ATTTAGAG 0.746611430237
ATTTAGCA 2.37496700645
ATTTAGCC -0.351187081883
ATTTAGCG 2.69763003776
ATTTAGGA 1.19389859168
ATTTAGGC 0.729574055186
ATTTAGGG 0.835011208045
ATTTAGTA 3.21962790018
ATTTAGTC 1.10859775405
ATTTAGTG 0.592916177848
ATTTATAA 1.96624565365
ATTTATAC 2.62786964279
ATTTATAG 2.35743614295
ATTTATCA 1.5178068449
ATTTATCC 0.910725614339
ATTTATCG 2.08573729481
ATTTATGA 1.47755564774
ATTTATGC 1.50810592843
ATTTATGG 0.776818255513
ATTTATTA 2.71707787387
ATTTATTC 0.89419531958
ATTTATTG 2.29446304383
ATTTCAAA 4.14403579543
ATTTCAAC 1.74374300359
ATTTCAAG 2.37661684707
ATTTCACA 2.27435312814
ATTTCACC 0.77612245771
ATTTCACG 1.89600248688
ATTTCAGA 4.07301841637
ATTTCAGC 3.18394460774
ATTTCAGG 2.38950086859
ATTTCATA 3.23478698709
ATTTCATC 1.78979321279
ATTTCATG 3.47996997944
ATTTCCAA 3.05059712829
ATTTCCAC 3.97471149201
ATTTCCAG 4.46856221612
ATTTCCCA 2.72385837379
ATTTCCCC 1.94038978639
ATTTCCCG 3.3850056029
ATTTCCGA 3.46596123292
ATTTCCGC 2.46008539973
ATTTCCGG 3.50794120794
ATTTCCTA 4.98964942506
ATTTCCTC 1.41716787396
ATTTCCTG 4.86038916959
ATTTCGAA 3.4300149906
ATTTCGAC 1.23851982772
ATTTCGAG 1.55632612763
ATTTCGCA 2.07720004919
ATTTCGCC 1.35348721487
ATTTCGCG 3.02956009209
ATTTCGGA 2.91313132045

ATTTCGGC	3.32853515507
ATTTCGGG	1.24250380597
ATTTCGTA	6.08686453346
ATTTCGTC	3.25127389251
ATTTCGTG	4.20754577234
ATTTCTAA	5.23767258981
ATTTCTAC	1.48845316915
ATTTCTAG	2.61701786316
ATTTCTCA	1.85019431703
ATTTCTCC	3.6611824405
ATTTCTCG	3.32940660131
ATTTCTGA	2.04835946551
ATTTCTGC	1.95406527138
ATTTCTGG	2.12487840435
ATTTCTTA	2.77460116675
ATTTCTTC	2.06941688947
ATTTCTTG	1.28540985598
ATTTGAAA	2.54052401695
ATTTGAAC	1.75356284869
ATTTGAAG	1.2970272223
ATTTGACA	2.45406996373
ATTTGACC	1.16474774805
ATTTGACG	1.55091134653
ATTTGAGA	1.92025190287
ATTTGAGC	0.115737940091
ATTTGAGG	1.27625522704
ATTTGATA	1.73555810036
ATTTGATC	2.03395603278
ATTTGATG	0.976879727456
ATTTGCAA	3.62679612354
ATTTGCAC	2.52787262504
ATTTGCAG	3.37123131567
ATTTGCCA	3.5966640534
ATTTGCCC	2.56986619191
ATTTGCCG	1.25971709083
ATTTGCGA	4.65377381557
ATTTGCGC	3.71144402995
ATTTGCGG	1.71300191433
ATTTGCTA	1.48649228443
ATTTGCTC	4.12112778978
ATTTGCTG	3.03323668559
ATTTGGAA	1.62931615357
ATTTGGAC	0.78975350783
ATTTGGAG	0.792802262741
ATTTGGCA	0.583380715922
ATTTGGCC	0.529029550057
ATTTGGCG	1.27471516669
ATTTGGGA	2.28833625927
ATTTGGGC	1.46633584282
ATTTGGGG	0.99950308855
ATTTGGTA	1.23666271149
ATTTGGTC	2.89613419816
ATTTGGTG	3.03917841198
ATTTGTAA	4.2060140762

ATTTGTAC 3.53183775878
ATTTGTAG 1.67606503578
ATTTGTCA 2.77901328806
ATTTGTCC 1.41779858775
ATTTGTCG 2.17445386711
ATTTGTGA 2.1363263954
ATTTGTGC 0.740105119602
ATTTGTGG 1.43758857236
ATTTGTTA 3.03769428729
ATTTGTTC 1.21635963481
ATTTGTTG 2.56573322615
ATTTTAAA 2.66806856363
ATTTTAAC 2.45741617089
ATTTTAAG 3.22854441049
ATTTTACA 4.29044085378
ATTTTACC 1.58124206668
ATTTTACG 3.66955710677
ATTTTAGA 2.36079489643
ATTTTAGC 1.4039393515
ATTTTAGG 1.71606217003
ATTTTATA 4.15433971924
ATTTTATC 2.34077829397
ATTTTATG 1.32664855281
ATTTTCAA 1.75716520981
ATTTTCAC 4.3091529007
ATTTTCAG 2.92446717879
ATTTTCCA 2.50634236177
ATTTTCCC 3.78206306345
ATTTTCCG 4.53051958636
ATTTTCGA 4.02343641907
ATTTTCGC 2.88794824941
ATTTTCGG 4.0060357236
ATTTTCTA 4.29422121577
ATTTTCTC 4.01660494973
ATTTTCTG 1.63456600288
ATTTTGAA 4.07475974055
ATTTTGAC 2.84999459645
ATTTTGAG 1.94144419974
ATTTTGCA 2.99083954554
ATTTTGCC 1.06821691162
ATTTTGCG 2.86775469161
ATTTTGGA 1.43284266677
ATTTTGGC 2.35162040248
ATTTTGGG 2.64660991892
ATTTTGTA 3.00330823171
ATTTTGTC 4.40635784026
ATTTTGTG 3.54007807491
ATTTTTAA 2.68497969138
ATTTTTAC 3.37088681473
ATTTTTAG 2.52148602718
ATTTTTCA 1.00928215812
ATTTTTCC 2.65448116424
ATTTTTCG 1.60333717557
ATTTTTGA 1.69375534205

ATTTTTGC 0.989480411333
ATTTTTGG 2.75631856979
ATTTTTTA 3.75136640907
ATTTTTTC 4.17660603307
ATTTTTTG 4.33622706758
CAAAAAAA 2.9870442848
CAAAAAAC 1.57491637073
CAAAAAAG 0.825452483157
CAAAAACA 0.899789408452
CAAAAACC 2.04593515122
CAAAAACG 1.02831910237
CAAAAAGA 0.402975139838
CAAAAAGC -0.8005195543
CAAAAAGG 0.110122940692
CAAAAATA 1.70600472906
CAAAAATC 6.51348242261
CAAAAATG -0.0435095801144
CAAAACAA 0.984658182308
CAAAACAC 0.0527107349816
CAAAACAG 0.975637119359
CAAAACCA -0.414531342866
CAAAACCC -0.148598049927
CAAAACCG 0.168382022767
CAAAACGA 1.1054431396
CAAAACGC 0.0628316916776
CAAAACGG 0.480399504519
CAAAACTA 0.503966453167
CAAAACTC 0.192458926904
CAAAACTG -0.186625412492
CAAAAGAA 0.415862297942
CAAAAGAC -0.170110800628
CAAAAGAG -0.481697002751
CAAAAGCA -0.609311338323
CAAAAGCC 0.628383830464
CAAAAGCG -0.431731297268
CAAAAGGA 0.627545318311
CAAAAGGC -0.336781819482
CAAAAGGG 0.113039697898
CAAAAGTA 0.728453250911
CAAAAGTC -0.158752724849
CAAAAGTG -0.367076468974
CAAAATAA 1.67875622067
CAAAATAC 2.82754113339
CAAAATAG 2.41551042824
CAAAATCA 4.82881950069
CAAAATCC 7.82338948434
CAAAATCG 7.7317807247
CAAAATGA 0.362591160823
CAAAATGC 0.477784904436
CAAAATGG 0.366653292173
CAAAATTA 1.41493541377
CAAAATTC 2.75464860279
CAAAATTG 1.27793225135
CAAACAAA 1.25291907834

CAAACAAC	0.909351269918
CAAACAAG	0.3937394826
CAAACACA	0.370637009045
CAAACACC	-0.0637812910081
CAAACACG	0.358647435335
CAAACAGA	0.645671609082
CAAACAGC	0.401288705794
CAAACAGG	-0.85540785934
CAAACATA	1.68624924682
CAAACATC	0.0520196420474
CAAACATG	-0.379679766666
CAAACCAA	1.64928021833
CAAACCAC	-0.458753449205
CAAACCAG	-0.007939204532
CAAACCCA	0.337989925208
CAAACCCC	-0.472830677706
CAAACCCG	-0.265075961311
CAAACCGA	0.410617414879
CAAACCGC	-0.480645725981
CAAACCGG	0.86824195698
CAAACCTA	-0.37948033251
CAAACCTC	-0.27713088042
CAAACCTG	-0.768212005154
CAAACGAA	1.08422601148
CAAACGAC	0.535957730584
CAAACGAG	0.797230589704
CAAACGCA	-0.258686226867
CAAACGCC	-0.671858385445
CAAACGCG	-0.777345984951
CAAACGGA	0.0672087878484
CAAACGGC	-0.624343916549
CAAACGGG	0.755382351001
CAAACGTA	1.27726651243
CAAACGTC	0.173414663975
CAAACGTG	0.328555356578
CAAACTAA	1.20222202716
CAAACTAC	1.00126793707
CAAACTAG	0.348283132375
CAAACTCA	0.48762670559
CAAACTCC	0.0103156859834
CAAACTCG	-0.316650209122
CAAACTGA	-0.41468451248
CAAACTGC	-0.0138239497362
CAAACTGG	0.0649407797585
CAAACTTA	1.31579964838
CAAACTTC	-0.013937389348
CAAACTTG	0.258124517822
CAAAGAAA	0.0805233048582
CAAAGAAC	0.841361735249
CAAAGAAG	-0.0345836600656
CAAAGACA	0.400137581255
CAAAGACC	0.176546538233
CAAAGACG	-0.915265289861
CAAAGAGA	-0.348423755672

CAAAGAGC -0.923557882308
CAAAGAGG -0.18708753515
CAAAGATA 1.37227950594
CAAAGATC 6.14770292911
CAAAGATG 0.289869312397
CAAAGCAA 1.22278491715
CAAAGCAC -0.690517110523
CAAAGCAG -0.123308335194
CAAAGCCA -0.626869908272
CAAAGCCC -1.1011117852
CAAAGCCG -0.349936632338
CAAAGCGA -0.410061717611
CAAAGCGC -0.778302902966
CAAAGCGG 0.0772063724321
CAAAGCTA 1.077240327
CAAAGCTC -0.693234956336
CAAAGCTG -1.33463428327
CAAAGGAA 0.920347070809
CAAAGGAC 0.108266608612
CAAAGGAG -0.811295271889
CAAAGGCA -0.776809891302
CAAAGGCC -1.61854906157
CAAAGGCG -0.714019759298
CAAAGGGA -0.602577102939
CAAAGGGC -0.90193744239
CAAAGGGG 0.298386693014
CAAAGGTA -0.0535531678586
CAAAGGTC -1.03154847196
CAAAGGTG -0.68366839
CAAAGTAA 1.24978223783
CAAAGTAC -0.0857660968679
CAAAGTAG -0.903863301974
CAAAGTCA -0.188384510619
CAAAGTCC -0.335526926449
CAAAGTCG -0.582591866272
CAAAGTGA 0.274819221604
CAAAGTGC -0.301779164721
CAAAGTGG -0.293236430089
CAAAGTTA 0.687891009649
CAAAGTTC 0.441005638982
CAAAGTTG -0.453785107869
CAAATAAA 1.97362550157
CAAATAAC 1.02829610078
CAAATAAG 0.848513919802
CAAATACA 2.44782973955
CAAATACC 1.60461637709
CAAATACG 2.80712540124
CAAATAGA 2.03395603278
CAAATAGC 0.426835620015
CAAATAGG 0.681057972022
CAAATATA 2.70634632977
CAAATATC 6.23375863678
CAAATATG 1.59186748985
CAAATCAA 3.30830239004

CAAATCAC	3.10366438777
CAAATCAG	3.9328222164
CAAATCCA	5.91625370985
CAAATCCC	5.2154954071
CAAATCCG	7.14747733175
CAAATCGA	3.05543843817
CAAATCGC	6.01939802336
CAAATCGG	3.94385042861
CAAATCTA	10.2618398068
CAAATCTC	12.4428438966
CAAATCTG	8.84121411571
CAAATGAA	2.24656434399
CAAATGAC	0.164116013773
CAAATGAG	0.752765398483
CAAATGCA	-0.0514571488575
CAAATGCC	0.295665449241
CAAATGCG	1.16849622148
CAAATGGA	-0.328141850896
CAAATGGC	-0.05610346807
CAAATGGG	-0.373017149839
CAAATGTA	0.0350180762746
CAAATGTC	0.9876972661
CAAATGTG	0.500724537258
CAAATTAA	0.546699991329
CAAATTAC	4.27946230408
CAAATTAG	1.29474326994
CAAATTCA	1.79713699005
CAAATTCC	1.01509685306
CAAATTCG	2.13782045259
CAAATTGA	0.150093152644
CAAATTGC	1.74444533593
CAAATTGG	0.512312106077
CAAATTTA	0.879397984788
CAAATTTC	2.59309569692
CAAATTTG	2.93493629577
CAACAAAA	2.27040757298
CAACAAAC	0.770388791067
CAACAAAG	0.985532503739
CAACAACA	0.421000537314
CAACAACC	-0.542292574893
CAACAACG	0.656721515963
CAACAAGA	0.256419525685
CAACAAGC	-0.32440017338
CAACAAGG	1.20634610595
CAACAATA	1.3593403329
CAACAATC	2.19662346976
CAACAATG	0.709930182881
CAACACAA	0.851983237697
CAACACAC	-0.33055963064
CAACACAG	-0.144337007184
CAACACCA	-0.0606295517493
CAACACCC	-0.714258662075
CAACACCG	-0.338173415087
CAACACGA	0.718412799746

CAACACGC	0.436111268637
CAACACGG	0.578511699592
CAACACTA	1.17638184279
CAACACTC	0.924391689592
CAACACTG	0.192103186554
CAACAGAA	0.101489506647
CAACAGAC	-1.11268131869
CAACAGAG	0.233752513865
CAACAGCA	0.223843537642
CAACAGCC	-1.26272010396
CAACAGCG	0.0487970683767
CAACAGGA	-0.0524561493092
CAACAGGC	-0.939247051098
CAACAGGG	-0.227235747967
CAACAGTA	0.84923350333
CAACAGTC	-0.616467182218
CAACAGTG	-0.497378852857
CAACATAA	0.717843772016
CAACATAC	1.66196349879
CAACATAG	0.301847646699
CAACATCA	0.947239054712
CAACATCC	2.0019386172
CAACATCG	-0.163349381558
CAACATGA	0.919864560387
CAACATGC	0.4395654264
CAACATGG	-0.368461791421
CAACATTA	0.160216984538
CAACATTC	0.160207052037
CAACATTG	0.686585669969
CAACCAAA	0.825566445532
CAACCAAC	0.732691030693
CAACCAAG	-0.0789568449662
CAACCACA	0.0969639457306
CAACCACC	-0.159496878246
CAACCACG	0.830006796048
CAACCAGA	0.385932537155
CAACCAGC	-0.722241255951
CAACCAGG	0.0142311822595
CAACCATA	0.206367041513
CAACCATC	0.217973429807
CAACCATG	-0.843574853204
CAACCCAA	1.38871308951
CAACCCAC	0.238093016613
CAACCCAG	-0.352050686669
CAACCCCA	0.0987510116878
CAACCCCC	-1.00654862941
CAACCCCG	-0.728095680891
CAACCCGA	-0.0725841003262
CAACCCGC	0.05163959321
CAACCCGG	-0.816712928185
CAACCCTA	1.10591963824
CAACCCTC	-0.724251803171
CAACCCTG	-0.339267035676
CAACCGAA	0.223034038846

CAACCGAC -0.13758813443
CAACCGAG -0.0383865008747
CAACCGCA 0.0296422410336
CAACCGCC -0.667598910991
CAACCGCG 0.311417872376
CAACCGGA 0.390893559806
CAACCGGC 0.267843208245
CAACCGGG -0.337548713078
CAACCGTA -0.809030923141
CAACCGTC 0.710271024479
CAACCGTG 0.127758095448
CAACCTAA -0.392313907387
CAACCTAC -0.402733100482
CAACCTAG -0.989150547762
CAACCTCA 0.0566408686269
CAACCTCC -1.09295092908
CAACCTCG -0.903076543376
CAACCTGA 0.451294664043
CAACCTGC -0.809608837845
CAACCTGG -0.991288126436
CAACCTTA 0.661690641444
CAACCTTC -0.972724021495
CAACCTTG -0.46431355847
CAACGAAA 0.282367922034
CAACGAAC -0.534006255605
CAACGAAG -0.456086834184
CAACGACA 1.00108915206
CAACGACC -0.695555763508
CAACGACG 0.568986954311
CAACGAGA 0.194388707211
CAACGAGC -0.797142242726
CAACGAGG -0.871859739621
CAACGATA 1.41499161081
CAACGATC 1.71616567714
CAACGATG -0.411593936514
CAACGCAA 1.13777029236
CAACGCAC -0.982394356323
CAACGCAG -0.681037061495
CAACGCCA 1.44204878788
CAACGCCC -0.917427961169
CAACGCCG -0.936900889912
CAACGCGA -0.106940097024
CAACGCGC 0.545453462508
CAACGCGG -0.669824575263
CAACGCTA -0.129797917407
CAACGCTC -0.870262698083
CAACGCTG -0.351404551369
CAACGGAA 0.258164247824
CAACGGAC -0.298231693729
CAACGGAG -0.61125915396
CAACGGCA -0.376040812117
CAACGGCC -0.830064038618
CAACGGCG -0.547380106236
CAACGGGA 0.423087407959

CAACGGGC	-1.32502589588
CAACGGGG	0.615571950259
CAACGGTA	0.554854574294
CAACGGTC	-0.822514031279
CAACGGTG	-1.13760196261
CAACGTAA	1.8035664545
CAACGTAC	-0.223968739425
CAACGTAG	1.45331720978
CAACGTCA	0.56018205394
CAACGTCC	-0.490589727338
CAACGTCG	-0.146860646473
CAACGTGA	-0.801204635458
CAACGTGC	-0.59449570682
CAACGTGG	-0.610423255623
CAACGTTA	0.154244415118
CAACGTTC	-0.586954325073
CAACGTTG	0.666614547909
CAACTAAA	0.911656394195
CAACTAAC	1.0789061119
CAACTAAG	-0.402515108233
CAACTACA	0.46910050098
CAACTACC	-0.348170999671
CAACTACG	0.242734369575
CAACTAGA	0.496202635684
CAACTAGC	0.108311304865
CAACTAGG	0.516391227231
CAACTATA	-0.191867420357
CAACTATC	0.130854421809
CAACTATG	0.440481046123
CAACTCAA	0.278621801031
CAACTCAC	-0.217050752781
CAACTCAG	-0.0173492032927
CAACTCCA	-0.58223900112
CAACTCCC	-0.311955272933
CAACTCCG	-1.12932897379
CAACTCGA	-0.201133136478
CAACTCGC	-0.718744492989
CAACTCGG	-0.14364591425
CAACTCTA	-0.342628664355
CAACTCTC	0.478870422195
CAACTCTG	-0.747400279887
CAACTGAA	0.273528519293
CAACTGAC	-0.704615510933
CAACTGAG	-0.375378732539
CAACTGCA	1.21083638035
CAACTGCC	0.167690407069
CAACTGCG	-0.268066166745
CAACTGGA	-0.446739567006
CAACTGGC	-0.237551433951
CAACTGGG	-0.882868609591
CAACTGTA	-0.375713039598
CAACTGTC	-1.03549245883
CAACTGTG	-0.310946862744
CAACTTAA	-0.246705017368

CAACTTAC 0.390560036893
CAACTTAG 1.01807346665
CAACTTCA 0.755652619569
CAACTTCC -0.0119341608126
CAACTTCG 1.43034438149
CAACTTGA 0.211896569131
CAACTTGC 1.17064138022
CAACTTGG -0.755078102825
CAACTTTA 0.246451999985
CAACTTTC 0.892085708736
CAAGAAAA 0.713741910664
CAAGAAAC 1.4979172739
CAAGAAAG -0.217577175311
CAAGAACA 0.556361961946
CAAGAACC -1.33567092267
CAAGAACG -1.28695906469
CAAGAAGA 0.194249390822
CAAGAAGC -1.09420137862
CAAGAAGG -1.12436716699
CAAGAATA 1.6091476884
CAAGAATC 8.27987048196
CAAGAATG -0.338041940146
CAAGACAA -0.264346706664
CAAGACAC -0.976735444816
CAAGACAG -0.744163330229
CAAGACCA -1.00918335588
CAAGACCC -1.39482575946
CAAGACCG -0.231759217831
CAAGACGA 0.784399367262
CAAGACGC -0.231999166134
CAAGACGG -0.401805718587
CAAGACTA -0.502793372573
CAAGACTC -0.357283546178
CAAGACTG 0.387949357533
CAAGAGAA 1.74438234296
CAAGAGAC -0.271855938474
CAAGAGAG -0.318683235159
CAAGAGCA -0.321624823617
CAAGAGCC -1.26721063975
CAAGAGCG 0.572621726756
CAAGAGGA -0.0858808433876
CAAGAGGC -1.46103162614
CAAGAGGG -0.353754110516
CAAGAGTA 0.246939738039
CAAGAGTC -0.771598465084
CAAGAGTG -0.303994112347
CAAGATAA 0.963544038537
CAAGATAC 3.84276240409
CAAGATAG 0.529216437897
CAAGATCA 2.14693901087
CAAGATCC 4.31643629882
CAAGATCG 4.05707884402
CAAGATGA 1.50780690788
CAAGATGC 0.119026381924

CAAGATGG -0.0595137137251
CAAGATTA 4.27658083338
CAAGATTC 8.92032282338
CAAGATTG 3.7350081034
CAAGCAAA -0.064339340711
CAAGCAAC 0.214385444668
CAAGCAAG -0.338996505726
CAAGCACA -0.161792331404
CAAGCACC -0.722659989265
CAAGCACG -0.066640805645
CAAGCAGA 1.28011086693
CAAGCAGC -0.333527618638
CAAGCAGG -0.071427748155
CAAGCATA 2.04778102804
CAAGCATC 0.752522313601
CAAGCATG -0.674467496515
CAAGCCAA -0.390314599576
CAAGCCAC -1.009079326
CAAGCCAG -0.909385249526
CAAGCCCA -0.990543973038
CAAGCCCC -0.805716604531
CAAGCCCG -0.751441239328
CAAGCCGA -1.00664324955
CAAGCCGC -1.13142577693
CAAGCCGG -0.684077974958
CAAGCCTA -0.295049634205
CAAGCCTC -1.08326778655
CAAGCCTG -0.743833728039
CAAGCGAA -0.634542242197
CAAGCGAC -1.42836520006
CAAGCGAG -0.566687057667
CAAGCGCA -0.185363462157
CAAGCGCC -1.00323849291
CAAGCGCG -0.030871257288
CAAGCGGA 0.341099059267
CAAGCGGC -0.714019759298
CAAGCGGG -1.06466787233
CAAGCGTA -0.748502264687
CAAGCGTC -1.02098212103
CAAGCGTG -0.465072349237
CAAGCTAA -0.360281070296
CAAGCTAC -0.316517165891
CAAGCTAG -0.136912724392
CAAGCTCA -0.0975073580642
CAAGCTCC -0.649997997224
CAAGCTCG -0.133297294186
CAAGCTGA -0.475517419109
CAAGCTGC -0.579473845239
CAAGCTGG -0.750524051315
CAAGCTTA -0.529264270729
CAAGCTTC 0.159839549516
CAAGCTTG -0.0408270208166
CAAGGAAA 0.792493048316
CAAGGAAC -1.5057660404

CAAGGAAG -0.132094938854
CAAGGACA -0.557413500098
CAAGGACC -0.871692194019
CAAGGACG -1.30627072099
CAAGGAGA 0.764003238729
CAAGGAGC -0.948492118073
CAAGGAGG -1.11689191479
CAAGGATA 2.88364799942
CAAGGATC 2.15540855866
CAAGGATG 1.09197806679
CAAGGCAA -0.763472634094
CAAGGCAC -1.0541598095
CAAGGCAG -0.711497949681
CAAGGCCA -1.16558390776
CAAGGCCC -1.29597124067
CAAGGCCG -1.35714028402
CAAGGCGA -0.750389962558
CAAGGCGC -0.524094404184
CAAGGCGG -1.45037823513
CAAGGCTA -0.633372559564
CAAGGCTC -1.36767579193
CAAGGCTG -0.720047218853
CAAGGGAA -0.0805295780165
CAAGGGAC -1.22450820599
CAAGGGAG -1.30084809844
CAAGGGCA -0.669348337999
CAAGGGCC -1.11224873215
CAAGGGCG -0.536653528387
CAAGGGGA -0.066640805645
CAAGGGGC -1.1330991419
CAAGGGGG -1.16484079989
CAAGGGTA 0.149329134245
CAAGGGTC -0.40034511824
CAAGGGTG -0.825905196078
CAAGGTAA 1.67484961136
CAAGGTAC 0.201368902675
CAAGGTAG -0.0876496126333
CAAGGTCA 0.116485752831
CAAGGTCC -0.832493057769
CAAGGTCG 0.0298691202571
CAAGGTGA -0.461827819512
CAAGGTGC -0.513174665337
CAAGGTGG -0.791561222934
CAAGGTTA -0.149644883211
CAAGGTTC -0.321501451504
CAAGTAAA 0.867933788081
CAAGTAAC 0.18228752356
CAAGTAAG 0.0858021675279
CAAGTACA -0.462717301076
CAAGTACC -0.474112754424
CAAGTACG 0.311129045715
CAAGTAGA 1.28972918682
CAAGTAGC -0.339294219362
CAAGTAGG 0.672968211692

CAAGTATA 2.64777358978
CAAGTATC 1.31778823954
CAAGTATG 1.44277046246
CAAGTCAA 0.110344592284
CAAGTCAC 0.102209351556
CAAGTCAG -0.583161939528
CAAGTCCA -0.181140058363
CAAGTCCC -0.0226236224752
CAAGTCCG -0.922285999472
CAAGTCGA 0.326788678385
CAAGTCGC -1.29541371373
CAAGTCGG -0.0701840945314
CAAGTCTA -0.127273755355
CAAGTCTC -0.798039304356
CAAGTCTG -0.913390138307
CAAGTGAA 0.291337492809
CAAGTGAC 0.341774469306
CAAGTGAG -0.481163261536
CAAGTGCA 0.189507667327
CAAGTGCC -0.647205919038
CAAGTGCG 1.59258132299
CAAGTGGA -0.400516323184
CAAGTGGC -0.639102305483
CAAGTGGG -0.74265646534
CAAGTGTA -0.237974349369
CAAGTGTC -0.808770587073
CAAGTGTG 0.561211636039
CAAGTTAA 0.0325216206716
CAAGTTAC 0.50324869931
CAAGTTAG -0.253392726828
CAAGTTCA 1.76111703813
CAAGTTCC -0.91112761923
CAAGTTCG -0.251541099617
CAAGTTGA 0.318124924075
CAAGTTGC 0.122046907622
CAAGTTGG -0.501231878932
CAAGTTTA -0.0144222522046
CAAGTTTC -0.199799044823
CAATAAAA 1.79768850522
CAATAAAC 1.08807093472
CAATAAAG 0.297052339978
CAATAACA -0.177253314062
CAATAACC -0.0171641451242
CAATAACG 1.42905472471
CAATAAGA 0.315298343519
CAATAAGC -0.0724685696617
CAATAAGG -0.589672170886
CAATAATA 2.52678423209
CAATAATC 2.43684465531
CAATAATG 0.572110464358
CAATACAA 1.17279621008
CAATACAC 1.08345206058
CAATACAG 0.00497095515209
CAATACCA 0.760156747194

CAATACCC 0.461576893182
CAATACCG -0.167904739976
CAATACGA 2.25815975426
CAATACGC 1.10856220615
CAATACGG 0.937214547824
CAATACTA 1.38773918169
CAATACTC 0.788398767029
CAATACTG -0.28100612393
CAATAGAA 0.410198681566
CAATAGAC -0.509455728019
CAATAGAG 1.27769805344
CAATAGCA 0.615655853751
CAATAGCC 0.503394550239
CAATAGCG 0.250963446295
CAATAGGA 0.914763959964
CAATAGGC -0.841830392447
CAATAGGG 0.987745360313
CAATAGTA 0.741938972865
CAATAGTC -0.318041281965
CAATAGTG 0.133284747869
CAATATAA 2.16778445436
CAATATAC 3.20926124478
CAATATAG 1.14785308734
CAATATCA 3.22867693095
CAATATCC 4.86128701537
CAATATCG 5.16247885565
CAATATGA 2.39720064759
CAATATGC 1.627336188
CAATATGG 1.90181404524
CAATATTA 3.03821914153
CAATATTC 4.05654667109
CAATATTG 1.12973908151
CAATCAAA 2.40328221313
CAATCAAC 1.47364720876
CAATCAAG 2.12177449792
CAATCACA 4.14630929253
CAATCACC 1.4738753949
CAATCACG 1.40036234439
CAATCAGA 2.12341858815
CAATCAGC 1.81664625084
CAATCAGG 2.26254704431
CAATCATA 3.55261236786
CAATCATC 1.08948396362
CAATCATG 2.00251966848
CAATCCAA 3.68098732387
CAATCCAC 4.5084723103
CAATCCAG 2.25944731999
CAATCCCA 2.72800937488
CAATCCCC 2.03944975112
CAATCCCG 3.80645780761
CAATCCGA 4.01355305824
CAATCCGC 5.57203558402
CAATCCGG 4.59353346101
CAATCCTA 2.63621372741

CAATCCTC 0.711036872549
CAATCCTG 5.05811493561
CAATCGAA 3.35777617536
CAATCGAC 0.947738293556
CAATCGAG 0.606204033935
CAATCGCA 2.25231212524
CAATCGCC 1.49154322235
CAATCGCG 4.2332327871
CAATCGGA 1.80953745563
CAATCGGC 2.42827787357
CAATCGGG 0.940366287083
CAATCGTA 3.15055258636
CAATCGTC 0.920357526073
CAATCGTG 1.14482837954
CAATCTAA 2.79576209783
CAATCTAC 4.17122392468
CAATCTAG 4.60191727563
CAATCTCA 2.70737329805
CAATCTCC 3.54823318064
CAATCTCG 7.86378600967
CAATCTGA 4.59828929911
CAATCTGC 4.57985876017
CAATCTGG 5.85395863458
CAATCTTA 3.75858890527
CAATCTTC 3.45224444963
CAATGAAA 0.677193706539
CAATGAAC 0.170492940519
CAATGAAG -0.250160482039
CAATGACA -0.355023640917
CAATGACC -0.527062914945
CAATGACG -0.35712148959
CAATGAGA 0.640817752884
CAATGAGC -0.989651877659
CAATGAGG -0.806110767974
CAATGATA 1.6498557806
CAATGATC 1.62523206616
CAATGATG 0.92853040575
CAATGCAA 0.682851572519
CAATGCAC 0.960753790023
CAATGCAG -0.49544096972
CAATGCCA -0.0792127375466
CAATGCCC -1.18487909702
CAATGCCG -0.195888253415
CAATGCGA -0.589469077388
CAATGCGC 0.717085765394
CAATGCGG -0.434783711521
CAATGCTA -0.206038746231
CAATGCTC -0.90387088204
CAATGCTG -0.119884497696
CAATGGAA 0.438268450931
CAATGGAC -0.81118078675
CAATGGAG -0.636239392886
CAATGGCA -0.424805469176
CAATGGCC -1.48614830625

CAATGGCG -0.217856330853
CAATGGGA 0.319446208032
CAATGGGC -1.19873023044
CAATGGGG 0.0544321941588
CAATGGTA 0.74770740326
CAATGGTC -0.365055205108
CAATGGTG -0.289226836439
CAATGTAA 1.07816404955
CAATGTAC 0.0661342481161
CAATGTAG 0.444938386443
CAATGTCA -0.225121693636
CAATGTCC -0.29473388524
CAATGTCG 1.07025647219
CAATGTGA 1.05393502133
CAATGTGC -0.0162401611899
CAATGTGG -0.370407777387
CAATGTTA 1.14220881321
CAATGTTC -0.337183562991
CAATTAAA 1.25834117812
CAATTAAC 0.588110154482
CAATTAAG -0.390838146908
CAATTACA 1.13322904855
CAATTACC 0.0732568965488
CAATTACG 3.76124794023
CAATTAGA 0.622362905449
CAATTAGC 0.748450511131
CAATTAGG -0.148448016892
CAATTATA 2.80908680872
CAATTATC 1.87853226391
CAATTATG 0.600107831021
CAATTCAA 0.267425259076
CAATTCAC -0.000327772518704
CAATTCAG -0.00245594145594
CAATTCCA 1.39276241316
CAATTCCC 2.61674707182
CAATTCCG 2.08505247504
CAATTCGA 0.0103156859834
CAATTCGC 2.22544941607
CAATTCGG 0.440980023586
CAATTCTA 0.405316857537
CAATTCTC 2.51753524439
CAATTCTG 0.33535310769
CAATTGAA 1.52749443092
CAATTGAC 0.147249843666
CAATTGAG 0.035044214434
CAATTGCA 0.386093025453
CAATTGCC 0.304600517645
CAATTGCG 1.38761476405
CAATTGGA 0.200053630495
CAATTGGC 0.276631641575
CAATTGGG -0.0111479249783
CAATTGTA 0.512659743597
CAATTGTC -0.306735482504
CAATTGTG -0.304573333959

CAATTTAA 0.419401143341
CAATTTAC 2.48736396701
CAATTTAG 2.28974432191
CAATTTCA 1.99581392369
CAATTTCC 2.15695358526
CAATTTCG 0.743232288991
CAATTTGA 0.771167185454
CAATTTGC 1.28699905607
CAATTTGG 1.83102040883
CAATTTTA 2.2569009405
CAATTTTC 0.951288378364
CACAAAAA 1.69892442445
CACAAAAC 0.218991249734
CACAAAAG 0.234253843762
CACAAACA 0.827602608148
CACAAACC 0.677463190962
CACAAACG -0.52513182773
CACAAAGA 1.04605933252
CACAAAGC 0.846679282395
CACAAAGG -0.555207439446
CACAAATA 0.295461832979
CACAAATC 4.3903644233
CACAAATG 1.16079409006
CACAACAA 1.49440927153
CACAACAC 2.00712495079
CACAACAG 0.167087922495
CACAACCA -0.591180342683
CACAACCC 0.0590792975161
CACAACCG -0.449735522835
CACAACGA 2.09985373055
CACAACGC -0.233721670837
CACAACGG -1.05175771265
CACAACTA 0.231187314903
CACAACTC -0.514768047533
CACAACTG -0.703274884738
CACAAGAA -0.276560023019
CACAAGAC -0.381889486661
CACAAGAG 0.411055229049
CACAAGCA -0.253423569856
CACAAGCC -0.9373384427
CACAAGCG 0.144361838435
CACAAGGA -0.773451660584
CACAAGGC -1.13609509773
CACAAGGG -0.90162744382
CACAAGTA 0.134745870979
CACAAGTC 0.0945310058549
CACAAGTG -1.1766458382
CACAATAA 1.05948990296
CACAATAC 0.835121511077
CACAATAG 0.545292712828
CACAATCA 2.67852879364
CACAATCC 5.30644313268
CACAATCG 2.32901925883
CACAATGA 0.603969221308

CACAATGC 0.141492129916
CACAATGG -0.142029530473
CACAATTA 1.23618333765
CACAATTC 1.75872069168
CACACAAA 1.40651213053
CACACAAC 0.276489972752
CACACAAG -0.307554129656
CACACACA 0.101820154363
CACACACC -0.26457724523
CACACACG -0.348366774485
CACACAGA 1.29775987491
CACACAGC 0.274784719233
CACACAGG -0.490287831597
CACACATA 0.89931839882
CACACATC -0.218063867839
CACACATG -1.00091768574
CACACCAA 1.06729580288
CACACCAC -0.325007362823
CACACCAG 1.12051361815
CACACCCA -0.629831623112
CACACCCC -0.971832448879
CACACCCG -0.72315452324
CACACCGA 1.35862440871
CACACCGC 0.293621445177
CACACCGG -1.00666206902
CACACCTA 1.2701509213
CACACCTC -0.758805404354
CACACCTG -0.257441527717
CACACGAA 0.448071044845
CACACGAC -0.543431153116
CACACGAG -0.46800505072
CACACGCA -0.219706389774
CACACGCC -0.352935201982
CACACGCG -0.855490978687
CACACGGA -0.86721864804
CACACGGC -0.929936115961
CACACGGG -0.745389209904
CACACGTA 0.252285514397
CACACGTC -0.523005227082
CACACGTG -0.888849543222
CACACTAA 1.68386962879
CACACTAC 0.229540088099
CACACTAG -0.0256360453445
CACACTCA 0.314784990068
CACACTCC -0.763058867031
CACACTCG 0.071341492229
CACACTGA -0.0446128718221
CACACTGC -0.267942533251
CACACTGG -0.996711794509
CACACTTA 0.18067061702
CACACTTC 1.58793265134
CACAGAAA -0.213180214139
CACAGAAC -0.456914368311
CACAGAAG -0.13993481838

CACAGACA 0.289726336665
CACAGACC -0.925454467153
CACAGACG 0.592323364393
CACAGAGA 0.81830448071
CACAGAGC -1.21701491846
CACAGAGG -0.144626095227
CACAGATA 1.88883200561
CACAGATC 1.27506463388
CACAGATG 1.41075435379
CACAGCAA 0.398379005891
CACAGCAC -0.158781999587
CACAGCAG 0.125189759907
CACAGCCA -0.126704989007
CACAGCCC -1.28374851457
CACAGCCG -0.960324601446
CACAGCGA 1.25260176108
CACAGCGC -0.688048361369
CACAGCGG 1.13987702801
CACAGCTA -1.01006551876
CACAGCTC -1.23822943276
CACAGCTG 0.127442085101
CACAGGAA 0.128727298398
CACAGGAC -0.720476930193
CACAGGAG 0.00322178952596
CACAGGCA -0.979897377957
CACAGGCC -1.33056143527
CACAGGCG -0.387147438803
CACAGGGA -0.253622742631
CACAGGGC -1.31523088203
CACAGGGG -0.962215435896
CACAGGTA 0.234346111465
CACAGGTC -0.310632682068
CACAGGTG -0.70518087932
CACAGTAA 0.40390043068
CACAGTAC -0.307047572127
CACAGTAG -0.589768359313
CACAGTCA 0.0346869057952
CACAGTCC -1.45922469518
CACAGTCG -0.574936522151
CACAGTGA 0.0486425918547
CACAGTGC -0.814831503472
CACAGTGG -0.649107208752
CACAGTTA 0.807874309588
CACAGTTC -0.0986976898427
CACATAAA -0.112465181155
CACATAAC 0.0116534369808
CACATAAG 0.364031896168
CACATACA 0.608978860936
CACATACC 0.547621622829
CACATACG 0.122335995665
CACATAGA -0.0165480687075
CACATAGC -0.825097004189
CACATAGG -0.828596903731
CACATATA -0.0667955435485

CACATATC 2.64171371891
CACATATG 1.58360181971
CACATCAA 0.838875473528
CACATCAC 0.382303515106
CACATCAG -0.158970717098
CACATCCA 0.730423022603
CACATCCC 0.693171963372
CACATCCG 0.839189654204
CACATCGA 0.0213872875363
CACATCGC -0.215414765385
CACATCGG 0.0720200388467
CACATCTA 0.53608816
CACATCTC 0.0223311364717
CACATCTG 0.352669899665
CACATGAA 1.48140396894
CACATGAC -0.651805973708
CACATGAG 0.173424596475
CACATGCA 0.211940219858
CACATGCC -1.05224335965
CACATGCG -0.963608077028
CACATGGA 0.153305009669
CACATGGC -0.205741816741
CACATGGG 0.81172838119
CACATGTA -0.397435418338
CACATGTC -0.603224806528
CACATGTG -0.578583840912
CACATTAA 0.164811027431
CACATTAC 1.97749969956
CACATTAG 0.51368854155
CACATTCA 0.56084936115
CACATTCC 1.02416052121
CACATTCG 0.429103889487
CACATTGA 0.99815671196
CACATTGC 0.4161440673
CACATTGG -0.040750174628
CACATTTA 0.338309333516
CACATTTC 1.16189032446
CACCAAAA 2.21888481734
CACCAAAC 0.213162962954
CACCAAAG 0.863164358138
CACCAACA 0.918674228608
CACCAACC -0.699416108267
CACCAACG -0.264478181606
CACCAAGA 0.155402858342
CACCAAGC 0.132097029907
CACCAAGG -0.651693841005
CACCAATA 1.24001100971
CACCAATC 0.962130486878
CACCAATG -0.488800047564
CACCACAA 1.15646482672
CACCACAC -0.377912304329
CACCACAG -0.525442871826
CACCACCA -1.08929158677
CACCACCC -0.366524169666

CACCACCG 0.587956984868
CACCACGA -0.458847023816
CACCACGC -0.727352834401
CACCACGG -0.888723557294
CACCACTA -0.295255080138
CACCACTC 0.385561898055
CACCACTG -1.06183371171
CACCAGAA -0.483374549821
CACCAGAC -0.429874965189
CACCAGAG -0.384412341805
CACCAGCA -0.146717147978
CACCAGCC -0.700252790749
CACCAGCG -0.93306642193
CACCAGGA 0.682943056077
CACCAGGC -0.18346818422
CACCAGGG -1.20915334426
CACCAGTA -0.654735277231
CACCAGTC -1.0332419633
CACCAGTG -1.15808460845
CACCATAA 1.18242995149
CACCATAC 0.516823291006
CACCATAG -0.0320302232752
CACCATCA -0.792111953952
CACCATCC 0.279040272963
CACCATCG -0.814099635009
CACCATGA 0.46910050098
CACCATGC 0.272513574564
CACCATGG -0.175760302398
CACCATTA 0.952139959597
CACCATTC -0.506965545575
CACCCAAA -0.849681511382
CACCCAAC 0.203064746456
CACCCAAG -0.936700410229
CACCCACA -0.148445925839
CACCCACC -0.234316052582
CACCCACG -0.346533705367
CACCCAGA -0.755008575321
CACCCAGC -0.824552023566
CACCCAGG -1.79077757587
CACCCATA 0.891915549318
CACCCATC -0.769705016818
CACCCATG -0.439329660202
CACCCCAA -0.0394717572525
CACCCCAC -0.582237955594
CACCCCAG -0.958426709693
CACCCCCA -0.355622204767
CACCCCCC -1.2583728053
CACCCCCG -0.426928149099
CACCCCGA 0.568604291658
CACCCCGC -0.86647736984
CACCCCGG -1.02479724677
CACCCCTA -0.937100062686
CACCCCTC -0.659523526649
CACCCCTG -0.991622172113

CACCCGAA −0.844918615978
CACCCGAC −1.10730496069
CACCCGAG −0.934649348862
CACCCGCA −0.384874987226
CACCCGCC −0.605022850512
CACCCGCG −0.720528160985
CACCCGGA −0.23842863058
CACCCGGC −0.276686270329
CACCCGGG −1.1681836091
CACCCGTA −0.590186569863
CACCCGTC −0.716721922215
CACCCGTG −0.328439041769
CACCCTAA −0.203592737276
CACCCTAC −0.567420233038
CACCCTAG −1.19328512908
CACCCTCA −0.375364879315
CACCCTCC −0.547954100216
CACCCTCG 0.574386575277
CACCCTGA −0.141795332565
CACCCTGC −0.527328740026
CACCCTGG −1.12161769401
CACCCTTA −0.59163253284
CACCCTTC −0.510581759926
CACCGAAA 1.43422563678
CACCGAAC −1.18178355481
CACCGAAG 0.248290296734
CACCGACA −0.761809724394
CACCGACC 0.0970813060663
CACCGACG −1.3455229177
CACCGAGA 1.54656561615
CACCGAGC −1.1760692304
CACCGAGG −1.53534136775
CACCGATA 1.62767180196
CACCGATC 0.705942545285
CACCGATG 0.657474033571
CACCGCAA 1.24215904365
CACCGCAC 0.0523881900948
CACCGCAG −0.131143248471
CACCGCCA −1.62784196138
CACCGCCC −0.182464478899
CACCGCCG −0.512311060551
CACCGCGA −0.0525144374046
CACCGCGC 0.388056262605
CACCGCGG −0.043845194081
CACCGCTA −0.945764862523
CACCGCTC −0.597232894871
CACCGCTG −0.395595291917
CACCGGAA −0.0939614553619
CACCGGAC −1.52517440789
CACCGGAG −1.00112809792
CACCGGCA −0.648545761088
CACCGGCC −1.44183628465
CACCGGCG −1.3135925422
CACCGGGA −1.06652185198

CACCGGGC -1.4792575033
CACCGGGG 0.642525620218
CACCGGTA -0.582892455105
CACCGGTC -0.400533835751
CACCGGTG -1.06283715565
CACCGTAA 1.22846944405
CACCGTAC 0.217842216247
CACCGTAG -1.18946503709
CACCGTCA -0.522952950763
CACCGTCC -0.203910577294
CACCGTCG -0.596176913231
CACCGTGA -0.245631523162
CACCGTGC -0.430799733268
CACCGTGG -0.490371212325
CACCGTTA 0.57436409646
CACCGTTC -0.89394439325
CACCTAAA -0.0208822982969
CACCTAAC 0.270026528699
CACCTAAG 0.026371311768
CACCTACA -0.879666685067
CACCTACC -0.865219340229
CACCTACG -0.5573481547
CACCTAGA 1.10684283803
CACCTAGC -0.0921328297312
CACCTAGG 0.86134278981
CACCTATA -0.135551449051
CACCTATC -0.677613485378
CACCTATG -0.251128639462
CACCTCAA 0.525737448883
CACCTCAC -0.693022191718
CACCTCAG -0.429972460523
CACCTCCA -0.438803499053
CACCTCCC -1.31622831419
CACCTCCG -0.409122312162
CACCTCGA -0.387488803165
CACCTCGC -0.653544945452
CACCTCGG -1.15249783827
CACCTCTA -0.903076543376
CACCTCTC -0.91241649187
CACCTCTG -0.359965321331
CACCTGAA 0.488094055879
CACCTGAC -0.811433542752
CACCTGAG -0.30101148698
CACCTGCA -0.211077137835
CACCTGCC -0.375364879315
CACCTGCG -0.625234705021
CACCTGGA -0.924277727218
CACCTGGC -0.658207993087
CACCTGGG -1.02422351417
CACCTGTA 0.920315705018
CACCTGTC 0.0254617038214
CACCTTAA 0.503587188474
CACCTTAC 0.433804576071
CACCTTAG 0.358122581094

CACCTTCA 0.31255488231
CACCTTCC -0.180607624056
CACCTTCG -1.04309918597
CACCTTGA -0.25002299532
CACCTTGC -0.347283086397
CACCTTGG -0.999474336574
CACCTTTA 0.785432608702
CACCTTTC -0.45837549142
CACGAAAA 1.05928968466
CACGAAAC -0.486333128082
CACGAAAG 0.482443508583
CACGAACA -0.0346388115819
CACGAACC -0.219324772647
CACGAACG -0.636478295663
CACGAAGA 0.358519358354
CACGAAGC -0.677982033426
CACGAAGG -0.86713160797
CACGAATA 1.03928223056
CACGAATC 4.37592831787
CACGAATG 0.422671549843
CACGACAA 0.535505801809
CACGACAC -0.417760973839
CACGACAG 0.213545102844
CACGACCA -0.686328470481
CACGACCC -1.06604953544
CACGACCG -0.533008562061
CACGACGA -0.525037730356
CACGACGC -0.247634228934
CACGACGG -0.485455670071
CACGACTA -0.324037375728
CACGACTC -0.373277485906
CACGACTG 0.259755277586
CACGAGAA -0.00715924185591
CACGAGAC -0.907478209417
CACGAGAG -0.317496562723
CACGAGCA -0.0375694220123
CACGAGCC -1.20538291477
CACGAGCG -0.460030559673
CACGAGGA -0.358120490041
CACGAGGC -1.25449259553
CACGAGGG 0.278243843246
CACGAGTA 1.204118612
CACGAGTC -0.653958189752
CACGAGTG 1.03834622307
CACGATAA 0.897445338318
CACGATAC 0.794765761274
CACGATAG -0.180274362524
CACGATCA 1.32147424278
CACGATCC 1.73957187611
CACGATCG 1.78594410744
CACGATGA 0.134212652527
CACGATGC 0.285095177585
CACGATGG 0.457143599968
CACGATTA 1.19326186612

CACGATTC 5.43865046562
CACGCAAA 0.278091196396
CACGCAAC 0.254205884967
CACGCAAG -0.2627585521
CACGCACA -0.317314118371
CACGCACC -0.877863152069
CACGCACG 0.0829395163119
CACGCAGA 0.603925047818
CACGCAGC -0.844977165455
CACGCAGG -0.565376751737
CACGCATA 0.262357069971
CACGCATC 0.25938202467
CACGCATG -0.973509473185
CACGCCAA 0.212619812002
CACGCCAC -1.0108530615
CACGCCAG -0.664383656005
CACGCCCA -1.10782511006
CACGCCCC -1.82370904289
CACGCCCG -1.07610932884
CACGCCGA -0.725643398777
CACGCCGC -1.00332840818
CACGCCGG -0.654175136475
CACGCCTA -1.00227059687
CACGCCTC -1.568079151
CACGCCTG -0.458981373955
CACGCGAA 0.167969562611
CACGCGAC -0.284227652075
CACGCGAG 0.390346226749
CACGCGCA 1.24285144349
CACGCGCC -0.972468651678
CACGCGCG 0.0949944354208
CACGCGGA -0.374574984138
CACGCGGC -0.889086354946
CACGCGGG -0.131019092213
CACGCGTA 0.714155154964
CACGCGTC 0.00247580645707
CACGCGTG 1.6928684743
CACGCTAA -0.195700842812
CACGCTAC 1.33596706801
CACGCTAG -0.193337430441
CACGCTCA -0.168999406091
CACGCTCC -0.302019635787
CACGCTCG -0.374527412688
CACGCTGA -0.806619416556
CACGCTGC -0.454993736359
CACGCTGG -1.35947180784
CACGCTTA 0.0492312232041
CACGCTTC -0.874177148832
CACGGAAA 0.602943559933
CACGGAAC -0.104084241729
CACGGAAG -0.784478043121
CACGGACA -0.617911838287
CACGGACC -0.54559643824
CACGGACG -0.868028408218

CACGGAGA	0.0709444535879
CACGGAGC	0.216343454188
CACGGAGG	−1.45307386351
CACGGATA	1.78740418502
CACGGATC	1.42130371492
CACGGATG	−0.0822790050242
CACGGCAA	0.18396193405
CACGGCAC	−1.01371048508
CACGGCAG	−0.578537053606
CACGGCCA	−0.675664885596
CACGGCCC	−1.35781726235
CACGGCCG	−0.448198599063
CACGGCGA	0.299945834222
CACGGCGC	−1.25708158022
CACGGCGG	−1.02473608348
CACGGCTA	0.0107469656133
CACGGCTC	−0.974958049978
CACGGCTG	−0.817010380438
CACGGGAA	−0.171784688356
CACGGGAC	−1.61764572678
CACGGGAG	−0.935000122961
CACGGGCA	−0.890079605003
CACGGGCC	−1.78155760153
CACGGGCG	−0.252683598564
CACGGGGA	−0.0836300864829
CACGGGGC	−0.706619523613
CACGGGGG	−1.31627902222
CACGGGTA	−0.209578375775
CACGGGTC	−0.992011107925
CACGGTAA	0.536014711772
CACGGTAC	0.732235965338
CACGGTAG	−0.788140783396
CACGGTCA	−0.0737545671035
CACGGTCC	−0.550087758167
CACGGTCG	0.0169257651106
CACGGTGA	0.194084981799
CACGGTGC	−1.51905258958
CACGGTGG	0.00114772657865
CACGGTTA	−0.256944641307
CACGGTTC	−0.471411898414
CACGTAAA	−0.0136216403826
CACGTAAC	−0.0529425804554
CACGTAAG	1.08056405535
CACGTACA	0.0210150801466
CACGTACC	−0.795017210368
CACGTACG	−0.33084009309
CACGTAGA	0.310143898488
CACGTAGC	−0.417758098642
CACGTAGG	−0.766494728082
CACGTATA	0.134864538222
CACGTATC	2.4966869257
CACGTATG	−0.244257440123
CACGTCAA	0.151012693091
CACGTCAC	−0.392110029744

CACGTCAG -0.291697153883
CACGTCCA -0.217886912499
CACGTCCC -0.298378067422
CACGTCCG -0.421389473125
CACGTCGA -0.759187282862
CACGTCGC -0.88402130242
CACGTCGG -1.06498153025
CACGTCTA -0.906419091198
CACGTCTC -0.50621198244
CACGTCTG -0.153965259575
CACGTGAA -0.209666722754
CACGTGAC -0.890361897124
CACGTGAG -0.275511360064
CACGTGCA -0.107854148457
CACGTGCC -0.277770742561
CACGTGCG -0.0743369252947
CACGTGGA -0.421370130888
CACGTGGC -0.381253283861
CACGTGGG -0.901787932118
CACGTGTA 0.336149537406
CACGTGTC 0.176158386566
CACGTTAA 0.509154616423
CACGTTAC 0.251160528017
CACGTTAG 0.635700946803
CACGTTCA -0.974955436162
CACGTTCC -0.301799029722
CACGTTCG -0.553565440273
CACGTTGA -0.220831898918
CACGTTGC 0.0334704358574
CACGTTGG -0.898679059441
CACGTTTA 0.742923858711
CACGTTTC 0.508465614542
CACTAAAA 1.97683814274
CACTAAAC 0.144956742943
CACTAAAG 0.0233737876496
CACTAACA 0.0594572553008
CACTAACC -0.339267035676
CACTAACG -0.204533972395
CACTAAGA 0.0317215316128
CACTAAGC -0.302960086762
CACTAAGG -0.833445532297
CACTAATA 1.65282141616
CACTAATC 0.919718186694
CACTAATG 0.75240573741
CACTACAA 0.752765398483
CACTACAC 0.105398468383
CACTACAG -0.502379082747
CACTACCA 0.353610612021
CACTACCC 0.111655420977
CACTACCG -0.943615783058
CACTACGA 0.507968728132
CACTACGC -0.504616247809
CACTACGG 1.03697606075
CACTACTA 1.27344955701

CACTACTC -0.789548584661
CACTACTG -0.585786994875
CACTAGAA 0.139150673599
CACTAGAC -0.171784688356
CACTAGAG -0.579535531295
CACTAGCA -0.503275098851
CACTAGCC -1.27045778329
CACTAGCG -0.537475834882
CACTAGGA -0.163919193433
CACTAGGC -0.0217981794018
CACTAGGG -0.302155815598
CACTAGTA -0.0873749005781
CACTAGTC -0.512812390448
CACTAGTG -0.337863677899
CACTATAA 0.106859852874
CACTATAC 0.165079989091
CACTATAG -0.3281162355
CACTATCA 0.86477838948
CACTATCC 1.85248794051
CACTATCG 1.59093252789
CACTATGA 0.562703340795
CACTATGC 0.57702234727
CACTATGG 0.323021646854
CACTATTA -0.193498441502
CACTATTC 0.670828803347
CACTCAAA 1.18900709653
CACTCAAC -0.143553385166
CACTCAAG 0.67268252161
CACTCACA 0.138202903939
CACTCACC 0.71778365425
CACTCACG -0.362789810834
CACTCAGA 0.234157916717
CACTCAGC 0.911492507935
CACTCAGG -0.610534342801
CACTCATA 0.9245634173
CACTCATC 0.836813695516
CACTCATG -0.983694991134
CACTCCAA -0.0426365655909
CACTCCAC 0.181283034095
CACTCCAG -0.584555364805
CACTCCCA 0.093276635586
CACTCCCC -0.649871227151
CACTCCCG -0.673775358054
CACTCCGA -0.590463634353
CACTCCGC 0.226038881649
CACTCCGG 0.0182297978824
CACTCCTA -0.558228749289
CACTCCTC -0.40320175768
CACTCCTG -0.824117345976
CACTCGAA 0.817723429426
CACTCGAC -0.434104380759
CACTCGAG -0.0286510820297
CACTCGCA -0.387258787362
CACTCGCC -1.27965135809

CACTCGCG 0.0762520682329
CACTCGGA 1.18791608976
CACTCGGC -0.798548475701
CACTCGGG -1.57275605185
CACTCGTA 0.436621485508
CACTCGTC -0.51674356962
CACTCTAA 0.572641068993
CACTCTAC -0.0842919046785
CACTCTAG -0.0865769025721
CACTCTCA 0.466277841148
CACTCTCC 0.663496788257
CACTCTCG -0.140576771575
CACTCTGA -0.050250088657
CACTCTGC -0.38124805623
CACTCTGG -0.116195357881
CACTCTTA 0.867593469246
CACTCTTC -1.54218015577
CACTGAAA 0.464810183498
CACTGAAC 0.00611946587552
CACTGAAG -1.02790690359
CACTGACA -0.20289641671
CACTGACC -0.105206614293
CACTGACG 0.374431224262
CACTGAGA 0.41062734738
CACTGAGC -0.957549251682
CACTGAGG -0.825387660522
CACTGATA 0.396489739731
CACTGATC 1.30206875049
CACTGATG -0.221025321297
CACTGCAA 0.738439073323
CACTGCAC 0.0833122464647
CACTGCAG -0.313856039884
CACTGCCA -0.561329519137
CACTGCCC -0.530765646604
CACTGCCG -0.889836520121
CACTGCGA 0.132067755168
CACTGCGC 0.109540321119
CACTGCGG -0.781723865267
CACTGCTA -0.157691515578
CACTGCTC -0.372429041253
CACTGCTG -0.83839688383
CACTGGAA -0.346250629101
CACTGGAC -0.638500082291
CACTGGAG -0.813931828026
CACTGGCA -1.19798450876
CACTGGCC -0.756681417522
CACTGGCG -0.812829320463
CACTGGGA -0.162058679248
CACTGGGC -1.34184475591
CACTGGGG -0.727570303887
CACTGGTA -0.463731200279
CACTGGTC -0.996711794509
CACTGTAA 0.772298706374
CACTGTAC -0.557519621025

CACTGTAG -0.0180849924794
CACTGTCA -0.682857845678
CACTGTCC -0.408817541224
CACTGTCG 0.156741654866
CACTGTGA -0.0217981794018
CACTGTGC -0.741559446791
CACTGTGG 1.34334613179
CACTGTTA 0.467362836144
CACTGTTC 0.0278243320483
CACTTAAA 0.81441799779
CACTTAAC -0.623920478367
CACTTAAG -0.523206490909
CACTTACA -0.00705834856068
CACTTACC -0.33482015062
CACTTACG 0.316916295585
CACTTAGA 1.54589125164
CACTTAGC -0.412749765922
CACTTAGG 0.0870021704253
CACTTATA 0.780158973664
CACTTATC 2.31862254455
CACTTATG 0.460835876363
CACTTCAA -0.741079027421
CACTTCAC -0.0228267159737
CACTTCAG -0.891234650266
CACTTCCA -0.28018120362
CACTTCCC -0.921170945593
CACTTCCG 0.315921215857
CACTTCGA 0.0113374266338
CACTTCGC -1.14950972389
CACTTCGG -0.39232906752
CACTTCTA -0.711678041599
CACTTCTC -0.513912284195
CACTTCTG -0.662616977812
CACTTGAA -0.0250625741276
CACTTGAC 0.128621700235
CACTTGAG 0.229586091259
CACTTGCA -0.640226246337
CACTTGCC -0.503476362678
CACTTGCG -0.739360443442
CACTTGGA 1.29576213539
CACTTGGC -0.524733482181
CACTTGGG -0.343844611529
CACTTGTA 0.226779375704
CACTTGTC -0.180274362524
CACTTTAA 0.733101138413
CACTTTAC 1.17745141627
CACTTTAG -0.863845518572
CACTTTCA 0.471729738432
CACTTTCC 0.131659477118
CACTTTCG -0.027462841304
CACTTTGA 0.113231813369
CACTTTGC -0.417044788272
CACTTTGG -0.177380345517
CACTTTTA 0.352149488911

CACTTTTC 0.320632357705
CAGAAAAA 0.169175315904
CAGAAAAC 1.60158879409
CAGAAAAG 0.583366601316
CAGAAACA 0.751809525994
CAGAAACC -0.259224411569
CAGAAACG 0.397539970975
CAGAAAGA -0.207775888304
CAGAAAGC -0.71954327514
CAGAAAGG -0.206028552349
CAGAAATA 2.76105689532
CAGAAATC 5.59799731083
CAGAAATG 0.607370318607
CAGAACAA -0.644191927879
CAGAACAC 0.682954556867
CAGAACAG -0.567718730818
CAGAACCA -0.0112618873532
CAGAACCC 0.467751771955
CAGAACCG 0.709671415103
CAGAACGA -0.557541838461
CAGAACGC -0.0504338399175
CAGAACGG 0.325991464524
CAGAACTA 1.43347364193
CAGAACTC 0.452527862402
CAGAACTG -0.0612589586273
CAGAAGAA 0.316351972724
CAGAAGAC -0.194259061941
CAGAAGAG -0.95562208519
CAGAAGCA -0.177008922272
CAGAAGCC -0.354082144416
CAGAAGCG 0.315576453535
CAGAAGGA -0.865789674867
CAGAAGGC -0.766280395175
CAGAAGGG -1.3984323027
CAGAAGTA 0.29364340123
CAGAAGTC -0.764530184023
CAGAATAA 1.20349312585
CAGAATAC 1.1209276466
CAGAATAG 0.138007390507
CAGAATCA 6.20509396291
CAGAATCC 10.0211489185
CAGAATCG 6.05726176105
CAGAATGA 0.308636249454
CAGAATGC 0.0510101863319
CAGAATGG -0.608815758821
CAGAATTA 1.97106291643
CAGAATTC 2.77090235582
CAGACAAA 0.320129459518
CAGACAAC 0.251750204892
CAGACAAG -0.641080702767
CAGACACA -0.188153972053
CAGACACC -0.548213652139
CAGACACG 0.874851513344
CAGACAGA 0.0720433018085

CAGACAGC -0.238979361598
CAGACAGG -1.48547655556
CAGACATA 0.527306522591
CAGACATC 0.486666389614
CAGACATG -0.158970717098
CAGACCAA -0.827351159055
CAGACCAC 0.0274555226194
CAGACCAG -1.27140137084
CAGACCCA -0.440168172355
CAGACCCC -0.909548874403
CAGACCCG -1.52890772119
CAGACCGA -0.177830444622
CAGACCGC 0.0326227753485
CAGACCGG 0.0254488961233
CAGACCTA -0.72215081792
CAGACCTC -1.14595963908
CAGACCTG -0.440436872633
CAGACGAA 2.17885579453
CAGACGAC -0.390189659174
CAGACGAG -0.994294014766
CAGACGCA -0.318683235159
CAGACGCC -0.928161334939
CAGACGCG -0.732756114709
CAGACGGA -0.839458877246
CAGACGGC -0.0590889686351
CAGACGGG -0.804419106299
CAGACGTA 0.574070303549
CAGACGTC 0.418211595707
CAGACTAA -0.853852116093
CAGACTAC -0.307644306306
CAGACTAG 0.655721208603
CAGACTCA -0.678328886801
CAGACTCC -1.15846152071
CAGACTCG -0.535700269714
CAGACTGA -0.673142291834
CAGACTGC -0.387780243642
CAGACTGG -0.881386575954
CAGACTTA -0.363638778251
CAGACTTC -0.424555849754
CAGAGAAA 0.207458571049
CAGAGAAC 0.583421752832
CAGAGAAG 0.348620314631
CAGAGACA 0.0801871681285
CAGAGACC -0.927968958086
CAGAGACG -0.798503256685
CAGAGAGA 0.491889316622
CAGAGAGC -0.396470920256
CAGAGAGG -0.824156814596
CAGAGATA 1.84561334321
CAGAGATC 4.04513971695
CAGAGATG -0.551600634832
CAGAGCAA -0.277138983249
CAGAGCAC -0.38618921388
CAGAGCAG -1.00666206902

CAGAGCCA −0.38442959299
CAGAGCCC −1.48970414146
CAGAGCCG −1.10961688089
CAGAGCGA −0.571170013383
CAGAGCGC −0.932651870722
CAGAGCGG −0.752251522269
CAGAGCTA −0.775830233088
CAGAGCTC −0.539985620946
CAGAGCTG −1.13348886185
CAGAGGAA −0.598884042399
CAGAGGAC −0.557425523652
CAGAGGAG −1.41251711126
CAGAGGCA −0.482881845516
CAGAGGCC −0.977704125003
CAGAGGCG −1.03159003163
CAGAGGGA −0.288357742639
CAGAGGGC −0.880421032346
CAGAGGGG −0.316734896759
CAGAGGTA −0.277727353217
CAGAGGTC −1.3848846333
CAGAGTAA 0.213096049266
CAGAGTAC −0.26616147907
CAGAGTAG 0.0413646827552
CAGAGTCA −0.766628294077
CAGAGTCC −0.329300032739
CAGAGTCG −0.700391322994
CAGAGTGA −0.388684885338
CAGAGTGC 0.0288405836853
CAGAGTGG −0.356022641369
CAGAGTTA 0.133322909582
CAGAGTTC −0.587961166973
CAGATAAA 0.337759648024
CAGATAAC 1.91807224176
CAGATAAG 0.37614954686
CAGATACA 2.14126598476
CAGATACC 3.71471705027
CAGATACG 7.68169347678
CAGATAGA −0.24214155612
CAGATAGC 2.57911491823
CAGATAGG 0.115140944531
CAGATATA 2.18943782836
CAGATATC 15.8497179906
CAGATATG 5.93136182735
CAGATCAA 0.79713806062
CAGATCAC 2.44888885777
CAGATCAG 0.597841391221
CAGATCCA 2.24586593237
CAGATCCC 2.86001204604
CAGATCCG 3.93229788492
CAGATCGA 0.425100568995
CAGATCGC 8.80074074419
CAGATCGG 3.32099011399
CAGATCTA 2.74989015087
CAGATCTC 7.30202129018

CAGATCTG 4.66681152947
CAGATGAA 1.72045128976
CAGATGAC -0.373772019882
CAGATGAG -0.577312219457
CAGATGCA -0.214281414794
CAGATGCC -0.406925661247
CAGATGCG 0.70482017272
CAGATGGA 0.354121351655
CAGATGGC -0.898455316797
CAGATGGG -1.05457226965
CAGATGTA 0.866341974175
CAGATGTC 1.85183134995
CAGATTAA 1.78012627592
CAGATTAC 17.5068647494
CAGATTAG 0.640397974044
CAGATTCA 4.1599523662
CAGATTCC 18.2922594577
CAGATTCG 12.3358706041
CAGATTGA 0.726692845877
CAGATTGC 16.8299530736
CAGATTGG 0.699715912956
CAGATTTA 4.55740764954
CAGATTTC 26.9467072763
CAGCAAAA 0.0292046882455
CAGCAAAC 1.3677769466
CAGCAAAG -0.28715747836
CAGCAACA -0.630334782681
CAGCAACC -0.839635571203
CAGCAACG -0.0505606099905
CAGCAAGA 0.226736770505
CAGCAAGC -0.704841606011
CAGCAAGG -0.612986363533
CAGCAATA 1.46476676911
CAGCAATC 5.16846344862
CAGCAATG -0.572902189206
CAGCACAA 0.71006453302
CAGCACAC -0.761872978739
CAGCACAG -0.472151346943
CAGCACCA 0.00759705602563
CAGCACCC -0.318659710816
CAGCACCG -0.141708553876
CAGCACGA -0.487985059755
CAGCACGC -0.541275800493
CAGCACGG 0.0383263831081
CAGCACTA -0.307139317066
CAGCACTC -0.826829180012
CAGCACTG -1.03482227642
CAGCAGAA -0.452909740911
CAGCAGAC -0.286884857358
CAGCAGAG -0.3770450402
CAGCAGCA 0.50336344583
CAGCAGCC -1.14357845276
CAGCAGCG -0.910188997927
CAGCAGGA -0.805878138356

CAGCAGGC -0.38143834203
CAGCAGGG -0.609327021219
CAGCAGTA -0.76562694119
CAGCAGTC -0.856291329127
CAGCATAA 0.632121064493
CAGCATAC -0.184416999405
CAGCATAG 0.132934496533
CAGCATCA 1.12548169811
CAGCATCC 0.513447547721
CAGCATCG 0.77286877963
CAGCATGA -0.274118718932
CAGCATGC -0.914481929224
CAGCATGG -1.30342845754
CAGCATTA 1.1664723438
CAGCATTC 0.137812922601
CAGCCAAA -0.888305869507
CAGCCAAC -0.256014906978
CAGCCAAG -1.26779169103
CAGCCACA -0.598943898784
CAGCCACC -1.49427204619
CAGCCACG -0.653163328325
CAGCCAGA 0.00302157122506
CAGCCAGC -0.512779456367
CAGCCAGG -1.17330485867
CAGCCATA 0.359198427734
CAGCCATC -0.847866477594
CAGCCATG -1.10721609095
CAGCCCAA 0.0436873195983
CAGCCCAC -1.49876911651
CAGCCCAG -0.652649452112
CAGCCCCA -1.00362742872
CAGCCCCC -1.16417479959
CAGCCCCG -1.00560295081
CAGCCCGA -0.224724132232
CAGCCCGC -0.503172637266
CAGCCCGG -1.54725775461
CAGCCCTA -0.55471944001
CAGCCCTC -1.11090679905
CAGCCCTG -1.65685348863
CAGCCGAA 0.202407633129
CAGCCGAC -1.08618585067
CAGCCGAG -0.599902907852
CAGCCGCA 0.317646072995
CAGCCGCC -1.45517511014
CAGCCGCG -0.503179433188
CAGCCGGA -0.306104507336
CAGCCGGC -1.48483878447
CAGCCGGG -1.12996935869
CAGCCGTA -0.954005701414
CAGCCGTC -0.883237680402
CAGCCTAA -0.835305523719
CAGCCTAC -0.113058778754
CAGCCTAG -0.854800931279
CAGCCTCA -0.0201410200967

CAGCCTCC −1.03227459003
CAGCCTCG −1.48837919816
CAGCCTGA −0.156394017346
CAGCCTGC −1.42353539097
CAGCCTGG −0.520111471456
CAGCCTTA −0.303983657084
CAGCCTTC −1.41605203594
CAGCGAAA 0.302169668822
CAGCGAAC −0.3969602266
CAGCGAAG −0.270201131603
CAGCGACA −0.512546826748
CAGCGACC −1.04619342128
CAGCGACG −0.343440254204
CAGCGAGA −0.566687841812
CAGCGAGC −0.461539515614
CAGCGAGG −1.35806087
CAGCGATA −0.0410695829357
CAGCGATC 2.8252503851
CAGCGATG −0.0852893368407
CAGCGCAA 0.792204221654
CAGCGCAC −0.510171390823
CAGCGCAG −0.729419055901
CAGCGCCA −0.606646291592
CAGCGCCC −0.520406048513
CAGCGCCG −0.956191897065
CAGCGCGA 0.61788439322
CAGCGCGC −0.564697159593
CAGCGCGG −0.353030344882
CAGCGCTA −0.664992675119
CAGCGCTC −0.218125815276
CAGCGCTG −0.364391557241
CAGCGGAA 2.05985319833
CAGCGGAC −0.19177123193
CAGCGGAG −0.555063679569
CAGCGGCA −0.556114694958
CAGCGGCC −1.11605836889
CAGCGGCG −1.35161990476
CAGCGGGA 0.125943845805
CAGCGGGC −1.35105113841
CAGCGGGG −0.867907911303
CAGCGGTA −0.796442524199
CAGCGGTC −0.785752278392
CAGCGTAA 0.462177548085
CAGCGTAC −0.306020342463
CAGCGTAG −0.0489068486461
CAGCGTCA −0.741955178524
CAGCGTCC 0.259358500327
CAGCGTCG −1.12611920781
CAGCGTGA −0.726284306445
CAGCGTGC −0.792452011405
CAGCGTGG −1.30020666801
CAGCGTTA 0.549184423379
CAGCGTTC −1.10017551633
CAGCTAAA −0.460375060613

CAGCTAAC -0.676023239761
CAGCTAAG -1.0037952357
CAGCTACA -0.746283657718
CAGCTACC -1.88896557161
CAGCTACG -0.831828625758
CAGCTAGA -0.117523176378
CAGCTAGC -1.06237189641
CAGCTAGG -1.29992803523
CAGCTATA 1.16905139599
CAGCTATC -0.231792151911
CAGCTATG 0.607308632551
CAGCTCAA -0.0574932340045
CAGCTCAC -1.16919986073
CAGCTCAG -1.40117628667
CAGCTCCA -1.28612865537
CAGCTCCC -1.08847555343
CAGCTCCG -1.23751847483
CAGCTCGA -0.497915207887
CAGCTCGC -0.75652040646
CAGCTCGG -0.365890319301
CAGCTCTA 0.0656770917084
CAGCTCTC 0.0748492332186
CAGCTGAA -0.159314695275
CAGCTGAC -0.285348717731
CAGCTGAG 0.0335331674399
CAGCTGCA -0.309648318986
CAGCTGCC -1.0183013914
CAGCTGCG -1.03603456425
CAGCTGGA 0.134864538222
CAGCTGGC -0.540659985458
CAGCTGGG -0.588267506201
CAGCTGTA 0.966058006707
CAGCTGTC -0.184667664354
CAGCTTAA -0.23404839783
CAGCTTAC -0.291697153883
CAGCTTAG -0.698253744319
CAGCTTCA -0.863747761855
CAGCTTCC -1.09843314663
CAGCTTCG -0.705614250003
CAGCTTGA -0.182859426487
CAGCTTGC -0.299360339451
CAGCTTGG -0.328338148473
CAGCTTTA -0.000626531680489
CAGCTTTC 0.0829395163119
CAGGAAAA 0.677980987899
CAGGAAAC -0.214921276936
CAGGAAAG -0.466767931636
CAGGAACA -0.674467496515
CAGGAACC -0.769794409323
CAGGAACG 0.0525047662857
CAGGAAGA -0.418725471921
CAGGAAGC -0.844464334768
CAGGAAGG -1.06951127327
CAGGAATA 1.55840620235

CAGGAATC 4.07523231847
CAGGAATG -0.214418117367
CAGGACAA -0.544775177272
CAGGACAC -0.418380186835
CAGGACAG -0.587805644925
CAGGACCA -0.867680247935
CAGGACCC -1.35451392177
CAGGACCG -1.30374838861
CAGGACGA -0.342514963361
CAGGACGC -0.980099164548
CAGGACGG -0.359654538616
CAGGACTA -0.0252047657146
CAGGACTC -1.31911867185
CAGGACTG -0.636459998951
CAGGAGAA 0.119949320332
CAGGAGAC -0.528832206954
CAGGAGAG -0.0513792571425
CAGGAGCA -1.134867127
CAGGAGCC -1.74705078766
CAGGAGCG -0.637049675827
CAGGAGGA -1.42728464855
CAGGAGGC -1.77961187695
CAGGAGGG -1.43803893285
CAGGAGTA 0.129961280903
CAGGAGTC -1.1407618047
CAGGATAA 0.983763995875
CAGGATAC 4.39116006887
CAGGATAG 0.42689207844
CAGGATCA 0.787712378964
CAGGATCC 3.59086216616
CAGGATCG 2.46241692354
CAGGATGA 0.861736691872
CAGGATGC -0.609714388741
CAGGATGG -1.07041330115
CAGGATTA 2.25484543565
CAGGATTC 8.13749200706
CAGGCAAA -0.64687500994
CAGGCAAC 0.224591089
CAGGCAAG -1.00323849291
CAGGCACA -0.961208855378
CAGGCACC -0.819846370732
CAGGCACG -0.373828478306
CAGGCAGA -0.587511067868
CAGGCAGC -1.43745500637
CAGGCAGG -1.30802563701
CAGGCATA 0.673044012355
CAGGCATC 0.760156747194
CAGGCATG -1.07122645929
CAGGCCAA -1.45478565157
CAGGCCAC -1.56138150903
CAGGCCAG -0.61438318677
CAGGCCCA -1.00934384418
CAGGCCCC -1.36940378564
CAGGCCCG -0.875773144844

CAGGCCGA -1.10545516315
CAGGCCGC -0.920937793211
CAGGCCGG -0.885428580922
CAGGCCTA -0.720447916836
CAGGCCTC -1.70594670235
CAGGCCTG -1.31526433887
CAGGCGAA -0.47626209527
CAGGCGAC 0.154935508052
CAGGCGAG -0.455179840054
CAGGCGCA -0.740408845015
CAGGCGCC -0.409868556613
CAGGCGCG -0.0825971064239
CAGGCGGA -0.0928401283242
CAGGCGGC -0.928871770111
CAGGCGGG -0.415573732662
CAGGCGTA -0.42140986089
CAGGCGTC -0.858779681901
CAGGCTAA -0.819556759926
CAGGCTAC -0.739136178034
CAGGCTAG -1.25884303078
CAGGCTCA -0.835226063715
CAGGCTCC -1.14913072058
CAGGCTCG -1.35760554326
CAGGCTGA -0.927235521334
CAGGCTGC -1.58326437607
CAGGCTGG -0.899745757725
CAGGCTTA -0.875981727356
CAGGCTTC -1.51518414199
CAGGGAAA 1.04894263288
CAGGGAAC -0.528038129672
CAGGGAAG -1.21651803205
CAGGGACA -0.82009206943
CAGGGACC -1.04315093953
CAGGGACG -0.308970295131
CAGGGAGA 0.183218826179
CAGGGAGC -0.787679183501
CAGGGAGG -1.48681665899
CAGGGATA 0.679782429844
CAGGGATC 0.923662957709
CAGGGATG -0.876906495435
CAGGGCAA -0.0658804465884
CAGGGCAC -0.955274447671
CAGGGCAG -0.530765646604
CAGGGCCA -1.16932976739
CAGGGCCC 0.0573847606431
CAGGGCCG -1.76967362599
CAGGGCGA -0.68933435881
CAGGGCGC -1.07307024505
CAGGGCGG -0.48280369242
CAGGGCTA -0.688745988843
CAGGGCTC -1.43677280041
CAGGGGAA -0.522952950763
CAGGGGAC -1.40299158184
CAGGGGAG -0.580283866798

CAGGGGCA	-1.22294645097
CAGGGGCC	-0.978366465962
CAGGGGCG	-0.524169943464
CAGGGGGA	-0.582413604025
CAGGGGGC	-0.817160674855
CAGGGGGG	-1.36094390898
CAGGGGTA	0.0889356100751
CAGGGGTC	-0.812917144679
CAGGGTAA	0.444282318642
CAGGGTAC	0.160419816654
CAGGGTAG	-0.0499897525895
CAGGGTCA	-0.429592150304
CAGGGTCC	-1.62717517693
CAGGGTCG	-0.979100164096
CAGGGTGA	-1.09808812293
CAGGGTGC	-1.03889172646
CAGGGTGG	-0.680371583957
CAGGGTTA	-0.533289547274
CAGGGTTC	-0.675044627074
CAGGTAAA	0.145879681351
CAGGTAAC	-0.653585720981
CAGGTAAG	-0.373777247514
CAGGTACA	-0.280398934488
CAGGTACC	-0.612384401722
CAGGTACG	0.688226884997
CAGGTAGA	-0.00558049702898
CAGGTAGC	-0.558103286124
CAGGTAGG	-0.758805404354
CAGGTATA	-0.560458595667
CAGGTATC	2.28289586277
CAGGTATG	0.355478967654
CAGGTCAA	-1.02215023537
CAGGTCAC	-0.895206866348
CAGGTCAG	-0.280395536527
CAGGTCCA	-1.23550635932
CAGGTCCC	-1.13971915352
CAGGTCCG	-0.67778756552
CAGGTCGA	-0.580130174421
CAGGTCGC	-1.4618358973
CAGGTCGG	-1.00546075922
CAGGTCTA	0.602688974261
CAGGTCTC	-0.774466866695
CAGGTGAA	0.385367952912
CAGGTGAC	-0.374032355949
CAGGTGAG	-0.597735793057
CAGGTGCA	-0.254453413336
CAGGTGCC	-1.57448692077
CAGGTGCG	0.0216894446588
CAGGTGGA	-0.623257353263
CAGGTGGC	-0.150759675708
CAGGTGGG	-0.732388612188
CAGGTGTA	0.653973088503
CAGGTGTC	-0.81438846167
CAGGTTAA	-0.351515115783

CAGGTTAC -0.0345481121689
CAGGTTAG 0.028468114914
CAGGTTCA 0.170492940519
CAGGTTCC -0.824245422957
CAGGTTCG -0.604564648578
CAGGTTGA -0.871180147477
CAGGTTGC -0.0138239497362
CAGGTTGG -0.0957367591474
CAGGTTTA 0.145036464329
CAGGTTTC -0.025998058852
CAGTAAAA 1.66260074711
CAGTAAAC 0.195861853874
CAGTAAAG 1.21645739152
CAGTAACA -0.425017449648
CAGTAACC -0.367415219519
CAGTAACG 0.132067755168
CAGTAAGA 2.08647909578
CAGTAAGC -0.629432232037
CAGTAAGG 0.694811871491
CAGTAATA 0.502751551518
CAGTAATC 7.58239251821
CAGTAATG 0.420934146389
CAGTACAA 2.09740144844
CAGTACAC -0.32942314347
CAGTACAG 0.0609928721648
CAGTACCA -0.161845391867
CAGTACCC -0.340112866514
CAGTACCG -0.417063607747
CAGTACGA 0.363403534817
CAGTACGC -0.235491224228
CAGTACGG -1.03863217453
CAGTACTA 0.175952679251
CAGTACTC 1.07681793434
CAGTACTG -0.0310056074272
CAGTAGAA 0.983959509307
CAGTAGAC -0.954238853796
CAGTAGAG -0.710558544232
CAGTAGCA -0.201520504
CAGTAGCC -0.951237670335
CAGTAGCG -0.987275657589
CAGTAGGA 0.293031768301
CAGTAGGC -0.973642255034
CAGTAGGG -0.562198874319
CAGTAGTA 0.538521622639
CAGTAGTC -0.585253253661
CAGTATAA 0.835121511077
CAGTATAC 0.865043953179
CAGTATAG 0.0810701151526
CAGTATCA 2.02825111811
CAGTATCC 3.34978391037
CAGTATCG 1.77642511256
CAGTATGA -0.194360739381
CAGTATGC -0.393863900239
CAGTATGG -0.602051203172

CAGTATTA 0.995121548892
CAGTATTC 0.672774527931
CAGTCAAA 0.575899974706
CAGTCAAC -0.596645309048
CAGTCAAG -1.06298745007
CAGTCACA -0.465229439575
CAGTCACC 0.31418172135
CAGTCACG -0.630783836259
CAGTCAGA -0.0347935494855
CAGTCAGC -0.178706857106
CAGTCAGG -1.09146053123
CAGTCATA 0.366592913025
CAGTCATC -0.603454299568
CAGTCATG 0.483909336562
CAGTCCAA -0.00263420370295
CAGTCCAC -1.15518614796
CAGTCCAG -0.628339918356
CAGTCCCA -0.682969717
CAGTCCCC -0.897302362586
CAGTCCCG -0.748491025278
CAGTCCGA -0.344947119092
CAGTCCGC -1.10487254358
CAGTCCGG -1.40379376195
CAGTCCTA -0.00540145063718
CAGTCCTC -1.0299260764
CAGTCGAA -0.26457724523
CAGTCGAC 0.0625386829109
CAGTCGAG -0.488105556669
CAGTCGCA 0.583641574753
CAGTCGCC -0.967309501778
CAGTCGCG -0.611119053426
CAGTCGGA -0.532763124744
CAGTCGGC -0.743736232704
CAGTCGGG -1.10959884556
CAGTCGTA -0.291828367443
CAGTCGTC -0.756672530548
CAGTCTAA -0.0145788197793
CAGTCTAC -0.523450098555
CAGTCTAG -0.105629006949
CAGTCTCA -0.56470813762
CAGTCTCC -1.10051949451
CAGTCTCG -0.7723423571
CAGTCTGA -0.815302513104
CAGTCTGC -0.635734403647
CAGTCTGG -0.415049139803
CAGTCTTA 1.13296531452
CAGTCTTC 0.637887665217
CAGTGAAA 0.347916152617
CAGTGAAC -0.653329305637
CAGTGAAG -1.67438226107
CAGTGACA -0.307464214388
CAGTGACC -1.12506897657
CAGTGACG -0.188920865649
CAGTGAGA -0.157691515578

CAGTGAGC	-0.550537073127
CAGTGAGG	-0.696486804745
CAGTGATA	0.716701534451
CAGTGATC	0.514839404708
CAGTGATG	-0.604167871319
CAGTGCAA	-0.407141039681
CAGTGCAC	0.0268266385045
CAGTGCAG	-1.06531348487
CAGTGCCA	-0.43146259699
CAGTGCCC	-1.16244183962
CAGTGCCG	-0.641009868355
CAGTGCGA	-0.0716316257982
CAGTGCGC	-0.031756818128
CAGTGCGG	-0.352257962273
CAGTGCTA	0.0504011672182
CAGTGCTC	-1.01135177758
CAGTGGAA	-0.307974431259
CAGTGGAC	-0.982776496214
CAGTGGAG	-0.425347574601
CAGTGGCA	-0.636608725078
CAGTGGCC	-0.870485133819
CAGTGGCG	-0.420305262274
CAGTGGGA	0.373315124856
CAGTGGGC	-0.403163857349
CAGTGGGG	-0.803860272451
CAGTGGTA	-0.0709023711513
CAGTGGTC	-0.790876925921
CAGTGTAA	1.66417635536
CAGTGTAC	-0.311336321319
CAGTGTAG	0.0122031224727
CAGTGTCA	-0.283422335384
CAGTGTCC	-0.161148287156
CAGTGTCG	-0.263915949797
CAGTGTGA	0.201575394134
CAGTGTGC	-0.586062491075
CAGTGTGG	-1.14997864247
CAGTGTTA	0.30751544518
CAGTGTTC	0.22219840189
CAGTTAAA	2.74345859537
CAGTTAAC	-0.88774389908
CAGTTAAG	0.652722116195
CAGTTACA	0.404588909798
CAGTTACC	0.174330022317
CAGTTACG	1.06757391289
CAGTTAGA	0.792975558738
CAGTTAGC	-0.221473329349
CAGTTAGG	-0.21058443353
CAGTTATA	0.61449296704
CAGTTATC	0.693202283636
CAGTTATG	0.332423020022
CAGTTCAA	0.055990812603
CAGTTCAC	-0.414583096421
CAGTTCAG	-1.40035188912
CAGTTCCA	0.141043076338

CAGTTCCC −1.1710478286
CAGTTCCG 0.54889925606
CAGTTCGA 0.153963952668
CAGTTCGC −1.43760922151
CAGTTCGG −0.671858385445
CAGTTCTA −0.00222357321899
CAGTTCTC −0.287472965944
CAGTTGAA 0.66607035143
CAGTTGAC −0.272400134952
CAGTTGAG −0.889168428767
CAGTTGCA −0.0697601335861
CAGTTGCC −1.0554549553
CAGTTGCG 0.487278283925
CAGTTGGA 0.291326514782
CAGTTGGC −0.08112709634
CAGTTGGG −0.416100677955
CAGTTGTA 1.22926744206
CAGTTGTC 0.191604731855
CAGTTTAA 1.03677244449
CAGTTTAC −0.25308795589
CAGTTTAG 0.430126414282
CAGTTTCA −0.500028216692
CAGTTTCC −0.0243733108645
CAGTTTCG 0.191650212252
CAGTTTGA −0.0594434020763
CAGTTTGC −0.029576372872
CAGTTTGG −0.164066089889
CAGTTTTA 1.11433168208
CAGTTTTC 0.528665184116
CATAAAAA 0.920315705018
CATAAAAC 1.59323425421
CATAAAAG 1.70538995955
CATAAACA 0.24174294919
CATAAACC 0.140182608131
CATAAACG 0.153568482316
CATAAAGA 1.13606268641
CATAAAGC −0.85353349193
CATAAAGG −0.771216325194
CATAAATA 0.758334394722
CATAAATC 2.16370219663
CATAAATG −0.494933366664
CATAACAA 1.1358342389
CATAACAC 0.150808292685
CATAACAG 1.10860716379
CATAACCA −0.0846317007506
CATAACCC −0.294617309049
CATAACCG −0.277614174987
CATAACGA 0.785255653363
CATAACGC −0.0336649037632
CATAACGG 0.0968005822344
CATAACTA 0.0750933636273
CATAACTC 0.884551907056
CATAAGAA 2.55015226934
CATAAGAC 0.233316006604

CATAAGAG 0.795641912377
CATAAGCA 0.689267967886
CATAAGCC -0.748608908377
CATAAGCG -0.306882117578
CATAAGGA 0.564341942007
CATAAGGC -0.215547808616
CATAAGGG -1.13005195528
CATAAGTA 0.583286095785
CATAAGTC -0.738976212499
CATAATAA 2.2377369648
CATAATAC 1.80692416245
CATAATAG 0.717212796849
CATAATCA 3.34772370064
CATAATCC 3.85912410772
CATAATCG 2.74870452396
CATAATGA 0.40795602749
CATAATGC 0.814551825167
CATAATGG 1.39041102434
CATAATTA 1.94580195367
CATAATTC 2.47335600463
CATACAAA 0.669375521685
CATACAAC 0.989462114621
CATACAAG 0.214886774565
CATACACA 0.825206000314
CATACACC 1.17911118939
CATACACG 0.233943845192
CATACAGA 2.17375362582
CATACAGC -1.24288725277
CATACAGG 0.143342972982
CATACATA 0.937512000078
CATACATC 0.118195711219
CATACATG 0.890598186085
CATACCAA 0.73943885792
CATACCAC -0.116915725553
CATACCAG -0.120951718744
CATACCCA 1.12629799282
CATACCCC -0.549853560259
CATACCCG 0.146733615018
CATACCGA 0.584314893739
CATACCGC -0.561090877742
CATACCGG -0.873266495359
CATACCTA 0.472994041202
CATACCTC -0.474077206527
CATACGAA 2.68302429567
CATACGAC 0.533195972664
CATACGAG -0.213366579215
CATACGCA 0.155003990029
CATACGCC -0.404072158387
CATACGCG -0.148028760816
CATACGGA 0.943915326364
CATACGGC -0.117085884971
CATACGGG -0.364498200932
CATACGTA 0.491139935593
CATACGTC 0.311457602379

CATACTAA	0.284328022607
CATACTAC	-0.207414136178
CATACTAG	-0.351646590725
CATACTCA	0.694502395684
CATACTCC	-0.927101693958
CATACTCG	-0.415003398024
CATACTGA	-1.26026128731
CATACTGC	0.568895209372
CATACTGG	-0.194360739381
CATACTTA	0.237356443282
CATACTTC	1.23411371819
CATAGAAA	0.0607320133341
CATAGAAC	0.129993692221
CATAGAAG	1.14637157647
CATAGACA	-0.201967989288
CATAGACC	-0.679055527632
CATAGACG	-0.444400463123
CATAGAGA	0.26515777375
CATAGAGC	-0.657610736145
CATAGAGG	0.436728129198
CATAGATA	3.32722484914
CATAGATC	2.06258359046
CATAGATG	-0.216282029513
CATAGCAA	0.264535162793
CATAGCAC	-1.33401585441
CATAGCAG	-0.525025968184
CATAGCCA	-0.621069327941
CATAGCCC	-0.750460535588
CATAGCCG	0.12657429821
CATAGCGA	-0.117848073699
CATAGCGC	-0.175689206605
CATAGCGG	-0.991340664137
CATAGCTA	0.600279035965
CATAGCTC	0.442299739253
CATAGGAA	0.678858707292
CATAGGAC	1.06757391289
CATAGGAG	-0.811465431306
CATAGGCA	-0.36055395268
CATAGGCC	-1.19102705349
CATAGGCG	-0.0459576801226
CATAGGGA	-0.190694862527
CATAGGGC	-0.570848775404
CATAGGGG	-0.69802268299
CATAGGTA	-0.612932518924
CATAGGTC	-0.168837872266
CATAGTAA	1.75072894944
CATAGTAC	0.871459825782
CATAGTAG	0.301847646699
CATAGTCA	-0.180485558852
CATAGTCC	-0.018273187227
CATAGTCG	-0.883349028961
CATAGTGA	0.323021646854
CATAGTGC	-0.799100513627
CATAGTGG	1.2324633548

CATAGTTA 0.65858725778
CATAGTTC 0.474408638388
CATATAAA 0.277127482459
CATATAAC 0.490557577402
CATATAAG 0.924307524719
CATATACA 1.64807002155
CATATACC 0.524226924652
CATATACG 0.838823197209
CATATAGA 0.420204891742
CATATAGC -0.329613167888
CATATAGG 0.353444373327
CATATATA 2.52249914224
CATATATC 3.31987035524
CATATATG 2.09426513069
CATATCAA 1.62469466561
CATATCAC 2.043998575
CATATCAG 2.36316144538
CATATCCA 6.89984310282
CATATCCC 3.48579643655
CATATCCG 6.76243349202
CATATCGA 2.95937835
CATATCGC 3.15167182235
CATATCGG 2.84123047161
CATATCTA 8.18727448405
CATATCTC 4.33314642411
CATATGAA 1.10528944722
CATATGAC 0.359516267753
CATATGAG 0.446011096504
CATATGCA 0.407335246205
CATATGCC 0.57795547956
CATATGCG 0.0504633760376
CATATGGA 0.511874553289
CATATGGC -0.096623888277
CATATGGG 0.709126173098
CATATGTA 1.44787236979
CATATGTC 0.463071995899
CATATTAA 0.269600476701
CATATTAC 1.96846007851
CATATTAG 1.16549007177
CATATTCA 0.340970198142
CATATTCC 3.82237908326
CATATTCG 1.87970273069
CATATTGA 1.23910662939
CATATTGC 2.45611632022
CATATTGG 0.23264791525
CATATTTA 2.90900724166
CATATTTC 3.34975463563
CATCAAAA 0.342511042637
CATCAAAC 0.209937252704
CATCAAAG 1.68640110952
CATCAACA 0.606288721571
CATCAACC -0.854061221369
CATCAACG 1.96800945665
CATCAAGA 1.59549990986

CATCAAGC -0.413090084757
CATCAAGG -0.00694543171213
CATCAATA 0.247671606502
CATCAATC 0.340660199571
CATCAATG 0.944674117131
CATCACAA 0.744400664716
CATCACAC -0.409916912208
CATCACAG -0.142978345659
CATCACCA 1.10739801254
CATCACCC -0.618630114907
CATCACCG 0.201254417537
CATCACGA -0.196185182906
CATCACGC 1.03108242858
CATCACGG -0.596951125512
CATCACTA -0.31041808778
CATCACTC 1.14971464706
CATCAGAA 1.43478786859
CATCAGAC -0.270720758212
CATCAGAG 0.528161240403
CATCAGCA 1.15852294539
CATCAGCC -0.731602899117
CATCAGCG -0.876043152031
CATCAGGA -0.211888466302
CATCAGGC -0.919448963652
CATCAGGG -0.701463510292
CATCAGTA 0.140900884751
CATCAGTC -0.0140868996197
CATCATAA 1.05532504864
CATCATAC 0.304837329369
CATCATAG -0.266801602593
CATCATCA 0.0266269429668
CATCATCC -0.197053492561
CATCATCG 1.04581102001
CATCATGA 0.588789485244
CATCATGC -0.635520332121
CATCATGG -0.310902689255
CATCATTA 0.621386383815
CATCATTC -0.628221773876
CATCCAAA 0.575069826764
CATCCAAC 0.926461309053
CATCCAAG 0.760045660017
CATCCACA 1.18167063796
CATCCACC 0.383782412164
CATCCACG 0.390346226749
CATCCAGA 0.687891009649
CATCCAGC -0.396685775926
CATCCAGG -0.121085284739
CATCCATA -0.256636995171
CATCCATC -0.747002457101
CATCCATG -0.116043233793
CATCCCAA 1.49807880772
CATCCCAC 0.247084543442
CATCCCAG 0.754885464591
CATCCCCA -0.298085320037

CATCCCCC -0.767689764729
CATCCCCG 1.0241762041
CATCCCGA 1.73504840625
CATCCCGC 0.180823525253
CATCCCGG -1.20295912325
CATCCCTA -0.928590784898
CATCCCTC -0.159289079879
CATCCGAA 0.482589098131
CATCCGAC 0.224335719183
CATCCGAG 0.301401206936
CATCCGCA -0.243537595213
CATCCGCC -0.657972749652
CATCCGCG -0.226000458555
CATCCGGA 0.910251990891
CATCCGGC -0.0554021812536
CATCCGGG 0.229540088099
CATCCGTA 0.175709071606
CATCCGTC -0.695546876533
CATCCTAA -0.350063663792
CATCCTAC -0.312621795998
CATCCTAG -0.181471751606
CATCCTCA 0.272185279282
CATCCTCC -0.948565566301
CATCCTCG 0.0187096944887
CATCCTGA -0.295091978024
CATCCTGC -0.398509173925
CATCCTGG 0.744970737972
CATCCTTA 0.701534083322
CATCCTTC -0.53416778943
CATCGAAA 0.649189282572
CATCGAAC -0.113692629119
CATCGAAG 0.217538229453
CATCGACA -0.338645731627
CATCGACC -1.0303678113
CATCGACG -0.608707808223
CATCGAGA 1.87878528129
CATCGAGC 1.224221209
CATCGAGG -0.474464835431
CATCGATA 0.911816621112
CATCGATC 1.05175326916
CATCGATG -0.479725140007
CATCGCAA 1.20896776333
CATCGCAC -0.0482641113068
CATCGCAG 0.44114469399
CATCGCCA -0.368531318925
CATCGCCC -1.07922525882
CATCGCCG -0.600350654522
CATCGCGA 0.550380244171
CATCGCGC 0.524919063112
CATCGCGG 0.0894040058913
CATCGCTA 0.108709127651
CATCGCTC -0.878845162717
CATCGGAA 0.512486708982
CATCGGAC -0.338671347024

CATCGGAG -1.10888265999
CATCGGCA 0.67421081979
CATCGGCC -0.812078893907
CATCGGCG -0.693783596301
CATCGGGA -0.407434832592
CATCGGGC -0.292474502743
CATCGGGG -0.0144983142484
CATCGGTA 0.799414432921
CATCGGTC 0.222746519093
CATCGTAA 0.908935411803
CATCGTAC 0.450282594511
CATCGTAG 0.360791809931
CATCGTCA 0.198100064462
CATCGTCC -0.379189937559
CATCGTCG 0.132067755168
CATCGTGA 0.435963849418
CATCGTGC 0.498167179744
CATCGTGG 0.162852495148
CATCGTTA 0.18348465126
CATCGTTC -0.30015049601
CATCTAAA 0.599453592892
CATCTAAC -0.0851594301886
CATCTAAG 1.16145643102
CATCTACA -0.459084096921
CATCTACC -0.182176959146
CATCTACG 0.477928925694
CATCTAGA 0.0796139582932
CATCTAGC -0.217642520709
CATCTAGG 0.976462562432
CATCTATA 1.30943683624
CATCTATC -0.592184570767
CATCTCAA 1.15694027984
CATCTCAC -0.381817345341
CATCTCAG -0.302446733312
CATCTCCA 0.625709896759
CATCTCCC -0.56902616155
CATCTCCG -0.562386546303
CATCTCGA 0.608245162802
CATCTCGC -0.101444287631
CATCTCGG -0.465159650689
CATCTCTA 1.01687686171
CATCTCTC 0.287821387609
CATCTGAA 0.53935333887
CATCTGAC 0.478071640044
CATCTGAG 1.47278125154
CATCTGCA 0.713150404117
CATCTGCC -1.10844745964
CATCTGCG 0.553578247971
CATCTGGA -0.484398381524
CATCTGGC 0.603117901456
CATCTGGG 0.666201042227
CATCTGTA 0.948271512008
CATCTGTC -0.323733650316
CATCTTAA 2.25213412437

CATCTTAC 0.26008383425
CATCTTAG 0.208917080343
CATCTTCA 0.91374326484
CATCTTCC -0.229023859451
CATCTTCG -0.49068852958
CATCTTGA 0.0835710142427
CATCTTGC 0.556031052848
CATCTTGG -0.0697928062854
CATCTTTA 0.771112295319
CATCTTTC 0.443362255432
CATGAAAA 1.60038042698
CATGAAAC 0.796770035336
CATGAAAG -0.196391151602
CATGAACA 0.462477614154
CATGAACC -0.385141073689
CATGAACG 1.32207672735
CATGAAGA 1.01660659314
CATGAAGC -0.848468178023
CATGAAGG -0.756279674012
CATGAATA 1.03791285239
CATGAATC 1.15682239674
CATGAATG 0.247368403853
CATGACAA 1.09287094631
CATGACAC 0.164223441608
CATGACAG -0.635489750475
CATGACCA -0.594662468276
CATGACCC -1.11564747702
CATGACCG -1.17004647572
CATGACGA -0.357929942859
CATGACGC -0.619360415081
CATGACGG -0.542512919577
CATGACTA -0.182775261614
CATGACTC -0.417995171748
CATGAGAA 1.20434549123
CATGAGAC -0.597489832977
CATGAGAG 0.839514812907
CATGAGCA 0.288784840164
CATGAGCC -1.35685903743
CATGAGCG -0.999263401628
CATGAGGA -0.507569075675
CATGAGGC -1.44626147503
CATGAGGG -0.470760274101
CATGAGTA 0.835222142991
CATGAGTC -0.421087577385
CATGATAA 1.10945221048
CATGATAC 2.55937015263
CATGATAG 0.694052035198
CATGATCA 1.28122252285
CATGATCC 1.35653962912
CATGATCG 0.824549148369
CATGATGA 0.284637237032
CATGATGC 0.374841331982
CATGATGG -0.747901609784
CATGATTA 1.57984576621

CATGATTC 2.01457066687
CATGCAAA -0.190826860232
CATGCAAC 0.357831140617
CATGCAAG 1.32414059642
CATGCACA -0.0888566728338
CATGCACC -0.195278450157
CATGCACG -0.564210728447
CATGCAGA -0.16793819682
CATGCAGC -0.992748726783
CATGCAGG -0.354262759098
CATGCATA 0.289757179693
CATGCATC -0.0387790960287
CATGCATG -0.379292399144
CATGCCAA 0.479078220562
CATGCCAC 0.154118690571
CATGCCAG -0.976611811322
CATGCCCA -0.711811607593
CATGCCCC -0.619267624615
CATGCCCG -0.915533467376
CATGCCGA 0.682722450012
CATGCCGC -0.346038387247
CATGCCGG -0.755888647148
CATGCCTA -0.439878300167
CATGCCTC -0.767852344081
CATGCGAA 1.07191467702
CATGCGAC -0.474911275193
CATGCGAG -0.0768051516855
CATGCGCA 0.531085577675
CATGCGCC -1.78062316233
CATGCGCG 0.0368710103935
CATGCGGA 0.211221943238
CATGCGGC -0.919448963652
CATGCGGG -0.497450471413
CATGCGTA -0.880551723143
CATGCGTC -0.855317159927
CATGCTAA 0.430670087997
CATGCTAC -0.563065092921
CATGCTAG 0.0344788460465
CATGCTCA -0.544136360656
CATGCTCC -0.697482668618
CATGCTCG 0.210691338602
CATGCTGA -0.291461387685
CATGCTGC -0.90955985243
CATGCTGG -0.909385249526
CATGCTTA -0.338316129438
CATGCTTC -0.0888566728338
CATGGAAA 0.836481218128
CATGGAAC -1.08847555343
CATGGAAG -0.996138584674
CATGGACA 0.0186584636963
CATGGACC -1.44095830387
CATGGACG 0.486666389614
CATGGAGA 0.513339858504
CATGGAGC -1.57679021537

CATGGAGG -0.773125979118
CATGGATA 3.26403271225
CATGGATC 2.28248575505
CATGGCAA -0.293795525318
CATGGCAC -0.413441904382
CATGGCAG -0.431603220287
CATGGCCA -0.446548497061
CATGGCCC -1.58116339082
CATGGCCG -0.358194722414
CATGGCGA -0.210839019203
CATGGCGC -0.551417406335
CATGGCGG -0.722749904533
CATGGCTA -0.561329519137
CATGGCTC 0.201770907567
CATGGGAA 0.210691338602
CATGGGAC -0.534653436431
CATGGGAG -0.408640847266
CATGGGCA -0.232543885375
CATGGGCC -1.54465178012
CATGGGCG -1.3242459332
CATGGGGA -0.559631584304
CATGGGGC -1.11560042833
CATGGGGG 0.0119252738384
CATGGGTA -0.39589588075
CATGGGTC -0.413946370859
CATGGTAA 1.34339919225
CATGGTAC -0.485157172291
CATGGTAG -0.785251994021
CATGGTCA -0.967139603742
CATGGTCC -1.40816615325
CATGGTCG -0.964901131773
CATGGTGA 0.275810119226
CATGGTGC -0.0144439468769
CATGGTGG -0.521089299999
CATGGTTA -0.137854220893
CATGGTTC -0.577999914431
CATGTAAA 1.21970897855
CATGTAAC 0.33948084582
CATGTAAG 0.5517331553
CATGTACA 0.488740713943
CATGTACC -0.646725499668
CATGTACG -0.796582363352
CATGTAGA 0.708595045699
CATGTAGC -0.854775054501
CATGTAGG 0.0412112517596
CATGTATA 1.08357255749
CATGTATC 0.218642043924
CATGTCAA 0.0796458468476
CATGTCAC 0.273291184806
CATGTCAG 0.119265807464
CATGTCCA 0.381410635581
CATGTCCC 0.221378709212
CATGTCCG -0.295971265705
CATGTCGA -0.00537714214895

CATGTCGC 0.471080727935
CATGTCGG -0.536624776412
CATGTCTA -0.443070814955
CATGTCTC -0.313528005984
CATGTGAA 1.07433742302
CATGTGAC -0.180306773842
CATGTGAG -1.33714668315
CATGTGCA 0.0566319816528
CATGTGCC -0.330717766504
CATGTGCG 0.639322911549
CATGTGGA 0.606120391825
CATGTGGC -1.4652498023
CATGTGGG -1.01508378398
CATGTGTA 0.118586738083
CATGTGTC 0.00852522206544
CATGTTAA 0.807325146859
CATGTTAC 0.00648592287012
CATGTTAG 0.546156840377
CATGTTCA -0.65076645911
CATGTTCC -0.67819270699
CATGTTCG -0.128882559065
CATGTTGA 1.06725607288
CATGTTGC -0.368461791421
CATGTTGG 0.71377249231
CATGTTTA 0.387202851701
CATGTTTC 0.0186584636963
CATTAAAA 0.142969720066
CATTAAAC 0.0475994179136
CATTAAAG 0.313327526301
CATTAACA 0.487836595009
CATTAACC 0.615386107946
CATTAACG 0.627593412524
CATTAAGA -0.0251381134082
CATTAAGC 0.198520888829
CATTAAGG -0.820598104196
CATTAATA 0.642808435103
CATTAATC 1.58150789176
CATTAATG 0.638740814739
CATTACAA 0.992479503741
CATTACAC 0.859084191458
CATTACAG 0.340701236482
CATTACCA 0.310809898789
CATTACCC -0.178998820346
CATTACCG -0.536640982071
CATTACGA 2.50585566925
CATTACGC 0.0165877987097
CATTACGG 0.185125604906
CATTACTA 1.41181190372
CATTACTC -0.617030982316
CATTAGAA 1.3836506508
CATTAGAC -0.489484605959
CATTAGAG 0.418207413602
CATTAGCA -0.419922599621
CATTAGCC -0.646790845067

CATTAGCG 0.314418533074
CATTAGGA 0.58976626826
CATTAGGC -1.07692091869
CATTAGGG -0.645396635645
CATTAGTA 0.0462995672473
CATTAGTC -0.527642136557
CATTATAA 0.291748384675
CATTATAC -0.131454553949
CATTATAG 1.38671874795
CATTATCA 2.01600695873
CATTATCC 1.12222671312
CATTATCG 1.16578804679
CATTATGA 2.23531813953
CATTATGC 0.356672958775
CATTATGG 0.50399494376
CATTATTA 1.48100719168
CATTATTC 2.73404755108
CATTCAAA 1.37191331033
CATTCAAC 0.326869706679
CATTCAAG 0.69399008776
CATTCACA 0.571694606242
CATTCACC 0.361746375511
CATTCACG 0.175438280275
CATTCAGA -0.0467057542442
CATTCAGC -0.737388319316
CATTCAGG 0.00158135864287
CATTCATA 2.11792957468
CATTCATC 0.785267676916
CATTCCAA 0.138202119794
CATTCCAC -0.913987133867
CATTCCAG -0.771085634397
CATTCCCA -0.124205396825
CATTCCCC -0.0027811001587
CATTCCCG -0.606346225522
CATTCCGA 0.752055486074
CATTCCGC -0.45977100775
CATTCCGG 0.443320695759
CATTCCTA 0.858586520903
CATTCCTC -0.422444932002
CATTCGAA 1.09413028283
CATTCGAC -0.799680780765
CATTCGAG 0.181283034095
CATTCGCA -0.69097583522
CATTCGCC -0.517221375173
CATTCGCG -0.363443787582
CATTCGGA 0.305904289035
CATTCGGC -0.696068594195
CATTCGGG 1.0072020834
CATTCGTA 0.187608730048
CATTCGTC -0.407371055483
CATTCTAA 0.26987754119
CATTCTAC 1.2930866334
CATTCTAG 0.278266322063
CATTCTCA -0.088495443471

CATTCTCC 0.446403430277
CATTCTCG 0.638610646705
CATTCTGA 0.072416293343
CATTCTGC -0.736425389524
CATTCTGG -0.441871857584
CATTCTTA 0.118230213589
CATTCTTC 0.733246727961
CATTGAAA 1.30266156394
CATTGAAC 1.02717947862
CATTGAAG -0.274714407584
CATTGACA 1.05012120249
CATTGACC 0.292174959436
CATTGACG -0.0921328297312
CATTGAGA 0.133550834332
CATTGAGC -0.955884250929
CATTGAGG -0.270875496115
CATTGATA 1.93369136028
CATTGATC -0.0911275561212
CATTGCAA -0.346489531878
CATTGCAC -0.0995309743639
CATTGCAG 1.10207732881
CATTGCCA -0.0703231495393
CATTGCCC -0.604720954772
CATTGCCG -0.852906698868
CATTGCGA 1.38599602784
CATTGCGC 0.214014282805
CATTGCGG 0.0322659894728
CATTGCTA -0.285567232743
CATTGCTC -0.428633402618
CATTGGAA 0.677144044036
CATTGGAC -0.434996214757
CATTGGAG 0.578803924214
CATTGGCA 0.565689886886
CATTGGCC -0.898866731425
CATTGGCG -0.672873591555
CATTGGGA 1.36113576307
CATTGGGC -1.49028101063
CATTGGGG -1.56339597698
CATTGGTA 0.759982144289
CATTGGTC 0.471270752354
CATTGTAA 0.0475183896195
CATTGTAC 0.643996153065
CATTGTAG -0.268472353741
CATTGTCA -0.401064701768
CATTGTCC -0.993506994786
CATTGTCG 0.206562293564
CATTGTGA -0.182353653103
CATTGTGC 0.196084812374
CATTGTGG -0.35712148959
CATTGTTA -0.443480399912
CATTGTTC -0.557562487607
CATTTAAA 1.52069354323
CATTTAAC 0.43164791654
CATTTAAG 1.04090854683

CATTTACA 1.55632612763
CATTTACC -0.081203681147
CATTTACG -0.0335577373097
CATTTAGA 1.72100724841
CATTTAGC -0.0348118461971
CATTTAGG 1.45554836306
CATTTATA 0.976732831
CATTTATC 1.38761476405
CATTTCAA 0.94732400373
CATTTCAC 0.107558003111
CATTTCAG 0.407116208429
CATTTCCA 0.0234537704173
CATTTCCC 0.982940382473
CATTTCCG 0.803814269291
CATTTCGA -0.124266560118
CATTTCGC 1.67854763815
CATTTCGG -0.134011650082
CATTTCTA -0.0110266439187
CATTTCTC 0.966988002418
CATTTGAA 1.38306332636
CATTTGAC -0.55105251763
CATTTGAG 0.420010423836
CATTTGCA 0.505907211501
CATTTGCC 0.247677356897
CATTTGCG 0.642036313875
CATTTGGA 0.634271712247
CATTTGGC -0.553758862653
CATTTGGG 1.3468052558
CATTTGTA 1.00361279135
CATTTGTC 0.128621700235
CATTTTAA 0.200817387512
CATTTTAC 1.625339494
CATTTTAG 1.33868909593
CATTTTCA -0.220831898918
CATTTTCC -0.682969717
CATTTTCG 0.880348106882
CATTTTGA 1.56923994665
CATTTTGC -0.614390505455
CATTTTGG 0.38666806496
CATTTTTA 1.39228878971
CATTTTTC 0.325740538194
CCAAAAAA 1.13850372911
CCAAAAAC 2.28181008363
CCAAAAAG 0.028468114914
CCAAAACA 1.04935038817
CCAAAACC -0.567158590062
CCAAAACG 0.703248223815
CCAAAAGA 0.185226498202
CCAAAAGC 0.361988414867
CCAAAAGG 0.0217427665039
CCAAAATA 3.13643144576
CCAAAATC 6.1255456114
CCAAACAA 0.209887328819
CCAAACAC 1.02669487714

CCAAACAG 0.947854085602
CCAAACCA -0.23842863058
CCAAACCC -0.539238069587
CCAAACCG -0.436516410107
CCAAACGA 1.03724031755
CCAAACGC -0.648104026195
CCAAACGG -0.813877460654
CCAAACTA 0.738146325938
CCAAACTC -0.705313922551
CCAAAGAA 0.00815066224143
CCAAAGAC -0.0751466854724
CCAAAGAG -0.365101992413
CCAAAGCA -0.261565606505
CCAAAGCC -1.27744111533
CCAAAGCG -0.722880072567
CCAAAGGA -0.669988984285
CCAAAGGC -1.27601501736
CCAAAGGG -1.11855430172
CCAAAGTA -0.348366774485
CCAAAGTC -0.151105222175
CCAAATAA 0.4161440673
CCAAATAC 0.897520093454
CCAAATAG -0.625359906805
CCAAATCA 1.9387621632
CCAAATCC 5.09609681778
CCAAATCG 2.95070335628
CCAAATGA 0.66212950114
CCAAATGC 1.89222656837
CCAAATGG -0.578661732627
CCAAATTA 1.44292703004
CCAAATTC 1.07009363146
CCAACAAA 2.77865885462
CCAACAAC 0.352790396579
CCAACAAG -0.730608603534
CCAACACA -0.0986976898427
CCAACACC -0.926407464444
CCAACACG -0.583594003303
CCAACAGA 0.233752513865
CCAACAGC 0.025240574993
CCAACAGG -0.760729695648
CCAACATA 1.59470687811
CCAACATC 0.591107939982
CCAACCAA -0.497640757214
CCAACCAC -0.460126748099
CCAACCAG -0.41943538433
CCAACCCA 0.274609593565
CCAACCCC -0.604731932798
CCAACCCG -1.10083079999
CCAACCGA -0.302429743508
CCAACCGC -0.872482089196
CCAACCGG -0.404904397382
CCAACCTA -0.730092113505
CCAACCTC -1.47091577111
CCAACGAA 0.139038802276

CCAACGAC -0.641291376332
CCAACGAG -1.206387927
CCAACGCA -0.534208564958
CCAACGCC -0.421674379063
CCAACGCG -0.0810298623871
CCAACGGA -0.413163010222
CCAACGGC -0.960480646257
CCAACGGG -0.593454885313
CCAACGTA 0.468767239448
CCAACGTC -0.33518425518
CCAACTAA 0.426721134877
CCAACTAC -0.155954634886
CCAACTAG -0.76298332775
CCAACTCA -0.266679276008
CCAACTCC -1.0768370152
CCAACTCG -0.523742323177
CCAACTGA -0.326961451619
CCAACTGC 0.251002653534
CCAACTGG 1.23902664663
CCAACTTA -0.378801524511
CCAACTTC 0.136532675554
CCAAGAAA 1.08532250726
CCAAGAAC -0.808239459675
CCAAGAAG -0.972147413699
CCAAGACA -0.442433305247
CCAAGACC -0.890910798471
CCAAGACG -0.562070797338
CCAAGAGA 0.352386562017
CCAAGAGC -0.762620007335
CCAAGAGG -0.460130407442
CCAAGATA 2.44186370467
CCAAGATC 3.83570954454
CCAAGCAA 0.254756615985
CCAAGCAC -0.114021969928
CCAAGCAG -0.25499682567
CCAAGCCA -1.15413591672
CCAAGCCC -1.15230127931
CCAAGCCG 0.232258456675
CCAAGCGA -0.668117753455
CCAAGCGC -0.666800651604
CCAAGCGG -0.392777075572
CCAAGCTA -0.120595717013
CCAAGCTC -0.346273107918
CCAAGGAA -0.132094938854
CCAAGGAC -1.60465218637
CCAAGGAG -1.30624850355
CCAAGGCA -0.768795931634
CCAAGGCC -1.44409174642
CCAAGGCG -0.801005201301
CCAAGGGA -1.10719021417
CCAAGGGC -1.45451459886
CCAAGGGG -0.693979632497
CCAAGGTA -0.561458903026
CCAAGGTC -0.796232112016

CCAAGTAA 0.250929205306
CCAAGTAC -0.335292989923
CCAAGTAG -0.255939629079
CCAAGTCA 0.458852774211
CCAAGTCC -0.236974303391
CCAAGTCG -0.586128359237
CCAAGTGA 0.345455767674
CCAAGTGC 0.278467585891
CCAAGTGG -0.98093584703
CCAAGTTA 0.337989925208
CCAAGTTC -1.00582930727
CCAATAAA 2.14745994439
CCAATAAC -0.096724258809
CCAATAAG 0.224648592951
CCAATACA -0.522896492339
CCAATACC 0.447744579235
CCAATACG -0.311538369291
CCAATAGA -0.286473965492
CCAATAGC 0.524340887027
CCAATAGG -0.386478301923
CCAATATA 1.14593454645
CCAATATC 3.99119552223
CCAATCAA -0.052199472584
CCAATCAC 1.04427226656
CCAATCAG 0.609686682292
CCAATCCA 1.4318481098
CCAATCCC -0.602577102939
CCAATCCG 1.58482273314
CCAATCGA 0.742810157717
CCAATCGC 0.239850023687
CCAATCGG 0.107666215091
CCAATCTA 2.60168574162
CCAATCTC 2.70988125444
CCAATGAA -0.0894787610271
CCAATGAC 0.0468087385922
CCAATGAG -1.09080603172
CCAATGCA 0.010612615474
CCAATGCC -0.430172940206
CCAATGCG 0.352981466524
CCAATGGA -0.248601602213
CCAATGGC -0.677159726932
CCAATGGG -0.865843780857
CCAATGTA 0.368114938046
CCAATGTC 0.789284589251
CCAATTAA 0.365596265007
CCAATTAC 0.948339993985
CCAATTAG -0.170009907333
CCAATTCA -0.319233704796
CCAATTCC 2.08505247504
CCAATTCG -0.207316902225
CCAATTGA -0.0111479249783
CCAATTGC 0.450282594511
CCAATTGG -0.0695599152852
CCAATTTA 0.906136276314

CCAATTTC 0.568408778226
CCACAAAA 0.833941111799
CCACAAAC 0.183874371216
CCACAAAG -1.30862106428
CCACAACA 1.57615531948
CCACAACC -0.423387996792
CCACAACG 0.97243937694
CCACAAGA -0.205186119472
CCACAAGC -0.758521543943
CCACAAGG -1.01320654137
CCACAATA 1.3538758893
CCACAATC 3.04682905124
CCACACAA 1.38481902652
CCACACAC -0.348366774485
CCACACAG 0.274784719233
CCACACCA 0.487976434162
CCACACCC -0.852084392374
CCACACCG 0.389595016048
CCACACGA -0.37370118547
CCACACGC -0.67212656296
CCACACGG -1.09220390048
CCACACTA 0.0290436771837
CCACACTC -0.218255721928
CCACAGAA -0.0436230197262
CCACAGAC -0.0928610388517
CCACAGAG -0.543239299026
CCACAGCA 0.232993461717
CCACAGCC -0.916750982841
CCACAGCG -1.06966810222
CCACAGGA 0.235683339699
CCACAGGC -0.473449367938
CCACAGGG -1.69063705963
CCACAGTA -0.728544473087
CCACAGTC -1.58277559249
CCACATAA 0.063335374009
CCACATAC 0.277727353217
CCACATAG -0.918744278875
CCACATCA -0.129797917407
CCACATCC 0.723828887753
CCACATCG -0.268918793504
CCACATGA 0.833721289879
CCACATGC -0.835751963482
CCACATGG 0.160825480888
CCACATTA 0.15268527391
CCACATTC 2.44131271227
CCACCAAA 1.08455221571
CCACCAAC -0.517951413965
CCACCAAG -0.989840072406
CCACCACA -0.289609760474
CCACCACC -0.32561533641
CCACCACG -0.929640231997
CCACCAGA -0.140031790951
CCACCAGC 0.368047501595
CCACCAGG -0.627401035671

CCACCATA 1.72255410468
CCACCATC -0.699563266105
CCACCCAA -0.760955790727
CCACCCAC -0.0228212269602
CCACCCAG -0.889426151018
CCACCCCA -0.498769925699
CCACCCCC -0.512564600697
CCACCCCG -0.468779524382
CCACCCGA -0.984937076469
CCACCCGC -1.07199439841
CCACCCGG 0.548723346247
CCACCCTA 0.621895293778
CCACCCTC -0.443480399912
CCACCGAA -0.0692190736868
CCACCGAC -1.29840104396
CCACCGAG -0.65076645911
CCACCGCA -0.70807541909
CCACCGCC -0.356004606039
CCACCGCG -0.149275551018
CCACCGGA 0.881358346742
CCACCGGC -1.23172625871
CCACCGGG -0.578682904536
CCACCGTA 0.520663248001
CCACCGTC -0.969157731029
CCACCTAA -0.15451050158
CCACCTAC -0.3360402799
CCACCTAG 0.479490942099
CCACCTCA 0.0523696320016
CCACCTCC -0.962118201943
CCACCTCG -1.19200566618
CCACCTGA 0.218781098932
CCACCTGC -0.484777646216
CCACCTGG -0.520878103671
CCACCTTA -0.0666397601186
CCACCTTC -1.38501349443
CCACGAAA -0.0859482798388
CCACGAAC -0.216594380518
CCACGAAG -0.237726298237
CCACGACA -0.00257879080505
CCACGACC -0.323033670408
CCACGACG -0.346258470549
CCACGAGA -0.252821346664
CCACGAGC -1.61210391423
CCACGAGG -0.548344342936
CCACGATA 0.569805601463
CCACGATC 1.7138487907
CCACGCAA 1.24253098965
CCACGCAC -0.583274856377
CCACGCAG -0.390838146908
CCACGCCA -0.916953292194
CCACGCCC -1.12360680793
CCACGCCG -1.02032683737
CCACGCGA -0.562003622268
CCACGCGC -0.36464718844

CCACGCGG	-0.540383705113
CCACGCTA	0.464544619799
CCACGCTC	-0.485625045344
CCACGGAA	0.955106640687
CCACGGAC	0.925630115584
CCACGGAG	-0.646725499668
CCACGGCA	-0.410195544987
CCACGGCC	-0.0367382285438
CCACGGCG	-1.23370909948
CCACGGGA	0.228185347298
CCACGGGC	-1.28898973829
CCACGGGG	-0.549560812874
CCACGGTA	-0.733021155646
CCACGGTC	-0.737330292602
CCACGTAA	0.312860960156
CCACGTAC	-0.366745821257
CCACGTAG	-0.619499470089
CCACGTCA	0.314776625857
CCACGTCC	-0.320983393185
CCACGTCG	-1.09630863703
CCACGTGA	-0.209666722754
CCACGTGC	-1.19962258721
CCACGTGG	0.284537389263
CCACGTTA	0.576737964096
CCACGTTC	-0.801168303416
CCACTAAA	0.105986315588
CCACTAAC	-0.371626076996
CCACTAAG	0.378023130124
CCACTACA	-0.32119249846
CCACTACC	-0.580391294633
CCACTACG	-0.296262183419
CCACTAGA	0.225165344362
CCACTAGC	-1.01651327992
CCACTAGG	-0.547212822016
CCACTATA	-0.438293543564
CCACTATC	0.22765108332
CCACTCAA	0.488281727864
CCACTCAC	1.16991866012
CCACTCAG	-0.49572143217
CCACTCCA	-0.552683800157
CCACTCCC	-1.24875370126
CCACTCCG	-0.578202485166
CCACTCGA	-0.351014047267
CCACTCGC	-0.368213217525
CCACTCGG	0.356913429841
CCACTCTA	-0.0546990647661
CCACTCTC	-0.511563770574
CCACTGAA	-0.240965338948
CCACTGAC	1.75146787521
CCACTGAG	0.41062734738
CCACTGCA	-0.444368313187
CCACTGCC	-0.568700480084
CCACTGCG	-0.657722346085
CCACTGGA	0.151669545037

CCACTGGC -0.803590003883
CCACTGGG 0.210825950123
CCACTGTA -0.00655701866365
CCACTGTC -0.985038492527
CCACTTAA -0.788733596851
CCACTTAC 0.4662799322
CCACTTAG -0.979516806357
CCACTTCA -0.305889390284
CCACTTCC -0.872029114894
CCACTTCG -0.901345674462
CCACTTGA 0.253246353136
CCACTTGC -0.317352280083
CCACTTTA 0.786615883178
CCACTTTC 0.0569712549616
CCAGAAAA 1.12775650212
CCAGAAAC -0.323794290846
CCAGAAAG -0.402916067598
CCAGAACA -0.610796508539
CCAGAACC 0.438378753963
CCAGAACG -0.252377520718
CCAGAAGA -0.0383948650857
CCAGAAGC -0.159041812891
CCAGAAGG -1.33760619199
CCAGAATA 0.259080913074
CCAGAATC 7.97288538004
CCAGACAA -0.531231689986
CCAGACAC -0.430096094017
CCAGACAG -1.08645298266
CCAGACCA -1.13475891502
CCAGACCC -0.945988082404
CCAGACCG -0.0468521279368
CCAGACGA -0.436725254001
CCAGACGC -1.0451962505
CCAGACGG -0.455751220218
CCAGACTA -0.73718888516
CCAGACTC -0.48721685925
CCAGAGAA 0.897813363602
CCAGAGAC -0.985411745443
CCAGAGAG -0.549053993963
CCAGAGCA -0.372645465212
CCAGAGCC -1.54111842373
CCAGAGCG -1.40049120551
CCAGAGGA -1.10878176669
CCAGAGGC -1.42983677844
CCAGAGGG -0.990726417391
CCAGAGTA 0.0107469656133
CCAGAGTC -1.20591900842
CCAGATAA 0.644196894129
CCAGATAC 4.05814998579
CCAGATAG 0.272471492128
CCAGATCA 1.2672780762
CCAGATCC 1.6712861961
CCAGATCG 3.91236074247
CCAGATGA 0.728818662379

CCAGATGC -0.00202152524694
CCAGATGG -0.660011526085
CCAGATTA 1.43619279465
CCAGATTC 20.1944987708
CCAGCAAA 0.4959297533
CCAGCAAC -0.590920529379
CCAGCAAG 0.66279210348
CCAGCACA 0.500531114879
CCAGCACC -1.09553181094
CCAGCACG -0.116091589388
CCAGCAGA 0.303232446383
CCAGCAGC -1.50857275595
CCAGCAGG -1.57570783419
CCAGCATA 0.628847521411
CCAGCATC -0.0835976751652
CCAGCCAA -0.422911759528
CCAGCCAC -0.64827706081
CCAGCCAG -1.16135318529
CCAGCCCA -1.73068856126
CCAGCCCC -0.260052207077
CCAGCCCG -0.991337266176
CCAGCCGA 0.363358054419
CCAGCCGC -0.799289231138
CCAGCCGG -1.74489778747
CCAGCCTA -0.576328117757
CCAGCCTC -1.29738557647
CCAGCGAA -0.794652321663
CCAGCGAC -1.55916865246
CCAGCGAG -0.375842162105
CCAGCGCA 0.346066093696
CCAGCGCC -0.995339018378
CCAGCGCG -0.655060697315
CCAGCGGA 0.812588849397
CCAGCGGC -0.950426341868
CCAGCGGG -0.838990742811
CCAGCGTA -0.320178860639
CCAGCGTC -0.645650959936
CCAGCTAA -0.417022832218
CCAGCTAC -0.997756275358
CCAGCTAG -0.805898264739
CCAGCTCA -0.741374388622
CCAGCTCC -1.85338892287
CCAGCTCG -0.456832555872
CCAGCTGA 0.284151328649
CCAGCTGC -1.03548514014
CCAGCTGG -0.612102109601
CCAGCTTA -0.62272047547
CCAGCTTC -1.35644527037
CCAGGAAA 0.20716843748
CCAGGAAC -1.27463178596
CCAGGAAG -0.545864615755
CCAGGACA -0.333588781931
CCAGGACC -1.48773881325
CCAGGACG -0.211416933907

CCAGGAGA 0.283510682363
CCAGGAGC −1.08495239093
CCAGGAGG −0.959782234638
CCAGGATA 2.40786841458
CCAGGATC 4.4066526787
CCAGGCAA −0.0779675156334
CCAGGCAC −1.78136156534
CCAGGCAG −0.754487903186
CCAGGCCA −1.09220912812
CCAGGCCC −1.00934384418
CCAGGCCG −0.669238296348
CCAGGCGA 0.368038876002
CCAGGCGC −1.2191558951
CCAGGCGG −0.474372044965
CCAGGCTA −0.0786518126462
CCAGGCTC −1.64831415196
CCAGGGAA −0.28440722123
CCAGGGAC −0.835631466567
CCAGGGAG −1.32356372724
CCAGGGCA −0.683676754211
CCAGGGCC −1.31442451981
CCAGGGCG −0.0435958360404
CCAGGGGA −0.921005229662
CCAGGGGC −1.04731422555
CCAGGGGG −1.18962029775
CCAGGGTA −0.178758872043
CCAGGGTC −1.38230767217
CCAGGTAA −0.156154069042
CCAGGTAC −0.67994082709
CCAGGTAG −0.635446883893
CCAGGTCA −0.542083208237
CCAGGTCC −1.2085762137
CCAGGTCG −1.04837203686
CCAGGTGA −0.835743599271
CCAGGTGC −1.02133263374
CCAGGTTA −0.398220608645
CCAGGTTC −0.810415984207
CCAGTAAA 0.200968204692
CCAGTAAC −0.347725866817
CCAGTAAG −0.946318468738
CCAGTACA 1.02721345822
CCAGTACC −0.239815521317
CCAGTACG −0.575685641799
CCAGTAGA −0.673376489742
CCAGTAGC −0.369776018075
CCAGTAGG −0.938543934611
CCAGTATA −0.580194474293
CCAGTATC 1.98655578764
CCAGTCAA −0.200295669851
CCAGTCAC −0.583194089464
CCAGTCAG −1.11913352334
CCAGTCCA −0.0949369314702
CCAGTCCC −0.987503320957
CCAGTCCG −0.862962310166

CCAGTCGA -0.189191134217
CCAGTCGC -0.592226914585
CCAGTCGG -1.31867380038
CCAGTCTA -0.798719942026
CCAGTCTC -0.910452731955
CCAGTGAA -0.702239813626
CCAGTGAC -0.742021569449
CCAGTGAG -1.08315748352
CCAGTGCA -0.270592942612
CCAGTGCC -1.08105388445
CCAGTGCG -0.368227593513
CCAGTGGA -0.756681417522
CCAGTGGC -0.54845647564
CCAGTGGG -0.252104115571
CCAGTGTA -0.48796127403
CCAGTGTC -0.317950059789
CCAGTTAA -0.00605046113474
CCAGTTAC 0.444061973959
CCAGTTAG 0.583721034757
CCAGTTCA -0.5068941884
CCAGTTCC -0.658228903614
CCAGTTCG -0.849231412277
CCAGTTGA -0.552892382669
CCAGTTGC -0.145850406612
CCAGTTTA 0.560114094726
CCAGTTTC -0.53996993805
CCATAAAA 0.942810727749
CCATAAAC 0.156465113139
CCATAAAG -0.117801025012
CCATAACA 0.0359101716545
CCATAACC -0.294617309049
CCATAACG 0.300595367482
CCATAAGA 0.206367041513
CCATAAGC -0.493099513402
CCATAAGG -0.326842784375
CCATAATA 1.2441350885
CCATAATC 4.51154981719
CCATACAA 0.696642326793
CCATACAC 0.642525620218
CCATACAG -0.388578764411
CCATACCA 0.272031064142
CCATACCC 0.030579032666
CCATACCG -0.715266549501
CCATACGA 0.545424710533
CCATACGC -0.62594958368
CCATACGG -0.373050606683
CCATACTA -0.750460535588
CCATACTC 0.0107469656133
CCATAGAA 0.86597708547
CCATAGAC -0.239459519586
CCATAGAG -0.692806813285
CCATAGCA -0.23842863058
CCATAGCC 0.192719524353
CCATAGCG -0.00282187568735

CCATAGGA 0.481505671424
CCATAGGC -0.633641521224
CCATAGGG -0.754823255771
CCATAGTA 0.0727202801366
CCATAGTC -0.746556540102
CCATATAA 1.07347460238
CCATATAC 1.35000561204
CCATATAG -0.0638597054863
CCATATCA 1.7288588901
CCATATCC 4.56001780614
CCATATCG 3.39254358669
CCATATGA 1.77376790727
CCATATGC -0.299985302842
CCATATGG 1.09001927312
CCATATTA 0.622726487246
CCATATTC 3.42558927745
CCATCAAA 0.91251163477
CCATCAAC 0.143896317817
CCATCAAG 0.026371311768
CCATCACA -0.297844326207
CCATCACC -0.768814228346
CCATCACG -0.342735046663
CCATCAGA 0.589794236091
CCATCAGC -0.83268177528
CCATCAGG -0.893564605794
CCATCATA 0.877259621969
CCATCATC -0.0909150528854
CCATCCAA 0.185070714772
CCATCCAC -0.0524734004944
CCATCCAG 0.197705901019
CCATCCCA -0.534191313773
CCATCCCC -0.312067405637
CCATCCCG -0.661119784043
CCATCCGA -0.465142660885
CCATCCGC -0.190136551442
CCATCCGG -0.157885199339
CCATCCTA -0.443259271084
CCATCCTC 0.150848284069
CCATCGAA -0.000360967981125
CCATCGAC -1.04932425001
CCATCGAG 0.337427432018
CCATCGCA 1.23839044383
CCATCGCC -0.536640982071
CCATCGCG 0.0704538403362
CCATCGGA -1.01658698952
CCATCGGC -1.04249225791
CCATCGGG -1.05073335818
CCATCGTA 0.14741451407
CCATCGTC -0.123746410746
CCATCTAA 0.712399193416
CCATCTAC -0.298463539203
CCATCTAG -0.877042413864
CCATCTCA 0.303898969447
CCATCTCC -0.643484367905

CCATCTCG	-0.447652834295
CCATCTGA	-0.032939047077
CCATCTGC	-0.821400545689
CCATCTTA	0.156572279593
CCATCTTC	-0.472624447628
CCATGAAA	0.802574536391
CCATGAAC	-0.183887440296
CCATGAAG	-0.382412249849
CCATGACA	-0.333596100616
CCATGACC	-1.00245434813
CCATGACG	-0.734827302459
CCATGAGA	0.201770907567
CCATGAGC	-0.736398728601
CCATGAGG	-0.899918008196
CCATGATA	1.13928996495
CCATGATC	0.849759403097
CCATGCAA	1.0167691725
CCATGCAC	-0.343585059607
CCATGCAG	-0.342596514419
CCATGCCA	-0.0739030318488
CCATGCCC	-0.711393919806
CCATGCCG	-0.614370640454
CCATGCGA	-0.224716029402
CCATGCGC	-1.66463325039
CCATGCGG	-1.03263738768
CCATGCTA	-0.639279260823
CCATGCTC	-0.544136360656
CCATGGAA	0.253531781836
CCATGGAC	-1.17187170339
CCATGGAG	-0.591853661669
CCATGGCA	0.0533343914646
CCATGGCC	-1.02723018665
CCATGGCG	-0.439399710469
CCATGGGA	0.513118468294
CCATGGGC	-1.14319814254
CCATGGGG	-0.820017575676
CCATGGTA	-0.326472145275
CCATGGTC	-0.624364304314
CCATGTAA	1.61001312286
CCATGTAC	0.0344788460465
CCATGTAG	-0.596176913231
CCATGTCA	0.457687012302
CCATGTCC	-0.660839844356
CCATGTCG	0.0182224791978
CCATGTGA	-0.271103420865
CCATGTGC	0.00737827963157
CCATGTTA	0.74221865117
CCATGTTC	-0.333355106786
CCATTAAA	-0.16231326492
CCATTAAC	0.646930422838
CCATTAAG	0.00323329031609
CCATTACA	0.866341974175
CCATTACC	0.368086970215
CCATTACG	0.266854924439

CCATTAGA 0.418207413602
CCATTAGC -1.20371294777
CCATTAGG -0.663146536921
CCATTATA 0.280873342081
CCATTATC 1.38656976044
CCATTCAA -0.02437749297
CCATTCAC -0.274149039197
CCATTCAG 0.466570327151
CCATTCCA 0.104718353476
CCATTCCC -0.859360471802
CCATTCCG -0.6252279091
CCATTCGA 0.59089282293
CCATTCGC -0.723359969173
CCATTCGG -0.284520660841
CCATTCTA 0.947987651597
CCATTCTC -0.410851874169
CCATTGAA 0.646747455722
CCATTGAC -0.611418073969
CCATTGAG -0.687142674146
CCATTGCA -0.477266323354
CCATTGCC -0.609723537096
CCATTGCG 0.488893360793
CCATTGGA -0.13863104699
CCATTGGC -0.609261937202
CCATTGGG -1.13695713422
CCATTGTA -0.254457595442
CCATTGTC -0.899470522907
CCATTTAA 0.196232231593
CCATTTAC -0.427586307953
CCATTTAG 1.25944839055
CCATTTCA 0.401288705794
CCATTTCC -0.324911435778
CCATTTCG 0.176546538233
CCATTTGA -0.51829826734
CCATTTGC 0.874027115797
CCATTTTA -0.518423207742
CCATTTTC -0.352935201982
CCCAAAAA 2.71281892218
CCCAAAAC -0.0319288072168
CCCAAAAG -0.163776479083
CCCAAACA 0.986093951403
CCCAAACC -1.00358351661
CCCAAACG 0.379700415812
CCCAAAGA 0.441545130591
CCCAAAGC -0.363027929466
CCCAAAGG -0.597599874628
CCCAAATA 0.573285897386
CCCAAATC 2.56483145965
CCCAACAA 0.492333142569
CCCAACAC -0.1419879708
CCCAACAG -0.37900801597
CCCAACCA -0.445349801072
CCCAACCC 0.0102529544009
CCCAACCG -0.391494476091

CCCAACGA	-0.710579977523
CCCAACGC	0.188000279676
CCCAACGG	-0.335909589103
CCCAACTA	0.257876466689
CCCAACTC	-0.714279049839
CCCAAGAA	-1.00763022645
CCCAAGAC	-0.562070797338
CCCAAGAG	-1.16769743933
CCCAAGCA	-0.666816334499
CCCAAGCC	-0.680261803687
CCCAAGCG	-0.748502264687
CCCAAGGA	-1.53751397155
CCCAAGGC	-1.46996198967
CCCAAGGG	-1.21192608021
CCCAAGTA	-0.575807445622
CCCAAGTC	-0.606079354915
CCCAATAA	1.78984209115
CCCAATAC	-0.0387869374765
CCCAATAG	0.0879214494909
CCCAATCA	0.881945932565
CCCAATCC	0.750540779737
CCCAATCG	-0.189390306992
CCCAATGA	-0.970914999484
CCCAATGC	-0.648564580563
CCCAATTA	0.259358500327
CCCAATTC	0.235016555253
CCCACAAA	0.388592356254
CCCACAAC	-0.290356789069
CCCACAAG	-0.911949402961
CCCACACA	0.353666024919
CCCACACC	-0.0788175285767
CCCACACG	-1.2049579083
CCCACAGA	0.273712531935
CCCACAGC	-0.565279256402
CCCACAGG	0.258153531179
CCCACATA	-0.954255843599
CCCACATC	0.167689361543
CCCACCAA	-0.883062293352
CCCACCAC	-0.339656494251
CCCACCAG	-0.483374549821
CCCACCCA	-0.224351663461
CCCACCCC	-0.654421096555
CCCACCCG	-0.377031448357
CCCACCGA	-0.182812116419
CCCACCGC	-0.0957362363842
CCCACCGG	-1.36711538979
CCCACCTA	-0.201153001479
CCCACCTC	-1.12747577829
CCCACGAA	-0.216594380518
CCCACGAC	-0.471978835091
CCCACGAG	-0.434187238724
CCCACGCA	-0.411455404269
CCCACGCC	-1.84200470893
CCCACGCG	-0.870483304148

CCCACGGA -0.597735793057
CCCACGGC -0.242469328639
CCCACGGG -1.03054973289
CCCACGTA -0.700013887973
CCCACGTC 0.29444688825
CCCACTAA -0.367375228135
CCCACTAC -0.478459268948
CCCACTAG -0.57608764669
CCCACTCA 0.445174152641
CCCACTCC -0.598345334934
CCCACTCG 0.203064746456
CCCACTGA 0.131507353031
CCCACTGC -0.654641441239
CCCACTTA -0.378730951481
CCCACTTC -1.43199369935
CCCAGAAA -0.0482907722294
CCCAGAAC -0.440927224504
CCCAGAAG -0.118315946752
CCCAGACA -0.652994214434
CCCAGACC -0.568108189393
CCCAGACG -0.80520481937
CCCAGAGA -0.720102108987
CCCAGAGC -0.832293884995
CCCAGAGG -1.30761265409
CCCAGATA 2.95890760174
CCCAGATC 2.61674707182
CCCAGCAA 0.372505887441
CCCAGCAC -0.642246987439
CCCAGCAG -0.197063947824
CCCAGCCA -1.28325581027
CCCAGCCC -1.74395054057
CCCAGCCG -1.0358152651
CCCAGCGA -0.82727169905
CCCAGCGC -0.776389066936
CCCAGCGG -1.42321755095
CCCAGCTA -1.06498153025
CCCAGCTC -1.00947113701
CCCAGGAA -0.915286723152
CCCAGGAC -0.96142083585
CCCAGGAG -0.959782234638
CCCAGGCA -0.813656593208
CCCAGGCC -1.47263958272
CCCAGGCG -1.61763997639
CCCAGGGA -0.183727213379
CCCAGGGC -0.728566951905
CCCAGGGG -0.973882203338
CCCAGGTA -0.464108112537
CCCAGGTC -1.33769166377
CCCAGTAA -0.319745489957
CCCAGTAC -0.222670457049
CCCAGTAG 0.299341781358
CCCAGTCA -1.28613336024
CCCAGTCC -0.960796917986
CCCAGTCG -0.482459975624

CCCAGTGA -0.674742992715
CCCAGTGC -0.288201175065
CCCAGTTA -0.828221559762
CCCAGTTC -0.728555189733
CCCATAAA -0.694574014241
CCCATAAC 0.0359101716545
CCCATAAG -0.389648860656
CCCATACA 0.820076386534
CCCATACC -0.374897529025
CCCATACG -0.48377629333
CCCATAGA 0.882028006385
CCCATAGC 0.0151021057302
CCCATAGG -0.528615521613
CCCATATA 0.0831109826375
CCCATATC 4.38452254468
CCCATCAA 0.0143958526636
CCCATCAC -0.477042319328
CCCATCAG -0.870435471316
CCCATCCA -0.147406672622
CCCATCCC -0.766835046917
CCCATCCG -0.824662065217
CCCATCGA -0.332572007531
CCCATCGC -0.107263687437
CCCATCGG -0.998374704209
CCCATCTA -0.598267965982
CCCATCTC -0.839503050735
CCCATGAA 0.33599924299
CCCATGAC -0.576513698688
CCCATGAG 0.298234046163
CCCATGCA 0.289740451271
CCCATGCC -0.616722290654
CCCATGCG -1.00358351661
CCCATGGA 0.148258515237
CCCATGGC -1.04187121524
CCCATGGG -1.15320958035
CCCATGTA 0.114907530768
CCCATGTC -0.371915687802
CCCATTAA -0.682227131892
CCCATTAC 0.507033243407
CCCATTAG 0.720394856372
CCCATTCA -0.622076692604
CCCATTCC -0.765698036984
CCCATTCG -0.910461618929
CCCATTGA -0.092634943773
CCCATTGC -0.522499976461
CCCATTTA -0.407076217046
CCCATTTC -0.389068593518
CCCCAAAA 0.571332592735
CCCCAAAC -0.00971738351505
CCCCAAAG -0.557625741952
CCCCAACA 0.171316031158
CCCCAACC -1.34231001515
CCCCAACG -0.998494416979
CCCCAAGA -0.287310125211

CCCCAAGC -0.32158274118
CCCCAAGG -0.593744496119
CCCCAATA 0.0873153055747
CCCCAATC 0.707646230513
CCCCACAA -0.757686168369
CCCCACAC -0.72315452324
CCCCACAG -0.118950058498
CCCCACCA -0.27310325144
CCCCACCC -0.654421096555
CCCCACCG -0.910509713142
CCCCACGA -0.503156954371
CCCCACGC -1.18587365399
CCCCACGG -0.91923201693
CCCCACTA -0.8409207845
CCCCACTC 0.11550975396
CCCCAGAA -0.597922158134
CCCCAGAC -1.50002714613
CCCCAGAG -0.903863301974
CCCCAGCA -0.64259148838
CCCCAGCC -0.992428795712
CCCCAGCG -1.05072551674
CCCCAGGA -1.11975508877
CCCCAGGC -1.09676866864
CCCCAGGG -1.12161769401
CCCCAGTA -0.251974993063
CCCCAGTC -1.39703443393
CCCCATAA -0.593507684395
CCCCATAC 0.398249622002
CCCCATAG -0.0622553452631
CCCCATCA -0.335965002001
CCCCATCC -1.21964232624
CCCCATCG 0.489199177258
CCCCATGA -0.0907145732029
CCCCATGC -0.771604999624
CCCCATTA 0.223597054799
CCCCATTC -0.834236995764
CCCCCAAA 0.1084712704
CCCCCAAC -0.623997063174
CCCCCAAG -1.0957283699
CCCCCACA -0.724553960294
CCCCCACC -1.22683502494
CCCCCACG -0.954277799653
CCCCCAGA -0.166181712509
CCCCCAGC -1.04015707475
CCCCCAGG -1.04127866317
CCCCCATA -0.443296910034
CCCCCATC -1.27880526587
CCCCCCAA -0.8667889367
CCCCCCAC -0.825991190622
CCCCCCAG -1.60065121831
CCCCCCCA -0.788804954026
CCCCCCCC -1.67995099593
CCCCCCCG -1.39586867202
CCCCCCGA -1.24367453413

CCCCCCGC	-0.612307555533
CCCCCCGG	-1.60753940746
CCCCCCTA	-1.33464839787
CCCCCCTC	-0.972061419155
CCCCCGAA	-0.514759160559
CCCCCGAC	-1.61609991604
CCCCCGAG	-0.607640064412
CCCCCGCA	0.0386792482599
CCCCCGCC	-1.26734525127
CCCCCGCG	0.202631375774
CCCCCGGA	-0.594637375643
CCCCCGGC	-1.77192542842
CCCCCGGG	-0.970624865915
CCCCCGTA	0.197535741601
CCCCCGTC	-0.837323651005
CCCCCTAA	-0.421999276384
CCCCCTAC	-0.540504724791
CCCCCTAG	-1.40816615325
CCCCCTCA	-0.903839516249
CCCCCTCC	-1.36602647407
CCCCCTCG	-1.10894120947
CCCCCTGA	-1.02572567419
CCCCCTGC	-0.820747091704
CCCCCTTA	-1.28168882761
CCCCCTTC	-0.945917509373
CCCCGAAA	-0.143890567422
CCCCGAAC	-0.773977560351
CCCCGAAG	-0.967071644528
CCCCGACA	-0.57299968454
CCCCGACC	-1.07786189243
CCCCGACG	-0.848931346207
CCCCGAGA	-0.642074736969
CCCCGAGC	-1.37723347129
CCCCGAGG	-0.489384496808
CCCCGATA	1.24437268437
CCCCGATC	1.36204066615
CCCCGCAA	0.108606666066
CCCCGCAC	-0.674500430596
CCCCGCAG	-0.49725966285
CCCCGCCA	-0.815378313766
CCCCGCCC	-1.01489794167
CCCCGCCG	-1.08262321954
CCCCGCGA	-0.316662755439
CCCCGCGC	-0.309820046693
CCCCGCGG	-0.290019606813
CCCCGCTA	-1.19177774143
CCCCGCTC	-0.839291593026
CCCCGGAA	0.234801438202
CCCCGGAC	-0.850800224603
CCCCGGAG	-1.23514669825
CCCCGGCA	-0.517162825696
CCCCGGCC	-1.00128440411
CCCCGGCG	-1.61624263039
CCCCGGGA	-0.83463168197

CCCCGGGC -1.32674395709
CCCCGGGG 0.318661017724
CCCCGGTA -0.939361274855
CCCCGGTC -1.08235870137
CCCCGTAA -0.150776926894
CCCCGTAC -0.747128704411
CCCCGTAG 0.0120993539799
CCCCGTCA 0.234755696423
CCCCGTCC -0.486502241973
CCCCGTCG -1.31653413065
CCCCGTGA -0.600949741135
CCCCGTGC -0.531713416263
CCCCGTTA -0.631054104827
CCCCGTTC -1.16197605762
CCCCTAAA -0.0529156581512
CCCCTAAC -0.216546286305
CCCCTAAG -0.421648240903
CCCCTACA 0.00646083023711
CCCCTACC -0.622424591505
CCCCTACG -0.429710033403
CCCCTAGA -0.32778663331
CCCCTAGC -1.12909059377
CCCCTAGG -1.69528991339
CCCCTATA -0.455381103881
CCCCTATC -0.819208599643
CCCCTCAA -0.878009002998
CCCCTCAC -0.941480295436
CCCCTCAG -0.921292488034
CCCCTCCA -1.31653099408
CCCCTCCC -1.59175797097
CCCCTCCG -1.25077627203
CCCCTCGA -0.805271733058
CCCCTCGC -0.296514416657
CCCCTCGG -0.762291712053
CCCCTCTA -0.546148998929
CCCCTCTC -0.741760449236
CCCCTGAA -0.829643475633
CCCCTGAC -1.16224737172
CCCCTGAG -1.10549593868
CCCCTGCA -0.839192006638
CCCCTGCC -0.934885637823
CCCCTGCG 0.333240621648
CCCCTGGA -1.14040031396
CCCCTGGC -1.00035571531
CCCCTGTA 0.402815174302
CCCCTGTC -0.76830976187
CCCCTTAA -0.477850249834
CCCCTTAC -1.38729692403
CCCCTTAG -1.23656234096
CCCCTTCA -1.17684553374
CCCCTTCC -1.36756052264
CCCCTTCG -1.10159377286
CCCCTTGA -0.461621589434
CCCCTTGC -1.2162475021

CCCCTTTA	−0.645180734449
CCCCTTTC	−0.692023452648
CCCGAAAA	0.666624480409
CCCGAAAC	−0.115476035734
CCCGAAAG	−1.21169815546
CCCGAACA	−0.556277535691
CCCGAACC	0.277622277816
CCCGAACG	−0.912892467752
CCCGAAGA	0.516819370282
CCCGAAGC	−1.19679051764
CCCGAAGG	−1.27141600821
CCCGAATA	1.54514579133
CCCGAATC	5.23253225938
CCCGACAA	−0.238055377664
CCCGACAC	−0.415003398024
CCCGACAG	−0.277338417405
CCCGACCA	−1.22314248717
CCCGACCC	−0.953935651147
CCCGACCG	−1.25194020427
CCCGACGA	−1.28739112846
CCCGACGC	−0.636767906469
CCCGACGG	−0.417733006009
CCCGACTA	−0.443563519259
CCCGACTC	0.194824953091
CCCGAGAA	1.31325535994
CCCGAGAC	−1.11639947187
CCCGAGAG	−0.885119627878
CCCGAGCA	−0.519139916072
CCCGAGCC	−1.16457053132
CCCGAGCG	−0.873156453708
CCCGAGGA	−0.0387127051039
CCCGAGGC	−0.315447592409
CCCGAGGG	−0.482943008809
CCCGAGTA	−0.36761047157
CCCGAGTC	−0.688878770692
CCCGATAA	0.469026268607
CCCGATAC	1.87934516067
CCCGATAG	0.206234782427
CCCGATCA	0.969436625189
CCCGATCC	1.57576769058
CCCGATCG	0.990976559576
CCCGATGA	−0.351227334648
CCCGATGC	−0.172338294571
CCCGATTA	3.26207156615
CCCGATTC	4.30354339032
CCCGCAAA	0.0852360149956
CCCGCAAC	−0.286725414586
CCCGCAAG	−1.07815176462
CCCGCACA	−0.0120700792414
CCCGCACC	−0.412465644129
CCCGCACG	−1.04063017544
CCCGCAGA	−0.466808445783
CCCGCAGC	−1.50666676137
CCCGCAGG	−0.553949148453

CCCGCATA -0.510993174555
CCCGCATC 0.530898689835
CCCGCCAA -0.267737348699
CCCGCCAC -0.0133338592477
CCCGCCAG -0.785910675637
CCCGCCCA -0.562954005743
CCCGCCCC -0.720340227619
CCCGCCCG 0.14036452972
CCCGCCGA -0.673291802106
CCCGCCGC -0.59846609323
CCCGCCGG -1.09082537396
CCCGCCTA 0.0183108261765
CCCGCCTC -0.346291927393
CCCGCGAA -0.774057020356
CCCGCGAC -1.18193933824
CCCGCGAG -0.586954325073
CCCGCGCA -0.0892448245006
CCCGCGCC -1.18151145657
CCCGCGCG -0.397998172909
CCCGCGGA -0.190506406398
CCCGCGGC -0.290019606813
CCCGCGGG -0.160375381783
CCCGCGTA 0.864534259071
CCCGCGTC -0.624287719506
CCCGCTAA -0.42644276348
CCCGCTAC -1.27558295358
CCCGCTAG -0.768250166866
CCCGCTCA -0.52749497872
CCCGCTCC -0.924056598389
CCCGCTCG -0.85993237473
CCCGCTGA -0.138399985661
CCCGCTGC -1.1042460119
CCCGCTTA -0.796235771358
CCCGCTTC -0.547617963487
CCCGGAAA 0.616211289638
CCCGGAAC -0.803780289683
CCCGGAAG -0.840962344174
CCCGGACA -0.0792294659686
CCCGGACC -1.00290078789
CCCGGACG 0.136585213255
CCCGGAGA 0.527138192844
CCCGGAGC -0.756195247757
CCCGGAGG -1.24059127685
CCCGGATA 2.14155899352
CCCGGATC 0.77393312548
CCCGGCAA -0.889375965752
CCCGGCAC -1.21249118722
CCCGGCAG -0.989840072406
CCCGGCCA -0.781928265673
CCCGGCCC -1.11531055615
CCCGGCCG -0.0335227121761
CCCGGCGA -0.798090012385
CCCGGCGC -1.02128192571
CCCGGCGG -1.09082537396

CCCGGCTA −0.904754613209
CCCGGCTC −1.64664967397
CCCGGGAA −0.277158064105
CCCGGGAC −1.29181318227
CCCGGGAG −1.15002830497
CCCGGGCA −0.542690659061
CCCGGGCC −1.8944514485
CCCGGGCG −1.19057224952
CCCGGGGA −0.758810631986
CCCGGGGC −1.0789528992
CCCGGGTA −0.220558755152
CCCGGGTC −1.53629384227
CCCGGTAA −0.0329293759581
CCCGGTAC −0.82176647992
CCCGGTAG −0.921617123974
CCCGGTCA −0.885159096498
CCCGGTCC −1.02026462855
CCCGGTCG −0.940647533678
CCCGGTGA −0.707900554804
CCCGGTGC −0.673775358054
CCCGGTTA −0.945427157503
CCCGGTTC −1.49312562652
CCCGTAAA 0.395172637879
CCCGTAAC 0.0847642212186
CCCGTAAG −0.78146692716
CCCGTACA −0.0364402535268
CCCGTACC −0.645435320121
CCCGTACG −0.749195448674
CCCGTAGA −0.787725186662
CCCGTAGC −1.52229502825
CCCGTAGG 0.254506735181
CCCGTATA −0.320863157652
CCCGTATC 1.86409354466
CCCGTCAA 0.209719521836
CCCGTCAC −0.889426151018
CCCGTCAG −0.571783737366
CCCGTCCA −0.922975001354
CCCGTCCC −0.631237856087
CCCGTCCG −0.590290076974
CCCGTCGA −1.3046559055
CCCGTCGC −1.30493244723
CCCGTCGG −1.2862985534
CCCGTCTA −1.11982879838
CCCGTCTC −0.647301061938
CCCGTGAA −0.494556193024
CCCGTGAC −0.371183819339
CCCGTGAG −1.07209973519
CCCGTGCA −0.0263425597927
CCCGTGCC −0.82665013362
CCCGTGCG −1.31627902222
CCCGTGGA −0.273639867852
CCCGTGGC −0.828970418029
CCCGTGTA −0.597109522758
CCCGTGTC −1.05124854131

CCCGTTAA 0.520110687312
CCCGTTAC -0.649141711122
CCCGTTAG -1.32202157584
CCCGTTCA 0.354186174291
CCCGTTCC -0.752708940059
CCCGTTCG -0.863818857649
CCCGTTGA -0.271345460221
CCCGTTGC -0.688531394554
CCCGTTTA -0.330717766504
CCCGTTTC 0.903271011282
CCCTAAAA 1.17813780434
CCCTAAAC -0.105609141948
CCCTAAAG -0.767009911204
CCCTAACA -0.248104715803
CCCTAACC -1.26628090542
CCCTAACG -0.260700433429
CCCTAAGA 0.386064273478
CCCTAAGC -1.14725347797
CCCTAAGG -1.5054549963
CCCTAATA 0.804684147235
CCCTAATC -0.0027811001587
CCCTACAA -0.377556563979
CCCTACAC -0.135633000108
CCCTACAG -0.754170063168
CCCTACCA -0.855490978687
CCCTACCC -0.389334941362
CCCTACCG -0.872145168322
CCCTACGA -0.470471186058
CCCTACGC -1.08974325416
CCCTACGG -0.935844124128
CCCTACTA -0.417754700681
CCCTACTC -1.17148485863
CCCTAGAA 2.22672809483
CCCTAGAC -0.47474346821
CCCTAGAG -0.149875160395
CCCTAGCA -0.859360471802
CCCTAGCC -1.03187546033
CCCTAGCG 0.133211299641
CCCTAGGA -0.446423033896
CCCTAGGC -1.09270104828
CCCTAGGG -1.52682607818
CCCTAGTA 0.0652541762895
CCCTAGTC -0.480344614385
CCCTATAA 0.582238216975
CCCTATAC 0.59944836526
CCCTATAG -1.16760517163
CCCTATCA 0.18905390888
CCCTATCC -0.728596488025
CCCTATCG -0.125519100715
CCCTATGA -0.810786361925
CCCTATGC -0.441963341142
CCCTATTA 0.127758095448
CCCTATTC 0.417093928012
CCCTCAAA -0.303713388516

CCCTCAAC	-0.275839916728
CCCTCAAG	-0.788780645538
CCCTCACA	-0.641990310714
CCCTCACC	-1.0035652199
CCCTCACG	-0.239207809111
CCCTCAGA	-0.866547420107
CCCTCAGC	-0.763389253365
CCCTCAGG	-0.805716604531
CCCTCATA	-0.0930847814961
CCCTCATC	-0.225144172453
CCCTCCAA	-0.309257553503
CCCTCCAC	-1.25789107902
CCCTCCAG	-1.15282613355
CCCTCCCA	-0.55847314108
CCCTCCCC	-0.351628294013
CCCTCCCG	-0.320863157652
CCCTCCGA	-0.822771230767
CCCTCCGC	-1.12162083058
CCCTCCGG	-1.1426774704
CCCTCCTA	-1.42999622121
CCCTCCTC	-1.58888015962
CCCTCGAA	-0.460375060613
CCCTCGAC	-1.23325272722
CCCTCGAG	-0.936847829448
CCCTCGCA	-0.0411636803095
CCCTCGCC	-1.55682798029
CCCTCGCG	-1.60755169239
CCCTCGGA	0.211251217976
CCCTCGGC	-0.485172855186
CCCTCGTA	-0.830371684754
CCCTCGTC	-0.905390293245
CCCTCTAA	-0.39568102508
CCCTCTAC	-0.873944519214
CCCTCTAG	-0.21997456729
CCCTCTCA	-0.967185606903
CCCTCTCC	-1.58676610529
CCCTCTCG	-0.658373709017
CCCTCTGA	-0.0918615156368
CCCTCTGC	-0.125735001912
CCCTCTTA	-0.658955021682
CCCTCTTC	-0.656224106789
CCCTGAAA	-0.104052353175
CCCTGAAC	-0.608393888928
CCCTGAAG	0.538962050624
CCCTGACA	-0.807238890933
CCCTGACC	-1.43520241979
CCCTGACG	-0.389557899862
CCCTGAGA	0.45922158364
CCCTGAGC	-0.90848400579
CCCTGAGG	-1.41314547261
CCCTGATA	1.0076777979
CCCTGATC	-0.463505366581
CCCTGCAA	0.315745044663
CCCTGCAC	-0.0807297963174

CCCTGCAG -1.03849076709
CCCTGCCA -0.574902803925
CCCTGCCC -0.168035430773
CCCTGCCG -1.36581240255
CCCTGCGA -0.871015999836
CCCTGCGC -0.339267035676
CCCTGCGG -0.0401497811069
CCCTGCTA -1.54864542949
CCCTGCTC -0.211449606606
CCCTGGAA -0.251216986441
CCCTGGAC -0.903556962746
CCCTGGAG -1.46971629097
CCCTGGCA -1.25746764084
CCCTGGCC -0.368915549868
CCCTGGCG -0.823449516003
CCCTGGGA 0.0671248843568
CCCTGGGC -1.10330346987
CCCTGGTA -1.16417479959
CCCTGGTC -1.96815739863
CCCTGTAA 0.105328940879
CCCTGTAC 0.0667903159166
CCCTGTAG -1.00640068743
CCCTGTCA -0.889240831468
CCCTGTCC -0.89153210252
CCCTGTCG -0.240297770357
CCCTGTGA -0.219310658041
CCCTGTGC -0.731317470417
CCCTGTTA 1.22266572714
CCCTGTTC -0.640975104604
CCCTTAAA 0.503967760075
CCCTTAAC -0.141301321353
CCCTTAAG -1.11125626624
CCCTTACA 0.577887781727
CCCTTACC -0.883404964622
CCCTTACG -0.393159476844
CCCTTAGA 1.15873544862
CCCTTAGC -1.09776035041
CCCTTAGG -1.8042125898
CCCTTATA -0.544341806589
CCCTTATC -0.457207638459
CCCTTCAA -0.529407507842
CCCTTCAC -1.65106859119
CCCTTCAG -0.396574427367
CCCTTCCA -0.156991535669
CCCTTCCC -1.54019182598
CCCTTCCG -0.572722620051
CCCTTCGA -0.91566363541
CCCTTCGC -1.48492138105
CCCTTCGG -1.12795044726
CCCTTCTA -0.801157064007
CCCTTCTC -0.522894139905
CCCTTGAA -0.452706124649
CCCTTGAC -0.896578596953
CCCTTGAG -0.848271619064

CCCTTGCA	-0.615740802769
CCCTTGCC	-1.1598363879
CCCTTGCG	-0.454436470801
CCCTTGGA	-0.501916437326
CCCTTGGC	-0.843528065899
CCCTTGTA	0.293169255019
CCCTTGTC	-0.89241269711
CCCTTTAA	-0.0230820857909
CCCTTTAC	-0.733575284625
CCCTTTAG	-1.30440184259
CCCTTTCA	-0.859356551079
CCCTTTCC	-1.41367973659
CCCTTTCG	-1.7675177506
CCCTTTGA	-1.03487298445
CCCTTTGC	-0.536180427702
CCCTTTTA	-0.969022596745
CCCTTTTC	-0.526893539672
CCGAAAAA	-0.00593414632547
CCGAAAAC	0.670898069469
CCGAAAAG	1.10712826673
CCGAAACA	0.207195621166
CCGAAACC	-0.757375647035
CCGAAACG	0.982624372126
CCGAAAGA	0.133682047892
CCGAAAGC	-1.21972361592
CCGAAAGG	-1.09902073245
CCGAAATA	2.66913996679
CCGAAATC	15.6951753391
CCGAACAA	-0.673575662516
CCGAACAC	-1.2897393807
CCGAACAG	-0.468169982506
CCGAACCA	-0.303475531265
CCGAACCC	-0.958449711273
CCGAACCG	0.0583142335909
CCGAACGA	-0.698217150896
CCGAACGC	-0.178240290961
CCGAACGG	-0.65564200998
CCGAACTA	-0.0607550149143
CCGAACTC	-0.00462593144819
CCGAAGAA	1.1587286527
CCGAAGAC	0.799797356956
CCGAAGAG	-0.864524326571
CCGAAGCA	0.191968313652
CCGAAGCC	-0.86871087556
CCGAAGCG	-1.06270228275
CCGAAGGA	-1.24919595892
CCGAAGGC	-1.10835257812
CCGAAGTA	-0.100832131938
CCGAAGTC	-0.754170063168
CCGAATAA	0.751175152866
CCGAATAC	1.5892027045
CCGAATAG	-0.388893729232
CCGAATCA	2.29521164071
CCGAATCC	6.92486202622

CCGAATCG 2.16554493687
CCGAATGA 0.363752217863
CCGAATGC 0.305904289035
CCGAATTA 0.727103999124
CCGAATTC 1.61626902993
CCGACAAA 0.189083967764
CCGACAAC -0.0162135002673
CCGACAAG -0.209067636141
CCGACACA -0.182775261614
CCGACACC -1.03891211422
CCGACACG -0.494303437023
CCGACAGA 1.2054195082
CCGACAGC -0.358881633243
CCGACAGG 0.267521970266
CCGACATA 0.532766261323
CCGACATC -0.45952008142
CCGACCAA -1.04899177262
CCGACCAC -1.39791372161
CCGACCAG -1.65409382176
CCGACCCA -0.464224950109
CCGACCCC -1.32937371731
CCGACCCG -1.38327347716
CCGACCGA -0.603584990365
CCGACCGC -1.05932993742
CCGACCGG -1.30677257365
CCGACCTA -0.52513182773
CCGACCTC -1.2344728565
CCGACGAA 0.441425679203
CCGACGAC -0.929213134473
CCGACGAG -0.923748168108
CCGACGCA -1.01703107685
CCGACGCC -1.60795108347
CCGACGCG -0.489576089517
CCGACGGA 0.143164449354
CCGACGGC -0.698074697928
CCGACGTA 2.10785854186
CCGACGTC -0.548165035163
CCGACTAA -0.287948157682
CCGACTAC -1.15233473615
CCGACTAG -0.689849541932
CCGACTCA 0.194824953091
CCGACTCC -0.831922200368
CCGACTCG -1.07327464546
CCGACTGA 0.281495430274
CCGACTGC -1.36740892132
CCGACTTA 0.179993377311
CCGACTTC -0.962878560999
CCGAGAAA 1.87134244041
CCGAGAAC 0.0997743206278
CCGAGAAG 0.184328129663
CCGAGACA -0.435123507593
CCGAGACC -0.836582634187
CCGAGACG -1.33525114383
CCGAGAGA -0.357617069092

CCGAGAGC	-0.516192838601
CCGAGAGG	-0.814323377653
CCGAGATA	4.40783726009
CCGAGATC	4.48918078039
CCGAGCAA	-0.298571489801
CCGAGCAC	-0.65436124017
CCGAGCAG	-0.495740251645
CCGAGCCA	-1.33929523985
CCGAGCCC	-1.36968032737
CCGAGCCG	-1.08841648119
CCGAGCGA	-0.074460297407
CCGAGCGC	-0.72848252565
CCGAGCGG	-0.728400451829
CCGAGCTA	-0.884507210803
CCGAGCTC	-0.75689104556
CCGAGGAA	0.991656674484
CCGAGGAC	-1.20429504458
CCGAGGAG	-0.510096897069
CCGAGGCA	-0.693421844175
CCGAGGCC	-0.969319264854
CCGAGGCG	-0.0908201713669
CCGAGGGA	-0.295585727854
CCGAGGGC	-0.528853640245
CCGAGGTA	0.1763510248
CCGAGGTC	-1.73445324036
CCGAGTAA	0.00648592287012
CCGAGTAC	-0.00120444638452
CCGAGTAG	-0.974156131248
CCGAGTCA	-0.42763832289
CCGAGTCC	-0.770117476973
CCGAGTCG	-1.24792329194
CCGAGTGA	-0.238055377664
CCGAGTGC	-1.13062594926
CCGAGTTA	0.356893303458
CCGAGTTC	-0.510241441091
CCGATAAA	0.324367239299
CCGATAAC	0.616648319663
CCGATAAG	0.339421250816
CCGATACA	-0.392246732318
CCGATACC	1.27077274811
CCGATACG	1.81518068424
CCGATAGA	0.580721680968
CCGATAGC	0.625801118935
CCGATAGG	-0.151928835578
CCGATATA	2.31045018764
CCGATATC	9.26809312507
CCGATCAA	0.0800392261464
CCGATCAC	1.38656976044
CCGATCAG	-0.306104507336
CCGATCCA	1.33760802166
CCGATCCC	1.22337407126
CCGATCCG	0.0857804728556
CCGATCGA	0.544444006792
CCGATCGC	3.64398666821

CCGATCGG 0.0448434103879
CCGATCTA 0.750947228116
CCGATCTC 2.98703644335
CCGATGAA 0.652357750253
CCGATGAC -0.293068361724
CCGATGAG -0.47002213248
CCGATGCA 0.99460296781
CCGATGCC 0.028963694416
CCGATGCG 0.51610893511
CCGATGGA -0.468867871361
CCGATGGC -0.726420747637
CCGATGTA 1.11301510299
CCGATGTC -0.564749958675
CCGATTAA 0.294352790876
CCGATTAC 10.2575954925
CCGATTAG 0.623158812403
CCGATTCA 0.704710131069
CCGATTCC 6.13564879414
CCGATTCG 3.62870159536
CCGATTGA 0.672025146902
CCGATTGC 7.34088115312
CCGATTTA 1.46209283541
CCGATTTC 6.56127029666
CCGCAAAA 0.0891123040326
CCGCAAAC -0.0424123001834
CCGCAAAG -0.408496303245
CCGCAACA 0.530952273062
CCGCAACC 0.408290595931
CCGCAACG -0.281804906081
CCGCAAGA -0.968482059608
CCGCAAGC 0.246373846888
CCGCAAGG -1.22010941515
CCGCAATA 2.49431384221
CCGCAATC 20.3124879906
CCGCACAA 0.152720037662
CCGCACAC 0.545292712828
CCGCACAG -0.292421442279
CCGCACCA -0.197359047644
CCGCACCC -0.96257692664
CCGCACCG -0.790005741068
CCGCACGA 0.917162920233
CCGCACGC -0.766494728082
CCGCACGG -1.32450888308
CCGCACTA -0.642124660853
CCGCACTC -1.01930718777
CCGCAGAA -0.446259931782
CCGCAGAC -0.49818129435
CCGCAGAG -0.601884180333
CCGCAGCA -1.07514091004
CCGCAGCC -1.55902619949
CCGCAGCG -0.852345512586
CCGCAGGA -0.558346632388
CCGCAGGC -0.512783377091
CCGCAGTA -0.851412380296

CCGCAGTC	-0.831954611686
CCGCATAA	-0.167941856163
CCGCATAC	1.08875549312
CCGCATAG	-0.637298772486
CCGCATCA	0.0136467330156
CCGCATCC	-0.410777380415
CCGCATCG	-1.00452814969
CCGCATGA	-0.694935504985
CCGCATGC	-0.674972224372
CCGCATTA	0.567244845988
CCGCATTC	0.209744353087
CCGCCAAA	0.0266157035583
CCGCCAAC	-0.652481122365
CCGCCAAG	-0.783952666118
CCGCCACA	-0.187552533005
CCGCCACC	-1.09220416187
CCGCCACG	0.0646281673722
CCGCCAGA	0.884237203617
CCGCCAGC	-0.763188773683
CCGCCAGG	-1.29776170458
CCGCCATA	-0.2501312073
CCGCCATC	-0.592229789782
CCGCCCAA	0.221602451856
CCGCCCAC	-0.979100686859
CCGCCCAG	-0.922127602226
CCGCCCCA	-0.893972099699
CCGCCCCC	-0.978419787807
CCGCCCCG	-0.997343553821
CCGCCCGA	-0.996402318702
CCGCCCGC	0.460768178531
CCGCCCGG	-1.03440014515
CCGCCCTA	-0.0830715140168
CCGCCCTC	0.0281387741057
CCGCCGAA	-0.331962465654
CCGCCGAC	-0.529301125533
CCGCCGAG	-0.997597355349
CCGCCGCA	-1.0974665575
CCGCCGCC	-0.196050832767
CCGCCGCG	-0.848617688295
CCGCCGGA	-0.604048158549
CCGCCGGC	-1.24875108744
CCGCCGTA	-0.185802583234
CCGCCGTC	-0.803361033607
CCGCCTAA	-0.503184922201
CCGCCTAC	0.200462169926
CCGCCTAG	-0.0278909843548
CCGCCTCA	-0.0185533882956
CCGCCTCC	-1.42415512673
CCGCCTCG	-1.28487402372
CCGCCTGA	-0.742487351449
CCGCCTGC	-1.13369195535
CCGCCTTA	-0.138022812021
CCGCCTTC	-0.900903416805
CCGCGAAA	0.859819980644

CCGCGAAC -0.883456718178
CCGCGAAG -0.438761416617
CCGCGACA -0.823908763464
CCGCGACC -1.02973944995
CCGCGACG -0.368804724072
CCGCGAGA -0.120259318902
CCGCGAGC -0.137786000297
CCGCGAGG -1.65368868029
CCGCGATA 2.57316979388
CCGCGATC 6.29315054668
CCGCGCAA 0.358838766661
CCGCGCAC -0.300234399501
CCGCGCAG -0.340941446166
CCGCGCCA -0.587469769577
CCGCGCCC -1.14278725067
CCGCGCCG -0.416033764267
CCGCGCGA -0.0143937616109
CCGCGCGC -0.168843884043
CCGCGCGG -0.222597008821
CCGCGCTA -0.135498388587
CCGCGCTC -1.32656700175
CCGCGGAA 0.497156155739
CCGCGGAC -0.486469307892
CCGCGGAG -0.383344336612
CCGCGGCA 0.242026809601
CCGCGGCC -0.315870769209
CCGCGGCG -1.33567092267
CCGCGGGA -0.117211348136
CCGCGGGC -0.743949520085
CCGCGGTA -0.506343196
CCGCGGTC -0.189969005841
CCGCGTAA 0.778801619048
CCGCGTAC -0.414900936439
CCGCGTAG -0.181778874978
CCGCGTCA -0.254523463603
CCGCGTCC -0.881715916762
CCGCGTCG 0.227028210982
CCGCGTGA 0.195473440826
CCGCGTGC -0.426872213438
CCGCGTTA -0.319405693885
CCGCGTTC -0.390110721933
CCGCTAAA 0.106938005971
CCGCTAAC 0.208325835178
CCGCTAAG -0.530553927513
CCGCTACA -0.401274068425
CCGCTACC -1.42987494015
CCGCTACG -0.64197645749
CCGCTAGA -1.06578397174
CCGCTAGC -0.0469106774138
CCGCTAGG -0.661119784043
CCGCTATA 0.232385749511
CCGCTATC 0.168716591207
CCGCTCAA 0.0671301119887
CCGCTCAC -0.883646742596

CCGCTCAG	0.579427580697
CCGCTCCA	-0.685975343948
CCGCTCCC	-1.59969247063
CCGCTCCG	0.433432368681
CCGCTCGA	0.199540277045
CCGCTCGC	-0.691191213653
CCGCTCTA	-0.444404645228
CCGCTCTC	-1.31565745679
CCGCTGAA	0.478045501885
CCGCTGAC	-1.15502121617
CCGCTGAG	-0.992001698187
CCGCTGCA	-1.00671539087
CCGCTGCC	-0.506857594976
CCGCTGCG	-1.00904011876
CCGCTGGA	-0.11398955861
CCGCTGGC	-1.32574626355
CCGCTGTA	-0.879666685067
CCGCTGTC	-0.204717200893
CCGCTTAA	0.385525304632
CCGCTTAC	0.0782937198626
CCGCTTAG	-0.230532815392
CCGCTTCA	-0.547617963487
CCGCTTCC	-0.971123581996
CCGCTTCG	-0.364047579064
CCGCTTGA	-0.700893175654
CCGCTTGC	-0.0346492668457
CCGCTTTA	-0.47376616243
CCGCTTTC	-0.166391079166
CCGGAAAA	0.680002251765
CCGGAAAC	-1.02098212103
CCGGAAAG	-0.511901736975
CCGGAACA	-0.365571695138
CCGGAACC	-0.891752185822
CCGGAACG	-0.586281528851
CCGGAAGA	-0.105609141948
CCGGAAGC	-1.19333087086
CCGGAAGG	-1.34002658555
CCGGAATA	1.52627717683
CCGGAATC	5.31545086517
CCGGACAA	-0.612164057038
CCGGACAC	-0.0410290687887
CCGGACAG	0.269030142063
CCGGACCA	-1.03332377574
CCGGACCC	-1.04531727018
CCGGACCG	-0.344646530259
CCGGACGA	-1.07764102498
CCGGACGC	-0.915910379635
CCGGACGG	0.314683312628
CCGGACTA	-1.13485771726
CCGGACTC	-0.657161682567
CCGGAGAA	0.816207416182
CCGGAGAC	0.227044939404
CCGGAGAG	-0.459430950296
CCGGAGCA	-1.02469373966

CCGGAGCC -0.683289909452
CCGGAGCG -0.348260392176
CCGGAGGA -0.846400649616
CCGGAGGC -0.533731804931
CCGGAGTA 0.0659209607355
CCGGAGTC -0.976380749993
CCGGATAA 1.64594812577
CCGGATAC 3.25289864049
CCGGATAG -0.00612992113928
CCGGATCA -0.19776340497
CCGGATCC 3.72718965717
CCGGATCG 0.52709820146
CCGGATGA 0.00522501806132
CCGGATGC 0.787739039886
CCGGATTA 2.34900449549
CCGGATTC 10.3892873809
CCGGCAAA 0.778122288285
CCGGCAAC 0.362550385294
CCGGCAAG -0.942893847096
CCGGCACA -0.274118718932
CCGGCACC -1.1913394045
CCGGCACG -0.711244932298
CCGGCAGA 0.086437324801
CCGGCAGC -0.865475755573
CCGGCAGG -1.0615982069
CCGGCATA -0.402473809941
CCGGCATC -0.45126643483
CCGGCCAA -0.994889703418
CCGGCCAC -1.39768945621
CCGGCCAG -0.40851355443
CCGGCCCA -1.05732592474
CCGGCCCC -1.2373357691
CCGGCCCG -1.68695549988
CCGGCCGA 1.44805873487
CCGGCCGC 0.0653359887284
CCGGCCGG -1.4840332064
CCGGCCTA -1.21878656291
CCGGCCTC -1.23122074671
CCGGCGAA 0.140482674201
CCGGCGAC -1.04328319861
CCGGCGAG -0.370517296275
CCGGCGCA -0.221908791084
CCGGCGCC -0.292429022345
CCGGCGCG -0.896514297081
CCGGCGGA -0.711897340756
CCGGCGGC -1.39705769689
CCGGCGTA 0.0736662201248
CCGGCGTC -0.82539550197
CCGGCTAA -0.690414126175
CCGGCTAC -1.04081941571
CCGGCTAG 0.144409148504
CCGGCTCA -0.782601061896
CCGGCTCC -0.9488737352
CCGGCTCG -1.05106766524

CCGGCTGA	−0.827566014725
CCGGCTGC	−1.76889261778
CCGGCTTA	−1.03880677744
CCGGCTTC	−0.576328117757
CCGGGAAA	0.434363409918
CCGGGAAC	−1.0257539034
CCGGGAAG	−0.935488906541
CCGGGACA	−0.833988683249
CCGGGACC	−0.338672653932
CCGGGACG	−1.52492635676
CCGGGAGA	−0.206916204242
CCGGGAGC	−1.10040579352
CCGGGAGG	−0.664284069618
CCGGGATA	0.86018330106
CCGGGATC	1.41873302695
CCGGGCAA	−0.129326385012
CCGGGCAC	−1.46814695588
CCGGGCAG	−1.5595756236
CCGGGCCA	−0.592226914585
CCGGGCCC	−1.30480175643
CCGGGCCG	−1.64066246718
CCGGGCGA	−0.549790044532
CCGGGCGC	−0.445993322556
CCGGGCTA	−1.72693355328
CCGGGCTC	−1.09082537396
CCGGGGAA	1.11204825247
CCGGGGAC	−2.02903604704
CCGGGGAG	−1.0441964659
CCGGGGCA	−0.714279049839
CCGGGGCC	−1.42900898293
CCGGGGCG	−0.850493885375
CCGGGGGA	−0.675892548964
CCGGGGGC	−0.903532915639
CCGGGGTA	0.678995671247
CCGGGGTC	−0.756298232105
CCGGGTAA	−0.326858728652
CCGGGTAC	−0.10100464379
CCGGGTAG	−0.771085634397
CCGGGTCA	−1.16081107986
CCGGGTCC	−0.874351751737
CCGGGTCG	−0.753224907325
CCGGGTGA	0.242539117525
CCGGGTGC	−0.713965391927
CCGGGTTA	−0.554077486816
CCGGGTTC	−0.881684550971
CCGGTAAA	0.547473680847
CCGGTAAC	−0.907573613698
CCGGTAAG	−0.286178081528
CCGGTACA	−0.286395289633
CCGGTACC	−0.735382215583
CCGGTACG	−0.814192164093
CCGGTAGA	−0.293922556773
CCGGTAGC	−0.844714215572
CCGGTAGG	−1.05752588166

CCGGTATA -0.214253969726
CCGGTATC 2.36583171974
CCGGTCAA -0.213145450387
CCGGTCAC -0.939127861091
CCGGTCAG -0.805463587148
CCGGTCCA -0.970521358804
CCGGTCCC -0.814323377653
CCGGTCCG 0.939252801493
CCGGTCGA -1.0303678113
CCGGTCGC -1.21215269805
CCGGTCTA -0.948952672441
CCGGTCTC -1.13206877565
CCGGTGAA -0.397193901745
CCGGTGAC -0.717301143827
CCGGTGAG -0.702906859453
CCGGTGCA -0.673775358054
CCGGTGCC -0.916437847691
CCGGTGCG -0.478488282305
CCGGTGGA -0.586281528851
CCGGTGGC -0.480609132557
CCGGTGTA -0.418070449647
CCGGTGTC -1.15013625557
CCGGTTAA -0.151085357174
CCGGTTAC -0.385028679603
CCGGTTAG -0.276644187892
CCGGTTCA -0.190786607466
CCGGTTCC -0.879895393961
CCGGTTCG -0.62759602634
CCGGTTGA -0.179853538158
CCGGTTGC -0.300502054253
CCGGTTTA 0.452528123784
CCGGTTTC -0.345648928672
CCGTAAAA 0.410667077382
CCGTAAAC 4.07755286431E-5
CCGTAAAG -0.406925661247
CCGTAACA 0.393606177987
CCGTAACC 0.155535640191
CCGTAACG -0.140777251257
CCGTAAGA 0.22219840189
CCGTAAGC 0.105916526702
CCGTAAGG -0.976351736636
CCGTAATA 2.0828887582
CCGTAATC 17.8755970707
CCGTACAA 0.147740195536
CCGTACAC -0.547796487115
CCGTACAG 0.122406307314
CCGTACCA -0.469463821395
CCGTACCC -0.533860143294
CCGTACCG 0.143171768039
CCGTACGA 0.180673230836
CCGTACGC -0.687014858547
CCGTACGG 0.250345540207
CCGTACTA -0.40253758705
CCGTACTC 0.381084431352

CCGTAGAA 1.32611690265
CCGTAGAC -1.44083362485
CCGTAGAG -0.601527917221
CCGTAGCA 0.168945822865
CCGTAGCC -1.07427416867
CCGTAGCG -1.18256142643
CCGTAGGA -1.32719222653
CCGTAGGC 0.752298048193
CCGTAGTA -0.142774990779
CCGTAGTC -0.901425134466
CCGTATAA -0.0288648921735
CCGTATAC 0.491889316622
CCGTATAG -0.185802583234
CCGTATCA 2.56050454875
CCGTATCC 5.52004573947
CCGTATCG 0.799693327082
CCGTATGA 0.761685568137
CCGTATGC -0.262532718402
CCGTATTA 0.886891272321
CCGTATTC 1.11348402157
CCGTCAAA 0.209719521836
CCGTCAAC -0.0119341608126
CCGTCAAG -0.690151437673
CCGTCACA -0.431643995816
CCGTCACC -0.805833703485
CCGTCACG 0.137935510569
CCGTCAGA -0.270269090818
CCGTCAGC -1.5158940544
CCGTCAGG -0.128069139545
CCGTCATA -0.0161774296074
CCGTCATC -0.351227334648
CCGTCCAA -1.24437660509
CCGTCCAC -0.267937044237
CCGTCCAG -0.485007139256
CCGTCCCA -0.154815795282
CCGTCCCC -1.30892557384
CCGTCCCG -0.403751443172
CCGTCCGA -0.579693928541
CCGTCCGC -0.0335849209955
CCGTCCTA -0.672213864412
CCGTCCTC -1.83260542681
CCGTCGAA 0.404439138145
CCGTCGAC -1.03187363066
CCGTCGAG -0.563810030463
CCGTCGCA -0.0900956215887
CCGTCGCC -1.37434442053
CCGTCGCG -0.795417124206
CCGTCGGA -0.689529872243
CCGTCGGC 0.246969535541
CCGTCGTA 0.458199320226
CCGTCGTC -1.11873517779
CCGTCTAA -0.790240200358
CCGTCTAC -1.09661628317
CCGTCTAG -1.48850387718

CCGTCTCA	-0.573943272094
CCGTCTCC	-0.577880201661
CCGTCTCG	-1.18190274481
CCGTCTGA	-0.0590889686351
CCGTCTGC	-0.999103436093
CCGTCTTA	-0.699468907349
CCGTCTTC	-0.695597845944
CCGTGAAA	0.216343454188
CCGTGAAC	-0.0602113411991
CCGTGAAG	-0.752651958871
CCGTGACA	-0.792162139218
CCGTGACC	-0.153890243058
CCGTGACG	-0.289006491755
CCGTGAGA	-0.392300576926
CCGTGAGC	-0.721287213134
CCGTGAGG	-0.954813631921
CCGTGATA	0.922589724884
CCGTGATC	2.76100932387
CCGTGCAA	0.445292819884
CCGTGCAC	-0.608778119871
CCGTGCAG	-0.584563729016
CCGTGCCA	-0.680371583957
CCGTGCCC	-0.675299212746
CCGTGCCG	-1.14353715447
CCGTGCGA	-0.607722660995
CCGTGCGC	0.140246908003
CCGTGCTA	-0.749481922901
CCGTGCTC	-0.132124997737
CCGTGGAA	0.0996935537153
CCGTGGAC	-1.23404209963
CCGTGGAG	0.0298722568362
CCGTGGCA	-1.21644092448
CCGTGGCC	-0.311950568065
CCGTGGCG	-0.165601968134
CCGTGGGA	-1.45011476249
CCGTGGGC	0.34433992965
CCGTGGTA	0.840543088097
CCGTGGTC	-0.0570130760166
CCGTGTAA	0.170947744492
CCGTGTAC	0.809056015774
CCGTGTAG	-1.06087705508
CCGTGTCA	-0.810730687646
CCGTGTCC	-1.23514669825
CCGTGTCG	-1.20817473158
CCGTGTGA	-0.603680133265
CCGTGTGC	-0.852647146946
CCGTGTTA	1.74114487054
CCGTGTTC	-0.975770423972
CCGTTAAA	0.904802184659
CCGTTAAC	-0.366701647768
CCGTTAAG	2.03244106506
CCGTTACA	0.583187554924
CCGTTACC	-0.740327293957
CCGTTACG	-0.708595829844

CCGTTAGA 0.195260676208
CCGTTAGC -0.54361046089
CCGTTAGG -1.25911512902
CCGTTATA 0.845419684494
CCGTTATC 1.31236143489
CCGTTCAA -0.278664667612
CCGTTCAC -0.478036614911
CCGTTCAG -0.624092728837
CCGTTCCA -0.173764653929
CCGTTCCC -0.821066500012
CCGTTCCG -1.29439746209
CCGTTCGA -0.693099299288
CCGTTCGC -1.08962249586
CCGTTCTA -1.00837411846
CCGTTCTC 0.0624299481678
CCGTTGAA -0.0897228914358
CCGTTGAC -0.915787007522
CCGTTGAG 0.324225047712
CCGTTGCA 1.09502420788
CCGTTGCC -0.87033614631
CCGTTGCG -0.299289243658
CCGTTGGA -0.232267866412
CCGTTGGC -1.13369195535
CCGTTGTA -0.376040812117
CCGTTGTC -0.521418118044
CCGTTTAA -0.181894928406
CCGTTTAC -0.497624028792
CCGTTTAG 0.417643613504
CCGTTTCA -0.451701373803
CCGTTTCC -0.427746796251
CCGTTTCG 0.56653336529
CCGTTTGA 0.724167115535
CCGTTTGC -0.0397511741763
CCGTTTTA -0.100524485802
CCGTTTTC 0.35501423118
CCTAAAAA 0.708595045699
CCTAAAAC 0.642605602986
CCTAAAAG -0.199284645846
CCTAAACA 0.165264001733
CCTAAACC -0.270582225967
CCTAAACG 0.454376353034
CCTAAAGA 1.23375797784
CCTAAAGC -1.11554004918
CCTAAAGG -0.596702290235
CCTAAATA 1.63294230042
CCTAAATC 0.732484800615
CCTAACAA 0.618805240575
CCTAACAC -0.609971065466
CCTAACAG -0.82205661349
CCTAACCA -0.654164158448
CCTAACCC -1.06162878854
CCTAACCG -0.816236690921
CCTAACGA -0.0362729693068
CCTAACGC 0.352289589445

CCTAACTA -0.411540614669
CCTAACTC 0.573208267052
CCTAAGAA 0.532031779045
CCTAAGAC -0.538507246651
CCTAAGAG 0.00841282798008
CCTAAGCA -0.979309007989
CCTAAGCC -0.503774860458
CCTAAGCG -0.689338018153
CCTAAGGA 0.455104562155
CCTAAGGC -0.41443698411
CCTAAGTA 0.132531446116
CCTAAGTC -0.420050153839
CCTAATAA 1.37636150229
CCTAATAC 0.795562190991
CCTAATAG -0.82848686208
CCTAATCA -0.322614675713
CCTAATCC 1.21119996214
CCTAATCG 0.652601357899
CCTAATGA 0.509517414076
CCTAATGC -1.4406582378
CCTAATTA 1.94368084203
CCTAATTC 1.58271939545
CCTACAAA -0.157620942547
CCTACAAC 0.25333731393
CCTACAAG -0.249186312838
CCTACACA -0.644404431115
CCTACACC -0.885353825786
CCTACACG -1.2018621047
CCTACAGA -0.25223689742
CCTACAGC -0.754170063168
CCTACAGG -0.879321661363
CCTACATA -0.313698165401
CCTACATC -0.667274797815
CCTACCAA -0.647483244909
CCTACCAC -0.87875707712
CCTACCAG -0.282735947319
CCTACCCA 0.0484206788815
CCTACCCC -0.385629857269
CCTACCCG -0.664921317944
CCTACCGA -0.494298993536
CCTACCGC -1.22142886944
CCTACCTA -0.236677635282
CCTACCTC -0.976987678054
CCTACGAA -0.634543026342
CCTACGAC -0.365508702174
CCTACGAG 1.42182700087
CCTACGCA 0.813577655966
CCTACGCC -0.96419383318
CCTACGCG -0.559115094274
CCTACGGA -0.369208297253
CCTACGGC -0.932142699377
CCTACGTA -0.403986686606
CCTACGTC -0.227963434325
CCTACTAA -0.701118486588

CCTACTAC -0.456623189215
CCTACTAG -0.507093361174
CCTACTCA 0.979838567099
CCTACTCC -0.670169598967
CCTACTCG -0.189883795441
CCTACTGA 0.249587533585
CCTACTGC -0.602345518846
CCTACTTA 1.12775467245
CCTACTTC -0.663021596519
CCTAGAAA 0.3503830721
CCTAGAAC -0.258390342903
CCTAGAAG -1.33915330964
CCTAGACA -0.967697914827
CCTAGACC -0.854219618614
CCTAGACG -0.24308279124
CCTAGAGA -0.0835835605592
CCTAGAGC -0.72633266204
CCTAGAGG 0.81959021677
CCTAGATA 1.32765068984
CCTAGATC 2.53932375267
CCTAGCAA -0.0562610811711
CCTAGCAC -0.35654435903
CCTAGCAG -1.10285154109
CCTAGCCA -1.05332103596
CCTAGCCC -0.0824402774676
CCTAGCCG -0.534247249434
CCTAGCGA -0.169918946539
CCTAGCGC -0.245879051531
CCTAGCTA 0.436734140975
CCTAGCTC -1.25058964558
CCTAGGAA -0.208145481878
CCTAGGAC 0.0442396189061
CCTAGGAG -0.613360139212
CCTAGGCA -0.698034183781
CCTAGGCC -0.13194020095
CCTAGGCG -0.917233493263
CCTAGGGA -0.0690240830178
CCTAGGGC -1.25411829709
CCTAGGTA -0.26952336913
CCTAGGTC -0.0807297963174
CCTAGTAA -0.105684419847
CCTAGTAC -0.839531018566
CCTAGTAG -0.0577692529676
CCTAGTCA 0.987880233215
CCTAGTCC -0.872432688075
CCTAGTCG -0.0216180874836
CCTAGTGA -0.635871890365
CCTAGTGC -0.229863678512
CCTAGTTA -0.532371575117
CCTAGTTC 0.0828503851884
CCTATAAA 1.03062631769
CCTATAAC 1.26171744417
CCTATAAG 0.222568256846
CCTATACA 0.18009662304

CCTATACC -0.342812938378
CCTATACG -0.638178321549
CCTATAGA 0.377307205939
CCTATAGC -0.829244868702
CCTATAGG -0.307604314922
CCTATATA 0.670126993767
CCTATATC 0.435455200836
CCTATCAA -0.0202440044447
CCTATCAC 0.421201801141
CCTATCAG 0.846139790785
CCTATCCA -0.795229974985
CCTATCCC -0.471415034993
CCTATCCG -0.30400116965
CCTATCGA 0.676445632417
CCTATCGC -0.108988283193
CCTATCTA 1.59527198511
CCTATCTC 1.26575657394
CCTATGAA 0.309839650313
CCTATGAC -0.010604251263
CCTATGAG -1.0424086158
CCTATGCA -0.364051238406
CCTATGCC -0.600618832037
CCTATGCG -0.355908940376
CCTATGGA -0.632105904361
CCTATGGC -0.616001138836
CCTATGTA 0.431409275145
CCTATGTC 0.201004798115
CCTATTAA -0.484890301683
CCTATTAC 0.252421955589
CCTATTAG -0.981733845036
CCTATTCA 0.562113141156
CCTATTCC 0.365648279945
CCTATTCG 0.650817689902
CCTATTGA -0.221535015405
CCTATTGC -0.225097646529
CCTATTTA -0.236259163351
CCTATTTC 1.6307513999
CCTCAAAA 1.41213288032
CCTCAAAC -0.215091959116
CCTCAAAG 0.0808617940223
CCTCAACA 0.318698918055
CCTCAACC -0.222579496254
CCTCAACG -0.658646068638
CCTCAAGA -0.0552748884174
CCTCAAGC -1.26504352495
CCTCAAGG -0.421871722166
CCTCAATA -0.443480399912
CCTCAATC 0.377566757862
CCTCACAA 0.676467327089
CCTCACAC -0.237510135659
CCTCACAG 0.293022881327
CCTCACCA -0.454958188462
CCTCACCC -0.288852799378
CCTCACCG -0.175511467121

CCTCACGA 0.0776188325872
CCTCACGC −1.00994345355
CCTCACTA −0.417865526477
CCTCACTC −0.635446883893
CCTCAGAA −0.00986741654992
CCTCAGAC −1.46326800705
CCTCAGAG −1.35238836665
CCTCAGCA −0.279951710581
CCTCAGCC −1.12504754328
CCTCAGCG −0.27140766904
CCTCAGGA −0.286555255168
CCTCAGGC −0.781297813269
CCTCAGTA 0.0396437463412
CCTCAGTC −0.536180427702
CCTCATAA −0.322183134701
CCTCATAC −0.0672780539708
CCTCATAG 0.299091377791
CCTCATCA −0.0833227017285
CCTCATCC −0.866872317429
CCTCATCG 0.0448818334822
CCTCATGA −0.872432688075
CCTCATGC −0.513688802932
CCTCATTA 1.02606991375
CCTCATTC −1.26358083355
CCTCCAAA −0.63029374577
CCTCCAAC −0.553985219113
CCTCCAAG −0.692041226597
CCTCCACA −1.1306594061
CCTCCACC −1.47815630264
CCTCCACG −0.967071644528
CCTCCAGA −0.531279784199
CCTCCAGC −0.900905507857
CCTCCAGG −1.19621809195
CCTCCATA −0.965039925399
CCTCCATC −0.751685631119
CCTCCCAA −0.22060763351
CCTCCCAC −0.772359085522
CCTCCCAG −1.2192651526
CCTCCCCA −1.19456250093
CCTCCCCC −1.30927216583
CCTCCCCG −0.318205429606
CCTCCCGA −0.141319879446
CCTCCCGC −0.648681679517
CCTCCCTA −0.645455446504
CCTCCCTC −0.332696163788
CCTCCGAA −0.636710402518
CCTCCGAC −1.17610268725
CCTCCGAG −1.09872537125
CCTCCGCA −0.517221375173
CCTCCGCC −1.19611380069
CCTCCGCG −0.198015899589
CCTCCGGA 1.29992751247
CCTCCGGC −1.28972369781
CCTCCGTA −0.683505287886

CCTCCGTC -1.287278473
CCTCCTAA -1.56827178923
CCTCCTAC -1.08775962924
CCTCCTAG -0.197050355982
CCTCCTCA -1.46900115093
CCTCCTCC -1.79727682921
CCTCCTCG -0.528461306472
CCTCCTGA -0.724961192817
CCTCCTGC -2.03535337878
CCTCCTTA -0.0653989816925
CCTCCTTC -0.896775417293
CCTCGAAA -0.827547979395
CCTCGAAC 0.513793616951
CCTCGAAG 0.257738979971
CCTCGACA -1.05722189487
CCTCGACC -0.961026933788
CCTCGACG -0.89132717935
CCTCGAGA 0.37004419559
CCTCGAGC -0.631573731435
CCTCGAGG -0.825905196078
CCTCGATA 0.291220393855
CCTCGATC -0.371814794507
CCTCGCAA 0.667421171507
CCTCGCAC -0.361785582751
CCTCGCAG -0.646725499668
CCTCGCCA -1.23665669971
CCTCGCCC -1.14936387296
CCTCGCCG -1.3843762461
CCTCGCGA -0.592796726459
CCTCGCGC -0.990930033653
CCTCGCTA -0.432928163587
CCTCGCTC -0.230668733821
CCTCGGAA -0.251178563347
CCTCGGAC -0.153523001919
CCTCGGAG -0.923218608999
CCTCGGCA -0.413865342565
CCTCGGCC -1.16768619992
CCTCGGCG -1.12934622497
CCTCGGGA -0.0376995900461
CCTCGGGC -0.193379251496
CCTCGGTA 0.224554234196
CCTCGGTC -1.28487402372
CCTCGTAA 2.03574597393
CCTCGTAC -0.367128222529
CCTCGTAG -0.593876493824
CCTCGTCA -0.776918103282
CCTCGTCC -1.51052579922
CCTCGTCG -1.61067781626
CCTCGTGA -0.249473048447
CCTCGTGC -1.342928444
CCTCGTTA -0.892295075392
CCTCGTTC -0.91037327195
CCTCTAAA 0.435433506164
CCTCTAAC -0.781787642376

CCTCTAAG 0.13111528064
CCTCTACA -0.376266645814
CCTCTACC -0.241344080878
CCTCTACG -0.692599799063
CCTCTAGA -0.317496562723
CCTCTAGC -0.604433173637
CCTCTATA 0.522219252629
CCTCTATC -0.814359971077
CCTCTCAA -0.663435624964
CCTCTCAC -0.157691515578
CCTCTCAG -1.57432277313
CCTCTCCA -0.734812142327
CCTCTCCC -1.49024023511
CCTCTCCG -1.11604033356
CCTCTCGA -0.0497105970473
CCTCTCGC -0.32950025104
CCTCTCTA -0.910602503608
CCTCTCTC -0.868568161209
CCTCTGAA -0.894058094243
CCTCTGAC -0.700296180094
CCTCTGAG 1.00477646221
CCTCTGCA -0.182465785807
CCTCTGCC -0.38644014021
CCTCTGCG -0.699292474773
CCTCTGGA -0.702570461342
CCTCTGGC -1.3048490665
CCTCTGTA -0.546897595814
CCTCTGTC -0.775494619122
CCTCTTAA -0.534208564958
CCTCTTAC -0.404866497051
CCTCTTAG -0.838589522065
CCTCTTCA -0.253573080128
CCTCTTCC -0.451789198018
CCTCTTCG -0.471351519266
CCTCTTGA -0.214400866182
CCTCTTGC -0.473358145762
CCTCTTTA 1.37342801666
CCTCTTTC -0.554220985311
CCTGAAAA 0.457416220971
CCTGAAAC -0.231331336161
CCTGAAAG -0.559547419431
CCTGAACA -0.537996507017
CCTGAACC -0.947728622437
CCTGAACG 0.0897077313034
CCTGAAGA -0.068531640095
CCTGAAGC -1.14841087567
CCTGAAGG -1.36940378564
CCTGAATA 1.51147252336
CCTGAATC 1.11895264727
CCTGACAA -0.832771690548
CCTGACAC -1.05748327646
CCTGACAG 0.236512703497
CCTGACCA -1.43520241979
CCTGACCC -0.449642732369

CCTGACCG -0.851886003744
CCTGACGA 0.0998801801733
CCTGACGC -0.847866477594
CCTGACTA -0.205181153222
CCTGACTC -0.0616418826623
CCTGAGAA 0.166201054747
CCTGAGAC -0.550654172081
CCTGAGAG -0.835864096185
CCTGAGCA -0.275034077274
CCTGAGCC -0.998948698189
CCTGAGCG -0.922467398298
CCTGAGGA -0.874924700191
CCTGAGGC -0.746548698654
CCTGAGTA -0.0476381023895
CCTGAGTC -0.854742643184
CCTGATAA 0.997447322314
CCTGATAC 1.73979509599
CCTGATAG 0.170990611073
CCTGATCA 1.23025572586
CCTGATCC 0.218781098932
CCTGATCG 0.706625274008
CCTGATGA -0.458038831927
CCTGATGC -0.651800484695
CCTGATTA 0.468947331366
CCTGATTC 5.13185538811
CCTGCAAA 0.150702171758
CCTGCAAC -0.771332378621
CCTGCAAG -0.663295001667
CCTGCACA -0.00202152524694
CCTGCACC -1.37862951038
CCTGCACG -0.0807297963174
CCTGCAGA -0.106478497129
CCTGCAGC -1.11414322595
CCTGCAGG -0.158383131275
CCTGCATA 1.5888022679
CCTGCATC 0.519196113115
CCTGCCAA -0.0966087281445
CCTGCCAC -0.879004344108
CCTGCCAG -0.856607600856
CCTGCCCA -1.03283786736
CCTGCCCC -0.69802268299
CCTGCCCG -1.89387170413
CCTGCCGA -0.640451818653
CCTGCCGC -0.997232466644
CCTGCCTA -0.570424291696
CCTGCCTC -0.458836568552
CCTGCGAA -0.046117645658
CCTGCGAC -1.53132445541
CCTGCGAG -0.692248763582
CCTGCGCA 0.375259281151
CCTGCGCC -1.16008992804
CCTGCGCG -0.376012844286
CCTGCGGA 0.78365625939
CCTGCGGC -0.724626885759

CCTGCGTA -0.568027945243
CCTGCGTC -1.22547061302
CCTGCTAA -0.52560911052
CCTGCTAC -0.397929952313
CCTGCTAG -1.06237189641
CCTGCTCA -1.08203249714
CCTGCTCC -1.32418424714
CCTGCTCG -1.10562898191
CCTGCTGA 0.0826849306395
CCTGCTGC -1.01370917818
CCTGCTTA -0.386744388385
CCTGCTTC -1.22516270551
CCTGGAAA 1.14645678687
CCTGGAAC -0.491718895823
CCTGGAAG -0.0398842174076
CCTGGACA -1.0100336302
CCTGGACC -0.321655143882
CCTGGACG -0.585091719836
CCTGGAGA 0.0471035770301
CCTGGAGC -1.29037715179
CCTGGATA 2.16874581586
CCTGGATC 2.24041194403
CCTGGCAA -1.28816272693
CCTGGCAC -1.32016393685
CCTGGCAG -1.03514377578
CCTGGCCA -0.54845647564
CCTGGCCC 0.306330079652
CCTGGCCG -0.175257665593
CCTGGCGA -0.166736887015
CCTGGCGC -0.782817224474
CCTGGCTA -0.829614462276
CCTGGCTC -0.461413791067
CCTGGGAA -0.545724515221
CCTGGGAC -1.48089871832
CCTGGGAG -0.411991497918
CCTGGGCA -1.68014572522
CCTGGGCC -0.973882203338
CCTGGGCG -1.08573470604
CCTGGGGA -0.308634158402
CCTGGGGC -1.36860709455
CCTGGGTA -0.810096837281
CCTGGGTC -1.19358807035
CCTGGTAA -0.549113588967
CCTGGTAC -0.712132061427
CCTGGTAG -0.897592234774
CCTGGTCA -0.231053487527
CCTGGTCC -1.35832983166
CCTGGTCG -1.15362596123
CCTGGTGA -0.0473236603321
CCTGGTGC -0.280008430387
CCTGGTTA -0.657787430102
CCTGGTTC -1.42356388156
CCTGTAAA 0.498167179744
CCTGTAAC 0.367901389284

CCTGTAAG -1.0815246327
CCTGTACA 0.0667903159166
CCTGTACC -0.788584870724
CCTGTACG -0.151105222175
CCTGTAGA -0.41619817329
CCTGTAGC -1.44189535689
CCTGTATA 1.23035923297
CCTGTATC 1.02997312509
CCTGTCAA 0.024243665594
CCTGTCAC -0.286342229169
CCTGTCAG -0.345254242465
CCTGTCCA -1.48086290904
CCTGTCCC -0.474464051286
CCTGTCCG -0.571430349451
CCTGTCGA -0.366174963856
CCTGTCGC -0.543578572335
CCTGTCTA 0.411013669376
CCTGTCTC -1.39113348307
CCTGTGAA -0.252546111846
CCTGTGAC -0.426188439189
CCTGTGAG -1.07296203307
CCTGTGCA 0.311626977651
CCTGTGCC -1.55165576131
CCTGTGCG -0.388879091862
CCTGTGGA -0.580283866798
CCTGTGGC -1.68837088122
CCTGTGTA 0.417492273561
CCTGTGTC -0.161277148282
CCTGTTAA 1.04410472096
CCTGTTAC -0.367397445571
CCTGTTAG 0.701013411187
CCTGTTCA -0.221015650178
CCTGTTCC -0.455373262433
CCTGTTCG -0.0297844326207
CCTGTTGA 0.343732478826
CCTGTTGC -0.901987627656
CCTGTTTA -0.114970785113
CCTGTTTC -0.255164632653
CCTTAAAA -0.149733230189
CCTTAAAC 0.325991464524
CCTTAAAG 0.687944331494
CCTTAACA -0.696857705227
CCTTAACC 0.0595359311606
CCTTAACG 0.167969562611
CCTTAAGA -0.466701540711
CCTTAAGC -1.28877958749
CCTTAAGG 0.636900426937
CCTTAATA 0.577764409615
CCTTAATC 0.109986238118
CCTTACAA 0.201007150549
CCTTACAC 0.188598843526
CCTTACAG 0.558217771263
CCTTACCA -0.445210484682
CCTTACCC -0.0151993396831

CCTTACCG 0.0695180942302
CCTTACGA 0.672457472058
CCTTACGC -0.791988059076
CCTTACTA -0.170832736591
CCTTACTC -0.936523193508
CCTTAGAA 1.13001797567
CCTTAGAC -0.695254651911
CCTTAGAG -0.436676375643
CCTTAGCA -0.276037782594
CCTTAGCC -1.19307367137
CCTTAGCG -0.103638586111
CCTTAGGA 0.177581609344
CCTTAGGC -0.487022914108
CCTTAGTA 0.256272106466
CCTTAGTC 0.0477486668037
CCTTATAA 0.291748384675
CCTTATAC -0.31894906024
CCTTATAG 0.232964709741
CCTTATCA 0.621537462376
CCTTATCC -0.545042570642
CCTTATCG -0.270511652937
CCTTATGA -0.848975781078
CCTTATGC 0.219150431124
CCTTATTA 1.26107575236
CCTTATTC 1.73850491644
CCTTCAAA 0.339057407638
CCTTCAAC -0.539238069587
CCTTCAAG -0.710580761668
CCTTCACA -0.941940327041
CCTTCACC -1.56504058997
CCTTCACG 0.0217427665039
CCTTCAGA -0.493222362751
CCTTCAGC -0.819164164772
CCTTCATA -0.282786655348
CCTTCATC 0.183871496019
CCTTCCAA -0.482459714242
CCTTCCAC -0.209492903994
CCTTCCAG 0.190769356281
CCTTCCCA -1.73515322027
CCTTCCCC -0.427443070839
CCTTCCCG -0.564803541902
CCTTCCGA -0.715155200942
CCTTCCGC -0.398311308059
CCTTCCTA -0.162328425053
CCTTCCTC -1.23281099232
CCTTCGAA -0.691660393614
CCTTCGAC -1.39904550392
CCTTCGAG -0.250158913749
CCTTCGCA 0.551633830295
CCTTCGCC -0.700296180094
CCTTCGCG -1.18550719699
CCTTCGGA -0.665462900606
CCTTCGGC -1.56328462842
CCTTCGTA -1.02147926882

CCTTCGTC −1.20858065719
CCTTCTAA −0.467530904509
CCTTCTAC −0.0868103163354
CCTTCTAG −0.253909216858
CCTTCTCA −0.206972924048
CCTTCTCC −1.16897115184
CCTTCTCG −0.587032216788
CCTTCTGA −0.38785369187
CCTTCTGC −0.552782079637
CCTTCTTA −0.263778985842
CCTTCTTC −1.04613016693
CCTTGAAA 1.39298171232
CCTTGAAC −1.22709248581
CCTTGAAG −0.603272900742
CCTTGACA −0.203905872425
CCTTGACC −0.270952080922
CCTTGACG −0.684277409114
CCTTGAGA 0.0218865263805
CCTTGAGC −1.446889575
CCTTGATA 0.409236013156
CCTTGATC 1.48236820564
CCTTGCAA −0.115611170018
CCTTGCAC −0.46946957179
CCTTGCAG −1.323138198
CCTTGCCA −0.977542591178
CCTTGCCC −0.724961192817
CCTTGCCG −0.850254982599
CCTTGCGA 0.302495873051
CCTTGCGC 0.166743944318
CCTTGCTA −0.0901123500107
CCTTGCTC −1.10779871052
CCTTGGAA 0.188595968328
CCTTGGAC −0.716074741389
CCTTGGAG −0.670031850867
CCTTGGCA −0.895145180292
CCTTGGCC −1.57527211108
CCTTGGCG −0.483107417832
CCTTGGGA −0.435644963873
CCTTGGGC −1.53042608687
CCTTGGTA −0.159233666981
CCTTGGTC −1.27065591054
CCTTGTAA 0.612768894047
CCTTGTAC −0.20301456119
CCTTGTAG −0.411292563536
CCTTGTCA −0.758522328088
CCTTGTCC −0.768345309767
CCTTGTCG −0.933914605202
CCTTGTGA −0.34576184552
CCTTGTGC −1.17621560409
CCTTGTTA 0.0958256288893
CCTTGTTC 0.0406265411341
CCTTTAAA 0.264377026929
CCTTTAAC 0.42625430735
CCTTTAAG −0.682647172113

CCTTTACA 1.4840956766
CCTTTACC -0.108873275292
CCTTTACG -0.367151485491
CCTTTAGA -0.701172331197
CCTTTAGC -0.42569991699
CCTTTATA 0.14215682331
CCTTTATC 0.143856326433
CCTTTCAA -0.101037839253
CCTTTCAC -0.908454208288
CCTTTCAG -0.479190614648
CCTTTCCA -0.909923695609
CCTTTCCC -0.33732601596
CCTTTCCG -1.22987044939
CCTTTCGA -0.888592343734
CCTTTCGC -0.630663862107
CCTTTCTA -0.448103194781
CCTTTCTC -0.453810723265
CCTTTGAA -0.295049634205
CCTTTGAC 0.00557056452841
CCTTTGAG -0.117982423838
CCTTTGCA 0.152345216457
CCTTTGCC -1.15408677698
CCTTTGCG -0.428276093979
CCTTTGGA -0.714019759298
CCTTTGGC -1.65136395239
CCTTTGTA 0.262796713812
CCTTTGTC 0.0301762436299
CCTTTTAA 1.38102507269
CCTTTTAC -0.90169644856
CCTTTTAG -0.607879489952
CCTTTTCA 0.00565342249367
CCTTTTCC -0.225929624143
CCTTTTCG -0.0159531641998
CCTTTTGA 0.236807541934
CCTTTTGC 0.0229380645326
CCTTTTTA 0.438452986336
CCTTTTTC -0.677825988614
CGAAAAAA 0.198833762596
CGAAAAAC 0.293070452777
CGAAAAAG 0.878494911381
CGAAAACA 1.75187772155
CGAAAACC 0.616293363458
CGAAAACG -0.32154823881
CGAAAAGA 1.15646404257
CGAAAAGC 0.450373032543
CGAAAATA 3.04473799848
CGAAAATC 8.95907055362
CGAAACAA 0.666614547909
CGAAACAC 0.100848337597
CGAAACAG -0.514116684601
CGAAACCA -0.992743760532
CGAAACCC -0.206990697996
CGAAACCG -0.895145180292
CGAAACGA 0.841171188067

CGAAACGC -1.00460813246
CGAAACTA -0.265380732249
CGAAACTC -0.322615721239
CGAAAGAA 0.860312423567
CGAAAGAC -0.0912556331022
CGAAAGAG -1.08744727824
CGAAAGCA -0.586062491075
CGAAAGCC -1.06377708386
CGAAAGCG -0.165302686209
CGAAAGGA -1.23376451238
CGAAAGGC -0.306099279704
CGAAAGTA 1.09001927312
CGAAAGTC -0.768945703288
CGAAATAA 2.30375777331
CGAAATAC 1.16839245299
CGAAATAG -0.00835872199015
CGAAATCA 11.0434484028
CGAAATCC 22.4198525212
CGAAATCG 14.0519633558
CGAAATGA -0.123717136007
CGAAATGC 0.154127054782
CGAAATTA 1.59307637972
CGAAATTC 4.85729963916
CGAACAAA 1.01331736717
CGAACAAC -0.294846279325
CGAACAAG -0.410845078247
CGAACACA -0.0366305393272
CGAACACC 0.868435640741
CGAACACG -0.802925571872
CGAACAGA 1.25327429593
CGAACAGC -0.0246634444337
CGAACATA 1.630111015
CGAACATC 0.277764469403
CGAACCAA -0.4051696997
CGAACCAC -0.0913722092931
CGAACCAG 0.00728784160009
CGAACCCA -0.323033670408
CGAACCCC -0.677704968936
CGAACCCG -0.277942208887
CGAACCGA 0.624637709461
CGAACCGC -0.407148619747
CGAACCTA -0.37742378213
CGAACCTC -1.41496207469
CGAACGAA 0.796862303039
CGAACGAC -1.04588655929
CGAACGAG -0.489954047301
CGAACGCA -0.158417895027
CGAACGCC -1.66296615858
CGAACGCG 0.426721134877
CGAACGGA -1.35065749773
CGAACGGC -0.616192470163
CGAACGTA 0.469559487059
CGAACGTC -0.559938446295
CGAACTAA 0.470989244377

CGAACTAC -0.0768652694521
CGAACTAG 0.458882571712
CGAACTCA -0.900905507857
CGAACTCC -0.13975603337
CGAACTCG -0.847011759782
CGAACTGA -0.535028257637
CGAACTGC -0.857375017216
CGAACTTA 0.570414620577
CGAACTTC -0.670056420737
CGAAGAAA 1.1587286527
CGAAGAAC -0.722868049014
CGAAGAAG -0.291430544657
CGAAGACA 0.382961935341
CGAAGACC -0.54807276746
CGAAGACG -0.570451214
CGAAGAGA 0.0288643694103
CGAAGAGC -0.642324356391
CGAAGATA 2.51477008851
CGAAGATC 3.72051031192
CGAAGCAA 0.75007055425
CGAAGCAC -0.19236482953
CGAAGCAG -1.04640827695
CGAAGCCA -0.637395222294
CGAAGCCC -1.60001867486
CGAAGCCG -0.0453230456127
CGAAGCGA -1.35687968657
CGAAGCGC -0.1590005146
CGAAGCTA -0.622170789978
CGAAGCTC -0.91923201693
CGAAGGAA 0.00707141764037
CGAAGGAC -0.892229729994
CGAAGGAG -0.861975594649
CGAAGGCA -1.39552835319
CGAAGGCC -1.60390097567
CGAAGGCG -0.0770006651177
CGAAGGGA -0.91566363541
CGAAGGGC -1.05803818959
CGAAGGTA -0.456086834184
CGAAGGTC -1.01184814123
CGAAGTAA 0.833357708082
CGAAGTAC -0.0835976751652
CGAAGTAG 0.222435474996
CGAAGTCA 0.317869815639
CGAAGTCC -0.170122301419
CGAAGTCG -0.754170063168
CGAAGTGA 0.211251217976
CGAAGTGC -0.619360415081
CGAAGTTA -0.658347570858
CGAAGTTC -0.146168508012
CGAATAAA 0.104731422555
CGAATAAC 0.371690376868
CGAATAAG 0.157127976861
CGAATACA 2.55944177119
CGAATACC 1.35311526886

CGAATACG 0.627294914744
CGAATAGA 0.953991586808
CGAATAGC -0.142748591238
CGAATATA 2.05668473065
CGAATATC 9.48407299746
CGAATCAA 1.95075225967
CGAATCAC 2.88861790905
CGAATCAG 4.59852245149
CGAATCCA 6.7704926707
CGAATCCC 6.59468375133
CGAATCCG 8.40235363547
CGAATCGA 3.37837905673
CGAATCGC 4.00833666577
CGAATCTA 8.80949755034
CGAATCTC 15.5954823081
CGAATGAA 0.227651867465
CGAATGAC 0.703564756925
CGAATGAG 1.05021948197
CGAATGCA 0.427845859876
CGAATGCC -0.824152632491
CGAATGCG -0.202241917199
CGAATGGA 0.225177106534
CGAATGGC -1.18739959973
CGAATGTA -0.0556930989676
CGAATGTC -0.169089059978
CGAATTAA 0.508717325017
CGAATTAC 2.22005606827
CGAATTAG 0.871701080994
CGAATTCA 0.684121887066
CGAATTCC 1.42461332866
CGAATTCG 2.67046203489
CGAATTGA 0.171568787159
CGAATTGC 1.28246565371
CGAATTTA 2.47003149214
CGAATTTC 2.54015782134
CGACAAAA 0.576906293842
CGACAAAC -0.482773894918
CGACAAAG -1.32162636687
CGACAACA -0.289536573628
CGACAACC 0.474513452407
CGACAACG -0.241344080878
CGACAAGA -0.761928130256
CGACAAGC -0.622696689745
CGACAATA 1.56841633325
CGACAATC 3.67747017314
CGACACAA 1.07786293796
CGACACAC -0.152978282677
CGACACAG -0.838184380594
CGACACCA -0.0622331278276
CGACACCC -0.589274348101
CGACACCG -1.38489012232
CGACACGA -0.0774201825759
CGACACGC -0.463409439537
CGACACTA 0.213781914568

CGACACTC 0.140182608131
CGACAGAA -0.0612589586273
CGACAGAC -0.381730566652
CGACAGAG -0.617097896004
CGACAGCA -0.650300677109
CGACAGCC -0.881764533739
CGACAGCG 0.555091908782
CGACAGGA 0.821631084255
CGACAGGC -1.23671551057
CGACAGTA 1.35926427086
CGACAGTC -0.864630970261
CGACATAA 1.97106291643
CGACATAC -0.141184222399
CGACATAG 0.172725662093
CGACATCA -0.163231237078
CGACATCC 0.999999452196
CGACATCG 0.051252748451
CGACATGA 0.910677781507
CGACATGC -0.450980483367
CGACATTA 0.262534286692
CGACATTC -0.0202633466826
CGACCAAA 0.261418971431
CGACCAAC -1.10051949451
CGACCAAG -0.64687500994
CGACCACA -0.134817489535
CGACCACC -1.45013697992
CGACCACG -0.381783888497
CGACCAGA 0.242888846097
CGACCAGC -1.29160041765
CGACCATA 1.25786598639
CGACCATC 0.138930590296
CGACCCAA -0.203136103631
CGACCCAC 0.00584867454428
CGACCCAG -1.18961297907
CGACCCCA -1.2369962344
CGACCCCC -0.717672044309
CGACCCCG -0.629492088422
CGACCCGA -0.430587752795
CGACCCGC -0.796846881525
CGACCCTA -0.472406455379
CGACCCTC -1.00895229455
CGACCGAA -0.953418638354
CGACCGAC -0.603584990365
CGACCGAG -1.46361564457
CGACCGCA -1.16913948159
CGACCGCC -0.884459900735
CGACCGCG 0.0922015730904
CGACCGGA 0.306632236774
CGACCGGC -0.63398523802
CGACCGTA -0.0929070420123
CGACCGTC -0.251530644354
CGACCTAA 0.133418575245
CGACCTAC -0.59251809368
CGACCTAG 0.239443313927

CGACCTCA −0.62904904662
CGACCTCC −1.22551949138
CGACCTCG −1.11559467794
CGACCTGA 0.0320662939351
CGACCTGC −0.48106053857
CGACCTTA −0.418628760731
CGACCTTC −0.21997456729
CGACGAAA −0.00863029746617
CGACGAAC −0.342373033156
CGACGAAG −0.474679952482
CGACGACA −0.427558601504
CGACGACC −1.40905563482
CGACGACG −0.338275353909
CGACGAGA −0.0110828409614
CGACGAGC −0.838589522065
CGACGATA 1.32703278375
CGACGATC 1.62307383834
CGACGCAA 0.508355311509
CGACGCAC −1.16758922735
CGACGCAG −0.475614653062
CGACGCCA −1.30816913551
CGACGCCC −0.524169943464
CGACGCCG −1.28559020928
CGACGCGA −0.238516977558
CGACGCGC −0.630865387316
CGACGCTA −0.340963924983
CGACGCTC −0.721287213134
CGACGGAA 0.883897668926
CGACGGAC −0.88774389908
CGACGGAG −0.306919495146
CGACGGCA −0.706619523613
CGACGGCC −0.0464752156784
CGACGGCG 0.212161348686
CGACGGGA −1.18981737948
CGACGGGC −1.44954024574
CGACGGTA −0.21046628905
CGACGGTC −0.821274821142
CGACGTAA 1.94943933993
CGACGTAC −0.367593743148
CGACGTAG −0.958496498578
CGACGTCA −0.0897354377523
CGACGTCC −0.766545436111
CGACGTCG 2.33639649293
CGACGTGA −0.77639795391
CGACGTGC −0.718827873717
CGACGTTA 1.26257451442
CGACGTTC 0.642242282571
CGACTAAA −0.0443841629275
CGACTAAC −0.24469316324
CGACTAAG −0.510117284833
CGACTACA 0.273100114861
CGACTACC −0.0111479249783
CGACTACG −0.474988382763
CGACTAGA −0.293253419892

CGACTAGC −1.35041179903
CGACTATA 0.154358900256
CGACTATC 0.0866615902085
CGACTCAA 0.576906293842
CGACTCAC −0.392110029744
CGACTCAG 0.0202126386534
CGACTCCA −0.6702472293
CGACTCCC −0.752839369474
CGACTCCG −0.89394439325
CGACTCGA −1.1461922687
CGACTCGC −0.575352118885
CGACTCTA −0.854972920368
CGACTCTC −0.569911983772
CGACTGAA −0.275628459018
CGACTGAC −0.523678284686
CGACTGAG 0.294400100945
CGACTGCA −0.696885411676
CGACTGCC −1.52381443946
CGACTGCG 0.390344135696
CGACTGGA 0.313213041163
CGACTGGC −1.14534330128
CGACTGTA −0.656562334572
CGACTGTC −0.0497079832313
CGACTTAA 1.13477302962
CGACTTAC −0.0274994347271
CGACTTAG 0.517975461071
CGACTTCA −0.466141661337
CGACTTCC −0.564823145521
CGACTTGA 0.194079492786
CGACTTGC −0.224078781077
CGACTTTA −0.0253796300009
CGACTTTC 1.52177775408
CGAGAAAA 1.45052722264
CGAGAAAC 0.919357480095
CGAGAAAG 0.690266445574
CGAGAACA 2.2433817617
CGAGAACC −0.976306256239
CGAGAACG −1.53800955106
CGAGAAGA 0.523393640131
CGAGAAGC −0.260885491598
CGAGAATA 1.84618367785
CGAGAATC 11.0242674373
CGAGACAA 0.270770943478
CGAGACAC −0.335445898155
CGAGACAG −0.325900765111
CGAGACCA −0.802320996245
CGAGACCC −0.544622269039
CGAGACCG −0.767087018774
CGAGACGA −0.721372423533
CGAGACGC −0.53307730542
CGAGACTA 0.324156827116
CGAGACTC −0.54949781991
CGAGAGAA 0.495268457868
CGAGAGAC −0.617030720934

CGAGAGAG -0.485647001398
CGAGAGCA 0.524226924652
CGAGAGCC -1.27447025214
CGAGAGCG -0.37498848982
CGAGAGGA 0.824712511865
CGAGAGGC -0.781716023819
CGAGAGTA 0.222881653377
CGAGAGTC -0.808384265078
CGAGATAA 0.50040512895
CGAGATAC 5.11349803601
CGAGATAG -0.010664107648
CGAGATCA 4.15446753484
CGAGATCC 8.3620031133
CGAGATCG 4.84237161357
CGAGATGA 0.367942687576
CGAGATGC 0.166166552377
CGAGATTA 6.37618076241
CGAGATTC 10.9412194476
CGAGCAAA 0.67421081979
CGAGCAAC -0.81883586949
CGAGCAAG -0.317352280083
CGAGCACA -0.586062491075
CGAGCACC -1.0411390854
CGAGCACG -1.14785334873
CGAGCAGA -0.297091285835
CGAGCAGC -0.309648318986
CGAGCATA 0.583947391218
CGAGCATC 0.312728439688
CGAGCCAA -0.744158886742
CGAGCCAC -1.25193288559
CGAGCCAG -0.950788355375
CGAGCCCA -1.06218056509
CGAGCCCC -1.38961381048
CGAGCCCG -0.539495269076
CGAGCCGA -0.0229560998626
CGAGCCGC 0.0223436827882
CGAGCCTA -0.589411312056
CGAGCCTC -1.28063781222
CGAGCGAA 0.0976882341272
CGAGCGAC -0.712470550591
CGAGCGAG 0.52552546841
CGAGCGCA -0.439631294561
CGAGCGCC -1.95881274527
CGAGCGCG 0.961417960653
CGAGCGGA -0.148874853035
CGAGCGGC -0.911617448337
CGAGCGTA 0.755655233385
CGAGCGTC -1.11811988552
CGAGCTAA 0.0805559775574
CGAGCTAC -0.571615146238
CGAGCTAG -1.05476778308
CGAGCTCA -0.839724963708
CGAGCTCC -0.985629737692
CGAGCTCG 0.683201823855

CGAGCTGA −0.544896981094
CGAGCTGC −1.28220270383
CGAGCTTA −1.58662652751
CGAGCTTC −0.999910059691
CGAGGAAA −0.36017677904
CGAGGAAC −0.226177413894
CGAGGAAG −0.499114949403
CGAGGACA −0.561874499761
CGAGGACC −0.701422996145
CGAGGACG −1.05594452302
CGAGGAGA 0.0984943349627
CGAGGAGC −1.14194534056
CGAGGATA 2.55861057772
CGAGGATC 4.76058426732
CGAGGCAA 1.30564340516
CGAGGCAC −0.738422344901
CGAGGCAG −0.858323309638
CGAGGCCA 0.17592131346
CGAGGCCC −0.653163328325
CGAGGCCG −1.368734126
CGAGGCGA 0.138868381477
CGAGGCGC −0.590305237107
CGAGGCTA 0.892808951606
CGAGGCTC −1.41049846121
CGAGGGAA 0.925991344947
CGAGGGAC −0.588134201588
CGAGGGAG −1.33738663145
CGAGGGCA −1.34990942361
CGAGGGCC −0.879901405738
CGAGGGCG −1.12948527998
CGAGGGGA 0.368364034705
CGAGGGGC −0.769604907667
CGAGGGTA −0.290961626077
CGAGGGTC −1.00422285599
CGAGGTAA 0.796676722107
CGAGGTAC −1.14387172291
CGAGGTAG −0.388785517252
CGAGGTCA −0.684077974958
CGAGGTCC −1.74008104746
CGAGGTGA 0.116717075542
CGAGGTGC −1.85138386466
CGAGGTTA −0.114315501458
CGAGGTTC −1.07325739427
CGAGTAAA 0.765239050905
CGAGTAAC 0.496829428746
CGAGTAAG −0.243644500285
CGAGTACA 0.696890377926
CGAGTACC −0.583438481254
CGAGTACG −0.577652799674
CGAGTAGA 1.36188253028
CGAGTAGC −0.936773858457
CGAGTATA 1.42100939925
CGAGTATC 2.30853582884
CGAGTCAA 0.192218194456

CGAGTCAC −0.590518524487
CGAGTCAG −0.130565333766
CGAGTCCA −0.996064090919
CGAGTCCC −1.22238291226
CGAGTCCG −0.672191646977
CGAGTCGA −0.674019488463
CGAGTCGC −1.0441170059
CGAGTCTA −0.208109411218
CGAGTCTC −0.958073844541
CGAGTGAA 0.00411153247147
CGAGTGAC −1.08005566815
CGAGTGAG 0.722005489754
CGAGTGCA −0.608778119871
CGAGTGCC −0.709654686681
CGAGTGCG −0.680585132719
CGAGTGGA −0.317352280083
CGAGTGGC −0.387258787362
CGAGTGTA 0.183873587072
CGAGTGTC 0.568128054394
CGAGTTAA 0.0272830107674
CGAGTTAC −1.07712662601
CGAGTTAG −0.0362729693068
CGAGTTCA 0.0011286457223
CGAGTTCC −0.696303576248
CGAGTTGA 0.208276434056
CGAGTTGC −0.455661827713
CGAGTTTA 0.564806417099
CGAGTTTC 0.392963963411
CGATAAAA 1.92153999136
CGATAAAC 0.675018227533
CGATAAAG 0.193819156718
CGATAACA 0.998237740254
CGATAACC 0.0217427665039
CGATAACG 1.62547933315
CGATAAGA 0.339421250816
CGATAAGC 0.450626311307
CGATAATA 0.889174701925
CGATAATC 5.10736654658
CGATACAA 0.118705405327
CGATACAC 1.77856687334
CGATACAG 0.296038179393
CGATACCA 0.92089989288
CGATACCC 2.65614355118
CGATACCG 2.31400184073
CGATACGA 1.64922454405
CGATACGC 3.64684356903
CGATACTA 1.87689915171
CGATACTC 2.43559864925
CGATAGAA 1.58516331335
CGATAGAC −0.351149442933
CGATAGAG −0.903076543376
CGATAGCA 0.0377534346544
CGATAGCC −0.769880665249
CGATAGCG −0.226001504081

CGATAGGA −0.14847232538
CGATAGGC 0.208755807899
CGATAGTA 1.47045364845
CGATAGTC −0.180274362524
CGATATAA 4.39946834421
CGATATAC 1.47229978665
CGATATAG 1.22174775498
CGATATCA 7.91926190053
CGATATCC 21.6768703744
CGATATCG 15.3960235821
CGATATGA 1.83956340484
CGATATGC 3.51877286119
CGATATTA 3.5197454621
CGATATTC 8.57336123634
CGATCAAA 2.05663951164
CGATCAAC −0.206906271741
CGATCAAG 1.08217416596
CGATCACA 1.73403450704
CGATCACC 1.58631809723
CGATCACG 1.62800114277
CGATCAGA 2.44280049631
CGATCAGC 0.316422284372
CGATCATA 1.40099331955
CGATCATC 1.77386670952
CGATCCAA 1.49589731694
CGATCCAC 2.71893420595
CGATCCAG 3.66758838061
CGATCCCA 1.89425593507
CGATCCCC 2.68191995844
CGATCCCG 1.31325535994
CGATCCGA 3.08729431993
CGATCCGC 3.32811276242
CGATCCTA 1.30825983492
CGATCCTC 4.34151743104
CGATCGAA 2.32991318388
CGATCGAC 1.64438663213
CGATCGAG 1.18976144381
CGATCGCA 4.04314694368
CGATCGCC 4.08963784226
CGATCGCG 7.35717411339
CGATCGGA 2.14376766799
CGATCGGC 0.486195902745
CGATCGTA 2.65811698221
CGATCGTC 2.3216608442
CGATCTAA 2.72642122032
CGATCTAC 2.07001310089
CGATCTAG 3.35517281469
CGATCTCA 2.40923570169
CGATCTCC 6.12480642425
CGATCTGA 2.97161492931
CGATCTGC 2.09214846255
CGATCTTA 2.10045255578
CGATCTTC 3.93106677762
CGATGAAA −0.458445803069

CGATGAAC -0.988425997983
CGATGAAG 0.345027886005
CGATGACA 0.347704956289
CGATGACC -0.868521896667
CGATGACG -0.540374556757
CGATGAGA 1.25581623193
CGATGAGC -0.520212626133
CGATGATA 1.81732793403
CGATGATC 1.22933461713
CGATGCAA 0.450282594511
CGATGCAC -0.563079468908
CGATGCAG 0.99460296781
CGATGCCA -0.840073123991
CGATGCCC 0.316454434308
CGATGCCG -0.565550309115
CGATGCGA 1.02123722946
CGATGCGC 1.90899210657
CGATGCTA 0.575642252454
CGATGCTC -0.68756480542
CGATGGAA 1.12790392134
CGATGGAC 0.357025562545
CGATGGAG -0.0153169614004
CGATGGCA -0.0571552676037
CGATGGCC -0.83477596461
CGATGGCG -0.376041596261
CGATGGGA -0.597968945439
CGATGGGC -0.889249718442
CGATGGTA -0.136254565539
CGATGGTC -1.16277980602
CGATGTAA 1.33350119406
CGATGTAC 1.30051797349
CGATGTAG 0.238891014619
CGATGTCA -0.1045845261
CGATGTCC 0.595838424067
CGATGTGA -0.0183777398645
CGATGTGC -0.353383210034
CGATGTTA 1.54847422455
CGATGTTC 0.819360462349
CGATTAAA 0.836439397073
CGATTAAC 0.536679143783
CGATTAAG 0.0693847896173
CGATTACA 8.17092480397
CGATTACC 8.04875164904
CGATTACG 13.5316006967
CGATTAGA -0.537020508145
CGATTAGC 0.514863974578
CGATTATA 2.41084136882
CGATTATC 3.55158487682
CGATTCAA 0.80126893533
CGATTCAC 1.14618442725
CGATTCAG 0.279021976251
CGATTCCA 2.679742127
CGATTCCC 16.3452831166
CGATTCCG 5.76550161421

CGATTCGA 3.20864098626
CGATTCGC 7.9471881714
CGATTCTA 3.6010889824
CGATTCTC 10.8297064796
CGATTGAA 1.81487042429
CGATTGAC 0.787879401802
CGATTGAG 0.545657078769
CGATTGCA 4.87077595137
CGATTGCC 3.64401071531
CGATTGCG 13.8488241156
CGATTGGA -0.162790547711
CGATTGGC 0.476854647343
CGATTGTA 1.66477178263
CGATTGTC 2.68461166609
CGATTTAA 2.11528491571
CGATTTAC 3.2957777682
CGATTTAG 1.38828494646
CGATTTCA 4.15084217213
CGATTTCC 18.6288191029
CGATTTGA 3.83620407852
CGATTTGC 6.86479130832
CGATTTTA 3.40620861641
CGATTTTC 9.45186686437
CGCAAAAA 2.19253624577
CGCAAAAC -0.0333499389426
CGCAAAAG 0.0995921376569
CGCAAACA -0.286655364318
CGCAAACC 0.134864538222
CGCAAACG 0.0301456619834
CGCAAAGA 0.176546538233
CGCAAAGC -0.457986555609
CGCAAATA 1.92129429266
CGCAAATC 10.5103948831
CGCAACAA 1.46107945897
CGCAACAC 0.898711470759
CGCAACAG -0.287478977721
CGCAACCA 0.477113937884
CGCAACCC 0.564835691838
CGCAACCG -0.373161432479
CGCAACGA 0.354268770874
CGCAACGC -0.211547886085
CGCAACTA -0.705554654999
CGCAACTC -0.378089782431
CGCAAGAA 0.084904321753
CGCAAGAC 0.802318643811
CGCAAGAG -0.392170931656
CGCAAGCA 0.160804570361
CGCAAGCC -0.0642554372194
CGCAAGCG -0.427759342568
CGCAAGGA -0.269884598493
CGCAAGGC -0.634639737531
CGCAAGTA 0.417114315776
CGCAAGTC -0.707898986515
CGCAATAA 2.79848177331

CGCAATAC 1.3190967158
CGCAATAG 0.126118710092
CGCAATCA 10.139934568
CGCAATCC 25.1404992994
CGCAATGA 0.964019753038
CGCAATGC 0.396453146308
CGCAATTA 2.13153657769
CGCAATTC 4.2563331696
CGCACAAA 1.01379360443
CGCACAAC -0.0205749135425
CGCACAAG -0.640647332085
CGCACACA 0.545292712828
CGCACACC -0.208046156872
CGCACACG 0.416346376654
CGCACAGA -0.000391026864419
CGCACAGC -0.196609927996
CGCACATA 0.710500256137
CGCACATC 1.19738699043
CGCACCAA -0.121664244969
CGCACCAC -0.803977371405
CGCACCAG -0.428223033515
CGCACCCA -0.734341655458
CGCACCCC -0.854808772727
CGCACCCG -0.920362753705
CGCACCGA 0.586390786357
CGCACCGC 0.154924791407
CGCACCTA -0.0770960693995
CGCACCTC -1.22356906193
CGCACGAA 0.657055300258
CGCACGAC 0.31740089706
CGCACGAG -0.279461097329
CGCACGCA 0.354193492975
CGCACGCC -0.912855351566
CGCACGCG 0.51178280835
CGCACGGA 0.0177864946992
CGCACGGC -0.602317551016
CGCACGTA 0.0998801801733
CGCACGTC -0.0175005432355
CGCACTAA 0.75240573741
CGCACTAC -0.18084103782
CGCACTAG -0.387258787362
CGCACTCA -0.48884683487
CGCACTCC -0.0690240830178
CGCACTGA -0.332086883293
CGCACTGC 0.284429700047
CGCACTTA -0.360392418855
CGCACTTC -0.152879480435
CGCAGAAA -0.458059742455
CGCAGAAC -0.846262117371
CGCAGAAG -0.173980555126
CGCAGACA -0.128639474183
CGCAGACC -0.208384384655
CGCAGACG 0.237818827321
CGCAGAGA 0.246454875182

CGCAGAGC -0.909452163214
CGCAGATA 2.88082194163
CGCAGATC 5.79959701345
CGCAGCAA -0.507216471905
CGCAGCAC 0.0278081263895
CGCAGCAG -1.01815632461
CGCAGCCA -0.22788397432
CGCAGCCC -1.55902619949
CGCAGCCG -1.15233473615
CGCAGCGA -1.14338503038
CGCAGCGC -0.290019606813
CGCAGCTA -0.630952688768
CGCAGCTC -1.26566038551
CGCAGGAA -0.391964701578
CGCAGGAC -1.19335073586
CGCAGGAG -0.365769038241
CGCAGGCA 0.068077881648
CGCAGGCC -1.03384784584
CGCAGGCG -0.273247272698
CGCAGGGA 0.164811027431
CGCAGGGC -1.44619508411
CGCAGGTA -0.0109231368075
CGCAGGTC -0.552757771148
CGCAGTAA -0.414961054206
CGCAGTAC -0.976470926643
CGCAGTAG -0.471254808076
CGCAGTCA -0.76572861863
CGCAGTCC -1.61933843399
CGCAGTGA -0.511672243935
CGCAGTGC 0.109540321119
CGCAGTTA 0.835283567665
CGCAGTTC -0.0314849812704
CGCATAAA 0.0153966827865
CGCATAAC 0.346066093696
CGCATAAG 0.618694937543
CGCATACA 1.41101547401
CGCATACC 0.0976924162327
CGCATACG 0.633998829863
CGCATAGA -0.0820769570521
CGCATAGC -0.49635946464
CGCATATA 0.939040298257
CGCATATC 3.53091560452
CGCATCAA 0.817038086887
CGCATCAC 0.64984482761
CGCATCAG 0.385417354033
CGCATCCA -0.534088590807
CGCATCCC -0.138758078445
CGCATCCG 0.829512784836
CGCATCGA 1.0941747177
CGCATCGC 1.31374048418
CGCATCTA 0.241698514319
CGCATCTC -0.335667288365
CGCATGAA -0.269757305657
CGCATGAC 0.281295734736

CGCATGAG −0.821816665186
CGCATGCA 0.226346266403
CGCATGCC −0.340312562051
CGCATGCG −0.0387869374765
CGCATGGA −1.04039519338
CGCATGGC −1.10424104564
CGCATGTA −0.0380511482898
CGCATGTC 0.106610756215
CGCATTAA 0.525859252705
CGCATTAC 0.147423923807
CGCATTAG 0.173030694413
CGCATTCA 0.98998174123
CGCATTCC −0.123046692219
CGCATTGA 0.849718366186
CGCATTGC −0.754277229622
CGCATTTA 0.500923187269
CGCATTTC 1.66737409778
CGCCAAAA 0.0718114563348
CGCCAAAC −0.606948971478
CGCCAAAG −0.963096291867
CGCCAACA 0.144975823799
CGCCAACC −0.0223638091709
CGCCAACG −0.487463080712
CGCCAAGA −0.00499735469307
CGCCAAGC −0.542019953891
CGCCAATA 0.517301096559
CGCCAATC 0.998712409228
CGCCACAA 0.421980456909
CGCCACAC 0.264622202864
CGCCACAG −1.45339118077
CGCCACCA −0.686830323141
CGCCACCC −0.858002855804
CGCCACCG −1.09895120495
CGCCACGA −0.348192432962
CGCCACGC −1.66279782883
CGCCACTA −0.264028082501
CGCCACTC −0.377031448357
CGCCAGAA −0.235100720127
CGCCAGAC −0.899841423389
CGCCAGAG −0.519828917953
CGCCAGCA −0.91455668436
CGCCAGCC −0.705109783526
CGCCAGCG 0.382303515106
CGCCAGGA −0.663427260753
CGCCAGGC −1.41789059407
CGCCAGTA 0.155447293213
CGCCAGTC −0.837704745369
CGCCATAA 0.253838905209
CGCCATAC −1.06105008969
CGCCATAG −0.16244761506
CGCCATCA 0.614177740837
CGCCATCC 0.0960786462722
CGCCATGA −0.0962945474687
CGCCATGC −1.21106352095

CGCCATTA -0.234182225206
CGCCATTC -0.788534424077
CGCCCAAA 0.831361014086
CGCCCAAC -0.203575224709
CGCCCAAG -0.389648860656
CGCCCACA -0.84865950935
CGCCCACC -0.46604207495
CGCCCACG -0.211673610632
CGCCCAGA -0.742917846934
CGCCCAGC -0.112465181155
CGCCCATA -0.630979872454
CGCCCATC -0.295574488446
CGCCCCAA 0.504290566343
CGCCCCAC -0.79378923964
CGCCCCAG -0.255137448967
CGCCCCCA -0.609302189967
CGCCCCCC -0.92474063402
CGCCCCCG -1.09104545726
CGCCCCGA -0.482737562876
CGCCCCGC -0.360456457346
CGCCCCTA -1.01359129508
CGCCCCTC -1.14221926847
CGCCCGAA 0.518653746307
CGCCCGAC -1.37200766908
CGCCCGAG -0.46865615227
CGCCCGCA -0.151775927345
CGCCCGCC -0.0596896235378
CGCCCGCG -0.502762790927
CGCCCGGA 0.276624322891
CGCCCGGC -1.0405554203
CGCCCGTA 0.879073087467
CGCCCGTC -1.28178057255
CGCCCTAA -0.759183362139
CGCCCTAC -0.250125979668
CGCCCTAG 0.130914016813
CGCCCTCA 0.252291787555
CGCCCTCC -0.597735793057
CGCCCTGA -1.03598333346
CGCCCTGC -1.11658008655
CGCCCTTA 0.0601156755358
CGCCCTTC -1.05803818959
CGCCGAAA 0.86896676814
CGCCGAAC -0.411678101387
CGCCGAAG -0.642324356391
CGCCGACA -0.0681048039522
CGCCGACC -1.10749054162
CGCCGACG -0.64208806743
CGCCGAGA -0.488058769364
CGCCGAGC -1.15735300137
CGCCGATA 1.3504917818
CGCCGATC 0.127431891219
CGCCGCAA 0.0653359887284
CGCCGCAC -0.369580504643
CGCCGCAG -0.650695886079

CGCCGCCA -1.43827417629
CGCCGCCC -0.368177408247
CGCCGCCG -0.603584990365
CGCCGCGA 0.256996656244
CGCCGCGC -0.49327960532
CGCCGCTA 1.05663666148
CGCCGCTC -0.98341217625
CGCCGGAA 0.905723293396
CGCCGGAC -0.268134648722
CGCCGGAG -0.540366976691
CGCCGGCA -1.01560576302
CGCCGGCC -0.603680133265
CGCCGGCG -0.622678915796
CGCCGGGA -0.852030286384
CGCCGGGC -1.09872537125
CGCCGGTA -0.0517864896657
CGCCGGTC 0.322275141022
CGCCGTAA 0.19004924999
CGCCGTAC -0.457749221121
CGCCGTAG -0.810162444061
CGCCGTCA 0.0234812154847
CGCCGTCC -1.25662546934
CGCCGTGA 0.690415433083
CGCCGTGC -1.45643366252
CGCCGTTA -0.227803991552
CGCCGTTC -0.567420233038
CGCCTAAA -0.217477327542
CGCCTAAC -0.658959465169
CGCCTAAG 0.0117051905364
CGCCTACA 0.364177747098
CGCCTACC -0.543343067519
CGCCTACG -0.412641031179
CGCCTAGA -0.268077928916
CGCCTAGC -0.555065770622
CGCCTATA -0.352397801425
CGCCTATC 0.594019469556
CGCCTCAA -0.331852162621
CGCCTCAC 0.386152359075
CGCCTCAG -0.603880874329
CGCCTCCA -0.732147095596
CGCCTCCC -1.47085277814
CGCCTCCG -1.55868300546
CGCCTCGA -0.390231480229
CGCCTCGC -1.08188298687
CGCCTCTA -0.919945065918
CGCCTCTC -0.760781710585
CGCCTGAA -1.45768019134
CGCCTGAC -1.07228400922
CGCCTGAG -0.742487351449
CGCCTGCA -0.442882358826
CGCCTGCC -0.432428663361
CGCCTGGA -0.269702415522
CGCCTGGC -1.26358083355
CGCCTGTA 0.339102888035

CGCCTGTC -1.18633891322
CGCCTTAA 0.211951197884
CGCCTTAC -0.817630116197
CGCCTTAG -0.693783596301
CGCCTTCA -0.857489763735
CGCCTTCC -1.52823414083
CGCCTTGA -0.799680780765
CGCCTTGC -1.05405525686
CGCCTTTA -1.18020088926
CGCCTTTC -0.830315487711
CGCGAAAA 1.69385153048
CGCGAAAC -0.697947405091
CGCGAAAG -0.143343234364
CGCGAACA -0.195405742993
CGCGAACC -0.583007985769
CGCGAACG -0.0120917739137
CGCGAAGA 0.426068203656
CGCGAAGC 0.0534159425219
CGCGAATA 2.65833601999
CGCGAATC 8.29054582902
CGCGACAA 0.285331989309
CGCGACAC -0.27595832259
CGCGACAG 0.469293923359
CGCGACCA 0.528324342517
CGCGACCC -0.476335543498
CGCGACCG -0.189969005841
CGCGACGA -0.257108004803
CGCGACGC -0.976202749128
CGCGACTA -0.0111479249783
CGCGACTC -0.794015334718
CGCGAGAA 0.187131970021
CGCGAGAC -0.274643573173
CGCGAGAG -0.932142699377
CGCGAGCA 0.622415965913
CGCGAGCC -0.355119045199
CGCGAGCG 0.577677892307
CGCGAGGA -0.554672391323
CGCGAGGC -1.0878639205
CGCGAGTA 2.55091942432
CGCGAGTC -0.572847560453
CGCGATAA 2.2196551089
CGCGATAC 2.06974910548
CGCGATAG 1.11949998033
CGCGATCA 5.4122315824
CGCGATCC 12.8699670364
CGCGATGA -0.000655283655815
CGCGATGC 2.01072809606
CGCGATTA 3.22688777394
CGCGATTC 6.05309011081
CGCGCAAA 1.15243615221
CGCGCAAC -0.250990107217
CGCGCAAG 1.49436928014
CGCGCACA 0.805305189902
CGCGCACC 0.323148678309

CGCGCACG 0.499083060849
CGCGCAGA 0.714464630771
CGCGCAGC -0.956790722297
CGCGCATA 2.80080153496
CGCGCATC 0.595006969217
CGCGCCAA 0.0301561172472
CGCGCCAC 0.00908405591312
CGCGCCAG -0.757380874667
CGCGCCCA -1.4620546737
CGCGCCCC -0.850423835108
CGCGCCCG -0.157781430846
CGCGCCGA -0.384675814451
CGCGCCGC -0.767447725373
CGCGCCTA 1.15646404257
CGCGCCTC 0.182398087974
CGCGCGAA 0.153540253104
CGCGCGAC 0.172200807853
CGCGCGAG 0.612705116938
CGCGCGCA 1.63563819018
CGCGCGCC -0.590715083446
CGCGCGCG 2.32315150343
CGCGCGGA 0.61181171465
CGCGCGGC -0.330694503542
CGCGCGTA 0.840052997609
CGCGCGTC 0.149860523025
CGCGCTAA 0.862159868673
CGCGCTAC -0.255939629079
CGCGCTAG 0.554249737286
CGCGCTCA -0.743126690828
CGCGCTCC -0.25619735133
CGCGCTGA 0.487269135569
CGCGCTGC -0.251797253579
CGCGCTTA 0.297590263298
CGCGCTTC -0.272484561207
CGCGGAAA 1.72662721406
CGCGGAAC -0.601176097596
CGCGGAAG -0.74668409432
CGCGGACA -0.432278630326
CGCGGACC -0.721473839592
CGCGGACG -0.162930648245
CGCGGAGA -0.15274094819
CGCGGAGC -0.0920536311083
CGCGGATA 4.15175674633
CGCGGATC 4.31376341064
CGCGGCAA 0.511488231293
CGCGGCAC -0.851964679604
CGCGGCAG -0.632860774404
CGCGGCCA -0.393302713957
CGCGGCCC -0.533632218544
CGCGGCCG -0.0859908850386
CGCGGCGA 0.259422538817
CGCGGCGC -0.906415954619
CGCGGCTA 0.453310438894
CGCGGCTC -1.41269380522

CGCGGGAA -0.117211348136
CGCGGGAC -0.313340072618
CGCGGGAG -1.08237647532
CGCGGGCA -0.551738382932
CGCGGGCC -0.940283690499
CGCGGGGA -0.352948793825
CGCGGGGC -0.524472100587
CGCGGGTA 0.307094359432
CGCGGGTC -0.448339745124
CGCGGTAA 0.88824078549
CGCGGTAC -0.594206618777
CGCGGTAG -0.679362651005
CGCGGTCA -0.326983669054
CGCGGTCC -0.554463808811
CGCGGTGA 1.11476426861
CGCGGTGC -0.0491339892512
CGCGGTTA 1.12350199392
CGCGGTTC 0.257390819688
CGCGTAAA 1.1310143623
CGCGTAAC 0.770149888291
CGCGTAAG 0.0962932405608
CGCGTACA 0.607308632551
CGCGTACC -0.871183806819
CGCGTACG -0.149985986191
CGCGTAGA 0.414679546229
CGCGTAGC 0.235120585128
CGCGTATA 0.812675889467
CGCGTATC 6.22244395035
CGCGTCAA -0.0929546134623
CGCGTCAC 0.111556618734
CGCGTCAG -0.316465150954
CGCGTCCA -0.319304016445
CGCGTCCC -0.646404523071
CGCGTCCG -0.915910379635
CGCGTCGA -0.377724370963
CGCGTCGC -0.138595499093
CGCGTCTA -4.07755286425E-5
CGCGTCTC -0.930441105201
CGCGTGAA 1.39224958247
CGCGTGAC 0.082961995129
CGCGTGAG 0.498089810792
CGCGTGCA 0.513524393909
CGCGTGCC -0.781478166569
CGCGTGGA 0.352691071574
CGCGTGGC -0.924526562495
CGCGTGTA -0.230413364004
CGCGTGTC -0.363631198185
CGCGTTAA 0.987603168726
CGCGTTAC -0.647634062089
CGCGTTAG -0.63574485891
CGCGTTCA 0.558559397006
CGCGTTCC 0.191641325278
CGCGTTGA 0.702717096416
CGCGTTGC -0.99847324507

CGCGTTTA 0.558559397006
CGCGTTTC -0.318105843218
CGCTAAAA 0.554015016615
CGCTAAAC -0.108957440165
CGCTAAAG 0.229403124144
CGCTAACA 1.07772440571
CGCTAACC 0.331955669732
CGCTAACG -0.134164558314
CGCTAAGA 1.42872172456
CGCTAAGC -1.02242285637
CGCTAATA 1.54912088261
CGCTAATC 1.00936370918
CGCTACAA 0.134541470572
CGCTACAC -0.40475148915
CGCTACAG -0.0728078429706
CGCTACCA 0.667183314257
CGCTACCC -1.50410365346
CGCTACCG -0.902119363979
CGCTACGA 0.122034099924
CGCTACGC -0.101298698083
CGCTACTA -0.342732955611
CGCTACTC -0.744395959847
CGCTAGAA 0.177306635907
CGCTAGAC -0.906285263822
CGCTAGAG 0.434715752307
CGCTAGCA 0.311459693431
CGCTAGCC -0.657378367908
CGCTAGCG 0.21260648154
CGCTAGGA -0.505861208341
CGCTAGGC -0.985928235472
CGCTAGTA -0.102857316527
CGCTAGTC -0.874266541337
CGCTATAA 0.74756965516
CGCTATAC -0.650459597118
CGCTATAG -0.857826161847
CGCTATCA 0.668213419118
CGCTATCC 2.74101781405
CGCTATGA -0.487867438037
CGCTATGC -1.16325316809
CGCTATTA -0.194360739381
CGCTATTC 1.69076200004
CGCTCAAA 0.161682289753
CGCTCAAC -0.214333952494
CGCTCAAG -0.0715511202673
CGCTCACA -0.524784712973
CGCTCACC -0.133808817965
CGCTCACG -0.515604991397
CGCTCAGA 0.888861044012
CGCTCAGC -1.02904835701
CGCTCATA 1.32307755747
CGCTCATC -0.570403119787
CGCTCCAA -0.330504479123
CGCTCCAC -1.02929771505
CGCTCCAG 0.215241469388

CGCTCCCA	0.396812545999
CGCTCCCC	-0.847524067706
CGCTCCCG	-1.18046514605
CGCTCCGA	-0.661584520516
CGCTCCGC	-0.401862699774
CGCTCCTA	0.623070465424
CGCTCCTC	-1.01145737575
CGCTCGAA	0.509629808161
CGCTCGAC	-1.01059141852
CGCTCGAG	-0.197270962047
CGCTCGCA	-0.745667581301
CGCTCGCC	-0.367235388983
CGCTCGGA	-0.648158916329
CGCTCGGC	-0.819391043995
CGCTCGTA	-0.0275109355173
CGCTCGTC	-0.927777888141
CGCTCTAA	-0.197068914075
CGCTCTAC	0.224789739012
CGCTCTAG	-0.30463737245
CGCTCTCA	-0.798933229407
CGCTCTCC	-1.30078615101
CGCTCTGA	-0.932651870722
CGCTCTGC	-0.663783785247
CGCTCTTA	-0.166290970016
CGCTCTTC	-0.675859353502
CGCTGAAA	1.3530010451
CGCTGAAC	0.407113855995
CGCTGAAG	-0.55841250055
CGCTGACA	-0.308336967529
CGCTGACC	-0.684077974958
CGCTGACG	-0.424462275143
CGCTGAGA	-0.691870283034
CGCTGAGC	-0.972258500877
CGCTGATA	1.64010284918
CGCTGATC	-0.203415520555
CGCTGCAA	0.741313225329
CGCTGCAC	-1.50029689193
CGCTGCAG	-0.757380874667
CGCTGCCA	-1.1081654289
CGCTGCCC	-0.814831503472
CGCTGCCG	-0.956729559004
CGCTGCGA	-0.391029739617
CGCTGCGC	-0.207836006071
CGCTGCTA	-0.0952992063593
CGCTGCTC	-0.261675386775
CGCTGGAA	-0.806303667591
CGCTGGAC	-0.567346523428
CGCTGGAG	0.101616799483
CGCTGGCA	-1.5141629241
CGCTGGCC	-0.865924025006
CGCTGGGA	-0.49280572049
CGCTGGGC	-1.39711284841
CGCTGGTA	0.161862120289
CGCTGGTC	-0.85614913754

CGCTGTAA 0.0986485501031
CGCTGTAC 0.422603852011
CGCTGTAG -0.288212414473
CGCTGTCA -0.699599336765
CGCTGTCC -0.607862238767
CGCTGTGA -0.148166247534
CGCTGTGC 0.0209377111948
CGCTGTTA -0.238690796318
CGCTGTTC -0.945809036012
CGCTTAAA 0.942874766239
CGCTTAAC -0.290309740382
CGCTTAAG -0.371041104989
CGCTTACA 1.06718445432
CGCTTACC -0.881797990583
CGCTTACG 0.632121064493
CGCTTAGA -0.111832114934
CGCTTAGC -0.248601602213
CGCTTATA 1.58847815473
CGCTTATC 0.185908181398
CGCTTCAA 0.898673309046
CGCTTCAC -0.787748188242
CGCTTCAG -0.838514244166
CGCTTCCA -0.667418034928
CGCTTCCC -0.681457363098
CGCTTCCG -0.63574485891
CGCTTCGA 0.103052568578
CGCTTCGC -0.614344240913
CGCTTCTA -0.0921652410489
CGCTTCTC -0.400435817653
CGCTTGAA -0.749468331058
CGCTTGAC -0.992801787246
CGCTTGAG -1.14948097191
CGCTTGCA 0.419368470642
CGCTTGCC -0.823908763464
CGCTTGGA -0.392777075572
CGCTTGGC -0.71959084659
CGCTTGTA -0.256458732924
CGCTTGTC -1.19585215771
CGCTTTAA 0.867529692137
CGCTTTAC -0.376319444896
CGCTTTAG -0.764986817667
CGCTTTCA -0.126221433058
CGCTTTCC -0.97616693985
CGCTTTGA 1.24723246038
CGCTTTGC -1.23732505245
CGCTTTTA 0.675531842365
CGCTTTTC 0.501604086321
CGGAAAAA -0.131767427717
CGGAAAAC 0.569933155681
CGGAAAAG 0.130197569864
CGGAAACA -0.10267905428
CGGAAACC -0.654198660819
CGGAAACG -0.837985469201
CGGAAAGA 0.321389057419

CGGAAAGC -0.833837343307
CGGAAATA 1.0408081763
CGGAAATC 19.4921016055
CGGAACAA -0.00265145488815
CGGAACAC 0.409670690746
CGGAACAG -0.424281660462
CGGAACCA -1.31584094667
CGGAACCC -0.371974237279
CGGAACCG -0.108548639352
CGGAACGA 0.471458162956
CGGAACGC -0.689529872243
CGGAACTA -0.292249975954
CGGAACTC 0.0383431115302
CGGAAGAA 0.63347188457
CGGAAGAC -0.719240595254
CGGAAGAG -0.466943057304
CGGAAGCA -0.251187450321
CGGAAGCC -1.73967067835
CGGAAGGA -0.172932153553
CGGAAGGC -1.88029685105
CGGAAGTA 0.00348709184373
CGGAAGTC -1.21177369474
CGGAATAA 0.882371984563
CGGAATAC 1.09611626018
CGGAATAG 1.49642948987
CGGAATCA 4.8888162476
CGGAATCC 16.4754704925
CGGAATGA -0.38854583033
CGGAATGC 0.678157943238
CGGAATTA 1.41135474732
CGGAATTC 2.52966779383
CGGACAAA 0.493169563669
CGGACAAC -0.0146718716267
CGGACAAG -0.952222556181
CGGACACA -0.253611241841
CGGACACC -1.0377649104
CGGACACG -0.47635227192
CGGACAGA 0.928873077019
CGGACAGC -0.58783936315
CGGACATA 0.461429473963
CGGACATC 0.239811861974
CGGACCAA 0.583041965376
CGGACCAC -1.52605343419
CGGACCAG -1.22918667514
CGGACCCA -0.999910059691
CGGACCCC -0.580509439114
CGGACCCG -1.55045863361
CGGACCGA -0.435164805885
CGGACCGC -0.629492088422
CGGACCTA -0.373017149839
CGGACCTC -1.31183083025
CGGACGAA -0.430490780224
CGGACGAC 0.270026528699
CGGACGAG -1.17780219037

CGGACGCA −1.08921421781
CGGACGCC −1.35767899149
CGGACGGA −0.718098357689
CGGACGGC −0.569097780107
CGGACGTA 0.133871288166
CGGACGTC −0.897044901716
CGGACTAA −0.766858832642
CGGACTAC 0.035485687946
CGGACTAG −0.310826888593
CGGACTCA 0.0507409632903
CGGACTCC −0.86987846714
CGGACTGA −1.11337476406
CGGACTGC −1.09553965239
CGGACTTA −0.000391026864419
CGGACTTC −0.36377469668
CGGAGAAA 0.940895323429
CGGAGAAC −1.10306953334
CGGAGAAG −0.397350730701
CGGAGACA 0.697512988882
CGGAGACC −0.787690684292
CGGAGACG −0.696783211472
CGGAGAGA 0.212221466453
CGGAGAGC −0.789266031158
CGGAGATA 2.21045238574
CGGAGATC 6.96308098149
CGGAGCAA 0.852407198643
CGGAGCAC −1.05050046718
CGGAGCAG −0.881386575954
CGGAGCCA 0.0285282326806
CGGAGCCC −1.09134996682
CGGAGCCG −0.345953176847
CGGAGCGA −0.841584432367
CGGAGCGC −0.220886789052
CGGAGCTA −0.00830879810572
CGGAGCTC −0.753818243543
CGGAGGAA −0.647542055768
CGGAGGAC −1.96351264771
CGGAGGAG −0.77251120961
CGGAGGCA −0.673733536999
CGGAGGCC −0.821856395189
CGGAGGGA −1.02477502933
CGGAGGGC −1.30041995539
CGGAGGTA −0.559981574258
CGGAGGTC −1.04557342414
CGGAGTAA −1.04910103013
CGGAGTAC 0.0944188731512
CGGAGTAG 0.18009662304
CGGAGTCA −0.116662969552
CGGAGTCC −0.318683235159
CGGAGTGA −0.109700548036
CGGAGTGC −0.328203014189
CGGAGTTA 0.885115445772
CGGAGTTC −0.572923361115
CGGATAAA −0.171854738623

CGGATAAC	1.59560603079
CGGATAAG	0.228910681221
CGGATACA	2.08170391544
CGGATACC	5.57747415084
CGGATACG	4.98307201863
CGGATAGA	0.766891505342
CGGATAGC	1.05923819248
CGGATATA	2.99024437965
CGGATATC	20.5305934181
CGGATCAA	−0.100127447161
CGGATCAC	2.54001745942
CGGATCAG	0.491889316622
CGGATCCA	1.50495575746
CGGATCCC	4.71386596676
CGGATCCG	6.93901218018
CGGATCGA	0.320067773462
CGGATCGC	5.96865340073
CGGATCTA	3.62425026682
CGGATCTC	6.51716790309
CGGATGAA	−0.619499470089
CGGATGAC	0.216900458364
CGGATGAG	−0.825079230241
CGGATGCA	0.644371235653
CGGATGCC	−0.243537595213
CGGATGGA	−0.00236707171402
CGGATGGC	−0.434460121108
CGGATGTA	0.144839905371
CGGATGTC	1.22838527918
CGGATTAA	1.14990441009
CGGATTAC	16.7020574914
CGGATTAG	0.455069537021
CGGATTCA	1.72135619283
CGGATTCC	9.84805392422
CGGATTGA	0.731051383954
CGGATTGC	14.4071490533
CGGATTTA	2.65977257323
CGGATTTC	25.5993837008
CGGCAAAA	0.597667572461
CGGCAAAC	1.02028684599
CGGCAAAG	−0.929213134473
CGGCAACA	−0.0758641779476
CGGCAACC	−0.566687841812
CGGCAACG	0.325580049895
CGGCAAGA	0.87395706553
CGGCAAGC	−1.66073657359
CGGCAATA	−0.479406515844
CGGCAATC	6.32309938833
CGGCACAA	−0.490391338708
CGGCACAC	−0.258132097888
CGGCACAG	−0.884870269837
CGGCACCA	−1.17495103994
CGGCACCC	−1.3428461088
CGGCACCG	−1.39717244341
CGGCACGA	−1.22992533953

CGGCACGC -0.62355532828
CGGCACTA -0.208171620037
CGGCACTC -0.368645804063
CGGCAGAA 0.146085650047
CGGCAGAC -0.371626076996
CGGCAGAG -1.43181752815
CGGCAGCA -0.525383276823
CGGCAGCC -0.882874359986
CGGCAGGA -1.47147591186
CGGCAGGC -0.640451818653
CGGCAGTA -0.585253253661
CGGCAGTC -0.766721868687
CGGCATAA 1.13177184616
CGGCATAC 0.0921179309804
CGGCATAG -0.251609058832
CGGCATCA -0.324327247916
CGGCATCC 0.23068337119
CGGCATGA 0.824148711767
CGGCATGC -0.0922399961847
CGGCATTA 1.07324641624
CGGCATTC -0.842906500469
CGGCCAAA 1.61094259581
CGGCCAAC -1.3734332443
CGGCCAAG -0.185301514719
CGGCCACA -1.19854883162
CGGCCACC -0.655860263611
CGGCCACG -1.10958943582
CGGCCAGA 0.585668327632
CGGCCAGC -0.752176767133
CGGCCATA -0.880360130435
CGGCCATC -0.820956196979
CGGCCCAA -1.26107052473
CGGCCCAC -0.658778589106
CGGCCCAG -1.1298783979
CGGCCCCA -0.562067137995
CGGCCCCC -1.53437033512
CGGCCCCG -1.58037532532
CGGCCCGA -0.985912029814
CGGCCCGC -1.38622473674
CGGCCCTA -1.25369747273
CGGCCCTC -0.32941660893
CGGCCGAA -0.21997456729
CGGCCGAC 0.746423235489
CGGCCGAG -0.0615906518699
CGGCCGCA -0.375364879315
CGGCCGCC -0.828221559762
CGGCCGGA 0.146021088793
CGGCCGGC -0.688596478571
CGGCCGTA 0.525997000805
CGGCCGTC -0.67317940802
CGGCCTAA -0.30028014128
CGGCCTAC -1.61667939903
CGGCCTAG -0.670904081246
CGGCCTCA -1.27203966469

CGGCCTCC -0.724028060527
CGGCCTGA -1.02136792026
CGGCCTGC -0.702403961267
CGGCCTTA -0.822589831941
CGGCCTTC -0.966020367757
CGGCGAAA -0.176274178612
CGGCGAAC -0.301740480245
CGGCGAAG -0.331996706643
CGGCGACA -0.193374808009
CGGCGACC -0.773568759538
CGGCGACG -0.49888807018
CGGCGAGA 0.501390014796
CGGCGAGC -0.763188773683
CGGCGATA 2.01860718282
CGGCGATC 4.75740429885
CGGCGCAA -0.424931716486
CGGCGCAC 0.986515298532
CGGCGCAG -0.494156540567
CGGCGCCA -0.518339042868
CGGCGCCC -0.889852203016
CGGCGCCG -0.00106094788949
CGGCGCGA 0.358332209132
CGGCGCGC -0.847063251956
CGGCGCTA 0.110149862996
CGGCGCTC -1.34293942203
CGGCGGAA -1.28029749339
CGGCGGAC -1.12457078325
CGGCGGAG -0.453935925049
CGGCGGCA -0.881562747148
CGGCGGCC -1.1541928979
CGGCGGGA 0.669973824153
CGGCGGGC -1.38965275634
CGGCGGTA -0.112598485768
CGGCGGTC -0.87987396067
CGGCGTAA -0.281227252759
CGGCGTAC 0.0686163277314
CGGCGTAG -1.0009778035
CGGCGTCA -0.446924363793
CGGCGTCC -0.960477509678
CGGCGTGA -0.477042319328
CGGCGTGC -0.749697301334
CGGCGTTA 0.200796738366
CGGCGTTC -0.86304333846
CGGCTAAA 0.266069211367
CGGCTAAC -0.847272618613
CGGCTAAG -0.978716717298
CGGCTACA -0.112331353779
CGGCTACC -1.57800354873
CGGCTACG -0.717416935874
CGGCTAGA -0.253909216858
CGGCTAGC 0.400811423004
CGGCTATA 0.459366911806
CGGCTATC 0.601336585894
CGGCTCAA -0.206045280771

CGGCTCAC -1.2203600801
CGGCTCAG -0.923748168108
CGGCTCCA -1.57679021537
CGGCTCCC -1.19328643599
CGGCTCGA 0.30052610136
CGGCTCGC -0.672213864412
CGGCTCTA -0.706689051117
CGGCTCTC -1.33658941759
CGGCTGAA -0.333498343899
CGGCTGAC -1.29185552609
CGGCTGAG -0.637750178498
CGGCTGCA -0.96379705592
CGGCTGCC -1.86384680044
CGGCTGGA 0.50290759633
CGGCTGGC -0.799469323056
CGGCTGTA -0.846229444672
CGGCTGTC -1.04540823097
CGGCTTAA -0.801757457529
CGGCTTAC -0.655342728055
CGGCTTAG -1.19863639445
CGGCTTCA -1.38087425551
CGGCTTCC -0.87188587778
CGGCTTGA -0.81466395787
CGGCTTGC -1.24855609678
CGGCTTTA -0.474094457712
CGGCTTTC -0.741370990662
CGGGAAAA 0.337427432018
CGGGAAAC 0.0680067858545
CGGGAAAG -0.977022441806
CGGGAACA -0.356433533235
CGGGAACC -0.828656760116
CGGGAACG -0.873952360661
CGGGAAGA 0.297253081042
CGGGAAGC -1.23383299436
CGGGAATA 0.922420349612
CGGGAATC 17.9242051602
CGGGACAA 0.126407275371
CGGGACAC -0.851554571883
CGGGACAG -0.453785107869
CGGGACCA -0.0758584275525
CGGGACCC -0.855055778333
CGGGACCG -1.390234069
CGGGACGA -0.649141711122
CGGGACGC -1.74045403899
CGGGACTA -0.628044295773
CGGGACTC -1.09430645403
CGGGAGAA -0.262848205986
CGGGAGAC -0.685384621546
CGGGAGAG -0.44976427481
CGGGAGCA -1.04847606674
CGGGAGCC -1.28572403666
CGGGAGGA -0.664284069618
CGGGAGGC -0.788235403533
CGGGAGTA 0.234019384473

CGGGAGTC	-0.0895101268184
CGGGATAA	0.787233789266
CGGGATAC	2.00197886997
CGGGATAG	0.346452938455
CGGGATCA	0.907464617574
CGGGATCC	5.36293605932
CGGGATGA	-0.79800061988
CGGGATGC	0.28326916577
CGGGATTA	1.47775795709
CGGGATTC	9.77499201834
CGGGCAAA	0.328608939805
CGGGCAAC	-0.599311924068
CGGGCAAG	-0.606231740384
CGGGCACA	-0.679844638664
CGGGCACC	-0.964868459073
CGGGCACG	-1.4714683318
CGGGCAGA	-0.816970650436
CGGGCAGC	-1.01874417182
CGGGCATA	-0.171759072959
CGGGCATC	-0.384509837139
CGGGCCAA	-0.479747096061
CGGGCCAC	-1.10558846776
CGGGCCAG	-1.14940151191
CGGGCCCA	-1.12637405487
CGGGCCCC	-1.03989020414
CGGGCCCG	-1.11639947187
CGGGCCGA	-0.98263482739
CGGGCCGC	-1.68524318906
CGGGCCTA	-0.699319135696
CGGGCCTC	-0.173021284676
CGGGCGAA	0.0852360149956
CGGGCGAC	-0.498120131057
CGGGCGAG	-0.964901131773
CGGGCGCA	-0.146924684963
CGGGCGCC	-1.55672055245
CGGGCGGA	-1.02824486999
CGGGCGGC	-1.09188684461
CGGGCGTA	-1.00046157485
CGGGCGTC	-1.50236154514
CGGGCTAA	-1.31279323728
CGGGCTAC	-1.25666258553
CGGGCTAG	0.504571812938
CGGGCTCA	-1.17800554525
CGGGCTCC	-1.49135006135
CGGGCTGA	0.120737385837
CGGGCTGC	-1.52288339822
CGGGCTTA	-1.09809570299
CGGGCTTC	-1.35187867254
CGGGGAAA	2.62203220765
CGGGGAAC	-0.313177493266
CGGGGAAG	-0.760465700238
CGGGGACA	-0.917102279703
CGGGGACC	-0.483374549821
CGGGGACG	-0.88064137703

CGGGGAGA −0.753706894984
CGGGGAGC −0.404880350276
CGGGGATA 0.66798575575
CGGGGATC 4.69941574672
CGGGGCAA −0.890580412137
CGGGGCAC −1.08235870137
CGGGGCAG 0.0616392688464
CGGGGCCA 0.0737025521664
CGGGGCCC −0.753065725934
CGGGGCGA −0.685027574288
CGGGGCGC −0.165866486307
CGGGGCTA 0.857818843162
CGGGGCTC −1.17899252215
CGGGGGAA −0.023494545946
CGGGGGAC −0.286433712727
CGGGGGAG −0.824028214852
CGGGGGCA −1.04647257682
CGGGGGCC −1.4650396515
CGGGGGGA −0.0925523471894
CGGGGGGC −0.964052687119
CGGGGGTA −1.60431160616
CGGGGGTC −1.22362944108
CGGGGTAA −0.301750674127
CGGGGTAC −0.242380197516
CGGGGTAG 0.395098405507
CGGGGTCA −1.1643891325
CGGGGTCC −1.01321490558
CGGGGTGA −0.514661926606
CGGGGTGC −0.179407359777
CGGGGTTA −0.645682587109
CGGGGTTC −0.380912180881
CGGGTAAA −0.467205484424
CGGGTAAC −0.886037600035
CGGGTAAG 0.217699240515
CGGGTACA −1.00796218107
CGGGTACC 0.465686595982
CGGGTACG 0.276936673895
CGGGTAGA 0.28128815467
CGGGTAGC −0.707223053713
CGGGTATA −0.195474486352
CGGGTATC 3.26379459362
CGGGTCAA −0.778200441382
CGGGTCAC −0.846895967736
CGGGTCAG −1.15058426362
CGGGTCCA −1.56878409715
CGGGTCCC −1.17540140043
CGGGTCGA −0.050879495535
CGGGTCGC −0.281432698692
CGGGTCTA −1.04950094397
CGGGTCTC −1.0117448955
CGGGTGAA 0.262193967857
CGGGTGAC −0.485952556481
CGGGTGAG −0.674630598629
CGGGTGCA −0.523008886424

CGGGTGCC −0.575430533363
CGGGTGGA 0.239077641077
CGGGTGGC −0.856188606161
CGGGTGTA −0.047287066909
CGGGTGTC −0.466701540711
CGGGTTAA 0.0274555226194
CGGGTTAC −0.211322052388
CGGGTTAG −1.16932976739
CGGGTTCA −0.450575341896
CGGGTTCC −0.702025219337
CGGGTTGA −0.166345337387
CGGGTTGC 0.717318917776
CGGGTTTA −0.539034453326
CGGGTTTC 0.7081491287
CGGTAAAA 0.127473712274
CGGTAAAC 0.127361318189
CGGTAAAG −0.0204797706424
CGGTAACA −0.119410612867
CGGTAACC −0.212897922017
CGGTAACG −0.935733559713
CGGTAAGA 0.152345216457
CGGTAAGC −0.420065836734
CGGTAATA 1.56259431963
CGGTAATC 6.13950992305
CGGTACAA −0.765393788809
CGGTACAC −0.0991015244052
CGGTACAG −0.573264464095
CGGTACCA −0.38846218822
CGGTACCC −0.282608393101
CGGTACCG 0.194211229109
CGGTACGA −0.575256975985
CGGTACGC −1.07520886925
CGGTACTA 0.639938726584
CGGTACTC −0.65698838657
CGGTAGAA 0.218638645963
CGGTAGAC −1.14346632005
CGGTAGAG −0.14277682045
CGGTAGCA −0.0648171462646
CGGTAGCC −1.33914311576
CGGTAGGA 0.181850493535
CGGTAGGC −0.0525873628693
CGGTAGTA −0.0958873149455
CGGTAGTC 0.338456229972
CGGTATAA 1.6894205897
CGGTATAC −0.0152171136315
CGGTATAG −0.016789062537
CGGTATCA 2.09426513069
CGGTATCC 3.59210085354
CGGTATGA −0.145410762772
CGGTATGC −1.05386967593
CGGTATTA 0.421682743274
CGGTATTC 1.43094399087
CGGTCAAA 0.0624181859961
CGGTCAAC −0.406925661247

CGGTCAAG −0.140494959136
CGGTCACA −0.189026463813
CGGTCACC −0.890356146729
CGGTCACG −0.487897758302
CGGTCAGA 1.41055152168
CGGTCAGC −0.958449711273
CGGTCATA 0.759789767436
CGGTCATC 0.537346712374
CGGTCCAA −0.760462302277
CGGTCCAC −0.570555243874
CGGTCCAG −0.319930286744
CGGTCCCA −0.409801120162
CGGTCCCC −1.87378923351
CGGTCCGA −0.399327559695
CGGTCCGC −0.894306668139
CGGTCCTA 0.334443238361
CGGTCCTC −0.948204859701
CGGTCGAA −0.886137709186
CGGTCGAC −1.03158114466
CGGTCGAG −1.18055924342
CGGTCGCA −0.188083399022
CGGTCGCC −0.996534577788
CGGTCGGA −0.596349163702
CGGTCGGC −1.34028927405
CGGTCGTA 0.469164016707
CGGTCGTC −1.81048548667
CGGTCTAA 0.144994904656
CGGTCTAC −0.0381583147433
CGGTCTAG −0.843808266968
CGGTCTCA −0.549413132273
CGGTCTCC −0.863327460253
CGGTCTGA −0.081206294963
CGGTCTGC −0.350819840743
CGGTCTTA 0.514063362756
CGGTCTTC −0.904237600416
CGGTGAAA 0.80357641204
CGGTGAAC −0.604973972154
CGGTGAAG −1.1878246062
CGGTGACA −0.578511176828
CGGTGACC −1.73361917169
CGGTGACG 0.434363409918
CGGTGAGA 0.658262099077
CGGTGAGC −0.791874880846
CGGTGATA 2.00829620171
CGGTGATC 1.65521462603
CGGTGCAA −0.143412239105
CGGTGCAC −0.948701223348
CGGTGCAG 0.166828893336
CGGTGCCA −1.06864975954
CGGTGCCC −0.860707893919
CGGTGCGA −0.269380916162
CGGTGCGC −0.0468521279368
CGGTGCTA −0.180697800706
CGGTGCTC −1.09384694518

CGGTGGAA 1.07368971943
CGGTGGAC -0.385141073689
CGGTGGAG -1.07149489818
CGGTGGCA -0.400843311558
CGGTGGCC -1.113174023
CGGTGGGA 0.30907667744
CGGTGGGC -0.982896731747
CGGTGGTA 0.215582049605
CGGTGGTC -1.05963679942
CGGTGTAA -0.122606786997
CGGTGTAC -0.251299060261
CGGTGTAG -0.074730565975
CGGTGTCA -0.909808426326
CGGTGTCC -0.743125122538
CGGTGTGA 0.799180235013
CGGTGTGC -1.48108429925
CGGTGTTA 0.144858202082
CGGTGTTC -0.0288648921735
CGGTTAAA 1.3666409822
CGGTTAAC 0.919791634922
CGGTTAAG -0.10111285577
CGGTTACA -0.47308552476
CGGTTACC 0.164732351571
CGGTTACG 2.04341987615
CGGTTAGA 0.430918923275
CGGTTAGC -0.614216948077
CGGTTATA -0.483532947066
CGGTTATC 0.288122499205
CGGTTCAA 0.619532665551
CGGTTCAC -0.30822561897
CGGTTCAG -0.871858694095
CGGTTCCA 0.0711467629416
CGGTTCCC -0.55324995269
CGGTTCGA -0.123362441184
CGGTTCGC -0.316985300326
CGGTTCTA -0.497450471413
CGGTTCTC 0.509426714662
CGGTTGAA -0.835413997081
CGGTTGAC 0.0305283246368
CGGTTGAG 0.143804572877
CGGTTGCA 0.0420662309531
CGGTTGCC -0.795132218269
CGGTTGGA -0.221273111048
CGGTTGGC -0.425672994686
CGGTTGTA 0.859819980644
CGGTTGTC -0.242470635547
CGGTTTAA -0.0781052637334
CGGTTTAC 1.11067312391
CGGTTTAG 0.00726091929592
CGGTTTCA 0.453310438894
CGGTTTCC -0.883404964622
CGGTTTGA 0.739130427639
CGGTTTGC -0.0410290687887
CGGTTTTA 0.393282064811

CGGTTTTC 0.0901390109332
CGTAAAAA 1.1310143623
CGTAAAAC 0.0431060069335
CGTAAAAG -0.234796733333
CGTAAACA 0.128628234774
CGTAAACC 1.13207740125
CGTAAACG -0.251974993063
CGTAAAGA -0.407419933841
CGTAAAGC 0.668965675346
CGTAAATA 1.57340689202
CGTAAATC 2.85094654822
CGTAACAA 0.729878041979
CGTAACAC 0.247084543442
CGTAACAG 0.851967554802
CGTAACCA -0.440076166034
CGTAACCC 0.560114094726
CGTAACGA 0.204572656871
CGTAACGC 0.100906625692
CGTAACTA 0.384797618274
CGTAACTC 0.235669486475
CGTAAGAA 1.17008411467
CGTAAGAC 0.431328508232
CGTAAGAG 0.175705673645
CGTAAGCA -0.326258073749
CGTAAGCC -0.753638935769
CGTAAGGA 0.202297330097
CGTAAGGC -1.15253443169
CGTAAGTA 0.735369930648
CGTAAGTC 0.592003171941
CGTAATAA 2.80580490143
CGTAATAC 1.11724870066
CGTAATAG 2.46158599146
CGTAATCA 10.9650467323
CGTAATCC 23.4778917563
CGTAATGA 0.720409493742
CGTAATGC 1.0762306099
CGTAATTA 2.82975895622
CGTAATTC 3.06131638746
CGTACAAA 1.27243539643
CGTACAAC 0.0112482955103
CGTACAAG -0.265469079228
CGTACACA 0.858587827811
CGTACACC -0.023620009111
CGTACACG 0.467763795509
CGTACAGA 0.496087627783
CGTACAGC 0.0490827584588
CGTACATA 1.65895865598
CGTACATC 2.23356792838
CGTACCAA 0.239883741913
CGTACCAC -0.432531386327
CGTACCAG -0.900994900363
CGTACCCA 0.405661097096
CGTACCCC -0.470660426332
CGTACCGA -0.0665631753116

CGTACCGC -1.3985175131
CGTACCTA -0.347801667479
CGTACCTC -0.954691043953
CGTACGAA -0.0612589586273
CGTACGAC 0.334936204047
CGTACGAG -0.36423054618
CGTACGCA 0.318489290017
CGTACGCC 0.0526778009008
CGTACGGA 0.505789067021
CGTACGGC 0.275844360215
CGTACGTA -0.403986686606
CGTACGTC -0.330763246901
CGTACTAA 1.10201877933
CGTACTAC 1.05347446696
CGTACTAG -0.466526937807
CGTACTCA -0.199129907942
CGTACTCC -0.2007366206
CGTACTGA -0.768596236097
CGTACTGC -0.0746704482084
CGTACTTA 2.15698364414
CGTACTTC -0.0835976751652
CGTAGAAA 2.14774432756
CGTAGAAC -0.636426803489
CGTAGAAG 0.286914393478
CGTAGACA -0.549793181111
CGTAGACC -0.951378293632
CGTAGACG -0.488636161305
CGTAGAGA 0.843703191567
CGTAGAGC -0.0485929293519
CGTAGATA 1.82660567371
CGTAGATC 1.96019231732
CGTAGCAA 1.02631535107
CGTAGCAC -0.00833336797554
CGTAGCAG -0.15468588863
CGTAGCCA -0.660139080302
CGTAGCCC -0.230668733821
CGTAGCGA -1.27807967057
CGTAGCGC -0.143006836253
CGTAGCTA 1.42172741449
CGTAGCTC -0.268626568882
CGTAGGAA 0.332882528864
CGTAGGAC -1.17915849946
CGTAGGAG -1.15669196732
CGTAGGCA 0.912565479378
CGTAGGCC -0.661588702622
CGTAGGGA -0.863836370216
CGTAGGGC -1.30162231072
CGTAGGTA 0.346956620786
CGTAGGTC -1.20914863939
CGTAGTAA 0.520663248001
CGTAGTAC -0.359461116236
CGTAGTAG -0.658560596857
CGTAGTCA -0.934220160285
CGTAGTCC -0.233811586106

CGTAGTGA 2.15793638005
CGTAGTGC 0.217706820581
CGTAGTTA -0.781723865267
CGTAGTTC -0.677973146452
CGTATAAA -0.295529008049
CGTATAAC 1.02926033748
CGTATAAG 0.72607180321
CGTATACA 1.09163931624
CGTATACC -0.415195513496
CGTATACG 1.39106186451
CGTATAGA 0.823805256352
CGTATAGC 0.206367041513
CGTATATA 0.409599856334
CGTATATC 1.84911585657
CGTATCAA 1.99803592862
CGTATCAC 2.37908402793
CGTATCAG 4.31452978147
CGTATCCA 5.80730515666
CGTATCCC 2.24468866967
CGTATCGA 4.17782825341
CGTATCGC 2.63621425017
CGTATCTA 6.44888248445
CGTATCTC 5.00516974134
CGTATGAA 0.436417085101
CGTATGAC 1.31135041088
CGTATGAG -0.372509546784
CGTATGCA -0.123717136007
CGTATGCC 0.218781098932
CGTATGGA 0.154143783204
CGTATGGC -0.896765746174
CGTATGTA 0.870389729537
CGTATGTC 0.133682047892
CGTATTAA 0.598035336364
CGTATTAC 2.47592146497
CGTATTAG 1.17083454122
CGTATTCA 1.12266870939
CGTATTCC 2.68110915273
CGTATTGA 2.48835695568
CGTATTGC 1.57553924307
CGTATTTA 1.72984246904
CGTATTTC 1.12423098718
CGTCAAAA 0.259723911795
CGTCAAAC 0.23068337119
CGTCAAAG -0.124636415073
CGTCAACA 0.159594896344
CGTCAACC 0.710547043442
CGTCAACG -0.228415624482
CGTCAAGA 0.241016831122
CGTCAAGC -0.16231326492
CGTCAATA 1.96187796722
CGTCAATC 0.414417380491
CGTCACAA 0.806477224969
CGTCACAC -0.0597366722247
CGTCACAG -0.550607123394

CGTCACCA -0.77366913007
CGTCACCC -1.11027321007
CGTCACGA -0.539282243077
CGTCACGC 0.23294641303
CGTCACTA 0.37162503147
CGTCACTC -0.477939380958
CGTCAGAA -0.316465150954
CGTCAGAC -0.77045152265
CGTCAGAG -1.26160008384
CGTCAGCA -0.788213447479
CGTCAGCC -1.67657263883
CGTCAGGA 0.135769179919
CGTCAGGC 0.0998801801733
CGTCAGTA 0.0269905247639
CGTCAGTC -0.996355270015
CGTCATAA 0.772269954398
CGTCATAC 0.723305079038
CGTCATAG -1.04768956952
CGTCATCA -0.619731054181
CGTCATCC -0.308970295131
CGTCATGA -0.64543349045
CGTCATGC -1.10275169332
CGTCATTA 0.829384446473
CGTCATTC 0.245663673098
CGTCCAAA -0.498543569239
CGTCCAAC -0.336772409744
CGTCCAAG -0.917060458648
CGTCCACA -0.844606526355
CGTCCACC -0.0605331019412
CGTCCACG -0.59190306279
CGTCCAGA -0.404194223592
CGTCCAGC -0.324206489619
CGTCCATA 0.302259061327
CGTCCATC -0.256014906978
CGTCCCAA -0.646404523071
CGTCCCAC -0.161182266764
CGTCCCAG -0.840913988579
CGTCCCCA -0.702263076588
CGTCCCCC -1.0320762014
CGTCCCGA 0.368372660297
CGTCCCGC -0.975578569882
CGTCCCTA -1.00423069744
CGTCCCTC -0.689529872243
CGTCCGAA -0.604274515009
CGTCCGAC -1.24790368832
CGTCCGAG -0.432614505674
CGTCCGCA 0.111655420977
CGTCCGCC -1.11429900938
CGTCCGGA 0.482043071981
CGTCCGGC -0.268134648722
CGTCCGTA -0.1825794868
CGTCCGTC -0.260533933354
CGTCCTAA 0.52374441423
CGTCCTAC 0.4395654264

CGTCCTAG 0.836616875176
CGTCCTCA -0.688515711659
CGTCCTCC -1.83144489254
CGTCCTGA -0.917546628412
CGTCCTGC -0.730790525124
CGTCCTTA 0.213878625758
CGTCCTTC -0.976993689831
CGTCGAAA 2.01644712533
CGTCGAAC -1.0231168245
CGTCGAAG -0.798556055767
CGTCGACA -0.393829920632
CGTCGACC -0.930871862067
CGTCGACG -1.1451470037
CGTCGAGA -0.38564972227
CGTCGAGC -1.64664967397
CGTCGATA 1.1100824015
CGTCGATC 0.758434242491
CGTCGCAA -0.0900956215887
CGTCGCAC -0.489642480441
CGTCGCAG -0.733919262802
CGTCGCCA -1.25094617007
CGTCGCCC -0.228273955658
CGTCGCGA -0.195888253415
CGTCGCGC -0.461018843479
CGTCGCTA 0.311342855859
CGTCGCTC -0.483502104038
CGTCGGAA 0.290562235002
CGTCGGAC -0.72524374632
CGTCGGAG -1.17024198915
CGTCGGCA -0.76196812164
CGTCGGCC -1.06555369456
CGTCGGGA 0.985965090277
CGTCGGGC -1.18737137052
CGTCGGTA -0.542648576624
CGTCGGTC -0.607568968618
CGTCGTAA 1.19178296906
CGTCGTAC -0.184391122628
CGTCGTAG 0.279597015758
CGTCGTCA -0.501620553362
CGTCGTCC -0.657489455085
CGTCGTGA 0.0425469117043
CGTCGTGC -0.70826230693
CGTCGTTA -0.377640990234
CGTCGTTC -0.552166787365
CGTCTAAA 0.269817162042
CGTCTAAC 0.0533343914646
CGTCTAAG -0.276217351749
CGTCTACA 0.00290447227101
CGTCTACC -0.944005764396
CGTCTAGA -0.46502373226
CGTCTAGC -0.62372914704
CGTCTATA -0.732756114709
CGTCTATC 0.29444688825
CGTCTCAA -0.455863614303

CGTCTCAC -0.531162162482
CGTCTCAG -0.701547152402
CGTCTCCA -0.852771564584
CGTCTCCC -1.13888508486
CGTCTCGA -1.01746627721
CGTCTCGC -0.102089377405
CGTCTCTA 0.104311120953
CGTCTCTC -0.572336298055
CGTCTGAA 0.492028633012
CGTCTGAC -0.805529978073
CGTCTGAG -1.19407763807
CGTCTGCA -0.839514812907
CGTCTGCC -1.46567428601
CGTCTGGA -0.403986686606
CGTCTGGC -0.8529119265
CGTCTGTA 1.08941757269
CGTCTGTC -0.352340820238
CGTCTTAA 0.293240612194
CGTCTTAC 0.297443889605
CGTCTTAG -0.900788147522
CGTCTTCA -0.822257093172
CGTCTTCC -0.277916854872
CGTCTTGA 0.627097048877
CGTCTTGC 0.0242661444111
CGTCTTTA 0.490011028489
CGTCTTTC 0.280873342081
CGTGAAAA 0.449829881591
CGTGAAAC 0.183321026382
CGTGAAAG 0.0829395163119
CGTGAACA 0.0822181031128
CGTGAACC -0.505211413698
CGTGAACG 0.560838121741
CGTGAAGA -0.145715010947
CGTGAAGC -1.00604494708
CGTGAATA 0.700500057737
CGTGAATC 2.87515675697
CGTGACAA 0.0359326504715
CGTGACAC -1.01085619808
CGTGACAG -0.741954394379
CGTGACCA -0.0718660850879
CGTGACCC -0.757488563884
CGTGACGA -0.858060882518
CGTGACGC -0.339468299503
CGTGACTA -0.287296533368
CGTGACTC -0.97011569457
CGTGAGAA 1.0673323963
CGTGAGAC -0.264431132919
CGTGAGAG 0.23429775587
CGTGAGCA 0.350092938531
CGTGAGCC -1.33749405928
CGTGAGGA -0.0633432154568
CGTGAGGC -0.769774805703
CGTGAGTA 0.123219465453
CGTGAGTC -0.565376751737

CGTGATAA 1.34648532473
CGTGATAC 1.19463516501
CGTGATAG 1.43929251898
CGTGATCA 1.84490839706
CGTGATCC 3.20236573695
CGTGATGA -0.652689182114
CGTGATGC -0.166366770678
CGTGATTA 1.01008878172
CGTGATTC 4.67530434022
CGTGCAAA 1.89352720319
CGTGCAAC -1.03567856252
CGTGCAAG -0.46946957179
CGTGCACA 0.196470088843
CGTGCACC -1.00584499016
CGTGCACG 2.798761713
CGTGCAGA -0.105293392982
CGTGCAGC -0.646771502829
CGTGCATA 0.413163271603
CGTGCATC 0.25747968943
CGTGCCAA -0.895145180292
CGTGCCAC -0.528165161127
CGTGCCAG -0.778226579541
CGTGCCCA -1.31296208979
CGTGCCCC -0.392302406597
CGTGCCGA -0.700252790749
CGTGCCGC -0.949318606673
CGTGCCTA -0.738667520836
CGTGCCTC -0.97850552097
CGTGCGAA -0.725021310584
CGTGCGAC 0.0620757761081
CGTGCGAG -0.636364856051
CGTGCGCA 0.657055300258
CGTGCGCC -0.0844419377134
CGTGCGGA 1.0702081166
CGTGCGGC -0.537573068834
CGTGCGTA -0.349936632338
CGTGCGTC -0.129326385012
CGTGCTAA -0.67492909641
CGTGCTAC 0.26552187831
CGTGCTAG 0.0705259816561
CGTGCTCA -0.516279355909
CGTGCTCC -0.627646995751
CGTGCTGA 0.203033903428
CGTGCTGC -0.0801369828625
CGTGCTTA 2.28950149841
CGTGCTTC 1.02423971983
CGTGGAAA 0.809133384726
CGTGGAAC -0.733824381284
CGTGGAAG -0.774237373655
CGTGGACA -0.334898303716
CGTGGACC -0.994128821598
CGTGGAGA -0.268186140896
CGTGGAGC -0.786646203443
CGTGGATA 2.79650964919

CGTGGATC 2.10896418601
CGTGGCAA -0.364685088771
CGTGGCAC 0.337112205816
CGTGGCAG -1.06885154613
CGTGGCCA -1.23809090052
CGTGGCCC -0.75355215708
CGTGGCGA -0.442302353069
CGTGGCGC -0.87814701248
CGTGGCTA -0.221132226369
CGTGGCTC -1.26169052187
CGTGGGAA -0.318005734068
CGTGGGAC -1.02644630325
CGTGGGAG -0.948270989245
CGTGGGCA -1.21419539521
CGTGGGCC -0.390838146908
CGTGGGGA 0.0180400348452
CGTGGGGC -0.942438781741
CGTGGGTA -0.720117530501
CGTGGGTC -0.645369974723
CGTGGTAA 0.753000903299
CGTGGTAC 0.149091015613
CGTGGTAG -0.613408233425
CGTGGTCA -0.942832683803
CGTGGTCC -0.931730239222
CGTGGTGA -0.24638404077
CGTGGTGC -1.15872264093
CGTGGTTA 0.623506188541
CGTGGTTC -0.279855522154
CGTGTAAA 0.709195177839
CGTGTAAC 0.899519662647
CGTGTAAG 0.0350165079851
CGTGTACA -0.611599472796
CGTGTACC -0.391538388198
CGTGTAGA -0.387651382516
CGTGTAGC -1.22283823899
CGTGTATA 0.41108659484
CGTGTATC 2.92787428787
CGTGTCAA -0.716452437792
CGTGTCAC -0.331177798109
CGTGTCAG -0.697983475751
CGTGTCCA -0.067277269826
CGTGTCCC -0.152431472383
CGTGTCGA -0.329141635493
CGTGTCGC -0.346067661985
CGTGTCTA -0.0701292043967
CGTGTCTC 0.620544473701
CGTGTGAA -0.0247857710197
CGTGTGAC -0.956288085492
CGTGTGAG -0.786930063853
CGTGTGCA 0.93459654978
CGTGTGCC -0.732345484225
CGTGTGGA -0.467324674431
CGTGTGGC -0.507414337771
CGTGTGTA -0.0643422159085

CGTGTGTC -0.751916953829
CGTGTTAA 1.57692639518
CGTGTTAC 0.318489290017
CGTGTTAG -0.869618131072
CGTGTTCA -0.41392363066
CGTGTTCC -1.27236037991
CGTGTTGA 0.752567271235
CGTGTTGC 0.560135528017
CGTGTTTA -0.250024824991
CGTGTTTC 0.29861226533
CGTTAAAA 1.58577599181
CGTTAAAC 0.33934963226
CGTTAAAG 0.577150947014
CGTTAACA 0.528295590542
CGTTAACC -0.267202561958
CGTTAACG 1.30739518461
CGTTAAGA 0.845418116204
CGTTAAGC 0.384089274155
CGTTAATA 1.29446934203
CGTTAATC 0.164891794344
CGTTACAA 2.58049161508
CGTTACAC -0.0469294968886
CGTTACAG 0.445763045372
CGTTACCA 1.61550579568
CGTTACCC 0.693478563981
CGTTACGA 1.35279638332
CGTTACGC -0.986797590654
CGTTACTA 0.148213818984
CGTTACTC -0.442375278534
CGTTAGAA -1.16085107124
CGTTAGAC -0.618424146211
CGTTAGAG -0.71708837921
CGTTAGCA -0.282142872482
CGTTAGCC 0.126003179427
CGTTAGGA 1.02997312509
CGTTAGGC -1.11242751716
CGTTAGTA 0.0108065606167
CGTTAGTC -0.0661969796986
CGTTATAA 1.29965280041
CGTTATAC 1.02291399239
CGTTATAG 0.0560025747747
CGTTATCA 2.59406855921
CGTTATCC 2.45143994213
CGTTATGA 0.16228869505
CGTTATGC 0.575642252454
CGTTATTA 0.529864402868
CGTTATTC 0.14359677451
CGTTCAAA 1.25196033065
CGTTCAAC -0.278664667612
CGTTCAAG -0.19504451363
CGTTCACA -0.674463314409
CGTTCACC 0.386152097694
CGTTCAGA -0.582608071931
CGTTCAGC -0.50252545644

CGTTCATA -0.00468291263565
CGTTCATC -1.04757560715
CGTTCCAA 0.468947331366
CGTTCCAC -0.65877257733
CGTTCCAG 0.113757451755
CGTTCCCA -0.468986015842
CGTTCCCC -0.18012119291
CGTTCCGA 0.628847521411
CGTTCCGC -0.650934788856
CGTTCCTA -0.745816830192
CGTTCCTC -0.98135248929
CGTTCGAA 0.591639328762
CGTTCGAC -0.353702618342
CGTTCGAG -0.961975748974
CGTTCGCA -0.82625649294
CGTTCGCC -0.565376751737
CGTTCGGA 0.323148678309
CGTTCGGC -0.773503675521
CGTTCGTA 0.517003644305
CGTTCGTC -0.759603140978
CGTTCTAA -0.640992355789
CGTTCTAC -0.533187085689
CGTTCTAG -0.223163422735
CGTTCTCA -0.114823365894
CGTTCTCC -0.40639636352
CGTTCTGA -0.0299679224996
CGTTCTGC 0.195601779188
CGTTCTTA 0.517065330362
CGTTCTTC -0.877275043483
CGTTGAAA 0.401288705794
CGTTGAAC -0.198188411441
CGTTGAAG -0.604880397544
CGTTGACA -0.725832639051
CGTTGACC -1.54230352788
CGTTGAGA 1.55429519264
CGTTGAGC -0.129326385012
CGTTGATA 1.22181336176
CGTTGATC 1.06640527579
CGTTGCAA 0.22462872795
CGTTGCAC -1.05144274783
CGTTGCAG 0.529765077863
CGTTGCCA -0.609430005567
CGTTGCCC -1.68837088122
CGTTGCGA 0.679053436579
CGTTGCGC 0.0140510903413
CGTTGCTA 0.11115252279
CGTTGCTC -0.87004418307
CGTTGGAA -0.304822692
CGTTGGAC -0.930745353376
CGTTGGAG -0.353107975216
CGTTGGCA -0.468053667696
CGTTGGCC -1.07098546546
CGTTGGGA 0.625709373996
CGTTGGGC -0.866595775702

CGTTGGTA 0.87060824455
CGTTGGTC -0.785082618748
CGTTGTAA -0.288396688497
CGTTGTAC -0.281227252759
CGTTGTAG -0.331969000194
CGTTGTCA -0.916247039128
CGTTGTCC -0.648681679517
CGTTGTGA 0.0567548310019
CGTTGTGC -0.641916862486
CGTTGTTA 0.0359101716545
CGTTGTTC 0.571580905249
CGTTTAAA 0.768018844156
CGTTTAAC -0.25629563081
CGTTTAAG 1.68390151734
CGTTTACA -0.275645971585
CGTTTACC -0.470729953836
CGTTTAGA 0.0764211821241
CGTTTAGC 0.536679143783
CGTTTATA 0.271930432228
CGTTTATC 0.833381755188
CGTTTCAA 0.808209400791
CGTTTCAC -0.470302856311
CGTTTCAG -0.193594368547
CGTTTCCA 0.0466681152947
CGTTTCCC -0.533110762264
CGTTTCGA 1.27779581015
CGTTTCGC -0.616035641207
CGTTTCTA -0.373836581136
CGTTTCTC 0.0683196596223
CGTTTGAA -0.207450729601
CGTTTGAC 0.0740933176492
CGTTTGAG -0.298457527426
CGTTTGCA 0.18067061702
CGTTTGCC -0.21087299881
CGTTTGGA 0.752803821577
CGTTTGGC -0.432038682023
CGTTTGTA 1.32200824537
CGTTTGTC -0.624343916549
CGTTTTAA 2.57748807919
CGTTTTAC -0.0349992567999
CGTTTTAG -0.0820492506032
CGTTTTCA 0.325950688995
CGTTTTCC 0.789573154531
CGTTTTGA 0.94576277147
CGTTTTGC -0.288313307768
CGTTTTTA 0.0286346149893
CGTTTTTC 0.38699113261
CTAAAAAA 1.83878396493
CTAAAAAC 1.07614069463
CTAAAAAG 0.438452986336
CTAAAACA 0.823886284647
CTAAAACC 0.0745329614901
CTAAAAGA 1.12847321045
CTAAAAGC 0.92980960727

CTAAAATA 0.983578676325
CTAAAATC 4.77223012423
CTAAACAA 1.15634955743
CTAAACAC -0.119716429332
CTAAACAG 0.435433506164
CTAAACCA 0.718579299821
CTAAACCC -0.657596360157
CTAAACGA 0.651944505953
CTAAACGC 0.647618901956
CTAAACTA 0.0504330557727
CTAAACTC 0.568468895992
CTAAAGAA 1.50743914398
CTAAAGAC 0.0560227011575
CTAAAGAG -0.260654430269
CTAAAGCA -0.597669924895
CTAAAGCC 0.0847728468112
CTAAAGGA -0.550320126404
CTAAAGGC -0.330763246901
CTAAAGTA 0.185202451095
CTAAAGTC 0.410162872287
CTAAATAA 1.85287452389
CTAAATAC 1.05925884163
CTAAATAG 0.593557346898
CTAAATCA 0.863527417172
CTAAATCC 3.39856503447
CTAAATGA 2.74805106998
CTAAATGC 0.705733440009
CTAAATTA 2.32363244556
CTAAATTC 2.74494324282
CTAACAAA 1.47177127307
CTAACAAC -0.161246566636
CTAACAAG -0.317474083906
CTAACACA -0.470302856311
CTAACACC -0.17468968339
CTAACAGA 0.491815607013
CTAACAGC -0.433809542321
CTAACATA 2.27902166479
CTAACATC 1.02223727544
CTAACCAA -0.426417670847
CTAACCAC -1.10752138465
CTAACCAG -0.286087643497
CTAACCCA 0.392625474247
CTAACCCC -0.549349616546
CTAACCGA -0.1145520518
CTAACCGC -0.470447400333
CTAACCTA 1.6088084151
CTAACCTC -0.750954024037
CTAACGAA 2.0885066328
CTAACGAC -0.0362729693068
CTAACGAG -0.767232608322
CTAACGCA 0.263164477715
CTAACGCC -0.0577951297454
CTAACGGA 0.122007700383
CTAACGGC -0.378898235701

CTAACGTA -0.300398024379
CTAACGTC -0.636133271959
CTAACTAA 1.08477752664
CTAACTAC -1.1710606363
CTAACTAG -1.32739924075
CTAACTCA 0.218586108263
CTAACTCC -0.735169712347
CTAACTGA 0.0358139832279
CTAACTGC -0.701269042386
CTAACTTA 1.03956792064
CTAACTTC -0.428021769688
CTAAGAAA 1.37106251324
CTAAGAAC 0.472241784975
CTAAGAAG -0.795891793181
CTAAGACA -0.19804726538
CTAAGACC -0.914590925349
CTAAGAGA 0.0272338710278
CTAAGAGC -1.08374036448
CTAAGATA 1.51936624749
CTAAGATC 2.64293437095
CTAAGCAA 0.0149055467716
CTAAGCAC 1.64380061459
CTAAGCAG -0.882352642325
CTAAGCCA -0.763938416094
CTAAGCCC -0.2819523253
CTAAGCGA -0.484388971786
CTAAGCGC -0.039986940374
CTAAGCTA -0.748298648425
CTAAGCTC -0.614362537624
CTAAGGAA 0.50108733491
CTAAGGAC -0.383344336612
CTAAGGAG 0.323839771243
CTAAGGCA -0.305128508465
CTAAGGCC -0.448413454733
CTAAGGGA 0.0657602110552
CTAAGGGC -1.88036350336
CTAAGGTA -0.0107281461385
CTAAGGTC -1.38157318989
CTAAGTAA 1.19663996184
CTAAGTAC 0.366423537752
CTAAGTAG -0.529833821222
CTAAGTCA -0.0444970797761
CTAAGTCC -0.670240956142
CTAAGTGA 0.900456454279
CTAAGTGC -0.696783211472
CTAAGTTA 1.42734136836
CTAAGTTC -0.0605636835876
CTAATAAA 2.61999290846
CTAATAAC 0.428736648347
CTAATAAG 1.26107575236
CTAATACA 1.12091875962
CTAATACC 0.185600796644
CTAATAGA 0.438529832525
CTAATAGC 1.04692685803

CTAATATA 1.29336944828
CTAATATC 2.93713765155
CTAATCAA 0.166318676465
CTAATCAC 0.0402647890082
CTAATCAG -0.485577996657
CTAATCCA 1.81681797854
CTAATCCC 0.628184134926
CTAATCGA -0.113082825861
CTAATCGC 0.83032359054
CTAATCTA 1.18441592884
CTAATCTC 2.29253509319
CTAATGAA 0.589471952586
CTAATGAC -0.521032318812
CTAATGAG -0.558842996035
CTAATGCA -0.324212501396
CTAATGCC -0.620576362255
CTAATGGA 0.105636325633
CTAATGGC -0.598746555681
CTAATGTA 1.11660596332
CTAATGTC 0.31683840387
CTAATTAA 1.9797102037
CTAATTAC 1.03205790468
CTAATTAG 0.138455398559
CTAATTCA 0.985767224411
CTAATTCC 1.94365679493
CTAATTGA 0.748450511131
CTAATTGC 0.730864757496
CTAATTTA 1.82580114116
CTAATTTC 0.760156747194
CTACAAAA 0.89135985205
CTACAAAC -0.280512635481
CTACAAAG 0.375496615638
CTACAACA 0.579664392421
CTACAACC 0.66205735982
CTACAAGA 0.268711256518
CTACAAGC -0.822502007725
CTACAATA 0.70482017272
CTACAATC 4.40677134595
CTACACAA 1.27606102052
CTACACAC -1.18153158295
CTACACAG 0.417492273561
CTACACCA -0.192699136588
CTACACCC 0.18294515965
CTACACGA 0.104731422555
CTACACGC -0.541316576022
CTACACTA -0.211705760568
CTACACTC -0.385846281229
CTACAGAA 0.116090021098
CTACAGAC -0.0865769025721
CTACAGAG -0.0412491520907
CTACAGCA 0.0169414480062
CTACAGCC -0.224157195555
CTACAGGA -0.821598150174
CTACAGGC -0.70074157433

CTACAGTA 0.0567882878459
CTACAGTC 0.164856246447
CTACATAA 0.57254697162
CTACATAC 0.42398943584
CTACATAG -0.389273516688
CTACATCA 0.589376286922
CTACATCC -0.541469745636
CTACATGA 2.71581644629
CTACATGC -0.215826441395
CTACATTA 0.101805255612
CTACATTC -0.050250088657
CTACCAAA 0.0727506004015
CTACCAAC 0.127542194252
CTACCAAG -0.528409291535
CTACCACA 0.319881408386
CTACCACC -0.608953768303
CTACCAGA 1.00531490829
CTACCAGC -0.459402459703
CTACCATA -0.724182537049
CTACCATC 0.068274179225
CTACCCAA 4.07755286431E-5
CTACCCAC -1.35576567822
CTACCCAG 0.369236003702
CTACCCCA -0.0566528921803
CTACCCCC -0.836968433419
CTACCCGA -0.235401308959
CTACCCGC -0.734341655458
CTACCCTA -0.360805663155
CTACCCTC -1.09325073377
CTACCGAA 0.325275278957
CTACCGAC -0.342851100091
CTACCGAG 1.13947267068
CTACCGCA -0.452036726388
CTACCGCC -1.07850881187
CTACCGGA 0.358732122971
CTACCGGC -0.427330676754
CTACCGTA 0.78928772583
CTACCGTC -1.31113398693
CTACCTAA -0.648919014004
CTACCTAC 0.957625313726
CTACCTAG -0.59612097757
CTACCTCA 0.265837104512
CTACCTCC -0.34264434725
CTACCTGA -0.290198391823
CTACCTGC -0.663295001667
CTACCTTA -0.541783926312
CTACCTTC -0.635453679815
CTACGAAA 0.753037758104
CTACGAAC -0.733422637774
CTACGAAG -0.523450098555
CTACGACA -0.735101753133
CTACGACC -0.444587089581
CTACGAGA 0.693367215422
CTACGAGC 0.119618672616

CTACGATA 1.40794162646
CTACGATC 3.03616677326
CTACGCAA 0.575877495889
CTACGCAC 0.275391647294
CTACGCAG 0.956237116081
CTACGCCA -0.475683657803
CTACGCCC -0.253772252903
CTACGCGA 0.874558243196
CTACGCGC 0.0998801801733
CTACGCTA 0.16226569347
CTACGCTC -0.998508008822
CTACGGAA 1.38987022583
CTACGGAC -1.22159458537
CTACGGAG -0.535611138591
CTACGGCA -0.728150571026
CTACGGCC -1.21718873722
CTACGGGA -0.468146719544
CTACGGGC -0.888155313709
CTACGGTA 0.212310858958
CTACGGTC -0.236907912466
CTACGTAA -0.0594765975388
CTACGTAC -0.766802374218
CTACGTAG 0.624532372678
CTACGTCA 0.317547270752
CTACGTCC 0.327240868542
CTACGTGA -0.55623310082
CTACGTGC -0.281127666371
CTACGTTA 0.443440147147
CTACGTTC 0.637435997823
CTACTAAA 1.19162718563
CTACTAAC 0.251621082385
CTACTAAG -0.74889799642
CTACTACA 0.23251356511
CTACTACC -0.792217552116
CTACTAGA 0.411933209823
CTACTAGC -0.468488345287
CTACTATA 2.31052259034
CTACTATC 1.12390530571
CTACTCAA 0.00893088629911
CTACTCAC 0.335574236518
CTACTCAG -0.656817965771
CTACTCCA 0.809529639222
CTACTCCC -0.350806510282
CTACTCGA 1.95618167814
CTACTCGC 0.17358926688
CTACTCTA -0.885608934221
CTACTCTC -0.862178165384
CTACTGAA 1.96468677383
CTACTGAC -0.0725841003262
CTACTGAG -1.68473401772
CTACTGCA -0.287478977721
CTACTGCC -0.585253253661
CTACTGGA 0.596077849607
CTACTGGC -0.431501804229

CTACTGTA 0.649751252999
CTACTGTC 0.0507393950007
CTACTTAA 1.15371273992
CTACTTAC 0.111507478995
CTACTTCA 0.611374946006
CTACTTCC 0.365373567889
CTACTTGA -0.0041355795781
CTACTTGC -0.793332344614
CTACTTTA 0.459289281472
CTACTTTC 0.416135180326
CTAGAAAA 2.20680088488
CTAGAAAC -0.625325665816
CTAGAAAG 0.191770709167
CTAGAACA 0.344294972016
CTAGAACC -0.485664252583
CTAGAAGA 0.737867693159
CTAGAAGC -1.15076174172
CTAGAATA 0.902487912027
CTAGAATC 5.74038467271
CTAGACAA -0.198045697091
CTAGACAC -0.355266464418
CTAGACAG -0.359101716545
CTAGACCA -0.465142660885
CTAGACCC -1.43098790297
CTAGACGA 0.159777340697
CTAGACGC -0.171784688356
CTAGACTA -1.34086169974
CTAGACTC -0.624751149072
CTAGAGAA 1.10327550204
CTAGAGAC 0.190312199873
CTAGAGAG -0.849392946102
CTAGAGCA -0.610182000412
CTAGAGCC -0.77732768824
CTAGAGGA 0.274502949875
CTAGAGGC -0.317496562723
CTAGAGTA 0.797211247466
CTAGAGTC -1.01983308754
CTAGATAA 1.24086990963
CTAGATAC 3.84764370535
CTAGATAG 0.503834716843
CTAGATCA 1.57268704711
CTAGATCC 3.85589238569
CTAGATGA 0.0746921428807
CTAGATGC 0.241698514319
CTAGATTA 2.6267433495
CTAGATTC 21.0535712001
CTAGCAAA 1.1413938254
CTAGCAAC -0.485881722069
CTAGCAAG -0.398155263247
CTAGCACA 0.939965066336
CTAGCACC -0.607640064412
CTAGCAGA 1.46171357072
CTAGCAGC -1.67389243197
CTAGCATA 0.925484264655

CTAGCATC 0.131174614262
CTAGCCAA 0.179598691104
CTAGCCAC -0.909007291741
CTAGCCAG -1.03815959661
CTAGCCCA -0.506478068902
CTAGCCCC -0.942522162469
CTAGCCGA 0.1763510248
CTAGCCGC -1.03815959661
CTAGCCTA -0.528615521613
CTAGCCTC -0.619906702612
CTAGCGAA 0.380117319455
CTAGCGAC -0.469532826136
CTAGCGAG -0.107117575126
CTAGCGCA -0.724745291621
CTAGCGCC -1.08605725092
CTAGCGGA 0.229403124144
CTAGCGGC -0.381817345341
CTAGCGTA 0.457251550567
CTAGCGTC -0.814438646936
CTAGCTAA -0.62929997295
CTAGCTAC 0.0705978615945
CTAGCTAG 0.323626745244
CTAGCTCA -0.557542361224
CTAGCTCC -0.531699301657
CTAGCTGA -0.185732532967
CTAGCTGC -0.859110590999
CTAGCTTA -0.106653622797
CTAGCTTC -1.07341945086
CTAGGAAA 1.59648479571
CTAGGAAC -0.544775177272
CTAGGAAG 0.0240693240709
CTAGGACA 0.183505561788
CTAGGACC -0.989150547762
CTAGGAGA -0.0962485443082
CTAGGAGC 0.415359661137
CTAGGATA 1.60332436788
CTAGGATC 1.84650596136
CTAGGCAA -0.293710837682
CTAGGCAC -0.76332939698
CTAGGCAG -0.267221642815
CTAGGCCA -1.14178798884
CTAGGCCC -1.472888418
CTAGGCGA -0.0358722713234
CTAGGCGC -0.614370640454
CTAGGCTA -0.955580786899
CTAGGCTC 0.0798886703483
CTAGGGAA 0.132934496533
CTAGGGAC -0.0274842745947
CTAGGGAG -0.864630970261
CTAGGGCA -0.017811064569
CTAGGGCC -0.805417322606
CTAGGGGA -0.760240127923
CTAGGGGC -1.32103747413
CTAGGGTA -0.183177789269

CTAGGGTC 0.0507393950007
CTAGGTAA -0.114817092736
CTAGGTAC -0.509856426003
CTAGGTCA -1.10135539285
CTAGGTCC -0.795122285768
CTAGGTGA 0.547621622829
CTAGGTGC -0.124730773828
CTAGGTTA -0.235132085918
CTAGGTTC -0.0932651347959
CTAGTAAA 0.723563846816
CTAGTAAC -0.323130381597
CTAGTAAG -0.588131065009
CTAGTACA -0.242400846662
CTAGTACC -0.42326462468
CTAGTAGA 0.266551199027
CTAGTAGC -0.541031408703
CTAGTATA 1.97071266509
CTAGTATC 1.84910540131
CTAGTCAA -0.266679276008
CTAGTCAC -0.0958873149455
CTAGTCAG 0.0838255999151
CTAGTCCA -0.271614683263
CTAGTCCC -1.5685462399
CTAGTCGA 0.185621968553
CTAGTCGC -1.01144901153
CTAGTCTA 0.00457496203739
CTAGTCTC -0.459704094062
CTAGTGAA -0.158968626045
CTAGTGAC -0.88909681021
CTAGTGAG -0.556103716931
CTAGTGCA 1.27525256725
CTAGTGCC -1.18867200533
CTAGTGGA 0.149075071336
CTAGTGGC -1.12253252958
CTAGTGTA 1.07433742302
CTAGTGTC -0.0534256136408
CTAGTTAA 0.160862335693
CTAGTTAC 0.581776355699
CTAGTTCA -0.544775177272
CTAGTTCC 0.150010033297
CTAGTTGA 0.799338893641
CTAGTTGC 0.367696727496
CTAGTTTA 0.638333059453
CTAGTTTC 0.678858707292
CTATAAAA 0.880923930533
CTATAAAC 0.733831438587
CTATAAAG -0.178810625599
CTATAACA 1.36697084577
CTATAACC 0.502641509867
CTATAAGA 2.01815211747
CTATAAGC 0.142942274999
CTATAATA 2.38232123897
CTATAATC 2.33770235537
CTATACAA 1.30553101108

CTATACAC 0.123062636496
CTATACAG 0.0854283918486
CTATACCA 0.789084109568
CTATACCC -0.201153001479
CTATACGA -0.175186308418
CTATACGC -0.160045518212
CTATACTA -0.00640071247052
CTATACTC -0.0325927164652
CTATAGAA 0.768461885957
CTATAGAC -0.940467180378
CTATAGAG -0.214402957235
CTATAGCA 0.145382794941
CTATAGCC -0.819984902977
CTATAGGA -0.307604314922
CTATAGGC -1.12548248225
CTATAGTA 0.660182469647
CTATAGTC -0.731345699629
CTATATAA 1.66752334667
CTATATAC 1.47567997342
CTATATAG 1.11896101148
CTATATCA 2.18568883216
CTATATCC 1.87245331218
CTATATGA 0.428301447994
CTATATGC 0.530493809746
CTATATTA 2.21321518919
CTATATTC 2.18006991204
CTATCAAA -0.176740483375
CTATCAAC 1.20584738986
CTATCAAG 0.297781071861
CTATCACA 0.695003202818
CTATCACC 0.121687507931
CTATCAGA 1.29883964228
CTATCAGC 0.425228123213
CTATCATA 0.972592546554
CTATCATC 0.367942687576
CTATCCAA 0.308594428399
CTATCCAC 0.276958368568
CTATCCAG 0.208917080343
CTATCCCA 0.808172545987
CTATCCCC 0.150953098088
CTATCCGA 1.00106222976
CTATCCGC 0.965045937176
CTATCCTA 0.614022480171
CTATCCTC -0.364598571464
CTATCGAA 0.356734906212
CTATCGAC -0.525149601678
CTATCGAG -0.326971645501
CTATCGCA 0.279089674084
CTATCGCC -0.778302902966
CTATCGGA 0.620577930545
CTATCGGC 0.536400511004
CTATCGTA 0.418229892419
CTATCGTC 0.216559878147
CTATCTAA 1.63438460406

CTATCTAC 1.29491159968
CTATCTCA 1.78104215703
CTATCTCC 1.06104120272
CTATCTGA 2.79644064445
CTATCTGC -0.639980286257
CTATCTTA 1.51332990096
CTATCTTC 0.0216612154466
CTATGAAA 1.03386509702
CTATGAAC -0.272084647369
CTATGAAG 0.511223190357
CTATGACA -0.635691014302
CTATGACC -0.624479312215
CTATGAGA 0.461283623034
CTATGAGC -0.863748546
CTATGATA 1.54160407073
CTATGATC 1.62305475749
CTATGCAA 0.825566445532
CTATGCAC -0.443054347914
CTATGCAG -0.431796381285
CTATGCCA -0.630010669504
CTATGCCC -0.94861784262
CTATGCGA 0.57127613431
CTATGCGC 0.484981001096
CTATGCTA -0.491736931153
CTATGCTC -0.61935544883
CTATGGAA 2.01960330808
CTATGGAC -1.02654275305
CTATGGAG -0.224542994787
CTATGGCA -0.5004953056
CTATGGCC -0.547811908629
CTATGGGA 0.457789473887
CTATGGGC -0.57359824839
CTATGGTA 0.407111503561
CTATGGTC -0.315300434572
CTATGTAA 0.550380244171
CTATGTAC -0.444910679994
CTATGTCA 1.33870451745
CTATGTCC 0.293194347652
CTATGTGA -0.123056886101
CTATGTGC -0.67778756552
CTATGTTA 1.78136862264
CTATGTTC -0.720472225324
CTATTAAA -0.0572995502435
CTATTAAC 0.727312320254
CTATTAAG -0.753521575434
CTATTACA 1.74513669024
CTATTACC 0.132067755168
CTATTAGA 2.96149031327
CTATTAGC -0.179429577213
CTATTATA 0.752777944799
CTATTATC 0.0287849094058
CTATTCAA 1.03166896887
CTATTCAC -0.129797917407
CTATTCAG 0.67678229191

CTATTCCA 0.385942469655
CTATTCCC 0.352682184599
CTATTCGA 0.998284527559
CTATTCGC 1.79691324741
CTATTCTA -0.360757046179
CTATTCTC 0.287639466019
CTATTGAA 0.464544619799
CTATTGAC 0.473814256643
CTATTGAG -0.175161477167
CTATTGCA 0.957946551705
CTATTGCC -0.365424798682
CTATTGGA 0.653214820499
CTATTGGC -0.633372559564
CTATTGTA 1.77018959325
CTATTGTC 0.223305091559
CTATTTAA -0.427526451568
CTATTTAC 0.247666901633
CTATTTCA -0.339121968891
CTATTTCC 0.671884262223
CTATTTGA 0.972078408958
CTATTTGC 1.83265718037
CTATTTTA 0.927589432012
CTATTTTC 2.40899941273
CTCAAAAA 2.31555026529
CTCAAAAC 0.563475462023
CTCAAAAG -0.590964441486
CTCAAACA -0.273472060869
CTCAAACC -1.04362665403
CTCAAAGA 0.561427798617
CTCAAAGC 0.957054979088
CTCAAATA -0.21181920018
CTCAAATC 2.99616179755
CTCAACAA 0.143489869438
CTCAACAC 0.314784990068
CTCAACAG -0.257964029523
CTCAACCA -0.722241255951
CTCAACCC -0.123355383881
CTCAACGA 0.952665859364
CTCAACGC -0.691199055101
CTCAACTA -0.151631383324
CTCAACTC -0.866753911566
CTCAAGAA 1.51758780713
CTCAAGAC -0.374577859336
CTCAAGAG -0.413459155568
CTCAAGCA -0.398058029294
CTCAAGCC -0.838940296164
CTCAAGGA -0.105046910139
CTCAAGGC -0.507896586812
CTCAAGTA 0.888861044012
CTCAAGTC -0.740551559365
CTCAATAA 0.334529494287
CTCAATAC 0.767057744035
CTCAATCA 0.268258804979
CTCAATCC 0.529454556529

CTCAATGA 0.261782030465
CTCAATGC 0.0870162850314
CTCAATTA 0.791150069686
CTCAATTC -0.195578516226
CTCACAAA -0.49725966285
CTCACAAC 0.957984452036
CTCACAAG -0.498079355528
CTCACACA 0.475339418244
CTCACACC 0.316801810447
CTCACAGA 0.508924600621
CTCACAGC -1.39449119102
CTCACATA -0.277158064105
CTCACATC -0.0322137131541
CTCACCAA 0.121650130363
CTCACCAC -0.318753285426
CTCACCAG -1.03976552512
CTCACCCA -0.382299855763
CTCACCCC -1.29053816285
CTCACCGA 0.306176125893
CTCACCGC -0.394504807907
CTCACCTA 0.270026528699
CTCACCTC -0.25308795589
CTCACGAA -0.324506294307
CTCACGAC 0.208128492074
CTCACGAG 0.825603038955
CTCACGCA -0.0160558871662
CTCACGCC -0.381817345341
CTCACGGA -0.869567945806
CTCACGGC -0.874756631826
CTCACGTA 0.134864538222
CTCACGTC -0.336311332613
CTCACTAA -0.717912515376
CTCACTAC -0.0770887507149
CTCACTCA 0.0116202415184
CTCACTCC 0.0805379422275
CTCACTGA -0.353383210034
CTCACTGC -0.311668275943
CTCACTTA -0.295586511999
CTCACTTC -0.368915549868
CTCAGAAA -0.0733457662907
CTCAGAAC 0.800016917495
CTCAGAAG -0.218823965513
CTCAGACA -0.108988283193
CTCAGACC -0.690070409379
CTCAGAGA 0.348620314631
CTCAGAGC -1.17445232386
CTCAGATA 1.16993957064
CTCAGATC 2.14366625193
CTCAGCAA 0.824678793639
CTCAGCAC 0.240953576776
CTCAGCAG -0.0509268056035
CTCAGCCA -0.853029025454
CTCAGCCC -0.0445085805662
CTCAGCGA -0.0181030278094

CTCAGCGC -0.358814458173
CTCAGCTA -0.273749125358
CTCAGCTC -0.31337980262
CTCAGGAA -0.00830879810572
CTCAGGAC -0.882352642325
CTCAGGAG -0.98542925801
CTCAGGCA 0.0559879374055
CTCAGGCC -0.922677026337
CTCAGGGA -0.372519217903
CTCAGGGC -0.182869358988
CTCAGGTA 0.0732568965488
CTCAGGTC -0.450659768151
CTCAGTAA 0.641165390404
CTCAGTAC -0.766834001391
CTCAGTCA -0.701463510292
CTCAGTCC -1.49306237218
CTCAGTGA -0.0467073225338
CTCAGTGC -0.488493969718
CTCAGTTA -0.00880019550218
CTCAGTTC -0.358194722414
CTCATAAA -0.255632767088
CTCATAAC -0.341806096479
CTCATAAG -0.825738696002
CTCATACA 0.90212929648
CTCATACC 0.26288819737
CTCATAGA 0.808953554189
CTCATAGC -0.077558453439
CTCATATA 0.930843110092
CTCATATC 1.78712973435
CTCATCAA 0.176407221843
CTCATCAC 0.477147133347
CTCATCAG 0.899832275033
CTCATCCA -0.403986686606
CTCATCCC -1.16679227487
CTCATCGA -0.0907072545183
CTCATCGC -0.0485751554035
CTCATCTA -0.35216569457
CTCATCTC -0.750866461203
CTCATGAA 0.247368403853
CTCATGAC 0.0413432494645
CTCATGAG -0.868902991031
CTCATGCA -0.0555117001415
CTCATGCC 0.247677356897
CTCATGGA -0.816474025408
CTCATGGC -0.848921413707
CTCATGTA 1.2125539188
CTCATGTC -0.0253796300009
CTCATTAA 0.583641574753
CTCATTAC 0.680103929205
CTCATTCA -0.884815902466
CTCATTCC 0.151069151515
CTCATTGA 0.866769071699
CTCATTGC 0.30266943043
CTCATTTA 1.23057252035

CTCATTTC 0.0113335059099
CTCCAAAA 0.028468114914
CTCCAAAC -0.16011400019
CTCCAAAG -1.42504643796
CTCCAACA 1.11021988822
CTCCAACC -0.512244931008
CTCCAAGA -0.143629708591
CTCCAAGC -0.64208806743
CTCCAATA 0.139556860595
CTCCAATC 0.0323896229667
CTCCACAA -0.571248689243
CTCCACAC -0.715266549501
CTCCACAG -1.56190923847
CTCCACCA -1.26549127162
CTCCACCC -0.0663951069467
CTCCACGA 0.332696947933
CTCCACGC -1.02929771505
CTCCACTA 0.660686151978
CTCCACTC -0.735864464624
CTCCAGAA -0.635734403647
CTCCAGAC -1.00316948817
CTCCAGAG -1.07195362288
CTCCAGCA -0.715091423833
CTCCAGCC -0.900905507857
CTCCAGGA -0.870262698083
CTCCAGGC -0.478334589928
CTCCAGTA -0.679561823779
CTCCAGTC 0.0731732544388
CTCCATAA 0.218974521312
CTCCATAC -0.956191897065
CTCCATCA -0.330053595874
CTCCATCC -0.766639272103
CTCCATGA -0.773125979118
CTCCATGC -1.08109413722
CTCCATTA 0.701860810315
CTCCATTC -0.0679830001294
CTCCCAAA -0.342848486275
CTCCCAAC -0.76298332775
CTCCCAAG -0.854403631256
CTCCCACA -0.326224094142
CTCCCACC -0.71152251955
CTCCCAGA -1.0108530615
CTCCCAGC -1.11378042829
CTCCCATA -0.890844407546
CTCCCATC -0.80561649538
CTCCCCAA -0.627372545077
CTCCCCAC -0.375325672076
CTCCCCAG -0.760724468016
CTCCCCCA -1.16725047681
CTCCCCCC -1.23217635781
CTCCCCGA -1.00980831927
CTCCCCGC -0.772858324366
CTCCCCTA -0.621458263753
CTCCCCTC -1.34140406655

CTCCCGAA -0.0522789325886
CTCCCGAC -0.367507748603
CTCCCGAG -1.06606155899
CTCCCGCA 0.0104406263853
CTCCCGCC -0.600011381213
CTCCCGGA 0.613653670742
CTCCCGGC -0.758064910298
CTCCCGTA -0.320863157652
CTCCCGTC -1.32476555981
CTCCCTAA -0.781660088158
CTCCCTAC -1.11735168501
CTCCCTCA -0.908346519072
CTCCCTCC -0.0443094077917
CTCCCTGA -0.804728059343
CTCCCTGC -0.386744388385
CTCCCTTA -0.0944774226282
CTCCCTTC -1.48147506474
CTCCGAAA 0.275477119075
CTCCGAAC -0.894148270893
CTCCGAAG -0.564801189467
CTCCGACA -0.692586468601
CTCCGACC -1.17610268725
CTCCGAGA 0.0748492332186
CTCCGAGC -0.892273903483
CTCCGATA 0.11135744596
CTCCGATC 1.77454891548
CTCCGCAA 0.0301456619834
CTCCGCAC -0.172806429006
CTCCGCAG -0.894293076296
CTCCGCCA 0.0573837151167
CTCCGCCC -0.475728092674
CTCCGCGA 0.632126814888
CTCCGCGC 0.21456292277
CTCCGCTA -1.3694299238
CTCCGCTC -0.896239846407
CTCCGGAA -0.890194351523
CTCCGGAC -0.524370684528
CTCCGGAG 2.13818429577
CTCCGGCA -0.725805455365
CTCCGGCC -1.23561143472
CTCCGGGA 0.297253081042
CTCCGGGC -1.04966509161
CTCCGGTA -0.262876435198
CTCCGGTC -0.114535061996
CTCCGTAA -0.198817818319
CTCCGTAC -0.794556394618
CTCCGTCA -0.632985192042
CTCCGTCC -1.21834456663
CTCCGTGA -0.159432316993
CTCCGTGC -0.543511135884
CTCCGTTA 0.728649287107
CTCCGTTC 0.196377559759
CTCCTAAA 0.380916101605
CTCCTAAC -0.111832114934

CTCCTAAG -0.218997000129
CTCCTACA -0.797968208562
CTCCTACC -0.875329057516
CTCCTAGA 0.112367424439
CTCCTAGC -0.545724515221
CTCCTATA 1.67726686834
CTCCTATC -0.154836183046
CTCCTCAA -0.105257060941
CTCCTCAC -0.862577033697
CTCCTCAG -1.21819714741
CTCCTCCA -1.20199619346
CTCCTCCC -1.59366448831
CTCCTCGA 0.100786651541
CTCCTCGC -0.17058494684
CTCCTCTA -0.214402957235
CTCCTCTC -0.958270664881
CTCCTGAA 0.379923897075
CTCCTGAC -0.244875346211
CTCCTGCA -0.589073607036
CTCCTGCC -1.47680809638
CTCCTGGA -0.357313605061
CTCCTGGC -0.96772797371
CTCCTGTA 0.0386382113496
CTCCTGTC -0.602692633603
CTCCTTAA 0.471972561933
CTCCTTAC -0.464582781511
CTCCTTCA -1.35687655
CTCCTTCC -1.02939756282
CTCCTTGA -0.256193169225
CTCCTTGC 0.033995812861
CTCCTTTA -0.0156073563512
CTCCTTTC -0.648234716991
CTCGAAAA 1.51037158408
CTCGAAAC 0.536606218319
CTCGAAAG 0.0181383143245
CTCGAACA 0.571580905249
CTCGAACC -0.486040119315
CTCGAAGA -0.0228860495955
CTCGAAGC -0.830167284347
CTCGAATA 1.00782809231
CTCGAATC 3.02546554942
CTCGACAA -0.0985215186484
CTCGACAC -0.748449988368
CTCGACAG -0.624343916549
CTCGACCA -0.373888596073
CTCGACCC -1.15845158821
CTCGACGA -0.427537168213
CTCGACGC -1.19102522382
CTCGACTA 0.196905027815
CTCGACTC -0.25654498885
CTCGAGAA 1.13947267068
CTCGAGAC -0.54949781991
CTCGAGAG -0.767087018774
CTCGAGCA 0.478268721766

CTCGAGCC -0.650042954858
CTCGAGGA 0.998612038696
CTCGAGGC -0.653113927204
CTCGAGTA 0.986077745744
CTCGAGTC -0.298526793549
CTCGATAA 0.866753911566
CTCGATAC 1.78959613107
CTCGATCA 0.69535606797
CTCGATCC 0.0157163524758
CTCGATGA 1.84187244984
CTCGATGC 1.39841714256
CTCGATTA 1.74624337991
CTCGATTC 3.51127591432
CTCGCAAA 0.533786695066
CTCGCAAC 0.196364490679
CTCGCAAG -0.42867470091
CTCGCACA -0.222582632833
CTCGCACC -0.552986218661
CTCGCAGA -0.691185201877
CTCGCAGC -0.255912445393
CTCGCATA 0.135791397354
CTCGCATC -0.333571530746
CTCGCCAA -0.807990101634
CTCGCCAC -0.252821346664
CTCGCCAG 0.447154118214
CTCGCCCA -0.754659892275
CTCGCCCC -0.689338018153
CTCGCCGA -0.732756114709
CTCGCCGC -0.979171521271
CTCGCCTA 0.0430788232478
CTCGCCTC -0.791074007643
CTCGCGAA 0.231057146869
CTCGCGAC -0.196967759398
CTCGCGAG -0.592796726459
CTCGCGCA -0.107391764418
CTCGCGCC -0.893394969139
CTCGCGGA 0.469576738244
CTCGCGGC -1.21064949251
CTCGCGTA 0.598373302765
CTCGCGTC -0.646404523071
CTCGCTAA 0.616293363458
CTCGCTAC -1.03670945153
CTCGCTCA -0.735021770365
CTCGCTCC -1.14151615198
CTCGCTGA -1.20265199988
CTCGCTGC 0.473858168751
CTCGCTTA -0.100148096307
CTCGCTTC -0.346976224405
CTCGGAAA 0.569933155681
CTCGGAAC -0.00893297735186
CTCGGAAG -0.49561975473
CTCGGACA 0.125513873083
CTCGGACC -0.814076110666
CTCGGAGA 1.11972346159

CTCGGAGC -0.795216905905
CTCGGATA 2.21615730041
CTCGGATC 2.66545710013
CTCGGCAA -0.238055377664
CTCGGCAC -0.124050658921
CTCGGCAG 0.108709127651
CTCGGCCA -0.606893035817
CTCGGCCC -0.58587795567
CTCGGCGA -0.584068672277
CTCGGCGC -0.86615430219
CTCGGCTA -0.294846279325
CTCGGCTC -0.945248895256
CTCGGGAA -0.458038831927
CTCGGGAC -0.525442871826
CTCGGGCA -0.90033517322
CTCGGGCC -0.353383210034
CTCGGGGA -0.969352983079
CTCGGGGC -0.273659471472
CTCGGGTA -0.310747428588
CTCGGGTC -1.51171351719
CTCGGTAA 0.679789748529
CTCGGTAC -0.886925251928
CTCGGTCA 0.144247614679
CTCGGTCC -0.913435095941
CTCGGTGA -0.524206275506
CTCGGTGC -0.0995309743639
CTCGGTTA -0.280062536376
CTCGGTTC 0.802305051968
CTCGTAAA 1.21157321506
CTCGTAAC 0.395664035276
CTCGTAAG -0.0883362620804
CTCGTACA -0.3714201083
CTCGTACC 0.395013456489
CTCGTAGA -0.471814948832
CTCGTAGC -0.285034275673
CTCGTATA -0.220263132569
CTCGTATC 1.34459370614
CTCGTCAA -0.0716073173099
CTCGTCAC -0.958200353232
CTCGTCAG -0.186936979352
CTCGTCCA -0.581961413867
CTCGTCCC -0.629803655282
CTCGTCGA -0.829634065896
CTCGTCGC 0.625709373996
CTCGTCTA -0.218823965513
CTCGTCTC -0.46034003548
CTCGTGAA -0.500069253602
CTCGTGAC -0.826904196529
CTCGTGCA -0.636459998951
CTCGTGCC -0.66683123325
CTCGTGGA -0.579486391555
CTCGTGGC -1.13694798587
CTCGTGTA 0.56018205394
CTCGTGTC -0.992066520823

CTCGTTAA -0.954799255933
CTCGTTAC 0.850456246426
CTCGTTCA -0.187838223087
CTCGTTCC -0.418759974291
CTCGTTGA -0.52195865518
CTCGTTGC -0.660011526085
CTCGTTTA 1.25373145233
CTCGTTTC 0.105049262574
CTCTAAAA 1.15874538112
CTCTAAAC 0.486930123642
CTCTAAAG 0.195779518672
CTCTAACA 0.269892178559
CTCTAACC -0.616582974264
CTCTAAGA -0.381220349781
CTCTAAGC -0.485932168717
CTCTAATA 0.622726487246
CTCTAATC 1.14426484082
CTCTACAA 1.49904147613
CTCTACAC 0.840371883153
CTCTACAG -0.467779478404
CTCTACCA -1.01638128221
CTCTACCC -0.729659788348
CTCTACGA -0.103991712645
CTCTACGC -0.596513572724
CTCTACTA -0.633372559564
CTCTACTC 0.229847734235
CTCTAGAA 1.43278202624
CTCTAGAC -0.296065101698
CTCTAGAG -0.317496562723
CTCTAGCA -0.255210635814
CTCTAGCC -0.963780327498
CTCTAGGA 1.36157488415
CTCTAGGC -0.626669689971
CTCTAGTA 0.427581603084
CTCTAGTC -0.704410587763
CTCTATAA 0.0551593577529
CTCTATAC 1.29082594399
CTCTATCA 0.387000280966
CTCTATCC -0.244788567521
CTCTATGA 0.562113141156
CTCTATGC -0.114964250574
CTCTATTA 1.19912779185
CTCTATTC 0.66853517986
CTCTCAAA 0.164722157689
CTCTCAAC -0.128753959321
CTCTCAAG -0.572336298055
CTCTCACA -0.263144351332
CTCTCACC -1.35739722213
CTCTCAGA 0.569541867435
CTCTCAGC -0.458701695649
CTCTCATA 0.585760333953
CTCTCATC -0.685700631893
CTCTCCAA -0.490906783211
CTCTCCAC -0.892895468914

CTCTCCAG -0.729452774126
CTCTCCCA -1.33332371595
CTCTCCCC -0.769878312815
CTCTCCGA 0.211736342214
CTCTCCGC -1.07145830476
CTCTCCTA 0.0270352210164
CTCTCCTC -1.05274730336
CTCTCGAA 0.571521310245
CTCTCGAC -0.427537168213
CTCTCGCA 0.0789727892434
CTCTCGCC -0.464605260328
CTCTCGGA -0.571773282102
CTCTCGGC -0.647900148551
CTCTCGTA 0.514063362756
CTCTCGTC -0.445889292681
CTCTCTAA 0.566689671483
CTCTCTAC -0.747291545144
CTCTCTCA 0.40795602749
CTCTCTCC -0.793664299238
CTCTCTGA -0.25356523868
CTCTCTGC -0.0380597738824
CTCTCTTA -0.974706600885
CTCTCTTC -1.12065554836
CTCTGAAA 0.375496615638
CTCTGAAC -0.535822334919
CTCTGAAG -0.245525663616
CTCTGACA -0.489747294461
CTCTGACC -1.50002714613
CTCTGAGA 1.06015930122
CTCTGAGC 0.0778875328657
CTCTGATA 1.29883964228
CTCTGATC 1.70233989774
CTCTGCAA 0.844467994111
CTCTGCAC -0.59449570682
CTCTGCAG -0.106478497129
CTCTGCCA -0.951187485069
CTCTGCCC -0.394496443696
CTCTGCGA 0.364979665828
CTCTGCGC -0.255521418529
CTCTGCTA -0.284420290309
CTCTGCTC -1.34086927981
CTCTGGAA -0.284382912741
CTCTGGAC -0.674174226366
CTCTGGCA 0.26740460993
CTCTGGCC 0.168409206452
CTCTGGGA -0.720102108987
CTCTGGGC -0.970599773282
CTCTGGTA -1.23041778245
CTCTGGTC -1.20591900842
CTCTGTAA 1.90028679258
CTCTGTAC -0.564263004765
CTCTGTCA -0.153965259575
CTCTGTCC -0.707975048558
CTCTGTGA 0.508924600621

CTCTGTGC −0.466583918994
CTCTGTTA 0.190981336754
CTCTGTTC −0.670202533048
CTCTTAAA 0.373243506299
CTCTTAAC 0.196974293938
CTCTTAAG −0.906419091198
CTCTTACA −0.111239824243
CTCTTACC −0.0384176052844
CTCTTAGA −0.38140070308
CTCTTAGC −0.679936122222
CTCTTATA 0.958786109384
CTCTTATC 0.290219040969
CTCTTCAA 0.524165499977
CTCTTCAC −0.268608794933
CTCTTCAG 0.495535589857
CTCTTCCA −0.550907712227
CTCTTCCC −0.62264676586
CTCTTCGA −0.172488850369
CTCTTCGC −0.139167402021
CTCTTCTA −0.367783767566
CTCTTCTC 0.173663237871
CTCTTGAA −0.0437252199294
CTCTTGAC −1.23093610215
CTCTTGCA −0.19768969536
CTCTTGCC −1.08068010877
CTCTTGGA −0.390652827358
CTCTTGGC 0.355271430668
CTCTTGTA 0.440611475538
CTCTTGTC −0.270574645901
CTCTTTAA 1.46625925801
CTCTTTAC 1.81297566912
CTCTTTCA 0.43517212457
CTCTTTCC −1.21311980995
CTCTTTGA 0.154483056513
CTCTTTGC −0.169308359135
CTCTTTTA 2.32992468467
CTCTTTTC 0.196934563935
CTGAAAAA 0.19193198161
CTGAAAAC 0.457416220971
CTGAAAAG −0.266678753244
CTGAAACA −0.0360343279116
CTGAAACC 1.66786288136
CTGAAAGA 0.638927179816
CTGAAAGC −1.09405291388
CTGAAATA 1.70418682008
CTGAAATC 8.05143656077
CTGAACAA −0.0896936166973
CTGAACAC −0.514293117177
CTGAACAG 0.221814955092
CTGAACCA −0.847336918485
CTGAACCC −0.611423562983
CTGAACGA 0.818303173802
CTGAACGC −0.385929139194
CTGAACTA 0.0607380251107

CTGAACTC	0.838173402567
CTGAAGAA	0.526820875589
CTGAAGAC	-0.592691651059
CTGAAGCA	-0.619860699451
CTGAAGCC	-0.434235855701
CTGAAGGA	-0.575742622986
CTGAAGGC	-1.42282312613
CTGAAGTA	1.12037038104
CTGAAGTC	0.493129833667
CTGAATAA	1.55093748469
CTGAATAC	0.259993918981
CTGAATCA	0.669751127035
CTGAATCC	1.6686334343
CTGAATGA	-0.737388319316
CTGAATGC	-0.425408476513
CTGAATTA	1.25946721003
CTGAATTC	0.358647435335
CTGACAAA	0.131974964702
CTGACAAC	-0.0559944719453
CTGACAAG	-1.14895298109
CTGACACA	-0.241166341394
CTGACACC	-1.09987152954
CTGACAGA	0.828339704243
CTGACAGC	-0.20289641671
CTGACATA	-0.53996993805
CTGACATC	0.209739386837
CTGACCAA	-0.893433914997
CTGACCAC	-0.802534545007
CTGACCAG	-0.734113730708
CTGACCCA	-0.306080721611
CTGACCCC	-1.12583142668
CTGACCGA	-0.289068700575
CTGACCGC	-0.806648952676
CTGACCTA	-1.08077577444
CTGACCTC	-0.74594124783
CTGACGAA	-0.0197191502042
CTGACGAC	0.0497058921786
CTGACGCA	0.941557925769
CTGACGCC	-1.23710313948
CTGACGGA	0.515283492036
CTGACGGC	-1.23504397528
CTGACGTA	-0.14220779272
CTGACGTC	-0.450089172132
CTGACTAA	-0.228009698867
CTGACTAC	-0.253414682882
CTGACTCA	-0.354188788106
CTGACTCC	-0.751547883019
CTGACTGA	-0.116662969552
CTGACTGC	-0.856970921271
CTGACTTA	0.893804292715
CTGACTTC	-0.724196390274
CTGAGAAA	0.635634555878
CTGAGAAC	-0.218823965513
CTGAGAAG	-0.20697292404 8

CTGAGACA -0.185802583234
CTGAGACC -0.559421433502
CTGAGAGA 0.755017985059
CTGAGAGC -0.942929133611
CTGAGATA 2.61693448243
CTGAGATC 3.78556270161
CTGAGCAA -0.182869358988
CTGAGCAC -0.750805036529
CTGAGCAG -0.778302902966
CTGAGCCA -0.410208352685
CTGAGCCC -1.37519103551
CTGAGCGA 0.124142665242
CTGAGCGC -0.669216863057
CTGAGCTA -0.687211678887
CTGAGCTC -1.02392266396
CTGAGGAA 0.806131155739
CTGAGGAC -0.813806887624
CTGAGGCA -0.164260296413
CTGAGGCC -1.7413058816
CTGAGGGA -0.598094931367
CTGAGGGC -1.23984686207
CTGAGGTA 0.265837104512
CTGAGGTC -1.19983718149
CTGAGTAA 0.509154616423
CTGAGTAC -0.203225757518
CTGAGTCA 0.24669560763
CTGAGTCC -0.870917720357
CTGAGTGA -0.0455311053614
CTGAGTGC -0.324037375728
CTGAGTTA 0.0762884002744
CTGAGTTC 0.542734309787
CTGATAAA 0.957625313726
CTGATAAC 1.15844792887
CTGATAAG 0.736492564594
CTGATACA 1.0194815293
CTGATACC 1.98234885089
CTGATAGA -0.332344082781
CTGATAGC 0.0137434442053
CTGATATA 2.66348105528
CTGATATC 8.83496918667
CTGATCAA 0.276358236428
CTGATCAC 1.27206841667
CTGATCAG 1.90494173739
CTGATCCA -0.121793628858
CTGATCCC 1.63655537819
CTGATCGA 0.703615203573
CTGATCGC 3.25175274359
CTGATCTA 1.17074619424
CTGATCTC 2.61577813026
CTGATGAA -0.915113949918
CTGATGAC -0.701463510292
CTGATGCA -0.37216295479
CTGATGCC -0.478893946539
CTGATGGA -0.330466840174

CTGATGGC	-0.047888767338
CTGATGTA	0.367193567928
CTGATGTC	0.993573647093
CTGATTAA	0.685017119025
CTGATTAC	7.44473956115
CTGATTCA	2.07771863028
CTGATTCC	7.12210109971
CTGATTGA	1.36050112856
CTGATTGC	7.56733118801
CTGATTTA	0.832582450274
CTGATTTC	9.90204333795
CTGCAAAA	0.873739857426
CTGCAAAC	0.165928433745
CTGCAAAG	-0.265949759979
CTGCAACA	0.640827162621
CTGCAACC	-0.321803608627
CTGCAAGA	0.617193300286
CTGCAAGC	-0.663295001667
CTGCAATA	1.44433143334
CTGCAATC	6.56878005123
CTGCACAA	1.73267793657
CTGCACAC	-0.810096837281
CTGCACAG	-0.27824436601
CTGCACCA	-1.01724645529
CTGCACCC	-1.00802961752
CTGCACGA	1.03649668691
CTGCACGC	-0.881864904271
CTGCACTA	0.981176318096
CTGCACTC	-0.645076181811
CTGCAGAA	1.11081217891
CTGCAGAC	0.185194086884
CTGCAGCA	-0.0263885629532
CTGCAGCC	-1.06543868665
CTGCAGGA	-0.379664606534
CTGCAGGC	-0.52517783089
CTGCAGTA	0.256749389257
CTGCAGTC	1.14460620519
CTGCATAA	0.932318870571
CTGCATAC	0.503801259999
CTGCATCA	0.233211976729
CTGCATCC	0.932754332306
CTGCATGA	-0.889596571817
CTGCATGC	-0.0790318614836
CTGCATTA	1.49531495875
CTGCATTC	0.934470563852
CTGCCAAA	0.555129024968
CTGCCAAC	0.0861586920219
CTGCCAAG	-0.434328907548
CTGCCACA	-0.575324935199
CTGCCACC	-0.788836058436
CTGCCAGA	0.325674931414
CTGCCAGC	-0.690640744017
CTGCCATA	-0.293640264651
CTGCCATC	-1.16349573021

CTGCCCAA -0.799903477883
CTGCCCAC -0.971832448879
CTGCCCAG -0.195177034098
CTGCCCCA -0.0180591157016
CTGCCCCC -1.04446830276
CTGCCCGA -0.863647129942
CTGCCCGC -0.99917348636
CTGCCCTA 0.759606277557
CTGCCCTC -0.530248895193
CTGCCGAA -0.949298741672
CTGCCGAC -0.759815382832
CTGCCGCA -0.787535423625
CTGCCGCC -0.93206454628
CTGCCGGA -0.559461163505
CTGCCGGC -0.637737893564
CTGCCGTA -0.615139363721
CTGCCGTC -1.11768729898
CTGCCTAA 0.408347315737
CTGCCTAC -1.00247578142
CTGCCTCA -0.729786819803
CTGCCTCC -0.693154712186
CTGCCTGA -0.253652017369
CTGCCTGC -0.529604850946
CTGCCTTA 0.0413432494645
CTGCCTTC -0.741161624005
CTGCGAAA 0.737027089953
CTGCGAAC -0.848050490236
CTGCGAAG -0.131676989685
CTGCGACA -0.654515193929
CTGCGACC -1.05841484046
CTGCGAGA -0.521267039483
CTGCGAGC -0.00696581947645
CTGCGATA 2.30456335138
CTGCGATC 2.90852054913
CTGCGCAA 1.20877773891
CTGCGCAC -0.349355319673
CTGCGCAG 0.4395654264
CTGCGCCA 0.0335023244118
CTGCGCCC 0.455494804874
CTGCGCGA 0.991536961714
CTGCGCGC -0.674481088358
CTGCGCTA -0.15109947178
CTGCGCTC -0.421586554847
CTGCGGAA -0.365945209435
CTGCGGAC -0.74508522311
CTGCGGCA -0.918213151477
CTGCGGCC -1.45709469657
CTGCGGGA -1.1813193411
CTGCGGGC -0.382824971385
CTGCGGTA -0.228214099273
CTGCGGTC -1.7314235663
CTGCGTAA 1.16786812151
CTGCGTAC -0.541718319532
CTGCGTCA -0.804900309813

CTGCGTCC -1.52346654055
CTGCGTGA 1.19062870794
CTGCGTGC -1.47670824861
CTGCGTTA 0.118079135028
CTGCGTTC -0.425320652297
CTGCTAAA 0.263054697445
CTGCTAAC -0.257271629681
CTGCTAAG -0.284420290309
CTGCTACA -0.0181607931416
CTGCTACC 0.0273572431401
CTGCTAGA 1.57487925454
CTGCTAGC -0.718252050066
CTGCTATA 0.463479228422
CTGCTATC 0.179349594445
CTGCTCAA -0.63358009655
CTGCTCAC 0.540592549007
CTGCTCCA -0.143629708591
CTGCTCCC -1.39138231835
CTGCTCGA -0.150923561968
CTGCTCGC -0.607032352206
CTGCTCTA -0.48115228351
CTGCTCTC -0.25356523868
CTGCTGAA -0.576845391931
CTGCTGAC -0.180787715975
CTGCTGCA -1.17978633805
CTGCTGCC -0.431603220287
CTGCTGGA -0.797287832273
CTGCTGGC -1.45058707903
CTGCTGTA 0.202360061679
CTGCTGTC -0.347919027814
CTGCTTAA -0.189098866515
CTGCTTAC 0.7054694446
CTGCTTCA -0.555063679569
CTGCTTCC -0.78508784638
CTGCTTGA -0.273300855925
CTGCTTGC -0.819556759926
CTGCTTTA -0.355414667782
CTGCTTTC -0.814373040156
CTGGAAAA 2.18943782836
CTGGAAAC -0.236743242062
CTGGAAAG -0.0941598439916
CTGGAACA -0.515844416937
CTGGAACC -0.437205150607
CTGGAAGA 1.19269231563
CTGGAAGC -0.702391937714
CTGGAATA 3.02584559826
CTGGAATC 4.9510752522
CTGGACAA 0.152586994431
CTGGACAC -0.247548495771
CTGGACAG -1.0851842364
CTGGACCA -0.699969453102
CTGGACCC -1.19837344457
CTGGACGA -0.390304928457
CTGGACGC -0.191808086735

CTGGACTA 0.256671497542
CTGGACTC -0.641660969906
CTGGAGAA 0.506135659014
CTGGAGAC -0.921243871058
CTGGAGCA -0.606555330797
CTGGAGCC -1.3040027129
CTGGAGGA -0.941879163748
CTGGAGGC -0.900896882265
CTGGAGTA 0.345321156153
CTGGAGTC -1.15351173747
CTGGATAA 0.67687743481
CTGGATAC 5.44139732479
CTGGATCA 1.26182539477
CTGGATCC 1.47836383963
CTGGATGA -0.400336231266
CTGGATGC -0.552262191646
CTGGATTA 3.21923007739
CTGGATTC 6.7704926707
CTGGCAAA -0.501665772378
CTGGCAAC -0.973392374231
CTGGCAAG 1.31020660503
CTGGCACA -1.24390245888
CTGGCACC -1.54698931571
CTGGCAGA -0.575691392194
CTGGCAGC -1.20730981988
CTGGCATA -0.123684201926
CTGGCATC 0.284684285719
CTGGCCAA -0.703957613461
CTGGCCAC -0.373263894063
CTGGCCAG -0.0521715047535
CTGGCCCA -0.853311056194
CTGGCCCC -0.515638971004
CTGGCCGA 0.588782166559
CTGGCCGC -0.781323951428
CTGGCCTA -1.14178798884
CTGGCCTC -0.1735761978
CTGGCGAA -0.41950099111
CTGGCGAC -0.681188662819
CTGGCGCA 0.128193557184
CTGGCGCC -0.672037170455
CTGGCGGA 0.331161069687
CTGGCGGC -0.886171427411
CTGGCGTA 0.554048996222
CTGGCGTC -1.08645298266
CTGGCTAA -0.87815694498
CTGGCTAC -0.371355285665
CTGGCTCA -0.150468757994
CTGGCTCC -1.72315815754
CTGGCTGA -0.357778602917
CTGGCTGC 0.0313668367899
CTGGCTTA -0.343574604343
CTGGCTTC -1.40897878863
CTGGGAAA 0.813777090122
CTGGGAAC -1.45133985802

CTGGGAAG -0.27817562265
CTGGGACA -0.952266468288
CTGGGACC -1.22296474768
CTGGGAGA -0.835236257597
CTGGGAGC -0.349747130682
CTGGGATA 1.42202251431
CTGGGATC 1.11889200674
CTGGGCAA -0.43391592463
CTGGGCAC -1.78350541717
CTGGGCCA -0.358822299621
CTGGGCCC -1.21979601862
CTGGGCGA -0.112465181155
CTGGGCGC -1.26602396731
CTGGGCTA -0.437598529906
CTGGGCTC -1.86116424114
CTGGGGAA -0.466887121643
CTGGGGAC -1.32506196654
CTGGGGCA -0.571987615009
CTGGGGCC -1.25751965577
CTGGGGGA 0.567657044762
CTGGGGGC -1.49828660609
CTGGGGTA -0.476149439803
CTGGGGTC -1.32353523665
CTGGGTAA 1.76112853892
CTGGGTAC -0.367515590051
CTGGGTCA -0.897129589353
CTGGGTCC -0.84518783902
CTGGGTGA -0.82727169905
CTGGGTGC -0.853392607251
CTGGGTTA 0.63486086636
CTGGGTTC 0.865043953179
CTGGTAAA 0.48811392088
CTGGTAAC 0.0100114378081
CTGGTAAG -0.175784088123
CTGGTACA -1.07004344619
CTGGTACC -1.01373139561
CTGGTAGA 0.900822388511
CTGGTAGC -0.954904331334
CTGGTATA 0.786249426183
CTGGTATC 1.66616599205
CTGGTCAA -0.102311551759
CTGGTCAC -0.832345115787
CTGGTCCA -0.699969453102
CTGGTCCC 0.44115619478
CTGGTCGA -0.603275775939
CTGGTCGC -0.460030559673
CTGGTCTA -0.946423544139
CTGGTCTC 0.92169527707
CTGGTGAA 0.464999423772
CTGGTGAC -0.537717874237
CTGGTGCA -0.757100150835
CTGGTGCC -0.0815860824189
CTGGTGGA -1.18407796244
CTGGTGGC -0.084050910849

CTGGTGTA −0.713861362052
CTGGTGTC −0.600862962446
CTGGTTAA −0.952847519572
CTGGTTAC 0.857767612369
CTGGTTCA 0.0562331133405
CTGGTTCC −1.45387029323
CTGGTTGA −0.106237241918
CTGGTTGC −0.245530891248
CTGGTTTA 0.44488872394
CTGGTTTC −0.0027811001587
CTGTAAAA −0.117211348136
CTGTAAAC 0.87347978274
CTGTAAAG −0.264855355246
CTGTAACA 0.0954372158409
CTGTAACC 0.147792210473
CTGTAAGA 2.13655196771
CTGTAAGC −0.514017882359
CTGTAATA 1.62966196142
CTGTAATC 12.8240284371
CTGTACAA 0.00597439909093
CTGTACAC −0.244889460817
CTGTACAG −0.496527271624
CTGTACCA −0.652649452112
CTGTACCC 0.140564748021
CTGTACGA −0.0607510941904
CTGTACGC 0.0686163277314
CTGTACTA 0.150010033297
CTGTACTC −0.284420290309
CTGTAGAA 0.838980026166
CTGTAGAC 0.382705781379
CTGTAGCA 0.560847531478
CTGTAGCC −0.916939177588
CTGTAGGA −0.322860635792
CTGTAGGC −0.521908469914
CTGTAGTA −0.125735001912
CTGTAGTC 0.0536109331909
CTGTATAA 0.988249304026
CTGTATAC 1.69779107386
CTGTATCA 1.0469414954
CTGTATCC 0.946472683879
CTGTATGA 0.592474704336
CTGTATGC −0.63847368275
CTGTATTA 0.373291861894
CTGTATTC 0.271035984414
CTGTCAAA 0.195160567058
CTGTCAAC −0.734769275745
CTGTCAAG 0.488629888147
CTGTCACA −0.31633655121
CTGTCACC −0.196643646221
CTGTCAGA −0.643096739001
CTGTCAGC −0.180157002189
CTGTCATA −0.415818385834
CTGTCATC 0.310143898488
CTGTCCAA −0.918072005416

CTGTCCAC -0.443480399912
CTGTCCCA -0.274778446075
CTGTCCCC -0.420065836734
CTGTCCGA 0.126407275371
CTGTCCGC -0.774717793025
CTGTCCTA 0.525809067439
CTGTCCTC -0.572693345312
CTGTCGAA 0.526443440568
CTGTCGAC -0.608778119871
CTGTCGCA 0.695442323896
CTGTCGCC -0.316919954927
CTGTCGGA -0.270201131603
CTGTCGGC -0.19119880624
CTGTCGTA -0.621530666454
CTGTCGTC -0.451053670213
CTGTCTAA -0.0966782556485
CTGTCTAC 0.0748492332186
CTGTCTCA -0.423370745607
CTGTCTCC -1.17213648294
CTGTCTGA -0.3050129778
CTGTCTGC -0.210496347933
CTGTCTTA 0.634613599372
CTGTCTTC -0.704596430076
CTGTGAAA 0.590564004885
CTGTGAAC 0.770083497366
CTGTGAAG 0.303570412784
CTGTGACA -0.629858022653
CTGTGACC -0.728499776835
CTGTGAGA -0.649107208752
CTGTGAGC -0.86579673217
CTGTGATA 1.29105569841
CTGTGATC 2.12174287075
CTGTGCAA 1.39657544785
CTGTGCAC 0.440218357621
CTGTGCCA -0.0901123500107
CTGTGCCC -1.17020121362
CTGTGCGA 0.568715901598
CTGTGCGC -0.199264258082
CTGTGCTA 0.0788407915385
CTGTGCTC -0.759122721609
CTGTGGAA -0.362968073081
CTGTGGAC -0.594708210055
CTGTGGCA -1.57029514415
CTGTGGCC -0.854552357383
CTGTGGGA 0.0836285181933
CTGTGGGC -1.16887339512
CTGTGGTA -0.423557372065
CTGTGGTC -0.417754700681
CTGTGTAA 1.56839359305
CTGTGTAC 0.131300077427
CTGTGTCA -0.120052043298
CTGTGTCC -0.15959907845
CTGTGTGA 0.982267324869
CTGTGTGC -0.0385226806851

CTGTGTTA 0.604982336365
CTGTGTTC 0.677144044036
CTGTTAAA 0.898591757989
CTGTTAAC -0.718790496149
CTGTTAAG 0.185324254918
CTGTTACA -0.720138179647
CTGTTACC -0.385897512021
CTGTTAGA -0.232037327846
CTGTTAGC 0.551677742402
CTGTTATA 1.71116570863
CTGTTATC 0.0912268811269
CTGTTCAA -0.535604342669
CTGTTCAC -0.623793446913
CTGTTCCA -0.295515677587
CTGTTCCC -0.135781464854
CTGTTCGA 1.1912215214
CTGTTCGC 0.881404088521
CTGTTCTA -0.234213068234
CTGTTCTC 0.0386248808883
CTGTTGAA 0.343732478826
CTGTTGAC -0.839291593026
CTGTTGCA -0.452863999132
CTGTTGCC -0.973085773621
CTGTTGGA 0.510915021458
CTGTTGGC -0.756681417522
CTGTTGTA -0.295785162011
CTGTTGTC -0.155038753781
CTGTTTAA 0.371690376868
CTGTTTAC -0.0511826981839
CTGTTTCA -0.360281070296
CTGTTTCC -0.448205394985
CTGTTTGA -0.656224106789
CTGTTTGC -0.0431049614071
CTGTTTTA 0.949621809322
CTGTTTTC 0.338460150696
CTTAAAAA 1.16652827946
CTTAAAAC 0.245949101798
CTTAAAAG -0.18084103782
CTTAAACA 0.286249961466
CTTAAACC 0.121951241959
CTTAAAGA 1.72230448526
CTTAAAGC 0.361419648519
CTTAAATA 0.737761049469
CTTAAATC 1.39217744116
CTTAACAA 0.866769071699
CTTAACAC -0.301799029722
CTTAACCA 0.947191483262
CTTAACCC 0.500063764589
CTTAACGA -0.503155386081
CTTAACGC -0.511672243935
CTTAACTA 0.945292545983
CTTAACTC 0.220800794508
CTTAAGAA 1.23937271586
CTTAAGAC -0.46817076665

CTTAAGCA 0.832326034931
CTTAAGCC -1.2698754251
CTTAAGGA -0.269161355623
CTTAAGGC -0.276428809459
CTTAAGTA -0.10669021622
CTTAAGTC 0.771316695726
CTTAATAA 0.865305857536
CTTAATAC 0.951237147572
CTTAATCA 0.846139790785
CTTAATCC 0.12650215689
CTTAATGA -0.180275408051
CTTAATGC -0.113637738985
CTTAATTA 1.1478253809
CTTAATTC -0.0343337792619
CTTACAAA 1.01099603723
CTTACAAC 0.144395556661
CTTACAAG 0.108186887226
CTTACACA 0.671026669213
CTTACACC 0.408910070308
CTTACAGA 4.65455430101
CTTACAGC -0.104244991409
CTTACATA 1.31214291988
CTTACATC 0.658578632187
CTTACCAA -0.425320652297
CTTACCAC 0.226186562249
CTTACCCA -0.278815223411
CTTACCCC 0.0670872454073
CTTACCGA 0.680737518188
CTTACCGC 0.568128054394
CTTACCTA -0.394995943922
CTTACCTC 0.112095326199
CTTACGAA 0.787429825461
CTTACGAC -0.349810123646
CTTACGCA -0.277919207307
CTTACGCC -1.11194343845
CTTACGGA 0.316916295585
CTTACGGC -0.677973146452
CTTACGTA 1.03921165753
CTTACGTC -0.293291058842
CTTACTAA 0.632126814888
CTTACTAC -0.0380707519093
CTTACTCA 0.947117250889
CTTACTCC -1.32719222653
CTTACTGA 1.71052767616
CTTACTGC 0.694811871491
CTTACTTA 1.20909662446
CTTACTTC 0.769489638385
CTTAGAAA -0.0172391616416
CTTAGAAC 0.344415207549
CTTAGAAG -0.394504807907
CTTAGACA -0.821733023076
CTTAGACC -0.120595717013
CTTAGAGA 0.00151522909962
CTTAGAGC -0.246526232357

CTTAGATA 2.17517606446
CTTAGATC 2.12928712769
CTTAGCAA 0.493841575747
CTTAGCAC -0.501709945867
CTTAGCCA -0.474043749683
CTTAGCCC -1.31279323728
CTTAGCGA 0.519408093587
CTTAGCGC -0.529764293718
CTTAGCTA 0.279089674084
CTTAGCTC -0.366720990006
CTTAGGAA 0.626819984388
CTTAGGAC -0.236065479589
CTTAGGCA -0.729969002774
CTTAGGCC -0.402408725924
CTTAGGGA -0.545724515221
CTTAGGGC -1.14725347797
CTTAGGTA 0.126003179427
CTTAGGTC -0.576071441031
CTTAGTAA 0.365141722416
CTTAGTAC 0.227628865884
CTTAGTCA -0.149578492286
CTTAGTCC -0.929624810483
CTTAGTGA -0.911933197303
CTTAGTGC -0.564823145521
CTTAGTTA 1.28917165988
CTTAGTTC 0.755461549624
CTTATAAA 0.082961995129
CTTATAAC 0.592072699444
CTTATAAG -0.309899245316
CTTATACA 1.99447172921
CTTATACC -0.30430933855
CTTATAGA 0.394857150296
CTTATAGC 0.385223931654
CTTATATA 1.31020660503
CTTATATC 1.61004056793
CTTATCAA 0.496260662398
CTTATCAC -0.357455012503
CTTATCCA -0.214096095244
CTTATCCC 0.0486274317223
CTTATCGA 0.187360417534
CTTATCGC -0.00368182113115
CTTATCTA 1.12328766101
CTTATCTC 0.671046795596
CTTATGAA 1.12543830876
CTTATGAC -0.428625038407
CTTATGCA 0.745264792265
CTTATGCC -0.259890673252
CTTATGGA -0.0897354377523
CTTATGGC 0.258964336883
CTTATGTA -0.367128222529
CTTATGTC -0.350294725121
CTTATTAA 1.15213373371
CTTATTAC 2.09303768273
CTTATTCA 1.36979847185

CTTATTCC 1.53548669591
CTTATTGA 0.00331301170221
CTTATTGC -0.197876844581
CTTATTTA 1.56492349101
CTTATTTC 0.51397214058
CTTCAAAA 0.58461678948
CTTCAAAC 0.555153594838
CTTCAAAG 0.293152526597
CTTCAACA -0.417626885082
CTTCAACC 0.0257617698911
CTTCAAGA 0.356332117176
CTTCAAGC -0.478878786406
CTTCAATA 2.33088552341
CTTCAATC 0.127929823156
CTTCACAA -0.0969273523075
CTTCACAC -0.0469227009671
CTTCACCA 0.29866976928
CTTCACCC -1.21272172578
CTTCACGA -0.814618216091
CTTCACGC -0.398586542877
CTTCACTA -0.611675012076
CTTCACTC -0.718854273258
CTTCAGAA -0.0299679224996
CTTCAGAC 0.225588521163
CTTCAGCA -0.197535741601
CTTCAGCC -0.887924775143
CTTCAGGA -0.374969147582
CTTCAGGC -1.17990683496
CTTCAGTA 0.641711939317
CTTCAGTC -0.815302513104
CTTCATAA 1.16794705875
CTTCATAC -0.609290950559
CTTCATCA -0.882530904572
CTTCATCC -0.619499470089
CTTCATGA -0.698483237359
CTTCATGC -0.311465443826
CTTCATTA -0.129453677848
CTTCATTC -0.591121793206
CTTCCAAA -0.489698416102
CTTCCAAC -0.672054160259
CTTCCAAG -0.156911552902
CTTCCACA -0.181870881299
CTTCCACC -1.07886977986
CTTCCAGA -0.564823145521
CTTCCAGC -0.260052207077
CTTCCATA 1.97545099062
CTTCCATC -0.553628171856
CTTCCCAA -1.01665808532
CTTCCCAC -0.851976964539
CTTCCCCA -0.983240448543
CTTCCCCC -1.60017027618
CTTCCCGA 0.702567063381
CTTCCCGC -1.51633474377
CTTCCCTA 0.100786651541

CTTCCCTC -0.386434912578
CTTCCGAA -0.367657520257
CTTCCGAC -1.03783443791
CTTCCGCA 0.238769472178
CTTCCGCC -0.87188587778
CTTCCGGA -0.136804512412
CTTCCGGC -0.922624227255
CTTCCGTA -0.158068427836
CTTCCGTC 0.594396120432
CTTCCTAA 0.492153312032
CTTCCTAC 0.198995557803
CTTCCTCA -0.748966217016
CTTCCTCC -1.54807404933
CTTCCTGA -0.0286003740005
CTTCCTGC -0.610638634057
CTTCCTTA -0.123857759305
CTTCCTTC -1.62385876727
CTTCGAAA 0.351176103856
CTTCGAAC 0.155003990029
CTTCGAAG 0.858498958069
CTTCGACA -0.0305034933854
CTTCGACC -1.09237327576
CTTCGAGA 0.710303697178
CTTCGAGC -1.24070785304
CTTCGATA 0.856540948549
CTTCGATC 1.14157626975
CTTCGCAA 1.00901345784
CTTCGCAC -0.48884683487
CTTCGCCA -0.301528761154
CTTCGCCC -0.821568352672
CTTCGCGA -0.624050123638
CTTCGCGC 0.0221696026467
CTTCGCTA -0.00414760313142
CTTCGCTC -1.21548400647
CTTCGGAA 0.255759014397
CTTCGGAC -0.964003024616
CTTCGGCA -0.585253253661
CTTCGGCC -1.08475870716
CTTCGGGA 0.681781214893
CTTCGGGC -1.08692346953
CTTCGGTA -0.609402299118
CTTCGGTC -1.31341610962
CTTCGTAA 0.0692703044792
CTTCGTAC 0.892574753698
CTTCGTCA -0.587671033404
CTTCGTCC -1.00273977683
CTTCGTGA 0.650052887358
CTTCGTGC -0.175725277265
CTTCGTTA -0.300590401232
CTTCGTTC -0.939127861091
CTTCTAAA -0.384473505098
CTTCTAAC -0.132352922487
CTTCTACA -0.614297453607
CTTCTACC -0.795694188696

CTTCTAGA -1.00245434813
CTTCTAGC -0.20803099674
CTTCTATA 0.526841001972
CTTCTATC -0.236605755344
CTTCTCAA 0.228225338682
CTTCTCAC -0.149183021934
CTTCTCCA -1.14126391874
CTTCTCCC -0.931378419596
CTTCTCGA 0.225267021802
CTTCTCGC -1.03869673579
CTTCTCTA -0.0829734959191
CTTCTCTC -0.444341652264
CTTCTGAA -0.548578279462
CTTCTGAC -0.241166341394
CTTCTGCA -0.630746720072
CTTCTGCC -1.47307687413
CTTCTGGA -0.501701581656
CTTCTGGC -0.407231216331
CTTCTGTA 0.543363978046
CTTCTGTC -1.32455279519
CTTCTTAA 0.220301817045
CTTCTTAC 2.03892437411
CTTCTTCA 0.399970297035
CTTCTTCC -1.36630406132
CTTCTTGA 0.541112175615
CTTCTTGC -1.20012470125
CTTCTTTA -0.179067302324
CTTCTTTC 0.31294198845
CTTGAAAA 0.772281193807
CTTGAAAC -0.349801759435
CTTGAAAG -0.286628964778
CTTGAACA -0.135502309311
CTTGAACC -0.881684550971
CTTGAAGA 0.594998082243
CTTGAAGC -1.30779300739
CTTGAATA -0.184205541696
CTTGAATC 0.690931923112
CTTGACAA 0.286150636461
CTTGACAC -0.18379464983
CTTGACCA 0.334164344201
CTTGACCC -0.805454700174
CTTGACGA -0.0285219595223
CTTGACGC -0.494727397968
CTTGACTA 0.0544321941588
CTTGACTC -1.23514669825
CTTGAGAA 1.20838488238
CTTGAGAC -0.433418254075
CTTGAGCA -0.456697421588
CTTGAGCC -1.32418424714
CTTGAGGA 0.899832275033
CTTGAGGC -1.17554960379
CTTGAGTA 1.27362781926
CTTGAGTC -0.287478977721
CTTGATAA 1.17648874786

CTTGATAC 2.71215449016
CTTGATCA -0.256564331088
CTTGATCC -0.0660273430442
CTTGATGA -0.471388374071
CTTGATGC 0.153496863759
CTTGATTA 1.46158000472
CTTGATTC 2.99667567377
CTTGCAAA 0.0750933636273
CTTGCAAC 0.529289886125
CTTGCAAG 0.844079581062
CTTGCACA 0.877168399792
CTTGCACC -0.885053236953
CTTGCAGA 1.52221086338
CTTGCAGC -1.27556152029
CTTGCATA 0.920315705018
CTTGCATC -0.810811454558
CTTGCCAA -0.0346492668457
CTTGCCAC -0.96475475808
CTTGCCCA -0.844715261098
CTTGCCCC -0.595322718183
CTTGCCGA 0.0153679308112
CTTGCCGC -0.640181550085
CTTGCCTA -0.673794961674
CTTGCCTC -0.335618148626
CTTGCGAA 0.459151271991
CTTGCGAC -0.138595499093
CTTGCGCA 1.65888912848
CTTGCGCC -1.29448946841
CTTGCGGA -0.519250480486
CTTGCGGC -0.162329993342
CTTGCGTA 1.37926022417
CTTGCGTC 0.19195655148
CTTGCTAA -0.213696704168
CTTGCTAC 0.204429158376
CTTGCTCA 0.10935996782
CTTGCTCC -0.49582154132
CTTGCTGA -0.388968745749
CTTGCTGC -1.31963960537
CTTGCTTA 0.0189057306841
CTTGCTTC -0.00199434156118
CTTGGAAA 0.378016334203
CTTGGAAC 0.375180866673
CTTGGACA 0.220301817045
CTTGGACC -0.944561723046
CTTGGAGA 1.12937157899
CTTGGAGC -0.802082877613
CTTGGATA 2.62972153138
CTTGGATC 0.906643356606
CTTGGCAA 0.318419762513
CTTGGCAC -0.974453060739
CTTGGCCA -0.773077884905
CTTGGCCC -1.77120218555
CTTGGCGA 0.0936653100161
CTTGGCGC -0.0595296580023

CTTGGCTA -0.233495575759
CTTGGCTC -1.08979709877
CTTGGGAA 0.0606671906988
CTTGGGAC -1.01920472619
CTTGGGCA -1.28054502176
CTTGGGCC -1.83280669064
CTTGGGGA 1.17569388643
CTTGGGGC -0.380729475147
CTTGGGTA -0.270322412663
CTTGGGTC -1.23517231364
CTTGGTAA 1.06707467405
CTTGGTAC -0.278293244368
CTTGGTCA -0.596229973695
CTTGGTCC -1.04743367694
CTTGGTGA -0.895375980239
CTTGGTGC -0.346273107918
CTTGGTTA 1.17424347997
CTTGGTTC -0.540006531473
CTTGTAAA 1.42464417169
CTTGTAAC 1.61749203441
CTTGTACA 0.390253697665
CTTGTACC -0.979511840106
CTTGTAGA -0.0112618873532
CTTGTAGC -0.181195732643
CTTGTATA 0.666305856246
CTTGTATC -0.115047892684
CTTGTCAA 0.950342699758
CTTGTCAC -0.0522679545616
CTTGTCCA -0.373632964874
CTTGTCCC -0.348463485675
CTTGTCGA -1.26241663993
CTTGTCGC -1.22764609203
CTTGTCTA -0.531194573799
CTTGTCTC 0.0593780566779
CTTGTGAA 1.12371972478
CTTGTGAC 0.309839650313
CTTGTGCA -0.0383932967962
CTTGTGCC -1.04892590446
CTTGTGGA -0.552510242779
CTTGTGGC -1.07427416867
CTTGTGTA -0.751937602975
CTTGTGTC -0.909385249526
CTTGTTAA 0.719112779655
CTTGTTAC 0.140255010833
CTTGTTCA -0.08112709634
CTTGTTCC -0.951845643922
CTTGTTGA -0.0875293771001
CTTGTTGC 1.62305475749
CTTGTTTA 0.214499668425
CTTGTTTC -0.22543064668
CTTTAAAA 0.997387727311
CTTTAAAC 0.485186447029
CTTTAAAG 0.597837731879
CTTTAACA -0.381889486661

CTTTAACC -0.0425482186122
CTTTAAGA -0.610796508539
CTTTAAGC 1.31646878526
CTTTAATA 0.563959017972
CTTTAATC 0.609523580177
CTTTACAA 1.22348176048
CTTTACAC 0.324634371288
CTTTACCA 0.92558358966
CTTTACCC 0.386004939856
CTTTACGA 0.728427112752
CTTTACGC 1.61796722614
CTTTACTA 0.124746979487
CTTTACTC -0.330875118223
CTTTAGAA -0.395947895687
CTTTAGAC -0.790892608816
CTTTAGCA 0.338098137188
CTTTAGCC -0.122790538257
CTTTAGGA 0.374136908587
CTTTAGGC -0.792020209012
CTTTAGTA 0.103704454273
CTTTAGTC -0.726524254749
CTTTATAA 0.1037054998
CTTTATAC 0.441724438365
CTTTATCA 0.50893427174
CTTTATCC 0.0409148450322
CTTTATGA 0.400581929964
CTTTATGC -0.160857369442
CTTTATTA 3.60425771147
CTTTATTC 1.18817956241
CTTTCAAA 0.863585966649
CTTTCAAC -0.271600045894
CTTTCACA -0.124369805847
CTTTCACC 0.701204219751
CTTTCAGA -0.274118718932
CTTTCAGC -0.398833809865
CTTTCATA 0.793517925545
CTTTCATC -0.571537515904
CTTTCCAA -0.676499477025
CTTTCCAC -0.182859426487
CTTTCCCA -0.508775090349
CTTTCCCC 0.608080231016
CTTTCCGA -0.805374717406
CTTTCCGC -0.158574723983
CTTTCCTA 0.827607313017
CTTTCCTC -0.936847045303
CTTTCGAA -0.642466025215
CTTTCGAC -0.494494245587
CTTTCGCA 0.425739124229
CTTTCGCC -0.902401656101
CTTTCGGA -0.792999344463
CTTTCGGC -1.19950130615
CTTTCGTA 0.418196696956
CTTTCGTC 0.289605316987
CTTTCTAA -0.540431799327

CTTTCTAC 0.650826576876
CTTTCTCA 0.582856907208
CTTTCTCC -0.28616370554
CTTTCTGA 0.168409206452
CTTTCTGC -0.458059742455
CTTTCTTA 0.605287107304
CTTTCTTC 0.451441037735
CTTTGAAA 0.901981354498
CTTTGAAC 0.106940619787
CTTTGACA -0.21253094226
CTTTGACC -0.349936632338
CTTTGAGA 1.58394684341
CTTTGAGC -0.876832263063
CTTTGATA 1.07124031251
CTTTGATC 0.74510325844
CTTTGCAA 1.08549318944
CTTTGCAC 0.294123297837
CTTTGCCA -0.756208316837
CTTTGCCC 0.236738798575
CTTTGCGA 0.448796901532
CTTTGCGC 1.06653178448
CTTTGCTA 1.60724561455
CTTTGCTC -0.219310658041
CTTTGGAA 0.128461211936
CTTTGGAC -0.410116346364
CTTTGGCA -0.531479479737
CTTTGGCC -0.863748546
CTTTGGGA 0.781392172024
CTTTGGGC -0.0951282627969
CTTTGGTA 0.372544310536
CTTTGGTC -0.168051113669
CTTTGTAA 1.73935283833
CTTTGTAC -0.597287262242
CTTTGTCA 0.261590699138
CTTTGTCC 0.613207492361
CTTTGTGA 1.06926269937
CTTTGTGC -1.34763958585
CTTTGTTA 0.15762146531
CTTTGTTC 0.17125904997
CTTTTAAA 1.3717604021
CTTTTAAC 1.25809129732
CTTTTACA 0.463038016292
CTTTTACC -0.426514120655
CTTTTAGA 0.850190421345
CTTTTAGC -0.236907912466
CTTTTATA 0.101818324692
CTTTTATC 0.337112205816
CTTTTCAA 0.714570228935
CTTTTCAC -0.403487447762
CTTTTCCA 0.157264418053
CTTTTCCC 0.337427432018
CTTTTCGA 0.603404114302
CTTTTCGC 0.407545658388
CTTTTCTA 0.67845330444

CTTTTCTC 0.577955740942
CTTTTGAA 0.0641895690577
CTTTTGAC 0.329272849053
CTTTTGCA 0.794922590231
CTTTTGCC -0.0511659697619
CTTTTGGA 0.563865966124
CTTTTGGC -0.0737545671035
CTTTTGTA 0.608976769883
CTTTTGTC -0.747221494877
CTTTTTAA 0.997387727311
CTTTTTAC 1.25966481451
CTTTTTCA -0.267949329172
CTTTTTCC -0.303544797388
CTTTTTGA 0.190852998391
CTTTTTGC -0.929600763377
CTTTTTTA 0.109523592697
CTTTTTTC 1.93873445675
GAAAAAAA -0.0602113411991
GAAAAAAC -0.0763035604069
GAAAAACA -0.28795939709
GAAAAACC 0.50399494376
GAAAAAGA -0.0688272626776
GAAAAAGC 0.376198425218
GAAAAATA 2.11113731258
GAAAAATC 9.10605953845
GAAAACAA 0.758638904279
GAAAACAC 0.637774748368
GAAAACCA 1.04826879114
GAAAACCC 0.453224705732
GAAAACGA 0.386152097694
GAAAACGC 0.36342705916
GAAAACTA 0.833621703492
GAAAACTC -0.354082144416
GAAAAGAA 1.1693522462
GAAAAGAC 0.856578326117
GAAAAGCA 0.153464975205
GAAAAGCC 0.209387567212
GAAAAGGA 0.18374054384
GAAAAGGC -0.385939594458
GAAAAGTA 1.22745083998
GAAAAGTC -0.558646175695
GAAAATAA 1.79908741951
GAAAATAC 1.68728405655
GAAAATCA 10.8220757053
GAAAATCC 12.8353381573
GAAAATGA 0.337827607239
GAAAATGC 0.56652683075
GAAAATTA 2.91251236883
GAAAATTC 2.96305520488
GAAACAAA 0.827983702512
GAAACAAC 0.666614547909
GAAACACA 0.187576841494
GAAACACC -0.143343234364
GAAACAGA 0.563627063348

GAAACAGC -1.25316739085
GAAACATA 2.25636902896
GAAACATC 0.224789739012
GAAACCAA 0.874796361828
GAAACCAC 0.37436378781
GAAACCCA 0.863981175619
GAAACCCC -0.675051422995
GAAACCGA -0.0401769647927
GAAACCGC -0.562859646988
GAAACCTA 0.127026749749
GAAACCTC -1.23958731014
GAAACGAA 1.09094142739
GAAACGAC 0.135789306301
GAAACGCA 0.332882528864
GAAACGCC -0.630017465426
GAAACGGA -0.12476057133
GAAACGGC -0.378393769225
GAAACGTA 1.82504731665
GAAACGTC -0.760218956013
GAAACTAA 1.66004286684
GAAACTAC 0.216343454188
GAAACTCA -0.557464469509
GAAACTCC -0.491677074768
GAAACTGA 0.334493162246
GAAACTGC -0.299342304121
GAAACTTA 1.65120503238
GAAACTTC -0.378120886841
GAAAGAAA 1.30649707745
GAAAGAAC 0.209719521836
GAAAGACA 1.17601852237
GAAAGACC -0.103924798957
GAAAGAGA 0.228960343723
GAAAGAGC -0.922381665136
GAAAGATA 2.40906136017
GAAAGATC 3.91031281768
GAAAGCAA 0.887402011955
GAAAGCAC -0.426828824094
GAAAGCCA -0.458545650838
GAAAGCCC -1.35999065031
GAAAGCGA 0.127026749749
GAAAGCGC -0.42124100838
GAAAGCTA -0.173442893187
GAAAGCTC -1.16340529218
GAAAGGAA -1.03669742797
GAAAGGAC 0.351068937402
GAAAGGCA -1.04232262126
GAAAGGCC -1.75673314604
GAAAGGGA -1.11576065525
GAAAGGGC -0.898866731425
GAAAGGTA 0.90710809308
GAAAGGTC -1.00978897703
GAAAGTAA 1.87237306803
GAAAGTAC 1.33654289166
GAAAGTCA -0.662572020178

GAAAGTCC -1.23375510264
GAAAGTGA 2.4107919677
GAAAGTGC -0.027462841304
GAAAGTTA 1.68482889924
GAAAGTTC -0.00951611968777
GAAATAAA 3.32193892917
GAAATAAC 1.33047910007
GAAATACA 2.01991879566
GAAATACC 1.32983061233
GAAATAGA 0.267285681305
GAAATAGC 0.568672250872
GAAATATA 2.80541884081
GAAATATC 9.26224654158
GAAATCAA 8.34709469133
GAAATCAC 12.4417126371
GAAATCCA 19.5252928858
GAAATCCC 17.7580929752
GAAATCGA 10.0582413191
GAAATCGC 14.0519633558
GAAATCTA 27.3653010119
GAAATCTC 26.58013266
GAAATGAA 1.23142096501
GAAATGAC 0.168589036989
GAAATGCA 0.435217604967
GAAATGCC -0.374032355949
GAAATGGA 0.841276786231
GAAATGGC 0.0359629707364
GAAATGTA 0.649189282572
GAAATGTC 0.372161386501
GAAATTAA 2.12004885664
GAAATTAC 4.85288046055
GAAATTCA 1.79649268442
GAAATTCC 4.69185240892
GAAATTGA 0.743232288991
GAAATTGC 3.64974359781
GAAATTTA 2.32002930029
GAAATTTC 5.72306605106
GAACAAAA 1.01331736717
GAACAAAC -0.421766646765
GAACAACA 0.137655048118
GAACAACC -0.253392726828
GAACAAGA 0.0413523978203
GAACAAGC -0.319675962453
GAACAATA 1.42671849602
GAACAATC 2.8784067757
GAACACAA -0.428488074452
GAACACAC 0.333272510202
GAACACCA -0.328299986761
GAACACCC -0.425070771493
GAACACGA 0.665737874043
GAACACGC -0.722081290416
GAACACTA -1.03984916723
GAACACTC 0.913158554215
GAACAGAA 1.31532707045

GAACAGAC 0.0663872654989
GAACAGCA −0.52566321651
GAACAGCC −0.799037520663
GAACAGGA 1.05745112653
GAACAGGC −0.455373262433
GAACAGTA −0.317060839606
GAACAGTC −0.461324921325
GAACATAA 0.989553336798
GAACATAC 0.176035537217
GAACATCA 0.468628707203
GAACATCC 0.726660434559
GAACATGA 0.481041980477
GAACATGC 0.0185899817187
GAACATTA 0.65679156623
GAACATTC 0.0445681755696
GAACCAAA 1.52779606528
GAACCAAC −1.21307642061
GAACCACA −0.953171371366
GAACCACC −0.590246164867
GAACCAGA 0.472393386299
GAACCAGC −0.0112618873532
GAACCATA −0.476746696745
GAACCATC −0.750265022156
GAACCCAA −0.164673540713
GAACCCAC −0.156116691475
GAACCCCA −0.361506427208
GAACCCCC −0.947000151935
GAACCCGA −0.29391889743
GAACCCGC −0.55522782721
GAACCCTA −0.634799180304
GAACCCTC −0.619499470089
GAACCGAA −0.0342660814291
GAACCGAC 0.153173273346
GAACCGCA 0.0717811360699
GAACCGCC −0.667471356773
GAACCGGA −0.057173041552
GAACCGGC −0.874025024745
GAACCGTA 1.66278371423
GAACCGTC −0.820579023339
GAACCTAA −0.566788996488
GAACCTAC −0.568170920975
GAACCTCA −0.637597531648
GAACCTCC −1.26710216639
GAACCTGA 0.294435910223
GAACCTGC −0.672709443914
GAACCTTA −0.111618827554
GAACCTTC −1.23208905636
GAACGAAA 0.830858377281
GAACGAAC 0.146200657948
GAACGACA 0.383086875742
GAACGACC −0.460882663669
GAACGAGA 1.35189226438
GAACGAGC −1.46774024612
GAACGATA 1.43848563399

GAACGATC 1.08455221571
GAACGCAA 0.266099008869
GAACGCAC -0.228939955959
GAACGCCA -0.647053272187
GAACGCCC -1.3351450229
GAACGCGA 1.92639280203
GAACGCGC 0.319964004969
GAACGCTA -0.257942334851
GAACGCTC -1.21730374512
GAACGGAA -0.907746909695
GAACGGAC -1.50117670238
GAACGGCA -0.676543911896
GAACGGCC -0.834660433946
GAACGGGA -0.149985986191
GAACGGGC -1.39843962138
GAACGGTA -0.467258806269
GAACGGTC -1.36581240255
GAACGTAA 0.663496788257
GAACGTAC -0.49285486023
GAACGTCA -0.109410675849
GAACGTCC -1.28528648387
GAACGTGA 0.435042479299
GAACGTGC -0.258696159368
GAACGTTA 0.740264300993
GAACGTTC 0.253367895577
GAACTAAA 0.910658177888
GAACTAAC 0.681231006638
GAACTACA 0.349478430403
GAACTACC -0.150476338061
GAACTAGA 1.59181730459
GAACTAGC 0.557801913147
GAACTATA 0.682851572519
GAACTATC 1.08422601148
GAACTCAA 0.864874316525
GAACTCAC -0.923296762096
GAACTCCA -0.37728446574
GAACTCCC -0.605666633378
GAACTCGA -0.614303204003
GAACTCGC -0.106369762386
GAACTCTA -0.658373709017
GAACTCTC -0.246253872737
GAACTGAA 0.747987342947
GAACTGAC -1.0526772531
GAACTGCA -0.553656401068
GAACTGCC -1.26264561021
GAACTGGA -0.493266797622
GAACTGGC 0.0629155951692
GAACTGTA 0.902682379933
GAACTGTC -0.0409428128627
GAACTTAA 1.66566204834
GAACTTAC -0.209082796273
GAACTTCA -0.442544653806
GAACTTCC -1.18947496959
GAACTTGA 0.884561578175

GAACTTGC -0.251541099617
GAACTTTA 0.445072736582
GAAGAAAA 0.0427638584272
GAAGAAAC 0.157940612237
GAAGAACA -0.504475885893
GAAGAACC -0.368930448619
GAAGAAGA 1.82364892512
GAAGAAGC -1.11280024732
GAAGAATA 2.06948354178
GAAGAATC 9.53307655729
GAAGACAA -0.239856819608
GAAGACAC 0.368364034705
GAAGACCA -0.840689984553
GAAGACCC -1.29910494459
GAAGACGA 0.0401670322921
GAAGACGC -0.29497331078
GAAGACTA 0.737867693159
GAAGACTC 0.213814848649
GAAGAGAA -0.59109460952
GAAGAGAC -0.328751131392
GAAGAGCA -0.173625860303
GAAGAGCC -0.804345658071
GAAGAGGA 0.495535589857
GAAGAGGC -0.68969506541
GAAGAGTA 0.301401206936
GAAGAGTC 0.0781410730117
GAAGATAA 1.93271144068
GAAGATAC 2.98264000494
GAAGATCA 4.37135048063
GAAGATCC 5.66109822555
GAAGATGA -0.686011414608
GAAGATGC -0.00394163443545
GAAGATTA 4.79992585654
GAAGATTC 8.10080474793
GAAGCAAA -0.0993124593515
GAAGCAAC -0.305715571525
GAAGCACA -0.928329926067
GAAGCACC -0.976637426719
GAAGCAGA -0.196551117137
GAAGCAGC -1.06678297219
GAAGCATA 0.545159408215
GAAGCATC -0.107391764418
GAAGCCAA -0.260131928463
GAAGCCAC -0.60519144164
GAAGCCCA -1.59400219333
GAAGCCCC -1.28743347228
GAAGCCGA 0.051252748451
GAAGCCGC -0.526024184491
GAAGCCTA -0.575678845877
GAAGCCTC -0.973470788709
GAAGCGAA 1.44976294286
GAAGCGAC -0.841972845416
GAAGCGCA 0.401503038701
GAAGCGCC -1.12101050456

GAAGCGGA −0.570787873493
GAAGCGGC −1.04934881988
GAAGCGTA 0.784422891605
GAAGCGTC −0.726893586941
GAAGCTAA −0.25002299532
GAAGCTAC −0.769538778124
GAAGCTCA −1.06455181891
GAAGCTCC −0.569030343656
GAAGCTGA 0.257254901259
GAAGCTGC −1.10891036644
GAAGCTTA −0.399727734916
GAAGCTTC −0.304050832153
GAAGGAAA −1.02872528936
GAAGGAAC 0.150953098088
GAAGGACA 0.327136315905
GAAGGACC −1.39904550392
GAAGGAGA −1.09990106566
GAAGGAGC −0.800392784227
GAAGGATA 0.565274812915
GAAGGATC 3.34189253867
GAAGGCAA −0.135502309311
GAAGGCAC −1.39388452434
GAAGGCCA −1.2332459313
GAAGGCCC −1.59939527976
GAAGGCGA −0.920561403716
GAAGGCGC −0.814076110666
GAAGGCTA −1.10835257812
GAAGGCTC −1.72638360641
GAAGGGAA −0.757083422413
GAAGGGAC −0.521723934509
GAAGGGCA −1.46737744847
GAAGGGCC −0.994703599723
GAAGGGGA −0.657867674252
GAAGGGGC −1.22357272127
GAAGGGTA −0.235448096265
GAAGGGTC −1.62497695773
GAAGGTAA −0.0894787610271
GAAGGTAC −0.709493675619
GAAGGTCA −0.993731260194
GAAGGTCC −1.49271212084
GAAGGTGA −0.329838478822
GAAGGTGC −0.695896605106
GAAGGTTA −0.110475544462
GAAGTAAA −0.0840624116391
GAAGTAAC −0.743125122538
GAAGTACA −0.0438177490136
GAAGTACC −0.646789015396
GAAGTAGA 0.0845768106158
GAAGTAGC 0.646170325163
GAAGTATA −0.487309126953
GAAGTATC 1.91085288213
GAAGTCAA −0.107859114708
GAAGTCAC −0.471815732977
GAAGTCCA −0.553628171856

GAAGTCCC -0.756682463048
GAAGTCGA 0.515718169627
GAAGTCGC -0.702758394708
GAAGTCTA -0.961323079134
GAAGTCTC -0.867969335978
GAAGTGAA -0.580035031521
GAAGTGAC 0.211251217976
GAAGTGCA -0.474074069948
GAAGTGCC -2.06325272734
GAAGTGGA 0.0632802224927
GAAGTGGC -1.04721490055
GAAGTGTA 1.31389888142
GAAGTGTC -0.880309422406
GAAGTTAA 2.00311117503
GAAGTTAC -0.265608656999
GAAGTTCA -0.162152776622
GAAGTTCC -0.314692983747
GAAGTTGA 0.101060579451
GAAGTTGC 0.626005780723
GAAGTTTA 0.317606342993
GAATAAAA 1.34947108668
GAATAAAC 0.302100664081
GAATAACA 0.478870422195
GAATAACC 0.263563084645
GAATAAGA 1.58010767056
GAATAAGC 0.233227136862
GAATAATA 3.71936650606
GAATAATC 3.20881558916
GAATACAA 2.7616606868
GAATACAC 1.14154725639
GAATACCA 1.02098264379
GAATACCC 0.223170741419
GAATACGA 2.86128575854
GAATACGC 2.52135376809
GAATACTA 0.165939673154
GAATACTC 1.14570688308
GAATAGAA 1.39395431323
GAATAGAC -0.794021346495
GAATAGCA 0.403904612786
GAATAGCC -0.545517500998
GAATAGGA 1.10462684488
GAATAGGC 0.141667255584
GAATAGTA 0.650523896991
GAATAGTC -0.257160019741
GAATATAA 1.7753409017
GAATATAC 2.96716621458
GAATATCA 6.38841315962
GAATATCC 11.0949442361
GAATATGA 4.28150500123
GAATATGC 2.29780010264
GAATATTA 3.01665254622
GAATATTC 6.80749437189
GAATCAAA 3.29547900904
GAATCAAC 0.250749636151

GAATCACA 4.08761370319
GAATCACC 3.53815639743
GAATCAGA 5.48295281611
GAATCAGC 1.95095038692
GAATCATA 4.96730469675
GAATCATC 2.82412644425
GAATCCAA 8.88371868359
GAATCCAC 9.77928076753
GAATCCCA 6.16608955595
GAATCCCC 5.13189198154
GAATCCGA 13.0864986853
GAATCCGC 7.08196177387
GAATCCTA 6.30176960474
GAATCCTC 9.02624353219
GAATCGAA 2.65472163531
GAATCGAC 1.53333500263
GAATCGCA 5.09348979776
GAATCGCC 4.19392622301
GAATCGGA 4.1099939794
GAATCGGC 2.67144535245
GAATCGTA 8.1828587034
GAATCGTC 4.76052153574
GAATCTAA 8.29309011745
GAATCTAC 8.61105978086
GAATCTCA 13.5588596603
GAATCTCC 12.5237362723
GAATCTGA 10.0013348868
GAATCTGC 14.8842073169
GAATCTTA 10.2031301028
GAATGAAA 0.783687102418
GAATGAAC -0.788777508959
GAATGACA 0.478695035146
GAATGACC -0.737388319316
GAATGAGA 1.30250813295
GAATGAGC -0.359985970477
GAATGATA 1.11112165472
GAATGATC -0.436354092137
GAATGCAA 1.10474995561
GAATGCAC -0.521011931047
GAATGCCA 1.08708683302
GAATGCCC -0.974003223016
GAATGCGA 2.43934189506
GAATGCGC -0.00183646707848
GAATGCTA -0.762568776542
GAATGCTC -1.23329010479
GAATGGAA 0.417185150188
GAATGGAC -0.335723224026
GAATGGCA -0.93487361427
GAATGGCC -0.884222043484
GAATGGGA -0.663332379235
GAATGGGC -0.765864537059
GAATGGTA 1.84840803522
GAATGGTC -0.410851874169
GAATGTAA 0.703994729647

GAATGTAC 1.03813973161
GAATGTCA 0.73626568537
GAATGTCC -0.0899570893439
GAATGTGA 0.174802338857
GAATGTGC 0.99037538191
GAATGTTA 1.4746407202
GAATTAAA 2.47378231801
GAATTAAC 0.704043869387
GAATTACA 3.83647042636
GAATTACC 2.29681181883
GAATTAGA 1.9061670943
GAATTAGC -0.183779489698
GAATTATA 2.17641632012
GAATTATC 1.65774636815
GAATTCAA 1.13113721165
GAATTCAC 1.31832537872
GAATTCCA 1.63025608178
GAATTCCC 1.85090919569
GAATTCGA 2.67046203489
GAATTCGC 2.61635865878
GAATTCTA 3.29874627897
GAATTCTC 2.58334930005
GAATTGAA 0.524407277952
GAATTGAC 0.69756997007
GAATTGCA 0.145237466775
GAATTGCC 0.266921838127
GAATTGGA 2.20895963546
GAATTGGC -0.207316902225
GAATTGTA 0.461932110768
GAATTGTC 0.508868142196
GAATTTAA 2.47003149214
GAATTTAC 3.02500081295
GAATTTCA 1.03324928199
GAATTTCC 5.10959665434
GAATTTGA 3.32449628669
GAATTTGC 3.73187413809
GAATTTTA 2.80438324694
GACAAAAA 0.989644297592
GACAAAAC 0.0907976925498
GACAAACA -0.00525952043171
GACAAACC 0.168315109079
GACAAAGA 0.0139630047442
GACAAAGC -1.03972213578
GACAAATA 2.50988199132
GACAAATC 3.5460454167
GACAACAA 0.643009960312
GACAACAC -0.190274299542
GACAACCA -0.308815557228
GACAACCC -0.868987940049
GACAACGA -0.791183265149
GACAACGC -0.440433213291
GACAACTA 0.331455646743
GACAACTC -0.449209623068
GACAAGAA -0.681540743826

GACAAGAC -1.00442255153
GACAAGCA -0.621771921666
GACAAGCC -1.16199069499
GACAAGGA 0.251161573543
GACAAGGC -1.44155529943
GACAAGTA -0.701902892751
GACAAGTC -0.739441994499
GACAATAA 0.831759359635
GACAATAC 0.987383608187
GACAATCA 3.06342782797
GACAATCC 6.25939389798
GACAATGA -0.486040119315
GACAATGC -0.991622172113
GACAATTA 1.74639733367
GACACAAA 0.878609135138
GACACAAC -0.226769443204
GACACACA 0.155877527316
GACACACC -0.114473114559
GACACAGA -0.147523248813
GACACAGC -0.309001399541
GACACATA 0.83428718103
GACACATC 0.368364034705
GACACCAA 0.103446993403
GACACCAC -0.526487875439
GACACCCA -1.29338722223
GACACCCC -1.044274619
GACACCGA -0.457454121302
GACACCGC -1.05466688979
GACACCTA -0.453166156255
GACACCTC -1.6704270348
GACACGAA 0.372075653338
GACACGAC -0.388156894519
GACACGCA -0.369545218128
GACACGCC -0.481529718531
GACACGGA -0.751441239328
GACACGGC -1.08252389454
GACACGTA 0.670899114995
GACACGTC -0.917034320488
GACACTAA 0.980962507952
GACACTAC -0.840589091258
GACACTCA 0.41980105718
GACACTCC -0.338207133313
GACACTGA 0.216059071014
GACACTGC -0.61423550617
GACACTTA 0.140482674201
GACAGAAA -0.0664667255034
GACAGAAC -0.738422344901
GACAGACA -0.222870152586
GACAGACC -0.429865032688
GACAGAGA 0.731248727058
GACAGAGC -0.185715543164
GACAGATA 1.29196530636
GACAGATC 2.154966301
GACAGCAA -0.284123883582

GACAGCAC 0.255529259976
GACAGCCA -0.282426471511
GACAGCCC -0.809523366064
GACAGCGA -0.501792019687
GACAGCGC -0.663493128915
GACAGCTA -0.5338841904
GACAGCTC -0.716610050893
GACAGGAA -0.0901123500107
GACAGGAC -0.212367840145
GACAGGCA -1.29390789436
GACAGGCC -1.31289047124
GACAGGGA -0.00297243148541
GACAGGGC -1.5511758647
GACAGGTA 0.310143898488
GACAGGTC -0.345126688247
GACAGTAA -0.316734896759
GACAGTAC 0.196084812374
GACAGTCA -0.676223196681
GACAGTCC -1.01712491284
GACAGTGA -0.755284594284
GACAGTGC -0.924983457521
GACAGTTA 1.1516141071
GACATAAA 0.343515270721
GACATAAC 1.1463966691
GACATACA 0.198725027853
GACATACC -0.305128508465
GACATAGA 0.92313679656
GACATAGC -0.942451066676
GACATATA 1.63006710289
GACATATC 2.23025648497
GACATCAA 1.78799020256
GACATCAC -0.505824092154
GACATCCA 1.36110282899
GACATCCC 0.0583142335909
GACATCGA 0.916629701781
GACATCGC -0.527812034593
GACATCTA 0.505310738704
GACATCTC 0.259412867698
GACATGAA 0.300337645231
GACATGAC -0.0817805503247
GACATGCA 0.344533874792
GACATGCC -0.629999691477
GACATGGA 0.155208390436
GACATGGC -0.388109323068
GACATGTA 0.326268529013
GACATGTC -0.599311924068
GACATTAA -1.13045526708
GACATTAC 1.49075646375
GACATTCA 0.671717762147
GACATTCC 1.23518459858
GACATTGA -0.152438268304
GACATTGC -0.126488042284
GACATTTA -0.0262325181417
GACCAAAA 0.185226498202

GACCAAAC 0.288255281055
GACCAACA −0.788756075668
GACCAACC −0.491422750477
GACCAAGA −0.287740097933
GACCAAGC −0.556585965972
GACCAATA −0.475576229968
GACCAATC −0.0260291632617
GACCACAA 0.571957033362
GACCACAC −0.0954000996545
GACCACCA −1.30862106428
GACCACCC −0.895203207006
GACCACGA −0.320358168413
GACCACGC −1.09420137862
GACCACTA −0.663502277271
GACCACTC −0.362789810834
GACCAGAA −0.473663439464
GACCAGAC −1.09589748379
GACCAGCA −1.40236635707
GACCAGCC −1.07285382109
GACCAGGA −0.970520313277
GACCAGGC −0.754070738163
GACCAGTA −0.396728381126
GACCAGTC −0.737388319316
GACCATAA 0.795641912377
GACCATAC 0.258153531179
GACCATCA −0.887962936856
GACCATCC −0.690953356403
GACCATGA −0.814076110666
GACCATGC −1.07948716318
GACCATTA 0.515329495197
GACCCAAA 0.0247010833832
GACCCAAC −1.19873781051
GACCCACA −1.41566257736
GACCCACC −0.448583091388
GACCCAGA 0.278849987163
GACCCAGC −1.24059127685
GACCCATA −0.380946683252
GACCCATC −0.790350503391
GACCCCAA −0.527695197021
GACCCCAC −1.25717175687
GACCCCCA −0.824402251913
GACCCCCC −1.38211686361
GACCCCGA −0.759122721609
GACCCCGC −0.815092100921
GACCCCTA −0.968841720682
GACCCCTC −1.12729019736
GACCCGAA −0.585096686086
GACCCGAC −1.06498153025
GACCCGCA −1.4099945175
GACCCGCC −1.33493121276
GACCCGGA −0.35087185568
GACCCGGC −1.55363964761
GACCCGTA −0.730031472975
GACCCGTC −1.52688436628

GACCCTAA -0.624343916549
GACCCTAC -0.471632765861
GACCCTCA -1.36360738742
GACCCTCC -0.787171057683
GACCCTGA -1.28528648387
GACCCTGC -0.791432361808
GACCCTTA -1.15732059006
GACCGAAA 0.539168542083
GACCGAAC -0.24877829617
GACCGACA -0.442911894946
GACCGACC -1.32831616738
GACCGAGA 0.0670469926418
GACCGAGC -1.12197160468
GACCGATA -0.289068700575
GACCGATC -0.0510415521232
GACCGCAA 0.112097678634
GACCGCAC -1.3514432108
GACCGCCA -0.999263401628
GACCGCCC -0.873023671858
GACCGCGA -0.183727213379
GACCGCGC 0.393037934402
GACCGCTA -0.466887121643
GACCGCTC -0.836116329423
GACCGGAA -1.15302086284
GACCGGAC -0.716883717422
GACCGGCA 0.224046108377
GACCGGCC -1.31552937981
GACCGGGA -0.681457363098
GACCGGGC -1.66938307671
GACCGGTA 0.024243665594
GACCGGTC -0.841569533616
GACCGTAA 0.0586895775596
GACCGTAC 0.448761876398
GACCGTCA -1.27282877573
GACCGTCC -0.951586092
GACCGTGA 0.132603587435
GACCGTGC -0.0737545671035
GACCGTTA 0.369902004003
GACCTAAA 0.163888089023
GACCTAAC -0.0287274054551
GACCTACA -1.03427625027
GACCTACC -0.1568391502
GACCTAGA -0.455518067836
GACCTAGC -0.0112198049166
GACCTATA 0.139217848668
GACCTATC -0.409214841247
GACCTCAA 0.129279336325
GACCTCAC -0.346669101033
GACCTCCA -0.371138861705
GACCTCCC -1.24210310799
GACCTCGA 0.592717005073
GACCTCGC -1.69370358849
GACCTCTA -0.534653436431
GACCTCTC -1.27974362579

GACCTGAA	-1.09826560103
GACCTGAC	-1.21237827037
GACCTGCA	0.129213990926
GACCTGCC	-1.48673353964
GACCTGGA	0.306351251561
GACCTGGC	-1.09045630315
GACCTGTA	-0.592621600792
GACCTTAA	-0.449650051054
GACCTTAC	-0.972815243672
GACCTTCA	-1.41356054659
GACCTTCC	-0.934296222329
GACCTTGA	-0.0304028614718
GACCTTGC	-0.397950340077
GACCTTTA	-0.463980819701
GACGAAAA	1.49075960033
GACGAAAC	-0.116730406003
GACGAACA	-0.0246634444337
GACGAACC	-0.183905475626
GACGAAGA	-0.414430972334
GACGAAGC	-1.41963662312
GACGAATA	1.9107909347
GACGAATC	2.4684046531
GACGACAA	-0.0120700792414
GACGACAC	-1.41139996633
GACGACCA	-0.295945650309
GACGACCC	-1.10943234548
GACGACGA	-0.681413189608
GACGACGC	-0.74233496598
GACGACTA	-0.404194223592
GACGACTC	-1.24994141922
GACGAGAA	0.586390786357
GACGAGAC	-0.453895410901
GACGAGCA	-1.14423765714
GACGAGCC	-1.29061474766
GACGAGGA	-0.735101753133
GACGAGGC	-1.34991151466
GACGAGTA	0.652722116195
GACGAGTC	-0.87004418307
GACGATAA	0.316230953046
GACGATAC	1.90140236923
GACGATCA	0.293598443596
GACGATCC	4.00946870946
GACGATGA	-0.48243148503
GACGATGC	0.320628175599
GACGATTA	1.9562959019
GACGCAAA	0.59640300831
GACGCAAC	0.647721363541
GACGCACA	-0.452076979153
GACGCACC	-0.756611890018
GACGCAGA	-0.727156536824
GACGCAGC	-0.735373067227
GACGCATA	-0.397691049536
GACGCATC	-0.209176632266
GACGCCAA	-0.970412624061

GACGCCAC −1.17774285675
GACGCCCA −0.671506565819
GACGCCCC −1.17853039949
GACGCCGA −0.761473064901
GACGCCGC −0.927557804839
GACGCCTA −1.31119802542
GACGCCTC −0.907369997437
GACGCGAA 0.508868142196
GACGCGAC −0.543817475112
GACGCGCA 0.0748565519033
GACGCGCC −0.999404024926
GACGCGGA −0.401862699774
GACGCGGC −0.973795947412
GACGCGTA −0.00211353156798
GACGCGTC 0.0112482955103
GACGCTAA −0.351404551369
GACGCTAC −1.12757771711
GACGCTCA −0.600764160204
GACGCTCC −0.789719528223
GACGCTGA 0.180083031197
GACGCTGC −1.13907380237
GACGCTTA −0.825714910277
GACGGAAA −0.131081301033
GACGGAAC 0.0650887217406
GACGGACA −0.596103465003
GACGGACC −1.31189617565
GACGGAGA −0.559547419431
GACGGAGC −0.921772123259
GACGGATA 2.3133959582
GACGGATC 0.776823221764
GACGGCAA −0.246805649281
GACGGCAC −0.853950656954
GACGGCCA −1.42124516545
GACGGCCC −0.403751443172
GACGGCGA −0.559393988435
GACGGCGC −1.00779254442
GACGGCTA −0.575565406266
GACGGCTC −0.57306032507
GACGGGAA −0.350748222186
GACGGGAC −1.04983185307
GACGGGCA −0.45256523997
GACGGGCC −0.998717375479
GACGGGGA 0.0854751791539
GACGGGGC −1.02654850345
GACGGGTA −0.36498175688
GACGGTAA 0.147036817667
GACGGTAC −0.61269727549
GACGGTCA −1.0245227961
GACGGTCC −1.25417580104
GACGGTGA 0.623115423058
GACGGTGC −0.896310419437
GACGGTTA −0.00468291263565
GACGTAAA 1.36946730137
GACGTAAC 0.470539668036

GACGTACA 0.809056015774
GACGTACC -0.483571631542
GACGTAGA 0.224060745747
GACGTAGC 0.481763393676
GACGTATA 0.552995105636
GACGTATC 2.56444121693
GACGTCAA 0.577483685783
GACGTCAC -0.0530113238146
GACGTCCA -1.01307114571
GACGTCCC -1.34906542244
GACGTCGA 0.142469697077
GACGTCGC -0.700252790749
GACGTCTA 0.211640676551
GACGTCTC -0.989011492754
GACGTGAA 2.03417402503
GACGTGAC -1.18867200533
GACGTGCA 0.295260830533
GACGTGCC -1.51654750838
GACGTGGA 0.285095177585
GACGTGGC -1.27560098891
GACGTGTA -0.454358317704
GACGTTAA -0.425139776234
GACGTTAC 0.523264779005
GACGTTCA -0.371975544187
GACGTTCC -1.53892334111
GACGTTGA -1.00238638891
GACGTTGC -0.370264278892
GACGTTTA -0.295404067647
GACTAAAA 1.86294817052
GACTAAAC 0.071341492229
GACTAACA -0.352385777872
GACTAACC -0.411052876615
GACTAAGA 0.873769916309
GACTAAGC -1.16422916696
GACTAATA 0.947999152387
GACTAATC 0.336957990676
GACTACAA -0.55250213995
GACTACAC -0.570226948593
GACTACCA 0.266848389899
GACTACCC -0.480279791749
GACTACGA 0.358541314407
GACTACGC -0.58947430502
GACTACTA 0.373243506299
GACTACTC 0.00893088629911
GACTAGAA 0.624689463016
GACTAGAC -1.16250143463
GACTAGCA -0.406595797676
GACTAGCC -0.571311159444
GACTAGGA 0.902066564897
GACTAGGC -0.293710837682
GACTAGTA -0.139626910863
GACTAGTC -0.105629006949
GACTATAA 0.361927774338
GACTATAC -0.016789062537

GACTATCA	-0.0375694220123
GACTATCC	0.0866615902085
GACTATGA	0.212161348686
GACTATGC	-1.40470598371
GACTATTA	0.63682227384
GACTCAAA	0.180572860304
GACTCAAC	-0.60096202607
GACTCACA	-0.419808114483
GACTCACC	-0.771860369441
GACTCAGA	-0.0149123426931
GACTCAGC	-0.402215042163
GACTCATA	0.0234924548932
GACTCATC	-0.856666150333
GACTCCAA	-0.217868615788
GACTCCAC	-0.753044554025
GACTCCCA	-1.08612416461
GACTCCCC	-1.7462407661
GACTCCGA	1.08434154214
GACTCCGC	-0.316919954927
GACTCCTA	-0.678689332019
GACTCCTC	-1.21819714741
GACTCGAA	-0.923671844683
GACTCGAC	-0.624215055423
GACTCGCA	-0.350138157546
GACTCGCC	-0.457749221121
GACTCGGA	-0.400924078471
GACTCGGC	-1.40963250399
GACTCGTA	-1.36311808108
GACTCTAA	-0.912373102525
GACTCTAC	-0.726420747637
GACTCTCA	0.155210481489
GACTCTCC	-1.24274453842
GACTCTGA	0.877766440879
GACTCTGC	-1.30724070808
GACTCTTA	-0.757151381628
GACTGAAA	0.567385207904
GACTGAAC	-0.590530809422
GACTGACA	0.0090764758469
GACTGACC	-0.962838308234
GACTGAGA	-0.393238414085
GACTGAGC	-0.548486795905
GACTGATA	0.326043740842
GACTGATC	0.0187311277794
GACTGCAA	0.176422120594
GACTGCAC	-0.959364024088
GACTGCCA	-0.415123110794
GACTGCCC	-1.52212852818
GACTGCGA	0.385367952912
GACTGCGC	-0.759849885203
GACTGCTA	-0.232742012624
GACTGCTC	-1.02902953754
GACTGGAA	1.15769750231
GACTGGAC	-1.09194879205
GACTGGCA	-0.776102069946

GACTGGCC -1.15733653433
GACTGGGA -0.556637196764
GACTGGGC -1.38982134747
GACTGGTA -0.862962310166
GACTGTAA -0.295404067647
GACTGTAC -0.623793446913
GACTGTCA 0.10932389716
GACTGTCC -0.398878506117
GACTGTGA -1.16250143463
GACTGTGC -0.281205296705
GACTGTTA 0.0662853266773
GACTTAAA 0.286249961466
GACTTAAC 0.724842786955
GACTTACA -0.314756760856
GACTTACC 0.0527415780097
GACTTAGA 0.368510408397
GACTTAGC -1.24216139608
GACTTATA -0.447586966134
GACTTATC -0.298302266759
GACTTCAA -0.634392470544
GACTTCAC -0.824307631776
GACTTCCA -0.422690107937
GACTTCCC -1.26649523833
GACTTCGA 0.629914481078
GACTTCGC -0.28628002035
GACTTCTA -0.268390802684
GACTTCTC -0.19067734996
GACTTGAA 0.539560091711
GACTTGAC -0.295529008049
GACTTGCA 0.158235189293
GACTTGCC -0.694170963823
GACTTGGA 0.556189188713
GACTTGGC -0.637395222294
GACTTGTA -1.26709746152
GACTTTAA -0.281697216864
GACTTTAC 0.547679910924
GACTTTCA -1.05607521382
GACTTTCC -0.588758903597
GACTTTGA -0.728314195903
GACTTTGC -0.262185080883
GACTTTTA 0.644274263081
GAGAAAAA 1.14330687728
GAGAAAAC -0.0500707808836
GAGAAACA 0.782254208521
GAGAAACC 0.0890056603423
GAGAAAGA 0.56158253652
GAGAAAGC 0.670855202888
GAGAAATA 2.02267271213
GAGAAATC 12.4818106646
GAGAACAA 0.816207416182
GAGAACAC 0.228275523948
GAGAACCA 0.598478900928
GAGAACCC -1.0941747177
GAGAACGA -0.862218940913

GAGAACGC −1.69607144435
GAGAACTA 0.722430496225
GAGAACTC −0.432022476364
GAGAAGAA 0.197897232346
GAGAAGAC −0.685700631893
GAGAAGCA 0.00381094363852
GAGAAGCC −1.17370738632
GAGAAGGA −0.754710077541
GAGAAGGC −0.607602425462
GAGAAGTA 0.769489638385
GAGAATAA 1.17850347719
GAGAATAC 1.78377307192
GAGAATCA 13.5424582267
GAGAATCC 25.936001634
GAGAATGA 0.80181835944
GAGAATGC 0.194081061075
GAGAATTA 4.64166086974
GAGACAAA 0.407008257831
GAGACAAC −0.898392585214
GAGACACA −0.0219246880932
GAGACACC −0.803176498202
GAGACAGA 0.825212796235
GAGACAGC −1.22294645097
GAGACATA 0.379550644159
GAGACATC 0.690073545958
GAGACCAA −0.555630877628
GAGACCAC −0.791561222934
GAGACCCA −0.240215696537
GAGACCCC −1.39365686098
GAGACCGA −0.898725062601
GAGACCGC −0.665857586813
GAGACCTA −0.0957973996772
GAGACCTC −0.76830976187
GAGACGAA −0.236777483051
GAGACGAC −1.33525114383
GAGACGCA −0.415565107069
GAGACGCC −1.60398827712
GAGACGGA −0.21813130429
GAGACGGC −1.43931578194
GAGACGTA −0.575685641799
GAGACTAA 0.506445134821
GAGACTAC −0.566402935874
GAGACTCA −0.180301023447
GAGACTCC −1.10089588401
GAGACTGA −0.336563565851
GAGACTGC 0.0292360540368
GAGACTTA −0.944945953989
GAGAGAAA 1.02123722946
GAGAGAAC −0.189191134217
GAGAGACA −0.257441527717
GAGAGACC −0.925131922266
GAGAGAGA −0.189191134217
GAGAGAGC −0.823197282765
GAGAGATA 0.990197381045

GAGAGATC 3.7596563877
GAGAGCAA 0.00842798811252
GAGAGCAC -0.244005991029
GAGAGCCA -1.10706292133
GAGAGCCC -1.89420836362
GAGAGCGA 0.249395156732
GAGAGCGC -0.894002419964
GAGAGCTA -0.0639532800969
GAGAGCTC -0.886925251928
GAGAGGAA -1.02361998407
GAGAGGAC 0.563475462023
GAGAGGCA -1.31325222336
GAGAGGCC -1.04286864741
GAGAGGGA 0.076128434739
GAGAGGGC -1.44156183397
GAGAGGTA -0.669895932438
GAGAGTAA 0.275220703732
GAGAGTAC -1.12161298914
GAGAGTCA -0.535566965102
GAGAGTCC -1.33219637714
GAGAGTGA 0.47565856517
GAGAGTGC -0.186907704613
GAGAGTTA 0.637087053395
GAGATAAA 0.0633458292727
GAGATAAC 2.43894903852
GAGATACA 2.53170291092
GAGATACC 3.73557974495
GAGATAGA 0.322767061182
GAGATAGC 0.63727655505
GAGATATA 3.77753645701
GAGATATC 15.2279672408
GAGATCAA 2.74270137289
GAGATCAC 4.63870098458
GAGATCCA 6.97217000366
GAGATCCC 5.4892027114
GAGATCGA 3.54092129193
GAGATCGC 9.04034271674
GAGATCTA 11.0905694924
GAGATCTC 11.3470313619
GAGATGAA 0.287821387609
GAGATGAC -0.147518021181
GAGATGCA -0.769674435171
GAGATGCC 0.0320545317634
GAGATGGA -0.397331649845
GAGATGGC -0.331877516636
GAGATGTA 0.733300833951
GAGATTAA 2.23101266192
GAGATTAC 15.7802942551
GAGATTCA 2.47974129559
GAGATTCC 15.6087939499
GAGATTGA 0.755375032316
GAGATTGC 19.025050859
GAGATTTA 4.81818623607
GAGCAAAA -0.0732223941784

GAGCAAAC	−0.268934737781
GAGCAACA	−0.736425389524
GAGCAACC	−0.586036352916
GAGCAAGA	0.392374547917
GAGCAAGC	0.325352647908
GAGCAATA	0.771992105764
GAGCAATC	6.85217154358
GAGCACAA	0.294435910223
GAGCACAC	−0.686138707444
GAGCACCA	−1.08929367782
GAGCACCC	−1.55091291482
GAGCACGA	−0.434787893627
GAGCACGC	−0.723190332519
GAGCACTA	0.141480629126
GAGCACTC	−0.5178784885
GAGCAGAA	0.345492099715
GAGCAGAC	−0.686958138741
GAGCAGCA	−0.513322607319
GAGCAGCC	−0.97905494508
GAGCAGGA	−1.32418424714
GAGCAGGC	−1.16913948159
GAGCAGTA	0.109001352273
GAGCATAA	0.811048266282
GAGCATAC	−0.325935006099
GAGCATCA	1.40465998055
GAGCATCC	−0.791053097115
GAGCATGA	−0.396520059996
GAGCATGC	−0.217523853466
GAGCATTA	−0.145971164909
GAGCCAAA	−0.245538994077
GAGCCAAC	−1.03487298445
GAGCCACA	−1.05153344724
GAGCCACC	−1.26348830447
GAGCCAGA	−0.476679783057
GAGCCAGC	−1.17330485867
GAGCCATA	−0.108846091606
GAGCCATC	0.285331989309
GAGCCCAA	−1.03567856252
GAGCCCAC	−1.08509876462
GAGCCCCA	−1.27336800595
GAGCCCCC	−0.998948698189
GAGCCCGA	−0.931104491686
GAGCCCGC	−1.25330330928
GAGCCCTA	−1.3689769495
GAGCCCTC	−1.3157907614
GAGCCGAA	−0.291110352204
GAGCCGAC	−1.17325963965
GAGCCGCA	−1.34720333997
GAGCCGCC	−0.909445105911
GAGCCGGA	−0.698148668919
GAGCCGGC	−0.567964690898
GAGCCGTA	−0.69534456718
GAGCCTAA	−0.00536956208273
GAGCCTAC	−0.0712656915668

GAGCCTCA -0.319743398904
GAGCCTCC -1.2491180672
GAGCCTGA -0.70568430027
GAGCCTGC -1.42924135116
GAGCCTTA -0.807510989173
GAGCGAAA 0.214856192919
GAGCGAAC -0.758256764388
GAGCGACA -0.66407783954
GAGCGACC -0.442500218935
GAGCGAGA -0.0451055761266
GAGCGAGC -0.667444695851
GAGCGATA 0.0150121904619
GAGCGATC 3.72825295749
GAGCGCAA 2.20238196765
GAGCGCAC -0.62337314531
GAGCGCCA -0.938777871137
GAGCGCCC -1.37609410892
GAGCGCGA 0.525735096448
GAGCGCGC 0.375075791272
GAGCGCTA -0.649510781933
GAGCGCTC -0.56839152704
GAGCGGAA -0.0715511202673
GAGCGGAC -0.739966587358
GAGCGGCA -0.682951158907
GAGCGGCC -0.899644080285
GAGCGGGA -0.522085163872
GAGCGGGC -1.31279323728
GAGCGGTA 0.819296423858
GAGCGTAA 1.57340689202
GAGCGTAC -0.640163514755
GAGCGTCA -1.7412264216
GAGCGTCC -1.00799119443
GAGCGTGA -0.166290970016
GAGCGTGC -0.451378306153
GAGCGTTA 1.52926999608
GAGCTAAA 0.0674944779305
GAGCTAAC -0.408428866794
GAGCTACA -0.265317216522
GAGCTACC -1.20745750048
GAGCTAGA -0.155084234179
GAGCTAGC -1.16773638519
GAGCTATA 0.0353032435935
GAGCTATC 0.286028048493
GAGCTCAA -0.751441239328
GAGCTCAC -1.4754970063
GAGCTCCA -1.17376253784
GAGCTCCC -1.23359931921
GAGCTCGA 1.01094951131
GAGCTCGC -0.150590823199
GAGCTCTA -1.31214344264
GAGCTGAA -0.378222825662
GAGCTGAC -0.633224878964
GAGCTGCA -0.748386734022
GAGCTGCC -1.10791241151

GAGCTGGA 0.16000578821
GAGCTGGC −1.44482021692
GAGCTGTA −0.178364969981
GAGCTTAA −0.79327274961
GAGCTTAC −0.527105258763
GAGCTTCA −1.29292666786
GAGCTTCC −1.07692091869
GAGCTTGA −0.96545003312
GAGCTTGC −0.723622396293
GAGCTTTA −0.923218608999
GAGGAAAA 0.791593111488
GAGGAAAC −0.102928150939
GAGGAACA −0.724223051196
GAGGAACC −0.817385463026
GAGGAAGA −0.528467056867
GAGGAAGC −1.41345364151
GAGGAATA 2.75509216735
GAGGAATC 6.82466139221
GAGGACAA −0.173576459181
GAGGACAC −0.506857594976
GAGGACCA −0.968593930931
GAGGACCC −1.27336800595
GAGGACGA −0.41276414191
GAGGACGC −0.0516667768957
GAGGACTA −0.734341655458
GAGGACTC −0.980735367347
GAGGAGAA 0.992198779909
GAGGAGAC −1.71251992667
GAGGAGCA −1.31634698143
GAGGAGCC −1.42454536945
GAGGAGGA −0.983721129294
GAGGAGGC −1.39962054342
GAGGAGTA −0.0789341047675
GAGGATAA 2.72113869831
GAGGATAC 1.9887759629
GAGGATCA 1.07108322217
GAGGATCC 6.43539362592
GAGGATGA −0.140201166224
GAGGATGC −0.690854292779
GAGGATTA 3.30664967422
GAGGCAAA 0.164894669541
GAGGCAAC −1.01804785125
GAGGCACA −0.664095352107
GAGGCACC −0.909211169384
GAGGCAGA −0.214772550809
GAGGCAGC −1.55169183197
GAGGCATA 0.0388541125462
GAGGCATC −0.253909216858
GAGGCCAA −0.838589522065
GAGGCCAC −0.599820049887
GAGGCCCA −0.984229777876
GAGGCCCC −1.44803730158
GAGGCCGA −0.0882330163508
GAGGCCGC −1.0509205074

GAGGCCTA -0.503964884877
GAGGCCTC -0.875344217649
GAGGCGAA -0.0748429600604
GAGGCGAC -0.348655339764
GAGGCGCA -0.925687096772
GAGGCGCC -1.25594064956
GAGGCGGA -0.956133870351
GAGGCGGC -0.719496226453
GAGGCGTA -1.19966989727
GAGGCTAA -0.546897595814
GAGGCTAC -1.22283823899
GAGGCTCA -1.20224372183
GAGGCTCC -0.685625353994
GAGGCTGA 0.231994461265
GAGGCTGC -1.38437415505
GAGGCTTA -0.383944730133
GAGGGAAA -0.0228860495955
GAGGGAAC -0.788780645538
GAGGGACA -1.22088049085
GAGGGACC -1.11393150686
GAGGGAGA -0.80673651551
GAGGGAGC -1.1157230163
GAGGGATA 1.18941642011
GAGGGATC 1.55741399782
GAGGGCAA 0.0247243463451
GAGGGCAC -1.51321280201
GAGGGCCA -1.48722702809
GAGGGCCC -1.60176104456
GAGGGCGA -1.06080308409
GAGGGCGC -0.789327978596
GAGGGCTA -1.21299094882
GAGGGGAA -0.301799029722
GAGGGGAC -1.09385635492
GAGGGGCA -0.379292399144
GAGGGGCC -0.878742962514
GAGGGGGA -0.825087071689
GAGGGGGC -1.21203455357
GAGGGGTA 0.0913750844906
GAGGGTAA 1.09400874039
GAGGGTAC -0.426825164751
GAGGGTCA -0.769911508277
GAGGGTCC -1.65725941424
GAGGGTGA -0.616938714613
GAGGGTGC -0.594514003531
GAGGGTTA -0.686358006601
GAGGTAAA 0.418207413602
GAGGTAAC -0.0730294945622
GAGGTACA -0.648314438378
GAGGTACC -0.472156313194
GAGGTAGA 1.15330210943
GAGGTAGC -0.702640772991
GAGGTATA -0.109738448367
GAGGTATC 0.742082209978
GAGGTCAA -0.5687030939

GAGGTCAC	−0.842274479775
GAGGTCCA	0.392914039527
GAGGTCCC	−0.807990101634
GAGGTCGA	−0.755319619418
GAGGTCGC	−0.939676762438
GAGGTCTA	−1.45392988823
GAGGTGAA	0.712431604734
GAGGTGAC	−0.446336255207
GAGGTGCA	−1.48984581028
GAGGTGCC	−0.714955505404
GAGGTGGA	−1.16798992534
GAGGTGGC	−1.07014747607
GAGGTGTA	0.788919700546
GAGGTTAA	−0.82671077415
GAGGTTAC	0.301066115733
GAGGTTCA	−1.14227154479
GAGGTTCC	−1.10386465615
GAGGTTGA	−0.852736278069
GAGGTTGC	−0.512662618795
GAGGTTTA	−0.942522162469
GAGTAAAA	2.37181422166
GAGTAAAC	0.00411153247147
GAGTAACA	−0.306700980133
GAGTAACC	−0.315537246296
GAGTAAGA	0.979254902
GAGTAAGC	0.429143358107
GAGTAATA	1.75000413828
GAGTAATC	5.28219998985
GAGTACAA	1.44690212132
GAGTACAC	0.5968277534
GAGTACCA	−0.682470216774
GAGTACCC	−0.12424564959
GAGTACGA	−0.320161348073
GAGTACGC	−0.639980286257
GAGTACTA	−0.0712656915668
GAGTACTC	−0.30246450726
GAGTAGAA	1.13826351943
GAGTAGAC	−0.0851769427554
GAGTAGCA	−0.35290697277
GAGTAGCC	−1.34925544686
GAGTAGGA	1.6894205897
GAGTAGGC	−1.14263199001
GAGTAGTA	1.59850684372
GAGTATAA	0.539286947945
GAGTATAC	0.898266860667
GAGTATCA	4.25032452952
GAGTATCC	3.28874398951
GAGTATGA	0.694502395684
GAGTATGC	−0.0689650107776
GAGTATTA	0.705739713168
GAGTCAAA	0.715142654625
GAGTCAAC	0.000475975882425
GAGTCACA	0.135287976404
GAGTCACC	−1.1429202939

GAGTCAGA	0.372407085199
GAGTCAGC	-0.246903144616
GAGTCATA	0.532066804178
GAGTCATC	-0.196391151602
GAGTCCAA	-0.127106993898
GAGTCCAC	-0.69395558539
GAGTCCCA	-0.870483304148
GAGTCCCC	-1.34727652682
GAGTCCGA	-0.525255199842
GAGTCCGC	-1.65584821502
GAGTCCTA	-1.31652106157
GAGTCGAA	-0.322183134701
GAGTCGAC	-0.829634065896
GAGTCGCA	-0.263241585285
GAGTCGCC	-1.3250227593
GAGTCGGA	-0.831922200368
GAGTCGGC	0.344698022433
GAGTCGTA	-0.105609141948
GAGTCTAA	0.0756297186579
GAGTCTAC	-0.0546094108794
GAGTCTCA	-0.476250855861
GAGTCTCC	-0.816474025408
GAGTCTGA	-0.251190064137
GAGTCTGC	-1.38266262838
GAGTCTTA	-0.579057987123
GAGTGAAA	-0.421761680515
GAGTGAAC	0.63486086636
GAGTGACA	-0.669714010849
GAGTGACC	-0.988700710039
GAGTGAGA	1.26357220796
GAGTGAGC	0.0658953453393
GAGTGATA	2.04411541257
GAGTGATC	1.4072395555
GAGTGCAA	0.70042843918
GAGTGCAC	-0.323829577361
GAGTGCCA	-0.394504807907
GAGTGCCC	-0.673182544599
GAGTGCGA	-0.449774468693
GAGTGCGC	-0.172497737344
GAGTGCTA	1.55468752642
GAGTGGAA	1.20728917074
GAGTGGAC	-0.735101753133
GAGTGGCA	-0.851976964539
GAGTGGCC	-1.34612461813
GAGTGGGA	1.04867863747
GAGTGGGC	-0.841915080083
GAGTGGTA	-0.286610145303
GAGTGTAA	0.320084763266
GAGTGTAC	0.501886117061
GAGTGTCA	0.803800677448
GAGTGTCC	-0.98599828574
GAGTGTGA	-0.514630299433
GAGTGTGC	-0.0654996136061
GAGTGTTA	-0.527376572858

GAGTTAAA 1.12400672177
GAGTTAAC -0.116223848474
GAGTTACA 1.47882674643
GAGTTACC -0.0192463109009
GAGTTAGA 0.257787858329
GAGTTAGC -0.576490174345
GAGTTATA 1.43690192292
GAGTTATC 1.16542969262
GAGTTCAA 0.114554404234
GAGTTCAC -0.352615270911
GAGTTCCA -1.0215519329
GAGTTCCC -0.717078969473
GAGTTCGA -1.54959607435
GAGTTCGC -0.604767219314
GAGTTCTA 0.0368710103935
GAGTTGAA 0.0104416719116
GAGTTGAC -0.723523594051
GAGTTGCA -0.467613762474
GAGTTGCC -0.838682573912
GAGTTGGA -0.218823965513
GAGTTGGC -0.787345399206
GAGTTGTA 0.235504816071
GAGTTTAA 0.0225844152361
GAGTTTAC 0.665492698108
GAGTTTCA -0.111832114934
GAGTTTCC -0.724148818823
GAGTTTGA 0.90513361652
GAGTTTGC 0.544138451709
GAGTTTTA -0.585592004206
GATAAAAA 1.51779952622
GATAAAAC 1.81826054356
GATAAACA 0.0634746903985
GATAAACC -0.32693374517
GATAAAGA 1.52487198939
GATAAAGC -0.697093994187
GATAAATA 0.587028034683
GATAAATC 1.70880517146
GATAACAA 1.24414005475
GATAACAC 1.85580147498
GATAACCA 0.472241784975
GATAACCC 0.201417519652
GATAACGA 3.03053818202
GATAACGC 2.50453438529
GATAACTA 0.367968564353
GATAAGAA -0.316064191589
GATAAGAC -0.0625815494923
GATAAGCA 0.627766708521
GATAAGCC -0.363044396507
GATAAGGA -0.238401446894
GATAAGGC 0.167690407069
GATAAGTA 1.36695777669
GATAATAA 1.20652044747
GATAATAC 1.28916042047
GATAATCA 3.2328428308

GATAATCC 5.09032838739
GATAATGA 1.1744815986
GATAATGC 1.10202479111
GATAATTA 2.16272044736
GATACAAA 1.02937116328
GATACAAC 0.490248362976
GATACACA 3.46774490091
GATACACC 1.06516554289
GATACAGA 0.554394542689
GATACAGC 0.69934109175
GATACATA 2.53932375267
GATACATC 1.08632908778
GATACCAA 1.76060133224
GATACCAC 1.3774904094
GATACCCA 4.03683274852
GATACCCC 4.77221836206
GATACCGA 3.44456871775
GATACCGC 1.84561334321
GATACCTA 2.33036955614
GATACGAA 3.23910710208
GATACGAC 1.66837937139
GATACGCA 10.1842089506
GATACGCC 5.78004357918
GATACGGA 6.51716790309
GATACGGC 5.00176995095
GATACGTA 4.70003365281
GATACTAA 0.300880534801
GATACTAC 1.59164505412
GATACTCA 5.70311976025
GATACTCC 4.12129428985
GATACTGA 1.59065441788
GATACTGC 3.59873837773
GATACTTA 2.42067898787
GATAGAAA 1.0145466448
GATAGAAC 0.129676113584
GATAGACA -0.757493791516
GATAGACC -0.822772799057
GATAGAGA 1.49257829346
GATAGAGC -1.15821869721
GATAGATA 1.94373233421
GATAGATC 1.6599424963
GATAGCAA 0.396489739731
GATAGCAC 0.478103528599
GATAGCCA 1.54988516239
GATAGCCC -0.0104782653348
GATAGCGA 1.12787830594
GATAGCGC 1.42979208218
GATAGCTA 1.17603315974
GATAGGAA 1.29315171741
GATAGGAC -0.893520955068
GATAGGCA -0.461147704605
GATAGGCC -0.556164618843
GATAGGGA 0.121872827481
GATAGGGC -0.379518494223

GATAGGTA 0.325032194074
GATAGTAA 1.16686023409
GATAGTAC 0.385720556682
GATAGTCA 0.640894599073
GATAGTCC -0.670728171433
GATAGTGA 1.53236344725
GATAGTGC 2.46278207363
GATAGTTA 1.99783623309
GATATAAA 3.85316565291
GATATAAC 1.34621322649
GATATACA 1.79796086484
GATATACC 2.7929621782
GATATAGA 2.87441234219
GATATAGC 1.425875279
GATATATA 3.43761831979
GATATATC 5.28423223174
GATATCAA 6.57570326551
GATATCAC 9.54007687914
GATATCCA 22.1608897526
GATATCCC 19.8923016569
GATATCGA 9.6793033534
GATATCGC 17.5924831656
GATATCTA 18.7691329245
GATATGAA 2.11259791292
GATATGAC 2.81764026
GATATGCA 6.68986036972
GATATGCC 3.6397251027
GATATGGA 3.5873071151
GATATGGC 2.08583243771
GATATGTA 3.5460454167
GATATTAA 3.78514318416
GATATTAC 6.55707982694
GATATTCA 7.46060829909
GATATTCC 12.2824737425
GATATTGA 3.30686740509
GATATTGC 5.6625389609
GATATTTA 7.77072318423
GATCAAAA 1.81913486499
GATCAAAC 0.99132158328
GATCAACA -0.612223913423
GATCAACC -0.298994927983
GATCAAGA 0.480771450527
GATCAAGC -0.478878786406
GATCAATA -0.118017710353
GATCAATC 0.755375032316
GATCACAA 1.61544829172
GATCACAC 2.08035884575
GATCACCA 1.59010865311
GATCACCC 0.690931923112
GATCACGA 2.64909095301
GATCACGC 2.65903913647
GATCACTA 0.302881933666
GATCAGAA 3.43621234819
GATCAGAC 1.00561340607

GATCAGCA 1.51077019101
GATCAGCC 0.537570977782
GATCAGGA 1.17748069101
GATCAGGC 0.28536178681
GATCAGTA 1.05118162762
GATCATAA 2.4259939212
GATCATAC 0.717833316753
GATCATCA 1.33080399739
GATCATCC 0.623944002711
GATCATGA 1.28122252285
GATCATGC 1.31358051864
GATCATTA 2.99750059408
GATCCAAA 1.62569993922
GATCCAAC 0.96588131275
GATCCACA 2.27822392816
GATCCACC 2.20625250629
GATCCAGA 2.70241515059
GATCCAGC 1.38345252355
GATCCATA 1.74237075022
GATCCATC 0.928696644443
GATCCCAA 1.44881778702
GATCCCAC 1.32862773424
GATCCCCA 6.67591017267
GATCCCCC 4.48807775006
GATCCCGA 4.59599828944
GATCCCGC 4.58587524169
GATCCCTA 2.71762154758
GATCCGAA 3.10869023306
GATCCGAC 1.61027398169
GATCCGCA 3.34425673518
GATCCGCC 3.69996571864
GATCCGGA 2.8382583015
GATCCGGC 1.36022510959
GATCCGTA 3.8552859804
GATCCTAA 2.40411680456
GATCCTAC 2.43836092993
GATCCTCA 5.14003401818
GATCCTCC 3.89094627125
GATCCTGA 2.54849693971
GATCCTGC 3.59086216616
GATCCTTA 2.57377123292
GATCGAAA 1.5260518659
GATCGAAC 0.679078790594
GATCGACA 0.0991352426308
GATCGACC 0.0407935639726
GATCGAGA 1.49138587063
GATCGAGC 0.672015998546
GATCGATA 3.01964797929
GATCGATC 1.36242594262
GATCGCAA 3.71986469938
GATCGCAC 6.91222187372
GATCGCCA 4.15781740134
GATCGCCC 6.95579183298
GATCGCGA 9.28285909407

GATCGCGC	12.0193529155
GATCGCTA	3.00139753226
GATCGGAA	1.84914486993
GATCGGAC	-0.387147438803
GATCGGCA	0.843286549306
GATCGGCC	0.135789306301
GATCGGGA	4.74271517604
GATCGGGC	1.68447812514
GATCGGTA	0.961436780127
GATCGTAA	2.84318612869
GATCGTAC	2.77479772571
GATCGTCA	3.58348989831
GATCGTCC	2.81148681452
GATCGTGA	1.60399376614
GATCGTGC	2.35344902812
GATCGTTA	2.04450068904
GATCTAAA	2.19736762316
GATCTAAC	2.59118917958
GATCTACA	3.68883007859
GATCTACC	3.36813734174
GATCTAGA	3.98438313375
GATCTAGC	2.3157930888
GATCTATA	3.63341535102
GATCTCAA	3.49829282917
GATCTCAC	5.4797762456
GATCTCCA	10.5096460248
GATCTCCC	5.46255381238
GATCTCGA	4.79290044206
GATCTCGC	9.20338340664
GATCTCTA	6.21909434522
GATCTGAA	3.24895805159
GATCTGAC	2.14337768665
GATCTGCA	4.73043442323
GATCTGCC	2.88739046108
GATCTGGA	2.72007487522
GATCTGGC	1.64558585088
GATCTGTA	3.5214865249
GATCTTAA	3.11072430462
GATCTTAC	6.67631113204
GATCTTCA	9.07492742233
GATCTTCC	4.37135048063
GATCTTGA	4.3600556592
GATCTTGC	3.77655575327
GATCTTTA	4.24273087145
GATGAAAA	1.69941137836
GATGAAAC	-0.22060763351
GATGAACA	0.195216502719
GATGAACC	-1.12297217342
GATGAAGA	-0.695546876533
GATGAAGC	-0.174667727336
GATGAATA	1.10791842329
GATGAATC	0.783436176088
GATGACAA	1.10516319991
GATGACAC	0.498167179744

GATGACCA −0.381775001523
GATGACCC −0.798477379907
GATGACGA −0.62113467334
GATGACGC −0.697851739428
GATGACTA 0.732352018765
GATGAGAA 0.591756950479
GATGAGAC −0.40371667942
GATGAGCA −0.594390108656
GATGAGCC −0.685980310198
GATGAGGA 0.310193560991
GATGAGGC −1.13081832611
GATGAGTA 1.42507231474
GATGATAA 1.03484580076
GATGATAC 0.679888550771
GATGATCA 0.703387017441
GATGATCC 1.29774497616
GATGATGA −0.0478472076646
GATGATGC 0.726712188115
GATGATTA 2.69323673593
GATGCAAA 1.97626310324
GATGCAAC 0.337918568033
GATGCACA −0.512609035568
GATGCACC 0.801127266506
GATGCAGA 1.5260518659
GATGCAGC −1.15820405984
GATGCATA 1.40437742705
GATGCATC 0.145631107455
GATGCCAA −0.282735947319
GATGCCAC −0.851066833829
GATGCCCA 0.0845234887707
GATGCCCC −0.105849090251
GATGCCGA −0.349193524466
GATGCCGC 0.10819891078
GATGCCTA 0.496235569765
GATGCGAA 1.10594734469
GATGCGAC 0.228930546222
GATGCGCA 2.32737307755
GATGCGCC 0.00140283501426
GATGCGGA 0.28326916577
GATGCGGC 0.100171882032
GATGCGTA 1.16101025263
GATGCTAA 0.347827021493
GATGCTAC 0.147049102602
GATGCTCA −0.162329993342
GATGCTCC 0.31288788246
GATGCTGA 0.901166105307
GATGCTGC 1.43713716635
GATGCTTA 0.402591170276
GATGGAAA 0.367606550846
GATGGAAC 0.721480635513
GATGGACA 0.152924438069
GATGGACC −0.959306520137
GATGGAGA 0.093112487945
GATGGAGC −0.766639272103

GATGGATA 2.74059254619
GATGGCAA -0.00343821348567
GATGGCAC -0.415214855734
GATGGCCA -0.836888712033
GATGGCCC -1.39192285548
GATGGCGA 1.90771551886
GATGGCGC -0.255912445393
GATGGCTA -0.465142660885
GATGGGAA 0.0936174771844
GATGGGAC -0.604048158549
GATGGGCA -0.350996011937
GATGGGCC -1.49659625132
GATGGGGA 0.152460224358
GATGGGGC -0.213120096372
GATGGGTA -0.178685685197
GATGGTAA 1.5044183569
GATGGTAC -0.42295462611
GATGGTCA 0.290742588302
GATGGTCC -1.06352458924
GATGGTGA 0.777370032058
GATGGTGC -0.0555854097509
GATGGTTA 0.675201194649
GATGTAAA 1.60947127882
GATGTAAC 0.460894687222
GATGTACA 0.141492129916
GATGTACC 0.552175412957
GATGTAGA 0.513802765307
GATGTAGC -0.103711772958
GATGTATA 1.24468974024
GATGTCAA 1.37347558811
GATGTCAC 0.257220398889
GATGTCCA 0.423421715018
GATGTCCC 0.390479531362
GATGTCGA 1.2166905439
GATGTCGC 0.929344348033
GATGTCTA 0.89169442049
GATGTGAA 1.23780599458
GATGTGAC -0.489954570064
GATGTGCA 1.0430092707
GATGTGCC -0.553428476318
GATGTGGA -0.114522254298
GATGTGGC -0.243203810918
GATGTGTA 0.926202018512
GATGTTAA 1.84661025261
GATGTTAC 1.4146708956
GATGTTCA 1.48879453351
GATGTTCC -0.175101620782
GATGTTGA 0.967767442331
GATGTTGC 0.290734485472
GATGTTTA 0.701534083322
GATTAAAA 1.53566182158
GATTAAAC -0.503155386081
GATTAACA 1.44679835282
GATTAACC 0.366975052915

GATTAAGA 1.09881110441
GATTAAGC -0.100567091002
GATTAATA 0.953561614086
GATTAATC 1.746801691
GATTACAA 9.67231035024
GATTACAC 12.7488663301
GATTACCA 12.3713931469
GATTACCC 7.4595361118
GATTACGA 16.2801342771
GATTACGC 14.6415006538
GATTACTA 8.66154746539
GATTAGAA 0.138842243318
GATTAGAC 0.64264402608
GATTAGCA 1.6238697453
GATTAGCC -0.0437056163098
GATTAGGA 0.375500274981
GATTAGGC 0.676813657701
GATTAGTA 0.321681543423
GATTATAA 2.55016220184
GATTATAC 2.90422395849
GATTATCA 5.06495581469
GATTATCC 5.09617497088
GATTATGA 3.50825094513
GATTATGC 4.41424110914
GATTATTA 2.39778091472
GATTCAAA 2.18417098924
GATTCAAC 0.739424481932
GATTCACA 4.27125283098
GATTCACC 1.65095253776
GATTCAGA 1.4684052009
GATTCAGC 0.802317859666
GATTCATA 1.24569240003
GATTCCAA 5.98334827394
GATTCCAC 6.22414293071
GATTCCCA 16.4508901675
GATTCCCC 28.160557125
GATTCCGA 8.68028199113
GATTCCGC 8.71169117174
GATTCCTA 8.91435757264
GATTCGAA 3.81765199713
GATTCGAC 3.77883630768
GATTCGCA 13.7221808127
GATTCGCC 13.1842449462
GATTCGGA 6.07299902406
GATTCGGC 4.01412339288
GATTCGTA 6.03395671676
GATTCTAA 5.35119218431
GATTCTAC 4.67342709761
GATTCTCA 21.7249104817
GATTCTCC 18.8195238979
GATTCTGA 6.74533207847
GATTCTGC 6.35920193683
GATTCTTA 8.69081776041
GATTGAAA 1.34923767292

GATTGAAC -0.295529008049
GATTGACA 2.52621154501
GATTGACC 0.402496811521
GATTGAGA 1.96253769436
GATTGAGC 0.0198367719215
GATTGATA 0.749849425422
GATTGCAA 7.42047184845
GATTGCAC 15.0499791831
GATTGCCA 6.35606195975
GATTGCCC 7.34088115312
GATTGCGA 15.4368798777
GATTGCGC 23.3257352574
GATTGCTA 8.71232920421
GATTGGAA 1.29518108411
GATTGGAC 0.458199320226
GATTGGCA 0.372161386501
GATTGGCC 0.121272172578
GATTGGGA 0.957418299503
GATTGGGC -0.245530891248
GATTGGTA 1.0600602376
GATTGTAA 1.45949888447
GATTGTAC 3.63624297711
GATTGTCA 5.15525113182
GATTGTCC 5.60437449896
GATTGTGA 4.06890583838
GATTGTGC 3.71767536715
GATTGTTA 3.09396713064
GATTTAAA 3.02071023409
GATTTAAC 2.81604374122
GATTTACA 3.98090911099
GATTTACC 2.8242492936
GATTTAGA 1.9615203972
GATTTAGC 2.03150531895
GATTTATA 3.8707121993
GATTTCAA 5.7333880102
GATTTCAC 9.26265638791
GATTTCCA 19.383233035
GATTTCCC 25.4970300666
GATTTCGA 17.2064353592
GATTTCGC 14.822049467
GATTTCTA 10.0582413191
GATTTGAA 3.22458055862
GATTTGAC 5.71901881846
GATTTGCA 7.9897379583
GATTTGCC 7.93398265052
GATTTGGA 4.67470446946
GATTTGGC 2.81721107142
GATTTGTA 5.71007799966
GATTTTAA 5.67942891673
GATTTTAC 5.30246647311
GATTTTCA 13.3607203886
GATTTTCC 11.6608176129
GATTTTGA 5.67922608461
GATTTTGC 18.3343552248

GATTTTTA 8.51648982915
GCAAAAAA 1.76108985444
GCAAAAAC 0.198211935785
GCAAAACA 0.0527107349816
GCAAAACC -0.213028090051
GCAAAAGA 0.651897718648
GCAAAAGC -0.403730271263
GCAAAATA 2.01834240327
GCAAACAA 1.21110586477
GCAAACAC -0.0288648921735
GCAAACCA -0.909713544807
GCAAACCC -0.320332291635
GCAAACGA 1.18111232687
GCAAACGC -0.258686226867
GCAAACTA 0.267634887115
GCAAAGAA 1.88842947796
GCAAAGAC 0.176546538233
GCAAAGCA -0.810303851503
GCAAAGCC -1.02479986059
GCAAAGGA 0.335574236518
GCAAAGGC -1.17106900051
GCAAAGTA 0.634610201411
GCAAATAA 1.43562847179
GCAAATAC 3.31183862162
GCAAATCA 5.65896404484
GCAAATCC 9.51839344626
GCAAATGA 0.531915464235
GCAAATGC 0.284263984116
GCAAATTA 1.25971709083
GCAACAAA 0.863757432974
GCAACAAC -0.352002069692
GCAACACA -0.429592150304
GCAACACC 0.0244057221822
GCAACAGA 0.314054689895
GCAACAGC -0.838168697698
GCAACATA 0.879186265697
GCAACCAA 0.783752709198
GCAACCAC 0.594824524865
GCAACCCA 0.173801508734
GCAACCCC -1.10285154109
GCAACCGA -0.577245044388
GCAACCGC -0.373161432479
GCAACCTA -0.132094938854
GCAACGAA 0.203562417011
GCAACGAC 0.212901058597
GCAACGCA -0.481057663373
GCAACGCC -0.756896273192
GCAACGGA 1.13527383676
GCAACGGC -0.686245873897
GCAACGTA 0.943601668452
GCAACTAA -0.395131078206
GCAACTAC -0.145646528969
GCAACTCA -0.856414178476
GCAACTCC -0.0102338735445

GCAACTGA −0.205838005167
GCAACTGC −0.687690268585
GCAACTTA −0.16437138359
GCAAGAAA 1.54305134062
GCAAGAAC −0.19236326124
GCAAGACA 0.120891862359
GCAAGACC −1.24635264994
GCAAGAGA 0.552132546376
GCAAGAGC −0.396477193414
GCAAGATA 1.84818167876
GCAAGCAA −0.258945517408
GCAAGCAC −0.617593998269
GCAAGCCA 0.348620314631
GCAAGCCC −1.31429984079
GCAAGCGA −0.745398619641
GCAAGCGC −0.479005556479
GCAAGCTA −1.6419317362
GCAAGGAA −0.675601369869
GCAAGGAC −0.579138231272
GCAAGGCA −1.0541598095
GCAAGGCC −0.337996198367
GCAAGGGA −0.127309564633
GCAAGGGC −0.945861835094
GCAAGGTA 0.251385054806
GCAAGTAA 0.272520631867
GCAAGTAC 0.144036941114
GCAAGTCA −0.00199434156118
GCAAGTCC −0.368531318925
GCAAGTGA −0.640226246337
GCAAGTGC −0.404757239545
GCAAGTTA 0.1763510248
GCAATAAA 0.758221739255
GCAATAAC 1.42905472471
GCAATACA −0.0177587882502
GCAATACC 0.866400785033
GCAATAGA 1.39731986263
GCAATAGC −0.0441044846221
GCAATATA 3.28050524168
GCAATCAA 6.32309938833
GCAATCAC 5.05162718307
GCAATCCA 18.3176432698
GCAATCCC 12.137147406
GCAATCGA 3.92083159716
GCAATCGC 5.32145924387
GCAATCTA 14.7840309914
GCAATGAA 1.34340886337
GCAATGAC −1.16492182819
GCAATGCA −0.00777976175974
GCAATGCC −1.46745952229
GCAATGGA 0.938331692756
GCAATGGC −1.46856778025
GCAATGTA 0.582768560229
GCAATTAA 1.29222904039
GCAATTAC 2.13854787756

GCAATTCA 1.39943862183
GCAATTCC 1.4791835323
GCAATTGA 0.296692156141
GCAATTGC 1.73653462199
GCAATTTA 4.40066181257
GCACAAAA 2.50528664152
GCACAAAC -0.241400539302
GCACAACA 0.267708335343
GCACAACC -0.663061849285
GCACAAGA -0.174549060092
GCACAAGC -0.430490780224
GCACAATA 2.36873148715
GCACACAA 0.545292712828
GCACACAC -0.572160126861
GCACACCA -0.091834331951
GCACACCC -1.58134661932
GCACACGA -0.0689712839358
GCACACGC 0.23264791525
GCACACTA 0.223481524135
GCACAGAA -0.597613727853
GCACAGAC 0.442863539351
GCACAGCA 0.494634868884
GCACAGCC -1.38989244326
GCACAGGA 0.00322178952596
GCACAGGC -1.50322070644
GCACAGTA -0.561892796472
GCACATAA 0.481094256796
GCACATAC 0.257402320478
GCACATCA 0.812298454446
GCACATCC 1.11749413798
GCACATGA 1.21927639201
GCACATGC -0.445934773079
GCACATTA 0.893692682775
GCACCAAA 0.998395091973
GCACCAAC -0.150073287643
GCACCACA -0.42140986089
GCACCACC -0.974156131248
GCACCAGA -0.239815521317
GCACCAGC -1.55828544405
GCACCATA 0.556453184122
GCACCCAA -0.223465841239
GCACCCAC -0.309982887426
GCACCCCA -0.861023120121
GCACCCCC -0.783345738057
GCACCCGA -0.553751543968
GCACCCGC -0.721599564139
GCACCCTA -0.678603598856
GCACCGAA -1.02760814443
GCACCGAC -0.923011072014
GCACCGCA -0.0929543520807
GCACCGCC -0.153828818383
GCACCGGA -0.884982141159
GCACCGGC -1.50261639219
GCACCGTA -0.0947225985632

GCACCTAA 0.268048654178
GCACCTAC -0.539067648788
GCACCTCA -0.471147641623
GCACCTCC -1.30535274883
GCACCTGA -0.458038831927
GCACCTGC -0.853329875669
GCACCTTA 0.275391647294
GCACGAAA 1.47676679809
GCACGAAC -1.02152161264
GCACGACA -0.0482907722294
GCACGACC -0.601858303556
GCACGAGA -0.264557380229
GCACGAGC -0.954420775385
GCACGATA 1.42350977557
GCACGCAA -0.2627585521
GCACGCAC 0.0678787088735
GCACGCCA -0.279003940921
GCACGCCC -1.28449815698
GCACGCGA 0.0532745350796
GCACGCGC 0.446491515874
GCACGCTA -0.161824219958
GCACGGAA 0.814523073191
GCACGGAC -1.08580815426
GCACGGCA -1.09186724099
GCACGGCC -1.04223505842
GCACGGGA -0.88062830795
GCACGGGC -0.814720154913
GCACGGTA 1.01636037168
GCACGTAA 1.98557325423
GCACGTAC -0.687737578654
GCACGTCA -0.291697153883
GCACGTCC -0.248791888013
GCACGTGA 0.444688505639
GCACGTGC 0.657055300258
GCACGTTA 0.7063058657
GCACTAAA -0.0623039622396
GCACTAAC -0.646791629212
GCACTACA 0.324484861017
GCACTACC -0.603272900742
GCACTAGA 0.356913429841
GCACTAGC -0.689033769978
GCACTATA -0.00833336797554
GCACTCAA -1.10811053876
GCACTCAC -0.946313241107
GCACTCCA 0.112018218629
GCACTCCC -0.997287095397
GCACTCGA 0.410208614066
GCACTCGC -1.18423060929
GCACTCTA 0.0909001541346
GCACTGAA -1.1771158023
GCACTGAC -0.522446393234
GCACTGCA 0.742082209978
GCACTGCC -0.656231425474
GCACTGGA -0.451255979567

GCACTGGC -0.726095066171
GCACTGTA -0.396699367769
GCACTTAA -0.147181100307
GCACTTAC -0.359754125003
GCACTTCA -1.02920570873
GCACTTCC -0.610638634057
GCACTTGA 0.158447169765
GCACTTTA -0.545657601533
GCAGAAAA 0.421682743274
GCAGAAAC 0.0839758943316
GCAGAACA 0.0969568884276
GCAGAACC -0.846799517928
GCAGAAGA 0.316351972724
GCAGAAGC -1.67280926664
GCAGAATA 1.11081217891
GCAGACAA 0.176198377949
GCAGACAC -0.247718132425
GCAGACCA -1.27366023058
GCAGACCC -0.795071577739
GCAGACGA 1.179397925
GCAGACGC -0.344246093658
GCAGACTA -0.551017753878
GCAGAGAA 0.40755088602
GCAGAGAC -0.567042013871
GCAGAGCA -0.870759584492
GCAGAGCC -1.48970414146
GCAGAGGA -0.0921328297312
GCAGAGGC -1.04255080739
GCAGAGTA -0.166588683651
GCAGATAA 2.89354861144
GCAGATAC 4.38657674262
GCAGATCA 2.76070403017
GCAGATCC 5.6720804346
GCAGATGA -0.16928457341
GCAGATGC 0.0161021517083
GCAGATTA 3.41101045768
GCAGCAAA 1.3677769466
GCAGCAAC -0.260901697257
GCAGCACA -0.628261503878
GCAGCACC 0.429407614899
GCAGCAGA -0.417184366043
GCAGCAGC -0.853691627795
GCAGCATA 0.0964333410951
GCAGCCAA -0.278279913906
GCAGCCAC -1.18098895476
GCAGCCCA -0.735373067227
GCAGCCCC -1.14357845276
GCAGCCGA -0.949400680493
GCAGCCGC -1.21171854323
GCAGCCTA -0.878544835266
GCAGCGAA -1.20885981273
GCAGCGAC -1.00218878443
GCAGCGCA -0.179102327457
GCAGCGCC -1.13350428337

GCAGCGGA -0.193330634519
GCAGCGGC -1.05487808612
GCAGCGTA 0.0765241664721
GCAGCTAA -1.26826531448
GCAGCTAC -0.981578584369
GCAGCTCA -1.30381294986
GCAGCTCC -1.14883013174
GCAGCTGA -1.01874417182
GCAGCTGC -1.06247069866
GCAGCTTA -0.407346485614
GCAGGAAA 0.140006436937
GCAGGAAC -1.35720092455
GCAGGACA -0.154197889194
GCAGGACC -1.17212864149
GCAGGAGA 0.663496788257
GCAGGAGC -0.895827909015
GCAGGATA 1.23583387046
GCAGGCAA 0.398707562555
GCAGGCAC -0.527092712447
GCAGGCCA -1.85251434006
GCAGGCCC -1.12153300637
GCAGGCGA 0.181433328511
GCAGGCGC -0.061436175348
GCAGGCTA -1.0884305958
GCAGGGAA -0.214086685506
GCAGGGAC -1.11913352334
GCAGGGCA -1.11089033201
GCAGGGCC -1.16911595724
GCAGGGGA -0.102649256779
GCAGGGGC -1.09854815453
GCAGGGTA -0.162153038003
GCAGGTAA 0.353354980822
GCAGGTAC -0.739130689021
GCAGGTCA -0.985016536473
GCAGGTCC -0.893588391519
GCAGGTGA -0.306684774475
GCAGGTTA -0.393658977069
GCAGTAAA 1.44567022987
GCAGTAAC -0.414961054206
GCAGTACA -0.191567615669
GCAGTACC -1.28739112846
GCAGTAGA 0.0549408427405
GCAGTAGC -0.868945334849
GCAGTATA 1.70224710727
GCAGTCAA -0.215376603672
GCAGTCAC -0.331522821813
GCAGTCCA -1.07063207754
GCAGTCCC -0.805769142231
GCAGTCGA 0.188646153594
GCAGTCGC -0.994146072783
GCAGTCTA -0.904252237785
GCAGTGAA -0.114522254298
GCAGTGAC -0.715192317128
GCAGTGCA -0.407141039681

GCAGTGCC −0.882100670469
GCAGTGGA −0.307974431259
GCAGTGGC −0.731751886626
GCAGTGTA 0.663739088995
GCAGTTAA 0.330564074127
GCAGTTAC 1.1698833736
GCAGTTCA −1.02920570873
GCAGTTCC −0.474019702576
GCAGTTGA 0.542684647284
GCAGTTTA 0.0711872770886
GCATAAAA 0.920315705018
GCATAAAC 0.369560639642
GCATAACA 0.729327310961
GCATAACC −0.729167868189
GCATAAGA 1.87083091663
GCATAAGC −0.779547863498
GCATAATA 1.19552543072
GCATACAA 0.183321026382
GCATACAC 0.762292757579
GCATACCA 0.534121786269
GCATACCC 0.944850026944
GCATACGA 2.16464055655
GCATACGC −0.757614027049
GCATACTA 0.264126623362
GCATAGAA 0.922262997892
GCATAGAC −0.422584248391
GCATAGCA −0.455960064111
GCATAGCC −1.51088493753
GCATAGGA 0.225786125648
GCATAGGC −0.0773616330988
GCATAGTA 2.32892437731
GCATATAA 1.08235242821
GCATATAC 0.372064413929
GCATATCA 2.68060939113
GCATATCC 6.68867997044
GCATATGA 0.412030443776
GCATATGC 1.03912095811
GCATATTA 0.840459707369
GCATCAAA 1.90058921109
GCATCAAC 0.928089716382
GCATCACA 0.273072931175
GCATCACC 0.230978993773
GCATCAGA 0.924865574422
GCATCAGC −0.000309475807133
GCATCATA 0.599453592892
GCATCCAA 0.199643261393
GCATCCAC 1.29446934203
GCATCCCA 0.33599924299
GCATCCCC 0.0419012991674
GCATCCGA 0.828963360726
GCATCCGC −0.291039256411
GCATCCTA −0.529076337363
GCATCGAA 1.57643081568
GCATCGAC −0.253561056575

GCATCGCA -0.287015548155
GCATCGCC -1.13348912324
GCATCGGA 1.73561508154
GCATCGGC 0.345364284116
GCATCGTA 0.631478327154
GCATCTAA 1.47900161071
GCATCTAC 0.71923170828
GCATCTCA 0.367355624516
GCATCTCC -0.17236600102
GCATCTGA 1.14629786686
GCATCTTA 0.34871859411
GCATGAAA 1.09651094639
GCATGAAC 0.578297366685
GCATGACA -0.434437642291
GCATGACC -1.44866984504
GCATGAGA 1.45011110315
GCATGAGC -0.895931938889
GCATGATA 1.17029923172
GCATGCAA 2.21741114792
GCATGCAC -0.248761567748
GCATGCCA -0.355782170303
GCATGCCC -0.619267624615
GCATGCGA 0.599810901531
GCATGCGC -0.531229860315
GCATGCTA -0.61588691508
GCATGGAA -0.697482668618
GCATGGAC -0.0889533840235
GCATGGCA -0.654717764664
GCATGGCC -1.20419859477
GCATGGGA -0.558646698458
GCATGGGC -1.20452584453
GCATGGTA 0.717843772016
GCATGTAA 0.683903633435
GCATGTAC -0.928060703025
GCATGTCA -0.276015303777
GCATGTCC -0.290458727891
GCATGTGA -0.313231076493
GCATGTTA -0.662019982252
GCATTAAA 0.697512988882
GCATTAAC 0.713938731004
GCATTACA 0.0973570636478
GCATTACC -0.523005227082
GCATTAGA 0.708228850086
GCATTAGC -0.454993736359
GCATTATA 0.153305009669
GCATTCAA 0.773949331139
GCATTCAC 0.709336846662
GCATTCCA -0.889168428767
GCATTCCC -0.607469382231
GCATTCGA 0.246969535541
GCATTCGC -0.923591861915
GCATTCTA 0.028468114914
GCATTGAA 0.496063057913
GCATTGAC 0.19115620104

GCATTGCA	-0.444284148314
GCATTGCC	-0.815676288783
GCATTGGA	-0.0769632875498
GCATTGGC	-0.0548145954306
GCATTGTA	0.222310795976
GCATTTAA	0.561949516278
GCATTTAC	1.66718799409
GCATTTCA	0.73505052234
GCATTTCC	-0.206389258949
GCATTTGA	2.10239828037
GCATTTTA	0.761328782261
GCCAAAAA	0.122747410294
GCCAAAAC	0.0718114563348
GCCAAACA	-0.680231483423
GCCAAACC	-0.252759137845
GCCAAAGA	-0.923557882308
GCCAAAGC	-1.26711262165
GCCAAATA	0.573042028358
GCCAACAA	-0.0425482186122
GCCAACAC	-0.626535601214
GCCAACCA	-0.587668419588
GCCAACCC	-0.136122306452
GCCAACGA	-0.503941099152
GCCAACGC	-0.626869908272
GCCAACTA	-0.395622998366
GCCAAGAA	0.101135595969
GCCAAGAC	-0.382710224866
GCCAAGCA	-0.38402784948
GCCAAGCC	-1.05449019583
GCCAAGGA	-0.797768251643
GCCAAGGC	-1.50996774952
GCCAAGTA	-0.293997834672
GCCAATAA	1.46418702474
GCCAATAC	-0.671978098215
GCCAATCA	0.31447368459
GCCAATCC	0.124360134728
GCCAATGA	-0.829862513409
GCCAATTA	-0.170009907333
GCCACAAA	0.428168143381
GCCACAAC	-0.0959505692912
GCCACACA	-0.398214335487
GCCACACC	0.215553036248
GCCACAGA	-0.158482194899
GCCACAGC	-1.33624334836
GCCACATA	-0.219706389774
GCCACCAA	-0.535839324722
GCCACCAC	-0.855429292631
GCCACCCA	-0.854502433499
GCCACCCC	-1.37059516295
GCCACCGA	-0.948758727299
GCCACCGC	-1.29571900743
GCCACCTA	-0.986794976838
GCCACGAA	0.500257971113
GCCACGAC	-0.501379036769

GCCACGCA 0.18118710705
GCCACGCC -1.55652216382
GCCACGGA -0.602837961769
GCCACGGC -0.846917662408
GCCACGTA -0.0136216403826
GCCACTAA 0.0233737876496
GCCACTAC -0.554230395048
GCCACTCA -0.460882663669
GCCACTCC -1.27728193394
GCCACTGA -0.31548706103
GCCACTTA -0.626644074575
GCCAGAAA 1.08307175036
GCCAGAAC 0.0722887391252
GCCAGACA -0.504096359819
GCCAGACC -0.440168172355
GCCAGAGA 0.477566128042
GCCAGAGC -0.571170013383
GCCAGATA 0.609805088154
GCCAGCAA -0.30262185898
GCCAGCAC -1.31634567453
GCCAGCCA -1.27265417282
GCCAGCCC -1.49142795307
GCCAGCGA -1.00642499592
GCCAGCGC -0.50780771707
GCCAGCTA -0.522777040951
GCCAGGAA -0.183779489698
GCCAGGAC -0.77713583415
GCCAGGCA -2.12602090329
GCCAGGCC -1.28387842122
GCCAGGGA -0.829769984324
GCCAGGGC -1.51014731868
GCCAGGTA -0.103345838726
GCCAGTAA -0.749274647297
GCCAGTAC -0.732054827893
GCCAGTCA -1.12753328224
GCCAGTCC -0.170426026831
GCCAGTGA -0.73740374083
GCCAGTTA 0.434515272625
GCCATAAA 0.00950801685836
GCCATAAC -0.489729259131
GCCATACA 0.169739638765
GCCATACC -1.33332371595
GCCATAGA 0.150125041198
GCCATAGC -0.620427374747
GCCATATA 0.144975823799
GCCATCAA 0.280606732855
GCCATCAC -0.587594709978
GCCATCCA -0.311067098277
GCCATCCC -1.0178426667
GCCATCGA -0.158780954061
GCCATCGC -0.826971632981
GCCATCTA 0.190896649117
GCCATGAA -0.217523853466
GCCATGAC -1.19802842087

GCCATGCA −1.10721609095
GCCATGCC −0.606367658813
GCCATGGA −0.0907584853107
GCCATGGC −0.118433045706
GCCATGTA 0.121127889938
GCCATTAA −0.10820387703
GCCATTAC −0.252821346664
GCCATTCA −0.246479967815
GCCATTCC −0.725872891817
GCCATTGA −0.754729419779
GCCATTTA 0.0194656100582
GCCCAAAA 0.972230794428
GCCCAAAC −0.692041226597
GCCCAACA −0.689616650932
GCCCAACC −0.65601264908
GCCCAAGA −0.347325952978
GCCCAAGC −1.28054502176
GCCCAATA 0.224751838681
GCCCACAA −0.390780120195
GCCCACAC −1.10019538134
GCCCACCA −1.64587180234
GCCCACCC −0.433111392084
GCCCACGA −0.471720851458
GCCCACGC −0.813626534324
GCCCACTA 0.314418533074
GCCCAGAA −0.0555854097509
GCCCAGAC −1.1300587512
GCCCAGCA −0.418030196881
GCCCAGCC −1.46694355503
GCCCAGGA −1.181900131
GCCCAGGC −1.59365507857
GCCCAGTA −0.414468872665
GCCCATAA 0.174837102609
GCCCATAC −0.513069851318
GCCCATCA −1.26020482889
GCCCATCC −0.546120246954
GCCCATGA −1.07045329253
GCCCATTA −1.32827434632
GCCCCAAA −0.783029204947
GCCCCAAC −1.2761663573
GCCCCACA −0.153965259575
GCCCCACC −0.527622271556
GCCCCAGA −0.213349850793
GCCCCAGC −0.989822821221
GCCCCATA −0.0585013828121
GCCCCCAA −1.01075739584
GCCCCCAC −0.751145616746
GCCCCCCA −1.50128177778
GCCCCCCC −1.2621445417
GCCCCCGA −1.45430732325
GCCCCCGC −0.705291705116
GCCCCCTA −1.11181771391
GCCCCGAA −0.155195582738
GCCCCGAC −0.42935533858

GCCCCGCA -0.90458915866
GCCCCGCC -1.01489794167
GCCCCGGA -0.714279049839
GCCCCGGC -1.36372448637
GCCCCGTA -0.88601041635
GCCCCTAA -0.937317532172
GCCCCTAC -0.307565369064
GCCCCTCA -0.822131630007
GCCCCTCC -2.07481781735
GCCCCTGA -0.965703050502
GCCCCTTA -1.38729692403
GCCCGAAA -0.385002541444
GCCCGAAC 0.212426912385
GCCCGACA -0.23005683951
GCCCGACC -1.22551949138
GCCCGAGA 0.709336846662
GCCCGAGC -0.996836996292
GCCCGATA -0.180778044856
GCCCGCAA -1.05405525686
GCCCGCAC -1.12805709095
GCCCGCCA -1.00789396048
GCCCGCCC -0.654120769104
GCCCGCGA -0.551738382932
GCCCGCGC -1.69236453059
GCCCGCTA -1.68372534615
GCCCGGAA -0.598250192034
GCCCGGAC -1.49085892534
GCCCGGCA -0.540604049797
GCCCGGCC -1.57439543721
GCCCGGGA -0.40652914537
GCCCGGGC -0.592226914585
GCCCGGTA -0.785708366284
GCCCGTAA 0.166900511893
GCCCGTAC -0.615915405673
GCCCGTCA -0.965207993764
GCCCGTCC -0.960231288217
GCCCGTGA -0.104712080317
GCCCGTTA -0.663427260753
GCCCTAAA -0.37441135926
GCCCTAAC -0.766345740574
GCCCTACA -0.214253969726
GCCCTACC -0.0486912088312
GCCCTAGA 0.714168746807
GCCCTAGC -0.468847483597
GCCCTATA 0.178745018818
GCCCTCAA -0.897129589353
GCCCTCAC -0.30171172827
GCCCTCCA -1.13593042732
GCCCTCCC -0.320863157652
GCCCTCGA -0.994982493884
GCCCTCGC -1.21299094882
GCCCTCTA -0.71956470843
GCCCTGAA 0.150394787003
GCCCTGAC -1.15652102376

GCCCTGCA −0.079773662447
GCCCTGCC −1.5966369198
GCCCTGGA 0.10503227277
GCCCTGTA −0.589451826203
GCCCTTAA 0.550986649468
GCCCTTAC −1.32988445694
GCCCTTCA −1.30146234519
GCCCTTCC −0.65315417997
GCCCTTGA −0.603272900742
GCCCTTTA −1.30440184259
GCCGAAAA 0.601873202306
GCCGAAAC −0.514116684601
GCCGAACA −0.698801861522
GCCGAACC −0.411678101387
GCCGAAGA 0.437322772324
GCCGAAGC −0.998975881875
GCCGAATA 0.0218865263805
GCCGACAA −0.85990937315
GCCGACAC −0.510430942746
GCCGACCA −1.25824760351
GCCGACCC −0.593089735226
GCCGACGA −0.631178783847
GCCGACGC −1.16691355593
GCCGACTA −1.04120521494
GCCGAGAA 1.08829258631
GCCGAGAC −0.754388839562
GCCGAGCA −0.57221580114
GCCGAGCC −1.22090872007
GCCGAGGA −0.663496265494
GCCGAGGC −0.834553267492
GCCGAGTA −0.626889773273
GCCGATAA 0.362321415018
GCCGATAC 0.572187571928
GCCGATCA 0.778200964145
GCCGATCC 0.374464681106
GCCGATGA 0.423446023506
GCCGATTA 1.45916536156
GCCGCAAA 0.0954249309059
GCCGCAAC −1.43043638781
GCCGCACA 0.203846800185
GCCGCACC −1.44189300445
GCCGCAGA −0.899452226196
GCCGCAGC −1.60674402327
GCCGCATA 0.475213955079
GCCGCCAA −0.682381347032
GCCGCCAC −1.08109413722
GCCGCCCA −0.808774769179
GCCGCCCC −0.857826161847
GCCGCCGA −0.595858027687
GCCGCCGC −1.10250285805
GCCGCCTA −0.200295669851
GCCGCGAA 0.181806581427
GCCGCGAC −0.827300973789
GCCGCGCA −0.0670503906025

GCCGCGCC -1.27672780496
GCCGCGGA -0.315870769209
GCCGCGGC -0.283967316007
GCCGCGTA -0.414900936439
GCCGCTAA 0.532125353655
GCCGCTAC -0.572515344447
GCCGCTCA -0.552953023199
GCCGCTCC -1.14738260048
GCCGCTGA -0.875990352949
GCCGCTTA 0.0117577282367
GCCGGAAA 0.439342990663
GCCGGAAC -0.402768648379
GCCGGACA -0.347335362715
GCCGGACC -1.53857204425
GCCGGAGA -0.220020047687
GCCGGAGC -1.49372445175
GCCGGATA 1.55559216811
GCCGGCAA 0.362550385294
GCCGGCAC -0.894278177545
GCCGGCCA -1.20679934163
GCCGGCCC -0.802766129099
GCCGGCGA -0.687624923187
GCCGGCGC -0.813299284569
GCCGGCTA -0.167202146252
GCCGGGAA -0.122183348814
GCCGGGAC -1.41963662312
GCCGGGCA -1.05652531292
GCCGGGCC -1.52098315403
GCCGGGGA -0.45126643483
GCCGGGTA -1.01244017054
GCCGGTAA -1.04219166908
GCCGGTAC -0.814192164093
GCCGGTCA -0.961534798225
GCCGGTCC -1.18462817069
GCCGGTGA -0.617943726842
GCCGGTTA -0.324780222218
GCCGTAAA -0.950637015432
GCCGTAAC -0.0633432154568
GCCGTACA -0.816730702133
GCCGTACC -1.38005769941
GCCGTAGA 0.045317033836
GCCGTAGC -1.14552888221
GCCGTATA 0.0463957556739
GCCGTCAA -0.155100439838
GCCGTCAC -0.519810621242
GCCGTCCA -1.19873781051
GCCGTCCC -0.720102108987
GCCGTCGA -1.40857521545
GCCGTCGC -1.09901681173
GCCGTCTA -1.0266514878
GCCGTGAA -0.132050503983
GCCGTGAC -0.52778040742
GCCGTGCA -1.04223505842
GCCGTGCC -0.825655315274

GCCGTGGA	−0.532234872543
GCCGTGTA	−0.462466636127
GCCGTTAA	−0.356057143739
GCCGTTAC	−0.538383874539
GCCGTTCA	−0.315806730719
GCCGTTCC	−1.32782581551
GCCGTTGA	−0.306294793137
GCCGTTTA	0.116416748091
GCCTAAAA	0.0861022335976
GCCTAAAC	−0.19460826775
GCCTAACA	−1.00934384418
GCCTAACC	−1.06828487083
GCCTAAGA	0.411706853362
GCCTAAGC	−1.1906378563
GCCTAATA	1.13253508042
GCCTACAA	−0.268472876505
GCCTACAC	−0.79345963745
GCCTACCA	−0.632384014376
GCCTACCC	−1.02202712464
GCCTACGA	0.684598647093
GCCTACGC	−0.482810488341
GCCTACTA	−0.607722660995
GCCTAGAA	−1.25733172241
GCCTAGAC	−0.817328220457
GCCTAGCA	−0.390563957616
GCCTAGCC	−1.32125703467
GCCTAGGA	−1.16651834696
GCCTAGGC	−1.75314489952
GCCTAGTA	−0.665309208229
GCCTATAA	1.86265803695
GCCTATAC	−0.942201708635
GCCTATCA	0.308968726842
GCCTATCC	−0.770480274625
GCCTATGA	−1.08072166845
GCCTATTA	−1.08892094767
GCCTCAAA	−0.0888566728338
GCCTCAAC	−0.882868609591
GCCTCACA	−0.046519911931
GCCTCACC	0.0186668279073
GCCTCAGA	−1.26224700328
GCCTCAGC	−0.261537900056
GCCTCATA	−0.77029417093
GCCTCCAA	−0.903979878164
GCCTCCAC	−1.15999739896
GCCTCCCA	−1.01804785125
GCCTCCCC	−1.34397135656
GCCTCCGA	−0.892411651583
GCCTCCGC	−1.01957013766
GCCTCCTA	−1.51003806117
GCCTCGAA	−0.495693987103
GCCTCGAC	−0.30108206001
GCCTCGCA	−0.773369064001
GCCTCGCC	−0.869618131072
GCCTCGGA	−0.675601369869

GCCTCGTA 0.518621857753
GCCTCTAA -0.423220189809
GCCTCTAC -0.474227239562
GCCTCTCA -0.67795328145
GCCTCTCC -1.52038903367
GCCTCTGA -0.959782234638
GCCTCTTA -0.629858022653
GCCTGAAA -0.791935782758
GCCTGAAC -0.693669372545
GCCTGACA -0.935682067539
GCCTGACC -1.48510539369
GCCTGAGA -0.481974590004
GCCTGAGC -0.954264991955
GCCTGATA 0.95552851058
GCCTGCAA 0.0228776853845
GCCTGCAC -1.40948534616
GCCTGCCA -0.547059652402
GCCTGCCC -1.04207875223
GCCTGCGA 0.511883701645
GCCTGCGC -0.988171412311
GCCTGCTA -0.656062050201
GCCTGGAA 0.0364580274752
GCCTGGAC -0.5736818905
GCCTGGCA -1.8885410879
GCCTGGCC -1.00934384418
GCCTGGGA -1.36094390898
GCCTGGTA -1.17649188444
GCCTGTAA -0.649001610588
GCCTGTAC -0.777004359208
GCCTGTCA -1.07623557615
GCCTGTCC -0.877989922142
GCCTGTGA -0.153905141809
GCCTGTTA -0.640737770116
GCCTTAAA 0.27145680878
GCCTTAAC -0.874106575802
GCCTTACA 0.239824408291
GCCTTACC -1.34513659571
GCCTTAGA -0.131873810025
GCCTTAGC -0.598645139622
GCCTTATA -0.333588781931
GCCTTCAA -1.18710789787
GCCTTCAC 0.0248639241162
GCCTTCCA -1.08702279453
GCCTTCCC -1.61576482484
GCCTTCGA -0.0289767634957
GCCTTCGC -1.03006408589
GCCTTCTA -0.318205429606
GCCTTGAA -1.05546070569
GCCTTGAC -1.01273840694
GCCTTGCA -1.15502121617
GCCTTGCC -1.4312346472
GCCTTGGA 0.0986780862232
GCCTTGTA -0.243477216065
GCCTTTAA -1.16376050977

GCCTTTAC	-0.360392418855
GCCTTTCA	-1.33083039693
GCCTTTCC	-1.31921956515
GCCTTTGA	-0.700964532829
GCCTTTTA	-0.095685528355
GCGAAAAA	-0.0926153401535
GCGAAAAC	-0.194629962422
GCGAAACA	-0.70573840626
GCGAAACC	-0.891047762427
GCGAAAGA	-0.114084178747
GCGAAAGC	-0.503651749728
GCGAAATA	2.46743492738
GCGAACAA	0.190566262783
GCGAACAC	-0.31551999511
GCGAACCA	-0.0279359419889
GCGAACCC	-0.514215486844
GCGAACGA	-1.01504457674
GCGAACGC	-0.226217928041
GCGAACTA	0.152736243321
GCGAAGAA	-0.249225258696
GCGAAGAC	-0.957749208601
GCGAAGCA	-0.414050400733
GCGAAGCC	-0.37438182314
GCGAAGGA	-0.723439951941
GCGAAGTA	0.346418697466
GCGAATAA	1.10382910825
GCGAATAC	1.26711889481
GCGAATCA	4.72949606331
GCGAATCC	18.7541554978
GCGAATGA	0.568308669075
GCGAATTA	1.97744010455
GCGACAAA	-0.634794214053
GCGACAAC	-0.363188940528
GCGACACA	-0.0212673133847
GCGACACC	-1.21980987185
GCGACAGA	0.24023425463
GCGACAGC	0.592677013689
GCGACATA	1.33063514488
GCGACCAA	-0.775450184251
GCGACCAC	-0.362789810834
GCGACCCA	-1.12247920774
GCGACCCC	-0.442582815519
GCGACCGA	-0.311465443826
GCGACCGC	-0.189969005841
GCGACCTA	-0.521985838866
GCGACGAA	0.0877497217837
GCGACGAC	-0.296611911992
GCGACGCA	-0.368804724072
GCGACGCC	-1.32078550228
GCGACGGA	-0.76562694119
GCGACGTA	0.460922655053
GCGACTAA	-0.624050123638
GCGACTAC	-0.204913237088
GCGACTCA	-0.778940674055

GCGACTCC -0.5240118076
GCGACTGA -0.430273572119
GCGACTTA 0.382303515106
GCGAGAAA 1.20879838806
GCGAGAAC 0.235829713392
GCGAGACA 0.16813658545
GCGAGACC -1.38551691538
GCGAGAGA -0.0333867937473
GCGAGAGC -0.0408824337145
GCGAGATA 1.31251565003
GCGAGCAA 0.325049706641
GCGAGCAC -0.679591098518
GCGAGCCA -0.370769790895
GCGAGCCC -0.817816481274
GCGAGCGA 0.577677892307
GCGAGCGC -1.16852628037
GCGAGCTA -0.455661827713
GCGAGGAA 0.0745329614901
GCGAGGAC -0.767462362743
GCGAGGCA -0.889836520121
GCGAGGCC -0.404461878344
GCGAGGGA -1.01738472615
GCGAGGTA -0.401307263887
GCGAGTAA 0.882930034266
GCGAGTAC 0.0890056603423
GCGAGTCA -0.195888253415
GCGAGTCC -1.02163871159
GCGAGTGA 0.195940006971
GCGAGTTA 0.450589456502
GCGATAAA 0.734231875188
GCGATAAC -0.278286448446
GCGATACA 0.264010047171
GCGATACC 2.06974910548
GCGATAGA -0.0246634444337
GCGATAGC -0.850498590244
GCGATATA 4.87467001436
GCGATCAA 1.13609196115
GCGATCAC 3.93672438221
GCGATCCA 6.22600370627
GCGATCCC 5.97818494193
GCGATCGA 5.101228784
GCGATCGC 8.72884172502
GCGATCTA 6.06706775293
GCGATGAA -0.795322504069
GCGATGAC -0.499774415164
GCGATGCA -0.599979492659
GCGATGCC -0.663050348495
GCGATGGA 0.0702013457166
GCGATGTA 0.93694819998
GCGATTAA 0.0781410730117
GCGATTAC 8.35759177614
GCGATTCA 2.76262727594
GCGATTCC 7.508727605
GCGATTGA 1.03190264402

GCGATTTA 3.62350768171
GCGCAAAA 0.106087470264
GCGCAAAC 0.349895856809
GCGCAACA 0.710547043442
GCGCAACC -0.298200850701
GCGCAAGA 0.270899543222
GCGCAAGC -0.805716604531
GCGCAATA 1.93342501244
GCGCACAA 0.322995770076
GCGCACAC -0.474751571039
GCGCACCA -0.317300003765
GCGCACCC -0.877903404834
GCGCACGA 0.51130395727
GCGCACGC 0.181516186477
GCGCACTA 0.356913429841
GCGCAGAA 0.489893929535
GCGCAGAC -0.364910661087
GCGCAGCA -0.584702784024
GCGCAGCC -1.15999739896
GCGCAGGA 0.061636916412
GCGCAGTA 0.880923930533
GCGCATAA 1.93722602357
GCGCATAC 1.30133740479
GCGCATCA 0.767528753667
GCGCATCC 0.361948162102
GCGCATGA 0.384627720238
GCGCATTA -0.111832114934
GCGCCAAA -0.088591370516
GCGCCAAC -0.179560790773
GCGCCACA -0.157422815299
GCGCCACC -0.559421433502
GCGCCAGA -0.442052210884
GCGCCAGC -0.708801275777
GCGCCATA -0.0782092936077
GCGCCCAA -0.0109231368075
GCGCCCAC -0.409131460518
GCGCCCCA -0.823571581208
GCGCCCCC -2.03903310886
GCGCCCGA -0.906977402283
GCGCCCGC -1.48818917374
GCGCCCTA -0.214253969726
GCGCCGAA 0.0504633760376
GCGCCGAC -0.979616915507
GCGCCGCA 0.0599321856569
GCGCCGCC -1.18046514605
GCGCCGGA 0.717872785373
GCGCCGTA 0.134349616482
GCGCCTAA 0.127255720025
GCGCCTAC 0.130542332186
GCGCCTCA -0.603880874329
GCGCCTCC -1.52916831864
GCGCCTGA -0.952030440709
GCGCCTTA -0.575248873155
GCGCGAAA 0.681057972022

GCGCGAAC	-0.140471696174
GCGCGACA	1.83346563364
GCGCGACC	-0.193870126129
GCGCGAGA	0.187131970021
GCGCGAGC	-0.0913324792908
GCGCGATA	3.04408794246
GCGCGCAA	2.7082771556
GCGCGCAC	0.0952401341191
GCGCGCCA	0.0544761062666
GCGCGCCC	-1.00005826306
GCGCGCGA	2.09499281705
GCGCGCGC	2.11123611482
GCGCGCTA	2.38861530775
GCGCGGAA	1.03249232089
GCGCGGAC	-1.09872510987
GCGCGGCA	-0.88103501771
GCGCGGCC	-1.15019794162
GCGCGGGA	0.032010358274
GCGCGGTA	-0.198536571724
GCGCGTAA	0.467961661375
GCGCGTAC	0.26542673541
GCGCGTCA	-0.0905415385878
GCGCGTCC	-0.785365172251
GCGCGTGA	0.305271222815
GCGCGTTA	0.417522332444
GCGCTAAA	0.833199310836
GCGCTAAC	0.181704642606
GCGCTACA	0.0949944354208
GCGCTACC	-1.52075052441
GCGCTAGA	-0.169353055388
GCGCTAGC	-0.555683153947
GCGCTATA	-0.539733910471
GCGCTCAA	-0.665597512127
GCGCTCAC	-0.508116147351
GCGCTCCA	-0.266060324393
GCGCTCCC	-1.74867527426
GCGCTCGA	-0.197270962047
GCGCTCTA	0.142360962334
GCGCTGAA	0.457129746744
GCGCTGAC	0.448139265441
GCGCTGCA	-0.228415624482
GCGCTGCC	-1.32476242323
GCGCTGGA	0.31740089706
GCGCTGTA	0.672988338075
GCGCTTAA	-0.148379012151
GCGCTTAC	0.0944471023633
GCGCTTCA	-0.323620210704
GCGCTTCC	-0.483532947066
GCGCTTGA	-0.935564707204
GCGCTTTA	-0.17861563493
GCGGAAAA	1.40543785217
GCGGAAAC	-0.159177208557
GCGGAACA	-0.204591999109
GCGGAACC	-1.27554191667

GCGGAAGA	0.452661167015
GCGGAAGC	-0.41937971005
GCGGAATA	1.89210763975
GCGGACAA	-0.756163097821
GCGGACAC	-0.874869810056
GCGGACCA	-0.594698538936
GCGGACCC	-1.6879223504
GCGGACGA	-0.265193321646
GCGGACGC	-0.431974904914
GCGGACTA	0.391701751695
GCGGAGAA	-0.777691531418
GCGGAGAC	-0.797142242726
GCGGAGCA	-0.881386575954
GCGGAGCC	-0.298820325078
GCGGAGGA	-0.726850720359
GCGGAGTA	-0.227792752144
GCGGATAA	1.28120631719
GCGGATAC	5.46460435098
GCGGATCA	0.867512963715
GCGGATCC	5.59538532456
GCGGATGA	-0.746548698654
GCGGATTA	3.31067547353
GCGGCAAA	-0.382618218545
GCGGCAAC	-1.40481497984
GCGGCACA	-0.85540785934
GCGGCACC	-1.55806823595
GCGGCAGA	-0.57479694438
GCGGCAGC	-1.13679925974
GCGGCATA	0.962894766658
GCGGCCAA	-0.30979495406
GCGGCCAC	-0.526596087419
GCGGCCCA	-1.14118811808
GCGGCCCC	-1.97709481947
GCGGCCGA	-0.776389066936
GCGGCCGC	-0.564826020719
GCGGCCTA	-0.918810147037
GCGGCGAA	-0.265291601126
GCGGCGAC	-1.02368114736
GCGGCGCA	0.56984114936
GCGGCGCC	-0.923745554292
GCGGCGGA	-0.448502063094
GCGGCGTA	0.219826102544
GCGGCTAA	-0.238866183368
GCGGCTAC	-0.816658560813
GCGGCTCA	-1.25458721567
GCGGCTCC	-1.59885814058
GCGGCTGA	-0.806971497563
GCGGCTTA	-0.899572984492
GCGGGAAA	-0.281846465755
GCGGGAAC	-0.717189272505
GCGGGACA	-0.20552696107
GCGGGACC	-0.733806868717
GCGGGAGA	0.0139522880988
GCGGGAGC	-0.678499046218

GCGGGATA	0.959153611905
GCGGGCAA	0.231110991478
GCGGGCAC	-0.781766209085
GCGGGCCA	-1.60927576539
GCGGGCCC	-1.49455329279
GCGGGCGA	-0.422079259152
GCGGGCTA	-1.61799754641
GCGGGGAA	-0.0351733369414
GCGGGGAC	-0.32327022075
GCGGGGCA	-0.281755243578
GCGGGGCC	-1.02596771355
GCGGGGGA	0.397381050966
GCGGGGTA	-0.128285824886
GCGGGTAA	0.772386791971
GCGGGTAC	-0.0228970276224
GCGGGTCA	-0.446864246027
GCGGGTCC	-1.36556853352
GCGGGTGA	0.220961021425
GCGGGTTA	0.205811344245
GCGGTAAA	-0.0797692189599
GCGGTAAC	-0.890254730671
GCGGTACA	0.360929819412
GCGGTACC	-0.157863243285
GCGGTAGA	0.245562518421
GCGGTAGC	-0.793723371478
GCGGTATA	0.948314639971
GCGGTCAA	-0.119948536187
GCGGTCAC	-1.08188298687
GCGGTCCA	-0.885570772509
GCGGTCCC	-1.00497406669
GCGGTCGA	-0.503826091251
GCGGTCTA	-0.711389214937
GCGGTGAA	-0.522052229791
GCGGTGAC	-0.91506663985
GCGGTGCA	-0.19668599004
GCGGTGCC	-0.376040812117
GCGGTGGA	-0.266060324393
GCGGTGTA	0.389645201314
GCGGTTAA	1.87740100437
GCGGTTAC	-0.489576089517
GCGGTTCA	0.263590268331
GCGGTTCC	-0.0151622234968
GCGGTTGA	0.0364580274752
GCGGTTTA	-0.410904934632
GCGTAAAA	2.78883600836
GCGTAAAC	0.0615600702234
GCGTAACA	0.334114681698
GCGTAACC	-0.316178153964
GCGTAAGA	0.528161240403
GCGTAAGC	-1.07903967789
GCGTAATA	3.13178747898
GCGTACAA	-0.559190372173
GCGTACAC	0.32818184228
GCGTACCA	-1.11045199508

GCGTACCC -0.0604544260814
GCGTACGA 1.38827658225
GCGTACGC 0.093276635586
GCGTACTA -0.30068345308
GCGTAGAA 0.199119975442
GCGTAGAC -0.352577631962
GCGTAGCA 0.0819561987558
GCGTAGCC 0.159864642149
GCGTAGGA 0.157371584507
GCGTAGTA 0.0998801801733
GCGTATAA 0.771761044435
GCGTATAC -0.15381836312
GCGTATCA 3.3700266079
GCGTATCC 9.34340840167
GCGTATGA 1.67089307818
GCGTATTA 0.919094530211
GCGTCAAA 0.517596980523
GCGTCAAC -0.622609388292
GCGTCACA -0.293096852318
GCGTCACC -0.651280073942
GCGTCAGA 0.320690907182
GCGTCAGC -1.52307002468
GCGTCATA -0.547617963487
GCGTCCAA -0.384675814451
GCGTCCAC -1.28896595257
GCGTCCCA -0.859805343275
GCGTCCCC -0.859360471802
GCGTCCGA -0.88042495307
GCGTCCTA 0.272138753359
GCGTCGAA -0.0969375461896
GCGTCGAC -1.12611920781
GCGTCGCA -0.721287213134
GCGTCGCC -1.02202712464
GCGTCGGA -1.50765687485
GCGTCGTA -0.0156081404959
GCGTCTAA 0.120394191804
GCGTCTAC -0.510873461785
GCGTCTCA -1.04822723146
GCGTCTCC -1.49852211091
GCGTCTGA -1.26781573814
GCGTCTTA -0.400448886733
GCGTGAAA 0.233787016236
GCGTGAAC -0.475283743964
GCGTGACA -0.412721798091
GCGTGACC -0.136686367932
GCGTGAGA 0.422118727772
GCGTGAGC -0.872137849637
GCGTGATA 2.38143567813
GCGTGCAA -0.739901241959
GCGTGCAC -0.488759010654
GCGTGCCA -0.907655426137
GCGTGCCC -1.70794365773
GCGTGCGA 0.534406169443
GCGTGCTA -0.0306778349085

GCGTGGAA -0.198638771927
GCGTGGAC -1.02260190276
GCGTGGCA 0.308960624012
GCGTGGCC -1.23153623429
GCGTGGGA -0.115540596988
GCGTGGTA 0.0492312232041
GCGTGTAA 0.619854949056
GCGTGTAC 0.121170233757
GCGTGTCA -0.147272845246
GCGTGTCC -0.563605630057
GCGTGTGA -0.331645148399
GCGTGTTA 0.162113046619
GCGTTAAA -0.30586900252
GCGTTAAC 2.57122119409
GCGTTACA -0.101444287631
GCGTTACC -0.169030771883
GCGTTAGA -0.967462148629
GCGTTAGC -0.380469661843
GCGTTATA 1.22566351264
GCGTTCAA -0.494977278772
GCGTTCAC -0.66695251431
GCGTTCCA -0.620808207729
GCGTTCCC 0.0666421125529
GCGTTCGA 0.0967948318394
GCGTTCTA -0.328299986761
GCGTTGAA -0.553026471427
GCGTTGAC -0.676142429768
GCGTTGCA -0.970047212592
GCGTTGCC -1.24701028603
GCGTTGGA 0.818950354628
GCGTTGTA -0.542931914272
GCGTTTAA 0.430737263067
GCGTTTAC 0.964340206872
GCGTTTCA 0.388209693601
GCGTTTCC -0.822269639489
GCGTTTGA -0.225536506225
GCGTTTTA 0.132638873951
GCTAAAAA 0.808288599414
GCTAAAAC -0.181378699758
GCTAAACA -0.203905872425
GCTAAACC -0.24880940058
GCTAAAGA 1.36417197166
GCTAAAGC -0.349167647688
GCTAAATA 2.00290076285
GCTAACAA 0.303467428436
GCTAACAC 0.164714839005
GCTAACCA -0.409363305992
GCTAACCC -0.590928370827
GCTAACGA 1.53855061096
GCTAACTA -0.52517783089
GCTAAGAA 0.888294368717
GCTAAGAC -0.908181325904
GCTAAGCA -0.66615059558
GCTAAGCC -0.663481105361

GCTAAGGA 0.153308669012
GCTAAGTA 0.0525993864226
GCTAATAA 1.42012331564
GCTAATAC 1.24033878223
GCTAATCA -0.217451189383
GCTAATCC 1.68326400763
GCTAATGA -0.238866183368
GCTAATTA 0.613762144103
GCTACAAA 1.66353178835
GCTACAAC -0.392393367392
GCTACACA -0.110533571176
GCTACACC -0.222154489782
GCTACAGA 0.711027985575
GCTACAGC 0.767967351982
GCTACATA 0.315678130975
GCTACCAA 0.0578683165917
GCTACCAC -0.704204880449
GCTACCCA -0.925820401384
GCTACCCC -0.844957823217
GCTACCGA 1.08404879476
GCTACCTA -0.865272400692
GCTACGAA 0.667117968859
GCTACGAC -0.383731704134
GCTACGCA 0.162286081235
GCTACGCC -0.816032290514
GCTACGGA -0.170588083419
GCTACGTA -0.0541749946704
GCTACTAA 0.0462995672473
GCTACTAC -1.02802374116
GCTACTCA -0.21546651894
GCTACTCC -1.27038616473
GCTACTGA -0.258394002245
GCTACTTA -0.00203511708982
GCTAGAAA 0.464549063286
GCTAGAAC 0.605159030323
GCTAGACA -0.359101716545
GCTAGACC -0.843065943241
GCTAGAGA 1.0286296237
GCTAGAGC -0.882164447577
GCTAGATA 1.24330807713
GCTAGCAA -0.254539669262
GCTAGCAC -0.0841586000657
GCTAGCCA -0.793537006402
GCTAGCCC -0.670820439136
GCTAGCGA 0.328938803376
GCTAGCTA -0.255510963265
GCTAGGAA -0.622698258034
GCTAGGAC -0.759467222549
GCTAGGCA -0.559190372173
GCTAGGCC -1.31129656628
GCTAGGGA -0.24954911049
GCTAGGTA 0.171449074389
GCTAGTAA 0.528569518452
GCTAGTAC -0.995623401552

GCTAGTCA 0.0960185285056
GCTAGTCC -0.409713034564
GCTAGTGA -0.570686457435
GCTAGTTA -0.623390657876
GCTATAAA 0.241134452839
GCTATAAC -0.0164691314661
GCTATACA -0.176780213377
GCTATACC 0.458545389456
GCTATAGA -0.314723565394
GCTATAGC -0.819984902977
GCTATATA 2.71144928262
GCTATCAA 0.297781071861
GCTATCAC 0.640565258264
GCTATCCA 1.53504391549
GCTATCCC 1.74849648925
GCTATCGA 0.0190745831938
GCTATCTA 1.11560591735
GCTATGAA 0.263590268331
GCTATGAC -0.923954136804
GCTATGCA -0.811265213005
GCTATGCC -0.838999368404
GCTATGGA 1.00634370624
GCTATGTA 0.135558244972
GCTATTAA -0.221124123539
GCTATTAC 0.61410769057
GCTATTCA 0.280264322967
GCTATTCC 0.593945237183
GCTATTGA 0.371583210415
GCTATTTA 0.689043702478
GCTCAAAA -0.482687900373
GCTCAAAC -0.740966110573
GCTCAACA -0.303503237714
GCTCAACC -0.674500430596
GCTCAAGA -0.622184120439
GCTCAAGC -0.751441239328
GCTCAATA 0.0257941812088
GCTCACAA -0.69371772814
GCTCACAC -0.788777508959
GCTCACCA -1.2175128504
GCTCACCC -1.03159003163
GCTCACGA -0.344501986238
GCTCACTA -0.71219479301
GCTCAGAA -0.532881269225
GCTCAGAC -0.167536714692
GCTCAGCA -0.805878138356
GCTCAGCC -1.07313872703
GCTCAGGA 0.30899146704
GCTCAGTA -0.469279808753
GCTCATAA -0.753505631157
GCTCATAC -0.101961823187
GCTCATCA -0.803713898759
GCTCATCC -0.707716542162
GCTCATGA -0.672846146488
GCTCATTA -0.140802082509

GCTCCAAA -0.348366774485
GCTCCAAC -0.728465797228
GCTCCACA -1.12161769401
GCTCCACC -1.31480012516
GCTCCAGA -1.07404833497
GCTCCAGC -1.51659194325
GCTCCATA -0.882559917929
GCTCCCAA -0.854403631256
GCTCCCAC -0.471978835091
GCTCCCCA -1.10021550772
GCTCCCCC -0.823264196453
GCTCCCGA -1.03089057449
GCTCCCTA -1.15998694369
GCTCCGAA -0.471254808076
GCTCCGAC -0.71367238316
GCTCCGCA -0.861935864647
GCTCCGCC -0.427055703317
GCTCCGGA -0.220886789052
GCTCCGTA -0.266091428803
GCTCCTAA 0.375500274981
GCTCCTAC -0.421888189207
GCTCCTCA -0.312790125744
GCTCCTCC -1.58007264543
GCTCCTGA -1.22854132399
GCTCCTTA 0.358122581094
GCTCGAAA 0.359674664998
GCTCGAAC -1.08868256765
GCTCGACA -0.621572226128
GCTCGACC -1.35560022367
GCTCGAGA -0.54949781991
GCTCGAGC 0.960637475213
GCTCGATA 0.939401266238
GCTCGCAA -0.971408487933
GCTCGCAC -0.854808772727
GCTCGCCA -0.167649108777
GCTCGCCC -1.42528142002
GCTCGCGA -0.150590823199
GCTCGCTA -0.461095428286
GCTCGGAA 0.53983872449
GCTCGGAC -1.64066246718
GCTCGGCA -0.710357803168
GCTCGGCC -0.33145172602
GCTCGGGA -1.15745232638
GCTCGGTA -1.13814694324
GCTCGTAA 0.470035985704
GCTCGTAC 0.399033505402
GCTCGTCA -0.579158880418
GCTCGTCC -1.28220270383
GCTCGTGA -0.0430811756821
GCTCGTTA -1.22464203337
GCTCTAAA 0.196364229298
GCTCTAAC -0.71708837921
GCTCTACA -0.0989062723546
GCTCTACC -1.23625626311

GCTCTAGA -0.223163422735
GCTCTATA -0.0638597054863
GCTCTCAA -0.264463021473
GCTCTCAC -0.625852872491
GCTCTCCA -1.513014152
GCTCTCCC -1.31383092221
GCTCTCGA -0.21519755728
GCTCTCTA -0.569874083441
GCTCTGAA -0.912294426665
GCTCTGAC -1.90093371203
GCTCTGCA -0.776068090339
GCTCTGCC -1.62425136242
GCTCTGGA -0.359101716545
GCTCTGTA 1.10327550204
GCTCTTAA -0.404566430981
GCTCTTAC 0.0806082538762
GCTCTTCA -0.348415914224
GCTCTTCC -0.595760793734
GCTCTTGA -0.49725966285
GCTCTTTA -0.338830528414
GCTGAAAA -0.0140022119832
GCTGAAAC 0.117934068243
GCTGAACA -0.00723739495247
GCTGAACC -1.24537874212
GCTGAAGA -0.268283113467
GCTGAAGC -0.457989169425
GCTGAATA 1.14033000231
GCTGACAA -0.280518124495
GCTGACAC 0.0713132630169
GCTGACCA -0.737012713965
GCTGACCC -1.12546261725
GCTGACGA -0.0197191502042
GCTGACTA -0.934066990671
GCTGAGAA -0.0748999412478
GCTGAGAC -0.701630794512
GCTGAGCA -0.233770549195
GCTGAGCC -0.923455420723
GCTGAGGA -0.298670814807
GCTGAGTA 0.27374442049
GCTGATAA 1.20016547678
GCTGATAC 1.46602506011
GCTGATCA 0.658630908506
GCTGATCC 0.762135928623
GCTGATGA -0.152545957521
GCTGATTA 2.03244106506
GCTGCAAA 0.407796584718
GCTGCAAC -0.569797498634
GCTGCACA -0.645319266694
GCTGCACC -1.70864050106
GCTGCAGA -0.213180214139
GCTGCAGC -0.766851775339
GCTGCATA 0.20626327302
GCTGCCAA 0.081341429247
GCTGCCAC -1.15265728104

GCTGCCCA −1.21084579008
GCTGCCCC −1.22357272127
GCTGCCGA −0.949400680493
GCTGCCTA −0.643788354698
GCTGCGAA −0.307238903454
GCTGCGAC −1.61736134361
GCTGCGCA −0.839393531847
GCTGCGCC 0.12491138851
GCTGCGGA −0.644283672819
GCTGCGTA 0.157116998834
GCTGCTAA −0.440069631494
GCTGCTAC −0.173109893036
GCTGCTCA −1.1235064374
GCTGCTCC −0.897433314765
GCTGCTGA 0.130824885689
GCTGCTTA 0.0625794584395
GCTGGAAA −0.297970834898
GCTGGAAC −0.266015889522
GCTGGACA −0.515469334349
GCTGGACC −0.968909941278
GCTGGAGA 0.573334775744
GCTGGATA 1.65398665531
GCTGGCAA −0.404904658764
GCTGGCAC −1.56039975977
GCTGGCCA −1.38649605083
GCTGGCCC −1.80817095266
GCTGGCGA −0.744929701062
GCTGGCTA −1.32506196654
GCTGGGAA −0.716188442382
GCTGGGAC −1.57254015066
GCTGGGCA −1.13210144835
GCTGGGCC −0.90978281093
GCTGGGGA −0.241344080878
GCTGGGTA 0.405816880526
GCTGGTAA 0.0916871741137
GCTGGTAC −0.803496429273
GCTGGTCA −1.31622831419
GCTGGTCC −1.2292980237
GCTGGTGA −0.758438685978
GCTGGTTA 0.609965315071
GCTGTAAA −0.316683927348
GCTGTAAC 0.169188646365
GCTGTACA 0.26552187831
GCTGTACC −0.384874987226
GCTGTAGA −0.0722500546493
GCTGTATA 0.885531042506
GCTGTCAA −0.0116126614521
GCTGTCAC −0.716610050893
GCTGTCCA −0.705655025531
GCTGTCCC −0.569243108273
GCTGTCGA −0.444259055681
GCTGTCTA −0.821733023076
GCTGTGAA 1.23966337219
GCTGTGAC −0.541275800493

GCTGTGCA 0.0944167820984
GCTGTGCC -0.933161826211
GCTGTGGA 0.101903796473
GCTGTGTA 0.48992764776
GCTGTTAA -0.385822756885
GCTGTTAC -0.742208457288
GCTGTTCA -1.01049026385
GCTGTTCC -1.02512292824
GCTGTTGA -0.265595326538
GCTGTTTA -0.197270962047
GCTTAAAA 0.676813657701
GCTTAAAC -0.261429426695
GCTTAACA -0.0177433667362
GCTTAACC -0.905731134843
GCTTAAGA 0.344837861586
GCTTAAGC -0.219899812154
GCTTAATA -0.0302967405447
GCTTACAA 0.351582290852
GCTTACAC 0.706728519737
GCTTACCA -0.745582632283
GCTTACCC -1.01038074496
GCTTACGA -0.352014093246
GCTTACTA -0.332663491089
GCTTAGAA -0.118418669718
GCTTAGAC -0.934395024571
GCTTAGCA -0.501709945867
GCTTAGCC -0.517438321896
GCTTAGGA 0.46080738577
GCTTAGTA -0.0499897525895
GCTTATAA 0.695767482599
GCTTATAC 0.306176125893
GCTTATCA 0.293833164268
GCTTATCC -0.448836108771
GCTTATGA -0.209452389847
GCTTATTA 0.073920283034
GCTTCAAA 0.546793043176
GCTTCAAC -0.74829943257
GCTTCACA -0.824245422957
GCTTCACC -1.03006408589
GCTTCAGA 0.209738341311
GCTTCATA 0.368385990759
GCTTCCAA -0.0447135037358
GCTTCCAC -0.769013401121
GCTTCCCA -1.07692091869
GCTTCCCC -1.44380187423
GCTTCCGA -0.342315529205
GCTTCCTA -0.490128911588
GCTTCGAA 0.536321835145
GCTTCGAC -0.739912219986
GCTTCGCA -0.2241153745
GCTTCGCC -0.398833809865
GCTTCGGA 0.0969568884276
GCTTCGTA -0.00199434156118
GCTTCTAA -0.0687742022141

GCTTCTAC -0.842939957313
GCTTCTCA -0.0888566728338
GCTTCTCC -0.518474438534
GCTTCTGA -0.218823965513
GCTTCTTA -0.481163261536
GCTTGAAA -0.305911084957
GCTTGAAC -0.112739109065
GCTTGACA 0.197787452076
GCTTGACC -1.43979672407
GCTTGAGA -0.887716715394
GCTTGATA 1.60383327784
GCTTGCAA -0.279934982159
GCTTGCAC -0.0873354319575
GCTTGCCA 0.159839549516
GCTTGCCC -0.887362281953
GCTTGCGA -0.0239049150483
GCTTGCTA -0.714019759298
GCTTGGAA 0.371551060479
GCTTGGAC -1.19122256692
GCTTGGCA -0.39457747199
GCTTGGCC -0.803396842885
GCTTGGGA -1.04315851959
GCTTGGTA -0.301740480245
GCTTGTAA 1.424750554
GCTTGTAC -1.13677887198
GCTTGTCA -0.584183680178
GCTTGTCC -0.829672750372
GCTTGTGA 0.629896707129
GCTTGTTA -0.354329934167
GCTTTAAA 0.838291808429
GCTTTAAC -0.791053097115
GCTTTACA 0.97697382483
GCTTTACC 0.554805434555
GCTTTAGA -0.877249689468
GCTTTATA -0.357170629329
GCTTTCAA -0.338173415087
GCTTTCAC 0.324229752581
GCTTTCCA -0.297970834898
GCTTTCCC -1.28972369781
GCTTTCGA -0.503017115218
GCTTTCTA 0.0607320133341
GCTTTGAA 1.08546522161
GCTTTGAC -0.281233787299
GCTTTGCA -0.454135881968
GCTTTGCC -1.73112140918
GCTTTGGA -0.139626910863
GCTTTGTA -0.034302152089
GCTTTTAA 0.478103528599
GCTTTTAC 0.542224354297
GCTTTTCA 0.129213990926
GCTTTTCC -0.14825250346
GCTTTTGA 0.345004361661
GCTTTTTA 0.288873709906
GGAAAAAA 1.26477011981

GGAAAAAC 0.781463790581
GGAAAACA -0.0382521507355
GGAAAACC -0.277338417405
GGAAAAGA 1.13163435945
GGAAAATA 3.98636257656
GGAAACAA 1.67969458059
GGAAACAC -0.10267905428
GGAAACCA 0.214235673015
GGAAACCC -0.162763364025
GGAAACGA -0.459718992813
GGAAACTA 0.538986097731
GGAAAGAA 0.296692156141
GGAAAGAC 0.662969058819
GGAAAGCA -0.259331578022
GGAAAGCC -0.845256843761
GGAAAGGA 0.740074276574
GGAAAGTA 0.561910570421
GGAAATAA 1.00367787537
GGAAATAC 1.32983061233
GGAAATCA 12.2207908229
GGAAATCC 29.2895238239
GGAAATGA 0.934205784297
GGAAATTA 2.06210839873
GGAACAAA 0.0603848985774
GGAACAAC -0.0340483505614
GGAACACA -0.91978640729
GGAACACC -0.855595269943
GGAACAGA 0.0663872654989
GGAACATA 0.31418172135
GGAACCAA -0.128882559065
GGAACCAC -0.95931017948
GGAACCCA -0.0827016590615
GGAACCCC -0.415573732662
GGAACCGA 0.483530856014
GGAACCTA -0.829322499036
GGAACGAA -0.480856660927
GGAACGAC -0.629720797317
GGAACGCA -0.140917613173
GGAACGCC -1.06966810222
GGAACGGA -1.0257539034
GGAACGTA -0.0119095909428
GGAACTAA -0.25840132093
GGAACTAC -0.411876751398
GGAACTCA -0.096062701995
GGAACTCC -1.19458184317
GGAACTGA -0.11130987451
GGAACTTA 0.0652489486576
GGAAGAAA 0.0447325845921
GGAAGAAC -0.716030045137
GGAAGACA -0.941466703593
GGAAGACC -1.1935760468
GGAAGAGA -0.639505355901
GGAAGATA 0.894742652637
GGAAGCAA -1.05988720298

GGAAGCAC	-0.243575756926
GGAAGCCA	-1.25434203974
GGAAGCCC	-1.14868846292
GGAAGCGA	0.234666303918
GGAAGCTA	0.276682872368
GGAAGGAA	-0.415624963454
GGAAGGAC	-1.06951127327
GGAAGGCA	-1.18670537022
GGAAGGCC	-1.72175506115
GGAAGGGA	-1.07598412706
GGAAGGTA	-0.023494545946
GGAAGTAA	0.643774501474
GGAAGTAC	0.00813419520102
GGAAGTCA	-0.268291216297
GGAAGTCC	-1.35105113841
GGAAGTGA	-0.543239299026
GGAAGTTA	0.878260974855
GGAATAAA	0.510389121691
GGAATAAC	0.454035250054
GGAATACA	1.20190000503
GGAATACC	0.759809893819
GGAATAGA	1.42637556337
GGAATATA	4.32773660926
GGAATCAA	6.34713656246
GGAATCAC	5.51610776438
GGAATCCA	17.2564619666
GGAATCCC	10.8791724235
GGAATCGA	4.00967886026
GGAATCTA	22.8938889522
GGAATGAA	0.0345962063821
GGAATGAC	-0.553195323936
GGAATGCA	0.0962887970737
GGAATGCC	-1.37434442053
GGAATGGA	0.216436506035
GGAATGTA	0.871832033172
GGAATTAA	0.433070093792
GGAATTAC	3.11305373738
GGAATTCA	0.930406341449
GGAATTCC	2.17913390455
GGAATTGA	2.70723607271
GGAATTTA	3.30814739075
GGACAAAA	0.15269834299
GGACAAAC	0.406530452278
GGACAACA	0.737971723033
GGACAACC	-0.87814701248
GGACAAGA	-0.055158050845
GGACAATA	0.825181430444
GGACACAA	0.30903407224
GGACACAC	-0.975750297589
GGACACCA	-0.432991417932
GGACACCC	-1.03901692824
GGACACGA	0.372075653338
GGACACTA	-0.654417175831
GGACAGAA	0.0141242771876

GGACAGAC -1.70543491719
GGACAGCA 0.0607320133341
GGACAGCC -0.864239159252
GGACAGGA -0.505410847854
GGACAGTA -0.293099988897
GGACATAA 1.22220674106
GGACATAC -0.479229821887
GGACATCA 0.352691071574
GGACATCC 0.687891009649
GGACATGA -0.0431943539123
GGACATTA -0.0905342199032
GGACCAAA -0.180301023447
GGACCAAC -0.730735634989
GGACCACA -0.119678267619
GGACCACC -1.40956715859
GGACCAGA -0.373232266891
GGACCATA 1.05820965591
GGACCCAA -1.17513270015
GGACCCAC -1.02854389054
GGACCCCA -1.39487908131
GGACCCCC -1.32291158016
GGACCCGA -0.775158482392
GGACCCTA -0.745389209904
GGACCGAA -0.0791821559001
GGACCGAC -0.28120947881
GGACCGCA -1.0763129451
GGACCGCC -1.20825053224
GGACCGGA -0.407582251811
GGACCGTA 0.358682460468
GGACCTAA -0.373017149839
GGACCTAC -0.140599511773
GGACCTCA -0.0296072159001
GGACCTCC -0.861020244924
GGACCTGA -1.52439653627
GGACCTTA -1.80227758186
GGACGAAA -0.129251368494
GGACGAAC -0.183905475626
GGACGACA -0.7319411269
GGACGACC -1.22954058582
GGACGAGA -0.412804917438
GGACGATA 0.704043869387
GGACGCAA 0.44236482327
GGACGCAC -0.467285467192
GGACGCCA -0.88774389908
GGACGCCC -1.58949780432
GGACGCGA -0.0189485972655
GGACGCTA -1.12673450009
GGACGGAA 1.92699188865
GGACGGAC -1.23656234096
GGACGGCA -0.721583881243
GGACGGCC -1.14338503038
GGACGGGA -0.892550967973
GGACGGTA -0.204771045501
GGACGTAA 0.237511181185

GGACGTAC -0.317539429305
GGACGTCA -0.790084155547
GGACGTCC -1.29291072358
GGACGTGA -0.258559195413
GGACGTTA 0.187005461329
GGACTAAA -0.16731819968
GGACTAAC -0.651942676282
GGACTACA -0.275660870336
GGACTACC -0.560119583739
GGACTAGA 0.073171163386
GGACTATA -0.419603191313
GGACTCAA 0.166798050308
GGACTCAC -0.870007328265
GGACTCCA -0.867775913599
GGACTCCC -1.10070664373
GGACTCGA -0.664246430668
GGACTCTA -1.31381131859
GGACTGAA 0.280795188984
GGACTGAC -1.02708642677
GGACTGCA -0.738422344901
GGACTGCC -1.26595156461
GGACTGGA -0.497753151299
GGACTGTA 0.0624566090904
GGACTTAA -0.79302025499
GGACTTAC -0.171341385172
GGACTTCA -0.270481332672
GGACTTGA 0.0713702442043
GGACTTTA -0.865148244435
GGAGAAAA -0.0949369314702
GGAGAAAC -1.19731014425
GGAGAACA 0.0661068030487
GGAGAACC -0.100980858065
GGAGAAGA -0.560234330259
GGAGAATA 2.4627243083
GGAGACAA -0.774782877042
GGAGACAC -0.654515193929
GGAGACCA -0.585785687967
GGAGACCC -1.24775182561
GGAGACGA -0.759603140978
GGAGACTA -0.894950189623
GGAGAGAA 0.212221466453
GGAGAGAC -0.847804268775
GGAGAGCA -1.05374316724
GGAGAGCC -1.46814695588
GGAGAGGA -1.20517250259
GGAGAGTA -0.66080455784
GGAGATAA 1.58516331335
GGAGATAC 3.73505175413
GGAGATCA 2.93665200455
GGAGATCC 6.71478885509
GGAGATGA -0.508858209696
GGAGATTA 2.93423971382
GGAGCAAA 0.60838108123
GGAGCAAC -0.872727265131

GGAGCACA −0.519849305718
GGAGCACC −1.95784772442
GGAGCAGA −0.281932198917
GGAGCATA −0.325935006099
GGAGCCAA −0.974156131248
GGAGCCAC −0.162914442586
GGAGCCCA −1.20231141966
GGAGCCCC −1.19955933286
GGAGCCGA −0.810619077705
GGAGCCTA −0.306104507336
GGAGCGAA 0.321626391906
GGAGCGAC −0.658955021682
GGAGCGCA 0.29592944465
GGAGCGCC −1.82088298509
GGAGCGGA 0.823572888116
GGAGCGTA −0.746595224578
GGAGCTAA 0.0298489938744
GGAGCTAC −1.1272057711
GGAGCTCA −1.42939060005
GGAGCTCC −0.0182998481495
GGAGCTGA −0.629416810523
GGAGCTTA −0.365403888154
GGAGGAAA −0.0171220626876
GGAGGAAC −0.941879163748
GGAGGACA −1.74016887167
GGAGGACC −1.56891165137
GGAGGAGA −0.291341152152
GGAGGATA 1.86794761627
GGAGGCAA −0.443812354537
GGAGGCAC −1.20300068293
GGAGGCCA −0.602975709869
GGAGGCCC −1.49178578447
GGAGGCGA −0.690199270505
GGAGGCTA −1.22283823899
GGAGGGAA 0.604641494767
GGAGGGAC −1.8388738802
GGAGGGCA −0.620754624502
GGAGGGCC −1.60176104456
GGAGGGGA −1.26601220514
GGAGGGTA −0.490405191932
GGAGGTAA 0.373362173543
GGAGGTAC −0.465998946987
GGAGGTCA −0.799980324072
GGAGGTGA 0.0186009597457
GGAGGTTA −0.904063520275
GGAGTAAA 0.0462316080329
GGAGTAAC −0.211077137835
GGAGTACA 0.380808935152
GGAGTACC −0.597896281356
GGAGTAGA 0.716573196089
GGAGTATA 0.542000611653
GGAGTCAA −1.14039587047
GGAGTCAC −0.299888330271
GGAGTCCA −0.633759927087

GGAGTCCC -1.23213558229
GGAGTCGA -0.322183134701
GGAGTCTA 0.0953621993234
GGAGTGAA -0.316408169766
GGAGTGAC -0.894148270893
GGAGTGCA -0.812556960842
GGAGTGCC -1.47699655251
GGAGTGGA -0.551840321754
GGAGTGTA 0.0439186423088
GGAGTTAA 0.142469697077
GGAGTTAC -0.809776122065
GGAGTTCA 0.0656350092718
GGAGTTGA -0.631643520321
GGAGTTTA 0.00678442065031
GGATAAAA 0.737125630814
GGATAAAC 0.333575190088
GGATAACA 1.85725946151
GGATAACC 0.350495204804
GGATAAGA 1.18687004062
GGATAATA 1.70938099511
GGATACAA 0.929879657537
GGATACAC 3.09507172925
GGATACCA 2.49280096554
GGATACCC 4.89134302347
GGATACGA 2.61716005474
GGATACTA 2.25231944392
GGATAGAA 0.241294418375
GGATAGAC -1.01897392624
GGATAGCA 0.281429039349
GGATAGCC 1.38765841478
GGATAGGA 1.80394911715
GGATAGTA 0.936131905262
GGATATAA 2.36173273359
GGATATAC 3.46754363709
GGATATCA 10.0395629904
GGATATCC 29.6211721089
GGATATGA 3.37719813469
GGATATTA 4.80724506393
GGATCAAA 0.99132158328
GGATCAAC 0.146774129165
GGATCACA 2.55316443083
GGATCACC 1.36635503073
GGATCAGA 1.71569675856
GGATCATA 1.32428670873
GGATCCAA 1.78711405146
GGATCCAC 3.96454505492
GGATCCCA 3.07329446038
GGATCCCC 7.57608198239
GGATCCGA 2.89313196918
GGATCCTA 3.86793109914
GGATCGAA 0.358541314407
GGATCGAC -0.144832325304
GGATCGCA 4.79992585654
GGATCGCC 5.06133515685

GGATCGGA	1.0290697903
GGATCGTA	2.48236791922
GGATCTAA	1.44821007481
GGATCTAC	3.47697637604
GGATCTCA	5.4892027114
GGATCTGA	3.41468548289
GGATCTTA	3.72927234571
GGATGAAA	1.73795915168
GGATGAAC	-0.141552509065
GGATGACA	-0.327444223422
GGATGACC	-0.0860324447121
GGATGAGA	-0.430915525314
GGATGATA	0.884017120315
GGATGCAA	0.0845768106158
GGATGCAC	0.410685374094
GGATGCCA	-0.522375820204
GGATGCCC	-0.297076387084
GGATGCGA	0.439334887834
GGATGCTA	-0.194596505578
GGATGGAA	0.13111528064
GGATGGAC	-0.256014906978
GGATGGCA	-0.337096784302
GGATGGCC	-1.42253064012
GGATGGGA	0.472082342202
GGATGGTA	0.129255811981
GGATGTAA	1.99370065351
GGATGTAC	0.889134449159
GGATGTCA	1.37347558811
GGATGTGA	1.80088102
GGATGTTA	1.11895264727
GGATTAAA	-0.413489737214
GGATTAAC	0.962459304923
GGATTACA	19.041406028
GGATTACC	13.0995612305
GGATTAGA	2.24525011734
GGATTATA	2.57181714413
GGATTCAA	1.64371279038
GGATTCAC	2.93832955162
GGATTCCA	8.43832026555
GGATTCCC	23.9767515973
GGATTCGA	5.07650417627
GGATTCTA	6.90252252554
GGATTGAA	0.151274597448
GGATTGAC	0.73551133809
GGATTGCA	16.3755341153
GGATTGCC	11.6122986545
GGATTGGA	1.50312817735
GGATTGTA	4.69816555856
GGATTTAA	1.76912289497
GGATTTAC	2.82090021124
GGATTTCA	12.3160194562
GGATTTGA	5.2154954071
GGATTTTA	8.61773233019
GGCAAAAA	-0.263144351332

GGCAAAAC -0.489729259131
GGCAAACA -0.490008414673
GGCAAACC -0.0781949176201
GGCAAAGA 0.370483316668
GGCAAATA 2.05778828374
GGCAACAA 0.196905027815
GGCAACAC -0.779026929981
GGCAACCA -0.478036614911
GGCAACCC -0.756724545485
GGCAACGA -0.576246566699
GGCAACTA 0.0624330847469
GGCAAGAA -0.207761250935
GGCAAGAC 0.314202631877
GGCAAGCA -0.889375965752
GGCAAGCC -1.40877543375
GGCAAGGA 0.0984943349627
GGCAAGTA -0.0571552676037
GGCAATAA 0.910318120434
GGCAATAC 0.50760932844
GGCAATCA 5.01056622573
GGCAATGA -0.490734794122
GGCAATTA 0.292749476179
GGCACAAA -0.764484703625
GGCACAAC -0.934886944731
GGCACACA -0.993593512094
GGCACACC -1.18293598625
GGCACAGA 0.55129037488
GGCACATA -0.586585254263
GGCACCAA -0.308673365641
GGCACCAC -0.851976964539
GGCACCCA -1.20772776905
GGCACCCC -2.01186092389
GGCACCGA -1.93363620876
GGCACCTA -0.482126191328
GGCACGAA -1.39960015566
GGCACGAC -1.04003736198
GGCACGCA -0.586062491075
GGCACGCC -0.948496822942
GGCACGGA -1.51005113025
GGCACGTA -0.289417906384
GGCACTAA -0.619885269321
GGCACTAC -0.490222224817
GGCACTCA -0.828583311888
GGCACTGA -0.798496722145
GGCACTTA -0.875773144844
GGCAGAAA 0.898697356153
GGCAGAAC 0.252615377968
GGCAGACA -0.504076233436
GGCAGACC -1.43846890557
GGCAGAGA 0.102414536107
GGCAGATA 2.7505216488
GGCAGCAA 0.670855202888
GGCAGCAC -1.2075751222
GGCAGCCA -0.592846388962

GGCAGCCC -1.34643618499
GGCAGCGA -0.347775006556
GGCAGCTA -1.10187031459
GGCAGGAA 0.140006436937
GGCAGGAC -1.19611902832
GGCAGGCA -0.239609291239
GGCAGGCC -0.786621894954
GGCAGGGA -0.50539699463
GGCAGGTA -0.890079605003
GGCAGTAA 0.999957631141
GGCAGTAC -0.127115358109
GGCAGTCA 0.244411916645
GGCAGTGA -0.724196390274
GGCAGTTA -0.70213055612
GGCATAAA 0.180673230836
GGCATAAC -0.460215617841
GGCATACA 1.05587499552
GGCATACC -0.595759748208
GGCATAGA -0.0893527750989
GGCATATA -1.27672780496
GGCATCAA -0.559377521395
GGCATCAC 0.802779982324
GGCATCCA 0.681335559275
GGCATCCC -0.3381689716
GGCATCGA 0.1206001605
GGCATCTA 0.337527279787
GGCATGAA 0.910658177888
GGCATGAC -1.28473444594
GGCATGCA -0.379292399144
GGCATGCC -1.23201377846
GGCATGGA 0.324673055764
GGCATGTA -0.310096849801
GGCATTAA 0.403386554467
GGCATTAC -0.218424574438
GGCATTCA 0.519816110255
GGCATTGA 0.746350832788
GGCATTTA 0.775350597864
GGCCAAAA 0.0265971454651
GGCCAAAC -0.424464627577
GGCCAACA -0.73794009586
GGCCAACC -1.12652565619
GGCCAAGA 0.355271430668
GGCCAATA -0.231447912352
GGCCACAA -0.295255080138
GGCCACAC -1.40470546095
GGCCACCA -1.30385398677
GGCCACCC -1.06493003807
GGCCACGA -0.657672422201
GGCCACTA -0.874345478579
GGCCAGAA 0.815929828929
GGCCAGAC -1.00156068446
GGCCAGCA -1.5446151867
GGCCAGCC -1.66016153408
GGCCAGGA -0.21602247759

GGCCAGTA	−0.734096479523
GGCCATAA	−0.431503372518
GGCCATAC	−1.1443659955
GGCCATCA	−1.6056770636
GGCCATGA	−0.643872780953
GGCCATTA	−0.591180342683
GGCCCAAA	−1.05243599789
GGCCCAAC	−1.2077682832
GGCCCACA	−1.39833663703
GGCCCACC	−1.19672360395
GGCCCAGA	−0.675492635126
GGCCCATA	−0.0200056244311
GGCCCCAA	−0.474148825084
GGCCCCAC	−0.906620877789
GGCCCCCA	−1.69929454079
GGCCCCCC	−1.49127922694
GGCCCCGA	−0.509076724708
GGCCCCTA	−1.48145964322
GGCCCGAA	−0.384449457991
GGCCCGAC	−1.29909004584
GGCCCGCA	−1.30439478529
GGCCCGCC	−1.44690447375
GGCCCGGA	−0.977823837773
GGCCCGTA	−0.773209098465
GGCCCTAA	−1.37125096937
GGCCCTAC	−1.60297594621
GGCCCTCA	−1.19429066407
GGCCCTGA	−0.527749564392
GGCCCTTA	−0.373275394854
GGCCGAAA	0.0114602759829
GGCCGAAC	−1.16095457836
GGCCGACA	−0.68113952308
GGCCGACC	−1.50870867438
GGCCGAGA	−0.481176853379
GGCCGATA	1.65944639404
GGCCGCAA	−0.216546286305
GGCCGCAC	−1.31752973315
GGCCGCCA	−1.13681389711
GGCCGCCC	−0.970599773282
GGCCGCGA	0.0325153475134
GGCCGCTA	−0.356057143739
GGCCGGAA	−0.385439310087
GGCCGGAC	−1.30774334489
GGCCGGCA	−0.643626820873
GGCCGGCC	−1.37200348698
GGCCGGGA	−0.929847768983
GGCCGGTA	−1.08259708138
GGCCGTAA	−0.858586259521
GGCCGTAC	−1.23306505523
GGCCGTCA	−0.707673152818
GGCCGTGA	0.0348583721208
GGCCGTTA	−0.316813834
GGCCTAAA	0.740889787147
GGCCTAAC	−1.0698602177

GGCCTACA -1.34217540363
GGCCTACC -1.35661281597
GGCCTAGA -0.430728114711
GGCCTATA 0.038323246529
GGCCTCAA -1.02444699543
GGCCTCAC -0.644404431115
GGCCTCCA -0.987112294093
GGCCTCCC -0.908066318003
GGCCTCGA -0.647751683806
GGCCTCTA -0.719092914653
GGCCTGAA -1.17524587838
GGCCTGAC -0.950426341868
GGCCTGCA -1.43299113151
GGCCTGGA -0.78750353507
GGCCTGTA -0.777004359208
GGCCTTAA -0.541316576022
GGCCTTAC -1.23673067071
GGCCTTCA -0.688973652211
GGCCTTGA -1.30608853802
GGCCTTTA -1.18409782744
GGCGAAAA 0.0677574278139
GGCGAAAC -0.26126632458
GGCGAACA -0.336375893866
GGCGAACC -0.639878347436
GGCGAAGA -0.309924337949
GGCGAATA 1.7138487907
GGCGACAA -0.778393602379
GGCGACAC 0.0719761267389
GGCGACCA -0.672238434282
GGCGACCC -1.43366601879
GGCGACGA -0.263241585285
GGCGACTA -1.18458922484
GGCGAGAA -0.411543228485
GGCGAGAC -0.558793072151
GGCGAGCA -0.964901131773
GGCGAGCC -1.32260628646
GGCGAGGA 0.0404438354
GGCGAGTA 0.5151988044
GGCGATAA 0.0346539717144
GGCGATAC 0.641846289456
GGCGATCA 2.37719371625
GGCGATGA -1.12457757917
GGCGATTA 1.38418439201
GGCGCAAA 0.606794233574
GGCGCAAC -0.518890558031
GGCGCACA -0.144967459588
GGCGCACC -1.38266681048
GGCGCAGA 0.44245996617
GGCGCATA 1.18765523093
GGCGCCAA -0.535604342669
GGCGCCAC -0.893915641274
GGCGCCCA 0.120100137511
GGCGCCCC -1.36959773079
GGCGCCGA 0.344698022433

GGCGCCTA -0.0763155839602
GGCGCGAA 0.113126215206
GGCGCGAC 0.072416293343
GGCGCGCA -0.277762901114
GGCGCGCC 0.0206088931497
GGCGCGGA -0.0610689342086
GGCGCGTA 0.259928312201
GGCGCTAA -0.421778408937
GGCGCTAC -1.2964720478
GGCGCTCA -1.1230079827
GGCGCTGA -0.520406048513
GGCGCTTA -0.636029242084
GGCGGAAA 0.2108319619
GGCGGAAC -1.10109819336
GGCGGACA -0.772359608285
GGCGGACC -1.54949387415
GGCGGAGA -1.02688385603
GGCGGATA 1.33405401613
GGCGGCAA -1.23690867157
GGCGGCAC -0.867275890609
GGCGGCCA -0.979038216658
GGCGGCCC -1.25436608684
GGCGGCGA -0.833642352638
GGCGGCTA -0.233457936809
GGCGGGAA 0.209184735095
GGCGGGAC -0.739054365595
GGCGGGCA -0.0922399961847
GGCGGGGA 0.614186889193
GGCGGGTA 0.316798412486
GGCGGTAA 0.568128054394
GGCGGTAC -1.46991363408
GGCGGTCA -0.145772514897
GGCGGTGA -0.188384510619
GGCGGTTA -0.413082766073
GGCGTAAA 0.76169105715
GGCGTAAC -0.419504389071
GGCGTACA 0.172143303902
GGCGTACC -1.41177008267
GGCGTAGA -0.529435737054
GGCGTATA -0.30203976217
GGCGTCAA -0.339594546813
GGCGTCAC -0.996850326754
GGCGTCCA -0.804872341983
GGCGTCCC -1.18353376596
GGCGTCGA -0.122654358447
GGCGTCTA -1.28486409122
GGCGTGAA -0.633302509297
GGCGTGAC -0.611861638534
GGCGTGCA -0.93224646787
GGCGTGGA -0.823053522889
GGCGTGTA -0.285957214081
GGCGTTAA -0.00730221758775
GGCGTTAC -1.19339752317
GGCGTTCA -1.33140282262

GGCGTTGA -0.925758715328
GGCGTTTA 0.325991464524
GGCTAAAA 0.331512105168
GGCTAAAC 0.5517331553
GGCTAACA 0.41782004608
GGCTAACC -1.37942201937
GGCTAAGA -0.902878938891
GGCTAATA -1.00115737266
GGCTACAA -0.33518425518
GGCTACAC -1.22283823899
GGCTACCA -1.56754619393
GGCTACCC -1.63115863242
GGCTACGA -0.197198559345
GGCTACTA -1.03924851233
GGCTAGAA -0.291479684396
GGCTAGAC -0.41468451248
GGCTAGCA -0.708201927782
GGCTAGCC -0.266765531933
GGCTAGGA -0.152869809316
GGCTAGTA -0.657373401658
GGCTATAA 0.283547014404
GGCTATAC 0.275054465038
GGCTATCA 0.607589356383
GGCTATGA 0.122059453939
GGCTATTA -0.27675710474
GGCTCAAA -0.231036497724
GGCTCAAC -0.978521203866
GGCTCACA -0.76823605226
GGCTCACC -1.2175128504
GGCTCAGA -0.677855001971
GGCTCATA -0.863748546
GGCTCCAA -0.542315576474
GGCTCCAC -0.190912593394
GGCTCCCA -0.826736128164
GGCTCCCC -1.22806273429
GGCTCCGA -1.13974973517
GGCTCCTA -1.26952935587
GGCTCGAA -1.14483020921
GGCTCGAC -1.40165827433
GGCTCGCA -0.854808772727
GGCTCGGA -0.569097780107
GGCTCGTA 0.236977962734
GGCTCTAA -0.771499924223
GGCTCTAC -0.620306093687
GGCTCTCA -1.41701235191
GGCTCTGA -0.640265453576
GGCTCTTA -0.530582156725
GGCTGAAA 0.0436873195983
GGCTGAAC -0.575678845877
GGCTGACA -0.586870421582
GGCTGACC -1.15504813848
GGCTGAGA -0.23594916478
GGCTGATA 1.18838683801
GGCTGCAA -0.321803608627

GGCTGCAC −1.98552359172
GGCTGCCA −1.17273295574
GGCTGCCC −1.49568089298
GGCTGCGA −1.09990106566
GGCTGCTA −1.01317047071
GGCTGGAA 0.13559875912
GGCTGGAC −0.635264962304
GGCTGGCA −1.21753349954
GGCTGGGA −0.654491146822
GGCTGGTA −0.485437373359
GGCTGTAA 0.313864404095
GGCTGTAC −0.563012816602
GGCTGTCA −0.598943898784
GGCTGTGA −1.0957283699
GGCTGTTA −1.09246109997
GGCTTAAA −0.161845130486
GGCTTAAC −0.905731134843
GGCTTACA −0.616991252314
GGCTTACC −1.19658716276
GGCTTAGA −0.54810726983
GGCTTATA −0.646725499668
GGCTTCAA −1.19180858446
GGCTTCAC −1.51121689216
GGCTTCCA −0.656721254581
GGCTTCCC −1.83614531774
GGCTTCGA −0.336781819482
GGCTTCTA −0.962140157997
GGCTTGAA −0.622684666191
GGCTTGAC −1.6458582105
GGCTTGCA −1.19621547813
GGCTTGGA −0.288511173635
GGCTTGTA −0.911143040744
GGCTTTAA −0.269702415522
GGCTTTAC 0.499936471753
GGCTTTCA −0.749468331058
GGCTTTGA 0.263374367135
GGCTTTTA 0.211001859936
GGGAAAAA 0.299982427645
GGGAAAAC 0.152894901949
GGGAAACA 0.089960225923
GGGAAACC 0.116177845314
GGGAAAGA 0.608080231016
GGGAAATA 1.86779104869
GGGAACAA 0.324803746561
GGGAACAC −1.51961351448
GGGAACCA −1.0359245226
GGGAACCC −0.952193804205
GGGAACGA 0.0838255999151
GGGAACTA −0.543609676745
GGGAAGAA −0.521985838866
GGGAAGAC −0.3933163058
GGGAAGCA −0.926691324855
GGGAAGGA −0.522952950763
GGGAAGTA 0.0998090843798

GGGAATAA 0.713363168734
GGGAATAC 1.26010654941
GGGAATCA 14.2962605259
GGGAATGA -0.175678228578
GGGAATTA 2.90686365121
GGGACAAA 0.90910713951
GGGACAAC -0.224399757674
GGGACACA -0.931674826324
GGGACACC -1.02219649991
GGGACAGA -1.46378292879
GGGACATA -0.89408867589
GGGACCAA -0.90277700007
GGGACCAC -0.849383013602
GGGACCCA -1.19873781051
GGGACCCC -1.74330911014
GGGACCGA -1.26243310697
GGGACCTA -0.467613762474
GGGACGAA -0.556307333193
GGGACGAC -0.845682895759
GGGACGCA -1.08718537388
GGGACGGA -0.758810631986
GGGACGTA -0.448502063094
GGGACTAA -1.30864092928
GGGACTAC -0.641192051326
GGGACTCA -1.23865574614
GGGACTGA -0.698048037005
GGGACTTA -0.0957362363842
GGGAGAAA 0.283360649328
GGGAGAAC -0.627372545077
GGGAGACA -0.753626389453
GGGAGACC -0.791255406469
GGGAGAGA -0.488460774256
GGGAGATA 2.28670157878
GGGAGCAA 0.712080569253
GGGAGCAC -0.988709858394
GGGAGCCA -0.557625741952
GGGAGCCC -1.57558968971
GGGAGCGA 0.679827126097
GGGAGCTA -1.0257539034
GGGAGGAA 0.253407102816
GGGAGGAC -0.896310419437
GGGAGGCA -1.10121163297
GGGAGGGA 1.93895323315
GGGAGGTA 0.657474033571
GGGAGTAA -0.300234399501
GGGAGTAC -0.600011381213
GGGAGTCA -0.204737588657
GGGAGTGA 0.227084930788
GGGAGTTA -0.21580108738
GGGATAAA 0.140566839074
GGGATAAC 0.794825094896
GGGATACA 0.490248362976
GGGATACC 2.75849065083
GGGATAGA 0.267870653312

GGGATATA 1.03970749841
GGGATCAA 1.10515954057
GGGATCAC 1.01303533643
GGGATCCA 1.63273894554
GGGATCCC 3.46492250246
GGGATCGA 1.73495221782
GGGATCTA 4.54010261974
GGGATGAA 1.20584738986
GGGATGAC -0.682693959418
GGGATGCA 0.0712693509091
GGGATGGA -0.758490439533
GGGATGTA 0.839256045129
GGGATTAA -0.231058976541
GGGATTAC 9.28336774265
GGGATTCA 1.726505933
GGGATTGA 0.529454556529
GGGATTTA 3.52263085352
GGGCAAAA -0.0625789356763
GGGCAAAC 0.0064059401024
GGGCAACA -0.652127995832
GGGCAACC -0.994982493884
GGGCAAGA -0.503880458622
GGGCAATA 0.666266387626
GGGCACAA -0.751335379783
GGGCACAC -0.753457536943
GGGCACCA -0.764516330798
GGGCACCC -1.4076428673
GGGCACGA -0.699292474773
GGGCACTA -1.60506595344
GGGCAGAA 1.54129093559
GGGCAGAC -1.30440184259
GGGCAGCA -0.299839190531
GGGCAGGA -0.734899705161
GGGCAGTA -0.198789066344
GGGCATAA -0.0288552210545
GGGCATAC -0.811025264702
GGGCATCA -0.537315085201
GGGCATGA -0.967948841157
GGGCATTA -0.922467398298
GGGCCAAA -0.789343661491
GGGCCAAC -1.1138201583
GGGCCACA -0.919510649709
GGGCCACC -1.65033175648
GGGCCAGA -0.424555849754
GGGCCATA -0.215239901098
GGGCCCAA -1.1950031903
GGGCCCAC -1.17924292571
GGGCCCCA -0.997214431314
GGGCCCCC -0.993047747326
GGGCCCGA -0.051358346615
GGGCCCTA -1.18068993422
GGGCCGAA -1.43520241979
GGGCCGAC -1.25926908278
GGGCCGCA -1.95626244505

GGGCCGGA −1.0874449258
GGGCCGTA −1.02886329884
GGGCCTAA −0.340938309587
GGGCCTAC −1.26306068418
GGGCCTCA −1.13081832611
GGGCCTGA −1.23211284209
GGGCCTTA −0.211822075377
GGGCGAAA −0.23095233285
GGGCGAAC −0.886921069823
GGGCGACA −0.306887867973
GGGCGACC −1.34335972363
GGGCGAGA −0.594945805924
GGGCGATA 0.559415160344
GGGCGCAA 0.356913429841
GGGCGCAC −0.463016321619
GGGCGCCA −0.604856611819
GGGCGCCC −0.339857496697
GGGCGCGA −0.136070552896
GGGCGCTA −1.25018345858
GGGCGGAA −0.246530675845
GGGCGGAC −1.60419293891
GGGCGGCA −1.2175128504
GGGCGGGA 0.283281973468
GGGCGGTA −0.703259463224
GGGCGTAA −0.0262468941294
GGGCGTAC −1.09872537125
GGGCGTCA −1.3082005013
GGGCGTGA −0.791206266729
GGGCGTTA −0.105462768255
GGGCTAAA −0.00199434156118
GGGCTAAC −0.292429022345
GGGCTACA −0.784937813345
GGGCTACC −1.44406613102
GGGCTAGA −0.571084541602
GGGCTATA −0.739317315479
GGGCTCAA −1.05528714831
GGGCTCAC −0.724553960294
GGGCTCCA −1.07581004692
GGGCTCGA −1.08868256765
GGGCTCTA −1.02693247301
GGGCTGAA −0.24161016734
GGGCTGAC −1.21972361592
GGGCTGCA −0.935861375313
GGGCTGGA 0.348479691333
GGGCTGTA −0.388578764411
GGGCTTAA −0.777737273197
GGGCTTAC −0.802819450944
GGGCTTCA −1.15024838827
GGGCTTGA −0.950426341868
GGGCTTTA −0.724336229426
GGGGAAAA 0.532066804178
GGGGAAAC 0.316848074989
GGGGAACA 0.238072106086
GGGGAACC −0.692626198604

GGGGAAGA −0.611018682894
GGGGAATA 2.57701288745
GGGGACAA −0.247442113462
GGGGACAC −0.971829312299
GGGGACCA −0.71834457915
GGGGACCC −0.645369974723
GGGGACGA −0.551194709217
GGGGACTA −1.49773430678
GGGGAGAA 0.0369753016495
GGGGAGAC −0.941940327041
GGGGAGCA −0.120052043298
GGGGAGGA −0.193964484884
GGGGAGTA −0.580283866798
GGGGATAA 0.508787375284
GGGGATAC 2.16986400632
GGGGATCA 2.86807227025
GGGGATGA −0.950423728052
GGGGATTA 1.70470618531
GGGGCAAA 0.0173583516484
GGGGCAAC −0.461293032771
GGGGCACA 0.123219465453
GGGGCACC −0.885233328871
GGGGCAGA 1.50268748799
GGGGCATA −0.597967899912
GGGGCCAA −0.0495352099978
GGGGCCAC −1.52299422401
GGGGCCCA −1.17187876069
GGGGCCCC −1.54462721025
GGGGCCGA −0.0597831981484
GGGGCCTA −1.00267181761
GGGGCGAA −0.177228482811
GGGGCGAC −1.26387462646
GGGGCGCA 0.140485549398
GGGGCGGA −0.878352197031
GGGGCGTA −0.0537980824121
GGGGCTAA 0.297862100155
GGGGCTAC −0.998773049758
GGGGCTCA −0.739130689021
GGGGCTGA −0.672609596146
GGGGCTTA −0.777737273197
GGGGGAAA 0.202631375774
GGGGGAAC −0.948155981343
GGGGGACA −0.424097386438
GGGGGACC −1.55305728941
GGGGGAGA −0.295403283502
GGGGGATA 1.37439277613
GGGGGCAA −0.0608637496574
GGGGGCAC −1.59175797097
GGGGGCCA −1.30243233228
GGGGGCGA 0.378355868893
GGGGGCTA −0.961774746528
GGGGGGAA −0.724251803171
GGGGGGAC −1.21582484807
GGGGGGCA −0.625125970278

GGGGGGGA -0.139870518508
GGGGGGTA -1.0621931114
GGGGGTAA -0.472979926596
GGGGGTAC -1.24163053006
GGGGGTCA -1.09688655174
GGGGGTGA 0.705977047655
GGGGGTTA -1.51147827375
GGGGTAAA -0.0446549542588
GGGGTAAC -0.549392744509
GGGGTACA -1.13102743138
GGGGTACC -0.400785807608
GGGGTAGA -0.260717945996
GGGGTATA -0.316662755439
GGGGTCAA -1.13436893368
GGGGTCAC -0.864919012778
GGGGTCCA -0.681351764934
GGGGTCGA -0.674140508141
GGGGTCTA -0.929914159908
GGGGTGAA 0.234666303918
GGGGTGAC -0.454545728307
GGGGTGCA -0.392177727577
GGGGTGGA 0.0587915163812
GGGGTGTA -0.492831335887
GGGGTTAA -0.167735364703
GGGGTTAC 0.0787161125182
GGGGTTCA -0.556590932222
GGGGTTGA -0.400843311558
GGGGTTTA -0.916951462523
GGGTAAAA 0.714319302605
GGGTAAAC -0.803780289683
GGGTAACA -0.694249378301
GGGTAACC -0.290356789069
GGGTAAGA 0.453534965684
GGGTAATA 1.63166701962
GGGTACAA -1.14026857764
GGGTACAC -0.259881786277
GGGTACCA -0.379923374312
GGGTACCC -0.932283322674
GGGTACGA 0.9876972661
GGGTACTA -0.349355319673
GGGTAGAA 1.55616590071
GGGTAGAC -0.672369125079
GGGTAGCA -1.1747314794
GGGTAGGA 0.0344584582822
GGGTAGTA -0.131685353896
GGGTATAA 0.909086228982
GGGTATAC -0.674142860575
GGGTATCA 2.19303914396
GGGTATGA -0.398470228068
GGGTATTA 1.29973435147
GGGTCAAA -0.360524939323
GGGTCAAC -1.11545849813
GGGTCACA -0.0385002018681
GGGTCACC -0.671038431385

GGGTCAGA -0.488150775685
GGGTCATA -0.832110395116
GGGTCCAA -0.987503320957
GGGTCCAC -1.20063988437
GGGTCCCA -1.46996198967
GGGTCCGA -1.30921230945
GGGTCCTA -1.19694969903
GGGTCGAA -0.654756971903
GGGTCGAC -0.0435519239327
GGGTCGCA 0.0757557045862
GGGTCGGA -1.15776310909
GGGTCGTA -0.938658158367
GGGTCTAA -0.517816018299
GGGTCTAC -1.2494432259
GGGTCTCA -0.871246538402
GGGTCTGA -0.300280664043
GGGTCTTA -0.688002358208
GGGTGAAA 0.779854986871
GGGTGAAC -0.722460032346
GGGTGACA 0.0604946788469
GGGTGACC -0.323971246185
GGGTGAGA -1.00312296224
GGGTGATA 0.668416512617
GGGTGCAA -0.18174960024
GGGTGCAC -0.521908469914
GGGTGCCA -0.828036240212
GGGTGCGA -0.111260473389
GGGTGCTA -0.690827631856
GGGTGGAA -0.126059899233
GGGTGGAC -0.504723152881
GGGTGGCA 0.301066115733
GGGTGGGA 0.768264804236
GGGTGGTA -0.407076217046
GGGTGTAA -0.0792127375466
GGGTGTAC -1.02140947993
GGGTGTCA -0.829672750372
GGGTGTGA -0.720595597437
GGGTGTTA 0.0037139710672
GGGTTAAA -0.402028938468
GGGTTAAC -0.366701647768
GGGTTACA 0.526877334014
GGGTTACC -0.388107232016
GGGTTAGA 0.491308003957
GGGTTATA 0.307814727105
GGGTTCAA -0.767001285611
GGGTTCAC -0.640021584549
GGGTTCCA -0.326666351799
GGGTTCGA -0.956288085492
GGGTTCTA -0.649510781933
GGGTTGAA -0.491024404928
GGGTTGAC -0.700922450393
GGGTTGCA 0.0473848236251
GGGTTGGA 0.434074844639
GGGTTGTA -0.260992658051

GGGTTTAA -0.459470680299
GGGTTTAC -0.117982423838
GGGTTTCA 1.07828872857
GGGTTTGA -0.60887953593
GGGTTTTA 0.625066898038
GGTAAAAA 1.96213412118
GGTAAAAC -0.225033608039
GGTAAACA 1.97564702682
GGTAAACC -0.786506102908
GGTAAAGA 0.916155294188
GGTAAATA 0.176537651258
GGTAACAA -0.0582342508231
GGTAACAC -0.218063867839
GGTAACCA -0.212380386462
GGTAACGA 0.696123222948
GGTAACTA 0.869123074333
GGTAAGAA 0.561557705269
GGTAAGAC -0.164115752392
GGTAAGCA -0.374938043172
GGTAAGGA 1.1901749495
GGTAAGTA 1.26152088521
GGTAATAA 2.59532528192
GGTAATAC 1.32592243474
GGTAATCA 4.55931782623
GGTAATGA -0.597232894871
GGTAATTA 1.82977780073
GGTACAAA -0.477010953536
GGTACAAC -0.596989287225
GGTACACA -0.194294348456
GGTACACC -0.268510776836
GGTACAGA -0.111061039232
GGTACATA -0.505579961746
GGTACCAA 0.582836780825
GGTACCAC 0.106241424023
GGTACCCA -1.22851518583
GGTACCGA -0.629492088422
GGTACCTA -0.390780120195
GGTACGAA 1.04637168353
GGTACGAC -0.469199825985
GGTACGCA -0.659226074395
GGTACGGA 0.0675587778026
GGTACGTA 1.24767576357
GGTACTAA 0.333240621648
GGTACTAC -0.177317613934
GGTACTCA -0.4990553544
GGTACTGA 0.0767730017495
GGTACTTA 0.592072699444
GGTAGAAA 1.50561574598
GGTAGAAC 0.225105226596
GGTAGACA -0.0623039622396
GGTAGACC -1.21718873722
GGTAGAGA 0.226745396097
GGTAGATA 3.29766912542
GGTAGCAA -0.397069484106

GGTAGCAC -0.405669722689
GGTAGCCA -1.38275045259
GGTAGCGA -0.79325968053
GGTAGCTA 0.156572279593
GGTAGGAA 0.339057407638
GGTAGGAC -0.039524817716
GGTAGGCA -0.612898016554
GGTAGGGA -0.343873363505
GGTAGGTA -0.269444693271
GGTAGTAA -0.306382355971
GGTAGTAC -0.0801929185236
GGTAGTCA 0.120962174008
GGTAGTGA -0.192088287804
GGTAGTTA -0.449380828012
GGTATAAA 1.22930691068
GGTATAAC 1.41534369182
GGTATACA -0.0861304628098
GGTATACC 0.337879099413
GGTATAGA 1.73118178833
GGTATATA 0.98999324202
GGTATCAA 2.00400117936
GGTATCAC 1.29259340633
GGTATCCA 4.19533716085
GGTATCGA 1.89644003967
GGTATCTA 6.15200187218
GGTATGAA 0.324834066826
GGTATGAC -0.465336606028
GGTATGCA 0.437808942089
GGTATGGA -0.388207079785
GGTATGTA -0.126916446716
GGTATTAA 1.68417570663
GGTATTAC 1.41566675947
GGTATTCA 0.0842560954002
GGTATTGA 1.0451891932
GGTATTTA 0.758334394722
GGTCAAAA 0.186944298036
GGTCAAAC 0.0740933176492
GGTCAACA -1.42295303278
GGTCAACC -0.85614913754
GGTCAAGA 0.308794123937
GGTCAATA -0.131614780866
GGTCACAA 0.264415450023
GGTCACAC -1.03472504247
GGTCACCA -0.976078070108
GGTCACGA -0.420050153839
GGTCACTA -0.832345115787
GGTCAGAA 1.05933777887
GGTCAGAC -0.980713149912
GGTCAGCA -0.356860630759
GGTCAGGA -0.125096969441
GGTCAGTA 0.513283661462
GGTCATAA 0.369990873745
GGTCATAC -0.194787836905
GGTCATCA -0.791013367113

GGTCATGA -0.671506565819
GGTCATTA -0.521032318812
GGTCCAAA -0.110475544462
GGTCCAAC -0.779365157764
GGTCCACA 0.195722276103
GGTCCACC -0.558793072151
GGTCCAGA -1.13317520394
GGTCCATA -0.486265953012
GGTCCCAA -0.516601900796
GGTCCCAC -1.44361577054
GGTCCCCA -1.68908628264
GGTCCCGA 0.30794672481
GGTCCCTA -0.725963329848
GGTCCGAA -0.022811033078
GGTCCGAC -1.55110189371
GGTCCGCA -0.594698538936
GGTCCGGA -0.545373218359
GGTCCGTA -0.872850898625
GGTCCTAA -0.341177735127
GGTCCTAC -1.26895483912
GGTCCTCA -1.26128799421
GGTCCTGA -0.867680247935
GGTCCTTA -0.394157170387
GGTCGAAA -0.553547404943
GGTCGAAC -0.489607193926
GGTCGACA -0.390299178062
GGTCGACC -1.16660381874
GGTCGAGA 0.30903407224
GGTCGATA 1.05824572657
GGTCGCAA -0.321142835958
GGTCGCAC -0.0175005432355
GGTCGCCA -0.476179498686
GGTCGCGA 0.0266112600712
GGTCGCTA -0.434969292453
GGTCGGAA -0.676480918932
GGTCGGAC -1.41331406374
GGTCGGCA -0.352014093246
GGTCGGGA -1.044487645
GGTCGGTA -0.52513182773
GGTCGTAA 0.307921893558
GGTCGTAC -0.394790236608
GGTCGTCA -0.337093386341
GGTCGTGA -0.289275192034
GGTCGTTA -0.645133162999
GGTCTAAA 0.194084197654
GGTCTAAC 0.375422383266
GGTCTACA 0.541618994526
GGTCTAGA -0.34809049414
GGTCTATA -0.718184352233
GGTCTCAA -0.495747831711
GGTCTCAC -0.540896797182
GGTCTCCA -0.725347514813
GGTCTCGA -0.435049797984
GGTCTCTA -0.419808114483

GGTCTGAA -0.173708456886
GGTCTGAC -1.15910478082
GGTCTGCA -0.579158880418
GGTCTGGA -0.652920766206
GGTCTGTA -0.834549085387
GGTCTTAA -0.499435403237
GGTCTTAC -0.976061341686
GGTCTTCA -0.87633825185
GGTCTTGA -0.374577859336
GGTCTTTA -0.216187670758
GGTGAAAA 0.742760233833
GGTGAAAC -0.439712584237
GGTGAACA -0.573836628404
GGTGAACC -1.51132249032
GGTGAAGA 0.614197605839
GGTGAATA 0.813865698483
GGTGACAA 0.458601325117
GGTGACAC 0.140482674201
GGTGACCA -1.25674309106
GGTGACGA -0.751091249374
GGTGACTA -0.879405564854
GGTGAGAA -0.017259549406
GGTGAGAC -0.685133433834
GGTGAGCA -0.0535069033165
GGTGAGGA -0.47468125939
GGTGAGTA 1.07370383403
GGTGATAA -0.219629543586
GGTGATAC 1.17283384903
GGTGATCA 0.21547462177
GGTGATGA 1.52417017981
GGTGATTA 0.821675519126
GGTGCAAA 1.24255372985
GGTGCAAC -0.659931543317
GGTGCACA -1.41556665032
GGTGCACC -0.246943397381
GGTGCAGA -0.00515575193895
GGTGCATA 0.414417380491
GGTGCCAA -0.648919014004
GGTGCCAC -0.851976964539
GGTGCCCA -0.250911692739
GGTGCCGA -0.346273107918
GGTGCCTA -0.947494424529
GGTGCGAA -0.2241153745
GGTGCGAC -0.959804190692
GGTGCGCA 0.384627720238
GGTGCGGA 1.11455986821
GGTGCGTA -0.234316052582
GGTGCTAA 0.29592944465
GGTGCTAC -0.129094800919
GGTGCTCA -0.904450626415
GGTGCTGA -0.0786343000794
GGTGCTTA -0.803931106863
GGTGGAAA 0.103761174079
GGTGGAAC -0.631580527357

GGTGGACA -0.443480399912
GGTGGAGA -0.58995969064
GGTGGATA 2.51533336584
GGTGGCAA 0.481160124957
GGTGGCAC -0.522841340823
GGTGGCCA -1.16478878496
GGTGGCGA -0.914543876662
GGTGGCTA -0.0992774342179
GGTGGGAA 0.613518275076
GGTGGGAC -0.682969717
GGTGGGCA -1.00025874274
GGTGGGGA -0.120052043298
GGTGGGTA -0.458847023816
GGTGGTAA 0.763482305213
GGTGGTAC -0.407076217046
GGTGGTCA -1.34164401485
GGTGGTGA 0.104731422555
GGTGGTTA -1.09370919708
GGTGTAAA -0.117982423838
GGTGTAAC 0.865054408443
GGTGTACA -0.885975129834
GGTGTAGA 1.40501964162
GGTGTATA 1.30351837281
GGTGTCAA -0.815113795593
GGTGTCAC -1.04953230976
GGTGTCCA -0.47002213248
GGTGTCGA -0.950592841943
GGTGTCTA -1.18434823101
GGTGTGAA 0.411916742782
GGTGTGAC -0.506313398498
GGTGTGCA -0.922410939874
GGTGTGGA -0.156051607458
GGTGTGTA 0.357035756427
GGTGTTAA 0.562080207075
GGTGTTAC -0.220831898918
GGTGTTCA -0.762415084165
GGTGTTGA 0.750996106474
GGTGTTTA 0.303811145232
GGTTAAAA 2.49935641592
GGTTAAAC -0.415526683975
GGTTAACA 0.666241556374
GGTTAACC -0.383729874463
GGTTAAGA 1.31526878236
GGTTAATA 0.166456163183
GGTTACAA 0.592286248207
GGTTACAC -0.69177618566
GGTTACCA -0.763472634094
GGTTACGA 0.71980413397
GGTTACTA -0.470884953121
GGTTAGAA 0.679485500354
GGTTAGAC 0.621895293778
GGTTAGCA -0.409363305992
GGTTAGGA 0.496950709806
GGTTAGTA -0.490023052042

GGTTATAA 0.0935009009935
GGTTATAC -0.24344036126
GGTTATCA 0.354126579287
GGTTATGA -0.280488588374
GGTTATTA 0.582101514402
GGTTCAAA 0.0626651916023
GGTTCAAC -0.718300144279
GGTTCACA -0.0180494445826
GGTTCAGA -0.46820030277
GGTTCATA 0.0820346132339
GGTTCCAA 0.206534848496
GGTTCCAC -0.446267250466
GGTTCCCA -0.989331423825
GGTTCCGA -0.588062060268
GGTTCCTA -0.671410900156
GGTTCGAA -0.281596846332
GGTTCGAC -0.966415053964
GGTTCGCA -0.322043818312
GGTTCGGA -0.530293330064
GGTTCGTA 0.0289189981634
GGTTCTAA 0.0537468516197
GGTTCTAC -0.662971411253
GGTTCTCA 0.176288031836
GGTTCTGA 1.0076777979
GGTTCTTA -0.263289156735
GGTTGAAA 0.239279689049
GGTTGAAC -1.41006378362
GGTTGACA -0.337438932809
GGTTGAGA 0.582856907208
GGTTGATA 0.295918989387
GGTTGCAA 1.65161278767
GGTTGCAC -0.0790318614836
GGTTGCCA -0.285516524714
GGTTGCGA 0.481794236704
GGTTGCTA 0.684921714743
GGTTGGAA 0.805305189902
GGTTGGAC -0.221705436204
GGTTGGCA -0.172903662959
GGTTGGGA 0.50760932844
GGTTGGTA -0.031756818128
GGTTGTAA 0.661042415091
GGTTGTAC -0.0383865008747
GGTTGTCA 0.236807541934
GGTTGTGA -0.189212306126
GGTTGTTA -0.416009717161
GGTTTAAA -0.027649467762
GGTTTAAC -0.31337980262
GGTTTACA 1.31329874929
GGTTTAGA 0.687250101981
GGTTTATA 0.653480122817
GGTTTCAA 0.261461838012
GGTTTCAC 0.169094026228
GGTTTCCA -0.792375426598
GGTTTCGA -0.0401769647927

GGTTTCTA 0.663198290477
GGTTTGAA 0.733300833951
GGTTTGAC 0.694313678174
GGTTTGCA -0.463836798442
GGTTTGGA -0.388578764411
GGTTTGTA 0.421993264607
GGTTTTAA 0.272262386852
GGTTTTAC -0.250712519965
GGTTTTCA 0.379709564168
GGTTTTGA -0.734281537691
GGTTTTTA 1.63635698956
GTAAAAAA 1.56505967082
GTAAAAAC 0.976470926643
GTAAAACA 1.09340625582
GTAAAAGA 1.50643517728
GTAAAATA 1.60947127882
GTAAACAA 0.525018910881
GTAAACAC -0.0947225985632
GTAAACCA 0.0462316080329
GTAAACGA -0.656624543391
GTAAACTA -0.0350674773959
GTAAAGAA -0.141898055532
GTAAAGAC -0.277338417405
GTAAAGCA 1.60668808761
GTAAAGGA 0.442863539351
GTAAAGTA 1.13161449444
GTAAATAA 2.33864176082
GTAAATAC 0.779519895667
GTAAATCA 2.78941261615
GTAAATGA 0.808627872723
GTAAATTA 3.13792315051
GTAACAAA 1.4940532698
GTAACAAC -0.0124208533404
GTAACACA 0.388026987866
GTAACAGA -0.252483118882
GTAACATA 1.13139990016
GTAACCAA -0.212380386462
GTAACCAC 1.16780565131
GTAACCCA 0.627886421291
GTAACCGA 0.663674004978
GTAACCTA 0.0314504789
GTAACGAA 1.13433965894
GTAACGAC 0.0660592315986
GTAACGCA 0.746516287337
GTAACGGA 0.240356058453
GTAACGTA 0.72380379512
GTAACTAA 0.0220781190888
GTAACTAC 0.55438983782
GTAACTCA 1.09571896016
GTAACTGA 0.446179687632
GTAACTTA 1.18842264729
GTAAGAAA 2.23834127905
GTAAGAAC 0.263521002209
GTAAGACA 0.568128054394

GTAAGAGA 0.773130161224
GTAAGATA 2.03341549564
GTAAGCAA 0.00748962819055
GTAAGCAC -0.536220680468
GTAAGCCA -0.256752003073
GTAAGCGA 2.61485048698
GTAAGCTA 0.160739224962
GTAAGGAA 0.112361674043
GTAAGGAC -0.124500235262
GTAAGGCA 0.201007150549
GTAAGGGA 1.96972046056
GTAAGGTA 0.660978115219
GTAAGTAA 0.920321978176
GTAAGTAC 0.656654863656
GTAAGTCA 0.522728162593
GTAAGTGA 0.576913089764
GTAAGTTA 1.41336268072
GTAATAAA 2.20138662654
GTAATAAC 0.609509726953
GTAATACA 1.29474326994
GTAATAGA 1.21593201452
GTAATATA 3.88984977546
GTAATCAA 7.67841967231
GTAATCAC 7.67119064157
GTAATCCA 16.4977574555
GTAATCGA 10.6703954436
GTAATCTA 13.4270112043
GTAATGAA -0.839991050171
GTAATGAC -0.14109744371
GTAATGCA 0.738424958717
GTAATGGA 1.7320270964
GTAATGTA 0.60073410132
GTAATTAA 1.03375165741
GTAATTAC 3.84061489291
GTAATTCA 1.66431305793
GTAATTGA 1.56804438724
GTAATTTA 3.12146395155
GTACAAAA 1.96538387854
GTACAAAC 0.741058639657
GTACAACA 0.0461443065806
GTACAAGA 0.556079931206
GTACAATA 1.204118612
GTACACAA 0.762149781847
GTACACAC 0.283535252233
GTACACCA -0.188503700625
GTACACGA -0.287692265101
GTACACTA 0.265741438849
GTACAGAA 0.284126497398
GTACAGAC -0.805398764513
GTACAGCA 0.207765955804
GTACAGGA -0.0857660968679
GTACAGTA 0.742639214155
GTACATAA 0.656946042752
GTACATAC -0.378343061195

GTACATCA 0.835121511077
GTACATGA -0.027649467762
GTACATTA 1.10305646426
GTACCAAA 1.5825866136
GTACCAAC -1.33159101737
GTACCACA -0.522720321145
GTACCAGA -1.03968554236
GTACCATA 1.31972664544
GTACCCAA -0.398489308924
GTACCCAC -0.405348223329
GTACCCCA -0.483571631542
GTACCCGA 0.266931247864
GTACCCTA -0.834379710114
GTACCGAA -0.733021155646
GTACCGAC -0.629492088422
GTACCGCA 0.241540901218
GTACCGGA 0.656688059119
GTACCGTA -0.0255746206699
GTACCTAA 0.802828337919
GTACCTAC 0.40291815865
GTACCTCA 0.529970001032
GTACCTGA 0.710339245075
GTACCTTA -0.595781181498
GTACGAAA 1.70005071774
GTACGAAC 0.363403534817
GTACGACA 0.427906239024
GTACGAGA 0.719498578887
GTACGATA 1.9160096796
GTACGCAA 0.957141757777
GTACGCAC -0.479747096061
GTACGCCA -0.111618827554
GTACGCGA 1.03772570317
GTACGCTA -0.163949252316
GTACGGAA -0.0766274122017
GTACGGAC -1.03257491748
GTACGGCA 0.301401206936
GTACGGGA 1.0977391785
GTACGGTA 0.304603131461
GTACGTAA 0.560329211778
GTACGTAC -0.625566136882
GTACGTCA -0.488759010654
GTACGTGA 0.442175060233
GTACGTTA -0.271179744291
GTACTAAA 0.422777409389
GTACTAAC -0.146217909133
GTACTACA 1.36635503073
GTACTAGA -0.42326462468
GTACTATA 1.31579180693
GTACTCAA -0.0709021097697
GTACTCAC -0.824063762749
GTACTCCA -0.417801226605
GTACTCGA -0.0394087642883
GTACTCTA 0.797211247466
GTACTGAA 0.28239667401

GTACTGAC	0.592677013689
GTACTGCA	0.618665662804
GTACTGGA	0.902617034534
GTACTGTA	-0.130791428845
GTACTTAA	2.09465485065
GTACTTCA	-0.102073694509
GTACTTGA	0.0014438719245
GTACTTTA	1.73781905114
GTAGAAAA	1.00383548847
GTAGAAAC	1.80747959834
GTAGAACA	-0.279586821876
GTAGAAGA	0.490345074166
GTAGAATA	1.29072348241
GTAGACAA	-0.554120614779
GTAGACAC	-0.631310258789
GTAGACCA	-1.27912049207
GTAGACGA	0.762398094361
GTAGACTA	0.521388320542
GTAGAGAA	0.273791469177
GTAGAGAC	-0.798310879832
GTAGAGCA	-0.780121334715
GTAGAGGA	-0.969146230238
GTAGAGTA	0.642726884046
GTAGATAA	1.92975050999
GTAGATAC	5.07735000711
GTAGATCA	1.54733956705
GTAGATGA	-0.221997138063
GTAGATTA	3.97071000119
GTAGCAAA	0.536427171927
GTAGCAAC	-0.287478977721
GTAGCACA	0.423844369055
GTAGCAGA	-0.12521485254
GTAGCATA	1.00908716745
GTAGCCAA	0.293360586346
GTAGCCAC	-0.992716838228
GTAGCCCA	-1.39482575946
GTAGCCGA	-0.897596155498
GTAGCCTA	-0.746655342345
GTAGCGAA	-0.873944519214
GTAGCGAC	-0.450114526146
GTAGCGCA	-0.17186911461
GTAGCGGA	-0.494245933073
GTAGCGTA	0.505693401357
GTAGCTAA	0.592656625925
GTAGCTAC	0.830043389472
GTAGCTCA	-0.183779489698
GTAGCTGA	0.332955715711
GTAGCTTA	-0.183738714169
GTAGGAAA	0.14741451407
GTAGGAAC	-0.158020856386
GTAGGACA	-0.767055130219
GTAGGAGA	-0.704030800307
GTAGGATA	4.8606489829
GTAGGCAA	-0.659952453844

GTAGGCAC −0.719547718627
GTAGGCCA −0.697851739428
GTAGGCGA −0.342083683731
GTAGGCTA −0.607413185188
GTAGGGAA −0.380300547952
GTAGGGAC −1.23568148499
GTAGGGCA −0.358673834876
GTAGGGGA 0.0819561987558
GTAGGGTA 0.122026519858
GTAGGTAA 0.819257216619
GTAGGTCA −1.03472504247
GTAGGTGA −0.443748838809
GTAGGTTA −0.456623189215
GTAGTAAA 0.194640417686
GTAGTAAC 0.370483316668
GTAGTACA −0.467835936829
GTAGTAGA −0.639049506402
GTAGTATA 1.83617982011
GTAGTCAA 0.466764272294
GTAGTCAC −0.895338079908
GTAGTCCA 0.225177106534
GTAGTCGA 0.111734880981
GTAGTCTA 0.495579240583
GTAGTGAA 0.893614268297
GTAGTGAC 0.415894186496
GTAGTGCA 0.171503180379
GTAGTGGA −0.568108189393
GTAGTGTA 0.584059785303
GTAGTTAA 1.87206594466
GTAGTTCA −0.952119049069
GTAGTTGA −0.581782628857
GTAGTTTA −0.0212346406854
GTATAAAA 0.82631713347
GTATAAAC −0.220597962391
GTATAACA 0.815929828929
GTATAAGA 1.28959849603
GTATAATA 3.98629200353
GTATACAA 0.67782206789
GTATACAC 0.805305189902
GTATACCA 0.719339397496
GTATACGA 1.8709278892
GTATACTA 2.6070482464
GTATAGAA 0.5674960337
GTATAGAC −0.232613935643
GTATAGCA 0.545217957692
GTATAGGA −0.535611138591
GTATAGTA 0.00705233678402
GTATATAA 0.715840543481
GTATATAC 0.948904055465
GTATATCA 0.735493825524
GTATATGA 1.68085720592
GTATATTA 2.85341503599
GTATCAAA 2.18104355847
GTATCAAC 1.23840455843

GTATCACA 1.67854763815
GTATCAGA 3.01181228187
GTATCATA 2.38902463133
GTATCCAA 5.0266817079
GTATCCAC 4.47407057183
GTATCCCA 1.75930801612
GTATCCGA 4.18810630044
GTATCCTA 3.34149759108
GTATCGAA 2.34435817628
GTATCGAC 0.386400932971
GTATCGCA 2.63621425017
GTATCGGA 0.648675929122
GTATCGTA 1.5696469178
GTATCTAA 4.58371884354
GTATCTCA 3.13590815981
GTATCTGA 3.78684791491
GTATCTTA 1.7907388914
GTATGAAA 0.999230467547
GTATGAAC 0.356024732422
GTATGACA −0.327634770604
GTATGAGA 2.04041921545
GTATGATA 1.65843118793
GTATGCAA −0.199986978189
GTATGCAC −0.00372573323892
GTATGCCA 0.218781098932
GTATGCGA −0.181450579697
GTATGCTA 0.134449725633
GTATGGAA 1.10900969144
GTATGGAC −0.459718992813
GTATGGCA −0.217345068455
GTATGGGA −0.484442816395
GTATGGTA −0.261763472372
GTATGTAA 1.7025704363
GTATGTCA −0.145345155991
GTATGTGA 1.44380004456
GTATGTTA 0.388486496708
GTATTAAA 0.563959017972
GTATTAAC 0.96955476967
GTATTACA 1.41566675947
GTATTAGA 1.29809757993
GTATTATA 0.963251291152
GTATTCAA 1.09844124946
GTATTCAC 1.08438127214
GTATTCCA 0.228429739088
GTATTCGA 1.49262142143
GTATTCTA 1.03890218172
GTATTGAA 1.55551898127
GTATTGAC 1.44959226068
GTATTGCA 0.615033504176
GTATTGGA 0.461576893182
GTATTGTA 1.26687842374
GTATTTAA 1.98090811554
GTATTTCA 1.06301071303
GTATTTGA 1.55091134653

GTATTTTA 1.51482082157
GTCAAAAA 0.527683957612
GTCAAAAC -0.650548205479
GTCAAACA 0.263636271492
GTCAAAGA 0.622988914367
GTCAAATA 1.48015744012
GTCAACAA 0.149441005567
GTCAACAC 0.643692166272
GTCAACCA 0.717657145558
GTCAACGA -0.570103315099
GTCAACTA -0.775576170179
GTCAAGAA 1.22838527918
GTCAAGAC -0.010604251263
GTCAAGCA 0.700031923303
GTCAAGGA -0.234658985233
GTCAAGTA 0.292663481635
GTCAATAA 0.972592546554
GTCAATCA 1.28639003696
GTCAATGA -0.158970717098
GTCAATTA -0.414850751173
GTCACAAA 1.08527571996
GTCACAAC -0.683846390866
GTCACACA 0.609288336743
GTCACAGA 0.839894338981
GTCACATA 0.30095738099
GTCACCAA -0.976470926643
GTCACCAC -0.678849558936
GTCACCCA -0.738657588336
GTCACCGA -0.431716137136
GTCACCTA -0.38141534045
GTCACGAA 0.321342531495
GTCACGAC -0.621623979683
GTCACGCA 0.266931247864
GTCACGGA 1.3868614623
GTCACGTA 0.35482394538
GTCACTAA -0.0792995162357
GTCACTCA -0.250331948364
GTCACTGA -0.426791185144
GTCACTTA 0.0829024001256
GTCAGAAA 0.485218335584
GTCAGAAC -0.461674127135
GTCAGACA -0.735373067227
GTCAGAGA -1.05159983817
GTCAGATA 3.27299653263
GTCAGCAA -0.357238327162
GTCAGCAC -0.603880874329
GTCAGCCA -0.892503919286
GTCAGCGA -0.302921140905
GTCAGCTA -0.305128508465
GTCAGGAA 0.653080208978
GTCAGGAC 0.100993143
GTCAGGCA -0.170943039623
GTCAGGGA -0.265144181907
GTCAGGTA -0.650459597118

GTCAGTAA 0.558782616887
GTCAGTCA -0.116662969552
GTCAGTGA -0.517519088808
GTCAGTTA 0.24129363423
GTCATAAA 0.782269630035
GTCATAAC -0.68440627024
GTCATACA -0.215547808616
GTCATAGA -0.195854012426
GTCATATA 0.686613899182
GTCATCAA 0.0198291918552
GTCATCAC -0.681245121244
GTCATCCA -0.0734199986634
GTCATCGA -0.406581160307
GTCATCTA -0.221273111048
GTCATGAA -0.234869658798
GTCATGAC -0.64543349045
GTCATGCA -0.261260835567
GTCATGGA 0.777810460044
GTCATGTA 0.0144381964818
GTCATTAA 0.514639186407
GTCATTCA -0.195747630118
GTCATTGA 0.460863844194
GTCATTTA 0.473541897023
GTCCAAAA -0.486502241973
GTCCAAAC 0.0770511117653
GTCCAACA 0.242539117525
GTCCAAGA -0.233510474509
GTCCAATA 0.388936857195
GTCCACAA -0.335310763871
GTCCACAC -0.766238051357
GTCCACCA -0.558793072151
GTCCACGA -0.334898303716
GTCCACTA -0.593300931554
GTCCAGAA -0.215805008104
GTCCAGAC -1.4369050595
GTCCAGCA -0.297516292307
GTCCAGGA -0.655368604833
GTCCAGTA 0.322874227635
GTCCATAA 0.349384071648
GTCCATCA -0.866085558831
GTCCATGA -0.298147528856
GTCCATTA -0.607348362553
GTCCCAAA 0.620851335692
GTCCCAAC 0.70696507008
GTCCCACA 0.115078997093
GTCCCAGA -0.348023841834
GTCCCATA -0.303953859582
GTCCCCAA -0.464490252427
GTCCCCAC -0.709162243758
GTCCCCCA -0.660868857712
GTCCCCGA -1.10449772237
GTCCCCTA -1.30480018814
GTCCCGAA -0.313684050795
GTCCCGAC -0.352385777872

GTCCCGCA	-0.730031472975
GTCCCGGA	-0.598344550789
GTCCCGTA	0.0112482955103
GTCCCTAA	-0.065276393725
GTCCCTCA	-1.24601416077
GTCCCTGA	-0.46341492855
GTCCCTTA	-0.406936116511
GTCCGAAA	0.216706774603
GTCCGAAC	-0.679422768771
GTCCGACA	0.539333212488
GTCCGAGA	-0.320983393185
GTCCGATA	0.118705405327
GTCCGCAA	-0.389149621812
GTCCGCAC	-0.432278630326
GTCCGCCA	-1.19484531582
GTCCGCGA	0.398443828527
GTCCGCTA	-1.12504336117
GTCCGGAA	-0.71679484768
GTCCGGAC	0.0313668367899
GTCCGGCA	-0.0792297273502
GTCCGGGA	-0.27824122943
GTCCGGTA	0.464648649673
GTCCGTAA	0.366742684678
GTCCGTCA	-0.510810730202
GTCCGTGA	-0.468305378171
GTCCGTTA	-0.608029522987
GTCCTAAA	0.353819194533
GTCCTAAC	-0.0835976751652
GTCCTACA	-0.187670416104
GTCCTAGA	-0.184583499481
GTCCTATA	-0.356706938382
GTCCTCAA	0.629588015467
GTCCTCAC	-1.01077412426
GTCCTCCA	-1.88102793537
GTCCTCGA	-0.514465367647
GTCCTCTA	0.269892178559
GTCCTGAA	0.619343425277
GTCCTGCA	0.971062418703
GTCCTGGA	-0.016789062537
GTCCTGTA	-0.409600117716
GTCCTTAA	-0.130645577916
GTCCTTCA	-0.465607658741
GTCCTTGA	-0.855488364871
GTCCTTTA	-0.544754266744
GTCGAAAA	1.04999234137
GTCGAAAC	-0.370253300865
GTCGAACA	0.441478216903
GTCGAAGA	0.0288643694103
GTCGAATA	0.436802622952
GTCGACAA	-0.639450465766
GTCGACAC	-0.175310203294
GTCGACCA	-1.10051949451
GTCGACGA	-0.829634065896
GTCGACTA	0.0421459523393

GTCGAGAA 1.67036351907
GTCGAGAC -0.861015540055
GTCGAGCA -0.551568223515
GTCGAGGA -0.400112488622
GTCGAGTA 0.0819561987558
GTCGATAA 0.358626002044
GTCGATCA -0.0817044882809
GTCGATGA -0.338645731627
GTCGATTA 0.705261384851
GTCGCAAA 0.660191618003
GTCGCAAC -0.480489419788
GTCGCACA 1.12606849979
GTCGCAGA -0.194479929387
GTCGCATA -0.478727969226
GTCGCCAA -0.702403961267
GTCGCCAC -1.05125167788
GTCGCCCA -0.35278882829
GTCGCCGA -1.32992706214
GTCGCCTA -0.884459900735
GTCGCGAA -0.369580504643
GTCGCGAC 0.0583884659635
GTCGCGCA 0.239945166587
GTCGCGGA -0.24621675655
GTCGCGTA 1.03122305187
GTCGCTAA 0.681663854557
GTCGCTCA -1.02284603317
GTCGCTGA 0.627694828583
GTCGCTTA 0.306861991195
GTCGGAAA -0.432038682023
GTCGGAAC -0.882164447577
GTCGGACA -0.0487021868581
GTCGGAGA -0.723599656095
GTCGGATA 1.01467994942
GTCGGCAA -0.829643475633
GTCGGCAC -0.653048843187
GTCGGCCA -0.777900898075
GTCGGCGA 0.0244990354112
GTCGGCTA 0.0892623370674
GTCGGGAA -0.44809639886
GTCGGGCA -0.620576362255
GTCGGGGA -0.331844843937
GTCGGGTA -0.42812370851
GTCGGTAA -0.4974023772
GTCGGTCA -1.11393150686
GTCGGTGA -0.188195270345
GTCGGTTA 0.0496651166499
GTCGTAAA 0.728427112752
GTCGTAAC 0.350847285811
GTCGTACA 0.252285514397
GTCGTAGA -0.0390953677573
GTCGTATA 0.457416220971
GTCGTCAA -0.85447629534
GTCGTCAC -1.16419989222
GTCGTCCA -0.767087018774

GTCGTCGA	-0.93649235048
GTCGTCTA	-0.571997286128
GTCGTGAA	-0.417754700681
GTCGTGCA	0.114665752793
GTCGTGGA	2.07174292428
GTCGTGTA	0.369113154353
GTCGTTAA	0.905198439155
GTCGTTCA	1.05545652358
GTCGTTGA	0.779764287458
GTCGTTTA	1.07054163951
GTCTAAAA	0.807320441991
GTCTAAAC	0.128119586193
GTCTAACA	0.427359690111
GTCTAAGA	0.276652552103
GTCTAATA	1.29336944828
GTCTACAA	0.20605495189
GTCTACAC	-0.522628837587
GTCTACCA	-0.382644879467
GTCTACGA	-0.50604312993
GTCTACTA	-0.711381112108
GTCTAGAA	-1.14455236058
GTCTAGAC	-0.317129582965
GTCTAGCA	0.0427256967145
GTCTAGGA	-0.709184722575
GTCTAGTA	-0.591945929371
GTCTATAA	0.623252648395
GTCTATCA	-0.189219102048
GTCTATGA	0.0507393950007
GTCTATTA	-0.701004785595
GTCTCAAA	0.348987294389
GTCTCAAC	0.221264224074
GTCTCACA	-1.11747427298
GTCTCAGA	-0.412301235107
GTCTCATA	0.966526402523
GTCTCCAA	-0.266229699666
GTCTCCAC	-0.749818321012
GTCTCCCA	-0.865843780857
GTCTCCGA	-0.577880201661
GTCTCCTA	-1.62469283594
GTCTCGAA	0.192509634933
GTCTCGCA	-0.827788973225
GTCTCGGA	-0.278667281428
GTCTCGTA	0.222230290445
GTCTCTAA	-0.135002809086
GTCTCTCA	-0.298302266759
GTCTCTGA	0.445174152641
GTCTCTTA	-0.620525654226
GTCTGAAA	0.286841206632
GTCTGAAC	0.119778899533
GTCTGACA	-0.373014536023
GTCTGAGA	-0.518522271366
GTCTGATA	2.04245851464
GTCTGCAA	-0.861556861336
GTCTGCAC	-0.414583096421

GTCTGCCA -0.665037371371
GTCTGCGA -0.148448016892
GTCTGCTA -0.322017941534
GTCTGGAA -0.0910995882907
GTCTGGCA -0.608763482502
GTCTGGGA -1.02102498761
GTCTGGTA -0.66248210491
GTCTGTAA 0.481026297581
GTCTGTCA -0.19519533081
GTCTGTGA 0.326788678385
GTCTGTTA -0.119410612867
GTCTTAAA -0.233519884247
GTCTTAAC -0.706131785559
GTCTTACA 0.112771520383
GTCTTAGA -0.192910071535
GTCTTATA -0.509266487745
GTCTTCAA 0.305217378207
GTCTTCAC -0.402220269795
GTCTTCCA -0.774196598127
GTCTTCGA 0.0723195821532
GTCTTCTA -0.324762971033
GTCTTGAA -0.482791146103
GTCTTGCA -0.900826047853
GTCTTGGA -0.910751229735
GTCTTGTA 0.362321415018
GTCTTTAA -0.0352358071423
GTCTTTCA 0.820201588318
GTCTTTGA -0.32060700369
GTCTTTTA 0.844327893576
GTGAAAAA -0.338275353909
GTGAAAAC 0.643291468288
GTGAAACA 0.601730749338
GTGAAAGA 0.701204219751
GTGAAATA 0.827022079628
GTGAACAA -0.181745940898
GTGAACAC -0.0787422506776
GTGAACCA -0.767097996801
GTGAACGA 1.87878136057
GTGAACTA -0.312661526
GTGAAGAA 0.481300225492
GTGAAGCA -0.617327389044
GTGAAGGA -1.0355695664
GTGAAGTA -0.308356048386
GTGAATAA 0.10016011986
GTGAATCA 1.24757068817
GTGAATGA 0.167690407069
GTGAATTA 1.74806102752
GTGACAAA -0.0783886013811
GTGACAAC 0.37923750901
GTGACACA 0.596463126077
GTGACAGA -0.89280790608
GTGACATA 0.205154230918
GTGACCAA -0.893410652035
GTGACCAC -1.23404209963

GTGACCCA −1.02880109002
GTGACCGA −0.281134462293
GTGACCTA −0.856351969657
GTGACGAA −0.718432926129
GTGACGCA −0.224078781077
GTGACGGA −0.598276068812
GTGACGTA 0.346638519386
GTGACTAA 0.0301456619834
GTGACTCA −0.646839200662
GTGACTGA 0.710271024479
GTGACTTA 0.58130090258
GTGAGAAA 0.61197899887
GTGAGAAC −0.307554129656
GTGAGACA −0.949106364819
GTGAGAGA −0.0889204499427
GTGAGATA 1.07087254861
GTGAGCAA −0.191222591965
GTGAGCAC −0.371006864
GTGAGCCA −1.03159003163
GTGAGCGA −0.240180410022
GTGAGCTA −0.839514812907
GTGAGGAA 1.37624571025
GTGAGGCA −0.145492836592
GTGAGGGA −0.187598013403
GTGAGGTA 0.512800889658
GTGAGTAA 0.70581394554
GTGAGTCA −0.45209449172
GTGAGTGA 1.21753323816
GTGAGTTA 0.437807635181
GTGATAAA 0.952827393189
GTGATAAC 0.426664415071
GTGATACA 0.113636432077
GTGATAGA 0.800105264474
GTGATATA 1.43758308335
GTGATCAA 0.36432908704
GTGATCAC 1.72787870913
GTGATCCA 1.58185108579
GTGATCGA 0.327859297393
GTGATCTA 1.80207448836
GTGATGAA 0.240036388763
GTGATGCA 0.398305819045
GTGATGGA −0.95644857379
GTGATGTA −0.596951125512
GTGATTAA 0.846139790785
GTGATTCA 1.16652932499
GTGATTGA 0.359516267753
GTGATTTA 1.55998991343
GTGCAAAA 0.00223585815391
GTGCAAAC −0.0459035741326
GTGCAACA −0.172911243025
GTGCAAGA −0.0737545671035
GTGCAATA 0.925630115584
GTGCACAA −0.122525758702
GTGCACAC −0.272543110684

GTGCACCA −0.488759010654
GTGCACGA 0.865554692814
GTGCACTA −0.289536573628
GTGCAGAA 0.153464975205
GTGCAGCA −0.374285896095
GTGCAGGA 0.00114432861793
GTGCAGTA 1.54898914629
GTGCATAA 0.435591642028
GTGCATCA −0.206311367234
GTGCATGA −0.375063767719
GTGCATTA 1.63249429237
GTGCCAAA −0.837143036324
GTGCCAAC −0.730092113505
GTGCCACA −0.844739046823
GTGCCAGA −0.606908718712
GTGCCATA −0.0193741265003
GTGCCCAA −0.448886294037
GTGCCCAC −1.06833087399
GTGCCCCA −0.466763749531
GTGCCCGA −0.38492804769
GTGCCCTA −1.30162231072
GTGCCGAA −0.464468034992
GTGCCGCA −1.54705858184
GTGCCGGA −0.586579503868
GTGCCGTA 0.106938005971
GTGCCTAA −1.24498039657
GTGCCTCA −1.10717296298
GTGCCTGA 0.206169959791
GTGCCTTA −0.274643573173
GTGCGAAA 0.272630673518
GTGCGAAC −0.325315008959
GTGCGACA 0.48172967545
GTGCGAGA 0.367942687576
GTGCGATA 0.707313230363
GTGCGCAA 1.52190060343
GTGCGCAC 1.82949315618
GTGCGCCA −0.0184990209241
GTGCGCGA 1.54347739262
GTGCGCTA −1.38373533844
GTGCGGAA 1.26636245648
GTGCGGCA −1.0142442263
GTGCGGGA 0.737528158469
GTGCGGTA 0.306143453194
GTGCGTAA 1.09904112022
GTGCGTCA 0.277017440808
GTGCGTGA 0.257220398889
GTGCGTTA 0.746516287337
GTGCTAAA 0.959832942668
GTGCTAAC 0.143977868874
GTGCTACA 0.26552187831
GTGCTAGA 2.04181107244
GTGCTATA 0.443923180332
GTGCTCAA −0.933424514713
GTGCTCCA −0.960188944398

GTGCTCGA 0.112441656811
GTGCTCTA -0.113005718291
GTGCTGAA -0.318798504442
GTGCTGCA -0.359810583427
GTGCTGGA -0.415652669903
GTGCTGTA -0.23890434508
GTGCTTAA 1.54984203443
GTGCTTCA -0.538744319757
GTGCTTGA 0.536915955507
GTGCTTTA 0.898391016924
GTGGAAAA 0.809133384726
GTGGAAAC 0.322700670257
GTGGAACA -0.544978009389
GTGGAAGA -0.50605463072
GTGGAATA 1.61899680824
GTGGACAA 0.307791464143
GTGGACAC -0.0741544809421
GTGGACCA -0.44418194811
GTGGACGA -0.67074411571
GTGGACTA -0.245536641643
GTGGAGAA -0.741374388622
GTGGAGCA -0.360730385256
GTGGAGGA -1.27420965469
GTGGAGTA 1.08591218414
GTGGATAA 0.431111822891
GTGGATCA 0.646091387922
GTGGATGA -0.261675386775
GTGGATTA 3.03817470666
GTGGCAAA 1.1921530854
GTGGCAAC -1.24852917447
GTGGCACA -0.764484703625
GTGGCAGA -1.14252325526
GTGGCATA -0.0215409799134
GTGGCCAA -0.858037358174
GTGGCCAC -1.3157878862
GTGGCCCA -0.628339918356
GTGGCCGA -0.655860263611
GTGGCCTA -0.516887852259
GTGGCGAA 0.672155837699
GTGGCGCA -0.580703645638
GTGGCGGA -0.615587371773
GTGGCGTA 0.693068979024
GTGGCTAA 0.162113046619
GTGGCTCA -0.841871429357
GTGGCTGA -0.180071791789
GTGGCTTA -0.987172934622
GTGGGAAA 0.73015536785
GTGGGAAC -0.602839007296
GTGGGACA -0.125290130439
GTGGGAGA 0.103301926619
GTGGGATA 0.814343765418
GTGGGCAA 0.209887328819
GTGGGCCA -0.384922558676
GTGGGCGA -0.61753178945

GTGGGCTA -0.885353825786
GTGGGGAA 0.321291823466
GTGGGGCA -0.464234621228
GTGGGGGA -0.714207692664
GTGGGGTA -0.316239055875
GTGGGTAA 0.0172747095384
GTGGGTCA -1.30482318972
GTGGGTGA -0.328420222294
GTGGGTTA -0.668522633544
GTGGTAAA -0.220946384056
GTGGTAAC 0.822569444177
GTGGTACA -0.249470957394
GTGGTAGA -0.182660515095
GTGGTATA 0.779430503162
GTGGTCAA 0.223693765989
GTGGTCCA -0.338871826706
GTGGTCGA 0.118586738083
GTGGTCTA -0.2493473239
GTGGTGAA 1.16189032446
GTGGTGCA -0.61257599443
GTGGTGGA 0.628764402065
GTGGTGTA -0.206273466903
GTGGTTAA -0.147653416847
GTGGTTCA -1.20663467122
GTGGTTGA 0.338977686252
GTGGTTTA 0.0462316080329
GTGTAAAA 0.744711447431
GTGTAAAC 0.125221648462
GTGTAACA 0.927839835579
GTGTAAGA 2.03032309
GTGTAATA 3.73043287998
GTGTACAA 0.235404968302
GTGTACAC -1.20545322642
GTGTACCA 0.00155156114117
GTGTACGA 0.548206072073
GTGTACTA -0.174860104189
GTGTAGAA 0.0276878908563
GTGTAGCA 0.315521040637
GTGTAGGA -0.757958789371
GTGTAGTA 0.608199159641
GTGTATAA 0.492027326104
GTGTATCA 2.45946435706
GTGTATGA 1.59876874808
GTGTATTA 0.88202879053
GTGTCAAA -0.742754744819
GTGTCAAC -0.781274288925
GTGTCACA -0.527538629446
GTGTCAGA 0.370590744503
GTGTCATA 0.239028762719
GTGTCCAA -0.0986224119437
GTGTCCCA -0.952266468288
GTGTCCGA 0.237654156917
GTGTCCTA -0.735864464624
GTGTCGAA 0.262392095105

GTGTCGCA −0.550049335073
GTGTCGGA 0.817153878933
GTGTCGTA 0.550497343125
GTGTCTAA −0.097593091227
GTGTCTCA 0.384797618274
GTGTCTGA −0.466701540711
GTGTCTTA −0.19804726538
GTGTGAAA −0.482628043988
GTGTGAAC −0.777294231396
GTGTGACA −0.558732431621
GTGTGAGA −0.0236043262154
GTGTGATA 1.14157626975
GTGTGCAA 0.508039039781
GTGTGCCA −0.809653272716
GTGTGCGA 0.869091185779
GTGTGCTA 0.203275681403
GTGTGGAA 0.640579111489
GTGTGGCA −0.647922104605
GTGTGGGA −0.126596515645
GTGTGGTA 0.457032774173
GTGTGTAA 2.33941440481
GTGTGTCA 0.479212047938
GTGTGTGA 0.530415133887
GTGTGTTA −0.218063867839
GTGTTAAA 0.0779544465537
GTGTTAAC 0.783051683764
GTGTTACA 0.575994594843
GTGTTAGA 0.175590404362
GTGTTATA 2.03909061281
GTGTTCAA −0.00214123801693
GTGTTCCA −1.02545801944
GTGTTCGA 0.554729372511
GTGTTCTA 0.174754506025
GTGTTGAA 0.997668712524
GTGTTGCA 0.422768522415
GTGTTGGA −0.422350311865
GTGTTGTA 0.858209870026
GTGTTTAA 0.109336443476
GTGTTTCA 0.302654270297
GTGTTTGA 0.22704755322
GTGTTTTA 1.93751877096
GTTAAAAA 2.32345810404
GTTAAAAC 0.832343024734
GTTAAACA 0.448147629652
GTTAAAGA 1.04355477409
GTTAAATA 1.70358146031
GTTAACAA 0.652864307782
GTTAACCA −0.765147305966
GTTAACGA 0.0377534346544
GTTAACTA 0.60583757694
GTTAAGAA 0.516298175384
GTTAAGCA 0.348724605887
GTTAAGGA 1.15001314484
GTTAAGTA 1.47037549535

GTTAATAA 1.75922254434
GTTAATCA 0.505564801613
GTTAATGA 1.17527175516
GTTAATTA 0.572641068993
GTTACAAA 2.32731400531
GTTACAAC -0.979957234342
GTTACACA 0.262162079302
GTTACAGA 0.70295547643
GTTACATA 2.30504167969
GTTACCAA 0.883109080658
GTTACCCA 0.0370461360614
GTTACCGA 0.247666901633
GTTACCTA 0.799386465091
GTTACGAA 0.855893767723
GTTACGCA 0.616762020656
GTTACGGA -0.275710532839
GTTACGTA 1.41552953413
GTTACTAA 0.466936522764
GTTACTCA -0.42388619011
GTTACTGA 0.132067755168
GTTACTTA 0.641966524989
GTTAGAAA 0.377862380444
GTTAGAAC 0.222381369006
GTTAGACA -0.00674730446398
GTTAGAGA 0.635771519833
GTTAGATA 2.58322017754
GTTAGCAA 0.54888775527
GTTAGCCA -0.542641780703
GTTAGCGA 0.684638115714
GTTAGCTA -0.460882663669
GTTAGGAA 0.358838766661
GTTAGGCA -0.446389315671
GTTAGGGA -0.609971065466
GTTAGGTA 0.958037773881
GTTAGTAA -0.20340480391
GTTAGTCA 0.396626180923
GTTAGTGA -0.0792995162357
GTTAGTTA -0.0696069639721
GTTATAAA 1.6262838657
GTTATAAC 1.35198923695
GTTATACA 0.0192716649155
GTTATAGA 0.502641509867
GTTATATA 1.3752252765
GTTATCAA 2.26836722826
GTTATCCA 2.04491628577
GTTATCGA 0.805200637264
GTTATCTA 0.665373508101
GTTATGAA 1.21619052091
GTTATGCA 1.21924607175
GTTATGGA -0.0380597738824
GTTATGTA 0.815573304435
GTTATTAA 1.03787207686
GTTATTCA 0.500609529357
GTTATTGA -0.100409739283

GTTATTTA 0.966771578458
GTTCAAAA -0.60628035736
GTTCAAAC -0.317440888444
GTTCAACA 0.35265108019
GTTCAAGA 0.462536686395
GTTCAATA 0.570112202073
GTTCACAA 0.0772168276958
GTTCACCA -0.607951108508
GTTCACGA -0.525655113681
GTTCACTA 0.0170669111713
GTTCAGAA 0.31369136948
GTTCAGCA -0.385615481281
GTTCAGGA 0.51479209464
GTTCAGTA 0.433683817774
GTTCATAA -0.102153677277
GTTCATCA -0.835626500317
GTTCATGA 0.387772140812
GTTCATTA -0.282448166184
GTTCCAAA 0.71278185607
GTTCCAAC -0.539985620946
GTTCCACA -0.0383674200184
GTTCCAGA 0.0840187609129
GTTCCATA 0.30659538197
GTTCCCAA -0.315725963806
GTTCCCCA -0.0504518752475
GTTCCCGA 0.324803746561
GTTCCCTA -0.359339835177
GTTCCGAA -0.443897042173
GTTCCGCA -0.128882559065
GTTCCGGA 1.00373877728
GTTCCGTA 0.491771172142
GTTCCTAA -0.336885326593
GTTCCTCA -0.196603654838
GTTCCTGA 0.574441465412
GTTCCTTA 0.251621082385
GTTCGAAA 0.733904625433
GTTCGAAC 0.33802625725
GTTCGACA 0.403515415592
GTTCGAGA 1.25260045418
GTTCGATA 0.15019090936
GTTCGCAA 1.04188585261
GTTCGCCA -0.72827760248
GTTCGCGA 1.14595179763
GTTCGCTA -0.857375017216
GTTCGGAA -0.423366040738
GTTCGGCA -0.279855522154
GTTCGGGA 0.277622277816
GTTCGGTA -0.781787642376
GTTCGTAA 0.474872852099
GTTCGTCA -0.733432047511
GTTCGTGA 0.55298935524
GTTCGTTA 0.914637451273
GTTCTAAA 0.0764211821241
GTTCTACA -0.261981203239

GTTCTAGA −0.284506807617
GTTCTATA 0.143341666075
GTTCTCAA 0.859239974888
GTTCTCCA 0.110448360776
GTTCTCGA −0.131796441074
GTTCTCTA 0.236156179003
GTTCTGAA −0.0299679224996
GTTCTGCA 1.54886943352
GTTCTGGA 0.708310401143
GTTCTGTA 0.474605458728
GTTCTTAA 1.30045576467
GTTCTTCA −0.601989255734
GTTCTTGA −0.240802498215
GTTCTTTA 0.120737385837
GTTGAAAA 0.980285268243
GTTGAAAC 1.04671827552
GTTGAACA 0.0486582747504
GTTGAAGA 1.12379238887
GTTGAATA 1.29841019232
GTTGACAA 0.249231009091
GTTGACCA −0.404753580202
GTTGACGA 1.44007823205
GTTGACTA −0.139187789785
GTTGAGAA 2.24414551872
GTTGAGCA −0.54090307034
GTTGAGGA −0.125256673595
GTTGAGTA 1.24424225495
GTTGATAA 0.26915273003
GTTGATCA −0.594206618777
GTTGATGA 0.362900113867
GTTGATTA 1.35444936052
GTTGCAAA 1.30910488161
GTTGCAAC 0.661764873816
GTTGCACA 0.23243149129
GTTGCAGA 0.124228921168
GTTGCATA 0.437808942089
GTTGCCAA 0.203091407379
GTTGCCCA −0.888723557294
GTTGCCGA −0.733773934636
GTTGCCTA −1.03288308638
GTTGCGAA 0.979423231746
GTTGCGCA 0.280156372369
GTTGCGGA 0.546217480907
GTTGCGTA 0.453405843176
GTTGCTAA 1.26442875544
GTTGCTCA −0.398490615832
GTTGCTGA 0.977001531279
GTTGCTTA 0.132730096127
GTTGGAAA 0.496055739228
GTTGGACA 0.0985040060816
GTTGGAGA 0.908761070279
GTTGGATA 4.02773091866
GTTGGCAA −0.741903424968
GTTGGCCA −0.756681417522

GTTGGCGA 0.083388047127
GTTGGCTA -0.748037528213
GTTGGGAA 0.708696984521
GTTGGGCA -0.799903477883
GTTGGGGA -0.336467377424
GTTGGGTA 0.579196780749
GTTGGTAA 0.710997142547
GTTGGTCA -0.862766012589
GTTGGTGA -0.546098813663
GTTGGTTA 0.836644843006
GTTGTAAA 1.31579964838
GTTGTACA 0.308058073369
GTTGTAGA 1.10080910532
GTTGTATA 0.904386587925
GTTGTCAA 0.833187287283
GTTGTCCA -0.676189478455
GTTGTCGA 0.124360134728
GTTGTCTA -0.471254808076
GTTGTGAA -0.313046541088
GTTGTGCA 0.312457648357
GTTGTGGA 0.97243937694
GTTGTGTA 1.95431515218
GTTGTTAA -0.165320198776
GTTGTTCA 0.278107402054
GTTGTTGA 0.245334855053
GTTGTTTA 0.542134700411
GTTTAAAA 0.935521317859
GTTTAAAC 1.32667939584
GTTTAACA 0.193769755597
GTTTAAGA 1.57671911958
GTTTAATA 0.258631336732
GTTTACAA -0.00639156411473
GTTTACCA 0.354693515964
GTTTACGA 0.0594880983289
GTTTACTA -0.486351424793
GTTTAGAA 1.17794176814
GTTTAGCA 0.00726091929592
GTTTAGGA 0.898371936068
GTTTAGTA 1.19162718563
GTTTATAA 0.653480122817
GTTTATCA 0.469975083793
GTTTATGA 0.181281988569
GTTTATTA 0.774326766161
GTTTCAAA 1.00536247974
GTTTCACA -0.14721612544
GTTTCAGA 0.644092341492
GTTTCATA 1.47755564774
GTTTCCAA 0.139514255396
GTTTCCCA -0.0699901493887
GTTTCCGA 0.571103361077
GTTTCCTA 0.571222551084
GTTTCGAA 1.53754795116
GTTTCGCA -0.314112716609
GTTTCGGA -0.0482588836749

GTTTCGTA 1.04637168353
GTTTCTAA -0.141324061551
GTTTCTCA 0.903303945363
GTTTCTGA 0.433750208699
GTTTCTTA 1.44240348271
GTTTGAAA 0.401288705794
GTTTGACA -0.228908067405
GTTTGAGA 0.159839549516
GTTTGATA 1.09622159696
GTTTGCAA 0.380808935152
GTTTGCCA -0.186703304207
GTTTGCGA 0.418211595707
GTTTGCTA -0.303216763487
GTTTGGAA 2.02252006528
GTTTGGCA -0.827713956707
GTTTGGGA -0.386617095549
GTTTGGTA -0.118529495514
GTTTGTAA 0.681009355046
GTTTGTCA -0.444095692184
GTTTGTGA 0.773368541237
GTTTGTTA 1.53631658247
GTTTTAAA 1.51322064346
GTTTTACA 0.39512846439
GTTTTAGA 0.232258456675
GTTTTATA 0.104718353476
GTTTTCAA 0.227450342256
GTTTTCCA 0.194036364823
GTTTTCGA 1.07772440571
GTTTTCTA 0.496202635684
GTTTTGAA 1.8622042785
GTTTTGCA -0.0263425597927
GTTTTGGA 0.00420928918757
GTTTTGTA 1.58516880237
GTTTTTAA 0.223767214217
GTTTTTCA -0.0695910196949
GTTTTTGA 1.10719413489
GTTTTTTA 0.0178487035185
TAAAAAAA 2.36755187201
TAAAAACA 1.01698716474
TAAAAAGA 1.71254815588
TAAAAATA 2.52509283179
TAAAACAA 1.5726290204
TAAAACCA 0.906165812434
TAAAACGA 1.35317773906
TAAAACTA 0.669751127035
TAAAAGAA 0.0551593577529
TAAAAGCA 0.87216294227
TAAAAGGA -0.459881833546
TAAAAGTA 0.661948886458
TAAAATAA 1.80501241748
TAAAATCA 3.84356928907
TAAAATGA 1.26686430914
TAAAATTA 1.63195323247
TAAACAAA 1.89968247834

TAAACACA 1.37692896173
TAAACAGA 0.455016476558
TAAACATA 0.952382521716
TAAACCAA -0.311317240463
TAAACCCA 0.118863802572
TAAACCGA 0.326869706679
TAAACCTA -0.35863854836
TAAACGAA 0.334452386717
TAAACGCA 0.427786264872
TAAACGGA 1.284969428
TAAACGTA 0.545726867655
TAAACTAA -0.167366816656
TAAACTCA 1.11563283965
TAAACTGA 1.08690517281
TAAACTTA 0.449434411239
TAAAGAAA 0.917192717734
TAAAGACA 0.320999860226
TAAAGAGA 1.3812861929
TAAAGATA 3.11018716544
TAAAGCAA 1.31278121373
TAAAGCCA 0.0200414337095
TAAAGCGA 0.137160514143
TAAAGCTA 0.0168842054372
TAAAGGAA -0.470126685117
TAAAGGCA -0.247902667831
TAAAGGGA 0.889365510488
TAAAGGTA 0.757410672169
TAAAGTAA 1.47327630828
TAAAGTCA 0.771897485627
TAAAGTGA 0.24669560763
TAAAGTTA 0.521032841575
TAAATAAA 2.20355923035
TAAATACA 1.06086267909
TAAATAGA 0.569966089762
TAAATATA 2.33634003451
TAAATCAA 1.87308742393
TAAATCCA 4.31045902453
TAAATCGA 1.10945221048
TAAATCTA 3.47115227137
TAAATGAA 2.61579146072
TAAATGCA 1.25778861743
TAAATGGA -0.465083850027
TAAATGTA 1.72060393661
TAAATTAA 0.95984967109
TAAATTCA 1.48097242793
TAAATTGA 0.887687440656
TAAATTTA 2.79028693758
TAACAAAA 1.36614278888
TAACAACA 1.17908766505
TAACAAGA 0.86873884339
TAACAATA 1.65468140758
TAACACAA 2.10899032417
TAACACCA -0.436144464099
TAACACGA 2.00989219772

TAACACTA 0.113991911044
TAACAGAA 1.09449883088
TAACAGCA 1.07575672507
TAACAGGA 0.124283027158
TAACAGTA 2.61990299319
TAACATAA 0.895250517074
TAACATCA 2.32852132689
TAACATGA 1.59921989271
TAACATTA 1.5803172986
TAACCAAA 0.588138645075
TAACCACA -0.410061717611
TAACCAGA 0.112097678634
TAACCATA 0.890081173292
TAACCCAA 0.45891498303
TAACCCCA 0.155535640191
TAACCCGA 0.560502507774
TAACCCTA -0.44107595063
TAACCGAA 1.16912641251
TAACCGCA 0.411996986932
TAACCGGA -0.18530987893
TAACCGTA 0.858209870026
TAACCTAA 0.695849556419
TAACCTCA 0.624590138011
TAACCTGA 0.701666865172
TAACCTTA 0.566672158916
TAACGAAA 3.26916755366
TAACGACA 2.06318058602
TAACGAGA 2.41081261685
TAACGATA 1.60025104309
TAACGCAA 2.58645189957
TAACGCCA -0.571879141647
TAACGCGA 0.615445702949
TAACGCTA -0.403599841847
TAACGGAA 0.634230675337
TAACGGCA -0.565101255537
TAACGGGA 0.41818911689
TAACGGTA 1.09793965818
TAACGTAA 2.16127552991
TAACGTCA 0.237511181185
TAACGTGA 1.03063807987
TAACGTTA 1.25059957808
TAACTAAA 0.480701138879
TAACTACA 1.52413802987
TAACTAGA 1.03144208965
TAACTATA -0.261231038065
TAACTCAA 0.820483096294
TAACTCCA 0.909350485774
TAACTCGA -0.270201131603
TAACTCTA 1.23067079983
TAACTGAA 1.7966790495
TAACTGCA 0.742760233833
TAACTGGA 0.773860722779
TAACTGTA 0.827069389697
TAACTTAA 1.05922956689

TAACTTCA 0.231994461265
TAACTTGA 0.00302157122506
TAAGAAAA 1.26870548108
TAAGAACA 0.680112554797
TAAGAAGA 1.42494136256
TAAGAATA 2.39934737462
TAAGACAA -0.191222591965
TAAGACCA -1.1236470607
TAAGACGA -0.435000396863
TAAGACTA 0.207087931949
TAAGAGAA 1.39784419411
TAAGAGCA 0.222115282543
TAAGAGGA -0.640673993007
TAAGAGTA 0.804558945452
TAAGATAA 2.28252496229
TAAGATCA 2.22175713968
TAAGATGA 1.47724120568
TAAGATTA 5.75864714329
TAAGCAAA 0.857976456263
TAAGCACA 0.335554371517
TAAGCAGA 0.397909564549
TAAGCATA -0.349810123646
TAAGCCAA -0.0897354377523
TAAGCCCA 0.0860729588591
TAAGCCGA -0.326739015882
TAAGCCTA -0.629141314323
TAAGCGAA 1.25304898499
TAAGCGCA 0.028391530107
TAAGCGGA -0.24617075339
TAAGCGTA 1.50134032725
TAAGCTAA -0.476455517649
TAAGCTCA 0.220695457726
TAAGCTGA -0.365945209435
TAAGCTTA 0.695767482599
TAAGGAAA 0.225177106534
TAAGGACA -0.528615521613
TAAGGAGA 0.351433564726
TAAGGATA 3.10115094236
TAAGGCAA 0.366362635841
TAAGGCCA -0.915798769694
TAAGGCGA 0.276795789216
TAAGGCTA -0.486931953313
TAAGGGAA 0.689904693448
TAAGGGCA -0.93311242509
TAAGGGGA -0.814076110666
TAAGGGTA 0.0060907139002
TAAGGTAA 1.5412339544
TAAGGTCA 0.275045578064
TAAGGTGA 0.470418386976
TAAGTAAA 2.94154297694
TAAGTACA 1.12629799282
TAAGTAGA 0.442088020162
TAAGTATA 1.27363539932
TAAGTCAA -0.803339077553

TAAGTCCA -0.442924179881
TAAGTCGA 0.422673379515
TAAGTCTA 0.458327135825
TAAGTGAA 1.06539137659
TAAGTGCA -0.105629006949
TAAGTGGA 0.0603289629164
TAAGTGTA 0.705942545285
TAAGTTAA 0.350353274598
TAAGTTCA 0.228387918033
TAAGTTGA 0.414417380491
TAATAAAA 2.0928066214
TAATAACA 1.4198669003
TAATAAGA 2.22706240189
TAATAATA 2.81046402834
TAATACAA 3.21800419771
TAATACCA 1.05655876977
TAATACGA 2.23998589204
TAATACTA 1.35212541676
TAATAGAA 0.501363092492
TAATAGCA 1.10335234823
TAATAGGA 0.220730482859
TAATAGTA 2.59331421193
TAATATAA 2.10366650386
TAATATCA 4.4422029279
TAATATGA 1.42558723648
TAATATTA 3.225239763
TAATCAAA 2.84951914333
TAATCACA 3.42782565837
TAATCAGA 3.95805599547
TAATCATA 2.53237701405
TAATCCAA 3.71936650606
TAATCCCA 2.50558252548
TAATCCGA 8.19755409937
TAATCCTA 3.57453104418
TAATCGAA 4.4388303212
TAATCGCA 2.23993884335
TAATCGGA 1.74475899384
TAATCGTA 2.59758388027
TAATCTAA 4.66253689488
TAATCTCA 4.59961764037
TAATCTGA 5.03647933556
TAATGAAA 0.709779627082
TAATGACA 1.42397477343
TAATGAGA -0.372429041253
TAATGATA 1.53048594326
TAATGCAA 0.889410468123
TAATGCCA -0.140471696174
TAATGCGA 0.913289245012
TAATGCTA 0.917408618931
TAATGGAA 1.72828149816
TAATGGCA 0.156320307736
TAATGGGA 0.130422619416
TAATGGTA 1.80447188034
TAATGTAA 3.21672055271

TAATGTCA 0.00604183554215
TAATGTGA 1.65424908243
TAATTAAA 1.59347629356
TAATTACA 2.4075286185
TAATTAGA 0.880760305655
TAATTATA 1.31683994712
TAATTCAA 1.74228135771
TAATTCCA 0.551633830295
TAATTCGA 0.647506507871
TAATTCTA 2.04778102804
TAATTGAA 0.944788340888
TAATTGCA 2.22124744557
TAATTGGA 2.0708976162
TAATTGTA 1.17279621008
TAATTTAA 2.84320128883
TAATTTCA 4.55487669157
TAATTTGA 2.19078890982
TACAAAAA 1.01263646811
TACAAACA 2.15728057363
TACAAAGA 0.422658742145
TACAAATA 3.30072049414
TACAACAA 0.998604981393
TACAACCA 0.155909938634
TACAACGA -0.356659628313
TACAACTA 0.596903554062
TACAAGAA 0.76446745244
TACAAGCA 0.16506509034
TACAAGGA 1.39229819945
TACAAGTA 0.136502355289
TACAATAA 0.349469282048
TACAATCA 3.56973077121
TACAATGA 0.0650887217406
TACACAAA 2.02910322211
TACACACA 0.096707530387
TACACAGA 4.20706692126
TACACATA 0.607438539203
TACACCAA 1.88789652089
TACACCCA -0.101613140141
TACACCGA 0.705942545285
TACACCTA -0.461792532997
TACACGAA 1.56479280022
TACACGCA 0.815002447034
TACACGGA 1.35935575441
TACACGTA 0.233943845192
TACACTAA 1.25608911431
TACACTCA -0.225588259781
TACACTGA 0.203846800185
TACAGAAA 0.668612026049
TACAGACA 0.242577279237
TACAGAGA 0.964546698331
TACAGATA 3.35910530077
TACAGCAA 0.369990873745
TACAGCCA -0.0881603522677
TACAGCGA 1.29283047943

TACAGCTA 0.0693847896173
TACAGGAA 1.73871637415
TACAGGCA -0.316650209122
TACAGGGA 0.170577105392
TACAGGTA -0.0219246880932
TACAGTAA 0.8142588164
TACAGTCA -0.522348375137
TACAGTGA 0.222749917053
TACATAAA 2.03908486241
TACATACA 2.15883161201
TACATAGA 0.714155154964
TACATATA 1.65895865598
TACATCAA 1.32319465643
TACATCCA 1.34784006553
TACATCGA 2.81589396957
TACATCTA 2.9108897119
TACATGAA 0.364177747098
TACATGCA 0.784422891605
TACATGGA -0.307238903454
TACATGTA 0.488740713943
TACATTAA 1.03299940118
TACATTCA 1.08314755102
TACATTGA 0.508107260377
TACCAAAA 1.77962651432
TACCAACA 0.770388791067
TACCAAGA 0.660738689679
TACCAATA 0.801986166423
TACCACAA 1.06464121141
TACCACCA 0.453224705732
TACCACGA -0.684792592236
TACCACTA 0.488740713943
TACCAGAA 1.43423034165
TACCAGCA 0.681007263993
TACCAGGA 0.745823626113
TACCAGTA 1.17758681194
TACCATAA 0.593608054927
TACCATCA 0.768387914966
TACCATGA 2.45209025953
TACCCAAA 0.38666806496
TACCCACA 0.344415207549
TACCCAGA 2.29837801734
TACCCATA 1.01920420342
TACCCCAA 0.00573993980123
TACCCCCA 0.23673461647
TACCCCGA -0.71129119684
TACCCCTA 0.377847743075
TACCCGAA 1.83251289773
TACCCGCA 0.363321722378
TACCCGGA 0.431574991075
TACCCGTA -0.028282795364
TACCCTAA 0.557499756024
TACCCTCA -0.434427448409
TACCCTGA 0.491133139671
TACCGAAA 1.71300191433

TACCGACA 1.2299739565
TACCGAGA 1.89616036136
TACCGATA 2.07125021997
TACCGCAA 1.95841413834
TACCGCCA 0.3486775572
TACCGCGA 0.718317918228
TACCGCTA 0.208325835178
TACCGGAA -0.169228899131
TACCGGCA -0.427330676754
TACCGGGA 0.148270277408
TACCGGTA 0.664448740022
TACCGTAA 2.49390817797
TACCGTCA 0.0722040514887
TACCGTGA 0.597500026859
TACCTAAA 1.74879760085
TACCTACA 0.900626352315
TACCTAGA -0.389943176331
TACCTATA 1.47734262174
TACCTCAA 1.50434203348
TACCTCCA -0.212716000428
TACCTCGA 0.927794355181
TACCTCTA 0.372228822952
TACCTGAA -0.273115274993
TACCTGCA 0.229306412954
TACCTGGA -0.414850751173
TACCTTAA -0.0769925622883
TACCTTCA 0.12188093031
TACCTTGA 0.386561159888
TACGAAAA 2.38090481212
TACGAACA 0.520006657437
TACGAAGA 2.35322450133
TACGAATA 1.67306568199
TACGACAA 1.43237374819
TACGACCA -0.420616567752
TACGACGA 1.57177377982
TACGACTA 1.15763346382
TACGAGAA 1.42444369201
TACGAGCA 0.63296349737
TACGAGGA 1.13698536343
TACGAGTA 0.592744450141
TACGATAA 1.9160096796
TACGATCA 3.18898561316
TACGATGA 0.98890589459
TACGCAAA 1.37064168887
TACGCACA 1.23610753699
TACGCAGA 2.1496069328
TACGCATA 1.70410866698
TACGCCAA 1.28340532054
TACGCCCA 0.127924072761
TACGCCGA -0.529411167184
TACGCCTA -0.556525848205
TACGCGAA 2.80712540124
TACGCGCA 0.665492698108
TACGCGGA 0.0535045508822

TACGCGTA 1.64842785295
TACGCTAA 0.701516309374
TACGCTCA -0.469279808753
TACGCTGA 1.20697760388
TACGGAAA 0.129313838695
TACGGACA -0.180281681209
TACGGAGA -0.182775261614
TACGGATA 2.73568667506
TACGGCAA 0.724570427334
TACGGCCA -0.183553133238
TACGGCGA 0.0246592623282
TACGGCTA 0.280666327858
TACGGGAA 1.54896248537
TACGGGCA -0.374606872693
TACGGGGA -0.312523255137
TACGGTAA 1.21812526747
TACGGTCA 1.83911069192
TACGGTGA 0.946080088725
TACGTAAA 0.310193560991
TACGTACA 0.903817298813
TACGTAGA 0.035750206119
TACGTATA 0.744203582994
TACGTCAA 0.499673260487
TACGTCCA 0.449389714986
TACGTCGA 0.830233675272
TACGTCTA 1.32609651488
TACGTGAA 0.491907613334
TACGTGCA 1.81173985694
TACGTGGA 0.425234919134
TACGTTAA 0.855100474586
TACGTTCA 1.4701235235
TACGTTGA 0.320334121306
TACTAAAA 1.69499690462
TACTAACA 0.100499654551
TACTAAGA 0.373370537754
TACTAATA 1.33285061527
TACTACAA 0.233371942265
TACTACCA -0.174598461213
TACTACGA 0.612108121377
TACTACTA 0.552960080502
TACTAGAA 0.91110304936
TACTAGCA -0.061436175348
TACTAGGA 1.58010767056
TACTAGTA 0.201893756916
TACTATAA 2.52896702978
TACTATCA 1.51545310365
TACTATGA 1.03386509702
TACTCAAA 2.0758664803
TACTCACA 0.439151397955
TACTCAGA 1.3649780725
TACTCATA 0.857891507245
TACTCCAA 0.426978857129
TACTCCCA 0.28095358623
TACTCCGA 0.55067037774

TACTCCTA -0.0109231368075
TACTCGAA 2.36908226124
TACTCGCA 0.177771895145
TACTCGGA 0.33948084582
TACTCTAA 1.09855913256
TACTCTCA 1.12059177125
TACTCTGA -0.706969513567
TACTGAAA 2.08602376904
TACTGACA 1.87591112929
TACTGAGA -0.570143567864
TACTGATA 2.7929621782
TACTGCAA 1.26006943322
TACTGCCA 0.574544711142
TACTGCGA 0.274097285641
TACTGCTA 0.948793229669
TACTGGAA 1.75332734387
TACTGGCA -0.0727989559964
TACTGGGA 0.522543888569
TACTGTAA 1.92328889561
TACTGTCA 0.0989488775544
TACTGTGA 0.215241469388
TACTTAAA 3.02573189727
TACTTACA 1.20929030822
TACTTAGA 0.308636249454
TACTTATA 1.90136917376
TACTTCAA 2.88364799942
TACTTCCA 0.646170325163
TACTTCGA 1.30451449806
TACTTCTA 0.52979748918
TACTTGAA 1.00894602139
TACTTGCA 1.51909806998
TACTTGGA 1.09526572447
TACTTTAA 1.97367673236
TACTTTCA 0.838875473528
TACTTTGA 0.423015789403
TAGAAAAA 0.0237752697778
TAGAAACA 0.305332908871
TAGAAAGA 1.46759648625
TAGAAATA 2.83734503422
TAGAACAA 0.966880574583
TAGAACCA -0.796267137149
TAGAACGA -0.223163422735
TAGAACTA -0.0749948227664
TAGAAGAA 0.29769716837
TAGAAGCA -0.211846645247
TAGAAGGA 0.642726884046
TAGAATAA 1.92370762892
TAGAATCA 3.53815639743
TAGAATGA 0.0611727027013
TAGACAAA -0.335544439016
TAGACACA -0.193000248185
TAGACAGA 0.126793074604
TAGACATA 0.273539235938
TAGACCAA -0.128160623103

TAGACCCA	−1.06767010132
TAGACCGA	0.300796369928
TAGACCTA	0.371709719106
TAGACGAA	0.508039039781
TAGACGCA	−0.354387438118
TAGACGGA	0.138045029456
TAGACTAA	0.346213774296
TAGACTCA	−0.204783591818
TAGACTGA	0.621895293778
TAGAGAAA	1.12441918193
TAGAGACA	0.625882669992
TAGAGAGA	0.590286417632
TAGAGATA	2.4708841189
TAGAGCAA	0.380113137349
TAGAGCCA	−0.84450563306
TAGAGCGA	−0.712987563384
TAGAGCTA	−0.506855503924
TAGAGGAA	0.646578603213
TAGAGGCA	0.462795976936
TAGAGGGA	−0.395123236758
TAGAGTAA	0.451013417448
TAGAGTCA	−0.260808645409
TAGAGTGA	−0.00742323726571
TAGATAAA	0.356229655591
TAGATACA	2.09240095717
TAGATAGA	0.358541314407
TAGATATA	4.20233434612
TAGATCAA	0.250359916195
TAGATCCA	1.21890862811
TAGATCGA	0.576572770928
TAGATCTA	1.16271236957
TAGATGAA	0.114847413001
TAGATGCA	0.366914151004
TAGATGGA	−0.902676629537
TAGATTAA	1.23070843878
TAGATTCA	3.77782319262
TAGATTGA	0.609166271538
TAGCAAAA	0.506077109537
TAGCAACA	1.37420484276
TAGCAAGA	0.134748484795
TAGCAATA	1.85287452389
TAGCACAA	0.169478257171
TAGCACCA	−0.342851100091
TAGCACGA	1.05333201399
TAGCACTA	0.485644910345
TAGCAGAA	0.166166552377
TAGCAGCA	−0.28702129855
TAGCAGGA	0.00420928918757
TAGCATAA	1.07595380679
TAGCATCA	0.909032384374
TAGCATGA	−0.124147631492
TAGCCAAA	1.11150954501
TAGCCACA	0.192978292131
TAGCCAGA	0.801986166423

TAGCCATA -0.213627176664
TAGCCCAA -0.52599778495
TAGCCCCA -0.00199434156118
TAGCCCGA -0.256397046868
TAGCCCTA -0.109806407582
TAGCCGAA 0.110763064215
TAGCCGCA -0.200267179257
TAGCCGGA 0.257254901259
TAGCCTAA 0.833426712823
TAGCCTCA -1.32949813495
TAGCCTGA -0.382238431089
TAGCGAAA 2.12273063179
TAGCGACA -0.604872033333
TAGCGAGA 1.67402416829
TAGCGATA 1.24908356483
TAGCGCAA 1.56012504771
TAGCGCCA 0.0832759144232
TAGCGCGA 0.253451014924
TAGCGCTA -0.596716666223
TAGCGGAA 2.46777132549
TAGCGGCA -0.372127406893
TAGCGGGA 0.500797985486
TAGCGTAA 2.29570591331
TAGCGTCA -0.484674139105
TAGCGTGA 0.733695781539
TAGCTAAA 0.315030688767
TAGCTACA 1.15294793737
TAGCTAGA 0.323626745244
TAGCTATA 0.847330906708
TAGCTCAA 0.227844244318
TAGCTCCA -0.398155263247
TAGCTCGA 1.80595025464
TAGCTGAA 1.42051538804
TAGCTGCA -0.226636661354
TAGCTGGA -0.392916653343
TAGCTTAA -0.295307095075
TAGCTTCA -0.938069788399
TAGCTTGA -0.0563232899904
TAGGAAAA -0.595830321238
TAGGAACA 0.627545318311
TAGGAAGA 0.80181835944
TAGGAATA 1.5366357294
TAGGACAA -0.356706938382
TAGGACCA -0.625853656635
TAGGACGA 0.261816532835
TAGGACTA -0.674157236563
TAGGAGAA 0.846124630652
TAGGAGCA 0.537348019282
TAGGAGGA -0.197050355982
TAGGATAA 0.964736984132
TAGGATCA 1.1655052319
TAGGATGA 0.366415957686
TAGGCAAA -0.158752724849
TAGGCACA 0.0132768780603

TAGGCAGA	-0.96791486155
TAGGCATA	-0.163364018928
TAGGCCAA	-0.534819936506
TAGGCCCA	-1.19697505304
TAGGCCGA	-0.902646832036
TAGGCCTA	-0.0603114503496
TAGGCGAA	-0.135025287903
TAGGCGCA	-0.718736128778
TAGGCGGA	-0.234006576775
TAGGCTAA	0.632268483712
TAGGCTCA	-0.186784593883
TAGGCTGA	-0.593445998339
TAGGGAAA	0.937927596812
TAGGGACA	-0.492296287764
TAGGGAGA	-0.86707096744
TAGGGATA	1.17228076558
TAGGGCAA	1.040315472
TAGGGCCA	-0.867870533735
TAGGGCGA	0.0361378350227
TAGGGGAA	0.532066804178
TAGGGGCA	0.183547905606
TAGGGGGA	-0.036014724292
TAGGGTAA	-0.20131558083
TAGGGTCA	0.369990873745
TAGGGTGA	-1.16746716215
TAGGTAAA	0.0947189392209
TAGGTACA	0.211916695514
TAGGTAGA	-0.106187840796
TAGGTATA	1.10716381463
TAGGTCAA	0.0379045132156
TAGGTCCA	-0.558793072151
TAGGTCGA	0.42811717397
TAGGTGAA	-0.824662065217
TAGGTGCA	-0.00918860855066
TAGGTGGA	-0.311340242043
TAGGTTAA	-0.456623189215
TAGGTTCA	-0.623754762437
TAGGTTGA	0.0871318156959
TAGTAAAA	1.36647500489
TAGTAACA	0.104432924775
TAGTAAGA	0.822309108109
TAGTAATA	0.364956664247
TAGTACAA	0.264305931135
TAGTACCA	-0.462231131311
TAGTACGA	1.3688094039
TAGTACTA	0.355818763726
TAGTAGAA	1.97408056693
TAGTAGCA	0.461492728309
TAGTAGGA	-0.103510770512
TAGTATAA	3.00155932747
TAGTATCA	0.397224744773
TAGTATGA	-0.323996600199
TAGTCAAA	1.41657401498
TAGTCACA	0.147469926968

TAGTCAGA -0.0481543310374
TAGTCATA 0.780265878736
TAGTCCAA 1.06638880875
TAGTCCCA -0.576513698688
TAGTCCGA -0.700391322994
TAGTCGAA 0.0596454500484
TAGTCGCA 0.0613517490931
TAGTCGGA 0.224115635881
TAGTCTAA 1.15873544862
TAGTCTCA -0.657764951285
TAGTCTGA 0.416346376654
TAGTGAAA 0.224193266215
TAGTGACA 0.654712537032
TAGTGAGA 0.197011148742
TAGTGATA 1.53902763236
TAGTGCAA 0.938936268383
TAGTGCCA -0.748581986073
TAGTGCGA 1.24019057886
TAGTGGAA 0.251655584755
TAGTGGCA -0.158093781851
TAGTGGGA -0.685872620981
TAGTGTAA 3.37736019128
TAGTGTCA 0.455571389681
TAGTGTGA 0.229540088099
TAGTTAAA -0.1622837288
TAGTTACA 0.79385981267
TAGTTAGA -0.780850850743
TAGTTATA 1.93587363521
TAGTTCAA 0.0577695143492
TAGTTCCA 0.459980374407
TAGTTCGA 0.717679362994
TAGTTGAA 1.15902636634
TAGTTGCA 0.128527602861
TAGTTGGA -0.497255480744
TAGTTTAA -0.07892992262
TAGTTTCA 0.233752513865
TAGTTTGA 1.1760650483
TATAAAAA 1.66888070129
TATAAACA 0.672604107132
TATAAAGA -0.0434228014253
TATAAATA 3.46942035693
TATAACAA 1.1525286813
TATAACCA 0.755652619569
TATAACGA 1.01316472032
TATAAGAA 1.93697535863
TATAAGCA 0.267753031595
TATAAGGA 1.22024951569
TATAATAA 2.80438716766
TATAATCA 2.64394644048
TATAATGA 1.89509392446
TATACAAA 1.78595247165
TATACACA -0.0637656081125
TATACAGA 2.94246408567
TATACATA 2.42584284264

TATACCAA 2.67055038187
TATACCCA 1.3929777916
TATACCGA 1.48611275836
TATACGAA 2.433770285
TATACGCA 0.51610893511
TATACGGA 0.47098976714
TATACTAA 1.59549990986
TATACTCA 0.880398030766
TATACTGA 1.52682843062
TATAGAAA 2.11990718781
TATAGACA 0.97078483145
TATAGAGA 1.46225227818
TATAGATA 4.32941598601
TATAGCAA 0.66255764419
TATAGCCA 0.0210401727796
TATAGCGA 0.451868396641
TATAGGAA 0.175881583458
TATAGGCA -0.891351487839
TATAGGGA 0.176198377949
TATAGTAA 0.606075434191
TATAGTCA 0.221115759328
TATAGTGA 0.849740060859
TATATAAA 2.22518332961
TATATACA 1.05998130036
TATATAGA 4.88546925629
TATATATA 2.12590406572
TATATCAA 1.54817363571
TATATCCA 3.41138371059
TATATCGA 1.28123716022
TATATGAA 2.20913763633
TATATGCA 2.59204807949
TATATGGA 2.05712019239
TATATTAA 1.65158769504
TATATTCA 1.62711192259
TATATTGA 2.6582534234
TATCAAAA 1.37347558811
TATCAACA 1.83531673809
TATCAAGA 1.90080171432
TATCAATA 5.9183348301
TATCACAA 2.14155899352
TATCACCA 0.579834551838
TATCACGA 3.08571060885
TATCAGAA 2.03580243236
TATCAGCA 1.52142227511
TATCAGGA 2.85460876573
TATCATAA 1.66078048569
TATCATCA 1.7633356451
TATCATGA 3.25930614889
TATCCAAA 3.25903875552
TATCCACA 2.88416004597
TATCCAGA 0.678677308466
TATCCATA 1.46365171523
TATCCCAA 2.72697691759
TATCCCCA 1.28401826038

TATCCCGA 1.54499314448
TATCCGAA 1.70446963496
TATCCGCA 4.09886801048
TATCCGGA 5.03487968021
TATCCTAA 2.9782710116
TATCCTCA 3.30153705024
TATCCTGA 2.65876311751
TATCGAAA 2.60701374403
TATCGACA 0.752055486074
TATCGAGA 0.980627416749
TATCGATA 3.52981780182
TATCGCAA 3.14173409415
TATCGCCA 0.0962197923329
TATCGCGA 2.2196551089
TATCGGAA 3.13178747898
TATCGGCA 0.32202055535
TATCGGGA 2.85133522265
TATCGTAA 1.91588343229
TATCGTCA 2.00655644582
TATCGTGA 1.20854432515
TATCTAAA 3.77948427265
TATCTACA 4.40366038222
TATCTAGA 2.40277800804
TATCTCAA 3.7225015169
TATCTCCA 3.7785903476
TATCTCGA 3.01399847752
TATCTGAA 3.9400219724
TATCTGCA 5.44791382931
TATCTGGA 3.05419661422
TATCTTAA 2.84689487213
TATCTTCA 2.57395838215
TATCTTGA 3.94945810932
TATGAAAA 1.26518519378
TATGAACA 0.168716591207
TATGAAGA 2.08666154013
TATGAATA 0.799693327082
TATGACAA 0.857302875896
TATGACCA 0.234314484292
TATGACGA 0.750258748998
TATGAGAA 0.524342978079
TATGAGCA -0.909002848254
TATGAGGA 1.78711405146
TATGATAA 2.41096866166
TATGATCA 2.55514883989
TATGATGA 0.69816199938
TATGCAAA 2.30385945075
TATGCACA 0.997851941021
TATGCAGA 0.762882957218
TATGCATA 1.60240613434
TATGCCAA 0.982380764481
TATGCCCA -0.332572007531
TATGCCGA 0.57795547956
TATGCGAA 1.32097578808
TATGCGCA 2.02206761374

TATGCGGA 2.20544353026
TATGCTAA 1.36614278888
TATGCTCA -0.597735793057
TATGCTGA 1.95536381514
TATGGAAA 1.74374300359
TATGGACA 0.15118625047
TATGGAGA -0.224542994787
TATGGCAA 1.21361852603
TATGGCCA -0.294846279325
TATGGCGA 0.656428768577
TATGGGAA 0.648773685838
TATGGGCA 0.152008034201
TATGGGGA 1.49075698652
TATGGTAA 0.600107831021
TATGGTCA 0.712536680135
TATGGTGA 0.190290505201
TATGTAAA 2.09626208607
TATGTACA 0.579202792526
TATGTAGA 1.11145831421
TATGTCAA 2.27040757298
TATGTCCA 0.92514891207
TATGTCGA 2.12152618541
TATGTGAA 1.76290122889
TATGTGCA -0.182775261614
TATGTGGA 1.35721085705
TATGTTAA 0.826945494821
TATGTTCA 0.189275821854
TATGTTGA 1.1121572486
TATTAAAA 2.24979711154
TATTAACA 1.65340351297
TATTAAGA 2.76956329791
TATTAATA 1.43992244862
TATTACAA 2.41738610255
TATTACCA 1.83920112995
TATTACGA 5.49345382164
TATTAGAA 2.58052637884
TATTAGCA 0.0900705289557
TATTAGGA 1.03484580076
TATTATAA 2.80933825781
TATTATCA 1.1181329546
TATTATGA 1.78162085588
TATTCAAA 2.79064895109
TATTCACA 1.23468091625
TATTCAGA 0.920529776543
TATTCCAA 1.97970053258
TATTCCCA 2.90210807449
TATTCCGA 2.07028859709
TATTCGAA 2.16099663575
TATTCGCA 2.8714101132
TATTCGGA 1.23328226334
TATTCTAA 2.38889550882
TATTCTCA 1.61961915782
TATTCTGA 2.45160565806
TATTGAAA 1.55551898127

TATTGACA 1.58791121805
TATTGAGA 2.45372572417
TATTGCAA 1.17567088485
TATTGCCA -0.208607343154
TATTGCGA 3.24182808447
TATTGGAA 1.17725172073
TATTGGCA 0.12830934923
TATTGGGA 0.737027089953
TATTGTAA 2.15906293472
TATTGTCA 1.04690385645
TATTGTGA 1.01859570707
TATTTAAA 3.46114815225
TATTTACA 0.889569910895
TATTTAGA 2.18039036587
TATTTCAA 2.10086187936
TATTTCCA 2.88702844758
TATTTCGA 0.541935527636
TATTTGAA 2.44782973955
TATTTGCA 2.87634682736
TATTTGGA 1.62569993922
TATTTTAA 2.01225587147
TATTTTCA 2.41741485453
TATTTTGA 2.54921077284
TCAAAAAA 2.83117198511
TCAAAACA -0.639776408614
TCAAAAGA -0.0582342508231
TCAAACAA 0.838895338529
TCAAACCA 0.208266501556
TCAAACGA 0.0740933176492
TCAAAGAA 1.44582705882
TCAAAGCA 1.07003847994
TCAAAGGA 0.93800914787
TCAAATAA 0.700502671553
TCAAATCA 3.00702115725
TCAAATGA 1.35935575441
TCAACAAA 2.06512291265
TCAACACA 0.728726133295
TCAACAGA 0.653568469796
TCAACCAA 1.2064603297
TCAACCCA -0.566790564778
TCAACCGA -0.13758813443
TCAACGAA -0.219899812154
TCAACGCA 0.527697026692
TCAACGGA 0.459573664647
TCAACTAA 1.52070086191
TCAACTCA 0.278621801031
TCAACTGA -0.226586476088
TCAAGAAA 1.18103469654
TCAAGACA 0.214637677906
TCAAGAGA 1.27573795287
TCAAGCAA 0.888257775294
TCAAGCCA -0.585874296328
TCAAGCGA -1.17102456564
TCAAGGAA -0.109539275593

TCAAGGCA −0.804215228656
TCAAGGGA −0.00152359331063
TCAAGTAA 2.31735013895
TCAAGTCA −0.188153972053
TCAAGTGA 0.623302833661
TCAATAAA 1.747599689
TCAATACA 1.761434094
TCAATAGA 0.793786625824
TCAATCAA 1.19802345462
TCAATCCA 0.387936549835
TCAATCGA 0.912796802089
TCAATGAA 0.32139742163
TCAATGCA 0.791285726734
TCAATGGA 0.129132178487
TCAATTAA 1.50169946556
TCAATTCA −0.230236147283
TCAATTGA 1.94284781889
TCACAAAA 0.642199415989
TCACAACA 2.00899670438
TCACAAGA 0.588651475762
TCACACAA 1.32947617889
TCACACCA 1.42619886941
TCACACGA −0.684167106081
TCACAGAA 2.2829423887
TCACAGCA 0.408374499422
TCACAGGA 0.420934146389
TCACATAA 1.491846425
TCACATCA 0.760241957594
TCACATGA 1.24850120664
TCACCAAA 1.2260574147
TCACCACA 0.731351972787
TCACCAGA 0.81750595994
TCACCCAA 2.06262541152
TCACCCCA −0.486122715898
TCACCCGA −0.825588401586
TCACCGAA −0.303216763487
TCACCGCA 1.2877256969
TCACCGGA −0.61148211246
TCACCTAA 0.304241379335
TCACCTCA 0.341409580601
TCACCTGA 0.404544736309
TCACGAAA 0.507259077105
TCACGACA 0.339833188208
TCACGAGA 0.151820884979
TCACGCAA 1.33428769127
TCACGCCA 0.527922337626
TCACGCGA 1.36475642091
TCACGGAA 0.296692156141
TCACGGCA −0.405080829958
TCACGGGA −0.468100455002
TCACGTAA 0.497900570518
TCACGTCA 0.505564801613
TCACGTGA 0.874145260278
TCACTAAA 0.651231718347

TCACTACA 0.138388484871
TCACTAGA -0.524537707367
TCACTCAA 2.16217102325
TCACTCCA 0.442251383658
TCACTCGA 0.553713382255
TCACTGAA 0.554544575724
TCACTGCA 0.550107100405
TCACTGGA 0.114984376956
TCACTTAA 0.517959255413
TCACTTCA 0.350526309213
TCAGAAAA 0.461010479268
TCAGAACA 0.711172790978
TCAGAAGA 0.885201701698
TCAGACAA 0.3225339088
TCAGACCA -0.732212179613
TCAGACGA 0.27018753976
TCAGAGAA 0.841005733518
TCAGAGCA -0.820948878295
TCAGAGGA -0.211300619097
TCAGATAA 1.82073112239
TCAGATCA 0.686132957049
TCAGATGA 0.614679070735
TCAGCAAA 1.11461057624
TCAGCACA 0.897820420905
TCAGCAGA 1.39181359798
TCAGCCAA 1.01610055838
TCAGCCCA 0.0485545062576
TCAGCCGA 0.43787481025
TCAGCGAA 1.60492794395
TCAGCGCA -0.029270033644
TCAGCGGA 0.47440079694
TCAGCTAA 0.6998544452
TCAGCTCA -0.0574932340045
TCAGCTGA 0.398477023989
TCAGGAAA 1.7762980811
TCAGGACA 0.746423235489
TCAGGAGA 0.718060718739
TCAGGCAA -0.16437138359
TCAGGCCA -0.285198684696
TCAGGCGA 0.347462132788
TCAGGGAA -0.124772594883
TCAGGGCA 0.994778093477
TCAGGGGA -0.162329993342
TCAGGTAA 0.570246290831
TCAGGTCA -0.668522633544
TCAGTAAA 2.66066179341
TCAGTACA 0.165375088911
TCAGTAGA 0.977924208305
TCAGTCAA -1.05270234573
TCAGTCCA -0.977424708079
TCAGTCGA 0.134748484795
TCAGTGAA -0.275034077274
TCAGTGCA -0.739648224576
TCAGTGGA 0.532597670195

TCAGTTAA 1.8780126373
TCAGTTCA -0.255608719981
TCATAAAA 1.52441640127
TCATAACA 1.11377990553
TCATAAGA 1.38947841482
TCATACAA -0.605063626041
TCATACCA 0.336754374414
TCATACGA 0.734911728714
TCATAGAA 0.678224334163
TCATAGCA -0.0313354709987
TCATAGGA 1.06757391289
TCATATAA 3.41710143296
TCATATCA 2.18527297404
TCATATGA 1.27526328389
TCATCAAA 1.08102931458
TCATCACA 0.447744579235
TCATCAGA 1.00736806071
TCATCCAA 1.2711648205
TCATCCCA 1.18632427586
TCATCCGA 0.774757261646
TCATCGAA 0.591756950479
TCATCGCA 0.413969372439
TCATCGGA -0.0638597054863
TCATCTAA 0.133643102034
TCATCTCA -0.600530485059
TCATGAAA 1.27071132343
TCATGACA 1.0927475742
TCATGAGA 3.04473799848
TCATGCAA 0.125113436482
TCATGCCA -0.48378936241
TCATGCGA 1.05098350037
TCATGGAA 1.00330802041
TCATGGCA -1.03764310658
TCATGGGA 0.455534534877
TCATGTAA 1.18939916893
TCATGTCA 0.626521747989
TCATTAAA 0.215772074024
TCATTACA 2.26850523774
TCATTAGA 1.08747446193
TCATTCAA 0.140422556434
TCATTCCA -0.333552711271
TCATTCGA 0.57436409646
TCATTGAA 1.10245580936
TCATTGCA 0.242673990427
TCATTGGA 0.62271394093
TCATTTAA 1.91471348828
TCATTTCA 0.304431403754
TCCAAAAA 1.65984944446
TCCAAACA 1.19978908728
TCCAAAGA 0.166201054747
TCCAACAA 1.30654883101
TCCAACCA 0.240219878642
TCCAACGA 0.0862449479479
TCCAAGAA 0.813422656681

TCCAAGCA 0.292372041158
TCCAAGGA 0.495264014381
TCCAATAA 0.388936857195
TCCAATCA 1.01466139132
TCCACAAA 0.863361178478
TCCACACA 0.171876694677
TCCACAGA −0.183324162961
TCCACCAA 0.0864621560524
TCCACCCA −0.513684882208
TCCACCGA −0.294642401682
TCCACGAA 0.399730610113
TCCACGCA −0.374967579292
TCCACGGA 0.702433236005
TCCACTAA 1.89710551721
TCCACTCA 0.303404958235
TCCAGAAA 0.171487758865
TCCAGACA −0.40923993388
TCCAGAGA 0.0801871681285
TCCAGCAA 2.65516807507
TCCAGCCA −0.0900501411913
TCCAGCGA −0.287195640073
TCCAGGAA −0.615757792572
TCCAGGCA 0.0471035770301
TCCAGGGA 0.73660678835
TCCAGTAA 0.582377010602
TCCAGTCA 0.462477614154
TCCATAAA 1.74374300359
TCCATACA 0.469609410943
TCCATAGA 0.639564689523
TCCATCAA 1.5723179763
TCCATCCA 0.494582069802
TCCATCGA 0.378459898768
TCCATGAA 0.519611448467
TCCATGCA 0.2037344061
TCCATGGA 0.383535406557
TCCATTAA 1.47240904415
TCCATTCA 0.00180771510316
TCCCAAAA 0.831662909827
TCCCAACA 0.706603317954
TCCCAAGA 0.228731112066
TCCCACAA 1.02831910237
TCCCACCA −0.162432454927
TCCCACGA 1.28569580745
TCCCAGAA 0.529560677456
TCCCAGCA 0.177323102948
TCCCAGGA 0.404363076101
TCCCATAA −0.683186663723
TCCCATCA 0.956384273918
TCCCCAAA 1.74114487054
TCCCCACA 0.250949331689
TCCCCAGA −0.495747831711
TCCCCCAA −0.288875278195
TCCCCCCA −0.664749067473
TCCCCCGA 0.890663531484

TCCCCGAA -0.252709998105
TCCCCGCA 1.45622560277
TCCCCGGA 1.20403575404
TCCCCTAA 3.39813689141
TCCCCTCA -0.705192902873
TCCCGAAA 0.670898069469
TCCCGACA -0.0918442644516
TCCCGAGA 1.17425132142
TCCCGCAA 0.934111948305
TCCCGCCA 0.523594903958
TCCCGCGA 0.449440423016
TCCCGGAA 0.524340887027
TCCCGGCA -0.633368900222
TCCCGGGA 0.733687155947
TCCCGTAA 0.877683844295
TCCCGTCA 0.0598514187444
TCCCTAAA 1.3098150554
TCCCTACA -0.642466025215
TCCCTAGA 0.00700319704437
TCCCTCAA -0.303713388516
TCCCTCCA -0.0331502434049
TCCCTCGA 0.0467995902364
TCCCTGAA 0.142671745049
TCCCTGCA -0.283402731764
TCCCTTAA -0.256629415105
TCCCTTCA 0.151314327451
TCCGAAAA 0.98688384658
TCCGAACA 1.14200781076
TCCGAAGA 1.05173340416
TCCGACAA 0.897425473317
TCCGACCA -0.332971398606
TCCGACGA 0.528595133848
TCCGAGAA 0.646502541169
TCCGAGCA -0.810074881227
TCCGAGGA 0.125513873083
TCCGATAA 1.21253562209
TCCGATCA 1.02612898599
TCCGCAAA 0.0534478310763
TCCGCACA 0.509778795669
TCCGCAGA -0.0659410871182
TCCGCCAA 0.78928772583
TCCGCCCA -0.101540737439
TCCGCCGA -0.37441135926
TCCGCGAA 1.60209456748
TCCGCGCA 1.14096097748
TCCGCGGA 3.18087807888
TCCGCTAA 0.615516537361
TCCGCTCA -0.412073571739
TCCGGAAA 2.87909734587
TCCGGACA 0.46088945959
TCCGGAGA 2.96716621458
TCCGGCAA 0.69054455559
TCCGGCCA -0.952034100051
TCCGGCGA 0.891881569711

TCCGGGAA -0.344639472956
TCCGGGCA -1.00373250412
TCCGGTAA 0.787719436267
TCCGGTCA 0.194249390822
TCCGTAAA 0.145389852244
TCCGTACA -0.0606295517493
TCCGTAGA 0.0647060590872
TCCGTCAA 0.501230572024
TCCGTCCA 0.0116576190863
TCCGTCGA 1.19403085077
TCCGTGAA 0.679674217864
TCCGTGCA 0.0123873964964
TCCGTTAA 0.311582281399
TCCGTTCA 0.392184000735
TCCTAAAA 0.768289374106
TCCTAACA 0.143635720368
TCCTAAGA 0.587137030808
TCCTACAA 2.02838494549
TCCTACCA -0.690070409379
TCCTACGA 0.401221007961
TCCTAGAA 0.392625474247
TCCTAGCA 0.111556618734
TCCTAGGA 1.89700985154
TCCTATAA 1.18059008645
TCCTATCA 0.806998419867
TCCTCAAA 1.84968645259
TCCTCACA 0.891192045066
TCCTCAGA 0.321142835958
TCCTCCAA -0.159054359208
TCCTCCCA -0.186366644714
TCCTCCGA -0.889605720173
TCCTCGAA -0.0423495686009
TCCTCGCA 0.736418593603
TCCTCTAA 1.9726651856
TCCTCTCA -0.263144351332
TCCTGAAA 0.807936257026
TCCTGACA -0.0278543909316
TCCTGAGA 1.23942263974
TCCTGCAA -0.203938806506
TCCTGCCA -0.957074059944
TCCTGCGA 0.333951840965
TCCTGGAA 1.10313043525
TCCTGGCA -0.0106800519252
TCCTGTAA 1.36567752964
TCCTGTCA 0.941230414632
TCCTTAAA 0.331417746412
TCCTTACA 0.0908732318304
TCCTTAGA 1.45809814051
TCCTTCAA 1.36697084577
TCCTTCCA -0.308815557228
TCCTTCGA 0.178407575181
TCCTTGAA 0.211008655857
TCCTTGCA 0.583286095785
TCCTTTAA 0.208650732499

TCCTTTCA 0.190852998391
TCGAAAAA 1.68915999225
TCGAAACA -0.0701804351891
TCGAAAGA 0.249646344443
TCGAACAA 0.365552614281
TCGAACCA 0.246885893431
TCGAACGA 0.703615203573
TCGAAGAA -0.0957362363842
TCGAAGCA 0.622260705246
TCGAATAA 0.238072106086
TCGAATCA 2.88543689505
TCGACAAA 0.576572770928
TCGACACA 0.507033243407
TCGACAGA 0.237783540806
TCGACCAA -0.275692497509
TCGACCCA -0.385753229381
TCGACCGA -1.31587283522
TCGACGAA -0.291413293472
TCGACGCA -0.245429997953
TCGACTAA 0.592677013689
TCGACTCA 1.56038381549
TCGAGAAA 3.10073482286
TCGAGACA 0.254546987947
TCGAGAGA 0.458882571712
TCGAGCAA 0.478268721766
TCGAGCCA -0.824177986505
TCGAGCGA 0.616623749793
TCGAGGAA -0.306768939348
TCGAGGCA 0.045736028531
TCGAGTAA 1.34085411967
TCGAGTCA 0.666500324152
TCGATAAA 1.83576657581
TCGATACA 0.344167417798
TCGATAGA 0.666862860423
TCGATCAA 1.46748095558
TCGATCCA 0.455767425877
TCGATCGA 2.52804513689
TCGATGAA 0.0826763050469
TCGATGCA -0.459889936375
TCGATTAA 0.606075434191
TCGATTCA 0.300398024379
TCGCAAAA 2.1929604681
TCGCAACA 0.979423231746
TCGCAAGA 1.30344623149
TCGCACAA 1.38449073124
TCGCACCA -0.729862097702
TCGCACGA 0.229526496256
TCGCAGAA 0.159839549516
TCGCAGCA 0.000206491459152
TCGCATAA 0.578942195077
TCGCATCA 2.24141512659
TCGCCAAA 0.122712907924
TCGCCACA 0.275311664526
TCGCCAGA -0.368531318925

TCGCCCAA 0.060275118308
TCGCCCCA 0.347704956289
TCGCCCGA -0.566074640592
TCGCCGAA 0.0628167929267
TCGCCGCA -0.0617195129957
TCGCCTAA 0.351767349021
TCGCCTCA 0.457948655278
TCGCGAAA 1.28762428085
TCGCGACA 0.557680632087
TCGCGAGA 1.87929445264
TCGCGCAA 0.296274991117
TCGCGCCA 0.0491541156339
TCGCGCGA 1.56394749214
TCGCGGAA 0.523899152133
TCGCGGCA 0.317289025738
TCGCGTAA 3.06131638746
TCGCGTCA 0.196232231593
TCGCTAAA 2.07043810736
TCGCTACA -0.0728078429706
TCGCTAGA 0.605159030323
TCGCTCAA -0.0696069639721
TCGCTCCA -0.899509730146
TCGCTGAA -0.215875842516
TCGCTGCA -0.246777942832
TCGCTTAA 1.06226682101
TCGCTTCA 1.01381320805
TCGGAAAA 0.697178420442
TCGGAACA -0.507414337771
TCGGAAGA 0.438268450931
TCGGACAA 0.208073863321
TCGGACCA -0.836959546445
TCGGACGA -0.29384335815
TCGGAGAA 0.766895164684
TCGGAGCA -0.120860235186
TCGGATAA 0.200199220043
TCGGATCA 1.11021988822
TCGGCAAA 1.09094142739
TCGGCACA -0.124050658921
TCGGCAGA -0.201153001479
TCGGCCAA -0.15852662977
TCGGCCCA -1.13233198692
TCGGCCGA 1.8079247312
TCGGCGAA 0.559415160344
TCGGCGCA -4.07755286425E-5
TCGGCTAA 0.219283997119
TCGGCTCA -0.421382677204
TCGGGAAA 0.0680067858545
TCGGGACA 0.212010008743
TCGGGAGA 0.134449464251
TCGGGCAA 1.7701875022
TCGGGCCA -0.556181347265
TCGGGGAA 0.920392812588
TCGGGGCA 0.126227706216
TCGGGTAA 1.18687004062

TCGGGTCA -0.653462087487
TCGGTAAA 1.07370383403
TCGGTACA -0.724381971205
TCGGTAGA 0.504229141669
TCGGTCAA -0.180222608969
TCGGTCCA -0.126192419701
TCGGTGAA -0.895810396448
TCGGTGCA -0.171937073825
TCGGTTAA 0.648063250666
TCGGTTCA -0.364292232236
TCGTAAAA 0.493064488268
TCGTAACA 0.336719087899
TCGTAAGA 0.898249870864
TCGTACAA 0.623951844158
TCGTACCA 0.426721134877
TCGTACGA -0.170692636056
TCGTAGAA -0.217109302258
TCGTAGCA 0.634613599372
TCGTATAA 0.384457822202
TCGTATCA 2.59665832805
TCGTCAAA 0.576906293842
TCGTCACA 0.484391324221
TCGTCAGA 0.200731654349
TCGTCCAA 0.163900373958
TCGTCCCA -0.591840592589
TCGTCGAA 0.614658160207
TCGTCGCA 0.539346281567
TCGTCTAA 0.396783009879
TCGTCTCA -0.407206385079
TCGTGAAA 1.0173925676
TCGTGACA 0.612820647602
TCGTGAGA 2.00312006201
TCGTGCAA -0.0482907722294
TCGTGCCA -0.411236105112
TCGTGGAA 1.24076143626
TCGTGGCA 0.352738120261
TCGTGTAA 2.2172467389
TCGTGTCA -0.195472918063
TCGTTAAA 1.50601539844
TCGTTACA 0.962130486878
TCGTTAGA 0.269988105604
TCGTTCAA 1.61723117558
TCGTTCCA -0.368533932741
TCGTTGAA -0.219899812154
TCGTTGCA 0.711205202295
TCGTTTAA 2.14865759485
TCGTTTCA 0.914339737637
TCTAAAAA 1.76339001247
TCTAAACA 1.06303136217
TCTAAAGA 0.276361895771
TCTAACAA 1.13556031098
TCTAACCA -0.173094994286
TCTAAGAA 1.99671229223
TCTAAGCA 1.46759648625

TCTAATAA 2.0928066214
TCTAATCA 1.29446934203
TCTACAAA 1.29334121907
TCTACACA 0.840371883153
TCTACAGA 1.81709661132
TCTACCAA 0.0603914331173
TCTACCCA 1.14877053674
TCTACGAA -0.0904921374666
TCTACGCA -0.298147528856
TCTACTAA -0.159837458463
TCTACTCA 0.234091003029
TCTAGAAA 2.03509199718
TCTAGACA 1.36288806527
TCTAGAGA 1.502014169
TCTAGCAA 1.86940089793
TCTAGCCA 1.01106530335
TCTAGGAA 2.69595588865
TCTAGGCA -0.414095096986
TCTAGTAA 1.69345344631
TCTAGTCA 0.541510521164
TCTATAAA 0.97078483145
TCTATACA 0.140049826281
TCTATAGA 0.343232717218
TCTATCAA 0.738788540514
TCTATCCA -0.187987471977
TCTATGAA 0.397381050966
TCTATGCA 0.0652588811582
TCTATTAA 0.695018362951
TCTATTCA 0.536455662521
TCTCAAAA 1.86719640556
TCTCAACA 0.942472499966
TCTCAAGA 1.26639225398
TCTCACAA 1.29749352707
TCTCACCA 0.127986020198
TCTCAGAA 1.27332644628
TCTCAGCA 0.931524270526
TCTCATAA 0.952081671502
TCTCATCA 1.00672506199
TCTCCAAA 0.226151014353
TCTCCACA -0.664476707853
TCTCCAGA 0.394007660116
TCTCCCAA -0.685619080835
TCTCCCCA -0.483481977656
TCTCCGAA -0.480901357179
TCTCCGCA -0.496033783175
TCTCCTAA 0.450796470725
TCTCCTCA -0.330744166045
TCTCGAAA 1.31470184568
TCTCGACA 0.18530491268
TCTCGAGA 0.827819032108
TCTCGCAA 1.24784775266
TCTCGCCA -0.272826448332
TCTCGGAA 0.657659614503
TCTCGGCA 0.965730756951

TCTCGTAA 1.68208831323
TCTCGTCA -0.224198232465
TCTCTAAA 0.929819539771
TCTCTACA 3.19690129335
TCTCTCAA 0.767057744035
TCTCTCCA -0.44652366581
TCTCTGAA 0.542601005174
TCTCTGCA 0.0970420988272
TCTCTTAA 0.56158253652
TCTCTTCA 0.107791678256
TCTGAAAA 0.73660678835
TCTGAACA 1.08148176612
TCTGAAGA 1.41116106355
TCTGACAA -0.433598084612
TCTGACCA -0.323129597453
TCTGAGAA 3.75434302266
TCTGAGCA 0.109671534679
TCTGATAA 1.11957212165
TCTGATCA 3.01484038763
TCTGCAAA 1.25341152126
TCTGCACA -0.232918706581
TCTGCAGA 2.01968302946
TCTGCCAA -0.0380597738824
TCTGCCCA -0.0624503359321
TCTGCGAA 1.7431904429
TCTGCGCA 1.94286376317
TCTGCTAA 0.509101294578
TCTGCTCA 0.345492099715
TCTGGAAA 2.39421357873
TCTGGACA -0.189367828175
TCTGGCAA 1.08220265656
TCTGGCCA 0.184684392776
TCTGGGAA 0.718697705683
TCTGGGCA 0.464142614907
TCTGGTAA 0.677084187651
TCTGGTCA 0.256217739095
TCTGTAAA 2.95939377151
TCTGTACA -0.496527271624
TCTGTCAA 0.326788678385
TCTGTCCA 0.0487168242274
TCTGTGAA 1.30191035324
TCTGTGCA 1.75216315025
TCTGTTAA 0.421665492089
TCTGTTCA -0.159314695275
TCTTAAAA 2.68687784451
TCTTAACA 0.557111865739
TCTTAAGA 1.87357751442
TCTTACAA 0.31189567793
TCTTACCA 1.33190650495
TCTTAGAA 1.5274946923
TCTTAGCA 2.2124524777
TCTTATAA 0.706970820475
TCTTATCA -0.0468732998459
TCTTCAAA 1.41733751062

TCTTCACA 0.995507870888
TCTTCCAA -0.343635767636
TCTTCCCA 0.0404817357311
TCTTCGAA 1.084459948
TCTTCGCA 0.534406169443
TCTTCTAA 0.174760779183
TCTTCTCA 0.122059453939
TCTTGAAA 0.574024823151
TCTTGACA 1.03827538866
TCTTGCAA 2.25804736017
TCTTGCCA -1.08194415016
TCTTGGAA 0.53469421196
TCTTGGCA 1.70243634754
TCTTGTAA 1.53420958544
TCTTGTCA -0.19236482953
TCTTTAAA 2.74895492753
TCTTTACA -0.0381496891507
TCTTTCAA 2.03356500591
TCTTTCCA 0.526233812529
TCTTTGAA 1.37227950594
TCTTTGCA 1.53527184024
TCTTTTAA 1.00680426061
TCTTTTCA 0.404109535955
TGAAAAAA 1.66662001188
TGAAAACA 1.24529510001
TGAAACAA 0.530675208572
TGAAACCA 0.400811423004
TGAAAGAA 1.48649228443
TGAAAGCA 1.11835460619
TGAAATAA 2.23248659273
TGAAATCA 7.59353207898
TGAACAAA 0.173801508734
TGAACACA 0.105553990431
TGAACCAA -0.335310763871
TGAACCCA -0.295049634205
TGAACGAA 1.55070694612
TGAACGCA 0.679933508406
TGAACTAA 0.671909093474
TGAACTCA -0.282300485583
TGAAGAAA 1.16794705875
TGAAGACA 0.739558832071
TGAAGCAA 0.720409493742
TGAAGCCA -0.0812002831863
TGAAGGAA 0.0469007449132
TGAAGGCA -1.04757560715
TGAAGTAA 0.471082818988
TGAAGTCA -0.638264054712
TGAATAAA 0.785255653363
TGAATACA 0.888218568055
TGAATCAA 0.300811007297
TGAATCCA 1.56088436124
TGAATGAA 0.0236341237171
TGAATGCA 0.773949331139
TGAATTAA 1.59254028608

TGAATTCA 1.1384030972
TGACAAAA 2.27772442794
TGACAACA 0.849061514241
TGACACAA 0.9607171966
TGACACCA -0.680786396546
TGACAGAA 0.277709056505
TGACAGCA 0.32499873723
TGACATAA 0.36524078604
TGACATCA 0.4959297533
TGACCAAA 0.628847521411
TGACCACA -0.381775001523
TGACCCAA 0.0247010833832
TGACCCCA -0.868521896667
TGACCGAA -0.0776810414065
TGACCGCA -0.778784890625
TGACCTAA -0.168729398905
TGACCTCA 0.431589628444
TGACGAAA 1.60311918332
TGACGACA -0.812525595051
TGACGCAA 1.41135344041
TGACGCCA -0.901901894493
TGACGGAA 0.876486977977
TGACGGCA -0.332572007531
TGACGTAA 1.04023392094
TGACGTCA 1.48649228443
TGACTAAA 0.071341492229
TGACTACA 0.375496615638
TGACTCAA -0.289068700575
TGACTCCA -0.16672460208
TGACTGAA 0.546195263471
TGACTGCA -0.117801025012
TGACTTAA 0.50701390117
TGAGAAAA 1.51581459439
TGAGAACA 0.292925908755
TGAGACAA -0.0957926948085
TGAGACCA 0.300675088869
TGAGAGAA -0.121249955143
TGAGAGCA 0.639395575632
TGAGATAA 0.58554181894
TGAGATCA 2.94564719072
TGAGCAAA 0.805483452149
TGAGCACA -0.137036619267
TGAGCCAA -1.12558389831
TGAGCCCA -1.11522142502
TGAGCGAA 0.435508784063
TGAGCGCA 0.577257067941
TGAGCTAA -0.814862869263
TGAGCTCA -0.285448042736
TGAGGAAA 0.679367878636
TGAGGACA 0.75831609801
TGAGGCAA -0.0252102547281
TGAGGCCA -1.07427416867
TGAGGGAA -0.133862662573
TGAGGGCA -0.552096998479

TGAGGTAA 0.964800238477
TGAGTAAA 0.351176103856
TGAGTACA 0.435186761939
TGAGTCAA 0.0687344722118
TGAGTCCA −0.557609536294
TGAGTGAA −0.262628122684
TGAGTGCA −0.24414713709
TGAGTTAA 0.215836373896
TGATAAAA 1.85057697968
TGATAACA 0.199244915844
TGATACAA 1.12269719999
TGATACCA 1.06597242787
TGATAGAA 0.287091087435
TGATAGCA 1.43773468468
TGATATAA 1.58110196615
TGATATCA 6.60107819064
TGATCAAA 1.75300218517
TGATCACA 1.28122252285
TGATCCAA 0.715403513456
TGATCCCA 0.948284058324
TGATCGAA 0.742440302762
TGATCGCA 2.9454799065
TGATCTAA 0.35171141336
TGATGAAA 0.231187314903
TGATGACA 0.776608104712
TGATGCAA 1.51780449247
TGATGCCA −0.440580109747
TGATGGAA 1.12792038838
TGATGGCA −0.047888767338
TGATGTAA 0.460894687222
TGATTAAA 0.471458162956
TGATTACA 6.71452512106
TGATTCAA 0.634982408801
TGATTCCA 3.58920683653
TGATTGAA −0.0304028614718
TGATTGCA 4.65426416744
TGATTTAA 1.02697298716
TGCAAAAA 0.512955888943
TGCAAACA 0.259066014323
TGCAACAA 0.684595510514
TGCAACCA 0.245623420332
TGCAAGAA 1.03953054307
TGCAAGCA −0.663295001667
TGCAATAA 0.530952273062
TGCACAAA 1.00495655412
TGCACACA −0.306207230303
TGCACCAA 0.213739309368
TGCACCCA −0.023620009111
TGCACGAA 0.694811871491
TGCACGCA −1.30650439613
TGCACTAA −0.0312586248101
TGCAGAAA 0.397539970975
TGCAGACA −0.182613205026
TGCAGCAA 0.180453147534

TGCAGCCA −1.06531348487
TGCAGGAA −0.16986667022
TGCAGGCA 0.852687661093
TGCAGTAA 2.44432565791
TGCATAAA 0.880563223933
TGCATACA −0.0714536249328
TGCATCAA 1.70255501479
TGCATCCA 0.0659834309364
TGCATGAA 0.707866575197
TGCATGCA 0.674519772834
TGCATTAA 1.41876021063
TGCCAAAA 0.0624566090904
TGCCAACA 0.40755088602
TGCCACAA 0.246706847039
TGCCACCA 0.448568454019
TGCCAGAA −0.478110585902
TGCCAGCA 0.515442412045
TGCCATAA 0.766883141131
TGCCCAAA 1.02996476088
TGCCCACA 0.11680202456
TGCCCCAA −0.554185698796
TGCCCCCA −0.853423450279
TGCCCGAA 0.401555053638
TGCCCGCA −1.04063017544
TGCCCTAA −0.280907844451
TGCCGAAA −1.04286864741
TGCCGACA −0.365234512882
TGCCGCAA −0.410582389746
TGCCGCCA −0.707023619557
TGCCGGAA −0.465154161676
TGCCGGCA −0.266046993932
TGCCGTAA 0.0951251262178
TGCCTAAA 0.417906563387
TGCCTACA −0.834391210904
TGCCTCAA −0.882868609591
TGCCTCCA −0.533458399784
TGCCTGAA 0.32499873723
TGCCTTAA 0.220441394816
TGCGAAAA 0.101420763288
TGCGAACA 0.137655048118
TGCGACAA 0.00302157122506
TGCGACCA −0.775450184251
TGCGAGAA 0.463408916773
TGCGAGCA −0.8029564149
TGCGATAA 0.460401198773
TGCGCAAA 1.99970537286
TGCGCACA 1.10855671714
TGCGCCAA 0.544642395422
TGCGCCCA −0.0164411636356
TGCGCGAA 0.435808327369
TGCGCGCA 0.565940813216
TGCGCTAA 1.3106770919
TGCGGAAA −0.297879351341
TGCGGACA −0.647907205854

TGCGGCAA 0.161862120289
TGCGGCCA 0.0590202252759
TGCGGGAA -0.551652649769
TGCGGTAA 0.137644854236
TGCGTAAA 0.928708406615
TGCGTACA -0.273267921844
TGCGTCAA 0.277017440808
TGCGTCCA -0.808365968366
TGCGTGAA 0.712431604734
TGCGTTAA 0.653364069389
TGCTAAAA 0.236843089831
TGCTAACA 0.191027339914
TGCTACAA 0.45816220404
TGCTACCA -0.213291301316
TGCTAGAA 1.38418439201
TGCTAGCA 0.364280208682
TGCTATAA 1.90652100498
TGCTCAAA -0.612272269018
TGCTCACA -0.769902882684
TGCTCCAA -0.239264006154
TGCTCCCA -0.473026713901
TGCTCGAA -0.124674315404
TGCTCTAA 0.184546906057
TGCTGAAA 0.602763206633
TGCTGACA 0.684481548139
TGCTGCAA -0.884712395354
TGCTGCCA -0.431603220287
TGCTGGAA -0.106369762386
TGCTGTAA 0.369990873745
TGCTTAAA 1.21782624693
TGCTTACA 0.023363855149
TGCTTCAA 0.29653611133
TGCTTCCA -0.994028189685
TGCTTGAA 0.514990221888
TGCTTTAA 0.803814269291
TGGAAAAA 1.50312817735
TGGAAACA -0.0907584853107
TGGAACAA 0.522691569169
TGGAACCA -0.576061508531
TGGAAGAA 0.390253697665
TGGAATAA 2.67104648413
TGGACAAA 1.41779858775
TGGACACA -0.00784040228952
TGGACCAA -0.998948698189
TGGACCCA -1.19837344457
TGGACGAA -0.123525804681
TGGACTAA 0.146203010383
TGGAGAAA 0.158239110017
TGGAGACA -0.919448963652
TGGAGCAA -0.49582154132
TGGAGCCA -1.30828492755
TGGAGGAA -0.868671929702
TGGAGTAA -0.462153500978
TGGATAAA 1.51546721826

TGGATACA 1.0869550967
TGGATCAA -0.432610062187
TGGATCCA 0.906643356606
TGGATGAA 0.156555551171
TGGATTAA 0.507578485412
TGGCAAAA 0.00564531966425
TGGCAACA -1.04491291285
TGGCACAA -0.764484703625
TGGCACCA -0.475770436492
TGGCAGAA -0.881893133483
TGGCATAA -0.317063714804
TGGCCAAA -0.388207079785
TGGCCACA -0.216626269072
TGGCCCAA -1.46765346744
TGGCCCCA -1.14521417877
TGGCCGAA 1.20787361998
TGGCCTAA 0.147996872261
TGGCGAAA 0.862846256738
TGGCGACA 0.213781914568
TGGCGCAA 1.04894263288
TGGCGCCA -0.473572478669
TGGCGGAA 0.2108319619
TGGCGTAA 1.1101406896
TGGCTAAA 0.331512105168
TGGCTACA -0.715571320439
TGGCTCAA -0.973635981876
TGGCTGAA 1.44092066492
TGGCTTAA -0.418082211818
TGGGAAAA 1.74418003361
TGGGAACA -0.79325968053
TGGGACAA 0.70696507008
TGGGACCA -0.820397885895
TGGGAGAA 0.562241218137
TGGGATAA 0.73000768725
TGGGCAAA 0.236738798575
TGGGCACA -0.794221564796
TGGGCCAA -0.747400279887
TGGGCCCA -1.23143873896
TGGGCGAA 0.20236842589
TGGGCTAA -0.357563747246
TGGGGAAA 0.486116965503
TGGGGACA 0.130389685335
TGGGGCAA -0.176104019194
TGGGGGAA -0.378706381611
TGGGGTAA 0.109152430834
TGGGTAAA 0.422412520684
TGGGTACA -0.483374549821
TGGGTCAA -0.415340057517
TGGGTGAA 0.401247146121
TGGGTTAA 0.124853361796
TGGTAAAA 0.0290013333655
TGGTAACA 0.851855944861
TGGTACAA 0.0623588523743
TGGTACCA 0.924373131499

TGGTAGAA 1.23793511709
TGGTATAA 0.890382023507
TGGTCAAA 0.695367307378
TGGTCACA -0.868327428762
TGGTCCAA 0.587954371052
TGGTCGAA -0.469163232562
TGGTCTAA 0.200699243032
TGGTGAAA 0.556259500361
TGGTGACA 0.576913089764
TGGTGCAA -0.128703251292
TGGTGGAA -0.349929836416
TGGTGTAA 3.63050852632
TGGTTAAA 1.26760741701
TGGTTACA -0.0930910546543
TGGTTCAA -0.10521602403
TGGTTGAA 0.955858896914
TGGTTTAA 0.218034070337
TGTAAAAA 1.28118828186
TGTAAACA -0.374285896095
TGTAACAA 0.605087150385
TGTAAGAA 1.70731660328
TGTAATAA 2.68063762034
TGTACAAA 1.68195553138
TGTACACA -0.370769790895
TGTACCAA 1.11375847224
TGTACGAA 0.690048976088
TGTACTAA 0.451763844004
TGTAGAAA 0.745226891934
TGTAGACA -0.366326826562
TGTAGCAA 0.906915193464
TGTAGGAA 0.582768560229
TGTAGTAA 0.40795602749
TGTATAAA 1.25825283114
TGTATACA 1.24459956359
TGTATCAA 1.63999986484
TGTATGAA 0.772283807623
TGTATTAA 1.52960273485
TGTCAAAA 0.943298465803
TGTCAACA 0.760537057413
TGTCACAA 1.43478786859
TGTCAGAA 0.715727626632
TGTCATAA 0.224638660451
TGTCCAAA -0.0835932316781
TGTCCACA 0.0020999397251
TGTCCCAA -0.269161355623
TGTCCGAA 0.516528713949
TGTCCTAA 0.772466251975
TGTCGAAA 0.562352043933
TGTCGACA -0.421403064968
TGTCGCAA 1.7633356451
TGTCGGAA 1.05647904838
TGTCGTAA 2.48131481278
TGTCTAAA 1.28475326542
TGTCTCAA 0.30725144977

TGTCTGAA 0.497124789947
TGTCTTAA -0.103964528959
TGTGAAAA 0.590564004885
TGTGAACA 0.116332060454
TGTGACAA -0.258900559774
TGTGAGAA 1.12667621199
TGTGATAA 0.926296638649
TGTGCAAA 0.310996002484
TGTGCACA 1.39906693721
TGTGCCAA -0.529264270729
TGTGCGAA 1.22440391474
TGTGCTAA 0.221675115939
TGTGGAAA 0.47042073941
TGTGGCAA -0.791047869483
TGTGGGAA 0.833941111799
TGTGGTAA 0.821752103933
TGTGTAAA 2.14014596463
TGTGTCAA -0.161277148282
TGTGTGAA -0.0790093826666
TGTGTTAA 0.347048104344
TGTTAAAA 1.20072823135
TGTTAACA 0.653106347138
TGTTACAA 0.850975873035
TGTTAGAA 0.575569588371
TGTTATAA 1.84018575442
TGTTCAAA 0.106940619787
TGTTCCAA -0.60178198013
TGTTCGAA 1.04884696722
TGTTCTAA 1.02519611508
TGTTGAAA 1.69461215091
TGTTGCAA 1.06725607288
TGTTGGAA 0.308005274287
TGTTGTAA 0.845568410621
TGTTTAAA 2.77146275796
TGTTTCAA 1.46748095558
TGTTTGAA 0.0474606242873
TGTTTTAA 1.88385294763
TTAAAAAA 2.07803124266
TTAAACAA 0.503967760075
TTAAAGAA 1.17441390077
TTAAATAA 1.06333744002
TTAACAAA 1.20728917074
TTAACCAA 0.963909450005
TTAACGAA 0.914637451273
TTAACTAA 0.574066644206
TTAAGAAA 1.25529529841
TTAAGCAA 1.96468677383
TTAAGGAA 0.331417746412
TTAAGTAA 3.21432420625
TTAATAAA 1.8152622353
TTAATCAA 0.880412668135
TTAATGAA 0.576219383013
TTAATTAA 0.07388682619
TTACAAAA 0.79713806062

TTACACAA 1.56835752239
TTACAGAA 4.88439916004
TTACATAA 3.2011069232
TTACCAAA 1.95677501436
TTACCCAA 1.31453220903
TTACCGAA 1.48879453351
TTACCTAA 0.917248914777
TTACGAAA 4.18424935364
TTACGCAA 1.198162771
TTACGGAA 0.340188928558
TTACGTAA 3.96692728677
TTACTAAA 1.71863730149
TTACTCAA 1.80280374301
TTACTGAA 2.46969901475
TTAGAAAA 1.09922042799
TTAGACAA 1.77369681148
TTAGAGAA 0.734377987499
TTAGATAA 0.396666433688
TTAGCAAA 0.600808595075
TTAGCCAA 0.224615136107
TTAGCGAA 2.26154360037
TTAGCTAA -0.0850577527485
TTAGGAAA 0.626819984388
TTAGGCAA 0.0655294111079
TTAGGGAA 1.12958382084
TTAGTAAA 0.0538163791237
TTAGTCAA 0.625311028446
TTAGTGAA -0.0835610817421
TTATAAAA 1.95199565192
TTATACAA 1.81218708085
TTATAGAA 1.52321430732
TTATATAA 2.68572933379
TTATCAAA 0.806559560171
TTATCCAA 2.58571637176
TTATCGAA 1.22749501347
TTATGAAA 1.79267912697
TTATGCAA 0.990278670721
TTATGGAA 1.34327686567
TTATTAAA 1.2420006464
TTATTCAA 0.689535099875
TTATTGAA 0.183779751079
TTCAAAAA 0.985573279268
TTCAACAA 0.326869706679
TTCAAGAA -0.0133087666147
TTCACAAA 1.97919841853
TTCACCAA 0.775745806834
TTCACGAA 0.650052887358
TTCAGAAA 0.528608725691
TTCAGCAA 1.18121818642
TTCAGGAA 2.26890645849
TTCATAAA 0.606942175556
TTCATCAA 0.352548879987
TTCATGAA -0.607375023476
TTCCAAAA 0.772774682256

TTCCACAA 0.765944781209
TTCCAGAA 0.893318122951
TTCCCAAA 1.25767204124
TTCCCCAA 1.48611275836
TTCCCGAA 1.23630906219
TTCCGAAA -0.031311423892
TTCCGCAA 0.929240056777
TTCCGGAA 3.20057919376
TTCCTAAA 0.969838630081
TTCCTCAA -0.0594434020763
TTCGAAAA 1.87851239891
TTCGACAA 0.588386173445
TTCGAGAA 3.53418444273
TTCGCAAA 1.51767536996
TTCGCCAA 2.73982643674
TTCGCGAA 2.58790021498
TTCGGAAA 1.70047990631
TTCGGCAA 0.818983811472
TTCGTAAA 1.59873267742
TTCGTCAA 0.794765761274
TTCTAAAA 1.00718091149
TTCTACAA 1.29334121907
TTCTAGAA -0.814562019049
TTCTCAAA 1.39349166781
TTCTCCAA 0.84052060928
TTCTGAAA 1.48840899566
TTCTGCAA 2.19045512552
TTCTTAAA 1.20465078493
TTCTTCAA 1.33444870233
TTGAAAAA 1.19078083203
TTGAACAA 0.532779069021
TTGACAAA 0.55742029602
TTGACCAA 0.232843951445
TTGAGAAA 2.17914305291
TTGAGCAA -0.0226042802373
TTGATAAA 1.00535228586
TTGATCAA -0.248755033208
TTGCAAAA 2.99083954554
TTGCACAA 1.64668051699
TTGCCAAA 0.0956886649341
TTGCCCAA 0.335685062314
TTGCGAAA 0.589579903184
TTGCGCAA 1.94719250375
TTGCTAAA 0.166936059789
TTGGAAAA 1.50312817735
TTGGACAA -0.0455311053614
TTGGCAAA 0.0956886649341
TTGGCCAA -0.770983956957
TTGGGAAA 1.18073436909
TTGGTAAA 0.96096655464
TTGTAAAA 1.54434700918
TTGTACAA 1.44240348271
TTGTCAAA 0.635634555878
TTGTGAAA 0.129213990926

TTGTTAAA 2.16591636011
TTTAAAAA 1.42169421902
TTTACAAA 0.569250426958
TTTAGAAA 1.86104661942
TTTATAAA 1.58232314095
TTTCAAAA 1.26107575236
TTTCCAAA 1.14877053674
TTTCGAAA 2.11530268966
TTTGAAAA 0.630613154078
TTTGCAAA 2.86273668777
TTTTAAAA 2.2667435258
