# Supplementary material for: The intracellular immune receptor Rx1 regulates the DNA-binding activity of a Golden2-like transcription factor
Source: J Biol Chem. 2017 Dec 7;293(9):3218–33. doi: 10.1074/jbc.RA117.000485 (PMC5836133; doi:10.1074/jbc.RA117.000485)
Supplement: Supporting Information [file supp_RA117.000485_133217_1_supp_23414_2z2jtw.pdf]

| #8mers | all |
|---|---|
| AAAAAAAA | 2.04307712851 |
| AAAAAAAC | 1.36946019616 |
| AAAAAAAG | 0.908960486355 |
| AAAAAAAT | 2.33051472676 |
| AAAAAACA | 1.06808882859 |
| AAAAAACC | 0.86230092858 |
| AAAAAACG | 0.717593283999 |
| AAAAAACT | 1.76573449744 |
| AAAAAAGA | 1.95481861176 |
| AAAAAAGC | 1.17728484684 |
| AAAAAAGG | -0.0813076617949 |
| AAAAAAGT | 0.000277709440698 |
| AAAAAATA | 2.07737695075 |
| AAAAAATC | 8.26942937609 |
| AAAAAATG | -0.559970068646 |
| AAAAAATT | 1.77131200219 |
| AAAAACAA | 0.810738779105 |
| AAAAACAC | -0.703789001381 |
| AAAAACAG | 0.591558997081 |
| AAAAACAT | 0.112871192514 |
| AAAAACCA | -0.572359157278 |
| AAAAACCC | 0.304869174184 |
| AAAAACCG | 1.18166324636 |
| AAAAACCT | 1.54881219755 |
| AAAAACGA | 1.00397125626 |
| AAAAACGC | -0.10733886239 |
| AAAAACGG | 0.926797593025 |
| AAAAACGT | -0.263380965897 |
| AAAAACTA | 2.40749374357 |
| AAAAACTC | 1.51709232516 |
| AAAAACTG | 0.541825274905 |
| AAAAACTT | 2.72320670116 |
| AAAAAGAA | 2.4438888773 |
| AAAAAGAC | -0.323434903825 |
| AAAAAGAG | -0.13101452901 |
| AAAAAGAT | 2.82400752555 |
| AAAAAGCA | -0.0131429011391 |
| AAAAAGCC | -0.567841694862 |
| AAAAAGCG | 2.27042685582 |
| AAAAAGCT | 0.371243604113 |
| AAAAAGGA | 0.427168123401 |
| AAAAAGGC | -0.39426038739 |
| AAAAAGGG | 0.0557833746097 |
| AAAAAGGT | 0.0769151061737 |
| AAAAAGTA | -0.27376113691 |
| AAAAAGTC | 1.41037674768 |
| AAAAAGTG | -0.0140411891726 |
| AAAAAGTT | 0.612392617761 |
| AAAAATAA | 3.26295227793 |
| AAAAATAC | 0.46155829489 |
| AAAAATAG | 0.356665940256 |
| AAAAATAT | 3.39764552023 |
| AAAAATCA | 4.71385212073 |

AAAAATCC 7.7557227025
AAAAATCG 7.89845786061
AAAAATCT 8.82180969152
AAAAATGA 1.00810587935
AAAAATGC 0.54361373203
AAAAATGG 0.562742999494
AAAAATGT 0.425530387142
AAAAATTA 1.63610705839
AAAAATTC 2.01303163171
AAAAATTG 0.689328333556
AAAAATTT 2.11333428616
AAAACAAA 1.82189467421
AAAACAAC 0.717858502761
AAAACAAG 0.607084078977
AAAACAAT 1.62195053861
AAAACACA 0.339717129076
AAAACACC 0.330461743751
AAAACACG 0.327104873637
AAAACACT 0.296985057289
AAAACAGA 1.24371652572
AAAACAGC 0.662429030736
AAAACAGG 0.0237135550149
AAAACAGT 0.774659451393
AAAACATA 1.3555626498
AAAACATC -0.0148872245301
AAAACATG 0.0798183564436
AAAACATT -0.289058055728
AAAACCAA 0.353206022037
AAAACCAC -0.395471150589
AAAACCAG -0.769742703589
AAAACCAT 0.287689909968
AAAACCCA -0.540543731189
AAAACCCC -1.11802073621
AAAACCCG 0.864250515471
AAAACCCT -0.332824147599
AAAACCGA 1.35288298269
AAAACCGC 0.222376563262
AAAACCGG 0.658074779866
AAAACCGT 0.805774774897
AAAACCTA 1.35774227337
AAAACCTC 0.369911056789
AAAACCTG 0.624493171696
AAAACCTT 1.85328125364
AAAACGAA 0.846116755163
AAAACGAC 0.315537047182
AAAACGAG -0.00212175008965
AAAACGAT 1.49344726071
AAAACGCA -0.165120953896
AAAACGCC -0.74903648784
AAAACGCG 0.774845978873
AAAACGCT 0.301592452598
AAAACGGA 0.803405292997
AAAACGGC 0.0984484130833
AAAACGGG 0.487086953813

AAAACGGT -0.451286376432
AAAACGTA 0.574126588426
AAAACGTC -0.269671272089
AAAACGTG -0.53124213035
AAAACGTT 0.335754252829
AAAACTAA 1.79852669466
AAAACTAC 1.22300697912
AAAACTAG 1.81893230139
AAAACTAT 1.28611905174
AAAACTCA 0.302414339308
AAAACTCC -0.149431411386
AAAACTCG 1.66138444647
AAAACTCT -0.258818537035
AAAACTGA -0.138701501347
AAAACTGC 0.166814273679
AAAACTGG -0.240415186228
AAAACTGT 0.381035464123
AAAACTTA 2.90718212757
AAAACTTC 0.604241824859
AAAACTTG -0.234726930787
AAAACTTT 1.9473210814
AAAAGAAA 1.20107917491
AAAAGAAC 0.133533274529
AAAAGAAG 0.416140727012
AAAAGAAT 2.85891835019
AAAAGACA 0.205111946068
AAAAGACC -1.17008043109
AAAAGACG 1.99416737483
AAAAGACT 0.250985007391
AAAAGAGA -0.0214721025801
AAAAGAGC -0.129999453123
AAAAGAGG -0.342764230338
AAAAGAGT -0.275384925244
AAAAGATA 2.54657308798
AAAAGATC 3.70282154877
AAAAGATG 0.913741710287
AAAAGATT 7.26393092608
AAAAGCAA 1.46922158783
AAAAGCAC 0.416250020457
AAAAGCAG 0.13469823857
AAAAGCAT -0.038945522299
AAAAGCCA -0.708118687249
AAAAGCCC -1.17347935315
AAAAGCCG -0.672098065731
AAAAGCCT -0.292739891686
AAAAGCGA 0.824394838993
AAAAGCGC 0.874098583538
AAAAGCGG -0.0415081933747
AAAAGCGT 2.22757445715
AAAAGCTA 1.01759400767
AAAAGCTC -0.721183105285
AAAAGCTG -0.454069508
AAAAGCTT 1.42190731028
AAAAGGAA 1.00844791579

AAAAGGAC 0.253748153873
AAAAGGAG 0.400173891418
AAAAGGAT 2.05586133897
AAAAGGCA -1.28334445547
AAAAGGCC -0.566930083437
AAAAGGCG -0.03066586722
AAAAGGCT -0.943650850507
AAAAGGGA -0.715821272936
AAAAGGGC -0.915499773425
AAAAGGGG -0.780554219401
AAAAGGGT -0.559017654335
AAAAGGTA 0.571561419215
AAAAGGTC -0.0813076617949
AAAAGGTG -0.0414453236213
AAAAGGTT 0.539653145741
AAAAGTAA 0.644125189009
AAAAGTAC -0.447080972825
AAAAGTAG -0.705766275945
AAAAGTAT 1.31092179401
AAAAGTCA -0.0249488832166
AAAAGTCC -0.329997923186
AAAAGTCG 2.42111129053
AAAAGTCT 0.314246759991
AAAAGTGA 1.36433568672
AAAAGTGC -0.173043375541
AAAAGTGG -0.233146651653
AAAAGTGT -0.152500579516
AAAAGTTA 0.414411600614
AAAAGTTC 0.0689510489298
AAAAGTTG 0.0471881216955
AAAAGTTT 2.01391826178
AAAATAAA 3.46546241546
AAAATAAC 2.00830619948
AAAATAAG 1.27229145319
AAAATAAT 3.0604421404
AAAATACA 1.77764727483
AAAATACC 1.79886644115
AAAATACG 3.7584061134
AAAATACT 1.85836100481
AAAATAGA 1.67336217539
AAAATAGC 1.12060838865
AAAATAGG 0.0134387220651
AAAATAGT -0.223407252498
AAAATATA 3.58136301411
AAAATATC 7.59491748655
AAAATATG 2.55894947775
AAAATATT 4.21768361021
AAAATCAA 5.17917448188
AAAATCAC 4.21847885014
AAAATCAG 5.26954038444
AAAATCAT 3.23138874721
AAAATCCA 10.6513215984
AAAATCCC 6.67097505468
AAAATCCG 10.1953372693

| | |
|---|---|
| AAAATCCT | 10.3737006334 |
| AAAATCGA | 5.89156768783 |
| AAAATCGC | 7.7127789137 |
| AAAATCGG | 4.89004751924 |
| AAAATCGT | 5.76883447932 |
| AAAATCTA | 10.9953911048 |
| AAAATCTC | 9.80891894683 |
| AAAATCTG | 18.908566733 |
| AAAATCTT | 10.3102232084 |
| AAAATGAA | 0.387444848126 |
| AAAATGAC | 0.971144917848 |
| AAAATGAG | -0.25548768917 |
| AAAATGAT | 2.43135614585 |
| AAAATGCA | 0.290827985012 |
| AAAATGCC | -0.0881048813963 |
| AAAATGCG | 1.76297863719 |
| AAAATGCT | -0.0397024574762 |
| AAAATGGA | 0.208941172045 |
| AAAATGGC | 0.586815245188 |
| AAAATGGG | 0.0636500045536 |
| AAAATGGT | 0.313567267026 |
| AAAATGTA | 0.0395296697432 |
| AAAATGTC | 0.574405546935 |
| AAAATGTG | 0.35471073256 |
| AAAATGTT | 1.21967529854 |
| AAAATTAA | 2.08320218731 |
| AAAATTAC | 1.93660470293 |
| AAAATTAG | 0.997186527343 |
| AAAATTAT | 1.14502329531 |
| AAAATTCA | 1.66462244695 |
| AAAATTCC | 2.27455523357 |
| AAAATTCG | 3.61374343526 |
| AAAATTCT | 2.31161965953 |
| AAAATTGA | 1.65234306812 |
| AAAATTGC | 1.05763537892 |
| AAAATTGG | 0.543686386149 |
| AAAATTGT | 0.464812325075 |
| AAAATTTA | 2.56910127751 |
| AAAATTTC | 2.81209537269 |
| AAAATTTG | 1.38153285424 |
| AAAATTTT | 3.32959671471 |
| AAACAAAA | 2.70294473724 |
| AAACAAAC | 0.289455467514 |
| AAACAAAG | 0.240434338603 |
| AAACAAAT | 2.13885399343 |
| AAACAACA | 0.625614002003 |
| AAACAACC | -0.0672693871143 |
| AAACAACG | 0.0537794532633 |
| AAACAACT | 1.76020195914 |
| AAACAAGA | 0.916146374267 |
| AAACAAGC | 0.40404392028 |
| AAACAAGG | -0.265566418452 |
| AAACAAGT | -0.780533817958 |
| AAACAATA | 1.6669271855 |

AAACAATC 1.74584558848
AAACAATG 0.0640796839281
AAACAATT 0.487226016711
AAACACAA -0.228300476189
AAACACAC 0.619677598396
AAACACAG 0.215584964466
AAACACAT -0.402748012283
AAACACCA 0.349456320054
AAACACCC -1.29562029527
AAACACCG 0.876176199893
AAACACCT 0.907943120511
AAACACGA 1.32198395612
AAACACGC 0.0168007966282
AAACACGG 0.135116676333
AAACACGT 0.911168838489
AAACACTA 3.02402722976
AAACACTC 0.535852231972
AAACACTG 0.546817799498
AAACACTT 2.06954009026
AAACAGAA 1.45343087082
AAACAGAC 0.389073840909
AAACAGAG 0.729361169504
AAACAGAT 1.47367076787
AAACAGCA -0.187851492435
AAACAGCC -0.655919304941
AAACAGCG 0.253028274378
AAACAGCT 0.78982063816
AAACAGGA 1.23354682265
AAACAGGC -0.735450375757
AAACAGGG 0.109780998408
AAACAGGT -0.467037539627
AAACAGTA 0.436863597001
AAACAGTC 0.487867621281
AAACAGTG -0.390107444637
AAACAGTT 0.310172300341
AAACATAA 0.815878069181
AAACATAC -0.127060188061
AAACATAG -0.303951941954
AAACATAT 1.32447085041
AAACATCA 0.394890125815
AAACATCC 0.40084422455
AAACATCG 1.74122133076
AAACATCT 1.23375645789
AAACATGA 1.70215235891
AAACATGC -0.13785338421
AAACATGG 0.36439891993
AAACATGT -0.827490445727
AAACATTA 0.381657916317
AAACATTC 1.00682204568
AAACATTG -0.20594507439
AAACATTT 2.62351713089
AAACCAAA 1.07432459215
AAACCAAC 0.923737584728
AAACCAAG 0.523137969317

AAACCAAT 1.04215567983
AAACCACA 0.816784684335
AAACCACC -0.822790203034
AAACCACG 0.221286543298
AAACCACT 0.79546746618
AAACCAGA -0.0588839776155
AAACCAGC 1.64177616145
AAACCAGG -1.42182029187
AAACCAGT -0.0949408221041
AAACCATA 1.29227487495
AAACCATC -0.538222338405
AAACCATG 0.643966973736
AAACCATT 0.287689909968
AAACCCAA -0.194941202114
AAACCCAC -0.797331908272
AAACCCAG -0.45671982201
AAACCCAT -1.07726115089
AAACCCCA -1.28586236828
AAACCCCC -1.48163649054
AAACCCCG 0.0387802289737
AAACCCCT -0.899303733862
AAACCCGA -0.238729360852
AAACCCGC -0.275827511654
AAACCCGG -0.585555768339
AAACCCGT 0.465440606254
AAACCCTA 0.20197116471
AAACCCTC -0.299982404011
AAACCCTG -0.550738207434
AAACCCTT -0.511505607685
AAACCGAA 1.26055021447
AAACCGAC -0.0937529584844
AAACCGAG -0.629055184203
AAACCGAT 1.23204565115
AAACCGCA 0.222376563262
AAACCGCC -0.572987438457
AAACCGCG 0.472619832469
AAACCGCT -0.539310901123
AAACCGGA -0.305192474605
AAACCGGC 0.464004594468
AAACCGGG -0.745959200769
AAACCGGT -0.26507428568
AAACCGTA 0.607190666109
AAACCGTC 0.109581147536
AAACCGTG -0.363310149034
AAACCGTT -0.224345927062
AAACCTAA 0.497795213341
AAACCTAC 0.595530200447
AAACCTAG 0.7176159754
AAACCTAT 0.642211825089
AAACCTCA 0.113633123963
AAACCTCC -0.829135884572
AAACCTCG 0.562772977124
AAACCTCT -0.866639566102
AAACCTGA 0.689161166629

| | |
|---|---|
| AAACCTGC | 0.313836649347 |
| AAACCTGG | -0.632617109638 |
| AAACCTGT | -0.147234300864 |
| AAACCTTA | 1.00287603185 |
| AAACCTTC | -0.348647340379 |
| AAACCTTG | 0.35415822817 |
| AAACCTTT | 0.268465505162 |
| AAACGAAA | 0.751613939553 |
| AAACGAAC | -0.126911548975 |
| AAACGAAG | -0.547773752835 |
| AAACGAAT | 1.37200454757 |
| AAACGACA | 0.16458468739 |
| AAACGACC | -0.53074562584 |
| AAACGACG | 0.672472577938 |
| AAACGACT | 0.925176302827 |
| AAACGAGA | 1.09672745853 |
| AAACGAGC | 0.089292120482 |
| AAACGAGG | -0.593347662384 |
| AAACGAGT | -0.358687556732 |
| AAACGATA | 1.70215235891 |
| AAACGATC | 1.83546163392 |
| AAACGATG | -0.464989276368 |
| AAACGATT | 2.15125807088 |
| AAACGCAA | 1.77216074386 |
| AAACGCAC | 0.373154053541 |
| AAACGCAG | 0.331940223847 |
| AAACGCAT | 0.500958061566 |
| AAACGCCA | -0.967812196382 |
| AAACGCCC | -1.30540237092 |
| AAACGCCG | -0.0459935963802 |
| AAACGCCT | -0.290490944844 |
| AAACGCGA | 0.798357393058 |
| AAACGCGC | 1.31656612389 |
| AAACGCGG | 0.176976690513 |
| AAACGCGT | 1.67728924503 |
| AAACGCTA | 0.51770847912 |
| AAACGCTC | 0.2136266341 |
| AAACGCTG | 1.07116153574 |
| AAACGCTT | -0.168681838441 |
| AAACGGAA | 0.253831008714 |
| AAACGGAC | -0.274918190187 |
| AAACGGAG | 0.468650710883 |
| AAACGGAT | 3.075226525 |
| AAACGGCA | -0.70700535135 |
| AAACGGCC | -0.819678774773 |
| AAACGGCG | -1.12011583952 |
| AAACGGCT | -1.01904459192 |
| AAACGGGA | 0.0298414823735 |
| AAACGGGC | 0.449298068434 |
| AAACGGGG | -0.635010323828 |
| AAACGGGT | -0.955801783517 |
| AAACGGTA | 0.911997803252 |
| AAACGGTC | -0.498640415986 |
| AAACGGTG | -0.878413072412 |

AAACGGTT -0.456948609623
AAACGTAA 0.533990704372
AAACGTAC -0.349569777059
AAACGTAG 0.127910803334
AAACGTAT 1.8569091715
AAACGTCA 1.15548153307
AAACGTCC -0.950616486104
AAACGTCG 1.87293450511
AAACGTCT -0.263380965897
AAACGTGA -0.152676073562
AAACGTGC -0.523349269979
AAACGTGG -0.354047477479
AAACGTGT 0.415644847036
AAACGTTA 0.842201759854
AAACGTTC -0.296586188258
AAACGTTG 0.173089382878
AAACGTTT 0.895388322198
AAACTAAA 2.19557312811
AAACTAAC 0.776433127881
AAACTAAG 0.627209894486
AAACTAAT 1.54032790351
AAACTACA 0.0167999639163
AAACTACC 0.943417899335
AAACTACG 1.7963791305
AAACTACT 1.96248768079
AAACTAGA 1.69893475987
AAACTAGC 0.692830303729
AAACTAGG 0.117725903277
AAACTAGT -0.553583584222
AAACTATA 1.07281904891
AAACTATC 1.97853445673
AAACTATG 0.892223184014
AAACTATT 1.26287264814
AAACTCAA 0.7718311452
AAACTCAC -0.116746634005
AAACTCAG 0.302414339308
AAACTCAT -0.597606359557
AAACTCCA -0.714704189833
AAACTCCC -1.13864159898
AAACTCCG -0.493704932163
AAACTCCT 0.50730103679
AAACTCGA 2.91684866443
AAACTCGC 0.638918657441
AAACTCGG -0.198732956052
AAACTCGT 0.93641270992
AAACTCTA 0.271574643465
AAACTCTC -0.0708263162775
AAACTCTG -0.675905016662
AAACTCTT 0.804810078084
AAACTGAA 0.551346087169
AAACTGAC -0.223330851175
AAACTGAG 0.401674646559
AAACTGAT 0.759669803291
AAACTGCA -0.944200648583

AAACTGCC -0.552481073579
AAACTGCG 0.166814273679
AAACTGCT -0.0962777411657
AAACTGGA -0.114068215966
AAACTGGC -0.78765641976
AAACTGGG -0.547026185667
AAACTGGT -0.373724877594
AAACTGTA 0.25894032116
AAACTGTC -0.288845714177
AAACTGTG 0.636439673918
AAACTGTT 0.172228150527
AAACTTAA 0.66922604216
AAACTTAC -0.341650061728
AAACTTAG 0.384692110544
AAACTTAT 0.895254463749
AAACTTCA -0.290230930532
AAACTTCC -0.479925214548
AAACTTCG 1.39178353855
AAACTTCT 0.186471688707
AAACTTGA -0.0966278965474
AAACTTGC 0.421897889368
AAACTTGG -1.04201016341
AAACTTGT 0.192946856955
AAACTTTA 0.379016762139
AAACTTTC 0.822202516564
AAACTTTG 0.844005622151
AAAGAAAA 0.495772555975
AAAGAAAC 0.400487407473
AAAGAAAG 1.10801216291
AAAGAAAT 2.79937819555
AAAGAACA 1.57590427319
AAAGAACC -0.437660085996
AAAGAACG -0.267879692294
AAAGAACT 0.540053680197
AAAGAAGA -0.144968075248
AAAGAAGC 0.406736702601
AAAGAAGG 0.415644847036
AAAGAAGT 1.06777885156
AAAGAATA 0.864971644033
AAAGAATC 8.47751285481
AAAGAATG -0.105394271771
AAAGAATT 2.26001067001
AAAGACAA 1.08463648078
AAAGACAC -0.325629516212
AAAGACAG 0.243437097953
AAAGACAT 0.593972404537
AAAGACCA -0.589328994436
AAAGACCC -1.10946795161
AAAGACCG -1.31782539256
AAAGACCT -0.926167854601
AAAGACGA 0.179246663333
AAAGACGC -0.433920168378
AAAGACGG -0.14614219912
AAAGACGT 1.35857873254

AAAGACTA 1.05105945252
AAAGACTC 0.236735848405
AAAGACTG 1.02060093058
AAAGACTT 0.946691081896
AAAGAGAA 0.314014016996
AAAGAGAC -0.533683641834
AAAGAGAG 0.361793780544
AAAGAGAT 3.22618887734
AAAGAGCA 0.0453744750334
AAAGAGCC -0.933062085146
AAAGAGCG -0.449859732656
AAAGAGCT -0.684721562781
AAAGAGGA -0.225482162539
AAAGAGGC -0.298112549291
AAAGAGGG -0.97665372386
AAAGAGGT -0.186755851665
AAAGAGTA -0.132210511571
AAAGAGTC 0.169282848303
AAAGAGTG 0.216582345224
AAAGAGTT 0.67630326116
AAAGATAA 1.50356429474
AAAGATAC 3.3597265236
AAAGATAG 1.13645281558
AAAGATAT 6.11477592217
AAAGATCA 1.38030689405
AAAGATCC 3.21233255022
AAAGATCG 2.86763018278
AAAGATCT 5.94546267988
AAAGATGA 0.528412783267
AAAGATGC 0.295653967211
AAAGATGG 0.496026741303
AAAGATGT 0.202609230254
AAAGATTA 3.6058543221
AAAGATTC 7.17763490325
AAAGATTG 4.86151401919
AAAGATTT 8.6980768123
AAAGCAAA 0.662309952925
AAAGCAAC 0.359920594976
AAAGCAAG 1.41805934828
AAAGCAAT 1.80888167614
AAAGCACA 0.497254366919
AAAGCACC -0.579009611395
AAAGCACG -0.621714828221
AAAGCACT 0.335004603882
AAAGCAGA -0.370489167072
AAAGCAGC 0.0908850984732
AAAGCAGG -0.179163392136
AAAGCAGT 0.858658021906
AAAGCATA 0.0485949885623
AAAGCATC -0.191096779145
AAAGCATG -0.038945522299
AAAGCATT -0.332700073516
AAAGCCAA -0.428926394718
AAAGCCAC -1.35422010993

AAAGCCAG -0.67679456122
AAAGCCAT -0.480425258084
AAAGCCCA -0.918470473364
AAAGCCCC -1.65925915736
AAAGCCCG -0.888131029235
AAAGCCCT -1.04141581524
AAAGCCGA -0.819141675555
AAAGCCGC -0.337341610015
AAAGCCGG -0.716797835894
AAAGCCGT -0.866846911381
AAAGCCTA -0.112181707006
AAAGCCTC -0.311435940749
AAAGCCTG -0.213981993931
AAAGCCTT -0.27784600546
AAAGCGAA 0.353892801231
AAAGCGAC -0.675229062724
AAAGCGAG -0.00345242381149
AAAGCGAT 3.01953828773
AAAGCGCA 0.348639013259
AAAGCGCC -0.920938215276
AAAGCGCG 1.29144549383
AAAGCGCT -0.0499131716046
AAAGCGGA -0.0415081933747
AAAGCGGC -0.625075237361
AAAGCGGG -0.598480498943
AAAGCGGT -0.0327872009536
AAAGCGTA 1.41764257594
AAAGCGTC 0.39569077837
AAAGCGTG -0.362447875793
AAAGCGTT 0.0537794532633
AAAGCTAA -0.687118524176
AAAGCTAC -0.635941503984
AAAGCTAG 1.04453536245
AAAGCTAT -0.353695864851
AAAGCTCA 0.0892596447153
AAAGCTCC -1.12748992033
AAAGCTCG -0.714363610639
AAAGCTCT -0.945082490555
AAAGCTGA 0.933611258688
AAAGCTGC -0.933677251111
AAAGCTGG -0.454571008782
AAAGCTGT 1.61424545478
AAAGCTTA 1.00939137845
AAAGCTTC 0.435595793032
AAAGCTTG 0.670246114318
AAAGCTTT -0.243069871976
AAAGGAAA -0.184735900614
AAAGGAAC -0.605426149452
AAAGGAAG -0.306248353378
AAAGGAAT 3.0868203737
AAAGGACA 1.00358695969
AAAGGACC -0.583803117828
AAAGGACG -0.549225169792
AAAGGACT -0.304585219404

AAAGGAGA 0.152989173262
AAAGGAGC 0.0742572977559
AAAGGAGG -0.994222280921
AAAGGAGT -0.000455909801446
AAAGGATA 2.05123853849
AAAGGATC 1.86836832904
AAAGGATG 0.381683730388
AAAGGATT 6.50460544919
AAAGGCAA -0.431774894176
AAAGGCAC -1.64464735231
AAAGGCAG 0.57237248067
AAAGGCAT 0.0474689538061
AAAGGCCA -0.914574214075
AAAGGCCC -1.07671655727
AAAGGCCG -0.869128958524
AAAGGCCT -0.541568175085
AAAGGCGA -0.53078809415
AAAGGCGC -0.858709233692
AAAGGCGG -0.754706839973
AAAGGCGT 0.354155938213
AAAGGCTA -0.101670383858
AAAGGCTC -0.681404038308
AAAGGCTG -0.348108575737
AAAGGCTT -0.438667667475
AAAGGGAA -0.0381190556726
AAAGGGAC -0.984445826083
AAAGGGAG 0.363327635985
AAAGGGAT 1.30751017308
AAAGGGCA -0.900160802653
AAAGGGCC -1.3277862931
AAAGGGCG -1.06062335763
AAAGGGCT -1.32942881745
AAAGGGGA -0.838930242717
AAAGGGGC -1.07726115089
AAAGGGGG -0.960116480569
AAAGGGGT -0.910064037888
AAAGGGTA -0.415377130139
AAAGGGTC -0.792713063174
AAAGGGTG -0.39234598258
AAAGGGTT -0.628371527679
AAAGGTAA 0.977096726626
AAAGGTAC -0.707890107814
AAAGGTAG 0.427356524483
AAAGGTAT 0.860720024912
AAAGGTCA 0.257828234329
AAAGGTCC -0.824254110671
AAAGGTCG 0.747115421334
AAAGGTCT -0.384619456425
AAAGGTGA 1.37277147529
AAAGGTGC -1.25067320966
AAAGGTGG -0.649247824847
AAAGGTGT 0.502584556214
AAAGGTTA 0.539653145741
AAAGGTTC 1.17246136278

AAAGGTTG -0.434662739274
AAAGTAAA 0.841962563342
AAAGTAAC -0.276480357836
AAAGTAAG 0.725715140161
AAAGTAAT 2.74867540482
AAAGTACA 0.427135439456
AAAGTACC -0.144276299782
AAAGTACG 0.889429643546
AAAGTACT 0.456473963802
AAAGTAGA -0.138469174709
AAAGTAGC -0.573832641102
AAAGTAGG -0.415690229838
AAAGTAGT 0.180078334409
AAAGTATA 1.91857586433
AAAGTATC 3.23571843308
AAAGTATG 0.13804199347
AAAGTATT 1.35895386928
AAAGTCAA -0.265200025187
AAAGTCAC -0.733443123562
AAAGTCAG 1.22928312921
AAAGTCAT -0.0249488832166
AAAGTCCA -0.2226523991
AAAGTCCC -0.565927290052
AAAGTCCG -0.805470210495
AAAGTCCT -0.918928256767
AAAGTCGA 0.469667868549
AAAGTCGC 0.728780352908
AAAGTCGG -0.362591726785
AAAGTCGT 1.58014173621
AAAGTCTA 0.521966551759
AAAGTCTC 0.241410485205
AAAGTCTG 0.293709376593
AAAGTCTT 0.496138116528
AAAGTGAA -0.210962163986
AAAGTGAC 1.68358038393
AAAGTGAG 0.0223004428084
AAAGTGAT 3.8888560637
AAAGTGCA -0.117598290168
AAAGTGCC -1.08950284951
AAAGTGCG -0.0791028486867
AAAGTGCT -0.446186640173
AAAGTGGA 0.0131048045667
AAAGTGGC -1.03324066552
AAAGTGGG 0.17184010675
AAAGTGGT -0.378057477954
AAAGTGTA 0.00788869681095
AAAGTGTC -0.25505967522
AAAGTGTG -0.190410624485
AAAGTGTT 1.26626803118
AAAGTTAA 0.161273616434
AAAGTTAC -0.565543826192
AAAGTTAG 0.408094647639
AAAGTTAT 0.0899008329292
AAAGTTCA 0.170138043492

AAAGTTCC 0.26129939416
AAAGTTCG 1.21381800257
AAAGTTCT 1.21488782927
AAAGTTGA 0.372666917041
AAAGTTGC -0.839717155525
AAAGTTGG -0.0688169823033
AAAGTTGT 0.160123016675
AAAGTTTA 0.531895601065
AAAGTTTC 0.82696875168
AAAGTTTG -0.0152819300021
AAATAAAA 1.81687612737
AAATAAAC 0.445399727365
AAATAAAG 2.81220633156
AAATAAAT 3.85157867164
AAATAACA 1.82493657102
AAATAACC 0.929250346121
AAATAACG 1.60121122256
AAATAACT 0.897611454969
AAATAAGA 1.44326845399
AAATAAGC 1.44835840588
AAATAAGG 1.00036207443
AAATAAGT 0.941869263256
AAATAATA 2.64658679138
AAATAATC 2.35508097882
AAATAATG 1.35047706964
AAATAATT 2.58210698935
AAATACAA 0.920274960195
AAATACAC -0.0895115400851
AAATACAG 2.39024848693
AAATACAT 1.8641935276
AAATACCA 0.111935432442
AAATACCC 0.18338982172
AAATACCG 1.87868729573
AAATACCT 0.707148577808
AAATACGA 1.82561564763
AAATACGC 2.05821999562
AAATACGG 3.06665375531
AAATACGT 3.43610182425
AAATACTA 1.36020460265
AAATACTC 2.12534532356
AAATACTG 3.43430170915
AAATACTT 1.78770393724
AAATAGAA 1.87947420853
AAATAGAC 0.11917565481
AAATAGAG 1.27981417309
AAATAGAT 3.69625707216
AAATAGCA 1.02331536342
AAATAGCC 0.55407405157
AAATAGCG 0.795541369367
AAATAGCT 0.0733481844669
AAATAGGA 0.051428291027
AAATAGGC 0.666133558096
AAATAGGG -0.0977583030414
AAATAGGT 0.131118826184

AAATAGTA 0.935966584484
AAATAGTC 0.141521064064
AAATAGTG -0.338606083135
AAATAGTT 1.76690695589
AAATATAA 3.31961083281
AAATATAC 2.58895500438
AAATATAG 2.57406007726
AAATATAT 4.52634703647
AAATATCA 4.08392758523
AAATATCC 9.17332801458
AAATATCG 7.40464301047
AAATATCT 8.97938960585
AAATATGA 1.1434967261
AAATATGC 4.35930897145
AAATATGG 3.81314464267
AAATATGT 2.76712892652
AAATATTA 3.4397449391
AAATATTC 4.11444814421
AAATATTG 2.15824556517
AAATATTT 5.29477197337
AAATCAAA 3.90120913753
AAATCAAC 3.47506233536
AAATCAAG 4.79635763869
AAATCAAT 4.292018142
AAATCACA 4.36552912166
AAATCACC 3.59595525042
AAATCACG 5.2730690014
AAATCACT 2.47146413406
AAATCAGA 2.93005443532
AAATCAGC 3.88377360621
AAATCAGG 4.71385212073
AAATCAGT 2.44143320971
AAATCATA 4.87436859381
AAATCATC 3.52704229806
AAATCATG 3.84038473286
AAATCATT 3.51204682098
AAATCCAA 5.25602047296
AAATCCAC 6.90526231355
AAATCCAG 6.41216213845
AAATCCAT 9.61030194592
AAATCCCA 6.96353216609
AAATCCCC 5.79710754871
AAATCCCG 6.67097505468
AAATCCCT 3.55318716384
AAATCCGA 5.79237274847
AAATCCGC 9.64386252811
AAATCCGG 5.78039501955
AAATCCGT 8.10290322075
AAATCCTA 6.42816540519
AAATCCTC 9.56798040113
AAATCCTG 7.80305280159
AAATCCTT 6.16629206431
AAATCGAA 4.68562901406
AAATCGAC 4.40495324516

| | |
|---|---|
| AAATCGAG | 2.15270053618 |
| AAATCGAT | 5.90937918861 |
| AAATCGCA | 5.03876425558 |
| AAATCGCC | 4.11772986207 |
| AAATCGCG | 8.67535938897 |
| AAATCGCT | 2.67244499606 |
| AAATCGGA | 1.28759794819 |
| AAATCGGC | 3.01944273403 |
| AAATCGGG | 3.92254259357 |
| AAATCGGT | 3.78671727936 |
| AAATCGTA | 5.62130456571 |
| AAATCGTC | 4.17778921263 |
| AAATCGTG | 4.37685712707 |
| AAATCGTT | 3.67632049045 |
| AAATCTAA | 9.42280872781 |
| AAATCTAC | 8.49640126034 |
| AAATCTAG | 8.53884250747 |
| AAATCTAT | 5.30007343411 |
| AAATCTCA | 7.65803913536 |
| AAATCTCC | 7.73898935554 |
| AAATCTCG | 12.6613908573 |
| AAATCTCT | 5.57085429489 |
| AAATCTGA | 15.3725886029 |
| AAATCTGC | 10.4626836096 |
| AAATCTGG | 14.7916804082 |
| AAATCTGT | 9.18687790369 |
| AAATCTTA | 13.341232728 |
| AAATCTTC | 9.12425817202 |
| AAATCTTG | 7.87884041576 |
| AAATGAAA | 2.40988841501 |
| AAATGAAC | -0.576478167019 |
| AAATGAAG | 0.773762204249 |
| AAATGAAT | 2.35175554359 |
| AAATGACA | 0.134051637728 |
| AAATGACC | 0.982931539373 |
| AAATGACG | 0.706654779613 |
| AAATGACT | 0.738053433363 |
| AAATGAGA | 0.716012380332 |
| AAATGAGC | -0.90956628431 |
| AAATGAGG | -1.02424238001 |
| AAATGAGT | 0.839001855946 |
| AAATGATA | 1.01593233095 |
| AAATGATC | 0.136509387096 |
| AAATGATG | 1.31470438811 |
| AAATGATT | 2.53222504444 |
| AAATGCAA | 1.05743906707 |
| AAATGCAC | -0.116835317829 |
| AAATGCAG | 1.38947567733 |
| AAATGCAT | 0.736982149418 |
| AAATGCCA | -0.476159691037 |
| AAATGCCC | 0.533707790481 |
| AAATGCCG | 1.83653666507 |
| AAATGCCT | -0.926753459291 |
| AAATGCGA | -0.359862305138 |

AAATGCGC 1.76411945258
AAATGCGG -0.184073478245
AAATGCGT 1.14725642063
AAATGCTA 0.924619010344
AAATGCTC 0.0706118929462
AAATGCTG -0.160767535737
AAATGCTT 0.997207969676
AAATGGAA -0.26226429915
AAATGGAC 0.0910391501869
AAATGGAG 0.208941172045
AAATGGAT 2.71910434566
AAATGGCA -0.584948096782
AAATGGCC -0.574741129857
AAATGGCG 0.681317644442
AAATGGCT -1.02141324111
AAATGGGA -0.223854418824
AAATGGGC -0.735961244548
AAATGGGG 0.41917471306
AAATGGGT -0.384941091422
AAATGGTA 0.969415375095
AAATGGTC -0.576212948257
AAATGGTG 0.267240377682
AAATGGTT 0.209268636025
AAATGTAA 1.420492949
AAATGTAC -0.635882381435
AAATGTAG 0.46665886386
AAATGTAT 0.837764862321
AAATGTCA 0.474930608175
AAATGTCC -0.387815613129
AAATGTCG 2.5175006151
AAATGTCT 0.0729434864513
AAATGTGA 0.730835069684
AAATGTGC 0.352361443926
AAATGTGG 1.01094730076
AAATGTGT 0.584418908327
AAATGTTA 1.36694249153
AAATGTTC 0.48209859278
AAATGTTG 0.980380526265
AAATTAAA 1.32294740387
AAATTAAC 0.628431691118
AAATTAAG 1.38751755514
AAATTAAT 2.06363012526
AAATTACA 2.54353348112
AAATTACC 4.17718466374
AAATTACG 3.9249505884
AAATTACT 2.01822817073
AAATTAGA 1.52979742799
AAATTAGC 1.16386944071
AAATTAGG 1.86436798076
AAATTAGT 1.11539665263
AAATTATA 2.47258579707
AAATTATC 1.89562132627
AAATTATG 0.683679423756
AAATTATT 2.48995242148

AAATTCAA 1.02288901489
AAATTCAC −0.183556155936
AAATTCAG 1.84884622971
AAATTCAT 2.02409254476
AAATTCCA 0.542750834255
AAATTCCC 3.38815260381
AAATTCCG 2.58124429975
AAATTCCT 1.40136493061
AAATTCGA 2.82132098857
AAATTCGC 4.25666244099
AAATTCGG 4.23460785639
AAATTCGT 1.57702531168
AAATTCTA 2.99520457048
AAATTCTC 3.18872891319
AAATTCTG 2.30990677102
AAATTCTT 4.02740143155
AAATTGAA 0.832363476152
AAATTGAC 0.583009335147
AAATTGAG 0.421121177281
AAATTGAT 2.48484997891
AAATTGCA 1.49646958879
AAATTGCC 0.711584226274
AAATTGCG 2.94995229593
AAATTGCT 1.37094950151
AAATTGGA 1.1059512008
AAATTGGC 0.676625520691
AAATTGGG 0.12073865517
AAATTGGT 0.370893656909
AAATTGTA 1.45113216944
AAATTGTC 1.180535338
AAATTGTG 0.252702892178
AAATTGTT 1.85586182802
AAATTTAA 1.23234209661
AAATTTAC 0.512736564149
AAATTTAG 1.21240155952
AAATTTAT 2.6225247464
AAATTTCA 1.67869028291
AAATTTCC 5.53966590091
AAATTTCG 5.24974723737
AAATTTCT 1.59156633621
AAATTTGA 0.998553424035
AAATTTGC 2.42329632673
AAATTTGG 1.62540150517
AAATTTGT 2.184857166
AAATTTTA 3.28926889055
AAATTTTC 3.17730014963
AAATTTTG 1.59322072671
AACAAAAA 0.784240635275
AACAAAAC 2.4933840275
AACAAAAG 0.201637871746
AACAAAAT 5.52935463681
AACAAACA 1.45009814935
AACAAACC 1.32573886256
AACAAACG 0.557908481996

AACAAACT 0.0603878554263
AACAAAGA 1.53474644338
AACAAAGC 0.00742945616164
AACAAAGG 0.291664860538
AACAAAGT 0.240434338603
AACAAATA 2.38205751568
AACAAATC 4.86572858262
AACAAATG 0.500013974374
AACAAATT 2.62763239343
AACAACAA 0.962698928554
AACAACAC -0.91271310283
AACAACAG -0.978278761262
AACAACAT 1.4247816238
AACAACCA -0.370721285532
AACAACCC -0.822464196299
AACAACCG -0.15918455029
AACAACCT -1.03319320094
AACAACGA 0.597141498102
AACAACGC -0.068331719405
AACAACGG 0.767942380318
AACAACGT 1.14806290217
AACAACTA 2.17510111261
AACAACTC 1.65913945501
AACAACTG 0.462576493446
AACAACTT 1.41270646758
AACAAGAA 1.22032065032
AACAAGAC 0.595138825823
AACAAGAG 0.368233142178
AACAAGAT 1.77362694145
AACAAGCA 1.14272646753
AACAAGCC 0.466228351773
AACAAGCG -0.00474062922307
AACAAGCT 0.433129300189
AACAAGGA 0.0935984904147
AACAAGGC -0.696000230006
AACAAGGG 0.0173853604285
AACAAGGT -1.09005035763
AACAAGTA 0.286497882788
AACAAGTC -0.130818841698
AACAAGTG -0.629397845177
AACAAGTT -0.100956333347
AACAATAA 3.17509783466
AACAATAC 0.750228723197
AACAATAG 0.808436746874
AACAATAT 1.94033400347
AACAATCA 1.74584558848
AACAATCC 2.18259760208
AACAATCG 1.29871090574
AACAATCT 2.59540831394
AACAATGA 1.01712394177
AACAATGC 0.24271805117
AACAATGG 2.48465554067
AACAATGT 1.02488023738
AACAATTA 1.40564756825

AACAATTC 0.987079277676
AACAATTG 0.419656436933
AACACAAA 0.681656558212
AACACAAC -0.423702584376
AACACAAG 0.492272251226
AACACAAT 2.54811568689
AACACACA 0.931620452555
AACACACC -0.543147413329
AACACACG 1.29131829708
AACACACT -0.885686186902
AACACAGA -0.268618307808
AACACAGC 0.189383682452
AACACAGG 0.0488660363073
AACACAGT -0.192757414983
AACACATA 1.21824428303
AACACATC 0.258378865117
AACACATG -0.445819414196
AACACATT 0.331666677966
AACACCAA 0.245445391037
AACACCAC -0.647850534168
AACACCAG -0.66932867391
AACACCAT 0.273927887482
AACACCCA 0.329845536896
AACACCCC -0.668248854668
AACACCCG -0.83842208024
AACACCCT -1.10873183423
AACACCGA -0.399635959488
AACACCGC 0.448800106679
AACACCGG 0.424821332903
AACACCGT -0.557919099074
AACACCTA 0.885712000973
AACACCTC 0.784861422046
AACACCTG -0.383832127261
AACACCTT 0.977472487901
AACACGAA 1.85927615526
AACACGAC -0.320806032148
AACACGAG -0.360357144224
AACACGAT 1.78587030549
AACACGCA 0.329667336535
AACACGCC -0.605426149452
AACACGCG 0.247009432287
AACACGCT -0.441242621052
AACACGGA -1.1137974293
AACACGGC 0.280805463788
AACACGGG -0.832009157211
AACACGGT -0.224148574326
AACACGTA 1.03414728067
AACACGTC -0.0322043025773
AACACGTG 0.377669434178
AACACGTT 0.109677742124
AACACTAA 2.65384554159
AACACTAC -0.415792861588
AACACTAG 0.309766977791
AACACTAT 0.176465821722

AACACTCA -0.308669671598
AACACTCC -0.17667337518
AACACTCG 0.227417385149
AACACTCT -0.339747731241
AACACTGA 1.26056520328
AACACTGC 0.267340719474
AACACTGG 0.30217784911
AACACTGT 0.539437056986
AACACTTA 2.0556369231
AACACTTC 0.50636610943
AACACTTG 0.0415061115948
AACAGAAA 1.83710207649
AACAGAAC 0.11871849594
AACAGAAG 0.241248106372
AACAGAAT 4.81361184698
AACAGACA 1.59321073416
AACAGACC 1.3137009702
AACAGACG 0.695124633374
AACAGACT -0.565429120118
AACAGAGA -0.0215052028808
AACAGAGC -0.562022287287
AACAGAGG -0.942228578469
AACAGAGT -0.24084195111
AACAGATA 3.04126915556
AACAGATC 3.87620196448
AACAGATG 0.125210526606
AACAGATT 6.19566348078
AACAGCAA 0.415499538798
AACAGCAC -0.90966787517
AACAGCAG -0.642920879328
AACAGCAT 0.236449187311
AACAGCCA -0.424880039096
AACAGCCC -0.606167263102
AACAGCCG -0.376707027323
AACAGCCT -1.06550034344
AACAGCGA -0.2460482745
AACAGCGC 0.866097054256
AACAGCGG -0.320000799677
AACAGCGT -0.0521744009486
AACAGCTA 0.752558859456
AACAGCTC 0.0939723780875
AACAGCTG -0.70160937781
AACAGCTT 0.480024723628
AACAGGAA 2.93647464458
AACAGGAC 0.119496248917
AACAGGAG 0.144942469355
AACAGGAT 2.78843719302
AACAGGCA -0.245372736918
AACAGGCC -0.620525299178
AACAGGCG -1.53399700261
AACAGGCT -0.912631913414
AACAGGGA -0.414773413964
AACAGGGC -0.348253259441
AACAGGGG -0.647075695683

AACAGGGT 0.0924505969694
AACAGGTA -0.131798943682
AACAGGTC -0.723713300594
AACAGGTG -1.5407186536
AACAGGTT -0.12505772396
AACAGTAA 0.792850044293
AACAGTAC -0.0626186907835
AACAGTAG -0.531583334078
AACAGTAT 1.96807059817
AACAGTCA 0.674502105177
AACAGTCC 0.26624341328
AACAGTCG 0.945857953574
AACAGTCT -0.0634060199475
AACAGTGA -0.787598129922
AACAGTGC 0.577786565695
AACAGTGG 0.362799280243
AACAGTGT -0.224759784909
AACAGTTA 1.06863800213
AACAGTTC -0.106468053851
AACAGTTG 0.876553418414
AACATAAA 1.75549796924
AACATAAC 0.666656709388
AACATAAG 0.463743539267
AACATAAT 2.27203711258
AACATACA 1.00141524688
AACATACC 0.0597874700988
AACATACG 0.376807785471
AACATACT 0.365398174289
AACATAGA 0.711966232888
AACATAGC -0.134730714337
AACATAGG 0.546271540448
AACATAGT -0.118897945369
AACATATA 0.964652679005
AACATATC 2.97529380284
AACATATG -0.422908385338
AACATATT 1.91708926529
AACATCAA -0.0705321607755
AACATCAC 0.315696095168
AACATCAG 1.1955628745
AACATCAT 0.474084156462
AACATCCA 0.866406406751
AACATCCC 0.233122711184
AACATCCG 1.39185286182
AACATCCT -0.122558963528
AACATCGA 1.38028607625
AACATCGC 0.69904504131
AACATCGG 1.33316831872
AACATCGT 1.07245765192
AACATCTA 1.6352830899
AACATCTC 1.25472747604
AACATCTG 1.08894701427
AACATCTT 1.81173433916
AACATGAA 1.20874387221
AACATGAC -0.402969721844

AACATGAG 1.70215235891
AACATGAT 1.73941705211
AACATGCA 0.14683459912
AACATGCC 0.215547700606
AACATGCG 0.0877337000374
AACATGCT -0.952480303662
AACATGGA 0.33363042096
AACATGGC 0.810872845731
AACATGGG -0.776941706714
AACATGGT -0.0949408221041
AACATGTA -0.523590964627
AACATGTC -1.03779851446
AACATGTG -0.451896546125
AACATGTT 1.33120749021
AACATTAA 1.61292893717
AACATTAC 0.999331801545
AACATTAG 0.354217142542
AACATTAT 1.19053662507
AACATTCA -0.338550291434
AACATTCC 1.23688578946
AACATTCG 0.315696095168
AACATTCT 0.517808196378
AACATTGA -0.300743502748
AACATTGC 0.741977380325
AACATTGG 0.114314698708
AACATTGT -0.370424631894
AACATTTA 1.59950520392
AACATTTC 2.12506345056
AACATTTG 0.244697823869
AACCAAAA 0.955096060126
AACCAAAC -0.238327993685
AACCAAAG -0.035530778704
AACCAAAT 1.455033425
AACCAACA 0.141127815838
AACCAACC -0.632268203324
AACCAACG 0.226451022912
AACCAACT 0.472619832469
AACCAAGA 0.323978248383
AACCAAGC 0.680985184189
AACCAAGG -0.268853548938
AACCAAGT 0.0906361175953
AACCAATA 0.325158825773
AACCAATC 1.45332053648
AACCAATG -0.192673935609
AACCAATT 0.823555048975
AACCACAA 0.263516281591
AACCACAC -0.0817186051502
AACCACAG -0.315598251512
AACCACAT 0.708921005228
AACCACCA 0.118424548616
AACCACCC -0.641652242648
AACCACCG -0.0481161791818
AACCACCT -1.07780366274
AACCACGA -0.106468053851

AACCACGC -0.369986833578
AACCACGG -0.4835579205
AACCACGT 0.267848465595
AACCACTA -0.0253000794883
AACCACTC 1.202226652
AACCACTG -0.106156619576
AACCACTT 0.946464792419
AACCAGAA 0.736424440579
AACCAGAC -1.16588064829
AACCAGAG -0.443445144203
AACCAGAT 2.75679247289
AACCAGCA 0.427911527009
AACCAGCC -0.668655426285
AACCAGCG -0.583065335026
AACCAGCT -0.832777750356
AACCAGGA -0.47657708791
AACCAGGC -0.834715054745
AACCAGGG -0.751449687118
AACCAGGT -0.844595598579
AACCAGTA 0.638396963395
AACCAGTC -0.942092430063
AACCAGTG -0.878894379929
AACCAGTT -0.0842234027443
AACCATAA 0.993230937326
AACCATAC -0.468718160552
AACCATAG 0.658638109511
AACCATAT 1.14056849447
AACCATCA -0.071157111106
AACCATCC 0.0300338388377
AACCATCG -0.711134353634
AACCATCT 0.544257626558
AACCATGA 0.972446863007
AACCATGC 0.571631783376
AACCATGG -0.45250650764
AACCATGT 0.35997826028
AACCATTA 0.193839107827
AACCATTC 0.287689909968
AACCATTG -0.306134480017
AACCCAAA -0.283746186096
AACCCAAC 0.233293833493
AACCCAAG 0.247986619779
AACCCAAT 0.353250155771
AACCCACA 0.0605306655285
AACCCACC -0.01351491521
AACCCACG -0.803427568042
AACCCACT -1.08984655137
AACCCAGA 0.918791067471
AACCCAGC -0.79075244285
AACCCAGG -0.49123219398
AACCCAGT -0.261215082073
AACCCATA 0.51325367828
AACCCATC -0.323999066182
AACCCATG -0.90092814673
AACCCATT -0.566702128536

AACCCCAA -1.02431003786
AACCCCAC -0.689624987194
AACCCCAG -1.0660057996
AACCCCAT -0.500262955252
AACCCCCA -1.45439077954
AACCCCCC -1.43036766385
AACCCCCG -0.887296651845
AACCCCCT -1.50533797123
AACCCCGA -0.770770686511
AACCCCGC -0.936156442812
AACCCCGG -0.159587999237
AACCCCGT -0.286614878819
AACCCCTA -0.493582523504
AACCCCTC 0.0591600216323
AACCCCTG -0.813765270745
AACCCCTT -0.338606083135
AACCCGAA 0.125509262024
AACCCGAC -0.992163192407
AACCCGAG -1.24329996155
AACCCGAT 0.425300974995
AACCCGCA 1.01841339625
AACCCGCC -0.256257531383
AACCCGCG -0.0390841688414
AACCCGCT -0.484719970049
AACCCGGA -0.593996553184
AACCCGGC -0.371797149393
AACCCGGG -0.621382576147
AACCCGGT -0.852202838745
AACCCGTA 0.326192429501
AACCCGTC -0.223761571439
AACCCGTG 0.541339387473
AACCCGTT -0.516799782187
AACCCTAA -0.171512850948
AACCCTAC -0.392092629965
AACCCTAG 0.107981091493
AACCCTAT -0.603068950054
AACCCTCA -0.267512674495
AACCCTCC -1.53540595126
AACCCTCG -0.810079479405
AACCCTCT -0.728564472331
AACCCTGA 0.350524689506
AACCCTGC -0.430016622859
AACCCTGG -1.09117347789
AACCCTGT -0.515863397581
AACCCTTA -0.170734473437
AACCCTTC -0.721054034931
AACCCTTG -0.374556756848
AACCGAAA 0.508070879003
AACCGAAC -0.303792477612
AACCGAAG 0.553343763176
AACCGAAT 2.98478463747
AACCGACA 1.52850526719
AACCGACC -0.455833191945
AACCGACG 0.994965059995

AACCGACT -0.188867401033
AACCGAGA 0.366527123537
AACCGAGC -1.04933865325
AACCGAGG -0.669610338732
AACCGAGT -0.207779330674
AACCGATA 2.62539281459
AACCGATC 1.18010232778
AACCGATG -0.164942753535
AACCGATT 1.57466707139
AACCGCAA -0.928456355262
AACCGCAC 0.300742878214
AACCGCAG -0.6908536537
AACCGCAT 0.217070730792
AACCGCCA -0.129893906882
AACCGCCC -0.871663317393
AACCGCCG -0.433816912095
AACCGCCT -1.33617378438
AACCGCGA 0.472619832469
AACCGCGC 0.83011661109
AACCGCGG -0.633326996589
AACCGCGT 0.832633483008
AACCGCTA -0.0563292173035
AACCGCTC -0.880856041143
AACCGCTG -1.11941011613
AACCGCTT -0.291056564447
AACCGGAA 0.747059629632
AACCGGAC -0.326384369609
AACCGGAG 0.264130406666
AACCGGAT 1.40043291774
AACCGGCA -0.380515227322
AACCGGCC -0.65186774487
AACCGGCG -0.343820941823
AACCGGCT -0.807394816027
AACCGGGA -1.15609149459
AACCGGGC -0.649188077763
AACCGGGG -1.31205553135
AACCGGGT -0.876439753231
AACCGGTA -0.655919304941
AACCGGTC -0.16742048799
AACCGGTG -1.40231672038
AACCGGTT -0.585672139836
AACCGTAA 2.15170357178
AACCGTAC -0.192733266336
AACCGTAG -0.418297034648
AACCGTAT 0.375116755646
AACCGTCA -0.382702345301
AACCGTCC -0.116093996001
AACCGTCG -0.817965053546
AACCGTCT 0.349578312357
AACCGTGA 0.0255009712502
AACCGTGC 0.474808615872
AACCGTGG -0.309540896492
AACCGTGT 0.212180421593
AACCGTTA 0.520988323377

AACCGTTC -0.292708248631
AACCGTTG -0.570283622703
AACCTAAA 1.09744858709
AACCTAAC 0.571056379407
AACCTAAG 0.497795213341
AACCTAAT 1.20588642109
AACCTACA 0.700727535838
AACCTACC -0.138239346206
AACCTACG -0.278128711173
AACCTACT -0.237100576246
AACCTAGA -0.0703579157966
AACCTAGC -0.975408819471
AACCTAGG -0.197463070304
AACCTAGT -0.112588070446
AACCTATA -0.171072970852
AACCTATC 0.011278667225
AACCTATG 0.323201328118
AACCTATT 0.0750927160359
AACCTCAA 0.51401956511
AACCTCAC -0.766304435881
AACCTCAG -0.480143801439
AACCTCAT -0.359589175613
AACCTCCA -1.10223522365
AACCTCCC -0.16835957891
AACCTCCG -1.43067576728
AACCTCCT -0.994222280921
AACCTCGA 1.06117357206
AACCTCGC -0.438533184493
AACCTCGG -0.716095651528
AACCTCGT 0.463581993145
AACCTCTA -0.0666150836869
AACCTCTC -0.687118524176
AACCTCTG -1.0786326275
AACCTCTT 0.926989741311
AACCTGAA 1.66531609602
AACCTGAC -0.546380209359
AACCTGAG 0.853583475185
AACCTGAT 0.484435182556
AACCTGCA 1.03211692072
AACCTGCC -0.836696909224
AACCTGCG -0.548743445919
AACCTGCT 0.00745069031677
AACCTGGA -0.689702429407
AACCTGGC 0.546271540448
AACCTGGG -0.942050378108
AACCTGGT -0.606961253962
AACCTGTA 0.212964628087
AACCTGTC -0.366370781865
AACCTGTG -0.984870925542
AACCTTAA 0.837228179459
AACCTTAC -1.29818671355
AACCTTAG -0.16742048799
AACCTTAT -0.0873454480832
AACCTTCA 0.483478604685

AACCTTCC 0.405371263154
AACCTTCG -0.702583234454
AACCTTCT -0.147902552217
AACCTTGA 0.186111957138
AACCTTGC -0.663383526828
AACCTTGG -0.401588669048
AACCTTGT -0.204737225684
AACCTTTA 0.671614051901
AACCTTTC 0.525304477675
AACCTTTG -0.336032586804
AACGAAAA 1.48237198338
AACGAAAC 1.98842374404
AACGAAAG 0.401813917635
AACGAAAT 3.31366610209
AACGAACA 0.279499771425
AACGAACC -0.917961894531
AACGAACG 0.399767111622
AACGAACT -0.25297831166
AACGAAGA -0.190635456716
AACGAAGC 0.608087496896
AACGAAGG -1.15473001052
AACGAAGT -0.564414252409
AACGAATA 0.531935154883
AACGAATC 4.9224997618
AACGAATG 0.427718337833
AACGAATT 1.08361765769
AACGACAA 0.231579279555
AACGACAC -1.14994545575
AACGACAG 0.112197528534
AACGACAT -0.55266239661
AACGACCA 0.455581712931
AACGACCC -0.994499782184
AACGACCG -0.0904333522315
AACGACCT -0.708918090736
AACGACGA 0.11115205866
AACGACGC -0.483992804324
AACGACGG -0.0167949676445
AACGACGT -0.18299594896
AACGACTA 0.377976704893
AACGACTC 1.40199841623
AACGACTG -0.249154498312
AACGACTT 2.169550671
AACGAGAA 1.13442849279
AACGAGAC 0.963639684898
AACGAGAG -0.202955430254
AACGAGAT 2.68000352258
AACGAGCA 0.816756788484
AACGAGCC -0.633926757382
AACGAGCG 0.308677166005
AACGAGCT 0.131950289082
AACGAGGA 0.160319953055
AACGAGGC 0.174987133448
AACGAGGG -1.39254338821
AACGAGGT 0.225898518522

AACGAGTA 0.546700178932
AACGAGTC -0.52238311592
AACGAGTG -0.812916737252
AACGAGTT 0.258729020498
AACGATAA 1.76828384512
AACGATAC 0.351765846692
AACGATAG 1.35288298269
AACGATAT 3.26981965351
AACGATCA 0.602028268275
AACGATCC 1.38710994264
AACGATCG 1.54547385528
AACGATCT 2.15983500413
AACGATGA 0.127313748855
AACGATGC 0.671572208124
AACGATGG -0.511001192411
AACGATGT -0.520597365109
AACGATTA 1.50148397207
AACGATTC 2.86632303317
AACGATTG 1.58376403326
AACGCAAA 0.156143278011
AACGCAAC 1.94500031315
AACGCAAG 0.378244838146
AACGCAAT 3.74766870895
AACGCACA 0.803730675198
AACGCACC -1.29415909395
AACGCACG -0.00466943234996
AACGCACT 0.427125655091
AACGCAGA 1.72336215722
AACGCAGC -0.493084769926
AACGCAGG -0.916528589059
AACGCAGT -0.0883653120637
AACGCATA 0.931067323631
AACGCATC 0.493441587004
AACGCATG -0.655746933564
AACGCATT 0.305093590059
AACGCCAA -0.463690870235
AACGCCAC -1.20996879151
AACGCCAG -1.16594830613
AACGCCAT -0.533083048329
AACGCCCA -0.0466172976428
AACGCCCC -1.37219482225
AACGCCCG 0.0299740917541
AACGCCCT -1.28362861843
AACGCCGA -0.398863202783
AACGCCGC -0.428145519071
AACGCCGG -0.595360327206
AACGCCGT -0.926037327
AACGCCTA -0.122567290648
AACGCCTC -0.166794288592
AACGCCTG -0.63849688883
AACGCCTT 0.428350782571
AACGCGAA 1.21278169253
AACGCGAC -0.0230249022189
AACGCGAG -0.0251932841787

AACGCGAT 3.23022086868
AACGCGCA 0.829783734481
AACGCGCC -0.527607134439
AACGCGCG 0.268091617489
AACGCGCT -0.0203831235064
AACGCGGA -0.0870997980532
AACGCGGC -0.117444030276
AACGCGGG -0.187201144389
AACGCGGT -0.0505110587963
AACGCGTA -0.295915855125
AACGCGTC 0.341662136051
AACGCGTG 0.215810213053
AACGCGTT 0.414876253891
AACGCTAA 0.759426026863
AACGCTAC -0.118017144287
AACGCTAG -0.201888518048
AACGCTAT 0.369296099002
AACGCTCA -0.392043291781
AACGCTCC 0.342052261607
AACGCTCG 0.263121367941
AACGCTCT -1.23567356901
AACGCTGA 0.800323217832
AACGCTGC 0.182537957379
AACGCTGG -0.0972416052665
AACGCTGT -0.145808281622
AACGCTTA -0.0120466358357
AACGCTTC 0.698311838424
AACGCTTG -0.549013244596
AACGGAAA 1.28577181085
AACGGAAC -0.163674325033
AACGGAAG -0.817971715242
AACGGAAT 1.9346592796
AACGGACA 0.941066945276
AACGGACC -0.219555126943
AACGGACG -0.253806027355
AACGGACT -0.618167891602
AACGGAGA 0.0782894972738
AACGGAGC -1.23653042963
AACGGAGG -1.11335546742
AACGGAGT 0.188497468743
AACGGATA 1.67693180341
AACGGATC 2.77870445556
AACGGATG 0.539615257347
AACGGATT 4.40089065166
AACGGCAA -0.151423258409
AACGGCAC -0.487330105707
AACGGCAG -0.614566828704
AACGGCAT -0.444451268436
AACGGCCA -0.802034857279
AACGGCCC -1.64328128833
AACGGCCG -0.493204472272
AACGGCCT -0.871022753713
AACGGCGA -0.0772419456204
AACGGCGC -0.563033824148

AACGGCGG -0.916750923154
AACGGCGT -0.0464411790619
AACGGCTA 0.750801004496
AACGGCTC 0.527524904133
AACGGCTG -1.32852324319
AACGGCTT -0.755371760478
AACGGGAA 0.0298414823735
AACGGGAC -1.29578392318
AACGGGAG 0.148781479697
AACGGGAT 1.06314376858
AACGGGCA 0.00242360817736
AACGGGCC -1.23476612115
AACGGGCG -0.398976867966
AACGGGCT -0.402460934833
AACGGGGA -1.49749257544
AACGGGGC 0.230732411486
AACGGGGG -0.666101290507
AACGGGGT -0.695484156765
AACGGGTA -0.0146873736583
AACGGGTC -1.32105673135
AACGGGTG -0.604417318905
AACGGTAA 0.700440458387
AACGGTAC 0.721576561689
AACGGTAG -0.393469311023
AACGGTAT 0.481056453754
AACGGTCA -0.433104110652
AACGGTCC -0.846700694429
AACGGTCG -0.211832347991
AACGGTCT -0.474796541549
AACGGTGA 0.0309183871237
AACGGTGC -0.672439893993
AACGGTGG -1.11448566574
AACGGTGT -0.62775573718
AACGGTTA 0.111644607788
AACGGTTC -0.895845481067
AACGGTTG 0.0338199719696
AACGTAAA 1.74636540893
AACGTAAC 2.23310116647
AACGTAAG 0.282149044545
AACGTAAT 3.02397227077
AACGTACA 0.0323916627697
AACGTACC -0.282359304317
AACGTACG 0.105848932504
AACGTACT 0.797920635632
AACGTAGA 0.765985090842
AACGTAGC -0.447779409986
AACGTAGG -0.0868714267965
AACGTAGT 0.118404563529
AACGTATA 2.07750956014
AACGTATC 4.57733503105
AACGTATG -0.24945427462
AACGTATT 2.43720469835
AACGTCAA 0.879985232604
AACGTCAC 0.283392908044

AACGTCAG 0.439466654607
AACGTCAT 0.157145238685
AACGTCCA -0.425102581369
AACGTCCC -0.595077205138
AACGTCCG -0.771215771057
AACGTCCT -1.00037810413
AACGTCGA 0.287689909968
AACGTCGC 0.159348802725
AACGTCGG -0.287369732217
AACGTCGT 1.46369779301
AACGTCTA -0.609288059373
AACGTCTC -0.0339925515246
AACGTCTG 0.0130806559196
AACGTCTT -0.0033687362589
AACGTGAA 0.31495581423
AACGTGAC -0.643650543189
AACGTGAG 0.456497071559
AACGTGAT 0.542864499439
AACGTGCA 0.238302387792
AACGTGCC -0.938948109679
AACGTGCG -0.194751760142
AACGTGCT 0.14830433574
AACGTGGA -0.309349997274
AACGTGGC -0.431863369822
AACGTGGG 0.0383932260875
AACGTGGT -1.26361168001
AACGTGTA -0.749576917906
AACGTGTC -0.448713712812
AACGTGTG -0.80486545343
AACGTTAA 1.19330913956
AACGTTAC 0.643306633147
AACGTTAG 0.373711554203
AACGTTAT 0.633304513366
AACGTTCA 0.108981178565
AACGTTCC -0.204730563988
AACGTTCG -0.0394064283722
AACGTTCT 0.109897994439
AACGTTGA 0.561178333709
AACGTTGC 0.294785864987
AACGTTGG 0.0360905693232
AACGTTGT 0.568492042908
AACGTTTA -0.171485371453
AACGTTTC 0.80539193557
AACGTTTG 0.824180415662
AACTAAAA 2.84016817485
AACTAAAC 1.30975266641
AACTAAAG 0.749400799325
AACTAAAT 1.2132425986
AACTAACA 0.519561471423
AACTAACC -0.638899921422
AACTAACG 0.921260058451
AACTAACT 0.745043633962
AACTAAGA 0.492938420798
AACTAAGC -0.37102002095

AACTAAGG -0.260493120798
AACTAAGT -0.241887004628
AACTAATA 0.519565634983
AACTAATC 0.485214184601
AACTAATG 0.416651179447
AACTAATT 1.74495666846
AACTACAA 1.1584788798
AACTACAC -0.735032146172
AACTACAG -0.655259380708
AACTACAT 0.603719922634
AACTACCA 0.927518721588
AACTACCC 0.664864713237
AACTACCG 0.787440955539
AACTACCT 0.296790619045
AACTACGA 0.110064953189
AACTACGC 1.10381009015
AACTACGG 0.444243714978
AACTACGT -0.0117845397444
AACTACTA 1.05090956437
AACTACTC 1.32557835732
AACTACTG 0.65134230362
AACTACTT 2.00922468078
AACTAGAA 3.16239377272
AACTAGAC -0.818086213138
AACTAGAG -0.669460034222
AACTAGAT 0.533605783265
AACTAGCA 0.570444960646
AACTAGCC 0.698109489416
AACTAGCG -0.0173045873677
AACTAGCT 0.0941782661211
AACTAGGA 0.531702411889
AACTAGGC 0.425771873612
AACTAGGG -0.353058632019
AACTAGGT 0.0907118943842
AACTAGTA -0.40404496117
AACTAGTC -0.537684822831
AACTAGTG -0.696006267168
AACTAGTT 1.6639173481
AACTATAA 1.10260515594
AACTATAC 0.180559017392
AACTATAG 1.07440453249
AACTATAT 1.28339108734
AACTATCA 1.26359981386
AACTATCC 1.30978264404
AACTATCG 0.452618923755
AACTATCT -0.103689710376
AACTATGA 1.31226745655
AACTATGC 1.21713011442
AACTATGG 0.261315007509
AACTATGT 0.265417571188
AACTATTA 0.986309019107
AACTATTC 1.00465657821
AACTATTG 0.864964357804
AACTCAAA 1.36288093891

AACTCAAC 0.503899824764
AACTCAAG -0.789456326675
AACTCAAT 0.42340572256
AACTCACA 0.572469283436
AACTCACC -0.749018584532
AACTCACG -0.241315556041
AACTCACT 0.10265173491
AACTCAGA 0.741851224462
AACTCAGC -0.311435940749
AACTCAGG 0.209655222555
AACTCAGT -0.0566658411158
AACTCATA 0.657366766517
AACTCATC -0.908320130853
AACTCATG -0.221909828205
AACTCATT 0.457360802046
AACTCCAA 0.624386584565
AACTCCAC -0.42361827229
AACTCCAG -1.27439009552
AACTCCAT -0.376691830329
AACTCCCA -0.883744718953
AACTCCCC -1.13864159898
AACTCCCG 0.537226623072
AACTCCCT -1.37389188924
AACTCCGA -0.177362236154
AACTCCGC -0.379331943618
AACTCCGG -0.453212647387
AACTCCGT 0.0196520024002
AACTCCTA 0.316179276286
AACTCCTC 0.489478086223
AACTCCTG -0.179057221361
AACTCCTT 0.947102441607
AACTCGAA 1.2947640592
AACTCGAC -0.592351114339
AACTCGAG -0.415442498028
AACTCGAT 0.33681658512
AACTCGCA 0.749698702031
AACTCGCC -0.919975600243
AACTCGCG 0.472619832469
AACTCGCT -0.315000780676
AACTCGGA -0.715024159406
AACTCGGC -0.0451275759354
AACTCGGG -0.102545980491
AACTCGGT -0.455110397958
AACTCGTA 0.547170453015
AACTCGTC 0.320281839965
AACTCGTG 0.399361164539
AACTCTAA 1.06953858012
AACTCTAC -0.294933671361
AACTCTAG 0.0710088883761
AACTCTAT -0.0285984515861
AACTCTCA -0.683533699162
AACTCTCC -0.432179384013
AACTCTCG 0.0729543117069
AACTCTCT -1.06594542799

AACTCTGA -0.00642478917452
AACTCTGC 0.710219203184
AACTCTGG -0.427719586901
AACTCTGT 0.390968676988
AACTCTTA 0.311568966486
AACTCTTC -0.331538023967
AACTCTTG 0.468968390498
AACTGAAA 0.570385629918
AACTGAAC 0.389627802545
AACTGAAG -0.562981155116
AACTGAAT 1.159303681
AACTGACA 0.235617932591
AACTGACC -0.708140754116
AACTGACG 1.20107917491
AACTGACT -0.845380429607
AACTGAGA -0.48214522465
AACTGAGC -0.296865354944
AACTGAGG -0.24266933752
AACTGAGT -0.67964077072
AACTGATA 1.9232261443
AACTGATC 0.372238070379
AACTGATG -0.228009027
AACTGATT 3.11472704982
AACTGCAA 0.309268391501
AACTGCAC -0.325084714408
AACTGCAG -0.680755772043
AACTGCAT -0.406567453894
AACTGCCA -1.09250352708
AACTGCCC -0.803659894681
AACTGCCG 0.545588716636
AACTGCCT -0.701003371677
AACTGCGA 0.0291848889882
AACTGCGC 0.640252245655
AACTGCGG 0.833892335322
AACTGCGT -0.614955288837
AACTGCTA 0.769330927522
AACTGCTC -0.134236916141
AACTGCTG -0.118738064672
AACTGCTT 0.379428538206
AACTGGAA 1.5697511563
AACTGGAC -0.330737787768
AACTGGAG -0.801976567441
AACTGGAT 1.43938343631
AACTGGCA -0.576131342485
AACTGGCC -0.387009547945
AACTGGCG -0.726497473053
AACTGGCT -0.191573298567
AACTGGGA -1.11012433681
AACTGGGC -0.328098715369
AACTGGGG -0.646913108672
AACTGGGT -0.321108098414
AACTGGTA -0.481139516773
AACTGGTC -0.667185065131
AACTGGTG -0.610326659373

AACTGTAA 1.34791065136
AACTGTAC -0.505828385678
AACTGTAG 0.3109662912
AACTGTAT 0.0317327794265
AACTGTCA -0.134869360879
AACTGTCC 0.0837666602309
AACTGTCG -0.797795937015
AACTGTCT -0.330352242127
AACTGTGA 0.218190520208
AACTGTGC 0.428120537712
AACTGTGG -0.23956810998
AACTGTGT -0.699580475104
AACTGTTA 0.908387580522
AACTGTTC 0.172228150527
AACTGTTG 0.428679287442
AACTTAAA 1.74043441795
AACTTAAC 0.462768225376
AACTTAAG 0.0806283770086
AACTTAAT 0.485076787126
AACTTACA 0.238051116956
AACTTACC -0.169910713125
AACTTACG 1.5697511563
AACTTACT 0.430898048475
AACTTAGA 0.281140838532
AACTTAGC 1.32667566352
AACTTAGG 0.88457534914
AACTTAGT 0.318355777188
AACTTATA 0.940268582679
AACTTATC 0.879076952027
AACTTATG 0.757257020369
AACTTATT 2.15098910492
AACTTCAA 0.51604492879
AACTTCAC -0.348887994137
AACTTCAG 0.555572933109
AACTTCAT -0.180512385522
AACTTCCA -1.07937290844
AACTTCCC -0.937666774141
AACTTCCG -0.445054984611
AACTTCCT -0.43682737403
AACTTCGA 0.241833294706
AACTTCGC 0.89488536417
AACTTCGG -0.463574290559
AACTTCGT 0.214145205477
AACTTCTA 0.0717672807992
AACTTCTC 0.988141609967
AACTTCTG -0.792513628658
AACTTCTT 1.37917211582
AACTTGAA 1.78821688781
AACTTGAC -0.481847946478
AACTTGAG -0.0565727855536
AACTTGAT 0.642079215709
AACTTGCA 0.635989384923
AACTTGCC -0.23750444155
AACTTGCG 0.218048126462

AACTTGCT 0.0977166674431
AACTTGGA -0.871960595565
AACTTGGC 0.557816259146
AACTTGGG -0.876471604463
AACTTGGT 0.182537957379
AACTTGTA 1.47443811195
AACTTGTC -0.201102437952
AACTTGTG -0.721999579368
AACTTTAA 0.960845519896
AACTTTAC 0.478474005769
AACTTTAG 0.291452935342
AACTTTAT 0.0655269373252
AACTTTCA 0.385971156124
AACTTTCC 0.7227342395
AACTTTCG 0.0713761143531
AACTTTCT 0.395892502844
AACTTTGA -0.0880010005785
AACTTTGC 0.859634793043
AACTTTGG 0.564179011279
AACTTTGT -0.384946087693
AACTTTTA 0.614177744038
AACTTTTC 1.13940144865
AACTTTTG -0.240053789234
AAGAAAAA 1.19785491418
AAGAAAAC -0.422650869163
AAGAAAAG 0.752851141356
AAGAAAAT 2.11065753355
AAGAAACA -0.162670907114
AAGAAACC 0.152215792023
AAGAAACG 0.0687024844079
AAGAAACT -0.354393885657
AAGAAAGA 0.244336218698
AAGAAAGC 0.0064524768474
AAGAAAGG -0.358613237189
AAGAAAGT 1.24612389601
AAGAAATA 3.66260156081
AAGAAATC 9.88878185336
AAGAAATG 0.319312771415
AAGAAATT 1.32597847542
AAGAACAA 2.1186186763
AAGAACAC -0.687429125739
AAGAACAG 0.145119628826
AAGAACAT 0.404553123648
AAGAACCA -0.256930987185
AAGAACCC -0.247073342931
AAGAACCG 0.30444157659
AAGAACCT -0.253861610878
AAGAACGA -0.117920549699
AAGAACGC -0.121474772548
AAGAACGG -0.609882823895
AAGAACGT -0.662137165192
AAGAACTA 1.13836576314
AAGAACTC -0.00501459145992
AAGAACTG -0.12885655595

AAGAACTT 0.326342317655
AAGAAGAA -0.983468846769
AAGAAGAC -0.852012980417
AAGAAGAG 0.00109501623544
AAGAAGAT 2.46818199984
AAGAAGCA -0.752483707201
AAGAAGCC 0.408535152269
AAGAAGCG 0.45580363067
AAGAAGCT 0.296399660777
AAGAAGGA -0.0605981151978
AAGAAGGC -0.761716192948
AAGAAGGG -0.704743289294
AAGAAGGT 0.0388147865203
AAGAAGTA 0.102019290172
AAGAAGTC 0.124255197803
AAGAAGTG -0.452678879017
AAGAATAA 1.10240468053
AAGAATAC 0.253609507331
AAGAATAG 0.550636200218
AAGAATAT 3.3562901295
AAGAATCA 4.30335072733
AAGAATCC 13.987879577
AAGAATCG 7.71904153222
AAGAATCT 8.03813946394
AAGAATGA 0.312902971055
AAGAATGC -0.63094086045
AAGAATGG 1.23057757996
AAGAATGT 0.0361080562745
AAGAATTA 1.00559129739
AAGAATTC 1.2248620532
AAGAATTG -0.311583538945
AAGACAAA 2.65286564778
AAGACAAC -0.628753534293
AAGACAAG 0.484076700055
AAGACAAT 0.87109041156
AAGACACA -0.264650851645
AAGACACC -0.655292897365
AAGACACG 0.54021814081
AAGACACT -0.470075897413
AAGACAGA 0.428991554429
AAGACAGC 0.797037336414
AAGACAGG 0.875105748661
AAGACAGT 1.93281232445
AAGACATA 0.53117780335
AAGACATC 0.412827157921
AAGACATG -0.0475391097893
AAGACATT 0.634931840726
AAGACCAA 0.657733159782
AAGACCAC -1.17903104383
AAGACCAG -0.0554956726254
AAGACCAT -0.60641728487
AAGACCCA -0.524414100405
AAGACCCC -0.98132315621
AAGACCCG -1.29964062865

AAGACCCT -1.45858702331
AAGACCGA -1.19326063409
AAGACCGC -1.37938237559
AAGACCGG -0.978374106782
AAGACCGT -0.384668794609
AAGACCTA -0.104472459624
AAGACCTC -0.800701893599
AAGACCTG -0.0315129434675
AAGACCTT -0.382482925698
AAGACGAA -0.558816762573
AAGACGAC -0.608316492687
AAGACGAG -0.348828663409
AAGACGAT 2.41490217376
AAGACGCA -0.196237526468
AAGACGCC -1.21962575218
AAGACGCG -0.0785642922226
AAGACGCT -0.0851901813369
AAGACGGA -1.16221754835
AAGACGGC -0.895811548054
AAGACGGG 0.153364726358
AAGACGGT 0.000473396752734
AAGACGTA 0.0264154971669
AAGACGTC 0.0393023393765
AAGACGTG -0.432237882029
AAGACTAA 0.865975478309
AAGACTAC -0.392774412887
AAGACTAG 0.513801394575
AAGACTAT 1.2098619962
AAGACTCA -0.278471372147
AAGACTCC -1.3461679934
AAGACTCG -0.239666161814
AAGACTCT -0.752970427346
AAGACTGA -0.292560858613
AAGACTGC -0.927819538786
AAGACTGG 1.14278683915
AAGACTGT -0.379170397497
AAGACTTA 1.31149199353
AAGACTTC 0.355036739295
AAGACTTG -0.245724557724
AAGAGAAA 0.971144917848
AAGAGAAC -0.881385645953
AAGAGAAG -0.488619768364
AAGAGAAT 2.92411386815
AAGAGACA -0.00960158532524
AAGAGACC -0.504157549118
AAGAGACG -1.05642378301
AAGAGACT -0.291419626864
AAGAGAGA -0.666551371325
AAGAGAGC -0.654025926108
AAGAGAGG -1.13450177144
AAGAGAGT -0.109942960886
AAGAGATA 3.65664767025
AAGAGATC 5.44145626798
AAGAGATG -0.0373194440072

AAGAGATT 4.76337579131
AAGAGCAA 0.251012903242
AAGAGCAC -0.0360905693232
AAGAGCAG 0.0450713678777
AAGAGCAT -0.347163655833
AAGAGCCA -0.633457732368
AAGAGCCC -1.10581442786
AAGAGCCG -0.370523100084
AAGAGCCT -0.375984441514
AAGAGCGA 0.170624763636
AAGAGCGC 0.324942112484
AAGAGCGG -0.74702049217
AAGAGCGT 1.17577763818
AAGAGCTA 0.0360239523659
AAGAGCTC -1.20473207413
AAGAGCTG -0.660798997063
AAGAGCTT -0.0821678532559
AAGAGGAA -0.271302763009
AAGAGGAC -0.751588750016
AAGAGGAG -0.704316108056
AAGAGGAT 3.527347487
AAGAGGCA -1.40607495766
AAGAGGCC -0.779207307796
AAGAGGCG -0.555492160049
AAGAGGCT -1.20759785236
AAGAGGGA -0.919433088397
AAGAGGGC -1.00949671652
AAGAGGGG -0.222878064043
AAGAGGGT -1.02825730076
AAGAGGTA -0.218713463323
AAGAGGTC -0.474727842812
AAGAGGTG -0.922366732654
AAGAGTAA 0.295008199082
AAGAGTAC -1.03715274633
AAGAGTAG 0.0845206809162
AAGAGTAT 2.16317293005
AAGAGTCA 0.31152504093
AAGAGTCC -0.789596430463
AAGAGTCG 0.668086059478
AAGAGTCT -0.80747017646
AAGAGTGA 0.102682961609
AAGAGTGC -0.139731566049
AAGAGTGG -0.564489404664
AAGAGTGT -0.329925060889
AAGAGTTA 0.153640354019
AAGAGTTC -0.348082969844
AAGAGTTG -0.393859852935
AAGATAAA 0.626190446861
AAGATAAC 0.452933688879
AAGATAAG 1.01660308043
AAGATAAT 1.92853676486
AAGATACA 0.497546232463
AAGATACC 3.75555782212
AAGATACG 4.7613516767

AAGATACT 4.00352487317
AAGATAGA 0.111217842906
AAGATAGC 1.70846972824
AAGATAGG 1.05810586118
AAGATAGT 0.0818859802554
AAGATATA 4.29054091098
AAGATATC 17.6906728136
AAGATATG 4.61670065653
AAGATATT 8.81897555635
AAGATCAA 3.12167332487
AAGATCAC 1.67451839596
AAGATCAG 1.42096821936
AAGATCAT 2.48940137434
AAGATCCA 4.2393703443
AAGATCCC 3.43610182425
AAGATCCG 4.06656720616
AAGATCCT 4.26540591663
AAGATCGA 2.25752086123
AAGATCGC 6.6254269592
AAGATCGG 1.31877176971
AAGATCGT 3.59904648542
AAGATCTA 5.30288800055
AAGATCTC 7.98511694586
AAGATCTG 5.27548365792
AAGATCTT 7.62246130843
AAGATGAA 0.381203880118
AAGATGAC 0.244328932468
AAGATGAG -0.367964176213
AAGATGAT 1.48870142703
AAGATGCA -0.633807887749
AAGATGCC -0.0199238828571
AAGATGCG 0.192833191772
AAGATGCT -0.446037168375
AAGATGGA -0.715777139201
AAGATGGC -0.314367503226
AAGATGGG -0.603305648431
AAGATGGT 0.5456297277
AAGATGTA 0.812583860643
AAGATGTC 1.18942412188
AAGATGTG -0.505304609851
AAGATTAA 0.580446872249
AAGATTAC 11.527924355
AAGATTAG 1.34861699929
AAGATTAT 3.15573061975
AAGATTCA 3.03654663782
AAGATTCC 15.1290263899
AAGATTCG 8.23012766125
AAGATTCT 9.82264453817
AAGATTGA 1.28346186786
AAGATTGC 11.4438044168
AAGATTGG 2.73143014818
AAGATTGT 3.33871241261
AAGATTTA 6.21147085203
AAGATTTC 15.3491583781

AAGATTTG 6.48141233915
AAGCAAAA 0.692474111186
AAGCAAAC -0.128384408265
AAGCAAAG 0.02643902128
AAGCAAAT 1.19001347377
AAGCAACA -0.678879880161
AAGCAACC -0.759115008944
AAGCAACG -0.0180432028817
AAGCAACT -0.354347670143
AAGCAAGA 1.54876743929
AAGCAAGC -0.688410893147
AAGCAAGG -0.160601201522
AAGCAAGT 1.5249391782
AAGCAATA 1.04001769185
AAGCAATC 5.19237879553
AAGCAATG 0.258749213763
AAGCAATT 1.25815804117
AAGCACAA 0.556050077066
AAGCACAC -1.54045218577
AAGCACAG -0.599303010188
AAGCACAT -0.00396995429843
AAGCACCA -0.669432138371
AAGCACCC -0.802955836713
AAGCACCG 0.0326693722104
AAGCACCT 0.212830353282
AAGCACGA 0.333410793179
AAGCACGC -0.636449458284
AAGCACGG -0.582815313259
AAGCACGT 0.270449649599
AAGCACTA 0.608138708682
AAGCACTC -0.817277025284
AAGCACTG 0.0867336129661
AAGCACTT -0.302637922471
AAGCAGAA 0.632542165561
AAGCAGAC -0.289929072444
AAGCAGAG -0.315701507796
AAGCAGAT 1.75256182685
AAGCAGCA -0.0759447885552
AAGCAGCC -0.64617532587
AAGCAGCG -0.168252159066
AAGCAGCT -0.918410934459
AAGCAGGA -0.437197514499
AAGCAGGC -0.934193740708
AAGCAGGG -0.738977535467
AAGCAGGT 1.53204012949
AAGCAGTA 0.500961184236
AAGCAGTC 0.360096089023
AAGCAGTG -0.577833405743
AAGCATAA -0.352322930997
AAGCATAC 0.472091060371
AAGCATAG 0.0973359098966
AAGCATAT 1.98971819479
AAGCATCA 0.83468028902
AAGCATCC 0.134886431474

AAGCATCG -0.0997557708701
AAGCATCT 0.196606626047
AAGCATGA 0.244335177808
AAGCATGC -0.417164546374
AAGCATGG -0.227826454902
AAGCATGT -0.893580088163
AAGCATTA 0.282123438652
AAGCATTC -0.485244786766
AAGCATTG -0.897469477579
AAGCCAAA -0.457612697416
AAGCCAAC -0.868236291297
AAGCCAAG -0.532000939129
AAGCCAAT -0.223297542696
AAGCCACA -0.292790062582
AAGCCACC -1.46933296306
AAGCCACG -0.895185348656
AAGCCACT -0.948081710879
AAGCCAGA -0.250454986225
AAGCCAGC -0.830996995816
AAGCCAGG -0.442747956109
AAGCCAGT 0.64582183964
AAGCCATA -0.488095784359
AAGCCATC -0.43830023332
AAGCCATG -0.376532365988
AAGCCATT 0.0965579487423
AAGCCCAA -0.287161554225
AAGCCCAC -1.11730044036
AAGCCCAG -0.735983311415
AAGCCCAT -0.744475516223
AAGCCCCA -0.608761160877
AAGCCCCC -1.3502740961
AAGCCCCG -1.51677422919
AAGCCCCT -1.12112841727
AAGCCCGA -0.932330131328
AAGCCCGC -1.47106042403
AAGCCCGG -1.25221039595
AAGCCCGT -0.310747704309
AAGCCCTA -0.490966767041
AAGCCCTC -0.879363404944
AAGCCCTG -0.613817387935
AAGCCCTT -1.67114882699
AAGCCGAA 0.0324145623488
AAGCCGAC -1.03082996438
AAGCCGAG -0.873128890453
AAGCCGAT 1.1584788798
AAGCCGCA -0.0185388746795
AAGCCGCC -1.47983387731
AAGCCGCG -0.772917209782
AAGCCGCT -1.26997026858
AAGCCGGA -0.891384434886
AAGCCGGC -1.25106395975
AAGCCGGG -1.2598111826
AAGCCGGT -0.200092774693
AAGCCGTA -0.745660465351

AAGCCGTC -1.14264111456
AAGCCGTG -0.530133166189
AAGCCTAA -0.866792993281
AAGCCTAC -0.363767724259
AAGCCTAG -0.5960906156
AAGCCTAT -0.348646924023
AAGCCTCA -0.599284482346
AAGCCTCC -1.39454668503
AAGCCTCG -0.854358938203
AAGCCTCT -1.89064379049
AAGCCTGA -0.424812173071
AAGCCTGC -1.11959893356
AAGCCTGG -0.508984155851
AAGCCTGT -0.00126988574832
AAGCCTTA -0.8124225227
AAGCCTTC -1.48887733744
AAGCCTTG -0.746204850799
AAGCGAAA 0.017666192539
AAGCGAAC -0.958095280449
AAGCGAAG -0.471844577629
AAGCGAAT 3.09175460846
AAGCGACA -0.0430012459299
AAGCGACC -0.457155538547
AAGCGACG -0.462182620686
AAGCGACT -0.950595251949
AAGCGAGA 0.0222304950033
AAGCGAGC -0.200315525144
AAGCGAGG -0.803962793658
AAGCGAGT 0.598711576514
AAGCGATA 1.99223111133
AAGCGATC 4.48194142181
AAGCGATG 0.604091312171
AAGCGATT 2.74634693399
AAGCGCAA 1.2221605274
AAGCGCAC -0.178535527314
AAGCGCAG -0.10124320262
AAGCGCAT 0.548675371716
AAGCGCCA -0.252877137156
AAGCGCCC -1.27332276696
AAGCGCCG -0.557502743091
AAGCGCCT -0.655206919854
AAGCGCGA 0.950596084661
AAGCGCGC 0.257599863072
AAGCGCGG 0.784628262695
AAGCGCGT 0.657050127791
AAGCGCTA -0.43879049249
AAGCGCTC -0.626280379754
AAGCGCTG -0.968933651222
AAGCGCTT 0.967779928793
AAGCGGAA -0.501185600111
AAGCGGAC -0.0227590589238
AAGCGGAG -0.802288001717
AAGCGGAT 2.65979485223
AAGCGGCA -0.986862772565

AAGCGGCC -1.1590671908
AAGCGGCG -0.763058316459
AAGCGGCT -1.48748774934
AAGCGGGA -0.362163296479
AAGCGGGC -0.687142672823
AAGCGGGG -0.262184775158
AAGCGGGT -0.0614299944519
AAGCGGTA 0.129235231716
AAGCGGTC -0.808158204722
AAGCGGTG -1.45209686625
AAGCGTAA 0.275349326808
AAGCGTAC -0.73203792212
AAGCGTAG 0.137581711931
AAGCGTAT 0.603499670319
AAGCGTCA 0.346219984998
AAGCGTCC -1.41701596019
AAGCGTCG 0.246105107092
AAGCGTCT 0.197779917207
AAGCGTGA -0.462312523753
AAGCGTGC -0.568588012962
AAGCGTGG -0.63355703327
AAGCGTGT -0.320806032148
AAGCGTTA 0.383660796774
AAGCGTTC -0.024251486945
AAGCGTTG -0.676935289542
AAGCTAAA -0.215801053221
AAGCTAAC -0.610991579878
AAGCTAAG -0.65727933176
AAGCTAAT -0.0838357753241
AAGCTACA -0.215826867292
AAGCTACC -0.608948104713
AAGCTACG -0.724129032043
AAGCTACT -0.995257966429
AAGCTAGA 0.695096321167
AAGCTAGC -0.832212338931
AAGCTAGG -0.0134441346929
AAGCTAGT 0.617982196833
AAGCTATA 1.56288627885
AAGCTATC 0.493662880209
AAGCTATG 0.775450944117
AAGCTATT 0.501392529034
AAGCTCAA 1.20461944984
AAGCTCAC -0.530794964024
AAGCTCAG 0.135548853843
AAGCTCAT -0.0698172775526
AAGCTCCA -0.188356115886
AAGCTCCC -1.26785351476
AAGCTCCG -0.274150429755
AAGCTCCT -1.15991614065
AAGCTCGA -0.415235985461
AAGCTCGC -1.20523732212
AAGCTCGG 0.121244111334
AAGCTCGT -0.340145767561
AAGCTCTA -0.55176369222

AAGCTCTC -0.522744721091
AAGCTCTG -0.97474972795
AAGCTGAA -0.940434500538
AAGCTGAC -0.653085586121
AAGCTGAG -0.701737199097
AAGCTGAT 0.947928700055
AAGCTGCA -0.29928084418
AAGCTGCC -1.27105258596
AAGCTGCG -0.897224243905
AAGCTGCT -0.985564158254
AAGCTGGA -0.417807400012
AAGCTGGC -1.1970280312
AAGCTGGG 0.753334947009
AAGCTGGT -0.832777750356
AAGCTGTA -0.0962608787484
AAGCTGTC 0.215365128507
AAGCTGTG -1.57959110542
AAGCTTAA 1.08493417531
AAGCTTAC -0.0181497900133
AAGCTTAG -0.106185764494
AAGCTTAT 0.217769584309
AAGCTTCA 0.0238642758808
AAGCTTCC -0.0304004402808
AAGCTTCG 1.09018234247
AAGCTTCT 0.438387668077
AAGCTTGA 0.574126588426
AAGCTTGC -0.859514049808
AAGCTTGG 0.43864122887
AAGCTTGT 0.49752166746
AAGCTTTA -0.0704923987791
AAGCTTTC 1.5778442839
AAGCTTTG -0.767040553259
AAGGAAAA 0.739144286038
AAGGAAAC -0.695900720926
AAGGAAAG -0.727362036252
AAGGAAAT 3.29267051893
AAGGAACA -0.368571431414
AAGGAACC -1.03117553984
AAGGAACG -0.582518451443
AAGGAACT -0.792892096247
AAGGAAGA 0.534877958972
AAGGAAGC -0.386892760092
AAGGAAGG -0.897176987501
AAGGAAGT 0.311229428182
AAGGAATA 1.37130069778
AAGGAATC 9.0287839979
AAGGAATG 0.474184706432
AAGGAATT 2.46744213526
AAGGACAA 1.21396580895
AAGGACAC -1.1215376952
AAGGACAG -0.35064355914
AAGGACAT -0.50164796343
AAGGACCA -0.855291783783
AAGGACCC -1.25727099474

AAGGACCG -1.14328896447
AAGGACCT -0.724010995122
AAGGACGA -0.522779278638
AAGGACGC -1.49607280154
AAGGACGG -0.93099904125
AAGGACGT -1.03324066552
AAGGACTA -0.0665511730435
AAGGACTC -1.0810608156
AAGGACTG -0.38517237717
AAGGACTT 0.386502842714
AAGGAGAA 0.453186833316
AAGGAGAC -0.952935172573
AAGGAGAG -0.795382321382
AAGGAGAT 1.56845212563
AAGGAGCA -1.44226690967
AAGGAGCC -1.44796619854
AAGGAGCG 0.112652189267
AAGGAGCT -0.654071308911
AAGGAGGA -0.408138781374
AAGGAGGC -1.02416160695
AAGGAGGG -0.599105449274
AAGGAGGT -1.01467826672
AAGGAGTA -0.40429581565
AAGGAGTC -0.504940506544
AAGGAGTG -0.925444019724
AAGGATAA 1.67880228267
AAGGATAC 0.930092426097
AAGGATAG 0.556557823187
AAGGATAT 5.48392999088
AAGGATCA 0.859261738082
AAGGATCC 1.48687195885
AAGGATCG 2.20692070225
AAGGATCT 4.47193805296
AAGGATGA 0.277841425544
AAGGATGC -0.337384286503
AAGGATGG -0.596432443862
AAGGATGT 0.438522567415
AAGGATTA 2.06215809868
AAGGATTC 3.68625432785
AAGGATTG 1.81619496898
AAGGCAAA 0.841669240552
AAGGCAAC -0.300000515497
AAGGCAAG -1.33633408143
AAGGCAAT 0.19603830013
AAGGCACA -0.740838438533
AAGGCACC -1.13658771492
AAGGCACG -1.31504038739
AAGGCACT -1.53034472793
AAGGCAGA -0.403385245115
AAGGCAGC 0.453650445703
AAGGCAGG -0.304439911166
AAGGCAGT 0.543851679474
AAGGCATA 0.285203848393
AAGGCATC -0.453996645703

AAGGCATG -0.0876167040062
AAGGCATT -0.62087337278
AAGGCCAA 0.69660103169
AAGGCCAC -1.16208618803
AAGGCCAG -1.2689797577
AAGGCCAT 0.280150744004
AAGGCCCA -1.12988063639
AAGGCCCC -1.37334604655
AAGGCCCG -1.13810866333
AAGGCCCT -0.874878210116
AAGGCCGA -0.64121985696
AAGGCCGC -1.13069835954
AAGGCCGG -0.873431581253
AAGGCCGT -0.636924728638
AAGGCCTA -0.738108808708
AAGGCCTC -1.27039141266
AAGGCCTG -0.417737452207
AAGGCCTT 0.842709922331
AAGGCGAA -0.371300436705
AAGGCGAC -0.653248797666
AAGGCGAG -1.42943669187
AAGGCGAT 1.79056138835
AAGGCGCA -0.976104966675
AAGGCGCC -1.08747686129
AAGGCGCG 0.54774731423
AAGGCGCT -0.917700214795
AAGGCGGA -0.431486775835
AAGGCGGC -0.316659959269
AAGGCGGG 0.268351007267
AAGGCGGT -1.19689563
AAGGCGTA -0.39426038739
AAGGCGTC -0.933846083462
AAGGCGTG -0.163284824011
AAGGCTAA -0.257478703481
AAGGCTAC -0.964884589287
AAGGCTAG -0.972304052905
AAGGCTAT -0.0838969796536
AAGGCTCA -0.943650850507
AAGGCTCC -1.91168017653
AAGGCTCG -0.876433924247
AAGGCTCT -0.366670974529
AAGGCTGA -0.560514245916
AAGGCTGC -0.652870330078
AAGGCTGG -0.689088720687
AAGGCTGT -0.439254729411
AAGGCTTA -0.89767765557
AAGGCTTC -0.945494058444
AAGGCTTG -1.10358046983
AAGGGAAA 1.0717956459
AAGGGAAC -0.179774186364
AAGGGAAG -0.5364422084
AAGGGAAT 1.51222491554
AAGGGACA -0.906106366091
AAGGGACC -0.927040120385

AAGGGACG -0.404791903804
AAGGGACT -1.02390304989
AAGGGAGA -0.866639566102
AAGGGAGC -0.646247979989
AAGGGAGG -1.42547423198
AAGGGAGT -0.893916295619
AAGGGATA 1.18145028027
AAGGGATC 1.04705369161
AAGGGATG 0.263715716107
AAGGGATT 3.62008599413
AAGGGCAA -0.112909913621
AAGGGCAC -0.869403337117
AAGGGCAG -0.675229062724
AAGGGCAT -0.742198465352
AAGGGCCA -0.936352754658
AAGGGCCC -1.07629603772
AAGGGCCG -1.38009309525
AAGGGCCT -1.43629053589
AAGGGCGA -1.23791418874
AAGGGCGC -0.488737180751
AAGGGCGG -1.31393329683
AAGGGCGT -0.998792204191
AAGGGCTA -0.391313003386
AAGGGCTC -1.10734578516
AAGGGCTG -1.36328397151
AAGGGGAA 0.713239865841
AAGGGGAC -0.841995039109
AAGGGGAG -0.814680421196
AAGGGGAT 2.12070295435
AAGGGGCA -1.19053204515
AAGGGGCC -1.65835420763
AAGGGGCG -1.21935949253
AAGGGGCT -1.23920614135
AAGGGGGA -0.522025049775
AAGGGGGC -0.982910721574
AAGGGGGG -1.74267878488
AAGGGGGT -1.14147802412
AAGGGGTA -0.170575217274
AAGGGGTC -1.20992382506
AAGGGGTG -1.22099660425
AAGGGTAA -0.40144127903
AAGGGTAC -0.442099689843
AAGGGTAG -0.99911154923
AAGGGTAT 0.344690709472
AAGGGTCA -0.346521010373
AAGGGTCC -1.00614005458
AAGGGTCG -0.45324553951
AAGGGTCT -0.879138156357
AAGGGTGA -0.275625995359
AAGGGTGC -0.145539315657
AAGGGTGG -0.498642914122
AAGGGTGT -0.274942338834
AAGGGTTA 0.0305097337263
AAGGGTTC -0.850864462438

AAGGGTTG -0.62926065588
AAGGTAAA 0.906729234642
AAGGTAAC -0.161103951371
AAGGTAAG 0.403157498392
AAGGTAAT 1.28264372835
AAGGTACA -0.334830567081
AAGGTACC -0.707890107814
AAGGTACG -0.895530924122
AAGGTACT 0.524369133959
AAGGTAGA 0.0368876828527
AAGGTAGC -0.461073864703
AAGGTAGG -0.391718325936
AAGGTAGT 0.346481456555
AAGGTATA 0.50719840504
AAGGTATC 1.74175509913
AAGGTATG 0.806971173814
AAGGTATT 0.626756066464
AAGGTCAA 0.304473219645
AAGGTCAC -0.406735661711
AAGGTCAG 0.00632736187449
AAGGTCAT -0.700382793084
AAGGTCCA -0.487313451467
AAGGTCCC -1.4293201122
AAGGTCCG -0.091870821263
AAGGTCCT -0.536657880799
AAGGTCGA -0.722598091094
AAGGTCGC -0.421050188586
AAGGTCGG -0.304203837324
AAGGTCGT -0.278978077378
AAGGTCTA -0.715299162533
AAGGTCTC -0.597993778799
AAGGTCTG -0.893129382811
AAGGTGAA -0.260493120798
AAGGTGAC 1.19499288315
AAGGTGAG 0.384989805072
AAGGTGAT 0.324593414348
AAGGTGCA -0.729589332583
AAGGTGCC -1.29091692991
AAGGTGCG -1.12775805359
AAGGTGCT -0.701213215092
AAGGTGGA -0.694876068852
AAGGTGGC -0.701572530305
AAGGTGGG -0.154774715895
AAGGTGGT -0.445365794353
AAGGTGTA -0.362148099485
AAGGTGTC 0.0893235553587
AAGGTGTG -0.757797034079
AAGGTTAA -0.288970620971
AAGGTTAC 0.151325206575
AAGGTTAG -0.269608818691
AAGGTTAT 0.0262395867641
AAGGTTCA -0.478385530123
AAGGTTCC -0.105852679708
AAGGTTCG 0.307039221568

AAGGTTCT -0.0603853572904
AAGGTTGA -0.0913770230671
AAGGTTGC -0.638083863695
AAGGTTGG -0.936824902343
AAGGTTGT -0.488384735412
AAGGTTTA -0.153415521788
AAGGTTTC -0.0490271660727
AAGGTTTG -0.383901242354
AAGTAAAA 3.69726236368
AAGTAAAC -0.142627738267
AAGTAAAG 0.122306651802
AAGTAAAT 0.870359290454
AAGTAACA -0.358296390286
AAGTAACC 0.466709242934
AAGTAACG 1.554396364
AAGTAACT -0.531267111709
AAGTAAGA 0.319698108877
AAGTAAGC 1.02562926179
AAGTAAGG 0.0711918768306
AAGTAAGT 0.447670116541
AAGTAATA 1.73294396564
AAGTAATC 3.89712614259
AAGTAATG -0.33569096672
AAGTAATT 3.24731103271
AAGTACAA 1.52273457327
AAGTACAC -0.42749475467
AAGTACAG -0.618675845901
AAGTACAT -0.288339425301
AAGTACCA -1.16006019982
AAGTACCC 0.0526619538048
AAGTACCG 0.163519648785
AAGTACCT -0.550357033532
AAGTACGA 0.625257601281
AAGTACGC 0.421701161166
AAGTACGG -0.852344399779
AAGTACGT 0.111280920837
AAGTACTA 0.506352994216
AAGTACTC -0.13709873899
AAGTACTG 0.179928238077
AAGTACTT 2.55217078599
AAGTAGAA 0.843890707899
AAGTAGAC -0.693779595371
AAGTAGAG -0.224105481481
AAGTAGAT 1.68432815928
AAGTAGCA -0.134837717824
AAGTAGCC -0.528352619827
AAGTAGCG -0.233864033012
AAGTAGCT -0.994722324457
AAGTAGGA -0.289016003774
AAGTAGGC 0.877314933507
AAGTAGGG -0.116463511936
AAGTAGGT -0.574912252166
AAGTAGTA 0.683156480641
AAGTAGTC 0.306725289157

AAGTAGTG -0.306706969494
AAGTATAA 2.74909529983
AAGTATAC -0.160526257445
AAGTATAG 0.55428139685
AAGTATAT 2.03139022425
AAGTATCA 1.67783550408
AAGTATCC 1.90698784461
AAGTATCG 2.76701172231
AAGTATCT 3.3629360037
AAGTATGA 0.549989183021
AAGTATGC 0.195792025566
AAGTATGG 0.0867336129661
AAGTATGT 0.784237720783
AAGTATTA 1.29041834362
AAGTATTC 0.188204562308
AAGTATTG 0.731072392595
AAGTCAAA 0.443529456289
AAGTCAAC 0.0112422360765
AAGTCAAG -0.134192782407
AAGTCAAT -0.43162417331
AAGTCACA -0.239684065121
AAGTCACC -0.97336284617
AAGTCACG -0.243007002222
AAGTCACT -0.686902019064
AAGTCAGA 0.0984111492229
AAGTCAGC -0.120779458057
AAGTCAGG -0.705581413888
AAGTCAGT 1.01967578759
AAGTCATA 2.03791181619
AAGTCATC -0.479370212023
AAGTCATG -0.952970146476
AAGTCATT 1.40861202285
AAGTCCAA 0.200280967597
AAGTCCAC -0.512536713277
AAGTCCAG -0.447816673847
AAGTCCAT -0.355382314761
AAGTCCCA -0.490544165718
AAGTCCCC -0.893102944207
AAGTCCCG 0.151073519384
AAGTCCCT -0.656571318411
AAGTCCGA 0.103085994201
AAGTCCGC -0.0339267672792
AAGTCCGG -0.709975634933
AAGTCCGT -0.647363397668
AAGTCCTA -0.254255067282
AAGTCCTC -1.30395595023
AAGTCCTG -0.453058595673
AAGTCGAA 0.606989982525
AAGTCGAC -1.13675425731
AAGTCGAG -1.30944268938
AAGTCGAT 0.453232216118
AAGTCGCA -0.770606850432
AAGTCGCC -1.48718485037
AAGTCGCG -0.328468023126

AAGTCGCT -1.23619089132
AAGTCGGA -0.0011941089594
AAGTCGGC -0.461973193627
AAGTCGGG -0.503803646532
AAGTCGGT -0.56591292577
AAGTCGTA 0.0757345287838
AAGTCGTC -0.911038102711
AAGTCGTG -0.55973128849
AAGTCTAA 1.24416993738
AAGTCTAC 0.20433336038
AAGTCTAG -0.334044070629
AAGTCTAT -0.297931018083
AAGTCTCA -0.218256928987
AAGTCTCC -0.801374933046
AAGTCTCG 0.180405382034
AAGTCTCT -0.879627166459
AAGTCTGA -0.68253361209
AAGTCTGC -0.501827204681
AAGTCTGG -0.494846996625
AAGTCTGT 0.642881533688
AAGTCTTA 1.04430574212
AAGTCTTC -0.417348575719
AAGTCTTG 0.496138116528
AAGTGAAA 0.732605415324
AAGTGAAC -0.400785102001
AAGTGAAG -0.275568121877
AAGTGAAT 1.29642802588
AAGTGACA 1.05354572228
AAGTGACC 0.897094340838
AAGTGACG 0.328680781033
AAGTGACT 0.00584855249398
AAGTGAGA 0.692003628925
AAGTGAGC 0.733279703839
AAGTGAGG 0.0159056312647
AAGTGAGT -0.119092799969
AAGTGATA 2.78413873386
AAGTGATC 0.969529040278
AAGTGATG 2.03586272022
AAGTGATT 3.65398299196
AAGTGCAA 1.97536140779
AAGTGCAC -0.238344231568
AAGTGCAG -0.607690709644
AAGTGCAT 0.681591814857
AAGTGCCA -0.636977189492
AAGTGCCC -0.498488446053
AAGTGCCG -0.493413899331
AAGTGCCT -0.776427923431
AAGTGCGA -0.0436299434643
AAGTGCGC 0.571064498349
AAGTGCGG -1.03076418013
AAGTGCGT 0.528717555846
AAGTGCTA -0.473078656763
AAGTGCTC -0.7270087582
AAGTGCTG -0.31180691393

AAGTGGAA 0.594471199004
AAGTGGAC 0.331789502981
AAGTGGAG -0.43747001949
AAGTGGAT 0.450225917743
AAGTGGCA -0.842631647407
AAGTGGCC -1.80504620482
AAGTGGCG -0.585050520354
AAGTGGCT -1.00259894694
AAGTGGGA -0.0502845611415
AAGTGGGC -0.458803059172
AAGTGGGG -0.841704422632
AAGTGGGT -0.88987389538
AAGTGGTA 0.24390383301
AAGTGGTC -0.796326200395
AAGTGGTG -0.134236916141
AAGTGTAA 0.99784291255
AAGTGTAC -0.868785048482
AAGTGTAG 0.177390964717
AAGTGTAT 0.429180580045
AAGTGTCA 0.403191015049
AAGTGTCC -0.532000939129
AAGTGTCG 0.502365969323
AAGTGTCT 0.215341396216
AAGTGTGA 0.509802919892
AAGTGTGC -0.408971909696
AAGTGTGG 0.206092672586
AAGTGTGT -0.846386137484
AAGTGTTA 1.02582952902
AAGTGTTC 0.013046306551
AAGTGTTG 0.527106258192
AAGTTAAA 2.88823314225
AAGTTAAC 0.668686444806
AAGTTAAG -0.513467060721
AAGTTAAT 0.983927046528
AAGTTACA -0.289926990665
AAGTTACC -0.170614771092
AAGTTACG 0.678423762182
AAGTTACT 0.013783464819
AAGTTAGA 0.755645306359
AAGTTAGC 0.0426494251243
AAGTTAGG -0.0613806562679
AAGTTAGT 0.557417390114
AAGTTATA 1.30882002901
AAGTTATC -0.219822635662
AAGTTATG 1.23734669553
AAGTTATT 0.909960989782
AAGTTCAA 1.51692724002
AAGTTCAC -0.465107521467
AAGTTCAG -0.267445016648
AAGTTCAT -0.290933323076
AAGTTCCA 0.144268389019
AAGTTCCC -0.663568388884
AAGTTCCG -0.178645028938
AAGTTCCT -0.226630472341

AAGTTCGA 0.886850734587
AAGTTCGC -0.652161275838
AAGTTCGG 0.0392488376327
AAGTTCGT 0.583982775435
AAGTTCTA 0.307048797756
AAGTTCTC -0.400382693943
AAGTTCTG 0.567027302559
AAGTTGAA 0.163349567366
AAGTTGAC -0.247036911782
AAGTTGAG 0.330861445495
AAGTTGAT 0.20280241943
AAGTTGCA -0.653062686542
AAGTTGCC -0.489520138178
AAGTTGCG 0.234883896993
AAGTTGCT -0.950103119177
AAGTTGGA 0.487065511479
AAGTTGGC -0.534496993247
AAGTTGGG 0.417238241383
AAGTTGGT -0.0688169823033
AAGTTGTA 0.509133835827
AAGTTGTC 0.275094933302
AAGTTGTG -0.481849195546
AAGTTTAA 0.364771142179
AAGTTTAC -0.395434511263
AAGTTTAG 0.00935551893925
AAGTTTAT 0.273530892052
AAGTTTCA 1.33336962683
AAGTTTCC 0.613521150653
AAGTTTCG 0.180405382034
AAGTTTCT -0.317839079413
AAGTTTGA 0.185507200072
AAGTTTGC -0.458234941433
AAGTTTGG -0.347496324264
AAGTTTGT -0.147313200323
AAGTTTTA 0.192946856955
AAGTTTTC -0.0151307927802
AAGTTTTG 0.453483070598
AATAAAAA 3.24013139014
AATAAAAC 0.503773668901
AATAAAAG 0.263829797647
AATAAAAT 2.92430018746
AATAAACA 0.392729030085
AATAAACC 0.085778492341
AATAAACG 0.984124399264
AATAAACT 1.145149243
AATAAAGA 1.80736926303
AATAAAGC 1.15421497817
AATAAAGG 2.04692633958
AATAAAGT 0.170138043492
AATAAATA 2.82537025868
AATAAATC 3.28113287828
AATAAATG -0.482506205287
AATAAATT 5.13096566349
AATAACAA 1.1268441522

| | |
|---|---|
| AATAACAC | 0.0740526587902 |
| AATAACAG | 0.416562703801 |
| AATAACAT | 0.538284167269 |
| AATAACCA | 0.105948025228 |
| AATAACCC | 0.381565693467 |
| AATAACCG | 1.46228426444 |
| AATAACCT | -0.387097815414 |
| AATAACGA | 1.57496310049 |
| AATAACGC | 0.0392353060632 |
| AATAACGG | 0.470833248946 |
| AATAACGT | 1.24855562313 |
| AATAACTA | 0.706881693623 |
| AATAACTC | 0.993099368835 |
| AATAACTG | 0.723616706006 |
| AATAAGAA | 1.04789223256 |
| AATAAGAC | -0.235627925134 |
| AATAAGAG | 0.8797431216 |
| AATAAGAT | 2.38651960275 |
| AATAAGCA | 1.21591164863 |
| AATAAGCC | 0.0724297031683 |
| AATAAGCG | 0.239988421345 |
| AATAAGCT | 0.995232776892 |
| AATAAGGA | 0.624386584565 |
| AATAAGGC | 1.47059181537 |
| AATAAGGG | -0.237155951592 |
| AATAAGGT | 0.498530081651 |
| AATAAGTA | 1.1830684478 |
| AATAAGTC | 0.799371428055 |
| AATAAGTG | 0.455090621049 |
| AATAATAA | 2.25402638547 |
| AATAATAC | 1.14629463831 |
| AATAATAG | 2.64384945897 |
| AATAATAT | 2.64658679138 |
| AATAATCA | 3.07385067665 |
| AATAATCC | 2.06269374066 |
| AATAATCG | 2.97261267848 |
| AATAATCT | 4.26221204989 |
| AATAATGA | 2.39051537112 |
| AATAATGC | 2.34137807889 |
| AATAATGG | 1.79031136658 |
| AATAATGT | 1.96494834465 |
| AATAATTA | 1.56829807392 |
| AATAATTC | 0.630503686668 |
| AATAATTG | 0.289290382366 |
| AATACAAA | 1.70382402818 |
| AATACAAC | -0.102590738759 |
| AATACAAG | -0.036586449299 |
| AATACAAT | 1.87425622718 |
| AATACACA | 0.0634447410539 |
| AATACACC | 0.735921066196 |
| AATACACG | 1.60650810337 |
| AATACACT | 0.731761669925 |
| AATACAGA | 1.66201480943 |
| AATACAGC | 1.40980842176 |

AATACAGG 1.84763047024
AATACAGT 0.763497780199
AATACATA 3.38645095691
AATACATC 0.506013247734
AATACATG 0.973859767036
AATACATT 1.92861087623
AATACCAA 0.509085122177
AATACCAC 1.61925317637
AATACCAG 0.178323602119
AATACCAT 0.544784733232
AATACCCA 1.88932644016
AATACCCC 0.172663242529
AATACCCG 0.473902208897
AATACCCT 0.127562521554
AATACCGA 0.903221019129
AATACCGC 1.48795469258
AATACCGG 0.847554224194
AATACCGT 0.331134991375
AATACCTA 1.7634457886
AATACCTC -0.0543325821868
AATACCTG 0.584308157636
AATACGAA 2.25618893844
AATACGAC -0.330115335573
AATACGAG 1.69979162049
AATACGAT 3.79153097905
AATACGCA 1.59062308173
AATACGCC 1.25524667195
AATACGCG 0.476855421885
AATACGCT 1.5675973468
AATACGGA 1.30117365138
AATACGGC 0.579608539477
AATACGGG 0.910876764767
AATACGGT 1.02131518927
AATACGTA 2.18149967135
AATACGTC 1.63108434798
AATACGTG 3.00470414859
AATACTAA 1.12200151576
AATACTAC 1.42724145495
AATACTAG 0.381459730869
AATACTAT 1.78727550693
AATACTCA 1.76722172101
AATACTCC 0.0769151061737
AATACTCG 1.84006236753
AATACTCT 0.470712922067
AATACTGA 2.84666041369
AATACTGC 1.05190465517
AATACTGG 0.817014304659
AATACTGT 3.04684686848
AATACTTA 0.929213706795
AATACTTC 0.721571149062
AATACTTG 1.35475825004
AATAGAAA 0.498977872511
AATAGAAC 0.566398605025
AATAGAAG -0.502466727471

AATAGAAT 3.35919379612
AATAGACA 0.961117816708
AATAGACC -0.120314804779
AATAGACG 2.40660045181
AATAGACT 0.158321444337
AATAGAGA 2.00492018445
AATAGAGC -0.174474182877
AATAGAGG 0.636417190695
AATAGAGT 0.350202429975
AATAGATA 3.16274954891
AATAGATC 3.21488314697
AATAGATG 2.84326898603
AATAGATT 6.1415596862
AATAGCAA 1.70098697851
AATAGCAC 0.588797307845
AATAGCAG 0.361596635986
AATAGCAT 0.556993747901
AATAGCCA -0.581716757997
AATAGCCC 0.064574314836
AATAGCCG 0.519565634983
AATAGCCT -0.228302557969
AATAGCGA 0.713645812924
AATAGCGC 0.746412820613
AATAGCGG -0.726988773113
AATAGCGT 0.356665940256
AATAGCTA -0.0176280959666
AATAGCTC -0.798492500575
AATAGCTG -0.620726399118
AATAGGAA 1.23335862975
AATAGGAC -0.93724188286
AATAGGAG -0.0993725151877
AATAGGAT 1.49168545037
AATAGGCA -0.574125963892
AATAGGCC -0.587737681868
AATAGGCG -0.976579196139
AATAGGCT -0.428102426227
AATAGGGA 0.704785341249
AATAGGGC -0.176254937417
AATAGGGG 0.153793781199
AATAGGGT -0.457886659653
AATAGGTA 0.193096328753
AATAGGTC -0.621632806093
AATAGGTG 0.182334567481
AATAGTAA 1.75185235625
AATAGTAC -0.36769396118
AATAGTAG -0.254817147859
AATAGTAT 0.958016380991
AATAGTCA -0.633676943793
AATAGTCC 0.486273810578
AATAGTCG 0.174697974218
AATAGTCT 0.700570153276
AATAGTGA 0.0955534899332
AATAGTGC 0.572942055654
AATAGTGG -0.119732530937

AATAGTGT −0.0752938159757
AATAGTTA 1.90573336403
AATAGTTC 0.541760323371
AATAGTTG 1.28445362781
AATATAAA 4.34239221968
AATATAAC 1.9139601419
AATATAAG 1.92188839253
AATATAAT 2.03265657097
AATATACA 2.05317542653
AATATACC 1.07556783111
AATATACG 3.12561205247
AATATACT 2.57689754329
AATATAGA 2.49197237254
AATATAGC 3.28615392326
AATATAGG 0.691079318642
AATATAGT 2.19345241891
AATATATA 2.8709425028
AATATATC 4.65331562622
AATATATG 3.422702656
AATATATT 6.95321777932
AATATCAA 3.72477162802
AATATCAC 4.84192093116
AATATCAG 3.15828829455
AATATCAT 2.84445393516
AATATCCA 5.11007312844
AATATCCC 4.92151965982
AATATCCG 10.3872428199
AATATCCT 6.92280339111
AATATCGA 3.21161308708
AATATCGC 5.50058797741
AATATCGG 7.19385155244
AATATCGT 2.98788253416
AATATCTA 4.98447726437
AATATCTC 6.58945047141
AATATCTG 7.08285979083
AATATGAA 4.99324447231
AATATGAC 0.375112800264
AATATGAG 1.27481165595
AATATGAT 3.01310121606
AATATGCA 1.37042739111
AATATGCC 0.583437557275
AATATGCG 1.62306304179
AATATGCT 0.820089718128
AATATGGA 1.67454421003
AATATGGC 0.765751931491
AATATGGG 3.19162341998
AATATGGT 1.40273619904
AATATGTA 1.28691928794
AATATGTC 1.55303071637
AATATGTG 1.36682216465
AATATTAA 3.14341980604
AATATTAC 2.30137168154
AATATTAG 2.32095685881
AATATTAT 1.22199231959

AATATTCA 0.938744927959
AATATTCC 4.22174911821
AATATTCG 2.83097295297
AATATTCT 3.78698686986
AATATTGA 1.46208025001
AATATTGC 2.90098779143
AATATTGG 1.93141420106
AATATTGT 1.7438808046
AATATTTA 2.86910429114
AATATTTC 4.78520179648
AATATTTG 3.1020181998
AATCAAAA 4.30042103845
AATCAAAC 2.78351524077
AATCAAAG 1.00314853684
AATCAAAT 2.72516086797
AATCAACA 3.56734618176
AATCAACC 0.825994895036
AATCAACG 2.4327234589
AATCAACT 3.30394793709
AATCAAGA 3.9832264781
AATCAAGC 1.21564705441
AATCAAGG 3.50093032441
AATCAAGT 2.7173918735
AATCAATA 3.67204243272
AATCAATC 2.39647009439
AATCAATG 1.27461513593
AATCAATT 4.0038858538
AATCACAA 3.2546919834
AATCACAC 2.51126839057
AATCACAG 3.21412662815
AATCACAT 1.89747993938
AATCACCA 3.01017048629
AATCACCC 0.804040027693
AATCACCG 2.25402638547
AATCACCT 1.8220574694
AATCACGA 4.86165412297
AATCACGC 2.263552194
AATCACGG 2.6881228806
AATCACGT 1.45335884124
AATCACTA 1.62369507017
AATCACTC 2.27092107037
AATCACTG 2.99025951047
AATCAGAA 4.06787144128
AATCAGAC 1.40484462573
AATCAGAG 1.72438618476
AATCAGAT 2.76405538666
AATCAGCA 2.72563030934
AATCAGCC 1.24488211429
AATCAGCG 3.55045795037
AATCAGCT 2.33127499278
AATCAGGA 4.94017740413
AATCAGGC 2.76710769237
AATCAGGG 3.88724830507
AATCAGGT 2.37311335645

AATCAGTA 2.22140780869
AATCAGTC 1.41617741924
AATCAGTG 2.95608417867
AATCATAA 3.55812577034
AATCATAC 2.73230699388
AATCATAG 4.52277990659
AATCATAT 2.11801371106
AATCATCA 2.04518243254
AATCATCC 1.41967439314
AATCATCG 2.46128048307
AATCATCT 2.59435160246
AATCATGA 3.8948530471
AATCATGC 0.602422973747
AATCATGG 0.587937324562
AATCATGT 5.1773978909
AATCATTA 1.95283987995
AATCATTC 1.85968730679
AATCATTG 2.79971211305
AATCCAAA 6.20284187428
AATCCAAC 4.22344264617
AATCCAAG 4.26109038687
AATCCAAT 5.41450013257
AATCCACA 6.18008781163
AATCCACC 5.43006476829
AATCCACG 5.08243229562
AATCCACT 2.81081778436
AATCCAGA 5.60843500228
AATCCAGC 4.6812424956
AATCCAGG 4.52342942192
AATCCAGT 6.59793101826
AATCCATA 5.29138741559
AATCCATC 6.94896595202
AATCCATG 6.93527866543
AATCCATT 6.92404871186
AATCCCAA 5.14710632771
AATCCCAC 2.48366919334
AATCCCAG 3.82299187802
AATCCCAT 2.43473903822
AATCCCCA 3.77358374623
AATCCCCC 1.29032882709
AATCCCCG 3.61631464164
AATCCCCT 2.55857392465
AATCCCGA 3.44681050013
AATCCCGC 2.88555888777
AATCCCGG 3.19282002707
AATCCCGT 5.98781774132
AATCCCTA 2.21428624777
AATCCCTC 3.89062516027
AATCCCTG 3.17547963309
AATCCGAA 8.4544121758
AATCCGAC 3.9635811374
AATCCGAG 3.94252143542
AATCCGAT 8.63981945044
AATCCGCA 7.47785712831

AATCCGCC 4.57728277837
AATCCGCG 7.86248054012
AATCCGCT 5.17053925879
AATCCGGA 8.77253916538
AATCCGGC 4.82001519382
AATCCGGG 2.99953300728
AATCCGGT 5.96183421349
AATCCGTA 5.28568583676
AATCCGTC 5.19120675344
AATCCGTG 2.11748389807
AATCCTAA 6.42816540519
AATCCTAC 4.11754375095
AATCCTAG 6.06665453854
AATCCTAT 3.56654407196
AATCCTCA 5.4325183541
AATCCTCC 3.50174700667
AATCCTCG 5.41470497972
AATCCTCT 3.90325282088
AATCCTGA 5.54466300542
AATCCTGC 4.20995437774
AATCCTGG 4.59516152064
AATCCTGT 4.98189835541
AATCCTTA 2.53173957337
AATCCTTC 2.15658243119
AATCCTTG 4.97108475782
AATCGAAA 5.50374811932
AATCGAAC 2.28368258943
AATCGAAG 3.31992039349
AATCGAAT 3.99699203961
AATCGACA 3.17465483189
AATCGACC 0.401134424671
AATCGACG 1.80079687566
AATCGACT 2.04549657313
AATCGAGA 3.70174152135
AATCGAGC 2.04974798408
AATCGAGG 2.09448522628
AATCGAGT 1.23806699138
AATCGATA 3.26513023608
AATCGATC 3.77619346553
AATCGATG 3.66745918606
AATCGATT 4.9076558383
AATCGCAA 3.15901983201
AATCGCAC 3.29657094178
AATCGCAG 1.25287302649
AATCGCAT 4.19051763139
AATCGCCA 2.47253375258
AATCGCCC 0.659384219432
AATCGCCG 2.58711491911
AATCGCCT 3.27090217907
AATCGCGA 5.01960938222
AATCGCGC 2.81617441226
AATCGCGG 3.80919467346
AATCGCGT 4.93477060534
AATCGCTA 2.54256024901

AATCGCTC 1.48062266372
AATCGCTG 1.40724491798
AATCGGAA 3.30283897293
AATCGGAC 1.20563244395
AATCGGAG -0.0942388459167
AATCGGAT 5.6482259354
AATCGGCA 1.62125897132
AATCGGCC 0.234607852976
AATCGGCG 1.3344094759
AATCGGCT 0.97454654623
AATCGGGA 3.26795104786
AATCGGGC 1.59539681125
AATCGGGG 2.372133879
AATCGGGT 2.73763052148
AATCGGTA 2.53415235629
AATCGGTC 1.80951183092
AATCGGTG 1.51652212564
AATCGTAA 4.93141935603
AATCGTAC 1.93591084568
AATCGTAG 3.47376018202
AATCGTAT 4.3230891236
AATCGTCA 2.27069727903
AATCGTCC 1.86656967119
AATCGTCG 4.42994397215
AATCGTCT 4.82794573441
AATCGTGA 1.98002938289
AATCGTGC 2.6178274182
AATCGTGG 1.55772721186
AATCGTGT 3.12209509348
AATCGTTA 3.6675174759
AATCGTTC 1.45394340504
AATCGTTG 2.87472030882
AATCTAAA 9.32651724689
AATCTAAC 4.27905219216
AATCTAAG 4.61641461997
AATCTAAT 6.31645751129
AATCTACA 4.85786465899
AATCTACC 5.62451800119
AATCTACG 5.11506024041
AATCTACT 6.37297512968
AATCTAGA 8.26929031319
AATCTAGC 5.92352738127
AATCTAGG 8.17160403974
AATCTAGT 5.63590658639
AATCTATA 6.81960851982
AATCTATC 4.30506673851
AATCTATG 4.62213680842
AATCTCAA 7.67798987318
AATCTCAC 4.9527392805
AATCTCAG 7.72982702578
AATCTCAT 5.95129228818
AATCTCCA 6.97615025051
AATCTCCC 5.54193462466
AATCTCCG 9.24976077235

AATCTCCT 5.36831667786
AATCTCGA 10.6596430973
AATCTCGC 4.97628212956
AATCTCGG 7.07872246143
AATCTCGT 10.6560822128
AATCTCTA 4.10100900579
AATCTCTC 5.51950094794
AATCTCTG 6.13373156919
AATCTGAA 13.7736492267
AATCTGAC 4.41561466464
AATCTGAG 8.8124691614
AATCTGAT 5.23180062907
AATCTGCA 4.58161267242
AATCTGCC 2.45968875414
AATCTGCG 6.77380415724
AATCTGCT 5.2618373824
AATCTGGA 10.198653961
AATCTGGC 7.1310933824
AATCTGGG 5.8945352651
AATCTGGT 9.5572371677
AATCTGTA 7.5316590648
AATCTGTC 6.82281508543
AATCTGTG 6.17059177255
AATCTTAA 5.89102684141
AATCTTAC 3.39700599744
AATCTTAG 4.3087539871
AATCTTAT 5.09391518545
AATCTTCA 7.98690436209
AATCTTCC 5.88656787701
AATCTTCG 8.0212433218
AATCTTCT 7.45849990595
AATCTTGA 5.74924388943
AATCTTGC 5.00803635132
AATCTTGG 5.40811656264
AATCTTGT 4.77928662702
AATCTTTA 4.72595121742
AATCTTTC 5.00041578776
AATCTTTG 5.36831667786
AATGAAAA 1.27923835277
AATGAAAC 0.610473424857
AATGAAAG -0.259908140642
AATGAAAT 2.27995578702
AATGAACA 0.399985490335
AATGAACC -0.591272544164
AATGAACG -0.376011921009
AATGAACT -0.57795289991
AATGAAGA 0.957424322783
AATGAAGC -0.579423677421
AATGAAGG 1.68779765368
AATGAAGT 0.444361543721
AATGAATA 0.403996039342
AATGAATC 2.7686444623
AATGAATG 0.0660382224724
AATGAATT 0.600971140434

AATGACAA 0.20443599213
AATGACAC -0.674400722495
AATGACAG -0.536420766067
AATGACAT -0.0261221743769
AATGACCA 0.0700456488093
AATGACCC -0.0331590068465
AATGACCG 1.14891310109
AATGACCT -0.505959537812
AATGACGA 0.213095363865
AATGACGC 0.0107813300032
AATGACGG -0.373701978015
AATGACGT 0.443529456289
AATGACTA 0.971144917848
AATGACTC 1.20589849542
AATGACTG 0.239974889776
AATGAGAA 0.696633923812
AATGAGAC -0.518638410208
AATGAGAG -0.397700736878
AATGAGAT 1.60420419754
AATGAGCA -0.00152261383003
AATGAGCC -0.844542305013
AATGAGCG -0.253688614967
AATGAGCT -0.933217802284
AATGAGGA 0.360436876395
AATGAGGC -0.738364451282
AATGAGGG 0.40607448841
AATGAGGT 0.318585189334
AATGAGTA 2.27995578702
AATGAGTC 1.48117475175
AATGAGTG -1.07322978409
AATGATAA 0.86456777873
AATGATAC 0.71925058899
AATGATAG 1.46444140479
AATGATAT 2.57594075724
AATGATCA 0.628214977829
AATGATCC 1.39675295538
AATGATCG 0.88531125834
AATGATCT 0.471650139385
AATGATGA 0.469511735056
AATGATGC -0.146032697497
AATGATGG 1.2356604538
AATGATGT 0.610759877773
AATGATTA 2.12885645357
AATGATTC 2.53222504444
AATGATTG 0.755253515379
AATGCAAA 0.564675723967
AATGCAAC 0.316715334614
AATGCAAG 1.46298249343
AATGCAAT 2.94086262028
AATGCACA 0.428120537712
AATGCACC -0.892944520755
AATGCACG -0.96898153216
AATGCACT 0.368345141937
AATGCAGA 0.918112615397

AATGCAGC 0.427778917628
AATGCAGG 2.76294059351
AATGCAGT 0.316602502143
AATGCATA 1.79482362455
AATGCATC 0.936255535536
AATGCATG −0.470230781839
AATGCATT 0.549989183021
AATGCCAA 0.0862764540967
AATGCCAC −0.426321255332
AATGCCAG −0.59328583352
AATGCCAT −0.4821400202
AATGCCCA 0.792192826373
AATGCCCC −0.459942625497
AATGCCCG −0.594000716743
AATGCCCT −0.285012324641
AATGCCGA 0.181907802599
AATGCCGC −0.680365646486
AATGCCGG −0.246834562774
AATGCCGT 0.835488435983
AATGCCTA 0.0519306245205
AATGCCTC −0.193400684977
AATGCCTG −0.810655091552
AATGCGAA 0.244212769149
AATGCGAC −0.896425673129
AATGCGAG −0.513024682489
AATGCGAT 0.880365573795
AATGCGCA 0.567188015969
AATGCGCC −0.851045160934
AATGCGCG −0.376220099001
AATGCGCT −0.112584323242
AATGCGGA −0.0218243397418
AATGCGGC −0.877367394361
AATGCGGG 0.00823010871705
AATGCGGT −0.226321952557
AATGCGTA 1.31064699906
AATGCGTC 0.706654779613
AATGCGTG −0.012530649666
AATGCTAA 2.20934909853
AATGCTAC −0.41406789875
AATGCTAG 0.597487073567
AATGCTAT 0.368516056068
AATGCTCA −0.0857018828401
AATGCTCC −0.269556357838
AATGCTCG 0.180926243369
AATGCTCT −0.350866101412
AATGCTGA 0.303568686271
AATGCTGC −0.551485774602
AATGCTGG −0.73346310865
AATGCTGT 0.601381251077
AATGCTTA 0.488144081654
AATGCTTC −0.229739402466
AATGCTTG 0.084648710381
AATGGAAA 0.345750127271
AATGGAAC −0.081599943695

AATGGAAG -1.13978699429
AATGGAAT 0.837302290823
AATGGACA 0.304715122471
AATGGACC -0.907713916542
AATGGACG -0.604294910246
AATGGACT -0.383964320285
AATGGAGA 0.450477604934
AATGGAGC 0.169787679932
AATGGAGG -0.184520228214
AATGGAGT -0.0895317333502
AATGGATA 4.81754016568
AATGGATC 2.22725990021
AATGGATG 0.880769439098
AATGGCAA 0.574072462148
AATGGCAC -0.481800898252
AATGGCAG -0.319979565521
AATGGCAT 0.276112715503
AATGGCCA 0.0694009215695
AATGGCCC -1.06710331398
AATGGCCG -0.495828972211
AATGGCCT -1.02457421573
AATGGCGA 0.598632468877
AATGGCGC -0.229683194408
AATGGCGG -0.253935514065
AATGGCGT 0.00399118845357
AATGGCTA -0.0663534039516
AATGGCTC 0.0709591338361
AATGGCTG -1.09408318168
AATGGGAA 0.323113477006
AATGGGAC -0.756914359395
AATGGGAG -1.0712404352
AATGGGAT 1.47797234971
AATGGGCA -0.358381951441
AATGGGCC -1.09314721343
AATGGGCG -1.4040664564
AATGGGCT -0.914071672403
AATGGGGA -0.0958438982313
AATGGGGC -0.314683517417
AATGGGGG -0.528509169677
AATGGGGT 0.61279794031
AATGGGTA 0.0900007583652
AATGGGTC -0.611269081141
AATGGGTG 0.480315131926
AATGGTAA 1.23348332836
AATGGTAC 0.266397048638
AATGGTAG 0.0957631251706
AATGGTAT 1.32109919966
AATGGTCA 0.448508241135
AATGGTCC 0.310682752776
AATGGTCG 0.0802292997988
AATGGTCT 0.0167406331887
AATGGTGA -0.0221742869456
AATGGTGC -0.0824253694315
AATGGTGG 0.0626172335376

AATGGTGT 0.828023797741
AATGGTTA -0.11209010869
AATGGTTC 0.194448652986
AATGGTTG -0.15293442245
AATGTAAA 1.95835076774
AATGTAAC 0.218365181543
AATGTAAG -0.000209427059477
AATGTAAT 2.75770679063
AATGTACA 1.16030293536
AATGTACC -0.351887630817
AATGTACG 0.332799790774
AATGTACT 0.801382427453
AATGTAGA 2.92324763953
AATGTAGC -0.420730219013
AATGTAGG 0.395089768509
AATGTAGT 0.816754290348
AATGTATA 1.82088105557
AATGTATC 1.79623049142
AATGTATG 1.22352617503
AATGTCAA 0.665865216665
AATGTCAC 0.301516259453
AATGTCAG 0.0404789613846
AATGTCAT 0.541308368952
AATGTCCA 0.291308459816
AATGTCCC -0.213981993931
AATGTCCG -0.682129746787
AATGTCCT -0.682331054905
AATGTCGA 0.978579370282
AATGTCGC 0.379893816017
AATGTCGG 0.504615124343
AATGTCGT 0.805117556977
AATGTCTA 0.443741797841
AATGTCTC 0.691027690501
AATGTCTG -0.820124900209
AATGTGAA 2.83276640636
AATGTGAC -0.188867401033
AATGTGAG 1.35913311053
AATGTGAT 0.786349270151
AATGTGCA 0.309890843696
AATGTGCC -0.294066610026
AATGTGCG -0.629353086909
AATGTGCT -0.177852287146
AATGTGGA 0.28498651057
AATGTGGC 0.178800746075
AATGTGGG -0.726552848399
AATGTGGT 0.710426756641
AATGTGTA 1.46010880443
AATGTGTC 1.11511582052
AATGTGTG 0.567676401537
AATGTTAA 2.82494078748
AATGTTAC 1.13795336254
AATGTTAG 1.3097243542
AATGTTAT 0.351573698406
AATGTTCA 0.154402910002

AATGTTCC 0.268263988866
AATGTTCG -1.09975103567
AATGTTCT 0.0935324979914
AATGTTGA 1.6939817891
AATGTTGC 0.908351149374
AATGTTGG 0.374042349031
AATGTTGT 1.87118830811
AATGTTTA 1.23688578946
AATGTTTC 0.784203579593
AATGTTTG 0.215810213053
AATTAAAA 1.3020919245
AATTAAAC -0.0666496412335
AATTAAAG 0.670818187439
AATTAAAT 1.1623447451
AATTAACA 0.997207969676
AATTAACC -0.168345214629
AATTAACG -0.034939969564
AATTAACT 0.762816413633
AATTAAGA 1.2025776401
AATTAAGC 0.986194104856
AATTAAGG 2.04934536784
AATTAAGT 1.19581039813
AATTAATA 2.47755271577
AATTAATC 1.2637432485
AATTAATG 0.673005721774
AATTAATT 1.70718984995
AATTACAA 1.92297966156
AATTACAC 1.89269038833
AATTACAG 0.917554698379
AATTACAT 1.45864073324
AATTACCA 1.88878684281
AATTACCC 2.13487279752
AATTACCG 1.71749507689
AATTACCT 1.07617133911
AATTACGA 2.07682278094
AATTACGC 1.01791460178
AATTACGG 3.38828458866
AATTACGT 2.2027413209
AATTACTA 2.89544671783
AATTACTC 2.11445907185
AATTACTG 0.419320021299
AATTAGAA 1.38578738786
AATTAGAC -0.0779818102023
AATTAGAG 0.151572522029
AATTAGAT 2.14788079932
AATTAGCA 0.466254998556
AATTAGCC 0.40021406977
AATTAGCG 0.724978606427
AATTAGCT 0.233863824834
AATTAGGA 0.471816057244
AATTAGGC 1.52906318421
AATTAGGG 0.717514592719
AATTAGGT 0.705008508056
AATTAGTA 1.98123161079

AATTAGTC 0.222372399702
AATTAGTG 1.77316894987
AATTATAA 2.90034306419
AATTATAC 0.135365449033
AATTATAG 1.43643397052
AATTATAT 2.01513277218
AATTATCA 1.10270633044
AATTATCC 2.16784777502
AATTATCG 2.11034276843
AATTATCT 1.52414726912
AATTATGA 1.06884118385
AATTATGC 1.65191817684
AATTATGG 0.373154053541
AATTATGT 1.58232011071
AATTATTA 0.76368014412
AATTATTC 1.09813890531
AATTATTG 0.249595419298
AATTCAAA 1.04407549726
AATTCAAC 0.557568527336
AATTCAAG 0.398407709338
AATTCAAT 1.14669683819
AATTCACA 1.56288627885
AATTCACC 0.9823523882
AATTCACG 1.2777236497
AATTCACT 0.00796988622764
AATTCAGA 1.09809914331
AATTCAGC 0.580446872249
AATTCAGG 2.32095685881
AATTCAGT 1.39036688731
AATTCATA 2.95034720958
AATTCATC 0.924041316417
AATTCATG 0.495216720738
AATTCCAA 1.19254283637
AATTCCAC 0.515668959337
AATTCCAG 0.510531126506
AATTCCAT 1.5608804839
AATTCCCA 1.47472914478
AATTCCCC 2.41037971507
AATTCCCG 1.87406262164
AATTCCCT 1.5507688625
AATTCCGA 0.0798714418314
AATTCCGC 1.4927719313
AATTCCGG 1.10453746406
AATTCCGT 1.93720696186
AATTCCTA 2.72791464644
AATTCCTC 1.32697231715
AATTCCTG 0.843697935079
AATTCGAA 0.465500353337
AATTCGAC -0.180811537295
AATTCGAG 0.0342421569364
AATTCGAT 1.4468428701
AATTCGCA 2.04013182629
AATTCGCC 0.891063008068
AATTCGCG 2.23937752474

AATTCGCT 0.993259874066
AATTCGGA 1.21854614112
AATTCGGC 1.93391504328
AATTCGGG 1.2782665779
AATTCGGT 1.09479181956
AATTCGTA 2.94636309918
AATTCGTC 0.23329008629
AATTCGTG 0.131183985895
AATTCTAA 1.09706720501
AATTCTAC 1.49747862751
AATTCTAG 1.7634457886
AATTCTAT 2.06226655942
AATTCTCA 2.61480467377
AATTCTCC 1.41097380216
AATTCTCG -0.0127454893533
AATTCTCT 2.06417534342
AATTCTGA 1.3140821441
AATTCTGC 1.78047828733
AATTCTGG 1.81213279183
AATTCTGT 1.7562644806
AATTCTTA 2.17791318092
AATTCTTC 1.42549754792
AATTCTTG 1.55308047091
AATTGAAA 2.34896512579
AATTGAAC 0.298065501065
AATTGAAG -0.0442698826103
AATTGAAT 0.753777533419
AATTGACA 0.9450073383
AATTGACC 0.296529147488
AATTGACG 1.26378030418
AATTGACT -0.255863658623
AATTGAGA 0.69133870842
AATTGAGC -0.158821487872
AATTGAGG 1.70039637755
AATTGAGT 0.296703184289
AATTGATA 2.31102697679
AATTGATC 1.96953096678
AATTGATG 0.040297430176
AATTGCAA 2.20871269841
AATTGCAC 2.07309181498
AATTGCAG 0.724489388147
AATTGCAT 2.66498847676
AATTGCCA 2.14088289614
AATTGCCC 1.2155458799
AATTGCCG 0.509471292352
AATTGCCT 0.19603830013
AATTGCGA 3.54662664262
AATTGCGC 2.6599947031
AATTGCGG 1.83860699519
AATTGCGT 2.75178724944
AATTGCTA 2.30676494877
AATTGCTC 1.34564088672
AATTGCTG -0.244114925493
AATTGGAA 1.25911857442

AATTGGAC 0.547268296671
AATTGGAG 0.658677246973
AATTGGAT 1.47646618194
AATTGGCA -0.428786290929
AATTGGCC -0.0897188853646
AATTGGCG 0.483919942027
AATTGGCT -0.223297542696
AATTGGGA -0.540091152235
AATTGGGC 0.0728974791152
AATTGGGG 0.75404483396
AATTGGGT 0.406005581494
AATTGGTA 1.33546223201
AATTGGTC 0.411099280591
AATTGGTG 0.581123867077
AATTGTAA 1.56137532299
AATTGTAC 0.328658922344
AATTGTAG 0.107548081271
AATTGTAT 0.450388504754
AATTGTCA 1.39476006747
AATTGTCC 1.88720843727
AATTGTCG 1.22565604406
AATTGTCT 0.895855057255
AATTGTGA 0.87813244848
AATTGTGC 0.947535035473
AATTGTGG 2.25167897043
AATTGTGT 0.93558561876
AATTGTTA 2.30420165316
AATTGTTC 0.77845536889
AATTGTTG 1.45368463979
AATTTAAA 3.76148756403
AATTTAAC 0.56333214321
AATTTAAG 1.11298116339
AATTTAAT 1.51452528235
AATTTACA 1.84371797306
AATTTACC 1.5691751278
AATTTACG 0.51747531977
AATTTACT 1.46596693311
AATTTAGA 2.0150805195
AATTTAGC 1.06004129197
AATTTAGG 2.25034538222
AATTTAGT 1.23353162566
AATTTATA 5.30007343411
AATTTATC 2.40002077821
AATTTATG 0.752725193672
AATTTCAA 2.51021188726
AATTTCAC 2.0640781243
AATTTCAG 1.01585447238
AATTTCAT 2.43821436161
AATTTCCA 2.92435785276
AATTTCCC 3.88717377735
AATTTCCG 2.78081371497
AATTTCCT 2.84360144629
AATTTCGA 2.95550273754
AATTTCGC 3.07929557202

AATTTCGG 4.68576266433
AATTTCGT 3.06873699247
AATTTCTA 0.814508466175
AATTTCTC 1.73665140749
AATTTCTG 0.753113653804
AATTTGAA 2.31880554745
AATTTGAC 0.556944409717
AATTTGAG 1.72234479138
AATTTGAT 2.24783371475
AATTTGCA 1.51592902655
AATTTGCC 0.887454034406
AATTTGCG 1.70752335109
AATTTGCT 0.915314078657
AATTTGGA 2.06995936074
AATTTGGC 1.88333257943
AATTTGGG 0.805810789689
AATTTGGT 1.16881574979
AATTTGTA 2.66929692847
AATTTGTC 0.767942380318
AATTTGTG 0.744658088322
AATTTTAA 1.28123644513
AATTTTAC 0.630437486066
AATTTTAG 3.62802319641
AATTTTAT 2.23542360014
AATTTTCA 3.06710633426
AATTTTCC 2.0527141041
AATTTTCG 2.09846038502
AATTTTCT 1.46228572169
AATTTTGA 1.97798257688
AATTTTGC 1.1146969664
AATTTTGG 1.94638573768
AATTTTGT 2.17476594604
AATTTTTA 2.51109039839
AATTTTTC 2.14867603925
AATTTTTG 1.04962968608
ACAAAAAA 1.72867215325
ACAAAAAC 0.534342525177
ACAAAAAG 1.55452085444
ACAAAAAT 2.34711150895
ACAAAACA 1.64052792621
ACAAAACC 1.02370611351
ACAAAACG -0.27426700943
ACAAAACT 0.469439705471
ACAAAAGA 1.24150734087
ACAAAAGC 1.133660316
ACAAAAGG 0.30094127184
ACAAAAGT -0.217060321892
ACAAAATA 5.72064044157
ACAAAATC 10.2376232156
ACAAAATG 0.251102003423
ACAAACAA 1.3295012634
ACAAACAC 0.598843977716
ACAAACAG 2.59580739115
ACAAACAT 1.72667739174

ACAAACCA 1.18733193307
ACAAACCC -0.166368772777
ACAAACCG -0.222260399942
ACAAACCT 0.601235110127
ACAAACGA 0.425856185699
ACAAACGC 0.529010254102
ACAAACGG -0.515034016463
ACAAACGT 0.199145148475
ACAAACTA 0.939139217075
ACAAACTC 0.152395033274
ACAAACTG -0.498367286461
ACAAAGAA 1.66701586932
ACAAAGAC 0.617572918902
ACAAAGAG 0.179600565919
ACAAAGAT 2.70487537994
ACAAAGCA 0.250287819298
ACAAAGCC -0.410398969828
ACAAAGCG -0.587941279944
ACAAAGCT -0.599117107241
ACAAAGGA 0.453186833316
ACAAAGGC -0.537435425597
ACAAAGGG -0.221392714074
ACAAAGGT -0.64313842533
ACAAAGTA 0.579210503157
ACAAAGTC 0.183124394781
ACAAAGTG -0.114575753909
ACAAATAA 1.90513256234
ACAAATAC 0.78174832836
ACAAATAG 1.57806953248
ACAAATAT 3.94782747607
ACAAATCA 3.6462668747
ACAAATCC 4.98972147616
ACAAATCG 4.0034526354
ACAAATCT 6.85159486004
ACAAATGA 1.37740426831
ACAAATGC -0.0418620959603
ACAAATGG -0.172766498813
ACAAATGT 0.182079133086
ACAAATTA 1.02560886035
ACAAATTC 2.87206645578
ACAAATTG 1.57648363254
ACAACAAA 1.19246955772
ACAACAAC -0.623993544517
ACAACAAG 0.253748153873
ACAACAAT 1.6557825849
ACAACACA 0.413148792918
ACAACACC 0.620710785768
ACAACACG -0.871254663996
ACAACACT 0.340150763833
ACAACAGA 0.027249874557
ACAACAGC -0.132963699545
ACAACAGG 0.338314217591
ACAACAGT 0.846864738686
ACAACATA 2.17409977647

ACAACATC 1.07013272011
ACAACATG 0.625614002003
ACAACCAA 0.37524998956
ACAACCAC -0.365883229009
ACAACCAG 0.413862427073
ACAACCAT -0.0530414622833
ACAACCCA 0.500540872871
ACAACCCC -0.840564856307
ACAACCCG -0.457533381601
ACAACCCT -0.553133919761
ACAACCGA 0.432198744566
ACAACCGC -0.129893906882
ACAACCGG -0.51821122897
ACAACCGT -0.0168865659608
ACAACCTA -0.506908413098
ACAACCTC -0.265348664273
ACAACCTG -0.494162923745
ACAACGAA 0.969942481769
ACAACGAC 0.249015435414
ACAACGAG 0.826916707182
ACAACGAT 1.73747350238
ACAACGCA 0.273818594036
ACAACGCC 0.390438655822
ACAACGCG 0.125451180364
ACAACGCT -0.00292823162883
ACAACGGA 0.829852016863
ACAACGGC -0.367242214938
ACAACGGG -0.55491259252
ACAACGGT -0.0231189986711
ACAACGTA 0.846803534357
ACAACGTC -0.454485863983
ACAACGTG -0.490735064936
ACAACTAA 1.3321705216
ACAACTAC 1.26413566401
ACAACTAG -0.554038661312
ACAACTAT 0.986989344784
ACAACTCA 0.244335177808
ACAACTCC 0.77635235482
ACAACTCG 1.65913945501
ACAACTCT -0.0572293789389
ACAACTGA 0.0578443367259
ACAACTGC -0.565795305205
ACAACTGG -0.233113967709
ACAACTGT 0.18198128943
ACAACTTA 0.593345788782
ACAACTTC 1.29957442805
ACAACTTG 0.882991114624
ACAAGAAA 0.234533533433
ACAAGAAC 0.803260609293
ACAAGAAG 0.0436545084674
ACAAGAAT 2.34667204521
ACAAGACA 0.169341970852
ACAAGACC 0.0778367101422
ACAAGACG -0.780914159148

ACAAGACT 0.686858718042
ACAAGAGA 2.89361995596
ACAAGAGC 0.358594292992
ACAAGAGG 0.138106736825
ACAAGAGT 0.629992401521
ACAAGATA 3.9674744822
ACAAGATC 3.71566051004
ACAAGATG -0.38897225005
ACAAGCAA 0.832570613254
ACAAGCAC 0.560551717955
ACAAGCAG 0.597780396358
ACAAGCAT -0.417164546374
ACAAGCCA -0.894179016245
ACAAGCCC -0.0534399149591
ACAAGCCG -1.20891665994
ACAAGCCT -0.518683168477
ACAAGCGA 0.916899978596
ACAAGCGC -0.792978906469
ACAAGCGG -0.234371779134
ACAAGCGT -1.16537873115
ACAAGCTA 0.514094301009
ACAAGCTC -0.0434051112335
ACAAGCTG -0.772788347605
ACAAGGAA 0.427150844628
ACAAGGAC -0.619602654319
ACAAGGAG -1.07402731397
ACAAGGAT 2.30598782033
ACAAGGCA 0.112787296784
ACAAGGCC -0.973456526266
ACAAGGCG -0.795901933648
ACAAGGCT -0.568599046395
ACAAGGGA 0.293792439611
ACAAGGGC -1.05976129257
ACAAGGGG -0.219897371561
ACAAGGGT 0.0968129667819
ACAAGGTA 0.284361143883
ACAAGGTC 0.316045834194
ACAAGGTG -1.06728109798
ACAAGTAA 1.44802365567
ACAAGTAC 0.48242293409
ACAAGTAG 0.286944632758
ACAAGTAT 1.83829785088
ACAAGTCA 0.301468586693
ACAAGTCC -0.221909828205
ACAAGTCG 0.003538193144
ACAAGTCT 0.731269745331
ACAAGTGA 1.00985228453
ACAAGTGC -0.767110501064
ACAAGTGG -0.569735281873
ACAAGTGT -0.862721239945
ACAAGTTA 1.47443811195
ACAAGTTC -0.652161275838
ACAAGTTG -0.201102437952
ACAATAAA 2.15765829505

ACAATAAC 0.639170760989
ACAATAAG 1.33754359556
ACAATAAT 2.06548790566
ACAATACA 1.06815419648
ACAATACC 1.21093099019
ACAATACG 0.469833370053
ACAATACT 0.751058312493
ACAATAGA 0.350202429975
ACAATAGC 0.722693228436
ACAATAGG -0.19583095485
ACAATAGT 1.04553815583
ACAATATA 2.86486308092
ACAATATC 4.93186152608
ACAATATG 0.920163584969
ACAATCAA 1.99776573141
ACAATCAC 1.23287919583
ACAATCAG 2.76405538666
ACAATCAT 2.73230699388
ACAATCCA 3.07639606896
ACAATCCC 3.39771276172
ACAATCCG 5.66016515139
ACAATCCT 4.02546412716
ACAATCGA 1.97045423617
ACAATCGC 2.08108085358
ACAATCGG 0.762576592587
ACAATCGT 1.71358466149
ACAATCTA 6.61527037135
ACAATCTC 6.44472783802
ACAATCTG 6.58089352325
ACAATGAA -0.186287243006
ACAATGAC 0.784237720783
ACAATGAG -0.085482671415
ACAATGAT 1.33963037175
ACAATGCA 0.750367786096
ACAATGCC -0.227554366267
ACAATGCG 0.501484543707
ACAATGCT 0.32748396576
ACAATGGA 1.34805741685
ACAATGGC -0.147539489799
ACAATGGG -0.191298919975
ACAATGGT 0.396678166584
ACAATGTA 1.64416937564
ACAATGTC 1.02488023738
ACAATGTG 0.252078150025
ACAATTAA 0.730105197646
ACAATTAC 2.25437570814
ACAATTAG 1.23363925368
ACAATTAT 1.14361871841
ACAATTCA 2.03331233164
ACAATTCC 1.53796529148
ACAATTCG 0.115450726007
ACAATTCT 1.09068696592
ACAATTGA 2.26893401144
ACAATTGC 0.777653259089

ACAATTGG 0.433941610711
ACAATTGT -0.0309795914532
ACAATTTA 2.68652636359
ACAATTTC 1.33970489947
ACAATTTG 0.966310608529
ACACAAAA 1.24032946979
ACACAAAC 0.209655222555
ACACAAAG -0.349808349038
ACACAAAT 2.48343957302
ACACAACA 0.733752892414
ACACAACC 0.029978047136
ACACAACG 0.388248206995
ACACAACT 0.800741655595
ACACAAGA 0.802513666659
ACACAAGC -0.315629061855
ACACAAGG -0.526612251818
ACACAAGT 0.808423215305
ACACAATA 1.75166166521
ACACAATC 6.09989265302
ACACAATG 0.663178263328
ACACACAA 1.12872399947
ACACACAC -0.614790411867
ACACACAG 1.34406039941
ACACACAT 1.72552783287
ACACACCA -0.162604081978
ACACACCC -0.274942338834
ACACACCG -0.632832573859
ACACACCT -0.824946718848
ACACACGA -0.342427398348
ACACACGC 0.577991204661
ACACACGG -0.263771091453
ACACACGT 0.392643677108
ACACACTA 0.540830392283
ACACACTC 0.157833475124
ACACACTG -1.39117024618
ACACAGAA 0.693360116718
ACACAGAC 0.256249204263
ACACAGAG -0.308461493606
ACACAGAT 2.21717492558
ACACAGCA 0.636667004285
ACACAGCC -0.57600851747
ACACAGCG -0.157498724914
ACACAGCT -0.254069997048
ACACAGGA 0.113417451564
ACACAGGC 0.228379583825
ACACAGGG 0.320717348323
ACACAGGT -0.0375623877233
ACACAGTA 0.937858922427
ACACAGTC -0.194349143906
ACACAGTG -1.01622877641
ACACATAA 1.44341313769
ACACATAC 1.37096115948
ACACATAG 0.757142522474
ACACATAT 1.97965153984

ACACATCA 0.467540705832
ACACATCC 1.15434321582
ACACATCG 1.26588789817
ACACATCT 0.294871842497
ACACATGA 2.06142156495
ACACATGC -0.540231256024
ACACATGG -0.48003596524
ACACATGT -0.909811101629
ACACATTA 2.21986229528
ACACATTC 1.55419068414
ACACATTG -0.181375075118
ACACCAAA 0.610677231111
ACACCAAC -0.888559459541
ACACCAAG -0.918214830791
ACACCAAT 0.37722768048
ACACCACA 1.12307217518
ACACCACC -0.002825808057
ACACCACG -0.702499130546
ACACCACT -0.150568479576
ACACCAGA -0.0531682426802
ACACCAGC -0.18364692154
ACACCAGG -1.16404597565
ACACCAGT -0.549009497392
ACACCATA 1.04536016365
ACACCATC 0.462098516778
ACACCATG -0.555718657703
ACACCCAA -0.267860956275
ACACCCAC -0.503489089587
ACACCCAG 0.49801296752
ACACCCAT 1.0799512269
ACACCCCA -0.776797855722
ACACCCCC -0.242974110099
ACACCCCG -0.513276369681
ACACCCCT -0.617789632191
ACACCCGA -0.295002994632
ACACCCGC -0.483387630903
ACACCCGG -1.3668538077
ACACCCGT -0.532472878635
ACACCCTA -0.0838824071942
ACACCCTC 0.390022716195
ACACCCTG -0.704246160251
ACACCGAA 1.29495933015
ACACCGAC -1.12066667848
ACACCGAG -0.908230197961
ACACCGAT 2.06048413945
ACACCGCA 0.377287219385
ACACCGCC 0.364496763586
ACACCGCG -0.294176736184
ACACCGCT -0.0610877498339
ACACCGGA 0.386977488535
ACACCGGC -0.515701643282
ACACCGGG -0.277344712857
ACACCGGT -0.835157432977
ACACCGTA 0.423530629355

ACACCGTC -0.416147388708
ACACCGTG -0.72190923012
ACACCTAA -0.277773559519
ACACCTAC 0.76027934845
ACACCTAG -1.34440639123
ACACCTAT 0.131118826184
ACACCTCA 1.75952433977
ACACCTCC 0.0867771221663
ACACCTCG 0.708598537519
ACACCTCT 0.0206866470181
ACACCTGA 0.861485703565
ACACCTGC -0.571984020537
ACACCTGG -0.90465328371
ACACCTGT -1.50704128355
ACACCTTA -0.0677340403914
ACACCTTC 0.0850719362377
ACACCTTG -0.682787172884
ACACGAAA 1.59847513921
ACACGAAC -0.732711169744
ACACGAAG 0.812748529435
ACACGAAT 2.30826216489
ACACGACA 0.147696456005
ACACGACC -0.317260344596
ACACGACG -0.801104718013
ACACGACT -1.10483078685
ACACGAGA 2.81791290667
ACACGAGC -0.80199738524
ACACGAGG 0.145052387335
ACACGAGT -0.501135637393
ACACGATA 2.66159600821
ACACGATC 4.15867181131
ACACGATG 0.418626580409
ACACGCAA 0.745984598484
ACACGCAC -0.388741172479
ACACGCAG -0.336546578265
ACACGCAT 1.46769314502
ACACGCCA -0.841838072903
ACACGCCC -0.392841862556
ACACGCCG 0.0433847097903
ACACGCCT -0.373472982224
ACACGCGA 0.414295229117
ACACGCGC -0.755371760478
ACACGCGG -0.714467699635
ACACGCGT 0.247009432287
ACACGCTA 0.323113477006
ACACGCTC 0.0136281640374
ACACGCTG -0.583953422338
ACACGGAA 0.169689628098
ACACGGAC -0.341990640922
ACACGGAG -0.64569443471
ACACGGAT 1.14753225647
ACACGGCA -0.926622098978
ACACGGCC 0.312701662938
ACACGGCG -0.424964143005

ACACGGCT -0.941766006972
ACACGGGA 0.0680392293269
ACACGGGC -0.797038169126
ACACGGGG -0.114275977601
ACACGGGT -0.786508734493
ACACGGTA 0.102494976883
ACACGGTC -1.07921885673
ACACGGTG -0.855721879514
ACACGTAA 1.92518031111
ACACGTAC 0.390770075184
ACACGTAG -0.100032855777
ACACGTAT 2.19866311404
ACACGTCA 0.248216031926
ACACGTCC -0.300902134378
ACACGTCG -0.0322043025773
ACACGTCT -0.476159691037
ACACGTGA 0.386973324975
ACACGTGC -0.77589935951
ACACGTGG -0.126264531777
ACACGTGT -0.564213360648
ACACGTTA -0.253076363494
ACACGTTC -0.497777310033
ACACGTTG -0.825673468217
ACACTAAA 1.54318015017
ACACTAAC -0.282561653324
ACACTAAG 0.159295092803
ACACTAAT 1.94838362186
ACACTACA 0.38227849491
ACACTACC 0.182888529117
ACACTACG -0.788475391979
ACACTACT 0.243177291819
ACACTAGA 0.00963260384597
ACACTAGC 0.0501731859161
ACACTAGG 0.187409738737
ACACTAGT 0.583685497263
ACACTATA 1.55049240212
ACACTATC 0.591884379282
ACACTATG -0.0269421874855
ACACTCAA 0.0360481010129
ACACTCAC 0.0614945296293
ACACTCAG -0.102285758001
ACACTCAT 0.437899490686
ACACTCCA -0.324336939062
ACACTCCC 0.30379372668
ACACTCCG 0.487277644853
ACACTCCT 0.429203479624
ACACTCGA -0.445819414196
ACACTCGC 0.157328851673
ACACTCGG -0.379202873263
ACACTCGT -0.980629923498
ACACTCTA 2.12591219223
ACACTCTC 0.117468387101
ACACTCTG -0.289970499865
ACACTGAA 1.54641273802

ACACTGAC 0.245266566142
ACACTGAG -0.394843493945
ACACTGAT 2.42550634429
ACACTGCA -0.46681020926
ACACTGCC -0.300126046825
ACACTGCG -0.87591431198
ACACTGCT 0.0954995718334
ACACTGGA -0.664009101692
ACACTGGC -0.768763850673
ACACTGGG 0.0657894497725
ACACTGGT -0.740543658497
ACACTGTA 0.50001855429
ACACTGTC -0.21825255725
ACACTGTG -0.432096529173
ACACTTAA 1.36255035226
ACACTTAC 1.00046866156
ACACTTAG -0.550936392882
ACACTTAT 0.908148800366
ACACTTCA 0.275453415804
ACACTTCC 1.2058135588
ACACTTCG 0.502365969323
ACACTTCT 0.178218264055
ACACTTGA -0.037963130357
ACACTTGC -0.270914719232
ACACTTGG -0.0663534039516
ACACTTTA 1.057976999
ACACTTTC -0.834565582947
ACACTTTG 0.0108775082353
ACAGAAAA 1.2033589321
ACAGAAAC 0.0977949423679
ACAGAAAG 0.251598507932
ACAGAAAT 2.24970606761
ACAGAACA -0.0805328233105
ACAGAACC -0.771404380317
ACAGAACG -0.562169260949
ACAGAACT 0.906361592309
ACAGAAGA 1.88333257943
ACAGAAGC -0.0697754337763
ACAGAAGG -0.978159683451
ACAGAAGT -0.42616491366
ACAGAATA 2.43663075162
ACAGAATC 11.5342286092
ACAGAATG -0.0771322358189
ACAGACAA 1.49907785084
ACAGACAC 0.101218637617
ACAGACAG 0.347954524023
ACAGACAT 0.180559017392
ACAGACCA -0.320806032148
ACAGACCC -1.05974526286
ACAGACCG 0.145479984929
ACAGACCT -0.869911291416
ACAGACGA -0.586890189265
ACAGACGC 0.461949877692
ACAGACGG -0.762946524878

ACAGACGT 0.649451631101
ACAGACTA 1.0973586542
ACAGACTC -1.28218573677
ACAGACTG -0.390847517397
ACAGAGAA 2.59663698045
ACAGAGAC 0.38227849491
ACAGAGAG -0.238452067767
ACAGAGAT 2.84360144629
ACAGAGCA -1.50524616473
ACAGAGCC -1.41053100757
ACAGAGCG -0.95380202573
ACAGAGCT -0.618657942594
ACAGAGGA 0.0564295590954
ACAGAGGC -0.657676535368
ACAGAGGG -1.45220969872
ACAGAGGT -0.905632552983
ACAGAGTA 0.268453430838
ACAGAGTC -0.626557881016
ACAGAGTG -1.06652083195
ACAGATAA 2.44277221055
ACAGATAC 2.84542758363
ACAGATAG -0.105688635451
ACAGATAT 7.58003380104
ACAGATCA 1.38090415671
ACAGATCC 2.3006740771
ACAGATCG 4.05641082649
ACAGATCT 5.72273263039
ACAGATGA 0.278504680625
ACAGATGC -0.378470919445
ACAGATGG -0.745240153986
ACAGATGT -0.6036757889
ACAGATTA 2.69626763634
ACAGATTC 6.88050495408
ACAGATTG 4.85096772214
ACAGCAAA 1.32623744884
ACAGCAAC 0.440982606741
ACAGCAAG 0.428991554429
ACAGCAAT 1.02946223497
ACAGCACA -0.345605235389
ACAGCACC -0.848951514873
ACAGCACG -0.552491482479
ACAGCACT 0.526948042918
ACAGCAGA 0.272151088324
ACAGCAGC -0.0430012459299
ACAGCAGG -0.858174424432
ACAGCAGT -0.859547150108
ACAGCATA 3.06850695579
ACAGCATC 1.2134299588
ACAGCATG -0.44371390199
ACAGCCAA 0.84029922119
ACAGCCAC -1.19572941689
ACAGCCAG -0.750061139914
ACAGCCAT -0.723335665718
ACAGCCCA -0.929307595069

ACAGCCCC -1.15872744432
ACAGCCCG -0.49290823499
ACAGCCCT -0.975283912676
ACAGCCGA 0.118906897023
ACAGCCGC -0.727843551946
ACAGCCGG -0.546039630165
ACAGCCGT -1.05544118289
ACAGCCTA -0.788464983079
ACAGCCTC -0.681404038308
ACAGCCTG -1.05826990544
ACAGCGAA 0.419320021299
ACAGCGAC -0.921621455444
ACAGCGAG -0.0514137185676
ACAGCGAT 1.14193830566
ACAGCGCA -0.748852458495
ACAGCGCC -0.584168470204
ACAGCGCG 0.866097054256
ACAGCGCT 0.290178053322
ACAGCGGA -0.345088329436
ACAGCGGC -0.605428855766
ACAGCGGG -0.331312567202
ACAGCGGT -1.13768314751
ACAGCGTA -0.525079853622
ACAGCGTC -1.3162103477
ACAGCGTG -1.02450801513
ACAGCTAA 0.296482515618
ACAGCTAC -0.23042514077
ACAGCTAG -0.359877293954
ACAGCTAT 0.724374057539
ACAGCTCA -0.491121859644
ACAGCTCC -0.124393427989
ACAGCTCG 1.19813345633
ACAGCTCT 0.234778767107
ACAGCTGA -0.540091152235
ACAGCTGC -0.950301720981
ACAGCTGG -0.457526928084
ACAGCTGT -1.41536385965
ACAGCTTA -0.240712880755
ACAGCTTC -0.254002963735
ACAGCTTG -0.564287680191
ACAGGAAA 1.6577954579
ACAGGAAC -0.292872292889
ACAGGAAG -0.698539585147
ACAGGAAT 3.2465411905
ACAGGACA -0.930581852555
ACAGGACC -0.130122694494
ACAGGACG 0.0325475880854
ACAGGACT -0.269266574073
ACAGGAGA 1.46906191531
ACAGGAGC 0.0334737719697
ACAGGAGG 0.388890227921
ACAGGAGT 0.106945197808
ACAGGATA 3.04196655183
ACAGGATC 3.81116403726

ACAGGATG -0.346874080247
ACAGGCAA 1.97943357748
ACAGGCAC -0.97871322873
ACAGGCAG -0.221610676431
ACAGGCAT 0.740034663308
ACAGGCCA -0.750135667635
ACAGGCCC -0.417987682153
ACAGGCCG -0.662447766756
ACAGGCCT 0.183231190091
ACAGGCGA -0.856767557565
ACAGGCGC -0.848666727381
ACAGGCGG -0.369038999183
ACAGGCGT -1.20523732212
ACAGGCTA -0.109799526249
ACAGGCTC -0.955917738658
ACAGGCTG -0.603482599723
ACAGGGAA 0.545138219462
ACAGGGAC -1.16179473885
ACAGGGAG -0.995152628365
ACAGGGAT 0.533923671058
ACAGGGCA -0.601835495455
ACAGGGCC -0.790590896729
ACAGGGCG -0.346311166958
ACAGGGCT -1.07387742582
ACAGGGGA 0.55768011074
ACAGGGGC -1.01926317881
ACAGGGGG -1.04083583136
ACAGGGGT -0.329952540384
ACAGGGTA 0.216605036625
ACAGGGTC -0.765566028545
ACAGGGTG -0.654869046974
ACAGGTAA 0.54794945506
ACAGGTAC -0.0139142005977
ACAGGTAG 0.047595734203
ACAGGTAT 1.17784318021
ACAGGTCA -0.944633450627
ACAGGTCC -1.12250197566
ACAGGTCG -0.851017889617
ACAGGTCT -0.464139493806
ACAGGTGA 0.0162258090156
ACAGGTGC -1.44537604797
ACAGGTGG -1.6021765439
ACAGGTTA 0.303372166247
ACAGGTTC -0.366370781865
ACAGGTTG -0.389437319683
ACAGTAAA 1.30354937862
ACAGTAAC 0.22787537673
ACAGTAAG 0.094944152952
ACAGTAAT 1.92854946372
ACAGTACA 0.00580691689567
ACAGTACC 0.2808062965
ACAGTACG 0.000329337582597
ACAGTACT 0.888925644629
ACAGTAGA 0.365801623237

ACAGTAGC -0.267595112979
ACAGTAGG -0.281517016163
ACAGTAGT 0.589378540797
ACAGTATA 1.97357107706
ACAGTATC 3.967391211
ACAGTATG 0.818512561664
ACAGTCAA 1.42470896969
ACAGTCAC -0.264630450202
ACAGTCAG -0.899469235365
ACAGTCAT 0.929204963319
ACAGTCCA -0.822300984754
ACAGTCCC -0.391580928461
ACAGTCCG 0.00648890799591
ACAGTCCT -0.419215099591
ACAGTCGA 0.342664096724
ACAGTCGC -0.609979418483
ACAGTCGG -0.161416218359
ACAGTCGT -0.520826777255
ACAGTCTA 0.881553021059
ACAGTCTC 0.0255009712502
ACAGTCTG -0.0974685192772
ACAGTGAA -0.560597517113
ACAGTGAC -0.835388510547
ACAGTGAG -1.40546457979
ACAGTGAT 2.18436669865
ACAGTGCA -0.0810536846453
ACAGTGCC -0.883531961046
ACAGTGCG 2.65806197863
ACAGTGCT -0.353760191851
ACAGTGGA -0.100402163534
ACAGTGGC -0.387426320285
ACAGTGGG -0.758492348571
ACAGTGGT -0.0291769782246
ACAGTGTA -0.18807382653
ACAGTGTC -0.797571937496
ACAGTGTG -0.39234598258
ACAGTTAA 1.12120086321
ACAGTTAC 0.567494870328
ACAGTTAG 0.0397520038382
ACAGTTAT 1.06863800213
ACAGTTCA -0.220085772643
ACAGTTCC -0.278824233842
ACAGTTCG 0.0953969400835
ACAGTTCT -0.10034137556
ACAGTTGA 0.09504178843
ACAGTTGC -0.38709719088
ACAGTTGG -0.432825360321
ACAGTTTA 0.184948658521
ACAGTTTC -0.0972416052665
ACAGTTTG -0.0433480704638
ACATAAAA 1.82645543765
ACATAAAC 0.418843710054
ACATAAAG 0.339482720658
ACATAAAT 1.97518445649

ACATAACA 0.113006091853
ACATAACC 0.375251863162
ACATAACG -0.0263553337274
ACATAACT 1.64121866079
ACATAAGA 0.411894104163
ACATAAGC 0.0720195925249
ACATAAGG -0.571835381451
ACATAAGT 0.406115707652
ACATAATA 0.708048323088
ACATAATC 4.39361254089
ACATAATG 0.536497583746
ACATACAA 1.2287922455
ACATACAC 1.57415870073
ACATACAG 0.528161720609
ACATACAT 0.904020214438
ACATACCA -0.91975618064
ACATACCC -0.0490138426812
ACATACCG -0.11307270881
ACATACCT 1.39119189669
ACATACGA 0.0288988524279
ACATACGC 0.623115866104
ACATACGG 0.505153888985
ACATACGT -0.0381944161056
ACATACTA 0.579459275857
ACATACTC -0.285404115621
ACATACTG 0.866419105609
ACATAGAA 0.635184152451
ACATAGAC 1.53428387188
ACATAGAG 0.0939723780875
ACATAGAT 3.32503033047
ACATAGCA 0.26379107654
ACATAGCC -0.593459453965
ACATAGCG -0.448269669156
ACATAGCT 0.49290303054
ACATAGGA 1.73558178897
ACATAGGC -0.19524847283
ACATAGGG -0.346194587283
ACATAGGT 0.194358720094
ACATAGTA 1.51320022943
ACATAGTC 0.487063637877
ACATAGTG -0.0273878965654
ACATATAA 2.09972069459
ACATATAC 0.623335702064
ACATATAG 0.286566789703
ACATATAT 3.30631700263
ACATATCA 2.35994172675
ACATATCC 1.94550202211
ACATATCG 2.74042885004
ACATATCT 4.79536504602
ACATATGA 0.844790245001
ACATATGC -0.219359855986
ACATATGG 0.125960383732
ACATATGT -0.650978200313
ACATATTA 1.84908584258

ACATATTC 1.00595685795
ACATATTG 0.81861040532
ACATCAAA 0.676575141617
ACATCAAC -0.491188476602
ACATCAAG 0.352799658598
ACATCAAT 0.0775063316696
ACATCACA -0.0367725604234
ACATCACC -0.0954125534329
ACATCACG 0.116059646633
ACATCACT 0.922838255804
ACATCAGA 1.02478738999
ACATCAGC 1.01118753816
ACATCAGG 0.275342040578
ACATCAGT 0.1962258685
ACATCATA 1.69216439523
ACATCATC -0.231133154119
ACATCATG 0.436608370783
ACATCCAA 1.40565152363
ACATCCAC 0.808300182112
ACATCCAG 1.06716993093
ACATCCAT 0.472580070473
ACATCCCA -0.191429239397
ACATCCCC -0.507044353326
ACATCCCG 0.0118218036049
ACATCCCT 0.382506241633
ACATCCGA 1.05450771278
ACATCCGC 1.29745434338
ACATCCGG 0.338953115847
ACATCCGT 0.563321942488
ACATCCTA 0.991876739491
ACATCCTC -0.398869448123
ACATCCTG -0.00396995429843
ACATCGAA 0.304439078454
ACATCGAC -0.111449336832
ACATCGAG 0.0258783979488
ACATCGAT 4.13318957608
ACATCGCA -0.556510983139
ACATCGCC -0.606131456488
ACATCGCG -0.0805328233105
ACATCGCT 0.42021435395
ACATCGGA 0.936076086108
ACATCGGC -0.295299856448
ACATCGGG -0.121446876697
ACATCGGT -0.438361645828
ACATCGTA 0.899751941078
ACATCGTC 0.291350303593
ACATCGTG -0.474975158265
ACATCTAA 1.86746233842
ACATCTAC 0.743909063908
ACATCTAG 1.62241852273
ACATCTAT 0.803090111518
ACATCTCA 1.44649646192
ACATCTCC 1.31249353784
ACATCTCG 1.16177870914

ACATCTCT 1.22149519054
ACATCTGA 0.382506241633
ACATCTGC 0.00903034491648
ACATCTGG 0.512414720974
ACATCTTA 0.942068281416
ACATCTTC 2.06663455004
ACATCTTG 0.413507275419
ACATGAAA 2.10341543758
ACATGAAC -0.514747979903
ACATGAAG 0.234184418941
ACATGAAT 0.837217354203
ACATGACA -0.44248148828
ACATGACC -0.944183786166
ACATGACG -0.170901432187
ACATGACT 1.01903335031
ACATGAGA 1.35755074962
ACATGAGC 0.326327537017
ACATGAGG -0.433355797843
ACATGAGT 0.227720492304
ACATGATA 1.16640421593
ACATGATC 0.831322586195
ACATGATG 0.235362290017
ACATGCAA 1.93783066312
ACATGCAC 0.582176623181
ACATGCAG 0.570038180851
ACATGCAT 0.826223266292
ACATGCCA -0.836259319086
ACATGCCC 0.162228945238
ACATGCCG -0.202937735125
ACATGCCT 0.787196762755
ACATGCGA 0.163291277528
ACATGCGC -0.711207215931
ACATGCGG -0.287694698061
ACATGCGT -1.00826388645
ACATGCTA 0.769580324756
ACATGCTC -0.545886202986
ACATGCTG -0.0582323805021
ACATGGAA 0.762544949532
ACATGGAC 0.186524774095
ACATGGAG -0.638767103863
ACATGGAT 1.12843379935
ACATGGCA -0.359296893713
ACATGGCC -0.246223144013
ACATGGCG -0.126904054567
ACATGGCT -0.288339425301
ACATGGGA -0.176353613785
ACATGGGC -0.802163719456
ACATGGGG -0.589443075975
ACATGGGT -1.27221151284
ACATGGTA 0.561061545856
ACATGGTC -0.310672343876
ACATGGTG -0.875635145293
ACATGTAA 0.634536718898
ACATGTAC 0.643700089551

ACATGTAG 0.0598948899424
ACATGTAT 1.44354616343
ACATGTCA -0.778468692282
ACATGTCC 0.541617929625
ACATGTCG -0.778468692282
ACATGTCT -0.149530295932
ACATGTGA 1.98156011566
ACATGTGC 0.191605357978
ACATGTGG 0.0563569049764
ACATGTTA -0.752514517544
ACATGTTC 0.804558182714
ACATGTTG -0.13785338421
ACATTAAA 0.985592886816
ACATTAAC 0.908387580522
ACATTAAG 0.294230446105
ACATTAAT 1.80416457103
ACATTACA 1.44451710558
ACATTACC 0.464753618881
ACATTACG 1.29484608132
ACATTACT 1.21027793583
ACATTAGA 1.64584250216
ACATTAGC 0.606842592507
ACATTAGG -0.107583887886
ACATTAGT 0.873051031885
ACATTATA 2.87578784556
ACATTATC 0.796595790894
ACATTATG 1.63128857059
ACATTCAA 0.398752452092
ACATTCAC 0.0630133962555
ACATTCAG 1.43657802969
ACATTCAT 0.541348130948
ACATTCCA -0.090986481155
ACATTCCC 0.390747591961
ACATTCCG 0.406522071091
ACATTCCT 1.5366058892
ACATTCGA -0.30335218116
ACATTCGC 0.214490780943
ACATTCGG -0.224534119966
ACATTCGT 0.630523671755
ACATTCTA 0.719875747499
ACATTCTC 1.04892687718
ACATTCTG 0.675377909987
ACATTGAA 0.262667331742
ACATTGAC -0.555958062394
ACATTGAG -0.226313209082
ACATTGAT 0.321396424932
ACATTGCA 0.149143501224
ACATTGCC -0.139692012231
ACATTGCG 1.21104590444
ACATTGCT 0.279284515382
ACATTGGA 0.443741797841
ACATTGGC -1.09147179695
ACATTGGG -0.596522584933
ACATTGGT -0.270511270285

ACATTGTA -0.0252773880873
ACATTGTC 0.338139348078
ACATTGTG 0.376128500684
ACATTTAA 0.40755047037
ACATTTAC 1.48619184135
ACATTTAG 1.04459802402
ACATTTAT 1.57536405131
ACATTTCA 1.73095274315
ACATTTCC 0.487361332405
ACATTTCG 1.4479666149
ACATTTCT 1.53919354163
ACATTTGA 1.41556995586
ACATTTGC -0.272409229033
ACATTTGG -0.185231988767
ACATTTTA 1.15617663939
ACATTTTC 1.24120964634
ACATTTTG -0.328967025772
ACCAAAAA 0.833892335322
ACCAAAAC 0.832096383789
ACCAAAAG -0.925666770175
ACCAAAAT 2.05404581871
ACCAAACA -0.403416471814
ACCAAACC -0.0214090246487
ACCAAACG -0.107061777483
ACCAAACT -0.784726522707
ACCAAAGA 1.29696096154
ACCAAAGC 0.0584289005261
ACCAAAGG -0.777459237201
ACCAAAGT -0.901833720993
ACCAAATA 1.38456559122
ACCAAATC 7.04378457364
ACCAAATG 0.656937503498
ACCAACAA 0.993054194211
ACCAACAC -0.26467416758
ACCAACAG -0.390847517397
ACCAACAT 0.575045069725
ACCAACCA 0.617115135499
ACCAACCC -0.99951999445
ACCAACCG 0.0645226866941
ACCAACCT -0.349809806283
ACCAACGA 0.906179852922
ACCAACGC -0.671393591408
ACCAACGG -1.25075856263
ACCAACGT -1.08649884109
ACCAACTA 0.0355101690828
ACCAACTC 0.0916162195794
ACCAACTG 0.876553418414
ACCAAGAA 1.73376793413
ACCAAGAC -0.0872563479028
ACCAAGAG -0.513371507023
ACCAAGAT 4.53754826148
ACCAAGCA -0.282272702272
ACCAAGCC -1.37943046471
ACCAAGCG -0.832944292749

ACCAAGCT 0.195004696402
ACCAAGGA -0.790078362513
ACCAAGGC 0.199592522979
ACCAAGGG -0.233992478833
ACCAAGGT -0.250209960729
ACCAAGTA -0.196686150039
ACCAAGTC -1.12051304313
ACCAAGTG 0.235239256824
ACCAATAA 1.46491771604
ACCAATAC 0.874049037176
ACCAATAG 0.30561278597
ACCAATAT 2.63877387136
ACCAATCA 0.433464466755
ACCAATCC 1.32349616105
ACCAATCG 1.55772721186
ACCAATCT 1.77531984488
ACCAATGA 0.290284432276
ACCAATGC -0.668927931276
ACCAATGG -0.729471712018
ACCAATTA 1.24602626053
ACCAATTC 0.534081678154
ACCAATTG 1.47375570449
ACCACAAA -0.0547491463478
ACCACAAC -0.132949127085
ACCACAAG -1.1551076454
ACCACAAT 2.78401465977
ACCACACA 0.187162006927
ACCACACC -0.891182502235
ACCACACG -0.774577429264
ACCACACT -0.217653004634
ACCACAGA 0.299735921269
ACCACAGC -0.506116920374
ACCACAGG 0.481457612744
ACCACAGT -0.192015676799
ACCACATA 0.342739665335
ACCACATC 1.21383278321
ACCACATG -0.207574691708
ACCACCAA -0.124511048555
ACCACCAC -0.149822786011
ACCACCAG -0.70450846452
ACCACCAT 0.209192651058
ACCACCCA -0.6735399065
ACCACCCC -0.66714676038
ACCACCCG -0.565933119036
ACCACCCT -0.741404890848
ACCACCGA -0.255054678948
ACCACCGC -0.834685077114
ACCACCGG -0.814935231058
ACCACCGT -0.93709053746
ACCACCTA -0.746204850799
ACCACCTC -1.00377057268
ACCACCTG -1.39034544498
ACCACGAA 0.890153270245
ACCACGAC -0.0565238637256

ACCACGAG -0.816017340258
ACCACGAT 1.12518081005
ACCACGCA -0.346406096122
ACCACGCC -0.696087664763
ACCACGCG 0.0162287235075
ACCACGCT -0.204819247812
ACCACGGA -0.641102028216
ACCACGGC -0.722210463673
ACCACGGG -1.13455652226
ACCACGGT -0.679655759535
ACCACGTA -0.0867236204225
ACCACGTC -1.02894033275
ACCACGTG 0.361652635865
ACCACTAA 1.68893284827
ACCACTAC -0.364006712594
ACCACTAG -0.801372018554
ACCACTAT 0.860565973199
ACCACTCA -0.142985388057
ACCACTCC -0.67506668389
ACCACTCG 1.52000619251
ACCACTCT 0.546271540448
ACCACTGA -0.130310679221
ACCACTGC -1.04001894092
ACCACTGG -0.863650546499
ACCACTTA 0.115714487523
ACCACTTC -0.667450700248
ACCACTTG -0.202496189605
ACCAGAAA 0.614843080899
ACCAGAAC -0.341014286141
ACCAGAAG -0.818398480125
ACCAGAAT 2.45521084555
ACCAGACA -0.899077860741
ACCAGACC -1.05126346696
ACCAGACG -1.01937517857
ACCAGACT -0.811051462448
ACCAGAGA 0.968569547915
ACCAGAGC -0.783096697212
ACCAGAGG 0.278585870042
ACCAGAGT -0.307116663781
ACCAGATA 2.50360639959
ACCAGATC 3.2183572213
ACCAGATG 0.491093963793
ACCAGCAA 0.749901051039
ACCAGCAC -0.292872292889
ACCAGCAG -0.690214963622
ACCAGCAT 0.489277402639
ACCAGCCA -0.59969042943
ACCAGCCC -1.14093030782
ACCAGCCG -0.501939620796
ACCAGCCT -0.0141704677053
ACCAGCGA 0.454790636563
ACCAGCGC -1.09329959972
ACCAGCGG -0.609426914094
ACCAGCGT -0.796242512843

ACCAGCTA −0.571425478986
ACCAGCTC −0.177107842648
ACCAGCTG −1.05127179408
ACCAGGAA −0.183844482454
ACCAGGAC −0.692004461637
ACCAGGAG −0.821559454748
ACCAGGAT 0.51746386998
ACCAGGCA −1.57160560585
ACCAGGCC −0.934929649908
ACCAGGCG −1.54783334464
ACCAGGCT −0.248514559166
ACCAGGGA −0.489186220679
ACCAGGGC −0.629658900378
ACCAGGGG −1.12819689279
ACCAGGGT −1.70369495783
ACCAGGTA −0.321687665942
ACCAGGTC −0.703303530305
ACCAGGTG −0.90465328371
ACCAGTAA 0.882283725809
ACCAGTAC −0.0303161281942
ACCAGTAG 0.113231756796
ACCAGTAT 0.130453489323
ACCAGTCA −0.726128165296
ACCAGTCC −1.31892998499
ACCAGTCG −0.816870037311
ACCAGTCT 0.107917389028
ACCAGTGA −0.800484347598
ACCAGTGC −0.545040792162
ACCAGTGG −0.78686346979
ACCAGTTA 0.215115314917
ACCAGTTC −1.26426306895
ACCAGTTG −0.128395441699
ACCATAAA 1.72879914183
ACCATAAC 0.299735921269
ACCATAAG −0.890607930978
ACCATAAT 0.869235545656
ACCATACA −0.00607421743679
ACCATACC −0.527064622594
ACCATACG −0.20384206032
ACCATACT 1.33904955516
ACCATAGA 0.337276033948
ACCATAGC 0.634078727316
ACCATAGG −0.0657671747274
ACCATAGT 0.35111195962
ACCATATA 1.28608137153
ACCATATC 4.34680600946
ACCATATG 0.458777869635
ACCATCAA 0.681031399703
ACCATCAC −0.302300674125
ACCATCAG −0.591969107724
ACCATCAT 1.39817668467
ACCATCCA 0.82475790141
ACCATCCC −0.855554088053
ACCATCCG −0.690341119485

ACCATCCT -0.235413918159
ACCATCGA -0.568366303401
ACCATCGC -1.27476752222
ACCATCGG -0.847215518602
ACCATCGT -0.334687548801
ACCATCTA 0.208648473788
ACCATCTC -0.164937340907
ACCATCTG 0.347534629014
ACCATGAA 0.211026699164
ACCATGAC -0.498331063491
ACCATGAG -1.23938142722
ACCATGAT 1.56407872239
ACCATGCA -0.577973301354
ACCATGCC -0.129906397561
ACCATGCG 0.25818879861
ACCATGCT 0.872694006629
ACCATGGA -0.887731327491
ACCATGGC -1.21962575218
ACCATGGG -0.809795316447
ACCATGGT -0.554841812003
ACCATGTA 0.669940300849
ACCATGTC -0.428143645469
ACCATGTG -0.329990636956
ACCATTAA 1.5786028845
ACCATTAC 1.41994960444
ACCATTAG 0.750435860299
ACCATTAT 0.714944843591
ACCATTCA 0.117390320354
ACCATTCC 0.0806287933646
ACCATTCG 0.207763717324
ACCATTCT 0.400154947221
ACCATTGA -0.044474521576
ACCATTGC -0.177920569527
ACCATTGG -0.502920555492
ACCATTTA 0.0834991515118
ACCATTTC -0.156557135858
ACCATTTG 0.218657047087
ACCCAAAA 0.353206022037
ACCCAAAC 0.833674581143
ACCCAAAG -0.700819134154
ACCCAAAT 0.867127118958
ACCCAACA -0.536034179536
ACCCAACC -0.372995421912
ACCCAACG 0.109554084397
ACCCAACT -1.00759209607
ACCCAAGA 0.0735222212678
ACCCAAGC -0.546039630165
ACCCAAGG -0.948290513405
ACCCAAGT -0.95646170775
ACCCAATA -0.0507408872989
ACCCAATC 0.360436876395
ACCCAATG -0.102767065518
ACCCACAA -0.377094030209
ACCCACAC 0.3805693536

ACCCACAG -1.05551196341
ACCCACAT -0.355244917286
ACCCACCA 0.122090146691
ACCCACCC -0.236591372879
ACCCACCG 0.516064081165
ACCCACCT -0.31309220485
ACCCACGA 0.266340216046
ACCCACGC -0.723259888929
ACCCACGG 0.0498831939739
ACCCACGT -1.23686809433
ACCCACTA -0.0867902373798
ACCCACTC -1.45811175296
ACCCACTG -0.631191090396
ACCCAGAA 1.07423882281
ACCCAGAC -0.533279984709
ACCCAGAG -0.119328249278
ACCCAGAT 2.64760478176
ACCCAGCA -0.933425772097
ACCCAGCC -0.783060057885
ACCCAGCG -0.873397856418
ACCCAGCT -0.825719059197
ACCCAGGA -0.00988366650375
ACCCAGGC -0.992480663844
ACCCAGGG -1.40860223848
ACCCAGGT -0.360383999185
ACCCAGTA -0.0550435100278
ACCCAGTC -1.02463208921
ACCCAGTG -0.308765433474
ACCCATAA 1.27178516431
ACCCATAC -0.186345116488
ACCCATAG -0.395369143373
ACCCATAT 2.23581206027
ACCCATCA 0.764319458732
ACCCATCC -0.645138183116
ACCCATCG 0.200633621115
ACCCATCT 0.338886707068
ACCCATGA -0.812450418551
ACCCATGC -1.08822775931
ACCCATGG -1.09170058456
ACCCATTA 1.24861495386
ACCCATTC -0.912332553462
ACCCATTG 0.401102781616
ACCCCAAA -1.07104037615
ACCCCAAC -0.343910874716
ACCCCAAG -1.28612529708
ACCCCAAT 0.330585817834
ACCCCACA -0.747335049115
ACCCCACC -0.513188518568
ACCCCACG 0.123716016805
ACCCCACT -0.62665489196
ACCCCAGA -1.03025872397
ACCCCAGC -0.265553303239
ACCCCAGG -0.737124751343
ACCCCAGT -0.0782332892161

ACCCCATA −0.554861588912
ACCCCATC −0.0397576246439
ACCCCATG −0.270792935107
ACCCCCAA −0.765314965887
ACCCCCAC −0.949503566561
ACCCCCAG −1.29287546846
ACCCCCAT −0.34689239991
ACCCCCCA −1.34862241192
ACCCCCCC −2.11903003601
ACCCCCCG −1.15475145286
ACCCCCCT −1.66139048363
ACCCCCGA −0.72927456746
ACCCCCGC −0.887296651845
ACCCCCGG −1.57479364361
ACCCCCGT −0.793665893841
ACCCCCTA −1.10499087572
ACCCCCTC −1.31602173844
ACCCCCTG −1.40108701299
ACCCCGAA −0.0477814289714
ACCCCGAC −0.302569223734
ACCCCGAG −1.18774870541
ACCCCGAT 1.04085269378
ACCCCGCA −0.418821226831
ACCCCGCC −0.793673180071
ACCCCGCG −0.313278315974
ACCCCGCT 0.135514920831
ACCCCGGA −1.0423659396
ACCCCGGC −0.252325881835
ACCCCGGG −0.324947941467
ACCCCGGT −0.968630544067
ACCCCGTA −0.169636750888
ACCCCGTC −1.22266639992
ACCCCGTG −1.27327780051
ACCCCTAA 0.249408267284
ACCCCTAC −0.591424514098
ACCCCTAG −1.14842138467
ACCCCTAT 0.197020067538
ACCCCTCA 0.0570445168824
ACCCCTCC −0.425509361165
ACCCCTCG −0.311463003888
ACCCCTCT −0.4917276576
ACCCCTGA −0.74828600618
ACCCCTGC −0.699052952074
ACCCCTGG −0.839209201226
ACCCCTTA 0.0633564735855
ACCCCTTC −1.55611820417
ACCCCTTG −0.771805747485
ACCCGAAA 1.31484594915
ACCCGAAC −0.514534805639
ACCCGAAG −1.3928036107
ACCCGAAT 1.82560482238
ACCCGACA −1.12891302508
ACCCGACC −0.356229599186
ACCCGACG −0.778266135096

ACCCGACT −1.03303831651
ACCCGAGA −0.443108104034
ACCCGAGC −0.990845842077
ACCCGAGG 0.11008764459
ACCCGAGT −1.15828235977
ACCCGATA 0.595318691608
ACCCGATC 0.347586048978
ACCCGATG −0.220738410646
ACCCGCAA 0.91104642983
ACCCGCAC −0.452525868193
ACCCGCAG −0.764436662941
ACCCGCAT 0.264251566257
ACCCGCCA −0.882190462069
ACCCGCCC −0.347057276879
ACCCGCCG −0.541018168832
ACCCGCCT 0.450740741916
ACCCGCGA −0.833483890103
ACCCGCGC −0.759824687717
ACCCGCGG −0.375848709464
ACCCGCGT −0.873145961049
ACCCGCTA −0.610127641213
ACCCGCTC −0.894770449919
ACCCGCTG −1.01884057749
ACCCGGAA −0.0363682787639
ACCCGGAC −1.44006584376
ACCCGGAG −1.51818505144
ACCCGGAT 1.38914862971
ACCCGGCA −0.232764645039
ACCCGGCC −1.43154511857
ACCCGGCG −1.25416081555
ACCCGGCT −0.527898167272
ACCCGGGA −0.852451403267
ACCCGGGC −0.955844251827
ACCCGGGG −0.515045674431
ACCCGGGT −1.27123370081
ACCCGGTA −0.493950998549
ACCCGGTC −1.3668538077
ACCCGGTG −0.769550138947
ACCCGTAA 1.08633937675
ACCCGTAC −0.623718125034
ACCCGTAG 0.159141873801
ACCCGTAT 0.924363159592
ACCCGTCA −0.947385563675
ACCCGTCC −1.36923890295
ACCCGTCG −0.870747750586
ACCCGTCT −0.714718554114
ACCCGTGA −1.21848556132
ACCCGTGC −0.912361698381
ACCCGTGG −0.856978441871
ACCCGTTA −0.172581636756
ACCCGTTC −1.20607544671
ACCCGTTG −1.12838550205
ACCCTAAA 0.12073865517
ACCCTAAC −0.342432186442

ACCCTAAG -0.936170807094
ACCCTAAT 0.606953967732
ACCCTACA 0.436593381968
ACCCTACC -0.39078922756
ACCCTACG -0.269065057778
ACCCTACT -0.693192741612
ACCCTAGA -0.131981307603
ACCCTAGC -0.43879049249
ACCCTAGG 0.464458838845
ACCCTAGT -0.353058632019
ACCCTATA 0.311730720785
ACCCTATC -0.452805451236
ACCCTATG -0.479281528198
ACCCTCAA -0.253444630361
ACCCTCAC -0.496789297286
ACCCTCAG -1.10571700056
ACCCTCAT -0.154499296412
ACCCTCCA -0.848473538205
ACCCTCCC -1.05416088824
ACCCTCCG 0.360438749997
ACCCTCCT -0.380344313191
ACCCTCGA -0.926927079736
ACCCTCGC -0.709975634933
ACCCTCGG -1.20019608387
ACCCTCGT -0.830593546869
ACCCTCTA -0.792569004004
ACCCTCTC -0.449206470118
ACCCTCTG -1.47457967298
ACCCTGAA -0.0247379989112
ACCCTGAC -0.66514637806
ACCCTGAG -0.784119475684
ACCCTGAT 0.00537848658911
ACCCTGCA -0.543156989517
ACCCTGCC -0.937304336258
ACCCTGCG -0.361373885535
ACCCTGCT 0.0996443956447
ACCCTGGA -1.1433992988
ACCCTGGC -1.140955081
ACCCTGGG -1.14662064504
ACCCTGTA -0.140155000084
ACCCTGTC -1.28801846774
ACCCTGTG -0.339743151325
ACCCTTAA -0.119424219332
ACCCTTAC -0.269227020255
ACCCTTAG -0.43364225076
ACCCTTAT -0.431006717387
ACCCTTCA -0.374623581984
ACCCTTCC -0.960689802758
ACCCTTCG -0.470411063979
ACCCTTCT -0.570428306407
ACCCTTGA -0.520131046408
ACCCTTGC -0.0360905693232
ACCCTTGG -0.767800611106
ACCCTTTA 1.00128659289

ACCCTTTC -1.16157594378
ACCCTTTG -0.353173338093
ACCGAAAA -0.0388445559731
ACCGAAAC -0.0309498220004
ACCGAAAG 0.206271081125
ACCGAAAT 5.28143484217
ACCGAACA -0.228528639267
ACCGAACC 0.420054056897
ACCGAACG -0.0609055940913
ACCGAACT -0.731014102757
ACCGAAGA 0.452933688879
ACCGAAGC -0.709205376365
ACCGAAGG 0.342337465456
ACCGAAGT -0.667145927669
ACCGAATA 1.95205629799
ACCGAATC 4.5160846942
ACCGAATG -0.0567876252409
ACCGACAA 0.19603830013
ACCGACAC -0.256863745694
ACCGACAG -0.705079704929
ACCGACAT 2.11850501112
ACCGACCA -0.74185039175
ACCGACCC -1.12066667848
ACCGACCG -0.562116591917
ACCGACCT -0.89718427373
ACCGACGA 0.0800115456197
ACCGACGC -1.14052477709
ACCGACGG -0.0554748548263
ACCGACGT -0.632087088471
ACCGACTA -0.476138040526
ACCGACTC -1.1150762667
ACCGACTG 0.184116362911
ACCGAGAA -0.0535371340811
ACCGAGAC -0.381862555283
ACCGAGAG -1.41497685676
ACCGAGAT 3.25432954552
ACCGAGCA -0.802964996545
ACCGAGCC -1.62450113536
ACCGAGCG -0.888275504761
ACCGAGCT -0.978440723739
ACCGAGGA -0.542976915554
ACCGAGGC -0.6084270352
ACCGAGGG -1.43729999097
ACCGAGGT -0.785910014589
ACCGAGTA 1.38758500481
ACCGAGTC -1.44702148682
ACCGAGTG -0.161945823169
ACCGATAA 1.73528097177
ACCGATAC 1.92487741213
ACCGATAG 0.977395045688
ACCGATAT 4.7663781343
ACCGATCA -0.152863017399
ACCGATCC 1.27197502264
ACCGATCG 1.2653058325

ACCGATCT 1.11637800368
ACCGATGA -0.378615603149
ACCGATGC -1.03140120478
ACCGATGG -0.629421577468
ACCGATTA 2.11409247041
ACCGATTC 1.17212952706
ACCGATTG 0.732445534627
ACCGCAAA -0.609005978195
ACCGCAAC -1.1123662056
ACCGCAAG -0.952698266019
ACCGCAAT 2.19540637754
ACCGCACA -0.0803219390051
ACCGCACC -0.298526407139
ACCGCACG -0.244575831566
ACCGCACT 0.678276372164
ACCGCAGA 0.38557145438
ACCGCAGC -0.00229620324655
ACCGCAGG -0.763684515858
ACCGCAGT -0.291347597279
ACCGCATA 0.205588049135
ACCGCATC 0.588187970864
ACCGCATG -0.524707631373
ACCGCCAA -0.296813726802
ACCGCCAC -0.685022171801
ACCGCCAG -0.124963211152
ACCGCCAT -0.292863549413
ACCGCCCA -0.605618505917
ACCGCCCC -0.871663317393
ACCGCCCG -0.504680492233
ACCGCCCT -0.603741156789
ACCGCCGA 0.0640963381674
ACCGCCGC -1.20953286679
ACCGCCGG -0.622217994427
ACCGCCGT -1.11160510687
ACCGCCTA -0.441067335183
ACCGCCTC -1.51797895523
ACCGCCTG -0.793455009536
ACCGCGAA -0.195865096041
ACCGCGAC -0.633226238441
ACCGCGAG -0.371979305135
ACCGCGAT 3.97923612236
ACCGCGCA -0.267391723082
ACCGCGCC -0.10635522138
ACCGCGCG -0.253007872935
ACCGCGCT 0.449628655085
ACCGCGGA -0.638950092318
ACCGCGGC -1.17081592393
ACCGCGGG -0.240803438182
ACCGCGGT -0.929475594708
ACCGCGTA -0.11946689582
ACCGCGTC -0.432284930255
ACCGCGTG -0.362554879281
ACCGCTAA 0.423510644268
ACCGCTAC -0.965226001193

| | |
|---|---|
| ACCGCTAG | -0.69983278683 |
| ACCGCTAT | 0.300932528365 |
| ACCGCTCA | -0.699277784305 |
| ACCGCTCC | -0.418821226831 |
| ACCGCTCG | -0.447051827906 |
| ACCGCTCT | -0.996830542977 |
| ACCGCTGA | -0.871941651368 |
| ACCGCTGC | -0.960261372451 |
| ACCGCTGG | -0.802270722943 |
| ACCGCTTA | -0.936910879854 |
| ACCGCTTC | -1.56228048089 |
| ACCGCTTG | -0.643813338378 |
| ACCGGAAA | 0.310494559872 |
| ACCGGAAC | -0.291880949293 |
| ACCGGAAG | -0.631511892681 |
| ACCGGAAT | 2.73577253291 |
| ACCGGACA | -0.518125251459 |
| ACCGGACC | -1.04072403978 |
| ACCGGACG | -0.970496443405 |
| ACCGGACT | -0.663039824964 |
| ACCGGAGA | -0.0896331160321 |
| ACCGGAGC | -0.409713023346 |
| ACCGGAGG | -0.558386666843 |
| ACCGGAGT | -0.0152475806335 |
| ACCGGATA | 2.89557266552 |
| ACCGGATC | 1.56369858937 |
| ACCGGATG | -0.328424513926 |
| ACCGGCAA | -0.690258889178 |
| ACCGGCAC | -1.03070651483 |
| ACCGGCAG | 0.202514509268 |
| ACCGGCAT | -0.0736204812798 |
| ACCGGCCA | -0.85656083682 |
| ACCGGCCC | -0.886993544689 |
| ACCGGCCG | -0.685131881603 |
| ACCGGCCT | -0.68139862568 |
| ACCGGCGA | -0.205185641077 |
| ACCGGCGC | -0.659279505903 |
| ACCGGCGG | -0.509795633662 |
| ACCGGCGT | -1.10141021427 |
| ACCGGCTA | -1.09968192058 |
| ACCGGCTC | -1.29322937104 |
| ACCGGCTG | -1.15967590325 |
| ACCGGGAA | -0.634505284021 |
| ACCGGGAC | -0.722634105886 |
| ACCGGGAG | -0.121486846872 |
| ACCGGGAT | 2.03791181619 |
| ACCGGGCA | -0.869232214808 |
| ACCGGGCC | -1.47522793925 |
| ACCGGGCG | -1.51270039408 |
| ACCGGGCT | -0.887115536992 |
| ACCGGGGA | 0.183291145352 |
| ACCGGGGC | -0.90962957042 |
| ACCGGGGG | -1.28099287688 |
| ACCGGGTA | -0.154749526358 |

ACCGGGTC -1.23253403672
ACCGGGTG -0.92146948551
ACCGGTAA 0.831515775371
ACCGGTAC -0.569328085722
ACCGGTAG 0.444371328087
ACCGGTAT 1.21416399439
ACCGGTCA 0.246380942931
ACCGGTCC -0.841020349752
ACCGGTCG -0.0887489841021
ACCGGTCT 0.230532352436
ACCGGTGA -0.985691563184
ACCGGTGC -1.02948263641
ACCGGTGG -1.07859432275
ACCGGTTA 0.0591737613797
ACCGGTTC -0.938924377388
ACCGGTTG -1.15953184408
ACCGTAAA 0.405582771994
ACCGTAAC 1.37733681865
ACCGTAAG 0.0207355688461
ACCGTAAT 6.16469950267
ACCGTACA -0.84519869022
ACCGTACC -0.447080972825
ACCGTACG -0.587935867316
ACCGTACT 0.106665406587
ACCGTAGA 0.288419782006
ACCGTAGC -0.759218889761
ACCGTAGG 0.13327513382
ACCGTAGT 0.174602212342
ACCGTATA 1.11125016339
ACCGTATC 2.71124125474
ACCGTATG 0.214934408243
ACCGTCAA -0.855141271095
ACCGTCAC -0.751989284472
ACCGTCAG -0.573955882473
ACCGTCAT 0.614065536101
ACCGTCCA 0.109752478023
ACCGTCCC 0.0697267201263
ACCGTCCG 0.0118336697504
ACCGTCCT -1.33496635203
ACCGTCGA -1.14924993308
ACCGTCGC -1.08699597013
ACCGTCGG -0.621170650952
ACCGTCGT -0.322949016393
ACCGTCTA -0.0963626777862
ACCGTCTC -1.0454278215
ACCGTCTG -0.745532435886
ACCGTGAA 0.53270145807
ACCGTGAC -0.518805368958
ACCGTGAG -0.561988354274
ACCGTGAT 1.74341115505
ACCGTGCA -0.135506593711
ACCGTGCC -0.283482632759
ACCGTGCG 0.198725045288
ACCGTGCT -0.109815764133

ACCGTGGA -0.760540403652
ACCGTGGC -0.97749538748
ACCGTGGG -0.460372721228
ACCGTGTA -0.44764222069
ACCGTGTC -0.207952326585
ACCGTGTG -0.347646836951
ACCGTTAA 0.107917389028
ACCGTTAC -0.156133910002
ACCGTTAG 0.236474376848
ACCGTTAT 0.769514956866
ACCGTTCA 0.259144960125
ACCGTTCC 0.549131489695
ACCGTTCG -0.229648636862
ACCGTTCT -0.0177854785282
ACCGTTGA -1.00964785374
ACCGTTGC 0.111418318312
ACCGTTGG -0.842328748429
ACCGTTTA 0.964431177622
ACCGTTTC -1.78151647098
ACCGTTTG 0.532997695352
ACCTAAAA -0.0209014867054
ACCTAAAC 0.288971037327
ACCTAAAG 0.621161074764
ACCTAAAT 2.56735383145
ACCTAACA 0.0528189200104
ACCTAACC -0.132561499665
ACCTAACG -0.151423258409
ACCTAACT -0.361201097802
ACCTAAGA 0.115427201894
ACCTAAGC -0.985406151158
ACCTAAGG 0.699315256343
ACCTAAGT -0.568286779408
ACCTAATA 1.0967428637
ACCTAATC 0.529992854222
ACCTAATG 1.82226648011
ACCTACAA 0.360851775132
ACCTACAC 1.02480987322
ACCTACAG -0.0666090465251
ACCTACAT 0.952424095604
ACCTACCA 0.208169039874
ACCTACCC -0.386283006755
ACCTACCG 0.325528966242
ACCTACCT -0.908115908244
ACCTACGA 1.07166074657
ACCTACGC -0.908220621773
ACCTACGG -0.563058180973
ACCTACGT -0.212236837828
ACCTACTA -0.456174603851
ACCTACTC -0.633971099295
ACCTACTG -0.622594796592
ACCTAGAA 1.62605331046
ACCTAGAC -0.860001810842
ACCTAGAG -0.212874278838
ACCTAGAT 2.39679360299

ACCTAGCA -0.138307212231
ACCTAGCC -0.568545336473
ACCTAGCG -0.948965426453
ACCTAGCT -0.359877293954
ACCTAGGA -0.755356355307
ACCTAGGC -0.198669461765
ACCTAGGG -0.693476280037
ACCTAGGT -0.77005975867
ACCTAGTA 0.259962683276
ACCTAGTC -0.287369732217
ACCTAGTG -0.508060053747
ACCTATAA 0.721331952549
ACCTATAC -0.0410079416611
ACCTATAG -0.251818760247
ACCTATAT 1.05791038205
ACCTATCA -0.0106612113021
ACCTATCC 0.330363067383
ACCTATCG 0.00677827540414
ACCTATCT 0.163240690276
ACCTATGA 0.140667117943
ACCTATGC -0.773237595711
ACCTATGG -0.343657313922
ACCTATTA 0.766954783926
ACCTATTC 0.0118307552585
ACCTATTG -0.0419205939759
ACCTCAAA -0.263534809433
ACCTCAAC -0.56008789739
ACCTCAAG -1.03319320094
ACCTCAAT 0.905366501509
ACCTCACA -0.650910958821
ACCTCACC -0.742363966855
ACCTCACG -0.259205123565
ACCTCACT -0.505215509671
ACCTCAGA -0.0477531167645
ACCTCAGC -1.09134147753
ACCTCAGG -0.784119475684
ACCTCAGT -0.546627316635
ACCTCATA 0.00141977390222
ACCTCATC -0.00840477005194
ACCTCATG -0.191390726469
ACCTCCAA 0.137396849874
ACCTCCAC -1.17900522976
ACCTCCAG -1.17329282567
ACCTCCAT 0.263000416528
ACCTCCCA 0.111569871889
ACCTCCCC -0.629858334894
ACCTCCCG -0.588971552824
ACCTCCCT -1.35083867482
ACCTCCGA -0.906790647149
ACCTCCGC -1.38148996958
ACCTCCGG -0.963690896684
ACCTCCGT -0.713063539082
ACCTCCTA -1.29144715926
ACCTCCTC -0.921412028384

ACCTCCTG 0.016609064698
ACCTCGAA 0.194924131519
ACCTCGAC -1.01644153431
ACCTCGAG 0.857604224913
ACCTCGAT 0.451835549973
ACCTCGCA -0.668913358817
ACCTCGCC -0.779796035156
ACCTCGCG -0.598301674048
ACCTCGCT -0.461045552496
ACCTCGGA -0.785188886026
ACCTCGGC -0.542976915554
ACCTCGGG -0.299835846705
ACCTCGTA -0.255338633728
ACCTCGTC -0.477681263977
ACCTCGTG -0.120123697383
ACCTCTAA 0.399417788953
ACCTCTAC -0.491144967401
ACCTCTAG 0.054793071904
ACCTCTAT 0.187809232303
ACCTCTCA -0.456291599882
ACCTCTCC -0.665847313357
ACCTCTCG -0.9031494059
ACCTCTCT -0.687118524176
ACCTCTGA 1.16707288364
ACCTCTGC -1.40543418581
ACCTCTGG -0.762940279538
ACCTCTTA 1.22289414665
ACCTCTTC -1.13206629712
ACCTCTTG -0.759205150014
ACCTGAAA 0.522410595415
ACCTGAAC -0.499215819955
ACCTGAAG -0.535447950312
ACCTGAAT 0.737351873531
ACCTGACA -0.162853062856
ACCTGACC -1.47671016654
ACCTGACG -0.590975265993
ACCTGACT -0.101462205867
ACCTGAGA 0.327620530523
ACCTGAGC 0.179457755816
ACCTGAGG 0.801173624928
ACCTGAGT -0.288263232156
ACCTGATA 2.13853444022
ACCTGATC 0.459728618522
ACCTGATG 0.0895560901752
ACCTGCAA 0.324713741227
ACCTGCAC -0.39963054686
ACCTGCAG 0.648126161829
ACCTGCAT 0.529300037867
ACCTGCCA -0.187671210294
ACCTGCCC -0.547621158367
ACCTGCCG -0.615124329366
ACCTGCCT -0.980531455308
ACCTGCGA -0.112966121679
ACCTGCGC -0.659728129474

ACCTGCGG −0.94347743824
ACCTGCGT −0.664818497723
ACCTGCTA −1.05026566984
ACCTGCTC −1.06687057098
ACCTGCTG −0.0808313505503
ACCTGGAA −0.0460527189297
ACCTGGAC −0.645668204283
ACCTGGAG −1.25283159907
ACCTGGAT 1.32694567037
ACCTGGCA −0.324573221083
ACCTGGCC −1.00176665133
ACCTGGCG −1.05388942414
ACCTGGCT −0.966211932161
ACCTGGGA −1.18941912561
ACCTGGGC −0.969450973531
ACCTGGGG −0.925325774625
ACCTGGTA −0.484150603242
ACCTGGTC −0.833123950356
ACCTGGTG −0.55664671519
ACCTGTAA 1.23105180942
ACCTGTAC −0.194532340539
ACCTGTAG 0.0922361736382
ACCTGTAT 1.00788292073
ACCTGTCA −0.636026648783
ACCTGTCC −0.6372334566
ACCTGTCG −1.15588248389
ACCTGTCT −0.73320392705
ACCTGTGA −1.0077469805
ACCTGTGC −0.601855064186
ACCTGTGG −0.63360470603
ACCTGTTA −0.60917543508
ACCTGTTC −1.16312999248
ACCTGTTG −0.0641438027495
ACCTTAAA 0.0591058953545
ACCTTAAC 0.0575978539839
ACCTTAAG 0.441307364408
ACCTTAAT 0.886122111617
ACCTTACA −0.106504484999
ACCTTACC −1.1061375201
ACCTTACG −0.118003612717
ACCTTACT −0.436348564649
ACCTTAGA −0.16742048799
ACCTTAGC −0.488638920739
ACCTTAGG 0.111478065395
ACCTTAGT 0.804413082654
ACCTTATA 0.48242293409
ACCTTATC −0.0196869763028
ACCTTATG 0.453431650634
ACCTTCAA −0.234853919362
ACCTTCAC −0.128111486918
ACCTTCAG −1.0315136209
ACCTTCAT 1.30381126653
ACCTTCCA 0.405854236095
ACCTTCCC −0.506907996742

| | |
|---|---|
| ACCTTCCG | -0.213371407882 |
| ACCTTCCT | -0.852150794247 |
| ACCTTCGA | -1.07195885745 |
| ACCTTCGC | -0.979925657353 |
| ACCTTCGG | -0.803113219275 |
| ACCTTCGT | -0.604472694251 |
| ACCTTCTA | 0.33539139859 |
| ACCTTCTC | -0.272693808348 |
| ACCTTCTG | -0.80927778596 |
| ACCTTGAA | -0.0457381619845 |
| ACCTTGAC | -0.0957239877082 |
| ACCTTGAG | -0.421153861226 |
| ACCTTGAT | 0.690453951956 |
| ACCTTGCA | 0.394886794967 |
| ACCTTGCC | -1.09330147332 |
| ACCTTGCG | -0.41504633531 |
| ACCTTGCT | 0.403996039342 |
| ACCTTGGA | -0.905685222014 |
| ACCTTGGC | -0.290321279781 |
| ACCTTGGG | -0.489941282254 |
| ACCTTGTA | 0.164887794545 |
| ACCTTGTC | -0.790323596187 |
| ACCTTGTG | 0.284361143883 |
| ACCTTTAA | 0.977096726626 |
| ACCTTTAC | -0.0671971493512 |
| ACCTTTAG | -0.215295597058 |
| ACCTTTAT | 0.857686247042 |
| ACCTTTCA | 0.0205935914559 |
| ACCTTTCC | 0.239565403666 |
| ACCTTTCG | 0.0596280057573 |
| ACCTTTCT | -0.323152198112 |
| ACCTTTGA | -0.221152060316 |
| ACCTTTGC | -0.745046132098 |
| ACCTTTGG | -0.31309220485 |
| ACCTTTTA | 0.420632583535 |
| ACCTTTTC | 0.28449312873 |
| ACCTTTTG | -0.0856594145298 |
| ACGAAAAA | 3.36472175451 |
| ACGAAAAC | 0.173587969167 |
| ACGAAAAG | 0.226799929226 |
| ACGAAAAT | 3.24739576116 |
| ACGAAACA | 0.0982789561982 |
| ACGAAACC | 0.0291848889882 |
| ACGAAACG | 0.84894256322 |
| ACGAAACT | 1.61117816026 |
| ACGAAAGA | 0.779348036118 |
| ACGAAAGC | -0.521393437748 |
| ACGAAAGG | 0.0630925038923 |
| ACGAAAGT | -0.447341819848 |
| ACGAAATA | 3.57191173329 |
| ACGAAATC | 14.7145204847 |
| ACGAAATG | 1.16030293536 |
| ACGAACAA | 0.610977423774 |
| ACGAACAC | 0.269058187904 |

ACGAACAG −0.153162377351
ACGAACAT 0.216190554243
ACGAACCA −1.21962575218
ACGAACCC −0.666078390928
ACGAACCG −0.480425258084
ACGAACCT −0.109274917711
ACGAACGA 1.29291814494
ACGAACGC −0.0314298804489
ACGAACGG −0.649570292556
ACGAACGT 0.0393152464119
ACGAACTA 0.132866272245
ACGAACTC −0.438740946128
ACGAACTG −0.160027671155
ACGAAGAA −0.35410951452
ACGAAGAC −0.353947552043
ACGAAGAG −0.633457732368
ACGAAGAT 4.61111836369
ACGAAGCA 0.396213929663
ACGAAGCC −0.514626820312
ACGAAGCG −0.00368017053422
ACGAAGCT −0.0520740591567
ACGAAGGA −0.6876943445
ACGAAGGC −1.03591325457
ACGAAGGG −1.04250562703
ACGAAGTA 0.0488660363073
ACGAAGTC −0.644346274036
ACGAAGTG −0.706369159408
ACGAATAA 0.503773668901
ACGAATAC 0.837904133397
ACGAATAG 0.167876397792
ACGAATAT 0.695729182261
ACGAATCA 2.94496976388
ACGAATCC 4.26954532782
ACGAATCG 4.70598091087
ACGAATCT 6.87777698968
ACGAATGA 0.655520019554
ACGAATGC −0.481800898252
ACGAATGG 0.174215417634
ACGAATTA 1.05777236004
ACGAATTC 1.30279785607
ACGAATTG 0.839455683968
ACGACAAA 0.398244081436
ACGACAAC 0.279599905039
ACGACAAG −0.209918775893
ACGACAAT 1.42117743824
ACGACACA −0.528908246887
ACGACACC 0.300634209303
ACGACACG −0.673997273548
ACGACACT −0.0761381859093
ACGACAGA 0.488546281533
ACGACAGC −0.856117834054
ACGACAGG −0.912478694412
ACGACAGT −1.16338730048
ACGACATA −0.560740951749

ACGACATC 1.18942412188
ACGACATG 0.147696456005
ACGACCAA -0.288652525
ACGACCAC -0.645285573134
ACGACCAG -0.455374784007
ACGACCAT -0.7697643541
ACGACCCA -1.10989492467
ACGACCCC -1.07650171758
ACGACCCG -1.04776857483
ACGACCCT -0.470230781839
ACGACCGA -0.258304753752
ACGACCGC -0.272320961565
ACGACCGG -0.582700190829
ACGACCGT -0.542605526017
ACGACCTA -0.514534805639
ACGACCTC -0.548164502925
ACGACCTG -1.12227297987
ACGACGAA 1.40576144161
ACGACGAC -0.0867902373798
ACGACGAG -0.860630924732
ACGACGAT 1.5566230358
ACGACGCA -1.19154004299
ACGACGCC -1.5369520892
ACGACGCG -0.454275187856
ACGACGCT -0.0384252854982
ACGACGGA 0.388692250651
ACGACGGC -1.10193065925
ACGACGGG -0.64721704854
ACGACGTA 0.61201123568
ACGACGTC -0.863380747822
ACGACGTG -0.18299594896
ACGACTAA -0.207733947872
ACGACTAC -0.174778330923
ACGACTAG -0.728323818573
ACGACTAT 0.111405203098
ACGACTCA 0.982437949355
ACGACTCC -0.775646215073
ACGACTCG -0.218124111429
ACGACTCT 0.658113084616
ACGACTGA 0.878352076261
ACGACTGC -0.843069237545
ACGACTGG -0.531158650975
ACGACTTA -0.686605157249
ACGACTTC 0.0922732293207
ACGACTTG -0.870243959847
ACGAGAAA 1.50655622883
ACGAGAAC -0.137701830632
ACGAGAAG -0.248727733429
ACGAGAAT 2.50505656748
ACGAGACA -0.595954467194
ACGAGACC -0.0960139796504
ACGAGACG -0.12485974669
ACGAGACT 1.1791921736
ACGAGAGA 0.829835362623

| | |
|---|---|
| ACGAGAGC | 0.17184010675 |
| ACGAGAGG | -0.535309720126 |
| ACGAGAGT | -0.492737320859 |
| ACGAGATA | 2.51375257854 |
| ACGAGATC | 7.02601928838 |
| ACGAGATG | 0.326266957222 |
| ACGAGCAA | -0.459400113651 |
| ACGAGCAC | -0.00320843920542 |
| ACGAGCAG | -0.592880510971 |
| ACGAGCAT | -0.0492632399151 |
| ACGAGCCA | -0.825467372005 |
| ACGAGCCC | -1.1100412738 |
| ACGAGCCG | -1.47919039914 |
| ACGAGCCT | -0.621330948005 |
| ACGAGCGA | -0.161999324913 |
| ACGAGCGC | -0.518475823197 |
| ACGAGCGG | -0.952485924468 |
| ACGAGCGT | 0.209603386236 |
| ACGAGCTA | 0.115608733103 |
| ACGAGCTC | -0.860151907174 |
| ACGAGCTG | -0.764673361317 |
| ACGAGGAA | 0.319251358907 |
| ACGAGGAC | 0.151851688716 |
| ACGAGGAG | -0.522931040394 |
| ACGAGGAT | 2.03397121499 |
| ACGAGGCA | 0.45288684883 |
| ACGAGGCC | -0.552024747422 |
| ACGAGGCG | -0.846879311146 |
| ACGAGGCT | -0.293424380922 |
| ACGAGGGA | -0.461605343116 |
| ACGAGGGC | -1.67742893246 |
| ACGAGGGG | -1.12670550566 |
| ACGAGGTA | -0.891955258939 |
| ACGAGGTC | -0.623862808738 |
| ACGAGGTG | -0.107961939118 |
| ACGAGTAA | 0.0945559009978 |
| ACGAGTAC | -0.0328546506229 |
| ACGAGTAG | -0.423170065074 |
| ACGAGTAT | 2.10321725213 |
| ACGAGTCA | -0.00993591917963 |
| ACGAGTCC | -1.04696729774 |
| ACGAGTCG | -0.389047194127 |
| ACGAGTCT | -0.528714224998 |
| ACGAGTGA | -0.0431144947573 |
| ACGAGTGC | 0.0101311901357 |
| ACGAGTGG | -0.568859268885 |
| ACGAGTTA | 0.408357160087 |
| ACGAGTTC | 0.320281839965 |
| ACGAGTTG | 0.0355478492993 |
| ACGATAAA | 0.804493023003 |
| ACGATAAC | 1.14669683819 |
| ACGATAAG | 0.442047645346 |
| ACGATAAT | 3.44453948642 |
| ACGATACA | 1.01269953492 |

ACGATACC 0.477885486587
ACGATACG 2.06087072598
ACGATACT 1.97973460285
ACGATAGA 1.57656045022
ACGATAGC 0.476514426335
ACGATAGG 0.760494188137
ACGATAGT 0.994166072863
ACGATATA 2.75569454216
ACGATATC 8.59895057168
ACGATATG 3.41862569821
ACGATCAA 0.556208292339
ACGATCAC 2.49938975437
ACGATCAG 1.34232981577
ACGATCAT 0.926742634035
ACGATCCA 0.814322771407
ACGATCCC 2.81981960889
ACGATCCG 2.76405455394
ACGATCCT 4.40548056001
ACGATCGA 0.577991204661
ACGATCGC 3.72734866338
ACGATCGG 0.647357776862
ACGATCGT 2.70907058282
ACGATCTA 3.49185189038
ACGATCTC 4.15867181131
ACGATCTG 4.3315170014
ACGATGAA -0.000572905832684
ACGATGAC 0.153690524915
ACGATGAG -0.945340839443
ACGATGAT 1.49652163329
ACGATGCA 1.26545988422
ACGATGCC 0.216893987677
ACGATGCG 1.21722795807
ACGATGCT 0.772730682301
ACGATGGA 0.00638814984802
ACGATGGC -0.524519438469
ACGATGGG 0.933311898736
ACGATGTA 0.582940428232
ACGATGTC -0.254865653331
ACGATGTG -0.337969683015
ACGATTAA 0.257440815087
ACGATTAC 8.50731186887
ACGATTAG 0.0307000084106
ACGATTAT 2.77628376188
ACGATTCA 2.240050356
ACGATTCC 7.00531078267
ACGATTCG 4.04785283744
ACGATTCT 5.34233502363
ACGATTGA 0.824445634423
ACGATTGC 3.41339251986
ACGATTGG 1.34402334373
ACGATTTA 1.92647538639
ACGATTTC 9.80138248718
ACGATTTG 3.34961261224
ACGCAAAA 1.36732200001

ACGCAAAC 0.000179657606688
ACGCAAAG -0.967541356815
ACGCAAAT 1.63611205466
ACGCAACA 1.58724352021
ACGCAACC 0.913037027785
ACGCAACG 0.666605497603
ACGCAACT 0.531170725298
ACGCAAGA 0.232905997895
ACGCAAGC -0.0520740591567
ACGCAAGG -0.353002423962
ACGCAAGT -0.546859643274
ACGCAATA 2.70597830694
ACGCAATC 17.9473377493
ACGCAATG 0.77836002337
ACGCACAA 0.432198744566
ACGCACAC 0.525971688138
ACGCACAG -0.383800692384
ACGCACAT 0.60347260718
ACGCACCA -1.37269111859
ACGCACCC -1.08553414428
ACGCACCG -0.253078653452
ACGCACCT -0.0192695794298
ACGCACGA 0.370628021792
ACGCACGC 0.523462726984
ACGCACGG -0.160264994066
ACGCACGT 0.0256825024588
ACGCACTA 0.52468722993
ACGCACTC -0.985869763545
ACGCACTG 0.161383742592
ACGCAGAA 1.29984464308
ACGCAGAC -0.185319423524
ACGCAGAG 0.0271980382371
ACGCAGAT 3.26490332207
ACGCAGCA -0.269087124645
ACGCAGCC -1.3006078236
ACGCAGCG -0.448080019006
ACGCAGCT 0.118085634846
ACGCAGGA -0.341710433345
ACGCAGGC -1.66935100185
ACGCAGGG -0.629323733812
ACGCAGTA -0.376667265326
ACGCAGTC -0.915113811429
ACGCAGTG -1.09504621307
ACGCATAA 1.00946882066
ACGCATAC 0.797720992938
ACGCATAG 0.205692762665
ACGCATAT 2.08611418106
ACGCATCA 0.579825252766
ACGCATCC 0.257900263914
ACGCATCG 0.612565613672
ACGCATCT -0.158270440729
ACGCATGA -0.269266574073
ACGCATGC -0.356485866294
ACGCATGG -0.00942192771855

ACGCATTA 0.358584508626
ACGCATTC 0.18507731252
ACGCATTG 0.252723918155
ACGCCAAA 0.946637371974
ACGCCAAC −1.0696031153
ACGCCAAG −1.0421852411
ACGCCAAT −0.138936742478
ACGCCACA −0.283332744605
ACGCCACC −0.821993714039
ACGCCACG −0.487313451467
ACGCCACT −0.585050520354
ACGCCAGA −0.59377817447
ACGCCAGC −0.908230197961
ACGCCAGG −1.04705369161
ACGCCAGT −0.822858069059
ACGCCATA −0.355757659679
ACGCCATC −0.742354807024
ACGCCATG −0.126904054567
ACGCCCAA 0.440215262664
ACGCCCAC −1.05624932985
ACGCCCAG −1.01990686516
ACGCCCAT −0.937121347803
ACGCCCCA −1.36599902887
ACGCCCCC −1.23681375987
ACGCCCCG −1.33103178799
ACGCCCCT −1.40485003836
ACGCCCGA −0.481849195546
ACGCCCGC −1.11414154752
ACGCCCGG −0.411008514987
ACGCCCGT −0.197896496882
ACGCCCTA −0.849567721728
ACGCCCTC −1.35375358305
ACGCCCTG −0.747147688923
ACGCCGAA 1.30183482468
ACGCCGAC −0.983354140695
ACGCCGAG −0.183537628094
ACGCCGAT 1.11605969953
ACGCCGCA −0.428454247033
ACGCCGCC −0.289464002811
ACGCCGCG 0.236786852013
ACGCCGCT −0.9011696332
ACGCCGGA −0.0992904930591
ACGCCGGC −0.741075969622
ACGCCGGG −0.164942753535
ACGCCGTA −0.994739811408
ACGCCGTC −1.5049986411
ACGCCGTG −0.882240424787
ACGCCTAA 0.751336854646
ACGCCTAC −0.893916295619
ACGCCTAG −0.185454947396
ACGCCTAT −0.620503648667
ACGCCTCA −0.605656186133
ACGCCTCC −0.839103238628
ACGCCTCG −0.83768721193

ACGCCTCT -1.20963112681
ACGCCTGA -0.0128955856852
ACGCCTGC -1.49639901645
ACGCCTGG -0.693885557968
ACGCCTTA 0.0823702022637
ACGCCTTC -0.931065241852
ACGCCTTG -0.555912263235
ACGCGAAA 1.97965153984
ACGCGAAC 0.303484166007
ACGCGAAG -0.49943815405
ACGCGAAT 2.93013479203
ACGCGACA 0.677705964467
ACGCGACC -0.636167793461
ACGCGACG 0.10398990304
ACGCGACT -0.913881814075
ACGCGAGA 1.18370255796
ACGCGAGC -0.273060826147
ACGCGAGG -0.0613933551254
ACGCGAGT 0.224335934518
ACGCGATA 2.32682498003
ACGCGATC 12.2293399191
ACGCGATG 0.575514719274
ACGCGCAA 0.591388707484
ACGCGCAC 0.355947934364
ACGCGCAG -0.232221716837
ACGCGCAT 0.278346048996
ACGCGCCA -1.13137910157
ACGCGCCC -0.436094171144
ACGCGCCG -1.00713014911
ACGCGCCT -0.0303161281942
ACGCGCGA 2.04838691637
ACGCGCGC 0.423374495862
ACGCGCGG 0.239109077509
ACGCGCGT 1.16722485358
ACGCGCTA 0.413598457379
ACGCGCTC -0.328439086385
ACGCGCTG -0.401557442349
ACGCGGAA 0.171968344393
ACGCGGAC -0.283032968297
ACGCGGAG -0.972304052905
ACGCGGAT 1.94035294766
ACGCGGCA -0.870318903924
ACGCGGCC -1.02347128873
ACGCGGCG -0.352278797263
ACGCGGCT -0.623459776146
ACGCGGGA -0.415003242466
ACGCGGGC -1.01705690846
ACGCGGGG -0.21825255725
ACGCGGTA -0.432284930255
ACGCGGTC -0.799979724146
ACGCGGTG -0.305978138345
ACGCGTAA 0.244884143172
ACGCGTAC 0.224998565065
ACGCGTAG 0.147106687755

ACGCGTAT 2.14234326475
ACGCGTCA -0.376995562019
ACGCGTCC 0.706279018338
ACGCGTCG 0.256619969266
ACGCGTCT -0.0499131716046
ACGCGTGA 0.741403849958
ACGCGTGC 0.0038920957296
ACGCGTGG 1.2550239215
ACGCGTTA 0.0657068031099
ACGCGTTC -0.464266482381
ACGCGTTG 0.25136659765
ACGCTAAA 0.845215136281
ACGCTAAC -0.159219524192
ACGCTAAG 0.718654991756
ACGCTAAT 0.300533242977
ACGCTACA -0.252769092779
ACGCTACC -0.376551102007
ACGCTACG -0.385558130989
ACGCTACT -0.0985437586035
ACGCTAGA -0.863607453655
ACGCTAGC -0.207574691708
ACGCTAGG -0.460172454
ACGCTAGT -0.371432629729
ACGCTATA 0.779348036118
ACGCTATC 0.808259587403
ACGCTATG 0.591759680665
ACGCTCAA 0.00648890799591
ACGCTCAC -1.26097614664
ACGCTCAG -1.10898789316
ACGCTCAT -0.79311463852
ACGCTCCA -0.518394009246
ACGCTCCC -0.749576917906
ACGCTCCG 0.145356951736
ACGCTCCT 0.368626598582
ACGCTCGA 0.0823048343744
ACGCTCGC 1.20254433162
ACGCTCGG -0.452880395313
ACGCTCTA -0.199485519491
ACGCTCTC -1.36734718954
ACGCTCTG -0.257999356638
ACGCTGAA -0.0801283334729
ACGCTGAC -0.094893982056
ACGCTGAG 0.36857184777
ACGCTGAT 3.8880472922
ACGCTGCA 0.684769027363
ACGCTGCC 0.237723861153
ACGCTGCG 0.23176747246
ACGCTGCT -0.0443036074449
ACGCTGGA 0.433863752143
ACGCTGGC -0.0563756409956
ACGCTGGG -0.851024967669
ACGCTGTA -0.919698307158
ACGCTGTC -1.2454344105
ACGCTGTG -0.340506956376

ACGCTTAA -0.0998942092345
ACGCTTAC 0.301592452598
ACGCTTAG -0.408133576924
ACGCTTAT -0.330806486505
ACGCTTCA -0.91656689381
ACGCTTCC -0.32832875205
ACGCTTCG -0.107359888367
ACGCTTCT -0.331654603642
ACGCTTGA 0.452969495493
ACGCTTGC -0.703968867166
ACGCTTGG -0.124578081868
ACGCTTTA 0.521762537327
ACGCTTTC -1.23839487172
ACGCTTTG -0.405967901278
ACGGAAAA 1.8306291982
ACGGAAAC -0.677137222194
ACGGAAAG 0.36886621145
ACGGAAAT 4.47472347449
ACGGAACA 0.648664718293
ACGGAACC 0.233717475706
ACGGAACG -0.760486277374
ACGGAACT -0.00550047889214
ACGGAAGA -0.0225729477993
ACGGAAGC -0.856098265323
ACGGAAGG -1.10110752347
ACGGAAGT -0.275372018209
ACGGAATA 2.99330473813
ACGGAATC 9.22463410513
ACGGAATG 0.152013443015
ACGGACAA 1.09737801475
ACGGACAC -0.137979956428
ACGGACAG -0.970119433062
ACGGACAT -0.296564954102
ACGGACCA -0.555291060109
ACGGACCC 0.321216767325
ACGGACCG -1.05365647297
ACGGACCT -1.51862264158
ACGGACGA -0.00965362982312
ACGGACGC -0.633646341628
ACGGACGG -0.164634233752
ACGGACGT -0.0842234027443
ACGGACTA 0.0597554106881
ACGGACTC -1.11261893369
ACGGACTG -1.0120319081
ACGGAGAA 1.45483357413
ACGGAGAC 0.506352994216
ACGGAGAG -0.430848710291
ACGGAGAT 3.00932486729
ACGGAGCA 0.26379107654
ACGGAGCC -0.985731741537
ACGGAGCG -0.554466258906
ACGGAGCT -1.59663463759
ACGGAGGA -0.183771620157
ACGGAGGC -0.799387041405

ACGGAGGG -0.731449611115
ACGGAGTA 1.2269563238
ACGGAGTC -0.733691896262
ACGGAGTG -0.45215093963
ACGGATAA 0.413129224186
ACGGATAC 2.27012354048
ACGGATAG 0.179731301697
ACGGATAT 7.14377891649
ACGGATCA 0.148221689078
ACGGATCC 1.47818947935
ACGGATCG 2.1303846882
ACGGATCT 2.77870445556
ACGGATGA -0.270278319112
ACGGATGC -0.100677791195
ACGGATGG 0.476634753214
ACGGATTA 2.68509971981
ACGGATTC 7.81405938018
ACGGATTG 1.43236450715
ACGGCAAA 1.03857876558
ACGGCAAC -0.232489017378
ACGGCAAG -0.385072243556
ACGGCAAT 2.04889029075
ACGGCACA -0.157810367367
ACGGCACC 0.237586047323
ACGGCACG -0.800777878566
ACGGCACT -0.656555496884
ACGGCAGA -0.190559055393
ACGGCAGC -1.12565087596
ACGGCAGG -0.953073194582
ACGGCAGT -0.669688613657
ACGGCATA 0.686338689419
ACGGCATC 0.509039114841
ACGGCATG 0.875911813844
ACGGCCAA 0.108778205023
ACGGCCAC -1.20307060558
ACGGCCAG -0.810871596663
ACGGCCAT -0.51964370173
ACGGCCCA -1.32304982744
ACGGCCCC -0.670031066453
ACGGCCCG -1.35061883886
ACGGCCCT -1.34873003994
ACGGCCGA -0.576478167019
ACGGCCGC -1.31988489743
ACGGCCGG 0.0584289005261
ACGGCCGT -0.543485286209
ACGGCCTA -0.633575977467
ACGGCCTC -0.502215664813
ACGGCCTG -1.67926277239
ACGGCGAA -0.655285194779
ACGGCGAC -0.960815542265
ACGGCGAG -1.31018650934
ACGGCGAT 0.716523249123
ACGGCGCA -0.475159395788
ACGGCGCC -0.393174530987

ACGGCGCG -0.437232072046
ACGGCGCT -1.12011583952
ACGGCGGA -0.836550768274
ACGGCGGC -1.51561405325
ACGGCGGG -1.06195757038
ACGGCGTA -0.732875422179
ACGGCGTC -1.51970017086
ACGGCGTG -1.24582869962
ACGGCTAA 0.648533774336
ACGGCTAC -0.922308859172
ACGGCTAG -1.07650171758
ACGGCTAT -1.01252549811
ACGGCTCA -0.386395839226
ACGGCTCC 0.585501433883
ACGGCTCG -0.385558130989
ACGGCTCT -0.391971678552
ACGGCTGA -0.0870219394844
ACGGCTGC -1.17603328075
ACGGCTGG -1.02364574189
ACGGCTTA 0.349981344948
ACGGCTTC -0.597671935624
ACGGCTTG -1.38751672243
ACGGGAAA 0.468016808899
ACGGGAAC -0.777374925114
ACGGGAAG -1.15666606585
ACGGGAAT 3.43716457289
ACGGGACA -0.280518178159
ACGGGACC -0.824254110671
ACGGGACG -1.13530159129
ACGGGACT -0.75007196517
ACGGGAGA -0.754251346528
ACGGGAGC -0.941179985926
ACGGGAGG 0.225239218823
ACGGGAGT -0.75895054833
ACGGGATA 1.26915129636
ACGGGATC 1.42263218604
ACGGGATG -1.12875980608
ACGGGCAA -0.459344738306
ACGGGCAC -1.05810148944
ACGGGCAG -0.614955288837
ACGGGCAT -0.594000716743
ACGGGCCA -0.818338941219
ACGGGCCC -1.01961916318
ACGGGCCG -1.23476612115
ACGGGCCT -1.93433410557
ACGGGCGA 0.507186122539
ACGGGCGC -0.990990942137
ACGGGCGG -1.00897210798
ACGGGCTA 0.2563718211
ACGGGCTC -0.272771875095
ACGGGCTG -1.16421376711
ACGGGGAA -0.386730173081
ACGGGGAC -1.25752830274
ACGGGGAG -1.54617499876

ACGGGGAT 0.38346739942
ACGGGGCA −1.09350069966
ACGGGGCC −1.18168864407
ACGGGGCG −0.460694564403
ACGGGGCT −1.30849776947
ACGGGGGA −0.0258261452729
ACGGGGGC −1.35881022647
ACGGGGGG −1.01934624183
ACGGGGTA −0.119889705321
ACGGGGTC −1.53371304783
ACGGGGTG −0.900411032599
ACGGGTAA −0.621793935858
ACGGGTAC 0.217469183467
ACGGGTAG −1.02563758891
ACGGGTAT 0.333602108753
ACGGGTCA −0.862887990516
ACGGGTCC −0.595815196118
ACGGGTCG −1.83399335455
ACGGGTCT −1.12501968029
ACGGGTGA −0.920574736502
ACGGGTGC −1.4737044927
ACGGGTGG −0.121147308568
ACGGGTTA 0.569070777724
ACGGGTTC −1.25200076071
ACGGGTTG −0.33956536732
ACGGTAAA 1.1220389878
ACGGTAAC −0.563289674899
ACGGTAAG −0.348912975496
ACGGTAAT 1.39650938713
ACGGTACA −0.4835579205
ACGGTACC 0.679499417864
ACGGTACG 0.200715643243
ACGGTACT 0.777590597514
ACGGTAGA −0.0696671812207
ACGGTAGC −0.257093366019
ACGGTAGG −0.301185464624
ACGGTAGT −0.0938961849426
ACGGTATA 0.764699799922
ACGGTATC 1.58015880681
ACGGTATG −1.36790781287
ACGGTCAA −0.367567805317
ACGGTCAC −0.392774412887
ACGGTCAG −1.06120417423
ACGGTCAT −0.875753598571
ACGGTCCA −0.0674348886175
ACGGTCCC −0.73346310865
ACGGTCCG −1.11040017265
ACGGTCCT −1.51313840057
ACGGTCGA −0.321364365521
ACGGTCGC −0.853848693946
ACGGTCGG −0.647733954493
ACGGTCTA 0.243101723208
ACGGTCTC −0.840573599782
ACGGTCTG −0.409331224909

ACGGTGAA 0.000145308238086
ACGGTGAC -0.712972357121
ACGGTGAG -1.01931730509
ACGGTGAT 2.21443009877
ACGGTGCA -0.193395688705
ACGGTGCC -1.05534687826
ACGGTGCG -0.512084550679
ACGGTGCT -0.691026857789
ACGGTGGA -0.0823048343744
ACGGTGGC -0.802224299251
ACGGTGGG -0.514151758135
ACGGTGTA 0.414395570909
ACGGTGTC -0.981252375693
ACGGTGTG -0.529920824637
ACGGTTAA 0.329967529199
ACGGTTAC 0.715821272936
ACGGTTAG -0.241229994886
ACGGTTAT 0.508404380145
ACGGTTCA -1.13450864132
ACGGTTCC -0.908682776914
ACGGTTCG -0.116532210673
ACGGTTCT -0.85404625486
ACGGTTGA -0.35550264164
ACGGTTGC -0.401182721965
ACGGTTGG 0.593402205018
ACGGTTTA -0.107994414885
ACGGTTTC -0.365645489743
ACGGTTTG -0.920723791944
ACGTAAAA 1.64381255856
ACGTAAAC -0.420909668442
ACGTAAAG 0.648139277042
ACGTAAAT 2.17222783997
ACGTAACA 1.4341460944
ACGTAACC 1.31074192822
ACGTAACG 0.461175455563
ACGTAACT 0.779590771656
ACGTAAGA 0.153364726358
ACGTAAGC 0.252265302039
ACGTAAGG -0.150319498698
ACGTAAGT 1.15428867318
ACGTAATA 2.14451810023
ACGTAATC 13.4356645549
ACGTAATG 0.975544551521
ACGTACAA 0.876597968504
ACGTACAC 0.244843964819
ACGTACAG -0.637069204165
ACGTACAT 0.0408586780411
ACGTACCA -0.522630431374
ACGTACCC 0.465219313049
ACGTACCG -0.642433951006
ACGTACCT -0.253216467283
ACGTACGA 0.908471684431
ACGTACGC -0.462709519183
ACGTACGG -0.214070053222

ACGTACGT 0.287499218927
ACGTACTA 1.33535980843
ACGTACTC -0.0350213671587
ACGTACTG -0.33706639871
ACGTAGAA 2.03704829388
ACGTAGAC 0.151577726479
ACGTAGAG 0.264130406666
ACGTAGAT 1.12352204781
ACGTAGCA 0.465090867228
ACGTAGCC -0.752448108765
ACGTAGCG -0.644288816911
ACGTAGCT -0.346034914763
ACGTAGGA 0.334132754453
ACGTAGGC -0.721455818454
ACGTAGGG -0.503044213219
ACGTAGTA 0.328537762753
ACGTAGTC -0.468203960913
ACGTAGTG -1.16788685959
ACGTATAA 2.3778556511
ACGTATAC 0.54858273251
ACGTATAG 0.151905814994
ACGTATAT 1.23837925837
ACGTATCA 2.22745079942
ACGTATCC 4.00456930215
ACGTATCG 1.04838852889
ACGTATCT 4.5798770925
ACGTATGA -0.129678026304
ACGTATGC -0.688757301325
ACGTATGG 0.0914167850635
ACGTATTA 1.98123161079
ACGTATTC 1.48399993527
ACGTATTG 1.827356432
ACGTCAAA 1.18600417384
ACGTCAAC -0.494846996625
ACGTCAAG 1.14626195436
ACGTCAAT 1.13436395761
ACGTCACA 0.638877854555
ACGTCACC 0.890494473973
ACGTCACG 0.331386470389
ACGTCACT -0.00724043054533
ACGTCAGA 0.316849817597
ACGTCAGC -0.0401371331225
ACGTCAGG -0.630898808496
ACGTCAGT -0.60470085733
ACGTCATA 0.395523611443
ACGTCATC -0.164345699055
ACGTCATG 0.457962436442
ACGTCCAA 0.123266560521
ACGTCCAC -0.527898167272
ACGTCCAG 0.301965507559
ACGTCCAT -0.213817741496
ACGTCCCA -0.908230197961
ACGTCCCC 0.306939712488
ACGTCCCG -1.07291231265

ACGTCCCT -0.116557192032
ACGTCCGA -0.876877551547
ACGTCCGC -0.0386032776809
ACGTCCGG -0.614376970376
ACGTCCTA -0.0650793546434
ACGTCCTC -0.758554801968
ACGTCCTG -0.213304166391
ACGTCGAA 0.553009845678
ACGTCGAC 0.40895275732
ACGTCGAG -0.785958936417
ACGTCGAT 0.562287089692
ACGTCGCA -0.406626160087
ACGTCGCC -0.426974309691
ACGTCGCG -1.11711162292
ACGTCGCT -0.232477983945
ACGTCGGA 0.0886954823583
ACGTCGGC -1.37237947613
ACGTCGGG -0.512776950679
ACGTCGTA 0.981794263005
ACGTCGTC 0.129918263707
ACGTCGTG -0.380948237544
ACGTCTAA 1.0777453729
ACGTCTAC -0.67964077072
ACGTCTAG -0.782512757945
ACGTCTAT 0.395523611443
ACGTCTCA -0.118003612717
ACGTCTCC -0.325029130884
ACGTCTCG 0.399747542891
ACGTCTCT -0.564081167623
ACGTCTGA 0.0256825024588
ACGTCTGC -1.08434961151
ACGTCTGG 0.360958362264
ACGTCTTA 0.104882778445
ACGTCTTC -0.351325758418
ACGTCTTG -0.093297256861
ACGTGAAA 1.36688669983
ACGTGAAC 0.996171659634
ACGTGAAG -0.504804774493
ACGTGAAT 0.965538684537
ACGTGACA 0.110059748739
ACGTGACC -0.354832308507
ACGTGACG -0.768083108641
ACGTGACT 0.447670116541
ACGTGAGA -0.18339502617
ACGTGAGC -1.10020340645
ACGTGAGG 0.308936972139
ACGTGAGT -0.158426366044
ACGTGATA 1.54368810447
ACGTGATC 2.52189899971
ACGTGATG -0.410841972594
ACGTGCAA 0.851361591481
ACGTGCAC -0.186066782513
ACGTGCAG 0.377669434178
ACGTGCAT -0.86146759208

ACGTGCCA -0.945641864818
ACGTGCCC 0.0447622235603
ACGTGCCG -0.810871596663
ACGTGCCT -0.938948109679
ACGTGCGA 0.966871856394
ACGTGCGC -0.672098065731
ACGTGCGG 0.252990385984
ACGTGCTA 0.755253515379
ACGTGCTC -0.00474062922307
ACGTGCTG -0.536015235339
ACGTGGAA -0.377276810486
ACGTGGAC 0.604125869717
ACGTGGAG -0.606584868153
ACGTGGAT 2.63029977803
ACGTGGCA -0.386199943736
ACGTGGCC 0.087964361252
ACGTGGCG -1.13816237325
ACGTGGCT -0.681278090623
ACGTGGGA -0.201398883411
ACGTGGGC -0.911234206379
ACGTGGGG 0.706768861152
ACGTGGTA 0.250549915389
ACGTGGTC -0.758665344482
ACGTGGTG -1.20494420751
ACGTGTAA 0.676702546548
ACGTGTAC -0.561549723246
ACGTGTAG 1.12830472899
ACGTGTAT 0.735294242263
ACGTGTCA -0.625935012466
ACGTGTCC -0.476943689353
ACGTGTCG -1.11821496628
ACGTGTCT 0.339842452227
ACGTGTGA -0.808230858841
ACGTGTGC 0.415644847036
ACGTGTGG -0.193914260082
ACGTGTTA 0.451178956588
ACGTGTTC 0.707472919119
ACGTGTTG -0.12928873346
ACGTTAAA 0.99390834851
ACGTTAAC 0.724584317311
ACGTTAAG 0.348772038996
ACGTTAAT 1.89547435261
ACGTTACA 0.650851628094
ACGTTACC 0.240593802944
ACGTTACG 0.732445534627
ACGTTACT 0.0360905693232
ACGTTAGA 0.732840031921
ACGTTAGC -0.219822635662
ACGTTAGG -0.207937337769
ACGTTAGT 0.923218180639
ACGTTATA 2.43952713202
ACGTTATC 1.45264145988
ACGTTATG -0.277569545087
ACGTTCAA -0.187756563271

ACGTTCAC -0.227076389598
ACGTTCAG -1.10256476941
ACGTTCAT -0.159441858287
ACGTTCCA -0.247584003544
ACGTTCCC -0.259557777083
ACGTTCCG -0.891944433684
ACGTTCCT 0.599168110849
ACGTTCGA -0.296586188258
ACGTTCGC -0.0203831235064
ACGTTCGG -0.473948632589
ACGTTCTA 1.32150223225
ACGTTCTC -0.392456733272
ACGTTCTG 0.344389684096
ACGTTGAA 0.954481726873
ACGTTGAC -0.269032165655
ACGTTGAG 0.0397018329422
ACGTTGAT 1.24315402878
ACGTTGCA 1.26146890394
ACGTTGCC -0.305660875086
ACGTTGCG 0.87477516201
ACGTTGCT -0.0180432028817
ACGTTGGA -0.430819565373
ACGTTGGC -0.900670838733
ACGTTGGG 0.0356829568158
ACGTTGTA 0.160339729964
ACGTTGTC 0.0590299103876
ACGTTGTG -0.409755908012
ACGTTTAA -0.418654268082
ACGTTTAC 0.398622965381
ACGTTTAG -0.390537956724
ACGTTTAT 0.0523600957171
ACGTTTCA 0.792491353613
ACGTTTCC 0.349720914281
ACGTTTCG 0.28292388303
ACGTTTCT 0.355525124863
ACGTTTGA 1.03377734838
ACGTTTGC -0.164345699055
ACGTTTGG 0.444767074449
ACGTTTTA 0.0962873173533
ACGTTTTC -0.21444747992
ACGTTTTG 0.234308493024
ACTAAAAA 2.07737695075
ACTAAAAC 1.17846271791
ACTAAAAG 0.325332654396
ACTAAAAT 2.66862992619
ACTAAACA 2.27827974601
ACTAAACC -0.104729767622
ACTAAACG 1.27019884802
ACTAAACT 0.0996323213212
ACTAAAGA 0.606560719506
ACTAAAGC -0.566781652529
ACTAAAGG 0.553947062995
ACTAAAGT -0.0543148870575
ACTAAATA 0.392729030085

ACTAAATC 2.47989784085
ACTAAATG 0.177484644813
ACTAACAA 0.308657597274
ACTAACAC 0.214490780943
ACTAACAG 0.68892051287
ACTAACAT 1.84310197439
ACTAACCA 0.2325323184
ACTAACCC -0.943703311361
ACTAACCG 0.273357687963
ACTAACCT -0.37308368938
ACTAACGA 0.42263775395
ACTAACGC -0.875335160808
ACTAACGG 0.961557280449
ACTAACTA 1.87586440216
ACTAACTC 0.572099351145
ACTAACTG 0.745043633962
ACTAAGAA 1.0662980815
ACTAAGAC -0.459597674565
ACTAAGAG 0.308132156024
ACTAAGAT 2.83528140468
ACTAAGCA 0.014276013947
ACTAAGCC -0.588176104718
ACTAAGCG -0.203987784914
ACTAAGCT -0.230335832412
ACTAAGGA -0.539993724935
ACTAAGGC 0.0626124454438
ACTAAGGG -0.119252056133
ACTAAGTA 0.188204562308
ACTAAGTC -0.676910724539
ACTAAGTG -0.162888036759
ACTAATAA 0.40026382431
ACTAATAC -0.0824058007003
ACTAATAG -0.385072243556
ACTAATAT 2.40064281405
ACTAATCA 1.62286381545
ACTAATCC -0.219169997658
ACTAATCG 0.498203242204
ACTAATCT 1.68195451382
ACTAATGA 0.625484098936
ACTAATGC -0.100114045194
ACTAATGG 0.178246159906
ACTAATTA 2.04934536784
ACTAATTC 1.74495666846
ACTAATTG 1.28123644513
ACTACAAA 0.631603907353
ACTACAAC 1.22300697912
ACTACAAG 0.0137297548972
ACTACAAT 2.02931802052
ACTACACA -0.172190262132
ACTACACC 0.53117780335
ACTACACG -0.5115310054
ACTACACT 0.818315833462
ACTACAGA -0.166869232669
ACTACAGC -0.239332244316

ACTACAGG −0.155100306274
ACTACAGT 0.3109662912
ACTACATA 0.306069112128
ACTACATC 0.149006728284
ACTACATG −0.251236694583
ACTACCAA 0.590105081988
ACTACCAC 0.784798968648
ACTACCAG −0.875949702239
ACTACCAT 0.207874884372
ACTACCCA 0.335959308151
ACTACCCC −0.704432271375
ACTACCCG 0.0130654589263
ACTACCCT −0.409379522203
ACTACCGA 0.440272095256
ACTACCGC −0.833483890103
ACTACCGG 0.849160317399
ACTACCTA 0.384520988235
ACTACCTC −0.461282250873
ACTACCTG −0.434473921836
ACTACGAA 0.772124884346
ACTACGAC −1.26822240617
ACTACGAG 0.621446694968
ACTACGAT 1.33469488793
ACTACGCA 0.497104478765
ACTACGCC −0.314232395709
ACTACGCG 0.556461436777
ACTACGCT −0.3637427429
ACTACGGA 0.165757562194
ACTACGGC −0.710277076665
ACTACGGG −0.436343984734
ACTACGTA −0.379176018302
ACTACGTC −0.625799904949
ACTACGTG −0.583038271888
ACTACTAA 0.744755307444
ACTACTAC 0.482916940464
ACTACTAG 0.487086953813
ACTACTAT 1.05734184795
ACTACTCA −0.0241380299396
ACTACTCC −0.466432782561
ACTACTCG −0.275414070163
ACTACTCT 0.155866817639
ACTACTGA 0.953832836073
ACTACTGC −0.085310300038
ACTACTGG 0.0935972413468
ACTACTTA 0.382145260995
ACTACTTC 0.739750083994
ACTACTTG 1.44873270991
ACTAGAAA 1.12131182208
ACTAGAAC 0.207763717324
ACTAGAAG 0.589041708807
ACTAGAAT 2.55450092225
ACTAGACA 0.821549878561
ACTAGACC 0.00279791220613
ACTAGACG −0.806322074836

ACTAGACT 0.520022793852
ACTAGAGA -0.387941144458
ACTAGAGC -0.244103683881
ACTAGAGG 0.489849059404
ACTAGAGT -0.354600398224
ACTAGATA 1.87165837402
ACTAGATC 0.987197106419
ACTAGATG -0.143360524798
ACTAGCAA 0.323089536537
ACTAGCAC -0.910409821532
ACTAGCAG 0.216362301086
ACTAGCAT -0.0545343066606
ACTAGCCA -0.8415108171
ACTAGCCC -0.455105401686
ACTAGCCG -0.618474329605
ACTAGCCT 0.303791228544
ACTAGCGA -0.00649369608972
ACTAGCGC -1.3294284011
ACTAGCGG 0.0112634702316
ACTAGCTA -0.236979416655
ACTAGCTC 0.0941782661211
ACTAGCTG -0.85605912786
ACTAGGAA 0.47771727877
ACTAGGAC -0.0592108170622
ACTAGGAG -0.273903947013
ACTAGGAT 2.01682588378
ACTAGGCA -0.190327561466
ACTAGGCC -0.690344866688
ACTAGGCG -0.489324242687
ACTAGGCT -0.236990241911
ACTAGGGA -0.546780327459
ACTAGGGC -0.823418484213
ACTAGGGG -0.828284852942
ACTAGGTA 0.524658085011
ACTAGGTC -0.287369732217
ACTAGGTG -0.221209101085
ACTAGTAA 1.3930192831
ACTAGTAC 0.228549040711
ACTAGTAG 0.691128656826
ACTAGTAT 0.289534158795
ACTAGTCA -1.15899037312
ACTAGTCC -0.637671463094
ACTAGTCG -1.15412379621
ACTAGTCT 0.00440400541077
ACTAGTGA -0.0176809731764
ACTAGTGC -0.681422357971
ACTAGTGG 0.566391526973
ACTAGTTA 0.366458632978
ACTAGTTC -0.444286599644
ACTAGTTG 0.466125720024
ACTATAAA 1.13864513801
ACTATAAC -0.196673034826
ACTATAAG 0.282428003054
ACTATAAT 2.78783181142

ACTATACA 0.451065915939
ACTATACC 0.10068445289
ACTATACG 1.61150395881
ACTATACT 0.805599697206
ACTATAGA 0.147287386252
ACTATAGC -0.152009487634
ACTATAGG 0.999589109543
ACTATAGT 0.49070217281З
ACTATATA 1.60537249243
ACTATATC 1.99740537531
ACTATATG 0.399184629602
ACTATCAA 0.472568412505
ACTATCAC 0.630365456481
ACTATCAG 0.470493086108
ACTATCAT 1.89535881382
ACTATCCA 1.68577749445
ACTATCCC 0.151881666347
ACTATCCG 0.87580647578
ACTATCCT 1.10581192972
ACTATCGA 2.40691251062
ACTATCGC -0.450489679258
ACTATCGG 0.730576720797
ACTATCTA 0.829266620351
ACTATCTC 0.830919137247
ACTATCTG 1.6153150733
ACTATGAA -0.110959702196
ACTATGAC -0.31278847316
ACTATGAG -0.163377255039
ACTATGAT 1.61064980451
ACTATGCA 1.08040297314
ACTATGCC 0.23882595544
ACTATGCG 1.12000758696
ACTATGCT 1.27270822553
ACTATGGA -0.330510873757
ACTATGGC -0.280286059699
ACTATGGG 0.269466841301
ACTATGTA 0.547336787231
ACTATGTC -0.729443816167
ACTATGTG 1.04064409943
ACTATTAA 0.881296337595
ACTATTAC 1.2208939725
ACTATTAG 0.686043076671
ACTATTAT 1.90694787443
ACTATTCA -0.0850386277591
ACTATTCC 0.83870499413
ACTATTCG 0.279397347853
ACTATTCT 0.435823747933
ACTATTGA 1.17734126307
ACTATTGC 0.000939715453755
ACTATTGG 0.172092834832
ACTATTTA 0.867055922085
ACTATTTC 0.046365402273
ACTATTTG 1.53130047309
ACTCAAAA 0.992737347308

ACTCAAAC 0.580505994799
ACTCAAAG -0.67020135605
ACTCAAAT 2.54545912754
ACTCAACA 0.823184908506
ACTCAACC -0.286662967935
ACTCAACG -0.191298919975
ACTCAACT 0.406185447279
ACTCAAGA -0.528838715437
ACTCAAGC -0.994739811408
ACTCAAGG 0.543238178933
ACTCAAGT -0.649623586122
ACTCAATA 1.21014157924
ACTCAATC 0.262176448038
ACTCAATG -0.405661463274
ACTCACAA -0.116746634005
ACTCACAC -0.594904001049
ACTCACAG -0.217126106137
ACTCACAT 1.45164512001
ACTCACCA -0.152897783123
ACTCACCC -1.13961170842
ACTCACCG -0.177956792498
ACTCACCT -0.348395445009
ACTCACGA -0.445689511129
ACTCACGC -0.790729543271
ACTCACGG 2.26749924872
ACTCACTA 0.987210846167
ACTCACTC -0.205889699045
ACTCACTG -0.302305670397
ACTCAGAA 1.32049298535
ACTCAGAC -0.0296859734138
ACTCAGAG 0.0673203907222
ACTCAGAT 2.32567542117
ACTCAGCA -1.00565125266
ACTCAGCC -0.580484760643
ACTCAGCG -1.05088499937
ACTCAGCT -0.292708248631
ACTCAGGA -0.153555001043
ACTCAGGC -0.457526928084
ACTCAGGG -0.577573183254
ACTCAGTA 0.38022419449
ACTCAGTC -0.365031781024
ACTCAGTG -0.633056781556
ACTCATAA 1.11135154607
ACTCATAC -0.635955243732
ACTCATAG -0.514534805639
ACTCATAT 1.17066374582
ACTCATCA 0.135558846387
ACTCATCC 0.0417563415406
ACTCATCG -0.480143801439
ACTCATCT -0.51359467383
ACTCATGA 0.981567973528
ACTCATGC -0.310244329926
ACTCATGG -0.220221504693
ACTCATTA 1.76296031752

ACTCATTC -0.0712114455618
ACTCATTG 0.363137361301
ACTCCAAA 1.04578130773
ACTCCAAC -0.525079853622
ACTCCAAG -0.338321295643
ACTCCAAT 1.21172831189
ACTCCACA 0.136712985172
ACTCCACC -0.389124636339
ACTCCACG -0.0395942049206
ACTCCACT -0.736563087121
ACTCCAGA -0.343106683134
ACTCCAGC -1.04933573875
ACTCCAGG -0.275652642141
ACTCCAGT -0.768502587294
ACTCCATA 0.948850928558
ACTCCATC -0.384043427922
ACTCCATG -0.772537493125
ACTCCCAA -0.0529242580741
ACTCCCAC -1.46685044051
ACTCCCAG -1.27107361194
ACTCCCAT -0.726542855855
ACTCCCCA -0.952382876362
ACTCCCCC -0.81375132282
ACTCCCCG -1.1721253635
ACTCCCCT -0.929408353217
ACTCCCGA 0.686858718042
ACTCCCGC -0.435824164289
ACTCCCGG -0.367527418786
ACTCCCTA -0.912442887797
ACTCCCTC -0.608365622693
ACTCCCTG -1.20149823721
ACTCCGAA -0.285815267154
ACTCCGAC -0.503193476839
ACTCCGAG 0.294540006779
ACTCCGAT 0.641199663694
ACTCCGCA -0.73876998201
ACTCCGCC -0.97379918724
ACTCCGCG 0.160006437
ACTCCGCT -0.547418184825
ACTCCGGA -0.910973151177
ACTCCGGC -1.13815820969
ACTCCGGG -0.439790787739
ACTCCGTA 1.22213887689
ACTCCGTC 0.0614945296293
ACTCCGTG -0.697941697955
ACTCCTAA -0.608761160877
ACTCCTAC -1.13335096351
ACTCCTAG -0.986393123016
ACTCCTAT 0.123716016805
ACTCCTCA -0.528924901126
ACTCCTCC -0.4200786219
ACTCCTCG -0.401845144334
ACTCCTCT 0.799824839721
ACTCCTGA -0.131840371102

ACTCCTGC -0.671431479802
ACTCCTGG -0.447165909445
ACTCCTTA 1.04344117892
ACTCCTTC -1.04416605469
ACTCCTTG -0.286952959878
ACTCGAAA 0.469571898495
ACTCGAAC -0.273066030597
ACTCGAAG 0.157833475124
ACTCGAAT 1.56369858937
ACTCGACA -0.56213491158
ACTCGACC -0.0322478117776
ACTCGACG -0.655547499049
ACTCGACT -0.294225033478
ACTCGAGA -0.59898970231
ACTCGAGC -0.80339051236
ACTCGAGG 0.418161718954
ACTCGAGT 0.263114914424
ACTCGATA 0.845274675187
ACTCGATC -0.310168553137
ACTCGATG -0.55629322896
ACTCGCAA 1.33706291258
ACTCGCAC -1.01369400118
ACTCGCAG 0.0728518881351
ACTCGCAT 0.711735155318
ACTCGCCA -0.507607682971
ACTCGCCC -0.579357684997
ACTCGCCG -0.862930250648
ACTCGCCT -0.836422946987
ACTCGCGA 0.530161894751
ACTCGCGC -0.376021080841
ACTCGCGG -0.142983930811
ACTCGCTA 0.4560492807
ACTCGCTC 0.22497629002
ACTCGCTG -0.292669111169
ACTCGGAA 0.132151180844
ACTCGGAC -1.06005065998
ACTCGGAG -0.776797855722
ACTCGGAT 2.20649539462
ACTCGGCA -0.992333690182
ACTCGGCC 0.302653743999
ACTCGGCG -0.315397151572
ACTCGGCT -0.127627056732
ACTCGGGA -0.645916560627
ACTCGGGC -1.49948671242
ACTCGGGG 0.00442961130372
ACTCGGTA -0.431271519792
ACTCGGTC -0.745290116704
ACTCGGTG -0.303796016638
ACTCGTAA 1.26567618115
ACTCGTAC 0.177562295204
ACTCGTAG 0.0612642847707
ACTCGTAT 0.81602337742
ACTCGTCA -0.800538890232
ACTCGTCC -0.329389210739

ACTCGTCG -0.191645744508
ACTCGTCT -0.389047194127
ACTCGTGA -0.129596212354
ACTCGTGC -0.665114110471
ACTCGTGG -0.0715428649244
ACTCGTTA 0.0101311901357
ACTCGTTC -0.588727776396
ACTCGTTG -0.01336773337
ACTCTAAA 0.830929962503
ACTCTAAC 0.803875150724
ACTCTAAG -0.748073872807
ACTCTAAT 1.8020016017
ACTCTACA 0.757756231193
ACTCTACC 0.624753394186
ACTCTACG -0.477308417195
ACTCTACT 0.814116675195
ACTCTAGA 0.14068918481
ACTCTAGC -0.568749350905
ACTCTAGG 0.556164366783
ACTCTATA 0.463420447024
ACTCTATC -0.196012902415
ACTCTATG -0.654865507948
ACTCTCAA -0.231077362418
ACTCTCAC -0.0406282250045
ACTCTCAG -1.09063200693
ACTCTCAT 0.581762973511
ACTCTCCA 0.171104405728
ACTCTCCC -0.91984528082
ACTCTCCG -0.729589332583
ACTCTCCT -0.912975615278
ACTCTCGA -0.48316508863
ACTCTCGC -0.288754324038
ACTCTCGG -0.234726930787
ACTCTCTA 0.583419862146
ACTCTCTC -0.196256054309
ACTCTCTG -1.05785250857
ACTCTGAA -0.551293418137
ACTCTGAC -0.835712435502
ACTCTGAG -0.00642478917452
ACTCTGAT 1.75549796924
ACTCTGCA -0.182552113482
ACTCTGCC 1.29307032306
ACTCTGCG 0.949813335413
ACTCTGCT 0.17184010675
ACTCTGGA -1.12589985683
ACTCTGGC -1.02654462042
ACTCTGGG -0.161486998876
ACTCTGTA -0.273353524403
ACTCTGTC -0.729921584658
ACTCTGTG -0.0573297207308
ACTCTTAA -0.0984777661802
ACTCTTAC -0.0552733385305
ACTCTTAG -0.275165921997
ACTCTTAT 1.38899936609

ACTCTTCA -0.362756395577
ACTCTTCC -0.4582134991
ACTCTTCG 0.357813417346
ACTCTTCT -0.344647824806
ACTCTTGA -0.074377832813
ACTCTTGC -0.513208503655
ACTCTTGG 0.503615661806
ACTCTTTA 1.60372830265
ACTCTTTC 0.139625395274
ACTCTTTG 0.181228101456
ACTGAAAA 2.28137410368
ACTGAAAC 0.716821776363
ACTGAAAG -0.228174320326
ACTGAAAT 3.13920461807
ACTGAACA 0.352031481809
ACTGAACC -0.00454390102107
ACTGAACG 0.168663726956
ACTGAACT 0.615216760394
ACTGAAGA 0.00109501623544
ACTGAAGC -0.404705926293
ACTGAAGG -0.267836391272
ACTGAAGT -0.937559146119
ACTGAATA 1.81395934553
ACTGAATC 1.68891057322
ACTGAATG 0.21154631143
ACTGACAA 0.184810428335
ACTGACAC -0.0391114401582
ACTGACAG -0.744267962766
ACTGACAT -0.223330851175
ACTGACCA -0.292757170459
ACTGACCC -0.973388660241
ACTGACCG -0.803247494079
ACTGACCT -1.07143383255
ACTGACGA 0.899802736508
ACTGACGC -0.211750742218
ACTGACGG 0.184116362911
ACTGACTA -0.0798144010617
ACTGACTC -0.373959286013
ACTGACTG -0.980489611532
ACTGAGAA 1.27654286413
ACTGAGAC 0.104599031843
ACTGAGAG -0.852417262076
ACTGAGAT 1.77531984488
ACTGAGCA -0.63089422858
ACTGAGCC -0.667719874391
ACTGAGCG -0.243285544375
ACTGAGCT 0.085212040026
ACTGAGGA -0.677366217985
ACTGAGGC -0.808654917409
ACTGAGGG -0.129335781686
ACTGAGTA 0.246530831085
ACTGAGTC -1.37034016453
ACTGAGTG -0.295657714415
ACTGATAA 0.315721492883

ACTGATAC 2.43050865325
ACTGATAG 0.112456710133
ACTGATAT 3.24615377126
ACTGATCA 0.162157748365
ACTGATCC -0.315320958427
ACTGATCG 1.98961535487
ACTGATCT 0.115468837493
ACTGATGA -0.287309568777
ACTGATGC 0.12601346912
ACTGATGG -0.384454371277
ACTGATTA 2.86302507743
ACTGATTC 2.22140780869
ACTGATTG 2.25703622287
ACTGCAAA 0.343815737373
ACTGCAAC -0.0377189375729
ACTGCAAG -0.727272311537
ACTGCAAT 1.0355601847
ACTGCACA -0.25805202567
ACTGCACC -0.944592231385
ACTGCACG 0.322492273879
ACTGCACT -0.0772914919824
ACTGCAGA 0.207156670301
ACTGCAGC -0.832551252701
ACTGCAGG -0.315227278331
ACTGCAGT -0.675724318165
ACTGCATA 0.459931592064
ACTGCATC -0.0135178297019
ACTGCATG -0.956176920258
ACTGCCAA -0.679133649133
ACTGCCAC -0.338321295643
ACTGCCAG -0.622110574583
ACTGCCAT -0.0596421618607
ACTGCCCA -0.838685425399
ACTGCCCC -1.20055706451
ACTGCCCG -0.705235422066
ACTGCCCT -1.15853258972
ACTGCCGA 0.498230097165
ACTGCCGC -1.07898611373
ACTGCCGG 0.388575879154
ACTGCCTA -0.549662551752
ACTGCCTC -0.947350589773
ACTGCCTG -0.401316372235
ACTGCGAA 0.488861463012
ACTGCGAC -0.106156619576
ACTGCGAG -0.533198795292
ACTGCGAT 0.81380524092
ACTGCGCA -0.365688582587
ACTGCGCC 0.623364430626
ACTGCGCG 0.210456083289
ACTGCGCT 0.111323597325
ACTGCGGA -0.747015912254
ACTGCGGC -0.859772190517
ACTGCGGG 1.17519578069
ACTGCGTA -0.465872367408

ACTGCGTC -0.442043689964
ACTGCGTG -0.758554801968
ACTGCTAA 0.369284024679
ACTGCTAC -0.48581332086
ACTGCTAG -0.404816885163
ACTGCTAT 0.490209623685
ACTGCTCA -0.221217011849
ACTGCTCC -0.669688613657
ACTGCTCG -0.171601951128
ACTGCTCT -0.286803279902
ACTGCTGA 0.00958867828976
ACTGCTGC -0.703100348585
ACTGCTGG -0.107197717711
ACTGCTTA 0.519953054225
ACTGCTTC -1.05205204519
ACTGCTTG 0.323978248383
ACTGGAAA 1.66190489145
ACTGGAAC 0.204686638432
ACTGGAAG -0.333925409174
ACTGGAAT 2.35721438688
ACTGGACA -0.670140776255
ACTGGACC -0.572986189389
ACTGGACG 0.416188816128
ACTGGACT -0.404450700076
ACTGGAGA 0.843826589078
ACTGGAGC -0.266532780689
ACTGGAGG -0.297588565287
ACTGGATA 2.52098239201
ACTGGATC 0.76897244502
ACTGGATG -0.278128711173
ACTGGCAA 0.903476453524
ACTGGCAC -1.32377262143
ACTGGCAG -1.198433649
ACTGGCAT -0.415506200494
ACTGGCCA -1.29674279101
ACTGGCCC -0.497481697286
ACTGGCCG -0.741388444787
ACTGGCCT -1.23679252572
ACTGGCGA 0.212440227726
ACTGGCGC -0.785162031065
ACTGGCGG -0.34808859065
ACTGGCTA -0.146393678134
ACTGGCTC -1.16765036939
ACTGGCTG -0.829135884572
ACTGGGAA -0.0972965642562
ACTGGGAC -0.408394007591
ACTGGGAG -0.973182564029
ACTGGGAT 1.38625016753
ACTGGGCA -1.58668102328
ACTGGGCC -0.710375753033
ACTGGGCG -1.11574389352
ACTGGGCT -0.777793779233
ACTGGGGA -0.646913108672
ACTGGGGC -0.949354927475

ACTGGGGG -0.709439576605
ACTGGGTA 0.554499983741
ACTGGGTC -1.35855583296
ACTGGGTG -1.02861016245
ACTGGTAA 1.19732655844
ACTGGTAC -1.11781422364
ACTGGTAG -0.666809720212
ACTGGTAT -0.072336855784
ACTGGTCA -0.602608252159
ACTGGTCC -0.710277076665
ACTGGTCG -0.864190560209
ACTGGTCT -0.484564669267
ACTGGTGA 0.0637212014267
ACTGGTGC -0.348038419754
ACTGGTGG -0.419031486602
ACTGGTTA -0.108066236292
ACTGGTTC -1.09496231734
ACTGGTTG 0.136120094252
ACTGTAAA 1.52007759756
ACTGTAAC 0.388719521968
ACTGTAAG -0.675747009566
ACTGTAAT 2.99029448437
ACTGTACA -0.0320535817115
ACTGTACC -0.547193144416
ACTGTACG 0.455369163201
ACTGTACT 0.467177227059
ACTGTAGA 0.770667638405
ACTGTAGC 0.67270552911
ACTGTAGG -0.376331266048
ACTGTATA 1.04554065397
ACTGTATC -0.00273171160482
ACTGTATG -0.166868191779
ACTGTCAA 0.202492234223
ACTGTCAC -0.916488202529
ACTGTCAG -0.796352847178
ACTGTCAT -0.200530364831
ACTGTCCA -0.213895808243
ACTGTCCC 0.0345912714282
ACTGTCCG 0.0991047982906
ACTGTCCT -0.103889769426
ACTGTCGA -0.0346812043205
ACTGTCGC 0.237328739325
ACTGTCGG -0.243272220983
ACTGTCTA 1.58925118876
ACTGTCTC -0.653248797666
ACTGTCTG 0.588051822457
ACTGTGAA 1.21255561123
ACTGTGAC 0.0722827295062
ACTGTGAG -0.808895571168
ACTGTGAT 2.45301040418
ACTGTGCA -0.433307500549
ACTGTGCC -0.0839944069536
ACTGTGCG -0.177656183478
ACTGTGCT 0.0364561298763

ACTGTGGA -0.148851011146
ACTGTGGC 0.124085324562
ACTGTGGG -0.666977095317
ACTGTGTA 0.00777836247544
ACTGTGTC -0.917757255565
ACTGTGTG 0.304621442375
ACTGTTAA 1.17454772261
ACTGTTAC 0.449491465789
ACTGTTAG 0.429584861705
ACTGTTAT 0.765653671479
ACTGTTCA -0.284915521875
ACTGTTCC 0.313567267026
ACTGTTCG 0.97493313276
ACTGTTCT 0.438324590145
ACTGTTGA 0.2808062965
ACTGTTGC -0.0626186907835
ACTGTTGG -0.309291707436
ACTGTTTA 0.309414948807
ACTGTTTC -0.680173706378
ACTGTTTG 0.294814801728
ACTTAAAA 1.00853160335
ACTTAAAC 0.860157527979
ACTTAAAG -0.256274601978
ACTTAAAT 1.2862452076
ACTTAACA 1.28992621085
ACTTAACC 0.361505245847
ACTTAACG 0.539437056986
ACTTAACT 0.253168586345
ACTTAAGA 0.354210689024
ACTTAAGC -0.0805765406887
ACTTAAGG -0.183485999952
ACTTAAGT -0.439059458455
ACTTAATA 1.61917073789
ACTTAATC 0.272727325005
ACTTAATG 1.33689199845
ACTTACAA 0.644148713122
ACTTACAC 0.18517890338
ACTTACAG -0.392938040788
ACTTACAT 0.616280966287
ACTTACCA 0.276310068239
ACTTACCC -0.503141848697
ACTTACCG -0.535506864684
ACTTACCT -0.0433480704638
ACTTACGA 2.82722075285
ACTTACGC 0.138278691846
ACTTACGG 0.527106258192
ACTTACTA 1.09277832203
ACTTACTC -0.210591190805
ACTTACTG 0.547221664801
ACTTAGAA 0.106158493178
ACTTAGAC 0.352596060522
ACTTAGAG -0.318018528841
ACTTAGAT 0.886409189067
ACTTAGCA -0.63914473874

ACTTAGCC -0.781185831427
ACTTAGCG 0.847554224194
ACTTAGCT 0.527011745384
ACTTAGGA 0.553544030404
ACTTAGGC -0.260223322121
ACTTAGGG -0.996080061318
ACTTAGTA 0.807880495281
ACTTAGTC -0.190002803799
ACTTAGTG 0.14201173959
ACTTATAA 0.939513521104
ACTTATAC 1.0805149729
ACTTATAG 0.930723829945
ACTTATAT 0.526576653381
ACTTATCA 0.432152945408
ACTTATCC 0.513497454708
ACTTATCG 1.01655311772
ACTTATCT 0.721049871371
ACTTATGA 1.75109292294
ACTTATGC 0.386168092504
ACTTATGG -0.284897410389
ACTTATTA 0.597733139953
ACTTATTC 0.509592660121
ACTTATTG 2.9276889088
ACTTCAAA 1.4474936345
ACTTCAAC 0.00486116428016
ACTTCAAG -0.799858148199
ACTTCAAT 0.648543558702
ACTTCACA 0.423479833925
ACTTCACC 0.0704430605951
ACTTCACG -0.570250106046
ACTTCACT -0.0176809731764
ACTTCAGA 0.632364381556
ACTTCAGC -0.454433194951
ACTTCAGG -0.0582444548256
ACTTCATA 0.858147569471
ACTTCATC -0.401184803745
ACTTCATG -0.322351961913
ACTTCCAA 0.811835668942
ACTTCCAC -0.408196238499
ACTTCCAG -0.772832897695
ACTTCCAT -0.48174302477
ACTTCCCA 0.152699805853
ACTTCCCC -0.771921494448
ACTTCCCG -0.36596816563
ACTTCCCT -0.300555518022
ACTTCCGA 1.70085312006
ACTTCCGC -0.48214522465
ACTTCCGG 0.00967028406244
ACTTCCTA 0.175275668145
ACTTCCTC 0.0252863397409
ACTTCCTG -0.414721369466
ACTTCGAA 0.541760323371
ACTTCGAC -0.154883592985
ACTTCGAG -0.340289618553

ACTTCGAT 1.45182415308
ACTTCGCA 1.21377116252
ACTTCGCC -1.13450114691
ACTTCGCG -0.277692370102
ACTTCGCT 0.187660385039
ACTTCGGA -0.433511098625
ACTTCGGC -0.631527922386
ACTTCGGG -0.317951703706
ACTTCGTA -0.156924986369
ACTTCGTC -0.570283622703
ACTTCGTG -0.0532569265046
ACTTCTAA 1.7842773275
ACTTCTAC -0.253746072093
ACTTCTAG -0.46246824089
ACTTCTAT 0.194448652986
ACTTCTCA 0.671020536447
ACTTCTCC -0.358559319089
ACTTCTCG 0.351935928111
ACTTCTCT 0.758553969256
ACTTCTGA 0.585323649879
ACTTCTGC -0.427463527971
ACTTCTGG 0.391275531348
ACTTCTTA -0.14989544013
ACTTCTTC -0.38114038583
ACTTCTTG 0.0667031429773
ACTTGAAA 1.92688757882
ACTTGAAC 0.799998876522
ACTTGAAG -0.0237516515874
ACTTGAAT 0.824445634423
ACTTGACA 0.161383742592
ACTTGACC -1.02571732108
ACTTGACG 0.0672360786356
ACTTGACT 0.0991047982906
ACTTGAGA 0.909960989782
ACTTGAGC -0.0814608807967
ACTTGAGG 0.1714991112
ACTTGATA 1.53811767777
ACTTGATC -0.392043291781
ACTTGATG -0.00516406325783
ACTTGCAA 0.299884560355
ACTTGCAC 0.0361080562745
ACTTGCAG -0.959256705464
ACTTGCAT 1.3664707602
ACTTGCCA -0.850387526659
ACTTGCCC -0.785184930645
ACTTGCCG -0.711708508535
ACTTGCCT 0.248082381656
ACTTGCGA 2.08674600126
ACTTGCGC 0.20280241943
ACTTGCGG -0.0184635142466
ACTTGCTA 0.372299274708
ACTTGCTC -0.0317679615071
ACTTGCTG 0.976625828009
ACTTGGAA 0.380374707178

ACTTGGAC -0.458328829707
ACTTGGAG -0.564031621261
ACTTGGAT 1.61546121425
ACTTGGCA -0.603233618846
ACTTGGCC 0.285526940636
ACTTGGCG -0.719214990553
ACTTGGCT 1.09909423411
ACTTGGGA 0.588418423901
ACTTGGGC -1.08503555799
ACTTGGGG -0.114221018611
ACTTGGTA -0.394453992923
ACTTGGTC -1.03160605193
ACTTGGTG -0.73683059584
ACTTGTAA 2.86499631483
ACTTGTAC 0.320145275203
ACTTGTAG 0.0673401676314
ACTTGTAT 0.287732794634
ACTTGTCA -0.742354807024
ACTTGTCC 0.291970882185
ACTTGTCG -0.54090013191
ACTTGTCT 0.122952419932
ACTTGTGA 0.848088825277
ACTTGTGC -0.301184631912
ACTTGTGG -0.663039824964
ACTTGTTA 0.558903364618
ACTTGTTC -0.567204253852
ACTTGTTG -0.0403742478548
ACTTTAAA 0.797532800034
ACTTTAAC 0.695885523933
ACTTTAAG 0.691789205594
ACTTTAAT 0.960845519896
ACTTTACA 1.27176726101
ACTTTACC -0.354040399427
ACTTTACG 1.65560521725
ACTTTACT 0.807066727512
ACTTTAGA 1.08203404771
ACTTTAGC 0.306919727401
ACTTTAGG 0.109690440982
ACTTTATA 0.666155624963
ACTTTATC -0.648033106266
ACTTTATG 0.76716379463
ACTTTCAA 0.23882595544
ACTTTCAC -0.0664816415943
ACTTTCAG -0.661671262847
ACTTTCAT 0.862477255339
ACTTTCCA 0.871554648481
ACTTTCCC 0.872302423827
ACTTTCCG -0.354067878922
ACTTTCCT -0.165034976385
ACTTTCGA 0.129928880784
ACTTTCGC 0.00857110426717
ACTTTCGG -0.515223874791
ACTTTCTA 0.395892502844
ACTTTCTC -0.670456998624

ACTTTCTG 0.939139217075
ACTTTGAA 0.755146720069
ACTTTGAC 0.426437210473
ACTTTGAG 0.185392493999
ACTTTGAT 0.0334400471351
ACTTTGCA -0.152843448668
ACTTTGCC 2.06945203097
ACTTTGCG -0.111000921438
ACTTTGCT 0.872109859185
ACTTTGGA 0.918203172823
ACTTTGGC 0.564179011279
ACTTTGGG -0.669887215461
ACTTTGTA 0.618510552576
ACTTTGTC -0.120224247353
ACTTTGTG 0.0117795434726
ACTTTTAA 0.610166362319
ACTTTTAC 0.251458404144
ACTTTTAG -0.0249488832166
ACTTTTAT 1.86983369392
ACTTTTCA -0.20714126513
ACTTTTCC -0.539116462879
ACTTTTCG 0.939122771014
ACTTTTCT 0.456803093207
ACTTTTGA 0.360936919931
ACTTTTGC -0.26108788532
ACTTTTGG -0.446500156228
ACTTTTTA 1.23727799679
ACTTTTTC -0.301879530048
ACTTTTTG 2.33906355598
AGAAAAAA 0.865717337599
AGAAAAAC 0.810738779105
AGAAAAAG 0.00543448646883
AGAAAAAT 3.06665375531
AGAAAACA 0.364269849575
AGAAAACC -0.379448523293
AGAAAACG 0.0336671693238
AGAAAACT -0.550633285726
AGAAAAGA 1.53337350952
AGAAAAGC 0.00857110426717
AGAAAAGG 0.638485230863
AGAAAATA 1.93232685338
AGAAAATC 14.4016964131
AGAAAATG 1.70782167015
AGAAACAA -0.631191090396
AGAAACAC 0.400487407473
AGAAACAG 0.436718288762
AGAAACAT 1.14845052959
AGAAACCA 0.246003932588
AGAAACCC -1.09705055077
AGAAACCG 0.472619832469
AGAAACCT -0.113069794318
AGAAACGA 0.353406705621
AGAAACGC 0.571287456978
AGAAACGG -0.763759043579

AGAAACTA 1.27144729143
AGAAACTC -0.323303959868
AGAAACTG -0.0247908761211
AGAAAGAA 0.0467472007095
AGAAAGAC 0.353206022037
AGAAAGAG -0.31180691393
AGAAAGAT 2.1591911096
AGAAAGCA -0.949397395785
AGAAAGCC -1.29580390826
AGAAAGCG -0.0135436437728
AGAAAGCT 0.245838222907
AGAAAGGA 0.569114078746
AGAAAGGC -0.375351372242
AGAAAGGG -1.26479163287
AGAAAGTA 1.71504274015
AGAAAGTC -0.594346500387
AGAAAGTG 3.0439844211
AGAAATAA 1.11218821342
AGAAATAC 1.02119798506
AGAAATAG 0.429763062066
AGAAATAT 7.06430571916
AGAAATCA 6.1317545028
AGAAATCC 14.9271322511
AGAAATCG 6.78053288628
AGAAATCT 15.3725886029
AGAAATGA 0.315279739185
AGAAATGC 0.848254951314
AGAAATGG -0.337676776581
AGAAATTA 1.19691436602
AGAAATTC 0.939566606492
AGAAATTG 1.27386111524
AGAACAAA 0.803924072552
AGAACAAC 0.651448474395
AGAACAAG -0.138576594552
AGAACAAT 2.82166031869
AGAACACA -0.368430494914
AGAACACC -1.28419756888
AGAACACG -0.971798388564
AGAACACT 0.341940678204
AGAACAGA 1.88485019699
AGAACAGC -0.0264552591633
AGAACAGG -0.0592054044345
AGAACATA 0.237717615813
AGAACATC 0.00903034491648
AGAACATG 0.167356785525
AGAACCAA -0.00533372832093
AGAACCAC -0.0946348004566
AGAACCAG -0.900766184253
AGAACCAT -0.386335675787
AGAACCCA -0.171743512162
AGAACCCC -0.348116902856
AGAACCCG -0.434694590507
AGAACCCT -0.329024899253
AGAACCGA 0.072190923012

AGAACCGC -0.321432647902
AGAACCGG -0.487506016109
AGAACCTA -0.152048625096
AGAACCTC -0.494425228014
AGAACCTG -0.101637491736
AGAACGAA 0.570852364975
AGAACGAC -0.391771203146
AGAACGAG -1.04119181573
AGAACGAT 0.271637721397
AGAACGCA -0.654440824846
AGAACGCC -0.703115961935
AGAACGCG -0.253007872935
AGAACGCT -0.549013244596
AGAACGGA 0.918791067471
AGAACGGC -1.20231158862
AGAACGGG -0.418571829597
AGAACGTA 0.512087881527
AGAACGTC -0.780184703466
AGAACGTG 0.722810224467
AGAACTAA 1.93857843846
AGAACTAC 0.246237508295
AGAACTAG 0.0662393224122
AGAACTAT 0.382564115114
AGAACTCA -0.240045878471
AGAACTCC -1.22906454232
AGAACTCG -0.63928359346
AGAACTCT 0.563472871532
AGAACTGA 0.300621302267
AGAACTGC -0.149541329366
AGAACTGG -1.05440716281
AGAACTTA 0.800279084098
AGAACTTC -0.777665541591
AGAACTTG 0.176116915408
AGAAGAAA -0.766996835881
AGAAGAAC -0.754543628428
AGAAGAAG -0.339513322822
AGAAGAAT 1.5375868239
AGAAGACA -0.739506515744
AGAAGACC -0.9983891716
AGAAGACG -0.939013893924
AGAAGACT -0.605663472363
AGAAGAGA 0.250403982617
AGAAGAGC 0.934543063378
AGAAGAGG -0.745598220132
AGAAGATA 1.88333257943
AGAAGATC 7.14816231228
AGAAGATG -0.265925525487
AGAAGCAA 0.127313748855
AGAAGCAC -0.254261728978
AGAAGCAG -0.344639914042
AGAAGCAT -0.0878927480229
AGAAGCCA -0.660457585157
AGAAGCCC -1.10743155449
AGAAGCCG -0.755371760478

| | |
|---|---|
| AGAAGCCT | 0.0573944640862 |
| AGAAGCGA | 0.504338455792 |
| AGAAGCGC | -0.849647453899 |
| AGAAGCGG | -0.62180725925 |
| AGAAGCTA | -0.0640988363033 |
| AGAAGCTC | -0.735047759521 |
| AGAAGCTG | 0.304302930047 |
| AGAAGGAA | 1.03014818145 |
| AGAAGGAC | -0.841250594611 |
| AGAAGGAG | -0.830319168276 |
| AGAAGGAT | 0.512460936488 |
| AGAAGGCA | -1.18675944359 |
| AGAAGGCC | -1.54153845853 |
| AGAAGGCG | -1.04076505084 |
| AGAAGGCT | -1.35661394866 |
| AGAAGGGA | -0.0521308917484 |
| AGAAGGGC | -1.03450909402 |
| AGAAGGGG | -1.22582175374 |
| AGAAGGTA | 0.541825274905 |
| AGAAGGTC | -0.825502554086 |
| AGAAGGTG | 1.2008938965 |
| AGAAGTAA | 1.55811067572 |
| AGAAGTAC | -0.0588250632439 |
| AGAAGTAG | -0.0634060199475 |
| AGAAGTAT | 1.2907458076 |
| AGAAGTCA | -1.07113572167 |
| AGAAGTCC | -0.160503566044 |
| AGAAGTCG | -0.693900130428 |
| AGAAGTCT | 1.18032403734 |
| AGAAGTGA | 0.0384013450291 |
| AGAAGTGC | -0.482590309196 |
| AGAAGTGG | -1.2138375713 |
| AGAAGTTA | 0.765631188256 |
| AGAAGTTC | -0.735035893376 |
| AGAAGTTG | 0.451240369095 |
| AGAATAAA | 1.58207279526 |
| AGAATAAC | 1.05703998986 |
| AGAATAAG | 0.0633564735855 |
| AGAATAAT | 2.22403189227 |
| AGAATACA | 1.26091744044 |
| AGAATACC | 0.369400604354 |
| AGAATACG | 2.341505692 |
| AGAATACT | 0.589696220413 |
| AGAATAGA | 1.72418071308 |
| AGAATAGC | -0.132561499665 |
| AGAATAGG | -0.642988953532 |
| AGAATATA | 2.78707674985 |
| AGAATATC | 10.2047539925 |
| AGAATATG | 1.76458597946 |
| AGAATCAA | 4.27273586372 |
| AGAATCAC | 8.38508515742 |
| AGAATCAG | 5.28514873754 |
| AGAATCAT | 4.68792500913 |
| AGAATCCA | 17.3505622281 |

AGAATCCC 9.92553775955
AGAATCCG 14.6151783635
AGAATCCT 10.1797643064
AGAATCGA 4.99969361831
AGAATCGC 6.72726263638
AGAATCGG 6.08222770955
AGAATCTA 20.278513233
AGAATCTC 16.4256548912
AGAATCTG 16.028171284
AGAATGAA 0.136653862622
AGAATGAC -0.457298556827
AGAATGAG 0.247794887849
AGAATGAT 1.62616572658
AGAATGCA 0.198867230857
AGAATGCC -1.46002178603
AGAATGCG -0.426481552385
AGAATGCT -0.604311356308
AGAATGGA -0.122070994316
AGAATGGC 1.96178466371
AGAATGGG -0.799945791134
AGAATGTA 2.88217932625
AGAATGTC -0.638632620881
AGAATGTG 1.04892687718
AGAATTAA 0.0730584007027
AGAATTAC 2.8778787853
AGAATTAG -0.0495680124947
AGAATTAT 0.743827458136
AGAATTCA 0.898160212155
AGAATTCC 1.08348879551
AGAATTCG 2.15101262903
AGAATTCT 4.90978591551
AGAATTGA 0.526085353321
AGAATTGC 1.31249353784
AGAATTGG -0.0715428649244
AGAATTTA 2.88388617761
AGAATTTC 3.79053068381
AGAATTTG 3.65736338618
AGACAAAA -0.108973059623
AGACAAAC 1.193715503
AGACAAAG -0.921665172822
AGACAAAT 1.56061130976
AGACAACA 0.258759414485
AGACAACC -0.215422377455
AGACAACG -0.366892475912
AGACAACT -0.0596908755107
AGACAAGA 1.79158125233
AGACAAGC -0.798777704423
AGACAAGG -0.386056925456
AGACAATA -0.54080686817
AGACAATC 4.13421089731
AGACAATG 1.2562904764
AGACACAA 0.865659047761
AGACACAC 0.0231175414252
AGACACAG -0.167329514208

AGACACAT 1.26254455963
AGACACCA -0.187198646253
AGACACCC -0.494105466619
AGACACCG 0.245266566142
AGACACCT -0.0427930679384
AGACACGA 0.200773516725
AGACACGC -0.826300708505
AGACACGG -0.315584511764
AGACACTA -0.148538952337
AGACACTC -0.131396535624
AGACACTG 0.259836319235
AGACAGAA -0.357347098645
AGACAGAC -0.452553555866
AGACAGAG 0.1491522447
AGACAGAT 2.48260332203
AGACAGCA 0.180279642527
AGACAGCC -0.882761702477
AGACAGCG -0.351317431298
AGACAGCT -0.119889705321
AGACAGGA -0.601855064186
AGACAGGC -0.395711387991
AGACAGGG -0.407781131584
AGACAGTA 1.59414253885
AGACAGTC -0.653248797666
AGACAGTG 1.94368046468
AGACATAA -0.337025804002
AGACATAC 0.50591831857
AGACATAG 0.125024415482
AGACATAT 1.09876343928
AGACATCA 0.00648890799591
AGACATCC -0.154248858288
AGACATCG 0.301606192345
AGACATCT 3.03347351431
AGACATGA -0.0475391097893
AGACATGC -0.227740061035
AGACATGG 0.152875924434
AGACATTA 0.10202678458
AGACATTC 0.068038396615
AGACATTG 0.959317077082
AGACCAAA -0.0213446976493
AGACCAAC 0.572388510375
AGACCAAG 0.156294623411
AGACCAAT 0.263120327052
AGACCACA -0.239856020142
AGACCACC -1.23728757298
AGACCACG -0.951193555496
AGACCACT -0.904763826224
AGACCAGA 0.115321447475
AGACCAGC -0.48424823872
AGACCAGG -0.321810282779
AGACCATA -0.652062183114
AGACCATC -0.67129387415
AGACCATG -0.654865507948
AGACCCAA -0.137729518305

AGACCCAC −0.501526387483
AGACCCAG −1.11762145082
AGACCCAT −0.693329098197
AGACCCCA −1.3568972789
AGACCCCC −1.62383788028
AGACCCCG −1.53371304783
AGACCCCT −0.749851712855
AGACCCGA −0.826185586076
AGACCCGC −1.64804106993
AGACCCGG −1.27041493677
AGACCCTA −0.950971429579
AGACCCTC −0.709975634933
AGACCCTG −1.11216468931
AGACCGAA 0.386851332672
AGACCGAC −0.37584496226
AGACCGAG −0.921976607097
AGACCGAT −0.785281108877
AGACCGCA −0.5815029592
AGACCGCC −1.77227253544
AGACCGCG −0.98967733901
AGACCGCT −0.249916846117
AGACCGGA −0.227767124174
AGACCGGC −1.17375206632
AGACCGGG −1.30602294951
AGACCGTA −0.281162072687
AGACCGTC −0.684717399221
AGACCGTG −0.293323622774
AGACCTAA −0.0639164723827
AGACCTAC −0.340512577182
AGACCTAG −0.309448257286
AGACCTAT 0.16998066093
AGACCTCA −1.25780788578
AGACCTCC −1.37556106038
AGACCTCG −0.251840618937
AGACCTCT 0.360936919931
AGACCTGA −0.234853919362
AGACCTGC −0.0793601566842
AGACCTGG −0.70209859609
AGACCTTA −0.374732667251
AGACCTTC −1.1249665949
AGACCTTG −0.117075138875
AGACGAAA −0.75007196517
AGACGAAC −0.83625890273
AGACGAAG −0.396115253295
AGACGAAT 0.0617006258409
AGACGACA −0.509212527108
AGACGACC −1.24014939583
AGACGACG −1.2521862473
AGACGACT −0.145052179157
AGACGAGA 1.06971344963
AGACGAGC −0.777793779233
AGACGAGG −0.823976817586
AGACGATA 0.873343730141
AGACGATC 1.95658666744

AGACGATG 0.970576175576
AGACGCAA 0.0768518200643
AGACGCAC -0.483086397349
AGACGCAG -0.61555921319
AGACGCAT -0.14019476208
AGACGCCA -0.592281791067
AGACGCCC -1.32230413387
AGACGCCG -0.931799485628
AGACGCCT -0.880377231762
AGACGCGA 0.773144748327
AGACGCGC -0.407667882757
AGACGCGG -0.855436883843
AGACGCTA -0.532799301726
AGACGCTC -1.01000550353
AGACGCTG -0.253911781774
AGACGGAA -0.371300436705
AGACGGAC -1.18895239055
AGACGGAG -0.266362074736
AGACGGAT -0.11701351819
AGACGGCA -0.954428641485
AGACGGCC -1.61332280993
AGACGGCG -1.57762444794
AGACGGCT -0.892645577159
AGACGGGA 0.193096328753
AGACGGGC -0.806322074836
AGACGGGG -0.89188156393
AGACGGTA -0.212618428087
AGACGGTC -1.22392629313
AGACGGTG -0.319829052834
AGACGTAA 0.140346940192
AGACGTAC 0.840960602669
AGACGTAG -0.943576530964
AGACGTAT 1.43123160252
AGACGTCA 0.188204562308
AGACGTCC -0.296677370218
AGACGTCG -0.245724557724
AGACGTCT 1.35847901528
AGACGTGA 0.0637434764718
AGACGTGC -0.208744235665
AGACGTGG -1.34142778053
AGACGTTA -0.510160153326
AGACGTTC -0.105460056016
AGACGTTG 0.0130806559196
AGACTAAA -0.455897518944
AGACTAAC 0.84285523057
AGACTAAG -0.308777091441
AGACTAAT -0.12751838782
AGACTACA -0.425883873371
AGACTACC -1.13706860608
AGACTACG -0.402969721844
AGACTACT 0.496026533125
AGACTAGA 1.0973586542
AGACTAGC -0.15131125865
AGACTAGG -0.245248454657

AGACTATA 1.28064938319
AGACTATC 0.757195399684
AGACTATG 0.859877736759
AGACTCAA -0.0695476870536
AGACTCAC -0.0896691308246
AGACTCAG -0.938115814069
AGACTCAT 1.27481165595
AGACTCCA 0.0399021001701
AGACTCCC -1.66167048303
AGACTCCG -0.387720683965
AGACTCCT -0.854810892623
AGACTCGA 0.163617908797
AGACTCGC -0.441392301028
AGACTCGG -1.09655425444
AGACTCTA -0.58730009173
AGACTCTC -0.832319758774
AGACTCTG -0.236979416655
AGACTGAA -0.0922263892726
AGACTGAC -0.469722203005
AGACTGAG -0.504151928312
AGACTGAT 0.309109759872
AGACTGCA -1.56498866839
AGACTGCC -0.990233590604
AGACTGCG -0.654718534286
AGACTGCT -0.687687266449
AGACTGGA 0.655994249018
AGACTGGC -0.0966853536731
AGACTGGG -0.958774981592
AGACTGTA 0.0897409522317
AGACTGTC -0.775374750972
AGACTGTG 1.21381800257
AGACTTAA -0.265668425668
AGACTTAC -0.0533814169435
AGACTTAG -0.542850551513
AGACTTAT 1.00338461068
AGACTTCA -0.780556301181
AGACTTCC -0.718458471732
AGACTTCG -0.277692370102
AGACTTGA -0.435880580525
AGACTTGC -0.593988850598
AGACTTGG -1.1416787077
AGACTTTA 0.458778910525
AGACTTTC -0.193395688705
AGACTTTG 1.17458165562
AGAGAAAA 0.441301743602
AGAGAAAC 0.582153515424
AGAGAAAG -0.318352862696
AGAGAAAT 3.43038775473
AGAGAACA 0.231723963259
AGAGAACC -0.672181336928
AGAGAACG -0.414168656898
AGAGAACT -1.11051342148
AGAGAAGA 0.558896286566
AGAGAAGC -0.368995906339

AGAGAAGG -0.501830119173
AGAGAATA 2.18046981483
AGAGAATC 15.2420664148
AGAGAATG -0.378214027803
AGAGACAA -0.623695433633
AGAGACAC 0.0471881216955
AGAGACAG -1.06244741319
AGAGACAT 0.151832119985
AGAGACCA 0.466474418159
AGAGACCC -1.15127383951
AGAGACCG -0.104127092336
AGAGACCT -0.852417262076
AGAGACGA -0.577789272009
AGAGACGC -1.13591072009
AGAGACGG -1.55746823844
AGAGACTA -0.267879692294
AGAGACTC -0.0759379186815
AGAGACTG -0.573036568463
AGAGAGAA -0.601058991546
AGAGAGAC -1.2603001927
AGAGAGAG -0.380948237544
AGAGAGAT 2.17642845548
AGAGAGCA -1.04089245577
AGAGAGCC -1.55743701174
AGAGAGCG -0.840146002188
AGAGAGCT -0.318459658005
AGAGAGGA 0.0981580047852
AGAGAGGC -1.77151310213
AGAGAGGG -0.949087626934
AGAGAGTA 0.640219769888
AGAGAGTC -0.610237767371
AGAGAGTG 0.166043598754
AGAGATAA -0.154930641211
AGAGATAC 2.2204068889
AGAGATAG 0.499432533244
AGAGATAT 6.96034204654
AGAGATCA 2.45521084555
AGAGATCC 5.87749423107
AGAGATCG 4.00788453667
AGAGATCT 10.003744402
AGAGATGA -0.788666499375
AGAGATGC -0.107611575558
AGAGATGG -0.250772874018
AGAGATTA 3.12865957009
AGAGATTC 6.59714431363
AGAGATTG 4.60085352328
AGAGCAAA -0.256661604864
AGAGCAAC -0.631622018838
AGAGCAAG -0.857610470253
AGAGCAAT 2.31326967829
AGAGCACA -0.805994402678
AGAGCACC -1.13339759538
AGAGCACG -1.02225969282
AGAGCACT -0.693476280037

AGAGCAGA -0.0996647970878
AGAGCAGC -1.84061195743
AGAGCAGG -1.18898403361
AGAGCATA -0.292997407862
AGAGCATC 0.116533459741
AGAGCATG -0.643861219316
AGAGCCAA -1.19145469001
AGAGCCAC -0.881142702237
AGAGCCAG -1.14722373668
AGAGCCAT -0.324197667986
AGAGCCCA -0.456174603851
AGAGCCCC -1.66734166788
AGAGCCCG 0.205608034222
AGAGCCCT -1.37103235635
AGAGCCGA -0.810072193176
AGAGCCGC -1.59144496844
AGAGCCGG -1.41053100757
AGAGCCTA -0.311891434195
AGAGCCTC -0.0343641492394
AGAGCCTG -0.775127435518
AGAGCGAA -0.0695722520566
AGAGCGAC -0.915372368494
AGAGCGAG 0.399361164539
AGAGCGAT 1.52603835799
AGAGCGCA -0.666215372046
AGAGCGCC -0.929624025616
AGAGCGCG -0.713604385504
AGAGCGCT 0.150406933455
AGAGCGGA -0.81601713208
AGAGCGGC -1.16429620559
AGAGCGGG -1.22793580125
AGAGCGTA 0.743777911774
AGAGCGTC -1.03257741044
AGAGCGTG -0.463489145761
AGAGCTAA 0.138133591786
AGAGCTAC 0.246171724049
AGAGCTAG 0.656692894358
AGAGCTAT 0.786729194986
AGAGCTCA -0.64115344818
AGAGCTCC -0.927755211786
AGAGCTCG -1.24304577623
AGAGCTCT -0.0453555308361
AGAGCTGA -0.29706999391
AGAGCTGC -0.521663236425
AGAGCTGG 0.105708204182
AGAGCTTA -0.886680861346
AGAGCTTC -0.50086646325
AGAGCTTG 0.39750463321
AGAGGAAA -0.3529528776
AGAGGAAC -0.922508293688
AGAGGAAG -0.297059376832
AGAGGAAT 1.82516993855
AGAGGACA 0.071649868412
AGAGGACC -1.41165600144

AGAGGACG -0.758554801968
AGAGGACT 0.110854780488
AGAGGAGA 0.29181391598
AGAGGAGC -1.70512659787
AGAGGAGG -0.885632060625
AGAGGATA 3.75838945916
AGAGGATC 7.00956115273
AGAGGATG -0.0998942092345
AGAGGCAA -1.08860685143
AGAGGCAC -0.706738467165
AGAGGCAG -1.22073742265
AGAGGCAT -0.150157119865
AGAGGCCA -1.00009498206
AGAGGCCC -1.92076797858
AGAGGCCG -1.21118101195
AGAGGCCT -0.75271499295
AGAGGCGA 0.54096966336
AGAGGCGC -0.790623164317
AGAGGCGG -0.797076057521
AGAGGCTA -0.113176173272
AGAGGCTC -0.621426709881
AGAGGCTG -0.852702257747
AGAGGGAA -0.928423254961
AGAGGGAC -1.31738738607
AGAGGGAG -0.949087626934
AGAGGGAT 0.0886342780288
AGAGGGCA -0.839690508742
AGAGGGCC -2.24020190958
AGAGGGCG -1.32808440399
AGAGGGCT -1.30120571079
AGAGGGGA -0.854940379334
AGAGGGGC -1.15688340367
AGAGGGGG -1.00092935945
AGAGGGTA -0.281284273168
AGAGGGTC -1.73809970177
AGAGGGTG -0.53117676246
AGAGGTAA -0.169219145837
AGAGGTAC 0.475179172697
AGAGGTAG -0.803296832263
AGAGGTAT 0.10068445289
AGAGGTCA -1.06268598517
AGAGGTCC -0.596929989262
AGAGGTCG -1.34444990043
AGAGGTGA -0.637129575782
AGAGGTGC -0.701457407876
AGAGGTGG -1.00377057268
AGAGGTTA -0.164232450228
AGAGGTTC -0.282251884473
AGAGGTTG -0.0184158414865
AGAGTAAA -0.682129746787
AGAGTAAC -0.0429142275295
AGAGTAAG -0.074377832813
AGAGTAAT 1.90752806649
AGAGTACA -0.29456582085

AGAGTACC -0.728928991994
AGAGTACG -0.1199126049
AGAGTACT 0.0303544329446
AGAGTAGA 1.34483190705
AGAGTAGC -0.994992955846
AGAGTAGG -1.00981772698
AGAGTATA 1.74742649215
AGAGTATC 3.33778976775
AGAGTATG 0.799172409895
AGAGTCAA -0.202753289424
AGAGTCAC -0.138469174709
AGAGTCAG -0.67571661558
AGAGTCAT 0.241188775644
AGAGTCCA -0.916611027544
AGAGTCCC -0.74755009698
AGAGTCCG -1.4323053846
AGAGTCCT -0.817876369722
AGAGTCGA 0.971347475034
AGAGTCGC -0.970064057716
AGAGTCGG -0.430323893575
AGAGTCTA -0.4582134991
AGAGTCTC -0.596096028228
AGAGTCTG -0.776414808217
AGAGTGAA -0.24084195111
AGAGTGAC -0.572090191313
AGAGTGAG -0.0011941089594
AGAGTGAT 0.552353252292
AGAGTGCA 0.0754863806179
AGAGTGCC -1.39237871942
AGAGTGCG -0.973143842923
AGAGTGCT -0.639032322625
AGAGTGGA 0.299735921269
AGAGTGGC -0.346496029014
AGAGTGGG -1.32460574975
AGAGTGTA 0.760766276772
AGAGTGTC 0.101218637617
AGAGTGTG 0.519314780503
AGAGTTAA -0.303681310565
AGAGTTAC 0.145783924797
AGAGTTAG -0.256099316109
AGAGTTAT 1.11527278672
AGAGTTCA -0.819577392091
AGAGTTCC -0.429610675776
AGAGTTCG -0.29509230299
AGAGTTGA -0.195124398747
AGAGTTGC 0.532919836783
AGAGTTGG -1.24411872559
AGAGTTTA 0.535258300162
AGAGTTTC 0.148120722752
AGAGTTTG 0.501182269263
AGATAAAA 1.09749542714
AGATAAAC -0.0758683872323
AGATAAAG -0.941310097171
AGATAAAT 1.97702454176

AGATAACA 0.796240431063
AGATAACC 0.239947202103
AGATAACG 1.58755120729
AGATAACT 0.606478072844
AGATAAGA 1.43964574058
AGATAAGC -0.634092675242
AGATAAGG 1.50404102234
AGATAATA 1.36943688022
AGATAATC 3.62077048337
AGATAATG 1.5989993314
AGATACAA 0.859515923409
AGATACAC 2.28531761937
AGATACAG 0.236317410642
AGATACAT 1.34277427578
AGATACCA 3.61564743117
AGATACCC 4.84250237229
AGATACCG 7.15535964998
AGATACCT 3.25720406723
AGATACGA 5.98403951896
AGATACGC 7.34985951475
AGATACGG 4.48441936444
AGATACTA 3.95769178202
AGATACTC 5.46850941434
AGATACTG 5.10859006843
AGATAGAA -0.468789357425
AGATAGAC -0.659173335127
AGATAGAG -0.345514261606
AGATAGAT 2.40193039493
AGATAGCA 0.848731678914
AGATAGCC 0.386168092504
AGATAGCG 2.06347898804
AGATAGCT 0.957020457479
AGATAGGA -0.636806899895
AGATAGGC 0.638793126112
AGATAGGG -0.270278319112
AGATAGTA 2.34336014155
AGATAGTC 0.889398000491
AGATAGTG 1.58175011937
AGATATAA 2.24657007434
AGATATAC 2.6102763861
AGATATAG 3.10486066209
AGATATAT 4.69143426553
AGATATCA 14.9484155362
AGATATCC 19.4275503024
AGATATCG 15.6340480859
AGATATCT 21.128823942
AGATATGA 3.60798627291
AGATATGC 9.09750605104
AGATATGG 7.18967466921
AGATATTA 5.36973353727
AGATATTC 11.3181698316
AGATATTG 4.98447726437
AGATCAAA 0.856728628281
AGATCAAC 1.14394118611

AGATCAAG 0.775118275686
AGATCAAT 2.14234576288
AGATCACA 2.06701718118
AGATCACC 1.18303930288
AGATCACG 2.48223609605
AGATCACT 1.67286129915
AGATCAGA 1.4626677283
AGATCAGC -0.0893860087562
AGATCAGG 1.01578285915
AGATCATA 0.868822104165
AGATCATC 2.12885645357
AGATCATG 1.20396348099
AGATCCAA 3.49676738911
AGATCCAC 4.04850276913
AGATCCAG 4.39815269471
AGATCCAT 2.28377960037
AGATCCCA 1.82384780013
AGATCCCC 6.57479973708
AGATCCCG 3.66538365149
AGATCCCT 3.43610182425
AGATCCGA 3.89657863447
AGATCCGC 3.90283084409
AGATCCGG 3.10139033498
AGATCCTA 4.84996971684
AGATCCTC 7.53575538314
AGATCCTG 3.96021698106
AGATCGAA 1.25732803551
AGATCGAC 0.947749875161
AGATCGAG 1.35006425269
AGATCGAT 3.65109889406
AGATCGCA 5.60263891064
AGATCGCC 4.67945965928
AGATCGCG 15.438001875
AGATCGCT 4.9385989986
AGATCGGA 2.73830585088
AGATCGGC 1.19835579043
AGATCGGG 2.91690861969
AGATCGTA 4.41487396735
AGATCGTC 4.24816003546
AGATCGTG 5.00775947459
AGATCTAA 4.97092279535
AGATCTAC 3.30906828297
AGATCTAG 4.57749491174
AGATCTAT 4.27376218121
AGATCTCA 8.98220000874
AGATCTCC 11.7287896783
AGATCTCG 9.73408520421
AGATCTGA 5.88625040557
AGATCTGC 10.2604403562
AGATCTGG 5.27548365792
AGATCTTA 4.53532034062
AGATCTTC 8.373530238
AGATCTTG 6.34748519186
AGATGAAA 0.492272251226

AGATGAAC -0.443146408785
AGATGAAG -0.145052179157
AGATGAAT 0.429191405301
AGATGACA -0.700578480396
AGATGACC 0.364536109226
AGATGACG -0.434498695017
AGATGACT 0.174641349804
AGATGAGA 0.326470138941
AGATGAGC -0.641040407531
AGATGAGG -0.265566418452
AGATGATA 2.38228193155
AGATGATC 1.77838172678
AGATGATG 0.272366760723
AGATGCAA 0.0687091461037
AGATGCAC 0.23358444997
AGATGCAG 0.313836649347
AGATGCAT 0.94284832435
AGATGCCA -0.703231708898
AGATGCCC 0.189559176499
AGATGCCG 1.11382636604
AGATGCCT -0.179724223646
AGATGCGA 0.517888969439
AGATGCGC 0.514363058796
AGATGCGG 0.811882300812
AGATGCTA 2.63498128471
AGATGCTC -0.335232766961
AGATGCTG 0.225453433977
AGATGGAA -0.274298860663
AGATGGAC -0.970900933242
AGATGGAG -0.20074291456
AGATGGAT 1.24172197238
AGATGGCA -0.720567314786
AGATGGCC -0.648078697247
AGATGGCG -0.353583240558
AGATGGCT -0.64770938949
AGATGGGA -0.288589655247
AGATGGGC -0.623390452875
AGATGGGG -0.258519593439
AGATGGTA -0.270601827711
AGATGGTC -0.36168427892
AGATGGTG -0.0557248765941
AGATGTAA 0.483979897289
AGATGTAC -0.306706969494
AGATGTAG 0.70838078334
AGATGTAT 1.29620527543
AGATGTCA 0.692643776249
AGATGTCC 0.203237095077
AGATGTCG 1.01698862607
AGATGTGA 0.230452203909
AGATGTGC -0.505304609851
AGATGTGG 0.409205485402
AGATGTTA 2.58210698935
AGATGTTC 0.99910946745
AGATGTTG 1.55091104807

AGATTAAA −0.172733398512
AGATTAAC −0.0696446979976
AGATTAAG 1.06381868162
AGATTAAT 1.45584865001
AGATTACA 14.2804735359
AGATTACC 15.8602859346
AGATTACG 16.2108056278
AGATTACT 8.46822561825
AGATTAGA 0.97572816451
AGATTAGC 2.09820474245
AGATTAGG 1.87596682573
AGATTATA 4.8675393148
AGATTATC 5.02645219281
AGATTATG 4.88567349146
AGATTCAA 1.21636714208
AGATTCAC 3.33680820852
AGATTCAG 3.05231070805
AGATTCAT 3.20847792653
AGATTCCA 10.1834927743
AGATTCCC 22.948273982
AGATTCCG 15.7295018586
AGATTCCT 12.2232661181
AGATTCGA 7.23959117167
AGATTCGC 16.2690609079
AGATTCGG 9.12942764791
AGATTCTA 9.5677224686
AGATTCTC 25.4485782623
AGATTCTG 13.5171756066
AGATTGAA 0.73754277275
AGATTGAC 0.73836674124
AGATTGAG 1.12129912322
AGATTGAT 1.10137690579
AGATTGCA 12.3596959812
AGATTGCC 10.6389041976
AGATTGCG 30.1083719713
AGATTGCT 9.5910246639
AGATTGGA 0.845491804832
AGATTGGC 2.04380408606
AGATTGGG 1.12040437421
AGATTGTA 5.60336253733
AGATTGTC 6.34186376156
AGATTGTG 11.1886073441
AGATTTAA 3.44778477313
AGATTTAC 4.72365230786
AGATTTAG 4.52698531019
AGATTTAT 4.66846119963
AGATTTCA 11.3082039348
AGATTTCC 32.9078132448
AGATTTCG 17.4456215035
AGATTTGA 2.94814718456
AGATTTGC 18.7427716988
AGATTTGG 6.14036620178
AGATTTTA 10.1184546389
AGATTTTC 23.8601964249

AGATTTTG 13.9487335793
AGCAAAAA 0.345205533645
AGCAAAAC 1.11386487897
AGCAAAAG 0.853286405191
AGCAAAAT 1.87272507805
AGCAAACA -0.47369049188
AGCAAACC 0.0539709770155
AGCAAACG -0.277797083632
AGCAAACT -0.511190634384
AGCAAAGA -0.199366649858
AGCAAAGC -0.21937713476
AGCAAAGG -0.35141569131
AGCAAATA 2.16606035879
AGCAAATC 7.00055495646
AGCAAATG 0.662309952925
AGCAACAA -0.351162130516
AGCAACAC 0.20270082857
AGCAACAG -0.65295401763
AGCAACAT -0.0968319109791
AGCAACCA -0.223862537765
AGCAACCC -0.858934482279
AGCAACCG -0.688680691824
AGCAACCT -0.445210701749
AGCAACGA -0.742002153506
AGCAACGC -0.782914957825
AGCAACGG -0.55151596041
AGCAACTA 0.622670781559
AGCAACTC -0.345278187764
AGCAACTG 0.0663280062366
AGCAAGAA -0.068921904011
AGCAAGAC 0.944293287789
AGCAAGAG -0.118275493174
AGCAAGAT 3.03449129651
AGCAAGCA -0.38345386785
AGCAAGCC -0.892341637292
AGCAAGCG 0.0830592714157
AGCAAGCT -1.49547075079
AGCAAGGA 0.240566323449
AGCAAGGC -0.947686589051
AGCAAGGG -0.362989346749
AGCAAGTA 2.20099179306
AGCAAGTC -0.141686773746
AGCAAGTG 0.574976787344
AGCAATAA 0.0322694622887
AGCAATAC 1.74451574747
AGCAATAG -0.0132151389022
AGCAATAT 3.0541934698
AGCAATCA 3.16423344163
AGCAATCC 6.79946396829
AGCAATCG 3.26471804365
AGCAATGA 0.125420786378
AGCAATGC -0.141835829188
AGCAATGG 0.200733130195
AGCAATTA 0.36015541975

AGCAATTC 0.89945112388
AGCAATTG 1.52609748054
AGCACAAA 0.388248206995
AGCACAAC 0.0696805046121
AGCACAAG 0.339676950724
AGCACAAT 0.73748260931
AGCACACA -0.861368915712
AGCACACC -1.04290803509
AGCACACG -1.40866011196
AGCACACT -0.485780845094
AGCACAGA -0.580313221978
AGCACAGC -1.16006019982
AGCACAGG -0.489520138178
AGCACATA 0.89139963188
AGCACATC -0.933626455681
AGCACATG 0.132065619689
AGCACCAA -0.784331400879
AGCACCAC -0.220598723214
AGCACCAG -0.949303923867
AGCACCAT -0.863958858105
AGCACCCA -0.168234255759
AGCACCCC -0.37745875805
AGCACCCG -1.50829451506
AGCACCCT -0.560119748622
AGCACCGA -0.553771152593
AGCACCGC -0.697533044558
AGCACCGG -0.739285222539
AGCACCTA 0.330998426613
AGCACCTC 0.0719563064155
AGCACCTG -0.349975932321
AGCACGAA 0.198261016545
AGCACGAC -0.122197774713
AGCACGAG 0.235160149187
AGCACGAT 0.00998504918563
AGCACGCA -0.479722449184
AGCACGCC -0.984050704255
AGCACGCG 0.476668061693
AGCACGCT -0.480339905107
AGCACGGA -0.130115408265
AGCACGGC -1.05272570917
AGCACGGG -0.0752546785133
AGCACGTA 0.556598626074
AGCACGTC -0.60470085733
AGCACGTG 0.361652635865
AGCACTAA -0.488716154774
AGCACTAC 0.442276016602
AGCACTAG -0.382275580418
AGCACTAT 1.53880466515
AGCACTCA -0.320018702984
AGCACTCC 0.0761015465828
AGCACTCG -0.863295186668
AGCACTGA 0.4916633306
AGCACTGC -0.817330943384
AGCACTGG -0.520876948151

AGCACTTA 0.236317410642
AGCACTTC 0.12820141981
AGCACTTG -0.733931092774
AGCAGAAA -0.0865574943853
AGCAGAAC -0.483463199514
AGCAGAAG -1.0779610453
AGCAGAAT 1.59807710289
AGCAGACA -0.174196681615
AGCAGACC -0.48499559771
AGCAGACG 0.318389502022
AGCAGACT -0.241852447081
AGCAGAGA 0.606745997919
AGCAGAGC -0.862377329903
AGCAGAGG -0.998415818383
AGCAGATA 1.97757538073
AGCAGATC 4.49387251886
AGCAGATG 0.464357247986
AGCAGCAA -0.629323733812
AGCAGCAC -0.752935245265
AGCAGCAG -0.356140290828
AGCAGCAT -0.200872193093
AGCAGCCA -0.335374744351
AGCAGCCC -1.5503620827
AGCAGCCG -0.778550922589
AGCAGCCT -1.53396848222
AGCAGCGA -0.55482349234
AGCAGCGC -0.792892096247
AGCAGCGG -0.204944154607
AGCAGCTA -0.620726399118
AGCAGCTC -0.748654897581
AGCAGCTG -0.614612211507
AGCAGGAA -0.673963340535
AGCAGGAC -0.984033217304
AGCAGGAG -1.03278579661
AGCAGGAT 0.875308722203
AGCAGGCA -1.49996385638
AGCAGGCC -1.1338618323
AGCAGGCG -1.08554975763
AGCAGGCT -0.94187717402
AGCAGGGA -0.576857883675
AGCAGGGC -0.953356108472
AGCAGGGG -0.486197825611
AGCAGGTA -0.436789485636
AGCAGGTC -0.267445016648
AGCAGGTG -0.683899884249
AGCAGTAA -0.293133764446
AGCAGTAC -0.79035440653
AGCAGTAG -0.494461242806
AGCAGTAT 3.24527047204
AGCAGTCA 0.348639013259
AGCAGTCC -0.672439893993
AGCAGTCG -1.00658763726
AGCAGTGA -0.503464316406
AGCAGTGC -0.954000627534

AGCAGTGG -0.106156619576
AGCAGTTA 0.659043640238
AGCAGTTC -0.222916993328
AGCAGTTG 0.836661935322
AGCATAAA -0.4508335893
AGCATAAC -0.101228421982
AGCATAAG -0.248937993201
AGCATAAT 0.848983157928
AGCATACA 0.0861944319681
AGCATACC -0.548973274422
AGCATACG -0.599315500867
AGCATACT -0.312769737141
AGCATAGA -0.110123034848
AGCATAGC -1.03025872397
AGCATAGG -0.69233171744
AGCATATA 3.94637314462
AGCATATC 4.30344919552
AGCATATG -0.0625928767126
AGCATCAA 0.536487591202
AGCATCAC 1.59343889724
AGCATCAG 0.0641254830862
AGCATCAT 0.916472381001
AGCATCCA 0.188923609091
AGCATCCC -0.344237297806
AGCATCCG -0.361698643202
AGCATCCT -0.0702871352795
AGCATCGA -0.117444030276
AGCATCGC 0.663776775054
AGCATCGG -0.42459566796
AGCATCTA 1.81206263585
AGCATCTC -0.177410533448
AGCATCTG 0.77365540894
AGCATGAA -0.614955288837
AGCATGAC -0.468252466385
AGCATGAG -0.746192984653
AGCATGAT -0.0903221851841
AGCATGCA -0.633660289553
AGCATGCC -0.728816575879
AGCATGCG -1.02819984363
AGCATGCT -0.370593880602
AGCATGGA 0.555301469008
AGCATGGC -0.868632037658
AGCATGGG -0.389814121847
AGCATGTA 1.45752573191
AGCATGTC -1.08473765528
AGCATGTG 0.531170725298
AGCATTAA 0.0918560406256
AGCATTAC -0.155416528643
AGCATTAG -0.0995165743579
AGCATTAT 1.87846537799
AGCATTCA 0.0507496307746
AGCATTCC -0.468224362356
AGCATTCG -0.345162232623
AGCATTGA -0.401014722326

| | |
|---|---|
| AGCATTGC | -0.352278797263 |
| AGCATTGG | -0.473078656763 |
| AGCATTTA | 1.30097754771 |
| AGCATTTC | 0.877950709093 |
| AGCATTTG | 0.0160376161113 |
| AGCCAAAA | -0.163291902062 |
| AGCCAAAC | -0.419205107047 |
| AGCCAAAG | -0.549783086809 |
| AGCCAAAT | 0.980422994575 |
| AGCCAACA | 0.573594069124 |
| AGCCAACC | -0.394453992923 |
| AGCCAACG | -1.33103366159 |
| AGCCAACT | -1.09147616869 |
| AGCCAAGA | -0.74032049169 |
| AGCCAAGC | -1.3042892432 |
| AGCCAAGG | -0.758144274969 |
| AGCCAATA | 0.587900477058 |
| AGCCAATC | 0.0820508572247 |
| AGCCAATG | -0.690454368312 |
| AGCCACAA | -0.821630651621 |
| AGCCACAC | -0.286940469198 |
| AGCCACAG | -0.235627925134 |
| AGCCACAT | -0.503085432461 |
| AGCCACCA | -0.5171695063 |
| AGCCACCC | -0.657672996342 |
| AGCCACCG | -1.32509746616 |
| AGCCACCT | -0.974156004318 |
| AGCCACGA | -0.253678206068 |
| AGCCACGC | -1.51221700478 |
| AGCCACGG | -1.02834973178 |
| AGCCACTA | -0.469544627178 |
| AGCCACTC | -1.28338192751 |
| AGCCACTG | -0.462087483344 |
| AGCCAGAA | -0.125263611994 |
| AGCCAGAC | -0.96170737678 |
| AGCCAGAG | -0.491067733367 |
| AGCCAGAT | 1.02692079805 |
| AGCCAGCA | -0.487004098972 |
| AGCCAGCC | -1.76607486845 |
| AGCCAGCG | -0.680186197058 |
| AGCCAGCT | -1.36231198846 |
| AGCCAGGA | -0.10092802114 |
| AGCCAGGC | -1.83945198966 |
| AGCCAGGG | -1.42930429067 |
| AGCCAGTA | -0.508906921817 |
| AGCCAGTC | -0.451534732775 |
| AGCCAGTG | -0.339974437074 |
| AGCCATAA | -0.192733266336 |
| AGCCATAC | -0.237115565062 |
| AGCCATAG | -0.813906623602 |
| AGCCATAT | 1.39149708563 |
| AGCCATCA | -0.42011068131 |
| AGCCATCC | -0.807587380669 |
| AGCCATCG | -0.966157805883 |

AGCCATGA -0.326870257041
AGCCATGC -0.826285095156
AGCCATGG -0.451341543599
AGCCATTA 0.176703560989
AGCCATTC 0.703014995609
AGCCATTG -0.506907996742
AGCCCAAA 0.155117585047
AGCCCAAC -1.03230469727
AGCCCAAG -0.918881416719
AGCCCAAT -0.748931566132
AGCCCACA -0.563390016691
AGCCCACC -1.12671653909
AGCCCACG -1.64091388821
AGCCCACT -1.16130468785
AGCCCAGA -0.62587755534
AGCCCAGC -1.63044919693
AGCCCAGG -1.88479836067
AGCCCATA 0.0781262857284
AGCCCATC -0.603129946206
AGCCCATG -1.05360380394
AGCCCCAA -0.547876592763
AGCCCCAC -0.86270729202
AGCCCCAG -1.32652556719
AGCCCCAT -0.54061825891
AGCCCCCA -1.46994063462
AGCCCCCC -1.6709198312
AGCCCCCG -1.62423029579
AGCCCCCT -0.921188445222
AGCCCCGA -1.10774569508
AGCCCCGC -1.22641610191
AGCCCCGG -1.17353993295
AGCCCCTA 0.0919917726761
AGCCCCTC -0.839201290462
AGCCCCTG -1.23013749168
AGCCCGAA -0.474125167526
AGCCCGAC -0.840561109103
AGCCCGAG -1.11906100163
AGCCCGAT -0.0995532136844
AGCCCGCA -0.745025730655
AGCCCGCC -0.806418253068
AGCCCGCG -1.20186921039
AGCCCGCT -1.42473894732
AGCCCGGA -0.459344738306
AGCCCGGC -1.29844589515
AGCCCGGG -0.677411600787
AGCCCGTA 0.407771971753
AGCCCGTC -0.71099487438
AGCCCGTG -0.83614898475
AGCCCTAA -1.37363041768
AGCCCTAC -0.331000300215
AGCCCTAG -0.869911291416
AGCCCTAT -0.722634105886
AGCCCTCA -0.606747455165
AGCCCTCC -1.85189811906

AGCCCTCG -1.27860923888
AGCCCTGA -0.298112549291
AGCCCTGC -1.18756196975
AGCCCTGG -1.18424090625
AGCCCTTA -1.04323570725
AGCCCTTC -1.19492168628
AGCCCTTG -0.91632332556
AGCCGAAA -0.0761381859093
AGCCGAAC -0.154465987934
AGCCGAAG -0.392541669892
AGCCGAAT 0.247986619779
AGCCGACA -0.398869448123
AGCCGACC -0.503114369202
AGCCGACG -0.417391460385
AGCCGACT -0.58017519997
AGCCGAGA -0.481030639683
AGCCGAGC -0.892687004579
AGCCGAGG -0.868273763335
AGCCGATA 2.59238307137
AGCCGATC 0.199306070063
AGCCGATG -0.578161910614
AGCCGCAA -0.370523100084
AGCCGCAC -0.0928496741792
AGCCGCAG -0.697267617619
AGCCGCAT -0.735961244548
AGCCGCCA -1.04530541284
AGCCGCCC -1.21480872163
AGCCGCCG -1.18826123962
AGCCGCCT -0.354000012897
AGCCGCGA 0.640189792258
AGCCGCGC -1.40001864353
AGCCGCGG -0.649294664895
AGCCGCTA -0.991650033658
AGCCGCTC -0.676356971082
AGCCGCTG -1.20808602975
AGCCGGAA -0.366093488781
AGCCGGAC -1.18727447594
AGCCGGAG -0.549006999257
AGCCGGAT 0.0299130956026
AGCCGGCA -0.646426804884
AGCCGGCC -1.39350558689
AGCCGGCG -1.24479738585
AGCCGGCT -1.64208551395
AGCCGGGA -0.772225434316
AGCCGGGC -1.55944280669
AGCCGGGG -0.779838503466
AGCCGGTA -0.537233701124
AGCCGGTC -0.827168186196
AGCCGGTG -1.3216171465
AGCCGTAA -0.334999191254
AGCCGTAC 0.248947985744
AGCCGTAG -1.04636087526
AGCCGTAT 0.0810653426128
AGCCGTCA -0.549009497392

AGCCGTCC −0.633971099295
AGCCGTCG −1.28519599053
AGCCGTGA 0.198644688584
AGCCGTGC −1.13277264505
AGCCGTGG −0.382139432012
AGCCGTTA −1.02021476041
AGCCGTTC 0.395328340487
AGCCGTTG −0.0597658195877
AGCCTAAA −0.808119899971
AGCCTAAC −1.39063356332
AGCCTAAG −0.266327725367
AGCCTAAT −0.250772874018
AGCCTACA −0.0224407547747
AGCCTACC −1.06076845769
AGCCTACG −0.688075102047
AGCCTACT −0.610337692806
AGCCTAGA 0.0567570230761
AGCCTAGC −1.09134147753
AGCCTAGG −1.18873463637
AGCCTATA 0.0297332298179
AGCCTATC 2.06152669484
AGCCTATG −0.232428645761
AGCCTCAA −0.581617665273
AGCCTCAC −0.310984194508
AGCCTCAG −0.731685684958
AGCCTCAT −1.28029256611
AGCCTCCA −0.601521146687
AGCCTCCC −1.33720697175
AGCCTCCG −1.31330064392
AGCCTCCT −1.17381764239
AGCCTCGA −0.13995223472
AGCCTCGC −1.26340454291
AGCCTCGG −0.8026939488
AGCCTCTA −0.289425698061
AGCCTCTC −1.37723564414
AGCCTCTG −0.65943730482
AGCCTGAA −0.10650344411
AGCCTGAC −0.42119070873
AGCCTGAG −0.526792533959
AGCCTGAT −0.580047586861
AGCCTGCA −0.470738111604
AGCCTGCC −1.37198227253
AGCCTGCG −1.79697243778
AGCCTGGA −0.122552301832
AGCCTGGC −1.34007816261
AGCCTGGG −0.969412460603
AGCCTGTA 0.348197259561
AGCCTGTC −0.712383421583
AGCCTGTG −0.705489399216
AGCCTTAA −1.31735074674
AGCCTTAC −0.72716738983
AGCCTTAG −1.04540846094
AGCCTTAT 0.51157659638
AGCCTTCA −0.890054593877

AGCCTTCC -1.28270222637
AGCCTTCG -0.618338181199
AGCCTTGA -0.506707521336
AGCCTTGC -1.25347924081
AGCCTTGG -1.00559213011
AGCCTTTA -0.118524057696
AGCCTTTC -0.437669245828
AGCCTTTG -0.101114964977
AGCGAAAA -0.496233045692
AGCGAAAC 0.716483695305
AGCGAAAG 0.286720633239
AGCGAAAT 2.43357615595
AGCGAACA -0.408591776683
AGCGAACC -1.06326909173
AGCGAACG -0.675293597901
AGCGAACT -0.121778920594
AGCGAAGA -0.342048514403
AGCGAAGC -0.932264763439
AGCGAAGG -1.3875235923
AGCGAATA 3.18741322828
AGCGAATC 11.66558684
AGCGAATG 0.222829558571
AGCGACAA -0.125495105921
AGCGACAC -0.277361575274
AGCGACAG -0.924536780037
AGCGACAT -0.36473887459
AGCGACCA -0.13239308367
AGCGACCC -0.72927456746
AGCGACCG -1.2986894634
AGCGACCT -0.701782998255
AGCGACGA 0.451116086834
AGCGACGC -0.356922415542
AGCGACGG -1.15317825178
AGCGACTA -0.0368046198341
AGCGACTC -1.13829831348
AGCGACTG 0.0433859588583
AGCGAGAA -0.585766236289
AGCGAGAC 1.46781014105
AGCGAGAG -0.401072803985
AGCGAGAT 1.02408041753
AGCGAGCA 0.0660382224724
AGCGAGCC -0.625318805611
AGCGAGCG -0.163476764119
AGCGAGCT -0.895348976557
AGCGAGGA -0.190364825327
AGCGAGGC -1.06571476677
AGCGAGGG -0.847935189919
AGCGAGTA 0.476668061693
AGCGAGTC -0.617956382762
AGCGAGTG 1.65177057865
AGCGATAA -0.399528123288
AGCGATAC 1.49412758638
AGCGATAG 0.831508697319
AGCGATAT 4.22371265303

AGCGATCA 1.8775581383
AGCGATCC 3.3337361259
AGCGATCG 2.25521216731
AGCGATGA 0.377443561057
AGCGATGC -0.0587894648074
AGCGATGG -0.998114168473
AGCGATTA 1.43406074142
AGCGATTC 1.17137259188
AGCGATTG 2.58182574088
AGCGCAAA 1.1347282691
AGCGCAAC 0.447817714737
AGCGCAAG -0.416764220097
AGCGCAAT 1.36951973506
AGCGCACA 0.159825322147
AGCGCACC -0.787633520181
AGCGCACG -0.975880758978
AGCGCACT -0.106222403821
AGCGCAGA 0.217794773846
AGCGCAGC -1.01932105229
AGCGCAGG -0.373235242958
AGCGCATA -0.0533959894029
AGCGCATC 0.556011355959
AGCGCATG -0.762785811468
AGCGCCAA -0.422752668201
AGCGCCAC -0.69880563662
AGCGCCAG -1.11051342148
AGCGCCAT -1.4419856612
AGCGCCCA -1.07722950784
AGCGCCCC -1.33021573026
AGCGCCCG -1.57651194475
AGCGCCCT -1.01859326203
AGCGCCGA -0.11249439035
AGCGCCGC -0.325432579832
AGCGCCGG -1.22909285452
AGCGCCTA -0.662658859239
AGCGCCTC -0.555492160049
AGCGCCTG -0.398333806151
AGCGCGAA -0.179555183116
AGCGCGAC -0.308827470515
AGCGCGAG -0.26487214485
AGCGCGAT 2.72866596081
AGCGCGCA 1.51630041608
AGCGCGCC -0.962585679727
AGCGCGCG -0.036758196142
AGCGCGCT -0.152720207297
AGCGCGGA 1.13462626188
AGCGCGGC -0.986176617905
AGCGCGGG -1.41223765075
AGCGCGTA 0.13327513382
AGCGCGTC -0.16159025516
AGCGCGTG 0.893582378121
AGCGCTAA 0.326077098893
AGCGCTAC 0.443970793631
AGCGCTAG -1.22451168964

AGCGCTAT 0.723973523084
AGCGCTCA 0.285487386817
AGCGCTCC -0.163410563518
AGCGCTCG -0.806383279166
AGCGCTGA 0.512920593493
AGCGCTGC -0.163410563518
AGCGCTGG 0.189586864172
AGCGCTTA -1.04438026984
AGCGCTTC 0.576128427993
AGCGCTTG -0.555449691738
AGCGGAAA -0.444617810829
AGCGGAAC -0.361024562865
AGCGGAAG -1.34942993435
AGCGGAAT 0.713790704806
AGCGGACA -0.861647874221
AGCGGACC -0.600869133218
AGCGGACG -0.922465200844
AGCGGACT -0.387090737362
AGCGGAGA 0.645307431823
AGCGGAGC -0.887096800973
AGCGGAGG -1.0662422898
AGCGGATA 2.9674573587
AGCGGATC 4.31003969437
AGCGGATG 0.552663021144
AGCGGCAA -0.865441293582
AGCGGCAC -0.495581032223
AGCGGCAG -1.06571851398
AGCGGCAT -0.604730834961
AGCGGCCA -0.948619642809
AGCGGCCC -2.07432672682
AGCGGCCG -0.755561410628
AGCGGCCT -0.907090631635
AGCGGCGA -0.519886853624
AGCGGCGC -0.809017147115
AGCGGCGG -1.13352666573
AGCGGCTA -1.06979214091
AGCGGCTC -0.98687359782
AGCGGCTG -0.598249213194
AGCGGGAA -1.07166657555
AGCGGGAC -1.36223496261
AGCGGGAG -0.621345104109
AGCGGGAT 1.54726814139
AGCGGGCA -0.621426709881
AGCGGGCC -1.19290423337
AGCGGGCG -0.5859827414
AGCGGGGA 1.07012605841
AGCGGGGC -1.02861682415
AGCGGGGG -0.835848792087
AGCGGGTA -0.0422834482151
AGCGGGTC -1.14411543109
AGCGGGTG -0.694107475707
AGCGGTAA 0.21154631143
AGCGGTAC -0.418821226831
AGCGGTAG -0.710819172155

AGCGGTAT 1.60338564168
AGCGGTCA -0.571260810195
AGCGGTCC -1.47571590846
AGCGGTCG -0.863278740606
AGCGGTGA -0.183682519976
AGCGGTGC -1.53711842341
AGCGGTGG -1.66380430745
AGCGGTTA 0.00578027011276
AGCGGTTC -0.111691239658
AGCGGTTG -0.688846401505
AGCGTAAA -0.0709966058746
AGCGTAAC 0.554456682719
AGCGTAAG -0.78371977394
AGCGTAAT 2.26893401144
AGCGTACA 0.427353193636
AGCGTACC -0.357500109468
AGCGTACG -0.72372516674
AGCGTACT -0.368945110909
AGCGTAGA 0.544257626558
AGCGTAGC -0.237020011364
AGCGTAGG -0.654694385639
AGCGTATA 0.826754120171
AGCGTATC 4.14912893218
AGCGTATG 0.395820889615
AGCGTCAA -0.635755601038
AGCGTCAC -0.0795964387046
AGCGTCAG 0.0567676401537
AGCGTCAT -0.432230803977
AGCGTCCA 0.254036688569
AGCGTCCC -1.17378121124
AGCGTCCG -1.05369123869
AGCGTCCT -1.17896484323
AGCGTCGA -0.236368206072
AGCGTCGC 0.194265664532
AGCGTCGG -1.06552449209
AGCGTCTA -1.04415627032
AGCGTCTC -0.969803418871
AGCGTCTG 0.520262614898
AGCGTGAA -0.776567402685
AGCGTGAC 0.301516259453
AGCGTGAG 0.00962469308229
AGCGTGAT 1.56944305287
AGCGTGCA -0.518093400227
AGCGTGCC -0.929742478893
AGCGTGCG -0.822763139895
AGCGTGGA 0.974428301131
AGCGTGGC -1.22426562325
AGCGTGGG -0.402904562133
AGCGTGTA -0.452880395313
AGCGTGTC -0.688630729106
AGCGTGTG 0.386168092504
AGCGTTAA -0.336632347598
AGCGTTAC 1.26674621603
AGCGTTAG -0.814025493235

AGCGTTAT 1.76287829539
AGCGTTCA 0.45505835346
AGCGTTCC -0.358591170322
AGCGTTCG 0.239210460191
AGCGTTGA 0.332058468946
AGCGTTGC -0.814318607847
AGCGTTGG -0.763324159754
AGCGTTTA -0.145808281622
AGCGTTTC 0.778906074242
AGCGTTTG 2.46280601139
AGCTAAAA -0.409755908012
AGCTAAAC -0.673222643241
AGCTAAAG -0.672958257192
AGCTAAAT -0.606581953661
AGCTAACA 0.118450154509
AGCTAACC -0.51560858772
AGCTAACG -0.690341119485
AGCTAACT -0.277344712857
AGCTAAGA -0.218571277755
AGCTAAGC -0.73914720053
AGCTAAGG -0.776790361314
AGCTAATA 1.3878802012
AGCTAATC -0.237115565062
AGCTAATG 0.131950289082
AGCTACAA -0.113304619093
AGCTACAC -0.0494085481532
AGCTACAG -0.709733315751
AGCTACAT -0.153854360994
AGCTACCA -0.816430365393
AGCTACCC -0.64110577542
AGCTACCG -0.618518046984
AGCTACCT -0.814825729434
AGCTACGA -0.481994503784
AGCTACGC -0.80339259414
AGCTACGG -0.556445198894
AGCTACTA -0.410279683838
AGCTACTC -0.937451101742
AGCTACTG -0.403666285404
AGCTAGAA -0.569489631843
AGCTAGAC -0.69262504023
AGCTAGAG -0.756632070039
AGCTAGAT 1.99997179359
AGCTAGCA 0.0868730922204
AGCTAGCC -0.706738467165
AGCTAGCG 0.0735222212678
AGCTAGCT -1.14200679622
AGCTAGGA 1.08018834163
AGCTAGGC -0.235599612928
AGCTAGGG -0.688491666208
AGCTAGTA 0.556639220782
AGCTAGTC -0.942823967525
AGCTAGTG -0.0479771162834
AGCTATAA 0.724374057539
AGCTATAC 0.557586014287

AGCTATAG -0.658331255151
AGCTATAT 1.79979866219
AGCTATCA 0.0445138672164
AGCTATCC 1.06165821043
AGCTATCG 0.143387587936
AGCTATGA 0.721049871371
AGCTATGC -0.557306223067
AGCTATGG -0.40558214746
AGCTATTA -0.328613955898
AGCTATTC -0.845675834177
AGCTATTG 0.501392529034
AGCTCAAA -0.503015484656
AGCTCAAC -0.446968556709
AGCTCAAG 0.15386310447
AGCTCAAT -0.203111980104
AGCTCACA -0.806189465456
AGCTCACC -0.700509781659
AGCTCACG -1.08468790074
AGCTCACT -0.701798611604
AGCTCAGA 0.46130952219
AGCTCAGC -0.190559055393
AGCTCAGG -0.392536673621
AGCTCATA -0.710239188271
AGCTCATC -1.23469534063
AGCTCATG -0.369068768636
AGCTCCAA 0.140667117943
AGCTCCAC -0.576730686922
AGCTCCAG -0.944053883099
AGCTCCAT -1.02162350088
AGCTCCCA -1.2368853731
AGCTCCCC -0.377094030209
AGCTCCCG -0.51064999614
AGCTCCCT -1.08136808631
AGCTCCGA -1.12981818299
AGCTCCGC -0.451030942036
AGCTCCGG -1.24363991621
AGCTCCTA -0.504799361866
AGCTCCTC -1.12054010626
AGCTCCTG -0.582140608388
AGCTCGAA 0.176976690513
AGCTCGAC -0.24398772874
AGCTCGAG -0.0243791000538
AGCTCGAT -0.489729981593
AGCTCGCA -0.963006615626
AGCTCGCC -0.863592673018
AGCTCGCG -0.590667162565
AGCTCGGA 1.8187799151
AGCTCGGC -0.791561214347
AGCTCGGG -1.02952593744
AGCTCGTA -0.167381558706
AGCTCGTC -0.57746784519
AGCTCGTG -1.33547222455
AGCTCTAA 0.251820009315
AGCTCTAC 0.257865498189

AGCTCTAG -0.514270627768
AGCTCTAT -0.151835034477
AGCTCTCA -0.827058684572
AGCTCTCC -0.80309614868
AGCTCTCG 0.178669593941
AGCTCTGA 0.115468837493
AGCTCTGC -0.428762974994
AGCTCTGG 0.0972187056874
AGCTCTTA -0.672760279922
AGCTCTTC -1.29824083983
AGCTCTTG -0.409796294542
AGCTGAAA -0.431109973671
AGCTGAAC -0.940434500538
AGCTGAAG -0.910973151177
AGCTGAAT -0.115020005743
AGCTGACA -0.807499945912
AGCTGACC -0.396021573199
AGCTGACG -0.954412195423
AGCTGACT -0.796813336895
AGCTGAGA -0.203248961222
AGCTGAGC -0.804744085661
AGCTGAGG -0.81463129119
AGCTGATA 2.65773180833
AGCTGATC 0.121330713378
AGCTGATG -0.951626565719
AGCTGCAA -0.790819476163
AGCTGCAC -0.595528535023
AGCTGCAG -0.0672098482087
AGCTGCAT -0.154481184927
AGCTGCCA -0.842631647407
AGCTGCCC -1.24565174832
AGCTGCCG -0.756478642859
AGCTGCCT -0.197039219913
AGCTGCGA -0.302808836602
AGCTGCGC -0.696466132351
AGCTGCGG -0.539348997696
AGCTGCTA -0.629250871515
AGCTGCTC -1.00267888729
AGCTGCTG 0.010885210821
AGCTGGAA -0.866712220221
AGCTGGAC -0.323095573699
AGCTGGAG -0.642242219076
AGCTGGAT 0.0238055696872
AGCTGGCA -0.719819123085
AGCTGGCC -1.44494387046
AGCTGGCG -0.56524134357
AGCTGGGA 1.46922408597
AGCTGGGC -0.805426909473
AGCTGGGG -0.498466171007
AGCTGGTA -0.317183735095
AGCTGGTC -1.06819166851
AGCTGGTG -0.113069794318
AGCTGTAA -0.160027671155
AGCTGTAC -0.163020437961

AGCTGTAG -0.700509781659
AGCTGTAT 1.36404340482
AGCTGTCA 0.0573447095462
AGCTGTCC 0.244577913346
AGCTGTCG -0.0576946567499
AGCTGTGA -0.459264381601
AGCTGTGC -0.934562840287
AGCTGTGG -0.728892352668
AGCTGTTA -0.584704944888
AGCTGTTC 0.192298174334
AGCTGTTG 0.455472419485
AGCTTAAA -0.651100400794
AGCTTAAC -0.535320545382
AGCTTAAG 0.112558092815
AGCTTAAT 0.769140652838
AGCTTACA -0.151832119985
AGCTTACC -0.146150109884
AGCTTACG -0.882319948779
AGCTTACT -1.28236227171
AGCTTAGA -0.103689710376
AGCTTAGC 0.301840808942
AGCTTAGG -0.928450109922
AGCTTATA 1.27727398524
AGCTTATC 0.167755030023
AGCTTATG -0.808456315605
AGCTTCAA -1.00065685446
AGCTTCAC -0.488361419477
AGCTTCAG -0.662031618951
AGCTTCAT -0.0196853108788
AGCTTCCA -0.959222147918
AGCTTCCC -0.836268478918
AGCTTCCG -0.114293048197
AGCTTCCT -1.06281568006
AGCTTCGA -0.44110917896
AGCTTCGC 0.907067732056
AGCTTCGG -0.683893847087
AGCTTCTA 0.244155728379
AGCTTCTC -0.57231960346
AGCTTCTG -0.171400851188
AGCTTGAA 0.280541077739
AGCTTGAC -0.615130782883
AGCTTGAG -0.483070784
AGCTTGAT 0.686889944741
AGCTTGCA -0.604612589861
AGCTTGCC -1.31596261589
AGCTTGCG -0.657603048537
AGCTTGGA 1.08306681872
AGCTTGGC -0.604826596837
AGCTTGGG -0.549053214771
AGCTTGTA 0.403153959366
AGCTTGTC -0.789769634552
AGCTTGTG 0.0517424316162
AGCTTTAA -0.884566813842
AGCTTTAC -0.159891939105

AGCTTTAG -1.52673159071
AGCTTTAT 0.882179012279
AGCTTTCA 1.27409531548
AGCTTTCC 0.406522071091
AGCTTTCG -0.191384481129
AGCTTTGA -0.616124000081
AGCTTTGC -0.785766996309
AGCTTTGG -0.675632928027
AGCTTTTA 0.0532835732875
AGCTTTTC 0.292144710808
AGCTTTTG -0.454069508
AGGAAAAA -0.158929324072
AGGAAAAC 0.383546298878
AGGAAAAG -0.681113838188
AGGAAAAT 1.45474613937
AGGAAACA 0.785674773459
AGGAAACC -0.791236248502
AGGAAACG -0.121619456252
AGGAAACT -0.331853205446
AGGAAAGA -0.540091152235
AGGAAAGC -0.814086281208
AGGAAAGG -0.858028699838
AGGAAATA 3.24811459976
AGGAAATC 23.7472313441
AGGAAATG -0.279926744486
AGGAACAA -0.439883426946
AGGAACAC -0.0476771317976
AGGAACAG -0.568754971711
AGGAACAT 0.551380644716
AGGAACCA 0.142390415357
AGGAACCC -1.3904701436
AGGAACCG -1.22061043408
AGGAACCT -0.306326420125
AGGAACGA -1.22318517948
AGGAACGC -1.05453810676
AGGAACGG -0.821689357815
AGGAACTA 0.00222750450935
AGGAACTC -0.61766639082
AGGAACTG -0.331423109716
AGGAAGAA -0.650160893518
AGGAAGAC -0.0475628420803
AGGAAGAG -0.24034752838
AGGAAGAT 2.02556686129
AGGAAGCA -0.377023874226
AGGAAGCC -0.853926136159
AGGAAGCG -0.449863063503
AGGAAGGA -0.372236613133
AGGAAGGC -1.30986071079
AGGAAGGG -1.48928952986
AGGAAGTA 0.261842114184
AGGAAGTC -1.11139713705
AGGAAGTG 0.826447057633
AGGAATAA 0.575383150783
AGGAATAC 1.21174163528

AGGAATAG 0.304335405814
AGGAATAT 4.76049127706
AGGAATCA 4.15155836934
AGGAATCC 8.19018267641
AGGAATCG 3.39600008138
AGGAATGA −0.680115832897
AGGAATGC 0.427150844628
AGGAATGG 0.0381427879637
AGGAATTA 1.52429299371
AGGAATTC 1.32697231715
AGGAATTG 1.58665062929
AGGACAAA 1.16523904372
AGGACAAC 0.508203280205
AGGACAAG 0.236599491821
AGGACAAT 1.48373325927
AGGACACA −0.5290622986
AGGACACC −0.373616625039
AGGACACG −0.703036854298
AGGACACT −0.352959331117
AGGACAGA −0.29928084418
AGGACAGC −0.994836822352
AGGACAGG 0.125419953666
AGGACATA 0.313409676287
AGGACATC −0.162711085466
AGGACATG −0.867216635494
AGGACCAA −1.06048033935
AGGACCAC −1.06653040814
AGGACCAG −0.70209859609
AGGACCAT −0.705293711904
AGGACCCA −1.09112455606
AGGACCCC −1.35609392003
AGGACCCG −1.19264796626
AGGACCCT −1.50153414297
AGGACCGA −0.893541575235
AGGACCGC −1.23562443901
AGGACCGG −0.886993544689
AGGACCTA −1.01375916089
AGGACCTC −1.03892912914
AGGACCTG −1.04231472781
AGGACGAA −0.411360335792
AGGACGAC −0.845427061477
AGGACGAG −0.582021114221
AGGACGAT 2.06339072057
AGGACGCA 0.416173410957
AGGACGCC −1.68361202698
AGGACGCG −0.548805482961
AGGACGGA −0.484648773176
AGGACGGC −1.22592230371
AGGACGGG −0.75242479283
AGGACGTA −0.199860031698
AGGACGTC −0.945109761872
AGGACGTG −0.17277711589
AGGACTAA −0.854957241751
AGGACTAC −0.39548759665

AGGACTAG −0.630114393824
AGGACTAT 0.274236615443
AGGACTCA −1.12377769039
AGGACTCC −1.16161945298
AGGACTCG −1.20349528868
AGGACTGA −0.0624946167006
AGGACTGC −0.64390847572
AGGACTGG −0.360741648975
AGGACTTA 0.459336411186
AGGACTTC −0.447455068675
AGGACTTG −0.719972550265
AGGAGAAA 0.499047195782
AGGAGAAC −0.279606358557
AGGAGAAG −0.854955784505
AGGAGAAT 1.40573895839
AGGAGACA 0.112807490049
AGGAGACC −0.981266531796
AGGAGACG −0.555590836417
AGGAGACT −0.880692829598
AGGAGAGA 0.29166673414
AGGAGAGC −0.701100798977
AGGAGAGG −0.883389983656
AGGAGATA 1.71334400773
AGGAGATC 6.12483071098
AGGAGATG 0.438204471444
AGGAGCAA −0.286913406059
AGGAGCAC −0.685663360015
AGGAGCAG −0.960261372451
AGGAGCAT −0.847128292024
AGGAGCCA −0.821630651621
AGGAGCCC −1.6600608508
AGGAGCCG −0.331052552891
AGGAGCCT −1.45868320155
AGGAGCGA −0.161943741389
AGGAGCGC −0.973490667457
AGGAGCGG −1.03834477351
AGGAGCTA −0.284968190906
AGGAGCTC −1.22223963504
AGGAGCTG −0.540937603949
AGGAGGAA −0.405880466521
AGGAGGAC −1.33325721072
AGGAGGAG −0.80048934387
AGGAGGAT 1.85545921179
AGGAGGCA −0.698352224954
AGGAGGCC −1.30284990056
AGGAGGCG −1.01094709258
AGGAGGGA −0.227173816898
AGGAGGGC −1.31561558318
AGGAGGGG −0.447801476853
AGGAGGTA −0.762722733537
AGGAGGTC −1.17070392417
AGGAGGTG −1.07800892624
AGGAGTAA −0.0993725151877
AGGAGTAC −0.252370015569

AGGAGTAG -0.883398935309
AGGAGTAT 0.839185468935
AGGAGTCA -0.8222485239
AGGAGTCC -0.813628914161
AGGAGTCG -1.24369945512
AGGAGTGA -0.220512745704
AGGAGTGC -0.852814257506
AGGAGTGG -0.894828531578
AGGAGTTA 0.238870713708
AGGAGTTC -0.131840371102
AGGAGTTG 0.343229716327
AGGATAAA 1.50395462847
AGGATAAC 1.23203336865
AGGATAAG 0.0393152464119
AGGATAAT 3.37415429931
AGGATACA 0.880271269165
AGGATACC 1.6940357072
AGGATACG 5.75154113357
AGGATACT 1.98993719804
AGGATAGA 0.660172797664
AGGATAGC 0.0888628574635
AGGATAGG -0.127725941278
AGGATATA 1.84211375346
AGGATATC 14.758772464
AGGATATG 4.32787430291
AGGATCAA 0.130343571343
AGGATCAC 2.69929246256
AGGATCAG 0.621446694968
AGGATCAT 2.6126685594
AGGATCCA 2.64578572247
AGGATCCC 2.53659449231
AGGATCCG 3.63797785161
AGGATCCT 4.19520621611
AGGATCGA 0.516808109307
AGGATCGC 5.39310568038
AGGATCGG 1.99611258998
AGGATCTA 3.94898577841
AGGATCTC 6.53179974022
AGGATCTG 4.83328945527
AGGATGAA -0.471515448224
AGGATGAC -0.133604471403
AGGATGAG 1.35366864643
AGGATGAT 1.02422364399
AGGATGCA -0.433947023339
AGGATGCC -0.556612157643
AGGATGCG 0.0444647372104
AGGATGGA -0.733761219533
AGGATGGC -0.935597068549
AGGATGGG -0.683893847087
AGGATGTA 0.321182834313
AGGATGTC -0.398869448123
AGGATGTG 0.671892594053
AGGATTAA -0.413685892136
AGGATTAC 11.8440172375

AGGATTAG -0.29928084418
AGGATTAT 4.76435943232
AGGATTCA 1.76538684019
AGGATTCC 9.60326781976
AGGATTCG 4.29202771819
AGGATTGA -0.706519463918
AGGATTGC 11.1146146405
AGGATTGG 0.0129567900147
AGGATTTA 3.42267434379
AGGATTTC 18.2223469133
AGGATTTG 5.92342995397
AGGCAAAA 0.0890864406264
AGGCAAAC -0.0318541471956
AGGCAAAG 1.07470888872
AGGCAAAT 2.06412017625
AGGCAACA 0.331154143751
AGGCAACC -0.78808651549
AGGCAACG -0.762218734619
AGGCAACT -0.491696639079
AGGCAAGA -0.509868079603
AGGCAAGC -1.31993257019
AGGCAAGG -1.28179394579
AGGCAATA -0.063050035582
AGGCAATC 7.43153627613
AGGCAATG 0.137839644462
AGGCACAA -1.23476612115
AGGCACAC -0.627646651912
AGGCACAG -0.511421920132
AGGCACAT -0.632643340065
AGGCACCA -0.229942584186
AGGCACCC -1.39458436524
AGGCACCG -1.16876120715
AGGCACCT -0.717933863194
AGGCACGA -0.639408292077
AGGCACGC -1.09754434896
AGGCACGG -0.796895983558
AGGCACTA -0.980219812855
AGGCACTC -1.50221988127
AGGCACTG -0.986788869378
AGGCAGAA -0.863612658105
AGGCAGAC -1.03727140779
AGGCAGAG -0.543331650852
AGGCAGAT 1.39775907961
AGGCAGCA 0.728262406065
AGGCAGCC -0.533944697036
AGGCAGCG -0.0880010005785
AGGCAGGA -0.40855888456
AGGCAGGC -1.52148134176
AGGCAGGG -1.05384570676
AGGCAGTA -0.236710867046
AGGCAGTC -0.963863268061
AGGCAGTG 0.864952075302
AGGCATAA 0.63363468366
AGGCATAC -0.442747956109

AGGCATAG 0.0153720710724
AGGCATAT 1.61530508076
AGGCATCA 0.91223429345
AGGCATCC −1.05482560057
AGGCATCG −0.756088725481
AGGCATGA −0.748073872807
AGGCATGC −0.590973392391
AGGCATGG −0.623619656844
AGGCATTA 0.858512921846
AGGCATTC −0.924237420085
AGGCATTG −0.143187737065
AGGCCAAA −1.07036567128
AGGCCAAC 0.268336851163
AGGCCAAG −0.502384288986
AGGCCAAT 0.0991331104975
AGGCCACA −0.772577671478
AGGCCACC −1.28818292835
AGGCCACG −1.16208618803
AGGCCACT −1.03135165842
AGGCCAGA −0.446670237647
AGGCCAGC −1.53541386202
AGGCCAGG −0.815716731238
AGGCCATA −0.674849346067
AGGCCATC −0.279363206662
AGGCCATG 0.347644338816
AGGCCCAA −1.27578009997
AGGCCCAC −0.681141942217
AGGCCCAG −1.35377981348
AGGCCCAT −1.46753992602
AGGCCCCA −0.901758776916
AGGCCCCC −1.91609979529
AGGCCCCG −1.42943669187
AGGCCCCT −1.07726115089
AGGCCCGA −1.37170685304
AGGCCCGC −1.05172437303
AGGCCCGG −1.42422474768
AGGCCCTA −0.839379490823
AGGCCCTC −1.47628673251
AGGCCCTG −0.659077156895
AGGCCGAA −0.655680316607
AGGCCGAC −0.865597427076
AGGCCGAG 0.611282196354
AGGCCGAT −0.632638343793
AGGCCGCA −0.887493380047
AGGCCGCC −1.14575816362
AGGCCGCG −1.36613434457
AGGCCGGA −0.495200066498
AGGCCGGC −1.3047715916
AGGCCGGG −1.45981735524
AGGCCGTA −0.820547709709
AGGCCGTC −1.06316167188
AGGCCGTG −0.0117845397444
AGGCCTAA −1.13723431576
AGGCCTAC −1.15644914438

AGGCCTAG -0.878833383777
AGGCCTAT -0.663159110953
AGGCCTCA -0.580395244107
AGGCCTCC -1.09306248498
AGGCCTCG -1.92873203582
AGGCCTGA -0.129446740556
AGGCCTGC -0.696769655863
AGGCCTGG -0.610913721309
AGGCCTTA -0.110153220657
AGGCCTTC -1.1668349362
AGGCCTTG -0.438419102953
AGGCGAAA 0.122497759199
AGGCGAAC -0.218218624237
AGGCGAAG -0.345346470145
AGGCGAAT 3.29099551881
AGGCGACA 0.66077443206
AGGCGACC -0.813171338936
AGGCGACG -0.772677596914
AGGCGACT -1.00485518002
AGGCGAGA -0.175844826773
AGGCGAGC -1.7604359512
AGGCGAGG -0.790167462694
AGGCGATA 0.336652124507
AGGCGATC 4.24612946733
AGGCGATG -0.340061039118
AGGCGCAA 1.40754885784
AGGCGCAC 0.00507746121336
AGGCGCAG -0.75271499295
AGGCGCAT -0.992640960898
AGGCGCCA -0.577884617529
AGGCGCCC -1.3610393964
AGGCGCCG -0.752876330893
AGGCGCCT -1.15656843037
AGGCGCGA 0.131636356671
AGGCGCGC 0.0823244031056
AGGCGCGG -0.356132588242
AGGCGCTA -0.336683143028
AGGCGCTC -0.0251932841787
AGGCGCTG -0.659298241922
AGGCGGAA -0.519160936967
AGGCGGAC -0.836257653662
AGGCGGAG -0.451030942036
AGGCGGAT 1.07118360261
AGGCGGCA -0.756639564446
AGGCGGCC -1.0901130192
AGGCGGCG -0.539249488616
AGGCGGGA 0.0608248210306
AGGCGGGC -1.37275190656
AGGCGGGG -1.22441384598
AGGCGGTA -0.797279863774
AGGCGGTC -1.66070890889
AGGCGGTG -0.198003084014
AGGCGTAA 0.02619233036
AGGCGTAC -0.797176815669

AGGCGTAG −0.859923744095
AGGCGTAT 1.41478262669
AGGCGTCA −0.411437153471
AGGCGTCC −1.81462947049
AGGCGTCG −1.57580164144
AGGCGTGA −0.0110950542365
AGGCGTGC −1.21673978068
AGGCGTGG −0.986721003352
AGGCGTTA −0.441488895616
AGGCGTTC −0.165230871875
AGGCGTTG 0.428350782571
AGGCTAAA −0.51888489295
AGGCTAAC −0.238166447563
AGGCTAAG −0.630393352332
AGGCTAAT 0.47727469236
AGGCTACA −0.793501016872
AGGCTACC −0.949268533609
AGGCTACG −0.738589283513
AGGCTACT −0.69225906332
AGGCTAGA −1.32494154085
AGGCTAGC −1.1758294745
AGGCTAGG −0.849260659191
AGGCTATA −0.349809806283
AGGCTATC −0.538958663961
AGGCTATG 0.999349288497
AGGCTCAA −0.923053720026
AGGCTCAC −1.22540831225
AGGCTCAG −0.242668921164
AGGCTCAT −0.94143146494
AGGCTCCA −0.423477752145
AGGCTCCC −1.7872474029
AGGCTCCG −1.16745926199
AGGCTCGA −0.250433960248
AGGCTCGC −1.35899300674
AGGCTCGG −1.276500604
AGGCTCTA −0.809795316447
AGGCTCTC −0.129263960279
AGGCTCTG 0.026439437636
AGGCTGAA −0.896369881428
AGGCTGAC −0.591043548374
AGGCTGAG −0.751184468356
AGGCTGAT −0.211679128989
AGGCTGCA −0.362473689864
AGGCTGCC −0.666618820994
AGGCTGCG −0.92052519014
AGGCTGGA −0.898201223219
AGGCTGGC −1.71473900846
AGGCTGGG −1.11811108546
AGGCTGTA −0.392841862556
AGGCTGTC −1.3895658184
AGGCTGTG −0.750864706961
AGGCTTAA −1.1260487041
AGGCTTAC −1.48936447394
AGGCTTAG −0.444715654485

AGGCTTAT -0.120133898105
AGGCTTCA -0.72716738983
AGGCTTCC -1.00224920792
AGGCTTCG -1.45555137184
AGGCTTGA -0.5648381028
AGGCTTGC -1.39174065388
AGGCTTGG -1.81208782539
AGGCTTTA -0.00329878845375
AGGCTTTC 0.0624902449628
AGGCTTTG -0.131900326364
AGGGAAAA -0.401682140966
AGGGAAAC 0.652577839999
AGGGAAAG -0.19583095485
AGGGAAAT 4.5436368432
AGGGAACA 0.652486658039
AGGGAACC -0.935133039806
AGGGAACG -0.660078493034
AGGGAACT -0.894777319792
AGGGAAGA -0.573091319274
AGGGAAGC -0.66121701847
AGGGAAGG -1.07427525396
AGGGAATA 1.02660561657
AGGGAATC 13.6358532997
AGGGAATG -0.31687230082
AGGGACAA -0.906106366091
AGGGACAC -1.03172700334
AGGGACAG -1.12320644998
AGGGACAT -0.154030479575
AGGGACCA -0.0618634210303
AGGGACCC -1.38834360541
AGGGACCG -1.05018302318
AGGGACCT -1.32069450164
AGGGACGA -0.954367437155
AGGGACGC -0.968819569683
AGGGACGG -1.06576223135
AGGGACTA -0.792569004004
AGGGACTC -0.74755009698
AGGGACTG -0.554536623067
AGGGAGAA -0.858159435617
AGGGAGAC -1.00600057533
AGGGAGAG -1.02687125169
AGGGAGAT 2.56728076097
AGGGAGCA -1.67077618838
AGGGAGCC -1.82121913663
AGGGAGCG -0.298473113573
AGGGAGGA 0.393441831528
AGGGAGGC -1.7852932361
AGGGAGGG -1.66286792284
AGGGAGTA 0.327422345075
AGGGAGTC -1.28917677008
AGGGAGTG -0.400155363576
AGGGATAA 0.457077055444
AGGGATAC 1.38014909513
AGGGATAG -0.35462163238

AGGGATAT 3.66485987566
AGGGATCA 1.07443159563
AGGGATCC 0.832850196297
AGGGATCG 0.613749938266
AGGGATGA −0.415346527974
AGGGATGC −0.035948591933
AGGGATGG −0.927116729886
AGGGATTA 1.22984625067
AGGGATTC 5.09519589646
AGGGATTG 2.96481329003
AGGGCAAA −0.0595751285474
AGGGCAAC 0.302038369856
AGGGCAAG −0.666101290507
AGGGCAAT 1.52082412384
AGGGCACA −0.925631379917
AGGGCACC −1.52336576894
AGGGCACG −0.801241699131
AGGGCACT −1.36871991522
AGGGCAGA −0.264407699751
AGGGCAGC −1.44791498676
AGGGCAGG −0.960866337695
AGGGCATA −0.822948834664
AGGGCATC −0.778325674002
AGGGCATG −0.82879613809
AGGGCCAA −0.532479332153
AGGGCCAC −0.673749125382
AGGGCCAG −1.04521714537
AGGGCCAT −1.15409673307
AGGGCCCA −1.08198637495
AGGGCCCC −1.29074351765
AGGGCCCG −1.17823413848
AGGGCCCT −0.801838337255
AGGGCCGA −0.895159118229
AGGGCCGC −1.61549785358
AGGGCCGG −1.46748038711
AGGGCCTA −0.94565726999
AGGGCCTC −1.1110259557
AGGGCCTG −1.16004167198
AGGGCGAA −0.73409222254
AGGGCGAC −1.1524373463
AGGGCGAG −0.699759091821
AGGGCGAT 0.528919904854
AGGGCGCA 0.150165655163
AGGGCGCC −0.912144360558
AGGGCGCG −0.203515637229
AGGGCGGA −0.0136820821372
AGGGCGGC −0.905523467715
AGGGCGGG 0.421701161166
AGGGCGTA 0.161555281257
AGGGCGTC −1.40889535309
AGGGCGTG −1.01220323858
AGGGCTAA −0.614327215836
AGGGCTAC −0.260219574918
AGGGCTAG −1.19464189506

AGGGCTAT -0.37430881686
AGGGCTCA -1.57821837975
AGGGCTCC -1.42755642825
AGGGCTCG -1.36161917211
AGGGCTGA -0.223054182624
AGGGCTGC -1.27860923888
AGGGCTGG -1.45426150101
AGGGCTTA -0.451294079017
AGGGCTTC -1.39730483524
AGGGCTTG -0.687299639028
AGGGGAAA -0.776725826137
AGGGGAAC -0.228373338486
AGGGGAAG -0.258988410276
AGGGGAAT 2.67812617345
AGGGGACA -0.310108597875
AGGGGACC -1.90338532446
AGGGGACG -1.08438729172
AGGGGACT -0.994165240151
AGGGGAGA 0.339676950724
AGGGGAGC -0.87605837115
AGGGGAGG -1.35000096658
AGGGGATA 2.09097034907
AGGGGATC 2.56078415039
AGGGGATG -0.102496642307
AGGGGCAA -0.665794019792
AGGGGCAC -1.29499034867
AGGGGCAG -0.987477313996
AGGGGCAT -0.659272011495
AGGGGCCA -1.26453848843
AGGGGCCC -1.3343763756
AGGGGCCG -1.11873437037
AGGGGCGA -1.09680261078
AGGGGCGC -0.909188649434
AGGGGCGG -1.13225573909
AGGGGCTA -0.578399441702
AGGGGCTC -1.35391263104
AGGGGCTG -0.878434514745
AGGGGGAA -0.721999579368
AGGGGGAC -1.16480291082
AGGGGGAG -0.161782403446
AGGGGGAT 0.0272613243465
AGGGGGCA -1.12589985683
AGGGGGCC -1.29074351765
AGGGGGCG -1.12931522496
AGGGGGGA -0.453613181843
AGGGGGGC -1.93453687094
AGGGGGGG -1.75160483262
AGGGGGTA -0.54153861381
AGGGGGTC -1.4199849947
AGGGGGTG -1.12748325864
AGGGGTAA 0.45505835346
AGGGGTAC -0.765478801967
AGGGGTAG -0.705713815091
AGGGGTAT 0.322842221083

AGGGGTCA -0.926842559471
AGGGGTCC -1.42461258328
AGGGGTCG -1.67425026271
AGGGGTGA 0.590058450118
AGGGGTGC -0.571640526851
AGGGGTGG -0.414830246555
AGGGGTTA 0.0591600216323
AGGGGTTC -0.362220545426
AGGGGTTG 0.229961112027
AGGGTAAA 0.000495463619836
AGGGTAAC 0.0815416538574
AGGGTAAG -0.621645713128
AGGGTAAT 2.69280480363
AGGGTACA -0.281284273168
AGGGTACC -1.26280977839
AGGGTACG 0.709861345216
AGGGTACT 0.618948975426
AGGGTAGA -0.0887333707527
AGGGTAGC -1.26922249324
AGGGTAGG -1.23672216156
AGGGTATA 0.22532665358
AGGGTATC 2.89999186792
AGGGTATG 0.573414203339
AGGGTCAA -1.02876046696
AGGGTCAC -0.384644229606
AGGGTCAG -1.90740669872
AGGGTCAT -0.680530315278
AGGGTCCA -0.764436662941
AGGGTCCC -1.37494526988
AGGGTCCG -0.907081471804
AGGGTCGA -0.890225716186
AGGGTCGC -1.00516598976
AGGGTCGG -0.926927079736
AGGGTCTA -0.235660817257
AGGGTCTC -1.19572941689
AGGGTCTG -0.546564238704
AGGGTGAA 0.570267801175
AGGGTGAC 0.568909231603
AGGGTGAG -0.803011628415
AGGGTGAT 0.00697562814011
AGGGTGCA -0.2555924027
AGGGTGCC -1.1675831279
AGGGTGCG 0.185231156055
AGGGTGGA -0.35359240039
AGGGTGGC -0.884855556716
AGGGTGGG -0.827647203754
AGGGTGTA 0.63418510627
AGGGTGTC -0.382623862198
AGGGTGTG 0.141521064064
AGGGTTAA -0.507309780266
AGGGTTAC 0.711943333309
AGGGTTAG -1.07606870736
AGGGTTAT -0.55993967466
AGGGTTCA -0.313108650911

AGGGTTCC -1.65654076914
AGGGTTCG -1.39082987517
AGGGTTGA 0.161736187932
AGGGTTGC -1.11459495918
AGGGTTGG -0.61913716833
AGGGTTTA -0.151002947044
AGGGTTTC -1.01140612505
AGGGTTTG 0.422558646313
AGGTAAAA 0.553544030404
AGGTAAAC 0.906729234642
AGGTAAAG 0.178541564476
AGGTAAAT 2.04896398576
AGGTAACA -0.412976421541
AGGTAACC -0.39662362395
AGGTAACG 0.173310884261
AGGTAACT -0.250534718396
AGGTAAGA 0.686043076671
AGGTAAGC 0.93139811846
AGGTAAGG 0.102523080912
AGGTAATA 0.266855248398
AGGTAATC 7.97481588248
AGGTAATG -0.377693999181
AGGTACAA -0.808895571168
AGGTACAC -0.82122865992
AGGTACAG -0.948112937578
AGGTACAT -0.380935330509
AGGTACCA 0.3805693536
AGGTACCC -1.13553058708
AGGTACCG -0.0931546549368
AGGTACCT -1.19055577744
AGGTACGA -0.153273128042
AGGTACGC -0.0628435230144
AGGTACGG -0.510017759579
AGGTACTA 0.356102818789
AGGTACTC -0.174752516852
AGGTACTG 0.336401894561
AGGTAGAA 1.15211716855
AGGTAGAC 0.0274099634325
AGGTAGAG -0.732178650442
AGGTAGAT 0.582940428232
AGGTAGCA 0.215151954244
AGGTAGCC -1.10530959623
AGGTAGCG -0.514966566794
AGGTAGGA 0.200783092913
AGGTAGGC -0.556059653254
AGGTAGGG -0.331172255236
AGGTAGTA 0.0692581114673
AGGTAGTC -0.145052179157
AGGTAGTG 0.319147686267
AGGTATAA 0.0556555533229
AGGTATAC 0.267585328614
AGGTATAG -0.897399113418
AGGTATAT 0.599133345124
AGGTATCA 1.36283180891

AGGTATCC 2.06765295677
AGGTATCG 0.251481720079
AGGTATGA -0.180512385522
AGGTATGC -0.218713463323
AGGTATGG -0.200189369281
AGGTATTA 0.426437626829
AGGTATTC -0.0115130756434
AGGTATTG 1.3295012634
AGGTCAAA 0.470833248946
AGGTCAAC 0.435767331697
AGGTCAAG -0.983412846889
AGGTCAAT 0.13027133358
AGGTCACA -0.989743123255
AGGTCACC -1.06246281837
AGGTCACG -0.70396636903
AGGTCACT -0.305022393186
AGGTCAGA -0.662969877158
AGGTCAGC -0.72691757624
AGGTCAGG -0.640386728638
AGGTCATA -0.793828689031
AGGTCATC -0.973377210452
AGGTCATG -0.827256245486
AGGTCCAA -0.720438036254
AGGTCCAC -0.256268356638
AGGTCCAG -1.53741278709
AGGTCCAT -1.16176247126
AGGTCCCA -0.7099106834
AGGTCCCC -1.95758321549
AGGTCCCG -1.528906218
AGGTCCGA 0.323840850908
AGGTCCGC -0.349038715003
AGGTCCGG -0.26756909073
AGGTCCTA -1.54276504326
AGGTCCTC -1.09388707801
AGGTCCTG -0.599049241216
AGGTCGAA -0.839523966349
AGGTCGAC -0.787160539784
AGGTCGAG -0.67571661558
AGGTCGAT 0.105229602979
AGGTCGCA -0.715332471011
AGGTCGCC -0.291642793671
AGGTCGCG -1.37290179471
AGGTCGGA 0.0540223969794
AGGTCGGC -1.12465203795
AGGTCGGG -0.841355516319
AGGTCGTA -0.371002325821
AGGTCGTC -0.580645682231
AGGTCGTG -0.839234390763
AGGTCTAA -0.252815932827
AGGTCTAC -0.776306972018
AGGTCTAG -0.746896418087
AGGTCTAT -0.340512577182
AGGTCTCA -1.16472775857
AGGTCTCC -0.688907605835

AGGTCTCG -0.847536737243
AGGTCTGA 0.638970493761
AGGTCTGC -0.6506850857
AGGTCTGG -0.529005049653
AGGTCTTA -0.382482925698
AGGTCTTC -0.537502250733
AGGTCTTG -0.146432815596
AGGTGAAA -0.0728150406306
AGGTGAAC -0.890756361886
AGGTGAAG -0.552404880434
AGGTGAAT 0.378858546865
AGGTGACA 0.352075615543
AGGTGACC -0.728521379487
AGGTGACG 0.67280920175
AGGTGACT -0.710920346659
AGGTGAGA 0.597481244584
AGGTGAGC -0.188384428093
AGGTGAGG -0.782300000038
AGGTGATA -0.16275417831
AGGTGATC 1.0254560577
AGGTGATG -0.670555050458
AGGTGCAA -0.183399814264
AGGTGCAC -0.509411753446
AGGTGCAG -1.14588619309
AGGTGCAT -0.525599257711
AGGTGCCA -1.11874602833
AGGTGCCC -1.64650763084
AGGTGCCG -1.32995405053
AGGTGCGA -0.183661702177
AGGTGCGC -0.644651046616
AGGTGCGG -0.93066637282
AGGTGCTA -0.286174582367
AGGTGCTC -0.901434227427
AGGTGCTG 0.50051922236
AGGTGGAA 0.336593418313
AGGTGGAC -0.501025511235
AGGTGGAG -0.668712675233
AGGTGGAT 1.59940153128
AGGTGGCA -1.53745442269
AGGTGGCC -1.49277817664
AGGTGGCG -0.918711959834
AGGTGGGA -0.28920502939
AGGTGGGC -0.778394164561
AGGTGGGG -1.05362462174
AGGTGGTA -0.419618548538
AGGTGGTC -1.1320352786
AGGTGGTG -0.730211784778
AGGTGTAA 0.505348327229
AGGTGTAC -0.4835579205
AGGTGTAG -0.725662679307
AGGTGTAT 0.301021628545
AGGTGTCA -0.718587125731
AGGTGTCC -0.28476875639
AGGTGTCG -0.668310267175

AGGTGTGA 0.0879930898149
AGGTGTGC -0.250283239382
AGGTGTGG 0.218592303732
AGGTGTTA 0.541660189758
AGGTGTTC -0.420615096584
AGGTGTTG 1.08611350363
AGGTTAAA -0.0664443777338
AGGTTAAC 0.145427107719
AGGTTAAG -0.0290068968055
AGGTTAAT -0.0181930910356
AGGTTACA 1.04854674417
AGGTTACC -0.670440136206
AGGTTACG 0.608094158592
AGGTTACT -0.261282323564
AGGTTAGA 0.909278374148
AGGTTAGC -0.77935719595
AGGTTAGG -0.514512114238
AGGTTATA 0.644907938258
AGGTTATC -0.267879692294
AGGTTATG 0.295384376712
AGGTTCAA -0.0969028996742
AGGTTCAC -0.39426871451
AGGTTCAG -0.8824588035
AGGTTCAT -0.0514137185676
AGGTTCCA -0.534971639068
AGGTTCCC -0.94520115201
AGGTTCCG -1.00679831339
AGGTTCGA 0.300927532093
AGGTTCGC -0.133817645666
AGGTTCGG 0.744656839254
AGGTTCTA 0.443631255327
AGGTTCTC -1.03420994225
AGGTTCTG -0.0209014867054
AGGTTGAA 0.419217181371
AGGTTGAC -0.337676776581
AGGTTGAG -0.419582741924
AGGTTGAT 0.516192735164
AGGTTGCA -0.474081658326
AGGTTGCC -0.703932019661
AGGTTGCG 0.718928121281
AGGTTGGA 0.543836274303
AGGTTGGC -0.579179276459
AGGTTGGG 0.155338253718
AGGTTGTA -0.0885709919193
AGGTTGTC -0.723785746535
AGGTTGTG -0.488384735412
AGGTTTAA -0.391798058107
AGGTTTAC -1.3667318154
AGGTTTAG -1.13277264505
AGGTTTAT 0.461373224655
AGGTTTCA 0.134202150416
AGGTTTCC 0.357165567436
AGGTTTCG -0.135166222695
AGGTTTGA 0.672684294955

AGGTTTGC 0.0591958282469
AGGTTTGG -0.363345122937
AGGTTTTA 0.673899221714
AGGTTTTC 0.508100023921
AGGTTTTG 0.990347255787
AGTAAAAA 1.5227266625
AGTAAAAC 1.28208497862
AGTAAAAG 0.949938866741
AGTAAAAT 3.83178573274
AGTAAACA 0.128054862504
AGTAAACC -0.288263232156
AGTAAACG -0.401408595085
AGTAAACT -0.132621038571
AGTAAAGA 0.491732237515
AGTAAAGC -0.265050969745
AGTAAAGG -0.626264766404
AGTAAATA 1.08144219768
AGTAAATC 2.29813763645
AGTAAATG 0.430061381127
AGTAACAA 0.823565041519
AGTAACAC 0.0414703049802
AGTAACAG -0.603482599723
AGTAACAT 1.04539451302
AGTAACCA 0.480453986646
AGTAACCC 0.0169802460569
AGTAACCG 0.406464613966
AGTAACGA 0.190750370967
AGTAACGC 0.280297509488
AGTAACGG -0.801081610256
AGTAACTA 0.824348207123
AGTAACTC -0.498854006606
AGTAACTG 0.691465488817
AGTAAGAA 1.3717299608
AGTAAGAC 0.811761349399
AGTAAGAG -1.0180724007
AGTAAGAT 1.93326906697
AGTAAGCA 0.94019592856
AGTAAGCC -0.994030548991
AGTAAGCG 0.423194005543
AGTAAGGA 0.115714487523
AGTAAGGC -0.75271499295
AGTAAGGG -1.06125434513
AGTAAGTA 1.25901302818
AGTAAGTC -0.664378409449
AGTAAGTG 0.725715140161
AGTAATAA 0.570976855414
AGTAATAC 2.62565033077
AGTAATAG -0.467810088153
AGTAATAT 3.68661593302
AGTAATCA 4.39240032045
AGTAATCC 6.94264150464
AGTAATCG 3.68774030235
AGTAATGA 0.32560994748
AGTAATGC -0.479250093321

AGTAATGG 0.00399118845357
AGTAATTA 3.20439805425
AGTAATTC 2.0182398287
AGTAATTG 1.33450919316
AGTACAAA 1.59205805262
AGTACAAC 1.19597694052
AGTACAAG 0.0308909076288
AGTACAAT 1.98123161079
AGTACACA -0.563934193961
AGTACACC 0.0502958027531
AGTACACG -0.607081164485
AGTACACT -0.0786916971534
AGTACAGA 0.130343571343
AGTACAGC 0.060494858914
AGTACAGG -0.685138959654
AGTACATA 1.45418655693
AGTACATC -0.430526867116
AGTACATG -0.385276674344
AGTACCAA 0.132866272245
AGTACCAC -0.971479876237
AGTACCAG -0.709109198133
AGTACCAT -0.754306305517
AGTACCCA -0.114716065875
AGTACCCC -0.727027494219
AGTACCCG 0.00109501623544
AGTACCGA -0.367964176213
AGTACCGC -0.0558304228358
AGTACCGG -0.612976765205
AGTACCTA -0.550357033532
AGTACCTC -0.754833828548
AGTACCTG 0.424149542524
AGTACGAA 1.28346186786
AGTACGAC -0.134766520951
AGTACGAG 0.462438679616
AGTACGAT 1.19832685369
AGTACGCA 0.592559084152
AGTACGCC -0.797176815669
AGTACGCG 0.659717720575
AGTACGGA 0.238490788874
AGTACGGC -0.762670272683
AGTACGGG -0.674804171443
AGTACGTA 0.955410408893
AGTACGTC -0.833035891065
AGTACGTG 0.2808062965
AGTACTAA 0.369570269417
AGTACTAC -0.373897873505
AGTACTAG -0.108195514825
AGTACTAT 0.046365402273
AGTACTCA -0.136624301348
AGTACTCC -0.575453514944
AGTACTCG -0.585562638213
AGTACTGA 0.333410793179
AGTACTGC -0.040543080206
AGTACTGG 0.534580888978

AGTACTTA 0.995448865647
AGTACTTC 0.628832017396
AGTACTTG 1.77777925967
AGTAGAAA 0.14879771758
AGTAGAAC 0.500066018872
AGTAGAAG -0.578614489568
AGTAGAAT 2.16923403227
AGTAGACA 0.659141483894
AGTAGACC 0.442281221052
AGTAGACG 0.10618618085
AGTAGACT 0.683156480641
AGTAGAGA 0.370548081443
AGTAGAGC -0.11912069582
AGTAGAGG 0.116154159441
AGTAGATA 3.0420566929
AGTAGATC 1.44615629908
AGTAGATG 0.977370688863
AGTAGCAA 0.159337352935
AGTAGCAC -0.211930191647
AGTAGCAG -0.111845707728
AGTAGCAT 0.313451520063
AGTAGCCA -0.832981348431
AGTAGCCC -1.16405909086
AGTAGCCG -0.443693084191
AGTAGCGA 0.827455471824
AGTAGCGC -0.475432941669
AGTAGCGG 0.169801627857
AGTAGCTA -0.314295057285
AGTAGCTC -0.531913088016
AGTAGCTG -0.761724520067
AGTAGGAA -0.245109391759
AGTAGGAC 1.35102083056
AGTAGGAG -0.304471970577
AGTAGGAT 0.447085552741
AGTAGGCA 0.10486674874
AGTAGGCC -1.31069612907
AGTAGGCG -0.333843595223
AGTAGGGA 0.327887831064
AGTAGGGC -0.328613955898
AGTAGGGG -0.543357048566
AGTAGGTA 0.259966846836
AGTAGGTC -1.55015910916
AGTAGGTG -0.646426804884
AGTAGTAA 0.708573139805
AGTAGTAC -0.0114468750421
AGTAGTAG 0.262978766017
AGTAGTAT 1.42170641851
AGTAGTCA -0.291328028548
AGTAGTCC 0.561170839301
AGTAGTCG -0.706916042992
AGTAGTGA 0.876553418414
AGTAGTGC -0.146510674165
AGTAGTGG 0.207914230012
AGTAGTTA 0.899480685155

AGTAGTTC 1.42282704064
AGTAGTTG 1.43543013625
AGTATAAA 1.19356270035
AGTATAAC 1.94201295897
AGTATAAG 0.345085623122
AGTATAAT 1.49207682499
AGTATACA 1.46874361117
AGTATACC 0.140162078136
AGTATACG 0.618712901584
AGTATACT 0.60494858914
AGTATAGA 0.843305519565
AGTATAGC -0.459127816838
AGTATAGG 0.304180521388
AGTATATA 2.48826409797
AGTATATC 2.36053753216
AGTATATG 1.06666780562
AGTATCAA 1.25097569228
AGTATCAC 1.29280052438
AGTATCAG 3.99807060979
AGTATCAT 1.71825346931
AGTATCCA 3.49715085297
AGTATCCC 0.505153888985
AGTATCCG 3.08369104214
AGTATCGA 2.15805029421
AGTATCGC 1.35096795335
AGTATCGG 2.13438441195
AGTATCTA 4.54124675168
AGTATCTC 2.46527437783
AGTATCTG 6.03982268539
AGTATGAA 0.93434487793
AGTATGAC 1.39000257583
AGTATGAG 0.359968059558
AGTATGAT 0.996171659634
AGTATGCA 0.818512561664
AGTATGCC 0.156225508318
AGTATGCG -0.0394747107535
AGTATGGA 0.280447397642
AGTATGGC -0.7483378425
AGTATGGG 0.848731678914
AGTATGTA 1.09853111264
AGTATGTC -0.285404115621
AGTATGTG 0.167135284142
AGTATTAA 7.16132290855E-5
AGTATTAC 3.31714433997
AGTATTAG 0.751445523558
AGTATTAT 2.26234871703
AGTATTCA 1.1597037991
AGTATTCC 0.938333568248
AGTATTCG 1.08725785805
AGTATTGA 1.26569679077
AGTATTGC 0.823155763587
AGTATTGG 0.547505619582
AGTATTTA 1.81432490608
AGTATTTC 2.60017683901

AGTATTTG 3.40363646647
AGTCAAAA 0.877534144932
AGTCAAAC 0.482412525191
AGTCAAAG 0.2325323184
AGTCAAAT 1.09399491421
AGTCAACA 0.289933027826
AGTCAACC -1.11802073621
AGTCAACG -0.349039964071
AGTCAACT -0.315524556503
AGTCAAGA 0.418211265316
AGTCAAGC -1.16058564107
AGTCAAGG -0.165604343192
AGTCAATA -0.476331229702
AGTCAATC -0.195530762186
AGTCAATG -0.321921241648
AGTCACAA -0.43521357824
AGTCACAC -0.108662249882
AGTCACAG -0.622370588895
AGTCACAT -0.784200456923
AGTCACCA -1.11357655245
AGTCACCC 0.40143711547
AGTCACCG -0.84613778114
AGTCACGA 0.538095141653
AGTCACGC -0.727321441543
AGTCACGG -1.09615892443
AGTCACTA -0.675229062724
AGTCACTC -0.735562791872
AGTCACTG 0.0508260320975
AGTCAGAA -0.115204243265
AGTCAGAC 0.324964803885
AGTCAGAG -0.912903169337
AGTCAGAT 1.07177690988
AGTCAGCA 0.425633643426
AGTCAGCC -1.58461756303
AGTCAGCG -0.0254347706489
AGTCAGGA 0.0328053124389
AGTCAGGC -1.12146275112
AGTCAGGG -0.895547162005
AGTCAGTA 0.960414591453
AGTCAGTC -0.819295310912
AGTCAGTG 0.44159319279
AGTCATAA 0.629000849747
AGTCATAC 0.868902044513
AGTCATAG -0.785275696249
AGTCATAT 0.757258685793
AGTCATCA 0.310162724153
AGTCATCC -0.821910442842
AGTCATCG -0.903518505479
AGTCATGA 0.852537797133
AGTCATGC -0.77664463672
AGTCATGG -0.955252193619
AGTCATTA 0.551739751751
AGTCATTC 0.88580568107
AGTCATTG 1.20916793078

AGTCCAAA -0.0826997480243
AGTCCAAC 0.0508066715443
AGTCCAAG 0.205923215701
AGTCCAAT -0.114346758118
AGTCCACA -1.60122392141
AGTCCACC -1.1098330958
AGTCCACG -0.550848125414
AGTCCACT -0.698586217017
AGTCCAGA 0.0708606656461
AGTCCAGC -0.217424217021
AGTCCAGG -0.872070513545
AGTCCATA 0.433365165853
AGTCCATC -0.970900933242
AGTCCATG -0.597681928168
AGTCCCAA -0.0596908755107
AGTCCCAC -0.561571581935
AGTCCCAG -0.26059866704
AGTCCCAT -0.574770274776
AGTCCCCA -1.18612283529
AGTCCCCC -0.337176733046
AGTCCCCG -1.46648279818
AGTCCCGA -0.324467466663
AGTCCCGC -1.32353675576
AGTCCCGG -1.32784749743
AGTCCCTA -0.612185688837
AGTCCCTC -0.400046486487
AGTCCCTG -0.0224784349912
AGTCCGAA 1.10824948582
AGTCCGAC 0.0944909494644
AGTCCGAG -0.924506802406
AGTCCGAT 0.091749453494
AGTCCGCA 0.390520261595
AGTCCGCC -0.950726404083
AGTCCGCG -0.830724074469
AGTCCGGA 0.368466926062
AGTCCGGC -1.06112298481
AGTCCGGG -1.45453338146
AGTCCGTA -0.429487434405
AGTCCGTC -0.977460413577
AGTCCGTG -0.490331824166
AGTCCTAA -0.108195514825
AGTCCTAC -0.461314934817
AGTCCTAG -0.757443756028
AGTCCTAT -0.93724188286
AGTCCTCA -0.509766280566
AGTCCTCC -1.27825825078
AGTCCTCG -1.6252016543
AGTCCTGA 1.24764317899
AGTCCTGC -1.07583096809
AGTCCTGG -0.350762428773
AGTCCTTA -0.162373004408
AGTCCTTC 0.264825721158
AGTCCTTG -0.62583196436
AGTCGAAA 1.7427949482

AGTCGAAC 0.303469385369
AGTCGAAG -0.368332234902
AGTCGAAT 0.943984976184
AGTCGACA 0.295241982966
AGTCGACC -0.567978467802
AGTCGACG -0.367193501288
AGTCGACT 0.0819107534364
AGTCGAGA 0.781459793664
AGTCGAGC -1.61287834991
AGTCGAGG -0.890497180287
AGTCGATA 1.09970711012
AGTCGATC 0.541680383023
AGTCGATG 0.0716544483278
AGTCGCAA 2.13427699211
AGTCGCAC -0.523590964627
AGTCGCAG -0.750142121153
AGTCGCAT -0.309457000762
AGTCGCCA -0.628081327558
AGTCGCCC -1.54833526178
AGTCGCCG -1.31366245727
AGTCGCGA 1.48951540298
AGTCGCGC 0.111181203579
AGTCGCGG -0.116379616206
AGTCGCTA 0.0249378497831
AGTCGCTC -0.53531659
AGTCGCTG -0.466055147685
AGTCGGAA 0.305994168051
AGTCGGAC 0.572967453369
AGTCGGAG -0.665847313357
AGTCGGAT -0.164229743914
AGTCGGCA -0.519257947911
AGTCGGCC -1.45209103727
AGTCGGCG -0.699624192483
AGTCGGGA 0.740008641059
AGTCGGGC -0.95665448057
AGTCGGGG -0.259009852609
AGTCGGTA -1.03893100274
AGTCGGTC -0.592709388662
AGTCGGTG -0.105373870327
AGTCGTAA -0.0772914919824
AGTCGTAC -0.413313253531
AGTCGTAG -1.27401891416
AGTCGTAT 0.988816314837
AGTCGTCA -0.764668573224
AGTCGTCC -0.392865594847
AGTCGTCG -1.0013394701
AGTCGTGA 0.0697746010643
AGTCGTGC 0.184810428335
AGTCGTGG 0.0911465700305
AGTCGTTA 1.92192628092
AGTCGTTC 0.086859768829
AGTCGTTG 0.377976704893
AGTCTAAA 0.123716016805
AGTCTAAC -0.306166539428

AGTCTAAG 0.525997918565
AGTCTAAT 0.44459907481
AGTCTACA 0.971323118209
AGTCTACC -0.359108284453
AGTCTACG -0.528015579659
AGTCTAGA 1.04855881849
AGTCTAGC -1.23492350371
AGTCTAGG -1.27433055662
AGTCTATA -0.528714224998
AGTCTATC -0.200547227248
AGTCTATG -0.446684810107
AGTCTCAA 1.81127551486
AGTCTCAC 0.0512536296921
AGTCTCAG -0.385997178373
AGTCTCAT -0.0347711372129
AGTCTCCA -0.570853197687
AGTCTCCC -1.0324358494
AGTCTCCG -0.534930003469
AGTCTCGA 0.703609343775
AGTCTCGC 0.911570830191
AGTCTCGG -1.23569438681
AGTCTCTA -0.589230734424
AGTCTCTC -1.02274453936
AGTCTCTG 0.565932910858
AGTCTGAA -0.0521744009486
AGTCTGAC 0.884371334708
AGTCTGAG 0.385546889377
AGTCTGAT 0.552577668167
AGTCTGCA 0.108241313983
AGTCTGCC -1.7739367103
AGTCTGCG -0.0930722164521
AGTCTGGA -0.327568902381
AGTCTGGC -1.03302582583
AGTCTGGG -0.701227163017
AGTCTGTA 0.636649100978
AGTCTGTC -0.472709349006
AGTCTGTG -0.597892187939
AGTCTTAA -0.347882494438
AGTCTTAC 0.841158996295
AGTCTTAG -0.417083356958
AGTCTTAT -0.0203146329472
AGTCTTCA 0.712135689774
AGTCTTCC -0.103913918073
AGTCTTCG -0.852968101042
AGTCTTGA 0.811918731961
AGTCTTGC 0.467103323872
AGTCTTGG -0.352883554328
AGTCTTTA 1.17728151599
AGTCTTTC 0.86922992485
AGTCTTTG 0.439404201209
AGTGAAAA 0.486326271432
AGTGAAAC -0.149822786011
AGTGAAAG 0.0591958282469
AGTGAAAT 1.89512211544

AGTGAACA -0.218740110106
AGTGAACC -1.06972011133
AGTGAACG -0.356292260762
AGTGAACT -0.464322482261
AGTGAAGA -0.101389135392
AGTGAAGC -1.37252415984
AGTGAAGG -1.39023302886
AGTGAATA 0.900688117506
AGTGAATC 1.65726980847
AGTGAATG -0.0649315482694
AGTGACAA -0.263373887845
AGTGACAC 0.0176609880892
AGTGACAG -0.625885257926
AGTGACAT 0.529048975209
AGTGACCA 0.878554841625
AGTGACCC -0.388205322329
AGTGACCG -0.782558973459
AGTGACGA 0.184116362911
AGTGACGC -1.09885753573
AGTGACGG -1.0617312809
AGTGACTA -0.268522962288
AGTGACTC -0.599476838811
AGTGACTG -0.322462712604
AGTGAGAA 0.0506442927109
AGTGAGAC -0.765231486513
AGTGAGAG 0.129785446148
AGTGAGAT 3.26139635562
AGTGAGCA 1.3236716551
AGTGAGCC -0.905814500547
AGTGAGCG -0.192020048537
AGTGAGGA 0.298293455966
AGTGAGGC -1.06286481007
AGTGAGGG -1.18710293728
AGTGAGTA 0.627505090878
AGTGAGTC -0.284286616162
AGTGAGTG -0.383144931711
AGTGATAA -0.539158514833
AGTGATAC 1.60285811865
AGTGATAG 0.657879092554
AGTGATAT 4.01729980269
AGTGATCA 0.948128967283
AGTGATCC 0.608042114094
AGTGATCG -0.0131424847831
AGTGATGA 1.07249554031
AGTGATGC 1.10210469605
AGTGATGG -0.322351961913
AGTGATTA 2.77342964162
AGTGATTC 4.16558019796
AGTGATTG 2.5251586507
AGTGCAAA 0.922270138065
AGTGCAAC 0.324007185124
AGTGCAAG 2.47586751493
AGTGCAAT 2.35070237113
AGTGCACA 0.230480932472

AGTGCACC -0.961281236432
AGTGCACG -0.692770556646
AGTGCACT 1.14764196627
AGTGCAGA 0.200071540537
AGTGCAGC -0.620009225937
AGTGCAGG -1.34731505413
AGTGCATA 0.0902155980524
AGTGCATC 0.727423865115
AGTGCATG 0.233039439988
AGTGCCAA -1.09147179695
AGTGCCAC -1.16038828833
AGTGCCAG -1.18350895243
AGTGCCAT -0.885301682152
AGTGCCCA -0.767142976831
AGTGCCCC -0.820271457515
AGTGCCCG -1.14796214402
AGTGCCGA -0.768198022892
AGTGCCGC -1.12240663014
AGTGCCGG -0.770821273763
AGTGCCTA -0.35981858776
AGTGCCTC -1.13310198263
AGTGCCTG -1.09222769124
AGTGCGAA 1.02596317929
AGTGCGAC 0.4849323116
AGTGCGAG 0.174360309516
AGTGCGAT 2.83301788538
AGTGCGCA 0.427353193636
AGTGCGCC 0.00507746121336
AGTGCGCG 0.485560176423
AGTGCGGA -0.424976217328
AGTGCGGC -0.982738766553
AGTGCGGG -0.896132350339
AGTGCGTA 0.717019753633
AGTGCGTC -0.336269077003
AGTGCGTG -0.239579976126
AGTGCTAA -0.472728917737
AGTGCTAC 0.884499572351
AGTGCTAG -0.473078656763
AGTGCTAT 0.516598682247
AGTGCTCA 0.108137224987
AGTGCTCC -0.561809529379
AGTGCTCG -0.748455671243
AGTGCTGA 0.769514956866
AGTGCTGC -0.409379522203
AGTGCTGG -0.342361197747
AGTGCTTA 0.4916633306
AGTGCTTC -0.877063870849
AGTGCTTG -0.701495712627
AGTGGAAA 0.246226683039
AGTGGAAC 0.025725387125
AGTGGAAG -0.888275504761
AGTGGAAT 1.71103968555
AGTGGACA 1.03015754946
AGTGGACC -0.239724243474

AGTGGACG -0.360650467014
AGTGGAGA 0.460262178714
AGTGGAGC -0.891325312337
AGTGGAGG -1.18762504768
AGTGGATA 2.06986172526
AGTGGATC 2.05635534535
AGTGGATG 0.225597076791
AGTGGCAA 0.431092486719
AGTGGCAC -1.03100670749
AGTGGCAG -0.121074654449
AGTGGCAT -0.773237595711
AGTGGCCA -0.725260895783
AGTGGCCC -1.50782611458
AGTGGCCG -0.98515612939
AGTGGCGA 0.16581293754
AGTGGCGC -1.11515620705
AGTGGCGG -0.548836917837
AGTGGCTA 0.212424822555
AGTGGCTC -1.28072724176
AGTGGCTG -0.99155760263
AGTGGGAA -0.571658846515
AGTGGGAC -0.287002089884
AGTGGGAG -0.497038278164
AGTGGGAT 1.98580361584
AGTGGGCA -0.367193501288
AGTGGGCC -1.11873437037
AGTGGGCG -0.798838284219
AGTGGGGA -0.577249050121
AGTGGGGC -1.1731487665
AGTGGGGG -1.15252290746
AGTGGGTA 0.166703522988
AGTGGGTC -1.14266380596
AGTGGGTG -1.00577740852
AGTGGTAA -0.450753232595
AGTGGTAC -0.556372752953
AGTGGTAG 0.288812405698
AGTGGTAT 0.914616057851
AGTGGTCA 0.352031481809
AGTGGTCC -0.84765102696
AGTGGTCG -0.906129057492
AGTGGTGA 1.11800616375
AGTGGTGC -0.590579311453
AGTGGTGG -0.146404087034
AGTGGTTA -0.000377634876631
AGTGGTTC 0.498134127111
AGTGGTTG -0.476299794826
AGTGTAAA 1.2761071476
AGTGTAAC 1.14151237349
AGTGTAAG 0.0149192839408
AGTGTAAT 3.16118155228
AGTGTACA 0.531540865767
AGTGTACC -0.935976993384
AGTGTACG -0.633646341628
AGTGTAGA 0.971323118209

AGTGTAGC -0.350696228171
AGTGTAGG -0.432447309088
AGTGTATA 1.85010279206
AGTGTATC 1.28772306317
AGTGTATG 0.308707559992
AGTGTCAA -0.440125121594
AGTGTCAC -0.612992586732
AGTGTCAG 0.436870050518
AGTGTCAT 0.150795809943
AGTGTCCA -0.381689351194
AGTGTCCC -0.739359125726
AGTGTCCG -1.32293741132
AGTGTCGA -1.00129242187
AGTGTCGC 0.66077443206
AGTGTCGG -0.89198523657
AGTGTCTA 0.534129350914
AGTGTCTC -0.0214537829169
AGTGTCTG -0.163803395388
AGTGTGAA -0.518104850016
AGTGTGAC 0.188559713962
AGTGTGAG -0.58779139179
AGTGTGAT 2.09667775688
AGTGTGCA 0.424042330858
AGTGTGCC -1.39769454444
AGTGTGCG -0.0543148870575
AGTGTGGA 1.50421859817
AGTGTGGC -0.758604764686
AGTGTGGG 0.166497426776
AGTGTGTA 0.483919942027
AGTGTGTC 0.138765620169
AGTGTGTG -1.12189763495
AGTGTTAA -0.116463511936
AGTGTTAC 1.20588642109
AGTGTTAG -0.495046014785
AGTGTTAT 0.527082942257
AGTGTTCA 0.677477385032
AGTGTTCC 0.301822697456
AGTGTTCG -0.618676678613
AGTGTTGA 0.877314933507
AGTGTTGC 0.00644373337175
AGTGTTGG 0.276932312255
AGTGTTTA 1.01113882451
AGTGTTTC 1.22872479583
AGTGTTTG 0.932909698856
AGTTAAAA 0.942203805288
AGTTAAAC -0.174778330923
AGTTAAAG -0.0228233859231
AGTTAAAT 0.979275101129
AGTTAACA 0.721091923325
AGTTAACC 0.19474155942
AGTTAACG 0.314246759991
AGTTAACT 1.13488565166
AGTTAAGA 0.201688667176
AGTTAAGC -0.0865574943853

AGTTAAGG -1.03123320514
AGTTAATA 0.925930323513
AGTTAATC -0.403773705247
AGTTAATG 0.405557790635
AGTTACAA 0.787248182719
AGTTACAC -0.376373942536
AGTTACAG 0.117670944287
AGTTACAT 0.916332485391
AGTTACCA 0.188985646133
AGTTACCC 0.140275535141
AGTTACCG 0.634244436998
AGTTACGA 0.246576838421
AGTTACGC 0.711712255739
AGTTACGG 0.681563919006
AGTTACTA 0.828430993893
AGTTACTC -0.0886309471809
AGTTACTG 0.151105786972
AGTTAGAA 0.567483212361
AGTTAGAC -0.15195910856
AGTTAGAG 0.282719868598
AGTTAGAT 2.94636309918
AGTTAGCA 2.31546595611
AGTTAGCC -0.357804049336
AGTTAGCG 0.437794568979
AGTTAGGA 0.885231317991
AGTTAGGC -0.602533724438
AGTTAGGG -1.00485518002
AGTTAGTA 1.9002757698
AGTTAGTC 0.241833294706
AGTTAGTG 1.99393379912
AGTTATAA 0.374913573926
AGTTATAC 0.737077494938
AGTTATAG -0.249792563856
AGTTATAT 1.80977788239
AGTTATCA 1.06863800213
AGTTATCC 0.494167711838
AGTTATCG -0.316256926677
AGTTATGA 1.42993486181
AGTTATGC 1.06136801031
AGTTATGG 0.151676819203
AGTTATTA 0.331295704785
AGTTATTC 1.45807990173
AGTTATTG 0.465302167889
AGTTCAAA 0.459551667229
AGTTCAAC -0.331607971772
AGTTCAAG 1.44451710558
AGTTCAAT 0.925423410103
AGTTCACA -0.106468053851
AGTTCACC -0.427044465674
AGTTCACG -0.0677340403914
AGTTCAGA -0.177656183478
AGTTCAGC -0.782754660771
AGTTCAGG -0.707481246239
AGTTCATA -0.262051957599

AGTTCATC -0.589735149697
AGTTCATG -0.346175851263
AGTTCCAA -0.658082482451
AGTTCCAC -0.501334863731
AGTTCCAG 0.385998219263
AGTTCCAT -0.0943843623327
AGTTCCCA -0.53173030774
AGTTCCCC 0.105010391554
AGTTCCCG -1.17248988316
AGTTCCGA -0.10737924892
AGTTCCGC -0.325216282898
AGTTCCGG -0.630042364239
AGTTCCTA 0.555035625713
AGTTCCTC -0.494885925909
AGTTCCTG -0.0718016301678
AGTTCGAA 0.386527824073
AGTTCGAC -0.18491868089
AGTTCGAG 0.886850734587
AGTTCGAT 1.31180426051
AGTTCGCA 0.270085962648
AGTTCGCC -0.180371240843
AGTTCGCG -0.610326659373
AGTTCGGA 0.610780487394
AGTTCGGC -0.526883091385
AGTTCGGG -0.830254633098
AGTTCGTA 0.961536670827
AGTTCGTC -0.462858366447
AGTTCGTG -0.475152109558
AGTTCTAA -0.174548086064
AGTTCTAC 0.18594479021
AGTTCTAG -0.00158923078732
AGTTCTAT 0.983748013455
AGTTCTCA -0.698489206073
AGTTCTCC -0.279606358557
AGTTCTCG -1.42597510823
AGTTCTGA 1.14453553428
AGTTCTGC 0.679003746066
AGTTCTGG -0.300630253921
AGTTCTTA 0.230471356284
AGTTCTTC 0.0823489681086
AGTTCTTG -0.260535589109
AGTTGAAA 1.30340989936
AGTTGAAC -0.800063828055
AGTTGAAG 0.0442261652321
AGTTGAAT -0.0937163191579
AGTTGACA 0.704296331147
AGTTGACC -0.477204328199
AGTTGACG -0.253568912622
AGTTGAGA 0.175901243009
AGTTGAGC -0.613753269113
AGTTGAGG -0.641113269828
AGTTGATA 1.08008487717
AGTTGATC 0.00704120420744
AGTTGATG -0.788575317415

AGTTGCAA 0.219391290863
AGTTGCAC -0.122697401892
AGTTGCAG 0.62343042305
AGTTGCAT 0.0412500526652
AGTTGCCA -0.0565211574117
AGTTGCCC -0.160594331648
AGTTGCCG -0.640631962312
AGTTGCGA 0.55226852385
AGTTGCGC -0.488894355135
AGTTGCGG 0.504921562347
AGTTGCTA 0.0903546609507
AGTTGCTC -0.769376518502
AGTTGCTG 0.256343300715
AGTTGGAA 0.674492737168
AGTTGGAC -0.840317124497
AGTTGGAG -0.159497649989
AGTTGGAT 2.33901088695
AGTTGGCA -0.656727035549
AGTTGGCC -0.241782915632
AGTTGGCG -1.22627662265
AGTTGGGA 0.576335148738
AGTTGGGC -0.394914898996
AGTTGGGG 0.743793525123
AGTTGGTA 2.36939946108
AGTTGGTC 0.270375746412
AGTTGGTG -1.28530840664
AGTTGTAA 0.567027302559
AGTTGTAC -0.740918795238
AGTTGTAG 0.643951152208
AGTTGTAT 1.07787943953
AGTTGTCA 0.0674833940896
AGTTGTCC -0.12541537375
AGTTGTCG 0.196520856714
AGTTGTGA 1.56740790483
AGTTGTGC -0.313328695048
AGTTGTGG -0.439059458455
AGTTGTTA 1.4226259407
AGTTGTTC 2.06438206417
AGTTGTTG 0.565347306168
AGTTTAAA 0.636439673918
AGTTTAAC 1.18595212934
AGTTTAAG 0.454254994591
AGTTTAAT 0.594959584572
AGTTTACA -0.00526648682966
AGTTTACC 0.267547440219
AGTTTACG -1.07468973634
AGTTTAGA 1.28304509552
AGTTTAGC -0.236331566746
AGTTTAGG 0.635820344393
AGTTTATA 2.3384579662
AGTTTATC -0.301285598238
AGTTTATG 0.402449693221
AGTTTCAA 0.00824926109227
AGTTTCAC -0.080078578933

AGTTTCAG 0.70825129663
AGTTTCAT 0.372420434299
AGTTTCCA 0.633515397671
AGTTTCCC 0.234435481599
AGTTTCCG -0.378411796895
AGTTTCGA -0.247584836256
AGTTTCGC 0.541680383023
AGTTTCGG -0.170476332728
AGTTTCTA 0.947535035473
AGTTTCTC -0.402732398934
AGTTTCTG 0.621810173742
AGTTTGAA -0.294933671361
AGTTTGAC -0.231654639988
AGTTTGAG 0.494153139379
AGTTTGAT 0.805315742425
AGTTTGCA 0.480278700778
AGTTTGCC -0.552876403585
AGTTTGCG -0.548208844837
AGTTTGGA 1.20101609698
AGTTTGGC -0.719212908773
AGTTTGGG 0.833674581143
AGTTTGTA 0.468538294767
AGTTTGTC 0.490702172813
AGTTTGTG -0.933062085146
AGTTTTAA -0.924493062659
AGTTTTAC 0.366458632978
AGTTTTAG 0.123086902915
AGTTTTAT 1.59274732995
AGTTTTCA 0.428496923521
AGTTTTCC 1.08431026586
AGTTTTCG -0.401258498754
AGTTTTGA 2.00766313766
AGTTTTGC 1.47480117436
AGTTTTGG 0.444996694774
AGTTTTTA 1.76427766785
AGTTTTTC 2.93806595715
AGTTTTTG 1.41580269885
ATAAAAAA 2.60830764683
ATAAAAAC 1.52869595823
ATAAAAAG 0.956487729999
ATAAAAAT 3.48368423506
ATAAAACA 1.18942412188
ATAAAACC 0.588675107364
ATAAAACG 0.996255555364
ATAAAAGA 1.78539024704
ATAAAAGC -0.224070507579
ATAAAAGG -0.589043582409
ATAAAATA 1.80370907758
ATAAAATC 7.47785712831
ATAAAATG -0.125049605019
ATAAACAA 1.36167912737
ATAAACAC 0.219176867532
ATAAACAG 0.178928151006
ATAAACAT 1.40247972375

ATAAACCA 2.64966574387
ATAAACCC -0.853387787873
ATAAACCG -0.896140469281
ATAAACGA 0.860363832369
ATAAACGC -0.145808281622
ATAAACGG 0.667888082208
ATAAACTA 0.601859435924
ATAAACTC 1.94057902896
ATAAACTG 0.446328617563
ATAAAGAA 1.59556418636
ATAAAGAC 2.66919533761
ATAAAGAG 2.37587879289
ATAAAGAT 2.77558303476
ATAAAGCA 0.728421870407
ATAAAGCC -0.526011658312
ATAAAGCG -0.244041438662
ATAAAGGA 1.80173804836
ATAAAGGC -0.116826366175
ATAAAGGG 0.438807146729
ATAAAGTA 0.889429643546
ATAAAGTC -0.215933038068
ATAAAGTG 0.476039988692
ATAAATAA 1.49574200671
ATAAATAC 1.29384037345
ATAAATAG 0.756581482787
ATAAATAT 2.39475304231
ATAAATCA 4.9271252686
ATAAATCC 3.2583119905
ATAAATCG 1.18291189795
ATAAATGA 1.02613034622
ATAAATGC 0.650508342586
ATAAATGG -0.192974128272
ATAAATTA 3.23834001852
ATAAATTC 3.75973949343
ATAAATTG 1.3788365329
ATAACAAA 0.952514653031
ATAACAAC 0.951651963434
ATAACAAG 0.48828439362
ATAACAAT 0.559916775081
ATAACACA 2.09972069459
ATAACACC 0.68253361209
ATAACACG 2.47243674163
ATAACAGA 2.34101355923
ATAACAGC 0.174927386365
ATAACAGG -0.414773413964
ATAACATA 1.08008487717
ATAACATC 1.43374826626
ATAACATG -0.293323622774
ATAACCAA 0.24507296061
ATAACCAC 0.203605570122
ATAACCAG -0.173415389612
ATAACCAT 0.276778260542
ATAACCCA 0.750466254286
ATAACCCC -0.347898315965

ATAACCCG 0.318033934013
ATAACCGA 1.00451418447
ATAACCGC 0.744395784053
ATAACCGG -0.26507428568
ATAACCTA 0.0219640271741
ATAACCTC 1.14945478022
ATAACCTG 0.289190040575
ATAACGAA 1.42458218929
ATAACGAC 0.580078605382
ATAACGAG 1.46298249343
ATAACGAT 2.77575082622
ATAACGCA 2.17066567232
ATAACGCC -0.659690657436
ATAACGCG 0.144015660937
ATAACGGA 0.251325378408
ATAACGGC -0.340176577904
ATAACGGG 0.305022393186
ATAACGTA 2.1182535321
ATAACGTC 1.0300767764
ATAACGTG 0.694093111426
ATAACTAA -0.335867085301
ATAACTAC 0.815454635147
ATAACTAG 1.17162885899
ATAACTAT 1.24552434339
ATAACTCA 1.01215056955
ATAACTCC -0.218117241555
ATAACTCG 0.762329693489
ATAACTGA 0.395228415051
ATAACTGC -0.124955508567
ATAACTGG 0.333051477965
ATAACTTA 1.75977269612
ATAACTTC 1.12219491312
ATAACTTG 1.61115526068
ATAAGAAA 0.97247559157
ATAAGAAC 1.59243714475
ATAAGAAG 1.33754359556
ATAAGAAT 2.99330473813
ATAAGACA 1.98123161079
ATAAGACC -0.986138313154
ATAAGACG 0.219863854904
ATAAGAGA 1.4691045918
ATAAGAGC -0.0986690817544
ATAAGAGG -0.0406323885643
ATAAGATA 4.05866435325
ATAAGATC 3.45495692129
ATAAGATG 1.01981693227
ATAAGCAA 1.23259586559
ATAAGCAC -0.16176845552
ATAAGCAG -0.243607179372
ATAAGCAT 1.10606361691
ATAAGCCA 0.44459907481
ATAAGCCC -1.04323570725
ATAAGCCG 0.128517434002
ATAAGCGA 0.462258605653

ATAAGCGC 0.713367270771
ATAAGCGG 0.0135463500867
ATAAGCTA 0.427149179204
ATAAGCTC 0.43413146904
ATAAGCTG 0.424752217809
ATAAGGAA 0.624386584565
ATAAGGAC 0.883901893337
ATAAGGAG 0.729790848879
ATAAGGAT 1.37345762995
ATAAGGCA 0.588511687641
ATAAGGCC −0.287014164207
ATAAGGCG −0.851315375967
ATAAGGGA 0.0141827502068
ATAAGGGC −0.739561474733
ATAAGGGG −0.394637605911
ATAAGGTA 0.539125206355
ATAAGGTC 0.498530081651
ATAAGGTG 0.752926918145
ATAAGTAA 0.930909108358
ATAAGTAC 1.41978909921
ATAAGTAG 0.224320112991
ATAAGTAT 0.812519325466
ATAAGTCA 0.627505090878
ATAAGTCC 0.199599809209
ATAAGTCG −0.499683179546
ATAAGTGA 0.259622520438
ATAAGTGC 0.327467727877
ATAAGTGG 0.421862707287
ATAAGTTA 1.3723930077
ATAAGTTC 1.99637197976
ATAAGTTG 0.310277222048
ATAATAAA 2.21640362612
ATAATAAC 1.22161801556
ATAATAAG 0.54218230016
ATAATAAT 1.04839061067
ATAATACA 2.44818192384
ATAATACC 1.04282060033
ATAATACG 1.33303237849
ATAATAGA 3.05105997468
ATAATAGC 0.514099089103
ATAATAGG 0.80722140376
ATAATATA 2.39174799301
ATAATATC 4.43868536602
ATAATATG 1.18725907077
ATAATCAA 2.19999128963
ATAATCAC 2.13503392729
ATAATCAG 2.53667672261
ATAATCAT 3.04667949338
ATAATCCA 5.55962933759
ATAATCCC 1.43478915621
ATAATCCG 5.09728225629
ATAATCGA 2.94593591794
ATAATCGC 2.28334034481
ATAATCGG 1.37128300265

ATAATCTA 4.58146569875
ATAATCTC 7.45866415838
ATAATCTG 4.78668589738
ATAATGAA 1.43213280504
ATAATGAC 1.25687878741
ATAATGAG 1.00414612578
ATAATGAT 1.59880489315
ATAATGCA 2.02787264073
ATAATGCC 0.593008748614
ATAATGCG 0.745904866313
ATAATGGA 1.2260813517
ATAATGGC 0.609538497497
ATAATGGG 0.28601470167
ATAATGTA 2.78591012039
ATAATGTC 2.6321379897
ATAATGTG 0.495185494039
ATAATTAA 0.881575296104
ATAATTAC 2.26893401144
ATAATTAG 0.769799536181
ATAATTAT 2.06438206417
ATAATTCA 2.03265657097
ATAATTCC 1.22646398285
ATAATTCG -0.0887648056295
ATAATTGA 0.375253736764
ATAATTGC 0.907514482026
ATAATTGG 1.65155053451
ATAATTTA 1.94496867009
ATAATTTC 3.63676563117
ATAATTTG 0.805810789689
ATACAAAA 3.43746414102
ATACAAAC 0.95443426229
ATACAAAG 0.54749229619
ATACAAAT 3.1927800569
ATACAACA 1.30303559533
ATACAACC -0.205154414379
ATACAACG 0.636667004285
ATACAAGA 2.20276380412
ATACAAGC 0.0192947689667
ATACAAGG -0.284925930774
ATACAATA 1.57988130554
ATACAATC 3.49445161713
ATACAATG 0.627224050589
ATACACAA 1.43415816872
ATACACAC 0.110639108089
ATACACAG 0.715917034812
ATACACAT 1.65649809266
ATACACCA 1.04278978999
ATACACCC 0.833674581143
ATACACCG 0.527607967151
ATACACGA 2.30438339255
ATACACGC 0.625414151131
ATACACGG 0.658772384315
ATACACTA 0.761802586814
ATACACTC 1.36795132208

ATACACTG 0.864295898273
ATACAGAA 1.66201480943
ATACAGAC 0.573212687043
ATACAGAG 1.4315022339
ATACAGAT 1.91547463679
ATACAGCA 0.906361592309
ATACAGCC -0.0280994489404
ATACAGCG -0.345088329436
ATACAGGA 1.50846001657
ATACAGGC 0.572396629317
ATACAGGG 0.662650532119
ATACAGTA 1.86382630162
ATACAGTC -0.0694723266206
ATACAGTG -0.0291769782246
ATACATAA 2.44102767898
ATACATAC 2.80455641491
ATACATAG 1.65719590528
ATACATAT 2.2961233062
ATACATCA 1.07190660477
ATACATCC 0.898496003255
ATACATCG -0.0322043025773
ATACATGA 1.63141972273
ATACATGC 1.61609011997
ATACATGG 0.367736013134
ATACATTA 2.24476184031
ATACATTC 2.18469187268
ATACATTG 2.1069538389
ATACCAAA 1.49655390088
ATACCAAC 0.0515619412975
ATACCAAG -0.186133607649
ATACCAAT 0.897773833802
ATACCACA 1.88568332531
ATACCACC 1.05342622811
ATACCACG 1.58232011071
ATACCAGA 0.109103170868
ATACCAGC 0.570730372672
ATACCAGG -0.223344174566
ATACCATA 1.4315022339
ATACCATC 0.260503113342
ATACCATG -0.158929324072
ATACCCAA 0.595855582648
ATACCCAC 2.19038845524
ATACCCAG 1.65123160583
ATACCCAT 1.42735741009
ATACCCCA 1.299294845
ATACCCCC -0.0559699020901
ATACCCCG 0.810031390289
ATACCCGA 0.929289483584
ATACCCGC 0.970431700049
ATACCCGG -0.156839425215
ATACCCTA 1.00682204568
ATACCCTC 1.03226930701
ATACCCTG 0.0924505969694
ATACCGAA 4.30634828223

ATACCGAC 0.632133720341
ATACCGAG 1.03986863641
ATACCGAT 3.36570893455
ATACCGCA 2.82597272579
ATACCGCC 0.54598009126
ATACCGCG 0.9503639662
ATACCGGA 0.370662371161
ATACCGGC 0.561437307131
ATACCGGG -0.147763489318
ATACCGTA 2.03127177097
ATACCGTC 0.342576870146
ATACCGTG -0.00574696163411
ATACCTAA 1.7226454004
ATACCTAC -0.183973136453
ATACCTAG 1.5562928655
ATACCTAT 0.85414472305
ATACCTCA 0.78528901964
ATACCTCC -0.129365134783
ATACCTCG -0.0911625997359
ATACCTGA 0.677177816902
ATACCTGC 1.61544331095
ATACCTGG 0.0299160100945
ATACCTTA 1.01127559745
ATACCTTC 1.03503370256
ATACCTTG 0.96000135814
ATACGAAA 3.17647243393
ATACGAAC 1.78280883995
ATACGAAG 0.658301902054
ATACGAAT 5.27151765901
ATACGACA 2.1874281642
ATACGACC 0.257599863072
ATACGACG 0.745276168779
ATACGAGA 2.82412181526
ATACGAGC 0.826916707182
ATACGAGG 0.624106793344
ATACGATA 2.94742126791
ATACGATC 3.73528565748
ATACGATG 3.01932490529
ATACGCAA 1.81567556489
ATACGCAC 1.62962481209
ATACGCAG 1.01291479096
ATACGCAT 2.80205807083
ATACGCCA 0.871495109576
ATACGCCC -0.218183858512
ATACGCCG 2.10015932561
ATACGCGA 0.829586798102
ATACGCGC 3.67217004583
ATACGCGG 1.14720604156
ATACGCTA 2.45216145433
ATACGCTC 2.5400054887
ATACGCTG 2.06371672731
ATACGGAA 0.656747436992
ATACGGAC 0.903160647511
ATACGGAG 1.28723238764

ATACGGAT 3.68652121203
ATACGGCA 1.62581015857
ATACGGCC -0.273915188624
ATACGGCG -0.111207225828
ATACGGGA 0.488492779789
ATACGGGC 0.30754280413
ATACGGGG -0.784218568408
ATACGGTA 2.33528033734
ATACGGTC 1.30468020147
ATACGGTG 1.1595145653
ATACGTAA 1.35030282467
ATACGTAC 0.646012322503
ATACGTAG 0.202739757855
ATACGTAT 2.11097875219
ATACGTCA 1.15919917565
ATACGTCC 0.533735478154
ATACGTCG 0.545050784706
ATACGTGA 1.36688669983
ATACGTGC 3.32529846372
ATACGTGG 0.629958468508
ATACGTTA 2.56646407871
ATACGTTC 1.15352278635
ATACGTTG 1.36785451931
ATACTAAA 1.92197332915
ATACTAAC 1.17494742435
ATACTAAG 0.813841047534
ATACTAAT 1.12946969303
ATACTACA 1.9944817236
ATACTACC 0.579459275857
ATACTACG 0.45467593049
ATACTAGA 2.13689878574
ATACTAGC 0.580360478383
ATACTAGG 0.214490780943
ATACTATA 1.55994888739
ATACTATC 1.92516761225
ATACTATG 0.763047283026
ATACTCAA 3.23214401696
ATACTCAC 1.77718387062
ATACTCAG 0.800323217832
ATACTCAT 3.90729105756
ATACTCCA 1.89542293264
ATACTCCC 0.634619781916
ATACTCCG 1.6270735908
ATACTCGA 1.53880466515
ATACTCGC 1.72288001699
ATACTCGG 1.58883712274
ATACTCTA 2.44522995992
ATACTCTC 1.74742649215
ATACTCTG 1.01076556137
ATACTGAA 1.89555804016
ATACTGAC 1.0319222743
ATACTGAG 1.84895489862
ATACTGAT 2.12145031334
ATACTGCA 1.30468020147

ATACTGCC 0.375116755646
ATACTGCG 0.356041406282
ATACTGGA 0.764255548088
ATACTGGC -0.37717272149
ATACTGGG -0.044055875635
ATACTGTA 3.73771051473
ATACTGTC 1.79710921072
ATACTGTG 1.93857365037
ATACTTAA 2.51167288041
ATACTTAC 0.872086335072
ATACTTAG -0.174778330923
ATACTTCA 0.964986596503
ATACTTCC 0.91502242129
ATACTTCG 0.549989183021
ATACTTGA 0.363427561421
ATACTTGC 2.78271771089
ATACTTGG 0.804449513803
ATACTTTA 1.40737565375
ATACTTTC 2.31826511738
ATACTTTG 0.918203172823
ATAGAAAA 1.44999676667
ATAGAAAC 0.216381037106
ATAGAAAG 0.335371205325
ATAGAAAT 0.947160315088
ATAGAACA 0.593128242781
ATAGAACC 0.514787533721
ATAGAACG 0.914896889962
ATAGAAGA 0.758790875811
ATAGAAGC -0.407585027916
ATAGAAGG -0.874337988228
ATAGAATA 1.76561208878
ATAGAATC 5.54587626675
ATAGAATG -0.258304337396
ATAGACAA 1.55689033634
ATAGACAC 0.236317410642
ATAGACAG 0.644707462852
ATAGACAT 1.62306304179
ATAGACCA -0.702102135116
ATAGACCC -0.98905342957
ATAGACCG 0.209698939934
ATAGACGA 1.28064938319
ATAGACGC -0.0573522039539
ATAGACGG 0.180689128636
ATAGACTA 0.323844598112
ATAGACTC -0.0492632399151
ATAGACTG -0.800182905866
ATAGAGAA 1.23224487749
ATAGAGAC 0.157690665022
ATAGAGAG 0.795267198952
ATAGAGAT 2.75672419051
ATAGAGCA -0.260177939319
ATAGAGCC -0.840877956006
ATAGAGCG -0.875571442828
ATAGAGGA 1.00940761634

ATAGAGGC -0.379552612289
ATAGAGGG -0.952545255195
ATAGAGTA 0.965960453147
ATAGAGTC -0.170655157623
ATAGAGTG 0.264916486762
ATAGATAA 1.23681042902
ATAGATAC 3.24975212784
ATAGATAG 1.15617768028
ATAGATAT 7.91347665363
ATAGATCA 0.759669803291
ATAGATCC 1.62037588028
ATAGATCG 3.17223621998
ATAGATGA 1.1817640045
ATAGATGC 1.37727936152
ATAGATGG 0.257290510577
ATAGATTA 4.24147939553
ATAGATTC 10.9945613073
ATAGATTG 2.64299114111
ATAGCAAA 1.19728825369
ATAGCAAC 0.181851802719
ATAGCAAG -0.976579196139
ATAGCAAT 2.56411562279
ATAGCACA 0.540892221147
ATAGCACC -0.382686523773
ATAGCACG 1.55917946153
ATAGCAGA 0.68323850277
ATAGCAGC -0.0891135037653
ATAGCAGG 0.146279180239
ATAGCATA -0.10539843533
ATAGCATC 0.877764597969
ATAGCATG -0.549943800218
ATAGCCAA 0.657145473312
ATAGCCAC 0.205439618227
ATAGCCAG -0.892439064592
ATAGCCAT -0.0832091595696
ATAGCCCA 0.56656806191
ATAGCCCC -0.055997381585
ATAGCCCG -0.0861309376806
ATAGCCGA 0.634978680774
ATAGCCGC 0.196110537893
ATAGCCGG -0.77108794977
ATAGCCTA -0.705489399216
ATAGCCTC 0.148230224375
ATAGCCTG -0.294933671361
ATAGCGAA 1.57488607464
ATAGCGAC 1.52885916978
ATAGCGAG 0.0301785225418
ATAGCGAT 2.3750302594
ATAGCGCA 1.18417845285
ATAGCGCC -0.150157119865
ATAGCGCG 1.56460416364
ATAGCGGA 0.470311763077
ATAGCGGC -0.834263724859
ATAGCGGG -0.860385066524

ATAGCGTA 2.14150451562
ATAGCGTC -0.169790178068
ATAGCGTG 0.516065330233
ATAGCTAA 0.401854512343
ATAGCTAC -0.389047194127
ATAGCTAG -0.481408482738
ATAGCTAT 0.30591443588
ATAGCTCA 0.200280967597
ATAGCTCC -0.770063297696
ATAGCTCG 0.84594084476
ATAGCTGA 0.173672905788
ATAGCTGC 0.422392103919
ATAGCTGG -1.16421043626
ATAGCTTA 1.25879569035
ATAGCTTC 0.501392529034
ATAGCTTG 1.57687542352
ATAGGAAA 1.16599410529
ATAGGAAC 0.653128054431
ATAGGAAG 0.0404556454495
ATAGGAAT 2.68062014579
ATAGGACA 0.263120327052
ATAGGACC -0.882761702477
ATAGGACG -0.245829479431
ATAGGAGA 0.886903611797
ATAGGAGC 0.256324981052
ATAGGAGG -0.164538680054
ATAGGATA 1.7993350498
ATAGGATC 2.43393588752
ATAGGATG -0.0702871352795
ATAGGCAA -0.160553112406
ATAGGCAC -0.433167813117
ATAGGCAG 0.118011107125
ATAGGCAT -0.512092045087
ATAGGCCA -0.469642470835
ATAGGCCC -0.776633811465
ATAGGCCG 0.221762230009
ATAGGCGA 0.907067732056
ATAGGCGC -0.338784075318
ATAGGCGG -0.861936408917
ATAGGCTA 0.22782041774
ATAGGCTC -0.0814608807967
ATAGGCTG 0.336030088668
ATAGGGAA 0.43750520157
ATAGGGAC -0.123558634243
ATAGGGAG 0.0628023037721
ATAGGGAT 1.52300145745
ATAGGGCA -0.281946695537
ATAGGGCC -0.892668893094
ATAGGGCG -0.404634104886
ATAGGGGA -0.715253363375
ATAGGGGC -0.608941026661
ATAGGGGG -0.764436454763
ATAGGGTA 0.959297924707
ATAGGGTC -1.04601821428

ATAGGGTG 0.462711392785
ATAGGTAA 1.9353762446
ATAGGTAC -0.212842427606
ATAGGTAG -0.212404837468
ATAGGTCA -0.687970388517
ATAGGTCC -1.20831211105
ATAGGTCG -0.801880181031
ATAGGTGA 0.908773126163
ATAGGTGC 1.47638124532
ATAGGTGG -0.736686953026
ATAGGTTA 0.00902847131456
ATAGGTTC -0.255033861149
ATAGGTTG 0.121332795158
ATAGTAAA 1.17742515881
ATAGTAAC 0.506078199268
ATAGTAAG 0.942068281416
ATAGTAAT 4.45375432994
ATAGTACA 0.822084479643
ATAGTACC -0.433511098625
ATAGTACG 0.321719933531
ATAGTAGA 3.52167880029
ATAGTAGC -0.00681970282445
ATAGTAGG 0.20649029255
ATAGTATA 0.690481639629
ATAGTATC 2.05373584168
ATAGTATG 0.358424419751
ATAGTCAA 0.95149478905
ATAGTCAC -0.613494712048
ATAGTCAG -0.29706999391
ATAGTCAT -0.0343208482172
ATAGTCCA 0.686043076671
ATAGTCCC -0.792569004004
ATAGTCCG -0.622806929965
ATAGTCGA 0.719859301437
ATAGTCGC -0.666038420754
ATAGTCGG -0.471641395909
ATAGTCTA 0.475812033791
ATAGTCTC 1.1817640045
ATAGTCTG 0.672903298202
ATAGTGAA 1.41369926843
ATAGTGAC -0.400354173558
ATAGTGAG 0.782695121866
ATAGTGAT 0.759138116701
ATAGTGCA 0.646420351366
ATAGTGCC 0.385971156124
ATAGTGCG 0.553947062995
ATAGTGGA 1.24233672199
ATAGTGGC -0.453805538307
ATAGTGGG -0.448441832355
ATAGTGTA 1.37123990981
ATAGTGTC 0.171023216312
ATAGTGTG 0.460228662058
ATAGTTAA 0.961159868663
ATAGTTAC 0.398863619139

ATAGTTAG 1.51199133984
ATAGTTCA 1.40980842176
ATAGTTCC -0.281671900589
ATAGTTCG 0.37524998956
ATAGTTGA 1.89815651785
ATAGTTGC 2.87253485626
ATAGTTGG 0.164376509398
ATAGTTTA 1.97853445673
ATAGTTTC 1.60034790843
ATAGTTTG 0.365904046808
ATATAAAA 2.42941155523
ATATAAAC 2.89997896088
ATATAAAG 1.69824340076
ATATAAAT 1.95385037592
ATATAACA 3.19312584054
ATATAACC 1.20829670588
ATATAACG 2.26604346003
ATATAAGA 3.1030311939
ATATAAGC 1.24240437983
ATATAAGG 0.681079072463
ATATAATA 2.32140693963
ATATAATC 3.92719495533
ATATAATG 2.6321379897
ATATACAA 2.28007986111
ATATACAC 1.14882150277
ATATACAG 2.62099297274
ATATACAT 4.6223177151
ATATACCA 0.508657940939
ATATACCC 0.595318691608
ATATACCG 0.622627896892
ATATACGA 2.15707851935
ATATACGC 1.78984692148
ATATACGG 0.820570817466
ATATACTA 1.59291595413
ATATACTC 2.74742904319
ATATACTG 2.46971543893
ATATAGAA 2.0615071261
ATATAGAC 1.09168788571
ATATAGAG 2.17642845548
ATATAGAT 3.89940610795
ATATAGCA 2.0305810364
ATATAGCC 0.0918995498259
ATATAGCG 0.779348036118
ATATAGGA 2.20450667027
ATATAGGC 0.907067732056
ATATAGGG 0.325754423006
ATATAGTA 1.65859236325
ATATAGTC 0.663190545829
ATATAGTG 2.14565849926
ATATATAA 1.45182415308
ATATATAC 2.40437919264
ATATATAG 1.38058855887
ATATATAT 2.83462314587
ATATATCA 3.5411330336

ATATATCC 2.59463347546
ATATATCG 1.9273124701
ATATATGA 3.08049217912
ATATATGC 2.2472666379
ATATATGG 1.93192319625
ATATATTA 2.86728148465
ATATATTC 4.34321285732
ATATATTG 5.20703556702
ATATCAAA 1.78369609455
ATATCAAC 2.5382934329
ATATCAAG 3.33145220515
ATATCAAT 4.00739531839
ATATCACA 7.02119955152
ATATCACC 2.68222894531
ATATCACG 4.65331562622
ATATCAGA 4.02246553137
ATATCAGC 4.3414073296
ATATCAGG 4.39771718635
ATATCATA 3.19877662395
ATATCATC 2.94256843075
ATATCATG 6.32817585044
ATATCCAA 6.99354227263
ATATCCAC 4.74293645975
ATATCCAG 6.22831245154
ATATCCAT 7.1363544566
ATATCCCA 4.56918007458
ATATCCCC 3.87991606803
ATATCCCG 3.90869584264
ATATCCGA 6.62298336593
ATATCCGC 7.21671449218
ATATCCGG 10.2047539925
ATATCCTA 6.07431652952
ATATCCTC 5.91810684273
ATATCCTG 7.03342126505
ATATCGAA 1.98703519684
ATATCGAC 3.89030789701
ATATCGAG 5.17314564725
ATATCGAT 4.18392463439
ATATCGCA 4.38948145683
ATATCGCC 4.2617465639
ATATCGCG 8.56536854715
ATATCGGA 3.90701147451
ATATCGGC 5.41346944334
ATATCGGG 4.07425896659
ATATCGTA 4.28616167875
ATATCGTC 4.9766535191
ATATCGTG 4.61011223946
ATATCTAA 5.59294156343
ATATCTAC 6.57032307755
ATATCTAG 4.79132014765
ATATCTCA 6.5854617811
ATATCTCC 5.36010655423
ATATCTCG 10.0211893013
ATATCTGA 5.27666548438

ATATCTGC 10.51508201
ATATCTGG 5.75981204517
ATATCTTA 8.14854166548
ATATCTTC 8.81897555635
ATATCTTG 10.5624795588
ATATGAAA 2.57366786993
ATATGAAC 1.51840696918
ATATGAAG 2.25040096574
ATATGAAT 4.01976317286
ATATGACA 1.19813345633
ATATGACC 0.175979517934
ATATGACG 0.878352076261
ATATGAGA 2.9576105397
ATATGAGC 0.940908938181
ATATGAGG 0.619786683664
ATATGATA 2.85623576859
ATATGATC 1.16984165093
ATATGATG 1.52422554404
ATATGCAA 1.51452528235
ATATGCAC 4.1282782409
ATATGCAG 1.95016042102
ATATGCAT 2.44345503436
ATATGCCA 2.55645383999
ATATGCCC 1.0567237675
ATATGCCG 2.15658243119
ATATGCGA 3.51990096024
ATATGCGC 1.5807585676
ATATGCGG 0.615993056124
ATATGCTA 1.53546299203
ATATGCTC 2.11204150084
ATATGCTG 3.72023105785
ATATGGAA 2.77213310908
ATATGGAC 1.61507192141
ATATGGAG 1.15268029002
ATATGGAT 1.90394469873
ATATGGCA 1.84972432448
ATATGGCC −0.376473659794
ATATGGCG 1.11238202713
ATATGGGA 2.38286441357
ATATGGGC 1.28720990442
ATATGGGG 0.651448474395
ATATGGTA 3.0267085623
ATATGGTC 0.321396424932
ATATGGTG 2.48229584313
ATATGTAA 0.807015723904
ATATGTAC 1.2223516348
ATATGTAG 1.63686024636
ATATGTCA 1.08657753237
ATATGTCC −0.354000012897
ATATGTCG 0.871824863514
ATATGTGA 2.05096436808
ATATGTGC 3.94451161702
ATATGTGG 2.17309219499
ATATGTTA 1.1155652768

ATATGTTC 0.309128495891
ATATGTTG 1.9511036755
ATATTAAA 2.21257148566
ATATTAAC 1.81288618799
ATATTAAG 4.62083819411
ATATTAAT 4.24476069704
ATATTACA 2.65601309083
ATATTACC 1.23850541423
ATATTACG 4.00177388808
ATATTAGA 3.21262399941
ATATTAGC 1.84742374949
ATATTAGG 0.797620442968
ATATTATA 3.18923936562
ATATTATC 2.43140652493
ATATTATG 0.993625226441
ATATTCAA 1.32678828781
ATATTCAC 1.13391887307
ATATTCAG 3.52404120414
ATATTCAT 0.488754251347
ATATTCCA 4.11432198835
ATATTCCC 6.06233713517
ATATTCCG 5.20862334056
ATATTCGA 2.91916672636
ATATTCGC 3.41312230483
ATATTCGG 2.01466312263
ATATTCTA 3.18368850765
ATATTCTC 4.73431643365
ATATTCTG 3.22062719412
ATATTGAA 1.8949172683
ATATTGAC 0.698489206073
ATATTGAG 1.37317929598
ATATTGAT 1.34407309827
ATATTGCA 3.36570893455
ATATTGCC 1.46208025001
ATATTGCG 5.60785834924
ATATTGGA 2.04962182821
ATATTGGC 1.40524557655
ATATTGGG 0.510806754167
ATATTGTA 2.50212646225
ATATTGTC 2.85091848633
ATATTGTG 2.04898188907
ATATTTAA 2.52208698443
ATATTTAC 3.02787873079
ATATTTAG 3.52094518105
ATATTTCA 3.96155140198
ATATTTCC 7.38098732894
ATATTTCG 5.74212107945
ATATTTGA 3.07337394905
ATATTTGC 7.03559526781
ATATTTGG 2.06778244348
ATATTTTA 4.32580043376
ATATTTTC 5.99614402827
ATATTTTG 3.58136301411
ATCAAAAA 2.4444072405

ATCAAAAC 2.71031736081
ATCAAAAG 0.528717555846
ATCAAAAT 3.30380762512
ATCAAACA 0.843798485049
ATCAAACC 0.468863676968
ATCAAACG 1.08495832395
ATCAAAGA 1.32742094073
ATCAAAGC 1.23804471634
ATCAAAGG 0.0928729901143
ATCAAATA 2.0788189997
ATCAAATC 4.55570054963
ATCAAATG 0.710805848764
ATCAACAA 2.45562095619
ATCAACAC 1.67377062061
ATCAACAG 2.12885645357
ATCAACAT 0.572967453369
ATCAACCA 0.933317103186
ATCAACCC 0.887103879025
ATCAACCG -0.304910185249
ATCAACGA 2.92826535366
ATCAACGC 0.550636200218
ATCAACGG 0.427949831759
ATCAACTA 0.957070836553
ATCAACTC 0.674428826524
ATCAACTG -0.584130373631
ATCAAGAA 0.833393332677
ATCAAGAC 2.22444096202
ATCAAGAG 1.40270413962
ATCAAGAT 3.53093481015
ATCAAGCA 1.21564705441
ATCAAGCC -0.713426393321
ATCAAGCG 0.523538503773
ATCAAGGA 2.76062294793
ATCAAGGC 0.111368563772
ATCAAGGG -0.238203295068
ATCAAGTA 0.558711632688
ATCAAGTC -0.0663534039516
ATCAAGTG 0.624386584565
ATCAATAA 2.96489801847
ATCAATAC 1.17165279946
ATCAATAG 1.26173537177
ATCAATCA 1.49137713876
ATCAATCC 1.65876889819
ATCAATCG 0.251292069929
ATCAATGA 1.99645504278
ATCAATGC 0.374149144341
ATCAATGG 0.0919093341915
ATCAATTA 1.84936854829
ATCAATTC 1.28197089708
ATCAATTG 1.18078515159
ATCACAAA 1.99142858517
ATCACAAC 2.11110698983
ATCACAAG 0.612353480298
ATCACAAT 1.9031673621

ATCACACA 3.87215227801
ATCACACC 2.43484000454
ATCACACG 0.34138442661
ATCACAGA 3.43329600128
ATCACAGC 0.907925841737
ATCACAGG -0.353730422398
ATCACATA 2.09864836975
ATCACATC 1.89747993938
ATCACATG 1.98156011566
ATCACCAA 1.46491771604
ATCACCAC 1.72291103551
ATCACCAG 0.0637212014267
ATCACCAT -0.0221742869456
ATCACCCA 1.06187804639
ATCACCCC 0.288812405698
ATCACCCG -0.436128104156
ATCACCGA 1.30468020147
ATCACCGC 1.65883031069
ATCACCGG 0.157637579634
ATCACCTA 0.267693789347
ATCACCTC 0.859869409639
ATCACCTG -0.365297624319
ATCACGAA 1.91417456523
ATCACGAC 2.42423437676
ATCACGAG 1.52568841079
ATCACGAT 2.01960464361
ATCACGCA 1.16030293536
ATCACGCC 1.17709249037
ATCACGCG 1.85518795586
ATCACGGA 2.94204340585
ATCACGGC 1.36793321059
ATCACGGG 0.311641620605
ATCACGTA 1.9198738541
ATCACGTC 1.0669744518
ATCACGTG 1.09285701331
ATCACTAA 1.17846271791
ATCACTAC 0.70832894702
ATCACTAG 1.39775907961
ATCACTCA 1.15073965478
ATCACTCC 0.529867114715
ATCACTCG 1.34748326195
ATCACTGA 0.560501963415
ATCACTGC 0.768362691683
ATCACTGG 0.690453951956
ATCACTTA 1.04741966852
ATCACTTC 1.17013060198
ATCACTTG 1.34187015876
ATCAGAAA 1.99319539179
ATCAGAAC 1.59360127608
ATCAGAAG 2.26699629069
ATCAGAAT 2.79846387781
ATCAGACA 1.0557201414
ATCAGACC 1.11447671408
ATCAGACG -0.202992277759

ATCAGAGA 2.37177810281
ATCAGAGC 0.0292188220009
ATCAGAGG -0.31403025488
ATCAGATA 3.23254163693
ATCAGATC 4.24816003546
ATCAGATG 0.701559831448
ATCAGCAA 0.944293287789
ATCAGCAC 1.20432924972
ATCAGCAG 1.55045555462
ATCAGCAT 1.65563873391
ATCAGCCA 2.10347997276
ATCAGCCC 0.975631569922
ATCAGCCG -0.710971974801
ATCAGCGA 2.19038845524
ATCAGCGC 0.343425403639
ATCAGCGG 0.362844663045
ATCAGCTA 3.05579477492
ATCAGCTC 0.835798413013
ATCAGCTG 1.22928312921
ATCAGGAA 2.48116731024
ATCAGGAC 1.99542185541
ATCAGGAG 0.450449709084
ATCAGGAT 2.95380962593
ATCAGGCA 1.98645729474
ATCAGGCC -0.392520435737
ATCAGGCG 0.947535035473
ATCAGGGA 1.18156123914
ATCAGGGC -0.0153110749209
ATCAGGGG 0.870912627556
ATCAGGTA 1.50646733683
ATCAGGTC 1.1606383101
ATCAGGTG 0.703568540888
ATCAGTAA 1.8817891478
ATCAGTAC 2.55971494822
ATCAGTAG 0.664796847212
ATCAGTCA 0.678644430853
ATCAGTCC -0.272738774794
ATCAGTCG 0.111016742966
ATCAGTGA 2.28431794866
ATCAGTGC 1.60897480439
ATCAGTGG 0.997429262881
ATCAGTTA 0.0802842587886
ATCAGTTC 2.44088695066
ATCAGTTG -0.33397870274
ATCATAAA 2.42947046961
ATCATAAC 1.86149637354
ATCATAAG 1.78110802576
ATCATAAT 1.22685161027
ATCATACA 1.78760984079
ATCATACC 1.26955557802
ATCATACG 0.608799881983
ATCATAGA 3.35446982114
ATCATAGC 1.65215258526
ATCATAGG 0.267288050442

ATCATATA 1.15701497216
ATCATATC 4.47792212933
ATCATATG 0.735294242263
ATCATCAA 1.74159625932
ATCATCAC 1.3071079732
ATCATCAG 1.46983946011
ATCATCAT 0.0687024844079
ATCATCCA 1.4696523081
ATCATCCC 0.718733683037
ATCATCCG 0.0512261501972
ATCATCGA 1.54323781548
ATCATCGC 1.18883664359
ATCATCGG 0.291085084831
ATCATCTA 1.87731248827
ATCATCTC 2.71856641373
ATCATCTG 1.78669656394
ATCATGAA 2.72602459845
ATCATGAC 2.54162094991
ATCATGAG 1.48544656414
ATCATGAT 1.31327420531
ATCATGCA 0.809177027812
ATCATGCC 1.09005889292
ATCATGCG 0.0724297031683
ATCATGGA 1.88347330775
ATCATGGC 0.11366664062
ATCATGGG -0.376721391604
ATCATGTA 1.10635090254
ATCATGTC 2.76934144222
ATCATGTG 0.790294034913
ATCATTAA 1.34192032966
ATCATTAC 1.63217832333
ATCATTAG 0.841837864725
ATCATTCA 1.5170390316
ATCATTCC -0.534197216939
ATCATTCG 2.87253485626
ATCATTGA 2.22234627507
ATCATTGC 1.71212783191
ATCATTGG 0.882516052447
ATCATTTA 0.60464485745
ATCATTTC 0.947648284301
ATCATTTG 1.34762565569
ATCCAAAA 2.08628696879
ATCCAAAC 3.67775233867
ATCCAAAG 2.07570736326
ATCCAAAT 2.39024848693
ATCCAACA 2.52163627908
ATCCAACC 4.03865678284
ATCCAACG 1.12073267091
ATCCAAGA 2.32265142766
ATCCAAGC 1.85549106302
ATCCAAGG -0.359862305138
ATCCAATA 3.40833421102
ATCCAATC 3.55301083708
ATCCAATG 0.807397730519

ATCCACAA 3.51251230697
ATCCACAC 2.41932449883
ATCCACAG 2.10005294666
ATCCACAT 1.37409465461
ATCCACCA 2.15125807088
ATCCACCC 2.39673489679
ATCCACCG 0.409527328577
ATCCACGA 4.4868261102
ATCCACGC 2.84553417076
ATCCACGG 2.42238533984
ATCCACTA 2.33512545291
ATCCACTC 0.848325731831
ATCCACTG 2.06878794318
ATCCAGAA 4.94398331417
ATCCAGAC 1.16995552429
ATCCAGAG 1.57488607464
ATCCAGAT 5.55530256621
ATCCAGCA 2.8774399461
ATCCAGCC 1.37269320037
ATCCAGCG 0.864177653174
ATCCAGGA 2.29374861985
ATCCAGGC 0.909829837648
ATCCAGGG 0.813861448977
ATCCAGTA 3.51742863842
ATCCAGTC 0.257290510577
ATCCAGTG 0.653862922741
ATCCATAA 2.94370799707
ATCCATAC 0.874311549623
ATCCATAG 2.28957048756
ATCCATCA 2.95217397145
ATCCATCC 0.728115640581
ATCCATCG 1.23994600593
ATCCATGA 2.335105676
ATCCATGC 1.06165821043
ATCCATGG 1.39248218388
ATCCATTA 3.29174329416
ATCCATTC 3.52462701701
ATCCATTG 3.59779700111
ATCCCAAA 2.13548817167
ATCCCAAC 0.917791813112
ATCCCAAG 1.57687542352
ATCCCAAT 1.57488607464
ATCCCACA 2.71566649431
ATCCCACC 1.25045941086
ATCCCACG 0.104126884158
ATCCCAGA 1.24416993738
ATCCCAGC 0.529910832094
ATCCCAGG 0.608218440853
ATCCCATA 3.25128327697
ATCCCATC 1.14161562977
ATCCCATG 0.946232882136
ATCCCCAA 3.815895923
ATCCCCAC 1.40926424449
ATCCCCAG 0.969337308348

ATCCCCAT 1.3171250818
ATCCCCCA 0.895809258096
ATCCCCCC -0.477705204446
ATCCCCCG 0.220743615096
ATCCCCGA 2.1216805582
ATCCCCGC 0.709281985866
ATCCCCGG 2.83601564845
ATCCCCTA 2.5230918596
ATCCCCTC 1.62886100703
ATCCCCTG 0.45270968936
ATCCCGAA 1.18078515159
ATCCCGAC 1.46466915151
ATCCCGAG 1.14771857577
ATCCCGAT 2.4264550114
ATCCCGCA 2.89073585806
ATCCCGCC 0.447832495374
ATCCCGCG 0.20256301474
ATCCCGGA 3.23417250331
ATCCCGGC -0.799160751928
ATCCCGGG 0.539141027882
ATCCCGTA 2.0765138448
ATCCCGTC 0.447855394953
ATCCCGTG 0.498280268061
ATCCCTAA 1.30978264404
ATCCCTAC 0.548040220664
ATCCCTAG 1.16799011587
ATCCCTCA 3.9972314443
ATCCCTCC 0.718733683037
ATCCCTCG 1.26293114616
ATCCCTGA 0.616543686912
ATCCCTGC 1.27234058319
ATCCCTGG 0.663602738253
ATCCCTTA 2.08960782411
ATCCCTTC 0.204318996099
ATCCCTTG 2.36829903222
ATCCGAAA 3.43055367259
ATCCGAAC 4.31985736845
ATCCGAAG 1.25644785897
ATCCGAAT 4.33449144855
ATCCGACA 1.19330913956
ATCCGACC 1.13943350806
ATCCGACG 1.02267063618
ATCCGAGA 3.61428219991
ATCCGAGC 1.03246374525
ATCCGAGG 0.0337525223003
ATCCGATA 5.44123788927
ATCCGATC 1.94361426408
ATCCGATG 1.32738950585
ATCCGCAA 2.93778366779
ATCCGCAC 3.51132236157
ATCCGCAG 1.44784441442
ATCCGCAT 2.57123135472
ATCCGCCA 2.40506243281
ATCCGCCC 1.08973892335

ATCCGCCG 0.343245954211
ATCCGCGA 3.08052174039
ATCCGCGC 1.99219676196
ATCCGCGG 0.922264309082
ATCCGCTA 4.26663833034
ATCCGCTC 3.00735467078
ATCCGCTG 2.24838122287
ATCCGGAA 3.34043945722
ATCCGGAC 2.29970792304
ATCCGGAG 0.706358542331
ATCCGGAT 7.66453220692
ATCCGGCA 2.63414524189
ATCCGGCC 1.96108622655
ATCCGGCG 1.11363130326
ATCCGGGA 1.57011754956
ATCCGGGC 0.972208291029
ATCCGGGG 2.96414816135
ATCCGGTA 4.61327009141
ATCCGGTC 0.776454362036
ATCCGGTG 1.53794842907
ATCCGTAA 2.89073585806
ATCCGTAC 2.90484199877
ATCCGTAG 1.45415158303
ATCCGTCA 0.969348133603
ATCCGTCC 0.181446271992
ATCCGTCG 0.641887067422
ATCCGTGA 4.18312398184
ATCCGTGC 0.524048123496
ATCCGTGG −0.00229620324655
ATCCGTTA 3.14714910658
ATCCGTTC 1.96054538013
ATCCGTTG 1.675554706
ATCCTAAA 2.48250714379
ATCCTAAC 2.95428989256
ATCCTAAG 1.40724491798
ATCCTAAT 2.63697729529
ATCCTACA 2.46744213526
ATCCTACC 2.6463396841
ATCCTACG 1.0303444933
ATCCTAGA 3.0439844211
ATCCTAGC 2.66572625956
ATCCTAGG 0.7160136294
ATCCTATA 4.4355545772
ATCCTATC 1.7244236568
ATCCTATG 0.19735356868
ATCCTCAA 4.73139236558
ATCCTCAC 1.37269320037
ATCCTCAG 1.59287306946
ATCCTCAT 1.07638034981
ATCCTCCA 2.65680375085
ATCCTCCC 2.63597679186
ATCCTCCG 1.57034050819
ATCCTCGA 1.47878361934
ATCCTCGC 0.772276646102

ATCCTCGG -0.107262669245
ATCCTCTA 4.07869107603
ATCCTCTC 0.450383092126
ATCCTCTG 3.89648703615
ATCCTGAA 1.86214359892
ATCCTGAC 5.45065065716
ATCCTGAG 2.51455781101
ATCCTGCA 4.08668053099
ATCCTGCC 1.73023577814
ATCCTGCG 2.42199771242
ATCCTGGA 1.99777509942
ATCCTGGC 1.14198743566
ATCCTGGG 1.40604914359
ATCCTGTA 2.0438709112
ATCCTGTC 3.98788550155
ATCCTGTG 1.40546062441
ATCCTTAA 3.29480226156
ATCCTTAC 1.70264449168
ATCCTTAG 0.48927219819
ATCCTTCA 2.0971780086
ATCCTTCC 1.43117664353
ATCCTTCG 1.57090425419
ATCCTTGA 1.56189493525
ATCCTTGC 1.21886756793
ATCCTTGG 0.99631405338
ATCCTTTA 1.65399121328
ATCCTTTC 1.33461557211
ATCCTTTG 2.18944332715
ATCGAAAA 1.17687681798
ATCGAAAC 2.52797821342
ATCGAAAG 0.330726546156
ATCGAAAT 5.09633025833
ATCGAACA 1.14019710494
ATCGAACC 0.114295338154
ATCGAACG 0.877506249081
ATCGAAGA 2.52768634787
ATCGAAGC 0.0996443956447
ATCGAAGG -0.129749639533
ATCGAATA 1.54217923039
ATCGAATC 4.85313818588
ATCGAATG 0.279284515382
ATCGACAA 0.731545164814
ATCGACAC 0.254648315508
ATCGACAG 1.24933316793
ATCGACAT 0.574405546935
ATCGACCA 0.537286578334
ATCGACCC -0.431409333623
ATCGACCG -0.120034389025
ATCGACGA 0.094944152952
ATCGACGC 1.076083488
ATCGACGG 0.194924131519
ATCGACTA 1.27142314279
ATCGACTC 0.0925242919784
ATCGACTG 0.41249865305

ATCGAGAA 2.2961233062
ATCGAGAC 0.565543201658
ATCGAGAG 1.63510697131
ATCGAGAT 4.13601704956
ATCGAGCA 0.113040233043
ATCGAGCC 0.102633831603
ATCGAGCG −0.0422505560925
ATCGAGGA 1.8013110753
ATCGAGGC 0.400427035855
ATCGAGGG 0.464466541431
ATCGAGTA 0.569070777724
ATCGAGTC −0.245393138361
ATCGAGTG −0.532479332153
ATCGATAA 1.70202911754
ATCGATAC 2.27032380771
ATCGATAG 1.33668132232
ATCGATCA 1.61436994522
ATCGATCC 1.37473730006
ATCGATCG 0.921429723514
ATCGATGA 0.729207950503
ATCGATGC 0.507643697764
ATCGATGG 1.31799942936
ATCGATTA 3.75006879302
ATCGATTC 5.20734825037
ATCGATTG 2.41548132493
ATCGCAAA 1.63788156759
ATCGCAAC 1.7662362064
ATCGCAAG 1.53204012949
ATCGCAAT 4.49234324333
ATCGCACA 1.83316438978
ATCGCACC 4.00469337623
ATCGCACG 2.59554862591
ATCGCAGA 1.73094337514
ATCGCAGC 0.356041406282
ATCGCAGG −0.00146807119625
ATCGCATA 4.88414130144
ATCGCATC 0.970161485016
ATCGCATG 1.72544414531
ATCGCCAA 2.46154445276
ATCGCCAC 2.35664606097
ATCGCCAG 2.07287947343
ATCGCCAT 1.36718023079
ATCGCCCA 0.898282828992
ATCGCCCC 1.26964592727
ATCGCCCG 0.791109259927
ATCGCCGA 1.1850794472
ATCGCCGC 1.03365722968
ATCGCCGG 0.617112012829
ATCGCCTA 3.54840302542
ATCGCCTC 1.21645187052
ATCGCCTG 1.1077588103
ATCGCGAA 2.34336014155
ATCGCGAC 4.04240315397
ATCGCGAG 3.60208130418

ATCGCGAT 8.12889944744
ATCGCGCA 3.23017964943
ATCGCGCC 2.10947529073
ATCGCGCG 4.8770266104
ATCGCGGA 3.16063029696
ATCGCGGC 1.29150586545
ATCGCGGG 1.45316773384
ATCGCGTA 3.4720397991
ATCGCGTC 2.95386208679
ATCGCGTG 3.11188417117
ATCGCTAA 1.8359754172
ATCGCTAC 1.67076723673
ATCGCTAG 0.674488781786
ATCGCTCA 1.71972299775
ATCGCTCC 0.251228159286
ATCGCTCG 2.08149887499
ATCGCTGA 0.886853232723
ATCGCTGC 0.904246087559
ATCGCTGG 0.790439551329
ATCGCTTA 2.94049039804
ATCGCTTC 1.53004078806
ATCGCTTG 1.1646376175
ATCGGAAA 3.46036621823
ATCGGAAC 1.24344714339
ATCGGAAG 1.0352887206
ATCGGAAT 3.54555681592
ATCGGACA 0.0411091161649
ATCGGACC 0.310183541952
ATCGGACG −0.3704406616
ATCGGAGA 1.40751138581
ATCGGAGC 0.169228722025
ATCGGAGG −0.638625126473
ATCGGATA 2.76351787108
ATCGGATC 1.99487247369
ATCGGATG 1.24612389601
ATCGGCAA 2.04071701462
ATCGGCAC 0.546101875385
ATCGGCAG 0.808015810975
ATCGGCAT 0.339213962871
ATCGGCCA 0.249826705047
ATCGGCCC 0.5792952316
ATCGGCCG −0.18178018949
ATCGGCGA 2.55412953271
ATCGGCGC 0.813789211214
ATCGGCGG −0.151802975066
ATCGGCTA 0.509014758016
ATCGGCTC 0.327521854155
ATCGGCTG 0.730710579246
ATCGGGAA 1.84530762021
ATCGGGAC −0.0631878494124
ATCGGGAG 0.699184520565
ATCGGGCA 0.967034859762
ATCGGGCC −0.893483077219
ATCGGGCG 0.557417390114

ATCGGGGA 1.69273813378
ATCGGGGC 1.41485090907
ATCGGGGG 0.08870256041
ATCGGGTA 2.44932065745
ATCGGGTC 0.358659660881
ATCGGGTG -0.617331432432
ATCGGTAA 1.40576144161
ATCGGTAC 0.732445534627
ATCGGTAG 0.573721682233
ATCGGTCA -0.590708381807
ATCGGTCC 0.207550959417
ATCGGTCG -0.0301121137625
ATCGGTGA 2.48666841367
ATCGGTGC -0.268394308289
ATCGGTGG 1.47601568477
ATCGGTTA 2.70906933375
ATCGGTTC 0.747485978159
ATCGGTTG 0.682794459114
ATCGTAAA 3.1899613269
ATCGTAAC 1.94313774466
ATCGTAAG 2.26898480687
ATCGTAAT 4.10693791499
ATCGTACA 1.24387307556
ATCGTACC 0.554451686447
ATCGTACG 1.3856668528
ATCGTAGA 1.49487452902
ATCGTAGC 1.25737300196
ATCGTAGG 0.613749938266
ATCGTATA 3.41312230483
ATCGTATC 4.60948791366
ATCGTATG 1.36983679014
ATCGTCAA 2.18909462902
ATCGTCAC 0.466688841491
ATCGTCAG 1.37900182622
ATCGTCAT 1.61088088208
ATCGTCCA 1.63419931527
ATCGTCCC 1.36100275707
ATCGTCCG 1.7277153672
ATCGTCGA 1.50459644122
ATCGTCGC 1.2651488663
ATCGTCGG 0.87266902527
ATCGTCTA 2.26187885931
ATCGTCTC 2.51796006393
ATCGTCTG 0.620610860332
ATCGTGAA 1.83826350151
ATCGTGAC 1.06528633647
ATCGTGAG 2.2264019987
ATCGTGCA 1.80021147915
ATCGTGCC 1.53491631662
ATCGTGCG 2.23028139557
ATCGTGGA 3.35488534441
ATCGTGGC 0.284742734142
ATCGTGGG 1.07332013334
ATCGTGTA 3.68087313495

ATCGTGTC 3.2136844581
ATCGTGTG 0.138189383488
ATCGTTAA 1.99634616569
ATCGTTAC 0.97877547395
ATCGTTAG 0.223908336923
ATCGTTCA 1.72610219595
ATCGTTCC -0.433153865192
ATCGTTCG 1.57479405997
ATCGTTGA 0.855509537962
ATCGTTGC 1.61874834474
ATCGTTGG 0.933311898736
ATCGTTTA 1.09419851228
ATCGTTTC 1.68470371237
ATCGTTTG 0.224123384789
ATCTAAAA 2.68619598511
ATCTAAAC 2.10199483097
ATCTAAAG 1.94666448801
ATCTAAAT 3.22922723512
ATCTAACA 1.24262900389
ATCTAACC 1.77957937476
ATCTAACG 1.16612817192
ATCTAAGA 2.67300416215
ATCTAAGC 1.07673695871
ATCTAAGG -0.235637501322
ATCTAATA 3.60575106581
ATCTAATC 1.9773959313
ATCTAATG 3.02299924684
ATCTACAA 4.43689107991
ATCTACAC 2.93647464458
ATCTACAG 1.65227665934
ATCTACAT 2.01212106117
ATCTACCA 3.17119533003
ATCTACCC 0.309074369613
ATCTACCG 1.52708486876
ATCTACGA 4.19145693048
ATCTACGC 1.89083926962
ATCTACGG 0.582767432321
ATCTACTA 4.37988049604
ATCTACTC 1.29589509022
ATCTACTG 1.30967980411
ATCTAGAA 3.32076205711
ATCTAGAC 2.22935271355
ATCTAGAG 3.78567430762
ATCTAGAT 4.74064837544
ATCTAGCA 3.97192178863
ATCTAGCC 1.62767793151
ATCTAGCG 1.11075594884
ATCTAGGA 4.00469920522
ATCTAGGC -0.564101985422
ATCTAGGG 2.28177193182
ATCTAGTA 1.92110959866
ATCTAGTC 2.22890658812
ATCTAGTG 0.95691158039
ATCTATAA 2.05812756459

ATCTATAC 3.41819060621
ATCTATAG 3.06270545152
ATCTATCA 3.13861797249
ATCTATCC 1.20008429229
ATCTATCG 1.68589615591
ATCTATGA 3.58212244742
ATCTATGC 1.37761327902
ATCTATGG 1.49203123401
ATCTATTA 6.07290549909
ATCTATTC 5.57534386145
ATCTATTG 2.21232167207
ATCTCAAA 4.49990926426
ATCTCAAC 4.30909248451
ATCTCAAG 1.10683429184
ATCTCAAT 1.46208025001
ATCTCACA 4.86284864829
ATCTCACC 2.61029012584
ATCTCACG 1.46109057184
ATCTCAGA 2.53955957144
ATCTCAGC 1.5262444542
ATCTCAGG 1.20497522603
ATCTCATA 3.02812250721
ATCTCATC 2.89741420803
ATCTCATG 2.42137068031
ATCTCCAA 2.99203651781
ATCTCCAC 6.97615025051
ATCTCCAG 3.74657202729
ATCTCCAT 2.96369870507
ATCTCCCA 3.7262871638
ATCTCCCC 2.52967486405
ATCTCCCG 1.92651285843
ATCTCCGA 3.61517174446
ATCTCCGC 3.85715471915
ATCTCCGG 1.95474179408
ATCTCCTA 4.02888865512
ATCTCCTC 3.29694149861
ATCTCCTG 3.18724397957
ATCTCGAA 5.99857263272
ATCTCGAC 6.44932003633
ATCTCGAG 2.95930760669
ATCTCGCA 2.63941172872
ATCTCGCC 3.45997567631
ATCTCGCG 3.12597449035
ATCTCGGA 4.78014182221
ATCTCGGC 2.01262630916
ATCTCGGG 1.02549228067
ATCTCGTA 6.03893792892
ATCTCGTC 2.96332752371
ATCTCGTG 7.31825538933
ATCTCTAA 2.53619499874
ATCTCTAC 2.26060231186
ATCTCTAG 3.14485394422
ATCTCTCA 3.59645904116
ATCTCTCC 1.46657939277

ATCTCTCG 2.19180031837
ATCTCTGA 4.20691789356
ATCTCTGC 2.92121020153
ATCTCTGG 2.50283697374
ATCTCTTA 5.12573123608
ATCTCTTC 3.70403023019
ATCTCTTG 4.61175164114
ATCTGAAA 2.5825491594
ATCTGAAC 3.3709979046
ATCTGAAG 0.908497082146
ATCTGAAT 5.20020170809
ATCTGACA 2.73879486099
ATCTGACC 0.513895491027
ATCTGACG 1.48571636281
ATCTGAGA 3.6841009347
ATCTGAGC 1.57780389737
ATCTGAGG 3.38173031277
ATCTGATA 4.00976979656
ATCTGATC 3.1230483405
ATCTGATG 1.33268763573
ATCTGCAA 3.96021698106
ATCTGCAC 4.18856263187
ATCTGCAG 2.16290104959
ATCTGCAT 2.66978531404
ATCTGCCA 2.28247432436
ATCTGCCC 1.56503425937
ATCTGCCG 0.936891727478
ATCTGCGA 3.85581904915
ATCTGCGC 2.44010378506
ATCTGCGG 1.53320238722
ATCTGCTA 5.04030831174
ATCTGCTC 1.33935037235
ATCTGCTG 2.61398112163
ATCTGGAA 3.15911559389
ATCTGGAC 6.25027980957
ATCTGGAG 2.86339126252
ATCTGGCA 2.08611418106
ATCTGGCC 1.71087709853
ATCTGGCG 1.46331911724
ATCTGGGA 5.04709345702
ATCTGGGC 0.903541821414
ATCTGGGG 1.93599411688
ATCTGGTA 3.10765815794
ATCTGGTC 1.93101783017
ATCTGGTG 3.06473435423
ATCTGTAA 3.23644143524
ATCTGTAC 2.26425646015
ATCTGTAG 1.97414439925
ATCTGTCA 1.46340571928
ATCTGTCC 0.305827417479
ATCTGTCG 1.16205454498
ATCTGTGA 6.08652616872
ATCTGTGC 1.23891510852
ATCTGTGG 1.08646927981

ATCTGTTA 3.9712079463
ATCTGTTC 2.42508311843
ATCTGTTG 3.83406611446
ATCTTAAA 1.97429262198
ATCTTAAC 4.43360957023
ATCTTAAG 1.8990735419
ATCTTAAT 3.98187082302
ATCTTACA 2.37630680684
ATCTTACC 1.82290517018
ATCTTACG 2.98256899911
ATCTTAGA 4.90883079488
ATCTTAGC 1.06436993695
ATCTTAGG 0.15254263147
ATCTTATA 4.60234657584
ATCTTATC 4.26153297328
ATCTTATG 2.20349950514
ATCTTCAA 3.48433749759
ATCTTCAC 4.27557332974
ATCTTCAG 3.62033310141
ATCTTCAT 3.48642302471
ATCTTCCA 4.01415506595
ATCTTCCC 3.34195957292
ATCTTCCG 3.55391974219
ATCTTCGA 5.23663785288
ATCTTCGC 1.69131544538
ATCTTCGG 3.78775858567
ATCTTCTA 2.40172263329
ATCTTCTC 3.17841244464
ATCTTCTG 3.79208244255
ATCTTGAA 2.37326345278
ATCTTGAC 2.72563030934
ATCTTGAG 2.89099649691
ATCTTGCA 3.5987979209
ATCTTGCC 2.35571154996
ATCTTGCG 2.77635370968
ATCTTGGA 2.45761030508
ATCTTGGC 0.816499688664
ATCTTGGG 0.726873442506
ATCTTGTA 3.40531521379
ATCTTGTC 3.75397962476
ATCTTGTG 4.05724249757
ATCTTTAA 3.9832264781
ATCTTTAC 1.76688051728
ATCTTTAG 1.94349747623
ATCTTTCA 1.3208793637
ATCTTTCC 0.6185018091
ATCTTTCG 1.77979254903
ATCTTTGA 2.18944332715
ATCTTTGC 0.839926790763
ATCTTTGG 3.03292329988
ATCTTTTA 3.08126639307
ATCTTTTC 2.65186535253
ATCTTTTG 3.62853864512
ATGAAAAA 0.827192334843

ATGAAAAC 1.67596398393
ATGAAAAG 1.489469812
ATGAAAAT 3.7794485366
ATGAAACA 1.74584558848
ATGAAACC -0.081280806834
ATGAAACG 1.11662011468
ATGAAAGA 0.5781052862
ATGAAAGC 1.95631437063
ATGAAAGG -0.349975932321
ATGAAATA 1.52202697627
ATGAAATC 12.3600111626
ATGAAATG 0.09671366588
ATGAACAA 0.645862434349
ATGAACAC 0.278240294576
ATGAACAG -0.138442736104
ATGAACAT 0.0557833746097
ATGAACCA 0.0467116022729
ATGAACCC -0.866312102121
ATGAACCG -1.20896828808
ATGAACGA 0.41009190729
ATGAACGC -0.841654043558
ATGAACGG 0.132338332858
ATGAACTA -0.558461194564
ATGAACTC -0.325291018797
ATGAACTG -1.17530195147
ATGAAGAA 1.58365744613
ATGAAGAC 0.135833016802
ATGAAGAG 0.165201310601
ATGAAGCA -0.761874408221
ATGAAGCC -1.3495221572
ATGAAGCG -0.0992511474187
ATGAAGGA 1.40573895839
ATGAAGGC -0.409878524849
ATGAAGGG -0.452494433316
ATGAAGTA -0.292728858253
ATGAAGTC -0.424068144929
ATGAAGTG 0.736531444067
ATGAATAA 2.18029327989
ATGAATAC 0.241019735115
ATGAATAG 0.217635517683
ATGAATCA 2.4142888814
ATGAATCC 2.54212182616
ATGAATCG 1.17212952706
ATGAATGA 1.00173334286
ATGAATGC -0.0251816262112
ATGAATGG 0.0288197447911
ATGAATTA 0.423462971508
ATGAATTC 1.22190779932
ATGAATTG 2.76397586266
ATGACAAA 0.536514654341
ATGACAAC -0.0615655183244
ATGACAAG 0.207004908545
ATGACAAT 1.65646228604
ATGACACA 1.09355461776

ATGACACC −0.671716267295
ATGACACG 0.224450432413
ATGACAGA −0.20034279646
ATGACAGC 0.515094388081
ATGACAGG −0.638329305547
ATGACATA −0.203495027608
ATGACATC 0.337856850544
ATGACATG 0.453606936503
ATGACCAA 0.670156181426
ATGACCAC 0.230692857667
ATGACCAG −0.600847899063
ATGACCAT −0.543526089096
ATGACCCA 0.566319081032
ATGACCCC −0.800700852709
ATGACCCG −0.307137897936
ATGACCGA −1.07150565396
ATGACCGC −0.901263313296
ATGACCGG 1.04219294369
ATGACCTA −0.716513672935
ATGACCTC −1.0016440345
ATGACCTG −0.986210759095
ATGACGAA 0.275036018931
ATGACGAC −0.151466975788
ATGACGAG 0.372878009525
ATGACGCA 0.237850225194
ATGACGCC −0.95616318051
ATGACGCG −0.305554079777
ATGACGGA 1.20207509843
ATGACGGC −0.101811944892
ATGACGGG −0.72819141737
ATGACGTA 0.443529456289
ATGACGTC −0.67020239694
ATGACGTG 0.895677897784
ATGACTAA 0.53579435849
ATGACTAC 0.276024656212
ATGACTAG 0.142593597077
ATGACTCA 0.060592494392
ATGACTCC −0.505154721697
ATGACTCG 0.0233758903126
ATGACTGA −0.373959286013
ATGACTGC −0.628180212104
ATGACTGG −0.85764710958
ATGACTTA −0.171432702421
ATGACTTC −0.479370212023
ATGACTTG 1.01489851904
ATGAGAAA 1.27472276395
ATGAGAAC 0.252631487126
ATGAGAAG 1.13948222171
ATGAGAAT 2.84511365122
ATGAGACA 0.946047811902
ATGAGACC −1.25119823455
ATGAGACG −0.0357524882649
ATGAGAGA 1.07014500261
ATGAGAGC −0.827826028649

ATGAGAGG −0.778532394747
ATGAGATA 1.36137726928
ATGAGATC 6.17326727609
ATGAGATG 0.06639087599
ATGAGCAA −0.127648915421
ATGAGCAC 0.115352674173
ATGAGCAG 0.659141483894
ATGAGCAT −0.0594454336587
ATGAGCCA 0.488928912682
ATGAGCCC −1.51147006215
ATGAGCCG −0.649214932724
ATGAGCGA 0.431532366816
ATGAGCGC −0.0223081453941
ATGAGCGG −0.860881154678
ATGAGCTA −0.779721091079
ATGAGCTC −1.05794806226
ATGAGCTG −0.459264381601
ATGAGGAA 0.119234777359
ATGAGGAC −0.382013900683
ATGAGGAG −0.116835317829
ATGAGGCA −0.481621032467
ATGAGGCC −1.08073126983
ATGAGGCG −0.232620377691
ATGAGGGA 1.29854269792
ATGAGGGC −0.871314411079
ATGAGGGG −0.631110317335
ATGAGGTA 0.093886608755
ATGAGGTC −0.503793237633
ATGAGGTG 1.03370198795
ATGAGTAA 1.76865523466
ATGAGTAC 0.319664384042
ATGAGTAG 0.465268651233
ATGAGTCA 0.616701694007
ATGAGTCC −1.33873853724
ATGAGTCG 0.38158193135
ATGAGTGA −0.501213079606
ATGAGTGC 0.182626016669
ATGAGTGG −0.424917511135
ATGAGTTA −0.221460996455
ATGAGTTC 0.256220267522
ATGAGTTG −0.00379716656546
ATGATAAA 1.72098122553
ATGATAAC 0.536259011767
ATGATAAG 0.545818545138
ATGATAAT 0.794326442609
ATGATACA 1.15707721738
ATGATACC 1.04436340743
ATGATACG 1.78696532173
ATGATAGA 2.13287761965
ATGATAGC 0.0445138672164
ATGATAGG −0.0394245398575
ATGATATA 0.83408427543
ATGATATC 9.04408008401
ATGATATG 2.33078327637

ATGATCAA 0.79080532006
ATGATCAC 0.324553860529
ATGATCAG 1.05683077098
ATGATCAT 1.50501696076
ATGATCCA 1.60100533452
ATGATCCC 0.879114840422
ATGATCCG 1.88918446277
ATGATCGA 1.05929289209
ATGATCGC 1.15701497216
ATGATCGG -0.22781833596
ATGATCTA 0.0558683112303
ATGATCTC 2.45521084555
ATGATCTG 0.174025559306
ATGATGAA 0.214823865729
ATGATGAC -0.341606344349
ATGATGAG 1.3524762029
ATGATGCA 0.447670116541
ATGATGCC -0.367136668696
ATGATGCG 0.177390964717
ATGATGGA 1.24668431116
ATGATGGC -0.128376289323
ATGATGGG -0.0362725168878
ATGATGTA 0.735333171547
ATGATGTC 0.520988323377
ATGATGTG 0.722155712862
ATGATTAA 2.48116044037
ATGATTAC 4.31642451337
ATGATTAG 1.52810473274
ATGATTCA 0.943566330243
ATGATTCC 2.89552645
ATGATTCG 2.26948505858
ATGATTGA -0.0503996835708
ATGATTGC 3.79857884496
ATGATTGG 0.045530816705
ATGATTTA 1.99799368631
ATGATTTC 6.72147362279
ATGATTTG 3.61850862949
ATGCAAAA 0.936928783161
ATGCAAAC 0.447645135182
ATGCAAAG -0.0292798181524
ATGCAAAT 3.07951873883
ATGCAACA 0.716860081113
ATGCAACC -0.58449426876
ATGCAACG 1.30812471451
ATGCAAGA 1.01106096594
ATGCAAGC -0.742311506001
ATGCAAGG 0.863525639704
ATGCAATA 0.741873707685
ATGCAATC 11.9067756157
ATGCAATG -0.413840152028
ATGCACAA -0.23869230517
ATGCACAC 0.832945541817
ATGCACAG 0.283099585254
ATGCACAT 0.763815459814

ATGCACCA 0.324924417354
ATGCACCC -0.199990351121
ATGCACCG 0.240593802944
ATGCACGA 0.16299462389
ATGCACGC -0.792023369488
ATGCACGG -0.318352862696
ATGCACTA 0.336394816509
ATGCACTC -0.125855253846
ATGCACTG 0.897356228751
ATGCAGAA 1.62900693981
ATGCAGAC 0.108241313983
ATGCAGAG 0.251820009315
ATGCAGCA -0.730024840941
ATGCAGCC -0.998855073945
ATGCAGCG -0.201756533201
ATGCAGGA 1.35263108732
ATGCAGGC -0.279093824341
ATGCAGGG 0.191696123582
ATGCAGTA 0.426324378001
ATGCAGTC -0.673352962664
ATGCAGTG 1.8775581383
ATGCATAA 1.68100459764
ATGCATAC 1.07028656364
ATGCATAG 0.938788228981
ATGCATCA 0.837667435021
ATGCATCC -0.035948591933
ATGCATCG -0.05621971568
ATGCATGA -0.741969261383
ATGCATGC -0.363935099365
ATGCATGG -0.898401906803
ATGCATTA 0.414472388588
ATGCATTC 0.698292894227
ATGCATTG 0.484676044493
ATGCCAAA 0.771735383324
ATGCCAAC 0.0584819859139
ATGCCAAG -0.718706619898
ATGCCAAT 0.873255879028
ATGCCACA 0.625614002003
ATGCCACC -0.725791541484
ATGCCACG -0.226276153399
ATGCCAGA 0.148066804652
ATGCCAGC -0.612953032914
ATGCCAGG -0.919128523995
ATGCCATA -0.768163881701
ATGCCATC -0.739618307325
ATGCCATG -1.24641367977
ATGCCCAA 0.643585175299
ATGCCCAC 0.976201144907
ATGCCCAG -0.645986092076
ATGCCCAT 1.20273627173
ATGCCCCA 0.557561032928
ATGCCCCC 0.157833475124
ATGCCCCG 0.0304089755784
ATGCCCGA -0.386730173081

ATGCCCGC -0.520738093431
ATGCCCGG -1.29041584549
ATGCCCTA -0.340159090952
ATGCCCTC -0.983412846889
ATGCCCTG -0.439732289724
ATGCCGAA 2.25776151499
ATGCCGAC -0.491817174136
ATGCCGAG 0.131434007663
ATGCCGCA 0.504921562347
ATGCCGCC -0.288607558554
ATGCCGCG 0.149868793347
ATGCCGGA 0.884895943247
ATGCCGGC 0.142497210667
ATGCCGGG 0.0453977909684
ATGCCGTA 1.07813237579
ATGCCGTC 0.895694135667
ATGCCGTG 0.230692857667
ATGCCTAA 1.2364048983
ATGCCTAC 1.06859699106
ATGCCTAG -0.63515729749
ATGCCTCA 0.056539477075
ATGCCTCC -0.995859809003
ATGCCTCG 0.377446683727
ATGCCTGA -0.0645655713603
ATGCCTGC -1.35916496176
ATGCCTGG -1.18931836746
ATGCCTTA -0.0528197527223
ATGCCTTC -0.882596825508
ATGCCTTG 0.874768292137
ATGCGAAA 1.91500686084
ATGCGAAC 0.332707151567
ATGCGAAG 0.462098516778
ATGCGAAT 3.05245643264
ATGCGACA 0.413522056056
ATGCGACC -0.937515845097
ATGCGACG -0.622809011745
ATGCGAGA -0.519411166913
ATGCGAGC 0.29003940678
ATGCGAGG -0.349453405562
ATGCGATA 0.813861032622
ATGCGATC 4.72372267202
ATGCGATG 0.730535293377
ATGCGCAA 2.38843525663
ATGCGCAC 0.554046155719
ATGCGCAG -0.111822808149
ATGCGCAT 1.30076562251
ATGCGCCA 0.358377163347
ATGCGCCC -0.275675333543
ATGCGCCG 0.721091923325
ATGCGCGA 1.02953509727
ATGCGCGC 0.627025032429
ATGCGCGG -0.376220099001
ATGCGCTA -0.231830758569
ATGCGCTC -0.42129958582

ATGCGCTG 0.140556783608
ATGCGGAA 0.825685126184
ATGCGGAC -0.206634976254
ATGCGGAG -0.638520204766
ATGCGGCA -0.273263591511
ATGCGGCC -1.27266159366
ATGCGGCG -0.962658750202
ATGCGGGA -0.547773752835
ATGCGGGC -0.667751725624
ATGCGGGG -0.867936098633
ATGCGGTA -0.199990351121
ATGCGGTC -0.648942219555
ATGCGGTG 0.485037233308
ATGCGTAA 2.68169559329
ATGCGTAC 0.698991331388
ATGCGTAG -0.0766881921629
ATGCGTCA 0.676868672585
ATGCGTCC -0.281464971665
ATGCGTCG 0.661453508668
ATGCGTGA -0.174300562432
ATGCGTGC -0.692770556646
ATGCGTGG -0.440413448112
ATGCGTTA 0.0479479713646
ATGCGTTC -0.794211736535
ATGCGTTG 0.690206636502
ATGCTAAA 0.778563621446
ATGCTAAC 0.863492331226
ATGCTAAG 0.188031774575
ATGCTAAT 1.24668431116
ATGCTACA 0.493950165837
ATGCTACC -0.666680441679
ATGCTACG 0.189875815224
ATGCTAGA 0.0205729818347
ATGCTAGC 0.0603149931293
ATGCTAGG 0.241190441068
ATGCTATA 0.083025130225
ATGCTATC 0.343519708269
ATGCTATG -0.332466081453
ATGCTCAA 0.00430303908488
ATGCTCAC 0.278979742802
ATGCTCAG -0.322810161672
ATGCTCCA 0.597857422214
ATGCTCCC -0.767391957709
ATGCTCCG -0.0702871352795
ATGCTCGA -0.989812654705
ATGCTCGC -0.348887994137
ATGCTCGG -0.62881702858
ATGCTCTA -0.00275794203176
ATGCTCTC -0.133977526363
ATGCTCTG -1.15567597132
ATGCTGAA 0.297977025419
ATGCTGAC -0.0378788182704
ATGCTGAG -0.374568414816
ATGCTGCA 0.150021595992

ATGCTGCC -0.787820880373
ATGCTGCG 0.427356524483
ATGCTGGA 0.629310826776
ATGCTGGC -0.892805041501
ATGCTGGG -0.783460175985
ATGCTGTA 0.888113334106
ATGCTGTC 0.892667644026
ATGCTGTG 1.86896038725
ATGCTTAA 2.3092580884
ATGCTTAC 1.2674729654
ATGCTTAG -0.0815672597504
ATGCTTCA -0.646684945593
ATGCTTCC -0.177465076082
ATGCTTCG 0.198022236389
ATGCTTGA -0.523661536966
ATGCTTGC 0.084648710381
ATGCTTGG 0.223563802347
ATGCTTTA -0.472322762475
ATGCTTTC -0.268394308289
ATGCTTTG -0.175549838559
ATGGAAAA 0.350176615905
ATGGAAAC 0.151344983485
ATGGAAAG 0.221447256708
ATGGAAAT 2.92058233671
ATGGAACA 0.240228242391
ATGGAACC -0.563035281394
ATGGAACG 0.00673372531395
ATGGAAGA -0.975033266374
ATGGAAGC -0.959222147918
ATGGAAGG -0.866239448002
ATGGAATA 0.877183156838
ATGGAATC 4.48355979752
ATGGAATG -0.539158514833
ATGGACAA 0.43972125629
ATGGACAC 0.0395692235616
ATGGACAG -0.30208250359
ATGGACAT 0.258759414485
ATGGACCA -0.104497857339
ATGGACCC -1.02227280803
ATGGACCG -0.59668142474
ATGGACGA 0.00509078460482
ATGGACGC -0.604294910246
ATGGACGG -0.321364365521
ATGGACTA 0.398602772116
ATGGACTC -0.681604097358
ATGGACTG -0.444584085994
ATGGAGAA 0.655364302416
ATGGAGAC -0.910869062181
ATGGAGAG -0.352839628772
ATGGAGCA -0.273803605221
ATGGAGCC -0.865795404346
ATGGAGCG -0.906146128088
ATGGAGGA 0.0490384076842
ATGGAGGC -0.452406374026

ATGGAGGG -0.82170434663
ATGGAGTA 0.0677465310709
ATGGAGTC -0.383409525938
ATGGAGTG -0.384043427922
ATGGATAA 2.28962607108
ATGGATAC 4.03510651537
ATGGATAG 1.78305865354
ATGGATCA 0.706398512505
ATGGATCC 3.62118933749
ATGGATCG 0.17106901547
ATGGATGA -0.152982511566
ATGGATGC -0.3435165856
ATGGATGG -0.520738093431
ATGGATTA 3.93021728341
ATGGATTC 8.65800109168
ATGGATTG 1.39870691401
ATGGCAAA 0.179038901697
ATGGCAAC -0.391927544817
ATGGCAAG -0.0103372863473
ATGGCAAT 2.60333906271
ATGGCACA 0.623304475365
ATGGCACC -0.859178258707
ATGGCACG -0.706369159408
ATGGCAGA 1.37790181371
ATGGCAGC -0.83896313484
ATGGCAGG -0.438969733741
ATGGCATA 0.22399993524
ATGGCATC -0.136020585172
ATGGCATG -0.720854600415
ATGGCCAA 0.115352674173
ATGGCCAC 0.914929365728
ATGGCCAG -0.714255358083
ATGGCCAT -0.879032818293
ATGGCCCA -0.298196028666
ATGGCCCC -1.21655013053
ATGGCCCG -1.18986629193
ATGGCCGA -0.551219098595
ATGGCCGC -0.922836174024
ATGGCCGG -0.524519438469
ATGGCCTA -0.487862833187
ATGGCCTC -0.669579112033
ATGGCCTG -0.766455573103
ATGGCGAA 0.278813616765
ATGGCGAC -0.259994326331
ATGGCGAG 0.0713467612563
ATGGCGCA 0.386590069293
ATGGCGCC -0.722209006428
ATGGCGCG -0.681436722253
ATGGCGGA -0.538694694268
ATGGCGGC -0.877034934108
ATGGCGGG -0.834950295875
ATGGCGTA -0.126904054567
ATGGCGTC -0.81409648193
ATGGCGTG -0.149687886672

ATGGCTAA 0.983748013455
ATGGCTAC -0.808363051865
ATGGCTAG 0.31406023251
ATGGCTCA 0.334100903221
ATGGCTCC -0.477803048102
ATGGCTCG -0.162513524552
ATGGCTGA -0.68149605298
ATGGCTGC -0.169346967124
ATGGCTGG -1.06308069064
ATGGCTTA 0.0149192839408
ATGGCTTC -0.518603852662
ATGGCTTG -0.498667062769
ATGGGAAA 1.65227665934
ATGGGAAC -0.609120892446
ATGGGAAG 0.322474162394
ATGGGAAT 1.92316577268
ATGGGACA -0.426674325205
ATGGGACC -0.735556546532
ATGGGACG -0.85482671415
ATGGGAGA -0.440280422375
ATGGGAGC -1.24699907629
ATGGGAGG -0.79993683948
ATGGGATA 2.20069618031
ATGGGATC 2.22246160568
ATGGGATG -0.601812595875
ATGGGCAA -1.02878232565
ATGGGCAC -0.476530664218
ATGGGCAG -0.833401659796
ATGGGCCA 0.347386406284
ATGGGCCC -1.64601841256
ATGGGCCG -0.737053346291
ATGGGCGA -0.2591849303
ATGGGCGC -0.572773015125
ATGGGCGG -0.932752108117
ATGGGCTA -0.206913518407
ATGGGCTC -0.404923264116
ATGGGCTG -0.975853487661
ATGGGGAA -0.139715328166
ATGGGGAC -0.78926917466
ATGGGGAG -0.218199055506
ATGGGGCA 0.0830592714157
ATGGGGCC -0.462988269513
ATGGGGCG -0.326331908755
ATGGGGGA 0.10731596281
ATGGGGGC -0.661976451783
ATGGGGGG -1.37259577307
ATGGGGTA 0.65748875882
ATGGGGTC -0.925029537343
ATGGGGTG 0.513364845327
ATGGGTAA 0.0325648668587
ATGGGTAC -0.617121589016
ATGGGTAG 0.037299250742
ATGGGTCA -0.395646020102
ATGGGTCC -0.918628688638

ATGGGTCG -1.27552362468
ATGGGTGA -0.600133848552
ATGGGTGC 0.180208237476
ATGGGTGG -0.106185764494
ATGGGTTA 0.430535402414
ATGGGTTC -0.380197131351
ATGGGTTG -0.603850033879
ATGGTAAA 1.28346082697
ATGGTAAC -0.174245603443
ATGGTAAG 0.0588617025704
ATGGTAAT 3.0485077125
ATGGTACA -0.361672412775
ATGGTACC -0.608365622693
ATGGTACG 1.71251712475
ATGGTAGA 1.77818812125
ATGGTAGC 1.0929175931
ATGGTAGG 0.564417375079
ATGGTATA 0.770418241172
ATGGTATC 1.55497260068
ATGGTATG -0.553217815491
ATGGTCAA 0.0529579829087
ATGGTCAC -0.515296320732
ATGGTCAG -0.194127226167
ATGGTCCA 0.470227450991
ATGGTCCC -0.362852573809
ATGGTCCG -0.497412165836
ATGGTCGA -1.06865590544
ATGGTCGC -0.216244472343
ATGGTCGG -1.29211624332
ATGGTCTA -0.899586231397
ATGGTCTC 0.920619702949
ATGGTCTG -0.591349986377
ATGGTGAA 1.04456867093
ATGGTGAC -1.0956124572
ATGGTGAG -0.212774561581
ATGGTGCA -0.169633003684
ATGGTGCC -0.539268016457
ATGGTGCG 0.0570445168824
ATGGTGGA 0.2872346247
ATGGTGGC -0.73078885417
ATGGTGGG -0.521105735764
ATGGTGTA 0.587020925043
ATGGTGTC 0.512540460481
ATGGTGTG 0.831508697319
ATGGTTAA -0.0782961589695
ATGGTTAC -0.133509750416
ATGGTTAG 0.423510644268
ATGGTTCA 0.516144646048
ATGGTTCC 0.609524341394
ATGGTTCG -0.0378217775007
ATGGTTGA -0.0084499446761
ATGGTTGC 0.207763717324
ATGGTTGG 0.030172693558
ATGGTTTA 0.3345674301

ATGGTTTC 0.307473064502
ATGGTTTG -0.292857304073
ATGTAAAA 1.01489851904
ATGTAAAC 0.941066945276
ATGTAAAG 1.06385094921
ATGTAAAT 1.99481584927
ATGTAACA 2.33293895947
ATGTAACC -0.183234104583
ATGTAACG -0.404780245836
ATGTAAGA 0.603560874648
ATGTAAGC -0.126400472006
ATGTAAGG -0.0106572559203
ATGTAATA 0.746705727047
ATGTAATC 11.5483130994
ATGTAATG 0.413296599292
ATGTACAA 0.495239620317
ATGTACAC 1.46014440287
ATGTACAG 0.670156181426
ATGTACAT 1.28865486786
ATGTACCA 0.504492923862
ATGTACCC 0.292235268235
ATGTACCG -0.583427356554
ATGTACGA 0.577275905082
ATGTACGC 0.482894665419
ATGTACGG -0.69451966813
ATGTACTA 0.656522188405
ATGTACTC 0.536827545862
ATGTACTG -0.0346812043205
ATGTAGAA 0.829982336285
ATGTAGAC -0.143499379518
ATGTAGAG 1.6872801232
ATGTAGCA 0.771771606294
ATGTAGCC -1.19813428905
ATGTAGCG -0.897787781728
ATGTAGGA 1.8240060154
ATGTAGGC 0.460154758871
ATGTAGGG 0.0801379096605
ATGTAGTA 2.14095450937
ATGTAGTC -0.225025836382
ATGTAGTG 0.361365766593
ATGTATAA 1.70577986041
ATGTATAC 2.12810284924
ATGTATAG 0.592539515421
ATGTATCA 2.14503188351
ATGTATCC 1.63979930324
ATGTATCG 0.405298817213
ATGTATGA 0.432993151782
ATGTATGC 0.976556712916
ATGTATGG -0.201186333682
ATGTATTA 1.50838319889
ATGTATTC 1.68549395603
ATGTATTG 0.962995374014
ATGTCAAA 0.150560152457
ATGTCAAC 0.0398096691418

ATGTCAAG -0.39279897789
ATGTCAAT -0.202256160381
ATGTCACA 2.90032162186
ATGTCACC -0.920574736502
ATGTCACG 0.597854924079
ATGTCAGA -0.0307543428664
ATGTCAGC -0.240027350629
ATGTCAGG -0.80879897658
ATGTCATA 0.833307355166
ATGTCATC 0.634707008495
ATGTCATG 0.240667497953
ATGTCCAA -0.424621898387
ATGTCCAC 1.44245780889
ATGTCCAG 0.777378047784
ATGTCCCA 0.525253057711
ATGTCCCC -0.698381786229
ATGTCCCG -0.551454339725
ATGTCCGA -0.737359367939
ATGTCCGC -0.438533184493
ATGTCCGG -0.604919444221
ATGTCCTA -0.10374008945
ATGTCCTC -0.484921902701
ATGTCCTG -0.863409684563
ATGTCGAA 0.640795590213
ATGTCGAC -0.564776898471
ATGTCGAG 0.382971519444
ATGTCGCA 0.794586248742
ATGTCGCC -0.0162674446139
ATGTCGCG 0.21966421221
ATGTCGGA 1.1274004038
ATGTCGGC 0.228970392965
ATGTCGGG -0.426159084676
ATGTCGTA 1.77418298487
ATGTCGTC -0.335776944231
ATGTCGTG 0.2020261237
ATGTCTAA 0.134601019448
ATGTCTAC 0.372235572243
ATGTCTAG 0.234308493024
ATGTCTCA 1.21302380353
ATGTCTCC 0.237739890858
ATGTCTCG 0.0543735932511
ATGTCTGA -0.159411880656
ATGTCTGC -0.263365977082
ATGTCTGG -0.947277935654
ATGTCTTA 0.633943411622
ATGTCTTC -0.256530660908
ATGTCTTG 1.25756973016
ATGTGAAA 1.14928365791
ATGTGAAC 1.91707157016
ATGTGAAG 0.641537744753
ATGTGAAT 0.921045843297
ATGTGACA 1.38515931485
ATGTGACC -0.648464867421
ATGTGACG 0.0206866470181

ATGTGAGA 0.724593893498
ATGTGAGC -0.0801435304663
ATGTGAGG -0.186021607889
ATGTGATA 2.15273197106
ATGTGATC 2.13660421388
ATGTGATG 0.138189383488
ATGTGCAA 0.459255638125
ATGTGCAC -0.0788507451389
ATGTGCAG -0.00106254046876
ATGTGCCA 0.387466290459
ATGTGCCC -0.432360915222
ATGTGCCG 0.758412408222
ATGTGCGA 0.966893506905
ATGTGCGC 0.162017020042
ATGTGCGG -0.0392001239826
ATGTGCTA 0.575863001054
ATGTGCTC -0.346010557938
ATGTGCTG 0.118456608027
ATGTGGAA 1.14565345009
ATGTGGAC -0.544039456023
ATGTGGAG 1.35540818173
ATGTGGCA 0.2464379837
ATGTGGCC -0.402115775723
ATGTGGCG -0.111291537915
ATGTGGGA 0.574159896905
ATGTGGGC -0.0398569255459
ATGTGGGG -0.481672660609
ATGTGGTA 0.351951333282
ATGTGGTC 0.0540375939728
ATGTGGTG 1.32398683658
ATGTGTAA 2.62108436288
ATGTGTAC 1.63757471323
ATGTGTAG 0.857320894667
ATGTGTCA 1.32902911571
ATGTGTCC -0.115987617048
ATGTGTCG -0.264510123323
ATGTGTGA 0.672724681485
ATGTGTGC 0.928344355502
ATGTGTGG -0.656137475477
ATGTGTTA 0.726350083035
ATGTGTTC 0.841656125338
ATGTGTTG -0.192468255753
ATGTTAAA 1.94585925554
ATGTTAAC 2.06778244348
ATGTTAAG 0.0201189456352
ATGTTAAT 2.19989989949
ATGTTACA 2.32340711377
ATGTTACC -0.522025049775
ATGTTACG -0.0772914919824
ATGTTAGA 1.33458705173
ATGTTAGC 0.723354818093
ATGTTAGG 0.393785949748
ATGTTATA 1.45182415308
ATGTTATC 1.51814674669

ATGTTATG 0.0791775845856
ATGTTCAA 0.272419846111
ATGTTCAC 0.875159458583
ATGTTCAG -0.287831054646
ATGTTCCA 1.77362694145
ATGTTCCC -0.563934193961
ATGTTCCG 0.600861222454
ATGTTCGA 0.10265173491
ATGTTCGC -0.983423672145
ATGTTCGG 0.00764054864505
ATGTTCTA 0.0716544483278
ATGTTCTC -0.0236025961454
ATGTTCTG -0.0931546549368
ATGTTGAA 0.6702500697
ATGTTGAC 1.17728318141
ATGTTGAG 0.97787572867
ATGTTGCA 0.734133858138
ATGTTGCC 0.331154143751
ATGTTGCG 1.22682787797
ATGTTGGA 0.68892051287
ATGTTGGC 0.79802243467
ATGTTGGG -0.354929943985
ATGTTGTA 1.93489243895
ATGTTGTC 0.10063365746
ATGTTGTG 2.15586962975
ATGTTTAA 1.00141524688
ATGTTTAC 0.130002575793
ATGTTTAG 1.54689487825
ATGTTTCA 3.17180341794
ATGTTTCC 0.702882594406
ATGTTTCG 0.119624070204
ATGTTTGA 0.215810213053
ATGTTTGC -0.286113586216
ATGTTTGG -0.430514584615
ATGTTTTA -0.163575856843
ATGTTTTC 0.532525547667
ATGTTTTG 0.686682807639
ATTAAAAA -0.0290499896497
ATTAAAAC 0.222376563262
ATTAAAAG 2.82390177113
ATTAAAAT 1.60705311335
ATTAAACA 0.474418490316
ATTAAACC 0.730105197646
ATTAAACG 0.272089467639
ATTAAAGA 2.89111703196
ATTAAAGC -0.313757541711
ATTAAAGG -0.692114171438
ATTAAATA 1.75386377201
ATTAAATC 3.71635228551
ATTAAATG 0.706647701561
ATTAACAA 2.50018541066
ATTAACAC 1.33777925305
ATTAACAG 2.31632802117
ATTAACCA 0.290178053322

ATTAACCC -0.498367286461
ATTAACCG -0.83482538908
ATTAACGA 0.659226628693
ATTAACGC -0.865561828639
ATTAACGG -0.0704815735235
ATTAACTA 0.287453419769
ATTAACTC 0.562835222344
ATTAACTG 0.253866815328
ATTAAGAA 1.23057757996
ATTAAGAC 0.945945804686
ATTAAGAG 2.28912186399
ATTAAGCA 2.18425927881
ATTAAGCC 0.0920550587855
ATTAAGCG 0.381565693467
ATTAAGGA 1.79966105654
ATTAAGGC 0.644428712521
ATTAAGGG 0.883592332664
ATTAAGTA 1.84577352255
ATTAAGTC 0.758241910447
ATTAAGTG 1.41140348153
ATTAATAA 0.674164024119
ATTAATAC 1.52378670484
ATTAATAG 1.52717500983
ATTAATCA 1.66947757407
ATTAATCC 1.54693068487
ATTAATCG 0.674428826524
ATTAATGA 1.29428358439
ATTAATGC 0.160201499778
ATTAATGG -0.446806594232
ATTAATTA 0.707148577808
ATTAATTC 0.822670500689
ATTAATTG 2.222167242
ATTACAAA 2.59864714713
ATTACAAC 3.02506437252
ATTACAAG 3.12524732463
ATTACAAT 3.33871241261
ATTACACA 3.6355205186
ATTACACC 3.65259444475
ATTACACG 2.16585530347
ATTACAGA 1.52000619251
ATTACAGC 1.26258681976
ATTACAGG 1.06422546142
ATTACATA 1.50263040827
ATTACATC 1.53981203845
ATTACATG 2.52387752334
ATTACCAA 2.86243239469
ATTACCAC 1.49079965301
ATTACCAG 1.88878684281
ATTACCCA 1.84375211426
ATTACCCC 3.05027680907
ATTACCCG 0.437297231757
ATTACCGA 1.71749507689
ATTACCGC 3.32384392409
ATTACCGG 0.955163717973

ATTACCTA 0.866268592921
ATTACCTC 1.605563808
ATTACCTG 2.52136960308
ATTACGAA 2.68130713316
ATTACGAC 3.87091882341
ATTACGAG 5.12669593289
ATTACGCA 4.53142449768
ATTACGCC 0.632020471514
ATTACGCG 3.19090603862
ATTACGGA 4.91208045333
ATTACGGC 1.41534595634
ATTACGGG 1.50997659324
ATTACGTA 3.42687537566
ATTACGTC 1.66534836361
ATTACGTG 3.89011158516
ATTACTAA 1.90672220949
ATTACTAC 2.93859743556
ATTACTAG 1.82509374541
ATTACTCA 2.97008289953
ATTACTCC 1.1215358216
ATTACTCG 1.81853863681
ATTACTGA 1.42963571003
ATTACTGC -0.000281040288562
ATTACTGG 0.817833276878
ATTACTTA 3.11756035229
ATTACTTC 1.79165702912
ATTACTTG 1.57007175041
ATTAGAAA 0.640189792258
ATTAGAAC 1.49884115247
ATTAGAAG 1.10387816436
ATTAGAAT 1.00983209126
ATTAGACA 0.622827747764
ATTAGACC 0.302458889399
ATTAGACG 0.230860857306
ATTAGAGA 0.446942950816
ATTAGAGC 0.656747436992
ATTAGAGG 0.271248428553
ATTAGATA 2.59631159825
ATTAGATC 3.02299924684
ATTAGATG 0.979327561983
ATTAGCAA 1.34855704403
ATTAGCAC 0.355610477839
ATTAGCAG 1.26308436516
ATTAGCCA 1.21986557323
ATTAGCCC -0.159612147884
ATTAGCCG 0.20831663807
ATTAGCGA 1.45316773384
ATTAGCGC -0.18085254836
ATTAGCGG -0.861860840306
ATTAGCTA -0.185757638196
ATTAGCTC 0.822721920653
ATTAGCTG 0.827299754687
ATTAGGAA 0.0879930898149
ATTAGGAC 0.603713677294

ATTAGGAG -0.574925991914
ATTAGGCA 1.75375302131
ATTAGGCC -0.436348564649
ATTAGGCG 0.505163048817
ATTAGGGA -0.765482340992
ATTAGGGC -0.310772893846
ATTAGGGG 0.093174223668
ATTAGGTA 1.82226648011
ATTAGGTC 1.00175124616
ATTAGGTG 1.24460607027
ATTAGTAA -0.069117799501
ATTAGTAC 1.86355150667
ATTAGTAG 1.16913967474
ATTAGTCA 0.716012380332
ATTAGTCC 0.159385858407
ATTAGTCG 0.240623572397
ATTAGTGA 0.330363067383
ATTAGTGC 0.172684060328
ATTAGTGG 1.09477225083
ATTAGTTA 0.539283421628
ATTAGTTC 0.399367201701
ATTAGTTG 0.846243119204
ATTATAAA 2.82003028502
ATTATAAC 2.1009256288
ATTATAAG 2.11332970625
ATTATAAT 6.84176552799
ATTATACA 0.637199107231
ATTATACC 0.853254970314
ATTATACG 1.5898590685
ATTATAGA 2.05640926345
ATTATAGC 0.848866786431
ATTATAGG 1.71972882673
ATTATATA 1.87846537799
ATTATATC 3.0465383487
ATTATATG 2.09418191094
ATTATCAA 1.34426462202
ATTATCAC 2.38670654659
ATTATCAG 2.72551497873
ATTATCCA 2.8567095817
ATTATCCC 3.80466680214
ATTATCCG 3.1300660206
ATTATCGA 0.971323118209
ATTATCGC 0.935074333612
ATTATCGG 0.989117340213
ATTATCTA 2.19999128963
ATTATCTC 2.42926832878
ATTATCTG 2.08611418106
ATTATGAA 1.1791921736
ATTATGAC 0.842554829728
ATTATGAG 1.34102807879
ATTATGCA 1.76776673099
ATTATGCC 1.18797895026
ATTATGCG 2.05768789267
ATTATGGA 3.1395826693

ATTATGGC 0.371101210367
ATTATGGG 0.384245985108
ATTATGTA 2.96593890843
ATTATGTC 1.09174367741
ATTATGTG 1.42470896969
ATTATTAA 3.17379380772
ATTATTAC 2.74545426676
ATTATTAG 3.18438715299
ATTATTCA 1.8433099442
ATTATTCC 2.91567349967
ATTATTCG 2.66725553509
ATTATTGA 2.21925399918
ATTATTGC 0.593790248794
ATTATTGG 1.11453812659
ATTATTTA 1.43183781683
ATTATTTC 1.9838867129
ATTATTTG 1.74466188842
ATTCAAAA 0.975778127228
ATTCAAAC -0.368309335323
ATTCAAAG 1.39170859447
ATTCAAAT 2.43487123124
ATTCAACA 0.93877282381
ATTCAACC 1.01916720876
ATTCAACG 1.56647568378
ATTCAAGA 0.191242711917
ATTCAAGC 0.0347536502616
ATTCAAGG -0.517135781466
ATTCAATA 1.26133504549
ATTCAATC 0.772509389096
ATTCAATG 0.794232346156
ATTCACAA 3.33832395247
ATTCACAC 1.28315751163
ATTCACAG 2.33898070114
ATTCACCA 1.16618958442
ATTCACCC 0.696913298677
ATTCACCG 0.974616077679
ATTCACGA 0.15602315931
ATTCACGC 0.303155869314
ATTCACGG 0.880055388588
ATTCACTA 1.01085882511
ATTCACTC 0.767030560715
ATTCACTG 0.111192028835
ATTCAGAA 1.67017372128
ATTCAGAC 1.45316773384
ATTCAGAG 1.98076154489
ATTCAGCA 0.99155552085
ATTCAGCC 0.339144431422
ATTCAGCG 0.118495329133
ATTCAGGA 2.5873647327
ATTCAGGC 0.0307000084106
ATTCAGGG 0.509085122177
ATTCAGTA 1.68990795398
ATTCAGTC 0.785677063417
ATTCAGTG 1.88310566542

ATTCATAA 1.92482162043
ATTCATAC 1.55316519936
ATTCATAG 1.08035842305
ATTCATCA 0.960894233546
ATTCATCC 0.946998560789
ATTCATCG 0.885074976319
ATTCATGA 0.755457113454
ATTCATGC 0.670478024601
ATTCATGG 0.756276918385
ATTCATTA -0.431510924482
ATTCATTC 0.811268592093
ATTCATTG 0.660363072349
ATTCCAAA 1.4702718458
ATTCCAAC 0.947706782316
ATTCCAAG 1.09554708931
ATTCCAAT 1.05311708379
ATTCCACA 1.46004114659
ATTCCACC 0.976264639194
ATTCCACG 2.45006676738
ATTCCAGA 0.00948063391216
ATTCCAGC 0.539166425597
ATTCCAGG 0.283936877136
ATTCCATA 3.20221801432
ATTCCATC 1.47427656582
ATTCCATG 1.40538755394
ATTCCCAA 1.91618265014
ATTCCCAC 4.2767135206
ATTCCCAG 0.691294574686
ATTCCCCA 1.32914944259
ATTCCCCC 3.38815260381
ATTCCCCG 0.363765226123
ATTCCCGA 1.5325385076
ATTCCCGC 1.08609060405
ATTCCCGG 1.30066611343
ATTCCCTA 3.54677507353
ATTCCCTC 3.59971994122
ATTCCCTG 2.75963243705
ATTCCGAA 1.00386258735
ATTCCGAC 1.04284141813
ATTCCGAG 2.05027384168
ATTCCGCA 1.58316031709
ATTCCGCC 1.37413129393
ATTCCGCG 3.17290592858
ATTCCGGA 2.19902825824
ATTCCGGC 1.19629233018
ATTCCGGG 0.60494858914
ATTCCGTA 3.08091373955
ATTCCGTC 1.60426456916
ATTCCGTG 2.99759611925
ATTCCTAA 2.32515705797
ATTCCTAC 1.37823614757
ATTCCTAG 1.76722172101
ATTCCTCA 4.04268232066
ATTCCTCC 0.858015168269

ATTCCTCG 2.72056450609
ATTCCTGA 1.10359483411
ATTCCTGC 0.961744640641
ATTCCTGG 0.762914049111
ATTCCTTA 2.10297472477
ATTCCTTC 0.938333568248
ATTCCTTG 2.88866740154
ATTCGAAA 1.97339620755
ATTCGAAC 1.14015630205
ATTCGAAG 0.604125869717
ATTCGAAT 0.972231190608
ATTCGACA 1.16371580535
ATTCGACC -0.302502814955
ATTCGACG 0.197224706503
ATTCGAGA 1.08035842305
ATTCGAGC 0.415649843308
ATTCGAGG 0.288650026865
ATTCGATA 1.81400826736
ATTCGATC 1.30863475059
ATTCGATG 0.634216332969
ATTCGCAA 4.77295697519
ATTCGCAC 3.09678023335
ATTCGCAG 2.39063319986
ATTCGCCA 1.85576814793
ATTCGCCC 1.31659027254
ATTCGCCG 2.6926717779
ATTCGCGA 3.71349816524
ATTCGCGC 1.18991084203
ATTCGCGG 2.21349537958
ATTCGCTA 4.18783338436
ATTCGCTC 0.735828426989
ATTCGCTG 3.761765898
ATTCGGAA 0.649512002718
ATTCGGAC 1.03652508969
ATTCGGAG 0.815466501292
ATTCGGCA 2.36437945699
ATTCGGCC 0.528554760657
ATTCGGCG 1.5312861088
ATTCGGGA 2.89207881428
ATTCGGGC 0.960769743107
ATTCGGGG 1.01832804327
ATTCGGTA 3.75620254936
ATTCGGTC 0.640920913364
ATTCGGTG 1.74495666846
ATTCGTAA 2.00629832275
ATTCGTAC 0.803260609293
ATTCGTAG 1.57837326417
ATTCGTCA 1.61240682676
ATTCGTCC 0.926221772701
ATTCGTCG 1.61655997769
ATTCGTGA 0.992131341174
ATTCGTGC 0.237384322849
ATTCGTGG 1.76185614146
ATTCGTTA 1.57496310049

ATTCGTTC 1.60408532791
ATTCGTTG 0.95552782128
ATTCTAAA 2.5382934329
ATTCTAAC 1.96476306624
ATTCTAAG 1.29432584452
ATTCTACA 1.54079672035
ATTCTACC 2.32049990812
ATTCTACG 1.30126254338
ATTCTAGA 2.92879100309
ATTCTAGC 1.85968730679
ATTCTAGG 0.764268246946
ATTCTATA 1.27010225343
ATTCTATC 2.37556007239
ATTCTATG 1.27922253124
ATTCTCAA 2.65945344032
ATTCTCAC 2.63029977803
ATTCTCAG 2.05348706898
ATTCTCCA 3.25270763079
ATTCTCCC 1.59963344156
ATTCTCCG 2.50829727428
ATTCTCGA 2.13179655134
ATTCTCGC 1.2828381666
ATTCTCGG 1.22282253342
ATTCTCTA 2.29932050379
ATTCTCTC 2.58476000967
ATTCTCTG 5.73176630615
ATTCTGAA 1.20686506583
ATTCTGAC 3.66022291908
ATTCTGAG 0.764276365888
ATTCTGCA 1.56552264493
ATTCTGCC 1.78047828733
ATTCTGCG 2.17098210287
ATTCTGGA 1.53015778409
ATTCTGGC 2.33202942982
ATTCTGGG 1.07423882281
ATTCTGTA 1.66201480943
ATTCTGTC 3.6827729673
ATTCTGTG 1.60033604228
ATTCTTAA 1.99925399587
ATTCTTAC 2.22490061903
ATTCTTAG 3.15949489419
ATTCTTCA 4.11135170477
ATTCTTCC 0.514294360059
ATTCTTCG 0.681011622794
ATTCTTGA 1.66947757407
ATTCTTGC 0.529300037867
ATTCTTGG 1.81415128564
ATTCTTTA 3.63447379966
ATTCTTTC 1.36179258437
ATTCTTTG 1.92255976655
ATTGAAAA 1.6976423909
ATTGAAAC 0.35860282829
ATTGAAAG 0.5669048939
ATTGAAAT 2.60393611719

ATTGAACA -0.461073864703
ATTGAACC 1.16437114967
ATTGAACG 1.28185556648
ATTGAAGA 0.30444157659
ATTGAAGC -0.238166447563
ATTGAAGG -0.908875133379
ATTGAATA 0.811042302616
ATTGAATC 1.4677491449
ATTGAATG 0.48438813433
ATTGACAA 2.60945283396
ATTGACAC -0.531583334078
ATTGACAG 0.431026494296
ATTGACCA 0.361652635865
ATTGACCC -0.585136706042
ATTGACCG 0.00803775225288
ATTGACGA 0.79051511994
ATTGACGC -0.34508166774
ATTGACGG -0.850206619984
ATTGACTA 0.476927243292
ATTGACTC -0.153739446743
ATTGACTG -0.359334782108
ATTGAGAA 0.817125055351
ATTGAGAC 0.926301504871
ATTGAGAG 0.431555890929
ATTGAGCA -0.0578522474895
ATTGAGCC -1.38018115454
ATTGAGCG 0.235217398135
ATTGAGGA 1.47623510437
ATTGAGGC 0.216735980581
ATTGAGGG 0.0166579865261
ATTGAGTA 0.736311191752
ATTGAGTC 0.0573230590351
ATTGAGTG 0.492903446896
ATTGATAA 1.90719144268
ATTGATAC 1.61939119838
ATTGATAG 1.29293396647
ATTGATCA 0.870801876864
ATTGATCC 0.351655512356
ATTGATCG 1.1602637979
ATTGATGA 0.18110236195
ATTGATGC -0.130819882588
ATTGATGG -0.384453954922
ATTGATTA 2.83511361322
ATTGATTC 4.44075965153
ATTGATTG 1.90410333035
ATTGCAAA 2.07812597517
ATTGCAAC 1.39350662778
ATTGCAAG 1.24195971164
ATTGCAAT 2.13562182194
ATTGCACA 1.10012783784
ATTGCACC 0.930069734696
ATTGCACG 3.87665475161
ATTGCAGA 2.39318796017
ATTGCAGC 0.848356542174

ATTGCAGG 0.385043098638
ATTGCATA 3.60085222132
ATTGCATC 1.164456919
ATTGCATG 2.30392852364
ATTGCCAA 2.34097192363
ATTGCCAC 1.87625286229
ATTGCCAG 1.95540962908
ATTGCCCA 2.23570880399
ATTGCCCC 1.20940900089
ATTGCCCG 0.638948218716
ATTGCCGA 0.742418093133
ATTGCCGC 0.0546169533232
ATTGCCGG -0.0879119003982
ATTGCCTA 1.16547636663
ATTGCCTC 0.895741600249
ATTGCCTG 2.33263439507
ATTGCGAA 3.95404866717
ATTGCGAC 3.82377879083
ATTGCGAG 1.50404102234
ATTGCGCA 2.52163627908
ATTGCGCC 2.00021536184
ATTGCGCG 3.94451161702
ATTGCGGA 2.90472146371
ATTGCGGC 2.01219891974
ATTGCGGG 1.42525397967
ATTGCGTA 3.58157785379
ATTGCGTC 2.07289529495
ATTGCGTG 4.35289209304
ATTGCTAA 1.55365233586
ATTGCTAC 2.4694110827
ATTGCTAG 0.87916792581
ATTGCTCA 2.77426235358
ATTGCTCC 0.585741879463
ATTGCTCG 1.18525931298
ATTGCTGA 1.8767645638
ATTGCTGC 0.142185776391
ATTGCTGG 0.151851688716
ATTGCTTA 1.36638957078
ATTGCTTC 1.45175836884
ATTGCTTG 1.8092509839
ATTGGAAA 1.57832684048
ATTGGAAC 1.76330630935
ATTGGAAG 1.41805934828
ATTGGACA 0.350971647654
ATTGGACC -0.804063759984
ATTGGACG 0.450554838969
ATTGGAGA 0.484201815028
ATTGGAGC 0.352545681448
ATTGGAGG -0.660306031579
ATTGGATA 4.36029844144
ATTGGATC 1.44800908321
ATTGGATG 0.267445849359
ATTGGCAA 1.02421739865
ATTGGCAC -0.448057327605

ATTGGCAG -0.317075274362
ATTGGCCA 0.182689719135
ATTGGCCC -0.359080804958
ATTGGCCG -0.673161855268
ATTGGCGA 1.78072227194
ATTGGCGC -0.173630645656
ATTGGCGG -1.1000100091
ATTGGCTA 0.121304066595
ATTGGCTC -0.66258308245
ATTGGCTG 0.907194304275
ATTGGGAA 0.487318447739
ATTGGGAC 0.508737048575
ATTGGGAG 0.170904971213
ATTGGGCA 0.331154143751
ATTGGGCC -0.787523602201
ATTGGGCG 0.33077276167
ATTGGGGA 0.757607175751
ATTGGGGC 0.537625283926
ATTGGGGG -0.710496912624
ATTGGGTA 0.215366585753
ATTGGGTC 0.12073865517
ATTGGGTG -0.153546465745
ATTGGTAA 1.77661096478
ATTGGTAC 1.60636508509
ATTGGTAG 1.01433623028
ATTGGTCA 0.619770862137
ATTGGTCC -0.577309213561
ATTGGTCG 1.08368385829
ATTGGTGA 1.09123447404
ATTGGTGC -0.126017840857
ATTGGTGG 0.412380616129
ATTGGTTA 1.56400044746
ATTGGTTC 0.231426268731
ATTGGTTG -0.104497857339
ATTGTAAA 1.10305232226
ATTGTAAC 2.27374084126
ATTGTAAG 1.68249140486
ATTGTACA 1.07797270327
ATTGTACC 1.68134330323
ATTGTACG 2.08795822171
ATTGTAGA 2.169550671
ATTGTAGC 0.0607992151376
ATTGTAGG 0.18507731252
ATTGTATA 2.23056639124
ATTGTATC 0.719227481233
ATTGTATG 1.00343165891
ATTGTCAA 2.69078318716
ATTGTCAC 1.25319133064
ATTGTCAG 1.45854975945
ATTGTCCA 1.88720843727
ATTGTCCC 1.48795469258
ATTGTCCG 1.64133357504
ATTGTCGA 1.27189966221
ATTGTCGC 0.109549088126

ATTGTCGG 0.836679630451
ATTGTCTA 2.00374647693
ATTGTCTC 0.246598072576
ATTGTCTG 1.97045423617
ATTGTGAA 1.44817520925
ATTGTGAC 1.51606267682
ATTGTGAG 1.75069238848
ATTGTGCA 2.22645508409
ATTGTGCC 1.33754359556
ATTGTGCG 3.39874698998
ATTGTGGA 2.63965966871
ATTGTGGC 0.420406710414
ATTGTGGG 1.64236717877
ATTGTGTA 1.36633461179
ATTGTGTC 1.90241854587
ATTGTGTG 2.68652636359
ATTGTTAA 1.38934494155
ATTGTTAC 2.38121689295
ATTGTTAG 2.26893401144
ATTGTTCA 0.336770369606
ATTGTTCC 0.702938802464
ATTGTTCG 1.68390597431
ATTGTTGA 1.17555738587
ATTGTTGC 1.57198677975
ATTGTTGG 1.77789750477
ATTGTTTA 0.927092789417
ATTGTTTC 1.02827291411
ATTGTTTG -0.205950070662
ATTTAAAA 2.27768414878
ATTTAAAC 1.3576227792
ATTTAAAG 3.29523485543
ATTTAAAT 1.11181141126
ATTTAACA 2.01794213417
ATTTAACC 0.738330726447
ATTTAACG 0.256183003662
ATTTAAGA 1.59982329989
ATTTAAGC 0.723312557961
ATTTAAGG -0.121350282109
ATTTAATA 2.69860380976
ATTTAATC 1.19546856986
ATTTAATG 1.93690635284
ATTTACAA 2.80548655417
ATTTACAC 2.4950440388
ATTTACAG 2.21181496684
ATTTACCA 1.97937632853
ATTTACCC 1.13031947559
ATTTACCG 0.663366456232
ATTTACGA 1.76534582913
ATTTACGC 1.82765787373
ATTTACGG 0.0224382566388
ATTTACTA 1.18564423409
ATTTACTC 0.987643231855
ATTTACTG 1.96186481224
ATTTAGAA 2.84974852602

ATTTAGAC 1.60769721606
ATTTAGAG 1.67180479584
ATTTAGCA 0.439397539513
ATTTAGCC -0.669610338732
ATTTAGCG 1.46781014105
ATTTAGGA 0.470493086108
ATTTAGGC 1.2364048983
ATTTAGGG 0.857744120524
ATTTAGTA 2.2160226604
ATTTAGTC 0.910264721472
ATTTAGTG 1.26335145752
ATTTATAA 4.22576008357
ATTTATAC 3.39416978048
ATTTATAG 1.21356360907
ATTTATCA 2.72363887867
ATTTATCC 1.91828399878
ATTTATCG 1.77348912762
ATTTATGA 2.82159994707
ATTTATGC -0.13753820273
ATTTATGG 0.360759344104
ATTTATTA 2.91919212408
ATTTATTC 3.22430486651
ATTTATTG 2.68051439137
ATTTCAAA 1.83484334529
ATTTCAAC 2.71887743165
ATTTCAAG 2.65158556131
ATTTCACA 3.97913120065
ATTTCACC 3.70025679592
ATTTCACG 2.34895138604
ATTTCAGA 2.49612510711
ATTTCAGC 1.68516420209
ATTTCAGG 0.729715904802
ATTTCATA 5.36226119645
ATTTCATC 2.50708401294
ATTTCATG 3.42687537566
ATTTCCAA 5.82728440583
ATTTCCAC 4.01983249613
ATTTCCAG 2.39288984929
ATTTCCCA 2.23764548385
ATTTCCCC 3.32960504183
ATTTCCCG 2.7346044462
ATTTCCGA 4.24957252313
ATTTCCGC 3.18611544668
ATTTCCGG 1.43442047299
ATTTCCTA 5.03086889707
ATTTCCTC 4.12240179256
ATTTCCTG 3.58351203551
ATTTCGAA 2.63597679186
ATTTCGAC 4.18815189669
ATTTCGAG 3.16119383478
ATTTCGCA 4.36653129051
ATTTCGCC 0.263786496624
ATTTCGCG 2.82384327311
ATTTCGGA 3.6665394557

ATTTCGGC 2.5400054887
ATTTCGGG 3.03546161413
ATTTCGTA 4.88836356747
ATTTCGTC 3.17072026785
ATTTCGTG 5.01153124344
ATTTCTAA 0.744028974432
ATTTCTAC 2.44351540598
ATTTCTAG 2.7085155803
ATTTCTCA 3.60389953076
ATTTCTCC 1.53919354163
ATTTCTCG 3.89076547223
ATTTCTGA 1.76690695589
ATTTCTGC 2.67789592859
ATTTCTGG 1.10541951421
ATTTCTTA 2.07301895268
ATTTCTTC 2.30676494877
ATTTCTTG 3.21684335094
ATTTGAAA 2.13419122278
ATTTGAAC 2.92092041776
ATTTGAAG 2.27482628131
ATTTGACA 0.843097341574
ATTTGACC 1.8817058766
ATTTGACG 1.41556995586
ATTTGAGA 2.09105965743
ATTTGAGC 0.854942044758
ATTTGAGG 0.985891622234
ATTTGATA 2.72363887867
ATTTGATC 0.984240354405
ATTTGATG 1.42019546265
ATTTGCAA 3.57241385861
ATTTGCAC 3.63380263382
ATTTGCAG 3.16376129395
ATTTGCCA 6.99361138773
ATTTGCCC 0.0183458936814
ATTTGCCG 2.63040261796
ATTTGCGA 2.12929862362
ATTTGCGC 2.38818648393
ATTTGCGG 1.31470438811
ATTTGCTA 1.83037959279
ATTTGCTC 1.50070309642
ATTTGCTG 1.83194113591
ATTTGGAA 1.42190731028
ATTTGGAC 1.49740867971
ATTTGGAG 0.869675217574
ATTTGGCA 2.21748698439
ATTTGGCC -0.799518401717
ATTTGGCG 1.00314853684
ATTTGGGA 2.05701297962
ATTTGGGC 1.00666632854
ATTTGGGG 0.113768023302
ATTTGGTA 2.65329428626
ATTTGGTC 0.78903393353
ATTTGGTG 1.62898029302
ATTTGTAA 2.26239097717

ATTTGTAC 1.77487018042
ATTTGTAG 4.04893598753
ATTTGTCA 3.05689915916
ATTTGTCC 0.444218109085
ATTTGTCG 0.97660084665
ATTTGTGA 2.20276380412
ATTTGTGC 0.855146891901
ATTTGTGG 0.630571136337
ATTTGTTA 3.42377331541
ATTTGTTC 1.48624451038
ATTTGTTG 1.88272719783
ATTTTAAA 2.63531603492
ATTTTAAC 1.85657421311
ATTTTAAG 2.15873082806
ATTTTACA 2.31220339062
ATTTTACC 1.68951949385
ATTTTACG 1.1402418632
ATTTTAGA 2.46524669016
ATTTTAGC 0.571631783376
ATTTTAGG 1.44388091364
ATTTTATA 2.35369867696
ATTTTATC 2.53243322244
ATTTTATG 2.14581588183
ATTTTCAA 3.51676892236
ATTTTCAC 4.14656709382
ATTTTCAG 2.80976565279
ATTTTCCA 3.20850915323
ATTTTCCC 1.81227601829
ATTTTCCG 3.4875563457
ATTTTCGA 1.73779451284
ATTTTCGC 1.34884849322
ATTTTCGG 1.5643027219
ATTTTCTA 5.07096834998
ATTTTCTC 2.51771587115
ATTTTCTG 3.53102953114
ATTTTGAA 1.14206237974
ATTTTGAC 1.97798257688
ATTTTGAG 2.69629220135
ATTTTGCA 1.76990638439
ATTTTGCC 1.25203615097
ATTTTGCG 3.07572240498
ATTTTGGA 4.55191275108
ATTTTGGC 1.19285447883
ATTTTGGG 2.19634463575
ATTTTGTA 3.98578623469
ATTTTGTC 3.88112579034
ATTTTGTG 2.72734611234
ATTTTTAA 1.96054538013
ATTTTTAC 4.57419966232
ATTTTTAG 2.12282178995
ATTTTTCA 3.51370599957
ATTTTTCC 0.97067110474
ATTTTTCG 3.68558482743
ATTTTTGA 1.80352504824

ATTTTTGC 0.313567267026
ATTTTTGG 1.14124299117
ATTTTTTA 2.72560865883
ATTTTTTC 3.36472175451
ATTTTTTG 2.41143871651
CAAAAAAA 0.755687150135
CAAAAAAC 1.75766676755
CAAAAAAG 1.30063405402
CAAAAACA 0.296604091565
CAAAAACC 0.200036566635
CAAAAACG -0.117169651683
CAAAAAGA 0.699595880276
CAAAAAGC 0.173177025812
CAAAAAGG 0.433125136629
CAAAAATA 1.43531938556
CAAAAATC 7.33450742877
CAAAAATG 0.948035079009
CAAAACAA 0.625614002003
CAAAACAC 0.841996079998
CAAAACAG 1.02277139432
CAAAACCA 0.353206022037
CAAAACCC -0.698650335838
CAAAACCG 0.551851959689
CAAAACGA 0.16458468739
CAAAACGC -0.465482658208
CAAAACGG -0.421175095381
CAAAACTA 2.41540471543
CAAAACTC -0.116746634005
CAAAACTG 0.863830620462
CAAAAGAA 0.0662393224122
CAAAAGAC 0.139087671521
CAAAAGAG -0.59599214741
CAAAAGCA 1.46711899012
CAAAAGCC -0.976549218508
CAAAAGCG 0.350580689386
CAAAAGGA 1.26466589336
CAAAAGGC -1.35056387987
CAAAAGGG -0.915499773425
CAAAAGTA -0.120779458057
CAAAAGTC -0.302143083385
CAAAAGTG -0.188070287504
CAAAATAA 2.63987721471
CAAAATAC 3.49901238057
CAAAATAG 1.60593415665
CAAAATCA 4.36552912166
CAAAATCC 7.51517553143
CAAAATCG 5.77415155341
CAAAATGA 1.58707989231
CAAAATGC -0.28192254689
CAAAATGG 0.0339132357098
CAAAATTA 1.01699112421
CAAAATTC 2.0677576703
CAAAATTG 2.01682588378
CAAACAAA 0.17106901547

CAAACAAC 0.205672985756
CAAACAAG 0.953090265177
CAAACACA -0.0577398313741
CAAACACC 0.100387174718
CAAACACG -0.324018010379
CAAACAGA 0.801711765036
CAAACAGC 0.253028274378
CAAACAGG 0.810485426489
CAAACATA -0.37676427627
CAAACATC 0.355567593173
CAAACATG 0.36439891993
CAAACCAA 0.335917464375
CAAACCAC 1.07549122161
CAAACCAG 0.788216002201
CAAACCCA -0.841967143258
CAAACCCC -0.883822161166
CAAACCCG -0.295779290362
CAAACCGA 0.261297936914
CAAACCGC -0.948654408534
CAAACCGG -0.396523698514
CAAACCTA 1.63283803938
CAAACCTC -0.106229065517
CAAACCTG 0.132883759196
CAAACGAA 0.729291638055
CAAACGAC 1.26917253052
CAAACGAG -0.277225843223
CAAACGCA 0.877159840903
CAAACGCC -0.719906766019
CAAACGCG 0.326272994383
CAAACGGA 0.648664718293
CAAACGGC -1.24804079896
CAAACGGG -0.179871613664
CAAACGTA 0.303309088316
CAAACGTC 0.515718089343
CAAACGTG 1.35877379532
CAAACTAA 1.28304509552
CAAACTAC 0.667101585756
CAAACTAG 0.741908889766
CAAACTCA 0.301729433716
CAAACTCC -0.941310097171
CAAACTCG 0.588485040858
CAAACTGA 0.397021243914
CAAACTGC -0.906106366091
CAAACTGG -1.22426874592
CAAACTTA -0.35850123743
CAAACTTC -0.720567314786
CAAACTTG 0.121986265873
CAAAGAAA 1.55699650711
CAAAGAAC 0.83409884789
CAAAGAAG 0.122306651802
CAAAGACA -0.0651665812219
CAAAGACC -0.385684078673
CAAAGACG -0.119139015483
CAAAGAGA 1.4907430286

CAAAGAGC −0.578090297385
CAAAGAGG 0.379142501646
CAAAGATA 3.02363835328
CAAAGATC 1.59339309808
CAAAGATG 0.497546232463
CAAAGCAA 0.369705168756
CAAAGCAC 0.221208476551
CAAAGCAG 0.438068531215
CAAAGCCA −0.480425258084
CAAAGCCC −0.49339537149
CAAAGCCG 0.213682425802
CAAAGCGA 0.885414722801
CAAAGCGC 0.0798150255957
CAAAGCGG −0.509505225364
CAAAGCTA −1.04888919696
CAAAGCTC −0.838537619025
CAAAGCTG −0.116418961846
CAAAGGAA −1.03493856522
CAAAGGAC 0.689010445763
CAAAGGAG −0.470929010822
CAAAGGCA −0.884938411557
CAAAGGCC −1.22983625813
CAAAGGCG −0.91659895322
CAAAGGGA −0.241342827358
CAAAGGGC −1.33823349743
CAAAGGGG −0.686237514916
CAAAGGTA −0.902291296219
CAAAGGTC −0.388140787151
CAAAGGTG −0.620712243014
CAAAGTAA 0.707594495066
CAAAGTAC 0.644802600194
CAAAGTAG 0.514548961743
CAAAGTCA 0.829783734481
CAAAGTCC −0.565927290052
CAAAGTCG 0.410421661229
CAAAGTGA 0.41589424427
CAAAGTGC −1.37748066964
CAAAGTGG −0.435880580525
CAAAGTTA −0.292276903833
CAAAGTTC 0.27834355086
CAAAGTTG 0.638725468265
CAAATAAA 0.183231190091
CAAATAAC 1.83086693747
CAAATAAG 0.82043154639
CAAATACA 1.07191368283
CAAATACC 0.0515619412975
CAAATACG 2.23837077597
CAAATAGA 1.30183690646
CAAATAGC −0.204737225684
CAAATAGG 0.0294979886875
CAAATATA 2.77645779868
CAAATATC 5.76215841931
CAAATATG 2.11208313643
CAAATCAA 2.40299355993

CAAATCAC 4.81292236147
CAAATCAG 2.78406961876
CAAATCCA 6.79983202698
CAAATCCC 3.35829488356
CAAATCCG 5.1106495733
CAAATCGA 3.20747575768
CAAATCGC 2.68821905883
CAAATCGG 2.63940610792
CAAATCTA 5.06396316056
CAAATCTC 7.73898935554
CAAATCTG 9.45067730735
CAAATGAA 0.489190592417
CAAATGAC 1.12919697986
CAAATGAG 0.48769358448
CAAATGCA 0.814116675195
CAAATGCC -0.591838788301
CAAATGCG -0.0418620959603
CAAATGGA 0.384422728223
CAAATGGC -0.631191090396
CAAATGGG -0.232744659952
CAAATGTA -0.283746186096
CAAATGTC 0.0535966729867
CAAATGTG 0.601161415118
CAAATTAA -0.0547491463478
CAAATTAC 2.94661062281
CAAATTAG 0.505163048817
CAAATTCA 3.00317258311
CAAATTCC 2.07850569183
CAAATTCG 3.27937835417
CAAATTGA 0.356199621555
CAAATTGC 1.3962581163
CAAATTGG 0.276281131498
CAAATTTA 1.23234209661
CAAATTTC 3.63737018006
CAAATTTG 1.94410931134
CAACAAAA 1.48073507983
CAACAAAC 1.95796251579
CAACAAAG 1.09649513189
CAACAACA -0.0493506746715
CAACAACC -0.92574296332
CAACAACG 0.719063645154
CAACAAGA 1.06363777495
CAACAAGC 0.388938941571
CAACAAGG -0.385008541091
CAACAATA 1.86685737318
CAACAATC 1.47976247226
CAACAATG 1.19979513306
CAACACAA 0.564093241947
CAACACAC -0.183771620157
CAACACAG -0.21189459321
CAACACCA -0.197900244086
CAACACCC -0.842229863883
CAACACCG -0.0498482200713
CAACACGA -0.0432477286719

CAACACGC -1.09840037687
CAACACGG -1.10060290002
CAACACTA 0.233209937763
CAACACTC -0.324167482177
CAACACTG 1.09674765179
CAACAGAA 1.0006337467
CAACAGAC 0.56333214321
CAACAGAG -1.0701252257
CAACAGCA -0.278204487962
CAACAGCC -1.07186996545
CAACAGCG 0.0130806559196
CAACAGGA -0.388341470735
CAACAGGC -1.09457718805
CAACAGGG -0.300202448148
CAACAGTA 0.179038901697
CAACAGTC -0.673556768918
CAACAGTG 0.122487974833
CAACATAA 1.10082065419
CAACATAC 0.568725826792
CAACATAG 0.615486350893
CAACATCA 0.677402024599
CAACATCC 0.734553128613
CAACATCG 1.00576450148
CAACATGA 0.733745606184
CAACATGC -0.210016411371
CAACATGG -0.353058632019
CAACATTA 2.00053324963
CAACATTC 0.248002649485
CAACATTG 0.612996125758
CAACCAAA 1.12750178648
CAACCAAC -0.662691751362
CAACCAAG 0.0478295180874
CAACCACA -0.224759784909
CAACCACC -0.901597022617
CAACCACG -0.839086792567
CAACCAGA 0.607475245423
CAACCAGC -0.898698560441
CAACCAGG -0.4566338445
CAACCATA 0.11307166792
CAACCATC -0.706032743774
CAACCATG 0.190137911316
CAACCCAA 0.329401701418
CAACCCAC -0.262162083757
CAACCCAG 0.224253496033
CAACCCCA -0.556191846278
CAACCCCC -1.10388211974
CAACCCCG -0.767547674846
CAACCCGA -0.439525985334
CAACCCGC -0.223862537765
CAACCCGG -0.351789578983
CAACCCTA -0.664938408246
CAACCCTC -0.68779905803
CAACCCTG -0.503171409972
CAACCGAA -0.132990554506

CAACCGAC 0.18358467632
CAACCGAG -0.569579981091
CAACCGCA -0.0391965849568
CAACCGCC -0.723141852008
CAACCGCG 1.57449615726
CAACCGGA 0.302038369856
CAACCGGC -0.467392274924
CAACCGGG -1.36079291366
CAACCGTA 0.470308640407
CAACCGTC -0.0939244971495
CAACCGTG -0.223908336923
CAACCTAA 1.37435112989
CAACCTAC -0.21444747992
CAACCTAG -0.686360339931
CAACCTCA -0.18203520753
CAACCTCC -1.24290192523
CAACCTCG 0.215669068375
CAACCTGA 0.660140738254
CAACCTGC -0.634255886787
CAACCTGG -0.807514102016
CAACCTTA -1.20445374016
CAACCTTC -0.305368801364
CAACCTTG -0.195435416666
CAACGAAA 0.555363714228
CAACGAAC 0.105694048078
CAACGAAG -0.528186285612
CAACGACA -0.474727842812
CAACGACC -0.429937931578
CAACGACG -0.778849033472
CAACGAGA 0.432326982209
CAACGAGC -0.605364528767
CAACGAGG 0.353176252584
CAACGATA 1.21219921051
CAACGATC 0.807518681932
CAACGATG 0.256686586223
CAACGCAA 1.79511320014
CAACGCAC 0.178747244331
CAACGCAG -0.177107842648
CAACGCCA -0.786341983921
CAACGCCC -0.35725633304
CAACGCCG -0.584248410552
CAACGCGA 0.0859414957084
CAACGCGC 0.194647462968
CAACGCGG 0.253609507331
CAACGCTA -0.644720786243
CAACGCTC -0.492525395663
CAACGCTG -0.180640414986
CAACGGAA 0.423227105844
CAACGGAC 0.429784296221
CAACGGAG -0.802294247056
CAACGGCA -0.615373518422
CAACGGCC -0.658946212938
CAACGGCG -1.16156324492
CAACGGGA 0.00967028406244

CAACGGGC -1.22634344779
CAACGGGG -0.633830787328
CAACGGTA -0.86102292389
CAACGGTC -0.743515191148
CAACGGTG -1.00964785374
CAACGTAA 1.31151031319
CAACGTAC -0.155689866346
CAACGTAG 0.121616749938
CAACGTCA 0.636649100978
CAACGTCC -0.439942757673
CAACGTCG -0.785958936417
CAACGTGA 0.257859669206
CAACGTGC -0.819661287821
CAACGTGG -1.07537630736
CAACGTTA 0.530813283687
CAACGTTC 0.192346679806
CAACGTTG 0.633345940786
CAACTAAA 1.38090415671
CAACTAAC 1.14950432658
CAACTAAG -0.243932145216
CAACTACA -0.407405162131
CAACTACC 0.64618365299
CAACTACG 1.62648590433
CAACTAGA 0.143470650955
CAACTAGC -0.424531965494
CAACTAGG 0.0907118943842
CAACTATA 1.48067054466
CAACTATC 0.628657147883
CAACTATG 1.17629475231
CAACTCAA 0.619477747525
CAACTCAC -0.23592686873
CAACTCAG -0.0127654744405
CAACTCCA -0.0578522474895
CAACTCCC -1.13932317373
CAACTCCG 0.0163613328881
CAACTCGA -0.21623239802
CAACTCGC 0.434353803135
CAACTCGG -0.455110397958
CAACTCTA -0.195124398747
CAACTCTC -0.352001296
CAACTCTG 0.168941852752
CAACTGAA 0.388410585828
CAACTGAC -0.0790816145315
CAACTGAG -0.303873250673
CAACTGCA -0.436136431276
CAACTGCC -0.655160287984
CAACTGCG -0.426561284556
CAACTGGA 0.238213912145
CAACTGGC -1.09939234499
CAACTGGG -0.451497677093
CAACTGTA -0.603797989381
CAACTGTC -0.438543593392
CAACTGTG 0.00853446494066
CAACTTAA 0.247540702522

CAACTTAC 0.410933362732
CAACTTAG -0.510478249297
CAACTTCA 1.408198165
CAACTTCC -0.714236622064
CAACTTCG 0.509342013819
CAACTTGA 0.0922571996153
CAACTTGC -1.12369004745
CAACTTGG 0.194002319373
CAACTTTA -0.183889865256
CAACTTTC -0.892385979204
CAAGAAAA 0.0358345103936
CAAGAAAC -0.59954428848
CAAGAAAG -0.196725079324
CAAGAACA 0.0353005338453
CAAGAACC 0.0743586804378
CAAGAACG -0.457511939268
CAAGAAGA -0.265765436612
CAAGAAGC 0.393465771997
CAAGAAGG 0.416258555755
CAAGAATA 0.657015570245
CAAGAATC 6.18692458505
CAAGAATG -0.476185088752
CAAGACAA 0.0848333642595
CAAGACAC -0.591424514098
CAAGACAG 0.985013319288
CAAGACCA 0.704324435175
CAAGACCC -0.66106046862
CAAGACCG -1.09715672154
CAAGACGA -0.66316972803
CAAGACGC -0.89154265016
CAAGACGG -0.689187605233
CAAGACTA 1.55778633441
CAAGACTC -0.827099903815
CAAGACTG -0.654718534286
CAAGAGAA -0.293061318505
CAAGAGAC -0.0756389750856
CAAGAGAG -0.596096028228
CAAGAGCA -0.366680134361
CAAGAGCC -0.955801783517
CAAGAGCG -0.337341610015
CAAGAGGA -0.0944603472997
CAAGAGGC -1.24921950274
CAAGAGGG -0.926366664583
CAAGAGTA 1.17608157805
CAAGAGTC -0.67964077072
CAAGAGTG -0.524506115078
CAAGATAA 1.23865217972
CAAGATAC 2.9061281224
CAAGATAG 1.88243449957
CAAGATCA 2.98133575269
CAAGATCC 5.25064406815
CAAGATCG 2.89767068331
CAAGATGA 0.769567001364
CAAGATGC -0.557807099314

CAAGATGG −0.998280502688
CAAGATTA 3.5538089915
CAAGATTC 6.59380388957
CAAGATTG 4.26528642247
CAAGCAAA 0.83148371596
CAAGCAAC −0.978159683451
CAAGCAAG 0.69011982628
CAAGCACA −1.02129624508
CAAGCACC 0.0326693722104
CAAGCACG −0.38345386785
CAAGCAGA 1.02223304604
CAAGCAGC −0.672111389122
CAAGCAGG 0.435786067716
CAAGCATA −0.125855253846
CAAGCATC 0.283209503234
CAAGCATG −0.885287317871
CAAGCCAA −1.03091781549
CAAGCCAC −1.00259894694
CAAGCCAG −1.09925099214
CAAGCCCA −0.362513660038
CAAGCCCC −0.44332627457
CAAGCCCG −1.23705982626
CAAGCCGA −0.753680314297
CAAGCCGC −1.58577669809
CAAGCCGG −1.29710543707
CAAGCCTA −0.5648381028
CAAGCCTC −1.38481623752
CAAGCCTG −0.927304506435
CAAGCGAA −0.467834028622
CAAGCGAC −0.322891142911
CAAGCGAG −0.647449375178
CAAGCGCA 0.446695219006
CAAGCGCC −0.932129447744
CAAGCGCG 0.825598315962
CAAGCGGA −1.19355270781
CAAGCGGC −1.28188262961
CAAGCGGG −0.494967531682
CAAGCGTA −0.408272431644
CAAGCGTC −0.510424955731
CAAGCGTG −0.934376520985
CAAGCTAA −0.3765871168
CAAGCTAC −0.774742098056
CAAGCTAG 0.174891996106
CAAGCTCA 0.0571627619816
CAAGCTCC −0.43364225076
CAAGCTCG −0.673569051419
CAAGCTGA −1.29884976046
CAAGCTGC −1.17489746163
CAAGCTGG −0.832777750356
CAAGCTTA 0.121030312536
CAAGCTTC −0.0520740591567
CAAGCTTG 0.00503353565715
CAAGGAAA −0.411315369346
CAAGGAAC −0.459384500302

CAAGGAAG 0.15386310447
CAAGGACA -0.620687053477
CAAGGACC -0.601560908684
CAAGGACG -0.514679281166
CAAGGAGA 0.956494183517
CAAGGAGC -1.42473894732
CAAGGAGG -0.743179608226
CAAGGATA 0.73266495423
CAAGGATC 1.44057587984
CAAGGATG -0.235413918159
CAAGGCAA 0.700538718399
CAAGGCAC -0.760288300104
CAAGGCAG 0.419935187263
CAAGGCCA -0.244158851049
CAAGGCCC -1.03344343088
CAAGGCCG -0.837333933878
CAAGGCGA -0.0928621648587
CAAGGCGC -0.986930430412
CAAGGCGG -0.646188857439
CAAGGCTA -1.01324308765
CAAGGCTC -0.845738495752
CAAGGCTG -0.975389042562
CAAGGGAA 0.913776059655
CAAGGGAC -0.660818773972
CAAGGGAG -0.68996223554
CAAGGGCA -0.514240025603
CAAGGGCC -1.46644719974
CAAGGGCG -1.0760087521
CAAGGGGA 0.310340091802
CAAGGGGC -1.27861506786
CAAGGGGG -1.15594743542
CAAGGGTA 0.0437148800849
CAAGGGTC -0.453996645703
CAAGGGTG -0.318927850308
CAAGGTAA 0.743567235646
CAAGGTAC -0.349570401593
CAAGGTAG -0.316279618078
CAAGGTCA -0.0957239877082
CAAGGTCC -0.533807507739
CAAGGTCG 1.02637120815
CAAGGTGA -0.244325185264
CAAGGTGC -1.39343501455
CAAGGTGG -0.468323038724
CAAGGTTA -1.12425858155
CAAGGTTC -0.31314404117
CAAGTAAA 0.845113961778
CAAGTAAC 0.872505397369
CAAGTAAG 0.242160134153
CAAGTACA 1.61115526068
CAAGTACC -0.854450744698
CAAGTACG 0.2808062965
CAAGTAGA 0.0306371386571
CAAGTAGC -0.84602786316
CAAGTAGG 0.0061404180381

CAAGTATA 1.47992235295
CAAGTATC 2.20538434868
CAAGTATG 0.835166176452
CAAGTCAA 0.115323945611
CAAGTCAC 0.318220669671
CAAGTCAG -0.472324636077
CAAGTCCA -0.0384767054621
CAAGTCCC -0.324467466663
CAAGTCCG -0.400728477587
CAAGTCGA -0.916566477454
CAAGTCGC -1.24015480846
CAAGTCGG -0.503803646532
CAAGTCTA -0.382143387394
CAAGTCTC -0.566516850124
CAAGTCTG -1.11846748618
CAAGTGAA 0.0538652225958
CAAGTGAC 0.44046757439
CAAGTGAG 0.331789502981
CAAGTGCA 0.233039439988
CAAGTGCC -0.402692845115
CAAGTGCG -0.306987801604
CAAGTGGA 0.0348333824324
CAAGTGGC -0.845956666287
CAAGTGGG -0.569735281873
CAAGTGTA -0.126470836167
CAAGTGTC -0.805356545312
CAAGTGTG -0.904276481546
CAAGTTAA 0.208089307703
CAAGTTAC 0.439346952261
CAAGTTAG 0.208873306019
CAAGTTCA 0.754091257652
CAAGTTCC -0.0583689452645
CAAGTTCG -0.395122244275
CAAGTTGA 0.0395721380535
CAAGTTGC -0.762499150374
CAAGTTGG -0.653140545111
CAAGTTTA -0.00607421743679
CAAGTTTC 0.194569812577
CAATAAAA 1.02276265085
CAATAAAC 1.18370255796
CAATAAAG 0.177392005607
CAATAACA 0.100672794923
CAATAACC -0.223165766028
CAATAACG -0.215696131514
CAATAAGA 0.719693799934
CAATAAGC 1.68679527665
CAATAAGG 0.651817365796
CAATAATA 1.00375079577
CAATAATC 2.93357472516
CAATAATG 1.97507058313
CAATACAA 0.408094647639
CAATACAC 1.02537861549
CAATACAG 1.32192878895
CAATACCA 1.84727011413

CAATACCC 0.329056125952
CAATACCG -0.209974567594
CAATACGA 0.127585629312
CAATACGC -0.211750742218
CAATACGG 0.360145219029
CAATACTA 0.241499793563
CAATACTC 0.414367883236
CAATACTG 1.39651854696
CAATAGAA 0.0661818652865
CAATAGAC 0.180990778546
CAATAGAG 0.00382756055223
CAATAGCA 0.859972041389
CAATAGCC -0.537207054341
CAATAGCG 0.444882196878
CAATAGGA -0.291605321632
CAATAGGC -0.824291166353
CAATAGGG -0.292932872684
CAATAGTA 0.917184349732
CAATAGTC 0.596834435564
CAATAGTG 0.385971156124
CAATATAA 1.7068365719
CAATATAC 1.11134550891
CAATATAG 2.98920321534
CAATATCA 3.69336776982
CAATATCC 6.62868536112
CAATATCG 3.06051791719
CAATATGA 1.2790630669
CAATATGC -0.513393990246
CAATATGG 0.785396439484
CAATATTA 1.22199231959
CAATATTC 1.68676259271
CAATATTG 2.29344884354
CAATCAAA 1.74622405607
CAATCAAC 1.3525045151
CAATCAAG 0.973412808888
CAATCACA 1.4113366564
CAATCACC 1.23287919583
CAATCACG 0.913037027785
CAATCAGA 1.40877252808
CAATCAGC 1.46800874286
CAATCAGG 1.67551848303
CAATCATA 2.73230699388
CAATCATC 0.534711000222
CAATCATG 0.240228242391
CAATCCAA 1.96781162475
CAATCCAC 2.29830209706
CAATCCAG 2.15258791189
CAATCCCA 2.48366919334
CAATCCCC 1.44670588898
CAATCCCG 3.14930041795
CAATCCGA 3.95305128641
CAATCCGC 1.55407389629
CAATCCGG 2.35719356908
CAATCCTA 1.71690343503

CAATCCTC 3.2909615858
CAATCCTG 4.39102010037
CAATCGAA 1.46732029824
CAATCGAC 1.22702189986
CAATCGAG 0.85433333231
CAATCGCA 1.51216121308
CAATCGCC 1.48231140358
CAATCGCG 1.35631708684
CAATCGGA 3.08414695194
CAATCGGC -0.518093400227
CAATCGGG 1.53431260044
CAATCGTA 2.38165781394
CAATCGTC 2.00463851963
CAATCGTG 1.24107911874
CAATCTAA 4.23677894466
CAATCTAC 3.53412784418
CAATCTAG 4.97108475782
CAATCTCA 4.39272882532
CAATCTCC 4.63536027445
CAATCTCG 4.8325154495
CAATCTGA 5.6773369212
CAATCTGC 1.82721383007
CAATCTGG 5.86271796541
CAATCTTA 2.89513153635
CAATCTTC 4.1117751388
CAATGAAA 0.0985610373768
CAATGAAC -0.690322591643
CAATGAAG 0.612385123353
CAATGACA 0.0635875511561
CAATGACC -0.21245438383
CAATGACG -0.47573958785
CAATGAGA -0.467564438123
CAATGAGC -0.873124726894
CAATGAGG -0.0755596592709
CAATGATA 1.45876897088
CAATGATC 1.7616675322
CAATGATG 0.972231190608
CAATGCAA 1.51964916726
CAATGCAC 0.152279286311
CAATGCAG 1.99672671506
CAATGCCA -0.122253358237
CAATGCCC -0.449837457611
CAATGCCG -0.553816743573
CAATGCGA -0.221527613412
CAATGCGC -1.28044162156
CAATGCGG 0.028601366078
CAATGCTA 0.083025130225
CAATGCTC -0.33914359871
CAATGCTG -0.141835829188
CAATGGAA 0.306770463781
CAATGGAC 0.109047795522
CAATGGAG 0.263507538116
CAATGGCA -0.0274520153868
CAATGGCC -0.189111593818

| | |
|---|---|
| CAATGGCG | -0.237961600419 |
| CAATGGGA | 0.200733130195 |
| CAATGGGC | -1.28365130983 |
| CAATGGGG | 0.660267310472 |
| CAATGGTA | -0.453613181843 |
| CAATGGTC | 0.530245374126 |
| CAATGGTG | 0.41917471306 |
| CAATGTAA | 0.939641134213 |
| CAATGTAC | 0.748336177076 |
| CAATGTAG | 0.152585932492 |
| CAATGTCA | 0.78381199679 |
| CAATGTCC | -0.21435837974 |
| CAATGTCG | -0.340159923664 |
| CAATGTGA | 0.841863470618 |
| CAATGTGC | -0.855850949869 |
| CAATGTGG | 0.0715903295064 |
| CAATGTTA | 0.998116042075 |
| CAATGTTC | -0.559557251689 |
| CAATTAAA | 0.200827226647 |
| CAATTAAC | 0.564614519637 |
| CAATTAAG | 1.5960282151 |
| CAATTACA | 1.90509404942 |
| CAATTACC | 0.263114914424 |
| CAATTACG | 1.69968919691 |
| CAATTAGA | 1.16114189266 |
| CAATTAGC | -0.120779458057 |
| CAATTAGG | 1.02946223497 |
| CAATTATA | 1.40510942814 |
| CAATTATC | 0.95552782128 |
| CAATTATG | 1.09174367741 |
| CAATTCAA | 1.54850700862 |
| CAATTCAC | 1.34406039941 |
| CAATTCAG | 1.85443122886 |
| CAATTCCA | 0.911423231995 |
| CAATTCCC | 2.5563818104 |
| CAATTCCG | 0.405340869167 |
| CAATTCGA | -0.28847016108 |
| CAATTCGC | 0.105876203821 |
| CAATTCGG | 0.177885179268 |
| CAATTCTA | 0.746279170342 |
| CAATTCTC | 0.792901672435 |
| CAATTCTG | 1.53015778409 |
| CAATTGAA | 0.722706135471 |
| CAATTGAC | 1.66749863408 |
| CAATTGAG | 0.893958972108 |
| CAATTGCA | 0.834378014577 |
| CAATTGCC | 0.722706135471 |
| CAATTGCG | 0.257466629158 |
| CAATTGGA | 0.778650223491 |
| CAATTGGC | -0.375506673024 |
| CAATTGGG | 0.395985350228 |
| CAATTGTA | 0.124459212235 |
| CAATTGTC | 0.667774625203 |
| CAATTGTG | 1.40379207781 |

CAATTTAA 0.786958607132
CAATTTAC 1.81119411727
CAATTTAG 0.775017517538
CAATTTCA 1.71507646498
CAATTTCC 1.85586182802
CAATTTCG 3.80596104472
CAATTTGA 1.52448077026
CAATTTGC 0.214133339331
CAATTTGG 0.0954995718334
CAATTTTA 0.58018560887
CAATTTTC 1.45474613937
CACAAAAA 0.675821537287
CACAAAAC 1.09762928558
CACAAAAG 1.12358450121
CACAAACA 0.908621572585
CACAAACC 0.124760653966
CACAAACG -0.389854092022
CACAAAGA 1.4119684766
CACAAAGC -0.625297987812
CACAAAGG 0.183575516489
CACAAATA 2.83619530606
CACAAATC 3.16618781662
CACAAATG -0.447503574148
CACAACAA 0.782802749888
CACAACAC 0.177597477284
CACAACAG 0.704352539204
CACAACCA 0.255685041906
CACAACCC -0.235783434094
CACAACCG 0.0519204237989
CACAACGA 0.524959943099
CACAACGC -0.554882614889
CACAACGG -1.04562683966
CACAACTA 0.868300410118
CACAACTC 0.478052861692
CACAACTG 0.268321862348
CACAAGAA -0.259908140642
CACAAGAC 0.537736034617
CACAAGAG -0.0821678532559
CACAAGCA -0.105852679708
CACAAGCC -1.02129624508
CACAAGCG -0.354393885657
CACAAGGA 0.00790264473638
CACAAGGC -1.04802963004
CACAAGGG -1.09274376448
CACAAGTA 1.74961215288
CACAAGTC 0.762927997036
CACAAGTG -0.246986949064
CACAATAA 1.57470246165
CACAATAC 2.00585240549
CACAATAG 0.860903013367
CACAATCA 5.60225877762
CACAATCC 6.4804470178
CACAATCG 3.7757633698
CACAATGA 0.640110684621

CACAATGC 0.549742283923
CACAATGG -0.836259319086
CACAATTA 1.23212267701
CACAATTC 1.44817520925
CACACAAA 1.12358450121
CACACAAC 0.993919590121
CACACAAG 0.0752909014838
CACACACA 0.515851739614
CACACACC -0.766996835881
CACACACG -0.926037327
CACACAGA 1.57536405131
CACACAGC 0.635929637839
CACACAGG -0.0454360957188
CACACATA 1.20817013366
CACACATC 0.104920875018
CACACATG 0.145271390582
CACACCAA -0.918214830791
CACACCAC -0.486005260969
CACACCAG -0.334580961669
CACACCCA -0.0883653120637
CACACCCC -1.13192827511
CACACCCG -0.536308558129
CACACCGA -0.608309830991
CACACCGC -1.52085181151
CACACCGG -0.535186478755
CACACCTA -0.822216672667
CACACCTC 0.761039198119
CACACCTG -0.245839263797
CACACGAA -0.118912517829
CACACGAC -1.05657825108
CACACGAG -0.374782421791
CACACGCA 0.856438428161
CACACGCC -0.607655527564
CACACGCG -0.518045311111
CACACGGA 1.13576083194
CACACGGC -1.53978268535
CACACGGG -1.20128464659
CACACGTA 1.26113561098
CACACGTC 0.705381563016
CACACGTG -0.222798956407
CACACTAA -0.382585765626
CACACTAC 0.265340545331
CACACTAG 0.138765620169
CACACTCA 0.866296280593
CACACTCC 0.592826592871
CACACTCG -0.782257739906
CACACTGA 1.06077303761
CACACTGC -1.19992274617
CACACTGG -0.929589676248
CACACTTA -0.53786114959
CACACTTC 1.13532990349
CACAGAAA 0.716750163134
CACAGAAC 0.15882273694
CACAGAAG -0.0697754337763

CACAGACA -0.0784631177187
CACAGACC -0.428329548416
CACAGACG -0.549053214771
CACAGAGA 2.37696922921
CACAGAGC -1.42755642825
CACAGAGG -1.11279234595
CACAGATA 2.27317834433
CACAGATC 3.23121845761
CACAGATG -0.923289793868
CACAGCAA 0.918116362601
CACAGCAC 0.37469956695
CACAGCAG 0.499036786882
CACAGCCA 0.146775892927
CACAGCCC -1.21734870131
CACAGCCG -0.697004480637
CACAGCGA 0.336100452829
CACAGCGC 0.186251228214
CACAGCGG -0.951867635833
CACAGCTA -0.0830651003994
CACAGCTC -0.532414588798
CACAGCTG -0.724728376481
CACAGGAA -0.0421364745531
CACAGGAC -0.0824792875313
CACAGGAG 1.48362125951
CACAGGCA -0.571274966298
CACAGGCC -0.662447766756
CACAGGCG -1.6175833807
CACAGGGA -0.0836827645003
CACAGGGC -1.16087334305
CACAGGGG -0.123183497503
CACAGGTA 0.234479823511
CACAGGTC -0.966692198788
CACAGGTG -0.854313347223
CACAGTAA 0.690670040711
CACAGTAC 0.448674367172
CACAGTAG 0.0255009712502
CACAGTCA -0.602009948611
CACAGTCC -0.101303574237
CACAGTCG -0.763979920428
CACAGTGA -0.856383469171
CACAGTGC -0.980151530474
CACAGTGG -0.94929330679
CACAGTTA 0.275568538233
CACAGTTC -0.0853396531348
CACATAAA 0.333410793179
CACATAAC 0.614858694248
CACATAAG 0.63418510627
CACATACA 2.83701469464
CACATACC -0.0750196455609
CACATACG 0.376766566228
CACATAGA 2.57287096458
CACATAGC -0.789713218316
CACATAGG -0.120256098586
CACATATA 4.19808615045

CACATATC 2.62866537262
CACATATG 0.83779192546
CACATCAA -0.369153913434
CACATCAC 0.417747028395
CACATCAG 1.45491205723
CACATCCA -0.166794288592
CACATCCC -0.13557716605
CACATCCG 0.103964505325
CACATCGA 0.574405546935
CACATCGC -0.600573936826
CACATCGG -0.769742703589
CACATCTA 0.549164798174
CACATCTC 0.0170123054676
CACATCTG -0.294632854163
CACATGAA 1.14891310109
CACATGAC 2.07731158287
CACATGAG 0.541680383023
CACATGCA 0.300588201967
CACATGCC -0.19323768161
CACATGCG -0.789390958785
CACATGGA 0.110535643627
CACATGGC -0.654679396824
CACATGGG -1.50390341669
CACATGTA 1.14095112562
CACATGTC -1.02121526384
CACATGTG -0.489406264816
CACATTAA 0.516708183871
CACATTAC 0.692387300963
CACATTAG 0.403881125091
CACATTCA 1.72461705415
CACATTCC 0.726350083035
CACATTCG 0.468033671316
CACATTGA 0.321396424932
CACATTGC 0.119202301593
CACATTGG -0.590924054207
CACATTTA 0.375034941695
CACATTTC 1.063813269
CACCAAAA 0.485446303061
CACCAAAC -0.249138676785
CACCAAAG -0.0461303693206
CACCAACA -0.265088858139
CACCAACC -0.61913716833
CACCAACG -1.55258854632
CACCAAGA 1.84772789754
CACCAAGC -0.653707621959
CACCAAGG -0.444138585092
CACCAATA 0.842460941454
CACCAATC 1.32349616105
CACCAATG -0.587447065392
CACCACAA 1.07915619515
CACCACAC -0.0645093633026
CACCACAG -0.220085772643
CACCACCA 0.367435404114
CACCACCC -0.163871677769

CACCACCG -0.362083147952
CACCACGA 1.20911234725
CACCACGC -0.703294786829
CACCACGG -1.13455652226
CACCACTA 1.17476839128
CACCACTC -0.149822786011
CACCACTG -0.941310097171
CACCAGAA -0.816295257876
CACCAGAC -0.715928692779
CACCAGAG -0.825428026365
CACCAGCA -0.575480578083
CACCAGCC -0.625318805611
CACCAGCG -1.12437412033
CACCAGGA 0.344389684096
CACCAGGC -1.32142374915
CACCAGGG -1.44239556367
CACCAGTA 0.53117780335
CACCAGTC -0.23729813716
CACCAGTG -0.54425845927
CACCATAA 0.252037138961
CACCATAC 0.852014437663
CACCATAG 0.281623603294
CACCATCA 0.256546690613
CACCATCC -0.908471684431
CACCATCG -0.68139862568
CACCATGA -0.358871794255
CACCATGC -0.820921389204
CACCATGG -0.954474232465
CACCATTA 0.546720580376
CACCATTC -0.201450511553
CACCCAAA 0.284966525482
CACCCAAC -0.665415135847
CACCCAAG -0.471641395909
CACCCACA -0.484592773296
CACCCACC -0.548605840267
CACCCACG -0.359906022517
CACCCAGA 1.19167286055
CACCCAGC -0.727587493017
CACCCAGG -0.370721285532
CACCCATA 0.828581298402
CACCCATC 1.04486532456
CACCCATG -1.32710346929
CACCCCAA -0.443019212032
CACCCCAC -0.273017316947
CACCCCAG 0.053195722175
CACCCCCA -0.850258039948
CACCCCCC -1.5476568097
CACCCCCG -1.06647086924
CACCCCGA 0.184544376861
CACCCCGC -0.34993346401
CACCCCGG -1.29146839341
CACCCCTA -0.0759112718986
CACCCCTC -0.0851125309461
CACCCCTG -0.372362977174

CACCCGAA -0.567169904483
CACCCGAC -0.208798153764
CACCCGAG -0.892883316425
CACCCGCA -0.584974327209
CACCCGCC -0.907984964287
CACCCGCG -0.867147312223
CACCCGGA -0.52843526649
CACCCGGC -1.47373613576
CACCCGGG -1.31407777236
CACCCGTA -0.31367468687
CACCCGTC -1.35021955347
CACCCGTG -1.18727447594
CACCCTAA -0.686597246485
CACCCTAC -0.938115814069
CACCCTAG 0.407651644873
CACCCTCA -0.338325042847
CACCCTCC -0.122070994316
CACCCTCG -0.28275900606
CACCCTGA -0.739625593555
CACCCTGC -1.02834973178
CACCCTGG -1.40207002946
CACCCTTA 0.147940856967
CACCCTTC -0.541285053017
CACCGAAA 0.541970166787
CACCGAAC -0.53677695861
CACCGAAG -0.456072388457
CACCGACA -0.579009611395
CACCGACC -0.647028855635
CACCGACG -0.775397650551
CACCGAGA 0.404938252932
CACCGAGC -1.47351921429
CACCGAGG -0.680645437707
CACCGATA 3.81823334549
CACCGATC 1.65617770673
CACCGATG -0.905306962604
CACCGCAA 0.221654601987
CACCGCAC -0.0857212433933
CACCGCAG 0.692289873663
CACCGCCA -0.561809529379
CACCGCCC -0.748312236607
CACCGCCG -0.945450549244
CACCGCGA 0.302355633115
CACCGCGC -0.210325139332
CACCGCGG -1.07456691133
CACCGCTA -0.0596536116502
CACCGCTC -0.699277784305
CACCGCTG -1.39224111377
CACCGGAA 0.134322477295
CACCGGAC -0.329997923186
CACCGGAG 0.178323602119
CACCGGCA -1.02766878157
CACCGGCC -0.704482858627
CACCGGCG -1.24635538994
CACCGGGA -0.326156414708

CACCGGGC −1.22562752368
CACCGGGG −1.17285044744
CACCGGTA 0.217986505776
CACCGGTC −1.34823270272
CACCGGTG −0.902201779682
CACCGTAA 0.507877481648
CACCGTAC −0.799891664856
CACCGTAG 0.161330865382
CACCGTCA 0.00975168165713
CACCGTCC −0.175321259125
CACCGTCG −0.982574722295
CACCGTGA 0.198177328993
CACCGTGC −0.31687230082
CACCGTGG −1.30650966966
CACCGTTA −0.250295313705
CACCGTTC −0.126249126606
CACCTAAA −0.22959388605
CACCTAAC −0.0261386204382
CACCTAAG −0.867768723528
CACCTACA 0.608579213312
CACCTACC 0.400346262795
CACCTACG 0.0998617334678
CACCTAGA 0.743827458136
CACCTAGC −0.943421854717
CACCTAGG −1.14252557577
CACCTATA 0.617198198517
CACCTATC 0.892717398566
CACCTATG −0.236172102404
CACCTCAA −0.584130373631
CACCTCAC 0.003710356343
CACCTCAG −0.154411237122
CACCTCCA −0.284417351941
CACCTCCC −0.445196962001
CACCTCCG −0.698800640348
CACCTCGA 0.394163168268
CACCTCGC −0.394775211563
CACCTCGG −0.931212423692
CACCTCTA 0.112871192514
CACCTCTC −0.466590165123
CACCTCTG −1.00005709367
CACCTGAA −0.671536817866
CACCTGAC −0.522025049775
CACCTGAG −0.526441337687
CACCTGCA −0.481172825251
CACCTGCC −0.462338546002
CACCTGCG −0.567534840502
CACCTGGA −0.491992876361
CACCTGGC −1.01147690557
CACCTGGG −1.26746880184
CACCTGTA 0.806351636111
CACCTGTC −1.4933215212
CACCTTAA −0.466886194227
CACCTTAC 0.765065776831
CACCTTAG −0.204979544866

CACCTTCA −0.481447828378
CACCTTCC −0.646097467301
CACCTTCG −0.553962884523
CACCTTGA 0.0776485172379
CACCTTGC −0.618077958709
CACCTTGG −1.17900522976
CACCTTTA 0.55148910545
CACCTTTC −0.786601790055
CACGAAAA −0.0957135788087
CACGAAAC 1.01856432529
CACGAAAG −0.000796280817593
CACGAACA −0.776876130647
CACGAACC −1.05551508608
CACGAACG −0.301285598238
CACGAAGA 2.022773529
CACGAAGC −0.464627046663
CACGAAGG −0.646083519376
CACGAATA 0.357114772006
CACGAATC 5.64137042596
CACGAATG 0.34138442661
CACGACAA 0.324063601359
CACGACAC 0.442956134101
CACGACAG −1.23715267364
CACGACCA −0.489735394221
CACGACCC −1.04776857483
CACGACCG 0.700541424713
CACGACGA −0.820207963227
CACGACGC −1.08607561523
CACGACGG −0.453076915337
CACGACTA 0.404485257622
CACGACTC −0.0912602352139
CACGACTG −0.531158650975
CACGAGAA −0.875601836815
CACGAGAC 0.235678720564
CACGAGAG −0.31687230082
CACGAGCA −0.441937102832
CACGAGCC −1.37786496621
CACGAGCG −0.386282174043
CACGAGGA 0.459293318342
CACGAGGC −0.565019425831
CACGAGGG −1.21083855916
CACGAGTA 0.173721619438
CACGAGTC −0.528714224998
CACGAGTG −0.76789762205
CACGATAA 1.14669683819
CACGATAC 2.84519942055
CACGATAG 1.26866998885
CACGATCA 1.48306584063
CACGATCC 3.54861182794
CACGATCG 3.06489465128
CACGATGA −0.906505235123
CACGATGC 0.668101672828
CACGATGG −0.49791970378
CACGATTA 1.95381061393

CACGATTC 5.2078984648
CACGCAAA 0.277477530415
CACGCAAC 0.909943086475
CACGCAAG -0.0343601938576
CACGCACA -0.399416123529
CACGCACC -1.27196440556
CACGCACG -0.319993721625
CACGCAGA 0.489844895844
CACGCAGC -0.530170221871
CACGCAGG -0.779082192823
CACGCATA -0.0135178297019
CACGCATC -0.285012324641
CACGCATG -0.641601447218
CACGCCAA -0.539432060714
CACGCCAC -0.129777743562
CACGCCAG -0.806322074836
CACGCCCA -0.984050704255
CACGCCCC -1.28620315565
CACGCCCG -0.542860335879
CACGCCGA 0.603525900746
CACGCCGC -1.23351372235
CACGCCGG -0.954451124708
CACGCCTA -0.748073872807
CACGCCTC -1.29051806088
CACGCCTG -0.991436026683
CACGCGAA 0.471444875885
CACGCGAC -1.13315361077
CACGCGAG 0.0656947287864
CACGCGCA -0.232221716837
CACGCGCC -0.905923169459
CACGCGCG 1.33307338955
CACGCGGA 0.384069450171
CACGCGGC -1.00170607154
CACGCGGG -1.07924508715
CACGCGTA 0.901732130133
CACGCGTC 0.174053246978
CACGCGTG 0.741403849958
CACGCTAA 0.0201166556773
CACGCTAC -0.46743307781
CACGCTAG -0.498497605884
CACGCTCA -0.746164464269
CACGCTCC -0.429627954549
CACGCTCG 0.268715943286
CACGCTGA 0.659129825927
CACGCTGC 0.259386654773
CACGCTGG -0.836998767312
CACGCTTA -0.138783523476
CACGCTTC -0.793308035874
CACGGAAA 0.376360410967
CACGGAAC 0.49885046758
CACGGAAG -0.0761381859093
CACGGACA 0.0660307280647
CACGGACC -1.04349426431
CACGGACG 0.615216760394

CACGGAGA 0.824191657273
CACGGAGC -0.221270721771
CACGGAGG -0.543148870575
CACGGATA 1.51222491554
CACGGATC 1.38710994264
CACGGATG -0.0933644983523
CACGGCAA 0.249552950988
CACGGCAC 0.816495316926
CACGGCAG -0.611733318062
CACGGCCA -1.02026659673
CACGGCCC -1.10060290002
CACGGCCG -0.253655098311
CACGGCGA -0.655285194779
CACGGCGC -0.609208951736
CACGGCGG -0.931946875646
CACGGCTA -0.418174625989
CACGGCTC -0.734415731139
CACGGCTG -0.894766910893
CACGGGAA 0.822009535566
CACGGGAC -0.824254110671
CACGGGAG 0.216893987677
CACGGGCA -1.51718662979
CACGGGCC -1.02254219035
CACGGGCG -1.47078771086
CACGGGGA -0.803712147357
CACGGGGC -1.50250258698
CACGGGGG -1.31800359292
CACGGGTA -1.18778201388
CACGGGTC -1.02834973178
CACGGTAA 0.0565253209715
CACGGTAC 0.163519648785
CACGGTAG -0.566341564255
CACGGTCA -1.26647704188
CACGGTCC -0.967286546953
CACGGTCG -0.12845539696
CACGGTGA -0.564072007791
CACGGTGC -1.33398791547
CACGGTGG -0.891182502235
CACGGTTA 0.329967529199
CACGGTTC 0.305040088315
CACGTAAA 1.34027447446
CACGTAAC 0.75886561171
CACGTAAG 1.10708410543
CACGTACA 0.451525364766
CACGTACC -0.503653133844
CACGTACG -0.439589895978
CACGTAGA 0.605658059735
CACGTAGC -0.524414100405
CACGTAGG -0.155891798998
CACGTATA 2.33048724726
CACGTATC 2.07616035857
CACGTATG -0.639383727074
CACGTCAA 0.245464959768
CACGTCAC 1.80868453158

CACGTCAG -0.479529468186
CACGTCCA 0.537569075868
CACGTCCC -1.06415218277
CACGTCCG -0.551454339725
CACGTCGA 0.0715903295064
CACGTCGC -0.564414252409
CACGTCGG -0.716440394282
CACGTCTA -0.321780305148
CACGTCTC -0.563878818615
CACGTCTG -0.428202143485
CACGTGAA 0.955915032344
CACGTGAC -0.182499652629
CACGTGAG 0.496982902818
CACGTGCA -0.50522800035
CACGTGCC -0.640422951608
CACGTGCG -0.366705948432
CACGTGGA 0.298807031071
CACGTGGC 0.235999106493
CACGTGGG -0.168397467305
CACGTGTA -0.218983262
CACGTGTC -0.684054560496
CACGTTAA -0.0958114224647
CACGTTAC -0.354312279884
CACGTTAG -0.164575943914
CACGTTCA -0.909377050516
CACGTTCC 0.207556372045
CACGTTCG -0.422699582813
CACGTTGA -0.269032165655
CACGTTGC 0.109677742124
CACGTTGG -1.1818889113
CACGTTTA -0.211137866211
CACGTTTC -0.963415685379
CACTAAAA 0.198022236389
CACTAAAC 0.787891244534
CACTAAAG -0.156365820284
CACTAACA 0.0665942658877
CACTAACC -0.480996498492
CACTAACG -0.502760050261
CACTAAGA 1.50263040827
CACTAAGC -0.102767065518
CACTAAGG -0.413203335551
CACTAATA 1.01215056955
CACTAATC 0.807767870987
CACTAATG 0.175465110117
CACTACAA 0.929420635718
CACTACAC 1.57920764156
CACTACAG -0.558208466482
CACTACCA 0.0412431827915
CACTACCC -1.02563758891
CACTACCG 0.198697149437
CACTACGA 0.429784296221
CACTACGC -0.804744085661
CACTACGG -0.620561938504
CACTACTA 0.30926131345

CACTACTC 0.477243882017
CACTACTG 0.607626174467
CACTAGAA 0.401337190034
CACTAGAC 0.507186122539
CACTAGAG 0.286791413756
CACTAGCA -0.910409821532
CACTAGCC -0.866628116312
CACTAGCG -0.952838577985
CACTAGGA 1.0858489094
CACTAGGC -1.06903333213
CACTAGGG -1.23707356601
CACTAGTA 0.932160466265
CACTAGTC -1.03140307839
CACTAGTG 1.25606023154
CACTATAA 0.958967338056
CACTATAC 1.24233672199
CACTATAG 0.612335993347
CACTATCA 0.949508771011
CACTATCC 0.639509258403
CACTATCG 1.50501696076
CACTATGA 0.844204223955
CACTATGC 0.816663108387
CACTATGG -0.28118247413
CACTATTA 0.641424287747
CACTATTC -0.150319498698
CACTCAAA -0.145459791664
CACTCAAC 0.465045484426
CACTCAAG -0.131189190345
CACTCACA -0.540050141171
CACTCACC -0.516649894033
CACTCACG -0.801694694441
CACTCAGA 1.41415934179
CACTCAGC -1.02995353503
CACTCAGG -1.41058617474
CACTCATA 0.207564699165
CACTCATC 0.290860877135
CACTCATG -0.156723053718
CACTCCAA -0.363101554687
CACTCCAC -0.358441282168
CACTCCAG -0.852090838986
CACTCCCA -0.327537259326
CACTCCCC -1.09160898625
CACTCCCG -0.855859276988
CACTCCGA 0.575962510133
CACTCCGC -1.28024135433
CACTCCGG -1.28730441723
CACTCCTA -1.27247569071
CACTCCTC -0.213389519367
CACTCCTG 0.524334992769
CACTCGAA -0.367954600025
CACTCGAC -0.590580976877
CACTCGAG -0.918940122913
CACTCGCA 1.18467766367
CACTCGCC -0.756246940754

CACTCGCG 0.0400382485765
CACTCGGA 0.430116756473
CACTCGGC -0.644355850224
CACTCGGG -0.892883316425
CACTCGTA 0.363458371764
CACTCGTC -0.839882032495
CACTCTAA 0.217325332475
CACTCTAC 0.492804770528
CACTCTAG 0.174836412583
CACTCTCA -0.340443878445
CACTCTCC -0.988352494272
CACTCTCG -1.12890511432
CACTCTGA 0.757257020369
CACTCTGC -0.219048421711
CACTCTGG -0.47922844281
CACTCTTA -0.0703633284244
CACTCTTC -0.438522775593
CACTGAAA 1.31799942936
CACTGAAC 0.261498828676
CACTGAAG 0.330585401478
CACTGACA -0.119027848436
CACTGACC -0.574770274776
CACTGACG -0.177486310237
CACTGAGA 1.30230010249
CACTGAGC -0.372815972483
CACTGAGG -0.769336756506
CACTGATA 3.90799365828
CACTGATC 1.48306584063
CACTGATG 0.546045459149
CACTGCAA -0.572798204662
CACTGCAC -0.0459935963802
CACTGCAG -0.610790479938
CACTGCCA -0.741634094817
CACTGCCC -1.50612738217
CACTGCCG -0.448846322193
CACTGCGA 0.656942083414
CACTGCGC 0.348639013259
CACTGCGG -1.11071077422
CACTGCTA -0.251471519358
CACTGCTC -0.774818499378
CACTGCTG 0.0862352348544
CACTGGAA 0.14084573466
CACTGGAC 0.304692222892
CACTGGAG 0.414290024667
CACTGGCA -0.97665372386
CACTGGCC -1.36311784547
CACTGGCG -1.52440915703
CACTGGGA -0.459080144078
CACTGGGC -1.29529886846
CACTGGGG -0.705713815091
CACTGGTA -0.376691830329
CACTGGTC -0.802955836713
CACTGTAA 0.304303762759
CACTGTAC -0.370593256068

CACTGTAG -0.317378173339
CACTGTCA -0.2061597059
CACTGTCC 0.0345912714282
CACTGTCG 0.0478497113526
CACTGTGA -0.406096971633
CACTGTGC -1.16752525442
CACTGTGG -1.16307045358
CACTGTTA 1.06077303761
CACTGTTC -0.116651496663
CACTTAAA -0.489205373054
CACTTAAC 1.12632828714
CACTTAAG 0.508184960542
CACTTACA 1.26906427796
CACTTACC -0.621793935858
CACTTACG 0.78446817382
CACTTAGA 0.678865099524
CACTTAGC -0.621733980597
CACTTAGG -1.03222225878
CACTTATA 1.11067288582
CACTTATC -0.239201924893
CACTTATG 0.509592660121
CACTTCAA 0.209291535604
CACTTCAC 2.02088618733
CACTTCAG -0.308620541592
CACTTCCA 0.489718115447
CACTTCCC -0.397883308977
CACTTCCG 0.0234071170114
CACTTCGA 1.18291189795
CACTTCGC -0.47205796007
CACTTCGG 0.325533754335
CACTTCTA -0.536454699079
CACTTCTC -0.534598167751
CACTTCTG 0.571458162931
CACTTGAA 0.114540571828
CACTTGAC -0.165387213547
CACTTGAG 0.0538652225958
CACTTGCA -0.449449205656
CACTTGCC -1.12791626886
CACTTGCG -0.0280673895297
CACTTGGA 0.778877762035
CACTTGGC -0.626330758828
CACTTGGG -0.793730220841
CACTTGTA 0.406015782216
CACTTGTC -0.569735281873
CACTTTAA -0.163168244335
CACTTTAC 0.184369090993
CACTTTAG 0.360436876395
CACTTTCA 0.612687814153
CACTTTCC 0.123257192512
CACTTTCG -0.336162906227
CACTTTGA 0.0216700798501
CACTTTGC -0.607539156066
CACTTTGG -0.302219484708
CACTTTTA 0.539283421628

CACTTTTC 0.0476467378109
CAGAAAAA 0.810738779105
CAGAAAAC 0.461401536862
CAGAAAAG 0.440003545647
CAGAAACA 0.930159251232
CAGAAACC -0.622370588895
CAGAAACG -0.521697793972
CAGAAAGA 1.26900640448
CAGAAAGC -0.962882957899
CAGAAAGG -0.959318950684
CAGAAATA 1.76690695589
CAGAAATC 9.23466786796
CAGAAATG 1.23694449565
CAGAACAA -0.0142712258532
CAGAACAC -1.03300209354
CAGAACAG -0.0709966058746
CAGAACCA -0.826856543743
CAGAACCC -0.591261302553
CAGAACCG -0.821735781507
CAGAACGA -0.646897495323
CAGAACGC -0.73763291382
CAGAACGG 0.6399283207
CAGAACTA 1.42491319229
CAGAACTC -0.219556167833
CAGAACTG 1.56656457578
CAGAAGAA -0.754543628428
CAGAAGAC -0.627342920222
CAGAAGAG -0.669192109147
CAGAAGCA -0.344639914042
CAGAAGCC -0.815746500691
CAGAAGCG -0.0697754337763
CAGAAGGA -0.678349234461
CAGAAGGC -1.04359543882
CAGAAGGG -0.99469297136
CAGAAGTA -0.0634060199475
CAGAAGTC -0.411797301397
CAGAATAA 1.64000997937
CAGAATAC 0.965417524945
CAGAATAG 0.306112621328
CAGAATCA 6.04622041142
CAGAATCC 12.5880522491
CAGAATCG 4.4606256609
CAGAATGA 0.154422270555
CAGAATGC -0.911204645104
CAGAATGG 0.0998384175328
CAGAATTA 1.44255211352
CAGAATTC 1.7930353756
CAGACAAA -0.00224124425679
CAGACAAC 0.150002235439
CAGACAAG 0.776332369733
CAGACACA -0.439340498744
CAGACACC -0.457717619124
CAGACACG -0.793308035874
CAGACAGA 0.362570700808

CAGACAGC -0.681404038308
CAGACAGG -0.649885057679
CAGACATA -0.556154998774
CAGACATC 0.462098516778
CAGACATG -0.769336756506
CAGACCAA 0.174644264296
CAGACCAC -0.853991920404
CAGACCAG -0.177107842648
CAGACCCA -0.433340392672
CAGACCCC -1.54219338649
CAGACCCG -1.04095324375
CAGACCGA -1.37141332207
CAGACCGC -1.62243267883
CAGACCGG -1.0786392892
CAGACCTA -0.179163392136
CAGACCTC -0.543111190358
CAGACCTG 0.261941206908
CAGACGAA -0.82319490105
CAGACGAC -1.24783220461
CAGACGAG -0.526856444602
CAGACGCA 0.76711820365
CAGACGCC -0.887146763691
CAGACGCG 0.159431033031
CAGACGGA 0.185964150764
CAGACGGC -1.92671645651
CAGACGGG -1.01937517857
CAGACGTA 0.0395296697432
CAGACGTC 0.604091312171
CAGACTAA -0.701227163017
CAGACTAC -0.167121128039
CAGACTAG -0.0216650835783
CAGACTCA -0.687481170237
CAGACTCC -1.12757860416
CAGACTCG -1.3777808623
CAGACTGA 0.335889776702
CAGACTGC -1.00257438194
CAGACTGG 0.0714223298673
CAGACTTA -0.204809255269
CAGACTTC -1.05345225036
CAGAGAAA 1.27386111524
CAGAGAAC -0.221542602228
CAGAGAAG 1.12881809592
CAGAGACA -0.125669142722
CAGAGACC -0.757418150135
CAGAGACG -0.72279544383
CAGAGAGA -0.545254590959
CAGAGAGC -1.16792807883
CAGAGAGG -0.509089077559
CAGAGATA 0.647817225689
CAGAGATC 4.52561216816
CAGAGATG 1.50446091735
CAGAGCAA -0.830047287819
CAGAGCAC -1.08541069473
CAGAGCAG -0.999512500042

CAGAGCCA −0.67138401522
CAGAGCCC −1.48699082848
CAGAGCCG −1.60874830674
CAGAGCGA 0.626873478852
CAGAGCGC −1.39587673421
CAGAGCGG −1.07079201981
CAGAGCTA 0.0784870581877
CAGAGCTC 0.052630727106
CAGAGCTG −0.300790342796
CAGAGGAA 0.0231860319844
CAGAGGAC −0.448560285633
CAGAGGAG −0.607081164485
CAGAGGCA −0.407693280472
CAGAGGCC −1.43641939806
CAGAGGCG −1.19987757155
CAGAGGGA −0.666806181186
CAGAGGGC −1.37582836092
CAGAGGGG −1.4438386535
CAGAGGTA −0.444240592308
CAGAGGTC −0.612072648188
CAGAGTAA −0.00368017053422
CAGAGTAC −0.898620701872
CAGAGTAG −0.406305774158
CAGAGTCA −0.308765433474
CAGAGTCC −0.93347323668
CAGAGTCG −0.777293735698
CAGAGTGA 0.848387352516
CAGAGTGC −1.014772155
CAGAGTGG −0.212218101809
CAGAGTTA 0.111564042906
CAGAGTTC −0.384562415655
CAGATAAA 0.908497082146
CAGATAAC 2.17658729529
CAGATAAG 1.45539232386
CAGATACA 2.40611123353
CAGATACC 4.41219971287
CAGATACG 4.4226052816
CAGATAGA 1.19974975026
CAGATAGC 0.902873361883
CAGATAGG −0.716115844794
CAGATATA 2.91882822895
CAGATATC 17.0878318184
CAGATATG 7.82885646364
CAGATCAA −0.251302478828
CAGATCAC 1.91802294358
CAGATCAG 0.969445769082
CAGATCCA 2.5563940929
CAGATCCC 3.45248001955
CAGATCCG 3.10139033498
CAGATCGA 1.83368192027
CAGATCGC 8.60412421112
CAGATCGG 1.56807365804
CAGATCTA 2.56097858863
CAGATCTC 12.6941185194

CAGATCTG 7.29474272607
CAGATGAA -0.21469208906
CAGATGAC 0.471633901501
CAGATGAG -0.228062736922
CAGATGCA -0.128395441699
CAGATGCC 0.0623582601161
CAGATGCG 1.23950570948
CAGATGGA -0.0904333522315
CAGATGGC -0.978130538532
CAGATGGG -0.523884703773
CAGATGTA -0.268618307808
CAGATGTC 0.0312200370334
CAGATTAA -0.330421565399
CAGATTAC 18.938425286
CAGATTAG 1.5893823409
CAGATTCA 3.78775858567
CAGATTCC 12.2232661181
CAGATTCG 7.90819996608
CAGATTGA 0.578698593476
CAGATTGC 19.944230174
CAGATTGG 1.02781096714
CAGATTTA 2.82159994707
CAGATTTC 22.0974717648
CAGCAAAA 1.10829195413
CAGCAAAC 0.256287092658
CAGCAAAG 0.279931740757
CAGCAACA -0.572394131181
CAGCAACC -0.767040553259
CAGCAACG -0.618755578072
CAGCAAGA 0.147523043738
CAGCAAGC -0.657903241201
CAGCAAGG -0.235413918159
CAGCAATA 0.941571152372
CAGCAATC 4.62457020097
CAGCAATG -0.253233121522
CAGCACAA -0.181375075118
CAGCACAC -1.41637373108
CAGCACAG -0.310388805452
CAGCACCA -0.7848599648
CAGCACCC -1.01618963894
CAGCACCG -0.871640625992
CAGCACGA 0.159494110963
CAGCACGC -0.254054175521
CAGCACGG -0.904276481546
CAGCACTA -0.0251528976483
CAGCACTC 0.534711000222
CAGCACTG -0.274285953627
CAGCAGAA -0.82647432895
CAGCAGAC -0.182454269826
CAGCAGAG -0.442473369338
CAGCAGCA -0.149327114213
CAGCAGCC -0.478436533731
CAGCAGCG -0.659457289907
CAGCAGGA -1.01689619505

CAGCAGGC −1.55373185985
CAGCAGGG −0.449728164165
CAGCAGTA −0.0412379783417
CAGCAGTC −1.03260634718
CAGCATAA 0.0522784899444
CAGCATAC 0.608062099181
CAGCATAG −0.296212925119
CAGCATCA 1.97936467056
CAGCATCC 0.428412819612
CAGCATCG 0.575734971589
CAGCATGA −0.565894606107
CAGCATGC −0.0780326056322
CAGCATGG 0.362462448253
CAGCATTA 0.600597460939
CAGCATTC −0.622964104349
CAGCCAAA 0.240434338603
CAGCCAAC −0.755876592107
CAGCCAAG 0.113789465635
CAGCCACA −0.170716778308
CAGCCACC −0.0591546090045
CAGCCACG −1.20420392657
CAGCCAGA −0.359080804958
CAGCCAGC −1.62691412646
CAGCCAGG −0.73634137756
CAGCCATA −0.075040047004
CAGCCATC −0.851338067368
CAGCCATG −1.21169750155
CAGCCCAA −0.957533199872
CAGCCCAC −1.51980967249
CAGCCCAG −0.86966314325
CAGCCCCA −1.11230083772
CAGCCCCC −1.17975841773
CAGCCCCG −1.35050517367
CAGCCCGA −0.115649119633
CAGCCCGC −0.737051680867
CAGCCCGG −0.508350670223
CAGCCCTA −1.16785313475
CAGCCCTC −0.63910435221
CAGCCCTG −1.01643362355
CAGCCGAA −0.404520439703
CAGCCGAC −0.912920864466
CAGCCGAG −0.334830567081
CAGCCGCA 0.185015691834
CAGCCGCC −0.0533959894029
CAGCCGCG −0.242367479432
CAGCCGGA −0.635755601038
CAGCCGGC −1.302079642
CAGCCGGG −0.975313057595
CAGCCGTA −0.0917677731572
CAGCCGTC −0.93085498208
CAGCCTAA −0.614481892084
CAGCCTAC −1.06418486671
CAGCCTAG −0.747847375152
CAGCCTCA −1.25661898127

CAGCCTCC −1.16861402531
CAGCCTCG −0.863418636217
CAGCCTGA −0.117337234967
CAGCCTGC −1.60444984757
CAGCCTGG −0.370721285532
CAGCCTTA −0.534438495231
CAGCCTTC −1.12482440933
CAGCGAAA 0.352261934846
CAGCGAAC −0.807167693838
CAGCGAAG 0.613736614874
CAGCGACA −1.26573655277
CAGCGACC 0.0224859293989
CAGCGACG −0.760163185131
CAGCGAGA 0.207524312634
CAGCGAGC −0.645585349442
CAGCGAGG −0.563035281394
CAGCGATA 0.161534047102
CAGCGATC 1.8775581383
CAGCGATG −0.387505427921
CAGCGCAA 1.9887022862
CAGCGCAC −0.684866454663
CAGCGCAG −0.704290293985
CAGCGCCA −1.38230810908
CAGCGCCC −1.71420211742
CAGCGCCG −1.15950269916
CAGCGCGA 0.47752450595
CAGCGCGC 0.171982500497
CAGCGCGG 0.709281985866
CAGCGCTA −0.175888960508
CAGCGCTC 0.295113537145
CAGCGCTG 1.47750790461
CAGCGGAA −0.64185729797
CAGCGGAC 0.634154295927
CAGCGGAG −0.392632851853
CAGCGGCA −1.30349379509
CAGCGGCC −1.24189954821
CAGCGGCG −1.12544498792
CAGCGGGA −0.47654398761
CAGCGGGC −0.746409697943
CAGCGGGG −0.849713029966
CAGCGGTA 0.131179405979
CAGCGGTC −1.1115867872
CAGCGTAA −0.314592751813
CAGCGTAC −0.45811294913
CAGCGTAG −0.654694385639
CAGCGTCA 0.479258628619
CAGCGTCC −0.407600849443
CAGCGTCG 0.23176747246
CAGCGTGA −0.255659852369
CAGCGTGC −0.851024967669
CAGCGTGG 0.158986989376
CAGCGTTA 0.709357346299
CAGCGTTC −0.403444367665
CAGCTAAA −0.606581953661

CAGCTAAC 0.427718337833
CAGCTAAG -0.554564102562
CAGCTACA -0.410279683838
CAGCTACC -0.568295314706
CAGCTACG -0.21623239802
CAGCTAGA -0.76461403059
CAGCTAGC -0.163814636999
CAGCTAGG -0.46976321407
CAGCTATA -0.185556330078
CAGCTATC 0.705257697111
CAGCTATG 0.729822283756
CAGCTCAA -0.298273262701
CAGCTCAC -0.747756193192
CAGCTCAG -0.603936427745
CAGCTCCA 0.0282959689644
CAGCTCCC -0.694535906014
CAGCTCCG -1.60161363061
CAGCTCGA 0.170763826534
CAGCTCGC -0.647449375178
CAGCTCGG -0.569714464074
CAGCTCTA 0.38199558102
CAGCTCTC -0.0603832755105
CAGCTGAA -0.614612211507
CAGCTGAC -0.7693311357
CAGCTGAG -0.747847375152
CAGCTGCA -0.830541086015
CAGCTGCC -0.188475193697
CAGCTGCG -0.544940034014
CAGCTGGA 0.0987225834982
CAGCTGGC -0.836259319086
CAGCTGGG -0.581120744408
CAGCTGTA -0.3197205921
CAGCTGTC -1.40607495766
CAGCTTAA -0.97379918724
CAGCTTAC -1.14231885503
CAGCTTAG -0.822678619631
CAGCTTCA -0.0614928642054
CAGCTTCC -0.878833383777
CAGCTTCG -0.506403789646
CAGCTTGA -1.45638637377
CAGCTTGC -1.31031099978
CAGCTTGG 0.154385631229
CAGCTTTA 0.483912863976
CAGCTTTC 1.00282253011
CAGGAAAA 0.964986596503
CAGGAAAC 0.942068281416
CAGGAAAG -0.460542594469
CAGGAACA -0.738809119472
CAGGAACC -0.754543628428
CAGGAACG -1.13575583567
CAGGAAGA 0.093198580493
CAGGAAGC -0.155429852034
CAGGAAGG -0.956482941905
CAGGAATA 0.621343438685

| | |
|---|---|
| CAGGAATC | 5.97261221264 |
| CAGGAATG | 0.772784808579 |
| CAGGACAA | 0.164888210901 |
| CAGGACAC | −0.748444429632 |
| CAGGACAG | −0.92481532219 |
| CAGGACCA | −0.483974692839 |
| CAGGACCC | −1.35482278522 |
| CAGGACCG | −1.24069378128 |
| CAGGACGA | 0.315905314049 |
| CAGGACGC | −1.04205367261 |
| CAGGACGG | −0.815753370564 |
| CAGGACTA | −0.429995596882 |
| CAGGACTC | −1.22370499992 |
| CAGGACTG | 1.00966534069 |
| CAGGAGAA | −0.914246333738 |
| CAGGAGAC | −0.823123704177 |
| CAGGAGAG | −0.0469006278892 |
| CAGGAGCA | −0.240123737039 |
| CAGGAGCC | −1.45868320155 |
| CAGGAGCG | −0.286432098543 |
| CAGGAGGA | −0.277464207024 |
| CAGGAGGC | −0.916868335541 |
| CAGGAGGG | −1.18835491972 |
| CAGGAGTA | −0.337447364435 |
| CAGGAGTC | −1.02767398602 |
| CAGGATAA | 1.70480371381 |
| CAGGATAC | 3.23039886086 |
| CAGGATAG | 0.60283308439 |
| CAGGATCA | 3.06879923769 |
| CAGGATCC | 3.18066493051 |
| CAGGATCG | 2.78747187168 |
| CAGGATGA | 0.831250140254 |
| CAGGATGC | −0.400177430444 |
| CAGGATGG | −0.55271631471 |
| CAGGATTA | 3.0874844615 |
| CAGGATTC | 5.8501063345 |
| CAGGCAAA | 0.209200561822 |
| CAGGCAAC | −0.435135303315 |
| CAGGCAAG | −0.433368288523 |
| CAGGCACA | −1.04802671554 |
| CAGGCACC | −0.874977927374 |
| CAGGCACG | −0.963415685379 |
| CAGGCAGA | −0.547349486088 |
| CAGGCAGC | −0.724104675218 |
| CAGGCAGG | −1.49118519865 |
| CAGGCATA | 1.04838852889 |
| CAGGCATC | −0.720936830721 |
| CAGGCATG | −0.59836371109 |
| CAGGCCAA | −0.126765199847 |
| CAGGCCAC | −1.38629534215 |
| CAGGCCAG | −1.0510313485 |
| CAGGCCCA | −1.20842036361 |
| CAGGCCCC | −1.32133027723 |
| CAGGCCCG | −1.45190575885 |

CAGGCCGA -0.687094791885
CAGGCCGC -1.38308669477
CAGGCCGG -1.1338618323
CAGGCCTA -1.16381822892
CAGGCCTC -1.36926388431
CAGGCCTG 1.22327136517
CAGGCGAA -0.650906378905
CAGGCGAC -1.01281007743
CAGGCGAG -0.679155507822
CAGGCGCA -0.42389494084
CAGGCGCC -0.544085255181
CAGGCGCG -0.758541478577
CAGGCGGA -0.149541329366
CAGGCGGC -0.396558256061
CAGGCGGG -1.26540888061
CAGGCGTA -0.289819987177
CAGGCGTC -1.20523732212
CAGGCTAA -0.255092567342
CAGGCTAC -0.981554650137
CAGGCTAG -1.40788777161
CAGGCTCA -1.25738653353
CAGGCTCC -1.29249887447
CAGGCTCG -0.995194472142
CAGGCTGA -0.403773705247
CAGGCTGC -0.677007319127
CAGGCTGG -0.380317250052
CAGGCTTA -0.459197348288
CAGGCTTC -0.122563543444
CAGGGAAA 0.135092735864
CAGGGAAC -0.60891937615
CAGGGAAG -0.423133425747
CAGGGACA -0.767040553259
CAGGGACC -1.29304679894
CAGGGACG -1.0979261474
CAGGGAGA -0.709028841428
CAGGGAGC -1.58983533621
CAGGGAGG -1.40979322477
CAGGGATA 1.09064241583
CAGGGATC 0.442273726644
CAGGGATG -0.465034867348
CAGGGCAA 0.168262359788
CAGGGCAC -0.597393601649
CAGGGCAG -1.43653847588
CAGGGCCA -0.867818061712
CAGGGCCC -1.16291307102
CAGGGCCG -1.24887226185
CAGGGCGA -0.131928638571
CAGGGCGC -0.115521714703
CAGGGCGG -0.0875604959485
CAGGGCTA -0.644572355335
CAGGGCTC -1.50051407081
CAGGGGAA 0.475478324471
CAGGGGAC -0.936619847021
CAGGGGAG -0.949741930361

CAGGGGCA −1.44405286866
CAGGGGCC −1.44718865374
CAGGGGCG −1.01468492842
CAGGGGGA 0.175919979028
CAGGGGGC −1.2651072307
CAGGGGGG −1.06782298529
CAGGGGTA −0.194081843365
CAGGGGTC −1.30885479473
CAGGGTAA 0.000495463619836
CAGGGTAC −0.444261826463
CAGGGTAG −1.2895785536
CAGGGTCA −0.70175343698
CAGGGTCC −1.3451577056
CAGGGTCG −1.08178943857
CAGGGTGA −0.382630732072
CAGGGTGC −1.27283979402
CAGGGTGG −0.925420287433
CAGGGTTA 0.150167528764
CAGGGTTC −0.768163881701
CAGGTAAA 0.294833537747
CAGGTAAC 0.205713164108
CAGGTAAG 0.0895163281789
CAGGTACA −0.807258459442
CAGGTACC −0.338704551325
CAGGTACG 0.120068322037
CAGGTAGA −0.236469588754
CAGGTAGC −1.24225011994
CAGGTAGG −0.813624958779
CAGGTATA 0.266083532583
CAGGTATC 1.74159625932
CAGGTATG 0.640233717814
CAGGTCAA 0.216605036625
CAGGTCAC −0.891571795079
CAGGTCAG −1.26833065872
CAGGTCCA −0.644598585762
CAGGTCCC −1.81885569189
CAGGTCCG −1.0672912987
CAGGTCGA −0.602518735623
CAGGTCGC −0.192183051905
CAGGTCGG −1.26268507977
CAGGTCTA −0.234853919362
CAGGTCTC −1.09133814668
CAGGTGAA −0.21209257048
CAGGTGAC −0.244103683881
CAGGTGAG −0.414775079387
CAGGTGCA −0.691970320446
CAGGTGCC −1.65424935399
CAGGTGCG −0.709504319961
CAGGTGGA −0.0544133552475
CAGGTGGC −1.79493354253
CAGGTGGG −0.96809844112
CAGGTGTA −0.72077944816
CAGGTGTC −1.03395846323
CAGGTTAA 0.497116136732

CAGGTTAC 0.895914804338
CAGGTTAG -1.07399234007
CAGGTTCA 0.694540902286
CAGGTTCC -0.593749862264
CAGGTTCG 0.300588201967
CAGGTTGA -0.250366926935
CAGGTTGC -0.314534461975
CAGGTTGG 0.58657355054
CAGGTTTA -0.288263232156
CAGGTTTC -0.485615759946
CAGTAAAA 1.61413491227
CAGTAAAC -0.413475216008
CAGTAAAG 0.597806834962
CAGTAACA 0.145982734779
CAGTAACC -0.570283622703
CAGTAACG -0.700505618099
CAGTAAGA 0.930200886831
CAGTAAGC -0.306134480017
CAGTAAGG -0.476992819359
CAGTAATA 0.732373088686
CAGTAATC 4.5941222961
CAGTAATG 0.45270968936
CAGTACAA 0.217629688699
CAGTACAC -0.561733960769
CAGTACAG -0.530705239309
CAGTACCA 0.0937796052674
CAGTACCC -0.794511304665
CAGTACCG -0.601521146687
CAGTACGA 0.595020788902
CAGTACGC -0.64121985696
CAGTACGG -0.768713055243
CAGTACTA 0.613218251675
CAGTACTC -0.54371115933
CAGTACTG 0.871379570791
CAGTAGAA -0.0241465652373
CAGTAGAC 0.0893568638374
CAGTAGAG -0.18324159899
CAGTAGCA -0.341720009533
CAGTAGCC -0.695592409321
CAGTAGCG 0.188985646133
CAGTAGGA 0.63707690675
CAGTAGGC -0.98367806565
CAGTAGGG -0.767391957709
CAGTAGTA 0.708573139805
CAGTAGTC 0.384601136762
CAGTATAA 1.25473101507
CAGTATAC 0.240672077869
CAGTATAG -0.0774505399679
CAGTATCA 1.51616739035
CAGTATCC 2.71977821782
CAGTATCG 1.45511024268
CAGTATGA 1.19042420895
CAGTATGC 0.224470001145
CAGTATGG -0.11925268066

CAGTATTA 1.30468020147
CAGTATTC 1.48727145241
CAGTCAAA 1.25409940304
CAGTCAAC 0.348639013259
CAGTCAAG 0.281074637931
CAGTCACA -0.622370588895
CAGTCACC -0.771371071839
CAGTCACG -0.54425845927
CAGTCAGA 0.326891491196
CAGTCAGC -0.69029490397
CAGTCAGG 0.193516848296
CAGTCATA -0.0150304509883
CAGTCATC -0.788666499375
CAGTCATG 0.0643836237957
CAGTCCAA -0.507698656754
CAGTCCAC -1.26256246293
CAGTCCAG -0.645138183116
CAGTCCCA -0.530032824397
CAGTCCCC -1.22898585103
CAGTCCCG -1.21972630215
CAGTCCGA 0.206767377457
CAGTCCGC -1.11083068474
CAGTCCGG -1.11040017265
CAGTCCTA -0.796867879529
CAGTCCTC -0.768554007257
CAGTCGAA 0.440007709207
CAGTCGAC -0.231522655141
CAGTCGAG -1.3668538077
CAGTCGCA 0.00963281202396
CAGTCGCC -0.510840062646
CAGTCGCG -0.446619442217
CAGTCGGA 0.28320367425
CAGTCGGC -0.89738016922
CAGTCGGG -0.98025041502
CAGTCGTA -0.295108749052
CAGTCGTC -0.39629511908
CAGTCTAA -0.667984052262
CAGTCTAC 0.0274099634325
CAGTCTAG -0.402151165981
CAGTCTCA -0.250680651168
CAGTCTCC -0.668597760981
CAGTCTCG 0.636456328158
CAGTCTGA 1.5010580399
CAGTCTGC -0.718713281594
CAGTCTGG -0.385581446924
CAGTCTTA -0.287946801609
CAGTCTTC -0.746048925483
CAGTGAAA -0.0654649002837
CAGTGAAC 0.601139764607
CAGTGAAG -0.674893063445
CAGTGACA -0.183311963152
CAGTGACC 0.0298198318624
CAGTGACG -1.42686590186
CAGTGAGA 0.62062668186

CAGTGAGC −1.28580949107
CAGTGAGG −1.01262812986
CAGTGATA 0.130153088481
CAGTGATC 0.849829193286
CAGTGATG 0.273144305522
CAGTGCAA 0.56704791218
CAGTGCAC −0.0296520404012
CAGTGCAG −0.484575078167
CAGTGCCA −1.11521782773
CAGTGCCC −0.836998767312
CAGTGCCG −0.963479387844
CAGTGCGA 2.30957618437
CAGTGCGC 1.09153612395
CAGTGCGG 0.109469564133
CAGTGCTA −0.607364702909
CAGTGCTC −0.523618235944
CAGTGGAA −0.650965501455
CAGTGGAC 0.512736564149
CAGTGGAG −0.587218485957
CAGTGGCA −0.773237595711
CAGTGGCC −1.09941961631
CAGTGGCG −0.683992107099
CAGTGGGA 0.242435553635
CAGTGGGC −1.79478323802
CAGTGGGG −0.78062583263
CAGTGGTA −0.290804669077
CAGTGGTC −0.855679203026
CAGTGTAA 0.15597236388
CAGTGTAC −0.355382314761
CAGTGTAG 0.079217138404
CAGTGTCA −0.422718735188
CAGTGTCC −0.803113219275
CAGTGTCG −1.1602117534
CAGTGTGA −0.237132219301
CAGTGTGC −1.23171048459
CAGTGTGG −0.0278463045027
CAGTGTTA 0.111273634607
CAGTGTTC 0.485560176423
CAGTTAAA 0.146035403811
CAGTTAAC 0.307953539307
CAGTTAAG −0.0608966424376
CAGTTACA 1.09853111264
CAGTTACC 0.29322557094
CAGTTACG 0.505817976778
CAGTTAGA 0.873035210357
CAGTTAGC 0.164688984563
CAGTTAGG −0.809195972009
CAGTTATA 0.393102085046
CAGTTATC −0.368982166591
CAGTTATG 1.71423542589
CAGTTCAA 0.00697687720806
CAGTTCAC −0.120630402615
CAGTTCAG −0.546932713749
CAGTTCCA −0.760753994271

CAGTTCCC -0.894777319792
CAGTTCCG -0.872267033569
CAGTTCGA 0.524772582907
CAGTTCGC -0.193304298567
CAGTTCGG -0.819401065332
CAGTTCTA -0.159258661654
CAGTTCTC -0.79280674327
CAGTTGAA 0.446268454124
CAGTTGAC 0.705957383341
CAGTTGAG -0.613637938506
CAGTTGCA 0.106218656617
CAGTTGCC -1.37443315202
CAGTTGCG -0.48214522465
CAGTTGGA -0.433314578601
CAGTTGGC -1.25355418488
CAGTTGGG -0.207914854546
CAGTTGTA -0.490654083697
CAGTTGTC -0.3998316468
CAGTTTAA -0.0896572646791
CAGTTTAC -0.163945580956
CAGTTTAG 0.333261321381
CAGTTTCA 0.221697694831
CAGTTTCC -0.447997788699
CAGTTTCG 0.282396359999
CAGTTTGA -0.248355719358
CAGTTTGC -1.14697080042
CAGTTTGG 0.0510635631858
CAGTTTTA -0.277691953747
CAGTTTTC -0.194424920695
CATAAAAA 1.39225256356
CATAAAAC 2.41037971507
CATAAAAG 0.595574334181
CATAAACA 0.806105777903
CATAAACC -0.272117571667
CATAAACG -0.532184343939
CATAAAGA 1.72879914183
CATAAAGC -0.958258491995
CATAAAGG 0.345554648137
CATAAATA 1.02287215247
CATAAATC 1.49589002126
CATAAATG -0.00938820288392
CATAACAA 0.054744982788
CATAACAC 1.18319918358
CATAACAG 0.174927386365
CATAACCA 0.166024030023
CATAACCC -0.309416614231
CATAACCG -0.373579153
CATAACGA 0.194887075836
CATAACGC 0.109469564133
CATAACGG -0.808548330278
CATAACTA 1.54346639491
CATAACTC 0.683687334519
CATAAGAA 0.798619072794
CATAAGAC 0.644051494

CATAAGAG 0.971144917848
CATAAGCA 0.0719384031083
CATAAGCC -0.993106446887
CATAAGCG -0.683930070057
CATAAGGA 1.19317569747
CATAAGGC -0.836257653662
CATAAGGG -1.00791352289
CATAAGTA 0.897738235366
CATAAGTC -0.116983124203
CATAATAA 0.392539588112
CATAATAC 2.77484775009
CATAATAG 0.485644280331
CATAATCA 2.52535371348
CATAATCC 4.81896347861
CATAATCG 1.90860746938
CATAATGA 0.538284167269
CATAATGC -0.192733266336
CATAATGG -0.367815537127
CATAATTA 1.74769358451
CATAATTC 0.381106036462
CATACAAA 1.76936637068
CATACAAC 1.00141524688
CATACAAG 2.15671066883
CATACACA 1.02683086516
CATACACC 0.240593802944
CATACACG -1.01950882884
CATACAGA 1.25007511429
CATACAGC -0.542145869012
CATACAGG -0.497035780028
CATACATA 0.236071344256
CATACATC 0.394935716795
CATACATG 0.78766682866
CATACCAA -1.22958248916
CATACCAC 0.218228200425
CATACCAG -0.850848849088
CATACCCA 0.608770737064
CATACCCC -0.263917648759
CATACCCG -0.762577008943
CATACCGA 1.96933923485
CATACCGC -0.93876970114
CATACCGG -0.171467468146
CATACCTA -0.358924671464
CATACCTC 0.279315117546
CATACGAA 0.0748770436367
CATACGAC 0.198951126587
CATACGAG 0.492859729518
CATACGCA 0.0267612808109
CATACGCC -0.757497257771
CATACGCG -0.386130620465
CATACGGA 0.175660589251
CATACGGC -1.10359733225
CATACGGG -1.07108867344
CATACGTA 0.219863854904
CATACGTC -0.361209841277

CATACTAA 0.735427684356
CATACTAC 0.317084850549
CATACTAG 1.1962798395
CATACTCA 0.800323217832
CATACTCC -0.0394747107535
CATACTCG 0.170138043492
CATACTGA 1.33904955516
CATACTGC 0.0591737613797
CATACTGG -0.356674267376
CATACTTA 1.01106096594
CATACTTC 0.153616621728
CATAGAAA 0.653018969163
CATAGAAC 0.906074306681
CATAGAAG 0.361229201831
CATAGACA 2.1746329203
CATAGACC -0.517804657352
CATAGACG 0.245746000057
CATAGAGA 0.703014995609
CATAGAGC -0.983567523137
CATAGAGG -0.436694972827
CATAGATA 3.60968562985
CATAGATC 2.17506364057
CATAGATG 0.937747339024
CATAGCAA 0.924958965004
CATAGCAC 0.543977627159
CATAGCAG 0.5849545503
CATAGCCA -0.919025059534
CATAGCCC 0.168185542109
CATAGCCG -0.991650033658
CATAGCGA 1.35592092412
CATAGCGC 0.318381591259
CATAGCGG -0.337001863533
CATAGCTA -0.690399409322
CATAGCTC 0.286045720191
CATAGGAA 1.66120499704
CATAGGAC -0.231041763981
CATAGGAG 1.0355601847
CATAGGCA -0.872498527495
CATAGGCC -0.660098269943
CATAGGCG -1.23036398934
CATAGGGA 0.139826078857
CATAGGGC -1.15539305743
CATAGGGG -0.819260753366
CATAGGTA -0.0816734305261
CATAGGTC -0.626058670193
CATAGTAA 0.708921005228
CATAGTAC -0.363028276033
CATAGTAG 0.841883872061
CATAGTCA 0.354564799788
CATAGTCC -0.354353290949
CATAGTCG -0.339501664855
CATAGTGA 0.555212577006
CATAGTGC 0.186696520938
CATAGTGG -0.255415867763

CATAGTTA -0.511001192411
CATAGTTC 0.164939422687
CATATAAA 1.45335884124
CATATAAC 2.11830474389
CATATAAG 1.02368363028
CATATACA 1.80571757885
CATATACC 0.326980383199
CATATACG 0.799269004483
CATATAGA 1.18397152393
CATATAGC -0.0851462557807
CATATAGG -0.0274062162286
CATATATA 1.2223516348
CATATATC 2.12491522783
CATATATG 5.25028933286
CATATCAA 2.42136401862
CATATCAC 3.08209265152
CATATCAG 2.56026224816
CATATCCA 5.86219502229
CATATCCC 2.90492755992
CATATCCG 3.36579761837
CATATCGA 2.12649342519
CATATCGC 4.2617465639
CATATCGG 1.97272025361
CATATCTA 5.36394327462
CATATCTC 5.20438150581
CATATGAA 0.807015723904
CATATGAC 0.832857690704
CATATGAG 0.557816259146
CATATGCA -0.0875846445955
CATATGCC 0.730446401374
CATATGCG 0.56798388043
CATATGGA 1.33098390705
CATATGGC -0.958792052187
CATATGGG -0.969268817789
CATATGTA 0.416140727012
CATATGTC -0.758982607741
CATATTAA 1.76759498415
CATATTAC 2.35670372627
CATATTAG 1.29837032654
CATATTCA 0.233547186109
CATATTCC 4.33253873899
CATATTCG 0.83409884789
CATATTGA 0.422095033926
CATATTGC 1.56807365804
CATATTGG -0.266828809793
CATATTTA 1.1434967261
CATATTTC 5.81926809591
CATCAAAA 1.7746934373
CATCAAAC 0.911997803252
CATCAAAG -0.429098974273
CATCAACA 1.59263824469
CATCAACC -0.81708154615
CATCAACG 0.138765620169
CATCAAGA 0.865359271454

CATCAAGC -1.02207961886
CATCAAGG -0.0524627274669
CATCAATA 0.954404076482
CATCAATC -0.492485841845
CATCAATG 0.0307574655362
CATCACAA -0.0681878684129
CATCACAC 1.46003386036
CATCACAG -0.297429309123
CATCACCA 0.218361226161
CATCACCC -0.944618253634
CATCACCG 0.111192028835
CATCACGA 1.90417535994
CATCACGC 0.0588298513377
CATCACGG -0.0835320436344
CATCACTA 0.70832894702
CATCACTC 0.337361595102
CATCAGAA 0.883592332664
CATCAGAC 0.0624933676326
CATCAGAG -0.0962577560785
CATCAGCA 0.156598355101
CATCAGCC -0.458693973904
CATCAGCG -0.436789485636
CATCAGGA 0.644428712521
CATCAGGC -0.640824318776
CATCAGGG -1.11566603495
CATCAGTA 0.688492082564
CATCAGTC -0.402151165981
CATCATAA 0.960894233546
CATCATAC 0.996171659634
CATCATAG 0.931972689717
CATCATCA 0.217066983588
CATCATCC -0.144868566168
CATCATCG 0.33072196624
CATCATGA 0.95552782128
CATCATGC -0.478545827176
CATCATGG -0.678799939812
CATCATTA 0.393219913789
CATCATTC -0.0856510874102
CATCCAAA 1.67250593931
CATCCAAC 1.58158774054
CATCCAAG 0.156344586129
CATCCACA 0.23333463638
CATCCACC 0.233163097715
CATCCACG 0.695971293265
CATCCAGA 0.909960989782
CATCCAGC -0.625297987812
CATCCAGG 0.274812435768
CATCCATA 0.278097068118
CATCCATC -0.520738093431
CATCCATG -0.163122445177
CATCCCAA -1.09921060561
CATCCCAC -0.802290916208
CATCCCAG -1.07802079238
CATCCCCA -0.28733517467

CATCCCCC -1.20250061424
CATCCCCG 0.339763136412
CATCCCGA 0.426911023582
CATCCCGC -0.342218179466
CATCCCGG -0.247949355919
CATCCCTA -0.363590564789
CATCCCTC -0.334027832746
CATCCGAA 0.26191809915
CATCCGAC -0.174752516852
CATCCGAG -0.287313732337
CATCCGCA 0.836598649212
CATCCGCC -0.313372828782
CATCCGCG 0.0427435215764
CATCCGGA 0.824661306822
CATCCGGC -0.643294975179
CATCCGGG -1.10399620128
CATCCGTA 0.720953276783
CATCCGTC -0.390035831408
CATCCTAA -0.0246776272937
CATCCTAC 0.180279642527
CATCCTAG -0.303340315015
CATCCTCA -0.428062456053
CATCCTCC -0.368741721011
CATCCTCG -0.919623571259
CATCCTGA 0.242820266564
CATCCTGC 0.0535429630649
CATCCTGG -0.386279675907
CATCCTTA 0.0794298963114
CATCCTTC 0.0218151799102
CATCGAAA 0.858658021906
CATCGAAC 0.481629359587
CATCGAAG -0.156943930567
CATCGACA -0.240712880755
CATCGACC -0.321432647902
CATCGACG -0.0901958211432
CATCGAGA 1.92280395933
CATCGAGC -1.23475529589
CATCGAGG -0.942964904025
CATCGATA 1.50237330845
CATCGATC 0.494564290912
CATCGATG 1.34462726808
CATCGCAA 0.395187195809
CATCGCAC 0.97787572867
CATCGCAG -0.445134092248
CATCGCCA -0.0757634655246
CATCGCCC -0.636026648783
CATCGCCG -0.593133863587
CATCGCGA 2.26986123621
CATCGCGC -0.0805328233105
CATCGCGG 0.0760678217482
CATCGCTA 0.0839409052098
CATCGCTC -0.0392373878431
CATCGGAA 0.580394827751
CATCGGAC -0.0516144021514

CATCGGAG -0.0096786111821
CATCGGCA -0.872590958523
CATCGGCC -0.711391245276
CATCGGCG -0.5051896956
CATCGGGA -0.155168796833
CATCGGGC -1.29605205643
CATCGGGG -0.23121392718
CATCGGTA 0.462098516778
CATCGGTC -1.07548622534
CATCGTAA 1.21926268976
CATCGTAC -0.0390304589195
CATCGTAG 0.364684123778
CATCGTCA 0.475710026576
CATCGTCC 1.37883819832
CATCGTCG -0.19381079562
CATCGTGA -0.441855288881
CATCGTGC -0.925092823453
CATCGTGG -0.934230171857
CATCGTTA 0.582940428232
CATCGTTC -0.520597365109
CATCTAAA 1.22199231959
CATCTAAC 0.858658021906
CATCTAAG 0.227164865245
CATCTACA 2.21059650105
CATCTACC 0.175262344753
CATCTACG 0.963639684898
CATCTAGA 1.27756064633
CATCTAGC -0.754409145445
CATCTAGG 0.123257192512
CATCTATA 1.81874202671
CATCTATC 0.51554738339
CATCTCAA 0.21121884745
CATCTCAC 0.508285510512
CATCTCAG -0.269087124645
CATCTCCA -0.369986833578
CATCTCCC 0.151344983485
CATCTCCG 0.287350996197
CATCTCGA 1.42372303872
CATCTCGC 0.122294993835
CATCTCGG -0.520011552241
CATCTCTA 0.115246087042
CATCTCTC -0.942952621524
CATCTGAA 0.805315742425
CATCTGAC -0.186489175658
CATCTGAG 0.223329810285
CATCTGCA 0.477178930484
CATCTGCC -0.167990895687
CATCTGCG 0.420791839699
CATCTGGA 1.70266364406
CATCTGGC -0.668536973008
CATCTGGG 0.294722995233
CATCTGTA 0.505079361264
CATCTGTC -0.747635033601
CATCTTAA -0.521105735764

CATCTTAC 0.942068281416
CATCTTAG -0.125846094014
CATCTTCA 1.46590406336
CATCTTCC 1.78208521325
CATCTTCG 0.263711136191
CATCTTGA 0.15386310447
CATCTTGC 0.0685026335361
CATCTTGG -0.817112564671
CATCTTTA 0.777533140388
CATCTTTC 0.193799762187
CATGAAAA 2.30412317006
CATGAAAC 1.73226093365
CATGAAAG 0.373307272543
CATGAACA 0.0301314743157
CATGAACC -0.239988421345
CATGAACG 0.132338332858
CATGAAGA 1.06010166359
CATGAAGC -0.781525786087
CATGAAGG 0.240770962415
CATGAATA 0.801161342426
CATGAATC 1.25646617863
CATGAATG 0.62710996905
CATGACAA -0.16526771938
CATGACAC -0.582143106524
CATGACAG -0.852324206514
CATGACCA 0.187162006927
CATGACCC -0.70175343698
CATGACCG -1.02305181008
CATGACGA -0.0809208670867
CATGACGC -1.22426562325
CATGACGG -0.144224047106
CATGACTA -0.01430057895
CATGACTC -0.506505796862
CATGAGAA 0.932613045219
CATGAGAC 0.0659391297484
CATGAGAG 0.101218637617
CATGAGCA -0.0274824093735
CATGAGCC -0.943650850507
CATGAGCG -0.815753995098
CATGAGGA -0.570424142847
CATGAGGC -0.944323473598
CATGAGGG -0.629685755339
CATGAGTA 1.81164003453
CATGAGTC -0.671685040596
CATGATAA 1.03835476606
CATGATAC 1.3905315561
CATGATAG 0.926454515695
CATGATCA 0.378877074706
CATGATCC 1.42283703319
CATGATCG 1.07528595811
CATGATGA 0.409797127254
CATGATGC -0.14728447176
CATGATGG 0.0558683112303
CATGATTA 3.54044229902

| | |
|---|---|
| CATGATTC | 3.74412739314 |
| CATGCAAA | 0.247551111421 |
| CATGCAAC | 0.490613280811 |
| CATGCAAG | 0.161383742592 |
| CATGCACA | -0.0404050581976 |
| CATGCACC | -0.172460893521 |
| CATGCACG | -1.07277616424 |
| CATGCAGA | 0.869649195325 |
| CATGCAGC | -1.22845853618 |
| CATGCAGG | 0.226740806676 |
| CATGCATA | -0.188251194179 |
| CATGCATC | -0.360916934843 |
| CATGCATG | -0.445819414196 |
| CATGCCAA | 0.0133683579039 |
| CATGCCAC | -0.52464267984 |
| CATGCCAG | -1.08502618998 |
| CATGCCCA | -0.115600405983 |
| CATGCCCC | 0.124441933461 |
| CATGCCCG | -1.22562752368 |
| CATGCCGA | 0.360473099365 |
| CATGCCGC | -0.914071672403 |
| CATGCCGG | 0.482229328558 |
| CATGCCTA | -0.324467466663 |
| CATGCCTC | -0.456349473364 |
| CATGCGAA | 0.680970195374 |
| CATGCGAC | -0.88022817632 |
| CATGCGAG | -0.340640398469 |
| CATGCGCA | 0.373307272543 |
| CATGCGCC | -0.155857033273 |
| CATGCGCG | -0.0433668064831 |
| CATGCGGA | 0.0585100899428 |
| CATGCGGC | -1.03658712673 |
| CATGCGGG | -0.756798196076 |
| CATGCGTA | 0.26781890432 |
| CATGCGTC | -0.311122008338 |
| CATGCTAA | 0.440626414197 |
| CATGCTAC | 0.386402500922 |
| CATGCTAG | -0.904904346368 |
| CATGCTCA | -0.204957061643 |
| CATGCTCC | -1.05109109558 |
| CATGCTCG | -0.41509608985 |
| CATGCTGA | 0.0928861053277 |
| CATGCTGC | -0.0903221851841 |
| CATGCTGG | -0.0996235778455 |
| CATGCTTA | 0.668910444325 |
| CATGCTTC | -0.601029846627 |
| CATGGAAA | 0.734516905643 |
| CATGGAAC | 0.461170459291 |
| CATGGAAG | -0.813533776819 |
| CATGGACA | -0.0740867999808 |
| CATGGACC | -0.571628868884 |
| CATGGACG | -0.179150485101 |
| CATGGAGA | 0.817957142783 |
| CATGGAGC | -1.01361052181 |

CATGGAGG -0.569414895944
CATGGATA 2.94186104193
CATGGATC 1.91127485398
CATGGCAA 0.0564179011279
CATGGCAC -0.276758275454
CATGGCAG -1.61478838299
CATGGCCA -1.06695675667
CATGGCCC -1.12548017
CATGGCCG -1.53570905841
CATGGCGA 0.0394309933752
CATGGCGC -1.34237041048
CATGGCGG -0.710229403905
CATGGCTA -0.159403969893
CATGGCTC -0.5484220191
CATGGGAA -0.289630128849
CATGGGAC -0.761968504673
CATGGGAG -0.94187717402
CATGGGCA -0.930842075045
CATGGGCC -1.16481914871
CATGGGCG -1.05483455222
CATGGGGA 0.521389066011
CATGGGGC -0.350451202675
CATGGGGG -1.35214936345
CATGGGTA -0.0194889990328
CATGGGTC -1.17889760174
CATGGTAA -0.00532144581943
CATGGTAC -0.580157921197
CATGGTAG 1.34776909033
CATGGTCA -0.780632910682
CATGGTCC 0.581149889326
CATGGTCG -1.2585313043
CATGGTGA -0.407727005306
CATGGTGC -0.798883458843
CATGGTGG -1.16349451215
CATGGTTA -0.176733538619
CATGGTTC -0.269480997405
CATGTAAA 0.273814430476
CATGTAAC 0.734133858138
CATGTAAG 0.232516913229
CATGTACA 1.34225653711
CATGTACC -0.613555291844
CATGTACG 0.308866816156
CATGTAGA 0.700209588995
CATGTAGC -0.845812607117
CATGTAGG 0.528804366069
CATGTATA 2.84339118652
CATGTATC 0.841863470618
CATGTCAA -0.343625879045
CATGTCAC 0.161258835797
CATGTCAG -1.06314501764
CATGTCCA 0.107417970026
CATGTCCC -0.630962510961
CATGTCCG -0.376219266289
CATGTCGA 0.250599253573

CATGTCGC -0.415235985461
CATGTCGG -0.254865653331
CATGTCTA 0.240580063197
CATGTCTC -0.0357549864008
CATGTGAA 0.194048118531
CATGTGAC -0.859696413728
CATGTGAG -0.6242585551
CATGTGCA 0.446794519908
CATGTGCC -0.399043693102
CATGTGCG -0.564668854093
CATGTGGA 0.112069290891
CATGTGGC -0.991396264686
CATGTGGG 0.0796280817593
CATGTGTA 0.32560994748
CATGTGTC -1.16768784143
CATGTTAA 0.300862996915
CATGTTAC -0.154652307236
CATGTTAG -0.389214361054
CATGTTCA 0.381212207237
CATGTTCC 0.724906993198
CATGTTCG 0.132338332858
CATGTTGA -0.144931435922
CATGTTGC -0.823411822517
CATGTTGG -0.31408000942
CATGTTTA 0.0906379911972
CATGTTTC -0.769358615195
CATTAAAA 0.246600154356
CATTAAAC 1.20824653498
CATTAAAG 0.742542583572
CATTAACA 2.41383338795
CATTAACC -0.55993967466
CATTAACG 0.0733989798968
CATTAAGA 1.84972432448
CATTAAGC -0.398991648604
CATTAAGG 1.23006129854
CATTAATA 1.23545061038
CATTAATC 1.35916829261
CATTAATG 0.889826847154
CATTACAA 0.502703217669
CATTACAC 0.363571620591
CATTACAG -0.483974692839
CATTACCA 1.44679040925
CATTACCC -0.311435940749
CATTACCG 0.0844873724375
CATTACGA 0.786458147241
CATTACGC 0.0538652225958
CATTACGG 0.126263490887
CATTACTA 0.924289672761
CATTACTC 0.117864549819
CATTAGAA -0.107301806707
CATTAGAC -0.586032079584
CATTAGAG 0.366673264487
CATTAGCA 0.113337927571
CATTAGCC 0.235198870294

CATTAGCG -0.292272948451
CATTAGGA 0.147037364484
CATTAGGC -0.807499945912
CATTAGGG -0.492535180029
CATTAGTA 0.548215090177
CATTAGTC 0.203298299406
CATTATAA 2.05543020235
CATTATAC 1.40025013746
CATTATAG 1.3658776611
CATTATCA 0.794326442609
CATTATCC 1.99185264374
CATTATCG 0.133132948252
CATTATGA 0.395660176205
CATTATGC -0.167314733571
CATTATGG 0.0443448266873
CATTATTA 3.18414067025
CATTATTC 2.27074849081
CATTCAAA 0.313116769853
CATTCAAC 1.01896007165
CATTCAAG 0.15677468186
CATTCACA 1.07266707898
CATTCACC 0.0825575624561
CATTCACG -0.732601668121
CATTCAGA 2.26753380627
CATTCAGC -0.918177775108
CATTCAGG 0.281753714539
CATTCATA 1.57687542352
CATTCATC 0.18110236195
CATTCCAA 0.00689839410525
CATTCCAC 0.620056065985
CATTCCAG -0.0697704375045
CATTCCCA 0.323113477006
CATTCCCC -0.584974327209
CATTCCCG -0.821340451501
CATTCCGA 0.406323885643
CATTCCGC -1.350670467
CATTCCGG -0.630906719259
CATTCCTA -0.676614695435
CATTCCTC 0.332220847779
CATTCGAA -0.0355778269301
CATTCGAC -0.29254045717
CATTCGAG 0.492808934088
CATTCGCA 1.62314048401
CATTCGCC 0.118109367137
CATTCGCG 0.170041032548
CATTCGGA 0.389058019382
CATTCGGC -0.65581167692
CATTCGGG -1.01730255849
CATTCGTA -0.281899230955
CATTCGTC 0.413817252448
CATTCTAA 0.295256763604
CATTCTAC -0.0500293349239
CATTCTAG 0.728488487364
CATTCTCA 0.872923626954

CATTCTCC 0.24601309242
CATTCTCG -0.162686312285
CATTCTGA 0.832947623597
CATTCTGC -0.524127855667
CATTCTGG 0.172018723467
CATTCTTA 0.552068256622
CATTCTTC 0.400890440065
CATTGAAA 1.14838016543
CATTGAAC 0.392026429363
CATTGAAG 0.615756565926
CATTGACA 1.29699364548
CATTGACC 0.490854350925
CATTGACG -0.443189085273
CATTGAGA 0.083025130225
CATTGAGC -1.35264690885
CATTGAGG -0.852272370194
CATTGATA 1.18760422988
CATTGATC 1.36086244511
CATTGCAA 0.0639785094242
CATTGCAC 0.222849543658
CATTGCAG -0.719931122844
CATTGCCA 0.164984597311
CATTGCCC -0.120615830155
CATTGCCG -0.611396277893
CATTGCGA 1.71212783191
CATTGCGC 0.360896741578
CATTGCGG 0.534377082724
CATTGCTA 0.367931700446
CATTGCTC 0.232053092664
CATTGGAA 1.67282757431
CATTGGAC 0.351751066054
CATTGGAG -0.0504879510392
CATTGGCA -0.914110809866
CATTGGCC -1.2667493387
CATTGGCG -1.59804545984
CATTGGGA 0.487318447739
CATTGGGC -1.58721937157
CATTGGGG -0.585258698345
CATTGGTA 0.0136810412472
CATTGGTC -0.310733964562
CATTGTAA 0.285526940636
CATTGTAC 0.65502705407
CATTGTAG -0.147539489799
CATTGTCA 1.19292255303
CATTGTCC 0.225957016538
CATTGTCG -0.0408732505005
CATTGTGA 1.28832115854
CATTGTGC -0.889196484196
CATTGTGG 0.419432021058
CATTGTTA 1.21285268123
CATTGTTC 0.42675780458
CATTTAAA 1.00716220852
CATTTAAC 2.28949949887
CATTTAAG 1.20359563048

CATTTACA 1.86036742429
CATTTACC 1.51752575174
CATTTACG 2.26760146412
CATTTAGA 1.16057190132
CATTTAGC -1.16030043722
CATTTAGG -0.31632187821
CATTTATA 1.28062232005
CATTTATC 1.36444477199
CATTTCAA 0.142443917101
CATTTCAC 0.589136637971
CATTTCAG -0.277773559519
CATTTCCA 0.837764862321
CATTTCCC -0.474749076967
CATTTCCG -0.160523967487
CATTTCGA 1.76124951079
CATTTCGC -0.462613965485
CATTTCGG 0.909847324599
CATTTCTA -0.112136532382
CATTTCTC 0.964484054831
CATTTGAA 0.971808172929
CATTTGAC 1.03307079228
CATTTGAG 0.277210021696
CATTTGCA 0.845148519324
CATTTGCC 0.701030226637
CATTTGCG -0.523958190604
CATTTGGA 0.242865441188
CATTTGGC 0.400991198212
CATTTGGG -0.74479715122
CATTTGTA 1.2266034621
CATTTGTC -0.35747304633
CATTTTAA 0.462594813109
CATTTTAC 1.14715482977
CATTTTAG 0.639608351127
CATTTTCA 1.42424431641
CATTTTCC 1.12386720692
CATTTTCG 0.247621475582
CATTTTGA 0.492889707148
CATTTTGC 0.412064810116
CATTTTGG -0.361753185835
CATTTTTA 1.41942811857
CATTTTTC 0.477567806972
CCAAAAAA 1.51841779444
CCAAAAAC -0.275078695419
CCAAAAAG 0.479929794464
CCAAAACA 0.702667130185
CCAAAACC -0.098800858423
CCAAAACG -0.388628964542
CCAAAAGA 0.506216013098
CCAAAAGC -0.577833405743
CCAAAAGG -0.785817583561
CCAAAATA 2.28658417427
CCAAAATC 6.58497381188
CCAAACAA -1.01435330088
CCAAACAC -0.372430635021

CCAAACAG -0.0343289671589
CCAAACCA 0.089978691498
CCAAACCC -0.423200875417
CCAAACCG -1.16154742339
CCAAACGA -0.275652642141
CCAAACGC 1.4339720576
CCAAACGG 0.0551176213928
CCAAACTA 0.470493086108
CCAAACTC 1.36675284138
CCAAAGAA 1.29696096154
CCAAAGAC 0.443972459055
CCAAAGAG 0.363427561421
CCAAAGCA -0.698650335838
CCAAAGCC -0.705885353756
CCAAAGCG -0.812211013861
CCAAAGGA -0.0829805801349
CCAAAGGC -0.666826790808
CCAAAGGG -1.04757705108
CCAAAGTA -0.566752091254
CCAAAGTC 0.474808615872
CCAAATAA -0.317183735095
CCAAATAC 0.101381432806
CCAAATAG 0.492049917131
CCAAATCA 3.21293585004
CCAAATCC 3.45762992671
CCAAATCG 2.92114212733
CCAAATGA 0.820399486979
CCAAATGC -0.300672722231
CCAAATGG -0.26226429915
CCAAATTA 1.83315169093
CCAAATTC 2.53605135593
CCAACAAA 1.22824307196
CCAACAAC -0.460439338185
CCAACAAG 0.505049383634
CCAACACA -0.303778321509
CCAACACC -0.698530633493
CCAACACG -0.660247741741
CCAACAGA -0.398750370312
CCAACAGC -0.80928194952
CCAACAGG -1.23523181531
CCAACATA 1.08674240934
CCAACATC 0.758091814115
CCAACCAA -0.12845539696
CCAACCAC -0.787598129922
CCAACCAG -0.531512553561
CCAACCCA -0.115765282953
CCAACCCC -1.10388211974
CCAACCCG -1.14936817818
CCAACCGA -0.75906567076
CCAACCGC 0.133073201168
CCAACCGG -0.736206061866
CCAACCTA 0.705008508056
CCAACCTC -1.10341205383
CCAACGAA 0.470032388213

CCAACGAC −0.282561653324
CCAACGAG −0.00713675790555
CCAACGCA 0.292991578878
CCAACGCC −0.477657948042
CCAACGCG 0.253609507331
CCAACGGA −0.19873940957
CCAACGGC −0.794840642248
CCAACGGG −1.11228293441
CCAACGTA 0.057373021753
CCAACGTC −0.842833163702
CCAACTAA −0.0272198969262
CCAACTAC −0.466515012868
CCAACTAG −0.553583584222
CCAACTCA −0.166794288592
CCAACTCC −0.266532780689
CCAACTCG 0.684637042517
CCAACTGA −0.207914854546
CCAACTGC −0.375848709464
CCAACTGG −0.880753201215
CCAACTTA 0.447670116541
CCAACTTC 0.61147954909
CCAAGAAA −0.152422512769
CCAAGAAC −0.550178000459
CCAAGAAG 0.778213049708
CCAAGACA 0.827361791728
CCAAGACC −1.03503474345
CCAAGACG 0.671572208124
CCAAGAGA −0.534598167751
CCAAGAGC −0.782899552654
CCAAGAGG −1.20059557744
CCAAGATA 1.95716831675
CCAAGATC 2.83624610149
CCAAGCAA 0.407764060989
CCAAGCAC −1.3042892432
CCAAGCAG −0.507646612256
CCAAGCCA −0.750531205819
CCAAGCCC −0.437865765852
CCAAGCCG −1.57431941414
CCAAGCGA −0.162613658166
CCAAGCGC −0.716982073416
CCAAGCGG −1.16577989014
CCAAGCTA 0.0765562073163
CCAAGCTC −0.364563796899
CCAAGGAA −0.315320958427
CCAAGGAC −1.09504621307
CCAAGGAG −0.967129164392
CCAAGGCA 0.464753618881
CCAAGGCC −0.711128941006
CCAAGGCG 0.120548380486
CCAAGGGA 0.0844873724375
CCAAGGGC −1.53434757435
CCAAGGGG −1.40485003836
CCAAGGTA −0.500379118572
CCAAGGTC −0.205984420031

CCAAGTAA 0.37319673003
CCAAGTAC -0.0485421113524
CCAAGTAG -0.624431551011
CCAAGTCA 0.212924241556
CCAAGTCC -0.394453992923
CCAAGTCG -0.993984125299
CCAAGTGA -0.493332709914
CCAAGTGC 0.336770369606
CCAAGTGG -0.376979532314
CCAAGTTA 0.130487422335
CCAAGTTC -0.70961007438
CCAATAAA 1.46491771604
CCAATAAC 0.172604952691
CCAATAAG 2.19347073858
CCAATACA 0.874049037176
CCAATACC 0.137682678257
CCAATACG 0.267330935108
CCAATAGA 0.716906712983
CCAATAGC -0.143202517702
CCAATAGG -0.296626574788
CCAATATA 2.68509971981
CCAATATC 2.76922236441
CCAATCAA 0.440806071804
CCAATCAC 0.404393451128
CCAATCAG -0.265525615566
CCAATCCA 0.421878528814
CCAATCCC 0.320678419039
CCAATCCG -0.101200734309
CCAATCGA 0.453232216118
CCAATCGC 0.712052002221
CCAATCGG 1.49391607754
CCAATCTA 1.53517903725
CCAATCTC 2.17801269
CCAATGAA 0.00334791845975
CCAATGAC -0.310733964562
CCAATGAG -0.848473538205
CCAATGCA 0.286605927166
CCAATGCC -0.548671416334
CCAATGCG 0.156344586129
CCAATGGA -0.998368978334
CCAATGGC -0.494354447497
CCAATGGG -1.18065129314
CCAATGTA -0.288652525
CCAATGTC -0.566053862271
CCAATTAA 1.35441683813
CCAATTAC 0.605148023656
CCAATTAG 0.631875163276
CCAATTCA 0.513446659278
CCAATTCC 0.770933065345
CCAATTCG -0.639032322625
CCAATTGA 0.120017734786
CCAATTGC 0.589533841579
CCAATTGG 0.359995955409
CCAATTTA 0.200280967597

| | |
|---|---|
| CCAATTTC | 1.04398847886 |
| CCACAAAA | 1.30654089635 |
| CCACAAAC | -0.490590381232 |
| CCACAAAG | 0.179600565919 |
| CCACAACA | 0.477358171734 |
| CCACAACC | -0.144169504473 |
| CCACAACG | -0.825467372005 |
| CCACAAGA | -0.746181534864 |
| CCACAAGC | -0.948676267223 |
| CCACAAGG | -0.918361596275 |
| CCACAATA | 1.25460506738 |
| CCACAATC | 6.79856359848 |
| CCACACAA | 1.30576022889 |
| CCACACAC | -0.801694694441 |
| CCACACAG | 0.636667004285 |
| CCACACCA | 0.0298414823735 |
| CCACACCC | -0.293343607862 |
| CCACACCG | -0.891783720274 |
| CCACACGA | -0.354010629974 |
| CCACACGC | -0.48214522465 |
| CCACACGG | -0.427554709931 |
| CCACACTA | -0.459296441012 |
| CCACACTC | 0.830471970921 |
| CCACAGAA | 1.09068696592 |
| CCACAGAC | -0.69305409507 |
| CCACAGAG | 0.458138971379 |
| CCACAGCA | 0.530894473104 |
| CCACAGCC | -1.47663168344 |
| CCACAGCG | -0.20207171468 |
| CCACAGGA | 0.192721608369 |
| CCACAGGC | -1.13864159898 |
| CCACAGGG | -1.01708938422 |
| CCACAGTA | 0.200163347032 |
| CCACAGTC | -0.101303574237 |
| CCACATAA | -0.0958114224647 |
| CCACATAC | -0.213637459355 |
| CCACATAG | -0.0813022491672 |
| CCACATCA | 1.45388386613 |
| CCACATCC | -0.0866095388832 |
| CCACATCG | -0.600573936826 |
| CCACATGA | 0.0193547242283 |
| CCACATGC | -0.207574691708 |
| CCACATGG | -0.395471150589 |
| CCACATTA | -0.560682661911 |
| CCACATTC | 1.51685916581 |
| CCACCAAA | 0.0512838155009 |
| CCACCAAC | -0.948569263735 |
| CCACCAAG | 0.495003338296 |
| CCACCACA | 0.406770635613 |
| CCACCACC | -1.48508266901 |
| CCACCACG | -0.591312097983 |
| CCACCAGA | -0.755981305637 |
| CCACCAGC | -0.970064057716 |
| CCACCAGG | -1.17430686067 |

CCACCATA 0.0774871792944
CCACCATC 0.0768426602327
CCACCCAA 0.165118872116
CCACCCAC -0.59969042943
CCACCCAG -1.06899752552
CCACCCCA 0.607665936463
CCACCCCC -1.78160244849
CCACCCCG -1.402134981
CCACCCGA -0.565933119036
CCACCCGC -0.903827441618
CCACCCGG -0.754952073647
CCACCCTA -0.700509781659
CCACCCTC -0.600775036766
CCACCGAA -0.349031220595
CCACCGAC -0.278537989104
CCACCGAG 0.155076782161
CCACCGCA -0.213335809446
CCACCGCC -1.41587764293
CCACCGCG -0.812129408088
CCACCGGA -0.0304564401605
CCACCGGC -1.66053549662
CCACCGGG -1.13961170842
CCACCGTA 0.161330865382
CCACCGTC -0.385008541091
CCACCTAA -0.284233322596
CCACCTAC 0.245314863436
CCACCTAG -0.534418718322
CCACCTCA -0.149687886672
CCACCTCC -1.44345498147
CCACCTCG -0.960116480569
CCACCTGA -1.00735623041
CCACCTGC -1.18977448544
CCACCTGG -1.27056357586
CCACCTTA -0.0334579504423
CCACCTTC -0.862819291779
CCACGAAA 0.341578864854
CCACGAAC -0.520826777255
CCACGAAG -0.0290841308403
CCACGACA 0.436046290206
CCACGACC -1.19024392681
CCACGACG -1.18439912152
CCACGAGA -0.310672135698
CCACGAGC -0.609872414995
CCACGAGG -0.914077085031
CCACGATA 2.49508275991
CCACGATC 2.86017741068
CCACGCAA 0.399361164539
CCACGCAC -0.644912518173
CCACGCAG -0.710551038902
CCACGCCA 0.282188182008
CCACGCCC -0.96672092735
CCACGCCG -1.10141021427
CCACGCGA -0.185603586482
CCACGCGC 0.250494956399

CCACGCGG -0.687935622792
CCACGCTA 0.202789096039
CCACGCTC -0.684828982625
CCACGGAA -0.517264227286
CCACGGAC -0.126859712655
CCACGGAG -1.15567597132
CCACGGCA 0.329876555417
CCACGGCC -1.04517363617
CCACGGCG -0.559424225953
CCACGGGA -0.719767494943
CCACGGGC -1.42943669187
CCACGGGG -2.03012554295
CCACGGTA -0.910409821532
CCACGGTC -0.760540403652
CCACGTAA 0.68126913897
CCACGTAC -0.486701408172
CCACGTAG -0.680169542818
CCACGTCA 0.498317948277
CCACGTCC -0.988947466972
CCACGTCG -0.569891831723
CCACGTGA 0.685089205114
CCACGTGC 0.298930896976
CCACGTGG 0.536151800102
CCACGTTA -0.707694836858
CCACGTTC -0.70209859609
CCACTAAA 0.678050290865
CCACTAAC 0.770555846824
CCACTAAG 1.91951474707
CCACTACA 0.387774185708
CCACTACC -0.618103980958
CCACTACG -0.858635330505
CCACTAGA -0.0190584869464
CCACTAGC -0.577877123122
CCACTAGG -0.218795693629
CCACTATA 1.07017789473
CCACTATC 1.53387542666
CCACTCAA 0.541533409361
CCACTCAC -0.767142976831
CCACTCAG -1.04035410749
CCACTCCA -0.478484414669
CCACTCCC -1.47703076065
CCACTCCG -1.52525186154
CCACTCGA -1.16943029122
CCACTCGC -0.0778154759871
CCACTCGG -0.714704189833
CCACTCTA 0.381798436462
CCACTCTC -0.983251717124
CCACTGAA 0.112652189267
CCACTGAC -0.626290580475
CCACTGAG -1.06913804566
CCACTGCA -0.14761193574
CCACTGCC -1.16768784143
CCACTGCG -0.434253877699
CCACTGGA 0.213183631334

CCACTGGC −1.21260994569
CCACTGGG −1.68839991261
CCACTGTA −0.724990264394
CCACTGTC 0.214823865729
CCACTTAA −0.0362725168878
CCACTTAC −0.750606358074
CCACTTAG 0.115714487523
CCACTTCA 0.743272039254
CCACTTCC −0.479001736978
CCACTTCG −0.931606920985
CCACTTGA 0.327609288911
CCACTTGC −1.02976409306
CCACTTTA −0.668698935485
CCACTTTC 0.746345370943
CCAGAAAA 2.87195570509
CCAGAAAC −0.878714305966
CCAGAAAG −0.536454699079
CCAGAACA 0.211745537768
CCAGAACC −0.900766184253
CCAGAACG −0.755044296497
CCAGAAGA −0.680103550395
CCAGAAGC −1.34424109791
CCAGAAGG −1.19350066331
CCAGAATA 1.08878005552
CCAGAATC 8.35163324414
CCAGACAA 0.351244985357
CCAGACAC −0.730289226991
CCAGACAG −0.311232342673
CCAGACCA −0.674893063445
CCAGACCC −1.05126346696
CCAGACCG −1.66870065381
CCAGACGA −0.387469413129
CCAGACGC −0.388202407837
CCAGACGG −0.885846900312
CCAGACTA −1.00661532493
CCAGACTC −0.95548743475
CCAGAGAA 0.16458468739
CCAGAGAC −0.419690994479
CCAGAGAG −1.05540724988
CCAGAGCA −1.02743812036
CCAGAGCC −1.27620415854
CCAGAGCG −1.14411543109
CCAGAGGA 0.100203769908
CCAGAGGC −1.88345540444
CCAGAGGG −1.0202507752
CCAGAGTA −0.292212576834
CCAGAGTC −1.04796051494
CCAGATAA 1.65056356265
CCAGATAC 6.1369928856
CCAGATAG 1.12082531011
CCAGATCA 0.737002342684
CCAGATCC 4.60109022166
CCAGATCG 2.42432139516
CCAGATGA −0.285631862343

CCAGATGC 1.21738638152
CCAGATGG -0.422115019013
CCAGATTA 3.95104465875
CCAGATTC 10.2079944912
CCAGCAAA 1.61750260764
CCAGCAAC -0.7738875274
CCAGCAAG -0.213905592608
CCAGCACA 0.451428978356
CCAGCACC -1.22582570912
CCAGCACG -0.920303272402
CCAGCAGA -0.648457373013
CCAGCAGC -0.659457289907
CCAGCAGG -1.29263460652
CCAGCATA 0.0618898596352
CCAGCATC 0.20973037481
CCAGCCAA -0.193174187322
CCAGCCAC -0.737677047554
CCAGCCAG -0.600984047469
CCAGCCCA -1.03230469727
CCAGCCCC -1.30020937092
CCAGCCCG -1.29673279846
CCAGCCGA -0.542976915554
CCAGCCGC 1.46424884015
CCAGCCGG -1.3132383987
CCAGCCTA -0.834955916681
CCAGCCTC -1.20641748315
CCAGCGAA 0.157328851673
CCAGCGAC 0.0511426708226
CCAGCGAG -0.529698074186
CCAGCGCA 0.515795531556
CCAGCGCC -1.49416276846
CCAGCGCG -0.901830390145
CCAGCGGA -0.413443364776
CCAGCGGC -1.24189954821
CCAGCGGG -1.06718096437
CCAGCGTA -0.612953032914
CCAGCGTC 0.805627176701
CCAGCTAA -0.259986415567
CCAGCTAC -0.869442890935
CCAGCTAG -0.802495346996
CCAGCTCA -0.113231548618
CCAGCTCC -0.773742635518
CCAGCTCG -0.841781448489
CCAGCTGA -0.873616235132
CCAGCTGC -0.861927873619
CCAGCTGG -1.01592920828
CCAGCTTA -0.283354395116
CCAGCTTC -0.844569784508
CCAGGAAA 1.61789814582
CCAGGAAC -0.589264042902
CCAGGAAG 0.189219430017
CCAGGACA -0.692004461637
CCAGGACC -0.916988037886
CCAGGACG -0.359862305138

CCAGGAGA −0.0265283296384
CCAGGAGC −0.72986641749
CCAGGAGG −1.13952094282
CCAGGATA 1.19843323264
CCAGGATC 1.53549380237
CCAGGCAA −0.942635774621
CCAGGCAC −1.12464204541
CCAGGCAG −1.43471067311
CCAGGCCA −1.12093814258
CCAGGCCC −1.44711808141
CCAGGCCG −1.44562544521
CCAGGCGA −0.544328407075
CCAGGCGC −1.07062318746
CCAGGCGG −1.09493088246
CCAGGCTA −1.11361652262
CCAGGCTC −0.0184495663211
CCAGGGAA −0.562606434731
CCAGGGAC −0.565927290052
CCAGGGAG −1.79746061517
CCAGGGCA −1.04758662727
CCAGGGCC −1.68208150239
CCAGGGCG −0.379688344339
CCAGGGGA −1.07996954656
CCAGGGGC −1.24213624658
CCAGGGGG −1.71762040004
CCAGGGTA −1.26277667809
CCAGGGTC −1.22495344334
CCAGGTAA 0.294833537747
CCAGGTAC −0.0381228028765
CCAGGTAG −0.81885397357
CCAGGTCA −0.765874756506
CCAGGTCC −1.71818477057
CCAGGTCG −0.67571661558
CCAGGTGA −0.624431551011
CCAGGTGC −1.2093559155
CCAGGTTA −0.662969877158
CCAGGTTC −0.704001759289
CCAGTAAA 1.67649296421
CCAGTAAC 0.241070114189
CCAGTAAG −0.190707278123
CCAGTACA 0.00463154395551
CCAGTACC −1.29635474723
CCAGTACG −0.63515729749
CCAGTAGA 0.600264167974
CCAGTAGC −0.402225693702
CCAGTAGG −0.24699152898
CCAGTATA −0.934445219722
CCAGTATC 1.35609558546
CCAGTCAA 1.04884069149
CCAGTCAC −0.62146210014
CCAGTCAG −0.097278660949
CCAGTCCA −0.712741904085
CCAGTCCC −1.0138763651
CCAGTCCG −1.26955724345

CCAGTCGA 0.137927495575
CCAGTCGC −0.152715002847
CCAGTCGG −0.79075244285
CCAGTCTA −0.932466487913
CCAGTCTC −0.065728245443
CCAGTGAA −0.067741951155
CCAGTGAC −0.365428360098
CCAGTGAG −0.508234506904
CCAGTGCA −0.187198646253
CCAGTGCC −1.0080269799
CCAGTGCG 0.14084573466
CCAGTGGA 0.152341115174
CCAGTGGC −1.04983370051
CCAGTGGG −1.40724200348
CCAGTGTA −0.690077357969
CCAGTGTC −1.04760640418
CCAGTTAA −0.646913108672
CCAGTTAC 0.554499983741
CCAGTTAG 0.526163628246
CCAGTTCA −0.201888518048
CCAGTTCC −1.29268498559
CCAGTTCG −0.648299782274
CCAGTTGA 0.379733727142
CCAGTTGC −0.953643810457
CCAGTTTA −0.0810921975737
CCAGTTTC 0.345254455473
CCATAAAA 0.983069561381
CCATAAAC 0.964431177622
CCATAAAG −0.433367872167
CCATAACA 0.275298739556
CCATAACC −0.773137253919
CCATAACG 0.0234491689657
CCATAAGA 1.02517772373
CCATAAGC −0.830996995816
CCATAAGG −0.290699331013
CCATAATA 1.22387299956
CCATAATC 3.87574418108
CCATACAA 0.697172272099
CCATACAC −0.928389946482
CCATACAG −0.607333892567
CCATACCA −0.400186382097
CCATACCC −0.478778361993
CCATACCG −0.42749475467
CCATACGA 0.60528125757
CCATACGC −0.629707197672
CCATACGG −0.781043854037
CCATACTA 0.988323141175
CCATACTC 0.494289912319
CCATAGAA 0.607775229909
CCATAGAC 0.172093667544
CCATAGAG −0.535394448569
CCATAGCA −0.206610827607
CCATAGCC −0.676535795976
CCATAGCG −0.522767412492

CCATAGGA 0.481740734812
CCATAGGC -0.941682527597
CCATAGGG -0.766164956627
CCATAGTA 0.22783290842
CCATAGTC -0.273045837332
CCATATAA 1.07665452023
CCATATAC 0.508657940939
CCATATAG -0.726210395603
CCATATCA 2.68222894531
CCATATCC 4.61204912749
CCATATCG 2.38286441357
CCATATGA 0.647538059003
CCATATGC 1.08062031096
CCATATGG -0.446607992428
CCATATTA 1.40273619904
CCATATTC 1.86217961371
CCATCAAA 0.984006154165
CCATCAAC -0.313955102625
CCATCAAG -0.568055285481
CCATCACA -0.244597898433
CCATCACC -0.991408963544
CCATCACG -0.490629726872
CCATCAGA -0.153523774344
CCATCAGC -1.07500845685
CCATCAGG -0.591969107724
CCATCATA 0.152989173262
CCATCATC 1.33558776333
CCATCCAA 0.966871856394
CCATCCAC -0.500449066377
CCATCCAG -0.994346563182
CCATCCCA -1.12858847559
CCATCCCC -0.844664297316
CCATCCCG -0.838140415417
CCATCCGA -1.04473458879
CCATCCGC 0.335001897568
CCATCCGG -1.16004937456
CCATCCTA -0.487661941425
CCATCCTC -0.581182573271
CCATCGAA 1.11632242016
CCATCGAC 0.46203710427
CCATCGAG -0.569474226672
CCATCGCA 0.517888969439
CCATCGCC -0.0381806763581
CCATCGCG -0.188356115886
CCATCGGA -0.122026652404
CCATCGGC -0.921441589659
CCATCGGG -1.1395646602
CCATCGTA 0.850339229365
CCATCGTC 0.0389892396772
CCATCTAA 0.279815993794
CCATCTAC 0.120064366656
CCATCTAG -0.868672840545
CCATCTCA 0.203845391168
CCATCTCC -0.223520709503

CCATCTCG -0.179127585522
CCATCTGA -0.100002045434
CCATCTGC -0.222197946545
CCATCTTA -0.3981980741
CCATCTTC 0.646835250103
CCATGAAA 1.46393449138
CCATGAAC 0.0769151061737
CCATGAAG -0.511586172567
CCATGACA -0.69709337264
CCATGACC -1.33686660074
CCATGACG -0.287963455848
CCATGAGA 0.101218637617
CCATGAGC -0.326870257041
CCATGAGG -0.612006655765
CCATGATA 1.04884069149
CCATGATC 0.161302344997
CCATGCAA -0.366486528829
CCATGCAC -0.570753272251
CCATGCAG -1.34237041048
CCATGCCA -0.629310618598
CCATGCCC -0.612327666228
CCATGCCG 2.14320886884
CCATGCGA 0.305056534377
CCATGCGC -0.142842577955
CCATGCGG -1.19877589362
CCATGCTA -0.212248703974
CCATGCTC -0.393695808677
CCATGGAA 0.215129887377
CCATGGAC -0.539249488616
CCATGGAG -1.07545499864
CCATGGCA -1.39347061299
CCATGGCC -1.44655266998
CCATGGCG -0.0200408788884
CCATGGGA -0.790078362513
CCATGGGC -1.16481914871
CCATGGGG -1.25131439787
CCATGGTA -1.12886285419
CCATGGTC -1.56731047753
CCATGTAA 0.709506818097
CCATGTAC -0.358924671464
CCATGTAG -0.363548304656
CCATGTCA -0.118912517829
CCATGTCC -0.630962510961
CCATGTCG -0.310672343876
CCATGTGA -0.311564802926
CCATGTGC -1.07193137795
CCATGTTA 0.0713467612563
CCATGTTC 0.189256902056
CCATTAAA 1.4201504962
CCATTAAC 1.68584536048
CCATTAAG 1.30469706388
CCATTACA 0.628832017396
CCATTACC -0.233450383343
CCATTACG 0.385043098638

CCATTAGA 0.566398605025
CCATTAGC 0.146188414634
CCATTAGG 0.0365997726905
CCATTATA 0.890797164772
CCATTATC 1.78456690309
CCATTCAA 0.580628611636
CCATTCAC 0.034768430899
CCATTCAG -0.978114717005
CCATTCCA 0.0999843503048
CCATTCCC -0.8450598355
CCATTCCG -0.159173725034
CCATTCGA 0.0311919330046
CCATTCGC 0.470588847984
CCATTCGG -0.147645244219
CCATTCTA -0.249621857903
CCATTCTC 0.107412349221
CCATTGAA 1.84375211426
CCATTGAC -0.000633485628218
CCATTGAG -1.17476755857
CCATTGCA -0.714255358083
CCATTGCC -0.0211458876674
CCATTGCG 0.143953415717
CCATTGGA -0.193794141381
CCATTGGC -1.78040563321
CCATTGGG -0.506907996742
CCATTGTA 0.144566916258
CCATTGTC 0.538928269975
CCATTTAA 1.18723450577
CCATTTAC 0.907070230192
CCATTTAG -0.934852624051
CCATTTCA -0.783183091078
CCATTTCC -0.832981348431
CCATTTCG -0.13548077964
CCATTTGA 0.17430493417
CCATTTGC 0.581309978202
CCATTTTA -0.376684752278
CCATTTTC 0.663579005962
CCCAAAAA 1.12615758119
CCCAAAAC -0.818338941219
CCCAAAAG -0.446500156228
CCCAAACA -0.15396261355
CCCAAACC -1.12273596772
CCCAAACG 1.39107490066
CCCAAAGA 0.750466254286
CCCAAAGC -1.22825056637
CCCAAAGG -0.99911154923
CCCAAATA 1.5159483871
CCCAAATC 4.8659217718
CCCAACAA -0.107344066839
CCCAACAC -0.992536871902
CCCAACAG -0.791827057642
CCCAACCA -1.02332244147
CCCAACCC -1.03441125036
CCCAACCG 0.184116362911

CCCAACGA 0.743397362405
CCCAACGC 0.143257476692
CCCAACGG -0.971754879364
CCCAACTA -0.69880563662
CCCAACTC -0.503803646532
CCCAAGAA -0.367303419267
CCCAAGAC -0.536324171479
CCCAAGAG -0.589328994436
CCCAAGCA -1.18612283529
CCCAAGCC -1.5149391402
CCCAAGCG -1.25436607905
CCCAAGGA -0.409755908012
CCCAAGGC -0.440661179922
CCCAAGGG -1.5916960311
CCCAAGTA -0.116463511936
CCCAAGTC -1.0853684346
CCCAATAA 0.680647519487
CCCAATAC -0.239005404869
CCCAATAG -0.206634976254
CCCAATCA 0.712279332588
CCCAATCC 0.354029574172
CCCAATCG 1.50295683136
CCCAATGA -0.127600826305
CCCAATGC -0.836696909224
CCCAATTA 0.806845642485
CCCAATTC 0.171454977466
CCCACAAA -0.889343457858
CCCACAAC -0.228630854661
CCCACAAG -0.826421243562
CCCACACA -0.274942338834
CCCACACC -0.861848557805
CCCACACG 0.3805693536
CCCACAGA -0.613940629306
CCCACAGC -0.938426207454
CCCACAGG -1.50146294609
CCCACATA -0.355244917286
CCCACATC -0.00642478917452
CCCACCAA 0.0916162195794
CCCACCAC -0.290658111771
CCCACCAG -0.728931906486
CCCACCCA -0.69233171744
CCCACCCC -1.62546375039
CCCACCCG 0.0964330419474
CCCACCGA 0.662650532119
CCCACCGC -0.605478610306
CCCACCGG -0.899300611192
CCCACCTA -0.879879270007
CCCACCTC -0.891830352144
CCCACGAA -0.294225033478
CCCACGAC -0.249895195606
CCCACGAG -0.506847416946
CCCACGCA -1.02819984363
CCCACGCC -0.976189695117
CCCACGCG 1.12197216267

CCCACGGA 0.0221509710105
CCCACGGC -0.448367929168
CCCACGGG -1.72465098717
CCCACGTA -0.206310218588
CCCACGTC -1.0110055906
CCCACTAA 0.00546050871777
CCCACTAC -0.837663271461
CCCACTAG -0.141198388178
CCCACTCA -0.803143196906
CCCACTCC -1.57378856027
CCCACTCG -0.884565772952
CCCACTGA -0.395646020102
CCCACTGC -0.669502086177
CCCACTTA -0.914444727364
CCCACTTC -0.479617943833
CCCAGAAA 0.355342552764
CCCAGAAC -0.437463357794
CCCAGAAG -0.473084485746
CCCAGACA -0.533279984709
CCCAGACC -1.15733452538
CCCAGACG 0.0226229105173
CCCAGAGA -0.252822178167
CCCAGAGC -0.94187717402
CCCAGAGG -1.06830387645
CCCAGATA 3.47552948677
CCCAGATC 3.39521608307
CCCAGCAA -0.245390223869
CCCAGCAC -1.01697530268
CCCAGCAG -0.885864387263
CCCAGCCA -0.851338067368
CCCAGCCC -0.904387232237
CCCAGCCG -0.919124152258
CCCAGCGA -0.255863658623
CCCAGCGC -1.04642062234
CCCAGCGG -1.10285205504
CCCAGCTA -0.610183641093
CCCAGCTC -0.474685374501
CCCAGGAA -0.456174603851
CCCAGGAC -1.3483813418
CCCAGGAG -0.310701072439
CCCAGGCA -1.23002445103
CCCAGGCC -1.23995849661
CCCAGGCG -0.74071436445
CCCAGGGA -1.14864288605
CCCAGGGC -1.02024598711
CCCAGGGG -1.65818995519
CCCAGGTA -0.840294224918
CCCAGGTC -1.25245625416
CCCAGTAA 0.241070114189
CCCAGTAC -1.35605145172
CCCAGTAG 0.578698593476
CCCAGTCA -0.84613778114
CCCAGTCC -1.50995244459
CCCAGTCG -0.251803355076

CCCAGTGA −0.36154230153
CCCAGTGC −0.764753509844
CCCAGTTA −0.261215082073
CCCAGTTC −1.09578815943
CCCATAAA 0.937617852313
CCCATAAC −0.269274693015
CCCATAAG 0.688078224717
CCCATACA 0.0804430985961
CCCATACC −0.54061825891
CCCATACG −0.719949442508
CCCATAGA 0.628243498214
CCCATAGC −1.06483396569
CCCATAGG −0.773735765645
CCCATATA −0.104472459624
CCCATATC 2.83367760143
CCCATCAA −0.0904333522315
CCCATCAC −0.833372306699
CCCATCAG −0.219977936443
CCCATCCA −0.758144274969
CCCATCCC −0.853788114151
CCCATCCG −0.159734972899
CCCATCGA 0.75855563468
CCCATCGC 1.16913967474
CCCATCGG −0.869706652451
CCCATCTA 0.231703353638
CCCATCTC 0.00154926061295
CCCATGAA −0.0859389975725
CCCATGAC −1.34764397536
CCCATGAG −0.502383456274
CCCATGCA −1.07238624687
CCCATGCC −0.607382814395
CCCATGCG −1.00600057533
CCCATGGA −1.03637749149
CCCATGGC −1.36341470728
CCCATGGG −1.17252527342
CCCATGTA −0.5282932891
CCCATGTC −0.00325257293963
CCCATTAA 0.89007499532
CCCATTAC −0.54061825891
CCCATTAG 0.821632733401
CCCATTCA −0.081599943695
CCCATTCC −1.03974227237
CCCATTCG 0.513417306181
CCCATTGA 0.710257091578
CCCATTGC 0.11115205866
CCCATTTA −0.482506205287
CCCATTTC −0.570736201656
CCCCAAAA −1.25904508759
CCCCAAAC −0.382297230929
CCCCAAAG −0.639002553172
CCCCAACA −0.836268478918
CCCCAACC −0.819029675795
CCCCAACG 0.18110236195
CCCCAAGA −0.75941082987

CCCCAAGC −1.28612529708
CCCCAAGG −1.21773591237
CCCCAATA −0.17827988474
CCCCAATC 1.2983640812
CCCCACAA −1.06862176425
CCCCACAC −0.566744388669
CCCCACAG −0.852260712227
CCCCACCA −1.21863815579
CCCCACCC −1.32497755564
CCCCACCG −0.662969877158
CCCCACGA 0.383546298878
CCCCACGC −0.160390733572
CCCCACGG −1.10691485672
CCCCACTA −1.10391209737
CCCCACTC −0.9983891716
CCCCAGAA −1.0245196731
CCCCAGAC −0.913133830551
CCCCAGAG −0.580669206344
CCCCAGCA −1.11099764349
CCCCAGCC −1.285180169
CCCCAGCG −0.324248255238
CCCCAGGA −0.666078390928
CCCCAGGC −1.58443582364
CCCCAGGG −1.26063098753
CCCCAGTA 0.484846750446
CCCCAGTC −0.640062387327
CCCCATAA 0.285295654887
CCCCATAC −0.603328339832
CCCCATAG −0.75727450732
CCCCATCA −0.35027654134
CCCCATCC −0.525047794212
CCCCATCG 1.24799291802
CCCCATGA −0.139715328166
CCCCATGC −0.456349473364
CCCCATTA −0.0591546090045
CCCCATTC −0.294893701186
CCCCCAAA −1.70532894688
CCCCCAAC −1.13611036278
CCCCCAAG −1.64022023914
CCCCCACA −1.13864159898
CCCCCACC −2.02764739214
CCCCCACG 0.486208442688
CCCCCAGA −1.13201425262
CCCCCAGC −1.26019214832
CCCCCAGG −1.29073685595
CCCCCATA −0.391591129183
CCCCCATC 0.451240369095
CCCCCCAA −1.62593922892
CCCCCCAC −1.16814749843
CCCCCCAG −1.11970010807
CCCCCCCA −1.35106100891
CCCCCCCC −0.563408960888
CCCCCCCG −1.1148331148
CCCCCCGA −0.561549723246

CCCCCCGC -0.870468167544
CCCCCCGG -1.42773129777
CCCCCCTA -1.39224756729
CCCCCCTC -1.43140938652
CCCCCGAA -0.467521553457
CCCCCGAC -1.0063621805
CCCCCGAG 0.0307574655362
CCCCCGCA -1.13951386477
CCCCCGCC -1.308207153
CCCCCGCG 0.024768601076
CCCCCGGA -0.693883059832
CCCCCGGC -0.932839959229
CCCCCGGG -1.7224070366
CCCCCGTA -0.742705378761
CCCCCGTC -1.26907676864
CCCCCTAA -1.38602783344
CCCCCTAC -0.813624958779
CCCCCTAG -0.726173131742
CCCCCTCA -1.05662946287
CCCCCTCC -1.9589877924
CCCCCTCG -1.08261548884
CCCCCTGA -1.02547645915
CCCCCTGC -0.964429512198
CCCCCTTA -0.988728047369
CCCCCTTC -1.44730398435
CCCCGAAA -1.19467312176
CCCCGAAC 1.54766180597
CCCCGAAG -0.365883229009
CCCCGACA -0.968179630537
CCCCGACC -1.64842286836
CCCCGACG 0.941571152372
CCCCGAGA -0.824920904777
CCCCGAGC -1.03387019576
CCCCGAGG -1.57096441763
CCCCGATA 1.81070593988
CCCCGATC 0.656522188405
CCCCGCAA -0.0160528131047
CCCCGCAC -0.280875619771
CCCCGCAG -0.11307270881
CCCCGCCA -0.895237601332
CCCCGCCC -0.924638370897
CCCCGCCG -0.722214835411
CCCCGCGA 0.478910555017
CCCCGCGC 0.0848033866287
CCCCGCGG -0.862168943733
CCCCGCTA -0.328057287949
CCCCGCTC -0.0878835881913
CCCCGGAA -0.869971454856
CCCCGGAC -1.45642696847
CCCCGGAG -1.47111850569
CCCCGGCA -0.810280787523
CCCCGGCC -1.30000702191
CCCCGGCG -0.0767306604732
CCCCGGGA -0.951290150084

CCCCGGGC −0.538450709662
CCCCGGGG −1.19144303204
CCCCGGTA −0.895178270604
CCCCGGTC −0.90962957042
CCCCGTAA −0.676294101328
CCCCGTAC −0.700819134154
CCCCGTAG −0.496171841363
CCCCGTCA 0.0334610731122
CCCCGTCC −1.30338366893
CCCCGTCG −1.25698745632
CCCCGTGA −1.06975799972
CCCCGTGC −0.483070784
CCCCGTTA −0.417301735671
CCCCGTTC −0.746170709608
CCCCTAAA −0.845628994129
CCCCTAAC −0.767081356145
CCCCTAAG −0.737040439256
CCCCTACA −0.406970902841
CCCCTACC −1.26190378777
CCCCTACG 0.398082743493
CCCCTAGA −0.228729739207
CCCCTAGC −0.198037017026
CCCCTAGG −1.28055924212
CCCCTATA −0.32901907027
CCCCTATC −0.124401963287
CCCCTCAA −1.00920485097
CCCCTCAC −0.730803842986
CCCCTCAG −0.800182905866
CCCCTCCA −0.877367394361
CCCCTCCC −2.06476760981
CCCCTCCG −1.61291290746
CCCCTCGA 0.312760577309
CCCCTCGC −1.20115828255
CCCCTCGG −1.41540278893
CCCCTCTA −0.629633502663
CCCCTCTC −0.666906939334
CCCCTGAA −1.19428840883
CCCCTGAC −0.813765270745
CCCCTGAG −1.21790245477
CCCCTGCA −0.780554219401
CCCCTGCC −1.25839786221
CCCCTGCG −0.844190692385
CCCCTGGA −1.09705055077
CCCCTGGC −0.889764601934
CCCCTGTA 0.772077003408
CCCCTGTC −0.942415522306
CCCCTTAA −0.776391908638
CCCCTTAC −0.135888600326
CCCCTTAG 0.591464067917
CCCCTTCA −1.90617553408
CCCCTTCC −2.00170091999
CCCCTTCG −1.00459495753
CCCCTTGA −0.830071436466
CCCCTTGC −0.362473689864

CCCCTTTA -0.838930242717
CCCCTTTC -1.14349880788
CCCGAAAA 0.120907695699
CCCGAAAC -1.45439723306
CCCGAAAG -0.506856160422
CCCGAACA 0.200827226647
CCCGAACC 0.401786021784
CCCGAACG -0.367790763946
CCCGAAGA -0.0856877267367
CCCGAAGC -1.29120359101
CCCGAAGG -1.47511552313
CCCGAATA 1.37917211582
CCCGAATC 4.58906190549
CCCGACAA -0.41667990801
CCCGACAC -1.30659127543
CCCGACAG -0.456991910645
CCCGACCA -1.44219154924
CCCGACCC -1.45454274947
CCCGACCG -0.884924463631
CCCGACGA 0.657516446492
CCCGACGC -0.514160917967
CCCGACGG -0.960004064454
CCCGACTA -0.349674490589
CCCGACTC 0.469044999999
CCCGAGAA -1.05248338999
CCCGAGAC -0.766996835881
CCCGAGAG -0.335690758542
CCCGAGCA -0.899016656412
CCCGAGCC -1.66909744106
CCCGAGCG -0.514702180745
CCCGAGGA -1.13858872177
CCCGAGGC -0.759685624818
CCCGAGGG -1.26599698344
CCCGAGTA -0.554477500518
CCCGAGTC -0.441728300307
CCCGATAA -0.295915855125
CCCGATAC 0.694831102406
CCCGATAG 0.0159347761835
CCCGATCA 0.362352738451
CCCGATCC -0.399462130865
CCCGATCG 0.999902833776
CCCGATGA -0.0769280132092
CCCGATGC -0.775076223732
CCCGATTA 1.91652406204
CCCGATTC 2.47357963881
CCCGCAAA -0.435893071204
CCCGCAAC -0.0990506720128
CCCGCAAG 0.895666447994
CCCGCACA 0.0591450328169
CCCGCACC -1.06647086924
CCCGCACG -0.37362890754
CCCGCAGA -0.309324183203
CCCGCAGC -0.831456652821
CCCGCAGG -1.47458466925

| | |
|---|---|
| CCCGCATA | −0.667751725624 |
| CCCGCATC | 0.246632630123 |
| CCCGCCAA | −1.2076438597 |
| CCCGCCAC | −0.641622681373 |
| CCCGCCAG | −0.102767065518 |
| CCCGCCCA | −0.874363802299 |
| CCCGCCCC | −1.65114375471 |
| CCCGCCCG | −1.09134147753 |
| CCCGCCGA | −0.334044070629 |
| CCCGCCGC | −1.33543745882 |
| CCCGCCGG | −0.735791371307 |
| CCCGCCTA | −0.819678774773 |
| CCCGCCTC | −0.666680441679 |
| CCCGCGAA | −0.376331266048 |
| CCCGCGAC | −0.752961475692 |
| CCCGCGAG | 0.126491445788 |
| CCCGCGCA | −0.248342604145 |
| CCCGCGCC | −0.428469860382 |
| CCCGCGCG | 0.102675883557 |
| CCCGCGGA | −0.226684806796 |
| CCCGCGGC | −0.536236528544 |
| CCCGCGGG | −0.893499731458 |
| CCCGCGTA | 0.435520432599 |
| CCCGCGTC | −1.10231495582 |
| CCCGCTAA | −0.580484760643 |
| CCCGCTAC | −0.812915488184 |
| CCCGCTAG | −0.506294704379 |
| CCCGCTCA | −0.342635159983 |
| CCCGCTCC | −1.22350556541 |
| CCCGCTCG | −0.521627429811 |
| CCCGCTGA | −0.59669183364 |
| CCCGCTGC | −1.34901149658 |
| CCCGCTTA | 0.180253203922 |
| CCCGCTTC | −0.238967100119 |
| CCCGGAAA | −0.483525861089 |
| CCCGGAAC | −0.717770235292 |
| CCCGGAAG | 0.142938548009 |
| CCCGGACA | −0.609310334418 |
| CCCGGACC | −1.13848837998 |
| CCCGGACG | −1.26717152366 |
| CCCGGAGA | −0.684700536804 |
| CCCGGAGC | −1.47703076065 |
| CCCGGAGG | −1.5708228566 |
| CCCGGATA | 2.55331722219 |
| CCCGGATC | 2.3890597906 |
| CCCGGCAA | −0.510338353686 |
| CCCGGCAC | −1.12590860031 |
| CCCGGCAG | −1.12875022989 |
| CCCGGCCA | −0.988472404795 |
| CCCGGCCC | −2.01670430784 |
| CCCGGCCG | −0.590580976877 |
| CCCGGCGA | −1.26053272751 |
| CCCGGCGC | −0.201233798264 |
| CCCGGCGG | −0.874133973797 |

CCCGGCTA −0.531939734799
CCCGGCTC −0.986602966431
CCCGGGAA −0.348088174294
CCCGGGAC −1.19312552657
CCCGGGAG −0.650710899771
CCCGGGCA −0.242216966744
CCCGGGCC −1.18460854858
CCCGGGCG −0.353643195819
CCCGGGGA −0.687481170237
CCCGGGGC −0.746714470522
CCCGGGTA −0.787842114528
CCCGGGTC −1.21408571947
CCCGGTAA −0.401465427677
CCCGGTAC −1.25656527135
CCCGGTAG −0.615198440731
CCCGGTCA −0.158992193825
CCCGGTCC −1.46573960275
CCCGGTCG −0.771568424575
CCCGGTGA −0.551637536357
CCCGGTGC −1.12613426525
CCCGGTTA −0.685504312029
CCCGGTTC −1.23052886631
CCCGTAAA −0.435871004337
CCCGTAAC −0.169636750888
CCCGTAAG 0.151170946684
CCCGTACA −0.151479050111
CCCGTACC −0.0313341185728
CCCGTACG −0.0859221351551
CCCGTAGA −0.200980445649
CCCGTAGC −1.19044127955
CCCGTAGG −1.38373163019
CCCGTATA −0.178082323826
CCCGTATC 1.60890256663
CCCGTCAA −0.362765763586
CCCGTCAC −0.797829453672
CCCGTCAG −0.055826259276
CCCGTCCA −0.486929154895
CCCGTCCC −1.63785783529
CCCGTCCG −1.46861912073
CCCGTCGA −0.236523090498
CCCGTCGC −1.34904147421
CCCGTCGG −1.28720574086
CCCGTCTA −0.637254898933
CCCGTCTC −0.216697259475
CCCGTGAA −0.824254110671
CCCGTGAC −0.495581032223
CCCGTGAG −0.804116220838
CCCGTGCA −0.597245170741
CCCGTGCC −1.12381995052
CCCGTGCG −0.594963539954
CCCGTGGA −1.07750909088
CCCGTGGC −1.24895053678
CCCGTGTA 0.216438494231
CCCGTGTC −0.169146699896

CCCGTTAA 0.321053347602
CCCGTTAC -1.19760093703
CCCGTTAG 1.47616848741
CCCGTTCA -0.974964983993
CCCGTTCC -1.05635050436
CCCGTTCG -1.0215608393
CCCGTTGA -0.0577192217529
CCCGTTGC -1.0809775444
CCCGTTTA -1.00031835705
CCCGTTTC 0.160628056483
CCCTAAAA 0.726212685561
CCCTAAAC -0.677580641316
CCCTAAAG -0.754706839973
CCCTAACA -0.923257109923
CCCTAACC -0.693125916477
CCCTAACG 0.480586387849
CCCTAAGA -0.693332012689
CCCTAAGC -1.18280489446
CCCTAAGG -0.725162011237
CCCTAATA -0.831884250416
CCCTAATC 0.990601024759
CCCTACAA -0.865012863276
CCCTACAC -0.452468619245
CCCTACAG -0.402969721844
CCCTACCA -1.08151755811
CCCTACCC -1.19625860534
CCCTACCG -0.672710317204
CCCTACGA 0.172018723467
CCCTACGC -0.406622412883
CCCTACGG -0.318927433952
CCCTACTA -0.361004994134
CCCTACTC -0.100124245915
CCCTAGAA -0.58159726383
CCCTAGAC -1.11364483483
CCCTAGAG 0.328623532086
CCCTAGCA -0.30066148062
CCCTAGCC -1.09478265973
CCCTAGCG -0.990789634019
CCCTAGGA -0.764952528004
CCCTAGGC -0.494299072151
CCCTAGGG -1.04380902944
CCCTAGTA -0.519125963065
CCCTAGTC -0.605679918424
CCCTATAA 0.468729402164
CCCTATAC -0.789044134252
CCCTATAG -1.15482827054
CCCTATCA 0.406826635493
CCCTATCC -0.803853916569
CCCTATCG 1.098121002
CCCTATGA -0.555295223669
CCCTATGC -0.258988410276
CCCTATTA 0.43750520157
CCCTATTC 0.164882381918
CCCTCAAA -0.0403779950587

CCCTCAAC -0.589937498705
CCCTCAAG -0.914710362481
CCCTCACA 0.118498659981
CCCTCACC -0.629728848183
CCCTCACG 0.0565253209715
CCCTCAGA -0.0670774470061
CCCTCAGC -0.807631930759
CCCTCAGG -0.691369935119
CCCTCATA -0.021720250746
CCCTCATC -0.265566418452
CCCTCCAA -0.877367394361
CCCTCCAC -1.22747822602
CCCTCCAG -1.53987261824
CCCTCCCA -1.00735623041
CCCTCCCC -1.57371236712
CCCTCCCG -0.490203170168
CCCTCCGA 0.287770058494
CCCTCCGC -1.33336671234
CCCTCCGG -1.51296186563
CCCTCCTA -0.625600678611
CCCTCCTC -0.450887299222
CCCTCGAA -0.59055266467
CCCTCGAC -0.890497180287
CCCTCGAG -0.0890731172349
CCCTCGCA -0.691078485931
CCCTCGCC -1.81548050212
CCCTCGCG -0.448106249433
CCCTCGGA -0.706570467526
CCCTCGGC -0.677367050697
CCCTCGTA 0.123539481868
CCCTCGTC -1.3023652622
CCCTCTAA -0.299459252719
CCCTCTAC -1.47808580671
CCCTCTAG -0.170575217274
CCCTCTCA -0.90956628431
CCCTCTCC -0.840330031532
CCCTCTCG -0.812029274474
CCCTCTGA -0.934445219722
CCCTCTGC -1.26727686173
CCCTCTTA -0.664559940658
CCCTCTTC -0.34808859065
CCCTGAAA -1.04705369161
CCCTGAAC -0.942861231385
CCCTGAAG -0.266066878344
CCCTGACA 0.375163803872
CCCTGACC -0.806893731601
CCCTGACG -0.857741622388
CCCTGAGA -0.939013893924
CCCTGAGC -0.298669633597
CCCTGAGG -0.628171052273
CCCTGATA 1.46417264701
CCCTGATC 0.218150550034
CCCTGCAA -0.864636477467
CCCTGCAC -0.786997120061

CCCTGCAG -0.546311094266
CCCTGCCA -0.632067103384
CCCTGCCC -1.48419853708
CCCTGCCG -1.12020369063
CCCTGCGA -0.677265668015
CCCTGCGC 0.191040362909
CCCTGCGG -0.993106446887
CCCTGCTA -0.748879729812
CCCTGCTC 0.191242711917
CCCTGGAA -1.17693968773
CCCTGGAC -0.790204518376
CCCTGGAG -1.20169621448
CCCTGGCA -0.408138781374
CCCTGGCC -1.27066599943
CCCTGGCG -0.489203915808
CCCTGGGA -1.14662064504
CCCTGGGC -1.20644517082
CCCTGGTA -0.688330952798
CCCTGGTC -1.25201262686
CCCTGTAA 0.431088947694
CCCTGTAC -1.38430703416
CCCTGTAG 0.30444157659
CCCTGTCA -0.932378844978
CCCTGTCC -1.43449812338
CCCTGTCG -0.841654043558
CCCTGTGA -0.377508304412
CCCTGTGC -0.581539390349
CCCTGTTA -0.112543936712
CCCTGTTC -1.01344252217
CCCTTAAA -0.416746524967
CCCTTAAC -0.928831492002
CCCTTAAG 0.14335303039
CCCTTACA 0.138205205015
CCCTTACC -0.28192254689
CCCTTACG -0.447116571261
CCCTTAGA -0.0543090580737
CCCTTAGC -0.315320958427
CCCTTAGG -0.218600630851
CCCTTATA -0.826461838271
CCCTTATC -0.172846439162
CCCTTCAA -0.988244658072
CCCTTCAC -1.91035429091
CCCTTCAG -0.58515148668
CCCTTCCA -1.37627844174
CCCTTCCC -1.3116974652
CCCTTCCG -1.06680645216
CCCTTCGA -0.383144931711
CCCTTCGC -0.945845671072
CCCTTCGG -0.978500470823
CCCTTCTA -0.578614489568
CCCTTCTC -0.336162906227
CCCTTGAA -0.456248715216
CCCTTGAC -0.633874296529
CCCTTGAG -0.578090297385

CCCTTGCA 0.415695642466
CCCTTGCC -0.855721879514
CCCTTGCG -1.07601957735
CCCTTGGA -1.2181083428
CCCTTGGC -0.705553518037
CCCTTGTA -0.127671398644
CCCTTGTC -0.000172371376985
CCCTTTAA -0.452472158271
CCCTTTAC 0.0740707702755
CCCTTTAG 0.166086899777
CCCTTTCA -1.06582926467
CCCTTTCC -1.05948316677
CCCTTTCG -0.748111136667
CCCTTTGA -0.491992876361
CCCTTTGC -0.423008102596
CCCTTTTA -0.537726250252
CCCTTTTC -0.943724961872
CCGAAAAA 0.686338689419
CCGAAAAC -0.954262307269
CCGAAAAG -0.28482059271
CCGAAACA -0.42459566796
CCGAAACC -0.341917362269
CCGAAACG -0.949496488509
CCGAAAGA 0.818952649938
CCGAAAGC -0.319166422287
CCGAAAGG -0.379717489258
CCGAAATA 1.8840716113
CCGAAATC 8.40367586842
CCGAACAA 0.854014403627
CCGAACAC -0.541363119764
CCGAACAG 0.0924505969694
CCGAACCA 0.918688435721
CCGAACCC -0.918494413833
CCGAACCG -0.603888963163
CCGAACGA -0.588727776396
CCGAACGC 0.383808603148
CCGAACGG -0.709543873779
CCGAACTA -0.733210380568
CCGAACTC 0.0879456252328
CCGAAGAA 0.134276678137
CCGAAGAC -0.477117726154
CCGAAGAG -0.0247769281956
CCGAAGCA -0.374726213733
CCGAAGCC -1.45050513733
CCGAAGCG -0.578181062989
CCGAAGGA 0.628218725033
CCGAAGGC -1.11137673561
CCGAAGTA 0.920367807579
CCGAAGTC -0.801374933046
CCGAATAA 0.976222795418
CCGAATAC -0.0137874202009
CCGAATAG 0.132456994313
CCGAATCA 3.85693321776
CCGAATCC 3.61758098836

CCGAATCG 2.64163652692
CCGAATGA -0.397825643673
CCGAATGC -0.320337631667
CCGAATTA 0.942481306551
CCGAATTC 1.21734203961
CCGACAAA 0.795515763474
CCGACAAC 0.321396424932
CCGACAAG -0.471878302464
CCGACACA -0.15046959503
CCGACACC -1.02789382198
CCGACACG -1.06714640682
CCGACAGA -0.205437744625
CCGACAGC 0.142482846385
CCGACAGG -0.727457381772
CCGACATA 0.904513804456
CCGACATC 1.01094938254
CCGACCAA -0.568366303401
CCGACCAC -0.7509996063
CCGACCAG -0.864190560209
CCGACCCA -1.17037958286
CCGACCCC -0.932264763439
CCGACCCG -1.67936124058
CCGACCGA -0.110439465395
CCGACCGC -0.646575027614
CCGACCGG -0.948202454114
CCGACCTA -1.17823413848
CCGACCTC -1.34664721913
CCGACGAA 0.161555281257
CCGACGAC -0.696604362538
CCGACGAG -0.353459166475
CCGACGCA -0.220598723214
CCGACGCC -2.01535177543
CCGACGCG -0.155636780958
CCGACGGA -0.837221517763
CCGACGGC -1.15455763915
CCGACGTA 0.414455317993
CCGACGTC -1.16936658875
CCGACTAA -0.962448074074
CCGACTAC -0.515932096319
CCGACTAG -0.808119899971
CCGACTCA 1.64563411599
CCGACTCC -1.17321746524
CCGACTCG -0.414874588467
CCGACTGA -0.64121985696
CCGACTGC 0.0427489342042
CCGACTTA -0.0891509758037
CCGACTTC -0.539116462879
CCGAGAAA 0.404938252932
CCGAGAAC -1.48981497111
CCGAGAAG -1.59081585455
CCGAGACA 0.113733882111
CCGAGACC -1.09575131192
CCGAGACG -1.08467541006
CCGAGAGA 1.10558647295

CCGAGAGC -0.332951968886
CCGAGAGG -1.01729506408
CCGAGATA 4.02997201339
CCGAGATC 4.33318492347
CCGAGCAA -0.326918554335
CCGAGCAC -0.483296448943
CCGAGCAG -0.891608018049
CCGAGCCA -0.548351863117
CCGAGCCC -1.62450113536
CCGAGCCG -1.11497384313
CCGAGCGA -0.138039703512
CCGAGCGC 0.231224127902
CCGAGCGG -1.49259248187
CCGAGCTA -0.163012527198
CCGAGCTC -0.865679241027
CCGAGGAA -0.778792825415
CCGAGGAC -1.1929171404
CCGAGGAG -0.720064981293
CCGAGGCA -0.270016223021
CCGAGGCC -0.610677647467
CCGAGGCG -0.318168208817
CCGAGGGA -0.685993530309
CCGAGGGC -0.780304405811
CCGAGGTA -0.319952918739
CCGAGGTC -0.439315517385
CCGAGTAA 1.17380119633
CCGAGTAC -0.545276449648
CCGAGTAG -0.526571865288
CCGAGTCA 0.203605570122
CCGAGTCC -1.33054839869
CCGAGTCG -0.746556671605
CCGAGTGA -0.247227186466
CCGAGTGC -0.383892707056
CCGAGTTA -0.470599881418
CCGAGTTC -0.419123917631
CCGATAAA 0.987347410929
CCGATAAC -0.295915855125
CCGATAAG 0.818952649938
CCGATACA 0.755457113454
CCGATACC 2.17711794099
CCGATACG 2.1874281642
CCGATAGA 1.58621095738
CCGATAGC 2.08533663626
CCGATAGG 1.1584788798
CCGATATA 1.60881534005
CCGATATC 11.8298921526
CCGATCAA 0.783461633231
CCGATCAC 0.459904112569
CCGATCAG -0.406406532306
CCGATCCA 0.662650532119
CCGATCCC 0.784237720783
CCGATCCG 0.357336273389
CCGATCGA -0.105711951386
CCGATCGC 3.08598100004

CCGATCGG -0.725083111779
CCGATCTA 0.522730564988
CCGATCTC 3.12965008097
CCGATGAA -0.823680372126
CCGATGAC -0.769050303589
CCGATGAG -0.87591431198
CCGATGCA 0.648711766519
CCGATGCC -0.752724985494
CCGATGCG -0.712519986346
CCGATGGA 0.00788869681095
CCGATGGC -0.686050987435
CCGATGTA -0.332068877845
CCGATGTC 0.19122064505
CCGATTAA 1.07886995041
CCGATTAC 6.68484220705
CCGATTAG 1.14817552646
CCGATTCA 0.12601346912
CCGATTCC 3.20176481084
CCGATTCG 2.37736560011
CCGATTGA -0.518093400227
CCGATTGC 4.67654933096
CCGATTTA 0.930352440409
CCGATTTC 9.72629726555
CCGCAAAA 0.684916209203
CCGCAAAC -1.024683301
CCGCAAAG -0.280825240697
CCGCAACA 0.162275993464
CCGCAACC -0.884905311256
CCGCAACG 0.217397778416
CCGCAAGA 0.911471737467
CCGCAAGC 0.0681474818825
CCGCAAGG -1.4293342683
CCGCAATA 1.03161604447
CCGCAATC 11.4075643756
CCGCACAA 0.32792655217
CCGCACAC -0.0752432287238
CCGCACAG -0.179515629298
CCGCACCA 0.126491445788
CCGCACCC -0.820311635867
CCGCACCG -0.95228357546
CCGCACGA -0.948138751649
CCGCACGC -1.01197611639
CCGCACGG -0.97758136499
CCGCACTA -0.621344687753
CCGCACTC -0.650968832303
CCGCAGAA -0.0618634210303
CCGCAGAC -0.588256253245
CCGCAGAG 0.136113432556
CCGCAGCA -0.41663535792
CCGCAGCC -0.951495205406
CCGCAGCG -0.169160023288
CCGCAGGA 0.333412042247
CCGCAGGC -1.15644914438
CCGCAGTA -0.501939620796

CCGCAGTC 0.586943066475
CCGCATAA 0.0585579708808
CCGCATAC -0.67601097926
CCGCATAG 0.348509526548
CCGCATCA 0.811503625045
CCGCATCC -0.020418513765
CCGCATCG -0.735961244548
CCGCATGA -0.50528295934
CCGCATGC -0.442923658334
CCGCATTA 0.176695233869
CCGCATTC -0.0942461321464
CCGCCAAA 0.211607099404
CCGCCAAC -1.2744356865
CCGCCAAG -0.602512698461
CCGCCACA -0.185603586482
CCGCCACC -1.4214813781
CCGCCACG -1.17725445285
CCGCCAGA 0.753413430112
CCGCCAGC 0.329231828177
CCGCCAGG -1.56124458721
CCGCCATA -1.18160953643
CCGCCATC -0.708764455379
CCGCCCAA -0.768165130769
CCGCCCAC -0.757627577194
CCGCCCAG -0.573357578926
CCGCCCCA -0.727791507448
CCGCCCCC -1.68078767617
CCGCCCCG -0.360198096239
CCGCCCGA -0.509001850981
CCGCCCGC -0.214915880401
CCGCCCGG -1.50728318638
CCGCCCTA -0.0666090465251
CCGCCCTC -0.705239793804
CCGCCGAA 0.603268176392
CCGCCGAC -0.847032113792
CCGCCGAG -0.73640050011
CCGCCGCA -1.14422680632
CCGCCGCC -1.33355490525
CCGCCGCG -0.980608689343
CCGCCGGA -0.190318193456
CCGCCGGC -1.20301981015
CCGCCGTA -1.51561405325
CCGCCGTC -1.16817247979
CCGCCTAA 0.276315897223
CCGCCTAC -1.06827077615
CCGCCTAG -0.422117100793
CCGCCTCA 0.0420723557317
CCGCCTCC -1.2943866325
CCGCCTCG -1.10489782016
CCGCCTGA -0.413502695503
CCGCCTGC -0.587250545368
CCGCCTTA -0.926772403488
CCGCCTTC 0.519035613816
CCGCGAAA 1.12407621763

CCGCGAAC −1.04680575162
CCGCGAAG −0.202945645889
CCGCGACA −0.206165951239
CCGCGACC −1.01529551447
CCGCGACG −0.578903648798
CCGCGAGA 0.654161658159
CCGCGAGC −0.261628523564
CCGCGAGG −1.36277747445
CCGCGATA 1.17575369771
CCGCGATC 5.04232951186
CCGCGCAA 0.87109041156
CCGCGCAC −0.544275529865
CCGCGCAG −0.702583234454
CCGCGCCA −0.308645314773
CCGCGCCC −0.389181468931
CCGCGCCG −0.267430027832
CCGCGCGA −0.184674279928
CCGCGCGC 0.0521048694994
CCGCGCGG −0.197463070304
CCGCGCTA −1.08497768451
CCGCGCTC −1.36192644282
CCGCGGAA −0.864834246559
CCGCGGAC −0.477200372817
CCGCGGAG 0.464083493927
CCGCGGCA 0.308390504911
CCGCGGCC −1.01654895416
CCGCGGCG 0.732947035409
CCGCGGGA −0.387097815414
CCGCGGGC −0.140840738388
CCGCGGTA 0.174572859245
CCGCGGTC −1.00769930774
CCGCGTAA 0.235310870053
CCGCGTAC 0.313567267026
CCGCGTAG −0.349038715003
CCGCGTCA −0.322825358666
CCGCGTCC −1.03747000959
CCGCGTCG −0.604969198761
CCGCGTGA −0.727975328615
CCGCGTGC −0.294176736184
CCGCGTTA −1.12801952514
CCGCGTTC −0.169906549565
CCGCTAAA −0.10374008945
CCGCTAAC −0.186952996223
CCGCTAAG 0.892943896221
CCGCTACA −0.800695440081
CCGCTACC −1.41912938316
CCGCTACG −1.02642429354
CCGCTAGA 1.8763019923
CCGCTAGC −0.494272841724
CCGCTAGG −1.0782135652
CCGCTATA −0.367699165629
CCGCTATC 1.40553994023
CCGCTCAA 0.0995105371961
CCGCTCAC −1.41546087059

| | |
|---|---|
| CCGCTCAG | 0.188143774335 |
| CCGCTCCA | -0.971154910392 |
| CCGCTCCC | -0.545276449648 |
| CCGCTCCG | -0.837162811569 |
| CCGCTCGA | -0.177362236154 |
| CCGCTCGC | -0.874977927374 |
| CCGCTCTA | -1.59588373957 |
| CCGCTCTC | -0.702771219181 |
| CCGCTGAA | -0.603475313494 |
| CCGCTGAC | -0.703654518399 |
| CCGCTGAG | -1.31318614602 |
| CCGCTGCA | -0.478385530123 |
| CCGCTGCC | -1.61538127391 |
| CCGCTGCG | -0.980313909307 |
| CCGCTGGA | -1.04933573875 |
| CCGCTGGC | -0.702480810882 |
| CCGCTGTA | -1.60177954847 |
| CCGCTGTC | 0.0451914865788 |
| CCGCTTAA | -0.454304540953 |
| CCGCTTAC | -0.129893906882 |
| CCGCTTAG | -0.578541627271 |
| CCGCTTCA | -0.309293997394 |
| CCGCTTCC | -1.38834360541 |
| CCGCTTCG | -0.861647874221 |
| CCGCTTGA | -0.803801663893 |
| CCGCTTGC | -1.17410555255 |
| CCGCTTTA | -0.843422723774 |
| CCGCTTTC | -0.378412421429 |
| CCGGAAAA | 0.821377507184 |
| CCGGAAAC | -0.80268978524 |
| CCGGAAAG | -0.464013129766 |
| CCGGAACA | 0.0519204237989 |
| CCGGAACC | -0.648943676801 |
| CCGGAACG | -1.10011388991 |
| CCGGAAGA | -0.618869035077 |
| CCGGAAGC | -0.464488191942 |
| CCGGAAGG | -0.484983315208 |
| CCGGAATA | 1.72625562312 |
| CCGGAATC | 7.20345188869 |
| CCGGACAA | 0.565905639541 |
| CCGGACAC | -1.24391554388 |
| CCGGACAG | -0.0285430762404 |
| CCGGACCA | -0.652178970968 |
| CCGGACCC | -1.24723806462 |
| CCGGACCG | -1.28867839197 |
| CCGGACGA | -0.341990640922 |
| CCGGACGC | -0.463866156104 |
| CCGGACGG | -1.03427073022 |
| CCGGACTA | -1.43909302801 |
| CCGGACTC | -0.891571795079 |
| CCGGAGAA | -0.27244482747 |
| CCGGAGAC | -0.449411317262 |
| CCGGAGAG | -1.04095324375 |
| CCGGAGCA | -0.505304609851 |

CCGGAGCC -1.64662337781
CCGGAGCG -0.633921552933
CCGGAGGA -1.3764878688
CCGGAGGC -1.08004324157
CCGGAGTA -0.808663660885
CCGGAGTC -0.563989985663
CCGGATAA 0.979696037028
CCGGATAC 3.46096202364
CCGGATAG 0.30444157659
CCGGATCA 1.85514278124
CCGGATCC 2.76682581937
CCGGATCG 1.46900216823
CCGGATGA 0.86087782383
CCGGATGC -0.324375451991
CCGGATTA 5.09950393181
CCGGATTC 7.94797341225
CCGGCAAA 0.356380736408
CCGGCAAC -0.650168596103
CCGGCAAG -0.965133153809
CCGGCACA 1.49436699107
CCGGCACC -1.38402807565
CCGGCACG -1.41934026746
CCGGCAGA -0.430452339395
CCGGCAGC -0.984273662884
CCGGCAGG -0.96151876752
CCGGCATA -0.48243958833
CCGGCATC 0.982555153564
CCGGCCAA -0.900599433681
CCGGCCAC -1.64611334173
CCGGCCAG -0.870656984982
CCGGCCCA -1.00266452301
CCGGCCCC -0.981461386397
CCGGCCCG -1.39350558689
CCGGCCGA -0.560364149584
CCGGCCGC -0.209915653223
CCGGCCGG -0.524707631373
CCGGCCTA -1.60155159357
CCGGCCTC -0.799448453912
CCGGCGAA 0.154432679455
CCGGCGAC -0.739134085317
CCGGCGAG -1.05855844013
CCGGCGCA -0.0819921510311
CCGGCGCC -1.1242098679
CCGGCGCG -0.108662666238
CCGGCGGA -0.793645076042
CCGGCGGC -0.629707197672
CCGGCGTA 0.584241957035
CCGGCGTC -0.753614321873
CCGGCTAA -0.796916593179
CCGGCTAC -1.35950970452
CCGGCTAG -0.622567525275
CCGGCTCA -0.32752435229
CCGGCTCC -0.991786182064
CCGGCTCG -1.45012875152

CCGGCTGA -0.815873905622
CCGGCTGC -1.04859295968
CCGGCTTA -0.887885379205
CCGGCTTC -0.277692370102
CCGGGAAA -0.164483512886
CCGGGAAC -1.01463871291
CCGGGAAG -1.38794640181
CCGGGACA -0.604919444221
CCGGGACC -1.50645859336
CCGGGACG -1.3774100973
CCGGGAGA 0.451414405896
CCGGGAGC -0.668922726826
CCGGGAGG -0.715869986586
CCGGGATA 0.99794242163
CCGGGATC 1.7849942925
CCGGGCAA -0.189500262128
CCGGGCAC -0.638274762914
CCGGGCAG -0.569444873575
CCGGGCCA -0.710417388631
CCGGGCCC -0.85605288252
CCGGGCCG -1.845600943
CCGGGCGA -1.07652794801
CCGGGCGC -0.873475090453
CCGGGCTA -0.9590208398
CCGGGCTC -0.535309720126
CCGGGGAA -0.439025109087
CCGGGGAC -1.57441101246
CCGGGGAG -1.26666523479
CCGGGGCA -0.157597193104
CCGGGGCC -1.37895269622
CCGGGGCG -1.09580065011
CCGGGGGA -0.294933671361
CCGGGGGC -1.45533320131
CCGGGGTA -0.151541503509
CCGGGGTC -0.699183271497
CCGGGTAA -0.577456187223
CCGGGTAC 0.00109501623544
CCGGGTAG -0.505507999749
CCGGGTCA -0.50994406457
CCGGGTCC -1.68452384659
CCGGGTCG -1.16647333103
CCGGGTGA -0.223819236743
CCGGGTGC -1.37910091895
CCGGGTTA -1.43722046698
CCGGGTTC 0.292861675811
CCGGTAAA 0.465434985448
CCGGTAAC -0.869232214808
CCGGTAAG -0.525883837026
CCGGTACA -0.740156863789
CCGGTACC -0.827903887218
CCGGTACG -1.50330948488
CCGGTAGA 0.612316632794
CCGGTAGC -1.13049330422
CCGGTAGG 0.623478512166

CCGGTATA 0.25112719296
CCGGTATC 2.88474491182
CCGGTCAA -0.158992193825
CCGGTCAC -1.18142613162
CCGGTCAG -0.462651437523
CCGGTCCA -0.571628868884
CCGGTCCC -1.05018302318
CCGGTCCG -1.09947748979
CCGGTCGA -0.247154740525
CCGGTCGC -0.722001244792
CCGGTCTA -0.566752091254
CCGGTCTC 0.110064953189
CCGGTGAA -0.342003131601
CCGGTGAC -0.73674565922
CCGGTGAG -1.40782511004
CCGGTGCA -0.408492892137
CCGGTGCC -1.22562752368
CCGGTGCG -1.17175147582
CCGGTGGA 0.00372721876031
CCGGTGGC -1.36355522743
CCGGTGTA -0.453212647387
CCGGTGTC -0.300302165406
CCGGTTAA 0.303029088917
CCGGTTAC -0.272873465955
CCGGTTAG 0.0777786284826
CCGGTTCA -0.21209257048
CCGGTTCC -1.14647450409
CCGGTTCG -1.32495507242
CCGGTTGA -0.630907135615
CCGGTTGC -1.31884879557
CCGGTTTA -1.30849402227
CCGGTTTC -0.0164038011984
CCGTAAAA 0.885692015886
CCGTAAAC -1.05229478073
CCGTAAAG -0.193395688705
CCGTAACA 0.256931611719
CCGTAACC 0.21479472081
CCGTAACG 0.782880816634
CCGTAAGA 1.69859605428
CCGTAAGC -1.13721266525
CCGTAAGG -0.661896095078
CCGTAATA 3.27653339374
CCGTAATC 11.7703295147
CCGTACAA 0.990347255787
CCGTACAC -0.633646341628
CCGTACAG -0.603744695815
CCGTACCA 0.640110684621
CCGTACCC -0.72417087582
CCGTACCG -1.51376647357
CCGTACGA -0.339849322101
CCGTACGC -0.368051194613
CCGTACGG -0.0793949224088
CCGTACTA -0.278799044305
CCGTACTC -0.0398148735916

CCGTAGAA 0.676564108183
CCGTAGAC -0.880377231762
CCGTAGAG -1.17322912321
CCGTAGCA -0.353831388724
CCGTAGCC -1.1184010774
CCGTAGCG -0.435960520873
CCGTAGGA -0.96461687239
CCGTAGGC -0.885728030679
CCGTAGTA 0.00339101130399
CCGTAGTC -0.226970010645
CCGTATAA 0.603666837246
CCGTATAC 0.208214839032
CCGTATAG 0.727341010275
CCGTATCA 2.02237299455
CCGTATCC 3.70909062081
CCGTATCG 1.74798836455
CCGTATGA -0.646843369045
CCGTATGC -0.729026419294
CCGTATTA 0.537984390961
CCGTATTC 1.70963677406
CCGTCAAA 0.461420689237
CCGTCAAC -1.06947654308
CCGTCAAG 0.0477502022727
CCGTCACA -0.380082425277
CCGTCACC 0.0539709770155
CCGTCACG -1.297128753
CCGTCAGA -0.140611118063
CCGTCAGC -0.818670360582
CCGTCAGG -0.857329429964
CCGTCATA 0.459513362479
CCGTCATC -0.0739027706363
CCGTCCAA 0.32562243816
CCGTCCAC -1.03807955475
CCGTCCAG -0.817089456914
CCGTCCCA -0.829223319328
CCGTCCCC -0.960815542265
CCGTCCCG -0.676084466091
CCGTCCGA -0.64121985696
CCGTCCGC -0.574925991914
CCGTCCTA -0.520131462764
CCGTCCTC -1.47958219012
CCGTCGAA -0.522667487056
CCGTCGAC -0.624387833633
CCGTCGAG -0.691026857789
CCGTCGCA -0.608757205495
CCGTCGCC -1.29602374422
CCGTCGCG -0.336134177664
CCGTCGGA -0.900742035606
CCGTCGGC -1.07626272925
CCGTCGTA -0.668913358817
CCGTCGTC -0.325136967084
CCGTCTAA -0.0779818102023
CCGTCTAC -0.601946662502
CCGTCTAG -0.652282851785

CCGTCTCA -0.108530265035
CCGTCTCC -1.11160510687
CCGTCTCG -1.39638656212
CCGTCTGA -0.385008541091
CCGTCTGC -0.734047256094
CCGTCTTA -0.189028947155
CCGTCTTC -0.562981155116
CCGTGAAA 0.312004683022
CCGTGAAC 0.054744982788
CCGTGAAG -0.547408608638
CCGTGACA -0.0389059684806
CCGTGACC -0.743746268719
CCGTGACG -0.412699544812
CCGTGAGA -0.108530265035
CCGTGAGC -0.31662456901
CCGTGAGG -0.392396569832
CCGTGATA 2.67684338067
CCGTGATC 1.49519845397
CCGTGCAA 0.447832495374
CCGTGCAC -0.959125761508
CCGTGCAG -0.629633502663
CCGTGCCA -0.103444476702
CCGTGCCC -0.538450709662
CCGTGCCG -0.567884163172
CCGTGCGA -0.753110739312
CCGTGCGC 0.289208776594
CCGTGCTA -0.277879105761
CCGTGCTC -0.0290841308403
CCGTGGAA -0.839709869296
CCGTGGAC -0.760540403652
CCGTGGAG -1.02883561922
CCGTGGCA -1.04003767694
CCGTGGCC -1.24895053678
CCGTGGCG 0.363488765751
CCGTGGGA -0.846002673623
CCGTGGGC -0.77979041435
CCGTGGTA 0.164147513608
CCGTGGTC -0.766921059092
CCGTGTAA -0.187985559061
CCGTGTAC -0.699439330426
CCGTGTAG 0.631329112404
CCGTGTCA 0.453015294651
CCGTGTCC -0.767040553259
CCGTGTCG -0.728922330298
CCGTGTGA 0.301771069315
CCGTGTGC -1.01733128705
CCGTGTTA 0.00334354672192
CCGTGTTC -0.895027549738
CCGTTAAA -0.0685111688337
CCGTTAAC 0.181394435672
CCGTTAAG 0.153411982762
CCGTTACA 0.0561310318556
CCGTTACC -0.54067529968
CCGTTACG -0.876201181252

CCGTTAGA −0.286777257653
CCGTTAGC −0.201450511553
CCGTTAGG 0.309471573221
CCGTTATA 0.729127593798
CCGTTATC 1.03072878987
CCGTTCAA −0.187201144389
CCGTTCAC −0.494311771009
CCGTTCAG 0.830014395696
CCGTTCCA 0.0874160204223
CCGTTCCC −0.71428887474
CCGTTCCG −1.05635050436
CCGTTCGA −0.204737225684
CCGTTCGC −0.351317431298
CCGTTCTA 0.257295923205
CCGTTCTC 0.292700546046
CCGTTGAA 0.287597062583
CCGTTGAC −0.481154921944
CCGTTGAG −0.457670987254
CCGTTGCA −0.4934482487
CCGTTGCC −0.716428111781
CCGTTGCG 0.217397778416
CCGTTGGA −0.373363896957
CCGTTGGC −0.875393450645
CCGTTGTA −0.00578505820656
CCGTTGTC 0.578022223182
CCGTTTAA 0.170817536456
CCGTTTAC −0.420186874455
CCGTTTAG 0.171142918657
CCGTTTCA −0.626058670193
CCGTTTCC −0.616032609943
CCGTTTCG −1.19507615435
CCGTTTGA −0.0775329784526
CCGTTTGC −0.673352962664
CCGTTTTA 0.119512694978
CCGTTTTC 0.915772070238
CCTAAAAA 0.116804923842
CCTAAAAC 0.0237135550149
CCTAAAAG 0.62326658697
CCTAAACA −0.0466376990859
CCTAAACC −0.277773559519
CCTAAACG −0.627378726837
CCTAAAGA 0.621161074764
CCTAAAGC −0.629909338502
CCTAAAGG 0.141948869837
CCTAAATA 1.43868749728
CCTAAATC 1.95631437063
CCTAACAA −0.151774038325
CCTAACAC −0.716209941246
CCTAACAG 0.11871849594
CCTAACCA −0.77174204502
CCTAACCC −1.19333599452
CCTAACCG −0.444170436325
CCTAACGA 0.686169024356
CCTAACGC −1.24637058693

CCTAACTA -0.520996025963
CCTAACTC -0.523779990243
CCTAAGAA 0.193766870064
CCTAAGAC -0.738809119472
CCTAAGAG -0.180371240843
CCTAAGCA -0.177410533448
CCTAAGCC -1.58567177638
CCTAAGCG -0.61477979479
CCTAAGGA 0.347539000752
CCTAAGGC -0.788475391979
CCTAAGTA -0.621235186129
CCTAAGTC -0.457331240772
CCTAATAA -0.543777984465
CCTAATAC -0.630646288592
CCTAATAG 0.80913164501
CCTAATCA 0.837472372243
CCTAATCC -0.360716043082
CCTAATCG 0.90962957042
CCTAATGA 0.134711145606
CCTAATGC -0.115864375677
CCTAATTA 0.960162279727
CCTAATTC 0.573560344289
CCTACAAA -0.278799044305
CCTACAAC -0.384750608559
CCTACAAG 0.0673401676314
CCTACACA 0.568036965818
CCTACACC -0.723815099632
CCTACACG -0.0117845397444
CCTACAGA 0.457077055444
CCTACAGC -0.645967147878
CCTACAGG 0.117014350902
CCTACATA 1.27986142949
CCTACATC 1.44518868778
CCTACCAA -0.208403864648
CCTACCAC 0.83870499413
CCTACCAG -0.990678258793
CCTACCCA -0.210325139332
CCTACCCC -0.984485171723
CCTACCCG -0.984897155969
CCTACCGA -0.0301121137625
CCTACCGC -0.851838110904
CCTACCTA -0.224187711788
CCTACCTC -0.743179608226
CCTACGAA 0.102523080912
CCTACGAC -1.29731632137
CCTACGAG -0.0269915256695
CCTACGCA -1.01211996739
CCTACGCC -1.41546087059
CCTACGCG 0.801132405686
CCTACGGA 0.309109759872
CCTACGGC -0.927561398076
CCTACGTA -0.631911386246
CCTACGTC -0.715976365539
CCTACTAA -0.190997686421

CCTACTAC -0.5648381028
CCTACTAG 0.0393294025154
CCTACTCA -0.518805368958
CCTACTCC -0.498228639919
CCTACTCG -0.105460056016
CCTACTGA -0.911375559235
CCTACTGC -0.995859809003
CCTACTTA 0.361630152642
CCTACTTC -0.560922482958
CCTAGAAA 0.433556481427
CCTAGAAC -0.00158923078732
CCTAGAAG -0.255863658623
CCTAGACA -0.693493766988
CCTAGACC -1.18796604323
CCTAGACG -0.809385830338
CCTAGAGA 0.356600156011
CCTAGAGC -0.968319526147
CCTAGAGG -0.522437658554
CCTAGATA 2.91612462137
CCTAGATC 1.35617719123
CCTAGCAA -0.0865574943853
CCTAGCAC -0.58581682354
CCTAGCAG -0.471641395909
CCTAGCCA 0.499047195782
CCTAGCCC -1.17018160559
CCTAGCCG -0.942952621524
CCTAGCGA -0.477552818157
CCTAGCGC -1.06975799972
CCTAGCTA -0.35462163238
CCTAGCTC 0.340664755294
CCTAGGAA -0.545564567989
CCTAGGAC -1.32497755564
CCTAGGAG -0.719778736554
CCTAGGCA -0.407700983057
CCTAGGCC -0.469211958748
CCTAGGCG 0.218173865969
CCTAGGGA -1.16381822892
CCTAGGGC -0.869911291416
CCTAGGTA -0.373241904654
CCTAGGTC -1.01223446528
CCTAGTAA 0.197384795379
CCTAGTAC -0.473523116775
CCTAGTAG 0.326455566482
CCTAGTCA -0.0317679615071
CCTAGTCC -0.867219966342
CCTAGTCG -0.367317367193
CCTAGTGA -0.683407126943
CCTAGTGC -0.989074663725
CCTAGTTA 0.0352936639716
CCTAGTTC 0.373896416259
CCTATAAA 0.364225924019
CCTATAAC -0.27888356457
CCTATAAG 0.825465706581
CCTATACA 0.12982874717

CCTATACC -0.93385357787
CCTATACG 0.585699411153
CCTATAGA 0.45816270367
CCTATAGC -0.774058233353
CCTATAGG 1.09399491421
CCTATATA 1.05042638325
CCTATATC 3.18511244512
CCTATCAA 0.99631405338
CCTATCAC -0.260957774076
CCTATCAG 0.274527648275
CCTATCCA 0.862532214329
CCTATCCC -0.0114797671648
CCTATCCG -0.353563671827
CCTATCGA -0.0925274146483
CCTATCGC 0.00208261262724
CCTATCTA -0.150996701705
CCTATCTC 0.365354873267
CCTATGAA -0.592741448073
CCTATGAC -0.834284126302
CCTATGAG -0.751958265951
CCTATGCA -0.536454699079
CCTATGCC 0.139826078857
CCTATGCG -0.0244392634934
CCTATGGA -0.495046014785
CCTATGGC -0.966612466617
CCTATGTA -0.0384889879636
CCTATGTC 0.230732411486
CCTATTAA 1.35275391234
CCTATTAC -0.297866066549
CCTATTAG 0.945945804686
CCTATTCA -0.163377255039
CCTATTCC -0.0522324826082
CCTATTCG 0.130840492209
CCTATTGA -0.266815278223
CCTATTGC -0.644749514806
CCTATTTA 0.292038748211
CCTATTTC -0.490767540703
CCTCAAAA -0.656571318411
CCTCAAAC -0.768213219885
CCTCAAAG 1.25654237177
CCTCAACA -0.244583325974
CCTCAACC -0.668816347873
CCTCAACG -0.092658150427
CCTCAAGA 0.208941172045
CCTCAAGC -1.30472766605
CCTCAAGG -0.946243291036
CCTCAATA 1.67442804671
CCTCAATC 0.255880937396
CCTCACAA 0.695729182261
CCTCACAC -0.918595796515
CCTCACAG -0.425164618411
CCTCACCA -0.276969367938
CCTCACCC -0.930008946723
CCTCACCG -0.571658846515

CCTCACGA 0.144815272602
CCTCACGC -0.0110950542365
CCTCACTA -1.10176411685
CCTCACTC -0.891707943485
CCTCAGAA -0.238436870774
CCTCAGAC -0.821630651621
CCTCAGAG 0.693997557728
CCTCAGCA -0.84613778114
CCTCAGCC -1.41934880276
CCTCAGCG -0.611005319625
CCTCAGGA -0.352756357576
CCTCAGGC -0.864247809157
CCTCAGTA -0.850228478673
CCTCAGTC -0.607333892567
CCTCATAA 0.079683457105
CCTCATAC -0.906924921954
CCTCATAG 0.389622598095
CCTCATCA 1.03246374525
CCTCATCC -0.378236302849
CCTCATCG -0.308620541592
CCTCATGA 0.353406705621
CCTCATGC -0.999811443638
CCTCATTA 0.362134359738
CCTCATTC -0.0917677731572
CCTCCAAA -0.471155508477
CCTCCAAC -0.889737538795
CCTCCAAG -0.255378395725
CCTCCACA -0.583318063108
CCTCCACC -1.4489569176
CCTCCACG -1.06151165312
CCTCCAGA -0.442152567053
CCTCCAGC -0.978101809969
CCTCCAGG -1.48024586155
CCTCCATA 0.77360773618
CCTCCATC -1.01749408224
CCTCCCAA -1.20224039175
CCTCCCAC -1.21945379716
CCTCCCAG 0.141690312771
CCTCCCCA -0.675776154485
CCTCCCCC -1.47958219012
CCTCCCCG -1.58567177638
CCTCCCGA -0.584948096782
CCTCCCGC -0.768715553379
CCTCCCTA -1.29888973063
CCTCCCTC -1.05080006275
CCTCCGAA -0.196390537291
CCTCCGAC -0.630829068869
CCTCCGAG -0.770582493607
CCTCCGCA -0.96206398568
CCTCCGCC -1.7395088586
CCTCCGCG -0.678151881725
CCTCCGGA -0.56424937544
CCTCCGGC -1.48913943353
CCTCCGTA -0.746777548454

CCTCCGTC -0.527913988799
CCTCCTAA -0.903039904276
CCTCCTAC -1.47626216751
CCTCCTAG -0.740015302755
CCTCCTCA -0.59954428848
CCTCCTCC -0.648421982755
CCTCCTCG -0.861338729903
CCTCCTGA -0.354981363949
CCTCCTGC -1.04155321272
CCTCCTTA -0.93792387396
CCTCCTTC -1.01467826672
CCTCGAAA 0.788590722586
CCTCGAAC -0.803052847657
CCTCGAAG -0.148784394189
CCTCGACA -0.059830979299
CCTCGACC -1.18356932405
CCTCGACG -0.901104265311
CCTCGAGA 0.681798535602
CCTCGAGC -0.908682776914
CCTCGAGG -0.0232638905532
CCTCGATA 1.61610614967
CCTCGATC 0.146867074887
CCTCGCAA -1.04141581524
CCTCGCAC -0.786601790055
CCTCGCAG -0.432102149978
CCTCGCCA 0.269466841301
CCTCGCCC -1.28829180544
CCTCGCCG -1.45686039505
CCTCGCGA 0.229068861155
CCTCGCGC -0.657603048537
CCTCGCTA -0.899948044746
CCTCGCTC 0.0823244031056
CCTCGGAA -0.902308366814
CCTCGGAC -1.32417607037
CCTCGGAG -0.424131431039
CCTCGGCA -0.878833383777
CCTCGGCC 0.335073510797
CCTCGGCG 0.11243422691
CCTCGGGA -1.04281393864
CCTCGGGC -1.09468606514
CCTCGGTA -0.780304405811
CCTCGGTC -1.15412379621
CCTCGTAA -0.11951456858
CCTCGTAC -0.88275837163
CCTCGTAG 0.815659274112
CCTCGTCA -0.235177636139
CCTCGTCC -0.583313899548
CCTCGTCG -0.365938187999
CCTCGTGA -0.955052134569
CCTCGTGC -0.364430354806
CCTCGTTA -0.722776083276
CCTCGTTC -1.08655754728
CCTCTAAA 0.503386874193
CCTCTAAC -0.476456344675

CCTCTAAG 0.213967005116
CCTCTACA -0.855709597012
CCTCTACC -1.33590065486
CCTCTACG -0.609544742837
CCTCTAGA 0.317801191018
CCTCTAGC -0.526961782666
CCTCTATA 0.242284208236
CCTCTATC 0.862239099717
CCTCTCAA 0.0502866429214
CCTCTCAC -0.483949295124
CCTCTCAG -0.450489679258
CCTCTCCA -0.704445594766
CCTCTCCC -0.779618251151
CCTCTCCG -0.681788543059
CCTCTCGA -0.589139136107
CCTCTCGC -1.37653200253
CCTCTCTA -0.726954423744
CCTCTCTC -0.93792387396
CCTCTGAA -0.570622536473
CCTCTGAC -1.32679182684
CCTCTGAG 0.306725289157
CCTCTGCA -1.05760415222
CCTCTGCC -0.642127721181
CCTCTGCG -0.448560285633
CCTCTGGA -1.00092935945
CCTCTGGC -0.996414811528
CCTCTGTA -0.357005894917
CCTCTGTC -0.758981358673
CCTCTTAA 0.417540932183
CCTCTTAC -1.43072031737
CCTCTTAG 0.546080849408
CCTCTTCA -1.02819984363
CCTCTTCC -1.78287046063
CCTCTTCG -0.0247769281956
CCTCTTGA -0.218713463323
CCTCTTGC -1.42506370498
CCTCTTTA -1.03502142006
CCTCTTTC -0.488536497168
CCTGAAAA -0.983971180262
CCTGAAAC 0.55812165626
CCTGAAAG 0.0876566741806
CCTGAACA -0.436902734463
CCTGAACC -1.35481862166
CCTGAACG -0.308587233113
CCTGAAGA 1.54199998914
CCTGAAGC -0.944669881776
CCTGAAGG -0.840264039109
CCTGAATA -0.807796391372
CCTGAATC 0.618196620165
CCTGACAA 1.25229720617
CCTGACAC -0.0885709919193
CCTGACAG -0.578810593236
CCTGACCA -1.02997976546
CCTGACCC -1.40337655454

CCTGACCG -0.423133425747
CCTGACGA 0.877687572112
CCTGACGC -0.832102837307
CCTGACTA -1.05015221284
CCTGACTC -1.14538927222
CCTGAGAA 0.069314735881
CCTGAGAC -0.617937646743
CCTGAGAG -0.596539030994
CCTGAGCA -0.298669633597
CCTGAGCC -1.00789790954
CCTGAGCG -0.834950295875
CCTGAGGA 0.0238601123209
CCTGAGGC -0.699038587793
CCTGAGTA -0.497932402637
CCTGAGTC -0.161418300139
CCTGATAA 0.357000898645
CCTGATAC 2.13853444022
CCTGATAG 0.450449709084
CCTGATCA -0.117075138875
CCTGATCC -0.0308432348687
CCTGATCG 0.980912212855
CCTGATGA 0.972446863007
CCTGATGC -0.131840371102
CCTGATTA 0.983273783991
CCTGATTC 5.50820854097
CCTGCAAA 0.762547655846
CCTGCAAC 0.0291848889882
CCTGCAAG -0.626601390217
CCTGCACA -0.136272896898
CCTGCACC -1.12874564998
CCTGCACG -0.613350028344
CCTGCAGA -0.212127128027
CCTGCAGC -0.164575943914
CCTGCAGG -0.749018584532
CCTGCATA -0.433367872167
CCTGCATC -0.562715103643
CCTGCCAA -0.875753598571
CCTGCCAC -0.107061777483
CCTGCCAG -0.813957835388
CCTGCCCA -0.816987033342
CCTGCCCC -1.51093004844
CCTGCCCG -0.380515227322
CCTGCCGA -0.116520969062
CCTGCCGC -1.17182912621
CCTGCCTA -1.01553866636
CCTGCCTC -1.23524409782
CCTGCGAA -0.104218274297
CCTGCGAC -0.997275835701
CCTGCGAG -0.241342827358
CCTGCGCA 0.746298947251
CCTGCGCC -1.124006478
CCTGCGCG 0.29801012572
CCTGCGGA 0.709719576004
CCTGCGGC -1.21875806631

CCTGCGTA -0.857960417457
CCTGCGTC -0.867219966342
CCTGCTAA -0.859313990758
CCTGCTAC -0.434840315101
CCTGCTAG -0.849631007838
CCTGCTCA -0.0578522474895
CCTGCTCC -1.09578815943
CCTGCTCG -0.754868386094
CCTGCTGA -0.0644235939701
CCTGCTGC -0.547554749588
CCTGCTTA -0.543627263599
CCTGCTTC -0.134236916141
CCTGGAAA 0.268465505162
CCTGGAAC -0.25353497961
CCTGGAAG -0.876305270248
CCTGGACA -0.210513332237
CCTGGACC -0.572905832684
CCTGGACG -0.293610492047
CCTGGAGA 0.602422973747
CCTGGAGC -0.604253691004
CCTGGATA 2.92468802306
CCTGGATC 1.19873384166
CCTGGCAA -0.0803741916809
CCTGGCAC -0.327926968526
CCTGGCAG -0.741634094817
CCTGGCCA -0.917266788217
CCTGGCCC -1.52110995222
CCTGGCCG -1.29944556587
CCTGGCGA 0.131333874049
CCTGGCGC -0.401069681315
CCTGGCTA -1.22495344334
CCTGGCTC -0.920946958751
CCTGGGAA -0.612327666228
CCTGGGAC -0.743784157113
CCTGGGAG -0.845956666287
CCTGGGCA -1.06012914308
CCTGGGCC -1.63419640078
CCTGGGCG -0.630229099897
CCTGGGGA -0.727272311537
CCTGGGGC -1.4427858974
CCTGGGTA -0.804066882654
CCTGGGTC -1.34970910103
CCTGGTAA 0.0966443426088
CCTGGTAC -1.29583513496
CCTGGTAG -1.12227297987
CCTGGTCA -0.888894626108
CCTGGTCC -1.71579051549
CCTGGTCG -0.517692449415
CCTGGTGA -0.681404038308
CCTGGTGC -0.958916542626
CCTGGTTA -0.806231933766
CCTGGTTC -0.678484550155
CCTGTAAA 0.946056971733
CCTGTAAC 0.718211572634

CCTGTAAG 0.268324152306
CCTGTACA -0.141199637245
CCTGTACC -1.3043412877
CCTGTACG -0.531939734799
CCTGTAGA 1.17609510962
CCTGTAGC -0.0868826684081
CCTGTATA 0.548514866485
CCTGTATC 1.17380119633
CCTGTCAA 0.442738796277
CCTGTCAC -0.722708217251
CCTGTCAG -0.644690392256
CCTGTCCA -1.06435161728
CCTGTCCC -0.754563197159
CCTGTCCG -1.29011794278
CCTGTCGA -0.602559122153
CCTGTCGC -0.245518253334
CCTGTCTA -0.616652980357
CCTGTCTC -1.45941182452
CCTGTGAA -0.58307324579
CCTGTGAC -1.29740979329
CCTGTGAG -0.380935330509
CCTGTGCA -0.571274966298
CCTGTGCC -0.606294876211
CCTGTGCG 0.464810243295
CCTGTGGA -0.649910039038
CCTGTGGC -1.16736058563
CCTGTGTA 0.454120511608
CCTGTGTC 0.194650169282
CCTGTTAA 0.891102561886
CCTGTTAC -0.714649230843
CCTGTTAG -0.0549879265041
CCTGTTCA -0.340383506827
CCTGTTCC -1.19877672633
CCTGTTCG -0.13139799287
CCTGTTGA 0.257328190793
CCTGTTGC -0.723463070649
CCTGTTTA -0.734070155673
CCTGTTTC 0.0746084940276
CCTTAAAA -0.112078034367
CCTTAAAC 0.744559620132
CCTTAAAG -0.488384735412
CCTTAACA -0.631033916012
CCTTAACC -0.768285874004
CCTTAACG -0.35462163238
CCTTAAGA -0.119539341761
CCTTAAGC 0.723698311779
CCTTAAGG 0.441307364408
CCTTAATA 0.585886771346
CCTTAATC 0.644428712521
CCTTACAA -0.120222373751
CCTTACAC -0.0269915256695
CCTTACAG 0.308843083865
CCTTACCA -0.275574783573
CCTTACCC -0.966722592774

CCTTACCG -0.569328085722
CCTTACGA 0.0253779380572
CCTTACGC -0.0701164293264
CCTTACTA -0.492587640883
CCTTACTC -0.297931018083
CCTTAGAA 0.0209489512874
CCTTAGAC -0.696989283644
CCTTAGAG -0.24383846512
CCTTAGCA -0.792892096247
CCTTAGCC -0.674559145947
CCTTAGCG -0.187499255273
CCTTAGGA -0.516339708826
CCTTAGGC 0.151409102306
CCTTAGTA 0.13041955631
CCTTAGTC -0.0294061821932
CCTTATAA 0.48242293409
CCTTATAC 0.0439638609628
CCTTATAG -0.127838357393
CCTTATCA -0.0155646357146
CCTTATCC -0.0408732505005
CCTTATCG 0.442047645346
CCTTATGA 0.14766585384
CCTTATGC -0.610002942596
CCTTATTA -0.0226478918763
CCTTATTC 0.0430949260261
CCTTCAAA 0.333700160587
CCTTCAAC -0.496887557298
CCTTCAAG -1.14674013921
CCTTCACA -0.37737382143
CCTTCACC -1.24009631044
CCTTCACG -0.278128294817
CCTTCAGA -0.443173263746
CCTTCAGC -0.865348029842
CCTTCATA -0.452494433316
CCTTCATC -0.710375753033
CCTTCCAA -0.567515688127
CCTTCCAC -0.628757489675
CCTTCCAG -0.877650516429
CCTTCCCA -0.602533724438
CCTTCCCC -1.40490249922
CCTTCCCG -1.04935114393
CCTTCCGA 0.301592452598
CCTTCCGC -0.630898808496
CCTTCCTA -1.07896883496
CCTTCCTC -1.46570754334
CCTTCGAA -0.543526089096
CCTTCGAC -1.23261585067
CCTTCGAG -0.538958663961
CCTTCGCA -0.483302486105
CCTTCGCC -1.54916922281
CCTTCGCG -0.600573936826
CCTTCGGA -1.03310826431
CCTTCGGC -0.949615149965
CCTTCGTA -0.0493796114124

CCTTCGTC -1.3365576646
CCTTCTAA 0.128957522276
CCTTCTAC -0.379617147466
CCTTCTAG -0.552538530705
CCTTCTCA -0.643961977464
CCTTCTCC -0.766696851395
CCTTCTCG -1.01205418314
CCTTCTGA -0.145808281622
CCTTCTGC -1.01472947851
CCTTCTTA -0.508489316766
CCTTCTTC -1.10516616159
CCTTGAAA -0.194349143906
CCTTGAAC -0.570045883436
CCTTGAAG -0.138307212231
CCTTGACA -0.0741288519351
CCTTGACC -0.771805747485
CCTTGACG 0.689156170357
CCTTGAGA 0.313223565162
CCTTGAGC -0.578090297385
CCTTGATA 0.514521690426
CCTTGATC -0.150139424736
CCTTGCAA 1.55128535209
CCTTGCAC -0.0913911791706
CCTTGCAG -0.171117312764
CCTTGCCA -0.176588230381
CCTTGCCC -1.16397810962
CCTTGCCG -0.0881098776681
CCTTGCGA -0.186268298809
CCTTGCGC 0.60494858914
CCTTGCTA 0.481812556219
CCTTGCTC -0.933745949848
CCTTGGAA -0.753859555547
CCTTGGAC -0.654208290029
CCTTGGAG -1.03709237472
CCTTGGCA -0.451757899582
CCTTGGCC 0.181555981793
CCTTGGCG -0.606001553421
CCTTGGGA -0.425208543967
CCTTGGGC -0.500379118572
CCTTGGTA -0.836788091184
CCTTGGTC -0.158033325996
CCTTGTAA 1.72283900593
CCTTGTAC -0.682787172884
CCTTGTAG 0.0882141748419
CCTTGTCA -0.406096971633
CCTTGTCC -0.354283967677
CCTTGTCG -0.220443214254
CCTTGTGA -0.506724175575
CCTTGTGC -0.181952769045
CCTTGTTA 0.855581983904
CCTTGTTC -1.39415218773
CCTTTAAA 1.19979513306
CCTTTAAC 0.65354586766
CCTTTAAG -0.263380965897

CCTTTACA 0.52701216174
CCTTTACC -1.00964785374
CCTTTACG 0.193839107827
CCTTTAGA 0.408814527134
CCTTTAGC -0.614639274645
CCTTTATA 0.102523080912
CCTTTATC 0.685079628927
CCTTTCAA 0.563326314226
CCTTTCAC -0.204899812695
CCTTTCAG -0.0172038292198
CCTTTCCA -0.687299639028
CCTTTCCC -1.13156396363
CCTTTCCG -0.285133276054
CCTTTCGA -0.683974828326
CCTTTCGC -0.506159388684
CCTTTCTA 0.38512782708
CCTTTCTC -0.669027232178
CCTTTGAA 0.594411035565
CCTTTGAC -1.05709744699
CCTTTGAG -0.354802539054
CCTTTGCA -0.94672272495
CCTTTGCC 0.13790417964
CCTTTGCG -0.803476281692
CCTTTGGA -0.390486744938
CCTTTGGC -0.949125515329
CCTTTGTA -0.594468700868
CCTTTGTC -0.976104966675
CCTTTTAA 1.39178353855
CCTTTTAC -0.405542801819
CCTTTTAG 0.154970611385
CCTTTTCA 0.283767003895
CCTTTTCC -0.695874074143
CCTTTTCG -0.318218587891
CCTTTTGA -0.0532061310746
CCTTTTGC -0.114068215966
CCTTTTTA 0.305752265225
CCTTTTTC -0.278505305159
CGAAAAAA 1.36946019616
CGAAAAAC 1.4861223099
CGAAAAAG 0.318355777188
CGAAAACA -0.532691881883
CGAAAACC -0.258263950865
CGAAAACG -0.291007642619
CGAAAAGA -0.0678483301087
CGAAAAGC 0.117881412236
CGAAAATA 1.53754789461
CGAAAATC 6.98600498027
CGAAACAA 0.580356523001
CGAAACAC -0.162800602002
CGAAACAG -0.65571216784
CGAAACCA 0.330093060528
CGAAACCC -0.833372306699
CGAAACCG 0.79408370707
CGAAACGA 0.446794519908

CGAAACGC 0.176976690513
CGAAACTA 0.959685968483
CGAAACTC 0.0500845020917
CGAAAGAA 0.817658199187
CGAAAGAC 0.132855030633
CGAAAGAG -0.353947552043
CGAAAGCA -0.415593843428
CGAAAGCC -0.81955678247
CGAAAGCG -0.488638712561
CGAAAGGA -0.557272498232
CGAAAGGC -0.524758218625
CGAAAGTA 0.0998796367751
CGAAAGTC -0.429498676016
CGAAATAA 2.92725777218
CGAAATAC 1.10233639815
CGAAATAG 1.13967915809
CGAAATCA 6.78143450516
CGAAATCC 18.9608677062
CGAAATCG 10.9131160399
CGAAATGA 0.270085962648
CGAAATGC 1.70900474568
CGAAATTA 2.64332464225
CGAAATTC 2.46407090087
CGAACAAA 0.194983254069
CGAACAAC 0.894248547694
CGAACAAG -0.281464971665
CGAACACA 0.0988349996136
CGAACACC -0.33333085283
CGAACACG 0.647683367241
CGAACAGA 0.970152117007
CGAACAGC -0.408591776683
CGAACATA -0.195875921296
CGAACATC 0.684576254543
CGAACCAA -0.367054646568
CGAACCAC -0.805415667861
CGAACCAG -0.699258215574
CGAACCCA 0.586062265392
CGAACCCC -0.816915420113
CGAACCCG 0.760203571661
CGAACCGA -0.203635547752
CGAACCGC -0.985673035343
CGAACCTA 0.655893699048
CGAACCTC -0.729281645512
CGAACGAA -0.246969462113
CGAACGAC -0.514534805639
CGAACGAG -0.52238311592
CGAACGCA 0.667456112876
CGAACGCC -0.989474990002
CGAACGCG 0.845465990761
CGAACGGA -0.554466258906
CGAACGGC -0.0772419456204
CGAACGTA -0.0203831235064
CGAACGTC -0.307133526198
CGAACTAA -0.1346516067

CGAACTAC 0.105322450364
CGAACTAG -0.184918889068
CGAACTCA 0.346745842604
CGAACTCC -0.374443508021
CGAACTCG -0.0073001776289
CGAACTGA -0.328467398592
CGAACTGC 0.125509262024
CGAACTTA 1.17614299056
CGAACTTC -0.991499729148
CGAAGAAA 0.551262399617
CGAAGAAC -0.442689249915
CGAAGAAG -0.626543308557
CGAAGACA -0.389162316556
CGAAGACC -1.03714275379
CGAAGACG -0.439162506561
CGAAGAGA -0.738246830717
CGAAGAGC 0.469511735056
CGAAGATA 2.13908861003
CGAAGATC 3.44567176652
CGAAGCAA -0.142149553421
CGAAGCAC 0.112558092815
CGAAGCAG -0.328137228297
CGAAGCCA -0.317082352413
CGAAGCCC -1.29578392318
CGAAGCCG -0.678126900366
CGAAGCGA 0.261288360726
CGAAGCGC -0.0946348004566
CGAAGCTA -0.63360470603
CGAAGCTC -1.03371156413
CGAAGGAA 0.549555756442
CGAAGGAC -0.670092270783
CGAAGGAG -1.31715755757
CGAAGGCA -0.569337870087
CGAAGGCC -1.68748955025
CGAAGGCG -0.309416614231
CGAAGGGA -1.01835822908
CGAAGGGC -0.578759797806
CGAAGGTA -0.65920310458
CGAAGGTC -1.47916541778
CGAAGTAA -0.581539598527
CGAAGTAC -0.0354381394977
CGAAGTAG 0.153634941391
CGAAGTCA -0.980313909307
CGAAGTCC -0.752365253924
CGAAGTCG -0.0869646905367
CGAAGTGA 0.287499218927
CGAAGTGC -0.232810652375
CGAAGTTA 0.222829558571
CGAAGTTC -1.03617222799
CGAATAAA 0.879865946615
CGAATAAC 0.511399020553
CGAATAAG -0.282974053926
CGAATACA 1.42163001719
CGAATACC 0.766551334979

CGAATACG 1.70963677406
CGAATAGA 0.0433859588583
CGAATAGC 0.62877351938
CGAATATA 1.95036048007
CGAATATC 5.54388088071
CGAATCAA 3.94945209711
CGAATCAC 3.1939700023
CGAATCAG 3.19584735143
CGAATCCA 6.69331671673
CGAATCCC 3.51676455062
CGAATCCG 9.77824829152
CGAATCGA 4.28962326239
CGAATCGC 3.18927975215
CGAATCTA 8.95001194404
CGAATCTC 11.5335595251
CGAATGAA 0.729790848879
CGAATGAC 0.0955141442928
CGAATGAG 0.367695626604
CGAATGCA -0.348116902856
CGAATGCC -0.881344218533
CGAATGCG 0.462442843176
CGAATGGA -0.248651123928
CGAATGGC 0.0870573297429
CGAATGTA 0.801382427453
CGAATGTC -0.40867712966
CGAATTAA 0.202691876917
CGAATTAC 1.09479181956
CGAATTAG 0.595568505198
CGAATTCA 0.491844029097
CGAATTCC 1.21854614112
CGAATTCG 2.82092503403
CGAATTGA 0.177885179268
CGAATTGC 1.91896911255
CGAATTTA 3.52691739127
CGAATTTC 3.40389398264
CGACAAAA 1.06991954584
CGACAAAC 0.640920913364
CGACAAAG -0.0764190180199
CGACAACA 0.741600786338
CGACAACC -0.423824160323
CGACAACG 0.25136659765
CGACAAGA -0.270286229876
CGACAAGC -0.757842833237
CGACAATA 0.278138703716
CGACAATC 2.96726000597
CGACACAA 0.265528530058
CGACACAC -0.854348321126
CGACACAG -0.280589375033
CGACACCA -0.760561221451
CGACACCC 0.577991204661
CGACACCG -0.375602643078
CGACACGA 1.18213830853
CGACACGC -1.01931730509
CGACACTA -0.945504467344

CGACACTC −0.379202873263
CGACAGAA 1.02140720394
CGACAGAC 0.0970844308829
CGACAGAG −0.516735455188
CGACAGCA −0.589566525524
CGACAGCC −0.757328217242
CGACAGCG 0.310494559872
CGACAGGA −0.0751435114658
CGACAGGC −0.0429646066034
CGACAGTA 0.385945133875
CGACAGTC −0.58517834164
CGACATAA 0.366084120771
CGACATAC 0.0510635631858
CGACATAG −0.581833962207
CGACATCA 0.589962480064
CGACATCC 1.87819224846
CGACATCG −0.181121930681
CGACATGA 0.412836734108
CGACATGC −0.335776944231
CGACATTA 1.80279642527
CGACATTC 0.193742929595
CGACCAAA −0.425349272289
CGACCAAC −0.636167793461
CGACCAAG −0.976549218508
CGACCACA −0.395089768509
CGACCACC −1.13472743639
CGACCACG −0.760540403652
CGACCAGA 0.0551176213928
CGACCAGC −0.257699996686
CGACCATA −1.06112902197
CGACCATC −0.815716731238
CGACCCAA −0.656517192133
CGACCCAC −0.987252273587
CGACCCAG −1.33410595239
CGACCCCA −0.436694972827
CGACCCCC −1.35312946543
CGACCCCG −0.379849265927
CGACCCGA 0.0437894078059
CGACCCGC −0.946589282858
CGACCCTA −0.640293256719
CGACCCTC −1.10175578973
CGACCGAA 0.580953369302
CGACCGAC −0.759494517422
CGACCGAG −0.970402555131
CGACCGCA −0.439254729411
CGACCGCC −0.656348151604
CGACCGCG −0.339837664133
CGACCGGA −0.0888861733985
CGACCGGC −0.96672821358
CGACCGTA −1.01145442235
CGACCGTC −0.668509909869
CGACCTAA 0.0153901825577
CGACCTAC −1.30568653388
CGACCTAG −0.693476280037

CGACCTCA -0.943000294284
CGACCTCC -0.506795996982
CGACCTCG -0.623862808738
CGACCTGA -0.808119899971
CGACCTGC -0.293653584891
CGACCTTA -0.824382972847
CGACCTTC -0.937515845097
CGACGAAA 1.13120631384
CGACGAAC -0.8275616426
CGACGAAG -0.881100025749
CGACGACA -0.434042785215
CGACGACC -1.26223354171
CGACGACG 0.527985185672
CGACGAGA 0.369499280722
CGACGAGC -0.598601034
CGACGATA 0.790762435394
CGACGATC 2.08454951528
CGACGCAA 0.0200825144867
CGACGCAC -1.10726834295
CGACGCAG -0.241238322006
CGACGCCA -0.800079025048
CGACGCCC -1.43303942019
CGACGCCG -0.570873807309
CGACGCGA 0.224335934518
CGACGCGC 0.0381746391964
CGACGCTA -0.484489517012
CGACGCTC -1.25363891333
CGACGGAA 1.05444296942
CGACGGAC -0.94029543764
CGACGGAG 0.208871224239
CGACGGCA -0.842969728465
CGACGGCC -0.7195522389
CGACGGCG -0.926037327
CGACGGGA -1.04647662222
CGACGGGC -0.514626820312
CGACGGTA -0.468566190618
CGACGGTC -0.992069928667
CGACGTAA 1.19677905032
CGACGTAC -0.554380073218
CGACGTAG -0.416095136032
CGACGTCA -0.397768186548
CGACGTCC -1.07947783015
CGACGTCG -0.785766996309
CGACGTGA -0.852150794247
CGACGTGC -0.351219171286
CGACGTTA 1.46369779301
CGACGTTC -0.593954293051
CGACTAAA -0.109938797326
CGACTAAC -0.188475193697
CGACTAAG -0.305507447906
CGACTACA -0.0658312935488
CGACTACC -0.00280998652964
CGACTACG 0.377976704893
CGACTAGA -0.173128312162

CGACTAGC −0.952656422243
CGACTATA 0.111405203098
CGACTATC 0.591210090767
CGACTCAA 1.09414188787
CGACTCAC −0.626725047944
CGACTCAG −0.277694451882
CGACTCCA −0.237641422668
CGACTCCC −0.518035110389
CGACTCCG 0.17352967933
CGACTCGA −0.086270000579
CGACTCGC −0.917289687796
CGACTCTA −0.552106145016
CGACTCTC −0.0115130756434
CGACTGAA 1.11788229785
CGACTGAC −0.704713311664
CGACTGAG 1.28593564693
CGACTGCA −0.291631135703
CGACTGCC −0.231522655141
CGACTGCG 0.10270148945
CGACTGGA −0.146626004773
CGACTGGC −0.542583875506
CGACTGTA −0.580336954269
CGACTGTC −1.30212835565
CGACTTAA −0.231581361335
CGACTTAC −1.22482104214
CGACTTAG −0.946751453513
CGACTTCA −0.975551837751
CGACTTCC −0.214982913715
CGACTTGA −0.557387620661
CGACTTGC −1.07352518866
CGACTTTA 0.811713676639
CGACTTTC −0.369222820349
CGAGAAAA 0.717440064998
CGAGAAAC 0.0380428625277
CGAGAAAG −0.717148407632
CGAGAACA −0.609940281021
CGAGAACC −0.469576270233
CGAGAACG −0.321836513206
CGAGAAGA −0.560652476103
CGAGAAGC −0.432426699467
CGAGAATA 0.714993973597
CGAGAATC 9.24617136742
CGAGACAA −0.0880334763452
CGAGACAC −0.497471288386
CGAGACAG −0.807086088065
CGAGACCA −0.322825358666
CGAGACCC −1.3336319311
CGAGACCG −0.263734660305
CGAGACGA −0.644079389851
CGAGACGC −1.00679831339
CGAGACTA 1.31065948974
CGAGACTC −0.631917631586
CGAGAGAA 1.21206347846
CGAGAGAC −1.04758662727

CGAGAGAG -0.519994065289
CGAGAGCA -0.335690758542
CGAGAGCC -0.0416603714865
CGAGAGCG 0.0725404538598
CGAGAGGA -0.896220825986
CGAGAGGC -1.32046779581
CGAGAGTA 1.22417839668
CGAGAGTC -1.14369657697
CGAGATAA 0.175268590093
CGAGATAC 3.95769178202
CGAGATAG 1.12224570855
CGAGATCA 1.96729242884
CGAGATCC 10.1352433612
CGAGATCG 3.91822748016
CGAGATGA -0.655654918892
CGAGATGC 0.326266957222
CGAGATTA 7.0407524612
CGAGATTC 11.8777004365
CGAGCAAA 1.51716706106
CGAGCAAC -0.588282275494
CGAGCAAG -0.739110769382
CGAGCACA -0.545524389636
CGAGCACC -0.949318496327
CGAGCACG 0.0459865183284
CGAGCAGA -0.739506515744
CGAGCAGC -0.862472675423
CGAGCATA -0.352745115964
CGAGCATC -1.15217941377
CGAGCCAA -0.922928396875
CGAGCCAC -1.16961910866
CGAGCCAG -1.23942160557
CGAGCCCA -0.82639147411
CGAGCCCC -1.17462370757
CGAGCCCG -0.309616881459
CGAGCCGA -0.651733261888
CGAGCCGC -1.58360977337
CGAGCCTA -0.621235186129
CGAGCCTC -1.25182172764
CGAGCGAA -0.500799221758
CGAGCGAC -0.584021288364
CGAGCGAG 0.185822173373
CGAGCGCA -0.503961029094
CGAGCGCC -0.226313209082
CGAGCGCG -0.111940220536
CGAGCGGA -0.862881745176
CGAGCGGC -1.11663281354
CGAGCGTA 0.973876629453
CGAGCGTC -1.2013110852
CGAGCTAA 0.211303575892
CGAGCTAC -0.912361698381
CGAGCTAG -0.399385313186
CGAGCTCA -1.07052617651
CGAGCTCC -0.597593660699
CGAGCTCG -0.0575060474896

CGAGCTGA -0.627440139344
CGAGCTGC -0.777793779233
CGAGCTTA -1.12610970025
CGAGCTTC -0.326927297811
CGAGGAAA 0.0637778258404
CGAGGAAC 0.0744107249356
CGAGGAAG -0.620726399118
CGAGGACA 0.115560435809
CGAGGACC -1.0153169568
CGAGGACG -0.350320675075
CGAGGAGA -0.877179825991
CGAGGAGC -0.745406280023
CGAGGATA 1.94475216498
CGAGGATC 2.01380876015
CGAGGCAA -0.49387313998
CGAGGCAC -1.02293314862
CGAGGCAG -0.913615762602
CGAGGCCA -0.571658846515
CGAGGCCC -1.11765684108
CGAGGCCG -0.384469568271
CGAGGCGA 0.0141827502068
CGAGGCGC -0.0306104918742
CGAGGCTA -0.135542192148
CGAGGCTC -1.26564308085
CGAGGGAA -0.33794532619
CGAGGGAC -0.914475954063
CGAGGGAG -0.544691261314
CGAGGGCA -1.01428605939
CGAGGGCC -1.72776137454
CGAGGGCG -1.3096373358
CGAGGGGA -0.833738283609
CGAGGGGC -0.588052030635
CGAGGGTA -0.543777984465
CGAGGGTC -1.58180591108
CGAGGTAA 0.494483309674
CGAGGTAC -0.907875670841
CGAGGTAG -1.00490347731
CGAGGTCA -0.642426040243
CGAGGTCC -0.970376949238
CGAGGTGA -1.2725410586
CGAGGTGC -0.101462205867
CGAGGTTA 0.347189261726
CGAGGTTC -0.439565122797
CGAGTAAA 0.63918512527
CGAGTAAC -0.481755099094
CGAGTAAG -0.00600801683549
CGAGTACA 0.130097504957
CGAGTACC -0.598175518185
CGAGTACG 0.860547029001
CGAGTAGA -0.447742354304
CGAGTAGC -0.859410377168
CGAGTATA 1.4607957918
CGAGTATC 3.88322443267
CGAGTCAA 0.0929169156705

CGAGTCAC −1.2551138544
CGAGTCAG −0.0432477286719
CGAGTCCA −0.556508693181
CGAGTCCC −0.524785489942
CGAGTCCG −0.727050393799
CGAGTCGA −0.564626593961
CGAGTCGC −0.917980422372
CGAGTCTA −0.604122122513
CGAGTCTC −0.533431538286
CGAGTGAA 0.406185655457
CGAGTGAC −0.834263724859
CGAGTGAG −0.431271519792
CGAGTGCA 0.416914524606
CGAGTGCC −1.04564474296
CGAGTGCG 0.915860337706
CGAGTGGA −1.34567565245
CGAGTGGC −0.920574736502
CGAGTGTA −0.443108104034
CGAGTGTC −0.769926732934
CGAGTTAA −0.0481540675762
CGAGTTAC −1.20937215339
CGAGTTAG −0.462850039327
CGAGTTCA −0.0715428649244
CGAGTTCC 0.078086315554
CGAGTTGA −0.900700816363
CGAGTTGC −0.909603548171
CGAGTTTA 2.52189899971
CGAGTTTC 0.390855636339
CGATAAAA 1.53511366936
CGATAAAC −0.191728391171
CGATAAAG 0.662624093515
CGATAACA 1.01089046817
CGATAACC 0.0649529906025
CGATAACG −0.0391559902484
CGATAAGA 0.557417390114
CGATAAGC 0.59517338337
CGATAATA 0.88270133086
CGATAATC 2.72706548841
CGATACAA 1.37269320037
CGATACAC 0.757849078577
CGATACAG 0.426410147334
CGATACCA 0.492005991574
CGATACCC 1.87198708707
CGATACCG 1.15500334823
CGATACGA 1.92436716787
CGATACGC 2.61134267377
CGATACTA 0.425545375957
CGATACTC 2.41490217376
CGATAGAA 3.0541934698
CGATAGAC −0.235021710823
CGATAGAG 0.657209592133
CGATAGCA 0.712740238661
CGATAGCC −0.00453036945162
CGATAGCG 1.13589448221

CGATAGGA -0.0306987593426
CGATAGGC -0.411241049803
CGATAGTA 2.00468744145
CGATAGTC 0.469071230426
CGATATAA 1.75617267411
CGATATAC 1.35228613639
CGATATAG 3.82811722018
CGATATCA 6.10526322885
CGATATCC 14.758772464
CGATATCG 10.3661787462
CGATATGA 3.19282002707
CGATATGC 2.75048717788
CGATATTA 3.21262399941
CGATATTC 5.37371848039
CGATCAAA 1.81988367481
CGATCAAC 0.728612145091
CGATCAAG 0.411565599292
CGATCACA 1.75735283514
CGATCACC 0.417579653289
CGATCACG 1.61127225671
CGATCAGA 0.28498130612
CGATCAGC -0.664764787801
CGATCATA 0.8388055441
CGATCATC 0.241609711543
CGATCCAA 2.04962182821
CGATCCAC 1.48363125205
CGATCCAG 0.755286615679
CGATCCCA 2.95495627031
CGATCCCC 1.79992148721
CGATCCCG 2.44966623292
CGATCCGA 2.24692460146
CGATCCGC 1.92151991748
CGATCCTA 1.37473730006
CGATCCTC 5.18062298435
CGATCGAA 0.708048323088
CGATCGAC 0.527916695113
CGATCGAG 0.482894665419
CGATCGCA 4.89445776999
CGATCGCC 2.91356070123
CGATCGCG 6.41418250586
CGATCGGA 1.02182834802
CGATCGGC -0.0469528805651
CGATCGTA 2.44830308343
CGATCGTC 1.79056138835
CGATCTAA 2.4647866168
CGATCTAC 1.9467090381
CGATCTAG 2.78603315358
CGATCTCA 3.91418362268
CGATCTCC 5.52432609743
CGATCTGA 3.6841009347
CGATCTGC 4.79574476268
CGATCTTA 3.58752758079
CGATCTTC 6.62858377026
CGATGAAA 0.509014758016

CGATGAAC -0.333238838158
CGATGAAG -0.303567437203
CGATGACA 0.774766038525
CGATGACC -1.09505745468
CGATGACG 0.121712303637
CGATGAGA -1.05501691614
CGATGAGC -0.728503059823
CGATGATA -0.0821678532559
CGATGATC 2.10619003385
CGATGCAA 0.706598987911
CGATGCAC -0.22925705406
CGATGCAG 0.600404896297
CGATGCCA -0.415506200494
CGATGCCC -0.583266851322
CGATGCCG 0.399899929181
CGATGCGA -0.23559003674
CGATGCGC 0.316094756022
CGATGCTA 0.730535293377
CGATGCTC -0.141568528646
CGATGGAA 1.07130434584
CGATGGAC -0.40295764752
CGATGGAG -0.0183194550764
CGATGGCA 0.47948762441
CGATGGCC -0.75113117479
CGATGGCG -0.380138008801
CGATGGGA 1.01661140755
CGATGGGC 0.399184629602
CGATGGTA -0.571229583496
CGATGGTC -1.20716609121
CGATGTAA 0.395187195809
CGATGTAC -0.708368709017
CGATGTAG -0.18910305852
CGATGTCA 0.0921895417681
CGATGTCC -0.52965831219
CGATGTGA -0.578597210794
CGATGTGC -0.755643849113
CGATGTTA 1.54461782738
CGATGTTC -0.0805328233105
CGATTAAA 0.779369686629
CGATTAAC -0.314690595469
CGATTAAG 0.945184705949
CGATTACA 7.56189317086
CGATTACC 6.16452921308
CGATTACG 10.7078219381
CGATTAGA 1.64798798454
CGATTAGC 0.851900147945
CGATTATA 1.75979122396
CGATTATC 2.65394234435
CGATTCAA 0.179246663333
CGATTCAC 1.04193875836
CGATTCAG 0.393441831528
CGATTCCA 2.04549657313
CGATTCCC 10.1432351061
CGATTCCG 6.15245072601

CGATTCGA 2.3522331039
CGATTCGC 7.17491484962
CGATTCTA 2.74649994481
CGATTCTC 8.37248247817
CGATTGAA -0.498526334447
CGATTGAC -0.133032398282
CGATTGAG 0.0526611210928
CGATTGCA 4.44575509061
CGATTGCC 4.29122290208
CGATTGCG 6.75084524744
CGATTGGA 0.130606708325
CGATTGGC 0.790296116693
CGATTGTA 0.743882417125
CGATTGTC 1.97045423617
CGATTTAA 0.995548166549
CGATTTAC 1.20681156409
CGATTTAG 1.68352563312
CGATTTCA 6.06816632711
CGATTTCC 16.8523456625
CGATTTGA 0.942732369209
CGATTTGC 3.6083788966
CGATTTTA 3.70480048876
CGATTTTC 7.84088727795
CGCAAAAA 0.813325182471
CGCAAAAC 0.595138825823
CGCAAAAG 0.340717632503
CGCAAACA 0.224316782143
CGCAAACC -0.68446675292
CGCAAACG -0.607081164485
CGCAAAGA -0.197896496882
CGCAAAGC 0.1723428566
CGCAAATA 1.24915913113
CGCAAATC 7.27730886017
CGCAACAA 1.48073507983
CGCAACAC -0.0936034866865
CGCAACAG 0.164191439164
CGCAACCA -0.344743170326
CGCAACCC 0.299369319826
CGCAACCG -0.163945580956
CGCAACGA 1.71988267027
CGCAACGC 0.33255559799
CGCAACTA 0.950984752971
CGCAACTC 0.611192887996
CGCAAGAA 0.459881421168
CGCAAGAC -0.175790492318
CGCAAGAG 0.0939580138061
CGCAAGCA 0.955915032344
CGCAAGCC -1.08923409172
CGCAAGCG -0.187756563271
CGCAAGGA -0.577788231119
CGCAAGGC 0.142632110005
CGCAAGTA 1.92035703522
CGCAAGTC -0.499933409492
CGCAATAA 0.941534096689

CGCAATAC 1.43404221358
CGCAATAG 0.426964733503
CGCAATCA 7.84144998306
CGCAATCC 16.2641087698
CGCAATGA 0.962290275157
CGCAATGC 0.405007576203
CGCAATTA 1.70349323335
CGCAATTC 3.92697345394
CGCACAAA 0.721786196927
CGCACAAC 0.93188213229
CGCACAAG 0.212568881725
CGCACACA 1.06130451602
CGCACACC 0.356264781267
CGCACACG 0.508336930476
CGCACAGA -0.399724643312
CGCACAGC -0.264051090852
CGCACATA 2.09343642556
CGCACATC 0.162017020042
CGCACCAA -1.20523732212
CGCACCAC -0.38974542311
CGCACCAG -0.00421768610835
CGCACCCA -0.568579685842
CGCACCCC -1.03025872397
CGCACCCG -0.39509892834
CGCACCGA -0.516649894033
CGCACCGC -0.810641559983
CGCACCTA 0.116905473812
CGCACCTC -0.457257545763
CGCACGAA 0.0410177260267
CGCACGAC -0.453076915337
CGCACGAG -0.630509515652
CGCACGCA 1.33378598282
CGCACGCC -1.26301150286
CGCACGCG 0.698679480757
CGCACGGA -0.160264994066
CGCACGGC -0.956828101015
CGCACGTA 0.982039913035
CGCACGTC 0.111676250843
CGCACTAA 0.346530586561
CGCACTAC 0.428568744928
CGCACTAG -0.713037308655
CGCACTCA -0.514534805639
CGCACTCC -0.961913056636
CGCACTGA 0.525644432335
CGCACTGC 1.01198590076
CGCACTTA -0.123558634243
CGCACTTC -0.104706243509
CGCAGAAA 0.503336911475
CGCAGAAC -0.662814160021
CGCAGAAG -0.757043221572
CGCAGACA -0.556431250968
CGCAGACC -0.431977659539
CGCAGACG -0.699703924653
CGCAGAGA -0.703496511303

CGCAGAGC -0.33702288951
CGCAGATA 2.86491554177
CGCAGATC 4.52698531019
CGCAGCAA -0.573558470687
CGCAGCAC -0.307086894328
CGCAGCAG -0.322891142911
CGCAGCCA -1.04227975391
CGCAGCCC -1.21625493414
CGCAGCCG -0.229405901324
CGCAGCGA -0.393965607354
CGCAGCGC -0.0215403849613
CGCAGCTA -0.201102437952
CGCAGCTC -0.019510233188
CGCAGGAA 0.235204699278
CGCAGGAC -1.32827759316
CGCAGGAG -0.376044188598
CGCAGGCA -0.728503059823
CGCAGGCC -1.20200973054
CGCAGGCG -0.810431716567
CGCAGGGA 0.0274228704679
CGCAGGGC -0.997275835701
CGCAGGTA -0.290947895535
CGCAGGTC -0.612390119625
CGCAGTAA -0.113613971588
CGCAGTAC -0.501663160423
CGCAGTAG -0.91814030307
CGCAGTCA -0.586186339475
CGCAGTCC -1.09250352708
CGCAGTGA -1.01937517857
CGCAGTGC 0.228780950993
CGCAGTTA 0.488861463012
CGCAGTTC 0.714309900717
CGCATAAA 1.00946882066
CGCATAAC -0.00212175008965
CGCATAAG 0.315721492883
CGCATACA 1.00511207166
CGCATACC 0.753866841777
CGCATACG 0.0579696598768
CGCATAGA 0.205692762665
CGCATAGC 0.657145473312
CGCATATA 1.59395309688
CGCATATC 4.2617465639
CGCATCAA 0.284188147972
CGCATCAC 0.346443359982
CGCATCAG 0.192818619313
CGCATCCA 0.304715122471
CGCATCCC 0.16299462389
CGCATCCG -0.0899093682269
CGCATCGA -0.211189702531
CGCATCGC -0.0700493960131
CGCATCTA 1.42200348851
CGCATCTC 0.0808430085179
CGCATGAA 0.0675999737648
CGCATGAC -0.954675124227

CGCATGAG −0.806628304662
CGCATGCA 1.02712002439
CGCATGCC −0.094083545135
CGCATGCG 0.759279677735
CGCATGGA −0.0424991206144
CGCATGGC −0.797728279168
CGCATGTA −0.662447766756
CGCATGTC −0.322825358666
CGCATTAA 0.465292383524
CGCATTAC −0.604013869958
CGCATTAG −0.275558545689
CGCATTCA −0.248397146779
CGCATTCC −0.0662359915643
CGCATTGA −0.79115464273
CGCATTGC −0.792193450907
CGCATTTA 0.430036607946
CGCATTTC 1.53351965047
CGCCAAAA 0.949680101498
CGCCAAAC −0.701323133072
CGCCAAAG −0.101114964977
CGCCAACA −0.964920395902
CGCCAACC −0.54760637773
CGCCAACG −0.905923169459
CGCCAAGA −1.01171964111
CGCCAAGC 0.071649868412
CGCCAATA 0.254759482556
CGCCAATC 0.415781203621
CGCCACAA 0.521260203834
CGCCACAC −1.12815650626
CGCCACAG −0.742899608827
CGCCACCA −1.07903961548
CGCCACCC −0.673203490866
CGCCACCG −0.807631930759
CGCCACGA −0.802906498529
CGCCACGC −0.388610853056
CGCCACTA 0.204042743904
CGCCACTC −0.677366217985
CGCCAGAA 0.541773438585
CGCCAGAC −0.775374750972
CGCCAGAG −0.560807776884
CGCCAGCA −0.307513451033
CGCCAGCC −1.1797105368
CGCCAGCG −0.214592163625
CGCCAGGA 0.595885560279
CGCCAGGC 0.207204343061
CGCCAGTA −0.0419520288526
CGCCAGTC 0.212440227726
CGCCATAA −0.297578780921
CGCCATAC −1.04218170208
CGCCATAG −0.865348029842
CGCCATCA −0.530705239309
CGCCATCC −1.16421043626
CGCCATGA −0.535888871298
CGCCATGC −0.509388229333

CGCCATTA -0.203876617867
CGCCATTC 0.257466629158
CGCCCAAA 0.664143584675
CGCCCAAC -0.860149825394
CGCCCAAG -0.768165130769
CGCCCACA -0.590240814038
CGCCCACC -0.621195632311
CGCCCACG -0.996144596495
CGCCCAGA -0.514138018388
CGCCCAGC -0.268389520195
CGCCCATA -0.163168244335
CGCCCATC 0.340790494801
CGCCCCAA 0.109549088126
CGCCCCAC -1.73684751116
CGCCCCAG -0.996694186393
CGCCCCCA -1.13349127547
CGCCCCCC -1.20263697083
CGCCCCCG -1.24274891441
CGCCCCGA -0.430932189666
CGCCCCGC -0.567943702078
CGCCCCTA -0.436094171144
CGCCCCTC -1.39917697991
CGCCCGAA -0.0346812043205
CGCCCGAC -1.44439719506
CGCCCGAG -0.707839312384
CGCCCGCA -1.01841631074
CGCCCGCC -1.07469431626
CGCCCGCG -0.779532689997
CGCCCGGA -0.814427276758
CGCCCGGC -0.936191416715
CGCCCGTA 0.566463140202
CGCCCGTC -0.803014126551
CGCCCTAA -0.133054256971
CGCCCTAC 0.0842084139289
CGCCCTAG -1.21813124238
CGCCCTCA -0.151379957387
CGCCCTCC -1.3531742237
CGCCCTGA -0.887451536271
CGCCCTGC -0.00791575994984
CGCCCTTA -0.778533019281
CGCCCTTC -0.821511157454
CGCCGAAA 1.24500264935
CGCCGAAC -0.300081288557
CGCCGAAG -0.339849322101
CGCCGACA -0.95389945303
CGCCGACC -1.41231655021
CGCCGACG -0.63251114704
CGCCGAGA -0.976866898123
CGCCGAGC 0.0855436675665
CGCCGATA 1.51980759071
CGCCGATC 1.78753323129
CGCCGCAA 0.687617943177
CGCCGCAC -0.642864671271
CGCCGCAG -0.565243217172

CGCCGCCA -1.28553906786
CGCCGCCC -0.59502099708
CGCCGCCG -0.458328829707
CGCCGCGA -0.230973065244
CGCCGCGC -0.248655703844
CGCCGCTA 0.584651026788
CGCCGCTC -0.983110364267
CGCCGGAA 0.263055375518
CGCCGGAC -0.891571795079
CGCCGGAG -0.693112384908
CGCCGGCA -0.969711820555
CGCCGGCC -1.22909285452
CGCCGGCG -0.741937826507
CGCCGGGA -0.596860665991
CGCCGGGC -0.769346957227
CGCCGGTA -0.428101385337
CGCCGGTC -0.830900817584
CGCCGTAA -0.926568805413
CGCCGTAC -0.794929742428
CGCCGTAG -1.04521714537
CGCCGTCA -0.630363374701
CGCCGTCC -0.624387833633
CGCCGTGA -1.00422877244
CGCCGTGC -0.985948454826
CGCCGTTA 0.0506980026327
CGCCGTTC 1.2591593773
CGCCTAAA 1.63672180799
CGCCTAAC -0.738592406183
CGCCTAAG -0.314564855962
CGCCTACA 0.72928601725
CGCCTACC -0.748073872807
CGCCTACG -1.50916011915
CGCCTAGA -0.556870090175
CGCCTAGC 0.115047693416
CGCCTATA 0.891064673491
CGCCTATC 0.368687386555
CGCCTCAA 0.0937862669631
CGCCTCAC -1.28975758668
CGCCTCAG 0.166623998995
CGCCTCCA -1.03637749149
CGCCTCCC -0.512400773048
CGCCTCCG -1.04665586347
CGCCTCGA 0.079390550671
CGCCTCGC -0.884245387023
CGCCTCTA -0.633858891357
CGCCTCTC 0.0672889558455
CGCCTGAA 0.370154416861
CGCCTGAC -0.630898808496
CGCCTGAG -0.531913088016
CGCCTGCA -0.134560008384
CGCCTGCC -0.991436026683
CGCCTGGA -0.852090838986
CGCCTGGC -0.601835495455
CGCCTGTA -0.731333447796

CGCCTGTC 0.622627896892
CGCCTTAA 0.311686587051
CGCCTTAC -0.974647720734
CGCCTTAG -1.1818889113
CGCCTTCA -0.427594680106
CGCCTTCC -0.0649790128515
CGCCTTGA -0.82529583334
CGCCTTGC 0.014571626695
CGCCTTTA 0.443463880222
CGCCTTTC 0.0729503563251
CGCGAAAA 2.16644257358
CGCGAAAC 0.83330069347
CGCGAAAG -0.105688635451
CGCGAACA 0.464812325075
CGCGAACC -1.01736147286
CGCGAACG -0.35191531849
CGCGAAGA -0.210026195736
CGCGAAGC 0.305275745802
CGCGAATA 2.24902595011
CGCGAATC 9.35306202259
CGCGACAA 0.124104685115
CGCGACAC -0.248820164458
CGCGACAG 0.277744830956
CGCGACCA -0.507289795178
CGCGACCC -0.0362725168878
CGCGACCG -0.499695878403
CGCGACGA 0.0349052038394
CGCGACGC 0.160006437
CGCGACTA 0.740909843584
CGCGACTC -0.0346812043205
CGCGAGAA 1.21986307509
CGCGAGAC -0.297225711048
CGCGAGAG 0.0694342300482
CGCGAGCA -0.562343505928
CGCGAGCC -0.811274212899
CGCGAGCG 1.15168249291
CGCGAGGA -0.706924994646
CGCGAGGC -0.562709482837
CGCGAGTA -0.0773674769493
CGCGAGTC -0.688850148709
CGCGATAA 0.833598387998
CGCGATAC 0.7651436354
CGCGATAG 0.615062292324
CGCGATCA 4.78654745901
CGCGATCC 12.4983579287
CGCGATGA 0.33072196624
CGCGATGC 1.87075342429
CGCGATTA 1.99966389834
CGCGATTC 4.8770266104
CGCGCAAA 2.10970407835
CGCGCAAC -0.153492964001
CGCGCAAG 0.498203242204
CGCGCACA 0.965744988926
CGCGCACC -0.812915488184

| | |
|---|---|
| CGCGCACG | -0.747382721875 |
| CGCGCAGA | -0.702583234454 |
| CGCGCAGC | -0.339501664855 |
| CGCGCATA | 0.787682858365 |
| CGCGCATC | 0.530852212971 |
| CGCGCCAA | -0.311471747364 |
| CGCGCCAC | -1.18383579188 |
| CGCGCCAG | -0.596976412954 |
| CGCGCCCA | -0.779207307796 |
| CGCGCCCC | -0.38803003646 |
| CGCGCCCG | -0.701152427119 |
| CGCGCCGA | -0.370088840794 |
| CGCGCCGC | -0.173321084982 |
| CGCGCCTA | 0.458675654241 |
| CGCGCCTC | -0.786601790055 |
| CGCGCGAA | 1.42163001719 |
| CGCGCGAC | 0.947685339983 |
| CGCGCGAG | 0.213232553162 |
| CGCGCGCA | 0.404951159967 |
| CGCGCGCC | -0.52680689824 |
| CGCGCGCG | 3.95547968268 |
| CGCGCGGA | 0.0432489777399 |
| CGCGCGGC | -0.076491255783 |
| CGCGCGTA | 1.92430950257 |
| CGCGCGTC | 0.423374495862 |
| CGCGCTAA | -0.269608818691 |
| CGCGCTAC | -0.723439338357 |
| CGCGCTAG | -1.06975799972 |
| CGCGCTCA | -0.632739518297 |
| CGCGCTCC | -0.530312823795 |
| CGCGCTGA | 0.729291638055 |
| CGCGCTGC | 0.579455320475 |
| CGCGCTTA | 0.954128448821 |
| CGCGCTTC | -0.752637342559 |
| CGCGGAAA | 1.51910457363 |
| CGCGGAAC | -0.482174369568 |
| CGCGGAAG | -0.668248854668 |
| CGCGGACA | -0.213009386355 |
| CGCGGACC | -0.460564453158 |
| CGCGGACG | 0.046365402273 |
| CGCGGAGA | -1.27638339979 |
| CGCGGAGC | -0.0523898651698 |
| CGCGGATA | 2.44776931506 |
| CGCGGATC | 1.8200285667 |
| CGCGGCAA | 0.117556862748 |
| CGCGGCAC | -1.22104427701 |
| CGCGGCAG | -0.922928396875 |
| CGCGGCCA | -1.06841004723 |
| CGCGGCCC | -1.01986044147 |
| CGCGGCCG | 1.5859136792 |
| CGCGGCGA | -0.352278797263 |
| CGCGGCGC | 1.11594790795 |
| CGCGGCTA | -0.446039041977 |
| CGCGGCTC | -1.10341205383 |

CGCGGGAA -0.109304478986
CGCGGGAC -1.09039218589
CGCGGGAG -0.212404837468
CGCGGGCA -0.0211458876674
CGCGGGCC -0.517353119289
CGCGGGGA 0.585092572308
CGCGGGGC -0.702583234454
CGCGGGTA -0.536164498959
CGCGGGTC -0.877996924607
CGCGGTAA 0.139422421732
CGCGGTAC -0.0558304228358
CGCGGTAG -0.726822647076
CGCGGTCA -0.820495248856
CGCGGTCC -1.26970796431
CGCGGTGA 0.146931818242
CGCGGTGC -0.227001861877
CGCGGTTA -0.348807637432
CGCGGTTC 0.490322872513
CGCGTAAA 0.699742229404
CGCGTAAC 0.467212825495
CGCGTAAG -0.24050511912
CGCGTACA 0.658189277761
CGCGTACC -0.0947097445335
CGCGTACG 1.42089910426
CGCGTAGA -0.516731291628
CGCGTAGC 0.45335129393
CGCGTATA 2.14234326475
CGCGTATC 4.42040067667
CGCGTCAA -0.0742995578882
CGCGTCAC -0.384911738325
CGCGTCAG -0.120466358357
CGCGTCCA 0.20188310542
CGCGTCCC -0.822790203034
CGCGTCCG 0.372235572243
CGCGTCGA -0.329997923186
CGCGTCGC 0.636232536817
CGCGTCTA 0.233750784185
CGCGTCTC -1.13591072009
CGCGTGAA 0.225513805594
CGCGTGAC -1.09848239899
CGCGTGAG -0.785677687951
CGCGTGCA 0.0680296531393
CGCGTGCC -1.09329959972
CGCGTGGA 0.164474144876
CGCGTGGC -0.79965392559
CGCGTGTA -0.102880106167
CGCGTGTC -1.02106225301
CGCGTTAA 0.215810213053
CGCGTTAC -0.564393642788
CGCGTTAG -0.554466258906
CGCGTTCA -0.26959237263
CGCGTTCC -1.04919959035
CGCGTTGA -0.0811948293235
CGCGTTGC 1.13262754499

CGCGTTTA 0.598897063104
CGCGTTTC 1.77774074674
CGCTAAAA 0.444173558995
CGCTAAAC -0.00216026301808
CGCTAAAG 1.29382038836
CGCTAACA 0.263055375518
CGCTAACC -0.862721656301
CGCTAACG -1.06094124543
CGCTAAGA 0.49290303054
CGCTAAGC 0.78240242361
CGCTAATA 0.263900369986
CGCTAATC -0.274945253326
CGCTACAA -0.425670282752
CGCTACAC -0.510634799146
CGCTACAG 0.188771639157
CGCTACCA -0.24084195111
CGCTACCC -0.215237099042
CGCTACCG -0.0499608443647
CGCTACGA -1.00931914069
CGCTACGC -0.337742977183
CGCTACTA 0.308657597274
CGCTACTC -1.08762820669
CGCTAGAA 0.661834682571
CGCTAGAC -1.18092671262
CGCTAGAG 0.312911506353
CGCTAGCA -0.399528123288
CGCTAGCC -0.718100197409
CGCTAGCG -0.141066195153
CGCTAGGA -0.76652010828
CGCTAGGC -1.41906588887
CGCTAGTA -0.630393352332
CGCTAGTC 0.496370443167
CGCTATAA 1.97045423617
CGCTATAC -0.741235433963
CGCTATAG 0.016217481896
CGCTATCA 0.0554282229562
CGCTATCC 1.33138506604
CGCTATGA 0.873970762251
CGCTATGC 0.487277644853
CGCTATTA -0.104561351627
CGCTATTC 1.42098633084
CGCTCAAA 0.283355644184
CGCTCAAC 0.743909063908
CGCTCAAG -0.0124350959679
CGCTCACA -1.37949895527
CGCTCACC -0.793061136776
CGCTCACG -1.31534287001
CGCTCAGA -0.364677462082
CGCTCAGC -0.858013502845
CGCTCATA -0.288607558554
CGCTCATC -0.894915549979
CGCTCCAA -0.108297522041
CGCTCCAC -0.994196883206
CGCTCCAG -0.767736492285

CGCTCCCA 0.0366511926544
CGCTCCCC -0.78371977394
CGCTCCCG -0.668922726826
CGCTCCGA -0.911695528808
CGCTCCGC -0.670491347992
CGCTCCTA -0.275977399808
CGCTCCTC -1.27510456239
CGCTCGAA -0.177362236154
CGCTCGAC -1.28096102565
CGCTCGAG -0.0587894648074
CGCTCGCA -0.510117685015
CGCTCGCC -1.46787613347
CGCTCGGA -0.193317830136
CGCTCGGC -0.611431668152
CGCTCGTA -0.509095322899
CGCTCGTC -0.996192477433
CGCTCTAA -0.453917538066
CGCTCTAC -0.348887994137
CGCTCTAG -0.424656455933
CGCTCTCA -1.62351728617
CGCTCTCC -1.57306118636
CGCTCTGA -0.291745217243
CGCTCTGC -0.317913190778
CGCTCTTA 0.201750287861
CGCTCTTC 0.629634751731
CGCTGAAA 1.25889311765
CGCTGAAC -0.902064382208
CGCTGAAG 0.293377332696
CGCTGACA 0.310126917538
CGCTGACC -0.564205033528
CGCTGACG -0.332126543149
CGCTGAGA 0.160909721305
CGCTGAGC -0.0761381859093
CGCTGATA 2.43720469835
CGCTGATC 0.507704693915
CGCTGCAA 0.824135241037
CGCTGCAC -0.757327800886
CGCTGCAG 0.197248230616
CGCTGCCA -1.40786029212
CGCTGCCC -1.49955291302
CGCTGCCG -1.12565087596
CGCTGCGA -0.313336189456
CGCTGCGC 0.427353193636
CGCTGCTA 0.784249795107
CGCTGCTC -0.966702607687
CGCTGGAA 0.0724936138117
CGCTGGAC 0.157833475124
CGCTGGAG -0.634146385164
CGCTGGCA -0.375716308261
CGCTGGCC -0.725015453931
CGCTGGGA -0.711525103724
CGCTGGGC -1.05756522294
CGCTGGTA -0.633506862374
CGCTGGTC -0.322087784042

CGCTGTAA -0.465651906915
CGCTGTAC -0.882065138918
CGCTGTAG -1.03149842391
CGCTGTCA -0.612976765205
CGCTGTCC -0.922274926159
CGCTGTGA 0.967776597945
CGCTGTGC -0.164483512886
CGCTGTTA 0.093174223668
CGCTGTTC -0.0515806773168
CGCTTAAA 0.556135013686
CGCTTAAC -0.754706839973
CGCTTAAG 0.0573230590351
CGCTTACA -0.0957239877082
CGCTTACC 0.319320474
CGCTTACG -0.137672269357
CGCTTAGA -0.382584100202
CGCTTAGC -0.279768945568
CGCTTATA 0.793010757702
CGCTTATC 0.960823453028
CGCTTCAA 1.87165837402
CGCTTCAC -1.27332276696
CGCTTCAG 0.485435894162
CGCTTCCA -0.638163387688
CGCTTCCC -0.849713029966
CGCTTCCG -0.246151114428
CGCTTCGA -0.0946348004566
CGCTTCGC -0.557262505689
CGCTTCTA -0.910826177515
CGCTTCTC 0.102729177123
CGCTTGAA -0.392167365864
CGCTTGAC -0.304742601966
CGCTTGAG -0.789456326675
CGCTTGCA -0.481125152491
CGCTTGCC -1.22143377804
CGCTTGGA 0.0423182139397
CGCTTGGC 0.0315806013147
CGCTTGTA -1.16278878875
CGCTTGTC -0.490655540943
CGCTTTAA -0.447642012512
CGCTTTAC -0.201888518048
CGCTTTAG 0.42713814577
CGCTTTCA -0.174436086305
CGCTTTCC -0.906934498142
CGCTTTGA 0.567676401537
CGCTTTGC 1.15585292261
CGCTTTTA -0.0415081933747
CGCTTTTC 1.41764257594
CGGAAAAA 0.33859109432
CGGAAAAC 0.82289845559
CGGAAAAG 0.155689658168
CGGAAACA 0.376883354082
CGGAAACC -0.766978932574
CGGAAACG -0.536164498959
CGGAAAGA 0.100508750665

CGGAAAGC -0.92967128202
CGGAAATA 2.13081520029
CGGAAATC 14.3117347922
CGGAACAA -0.470245354298
CGGAACAC -0.299021038046
CGGAACAG 1.32007850297
CGGAACCA 0.498677055313
CGGAACCC -1.20217044395
CGGAACCG -0.908682776914
CGGAACGA -0.359221325102
CGGAACGC -1.2555195933
CGGAACTA -0.340103715606
CGGAACTC -0.141596424497
CGGAAGAA -0.0160617647583
CGGAAGAC -0.885079348057
CGGAAGAG -0.805925079406
CGGAAGCA -0.0248066976484
CGGAAGCC -0.691644521889
CGGAAGGA -0.558920018857
CGGAAGGC -0.64121985696
CGGAAGTA 1.72739394038
CGGAAGTC -0.609613441574
CGGAATAA 1.16256832826
CGGAATAC 1.30551374614
CGGAATAG 0.258434240462
CGGAATCA 3.23656301119
CGGAATCC 9.55101805838
CGGAATGA -1.11411760705
CGGAATGC 0.0534342941533
CGGAATTA 1.19750496698
CGGAATTC 1.19617241965
CGGACAAA 0.490702172813
CGGACAAC 0.242743032529
CGGACAAG -0.328613955898
CGGACACA -0.110331837374
CGGACACC -0.788903405929
CGGACACG -0.261607081231
CGGACAGA -0.00746338917426
CGGACAGC -0.962311092956
CGGACATA -0.500824619473
CGGACATC -0.0953634234269
CGGACCAA -0.26467416758
CGGACCAC -1.16715740391
CGGACCAG -1.11453250578
CGGACCCA -0.00344701118371
CGGACCCC -0.621151498576
CGGACCCG 0.272700261866
CGGACCGA -0.976890214059
CGGACCGC -1.47571590846
CGGACCTA -0.302214072081
CGGACCTC -0.785362298293
CGGACGAA -0.630723522627
CGGACGAC -0.460172454
CGGACGAG -1.1100412738

CGGACGCA -0.336269077003
CGGACGCC -1.11246113477
CGGACGGA -0.514879964749
CGGACGGC -0.414338530139
CGGACGTA -0.482167291517
CGGACGTC -1.02920138795
CGGACTAA -0.0580316969182
CGGACTAC -0.730666861867
CGGACTAG -1.03085182306
CGGACTCA -0.317779540507
CGGACTCC -0.748267270161
CGGACTGA -1.11040017265
CGGACTGC -1.01455669078
CGGACTTA 0.496945430779
CGGACTTC -0.863612658105
CGGAGAAA 0.644686436875
CGGAGAAC 0.155076782161
CGGAGAAG -0.638850791416
CGGAGACA -0.619805003327
CGGAGACC -0.639182002601
CGGAGACG -0.269671272089
CGGAGAGA -1.0194278476
CGGAGAGC -1.58488715353
CGGAGATA 1.52741483087
CGGAGATC 3.85715471915
CGGAGCAA 0.383514655824
CGGAGCAC -0.505304609851
CGGAGCAG -0.0802438722582
CGGAGCCA -0.887096800973
CGGAGCCC -1.32492259665
CGGAGCCG -0.3699431162
CGGAGCGA -0.821749521254
CGGAGCGC -0.716513672935
CGGAGCTA -0.798492500575
CGGAGCTC -1.05427038987
CGGAGGAA -0.0785642922226
CGGAGGAC -1.50341836197
CGGAGGAG -0.244321438061
CGGAGGCA -1.08434961151
CGGAGGCC -1.37895269622
CGGAGGGA -0.731449611115
CGGAGGGC -1.3435601477
CGGAGGTA -0.114757701474
CGGAGGTC -1.35914247854
CGGAGTAA -0.318325799557
CGGAGTAC -1.09348779262
CGGAGTAG 0.137839644462
CGGAGTCA 0.477842185565
CGGAGTCC -0.691418232413
CGGAGTGA -0.569962404062
CGGAGTGC -0.824444593533
CGGAGTTA 0.560715137678
CGGAGTTC -0.603868978076
CGGATAAA 0.0910412319668

CGGATAAC 0.678276372164
CGGATAAG -0.365317401228
CGGATACA 1.79785115708
CGGATACC 4.38825612117
CGGATACG 6.43423816538
CGGATAGA 0.350930220234
CGGATAGC 0.905698337228
CGGATATA 1.94156662535
CGGATATC 13.4082622941
CGGATCAA 0.222546852859
CGGATCAC 2.05180686441
CGGATCAG 1.23547017911
CGGATCCA 1.52602149558
CGGATCCC 1.91461340444
CGGATCCG 3.95654451311
CGGATCGA 0.455962262299
CGGATCGC 5.68718582198
CGGATCTA 2.99592111913
CGGATCTC 3.90283084409
CGGATGAA 0.511089043524
CGGATGAC -0.291769574068
CGGATGAG 0.170469671032
CGGATGCA -0.174752516852
CGGATGCC -0.477204328199
CGGATGGA -0.82170434663
CGGATGGC -0.867599891176
CGGATGTA -0.493324382795
CGGATGTC 0.902185125443
CGGATTAA -0.277773559519
CGGATTAC 9.95281282364
CGGATTAG 0.0215131136445
CGGATTCA 3.11942000629
CGGATTCC 12.3296300829
CGGATTGA 0.277163181648
CGGATTGC 8.49422517579
CGGATTTA 3.65001886664
CGGATTTC 18.0931210496
CGGCAAAA 0.224586164464
CGGCAAAC 0.00316347275925
CGGCAAAG 1.11632242016
CGGCAACA -1.03503474345
CGGCAACC -0.43364225076
CGGCAACG -0.28192254689
CGGCAAGA -0.0777299148325
CGGCAAGC -0.899972609749
CGGCAATA 0.629575421004
CGGCAATC 4.97976869456
CGGCACAA 1.36778332244
CGGCACAC -0.920970066508
CGGCACAG -0.114154609832
CGGCACCA -1.02225219841
CGGCACCC -1.0595822595
CGGCACCG -0.83812667567
CGGCACGA -1.24413600437

CGGCACGC −0.969242171006
CGGCACTA −0.597684218125
CGGCACTC −1.12984441342
CGGCAGAA 0.528248322653
CGGCAGAC −1.27450396888
CGGCAGAG 0.0607994233156
CGGCAGCA −1.024683301
CGGCAGCC −0.918364302588
CGGCAGGA −0.364291916442
CGGCAGGC −1.6471550644
CGGCAGTA −0.0905330694895
CGGCAGTC −0.916798179558
CGGCATAA 1.02287215247
CGGCATAC −0.548168041951
CGGCATAG 0.32277831044
CGGCATCA 0.412079590753
CGGCATCC 0.617795669353
CGGCATGA −0.914710362481
CGGCATGC 0.836756656308
CGGCATTA −0.534825498118
CGGCATTC −0.611057364123
CGGCCAAA −0.577567978804
CGGCCAAC 0.142313597678
CGGCCAAG −0.106630640862
CGGCCACA −0.906732357312
CGGCCACC −1.50539272204
CGGCCACG −1.44239493913
CGGCCAGA −0.945118088992
CGGCCAGC −0.564041405627
CGGCCATA −0.136675513134
CGGCCATC −0.550987812846
CGGCCCAA −0.743256217727
CGGCCCAC −1.68523165176
CGGCCCAG −1.03344343088
CGGCCCCA −0.157780597915
CGGCCCCC −0.941728118578
CGGCCCCG −0.595019748012
CGGCCCGA −1.39350558689
CGGCCCGC −0.259167859704
CGGCCCTA −0.661273851061
CGGCCCTC −2.02450848438
CGGCCGAA 0.746101178159
CGGCCGAC −1.15339704685
CGGCCGAG −0.419388928214
CGGCCGCA −0.948036744433
CGGCCGCC −1.23053136444
CGGCCGGA −0.18178018949
CGGCCGGC 0.262730617851
CGGCCGTA 0.214015510588
CGGCCGTC −1.39416509477
CGGCCTAA −0.435276239815
CGGCCTAC −1.00751944195
CGGCCTAG −1.22527278838
CGGCCTCA −0.937688841008

CGGCCTCC −0.735961244548
CGGCCTGA −0.99075112109
CGGCCTGC −1.29434083334
CGGCCTTA −0.609185843979
CGGCCTTC −1.36076668323
CGGCGAAA −0.314592751813
CGGCGAAC −0.947202783399
CGGCGAAG −0.83442839365
CGGCGACA −0.6061322892
CGGCGACC −1.39918176801
CGGCGACG −0.616155018602
CGGCGAGA −0.341835131962
CGGCGAGC −0.501738729034
CGGCGATA 1.00128659289
CGGCGATC 1.63822464492
CGGCGCAA 1.68735236096
CGGCGCAC −0.563058180973
CGGCGCAG 1.21729166054
CGGCGCCA −0.0554748548263
CGGCGCCC −0.830939746868
CGGCGCCG −0.326451194744
CGGCGCGA 0.176903828216
CGGCGCGC −0.356793553365
CGGCGCTA −0.11249439035
CGGCGCTC −1.12011583952
CGGCGGAA 0.166259895688
CGGCGGAC −1.62959108725
CGGCGGAG −0.891571795079
CGGCGGCA −0.847032113792
CGGCGGCC −1.47175844484
CGGCGGGA −0.466790015995
CGGCGGGC −0.650970914083
CGGCGGTA 0.105431743809
CGGCGGTC −1.4214813781
CGGCGTAA −0.164942753535
CGGCGTAC −1.51561405325
CGGCGTAG −0.371266295514
CGGCGTCA −0.428202143485
CGGCGTCC −0.15849006851
CGGCGTGA −1.3142788723
CGGCGTGC −0.971754879364
CGGCGTTA −0.213304166391
CGGCGTTC −1.03226056353
CGGCTAAA −0.642948150645
CGGCTAAC −1.05794806226
CGGCTAAG −0.129049953304
CGGCTACA −0.798249765037
CGGCTACC −1.25026996889
CGGCTACG −0.235182424233
CGGCTAGA −0.452712603852
CGGCTAGC −0.927232685028
CGGCTATA −0.469211958748
CGGCTATC 0.225597076791
CGGCTCAA −1.16992762844

CGGCTCAC -0.963690896684
CGGCTCAG -0.309124748687
CGGCTCCA -0.507634954288
CGGCTCCC -0.978031029452
CGGCTCGA -0.802700610496
CGGCTCGC -0.98687359782
CGGCTCTA -0.844022692746
CGGCTCTC -1.52335161283
CGGCTGAA -0.0172038292198
CGGCTGAC -0.739442813278
CGGCTGAG -0.570913569305
CGGCTGCA -0.855189984746
CGGCTGCC -0.967578828854
CGGCTGGA -1.21202975362
CGGCTGGC -0.780055008577
CGGCTGTA 0.211607099404
CGGCTGTC -0.729255206907
CGGCTTAA -0.662704450219
CGGCTTAC -1.23966267569
CGGCTTAG -0.718474085082
CGGCTTCA -0.433467381247
CGGCTTCC -0.508060053747
CGGCTTGA -1.13933982797
CGGCTTGC -1.3249155186
CGGCTTTA -0.743059905881
CGGCTTTC -1.00123413203
CGGGAAAA -0.273263591511
CGGGAAAC 0.0298414823735
CGGGAAAG -0.734919105522
CGGGAACA -0.931559456403
CGGGAACC -0.848840764182
CGGGAACG -0.038134044488
CGGGAAGA -1.38794640181
CGGGAAGC -1.10111085432
CGGGAATA 2.3671896517
CGGGAATC 13.5568108226
CGGGACAA 0.670246114318
CGGGACAC -0.606297166169
CGGGACAG -0.982574722295
CGGGACCA -0.731702339197
CGGGACCC -1.32784749743
CGGGACCG -0.537207054341
CGGGACGA -1.537037234
CGGGACGC -1.39304426446
CGGGACTA -1.29352061205
CGGGACTC -1.17481002688
CGGGAGAA 0.103415748139
CGGGAGAC -0.754251346528
CGGGAGAG -1.00113212482
CGGGAGCA 0.144014828225
CGGGAGCC -0.784145081577
CGGGAGGA 0.164147513608
CGGGAGGC -1.01645548224
CGGGAGTA 0.756527356509

CGGGAGTC -1.01746139829
CGGGATAA 1.0922195723
CGGGATAC 1.7804293655
CGGGATAG -0.162684855039
CGGGATCA 0.0197821136449
CGGGATCC 2.42475232361
CGGGATGA -0.839396977774
CGGGATGC 0.00346824533885
CGGGATTA 2.42941217977
CGGGATTC 4.70229740949
CGGGCAAA 0.414472388588
CGGGCAAC -0.755041590183
CGGGCAAG -0.924504304271
CGGGCACA -0.887636398327
CGGGCACC -1.72619941507
CGGGCACG 0.253096973115
CGGGCAGA -0.9267193181
CGGGCAGC -0.108629149581
CGGGCATA 0.706598987911
CGGGCATC -0.775374750972
CGGGCCAA -0.670699109628
CGGGCCAC -1.06863342221
CGGGCCAG -1.12440742881
CGGGCCCA -1.22909285452
CGGGCCCC -1.47434026829
CGGGCCCG -0.342860200392
CGGGCCGA -0.413502903681
CGGGCCGC -1.83796726423
CGGGCCTA -1.33765247265
CGGGCCTC -1.75949415396
CGGGCGAA 0.422723939638
CGGGCGAC -1.01328555596
CGGGCGAG -1.24353770082
CGGGCGCA 0.132456994313
CGGGCGCC -0.906051198924
CGGGCGGA -0.598480498943
CGGGCGGC -0.967540940459
CGGGCGTA -0.353643195819
CGGGCGTC -1.18812009494
CGGGCTAA 0.362103549395
CGGGCTAC -0.795177890594
CGGGCTAG -0.465457676849
CGGGCTCA -0.945912288029
CGGGCTCC -1.20996088075
CGGGCTGA -0.570775339119
CGGGCTGC -0.648579365316
CGGGCTTA -0.750878030353
CGGGCTTC -1.22907203672
CGGGGAAA 1.42991217041
CGGGGAAC -0.13203647477
CGGGGAAG -1.16010641533
CGGGGACA -0.741885157475
CGGGGACC -1.50822997989
CGGGGACG 0.571458162931

CGGGGAGA −0.855034475786
CGGGGAGC −0.612050164965
CGGGGATA 2.26893401144
CGGGGATC 3.33907589138
CGGGGCAA 1.25737300196
CGGGGCAC −0.952390578948
CGGGGCAG −1.38231477078
CGGGGCCA −1.4522117805
CGGGGCCC −0.79227922024
CGGGGCGA −1.09580065011
CGGGGCGC −0.051108321454
CGGGGCTA 0.00221168298199
CGGGGCTC −1.34256609779
CGGGGGAA 0.89325699592
CGGGGGAC −0.728167893257
CGGGGGAG −0.260535589109
CGGGGGCA −1.19591344623
CGGGGGCC −1.53831128331
CGGGGGGA −0.25647757552
CGGGGGGC −1.39505151666
CGGGGGTA −0.137216151378
CGGGGGTC −1.58642912791
CGGGGTAA 0.824878644645
CGGGGTAC −0.278537989104
CGGGGTAG −0.635010323828
CGGGGTCA −1.20592909758
CGGGGTCC −0.990544816701
CGGGGTGA −0.327372174179
CGGGGTGC −0.272372173351
CGGGGTTA −0.316461357465
CGGGGTTC −0.816915420113
CGGGTAAA 0.267481655974
CGGGTAAC −0.0995074145263
CGGGTAAG −0.815221683974
CGGGTACA −0.0896331160321
CGGGTACC −0.521675727105
CGGGTACG 0.215761499403
CGGGTAGA −0.164942753535
CGGGTAGC −0.382093216498
CGGGTATA −0.329284289031
CGGGTATC 2.3161073525
CGGGTCAA −0.429161011314
CGGGTCAC −0.972635472268
CGGGTCAG −0.558664792639
CGGGTCCA −1.01619588428
CGGGTCCC −1.505315488
CGGGTCGA −1.16636237216
CGGGTCGC −0.874337988228
CGGGTCTA −1.06491203244
CGGGTCTC −1.457649806
CGGGTGAA 0.237491534515
CGGGTGAC −0.0832890999183
CGGGTGAG −0.748111761201
CGGGTGCA −1.31853236502

CGGGTGCC -1.38493885436
CGGGTGGA -0.327781243932
CGGGTGGC -0.821993714039
CGGGTGTA 0.0159872370374
CGGGTGTC -0.946855750687
CGGGTTAA -0.0390841688414
CGGGTTAC -0.118602748977
CGGGTTAG -1.1395646602
CGGGTTCA -0.634080184562
CGGGTTCC -1.02809408921
CGGGTTGA 0.0489166235592
CGGGTTGC 0.00707263908416
CGGGTTTA 0.256475701918
CGGGTTTC -1.04944024411
CGGTAAAA 1.72563067279
CGGTAAAC 0.585420452645
CGGTAAAG 0.32748396576
CGGTAACA -0.517721594334
CGGTAACC -0.57397794934
CGGTAACG -0.582651893535
CGGTAAGA 0.507186122539
CGGTAAGC -0.54724144171
CGGTAATA 0.154385631229
CGGTAATC 5.88656787701
CGGTACAA 1.49639547742
CGGTACAC -0.730742846834
CGGTACAG -0.641543365558
CGGTACCA -0.147539489799
CGGTACCC -0.827903887218
CGGTACCG 0.576067223663
CGGTACGA -0.625318805611
CGGTACGC -0.932330131328
CGGTACTA -0.0663783853105
CGGTACTC -0.550633285726
CGGTAGAA 1.42190731028
CGGTAGAC -0.216346271381
CGGTAGAG -0.643098246977
CGGTAGCA -0.21725122111
CGGTAGCC -1.35852960253
CGGTAGGA 0.744121821816
CGGTAGGC -1.21095139163
CGGTAGTA 0.078996469733
CGGTAGTC -0.78371977394
CGGTATAA 1.66204811791
CGGTATAC 0.510020257715
CGGTATAG -0.36109555156
CGGTATCA 1.47388310942
CGGTATCC 4.0110113701
CGGTATGA 0.0124007465993
CGGTATGC -0.0782332892161
CGGTATTA 0.617198198517
CGGTATTC 0.485075954414
CGGTCAAA 0.465433320024
CGGTCAAC -0.422386066758

CGGTCAAG -0.468114860733
CGGTCACA -0.896693390026
CGGTCACC -1.27739056491
CGGTCACG -0.247232182738
CGGTCAGA -0.90465328371
CGGTCAGC -0.653519220877
CGGTCATA 0.118463061545
CGGTCATC -1.16594830613
CGGTCCAA -0.853570984505
CGGTCCAC -0.814025493235
CGGTCCAG -0.579097878864
CGGTCCCA -0.113018998888
CGGTCCCC -1.15437277709
CGGTCCGA -1.37862502406
CGGTCCGC -1.33752132052
CGGTCCTA -1.32047945378
CGGTCCTC -0.918637432113
CGGTCGAA -0.176895501097
CGGTCGAC -0.601770543921
CGGTCGAG -0.985869763545
CGGTCGCA -0.478545827176
CGGTCGCC -0.841306178135
CGGTCGGA 0.15923326394
CGGTCGGC -0.827822281445
CGGTCGTA -0.717770235292
CGGTCGTC -1.57837992587
CGGTCTAA 0.119271208508
CGGTCTAC 0.0278590033602
CGGTCTAG -0.704727675945
CGGTCTCA -0.395677663157
CGGTCTCC -0.804577543267
CGGTCTGA -0.413025135191
CGGTCTGC -0.787636434673
CGGTCTTA -0.859425990517
CGGTCTTC -1.1186186234
CGGTGAAA 1.03072878987
CGGTGAAC -0.556372752953
CGGTGAAG 0.390656201823
CGGTGACA 0.0834831218064
CGGTGACC -1.05860403111
CGGTGACG -0.354257945428
CGGTGAGA -0.603741156789
CGGTGAGC -0.497985279847
CGGTGATA 1.45264145988
CGGTGATC 0.84123310766
CGGTGCAA 0.829950485053
CGGTGCAC -0.240219707094
CGGTGCAG -0.42021227217
CGGTGCCA -1.2812603856
CGGTGCCC -1.14842138467
CGGTGCGA -0.57301304435
CGGTGCGC -1.22699858393
CGGTGCTA -0.604236620409
CGGTGCTC -0.769642361797

CGGTGGAA 1.54145060742
CGGTGGAC -1.58253286862
CGGTGGAG -0.40843356141
CGGTGGCA -0.843069237545
CGGTGGCC -1.27043367279
CGGTGGGA -0.221714140893
CGGTGGGC -0.624386792743
CGGTGGTA -0.203876617867
CGGTGGTC -1.30642972931
CGGTGTAA 1.60067266609
CGGTGTAC -0.153048712167
CGGTGTAG 0.0867798284802
CGGTGTCA 0.0870787720761
CGGTGTCC -0.217611369036
CGGTGTGA -0.65634752707
CGGTGTGC -0.0398392304166
CGGTGTTA 0.615190738145
CGGTGTTC -0.939147127839
CGGTTAAA 1.38530295767
CGGTTAAC 0.0432837434644
CGGTTAAG 0.273429301192
CGGTTACA 1.43817079951
CGGTTACC -0.288947721392
CGGTTACG 1.07111969197
CGGTTAGA -0.0614928642054
CGGTTAGC 0.202317572888
CGGTTATA -0.223819236743
CGGTTATC 0.657069696523
CGGTTCAA 1.17862884395
CGGTTCAC -0.0519256282487
CGGTTCAG -0.303792477612
CGGTTCCA -0.411842476021
CGGTTCCC -0.907923135423
CGGTTCGA -0.871097073256
CGGTTCGC -1.32218214157
CGGTTCTA -0.633226238441
CGGTTCTC -0.200092774693
CGGTTGAA 0.592558667796
CGGTTGAC -0.950488456639
CGGTTGAG -0.640060305547
CGGTTGCA -0.0749145156751
CGGTTGCC -1.00095517352
CGGTTGGA -0.969329189406
CGGTTGGC -0.448058993029
CGGTTGTA -0.0517776136968
CGGTTGTC -0.169614892199
CGGTTTAA 0.659717720575
CGGTTTAC -0.373960326903
CGGTTTAG -0.127882074771
CGGTTTCA -0.155592647224
CGGTTTCC 0.146628919265
CGGTTTGA -0.659410658037
CGGTTTGC -0.0625408322147
CGGTTTTA 0.484679167163

| | |
|---|---|
| CGGTTTTC | 0.645903445413 |
| CGTAAAAA | 2.46034222486 |
| CGTAAAAC | 0.286008039974 |
| CGTAAAAG | 0.728563431441 |
| CGTAAACA | -0.193788312397 |
| CGTAAACC | -0.611015520347 |
| CGTAAACG | -0.264995594399 |
| CGTAAAGA | 0.309991810022 |
| CGTAAAGC | -0.27784600546 |
| CGTAAATA | 0.464810243295 |
| CGTAAATC | 2.26239597344 |
| CGTAACAA | 2.24352172401 |
| CGTAACAC | 1.05265326323 |
| CGTAACAG | 0.872547657501 |
| CGTAACCA | 0.54823653251 |
| CGTAACCC | 1.22289414665 |
| CGTAACGA | 0.0127471547772 |
| CGTAACGC | 0.773955809782 |
| CGTAACTA | 0.681563919006 |
| CGTAACTC | 0.602584519868 |
| CGTAAGAA | 1.69859605428 |
| CGTAAGAC | -0.729241258981 |
| CGTAAGAG | -0.692004461637 |
| CGTAAGCA | 0.0935972413468 |
| CGTAAGCC | -0.854967026117 |
| CGTAAGGA | 0.514884961021 |
| CGTAAGGC | -0.606383560035 |
| CGTAAGTA | 0.838587998099 |
| CGTAAGTC | 0.44940423921 |
| CGTAATAA | 3.45352923663 |
| CGTAATAC | 3.93286364204 |
| CGTAATAG | 0.96717704533 |
| CGTAATCA | 6.00918721215 |
| CGTAATCC | 23.9993259401 |
| CGTAATGA | 0.0785407681095 |
| CGTAATGC | 0.35958542841 |
| CGTAATTA | 1.08810847332 |
| CGTAATTC | 2.0748538335 |
| CGTACAAA | 1.82863860025 |
| CGTACAAC | -0.0493506746715 |
| CGTACAAG | 0.0848333642595 |
| CGTACACA | 0.108695974716 |
| CGTACACC | -0.216769497238 |
| CGTACACG | 0.0576324115305 |
| CGTACAGA | -0.637069204165 |
| CGTACAGC | -0.459769837764 |
| CGTACATA | 0.657128402716 |
| CGTACATC | -0.673203490866 |
| CGTACCAA | -0.0824064252342 |
| CGTACCAC | -0.175571489071 |
| CGTACCAG | -1.13335096351 |
| CGTACCCA | -0.0492632399151 |
| CGTACCCC | -0.268546278223 |
| CGTACCGA | -1.25904508759 |

CGTACCGC -0.0947097445335
CGTACCTA -0.487955888749
CGTACCTC -0.240759512626
CGTACGAA 0.443400385935
CGTACGAC -0.938818831146
CGTACGAG -0.239306846601
CGTACGCA 0.339359271109
CGTACGCC -0.843069237545
CGTACGGA 0.757024277375
CGTACGGC -0.378160734238
CGTACGTA 0.666747058637
CGTACGTC 0.670478024601
CGTACTAA 2.83059948165
CGTACTAC 0.740999984655
CGTACTAG -0.0801724672071
CGTACTCA -0.309447216396
CGTACTCC -0.39678641914
CGTACTGA -0.314578179353
CGTACTGC -0.655547499049
CGTACTTA -0.0306987593426
CGTACTTC -0.553374157163
CGTAGAAA 1.40415222573
CGTAGAAC -0.524519438469
CGTAGAAG 0.288971037327
CGTAGACA 0.372235572243
CGTAGACC -1.12290375918
CGTAGACG -0.568304682715
CGTAGAGA 0.434353803135
CGTAGAGC -0.309718472319
CGTAGATA 1.93907515115
CGTAGATC 1.75636565511
CGTAGCAA 1.83383264114
CGTAGCAC -0.151890826178
CGTAGCAG -0.153739446743
CGTAGCCA -0.540543731189
CGTAGCCC -0.908752308364
CGTAGCGA -0.645772501456
CGTAGCGC -0.112584323242
CGTAGCTA -0.375482732555
CGTAGCTC -0.497844343347
CGTAGGAA 0.263119702518
CGTAGGAC -1.04082708788
CGTAGGAG -0.972741851221
CGTAGGCA 0.340671625167
CGTAGGCC -2.16772495001
CGTAGGGA -0.687271743177
CGTAGGGC -0.498982660604
CGTAGGTA -0.187433887384
CGTAGGTC -1.26346595542
CGTAGTAA 0.697786188996
CGTAGTAC 0.586840018369
CGTAGTAG -0.167957170852
CGTAGTCA 0.565678100996
CGTAGTCC -0.190559055393

CGTAGTGA -1.04018007069
CGTAGTGC -0.303613028184
CGTAGTTA -0.123527823901
CGTAGTTC -0.234290589717
CGTATAAA 2.33048724726
CGTATAAC -0.467219279013
CGTATAAG 0.793010757702
CGTATACA 1.22550511502
CGTATACC 0.336851975379
CGTATACG 3.22761614565
CGTATAGA 0.736631161325
CGTATAGC 0.744266089164
CGTATATA 1.27923377285
CGTATATC 1.64873451082
CGTATCAA 2.6018181143
CGTATCAC 2.3595921959
CGTATCAG 3.19150954662
CGTATCCA 5.78647131877
CGTATCCC 2.32748677787
CGTATCGA 0.71787932056
CGTATCGC 1.98528358722
CGTATCTA 6.32087837912
CGTATCTC 5.37658800582
CGTATGAA 1.7247802657
CGTATGAC -0.602009948611
CGTATGAG -0.801241699131
CGTATGCA 0.133986686195
CGTATGCC -1.32304982744
CGTATGGA 0.492859729518
CGTATGGC -0.18797119478
CGTATGTA 1.05646541861
CGTATGTC -0.479182019118
CGTATTAA 2.25274692353
CGTATTAC 2.62284908772
CGTATTAG 0.656747436992
CGTATTCA 0.898267631998
CGTATTCC 1.77652998354
CGTATTGA 1.02660561657
CGTATTGC 0.453178922553
CGTATTTA 0.821549878561
CGTATTTC 1.99724008198
CGTCAAAA 1.36433568672
CGTCAAAC -0.379540954321
CGTCAAAG 0.428120537712
CGTCAACA 0.332015167924
CGTCAACC -0.607342219686
CGTCAACG -0.371705967432
CGTCAAGA 0.308973611465
CGTCAAGC 0.0364592525462
CGTCAATA -0.48989194407
CGTCAATC -0.293647131373
CGTCACAA 0.254741162893
CGTCACAC -0.29346705741
CGTCACAG -0.0550457999857

CGTCACCA −0.680163505657
CGTCACCC 0.568909231603
CGTCACGA −1.0277329004
CGTCACGC −0.689383084368
CGTCACTA −0.244558969149
CGTCACTC −0.596096028228
CGTCAGAA 1.09947582437
CGTCAGAC −1.08024205155
CGTCAGAG −0.837487361058
CGTCAGCA 0.487952974257
CGTCAGCC −1.21734870131
CGTCAGGA 0.954404076482
CGTCAGGC 0.494466447256
CGTCAGTA 0.203664068137
CGTCAGTC −0.573955882473
CGTCATAA 2.3106428884
CGTCATAC −0.62758669665
CGTCATAG −0.333101024327
CGTCATCA −0.13203647477
CGTCATCC 0.253748153873
CGTCATGA 0.372878009525
CGTCATGC −0.809002782833
CGTCATTA 0.157145238685
CGTCATTC −0.806837107187
CGTCCAAA 0.605784215598
CGTCCAAC −0.732486337513
CGTCCAAG −1.10739907873
CGTCCACA 0.354137410371
CGTCCACC −0.421945978484
CGTCCACG 0.169703367845
CGTCCAGA −1.10850158937
CGTCCAGC 0.166024030023
CGTCCATA 1.113418129
CGTCCATC −1.03874947153
CGTCCCAA 0.389901348426
CGTCCCAC −0.403881541447
CGTCCCAG −1.03943187899
CGTCCCCA −0.78062583263
CGTCCCCC −0.237100576246
CGTCCCGA −0.973182564029
CGTCCCGC −1.35783970067
CGTCCCTA −0.0912119379199
CGTCCCTC −0.976996176656
CGTCCGAA −0.518006173648
CGTCCGAC −0.689234028926
CGTCCGAG −1.31284910585
CGTCCGCA 0.0606843008863
CGTCCGCC −1.10495444457
CGTCCGGA 0.0452583117141
CGTCCGGC −0.710043500959
CGTCCGTA −0.633646341628
CGTCCGTC −0.969899597103
CGTCCTAA 0.649076286182
CGTCCTAC 0.040297430176

CGTCCTAG -1.22864652091
CGTCCTCA -1.11273863603
CGTCCTCC -1.6792048989
CGTCCTGA 0.0479850270471
CGTCCTGC -0.92967128202
CGTCCTTA -0.438042717145
CGTCCTTC -1.36328417968
CGTCGAAA 1.03499352421
CGTCGAAC 0.244212769149
CGTCGAAG -1.21735328122
CGTCGACA -0.378057477954
CGTCGACC -0.551250325293
CGTCGACG 0.300316737866
CGTCGAGA -0.67173125611
CGTCGAGC -0.352745115964
CGTCGATA 0.394006826597
CGTCGATC -0.539466618261
CGTCGCAA 1.24933504153
CGTCGCAC -0.406626160087
CGTCGCAG -0.124406751381
CGTCGCCA -0.414546291775
CGTCGCCC -1.15725312778
CGTCGCGA -0.248342604145
CGTCGCGC 0.613029226059
CGTCGCTA 0.147245542475
CGTCGCTC -1.02262483702
CGTCGGAA 0.559167334311
CGTCGGAC -0.668486593934
CGTCGGAG -0.777710091681
CGTCGGCA -0.600143008383
CGTCGGCC -1.79412497921
CGTCGGGA -0.390847517397
CGTCGGGC -0.819029675795
CGTCGGTA 0.0233255112387
CGTCGGTC -1.50773722258
CGTCGTAA 2.21567791765
CGTCGTAC -0.623961901462
CGTCGTAG -1.14366181125
CGTCGTCA 0.278873988382
CGTCGTCC -0.443989321473
CGTCGTGA -0.348641719573
CGTCGTGC -0.621233937061
CGTCGTTA -0.565795305205
CGTCGTTC 0.11115205866
CGTCTAAA 1.98886466503
CGTCTAAC -0.155589316376
CGTCTAAG -0.383805480478
CGTCTACA -0.325640966001
CGTCTACC -0.482555335293
CGTCTAGA 0.222970078716
CGTCTAGC -0.428100552625
CGTCTATA -0.0429587776197
CGTCTATC 0.758412408222
CGTCTCAA 0.405371263154

CGTCTCAC -1.06951817868
CGTCTCAG -0.434294264229
CGTCTCCA -1.09381234211
CGTCTCCC -0.963721082493
CGTCTCGA -0.924448304391
CGTCTCGC -0.520286555367
CGTCTCTA -0.983374125783
CGTCTCTC -1.41989110643
CGTCTGAA 1.04080127381
CGTCTGAC -0.948761203843
CGTCTGAG -0.702448751472
CGTCTGCA -0.550848125414
CGTCTGCC -1.51194928788
CGTCTGGA -0.65600653152
CGTCTGGC -0.259167859704
CGTCTGTA -0.559839749224
CGTCTGTC -0.243954420262
CGTCTTAA 0.691058292665
CGTCTTAC 0.432909880585
CGTCTTAG -0.627440139344
CGTCTTCA -0.230490092304
CGTCTTCC -0.772935113089
CGTCTTGA -1.23730859896
CGTCTTGC -0.00574696163411
CGTCTTTA -0.354000012897
CGTCTTTC -0.993645627885
CGTGAAAA 3.07936239716
CGTGAAAC -0.443156817684
CGTGAAAG 0.619547278974
CGTGAACA 0.235217398135
CGTGAACC -0.398863202783
CGTGAACG 0.0939140882499
CGTGAAGA -0.861495071575
CGTGAAGC -0.467328364281
CGTGAATA -0.0214585710107
CGTGAATC 1.41371592266
CGTGACAA 0.485933855917
CGTGACAC -1.17839735003
CGTGACAG -1.11216468931
CGTGACCA -0.709971055018
CGTGACCC -0.327017230703
CGTGACGA -1.0484414061
CGTGACGC -0.618966878733
CGTGACTA 0.382781869294
CGTGACTC -0.3449344859
CGTGAGAA 0.607032867191
CGTGAGAC -1.06181205396
CGTGAGAG -0.381476801465
CGTGAGCA 1.38460826771
CGTGAGCC -1.26915712535
CGTGAGGA -0.0342871233826
CGTGAGGC 0.013588193863
CGTGAGTA 0.878626454854
CGTGAGTC -0.497707778584

CGTGATAA 2.18259760208
CGTGATAC 2.79439649621
CGTGATAG -0.0197011324062
CGTGATCA 0.733752892414
CGTGATCC 4.59931696153
CGTGATGA -0.385530235138
CGTGATGC 1.00157908296
CGTGATTA 2.0790885902
CGTGATTC 1.93743241862
CGTGCAAA 0.427150844628
CGTGCAAC -0.142627738267
CGTGCAAG -0.263852905404
CGTGCACA 0.244955131867
CGTGCACC -1.04459844038
CGTGCACG -0.812514121016
CGTGCAGA -0.0474100394345
CGTGCAGC -0.0532569265046
CGTGCATA 1.47596738747
CGTGCATC -0.327781243932
CGTGCCAA -0.430374689005
CGTGCCAC -0.919503660736
CGTGCCAG -1.09837435462
CGTGCCCA 0.446728319307
CGTGCCCC -0.498416832823
CGTGCCGA -0.992333690182
CGTGCCGC 0.0196520024002
CGTGCCTA -0.19828120981
CGTGCCTC -0.733212045992
CGTGCGAA 0.709951486286
CGTGCGAC -0.493203015026
CGTGCGAG -0.92732053614
CGTGCGCA 0.712457116592
CGTGCGCC -0.370605122213
CGTGCGGA -0.866278793642
CGTGCGGC -0.980057434022
CGTGCGTA 0.870886813485
CGTGCGTC -0.53211793516
CGTGCTAA -0.121887381327
CGTGCTAC 0.522218447129
CGTGCTAG -0.373390960096
CGTGCTCA 0.533258126019
CGTGCTCC 0.0663446604759
CGTGCTGA -0.443787388821
CGTGCTGC -0.0739458634806
CGTGCTTA 0.0935972413468
CGTGCTTC -0.294176736184
CGTGGAAA 0.53687854947
CGTGGAAC -0.582654599849
CGTGGAAG -0.849567721728
CGTGGACA 0.747987687118
CGTGGACC -1.25345446762
CGTGGAGA -0.606584868153
CGTGGAGC -0.739512136549
CGTGGATA 4.32791968571

CGTGGATC 3.38088885733
CGTGGCAA 1.36043942743
CGTGGCAC -0.507460084775
CGTGGCAG -0.827640750237
CGTGGCCA -0.827379903213
CGTGGCCC -0.89188156393
CGTGGCGA 0.0885077058099
CGTGGCGC -0.186196477402
CGTGGCTA -0.821838413257
CGTGGCTC -1.14790676867
CGTGGGAA 0.318355777188
CGTGGGAC -0.729557273172
CGTGGGAG -0.948271569207
CGTGGGCA 0.552828106291
CGTGGGCC -0.867305735674
CGTGGGGA -0.377094030209
CGTGGGGC -0.126249126606
CGTGGGTA 0.42396030873
CGTGGGTC -1.01687246276
CGTGGTAA 0.868300410118
CGTGGTAC 0.609100074647
CGTGGTAG 0.102713355596
CGTGGTCA 0.118109367137
CGTGGTCC -0.860801422507
CGTGGTGA -1.07996455029
CGTGGTGC -0.285404115621
CGTGGTTA -0.278904382369
CGTGGTTC -0.0946348004566
CGTGTAAA 1.38951481479
CGTGTAAC -0.508138745028
CGTGTAAG -0.674388648172
CGTGTACA 1.28307632222
CGTGTACC -0.361672412775
CGTGTAGA 0.135273434361
CGTGTAGC 1.67620047413
CGTGTATA 1.45030549463
CGTGTATC 1.24612389601
CGTGTCAA 1.39898212531
CGTGTCAC 0.283737026264
CGTGTCAG 0.539967494508
CGTGTCCA 0.0866174496468
CGTGTCCC -0.758554801968
CGTGTCGA -1.01931730509
CGTGTCGC -0.445475504154
CGTGTCTA 0.111575284517
CGTGTCTC -0.617507967369
CGTGTGAA -0.10102544844
CGTGTGAC -0.281899230955
CGTGTGAG -0.901830390145
CGTGTGCA 0.315266623971
CGTGTGCC -1.02036319132
CGTGTGGA -0.0472965824291
CGTGTGGC -0.406068451248
CGTGTGTA 0.491998705345

CGTGTGTC -0.393212003025
CGTGTTAA 0.20344818756
CGTGTTAC 0.0574025830278
CGTGTTAG 0.251215044072
CGTGTTCA 0.341357988005
CGTGTTCC -0.263079107809
CGTGTTGA -0.222943848289
CGTGTTGC -0.470366930245
CGTGTTTA 0.397123667486
CGTGTTTC 0.453932943238
CGTTAAAA 2.0365865551
CGTTAAAC -0.405910860508
CGTTAAAG 0.515217629452
CGTTAACA 0.180926243369
CGTTAACC -0.85311028661
CGTTAACG 0.0101311901357
CGTTAAGA 0.170450310479
CGTTAAGC 0.0506980026327
CGTTAATA 0.676746680282
CGTTAATC 0.970988576177
CGTTACAA 1.5453393723
CGTTACAC -0.897532971866
CGTTACAG 0.670018783952
CGTTACCA 0.240593802944
CGTTACCC 1.03750831434
CGTTACGA -0.542435028242
CGTTACGC -0.432368825985
CGTTACTA -0.00707513722006
CGTTACTC -0.284657381165
CGTTAGAA 1.87943798556
CGTTAGAC -0.603741156789
CGTTAGAG -0.92172554444
CGTTAGCA -0.0163138683061
CGTTAGCC 1.49072720707
CGTTAGGA 1.01282756438
CGTTAGGC -0.977631952242
CGTTAGTA 0.524825876473
CGTTAGTC 0.287961374069
CGTTATAA 2.01917746238
CGTTATAC 1.29837532281
CGTTATAG 2.67666309853
CGTTATCA 1.03072878987
CGTTATCC 2.58870165177
CGTTATGA 0.288650026865
CGTTATGC -0.540863076228
CGTTATTA 0.749700783811
CGTTATTC 1.35046915888
CGTTCAAA 0.373321845003
CGTTCAAC -0.187201144389
CGTTCAAG -0.855702102605
CGTTCACA -0.719381532947
CGTTCACC -0.16678304698
CGTTCAGA 0.405381672054
CGTTCAGC 0.572942055654

CGTTCATA 0.147940856967
CGTTCATC -0.152331747164
CGTTCCAA -0.325629516212
CGTTCCAC -0.281517224341
CGTTCCAG -0.660348291711
CGTTCCCA 0.234390515153
CGTTCCCC -0.243701484002
CGTTCCGA -0.983864176775
CGTTCCGC -0.299021038046
CGTTCCTA -0.902177631035
CGTTCCTC -0.567566483557
CGTTCGAA -0.342202774295
CGTTCGAC 0.0566833280671
CGTTCGAG -0.803052847657
CGTTCGCA 0.690645475708
CGTTCGCC -0.793577834551
CGTTCGGA -0.392026429363
CGTTCGGC -0.899978230555
CGTTCGTA 1.11255981114
CGTTCGTC -0.275023736429
CGTTCTAA 0.390520261595
CGTTCTAC 0.157947140308
CGTTCTAG 1.69706657058
CGTTCTCA 0.0572922486923
CGTTCTCC 0.287848541597
CGTTCTGA 0.767158798358
CGTTCTGC 0.187834838196
CGTTCTTA 0.113102061907
CGTTCTTC -0.579245893416
CGTTGAAA 0.967779928793
CGTTGAAC 0.206826083651
CGTTGAAG -0.528186285612
CGTTGACA -0.176360067303
CGTTGACC -0.362412069179
CGTTGAGA 0.235265487251
CGTTGAGC -0.595954467194
CGTTGATA 0.595774185053
CGTTGATC -0.0310992937983
CGTTGCAA 0.279499771425
CGTTGCAC 0.112485230518
CGTTGCAG 0.875061614927
CGTTGCCA -0.0189766729957
CGTTGCCC -1.48928952986
CGTTGCGA 1.17457770024
CGTTGCGC 0.517888969439
CGTTGCTA -0.263783998489
CGTTGCTC -0.839222524617
CGTTGGAA -0.171874247941
CGTTGGAC -0.204214698925
CGTTGGAG -0.71846076169
CGTTGGCA -0.251485259105
CGTTGGCC -0.760239378276
CGTTGGGA 0.301827277372
CGTTGGGC -1.02853605109

CGTTGGTA -0.123527823901
CGTTGGTC -0.606270935742
CGTTGTAA 0.939472301861
CGTTGTAC 0.555100369069
CGTTGTAG 0.445430121352
CGTTGTCA 1.73747350238
CGTTGTCC 0.597557021373
CGTTGTGA 0.426165746372
CGTTGTGC -0.937433406612
CGTTGTTA 1.41631564942
CGTTGTTC 0.613924807779
CGTTTAAA 0.514531058436
CGTTTAAC -0.433923291048
CGTTTAAG 0.488784228977
CGTTTACA 1.06617067657
CGTTTACC 0.351357817828
CGTTTAGA 0.516581611652
CGTTTAGC -0.601521146687
CGTTTATA 1.05845268572
CGTTTATC -0.0478757336016
CGTTTCAA 0.53737817665
CGTTTCAC -0.625400203205
CGTTTCAG 0.991670851457
CGTTTCCA 0.0258709035411
CGTTTCCC 0.0606499515177
CGTTTCGA 0.965960869503
CGTTTCGC -0.385558130989
CGTTTCTA 0.57565086768
CGTTTCTC 0.718348137397
CGTTTGAA 0.729291638055
CGTTTGAC 0.482412525191
CGTTTGAG 0.776340696853
CGTTTGCA 0.343106474956
CGTTTGCC 0.628457088833
CGTTTGGA 0.316591260531
CGTTTGGC -0.380797516678
CGTTTGTA 0.163466563397
CGTTTGTC 0.101127455656
CGTTTTAA -0.281648792831
CGTTTTAC -0.429430393635
CGTTTTAG 0.316715334614
CGTTTTCA 1.66353846415
CGTTTTCC 0.121215382771
CGTTTTGA 0.442738796277
CGTTTTGC 0.015725349124
CGTTTTTA 0.73512978165
CGTTTTTC 0.981373118928
CTAAAAAA 0.693900130428
CTAAAAAC 0.112871192514
CTAAAAAG 0.392430294667
CTAAAACA -0.0941895077327
CTAAAACC 0.49801296752
CTAAAAGA 1.00358695969
CTAAAAGC 0.216114569277

CTAAAATA 2.43032212577
CTAAAATC 2.42941217977
CTAAACAA -0.0466376990859
CTAAACAC 1.65577675592
CTAAACAG -0.0028183136493
CTAAACCA -0.274153552425
CTAAACCC 0.119724411995
CTAAACGA 0.43353545545
CTAAACGC 0.381926257748
CTAAACTA 0.795884446697
CTAAACTC -0.288652525
CTAAAGAA 0.944418194584
CTAAAGAC 0.00580691689567
CTAAAGAG -0.466055147685
CTAAAGCA -0.210595146187
CTAAAGCC -0.321256321144
CTAAAGGA 0.269466841301
CTAAAGGC 0.553947062995
CTAAAGTA 2.63641313293
CTAAAGTC -0.195530762186
CTAAATAA 1.08399029629
CTAAATAC 2.00756113045
CTAAATAG -0.587666484995
CTAAATCA 1.2810116129
CTAAATCC 3.24313914576
CTAAATGA -0.282520225904
CTAAATGC 0.486599400956
CTAAATTA 2.25034538222
CTAAATTC 1.62489542447
CTAACAAA 1.38435366603
CTAACAAC 0.201163642281
CTAACAAG -0.137853592388
CTAACACA -0.383799859672
CTAACACC 0.335979709594
CTAACAGA 0.180696414866
CTAACAGC -0.962448074074
CTAACATA 1.90568756487
CTAACATC 1.87104549801
CTAACCAA 0.182570433146
CTAACCAC -0.276480357836
CTAACCAG -0.313329111404
CTAACCCA -1.32710346929
CTAACCCC -0.786473344234
CTAACCGA 0.842327707539
CTAACCGC -0.356922415542
CTAACCTA 0.0327688812904
CTAACCTC -0.979973746469
CTAACGAA 2.60884578694
CTAACGAC 0.176755605486
CTAACGAG -0.24562442411
CTAACGCA 0.050690300047
CTAACGCC -0.972956899087
CTAACGGA 0.34807401819
CTAACGGC -0.0579565446633

CTAACGTA 2.03704704481
CTAACGTC -0.644291315047
CTAACTAA 0.46296578629
CTAACTAC 0.732628106726
CTAACTAG 1.15559894546
CTAACTCA 0.205329700247
CTAACTCC 1.32557835732
CTAACTGA 0.167843922025
CTAACTGC -0.109894247236
CTAACTTA 1.3723930077
CTAACTTC 0.202702285816
CTAAGAAA 0.215305589602
CTAAGAAC 0.678978972885
CTAAGAAG -0.689993254061
CTAAGACA -0.569735281873
CTAAGACC -0.038528125426
CTAAGAGA 0.462393504992
CTAAGAGC -0.169122551249
CTAAGATA 3.09346541519
CTAAGATC 1.51506696148
CTAAGCAA 0.255822231203
CTAAGCAC -0.697941697955
CTAAGCAG -0.891571795079
CTAAGCCA -0.516177121815
CTAAGCCC -0.588176104718
CTAAGCGA -0.495670340581
CTAAGCGC -0.310733964562
CTAAGCTA 1.85936608815
CTAAGCTC -0.535606998298
CTAAGGAA 0.796428623967
CTAAGGAC -0.869727678428
CTAAGGAG -0.651614808611
CTAAGGCA -0.369314210488
CTAAGGCC 0.419665388586
CTAAGGGA -0.266822564453
CTAAGGGC -0.660075370364
CTAAGGTA 1.86108917739
CTAAGGTC -0.8241308693
CTAAGTAA 1.1966978609
CTAAGTAC -0.288339425301
CTAAGTAG 0.361630152642
CTAAGTCA -0.923472990501
CTAAGTCC 0.72213822591
CTAAGTGA -0.148808751014
CTAAGTGC -0.557467769188
CTAAGTTA 1.8004048765
CTAAGTTC 0.931579441491
CTAATAAA -0.202939192371
CTAATAAC 1.39733856007
CTAATAAG 0.329943172374
CTAATACA 1.15394455496
CTAATACC -0.0418752111738
CTAATAGA 1.8146690243
CTAATAGC -0.351373014822

CTAATATA 2.8228369407
CTAATATC 1.92390792722
CTAATCAA 1.16413507583
CTAATCAC 0.0216700798501
CTAATCAG -0.360545961663
CTAATCCA 0.0980106147672
CTAATCCC 0.45270968936
CTAATCGA 1.19771418586
CTAATCGC 0.890373106204
CTAATCTA 2.2034791037
CTAATCTC 1.43878659001
CTAATGAA 0.74779470612
CTAATGAC -0.396320100439
CTAATGAG 0.457360802046
CTAATGCA -0.100114045194
CTAATGCC 0.0431615429834
CTAATGGA -0.279702120433
CTAATGGC -0.66598491901
CTAATGTA 2.17848150683
CTAATGTC -0.389134628883
CTAATTAA 0.703422608116
CTAATTAC 1.16698961245
CTAATTAG 0.303004523914
CTAATTCA 0.0629230470072
CTAATTCC 1.05567309317
CTAATTGA 0.0166579865261
CTAATTGC 1.87868729573
CTAATTTA 2.25667711583
CTAATTTC 1.25719688338
CTACAAAA 0.157742501342
CTACAAAC -0.55664671519
CTACAAAG -0.930842075045
CTACAACA 1.45953444135
CTACAACC -0.298206645744
CTACAAGA 0.756107669678
CTACAAGC -0.505215093315
CTACAATA 1.88828138664
CTACAATC 1.24397383371
CTACACAA 0.680638359655
CTACACAC 0.663602738253
CTACACAG -0.383633733635
CTACACCA 0.512886035947
CTACACCC -0.264457662469
CTACACGA 0.716400424108
CTACACGC -0.949807090073
CTACACTA 1.02480987322
CTACACTC -0.435973427909
CTACAGAA 0.0274099634325
CTACAGAC 1.00873395235
CTACAGAG -0.925069715696
CTACAGCA -0.111845707728
CTACAGCC -0.431306077339
CTACAGGA 0.407002337718
CTACAGGC -0.128801805138

CTACAGTA 1.63822464492
CTACAGTC -0.467935203126
CTACATAA 0.0204303799105
CTACATAC 0.684279184549
CTACATAG 0.00999171088136
CTACATCA 0.494307191093
CTACATCC 1.09292446298
CTACATGA 0.275312479303
CTACATGC -0.375984441514
CTACATTA 1.92651285843
CTACATTC 0.73398334545
CTACCAAA 0.0584289005261
CTACCAAC 1.00616690954
CTACCAAG -0.722232530541
CTACCACA 0.215968428327
CTACCACC -0.192986202596
CTACCAGA -0.443200951419
CTACCAGC -1.18431189494
CTACCATA 1.92354424027
CTACCATC 0.827852675432
CTACCCAA -0.808955526429
CTACCCAC 0.137362708684
CTACCCAG -0.569948456136
CTACCCCA -0.794042071472
CTACCCCC -0.705713815091
CTACCCGA 1.70202911754
CTACCCGC -0.641652450826
CTACCCTA -0.107994414885
CTACCCTC -0.979210982308
CTACCGAA 0.201416994897
CTACCGAC -0.474672467466
CTACCGAG -0.838396474347
CTACCGCA -0.505966824042
CTACCGCC -0.334122137376
CTACCGGA 0.146191537304
CTACCGGC 0.0660382224724
CTACCGTA 1.84751347421
CTACCGTC -1.29529886846
CTACCTAA -0.431751161885
CTACCTAC 0.513861974371
CTACCTAG 0.196702179745
CTACCTCA 0.174641349804
CTACCTCC -0.129178399125
CTACCTGA 0.640199160267
CTACCTGC -1.32224563586
CTACCTTA 0.67748279766
CTACCTTC -0.610913721309
CTACGAAA 1.48039096161
CTACGAAC 0.110064953189
CTACGAAG -0.486717646056
CTACGACA -0.192105609692
CTACGACC -1.43285747263
CTACGAGA 0.375937809644
CTACGAGC -0.938000067105

CTACGATA 1.1616977279
CTACGATC 1.55322515462
CTACGCAA 0.395570243313
CTACGCAC −0.485884934089
CTACGCAG −0.684314782986
CTACGCCA −0.767227080739
CTACGCCC −0.253826012442
CTACGCGA 1.05140148896
CTACGCGC −0.100651560768
CTACGCTA −0.187433887384
CTACGCTC 0.0998617334678
CTACGGAA 0.567676401537
CTACGGAC −0.256538779849
CTACGGAG −0.67930393873
CTACGGCA −0.517353119289
CTACGGCC −0.506719803838
CTACGGGA −1.22900167256
CTACGGGC −0.986908571723
CTACGGTA 1.7995261572
CTACGGTC −0.445672024178
CTACGTAA 0.236991907335
CTACGTAC −0.0582444548256
CTACGTAG −0.691556462599
CTACGTCA −0.432285971145
CTACGTCC 0.0241024315031
CTACGTGA 0.129052867796
CTACGTGC −0.507105765834
CTACGTTA 0.47044728695
CTACGTTC −0.564287680191
CTACTAAA 0.136509387096
CTACTAAC 0.782476951331
CTACTAAG 0.0206866470181
CTACTACA 1.30567425138
CTACTACC −0.100026402259
CTACTAGA 0.625420812827
CTACTAGC 0.334963800996
CTACTATA 2.03997902364
CTACTATC 1.2177375778
CTACTCAA −0.762946524878
CTACTCAC 0.426165746372
CTACTCAG −0.706772608356
CTACTCCA −0.221289041434
CTACTCCC −0.379356300443
CTACTCGA 0.285078108886
CTACTCGC −0.484948133128
CTACTCTA 0.246171724049
CTACTCTC −1.29630436816
CTACTGAA 0.0341070494199
CTACTGAC −0.582655224383
CTACTGAG −1.25397699438
CTACTGCA 0.0964967444128
CTACTGCC −0.0002874938063
CTACTGGA −0.266118506486
CTACTGGC −0.510836731798

CTACTGTA 0.945415575342
CTACTGTC 0.860582002904
CTACTTAA 0.271821750741
CTACTTAC 0.960195588206
CTACTTCA −0.555395565461
CTACTTCC 0.113232173152
CTACTTGA 0.323258785244
CTACTTGC 1.85816510932
CTACTTTA 2.46518631854
CTACTTTC −0.978708024281
CTAGAAAA 0.169689628098
CTAGAAAC 0.189228798027
CTAGAAAG −0.373724877594
CTAGAACA 0.227386574806
CTAGAACC −0.225611857428
CTAGAAGA 0.694282761576
CTAGAAGC −0.185362516368
CTAGAATA 1.79620592641
CTAGAATC 4.47813093185
CTAGACAA 0.258759414485
CTAGACAC 0.318367851511
CTAGACAG −1.054812069
CTAGACCA −0.573952551625
CTAGACCC −0.267445016648
CTAGACGA 0.093174223668
CTAGACGC −0.565243217172
CTAGACTA −0.0902867949255
CTAGACTC −0.934960668429
CTAGAGAA 1.44216427792
CTAGAGAC −0.983726987478
CTAGAGAG −0.467037123271
CTAGAGCA −0.799605003762
CTAGAGCC −1.1068544851
CTAGAGGA 0.344517921739
CTAGAGGC −0.819678774773
CTAGAGTA 0.246171724049
CTAGAGTC −1.25143534929
CTAGATAA 2.0064028281
CTAGATAC 3.61667999401
CTAGATAG −0.393212003025
CTAGATCA −0.175418894603
CTAGATCC 2.15774448074
CTAGATGA −0.561644860588
CTAGATGC 0.381565693467
CTAGATTA 5.02645219281
CTAGATTC 9.47506265275
CTAGCAAA 1.12120086321
CTAGCAAC −0.101990145253
CTAGCAAG −0.838658778616
CTAGCACA 0.195798687261
CTAGCACC −0.560678290174
CTAGCAGA 0.12886071951
CTAGCAGC −0.862633597011
CTAGCATA −0.0451275759354

CTAGCATC 0.421392016848
CTAGCCAA 0.070003180499
CTAGCCAC -0.128245761723
CTAGCCAG -0.764604246224
CTAGCCCA -1.46981947503
CTAGCCCC 0.228239688215
CTAGCCGA -0.0451275759354
CTAGCCGC -1.16006019982
CTAGCCTA -0.533623894751
CTAGCCTC -1.31330064392
CTAGCGAA -0.0974674783873
CTAGCGAC -0.303933830469
CTAGCGAG 0.0132950792509
CTAGCGCA -1.06118814452
CTAGCGCC -1.3294284011
CTAGCGGA 1.81832046628
CTAGCGGC -0.084673275384
CTAGCGTA -0.207574691708
CTAGCGTC -0.787022309598
CTAGCTAA -0.376532365988
CTAGCTAC 0.473837049186
CTAGCTAG -0.842950159734
CTAGCTCA -0.356144038032
CTAGCTCC 0.00777836247544
CTAGCTGA -0.0933286917377
CTAGCTGC -1.13453632899
CTAGCTTA 1.22789770467
CTAGCTTC -1.22589648964
CTAGGAAA 0.381370422511
CTAGGAAC -0.407717637297
CTAGGAAG -0.0646236530199
CTAGGACA 0.166814273679
CTAGGACC -1.59770612971
CTAGGAGA 0.600732360277
CTAGGAGC 0.0150718784086
CTAGGATA 1.75061723623
CTAGGATC 2.56588513572
CTAGGCAA -0.169122551249
CTAGGCAC -0.631738598514
CTAGGCAG -1.12763460404
CTAGGCCA -1.09487717254
CTAGGCCC -0.615733041813
CTAGGCGA 1.04510722739
CTAGGCGC -0.808313921859
CTAGGCTA -0.545692181097
CTAGGCTC -0.76961404959
CTAGGGAA 0.433263366815
CTAGGGAC -1.25965234279
CTAGGGAG -1.21449957732
CTAGGGCA -1.15804212237
CTAGGGCC -1.05827177904
CTAGGGGA -0.25843028508
CTAGGGGC -0.56096474309
CTAGGGTA 0.856000005311

CTAGGGTC −1.54619831469
CTAGGTAA 0.607790010546
CTAGGTAC 0.270368668361
CTAGGTCA −0.812883428773
CTAGGTCC −0.28521966992
CTAGGTGA −0.124962586618
CTAGGTGC −0.590496456612
CTAGGTTA −0.72214447125
CTAGGTTC −0.055998422475
CTAGTAAA 0.487062805166
CTAGTAAC 0.4951688398
CTAGTAAG −0.720076847438
CTAGTACA −0.285162212794
CTAGTACC −0.919625653039
CTAGTAGA 1.46121277232
CTAGTAGC 0.332765857761
CTAGTATA 1.31434673832
CTAGTATC 0.513935461202
CTAGTCAA −0.0318070989695
CTAGTCAC −0.376023787155
CTAGTCAG −0.513701260961
CTAGTCCA −0.539653978453
CTAGTCCC −1.25245625416
CTAGTCGA 0.760203571661
CTAGTCGC −0.806540869905
CTAGTCTA −0.0984013648573
CTAGTCTC −0.162073019922
CTAGTGAA −0.0649315482694
CTAGTGAC −1.10239572888
CTAGTGAG −0.861382655459
CTAGTGCA 0.73728983649
CTAGTGCC −1.19840741857
CTAGTGGA 1.49374870244
CTAGTGGC 0.070003180499
CTAGTGTA 1.20211298682
CTAGTGTC −1.10299382425
CTAGTTAA 0.330104926673
CTAGTTAC 0.97454862801
CTAGTTCA 0.0222304950033
CTAGTTCC 0.221550096635
CTAGTTGA −0.13203647477
CTAGTTGC 1.01997140034
CTAGTTTA −0.360636727267
CTAGTTTC −0.545560820785
CTATAAAA 0.626865776266
CTATAAAC 0.731213745451
CTATAAAG −0.54842930533
CTATAACA 0.0158052894728
CTATAACC 0.381751388235
CTATAAGA 1.31774690946
CTATAAGC 1.06308506238
CTATAATA 2.34690624545
CTATAATC 2.14088289614
CTATACAA 0.898076524602

CTATACAC 0.948966883699
CTATACAG -0.996144596495
CTATACCA -1.22570746402
CTATACCC -0.220598723214
CTATACGA 2.12153941352
CTATACGC 0.995947243759
CTATACTA 0.1810867486
CTATACTC 0.270694675095
CTATAGAA 0.360935462685
CTATAGAC -0.459127816838
CTATAGAG -0.0150443989137
CTATAGCA -0.316659959269
CTATAGCC -1.192894449
CTATAGGA 1.67047849385
CTATAGGC -0.248727733429
CTATAGTA 1.42885004629
CTATAGTC -0.879355910536
CTATATAA 1.60539351841
CTATATAC 1.60788686621
CTATATAG 1.39230627348
CTATATCA 1.99740537531
CTATATCC 2.6678305227
CTATATGA 0.24748491082
CTATATGC 1.27010225343
CTATATTA 2.35372657281
CTATATTC 0.177131574939
CTATCAAA 1.35861120831
CTATCAAC -0.271065856454
CTATCAAG -0.79100642
CTATCACA 0.200020953286
CTATCACC 0.0361609334843
CTATCAGA 0.0343189746153
CTATCAGC -0.110038722762
CTATCATA 1.08143969954
CTATCATC 0.628657147883
CTATCCAA 1.75553793941
CTATCCAC 0.882426952267
CTATCCAG 0.351860775856
CTATCCCA -0.389437319683
CTATCCCC 1.06552178577
CTATCCGA 0.68323850277
CTATCCGC 0.440008750097
CTATCCTA 0.90933208407
CTATCCTC 0.300588201967
CTATCGAA 1.99441635571
CTATCGAC -0.00558562369068
CTATCGAG 0.399184629602
CTATCGCA 0.00208261262724
CTATCGCC 0.13633285216
CTATCGGA 1.50501696076
CTATCGGC 1.73548894158
CTATCGTA 1.55359571144
CTATCGTC -0.423752130738
CTATCTAA 0.5462142915

CTATCTAC 0.821232198946
CTATCTCA 0.439574282628
CTATCTCC 0.726704401977
CTATCTGA 0.642779942828
CTATCTGC -0.324877993662
CTATCTTA 1.109046183
CTATCTTC -0.000663046903015
CTATGAAA 0.485722555256
CTATGAAC -0.211907916602
CTATGAAG -0.833187860999
CTATGACA -0.0774332611946
CTATGACC -0.673719564107
CTATGAGA 0.398511590155
CTATGAGC -0.423116771508
CTATGATA 2.29903904715
CTATGATC 0.668987886538
CTATGCAA 1.47766736895
CTATGCAC 0.0328798401599
CTATGCAG -0.52847565302
CTATGCCA -0.270455478583
CTATGCCC 1.5749166768
CTATGCGA 1.05659677892
CTATGCGC -0.399441104888
CTATGCTA -0.310108597875
CTATGCTC -0.601666454925
CTATGGAA 1.29399484152
CTATGGAC -0.703281463438
CTATGGAG -0.715286880031
CTATGGCA -0.85690287326
CTATGGCC -0.644096044091
CTATGGGA -0.0730937909612
CTATGGGC -0.806730728234
CTATGGTA 3.08808026691
CTATGGTC -1.07945243243
CTATGTAA 1.50263040827
CTATGTAC 0.886903611797
CTATGTCA -0.671647360379
CTATGTCC 0.642308627855
CTATGTGA 1.08505949846
CTATGTGC 0.729267489408
CTATGTTA 1.23990395398
CTATGTTC -0.595623047831
CTATTAAA 1.67454421003
CTATTAAC 0.676746680282
CTATTAAG 0.488410133127
CTATTACA 1.04013427153
CTATTACC 0.196176738494
CTATTAGA 0.658908116366
CTATTAGC 0.458324249791
CTATTATA 1.05937075066
CTATTATC 1.14522939153
CTATTCAA 0.233163097715
CTATTCAC 0.0906342439934
CTATTCAG -0.944988602281

CTATTCCA 0.0542418165825
CTATTCCC 0.918757342636
CTATTCGA -0.144343749452
CTATTCGC 0.791307029019
CTATTCTA 1.31863562131
CTATTCTC -0.672835015821
CTATTGAA 0.960062978825
CTATTGAC -0.785425584403
CTATTGAG 0.445383489482
CTATTGCA -0.218713463323
CTATTGCC -0.49987241334
CTATTGGA 0.716906712983
CTATTGGC 0.053713044484
CTATTGTA 2.48984458528
CTATTGTC -0.685821158932
CTATTTAA 0.292038748211
CTATTTAC 1.38092809718
CTATTTCA 0.509014758016
CTATTTCC 0.704051722007
CTATTTGA -0.32478701988
CTATTTGC 1.44555966096
CTATTTTA 0.468729402164
CTATTTTC 0.844883300563
CTCAAAAA 0.608341890402
CTCAAAAC -0.157955051072
CTCAAAAG -0.656571318411
CTCAAACA -0.261215082073
CTCAAACC -0.609959225218
CTCAAAGA 0.371766963584
CTCAAAGC 1.41805934828
CTCAAATA 0.929213706795
CTCAAATC 1.64606212994
CTCAACAA -0.24444384672
CTCAACAC -0.468640926517
CTCAACAG -0.0428871643906
CTCAACCA -0.821563201952
CTCAACCC 0.330585817834
CTCAACGA -0.88191358534
CTCAACGC 0.348639013259
CTCAACTA 1.42544175622
CTCAACTC -1.0595822595
CTCAAGAA -0.178164137777
CTCAAGAC -0.774058233353
CTCAAGAG -1.3207856836
CTCAAGCA -0.523661536966
CTCAAGCC -1.20368514701
CTCAAGGA 0.398762444635
CTCAAGGC 0.0259439740161
CTCAAGTA 0.576469839899
CTCAAGTC -0.292560858613
CTCAATAA 1.92796219361
CTCAATAC 0.964652054471
CTCAATCA 0.639991190454
CTCAATCC 0.596818822215

CTCAATGA 0.117864549819
CTCAATGC -0.444268904515
CTCAATTA 0.384742905974
CTCAATTC 0.329311560348
CTCACAAA 0.0951348439922
CTCACAAC -0.116746634005
CTCACAAG -0.637166839643
CTCACACA -0.918595796515
CTCACACC 0.324903183199
CTCACAGA -0.222488146665
CTCACAGC -0.398869448123
CTCACATA 1.26709054243
CTCACATC -0.219941921651
CTCACCAA 0.43771254685
CTCACCAC -0.629728848183
CTCACCAG -0.055997381585
CTCACCCA -1.41649135165
CTCACCCC -0.394512074582
CTCACCGA 0.273399323561
CTCACCGC -0.693375105533
CTCACCTA 0.692789292665
CTCACCTC -0.89086274084
CTCACGAA 0.144815272602
CTCACGAC -0.0620649373261
CTCACGAG -0.974156004318
CTCACGCA -0.490735064936
CTCACGCC -1.21792077443
CTCACGGA 2.07481053248
CTCACGGC -0.171528880653
CTCACGTA -0.18339502617
CTCACGTC -0.434203914981
CTCACTAA 0.266083532583
CTCACTAC -0.481155754656
CTCACTCA -1.00018075139
CTCACTCC -0.207328417144
CTCACTGA 0.713773217855
CTCACTGC -0.868972200497
CTCACTTA 0.384885716076
CTCACTTC 0.364684123778
CTCAGAAA 0.569114078746
CTCAGAAC -0.129777743562
CTCAGAAG -1.03490442403
CTCAGACA 0.0724521863913
CTCAGACC -0.78957082457
CTCAGAGA 1.03593719504
CTCAGAGC -0.495410742626
CTCAGATA 2.40733698555
CTCAGATC 2.24794071824
CTCAGCAA -0.675388943421
CTCAGCAC -1.46678798711
CTCAGCAG -1.34696115155
CTCAGCCA -1.04714216726
CTCAGCCC -0.869358787027
CTCAGCGA 0.193457725747

CTCAGCGC -0.494146269505
CTCAGCTA -0.0143828092566
CTCAGCTC -1.10815393212
CTCAGGAA -0.384756645721
CTCAGGAC -1.31037532678
CTCAGGAG -0.374388965387
CTCAGGCA -1.03695185457
CTCAGGCC -1.02246745446
CTCAGGGA -0.355712693233
CTCAGGGC -0.374200980661
CTCAGGTA 0.440294370301
CTCAGGTC -0.595894095576
CTCAGTAA -0.747973947371
CTCAGTAC 0.133496218847
CTCAGTCA -0.548743445919
CTCAGTCC -1.08925261956
CTCAGTGA 0.589634599727
CTCAGTGC 0.0531928076832
CTCAGTTA 0.192629801874
CTCAGTTC -1.19940209301
CTCATAAA 1.24668431116
CTCATAAC 0.469735734575
CTCATAAG -0.349453405562
CTCATACA -1.0905651818
CTCATACC -0.88191358534
CTCATAGA -0.574701159683
CTCATAGC 0.386821771397
CTCATATA 1.08671201535
CTCATATC 2.41872931795
CTCATCAA 1.34174754193
CTCATCAC -0.975872640036
CTCATCAG 0.135558846387
CTCATCCA -0.902625630073
CTCATCCC -0.852202838745
CTCATCGA 0.932160258087
CTCATCGC -0.575681678023
CTCATCTA 0.355610477839
CTCATCTC -1.05551196341
CTCATGAA 1.01336237364
CTCATGAC 0.0619117183243
CTCATGAG -0.675229062724
CTCATGCA -0.198732956052
CTCATGCC -1.33509084247
CTCATGGA 0.149006728284
CTCATGGC 0.0385083485168
CTCATGTA 1.09959448582
CTCATGTC -0.516375931797
CTCATTAA 0.299879564084
CTCATTAC 0.683156480641
CTCATTCA -0.997850406958
CTCATTCC -0.401874289253
CTCATTGA 1.22325804178
CTCATTGC -0.548602093063
CTCATTTA 0.415682527253

CTCATTTC -0.889428810834
CTCCAAAA 0.509233344907
CTCCAAAC -0.383800692384
CTCCAAAG -0.128701879702
CTCCAACA -0.181330941384
CTCCAACC -0.791329512242
CTCCAAGA 0.413507275419
CTCCAAGC -0.108297522041
CTCCAATA 0.971439906062
CTCCAATC 0.00638814984802
CTCCACAA -0.196306433383
CTCCACAC -0.5625329479
CTCCACAG -0.623769753176
CTCCACCA -1.26942671585
CTCCACCC -1.00531983329
CTCCACGA -0.0472101885626
CTCCACGC 0.0351675081088
CTCCACTA 0.234320983704
CTCCACTC -1.23995849661
CTCCAGAA -0.421268567299
CTCCAGAC -0.618518046984
CTCCAGAG -0.975841621515
CTCCAGCA -0.554681098594
CTCCAGCC -1.4425315039
CTCCAGGA 0.477178930484
CTCCAGGC -0.906686349976
CTCCAGTA 0.256899968665
CTCCAGTC -0.825559803033
CTCCATAA 0.32562243816
CTCCATAC -0.639969123587
CTCCATCA -0.376691830329
CTCCATCC -0.871325652691
CTCCATGA 0.0769151061737
CTCCATGC -0.195082763149
CTCCATTA 0.491738899211
CTCCATTC -0.298410035641
CTCCCAAA -0.426103501152
CTCCCAAC -0.588304342361
CTCCCAAG -0.171683140545
CTCCCACA -1.50310005782
CTCCCACC -0.963651967399
CTCCCAGA -0.226519513471
CTCCCAGC -0.971546701372
CTCCCATA -0.102511214766
CTCCCATC -1.06535794969
CTCCCCAA 0.124520000208
CTCCCCAC -0.613934800322
CTCCCCAG -0.356990281567
CTCCCCCA -1.07518062005
CTCCCCCC -1.13722307415
CTCCCCGA -1.12453316832
CTCCCCGC -0.708710537279
CTCCCCTA -0.713069159888
CTCCCCTC -1.29886266749

CTCCCGAA -0.188463119374
CTCCCGAC -1.15455763915
CTCCCGAG -0.93026417294
CTCCCGCA -0.405800734351
CTCCCGCC -0.563475786024
CTCCCGGA 0.809367094318
CTCCCGGC -1.13073541522
CTCCCGTA -0.358123394375
CTCCCGTC -1.30338741614
CTCCCTAA -1.34017350813
CTCCCTAC -1.20313368351
CTCCCTCA -1.20410358478
CTCCCTCC -1.16641837204
CTCCCTGA -0.367060683729
CTCCCTGC -1.12063420272
CTCCCTTA -0.751037078338
CTCCCTTC -1.53740237819
CTCCGAAA -0.181621974216
CTCCGAAC -0.787648508996
CTCCGAAG -0.69845215039
CTCCGACA -0.40675856129
CTCCGACC -0.970402555131
CTCCGAGA 0.451240369095
CTCCGAGC -0.00474062922307
CTCCGATA 0.581952415484
CTCCGATC 0.13141881067
CTCCGCAA 0.356276647412
CTCCGCAC -1.02563758891
CTCCGCAG -0.904286057733
CTCCGCCA -1.12405581619
CTCCGCCC -0.71192147462
CTCCGCGA 0.160006437
CTCCGCGC -0.549590313989
CTCCGCTA -0.0649315482694
CTCCGCTC -1.03834477351
CTCCGGAA -0.265912410274
CTCCGGAC -1.29041126557
CTCCGGAG -1.13922845274
CTCCGGCA -1.3250687376
CTCCGGCC -1.48571990184
CTCCGGGA -0.777557080857
CTCCGGGC -0.697802218701
CTCCGGTA -0.50031916331
CTCCGGTC -0.736060753628
CTCCGTAA 0.604227668755
CTCCGTAC 0.315752927759
CTCCGTCA -0.687687266449
CTCCGTCC -1.41151423223
CTCCGTGA 0.712918439022
CTCCGTGC 0.470141265302
CTCCGTTA -0.467210535537
CTCCGTTC -0.609882823895
CTCCTAAA -0.456991910645
CTCCTAAC -0.506505796862

CTCCTAAG -0.699052952074
CTCCTACA -0.499254749239
CTCCTACC -1.23964435602
CTCCTAGA -0.505774675756
CTCCTAGC 0.454422161518
CTCCTATA 0.184635975178
CTCCTATC -0.76130962133
CTCCTCAA -0.58219119564
CTCCTCAC -0.566752091254
CTCCTCAG -0.911051217924
CTCCTCCA -0.892964089486
CTCCTCCC -0.376134537846
CTCCTCGA -0.635631110599
CTCCTCGC -0.746294991869
CTCCTCTA -0.00514657630655
CTCCTCTC -1.1050431284
CTCCTGAA -0.209111253464
CTCCTGAC -0.850440403869
CTCCTGCA -0.490597667462
CTCCTGCC -1.22618210984
CTCCTGGA -0.151832119985
CTCCTGGC -0.778328796672
CTCCTGTA 0.0310855540509
CTCCTGTC -1.12535505503
CTCCTTAA -0.49325443499
CTCCTTAC 0.124459212235
CTCCTTCA -1.27439696539
CTCCTTCC -1.30438771139
CTCCTTGA -0.103469458061
CTCCTTGC -1.01623606264
CTCCTTTA -0.737677047554
CTCCTTTC -0.741086170343
CTCGAAAA 0.663579005962
CTCGAAAC -0.199940596581
CTCGAAAG -0.994219158251
CTCGAACA -0.0824351537971
CTCGAACC -0.737008796201
CTCGAAGA -0.847100604351
CTCGAAGC 0.0762345723194
CTCGAATA 1.31254641505
CTCGAATC 2.05373584168
CTCGACAA -1.02737379336
CTCGACAC -0.788064032267
CTCGACAG -0.45008414853
CTCGACCA -0.984159164989
CTCGACCC -1.18370255796
CTCGACGA -0.419005672531
CTCGACGC -0.329997923186
CTCGACTA 0.285219253564
CTCGACTC -0.577603785419
CTCGAGAA 0.268336851163
CTCGAGAC -0.820808348555
CTCGAGAG -0.926586292364
CTCGAGCA -0.430645112216

CTCGAGCC -1.16765036939
CTCGAGGA -0.310778098296
CTCGAGGC 0.323006473518
CTCGAGTA 0.54146096342
CTCGAGTC 0.402463849325
CTCGATAA 0.237129929343
CTCGATAC 0.845274675187
CTCGATCA -0.524785489942
CTCGATCC 0.297564624818
CTCGATGA -1.09630090182
CTCGATGC -0.399724643312
CTCGATTA 2.10218385658
CTCGATTC 3.18516532233
CTCGCAAA 0.0467655203727
CTCGCAAC -0.969668103176
CTCGCAAG -1.16762309807
CTCGCACA 0.484566126513
CTCGCACC -0.596671640375
CTCGCAGA -0.883215946855
CTCGCAGC -0.432102149978
CTCGCATA 1.18289316193
CTCGCATC -0.477014261692
CTCGCCAA -0.325194007853
CTCGCCAC -0.736220217969
CTCGCCAG 0.585560140077
CTCGCCCA -0.484648773176
CTCGCCCC -1.10231495582
CTCGCCGA -0.209324427727
CTCGCCGC -1.02063298999
CTCGCCTA -0.550848125414
CTCGCCTC -0.846879311146
CTCGCGAA 0.570168083917
CTCGCGAC -0.571225628114
CTCGCGAG 0.440138861341
CTCGCGCA -0.85975949166
CTCGCGCC -0.946812449665
CTCGCGGA 0.340249648379
CTCGCGGC -0.807587380669
CTCGCGTA 0.231906535358
CTCGCGTC 0.224335934518
CTCGCTAA 0.723354818093
CTCGCTAC -0.903753538431
CTCGCTCA -0.490597667462
CTCGCTCC -0.612591011387
CTCGCTGA -0.625318805611
CTCGCTGC -0.456584298138
CTCGCTTA 0.342341212659
CTCGCTTC -0.017371204325
CTCGGAAA 0.614858694248
CTCGGAAC -0.958774981592
CTCGGAAG 0.399417788953
CTCGGACA -1.18685853631
CTCGGACC -1.09947748979
CTCGGAGA 0.176116915408

CTCGGAGC -0.798492500575
CTCGGATA 2.1230010312
CTCGGATC 3.34974792794
CTCGGCAA -0.116404389387
CTCGGCAC -1.49859383701
CTCGGCAG -0.646495503621
CTCGGCCA -0.689628318042
CTCGGCCC -0.974845281648
CTCGGCGA 0.0634447410539
CTCGGCGC -0.323878947481
CTCGGCTA -0.549752484644
CTCGGCTC -0.713711597169
CTCGGGAA 0.00311933902505
CTCGGGAC -1.60211492322
CTCGGGCA -1.74307765391
CTCGGGCC -1.57647988534
CTCGGGGA -0.725277966379
CTCGGGGC -1.43798114936
CTCGGGTA 0.356878281808
CTCGGGTC 0.687541333676
CTCGGTAA 0.285230911532
CTCGGTAC -1.21269550684
CTCGGTCA -1.1637484893
CTCGGTCC -0.461168377511
CTCGGTGA -0.484674170891
CTCGGTGC -0.645195223886
CTCGGTTA 0.158174054319
CTCGGTTC -0.904636629471
CTCGTAAA -0.289058055728
CTCGTAAC -0.11951456858
CTCGTAAG -0.0598353510368
CTCGTACA -1.13147965154
CTCGTACC -0.978932440155
CTCGTAGA 0.207874884372
CTCGTAGC -0.184513150163
CTCGTATA 1.53268298313
CTCGTATC 0.936891727478
CTCGTCAA 0.279981078941
CTCGTCAC -1.30815719028
CTCGTCAG -0.09936502078
CTCGTCCA -0.622492581198
CTCGTCCC -1.17345229001
CTCGTCGA 0.04796400107
CTCGTCGC 0.386402500922
CTCGTCTA 0.88142644884
CTCGTCTC -0.779166088554
CTCGTGAA -0.505037101132
CTCGTGAC -0.971819206363
CTCGTGCA -0.276508253687
CTCGTGCC -1.27125680857
CTCGTGGA -0.373355569837
CTCGTGGC -0.304471970577
CTCGTGTA 1.23722158056
CTCGTGTC 1.18750388809

CTCGTTAA 0.200304699888
CTCGTTAC 0.939472301861
CTCGTTCA 0.823457621675
CTCGTTCC 0.339622824446
CTCGTTGA -0.511852432219
CTCGTTGC -0.0855422103206
CTCGTTTA 0.27712570961
CTCGTTTC -0.438742403374
CTCTAAAA -0.847002760695
CTCTAAAC -0.211137866211
CTCTAAAG -0.483949295124
CTCTAACA -0.80927778596
CTCTAACC -0.965715219473
CTCTAAGA -0.660798997063
CTCTAAGC -0.592447708927
CTCTAATA 1.43067951448
CTCTAATC 0.632020471514
CTCTACAA -0.804221558902
CTCTACAC -0.283191599927
CTCTACAG -0.223437854663
CTCTACCA -0.443684757071
CTCTACCC -0.881443727613
CTCTACGA -0.261071855615
CTCTACGC -0.374132073746
CTCTACTA 2.14792285128
CTCTACTC -0.461998591342
CTCTAGAA -0.0545451319161
CTCTAGAC -0.659728129474
CTCTAGAG -0.644154750284
CTCTAGCA -1.06733355883
CTCTAGCC -0.970715238474
CTCTAGGA 0.586558769902
CTCTAGGC -0.399011841869
CTCTAGTA 1.4893846672
CTCTAGTC -0.661898801392
CTCTATAA 0.242284208236
CTCTATAC -0.380367004592
CTCTATCA -0.0944237079731
CTCTATCC 0.660172797664
CTCTATGA -0.654865507948
CTCTATGC -0.436694972827
CTCTATTA 0.966424065535
CTCTATTC -0.813533776819
CTCTCAAA -0.151880417279
CTCTCAAC -1.27982874555
CTCTCAAG -0.513051329272
CTCTCACA -1.03375944507
CTCTCACC -0.244090152312
CTCTCAGA -1.24225011994
CTCTCAGC -0.49217232579
CTCTCATA 0.581762973511
CTCTCATC -0.827826028649
CTCTCCAA -0.809662498888
CTCTCCAC -1.05369123869

CTCTCCAG 0.375585156126
CTCTCCCA -1.51718662979
CTCTCCCC -0.396216844155
CTCTCCGA -0.667713212695
CTCTCCGC -1.54704643183
CTCTCCTA -0.585808912777
CTCTCCTC -1.00226711123
CTCTCGAA -0.42970456405
CTCTCGAC -1.12178792515
CTCTCGCA -0.93792387396
CTCTCGCC -1.30805976298
CTCTCGGA 0.522401851939
CTCTCGGC 1.04905178397
CTCTCGTA 3.93289195424
CTCTCGTC -0.343657313922
CTCTCTAA -0.738647573351
CTCTCTAC -0.327964648743
CTCTCTCA -1.15127383951
CTCTCTCC -0.692308401504
CTCTCTGA -1.09093761223
CTCTCTGC -0.902060218648
CTCTCTTA -0.440786086717
CTCTCTTC -0.914102899102
CTCTGAAA 0.753959480983
CTCTGAAC -0.646279414866
CTCTGAAG -0.567841694862
CTCTGACA -1.1017428827
CTCTGACC -1.32605258679
CTCTGAGA -0.0339925515246
CTCTGAGC 0.461387797114
CTCTGATA 1.15539722099
CTCTGATC 0.249174483399
CTCTGCAA 0.530428154403
CTCTGCAC -0.755339701067
CTCTGCAG -1.1127067848
CTCTGCCA -0.982018887058
CTCTGCCC -0.392289982701
CTCTGCGA -0.539436016096
CTCTGCGC -0.399628256902
CTCTGCTA 0.818988664731
CTCTGCTC -1.05079173563
CTCTGGAA -0.243993765902
CTCTGGAC -1.82953126748
CTCTGGCA -1.24532719884
CTCTGGCC -0.989701904013
CTCTGGGA -0.305347567209
CTCTGGGC -1.14106062725
CTCTGGTA 0.152051955944
CTCTGGTC 0.106313585781
CTCTGTAA 0.366053934962
CTCTGTAC -0.734868726448
CTCTGTCA -1.0871533527
CTCTGTCC -0.61103696268
CTCTGTGA -0.399792301159

CTCTGTGC -1.72917969119
CTCTGTTA -0.159219524192
CTCTGTTC -0.764744350012
CTCTTAAA 0.481658504505
CTCTTAAC 0.0274828257295
CTCTTAAG -0.190720601514
CTCTTACA 0.399602651009
CTCTTACC -0.82899619714
CTCTTAGA -0.0703633284244
CTCTTAGC 0.295008199082
CTCTTATA 0.353158973811
CTCTTATC 0.0988387468175
CTCTTCAA -0.382297230929
CTCTTCAC -1.05636549317
CTCTTCAG -0.86963732918
CTCTTCCA -0.270090542564
CTCTTCCC -0.768083108641
CTCTTCGA 0.486761363434
CTCTTCGC 0.215151954244
CTCTTCTA 1.61929335472
CTCTTCTC -0.706941440707
CTCTTGAA -0.607318487395
CTCTTGAC -0.214848847088
CTCTTGCA -0.0756389750856
CTCTTGCC -1.17075263782
CTCTTGGA -0.511803302213
CTCTTGGC -0.366680134361
CTCTTGTA 2.59776259885
CTCTTGTC 0.243781007995
CTCTTTAA -0.329925060889
CTCTTTAC 0.726056760245
CTCTTTCA 0.0717558310097
CTCTTTCC -0.507309780266
CTCTTTGA 0.22314682183
CTCTTTGC -0.197896496882
CTCTTTTA 0.146082035681
CTCTTTTC 0.226799929226
CTGAAAAA 1.22531359126
CTGAAAAC 0.486273810578
CTGAAAAG 0.016217481896
CTGAAACA -0.057078449895
CTGAAACC -0.48470019314
CTGAAAGA -0.529543606117
CTGAAAGC -0.341273675919
CTGAAATA 1.17784318021
CTGAAATC 6.83198928133
CTGAACAA 0.308713805332
CTGAACAC 0.524048123496
CTGAACAG -0.0820729240918
CTGAACCA -0.780957251993
CTGAACCC -0.895547162005
CTGAACGA 1.56680169051
CTGAACGC -0.11012907201
CTGAACTA 1.30340989936

CTGAACTC -0.989407540333
CTGAAGAA 0.706928741849
CTGAAGAC 0.475179172697
CTGAAGCA -0.701278791159
CTGAAGCC -1.05388942414
CTGAAGGA -0.396021573199
CTGAAGGC -0.685443524056
CTGAAGTA -0.0551588406351
CTGAAGTC -1.05369123869
CTGAATAA 0.229701097716
CTGAATAC 0.738053433363
CTGAATCA 0.00963281202396
CTGAATCC 1.92726396462
CTGAATGA 0.348789734125
CTGAATGC -0.415007406026
CTGAATTA 2.21160553978
CTGAATTC 0.661803872228
CTGACAAA 1.11677562364
CTGACAAC 0.288275931014
CTGACAAG -0.475453759468
CTGACACA 0.0975034931798
CTGACACC 0.532919836783
CTGACAGA -0.0958438982313
CTGACAGC -1.11628848714
CTGACATA 0.375679044401
CTGACATC -0.304220699741
CTGACCAA -0.088628865401
CTGACCAC -0.339958407368
CTGACCAG -0.218598549071
CTGACCCA -1.31055373532
CTGACCCC -1.04815932492
CTGACCGA -0.675287977095
CTGACCGC -1.18628375688
CTGACCTA -0.662969877158
CTGACCTC -1.09093761223
CTGACGAA 0.717128422544
CTGACGAC -0.367681054144
CTGACGCA -1.05715490412
CTGACGCC -0.747746825182
CTGACGGA 0.256287092658
CTGACGGC -0.814171634185
CTGACGTA 0.404652216371
CTGACGTC -0.310800581519
CTGACTAA -0.213579377696
CTGACTAC 0.070003180499
CTGACTCA -0.563790967503
CTGACTCC -0.676935289542
CTGACTGA 0.655520019554
CTGACTGC 0.222990271981
CTGACTTA 1.67264312861
CTGACTTC -0.99321282584
CTGAGAAA 1.24343839992
CTGAGAAC 0.105940114464
CTGAGAAG -0.145448966409

CTGAGACA -0.980491276956
CTGAGACC -0.479617943833
CTGAGAGA 0.147863206576
CTGAGAGC -1.03356209234
CTGAGATA 1.02750286371
CTGAGATC 2.45840804314
CTGAGCAA -0.135119382647
CTGAGCAC -0.669490636387
CTGAGCAG -0.436343984734
CTGAGCCA -0.806790475317
CTGAGCCC -1.05127096136
CTGAGCGA -0.441676880343
CTGAGCGC -0.674503770601
CTGAGCTA 0.843254724135
CTGAGCTC -0.273129733062
CTGAGGAA 1.47036344412
CTGAGGAC -0.687651043478
CTGAGGCA -1.00925731183
CTGAGGCC -1.27216779546
CTGAGGGA 0.334963800996
CTGAGGGC -0.888273006625
CTGAGGTA -0.0628435230144
CTGAGGTC -1.43023630354
CTGAGTAA 0.132456994313
CTGAGTAC -0.856082027439
CTGAGTCA -1.16335315929
CTGAGTCC -0.381096668452
CTGAGTGA -0.859293381137
CTGAGTGC -0.707273484603
CTGAGTTA 0.876172869046
CTGAGTTC -0.424847146974
CTGATAAA 1.45113216944
CTGATAAC 2.43720469835
CTGATAAG 0.497072627532
CTGATACA 1.36989362274
CTGATACC 1.52989048355
CTGATAGA 0.450449709084
CTGATAGC -0.311967210984
CTGATATA 3.89531291228
CTGATATC 7.50621763245
CTGATCAA -0.117075138875
CTGATCAC 0.329206014106
CTGATCAG -0.0655977178423
CTGATCCA -0.44219336994
CTGATCCC -0.244583325974
CTGATCGA -1.19927239813
CTGATCGC 2.69737306148
CTGATCTA 0.440009374631
CTGATCTC 2.3923477538
CTGATGAA 1.06953858012
CTGATGAC -0.449407986414
CTGATGCA -0.17898061186
CTGATGCC 0.12601346912
CTGATGGA -0.0921358318463

CTGATGGC -0.530705239309
CTGATGTA -0.413351350103
CTGATGTC -0.305497455363
CTGATTAA 1.57514879526
CTGATTAC 6.81913220858
CTGATTCA 0.0726432937876
CTGATTCC 7.12611459755
CTGATTGA -0.0571871188066
CTGATTGC 6.29782308292
CTGATTTA 2.49172422437
CTGATTTC 5.83790044251
CTGCAAAA -0.903971917144
CTGCAAAC 0.762547655846
CTGCAAAG -0.808117818191
CTGCAACA 0.276673130656
CTGCAACC -0.147902552217
CTGCAAGA -0.0591346239173
CTGCAAGC -0.727272311537
CTGCAATA 1.0355601847
CTGCAATC 3.66451700651
CTGCACAA -0.0295737654764
CTGCACAC 0.911423231995
CTGCACAG -0.601567778557
CTGCACCA -0.714702940765
CTGCACCC -0.499252875637
CTGCACGA 0.319003002563
CTGCACGC -0.205912806802
CTGCACTA 0.465366911245
CTGCACTC -0.927198543837
CTGCAGAA 1.56055426899
CTGCAGAC 0.560639360889
CTGCAGCA -0.281170191629
CTGCAGCC -0.283746186096
CTGCAGGA -0.0475628420803
CTGCAGGC -0.903753538431
CTGCAGTA 0.423542495501
CTGCAGTC -1.59972379081
CTGCATAA -0.0661187873551
CTGCATAC 0.694974953398
CTGCATCA -0.562715103643
CTGCATCC -0.398148944094
CTGCATGA -0.686267700724
CTGCATGC 0.426324378001
CTGCATTA 0.18439219875
CTGCATTC 0.551133745618
CTGCCAAA -0.123185162927
CTGCCAAC -0.660247741741
CTGCCAAG -0.650123837835
CTGCCACA -1.05128178662
CTGCCACC -0.826185586076
CTGCCAGA -0.536164498959
CTGCCAGC -0.667247726706
CTGCCATA -0.438969733741
CTGCCATC 0.0557812928298

CTGCCCAA -0.538294992524
CTGCCCAC -0.139165321912
CTGCCCAG -0.885864387263
CTGCCCCA -1.18023139813
CTGCCCCC -1.50271701031
CTGCCCGA 0.755270169618
CTGCCCGC -1.31774545221
CTGCCCTA -0.688408186833
CTGCCCTC -1.63284241112
CTGCCGAA 0.602213963043
CTGCCGAC -0.0522851516401
CTGCCGCA -0.918096585691
CTGCCGCC -1.0543751034
CTGCCGGA 1.14455635208
CTGCCGGC -0.84078219413
CTGCCGTA -0.517353119289
CTGCCGTC -1.28357928024
CTGCCTAA -0.402179894544
CTGCCTAC 0.187985142705
CTGCCTCA -0.640360914567
CTGCCTCC -0.698352224954
CTGCCTGA -0.298273262701
CTGCCTGC -0.833879428287
CTGCCTTA 0.316775289876
CTGCCTTC -0.804329186923
CTGCGAAA 1.21967529854
CTGCGAAC -0.0386141029365
CTGCGAAG -0.403799935674
CTGCGACA -0.754543628428
CTGCGACC -0.560576491136
CTGCGAGA -0.633641137178
CTGCGAGC -1.10141021427
CTGCGATA 0.497795213341
CTGCGATC 2.89767068331
CTGCGCAA -0.450303776312
CTGCGCAC -0.3197205921
CTGCGCAG -0.423287061105
CTGCGCCA -0.209586107462
CTGCGCCC 0.663746172889
CTGCGCGA 0.296529147488
CTGCGCGC -0.760516671361
CTGCGCTA -0.170100363275
CTGCGCTC -0.202663148354
CTGCGGAA 0.0842623320287
CTGCGGAC -0.072952646283
CTGCGGCA -0.41037794385
CTGCGGCC -1.31624657068
CTGCGGGA 0.34301383575
CTGCGGGC -1.38839564991
CTGCGGTA 0.984124399264
CTGCGGTC -1.05230935319
CTGCGTAA 1.14791343037
CTGCGTAC -0.0628435230144
CTGCGTCA -0.875788572473

CTGCGTCC -0.308716719824
CTGCGTGA -0.479031714608
CTGCGTGC -0.928840651834
CTGCGTTA -0.664818497723
CTGCGTTC -0.642596954374
CTGCTAAA 0.168293794665
CTGCTAAC 0.443493233319
CTGCTAAG -1.156034662
CTGCTACA -0.325078677246
CTGCTACC -0.964429512198
CTGCTAGA -1.10370308667
CTGCTAGC -0.465985616235
CTGCTATA 1.21356360907
CTGCTATC 0.13633285216
CTGCTCAA -0.930581852555
CTGCTCAC 0.891293044748
CTGCTCCA -0.521868083569
CTGCTCCC -1.50271367947
CTGCTCGA -0.403799935674
CTGCTCGC -0.636277919619
CTGCTCTA -1.17571518478
CTGCTCTC -1.03356209234
CTGCTGAA 1.02582286732
CTGCTGAC 0.574046856255
CTGCTGCA -0.679005827846
CTGCTGCC -0.849500896593
CTGCTGGA -0.19569210013
CTGCTGGC -1.03605439925
CTGCTGTA 0.107119650964
CTGCTGTC -1.09823508354
CTGCTTAA 0.106218656617
CTGCTTAC -0.108821297867
CTGCTTCA -0.522979337688
CTGCTTCC -0.534752635821
CTGCTTGA 0.526737158613
CTGCTTGC 0.234607852976
CTGCTTTA 0.678792029049
CTGCTTTC -1.05058647213
CTGGAAAA -0.569278123004
CTGGAAAC 0.695344052977
CTGGAAAG -0.678435003793
CTGGAACA 1.15313682436
CTGGAACC -0.360741648975
CTGGAAGA -0.435866216243
CTGGAAGC -0.281116065351
CTGGAATA 1.05455143015
CTGGAATC 3.84497422486
CTGGACAA -0.190636289427
CTGGACAC 0.480453986646
CTGGACAG -0.299021038046
CTGGACCA -0.456584298138
CTGGACCC -0.767573905273
CTGGACGA -0.0847652900562
CTGGACGC -0.946581163916

CTGGACTA −0.0992043073706
CTGGACTC −0.497265400352
CTGGAGAA 0.816224269181
CTGGAGAC −0.433923291048
CTGGAGCA −0.258818537035
CTGGAGCC −1.19410021593
CTGGAGGA −0.855877180296
CTGGAGGC −0.324885904426
CTGGAGTA −0.0533814169435
CTGGAGTC −0.820801895037
CTGGATAA 0.755608250676
CTGGATAC 3.56738448651
CTGGATCA 0.0662147574092
CTGGATCC 3.96956542194
CTGGATGA −0.2043851967
CTGGATGC 0.0222585990321
CTGGATTA 3.57315663768
CTGGATTC 10.835910109
CTGGCAAA 1.37289346759
CTGGCAAC −0.117056611034
CTGGCAAG −1.11390006105
CTGGCACA −0.7605229167
CTGGCACC −0.97665372386
CTGGCAGA 0.190297167479
CTGGCAGC −0.79075244285
CTGGCATA 0.177488183839
CTGGCATC −0.568859268885
CTGGCCAA −1.11582362569
CTGGCCAC −0.705409042511
CTGGCCAG −0.952862518454
CTGGCCCA −1.47329542295
CTGGCCCC −0.842833163702
CTGGCCGA −0.791858908875
CTGGCCGC −1.38817956116
CTGGCCTA −0.819333615663
CTGGCCTC −1.00462410245
CTGGCGAA 0.213981577575
CTGGCGAC 0.0984554911351
CTGGCGCA −0.187756563271
CTGGCGCC −0.695041362177
CTGGCGGA 0.698311838424
CTGGCGGC −1.17075263782
CTGGCGTA 0.528247489941
CTGGCGTC −1.04476040286
CTGGCTAA −0.550633285726
CTGGCTAC −0.931018609981
CTGGCTCA −1.41196597847
CTGGCTCC 0.160283105551
CTGGCTGA −1.03652800418
CTGGCTGC −1.31151759942
CTGGCTTA 0.80539193557
CTGGCTTC −0.454669060616
CTGGGAAA 0.0984044875271
CTGGGAAC −1.10065494451

CTGGGAAG -0.0426510905482
CTGGGACA -0.715438641787
CTGGGACC -0.267445016648
CTGGGAGA -0.691673874986
CTGGGAGC -0.953309060246
CTGGGATA 0.972863635346
CTGGGATC 0.488119724829
CTGGGCAA -1.0701252257
CTGGGCAC -0.939083841729
CTGGGCCA -1.10414983664
CTGGGCCC -0.927433576789
CTGGGCGA -0.0658312935488
CTGGGCGC -1.11926251793
CTGGGCTA -1.15362895713
CTGGGCTC -1.51202256654
CTGGGGAA -0.0871391436936
CTGGGGAC -1.60431765455
CTGGGGCA -0.197428929113
CTGGGGCC -0.426796317508
CTGGGGGA -0.698479838063
CTGGGGGC -1.64216212345
CTGGGGTA 0.0345912714282
CTGGGGTC -1.06283941235
CTGGGTAA -0.043588724222
CTGGGTAC -0.851322037663
CTGGGTCA -0.84613778114
CTGGGTCC -1.02154564231
CTGGGTGA 0.0726432937876
CTGGGTGC -1.13131623182
CTGGGTTA -0.251803355076
CTGGGTTC -0.468319083342
CTGGTAAA 0.586840018369
CTGGTAAC 1.02900819877
CTGGTAAG -0.54469792301
CTGGTACA -0.490393444852
CTGGTACC -0.50891899614
CTGGTAGA -0.317183735095
CTGGTAGC -1.38942404919
CTGGTATA 0.267585328614
CTGGTATC 0.612687814153
CTGGTCAA -0.614939467309
CTGGTCAC -1.03268337303
CTGGTCCA -0.065751977734
CTGGTCCC -0.821559454748
CTGGTCGA -0.158123050711
CTGGTCGC -1.09134147753
CTGGTCTA -0.227363258871
CTGGTCTC -0.816395599668
CTGGTGAA 0.709522431446
CTGGTGAC -1.16739701678
CTGGTGCA -0.535071772682
CTGGTGCC -1.17329282567
CTGGTGGA -0.979366907624
CTGGTGGC -1.12478985178

CTGGTGTA 1.14765695508
CTGGTGTC -0.936488694887
CTGGTTAA -0.39878576057
CTGGTTAC 1.2855140865
CTGGTTCA -0.99509413035
CTGGTTCC 0.226512643597
CTGGTTGA -0.845854450893
CTGGTTGC -0.223862537765
CTGGTTTA 0.733175406665
CTGGTTTC -0.229554124054
CTGTAAAA 0.310154605211
CTGTAAAC -0.0972416052665
CTGTAAAG 0.338682068102
CTGTAACA 1.02946223497
CTGTAACC 1.62473179657
CTGTAAGA -0.291990242739
CTGTAAGC -0.488638712561
CTGTAATA 1.28162344801
CTGTAATC 6.40949641927
CTGTACAA -0.480645926755
CTGTACAC -0.484416030181
CTGTACAG -0.378470919445
CTGTACCA -0.878061043429
CTGTACCC -0.87773337127
CTGTACGA -0.30536297238
CTGTACGC 0.617572918902
CTGTACTA 1.06282171722
CTGTACTC -0.539688119644
CTGTAGAA 1.01421569523
CTGTAGAC 0.101358949583
CTGTAGCA 0.158961591661
CTGTAGCC 0.22782041774
CTGTAGGA 0.882333688527
CTGTAGGC -0.446568646788
CTGTAGTA 2.15292765837
CTGTAGTC -0.819295310912
CTGTATAA 1.00726255031
CTGTATAC 0.797027760227
CTGTATCA 0.830929962503
CTGTATCC 2.28327601781
CTGTATGA -0.497035780028
CTGTATGC 0.107443992275
CTGTATTA 3.26429794047
CTGTATTC 1.05930663184
CTGTCAAA -0.0671763315521
CTGTCAAC -0.126983370382
CTGTCAAG -0.20881834703
CTGTCACA -0.438195103434
CTGTCACC 0.176720631584
CTGTCAGA -0.640691293039
CTGTCAGC -0.766723081822
CTGTCATA 0.933617712206
CTGTCATC -0.187671210294
CTGTCCAA -0.268853548938

CTGTCCAC 0.242842541609
CTGTCCCA -0.25003779753
CTGTCCCC -0.315223114771
CTGTCCGA -0.613753269113
CTGTCCGC -0.366370781865
CTGTCCTA 0.0414703049802
CTGTCCTC -0.751663277737
CTGTCGAA 1.5089080156
CTGTCGAC -0.667893286658
CTGTCGCA 0.0291848889882
CTGTCGCC -0.672703030974
CTGTCGGA 0.558903364618
CTGTCGGC -0.332440267382
CTGTCGTA 0.01493926902 8
CTGTCGTC -1.43098990787
CTGTCTAA 0.0433974086478
CTGTCTAC 0.124245621615
CTGTCTCA -0.0350192853788
CTGTCTCC -0.922308859172
CTGTCTGA 0.140476635081
CTGTCTGC -0.726970869806
CTGTCTTA 2.77123607012
CTGTCTTC 0.597481244584
CTGTGAAA 1.5270163782
CTGTGAAC -0.248820164458
CTGTGAAG -0.709971055018
CTGTGACA -0.535972142495
CTGTGACC -0.854496543856
CTGTGAGA 0.34488993581
CTGTGAGC -0.764844899982
CTGTGATA 3.11514215674
CTGTGATC 0.361704472185
CTGTGCAA -0.0795889442969
CTGTGCAC -0.335825866059
CTGTGCCA 0.00256121382976
CTGTGCCC -0.272487920314
CTGTGCGA 0.759314443459
CTGTGCGC 0.0443818823697
CTGTGCTA -0.311676178151
CTGTGCTC -0.93385357787
CTGTGGAA -0.0462384136982
CTGTGGAC -0.638517914808
CTGTGGCA -0.6120091539
CTGTGGCC -0.510145580866
CTGTGGGA -1.25379171597
CTGTGGGC -1.09300815053
CTGTGGTA 1.50676981945
CTGTGGTC -0.0975047422478
CTGTGTAA 0.754496580202
CTGTGTAC -0.128376289323
CTGTGTCA -0.655388659241
CTGTGTCC 0.287297286276
CTGTGTGA 0.84427833532
CTGTGTGC -0.0808313505503

CTGTGTTA -0.0650874735851
CTGTGTTC 0.296653637927
CTGTTAAA 0.891135037653
CTGTTAAC 0.821204511273
CTGTTAAG 0.308347412067
CTGTTACA 0.244586240466
CTGTTACC -0.70160937781
CTGTTAGA -0.159219524192
CTGTTAGC 0.318554587169
CTGTTATA 3.93857292345
CTGTTATC 1.56297246454
CTGTTCAA -0.720092668966
CTGTTCAC -0.623619656844
CTGTTCCA 0.475555974862
CTGTTCCC -0.369068768636
CTGTTCGA 0.430232711614
CTGTTCGC 0.161916261894
CTGTTCTA 2.08475810962
CTGTTCTC 0.0633429420161
CTGTTGAA 0.2808062965
CTGTTGAC 0.175516738259
CTGTTGCA -0.0789356817595
CTGTTGCC -0.980489611532
CTGTTGGA -0.528503757049
CTGTTGGC -1.29179190201
CTGTTGTA 2.09002501281
CTGTTGTC 0.298273262701
CTGTTTAA -0.462431601564
CTGTTTAC 0.18565459009
CTGTTTCA -0.334829526191
CTGTTTCC 0.317230991499
CTGTTTGA 0.298443135942
CTGTTTGC 0.0981742426685
CTGTTTTA 1.33240493002
CTGTTTTC -0.252370015569
CTTAAAAA 0.50902683234
CTTAAAAC -0.352923732681
CTTAAAAG 0.621517475485
CTTAAACA -0.333101024327
CTTAAACC 1.8079323845
CTTAAAGA -0.491972058562
CTTAAAGC -0.592912154026
CTTAAATA 0.125516548254
CTTAAATC 1.29032882709
CTTAACAA 0.778403740749
CTTAACAC -0.0322043025773
CTTAACCA -0.595174840616
CTTAACCC 0.370954236705
CTTAACGA 0.465111476849
CTTAACGC -0.0833748692508
CTTAACTA 1.14576732346
CTTAACTC -1.13069835954
CTTAAGAA 0.0480100084061
CTTAAGAC -0.29617649397

CTTAAGCA 0.803057219395
CTTAAGCC -0.836259319086
CTTAAGGA 0.621041580597
CTTAAGGC -0.265668425668
CTTAAGTA -0.463592402045
CTTAAGTC -0.55448561946
CTTAATAA 0.633304513366
CTTAATAC 1.41140348153
CTTAATCA 1.27611922192
CTTAATCC -0.0532569265046
CTTAATGA 0.353158973811
CTTAATGC 0.937929911122
CTTAATTA 1.76697836094
CTTAATTC 0.298617172743
CTTACAAA 1.01456168705
CTTACAAC 0.567027302559
CTTACAAG 1.29529637032
CTTACACA 0.215111359535
CTTACACC 1.61115526068
CTTACAGA -0.0820113034063
CTTACAGC -1.04555793274
CTTACATA 0.232074534997
CTTACATC 0.30782571802
CTTACCAA 1.58596530735
CTTACCAC -0.658581485097
CTTACCCA -1.03400009883
CTTACCCC -0.220684076191
CTTACCGA -0.451537647267
CTTACCGC -0.609185843979
CTTACCTA 1.67880228267
CTTACCTC -0.873982836575
CTTACGAA 1.09910172852
CTTACGAC -0.480573064458
CTTACGCA 0.180299835792
CTTACGCC 0.273396825425
CTTACGGA 0.899378469761
CTTACGGC -0.138783523476
CTTACGTA 0.099267801658
CTTACGTC -0.498761991933
CTTACTAA -0.652458762188
CTTACTAC -0.371885000505
CTTACTCA 0.173460772414
CTTACTCC -0.20365345106
CTTACTGA 0.653749257558
CTTACTGC -0.2067007605
CTTACTTA 0.480761257362
CTTACTTC -0.234651153998
CTTAGAAA 1.33079862864
CTTAGAAC -1.20342284274
CTTAGAAG -0.345132046814
CTTAGACA 0.0845829261356
CTTAGACC -0.833888588118
CTTAGAGA -0.479738270712
CTTAGAGC -1.00794953768

CTTAGATA 3.16193869563
CTTAGATC 0.91026597054
CTTAGCAA 0.900759106201
CTTAGCAC -0.904970130614
CTTAGCCA -0.705909294225
CTTAGCCC 0.00342327889268
CTTAGCGA 1.29343983899
CTTAGCGC -0.489520138178
CTTAGCTA -0.299869155184
CTTAGCTC 0.138133591786
CTTAGGAA 0.639920826293
CTTAGGAC -0.784993823248
CTTAGGCA 0.505153888985
CTTAGGCC -0.526961782666
CTTAGGGA -0.943345661572
CTTAGGGC -1.09358480357
CTTAGGTA -0.145448758231
CTTAGGTC -0.106185764494
CTTAGTAA -0.265765436612
CTTAGTAC 0.778973732089
CTTAGTCA 0.014276013947
CTTAGTCC -0.169122551249
CTTAGTGA -0.394711717276
CTTAGTGC -0.289016003774
CTTAGTTA -0.200472283171
CTTAGTTC -0.37102002095
CTTATAAA 1.43382674936
CTTATAAC 1.57761362268
CTTATAAG 0.963185024164
CTTATACA 0.187873559302
CTTATACC 0.334132754453
CTTATAGA 0.0603528815238
CTTATAGC 2.18984927424
CTTATATA 0.844535226961
CTTATATC 1.09738155377
CTTATCAA 0.0979310907744
CTTATCAC -0.0254347706489
CTTATCCA 0.913149027545
CTTATCCC 0.530725224397
CTTATCGA 1.39826911569
CTTATCGC 0.0403844485764
CTTATCTA 1.19785491418
CTTATCTC -0.354040399427
CTTATGAA 0.453431650634
CTTATGAC 0.36061986485
CTTATGCA 0.492859729518
CTTATGCC 0.581309978202
CTTATGGA -0.410165185943
CTTATGGC -0.641126801397
CTTATGTA 0.403904441026
CTTATGTC -0.347054362388
CTTATTAA 0.116700418491
CTTATTAC 1.83162949345
CTTATTCA 0.636067451669

CTTATTCC 0.527335253983
CTTATTGA 1.34909684956
CTTATTGC 1.19944414497
CTTATTTA 1.66615317972
CTTATTTC 1.21300569205
CTTCAAAA 0.433457805059
CTTCAAAC 0.261918515506
CTTCAAAG 0.562642241346
CTTCAACA 0.164706055159
CTTCAACC 0.396266390517
CTTCAAGA -1.21364230035
CTTCAAGC -0.87773337127
CTTCAATA 0.648543558702
CTTCAATC -1.26598324369
CTTCACAA 0.906074306681
CTTCACAC -0.55785622932
CTTCACCA -0.481604794584
CTTCACCC -0.819295310912
CTTCACGA 0.090274304246
CTTCACGC -0.591006076335
CTTCACTA -0.178759318655
CTTCACTC -1.05636549317
CTTCAGAA 1.08888747536
CTTCAGAC -0.195991876438
CTTCAGCA 0.0453224305355
CTTCAGCC -1.19064029771
CTTCAGGA 0.0921133486232
CTTCAGGC -0.863612658105
CTTCAGTA -0.622567525275
CTTCAGTC -1.08628962221
CTTCATAA 0.0288994769618
CTTCATAC 0.00507746121336
CTTCATCA 1.12615758119
CTTCATCC -0.397950966824
CTTCATGA 0.533915760295
CTTCATGC -0.182647875359
CTTCATTA 1.55996928883
CTTCATTC 0.581489011275
CTTCCAAA -0.221152060316
CTTCCAAC -0.326753677366
CTTCCAAG -0.0460527189297
CTTCCACA -0.226997073784
CTTCCACC -0.408196238499
CTTCCAGA -0.432416706924
CTTCCAGC -1.08614889389
CTTCCATA 0.0591600216323
CTTCCATC -0.398672719921
CTTCCCAA -0.355048397262
CTTCCCAC -0.294176736184
CTTCCCCA -1.00003606769
CTTCCCCC -1.01491225879
CTTCCCGA -0.777326835998
CTTCCCGC -0.611057364123
CTTCCCTA -0.333925409174

CTTCCCTC -1.06913804566
CTTCCGAA 0.429060253166
CTTCCGAC 0.306725289157
CTTCCGCA 0.337426962991
CTTCCGCC -0.460408319664
CTTCCGGA 0.779177330165
CTTCCGGC -0.406211677706
CTTCCGTA -0.0558304228358
CTTCCGTC -0.64121985696
CTTCCTAA -0.446556156108
CTTCCTAC -0.464438853758
CTTCCTCA -0.406735661711
CTTCCTCC -1.29683980195
CTTCCTGA 0.021156712923
CTTCCTGC -0.617192994067
CTTCCTTA 0.145081324075
CTTCCTTC -0.342413866778
CTTCGAAA 0.676746680282
CTTCGAAC 0.172226901459
CTTCGAAG -0.485011002881
CTTCGACA -0.193695256835
CTTCGACC -0.80023640761
CTTCGAGA -0.443080208184
CTTCGAGC -0.984478718206
CTTCGATA 1.76137067038
CTTCGATC -0.314272157706
CTTCGCAA 0.40666633844
CTTCGCAC -0.155459205131
CTTCGCCA -0.239727574322
CTTCGCCC -1.13450114691
CTTCGCGA 0.329967529199
CTTCGCGC -0.535492500403
CTTCGCTA -0.349809806283
CTTCGCTC -0.672736755809
CTTCGGAA -0.539116462879
CTTCGGAC -1.80435297211
CTTCGGCA 0.903504765731
CTTCGGCC -0.719242053692
CTTCGGGA -0.121887381327
CTTCGGGC -1.31838913856
CTTCGGTA 0.370285777174
CTTCGGTC -0.301511055003
CTTCGTAA 0.611034464544
CTTCGTAC -0.0493506746715
CTTCGTCA -0.559120286085
CTTCGTCC -1.32025462155
CTTCGTGA 0.785001317656
CTTCGTGC -0.727627879547
CTTCGTTA 0.70041630974
CTTCGTTC -0.488558772213
CTTCTAAA 1.26254851501
CTTCTAAC 0.802511584879
CTTCTACA 0.144304403811
CTTCTACC -0.936910879854

CTTCTAGA 0.0802842587886
CTTCTAGC -0.581716757997
CTTCTATA 0.104863209714
CTTCTATC -0.644527805245
CTTCTCAA 0.62515101415
CTTCTCAC -0.618226181439
CTTCTCCA -0.436915641498
CTTCTCCC -0.83178432498
CTTCTCGA 0.00948146662412
CTTCTCGC -0.675293597901
CTTCTCTA 0.749722434322
CTTCTCTC -0.41524535347
CTTCTGAA -0.171400851188
CTTCTGAC 0.129809386617
CTTCTGCA 1.03548190977
CTTCTGCC -0.576634716868
CTTCTGGA -0.911915156589
CTTCTGGC -1.34424109791
CTTCTGTA -0.0752825743642
CTTCTGTC -1.20401240282
CTTCTTAA -0.244091817736
CTTCTTAC -0.116257415725
CTTCTTCA 0.406328049203
CTTCTTCC -1.28465847495
CTTCTTGA -0.201560845889
CTTCTTGC 0.351655512356
CTTCTTTA 0.364257775251
CTTCTTTC -0.0896922385817
CTTGAAAA -0.218068736083
CTTGAAAC -0.0831367136285
CTTGAAAG -0.085850730104
CTTGAACA -0.46017661756
CTTGAACC 0.595707568096
CTTGAAGA 0.359341651982
CTTGAAGC -0.136272064186
CTTGAATA 0.339144431422
CTTGAATC -0.522785523978
CTTGACAA 0.913618260738
CTTGACAC -0.517127870702
CTTGACCA -0.84004524404
CTTGACCC -1.46331932542
CTTGACGA 0.583724010192
CTTGACGC -0.656836120816
CTTGACTA 0.0483672418396
CTTGACTC -1.16058564107
CTTGAGAA 0.909960989782
CTTGAGAC 0.854450952876
CTTGAGCA 0.2325323184
CTTGAGCC -1.35062258606
CTTGAGGA -0.582314228833
CTTGAGGC -0.681640736685
CTTGAGTA 0.429460787622
CTTGAGTC -0.695041362177
CTTGATAA 1.3491532658

CTTGATAC 1.3828139816
CTTGATCA -0.177895588168
CTTGATCC 0.166700816674
CTTGATGA 0.754874215078
CTTGATGC -0.395135567667
CTTGATTA 2.27106637861
CTTGATTC 3.23050003536
CTTGCAAA 1.27789123298
CTTGCAAC 0.474171591218
CTTGCAAG 0.855064453417
CTTGCACA -0.293424380922
CTTGCACC 0.016217481896
CTTGCAGA -0.0589988918669
CTTGCAGC -0.508596112075
CTTGCATA 0.636930973978
CTTGCATC -0.612388662379
CTTGCCAA 0.932021611545
CTTGCCAC -0.935082452554
CTTGCCCA -1.3257677993
CTTGCCCC -1.16397810962
CTTGCCGA -0.116404389387
CTTGCCGC -0.0598153659496
CTTGCCTA -0.233113967709
CTTGCCTC -1.22086836661
CTTGCGAA 1.72503195289
CTTGCGAC -0.167222927076
CTTGCGCA 0.810761886862
CTTGCGCC -0.29465075747
CTTGCGGA 0.742055238894
CTTGCGGC -0.40861176177
CTTGCGTA -0.40979754361
CTTGCGTC -0.0693840591522
CTTGCTAA 1.38704519928
CTTGCTAC 0.605592691846
CTTGCTCA 0.718923541365
CTTGCTCC -0.654816169764
CTTGCTGA 0.636678245896
CTTGCTGC -0.274150429755
CTTGCTTA 0.130194515901
CTTGCTTC -0.128396066233
CTTGGAAA 1.79827479929
CTTGGAAC -0.685714571801
CTTGGACA -0.584584409831
CTTGGACC 0.195191015704
CTTGGAGA -0.689003784067
CTTGGAGC -0.27510138682
CTTGGATA 1.62581015857
CTTGGATC 1.46657939277
CTTGGCAA 0.490363259043
CTTGGCAC -0.618133958589
CTTGGCCA -0.123993101712
CTTGGCCC -0.203580172407
CTTGGCGA 0.31047707292
CTTGGCGC -0.986478892348

CTTGGCTA -0.422699582813
CTTGGCTC -0.880856041143
CTTGGGAA 0.232053092664
CTTGGGAC -0.0863572271574
CTTGGGCA -0.558386666843
CTTGGGCC -1.21253958153
CTTGGGGA 0.217010150996
CTTGGGGC -1.09154299382
CTTGGGTA -0.34036143996
CTTGGGTC -1.1485941724
CTTGGTAA 1.01873898663
CTTGGTAC -0.694757615575
CTTGGTCA -0.601191809105
CTTGGTCC -0.250331953032
CTTGGTGA 0.507926819832
CTTGGTGC -0.788048627096
CTTGGTTA -0.858884935917
CTTGGTTC -0.00519466542259
CTTGTAAA 2.80068430427
CTTGTAAC 1.79360890597
CTTGTACA 0.531540865767
CTTGTACC -0.0452439474327
CTTGTAGA 0.504693399268
CTTGTAGC -0.147794716017
CTTGTATA 0.988071662161
CTTGTATC 0.708219861753
CTTGTCAA 0.373993427203
CTTGTCAC -0.765476095653
CTTGTCCA -0.151832119985
CTTGTCCC 0.300996855364
CTTGTCGA -0.738589283513
CTTGTCGC -0.636924728638
CTTGTCTA -0.00585084245188
CTTGTCTC -0.566766663714
CTTGTGAA 0.464180713049
CTTGTGAC 0.0856131990157
CTTGTGCA -0.220332463563
CTTGTGCC -0.992126761258
CTTGTGGA 0.102295958723
CTTGTGGC -0.861647874221
CTTGTGTA 0.541822776769
CTTGTGTC -0.0160617647583
CTTGTTAA 1.33458705173
CTTGTTAC 0.100232290293
CTTGTTCA -0.978261274311
CTTGTTCC -1.29992416707
CTTGTTGA 0.723354818093
CTTGTTGC 0.237972217497
CTTGTTTA 0.219931304573
CTTGTTTC -0.867458330142
CTTTAAAA 0.990301456629
CTTTAAAC 0.130326917104
CTTTAAAG -0.445244842939
CTTTAACA 0.622827747764

CTTTAACC 0.812914447294
CTTTAAGA -0.420838263391
CTTTAAGC 0.0516096140576
CTTTAATA 1.11752339899
CTTTAATC -0.406970902841
CTTTACAA 0.844175079036
CTTTACAC 1.37974522983
CTTTACCA -0.652944025087
CTTTACCC -0.95548743475
CTTTACGA 0.18989392671
CTTTACGC 0.571631783376
CTTTACTA 0.936344635717
CTTTACTC -0.846555386191
CTTTAGAA 0.601139764607
CTTTAGAC 0.990453010207
CTTTAGCA 0.681460038188
CTTTAGCC -1.19507657071
CTTTAGGA 0.180062096526
CTTTAGGC -0.51888489295
CTTTAGTA 1.10139272732
CTTTAGTC -1.11453729388
CTTTATAA 1.00281649295
CTTTATAC 1.78369609455
CTTTATCA 0.573233713021
CTTTATCC 0.801116584158
CTTTATGA 0.234007467648
CTTTATGC 0.426443247635
CTTTATTA 1.64579462122
CTTTATTC 0.715517333068
CTTTCAAA 1.11272801895
CTTTCAAC 0.411240217091
CTTTCACA -0.0615357488716
CTTTCACC -0.391385657505
CTTTCAGA -0.360577188361
CTTTCAGC -0.686091998499
CTTTCATA 1.12944929159
CTTTCATC 1.17470968508
CTTTCCAA -0.306585393547
CTTTCCAC -0.5773258678
CTTTCCCA -0.628388598274
CTTTCCCC 0.270375746412
CTTTCCGA 0.0191215648778
CTTTCCGC -0.616477902667
CTTTCCTA 0.341702522581
CTTTCCTC -1.26082958933
CTTTCGAA 0.0585579708808
CTTTCGAC -0.25353497961
CTTTCGCA 0.748348459578
CTTTCGCC -0.27239507293
CTTTCGGA -0.656555496884
CTTTCGGC 0.818952649938
CTTTCGTA 0.0700456488093
CTTTCGTC -1.05651912853
CTTTCTAA 0.379383155404

CTTTCTAC 0.535113200102
CTTTCTCA -0.678326334881
CTTTCTCC -0.107167531902
CTTTCTGA 1.82765787373
CTTTCTGC 0.315384036359
CTTTCTTA 0.236735848405
CTTTCTTC -0.0166009457564
CTTTGAAA 0.778403740749
CTTTGAAC -0.11431553142
CTTTGACA -0.135542192148
CTTTGACC -0.0222550600063
CTTTGAGA 0.279210820373
CTTTGAGC -0.108424510615
CTTTGATA 0.426345820335
CTTTGATC 0.564358044352
CTTTGCAA 1.41580269885
CTTTGCAC -0.769742703589
CTTTGCCA -0.0643172150164
CTTTGCCC 0.410253245234
CTTTGCGA -0.111000921438
CTTTGCGC -0.135087531414
CTTTGCTA 0.102416285602
CTTTGCTC -0.536657880799
CTTTGGAA -0.221152060316
CTTTGGAC 0.247551111421
CTTTGGCA -0.349975932321
CTTTGGCC -0.715777139201
CTTTGGGA 0.805824737615
CTTTGGGC -0.770330181881
CTTTGGTA 0.256717812922
CTTTGGTC -0.187084148358
CTTTGTAA 0.50671647299
CTTTGTAC 0.690487676791
CTTTGTCA 0.514671786758
CTTTGTCC -0.500030212258
CTTTGTGA 0.37804332185
CTTTGTGC 0.142390415357
CTTTGTTA 0.308657597274
CTTTGTTC -0.871053772234
CTTTTAAA 2.55522288352
CTTTTAAC -0.236431908538
CTTTTACA -0.519411166913
CTTTTACC -0.0162751471996
CTTTTAGA -0.24365922387
CTTTTAGC 0.241188775644
CTTTTATA 1.68763777298
CTTTTATC -0.358661118127
CTTTTCAA -0.0694664976369
CTTTTCAC -0.235637501322
CTTTTCCA -0.331117712602
CTTTTCCC -0.0278292339074
CTTTTCGA 0.914545901868
CTTTTCGC -0.594981026905
CTTTTCTA 0.54420558206

CTTTTCTC -0.251039758203
CTTTTGAA 0.369173690343
CTTTTGAC 0.180663106387
CTTTTGCA -0.227662202467
CTTTTGCC -1.27683389696
CTTTTGGA 0.0537794532633
CTTTTGGC -0.196955948716
CTTTTGTA 1.0711448815
CTTTTGTC -0.682220096035
CTTTTTAA -0.202075461884
CTTTTTAC 1.41037674768
CTTTTTCA -0.582140608388
CTTTTTCC -1.05391960995
CTTTTTGA 0.486562553452
CTTTTTGC 0.699595880276
CTTTTTTA 1.73495996131
CTTTTTTC 0.377317613372
GAAAAAAA 1.86896038725
GAAAAAAC 0.0357549864008
GAAAAACA -0.528273928546
GAAAAACC 0.933901875164
GAAAAAGA -0.197607962186
GAAAAAGC -0.426320214442
GAAAAATA 2.69603239521
GAAAAATC 6.79232117323
GAAAACAA -0.21086432033
GAAAACAC -0.120466358357
GAAAACCA -0.988633950917
GAAAACCC -0.499024088025
GAAAACGA 0.395805068088
GAAAACGC 0.432993151782
GAAAACTA 0.450477604934
GAAAACTC -0.386046100201
GAAAAGAA 0.0645226866941
GAAAAGAC -0.664009101692
GAAAAGCA -0.477465799756
GAAAAGCC -0.262925888807
GAAAAGGA 1.00844791579
GAAAAGGC -0.567396610316
GAAAAGTA 0.441952508003
GAAAAGTC -0.262876550623
GAAAATAA 1.27923835277
GAAAATAC 2.99179752947
GAAAATCA 6.98600498027
GAAAATCC 17.1039412559
GAAAATGA 0.415878214565
GAAAATGC 1.29002467904
GAAAATTA 2.2879308777
GAAAATTC 2.0527141041
GAAACAAA 0.507689288744
GAAACAAC -0.0704586739445
GAAACACA -0.721999579368
GAAACACC 0.907943120511
GAAACAGA 0.229009946784

GAAACAGC -0.457526928084
GAAACATA 2.03032768378
GAAACATC 1.43978438712
GAAACCAA 1.21181449758
GAAACCAC -0.650968832303
GAAACCCA -1.07816047982
GAAACCCC -1.36101941131
GAAACCGA 0.317102962035
GAAACCGC 0.26737423613
GAAACCTA 0.532997695352
GAAACCTC -0.799266298169
GAAACGAA 0.748822480864
GAAACGAC -0.733916312137
GAAACGCA 0.0685421873545
GAAACGCC -0.521697793972
GAAACGGA 0.367875492388
GAAACGGC -1.01341837352
GAAACGTA 0.757001585974
GAAACGTC 0.222380726822
GAAACTAA 0.947535035473
GAAACTAC 1.14928365791
GAAACTCA -1.12498033465
GAAACTCC -0.616464162919
GAAACTGA 0.534047745141
GAAACTGC -0.266066878344
GAAACTTA -0.310908834075
GAAACTTC 0.414372254974
GAAAGAAA 0.756001082546
GAAAGAAC -0.859251329182
GAAAGACA -0.264476398488
GAAAGACC -0.94393771978
GAAAGAGA 0.350031307666
GAAAGAGC -0.782899552654
GAAAGATA 1.13645281558
GAAAGATC 2.48430954885
GAAAGCAA 1.52609748054
GAAAGCAC -1.13722307415
GAAAGCCA -1.16455392995
GAAAGCCC -1.62120317962
GAAAGCGA -0.0809962275196
GAAAGCGC 0.279057185015
GAAAGCTA 0.795884446697
GAAAGCTC -1.13616323999
GAAAGGAA -0.0842092466409
GAAAGGAC -1.11283481426
GAAAGGCA -0.431774894176
GAAAGGCC -0.451221841254
GAAAGGGA 0.28394270612
GAAAGGGC -1.20881735904
GAAAGGTA 0.427356524483
GAAAGGTC 0.0205935914559
GAAAGTAA 0.370098208804
GAAAGTAC 0.427135439456
GAAAGTCA -0.828284852942

GAAAGTCC −0.566219571952
GAAAGTGA 0.917733106918
GAAAGTGC 0.682415158813
GAAAGTTA 0.0499462719053
GAAAGTTC 0.939139217075
GAAATAAA 2.98891343158
GAAATAAC 0.987944257231
GAAATACA 0.275088063428
GAAATACC 0.341459370687
GAAATAGA 1.4506804232
GAAATAGC 1.15675225153
GAAATATA 5.80326524552
GAAATATC 8.32566803597
GAAATCAA 7.2030451089
GAAATCAC 8.09691706261
GAAATCCA 14.7293775234
GAAATCCC 19.0921418332
GAAATCGA 6.66183479178
GAAATCGC 9.40217433346
GAAATCTA 17.3799315628
GAAATCTC 18.3524796003
GAAATGAA 0.418758773433
GAAATGAC −0.0215335150876
GAAATGCA 0.678869679439
GAAATGCC −0.151083720105
GAAATGGA −0.709504319961
GAAATGGC −0.858709233692
GAAATGTA 1.46381874442
GAAATGTC 0.469888120865
GAAATTAA 2.3935897437
GAAATTAC 4.33036411169
GAAATTCA −0.00407237787026
GAAATTCC 1.72910974339
GAAATTGA 1.92554441441
GAAATTGC 2.52258640344
GAAATTTA 1.74465002228
GAAATTTC 7.02646229115
GAACAAAA 0.433941610711
GAACAAAC −0.158933487632
GAACAACA 0.578730861065
GAACAACC −0.746310605219
GAACAAGA 0.246237508295
GAACAAGC 0.714258272575
GAACAATA 1.70680971694
GAACAATC 3.25726527156
GAACACAA 0.750914253323
GAACACAC −0.689215292906
GAACACCA −0.792941642609
GAACACCC −0.592954622336
GAACACGA 0.0743361972147
GAACACGC 0.665912264891
GAACACTA 1.33102866532
GAACACTC −0.383413897676
GAACAGAA 0.930159251232

GAACAGAC 0.310172300341
GAACAGCA -0.378004808922
GAACAGCC -0.38974542311
GAACAGGA 1.95362304556
GAACAGGC -0.876201181252
GAACAGTA 1.10051296712
GAACAGTC 0.235217398135
GAACATAA 0.76716379463
GAACATAC 0.301818117541
GAACATCA -0.182728856597
GAACATCC 0.44139105196
GAACATGA 0.814010296241
GAACATGC -0.365522040194
GAACATTA 0.534711000222
GAACATTC 0.118647090889
GAACCAAA 0.186557874395
GAACCAAC -0.138507895815
GAACCACA -0.44287640193
GAACCACC -0.282047870041
GAACCAGA 1.08515609304
GAACCAGC -0.893564474814
GAACCATA 0.514160501611
GAACCATC 0.550842088252
GAACCCAA 0.263055375518
GAACCCAC -0.731094875818
GAACCCCA -1.28612529708
GAACCCCC -1.29616613797
GAACCCGA -0.140545333818
GAACCCGC -0.530507470217
GAACCCTA -0.392092629965
GAACCCTC -1.18498160354
GAACCGAA 0.352596060522
GAACCGAC -0.38586789984
GAACCGCA -0.928456355262
GAACCGCC -1.04106607622
GAACCGGA 0.0383459696834
GAACCGGC -0.882190462069
GAACCGTA -0.699277784305
GAACCGTC -0.751989284472
GAACCTAA 0.0131048045667
GAACCTAC -0.953975854353
GAACCTCA -0.929043417198
GAACCTCC -1.22829324286
GAACCTGA 0.853583475185
GAACCTGC 0.00745069031677
GAACCTTA -0.383253600622
GAACCTTC -0.174768962913
GAACGAAA 1.14354939513
GAACGAAC -0.420760404822
GAACGACA -1.11632158744
GAACGACC -0.7697643541
GAACGAGA 0.927077384246
GAACGAGC -0.486507386284
GAACGATA 1.54145060742

GAACGATC 0.908300562122
GAACGCAA 0.65855525467
GAACGCAC -0.624387833633
GAACGCCA -1.36797318076
GAACGCCC -1.37991135587
GAACGCGA 1.08632730242
GAACGCGC -0.116244092333
GAACGCTA 0.356665940256
GAACGCTC 0.036758612498
GAACGGAA 1.17295890817
GAACGGAC -0.489963973655
GAACGGCA -0.244435727778
GAACGGCC -0.923392425618
GAACGGGA -0.347054362388
GAACGGGC -1.33975611126
GAACGGTA 0.15720581848
GAACGGTC -0.0177854785282
GAACGTAA 1.64541719452
GAACGTAC -0.0857618381017
GAACGTCA -0.771641078694
GAACGTCC -1.09293632912
GAACGTGA -0.172930126714
GAACGTGC 0.608770737064
GAACGTTA 0.812280128954
GAACGTTC -0.180978704223
GAACTAAA 0.805038865697
GAACTAAC 0.273357687963
GAACTACA -0.635263884622
GAACTACC -0.11337123605
GAACTAGA 0.0222304950033
GAACTAGC 0.125509262024
GAACTATA 0.909368307041
GAACTATC -0.145012833516
GAACTCAA -0.279606358557
GAACTCAC -0.387426320285
GAACTCCA -0.727241084839
GAACTCCC -1.35704612617
GAACTCGA -0.501939620796
GAACTCGC -0.570285288127
GAACTCTA 0.121875515182
GAACTCTC -0.494406075639
GAACTGAA 0.301818117541
GAACTGAC -0.782754660771
GAACTGCA -0.580778707967
GAACTGCC -1.1123662056
GAACTGGA -0.159258661654
GAACTGGC -0.638192532607
GAACTGTA -0.180371240843
GAACTGTC -0.248200210399
GAACTTAA 0.300927532093
GAACTTAC 0.655520019554
GAACTTCA -0.664367792372
GAACTTCC -1.495459301
GAACTTGA 0.817886154088

GAACTTGC 0.861682431767
GAACTTTA 1.33524614325
GAAGAAAA 1.45723199277
GAAGAAAC -0.156385389015
GAAGAACA 0.280092662345
GAAGAACC -0.532804506176
GAAGAAGA 0.263715716107
GAAGAAGC -0.629805665862
GAAGAATA 0.645562033507
GAAGAATC 8.43908278122
GAAGACAA -0.455482828385
GAAGACAC -0.733954825066
GAAGACCA -0.9983891716
GAAGACCC -1.28217262156
GAAGACGA -0.0944699234873
GAAGACGC -0.837609145183
GAAGACTA 0.184487127914
GAAGACTC -0.348305095761
GAAGAGAA -0.0350213671587
GAAGAGAC -0.633102788892
GAAGAGCA 0.236290347503
GAAGAGCC -0.225219650092
GAAGAGGA -0.405880466521
GAAGAGGC -1.34221927325
GAAGAGTA 0.290875241416
GAAGAGTC -0.602614705677
GAAGATAA -0.266071458259
GAAGATAC 2.08608045622
GAAGATCA 3.48433749759
GAAGATCC 5.59587666494
GAAGATGA 0.381203880118
GAAGATGC 0.309327930407
GAAGATTA 6.4410920094
GAAGATTC 9.8410807811
GAAGCAAA 0.127313748855
GAAGCAAC -1.08310429076
GAAGCACA -0.983509857833
GAAGCACC -0.312967298055
GAAGCAGA -0.155050135378
GAAGCAGC -0.454069508
GAAGCATA 1.06136801031
GAAGCATC -0.0878927480229
GAAGCCAA -0.975016612135
GAAGCCAC -0.777011029985
GAAGCCCA -1.11768765142
GAAGCCCC -1.55261477675
GAAGCCGA -0.264382302036
GAAGCCGC -1.21113417191
GAAGCCTA -0.427685029354
GAAGCCTC -1.05388942414
GAAGCGAA -0.00964571905944
GAAGCGAC -0.665720949317
GAAGCGCA -0.0553482826074
GAAGCGCC -0.439883426946

GAAGCGGA -0.0115913505683
GAAGCGGC -1.33160011391
GAAGCGTA -0.367193501288
GAAGCGTC -0.883567559483
GAAGCTAA -0.959222147918
GAAGCTAC -1.03864392529
GAAGCTCA -0.847345421669
GAAGCTCC -1.2056971873
GAAGCTGA -0.242810690376
GAAGCTGC -0.969424534926
GAAGCTTA 0.0285426598844
GAAGCTTC 0.947685339983
GAAGGAAA 0.0786704629983
GAAGGAAC -0.983953276955
GAAGGACA -0.615480313731
GAAGGACC -1.20976186259
GAAGGAGA -0.681843502048
GAAGGAGC -1.53003495907
GAAGGATA 1.47794820106
GAAGGATC 2.2121293156
GAAGGCAA -0.955656267101
GAAGGCAC -1.23920614135
GAAGGCCA -1.42417998941
GAAGGCCC -1.45286171219
GAAGGCGA 0.253354281113
GAAGGCGC -1.03591325457
GAAGGCTA -0.657918438194
GAAGGCTC -0.991236175811
GAAGGGAA -0.086244394686
GAAGGGAC -0.997534392766
GAAGGGCA -1.01350622463
GAAGGGCC -0.887753394358
GAAGGGGA 0.168623964959
GAAGGGGC -2.00444470592
GAAGGGTA -0.0500293349239
GAAGGGTC -1.1768435095
GAAGGTAA 0.337686352769
GAAGGTAC -0.959309166318
GAAGGTCA -0.487059890674
GAAGGTCC -1.00483665218
GAAGGTGA 0.865683196408
GAAGGTGC -1.16284832766
GAAGGTTA -0.109875927572
GAAGTAAA 0.788027809297
GAAGTAAC -0.267309492775
GAAGTACA -0.344963006285
GAAGTACC -0.964668500532
GAAGTAGA -0.0634060199475
GAAGTAGC -0.0267273477983
GAAGTATA 0.802765978385
GAAGTATC 1.51552141404
GAAGTCAA -0.501731859161
GAAGTCAC -0.982285771243
GAAGTCCA -0.746123453204

GAAGTCCC -0.638641780713
GAAGTCGA 0.00760078664866
GAAGTCGC -0.97665372386
GAAGTCTA 0.211698281364
GAAGTCTC -0.801374933046
GAAGTGAA 0.48558432507
GAAGTGAC 0.0687770121289
GAAGTGCA 0.460930430067
GAAGTGCC -0.531451349231
GAAGTGGA -0.0489445194101
GAAGTGGC -1.32123451535
GAAGTGTA 0.785984750488
GAAGTGTC 0.502365969323
GAAGTTAA -0.00150575141272
GAAGTTAC -0.339513322822
GAAGTTCA -0.69448344516
GAAGTTCC -0.975303481407
GAAGTTGA 0.733012819654
GAAGTTGC -0.41037794385
GAAGTTTA -0.154294449269
GAATAAAA 1.15147910301
GAATAAAC 0.5164875152
GAATAACA 0.117853724564
GAATAACC 0.744395784053
GAATAAGA 0.453232216118
GAATAAGC -0.0652277855514
GAATAATA 3.0886535891
GAATAATC 3.84363688944
GAATACAA 0.950974968605
GAATACAC 0.621667155461
GAATACCA 0.472055670112
GAATACCC 0.771965420005
GAATACGA 1.00243989896
GAATACGC 2.17454298741
GAATACTA 1.59235553897
GAATACTC 0.650610349801
GAATAGAA 0.606030490162
GAATAGAC 0.189980320576
GAATAGCA 0.24722572922
GAATAGCC 1.19791382855
GAATAGGA 0.108981178565
GAATAGGC -1.03304102282
GAATAGTA 0.31833787388
GAATAGTC -0.550942638222
GAATATAA 1.9139601419
GAATATAC 3.08916820509
GAATATCA 4.08891178271
GAATATCC 11.8089432013
GAATATGA 1.49323741729
GAATATGC 1.49915466852
GAATATTA 2.28362804679
GAATATTC 3.57052485151
GAATCAAA 3.92773996531
GAATCAAC 3.34172787081

GAATCACA 5.44373956419
GAATCACC 2.35508097882
GAATCAGA 2.22738751331
GAATCAGC 2.27895486724
GAATCATA 3.32290691495
GAATCATC 2.58966135231
GAATCCAA 9.0287839979
GAATCCAC 5.28829930326
GAATCCCA 5.21568494621
GAATCCCC 3.41448212347
GAATCCGA 12.3296300829
GAATCCGC 9.22463410513
GAATCCTA 6.38467743912
GAATCCTC 6.64529130315
GAATCGAA 6.05337215814
GAATCGAC 2.55460709302
GAATCGCA 2.81325429957
GAATCGCC 3.44824526285
GAATCGGA 5.65337938158
GAATCGGC 1.0222372096
GAATCGTA 4.85184560873
GAATCGTC 4.42674677456
GAATCTAA 9.19966273869
GAATCTAC 10.7732293812
GAATCTCA 12.7375669316
GAATCTCC 13.4125174522
GAATCTGA 9.81703955392
GAATCTGC 7.66112162688
GAATCTTA 7.14262727584
GAATGAAA 0.158320819803
GAATGAAC -0.503961653628
GAATGACA -0.479249885144
GAATGACC 0.539699985789
GAATGAGA -0.0283944371544
GAATGAGC -0.680115832897
GAATGATA 0.844448624917
GAATGATC 0.796335568405
GAATGCAA 1.04732473936
GAATGCAC -0.938241969931
GAATGCCA -0.891776225867
GAATGCCC -0.660798997063
GAATGCGA -0.108036050483
GAATGCGC -0.479915846538
GAATGCTA 1.19140826632
GAATGCTC -0.524696389762
GAATGGAA -0.0741044951101
GAATGGAC -0.987937387357
GAATGGCA 0.251564366742
GAATGGCC -0.546854022468
GAATGGGA -0.327379044052
GAATGGGC -1.06993016292
GAATGGTA 0.812728960704
GAATGGTC -0.199827139575
GAATGTAA 3.3317638476

GAATGTAC 0.414403273495
GAATGTCA -0.50740262765
GAATGTCC -0.930581852555
GAATGTGA 0.802475361909
GAATGTGC -0.138725233638
GAATGTTA 1.81020714541
GAATTAAA 0.977096726626
GAATTAAC 0.102582619817
GAATTACA 1.13910229688
GAATTACC 1.48306084435
GAATTAGA 0.39544512834
GAATTAGC 0.448009030311
GAATTATA 1.53333916016
GAATTATC 0.787856686988
GAATTCAA 0.471669708116
GAATTCAC 1.29589509022
GAATTCCA 0.503254056635
GAATTCCC 1.68598546427
GAATTCGA 1.25708863082
GAATTCGC 3.03328365598
GAATTCTA 2.34477720913
GAATTCTC 1.41332309079
GAATTGAA 0.494040931442
GAATTGAC -0.199082695078
GAATTGCA 1.90598692482
GAATTGCC 1.30351919281
GAATTGGA 0.514050999987
GAATTGGC -0.437400279863
GAATTGTA 1.17271804624
GAATTGTC 1.08172989966
GAATTTAA 1.50100932625
GAATTTAC 1.80953389779
GAATTTCA 1.03873115187
GAATTTCC 3.73862649789
GAATTTGA 3.00367366753
GAATTTGC 3.00812243121
GAATTTTA 3.19432140675
GACAAAAA 1.64838622904
GACAAAAC 1.05159093094
GACAAACA 0.803724429858
GACAAACC 1.22369646463
GACAAAGA 1.2890753874
GACAAAGC -0.630777232549
GACAAATA 1.10714177073
GACAAATC 3.78119244364
GACAACAA 0.214686468255
GACAACAC 0.527106258192
GACAACCA 0.35220343683
GACAACCC -0.169636750888
GACAACGA 0.800568451506
GACAACGC -0.00292823162883
GACAACTA 0.4535823715
GACAACTC -0.0573711481511
GACAAGAA 1.29322541566

GACAAGAC -0.305507447906
GACAAGCA 0.00351570992092
GACAAGCC 0.0787803809778
GACAAGGA -0.881776187865
GACAAGGC -0.433705953225
GACAAGTA 1.07725032564
GACAAGTC -0.848868660033
GACAATAA 0.713442839382
GACAATAC -0.0499308667339
GACAATCA 0.873225901397
GACAATCC 6.52497191845
GACAATGA -0.149687886672
GACAATGC 1.18575269482
GACAATTA 2.23440373616
GACACAAA 2.14766595964
GACACAAC 0.193484788886
GACACACA -0.506162303176
GACACACC 0.10618513996
GACACAGA 0.525196641476
GACACAGC -0.05621971568
GACACATA 0.882426952267
GACACATC 0.246381151109
GACACCAA -0.489368376422
GACACCAC -0.923363488877
GACACCCA 0.263120327052
GACACCCC -0.252325881835
GACACCGA 1.26909342288
GACACCGC 0.0628023037721
GACACCTA -0.0867902373798
GACACCTC -0.337136762871
GACACGAA 0.565786978086
GACACGAC -0.706119762174
GACACGCA -0.324014263175
GACACGCC 0.0754541130292
GACACGGA -0.166521783601
GACACGGC -1.01590276967
GACACGTA 0.215968428327
GACACGTC -1.41639871244
GACACTAA -0.17950917578
GACACTAC -0.651389560024
GACACTCA -0.905632552983
GACACTCC 0.0900007583652
GACACTGA 0.296967778516
GACACTGC -0.256863745694
GACACTTA 1.31580731511
GACAGAAA 0.373154053541
GACAGAAC -0.0209014867054
GACAGACA 0.280743843102
GACAGACC -0.320806032148
GACAGAGA 2.8203454665
GACAGAGC -1.39587673421
GACAGATA 2.61720892139
GACAGATC 0.921266511968
GACAGCAA 1.27005687063

GACAGCAC -0.906505235123
GACAGCCA -1.21900683901
GACAGCCC -0.80568838103
GACAGCGA 0.0197893998746
GACAGCGC -0.662658859239
GACAGCTA 0.435695093934
GACAGCTC 0.0562143030522
GACAGGAA -0.365317401228
GACAGGAC -0.879750824186
GACAGGCA 1.00138984917
GACAGGCC 0.474052929763
GACAGGGA -0.547028059269
GACAGGGC -1.35021247542
GACAGGTA 0.180279642527
GACAGGTC -1.23308071213
GACAGTAA 0.585155650239
GACAGTAC -0.74899193775
GACAGTCA -0.647728958221
GACAGTCC -0.371687855947
GACAGTGA 0.442376982928
GACAGTGC 1.15029727655
GACAGTTA 0.083025130225
GACATAAA -0.574161145973
GACATAAC 0.117031629675
GACATACA -0.412155159364
GACATACC 0.0437148800849
GACATAGA 0.876967692617
GACATAGC -0.18000255762
GACATATA 0.369199296236
GACATATC 1.7892300901
GACATCAA 0.954404076482
GACATCAC -0.443200951419
GACATCCA 1.04855881849
GACATCCC 0.426911023582
GACATCGA -0.181121930681
GACATCGC -0.305022393186
GACATCTA 2.0441044869
GACATCTC 2.09415151696
GACATGAA 0.354029574172
GACATGAC -0.580360062027
GACATGCA 0.408345085763
GACATGCC -0.0626186907835
GACATGGA 0.274827008227
GACATGGC -1.16959724997
GACATGTA 1.45113216944
GACATGTC -0.0205300971685
GACATTAA 0.0918560406256
GACATTAC 1.37880822069
GACATTCA -0.10374008945
GACATTCC -0.581140104961
GACATTGA -0.366152194974
GACATTGC 0.279284515382
GACATTTA 1.48619184135
GACCAAAA -0.759368153381

GACCAAAC 0.0551176213928
GACCAACA 0.547505619582
GACCAACC 0.452588946125
GACCAAGA -0.722858521761
GACCAAGC -1.2082350852
GACCAATA 1.12672778071
GACCAATC 0.161268203807
GACCACAA -0.318494007374
GACCACAC -0.700043462958
GACCACCA -0.761702869556
GACCACCC -0.868235874941
GACCACGA -0.445672024178
GACCACGC -0.384756645721
GACCACTA 0.543434282601
GACCACTC -0.888790120756
GACCAGAA -0.182849183476
GACCAGAC -1.03336765409
GACCAGCA -0.718855467162
GACCAGCC -0.0582444548256
GACCAGGA -0.208818971563
GACCAGGC -1.31069154915
GACCAGTA -0.128432081025
GACCAGTC -0.946285134812
GACCATAA -0.7697643541
GACCATAC 0.0070593156927
GACCATCA 0.176319680772
GACCATCC -0.66538266008
GACCATGA -0.825838345186
GACCATGC 0.140189141275
GACCATTA 1.54205369906
GACCCAAA 0.396215595087
GACCCAAC -0.496657312439
GACCCACA -0.590604084634
GACCCACC 0.516064081165
GACCCAGA 0.573193118312
GACCCAGC -1.54174455474
GACCCATA -0.325585382477
GACCCATC -1.17735104744
GACCCCAA -0.489968969927
GACCCCAC -1.25239796432
GACCCCCA -1.03336765409
GACCCCCC -1.38554361143
GACCCCGA -1.26006453521
GACCCCGC -0.311074751934
GACCCCTA -0.64843905335
GACCCCTC -1.50992371603
GACCCGAA -0.287354327045
GACCCGAC -1.07173485793
GACCCGCA -0.269546365294
GACCCGCC -0.797076057521
GACCCGGA -1.04095324375
GACCCGGC -1.58002349111
GACCCGTA -0.159147494607
GACCCGTC -1.72048159835

GACCCTAA -0.805671310435
GACCCTAC -0.402969721844
GACCCTCA -1.0120319081
GACCCTCC -0.166587359668
GACCCTGA -0.811001916086
GACCCTGC -1.01523056294
GACCCTTA -0.346521010373
GACCGAAA 0.0763359550013
GACCGAAC 0.00879572832003
GACCGACA 0.1741504661
GACCGACC -1.28775241626
GACCGAGA -0.381862555283
GACCGAGC -1.50996805794
GACCGATA 1.53511366936
GACCGATC -0.646775711198
GACCGCAA -1.11923795293
GACCGCAC -0.714546807271
GACCGCCA -0.983653084291
GACCGCCC -1.46163891267
GACCGCGA -0.524581059155
GACCGCGC -1.09747315209
GACCGCTA -0.580441459621
GACCGCTC -0.863278740606
GACCGGAA -0.441531363927
GACCGGAC -1.16447190782
GACCGGCA -0.770821273763
GACCGGCC -0.934162930365
GACCGGGA 0.501002403478
GACCGGGC -1.59770612971
GACCGGTA 0.949313083699
GACCGGTC -0.940434500538
GACCGTAA -0.276311317307
GACCGTAC -0.991178510507
GACCGTCA -1.01404998555
GACCGTCC -0.321800706591
GACCGTGA 0.342769018432
GACCGTGC -0.739746753146
GACCGTTA -0.421139496944
GACCTAAA -0.145625917701
GACCTAAC -0.510114354167
GACCTACA 0.268352256335
GACCTACC -0.38469460868
GACCTAGA 0.115701164131
GACCTAGC -0.954508998189
GACCTATA 0.0467315873601
GACCTATC -0.942952621524
GACCTCAA -1.5239686524
GACCTCAC -0.945058966442
GACCTCCA -1.19821589482
GACCTCCC -1.05080006275
GACCTCGA -0.589107493053
GACCTCGC -0.987624287658
GACCTCTA -0.387878274704
GACCTCTC -1.07465101524

GACCTGAA -1.37126135214
GACCTGAC -0.699422676187
GACCTGCA -0.557807099314
GACCTGCC -0.187671210294
GACCTGGA -0.150189179276
GACCTGGC -1.3066104278
GACCTGTA 0.451065915939
GACCTTAA 0.139831283307
GACCTTAC -0.893706452204
GACCTTCA -0.468318458808
GACCTTCC -0.875949702239
GACCTTGA -0.518805368958
GACCTTGC 0.214522423997
GACCTTTA -0.223220933195
GACGAAAA 0.568897573635
GACGAAAC 0.126543282108
GACGAACA -0.462758857367
GACGAACC -0.0809208670867
GACGAAGA -0.422767448838
GACGAAGC -0.911175083829
GACGAATA 0.958016380991
GACGAATC 2.21870066208
GACGACAA -0.180788845894
GACGACAC 0.0393304434053
GACGACCA -1.35403732966
GACGACCC -0.919482634759
GACGACGA 0.432878029353
GACGACGC -1.35066630344
GACGACTA -0.314737227339
GACGACTC -0.758272096256
GACGAGAA -0.00370848274107
GACGAGAC -0.128111486918
GACGAGCA -0.479708084903
GACGAGCC -0.988062085974
GACGAGGA -0.444715654485
GACGAGGC -0.406563914868
GACGAGTA 1.59191586706
GACGAGTC -1.26968839558
GACGATAA 0.520529915439
GACGATAC 0.918042459414
GACGATCA 0.326874836957
GACGATCC 2.30538223055
GACGATGA 0.580881547895
GACGATGC 0.222440265727
GACGATTA 2.85611002909
GACGCAAA 0.322980243091
GACGCAAC -0.153273128042
GACGCACA -1.13285008726
GACGCACC -0.854470521607
GACGCAGA 0.335245673996
GACGCAGC -1.40600147083
GACGCATA 0.188855743066
GACGCATC -0.115943691491
GACGCCAA -0.95616318051

GACGCCAC −0.246456927898
GACGCCCA −1.14944728581
GACGCCCC −1.80771109129
GACGCCGA −0.0271158079304
GACGCCGC −0.240053789234
GACGCCTA −0.918630978596
GACGCCTC −1.15312953813
GACGCGAA 0.337919928475
GACGCGAC −0.406945921482
GACGCGCA −0.833797614336
GACGCGCC −0.720092668966
GACGCGGA −0.491972058562
GACGCGGC −1.28866090502
GACGCGTA −0.108934338517
GACGCGTC −0.329818057401
GACGCTAA 0.258059103722
GACGCTAC −0.998427268172
GACGCTCA −1.61478963205
GACGCTCC −0.955672504984
GACGCTGA 0.924827396513
GACGCTGC −0.0880010005785
GACGCTTA 0.0284348236848
GACGGAAA 0.984744145145
GACGGAAC 0.444996694774
GACGGACA −1.18735816349
GACGGACC −0.973666786038
GACGGAGA 0.803219598229
GACGGAGC −1.11886448161
GACGGATA 0.266992021338
GACGGATC 0.195701468139
GACGGCAA −0.798970477244
GACGGCAC −0.817889901292
GACGGCCA −0.504430886821
GACGGCCC −1.27770220737
GACGGCGA −0.622567525275
GACGGCGC −0.54067529968
GACGGCTA −1.14456925911
GACGGCTC −0.386395839226
GACGGGAA −0.0989744788679
GACGGGAC −0.465582583644
GACGGGCA −0.885846900312
GACGGGCC −1.40410892471
GACGGGGA −0.453430817922
GACGGGGC −1.2545888295
GACGGGTA −0.0896331160321
GACGGTAA −0.190335264052
GACGGTAC −0.564552482596
GACGGTCA −1.24631417069
GACGGTCC −1.09590786177
GACGGTGA 0.27845346884
GACGGTGC −0.678552624358
GACGGTTA −0.336214534369
GACGTAAA 0.107443992275
GACGTAAC 1.19754389626

GACGTACA -0.269032165655
GACGTACC 0.319592770813
GACGTAGA 1.06296348644
GACGTAGC -0.734465693857
GACGTATA 0.671058216663
GACGTATC 2.37393066325
GACGTCAA -0.414529637535
GACGTCAC -0.016094240525
GACGTCCA -0.135888600326
GACGTCCC -1.00006958435
GACGTCGA 0.289914291807
GACGTCGC -0.801674501175
GACGTCTA 0.485200444853
GACGTCTC -1.11265390759
GACGTGAA 0.782944935456
GACGTGAC 0.0672360786356
GACGTGCA -0.340341663051
GACGTGCC -0.141252306277
GACGTGGA 0.634383708074
GACGTGGC -0.780841296851
GACGTGTA 0.293160827585
GACGTTAA 0.286788499264
GACGTTAC 0.388075211084
GACGTTCA -0.942952621524
GACGTTCC -1.06695675667
GACGTTGA 0.482914442328
GACGTTGC 0.257535327895
GACGTTTA 0.522625851458
GACTAAAA 1.7153042117
GACTAAAC 0.380200254021
GACTAACA -0.0902878358155
GACTAACC -0.289726723437
GACTAAGA -0.169122551249
GACTAAGC -0.324859882177
GACTAATA 0.8023373399
GACTAATC 0.429784296221
GACTACAA 0.564542073696
GACTACAC -0.252315264757
GACTACCA -0.949503566561
GACTACCC -0.00280998652964
GACTACGA 0.593978025342
GACTACGC 0.500961184236
GACTACTA 0.874177274819
GACTACTC -1.16765036939
GACTAGAA 1.2017480508
GACTAGAC 0.106371251085
GACTAGCA -0.602492713374
GACTAGCC -0.633569940305
GACTAGGA -0.194738853107
GACTAGGC -0.628193327318
GACTAGTA -0.106515934789
GACTAGTC -1.42109000348
GACTATAA 0.863499409277
GACTATAC 0.765657418683

GACTATCA -0.892868743966
GACTATCC 0.884895943247
GACTATGA -0.701530686529
GACTATGC 1.06426626431
GACTATTA 1.24764317899
GACTCAAA 0.525594885973
GACTCAAC -0.88191358534
GACTCACA -0.658932889547
GACTCACC -0.462180747084
GACTCAGA 0.458089841373
GACTCAGC -1.13783220295
GACTCATA 0.612406357508
GACTCATC -0.0712260180212
GACTCCAA 0.187779671028
GACTCCAC -1.23524409782
GACTCCCA -1.45966621802
GACTCCCC -1.0033787817
GACTCCGA 0.057373021753
GACTCCGC 0.102118382896
GACTCCTA -1.07440265889
GACTCCTC -1.25986322709
GACTCGAA 0.729680098188
GACTCGAC -0.313850805451
GACTCGCA -1.11115856508
GACTCGCC -0.441392301028
GACTCGGA -0.636922022325
GACTCGGC -1.32009869623
GACTCGTA 0.281714785255
GACTCTAA -0.122665342482
GACTCTAC 0.0710934086406
GACTCTCA -0.870390517153
GACTCTCC -0.851338067368
GACTCTGA -0.395219879753
GACTCTGC 0.949813335413
GACTCTTA -0.166447464058
GACTGAAA 1.90752640107
GACTGAAC 0.30108949457
GACTGACA -0.726280343408
GACTGACC -0.669107588883
GACTGAGA -0.369886908142
GACTGAGC -0.706180758326
GACTGATA 1.00966534069
GACTGATC -0.6448952394
GACTGCAA -0.599180809707
GACTGCAC -1.36158190825
GACTGCCA -0.991122094271
GACTGCCC -0.507698656754
GACTGCGA 0.0433859588583
GACTGCGC -0.669707766032
GACTGCTA -0.403443951309
GACTGCTC -0.750702536306
GACTGGAA 2.23933984452
GACTGGAC -0.367434571402
GACTGGCA -0.79075244285

GACTGGCC -0.637064207893
GACTGGGA 0.0984044875271
GACTGGGC -1.58628257061
GACTGGTA 0.174476889191
GACTGTAA 0.340016697206
GACTGTAC -0.249457605468
GACTGTCA -1.43949377064
GACTGTCC -0.158855212707
GACTGTGA -0.444584085994
GACTGTGC -0.72480207149
GACTGTTA 0.615770513851
GACTTAAA -0.0954421147077
GACTTAAC 0.45580363067
GACTTACA -0.324197667986
GACTTACC 0.0127835859257
GACTTAGA -0.12457371013
GACTTAGC -0.721485379729
GACTTATA 0.478910555017
GACTTATC 0.938771158386
GACTTCAA -0.641925996707
GACTTCAC -0.623580311204
GACTTCCA -0.947385563675
GACTTCCC -0.423133425747
GACTTCGA 0.13469823857
GACTTCGC -0.338444745192
GACTTCTA 0.108103916508
GACTTCTC -1.0178690108
GACTTGAA 0.595707568096
GACTTGAC -0.686157574567
GACTTGCA -0.0475642993262
GACTTGCC -0.943516159347
GACTTGGA 0.151300433394
GACTTGGC -0.985805644724
GACTTGTA 0.873677231283
GACTTTAA 0.574127837494
GACTTTAC 0.921699522191
GACTTTCA -0.303778321509
GACTTTCC 0.0297332298179
GACTTTGA 0.513417306181
GACTTTGC 0.610506525158
GACTTTTA 0.314246759991
GAGAAAAA 0.92898304558
GAGAAAAC -0.0798152337737
GAGAAACA 0.263008951826
GAGAAACC 0.239720912626
GAGAAAGA 0.574976787344
GAGAAAGC -0.189420529957
GAGAAATA 3.12842745163
GAGAAATC 10.1630217997
GAGAACAA 0.246237508295
GAGAACAC 0.133103803333
GAGAACCA -0.619348052636
GAGAACCC -0.171743512162
GAGAACGA -0.548101841349

GAGAACGC -0.176414609936
GAGAACTA -0.714007001739
GAGAACTC -0.886642556595
GAGAAGAA -0.0011941089594
GAGAAGAC -1.18321084155
GAGAAGCA -0.0779214385848
GAGAAGCC -1.25339222241
GAGAAGGA -0.801280836593
GAGAAGGC -1.65208680102
GAGAAGTA 1.50591878782
GAGAATAA 1.43847661298
GAGAATAC 1.01106096594
GAGAATCA 6.52100737678
GAGAATCC 20.7948325404
GAGAATGA -0.400530083961
GAGAATGC -0.61566392672
GAGAATTA 0.90643195647
GAGACAAA -0.418297034648
GAGACAAC -0.385881223231
GAGACACA 0.418087607589
GAGACACC 0.0300246790061
GAGACAGA 0.646937257319
GAGACAGC -1.3977440908
GAGACATA 0.506927357295
GAGACATC 0.14286901656
GAGACCAA 0.235094781298
GAGACCAC -1.06636761295
GAGACCCA -1.09642476773
GAGACCCC -1.49014097785
GAGACCGA -0.272739399328
GAGACCGC -0.419005672531
GAGACCTA -0.250049455497
GAGACCTC -1.25700619234
GAGACGAA -1.06634679515
GAGACGAC -1.36547233855
GAGACGCA -1.00349598591
GAGACGCC -1.41273623704
GAGACGGA -1.43421437678
GAGACGGC -1.50231147959
GAGACGTA 0.988224464807
GAGACTAA -0.134277302671
GAGACTAC -0.591547339113
GAGACTCA -0.0761760743038
GAGACTCC -0.406970902841
GAGACTGA 0.164147513608
GAGACTGC -0.981294427647
GAGACTTA -0.445054984611
GAGAGAAA 0.777791905631
GAGAGAAC -0.726482067882
GAGAGACA -1.66912804322
GAGAGACC -0.852417262076
GAGAGAGA -0.384472690941
GAGAGAGC -1.50909017135
GAGAGATA 2.19180031837

GAGAGATC 2.62355023119
GAGAGCAA -0.332951968886
GAGAGCAC -1.57737671613
GAGAGCCA -1.17531465033
GAGAGCCC -0.897168035847
GAGAGCGA -0.845751402788
GAGAGCGC -1.14796214402
GAGAGCTA 0.629062262254
GAGAGCTC -0.93878115093
GAGAGGAA -0.495824184117
GAGAGGAC -0.794097446818
GAGAGGCA -1.30649051728
GAGAGGCC -1.24411872559
GAGAGGGA -0.863679275062
GAGAGGGC -1.93912469751
GAGAGGTA -0.285725958796
GAGAGTAA 0.285408903714
GAGAGTAC -0.135719143441
GAGAGTCA -0.851338067368
GAGAGTCC -1.54795367152
GAGAGTGA -0.412027338077
GAGAGTGC -1.31199432702
GAGAGTTA -0.729034330058
GAGATAAA -0.0390841688414
GAGATAAC -0.28847016108
GAGATACA 0.715917034812
GAGATACC 4.8201752827
GAGATAGA -0.0449279332415
GAGATAGC 0.936406048224
GAGATATA 3.4413841326
GAGATATC 16.8330608861
GAGATCAA 1.1646376175
GAGATCAC 2.00260815967
GAGATCCA 4.85049786441
GAGATCCC 7.47429728466
GAGATCGA 1.01168591627
GAGATCGC 8.22917816143
GAGATCTA 8.29841274878
GAGATCTC 15.6187611596
GAGATGAA -0.549783086809
GAGATGAC -1.07456691133
GAGATGCA -0.210257273307
GAGATGCC -0.477264075282
GAGATGGA -0.0965798074314
GAGATGGC -0.326613573577
GAGATGTA 1.46208025001
GAGATTAA 1.01924256919
GAGATTAC 14.7662926858
GAGATTCA 2.05957960608
GAGATTCC 17.4045796288
GAGATTGA 1.03750831434
GAGATTGC 16.4650005316
GAGATTTA 4.51394337538
GAGCAAAA 0.641148035553

GAGCAAAC -0.869899425271
GAGCAACA -0.439403993031
GAGCAACC -0.603185321552
GAGCAAGA -0.734516905643
GAGCAAGC -1.02059801609
GAGCAATA 0.768877932212
GAGCAATC 4.11083896238
GAGCACAA 1.02833973924
GAGCACAC -0.849310830087
GAGCACCA -1.02737379336
GAGCACCC -0.608828610546
GAGCACGA -0.420298249681
GAGCACGC -0.325754631184
GAGCACTA -0.526619954404
GAGCACTC -0.549590313989
GAGCAGAA -0.991561974367
GAGCAGAC -0.687481170237
GAGCAGCA -0.882844973674
GAGCAGCC -1.70211467869
GAGCAGGA -0.0732692850081
GAGCAGGC -1.55079217843
GAGCAGTA 1.09113496496
GAGCATAA 0.00430303908488
GAGCATAC -0.931912109921
GAGCATCA 0.034693486822
GAGCATCC -0.389134628883
GAGCATGA -0.525390663364
GAGCATGC -0.967352539377
GAGCATTA 0.240603379132
GAGCCAAA -0.320899295888
GAGCCAAC -1.06508627742
GAGCCACA -0.821630651621
GAGCCACC -0.933888551773
GAGCCAGA -0.306326420125
GAGCCAGC -1.50176501236
GAGCCATA 0.388557767668
GAGCCATC -0.600775036766
GAGCCCAA -0.803052847657
GAGCCCAC -1.31731160928
GAGCCCCA -1.3963838558
GAGCCCCC -1.79468830886
GAGCCCGA -0.388340429845
GAGCCCGC -1.54423498805
GAGCCCTA -1.11364483483
GAGCCCTC -1.64643497672
GAGCCGAA -0.794016465579
GAGCCGAC -1.13804537722
GAGCCGCA -1.52127732733
GAGCCGCC -1.87857675321
GAGCCGGA -0.358700671946
GAGCCGGC -1.39350558689
GAGCCGTA 0.41617819905
GAGCCTAA -0.311891434195
GAGCCTAC -0.45740909934

GAGCCTCA -0.845812607117
GAGCCTCC -1.49117291615
GAGCCTGA -0.885019600973
GAGCCTGC -1.67046725224
GAGCCTTA -0.991151655546
GAGCGAAA -7.16132290855E-5
GAGCGAAC -0.495445924706
GAGCGACA -0.521628470701
GAGCGACC -1.33452314109
GAGCGAGA -0.390192381258
GAGCGAGC -0.654832615826
GAGCGATA 0.708219861753
GAGCGATC 3.79183429439
GAGCGCAA -0.0703633284244
GAGCGCAC -0.392234190999
GAGCGCCA -1.4146889466
GAGCGCCC -0.307765346403
GAGCGCGA -0.0498205323984
GAGCGCGC 0.226335692305
GAGCGCTA 0.590667162565
GAGCGCTC -0.359877293954
GAGCGGAA -0.973999454468
GAGCGGAC -1.47606981104
GAGCGGCA -0.661991024242
GAGCGGCC -1.11116855762
GAGCGGGA -0.408768519798
GAGCGGGC -1.39165030463
GAGCGGTA 0.796240431063
GAGCGTAA 0.00648890799591
GAGCGTAC 0.386431021307
GAGCGTCA -1.2976262984
GAGCGTCC -1.26972711669
GAGCGTGA 0.278920620252
GAGCGTGC -1.10680077518
GAGCGTTA 0.109469564133
GAGCTAAA -0.687934165546
GAGCTAAC -0.733595926208
GAGCTACA -0.384453954922
GAGCTACC -1.09754434896
GAGCTAGA -0.464246080938
GAGCTAGC -0.535415682724
GAGCTATA 0.00881966878906
GAGCTATC -0.221270721771
GAGCTCAA -1.23701048807
GAGCTCAC -0.676429833379
GAGCTCCA -0.864203467245
GAGCTCCC -0.331654603642
GAGCTCGA -0.517918322536
GAGCTCGC -1.12544602881
GAGCTCTA -0.194941202114
GAGCTGAA -0.709504319961
GAGCTGAC -1.16785313475
GAGCTGCA -0.393043795208
GAGCTGCC -0.842631647407

GAGCTGGA −0.0348868841762
GAGCTGGC −0.986578401428
GAGCTGTA 0.752066934862
GAGCTTAA 0.431555890929
GAGCTTAC −0.68996223554
GAGCTTCA −0.510239677318
GAGCTTCC −0.99031373913
GAGCTTGA −0.310672343876
GAGCTTGC −0.620536748967
GAGCTTTA −0.97474972795
GAGGAAAA 0.212249536686
GAGGAAAC −1.31284910585
GAGGAACA 0.185822173373
GAGGAACC −1.18197613788
GAGGAAGA −0.367425619749
GAGGAAGC −1.1622350353
GAGGAATA 2.19358315469
GAGGAATC 6.96127385123
GAGGACAA −0.442099689843
GAGGACAC 0.124390721676
GAGGACCA −1.38077633542
GAGGACCC −1.53394121091
GAGGACGA −0.7575792799
GAGGACGC −1.1574421534
GAGGACTA −0.509766280566
GAGGACTC −1.07336364254
GAGGAGAA −0.310778098296
GAGGAGAC −0.692381263801
GAGGAGCA −0.947904967764
GAGGAGCC −0.901947386177
GAGGAGGA −0.35358303238
GAGGAGGC −0.919058992546
GAGGAGTA 0.372878009525
GAGGATAA 1.96662542655
GAGGATAC 3.63133364284
GAGGATCA 1.4995843479
GAGGATCC 4.3776598614
GAGGATGA −0.595858288962
GAGGATGC −0.408138781374
GAGGATTA 0.182570433146
GAGGCAAA 0.120084559921
GAGGCAAC −1.00307463365
GAGGCACA −1.14009426501
GAGGCACC −1.26823302324
GAGGCAGA 0.00584043355231
GAGGCAGC −0.486595861931
GAGGCATA −0.150157119865
GAGGCATC −0.158543778432
GAGGCCAA −0.61103696268
GAGGCCAC −1.16920504263
GAGGCCCA −1.4395641348
GAGGCCCC −1.6480791665
GAGGCCGA −0.716307784902
GAGGCCGC −1.1804624757

GAGGCCTA −0.831351939292
GAGGCCTC −0.757627577194
GAGGCGAA 1.08931049304
GAGGCGAC −0.779166088554
GAGGCGCA −0.221141026882
GAGGCGCC −0.582126868641
GAGGCGGA −0.357347098645
GAGGCGGC −1.03637749149
GAGGCGTA −0.48499559771
GAGGCTAA 0.0807865922821
GAGGCTAC −0.213357043601
GAGGCTCA −1.40119984546
GAGGCTCC −1.58948851168
GAGGCTGA −1.15165459706
GAGGCTGC −1.20831211105
GAGGCTTA −1.22780298369
GAGGGAAA 1.01766811904
GAGGGAAC −1.53429011722
GAGGGACA −1.36275977932
GAGGGACC −0.823680372126
GAGGGAGA −0.619909300501
GAGGGAGC −1.76826906449
GAGGGATA 1.2177375778
GAGGGATC 2.02762657434
GAGGGCAA 0.219607379618
GAGGGCAC −1.70181511056
GAGGGCCA −1.52110995222
GAGGGCCC −1.09145451818
GAGGGCGA −0.44718818449
GAGGGCGC −1.28306528878
GAGGGCTA −1.60327614005
GAGGGGAA −0.389437319683
GAGGGGAC −1.83895527697
GAGGGGCA −0.716315071131
GAGGGGCC −0.921193857849
GAGGGGGA −0.889005584977
GAGGGGGC −1.32335022828
GAGGGGTA 0.127882699305
GAGGGTAA 0.561088192639
GAGGGTAC −0.837020001467
GAGGGTCA −1.48825030533
GAGGGTCC −1.47174678687
GAGGGTGA −0.363821850537
GAGGGTGC −0.865124030323
GAGGGTTA −1.13002719369
GAGGTAAA 0.418041183897
GAGGTAAC −0.195906939817
GAGGTACA −1.11418776303
GAGGTACC −0.962273620917
GAGGTAGA −0.61005540345
GAGGTAGC −0.7483378425
GAGGTATA −0.0723308186223
GAGGTATC 1.33271740519
GAGGTCAA −0.386901087212

GAGGTCAC −1.23349706811
GAGGTCCA −1.15587457312
GAGGTCCC −1.73116383563
GAGGTCGA −0.614899497135
GAGGTCGC −1.12238914319
GAGGTCTA −0.619167145961
GAGGTGAA −0.474500928801
GAGGTGAC −1.35054889105
GAGGTGCA −0.0806477375618
GAGGTGCC −1.16037996121
GAGGTGGA 0.703651395729
GAGGTGGC −1.42705763379
GAGGTGTA 0.593402205018
GAGGTTAA 0.347189261726
GAGGTTAC 0.572263187224
GAGGTTCA −1.12756257445
GAGGTTCC −0.962115613822
GAGGTTGA −0.705262901561
GAGGTTGC −0.334111936654
GAGGTTTA −0.999811443638
GAGTAAAA 1.01759400767
GAGTAAAC −0.374695819747
GAGTAACA −0.320806032148
GAGTAACC 0.166669798153
GAGTAAGA 1.15268029002
GAGTAAGC −0.570591934308
GAGTAATA 2.00742165119
GAGTAATC 7.30126660797
GAGTACAA 1.02585492673
GAGTACAC −0.563934193961
GAGTACCA −0.768113086271
GAGTACCC −0.278537989104
GAGTACGA −0.30905604995
GAGTACGC 0.00256808370348
GAGTACTA 0.238267830245
GAGTACTC −0.630723522627
GAGTAGAA 0.105694048078
GAGTAGAC 0.740325279784
GAGTAGCA −0.549013244596
GAGTAGCC −0.760079497578
GAGTAGGA −0.000352445339656
GAGTAGGC −0.777142806654
GAGTAGTA −0.39339603237
GAGTATAA 1.2135415422
GAGTATAC 1.46057054322
GAGTATCA 2.17212208555
GAGTATCC 4.0677407055
GAGTATGA 0.538827095471
GAGTATGC 0.206767377457
GAGTATTA 1.80364204427
GAGTCAAA 1.12326515617
GAGTCAAC −1.04959346311
GAGTCACA −1.41509031376
GAGTCACC 0.445650373667

GAGTCAGA 0.907200965971
GAGTCAGC -0.67291287439
GAGTCATA 0.94019592856
GAGTCATC -0.593152807784
GAGTCCAA -0.128637136347
GAGTCCAC -1.61853142327
GAGTCCCA -0.615514871278
GAGTCCCC -1.27932453845
GAGTCCGA -0.0177854785282
GAGTCCGC -0.736060753628
GAGTCCTA -1.07642427537
GAGTCGAA 0.405539679149
GAGTCGAC -0.504680492233
GAGTCGCA -0.906608491407
GAGTCGCC -0.933140151893
GAGTCGGA -0.0773118934256
GAGTCGGC -0.729589332583
GAGTCGTA 0.607957802007
GAGTCTAA 0.389371951793
GAGTCTAC -0.703382429764
GAGTCTCA -0.115987617048
GAGTCTCC -0.852417262076
GAGTCTGA 0.269926290129
GAGTCTGC -1.11763227608
GAGTCTTA -0.40075408348
GAGTGAAA 0.54021814081
GAGTGAAC -1.10223584818
GAGTGACA -0.834263724859
GAGTGACC -0.419151188947
GAGTGAGA 1.06898815751
GAGTGAGC -0.517097060359
GAGTGATA 1.60011391636
GAGTGATC 0.496520955855
GAGTGCAA 1.32593767254
GAGTGCAC -0.483070784
GAGTGCCA -1.28126330009
GAGTGCCC -1.22538520449
GAGTGCGA -0.191645744508
GAGTGCGC 0.00436778244024
GAGTGCTA 0.160941572538
GAGTGGAA 0.987767930472
GAGTGGAC -1.0809727563
GAGTGGCA -0.549867190718
GAGTGGCC -0.657351985879
GAGTGGGA -0.687092293749
GAGTGGGC -1.11227439911
GAGTGGTA 1.02372484952
GAGTGTAA 1.71283105716
GAGTGTAC -0.503867348998
GAGTGTCA -0.608365622693
GAGTGTCC -1.41058617474
GAGTGTGA 0.369567771281
GAGTGTGC 0.521535831495
GAGTGTTA -0.368752754445

| | |
|---|---|
| GAGTTAAA | -0.0930722164521 |
| GAGTTAAC | -0.419015248719 |
| GAGTTACA | 0.200984609208 |
| GAGTTACC | -0.0844111792926 |
| GAGTTAGA | 0.14836658096 |
| GAGTTAGC | -0.277344712857 |
| GAGTTATA | 0.730740556876 |
| GAGTTATC | 0.430067001933 |
| GAGTTCAA | 0.624386584565 |
| GAGTTCAC | -0.426259426468 |
| GAGTTCCA | -0.697451855141 |
| GAGTTCCC | -0.915454390623 |
| GAGTTCGA | 0.402840859667 |
| GAGTTCGC | -0.248101534031 |
| GAGTTCTA | -0.805831607488 |
| GAGTTGAA | -0.204456185395 |
| GAGTTGAC | -0.423344726409 |
| GAGTTGCA | -0.508988111233 |
| GAGTTGCC | -0.550818772317 |
| GAGTTGGA | -0.117414260823 |
| GAGTTGGC | -0.266532780689 |
| GAGTTGTA | 0.757065080261 |
| GAGTTTAA | 1.80455303116 |
| GAGTTTAC | 0.267547440219 |
| GAGTTTCA | -0.99198978014 |
| GAGTTTCC | -0.960866337695 |
| GAGTTTGA | 0.539653145741 |
| GAGTTTGC | -0.448773876252 |
| GAGTTTTA | 0.231314268972 |
| GATAAAAA | 1.16666214847 |
| GATAAAAC | 0.80190724417 |
| GATAAACA | 0.174625111921 |
| GATAAACC | 0.60528125757 |
| GATAAAGA | 1.84933274167 |
| GATAAAGC | -0.247232182738 |
| GATAAATA | 1.73578850971 |
| GATAAATC | 3.23549110271 |
| GATAACAA | 0.642759333207 |
| GATAACAC | 1.04281560406 |
| GATAACCA | 0.221776386112 |
| GATAACCC | -0.196009155211 |
| GATAACGA | 1.69203365945 |
| GATAACGC | 1.26946189793 |
| GATAACTA | 1.00159178182 |
| GATAAGAA | 1.47164373877 |
| GATAAGAC | -0.38782685474 |
| GATAAGCA | -0.529626460957 |
| GATAAGCC | 0.310513712247 |
| GATAAGGA | 0.88580568107 |
| GATAAGGC | 0.799159086504 |
| GATAAGTA | 1.41418598857 |
| GATAATAA | 1.30117365138 |
| GATAATAC | 1.79633582948 |
| GATAATCA | 2.2117518889 |

| | |
|---|---|
| GATAATCC | 8.13196445201 |
| GATAATGA | −0.349975932321 |
| GATAATGC | 0.954478187847 |
| GATAATTA | 2.11310112681 |
| GATACAAA | 2.54986146753 |
| GATACAAC | 1.08236484253 |
| GATACACA | 0.779753983202 |
| GATACACC | 1.32649017693 |
| GATACAGA | 1.41018772206 |
| GATACAGC | 0.440294370301 |
| GATACATA | 2.40618784303 |
| GATACATC | 0.691719674144 |
| GATACCAA | 1.483213647 |
| GATACCAC | 1.83037959279 |
| GATACCCA | 3.25646232904 |
| GATACCCC | 3.8653731699 |
| GATACCGA | 5.04161629406 |
| GATACCGC | 3.18027563766 |
| GATACCTA | 2.20956373004 |
| GATACGAA | 3.94278748689 |
| GATACGAC | 2.66173507111 |
| GATACGCA | 4.09145176238 |
| GATACGCC | 4.31554350411 |
| GATACGGA | 3.89066846129 |
| GATACGGC | 3.46096202364 |
| GATACGTA | 3.19478564367 |
| GATACTAA | 2.08859274823 |
| GATACTAC | 2.01255532047 |
| GATACTCA | 4.16331376417 |
| GATACTCC | 4.08364716948 |
| GATACTGA | 2.57808707233 |
| GATACTGC | 2.90333937002 |
| GATACTTA | 2.32992766482 |
| GATAGAAA | 0.451143774507 |
| GATAGAAC | 0.30444157659 |
| GATAGACA | −0.615485726359 |
| GATAGACC | −0.524020644001 |
| GATAGAGA | 1.76147475938 |
| GATAGAGC | −0.342531070988 |
| GATAGATA | 3.63923899389 |
| GATAGATC | 1.7668434616 |
| GATAGCAA | 1.91857586433 |
| GATAGCAC | 0.329451039602 |
| GATAGCCA | 0.394006826597 |
| GATAGCCC | 0.780923110802 |
| GATAGCGA | 1.21533104021 |
| GATAGCGC | 1.30738276815 |
| GATAGCTA | 0.512104952122 |
| GATAGGAA | −0.818232354088 |
| GATAGGAC | −1.14411543109 |
| GATAGGCA | −0.357097909589 |
| GATAGGCC | 0.0575428949941 |
| GATAGGGA | 0.53151671712 |
| GATAGGGC | −0.0140611742597 |

GATAGGTA 1.78107013736
GATAGTAA 3.06024395495
GATAGTAC 0.276315897223
GATAGTCA -0.29706999391
GATAGTCC 0.496276763071
GATAGTGA 0.996577606717
GATAGTGC 1.82124578341
GATAGTTA 0.951931962832
GATATAAA 1.72832865956
GATATAAC 2.97635551059
GATATACA 1.78102100736
GATATACC 2.22658186449
GATATAGA 3.82811722018
GATATAGC 1.29415513857
GATATATA 2.6321379897
GATATATC 5.71325387008
GATATCAA 6.15475275824
GATATCAC 7.48292418062
GATATCCA 18.576395848
GATATCCC 14.9198847425
GATATCGA 7.24780712428
GATATCGC 14.3286602875
GATATCTA 14.9544812184
GATATGAA 2.20989056948
GATATGAC 3.39766217447
GATATGCA 5.9189158224
GATATGCC 5.34555886801
GATATGGA 5.74482052347
GATATGGC 5.19840138482
GATATGTA 5.59249481346
GATATTAA 3.52050571731
GATATTAC 4.64028784751
GATATTCA 4.30831806238
GATATTCC 9.75302086615
GATATTGA 1.97429262198
GATATTGC 7.16765401763
GATATTTA 6.50630001804
GATCAAAA 0.748847878579
GATCAAAC -0.180512385522
GATCAACA 0.927518721588
GATCAACC 0.471643894045
GATCAAGA 1.20851071286
GATCAAGC 0.539141027882
GATCAATA 0.846133409402
GATCAATC -0.195881542102
GATCACAA 1.75735283514
GATCACAC 4.98657736395
GATCACCA 0.119449408869
GATCACCC 1.29359118439
GATCACGA 2.2365373524
GATCACGC 2.39645718735
GATCACTA 1.39775907961
GATCAGAA 1.36843450319
GATCAGAC 0.621446694968

GATCAGCA 0.311641620605
GATCAGCC -0.187369560384
GATCAGGA 1.73014168169
GATCAGGC 0.300509302508
GATCAGTA 0.872763746256
GATCATAA 1.12109302701
GATCATAC 0.987210846167
GATCATCA 1.51509090195
GATCATCC 1.50669341813
GATCATGA 1.42283703319
GATCATGC 0.894873498024
GATCATTA 1.2429429363
GATCCAAA 2.35707969572
GATCCAAC 2.44942433009
GATCCACA 0.945856704506
GATCCACC 1.58807269315
GATCCAGA 4.59237755636
GATCCAGC 0.61417129052
GATCCATA 1.36324774853
GATCCATC 0.680883176973
GATCCCAA 2.21973905391
GATCCCAC 3.27184231088
GATCCCCA 3.74783067143
GATCCCCC 2.51541425527
GATCCCGA 2.42475232361
GATCCCGC 1.32494362263
GATCCCTA 1.56928650302
GATCCGAA 3.7231467988
GATCCGAC 1.1962798395
GATCCGCA 2.18465981326
GATCCGCC 2.39499785963
GATCCGGA 4.67224774912
GATCCGGC 1.06808882859
GATCCGTA 2.90484199877
GATCCTAA 2.11611929133
GATCCTAC 3.6799219697
GATCCTCA 3.88502538047
GATCCTCC 6.933971724
GATCCTGA 2.95380962593
GATCCTGC 4.11348136562
GATCCTTA 2.71102245967
GATCGAAA 0.615571079336
GATCGAAC 0.173587969167
GATCGACA -0.560414736836
GATCGACC -0.726189785982
GATCGAGA 2.2961233062
GATCGAGC -0.830180521733
GATCGATA 1.56107471397
GATCGATC 0.995191974006
GATCGCAA 2.96389293513
GATCGCAC 5.5674622427
GATCGCCA 2.27177439196
GATCGCCC 4.57886284933
GATCGCGA 8.07190135425

GATCGCGC 10.7495216552
GATCGCTA 2.73683278342
GATCGGAA 1.30615618343
GATCGGAC -0.883985372712
GATCGGCA 0.815645534365
GATCGGCC 0.288496391507
GATCGGGA 2.42475232361
GATCGGGC -0.079266892944
GATCGGTA 2.30811810572
GATCGTAA 3.78775858567
GATCGTAC 2.19064742866
GATCGTCA 1.85995481551
GATCGTCC 2.68000352258
GATCGTGA 2.56688543096
GATCGTGC 3.74378015225
GATCGTTA 1.75768383815
GATCTAAA 2.27773889959
GATCTAAC 2.65212370142
GATCTACA 3.47901854991
GATCTACC 1.30723225546
GATCTAGA 4.52019725042
GATCTAGC 2.66710189973
GATCTATA 3.79123037003
GATCTCAA 2.96971150999
GATCTCAC 5.70750336942
GATCTCCA 7.62507685671
GATCTCCC 9.37367185193
GATCTCGA 4.87972959344
GATCTCGC 6.34748519186
GATCTCTA 4.4033051
GATCTGAA 2.05675317349
GATCTGAC 2.26034625293
GATCTGCA 4.9598618823
GATCTGCC 2.45174676377
GATCTGGA 4.51355408254
GATCTGGC 2.27076327145
GATCTGTA 3.71122048984
GATCTTAA 3.91803012743
GATCTTAC 3.98009673018
GATCTTCA 5.83881871563
GATCTTCC 4.26540591663
GATCTTGA 5.28162386778
GATCTTGC 5.25064406815
GATCTTTA 3.74080695417
GATGAAAA 0.492272251226
GATGAAAC -0.783511595949
GATGAACA -0.116746634005
GATGAACC -0.404043503924
GATGAAGA 0.928364756945
GATGAAGC -0.411702580411
GATGAATA 1.18173277781
GATGAATC 2.58848889386
GATGACAA 0.319746614349
GATGACAC 0.332164223366

GATGACCA 0.347272324745
GATGACCC -0.670747823278
GATGACGA 0.799346446696
GATGACGC -0.341606344349
GATGACTA 0.1379849527
GATGAGAA 1.65754689338
GATGAGAC -0.215622020149
GATGAGCA -0.540843507497
GATGAGCC -0.867219966342
GATGAGGA 0.524298769798
GATGAGGC -0.575113560284
GATGAGTA 0.144274009824
GATGATAA -0.17086375197
GATGATAC 2.11615593066
GATGATCA 1.64743901918
GATGATCC 2.90622305156
GATGATGA -0.000203389897722
GATGATGC 0.110629531902
GATGATTA 2.39391575043
GATGCAAA 0.712913234572
GATGCAAC -0.21008073837
GATGCACA -0.192504895079
GATGCACC -0.0037740588084
GATGCAGA 1.73555659943
GATGCAGC -0.379552612289
GATGCATA 0.950749720018
GATGCATC 0.308625121508
GATGCCAA -0.481994503784
GATGCCAC -0.417164546374
GATGCCCA -0.394302439345
GATGCCCC 0.555328740325
GATGCCGA 0.901629082028
GATGCCGC 0.119746062507
GATGCCTA 0.301516259453
GATGCGAA 1.21812791153
GATGCGAC 0.00290783018566
GATGCGCA 0.345424536892
GATGCGCC 0.13641154344
GATGCGGA 1.59056937181
GATGCGGC -0.867646939403
GATGCGTA 1.94666448801
GATGCTAA 0.438865436567
GATGCTAC 0.189875815224
GATGCTCA 0.00998504918563
GATGCTCC 0.0673401676314
GATGCTGA 0.636592684742
GATGCTGC 0.560888341767
GATGCTTA 1.93381865687
GATGGAAA -0.0917677731572
GATGGAAC -0.708764455379
GATGGACA -0.712282871614
GATGGACC -1.12610970025
GATGGAGA 0.235678720564
GATGGAGC -0.512364966434

GATGGATA 3.08315061207
GATGGCAA -0.486907504384
GATGGCAC -0.83577176623
GATGGCCA -0.842018563222
GATGGCCC -0.623852399838
GATGGCGA -0.19739395521
GATGGCGC -0.604598641936
GATGGCTA -0.421215273733
GATGGGAA 1.59327402027
GATGGGAC -0.78962370178
GATGGGCA -0.780038354338
GATGGGCC -0.422115019013
GATGGGGA -0.49325443499
GATGGGGC -0.696936198256
GATGGGTA 1.50738852444
GATGGTAA 0.790260310078
GATGGTAC -0.899791911252
GATGGTCA -0.899616209027
GATGGTCC -0.201349337049
GATGGTGA -0.0254876478587
GATGGTGC -0.916756960316
GATGGTTA 0.772937611225
GATGTAAA 0.647770385641
GATGTAAC -0.0748945305879
GATGTACA 0.195669200551
GATGTACC -0.321302953014
GATGTAGA 0.914929365728
GATGTAGC -0.389913214571
GATGTATA 1.98798969293
GATGTCAA -0.195124398747
GATGTCAC 0.693777721769
GATGTCCA 0.922072368974
GATGTCCC 0.516125077317
GATGTCGA -0.395708473499
GATGTCGC 0.488861463012
GATGTCTA 1.60229978527
GATGTGAA -0.0908753141076
GATGTGAC -0.605186744762
GATGTGCA -0.755643849113
GATGTGCC 0.378908093227
GATGTGGA 0.0317504745558
GATGTGGC -0.156657685828
GATGTGTA 2.8180459324
GATGTTAA 1.97929805361
GATGTTAC 1.52060074885
GATGTTCA -0.0237639340889
GATGTTCC 0.304302930047
GATGTTGA 1.49996406455
GATGTTGC 0.280771947131
GATGTTTA 2.1457829897
GATTAAAA -0.0918381373184
GATTAAAC 0.0109503705323
GATTAACA 1.33777925305
GATTAACC -0.151723034717

GATTAAGA 1.0100331912
GATTAAGC 0.945184705949
GATTAATA 2.54346790505
GATTAATC 0.900946882749
GATTACAA 7.34878469178
GATTACAC 11.8125326062
GATTACCA 11.1083988621
GATTACCC 12.8155579017
GATTACGA 15.9283160041
GATTACGC 14.8593544933
GATTACTA 7.47961539963
GATTAGAA 0.35912410598
GATTAGAC 1.3705693685
GATTAGCA 0.920367807579
GATTAGCC 0.576017677301
GATTAGGA 1.84830038701
GATTAGGC 0.423586004701
GATTAGTA 2.61732945645
GATTATAA 1.87527734022
GATTATAC 2.37393066325
GATTATCA 3.32198322921
GATTATCC 8.72137838307
GATTATGA 1.96219560707
GATTATGC 4.52827767916
GATTATTA 3.56526606727
GATTCAAA 1.27708121242
GATTCAAC 0.179246663333
GATTCACA 2.90448705529
GATTCACC 2.19602799703
GATTCAGA 2.42112211579
GATTCAGC 0.72795097179
GATTCATA 2.63549923155
GATTCCAA 3.68895751907
GATTCCAC 6.36673749252
GATTCCCA 13.3063010856
GATTCCCC 22.5365949257
GATTCCGA 9.47506265275
GATTCCGC 9.46679049208
GATTCCTA 6.8498888414
GATTCGAA 3.82331538662
GATTCGAC 1.35108932112
GATTCGCA 11.0548419511
GATTCGCC 6.58365437977
GATTCGGA 3.92773996531
GATTCGGC 3.10486066209
GATTCGTA 6.42677352714
GATTCTAA 3.81556929174
GATTCTAC 5.7347445005
GATTCTCA 13.2518910612
GATTCTCC 20.709561586
GATTCTGA 6.1418359384
GATTCTGC 10.2107024705
GATTCTTA 9.54498984828
GATTGAAA 0.62362215498

GATTGAAC 0.0880792755034
GATTGACA -0.556986878028
GATTGACC -0.176540557621
GATTGAGA 1.04986159636
GATTGAGC 0.0961111987725
GATTGATA 2.57837290072
GATTGCAA 4.40569081979
GATTGCAC 7.92474782645
GATTGCCA 5.98220484632
GATTGCCC 10.9430980424
GATTGCGA 14.7718772686
GATTGCGC 16.7732176243
GATTGCTA 8.19927922193
GATTGGAA 0.865217710419
GATTGGAC -0.875310803983
GATTGGCA 0.680638359655
GATTGGCC 0.315279739185
GATTGGGA 1.65914736578
GATTGGGC 0.0341351534488
GATTGGTA 1.95185124267
GATTGTAA 3.33871241261
GATTGTAC 2.63368308675
GATTGTCA 2.69639129407
GATTGTCC 4.10601631102
GATTGTGA 5.49654349539
GATTGTGC 5.67666554718
GATTGTTA 2.59540831394
GATTTAAA 1.0685976156
GATTTAAC 1.21243736613
GATTTACA 2.67813075337
GATTTACC 2.04934536784
GATTTAGA 3.46471547283
GATTTAGC 0.941655880815
GATTTATA 5.586263838
GATTTCAA 5.96268316334
GATTTCAC 11.0342233783
GATTTCCA 17.531890255
GATTTCCC 24.2869937832
GATTTCGA 9.60233393329
GATTTCGC 14.8256925285
GATTTCTA 9.53929326572
GATTTGAA 3.55278288218
GATTTGAC 2.87237414285
GATTTGCA 7.07309207947
GATTTGCC 8.90246950341
GATTTGGA 3.06537241977
GATTTGGC 3.00288217481
GATTTGTA 7.33252390887
GATTTTAA 4.4615809897
GATTTTAC 8.2313615322
GATTTTCA 15.0748249599
GATTTTCC 12.0056022882
GATTTTGA 4.13051698702
GATTTTGC 6.4196796539

GATTTTTA 7.41911637715
GCAAAAAA 2.41536870064
GCAAAAAC -0.167261856361
GCAAAACA -0.288098563365
GCAAAACC -0.0222550600063
GCAAAAGA 0.165123452032
GCAAAAGC -0.393254887691
GCAAAATA 1.67145484864
GCAAACAA 0.884241639819
GCAAACAC 0.447645135182
GCAAACCA 0.30094127184
GCAAACCC -0.502579976298
GCAAACGA 0.138045948852
GCAAACGC -0.47241665075
GCAAACTA 0.762547655846
GCAAAGAA 0.390748424673
GCAAAGAC -0.959463842566
GCAAAGCA 0.246612645035
GCAAAGCC -0.150538501945
GCAAAGGA -0.15564385901
GCAAAGGC -1.30390619569
GCAAAGTA 0.905212449796
GCAAATAA 1.85462691618
GCAAATAC 3.4485427492
GCAAATCA 3.02432409158
GCAAATCC 9.61646963527
GCAAATGA 0.662309952925
GCAAATGC 0.757573450916
GCAAATTA 0.819662953245
GCAACAAA 1.48073507983
GCAACAAC 0.430227090809
GCAACACA -0.399385313186
GCAACACC 0.383643101645
GCAACAGA 0.0467472007095
GCAACAGC -0.548658717477
GCAACATA 0.784966968287
GCAACCAA 0.131950289082
GCAACCAC -1.015504317
GCAACCCA 0.195375461405
GCAACCCC -0.887296651845
GCAACCGA -0.605805657931
GCAACCGC -0.480763339142
GCAACCTA 0.197384795379
GCAACGAA -0.533021635821
GCAACGAC -0.825552308626
GCAACGCA 0.713807775401
GCAACGCC -0.816752208568
GCAACGGA -0.0189766729957
GCAACGGC -0.617389722269
GCAACGTA 0.845274675187
GCAACTAA 1.13900382869
GCAACTAC 0.80913164501
GCAACTCA 0.397029362856
GCAACTCC -0.774460433233

GCAACTGA −0.0553961635455
GCAACTGC −0.125263611994
GCAACTTA −0.399527290576
GCAAGAAA 0.00857110426717
GCAAGAAC −0.426465106324
GCAAGACA 0.880286466158
GCAAGACC −0.175790492318
GCAAGAGA 0.323859795106
GCAAGAGC −0.505017324223
GCAAGATA 3.088792652
GCAAGCAA 0.568680235812
GCAAGCAC −0.830725531715
GCAAGCCA −1.05092496954
GCAAGCCC −1.11071077422
GCAAGCGA −0.137858796837
GCAAGCGC 0.377901552638
GCAAGCTA −1.0099442992
GCAAGGAA −0.401816207593
GCAAGGAC −0.162888036759
GCAAGGCA −0.125605648434
GCAAGGCC −0.675518013776
GCAAGGGA −0.74755009698
GCAAGGGC −1.04405488764
GCAAGGTA 0.2808062965
GCAAGTAA 1.99778134476
GCAAGTAC 0.264444339078
GCAAGTCA 0.248415466442
GCAAGTCC −0.884867839218
GCAAGTGA 0.030147712199
GCAAGTGC −0.266407873894
GCAAGTTA 0.644050244932
GCAATAAA −0.128801805138
GCAATAAC −0.332531032987
GCAATACA 1.6762508532
GCAATACC 1.2522051915
GCAATAGA 0.672684294955
GCAATAGC 0.467553821046
GCAATATA 4.6285341181
GCAATCAA 7.09880955583
GCAATCAC 2.7508454522
GCAATCCA 9.90782264518
GCAATCCC 6.69816497397
GCAATCGA 2.76345958124
GCAATCGC 5.21787206419
GCAATCTA 12.9559620908
GCAATGAA −0.0769355076169
GCAATGAC −0.475492272396
GCAATGCA 1.01846668981
GCAATGCC −0.326918554335
GCAATGGA 0.267848465595
GCAATGGC −0.517092064087
GCAATGTA 0.657516446492
GCAATTAA 1.00692342836
GCAATTAC 0.692744742575

| | |
|---|---|
| GCAATTCA | 3.24605280493 |
| GCAATTCC | 1.8187799151 |
| GCAATTGA | 0.820399486979 |
| GCAATTGC | 1.69949600774 |
| GCAATTTA | 2.19279769913 |
| GCACAAAA | 1.40461771172 |
| GCACAAAC | 0.11871849594 |
| GCACAACA | 0.0151468224856 |
| GCACAACC | -0.344647824806 |
| GCACAAGA | 0.361098049696 |
| GCACAAGC | -0.209807608845 |
| GCACAATA | 2.02580064518 |
| GCACACAA | 2.18140349312 |
| GCACACAC | -1.05617730027 |
| GCACACCA | -0.859036489495 |
| GCACACCC | -0.759608390783 |
| GCACACGA | -0.464895596272 |
| GCACACGC | -0.136483781203 |
| GCACACTA | -0.171680850587 |
| GCACAGAA | 0.358581594134 |
| GCACAGAC | -1.23314920269 |
| GCACAGCA | -0.302495528725 |
| GCACAGCC | -0.222562674386 |
| GCACAGGA | -0.0468204793625 |
| GCACAGGC | -0.78135861916 |
| GCACAGTA | 0.629353503265 |
| GCACATAA | 0.9450073383 |
| GCACATAC | 0.303842023974 |
| GCACATCA | 0.0730550698548 |
| GCACATCC | -0.071453348388 |
| GCACATGA | 0.491707672512 |
| GCACATGC | 0.137393310848 |
| GCACATTA | 0.391065063398 |
| GCACCAAA | 0.723973523084 |
| GCACCAAC | -1.2746580206 |
| GCACCACA | -0.310943807977 |
| GCACCACC | -0.0888160174154 |
| GCACCAGA | -0.792513628658 |
| GCACCAGC | -0.660798997063 |
| GCACCATA | 0.333323358422 |
| GCACCCAA | -0.135087531414 |
| GCACCCAC | -1.13575583567 |
| GCACCCCA | -0.0645033261408 |
| GCACCCCC | -1.40425527384 |
| GCACCCGA | -1.5197130779 |
| GCACCCGC | -1.05717030929 |
| GCACCCTA | -0.269065057778 |
| GCACCGAA | -0.141686773746 |
| GCACCGAC | -0.926927079736 |
| GCACCGCA | 0.723285286644 |
| GCACCGCC | -0.628364033271 |
| GCACCGGA | 0.367931700446 |
| GCACCGGC | -1.28398626822 |
| GCACCGTA | 0.573414203339 |

GCACCTAA 0.495277508711
GCACCTAC -0.991697914596
GCACCTCA -0.555295223669
GCACCTCC -0.698800640348
GCACCTGA -0.548853363899
GCACCTGC -0.393920849086
GCACCTTA -0.074377832813
GCACGAAA 0.653813376379
GCACGAAC -1.35380021492
GCACGACA -0.854348321126
GCACGACC -0.506859907626
GCACGAGA -0.579097878864
GCACGAGC -0.945430980513
GCACGATA 0.398396884082
GCACGCAA 1.29981300002
GCACGCAC -0.595999849996
GCACGCCA -1.10749671421
GCACGCCC -1.36599902887
GCACGCGA 0.584301287762
GCACGCGC 1.45348666252
GCACGCTA -0.603482599723
GCACGGAA 0.304799850913
GCACGGAC -0.999134656987
GCACGGCA -0.162185644215
GCACGGCC -0.979369613938
GCACGGGA 0.851019346863
GCACGGGC -1.05951168716
GCACGGTA 0.999534983265
GCACGTAA 1.71806090467
GCACGTAC -0.351587229975
GCACGTCA 0.180926243369
GCACGTCC -0.148157986612
GCACGTGA -0.823881888422
GCACGTGC 0.560551717955
GCACGTTA -0.178378144752
GCACTAAA -0.159506185286
GCACTAAC -0.742622523921
GCACTACA 0.410597571632
GCACTACC 0.201579165552
GCACTAGA 0.161916261894
GCACTAGC -0.492365931322
GCACTATA 1.20991882879
GCACTCAA 0.0583298078021
GCACTCAC -1.04469087141
GCACTCCA -0.721774330781
GCACTCCC -0.950963726994
GCACTCGA -1.04564474296
GCACTCGC 1.74984302228
GCACTCTA -0.191645744508
GCACTGAA 0.541680383023
GCACTGAC -0.155416528643
GCACTGCA -0.305991878093
GCACTGCC -0.679133649133
GCACTGGA 0.20344818756

GCACTGGC -1.18350895243
GCACTGTA 0.874507653291
GCACTTAA 0.506391298967
GCACTTAC -0.65319134054
GCACTTCA -0.104706243509
GCACTTCC 0.0433761744927
GCACTTGA -0.437885750939
GCACTTTA -0.63515729749
GCAGAAAA 0.380771702607
GCAGAAAC -1.04879530869
GCAGAACA -0.426082891531
GCAGAACC -0.734012906725
GCAGAAGA -0.603705350174
GCAGAAGC -0.476385564158
GCAGAATA 0.328737197269
GCAGACAA 1.21967529854
GCAGACAC -0.726925487004
GCAGACCA -0.37909024897
GCAGACCC -0.702583234454
GCAGACGA -0.756141810869
GCAGACGC -0.139868130812
GCAGACTA 0.107981091493
GCAGAGAA 1.40111303524
GCAGAGAC -0.980126965471
GCAGAGCA -1.00511998242
GCAGAGCC -0.379448523293
GCAGAGGA 0.302252168653
GCAGAGGC -0.325101785003
GCAGAGTA 0.064574314836
GCAGATAA 1.8691972938
GCAGATAC 3.70849981167
GCAGATCA 0.924251992545
GCAGATCC 4.00435987509
GCAGATGA 0.0407229459907
GCAGATGC 0.64587575774
GCAGATTA 2.31969696561
GCAGCAAA 0.84446902636
GCAGCAAC -0.684122426522
GCAGCACA -1.43715051917
GCAGCACC -0.0552231676345
GCAGCAGA -1.03319320094
GCAGCAGC -0.2591849303
GCAGCATA 0.478735893682
GCAGCCAA 0.215365128507
GCAGCCAC -0.146951178795
GCAGCCCA -0.869967499474
GCAGCCCC -0.847897926058
GCAGCCGA -0.498324818151
GCAGCCGC 0.0906361175953
GCAGCCTA -0.711952909497
GCAGCGAA -0.75773270708
GCAGCGAC -1.31253329984
GCAGCGCA -0.583953422338
GCAGCGCC -1.78398338018

GCAGCGGA -0.339501664855
GCAGCGGC -1.32010327615
GCAGCGTA 0.685016759173
GCAGCTAA 1.72824934375
GCAGCTAC -0.528444218143
GCAGCTCA -0.244416159047
GCAGCTCC -0.696329983945
GCAGCTGA -0.774980461856
GCAGCTGC 0.33300130707
GCAGCTTA -0.954666380751
GCAGGAAA 0.700771253216
GCAGGAAC -1.33398791547
GCAGGACA -1.04356400394
GCAGGACC -1.22497342843
GCAGGAGA -0.748884934262
GCAGGAGC -0.41037794385
GCAGGATA 2.55374440343
GCAGGCAA -0.146690123594
GCAGGCAC -1.5091463794
GCAGGCCA -0.877367394361
GCAGGCCC -1.90597984677
GCAGGCGA -0.887058496223
GCAGGCGC -0.445134092248
GCAGGCTA -0.838685425399
GCAGGGAA 0.882442773794
GCAGGGAC -1.25521419619
GCAGGGCA -0.787858352412
GCAGGGCC -1.25099047292
GCAGGGGA 0.051182224641
GCAGGGGC -1.20994922278
GCAGGGTA 0.100276424027
GCAGGTAA 2.23199095324
GCAGGTAC -0.43121364631
GCAGGTCA -0.910410237888
GCAGGTCC -1.05769366876
GCAGGTGA -0.406324510177
GCAGGTTA -0.377490401105
GCAGTAAA 0.446122521352
GCAGTAAC -0.190559055393
GCAGTACA -0.979162685014
GCAGTACC -0.433511098625
GCAGTAGA -0.467328364281
GCAGTAGC -0.190766817028
GCAGTATA 0.619706326959
GCAGTCAA 0.348639013259
GCAGTCAC -0.978101809969
GCAGTCCA -0.765874756506
GCAGTCCC -1.14765237517
GCAGTCGA -0.231522655141
GCAGTCGC 0.0265093854411
GCAGTCTA -0.961360552247
GCAGTGAA 0.284361143883
GCAGTGAC -0.668712675233
GCAGTGCA -0.0459935963802

GCAGTGCC -1.33484893964
GCAGTGGA -0.128777864669
GCAGTGGC -0.492640518093
GCAGTGTA -0.0972008023801
GCAGTTAA 0.868721970551
GCAGTTAC 0.659043640238
GCAGTTCA -0.784119475684
GCAGTTCC -0.946285134812
GCAGTTGA -0.248986706851
GCAGTTTA -0.104371701476
GCATAAAA 1.16759582676
GCATAAAC -0.364378518487
GCATAACA 0.134133451679
GCATAACC -0.0766881921629
GCATAAGA 2.81288853084
GCATAAGC -0.59954428848
GCATAATA 0.866097054256
GCATACAA 2.11065753355
GCATACAC 0.462098516778
GCATACCA -0.622809011745
GCATACCC -0.522669777014
GCATACGA 0.252015280271
GCATACGC -0.931912109921
GCATACTA 0.215151954244
GCATAGAA 1.6531226947
GCATAGAC -0.65984054559
GCATAGCA 0.377446683727
GCATAGCC -0.192733266336
GCATAGGA 0.499687134928
GCATAGGC -1.04606609522
GCATAGTA 0.501490580868
GCATATAA 3.17852319533
GCATATAC 1.59395309688
GCATATCA 3.15995871476
GCATATCC 6.93577496176
GCATATGA 0.943841957904
GCATATGC 0.678927761099
GCATATTA -0.188251194179
GCATCAAA 0.124004967857
GCATCAAC -0.770660352176
GCATCACA 1.00578407021
GCATCACC -0.394302439345
GCATCAGA 1.11212804998
GCATCAGC -0.511422961022
GCATCATA 0.547736280796
GCATCCAA 0.304715122471
GCATCCAC 0.0252438714306
GCATCCCA -0.354047477479
GCATCCCC -0.0810536846453
GCATCCGA -0.296075735822
GCATCCGC -0.0111872770867
GCATCCTA -0.104241382054
GCATCGAA -0.288844881465
GCATCGAC -0.432284930255

GCATCGCA 1.10952873958
GCATCGCC -1.42140060504
GCATCGGA -0.0219294696275
GCATCGGC -0.370189598942
GCATCGTA 0.891284093095
GCATCTAA 1.73555659943
GCATCTAC 2.33287400793
GCATCTCA 0.00392103247042
GCATCTCC -0.273698683513
GCATCTGA -0.0765861849471
GCATCTTA -0.614959452396
GCATGAAA 1.489469812
GCATGAAC -0.434727274451
GCATGACA -0.917700214795
GCATGACC -0.911526071923
GCATGAGA -0.198732956052
GCATGAGC -1.17870420439
GCATGATA 0.460038387373
GCATGCAA 0.945936228498
GCATGCAC -0.307116663781
GCATGCCA -0.643188388048
GCATGCCC -1.39367754191
GCATGCGA -0.0595751285474
GCATGCGC 0.971808172929
GCATGCTA -1.05153659648
GCATGGAA 0.555301469008
GCATGGAC -0.186133607649
GCATGGCA -0.263079107809
GCATGGCC -1.5690625035
GCATGGGA 0.261733861628
GCATGGGC -1.05483455222
GCATGGTA 0.313985080255
GCATGTAA 0.987465031494
GCATGTAC 0.983215910509
GCATGTCA 0.2325323184
GCATGTCC -0.849168852697
GCATGTGA -0.436692058336
GCATGTTA -0.293323622774
GCATTAAA 1.01540730605
GCATTAAC -0.91133662995
GCATTACA -0.276355034685
GCATTACC -0.255863658623
GCATTAGA 0.165098262495
GCATTAGC -0.00900994347331
GCATTATA 2.27750948744
GCATTCAA 0.122306651802
GCATTCAC -0.348305095761
GCATTCCA -0.43948081071
GCATTCCC -0.744272542682
GCATTCGA -0.0974685192772
GCATTCGC 0.961651585079
GCATTCTA -0.19858702328
GCATTGAA -0.320000799677
GCATTGAC -0.419185746494

GCATTGCA −0.345243838395
GCATTGCC −0.965910906785
GCATTGGA 0.932923022248
GCATTGGC −0.84116878066
GCATTGTA 0.427865727851
GCATTTAA 2.21774928866
GCATTTAC 1.04661859961
GCATTTCA 0.140659415357
GCATTTCC 1.51722826539
GCATTTGA 0.336401894561
GCATTTTA 0.0674007474269
GCCAAAAA 0.526734452299
GCCAAAAC −0.0538400330588
GCCAAACA −1.02512089114
GCCAAACC −0.292857304073
GCCAAAGA −0.0979935441719
GCCAAAGC −0.909493838369
GCCAAATA −0.382041796534
GCCAACAA 0.65191645852
GCCAACAC −0.694580456104
GCCAACCA −0.587250545368
GCCAACCC −0.443684757071
GCCAACGA −0.202955430254
GCCAACGC −1.14915542027
GCCAACTA −0.701577318399
GCCAAGAA 0.013980401199
GCCAAGAC −1.143249827
GCCAAGCA −0.725654144009
GCCAAGCC −1.43957121286
GCCAAGGA −0.422699582813
GCCAAGGC −1.01433206672
GCCAAGTA 0.122801074532
GCCAATAA 0.706881693623
GCCAATAC 0.632651250828
GCCAATCA −0.165955539464
GCCAATCC −0.353831388724
GCCAATGA −0.803659894681
GCCAATTA −0.177046638319
GCCACAAA 0.63633891577
GCCACAAC −0.187671210294
GCCACACA 0.0944328678048
GCCACACC −0.584130373631
GCCACAGA −0.348143133283
GCCACAGC −1.25560328085
GCCACATA −0.126771653365
GCCACCAA −0.831191642238
GCCACCAC −0.620598577831
GCCACCCA −0.69383559525
GCCACCCC −1.69392433197
GCCACCGA −0.841355516319
GCCACCGC −1.23182748062
GCCACCTA −0.538545847004
GCCACGAA 0.315279739185
GCCACGAC −1.22465949601

GCCACGCA −0.612390119625
GCCACGCC −1.16087334305
GCCACGGA −0.399364703565
GCCACGGC −1.08312011229
GCCACGTA −0.139864799964
GCCACTAA 0.193306588525
GCCACTAC −0.267117344489
GCCACTCA −0.132949127085
GCCACTCC −1.45365965843
GCCACTGA −0.740182261504
GCCACTTA −0.825680546268
GCCAGAAA 0.355525124863
GCCAGAAC −0.574153027031
GCCAGACA −0.82879613809
GCCAGACC −1.35911291727
GCCAGAGA −0.219013864165
GCCAGAGC −1.68384976625
GCCAGATA 3.01028873139
GCCAGCAA −0.680548426763
GCCAGCAC −0.463558260854
GCCAGCCA −0.588431330936
GCCAGCCC −1.74980679931
GCCAGCGA −0.547273917477
GCCAGCGC −0.626440468629
GCCAGCTA −1.42606878833
GCCAGGAA 0.671676921654
GCCAGGAC −1.15018090505
GCCAGGCA −1.14862914631
GCCAGGCC −1.59458679069
GCCAGGGA 0.0964967444128
GCCAGGGC −1.53208467958
GCCAGGTA −0.000833336500085
GCCAGTAA −0.256108475941
GCCAGTAC −1.36801960446
GCCAGTCA −0.704036316835
GCCAGTCC −1.198433649
GCCAGTGA −0.97665372386
GCCAGTTA 0.320570999195
GCCATAAA −0.0397361823108
GCCATAAC −1.33598663237
GCCATACA −1.06249321235
GCCATACC −0.227173816898
GCCATAGA 0.133734166291
GCCATAGC −1.24692871212
GCCATATA 0.537226623072
GCCATCAA −0.491992876361
GCCATCAC −0.448463274688
GCCATCCA −0.260055947016
GCCATCCC −1.13571420007
GCCATCGA −0.0974560285977
GCCATCGC −0.723335665718
GCCATCTA −0.587864670443
GCCATGAA −0.278313989585
GCCATGAC −1.22880515254

GCCATGCA −1.71959413557
GCCATGCC 0.0241367808717
GCCATGGA 0.648033522622
GCCATGGC −1.33113733423
GCCATGTA −0.44256538401
GCCATTAA 1.0818458548
GCCATTAC −0.193575762668
GCCATTCA 0.156225508318
GCCATTCC −0.0254029194162
GCCATTGA −0.0878844209033
GCCATTTA −0.632052114569
GCCCAAAA 1.41412665784
GCCCAAAC −0.950299431023
GCCCAACA −1.47111850569
GCCCAACC −0.406471275661
GCCCAAGA −0.244554805589
GCCCAAGC −1.47322318519
GCCCAATA −0.435454856532
GCCCACAA 0.497003304261
GCCCACAC −1.19386580751
GCCCACCA −0.838685425399
GCCCACCC −0.794564181875
GCCCACGA −0.454907840772
GCCCACGC −0.940148463978
GCCCACTA −0.575651284036
GCCCAGAA −0.217610119968
GCCCAGAC −0.684314782986
GCCCAGCA −1.01697530268
GCCCAGCC −0.869967499474
GCCCAGGA −0.447165909445
GCCCAGGC −1.58427198756
GCCCAGTA −1.23343419836
GCCCATAA 0.763578136904
GCCCATAC −0.773137253919
GCCCATCA −0.990751953802
GCCCATCC −0.845321098879
GCCCATGA −0.95380202573
GCCCATTA 0.093174223668
GCCCCAAA 0.0850719362377
GCCCCAAC −0.547876592763
GCCCCACA −0.912007795795
GCCCCACC −0.992753377013
GCCCCAGA 0.00963281202396
GCCCCAGC −0.871663317393
GCCCCATA −0.310769562998
GCCCCCAA −1.32671001289
GCCCCCAC −1.64273169843
GCCCCCCA −1.48949396065
GCCCCCCC −1.62543876903
GCCCCCGA −1.41872426878
GCCCCCGC −1.739917512
GCCCCCTA −1.02525183509
GCCCCGAA −0.515900661442
GCCCCGAC −1.09186900056

| | |
|---|---|
| GCCCCGCA | -0.510745549838 |
| GCCCCGCC | 0.126472085235 |
| GCCCCGGA | -1.71448253317 |
| GCCCCGGC | -1.23050763215 |
| GCCCCGTA | -0.851838110904 |
| GCCCCTAA | -0.767081356145 |
| GCCCCTAC | -1.25046107628 |
| GCCCCTCA | -0.924984779075 |
| GCCCCTCC | -1.16291307102 |
| GCCCCTGA | -1.44405286866 |
| GCCCCTTA | -0.237155951592 |
| GCCCGAAA | -0.0762389440572 |
| GCCCGAAC | -0.636166128037 |
| GCCCGACA | -0.944183786166 |
| GCCCGACC | -1.43319139013 |
| GCCCGAGA | -1.27252294711 |
| GCCCGAGC | -1.74307765391 |
| GCCCGATA | -0.203781272347 |
| GCCCGCAA | 0.0955920028616 |
| GCCCGCAC | -1.01841631074 |
| GCCCGCCA | -0.882069302478 |
| GCCCGCCC | -1.63109829591 |
| GCCCGCGA | -0.294049331253 |
| GCCCGCGC | -1.19110557552 |
| GCCCGCTA | -0.717171099033 |
| GCCCGGAA | -0.574705739599 |
| GCCCGGAC | -0.559886589272 |
| GCCCGGCA | -1.33926397849 |
| GCCCGGCC | -1.5160212494 |
| GCCCGGGA | -1.16846288809 |
| GCCCGGGC | -0.702718758327 |
| GCCCGGTA | -1.34582241793 |
| GCCCGTAA | 0.270344103358 |
| GCCCGTAC | -0.0793949224088 |
| GCCCGTCA | -1.3779596872 |
| GCCCGTCC | -1.29837594735 |
| GCCCGTGA | -1.01961916318 |
| GCCCGTTA | -0.0494249942145 |
| GCCCTAAA | -0.183889865256 |
| GCCCTAAC | -0.970629885497 |
| GCCCTACA | -0.196807725986 |
| GCCCTACC | -0.75639766162 |
| GCCCTAGA | -1.2161531351 |
| GCCCTAGC | -1.2828277577 |
| GCCCTATA | -1.14121925887 |
| GCCCTCAA | 0.134175711811 |
| GCCCTCAC | -0.754786155788 |
| GCCCTCCA | -1.82683473795 |
| GCCCTCCC | -1.57371236712 |
| GCCCTCGA | -0.653085586121 |
| GCCCTCGC | -1.36895557271 |
| GCCCTCTA | -1.63960715496 |
| GCCCTGAA | -0.276948966495 |
| GCCCTGAC | -1.31370305197 |

| | |
|---|---|
| GCCCTGCA | −1.15409673307 |
| GCCCTGCC | −1.1675292098 |
| GCCCTGGA | −1.10528274127 |
| GCCCTGTA | −0.163302935496 |
| GCCCTTAA | 0.570948126852 |
| GCCCTTAC | −1.07845775799 |
| GCCCTTCA | −1.0560786239 |
| GCCCTTCC | −1.29074351765 |
| GCCCTTGA | −0.644240103261 |
| GCCCTTTA | −0.623852399838 |
| GCCGAAAA | 0.961198589769 |
| GCCGAAAC | −0.21877487583 |
| GCCGAACA | −0.312039032391 |
| GCCGAACC | −0.0733265339558 |
| GCCGAAGA | 0.253722131624 |
| GCCGAAGC | −0.702522862837 |
| GCCGAATA | 0.246598072576 |
| GCCGACAA | 0.102938812361 |
| GCCGACAC | −0.838564682164 |
| GCCGACCA | −0.847032113792 |
| GCCGACCC | −1.36422451967 |
| GCCGACGA | −1.04829693058 |
| GCCGACGC | −1.61132555027 |
| GCCGACTA | −0.919883793749 |
| GCCGAGAA | −0.681422357971 |
| GCCGAGAC | −1.32440215167 |
| GCCGAGCA | −0.810072193176 |
| GCCGAGCC | −0.73640050011 |
| GCCGAGGA | −0.817510600991 |
| GCCGAGGC | −0.0706743463437 |
| GCCGAGTA | 0.248422128138 |
| GCCGATAA | 0.967518457236 |
| GCCGATAC | 1.07000240069 |
| GCCGATCA | −0.648033106266 |
| GCCGATCC | 1.99611258998 |
| GCCGATGA | −1.06203251446 |
| GCCGATTA | 1.27650789023 |
| GCCGCAAA | −0.273915188624 |
| GCCGCAAC | −0.420492271569 |
| GCCGCACA | −0.297560461258 |
| GCCGCACC | −1.11040017265 |
| GCCGCAGA | −0.436902734463 |
| GCCGCAGC | −0.628339051912 |
| GCCGCATA | 0.00336478087706 |
| GCCGCCAA | −0.984818048332 |
| GCCGCCAC | −1.0038498885 |
| GCCGCCCA | −1.12990311961 |
| GCCGCCCC | −0.493413899331 |
| GCCGCCGA | −1.22562752368 |
| GCCGCCGC | −1.27556775842 |
| GCCGCCTA | −0.884832240781 |
| GCCGCGAA | −0.0920359064103 |
| GCCGCGAC | −0.788802439603 |
| GCCGCGCA | −0.942899744314 |

| | |
|---|---|
| GCCGCGCC | −0.683905088698 |
| GCCGCGGA | 0.0906361175953 |
| GCCGCGGC | 0.46592087288 |
| GCCGCGTA | 0.237696381658 |
| GCCGCTAA | −0.0966424690068 |
| GCCGCTAC | −1.41002680048 |
| GCCGCTCA | −0.316623736298 |
| GCCGCTCC | −0.580617161846 |
| GCCGCTGA | −1.54923771337 |
| GCCGCTTA | −0.802288001717 |
| GCCGGAAA | −0.104241382054 |
| GCCGGAAC | −0.697898188755 |
| GCCGGACA | −0.821244065091 |
| GCCGGACC | −1.46472077966 |
| GCCGGAGA | −0.97856604689 |
| GCCGGAGC | −0.791329512242 |
| GCCGGATA | 1.28339108734 |
| GCCGGCAA | −0.0868714267965 |
| GCCGGCAC | −1.00466344809 |
| GCCGGCCA | −0.846431936642 |
| GCCGGCCC | −0.802700610496 |
| GCCGGCGA | −1.00272676823 |
| GCCGGCGC | −1.09785078697 |
| GCCGGCTA | −1.2177309161 |
| GCCGGGAA | −0.456584298138 |
| GCCGGGAC | −1.53377508487 |
| GCCGGGCA | −1.68234255759 |
| GCCGGGCC | −1.33484893964 |
| GCCGGGGA | −0.734707180327 |
| GCCGGGTA | −0.161936038804 |
| GCCGGTAA | −0.330158012061 |
| GCCGGTAC | −0.844168625518 |
| GCCGGTCA | −0.469627065663 |
| GCCGGTCC | −0.608275273444 |
| GCCGGTGA | −1.01585697051 |
| GCCGGTTA | −0.0730203041302 |
| GCCGTAAA | −0.462018160073 |
| GCCGTAAC | −0.820266253065 |
| GCCGTACA | −0.712519986346 |
| GCCGTACC | −0.339144015066 |
| GCCGTAGA | −1.17322912321 |
| GCCGTAGC | −1.04636087526 |
| GCCGTATA | 0.00869663559607 |
| GCCGTCAA | −0.174749394182 |
| GCCGTCAC | −1.01360781549 |
| GCCGTCCA | −1.35020956093 |
| GCCGTCCC | −0.980223143703 |
| GCCGTCGA | −0.29838942602 |
| GCCGTCGC | −1.1319586691 |
| GCCGTCTA | −1.18862867378 |
| GCCGTGAA | 0.560968073938 |
| GCCGTGAC | −1.02139304784 |
| GCCGTGCA | −1.04459844038 |
| GCCGTGCC | −0.569714464074 |

GCCGTGGA -0.0787277119459
GCCGTGTA 0.725366233847
GCCGTTAA -0.0539851331189
GCCGTTAC -0.982018262524
GCCGTTCA 0.403544293101
GCCGTTCC -0.71316533812
GCCGTTGA -0.595365948012
GCCGTTTA -0.999550804792
GCCTAAAA 0.840006106577
GCCTAAAC -0.300271979598
GCCTAACA -0.507123252785
GCCTAACC -0.884938411557
GCCTAAGA 0.156430771818
GCCTAAGC -0.463010752736
GCCTAATA -0.375352413132
GCCTACAA 0.517274636186
GCCTACAC -0.698001861395
GCCTACCA -0.156365820284
GCCTACCC -0.72587689446
GCCTACGA -1.10884903844
GCCTACGC -1.41546087059
GCCTACTA -0.591424514098
GCCTAGAA -0.44764222069
GCCTAGAC -0.932505000841
GCCTAGCA -0.810021397746
GCCTAGCC -1.13079391324
GCCTAGGA -1.1655769166
GCCTAGGC -1.02457421573
GCCTAGTA -0.324467466663
GCCTATAA 1.27727398524
GCCTATAC 0.00580691689567
GCCTATCA 0.625420812827
GCCTATCC 0.382461899721
GCCTATGA -1.20237820558
GCCTATTA -0.638307863214
GCCTCAAA -0.888304233324
GCCTCAAC -0.343905878444
GCCTCACA -1.06454772095
GCCTCACC -0.837118261479
GCCTCAGA -0.0743559741239
GCCTCAGC -1.53910298421
GCCTCATA -0.780145566004
GCCTCCAA -0.496532405644
GCCTCCAC -1.19796857937
GCCTCCCA -0.490775451466
GCCTCCCC -1.50146294609
GCCTCCGA -1.31583479461
GCCTCCGC -1.0686136453
GCCTCCTA -0.879259524126
GCCTCGAA -0.345236552166
GCCTCGAC -1.10175578973
GCCTCGCA -1.15853258972
GCCTCGCC -1.37405343536
GCCTCGGA -0.658177411616

GCCTCGTA −0.428663674092
GCCTCTAA −0.604112546326
GCCTCTAC −1.13783220295
GCCTCTCA 0.304453442735
GCCTCTCC −0.927790185689
GCCTCTGA −1.32605258679
GCCTCTTA −1.45966621802
GCCTGAAA 0.267079456094
GCCTGAAC −0.87969857151
GCCTGACA −0.207069651901
GCCTGACC −1.12292998961
GCCTGAGA −0.43948081071
GCCTGAGC −1.14106062725
GCCTGATA 0.400487407473
GCCTGCAA 0.271248428553
GCCTGCAC −1.56826060188
GCCTGCCA −1.32918087747
GCCTGCCC −1.18023139813
GCCTGCGA −1.15445604829
GCCTGCGC −0.861368915712
GCCTGCTA −0.681185035061
GCCTGGAA −0.793455009536
GCCTGGAC −1.25718605812
GCCTGGCA −0.7483378425
GCCTGGCC −1.17838881473
GCCTGGGA −0.506956502214
GCCTGGTA −1.54783334464
GCCTGTAA 0.718211572634
GCCTGTAC −0.178533653712
GCCTGTCA 0.885074976319
GCCTGTCC 0.0729547280629
GCCTGTGA −1.50508524315
GCCTGTTA −0.705602648043
GCCTTAAA −0.629633502663
GCCTTAAC −1.10263263543
GCCTTACA −0.604653809104
GCCTTACC −0.615086232793
GCCTTAGA −0.989572000946
GCCTTAGC −1.81250147506
GCCTTATA 0.0439638609628
GCCTTCAA −0.831507240073
GCCTTCAC −1.09560766911
GCCTTCCA −1.16807088893
GCCTTCCC −1.42943669187
GCCTTCGA −0.873690346497
GCCTTCGC −0.68593648954
GCCTTCTA −1.42274564305
GCCTTGAA −0.972304052905
GCCTTGAC −0.6708015332
GCCTTGCA 0.117864549819
GCCTTGCC −0.271089380567
GCCTTGGA −0.642378159305
GCCTTGTA 0.266397048638
GCCTTTAA −0.0668218044325

GCCTTTAC -0.626264766404
GCCTTTCA 0.787291483741
GCCTTTCC -0.976425560781
GCCTTTGA -0.444021797239
GCCTTTTA -0.849502353839
GCGAAAAA 2.00845712852
GCGAAAAC -0.194731358699
GCGAAACA 0.0944328678048
GCGAAACC 0.38812642287
GCGAAAGA 0.666535133441
GCGAAAGC -0.111664384697
GCGAAATA 1.05690488235
GCGAACAA 0.475555974862
GCGAACAC -0.574770274776
GCGAACCA -0.369431414697
GCGAACCC -0.442099689843
GCGAACGA -0.576447564854
GCGAACGC 0.414876253891
GCGAACTA -0.0676237060559
GCGAAGAA 0.342782758179
GCGAAGAC -0.624431551011
GCGAAGCA -0.488716154774
GCGAAGCC -1.43789329824
GCGAAGGA -0.581735910373
GCGAAGTA -0.0695722520566
GCGAATAA 1.2548773642
GCGAATAC 3.08547637659
GCGAATCA 5.34201297228
GCGAATCC 11.1791835427
GCGAATGA 3.69717867613
GCGAATTA -0.0306858523071
GCGACAAA 0.607513966529
GCGACAAC 0.151254634236
GCGACACA -0.308827470515
GCGACACC -1.07664202955
GCGACAGA -0.669659052382
GCGACAGC -0.5195389882
GCGACATA -0.076491255783
GCGACCAA -0.65608938636
GCGACCAC -0.505215509671
GCGACCCA -0.564041613805
GCGACCCC -0.72927456746
GCGACCGA -0.498666230057
GCGACCGC -0.847581495511
GCGACCTA -0.834520616501
GCGACGAA -0.397704900438
GCGACGAC -0.367681054144
GCGACGCA 0.831844696598
GCGACGCC -0.683228718404
GCGACGGA -0.071993570276
GCGACGTA -0.663559020874
GCGACTAA -0.309457000762
GCGACTAC 0.0964967444128
GCGACTCA -0.531383483206

GCGACTCC −0.687429125739
GCGACTGA 0.0672889558455
GCGACTTA −1.49367063569
GCGAGAAA −0.246986949064
GCGAGAAC −0.66730539201
GCGAGACA −0.319166422287
GCGAGACC −0.286768514177
GCGAGAGA −0.992755250615
GCGAGAGC −0.467715991701
GCGAGATA 1.601167297
GCGAGCAA 0.911164258573
GCGAGCAC −0.854770922449
GCGAGCCA −0.995895615617
GCGAGCCC −0.803853916569
GCGAGCGA −0.523395693671
GCGAGCGC −0.0604036769537
GCGAGCTA −0.448269669156
GCGAGGAA 1.30610663706
GCGAGGAC −1.17070392417
GCGAGGCA −1.01581283678
GCGAGGCC −1.56864489845
GCGAGGGA −0.259167859704
GCGAGGTA −0.590667162565
GCGAGTAA 0.662074295439
GCGAGTAC −0.598175518185
GCGAGTCA −0.779617002083
GCGAGTCC −0.658035642403
GCGAGTGA 0.0834394044282
GCGAGTTA −0.976936013217
GCGATAAA 1.60446483639
GCGATAAC 0.256743002459
GCGATACA 0.341552218072
GCGATACC 1.15500334823
GCGATAGA 0.432438773791
GCGATAGC 0.527082942257
GCGATATA 2.41324715873
GCGATCAA 2.32156765304
GCGATCAC 1.8270489531
GCGATCCA 4.27737240394
GCGATCCC 3.0168721522
GCGATCGA 1.60100533452
GCGATCGC 8.17398726138
GCGATCTA 4.93934989662
GCGATGAA 1.38681995069
GCGATGAC −0.164937340907
GCGATGCA 0.767030560715
GCGATGCC −0.640628631464
GCGATGGA −0.0850386277591
GCGATGTA −0.865869723889
GCGATTAA 1.17099100162
GCGATTAC 4.81127837987
GCGATTCA 1.01208082992
GCGATTCC 1.93126368838
GCGATTGA 0.440602890084

GCGATTTA 1.20681156409
GCGCAAAA 2.59998281713
GCGCAAAC -0.0703633284244
GCGCAACA 0.94028815141
GCGCAACC 0.397680543613
GCGCAAGA 0.151316254922
GCGCAAGC -0.651929781912
GCGCAATA 1.54324343628
GCGCACAA 1.85170034997
GCGCACAC -0.0494085481532
GCGCACCA -0.448560285633
GCGCACCC -0.161669362796
GCGCACGA -0.0586316658898
GCGCACGC -0.739393683272
GCGCACTA -0.280507352904
GCGCAGAA 0.546766587712
GCGCAGAC -0.970813498486
GCGCAGCA -0.475823899937
GCGCAGCC -1.08972830627
GCGCAGGA 0.138713783849
GCGCAGTA -0.367785559496
GCGCATAA 0.842179901165
GCGCATAC 0.815591408087
GCGCATCA 0.239005404869
GCGCATCC 0.0484717471913
GCGCATGA -0.572090191313
GCGCATTA -0.112584323242
GCGCCAAA 0.897454488763
GCGCCAAC -0.895661243544
GCGCCACA -0.283327540155
GCGCCACC -0.994163158372
GCGCCAGA -0.792326268466
GCGCCAGC -1.04423058987
GCGCCATA -0.722209006428
GCGCCCAA -0.560688282717
GCGCCCAC -0.926842559471
GCGCCCCA -0.335367874477
GCGCCCCC -1.21564060089
GCGCCCGA -0.789114914769
GCGCCCGC -0.733931092774
GCGCCCTA -0.18448129893
GCGCCGAA 0.777221081579
GCGCCGAC -0.717917833488
GCGCCGCA 1.17434016915
GCGCCGCC -0.456584298138
GCGCCGGA -0.650903048058
GCGCCGTA -0.500145126509
GCGCCTAA 0.237328739325
GCGCCTAC -0.248655703844
GCGCCTCA -0.14339862137
GCGCCTCC -0.582126868641
GCGCCTGA -0.220863525619
GCGCCTTA -0.866373098272
GCGCGAAA 0.829757920411

GCGCGAAC -0.914954138909
GCGCGACA 0.237328739325
GCGCGACC -0.450910198801
GCGCGAGA -0.2904374431
GCGCGAGC -1.07148587705
GCGCGATA 1.3605920219
GCGCGCAA 2.18944332715
GCGCGCAC -0.188251194179
GCGCGCCA -0.52680689824
GCGCGCCC -0.914475954063
GCGCGCGA 0.448533847028
GCGCGCGC 0.680262806559
GCGCGCTA -0.945634578589
GCGCGGAA 0.332568921381
GCGCGGAC -0.363039934001
GCGCGGCA -1.11435680356
GCGCGGCC 0.0521048694994
GCGCGGGA -0.548208844837
GCGCGGTA 0.284956116583
GCGCGTAA 0.762503938468
GCGCGTAC 0.120548380486
GCGCGTCA -0.0949120935413
GCGCGTCC -0.99224583907
GCGCGTGA 0.235217398135
GCGCGTTA -0.476269609017
GCGCTAAA 0.918751097297
GCGCTAAC -0.758545642137
GCGCTACA -0.440213180884
GCGCTACC -0.328424513926
GCGCTAGA -0.768934348448
GCGCTAGC -0.970610733122
GCGCTATA 0.576128427993
GCGCTCAA 1.10051296712
GCGCTCAC -0.904847930133
GCGCTCCA -0.578181062989
GCGCTCCC 0.246722563015
GCGCTCGA -1.39930167853
GCGCTCTA -0.580441459621
GCGCTGAA 1.37277147529
GCGCTGAC -0.567456981934
GCGCTGCA -0.188455833144
GCGCTGCC -1.27966470129
GCGCTGGA 0.570852364975
GCGCTGTA -0.194744265734
GCGCTTAA 0.627788629302
GCGCTTAC -1.02734131759
GCGCTTCA 0.135839053964
GCGCTTCC -0.39423249154
GCGCTTGA -0.0639562343791
GCGCTTTA -0.0936975831387
GCGGAAAA 1.16958642471
GCGGAAAC -0.473078656763
GCGGAACA -0.624227744757
GCGGAACC -1.4293238594

GCGGAAGA -0.713923522365
GCGGAAGC -0.382093216498
GCGGAATA 1.29704756358
GCGGACAA 0.7302115766
GCGGACAC -1.03722810677
GCGGACCA -1.14157899044
GCGGACCC -0.505395791811
GCGGACGA -0.517899170161
GCGGACGC -0.518093400227
GCGGACTA -0.796242512843
GCGGAGAA 0.402237143492
GCGGAGAC -1.11857344878
GCGGAGCA -0.0449635316781
GCGGAGCC -0.581617665273
GCGGAGGA -0.712813100958
GCGGAGTA -0.355105438032
GCGGATAA 1.4940574304
GCGGATAC 3.2761024653
GCGGATCA 1.61587423939
GCGGATCC 2.9188003331
GCGGATGA -0.725176375519
GCGGATTA 2.65979485223
GCGGCAAA 0.875263963935
GCGGCAAC -0.754396446588
GCGGCACA -0.35248947339
GCGGCACC -1.24718643648
GCGGCAGA -0.576634716868
GCGGCAGC -0.893133130015
GCGGCATA 0.390957851733
GCGGCCAA -0.587250545368
GCGGCCAC -1.44826118676
GCGGCCCA -1.33624040134
GCGGCCCC -1.31157588926
GCGGCCGA -0.923316857007
GCGGCCGC -0.344117387283
GCGGCCTA -0.523006400827
GCGGCGAA 0.356851218669
GCGGCGAC -1.49963472697
GCGGCGCA 1.33502755636
GCGGCGCC -0.628298040848
GCGGCGGA -1.26307499715
GCGGCGTA -0.246079501199
GCGGCTAA -0.4360510783
GCGGCTAC -0.642067349563
GCGGCTCA -1.28668529588
GCGGCTCC -1.27234183226
GCGGCTGA -0.485002675761
GCGGCTTA -1.15286202941
GCGGGAAA -0.386474114151
GCGGGAAC -0.439640691408
GCGGGACA -0.170450310479
GCGGGACC -0.73640050011
GCGGGAGA -0.0749632293251
GCGGGAGC -0.458315714493

GCGGGATA 2.10740579332
GCGGGCAA -0.385592272179
GCGGGCAC -1.125955024
GCGGGCCA -1.16139045719
GCGGGCCC -1.49780192793
GCGGGCGA -1.11597372202
GCGGGCTA -0.0835022741816
GCGGGGAA 0.693360116718
GCGGGGAC -1.00498487491
GCGGGGCA -0.0178743705305
GCGGGGCC -1.09675119082
GCGGGGGA -0.237100576246
GCGGGGTA -0.0112836634968
GCGGGTAA 1.19190997528
GCGGGTAC -0.521675727105
GCGGGTCA -0.592098594435
GCGGGTCC -0.824583448253
GCGGGTGA -1.01276760912
GCGGGTTA 0.515183904617
GCGGTAAA 0.431623548776
GCGGTAAC 0.329534518977
GCGGTACA 0.0880155730379
GCGGTACC 0.241190441068
GCGGTAGA -0.23559003674
GCGGTAGC 0.0298498094932
GCGGTATA 0.244587489534
GCGGTCAA 0.0521048694994
GCGGTCAC -0.965226001193
GCGGTCCA -1.24510923648
GCGGTCCC -1.23555220124
GCGGTCGA -0.783009054277
GCGGTCTA -0.594887763165
GCGGTGAA 0.8124329316
GCGGTGAC -0.992753377013
GCGGTGCA 0.134211934782
GCGGTGCC -0.795274068826
GCGGTGGA -1.12051304313
GCGGTGTA 1.31673412353
GCGGTTAA -0.391484750229
GCGGTTAC -0.269154157958
GCGGTTCA -0.10650344411
GCGGTTCC -0.430323893575
GCGGTTGA -0.739510679303
GCGGTTTA -0.491321710516
GCGTAAAA 1.51244391879
GCGTAAAC -0.125193039655
GCGTAACA 0.56891984868
GCGTAACC 0.778177867628
GCGTAAGA 0.555106822586
GCGTAAGC -0.997810436783
GCGTAATA 2.44237125974
GCGTACAA 0.44065722454
GCGTACAC -0.779789581638
GCGTACCA -0.0175425348121

GCGTACCC 0.268704909852
GCGTACGA -0.209134777577
GCGTACGC -0.289402590304
GCGTACTA 0.21933050289
GCGTAGAA 0.149382697736
GCGTAGAC -0.841499367311
GCGTAGCA 0.742894404378
GCGTAGCC -0.484575078167
GCGTAGGA 0.00995715333476
GCGTAGTA 0.723234074858
GCGTATAA 0.851670527621
GCGTATAC 2.24446726845
GCGTATCA 2.49196425359
GCGTATCC 9.02690852238
GCGTATGA -0.267401715625
GCGTATTA 1.22387299956
GCGTCAAA 0.78132655975
GCGTCAAC -0.0776961899979
GCGTCACA 0.137713904955
GCGTCACC -0.578725448437
GCGTCAGA 0.0680296531393
GCGTCAGC -1.03694810737
GCGTCATA -0.101462205867
GCGTCCAA -0.327177319579
GCGTCCAC 0.266834638776
GCGTCCCA -0.819744559018
GCGTCCCC -0.349674490589
GCGTCCGA -0.82686299726
GCGTCCTA -1.03326148332
GCGTCGAA -0.620161404049
GCGTCGAC -0.367193501288
GCGTCGCA 0.831844696598
GCGTCGCC 0.0850550738204
GCGTCGGA -0.950884203001
GCGTCGTA 0.0708512976365
GCGTCTAA 0.352596060522
GCGTCTAC 0.259966846836
GCGTCTCA -0.981161401911
GCGTCTCC -1.11111047596
GCGTCTGA -0.221339420508
GCGTCTTA -0.84078219413
GCGTGAAA 1.51720786395
GCGTGAAC -1.00593978735
GCGTGACA -0.668248854668
GCGTGACC -0.989426692708
GCGTGAGA -1.04656801236
GCGTGAGC -1.0695822975
GCGTGATA 1.35881522274
GCGTGCAA -0.376371236222
GCGTGCAC -0.714255358083
GCGTGCCA -1.33305860892
GCGTGCCC -0.769264102387
GCGTGCGA -0.0771940646824
GCGTGCTA -0.143946545844

GCGTGGAA 0.316715334614
GCGTGGAC -0.464266482381
GCGTGGCA 0.475625922667
GCGTGGCC -0.972304052905
GCGTGGGA -0.342108469665
GCGTGGTA 0.941436669389
GCGTGTAA 0.474418490316
GCGTGTAC -0.163476764119
GCGTGTCA 1.19167286055
GCGTGTCC -0.320806032148
GCGTGTGA -0.389483951553
GCGTGTTA 0.519944727105
GCGTTAAA 1.1434967261
GCGTTAAC -0.595717976995
GCGTTACA 0.233931066325
GCGTTACC 0.682197612812
GCGTTAGA 0.40404392028
GCGTTAGC -0.690341119485
GCGTTATA 0.316314175625
GCGTTCAA -1.12320644998
GCGTTCAC -0.970813498486
GCGTTCCA -1.30873925594
GCGTTCCC -0.524696806118
GCGTTCGA -0.204944154607
GCGTTCTA -0.765044750854
GCGTTGAA 0.181086956778
GCGTTGAC -0.35725633304
GCGTTGCA 1.30758032907
GCGTTGCC -0.640132126954
GCGTTGGA 0.253609507331
GCGTTGTA 0.445430121352
GCGTTTAA -0.00389355297554
GCGTTTAC -0.264995594399
GCGTTTCA 1.4686657526
GCGTTTCC 0.15780183207
GCGTTTGA 0.453619427183
GCGTTTTA 0.0824769975734
GCTAAAAA 0.508300499327
GCTAAAAC -0.338875257278
GCTAAACA -0.216265081964
GCTAAACC -0.832670746868
GCTAAAGA 0.533107613332
GCTAAAGC -0.745532435886
GCTAAATA 0.933817979434
GCTAACAA 1.79508343068
GCTAACAC -0.359893948193
GCTAACCA -0.641371826893
GCTAACCC -1.11507251949
GCTAACGA 0.130606708325
GCTAACTA 0.984510777616
GCTAAGAA 0.849864167188
GCTAAGAC -0.458141261337
GCTAAGCA -1.27603366076
GCTAAGCC -0.210550804275

GCTAAGGA -0.792892096247
GCTAAGTA 0.913456714616
GCTAATAA 0.933901875164
GCTAATAC 0.751445523558
GCTAATCA -0.344302041162
GCTAATCC -0.29928084418
GCTAATGA 0.0364561298763
GCTAATTA 0.033049297045
GCTACAAA -0.524519438469
GCTACAAC 0.350354816265
GCTACACA -0.0616771017279
GCTACACC -0.277773559519
GCTACAGA -0.149962473443
GCTACAGC -1.26902347508
GCTACATA -0.0485473158022
GCTACCAA -0.40764061144
GCTACCAC -1.04001894092
GCTACCCA -0.861848557805
GCTACCCC -0.794042071472
GCTACCGA -0.648703439399
GCTACCTA -0.389711073742
GCTACGAA 1.00389985121
GCTACGAC -1.43285747263
GCTACGCA -0.513214332639
GCTACGCC -0.622933085828
GCTACGGA -0.311676178151
GCTACGTA -0.42011068131
GCTACTAA -0.0833736201829
GCTACTAC -1.09675119082
GCTACTCA -0.873516517874
GCTACTCC -0.550265227037
GCTACTGA -0.462651437523
GCTACTTA -0.227554366267
GCTAGAAA 0.152307598517
GCTAGAAC -0.458416264463
GCTAGACA -0.409755908012
GCTAGACC -1.36182193747
GCTAGAGA -0.953643810457
GCTAGAGC -1.48867915199
GCTAGATA 1.42460592158
GCTAGCAA -0.230384962418
GCTAGCAC -0.059586370159
GCTAGCCA -0.573055720838
GCTAGCCC -1.53038927802
GCTAGCGA -0.0663708909028
GCTAGCTA -0.699602125616
GCTAGGAA 1.00158095657
GCTAGGAC -1.25392682349
GCTAGGCA -1.25641413413
GCTAGGCC -1.24576083359
GCTAGGGA -0.711952909497
GCTAGGTA 1.5562928655
GCTAGTAA -0.731094459462
GCTAGTAC -1.03168828223

GCTAGTCA 0.421856670125
GCTAGTCC -1.33521991282
GCTAGTGA -1.17566272393
GCTAGTTA 1.53491631662
GCTATAAA 0.415499538798
GCTATAAC 0.39176662323
GCTATACA 0.205713164108
GCTATACC -1.08361536773
GCTATAGA -0.0500293349239
GCTATAGC -0.658331255151
GCTATATA 1.31891999244
GCTATCAA -0.256133249122
GCTATCAC -0.112782092334
GCTATCCA 1.16329362038
GCTATCCC 0.220560210286
GCTATCGA 0.184390949682
GCTATCTA 0.883454935189
GCTATGAA 0.947007928799
GCTATGAC -0.549943800218
GCTATGCA -0.191999230738
GCTATGCC -0.199153891951
GCTATGGA 0.63993352515
GCTATGTA 1.46596693311
GCTATTAA -0.218437211128
GCTATTAC 0.196176738494
GCTATTCA -0.812689615063
GCTATTCC 0.375748992206
GCTATTGA 0.830014395696
GCTATTTA -0.233776806434
GCTCAAAA 0.607626174467
GCTCAAAC -0.192037535488
GCTCAACA -0.660276470304
GCTCAACC -0.720443032525
GCTCAAGA -0.547859522167
GCTCAAGC -0.256402631443
GCTCAATA -0.192232598267
GCTCACAA 1.48930326961
GCTCACAC -1.67499658081
GCTCACCA -0.700509781659
GCTCACCC -1.31407777236
GCTCACGA -0.382169409642
GCTCACTA -0.47760736079
GCTCAGAA -0.365645489743
GCTCAGAC -0.598324157271
GCTCAGCA -1.38981250932
GCTCAGCC -0.683893847087
GCTCAGGA -0.619040990098
GCTCAGTA -0.353760191851
GCTCATAA 0.681049303011
GCTCATAC -0.814935231058
GCTCATCA -1.2500059992
GCTCATCC -1.39651146891
GCTCATGA -0.342249822521
GCTCATTA 0.610720323955

GCTCCAAA 0.308523530648
GCTCCAAC -0.642238055516
GCTCCACA -0.600573936826
GCTCCACC -1.16430661449
GCTCCAGA -0.541813408759
GCTCCAGC -1.22838962927
GCTCCATA -0.707273484603
GCTCCCAA -0.477803048102
GCTCCCAC -0.98515612939
GCTCCCCA -0.503897326628
GCTCCCCC -0.709105034573
GCTCCCGA -0.545276449648
GCTCCCTA -1.28436161314
GCTCCGAA -0.663478247814
GCTCCGAC -1.24648279487
GCTCCGCA -0.194941202114
GCTCCGCC -0.44709387986
GCTCCGGA -1.17256586813
GCTCCGTA 0.0874624441145
GCTCCTAA -0.363768973327
GCTCCTAC -1.03834477351
GCTCCTCA -1.01657913997
GCTCCTCC -1.57186666105
GCTCCTGA -1.03091406828
GCTCCTTA -0.0874845109816
GCTCGAAA -0.515981642681
GCTCGAAC -0.967541356815
GCTCGACA -1.04809437339
GCTCGACC -1.32336750705
GCTCGAGA -0.310312612307
GCTCGAGC -0.152009487634
GCTCGATA 0.657209592133
GCTCGCAA -0.673650240836
GCTCGCAC -0.750321986937
GCTCGCCA -0.560249859867
GCTCGCCC -1.37094575431
GCTCGCGA -0.200117339696
GCTCGCTA -0.02000132507
GCTCGGAA 1.09021731637
GCTCGGAC -0.689323545462
GCTCGGCA -1.15386440644
GCTCGGCC -0.973143842923
GCTCGGGA -0.672481945947
GCTCGGTA 0.518973368597
GCTCGTAA -0.332466081453
GCTCGTAC -1.39657246506
GCTCGTCA -0.834492304294
GCTCGTCC -0.970095700771
GCTCGTGA -1.13611723266
GCTCGTTA 1.79795024981
GCTCTAAA -0.482489967404
GCTCTAAC -1.21579028086
GCTCTACA 0.18540789917
GCTCTACC -1.13740252358

GCTCTAGA −0.738726889165
GCTCTATA −0.329109835874
GCTCTCAA −0.592628199245
GCTCTCAC −1.02694473852
GCTCTCCA −1.18592111082
GCTCTCCC −1.43441547672
GCTCTCGA 0.135155397439
GCTCTCTA −0.483958246778
GCTCTGAA 0.115468837493
GCTCTGAC −1.33214304211
GCTCTGCA −0.715740499875
GCTCTGCC −0.589971639895
GCTCTGGA −0.758018951818
GCTCTGTA −0.456998572341
GCTCTTAA 0.358449609288
GCTCTTAC −1.01961312601
GCTCTTCA −0.382297230929
GCTCTTCC −0.464643284546
GCTCTTGA −1.26873369131
GCTCTTTA −0.364211351559
GCTGAAAA 2.27551430957
GCTGAAAC −0.166000297732
GCTGAACA −0.955891300053
GCTGAACC −0.902064382208
GCTGAAGA 0.307041303348
GCTGAAGC −1.10887714247
GCTGAATA 0.120603547654
GCTGACAA −0.491163495243
GCTGACAC −0.807499945912
GCTGACCA −0.387716104049
GCTGACCC −0.970900933242
GCTGACGA −0.490230857841
GCTGACTA −0.616945262258
GCTGAGAA 1.16200687222
GCTGAGAC −1.34599812016
GCTGAGCA −1.07088840622
GCTGAGCC −0.511579302694
GCTGAGGA −0.315171902985
GCTGAGTA −0.268527125847
GCTGATAA 2.49971971649
GCTGATAC 1.82413924932
GCTGATCA −0.0247769281956
GCTGATCC 0.436703091769
GCTGATGA −0.13203647477
GCTGATTA 1.54329027633
GCTGCAAA −0.138036580842
GCTGCAAC −0.147902552217
GCTGCACA −0.665119523099
GCTGCACC −0.265623875578
GCTGCAGA −0.0336398980069
GCTGCAGC 0.112849958359
GCTGCATA −0.950911890674
GCTGCCAA −0.206707005839
GCTGCCAC −1.05128178662

GCTGCCCA -0.756317721271
GCTGCCCC -0.93478746434
GCTGCCGA -0.864927926655
GCTGCCTA 0.629908297612
GCTGCGAA 0.467122892603
GCTGCGAC -0.544940034014
GCTGCGCA -0.780947051271
GCTGCGCC -0.539525948988
GCTGCGGA -0.238955025795
GCTGCGTA 0.368888902851
GCTGCTAA -0.0453661479137
GCTGCTAC -1.16156324492
GCTGCTCA -1.24130832271
GCTGCTCC -0.67930393873
GCTGCTGA 0.209461825201
GCTGCTTA -0.568749350905
GCTGGAAA 0.117725903277
GCTGGAAC -0.691097638306
GCTGGACA -0.0644235939701
GCTGGACC -1.33054839869
GCTGGAGA -0.264880055614
GCTGGATA 1.32812603958
GCTGGCAA 0.309136406655
GCTGGCAC -1.33209141397
GCTGGCCA -0.751753418807
GCTGGCCC -1.39931375285
GCTGGCGA -0.142730370017
GCTGGCTA -0.806487784518
GCTGGGAA 0.459601213591
GCTGGGAC -0.841004320047
GCTGGGCA -0.729524797406
GCTGGGCC -1.35377981348
GCTGGGGA -0.995054576531
GCTGGGTA -0.0479152874199
GCTGGTAA 1.02786259528
GCTGGTAC -0.37972664909
GCTGGTCA -0.626052424853
GCTGGTCC -0.855291783783
GCTGGTGA -0.56424937544
GCTGGTTA -0.0895219489846
GCTGTAAA -0.00224290968072
GCTGTAAC 0.483629533729
GCTGTACA -0.4295584231
GCTGTACC -0.437819966694
GCTGTAGA -0.111398957758
GCTGTATA 0.449254351056
GCTGTCAA 0.0640059889191
GCTGTCAC -1.05352990075
GCTGTCCA 0.0061697711349
GCTGTCCC -1.18221262808
GCTGTCGA 0.0279718358316
GCTGTCTA -0.0866428473618
GCTGTGAA 2.00471450459
GCTGTGAC -0.665847313357

GCTGTGCA 0.336100452829
GCTGTGCC -0.258263950865
GCTGTGGA 0.0201166556773
GCTGTGTA -0.128376289323
GCTGTTAA 0.732316464272
GCTGTTAC -0.66760704192
GCTGTTCA -0.877996924607
GCTGTTCC 0.397922238261
GCTGTTGA 0.0813830222279
GCTGTTTA -0.587402098946
GCTTAAAA 1.28811381326
GCTTAAAC 0.520988323377
GCTTAACA -1.04124136209
GCTTAACC -0.746846455369
GCTTAAGA 0.09671366588
GCTTAAGC -0.0998942092345
GCTTAATA 0.549131489695
GCTTACAA 1.72283900593
GCTTACAC -0.861091622627
GCTTACCA 0.230901660193
GCTTACCC -1.32227415624
GCTTACGA -0.207894453103
GCTTACTA -0.720076847438
GCTTAGAA 0.588043703516
GCTTAGAC -0.966060378583
GCTTAGCA -0.0929452278773
GCTTAGCC -0.0495680124947
GCTTAGGA 0.272403608228
GCTTAGTA -0.588176104718
GCTTATAA 2.63557084478
GCTTATAC 0.295086890363
GCTTATCA 0.268784225667
GCTTATCC 0.32154173317
GCTTATGA -0.351689029013
GCTTATTA 0.44459907481
GCTTCAAA 0.228246141733
GCTTCAAC -0.107067398289
GCTTCACA -0.488361419477
GCTTCACC -0.943421854717
GCTTCAGA -0.332531032987
GCTTCATA -0.340530480489
GCTTCCAA -0.736181913219
GCTTCCAC -1.44391713661
GCTTCCCA -0.0745445833842
GCTTCCCC -1.00003606769
GCTTCCGA 0.409059136274
GCTTCCTA -0.772631173221
GCTTCGAA 0.172226901459
GCTTCGAC -0.929197052556
GCTTCGCA -0.528604931553
GCTTCGCC -0.908214584612
GCTTCGGA -0.607716107359
GCTTCGTA 0.189875815224
GCTTCTAA 0.140667117943

GCTTCTAC -0.308181910564
GCTTCTCA -0.559976313986
GCTTCTCC -0.948271569207
GCTTCTGA -0.068503882604
GCTTCTTA -0.0639331266221
GCTTGAAA -0.251554374198
GCTTGAAC -0.420876359963
GCTTGACA 0.386723095029
GCTTGACC -1.2484074004
GCTTGAGA -0.507650151282
GCTTGATA 0.285604799204
GCTTGCAA 0.711500746899
GCTTGCAC -0.271036503357
GCTTGCCA -1.11390006105
GCTTGCCC -1.6111342347
GCTTGCGA 0.0561310318556
GCTTGCTA -0.997922852899
GCTTGGAA 1.05767076918
GCTTGGAC 0.223563802347
GCTTGGCA -1.01921633876
GCTTGGCC -0.935689499577
GCTTGGGA -0.565709535873
GCTTGGTA -0.508586744066
GCTTGTAA 0.225610816538
GCTTGTAC -0.912642738669
GCTTGTCA 0.193602617629
GCTTGTCC -0.400683511141
GCTTGTGA -0.243489975162
GCTTGTTA -0.197080855511
GCTTTAAA 0.0195029469583
GCTTTAAC -0.442207109687
GCTTTACA -0.151001281621
GCTTTACC -0.159891939105
GCTTTAGA 0.127756751621
GCTTTATA 1.17264539212
GCTTTCAA 0.665057486058
GCTTTCAC -0.583955087762
GCTTTCCA -0.680115832897
GCTTTCCC -0.938026089354
GCTTTCGA -0.263457783576
GCTTTCTA -0.69526494534
GCTTTGAA -0.316764048264
GCTTTGAC -0.0810832459201
GCTTTGCA 0.401223316673
GCTTTGCC 0.161868380956
GCTTTGGA -0.363171502492
GCTTTGTA -0.519046439072
GCTTTTAA 1.25455073293
GCTTTTAC -0.740245963969
GCTTTTCA 0.321055845738
GCTTTTCC -0.034768639077
GCTTTTGA 0.201579165552
GCTTTTTA -0.0209014867054
GGAAAAAA -0.0813076617949

GGAAAAAC -0.302219484708
GGAAAACA -0.522979337688
GGAAAACC -0.315209999558
GGAAAAGA 0.295653967211
GGAAAATA 1.31389374302
GGAAACAA 0.370022223837
GGAAACAC 1.06322079443
GGAAACCA -0.0352128909109
GGAAACCC -0.803853916569
GGAAACGA 0.232071204149
GGAAACTA 0.360722912955
GGAAAGAA -0.251485259105
GGAAAGAC -1.24567672968
GGAAAGCA -0.517796954767
GGAAAGCC -0.946929653874
GGAAAGGA -0.55629322896
GGAAAGTA -0.354067878922
GGAAATAA 1.9838867129
GGAAATAC 1.18185497829
GGAAATCA 9.85215834838
GGAAATCC 24.9786489222
GGAAATGA -0.0826512425523
GGAAATTA 3.38392346792
GGAACAAA -0.00617372651674
GGAACAAC 0.191649491712
GGAACACA 0.208145932117
GGAACACC -0.900742035606
GGAACAGA 1.20057205333
GGAACATA 0.241400909017
GGAACCAA -0.288763067514
GGAACCAC 0.0752909014838
GGAACCCA -1.08721351613
GGAACCCC -1.33439927518
GGAACCGA -0.400159735314
GGAACCTA -0.854700974643
GGAACGAA -0.325629516212
GGAACGAC -1.39526219278
GGAACGCA -0.983864176775
GGAACGCC -1.64578421232
GGAACGGA -0.695027830608
GGAACGTA -0.415525352869
GGAACTAA -0.178283423766
GGAACTAC 0.170586042529
GGAACTCA -0.457526928084
GGAACTCC -1.06257356906
GGAACTGA -0.096786111821
GGAACTTA 0.423535833805
GGAAGAAA -0.0772419456204
GGAAGAAC -0.614052629065
GGAAGACA -0.203622016183
GGAAGACC -1.6788114425
GGAAGAGA 0.490329117853
GGAAGATA 1.41220330138
GGAAGCAA -0.35141569131

GGAAGCAC -0.65256639021
GGAAGCCA -0.888370433925
GGAAGCCC -1.01657268645
GGAAGCGA -0.628160851551
GGAAGCTA -0.959222147918
GGAAGGAA -0.571685076942
GGAAGGAC -1.23138531057
GGAAGGCA -1.26409465295
GGAAGGCC -1.11902290506
GGAAGGGA -1.25461235361
GGAAGGTA -0.140052992868
GGAAGTAA 0.359286276636
GGAAGTAC -0.00212175008965
GGAAGTCA -0.177977610297
GGAAGTCC -1.31472270778
GGAAGTGA 0.515668959337
GGAAGTTA -0.506999595058
GGAATAAA 0.742782404618
GGAATAAC 1.0835144014
GGAATACA 0.910972734821
GGAATACC 1.03912877183
GGAATAGA 0.586348093775
GGAATATA 2.89091447478
GGAATCAA 4.08136054242
GGAATCAC 4.04171221122
GGAATCCA 10.999450992
GGAATCCC 5.78644217385
GGAATCGA 5.02411227218
GGAATCTA 14.7093435144
GGAATGAA -0.742777824702
GGAATGAC -0.507027907265
GGAATGCA 0.994877625238
GGAATGCC -0.584948096782
GGAATGGA 0.165842290637
GGAATGTA 0.390747591961
GGAATTAA 1.22051175771
GGAATTAC 1.65041950348
GGAATTCA 0.580446872249
GGAATTCC 1.69860875314
GGAATTGA 1.50757005565
GGAATTTA 1.61559153368
GGACAAAA 2.06588719105
GGACAAAC -0.215295597058
GGACAACA 0.358276197021
GGACAACC -0.507398047734
GGACAAGA 0.868822104165
GGACAATA 0.329781209897
GGACACAA 0.0557833746097
GGACACAC -0.0567876252409
GGACACCA -0.704496598374
GGACACCC -0.864794484563
GGACACGA 0.552237921685
GGACACTA -0.718622932346
GGACAGAA -0.105137796485

GGACAGAC -0.54877446444
GGACAGCA -0.81419911368
GGACAGCC -1.48749253744
GGACAGGA 0.254648315508
GGACAGTA -0.170489864297
GGACATAA -0.728614435049
GGACATAC -0.754597338349
GGACATCA 0.194816711675
GGACATCC 0.0434802634884
GGACATGA -0.803011628415
GGACATTA 0.752558859456
GGACCAAA 0.153247938505
GGACCAAC -0.183610074035
GGACCACA -0.784726522707
GGACCACC -1.40204463175
GGACCAGA -0.340563788968
GGACCATA -0.725017119355
GGACCCAA -0.768198022892
GGACCCAC -1.21460928712
GGACCCCA -1.06442198144
GGACCCCC -1.59779689532
GGACCCGA 0.130692269479
GGACCCTA -1.22515891502
GGACCGAA -1.19410021593
GGACCGAC -1.33720884535
GGACCGCA -1.18083095075
GGACCGCC -1.50367754357
GGACCGGA -0.581140104961
GGACCGTA -0.732043751103
GGACCTAA -0.256268356638
GGACCTAC -0.531047692105
GGACCTCA -1.18786028881
GGACCTCC -1.19821589482
GGACCTGA -0.971546701372
GGACCTTA -0.189500262128
GGACGAAA -0.00407237787026
GGACGAAC -0.7575792799
GGACGACA -0.390847517397
GGACGACC -1.65610005634
GGACGAGA -0.424253215164
GGACGATA 1.24205172632
GGACGCAA 0.808772746153
GGACGCAC -0.310778098296
GGACGCCA -0.921404950333
GGACGCCC -1.70762556649
GGACGCGA 0.279603027709
GGACGCTA -0.73758836373
GGACGGAA 0.31996207857
GGACGGAC -1.11261893369
GGACGGCA -0.770390137143
GGACGGCC -1.55441759815
GGACGGGA -0.836257653662
GGACGGTA 0.0607419661899
GGACGTAA -0.315106535096

GGACGTAC −0.72607278995
GGACGTCA −0.537726250252
GGACGTCC −0.962562363792
GGACGTGA 0.306939712488
GGACGTTA −0.771215771057
GGACTAAA 0.410872782936
GGACTAAC −0.0669356777939
GGACTACA −0.683336554604
GGACTACC −1.16417587871
GGACTAGA 0.229300771438
GGACTATA −0.50057730402
GGACTCAA −1.04768405457
GGACTCAC −0.655934710113
GGACTCCA −0.819899443444
GGACTCCC −1.40613366386
GGACTCGA 0.13607262967
GGACTCTA −0.413669029719
GGACTGAA −1.11655079141
GGACTGAC −0.699227821587
GGACTGCA −0.875253763213
GGACTGCC −1.43794159554
GGACTGGA −0.507698656754
GGACTGTA −0.765978429146
GGACTTAA −0.282533757473
GGACTTAC 0.0373450499001
GGACTTCA −0.608309830991
GGACTTGA 0.767279957949
GGACTTTA −1.23667844418
GGAGAAAA 0.450477604934
GGAGAAAC −0.44926746627
GGAGAACA −0.0797700591495
GGAGAACC −0.15977327765
GGAGAAGA −0.667997375654
GGAGAATA 1.80903468696
GGAGACAA −0.281899230955
GGAGACAC 0.60011844338
GGAGACCA −0.605697613553
GGAGACCC −1.46944288104
GGAGACGA −0.407268597369
GGAGACTA −0.705581413888
GGAGAGAA −0.82573946064
GGAGAGAC −1.32592559821
GGAGAGCA −0.349453405562
GGAGAGCC −0.599105449274
GGAGAGGA −0.809662498888
GGAGAGTA −0.715253363375
GGAGATAA 0.960954188807
GGAGATAC 2.35014965856
GGAGATCA 3.60477533557
GGAGATCC 6.41075069167
GGAGATGA 0.194887075836
GGAGATTA 4.93715132885
GGAGCAAA −0.74498992404
GGAGCAAC −0.796783775621

GGAGCACA 0.0536104127342
GGAGCACC -1.02387140683
GGAGCAGA -0.196850402475
GGAGCATA 0.553600654818
GGAGCCAA -0.00702808899397
GGAGCCAC -1.14577002977
GGAGCCCA -1.05360380394
GGAGCCCC -1.68973058633
GGAGCCGA -0.195875921296
GGAGCCTA -0.838269485772
GGAGCGAA -0.266468245511
GGAGCGAC -0.410055476142
GGAGCGCA 0.408094647639
GGAGCGCC -1.23133243336
GGAGCGGA -0.559783541166
GGAGCGTA 0.419693908971
GGAGCTAA -0.618474329605
GGAGCTAC -0.891770813239
GGAGCTCA -0.72716738983
GGAGCTCC -1.13653504589
GGAGCTGA -0.807499945912
GGAGCTTA 0.139758004654
GGAGGAAA -0.640997939221
GGAGGAAC -0.793076541947
GGAGGACA -0.937149035476
GGAGGACC -1.64661130348
GGAGGAGA 0.0222040563983
GGAGGATA 1.8760078368
GGAGGCAA -0.553553814769
GGAGGCAC -1.18095544119
GGAGGCCA -0.724104675218
GGAGGCCC -1.4290107597
GGAGGCGA -1.10502584962
GGAGGCTA -0.380399688537
GGAGGGAA -0.740015302755
GGAGGGAC -0.858565799056
GGAGGGCA -1.05268178361
GGAGGGCC -1.74095174026
GGAGGGGA -0.808357639237
GGAGGGTA 0.116130635328
GGAGGTAA -0.0593842293292
GGAGGTAC -0.845956666287
GGAGGTCA -0.619167145961
GGAGGTGA -0.518681503053
GGAGGTTA -0.787121194144
GGAGTAAA -1.01730672205
GGAGTAAC -0.501076098487
GGAGTACA -0.00667085556051
GGAGTACC -0.837878111148
GGAGTAGA 0.36439891993
GGAGTATA 0.709316543412
GGAGTCAA -0.801280836593
GGAGTCAC 0.275850411233
GGAGTCCA -0.453719144441

GGAGTCCC -1.28574807856
GGAGTCGA 0.17352967933
GGAGTCTA -0.667219622678
GGAGTGAA -1.10714510158
GGAGTGAC -0.905306962604
GGAGTGCA -0.171974589733
GGAGTGCC -1.53562537086
GGAGTGGA -0.578622400331
GGAGTGTA 0.503773668901
GGAGTTAA -0.80048934387
GGAGTTAC 0.335331651507
GGAGTTCA -0.101199485241
GGAGTTGA 0.890784465915
GGAGTTTA -0.207350275833
GGATAAAA 0.843008657749
GGATAAAC -0.181925081372
GGATAACA 1.59182260332
GGATAACC -0.195549914562
GGATAAGA 0.212782472344
GGATAATA 3.02552007414
GGATACAA 0.18198128943
GGATACAC 1.65649809266
GGATACCA 1.66704085068
GGATACCC 3.62668544464
GGATACGA 4.31747268956
GGATACTA 1.50832948897
GGATAGAA 0.00343077330037
GGATAGAC -0.25224344335
GGATAGCA 0.158060389135
GGATAGCC 0.800264719817
GGATAGGA -0.190766817028
GGATAGTA 0.513211418147
GGATATAA 2.32140693963
GGATATAC 1.70447729072
GGATATCA 8.42441414358
GGATATCC 16.7507473082
GGATATGA 3.61111331452
GGATATTA 3.14319830466
GGATCAAA 0.0926233847024
GGATCAAC -0.327016397991
GGATCACA 2.79804523187
GGATCACC 1.65399121328
GGATCAGA 0.433768822978
GGATCATA 1.72234479138
GGATCCAA 1.81224062803
GGATCCAC 1.675554706
GGATCCCA 1.05121204699
GGATCCCC 6.85231765403
GGATCCGA 2.2653854094
GGATCCTA 3.30466032218
GGATCGAA 0.516808109307
GGATCGAC -0.738400674253
GGATCGCA 3.87969852203
GGATCGCC 1.8088564866

GGATCGGA 0.247078131024
GGATCGTA 3.06734823709
GGATCTAA 1.81216318582
GGATCTAC 2.12881648339
GGATCTCA 4.96038232728
GGATCTGA 2.36771967287
GGATCTTA 3.16014732402
GGATGAAA -0.0586583126727
GGATGAAC -0.570154552348
GGATGACA 0.33314848891
GGATGACC -1.16594830613
GGATGAGA 0.395523611443
GGATGATA 1.19346527305
GGATGCAA -0.549459161854
GGATGCAC -0.0655714874154
GGATGCCA -0.554221857944
GGATGCCC -0.331681250425
GGATGCGA 0.149737641212
GGATGCTA -0.098800858423
GGATGGAA -0.429402081428
GGATGGAC -1.40989648105
GGATGGCA -0.57325036726
GGATGGCC -0.930932007937
GGATGGGA -0.383532975487
GGATGGTA 0.790260310078
GGATGTAA 0.999860781822
GGATGTAC -0.0786883663055
GGATGTCA 0.794421163595
GGATGTGA 0.0703112839265
GGATGTTA 1.48625138025
GGATTAAA -0.621336776989
GGATTAAC -0.234783555201
GGATTACA 13.0638467959
GGATTACC 11.0556082543
GGATTAGA 1.05986892059
GGATTATA 2.68509971981
GGATTCAA 1.20235197515
GGATTCAC 1.97679929317
GGATTCCA 6.22900235341
GGATTCCC 17.6911607828
GGATTCGA 3.26964832303
GGATTCTA 7.73607902722
GGATTGAA -0.540082200582
GGATTGAC -0.491163911599
GGATTGCA 9.03359457493
GGATTGCC 6.93287379327
GGATTGGA 0.258728187786
GGATTGTA 3.18551089779
GGATTTAA 2.11994893367
GGATTTAC 3.65196907807
GGATTTCA 12.605673267
GGATTTGA 4.95528050924
GGATTTTA 7.02597723642
GGCAAAAA 0.640083621482

GGCAAAAC −0.710228779371
GGCAAACA 0.165535852633
GGCAAACC −1.07991854296
GGCAAAGA 0.0206058739574
GGCAAATA 2.11208313643
GGCAACAA 0.882283725809
GGCAACAC −0.899186321475
GGCAACCA −0.317183735095
GGCAACCC −0.11853717291
GGCAACGA −0.524793608884
GGCAACTA 0.247781772636
GGCAAGAA 0.152051955944
GGCAAGAC −0.798014732084
GGCAAGCA −1.01067208946
GGCAAGCC −1.74958321614
GGCAAGGA −0.364868361301
GGCAAGTA 0.867127118958
GGCAATAA 1.00633844821
GGCAATAC 0.227421965064
GGCAATCA 4.92826462675
GGCAATGA 0.258378865117
GGCAATTA 0.263114914424
GGCACAAA 0.663128925144
GGCACAAC −1.10806566466
GGCACACA 0.612151339469
GGCACACC −0.843977518122
GGCACAGA −0.38492360447
GGCACATA 0.717699454775
GGCACCAA −0.517456791929
GGCACCAC −0.441653148052
GGCACCCA −0.892805041501
GGCACCCC −1.16557233668
GGCACCGA −1.15513096134
GGCACCTA −0.815230843806
GGCACGAA −0.77570658669
GGCACGAC −0.942116786888
GGCACGCA −0.675847351358
GGCACGCC −1.31647744007
GGCACGGA −0.221270721771
GGCACGTA −0.874818879389
GGCACTAA −0.316944330405
GGCACTAC −1.1414574145
GGCACTCA −0.409396176442
GGCACTGA −0.768284000402
GGCACTTA −0.625470151011
GGCAGAAA 0.295522398721
GGCAGAAC −0.757474774548
GGCAGACA −0.267911543527
GGCAGACC −1.23370024983
GGCAGAGA −0.115853966777
GGCAGATA 2.01089572552
GGCAGCAA −0.156942473321
GGCAGCAC −0.605937226422
GGCAGCCA 0.792053555297

GGCAGCCC -0.827060349996
GGCAGCGA -1.10384423134
GGCAGCTA -0.395737618418
GGCAGGAA -0.960866337695
GGCAGGAC -1.13011213031
GGCAGGCA -1.03536428921
GGCAGGCC -1.90597984677
GGCAGGGA -1.10883633958
GGCAGGTA -0.313896188253
GGCAGTAA -0.125495105921
GGCAGTAC -0.598453643982
GGCAGTCA -0.545100955601
GGCAGTGA 0.0847101228885
GGCAGTTA 0.63610679731
GGCATAAA 1.02287215247
GGCATAAC -0.221305279317
GGCATACA 0.706598987911
GGCATACC -0.918595796515
GGCATAGA -0.628143780956
GGCATATA 1.51117382486
GGCATCAA -0.56047344303
GGCATCAC 0.315220408457
GGCATCCA -0.300979784769
GGCATCCC -0.521425497159
GGCATCGA -0.161556113969
GGCATCTA 1.11382636604
GGCATGAA 0.505574616706
GGCATGAC -1.3437606231
GGCATGCA 0.0255009712502
GGCATGCC -1.11435680356
GGCATGGA 0.263000416528
GGCATGTA 0.169961508555
GGCATTAA 1.52060074885
GGCATTAC -0.852541127981
GGCATTCA -0.660798997063
GGCATTGA -0.220891837826
GGCATTTA 0.326261544594
GGCCAAAA -0.275437802454
GGCCAAAC -1.02512089114
GGCCAACA 0.599916510729
GGCCAACC -0.587250545368
GGCCAAGA 0.288496391507
GGCCAATA 0.679248147028
GGCCACAA -0.292181558313
GGCCACAC -0.675518013776
GGCCACCA -1.18080701028
GGCCACCC -1.21754605404
GGCCACGA -1.16208618803
GGCCACTA -0.565220525771
GGCCAGAA -0.106574224627
GGCCAGAC -1.22372935675
GGCCAGCA -1.01283339336
GGCCAGCC -1.12073995714
GGCCAGGA -0.81488214567

GGCCAGTA -1.44448254803
GGCCATAA -0.646256515287
GGCCATAC -0.128376289323
GGCCATCA -0.0762389440572
GGCCATGA -0.261208836733
GGCCATTA -0.638682167243
GGCCCAAA -0.878561919676
GGCCCAAC -1.60301008858
GGCCCACA -0.114068215966
GGCCCACC -1.40599834816
GGCCCAGA -0.802812818433
GGCCCATA -0.685193918644
GGCCCCAA 0.0850719362377
GGCCCCAC -0.891830352144
GGCCCCCA -1.29692036683
GGCCCCCC -1.69703201303
GGCCCCGA -1.24895053678
GGCCCCTA -1.24758593004
GGCCCGAA -0.108662249882
GGCCCGAC -1.10086499611
GGCCCGCA -0.651685797306
GGCCCGCC -1.23340297166
GGCCCGGA -0.670699109628
GGCCCGTA -1.26025043816
GGCCCTAA -0.811728873632
GGCCCTAC -0.634699514087
GGCCCTCA -1.04802963004
GGCCCTGA -0.287831054646
GGCCCTTA -0.655843319975
GGCCGAAA 0.201577916484
GGCCGAAC -0.6189558453
GGCCGACA -0.0933644983523
GGCCGACC -1.13343819009
GGCCGAGA -0.449120492608
GGCCGATA 0.995112658191
GGCCGCAA -0.575882777963
GGCCGCAC -1.3970412819
GGCCGCCA -0.706536742691
GGCCGCCC -1.66775177852
GGCCGCGA -0.857983108858
GGCCGCTA -1.335106664
GGCCGGAA -0.358506233702
GGCCGGAC -1.07336593249
GGCCGGCA -0.251288530903
GGCCGGCC -0.251234196447
GGCCGGGA -1.57939562629
GGCCGGTA -0.806052067981
GGCCGTAA -0.445986164767
GGCCGTAC -0.980467961021
GGCCGTCA -0.543331650852
GGCCGTGA -0.129116778439
GGCCGTTA -1.37753417138
GGCCTAAA 0.191040362909
GGCCTAAC -0.768316267991

GGCCTACA -0.819333615663
GGCCTACC -1.09388707801
GGCCTAGA -0.986176617905
GGCCTATA -0.462220925437
GGCCTCAA -0.688567859353
GGCCTCAC -1.35511277716
GGCCTCCA -0.571209390231
GGCCTCCC -1.0482011687
GGCCTCGA -0.906718825742
GGCCTCTA -1.57112450651
GGCCTGAA -0.365179795576
GGCCTGAC -1.13427673104
GGCCTGCA -0.903753538431
GGCCTGGA -0.772832897695
GGCCTGTA -0.699183271497
GGCCTTAA -0.332702571652
GGCCTTAC -0.291880949293
GGCCTTCA -0.88682554505
GGCCTTGA -0.526795240273
GGCCTTTA -0.731070310815
GGCGAAAA 0.215305589602
GGCGAAAC -0.610687848188
GGCGAACA 0.216637096035
GGCGAACC -0.829361341337
GGCGAAGA 0.680244486895
GGCGAATA 0.886774541442
GGCGACAA -0.138926333578
GGCGACAC -0.660806491471
GGCGACCA -0.916868335541
GGCGACCC -1.43928850714
GGCGACGA 0.680007996697
GGCGACTA -1.3687153353
GGCGAGAA -0.736220217969
GGCGAGAC -0.871888982336
GGCGAGCA -0.23574242303
GGCGAGCC -1.62495537974
GGCGAGGA -0.0822186486859
GGCGAGTA -0.603482599723
GGCGATAA 0.834940303332
GGCGATAC 0.180805083778
GGCGATCA 1.51039711278
GGCGATGA -0.331090857641
GGCGATTA 0.585592615844
GGCGCAAA 0.575573633645
GGCGCAAC 0.751246089042
GGCGCACA -0.252815932827
GGCGCACC -1.19260924515
GGCGCAGA 2.52918606212
GGCGCATA 0.766862769254
GGCGCCAA -0.491992876361
GGCGCCAC -0.185603586482
GGCGCCCA 0.0932266845219
GGCGCCCC -1.03498936065
GGCGCCGA -0.0659160219914

| | |
|---|---|
| GGCGCCTA | -1.28342855938 |
| GGCGCGAA | -0.80927778596 |
| GGCGCGAC | 0.12492615547 |
| GGCGCGCA | -0.227202753639 |
| GGCGCGCC | -0.437232072046 |
| GGCGCGGA | -0.187694942585 |
| GGCGCGTA | -0.129460480303 |
| GGCGCTAA | -0.805125675919 |
| GGCGCTAC | -0.626765018118 |
| GGCGCTCA | 0.00697687720806 |
| GGCGCTGA | -0.666366092912 |
| GGCGCTTA | -0.463010752736 |
| GGCGGAAA | 0.254741162893 |
| GGCGGAAC | -1.28325306533 |
| GGCGGACA | -0.588557903155 |
| GGCGGACC | -1.5250366055 |
| GGCGGAGA | -0.481699931926 |
| GGCGGATA | 0.470895494166 |
| GGCGGCAA | -0.764485792947 |
| GGCGGCAC | -0.430932189666 |
| GGCGGCCA | -0.890688704039 |
| GGCGGCCC | -1.75048712498 |
| GGCGGCGA | -0.121069241821 |
| GGCGGCTA | -1.05794806226 |
| GGCGGGAA | -0.464483403848 |
| GGCGGGAC | -0.778039637441 |
| GGCGGGCA | -0.794106398471 |
| GGCGGGGA | -1.3840994807 |
| GGCGGGTA | -0.721272413644 |
| GGCGGTAA | 0.367371285293 |
| GGCGGTAC | 0.117390320354 |
| GGCGGTCA | -0.181121930681 |
| GGCGGTGA | -0.145422735982 |
| GGCGGTTA | -0.719778736554 |
| GGCGTAAA | 0.177702398992 |
| GGCGTAAC | -0.318352862696 |
| GGCGTACA | -0.494926728795 |
| GGCGTACC | -1.05590875066 |
| GGCGTAGA | 0.857840715112 |
| GGCGTATA | -0.43162417331 |
| GGCGTCAA | -0.91079765713 |
| GGCGTCAC | -0.64342654367 |
| GGCGTCCA | -0.892850007947 |
| GGCGTCCC | -0.95380202573 |
| GGCGTCGA | -0.641762160628 |
| GGCGTCTA | -0.451920278416 |
| GGCGTGAA | 0.0304526929566 |
| GGCGTGAC | -1.43949377064 |
| GGCGTGCA | -0.805820157699 |
| GGCGTGGA | 0.242880221825 |
| GGCGTGTA | -0.806322074836 |
| GGCGTTAA | 0.407455124849 |
| GGCGTTAC | 0.0506980026327 |
| GGCGTTCA | -1.32168626159 |

GGCGTTGA 0.0488154490553
GGCGTTTA -0.545243557526
GGCTAAAA 0.446717910407
GGCTAAAC -0.488894355135
GGCTAACA 0.441633371142
GGCTAACC -1.05794806226
GGCTAAGA 1.042171085
GGCTAATA 0.18625684902
GGCTACAA -0.117075138875
GGCTACAC 0.289516047309
GGCTACCA -1.04322404928
GGCTACCC -1.02463208921
GGCTACGA 0.123759317827
GGCTACTA -1.16365959729
GGCTAGAA -0.425167532903
GGCTAGAC -1.07650171758
GGCTAGCA -0.942202764398
GGCTAGCC -1.31076649323
GGCTAGGA -0.348642760463
GGCTAGTA -1.00559213011
GGCTATAA 0.800279084098
GGCTATAC -0.40133552461
GGCTATCA -0.613536764002
GGCTATGA 0.0380878289739
GGCTATTA -0.3126329642
GGCTCAAA -0.163690562916
GGCTCAAC -1.36148198281
GGCTCACA -0.644291315047
GGCTCACC -1.0457134417
GGCTCAGA -0.222262273544
GGCTCATA -0.286585317545
GGCTCCAA -0.185050249381
GGCTCCAC -0.785954148323
GGCTCCCA -0.947679510999
GGCTCCCC -1.29045206846
GGCTCCGA -0.747656684112
GGCTCCTA -0.913867657971
GGCTCGAA -0.0995532136844
GGCTCGAC -1.4210671039
GGCTCGCA -0.980320154647
GGCTCGGA -0.0967494724945
GGCTCGTA -0.69188392658
GGCTCTAA -0.366670974529
GGCTCTAC -1.22063333366
GGCTCTCA -0.590865764369
GGCTCTGA -0.846320977772
GGCTCTTA -0.533994451576
GGCTGAAA -0.13785338421
GGCTGAAC -0.723463070649
GGCTGACA -0.561269307491
GGCTGACC -1.10342350362
GGCTGAGA -0.660164886901
GGCTGATA 0.912351081303
GGCTGCAA -0.362473689864

GGCTGCAC 0.0563823026914
GGCTGCCA 0.000138854720349
GGCTGCCC -0.692756400542
GGCTGCGA -0.230842329465
GGCTGCTA -1.29057572618
GGCTGGAA -1.17329282567
GGCTGGAC -0.957230300895
GGCTGGCA -1.2692493482
GGCTGGGA -1.05205204519
GGCTGGTA -0.726224343528
GGCTGTAA 0.0686974881362
GGCTGTAC -0.298669633597
GGCTGTCA -0.700876383102
GGCTGTGA -0.631110317335
GGCTGTTA -0.436789485636
GGCTTAAA 1.04461571915
GGCTTAAC -1.07646799275
GGCTTACA -0.975515822958
GGCTTACC -1.45668281923
GGCTTAGA 0.224088619064
GGCTTATA 0.547197516154
GGCTTCAA -0.339101130399
GGCTTCAC -0.674259161461
GGCTTCCA -1.13334430181
GGCTTCCC -1.00705603774
GGCTTCGA -0.643444030621
GGCTTCTA -0.662749624843
GGCTTGAA -0.559454411761
GGCTTGAC -1.38562438449
GGCTTGCA -0.731627811476
GGCTTGGA -0.931631069633
GGCTTGTA -0.986319011651
GGCTTTAA -0.26457986295
GGCTTTAC -1.18344025369
GGCTTTCA -0.451245781723
GGCTTTGA 0.607397178676
GGCTTTTA -0.613817387935
GGGAAAAA -0.0694970998016
GGGAAAAC -0.426556080106
GGGAAACA 1.09399491421
GGGAAACC -0.02879809428
GGGAAAGA -0.206145757974
GGGAAATA 1.42991217041
GGGAACAA 0.399899929181
GGGAACAC -0.93006723656
GGGAACCA -1.38980251678
GGGAACCC -1.34185308817
GGGAACGA 0.347189261726
GGGAACTA -0.0156635202605
GGGAAGAA -1.06904145108
GGGAAGAC -1.029119574
GGGAAGCA -0.229739402466
GGGAAGGA -1.06235394128
GGGAAGTA 0.00649223884378

GGGAATAA 1.30654089635
GGGAATAC 0.947754871432
GGGAATCA 10.6219647544
GGGAATGA -0.441488895616
GGGAATTA 0.679422600185
GGGACAAA 0.519035613816
GGGACAAC -0.238955025795
GGGACACA 0.194445738494
GGGACACC -0.798883458843
GGGACAGA -0.914097070118
GGGACATA -0.789251479531
GGGACCAA 0.156027114692
GGGACCAC -1.37141332207
GGGACCCA -1.13684627198
GGGACCCC -0.930081392664
GGGACCGA -0.456723569214
GGGACCTA -0.756914359395
GGGACGAA -0.326437246819
GGGACGAC -1.31017859858
GGGACGCA 0.0878594395443
GGGACGGA -0.573955882473
GGGACGTA 0.312004683022
GGGACTAA -0.169122551249
GGGACTAC -0.87846511691
GGGACTCA -1.22318517948
GGGACTGA -1.68033821989
GGGACTTA 0.144137028706
GGGAGAAA -0.290699331013
GGGAGAAC -0.645181067782
GGGAGACA 0.370472721011
GGGAGACC -1.03575337387
GGGAGAGA -1.18710293728
GGGAGATA 1.40767334828
GGGAGCAA -0.952463857601
GGGAGCAC -0.5329906173
GGGAGCCA 0.0639785094242
GGGAGCCC -1.28173836227
GGGAGCGA -0.266468245511
GGGAGCTA -0.62775573718
GGGAGGAA -1.17805926897
GGGAGGAC -1.51074643545
GGGAGGCA -1.37299714023
GGGAGGGA -0.17827988474
GGGAGGTA -0.715869986586
GGGAGTAA -0.124038692692
GGGAGTAC 0.0495911202517
GGGAGTCA -0.438881466272
GGGAGTGA -0.762584086994
GGGAGTTA 0.0743361972147
GGGATAAA 0.546350231729
GGGATAAC 0.658962659
GGGATACA 2.45219809365
GGGATACC 1.65914736578
GGGATAGA -0.509766280566

GGGATATA 2.15069099403
GGGATCAA -0.394405695628
GGGATCAC 2.17878086678
GGGATCCA 3.56497316083
GGGATCCC 1.25045941086
GGGATCGA 1.88367045231
GGGATCTA 1.56928650302
GGGATGAA -0.852435165384
GGGATGAC -1.28357928024
GGGATGCA -0.0655714874154
GGGATGGA -1.09671496785
GGGATGTA -0.0212112555567
GGGATTAA -0.092092114468
GGGATTAC 9.10901683673
GGGATTCA 0.954289370408
GGGATTGA 1.56520288354
GGGATTTA 2.0275909759
GGGCAAAA -0.650970914083
GGGCAAAC -0.651401842525
GGGCAACA 0.179038901697
GGGCAACC -1.13227280969
GGGCAAGA -0.334267237436
GGGCAATA -0.174436086305
GGGCACAA -0.887636398327
GGGCACAC -1.12365028546
GGGCACCA -1.45858702331
GGGCACCC -1.80810204956
GGGCACGA -0.438749897782
GGGCACTA -1.49500547297
GGGCAGAA -0.392119901282
GGGCAGAC -1.37854883091
GGGCAGCA -0.629086410901
GGGCAGGA -1.13103810602
GGGCAGTA -0.979162685014
GGGCATAA 0.120548380486
GGGCATAC -0.419968912098
GGGCATCA -0.847879814573
GGGCATGA 0.227456938967
GGGCATTA -0.146985319986
GGGCCAAA -0.670699109628
GGGCCAAC -0.701577318399
GGGCCACA 0.0265485229035
GGGCCACC -1.5501720162
GGGCCAGA -0.835456792929
GGGCCATA -1.03222225878
GGGCCCAA -1.3869750433
GGGCCCAC -0.853469810002
GGGCCCCA -1.11117792563
GGGCCCCC -1.67636118754
GGGCCCGA -1.17823413848
GGGCCCTA -1.14027871071
GGGCCGAA 0.528554760657
GGGCCGAC -0.968074500651
GGGCCGCA -1.17994203072

GGGCCGGA −1.00786605831
GGGCCGTA −1.17322912321
GGGCCTAA −0.787908106952
GGGCCTAC −2.01100834981
GGGCCTCA −1.42738239145
GGGCCTGA −0.855512660632
GGGCCTTA −0.972476840638
GGGCGAAA −0.950103119177
GGGCGAAC −0.28218797383
GGGCGACA −0.262046128615
GGGCGACC −0.560693695345
GGGCGAGA −1.08942145191
GGGCGATA 0.858601813849
GGGCGCAA −0.565949356919
GGGCGCAC −0.545564567989
GGGCGCCA −0.36061778307
GGGCGCCC −0.922106301986
GGGCGCGA 0.0511426708226
GGGCGCTA −1.40113364486
GGGCGGAA −1.19721122783
GGGCGGAC −0.678484966511
GGGCGGCA −0.0666090465251
GGGCGGGA −0.561169173878
GGGCGGTA 0.0316932256081
GGGCGTAA −0.420428360925
GGGCGTAC −0.602427137306
GGGCGTCA −1.04018714874
GGGCGTGA −0.854570238865
GGGCGTTA −0.283995166974
GGGCTAAA −0.470216001201
GGGCTAAC 0.361300398704
GGGCTACA 0.576590583134
GGGCTACC −0.651592533566
GGGCTAGA −0.169910713125
GGGCTATA −1.03799232817
GGGCTCAA −1.65186446692
GGGCTCAC −1.05126346696
GGGCTCCA −1.22252712885
GGGCTCGA −0.947977621883
GGGCTCTA −1.49022299997
GGGCTGAA −0.924319442214
GGGCTGAC −1.58619388678
GGGCTGCA 0.00786454816393
GGGCTGGA −1.02627773623
GGGCTGTA −0.556914640265
GGGCTTAA 0.332164223366
GGGCTTAC −0.273547754469
GGGCTTCA −1.55261477675
GGGCTTGA −0.791097185604
GGGCTTTA −1.36142785653
GGGGAAAA 0.0987225834982
GGGGAAAC 0.0802336715367
GGGGAACA 0.378908093227
GGGGAACC −0.915394019005

GGGGAAGA 0.216438494231
GGGGAATA 1.39204001383
GGGGACAA 0.167823520582
GGGGACAC -0.802984773454
GGGGACCA -1.64382900463
GGGGACCC -1.44995346565
GGGGACGA -0.19825227307
GGGGACTA -0.937433406612
GGGGAGAA 0.585782057816
GGGGAGAC -1.14997938876
GGGGAGCA -0.5329906173
GGGGAGGA -1.4807051022
GGGGAGTA -0.554294720241
GGGGATAA 1.54822097206
GGGGATAC 1.13785177168
GGGGATCA 0.128920050237
GGGGATGA -0.201177382028
GGGGATTA 2.05640926345
GGGGCAAA -0.567134097869
GGGGCAAC -0.442984446307
GGGGCACA -0.497800834147
GGGGCACC -1.7440198675
GGGGCAGA 0.395985350228
GGGGCATA 0.170966175542
GGGGCCAA -1.17844897817
GGGGCCAC -1.24895053678
GGGGCCCA -1.32089934879
GGGGCCCC -0.740105652003
GGGGCCGA -0.59669183364
GGGGCCTA -0.782512757945
GGGGCGAA -0.62803761018
GGGGCGAC -1.18098854149
GGGGCGCA 0.287848541597
GGGGCGGA -0.479182019118
GGGGCGTA -0.521662611891
GGGGCTAA -0.450489679258
GGGGCTAC -0.935188831508
GGGGCTCA -1.04982329161
GGGGCTGA -1.1708833736
GGGGCTTA -1.01277114814
GGGGGAAA 0.258948648279
GGGGGAAC -0.71316533812
GGGGGACA 0.130606708325
GGGGGACC -1.89610554828
GGGGGAGA 0.087301106171
GGGGGATA 0.414411600614
GGGGGCAA -1.62071979032
GGGGGCAC -1.59608067595
GGGGGCCA -0.803811031903
GGGGGCGA -0.657561412939
GGGGGCTA -0.976546928551
GGGGGGAA 0.0394309933752
GGGGGGAC -0.798883458843
GGGGGGCA -0.970813498486

GGGGGGGA −0.991257409966
GGGGGGTA −1.1148331148
GGGGGTAA −0.599616109887
GGGGGTAC −0.800103590051
GGGGGTCA −1.00135695705
GGGGGTGA −0.909279831394
GGGGGTTA −1.11765684108
GGGGTAAA −0.678435003793
GGGGTAAC 0.824878644645
GGGGTACA −0.336162906227
GGGGTACC −0.800103590051
GGGGTAGA −0.397384930865
GGGGTATA −0.627718473319
GGGGTCAA −1.30158979918
GGGGTCAC −0.106418507489
GGGGTCCA −1.40728572086
GGGGTCGA 0.327610537979
GGGGTCTA −1.07650171758
GGGGTGAA 0.286731874851
GGGGTGAC −0.821984554207
GGGGTGCA −0.546832371957
GGGGTGGA 0.0292906434079
GGGGTGTA −0.0616356743076
GGGGTTAA −0.515223874791
GGGGTTAC −0.321301079412
GGGGTTCA −1.12969827247
GGGGTTGA 0.142820719265
GGGGTTTA −0.774880120064
GGGTAAAA 0.630437486066
GGGTAAAC −0.982777071303
GGGTAACA −0.0264331922962
GGGTAACC −0.310778098296
GGGTAAGA −0.236794138243
GGGTAATA 1.19972518526
GGGTACAA 0.152060907597
GGGTACAC −0.385592272179
GGGTACCA −1.2049404603
GGGTACCC −1.42994318893
GGGTACGA 1.01619088801
GGGTACTA −0.100032855777
GGGTAGAA 1.03569758217
GGGTAGAC −0.893706452204
GGGTAGCA −1.22928479463
GGGTAGGA −0.928077887673
GGGTAGTA −0.689951410284
GGGTATAA 0.230692857667
GGGTATAC −0.0771940646824
GGGTATCA 2.12848048412
GGGTATGA 0.128957522276
GGGTATTA 0.77713177322
GGGTCAAA 0.307681242494
GGGTCAAC −1.03802875932
GGGTCACA −0.491789070107
GGGTCACC −0.776085262457

GGGTCAGA -0.360115865932
GGGTCATA -0.194751760142
GGGTCCAA -0.935116385567
GGGTCCAC -1.22588045994
GGGTCCCA -1.37494526988
GGGTCCGA -0.168748871754
GGGTCCTA -1.34517769069
GGGTCGAA -1.08820256977
GGGTCGAC -0.749407461021
GGGTCGCA -1.05557108596
GGGTCGGA -0.62425959599
GGGTCGTA -0.73093395423
GGGTCTAA -0.815359914161
GGGTCTAC -0.689088720687
GGGTCTCA -1.29828705535
GGGTCTGA -0.433340392672
GGGTCTTA -0.830071436466
GGGTGAAA -0.376691830329
GGGTGAAC 0.378286473745
GGGTGACA -0.235360208237
GGGTGACC -1.23349706811
GGGTGAGA 0.125172013678
GGGTGATA 0.286274715981
GGGTGCAA -0.482459989773
GGGTGCAC -0.975016612135
GGGTGCCA -0.932243321106
GGGTGCGA -0.212963379019
GGGTGCTA -0.434936701511
GGGTGGAA -0.430627417086
GGGTGGAC -0.243623000899
GGGTGGCA -0.55767594718
GGGTGGGA -0.534075016458
GGGTGGTA -0.896443368259
GGGTGTAA 0.685698750273
GGGTGTAC -0.38881778198
GGGTGTCA -0.644154750284
GGGTGTGA 0.216959147388
GGGTGTTA 0.116154159441
GGGTTAAA -0.316461357465
GGGTTAAC -0.0390841688414
GGGTTACA 0.541617929625
GGGTTACC -1.53467774464
GGGTTAGA -1.00144480816
GGGTTATA -0.0944237079731
GGGTTCAA -0.473078656763
GGGTTCAC -0.452805451236
GGGTTCCA -0.979479948273
GGGTTCGA 0.411522090092
GGGTTCTA -0.276467034444
GGGTTGAA 0.264886509132
GGGTTGAC -0.996192477433
GGGTTGCA -0.386279675907
GGGTTGGA 0.238213912145
GGGTTGTA -0.145539315657

GGGTTTAA 0.147772440972
GGGTTTAC −0.462651437523
GGGTTTCA −1.36101941131
GGGTTTGA −0.528527905696
GGGTTTTA −0.504701934566
GGTAAAAA 2.17335533197
GGTAAAAC 0.0790149975742
GGTAAACA 1.25114348374
GGTAAACC 0.072190714834
GGTAAAGA −0.0551588406351
GGTAAATA 1.09772962738
GGTAACAA 1.17255545923
GGTAACAC −0.515897122416
GGTAACCA −0.44032143344
GGTAACGA 1.0183088909
GGTAACTA 0.639178047219
GGTAAGAA −0.0398521374521
GGTAAGAC −0.445042493932
GGTAAGCA 0.0385320808078
GGTAAGGA −0.61999132263
GGTAAGTA −0.559783541166
GGTAATAA 1.03875988043
GGTAATAC 0.502365969323
GGTAATCA 4.42543691864
GGTAATGA 1.30310471043
GGTAATTA 1.85968730679
GGTACAAA 1.63563282892
GGTACAAC −0.12822431939
GGTACACA 1.07380206539
GGTACACC −0.826421243562
GGTACAGA −0.71501437504
GGTACATA 0.644051494
GGTACCAA −0.207040090626
GGTACCAC −0.584489480666
GGTACCCA −1.27786812523
GGTACCGA −0.0500709705222
GGTACCTA −0.328193019999
GGTACGAA 0.702403785026
GGTACGAC −0.273421806784
GGTACGCA 0.294388453201
GGTACGGA −0.776209128362
GGTACGTA 0.174710464898
GGTACTAA 0.556135013686
GGTACTAC 0.0033343868903
GGTACTCA 0.0111335671649
GGTACTGA 1.14720604156
GGTACTTA 0.605851248911
GGTAGAAA 2.35508097882
GGTAGAAC 0.242284208236
GGTAGACA 0.679923476432
GGTAGACC −0.884730025387
GGTAGAGA −0.110649516989
GGTAGATA 0.975767718328
GGTAGCAA −0.63256610603

GGTAGCAC -1.05640046707
GGTAGCCA -1.38942404919
GGTAGCGA 0.846803534357
GGTAGCTA -0.329328006409
GGTAGGAA 0.24585446079
GGTAGGAC -0.871778023466
GGTAGGCA 0.546898156202
GGTAGGGA -0.506187492713
GGTAGGTA -0.459474641372
GGTAGTAA 1.5397945515
GGTAGTAC -0.362940008565
GGTAGTCA -0.145052179157
GGTAGTGA 0.136046815599
GGTAGTTA 0.745591766614
GGTATAAA 3.70035089237
GGTATAAC -0.569328085722
GGTATACA 1.6786430265
GGTATACC -0.186133607649
GGTATAGA -0.396677958406
GGTATATA 0.152051955944
GGTATCAA 1.19610122278
GGTATCAC 1.89453463715
GGTATCCA 3.70735733085
GGTATCGA 1.27379200015
GGTATCTA 5.57596673001
GGTATGAA 0.0545336821266
GGTATGAC -0.162027845298
GGTATGCA 0.890379143365
GGTATGGA 0.169124216673
GGTATGTA -0.0830651003994
GGTATTAA 2.36656865675
GGTATTAC 1.51211832841
GGTATTCA 0.035481856876
GGTATTGA 0.814515336049
GGTATTTA 0.22855736783
GGTCAAAA 1.45640677521
GGTCAAAC 0.981341892229
GGTCAACA 1.15292323374
GGTCAACC -1.15845722929
GGTCAAGA -0.138005354143
GGTCAATA -0.613781373142
GGTCACAA -0.144224047106
GGTCACAC -1.37837229598
GGTCACCA -0.989474573646
GGTCACGA -0.346521010373
GGTCACTA -0.682806741615
GGTCAGAA 0.847776766467
GGTCAGAC -0.425883873371
GGTCAGCA -0.222642198379
GGTCAGGA -0.26359830372
GGTCAGTA -0.948724148161
GGTCATAA -0.707694836858
GGTCATAC -0.515239904497
GGTCATCA 0.0532540120127

GGTCATGA 0.734551879545
GGTCATTA -0.23476398647
GGTCCAAA 0.847554224194
GGTCCAAC -0.971668901853
GGTCCACA 0.314999115252
GGTCCACC -1.43043240721
GGTCCAGA -0.992844975329
GGTCCATA -0.764436662941
GGTCCCAA -0.566752091254
GGTCCCAC -0.723779917552
GGTCCCCA -1.39117024618
GGTCCCGA -0.676415885453
GGTCCCTA -0.885864387263
GGTCCGAA -0.956782718213
GGTCCGAC -0.266934356034
GGTCCGCA -0.206634976254
GGTCCGGA -0.205185641077
GGTCCGTA 0.240706010882
GGTCCTAA -1.15620932333
GGTCCTAC -1.2494932568
GGTCCTCA -0.485200028497
GGTCCTGA -1.19619906644
GGTCCTTA -1.25727099474
GGTCGAAA 0.630091494245
GGTCGAAC -1.16988786644
GGTCGACA -0.163022936097
GGTCGACC -1.80378631162
GGTCGAGA -0.67571661558
GGTCGATA 0.109138769304
GGTCGCAA -1.07151127477
GGTCGCAC -0.505215509671
GGTCGCCA -0.543485286209
GGTCGCGA 0.419977655574
GGTCGCTA -0.881722061588
GGTCGGAA 0.150137342956
GGTCGGAC -0.908341781364
GGTCGGCA -0.862680853415
GGTCGGGA -0.99692297400
GGTCGGTA -0.693476280037
GGTCGTAA -0.522823412372
GGTCGTAC -0.611170612951
GGTCGTCA -0.625155594065
GGTCGTGA -0.0369114151438
GGTCGTTA -0.605742163644
GGTCTAAA 1.78259670657
GGTCTAAC -0.431294627549
GGTCTACA -0.00325257293963
GGTCTAGA 0.0313545200159
GGTCTATA -0.578181062989
GGTCTCAA -0.765864763963
GGTCTCAC -0.67626079285
GGTCTCCA -0.742176814841
GGTCTCGA 0.215123225681
GGTCTCTA -0.476331229702

GGTCTGAA -0.233452465123
GGTCTGAC -0.633575977467
GGTCTGCA 0.117740475736
GGTCTGGA -0.432406089846
GGTCTGTA -1.42805896992
GGTCTTAA -0.478788146358
GGTCTTAC -0.0252367933789
GGTCTTCA -0.903538282388
GGTCTTGA 0.248713577326
GGTCTTTA -0.615656432312
GGTGAAAA 0.707148577808
GGTGAAAC -1.18043291443
GGTGAACA 0.941803687189
GGTGAACC -0.0519256282487
GGTGAAGA 0.693322852857
GGTGAATA 0.929009276007
GGTGACAA -0.280179264389
GGTGACAC -0.710512734152
GGTGACCA -0.809553205443
GGTGACGA -0.167099061171
GGTGACTA -0.0185370010776
GGTGAGAA 0.180279642527
GGTGAGAC -0.055997381585
GGTGAGCA 0.151858766768
GGTGAGGA -0.538856032212
GGTGAGTA 0.164921103024
GGTGATAA -0.231379428683
GGTGATAC 0.574072462148
GGTGATCA 1.1200034234
GGTGATGA -0.774980461856
GGTGATTA 1.30348671704
GGTGCAAA -0.595393219329
GGTGCAAC 0.294923054283
GGTGCACA 0.882503561768
GGTGCACC -0.685051524898
GGTGCAGA -0.108036050483
GGTGCATA 0.713503211
GGTGCCAA -1.22281628808
GGTGCCAC -0.615038143677
GGTGCCCA -1.17437930661
GGTGCCGA -0.916162195794
GGTGCCTA -0.0608106649271
GGTGCGAA -0.413750219135
GGTGCGAC -0.761133502749
GGTGCGCA 0.0482504539863
GGTGCGGA 0.735868605342
GGTGCGTA 0.533915760295
GGTGCTAA -0.630823239885
GGTGCTAC -1.3406808379
GGTGCTCA -0.0631795222927
GGTGCTGA -0.403082970671
GGTGCTTA -0.395871685045
GGTGGAAA 0.455217401446
GGTGGAAC 0.142777626421

GGTGGACA -0.381128519685
GGTGGAGA 0.0373429681202
GGTGGATA 2.83561782031
GGTGGCAA -0.855721879514
GGTGGCAC -0.713064371794
GGTGGCCA -0.621793935858
GGTGGCGA -0.601404983368
GGTGGCTA -0.857951257625
GGTGGGAA -0.21028600187
GGTGGGAC -0.298335716098
GGTGGGCA 0.152051955944
GGTGGGGA -0.622386826778
GGTGGGTA 0.122090146691
GGTGGTAA -0.402917885524
GGTGGTAC -0.937289763798
GGTGGTCA -0.583637616325
GGTGGTGA 0.315696095168
GGTGGTTA -0.571450252167
GGTGTAAA 0.79600227544
GGTGTAAC -0.466592663259
GGTGTACA 0.939751676726
GGTGTAGA 0.757756231193
GGTGTATA 0.215778153642
GGTGTCAA -0.91381290716
GGTGTCAC -0.152101918662
GGTGTCCA -0.124974452764
GGTGTCGA -0.264022154111
GGTGTCTA 0.11917565481
GGTGTGAA 0.0181735223044
GGTGTGAC -0.913290796757
GGTGTGCA 0.540892221147
GGTGTGGA -0.0249755299995
GGTGTGTA -0.685714571801
GGTGTTAA 0.287171963125
GGTGTTAC 0.256931611719
GGTGTTCA -0.459222746003
GGTGTTGA -0.301778980078
GGTGTTTA -0.0163215708917
GGTTAAAA 0.302303380439
GGTTAAAC -0.876912525449
GGTTAACA 0.778517197754
GGTTAACC -0.95946654888
GGTTAAGA 0.943906493081
GGTTAATA 0.299735921269
GGTTACAA 1.5251727539
GGTTACAC 0.505348327229
GGTTACCA -0.921293366929
GGTTACGA 1.18045331587
GGTTACTA 0.470833248946
GGTTAGAA 0.0995221951636
GGTTAGAC -0.509212527108
GGTTAGCA -0.395135567667
GGTTAGGA -1.05635050436
GGTTAGTA 0.282413222416

GGTTATAA 0.41865135359
GGTTATAC -0.931181196993
GGTTATCA 0.133099015239
GGTTATGA 0.532099407319
GGTTATTA 0.255375689411
GGTTCAAA 0.423715283234
GGTTCAAC -0.750636127527
GGTTCACA 0.784237720783
GGTTCAGA -0.274843662466
GGTTCATA -0.127762372426
GGTTCCAA -0.203675726105
GGTTCCAC 0.0383459696834
GGTTCCCA -1.63003409002
GGTTCCGA -0.430323893575
GGTTCCTA -0.591261302553
GGTTCGAA 0.884611155754
GGTTCGAC -0.342484647296
GGTTCGCA 0.267848465595
GGTTCGGA -0.657336997064
GGTTCGTA -0.299626211468
GGTTCTAA -0.0320927191739
GGTTCTAC -0.373948877113
GGTTCTCA 0.0288988524279
GGTTCTGA -0.253764391756
GGTTCTTA -0.120169288363
GGTTGAAA 0.0903067800127
GGTTGAAC -1.31318052522
GGTTGACA -0.630907135615
GGTTGAGA -0.292708248631
GGTTGATA 1.1719115647
GGTTGCAA -0.310158352415
GGTTGCAC -0.833905242358
GGTTGCCA -0.317183735095
GGTTGCGA 0.224253496033
GGTTGCTA -0.0662588911434
GGTTGGAA 0.0141003117221
GGTTGGAC -1.26970796431
GGTTGGCA 0.0303794143036
GGTTGGGA -1.04499272949
GGTTGGTA -0.0316047499618
GGTTGTAA 1.74252161049
GGTTGTAC -0.038945522299
GGTTGTCA 0.0856131990157
GGTTGTGA 0.255685041906
GGTTGTTA -0.419875648358
GGTTTAAA 0.725000673294
GGTTTAAC -0.749534657773
GGTTTACA -0.483033728318
GGTTTAGA 0.140667117943
GGTTTATA 0.606884644461
GGTTTCAA -0.0397278551912
GGTTTCAC -1.06920695258
GGTTTCCA 0.673935652862
GGTTTCGA -0.594314024621

GGTTTCTA 0.0791775845856
GGTTTGAA -0.597264114939
GGTTTGAC 0.343425403639
GGTTTGCA 0.708921005228
GGTTTGGA -0.638312234952
GGTTTGTA -0.0179024745594
GGTTTTAA 0.466482328923
GGTTTTAC 0.304772995952
GGTTTTCA 0.466611399278
GGTTTTGA 1.25967149516
GGTTTTTA 1.10694171168
GTAAAAAA 2.48676958817
GTAAAAAC 1.56049431373
GTAAAACA 1.745916369
GTAAAAGA 0.821559454748
GTAAAATA 2.18820196179
GTAAACAA -0.210776052862
GTAAACAC 1.39957564077
GTAAACCA 0.223532159293
GTAAACGA 0.370098208804
GTAAACTA -0.102399006829
GTAAAGAA 0.124255197803
GTAAAGAC -0.322550980073
GTAAAGCA 0.530725224397
GTAAAGGA -0.218550251777
GTAAAGTA 1.40474178581
GTAAATAA 1.26611439582
GTAAATAC 1.92586667395
GTAAATCA 2.66743144549
GTAAATGA 2.31343164077
GTAAATTA 1.73528097177
GTAACAAA 2.42227250737
GTAACAAC 1.00957603233
GTAACACA 1.35893783958
GTAACAGA 0.732157416287
GTAACATA 0.87788367578
GTAACCAA 0.0903546609507
GTAACCAC -0.106468053851
GTAACCCA 0.281749342801
GTAACCGA 2.29005783224
GTAACCTA 0.123716016805
GTAACGAA 0.534877958972
GTAACGAC -0.746556671605
GTAACGCA 0.773955809782
GTAACGGA 0.481416809857
GTAACGTA 1.70218254472
GTAACTAA 0.216729110708
GTAACTAC 0.771489733294
GTAACTCA 0.774518723071
GTAACTGA 1.11231582653
GTAACTTA 0.678423762182
GTAAGAAA 0.343245954211
GTAAGAAC 0.151409102306
GTAAGACA 0.301606192345

GTAAGAGA 0.717252496627
GTAAGATA 1.83607221997
GTAAGCAA -0.154652723592
GTAAGCAC 0.0935972413468
GTAAGCCA -0.490383452308
GTAAGCGA 0.975544551521
GTAAGCTA -0.621114026538
GTAAGGAA 0.212897802951
GTAAGGAC -0.800372556016
GTAAGGCA -0.375655936644
GTAAGGGA 1.09296068595
GTAAGGTA -0.190416869824
GTAAGTAA 0.838587998099
GTAAGTAC 0.02162240709
GTAAGTCA 0.676702546548
GTAAGTGA 2.48589836328
GTAAGTTA 0.61481914043
GTAATAAA 1.57852689953
GTAATAAC 0.241714633251
GTAATACA 3.45900764865
GTAATAGA 1.22800033642
GTAATATA 2.24672329334
GTAATCAA 4.42543691864
GTAATCAC 7.49194245122
GTAATCCA 16.7282388956
GTAATCGA 6.16452921308
GTAATCTA 15.0110550448
GTAATGAA -0.350320675075
GTAATGAC -0.451674628386
GTAATGCA 1.63439854161
GTAATGGA 0.0990789842197
GTAATGTA 1.31064699906
GTAATTAA 0.677621027846
GTAATTAC 4.41664035761
GTAATTCA 1.44228481298
GTAATTGA 1.4281840849
GTAATTTA 3.38219121885
GTACAAAA 1.76185614146
GTACAAAC 0.0497262277682
GTACAACA 1.46755949475
GTACAAGA 0.720012104083
GTACAATA 2.91213072661
GTACACAA 0.0920775420086
GTACACAC 0.301228557469
GTACACCA 0.684506514916
GTACACGA 0.880962003741
GTACACTA 0.198121329113
GTACAGAA -0.591261302553
GTACAGAC -0.626567457204
GTACAGCA 0.297741576111
GTACAGGA -0.514710716042
GTACAGTA 1.95340654044
GTACATAA 0.644051494
GTACATAC 1.52784409389

GTACATCA -0.549783086809
GTACATGA 0.526163628246
GTACATTA 2.4453209337
GTACCAAA -0.114603233404
GTACCAAC -0.127732186618
GTACCACA -0.197238238073
GTACCAGA 0.169717523949
GTACCATA 2.11017622603
GTACCCAA -0.787598129922
GTACCCAC -0.461139648949
GTACCCCA -0.26786553619
GTACCCGA -0.0795104611941
GTACCCTA 0.474052929763
GTACCGAA 0.393015066645
GTACCGAC -1.56493808113
GTACCGCA 0.435615361763
GTACCGGA -0.948506393982
GTACCGTA 1.76405075384
GTACCTAA 0.0511903435827
GTACCTAC -0.747760981286
GTACCTCA -1.1868785214
GTACCTGA 0.183231190091
GTACCTTA 0.556135013686
GTACGAAA 1.35710483236
GTACGAAC 0.880055388588
GTACGACA 0.128957522276
GTACGAGA 1.51397361067
GTACGATA 2.40197765133
GTACGCAA 1.68598629698
GTACGCAC 0.706184089174
GTACGCCA -0.693038273543
GTACGCGA 1.06952192588
GTACGCTA 0.047595734203
GTACGGAA 0.20604666525
GTACGGAC -0.0274520153868
GTACGGCA -0.385072243556
GTACGGGA 0.5891212328
GTACGGTA 1.32352634686
GTACGTAA 0.370628021792
GTACGTAC 0.757024277375
GTACGTCA -0.100651560768
GTACGTGA 0.047595734203
GTACGTTA 1.70718984995
GTACTAAA 0.703494429523
GTACTAAC 0.516588481526
GTACTACA 1.81673102731
GTACTAGA 1.39149708563
GTACTATA 1.1691003291
GTACTCAA 0.401855553233
GTACTCAC 0.630523671755
GTACTCCA 0.0318143851992
GTACTCGA 0.108902279106
GTACTCTA 0.476082873358
GTACTGAA 0.560927271051

GTACTGAC 0.0573230590351
GTACTGCA −0.0769188533776
GTACTGGA 0.612687814153
GTACTGTA 2.72508467482
GTACTTAA −0.608116433637
GTACTTCA −0.322351961913
GTACTTGA 0.301995693367
GTACTTTA 1.09574860561
GTAGAAAA 2.35508097882
GTAGAAAC 1.02090382956
GTAGAACA 0.38227849491
GTAGAAGA 0.757195399684
GTAGAATA 1.35609558546
GTAGACAA 0.30405499006
GTAGACAC 0.528247489941
GTAGACCA 0.0540375939728
GTAGACGA 0.548285662516
GTAGACTA 1.2895067322
GTAGAGAA 0.379737266167
GTAGAGAC 0.038676139978
GTAGAGCA −0.405060869769
GTAGAGGA −0.291745217243
GTAGAGTA 1.69695540353
GTAGATAA 0.306757973102
GTAGATAC 4.87537908978
GTAGATCA 0.294496497579
GTAGATGA 1.08581435185
GTAGATTA 4.18941220625
GTAGCAAA 0.127367458776
GTAGCAAC −0.099439964857
GTAGCACA −0.12099783677
GTAGCAGA 1.84751347421
GTAGCATA −0.310108597875
GTAGCCAA −0.481621657001
GTAGCCAC −0.94244924714
GTAGCCCA −1.12029612166
GTAGCCGA 0.0613129984207
GTAGCCTA −0.666410643002
GTAGCGAA 0.820020811213
GTAGCGAC 0.0542578462878
GTAGCGCA −0.56342436606
GTAGCGGA −0.147794716017
GTAGCGTA 0.532927747547
GTAGCTAA −0.522518431615
GTAGCTAC 0.0910666296818
GTAGCTCA −0.641040407531
GTAGCTGA 0.432644870002
GTAGCTTA 0.353787671346
GTAGGAAA 0.341702522581
GTAGGAAC −0.347863966597
GTAGGACA −0.0153926806935
GTAGGAGA 0.481440542148
GTAGGATA 1.06671277206
GTAGGCAA 0.197384795379

GTAGGCAC 0.003685583162
GTAGGCCA −1.26858255409
GTAGGCGA 0.978167594215
GTAGGCTA −1.20968900029
GTAGGGAA −0.387707152395
GTAGGGAC −0.589127686318
GTAGGGCA −1.33455998859
GTAGGGGA 0.670540894354
GTAGGGTA 0.318696356382
GTAGGTAA 0.830356432136
GTAGGTCA −1.00849850305
GTAGGTGA 0.692789292665
GTAGGTTA 0.465268651233
GTAGTAAA 0.948437278889
GTAGTAAC 0.554499983741
GTAGTACA 0.456253086953
GTAGTAGA 1.72483897189
GTAGTATA 1.29746391957
GTAGTCAA 0.206372671985
GTAGTCAC 0.68323850277
GTAGTCCA −0.469627065663
GTAGTCGA 0.825994895036
GTAGTCTA 0.0723734951105
GTAGTGAA −0.341014286141
GTAGTGAC 0.177702398992
GTAGTGCA −0.036979072991
GTAGTGGA −0.0168045438321
GTAGTGTA 1.07078993803
GTAGTTAA 0.506391298967
GTAGTTCA −0.372859273505
GTAGTTGA 1.2145507891
GTAGTTTA 1.52448077026
GTATAAAA 1.7842773275
GTATAAAC 2.61540776541
GTATAACA 1.84006236753
GTATAAGA 1.63342926488
GTATAATA 0.847554224194
GTATACAA 0.725519869205
GTATACAC 1.72702379991
GTATACCA −0.0494085481532
GTATACGA 3.46822410469
GTATACTA 1.47646618194
GTATAGAA −0.091455297992
GTATAGAC 1.29894573051
GTATAGCA −0.127732811152
GTATAGGA 0.535916142615
GTATAGTA 1.13132330987
GTATATAA 1.23837925837
GTATATAC 2.11952341785
GTATATCA 1.71193922265
GTATATGA 2.02793946586
GTATATTA 2.24672329334
GTATCAAA 1.41168910174
GTATCAAC 1.49437282006

GTATCACA 1.71251712475
GTATCAGA 2.40559974021
GTATCATA 1.98170584026
GTATCCAA 3.55301083708
GTATCCAC 3.38110806876
GTATCCCA 3.20674026483
GTATCCGA 4.39870499092
GTATCCTA 1.87424644281
GTATCGAA 0.764062775268
GTATCGAC 0.27240340005
GTATCGCA 1.56807011902
GTATCGGA 2.65207602866
GTATCGTA 1.56982901487
GTATCTAA 3.06699641628
GTATCTCA 2.70670901169
GTATCTGA 5.38736246595
GTATCTTA 3.09346541519
GTATGAAA 2.01483778396
GTATGAAC -0.614701728043
GTATGACA 0.436104580043
GTATGAGA 0.10618618085
GTATGATA 0.644185560627
GTATGCAA -0.436789485636
GTATGCAC 1.02366489426
GTATGCCA -0.326009232868
GTATGCGA 2.21815232125
GTATGCTA -0.694107475707
GTATGGAA 0.953149387727
GTATGGAC 0.281421878821
GTATGGCA -0.962216788326
GTATGGGA 1.42016839951
GTATGGTA 0.576906180969
GTATGTAA -0.0639148069588
GTATGTCA -0.36006423779
GTATGTGA 1.36902968407
GTATGTTA 1.40116487156
GTATTAAA 1.76722172101
GTATTAAC 1.03976413106
GTATTACA 2.69219192763
GTATTAGA 1.86241443848
GTATTATA 2.27442949406
GTATTCAA 0.035481856876
GTATTCAC 1.40336989284
GTATTCCA 1.46817237076
GTATTCGA 1.47443811195
GTATTCTA 1.12591963374
GTATTGAA -0.187756563271
GTATTGAC 0.171999154736
GTATTGCA 0.388601901403
GTATTGGA 0.740700832881
GTATTGTA 1.06815419648
GTATTTAA -0.345167437072
GTATTTCA 1.62230714751
GTATTTGA 1.82171564114

GTATTTTA 3.40101342377
GTCAAAAA 0.864250515471
GTCAAAAC 1.06953858012
GTCAAACA 1.19751475134
GTCAAAGA 0.865644475302
GTCAAATA 1.01851207262
GTCAACAA 1.12422048498
GTCAACAC -0.328193019999
GTCAACCA -0.676309090144
GTCAACGA -0.432341138313
GTCAACTA -0.670533399947
GTCAAGAA 0.359786111993
GTCAAGAC -0.491643553691
GTCAAGCA -0.549529109659
GTCAAGGA 0.0989043228848
GTCAAGTA 0.161383742592
GTCAATAA -0.419987856295
GTCAATCA -0.429885470725
GTCAATGA 1.2356604538
GTCAATTA -0.199875853225
GTCACAAA 0.233039439988
GTCACAAC 0.440160095496
GTCACACA 0.39927289707
GTCACAGA 0.61488034476
GTCACATA 0.0766898575869
GTCACCAA -0.540439642193
GTCACCAC -0.730211784778
GTCACCCA 0.317779540507
GTCACCGA -0.64342654367
GTCACCTA -0.104304876341
GTCACGAA -0.298052802208
GTCACGAC -0.632971844935
GTCACGCA -0.127994490887
GTCACGGA -0.0761381859093
GTCACGTA -0.40069600182
GTCACTAA -0.299536694931
GTCACTCA -0.674977791888
GTCACTGA 0.352031481809
GTCACTTA -0.00738282429153
GTCAGAAA 0.86352397428
GTCAGAAC 0.279056560481
GTCAGACA 0.302824449952
GTCAGAGA -0.64729678071
GTCAGATA 1.4303189502
GTCAGCAA -0.016831190615
GTCAGCAC 0.13907434813
GTCAGCCA -1.67070207702
GTCAGCGA 0.788147303464
GTCAGCTA -0.931503664702
GTCAGGAA 0.228292565425
GTCAGGAC -0.608941026661
GTCAGGCA 0.000329337582597
GTCAGGGA 0.178246159906
GTCAGGTA -1.23298890563

GTCAGTAA 0.34935410466
GTCAGTCA -0.833372306699
GTCAGTGA -0.25524932537
GTCAGTTA 0.381013605434
GTCATAAA 0.571561419215
GTCATAAC 0.00554024088852
GTCATACA -0.378214027803
GTCATAGA -0.187265679567
GTCATATA 0.603826509765
GTCATCAA -0.542559310503
GTCATCAC 0.599752882827
GTCATCCA 0.230176576248
GTCATCGA -0.0381228028765
GTCATCTA -0.373959286013
GTCATGAA -0.10374008945
GTCATGAC 1.40354413782
GTCATGCA -0.725302947738
GTCATGGA -0.0758683872323
GTCATGTA -0.121966697142
GTCATTAA 0.0700456488093
GTCATTCA 0.741082423139
GTCATTGA 0.759036525841
GTCATTTA 0.587300508086
GTCCAAAA 2.0243011391
GTCCAAAC 0.50303172254
GTCCAACA 0.208873306019
GTCCAAGA -0.470075897413
GTCCAATA 0.350971647654
GTCCACAA 0.153602465625
GTCCACAC -0.459281868552
GTCCACCA -0.825300205078
GTCCACGA -0.624364101342
GTCCACTA 0.649148107589
GTCCAGAA 0.268091617489
GTCCAGAC -0.83606092546
GTCCAGCA -0.251172575762
GTCCAGGA 0.427718337833
GTCCAGTA -0.311554602204
GTCCATAA 0.0453224305355
GTCCATCA -0.321364365521
GTCCATGA -0.104127508692
GTCCATTA -0.604294910246
GTCCCAAA 1.00255710317
GTCCCAAC -0.555380993001
GTCCCACA -0.0566073431002
GTCCCAGA 0.28836440666
GTCCCATA -1.01359553299
GTCCCCAA -0.809656669905
GTCCCCAC -1.41587764293
GTCCCCCA -0.870066592198
GTCCCCGA -0.329997923186
GTCCCCTA -1.07449675534
GTCCCGAA -0.515223874791
GTCCCGAC -0.599180809707

GTCCCGCA −0.758554801968
GTCCCGGA −1.09425846755
GTCCCGTA −0.99139480744
GTCCCTAA −1.27328696034
GTCCCTCA −0.242736370833
GTCCCTGA 0.11150866756
GTCCCTTA −0.933280672037
GTCCGAAA −0.46155309044
GTCCGAAC 0.284374883631
GTCCGACA 0.800568451506
GTCCGAGA −1.14957968701
GTCCGATA 0.5437577912
GTCCGCAA 0.657516446492
GTCCGCAC −1.07270642462
GTCCGCCA −0.923665763321
GTCCGCGA 0.0779732749046
GTCCGCTA −0.633971099295
GTCCGGAA −0.698435496151
GTCCGGAC 0.260886369024
GTCCGGCA −0.710570191277
GTCCGGGA 0.0130315259136
GTCCGGTA −1.53796404242
GTCCGTAA 0.800689194742
GTCCGTCA −1.42327774599
GTCCGTGA 0.953466026452
GTCCGTTA −0.651361247817
GTCCTAAA −0.0125851922998
GTCCTAAC −0.27259617287
GTCCTACA 0.497116136732
GTCCTAGA −1.15383609423
GTCCTATA −0.0358830158656
GTCCTCAA −0.614932805613
GTCCTCAC −0.186952996223
GTCCTCCA −1.19596528255
GTCCTCGA −0.275407200289
GTCCTCTA −0.592880510971
GTCCTGAA −0.187187404642
GTCCTGCA −0.849168852697
GTCCTGGA 0.153073693526
GTCCTGTA −0.490843733848
GTCCTTAA −0.192908552205
GTCCTTCA −0.39662362395
GTCCTTGA −0.387815613129
GTCCTTTA 0.506299908829
GTCGAAAA 1.53145889654
GTCGAAAC −0.223529036623
GTCGAACA 0.445296679259
GTCGAAGA −0.443633128929
GTCGAATA 0.524298769798
GTCGACAA −0.108020228956
GTCGACAC −0.662447766756
GTCGACCA −0.847032113792
GTCGACGA −0.0553391227758
GTCGACTA 0.494509123744

GTCGAGAA -0.65930677722
GTCGAGAC -0.658035642403
GTCGAGCA -0.97670556018
GTCGAGGA -0.48630358003
GTCGAGTA 1.16373454137
GTCGATAA 0.258813540763
GTCGATCA -0.647538267181
GTCGATGA 0.0818387238513
GTCGATTA 1.25840785476
GTCGCAAA 0.761059391384
GTCGCAAC 1.02867719576
GTCGCACA -0.318858527037
GTCGCAGA -0.584590238814
GTCGCATA 0.441307364408
GTCGCCAA -0.687429125739
GTCGCCAC -0.601191809105
GTCGCCCA -0.253806027355
GTCGCCGA -0.83482538908
GTCGCCTA 1.05520594176
GTCGCGAA 0.494321139018
GTCGCGAC -1.21165086968
GTCGCGCA -0.219043217261
GTCGCGGA -0.378411796895
GTCGCGTA -0.228429338365
GTCGCTAA -0.808456315605
GTCGCTCA -1.49102781609
GTCGCTGA 0.569969690291
GTCGCTTA -0.987780421151
GTCGGAAA 1.06296348644
GTCGGAAC -0.319500547963
GTCGGACA -0.697884032652
GTCGGAGA -0.69771832297
GTCGGATA 2.51068570037
GTCGGCAA -0.332440267382
GTCGGCAC -1.03960508307
GTCGGCCA -1.45209103727
GTCGGCGA -0.782373486869
GTCGGCTA 0.0598948899424
GTCGGGAA -0.237740931748
GTCGGGCA -0.728680843828
GTCGGGGA 0.234435481599
GTCGGGTA -0.382093216498
GTCGGTAA -0.436343984734
GTCGGTCA -1.0138763651
GTCGGTGA 0.0825575624561
GTCGGTTA 1.62957609844
GTCGTAAA -0.0154926061295
GTCGTAAC 0.0556555533229
GTCGTACA -0.273941002695
GTCGTAGA -0.290115599925
GTCGTATA 0.875042046196
GTCGTCAA -0.633154208856
GTCGTCAC -0.759198488318
GTCGTCCA -0.273441167337

GTCGTCGA -0.434042785215
GTCGTCTA 0.146149693528
GTCGTGAA -1.1337190222
GTCGTGCA 0.0495309568122
GTCGTGGA 0.148510848308
GTCGTGTA -0.592880510971
GTCGTTAA -0.626643025815
GTCGTTCA 0.743827458136
GTCGTTGA -0.385276674344
GTCGTTTA 0.388109768631
GTCTAAAA 0.417485556837
GTCTAAAC 0.155156722509
GTCTAACA -0.148460677412
GTCTAAGA -0.250844903603
GTCTAATA 1.47967087394
GTCTACAA -0.325640966001
GTCTACAC 0.15488505023
GTCTACCA -0.29096704791
GTCTACGA -0.425392365133
GTCTACTA 2.09147642977
GTCTAGAA 0.00903034491648
GTCTAGAC 0.76548567184
GTCTAGCA -0.460542594469
GTCTAGGA -0.893491196161
GTCTAGTA 0.34837983166
GTCTATAA -0.578181062989
GTCTATCA 0.540447136601
GTCTATGA -0.131253309166
GTCTATTA 0.977364651701
GTCTCAAA 0.499191879486
GTCTCAAC -0.282193594635
GTCTCACA -0.203125928029
GTCTCAGA 0.142177241094
GTCTCATA 0.105768159443
GTCTCCAA -1.08340781427
GTCTCCAC -0.786615529802
GTCTCCCA -0.617937646743
GTCTCCGA -0.59982928415
GTCTCCTA -0.474796541549
GTCTCGAA -0.238778490858
GTCTCGCA -0.2987916259
GTCTCGGA -0.958774981592
GTCTCGTA -0.268870203178
GTCTCTAA -0.566702128536
GTCTCTCA -0.543322907376
GTCTCTGA 0.733337160965
GTCTCTTA -0.741776280385
GTCTGAAA 0.700135061274
GTCTGAAC -0.428762974994
GTCTGACA -0.134192782407
GTCTGAGA 1.05191090051
GTCTGATA 1.18996163745
GTCTGCAA 0.00232576452135
GTCTGCAC 0.209614419669

GTCTGCCA −0.65561578143
GTCTGCGA −0.373872267612
GTCTGCTA 0.607862248309
GTCTGGAA −1.04095324375
GTCTGGCA −0.538958663961
GTCTGGGA −0.237531921045
GTCTGGTA −1.25827566173
GTCTGTAA −0.883855053289
GTCTGTCA −0.461073864703
GTCTGTGA 0.399485654978
GTCTGTTA 1.05261246034
GTCTTAAA −0.447816673847
GTCTTAAC 0.474419323028
GTCTTACA 0.80722140376
GTCTTAGA −0.425493955993
GTCTTATA 1.28376330959
GTCTTCAA −0.429161011314
GTCTTCAC −0.304426795952
GTCTTCCA −0.483858737698
GTCTTCGA −1.0512715859
GTCTTCTA −0.402627685404
GTCTTGAA −0.740201830235
GTCTTGCA 0.438068531215
GTCTTGGA 1.77432870946
GTCTTGTA 0.206235066333
GTCTTTAA −0.325629516212
GTCTTTCA 0.0285401617485
GTCTTTGA 0.332624921261
GTCTTTTA 1.11323972046
GTGAAAAA 1.81396767265
GTGAAAAC 0.882333688527
GTGAAACA 0.391275531348
GTGAAAGA 0.955915032344
GTGAAATA 2.41244275897
GTGAACAA −0.186005578184
GTGAACAC 1.18520976662
GTGAACCA −0.313925749528
GTGAACGA 1.04510722739
GTGAACTA −0.0677340403914
GTGAAGAA 0.586318324322
GTGAAGCA −0.833797822514
GTGAAGGA −0.278925200168
GTGAAGTA 0.284884711532
GTGAATAA 0.729155073293
GTGAATCA 0.642881533688
GTGAATGA 1.32150223225
GTGAATTA 1.28315751163
GTGACAAA 1.2338320265
GTGACAAC −0.245897761812
GTGACACA 0.0330307692037
GTGACAGA 0.419320021299
GTGACATA 0.887547506325
GTGACCAA −0.15500329533
GTGACCAC 0.0504527689587

GTGACCCA -0.458876337825
GTGACCGA -0.247232182738
GTGACCTA -1.01573768452
GTGACGAA -0.0654061940901
GTGACGCA -0.320891385124
GTGACGGA -0.495581032223
GTGACGTA 0.23411613656
GTGACTAA 0.390052693826
GTGACTCA -0.490735064936
GTGACTGA -0.322462712604
GTGACTTA -0.756199476172
GTGAGAAA 0.369400604354
GTGAGAAC 0.191170057798
GTGAGACA 0.151170946684
GTGAGAGA 0.0195656085337
GTGAGATA 1.84836492219
GTGAGCAA 0.768877932212
GTGAGCAC 2.19549506137
GTGAGCCA -0.600775036766
GTGAGCGA 0.273651010753
GTGAGCTA -0.7871755286
GTGAGGAA 0.695729182261
GTGAGGCA -0.458266376309
GTGAGGGA 0.203714655389
GTGAGGTA 0.220362441194
GTGAGTAA 0.539670632693
GTGAGTCA -0.660306031579
GTGAGTGA -0.455983496454
GTGAGTTA 0.837565635983
GTGATAAA -0.447816673847
GTGATAAC 0.890585655933
GTGATACA 2.68796050177
GTGATAGA 0.657879092554
GTGATATA 2.19710136275
GTGATCAA 0.733752892414
GTGATCAC 4.41115840655
GTGATCCA 0.654822623282
GTGATCGA 0.118383953908
GTGATCTA 0.741403849958
GTGATGAA -0.0798520812782
GTGATGCA 0.84285523057
GTGATGGA -0.251237943651
GTGATGTA 0.892461755993
GTGATTAA 0.427718337833
GTGATTCA 0.857177043675
GTGATTGA 0.681031399703
GTGATTTA 0.610683060094
GTGCAAAA 0.785633970572
GTGCAAAC 0.270494199689
GTGCAACA -0.197900244086
GTGCAAGA 1.35475242106
GTGCAATA 1.52394200562
GTGCACAA 0.569830627393
GTGCACAC 0.814432064852

GTGCACCA -0.825415327507
GTGCACGA -0.531117848089
GTGCACTA -0.431306077339
GTGCAGAA -0.449310767292
GTGCAGCA -0.158967420644
GTGCAGGA 0.177085775781
GTGCAGTA 0.377669434178
GTGCATAA 0.315233731849
GTGCATCA -0.713982436736
GTGCATGA -0.121750192031
GTGCATTA 0.138063019447
GTGCCAAA 0.100387174718
GTGCCAAC -1.42560621683
GTGCCACA -0.809197012899
GTGCCAGA -0.00301670727523
GTGCCATA -0.390665569833
GTGCCCAA -0.569130732986
GTGCCCAC -0.434203914981
GTGCCCCA 0.534152666849
GTGCCCGA -0.926753459291
GTGCCCTA -1.34856099941
GTGCCGAA -0.366201116802
GTGCCGCA -0.641667647819
GTGCCGGA 0.226799929226
GTGCCGTA -0.248729190675
GTGCCTAA 0.570644186984
GTGCCTCA -1.3163891726
GTGCCTGA -0.545160286329
GTGCCTTA -1.15466651624
GTGCGAAA 0.284966525482
GTGCGAAC 0.889968824544
GTGCGACA 0.442376982928
GTGCGAGA 0.300404797156
GTGCGATA 2.43117607189
GTGCGCAA 0.927234350451
GTGCGCAC 2.04365128342
GTGCGCCA -1.1674961095
GTGCGCGA -0.43432632364
GTGCGCTA -0.254865653331
GTGCGGAA -0.523218742378
GTGCGGCA -0.972685434986
GTGCGGGA -0.139671610788
GTGCGGTA -0.0221189115998
GTGCGTAA 1.32924832714
GTGCGTCA -0.725757192115
GTGCGTGA 0.235728683282
GTGCGTTA 1.08065653394
GTGCTAAA -0.160767535737
GTGCTAAC -0.108414101716
GTGCTACA 0.819876543865
GTGCTAGA 0.0307574655362
GTGCTATA -0.278978077378
GTGCTCAA 0.180711611859
GTGCTCCA -0.329389418917

GTGCTCGA -1.06865590544
GTGCTCTA -0.869818860388
GTGCTGAA 0.693955089417
GTGCTGCA -0.126641750298
GTGCTGGA -0.141051830871
GTGCTGTA 0.670156181426
GTGCTTAA 0.758384096015
GTGCTTCA -0.651035241082
GTGCTTGA -0.232957209681
GTGCTTTA -0.353002423962
GTGGAAAA 0.475412748404
GTGGAAAC 0.0527221172443
GTGGAACA -0.180788845894
GTGGAAGA 0.724030147497
GTGGAATA 1.85545921179
GTGGACAA 0.403438955037
GTGGACAC 0.0866174496468
GTGGACCA 0.169474163877
GTGGACGA 0.726762899992
GTGGACTA -0.176890088469
GTGGAGAA 0.187341664534
GTGGAGCA -0.85606745498
GTGGAGGA -0.841110699001
GTGGAGTA -0.0555685349225
GTGGATAA 1.99394233442
GTGGATCA 0.878626454854
GTGGATGA 0.624413231348
GTGGATTA 4.58442453255
GTGGCAAA 0.19735356868
GTGGCAAC 0.0600291647469
GTGGCACA -0.153037886912
GTGGCAGA -0.636611628939
GTGGCATA -0.0106612113021
GTGGCCAA -0.932129447744
GTGGCCAC -0.558291737679
GTGGCCCA -0.763470925238
GTGGCCGA 0.32738862024
GTGGCCTA -0.969795924463
GTGGCGAA -0.725331884478
GTGGCGCA -1.11960184806
GTGGCGGA -0.185603586482
GTGGCGTA -0.807631930759
GTGGCTAA -0.509505225364
GTGGCTCA -0.675293597901
GTGGCTGA -0.628908626897
GTGGCTTA -0.54425845927
GTGGGAAA -0.467255710162
GTGGGAAC -0.912136657972
GTGGGACA 0.865717337599
GTGGGAGA 0.104126884158
GTGGGATA 0.662650532119
GTGGGCAA 0.524413892227
GTGGGCCA -0.635160836516
GTGGGCGA -0.536194892946

GTGGGCTA -0.488894355135
GTGGGGAA 0.350202429975
GTGGGGCA -0.663309623641
GTGGGGGA 0.0859910420703
GTGGGGTA -0.26786553619
GTGGGTAA -0.437044503675
GTGGGTCA -0.642106487026
GTGGGTGA 0.40143711547
GTGGGTTA 0.214412089662
GTGGTAAA -0.254423899633
GTGGTAAC 0.165118872116
GTGGTACA -0.0822042844045
GTGGTAGA -0.00276460372749
GTGGTATA 0.903176677217
GTGGTCAA -0.0721188934269
GTGGTCCA -0.385072243556
GTGGTCGA -0.254331052249
GTGGTCTA -0.517247573047
GTGGTGAA 1.16437885225
GTGGTGCA -0.0416276875418
GTGGTGGA 0.0912392092367
GTGGTGTA 1.05450771278
GTGGTTAA -0.3605503334
GTGGTTCA 0.051182224641
GTGGTTGA -0.39450916009
GTGGTTTA 0.752944405097
GTGTAAAA 0.308975693245
GTGTAAAC 0.123257192512
GTGTAACA 1.7073709648
GTGTAAGA 1.01228421982
GTGTAATA 2.69506644933
GTGTACAA -0.0950811340704
GTGTACAC 1.04261950039
GTGTACCA 0.0968718811535
GTGTACGA -0.308716719824
GTGTACTA -0.13975529834
GTGTAGAA 0.76027934845
GTGTAGCA 0.717367619057
GTGTAGGA -0.317260344596
GTGTAGTA 0.603560874648
GTGTATAA 1.18867176662
GTGTATCA 1.06989976894
GTGTATGA -0.0150300346323
GTGTATTA 1.41140348153
GTGTCAAA 0.717059099273
GTGTCAAC 0.580966692694
GTGTCACA 0.814134370324
GTGTCAGA 1.50356429474
GTGTCATA 0.550775887651
GTGTCCAA -0.282561653324
GTGTCCCA -0.312889855842
GTGTCCGA -0.40295764752
GTGTCCTA 0.263120327052
GTGTCGAA -0.3307261298

GTGTCGCA -0.277344712857
GTGTCGGA -0.172190262132
GTGTCGTA -0.00466984870595
GTGTCTAA 0.289919079901
GTGTCTCA 0.0712836833249
GTGTCTGA 0.615216760394
GTGTCTTA 0.25136659765
GTGTGAAA -0.181925081372
GTGTGAAC -0.769346957227
GTGTGACA 1.05095411446
GTGTGAGA 0.310183541952
GTGTGATA 0.821169329192
GTGTGCAA -0.102767065518
GTGTGCCA -0.238452067767
GTGTGCGA 0.600396152821
GTGTGCTA 0.268019171548
GTGTGGAA 0.571545597687
GTGTGGCA -0.0736358864512
GTGTGGGA 0.911168838489
GTGTGGTA 0.0461851201324
GTGTGTAA 0.8124329316
GTGTGTCA -0.0810449411696
GTGTGTGA -0.162604081978
GTGTGTTA 1.18015562134
GTGTTAAA -0.134236916141
GTGTTAAC 0.616768102787
GTGTTACA 2.73839953098
GTGTTAGA 0.390520261595
GTGTTATA 2.03284892744
GTGTTCAA -0.503022562708
GTGTTCCA -0.081599943695
GTGTTCGA 0.265842670647
GTGTTCTA 0.22262762592
GTGTTGAA -0.150706085229
GTGTTGCA 0.584308157636
GTGTTGGA 0.269510975035
GTGTTGTA 0.822461698163
GTGTTTAA -0.0824792875313
GTGTTTCA 0.373198603632
GTGTTTGA 0.812852618431
GTGTTTTA 0.641059768084
GTTAAAAA 1.12120086321
GTTAAAAC 0.530805372923
GTTAAACA -0.0480674655318
GTTAAAGA -0.0813517955291
GTTAAATA 0.836213103572
GTTAACAA 0.616280966287
GTTAACCA 0.677948700005
GTTAACGA 0.316591260531
GTTAACTA 1.32557835732
GTTAAGAA -0.559162754395
GTTAAGCA 0.488144081654
GTTAAGGA 0.718654991756
GTTAAGTA 0.662036615222

GTTAATAA 0.347957230337
GTTAATCA 1.77479398727
GTTAATGA 1.99864674067
GTTAATTA 0.411056187747
GTTACAAA -0.205017016904
GTTACAAC 1.2638369286
GTTACACA 0.567027302559
GTTACAGA 1.28234416022
GTTACATA 0.978736336487
GTTACCAA -0.327311178027
GTTACCCA -0.205154414379
GTTACCGA 0.226070265365
GTTACCTA -0.161561318419
GTTACGAA 0.0340870643327
GTTACGCA 0.349140722219
GTTACGGA 0.770555846824
GTTACGTA 0.647910281251
GTTACTAA -0.424068144929
GTTACTCA 0.345732848497
GTTACTGA -0.510622308467
GTTACTTA 0.564880987466
GTTAGAAA 0.240672077869
GTTAGAAC -0.348887994137
GTTAGACA -0.201073501211
GTTAGAGA -0.338123942907
GTTAGATA 1.2205941962
GTTAGCAA 0.813780259561
GTTAGCCA 0.207524312634
GTTAGCGA 0.138029710968
GTTAGCTA -0.763632263182
GTTAGGAA -0.158929324072
GTTAGGCA -0.367709366351
GTTAGGGA 0.492938420798
GTTAGGTA -0.333201366119
GTTAGTAA 0.515224082969
GTTAGTCA 0.557417390114
GTTAGTGA 0.942068281416
GTTAGTTA -0.0764566982364
GTTATAAA 0.689161166629
GTTATAAC 0.635410441928
GTTATACA 1.55532275606
GTTATAGA 0.72135672573
GTTATATA 1.33240493002
GTTATCAA 1.03072878987
GTTATCCA 2.55466767282
GTTATCGA 0.935966584484
GTTATCTA 0.968216478042
GTTATGAA 0.21435421618
GTTATGCA 1.35003968768
GTTATGGA 0.684185296275
GTTATGTA 0.2660464769
GTTATTAA -0.189505674756
GTTATTCA 1.13828061835
GTTATTGA 0.170966175542

GTTATTTA 0.805627176701
GTTCAAAA 0.287689909968
GTTCAAAC -0.13849394789
GTTCAACA -0.346013888786
GTTCAAGA -0.117075138875
GTTCAATA 1.22452418032
GTTCACAA -0.0848770816377
GTTCACCA 1.96305975391
GTTCACGA 0.00565161611399
GTTCACTA 0.0796971968525
GTTCAGAA -0.345432864012
GTTCAGCA 0.344956344589
GTTCAGGA -0.378957015055
GTTCAGTA 0.771907754701
GTTCATAA -0.127762372426
GTTCATCA -0.0805328233105
GTTCATGA 1.16329362038
GTTCATTA 0.32560994748
GTTCCAAA 0.179224388288
GTTCCAAC -0.521581630653
GTTCCACA -0.116184761606
GTTCCAGA 0.304508193547
GTTCCATA 0.196686982751
GTTCCCAA -0.58689997363
GTTCCCCA -0.142287367251
GTTCCCGA -0.237740931748
GTTCCCTA -0.14714249437
GTTCCGAA -0.6692653878
GTTCCGCA -0.723785746535
GTTCCGGA -0.864310470732
GTTCCGTA -0.649538024967
GTTCCTAA -0.309124748687
GTTCCTCA 0.298605306597
GTTCCTGA 0.711373133791
GTTCCTTA 0.210982565429
GTTCGAAA -0.294952615558
GTTCGAAC 0.311007094087
GTTCGACA -0.39339603237
GTTCGAGA 1.34762565569
GTTCGATA 1.461136371
GTTCGCAA 0.00738782056333
GTTCGCCA -0.787598129922
GTTCGCGA 0.318595181878
GTTCGCTA -0.0867902373798
GTTCGGAA 0.363661345306
GTTCGGCA -0.804931654031
GTTCGGGA -0.264382302036
GTTCGGTA 0.282428003054
GTTCGTAA 1.29290565427
GTTCGTCA 0.642172895805
GTTCGTGA -0.0629093072597
GTTCGTTA 0.332277472193
GTTCTAAA 0.387196491782
GTTCTACA 0.257440815087

GTTCTAGA 0.902852752262
GTTCTATA 1.01178667442
GTTCTCAA -0.485653440163
GTTCTCCA -0.154657303508
GTTCTCGA -0.83557691163
GTTCTCTA 0.0733829501915
GTTCTGAA -0.262886543167
GTTCTGCA 0.124550602373
GTTCTGGA -0.30182852644
GTTCTGTA 0.0357139753365
GTTCTTAA -0.827627218667
GTTCTTCA 0.970064265894
GTTCTTGA 0.0720331240944
GTTCTTTA 0.278979742802
GTTGAAAA 1.23902523468
GTTGAAAC 0.453178922553
GTTGAACA -0.108876673213
GTTGAAGA 0.51325367828
GTTGAATA 1.09532829424
GTTGACAA 1.39476006747
GTTGACCA -0.188116503018
GTTGACGA 0.127995115421
GTTGACTA -0.544092749589
GTTGAGAA 0.467643129404
GTTGAGCA -0.609263702548
GTTGAGGA -0.295759721631
GTTGAGTA 0.384740407838
GTTGATAA 0.177168006088
GTTGATCA -0.195137305782
GTTGATGA 1.20299357972
GTTGATTA 1.35916829261
GTTGCAAA 1.03066571194
GTTGCAAC -0.470149592422
GTTGCACA -0.12845643785
GTTGCAGA 1.41732739446
GTTGCATA 0.207763717324
GTTGCCAA 0.46626290932
GTTGCCCA -1.0800315836
GTTGCCGA -0.237943280756
GTTGCCTA -0.105460056016
GTTGCGAA 0.437755639694
GTTGCGCA 0.519414081405
GTTGCGGA 0.665191344506
GTTGCGTA 1.43834088093
GTTGCTAA 0.0191981743787
GTTGCTCA -0.859008177288
GTTGCTGA -0.628143780956
GTTGCTTA 0.0616088193466
GTTGGAAA 0.628098606332
GTTGGACA -0.661312155812
GTTGGAGA -0.00350571737732
GTTGGATA 2.81756962116
GTTGGCAA 0.552346798775
GTTGGCCA -0.777805853557

GTTGGCGA -0.54760637773
GTTGGCTA 0.206533385395
GTTGGGAA 0.283616283029
GTTGGGCA -0.12323762378
GTTGGGGA 0.126434821374
GTTGGGTA -0.803891804963
GTTGGTAA 0.464961588695
GTTGGTCA 0.611889659733
GTTGGTGA -0.106156619576
GTTGGTTA 0.23033395881
GTTGTAAA 1.14955553837
GTTGTACA 0.273194892774
GTTGTAGA 0.836661935322
GTTGTATA 1.13833536916
GTTGTCAA 1.26056520328
GTTGTCCA 0.164376509398
GTTGTCGA -0.438351861462
GTTGTCTA 0.258759414485
GTTGTGAA 0.67045637409
GTTGTGCA -0.668329003194
GTTGTGGA 0.0313545200159
GTTGTGTA 0.680638359655
GTTGTTAA 0.0963122987123
GTTGTTCA 0.279057185015
GTTGTTGA 1.15674329988
GTTGTTTA 0.399767111622
GTTTAAAA 0.659717720575
GTTTAAAC 1.13533281799
GTTTAACA 1.05404389221
GTTTAAGA 0.286497882788
GTTTAATA 1.75833106353
GTTTACAA 0.571864734548
GTTTACCA 0.591767175072
GTTTACGA 0.45141919399
GTTTACTA 0.0893845515102
GTTTAGAA 0.126169186257
GTTTAGCA -0.0781229548806
GTTTAGGA 0.543092246161
GTTTAGTA 0.375163803872
GTTTATAA 0.308657597274
GTTTATCA 0.701559831448
GTTTATGA 0.829266620351
GTTTATTA 0.282595378159
GTTTCAAA 0.318381591259
GTTTCACA -0.31110930948
GTTTCAGA 0.750661941598
GTTTCATA 0.811658093115
GTTTCCAA 0.827436943983
GTTTCCCA 0.00460427263862
GTTTCCGA 1.39974343223
GTTTCCTA -0.0902867949255
GTTTCGAA -0.50740262765
GTTTCGCA -0.577359176279
GTTTCGGA -0.0308232497816

GTTTCGTA 0.281106697342
GTTTCTAA 1.09247354945
GTTTCTCA 0.297602513212
GTTTCTGA 0.724374890251
GTTTCTTA 0.0197683738975
GTTTGAAA 0.318381591259
GTTTGACA -0.0534469930108
GTTTGAGA 0.432993151782
GTTTGATA 1.02719684207
GTTTGCAA 1.347707886
GTTTGCCA -0.260223322121
GTTTGCGA 0.0528513957771
GTTTGCTA -0.42891057319
GTTTGGAA -0.441552598082
GTTTGGCA -0.123185162927
GTTTGGGA 1.42575360685
GTTTGGTA -1.17411596145
GTTTGTAA 0.487908632345
GTTTGTCA 1.78355703165
GTTTGTGA 0.73188969939
GTTTGTTA 1.42480847877
GTTTTAAA -0.175928722504
GTTTTACA 0.945349166562
GTTTTAGA 0.258201913824
GTTTTATA 0.230002123091
GTTTTCAA 0.592611961362
GTTTTCCA -0.0862427292621
GTTTTCGA -0.373724877594
GTTTTCTA -0.229862227481
GTTTTGAA 0.30040333991
GTTTTGCA 0.015725349124
GTTTTGGA 0.441707482507
GTTTTGTA 0.813943887462
GTTTTTAA 0.671572208124
GTTTTTCA 0.833406031534
GTTTTTGA 1.06721677098
GTTTTTTA 1.66812108628
TAAAAAAA 1.447263806
TAAAAACA 1.08119404951
TAAAAAGA 2.18294442661
TAAAAATA 1.07000240069
TAAAACAA 1.47769089306
TAAAACCA 0.844209012048
TAAAACGA 0.719921130301
TAAAACTA 0.366458632978
TAAAAGAA 1.47886647418
TAAAAGCA 1.59418500716
TAAAAGGA 1.15710115785
TAAAAGTA -0.162875129723
TAAAATAA 1.47977038302
TAAAATCA 2.95313221475
TAAAATGA 1.47564804243
TAAAATTA 1.16386944071
TAAACAAA 1.26875721543

TAAACACA 0.447670116541
TAAACAGA 2.17374087761
TAAACATA -0.106229065517
TAAACCAA 1.75019796575
TAAACCCA 0.107283695222
TAAACCGA -0.368752754445
TAAACCTA -0.268372241422
TAAACGAA 0.662985698686
TAAACGCA 1.72937912571
TAAACGGA 1.25019710659
TAAACGTA -0.181925081372
TAAACTAA 0.635909236396
TAAACTCA 0.704850084604
TAAACTGA 1.55955876183
TAAACTTA -0.0815370739416
TAAAGAAA 0.621161074764
TAAAGACA 0.83148371596
TAAAGAGA 0.766036718984
TAAAGATA 2.28382997944
TAAAGCAA 0.626362193704
TAAAGCCA -0.355412292391
TAAAGCGA 0.130919391668
TAAAGCTA -0.464709068791
TAAAGGAA 0.972931085016
TAAAGGCA 0.616280966287
TAAAGGGA 0.709093376605
TAAAGGTA 0.557816259146
TAAAGTAA 2.23702532161
TAAAGTCA 0.343425403639
TAAAGTGA 1.57359766105
TAAAGTTA -0.0390841688414
TAAATAAA 1.56740790483
TAAATACA 1.22059461255
TAAATAGA 0.555930374721
TAAATATA 0.905449980884
TAAATCAA 1.83142547902
TAAATCCA 3.54901340329
TAAATCGA 1.18291189795
TAAATCTA 4.37569944926
TAAATGAA 1.40587364955
TAAATGCA 0.85857058715
TAAATGGA 1.20159379091
TAAATGTA 0.23769305081
TAAATTAA 1.23572249084
TAAATTCA 1.55250860597
TAAATTGA 0.739615601011
TAAATTTA 0.536274833295
TAACAAAA 2.38484814166
TAACAACA -0.249138676785
TAACAAGA 1.4479666149
TAACAATA 1.19492314353
TAACACAA 1.59182260332
TAACACCA 0.436718288762
TAACACGA 1.24997269072

TAACACTA 0.840141838628
TAACAGAA 1.61446133536
TAACAGCA 0.710588094585
TAACAGGA 1.84211375346
TAACAGTA 0.45270968936
TAACATAA 1.5880674887
TAACATCA 1.01745244664
TAACATGA 0.45368188058
TAACATTA 1.10037098973
TAACCAAA 0.650268521539
TAACCACA -0.0237562315032
TAACCAGA 0.241190441068
TAACCATA 0.00377343427443
TAACCCAA 0.144800491965
TAACCCCA -0.477239302101
TAACCCGA -0.498367286461
TAACCCTA -0.280850013878
TAACCGAA 1.0907723189
TAACCGCA 0.905212449796
TAACCGGA 0.303029088917
TAACCGTA 0.61289849028
TAACCTAA 1.12878312202
TAACCTCA 0.505348327229
TAACCTGA 0.484435182556
TAACCTTA -0.181947564595
TAACGAAA 2.4977634679
TAACGACA -0.623242854679
TAACGAGA 0.833156426122
TAACGATA 0.705608060671
TAACGCAA 1.84070938473
TAACGCCA -0.859145991119
TAACGCGA 0.912633995194
TAACGCTA 0.168568381436
TAACGGAA 1.02219349222
TAACGGCA 0.521224813575
TAACGGGA 0.381718496113
TAACGGTA 0.5792952316
TAACGTAA 3.10877336745
TAACGTCA 0.769514956866
TAACGTGA 0.240593802944
TAACGTTA 0.524825876473
TAACTAAA 1.17000569519
TAACTACA 0.818512561664
TAACTAGA 0.264130406666
TAACTATA 0.0891534739396
TAACTCAA 0.486530702219
TAACTCCA -0.644965811739
TAACTCGA -0.800979811218
TAACTCTA 1.10314433694
TAACTGAA 1.00595685795
TAACTGCA 0.221631286052
TAACTGGA 0.507864990969
TAACTGTA 0.869810741446
TAACTTAA 1.59768510374

TAACTTCA 0.133928812713
TAACTTGA 0.721091923325
TAAGAAAA 0.909224039692
TAAGAACA 0.291298050917
TAAGAAGA 0.163650384564
TAAGAATA 2.17082222217
TAAGACAA 0.607385937065
TAAGACCA -0.269154157958
TAAGACGA 0.451155432475
TAAGACTA -0.392774412887
TAAGAGAA 0.971144917848
TAAGAGCA -0.226166235419
TAAGAGGA 0.43750520157
TAAGAGTA 0.398700615772
TAAGATAA 1.6813341434
TAAGATCA 1.61424545478
TAAGATGA 0.557417390114
TAAGATTA 1.38568225797
TAAGCAAA 0.203845391168
TAAGCACA -0.00396995429843
TAAGCAGA -0.0865574943853
TAAGCATA 1.27270822553
TAAGCCAA 0.301173806657
TAAGCCCA 0.455673311247
TAAGCCGA -0.358700671946
TAAGCCTA -0.911674919187
TAAGCGAA 0.591139934784
TAAGCGCA 0.00638814984802
TAAGCGGA -0.129893906882
TAAGCGTA -0.171485371453
TAAGCTAA 0.259622520438
TAAGCTCA 0.686803342696
TAAGCTGA -0.517419944424
TAAGCTTA -0.437595134463
TAAGGAAA 0.996858438828
TAAGGACA -0.251284575521
TAAGGAGA -0.478365753213
TAAGGATA 1.22773261953
TAAGGCAA 0.908478970661
TAAGGCCA 0.883592332664
TAAGGCGA -1.13456755569
TAAGGCTA -0.347312711275
TAAGGGAA 0.235395598496
TAAGGGCA -0.0673730597541
TAAGGGGA 1.14249247547
TAAGGGTA -0.49372595814
TAAGGTAA 0.922178956105
TAAGGTCA 0.0278556725123
TAAGGTGA 0.504642603838
TAAGTAAA 0.381855060875
TAAGTACA -1.09355191144
TAAGTAGA 0.943727460008
TAAGTATA -0.202816783712
TAAGTCAA 0.502458400351

TAAGTCCA -0.493232784478
TAAGTCGA -0.908357602892
TAAGTCTA 0.45580363067
TAAGTGAA -0.0928394734576
TAAGTGCA 0.177416154254
TAAGTGGA 0.332961128717
TAAGTGTA 0.621199379514
TAAGTTAA 2.2465061637
TAAGTTCA 0.632083341267
TAAGTTGA -0.176890088469
TAATAAAA 1.96632231939
TAATAACA 0.983748013455
TAATAAGA 0.589795937671
TAATAATA 2.31383633879
TAATACAA 2.01904964109
TAATACCA 1.69068071069
TAATACGA 2.94006613129
TAATACTA 1.05478334044
TAATAGAA 0.53389556703
TAATAGCA 0.723783040221
TAATAGGA 1.24445305945
TAATAGTA 0.936891935656
TAATATAA 2.36383486337
TAATATCA 2.92684620429
TAATATGA 2.82456252807
TAATATTA 2.12789529578
TAATCAAA 2.78571047769
TAATCACA 3.21412662815
TAATCAGA 2.23476617405
TAATCATA 2.51851735641
TAATCCAA 4.31580789016
TAATCCCA 1.76066723695
TAATCCGA 0.831363597259
TAATCCTA 1.6112978626
TAATCGAA 4.45124245429
TAATCGCA 3.54761819439
TAATCGGA 1.53245065649
TAATCGTA 2.85015697124
TAATCTAA 3.61581688806
TAATCTCA 5.09355066579
TAATCTGA 5.57961317571
TAATGAAA 1.46725180768
TAATGACA -0.482687944674
TAATGAGA 0.319592770813
TAATGATA 1.21416399439
TAATGCAA 0.929213706795
TAATGCCA 1.1722644264
TAATGCGA -0.226841564824
TAATGCTA 0.35958542841
TAATGGAA -0.467454520144
TAATGGCA 0.179038901697
TAATGGGA 1.08941270844
TAATGGTA 1.48619184135
TAATGTAA 0.337540211819

TAATGTCA 1.37880822069
TAATGTGA 0.886173323403
TAATTAAA 0.670818187439
TAATTACA 1.22872479583
TAATTAGA 0.380265830088
TAATTATA 0.881575296104
TAATTCAA 0.355438106462
TAATTCCA 1.56757028366
TAATTCGA -0.294821671601
TAATTCTA 0.145081324075
TAATTGAA 0.439423561762
TAATTGCA 2.97008289953
TAATTGGA 2.29610852556
TAATTGTA 0.262604461989
TAATTTAA 1.97381339624
TAATTTCA 2.64855657153
TAATTTGA 0.505163048817
TACAAAAA 2.41518862667
TACAAACA 1.94603870497
TACAAAGA 1.71150662878
TACAAATA 2.15186553426
TACAACAA 1.76828384512
TACAACCA 0.263516281591
TACAACGA 2.48430954885
TACAACTA 0.41048786183
TACAAGAA 1.76062372775
TACAAGCA 0.848356542174
TACAAGGA 0.0893179345529
TACAAGTA 0.808469430819
TACAATAA 1.57988130554
TACAATCA 2.43689472132
TACAATGA 0.889357613961
TACACAAA 0.463707316296
TACACACA 2.47637921644
TACACAGA 0.112266227271
TACACATA 1.89404250438
TACACCAA 0.657069696523
TACACCCA 0.342571665696
TACACCGA -0.314272157706
TACACCTA -0.112865779887
TACACGAA 1.54367207477
TACACGCA 1.12000758696
TACACGGA 0.0962873173533
TACACGTA 1.85612288322
TACACTAA 0.61289849028
TACACTCA 0.115321447475
TACACTGA 0.833775755647
TACAGAAA 0.484108759466
TACAGACA 1.07995247597
TACAGAGA 1.69110102205
TACAGATA 1.98401515872
TACAGCAA 0.440982606741
TACAGCCA -0.244714061753
TACAGCGA 0.36103955168

TACAGCTA -0.160027671155
TACAGGAA 0.0415061115948
TACAGGCA 1.09497543255
TACAGGGA 0.443317531094
TACAGGTA 1.20182028856
TACAGTAA 1.37805857174
TACAGTCA 0.358448568398
TACAGTGA -0.567007109294
TACATAAA 1.7329121144
TACATACA 1.17254609122
TACATAGA 1.9611055871
TACATATA 0.531651616459
TACATCAA 0.884499572351
TACATCCA 0.914929365728
TACATCGA -0.0376027742536
TACATCTA 0.36439891993
TACATGAA 0.280263368298
TACATGCA 1.74494063875
TACATGGA 0.711710173959
TACATGTA 0.0804899386442
TACATTAA 1.23006129854
TACATTCA 1.42885004629
TACATTGA 1.07160412215
TACCAAAA 1.7332687233
TACCAACA 0.496781386522
TACCAAGA 0.695633628563
TACCAATA 1.89879187708
TACCACAA 0.606651901466
TACCACCA 0.108147425709
TACCACGA 1.09479181956
TACCACTA 0.152487047946
TACCAGAA 0.232556467047
TACCAGCA -0.22541367198
TACCAGGA -0.430792085878
TACCAGTA -0.691097638306
TACCATAA 0.869235545656
TACCATCA 0.478525009377
TACCATGA 0.303710247306
TACCCAAA 0.865390081796
TACCCACA -0.0909985554785
TACCCAGA 1.14177363687
TACCCATA 1.34943763693
TACCCCAA 0.23612796867
TACCCCCA -0.449329711489
TACCCCGA -0.143317223775
TACCCCTA -0.45752838533
TACCCGAA 0.929289483584
TACCCGCA 0.42021435395
TACCCGGA -0.479503445937
TACCCGTA 0.51265141935
TACCCTAA 0.510016510511
TACCCTCA 0.338108329558
TACCCTGA -0.590667162565
TACCGAAA 0.541970166787

TACCGACA -0.155429852034
TACCGAGA 0.134927858895
TACCGATA 2.15824285885
TACCGCAA 0.882046611076
TACCGCCA 0.838891521611
TACCGCGA -0.0011941089594
TACCGCTA -0.0156212601282
TACCGGAA 0.310494559872
TACCGGCA 0.538932641713
TACCGGGA 0.423693840901
TACCGGTA 1.62768875676
TACCGTAA 3.03981919585
TACCGTCA -0.370593880602
TACCGTGA -0.0525661919287
TACCTAAA 1.02061925025
TACCTACA 0.582940428232
TACCTAGA 0.620706414031
TACCTATA 1.00774260876
TACCTCAA 0.602085100866
TACCTCCA 0.152487047946
TACCTCGA -0.570868186503
TACCTCTA -0.169910713125
TACCTGAA 1.44167297786
TACCTGCA 2.24693230405
TACCTGGA 0.270375746412
TACCTTAA 0.682197612812
TACCTTCA -0.520922955487
TACCTTGA 0.591482179402
TACGAAAA 2.7824697709
TACGAACA 1.49178475127
TACGAAGA 0.35958542841
TACGAATA 0.872547657501
TACGACAA 1.37853925472
TACGACCA -0.181803713603
TACGACGA -0.143443379638
TACGACTA -0.908545795796
TACGAGAA 2.04787521486
TACGAGCA -0.511001192411
TACGAGGA 0.815659274112
TACGAGTA 0.447710711249
TACGATAA 1.1717081748
TACGATCA 1.18045331587
TACGATGA 0.153690524915
TACGCAAA 1.14005596026
TACGCACA 1.33183889406
TACGCAGA 1.05586690688
TACGCATA 1.90156564064
TACGCCAA 0.832258346267
TACGCCCA -0.388352920525
TACGCCGA 0.673553021714
TACGCCTA 0.704052971074
TACGCGAA 2.06206108774
TACGCGCA 0.879064461348
TACGCGGA 0.38420018595

TACGCGTA 1.11504358275
TACGCTAA 1.35288173362
TACGCTCA 0.00648890799591
TACGCTGA -0.539116462879
TACGGAAA 1.12513938263
TACGGACA 0.962129353569
TACGGAGA 1.37103298088
TACGGATA 1.3137009702
TACGGCAA 0.737777805702
TACGGCCA -0.592709388662
TACGGCGA -0.963863268061
TACGGCTA -0.922308859172
TACGGGAA -0.179909085702
TACGGGCA -0.459344738306
TACGGGGA -1.08734133742
TACGGTAA 1.46253511892
TACGGTCA 0.501040291873
TACGGTGA -0.05086204689
TACGTAAA 2.03336687428
TACGTACA 0.745009076416
TACGTAGA 0.65418643134
TACGTATA 0.0899830632359
TACGTCAA 1.67917408856
TACGTCCA -0.696659529705
TACGTCGA 0.437896576194
TACGTCTA -0.335690758542
TACGTGAA 2.93569460165
TACGTGCA 1.08361765769
TACGTGGA 1.18597003265
TACGTTAA 1.88522741551
TACGTTCA 0.572722636051
TACGTTGA 1.02182647442
TACTAAAA 0.904321447992
TACTAACA 1.02421739865
TACTAAGA 1.77875207543
TACTAATA -0.16659027416
TACTACAA 1.39477172544
TACTACCA 1.41687169284
TACTACGA 0.503773668901
TACTACTA 0.402597915951
TACTAGAA 1.79244914638
TACTAGCA 0.0868730922204
TACTAGGA 0.510437862766
TACTAGTA 1.3930192831
TACTATAA 1.6931673968
TACTATCA 1.92516761225
TACTATGA 0.272868261505
TACTCAAA 1.23837925837
TACTCACA 1.06207581548
TACTCAGA 0.121655887401
TACTCATA 0.189766105423
TACTCCAA 0.399207945537
TACTCCCA -0.407516745535
TACTCCGA -0.00667085556051

TACTCCTA -0.608761160877
TACTCGAA 0.917350059414
TACTCGCA 0.915860337706
TACTCGGA -0.424322746613
TACTCTAA 0.965195399029
TACTCTCA 0.59201407417
TACTCTGA 0.0435341815882
TACTGAAA 0.805977123904
TACTGACA 0.978789630053
TACTGAGA 1.13616823627
TACTGATA 2.0808337463
TACTGCAA 0.413334487686
TACTGCCA 0.743909063908
TACTGCGA -0.229164623031
TACTGCTA -0.339701515727
TACTGGAA -0.0731064898187
TACTGGCA -0.25268915243
TACTGGGA 1.09107438517
TACTGTAA 2.32184203163
TACTGTCA -0.0614268717821
TACTGTGA 1.52309763569
TACTTAAA 0.89582237331
TACTTACA 1.28346186786
TACTTAGA 0.104213069847
TACTTATA 0.421814409993
TACTTCAA 0.0395650600018
TACTTCCA 0.609445233757
TACTTCGA 0.153634941391
TACTTCTA 0.406141938079
TACTTGAA 0.917050074928
TACTTGCA 1.61664116711
TACTTGGA 0.459514403369
TACTTTAA 2.02155194055
TACTTTCA 0.00697666903006
TACTTTGA 2.11830578478
TAGAAAAA 0.524334992769
TAGAAACA 1.22872479583
TAGAAAGA 0.103265443629
TAGAAATA 0.931298401202
TAGAACAA 1.53713611854
TAGAACCA 0.00478913469509
TAGAACGA 1.58119761498
TAGAACTA 0.531702411889
TAGAAGAA 0.390901435497
TAGAAGCA 1.45140904617
TAGAAGGA 0.104863209714
TAGAATAA 0.496026533125
TAGAATCA 3.85608072889
TAGAATGA 1.05436032276
TAGACAAA 0.111608801174
TAGACACA 1.14287677204
TAGACAGA -0.790176206169
TAGACATA 0.341308649821
TAGACCAA 0.101486146336

TAGACCCA -0.457518184608
TAGACCGA 0.488522549242
TAGACCTA -0.326023180794
TAGACGAA 0.23329008629
TAGACGCA 1.36795132208
TAGACGGA 0.497254366919
TAGACTAA 0.180990778546
TAGACTCA 0.123704150659
TAGACTGA -0.51889113829
TAGAGAAA 0.888586939036
TAGAGACA 0.215584964466
TAGAGAGA 0.117534795881
TAGAGATA 2.26060231186
TAGAGCAA 0.221052343058
TAGAGCCA -1.23000134328
TAGAGCGA 0.399361164539
TAGAGCTA 0.138133591786
TAGAGGAA 0.882619725087
TAGAGGCA 0.815629296482
TAGAGGGA 0.319363358667
TAGAGTAA 2.14565849926
TAGAGTCA 0.331983941225
TAGAGTGA -0.0634060199475
TAGATAAA 0.489393565959
TAGATACA 1.21243736613
TAGATAGA -0.0420061551304
TAGATATA 1.76329548409
TAGATCAA 0.822227289745
TAGATCCA 1.03601193094
TAGATCGA 0.818749051863
TAGATCTA 1.29134806653
TAGATGAA -0.0642487244572
TAGATGCA 2.60449445056
TAGATGGA -0.12473442354
TAGATTAA 0.534047745141
TAGATTCA 1.92262409355
TAGATTGA 0.725715140161
TAGCAAAA 1.73250387736
TAGCAACA 0.263272713341
TAGCAAGA 0.684097028807
TAGCAATA 1.56807365804
TAGCACAA 0.0608004642055
TAGCACCA -1.01861990882
TAGCACGA 0.152031970857
TAGCACTA 0.787891244534
TAGCAGAA -0.240173699757
TAGCAGCA -0.200872193093
TAGCAGGA -0.234651153998
TAGCATAA -0.209734330192
TAGCATCA 0.403073602662
TAGCATGA -0.27077711358
TAGCCAAA 0.499047195782
TAGCCACA -0.373959286013
TAGCCAGA -0.497049519775

TAGCCATA -0.196807725986
TAGCCCAA 0.064574314836
TAGCCCCA 0.517947675633
TAGCCCGA -0.650160268984
TAGCCCTA -0.126641750298
TAGCCGAA 0.921192400603
TAGCCGCA -0.413685892136
TAGCCGGA -0.753268330052
TAGCCTAA -0.784993823248
TAGCCTCA 0.361081187279
TAGCCTGA -0.255092567342
TAGCGAAA 1.29239249552
TAGCGACA 1.03064135511
TAGCGAGA 1.46048893745
TAGCGATA 1.95917723437
TAGCGCAA 0.60494858914
TAGCGCCA -0.778048797273
TAGCGCGA -0.283865680263
TAGCGCTA 0.364023783189
TAGCGGAA 0.367746422033
TAGCGGCA -0.222197946545
TAGCGGGA -0.860385066524
TAGCGTAA 1.17739039308
TAGCGTCA -0.240305892782
TAGCGTGA 0.36684292955
TAGCTAAA -0.501850728794
TAGCTACA -0.0494085481532
TAGCTAGA -0.617166555463
TAGCTATA 0.132667045907
TAGCTCAA -0.446968556709
TAGCTCCA -1.03217500238
TAGCTCGA 0.295008199082
TAGCTGAA 0.475179172697
TAGCTGCA 0.307816558188
TAGCTGGA -0.855034475786
TAGCTTAA 0.275392003296
TAGCTTCA -1.33517224006
TAGCTTGA 0.433007724241
TAGGAAAA 0.668910444325
TAGGAACA 0.744121821816
TAGGAAGA 0.874177274819
TAGGAATA 1.85391265749
TAGGACAA 0.669088436507
TAGGACCA -0.404681985824
TAGGACGA 0.607315989259
TAGGACTA -0.344439646814
TAGGAGAA 1.31312431716
TAGGAGCA 0.0319971654758
TAGGAGGA -0.80048934387
TAGGATAA 1.2519668277
TAGGATCA 1.28689742925
TAGGATGA 0.53250660347
TAGGCAAA 0.543210074904
TAGGCACA 0.23318329098

TAGGCAGA 0.119584516385
TAGGCATA 0.598400766772
TAGGCCAA -0.0359435956612
TAGGCCCA -0.817811001833
TAGGCCGA 0.197847158698
TAGGCCTA -0.619135502906
TAGGCGAA 0.894005812156
TAGGCGCA 0.972446863007
TAGGCGGA -0.430394257736
TAGGCTAA -0.485576414306
TAGGCTCA 0.935146779553
TAGGCTGA -0.178030279329
TAGGGAAA 0.00399118845357
TAGGGACA -0.888131029235
TAGGGAGA 0.2295555813
TAGGGATA -0.202206197663
TAGGGCAA 0.640486862251
TAGGGCCA -0.429370438373
TAGGGCGA -0.699759091821
TAGGGGAA 0.320269349286
TAGGGGCA -0.442152567053
TAGGGGGA -0.470659212145
TAGGGTAA 1.54912175823
TAGGGTCA -1.04407841176
TAGGGTGA 0.340735119455
TAGGTAAA 2.35120158195
TAGGTACA -0.167314733571
TAGGTAGA 0.567809427273
TAGGTATA -0.36769396118
TAGGTCAA -0.0180432028817
TAGGTCCA -0.526991760297
TAGGTCGA -0.544039456023
TAGGTGAA -0.238302387792
TAGGTGCA 0.397729049085
TAGGTGGA 0.342178625648
TAGGTTAA 0.384774549028
TAGGTTCA -0.432237882029
TAGGTTGA 0.83330069347
TAGTAAAA 1.24495976468
TAGTAACA 1.1940843944
TAGTAAGA 0.478525009377
TAGTAATA 0.452748410466
TAGTACAA 0.299428234198
TAGTACCA -0.458702509202
TAGTACGA 3.31611011171
TAGTACTA -0.300714774186
TAGTAGAA 2.11940059284
TAGTAGCA 0.600813341516
TAGTAGGA 0.783772234794
TAGTATAA 0.556120649405
TAGTATCA 1.12946969303
TAGTATGA 0.613749938266
TAGTCAAA 1.01851207262
TAGTCACA -0.236940071015

TAGTCAGA 0.286440842018
TAGTCATA 0.306759846703
TAGTCCAA 0.100145688248
TAGTCCCA 0.293481421692
TAGTCCGA -0.142285910005
TAGTCGAA 0.760203571661
TAGTCGCA 0.594515540916
TAGTCGGA 0.236474376848
TAGTCTAA -0.440672005178
TAGTCTCA 0.705429443954
TAGTCTGA -0.15131125865
TAGTGAAA 0.330363067383
TAGTGACA 0.167423818838
TAGTGAGA -0.112750865635
TAGTGATA 0.337929921019
TAGTGCAA 0.811503625045
TAGTGCCA -0.346288267379
TAGTGCGA 1.7450811589
TAGTGGAA 1.77235497392
TAGTGGCA 2.28951490404
TAGTGGGA 1.31631360399
TAGTGTAA 2.17274745223
TAGTGTCA -0.0764566982364
TAGTGTGA 0.288239499865
TAGTTAAA 0.719693799934
TAGTTACA -0.331208686384
TAGTTAGA 2.59475151238
TAGTTATA -0.664433160261
TAGTTCAA 0.929618821166
TAGTTCCA 0.218460735241
TAGTTCGA 0.355318195939
TAGTTGAA -0.00533622645683
TAGTTGCA 2.19165459378
TAGTTGGA 0.664348431818
TAGTTTAA 0.740512223621
TAGTTTCA 0.70825129663
TAGTTTGA 1.28304509552
TATAAAAA 2.95255139815
TATAAACA 2.32326638545
TATAAAGA 2.6830529138
TATAAATA 0.50168730907
TATAACAA 1.71377368711
TATAACCA 0.58136098181
TATAACGA 3.13354967111
TATAAGAA 2.96573177133
TATAAGCA 1.28064938319
TATAAGGA 0.931424765243
TATAATAA 1.29997246437
TATAATCA 2.19999128963
TATAATGA 1.18202630877
TATACAAA 1.55532275606
TATACACA 1.52877902125
TATACAGA 1.43273818664
TATACATA 2.12412123697

TATACCAA −0.0494085481532
TATACCCA 1.01757173263
TATACCGA 2.17175194508
TATACGAA 2.50267667668
TATACGCA 1.92911862235
TATACGGA 0.984619446528
TATACTAA 0.807880495281
TATACTCA 2.99811947872
TATACTGA 1.50800598037
TATAGAAA 0.571130907128
TATAGACA 1.50800598037
TATAGAGA 0.530852212971
TATAGATA 2.87253485626
TATAGCAA 1.02422843209
TATAGCCA 0.647291159905
TATAGCGA 2.16500572908
TATAGGAA 1.7560535963
TATAGGCA 1.06866818794
TATAGGGA 0.512056654828
TATAGTAA 1.39150749453
TATAGTCA 0.1227153052
TATAGTGA 1.00128659289
TATATAAA 2.34136829452
TATATACA 2.99818318118
TATATAGA 1.70387003552
TATATATA 0.628366739585
TATATCAA 2.19180031837
TATATCCA 3.00442727186
TATATCGA 1.54678537663
TATATGAA 2.76200753975
TATATGCA 3.13168835168
TATATGGA 1.01907561044
TATATTAA 1.7435241957
TATATTCA 0.680106256709
TATATTGA 2.75873352448
TATCAAAA 2.68735553653
TATCAACA 1.91857586433
TATCAAGA 2.57394995111
TATCAATA 1.8513349976
TATCACAA 3.20254776826
TATCACCA 1.26326402276
TATCACGA 2.42672876546
TATCAGAA 2.65186535253
TATCAGCA 1.86836832904
TATCAGGA 2.62099297274
TATCATAA 1.82912011594
TATCATCA 1.66947757407
TATCATGA 2.79830607889
TATCCAAA 3.67878490151
TATCCACA 1.59191586706
TATCCAGA 2.29332872484
TATCCATA 2.86431057653
TATCCCAA 2.0853514169
TATCCCCA 2.12491522783

TATCCCGA 1.01097228212
TATCCGAA 4.19371357991
TATCCGCA 3.04538837347
TATCCGGA 2.54371688593
TATCCTAA 1.72774576119
TATCCTCA 2.0487772501
TATCCTGA 2.51395242942
TATCGAAA 1.75522733785
TATCGACA 1.95979739661
TATCGAGA 1.14198743566
TATCGATA 1.75140768806
TATCGCAA 1.89617112434
TATCGCCA 2.15586962975
TATCGCGA 2.55971494822
TATCGGAA 3.75563692976
TATCGGCA 2.60394777515
TATCGGGA 0.171023216312
TATCGTAA 2.26898480687
TATCGTCA 1.11563439189
TATCGTGA 2.13503392729
TATCTAAA 1.24672448951
TATCTACA 2.80252980216
TATCTAGA 2.79508681443
TATCTCAA 0.69133870842
TATCTCCA 3.83837290075
TATCTCGA 3.07526649517
TATCTGAA 2.52258640344
TATCTGCA 4.73435890196
TATCTGGA 3.78802921706
TATCTTAA 4.2512820808
TATCTTCA 1.69910588218
TATCTTGA 2.70107300892
TATGAAAA 3.9112293688
TATGAACA 0.199051884735
TATGAAGA 1.67016227149
TATGAATA 0.938744927959
TATGACAA 0.939849104026
TATGACCA -0.664285145709
TATGACGA 0.472153097412
TATGAGAA 2.29416122863
TATGAGCA 0.718923541365
TATGAGGA 0.245662937038
TATGATAA 1.22595935939
TATGATCA 0.79080532006
TATGATGA 0.886198929296
TATGCAAA 1.33098390705
TATGCACA 1.71090353714
TATGCAGA 0.995333743218
TATGCATA 2.16751718837
TATGCCAA 1.31472104235
TATGCCCA 1.27763933761
TATGCCGA 0.288402503233
TATGCGAA 2.69628262516
TATGCGCA 2.38843525663

TATGCGGA 0.0667443622196
TATGCTAA -0.265088858139
TATGCTCA -0.37838639918
TATGCTGA -0.259904393438
TATGGAAA 1.75522733785
TATGGACA 1.62979260355
TATGGAGA 0.0443448266873
TATGGCAA 1.03483780707
TATGGCCA -0.128376289323
TATGGCGA 0.977082570523
TATGGGAA 1.10892210891
TATGGGCA 1.57593487536
TATGGGGA 0.298023032755
TATGGTAA 1.5697511563
TATGGTCA 0.0539994974003
TATGGTGA 1.42864207648
TATGTAAA 1.30239565619
TATGTACA 1.88636781454
TATGTAGA 0.911085983649
TATGTCAA -0.226630472341
TATGTCCA 0.236944650931
TATGTCGA 0.465063804089
TATGTGAA 1.79439394518
TATGTGCA 1.87424644281
TATGTGGA 0.718486992117
TATGTTAA 1.40840113854
TATGTTCA 0.0834756273987
TATGTTGA 0.419017746855
TATTAAAA 3.34183362523
TATTAACA 2.56838514522
TATTAAGA 3.07128529926
TATTAATA 1.15217795653
TATTACAA 3.10463124995
TATTACCA 1.14810953404
TATTACGA 3.77194684268
TATTAGAA 3.28350798099
TATTAGCA 1.88969200071
TATTAGGA 1.20945709001
TATTATAA 3.05236816517
TATTATCA 1.41897366602
TATTATGA 0.964057498127
TATTCAAA 1.02442016402
TATTCACA 2.91200082354
TATTCAGA 1.43522695453
TATTCCAA 1.4312619965
TATTCCCA 2.45224992997
TATTCCGA 2.02406610615
TATTCGAA 0.94501150186
TATTCGCA 3.41118291866
TATTCGGA 0.606579039169
TATTCTAA -0.114456676098
TATTCTCA 2.86321306216
TATTCTGA 1.31513656562
TATTGAAA 1.20829962037

TATTGACA 1.32065494782
TATTGAGA 2.08608045622
TATTGCAA 0.279499771425
TATTGCCA 0.848576794489
TATTGCGA 2.19331876864
TATTGGAA 1.28399105631
TATTGGCA 1.02421739865
TATTGGGA 0.170887900617
TATTGTAA 2.22746391464
TATTGTCA 2.2828230225
TATTGTGA 2.46966381079
TATTTAAA 1.447263806
TATTTACA 4.49985097442
TATTTAGA 1.44223963835
TATTTCAA 1.30469435757
TATTTCCA 2.6321379897
TATTTCGA 3.50110061401
TATTTGAA 2.52122575208
TATTTGCA 5.80067925851
TATTTGGA 0.285487386817
TATTTTAA 3.36269285181
TATTTTCA 4.21409295621
TATTTTGA 2.12492001593
TCAAAAAA 1.32316307627
TCAAAACA 0.706752623269
TCAAAAGA 0.491738899211
TCAAACAA 0.936813036198
TCAAACCA −0.498982660604
TCAAACGA 1.53971815017
TCAAAGAA 0.965303859762
TCAAAGCA 1.25545818079
TCAAAGGA 0.847915204832
TCAAATAA 1.09399491421
TCAAATCA 2.42234432878
TCAAATGA 1.11489723363
TCAACAAA 3.10426631393
TCAACACA 0.024399709675
TCAACAGA 1.83710207649
TCAACCAA −0.450259850755
TCAACCCA −0.360383999185
TCAACCGA 0.216153706739
TCAACGAA −0.0108922888727
TCAACGCA 0.54431029559
TCAACGGA 1.38772219411
TCAACTAA −0.201177382028
TCAACTCA −0.383737822631
TCAACTGA −0.209621497721
TCAAGAAA −0.348860930998
TCAAGACA 0.297102886033
TCAAGAGA −0.5444397823
TCAAGCAA −0.12522468271
TCAAGCCA −1.18521601196
TCAAGCGA 0.782795463658
TCAAGGAA 2.2234129791

TCAAGGCA 0.380543955885
TCAAGGGA 1.18056031936
TCAAGTAA 0.831626109707
TCAAGTCA 0.0163756971695
TCAAGTGA 0.636067451669
TCAATAAA 0.990862496316
TCAATACA 0.894410301993
TCAATAGA 0.498282766197
TCAATCAA 0.570089808993
TCAATCCA 0.0763359550013
TCAATCGA −0.250869260428
TCAATGAA 0.384740407838
TCAATGCA 1.05588918193
TCAATGGA 1.91106084701
TCAATTAA 0.431555890929
TCAATTCA 0.65874906838
TCAATTGA 1.12112133922
TCACAAAA 1.6637035493
TCACAACA 2.02351380994
TCACAAGA 1.79409375251
TCACACAA 0.882333688527
TCACACCA 1.00400831195
TCACACGA 0.00311725724513
TCACAGAA 0.414876253891
TCACAGCA 2.55496120379
TCACAGGA 1.54425330772
TCACATAA 1.23743829385
TCACATCA 0.274677536429
TCACATGA 1.24647842313
TCACCAAA −0.111095226069
TCACCACA 1.30576022889
TCACCAGA 1.88521825568
TCACCCAA −0.287963455848
TCACCCCA −0.124606185897
TCACCCGA 0.093886608755
TCACCGAA 0.801803155174
TCACCGCA 0.95616942585
TCACCGGA 0.578243308209
TCACCTAA −0.375295788718
TCACCTCA 1.00844791579
TCACCTGA 0.157401297614
TCACGAAA 0.651003181671
TCACGACA −0.346521010373
TCACGAGA 0.180405382034
TCACGCAA 0.827068677116
TCACGCCA 0.584368737431
TCACGCGA 0.151316254922
TCACGGAA 1.05530003821
TCACGGCA 1.18784654906
TCACGGGA 0.367468712593
TCACGTAA 1.00032980684
TCACGTCA 1.10952873958
TCACGTGA 1.02438602283
TCACTAAA 0.681559547268

TCACTACA 0.0274099634325
TCACTAGA 1.11376287175
TCACTCAA -0.207254097601
TCACTCCA -0.206143468016
TCACTCGA -0.245897761812
TCACTGAA 0.534687476109
TCACTGCA -0.495652229096
TCACTGGA 1.65133819296
TCACTTAA -0.0928394734576
TCACTTCA 2.00374647693
TCAGAAAA 0.513417306181
TCAGAACA -0.774891778031
TCAGAAGA 0.7901191654
TCAGACAA 0.0525499540453
TCAGACCA -0.309448257286
TCAGACGA -0.715848960608
TCAGAGAA 1.12881809592
TCAGAGCA -0.805994402678
TCAGAGGA -0.0197390208007
TCAGATAA 0.983256505217
TCAGATCA 0.185822173373
TCAGATGA 0.98162376523
TCAGCAAA 0.256287092658
TCAGCACA -0.292509646827
TCAGCAGA 1.24343839992
TCAGCCAA -1.11632158744
TCAGCCCA -0.891182502235
TCAGCCGA -0.576506271047
TCAGCGAA 2.07574795797
TCAGCGCA 0.153640354019
TCAGCGGA 1.09013779238
TCAGCTAA -0.464522333133
TCAGCTCA -1.23925589589
TCAGCTGA 1.12223634054
TCAGGAAA 0.964986596503
TCAGGACA -0.637332757502
TCAGGAGA 0.336770369606
TCAGGCAA 0.503788241361
TCAGGCCA -0.935609351051
TCAGGCGA 0.0249189055859
TCAGGGAA 0.27780457804
TCAGGGCA -0.583266851322
TCAGGGGA -0.424880039096
TCAGGTAA 0.201814406683
TCAGGTCA -0.505959537812
TCAGTAAA 0.0906361175953
TCAGTACA 0.221621293508
TCAGTAGA 0.127143875614
TCAGTCAA -0.0498038781591
TCAGTCCA -0.655285194779
TCAGTCGA -0.389124636339
TCAGTGAA 0.150790397315
TCAGTGCA 1.46171968573
TCAGTGGA 0.870991318836

TCAGTTAA −0.0394747107535
TCAGTTCA −0.782754660771
TCATAAAA 1.56055072996
TCATAACA 0.442129459296
TCATAAGA 0.806646624325
TCATACAA 0.219488301807
TCATACCA 0.260193760847
TCATACGA 0.723461405225
TCATAGAA 0.517888969439
TCATAGCA 0.947007928799
TCATAGGA −0.0115913505683
TCATATAA 1.33303237849
TCATATCA 1.42386834696
TCATATGA 0.664134008487
TCATCAAA 1.98889297724
TCATCACA 0.310162724153
TCATCAGA 0.883592332664
TCATCCAA 0.682748659956
TCATCCCA −1.09862687452
TCATCCGA 0.146027076691
TCATCGAA 1.00554924544
TCATCGCA −0.00609003896415
TCATCGGA 0.514727162104
TCATCTAA 0.349175071587
TCATCTCA −1.11001337794
TCATGAAA 2.07731158287
TCATGACA 0.790823847901
TCATGAGA 1.07720702462
TCATGCAA −0.0665734480886
TCATGCCA −0.510145580866
TCATGCGA −0.233830932712
TCATGGAA 1.54985766743
TCATGGCA −0.0685769530791
TCATGGGA −0.658362898206
TCATGTAA 0.144907287275
TCATGTCA 0.315492288914
TCATTAAA 0.246600154356
TCATTACA 0.376848380179
TCATTAGA 0.159152907235
TCATTCAA 0.676273699885
TCATTCCA −0.0697704375045
TCATTCGA 0.799670371651
TCATTGAA −0.164949207053
TCATTGCA −0.386802410844
TCATTGGA 1.53546507381
TCATTTAA 0.26482030853
TCATTTCA 0.265583697225
TCCAAAAA 0.903793508605
TCCAAACA 0.398629418899
TCCAAAGA 1.98720007381
TCCAACAA 0.22571948545
TCCAACCA −0.289726723437
TCCAACGA −0.0797879624568
TCCAAGAA −0.0887960323282

TCCAAGCA 1.19152588688
TCCAAGGA 0.468565149728
TCCAATAA 1.46298249343
TCCAATCA -0.326511358184
TCCACAAA 1.01328555596
TCCACACA 0.0159851552574
TCCACAGA 1.08646927981
TCCACCAA 0.318501918138
TCCACCCA -0.265017661266
TCCACCGA 0.756988054404
TCCACGAA 0.446309465188
TCCACGCA 0.319664384042
TCCACGGA -0.128420423058
TCCACTAA 2.12400882086
TCCACTCA -0.699183271497
TCCAGAAA 0.686043076671
TCCAGACA -0.192340226288
TCCAGAGA 0.246786890014
TCCAGCAA 1.44908244893
TCCAGCCA -0.819899443444
TCCAGCGA 0.893767864711
TCCAGGAA 1.62979260355
TCCAGGCA -0.539347956806
TCCAGGGA 0.264656680629
TCCAGTAA 1.22059461255
TCCAGTCA 1.49996406455
TCCATAAA 0.0453224305355
TCCATACA 0.418087607589
TCCATAGA 0.734516905643
TCCATCAA -0.527583402148
TCCATCCA -0.520738093431
TCCATCGA 2.7056768652
TCCATGAA 1.54420396953
TCCATGCA -0.535086553319
TCCATGGA 0.313056814591
TCCATTAA 1.50715453238
TCCATTCA -0.262467897226
TCCCAAAA 1.67336217539
TCCCAACA 0.570690402498
TCCCAAGA 1.8133826925
TCCCACAA 0.347580219994
TCCCACCA -0.21028600187
TCCCACGA 0.010541508957
TCCCAGAA 0.28836440666
TCCCAGCA 0.411047236093
TCCCAGGA 0.274504540518
TCCCATAA -0.206754886777
TCCCATCA 0.347275031059
TCCCCAAA 3.06394161244
TCCCCACA -0.240070651652
TCCCCAGA 0.780744077729
TCCCCCAA -1.55215095618
TCCCCCCA -1.0399566957
TCCCCCGA 1.58621095738

TCCCCGAA 0.56868044399
TCCCCGCA 0.683996270659
TCCCCGGA −0.539620045441
TCCCCTAA 0.141219830511
TCCCCTCA −1.2473442354
TCCCGAAA 0.0211410995736
TCCCGACA 0.947007928799
TCCCGAGA 1.27355946533
TCCCGCAA −0.244688039504
TCCCGCCA −0.00641958472474
TCCCGCGA 0.735412487362
TCCCGGAA 2.79610980108
TCCCGGCA −0.958446893077
TCCCGGGA 1.53434882342
TCCCGTAA −0.0188038852627
TCCCGTCA 0.12183637772
TCCCTAAA 0.489004064937
TCCCTACA −0.275242947854
TCCCTAGA 0.896313048836
TCCCTCAA −0.0694723266206
TCCCTCCA −0.823680372126
TCCCTCGA 1.8764722819
TCCCTGAA 0.100232290293
TCCCTGCA −0.46921487324
TCCCTTAA 0.039658323742
TCCCTTCA −0.0376629376932
TCCGAAAA 0.399862040786
TCCGAACA 0.377959634298
TCCGAAGA 0.0326202422044
TCCGACAA 0.845252816498
TCCGACCA −0.631114480895
TCCGACGA 0.789893292279
TCCGAGAA 0.393843406873
TCCGAGCA 0.726244120437
TCCGAGGA −0.446345063625
TCCGATAA −0.129596212354
TCCGATCA 0.730835069684
TCCGCAAA 0.670246114318
TCCGCACA −0.0393962276507
TCCGCAGA 0.983215910509
TCCGCCAA −0.788903405929
TCCGCCCA −0.544468510863
TCCGCCGA 0.563423949704
TCCGCGAA 1.46363409054
TCCGCGCA 0.0109387125648
TCCGCGGA 0.340770301535
TCCGCTAA −0.0905124598683
TCCGCTCA −1.367233108
TCCGGAAA 0.849315201825
TCCGGACA −0.0970259328672
TCCGGAGA −0.107957775558
TCCGGCAA −0.427375885037
TCCGGCCA −0.326179522465
TCCGGCGA 1.0279040227

TCCGGGAA -0.252822178167
TCCGGGCA 0.514050999987
TCCGGTAA -0.243317811963
TCCGGTCA -0.823740327387
TCCGTAAA -0.113056054571
TCCGTACA 2.09317391311
TCCGTAGA 0.807398771409
TCCGTCAA -0.571451917591
TCCGTCCA -0.163122445177
TCCGTCGA 0.17352967933
TCCGTGAA 1.07839946815
TCCGTGCA 0.822227289745
TCCGTTAA 0.271821750741
TCCGTTCA 1.28577181085
TCCTAAAA 1.59115830735
TCCTAACA 1.41013651028
TCCTAAGA -0.309124748687
TCCTACAA -0.351155052465
TCCTACCA 0.643652000435
TCCTACGA -0.446469554064
TCCTAGAA 0.169689628098
TCCTAGCA 1.06305987285
TCCTAGGA -0.23209077288
TCCTATAA 0.536598133716
TCCTATCA -0.474796541549
TCCTCAAA 0.99479248044
TCCTCACA 0.178776181072
TCCTCAGA 1.06955689978
TCCTCCAA -0.580778707967
TCCTCCCA -0.932398621887
TCCTCCGA 0.121387754148
TCCTCGAA 0.253429641546
TCCTCGCA 0.0858875776086
TCCTCTAA 0.805038865697
TCCTCTCA -0.632954357984
TCCTGAAA 0.177885179268
TCCTGACA 0.0338872134609
TCCTGAGA 0.309123499619
TCCTGCAA -0.415213085882
TCCTGCCA -0.471305812987
TCCTGCGA 0.477086707634
TCCTGGAA -0.202953973008
TCCTGGCA 0.0697104822429
TCCTGTAA 0.452388470719
TCCTGTCA -0.657819137292
TCCTTAAA 0.430036607946
TCCTTACA 1.09617120693
TCCTTAGA 0.498677055313
TCCTTCAA 0.323081001239
TCCTTCCA -0.927793932893
TCCTTCGA 0.596511551499
TCCTTGAA 0.115478830036
TCCTTGCA 0.985750685734
TCCTTTAA 0.135358579159

TCCTTTCA -0.305022393186
TCGAAAAA 0.447954071321
TCGAAACA 0.788590722586
TCGAAAGA 0.0588294349818
TCGAACAA 0.537849699801
TCGAACCA -0.375352413132
TCGAACGA 0.173587969167
TCGAAGAA 0.495244824767
TCGAAGCA 0.329401701418
TCGAATAA 0.196520856714
TCGAATCA 1.26020547171
TCGACAAA 0.486686211179
TCGACACA -0.662447766756
TCGACAGA 1.83829785088
TCGACCAA -0.533438199982
TCGACCCA -1.05092746768
TCGACCGA -0.100214386985
TCGACGAA 0.0543671397334
TCGACGCA 0.188391506145
TCGACTAA 1.01959709631
TCGACTCA -0.443411003012
TCGAGAAA 0.268336851163
TCGAGACA -0.221134157008
TCGAGAGA 0.187576072952
TCGAGCAA -0.113465957036
TCGAGCCA -1.32896041697
TCGAGCGA -0.194884785879
TCGAGGAA 0.238039042632
TCGAGGCA -0.885465518231
TCGAGTAA 0.530232258913
TCGAGTCA -0.471832295127
TCGATAAA 0.259136633006
TCGATACA 0.454790636563
TCGATAGA 1.68589615591
TCGATCAA -0.460312765966
TCGATCCA -0.738400674253
TCGATCGA 0.477842185565
TCGATGAA 0.885074976319
TCGATGCA -0.482117745155
TCGATTAA 0.300927532093
TCGATTCA 1.24343839992
TCGCAAAA 0.294335992347
TCGCAACA 1.05271092853
TCGCAAGA 1.39735396524
TCGCACAA 1.23242162061
TCGCACCA 0.118364593355
TCGCACGA 2.08813184215
TCGCAGAA -0.719819955797
TCGCAGCA 0.0580845741281
TCGCATAA 0.467330654239
TCGCATCA 0.291083835763
TCGCCAAA 0.0159851552574
TCGCCACA -0.249567939803
TCGCCAGA 1.9062567235

TCGCCCAA 0.173938124549
TCGCCCCA -0.244597898433
TCGCCCGA -0.707839312384
TCGCCGAA -0.38424161337
TCGCCGCA -0.601946662502
TCGCCTAA -0.078579489216
TCGCCTCA -0.758566459936
TCGCGAAA 0.561743536956
TCGCGACA 0.0783869245738
TCGCGAGA 0.541222807797
TCGCGCAA 0.691280210404
TCGCGCCA -0.846549973563
TCGCGCGA 1.82341333266
TCGCGGAA 1.35350543489
TCGCGGCA 0.482229328558
TCGCGTAA 0.449132983287
TCGCGTCA 0.331154143751
TCGCTAAA 0.571863277302
TCGCTACA 0.0783715194024
TCGCTAGA 0.309013789818
TCGCTCAA -0.211284631695
TCGCTCCA -0.448065446547
TCGCTGAA -0.126756456371
TCGCTGCA -0.236625097714
TCGCTTAA 0.427689817448
TCGCTTCA 0.432152945408
TCGGAAAA 0.539653145741
TCGGAACA 0.417660218172
TCGGAAGA 0.534342733355
TCGGACAA -0.059241211049
TCGGACCA -0.173031717574
TCGGACGA 0.0619743798998
TCGGAGAA -0.124668222938
TCGGAGCA 0.646937257319
TCGGATAA 0.0187391419074
TCGGATCA 1.33952274373
TCGGCAAA 0.884622813722
TCGGCACA -0.202937735125
TCGGCAGA 0.830919137247
TCGGCCAA -0.663643124783
TCGGCCCA -0.234726930787
TCGGCCGA 1.88663136788
TCGGCGAA 0.110927226429
TCGGCGCA 0.991158733598
TCGGCTAA -0.00989365904735
TCGGCTCA -1.22032918561
TCGGGAAA 0.991442688378
TCGGGACA -0.155168796833
TCGGGAGA -0.445210701749
TCGGGCAA -0.327273705989
TCGGGCCA -1.16745926199
TCGGGGAA 0.280092662345
TCGGGGCA 0.796318289631
TCGGGTAA -0.0516806027527

TCGGGTCA −0.238230150029
TCGGTAAA 0.585014505561
TCGGTACA −0.229330957247
TCGGTAGA −0.402393068807
TCGGTCAA −0.0725460746655
TCGGTCCA −0.662193997784
TCGGTGAA 0.783839892641
TCGGTGCA −0.520797632337
TCGGTTAA 0.296807689641
TCGGTTCA −0.288776807261
TCGTAAAA 1.23766312608
TCGTAACA 0.772945938345
TCGTAAGA 1.88600475213
TCGTACAA 1.00155659974
TCGTACCA −0.19827912803
TCGTACGA −0.888421645711
TCGTAGAA 0.136346591907
TCGTAGCA 0.636456328158
TCGTATAA 1.00340563666
TCGTATCA 2.64722069336
TCGTCAAA 1.14629463831
TCGTCACA −0.288219514778
TCGTCAGA 0.326617528959
TCGTCCAA 0.0229678614493
TCGTCCCA −0.266071458259
TCGTCGAA 0.443502809506
TCGTCGCA 0.406702977766
TCGTCTAA 0.223394969996
TCGTCTCA −0.816268611094
TCGTGAAA 0.929809512207
TCGTGACA −0.642192464536
TCGTGAGA 1.46109057184
TCGTGCAA 0.901613468678
TCGTGCCA −0.753268330052
TCGTGGAA 0.612969270797
TCGTGGCA 0.939498532288
TCGTGTAA 1.02655877652
TCGTGTCA 0.794225892639
TCGTTAAA 0.469667868549
TCGTTACA 0.381203880118
TCGTTAGA 1.87216070751
TCGTTCAA 0.743827458136
TCGTTCCA −0.0904333522315
TCGTTGAA 0.644386452389
TCGTTGCA 0.575112311216
TCGTTTAA 0.579361848557
TCGTTTCA 0.31495581423
TCTAAAAA 1.0179608173
TCTAAACA 0.947007928799
TCTAAAGA 0.988364984952
TCTAACAA 0.753201296738
TCTAACCA 1.24018332884
TCTAAGAA 0.203237095077
TCTAAGCA −0.20001012803

TCTAATAA 2.92961851061
TCTAATCA 0.699586304088
TCTACAAA -0.220891837826
TCTACACA 0.172797309156
TCTACAGA 1.04655323172
TCTACCAA 1.30256344765
TCTACCCA -0.369260292388
TCTACGAA -0.0166779716132
TCTACGCA 0.434412717506
TCTACTAA 3.03915240174
TCTACTCA 0.664745427248
TCTAGAAA 0.428639317267
TCTAGACA 0.822227289745
TCTAGAGA 0.676786650456
TCTAGCAA 0.240979348585
TCTAGCCA 0.387059718841
TCTAGGAA 0.165662841208
TCTAGGCA -0.63515729749
TCTAGTAA 1.58727224878
TCTAGTCA -0.297187198119
TCTATAAA 0.414908313302
TCTATACA 1.79188290224
TCTATAGA 0.423136132061
TCTATCAA 0.342815233946
TCTATCCA 0.395523611443
TCTATGAA 0.0303294515857
TCTATGCA 0.748416325603
TCTATTAA 0.796515434189
TCTATTCA 0.400537786547
TCTCAAAA 2.5306316501
TCTCAACA 0.877314933507
TCTCAAGA 0.339909693718
TCTCACAA 0.19229546802
TCTCACCA 0.180279642527
TCTCAGAA -0.550909121565
TCTCAGCA -0.22532353091
TCTCATAA 0.997829797336
TCTCATCA 1.6577954579
TCTCCAAA -0.361240235264
TCTCCACA 0.966166757537
TCTCCAGA 1.71450605728
TCTCCCAA -0.0580404403939
TCTCCCCA 0.930723829945
TCTCCGAA -0.017815247981
TCTCCGCA -0.491618155976
TCTCCTAA -0.372076732435
TCTCCTCA -0.145299494611
TCTCGAAA 0.441474739513
TCTCGACA -0.602559122153
TCTCGAGA 1.89023180625
TCTCGCAA 0.222106972763
TCTCGCCA 0.254759482556
TCTCGGAA 1.46935294815
TCTCGGCA 0.495753611778

TCTCGTAA 2.36299590606
TCTCGTCA 0.684637042517
TCTCTAAA -0.810280787523
TCTCTACA 0.76239068964
TCTCTCAA -1.28775241626
TCTCTCCA -0.435880580525
TCTCTGAA 1.00924253119
TCTCTGCA 0.866385588952
TCTCTTAA 0.317102962035
TCTCTTCA -0.291419626864
TCTGAAAA 0.872043450406
TCTGAACA 1.10863253333
TCTGAAGA 0.532160819826
TCTGACAA 1.34616362166
TCTGACCA 1.32567745005
TCTGAGAA 1.39663804113
TCTGAGCA 0.804362287224
TCTGATAA 3.1553565239
TCTGATCA 0.83779192546
TCTGCAAA 1.47018461922
TCTGCACA 2.50457151276
TCTGCAGA 2.29214335936
TCTGCCAA 0.129326413677
TCTGCCCA -0.12323762378
TCTGCGAA 0.770933065345
TCTGCGCA -0.540439642193
TCTGCTAA 1.27065621506
TCTGCTCA 0.169124216673
TCTGGAAA 0.509398430055
TCTGGACA 2.90309538542
TCTGGCAA -0.534141008882
TCTGGCCA -0.454534993989
TCTGGGAA 0.640352171091
TCTGGGCA 0.124398840617
TCTGGTAA 1.46709275969
TCTGGTCA -0.798533303461
TCTGTAAA 0.360582392811
TCTGTACA 0.636706349925
TCTGTCAA -0.628660686909
TCTGTCCA 0.305827417479
TCTGTGAA 0.0848658400261
TCTGTGCA -0.0498482200713
TCTGTTAA 2.07472184865
TCTGTTCA -0.378470919445
TCTTAAAA 0.448163706559
TCTTAACA 0.816495316926
TCTTAAGA 0.941557620803
TCTTACAA 1.76695233869
TCTTACCA 1.08677238697
TCTTAGAA 0.9881526434
TCTTAGCA -0.256786928015
TCTTATAA 1.44611570438
TCTTATCA 1.18600417384
TCTTCAAA 1.64328961545

TCTTCACA 1.03986863641
TCTTCCAA 0.661166639396
TCTTCCCA 0.00141977390222
TCTTCGAA 0.944088857002
TCTTCGCA 0.616484564362
TCTTCTAA 1.50739060622
TCTTCTCA -0.0011941089594
TCTTGAAA 0.0975311808527
TCTTGACA 0.601693726242
TCTTGCAA 1.13443619538
TCTTGCCA 0.472078153335
TCTTGGAA 2.52209135617
TCTTGGCA 0.680057334881
TCTTGTAA 2.5579473089
TCTTGTCA 0.686858718042
TCTTTAAA 1.37676391281
TCTTTACA 1.89094273409
TCTTTCAA 0.265694864273
TCTTTCCA -0.245488483881
TCTTTGAA 1.97536140779
TCTTTGCA 0.640076959786
TCTTTTAA 1.37676391281
TCTTTTCA 1.31508139846
TGAAAAAA 2.10582759597
TGAAAACA 0.592611961362
TGAAACAA 0.621968805371
TGAAACCA 0.030296351285
TGAAAGAA -0.198154221236
TGAAAGCA -0.148851011146
TGAAATAA 1.09809872696
TGAAATCA 3.09670757923
TGAACAAA 0.442771272044
TGAACACA 0.532470172322
TGAACCAA 0.67280920175
TGAACCCA -0.632116649746
TGAACGAA 0.450878972102
TGAACGCA -0.740179138834
TGAACTAA -0.0747294454407
TGAACTCA -0.749018584532
TGAAGAAA 1.45723199277
TGAAGACA 0.390544410242
TGAAGCAA -0.364097478198
TGAAGCCA -0.532000939129
TGAAGGAA -0.396021573199
TGAAGGCA -1.12362863495
TGAAGTAA 0.0771486818802
TGAAGTCA -0.88231162166
TGAATAAA 1.19376962928
TGAATACA -0.0752825743642
TGAATCAA 0.442066589543
TGAATCCA 2.0139842542
TGAATGAA 0.957424322783
TGAATGCA 0.781468953496
TGAATTAA 0.531758619947

TGAATTCA 2.05718077108
TGACAAAA 3.12527459594
TGACAACA -0.235864623511
TGACACAA 0.607315989259
TGACACCA 0.0749563594514
TGACAGAA 0.261935169746
TGACAGCA 0.886173323403
TGACATAA 0.763704292767
TGACATCA -0.638307863214
TGACCAAA 0.463851791822
TGACCACA -0.184735900614
TGACCCAA 0.775119108398
TGACCCCA -0.973388660241
TGACCGAA 0.319005500699
TGACCGCA -1.14162437325
TGACCTAA 0.41539982154
TGACCTCA -1.23667219884
TGACGAAA 0.942712384122
TGACGACA -0.205947572526
TGACGCAA 0.955915032344
TGACGCCA -1.11932684493
TGACGGAA 1.48759038109
TGACGGCA -0.360383999185
TGACGTAA 0.96717704533
TGACGTCA -0.630898808496
TGACTAAA 0.466473585447
TGACTACA 0.286440842018
TGACTCAA -0.625275296411
TGACTCCA -0.687922507579
TGACTGAA 0.655738606445
TGACTGCA -0.681527696035
TGACTTAA 1.34856099941
TGAGAAAA 0.995214873585
TGAGAACA 0.103143243148
TGAGACAA -0.418297034648
TGAGACCA -0.664938408246
TGAGAGAA -0.392456733272
TGAGAGCA -1.07892241127
TGAGATAA 0.0501159369684
TGAGATCA 1.38647583247
TGAGCAAA 0.819038002915
TGAGCACA 0.110225666598
TGAGCCAA -0.482280540344
TGAGCCCA -1.77912200772
TGAGCGAA 0.37004366617
TGAGCGCA 0.39594787819
TGAGCTAA -0.10024186648
TGAGCTCA -0.98546090197
TGAGGAAA 0.693240622551
TGAGGACA -0.186952996223
TGAGGCAA -0.114645701714
TGAGGCCA -1.31240735216
TGAGGGAA 0.76348528952
TGAGGGCA -0.983412846889

TGAGGTAA 0.838865291184
TGAGTAAA 1.01759400767
TGAGTACA 0.630523671755
TGAGTCAA -0.806791308029
TGAGTCCA -0.681604097358
TGAGTGAA -0.869531366582
TGAGTGCA -0.485920740704
TGAGTTAA 0.108647677422
TGATAAAA 1.98194586948
TGATAACA 0.844448624917
TGATACAA 1.36886064354
TGATACCA 1.03071442559
TGATAGAA 0.0238642758808
TGATAGCA -0.326437246819
TGATATAA 2.36558189307
TGATATCA 4.45377035964
TGATCAAA 0.491738899211
TGATCACA 2.90339703533
TGATCCAA 0.699590884004
TGATCCCA 1.38625016753
TGATCGAA -0.649933146795
TGATCGCA 1.70138376577
TGATCTAA 1.12950903867
TGATGAAA 2.18808704754
TGATGACA -0.495983648458
TGATGCAA 0.547736280796
TGATGCCA 0.0141003117221
TGATGGAA -0.48321067961
TGATGGCA -0.89916612821
TGATGTAA 1.12165510759
TGATTAAA 0.574051852527
TGATTACA 6.28506926644
TGATTCAA -0.0532061310746
TGATTCCA 3.08768805957
TGATTGAA -0.283383123679
TGATTGCA 3.79857884496
TGATTTAA 0.971323118209
TGCAAAAA 1.07357327777
TGCAAACA 0.714622167704
TGCAACAA 1.04663358842
TGCAACCA -0.342424692034
TGCAAGAA -0.0456095079858
TGCAAGCA 0.244335177808
TGCAATAA -0.576993615726
TGCACAAA 0.369572975731
TGCACACA 0.902465749375
TGCACCAA 0.581123867077
TGCACCCA -0.275988849598
TGCACGAA -0.41254632581
TGCACGCA -0.692770556646
TGCACTAA -0.353816399908
TGCAGAAA 2.13548817167
TGCAGACA 0.994564941895
TGCAGCAA -0.235413918159

TGCAGCCA -1.01649482788
TGCAGGAA 0.326421633469
TGCAGGCA -1.02377668584
TGCAGTAA 0.446122521352
TGCATAAA -0.0295113120789
TGCATACA 1.19004365958
TGCATCAA -0.686419878836
TGCATCCA -0.327781243932
TGCATGAA 0.0464436771978
TGCATGCA 0.278906464149
TGCATTAA 0.133424189262
TGCCAAAA 0.815591408087
TGCCAACA -0.728913170467
TGCCACAA 0.371502785713
TGCCACCA -0.60317512083
TGCCAGAA -0.566766663714
TGCCAGCA -1.17903458286
TGCCATAA 0.369048367192
TGCCCAAA -0.145931939349
TGCCCACA 0.684901844922
TGCCCCAA -0.222948011848
TGCCCCCA -1.34060631018
TGCCCGAA -0.498488446053
TGCCCGCA -1.34621379256
TGCCCTAA -0.21245438383
TGCCGAAA 1.43909968971
TGCCGACA -0.514240025603
TGCCGCAA 0.865047629
TGCCGCCA -1.00885074021
TGCCGGAA -0.202256160381
TGCCGGCA -0.747326930173
TGCCGTAA 1.0752888726
TGCCTAAA 0.142952079578
TGCCTACA 0.414678276621
TGCCTCAA -0.367641292148
TGCCTCCA -0.553553814769
TGCCTGAA -0.575307165816
TGCCTTAA -0.47647445616
TGCGAAAA 1.56083739106
TGCGAACA -0.0296455868835
TGCGACAA 1.08612870062
TGCGACCA -0.542712945861
TGCGAGAA -0.794482992458
TGCGAGCA -0.422988117509
TGCGATAA 0.935074333612
TGCGCAAA 0.877379468684
TGCGCACA 1.03572297989
TGCGCCAA -0.172675941386
TGCGCCCA -0.82381568782
TGCGCGAA 0.355944395338
TGCGCGCA 0.738330726447
TGCGCTAA 0.517050844845
TGCGGAAA 0.76239068964
TGCGGACA -0.222027240592

TGCGGCAA 0.268370159642
TGCGGCCA -1.83566564835
TGCGGGAA 0.156326266466
TGCGGTAA 0.549230582419
TGCGTAAA 1.87734600493
TGCGTACA 0.239440496871
TGCGTCAA 0.300821361317
TGCGTCCA 0.0578443367259
TGCGTGAA -0.467328364281
TGCGTTAA -0.341710433345
TGCTAAAA 0.382201469053
TGCTAACA 1.00595685795
TGCTACAA 0.559629073096
TGCTACCA -1.20237820558
TGCTAGAA 0.177131574939
TGCTAGCA -0.0688228112871
TGCTATAA 0.86560429695
TGCTCAAA 0.539840505934
TGCTCACA 1.29223990105
TGCTCCAA -0.237402434334
TGCTCCCA -1.24699907629
TGCTCGAA -0.702583234454
TGCTCTAA -1.02111929378
TGCTGAAA 1.80041695083
TGCTGACA -0.450948919907
TGCTGCAA 0.214485576493
TGCTGCCA -0.809196596543
TGCTGGAA 1.00853160335
TGCTGTAA 1.2208939725
TGCTTAAA 1.23790377984
TGCTTACA -0.134277302671
TGCTTCAA -0.0660513376858
TGCTTCCA -0.476979704146
TGCTTGAA -0.248937993201
TGCTTTAA -0.384384007116
TGGAAAAA -0.104538660225
TGGAAACA 0.508657940939
TGGAACAA 0.469977429223
TGGAACCA 0.763880827704
TGGAAGAA -0.354393885657
TGGAATAA 1.0999021729
TGGACAAA -0.591838788301
TGGACACA 0.802291332564
TGGACCAA -0.103521918915
TGGACCCA -1.21460928712
TGGACGAA 0.171319661772
TGGACTAA 0.405606504285
TGGAGAAA 0.450477604934
TGGAGACA -0.565927290052
TGGAGCAA -0.891325312337
TGGAGCCA -0.195082763149
TGGAGGAA -0.629707197672
TGGAGTAA -0.073590711827
TGGATAAA 2.04339813898

TGGATACA 1.46300456029
TGGATCAA 0.305056534377
TGGATCCA 3.43610182425
TGGATGAA 0.45505835346
TGGATTAA 1.36873365497
TGGCAAAA 0.175991175902
TGGCAACA 0.120965985537
TGGCACAA -0.371393700445
TGGCACCA -0.780121209179
TGGCAGAA 0.433948688763
TGGCATAA 1.18797895026
TGGCCAAA -0.541996189036
TGGCCACA -0.83090019305
TGGCCCAA -0.782846259088
TGGCCCCA 0.0480200009497
TGGCCGAA 0.246598072576
TGGCCTAA 0.254479066801
TGGCGAAA 0.21829606645
TGGCGACA -0.611620901946
TGGCGCAA -0.188490390691
TGGCGCCA -0.13306778854
TGGCGGAA -0.207479346188
TGGCGTAA 1.75187442312
TGGCTAAA 0.0777786284826
TGGCTACA -0.152898824013
TGGCTCAA -0.772260616397
TGGCTGAA -0.194731358699
TGGCTTAA 0.415499538798
TGGGAAAA 1.11628411541
TGGGAACA -0.973658875274
TGGGACAA 0.910634237407
TGGGACCA -0.199827139575
TGGGAGAA -0.253914904444
TGGGATAA 1.03161687718
TGGGCAAA 0.0356829568158
TGGGCACA -0.558386666843
TGGGCCAA 0.557816259146
TGGGCCCA -1.29348563815
TGGGCGAA -0.458803059172
TGGGCTAA 0.843323422872
TGGGGAAA 1.01833595404
TGGGGACA -1.39209080926
TGGGGCAA -0.121311144647
TGGGGGAA -0.829329698282
TGGGGTAA 0.903349881305
TGGGTAAA 0.233163097715
TGGGTACA -0.447302890564
TGGGTCAA -0.585136706042
TGGGTGAA -0.482472896808
TGGGTTAA -0.223908336923
TGGTAAAA 1.19739421629
TGGTAACA -0.138725233638
TGGTACAA 2.46538721031
TGGTACCA -0.540697782903

TGGTAGAA 1.52000619251
TGGTATAA 1.74909025066
TGGTCAAA 2.46280601139
TGGTCACA -0.617331432432
TGGTCCAA 1.41700180408
TGGTCGAA -0.254331052249
TGGTCTAA 0.0201666183953
TGGTGAAA 1.21533104021
TGGTGACA -1.30344612233
TGGTGCAA 0.246600154356
TGGTGGAA 0.558813015369
TGGTGTAA 1.61115526068
TGGTTAAA 0.480586387849
TGGTTACA -0.159933158347
TGGTTCAA 0.996050291865
TGGTTGAA -0.830254633098
TGGTTTAA 2.11288753619
TGTAAAAA 1.66138444647
TGTAAACA 0.992918253982
TGTAACAA 2.18759512295
TGTAAGAA 0.189564797305
TGTAATAA 1.24552434339
TGTACAAA 0.654499114683
TGTACACA 0.211607099404
TGTACCAA 0.611058196835
TGTACGAA 0.467229896091
TGTACTAA 0.313056814591
TGTAGAAA 1.41174635069
TGTAGACA -0.856117834054
TGTAGCAA 0.679653677755
TGTAGGAA 0.18442946261
TGTAGTAA 0.545023721567
TGTATAAA 2.90280081356
TGTATACA 0.294042461379
TGTATCAA 1.29309093268
TGTATGAA 0.271804471968
TGTATTAA 1.51860328103
TGTCAAAA 1.151647519
TGTCAACA 0.524334992769
TGTCACAA 3.56833981531
TGTCAGAA 1.27773489131
TGTCATAA 1.22353741664
TGTCCAAA 1.06315459383
TGTCCACA 0.0313545200159
TGTCCCAA 1.93824368825
TGTCCGAA -0.320337631667
TGTCCTAA 0.266083532583
TGTCGAAA 2.61146841327
TGTCGACA -0.960799512559
TGTCGCAA 1.91077293685
TGTCGGAA 1.1274004038
TGTCGTAA 1.3111716076
TGTCTAAA 0.691942424595
TGTCTCAA 0.875594550585

TGTCTGAA -0.214630676553
TGTCTTAA 1.64392414197
TGTGAAAA 0.882333688527
TGTGAACA 0.00865312639584
TGTGACAA 1.14677303133
TGTGAGAA 0.286603637208
TGTGATAA 1.74991255373
TGTGCAAA 0.358739809408
TGTGCACA 0.388848175967
TGTGCCAA 0.110629531902
TGTGCGAA 1.3964879448
TGTGCTAA -0.130000494013
TGTGGAAA 1.581626878
TGTGGCAA 0.845372935199
TGTGGGAA 0.187162006927
TGTGGTAA 1.14810953404
TGTGTAAA 1.70280000064
TGTGTCAA 0.811568992935
TGTGTGAA 0.291298050917
TGTGTTAA 2.22142529564
TGTTAAAA 0.241238738362
TGTTAACA 2.96669147187
TGTTACAA 2.0285737842
TGTTAGAA 0.351357817828
TGTTATAA 1.584718113
TGTTCAAA 0.137839644462
TGTTCCAA 0.468664242452
TGTTCGAA -0.00856256896952
TGTTCTAA 0.844680118843
TGTTGAAA 1.6939817891
TGTTGCAA 0.969316282371
TGTTGGAA 0.226095246724
TGTTGTAA 2.48323535041
TGTTTAAA 1.23614863119
TGTTTCAA -0.0198843290387
TGTTTGAA 0.951933836434
TGTTTTAA 0.590715043503
TTAAAAAA 1.447263806
TTAAACAA 1.22032065032
TTAAAGAA 0.0994214370157
TTAAATAA 1.7029242829
TTAACAAA 2.59998281713
TTAACCAA 1.26192543828
TTAACGAA 0.955268847858
TTAACTAA 1.05027503785
TTAAGAAA 2.00455920381
TTAAGCAA 1.57479405997
TTAAGGAA 1.83880330704
TTAAGTAA 1.9139601419
TTAATAAA 3.25887427925
TTAATCAA 1.30780870032
TTAATGAA 2.47000376545
TTAATTAA 2.14276836421
TTACAAAA 3.12127341495

TTACACAA 0.845073991603
TTACAGAA 1.53291281163
TTACATAA 1.19526705357
TTACCAAA 1.70212009132
TTACCCAA 0.181446271992
TTACCGAA 0.6725760424
TTACCTAA 1.13132976339
TTACGAAA 0.788022604847
TTACGCAA 1.50776615932
TTACGGAA 1.54163005685
TTACGTAA 2.30704952809
TTACTAAA 1.06321538181
TTACTCAA 1.18723117492
TTACTGAA 0.832363476152
TTAGAAAA 0.403230777045
TTAGACAA 0.581539390349
TTAGAGAA 0.426238192313
TTAGATAA 1.44746074238
TTAGCAAA 1.53583979419
TTAGCCAA 1.21537017768
TTAGCGAA 0.601139764607
TTAGCTAA 0.0517426397942
TTAGGAAA 1.20773025357
TTAGGCAA 2.03267655606
TTAGGGAA 0.646246314565
TTAGTAAA 1.35542608504
TTAGTCAA 0.92790718172
TTAGTGAA 0.468729402164
TTATAAAA 2.52942421775
TTATACAA 1.77378682215
TTATAGAA 1.05937075066
TTATATAA 2.06438206417
TTATCAAA 1.23587779162
TTATCCAA 2.60656103348
TTATCGAA 1.60372830265
TTATGAAA 1.79610974818
TTATGCAA 1.41754723042
TTATGGAA 0.0974214710511
TTATTAAA 3.4426175872
TTATTCAA 1.23688578946
TTATTGAA 0.768877932212
TTCAAAAA 1.42117743824
TTCAACAA 2.3204867929
TTCAAGAA -0.555912263235
TTCACAAA 1.61250633584
TTCACCAA 1.04183008945
TTCACGAA 0.31888621471
TTCAGAAA 0.655977178423
TTCAGCAA 1.80131877789
TTCAGGAA 0.529599814174
TTCATAAA 1.64861189398
TTCATCAA 2.17642845548
TTCATGAA 0.755457113454
TTCCAAAA 1.52250058121

TTCCACAA 1.19638538574
TTCCAGAA -0.524785489942
TTCCCAAA 2.05701297962
TTCCCCAA 0.559997339963
TTCCCGAA 1.25997252054
TTCCGAAA 0.85414472305
TTCCGCAA 0.398838013246
TTCCGGAA 1.59360856231
TTCCTAAA 1.61281548016
TTCCTCAA 0.388328980056
TTCGAAAA 0.873970762251
TTCGACAA 0.769769350372
TTCGAGAA -0.250806182497
TTCGCAAA 0.118085634846
TTCGCCAA 0.215305589602
TTCGCGAA 0.737696408107
TTCGGAAA 0.396545765381
TTCGGCAA 1.05418253875
TTCGTAAA 1.2362052556
TTCGTCAA 1.07901983857
TTCTAAAA 1.14011778912
TTCTACAA 0.911236288159
TTCTAGAA 0.832947623597
TTCTCAAA 1.19608914846
TTCTCCAA -0.627637075724
TTCTGAAA -0.145808281622
TTCTGCAA 0.294139680501
TTCTTAAA 0.286497882788
TTCTTCAA 1.70913652235
TTGAAAAA 3.67987367241
TTGAACAA 0.0728173305885
TTGACAAA 3.39533349545
TTGACCAA 0.937858922427
TTGAGAAA 0.609014305314
TTGAGCAA 1.47856857148
TTGATAAA 2.1442412235
TTGATCAA 0.419017746855
TTGCAAAA 1.82421752424
TTGCACAA 0.355957302373
TTGCCAAA 0.847730342775
TTGCCCAA 0.359392239233
TTGCGAAA 1.16523925189
TTGCGCAA 2.32768308972
TTGCTAAA 1.09949456039
TTGGAAAA 0.955154766319
TTGGACAA 0.574072462148
TTGGCAAA 0.701979518279
TTGGCCAA 0.790296116693
TTGGGAAA 1.06651583568
TTGGTAAA 1.31795321385
TTGTAAAA 1.20202513571
TTGTACAA 0.965613836791
TTGTCAAA 2.25402638547
TTGTGAAA 0.651943938015

TTGTTAAA 2.51556289436
TTTAAAAA 2.12882626776
TTTACAAA 1.84775412796
TTTAGAAA 0.498282766197
TTTATAAA 1.17336152441
TTTCAAAA 2.325298619
TTTCCAAA 1.42190731028
TTTCGAAA 0.751773195716
TTTGAAAA 3.51676892236
TTTGCAAA 2.87121250966
TTTTAAAA 1.92713780876
